# Provenance and family variations in early growth of Manchurian walnut (*Juglans mandshurica* Maxim.) and selection of superior families

**DOI:** 10.1371/journal.pone.0298918

**Published:** 2024-03-07

**Authors:** Qinhui Zhang, Su Chen, Guanzheng Qu, Yuchun Yang, Zhiming Lu, Jun Wang, Mulualem Tigabu, Jifeng Liu, Lianfeng Xu, Fang Wang

**Affiliations:** 1 State Key Laboratory of Tree Genetics and Breeding, Northeast Forestry University, Harbin, China; 2 Jilin Provincial Academy of Forestry Sciences, Changchun, China; 3 Southern Swedish Forest Research Center, Swedish University of Agricultural Sciences, Alnarp, Sweden; 4 Wanrenhuan Forest Farm of Binxian County, Harbin, China; 5 Qiqihar Branch of Heilongjiang Academy of Forestry, Qiqihar, China; Government College University Faisalabad, PAKISTAN

## Abstract

This study, conducted in China in November 2020, was aimed at exploring the variations in growth traits among different provenances and families as well as to select elite materials of *Juglans mandshurica*. Thus, seeds of 44 families from six *J*. *mandshurica* provenances in Heilongjiang and Jilin provinces were sown in the nursery and then transplanted out in the field. At the age of 5 years, seven growth traits were assessed, and a comprehensive analysis was conducted as well as selection of provenance and families. Analysis of variance revealed statistically significant (*P* < 0.01) differences in seven growth traits among different provenances and families, thereby justifying the pursuit of further breeding endeavors. The genetic coefficient of variation (GCV) for all traits ranged from 5.44% (branch angle) to 21.95% (tree height) whereas the phenotypic coefficient of variation (PCV) ranged from 13.74% (tapering) to 38.50% (branch number per node), indicating considerable variability across the traits. Further, all the studied traits except stem straightness degree, branch angle and branch number per node, showed high heritability (Tree height, ground diameter, mean crown width and tapering, over 0.7±0.073), indicating that the variation in these traits is primarily driven by genetic factors. Correlation analysis revealed a strong positive correlation (*r* > 0.8) between tree height and ground diameter (*r* = 0.86), tree height and mean crown width (*r* = 0.82), and ground diameter and mean crown width (*r* = 0.83). This suggests that these relationships can be employed for more precise predictions of the growth and morphological characteristics of trees, as well as the selection of superior materials. There was a strong correlation between temperature factors and growth traits. Based on the comprehensive scores in this study, Sanchazi was selected as elite provenance. Using the top-percentile selection criteria, SC1, SC8, DJC15, and DQ18 were selected as elite families. These selected families exhibit genetic gains of over 10% in tree height, ground diameter and mean crown width, signifying their significant potential in forestry for enhancing timber production and reducing production cycles, thereby contributing to sustainable forest management. In this study, the growth traits of *J*. *mandshurica* were found to exhibit stable variation, and there were correlations between these traits. The selected elite provenance and families of *J*. *mandshurica* showed faster growth, which is advantageous for the subsequent breeding and promotion of improved *J*. *mandshurica* varieties.

## 1. Introduction

The genus *Juglans* is one of the most important genera of Juglandaceae family, comprises about 21 species, and *Juglans mandshurica* Maxim. is a deciduous fruit tree belonging to the Juglandaceae family [1, 2]. This tree is native to northern and northeastern China and is mainly distributed in the Changbai and Xiaoxingan Mountains at an altitude ranging from 500 m to 1000 m [3]. The species also grows naturally in Russia, Japan, and Korea and can tolerate temperatures of -50°C, and has important economic values [4, 5]. The *J*. *mandshurica* is a versatile wood that is rigid, wear and corrosion-resistant, easily processed, and produces high quality wood suitable for joinery in luxurious furnishings [6, 7]. Besides, its seeds are rich in nutrition and are considered a premium forest food product [8]. Due to its high economic returns and nutritional value, its production and consumption are increasing all over the world [9, 10]. Furthermore, *J*. *mandshurica* has high medicinal and health values [11]. For instance, its husk, bark, roots and leaves contain juglone, which has good antitumor [12], antifungal [13], antiviral [14], antioxidant [15], anthelmintic [16] and hypoglycemic activities [17]. They are widely used in pharmaceutical, food, and cosmetic industries.

Understanding and leveraging the variation and interplay of genetic and phenotypic traits hold decisive importance in optimizing the improvement of biological characteristics across various research domains. Against this backdrop, techniques such as correlation analysis, general combining ability analysis, phenotypic coefficient of variation analysis, genetic coefficient of variation analysis, and family heritability analysis have been widely employed to interpret and exploit genetic diversity. Prior studies have laid the foundation for plant breeding by exploring genetic associations between traits, evaluating parental combining effects, and analyzing phenotypic and genetic variations [18, 19]. Recently, researchers have delved deeper into understanding the specific roles of genotype and environmental factors in phenotypic expression and genetic variation using more advanced and refined methods [20]. While these studies have offered valuable insights, there remain unresolved issues and challenges in the specific application of *Juglans mandshurica*.

Provenance tests are crucial for species that have high economic or ecological value [21]. Systematic provenance test can explore the adaptability of different provenance materials to the local environment, quantify the genetic population differentiation, and provide materials for forest breeding and *ex-situ* protection [22, 23]. Provenance test for *J*. *mandshurica* began in the 1980s ~ 1990s [24] by investigating and analyzing phenotypic growth traits of eight *J*. *mandshurica* provenances, which resulted in selection of two best provenances in Kuandian and Shulan. Based on a geographical and climatic variation study, Liu profiled the differences in growth and physiological performance of the provenances, and preliminarily classified *J*. *mandshurica* into four major provenance zones [25]. Since then, there have been several studies on the provenance of *J*. *mandshurica* in China [8, 26–34]. Most of these studies, however, concentrated on seed traits or a few growth traits, other growth data of *J*. *mandshurica* seedlings are relatively scarce. In addition, due to challenges posed by late genetic improvement of the *J*. *mandshurica*, coupled with long sexual reproduction cycle and low survival rate of vegetative propagation such as cuttings and grafting. These led to a small number and usage rate of improved varieties of *J*. *mandshurica*. In addition, excessive logging and man-made destruction of natural forests in recent decades have led to a large reduction in the number of trees [32]. Some available natural distribution areas are even mostly secondary forests, and *J*. *mandshurica* trees are on the verge of depletion [35]. At the same time, climate and environmental changes include warming temperatures, late spring frost, drought stress, high temperature stress and salt stress also negatively affected the growth of genus *Juglans* [36–38]. It is, therefore, important to explore variation patterns of provenances and families to guide collection and conservation of *J*. *mandshurica* resources, protection of biodiversity and maintenance of ecological balance. Recent finding shown that although *J*. *mandshurica* natural forest is seriously damaged, there is still a high degree of genetic diversity [6]. The result showed that the existing resources of *J*. *mandshurica* may still have rich variation, which provided the possibility for the selection of improved varieties of *J*. *mandshurica*. On the other hand, the main breeding goal of genus *Juglans* plants is to cultivate productive new varieties and rootstocks with superior resistance [39–44]. Due to its good drought and cold tolerance and well-developed root system, *J*. *mandshurica* is often used as an excellent rootstock for walnuts, which can effectively improve walnut yield and stress resistance [45, 46]. Therefore, preliminary selecting of *J*. *mandshurica* resources can provide basic materials for the breeding of high-yield and high-resistance *J*. *mandshurica* varieties. This study employed the provenance test to profile 5-year-old *J*. *mandshurica* tress in Wanrenhuan forest farm of Binxian county, Heilongjiang Province. The objectives were to: (1) evaluate the variations among different *J*. *mandshurica* provenances and families. (2) select elite provenance and families. The existing studies have primarily focused on preliminary insights into the economic, medicinal, and timber value of *J*. *mandshurica*, as well as the presence of certain bioactive components. However, these studies have limitations in the in-depth exploration of diversity, the selection of genetic sources, and the evaluation of families for *J*. *mandshurica*. They also do not provide comprehensive information on growth characteristics. Therefore, the primary strength of this study lies in the in-depth investigation of the growth traits of *J*. *mandshurica*. It involves the assessment of genetic variations and the selection of elite provenance and families, thereby providing robust support for the forest management and timber production of this species.

## 2. Materials and methods

### 2.1. Experimental site and plant materials

**Fig 1:**
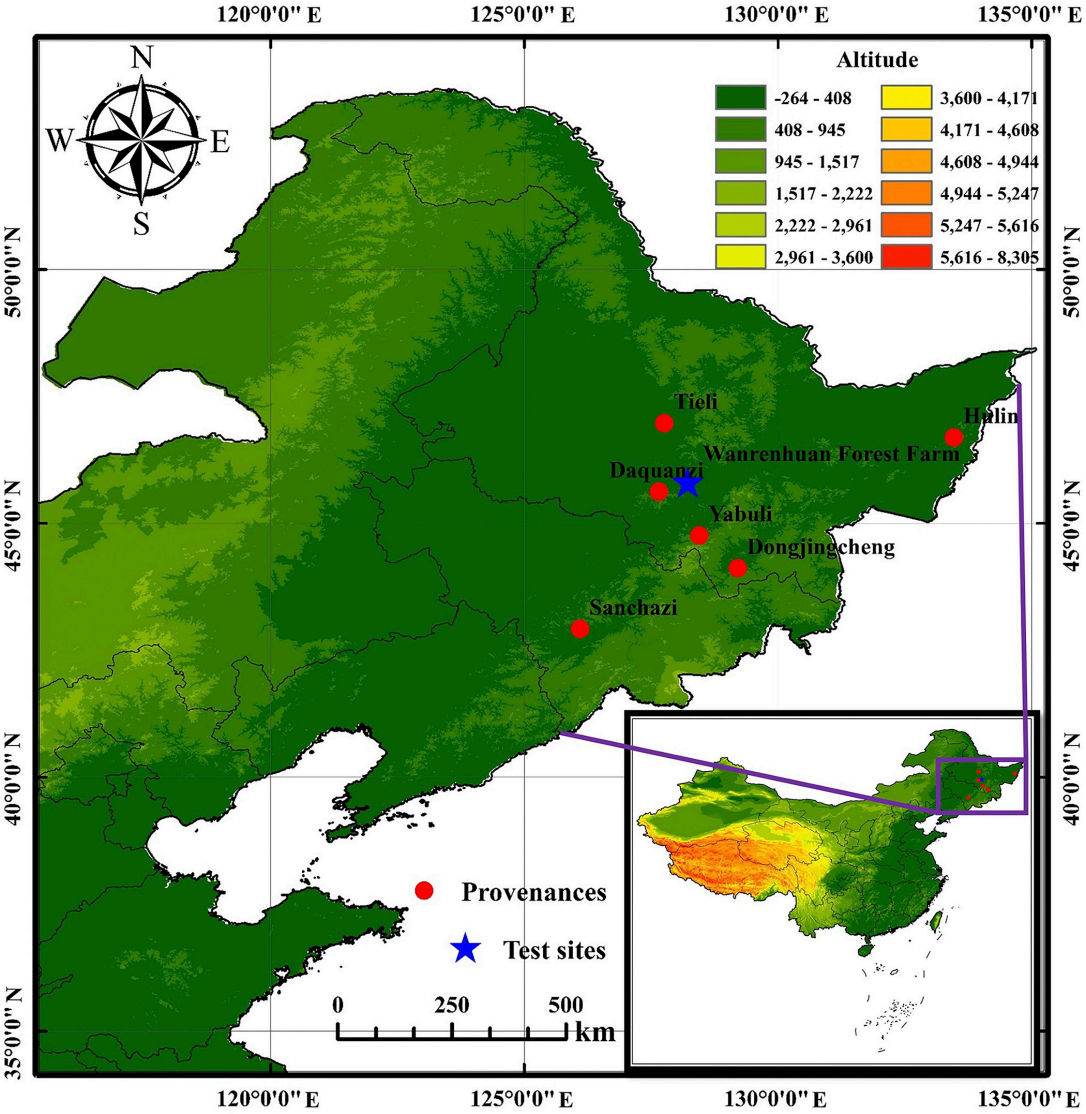
Provenance distribution of *Juglans mandshurica* collected in Northeast China.

**Fig 2:**
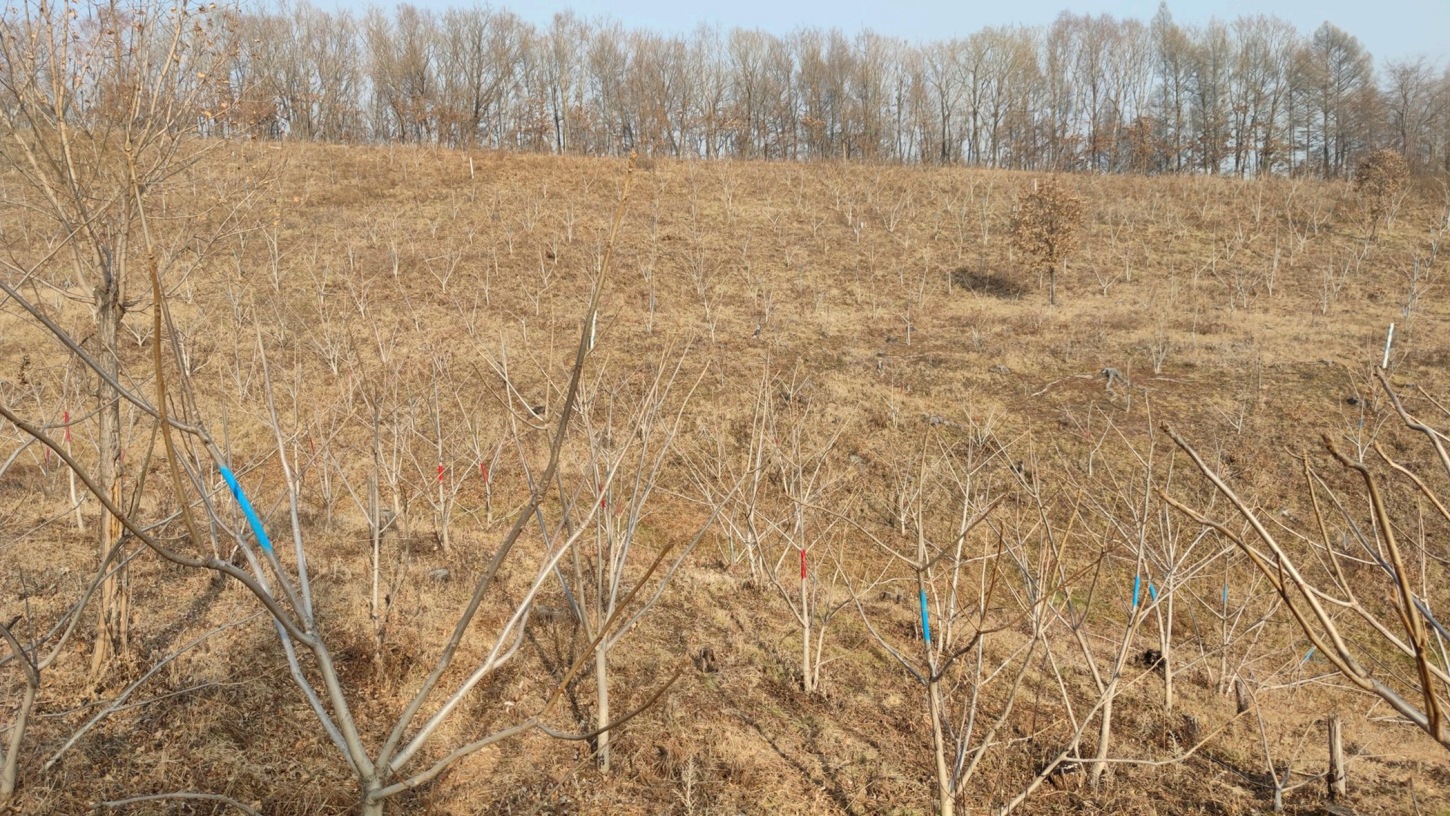
Test forest of *Juglans mandshurica* in Northeast China.

Seeds of 44 half-sib families (44 individual trees, each tree collected 120 seeds) from six *J*. *mandshurica* provenances in Heilongjiang and Jilin provinces, China, were collected. The provenances were Sanchazi (15 families), Hulin (3 families), Daquanzi (9 families), Dongjingcheng (10 families), Yabuli (4 families) and Tieli (3 families) (Table 1). The seeds from open-pollination were sown in autumn 2015, with a sowing area of 1.69 hm^2^, each family selects 100 healthy seeds for sowing in the nursery, with a germination rate of 74%. 32 seedlings with consistent growth of *J*. *mandshurica* were selected per family for afforestation, planted the following year in a randomized complete block design, with 8 trees and 4 blocks at a spacing of 3 m × 4 m (plant spacing and row spacing) in Wanrenhuan Forest Farm of Binxian County, Heilongjiang province (E45°44′20″ ~ 45°57′18″, E128°04′29″ ~ 128°19′13″), and the distance between families is 4 m. The altitude of experimental area is 340 m, with a mean annual temperature of 2.4°C, mean annual rainfall of 577 mm, mean annual sunshine hours of 2090 h, and a frost-free period of 130 d. The geographical location and environmental factors of the provenances are given in Fig 1 and Table 1, and the test forest of *Juglans mandshurica* given in Fig 2.

**Table 1:** Family numbers and environmental factors of different provenance of *J*. *mandshurica* collected in Northeast China.

Provenances	Sanchazi	Hulin	Daquanzi	Dongjingcheng	Yabuli	Tieli
Family number	SC1, SC6, SC8, SC11, SC13, SC18, SC20, SC22, SC23, SC25	HL4, HL6, HL12, HL14, HL15, HL23, HL24, HL26	CK, DQ1, DQ6, DQ8, DQ10, DQ17, DQ18, DQ21, DQ23	DJC3, DJC4, DJC5, DJC8, DJC10, DJC12, DJC13, DJC15, DJC17, DJC23	YBL6, YBL13, YBL14, YBL16	TL3, TL16, TL30
Longitude (E)	126.93	132.93	127.63	129.42	128.6	128.75
Latitude (N)	41.25	45.58	45.65	43.75	44.92	46.9
Climate	Temperate continental monsoon climate	Cold-temperate continental monsoon climate	Mid-temperate continental monsoon climate	Mid-temperate continental monsoon climate	Temperate continental monsoon climate	Cold-temperate continental monsoon climate
Altitude (m)	520	70	500	351	834	500
Annual temperature (°C)	2.5	3.7	3.5	3.5	2.3	1.4
January mean temperature (°C)	-16.3	-17.6	-16.5	-17.1	-18.7	-20.9
July mean temperature (°C)	21.2	21.7	23	22.2	22.3	22.1
Summer mean temperature (°C)	19.8	20.6	21.7	21.1	21	20.5
Winter mean temperature (°C)	-13.6	-15.1	-13.9	-14.2	-16	-18.2
Annual rainfall (mm)	726	587	575	424	666	630
January mean rainfall (mm)	7.8	8.2	3.8	5.1	6.3	5
July mean rainfall (mm)	207.4	120	164.9	125.5	183.8	153.3
Summer mean rainfall (mm)	163	105.7	128.2	107.4	141.9	128.4
Winter mean rainfall (mm)	10.3	9.3	23.8	5.5	8	6.1
Annual sunshine hours (h)	2300	2295	2704	2500	2525	2420
Frost-free days (d)	110	141	135	140	120	128

### 2.2. Trait measurements

In November 2020, we assessed the growth and stem traits of a total of 1408 individual tree (The test forest consists of 44 families, with 32 individual trees per families) in a 5-year-old *J*. *mandshurica* provenance test forest. The traits included tree height (m), ground diameter (cm), mean crown width (m), stem straightness degree, tapering, branch angle (°) and branch number per node. The tree height was measured using a Box staff. The ground diameter was measured using a tree caliper. The east-west and north-south crown width were measured using a stainless-steel measuring tape, and the average of two crown width was taken as the mean crown width value. The stem straightness degree was meas ured using grading method and a score from 1 to 5 was assigned, where score 1 was assigned if the stem had more than two obviously bend points in the stem and a score of 5 if the stem was completely straight [47]. Stem straightness degree necessitates the square root transformation of data during the process of conducting analysis of variance (ANOVA). The ratio of ground diameter to tree height was used to calculate tapering [48]. The branch angle was measured using a protractor. Climatic factors were obtained from the National Meteorological Information Center (http://data.cma.cn/). In this study, field site access and sample collection did not require specific permits. The research was conducted with the full consent and cooperation of the relevant site managers and authorities. No permits were necessary due to the non-sensitive nature of the work and the site’s accessibility to researchers. In addition, we collected growth measurement data from the Experimental Forest. These data, referred to as ’Experimental Forest Growth Measurement Data,’ have been included as supporting information in this study.

### 2.3. Statistical analysis

Mixed linear model was applied to investigate into the differences of provenance, family and block effects on growth traits. The linear model was applied using the following formula [49]. All data were tested for normal distribution and homogeneity of variances.

$$X_{\mathrm{ijkl}}=\mu +B_{k}+P_{i}+F_{j(i)}+{BP}_{ik}+{BF}_{j(i)k}+e_{ijkl}-$$ (1)

where *X*_*ijkl*_ is the observed value of an individual tree *l* in family *j* within provenance *i* growing in block *k*, *μ* is the family mean, *B*_*k*_ is the fixed effect of block *k*, *P*_*i*_ is the fixed effect of provenance *i*, *F*_*j*(*i*)_ is the random effect of family *j* within provenance *i*, *BP*_*ik*_ is the fixed interactive effect of block *k* and provenance *i*, *BF*_*j*(*i*)*k*_ is the random interactive effect of block *k* and family *j* within provenance *i*, and *e*_*ijkl*_ is the random error.

Heritability (*h*^*2*^) among families for each trait was calculated following a formula described by Zhang [50]:

h2=σF2σF2+σFB2B+σe2BN (2)

where $\sigma_{F}^{2},\;\sigma_{FB}^{2}$ and $\sigma_{e}^{2}$ denotes the variance components of family, family by block interaction, and residual error, respectively. B and N represent the number of blocks and replicates, respectively.

The coefficient of variation was calculated by using the following formula [51]:

phenotypic coefficient of variation (PCV):

$$PCV=\frac{\sqrt{\sigma_{P}^{2}}}{\bar{X}}\times 100\%$$ (3)

genetic coefficient of variation (GCV):

$$GCV=\frac{\sqrt{\sigma_{g}^{2}}}{\bar{X}}\times 100\%$$ (4)

where $\sigma_{P}^{2}$ denotes the phenotypic variance of a trait, $\sigma_{g}^{2}$ denotes the additive genetic variance component in maternal half-sib of a trait ($4\sigma_{F}^{2}$), $\bar{X}$ is the mean value of a growth trait.

The Pearson correlation coefficient was used to measure the linear relationship between two variables. The Pearson correlation coefficient (*r*) is a standard statistical method employed to quantify the correlation between two variables. Its calculation formula is as follows:

$$r_{A(xy)}=\frac{\sigma_{a(xy)}}{\sqrt{\sigma_{a(x)}^{2}∙\sigma_{a(y)}^{2}}}$$ (5)

where *σ*_*a*(*xy*)_ is the covariance component between trait or environmental factor *x* and *y*, $\sigma_{a(x)}^{2}$ and $\sigma_{a(y)}^{2}$ are the variance component for trait or environmental factor *x* and *y*, respectively.

The general combining ability (GCA) was calculated by the formula [52]:

g=x‐μ (6)

where *g* is the general combining ability of parents, *x* is the offspring mean of a hybrid combination of the parents for a particular trait, and *μ* is the offspring mean of all combinations for this trait.

The principal component values and comprehensive score were calculated according to Chen [52]:

$$Y_{i}=\sum_{j=1}^{n}\alpha_{ij}X_{j}(j=1,2,3,\cdots n)$$ (7)

$$W=\sum_{i=1}^{p}Y_{i}\omega_{i}\;(i=1,2,3,\cdots ,p)$$ (8)

where *Y*_*i*_ is the value of principal component *i*, *α*_*ij*_ is the eigenvalue of trait *j* within principal component *i*, *X*_*j*_ is the mean of trait *j*, *W* is the comprehensive score, *ω*_*i*_ the contribution rate of principal component *i*, *n* is the number of traits, and *p* is the number of extracted principal components.

Selecting elite provenance and families with a selection rate of 10%. The real gains of elite provenance and genetic gain of elite families were computed using the following formula [49]:

genetic gains (Δ*G*_1_):

$$\Delta G_{1}=h^{2}W/\bar{X}$$ (9)

real gains (Δ*G*_2_):

$$\Delta G_{2}=W/\bar{X}$$ (10)

where *h*^2^ is the heritability of family, *W* the selection differential, $\bar{X}$ is the family or provenance mean.

The mean analysis, ANOVA, principal component analysis and Pearson correlation analysis were conducted using IBM SPSS Statistics, version 26.0 (IBM Corp., Armonk, NY, USA) [53]. Additionally, variance parameters and general combining ability were calculated. For all statistical analyses, *P*-values less than 0.05 were considered statistically significant. Provenance distribution map was drawn using ArcGIS, version 10.7 (Esri, Redlands, CA, USA) [54]. Custom data visualization was carried out in the R environment, version 4.0.2, using "corrplot" packages (R Core Team, Vienna, Austria) [55].

## 3. Results

### 3.1. Variations in early growth among provenances and families

Significant variations (*P*<0.01) in growth traits were detected among different provenances and families within provenances (Table 2). Description statistics of growth and stem traits together with GCV, PCV and heritability are shown in Table 3. The *GCVs* of all the traits ranged from 5.44 to 21.95%, among which tree height, ground diameter and mean crown width were higher than 15%. The *PCVs* of all the traits ranged from 13.74% to 38.50%, among which the stem straightness degree and branch number per node were more than 30%. The heritability of all traits in the families ranged from 0.260 to 0.908, among which the heritability of all the traits was higher than 0.7, except for the stem straightness degree, branch angle and branch number per node. In addition, the mean value of all growth traits for different provenances and families are shown in S1 and S2 Tables. The results in S1 Table showed that Daquanzi and Sanchazi provenance had more elite growth traits, which was also reflected in S2 Table. Most of the families with elite growth traits from the Daquanzi and Sanchazi provenance.

**Table 2:** Variance analysis of different traits among *J*. *mandshurica* families in Northeast China.

Traits	Variance sources	*df*	*MS*	*F*	*EMS*	*σ* ^*2*^
Tree height	Block	3	0.378	5.921**	$\sigma^{2}+432\sigma_{B}^{2}$	0.315
	Provenance	5	169.158	23.186**	$\sigma^{2}+32\sigma_{F/P}^{2}+288\sigma_{P}^{2}$	162.474
	Family/Provenance	48	7.296	10.797**	$\sigma^{2}+8\sigma_{BF/P}^{2}+32\sigma_{F/P}^{2}$	6.620
	Block×Provenance	15	2.257	35.304**	$\sigma^{2}+72\sigma_{BP}^{2}$	2.193
	Block×Family/Provenance	144	0.676	10.572**	$\sigma^{2}+8\sigma_{BF/P}^{2}$	0.612
	Random error					0.064
Ground diameter	Block	3	2.827	5.681**	$\sigma^{2}+432\sigma_{B}^{2}$	2.329
	Provenance	5	998.889	21.229**	$\sigma^{2}+32\sigma_{F/P}^{2}+288\sigma_{P}^{2}$	955.530
	Family/Provenance	48	47.053	11.227**	$\sigma^{2}+8\sigma_{BF/P}^{2}+32\sigma_{F/P}^{2}$	42.862
	Block×Provenance	15	10.137	20.372**	$\sigma^{2}+72\sigma_{BP}^{2}$	9.639
	Block×Family/Provenance	144	4.191	8.423**	$\sigma^{2}+8\sigma_{BF/P}^{2}$	3.694
	Random error					0.498
Mean crown width	Block	3	0.135	1.331	$\sigma^{2}+432\sigma_{B}^{2}$	0.034
	Provenance	5	74.345	20.779**	$\sigma^{2}+32\sigma_{F/P}^{2}+288\sigma_{P}^{2}$	71.591
	Family/Provenance	48	3.578	3.868**	$\sigma^{2}+8\sigma_{BF/P}^{2}+32\sigma_{F/P}^{2}$	2.653
	Block×Provenance	15	2.295	22.566**	$\sigma^{2}+72\sigma_{BP}^{2}$	2.193
	Block×Family/Provenance	144	0.925	9.095**	$\sigma^{2}+8\sigma_{BF/P}^{2}$	0.823
	Random error					0.102
Stem straightness degree	Block	3	2.689	3.110*	$\sigma^{2}+432\sigma_{B}^{2}$	1.824
	Provenance	5	524.602	18.995**	$\sigma^{2}+32\sigma_{F/P}^{2}+288\sigma_{P}^{2}$	497.854
	Family/Provenance	48	27.617	15.930**	$\sigma^{2}+8\sigma_{BF/P}^{2}+32\sigma_{F/P}^{2}$	25.884
	Block×Provenance	15	1.300	1.504	$\sigma^{2}+72\sigma_{BP}^{2}$	0.436
	Block×Family/Provenance	144	1.734	2.005**	$\sigma^{2}+8\sigma_{BF/P}^{2}$	0.869
	Random error					0.865
Tapering	Block	3	0.331	8.421**	$\sigma^{2}+432\sigma_{B}^{2}$	0.292
	Provenance	5	199.650	14.973**	$\sigma^{2}+32\sigma_{F/P}^{2}+288\sigma_{P}^{2}$	186.463
	Family/Provenance	48	13.334	71.423**	$\sigma^{2}+8\sigma_{BF/P}^{2}+32\sigma_{F/P}^{2}$	13.148
	Block×Provenance	15	0.257	6.534**	$\sigma^{2}+72\sigma_{BP}^{2}$	0.218
	Block×Family/Provenance	144	0.187	4.744**	$\sigma^{2}+8\sigma_{BF/P}^{2}$	0.147
	Random error					0.039
Branch angle	Block	3	1728.047	34.443**	$\sigma^{2}+432\sigma_{B}^{2}$	1677.875
	Provenance	5	75332.383	17.989**	$\sigma^{2}+32\sigma_{F/P}^{2}+288\sigma_{P}^{2}$	71214.599
	Family/Provenance	48	4187.668	34.881**	$\sigma^{2}+8\sigma_{BF/P}^{2}+32\sigma_{F/P}^{2}$	4067.613
	Block×Provenance	15	231.455	4.613**	$\sigma^{2}+72\sigma_{BP}^{2}$	181.284
	Block×Family/Provenance	144	120.056	2.393**	$\sigma^{2}+8\sigma_{BF/P}^{2}$	69.884
	Random error					50.172
Branch number per node	Block	3	0.976	1.928	$\sigma^{2}+432\sigma_{B}^{2}$	0.470
	Provenance	5	155.634	17.655**	$\sigma^{2}+32\sigma_{F/P}^{2}+288\sigma_{P}^{2}$	146.921
	Family/Provenance	48	8.815	14.503**	$\sigma^{2}+8\sigma_{BF/P}^{2}+32\sigma_{F/P}^{2}$	8.207
	Block×Provenance	15	0.612	1.029	$\sigma^{2}+72\sigma_{BP}^{2}$	0.106
	Block×Family/Provenance	144	0.608	1.201*	$\sigma^{2}+8\sigma_{BF/P}^{2}$	0.102
	Random error					0.506

Note: *df*: degree of freedom; *MS*: mean square; *F*: f value; *EMS*: expected mean square; *σ*^*2*^: variance component

** significant at the 0.01 level

* significant at the 0.05 level.

**Table 3:** Variation parameters of different traits among *J*. *mandshurica* families in Northeast China.

Traits	Mean	Range	SD	GCV (%)	PCV (%)	*h* ^*2*^
Tree Height (m)	2.07	1.68~2.64	0.24	21.95	29.56	0.908
Ground diameter (cm)	5.13	4.14~6.60	0.54	19.67	28.46	0.880
Mean crown width (m)	1.33	0.80~1.96	0.53	17.21	19.12	0.741
Stem straightness degree	3.83	3.22~4.50	0.30	11.83	30.53	0.585
Tapering	2.49	2.14~2.81	1.99	8.99	13.74	0.857
Branch angle (°)	46.85	42.95~50.33	0.16	5.44	19.23	0.411
Branch number per node	2.11	1.84~2.44	0.12	7.90	38.50	0.260

Note: SD: standard deviation; GCV: genetic coefficients of variation; PCV: phenotype coefficients of variation; *h*^*2*^: heritability.

### 3.2. Correlation analysis

**Fig 3:**
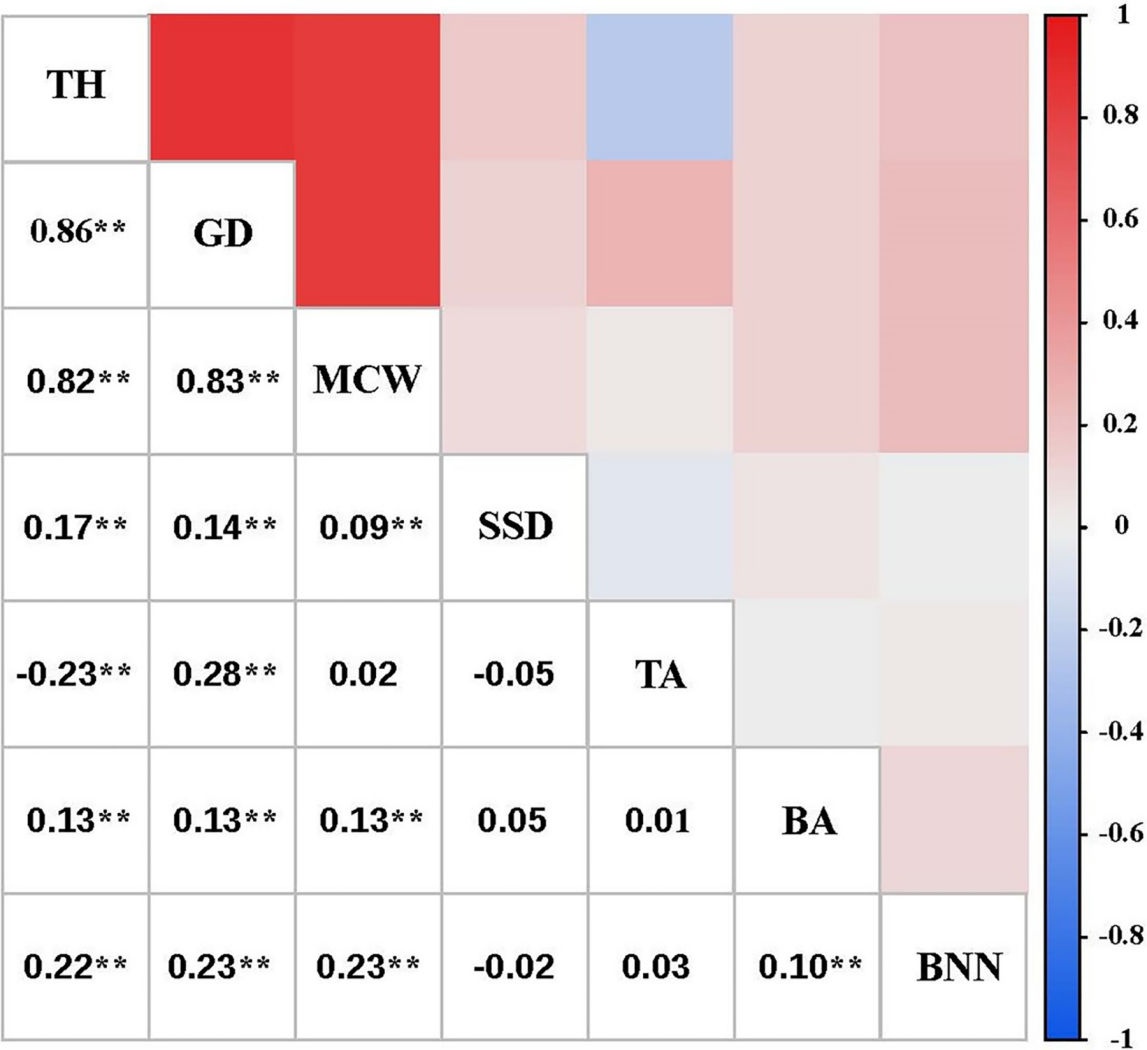
Correlation analysis among different growth traits of *J*. *mandshurica* in Northeast China. TH: Tree height; GD: Ground diameter; MCW: Mean crown width; SSD: Stem straightness degree; TA: Tapering; BA: Branch angle; BNN: Branch number per node. **correlation is significant at 1% level; Numbers are the correlation coefficients among different traits.

**Fig 4:**
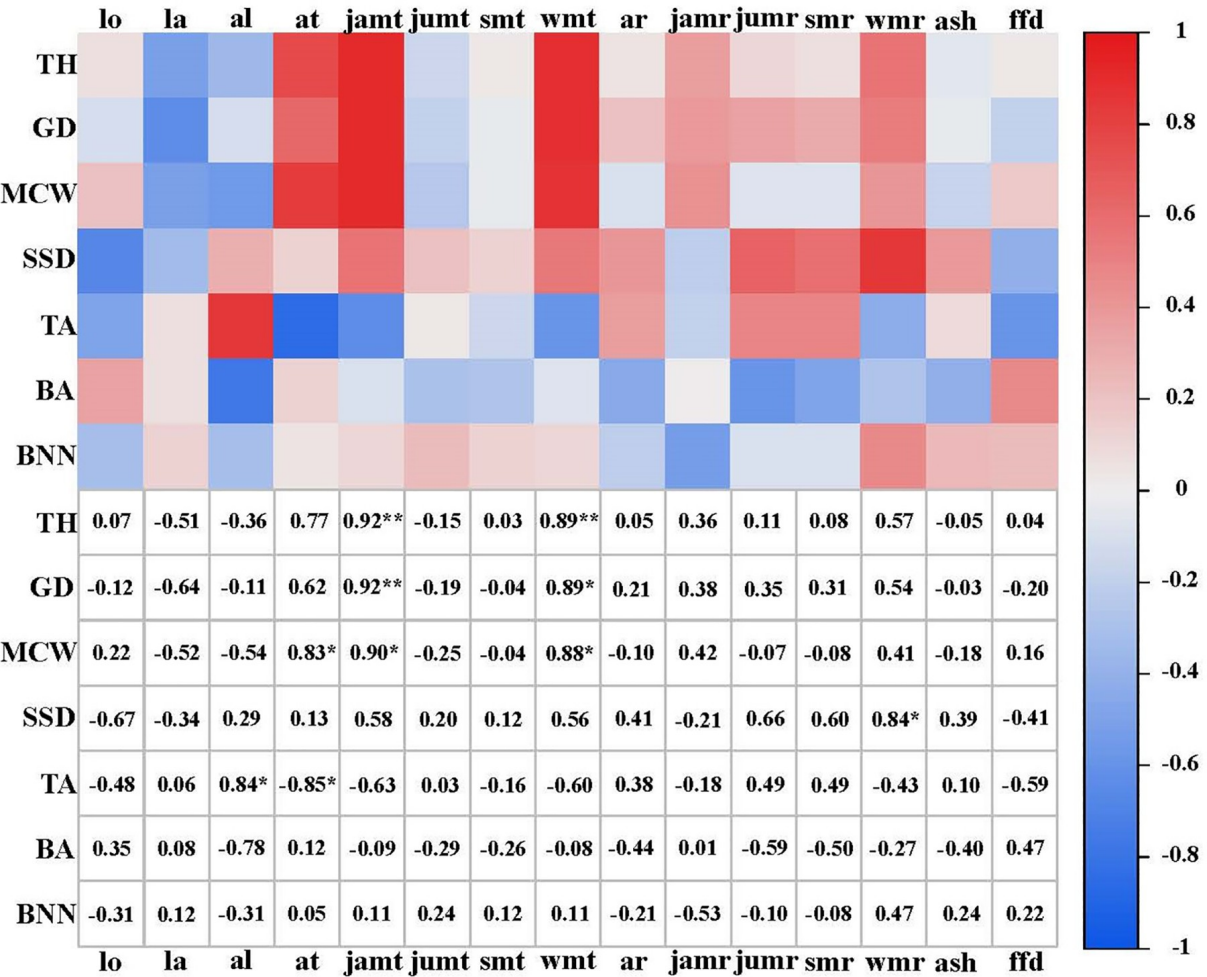
Correlation analysis between different growth traits and environmental factors of *J*. *mandshurica* in Northeast China. TH: Tree height; GD: Ground diameter; MCW: Mean crown width; SSD: Stem straightness degree; TA: Tapering; BA: Branch angle; BNN: Branch number per node; lo: Longitude; la: Latitude; al: Altitude; at: Annual temperature; jamt: January mean temperature; jumt: July mean temperature; smt: Summer mean temperature; wmt: Winter mean temperature; ar: Annual rainfall; jamr: January mean rainfall; jumr: July mean rainfall; smr: Summer mean rainfall; wmr: Winter mean rainfall; ash: Annual sunshine hours; ffd: Frost-free days. **correlation is significant at 1% level, *correlation is significant at 5% level; Numbers are the correlation coefficients between traits and environmental factors.

The correlation among the tree height, ground diameter and mean crown width were significant and positively correlated with a coefficient exceeding 0.8 (Fig 3). Stem straightness degree, branch angle and branch number per node had a weak positive correlation with tree height, ground diameter and mean crown width, with a correlation coefficient of not more than 0.3. Among environmental factors, annual temperature had a strong positive correlation with mean crown width, while annual temperature had a negative correlation with tapering, with correlation coefficients of more than 0.8 (Fig 4). Additionally, it was observed that January mean temperature and winter mean temperature had a strong positive correlation with tree height, ground diameter and mean crown width, the magnitude of the correlation coefficients exceeded 0.9. Tapering was strongly positively correlated with altitude (*r* = 0.84). In addition, stem straightness degree was strongly positively correlated with winter mean rainfall (*r* = 0.84). No correlation was observed between the remaining traits and the environmental factors.

### 3.3. General combining ability

We evaluated the general combining ability (GCA) of different traits among the families (Table 4). The GCAs for tree height ranged from -0.395 (DQ1) to 0.570 (SC1), while that for ground diameter ranged from -0.984 (DQ1) to 1.469 (SC1), from -0.528 (DQ1) to 0.625 (SC1) for mean crown width. In addition, the GCAs for the stem straightness degree ranged from -0.610 (DJC5) to 0.670 (DJC8), from -0.349 (SC13) to 0.322 (YBL13) for tapering, from -3.894 (SC13) to 3.481 (SC8) for branch angle and -0.270 (SC20) to 0.329 (DQ23) for branch number per node.

**Table 4:** General combining ability values of different traits among *J*. *mandshurica* families in Northeast China.

Families	Tree height	Families	Ground diameter	Families	Mean crown width	Families	Stem straightness degree	Families	Tapering	Families	Branch angle	Families	Branch number per node
SC1	0.570	SC1	1.469	SC1	0.625	DJC8	0.670	YBL13	0.322	SC8	3.481	DQ23	0.329
DQ21	0.448	SC6	0.988	DQ8	0.474	DQ8	0.510	SC8	0.234	DQ18	3.419	HL4	0.309
DQ8	0.437	DQ21	0.810	SC6	0.422	SC1	0.510	DJC10	0.189	DJC15	3.247	SC6	0.283
SC13	0.351	SC8	0.782	DQ21	0.399	DQ21	0.420	TL3	0.150	DJC10	3.200	DJC5	0.267
SC6	0.341	SC22	0.733	HL24	0.341	DQ18	0.390	TL30	0.130	HL6	2.809	DJC17	0.246
DQ6	0.335	HL14	0.722	HL14	0.264	DQ1	0.360	YBL6	0.114	DJC13	2.262	DQ10	0.225
SC22	0.280	DQ8	0.701	DJC10	0.213	DQ17	0.300	SC23	0.103	TL16	2.200	DQ21	0.194
HL14	0.276	DJC15	0.494	HL6	0.200	HL12	0.260	SC25	0.101	DQ6	2.122	SC1	0.184
HL6	0.237	DQ6	0.485	SC8	0.189	SC25	0.260	SC11	0.085	SC1	1.856	SC13	0.126
HL24	0.196	HL24	0.432	SC22	0.171	SC23	0.230	YBL14	0.084	TL30	1.700	TL16	0.105
DJC15	0.181	HL6	0.432	HL4	0.163	HL4	0.200	DQ18	0.080	HL12	1.341	DJC23	0.074
HL26	0.151	DQ18	0.404	HL26	0.141	DQ6	0.200	DJC12	0.069	SC11	1.106	SC8	0.074
CK	0.104	CK	0.401	DQ18	0.139	SC11	0.200	SC6	0.059	DJC5	1.012	HL12	0.059
DQ18	0.103	DJC10	0.244	DJC15	0.132	SC20	0.200	CK	0.056	YBL16	0.887	HL23	0.059
SC8	0.102	HL26	0.076	DJC13	0.129	DJC23	0.110	DJC5	0.050	DJC4	0.841	DJC15	0.048
DJC17	0.057	SC13	0.057	CK	0.100	SC22	0.110	DQ10	0.047	DJC17	0.653	DQ18	0.048
YBL16	0.043	SC23	0.013	SC13	0.090	YBL16	0.080	DJC13	0.037	SC18	0.653	SC22	0.043
DQ23	-0.012	DQ23	0.007	HL23	0.088	DJC13	0.040	DJC15	0.032	HL23	0.637	HL6	0.038
HL4	-0.014	YBL14	-0.003	DJC17	0.058	DQ10	0.010	SC18	0.021	HL14	0.622	HL14	0.027
HL23	-0.030	DJC17	-0.021	DQ6	0.038	DQ23	-0.020	HL14	0.020	HL15	0.434	SC25	0.022
HL12	-0.046	DJC13	-0.021	DJC12	-0.021	SC13	-0.020	DJC8	0.017	SC20	0.325	DQ8	0.017
DJC13	-0.049	HL4	-0.040	DQ17	-0.029	SC6	-0.020	SC1	0.015	DQ21	0.200	TL30	0.017
DJC3	-0.049	YBL6	-0.074	SC11	-0.057	SC8	-0.020	HL4	0.014	DQ10	0.169	SC23	-0.004
DJC10	-0.063	DJC12	-0.093	DJC3	-0.062	TL30	-0.020	SC22	0.002	HL24	0.044	DJC12	-0.009
SC20	-0.065	YBL16	-0.109	YBL14	-0.067	HL14	-0.050	DQ17	-0.009	SC23	-0.003	CK	-0.020
YBL14	-0.072	SC25	-0.137	YBL16	-0.077	DJC15	-0.050	DQ23	-0.014	SC6	-0.347	TL3	-0.020
SC23	-0.073	HL23	-0.178	DJC4	-0.080	HL15	-0.080	HL24	-0.026	DJC3	-0.378	DJC3	-0.030
DJC23	-0.078	SC11	-0.181	DQ23	-0.096	YBL14	-0.080	DQ1	-0.029	SC25	-0.394	DQ17	-0.040
DQ17	-0.097	DQ17	-0.259	YBL13	-0.133	DJC3	-0.110	TL16	-0.045	HL4	-0.503	YBL16	-0.046
DJC12	-0.107	YBL13	-0.281	SC25	-0.140	SC18	-0.110	HL23	-0.052	DJC8	-0.784	YBL6	-0.056
YBL6	-0.113	DJC23	-0.293	SC20	-0.150	TL16	-0.110	DJC23	-0.056	YBL6	-1.269	DJC10	-0.061
HL15	-0.141	DQ10	-0.321	HL12	-0.155	YBL13	-0.110	HL6	-0.069	DQ8	-1.300	YBL14	-0.066
SC25	-0.144	SC20	-0.328	SC23	-0.155	HL26	-0.140	DJC4	-0.088	DQ17	-1.331	DQ1	-0.077
SC11	-0.150	HL12	-0.378	DJC23	-0.160	YBL6	-0.140	DJC17	-0.090	DQ1	-1.738	DJC8	-0.103
DJC8	-0.159	DJC3	-0.399	DJC8	-0.176	DJC17	-0.210	SC20	-0.094	YBL14	-1.769	SC11	-0.155
DQ10	-0.174	DJC8	-0.449	TL3	-0.188	CK	-0.240	YBL16	-0.102	DJC12	-1.847	DQ6	-0.176
DJC4	-0.245	DJC5	-0.559	DQ10	-0.206	TL3	-0.240	DQ21	-0.122	CK	-1.847	DJC13	-0.202
TL16	-0.256	TL3	-0.574	TL16	-0.216	HL6	-0.270	HL12	-0.128	TL3	-2.206	HL26	-0.228
DJC5	-0.274	TL30	-0.653	DJC5	-0.231	HL24	-0.390	HL26	-0.134	HL26	-2.347	DJC4	-0.228
TL3	-0.342	TL16	-0.674	YBL6	-0.258	DJC12	-0.390	DQ6	-0.137	DJC23	-2.863	YBL13	-0.228
TL30	-0.350	HL15	-0.690	HL15	-0.312	DJC10	-0.450	DJC3	-0.141	DQ23	-3.159	HL15	-0.243
YBL13	-0.355	DJC4	-0.734	TL30	-0.331	DJC4	-0.550	DQ8	-0.162	SC22	-3.488	SC18	-0.264
SC18	-0.358	SC18	-0.818	SC18	-0.357	HL23	-0.580	HL15	-0.186	YBL13	-3.769	HL24	-0.270
DQ1	-0.395	DQ1	-0.984	DQ1	-0.528	DJC5	-0.610	SC13	-0.349	SC13	-3.894	SC20	-0.270

### 3.4. Principal components analysis

**Fig 5:**
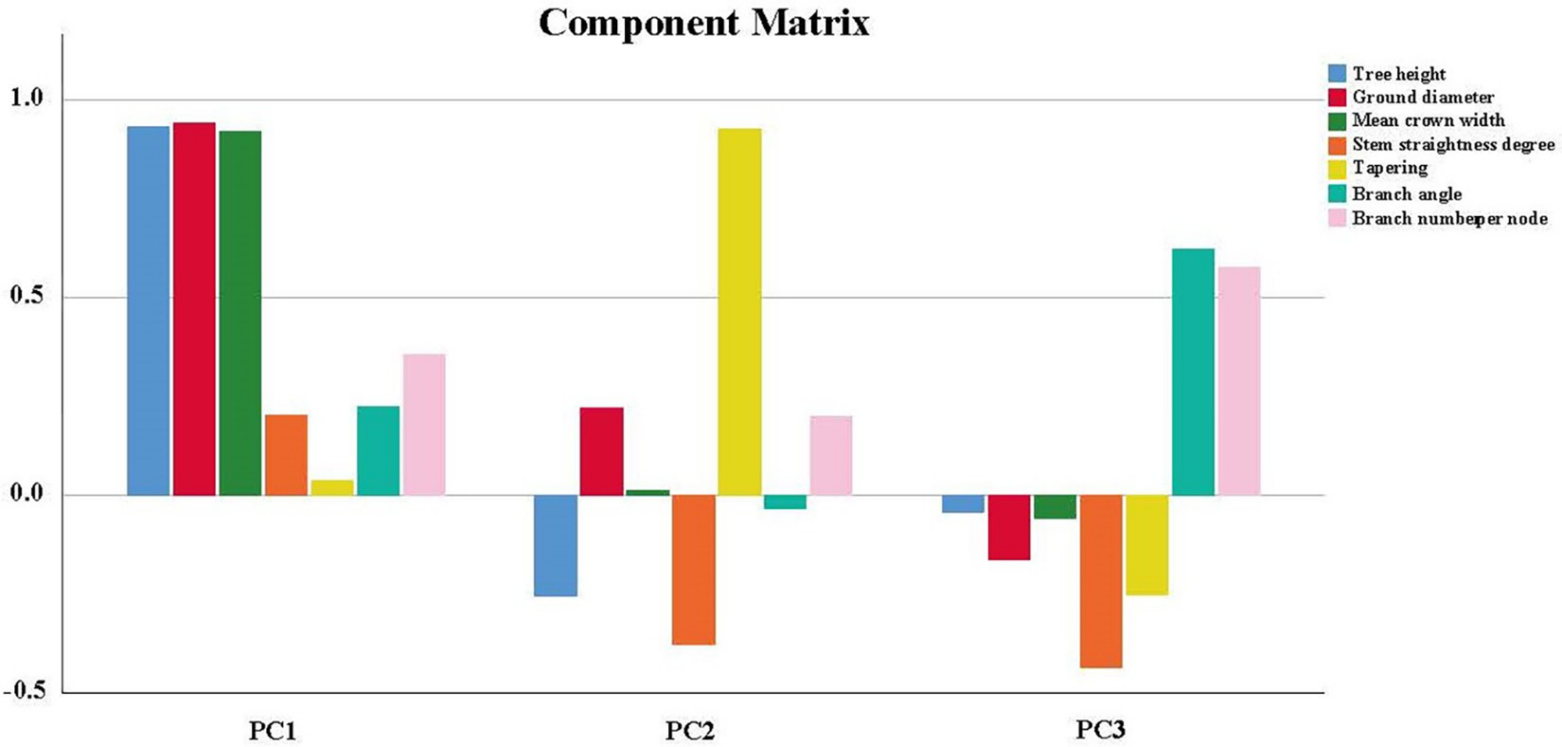
Component matrix of principal component analysis. PC1: principal component 1; PC2: principal component 2; PC3: principal component 3.

We performed principal components analysis (PCA) and three principal components were extracted based on eigenvalues greater than 1 (Table 5 and Fig 5). The eigenvalue for PC1 was 2.826 and the contribution rate was 40.37%. The absolute eigenvalues for the tree height, ground diameter and mean crown width were relatively high at 0.934, 0.942 and 0.920, respectively. On the other hand, the eigenvalue for PC2 was 1.154 and the contribution rate was 16.49%. The absolute eigenvalue for tapering was relatively high, at 0.926. The eigenvalue for PC3 was 1.007 while the contribution rate was 14.39%. We found that the absolute eigenvalues for the branch angle and branch number per node were relatively high, at 0.623 and 0.578, respectively. The cumulative contribution rate of the three principal components was 71.25%, which covered most of the information of growth and stem form traits for the tested families.

**Table 5:** Principal component analysis of different traits among *J*. *mandshurica* families in Northeast China.

Principal component factor	PC1	PC2	PC3
Eigenvalue	2.826	1.154	1.007
Contribution (%)	40.37	16.49	14.39
Cumulative contribution (%)	40.37	56.86	71.25
Tree height	0.934	-0.254	-0.042
Ground diameter	0.942	0.220	-0.163
Mean crown width	0.920	0.011	-0.059
Stem straightness degree	0.203	-0.377	-0.436
Tapering	0.036	0.926	-0.251
Branch angle	0.223	-0.033	0.623
Branch number per node	0.357	0.198	0.578

Note: PC1: principal component 1; PC2: principal component 2; PC3: principal component 3.

### 3.5. Selection of elite provenance and family

**Fig 6:**
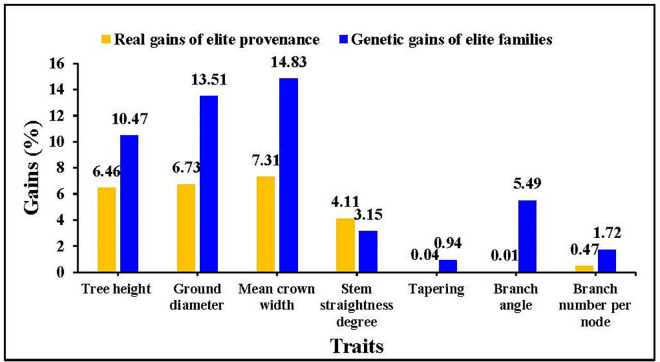
Genetic gains of different *J*. *mandshurica* traits in Northeast China.

The linear equation for each principal component score was obtained from the results of principal component analysis,

$$Y_{1}=0.934x_{1}+0.942x_{2}+0.920x_{3}+0.203x_{4}+0.036x_{5}+0.223x_{6}+0.357x_{7},$$

$$Y_{2}=‐0.254x_{1}+0.220x_{2}+0.011x_{3}‐0.377x_{4}+0.926x_{5}‐0.033x_{6}+0.198x_{7}$$

and $Y_{3}=‐0.042x_{1}‐0.163x_{2}‐0.059x_{3}‐0.436x_{4}‐0.251x_{5}+0.623x_{6}+0.578x_{7}$, where x_1_, x_2_, x_3_, x_4_, x_5_, x_6_ and x_7_ denote the tree height, ground diameter, mean crown width, stem straightness degree, tapering, branch angle and branch number per node, respectively. The contribution rate of each principal component was used as the weight to calculate the comprehensive score, using the following formula: $W=40.37\%Y_{1}+16.49\%Y_{2}+14.39\%Y_{3}$. The final comprehensive scores and ranking of provenances and families are given in Tables 6 and 7. The provenance was selected based of tree height, ground diameter and mean crown width, with a real gain of 5% or more. The real gain for each trait was 6.46%, 6.73%, 7.31%, 4.11%, 0.04%, 0.01% and 0.47% for tree height, ground diameter, mean crown width, stem straightness degree, tapering, branch angle and branch number per node, respectively (Fig 6). We then applied 10% inclusion rate and selected four families: SC1, SC8, DJC15 and DQ18. The mean values of the selected elite families for tree height, ground diameter, mean crown width, stem straightness, tapering, branch angle, and branch number per node were 2.31 m, 5.91 cm, 1.60 m, 4.04, 2.58, 49.85° and 2.20, respectively. The genetic gain achieved after selecting these superior families were 10.47%, 13.51%, 14.83%, 3.51%, 0.94%, 5.49% and 1.72%, for tree height, ground diameter, mean crown width, stem straightness, tapering, branch angle, and branch number per node, respectively (Fig 6).

**Table 6:** Comprehensive scores and rankings of different *J*. *mandshurica* provenances in Northeast China.

Provenances	Comprehensive scores	Rankings
Sanchazi	1218.74352	1
Hulin	1218.147304	2
Daquanzi	1207.675783	3
Dongjingcheng	1204.402831	4
Tieli	1172.938939	5
Yabuli	1164.684839	6

**Table 7:** Comprehensive scores and rankings of different *J*. *mandshurica* families in Northeast China.

Families	Comprehensive scores	Rankings	Families	Comprehensive scores	Rankings
SC1	1339.36	1	DJC5	1193.35	23
SC8	1311.61	2	SC22	1189.19	24
DJC15	1293.54	3	DQ10	1188.47	25
DQ18	1289.43	4	TL30	1187.51	26
HL6	1287.15	5	SC25	1183.67	27
DJC10	1278.76	6	SC20	1181.02	28
SC6	1272.60	7	DJC3	1176.64	29
DQ21	1271.21	8	DJC4	1175.04	30
DQ6	1266.13	9	HL26	1170.07	31
HL14	1264.10	10	YBL14	1168.95	32
DJC13	1241.63	11	YBL6	1167.44	33
DQ8	1237.65	12	DJC12	1167.19	34
HL24	1235.89	13	DQ17	1164.91	35
DJC17	1225.62	14	HL15	1160.92	36
HL23	1214.62	15	DJC8	1156.64	37
YBL16	1211.85	16	DQ23	1154.78	38
HL4	1207.45	17	SC18	1153.23	39
SC11	1206.31	18	SC13	1153.03	40
HL12	1204.98	19	DJC23	1135.89	41
TL16	1203.04	20	TL3	1128.34	42
SC23	1197.60	21	YBL13	1110.55	43
CK	1196.89	22	DQ1	1099.82	44

## 4. Discussion

Genetic variation drives evolution of different species, thereby enabling them to adapt to the prevailing environmental conditions [56]. Thus, assessment of genetic variation in different populations is important as a first step in breeding improved genotypes and for effective conservation of forest genetic resources, and the results will be helpful for the breeding of high-quality commercial varieties [57, 58]. The variation in phenotypic traits is the result of the interaction between genetic variation and environmental factors, reflecting the variability of genotypes, populations, ecotypes, and provenance [59]. Our results demonstrated that most of the traits varied significantly among different provenances and families, while the differences among blocks were small, which was consistent with data from previous studies [27, 33, 34, 60, 61]. Similar research results were also obtained in *Eucalyptus urophylla* and some subtropical pine species [62, 63]. The results also demonstrated that the accessions had comparatively large variation for genetic improvement, and the differences mainly resulted from genetic rather than environmental stimuli. It should be noted that in natural environments different populations often have significant differences due to interaction between genetic and environmental diversity. Thus, analyses of the variations have contributed to a better understanding of the inherent patterns of genetic variation [64].

In this study, the mean values of tree height and ground diameter for 5-year-old trees were higher than previous findings that used 7-year-old *J*. *mandshurica* [33] and 5-year-old *J*. *mandshurica* [65, 66]. The accessions showed a distinct growth advantage. In comparison to the 1-9-year-old walnut trees in the edge habitats of natural forests, our research material did not exhibit a significant advantage in tree height growth [67]. This phenomenon appears to be influenced by a variety of interrelated factors. Notably, the high-density planting method in artificial forests may exacerbate competition among trees, thereby imposing certain limitations on their growth. Additionally, it is worth noting that nutrient supply in artificial forests is typically relatively inadequate, necessitating supplementary fertilization and management interventions. Moreover, adverse growth conditions such as limited light exposure and water availability should be considered, as they may significantly impact tree height. By enhancing management practices and nutrient supply, there is the potential to elevate the tree height of walnut trees in artificial forests, gradually reducing the disparity in tree height compared to natural forests. The crown is a major primary site of photosynthesis and energy retention for trees, which reflects the viability and competitiveness of tree growth [68]. The mean crown width in this study were 1.33 m, which was slightly lower than the results by Zhao on a 6-year-old *J*. *mandshurica* [69]. This variation could be associated with the variety used and the soil fertility [70, 71]. In addition, there are few studies on the traits reflecting the stem form characteristics such as stem straightness degree, tapering, branch angle and branch number per node of the *J*. *mandshurica*. Therefore, our study investigated and analyzed these traits to gain a deeper understanding of the growth variation pattern of the *J*. *mandshurica*. The form quality of the trees is controlled by their own genetic and environment conditions, which could directly affect the processing, quality, yield, and commercial value of the wood [72]. Larger stem straightness degree and smaller tapering have been shown to be beneficial for the selection of large-size timber, while larger branch angle and branch number per node could be conducive in improving fruit production [73, 74]. ANOVA results showed that the significant differences in stem form traits among the different provenances and families in this study, which could provide materials for directional breeding of *J*. *mandshurica* for diverse objectives.

The coefficient of variation is a small-scale variability measure used to reflect the genetic variability of traits in population, and the larger the coefficient of variation, the more favorable the selection of the elite materials [75, 76]. In this study, the phenotypic coefficient of variation (PCV) and genetic coefficient of variation (GCV) were higher than a previous report by You in Hebei province [30], thus indicating obvious differences in geographical provenance. Among the analyzed traits, the coefficient of variation was relatively large for tree height, ground diameter, mean crown width and stem straightness degree, demonstrating that using these traits as the selection index could yield more selection potential. In addition, the genetic coefficients of variation for traits such as tree height, ground diameter, mean crown width, and tapering accounted for more than 65% of the phenotypic coefficient of variation, which was consistent with the findings for *Pinus koraiensis* [77]. This data indicated that growth variation among families is more controlled by genetic factors, which provides a basis for the selection of elite provenances and families. Besides, heritability is an important indicator in forest breeding research, which reflects the relative roles of genetics and environment in the expression of various traits, while helping to rank the importance of each trait in hybrid breeding, and provide support for early selection of trees [78, 79]. In this study, except for the stem straightness degree, branch angle and branch number per node, the heritability of all the traits in the family was high and could be inherited more stably. The outcomes were better than for 4-year-old, 5-year-old, 6-year-old and 7-year-old *J*. *mandshurica* [61, 65, 80]. It has been shown that high genetic control could maximize the genetic gain and facilitate the selection of elite families [81].

In tree breeding, it is often desired to improve comprehensive traits in the target material [82]. Thus, the correlation between the traits is particularly important [83]. Correlation analyses and coefficients could effectively and quantitatively characterize the degree of association among traits and provide space for selection of comprehensive traits [84]. However, data on the correlations among traits in the provenances of *J*. *mandshurica* remains limited. Here, we analyzed the correlation among and between traits as well as environmental factors in juvenile *J*. *mandshurica*. The data showed large correlation coefficients (*r* > 0.8) among some growth traits. This finding was similar to that observed in a previous study with growth traits of *P*. *sylvestris* clones [85], which indicated strong associations among these traits and were favorable for comprehensive selection. The correlation coefficients among stem straightness degree, tapering, branch angle and branch number per node were small (*r* < 0.3), and weakly correlated, as in the case of previous findings on *P*. *koraiensis* and *P*. *Sylvestris* [77, 86]. These data demonstrate that the genetic independence between growth and stem form traits and could be used for the evaluation and selection based on different breeding objectives to improve breeding efficiency. Furthermore, it was also observed in this study that the January mean temperature and winter mean temperature has a strong positive correlation with the growth traits. This indicates that the temperature in cold season has a significant effect on the growth of *J*. *mandshurica* at seedling. Similar results were reported by Liu, who studied *P*. *sylvestris*. The investigation may provide references for cultivation and promotion of *J*. *mandshurica* [87].

Phenotypic variation is an effect of genetic and environmental interactions, and environmental factors play a crucial role in shaping the plant phenotypes, such as phenotypes and nutritional traits in the fruit [88, 89]. The correlations between most traits and geographic factors, such as longitude, latitude and altitude, in our study were insignificant, which is consistent with results by Xia [29]. The study demonstrated that the growth of different *J*. *mandshurica* provenances is weakly associated with geographic factors and their changes show a random variation. On the other hand, annual temperature had a strong positive correlation (*r* > 0.8) with mean crown width. This finding was consistent with the findings on *Xanthoceras sorbifolium* [90], which indicated that the increase in temperature is conducive to physiological activities such as photosynthesis of *J*. *mandshurica*, thus promoting the growth and development of the crown. In addition, the annual temperature showed a strong negative correlation (*r* = -0.85) with tapering, indicating that the higher the temperature, the relatively more use of biomass, and the tree height growth and tree morphology tends to be slender and tall, which is different from studies that evaluated coniferous tree species such as *Larix* Mill. [91] and might be due to the difference of the tree species.

Combining ability is one of the breeding objectives, which refers to the relative potential of parental dominance hybrid to progeny [92]. It is widely used in the breeding of cross-pollinated plants, and the data could help in the selection of elite hybrid parents [93]. In this study, the data showed that the general combining ability of different traits varied widely among different families, which was similar with the results from *P*. *koraiensis* [94], it was difficult to perform comprehensive selection. Therefore, it was necessary to carry out further selection in combination with PCA. PCA is a common multivariate analysis tool that helps reduce data dimensionality and retain data trends and patterns [95], and is widely used in trait analysis of walnut tree species [96–98]. In our study, the higher eigenvalues for tree height, ground diameter, mean crown width in PC1 could reflect growth patterns. In addition, the higher eigenvalues for tapering in PC2 could represent stem form, while that of branch angle and branch number per node in PC3 could represent the branching features. This result is consistent with the data from Yang on *Castanopsis hystrix* [99], suggesting that the differences in *J*. *mandshurica* family are mainly in growth, stem form and branching. Based on the PCA analysis, we primarily selected one elite provenance (Sanchazi) and four elite families (SC1, SC68, DJC15 and DQ18). The selected families, whose genetic gain in tree height was higher than the results of Chu which used 15-year-old *J*. *mandshurica* [31], had obvious advantages in growth and had high genetic gain. The differentiation of materials in our study was large, which has a large potential for genetic improvement and is conducive for early selection new *J*. *mandshurica* varieties. The selected families could provide material for regional afforestation.

## 5. Conclusions

Taken together, our study successfully investigated the variations in growth and stem form traits of 5-year-old *J*. *mandshurica* provenances and families within provenances, revealing significant genetic factors at play. We identified elite families, with particular focus on tree height, ground diameter, and mean crown width. This provides a robust foundation for comprehensive multi-trait selection, offering potential for the breeding and promotion of *J*. *mandshurica* varieties. This research contributes to the theoretical and breeding basis for the conservation and utilization of valuable resources, as well as the development of the local *J*. *mandshurica* cultivation industry.

## Supporting information

S1 Table Average values of different traits among *J*. *mandshurica* provenance in Northeast China. (DOCX)

S2 Table Average values of different traits among *J*. *mandshurica* families in Northeast China. (DOCX)

S1 Data Test forest growth measurement data. (XLSX)

S1 File (ZIP)

S2 File (ZIP)
