# Supplementary material for: Provenance and family variations in early growth of Manchurian walnut (Juglans mandshurica Maxim.) and selection of superior families
Source: PLoS One. 2024 Mar 7;19(3):e0298918. doi: 10.1371/journal.pone.0298918 (PMC10919699; doi:10.1371/journal.pone.0298918)
Supplement: S1 Table — (DOCX) [file pone.0298918.s001.docx]

Table S1. Average values of different traits among *J. mandshurica* provenance in Northeast China.

| Provenances | Tree height | Ground diameter | Mean crown width | Stem straightness degree | Tapering | Branch angle | Branch number per node |
| --- | --- | --- | --- | --- | --- | --- | --- |
| Hulin | 2.15±0.47 | 5.17±1.16 | 1.42±0.57 | 3.70±1.05 | 2.42±0.27 | 47.23±9.13 | 2.08±0.87 |
| Dongjingcheng | 1.99±0.41 | 4.94±1.04 | 1.31±0.47 | 3.68±1.15 | 2.49±0.25 | 47.38±8.40 | 2.11±0.73 |
| Daquanzi | 2.15±0.52 | 5.27±1.29 | 1.36±0.64 | 4.05±1.07 | 2.46±0.29 | 46.46±8.97 | 2.17±0.82 |
| Sanchazi | 2.16±0.46 | 5.39±1.21 | 1.39±0.55 | 3.97±1.10 | 2.51±0.29 | 46.78±9.05 | 2.12±0.84 |
| Tieli | 1.75±0.29 | 4.49±0.81 | 1.09±0.38 | 3.71±1.05 | 2.57±0.26 | 47.41±7.50 | 2.15±0.72 |
| Yabuli | 1.95±0.41 | 5.01±1.05 | 1.20±0.51 | 3.77±1.09 | 2.60±0.31 | 45.37±8.15 | 2.01±0.73 |
