# Supplementary material for: Provenance and family variations in early growth of Manchurian walnut (Juglans mandshurica Maxim.) and selection of superior families
Source: PLoS One. 2024 Mar 7;19(3):e0298918. doi: 10.1371/journal.pone.0298918 (PMC10919699; doi:10.1371/journal.pone.0298918)
Supplement: S2 Table — (DOCX) [file pone.0298918.s002.docx]

Table S2. Average values of different traits among *J. mandshurica* families in Northeast China.

| Families | Tree height | Ground diameter | Mean crown width | Stem straightness degree | Tapering | Branch angle | Branch number per node |
| --- | --- | --- | --- | --- | --- | --- | --- |
| HL12 | 2.02±0.44 | 4.75±1.06 | 1.17±0.44 | 4.09±0.89 | 2.36±0.26 | 48.19±11.52 | 2.17±0.98 |
| HL14 | 2.35±0.39 | 5.85±0.84 | 1.59±0.54 | 3.78±1.01 | 2.51±0.23 | 47.47±8.13 | 2.14±1.05 |
| HL15 | 1.93±0.26 | 4.44±0.56 | 1.02±0.31 | 3.75±1.05 | 2.30±0.13 | 47.28±9.35 | 1.87±0.63 |
| HL23 | 2.04±0.53 | 4.95±1.22 | 1.42±0.54 | 3.25±1.02 | 2.44±0.23 | 47.48±8.97 | 2.17±0.85 |
| HL24 | 2.27±0.66 | 5.56±1.58 | 1.67±0.70 | 3.44±1.05 | 2.47±0.26 | 46.89±7.29 | 1.84±0.57 |
| HL26 | 2.22±0.48 | 5.20±1.07 | 1.47±0.55 | 3.69±1.18 | 2.36±0.20 | 44.50±7.74 | 1.89±0.63 |
| HL4 | 2.06±0.50 | 5.09±1.36 | 1.49±0.72 | 4.03±0.97 | 2.51±0.50 | 46.34±11.28 | 2.42±1.22 |
| HL6 | 2.31±0.28 | 5.56±0.56 | 1.53±0.33 | 3.56±1.08 | 2.42±0.20 | 49.66±7.95 | 2.15±0.72 |
| DJC10 | 2.01±0.36 | 5.37±1.00 | 1.54±0.47 | 3.38±1.16 | 2.68±0.19 | 50.05±8.62 | 2.05±0.77 |
| DJC12 | 1.96±0.30 | 5.03±0.86 | 1.31±0.38 | 3.44±1.13 | 2.56±0.13 | 45.00±6.74 | 2.10±0.83 |
| DJC13 | 2.02±0.41 | 5.11±1.11 | 1.46±0.54 | 3.87±1.13 | 2.53±0.26 | 49.11±11.39 | 1.91±0.61 |
| DJC15 | 2.25±0.59 | 5.62±1.32 | 1.46±0.58 | 3.78±1.10 | 2.52±0.20 | 50.09±5.96 | 2.16±0.76 |
| DJC17 | 2.13±0.36 | 5.11±0.94 | 1.39±0.48 | 3.62±1.04 | 2.40±0.17 | 47.50±7.26 | 2.36±0.77 |
| DJC23 | 1.99±0.40 | 4.83±0.99 | 1.17±0.36 | 3.94±1.05 | 2.44±0.24 | 43.98±8.98 | 2.19±0.61 |
| DJC3 | 2.02±0.29 | 4.73±0.67 | 1.27±0.30 | 3.72±1.02 | 2.35±0.24 | 46.47±6.70 | 2.08±0.82 |
| DJC4 | 1.83±0.24 | 4.39±0.76 | 1.25±0.38 | 3.28±1.44 | 2.40±0.22 | 47.69±8.85 | 1.89±0.62 |
| DJC5 | 1.80±0.23 | 4.57±0.80 | 1.10±0.30 | 3.22±1.13 | 2.54±0.28 | 47.86±9.54 | 2.38±0.74 |
| DJC8 | 1.91±0.60 | 4.68±1.26 | 1.15±0.61 | 4.50±0.76 | 2.51±0.34 | 46.06±7.38 | 2.01±0.69 |
| CK | 2.17±0.36 | 5.53±1.02 | 1.43±0.44 | 3.59±0.95 | 2.55±0.28 | 45.00±9.19 | 2.09±0.51 |
| DQ1 | 1.68±0.33 | 4.14±0.99 | 0.80±0.45 | 4.19±0.93 | 2.46±0.25 | 45.11±8.96 | 2.04±0.78 |
| DQ10 | 1.90±0.38 | 4.81±1.05 | 1.12±0.41 | 3.84±1.14 | 2.54±0.20 | 47.02±7.79 | 2.34±0.87 |
| DQ17 | 1.97±0.46 | 4.87±1.18 | 1.30±0.56 | 4.13±1.04 | 2.48±0.32 | 45.52±8.56 | 2.07±0.64 |
| DQ18 | 2.17±0.44 | 5.53±1.05 | 1.47±0.73 | 4.22±0.98 | 2.57±0.34 | 50.27±12.14 | 2.16±1.20 |
| DQ21 | 2.52±0.47 | 5.94±1.23 | 1.73±0.58 | 4.25±1.05 | 2.37±0.32 | 47.05±7.89 | 2.31±0.90 |
| DQ23 | 2.06±0.53 | 5.13±1.54 | 1.23±0.67 | 3.81±1.15 | 2.48±0.21 | 43.69±7.99 | 2.44±0.87 |
| DQ6 | 2.41±0.42 | 5.61±0.97 | 1.37±0.35 | 4.03±1.09 | 2.35±0.32 | 48.97±8.38 | 1.94±0.55 |
| DQ8 | 2.51±0.60 | 5.83±1.47 | 1.80±0.83 | 4.34±1.15 | 2.33±0.23 | 45.55±8.21 | 2.13±0.82 |
| SC1 | 2.64±0.33 | 6.60±1.04 | 1.96±0.44 | 4.34±0.87 | 2.51±0.29 | 48.70±8.25 | 2.30±0.65 |
| SC11 | 1.92±0.34 | 4.95±0.96 | 1.27±0.48 | 4.03±1.03 | 2.58±0.20 | 47.95±8.94 | 1.96±0.84 |
| SC13 | 2.42±0.61 | 5.18±1.43 | 1.42±0.71 | 3.81±1.20 | 2.14±0.20 | 42.95±8.01 | 2.24±0.80 |
| SC18 | 1.71±0.20 | 4.31±0.64 | 0.97±0.31 | 3.72±1.14 | 2.51±0.17 | 47.50±10.68 | 1.85±0.71 |
| SC20 | 2.01±0.42 | 4.80±1.13 | 1.18±0.54 | 4.03±1.20 | 2.40±0.28 | 47.17±7.93 | 1.84±0.82 |
| SC22 | 2.35±0.39 | 5.86±1.08 | 1.50±0.50 | 3.94±1.08 | 2.49±0.18 | 43.36±8.75 | 2.16±0.76 |
| SC23 | 2.00±0.36 | 5.14±0.89 | 1.17±0.37 | 4.06±0.98 | 2.59±0.29 | 46.84±6.79 | 2.11±0.96 |
| SC25 | 1.93±0.31 | 4.99±0.89 | 1.19±0.39 | 4.09±1.03 | 2.59±0.20 | 46.45±9.33 | 2.14±0.95 |
| SC6 | 2.41±0.38 | 6.12±0.94 | 1.75±0.50 | 3.81±1.12 | 2.55±0.26 | 46.50±11.14 | 2.40±0.88 |
| SC8 | 2.17±0.31 | 5.91±1.07 | 1.52±0.36 | 3.81±1.31 | 2.73±0.35 | 50.33±8.57 | 2.19±0.90 |
| TL16 | 1.81±0.24 | 4.45±0.74 | 1.11±0.30 | 3.72±1.05 | 2.45±0.12 | 49.05±7.94 | 2.22±0.92 |
| TL3 | 1.73±0.34 | 4.55±1.04 | 1.14±0.53 | 3.59±0.95 | 2.64±0.35 | 44.64±6.93 | 2.09±0.62 |
| TL30 | 1.72±0.29 | 4.48±0.63 | 1.00±0.25 | 3.81±1.15 | 2.62±0.22 | 48.55±7.01 | 2.13±0.60 |
| YBL13 | 1.72±0.29 | 4.85±1.22 | 1.20±0.59 | 3.72±1.05 | 2.81±0.38 | 43.08±8.10 | 1.89±0.60 |
| YBL14 | 2.00±0.34 | 5.12±0.88 | 1.26±0.45 | 3.75±1.19 | 2.58±0.26 | 45.08±7.53 | 2.05±0.81 |
| YBL16 | 2.11±0.31 | 5.02±0.69 | 1.25±0.26 | 3.91±1.03 | 2.39±0.24 | 47.73±7.72 | 2.07±0.69 |
| YBL6 | 1.96±0.55 | 5.05±1.33 | 1.07±0.66 | 3.69±1.12 | 2.61±0.20 | 45.58±8.88 | 2.06±0.81 |
