# Supplementary material for: Provenance and family variations in early growth of Manchurian walnut (Juglans mandshurica Maxim.) and selection of superior families
Source: PLoS One. 2024 Mar 7;19(3):e0298918. doi: 10.1371/journal.pone.0298918 (PMC10919699; doi:10.1371/journal.pone.0298918)
Supplement: S1 File — (ZIP) [file pone.0298918.s004.zip › A superior genetic source for late leafing in walnut ‘Ahir Nut’.pdf]

https://doi.org/10.17221/22/2022-HORTSCI

A superior genetic source for late leafing in walnut 'Ahir Nut'

Mehmet Sütyemez[1]*, *Akide Özcan*[2], *Şakir Burak Bükücü*[3]

[1]*Department of Horticulture, Agricultural Faculty, Kahramanmaras Sutcu Imam University, Kahramanmaras, Turkey*
[2]*Department of Organic Farming, Goksun Vocational School, Kahramanmaras Sutcu Imam University, Kahramanmaras, Turkey*
[3]*Department of Plant and Animal Production, Silifke Tasucu Vocational School, Selcuk University, Mersin, Turkey*

**Corresponding author: sutyemezmehmet@gmail.com*

Citation: Sütyemez M., Özcan A., Bükücü Ş.B. (2022): A superior genetic source for late leafing in walnut 'Ahir Nut'. Hort. Sci. (Prague), 49: 205–212.

Abstract: One of the main objectives of walnut crossbreeding programmes is to produce cultivars with late leafing dates. Sixteen years ago, a walnut crossbreeding programme was initiated by Prof. Mehmet Sütyemez, and now a new walnut genotype, namely 'Ahir Nut', has been generated with a leafing date that starts later than all commercially-famous walnut cultivars in the world. The present study describes this walnut genotype. For a detailed analysis, 'Ahir Nut' was compared with two French-origin walnut cultivars, i.e., 'Franquette' and 'Ronde de Montignac', which have had the latest leafing dates until now. In an experimental orchard in Turkey, the leafing date in 'Ahir Nut' was 14 May, whereas the leafing date in 'Franquette' and 'Ronde de Montignac' were 23 April and 25 April, respectively. This approximately 3-week difference between 'Franquette' and 'Ronde de Montignac' to 'Ahir Nut' reveals how valuable this genotype is for walnut breeding studies. The harvest dates for 'Ahir Nut', 'Franquette' and 'Ronde de Montignac' were 4 October, 3 October, and 7 October, respectively. The defoliation date for 'Ahir Nut' was 4 December. The nut weight and kernel percentage of 'Ahir Nut' were calculated as 11.61 g and 49.84%, respectively. Remarkable phenological traits were found in 'Ahir Nut', making it a promising option for future walnut cultivation. In the present study, the late leafing date of 'Ahir Nut' prompted further research into the molecular and walnut crossbreeding potentials. This new genotype has been conserved as an important genetic resource for future walnut breeding programmes around the world.

Keywords: *Juglans regia* L.; late leafing; plant breeding; crossbreeding; phenological traits

Plant production is usually considered the pinnacle of success when fruitful and high-quality cultivars are bred. In all fruit species, crossbreeding programmes mainly focus on producing high-quality, fruitful cultivars, with physiological resistance to biotic and abiotic stresses, and walnut crossbreeding is no exception in this regard (Arab et al. 2019; Vahdati et al. 2019). Growers usually encounter a variety of common problems in their agricultural activities. One of the most problematic issues in walnut cultivation is the early leafing date, which limits the yield of orchards in many regions where a risk of late spring frost is common (Vahdati 2014; Akca et al. 2016). In addition, the early leafing date can potentially pose threats to walnut cultivation in terms of plant diseases (Khodadadi et al. 2020). Other recurrent risks in walnut cultivation include a late harvest date and a late defoliation date. These factors are heavily affected by early autumn frost events. Global warming significantly increases these risks.

Supported by the Scientific and Technological Research Council of Turkey, Turkey, Project No. 104 0 318.

https://doi.org/10.17221/22/2022-HORTSCI

Therefore, it is vital to obtain new cultivars with early harvest and early defoliation dates (Rezaee et al. 2008; Hajinia et al. 2021).

In this regard, walnut crossbreeding studies usually aim at producing new, high-quality cultivars with a late leafing early harvest and defoliation dates, as well as relatively larger nuts and higher kernel percentages (McGranahan, Leslie 2009; Vahdati 2014; Sütyemez et al. 2021). In walnut crossbreeding, a great deal of priority is given to cultivars that are resistant to early spring frost events and are adaptable to changes in climatic conditions. Thus, the phenology and tree development are decisive factors in the plant productivity, as they bear the utmost importance in the genetic development and sustainable cultivation for walnut production (Vahdati 2014).

Various studies in the current literature reported that 'Franquette' is among walnut cultivars with the latest leafing date (Germain 1997; Ramos 1997; Sütyemez, Kaska 2004). Prof. Mehmet Sütyemez initiated a walnut crossbreeding programme to obtain high-quality walnut cultivars from different combinations with a late leafing date, an early harvest date, and an early defoliation date. Within the framework of this programme, important cultivars have, thus far, been selected from different combinations, among which 'Ahir Nut' is a promising one.

The present study aims to characterise the phenological and pomological traits of the 'Ahir Nut' genotype. In addition, by comparing it with the world's latest walnut cultivars, such as 'Franquette' and 'Ronde de Montignac', it has been revealed how important this genotype is for world literature.

MATERIAL AND METHODS

Material. The present study was conducted for three consecutive years, i.e., from 2018 to 2020, in crossbreeding plots affiliated with the Nut Application and Research Center (SEKAMER) at Kahramanmaraş Sütcü Imam University. The study area (SEKAMER) is located in Kahramanmaraş province, Turkey (37°35'27"N and 37°03'28"E) with a mild climate and an altitude of 930 m above sea level. While the average annual precipitation in this region, between 2003 and 2020, was 750.9 mm and the average temperature was 23.5 °C (Turkish State Meteorological Service 2021). The mean monthly climate values in Kahramanmaraş between these years is presented in Figures 1 and 2. As a result of the analysis made on the soil samples taken at a depth of 0–30 cm, 30–60 cm, and 60–90 cm from the experimental area; it was determined that mechanical soil composition was sandy loam, the pH was 7.1–7.8, the electrical conductivity was 0.40–0.60 dS/m, the total salts were 0.028 0.034%, the organic matter was 1.2–1.6% and the calcium carbonate was ($CaCO_3$) 2.9–3.7% (Tüzüner 1990).

Sütyemez, Baymış (2005), initiated the first walnut crossbreeding studies on various combinations in 2005. 'Ahir Nut' was selected from 1 150 different walnut genotypes which resulted from the 'KSÜ-2008/1 × Franquette' combination, within the said framework of the crossbreeding programme. The present study benefited from 'Franquette' and 'Ronde de Montignac' as reference cultivars for 'Ahir Nut'. Specifically, 'Franquette' and 'Ronde de Mon-

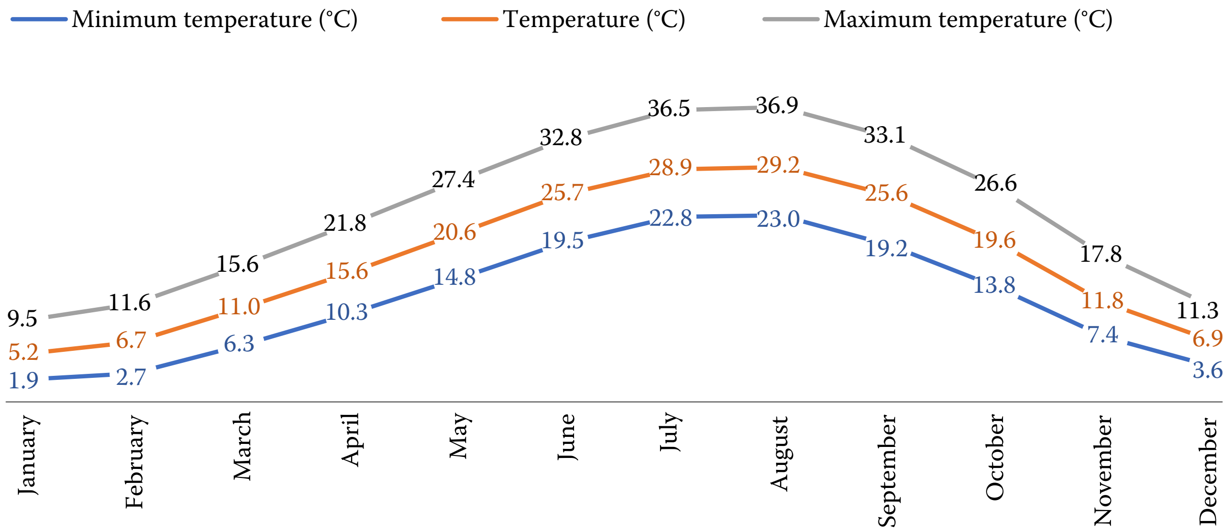

Figure 1. Mean minimum, mean maximum, and mean temperature values of Kahramanmaras between 2003 and 2020 (Turkish State Meteorological Service 2021)

https://doi.org/10.17221/22/2022-HORTSCI

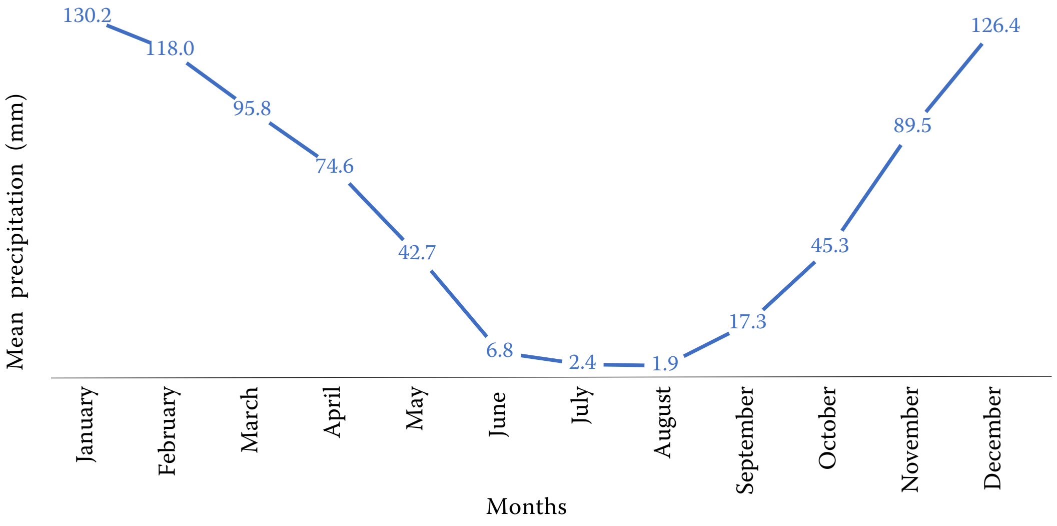

Figure 2. Mean precipitation values of Kahramanmaras between 2003 and 2020 (Turkish State Meteorological Service 2021)

tignac' are of French origin and have been widely reported as cultivars having the latest leafing dates. However, their lateral bud flowering percentages are relatively low (McGranahan et al. 1990; Germain 1997; Ramos 1997; Sütyemez et al. 2021).

'Ahir Nut' and the reference cultivars (i.e., 'Franquette' and 'Ronde de Montignac') were grafted onto seedlings of 'Maraş 18' in the same year (2010) to minimise the effects of the rootstocks on the sapling performance. Later, these saplings were planted in a crossbreeding plot in 2012, and regular cultivation practices were applied to all the plants. Phenological observations were performed on ten plant samples from each genotype.

Method. The mean values of the phenological and pomological traits were recorded through the course of three consecutive years (2018–2020). The characterisation of 13 phenological and 12 pomological traits was based on the International Plant Genetic Resources Institute (IPGRI 1994) and the International Union for the Protection of New Varieties of Plants (UPOV 2014) criteria. The phenological traits were observed every other day, while the pomological analyses focused on the nut samples during the harvest maturity period to represent the trees. The harvest data were recorded for statistical analysis. The nuts were dried after their green shells were removed. Fifty fruit samples from each genotype were used in the pomological analysis. A digital calliper with a sensitivity of 0.01 mm was used to measure the shell thickness. An electronic scale with a sensitivity of 0.01 g was used to measure the shell and kernel weight. The phenological and pomological traits are described in Tables 1 and 2, whereas the data and mean values are presented below.

The leafing date, harvest date, defoliation date, shelled nut weight, kernel weight, and kernel percentage were calculated using a variance analysis. The mean values of all the genotypes were compared using multiple range tests ($P \leq 0.05\%$) (LSD). The JMP (13) package program was used for the statistical data analysis.

RESULTS AND DISCUSSION

The present study mainly aims to offer insights into the details of the 'Ahir Nut' walnut genotype and its performance. This new genotype was compared with 'Franquette' and 'Ronde de Montignac' in many respects. The mean values of the plant samples were obtained from the phenological observations during three consecutive years (2018–2020) (Tables 3 and 4).

The most striking phenological trait of 'Ahir Nut' was its later leafing date in comparison with all the well-known walnut cultivars, including 'Franquette' and 'Ronde de Montignac', and genotypes that had resulted from the previous walnut crossbreeding lines. The leafing date of 'Ahir Nut' was 14 May, whereas 'Franquette' and 'Ronde de Montignac' had leafing dates on 23 April and 25 April, respectively. As can be seen, the leafing date of 'Ahir Nut' was 21 to 19 days later than those of 'Franquette' and 'Ronde de Montignac' which have, so far, been

https://doi.org/10.17221/22/2022-HORTSCI

Table 1. Description of the phenological traits

Traits	Description
Leafing date	date when 50% of terminal buds had enlarged and the bud scales had split exposing the green leaves
First male bloom date	date when the first pollen shedding occurred
Last male bloom date	date when the last pollen shedding occurred
First female bloom date	date of initial pistillate flower receptivity
Last female bloom date	date of last pistillate flower receptivity
Male flowering times	duration of catkins receptiveness
Female flowering times	duration of female flower receptiveness
Female abundance	female flower abundance: 3 – light; 5 – intermediate; 7 – high
Catkin abundance	male flower abundance: 3 – light; 5 – intermediate; 7 – high
Lateral bud flowering	percentage of lateral buds with female flowers
Dichogamy	overlapping duration of female flowers and catkins receptiveness, status 1 protandrous; 2 protogynous; 3 unknown
Harvest date	the date when 50% of the green husk begins to crack on the trees
Defoliation date	the date when 75% of the leaves on the trees fall

regarded as the walnut cultivars with the latest leafing dates (Table 3 and Figure 3). Despite being 7-, 8- and 9-year-old trees, no male flowers were found in the 'Ahir Nut' genotype. Therefore, artificial pollination was used to produce nuts. It was observed that the first and last female bloom dates in 'Ahir Nut' were 27 May and 5 June, respectively. The first and last male bloom dates in 'Franquette' and 'Ronde de Montignac' were 27 April–5 May and 7 May–15 May, respectively, whereas the first and last female bloom dates were 1 May–10 May and 4 May–12 May, respectively (Figure 3).

Many studies in the available literature have claimed that 'Franquette' and 'Ronde de Montignac' are walnut cultivars with the latest leafing dates (Germain 1997; Ramos 1997; Sütyemez, Kaska 2004; Mahmoodi et al. 2016). Soleimani et al. (2019) reported that 'Ronde de Montignac' had its leafing date on 13 April under the ecological conditions of Iran. Meanwhile, the fact that 'Ahir Nut' has a much later leafing date (21 to 19 days) compared to 'Franquette' and 'Ronde de Montignac' bears evidence that the recent genotype can be an important genetic resource for conventional and molecular breeding studies.

The harvest date of 'Ahir Nut' is also worthy of consideration. It was observed that the harvest date of 'Ahir Nut' was 4 October, whereas 'Franquette' and 'Ronde de Montignac' had harvest dates on 3 Octo-

Table 2. Description of the pomological traits

Traits	Description
Nut shape	1 – round; 2 – triangular; 3 – broad ovate; 4 – ovate; 5 – short trapezoid; 6 – long trapezoid; 7 – broad elliptic; 8 – elliptic; 9 – cordate
Shell texture	1 – very smooth; 3 – smooth; 5 – medium; 7 – rough; 9 – very rough
Shell colour	1 – very light; 3 – light; 5 – medium; 7 – dark; 9 – very dark
Shell strength	1 – paper; 3 – weak; 5 – intermediate; 7 – strong
Shell thickness (mm)	near center of half-shell was measured with a digital caliper
Shelled nut weight (g)	average of total 75 nuts
Kernel weight (g)	average of total 75 nuts
Kernel percentage (%)	kernel weight/nut weight × 100
Kernel colour	1 – extra light; 2 – light; 3 – light amber; 4 – amber
Kernel fill	3 – poor; 5 – moderate; 7 – well
Kernel flavour	1 – satisfactory; 2 – unsatisfactory
Ease of removal of kernel halves	1 – very easy; 3 – easy; 5 – moderate; 7 – difficult; 9 – very difficult

https://doi.org/10.17221/22/2022-HORTSCI

Table 3. Phenological traits of 'Ahir Nut', 'Franquette' and 'Ronde de Montignac'

Phenological traits	Genotypes		
	'Ahir Nut'	'Franquette'	'Ronde de Montignac'
Leafing date	14 May	23 April	25 April
First male bloom date	–	27 April	7 May
Last male bloom date	–	5 May	15 May
First female bloom date	27 May	1 May	4 May
Last female bloom date	5 June	10 May	12 May
Male flowering time	–	9 days	9 days
Female flowering time	10 days	10 days	9 days
Female abundance	high	intermediate	intermediate
Catkin abundance	–	high	intermediate
Lateral bud flowering (%)	73	32	34
Dichogamy	–	protandrous	protogynous
Harvest date	4 October	3 October	7 October
Defoliation date	4 December	2 December	8 December

ber and 7 October, respectively. Accordingly, 'Ahir Nut' cannot be said to have a later harvest date compared to the other two cultivars (Figure 4).

As stated in Sütyemez et al. (2021), the harvest date of the 'Chandler' cultivar occurred on 5 October, on average. The earlier harvest date of 'Ahir Nut', observed in the same study area, as compared to 'Chandler', can be another notable aspect in walnut cross-breeding studies.

The defoliation dates of 'Ahir Nut', 'Franquette' and 'Ronde de Montignac' were on 4 December, 2 December, and 8 December, respectively (Figure 4). Thus, the defoliation date of 'Ahir Nut' can be considered as an intermediate value. Statistical differences between the leafing date, harvest date, and defoliation for each genotype are shown in Figure 5.

The most important pomological traits which determine the nut quality in a walnut cultivar are the shelled nut weight, kernel weight, yield per tree, kernel colour, kernel percentage, and ease of removal of the kernel halves (Germain 1997; Ramos 1997; Sütyemez, Kaska 2004; McGranahan, Leslie 2009; Ber-

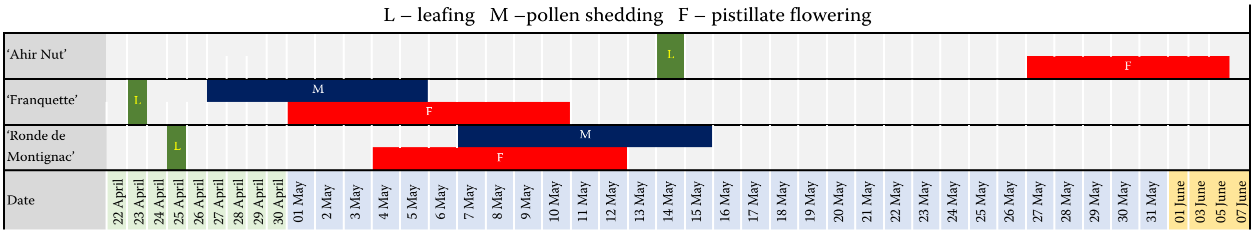

Figure 3. (L) – leafing date; (M) – pollen shedding period; (F) – female flower receptivity period, 'Ahir Nut' reference cultivars: 'Franquette' and 'Ronde de Montignac'

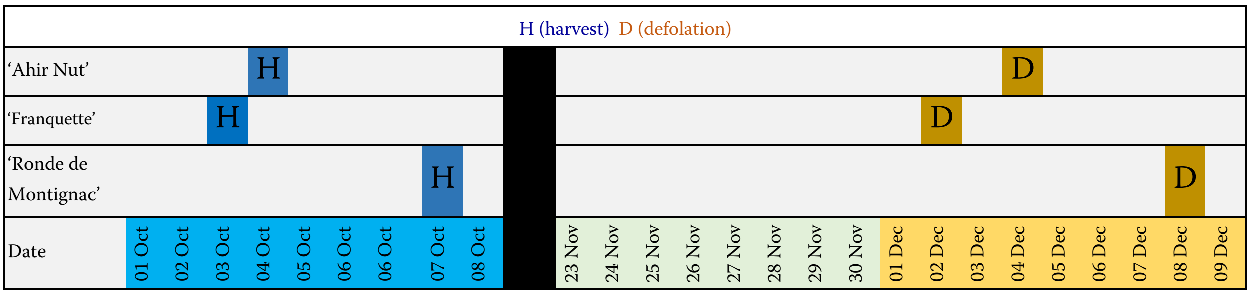

Figure 4. Harvest (fruit ripening) (H) and defoliation dates (D) of 'Ahir Nut', along with the reference cultivars: 'Franquette' and 'Ronde de Montignac'

https://doi.org/10.17221/22/2022-HORTSCI

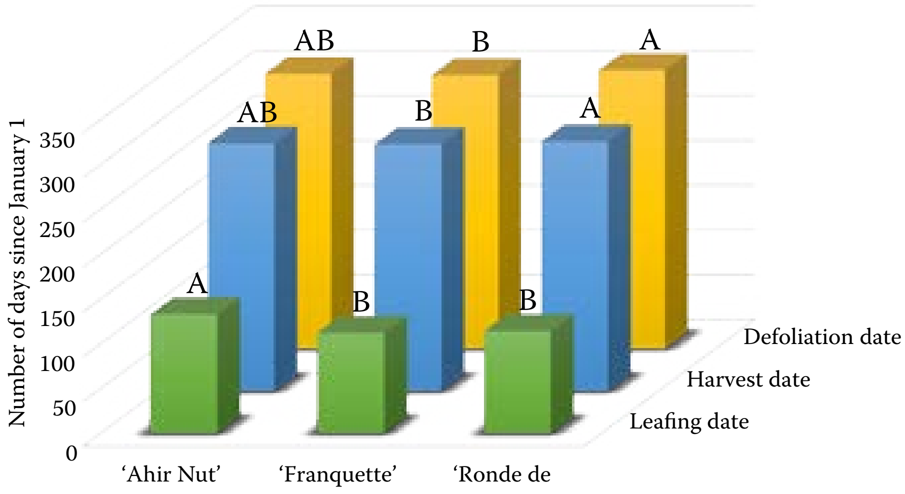

Figure 5. Comparison of the three genotypes in terms of the leafing date, harvest date, and defoliation date

The different letters over the columns represent significant differences based on the LSD multiple range test (P = 0.05)

nard et al. 2018; Sütyemez et al. 2018; Cosmulescu, Lonescu 2021; Sarikhani et al. 2021).

In the present study, 12 pomological traits were observed in three consecutive years, with comparisons of the pomological traits in 'Ahir Nut', 'Franquette' and 'Ronde de Montignac' (Table 4). It was demonstrated that 'Ahir Nut' had a round nut shape, a medium smooth shell texture, and a very light shell colour. The shell thicknesses of 'Ahir Nut', 'Franquette' and 'Ronde de Montignac' were calculated as 1.19 mm, 1.34 mm, and 1.36 mm, respectively. For this reason, 'Ahir Nut' had a weaker shell strength, compared to the other two cultivars (Table 4).

The shelled nut weight, kernel weight, and kernel percentage of 'Ahir Nut' were calculated as 11.61 g, 5.79 g, and 49.84%, respectively. The shelled nut weight, kernel weight, and kernel percentage were 11.75 g, 5.70 g, and 48.54% in 'Franquette', whereas in 'Ronde de Montignac' they were 9.83 g, 4.64 g, and 47.24%, respectively. Thus, it can be said that 'Ahir Nut' displayed pomological traits similar to the standard criteria in the existing literature. The statistical differences between the shelled nut weight, kernel weight, and kernel percentage for each genotype are shown in Figure 6.

The kernel fill of 'Ahir Nut' was determined descriptively as 'good'. The kernel colour and ease of removal of the kernel halves can be classified as 'light' and 'very easy', respectively. A visual overview of the 'Ahir Nut' nuts and the tree is shown in Figures 7 and 8.

Chandler is the most frequently cultivated walnut cultivar in the world. Various studies have reported

Table 4. Pomological traits of 'Ahir Nut', 'Franquette' and 'Ronde de Montignac'

Pomological traits	Genotypes		
	'Ahir Nut'	'Franquette'	'Ronde de Montignac'
Nut shape	round	elliptic	round
Shell texture	medium	rough	rough
Shell colour	very light	light	light
Shell strength	weak	intermediate	intermediate
Shell thickness (mm)	1.19 ± 0.16	1.34 ± 0.18	1.36 ± 0.21
Shelled nut weight (g)	11.61 ± 0.87	11.75 ± 0.81	9.83 ± 0.80
Kernel weight (g)	5.79 ± 0.47	5.70 ± 0.40	4.64 ± 0.43
Kernel percentage (%)	49.84 ± 1.52	48.54 ± 1.36	47.24 ± 1.70
Kernel colour	light	light	light
Kernel fill	good	good	good
Ease of removal of kernel halves	very easy	easy	easy
Kernel flavour	satisfactory	satisfactory	satisfactory

https://doi.org/10.17221/22/2022-HORTSCI

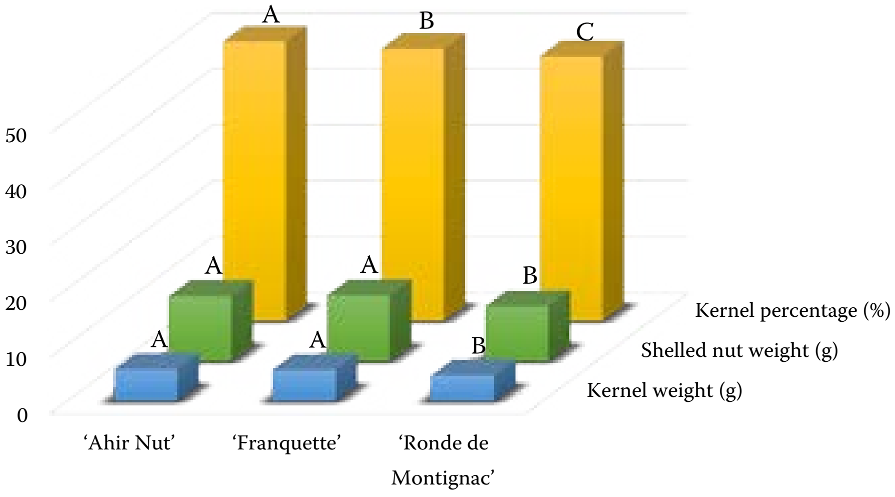

Figure 6. Comparing the three genotypes in terms of the kernel weight, shelled nut weight, and kernel percentage
Different letters over the columns represent significant differences based on the LSD multiple range test (P = 0.05)

that the nut weight of the 'Chandler' cultivar usually varied between 7.7 and 14.0 g, whereas its kernel weight varied between 3.32 and 7.00 g, respectively (Mahmoodi et al. 2016; Sütyemez 2016; Hassani et al. 2020; Manthos, Rouskas 2021). Furthermore, Bobokasvili et al. (2017) reported that the shelled nut weight, kernel weight, kernel percentage, and shell thickness of the 'Franquette' cultivar were 12.20 g, 5.87 g, 48.14%, and 1.44 mm, respectively, which are in line with the findings of the current research.

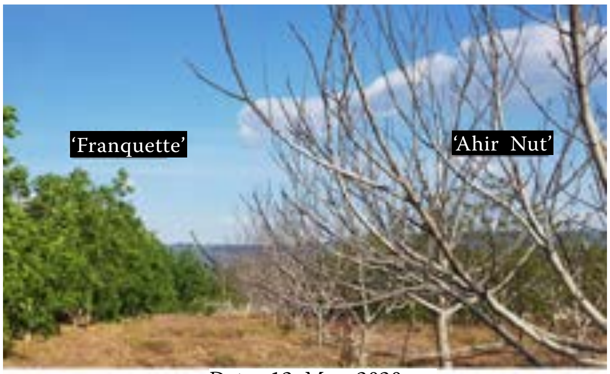

Date: 12 May 2020

Figure 7. A visual overview of the 'Ahir Nut' and 'Franquette' trees on the same date

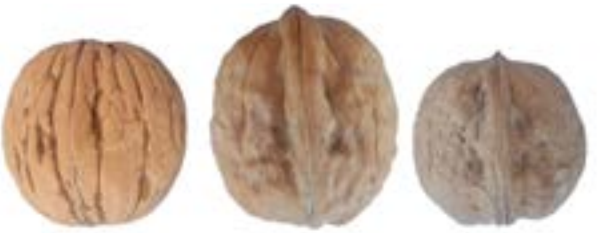

'Ahir Nut' 'Franquette' 'Ronde de Montignac'

Figure 8. A visual preview of 'Ahir Nut', 'Franquette' and 'Ronde de Montignac' nuts

CONCLUSION

Plant breeding is increasingly becoming important in the light of opportunities for economic investment and through the motive to maintain genetic diversity. Walnut breeding research has an important position in agriculture and can be a precursor of high-quality cultivars with late leafing and early defoliation dates. It could be said that the initial work by Sütyemez and Baymış (2005) laid an important background for walnut crossbreeding from which important results are continuously arising. As a walnut genotype with superior phenological traits, 'Ahir Nut' was selected from among thousands of walnut genotypes that resulted from the cross of 'KSÜ-2008/1 × 'Franquette'. The most significant phenological traits of this genotype are its late leafing date, early harvest date, and lateral fruitfulness. Specifically, the leafing date of the 'Ahir Nut' genotype was 21 to 19 days later than those of 'Franquette' and 'Ronde de Montignac'. In addition, its lateral fruitfulness was greater than that of 'Franquette' and 'Ronde de Montignac', making it superior in its phenological performance compared to the other two walnut cultivars. The walnut variety 'Ahir Nuts' a very important genetic resource for future walnut breeding research.

REFERENCES

Akca Y., Sütyemez M., Yılmaz S., Karadag H. (2016): The new walnut variety breeding program in Turkey. VIIth International Scientific Agricultural Symposium. Jahorina, Bosnia and Herzegovina: 461–466.

Arab M.M., Marrano A., Abdollahi-Arpanahi R., Leslie C.A., Askari H., Neale D.B., Vahdati K. (2019): Genome-wide patterns of population structure and association mapping of nut-related traits in Persian walnut populations from Iran using the Axiom *J. regia* 700K SNP array. Scientific Reports, 9: 1–14.

Bernard A., Lheureux F., Dirlewanger E. (2018): Walnut: past and future of genetic improvement. Tree Genetics and Genomes, 14: 1–28.

Bobokasvili Z., Maglakelidze E., Shengelia N., Maghradze D. (2017): Evaluation of some introduced walnut (*Juglans regia* L.) cultivars in Shida Kartli region of Georgia. International Journal of Minor Fruits, Medicinal and Aromatic Plants, 3: 16–20.

Cosmulescu S., Lonescu M. (2021): Phenological and pomological properties of promising walnut (*Juglans regia* L.) genotype with cluster fruiting from selected native population in Oltenia, Romania. Genetic Resources and Crop Evolution, 68: 2289–2297.

Germain E. (1997): Genetic improvement of the Persian walnut (*Juglans regia* L.). Acta Horticultirae (ISHS), 442: 21–32.

Hajinia Z., Sarikhan S., Vahdati K. (2021): Exploring low-chill genotypes of Persian walnut (*Juglans regia* L.) in west of Iran. Genetic Resources and Crop Evolution, 3: 1–12.

Hassani D., Sarikhani S., Dastjerdi R., Mahmoudi R., Soleimani A., Vahdati K. (2020): Situation and recent trends on cultivation and breeding of Persian walnut in Iran. Scientia Horticulturae, 270: 109369.

IPGRI (1994): Descriptors for walnut (*Juglans* spp.); International Plant Genetic Resources Institute: Rome, Italy, 54.

Khodadadi F., Tohidfar M., Vahdati K., Dandekar A.M., Leslie C.A. (2020): Functional analysis of walnut polyphenol oxidase gene (JrPPO1) in transgenic tobacco plants and PPO induction in response to walnut bacterial blight. Plant Pathology, 69: 756–764.

Mahmoodi R., Hassani D., Amiri M.E., Jaffaraghaei M. (2016): Phenological and pomological characteristics of five promised walnut genotypes in Karaj. Journal of Nuts, 7: 1–8.

Manthos I., Rouskas D. (2021): 'Ourania' walnut. HortScience, 56: 521–522.

McGranahan G.H., Leslie C. (2009): Breeding walnuts *(Juglans regia).* In Breeding Plantation Tree Crops: Temperate Species, Springer, New York, NY, USA, 249–273.

McGranahan G.H., Leslie C.A., Ballington J.R., Moore J.N. (1990): Walnuts (Juglans). In: Genetic Resources of Temperate Fruit and Nut Crops, II. ISHS, Secretariat, Netherlands: 907–951.

Ramos D.E. (Ed.) (1997): Walnut Production Manual; UCANR Publications: California, United States of America, 3373.

Rezaee R., Vahdati K., Grigoorian W., Valizadeh M. (2008): Walnut grafting success and bleeding rate as affected by different grafting methods and seedling vigor. The Journal of Horticultural Science and Biotechnology, 83: 94–99.

Sarikhani S., Vahdati K., Ligterink W. (2021): Biochemical properties of superior Persian walnut genotypes originated from southwest of Iran. International Journal of Horticultural Science and Technology, 8: 13–24.

Soleimani A., Rabiei V., Hassani D., Mozaffari M.R. (2019): Phenological characteristics of walnut (*Juglans regia* L.) genotypes under environmental conditions of Karaj in Iran. Crop Breeding Journal, 9: 1122.

Sütyemez M., Kaska N. (2004): Comparison of the behaviors of some locally selected genotypes and local and foreign walnut cultivars under ecological conditions of Kahramanmaraş. Acta Horticulturae (ISHS), 705: 151–157.

Sütyemez M., Baymış M. (2005): Kalite ve verim bakımından üstün özelliklere sahip bazı ceviz (*Juglans regia* L.) tip ve çeşitlerinin karşılıklı melezlenmesi suretiyle yeni çeşit eldesi üzerine araştırmalar. Tübitak Project No: 104O318 (in Turkish).

Sütyemez M. (2016): New walnut cultivars: Maras 18, Sutyemez 1, and Kaman 1. HortScience, 51: 1301–1303.

Sütyemez M., Özcan A., Bükücü Ş.B. (2018): Walnut cultivars through cross-breeding: 'Dirilis' and '15 Temmuz'. Journal of the American Pomological Society, 73: 173–180.

Sütyemez M., Bükücü Ş.B., Özcan A. (2021): 'Helete Güneşi', a new walnut cultivar with late leafing, early harvest date, and superior nut traits. Agriculture, 11: 991.

Turkish State Meteorological Service (2021): Available at: https://mgm.gov.tr/veridegerlendirme/il-ve-ilceler-istatistik.aspx?m=K.MARAS (Accessed on 27 Dec. 2021).

Tüzüner A. (1990): Toprak ve Su Analiz Laboratuvarları El Kitabı. Tarım Orman ve Köy İşleri Bakanlığı, Köy Hizmetleri Genel Müdürlüğü, Ankara, Turkey, 374.

UPOV (Union Internationale Pour le Protection des Obtentions Vegetales) (2014): Draft Guidelines for the Conduct of Tests for Distinctness Homogeneity and Stability. Walnut *(Juglans regia L.)*, Geneva, Switzerland, TG/125/7, 28.

Vahdati K. (2014): Traditions and folks for walnut growing around the Silk Road. Acta Horticulturae (ISHS), 1032: 19–24.

Vahdati K., Arab M.M., Sarikhani S., Sadat-Hosseini M., Leslie C.A., Brown P.J. (2019): Advances in Persian walnut (*Juglans regia* L.) breeding strategies. In: Advances in Plant Breeding Strategies: Nut and Beverage Crops, Springer, New York, USA, 401–472.

Received: February 15, 2022
Accepted: July 14, 2022
