# Supplementary material for: Provenance and family variations in early growth of Manchurian walnut (Juglans mandshurica Maxim.) and selection of superior families
Source: PLoS One. 2024 Mar 7;19(3):e0298918. doi: 10.1371/journal.pone.0298918 (PMC10919699; doi:10.1371/journal.pone.0298918)
Supplement: S1 File — (ZIP) [file pone.0298918.s004.zip › Advances in Persian walnut (Juglans regia L.) breeding strategies.pdf]

Chapter 11
Advances in Persian Walnut (*Juglans regia* L.) Breeding Strategies

Kourosh Vahdati, Mohammad M. Arab, Saadat Sarikhani, Mohammad Sadat-Hosseini, Charles A. Leslie, and Patrick J. Brown

Abstract Walnut (*Juglans regia* L.) is one of the oldest trees with harvestable products known to humans and has a history dating to 7000 BC in Persia. Walnut breeding programs aim to release productive scion cultivars with disease resistance and high-quality nuts, along with rootstocks resistant to biotic and abiotic stresses. Genetic improvement of walnut began with the selection of superior trees in their main centers of origin, primarily from the Persian plateau. The first selection and grafting of superior walnut genotypes began in France. The first organized walnut-breeding program employing targeted hybridization began in the USA in 1948, primarily using introduced French cultivars and selected local genotypes derived from seed imported from centers of origin (Iran, Afghanistan, China). Currently, both conventional hybridization with phenotypic evaluation and molecular breeding approaches are used in the USA programs as well as those in France, China, Iran, Spain and Italy. Recent advances in biotechnology and genomics show potential to accelerate cultivar development. In addition, the exploration, description, and preservation of biodiverse germplasm can provide a gene bank of desirable traits and enable biotechnologists to conduct breeding more accurately and rapidly in the future. Recent advancements have opened up new avenues to enhance the efficiency of walnut breeding to release new scions and rootstocks. These include next-generation sequencing (NGS) techniques, bioinformatics tools, high-throughput genotyping platforms and genomics-based approaches such as genome wide association studies (GWAS), marker-assisted selection (MAS), genomic selection (GS) and genome editing with the CRISPR-Cas9 system. In this chapter, we describe the

K. Vahdati (✉) · M. M. Arab · S. Sarikhani
Department of Horticulture, College of Aburaihan, University of Tehran, Tehran, Iran
e-mail: kvahdati@ut.ac.ir; mm.arab@ut.ac.ir; saadat.sarikhani@ut.ac.ir

M. Sadat-Hosseini
Department of Horticulture, College of Aburaihan, University of Tehran, Tehran, Iran

Department of Horticulture, Faculty of Agriculture, University of Jiroft, Jiroft, Iran
e-mail: m.hosseini@ujiroft.ac.ir

C. A. Leslie · P. J. Brown
Department of Plant Sciences, University of California, Davis, CA, USA
e-mail: caleslie@ucdavis.edu; pjbrown@ucdavis.edu

© Springer Nature Switzerland AG 2019

J. M. Al-Khayri et al. (eds.), *Advances in Plant Breeding Strategies: Nut and Beverage Crops*, https://doi.org/10.1007/978-3-030-23112-5_11

background and development of conventional walnut breeding programs in the leading walnut producing countries of the USA, France, China, Iran and Turkey, and finally focus on the current use and status of molecular breeding and biotechnology in walnut breeding.

Keywords Bioinformatics · Biotechnology · Genetic diversity · Genomics · *Juglans regia* · Molecular · Rootstock · Cultivar · Selection · Marker

11.1 Introduction

Walnuts (*Juglans* spp.) are among the oldest tree foods known to humanity, with historical references dating back to 7000 BC in Persia (Dreher et al. 1996) (Fig. 11.1). *Juglans* is the most important genus in the Juglandaceae, containing 21 species that produce edible nuts (Karimi et al. 2010; Vischi et al. 2017). The genus *Juglans* is classified into four sections; *Rhysocaryon*, *Cardiocaryon*, *Trachycaryon* and *Dioscaryon* (McGranahan and Leslie 1991). Persian (or English) walnut is the only species in section *Dioscaryon* and the most widely cultivated *Juglans* species,

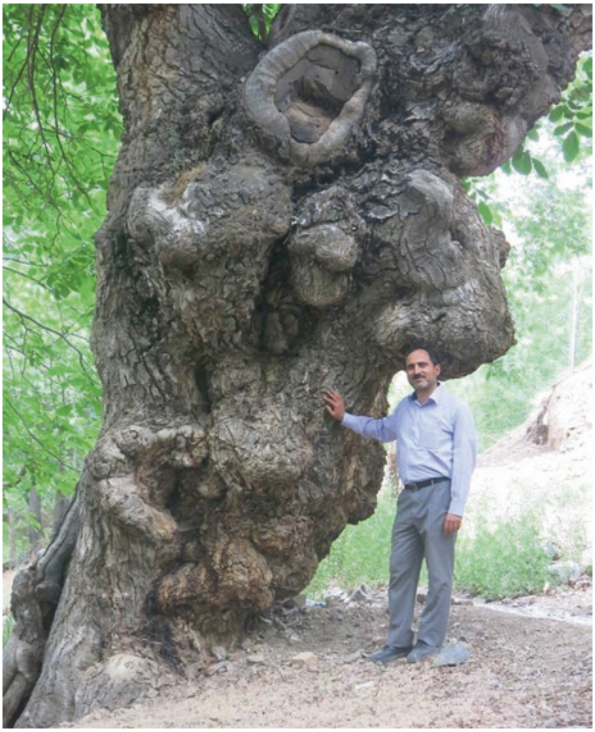

Fig. 11.1 Kourosh Vahdati stands next to one of the historic walnut trees in Rabor, Kerman, Iran estimated to be many hundreds of years old

grown throughout the temperate and semiarid regions worldwide for edible nuts (Amiri et al. 2010; Arzani et al. 2008; McGranahan and Leslie 1991; Molnar et al. 2011).

Persian walnut is a large deciduous tree with smooth bark and alternately arranged leaves. The male flowers develop as drooping catkins 5–10 cm long. Female flowers are borne terminally in clusters of 2–5. The characteristic of this species is having four-chambered nuts with hulls that dehisce and separate from the shells at maturity (Germain 1999; Ramos 1997). It is a diploid species (2n = 2x = 32) with an estimated genome size of 1315 Mbp (1.35 pg; monoploid genome size (1C DNA) = 657.80 Mbp). In comparison, the human genome (6153 Mbp) is approximately 4.7 times larger than walnut genome size (IHGSC 2004; Sarikhani Khorami et al. 2018).

Persian walnut, as its name suggests, originated in Persia (now Iran) and was distributed along the Silk Road eastward to Pakistan, Afghanistan, India, Uzbekistan and China, and westward to Turkey and Europe (Vahdati et al. 2014). Walnut has a long history of cultivation and widespread use from China to Western Europe. According to molecular phylogeographic studies and paleontology, its evolutionary history dates back to the Holocene in Eurasia (Pollegioni et al. 2017). Currently it is grown from 30 to 55° of latitude in the Northern Hemisphere in many countries of Asia, Europe and North America and from 30 to 40° in the Southern Hemisphere in Australia, New Zealand, South Africa, Chile and Argentina) (Fig. 11.2).

Walnut (*Juglans regia* L.) is an important nut and timber species and a valuable dietary source contributing to reduction of coronary heart disease (CHD) (Maguire et al. 2004; Zhang et al. 2009). Walnuts contain antioxidants that stimulate the immune

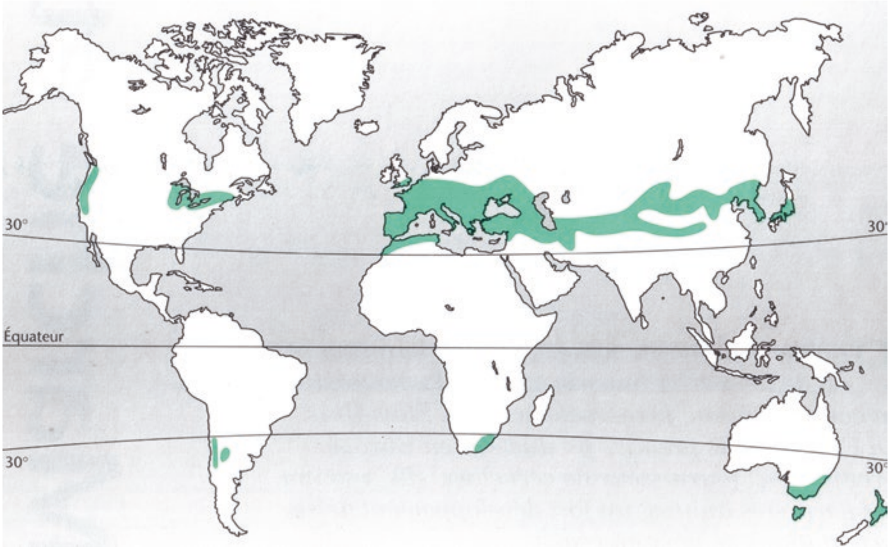

Fig. 11.2 Walnut distribution in the world; green areas on the map indicate walnut-producing areas. (Source: Germain 1999)

system and appear to have anti-cancer properties (Jahanbani et al. 2016a, b; Milind and Deepa 2011). Bioactive peptides in walnuts also appear to have value for prevention and treatment of hypertension (Huang et al. 2013; Jahanbani et al. 2018).

Due to their high content of unsaturated fatty acids (UFAs), antioxidants, proteins, tocopherols and other bioactive constituents, walnut kernels have been implicated in the control of heart disease (Pereira et al. 2008; Ros et al. 2004; Siqueira et al. 2015). Their high UFA content can also play an important role in decreasing total and LDL-cholesterol and increasing HDL-cholesterol. Dietary intervention studies have demonstrated that omega-3 fatty acid, found in abundance in walnut kernels, can help prevent some cognitive disorders including depression, dementia and Alzheimer's disease (Bourre 2005; Dogan and Akgul 2005). Due to its high nutrient value, walnut is a strategic product for human health and nutrition on the FAO list of priority crops (Gandev 2007).

With uses ranging from landscaping to timber production to human nutrition, walnut production plays a significant role in creating employment and generating income. According to FAO statistics (FAO 2016), the world's annual walnut production (shelled + in shell) is approximately 3.74 million mt with a commercial value of 2.77 (1.62 + 1.15) million USD. China, the USA and Iran, the largest walnut producers, account for more than 74.7% of world's production. The USA is also the largest walnut exporter (Table 11.1). The USA exports more than 50% of its walnut production, of which half is shelled walnut kernels, accounting for about 53.8% of the world's total walnut exports (FAO 2016).

Despite the high economic value and ancient cultivation of walnut, breeding has been limited, relative to other temperature-zone fruits, particularly in countries of the crop's origin. The first established walnut breeding programs dates back to the mid-twentieth century (Bernard et al. 2018). Prior to that, the genetic improvement of walnut involved selection of elite trees from natural populations. As in other tree crops, high yield and quality (large nut size, high kernel percentage, light kernel color) have been primary walnut breeding objectives.

Table 11.1 Leading nations for Persian walnut total production, area cultivated, export and import value in international trade (2016)

		World	Top three countries
Production (mt)		3,747,549	China, USA, Iran
Area harvested (ha)		1,186,399	China, Iran, USA
Export value (1000 USD)	(Shelled)	1,736,902	USA, Mexico[a], Chile
	(In shell)	1,218,144	USA, Mexico[a], France
Import value (1000 USD)	(Shelled)	1,401,865	Germany, Japan, Spain
	(In shell)	843,032	Italy, Turkey, Vietnam

Source: FAO (2016)

[a]Mexico data on walnut export is sometimes misleading and confused with data of pecan (Avanzato et al. (2014))

In this chapter, we discuss walnut cultivation, traditional breeding programs, germplasm biodiversity and conservation, molecular breeding, genetic engineering, mutation breeding, and hybridization.

11.2 Cultivation and Traditional Breeding

11.2.1 Current Cultivation Practices

Walnut cultivation is of great interest in suitable regions because walnut orchards require relatively low maintenance, are productive for at least 40 years, and nuts can be stored for up to 2 years under simple storage conditions (Adem 2009). Walnut trees require a temperate climate with 600–1000 chill hours (Aslani Aslamarz et al. 2009), can be seriously damaged by frost during late spring or early fall, and most cultivars are sensitive to both extremely high (>38 °C) and very low (< −20 °C) temperature. Walnut trees are very demanding in terms of soil texture and structure, requiring good drainage to allow water, air and nutrients to move to the roots. Due to their huge and spreading root systems, walnut trees prefer a deep and sandy loam soil. For commercial walnut production, soil pH of 7–7.5 is best and electrical conductivity (EC) should be lower than 2.5 mmhos.cm^{-1} (Germain 1999; Ramos 1997).

Most walnut trees in the past were grown from seed, but nowadays seedlings are mainly to provide rootstock. Typically, modern walnut orchards are established using one or two commercial scion cultivars, propagated by grafting or micropropagation. Tree spacing in walnut orchards ranges from 10 × l0 m to 6 × 3 m, giving approximately 100–550 trees per hectare, respectively (Adem 2009). Several walnut cultivars have been released to growers in the leading walnut producing countries, depending on the breeding objectives and the production areas. Chandler is the world's most common walnut cultivar, having a lateral bearing habit, strong yield and light kernel color (Tulecke and McGranahan 1994). Franquette, a late-leafing cultivar, is another common walnut cultivar in the world. It has long been the dominant walnut cultivar in France (Germain 1999); but due to its terminal bearing habit, it is being replaced by new lateral bearing cultivars such as Chandler and Fernor in modern orchards.

11.2.2 Walnut Production Challenges and Breeding Objectives

Leading challenges always dictate specific objectives in plant breeding, but high yield and crop quality are the main objectives for all crops. All targeted breeding traits also require high heritability, indicating the control of a high proportion of total phenotypic variance is dependent on genetic factors (Hansche et al. 1972; Ramos 1997). In initial walnut breeding work, nut size and kernel color were

primary targets. High kernel percentage, ease of kernel removal and reduced shell thickness were additional traits of interest. Lateral bearing, as a major component of yield, became one of the main traits pursued in walnut breeding programs beginning in the mid-twentieth century, leading to the introduction of the Chandler cv. Other commonly targeted traits are disease resistance (blight, anthracnose) and late leafing date. Some traits in walnut breeding depend on the production area and climatic conditions (Avanzato et al. 2014; Bernard et al. 2018; Ramos 1997). For example, due to late-spring frost, late leafing is an important trait in walnut breeding in France, Iran and Turkey. In addition, late leafing is helpful to avoid walnut blight (*Xanthomonas arboricola* pv. *juglandis*) which spreads by spring rains. Early harvest date is an important breeding trait in the US in order to widen the harvest window and avoid autumn rain (Akca and Ozongun 2004; Bernard et al. 2018; Ebrahimi et al. 2015; Germain 1990; Leslie and McGranahan 2014). Current and future breeding objectives are keeping in mind climate change and global warming, including rootstocks tolerant to abiotic stresses, especially drought, and with disease resistance.

11.2.3 The World's Walnut Breeding Programs

11.2.3.1 Cultivars

Although organized walnut breeding programs began during the last century, genetic improvement of walnut began much earlier by farmers selecting and propagating superior trees from their natural seedling populations. Crows have been the other early walnut breeders, selecting nuts from the highest parts of trees where nuts are larger because of more light and ventilation. Nuts hidden in the soil by crows as future food start to germinate after the cold season fulfills the chilling requirement. Walnut growers have used these seedlings to develop stronger trees. In Iran, walnut seedlings *planted* by crows are called Kalagh-kar, meaning *crow planted seedlings*, and were used extensively in the past to establish walnut orchards (Vahdati et al. 2014). Old literature reports that superior walnut genotypes, in terms of shell thickness, nut size and kernel color, were selected in the centers of walnut origin, such as Iran, China and other Silk Road countries, and used to establish new orchards. Abounasri Harvi (1515) in his book entitled *Agricultural Guidance* (*Ershad Al-Zerae* in Persian) reported that growers used thin-shelled walnut to establish orchards in the 1500s.

Development in walnut genetic improvement has occurred in 4 phases: 1700–1948, 1948–1979, 1979–2009 and 2009 to present. The first organized efforts at genetic improvement of walnut began in France with development of grafting methods that allowed selection and propagation of superior genotypes, leading to establishment of cultivars such as Franquette, Mayette, Crone, Grandjean and Parisienne. Walnut breeding in the USA, where Persian walnut is not native, began with the introductions of French cultivars and selection of superior local genotypes

grown from imported seeds from walnut centers of origin such as Iran and Afghanistan. The first breeding and selection efforts in the USA were by Felix Gillet (1835–1908), the father of the Northern California walnut industry and Josef Sexton (1842–1917), a walnut grower in Southern California. Their work, and additional seedling selections by other California walnut growers, led to the development and use of many terminal bearing cvs. including Eureka, Waterloo, Poe and Hartley. Discovery by California farmer George Payne of a highly productive seedling in his orchard fencerow led to widespread propagation and planting of this selection as the cultivar Payne. The precocity and abundant yield of this early-leafing selection was later determined to be due to its lateral bearing habit. Because cv. Payne was for many years the sole source of this trait in California, it became the most important parent in the University of California walnut-breeding program and almost all the University of California walnut releases have cv. Payne in their background (Ramos 1997; Tulecke and McGranahan 1994). The heritage of many current cultivars in the USA also includes Eureka (with a parent thought to be from Iran) and Pl159568 (originating from Afghanistan). In general, the objectives during this early phase of walnut genetic improvement were large nuts, high kernel percentage, thin shell and light kernel color (Table 11.2).

The walnut growers of the USA and France led the second phase of the world's walnut breeding. Work continued in the USA based on hybridization between French cultivars and genotypes originating from Silk Road countries including Iran and Afghanistan, but now strongly emphasizing incorporation of the lateral bearing trait. This breeding phase was led by Eugene F. Serr and Harold I. Forde, from 1948 to 1979. A total of 13 cultivars, namely Midland, Vina, Pioneer, Pedro, Gustine, Lompoc, Amigo, Chico, Tehama, Serr, Chandler, Howard and Sunland were released (Table 11.3). Chandler, the world's most prominent walnut cultivar, Serr and Howard were the most important of these. Chandler and Serr account for 75% and 12% of current California walnut acreage, respectively. Major breeding objectives were late leafing, lateral bearing, precocious production, moderate tree vigor, kernel quality and disease tolerance. Pedigrees of the Serr/Forde cultivars show that cv. Payne is a parent or ancestor of all of these (Fig. 11.3). More information about the California Walnut Improvement Program is available from the Walnut Research Reports Database at the University of California, Fruit and Nut Research Information Center website; http://ucanr.edu/sites/cawalnut/ (Ramos 1997; Tulecke and McGranahan 1994; Bernard et al. 2018).

The second phase of the French walnut-breeding program included 28 crosses (1900 intraspecific hybrids) between French and Californian cultivars (Fig. 11.4), led by Eric Germain at INRA (Bernard et al. 2018; Germain 1999; Ramos and Doyle 1984). In this phase, hybrids were evaluated. Lara, a natural seedling of Payne, served as a main parent in these crosses. The main objective was to obtain lateral bearing and late-leafing cultivars. Therefore, one of the main crosses was between Franquette (a late-leafing cultivar) and Lara (a lateral-bearing cultivar), led to release of Fernor in 1987 (Germain 1997, 1999).

The third and fourth walnut breeding phases cover the periods from 1979 to 2009, and 2009 until today. In addition to the USA and France, other walnut growing

Table 11.2 Walnut breeding programs and released cultivars and rootstocks in the world from past to present

Class	Country	1950–1700	1700–1948		1948–1979		1978–2009			2009 until now		
		Center of origin countries	France	United States	France	United States	France	United States	Other countries	France	United States	Other countries
Breeding for cultivars	Strategy	Selection	Germplasm evaluation and selection, introduction		Hybridization		Germplasm evaluation and selection, hybridization, molecular breeding			Germplasm evaluation and selection, hybridization, molecular breeding		
	Objectives	Large nut with thin shell, high kernel percentage, light kernel color			Lateral bearing, early production, kernel quality, disease tolerance, late leafing		Lateral bearing, No PFA, Precocity, Late leafing, Early harvest, Disease tolerance, kernel quality,			Early harvest, late-leafing, disease resistance, reduced water use, adapted to climatic conditions, winter hardiness		
	Released cultivars	-	Franquette, Crone, Marbot, Grandjean, Mayette, Parisienne	Eureka, Payne, Pl159568, Waterloo, Ashley, Adams, Olmo13-1048, Poe, Hartley	Lara	Midland, Vina, Pioneer, Gustine, Lompoc, Pedro, Amigo, Chico, Tehama, Serr, Chandler, Howard, Sunland	Fernor, Fernette, Ferjean	Cisco, Tulare, Robert Livermore, Sexton, Gillet, Forde	Jamal, Damavand, Baokexiang, Beijing 861, Jinglong 1,2, Lipin 1,2, Xilin 1, Xinzaofeng, Sebin, Bilecik, Maras 10, Yalova 1, Sutymez, Kaman 1	Feradam, Ferbel, Ferouette, Fertignac	Ivanhoe, Solano, Durham.	Dirilish, 15 Temmuz, Maras 12, Luguo series, Zanmei, Shuang zao, Jinbaoxiang, Rili, Persia, Caspian, Chaldoran, Alvand

Breeding for rootstock	Strategy	-	Selection	Inter-species hybridization	Germplasm evaluation and Selection, Inter-species hybridization			Germplasm evaluation and selection, interspecies hybridization		
	Objectives		Uniform orchards	Vigor and disease resistance	Disease resistance especially *Armillaria*, *Phytophthora*, Nematode and CLRV			Disease resistance, drought tolerance, dwarfness		
	Released rootstock		*J. regia* seedling	*J. regia* seedling, Paradox and Paradox series	*J. regia* seedling	Vlach, RX1, VX211	*J. regia* seedling	---	---	Jin RS-1, Jin RS-2, Jin RS3

- Cells with (---) indicate that there was no released cultivar or breeding program at that time

Table 11.3 Characteristics and phylogeny of some major walnut cultivars in the leading walnut producer countries

Cultivar	Parent	Origin	Leafing date	Tree vigor	Yield	Bearing habit	Nut weight (g)	Kernel weight (g)	Kernel percentage	Shell	Kernel color
Eureka	Superior genotype	USA	28-Mar	Vigorous	Fair	Terminal	15.2	7.40	49	Strength	Poor
Hartley	Franquette × Mayette	USA	3-Apr	Vigorous	Moderate	Terminal	14.3	6.50	45	Thin	Medium
Payne	Chance seedling	USA	18-Mar	Moderate	Moderate	Lateral	12.9	6.40	50	Strength	Light
Vina	Franquette × Payne	USA	26-Mar	Moderate	Strong	Lateral	12.6	6.20	49	Strength	Medium
Pedro	Conway Mayette × Payne	USA	5-Apr	Small	Strong	Lateral	13.5	6.50	48	Strength	Medium
Tehama	Waterloo × Payne	USA	31-Mar	Vigorous	Heavy	Lateral	14.1	6.80	48	Strength	Light
Serr	Payne × Pl159568	USA	20-Mar	Vigorous	Variable	Moderate	14.4	8.10	56	Thin	Light
Chandler	Pedro × 56-224	USA	4-Apr	Moderate	Strong	Lateral	13.2	6.50	49	Thin	Extra light
Howard	Pedro × 56-224	USA	2-Apr	Moderate	Strong	Lateral	14.3	7.20	51	Strength	Light
Sunland	Lumpoc × Pl159568	USA	20-Mar	Vigorous	Variable	Lateral	17.9	9.80	55	Strength	Medium
Cisco	Meylan × Pedro	USA	14-Apr	Moderate	Moderate	Terminal	14.2	6.20	44	Strength	Medium
Tulare	Tehama × Serr	USA	1-Apr	Vigorous	Strong	Lateral	14.1	7.60	53	Strength	Light
Robert Livermore	UC86-11 × Howard	USA	3-Apr	Moderate	Moderate	Lateral	12.9	6.40	50	Strength	Red skin
Sexton	Chandler × 85-8	USA	26-Mar	Small	Good	Lateral	15.6	8.30	53	Strength	Light
Gillet	Chico × 76-80	USA	25-Mar	Moderate	Strong	Lateral	15.2	7.70	51	Thin	Light
Forde	Chico × Lara	USA	30-Mar	Moderate	Good	Lateral	15.5	8.10	52	Strength	Light
Ivanhoe	UC 67-13 × Chico	USA	17-Mar	Moderate	Strong	Lateral	12.8	7.30	57	Thin	Extra light
Solano	UC67-13 × Chico	USA	28-Mar	Moderate	Strong	Lateral	14.6	7.90	54	Strength	Extra light
Durham	Chandler × Pl159568	USA	30-Mar	Moderate	Strong	Lateral	15.1	8.30	55	Strength	Light

Franquette	Superior genotype	France	23-Apr	Vigorous	Fair	Terminal	11.0	5.50	50	Medium	Extra light
Lara	UC49-46 × Franquette	USA	13-Apr	Moderate	Strong	Lateral	11.5	5.50	48	Thin	Medium
Fernor	Franquette × Lara	France	21-Apr	Moderate	Strong	Lateral	11.0	4.90	44	Medium	Extra light
Fernette	Franquette × Lara	France	15-Apr	Moderate	Good	Lateral	14.9	7.50	50	Medium	Extra light
Ferjean	Grosvert × Lara	France	15-Apr	Moderate	Strong	Lateral	11.9	5.95	50	Thin	Extra light
Jamal	Superior genotype	Iran	Early Apr	Vigorous	Moderate	Moderate	11.42	5.80	50.77	Medium	Medium
Damavand	Superior genotype	Iran	Early Apr	Vigorous	Moderate	Moderate	13.09	6.27	47.07	Medium	Medium
Yalova 1	Superior genotype	Turkey	Early Apr	Vigorous	Low	Terminal	17.16	7.98	47.76	Medium	Dark
Yalova 3	Superior genotype	Turkey	Early Apr	Vigorous	Low	Terminal	13.42	7.28	53	Thin	Light
Sebin	Superior genotype	Turkey	8–16 Apr	Moderate	Strong	Moderate	12.68	6.34	61.24	Thin	Light
Bilecik	Superior genotype	Turkey	9–13 Apr	Vigorous	Moderate	Terminal	12.87	4.80	50.11	Medium	Medium
Maras 18	Superior genotype	Turkey	4–8 Apr	Vigorous	Moderate	Lateral	13–15	7–9	53–57	Medium	Light
Sütyemez 1	Superior genotype	Turkey	2–6 Apr	Vigorous	Moderate	Lateral	25–27	12–14	49–51	Medium	Light
Kaman 1	Superior genotype	Turkey	6–10 Apr	Vigorous	Strong	Lateral	13–14	7–8	52–57	Medium	Light

(continued)

Table 11.3 (continued)

Cultivar	Parent	Origin	Leafing date	Tree vigor	Yield	Bearing habit	Nut weight (g)	Kernel weight (g)	Kernel percentage	Shell	Kernel color
Zha 343	Superior genotype	China	Early Apr	Vigorous	Strong	Lateral	12.13	6.48	53	Thin	Light
Jinlong 1	Superior genotype	China	Early Apr	Vigorous	Moderate	Terminal	14.85	9.10	61	Thin	Light
Wen 185	Superior genotype	China	Early Apr	Moderate	Strong	Lateral	15.2	9.86	65	Thin	Light
Xiangling	Shangsong5 × Akesu9	China	Early Apr	Vigorous	Strong	Lateral	12.2	8.00	65	Thin	Light
Zhonglin 1	Jian92723 × Fenyangchuanzi	China	Early Apr	Vigorous	Strong	Lateral	14.0	6.29	56	Thin	Extra light
Liaoning 1	Dabopi10103 × Xinjiangzhipi11001	China	Early Apr	Vigorous	Strong	Lateral	9.4	7.29	65	Thin	Extra light
Jinboxiang1	Superior genotype	China	Early Apr	Moderate	Strong	Lateral	11.5	5.40	47	Thin	Light
Luguo 2	Shangsong6 × Luxiang	China	Late Mar	Vigorous	Strong	Lateral	14.5	10.01	60	Thin	Light

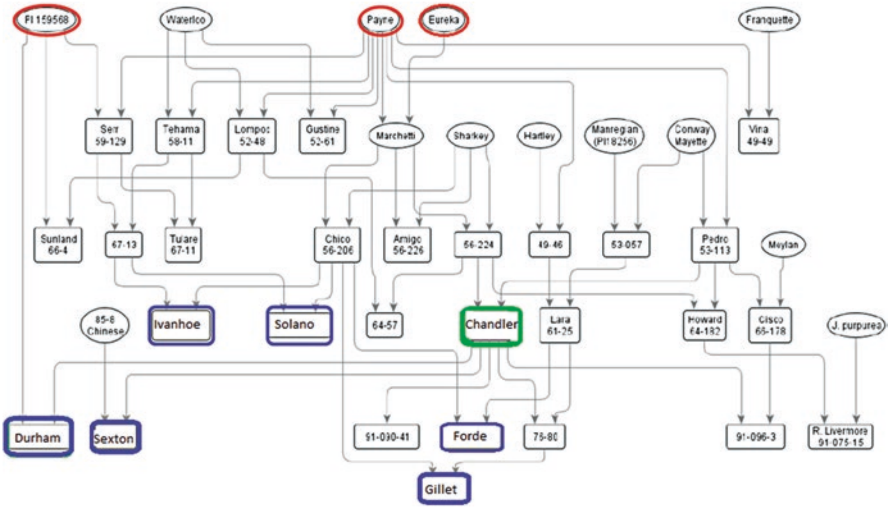

Fig. 11.3 The phylogeny of main California walnut cultivars; red, blue and green lines indicate main parents, new cultivars and the most common cultivar, respectively. (Source: Leslie 2016)

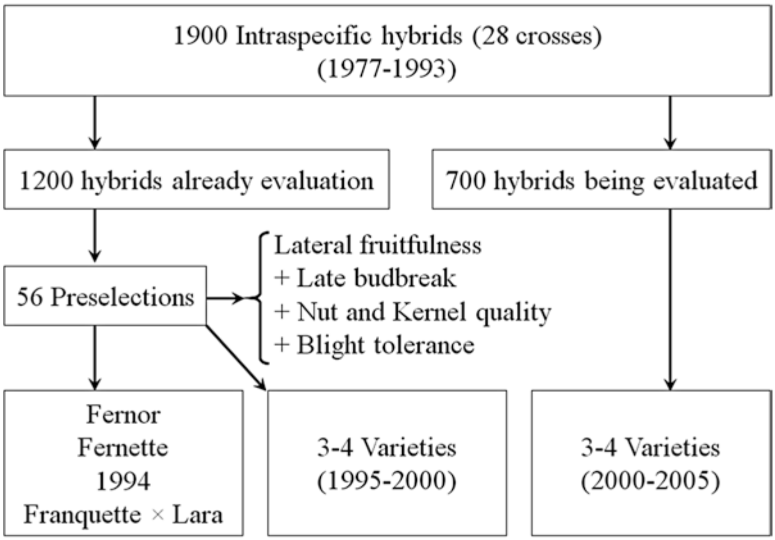

Fig. 11.4 The French walnut breeding program scheme in the second and third breeding phase. (Source: Germain 1997)

countries including China, Turkey and Iran started breeding programs in these phases. Walnut genetic improvement also started in some Asian (a primary center of diversity) and European (a secondary center of diversity) countries. Exploitation of genetic diversity and identification of superior genotypes were the main breeding strategies in these countries (Avanzato et al. 2014).

After the retirement of Harold Forde, the walnut breeding program in the USA continued under Gale McGranahan with the assistance of Charles Leslie (as the third phase). In addition to previous breeding objectives, by making 300 controlled crosses, late-leafing walnut cultivars with the additional trait of early harvest were released to growers. In addition, resistance to blight and blackline (CLRV) and absence of pistillate flower abscission (PFA) were other breeding objectives. Tulare, Robert Livermore, Sexton, Gillet and Forde were the main commercial cultivars obtained from this third phase of the walnut breeding program in the USA (Fig. 11.3; Table 11.2). Application of molecular markers along with traditional breeding was the most significant development in the third breeding phase (Leslie et al. 2009; McGranahan and Leslie 2004, 2005). The fourth walnut breeding phase continued under the leadership of Charles Leslie after the retirement of Gale McGranahan in 2009 in the USA. The main objectives are still early harvest dates, high yield and quality, and resistance to blight and blackline (CLRV). Ivanhoe (2010), Solano (2012) and Durham (2016) are three new cultivars with harvest dates earlier than cv. Chandler.

Modern biotechnology-based methods of plant breeding were also developed and introduced during this phase. A cooperative effort involving multiple laboratories, notably those of Abhaya Dandekar, Jan Dvorak and MingCheng Luo (UC Davis), and Malli Aradhya (USDA-ARS), with the participation of the UC Davis Genome Center, worked for several years to develop integrated physical, functional and genetic maps of the walnut genome (Leslie and McGranahan 2014). A major recent development has been the first sequencing of the Persian walnut genome by a group led by David Neale at UC Davis. This accumulating genetic information should accelerate the rate of breeding and varietal improvement in walnuts and help breeders select for desired traits (Martínez-García et al. 2016).

The third phase of the French breeding program continued under the leadership of E. Germain and F. Delort at INRA, Bordeaux. Additional hybrids, obtained from 28 crosses, were tested for late leafing and lateral bearing as the main objectives and more cultivars (e.g. Ferjean) were released. Germain (1997) described crosses carried out using the best previous hybrids with new sources from the Mediterranean (for lateral bearing) and Iran or Central Asia (for precocity). The French walnut breeding was then inactive for several years. In 2009, Fabrice Lheureux started a new phase of French walnut breeding (the fourth phase) and four cultivars (Feradam, Ferbel, Ferouette, Fertignac) were released (Bernard et al. 2018).

The walnut breeding program in Iran, initially led by Jamal Atefi at Horticulture Science Research Institute (HSRI), started to release new cultivars in 1983. Iran is one of the main centers of origin of walnut in the world and its traditional orchards contain great genetic diversity (Atefi 1993; Vahdati 2000). The main breeding strategy has been evaluation and selection from among this resource (Atefi 1990, 1993, 1997; Hassani et al. 2014). For this purpose, four collections of the selected superior genotypes are maintained in Karaj, Shahrood, Mashhad and Uremia. In 1994, seven genotypes from the Karaj collection (K72, Z63, Z30, Z60, Z67, Z53, B21) were selected and planted with eight French/Californian commercial cultivars (Chandler, Pedro, Hartley, Lara, Serr, Vina, Franquette, Ronde de Montignac) in Karaj. Among these genotypes, two promising genotypes, Jamal (Z63) and Damavad (Z30), were

released as the first Iranian walnut cultivars in 2009–2010 (Hassani et al. 2014). Currently, the fourth phase of the walnut-breeding program at HSRI continues based on germplasm evaluation and hybridization. Recently, four commercial walnut cultivars (Persia, Caspian, Chaldoran and Alvand) have been released which are characterized by high yield, lateral fruitfulness and late-leafing date.

In addition to HSRI, walnut breeding is in progress at the University of Tehran (Aburaihan Campus) under the management of Kourosh Vahdati. Germplasm evaluation in the walnut plantation areas (such as Kerman, Ilam, Fars, Qazvin, Alborz, Yazd, Kohgilooye and Boyer-Ahmad, Mazandaran etc.), hybridization, and molecular breeding are the main walnut breeding strategies used at the University of Tehran to release commercial cultivars and rootstocks. High yield, late leafing, lateral bearing, early harvest, dwarfing and drought stress tolerance are the most important objectives (Karimi et al. 2014; Vahdati and Mohseniazar 2016; Vahdati and Rezaee 2014; Vahdati et al. 2015).In China, walnut genetic improvement is based on germplasm evaluation and hybridization. The majority of germplasm evaluation is conducted in Xinjiang province, where there are extensive germplasm resources for walnut. About 80% of Chinese walnut cultivars are selected or originate from Xinjiang germplasm. Although the walnut breeding program in China began at the Liaoning Economic Forest Institute and China Academy of Forestry in the early 1960s, most of the current walnut cultivars were released after establishing uniform national selection criteria. Since the 1980s, based on these national standards, excellent progress has been made in selection of superior genotypes. Approximately 26 cultivars, including Baokexiang, Beijing 861, Jinglong 1, Jinglong 2, Lipin 1, Lipin 2, Lubo, Xifu 1, Xilin 1 and Xinzaofeng, were released (1979–2006) as a result of this work. In addition to selection, controlled crosses in China led to release of 16 cultivars, including Liaoning 1-8, Xiangling, Fenghui and Zhonglin 1-6. Most of the controlled crosses were between Chinese cultivars, especially Xinjiang walnuts (Baojun et al. 2010; Chen et al. 2014; Wu et al. 2010). The fourth phase of walnut breeding in China produced about 20 additional cultivars from controlled crosses. Chen et al. (2014) and Zhang et al. (2014) reported details about these cultivars.

Most Turkish walnut trees are early leafing and terminal bearing. Therefore, late leafing and lateral bearing are main breeding objectives. The walnut breeding program in Turkey is based on germplasm evaluation and selection of superior genotypes. In addition to Turkey being a center of origin, most walnut orchards in Turkey are seed propagated. Therefore, high genetic variation is retained in traditional orchards. Although many trees have been cut for timber, large seedling walnut populations remains.

The first walnut breeding program in Turkey began using identification of superior genotypes from 20 different sites in the Marmara region of northwest Turkey in 1971 (Ölez 1971). Subsequently, germplasm evaluation and selection of superior genotypes were continued by other researchers in different regions of Turkey (Akça and Polat 2007; Ertürk and Akça 2014). All Turkish walnut cultivars have been selected based on germplasm evaluation (Aslantaş 2006). Sebin, Bilecik, Acka 1, Maras 10, Bursa 95, Yalova 1 and Yalova 3 are some Turkish walnut cultivars released, based on selection from native walnut populations (Akça and Polat 2007). The leading cultivars are Yalova 1, Yalova 3, Yalova 4, Sebin and Bilecik. In addition

to germplasm evaluation, some controlled crosses between local and foreign cultivars were conducted in a new Turkish walnut breeding program since 2008. These controlled crosses produced about 1340 hybrids, which are under evaluation (Akça et al. 2016). Maras 18, Sutyemez 1 and Kaman 1 are new Turkish walnut cultivars released based on germplasm evaluation in 2009 and 2010. Also, Dirilis, 15 Temmuz, Maras 12 and Bayrak are Turkish walnut cultivars recently released (Ozcan et al. 2017). In addition to late leafing, lateral bearing and early harvest, the aim of the new Turkish walnut breeding program is to search for new promising rootstock candidates having tolerance to salt, lime, drought and disease stresses (Ertürk and Akça 2014).

In addition to these leading countries, walnut breeding is also conducted in other walnut growing countries including Spain, Germany (Bollersen 2017), Georgia, Italy, Hungary, Greece, Romania, Ukraine, Serbia and Azerbaijan. Walnut breeding in some of these countries dates back to the onset of the second phase of the world's walnut breeding period and most breeding objectives are the same in these countries. Some local cultivars were released based on genetic diversity evaluation and hybridization between local superior genotypes and French/Californian commercial cultivars. A brief summary of the released cultivars is contained in the book *Following Walnut Footprints* (Avanzato et al. 2014).

11.2.3.2 Rootstock

Rootstocks can play a crucial role in determining orchard efficiency. Combining the desirable attributes of two different plants by budding or grafting can produce different growth effects (Nimbolkar et al. 2016). Rootstocks are bred to grow in different soil types and conditions, and to provide the best anchorage, vigor, and resistance or tolerance to soil-borne pests and diseases and abiotic stresses. However, no individual rootstock is tolerant to all the factors impacting walnut production. The strengths and weaknesses of each rootstock needs to be considered in the context of a specific orchard location. Traditionally, the main walnut rootstocks were Persian walnut (*Juglas regia*) seedlings. Seedlings of Northern California black walnut (*J. hindsii*) were also traditionally used as rootstocks for Persian walnut scions in California.

The USA is a leading country in walnut rootstock breeding, having used Paradox cv., as walnut rootstocks for a long time. Paradox cv. rootstocks are hybrids of *Juglans hindsii* × *J. regia*. Other *Juglans* hybrids include Royal *(J. hindsii* × *J. nigra)* which is less vigorous than Paradox, perhaps due to their crop load, and not used as rootstock (Forde 1975). Because they display vigor and disease resistance superior to either parent, Paradox cv. hybrids are the most common rootstocks for *J. regia* in California. Currently, Paradox cv. rootstock accounts for 80% of walnut orchards (Baumgartner et al. 2013). Different types of Paradox have been introduced over the years (Tulecke and McGranahan 1994). Paradox is more vigorous, more resistant to some *Phytophthora* species, and more tolerant to soil salinity than *J. regia* (McGranahan and Catlin 1987). Despite its widespread use, the resistance of seedling Paradox root-

stock to *Armillaria* root disease, crown gall, and *Phytophthora* remains insufficient as evidenced by serious losses to the walnut industry from these root diseases (McGranahan and Leslie 1991). In addition, Paradox is susceptible to blackline disease caused by a hypersensitive response scions infected with cherry leaf roll virus. Therefore, walnut rootstock breeding in California was directed toward genetic tolerance to CLRV and resistance to soil-borne pathogens. In recent years, three clonal rootstock cvs. have become available (Vlach, RX1 and VX211). In addition, a few walnut growers use own-rooted English walnuts in areas where blackline disease (CLRV) is prevalent. Vlach cv. is vigorous but in trials has not demonstrated resistance to pathogens. RX1 cv., a *J. microcarpa* × *J. regia* hybrid with tolerance to *Phytophthora*, and VX211, a very vigorous *J. hindsii* × *J. regia* hybrid exhibiting nematode tolerance, have been released commercially (Leslie and McGranahan 2014).

Rootstock improvement work has also been proceeding in China. The Shanxi Academy of Forestry Sciences released the Jin RS-1 cv. rootstock series in 2011; Jin RS-2 and Jin RS-3, have been evaluated for cold, disease, and pest resistance, and seem to be ideal for northern Chinese areas subject to frost (Bernard et al. 2018; Wang et al. 2014). Walnut rootstock breeding is also proceeding, based on germplasm evaluation in other countries such as Turkey and Iran. Some of the main objectives in these countries are dwarfing, tolerance to salt and drought stress, and resistance to soil-borne disease such as *Agrobacterium*, *Phytophthora* and *Armillaria*.

Walnut rootstock breeding in Iran began at the University of Tehran (directed by Kourosh Vahdati) in 2003 with drought tolerance and dwarfing as the main objectives. Walnut germplasm from different regions of Iran were evaluated and superior genotypes were selected and planted in the Walnut Research Orchard at Aburaihan Campus, University of Tehran, Pakdasht, Tehran, Iran. Compatibility evaluation and molecular studies of these selected genotypes are ongoing.

11.3 Germplasm Biodiversity and Conservation

11.3.1 Genetic Resources and Biodiversity Conservation

Walnut, like other woody plants, typically has a long breeding cycle. Therefore, introduction of new cultivars may require many breeding cycles and dozens of years. Walnut breeders have been able to bypass long breeding cycles by using biotechnology alongside exploiting biodiversity. Recent advances in biotechnology and genomics have the potential to accelerate cultivar development greatly (van Nocker and Gardiner 2014), but exploitation of biodiversity also can be considered as a short cut to obtain commercial cultivars with desirable characteristics. Selection of superior genotypes provides a gene bank of desirable genes and enables biotechnologists to conduct breeding programs more confidently and rapidly.

The primary center of origin of walnut is the Persian plateau (including modern Iran and some central Asian countries). It is easy to find wild walnut populations in these countries. The existence of different types of walnut trees with a variety of

characteristics enables walnut breeders to implement any breeding program based on these populations. In addition, walnuts dispersed to other regions in the world, especially Europe and Eastern Asian countries using seed, so high genetic diversity exists in the secondary walnut diversity centers. Walnut genetic resources have played a key role in producing the current walnut cultivars. Many commercial walnut cultivars released in California originated from seedlings collected in Iran (cvs. Eureka and Olmo), Afghanistan (cv. Pl159568) and other countries of walnut origin such as China (Tulecke and McGranahan 1994).

Biodiversity conservation is a global concern. A large proportion of walnut genetic resources are threatened by destruction, from urbanization, the commercialization of traditional orchards and timber use. Currently, the only method of walnut germplasm conservation in center of origin countries is the identification of superior genotypes and their transfer to collections. Due to the existence of a huge genetic diversity in these countries (Iran is estimated to have more than 20 million walnut genotypes), this method is not efficient. On the other hand, classical methods of biodiversity conservation have certain limitations in terms of rapid production of plants and their long-term conservation. Therefore, it is necessary to conserve walnut biodiversity as international capital in the framework of an international project using new germplasm conservation techniques that make use of biotechnology. Biotechnological methods such as plant tissue culture, plant cell culture, anther culture, embryo culture etc. are quite applicable and useful techniques for ex situ conservation. It is axiomatic that modern biotechnology can help to counteract trends of genetic erosion in all agricultural sectors (Ogbu 2014; Pathak and Abido 2014).

11.3.2 Cultivar Characterization and Phylogeny

For a long time, walnut growers have used seeds of superior genotypes to establish new orchards, which has led to high genetic diversity in the walnut populations of the world. Following development of grafting techniques and walnut breeding programs in France and California, walnut cultivars were introduced. In addition to France and California, walnut cultivars were released in other countries based on exploitation of local genetic diversity and hybridization, although most are only locally cultivated. Several French/Californian cultivars are cultivated worldwide. The first of these was Franquette. Franquette, the main cultivar in French orchards, is late leafing with good nut characteristics but is terminal bearing and hence only moderate yield (Germain 1999; Tulecke and McGranahan 1994).

Nowadays cv. Chandler, a late-leafing and laterally-fruitful cultivar is the premier cultivar planted worldwide. Chandler is a cross between cvs. Pedro and UC 56-224 (Fig. 11.3) patented and released by the University of California, Davis in 1979. Chandler is highly lateral fruitful (therefore high yield) and quite vigorous with good nut characteristics (good shell thickness, strong seal, ease of kernel removal) and light kernel color. Table 11.3 presents important walnut cultivars in the leading walnut growing countries.

11.4 Molecular Breeding

Recent advancements in next-generation sequencing (NGS) techniques, bioinformatics tools, high-throughput genotyping platforms, and genomics-based approaches such as genome wide association studies (GWAS), marker-assisted selection (MAS), genomic selection (GS), and genome editing using CRISPR-Cas9 system have opened up new avenues to enhance the efficiency of fruit trees breeding, leading to release of new scions and rootstocks. Hindering walnut genetic improvement is the long juvenile phase and high degree of heterozygosity. In this section, we describe the potential of molecular breeding using novel genomic technologies in walnut genetic improvement to overcome conventional breeding barriers.

We first introduce molecular marker systems and whole nuclear and chloroplast genome sequence information that is available for walnut breeding. Next, we review QTL mapping, GWAS and GS studies conducted on walnut. We then review functional genomic studies including transcriptomics, proteomics and metabolomics done on walnut. Then we briefly review bioinformatics-assisted walnut breeding. Since fruit and nut tree crops, including walnut, have a long juvenile period, development of a new variety or rootstock may take 15–20 years via classical breeding and MAS and GS alone cannot accelerate the genetic improvement of walnut trees. Therefore, we note the future prospects of molecular breeding in walnut using novel technologies for rapid generation advancement.

11.4.1 Molecular Markers in Fruit and Nut Breeding

Many studies have described genetic variability in fruit crops. Additional and more direct indicators of genetic diversity are needed despite the considerable quantity of morphological descriptors, the large amount of walnut germplasm resources, and the prolific development of new scion and rootstock. Nowadays, genetic markers systems are widely used in germplasm characterization in order to assist phenotypic evaluation and accelerate breeding. Molecular markers have been widely used to analyze genetic relationships between wild and cultivated individuals and related species, interspecific hybrids identification, germplasm variability evaluation and for cultivar identification.

11.4.2 Molecular Marker Systems

Isozymes markers were once among the most widely used molecular markers in genetic studies. Isozymes have been used in different aspects of walnut genetic analysis including evaluation, interspecific hybrid identification, genetic diversity or relationship analysis and determination of genotype origin (Aletà et al. 1990;

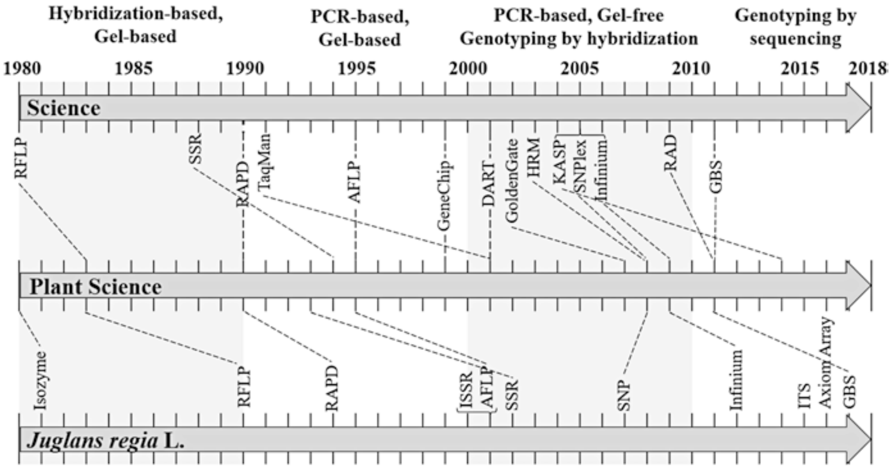

Fig. 11.5 Timeline for the first scientific documentation for the application of different commonly used molecular markers in science, plant science and Persian walnut genotypes. (Adopted from Obermeier and Friedt 2015)

Arulsekar et al. 1986; Busov et al. 2002; Cheng and Yang 1987; Fornari et al. 2001; Malvolti et al. 1993, 1994; McGranahan et al. 1986; Ninot and Aletà 2003; Solar et al. 1994; Vyas et al. 2003).

Isozymes were superseded by an emerging variety of more informative and robust DNA markers with higher rates of polymorphism, including restriction fragment length polymorphisms (RFLPs), random amplified polymorphic DNAs (RAPDs), amplified fragment length polymorphisms (AFLPs) and simple sequence repeats (SSRs). Some of these are still viable, cheap and quick methods to identify genetic variability, and RFLPs, RAPDs, AFLPs and SSRs consecutively contributed to the identification of walnut germplasm, evaluation of its genetic diversity, and assessment of uniformity and stability of cultivars (Fig. 11.5).

11.4.3 Overview of the Diversity and History of Fingerprinting Techniques in Walnut

11.4.3.1 Hybridization-Based Markers

Restriction Fragment Length Polymorphism (RFLP) The first DNA marker technology used in plants was RFLPs (Tanksley et al. 1989). Aly et al. (1992), for the first time, used a combination of isozyme and RFLP markers in *Juglans regia*, to determine the origin of somatic embryos derived from ovule tissues. RFLPs have been applied for different purposes in walnut, including genetic mapping (Fjellstrom and Parfitt 1994a, b) and genetic diversity assessment (Fjellstrom et al. 1994). Limitations of the RFLP technique include a requirement for high molecular weight

DNA, low levels of polymorphism and complexity of radioactivity or other staining techniques, which motivated the development of several alternative technologies (Bernard et al. 2018).

11.4.3.2 PCR-Based Markers for Walnut Genome Profiling

Random Amplified Polymorphic DNA (RAPD) RAPD markers are based on polymerase chain reaction (PCR) amplification of random genomic locations, and have been widely used for genetic diversity and mapping studies because they are easy to develop and are cost- and time-effective (Welsh and McClelland 1990; Williams et al. 1990). The first applications in walnut (Fjellstrom and Parfitt 1994a) identified RAPDs in a backcross population of [*Juglans hindsii* × *J. regia*] × *J. regia* and in the next step both RAPDs and RFLPs were used to construct a revised genetic map of walnut. In other studies, RAPD markers have been used for association mapping (Woeste et al. 1996a; Yang et al. 2002), assessment of genetic and morphological diversity (Erturk and Dalkilic 2011; Fatahi et al. 2010; Francesca et al. 2010; Nicese et al. 1998; Qianwen et al. 2010; Woeste et al. 1998), genetic mapping (Malvolti et al. 2001) and identification of interspecific hybrids (Emilia et al. 1995; Ross-Davis et al. 2008).

Sequence Characterized Amplified Region (SCAR) RAPDs markers are easy to develop and simple, but a lack of reproducibility makes them less reliable for genetic analysis in plant breeding. Therefore, there is an option to improve the reproducibility of RAPD markers by converting RAPD amplicons into sequence characterized amplified region (SCAR) markers. Recently, several studies have identified molecular markers linked to economically-important traits in walnut, including a RAPD marker correlated with precocity (Keqiang et al. 2002), a SCAR marker correlated with shell thickness (Li et al. 2007) and a SCAR marker correlated with precocity (Li et al. 2010).

Inter-Simple Sequence Repeat (ISSR) ISSRs are amplification reactions with a single long oligonucleotide primer anchored at both ends by a simple sequence repeat (Godwin et al. 1997). ISSR markers have been applied in several genetic studies in walnut, including genetic diversity studies (Christopoulos et al. 2010; Ji et al. 2014; Pollegioni et al. 2003; Potter et al. 2002), genetic mapping studies (Malvolti et al. 2001) and studies to determine the origin of a genotype (Malvolti et al. 2010). In total, RAPD and ISSR markers have been widely used for local *Juglans regia* germplasm characterization by Iranian, Romanian, Chinese, Greek and Turkish walnut researchers (Christopoulos et al. 2010; Erturk and Dalkilic 2011; Fatahi et al. 2010; Ji et al. 2014; Pop et al. 2010; Qianwen et al. 2010).

Amplified Fragment Length Polymorphism (AFLP) Another high-performing molecular marker based on the selective amplification of a subset of genomic restriction fragments is AFLP (Vos et al. 1995). Like RAPDs, informative frag-

ments from AFLP are generally sequenced to generate SCAR markers (Lecouls et al. 2004). Sütyemez (2006) first stressed the high potential of AFLPs for the study of genetic diversity in walnut. AFLP markers have been widely employed in walnut genetic studies for characterization of superior genotypes (Kafkas et al. 2005), assessment of genetic diversity (Ali et al. 2016; Bayazit et al. 2007; Qing Guo et al. 2010), evolutionary studies (Chen et al. 2008, 2009; Wang et al. 2010) and for fingerprinting walnut cultivars (He et al. 2010; Ma et al. 2011; Xu et al. 2012).

Simple Sequence Repeat (SSR) or Microsatellite Technique Since the late 1990s to early 2000s, microsatellites or simple sequence repeats (SSRs) have been a revolutionary tool for efficient genetic investigations, including genetic variability, population structure, genetic mapping and marker-assisted selection (MAS). SSRs have been the most widely used markers for marker-assisted breeding of fruit and nut tree crops over the past 10 years because they are highly informative, reproducible and codominant (Bernard et al. 2018; Singh et al. 2008; Wani et al. 2010).

The excellent properties of simple sequence repeats (SSRs) have made them the markers of choice for many uses in walnut genetic studies and breeding. In walnut breeding, SSRs are primarily used to evaluate genetic diversity of collected material before choosing the parental material that will help to maximize this diversity for subsequent optimal combinations. SSRs markers have been applied in numerous walnut genetic studies with different purposes as follows: fingerprint clones of *Juglans nigra* accessions with high-quality timber (Woeste et al. 2002), assessment of genetic diversity (Gunn et al. 2010; Victory et al. 2006; Wang et al. 2008 Pop et al. 2013), hybrid identification (Pollegioni et al. 2008) and identification of parentage among progenies (Pollegioni et al. 2009; Robichaud et al. 2006).

SSRs were originally developed from both coding and non-coding regions of plant genomes; in 2002–2018, hundreds of SSRs were developed in various walnut species. They were developed from several sources including a variety of DNA libraries (genomic, genomic-enriched for SSR, bacterial artificial chromosome and cDNA libraries), as well as public databases, including expressed sequence tags (ESTs) from NCBI. The advances made in next-generation sequencing (NGS) technology to sequence the genome or transcriptome especially RNA-seq have also provided a new scenario for detecting SSRs markers.

The development of SSR markers in walnuts was accomplished using enriched SSR libraries from genomic DNA in *Juglans nigra*, *J. cinerea* and *J. regia* (Dangl et al. 2005; Hoban et al. 2008; Topçu et al. 2015; Woeste et al. 2002), from expressed sequence tag (EST) collections from the NCBI database in *J. regia* and *J. hindsii* × *J. regia* (Qi et al. 2011; Yi et al. 2011; Zhang et al. 2010, 2013), from BAC-end sequences from the NCBI database in *J. regia* (Ikhsan et al. 2016; Wu et al. 2012b), from fast isolation by AFLP of sequences containing repeats (FIASCO) in *J. mandshurica* and *J. regia* (Chen et al. 2013; Najafi et al. 2014) and from next-generation sequencing of transcriptomes in *J. cathayensis*, *J. hopeiensis* and *J. regia* (Dang et al. 2015, 2016; Hu et al. 2015).

SSRs have been broadly used for landrace/cultivar identification and studies of genetic diversity in cultivated germplasm in walnut, as well as for studying evolution and relationships between cultivated and wild relatives (Aradhya et al. 2009; Bai et al. 2010; Dang et al. 2015; Dangl et al. 2005; Ebrahimi et al. 2011, 2017; Foroni et al. 2005, 2007; Gunn et al. 2010; Han et al. 2016; Hu et al. 2015; Karimi et al. 2010; Mahmoodi et al. 2013; Mohsenipoor et al. 2010; Najafi et al. 2014; Noor Shah et al. 2016; Pollegioni et al. 2011, 2017; Pop et al. 2013; Qi et al. 2011; Robichaud et al. 2006; Roor et al. 2017; Ruiz-Garcia et al. 2011; Victory et al. 2006; Vischi et al. 2017; Wang et al. 2008, 2015; Zhang et al. 2013).

11.4.4 High-Throughput SNP Assays in Walnut

Recent advances in next-generation sequencing technologies and the continuous decrease in cost have resulted in the enormous generation of sequence data sets, allowing the rapid discovery of single nucleotide polymorphism (SNP) markers. SNP markers are distributed throughout the genome and are broadly used in animal and human genetic analysis, but their application in plants, and particularly in walnut, is in the early stages. So far, the recent availability of genomic and transcriptomic databases has made possible the discovery of SNPs in silico, using bioinformatics tools.

A walnut genome sequence (cv. Chandler v1.0) has recently been released (Martínez-García et al. 2016; available at https://www.hardwoodgenomics.org/english-walnut-genome), 27 genomes of most important founders in the walnut improvement program at the UC Davis have been resequenced and, finally, a novel Axiom® Walnut700K SNP array has been designed by UC Davis for walnut variability, linkage mapping and association mapping analysis (Marrano et al. 2019; Neale et al. 2017). The availability of a high-density genotyping array in walnut opens new opportunities to apply GWAS and genomic selection in walnut-breeding programs and other walnut populations (Bernard et al. 2018; Marrano et al. 2019; Neale et al. 2017).

11.4.5 Nuclear Ribosomal Internal Transcribed Spacer (ITS)

The nuclear ribosomal internal transcribed spacer (ITS) region is an effective genetic marker for molecular identification of plants, because of its relatively high variability and facility of amplification. Sequencing of PCR amplicons from this region have been used for SNP discovery and characterization of English walnut (*Juglans regia*) cultivars. In one study, the first and second internal transcribed spacers (ITS1 and ITS2), as well as the intervening 5.8S coding region of the rRNA gene, were amplified and sequenced, and alignment of the ITS1-5.8S-ITS2 sequences from 18 walnut cultivars showed 244 SNPs and 1 short insertion-deletion

(indel) (Ciarmiello et al. 2011). Phylogenetic analysis of the ITS1-5.8 S-ITS2 region clustered the sequences into two groups that indicated these regions could be used to differentiate these walnut cultivars (Ciarmiello et al. 2011). In another study, a total of 32.6X walnut genome (cv. Chandler) equivalents of ABI SOLiD reads were mapped to 48,661 Chandler cv. bacterial artificial chromosome (BAC) end sequences (BESs) created by Sanger sequencing and 22,799 SNPs were discovered. Finally, 6000 SNPs were selected to construct an Infinium BeadChip that was used to genotype a walnut mapping population (You et al. 2012).

Recently, a new nuclear DNA marker from the sequence of the ubiquitin ligase gene (*UBE3*) region of nuclear DNA was developed for genetic diversity assessment of walnut genetic resources. The results showed that all walnut taxa (species/variety/cultivars) were distinguished using the ubiquitin ligase gene (*UBE3*) sequence (Suo et al. 2015). In another study sequences of 2 maternally-inherited mitochondrial DNA (mtDNA) markers (3–9 and nad5) 2 maternally inherited chloroplast DNA (cpDNA) intergenic spacers (trnL-F and trnS-G), 3 nuclear DNA sequences (15R-8, ITS and Jr5680) and 11 microsatellites (EST-SSRs) were obtained from 108 individuals of *Juglans hopeiensis*, *J. regia* and *J. mandshurica*. It was previously suggested that *J. hopeiensis* was simply a hybrid of *J. regia* and *J. mandshurica*, but results from this study showed that *J. hopeiensis* haplotypes are different from haplotypes found in both *J. regia* and *J. mandshurica* (Hu et al. 2017a).

11.4.6 Emerging Marker Technology: Genotyping by Sequencing (GBS)

Next-generation sequencing (NGS) has facilitated discovery of whole genome single nucleotide polymorphisms (SNPs) and development of high-throughput genotyping technology. New methods such as restriction site-associated DNA sequencing (RAD-seq) and genotyping-by-sequencing (GBS) have been established as powerful tools for reduced-representation sequencing of multiplexed samples that integrate genome-wide molecular-marker discovery and genotyping (Scheben et al. 2017).

In plant breeding programs, GBS was for simultaneous SNP discovery and genotyping in plants with and without reference genome sequences. The flexibility and low cost of the GBS method makes it a powerful tool for plant breeding (He et al. 2014; Scheben et al. 2017), that could be applied to various approaches for walnut genetic improvement, including genomic diversity studies, linkage maps, genome-wide association studies (GWAS), marker-assisted selection (MAS) and genomic selection (GS).

Whole chloroplast genomes, transcriptomes and genotyping-by-sequencing (GBS) are used in China to determine population genetics, phylogenomics, and hybrid speciation of *Juglans*. Reconstruction of the evolutionary history of *Juglans* through genomic and transcriptomic analysis revealed that climatic variation over the past years, associated with glacial advances and population isolation, have shaped Chinese walnut demography and evolution (Zhao et al. 2018).

11.4.7 Physical Mapping

A physical map indicates the physical distance (number of base pairs) between loci. The cloning of exogenous DNA into bacterial artificial chromosomes (BACs) is a new method for genome analysis (Choi and Wing 2000). To construct the physical map of the walnut genome, two bacterial artificial chromosome (BAC) libraries were built from genomic DNA isolated from in-vitro micropropagated shoots of Persian walnut (*Juglans regia* cv. Chandler) and fragmented with either HindIII or MboI restriction endonucleases. The average insert size for the HindIII and MboI libraries were around 135 kb and 120 kb, respectively. A total of 129,024 clones, 64,512 per BAC library, were arranged in 336 plates (Wu et al. 2012b). These BAC libraries represent around 27× genome equivalents assuming the walnut genome size is approximately 606 Mb. In this study BAC fingerprinting and BAC-end sequencing were performed using a fluorescence-based, high-throughput BAC DNA fingerprinting method (Luo et al. 2003). In total, 52,840 BAC clones from HindIII and MboI libraries of Persian walnut (*J. regia* cv. Chandler) were sequenced and the resulting 48,218 walnut BESs were deposited at GenBank (Wu et al. 2012b). The average GC content of the BES was 37.7%, which is slightly higher than papaya, poplar and *Arabidopsis* genomes with GC contents of 33–35%, but lower than that of the rice genome at 43%. Based on Blast2GO analysis, 1330 unique GO terms were assigned to 6396 BESs. Distribution of GO terms in the categories of biological process, molecular function and cellular component showed that the walnut genes cover a broad range of functional categories and biological processes. Through aligning of BES with ESTs and whole walnut genome shotgun sequences. Approximately 4000 SNPs were discovered and genetically mapped in a population of cvs. Chandler × Idaho, which ultimately led to anchoring of BAC contigs onto a linkage map.

The physical map of walnut enabled the discovery of two markers flanking the LB1 locus associated with lateral bearing. Recently, a 2-year project at the University of California, Davis was initiated to discover the causative mutation at the LB1 locus, develop a predictive SNP marker, and implement the marker in walnut genetic improvement. During the first phase of this project, over 700 progenies from a selfed cv. Chandler population were identified that carry recombination events in the LB1 region. These progenies are being phenotyped for bearing habit and genotyped with additional SNP markers in the LB1 region. Preliminary results from this study suggested three LB1 candidate genes (Dvorak et al. 2015).

11.4.8 Genetic Mapping and QTL Detection

Genetic mapping or linkage mapping in plants determines the relative positions of genetic markers and genes along chromosomes based on recombination frequencies. The procedure of constructing a genetic map is as follows: (1) grouping of

markers into linkage groups, (2) ordering of markers within these groups and (3) estimating genetic distances between the markers (Cheema and Dicks 2009; Paterson 1996). Distances on genetic maps are usually expressed as centimorgans (cM), and are related to recombination frequencies with possible correction for unobserved double recombinants and interference between crossovers. Applications of genetic maps in plant genetic improvement include: (1) identification of genomic regions or quantitative trait loci (QTLs) linked to agronomic traits of interest; (2) map-based cloning of major genes involved in important agronomic traits and the development of markers for MAS; (3) understanding chromosome evolution and phylogenetic relationships within and between species and (4) assisting with genome assembly (Luo et al. 2015; Semagn et al. 2006; You et al. 2012; Zhu et al. 2015).

Several genetic and QTL mapping studies have been conducted in walnut. Forty-two RFLP markers were applied for the first time by Fjellstrom and Parfitt (1994a) to construct a genetic linkage map from a progeny of 63 individuals from an inter-specific backcross of [*Juglans hindsii* × *J. regia*] × *J. regia*. A few years later, 66 RAPD markers were used to investigate the same set of progeny and a new genetic map was constructed including the previously found RFLPs, along with the new RAPD markers (Woeste et al. 1996b). In another study, 120 RAPD and 4 isozyme markers were used for genetic map construction using 82 progenies from an intra-specific cross of walnut *J. regia* cv. Lara 480 and *J. regia* cv. Chandler 1036 (Malvolti et al. 2001).

11.4.9 Next-Generation Mapping

Integration of genetic linkage mapping and comparative genomics has been proposed as a powerful tool for map-based cloning and molecular marker development from functional genes involved in the trait(s) of interest. Establishment of segregating mapping populations by crossing two parents with phenotypic difference(s) in at least one trait of interest is required for QTL mapping. A 6 K Infinium SNP iSelect assay was used to genotype 425 F_1 progeny from a cross of cv. Chandler with cv. Idaho, and 1525 SNPs markers were mapped into 16 linkage groups (LGs) corresponding to the 16 walnut chromosomes. The LG lengths ranged from 37.7 cM (LG15) to 97.3 cM (LG7), and the total length of the genetic map was 1049.5 cM (Luo et al. 2015; You et al. 2012). This genetic map was used to construct a walnut bacterial artificial chromosome (BAC) clone-based physical map with 15,203 exonic BAC-end sequences (Luo et al. 2015). In another study, specific length amplified fragment sequencing (SLAF-seq) technique was used to generate large numbers of molecular markers to construct high-density genetic maps for walnut molecular breeding. In this research a F_1 population of 84 individuals was created from an intraspecific cross between cv. Yuan Lin (maternal line susceptible to anthracnose) and cv. Qing Lin (paternal line with resistance to anthracnose) and 2577 SLAF markers were used to construct genetic linkage maps. A total of 2395 of

these markers were assigned into 16 linkage groups (LGs) for the female map; likewise, 448 markers were used for the male map (Zhu et al. 2015). Finally, a QTL linked to walnut anthracnose resistance was identified on LG14. The 95% confidence interval for the QTL ranged from 165.51 to 176.33 cM on LG14, and 10 markers in this interval were considered to be linked markers to the anthracnose resistance trait. The phenotypic variance explained by each marker was 16.2–19.9% with LOD scores of 3.22–4.04. These results will assist molecular marker-assisted breeding and walnut-anthracnose-resistance gene identification (Zhu et al. 2015).

11.4.10 Comparative Mapping

Comparative mapping and comparative sequence analysis are valuable methods to identify similarities and differences between genomes, assist in the reconstruction of ancestral genomes, and consolidate genetic maps and to identify candidate genes underlying QTL (Luo et al. 2015).

A genetic map for walnut was constructed initially with walnut 6 K Infinium SNP assay, then used to construct a walnut bacterial artificial chromosome (BAC) clone-based physical map, and synteny was quantified with other plant genomes. These included three long-lived woody perennials, *Vitis vinifera*, *Populus trichocarpa* and *Malus domestica*, and three short-lived herbs, *Cucumis sativus*, *Medicago truncatula* and *Fragaria vesca*. The results of synteny analysis revealed that long-lived woody perennials were less diverged from the walnut genome than short-lived herbaceous annuals (Luo et al. 2015).

11.4.11 Walnut Genome Sequencing

11.4.11.1 Chloroplast Genome Sequencing

The advent of high-throughput sequencing technologies has facilitated a rapid improvement in the field of chloroplast genetics and genomics. Currently, more than 800 complete chloroplast genomes, including 300 from crop and tree genomes, have been sequenced, and the genomic information is available in the National Center for Biotechnology Information (NCBI) organelle genome database (Daniell et al. 2016). The genetic information gained from complete chloroplast genome sequences has improved our understanding of plant biology, diversity, phylogeny and evolution. In addition, considerable variation within and between plant species, in terms of both sequence and structural variation, have been revealed by chloroplast genome sequences (Daniell et al. 2016; Hu et al. 2016, 2017b). The information obtained from chloroplast genome sequences has been especially valuable for understanding plant adaptability to severe environmental conditions, assisting breeding of closely related species (Daniell et al. 2016).

The first complete chloroplast genome of walnut was sequenced using the Illumina MiSeq platform and was assembled and annotated using SPAdes and CpGAVAS software, respectively (Liu et al. 2012). The length of the chloroplast genome of walnut was 160,367 bp (GenBank accession number KT963008) with 36.11% GC content. A total of 137 functional genes included: (1) 86 protein-coding genes; (2) 3 pseudo genes (2 ycf15 and 1 infA); (3) 40 transfer RNA genes and 8 ribosomal RNA genes. Also the results of this study showed that there were 12 protein-coding genes, 14 transfer RNA and all 8 ribosomal RNA genes duplicated in the inverted repeat (IR) regions (Hu et al. 2016). Phylogenetic analysis of the walnut chloroplast genome with 11 chloroplast genomes from other species revealed that walnut was most closely related to the Fagaceae family and the genus *Populus* (Hu et al. 2016). Recently, the chloroplast genomes of 5 *Juglans,* including *J. regia* (common walnut), *J. sigillata* (iron walnut), *J. cathayensis* (Chinese walnut), *J. hopeiensis* (ma walnut), and *J. mandshurica* (Manchurian walnut), were sequenced to evaluate the structural patterns of their whole chloroplast genomes, to discover potential simple sequence repeats (SSRs) and divergence hotspots, and to determine their phylogenetic relationships (Hu et al. 2017b).

A combination of de novo and reference-based assembly strategies were used to reconstruct each species' chloroplast genome. Then, genome annotation and analysis were done using the online program Dual Organellar Genome Annotator (DOGMA, Wyman et al. 2004), and genomic sequences were analyzed using MISA software (http://pgrc.ipk-gatersleben.de/misa/) to identify potential simple sequence repeats (SSRs) (Hu et al. 2017b). Phylogenetic analysis powerfully supported division of the 5 walnut species into 2 previously documented sections including *Juglans*/*Dioscaryon* and *Cardiocaryon* (Hu et al. 2017a, b). In the other study, the complete chloroplast genomes and 2 nuclear DNA regions (the internal transcribed spacer and ubiquitin ligase gene) of 10 representative taxa of *Juglans* were used for phylogenetic analysis of the *Juglans* genus (Dong et al. 2017). The result of this study revealed that all 10 chloroplast genomes possessed 112 unique genes, including 78 protein coding, 30 transfer RNA and 4 ribosomal RNA genes. Also, based on 2 nuclear DNA regions, *Juglans* could be classified into 3 branches; *Juglans*, *Cardiocaryon* and *Rhysocaryon*.

11.4.11.2 Nuclear Genome Sequencing

The first complete high-quality draft genome of *Juglans regia,* from cv. Chandler, was obtained using the Illumina sequencing platform, resulting in 500 million reads and 120x genome coverage (Martínez-García et al. 2016). The nuclear genome of *J. regia* was 667 Mbp in length, with an N50 scaffold size of 464,955 bp (based on a genome size of 606 Mbp estimated by flow cytometry), 221,640 contigs and 37% GC content (Martínez-García et al. 2016). The genome assembly was performed using two different methods: SOAPdenovo2 (Luo et al. 2012) and MaSuRCA (Zimin et al. 2013). Extra scaffolding was done using RNA-seq transcripts from 19 different tissues that were assembled separately. The assembled genome was anno-

tated with MAKER-P and other genomic resources including expressed sequence tags (ESTs) and protein sequences from related species and the assembled *J. regia* transcriptome, which ultimately yielded 32,498 gene models. More than 1.2 million SNPs were discovered in the draft consensus sequence genome of *J. regia* (Martínez-García et al. 2016).

The availability of the first release of the walnut genome sequence (cv. Chandler v1.0) has helped foster genomic research in walnut breeding programs. However, short-read sequencing technologies were used for the first walnut genome sequence (cv. Chandler v1.0). To overcome the limitations of these technologies, the latest sequencing and optical mapping approaches have been used to improve the quality of the first genome assembly. In the first step, a second genome sequence of cv. Chandler was obtained using the Oxford Nanopore MinION sequencing platform, resulting in over 7 million reads and 35X genome coverage. In the next steps, chromosome-scale assembly and gene annotation will be done by optical mapping technology and Isoform Sequencing (Iso-Seq) to release walnut genome (cv. Chandler v2.0). The improved high quality walnut genome assembly (cv. Chandler v2.0) will provide a valuable genomic tool for genetic and genome-wide studies in walnut genetic improvement programs (Neale et al. 2017). Genome assemblies of tree crops are typically mosaics of the two distinct haplotypes found in a heterozygous diploid individual. To circumvent this problem, Zhu et al. (2019) generated a genome assembly from an interspecific hybrid of *Juglans microcarpa* and *J. regia* cv Serr, using BioNano and PacBio technologies. Due to the high divergence between *J. microcarpa* and *J. regia*, sequence reads for each species assembled separately, generating high quality genome assemblies from a perfectly phased gamete/haplotype from each parental species. This strategy can be efficiently applied to any heterozygous tree crop for which interspecific hybrids are already available or can be generated.

11.4.11.3 Walnut Genome Resequencing

To identify allelic variants at each genetic locus, the genetic variation in related *Juglans* species and in *J. regia* was discovered through sequencing and resequencing methodologies. A genome resequencing project was conducted at UC Davis to discover whole genome sequence variation in 27 accessions representing most important selections of their walnut improvement program. This genomic information will become an important resource for future genetic and population studies to identify alleles associated with phenotypes across walnut germplasm (Marrano et al. 2019).

SNP Discovery and SNP Genotyping Array Design After genome resequencing of the selected walnut accessions, BWA-MEN was used to align reads to v1.0 reference genome, and SNP calling, and yielded 17,800,528 SNPs, of which 609,658 SNPs covering 622 MB on 8079 scaffolds, were used for final array design (Marrano et al. 2019).

SNP Genotyping The novel Axiom® Walnut700K SNP array has been applied to genotype a total of 1284 trees in the walnut-breeding program at UC Davis and also a population of 95 walnut trees collected from different parts of Iran (Arab et al. 2019; Marrano et al. 2019). The results showed that a large majority of SNPs for both of California and Iran walnut trees (55.7 and 53%, respectively) fell in the class of PolyHigh Resolution (PHR) polymorphisms. Therefore, the Axiom® Walnut700K SNP array is a valid genomic tool for walnut genetic research including diversity and GWA studies worldwide (Marrano et al. 2019; Neale et al. 2017).

11.4.11.4 Phylogeographic Study in Walnut Using Whole Genome Sequencing and Resequencing

Demographic responses to climate change and diversification of the walnut genus *Juglans* were revealed by applying the pairwise sequentially Markovian coalescent approach to whole-genome sequences of 11 temperate *Juglans* species. Genome sequencing of 3 walnut species was done using the Illumina HiSeq 2500 sequencing platform to a depth of 90× for *J. mandshurica*, 57× for *J. regia* and 53× for *J. nigra,* and ALLPATHSLG (v.474117) was used for assembly. Then the genomes of 31 individuals from populations of 11 *Juglans* species (*Rhysocaryon*: *J. californica*, *J. hindsii*, *J. microcarpa*, *J. major*, *J. nigra*; *Cardiocaryon*: *J. cinerea*, *J. mandshurica*, *J. ailantifolia*, *J. cathayensis*; *Dioscaryon*: *J. regia,* J. *sigillata*) were resequenced to an average depth of 30–40X and >80% coverage using Illumina HiSeq 4000 paired-end sequencing libraries with insert sizes of 350 bp. Results indicated that the population histories of walnut species were not driven by extrinsic environmental changes alone, and possibly interactions with specialized pathogens have played a key role (Bai et al. 2018).

11.4.11.5 Walnut Rootstock Genome Sequencing

Recently the genomes of related *Juglans* species including*; J. sigillata*, *J. nigra*, *J. microcarpa*, *J. hindsii*, *J. cathayensis* and *Pterocarya* sp. were sequenced, assembled and annotated (Stevens et al. 2018). The main purpose of this genome-sequencing project was to resequence representative samples of *J. hindsii* and *J. microcarpa* genomes. In total, 34 *J. hindsii*, 13 *J. microcarpa* and 3 hybrid individuals were sequenced. Analysis of patterns of genomic variation in this study showed significant geographic structure (Stevens et al. 2018).

11.4.12 Genomics-Assisted Breeding in Walnut

Recent advancements in next-generation sequencing (NGS) techniques, bioinformatics tools, high-throughput genotyping platforms, and genomics-based approaches such as genome wide association studies (GWAS), marker-assisted

selection (MAS), genomic selection (GS), and genome editing using CRISPR-Cas9 system have opened up new avenues to enhance the efficiency of fruit and nut tree breeding to release new scion and rootstock.

The advent of next-generation sequencing technologies has accelerated the discovery of single nucleotide polymorphism (SNP) markers, facilitated genome-wide association studies (GWAS), and enabled marker-assisted selection (MAS), and genomic selection (GS) in fruit and nut crop breeding (Iwata et al. 2016; Laurens et al. 2018; Ru et al. 2015; van Nocker and Gardiner 2014).

Genome-wide association studies (GWAS) have become a vital methodology for detection of candidate genomic regions associated with simple and complex traits. Genomic selection goes a step further, selecting genotypes predicted to be superior based on their genomic estimated breeding values (Iwata et al. 2016; Laurens et al. 2018; Ru et al. 2015; van Nocker and Gardiner 2014).

Hampering conventional genetic improvement of fruit and nut tree crops is their long juvenile phase and high degree of heterozygosis. In addition, tree crops are affected by numerous biotic and abiotic stresses that complicate genetic improvement (Rikkerink et al. 2007). Selecting promising progeny during the juvenile period through genomics-based approaches, for example GWAS and GS, will speed the genetic improvement of trees through acceleration of the breeding cycle and increased selection intensity. Thus, genomic-based approaches have great potential for promoting the efficiency of fruit and nut tree genetic improvement (Ru et al. 2015).

11.4.12.1 Genetic and Association Mapping of Economically-Important Traits in Walnut

The University of California, Davis launched a walnut genome analysis in 2007 to develop new genomic tools in order to accelerate walnut breeding. The main objectives of the project included physical and functional mapping of the walnut genome and association mapping of horticulturally important traits in walnut. As a first step, genotyping of mapping populations from the cross (cvs. Chandler × Idaho) were done using 15 microsatellite markers. The result showed that 7 out of 265 F_1 individuals were half-sib origin. For association mapping, phenotypic data included lateral vs. terminal bearing, leafing and harvest dates, nut size, shell thickness, seal strength, kernel plumpness, percent kernel (kernel/nut ratio), kernel color and yield (Dvorak et al. 2008).

Evaluation of genetic structure and differentiation within the Persian walnut germplasm collection of 399 trees, from 204 diverse accessions at the USDA germplasm repository, and 62 elite germplasm frequently used in walnut breeding program at the UC Davis, was done using 14 polymorphic microsatellite loci. This analysis provides valuable information on the genetic diversity, which is key to the association genetic analysis, and helps with identification of diverse genotypes in this population for a resequencing panel to identify SNPs in the walnut genome (Dvorak et al. 2008). The results of this study showed that observed heterozygosity was consistently lower than the expected for all loci, which a range of 0.33–0.64,

with an average of 0.52, while the expected levels ranged from 0.41 to 0.85, for with an average of 0.69. Also, the analysis of molecular variance (AMOVA) revealed ~87% of the variation could be attributed to within populations, with only 13% accounting for variation among groups suggesting significant genetic differentiation within cultivated walnut (Dvorak et al. 2008).

Single nucleotide polymorphisms (SNPs) were discovered by comparing different sources of sequence information including; BAC-end sequences, SOLiD shotgun genome sequences and RNAseq data obtained from various walnut tissues. This comparison discovered ~6000 SNPs that were then processed by Illumina to generate an Infinium array. These SNPs were used to create the genetic map by analyzing 352 progenies from a cv. Chandler × cv. Idaho cross and this map has been used to align any phenotypic trait to linkage groups (Dvorak et al. 2011). Lateral bearing is one of the key determinants of yield and the most important breeding goal in walnut genetic improvement program. Therefore, the molecular markers discovered in a previous project by Dvorak et al. (2011) were used in developing a high-throughput genotyping platform (KASPTM genotyping) for lateral bearing (Martínez-García et al. 2014). In this project, an allele specific PCR (KASP) genotyping service was used because of its ability to genotype thousands of individuals using a small number of markers at low cost and high accuracy.

11.4.12.2 QTL Mapping, GWAS and GS in Walnut

Most economically- and horticulturally-important traits in tree crops, such as fruit and nut quality, are quantitative and controlled by multiple genes or QTLs. Due to the long juvenile phase of most tree species, generating segregating populations derived from biparental crosses is difficult and costly (Rikkerink et al. 2007). Therefore, genome wide association studies (GWAS) are more realistic for QTL detection in fruit trees compared to traditional QTL mapping in biparental populations.

High-throughput genotyping technologies are indispensable for genomics-assisted breeding, and various marker systems exist for molecular breeding in walnut. The availability of walnut genome sequences enables breeders to develop genome-wide markers for high-throughput genotyping and to construct high-density genetic maps (Bernard et al. 2018; Marrano et al. 2019).

Single nucleotide polymorphism (SNP) markers are cost-effective in terms of cost per marker and allow for higher-throughput genotyping and higher-density mapping, compared to SSR markers. To date, the walnut Axiom 700 k SNP array has been used to create linkage maps and to assist GWAS and GS (Marrano et al. 2019; Neale et al. 2017). So far, a few genome-wide association studies have been conducted on walnut. For example, assessment of water use efficiency (WUE) has been done on 260 individual clones of 64 cultivars located within the walnut improvement program in UC Davis. This panel has been genotyped with the Axiom® Walnut700K SNP array and then a two-step association genetics approach identified four loci associated with $\Delta^{13}C$, which were related to abiotic stress response

(Famula et al. 2019). The walnut breeding program population at UC Davis, genotyped with the novel Axiom® Walnut700K SNP array, was phenotyped for economically-important traits such as yield, harvest date, seal strength and kernel color, over the years, and this genotype-phenotype information is used for association analysis. Also, the seedlings of 95 trees from Iran, genotyped with the novel Axiom® Walnut700K SNP array, have been extensively phenotyped over 2 years for morpho-physiological and biochemical drought tolerance-related traits and will be used to carry out marker-trait association analysis for drought tolerance (Arab et al. 2019; Neale et al. 2017). Selecting the promising progeny during the juvenile period through GS will accelerate walnut genetic improvement via reduced breeding cycles and improved selection intensity. To the best of our knowledge, there has not been any scientific report on genomic selection research in walnut. Walnut breeding began at the UC Davis using the genomics-based approach. The genetic value of each individual in a breeding population is defined by breeding values (BVs) and can be used for choosing the best candidates to produce the next generation of offspring. Estimation of BVs for the four most important traits of interest: yield, harvest date, lateral bearing and leafing date have been done using 15 different families and phenotypic data collected for almost 16 years from walnut improvement program (WIP) at UC Davis. Linear mixed model (asreml-R or lmer) and Bayesian approaches (Rjags, MCMCglmm) have been used to estimate heritability and variance components for important traits such as lateral bearing, yield, harvest date and kernel color. Repeatability estimates for yield, harvest date, kernel color and lateral bearing, were 0.44, 0.47, 0.39 and 0.78, respectively, and average narrow-sense heritability estimates were 0.26, 0.38, 0.28 and 0.58, respectively, among different locations. Finally, a ranking of individuals, based on their breeding values (BVs) will help breeders with future selections. The results will guide future crossing designs in the walnut breeding program to implement genomic selection methods in walnut in the future (Martínez-García et al. 2017).

The walnut breeding population at UC Davis was genotyped with the novel Axiom® Walnut700K SNP array, enabling selection of superior genotypes based on their genomic estimated breeding values (GEBV).

11.4.12.3 High-Throughput Phenotyping in Walnut Breeding

Tree crop breeders need to phenotype large number of trees rapidly and accurately to identify the best progeny for traits of interest. So far, few studies have been done on walnut, but more attention will need to be paid to high-throughput phenotyping in walnut breeding programs. Two economically important traits of interest studied in walnut breeding programs are seal strength and kernel color. Recently, two accurate and consistent phenotyping methods including a texturometer and a virtual camera system were used for measuring seal strength and kernel color within the walnut improvement program in UC Davis (Neale et al. 2017). High-throughput phenotyping will be one of the most challenging future goals of fruit and nut tree genetic improvement programs.

Since fruit tree crops, especially walnut, have a long juvenile period, development of a new variety or rootstock may take 15–20 years via classical breeding. In summary, production of early-flowering walnut using continually flowering transgenic intermediates along with deployment of high throughput genotyping and phenotyping to select progeny will accelerate walnut genetic improvement.

11.4.12.4 Genomics-Assisted Walnut Rootstock Breeding

An active walnut rootstock-breeding program is ongoing in California and includes researchers from the USDA, UC Riverside and UC Davis. The main goals are to dissect the underlying genetic basis of resistance to crown gall, *Phytophthora*, root lesion nematode and *Armillaria* in walnut through genomic-based tools, and to use this information to release improved rootstock. This project focuses on identification and deployment of resistance QTLs for rapid screening to accelerate rootstock breeding. Several genomic and genetic resources such as transcriptome profiles, a 6 K Illumina Infinium SNP array, a SNP-based genetic map and a physical map have been developed by this working group.

Preliminary results show that resistance to both crown gall (CG) and *Phytophthora* spp. (PHY) is expressed in hybrids of wild *Juglans* spp. and in *J. regia*, including the commercially-released rootstock cv. RX1 (*J. microcarpa* × *J. regia*). Some mother trees of *J. microcarpa* showed significantly greater half-sib family mean resistance to PHY and CG than the population mean. Similarly, some mother trees of *J. cathayensis* showed significantly greater half-sib family mean resistance to lesion nematode (NEM). These mother trees were used for QTL mapping of resistance genes (Kluepfel et al. 2015).

Genotyping by sequencing (GBS), a series of genetic analyses that includes single SNP discovery and genotyping using NGS technology, have opened new possibilities in walnut breeding and genetic studies. GBS can simultaneously perform SNP discovery and genotyping with or without reference genome sequences. Therefore, GBS and the newly designed Axiom *J. regia* 700K SNP array can be applied to walnut breeding and genetics studies, including genotyping and genetic map construction, genome-wide association studies, genomic selection and population genetic studies (Fig. 11.6) (Arab et al. 2019; He et al. 2014; Marrano et al. 2019; Scheben et al. 2017).

The reference genome sequences of *Juglans regia*, *J. microcarpa* and *J. cathayensis* is facilitated application of GBS and discovery of genes controlling targeted traits. In a study, GBS information from 600 interspecific hybrid progeny was used to generate a genetic map for *J. microcarpa* × *J. regia*. Also, this group has identified QTLs for *Agrobacterium tumefaciens* (crown gall) and *Phytophthora* resistance. Detailed information about the rootstock program is available from the walnut rootstock website (http://www.rootstocks.net/).

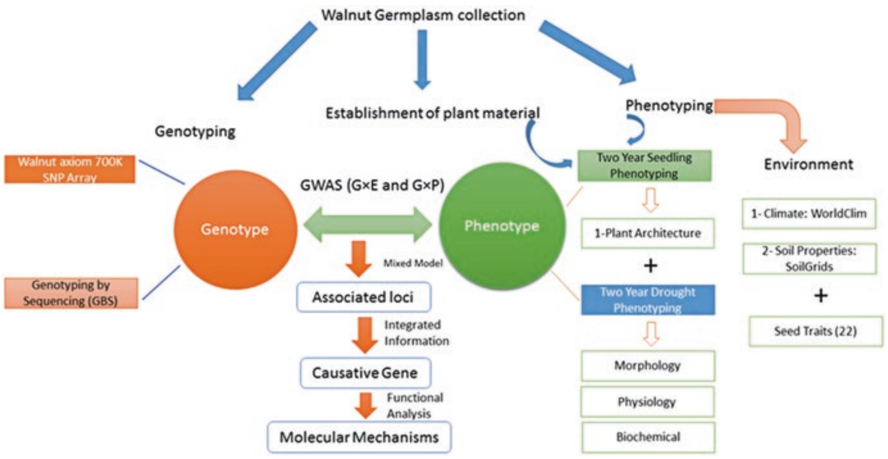

Fig. 11.6 Schematic representation of genomics-assisted breeding in walnut; an ongoing project by the University of Tehran and the University of California, Davis, on drought-resistant rootstock

11.4.13 Application of Functional Genomics in Genomics-Assisted Breeding

Functional genomics includes genome scale approaches to define gene function and gene networks at the transcript or protein level. Functional genomics is a most efficient tool and could be integrated into breeding at various stages (Dvorak et al. 2008). Functional genomics methods can be employed to produce useful molecular markers to increase the efficiency of variety development in fruit crop breeding program and to identify new genes of interest.

Functional mapping integrates mathematical and molecular genetics with developmental mechanisms underlying biological processes to shed light on the genetic basis of complex traits. In 2008, over 95% of more than 18,000 walnut sequences in the NCBI GenBank (http://www.ncbi.nlm.nih.gov/) were produced by the Dvorak (2008) Lab.

For functional mapping, 18 samples of walnut tissue were collected from cv. Chandler trees at UC Davis in 2008 and paired-end (85 bp × 85 bp) sequencing conducted on an Illumina Genome Analyzer. In the next step, to generate a Unigene set, these new sequences were combined with the 18,000 walnut ESTs and this set of genes was annotated using Blast2GO (http://blast2go.bioinfo.cipf.es/). A total of 21 cDNA libraries were constructed for the characterization of the walnut transcriptome using next-generation DNA sequencing. Over a billion reads were handled to obtain consensus sequences or genes that state the gene space in the walnut genome (Dvorak et al. 2008). The trimmed Illumina reads derived from each sample were assembled using Velvet v. 0.1.12, Oases v. 0.1.15 and CAP3 (Zerbino et al. 2009). The walnut contigs have also been annotated using Blast2GO software (Conesa and Gotz 2008; Gotz et al. 2008).

11.4.13.1 Functional Genomics: Transcriptomics

Several genomic techniques are employed to study transcriptome variation during development or as a response to biotic and abiotic stress.

cDNA-amplified fragment length polymorphism (AFLP) analysis by Bâaziz et al. (2012) was conducted on walnut leaves maintained under irradiance or in darkness to identify the early molecular events occurred during light-induced leaf hydraulic conductance (Kleaf). The results of this study showed that the most of transcript-derived fragments (TDFs) obtained via cDNA-AFLP correspond to genes whose protein products are involved in cellular regulation and global metabolism, respectively, 57.9 and 39.8% (Bâaziz et al. 2012). Availability of transcriptome information in walnut has increased the development of molecular markers like EST-SSRs that result from cDNA libraries. Available ESTs are used to both develop molecular markers and transcriptomics tools.

The first construction of walnut ESTs in *Juglans regia* from seed coat tissues by Muir et al. (2004) was submitted to the NCBI database (https://www.ncbi.nlm.nih.gov). In another study, functional genomic analysis to identify the genes involved in interactions between the walnut root and the nematode was done at UC Davis. In this project, at the first step, 13,559 expressed sequence tags (ESTs) were generated by sequencing cDNA libraries provided from *Pratylenchus vulnus* and infected and uninfected walnut (*J. hindsii* × *J. regia*) leaves and roots (Britton et al. 2007). In the next step, to identify and validate nematode and walnut genes associated with the infection analysis of gene expression between uninfected and infected plants. Walnut gene chips were used and confirmed by Taqman® real-time quantitative RT-PCR. Finally, functional analysis of nematode genes were done using RNA interference in vitro and *Medicago* root assays (Britton et al. 2009). Also in this project, 2733 *P. vulnus* genes and 8622 walnut genes were sequenced and displayed valuable information on the paths involved in the interaction between *P. vulnus* and a susceptible walnut rootstock. The result of gene expression indicated that a greater response was identified in the leaves than in the roots of plants inoculated with *P. vulnus*.

Zhang et al. (2010) obtained a total of 5025 walnut ESTs from the NCBI database and along with the SSR Hunter software, were used to analyze SSR motifs. Subsequently, a total of 123 primer pairs were designed from the non-redundant SSR-containing unigenes. The efficiency of candidate markers was examined by 7 DNA pools collected from different walnut accessions. Results revealed that 41 SSR primer sets with high polymorphic amplification products could be used for future genetic study in walnut (Zhang et al. 2010). In another study, 5213 EST sequences of walnut (*Juglans regia*) in NCBI were used for development of walnut EST-SSR markers, and 207 SSRs were obtained from the EST sequences (Feng et al. 2011)

A total of 7262 unigenes were obtained from 13,559 ESTs retrieved from the NCBI database and 309 EST-SSR primers were randomly designed. Finally, 13 highly polymorphic EST-SSRs were used for genetic analyses in *Juglans regia*, *J. nigra*, *Carya cathayensis*, *C. dabieshanensis* and an endangered species *Annamocarya sinensis* (Zhang et al. 2013). Also 40 polymorphic EST-SSR markers were developed by Zhao et al. (2015) in *J. regia*. Recently several transcriptome analysis have been performed in walnut which includes expression of the transcrip-

tion factor gene JrCBF involved in cold resistance mechanisms (Xu et al. 2014). In addition, expression of a large family of NBSLRR resistance genes in *J. regia* involved in plant-microbe interactions (Chakraborty et al. 2016) and transcriptome analysis of buds, leaves, female flowers and male flowers in *J. regia* were used to identify new EST-SSR markers (Dang et al. 2016). Also, Li et al. (2017) carried out comparative transcriptome analysis of genes involved in anthocyanin biosynthesis in leaf and peel color change in red and green *J. regia*.

11.4.13.2 Proteomics and Metabolomics

Transcriptomic information has facilitated the identification of candidate genes linked to agronomic traits of interest. However, the mechanisms of plant development stages and response to biotic and abiotic stress are complex due to the influence of multi-genes and post-transcriptional regulations. Therefore, functional genomics involving various proteomics and metabolomics approaches have been obligatory for understanding complex mechanisms. These approaches, integrated with genomics information, will accelerate identification of candidate genes and pathways involved in important agronomic traits that can be employed in plant genetic improvement programs.

Several proteomics and metabolomics studies have focused on nutritional properties and the beneficial effects of walnut consumption on health. Proteomics and metabolomics research carried out on *Juglans regia* has the following breeding purposes. One, proteomics studies such as protein markers development and assessment the genetic structure of Pakistan *J. regia* germplasm (Khan et al. 2010) and isolation of tyrosinase from *J. regia* leaves and identification as a PPO corresponding to the known *Jr*PPO1 sequence (Zekiri et al. 2014). And, two, metabolomics studies such as investigation of metabolite changes during kernel maturation using gas chromatography-mass spectrometry (GC-MS) (Rao et al. 2016), the study of biosynthesis of nonstructural polyphenols involved in pathogen resistance in *J. regia* (Colaric et al. 2005; Farooqui et al. 2015; Solar et al. 2006), identification of novel functions for the polyphenol oxidase enzyme in secondary metabolism and the regulation of walnut cell death by metabolite profiling (Araji et al. 2014; Escobar 2013).

Only a single PPO gene, *Jr*PPO1 has been identified in walnut by metabolite profiling. Metabolomics data integrated with information obtained from genome sequence, allow identification of second PPO gene, *Jr*PPO2 (Martínez-García et al. 2016). Also, PPO may be involved in pellicle color as an important commercial trait. Therefore, the results of this study are applicable to future walnut breeding.

11.4.14 Bioinformatics as a Tool for Walnut Research

Walnut databases that house genomics, transcriptomics, proteomics, metabolomics and phenomics resources provide an effective platform for walnut breeding programs. It is important for a breeder to understand how these data can be used for

genetic improvement of walnut. More recently, advances in NGS-Based genotyping and high-throughput phenotyping technology have revolutionized plant breeding, especially fruit tree genetic improvement, and turned it into an information-based science. All of this large-scale information requires appropriate analysis, storage and combination to enhance our understanding of genes underlying important traits to be utilized in further plant breeding program. Therefore, bioinformatic information and web sites have become an essential and integral part of plant genetic improvement programs (Mochida and Shinozaki 2010).

The walnut genome v1.0 sequencing projects was released by UC Davis on 2015 through NCBI (https://www.ncbi.nlm.nih.gov/) and (http://ucanr.edu/sites/wgig/) at Davis, California, providing access to walnut genomics and genetics information. Also, the Hardwood Genomics Project (https://www.hardwoodgenomics.org/organism/Juglans/regia) is a central repository of walnut genetics data.

A research report database for walnut (http://ucanr.edu/sites/cawalnut/) is under development by UC Davis to house and integrate growth and development, physiology, genomic, genetic, and breeding data for walnut management and genetic improvement. Initial transcriptome analyses in walnut focused on generating ESTs for identification of candidate genes involved in different stages of plant development and response to abiotic and biotic stresses. In 2004, the first walnut ESTs became available in the National Center for Biotechnology (NCBI) dbEST repository (https://www.ncbi.nlm.nih.gov), rising to over 21,000 sequenced ESTs by 2018. As of May 1, 2018, this search returned 1 genome match for the Persian walnut, 2 genome assembly information, 93 high-throughput DNA and RNA sequence read archive (SRA), 49,405 genome survey sequences (GSS), 173,178 DNA and RNA sequences (Nucleotide), 43,454 genes, 21,334 expressed sequence tag sequences (ESTs), 97 gene expression omnibus (GEO) datasets of expression and molecular abundance profiles on Persian walnut (https://www.ncbi.nlm.nih.gov/). Whole transcriptome shotgun sequencing (WTSS) or RNA-Seq is the latest powerful tool for transcriptome analysis. In walnut, RNA Seq technology is used to study walnut response to drought stress. Proteomics and metabolomics allow the parallel assessment of large-scale of proteins and metabolites in a biological sample. Many different methods are being taken to generate proteomics data; UniProt database (http://www.uniprot.org/) provides a comprehensive, high-quality and accessible resource of protein sequence and functional information. In total, 46,591 protein entries are available on the UniProt database for *Juglans* as follows: English walnut (45,744), *Juglans cathayensis* (103), *J. cinerea* (108), *J. mandshurica* (108) and *J. sigillata* (110). Currently (searched in May 2018), 45,764 protein entries are available for *J. regia* including; English walnut (45,744), *J. nigra* × *J. regia* (8) and *J. mandshurica* × *J. regia* (3) that all entries are in both Swiss-Prot and in the Translated European Molecular Biology Laboratory Nucleotide Sequence Database (TrEMBL). Since May 2018, 93,270 protein sequences from J. *regia* and 115 bioactivity screening studies of *J. mandshurica* and *J. regia* became available on the NCBI database (https://www.ncbi.nlm.nih.gov).

11.5 Genetic Engineering

Sometimes it is impossible to introduce specific traits into an existing cultivar by conventional methods, such as selection or hybridization. Consequently, an alternative method is direct gene transfer. Production of somatic embryos in walnuts was successfully reported in the 1980s (Tulecke and McGranahan 1985) and genetic engineering has been used successfully for walnut breeding. Walnut is one of the first woody plants to be transformed and to express foreign genes (Dandekar et al. 1988; McGranahan et al. 1988).

11.5.1 Tissue Culture

Traditionally, walnuts have been propagated by seeds and grafting onto seedling rootstock. In vitro propagation is important for the production of cultivars on their own roots, in vitro breeding (haploid induction etc.), disease-resistant rootstocks and development of transgenic walnuts (Dandekar et al. 2005; Sadat Hosseini Grouh et al. 2011; Vahdati et al. 2004). In addition, mature self-rooted clones in cv. Chandler exhibited more yield and superior vigor than grafted trees (Hasey et al. 2001).

The first reports of walnut micropropagation were published in the early 1980s (Chalupa 1981; Cossio and Minolta 1983; Driver and Kuniyuki 1984; Rodriguez 1982). Researchers have developed methods for rooting and acclimation of tissue-culture seedlings (Jay-Allemand et al. 1992; Navatel and Bourrain 2001; Ripetti et al. 1994; Vahdati et al. 2004). Driver and Kuniyuki (1984) developed DKW medium specifically for walnut micropropagation. The best explants for micropropagation of walnut are shoot buds, nodal segments and shoot tips (Gruselle et al. 1987; Rodriguez et al. 1993; Saadat and Hennerty 2002). A wide range of media (MS, DKW, WPM) and hormones (BAP, IBA, Kn, GA3, IAA) have been used for microshoot multiplication (Chalupa 1981; Revilla et al. 1989; Sommers et al. 1982). In the 1990–2000s, investigation of walnut micropropagation focused on rooting of microshoots (Jay-Allemand et al. 1992; Navatel and Bourrain 2001; Vahdati et al. 2004).

Rooting of commercial walnut cultivars, Chandler, Vina and Sunland, was accomplished using a two-phase procedure consisting of root induction (MS medium with 15 µM IBA in dark conditions) and root development (one-quarter of DKW medium and vermiculite (1:1.25, v/v). There was a positive relationship between vigor of cultivars and rooting ability, that is, the microshoots with expanded shoots root better. Rooting percentages were Chandler 55%, Vina 27% and Sunland 94%, and rooted plants were acclimatized successfully (Vahdati et al. 2004). This technique was extended to some commercial companies, (Fig. 11.7) but ex-vitro rooting methods are more commonly used commercially to reduce costs.

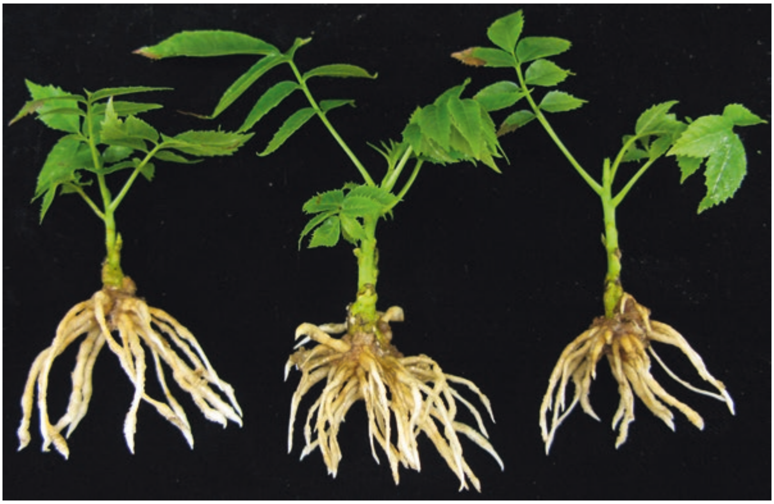

Fig. 11.7 In vitro rooting of Persian walnut cv. Chandler. (Photo by Kourosh Vahdati)

Acclimatization of micropropagated walnut is the most difficult phase because of susceptibility to abiotic stresses and rapid desiccation (Vahdati et al. 2004). Driver (1985, 1986) patented a method for the acclimatization of tissue-cultured cv. Paradox walnut propagules, which allows the direct field rooting and acclimatization of the propagules simultaneously (Driver 1985, 1986). He used polystyrene cups for acclimatization. The polystyrene cup(s) provide covering holding proper humidity (passive humidity) and adequate light. This system can be adapted to the greenhouse or directly in the field. It has shown value in acclimatizing small quantities rather than large, because of the logistics of handling larger quantities of cups. Clearly, walnuts require passive acclimatization and much in this area needs investigation.

Many recent studies have focused on acclimatization of micropropagated walnut (Fig. 11.8); Asayesh et al. (2017a, b) and Vahdati et al. (2017). Asayesh et al. 2017a compared the leaves of in vitro plants with those of greenhouse-grown plants. They reported that in-vitro plants had higher stomatal and epidermal densities and thinner leaves with larger stomata and pore area. Vahdati et al. (2017) also showed that increasing the CO_2 concentration of culture vessel headspace could be an efficient tool for improving acclimation of in vitro-grown cv. Chandler, because a higher CO_2 concentration resulted in a lower transpiration rate and a higher relative water content (RWC) during acclimatization. The results of this study and experiences at a commercial scale have consistently showed that using high-quality shoots with expanded leaves during the elongation stage will result in better rooting in walnut.

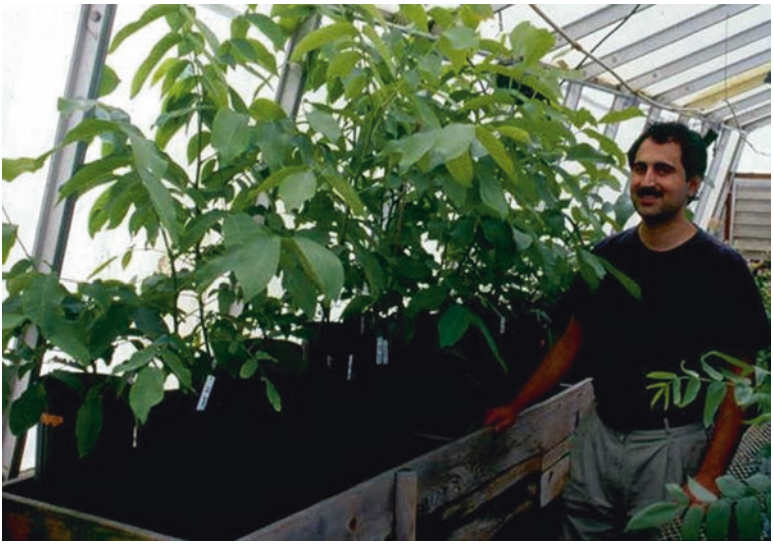

Fig. 11.8 Successful acclimatization of Persian walnut. (Photo by Kourosh Vahdati)

Fig. 11.9 Somatic embryogenesis in walnut on DKW medium under dark condition. (Source: Bahrami Sirmandi and Vahdati 2009)

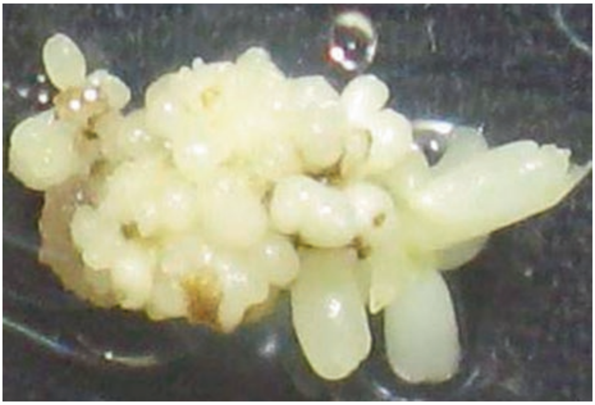

11.5.2 Somatic Embryogenesis

Walnut somatic embryos are important for genetic improvement and mass clonal propagation (McGranahan et al. 1990; Vahdati et al. 2008), intergeneric hybridization (McGranahan et al. 1986) and generating triploid plants (Tulecke et al. 1988) (Fig. 11.9). The first somatic embryos of walnut were obtained from cotyledons and endosperm of *Juglans hindsii*, *J. regia*, and *Pterocarya* sp. (Cornu 1988, 1989; Cornu and Jay-Allemand 1989; Long et al. 1995; Tulecke and McGranahan 1985; Tulecke et al. 1988; Vahdati et al. 2006). Generally, somatic embryogenesis consists of four stages: induction, proliferation, maturation and germination (Hartmann

et al. 1997). For induction of somatic embryos, immature cotyledonary explants are harvested from developing nuts and cultured on conditioning medium for 2 to 4 weeks and then transferred to basal DKW medium (Polito et al. 1989; Tulecke and McGranahan 1985). For induction and maintenance, all cultures are maintained at room temperature in the dark. Germination efficiency of walnut somatic embryos is low (0–45%) (Deng and Cornu 1992; Lee et al. 1988; Vahdati et al. 2006). To solve this problem, studies have been conducted by various researchers. For example, gibberellic acid (GA_3), cold, and desiccation storage pretreatments and liquid germination medium were tested for promoting germination (Deng and Cornu 1992; Tang et al. 2001; Tulecke and McGranahan 1985). Vahdati et al. (2008) studied the effect of sucrose and abscisic acid (ABA) on maturation and germination of walnut somatic embryos; results showed that the best treatment was 2 mgl^{-1} ABA; sucrose had little influence on maturation of walnut somatic embryos.

11.5.3 Selection of Transformed Somatic Embryos

In most early *Juglans regia* transformation studies, β-glucuronidase (GUS) is used for marker selection. Escobar et al. (2000) used green fluorescent protein (GFP); while Zhang et al. (2015) employed a new red fluorescent protein from *Discosoma* sp. (DsRED), which was more stable and reliable. Liu et al. (2017) used this marker in confirming that genes were not translocated from rootstock to scion.

11.5.4 Rootstock Transformation

Tolerance to biotic and abiotic stresses is a primary goal in walnut rootstock improvement. Walnuts are quite susceptible to crown gall disease caused by *Agrobacterium tumefaciens*. Escobar et al. (2001) reported a strategy of gene silencing for production of plants resistant to crown gall disease. Their report was the first use of gene silencing for resistance to bacterial disease; Escobar et al. (2002) then used this method to produce gall-resistant walnut. In another study, in order to increase the rooting potential of Paradox hybrid (*J. hindsii* × *J. regia*), *rol*ABC genes (*rol*A + *rol*B + *rol*C), derived from the bacteria *A. rhizogenes,* were inserted to somatic embryos of walnut (Vahdati et al. 2002). Although the *rol*ABC genes induced a shorter internode length and a more fibrous root system, they did not increase rooting potential. Recently, Walawage et al. (2013) researched co-transformation using an RNAi inducing construct inserted into *A. rhizogenes* to silence Pv010 and a construct to silence the *iaaM* and *ipt* genes inserted into *A. tumefaciens.* The objective was to produce concurrent resistance to both the nematode *Pratylenchus vulnus* and crown gall in the same rootstock; combining the two bacterial strains at a 1:1 rather than a 1:3 ratio increased the cotransformation efficiency.

Flavonoid effects on the rooting ability of hybrid walnut were also investigated. Somatic embryos of hybrid walnut (*Juglans nigra* × *J. regia*) were transformed with an antisense construct including a 400 bp cDNA fragment of a walnut *chs* gene and CaMV-35S promoter. Resulting decreased flavonoid content in stems of antisense *chs* transformed lines was linked to improved adventitious rooting ability. Auxin content was determined during the latter phase of the in vitro propagation and no differences were identified between the control and antisense *chs* transformed lines. Transformed plantlets low in flavonoids were more sensitive to exogenous application of auxin (El Euch et al. 1998).

11.5.5 Scion Transformation

To produce plants resistant to the codling moth (*Cydia pomonella* L.), a major pest of walnut in California, Dandekar et al. (1994) inserted the *cry1A*(c) gene of *Bacillus thuringiensis* into walnut but, due to the low expression, this transformation was unsuccessful. In 1998, a synthetic *cry1A*(c) gene with altered codon bias corrected the problem. This resulted in levels of expression sufficient to obtain efficacious control of *C. pomonella* when fed on transformed embryos (Dandekar et al. 1998). Sheikh Beig Goharrizi et al. (2016) reported that transgenic walnut expressing the *fld* gene had increased tolerance to osmotic stress. A significant difference was observed between transgenic and non-transgenic somatic embryos exposed to 50 and 100 mM NaCl and 5 and 10% PEG (Fig. 11.10). Walnut transformation with a betaine aldehyde dehydrogenase (*badh*) gene is also under study Vahdati (2014).

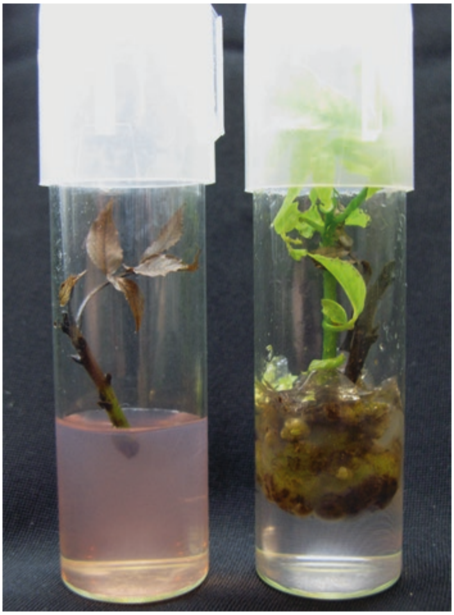

Fig. 11.10 Wild (left) vs. transgenic (right) walnut, expressing the *fld* gene after 45 days on DKW medium containing 200 mM NaCl. (Source: Sheikh Beig Goharrizi et al. 2016)

11.5.6 Promoter Isolation

Studies of genetic engineering in walnut generally use 35S as a promoter to enhance expression of foreign genes in the gene cassette. Xu et al. (2018) tested a 1200 bp promoter fragment of vacuolar H^+-ATPase (V-ATPase). The G subunit of the *Juglans regia* (JrVHAG1) gene was identified from *J. regia* DNA and amplified by a PCR reaction. Cis-elements of this promoter were analyzed using the PLANTCARE database. Finally, to check the expression activity of the JrVHAG1 promoter, it was inserted into a pCAMBIA1301 vector to drive the expression of the GUS gene. Results showed that expression activity was enhanced significantly in *Arabidopsis* when subjected to the cadmium stress.

11.6 Mutation Breeding

Chemical and physical mutagenesis have successfully assisted in the development of improved and new cultivars in plant breeding programs (Parry et al. 2009). The number of physical and chemical mutagens used in mutation breeding is large and continues to increase (Mba 2013; Mba et al. 2010). The most powerful, effective, reliable and frequently used chemical is ethyl methane sulphonate (EMS). Gamma rays can successfully be used to develop new mutant varieties, especially in ornamental plants (Taheri et al. 2014).

11.6.1 Haploid Mutagenesis

Haploid refers to plants that contain a gametophytic chromosome number (n) in their somatic tissues; doubled haploid (DH) plants are generated by the spontaneous or induced doubling of their chromosome numbers. Szarejko (2012) found that haploid somatic embryos or callus can be induced in vitro from male or female gametes and regenerated into haploid plants with only one set of homologous chromosomes.

Haploid plants are normally sterile so their chromosomes are doubled to provide completely homozygous lines from heterozygous plant material. Therefore, *doubled haploidy* techniques are integrated into breeding programs of many horticultural crops, including major ornamental crops, vegetables, fruit crops and medicinal plants (Szarejko 2012). Researchers have tried to produce haploid plants in more than 250 plant species, but efficient and reproducible doubled haploidy production protocols are available for fewer than 30 of them (Maluszynski et al. 2003).

Szarejko (2012) described the development of three main methods of doubled haploid production: (1) androgenesis, (2) wide crossing followed by chromosome elimination and (3) gynogenesis.

One of the most important applications of radiation techniques in plant breeding has been irradiation to destroy pollen or egg cell nuclei. Parthenogenesis, the production of an embryo from an egg cell without the participation of the male gamete, is commonly used for haploid production in fruit crops, especially in those species in which in vitro pollen embryogenesis has not been applied successfully (Germanà 2012).

11.6.2 Mutant Selection

The ultimate goal of any mutation breeding program is selecting desired mutants. Characteristics of fruit crops, such as a long juvenile phase, a high degree of heterozygosis, and self-incompatibility, make it impossible to obtain homozygous lines by conventional inbreeding approaches. Therefore, techniques to produce doubled haploids are particularly useful for fruit trees breeding (Germanà 2012).

11.6.2.1 Ploidy Determination

In fruit crops, haploid, diploid, triploid or hexaploid regenerants are produced through parthenogenesis induced using irradiated pollen. Various methods, including chromosome counting, flow cytometry analysis, stomata size, number of chloroplast guard cells and nucleus size, have been used to determine ploidy level of mutants. Among these techniques, flow cytometry is gaining in importance because it allows rapid analysis of a large number of samples (Germanà 2012).

11.6.2.2 Molecular Techniques for Mutation Detection and Screening

Screening of novel induced mutations in plants has long been a major challenge. Mutant screening and confirmation (mutant validation) are the two major steps for identifying and selecting mutant plants with improved traits (Shu et al. 2012a; Wu et al. 2012a, b).

Since plant mutagenesis induced by chemical or physical mutagens is a random process, occurring at an extremely low frequency, previous studies have revealed that DNA markers, with the exception of functional markers, are not useful for screening or selecting induced mutants, (Wu et al. 2012a).

Techniques developed for detection of mutations in a gene of interest include: single-strand conformation polymorphism (SSCP), denaturing high-performance liquid chromatography (DHPLC), temperature/denaturing gradient gel electrophoresis (TGGE/DGGE), conformation-sensitive gel/capillary electrophoresis (CSGE/CSCE), mismatch cleavage and matrix-assisted laser desorption/ionization time of flight mass spectrometry (MALDI-TOF) (Gady et al. 2009; Hestekin et al. 2006; McCallum et al. 2000; Shu et al. 2012a, b).

The advent of next-generation sequencing as an important tool for whole-genome sequencing and re-sequencing has revolutionized plant breeding. Recently, the efficiency of identifying DNA changes that generate a new trait has been increased tremendously by use of high-throughput mutation detection technologies (Quail et al. 2012) and reverse genetic techniques such as TILLING (targeting induced local lesions in genomes) (Taheri et al. 2017).

11.6.3 Practical Examples in Walnut

So far, few limited reports are available on walnut mutation breeding. Sadat Hosseini Grouh et al. (2011) reported the first successful production of haploid lines in Persian walnut through parthenogenesis, induced by gamma ray-irradiated pollen. In their study, female flowers of cvs. Hartley and Pedro and two native Iranian selections (Z63 and Z67) were pollinated with gamma ray-irradiated pollen from selections Z53 and Z30. The results revealed that using pollen irradiated at 300 and 600 Gy successfully generated haploid lines in Persian walnut. Simple sequence repeat (SSR) markers confirmed haploid plantlets. The techniques used in other fruit and nut breeding programs to induce haploids and other ploidy changes could be applied to Persian walnut to accelerate genome analysis (Sadat Hosseini Grouh et al. 2011). (Fig. 11.11).

Triploid walnut plants are produced by culturing endosperm from immature open-pollinated seed of *Juglans regia* cv. Manregian and inducing somatic embryos. The resulting plantlets were evaluated morphologically, and the roots were used for chromosome counting (Tulecke et al. 1988). When planted in the field, the resulting triploid trees produce abundant male flowers that mostly fail to produce pollen and rarely produce nuts. The few nuts observed have been very small and contained no kernel. These triploid plants represent novel germplasm that of possible use for breeding, following further ploidy manipulation.

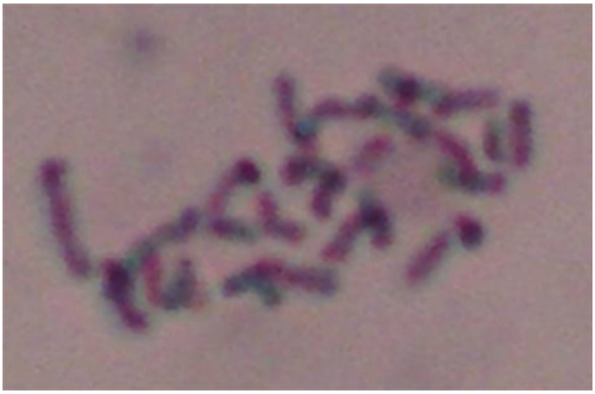

Fig. 11.11 Haploid set of chromosomes (n = x = 16) observed in a root apex of an embryo collected from Z63 genotype of Persian walnut

11.7 Hybridization

In natural populations, hybridization can be useful for the production of new lineages with adaptation to environmental changes, disease resistance and production of plants with uniform growth. Hybridization has always been an important walnut breeding strategy, leading to the release many varieties and rootstocks. A complete description of walnut breeding programs and varieties released based on hybridization is presented in Sect. 11.2.

11.8 Conclusions and Prospects

The high nutritional and economic value of walnut, along with significant improvements generated by walnut breeding and the release of high-quality and productive varieties, have led to its increased cultivation in recent years. High yield and quality have always been primary walnut breeding objectives. Lateral bearing, high nut weight and size, high kernel percentage, late leafing, early harvest date and light kernel color are the primary traits targeted for scion cultivars. Dwarfing, salt and drought tolerance and resistance to soil-borne pathogens such as *Agrobacterium*, *Phytophthora* nematodes and *Armillaria* are targeted traits for rootstocks. Breeders have used several strategies to achieve these goals, such as germplasm evaluation and selection, hybridization, genetic engineering, mutation breeding, genome sequencing, bioinformatics, marker-assisted selection, haploid and polyploid induction, proteomics and metabolomics. Current walnut breeding strategies focus on integrating molecular breeding (usage of genomic, transcriptomic, proteomic and metabolomics information) into traditional breeding programs. Climate change and global warming can severely impact global walnut production. Therefore, walnut breeding programs, especially in the leading and most active countries, are striving to generate cultivars quickly and efficiently that maintain and continually improve walnut production and quality. Accordingly, the topics listed below are priorities for future walnut breeding programs:

(a) Use of CRISPR-Cas9 systems for targeted-genome editing;
(b) Production of haploid, doubled haploid and wide-hybrid plants to enhance genome assembly quality;
(c) Combining of high-throughput phenotyping with GWAS and genomic selection to unlock genetic information coded in the walnut genome that controls complex traits;
(d) Simultaneous use of transcriptomics, metabolomics and proteomics for understanding drought and salinity tolerance mechanisms in walnut;
(e) Continued evaluation of genetic diversity to release and develop new cultivars and rootstocks;
(f) Release of new cultivars with low chilling requirement, early harvest and late-leafing date.

Acknowledgments We would like to acknowledge Neus Aleta Soler, Fabrice Lheureux, Damiano Avanzato, Geza Bujdosó, Mehmet Sutyemez, Gamalier Lemus, Iosif Kiss, Joao Martins, Shugang Zhao, Hongxia Wang, Baojun Zhao, Yasar Akca, David L. McNeil, Mihai Botu, Dragan Milatovic, Anita Solar, Alexandros Papachatzis, Oleg Tirsina, Heini Gubler, Christof Gubler, David Zaurov, Zviad Bobokashvili, Endrit Kullaj, Sergey Khokhlov, Stefan Gandev, Basharat Ali Saleem, David Maghradze, Dietrich Darr and Davlet Mamadjanov who helped us to complete information of the walnut cultivars and research institutions inventory of their countries.

Appendices

Appendix I: Some Important Research Institutes Relevant to Walnut

Country	Institution	Specialization and research activities	Contact information and website
Albania	Agricultural University of Tirana	Walnut breeding program based on selection	Prof. Endrit Kullaj<br>ekullaj@ubt.edu.al<br>http://ubt.edu.al/
Argentina	National Research Institute of Argentina (INTA-Catamarca)	Variety walnut breeding program based on hybridization and selection	Dr. Dante Carabajal<br>Carabajal.dante@inta.gob.ar<br>https://inta.gob.ar/
Belarus	Belarusian Research Institute for Fruit Growing	Walnut breeding program for early bearing, high yield, winter resistant, diseases tolerant	Dr. Vyacheslav A. Samus<br>director@belsad.by<br>http://www.belsad.by
Bulgaria	Fruit Growing Institute	Breeding and studies of walnut cultivars	Dr. Stefan Gandev<br>sgandev@yahoo.com<br>http://www.fruitgrowinginstitute.com/
China	Hebei Agricultural University (College of Life Science)	Walnut breeding based on classic and molecular breeding	Dr. Shugang Zhao<br>zshug@126.com<br>http://life-auh.com/Index.html
China	Mountainous Area Research Institute of Hebei	Walnut breeding based on selection and hybridization	Dr. Hongxia Wang<br>whx@hebau.edu.cn<br>http://shanyansuo.hebau.edu.cn/
China	Chinese Academy of Forestry (Research Institute of Forestry)	Walnut improvement program	Prof. Pei Dong<br>peigu@caf.ac.cn<br>http://www.caf.ac.cn
China	Pomology Research Institute, Shanxi	Walnut breeding program based on selection and hybridization	Prof. Jianbao Tian<br>tianjb-001@163.com<br>http://www.gfar.net/organizations/pomology-research-institute-shanxi-academy-agriculture-science
China	Liaoning Institute of Economic Forestry	Walnut breeding program for high yield, blight and coldness tolerant and rootstock selection	Prof. Baojun Zhao<br>agroforestry@163.com<br>liufeng0427@sina.cn<br>http://www.lnly.gov.cn/lnly/kyyszw/sjjls/

Country	Institution	Specialization and research activities	Contact information and website
Chile	Instituto de Investigaciones Agropecuarias	Orchard management and plant material evaluation	Dr. Gamalier Lemus glemus@inia.cl http://www.inia.cl
France	French National Institute for Agricultural Research (INRA)	Walnut breeding program for late flowering, frost resistance, early bearing, high yield, blight tolerant, systematics and ecology of plant pathogenic bacteria	Dr. Sophie Cesbron sophie.cesbron@angers.inra.fr http://www.inra.fr
France	Centre Technique Interprofessionnel des Fruits et Legumes (CTIFL)	Walnut breeding program and study of the behavior of new INRA walnut varieties and rootstocks	Dr. Fabrice Lheureux lheureux@ctifl.fr http://www.ctifl.fr/
France	Station Expérimentale de la Noix de Creysse	Study on walnut orchard management and walnut quality, improving the efficiency of the walnut industry in the south west of France	Dr. Eloise Tranchand e.tranchand.creysse@orange.fr Dr. Fabrice Lheureux lheureux@ctifl.fr http://www.noixsudouest.fr
France	Station d'Expérimentation Nucicole Rhône-Alpes	Orchard management and plant material evaluation walnut industry efficiency in the south east of France	Dr. Agnès Verhaeghe Averhaeghe@senura.com http://senura.com/
Georgia	Georgian Research Institute of Horticulture, Viticulture and Oenology	Breeding new species and varieties of walnut through hybridization of different species and varieties	Dr. Zviad Bobokashvili bobokashvili@hotmail.com http://agruni.edu.ge
Georgia	Scientific-Research Center of Agriculture Georgia	Selecting and preservation of walnut varieties	Dr. Zviad Bobokashvili bobokashvili@hotmail.com Dr. Nugzar Shengelia shengelianugzar@gmail.com http://agruni.edu.ge
Germany	Hochschule Geisenhiem University	Breeding, selection and preservation of walnut varieties	Prof. Joachim Heller (Head) Joachim.Heller@hs-gm.de https://www.hs-geisenheim.de/en/research/departments/pomology/department-of-pomology/
Germany	State Education and Research Institute for Viticulture and Pomology Weinsberg	Breeding, selection and preservation of walnut varieties	obstbau@lvwo.bwl.de http://www.lvwo-bw.de/pb/,Lde/Startseite
Greece	Technological Educational Institute of Thessaly (TEI)	Walnut breeding program based on crossing of local genetical material with foreign cultivars	Prof. Alexandros Papachatzis papachad@teilar.gr http://www.teilar.gr

(continued)

Country	Institution	Specialization and research activities	Contact information and website
Greece	Technological Educational Institute of Peloponnese (TEI)	Walnut breeding program based on crossing of local genetical material with foreign cultivars	Prof. George Zakynthinos gzakyn@yahoo.gr http://www.teipel.gr
Greece	National Agricultural Research Foundation (NAGREF)	Walnut breeding program based on selection	Dr. Pavlina Drogoudi drogoudi@otenet.gr http://www.elgo.gr
Hungary	NARIC Fruitculture Research Institute	Walnut breeding program based on selection and hybridization	Dr. Geza Bujdosó resinfru@yahoo.com https://fruitresearch.naik.hu/en
Iran	University of Tehran, Aburaihan Campus, Center of Excellence in Walnut Improvement and Technology	Walnut breeding for late leafing, lateral bearing and high yield; rootstock breeding for drought tolerance and dwarfing using classic breeding and biotechnology; giving consultation for walnut commercial micropropagation labs and establishment of modern walnut orchards and nurseries	Prof. Kourosh Vahdati kvahdati@ut.ac.ir http://walnut.ut.ac.ir/ https://rtis2.ut.ac.ir/cv/kvahdati/?lang=en-gb
Iran	Horticultural Science Research Institute (HSRI)	Walnut breeding program for late leafing, lateral bearing, high yield.	Dr. Darab Hassani hassanida@gmail.com http://www.hsri.ir
Italy	University of Turin	Evaluation of the performance of Italian and foreign walnut cultivars and selections obtained by intraspecific hybridization	Prof. Roberto Botta roberto.botta@unito.it http://www.disafa.unito.it/do/home.pl
Italy	Istituto Sperimentale per la Frutticoltura	Evaluation of the performance of Italian and foreign walnut cultivars and selections obtained by intraspecific hybridization; improving rooting and disease resistance in walnut through tissue culture techniques	Dr. Pasquale Piccirillo pasquale.piccirillo@entecra.it http://sito.entecra.it/portale/index2.php
Kyrgyzstan	National Academy of Kyrgyzstan (Jalal-Abad Research Center)	Walnut breeding based on selection	Dr. D.K. Mamadjanov jangak@mail.ru http://www.nas.aknet.kg
Moldova	State Agrarian University of Moldova	Creation of the walnut assortment in the republic of Moldova	Dr. Valerian Balan v.balan@uasm.md http://www.uasm.md

Country	Institution	Specialization and research activities	Contact information and website
Moldova	Iargara State Forestry Service	Forestry management; walnut varieties improvement program	Ana Petrenco iargara@moldsilva.gov.md http://iargara.silvicultura.md/
Moldova	Institute for Horticulture and Food Technologies	Selection of walnut varieties within the existing local genetic resources	Constantin Dadu www.isphta.md
Morocco	Institut National de Recherches Agronomiques	Prospection in local walnut populations in the south of Morocco	Dr. Abdellah Kajji kajjiabdellah03@yahoo.fr https://www.inra.org.ma/
Pakistan	Arid Agriculture University Rawalpindi, Department of Horticulture	Walnut breeding based on selection within local genetic resource	Prof. Nadeem Akhtar Abbasi nadeemabbasi65@yahoo.com http://www.uaar.edu.pk/homeUaar.php
Pakistan	Ayub Agriculture Research Institute (AARI), Hill Fruit Research Station	Walnut breeding based on selection within local genetic resource	Dr. Muhammad Afzal mafzal834@gmail.com www.aari.punkab.gov.pk
Pakistan	University of Azad Jammu and Kashmir, Department of Botany	Walnut breeding based on selection within local genetic resource	Prof. Dr. Muhammad Qayyum Khan mqkhan2004@yahoo.com www.ajku.edu.pk
Pakistan	Agriculture Research Institute (North) Mangora	Walnut breeding based on selection within local genetic resource	Dr Khalil Ur Rehman khalilswat66@gmail.com http://agrires.kp.gov.pk
Pakistan	Hazara Agriculture Research Station	Walnut breeding based on selection within local genetic resource	Mr Akhtar Nawaz akhtarsaeed5650@gmail.com http://agrires.kp.gov.pk/page/hazaraagricultureresearchstation
Portugal	Direcção Regional de Agricultura da Beira Litoral	Behavior of some walnut-tree varieties in Região Agrária Da Beira Litoral	drapc@drapc.gov.pt http://www.drapc.min-agricultura.pt/drapc/contactos.htm
Romania	University of Craiova (Fruit Growing Research Station – SCDP Vâlcea)	Breeding new cultivars for high yield, intensive growing, nut quality, resistance to diseases, adapted to environmental conditions and rootstock selection; evaluation of the performance of Romanian and foreign walnut cultivars	Prof. Mihai Botu btmihai2@yahoo.com http://horticultura.ucv.ro/horticultura/en

(continued)

Country	Institution	Specialization and research activities	Contact information and website
Romania	Fruit Growing Research Station – SCDP Iaşi	Selection of walnut with high yield and fruit quality, resistance to late spring and winter frosts, resistance to diseases and pests, reduced vigor	Dr. Gelu Corneanu office@pomicolaiasi.ro www.pomicolaiasi.ro
Romania	Via Roots Srl	Study on walnut orchard management and walnut quality, study on lateral bearing in Romanian climate conditions	Iosif Kiss iosif.kiss@nucifere.com www.nucifere.com
Russia Federation	Nikita Botanical Gardens	Fundamental bases of management of selection process of creation of new plant genotypes with high economically valuable characteristics of productivity, resistance to biotic and abiotic stress	Dr. Sergei Khokhlov ocean-10@mail.ru http://www.nbgnscpro.com
Serbia	University of Priština, Faculty of Agriculture in Lešak	Biology of walnut flowering	Dr. Dragan Jankovic draganjankovickv@gmail.com Dr. Sladana Jankovic https://www.uni-pr.edu/
Slovenia	University of Ljubljana	Selection of walnut populations in Slovenia, quantitative analysis of genotypic diversity in tree architecture constitution, evaluation of the performance of foreign walnut cultivars	Dr. Anita Solar anita.solar@email.si https://www.uni-lj.si/academies_and_faculties/faculties/2013052914461802/
Spain	Institute of Agrifood Research and Technology (IRTA.)	Breeding and selection for walnut varieties and rootstocks, selection of basic materials for woodland.	Dr. Neus Aleta neus.Aleta@irta.es http://www.irta.cat
Spain	Bosques Naturales S. A.	Selection of genotypes for timber production, genotype × environment studies, genotyping by SSR markers	Dr. Ricardo Julian Licea-Moreno ricardolicea@bosquesnaturales.es https://bosquesnaturales.com
Switzerland	Nuss-Baumschule Gubler GmbH	Walnut breeding for high yield, lateral bearing, late leafing and disease resistance	Dr. Heini Gubler heini.gubler@skigubler.ch www.nussbaeume.ch
Tajikistan	Tajikistan Forestry Institute	Walnut breeding based on selection	Prof. Hafiz Muminjanov

Country	Institution	Specialization and research activities	Contact information and website
Turkey	Kahramanmaraş Sütçü imam University	Walnut cultivar and rootstock breeding based on classic (selection, hybridization) and molecular breeding program for late leafing, lateral bearing, high yield, nut quality	Dr. Mehmet Sütyemez sutyemez@ksu.edu.tr Dr. Akide Özcan akideozcan@ksu.edu.tr http://www.ksu.edu.tr
Turkey	University of Gaziosmanpasa	Walnut breeding for late leafing, lateral bearing, nut quality and blight resistance using intraspecific crosses, rootstock breeding for salt stress	Prof. Yasar Akca akcanut@gmail.com https://ziraat.gop.edu.tr http://www.ceviz.gen.tr/
Ukraine	Institute of Horticulture of the National Academy of Agrarian Sciences of Ukraine	Creation of the national genetic collection of Persian walnut of promising cultivars (breeding to combine precocity, high productivity, tolerance to diseases and high nut quality)	Dr. Igor V. Grynyk (Director) sad-institut@ukr.net http://sad-institut.com.ua/o_nas.html
United States	University of California, Davis, Department of Plant Science	Walnut cultivar and rootstock breeding based on classic and molecular breeding	Dr. Pat J. Brown pjbrown@ucdavis.edu https://pjblab.faculty.ucdavis.edu/ http://fruitsandnuts.ucdavis.edu/
Uzbekistan	Schroeder Uzbek Research Institute	Walnut breeding based on selection	Aziz Nurbekov a.nurbekov@cgiar.org
Uzbekistan	Uzbek Scientific Research Institute of Plant Industry (VIR)	Walnut breeding based on selection	Aziz Nurbekov a.nurbekov@cgiar.org

Appendix II: Some Walnut Genetic Resources

Cultivar	Important traits	Cultivation location
Gizavezhda	High yield, average nut, tender shell, very light kernel color, cold-hardy and resistant to pest and diseases	Albania
Leshnica	Medium yield, lateral bearing, large nut, light kernel color, aromatic	Albania
Smokthina	Medium yield, lateral bearing, average nut, light tasty kernel	Albania
Trompito	High yield, early leafing, medium kernel color	Argentina
Ivarto	Low yield, pollinizer for medium and late varieties	Argentina
Rote Donaunuss	Moderate yield, early to mid-early leafing, medium nut size	Austria
Weinberg 2	Early leafing, large nut, thin shell, light kernel color, good kernel quality	Austria
Axel	Medium yield, late leafing, large nut	Belgium/ Netherland
Dryanovski	Early flowering, medium nut size, light kernel color, high kernel quality	Bulgaria
Izvor 10	Moderate yield, medium nut size, thin shell, light kernel color	Bulgaria
Plovdivski	Large nut, high kernel percentage, high kernel quality, resistance to bacterial disease	Bulgaria
Proslavski	Large nut, good kernel quality, resistance to bacterial disease	Bulgaria
Silistrenski	Late flowering, medium nut size, frost resistant	Bulgaria
Broadview	Early leafing, precocious, homogamous, large nut, good kernel quality	Canada
Zha 343	High yield, lateral bearing, thin shell, light kernel color	China
Zanmei	High yield, lateral bearing, light kernel color	China
Jinlong 1	Moderate yield, terminal bearing, large nut, thin shell, light kernel color	China
Wen 185	High yield, lateral bearing, large nut, thin shell, light kernel color	China
Xiangling	High yield, lateral bearing, thin shell, light kernel color	China
Zhonglin 1	High yield, lateral bearing, thin shell, extra light kernel color	China
Liaoning 1	High yield, lateral bearing, thin shell, extra light kernel color	China
Jinboxiang1	High yield, lateral bearing, thin shell, light kernel color	China
Luguo 2	High yield, lateral bearing, thin shell, light kernel color	China
Luguo 7	High yield, thin shell, light kernel color, lateral bearing	China
Daixiang	High yield, thin shell, light kernel color, lateral bearing, dwarf	China

Cultivar	Important traits	Cultivation location
Xinfeng	High yield, thin shell, light kernel color, lateral bearing	China
Xinxin 2	High yield, thin shell, light kernel color, lateral bearing	China
Mars	High yield, late leafing, thin shell, good kernel quality	Czech Republic
Jupiter	High yield, large nut, thin shell, late leafing,	Czech Republic
Saturn	High yield, large nut, good kernel color	Czech Republic
Apollo	Good yield, terminal bearing, early flowering, large nut, good kernel color	Czech Republic
Sychrov	Medium nut size, red kernel color, thin shell, high kernel quality	Czech Republic
Franquette	Fair yield, terminal bearing, late leafing, good kernel quality, extra light kernel color, used as pollinizer for 'Chandler'	France
Lara	High yield, lateral bearing, medium leafing	France
Fernor	High yield, lateral bearing, late leafing, extra light kernel color	France
Fernette	Good yield, lateral bearing, extra light kernel color, used as pollinizer for 'Chandler'	France
Ferbel	High yield, lateral bearing, large nut, thin shell, good kernel quality	France
Ferouette	High yield, lateral bearing, extra light kernel color, large nut	France
Feradam	High yield, lateral bearing, extra light kernel color, medium leafing	France
Ferjean	High yield, lateral bearing, thin shell, extra light kernel color	France
Meylanaise	Moderate yield, late leafing, good kernel quality, Used as pollinizer	France
Ronde de Montignac	Late leafing, terminal bearing, high kernel quality, used as pollinizer,	France
Rubis	Early leafing, good kernel quality, red kernel color	France
Akura	Moderate yield, moderate lateral bearing, light kernel color	Georgia
Kaspura	High yield, moderate lateral bearing	Georgia
Avenisuri	Moderate yield, terminal bearing, large nut, thin shell	Georgia
Alazani	High yield, lateral bearing	Georgia
Aragvi	Moderate yield, terminal bearing, thin shell	Georgia
Atskuri	High yield, moderate lateral bearing, thin shell, frost resistance	Georgia
Drianovski	High yield, moderate lateral bearing, large nut	Georgia
Aufhausener Baden	Large nut, mid-early flowering, old favorite German cultivar	Germany

(continued)

Cultivar	Important traits	Cultivation location
Finkenwerder Deichnuss Royal	Large nut, precocious, thin shell, high kernel quality	Germany
Geisenheimer	Moderate yield, medium nut size, medium shell thickness	Germany
Kurmarker	Medium nut size, good kernel quality	Germany
Ledema	Protogynous, large and heavy nut,	Germany
Moselaner	Large nut, light kernel color, good kernel quality	Germany
Ockerwitzer Lange	High yield, large nut, frost resistant.	Germany
Seifersdorfer Runde	Good yield, early flowering, thin shell, light kernel color, frost resistant	Germany
Spreewalder	High yield, early leafing, precocious, light kernel color, good kernel quality	Germany
Weinheimer	Medium nut size, late leafing, high kernel quality, light kernel color	Germany
Weinsberg 1	Moderate to high yield, early flowering, large nut	Germany
Wunder von Monrepos	Medium nut size, late leafing, high kernel quality	Germany
ZP–1, 2, 3, 4, 5	'Chandler' hybrids, lateral bearing, blight resistant, moderate to late leafing, light kernel color.	Greece
Milotai Kései℗	Late leafing and flowering, lateral bearing, nut like Milotai 10, higher tolerance to blight compared to Milotai 10	Hungary
Alsószentiváni 117	Moderate yield, terminal bearing, light kernel color	Hungary
Alsószentiváni 118	High yield, mid-early flowering, large nut	Hungary
Milotai 10	High yield, moderate lateral bearing, light kernel color	Hungary
Tiszacsécsi 83	High yield, moderate lateral bearing	Hungary
Esterhazy II	Early leafing, medium to large nut size, light kernel color, good kernel quality	Hungary
Jamal	Moderate yield, terminal bearing, medium light kernel color	Iran
Damavand	Early leafing, used as pollinizer for Jamal	Iran
Sorrento	Moderate yield, terminal bearing	Italy
Malizia	High yield, moderate lateral bearing	Italy
Qingxiang	High yield, terminal bearing, light kernel color	Japan
Kyrgyzskya Bomba	Large nut, great kernel quality, light kernel color	Kyrgyzstan
Ak Terek	Medium nut, great kernel quality, light kernel color	Kyrgyzstan
Oshsky	Medium nut size, great kernel quality	Kyrgyzstan
Uygursky	Large nut, great kernel quality, light kernel color	Kyrgyzstan
Ostrovershinny	Large nut, great kernel quality	Kyrgyzstan
Immuniy	Medium nut, great kernel quality, light kernel color	Kyrgyzstan
Desertniy	Large nut, great kernel quality, light kernel color	Kyrgyzstan
Pescianski	High yield, partial lateral bearing, thin shell, frost resistant, extra light kernel	Moldova
Calarasi	High yield, terminal bearing, frost resistant, light kernel	Moldova

Cultivar	Important traits	Cultivation location
Ovata	Large nut, terminal bearing, very cold resistant	Moldova
Carpatica	Huge nut, terminal bearing	Moldova
Amphyon	High yield, high kernel quality, low susceptible to disease	Netherland
Dionym	High yield, high kernel quality, low susceptible to disease	Netherland
Big & Easy	Late flowering, thin shell, good kernel quality	Netherland
Blanco	Large nut, homogamous	Netherland
Coenen	Early flowering, large nut, thin shell	Netherland
Lange van Lod	Large nut, late leafing, high kernel quality, slight tolerance to late spring frost	Netherland
Rex	High yield, late leafing, light color	New Zealand
Shannon	High yield, light kernel color, blight resistant	New Zealand
Meyric	High yield, late leafing, thin shell, high kernel quality	New Zealand
Wilsons Wonder	Large nut, light kernel color	New Zealand
Valcor	High yield, terminal bearing, thin shell, light kernel color	Romania
Valmit (Verisval)	Thin shell, terminal bearing, light kernel color,	Romania
Valrex	High yield, terminal bearing, large nut, thin shell	Romania
Sibişel 44	Moderate yield, terminal bearing, large nut	Romania
Jupâneşti	Precocious, high yield, terminal bearing, thin shell	Romania
Velniţa	Precocious, high yield, terminal bearing	Romania
Valstar	Precocious, high yield, terminal bearing light kernel	Romania
Valcris	Precocious, high yield, terminal bearing light kernel	Romania
Timval	High yield, terminal bearing, large nut	Romania
Miroslava	Terminal bearing, large nut	Romania
Ovidiu	Terminal bearing, large nut	Romania
Anica	Terminal bearing, large nut	Romania
Sibişel 252	Precocious, high yield, terminal bearing	Romania
Ciprian	Precocious, high yield, terminal bearing, large nut	Romania
Claudia	Precocious, high yield, terminal bearing	Romania
Germisara	Moderate yield, terminal bearing, large nut	Romania
Şuşiţa	Precocious, high yield, terminal bearing, light kernel	Romania
Ronutex	High yield, terminal bearing, large nut	Romania
Belbeksky 70	Late leafing, light kernel color, moderate shell thickness	Russia Federation
Vynoslivy	High yield, light kernel color, resistance to low temperature	Russia Federation
Pervomaysky	Late leafing, thin shell, light kernel color	Russia Federation
Krymsky Urozhayany	High yield, thin shell, light kernel color	Russia Federation
Elit	Late leafing, precocious, light kernel color	Slovenia
Krka	Homogenous flowering, high yield, bright kernel	Slovenia

(continued)

Cultivar	Important traits	Cultivation location
Sava	Intermediate fruit-bearing, late leafing, moderate yield	Slovenia
Fischenthal	Medium nut size, thin shell, cluster bearing, homogamous	Switzerland
Giswill	Lateral bearing, interesting for wood production	Switzerland
Nyffenegger	Terminal bearing, thin shell, red kernel color	Switzerland
Rote Gubler	Medium nut size, light kernel color, forest resistant	Switzerland
Yalova 1	Fair yield, terminal bearing, large nut, thin shell	Turkey
Yalova 3	Fair yield, terminal bearing, thin shell, light kernel color	Turkey
Sebin	High yield, moderate lateral bearing, thin shell, light kernel color	Turkey
Bilecik	Moderate yield, terminal bearing	Turkey
Maras 18	Moderate yield, lateral bearing, light kernel color, high kernel percentage, very early harvest,	Turkey
Sütyemez 1	Moderate yield, lateral bearing, extra-large nut, light kernel color, very early harvest	Turkey
Kaman 1	High yield, lateral bearing, thin shell, light kernel color	Turkey
Maraş 12	High yield, terminal bearing, moderate nut, thin shell, light kernel color, extra high kernel percentage	Turkey
Diriliş	High yield, lateral bearing, late leafing, light kernel color, high kernel percentage, thin shell, early harvest	Turkey
15 Temmuz	High yield, lateral bearing, very late leafing, light kernel color, high kernel percentage thin shell, early harvest	Turkey
Bayrak	High yield, lateral bearing, thin shell, extra light kernel color, high kernel percentage	Turkey
Akça	High yield, lateral bearing, late leafing, good kernel quality, extra light kernel color, used as pollinizer for 'Chandler'	Turkey
Niksar 1	Moderate yield, lateral bearing, late leafing, used as pollinizer for cv. Chandler	Turkey
Eureka	Fair yield, terminal bearing, poor kernel color	USA
Scharsch Franquette	Late leafing, medium thin shell, light kernel color	USA
Hartley	Moderate yield, terminal bearing, light kernel color	USA
Payne	High yield, lateral bearing, precocious, early leafing, early harvest, light kernel color	USA
Vina	High yield, lateral bearing, poor color	USA
Pedro	High yield, lateral bearing	USA
Tehama	Moderate yield, lateral bearing, light kernel color, used as pollinizer for cv. Serr	USA
Serr	Moderate yield, moderate lateral bearing, light kernel color, thin shell, excellent kernel quality, susceptible to pistillate flower abscission (PFA)	USA
Chandler	High yield, lateral bearing, medium leafing, extra light kernel color, thin shell	USA
Howard	High yield, lateral bearing, medium leafing, large nut, thin shell, light kernel color	USA

Cultivar	Important traits	Cultivation location
Sunland	High yield but susceptible to nut drop, lateral bearing, large nut, thin shell, light kernel color	USA
Cisco	Moderate yield, terminal bearing, medium light kernel color, high susceptibility to blight, used as pollinizer for cv. Chandler	USA
Tulare	High yield, lateral bearing, light kernel color, susceptible to winter cold	USA
Robert Livermore	Moderate yield, lateral bearing, red kernel color	USA
Sexton	High yield, lateral bearing, very precocious, light kernel color	USA
Gillet	High yield, lateral bearing, large nut, light kernel color, low susceptibility to blight	USA
Forde	Good yield, lateral bearing, light kernel color, low susceptibility to blight	USA
Ivanhoe	High yield, lateral bearing, very precocious, very early harvest, thin shell, extra light kernel color	USA
Solano	High yield, lateral bearing, extra light kernel color	USA
Durham	Good yield, early harvest, lateral bearing, large nut, light kernel color	USA
Ideal	Small nut from secondary flower, high kernel quality, precocious, cluster bearing habit	Uzbekistan
Hybridiy	Medium size nut	Uzbekistan
Pioner	Large size nut	Uzbekistan
Kazahstansky	High kernel quality	Uzbekistan
Bostonliksky	Large nut	Uzbekistan
Rodina	Large nut, high kernel quality, high frost resistance, low susceptibility to anthracnose	Uzbekistan
Parkent	Large nut	Uzbekistan
Nani	Large nut	Uzbekistan
Gvardiesky	High kernel quality	Uzbekistan
Panfilovets	High kernel quality	Uzbekistan
Tonkoskorlupnii	High kernel quality	Uzbekistan
Ubilini	High yield, high kernel quality, resistance to spring frosts, precocious	Uzbekistan
Bostandik	Large nut, high kernel quality, resistance to spring frosts, precocious	Uzbekistan

References

Abounasri Harvi G (1515) Agriculture guidance (Arshad Al-Zerae). The old manuscript. http://dl.nlai.ir/UI/cf0c4112-7703-4523-93f0-6811d1e20591/LRRView.aspx

Adem HH (2009) Best practice management for establishing a walnut orchard. Department of Primary Industries, Melbourne

Akça Y, Ozongun S (2004) Selection of late leafing, late flowering, laterally fruitful walnut (*Juglans regia*) types in Turkey. N Z J Crop Hortic Sci 32(4):337–342

Akça Y, Polat AA (2007) Present status and future of walnut production in Turkey. Eur J Plant Sci Biotechnol 1(1):57–64

Akça Y, Sütyemez M et al (2016) The new walnut variety breeding program in Turkey. VII international scientific agricultural symposium, Jahorina, Bosnia and Herzegovina, pp 461–466

Aletà N, Olarte C, Truco MJ, Arus P (1990) Identification of walnut cultivars by isozyme analysis. Acta Hortic 284:91–96

Ali AM, Zubair SJ, Abbas AM et al (2016) Genetic diversity among walnuts (*Juglans regia*) population in Kurdistan region-Iraq using AFLP-PCR. ZANCO J Pure Appl Sci 28:50–55

Aly MA, Fjellstrom RG, McGranahan GH et al (1992) Origin of walnut somatic embryos determined by RFLP and isozyme analysis. HortScience 27(1):61–63

Amiri R, Vahdati K, Mohsenipoor S et al (2010) Correlations between some horticultural traits in walnut. HortScience 45:1690–1694

Araji S, Grammer TA, Gertzen R et al (2014) Novel roles for the polyphenol oxidase enzyme in secondary metabolism and the regulation of cell death in walnut. Plant Physiol 164(3):1191–1203

Arab MM, Marrano A, Abdollahi-Arpanahi R et al (2019) Genome-wide patterns of population structure and association mapping of nut-related traits in Persian walnut populations from Iran using the Axiom *J. regia* 700K SNP array. Sci Rep 9(1):6376

Aradhya M, Woeste K, Velasco D (2009) Genetic diversity, structure and differentiation in cultivated walnut (Juglans regia L.). In: VI international Walnut symposium, vol 861, pp 127–132

Arulsekar S, McGranahan GH, Parfitt DE (1986) Inheritance of phosphoglucomutase and esterase isozymes in Persian walnut. J Hered 77(3):220–221

Arzani K, Mansouri-Ardakan H, Vezvaei A et al (2008) Morphological variation among Persian walnut (*Juglans regia*) genotypes from central Iran. N Z J Crop Hortic Sci 36(3):159–168

Asayesh ZM, Vahdati K, Aliniaeifard S (2017a) Investigation of physiological components involved in low water conservation capacity of in vitro walnut plants. Sci Hortic 224:1–7

Asayesh ZM, Vahdati K, Aliniaeifard S et al (2017b) Enhancement of ex vitro acclimation of walnut plantlets through modification of stomatal characteristics in vitro. Sci Hortic 220:114–121

Aslani Aslamarz A, Vahdati K, Rahemi M et al (2009) Estimation of chilling and heat requirements of some Persian walnut cultivars and genotypes. HortScience 44(3):697–701

Aslantaş R (2006) Identification of superior walnut (*Juglans regia*) genotypes in north-eastern Anatolia, Turkey. N Z J Crop Hortic Sci 34(3):231–237

Atefi J (1990) Preliminary research of Persian walnut and correlation between pair characters. Acta Hortic 284:97–104

Atefi J (1993) Evaluation of walnut genotypes in Iran. Acta Hortic 311:24–33

Atefi J (1997) Study on phonological and pomological characters on walnut promising clones in Iran. Acta Hortic 442:101–108

Avanzato D, McGranahan GH, Vahdati K et al (eds) (2014) Following walnut footprints (*Juglans regia* L.): cultivation and culture, folklore and history, traditions and uses. Scripta Horticulturae 17, ISHS

Bâaziz KB, Lopez D, Bouzid S (2012) Early gene expression in the walnut tree occurring during stimulation of leaf hydraulic conductance by irradiance. Biol Plant 56(4):657–666

Bahrami Sirmandi H, Vahdati K (2009) Effect of carbohydrate source and polyethylene glycol on maturation and germination of somatic embryos in walnut (*Juglans regia* L.). Acta Hortic 839:165–172

Bai WN, Liao WJ, Zhang DY (2010) Nuclear and chloroplast DNA phylogeography reveal two refuge areas with asymmetrical gene flow in a temperate walnut tree from East Asia. New Phytol 188(3):892–901

Bai WN, Yan PC, Zhang BW (2018) Demographically idiosyncratic responses to climate change and rapid Pleistocene diversification of the walnut genus *Juglans* (Juglandaceae) revealed by whole genome sequences. New Phytol 217(4):1726–1736

Baojun Z, Yonghong G, Liqun H (2010) Overview of walnut culture in China. Acta Hortic 861:39–44

Baumgartner K, Fujiyoshi P, Browne GT et al (2013) Evaluating paradox walnut rootstocks for resistance to *Armillaria* root disease. HortScience 48(1):68–72

Bayazit S, Kazan K, Gülbitti S et al (2007) AFLP analysis of genetic diversity in low chill requiring walnut (*Juglans regia* L.) genotypes from Hatay, Turkey. Sci Hortic 111(4):394–398

Bernard A, Lheureux F, Dirlewanger E (2018) Walnut: past and future of genetic improvement. Tree Genet Genomes 14(1):1–28

Bollersen V (2017) Revival der walnuss: neues und altes wissen zum walnussanbau in Deutschland. OLV Organic Farming Publication, Germany

Bourre JM (2005) Dietary omega-3 fatty acids and psychiatry: mood, behavior, stress, depression, dementia and aging. J Nutr Health Aging 9(1):31–38

Britton M, Leslie C, McGranahan G et al (2007) Analysis of genes expressed in nematode–infected walnut plants (Unpublished raw data)

Britton MT, Leslie CA, McGranahan GH et al (2009) Functional genomic analysis of walnut-nematode interactions. Walnut Research Reports Database

Busov VB, Rink G, Woeste K (2002) Allozyme variation and mating system of black walnut (*Juglans nigra* L.) in the central hardwood region of the United States. For Genet 9(4):315–322

Chakraborty S, Britton M, Martínez-García PJ et al (2016) Deep RNA-Seq profile reveals biodiversity, plant-microbe interactions and a large family of NBS-LRR resistance genes in walnut (*Juglans regia*) tissues. AMB Express 6(1):12

Chalupa V (1981) Clonal propagation of broad-leaved forest trees in vitro. Commun Inst Cech 12:255–271

Cheema J, Dicks J (2009) Computational approaches and software tools for genetic linkage map estimation in plants. Brief Bioinform 10(6):595–608

Chen L-H, Hu T-X, Zhang F, Li G–H (2008) Genetic diversities of four *Juglans* populations revealed by AFLP in Sichuan province, China. J Plant Ecol 32:1362–1372

Chen L-H, Hu T-X, Zhang F (2009) AFLP analysis on genetic diversity of *Juglans* populations in dry and dry-hot valleys of Sichuan province. J Fruit Sci 26:48–54

Chen C-M, Han S-J, Yuan S-S et al (2013) Isolation and characterization of 20 polymorphic microsatellite markers for *Juglans mandshurica* (Juglandaceae). Appl Plant Sci 1:1–4

Chen X, Xu L, Zhang SL, Liu ZQ (2014) Walnut genebank in China national clonal plant germplasm repository. Acta Hortic 1050:89–94

Cheng SZ, Yang WH (1987) Taxonomic studies of ten species of the genus *Juglans* based on isozymic zymograms. Acta Hortic Sin 14(2):90–96

Choi S, Wing RA (2000) The construction of bacterial artificial chromosome (BAC) libraries. Plant Mol Biol Man H5:1–28

Christopoulos MV, Rouskas D, Tsantili E et al (2010) Germplasm diversity and genetic relationships among walnut (*Juglans regia* L.) cultivars and Greek local selections revealed by inter-simple sequence repeat (ISSR) markers. Sci Hortic 125(4):584–592

Ciarmiello LF, Piccirillo P, Pontecorvo G et al (2011) A PCR based SNPs marker for specific characterization of English walnut (*Juglans regia* L.) cultivars. Mol Biol Rep 38(2):1237–1249

Colaric M, Veberic R, Solar A et al (2005) Phenolic acids, syringaldehyde, and juglone in fruits of different cultivars of *Juglans regia* L. J Agr Food Chem 53(16):6390–6396

Cornu D (1988) Somatic embryogenesis in tissue culture of walnut (*Juglans regia*, *J. major* and hybrids *J. nigra* x *J. regia*). In: Ahuja MR (ed) Somatic cell genetics of woody plants. Kluwer Academic Publishers, Boston, pp 45–49

Cornu D (1989) Walnut somatic embryogenesis, physiological and histological aspects. Ann Sci For 46S:133–135

Cornu D, Jay-Allemand C (1989) Micropropagation of hybrid walnut trees (*Juglans nigra* x *Juglans regia*) through culture and multiplication of embryos. Ann Sci For 46S:113–135

Cossio F, Minolta G (1983) Prove preliminary di coltura in vitro di embrioni isolati di noce (*Juglans regia* L). e confronto tra differenti combinazioni di Sali minerali. Rivista Ortoflorofrutticoltora Italiana 67:287–298

Conesa A, Götz S (2008) Blast2GO: A comprehensive suite for functional analysis in plant genomics. Int J Plant Genomics 2008: 619832

Dandekar AM, Martin LA, McGranahan G (1988) Genetic transformation and foreign gene expression in walnut tissue. J Am Soc Hortic Sci (USA)

Dandekar AM, McGranahan GH, Vail PV et al (1994) Low levels of expression of wild type *Bacillus thuringiensis* var. *Kurstaki* cryIA (c) sequences in transgenic walnut somatic embryos. Plant Sci 1;96(1-2):151–162

Dandekar AM, McGranahan GH, Vail PV et al (1998) High levels of expression of full-length *cry*IA (c) gene from *Bacillus thuringiensis* in transgenic somatic walnut embryos. Plant Sci 131(2):181–193

Dandekar A, Leslie C, McGranahan G (2005) *Juglans regia* walnut. In: Litz RE (ed) Biotechnology of fruit and nut crops. CABI Publisher, Cambridge, pp 307–324

Dang M, Liu Z-X, Chen X et al (2015) Identification, development, and application of 12 polymorphic EST-SSR markers for an endemic Chinese walnut (*Juglans cathayensis* L.) using next-generation sequencing technology. Biochem Syst Ecol 60:74–80

Dang M, Zhang T, Hu Y et al (2016) De Novo assembly and characterization of bud, leaf and flowers Transcriptome from *Juglans regia* L. for the identification and characterization of new EST-SSRs. Forests 7(10):247–263

Dangl GS, Woeste K, Aradhya MK et al (2005) Characterization of 14 microsatellite markers for genetic analysis and cultivar identification of walnut. J Am Soc Hortic Sci 130:348–354

Daniell H, Lin CS, Yu M et al (2016) Chloroplast genomes: diversity, evolution, and applications in genetic engineering. Genome Biol 17(1):134

Deng MD, Cornu D (1992) Maturation and germination of walnut somatic embryos. Plant Cell Tissue Organ Cult 28:195–202

Dogan M, Akgul A (2005) Fatty acid composition of some walnut (*Juglans regia* L.) cultivars from east Anatolia. Grasas Aceites 56(4):328–331

Dong W, Xu C, Li W et al (2017) Phylogenetic resolution in *Juglans* based on complete chloroplast genomes and nuclear DNA sequences. Front Plant Sci 30(8):1148

Dreher ML, Maher CV, Kearney P (1996) The traditional and emerging role of nuts in healthful diets. Nutr Rev 54(8):241–245

Driver JA (1985) Direct field rooting and acclimatization of tissue–culture cuttings. In Vitro Cell Dev Biol 21(3):57

Driver JA (1986) Method for acclimatizing and propagating plant tissue culture shoots. U.S. Patent No. 4,612,725. 23 Sep 1986

Driver JA, Kuniyuki AH (1984) *In vitro* propagation of Paradox walnut *Juglans hindsii* × *Juglans regia* rootstock. HortScience 19:507–509

Dvorak J, Luo MC, Aradhya M et al (2008) Walnut genome analysis. Walnut Research Reports Database

Dvorak J, Luo MC, Aradhya M et al (2011) Walnut genome analysis. Walnut Research Reports Database

Dvorak J, Aradhaya M, Leslie C et al (2015) Discovery of the causative mutation of the lateral bearing phenotype in walnut. Walnut Research Reports Database

Ebrahimi A, Fatahi R, Zamani Z (2011) Analysis of genetic diversity among some Persian walnut genotypes (*Juglans regia* L.) using morphological traits and SSRs markers. Sci Hortic 130(1):146–151

Ebrahimi A, Khadivi–Khub A, Nosrati Z et al (2015) Identification of superior walnut (*Juglans regia*) genotypes with late leafing and high kernel quality in Iran. Sci Hortic 193:195–201

Ebrahimi A, Zarei A, McKenna JR et al (2017) Genetic diversity of Persian walnut (*Juglans regia*) in the cold temperate zone of the United States and Europe. Sci Hortic 220:36–41
El Euch C, Jay-Allemand C, Pastuglia et al (1998) Expression of antisense chalcone synthase RNA in transgenic hybrid walnut microcuttings. Effect on flavonoid content and rooting ability. Plant Mol Biol 38:467–479
Emilia M, Spada M, Beritognolo I et al (1995) Differentiation of walnut hybrids (*Juglans nigra* L. X *Juglans regia* L.) through RAPD markers. III Int Walnut Congr 442(13):43–52
Ertürk U, Akça Y (2014) Overview of walnut culture in Turkey. Acta Hortic 1050:369–372
Erturk UM, Dalkilic ZE (2011) Determination of genetic relationship among some walnut (Juglans regia L.) genotypes and their early-bearing progenies using RAPD markers. Rom Biotechnol Lett 16(1):5944–5952
Escobar MA, Park JI, Polito VS et al (2000) Using GFP as a scorable marker in walnut somatic embryo transformation. Ann Bot 85(6):831–835
Escobar MA, Civerolo EL, Summerfelt KR et al (2001) RNAi-mediated oncogene silencing confers resistance to crown gall tumorigenesis. Proc Natl Acad Sci 98(23):13437–13442
Escobar MA, Leslie CA, McGranahan GH et al (2002) Silencing crown gall disease in walnut (*Juglans regia* L.). Plant Sci 163(3):591–597
Escobar MA (2013) RNA-seq and metabolite profiling reveal novel functions for the polyphenol oxidase enzyme in walnut (*Juglans regia*). Plant and animal genome XXI conference, San Diego, CA
FAO (2016) FAO statistical yearbook. Agricultural production. Food and Agriculture Organization of the United Nations. http://www.fao.org/faostat/en/#data/QC
Fatahi R, Ebrahimi A, Zamani Z (2010) Characterization of some Iranians and foreign walnut genotypes using morphological traits and RAPD markers. Hortic Environ Biotechnol 51(1):51–60
Famula RA, Richards JH, Famula TR et al (2019) Association genetics of carbon isotope discrimination and leaf morphology in a breeding population of *Juglans regia* L. Tree Genet Genomes 15(1):6
Farooqui A, Khan A, Borghetto I et al (2015) Synergistic antimicrobial activity of *Camellia sinensis* and *Juglans regia* against multidrug-resistant bacteria. PloS one 10(2):e0118431
Feng Y, Zhang Z, Zhang S et al (2011) Development of walnut EST-SSR markers and primer design. Agric Sci Tech-Hunan 12(12):1810–1813
Fjellstrom RG, Parfitt DE (1994a) RFLP inheritance and linkage in walnut. Theor Appl Genet 89(6):665–670
Fjellstrom RG, Parfitt DE (1994b) Walnut (*Juglans* spp.) genetic diversity determined by restriction fragment length polymorphisms. Genome 37(4):690–700
Fjellstrom RG, Parfitt DE, McGranahan GH (1994) Genetic relationships and characterization of Persian walnut (*Juglans regia* L.) cultivars using restriction fragment length polymorphisms (RFLPs). J Am Soc Hortic Sci 119(4):833–839
Fornari B, Malvolti ME, Taurchini D et al (2001) Isozyme and organellar DNA analysis of genetic diversity in natural/naturalised European and Asiatic walnut (*Juglans regia* L.) populations. Acta Hortic 544:167–178
Forde HI (1975) Walnuts. In: Janick J, Moore JN (eds) Advances in Fruit Breeding. Purdue University Press, West Lafayette, pp 439–455
Foroni I, Rao R, Woeste K et al (2005) Characterization of *Juglans regia* L. with SSR markers and evaluation of genetic relationships among cultivars and the 'Sorrento' landrace. J Hortic Sci Biotechnol 80(1):49–53
Foroni I, Woeste K, Monti LM et al (2007) Identification of "Sorrento" walnut using simple sequence repeats (SSRs). Genet Resour Crop Evol 54(5):1081–1094
Francesca PI, Pamfil DO, Raica P et al (2010) Assessment of the genetic variability among some *Juglans* cultivars from the Romanian National Collection at SCDP Valcea using RAPD markers. Rom Biotechnol Lett 15(1):41–49
Gady AL, Hermans FW, Van de Wal MH et al (2009) Implementation of two high through-put techniques in a novel application: detecting point mutations in large EMS mutated plant populations. Plant Methods 5(1):13

Gandev S (2007) Budding and grafting of the walnut (*Juglans regia* L.) and their effectiveness in Bulgaria (Review). Bulgarian J Agric Sci 13:683–689

Germain E (1990) Inheritance of late leafing and lateral bud fruitfulness in walnut, phenotypic correlations among some traits of the trees. Acta Hortic 284:125–134

Germain E (1997) Genetic improvement of the Persian walnut (*Juglans regia* L.). Acta Hortic 442:21–32

Germain E (1999) Le Noyer. Centre Technique Interpr. des Fruits et Legumes (CTIFL) Publication 280

Germanà M (2012) Use of irradiated pollen to induce parthenogenesis and haploid production in fruit crops. In: Shu QY, Brian PF, Hitoshi N, Hitoshi N (eds) Plant mutation breeding and biotechnology. CABI Press, Wallingford, pp 409–419

Godwin ID, Aitken EA, Smith LW (1997) Application of inter simple sequence repeat (ISSR) markers to plant genetics. Electrophoresis 18(9):1524–1528

Götz S, García-Gómez JM, Terol J et al (2008) High-throughput functional annotation and data mining with the Blast2GO suite. Nucleic Acids Res 36(10):3420–3435

Gruselle R, Badia N, Boxus P (1987) Walnut micropropagation: first results. Acta Hortic 212:511–516

Gunn BF, Aradhya M, Salick JM et al (2010) Genetic variation in walnuts (*Juglans regia* and *J. sigillata*; Juglandaceae): species distinctions, human impacts, and the conservation of agrobiodiversity in Yunnan, China. Am J Bot 97(4):660–671

Han H, Woeste KE, Hu Y et al (2016) Genetic diversity and population structure of common walnut (*Juglans regia*) in China based on EST-SSRs and the nuclear gene phenylalanine ammonia-lyase (PAL). Tree Genet Genomes 12(6):111

Hansche PE, Beres V, Forde HI (1972) Estimates of quantitative genetic properties of walnut and their implications for cultivar improvement. J Am Soc Hortic Sci 97:279–285

Hartmann HT, Kester DE, Davies FT, Geneve RL (1997) Plant propagation: principles and practices, 6th edn. Prentice Hall International, New York

Hasey JK, Westerdahl BB, Micke W et al (2001) Yield performance of own-rooted 'Chandler' walnut versus 'Chandler' walnut on Paradox rootstock. Acta Hortic 544:489–493

Hassani D, Dastjerdi R, Haghjooyan R et al (2014) Genetic improvement of Persian walnut (*Juglans regia* L.) in Iran. Acta Hortic 1050:95–102

He F, Wang H, Zhang Z et al (2010) Identification of walnut cultivars with AFLP fingerprinting. Acta Hortic 861:151–154

He J, Zhao X, Laroche A et al (2014) Genotyping-by-sequencing (GBS), an ultimate marker–assisted selection (MAS) tool to accelerate plant breeding. Front Plant Sci 30(5):484

Hestekin CN, Jakupciak JP, Chiesl TN et al (2006) An optimized microchip electrophoresis system for mutation detection by tandem SSCP and heteroduplex analysis for p53 gene exons 5–9. Electrophoresis 27(19):3823–3835

Hoban S, Anderson R, McCleary T et al (2008) Thirteen nuclear microsatellite loci for butternut (*Juglans cinerea* L.). Mol Ecol Resour 8(3):643–646

Hu YH, Zhao P, Zhang Q et al (2015) De novo assembly and characterization of transcriptome using Illumina sequencing and development of twenty five microsatellite markers for an endemic tree *Juglans hopeiensis* Hu in China. Biochem Syst Ecol 63:201–211

Hu Y, Woeste KE, Dang M et al (2016) The complete chloroplast genome of common walnut (*Juglans regia*). Mitochondrial DNA B 1(1):189–190

Hu Y, Dang M, Feng X et al (2017a) Genetic diversity and population structure in the narrow endemic Chinese walnut *Juglans hopeiensis* Hu: implications for conservation. Tree Genet Genomes 13(4):91

Hu Y, Woeste KE, Zhao P (2017b) Completion of the chloroplast genomes of five Chinese *Juglans* and their contribution to chloroplast phylogeny. Front Plant Sci 6(7):1955

Huang W-Y, Davidge ST, Wu J (2013) Bioactive natural constituents from food sources-potential use in hypertension prevention and treatment. Crit Rev Food Sci Nutr 53:615–630

IHGSC (2004) International human genome sequencing consortium: finishing the euchromatic sequence of the human genome. Nature 431(7011):931–945
Ikhsan AS, Topçu H, Sütyemez M et al (2016) Novel 307 polymorphic SSR markers from BAC-end sequences in walnut (*Juglans regia* L.): effects of motif types and repeat lengths on polymorphism and genetic diversity. Sci Hortic 213:1–4
Iwata H, Minamikawa MF, Kajiya-Kanegae H et al (2016) Genomics-assisted breeding in fruit trees. Breed Sci 66(1):100–115
Jahanbani R, Ghaffari SM, Salami M et al (2016a) Antioxidant and anticancer activities of walnut (*Juglans regia* L.) protein hydrolysates using different proteases. Plant Foods Hum Nutr 71(4):402–409
Jahanbani R, Ghaffari SM, Vahdati K et al (2016b) Kinetics study of protein hydrolysis and inhibition of angiotensin converting enzyme by peptides hydrolysate extracted from walnut. Int J Pept Res Ther 24:77–85
Jahanbani R, Ghaffari M, Vahdati K et al (2018) Kinetics study of protein hydrolysis and inhibition of angiotensin converting enzyme by peptides hydrolysate extracted from walnut. Int J Pept Res Ther 24(1):77–85
Jay-Allemand C, Capelli P, Cornu D (1992) Root development of in vitro hybrid walnut microcuttings in a vermiculite–containing gelrite medium. Sci Hortic 51(3–4):335–342
Ji A, Wang Y, Wu G et al (2014) Genetic diversity and population structure of North China mountain walnut revealed by ISSR. Am J Plant Sci 5(21):3194–3202
Kafkas S, Ozkan H, Sütyemez M (2005) DNA polymorphism and assessment of genetic relationships in walnut genotypes based on AFLP and SAMPL markers. J Am Soc Hortic Sci 130:585–590
Karimi R, Ershadi A, Vahdati K et al (2010) Molecular characterization of Persian walnut populations in Iran with microsatellite markers. HortScience 45:1403–1406
Karimi R, Ershadi A, Ehteshamnia A et al (2014) Morphological and molecular evaluation of Persian walnut populations in northern and western regions of Iran. J Nuts 2:21–31
Keqiang Y, Yuejin W, Yindong Z et al (2002) RAPD analysis for the identification of the precocious trait in walnuts. Acta Hortic Sin 29:573–574
Khan MW, Khan IA, Ahmad H et al (2010) Estimation of genetic diversity in walnut. Pak J Bot 42:1791–1796
Kluepfel D, Leslie C, Aradhya M et al (2015) Development of disease-resistant walnut rootstocks: integration of conventional and genomic approaches. Walnut Research Reports Database
Laurens F, Aranzana MJ, Arus P et al (2018) An integrated approach for increasing breeding efficiency in apple and peach in Europe. Hortic Res 5(1):1–14
Lecouls AC, Bergougnoux V, Rubio-Cabetas MJ (2004) Marker-assisted selection for the wide-spectrum resistance to root-knot nematodes conferred by the Ma gene from Myrobalan plum (*Prunus cerasifera*) in interspecific *Prunus* material. Mol Breed 13(2):113–124
Lee BC, Shim SY, Lee SK (1988) Mass propagation and germination of somatic embryos in *Juglans regia* L. (English walnut). Res Rep Inst Genet Korea 24:99–106
Leslie CA (2016) New walnut varieties. UC Davis walnut improvement program. Department of Plant Science, Sacramento Solano Yolo Walnut Day, February 23rd 2016. http://ccfruitandnuts.ucanr.edu/files/239180.pdf
Leslie CA, McGranahan GH (2014) The California walnut improvement program: scion breeding and rootstock development. Acta Hortic 1050:81–88
Leslie CA, McGranahan GH, Hackett W et al (2009) Walnut improvement programs. Walnut Research Reports, University of California, Davis
Li Z, Lanying Z, Qianwen X (2007) Identification of RAPD markers linked to thickness gene of shuck in walnut. Adv Biol Res 1:137–140
Li W, Ma M, Sun C et al (2010) Development of a SCAR marker linked to precocious trait in walnut (*Juglans regia*). Sci Silvae Sin 46(3):56–61
Li Y, Luo X, Wu C (2017) Comparative transcriptome analysis of genes involved in anthocyanin biosynthesis in red and green walnut (*Juglans regia* L.). Molecules 23(1):25

Liu C, Shi L, Zhu Y et al (2012) CpGAVAS, an integrated web server for the annotation, visualization, analysis, and GenBank submission of completely sequenced chloroplast genome sequences. BMC Genomics 13(1):715

Liu X, Walawage SL, Leslie CA et al (2017) In vitro gene expression and mRNA translocation from transformed walnut (*Juglans regia*) rootstocks expressing DsRED fluorescent protein to wild-type scions. Plant cell Rep 36(6):877–885

Long LM, Preece JE, Van Sambeek JW (1995) Adventitious regeneration of *Juglans nigra* L. (eastern black walnut). Plant Cell Rep 8:512–516

Luo MC, Thomas C, You FM et al (2003) High-throughput fingerprinting of bacterial artificial chromosomes using the snapshot labeling kit and sizing of restriction fragments by capillary electrophoresis. Genomics 82(3):378–389

Luo R, Liu B, Xie Y et al (2012) SOAPdenovo2: an empirically improved memory-efficient short-read de novo assembler. Gigascience 1(1):18

Luo M-C, You FM, Li P et al (2015) Synteny analysis in Rosids with a walnut physical map reveals slow genome evolution in long-lived woody perennials. BMC Genomics 16(1):707

Ma Q, Zhang J, Pei D (2011) Genetic analysis of walnut cultivars in China using fluorescent amplified fragment length polymorphism. J Am Soc Hortic Sci 136:422–428

Maguire LS, OSullivan SM, Galvin K et al (2004) Fatty acid profile, tocopherol, squalene and phytosterol content of walnuts, almonds, peanuts, hazelnuts and the macadamia nut. Int J Food Sci Nutr 55(3):171–178

Mahmoodi R, Rahmani F, Rezaee R (2013) Genetic diversity among *Juglans regia* L. genotypes assessed by morphological traits and microsatellite markers. Span J Agric Res 11(2):431–437

Maluszynski M, Kasha KJ, Forster BP et al (2003) Doubled haploid production in crop plants: a manual. Kluwer Academic Publishers, Dordrecht

Malvolti ME, Paciucci M, Cannata F et al (1993) Genetic variation in Italian populations of *Juglans regia* L. Acta Hortic 311:86–94

Malvolti ME, Fineschi S, Pigliucci M (1994) Morphological integration and genetic variability in *Juglans regia* L. J Hered 85(5):389–394

Malvolti ME, Fornari B, Maccaglia E et al (2001) Genetic linkage mapping in an intraspecific cross of walnut (*Juglans regia* L.) using molecular markers. Acta Hortic 544:179–185

Malvolti ME, Pollegioni P, Bertani A et al (2010) *Juglans regia* provenance research by molecular, morphological and biochemical markers: a case study in Italy. Biorem Biodiv Bioavail 4:84–92

Martínez-García PJ, Crepeau M, Puiu D et al (2014) Application of marker breeding in the walnut improvement program. Walnut Research Reports Database

Martínez-García PJ, Crepeau MW, Puiu D et al (2016) The walnut (*Juglans regia*) genome sequence reveals diversity in genes coding for the biosynthesis of non-structural polyphenols. Plant J 87(5):507–532

Martínez-García PJ, Famula RA, Leslie C et al (2017) Predicting breeding values and genetic components using generalized linear mixed models for categorical and continuous traits in walnut (*Juglans regia*). Tree Genet Genomes 13(5):109

Marrano A, Martínez-García PJ, Bianco L et al (2019) A new genomic tool for walnut (Juglans regia L.): development and validation of the high-density Axiom™ J. regia 700K SNP genotyping array. Plant Biotechnol J 17(6):1027–1036

Mba C (2013) Induced mutations unleash the potentials of plant genetic resources for food and agriculture. Agronomy 3(1):200–231

Mba C, Afza R, Bado S et al (2010) Induced mutagenesis in plants using physical and chemical agents. In: Plant cell culture, essential methods, vol 20. Chichester, Wiley, pp 111–130

McCallum CM, Comai L, Greene EA et al (2000) Targeting induced local lesions in genomes (TILLING) for plant functional genomics. Plant Physiol 123(2):439–442

McGranahan GH, Catlin PB (1987) *Juglans* rootstocks. In: Rom RC, Carlson RF (eds) Rootstocks for fruit crops. Wiley, New York, pp 411–450

McGranahan GH, Leslie CA, Uratsu SL et al (1988) Agrobacterium-mediated transformation of walnut somatic embryos and regeneration of transgenic plants. Bio/technology 6(7):800

McGranahan GH, Leslie CA (1991) Walnuts (Juglans). Acta Hortic 290:905–951
McGranahan GH, Leslie CA (2004) Three new walnut varieties: Sexton, Gillet and Forde. Walnut Research Reports, University of California, Davis
McGranahan GH, Leslie CA (2005) Advances in genetic improvement of walnut at the University of California, Davis. Acta Hortic 705:117–122
McGranahan GH, Tulecke W, Arulsekar S, Hansen JJ (1986) Intergeneric hybridization in the Juglandaceae: *Pterocarya* sp × *Juglans regia*. J Am Soc Hortic Sci 111:627–630
McGranahan G, Leslie C, Uratsu S et al (1990) Improved efficiency of the walnut somatic embryo gene transfer system. Plant Cell Rep 8:512–516
Milind P, Deepa K (2011) Walnut: not a hard nut to crack. Int Res J Pharm 2(5):8–17
Mochida K, Shinozaki K (2010) Genomics and bioinformatics resources for crop improvement. Plant Cell Physiol 51(4):497–523
Mohsenipoor S, Vahdati K, Amiri R et al (2010) Study of the genetic structure and gene flow in Persian walnut (*Juglans regia* L.) using SSR markers. Acta Hortic 861:133–142
Molnar TJ, Zaurov DE, Capik JM et al (2011) Persian walnut (*Juglans regia* L.) in Central Asia. Annu Rep North Nut Grow Assoc 101:56–69
Muir R, Baek J, Leslie A et al (2004) Analysis of genes expressed in walnut seed coat tissue (Unpublished raw data)
Najafi F, Mardi M, Fakheri B et al (2014) Isolation and characterization of novel microsatellite markers in walnut (*Juglans regia* L.). Am J Plant Sci 5(03):409–415
Navatel JC, Bourrain L (2001) Plant production of walnut *Juglans regia* L. by in vitro multiplication. Acta Hortic 544:465–471
Neale DB, Marrano A, Sideli GM et al (2017) Application of marker breeding in the walnut improvement program (WIP). Walnut Research Reports Database
Nicese FP, Hormaza JI, McGranahan GH (1998) Molecular characterization and genetic relatedness among walnut (*Juglans regia* L.) genotypes based on RAPD markers. Euphytica 101(2):199–206
Nimbolkar PK, Awachare C, Reddy YTN et al (2016) Role of rootstocks in fruit production–a review. J Agric Eng Food Technol 3:183–188
Ninot A, Aleta N (2003) Identification and genetic relationship of Persian walnut genotypes using isozyme markers. J Am Pomol Soc 57(3):106
Noor Shah U, Mir JI, Ahmed N, Fazili KM (2016) Assessment of germplasm diversity and genetic relationships among walnut (*Juglans regia* L.) genotypes through microsatellite markers. J Saudi Soc Agric Sci. https://www.sciencedirect.com/science/article/pii/S1658077X16300662
Obermeier C, Friedt W (2015) Applied oilseed raps marker technology and genomics. In: Poltronieri P, Hong Y (eds) Applied plant genomics and biotechnology (No. 72). Woodhead Publishing, Cambridge, pp 253–295
Ogbu JU (2014) Genetic resources and biodiversity conservation in Nigeria through biotechnology approaches. In: Ahuja M, Ramawat K (eds) Biotechnology and biodiversity. Sustainable development and biodiversity, vol 4. Springer, Cham, pp 271–285
Ölez H (1971) Studies on the selection of walnut (Juglans regia L.) in the Marmara region. Bahçe 4:7–21
Ozcan A, Bukucu SB, Sutyemez M (2017) Determination of pollen quality and production in new walnut cultivars. Asian J Agric Res 11:93–97
Parry MA, Madgwick PJ, Bayon C et al (2009) Mutation discovery for crop improvement. J Exp Bot 60(10):2817–2825
Paterson AH (1996) Making genetic maps. In: Paterson AH (ed) Genome mapping in plants. Academic, Austin, pp 23–39
Pathak MR, Abido MS (2014) The role of biotechnology in the conservation of biodiversity. J Exp Biol 2(4):352–363
Pereira JA, Oliveira I, Sousa A et al (2008) Bioactive properties and chemical composition of six walnut (*Juglans regia* L.) cultivars. Food Chem Toxicol 46(6):2103–2111

Polito VS, McGranahan GH, Pinney K, Leslie CA (1989) Origin of somatic embryos from repetitively embryogenic cultures of walnut (*Juglans regia* L.): implications for *Agrobacterium*-mediated transformation. Plant Cell Rep 8:219–221
Pollegioni P, Bartoli S, Cannata F et al (2003) Genetic differentiation of four Italian walnut (*Juglans regia* L.) varieties by intersimple sequence repeat (ISSR). J Genet Breed 57:231–240
Pollegioni P, Woeste K, Major A et al (2008) Characterization of Juglans nigra L., Juglans regia L. and Juglans ×intermedia (Carr.) by SSR markers:a case study in Italy. Silvae Genet 57:68–78
Pollegioni P, Woeste K, Mugnozza GS et al (2009) Retrospective identification of hybridogenic walnut plants by SSR fingerprinting and parentage analysis. Mol Breed 24(4):321–335
Pollegioni P, Woeste K, Olimpieri I et al (2011) Long-term human impacts on genetic structure of Italian walnut inferred by SSR markers. Tree Genet Genomes 7(4):707–723
Pollegioni P, Woeste K, Chiocchini F et al (2017) Rethinking the history of common walnut (*Juglans regia* L.) in Europe: its origins and human interactions. PLoS One 12(3):0172541
Pop IF, Pamfil D, Raica P et al (2010) Assessment of the genetic variability among some *Juglans* cultivars from the Romanian National Collection at S.C.D.P. Vâlcea using RAPD markers. Rom Biotechnol Lett 15:41–49
Pop IF, Vicol AC, Botu M et al (2013) Relationships of walnut cultivars in a germplasm collection: comparative analysis of phenotypic and molecular data. Sci Hortic 153:124–135
Potter D, Gao F, Aiello G et al (2002) Intersimple sequence repeat markers for fingerprinting and determining genetic relationships of walnut (*Juglans regia*) cultivars. J Am Soc Hortic Sci 127:75–81
Qi J, Hao Y, Zhu Y et al (2011) Studies on germplasm of *Juglans* by ESTSSR markers. Acta Hortic Sin 38:441–448
Qianwen X, Kaizhi W, Lanying Z et al (2010) RAPD markers and heterotic effect of walnut quality in Sichuan of China. Adv Biol Res 4(2):81–85
Qing Guo M, Jing Q, Dong P (2010) FISH–AFLP analysis of genetic diversity of early-fruiting walnut cultivars. For Res 23:631–636
Quail MA, Smith M, Coupland P et al (2012) A tale of three next generation sequencing platforms: comparison of ion torrent, pacific biosciences and Illumina MiSeq sequencers. BMC Genomics 13(1):341
Ramos DE (1997) Walnut production manual, vol 3373. UCANR Publications, Oakland
Ramos D, Doyle J (1984) Walnut research and industry survey – France. Walnut Research Reports, University of California, Davis, pp 49–55
Rao G, Sui J, Zhang J (2016) Metabolomics reveals significant variations in metabolites and correlations regarding the maturation of walnuts (*Juglans regia* L.). Biol Open 5(6):829–836
Revilla MA, Majada J, Rodriguez R (1989) Walnut (*Juglans regia* L.) micropropagation. For Tree Physiol 46:149–151
Rikkerink EH, Oraguzie NC, Gardiner SE (2007) Prospects of association mapping in perennial horticultural crops. In: Association mapping in plants. Springer, New York, pp 249–269
Ripetti V, Kevers CL, Gaspar T (1994) Two successive media for the rooting of walnut shoots *in vitro*. Changes in peroxidases activity and in ethylene production. Adv Hortic Sci 8:29–32
Robichaud RL, Glaubitz JC, Rhodes OE et al (2006) A robust set of black walnut microsatellites for parentage and clonal identification. New For 32(2):179–196
Rodriguez R (1982) Stimulation of multiple shoot-bud formation in walnut seeds [Vegetative propagation, *Juglans regia*]. HortScience (USA)
Rodriguez R, Lopez C, Diaz-Sala C, Berros B (1993) Simultaneous shoot-bud development on walnut tissues of different ages: macro morphological and histological analyses. Acta Hortic 311:141–152
Roor W, Konrad H, Mamadjanov D et al (2017) Population differentiation in common walnut (*Juglans regia* L.) across major parts of its native range-insights from molecular and morphometric data. J Hered 108(4):391–404
Ros E, Nunez I, Perez-Heras A, Serra M, Gilabert R et al (2004) A walnut diet improves endothelial function in hypercholesterolemic subjects: a randomized crossover trial. Circulation 109:1609–1614

Ross-Davis A, Huang Z, McKenna J (2008) Morphological and molecular methods to identify butternut (*Juglans cinerea*) and butternut hybrids: relevance to butternut conservation. Tree Physiol 28(7):1127–1133
Ru S, Main D, Evans K et al (2015) Current applications, challenges, and perspectives of marker–assisted seedling selection in Rosaceae tree fruit breeding. Tree Genet Genomes 11(1):8
Ruiz-Garcia L, Lopez-Ortega G, Denia AF, Tomas DF (2011) Identification of a walnut (*Juglans regia* L.) germplasm collection and evaluation of their genetic variability by microsatellite markers. Span J Agric Res 9(1):179–192
Saadata YA, Hennerty MJ (2002) Factors affecting the shoot multiplication of Persian walnut (*Juglans regia* L.). Sci Hortic 95:251–260
Sadat Hosseini Grouh MS, Vahdati K, Lotfi M et al (2011) Production of haploids in Persian walnut through parthenogenesis induced by gamma-irradiated pollen. J Am Soc Hortic Sci 136(3):198–204
Sarikhani Khorami S, Arzani K, Karimzadeh G et al (2018) Genome size; a novel predictor of nut weight and nut size of walnut trees. HortScience 53(3):275–282
Scheben A, Batley J, Edwards D (2017) Genotyping-by-sequencing approaches to characterize crop genomes: choosing the right tool for the right application. Plant Biotechnol J 15(2):149–161
Semagn K, Bjørnstad A, Ndjiondjop MN (2006) Principles, requirements and prospects of genetic mapping in plants. Afr J Biotechnol 5:2569–2587
Sheikh Beig Goharrizi MA, Dejahang A, Tohidfar M et al (2016) Agrobacterium mediated transformation of somatic embryos of Persian walnut using *fld* gene for osmotic stress tolerance. J Agric Sci Technol 18:423–435
Shu QY, Forster BP, Nakagawa H et al (2012a) Principles and applications of plant mutation breeding. In: Plant mutation breeding and biotechnology. CABI, Wallingford, pp 301–325. https://www.cabi.org/bookshop/book/9781780640853
Shu QY, Shirasawa K, Hoffmann M et al (2012b) Molecular techniques and methods for mutation detection and screening in plants. In: Plant mutation breeding and biotechnology. CABI/FAO, Oxfordshire, pp 241–256
Singh RK, Mishra GP, Thakur AK et al (2008) Molecular markers in plants. In: Singh RK, Singh R, Ye G et al (eds) Molecular plant breeding: principle, method and application. Studium Press LLC, Houston, pp 35–78
Siqueira APS, Pacheco MTB, Naves MMV (2015) Nutritional quality and bioactive compounds of partially defatted baru almond flour. Food Sci Technol 35:127–132
Solar A, Colarič M, Usenik V et al (2006) Seasonal variations of selected flavonoids, phenolic acids and quinones in annual shoots of common walnut (*Juglans regia* L.). Plant Sci 170(3):453–461
Solar A, Smole J, Stampar F, Virščček-Marn M (1994) Characterization of isozyme variation in walnut (*Juglans regia* L.). Euphytica 77(1–2):105–112
Sommers PW, Van Sambeek JW, Preece JE et al (1982) *In vitro* micropropagation of black walnut. In: Proceedings of the 7th North America forest biology. University Kentucky Press, Lexington, pp 224–230
Stevens KA, Woeste K, Chakraborty S et al (2018) Genomic variation among and within six *Juglans* species. G3: Genes Genomes Genet 8:2153–2165
Suo Z, Chen L, Pei D et al (2015) A new nuclear DNA marker from ubiquitin ligase gene region for genetic diversity detection of walnut germplasm resources. Biotechnol Rep 5(1):40–45
Sütyemez M (2006) Comparison of AFLP polymorphism in progeny derived from dichogamous and homogamous walnut genotypes. Pak J Biol Sci 9:2303–2307
Szarejko I (2012) Haploid mutagenesis. In: Plant mutation breeding and biotechnology. CABI, Wallingford, pp 387–410
Taheri S, Abdullah TL, Ahmad Z et al (2014) Effect of acute gamma irradiation on *Curcuma alismatifolia* varieties and detection of DNA polymorphism through SSR marker. Biomed Res Int 2014:631813
Taheri S, Abdullah TL, Jain SM et al (2017) TILLING, high-resolution melting (HRM), and next–generation sequencing (NGS) techniques in plant mutation breeding. Mol Breed 37(3):40

Tang H, Ren Z, Reustle G, Krczal G (2001) Optimizing secondary somatic embryo production in English walnut (*Juglans regia* L.). Acta Hortic 544:187–194
Tanksley SD, Young ND, Paterson AH et al (1989) RFLP mapping in plant breeding: new tools for an old science. Nat Biotechnol 7(3):257
Topçu H, Ikhsan AS, Sütyemez M et al (2015) Development of 185 polymorphic simple sequence repeat (SSR) markers from walnut (*Juglans regia* L.). Sci Hortic 194:160–167
Tulecke W, McGranahan GH (1985) Somatic embryogenesis and plant regeneration from cotyledons of walnut, *Juglans regia* L. Plant Sci 40:57–63
Tulecke W, McGranahan G, Ahmadi H (1988) Regeneration by somatic embryogenesis of triploid plants from endosperm of walnut, *Juglans regia* L. cv Manregian. Plant cell Rep 7(5):301– 304
Tulecke W, McGranahan G (1994) The walnut germplasm collection of the University of California, Davis. A description of the collection and a history of the breeding program of Eugene F Serr and Harold I Forde. Report no. 13. University of California Genetic Resources Conservation Program, Davis, CA
Tulecke W, McGranahan G, Ahmadi H (1988) Regeneration by somatic embryogenesis of triploid plants from endosperm of walnut, *Juglans regia* L. cv Manregian. Plant Cell Rep 7(5):301–304
Vahdati K (2000) Walnut situation in Iran. Nucis Newsl 9:32–33
Vahdati K (2014) Walnut tolerance to abiotic stresses: approaches and prospects. Acta Hortic 1050:399–406
Vahdati K, Mohseniazar M (2016) Early bearing genotypes of walnut: a suitable material for breeding and high–density orchards. Acta Hortic 1139(2):101–106
Vahdati K, Rezaee R (2014) Behavior of some early mature and dwarf persian walnut trees in Iran. Acta Hortic 1050:189–196
Vahdati K, Leslie C, Zamani Z, McGranahan G (2004) Rooting and acclimatization of in vitro-grown shoots from mature trees of three Persian walnut cultivars. HortScience 39(2):324–327
Vahdati K, Jariteh M, Niknam V et al (2006) Somatic embryogenesis and embryo maturation in Persian walnut. Acta Hortic 705:199–205
Vahdati K, Bayat S, Ebrahimzadeh H et al (2008) Effect of exogenous ABA on somatic embryo maturation and germination in Persian walnut (Juglans regia L.). Plant Cell Tissue Organ Cult 93(2):163–171
Vahdati K, Hassani D, Rezaee R et al (2014) Following walnut footprints (*Juglans regia* L.) cultivation and culture, folklore and history, traditions and uses. In: Avanzato D, McGranahan GH, Vahdati K et al (eds) Walnut footprint in Iran. Scripta Horticulturae (ISHS) 17:187–201
Vahdati K, Pourtaklu SM, Karimi R et al (2015) Genetic diversity and gene flow of some Persian walnut populations in southeast of Iran revealed by SSR markers. Plant Syst 301(2):691–699
Vahdati K, Asayesh ZM, Aliniaeifard S et al (2017) Improvement of *ex vitro* desiccation through elevation of CO_2 concentration in the atmosphere of culture vessels during *in-vitro* growth. HortScience 52(7):1006–1012
van Nocker S, Gardiner SE (2014) Breeding better cultivars, faster: applications of new technologies for the rapid deployment of superior horticultural tree crops. Hortic Res 1:14022
Vahdati K, McKenna JR, Dandekar AM et al (2002) Rooting and other characteristics of a transgenic walnut hybrid (*Juglans hindsii*× *J. regia*) rootstock expressing rolABC. J Am Soc Hortic Sci 127(5):724–728
Victory ER, Glaubitz JC, Rhodes OE Jr et al (2006) Genetic homogeneity in *Juglans nigra* (Juglandaceae) at nuclear microsatellites. Am J Bot 93(1):118–126
Vischi M, Chiabà C, Raranciuc S et al (2017) Genetic diversity of walnut (*Juglans regia* L.) in the Eastern Italian Alps. Forests 8(3):81
Vos P, Hogers R, Bleeker M et al (1995) AFLP: a new technique for DNA fingerprinting. Nucleic Acids Res 23(21):4407–4414
Vyas D, Sharma SK, Sharma DR (2003) Genetic structure of walnut genotype using leaf isozymes as variability measure. Sci Hortic 97(2):141–152
Wang H, Pei D, Gu R et al (2008) Genetic diversity and structure of walnut populations in central and southwestern China revealed by microsatellite markers. J Am Soc Hortic Sci 133:197–203
Wang H, Zhao S, Zhang Z et al (2010) Genetic relationship and diversity of eight *Juglans* species in China estimated through AFLP analysis. Acta Hortic 861:143–150

Wang G, Wang JY, He Q et al (2014) Agronomic evaluation and heritability of Jin RS–2 and Jin RS–3 walnut rootstocks. Acta Hortic 1050:113–121

Wang H, Wu W, Pan G et al (2015) Analysis of genetic diversity and relationships among 86 Persian walnut (*Juglans regia* L.) genotypes in Tibet using morphological traits and SSR markers. J Hortic Sci Biotechnol 90(5):563–570

Wani N, Bhat MA, Ahmad MF et al (2010) Molecular markers and their application in walnut improvement. Int J Curr Res 3:6–11

Walawage SL, Britton MT, Leslie CA et al (2013) Stacking resistance to crown gall and nematodes in walnut rootstocks. BMC genomics 14(1):668

Welsh J, McClelland M (1990) Fingerprinting genomes using PCR with arbitrary primers. Nucleic Acids Res 18(24):7213–7218

Williams JG, Kubelik AR, Livak KJ et al (1990) DNA polymorphisms amplified by arbitrary primers are useful as genetic markers. Nucleic Acids Res 18(22):6531–6535

Woeste K, McGranahan G, Bernatzky R (1996a) The identification and characterization of a genetic marker linked to hypersensitivity to the cherry leafroll virus in walnut. Mol Breed 2(3):261–266

Woeste K, McGranahan GH, Bernatzky R (1996b) Randomly amplified polymorphic DNA loci from a walnut backcross [(*Juglans hindsii* × *J. regia*) × *J. regia*]. J Am Soc Hortic Sci 121(3):358–361

Woeste K, McGranahan G, Bernatzky R (1998) Low correlation between genomic and morphological introgression estimates in a walnut backcross. J Am Soc Hortic Sci 123(2):258–263

Woeste K, Burns R, Rhodes O et al (2002) Thirty polymorphic nuclear microsatellite loci from black walnut. J Hered 93(1):58–60

Wu GL, Meng HJ, Hao YY et al (2010) Thirty years of breeding walnut in China. Acta Hortic 861:109–118

Wu D, Shu QY, Li C (2012a) Applications of DNA marker techniques in plant mutation research. CABI Publishing, Wallingford

Wu J, Gu YQ, Hu Y et al (2012b) Characterizing the walnut genome through analyses of BAC end sequences. Plant Mol Biol 78(1–2):95–107

Wyman SK, Jansen RK, Boore JL (2004) Automatic annotation of organellar genomes with DOGMA. Bioinformatics 20(17):3252–3255

Xu Z, Hu T, Zhang F (2012) Genetic diversity of walnut revealed by AFLP and RAPD markers. J Agric Sci 4:271–276

Xu L, Chen X, Zhang LS et al (2014) Molecular cloning and expression analysis of the transcription factor gene *Jr*CBF from *Juglans regia* L. Acta Hortic 1050:41–47

Xu Z, Ge Y, Zhang W et al (2018) The walnut JrVHAG1 gene is involved in cadmium stress response through ABA-signal pathway and MYB transcription regulation. BMC plant Biol 18(1):19

Yang K, Wang Y, Zhang Y (2002) Analysis for the identification of the precocious trait in walnuts. Acta Hortic Sin 29(6):573–574

Yi F, Zhijun Z, Shelong Z (2011) Development of walnut EST-SSR markers and primer design. Agric Sci Technol 12:1810–1813

You FM, Deal KR, Wang J et al (2012) Genome–wide SNP discovery in walnut with an AGSNP pipeline updated for SNP discovery in allogamous organisms. BMC Genomics 13(1):354

Zekiri F, Molitor C, Mauracher SG et al (2014) Purification and characterization of tyrosinase from walnut leaves (*Juglans regia*). Phytochemistry 101:5–15

Zerbino DR, McEwen GK, Margulies EH et al (2009) Pebble and rock band: heuristic resolution of repeats and scaffolding in the velvet short-read de novo assembler. PloS one 4(12):e8407

Zhang Z, Liao L, Moore J et al (2009) Antioxidant phenolic compounds from walnut kernels (*Juglans regia* L.). Food Chem 113:160–165

Zhang R, Zhu A, Wang X et al (2010) Development of *Juglans regia* SSR markers by data mining of the EST database. Plant Mol Biol Report 28(4):646–653

Zhang ZY, Han JW, Jin Q et al (2013) Development and characterization of new microsatellites for walnut (*Juglans regia*). Genet Mol Res 12(4):4723–4734

Zhang MY, Xu Y et al (2014) Review of walnut breeding research at the Shandong Institute of Pomology. Acta Hortic 1050:55–60

Zhang Q, Walawage SL, Tricoli DM et al (2015) A red fluorescent protein (DsRED) from *Discosoma sp.* as a reporter for gene expression in walnut somatic embryos. Plant cell Rep 34(5):861–869

Zhao P, Zhang T, Zhou HJ (2015) Identification, development, and application of 40 polymorphic EST-SSR markers for common walnut (*Juglans regia* L.). NCBI, EST (unpublished raw data)

Zhao P, Zhou HJ, Potter D et al (2018) Population genetics, phylogenomics and hybrid speciation of *Juglans* in China determined from whole chloroplast genomes, transcriptomes, and genotyping-by-sequencing (GBS). Mol Phylogenet Evol 126:250–265

Zhu Y, Yin Y, Yang K et al (2015) Construction of a high-density genetic map using specific length amplified fragment markers and identification of a quantitative trait locus for anthracnose resistance in walnut (*Juglans regia* L.). BMC Genomics 16(1):614

Zhu T, Wang L, You FM et al (2019) Sequencing a *Juglans regia*× *J. microcarpa* hybrid yields high-quality genome assemblies of parental species. Hortic Res 6(1):55

Zimin AV, Marçais G, Puiu D et al (2013) Genome assembler. Bioinformatics 29(21):2669–2677
