# Supplementary material for: Provenance and family variations in early growth of Manchurian walnut (Juglans mandshurica Maxim.) and selection of superior families
Source: PLoS One. 2024 Mar 7;19(3):e0298918. doi: 10.1371/journal.pone.0298918 (PMC10919699; doi:10.1371/journal.pone.0298918)
Supplement: S1 File — (ZIP) [file pone.0298918.s004.zip › Advances in rootstock breeding of nut trees objectives and strategies.pdf]

plants

Review

Advances in Rootstock Breeding of Nut Trees: Objectives and Strategies

Kourosh Vahdati [1,*], Saadat Sarikhani [1], Mohammad Mehdi Arab [1], Charles A. Leslie [2], Abhaya M. Dandekar [2], Neus Aletà [3], Beatriz Bielsa [4], Thomas M. Gradziel [2], Álvaro Montesinos [4], María José Rubio-Cabetas [4,5], Gina M. Sideli [2], Ümit Serdar [6], Burak Akyüz [6], Gabriele Loris Beccaro [7], Dario Donno [7], Mercè Rovira [8], Louise Ferguson [2], Mohammad Akbari [9], Abdollatif Sheikhi [10], Adriana F. Sestras [11], Salih Kafkas [12], Aibibula Paizila [12], Mahmoud Reza Roozban [1], Amandeep Kaur [13], Srijana Panta [13], Lu Zhang [13], Radu E. Sestras [11,*] and Shawn A. Mehlenbacher [14]

[1] Department of Horticulture, College of Aburaihan, University of Tehran, Tehran 3391653755, Iran; saadat.sarikhani@ut.ac.ir (S.S.); mm.arab@ut.ac.ir (M.M.A.); m.roozban@ut.ac.ir (M.R.R.)
[2] Department of Plant Sciences, University of California Davis, One Shields, Avenue, Davis, CA 95616, USA; caleslie@ucdavis.edu (C.A.L.); amdandekar@ucdavis.edu (A.M.D.); tmgradziel@ucdavis.edu (T.M.G.); gsideli@ucdavis.edu (G.M.S.); lferguson@ucdavis.edu (L.F.)
[3] Institut de Recerca i Tecnologia Agroalimentàries, IRTA Fruit Production, Torre Marimon, 08140 Caldes de Montbui, Spain; neus.aleta@irta.cat
[4] Unidad de Hortofruticultura, Centro de Investigación y Tecnología Agroalimentaria de Aragón, Instituto Agroalimentario de Aragón-IA2 (CITA-Universidad de Zaragoza), Av. Montañana 930, 50059 Zaragoza, Spain; bbielsa@cita-aragon.es (B.B.); amontesinos@cita-aragon.es (Á.M.); mjrubioc@cita-aragon.es (M.J.R.-C.)
[5] Instituto Agroalimentario de Aragón–IA2 (CITA-Universidad de Zaragoza), 50059 Zaragoza, Spain
[6] Department of Horticulture, Faculty of Agriculture, Ondokuz Mayıs University, Samsun 55139, Turkey; userdar@omu.edu.tr (Ü.S.); burak.akyuz@omu.edu.tr (B.A.)
[7] Department of Agricultural, Forest and Food Sciences, University of Torino, 10124 Torino, Italy; gabriele.beccaro@unito.it (G.L.B.); dario.donno@unito.it (D.D.)
[8] Institut de Recerca i Tecnologia Agroalimentàries, IRTA Fruit Production, Mas Bové, Ctra. Reus-El Morell, Km. 3.8, 43120 Constantí, Spain; merce.rovira@irta.cat
[9] Nazari Business Group, Tehran 1193653471, Iran; mohammad_akbari@ut.ac.ir
[10] Department of Horticultural Sciences, College of Agriculture, Vali-e-Asr University of Rafsanjan, Rafsanjan 7718897111, Iran; sheikhi.abdollatif@gmail.com
[11] Faculty of Horticulture, University of Agricultural Sciences and Veterinary Medicine, 400372 Cluj-Napoca, Romania; adriana.sestras@usamvcluj.ro
[12] Department of Horticulture, Faculty of Agriculture, Cukurova University, Adana 01380, Turkey; salihkafkas@gmail.com (S.K.); aibibulapaizila@gmail.com (A.P.)
[13] Department of Horticulture and Landscape Architecture, Oklahoma State University, Stillwater, OK 74078, USA; amandeep.kaur@okstate.edu (A.K.); srijana.panta@okstate.edu (S.P.); luzhang@okstate.edu (L.Z.)
[14] Department of Horticulture, Oregon State University, Corvallis, OR 97331, USA; shawn.mehlenbacher@oregonstate.edu
* Correspondence: kvahdati@ut.ac.ir (K.V.); rsestras@usamvcluj.ro (R.E.S.)

check for updates

Citation: Vahdati, K.; Sarikhani, S.; Arab, M.M.; Leslie, C.A.; Dandekar, A.M.; Aletà, N.; Bielsa, B.; Gradziel, T.M.; Montesinos, Á.; Rubio-Cabetas, M.J.; et al. Advances in Rootstock Breeding of Nut Trees: Objectives and Strategies. *Plants* **2021**, *10*, 2234. https://doi.org/10.3390/plants10112234

Academic Editor: Bruno Mezzetti

Received: 12 September 2021
Accepted: 15 October 2021
Published: 20 October 2021

Publisher's Note: MDPI stays neutral with regard to jurisdictional claims in published maps and institutional affiliations.

Copyright: © 2021 by the authors. Licensee MDPI, Basel, Switzerland. This article is an open access article distributed under the terms and conditions of the Creative Commons Attribution (CC BY) license (https://creativecommons.org/licenses/by/4.0/).

Abstract: The production and consumption of nuts are increasing in the world due to strong economic returns and the nutritional value of their products. With the increasing role and importance given to nuts (i.e., walnuts, hazelnut, pistachio, pecan, almond) in a balanced and healthy diet and their benefits to human health, breeding of the nuts species has also been stepped up. Most recent fruit breeding programs have focused on scion genetic improvement. However, the use of locally adapted grafted rootstocks also enhanced the productivity and quality of tree fruit crops. Grafting is an ancient horticultural practice used in nut crops to manipulate scion phenotype and productivity and overcome biotic and abiotic stresses. There are complex rootstock breeding objectives and physiological and molecular aspects of rootstock–scion interactions in nut crops. In this review, we provide an overview of these, considering the mechanisms involved in nutrient and water uptake, regulation of phytohormones, and rootstock influences on the scion molecular processes, including long-distance gene silencing and trans-grafting. Understanding the mechanisms resulting from

rootstock × scion × environmental interactions will contribute to developing new rootstocks with resilience in the face of climate change, but also of the multitude of diseases and pests.

Keywords: almond; Persian walnut; pistachio; hazelnut; pecan; chestnut; grafting; graft compatibility

1. Introduction

Nut trees are among of the most important horticultural tree crops. Both production and consumption are increasing dramatically due to strong economic returns and the nutritional value of their products. The world's tree nut production has increased by 48% over the last 10 years (ca. 4.5 million metric tons). The world-wide export value of tree nut crops amounted to approximately 34.5 billion dollars in 2019, an increase of ~107% compared to the prior 10-year period [1]. Technical knowledge regarding nut tree production has also rapidly increased as a result of the demand for higher production and quality, multiple destinations of nuts fruit in current consumption and food industry, but also of the growing importance accorded to the nuts in a balanced and healthy diet and in the prevention of various diseases [2–7]. Among the areas of interest and progress has been the use of rootstocks to adapt to climate and edaphic factors including soil borne diseases and abiotic stresses, control scion vigor, increase yield, and improve fruit quality. the selection of the scion cultivar is the grower's top consideration for long-term productivity and profitability, rootstock selection is becoming more important. Now, the rootstock scion and interaction per se is considered when planting an orchard.

The advantages of selected rootstocks have been recognized and utilized in the nut trees' production, but they do not have a long history of use in many species. Although nut trees are grown around the world, rootstock studies are limited to only a few tree nut species. Initially, most rootstocks were open-pollinate seedling, or seedstock. Seedstocks are not as genetically uniform as clonal rootstocks, but they have advantages such as deep root system and tolerance to edaphic abiotic stresses. However, seedstocks have high heterozygosity in terms of different traits. Hence, the type of seed and location in which it is grown is important for choosing seedstocks. Seedstocks should be uniform, vigorous, disease resistant, and readily available [8]. Therefore, several studies have been performed to study the growth vigor of seedstocks and improve seed germination in nut trees [9]. In addition to seedstocks, a wide range of clonal rootstocks are now being developed. Numerous rootstock breeding programs have begun to introduce clonal rootstocks to meet important challenges, including excess vigor, low yield, poor nut quality, poor soil, climate change, drought and salt stress, suckering, diseases, and graft incompatibility. Common tree nut rootstocks, especially clonal rootstocks, and their main characteristics are listed in Table 1.

Table 1. The main characteristics of commercial rootstocks of nut tree crops around the world.

Rootstocks	Main Characteristics
**Almond**	
Almond seedlings	Ability to grow in poor, high limestone content soils, deep root system, suitable for growing rainfed almonds, susceptible to soil pathogens such as nematodes, *Agrobacterium*, *Phytophthora*, *Armillaria*, etc. and sensitive to neck and root asphyxia
Peach seedlings	The most important cultivars are INRA's 'GF-305′, 'Montclar', and the U.S. cultivars 'Lovell', 'Nemaguard' and 'Nemared'. Adapted to cultivation in irrigated soils, tolerant to certain species of nematodes, highly sensitive to some of the common pathogens: *Agrobacterium*, *Armillaria*, *Phytophthora*, etc. Some cultivars (e.g., 'Nemaguard' and 'Nemared') are resistant to RKN (*Meloidogyne* spp.)
'Hansen 536′ (*P. persica* × *P. dulcis*)	High vigor rootstock with good anchorage, requires very well-drained soils, tolerant to drought, salinity, alkalinity, and boron soils, very susceptible to wet soil, moderately resistant to root-knot nematodes, but some tolerance to *Phytopthora syringae*, some tolerance to iron chlorosis

Table 1. *Cont.*

Rootstocks	Main Characteristics
'Nickels' (*P. persica* × *P. dulcis*)	Vigorous, highly compatible with almond cultivars, adapted to wide range of California almond production area possibly because of its greater winter chilling requirements, resistance to nematode species from its parent 'Nemaguard'
'Krymsk 86' (*P. domestica* × *P. persica*)	Excellent root system, compatible with Nonpareil, tolerant to wet and heavy soils, cold hardy, tolerant to high pH soils
Bright's Hybrid®5	Vigor and productivity are superior to 'Nemaguard', deep rooting, well anchored, and drought tolerant, needs deep well drained soil, resistant to nematode
Viking	Vigorous, productive, early blooming, somewhat tolerant to wet soil conditions, tolerant to saline, and alkaline soil conditions, intolerant of dehydration during transplanting, resistant to root knot nematodes and less susceptible to bacterial canker
'GF-677'	High vigor, high yield, ability to perform in non-irrigated soils due to deep roots system, resistance to chlorosis, moderately tolerant to salinity, sensitive to waterlogging, highly susceptible to *Phythophtora*, *Armillaria*, *Agrobacterium* and *RKN*
GN series; 'Garnem', 'Felinem' and 'Monegro' are three almond × peach- [*P. amygdalus* Batsch. × *P. persica* (L.) Batsch.]	Vigorous, high yield, suitable for rainfed or irrigated conditions, tolerant to iron chlorosis and drought, good adaptation to poor soils, very resistant to the main root-knot nematode species attacking *Prunus*, low tolerance to root asphyxia, susceptible to the root lesion nematode (*Pratylenchus vulnus*) and crown gall caused by *Agrobacterium* tumefaciens, more tolerant to *Phythophtora*, than other almond × peach hybrids
'Root-Pac 40' (*P. dulcis* × *P. persica*) × (*P. dulcis* × *P. persica*)	Medium vigor, around 25–30% less than GF–677, erect structure similar to Garnem, high productivity, adaptable to warm conditions (low chilling areas)
'Root-Pac 20' (*P. cerasifera* × *P. besseyi*)	Low vigor, around 40–50% less than GF–677, high productivity, very adaptable to warm and colder climates, good adaptation to heavy soils, moderately tolerant to chlorosis, salinity, and root-knot nematodes, tolerant to root asphyxia and *Rosellinia necatrix*
'Root-Pac R' (*P. cerasifera* × *P. dulcis*)	High vigor, high productivity, compatibility with several *Prunus* species, and outstanding adaptation to poor, heavy soils with high lime content, ideal for replanting sites, adapts well to dense and asphyxiating soils
**Walnut**	
J. regia seedlings and clonal	Moderate vigor, less susceptible to crown gall (CG), susceptible to *Phytophthora*; resistance to blackline, moderate tolerance to calcareous soil
J. hindsii seedling and clonal	Moderate vigor, moderately tolerant to salinity, some resistance to CG, susceptible to blackline
Paradox (seedling)	Hybrid (*J. hindsii* × *J. regia*). Vigorous, intermediate in salt sensitivity between *J. hindsii* and *J. regia*, low susceptibility to *Phytophthora*; susceptible to CG and blackline
Vlach (clonal Paradox)	Hybrid (*J. hindsii* × *J. regia*); vigorous, not resistant to CG or *Phytophthora*, susceptible to nematodes and blackline. Tolerant to calcareous soil
RX1 (clonal Paradox)	Hybrid (*J. microcarpa* × *J. regia*); moderately vigor, moderate resistance to CG, resistant to *Phytophthora citricola* and *P. cinnamomi*; susceptible to nematodes and blackline, excellent survival in orchard replant trials
VX211 (clonal Paradox)	Hybrid (*J. hindsii* × *J. regia*); highly vigorous, some tolerance to root knot and root lesion nematodes, susceptible to CG, *Phytophthora*, and blackline
Ng209 × Ra seedlings	Hybrid progeny of *Juglans nigra* 209 × *J. regia*, highly vigorous, susceptible to CG, tolerant to *Phytophthora* and *Armillaria*, tolerant to calcareous soil, susceptible to blackline
Grizzly	The mother tree of Grizzly is a 'Tulare' variety grafted onto a seedling paradox rootstock. high vigorous, resistance to crown gall, tolerant to nematode, pest resistance; best rootstock for poor soil
**Pistachio**	
Badami-Riez Zarand seedling (*P. vera*)	Vigorous, tolerant to *Phytophthora* spp.; salinity tolerant
Sarakhs seedling (*P. vera*)	Salinity tolerant, susceptible to *Phytophthora* spp.
Qazvini seedling (*P. vera*)	Salinity tolerant
Beneh (*P. atlantica Desf.* ssp. *mutica* F&M)	Resistant to root-knot nematode, less vigorous and difficult to bud than 'Badami-Riez Zarand', scion-rootstock incompatibility, negative effects on yield
Terebinthus (*P. terebinthus*)	Cold resistant, less vigorous, and less uniform than other common rootstocks, efficient zinc and copper absorption, resistant to *Armillaria* root rot, *Verticillium dahliae* susceptible, high rusticity
Atlantica (*P. atlantica*)	Higher cold tolerance and less vigorous than *P. integerrima*, susceptible to *Verticillium dahliae*, tolerant to root asphyxia.

Table 1. *Cont.*

Rootstocks	Main Characteristics
Integerrima (*P. integerrima*)	Vigorous, buds easily, least cold tolerant of the commonly used rootstocks, tolerant to Verticillium wilt
Khinjuk (*P. khinjuk*)	Drought tolerant, susceptible to *Phytophthora* spp.; more vigorous than 'Beneh'
Pioneer Gold I (PGI) (*P. integerrima* × *P. integerrima*)	Resistant to *Verticillium dahliae*; sensitive to frost
Pioneer Gold II (PGII) (*P. integerrima* × *P. atlantica*)	Vigorous, susceptible to Verticillium wilt, no longer commercially available
UCB-1 selected seedling	Highly vigorous, positive effect on yield, salinity tolerance, moderately resistant to Verticillium wilt
**Hazelnut**	
C. colurna seedling	Compatibility with cultivars of *C. avellana*, non-suckering rootstock, drought tolerance due to deep taproot, seeds of this species are difficult to germinate
'MB-69′ ('Tonda Bianca' seedling')	High vegetative growth, emission of few suckers
Dundee	Open pollinated *C. colurna* seedling, probably *C. colurna* × *C. avellana*, high vegetative growth and high yield performance, emission of few suckers
Newberg	Open pollinated *C. colurna* seedling, probably *C. colurna* × *C. avellana*, high vegetative growth and high yield performance, emission of few suckers
**Pecan**	
Elliott	Positive effect on nut quality, susceptible to spring frost, very resistant to pecan scab, very susceptible to black aphid
Riverside	Common rootstock for western pecan regions because of salt tolerance, drought tolerance, nut germination limited by poor quality, very susceptible to pecan scab
Stuart	Cold hardy rootstock, moderate resistance to spring frost, susceptible to pecan scab
'VC1-68′	Used as rootstock in the west, especially in California, drought tolerance, frost susceptibility limits use in the southern parts of southeast and southwest
Apache (Burkett × Schley)	Growth initiation in spring not as early as in 'Elliott' and 'Curtis', very susceptible to pecan scab
Colby	Cold hardy rootstock, only recommended for northern areas of Oklahoma.
Giles	Cold hardy rootstock, adapted to northern Oklahoma also used as seed stock in Kansas and far north Texas
Peruque	Best suited for northern Oklahoma, cold hardy rootstock
Kanza	Adapted to all of Oklahoma, cold hardy trees
Moore	Vigorous, productive, some resistance to scab
San Felipe	Drought tolerance
**Chestnut**	
PO-11 (clonal)	Natural hybrid of *C. sativa* and Asian chestnut. resistant to *Phytophthora* spp. and highly compatible with *C. sativa*
Ferosacre	Resistance to the *Phytophthora* spp.; sensitive to temperatures less than −10 °C
Marigoule (*Castanea crenata* × *Castanea sativa*)	Vigorous but very demanding on the quality of the soil, incompatible with many chestnut cultivars, moderately productive, resistant to *Phytophthora* spp. and canker, sensitive to cold weather and root asphyxia
Marsol (*C. crenata* × *C. sativa*)	Vigorous, good graft compatibility with many cultivars, resistance to *Phytophthora* spp.
Maraval (*C. crenata* × *C. sativa*)	Moderate vigor, good graft compatibility with many cultivars, sensitive to spring frosts, resistance to *Phytophthora* spp.
Marlhac (*C. crenata* × *C. sativa*)	Resistance to *Phytophthora* spp.; able to grow at temperatures less than −10 °C

Advances in the development of temperate nut trees rootstocks until 2003 were last reviewed by Grauke and Thompson [10]. Given the recent advances in rootstock breeding for tree nut crops, this review will focus on the physiological and molecular effects of rootstocks on scions under different edaphic and climatic conditions.

The main purpose of this review paper is to present studies on various aspects of breeding and physiology of nut trees rootstock, as well as, draw a comprehensive vision to accelerate future research in this field using combination of traditional and modern methods. To this end, we first provide overall information on vigor, rootstock-scion com-

patibility, suckering, and rooting ability which can be useful for tree nut crops researchers and growers. Next, we review water and nutrient uptake on nut trees. In the following, we review phenology and yield related traits which are important in industry and marketing. Then, we comprehensively review abiotic and biotic stresses studies on tree nut crops. Finally, we briefly review rootstock-scion transfer of macromolecules and small interfering RNAs in nut trees. Since nut tree crops have a long juvenile period, development of a new variety or rootstock may take more than 20 years via classical breeding. Therefore, in the conclusion and perspectives section, we note the future prospects of molecular breeding in nut tree crops using novel technologies for rapid generation advancement.

2. Vigor

The nut trees growth is strongly controlled by the distribution of organic and inorganic constituents within the tree trunk, canopy, and the root system. The vascular system plays a role in this long-distance signaling. Hypothetically, rootstocks impact scion vigor by controlling water and nutrient transfer and hormones signaling and RNAs which move up through the graft union [11]. Numerous studies have been conducted regarding the effect of rootstocks on the growth of nut trees [12–15].

Pistachio growers and breeders are seeking vigorous rootstocks. Kallsen and Parfitt [13] reported 'Kerman', the previously primary female pistachio cultivar in California, has a rapid growth habit that produces trunk circumferences larger than that of the rootstocks. Matching the scion and rootstock growth rates produces stronger graft unions. Highly vigorous rootstocks produce more uniform graft unions and reduce bark damage from trunk shaking harvesters by uneven graft unions. They report that UCB1 is a better rootstock for 'Kerman' as it produces a smoother trunk compared to Pistacia integerrima rootstocks [13]. Caruso et al. [12] evaluated one seedling (*P. terebinthus*) and eight clonal (*P. atlantica* and *P. integerrima*) pistachio rootstocks and reported that rootstock had a significant effect on growth rate of the scion and nut yield. Clones of *P. integerrima* and *P. atlantica* are highly to intermediately vigorous rootstocks.

The pistachio cultivar 'Bianca' onto *P. integerrima* seedling rootstock had significantly better growth than on *P. terebinthus* or *P. atlantica* clonal rootstocks. Scions grown on *P. terebinthus* rootstocks had the least vigor. When 'Bianca' scions were budded onto eight in vitro propagated clonal rootstocks and observed for 4 years, trunk cross-sectional areas on *P. integerrima* were three times higher than on *P. terebinthus* rootstocks [16].

Ak and Turker [17] reported the cultivars, 'Kirmizi' and 'Siirt', grafted onto *P. vera*, *P. khinjuk*, and *P. atlantica* demonstrated different budbreak, flowering time and vegetative growth. *P. vera* flowered earlier and *P. atlantica* and *P. khinjuk* had greater stem diameters. Rahemi and Tavallali [18] studied the effect of 'Badami' (*P. vera*), 'Sarakhs' (wild *P. vera*), and 'Beneh' (*P. mutica*) seedling rootstocks on growth, yield, and nut quality of the Iranian cultivars, 'Ohadi', 'Kalleh- Ghouchi', and 'Ahmad-Aghaei'. 'Sarakhs' seedlings had the least vigor, while 'Badami' rootstocks produced the highest yields and best nut quality. Ghazvini et al. [19] evaluated the ecophysiological characteristics of four seedling rootstocks, 'Badami', 'Sarakhs', *P. mutica,* and *P. atlantica*. Photosynthesis, stomatal conductance, and transpiration was highest in trees on the 'Sarakhs' rootstock and lowest on the *P. mutica* rootstock. *P. integerrima* is the most vigorous rootstock now commonly used in pistachio cultivation but is also the least cold tolerant [20]. It is rapidly being replaced by the more cold- and salinity-tolerant hybrids, available as both a seedling and a clone, and *P. integerrima* × *P. atlantica*, now available as a clone (Ferguson; personal communication 2021).

In contrast to pistachio, there is no a specific walnut breeding program to select high vigorous rootstock. Nevertheless, the major walnut clonal rootstocks introduced in the last few years are vigorous. Among the clones of 'Paradox' ('Vlach', 'RX1′, and 'Vx211′) which was introduced by the University of California-Davis, 'VX211′ (*J. hindsii* × *J. regia*) is highly vigorous and nematodes-tolerant rootstock [21,22]. Furthermore, 'Grizzly' clonal walnut rootstock has been recently introduced as a highly vigorous rootstock. The mother tree of 'Grizzly' is a Tulare variety grafted on a seedling Paradox rootstock. This rootstock shows

good performance in poor soil structure with low nutrition and heavy populations of lesion nematodes. In addition, high vigorous trees are very important for the wood industry. Numerous interspecific hybrids were carried out in Juglans genus between *J. regia* with *J. cinerea*, *J. nigra*, and *J. major*. Compared to the parent, most of them such as 'NG23′, 'NG38′ (*J. nigra* × *J. regia*), and 'MJ209′ (*J. major* × *J. regia*) show high vigor, disease resistance, greater winter-hardiness, and high wood quality [23–25].

Walnuts are highly vigorous trees with an extended juvenility phase [14]. Dwarf walnut trees could potentially decrease labor costs and increase yields per hectare by allowing increased plant density [26]. Although dwarfing has not generally been the most important objective of walnut rootstock breeding programs, identifying sources of this trait is of great interest in countries with high genetic diversity such as Iran, China, Turkey, and Central Asian countries [27–29]. In these countries, traditional orchards of giant walnut trees are difficult to harvest mechanically. Harvest injuries and death of laborers during manual harvesting have precipitated interest in dwarfing rootstocks [30]. Reportedly dwarf walnut trees have a short life span. Therefore, in some countries, breeders are attempting to combine slow-growing scions with vigorous rootstocks. Juvenile and mature walnut tree vigor is highly heritable [31,32]. Wang et al. [29] evaluated Persian walnuts in China and selected six dwarf walnut rootstocks; 'Xinwen 609′, 'Xinwen 724′, 'Xinwen 908′, 'Xinwen 915′, 'Xin 916′, and 'Xinwen 917′ as potential rootstocks for breeding. Analysis of growth traits of 'semi-cultivated' local genotypes of Juglans regia on their own roots, in the sands area of south-west Romania, showed that climatic and edaphic factors significantly influenced the annual growth ring width of the trees, but also their adaptability to environmental factors [33].

Precocious and dwarf walnut trees have been evaluated in Iran [14,28,34]. These genotypes induce dwarfing and precocity in scions in preliminary experiments, apparently due to a slower growth rate. They have fewer nodes, shorter internodes, and smaller shoot length, smaller root system, and lower sap flow and hydraulic conductivity which are the typic traits of dwarf rootstocks in other fruit trees. They also have a better rooting ability and higher grafting success [35–39].

Dwarfing is a desirable trait for other tree nuts. In China, dwarfing chestnut rootstocks are being evaluated [40]. In the USA, Anagnostakis et al. [41] attempted to breed dwarfing chestnut rootstocks and suggested that hybrids with Castanea seguinii could be a source of dwarfing. Researchers at the University of Missouri identified various chestnut cultivars as potential sources of dwarfing. Studies of graft compatibility, vegetative growth, and productivity of these trees are continuing to determine if dwarf chestnut rootstocks are feasible.

Knowledge of rootstock effect on almond vigor is limited. Almond rootstocks have been shown to alter root, shoot, trunk, and fruit development, probably by affecting the allocation of carbon assimilates between these tissues [42]. Khadivi-Khub and Anjam [42] evaluated the Iranian cultivar 'Rabiee' grown on *P. scoparia* (a wild almond species) and 'Estahban' (*P. dulcis*) rootstock under normal and rainfed conditions. They reported significant differences in tree height, trunk diameter, annual growth, and internode length, observing reduced scion growth when grafted on *P. scoparia* rootstock. *P. scoparia*, suggesting potential as a dwarfing rootstock. Parvaneh et al. [43] evaluated three Iranian cultivars on bitter almond, sweet almond, and peach rootstocks and found that cultivars grafted on peach had greater vegetative growth, while scions grown on both bitter and sweet almonds had reduced growth, resulting in smaller trees. The magnitude of the effect varied with cultivar.

In a regional rootstock trial at California State University, Fresno, significant differences among rootstocks were found in canopy growth, tree height, and tree circumference [44]. Almonds grafted on peach rootstock had larger scion diameters than on almond rootstocks [45]. Preliminary results from a vigor study showed that trunk diameter of the scion cultivar depends on the scion-rootstock interaction. The rootstock effect differed depending on the cultivar grafted and scion vigor itself. Lordan et al. [15] studied the performance of two Spanish almond cultivars, 'Marinada' and 'Vairo', grafted onto different rootstock

genotypes and reporting strong rootstock effects on vigor, bloom, and ripening dates, yield, and kernel weight.

The effect of rootstock on tree architecture is less clear. Rootstock effects on shoot length and shoot diameter have been reported, but the magnitude of the effect varied as a function of specific scion-rootstock combinations [46,47]. Similarly, the scion can influence root structure, primarily by altering auxin and cytokinin responses [48]. This suggests the regulatory feedback between the rootstock and scion ultimately modulates final tree architecture. The underlying molecular mechanisms of these interactions remains unknown.

Studies of the effect of rootstock on pecan (*Carya illinoinensis*) scion vigor have demonstrated that common pecan rootstocks vary by geographic region and have a diverse effect on scion growth. Before introducing clonal rootstocks, open-pollinated seedstocks widely used for the vegetative propagation of commercial pecan cultivars had different growth responses. Grauke and Pratt [49] evaluated bud growth of three pecan cultivars ('Cape Fear', 'Stuart', and 'Candy') on seven open-pollinated seedstocks including 'Curtis', 'Burkett', 'Elliott', 'Moore', 'Riverside', 'Apache', and 'Sioux'. They reported that scion growth was significantly influenced by rootstock, with bud growth of 'Candy' on 'Elliot', and 'Curtis' rootstocks were more than 'Sioux', 'Riverside', 'Apache', and 'Burkett' rootstocks [49]. Liu et al. [50] studied the grafting-responsive MicroRNAs (miRNAs) which are involved in growth regulation of grafted pecan and identified some miRNAs that regulate grafted pecan by regulating inorganic phosphate (Pi) acquisition, auxin transport, and cell activity.

The rootstock effect on vigor of other nut trees has been less studied. In hazelnut, new rootstocks have produced superior vigor compared to own-rooted varieties. This is an important improvement when trees are trained to a trunk, and not grown as bushes with many stems [51,52].

3. Rootstock-Scion Compatibility

Graft success depends on the rootstock-scion physiological compatibility and the proper alignment of tissues in the graft union [53–55]. Graft incompatibility is a complex physiological process defined by the adjustment of the metabolisms of the cultivar–rootstock combinations, growth conditions, the presence or absence of viruses, environmental conditions, the nutritional status of trees, and as other stresses. Graft incompatibility can be detected by a variety of symptoms including poor graft success, yellow-colored leaves, slow vegetative growth, drying of the scion, a generally diseased appearance, symptoms of water stress, overgrowth in the graft area, thicker bark tissues of scion, and excessive sprouting on the rootstock (Figure 1).

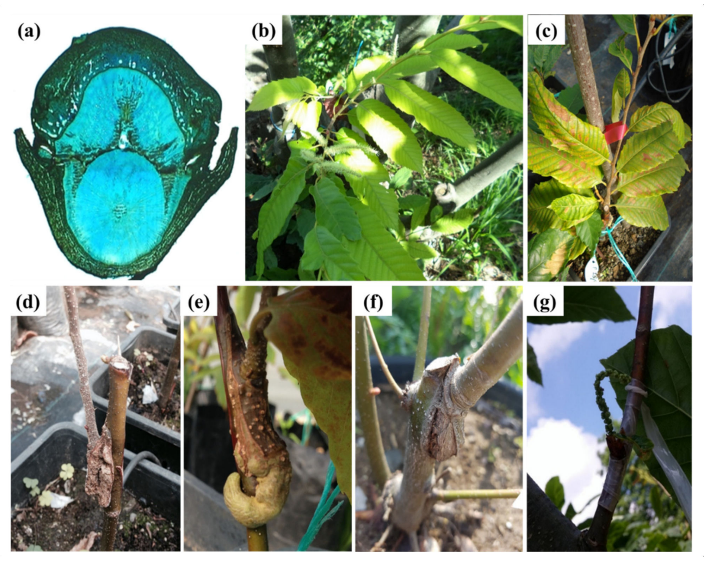

Figure 1. Some graft incompatibility symptoms in chestnut: (**a**) Interruption of the cambial

connection; (**b**) Yellow or gold color leaves during the growth period; (**c**) disease appearance; (**d**) Drying of the scion; (**e**) Overgrowth in the graft area; (**f**) Rootstock tend to give lots of suckers and (**g**) scion shoots become short and turns into fruiting branches.

In pistachio, *P. terebinthus*, *P. atlantica*, *P. integerrima*, *P. vera* and their interspecific hybrids (ex. UCB1) are commonly used rootstocks [56]. *P. terebinthus* is more difficult to bud than *P. atlantica* or *P. integerrima* due to scion-rootstock incompatibility problems. Although rootstock-scion incompatibility is not a serious problem in pistachio production, some evidence of incompatibility between *P. vera* (cv. 'Kerman') as a scion and UCB1 as a rootstock was observed in the late 1980s in the USA. This incompatibility appeared to be related to a single paternal *P. integerrima* tree used to produce the first UCB1 seedlings at the University of California, Berkeley. There have been fewer reports of rootstock-scion incompatibility since removal of this paternal tree [20]. When facing rootstock-scion incompatibility problems in pistachio it is worth testing different individuals within a single species to find a compatible genotype.

The success of walnut grafting mainly depends on several factors such as rootstock, scion, grafting methods, and environmental conditions [57–59]. The specific graft incompatibility between different *Juglans* species has not been reported. Nevertheless, some literatures refer to blackline disease as a delayed graft incompatibility in walnuts [60]. California black walnut and its hybrids are considered as interesting rootstocks for Persian walnut specially in California due to high vigor, resistance to soil-borne pests, and tolerance to saline and saturated soil. However, if Persian walnut was grafted on California black walnut and its hybrids and the tree was infected with CLRV virus, the symptoms of blackline disease would appear, which is similar to a graft incompatibility. Therefore, in regions where there is a possibility of infection with the CLRV virus, Persian walnut is a more suitable rootstock that can be used to avoid blackline disease [61,62]. Andrews and Marquez [63] reported that blackline disease has a long-delayed incompatibility where a CLRV virus migrates to a graft union.

In almond, graft incompatibility appears to be genetically dependent. For example, 'Nonpareil' shows distinct graft-incompatibility on plum rootstocks while the closely related 'Carmel' cultivar does not [64]. Graft-incompatibilities can produce both slow general tree deterioration over time and distinct localized deterioration such as the stem-pitting decline seen on almond-Myrobalan plum combinations [64]. These more localized types of graft-incompatibility can often be observed as a weakness and occasional breakage at the graft-scion union [65–68]. Because this often occurs at a critical time, when the tree is coming into bearing, several studies have pursued earlier physiological and molecular predictors of graft-compatibility as an aid to both breeding and orchard management [69–72]. These studies generally involve anatomical, physiological, or molecular aspects of compatible graft union formation [72,73] such as the similarities/differences in scion vs. rootstock vascular size and configuration [74,75]. Related studies have identified several molecular candidates that may contribute to compatible graft formation [69,71,76], however, the specific cause and effect relationships remain vague. Studies have identified several metabolic pathways, including the phenylpropanoid pathway, cell wall biosynthesis, oxidative stress, and auxin signaling, that appear to be associated with graft-incompatibility [69,77,78], supporting the complex genetic control commonly encountered when breeding for this trait [79].

Japanese and Chinese chestnuts are used in chestnut rootstock breeding programs due to their root-rot resistance. The potential use of hybrid chestnut cultivars also has been evaluated [80,81]; while incompatibility has been observed in the hybrids. Tokar and Kovalovsky [82] grafted Chinese, European, and Chinese × Japanese hybrid chestnut cultivars onto European chestnut rootstocks. The least successful grafting combinations were the Chinese × Japanese hybrid on European rootstocks. Viéitez and Viéitez [83], used Chinese and European chestnuts for European, Chinese, and European × Chinese chestnut hybrid scions. The least successful grafting combinations were the Chinese rootstocks with European chestnut cultivars.

Soylu [84] suggested that scions and rootstocks of the same species should have better graft compatibility, but genetic intraspecies graft incompatibility was reported in Chinese [85] and European [86,87] chestnuts.

Although graft compatibility in chestnut may be mostly controlled by genetic factors [88,89], graft success can also be affected by environmental factors, stress, and their interactions with genotype [90,91]. Oraguzie et al. [90] suggested that growing the rootstock and the scion plant under the same environmental conditions would produce better graft compatibility. Oraguzie et al. [90] divided graft incompatibility into two groups, early and late. Early graft incompatibility can be seen in the first two years and late incompatibility in 5 to 7 years.

Chestnut mosaic virus can also induce graft incompatibility [92]. The first hypothesis was suggested by Santamour et al. [93]. They identified four different cambial peroxidase isozymes patterns (A, B, AB, and BC) in ten chestnut genotypes. They found that *C. dentata*, *C. alnifolia*, *C. ashei*, *C. ozarkensis*, and *C. pumila* species have A cambial peroxidase isozymes, *C. crenata* and *C. seguinii* have B pattern, *C. sativa* has A, B, and AB isozymes, *C. henryii* has A and B and *C. mollissima* has A, AB, B, and BC isozymes. Grafting plants with different isoenzyme bands could lead to graft incompatibility. Santamour [94] tested his hypothesis with 200 Chinese chestnut seedlings. If the scion and the rootstock belonged to the same cambial peroxidase isozymes group, the cambium layer in the graft area united and cambial continuity occurred. If the scion and the rootstock cambial peroxidase isozymes groups were different, cambial continuity was interrupted. Thus, he suggested that cambial peroxidase isozymes groups could be used to predict graft incompatibility in Chinese chestnut. However, this hypothesis was not confirmed in subsequent study [85].

The other hypothesis of graft incompatibility in Chinese chestnut is a mismatch of phloem fiber bundles. Young chestnut branches have a particularly channeled structure. A very important aspect of this anatomical structure is the presence of a fiber bundle in four or more places in the branch. When the seedlings are 2–3 years old, phloem fiber bundles can be better distinguished. This situation should be considered when grafting, as the cambium of the rootstock and scion may not combine uniformly [85,95,96].

Given the importance of early detection of graft incompatibility, it is important to find specific markers for prediction in different rootstock-scion combinations. Many studies have addressed strategies for compatibility detection such as phenolic marker identification and peroxidase isozyme studies. Phenolic compounds, whose biosynthesis is triggered by wounding and infections, are produced and accumulated during the callusing phase. This suggests that quantitative and qualitative differences in phenolic patterns between scion and rootstock may predict graft union dysfunctions and could be potential markers of graft incompatibility [73,97,98].

Research at the University of Torino Chestnut R&D Center, demonstrated different chemical markers: six phenolic acids, five flavonols, two catechins, and two tannins. Chromatographic methods were used to identify and quantify the main bioactive compounds, benzoic acids, binnamic acids, batechins, flavonols, and tannins and obtained specific phytochemical profiles. Benzoic acids (gallic and ellagic), catechins (catechin and epicatechin), and tannins (castalagina and vescalagina) were used to establish specific profiles for distinguishing compatible and incompatible chestnut scion-rootstock combinations [99,100]. Another promising technique is the analysis of peroxidase isozyme profiles of rootstocks and scions. It appears peroxidases play an important role in grafting, as these enzymes are involved in lignin formation and lignin–carbohydrate bonding [93]. Differences in peroxidase isozymes in rootstock and scion graft performance have been reported in Chinese chestnut [93,94] and peach–plum combinations [101]. Other strategies for evaluating rootstock–scion compatibility include describing the phenylalanine ammonia-lyase (PAL) transcriptomic-level [102] and phenotypic evaluation (e.g., photosynthetic efficiency and morpho-phenological parameters of the grafted trees) [103].

4. Suckering

Another important trait in rootstock selection is suckering. Suckers not only divert water and nutrients from the main trunk, but also increase orchard management costs incurred in removing them. Suckering is an important issue in hazelnut cultivation, requiring four to five herbicide sprays per year in commercial orchards and occasional hand-removal in winter [104]. This situation could be improved by use of non-suckering rootstocks. Currently, three types of hazelnut rootstocks are in use: *C. colurna* seedlings, *C. avellana* seedlings, and two clonal selections from open pollinated *C. colurna*: 'Dundee' and 'Newberg' [105]. A hazelnut rootstock trial in IRTA-Mas Bové, Spain in 1989 led to selection of a clonal *C. avellana* rootstock ('MB-69′), which is a seedling of 'Tonda Bianca' [106].

One of the first European hazelnut rootstock trials was conducted in Nebrosi, Sicilia (Italy) in 1970 to compare self-rooted trees grafted on *C. avellana* rootstock (cv. Sicilian). After 12 years of evaluation, self-rooted trees showed better vegetative and productive behavior than grafted ones [107]. Experience with *C. colurna* in the U.S.A. has demonstrated that members of this species are more drought tolerant and cold hardy than *C. avellana* cultivars. The *C. colurna* was non-suckering, deeply-rooted, and graft-compatible with all *C. avellana* cultivars and *Corylus* species, suggesting its potential use as a rootstock. Due to differences in bark color and texture, the union between the Turkish (*C. colurna*) and European (*C. avellana*) hazelnut is readily evident. However, the Turkish hazelnut is difficult to propagate and its seedlings often require two additional years before reaching sufficient size for grafting. In addition, hazelnut trees on *C. colurna* rootstocks are frequently more variable in size and yield than self-rooted trees of *C. avellana*. In a trial using 'Barcelona' as a scion cultivar, the graft unions were overgrown and nut yields declined with age, at ~20–25 years. Due to these disadvantages, the Faculty of Agriculture, at Novi Sad in Serbia, has focused on identifying non-suckering selections of *C. avellana* [108]. Currently, seeds of selected *C. colurna* are used as hazelnut rootstock as it has been demonstrated to be long-living, resistant to frost and drought, has wide adaptability to soil conditions, and the trees are more vigorous and productive than self-rooted trees [109–111].

Hazelnut rootstock breeding started in Oregon in 1968. In nursery rows, open-pollinated seedlings of *C. colurna* seedlings whose traits were intermediate between *C. colurna* and *C. avellana* were selected and propagated. During twenty years, approximately 150 potential rootstocks were selected from 20,000 seedlings investigated. Two non-suckering clonal rootstocks ('Newberg' and 'Dundee') that impart vigor to scions were released [105]. Both rootstocks are thought to be interspecific hybrids because their nut and husk characteristics differ from those of the maternal parent. In 2000, a rootstock trial was established at the IRTA-Mas Bové Research Center (Constantí, Tarragona, Spain), with Spanish cultivar 'Negret' grafted onto four different rootstocks, 'Dundee', 'Newberg', and two open pollinated *C. colurna* seedlings, compared to own-rooted 'Negret' as the control. The results showed that 'Dundee' and 'Newberg' rootstocks improved agronomic performance, solving the problem of suckering, increasing productivity and vigor, and producing increased yield at lower cost [51]. However, the search for additional non-suckering rootstocks necessary for commercial hazelnut orchards remains topical and is continuing [52].

5. Rooting Ability

Rootstocks can be vegetatively propagated by micropropagation, layering, or cuttings. The rooting ability of rootstocks and the most effective propagation methods vary by species and genotype. Among walnut rootstocks, Persian walnut is more difficult to root than black walnut × Persian hybrids. Japanese × European chestnut hybrids (*C. crenata* × *C. sativa*) are more easily propagated by cuttings or layering than European chestnuts (*C. sativa*) [112,113].

Many efforts have been made to propagate walnuts by layering [114,115], cuttings [114–118], and micropropagation [39,119–127]. In vitro propagation of walnuts obtained seems quite dif-

ficult [128]. Generally, the effects of genotypes, but also the culture medium in vitro on proliferation, rooting, and survival rates are significant [128,129]. Along with genotypes, nutritive support in vitro has an important influence on all stages of micropropagation. In addition to the well-known Murashige and Skoog (MS) culture medium, the Driver and Kuniyuki (DKW) medium is also widely used for tissue culture of walnuts [120]. However, depending on nutritive improvements of the medium, large variations of the results can be obtained regarding the success of in vitro culture [128]. Vahdati et al. [125] reported in-vitro rooting of Persian walnut cultivars 'Sunland' (95%), 'Chandler' (55%), and 'Vina' (27%).

Vahdati and Khalighi [115] and Vahdati et al. [130] evaluated stool layering of Persian walnut and found the greatest root number and root length was obtained using 5000 and 10,000 ppm IBA + IAA + NAA, respectively. Vahdati et al. [118] obtained up to 81% and 82% rooting of Paradox walnut semi-hardwood and hardwood cuttings respectively, using 8000 ppm of IBA. Dong et al. [124] reported a range of 60.5 to 87.5% rooting in a study of six cultivars.

Currently, the nursery pathogen problems have demonstrated it is better to produce plants in inert medium, and micropropagate the rootstocks and graft the material in the nursery [131–134]. Vahdati et al. [130] found rooting success of low-vigor (dwarf) walnuts was better than more vigorous ones in response to stool layering. Peixe et al. [127] achieved ex-vitro rooting rates exceeding 80% for microcuttings of 'Vlach' hybrid walnut. RolABC genes (rolA + rolB + rolC), derived from the bacterium *A. rhizogenes*, were inserted into somatic embryos of Px1 (a Paradox somatic embryo culture) to increase the rooting potential. In a field trial, the rolABC genes produced shorter internodes and a more fibrous root system [118].

6. Water and Nutrient Uptake

Water and nutrient uptake are one direct effect of rootstock on nut tree yield. These are regulated by complex interactions between the scion and rootstock. Hormones, macromolecules, and miRNAs act as long-distance signaling molecules that regulate nutrient uptake [135]. Water and nutrient uptake are enhanced by rootstock vigor. In addition, the rate of vascular bundle development in a graft union determines the transfer potential of water and nutrient to the scion. Insufficient vascular bundle connection in a graft union leads to decreased water flow and subsequently altered nutrient translocation and hormonal signaling [136]. Vertical and lateral root development plays an important role in water and nutrient uptake. Rootstocks with a vigorous root system, i.e., long roots with many lateral branches and root hairs, are able to exploit water and nutrients from different soil depths and textures [135]. Water uptake by roots is both parallel symplastic and apoplastic pathways. Root system hydraulic conductivity defines the root's ability to conduct water across a water-potential gradient between the root surface and the stem xylem. Rootstock effects on the canopy nutrient content are influenced not only by the roots physical characteristics but also depending on the chemical composition of the soil and environmental conditions [136].

Pistachio rootstocks differ in efficiency of macro- and micronutrient uptake [20]. The *P. integerrima* rootstock is less efficient in zinc (Zn) and copper (Cu) uptake than *P. terebinthus* or *P. atlantica*. Trees on *P. integerrima* rootstocks have much higher sodium (Na), chloride (Cl) and boron (B) uptake than the reciprocal hybrids of the latter rootstock species. This tendency to absorb and translocate Na and Cl ions to the leaves can be harmful to scions on *P. integerrima* in saline environments. The PGII rootstock (*P. integerrima* × *P. atlantica*) is more efficient in Zn and Cu uptake than *P. terebinthus*; *P. atlantica* is intermediate; UCB1 and *P. integerrima* are the least efficient. Boron uptake by PGII is somewhat less efficient than *P. integerrima* rootstocks and slightly more efficient than UCB1. Boron uptake by UCB1 is similar to *P. atlantica* and *P. terebinthus*. PGII is less efficient than *P. integerrima* rootstocks and a bit more efficient than UCB1.

The 'Kerman' scion onto different pistachio rootstocks demonstrated that leaves of trees on *P. terebinthus* often have the highest nutrient levels. *P. terebinthus* was more

efficient than other rootstocks in absorbing Cu, Zn, and other micronutrients that are often deficient in pistachio orchards. PGII and *P. atlantica* rootstocks were superior to UCB1 and *P. integerrima* in absorbing Cu [137,138]. A study of 'Bianca' scion budded onto various in vitro propagated clonal rootstocks, revealed that *P. terebinthus* was the most efficient at K uptake, but less efficient in uptake of Mg. The *P. atlantica* and *P. integerrima* clones seemed to be deficient in K uptake and the most efficient in Mg uptake [16].

A four-year study of two pistachio cultivars (Akbari and Barg-Seyah) budded on six *P. vera* seedling rootstocks (Akbari, Sarakhsi, Badami-sefid, Kalle-Ghouchi, Daneshmandi, Barg-Seyah) demonstrated that, K, P, and Fe absorption differed significantly among these rootstocks. Scions on 'Badami' and 'Daneshmandi' seedlings had the maximum and minimum K absorption, respectively. 'Akbari' budded on 'Badami' was the most efficient in uptake of K and Zn. 'Kalle-Ghouchi' and 'Daneshmandi' had the maximum and minimum Fe content, respectively. The minimum K and Zn uptake occurred in 'Akbari' budded on 'Daneshmandi'. 'Barg-Seyah' budded onto 'Kalle-Ghouchi' gave the maximum Fe and Cu uptake [139]. These results indicate that selecting the appropriate rootstock and scion for a particular environment is an important decision that can affect orchard growth and yield. Tavallali and Rahemi [140] reported that leaves of pistachio cultivars grafted on 'Beneh' rootstock had higher K, P, and Zn uptake than trees on 'Badami' and 'Sarakhs' rootstocks. Leaves of pistachios on 'Badami' and 'Sarakhs' had the highest Ca and Cu content, respectively. Kernels of cultivars grafted on 'Sarakhs' rootstock had greater K, P, Mg, Cu, Fe, and Zn content than cultivars on other rootstocks. Trees grafted on *P. atlantica* seedling rootstocks were less likely to show B, Ca, or Zn deficiency [140].

These studies identified the effect of pistachio rootstocks on nutrient uptake and yield but our knowledge on the effect on nutrient uptake of different cultivars grafted on the same rootstock is limited. Surucu et al. [141] grafted 14 pistachio cultivars of different origins on a single source of *P. khinjuk* seedling rootstocks and evaluated nutrient uptake and yield. Scion cultivar 'Haciserifi' had the greatest N, P, and K accumulation, while 'Mumtaz' had the greatest uptake of Ca, Mg, and Cu. 'Vahidi' accumulated the most Fe and Zn, and 'Sel-15′ accumulated the most Mn. 'Sel-2′, 'Sel-5′, and 'Siirt' scions had the highest percentage nut split and 'Mumtaz' had the highest yield.

Knipfer et al. [142] reported that the root hydraulic conductance of 'RX1′ and 'Vlach' walnut rootstocks was more than 50% greater than 'VX211′ and possibly, one reason for the tolerance of these two rootstocks to drought stress. Under drought stress, 'Vlach' and 'RX1′ decreased root hydraulic conductivity to maintain root biomass [142].

Walnut roots selectively absorb ions when they are under stress [143]. A study of the response of own-rooted walnut varieties to salt stress showed that the tolerant varieties accumulate and translocate more K and Ca in shoots than the less tolerant varieties. In other words, the roots of salt-tolerant walnuts not only absorb more K and Ca, but also translocate more to the leaves [144]. A comparison of nutrient uptake between two walnut rootstocks, *J. hindsii* and Paradox, showed that N, P, Ca, Mg, and Mn uptake were significantly higher with Paradox rootstock [143].

In almonds, the impact of rootstock choice on concentrations of lime, alkali, B, Zn, and K has been well studied. Jiménez et al. [145] reported that high levels of sucrose, organic acids, amino acids, and PEP carboxylase activity in the roots of *Prunus* rootstocks lead to root growth and iron uptake under iron deficient conditions [145]. Trees on almond or almond × peach hybrids show reduced levels of chlorosis from iron (Fe) deficiency in high-lime soils. Somewhat less tolerant are the Myrobalan rootstocks, which will often develop some chlorotic leaves at the shoot tips by late summer. The three-way, and similarly complex, hybrids tend to show more intermediate tolerance to calcareous soils. In general, almond trees on peach perform poorly on calcareous soils, whereas trees on almond rootstocks typically perform better. All *Prunus* rootstocks are generally sensitive to alkaline soils or water containing an excess of alkali salt. Trees on almond rootstocks appear to be the most tolerant, followed by Myrobalan plum, and peach, with little difference

among the latter two. Some peach × almond hybrids have also demonstrated greater tolerance to alkali than peach or Myrobalan.

The *Prunus* scion also appears to have considerable influence on sensitivity to alkaline soils, but the extent of this influence has not been well characterized. Marianna plum (*P. cerasifera* × *P. munsoniana*) and peach show greater tolerance to excess boron than almond, which, in turn, is more tolerant than Myrobalan rootstocks. For this reason, almond rootstocks are recommended for locations where excess B is a problem. If boron is low, more vigorous rootstocks and Marianna plum are generally preferred. Almond and peach rootstocks are more likely to experience Zn deficiency than trees on Marianna. Almond trees on almond or Myrobalan rootstocks are more susceptible to K deficiency than peach, with tree death possible if not treated [146].

Reid [147] performed a leaf analysis of two pecan scions, 'Posey' and 'Pawnee', grown on 10 rootstocks: 'Chickasaw', 'Colby', 'Dooley', 'Giles', 'Greenriver', 'Major', 'Mohawk', 'Peruque', 'Posey', and 'Shoshoni'. He concluded that rootstock influenced K and Zn concentration. The greatest K accumulation was seen in trees on 'Posey' seedlings while scions on 'Greenriver' seedlings showed the least. Trees on 'Chickasaw' seedling rootstocks contained the highest amount of Zn while those on 'Major' seedlings had the least. A study of hazelnut rootstocks showed that 'Dundee' and 'Newberg' are more resistant to iron chlorosis and maintain leaves on the tree for a longer period during the season, an important aspect to be considered, as these trees can then absorb soil nutrients up for a longer period [51].

7. Precocity and Phenology

In fruit trees, there is a lag between planting and fruiting, leading to a delay in the profitability of commercial orchards. Rootstocks are not only able to induce precocity, but also increase the quality of flowers and ability to set fruits [148]. Previous results on pistachio showed that flowering time of pistachio (M1 promising genotype) can be delayed when 'Badami-e-Zarand' is used as rootstock and 'Akbari' as interstock. In contrast, 'Badami-e-Zarand' and 'Fandoghi' as rootstock without interstock had no significant effect on flowering time. In addition to flowering time, pollen tube length and growth rate were significantly affected by grafting combinations [149]. The phenological traits of two commercial pistachio varieties in Turkey ('Kirmizi' and 'Siirt') on three rootstocks (*P. vera*, *P. khinjuk*, and *P. atlantica*) were evaluated and the results showed that rootstock changed flowering time (budbreak, start, full, and end of flowering) of the studied varieties [17].

Precocious walnut genotypes have been selected from regions in Iran [14,28] and China [29,150]. Vahdati and Mohseniazar [14] reported that selected precocious genotypes had cluster bearing habit, low vigor, and good rooting ability. The use of these precocious genotypes as rootstocks is currently being studied.

Bloom time is important as it is when the crop is most vulnerable to cold and precipitation. In almond, the choice of rootstock does not have a large effect on bloom, even when late-flowering peach or plum species are used as rootstocks for early-flowering varieties [15,44]. Reighard [151] conducted a five-year study of almond grafted on peach rootstocks across multiple environments (20 locations, 17 U.S. states). They found that over a three-year evaluation period, bloom date was not significantly affected, with only a 1–2-day average difference between rootstocks/scions. Similar studies by Barbera et al. [45] also showed that bloom time was not significantly changed by rootstock species, though they did report some scion-dependent variation. For example, 'Marinada' showed significant differences in bloom on different rootstock species while no significant differences were observed for 'Vairo' [152]. We found almond cultivars bloomed earlier on 'Garrigues' and the plum-based rootstocks 'Montizo', 'Root-Pac 20′, and 'Rootpac R', but flowering was delayed on almond × peach hybrids 'GF-677′ and 'Garnem' and Monegro'. Rootstocks can have a more significant effect on the time of nut maturity. Almond scion matured earlier in plum based rootstock than in almond peach rootstock [15]. Similar results have been found

in California that 'Nonpareil' matured earlier on a plum based rootstock ('Rootpac-R') than on more vigorous rootstocks like almond peach hybrids [44].

There are some reports that confirm the effect of rootstock on flowering and bud growth in the other nut trees. Grauke and Pratt [49] reported that bud growth of pecan trees was influenced by rootstock and scion. So that, among three studied pecan cultivars ('Candy', 'Cape Fear', and 'Stuart'), bud growth of 'Candy' trees was more advanced than 'Cape Fear', and 'Stuart'. In addition, the different studied rootstocks had significant effect on 'Candy' cultivar and thus on the severity of freeze damage [49].

8. Yield

Rootstock choice is an influential factor in determining orchard performance by increasing water and nutrient uptake, promoting scion growth, alleviating biotic and abiotic stresses, and conferring adaptability to environmental conditions [148]. In pistachio it is possible to increase productivity and yield with vigorous rootstocks. The effect of four different rootstocks on the marketable yield of pistachio trees (*P. vera* cv. 'Kerman') in three identical rootstock trials grown in three different micro-climates in California was investigated during the first 5 years of production, from 1989 through 2001. The pistachio trees on UCB1 seedling rootstocks produced 45.3% more marketable yield than trees on *P. atlantica*, 19.1% more than trees on *P. integerrima*, and 15.1% more than trees grown on PGII [153]. An analysis of the components of yield in pistachio (clusters per tree, nuts per cluster, and nut size) showed that trees on UCB1 seedling rootstock had greater yield due to larger trees, resulting in more clusters per tree, not a higher density of clusters and not more or bigger nuts per cluster. This suggests that trees on different rootstocks, when pruned to the same size of canopy, may yield equally, or trees on less vigorous but more efficient rootstocks planted at high densities could potentially be more productive than trees on UCB1. New pistachio rootstock investigations are needed to evaluate these suggestions [20].

Rahemi and Tavallali [18] demonstrated that increased vegetative growth in pistachio is not necessarily an advantage, as it may not be associated with increased yield. They concluded that the interaction of rootstock and scion influences scion vigor, shell split, blanks, nut weight, and overall yield [18].

Yield efficiency of walnut is affected significantly by rootstock. A five-year study of clonal rootstocks showed that 'Chandler' grafted on RX1 had the highest yield while own-rooted 'Chandler' trees had the least. In general, the yield on Paradox, seedling or clonal, exceeded yield of own-rooted 'Chandler' [154]. Connell et al. [155] reported that own-rooted 'Chandler' trees had fewer catkins, lower yield efficiency, and better nut quality than 'Chandler' grafted on Paradox or *J. regia* cv. Waterloo. The grafted Chandler trees, especially on 'Trinta' paradox, had highest yield efficiency [155]. Another study comparing micropropagated ungrafted 'Chandler' to 'Chandler' grafted on Paradox, showed that although own-rooted 'Chandler' had greater trunk diameter and yield than grafted trees in the early years, after six years there was no significant difference in these parameters. The own-rooted trees were more sensitive to nematodes and showed more dieback [156,157]. Rootstocks also can indirectly improve yield by resistance to biotic or abiotic stresses. For example, the 'RX1′ clonal rootstock is resistant to *Phytophthora* [158].

Many reports demonstrate the influence of rootstock on almond yields. In a current regional rootstock trial in California, the four-year average cumulative yield over two locations was consistently higher for 'Kester' on peach × almond 'Hansen' rootstock than 'Kester' on 'Nemaguard'. In a separate 13-year rootstock study, the survival rate of almond on hybrid 'GF 677′ and pure almond rootstocks was higher than on 'GF 305′ peach rootstock. While trees on 'GF 677′ and almond rootstocks differed in shoot vigor, there was no difference in final yield [159]. Similarly, differences in yield were observed for the cultivar 'Marinada' when grafted onto 10 rootstocks of different vigor [152]. Trees on less vigorous rootstocks, such as the Rootpac® series, produced kernels with lower quality,

more breakage, and stronger shells than trees on more vigorous rootstocks, including 'Garnem', 'Cadaman', and 'GF-677' [152].

Higher yields are often correlated with larger tree size. In almonds, greater cumulative yield was found at the 10th leaf on both 'Nickels' and 'Empyrean 1', which also developed significantly larger trees [44]. Intermediate yields were found on the rootstocks 'Lovell' and 'Krymsk 86', which developed smaller tree sizes. *P. scorparia* rootstock exhibited a significantly higher nut yield than *P. dulcis* cv. 'Estahban' under non-irrigated conditions, probably due to the greater tolerance of *P. scorparia* to drought stress [42]. Prune hybrid rootstocks (plum × prune) and (peach × prune) produced greater fruit set than trees on the more traditional almond and peach rootstocks [160]. Preliminary results for two almond cultivars grown on hybrid rootstocks of differing vigor, and under either rainfed or irrigated conditions, showed that the almond × peach hybrid 'Monegro', when grafted with the scion cultivar 'Vialfas', had higher yield potential than trees on 'Garnem' or 'GF-677' under either water regime and these trees had greater vigor than those on plum rootstocks ('Rootpac R' and 'Montizo') when irrigated. The effect of rootstock on the yield of other nut trees has also been studied. In a trial conducted in IRTA-Mas de Bover, Spain, 'Negret' was grafted on four clonal rootstocks ('Newberg', 'Dundee', 'Tonda Bianca', and 'MB-69'). Results showed that rootstocks had a significant effect on yield of scion and the highest yield was obtained when 'Dundee' was used as rootstock [51].

9. Nut Quality

Nut quality is a complex trait which is ultimately defined by consumer preference. Nut quality is foremost a scion trait, so manipulating it by rootstock is difficult and not a straight-forward process. Rootstocks may affect nut quality through their impact on water and nutrient uptake, photosynthesis rate and subsequent assimilation into the crop, but effects of rootstock on commercial nut quality have not been studied extensively.

Pistachio quality factors include nut size, nut weight, percentage of split nuts, and frequency of blanks [56]. Long-term pistachio field trials in California have found little effect of rootstocks on nut characteristics and suggest that quality improvement comes mainly from scion cultivar breeding [20]. Another study found that nut quality of the cultivar 'Kerman', including splits, nut size, oil content, color, flavor, and aftertaste, was significantly influenced by rootstock. Use of *P. atlantica* rootstock increased kernel mineral content, sensory attributes, and consumer satisfaction relative to *P. integerrima* or *P. terebinthus* [161]. Nut split is very important commercially in pistachio. This trait is largely controlled by genetic factors of the scion cultivar, also affected by cultural practices and rootstock. Turker and Ak [162] investigated the effect of pistachio rootstocks (*P. vera*, *P. khinjuk*, and *P. atlantica*) on nut split, blanks, and total filled nuts, by budding cultivars 'Siirt' and 'Ohadi' onto these rootstocks. Cultivars on *P. atlantica* produced nuts with the greatest number of splits and filled nuts, and the fewest blanks.

In walnuts, Buchner et al. [163] investigated effects of deficit irrigation on quality of trees on either *J. hindsii* or Paradox rootstocks. Connell et al. [155] found that own-rooted 'Chandler' trees had better nut quality (higher edible kernel percentage and light kernel color) than 'Chandler' trees grafted on clonal Paradox [161].

Almond kernel quality is determined by both physical and chemical parameters. Rootstock effect on kernel size appears to be a result of effect on tree size. In the current U.S. regional rootstock trial, nut size was significantly smaller when trees were grown on dwarfing rootstocks [44]. In another study, the cultivar 'Marinada' was evaluated on 10 different rootstocks and significant differences were found in shell thickness, kernel weight, length, width, and thickness, and in yellow pigmentation of the almond pellicle [152]. Khadivi-Khub and Anjam [42] reported no significant differences in kernel thickness, weight, kernel percentage, doubles, shrivel or pellicle color when trees were grown on *P. scoparia* rootstock vs. 'Estahban' almond rootstock, although nut length and width was greater on *P. scoparia* rootstock. Differences in kernel weights, and to a lesser degree, shelling percentage, were reported in a study of almond cultivars 'Marinada' and 'Vairo'

grown on a genetically diverse set of rootstocks including ‘Cadaman’, ‘Garnem’, ‘GF-677′, ‘IRTA-1′, ‘IRTA-2′, ‘Ishtara’, ‘Adesoto’, ‘Rootpac 20′, ‘Rootpac 40′, and ‘Rootpac R’ [15]. In a different study by Reighard [151], nut size and maturity were generally not affected, except for a peach × plum hybrid rootstock which produced smaller fruit and a peach × plum hybrid that produced the largest fruit.

Rootstocks also can significantly influence oil content, fatty acid profile, total phenol content, and radical-scavenging activity of kernels. Čolić et al. [164] examined the influence of non-irrigated rootstocks ‘GF-677′ and Myrobalan plum, on fatty acid (oleic and linoleic) content, total phenolics content (TPC), and radical scavenging activity (RSA) in kernels of almond cultivars ‘Marcona’, ‘Texas’, and ‘Troito’. Myrobalan plum rootstock gave a significantly higher oil content in ‘Marcona’ and ‘Texas’ scions, while oleic acid was significantly higher in ‘Texas’ on rootstock ‘GF-677′. In addition, the oleic/linoleic ratio, which is an indicator of vulnerability to rancidity through lipid oxidation, was found to be significantly higher in ‘Texas’ on ‘GF-677′ rootstock. By comparison, Barbera et al. [45] examined almond cultivars ‘Tuono’ and ‘Ferragnes’ on both peach and almond rootstocks and failed to find any significant difference in the fatty acid composition of kernels but did find significant differences in kernel moisture, oil, ash, and nutrients (K, Ca, Z, Fe, Mn).

10. Alleviation of Abiotic Stresses

Climate change, higher and erratic temperatures, and altered precipitation regimes have simultaneously led to an increase of abiotic stresses and potentially serious drops in crop production [165,166]. Several studies of abiotic stress-tolerance in nut trees have led to release of stress-tolerant rootstocks. Nevertheless, our knowledge about the physiological and molecular mechanisms involved in abiotic stress tolerance in nut trees remains limited.

Drought stress greatly limits nut production by upsetting the carbon/nitrogen (C/N) balance by reducing photosynthesis as a result of stomata closure and a drop in leaf water potential [167,168]. Drought stress also affects root architecture and anatomical parameters as well as mineral elements in the roots [169,170]. Alleviating oxidative stress, increasing the accumulation of osmoregulators, and alteration of hormonal signaling and the mobility of genetic components, are additional mechanisms which may play a role in the drought stress tolerance of rootstocks [168,170–172].

Pistachio is a drought and salinity tolerant species [173]. Although pistachio orchards are irrigated in California and in many parts of Iran, pistachio is cultivated under unirrigated or deficit irrigated conditions in Turkey, Syria, and Spain. Thus, drought stress is one of the main stresses affecting pistachio cultivation and yield in unirrigated areas. Gijón et al. [174] studied the drought resistance of pistachio cultivar ‘Kerman’ on three rootstocks (*P. terebinthus*, *P. atlantica,* and hybrid *P. atlantica* × *P. vera*). The *P. atlantica* was highly sensitive to water stress with low stomatal control of transpiration, while *P. terebinthus* had the greatest resistance to water stress with better stomatal control. Moriana et al. [175] investigated the effect of water stress on ‘Kerman’ grafted onto three pistachio rootstocks (UCB1, *P. terebinthus* and *P. atlantica*). All three rootstocks showed dehydration leading to reduction in vegetative growth and number of leaves, while root weight was promoted. UCB1 was least affected by drought stress and *P. atlantica* also showed good tolerance. Drought stress also affects the foliar epidermal anatomy of pistachio trees. Regulated deficit irrigation (RDI) of ‘Kerman’ grafted onto *P. atlantica*, *P. integerrima*, or *P. terebinthus* rootstocks and grown on shallow soils was studied by Memmi et al. [176]. The RDI irrigation regime decreased by 40% water compared to normal irrigation. The *P. integerrima* rootstocks had less tolerance to drought than *P. atlantica* or *P. terebinthus*. Carbonell-Barrachina et al. [161] investigated the performance of the same rootstocks under RDI. Yield, nut weight, mineral content, and consumer satisfaction were all greater for the trees grown on *P. atlantica*. In another study, nuts produced under RDI on *P. terebinthus* and *P. atlantica* rootstocks had higher polyphenol and tri-terpenoid content than those produced on *P. integerrima* [177]. Noguera-Artiaga et al. [178] also studied the effect of RDI on ‘Kerman’ trees budded on

P. atlantica, *P. integerrima*, and *P. terebinthus* rootstocks. Nuts produced on *P. terebinthus* rootstock had the largest size, greatest weight, and most oleic acid.

Soil salinization is a serious obstacle to pistachio production in the majority of growing areas in Iran and in many parts of the world [179]. Salinity affects the ionic balance. Hyperosmotic stress in plants leads to competition between Na and K ions. A decrease of K causes the inhibition of important metabolic enzymes [180]. In the United States, hybrid seedling rootstock 'UCB1′' is generated from a controlled cross of *P. atlantica* × *P. integerrima*. This rootstock is favored due to its high vigor and resistance to many biotic and abiotic stresses [181]. According to Ferguson et al. [20], *P. atlantica* is the most salt tolerant rootstock, followed by 'UCB1′' and *P. integerrima*.

Salinity stress is the main research objective of Iranian pistachio researchers [179,181–186]. The cultivars 'Ghazvini', 'Badami', and 'Kaleh-Ghouchi' are the most favorable *P. vera* rootstocks in Iran for tolerance to salinity and drought stress [181]. Hokmabadi et al. [182] studied the effect of salinity on three *P. vera* rootstocks ('Ghazvini', 'Badami', and 'Sarakhs') under different salinity treatments (0, 75, 150, and 225 Mm NaCl) and detected a decrease in K ions in the roots and stems of all rootstocks. However, the decrease in 'Ghazvini' was less than the other two, suggesting greater salt tolerance. 'Ghazvini' also proved to be more salt tolerant than the other two in *Verticillium* infected soil conditions [183] and 'Ghazvini' was more calcium-tolerant than 'Badami' [184]. Karimi and Roosta [187] and Karimi and Maleki Kuhbanani [188] suggested 'Badami Zarand' and an inter-specific hybrid of *P. atlantica* × *P. vera*, were more salt tolerant than 'Ghazvini'. With the increasing popularity of UCB1 seedling rootstock worldwide, researchers in Iran have initiated investigations of its possible use in Iran for saline and drought stress conditions. Salinity tolerance of five rootstocks ('Akbari', 'Badami', 'Ghazvini', 'Kaleh-Ghouchi', and UCB-1) were compared for ion homeostasis, osmoregulation, and physiological changes [179] and antioxidative activities [185] in leaves and roots. In both studies, UCB1 appeared to be the most salt-tolerant, followed by 'Badami', 'Ghazvini', 'Kale-Ghouchi', and 'Akbari'. In most of the pistachio growing areas of Iran, salt and drought stress occur together. The physiological and biochemical responses of plant to these stresses combined cannot be directly assessed from their response to each single stress [181,186,189]. Goharrizi et al. [181,186] studied the effect of salt, drought, and salt + drought stress on four pistachio seedling rootstocks ('Badami', 'Ghazvini', 'Kale-Ghouchi', and 'UCB1′'). The effect of these three stresses, in order from strong to weak, was drought + salinity > salinity > drought. Tolerance of the four rootstocks, to all three types of stress in order from high to low, was UCB1, 'Badami', 'Ghazvini', and 'Kaleh-Ghouchi'.

Cold stress is an additional concern for pistachio production, notably in some pistachio growing regions in Iran. Cold tolerance is important for newly established young orchards, especially when the *P. integerrima* or UCB1 rootstocks are used, but not as damaging to mature orchards [56]. Among the common pistachio rootstocks, *P. terebinthus* is the most cold-hardy, followed by *P. atlantica*, and UCB1, with *P. integerrima* being the least cold hardy. In California, the main scion cultivar 'Kerman' is more cold-tolerant than its rootstocks (PG1 and UCB1) [20].

The selection of drought-tolerant walnut rootstocks is especially important in arid and semi-arid regions and resistance to drought stress can be genotype dependent. A study of stem xylem anatomy in walnut species and hybrids found *J. microcarpa* had greater resistance to drought-induced embolism than *J. ailantifolia* or *J. hindsii* [190,191]. Hybrids of *J. microcarpa* × *J. regia* ('RX1′') and *J. hindsii* × *J. regia* ('Vlach' and 'VX211′'), which are common clonal walnut rootstocks, have a better response to drought stress and are able to preserve their root biomass under drought stress. Drought tolerance in RX1 and VX211 was accompanied by greater leaf water use efficiency and leaf turgor, and reduced hydraulic conductivity in the root system hydraulic conductance (Kro) [142]. Liu et al. [192] reported that *J. mandshurica* and *J. regia* cv. Jizhaomian were more tolerant than *J. nigra* and associated with increased WUE, greater chlorophyll fluorescence, and better gas exchange. It seems

that leaf water use efficiency, Kro, and leaf turgor are useful canopy traits for selecting drought-tolerant rootstocks [142,192,193].

Given that of the area of Persian walnut origin includes arid and semi-arid regions, utilization of genetic diversity can be an effective strategy in the development of drought-tolerant rootstocks. A walnut rootstock breeding program based on exploration of genetic diversity started at the University of Tehran, Iran in collaboration with University of California-Davis in 2008. Preliminary studies led to identification of some drought-tolerant candidate genotypes and to understanding of some physiological mechanisms involved in drought tolerance [167]. Accordingly, several physiological processes, including cavitation resistance via stomatal regulation, maintenance of net assimilation and photosynthetic rate, increasing antioxidative enzyme activity (POD, APX, CAT, SOD, and LOX), accumulation of proline and total soluble sugars, and improved WUE, are responsible for drought tolerance in walnut genotypes [168,172,194,195]. WUE differences were studied also in a wild population of *J. regia*, examining variation in δ13C (carbon isotope composition) as a surrogate for intrinsic water-use efficiency (WUEi) [32].

New and advanced biotechnology techniques have accelerated the understanding of the molecular mechanisms involved in drought tolerance in walnut. Considering that WUE is associated with drought tolerance, a natural Persian walnut population that was diverse in WUE was used to study the relationship between phenotypic and genotypic traits, using association analysis and a large data set of SNPs. This study led to identification of drought stress-responsive genes involved in ABA signaling, antioxidant responses, stomatal regulation, osmotic adjustment, transduction of environmental signals, and leaf development [193]. In addition to exploiting genetic diversity, genetic transformation has been used successfully to induce drought and salt tolerance in walnut. Sheikh Beig Goharrizi et al. [196] reported that Persian walnut genetically transformed with a flavodoxin (*fld*) gene had better growth under both osmotic and salinity stress.

In contrast to drought stress, studies of salinity-tolerant rootstocks are rare in walnut. Salinity is an important environmental stress that mostly affects growth and physiological aspects of nut trees. An examination of the response of *Juglans* species to salinity stress showed that *J. hindsii* and its hybrid (Paradox) are more tolerant than Persian walnut [197].

Waterlogging can result in root asphyxiation and later in *Phytophthora* damage; particularly with spring rains and poorly drained soils. Unlike Chinese wingnut (*Pterocarya stenoptera*) that is very tolerant, Juglans species are highly sensitive to waterlogging, probably due to a shift in cellular metabolism towards production of acetaldehyde and ethanol under anaerobic conditions. Ethanol production and accumulation in roots is the start of events leading to cell death. The ethanol produced in roots moves up to the leaves and is released to the external environment [198,199]. During waterlogging, transfer of ABA to the leaves leads to an increase in leaf ABA content and plays a critical role in reducing growth [200].

Almond seedlings have traditionally been used as rootstocks in arid and semi-arid regions due to their performance on calcareous soils under limited rainfed conditions. However, almond rootstocks are susceptible to fungal diseases and nematodes, as well as to root asphyxia in wet and poorly drained soils. For this reason, other rootstock species have been utilized, particularly peach and plum, as well as their interspecific hybrids. In recent years, knowledge of the physiological behavior of hybrid *Prunus* rootstocks under drought stress has improved. In a long-term drought experiment, the almond × peach hybrid, 'Garnem' consumed its water reserves during the first days of drought stress in order to maintain shoot growth rate. As water stress became more severe, water consumption diminished in response to the loss of hydraulic conductivity [201]. In shorter-term drought experiments, 'Garnem' was able to maintain high leaf water content rates under low water potential, as well as preserve a high cell membrane stability, indicating osmotic adjustment as part of its drought tolerance mechanism [202,203]. In addition, abscisic acid (ABA) was demonstrated to be involved in rapid long-distance hydraulic signaling from root to shoot for inducing stomatal closure in drought stressed 'Garnem' [202]. Recent research

has also provided insights into the genetic response of *Prunus* species under drought, identifying key drought-responsive genes, including those directly related to water use efficiency (WUE). These include ERF023TF; LRR receptor-like serine/threonine-kinase ERECTA; and NF-YB3TF [203] as well as the gene ppa008651m coding for a LEA protein homolog to LEA D29 and PpDhn1 [201], and PpDhn2 and DREB2B [204]. No less important has been the characterization of natural sources of drought tolerance. Bielsa et al. [204] investigated differences in 48 *Prunus* species by evaluating leaf ash content and carbon isotope discrimination (Δ^{13}C), which are strongly correlated with WUE. Almond and wild peach species showed the lowest Δ13C ratios, and therefore, greater WUE than hybrid genotypes, although, among the GN serie ′Monegro′ showed the greatest WUE [204].

An important abiotic limitation to almond production is root asphyxia on heavy soils. *Prunus* rootstocks vary in their response, demonstrating different levels of susceptibility. European plum (*P. domestica* L.) and Myrobalan plum rootstocks are considered root-asphyxia tolerant, while almond, peach, and their hybrids, are more susceptible to waterlogging damage [205]. The physiological response to hypoxia has previously been shown to be under genetic control. Both gas exchange parameters and photosynthetic activity were strongly affected in sensitive genotypes relative to more tolerant genotypes [205]. In addition, morpho-anatomical changes were shown to be important factors in conferring tolerance [206].

Recent studies of alterations in metabolism and regulatory processes in *Prunus* under waterlogging stress have led to the identification of candidate genes involved and to clarifying their roles in waterlogging response. Arismendi et al. [207] found groups of differentially expressed genes coding for key enzymes that were upregulated under hypoxia in tolerant, but not in sensitive, genotypes. These were associated with post-transcriptional protein modifications, such ashexokinases (HXK) and fructokinases (FRK), as well as genes coding for proteins involved in transcription regulation, including AP2 domain-containing, ARR6 (Response regulator 6), Sin3-like2, and zinc finger (GATA type) proteins. Other strategies have also been demonstrated in tolerant and sensitive genotypes under hypoxia conditions. Rubio-Cabetas et al. [208] demonstrated that the tolerant Myrobalan 'P.2175′ plum represses secondary metabolism gene expression as a strategy to prevent the waste of resources/energy. At the same time, they reported the upregulation of protein degradation genes, which led to structural adaptations conferring long-term tolerance to hypoxia. The more sensitive almond-peach hybrid 'Felinem' (*P. amygdalus* × *P. persica*) was found to upregulate a group of signal transduction and transcription factor genes [209]. In addition, three candidate genes involved in the oxygen sensing mechanism were identified as possible biomarkers for hypoxia-tolerant selection, including the genes *ERF74/RAP2.12*, *ACBP1/2*, and *HCR1* [208].

The temperature, especially low or freezing conditions, is an important abiotic factor that affects the growth of pecan trees at various growth stages, and is affected by rootstock [210]. The pecan rootstocks most used in Georgia are seedlings of 'Curtis' and 'Elliott'. Both give good germination and quickly develop large stem calipers, but 'Curtis' is more resistant to cold [211]. Among eleven rootstocks, 'Apache', 'Giles', and 'Peruque' were in the group of the least damaged after a freeze event on 8–9 October 2000 (−2 °C and −5 °C, respectively). Among the scion cultivars, 'Kanza' was less damaged than 'Mohawk', 'Mount', or 'Creek' [212]. 'Kanza' exhibited no injury when other cultivars were severely injured during an autumn and winter freeze in Oklahoma, and is considered most cold hardy [213,214]. 'Pawnee' is resistant to both fall and midwinter freeze damage, but is one of the first cultivars to break bud in the spring, making it highly susceptible to spring frost damage [212]. Smith [212] reported that 'Pawnee' grafts (six and seven years old) showed significant damage after freeze events in October 2000, although 1-year-old 'Pawnee' grafts were not damaged by a freeze in November 1991 [214]. After the same freeze event (7 October 2000) trees grafted to 'Kanza' and 'Pawnee' in southwest Missouri experienced the most death, while 'Posey' and 'Dooley' suffered the least damage [215]. It was concluded that scion cultivar impacts the cold hardiness of the above-ground part of the rootstock

and that 'Kanza' and 'Pawnee' scions decreased the cold resistance of the rootstock during this early autumn freeze event because they enter into dormancy later in the fall [215].

In addition to autumn freezes, very cold winters can cause serious damage to pecan trees. Symptoms typically are death and browning of the cambium, inner bark, and phloem, as well as splitting and browning of the rootstock inner bark and phloem, and delayed bud break [216,217]. Some rootstocks impart sufficient cold resistance for a cultivar to reduce or escape damage on one rootstock type, while being severely damaged on another type [212,218]. Cultivars ('Choctaw' and 'Wichita') grafted onto 'Apache' seedling rootstock showed one third of the damage from a fall freeze on 14 November 1976 (14 °F) than the same cultivars grafted onto 'Riverside' seedling rootstock [219]. The extent of freeze damage was evaluated by observing the extent of cambium discoloration or browning [219]. 'Apache' rootstock produced cold-hardy and fast-growing trees [210]. Similar findings were observed by Hinrichs [218]. He observed that 'Stuart' scion was killed on some rootstocks by cold fall temperatures, while the same scion was not damaged on the 'Giles', 'Major', and 'Indiana' rootstocks [218]. 'Stuart' exhibited less injury during both fall and winter freeze [213,220], and early autumn freezes [212]. 'Desirable' and 'Mohawk' budded on 'Apache' were the most damaged by this freeze, while 'Wichita' and 'Choctaw' budded to same seeding rootstock ('Apache') were the least damaged [220]. Variation in cold injury also was observed among different scions on 'Curtis' seedling rootstock [221].

The 'Pawnee' scion was the most resistant to early fall freeze injury (30 October–1 November 1993) among the nine scion cultivars and un-grafted 'Elliott' rootstock seedlings [222]. Similarly, freeze resistance by 'Pawnee' and 'Elliott' was observed by Goff and Tyson [223]. These observations indicate that the scion can also increase the cold resistance of a juvenile rootstock, just as the rootstock impacts the cultivar susceptibility [219,222]. The 'Kanza' cultivar exhibited much less low winter temperature injury than other cultivars and is one of the last cultivars to break bud dormancy in the spring [214]. The selection of rootstocks and cultivars resistant to winter freeze damage is an important aspect to avoiding loss during freeze events [220].

Late-spring frost is another aspect of low temperature limiting tree nut cultivation. Damage caused by a spring freeze (−5 °C on 22 May 1986) to one-year old grafted trees was significantly influenced by rootstock and scion, and was directly correlated with stage of bud growth at the time of the freeze event [49]. Pecan rootstocks such as 'Giles', 'Peruque', or 'Colby' (northern origin) are considered hardier than 'Riverside' or 'Moore' (southern origin) to late-spring frost [210]. The southern seedling rootstock break bud dormancy earlier in the spring and are more susceptible to spring freeze damage [224]. Scion growth has been observed to vary as a function of rootstock, with early leafing rootstock also forcing early spring growth in scions [49]. The 'Stuart' seedlings tend to begin growth later in the spring, offering some protection from spring freeze. 'Elliott' seedlings (known for excellent nut quality) have early spring growth, making them more susceptible to freeze damage than 'Moore' (rootstock) [225].

Twelve pecan rootstocks including '87MX1-2.2′, '87MX5-1.7′, 'Elliott', 'Frutoso', 'Giles', 'Major', 'Moore', 'Peruque', 'Posey', 'Riverside', 'San Felipe', and 'VC1-68′ were tested under drought conditions. Among them, 'Posey' had the highest resistance and greatest water content under environmental pressure followed by 'Perque' with lowest $\hat{\varepsilon}$max (Bulk Elastic Modulus) value in PV test while 'Frutoso' with lowest Va/Vp and shoot tissue water content in transpiration test had the lowest resistance [226].

The physiological roles of mycorrhizal fungi, including arbuscular mycorrhizal fungi on seedlings and nut trees, are increasingly studied. Mycorrhizal fungi provide beneficial symbiosis in the roots of nut trees, accelerate plant growth and biomass production, nutrient acquisition, and increase potential tolerance to abiotic stress, e.g., drought and salt tolerance [166,227–230]. Consequently, future outlooks in this field seem optimistic.

11. Resistance to Biotic Stresses

In pistachio, several fungal and bacterial diseases can infest both the above-ground and under-ground tree parts [231]. Among these, *Phytophthora* root and crown rot (*Phytophthora* spp.), *Armillaria* root rot (*Armillaria mellea* Vahl.), and Verticillium wilt (*Verticillium dahlia*) are the three most serious soilborne fungal diseases of pistachio trees worldwide [56]. The *P. vera* is the only pistachio species that produces edible nuts large enough for commercial use [232,233]. Initial evaluations demonstrated that the *P. vera* seedling trees were susceptible to soilborne pathogens *Phytophthora* spp.; *Verticillium dahlia*, and nematodes [96]. The best defense against soilborne diseases is the use of resistant or tolerant rootstocks. Therefore, other available *Pistacia* spp. were used as rootstocks. Verticillium wilt has killed a majority of the trees in the late 1970s and caused growers the most severe economic losses ever experienced in California. A small number of *P. integerrima* seedlings from an Iranian seedling tree selected and planted at the USDA Plant Introduction Station in Chico (California) were found to be tolerant to Verticillium wilt; the trees can be infested but exhibit few symptoms and no mortality. This *P. integerrima* seedling rootstock was quickly commercialized as Pioneer Gold 1 (PG1). *Verticillium*-tolerant *P. integerrima* was then used to produce UCB1 (University of California Berkeley 1) which is moderately resistant to this disease; it exhibits mild symptoms when infested but, as with PGI, no mortality. The *P. atlantica* and *P. terebinthus* rootstocks are susceptible to Verticillium wilt [20]. *Armillaria* root rot occasionally affects pistachio and resistant rootstocks would offer the best protection. Field trials indicate that *P. terebinthus* and UCB1 are tolerant, whereas *P. atlantica* and *P. integerrima* are susceptible to this pathogen [234]. Root and crown rot caused by *Phytophthora* spp. also can affect pistachio trees. According to Ferguson et al. [20], UCB1 and *P. atlantica* are more tolerant to *Phytophthora* root and crown rot than *P. integerima*. Epstein et al. [235] studied the resistance of four rootstocks (UCB1, PGII, *P. atlantica,* and *P. integerrima*) to *Verticillium dahlia*. Yield, growth, incidence of *Verticillium* symptoms, and mortality rates were studied for 10 consecutive years. UCB1 and *P. integerrima* showed the greatest tree vigor, and UCB1 had the fewest symptoms. UCB1 is also resistant to *Phytophthora* [20]. Thus, UCB1 has become the major rootstock in California. However, a stunted and difficult-to-graft phenotype has emerged in California in clonally reproduced UCB1 rootstocks from multiple sources. This has been at times, since 2010, a serious production problem which has been variously attributed to either somaclonal mutation during in vitro propagation or to *Rhodoccus* sp. bacterial infection [236], Chang et al. [237]. The syndrome was identified by its appearance as Pistachio Bushy Top Syndrome, PBTS. However, as this problem proved to be non-transmissible in the field, and nurseries can now identify it in young rootstocks, it is no longer a problem. Nouri et al. [238] reported a new pathogen, *Macrophomina phaseolina* in Kern County of California which is characterized by wilted foliage combined with crown rot of the rootstock. UCB1 is highly susceptible to *M. phaseolina* and this pathogen is now an emerging threat to pistachio production in California.

One important biotic stress in Persian walnut is blackline disease caused by *Cherry leaf roll virus* (CLRV) (Figure 2) [239]. Persian walnut tolerates this virus and is generally symptomless. In contrast, *J. hindsii* or its hybrids are resistant to CLRV. Blackline symptoms occur when a hypersensitive rootstock [Northern California Black walnut (*Juglans hindsii*), other black walnuts, or hybrids of these with *J. regia*, are used as rootstock for Persian walnut [240,241]. The virus is transmitted through infected pollen and scions. The pollen-borne virus enters through flowers during pollination and is systemically transported to the graft union. The resulting hypersensitive reaction of the rootstock and death of tissue at the graft union blocks nutrient and water transport between the rootstock and scion [61]. The hypersensitive response to this virus is controlled by a single dominant gene (*R* gene) [240]. To develop CLRV-resistant scion cultivars capable of blocking the virus at the pistillate flower and/or movement toward the graft union, a breeding program was initiated in 1984 the University of California-Davis (UC-Davis) to backcross resistance from Paradox into scion cultivars with commercially acceptable horticultural traits. This program is still ongoing [240]. A DNA marker related to CLRV-resistance that maps to ~6.2 Mb on

chromosome 14 has been developed in order to accelerate selection of CLRV-resistant offspring [242–245]. In continuation of work started by E. Germain (INRA-Bordeaux), a hybrid resistant to blackline is in evaluation to be registered in France.

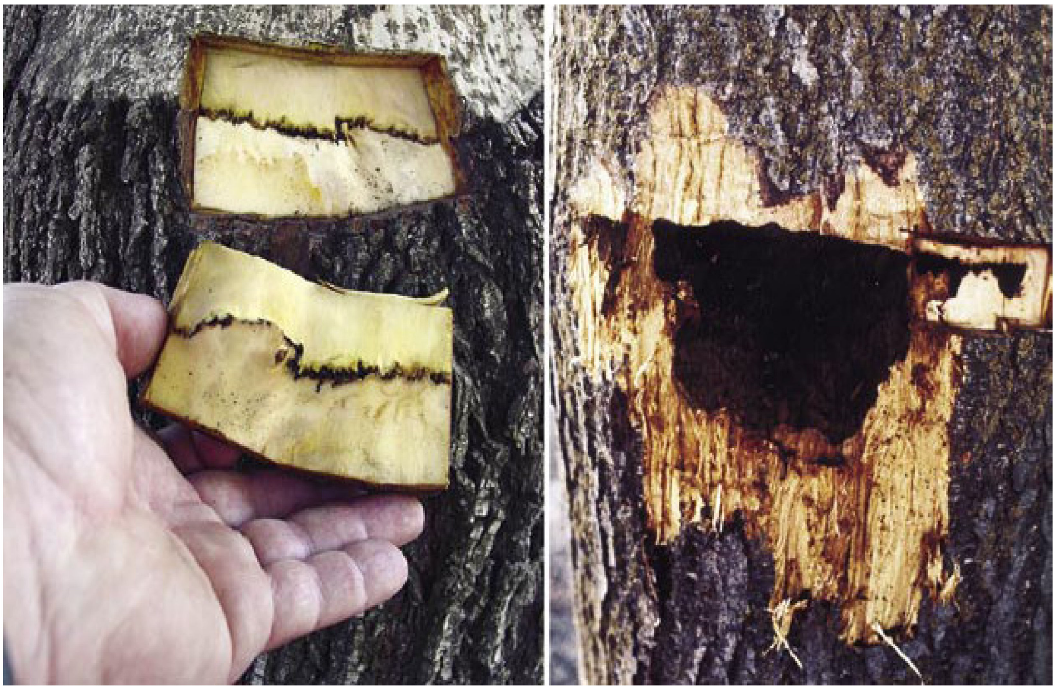

Figure 2. Symptoms of blackline disease in walnut at graft union.

In California, screening of a huge multi-species *Juglans* population, *J. regia*, *J. microcarpa*, *J. major*, *J. cathayensis*, and others and targeted interspecies hybridization between the selected superior genotypes to produce rootstocks resistant to the soil borne pathogens, *Agrobacterium tumefaciens*, *Phytophthora* spp.; *Pratylenchus vulnus*, and *Armillaria mellea*, has been in progress for several years and is continuing [246]. Crown gall (*Agrobacterium tumefaciens*) is a major rootstock issue in walnuts, particularly when using Paradox hybrid rootstocks. This bacterial disease can significantly reduce production and increase management costs. The RNAi technology, RNA interference has been used experimentally to suppress genes involved in the plant response to the bacterium [247]. Silencing of tryptophan monooxygenase (*iaaM*) and isopentenyl transferase (*ipt*) genes blocks bacterial induction of de novo auxin and cytokinin and therefore prevents gall development [248].

Using RNAi-mediated silencing technology, walnut researchers at UC-Davis were also able to develop apparent nematode resistance in Paradox microshoots evaluated in vitro but this work has not been confirmed in the greenhouse or field trials [249].

Nematodes are another serious problem for nut growers. Three separate root-knot nematode (*RKN*) resistance genes have been identified in *Prunus* species, *Ma* in the Myrobalan plum clones 'P2980′ and 'P2175′, *RMia* in the peach rootstock 'Nemared', and *RMja* in the bitter almond 'Alnem'. Pyramiding of these three genes by interspecific crosses of almond × peach × Myrobalan is the main objective of the French rootstock breeding program [250]. To ensure the presence of the three genes in the same rootstock, it has been necessary to develop effective molecular markers. The identification of intra-gene markers for nematode-resistance genes *Ma* and *RMia* has allowed the application of marker-assisted selection for these two genes [250,251]. The *RMja* gene is located on linkage group 7 of the *Prunus* genome in the same region as the *Ma* gene [252,253].

Chestnut cultivation has been threatened by chestnut blight (*Cryphonectria parasitica*) and root rot (*Phytophthora* spp.) diseases. The first pandemic disease for chestnut was root rot [254]. Today two species of root rot (*P. cinnamomi* and *P. cambivora*) are widely

spread in Europe and Asia Minor [255]. The most effective method against root rot disease is using resistant rootstocks. Due to the resistance to root rot, *C. crenata* and *C. mollissima* trees were imported into Europe at the beginning of the 19th century [254]. The following years showed that their nut quality was low, and they were sensitive to spring frosts [256]. Therefore, they were used as rootstocks. However, graft incompatibility was observed [85,256–258]. In France, two of these genotypes were registered as 'Ipharra' and 'Marki'. Schad et al. [259] planted some superior genotypes in orchards infected with *Phytophthora* spp. in 1946. As a result of this study, natural hybrids of *C. crenata* × *C. sativa* ('Marigoule', 'Ferosacre', 'Marsol', 'Maraval', and 'Précoce Migoule') were obtained [260,261]. Amongst those, 'Marsol' and 'Maraval' have been used as resistant rootstocks. 'Marigoule' has been used in forest areas due to its fast-growing characteristic [254]. Now, 'Marigoule' is also used as rootstocks in many countries [113] due to its resistance to root rot (*Phytophthora* spp.) and tolerance to the chestnut blight (*C. parasitica*). However, seedlings of the 'Marigoule' are not tolerant or resistant to these diseases as a scion cultivar. Ten years of observation have demonstrated that 'Marigoule' seedling survival from root rot (*Phytophthora* spp.) is only 10% greater than European chestnut seedlings.

As a continuation of these studies, in 1980, a new breeding program was initiated in France. Early results showed that 'Maridonne' and 'Marlhac' rootstocks could also be used against root rot [254,256]. This breeding program is continuing. A similar program was also initiated in Spain by Gallasteguie in 1926 and continued by Urquijo. They imported some chestnut genotypes from Korea and Japan between 1917 and 1940 [262]. In this study, 263,000 genotypes were tested and 12,000 of these were found resistant to *Phytophthora*. As a result of this study, genotypes 111-1, 7521, and 1483 were selected for both resistance to root rot and better graft compatibility with chestnut cultivars. Genotypes 'CHR-151′ ('HS'), 'CHR-137′ ('125′), 'CHR-168′ ('110′), 'CHR-161′ ('100′), 'CHR-31′ ('2′), 'CHR-149′ ('90025′), 'CHR-147′ ('431′), 'CHR-167′ ('19′), and '776′ also were found promising [262]. Hybridization has been undertaken in several countries, including Portugal [256,263], Italy [99], Australia [264,265], and USA [10,266,267], to obtain root rot-resistant rootstocks. A limited number of resulting hybrids were used commonly but most of them exhibited graft incompatibility problems. One example is 'Menzies' (*C. sativa* × *C. crenata*), commonly used as a seedling rootstock source in Australia for its resistance to root rot [264,265]. In Asia, chestnut production is from *C. crenata* and *C. mollissima* trees which are naturally resistant to chestnut blight and root rot but are sensitive to the Asian chestnut gall wasp (*D. kuriphilus*). In Japan, seedlings of 'Shibaguri' have been used as scions for production but devastation from the gall wasp has reduced yield. In recent years, due to graft incompatibility problems, they have started using seedlings of the chosen scion cultivar as seedling rootstocks [268].

12. Rootstock-Scion Transfer of Macromolecules and Small Interfering RNAs

Scions and rootstocks can interact at trans-graft-union movement at the molecular level in different ways. In some cases, mobile macromolecules and large signaling molecules (e.g., RNA and protein) can move through the graft union via the vascular system and regulate various physiological processes in scion including vigor, yield, water use efficiency, biotic and abiotic resistance, etc. [11,269,270]. RNAs and proteins can be targeted to move up through the graft union and this process has been studied in various vegetable and fruit trees. In addition, some studies have focused on protein production in transgenic rootstocks with targeted delivery to scions to control disease [271]. Transgenic rootstocks expressing a polygalacturonidase inhibitory protein (PGIP), were able to protect wild type scion from both a bacterial disease caused by *Xylella fastidiosa* and a fungal disease caused by Botrytis cinereal, both pathogens use polygalacturonase as a virulence factor [272]. Recently, the strategy of delivering therapeutic proteins from a rootstock to a scion was validated in the field where transgenic rootstocks were able to transgraft protected a sensitive wild type scion variety from succumbing to Pierces Disease [273].

Transgrafting also holds great promise for the improvement of nut tree rootstocks. Commercially accepted scion cultivars grafted onto transgenic rootstocks could benefit from the rootstock-mediated increase in productivity and/or disease resistance while avoiding potential consumer concerns regarding use of any transgenic scion [248].

Rootstocks can also be improved with enhanced features while simultaneously designed to avoid transmission of macromolecules or products to the scion. As discussed in the biotic stress section, a crown gall resistant rootstock was generated by silencing *ipt* and *iaaM* genes responsible for tumor formation [247]. Examination for movement from the transgenic rootstock to a standard untransformed scion showed that none of the genes or their products (small RNAs, protein, and metabolite) transfer through graft union [274,275]. This method can produce rootstocks with enhanced disease resistance or other features while avoiding concerns about changes in the scion or food product.

13. Conclusions and Perspectives

The technique of producing trees on rootstocks means two species are genetically joined and therefore can affect one another's performance. The selection of rootstock is an important aspect of orchard management. In nut tree crops, rootstocks influence vigor, rooting ability, water and nutrient uptake, bud break timing, yield, nut quality, susceptibility to abiotic factors including temperatures, drought, waterlogging and salinity, and biotic factors, including crown gall, root rot, root-knot nematodes and soil borne fungal infections, harvest efficiency and postharvest nut quality. And now, producing sustainable orchards which can meet the challenges of climate change and economic production, producing better rootstocks is even more important.

Breeding tree nut crop rootstocks began many years ago when local growers near the centers of a species origins started collecting and domesticating the best wild species tolerant to abiotic and biotic stresses and that also produced good nuts. Later traditional breeding programs were started for both scions and rootstocks. The traditional rootstock breeding programs have produced the interspecific hybrid 'GF-677′, GN series, 'Root-Pac 40′, 'Vlach', 'RX1′, 'VX211′, 'UCB1′, 'Newberg', and 'Apache' rootstocks in different nut trees. However, for tree nut crops, which have long extended juvenility, long productive lives and high heterozygosity, the traditional breeding approaches employed in annual crops are too slow, and costly. Understanding how rootstocks and scion interact can provide modern breeders new techniques to improve tree nut crops productivity.

Incorporating the newly emerging technologies including high-throughput phenotyping and genotyping as well as genome-wide transcriptome analysis into investigations of the genetic and domestication processes of nut trees rootstock species will address pertinent questions for rootstock biology and breeding. Among these questions are how the rootstock/scion interactions affect graft compatibility, vigor, water and nutrient uptake and efficiency, biotic and abiotic stresses, yield, and quality. Of particular value in rootstock breeding programs is germplasm collection and construction of grafting experiments to identify the genes associated with phenotypic variation in both the rootstock and the scion.

The collection of genomic data for nut trees is accelerating as the cost of next generation sequencing (NGS) decreases. The almond, hazelnut, walnut, pistachio, and pecan genomes have been fully sequenced and are available. In the near future reliable phenotypic data will be the rate limiting step in rootstock improvement. As tree nut crops are highly heterozygous with long juvenility periods and productive lives, genomic based approaches, such as marker-assisted selection (MAS), genome-wide association study (GWAS), genomic selection (GS), and genetic transformation offer promise for rootstock breeding. Comprehensive germplasm collections, coupled with genomic approaches, has the potential to yield significant advances in grafted tree nut crops.

Author Contributions: The authors have equal contributions to this work. All authors have read and agreed to the published version of the manuscript.

Funding: This research is part of the I+D+I project no. RTI2018-094210-R100 funded by MCIN/AEI/10.13039/501100011033, as well as by Gobierno de Aragón—European Social Fund, European Union (Grupo Consolidado A12).

Acknowledgments: This work has been supported by the Center of International Scientific Studies & Collaboration (CISSC), Ministry of Science Research and Technology. We would like to appreciate Patrick J. Brown for his collaboration in the ICRP project of CISSC titled "Association mapping of drought tolerance related traits in Persian walnut" that some part of its results has been reported in this paper. We also would like to thank University of California Davis, Iran National Science Foundation (INSF), Center of Excellence for Walnut Improvement and Technology of Iran, and the University of Tehran for their supports.

Conflicts of Interest: The authors declare no conflict of interest.

References

1. FAO. *FAO Statistical Yearbook*; Food and Agriculture Organization of the United Nations: Rome, Italy, 2019; Available online: http://www.fao.org/faostat/en/#data/QC (accessed on 25 September 2020).
2. Ros, E. Health benefits of nut consumption. *Nutrients* **2010**, *2*, 652–682. [CrossRef]
3. Vadivel, V.; Kunyanga, C.N.; Biesalski, H.K. Health benefits of nut consumption with special reference to body weight control. *Nutrition* **2015**, *28*, 1089–1097. [CrossRef]
4. Grosso, G.; Estruch, R. Nut consumption and age-related disease. *Maturitas* **2016**, *84*, 11–16. [CrossRef] [PubMed]
5. Relja, A.; Miljković, A.; Gelemanović, A.; Bošković, M.; Hayward, C.; Polašek, O.; Kolčić, I. Nut Consumption and Cardiovascular Risk Factors: A Cross-Sectional Study in a Mediterranean Population. *Nutrients* **2017**, *9*, 1296. [CrossRef]
6. Rusu, M.E.; Mocan, A.; Ferreira, I.C.F.R.; Popa, D.-S. Health Benefits of Nut Consumption in Middle-Aged and Elderly Population. *Antioxidants* **2019**, *8*, 302. [CrossRef] [PubMed]
7. Gervasi, T.; Barreca, D.; Laganà, G.; Mandalari, G. Health Benefits Related to Tree Nut Consumption and Their Bioactive Compounds. *Int. J. Mol. Sci.* **2021**, *22*, 5960. [CrossRef]
8. Casales, F.G.; Van der Watt, E.; Coetzer, G.M. Propagation of pecan (*Carya illinoensis*): A review. *Afr J. Biotechnol.* **2018**, *17*, 586–605. [CrossRef]
9. Raoufi, A.; Vahdati, K.; Karimi, S.; Roozban, M. Optimizing Seed Germination and Growth of Seedlings in Persian Walnut. *J. Nuts* **2020**, *11*, 185–193. [CrossRef]
10. Grauke, L.J.; Thompson, T.E. Rootstock development in temperate nut crops. *Acta Hortic.* **2003**, *622*, 553–566. [CrossRef]
11. Albacete, A.; Martínez-Andújar, C.; Martínez-Pérez, A.; Thompson, A.J.; Dodd, I.C.; Pérez-Alfocea, F. Unravelling rootstock × scion interactions to improve food security. *J. Exp. Bot.* **2015**, *66*, 2211–2226. [CrossRef]
12. Caruso, T.; Barone, E.; Marra, F.P.; Sottile, F.; La Mantia, M.; De Pasquale, C. Effect of rootstock on growth, yield and fruit characteristics in cv. Bianca pistachio (*Pistacia vera* L.) trees. In Proceedings of the XIII GREMPA Meeting on Pistachios and Almond, Palermo, Italy, 1–5 June 2003; pp. 1–5.
13. Kallsen, C.E.; Parfitt, D.E. Comparisons of scion/rootstock growth rates among US pistachio cultivars. *HortScience* **2011**, *46*, 197–200. [CrossRef]
14. Vahdati, K.; Mohseniazar, M. Early bearing genotypes of walnut: A suitable material for breeding and high-density orchards. *Acta Hortic.* **2016**, *1139*, 101–106. [CrossRef]
15. Lordan, J.; Zazurca, L.; Maldonado, M.; Torguet, L.; Alegre, S.; Miarnau, X. Horticultural performance of 'Marinada' and 'Vairo' almond cultivars grown on a genetically diverse set of rootstocks. *Sci. Hortic.* **2019**, *256*, 108558. [CrossRef]
16. Barone, E.; Sottile, F.; Palazzolo, E.; Caruso, T. Effect of rootstock on trunk growth and foliar mineral content in cv. Bianca pistachio (*Pistacia vera* L.) trees. *Acta Hortic.* **1997**, *470*, 394–401. [CrossRef]
17. Ak, B.E.; Turker, S. Effects of different rootstocks on phenological stages and vegetative growth of some pistachio cultivars. *Acta Hortic.* **2006**, *726*, 208–214. [CrossRef]
18. Rahemi, M.; Tavallali, V. Effects of rootstock on Iranian pistachio scion cultivars. *Fruits* **2007**, *62*, 317–323. [CrossRef]
19. Ghazvini, R.F.; Sajadian, H.; Hokmabadi, H.; Ahmad, S.A.E.E.D. Effects of pistachio rootstocks on ecophysiological characteristics of commercial pistachio cultivars. *Int. J. Agr. Biol.* **2007**, *9*, 352–354.
20. Ferguson, L.; Beede, R.H.; Reyes, H.; Sanden, B.L.; Grattan, S.R.; Epstein, L. Pistachio rootstocks. In *Pistachio Production Manual*; Ferguson, L., Haviland, D., Eds.; UCANR Publications: Davis, CA, USA, 2016; Volume 3545, pp. 65–74.
21. Buzo, T.; McKenna, J.; Kaku, S.; Anwar, S.A.; McKenry, M.V. VX211, a vigorous new walnut hybrid clone with nematode tolerance and a useful resistance mechanism. *J. Nematol.* **2009**, *41*, 211.
22. McGranahan, G.; Leslie, C. Walnut. In *Fruit Breeding*; Badenes, M.L., Byrne, D.H., Eds.; Springer: Boston, MA, USA, 2012; pp. 827–846. [CrossRef]
23. Ross-Davis, A.; Huang, Z.; McKenna, J.; Ostry, M.; Woeste, K. Morphological and molecular methods to identify butternut (*Juglans cinerea*) and butternut hybrids: Relevance to butternut conservation. *Tree Physiol.* **2008**, *28*, 1127–1133. [CrossRef] [PubMed]
24. Pollegioni, P.; Woeste, K.; Mugnozza, G.S.; Malvolti, M.E. Retrospective identification of hybridogenic walnut plants by SSR fingerprinting and parentage analysis. *Mol. Breed.* **2009**, *24*, 321–335. [CrossRef]

25. Clark, J.; Hemery, G. Walnut hybrids in the UK: Fast growing quality hardwoods. *Q. J. Forestry* **2010**, *104*, 43–46.
26. Mohseniazar, M.; Vahdati, K.; Aliniaeifard, S.; Wang, Y. Cloning and in silico characterization of GAI gene and its promoter region from dwarf/precocious and vigorous/non-precocious Persian walnut genotypes. *Acta Hortic.* **2021**, *1315*, 313–318. [CrossRef]
27. Germain, E.; Delort, F.; Kanivets, V. Precocious maturing walnut population originating from central Asia: Their behavior in France. *Acta Hortic.* **1997**, *442*, 83–90. [CrossRef]
28. Rezaee, R.; Grigoorian, W.; Vahdati, K.; Valizadeh, M. Evaluation of morphological traits associated with the vigor of Persian walnut (*Juglans regia* L.) seedlings. *Iran. J. Hortic. Sci. Technol.* **2006**, *7*, 25–31.
29. Wang, G.A.; Zhang, Q.; Huang, M.M.; Yakup, A. The breeding of six Xinjiang dwarf walnut cultivars. *Acta Hortic.* **2014**, *1050*, 151–159. [CrossRef]
30. Vahdati, K.; Hassani, D.; Rezaee, R.; Jafari Sayadi, M.H.; Sarikhani Khorami, S. Walnut footprint in Iran. In *Following Walnut Footprints (Juglans regia L.) Cultivation and Culture, Folklore and History, Traditions and Uses*; Avanzato, D., McGranahan, G.H., Vahdati, K., Botu, M., Iannamico, L., Assche, J.V., Eds.; International Society for Horticultural Science (ISHS): Leuven, Belgium, 2014; p. 442.
31. Rezaee, R.; Vahdati, K. Morphological variation, heritability and phenotypic correlation of traits related to the vigor in Persian walnut (*Juglans regia* L.). *J. Crop. Prod. Process.* **2014**, *12*, 259–270.
32. Aletà, N.; Vilanova, A.; Díaz, R.; Voltas, J. Genetic variation for carbon isotope composition in *Juglans regia* L: Relationship with growth, phenology and climate of origin. *Ann. Sci.* **2009**, *66*, 413–423. [CrossRef]
33. Cosmulescu, S.N.; Bîrsanu Ionescu, M.; Netoiu, C. Impact of Climatic Factors on Radial Growth in Walnut (*Juglans regia* L.). *Not. Sci. Biol.* **2019**, *11*, 304–308. [CrossRef]
34. Rezaee, R.; Vahdati, K.; Valizadeh, M. Variability of seedling vigour in Persian walnut as influenced by the vigour and bearing habit of the mother tree. *J. Hortic. Sci. Biotechnol.* **2009**, *84*, 228–232. [CrossRef]
35. Rezaee, R.; Grigoorian, W.; Vahdati, K.; Valizadeh, M. Effects on walnut seedling vigor on root pressure, grafting success and scion growth. *J. Hortic. Sci. Technol.* **2007**, *8*, 21–30.
36. Rezaee, R.; Vahdati, K.; Grigoorian, V.; Valizadeh, M. Walnut grafting success and bleeding rate as affected by different grafting methods and seedling vigour. *J. Hortic. Sci. Biotech.* **2008**, *83*, 94–99. [CrossRef]
37. Vahdati, K.; Mirmasoumi, M.; Rezaee, R. Rooting and multiplication ability of Persian walnut as influenced by motherstock vigor and precocity. *Acta Hortic.* **2009**, *839*, 223–228. [CrossRef]
38. Vahdati, K.; Razaee, R.; Mirmasoomi, M. Micropropagation of Some Dwarf and Early Mature Walnut Genotypes. *Biotechnology* **2009**, *8*, 171–175. [CrossRef]
39. Raufi, A.; Vahdati, K.; Karimi, S.; Roozban, M.R. Optimizing early grafting of Persian walnut by evaluating different rootstocks, covering materials and grafting methods. *J. Nuts* **2017**, *8*, 97–106. [CrossRef]
40. Warmund, M.R. Chinese chestnut (*Castanea mollissima*) as a niche crop in the central region of the United States. *HortScience* **2011**, *46*, 345–347. [CrossRef]
41. Anagnostakis, S.L.; Gordon, P.; Hebard, F.V. Identification of chestnut trees. *Annu. Rep. North. Nut Grow. Assn* **1998**, *89*, 1–4.
42. Khadivi-Khub, A.; Anjam, K. *Prunus scoparia*, a suitable rootstock for almond (*Prunus dulcis*) under drought condition based on vegetative and fruit characteristics. *Sci. Hortic.* **2016**, *210*, 220–226. [CrossRef]
43. Parvaneh, T.; Afshari, H.; Ebadi, A. A study of the influence of different rootstocks on the vegetative growth of almond cultivars. *Afr. J. Biotechnol.* **2011**, *10*, 16793–16807. [CrossRef]
44. Duncan, R.; Connell, J.; Jarvis-Shean, K.; Brar, G.; Yaghmour, M. Field Evaluation of Almond Rootstock. In *Annual Research Report 2019–2020*; Almond Board of California: Sacramento, CA, USA, 2019; p. 24.
45. Barbera, G.; Di Marco, L.; La Mantia, T.; Schirra, M. Effect of rootstock on productive and qualitative response of two almond varieties. *Acta Hortic.* **1993**, *373*, 129–134. [CrossRef]
46. Tworkoski, T.; Miller, S. Rootstock effect on growth of apple scions with different growth habits. *Sci. Hortic.* **2007**, *111*, 335–343. [CrossRef]
47. Van Hooijdonk, B.M.; Woolley, D.J.; Warrington, I.J.; Tustin, D.S. Initial alteration of scion architecture by dwarfing apple rootstocks may involve shoot-root-shoot signalling by auxin, gibberellin, and cytokinin. *J. Hortic. Sci. Biotechnol.* **2010**, *85*, 59–65. [CrossRef]
48. Li, G.; Ma, J.; Tan, M.; Mao, J.; An, N.; Sha, G.; Zhang, D.; Zhao, C.; Han, M. Transcriptome analysis reveals the effects of sugar metabolism and auxin and cytokinin signaling pathways on root growth and development of grafted apple. *BMC Genom.* **2016**, *17*, 150. [CrossRef]
49. Grauke, L.J.; Pratt, J.W. Pecan bud growth and freeze damage are influenced by rootstock. *J. Am. Soc. Hortic. Sci.* **1992**, *117*, 404–406. [CrossRef]
50. Liu, Z.; Li, F.; Peng, F.; Tan, P.; Zhu, K.; Feng, G.; Mo, Z.; Li, Y. Identification of grafting-responsive micrornas associated with growth regulation in pecan [*Carya illinoinensis* (Wangenh.) K. Koch]. *Forests* **2020**, *11*, 196. [CrossRef]
51. Rovira, M.; Cristofori, V.; Silvestri, C.; Celli, T.; Hermoso, J.F.; Tous, J.; Romero, A. Last results in the evaluation of 'Negret' hazelnut cultivar grafted on non-suckering rootstocks in Spain. *Acta Hortic.* **2014**, *1052*, 145–150. [CrossRef]
52. Rovira, M. Advances in Hazelnut (*Corylus avellana* L.) Rootstocks Worldwide. *Horticulturae* **2021**, *7*, 267. [CrossRef]
53. Dehghan, B.; Vahdati, K.; Hassani, D.; Rezaee, R. Bench grafting of Persian walnut as affected by pre- and post-grafting heating and chilling treatments. *J. Hortic. Sci. Biotechnol.* **2009**, *85*, 48–52. [CrossRef]

54. Dehgan, B.; Vahdati, K.; Rezaee, R.; Hassani, D. Mature walnut grafting (topworking) as affected by grafting cover and scion cultivar. *Acta Hortic.* **2010**, *861*, 353–360. [CrossRef]
55. Ajamgard, F.; Rahemi, M.; Vahdati, K. Development of improved techniques for grafting of pecan. *Sci. Hortic.* **2016**, *204*, 65–69. [CrossRef]
56. Sheikhi, A.; Arab, M.M.; Brown, P.J.; Ferguson, L.; Akbari, M. Pistachio (*Pistacia* spp.) breeding. In *Advances in Plant Breeding Strategies: Nut and Beverage Crops*; Al-Khayri, J., Jain, S., Johnson, D., Eds.; Springer: Cham, Switzerland, 2019.
57. Dehghan, B.; Vahdati, K.; Hassani, D.; Rezaee, R. Persian walnut (*Juglans regia* L.) grafting as influenced by different bench grafting methods and scion cultivars. *J. Appl. Hortic.* **2009**, *11*, 56–58. [CrossRef]
58. Sadeghi Majd, R.; Vahdati, K.; Roozban, M.R.; Arab, M. Exploring combinations of graft cover and grafting method in commercial walnut cultivars. *Int. J. Fruit Sci.* **2019**, *19*, 359–371. [CrossRef]
59. Rezaee, R.; Hassani, D.; Vahdati, K. Long term trials on topworking of walnut trees in Iran. *Acta Hortic.* **2014**, *1050*, 197–201. [CrossRef]
60. Serr, E. Blackline in walnuts: Delayed failure of unions killing many walnut trees in central coastal counties. *Calif. Agric.* **1959**, *13*, 8–9.
61. Grant, J.; McGranahan, G. English walnut rootstocks help avoid blackline disease, but produce less than 'Paradox' hybrid. *Cali. Agri.* **2005**, *59*, 249–251. [CrossRef]
62. Vahdati, K.; Arab, M.M.; Sarikhani, S.; Sadat-Hosseini, M.; Leslie, C.A.; Brown, P.J. Advances in Persian Walnut (*Juglans regia* L.) Breeding Strategies. In *Advances in Plant Breeding Strategies: Nut and Beverage Crops*; Al-Khayri, J., Jain, S., Johnson, D., Eds.; Springer: Cham, Switzerland, 2019.
63. Andrews, P.K.; Marquez, C.S. Graft incompatibility. *Hortic. Rev.* **1993**, *15*, 183–232.
64. Kester, D.E.; Grasselly, C. Almond rootstocks. In *Rootstocks for Fruit Crops*; Rom, R.C., Carlson, R.F., Eds.; John Wiley: New York, NY, USA, 1987; pp. 265–293.
65. Gainza, F.; Opazo, I.; Muñoz, C. Graft incompatibility in plants: Metabolic changes during formation and establishment of the rootstock/scion union with emphasis on *Prunus species*. *Chil. J. Agric. Res.* **2015**, *75*, 28–34. [CrossRef]
66. Pina, A.; Cookson, S.; Calatayud, A.; Trinchera, A.; Errea, P. Physiological and Molecular Mechanisms Underlying Graft Compatibility. In *Vegetable Grafting: Principles and Practices*; Colla, G., Perez-Alfocea, F., Schwarz, D., Eds.; CABI Publishing: Oxfordshire, UK, 2017; pp. 132–154.
67. Mo, Z.; Feng, G.; Su, W.; Liu, Z.; Peng, F. Identification of miRNAs associated with graft union development in pecan [*Carya illinoinensis* (Wangenh.) K. Koch]. *Forests* **2018**, *9*, 472. [CrossRef]
68. Mo, Z.; He, H.; Su, W.; Peng, F. Analysis of differentially accumulated proteins associated with graft union formation in pecan (*Carya illinoensis*). *Sci. Hortic.* **2017**, *224*, 126–134. [CrossRef]
69. Pina, A.; Errea, P. Influence of graft incompatibility on gene expression and enzymatic activity of UDP-glucose pyrophosphorylase. *Plant Sci.* **2008**, *174*, 502–509. [CrossRef]
70. Pina, A.; Errea, P.; Martens, H.J. Graft union formation and cell-to-cell communication via plasmodesmata in compatible and incompatible stem unions of *Prunus* spp. *Sci. Hortic.* **2012**, *143*, 144–150. [CrossRef]
71. Irisarri, P.; Zhebentyayeva, T.; Errea, P.; Pina, A. Differential expression of phenylalanine ammonia lyase (PAL) genes implies distinct roles in development of graft incompatibility symptoms in *Prunus*. *Sci. Hortic.* **2016**, *204*, 16–24. [CrossRef]
72. Reig, G.; Zarrouk, O.; Font i Forcada, C.; Moreno, M.A. Anatomical graft compatibility study between apricot cultivars and different plum based rootstocks. *Sci. Hortic.* **2018**, *237*, 67–73. [CrossRef]
73. Usenik, V.; Krška, B.; Vičan, M.; Štampar, F. Early detection of graft incompatibility in apricot (*Prunus armeniaca* L.) using phenol analyses. *Sci. Hortic.* **2006**, *109*, 332–338. [CrossRef]
74. Pina, A.; Errea, P. A review of new advances in mechanism of graft compatibility-incompatibility. *Sci. Hortic.* **2005**, *106*, 1–11. [CrossRef]
75. Aloni, B.; Cohen, R.; Karni, L.; Aktas, H.; Edelstein, M. Hormonal signaling in rootstock-scion interactions. *Sci. Hortic.* **2010**, *127*, 119–126. [CrossRef]
76. He, W.; Wang, Y.; Chen, Q.; Sun, B.; Tang, H.-R.; Pan, D.-M.; Wang, X.-R. Dissection of the mechanism for compatible and incompatible graft combinations of *Citrus grandis* (L.) Osbeck ('Hongmian Miyou'). *Int. J. Mol. Sci.* **2018**, *19*, 505. [CrossRef]
77. Irisarri, P.; Binczycki, P.; Errea, P.; Martens, H.J.; Pina, A. Oxidative stress associated with rootstock–scion interactions in pear/quince combinations during early stages of graft development. *J. Plant Physiol.* **2015**, *176*, 25–35. [CrossRef]
78. Cookson, S.J.; Clemente Moreno, M.J.; Hevin, C.; Nyamba Mendome, L.Z.; Delrot, S.; Magnin, N.; Trossat-Magnin, C.; Ollat, N. Heterografting with nonself rootstocks induces genes involved in stress responses at the graft interface when compared with autografted controls. *J. Exp. Bot.* **2014**, *65*, 2473–2481. [CrossRef]
79. Irisarri, P.; Zhebentyayeva, T.; Errea, P.; Pina, A. Inheritance of self- and graft-incompatibility traits in an F1 apricot progeny. *PLoS ONE* **2019**, *14*, e0216371. [CrossRef]
80. Akyüz, B. Determination of Seedling Rootstock Potential and Graft Compatibility of Some Hybrid Chestnut Genotypes. Ph.D. Thesis, Ondokuz Mayıs University, Samsun, Turkey, 2019. (In Turkish).
81. Akyüz, B.; Serdar, Ü. Generative rootstock potential of some hybrid chestnut genotypes. *Anadolu J. Agri. Sci.* **2020**, *35*, 185–191. [CrossRef]
82. Tokar, F.; Kovalovsky, D. Grafting of Castanea sativa in the open air. *Pol'nohospodarstvo AgrıcultureRoc. XviiCislo* **1971**, *3*, 164–172.
83. Viéitez, M.L.; Viéitez, A.M. Observaciones sobre el injerto juvenil del castano. *An. De Edafol. Y Agrobiol.* **1982**, *41*, 1999–2002.

84. Soylu, A. *Meyve Yetiştirme Ilkeleri*; Uludağ Üniversitesi Ziraat Fakültesi: Bursa, Turkey, 1990; Volume 20, p. 178.
85. Huang, H.; Norton, J.; Boyhan, G.; Abrahams, B. Graft compatibility among chestnut (*Castanea*) species. *J. Am. Soci. Hortic. Sci.* **1994**, *119*, 1127–1132. [CrossRef]
86. Serdar, Ü.; Soylu, A. The effect of grafting time and methods on chestnut nursery tree production. *Acta Hortic.* **2005**, *693*, 187–194. [CrossRef]
87. Serdar, Ü.; Demirsoy, H.; Macit, I.; Ertürk, U. Graft compatibility in some Turkish chestnut genotypes (*C. sativa* Mill.;). *Acta Hortic.* **2010**, *866*, 285–290. [CrossRef]
88. Bounous, G.; Paglietta, R.; Craddock, J.H.; Bellini, E. An overview of chestnut breeding. In Proceedings of the International Congress on Chestnut, Spoleto, Italy, 20–23 October 1993; pp. 20–23.
89. MacDaniels, L.H. Stock-scion incompatibility in nut trees. In *Annual Report of the Northern Nut Growers Association*; East Lansing: Michigan, MI, USA, 1955; pp. 92–97.
90. Oraguzie, N.; McNeil, D.; Thomas, M. Examination of graft failure in New Zealand chestnut (*Castanea* spp.) selections. *Sci. Hortic.* **1998**, *76*, 89–103. [CrossRef]
91. Ebrahimi, A.; Vahdati, K.; Fallah, E. Improved success of Persian walnut grafting under environmentally controlled conditions. *Int. J. Fruit. Sci.* **2007**, *6*, 3–12. [CrossRef]
92. Desvignes, J. Sweet chestnut incompatibility and mosaics caused by the chestnut mosaic virus (ChMV). *Acta Hortic.* **1999**, *494*, 451–458. [CrossRef]
93. Santamour, F.S., Jr.; Mcardle, A.; Jaynes, R. Cambial isoperoxidase patterns in *Castanea*. *J. Environ. Hortic.* **1986**, *4*, 14–16. [CrossRef]
94. Santamour, F.S., Jr. Graft incompatibility related to cambial peroxidase isozymes in Chinese chestnut. *J. Environ. Hortic.* **1988**, *6*, 33–39. [CrossRef]
95. Stoke, H.F. Topworking Chinese chestnut. *Ann. Report Northern Nut Growers Asso.* **1961**, *51*, 42–44.
96. Woodruff, J.G. *Tree nuts: Production, processing, products*, 2nd ed.; AVI Publishing Co. Inc.: Westport, CT, USA, 1979.
97. Errea, P.; Garay, L.; Marín, J.A. Early detection of graft incompatibility in apricot (*Prunus armeniaca*) using in vitro techniques. *Phys. Plant* **2001**, *112*, 135–141. [CrossRef] [PubMed]
98. Assunção, M.; Canas, S.; Cruz, S.; Brazão, J.; Zanol, G.C.; Eiras-Dias, J.E. Graft compatibility of *Vitis* spp. the role of phenolic acids and flavanols. *Sci. Hortic.* **2016**, *207*, 140–145. [CrossRef]
99. Beccaro, G.; Alma, A.; Bounous, G.; Gomes-Laranjo, J. *The Chestnut Handbook: Crop & Forest Management*; CRC Press: Boca Raton, FL, USA, 2019; 378p.
100. Donno, D.; Mellano, M.G.; Beccaro, G.L. L'analisi dei composti polifenolici come metodo predittivo per valutare la compatibilita'di innesto di *Castanea* spp. su portinnesti clonali. In Proceedings of the VII Convegno Nazıonale Castagno, Torino, Italy, 11–14 June 2019; 2020; Volume 25, pp. 168–170.
101. Zarrouk, O.; Testillano, P.S.; Risueño, M.C.; Moreno, M.Á.; Gogorcena, Y. Changes in cell/tissue organization and peroxidase activity as markers for early detection of graft incompatibility in peach/plum combinations. *J. Am. Soci. Hortic. Sci.* **2010**, *135*, 9–17. [CrossRef]
102. Pina, A.; Errea, P. Differential induction of phenylalanine ammonia-lyase gene expression in response to in vitro callus unions of *Prunus* spp. *J. Plant Physiol.* **2008**, *165*, 705–714. [CrossRef] [PubMed]
103. Gamba, G.; Donno, D.; Mellano, M.G.; Beccaro, G.L. Definizione della dose ottimale di fertilizzante su castagno utilizzando strumenti non distruttivi e routinari per la misurazione del contenuto di clorofilla e fluorescenza fogliare. In Proceedings of the VII Convegno Nazıonale Castagno, Torino, Italy, 13 September 2020; Volume 25, pp. 179–181.
104. Tous, J.; Romero, A.; Plana, J.; Rovira, M.; Vargas, F.J. Performance of 'Negret' Hazelnut cultivar on several rootstocks. *Acta Hortic.* **1997**, *445*, 433–439. [CrossRef]
105. Lagerstedt, H.B. Newberg and Dundee, two new filbert rootstocks. *Proc. Nut Grow. Soc. Or.* **1993**, *78*, 94–101.
106. Tous, J.; Girona, J.; Tasias, J. Cultural practices and costs in hazelnut production. *Acta Hortic.* **1994**, *351*, 395–418. [CrossRef]
107. Baratta, B.; Picciotto, F.; Raimondo, A. Confronto di quattro cultivar di nocciolo con piante innestate e franche di piedi del territorio dei Nebrodi. *Agric. Ric.* **1990**, *108*, 43–46.
108. Cerovic, S.; Ninic-Todorovic, J.; Golosin, B.; Ognjanov, V.; Bileluic, S. Production technology of young hazelnut trees grafted on Turkish filbert (*Corylus colurna* L.). *Acta Hortic.* **2007**, *732*, 257–355. [CrossRef]
109. Miletic, R.; Mitrovic, M.; Rakicevic, M. Contrasting fruit properties of hazelnut cultivars grown on different rootstocks. *Acta Hortic.* **2009**, *845*, 283–285. [CrossRef]
110. Ninit-Todorovic, J.; Cerovic, S.; Ognjanov, V.; Golosin, B.; Bijelic, S.; Jacimovic, G.; Kurkajov, A. Rootstocks of *Corylus colurna* L. for nursery production. *Acta Hortic.* **2009**, *845*, 273–278. [CrossRef]
111. Ninit-Todorovic, J.; Ognjanov, V.; Keserovic, S.; Cerovic, S.; Golosin, B.; Bijelic, S.; Cukanovic, A.; Kurkajov, A.; Cabilovski, R. Turkish hazel (*Corylus colurna* L.) offspring variability as a foundation for grafting rootstock production. *Bul. J. Agri. Sci.* **2012**, *18*, 883–888.
112. Chapa, J.; Chazerans, P.; Coulie, J. Multiplication végétative du châtaignier. *L'Arboriculture Fruitière* **1990**, *43*, 41–48.
113. Bounous, G.; Craddock, J.H.; Peano, C.; Salarin, P. Phenology of blooming and fruiting habits in Euro-Japanese hybrid chestnut. In Proceedings of the International Congress on Chestnut, Morgantown, WV, USA, 10–14 July 1992; pp. 117–128.
114. Gutenev, V.L.; Bogoroditskii, I.I. *Vegetative Propagation of Walnut by Cutting and Layering in the Rostovskaya Oblast*; Trudy Novocherkas, Inzh-Melior Institute: Rostov, Russia, 1974; Volume 3, pp. 115–121.

115. Vahdati, K.; Khalighi, A. Persian walnut stooling in Iran. *Acta Hortic.* **2001**, *544*, 527–530. [CrossRef]
116. Güneş, T. An investigation on rooting of Juglans regia L. hardwood cuttings. *Turk. J. Bot.* **1999**, *23*, 367–372.
117. Pijut, P.M.; Moore, M.J. Early season softwood cuttings effective for vegetative propagation of *Juglans cinerea*. *HortScience* **2002**, *37*, 697–700. [CrossRef]
118. Vahdati, K.; McKenna, J.R.; Dandekar, A.M.; Leslie, C.A.; Uratsu, S.L.; Hackett, W.P.; Negri, P.; McGranahan, G.H. Rooting and other characteristics of a transgenic walnut hybrid (*Juglans hindsii* × *J. regia*) rootstock expressing rolABC. *J. Am. Soci. Hortic. Sci.* **2002**, *127*, 724–728. [CrossRef]
119. Chalupa, V. Clonal propagation of broad–leaved forest trees in vitro. *Commun. Inst. Cech.* **1982**, *12*, 255–271.
120. Driver, J.A.; Kuniyuki, A.H. In vitro propagation of paradox walnut rootstock. *HortScience* **1984**, *19*, 507–509.
121. Gruselle, R.; Badia, N.; Boxus, P. Walnut micropropagation: First results. *Acta Hortic.* **1987**, *212*, 511–515. [CrossRef]
122. McGranahan, G.; Leslie, C.A.; Driver, J.A. In vitro propagation of mature Persian walnut cultivars. *HortScience* **1988**, *23*, 220.
123. Jay-Allemand, C.; Capelli, P.; Cornu, D. Root development of *in vitro* hybrid walnut microcuttings in a vermiculite–containing gelrite medium. *Sci. Hortic.* **1992**, *51*, 335–342. [CrossRef]
124. Dong, P.; Lichai, Y.; Qingming, W.; Ruisheng, G. Factors affecting rooting of *in vitro* shoots of walnut cultivars. *J. Hortic. Sci. Biotechnol.* **2007**, *82*, 223–226. [CrossRef]
125. Vahdati, K.; Leslie, C.; Zamani, Z.; McGranahan, G. Rooting and acclimatization of *in vitro*-grown shoots from mature trees of three Persian walnut cultivars. *HortScience* **2004**, *39*, 324–327. [CrossRef]
126. Licea-Moreno, R.J.; Contreras, A.; Morales, A.V.; Urban, I.; Daquinta, M.; Gomez, L. Improved walnut mass micropropagation through the combined use of phloroglucinol and FeEDDHA. *Plant Cell Tissue Organ. Cul.* **2015**, *123*, 143–154. [CrossRef]
127. Peixe, A.; Alpendre, P.; Barroso, J.; Carlos, R.; Soto, M.G. New strategies for *in vitro* rooting and plantlet acclimatization of the 'Paradox' (*Juglans regia* × *Juglans hindsii*) rootstock. *Acta Hortic.* **2015**, *1083*, 287–293. [CrossRef]
128. Yegizbayeva, T.K.; García-García, S.; Yausheva, T.V.; Kairova, M.; Apushev, A.K.; Oleichenko, S.N.; Licea-Moreno, R.J. Unraveling Factors Affecting Micropropagation of Four Persian Walnut Varieties. *Agronomy* **2021**, *11*, 1417. [CrossRef]
129. Gotea, R.; Gotea, I.; Sestras, R.E.; Vahdati, K. In vitro propagation of several walnut cultivars. *Bull. Univ. Agri. Sci. Vet. Med. Cluj-Napoca. Hortic.* **2012**, *69*, 167–171.
130. Vahdati, K.; Rezaee, R.; Grigoorian, V.; Valizadeh, M.; Motallebi Azar, A. Rooting ability of Persian walnut as affected by seedling vigour in response to stool layering. *J. Hortic. Sci. Biotechnol.* **2008**, *83*, 334–338. [CrossRef]
131. Bacchetta, L.; Aramini, M.; Bernardini, C.; Rugini, E. *In vitro* propagation of traditional Italian hazelnut cultivars as a tool for the valorization and conservation of local genetic resources. *HortScience* **2008**, *43*, 562–566. [CrossRef]
132. Cuenca, B.; Ocaña, L.; Salinero, C.; Pintos, C.; Mansilla, J.P.; Rial, C. Selection of *Castanea sativa* Mill. for resistance to *Phytophthora cinnamomi*: Micropropagation and testing of selected clones. *Acta Hortic.* **2009**, *844*, 395–404. [CrossRef]
133. Viéitez, A.M.; Sänchez, M.C.; García-Nimo, M.L.; Ballester, A. Protocol for micropropagation of *Castanea sativa*. In *Protocols for Micropropagation of Woody Trees and Fruits*; Springer: Dordrecht, The Netherlands, 2007; pp. 299–312.
134. Silvestri, C.; Cristofori, V.; Ceccarelli, M.; Caceres, M.E.; Escribà-Lacuesta, J.; Rugini, E. Adventitious shoot organogenesis from leaf and petiole explants of European hazelnut. *Plant Cell Tissue Organ. Cul.* **2016**, *126*, 59–65. [CrossRef]
135. Nawaz, M.A.; Imtiaz, M.; Kong, Q.; Cheng, F.; Ahmed, W.; Huang, Y.; Bie, Z. Grafting: A technique to modify ion accumulation in horticultural crops. *Front. Plant Sci.* **2016**, *7*, 1457. [CrossRef]
136. Martínez-Ballesta, M.C.; Alcaraz-López, C.; Muries, B.; Mota-Cadenas, C.; Carvajal, M. Physiological aspects of rootstock-scion interactions. *Sci. Hortic.* **2010**, *127*, 112–118. [CrossRef]
137. Brown, P.H.; Zhang, Q.; Ferguson, L. Influence of rootstock on nutrient acquisition by pistachio. *J. Plant Nut.* **1994**, *17*, 1137–1148. [CrossRef]
138. Brown, P.; Zhang, Q.; Ferguson, L. Nutrient uptake efficiencies of various pistachio rootstocks. In *Annual Report Fresno*; California Pistachio Industry, California Pistachio Commission: California, CA, USA, 1991; pp. 158–162.
139. Hokmabadi, H.; Sharafati, A. Effect of Some Pistachio Rootstocks on Nutrients Element Uptake of Two Pistachio Cultivars (Akbari and Bargsiyah). *J. Pist. Sci. Tech.* **2018**, *1*, 32–43.
140. Tavallali, V.; Rahemi, M. Effects of Rootstock on Nutrient Acquisition by Leaf, Kernel and Quality of Pistachio (*Pistacia vera* L.). American-Eurasian. *J. Agric. Environ. Sci.* **2007**, *2*, 240–246.
141. Surucu, A.; Acar, I.; Demirkiran, A.R.; Farooq, S.; Gokmen, V. Variations in nutrient uptake, yield and nut quality of different pistachio cultivars grafted on Pistacia khinjuk rootstock. *Sci. Hortic.* **2020**, *260*, 108913. [CrossRef]
142. Knipfer, T.; Reyes, C.; Momayyezi, M.; Brown, P.J.; Kluepfel, D.; McElrone, A.J. A comparative study on physiological responses to drought in walnut genotypes (RX1, Vlach, VX211) commercially available as rootstocks. *Trees* **2020**, *34*, 665–678. [CrossRef]
143. Reil, W.; Sibbett, S.; Ramos, D. Walnut cultivar nutritional evaluation. In *Annual Walnut Research Report*; California Walnut Board, University of California-Davis: Davis, CA, USA, 1992; pp. 1–13.
144. Lotfi, N.; Vahdati, K.; Kholdebarin, B.; Ashrafi, E.N. Germination, mineral composition, and ion uptake in walnut under salinity conditions. *HortScience* **2009**, *44*, 1352–1357. [CrossRef]
145. Jiménez, S.; Ollat, N.; Deborde, C.; Maucourt, M.; Rellán-Álvarez, R.; Moreno, M.Á.; Gogorcena, Y. Metabolic response in roots of *Prunus* rootstocks submitted to iron chlorosis. *J. Plant Physiol.* **2011**, *168*, 415–423. [CrossRef]
146. Day, L.H. Rootstock for stone fruits: Observations and Experiment with Plum, Peach, Apricot and Almond Roots for Stone Fruits. *Calif. Agric. Exp. Sta. Bul.* **1953**, *736*.

147. Reid, W. Rootstock influences yield, nut quality, and leaf analysis of pecan trees. *HortScience* **1997**, *32*, 474–475. [CrossRef]
148. Webster, A.D. Rootstock and interstock effects on deciduous fruit tree vigour, precocity, and yield productivity. *N. Zldn. J. Crop. Hortic. Sci.* **1995**, *23*, 373–382. [CrossRef]
149. Meimand, M.J.M.; Shamshiri, M.H. Effects of rootstock and interstock on pollen traits and flowering synchronization in pistachio (*Pistacia vera* L.). *Erwerbs-Obstbau* **2019**, *61*, 267–271. [CrossRef]
150. Chen, L.; Dong, R.; Ma, Q.; Zhang, Y.; Xu, S.; Ning, D.; Chen, Q.; Pei, D. Precocious genotypes and homozygous tendency generated by self-pollination in walnut. *BMC Plant Biol.* **2018**, *18*, 323. [CrossRef]
151. Reighard, G.L. Five-year performance of 19 peach rootstocks at 20 sites in North America. *Acta Hortic.* **2001**, *557*, 97–102. [CrossRef]
152. Romero, A.; Batlle, I.; Miarnau, X. Almond physical traits affected by rootstocks in 'Marinada' cultivar. *Acta Hortic.* **2018**, *1219*, 31–36. [CrossRef]
153. Ferguson, L.; Beede, R.H.; Reyes, H.; Metheney, P. California pistachio rootstocks evaluations. *Acta Hortic.* **2002**, *591*, 63–66. [CrossRef]
154. Grant, J. Performance of clonal paradox and blackline tolerant walnut rootstocks in San Joaquin County. In *Walnut Research Report*; California Walnut Board, University of California-Davis: Davis, CA, USA, 2015; pp. 1–3.
155. Connell, J.H.; Olson, W.H.; Limberg, J.; Metcalf, S.G. Effects of various roots on 'Chandler' walnut catkin and pistillate bloom, tree growth, yield, and nut quality. *Acta Hortic.* **2010**, *861*, 237–244. [CrossRef]
156. Hasey, J.K.; Westerdahl, B.B.; Lampinen, B. Long-term performance of own-rooted 'Chandler' walnut compared to 'Chandler' walnut on paradox rootstock. *Acta Hortic.* **2004**, *636*, 83–87. [CrossRef]
157. Hasey, J.K.; Westerdahl, B.B.; Micke, W.C.; Ramos, D.E.; Yeager, J.T. Yield performance of own-rooted 'Chandler' walnut versus 'Chandler' walnut on paradox rootstock. *Acta Hortic.* **2001**, *544*, 489–493. [CrossRef]
158. Browne, G.T.; Leslie, C.A.; Grant, J.A.; Bhat, R.G.; Schmidt, L.S.; Hackett, W.P.; Kluepfel, D.A.; Robinson, R.; McGranahan, G.H. Resistance to species of *Phytophthora* identified among clones of *Juglans microcarpa* × *J. regia*. *HortScience* **2015**, *50*, 1136–1142. [CrossRef]
159. Godini, A.; Palasciano, M. Growth and yield of four self-fruitful and four self-unfruitful almonds on three rootstocks: A thirteen-year study. *Acta Hortic.* **1997**, *470*, 200–207. [CrossRef]
160. Dejampour, J.; Rahnemoun, H.; Hassani, D. Breeding almond interspecific hybrid rootstocks in Iran. *Acta Hortic.* **2006**, *726*, 45–50. [CrossRef]
161. Carbonell-Barrachina, Á.A.; Memmi, H.; Noguera-Artiaga, L.; Gijón-López, M.D.C.; Ciapa, R.; Pérez-López, D. Quality attributes of pistachio nuts as affected by rootstock and deficit irrigation. *J. Sci. Food Agri.* **2015**, *95*, 2866–2873. [CrossRef]
162. Turker, S.; Ak, B.E. Effects of different rootstocks on physical traits of Siirt and Ohadi pistachio cultivars. *Options Méditerranéennes* **2010**, *94*, 245–250.
163. Buchner, R.P.; Fulton, A.E.; Gilles, C.K.; Lampinen, B.D.; Shackel, K.A.; Metcalf, S.G.; Little, C.C.; Prichard, T.L.; Schwankl, L.J. Effects of regulated deficit irrigation on walnut grafted on 'northern California black' or 'Paradox' rootstock. *Acta Hortic.* **2008**, *792*, 141–146. [CrossRef]
164. Čolić, S.; Zec, G.; Bakić, I.; Janković, Z.; Rahović, D.; Fotirić Akšić, M. Rootstock effect on some quality characteristics of almond cultivars 'Troito', 'Marcona' and 'Texas'. *Acta Hortic.* **2018**, *1219*, 19–24. [CrossRef]
165. Nimbolkar, P.K.; Awachare, C.; Reddy, Y.T.N.; Chander, S.; Hussain, F. Role of rootstocks in fruit production-a review. J. Agricultural Engine. *Food Technol.* **2016**, *3*, 183–188.
166. Behrooz, A.; Vahdati, K.; Rejali, F.; Lotfi, M.; Sarikhani, S.; Leslie, C. Arbuscular mycorrhiza and plant growth-promoting bacteria alleviate drought stress in walnut. *HortScience* **2019**, *54*, 1087–1092. [CrossRef]
167. Vahdati, K.; Lotfi, N.; Kholdebarin, B.; Hassani, D.; Amiri, R.; Mozaffari, M.R.; Leslie, C. Screening for drought-tolerant genotypes of Persian walnuts (*Juglans regia* L.) during seed germination. *HortScience* **2009**, *44*, 1815–1819. [CrossRef]
168. Vahdati, K.; Lotfi, N. Abiotic stress tolerance in plants with emphasizing on drought and salinity stresses in walnut. In *Abiotic Stress-Plant Responses and Applications in Agriculture*; Vahdati, K., Leslie, C., Eds.; IntechOpen Press: London, UK, 2013; pp. 307–365.
169. Sadat-Hosseini, M.; Vahdati, K.; Boroomand, N.; Arab, M.M.; Asadi-Yekta, S. How walnut roots respond to drought stress: A morphological and anatomical study. *S.W. J. Hortic. Biol. Environ.* **2019**, *10*, 51–64.
170. López-Serrano, L.; Canet-Sanchis, G.; Vuletin Selak, G.; Penella, C.; San Bautista, A.; López-Galarza, S.; Calatayud, Á. Pepper rootstock and scion physiological responses under drought stress. *Front. Plant Sci.* **2019**, *10*, 38. [CrossRef]
171. Yadollahi, A.; Kholdebarin, B.; Lotfi, N.; Vahdati, K.; Amiri, R.; Hassani, D. Morphological and physiological responses to water stress for seedlings of different walnut genotypes. *Acta Hortic.* **2010**, *861*, 253–262. [CrossRef]
172. Lotfi, N.; Vahdati, K.; Amiri, R.; Kholdebarin, B. Drought-induced accumulation of sugars and proline in radicle and plumule of tolerant walnut varieties during germination phase. *Acta Hortic.* **2010**, *861*, 289–296. [CrossRef]
173. Behboudian, M.H.; Walker, R.R.; Törökfalvy, E. Effects of water stress and salinity on photosynthesis of pistachio. *Sci. Hortic.* **1986**, *29*, 251–261. [CrossRef]
174. Gijón, M.C.; Gimenez, C.; Perez-López, D.; Guerrero, J.; Couceiro, J.F.; Moriana, A. Rootstock influences the response of pistachio (Pistacia vera L. cv. Kerman) to water stress and rehydration. *Sci. Hortic.* **2010**, *125*, 666–671. [CrossRef]

175. Moriana, A.; Memmi, H.; Centeno, A.; Martín-Palomo, M.J.; Corell, M.; Torrecillas, A.; Pérez-López, D. Influence of rootstock on pistachio (*Pistacia vera* L. cv Kerman) water relations. *Agri. Water Manag.* **2018**, *202*, 263–270. [CrossRef]
176. Memmi, H.; Gijón, M.C.; Couceiro, J.F.; Pérez-López, D. Water stress thresholds for regulated deficit irrigation in pistachio trees: Rootstock influence and effects on yield quality. *Agri. Water Manag.* **2016**, *164*, 58–72. [CrossRef]
177. Noguera-Artiaga, L.; Pérez-López, D.; Burgos-Hernández, A.; Wojdyło, A.; Carbonell-Barrachina, Á.A. Phenolic and triterpenoid composition and inhibition of α-amylase of pistachio kernels (*Pistachia vera* L.) as affected by rootstock and irrigation treatment. *Food Chem.* **2018**, *261*, 240–245. [CrossRef]
178. Noguera-Artiaga, L.; Sánchez-Bravo, P.; Hernández, F.; Burgos-Hernández, A.; Pérez-López, D.; Carbonell-Barrachina, Á.A. Influence of regulated deficit irrigation and rootstock on the functional, nutritional and sensory quality of pistachio nuts. *Sci. Hortic.* **2020**, *261*, 108994. [CrossRef]
179. Akbari, M.; Mahna, N.; Ramesh, K.; Bandehagh, A.; Mazzuca, S. Ion homeostasis, osmoregulation, and physiological changes in the roots and leaves of pistachio rootstocks in response to salinity. *Protoplasma* **2018**, *255*, 1349–1362. [CrossRef] [PubMed]
180. Kurum, R.; Ulukapi, K.; Aydinşakir, K.; Onus, A.N. The influence of salinity on seedling growth of some pumpkin varieties used as rootstock. *Not. Bot. Horti. Agrobo.* **2013**, *41*, 219–225. [CrossRef]
181. Goharrizi, K.J.; Amirmahani, F.; Salehi, F. Assessment of changes in physiological and biochemical traits in four pistachio rootstocks under drought, salinity and drought+ salinity stresses. *Physiol. Plant* **2020**, *168*, 973–989. [CrossRef]
182. Hokmabadi, H.; Arzani, K.; Grierson, P.F. Growth, chemical composition, and carbon isotope discrimination of pistachio (Pistacia vera L.) rootstock seedlings in response to salinity. *Aust. J. Agri. Res.* **2005**, *56*, 135–144. [CrossRef]
183. Mohammadi, A.H.; Banihashemi, Z.; Maftoun, M. Interaction between salinity stress and Verticillium wilt disease in three pistachio rootstocks in a calcareous soil. *J. Plant Nut.* **2007**, *30*, 241–252. [CrossRef]
184. Tavallali, V.; Rahemi, M.; Panahi, B. Calcium induces salinity tolerance in pistachio rootstocks. *Fruits* **2008**, *63*, 285–296. [CrossRef]
185. Akbari, M.; Katam, R.; Husain, R.; Farajpour, M.; Mazzuca, S.; Mahna, N. Sodium Chloride Induced Stress Responses of Antioxidative Activities in Leaves and Roots of Pistachio Rootstock. *Biomolecules* **2020**, *10*, 189. [CrossRef] [PubMed]
186. Goharrizi, K.J.; Baghizadeh, A.; Kalantar, M.; Fatehi, F. Combined effects of salinity and drought on physiological and biochemical characteristics of pistachio rootstocks. *Sci. Hortic.* **2020**, *261*, 108970. [CrossRef]
187. Karimi, H.R.; Roosta, H.R. Evaluation of inter-specific hybrid of *P. atlantica* and *P. vera* L. cv. 'Badami-Riz-e-Zarand' as Pistachio rootstock to salinity stress according to some growth indices and eco-physiological and biochemical parameters. *J. Stress Physio. Biochem.* **2014**, *10*, 5–17.
188. Karimi, H.R.; Maleki Kuhbanani, A. The evaluation of inter-specific hybrid of *P. atlantica*× *P. vera* cv. 'Badami Zarand' as a pistachio rootstock to salinity stress. *J. Nuts* **2015**, *6*, 113–122. [CrossRef]
189. Mittler, R. Abiotic stress, the field environment and stress combination. *Trends Plant. Sci.* **2006**, *11*, 15–19. [CrossRef]
190. Knipfer, T.; Brodersen, C.R.; Zedan, A.; Kluepfel, D.A.; McElrone, A.J. Patterns of drought-induced embolism formation and spread in living walnut saplings visualized using X-ray microtomography. *Tree Physiol.* **2015**, *35*, 744–755. [CrossRef]
191. Knipfer, T.; Barrios-Masias, F.H.; Cuneo, I.F.; Bouda, M.; Albuquerque, C.P.; Brodersen, C.R.; Kluepfel, D.A.; McElrone, A.J. Variations in xylem embolism susceptibility under drought between intact saplings of three walnut species. *Tree Physiol.* **2018**, *38*, 1180–1192. [CrossRef] [PubMed]
192. Liu, B.; Liang, J.; Tang, G.; Wang, X.; Liu, F.; Zhao, D. Drought stress affects on growth, water use efficiency, gas exchange and chlorophyll fluorescence of *Juglans* rootstocks. *Sci. Hortic.* **2019**, *250*, 230–235. [CrossRef]
193. Arab, M.M.; Marrano, A.; Abdollahi-Arpanahi, R.; Leslie, C.A.; Cheng, H.; Neale, D.B.; Vahdati, K. Combining phenotype, genotype, and environment to uncover genetic components underlying water use efficiency in Persian walnut. *J. Exp. Bot.* **2020**, *71*, 1107–1127. [CrossRef] [PubMed]
194. Lotfi, N.; Soleimani, A.; Vahdati, K.; Çakmakçı, R. Comprehensive biochemical insights into the seed germination of walnut under drought stress. *Sci. Hortic.* **2019**, *250*, 329–343. [CrossRef]
195. Lotfi, N.; Vahdati, K.; Hassani, D.; Kholdebarin, B.; Amiri, R. Peroxidase, guaiacol peroxidase and ascorbate peroxidase activity accumulation in leaves and roots of walnut trees in response to drought stress. *Acta Hortic.* **2010**, *861*, 309–316. [CrossRef]
196. Sheikh Beig Goharrizi, M.A.; Dejahang, A.; Tohidfar, M.; Izadi Darbandi, A.; Carrillo, N.J.; Hajirezaei, M.R.; Vahdati, K. *Agrobacterium* mediated transformation of somatic embryos of Persian walnut using *fld* gene for osmotic stress tolerance. *J. Agri. Sci. Technol.* **2016**, *18*, 423–435.
197. Caprile, J.; Grattan, S. Evaluation of the salt tolerance of walnut rootstocks. *HortScience* **2006**, *41*, 1040A. [CrossRef]
198. Catlin, P.B. Differential sensitivity to waterlogging. In *Walnut Research Report*; California Walnut Board, University of California-Davis: Davis, CA, USA, 1978.
199. Catlin, P.B. Differential sensitivity of walnut rootstocks to root environments saturated with water. In *Walnut Research Report*; California Walnut Board, University of California-Davis: Davis, CA, USA, 1981.
200. Shaybany, B.; Martin, G.C. Abscisic acid identification and its quantitation in leaves of *Juglans* seedlings during waterlogging. *J. Am. Soci. Hortic. Sci.* **1977**, *12*, 300–302.
201. Bielsa, B.; Leida, C.; Rubio-Cabetas, M.J. Physiological characterization of drought stress response and expression of two transcription factors and two LEA genes in three *Prunus* genotypes. *Sci. Hortic.* **2016**, *213*, 260–269. [CrossRef]
202. Bielsa, B.; Sanz, M.; Rubio-Cabetas, M. Uncovering early response to drought by proteomic, physiological and biochemical changes in the almond × peach rootstock 'Garnem'. *Funct. Plant Biol.* **2019**, *46*, 994–1008. [CrossRef] [PubMed]

203. Bielsa, B.; Hewitt, S.; Reyes-Chin-Wo, S.; Dhingra, A.; Rubio-Cabetas, M.J. Identification of water use efficiency related genes in 'Garnem' almond-peach rootstock using time-course transcriptome analysis. *PLoS ONE* **2018**, *13*, e0205493. [CrossRef]
204. Bielsa, B.; Bassett, C.; Glenn, D.M.; Rubio-Cabetas, M.J. Assessing field *Prunus* genotypes for drought responsive potential by carbon isotope discrimination and promoter analysis. *Agronomy* **2018**, *8*, 42. [CrossRef]
205. Amador, M.L.; Sancho, S.; Bielsa, B.; Gomez-Aparisi, J.; Rubio-Cabetas, M.J. Physiological and biochemical parameters controlling waterlogging stress tolerance in *Prunus* before and after drainage. *Physiol. Plant* **2012**, *144*, 357–368. [CrossRef]
206. Pimentel, P.; Pinto, M. Physiological and morphological responses of *Prunus* species with different degree of tolerance to long-term root hypoxia. *Sci. Hortic.* **2014**, *180*, 14–23. [CrossRef]
207. Arismendi, M.J.; Almada, R.; Pimentel, P.; Bastias, A.; Salvatierra, A.; Rojas, P.; Hinrichsen, P.; Pinto, M.; Di Genova, A.; Travisany, D.; et al. Transcriptome sequencing of *Prunus* sp. rootstocks roots to identify candidate genes involved in the response to root hypoxia. *Tree Genet. Genomes* **2015**, *11*, 11. [CrossRef]
208. Rubio-Cabetas, M.J.; Pons, C.; Bielsa, B.; Amador, M.L.; Marti, C.; Granell, A. Preformed and induced mechanisms underlies the differential responses of *Prunus* rootstock to hypoxia. *J. Plant Physiol.* **2018**, *228*, 134–149. [CrossRef] [PubMed]
209. Rubio-Cabetas, M.J.; Felipe, A.J.; Reighard, G.L. Rootstock Development. In *Almonds. Botany, Production and Uses*; Rafael Socias Company, Gradziel, T.M., Eds.; CABI: Boston, MA, USA, 2017; pp. 209–227.
210. Carroll, B.; Smith, M.W. *Pecan varieties for Oklahoma*; HLA6201; Division of Agricultural Sciences and Natural Resources, Oklahoma State University: Stillwater, OK, USA, 2017.
211. Wells, L. Budding and Grafting of Pecan. *Univ. Ga. Ext. Bullet.* **2017**, *1376*, 1–8.
212. Smith, M.W.; Cheary, B.S.; Carroll, B.L. Rootstock and scion affect cold injury of young pecan trees. *J. Amer. Pomol. Soc.* **2001**, *55*, 124–128.
213. Smith, M.W.; Anderson, J.A.; Parker, B.S. Cultivar and crop load influence cold damage of pecan. *Fruit Var. J.* **1993**, *47*, 214–218.
214. Smith, M.W. Damage by early autumn freeze varies with pecan cultivars. *HortScience* **2002**, *37*, 398–401. [CrossRef]
215. Thomas, A.L.; Reid, W.R. Hardiness of black walnut and pecan cultivars in response to an early hard freeze. *J. Amer. Pomol. Soc.* **2006**, *60*, 90–94.
216. Wood, B.W.; Reilly, C.C. Atypical Symptoms of Cold Damage to Pecan. *HortScience* **2001**, *36*, 298–301. [CrossRef]
217. Sparks, D.; Payne, J.A.; Horton, B.D. Effect of subfreezing temperatures on bud break of pecan. *HortScience* **1976**, *11*, 415–416.
218. Hinrichs, H.A. Pecan investigations in Oklahoma. *North. Nut Grower's Assn. Proc.* **1965**, *56*, 44–51.
219. Madden, G. Effect of winter injury. *Pecan Q.* **1978**, *12*, 17.
220. Smith, M.W.; Couch, G. Early fall or late spring freezes pose threat of injury to pecan trees. *Pecan South.* **1984**, *18*, 11–14.
221. Wood, B.W. Cold injury susceptibility of pecan as influenced by cultivar, carbohydrates, and crop load. *HortScience* **1986**, *21*, 285–286.
222. Sanderlin, S. Pecan scion cultivar effects on freeze susceptibility of the rootstock. *J. Amer. Pomol. Soc.* **2000**, *54*, 188–193.
223. Goff, W.D.; Tyson, T.W. Fall freeze damage to 30 genotypes of young pecan trees. *Fruit Var. J.* **1991**, *45*, 176–179.
224. Grauke, L.J. Pecan seed stock selection—Regional implications. *Proc. SE Pecan Grow Assoc.* **2010**, *103*, 42–50.
225. Grauke, L.J. Family Trees: Roots & Resilience. *Pecan South. Mag.* **2019**, *52*, 12–21.
226. Cao, F.; Wei, Y.; Wang, X.; Li, Y.; Peng, F. A study of the evaluation of the pecan drought resistance of grafted "Pawnee" trees from different seedling rootstocks. *HortScience* **2019**, *54*, 2139–2145. [CrossRef]
227. Luciani, E.; Palliotti, A.; Tombesi, S.; Gardi, T.; Micheli, M.; Berrios, J.G.; Zadra, C.; Farinelli, D. Mitigation of multiple summer stresses on hazelnut (*Corylus avellana* L.): Effects of the new arbuscular mycorrhiza *Glomus iranicum* tenuihypharum sp. nova. *Sci. Hortic.* **2019**, *257*, 108659. [CrossRef]
228. Cheng, W.-J.; Xu, Y.-J.; Huang, G.-M.; Rahman, M.M.; Xiao, Z.-Y.; Wu, Q.-S. Effects of five mycorrhizal fungi on biomass and leaf physiological activities of walnut. *Not. Bot. Horti. Agrobo.* **2020**, *48*, 2021–2031. [CrossRef]
229. Mortier, E.; Lamotte, O.; Martin-Laurent, F.; Recorbet, G. Forty years of study on interactions between walnut tree and arbuscular mycorrhizal fungi. A review. *Agron. Sustain. Dev.* **2020**, *40*, 43. [CrossRef]
230. Ma, W.-Y.; Wu, Q.-S.; Xu, Y.-J.; Kuča, K. Exploring mycorrhizal fungi in walnut with a focus on physiological roles. *Not. Bot Horti Agrobo* **2021**, *49*, 12363. [CrossRef]
231. Sheikhi, A.; Mirdehghan, S.H.; Arab, M.M.; Eftekhari, M.; Ahmadi, H.; Jamshidi, S.; Gheysarbigi, S. Novel organic-based postharvest sanitizer formulation using Box Behnken design and mathematical modeling approach: A case study of fresh pistachio storage under modified atmosphere packaging. *Postharvest Biol. Technol.* **2020**, *160*, 111047. [CrossRef]
232. Sheikhi, A.; Mirdehghan, S.H.; Ferguson, L. Extending storage potential of de-hulled fresh pistachios in passive-modified atmosphere. *J. Sci. Food Agri.* **2019**, *99*, 3426–3433. [CrossRef] [PubMed]
233. Sheikhi, A.; Mirdehghan, S.H.; Karimi, H.R.; Ferguson, L. Effects of Passive-and Active-Modified Atmosphere Packaging on Physio-Chemical and Quality Attributes of Fresh In-Hull Pistachios (*Pistacia vera* L. cv. Badami). *Foods* **2019**, *8*, 564. [CrossRef] [PubMed]
234. Holtz, B.A.; Teviotdale, B.L. Soil-borne diseases. In *Pistachio Production Manual*; Ferguson, L., Haviland, D., Eds.; University of California Agriculture and Natural Resources: Oakland, CA, USA, 2016; pp. 257–264.
235. Epstein, L.; Beede, R.; Kaur, S.; Ferguson, L. Rootstock effects on pistachio trees grown in *Verticillium dahliae*-infested soil. *Phytopathology* **2004**, *94*, 388–395. [CrossRef] [PubMed]

236. Stamler, R.A.; Kilcrease, J.; Kallsen, C.; Fichtner, E.J.; Cooke, P.; Heerema, R.J.; Randall, J.J. First report of *Rhodococcus* isolates causing pistachio bushy top syndrome on 'UCB-1' rootstock in California and Arizona. *Plant Dis.* **2015**, *99*, 1468–1476. [CrossRef] [PubMed]
237. Chang, J.H.; Putnam, M.L.; Grünwald, N.J.; Savory, E.A.; Fuller, S.L.; Weisberg, A.J. Response to comments on "Evolutionary transitions between beneficial and phytopathogenic *Rhodococcus* challenge disease management". *eLife* **2018**, *7*, e35852. [CrossRef]
238. Nouri, M.T.; Lawrence, D.P.; Kallsen, C.E.; Trouillas, F.P. Macrophomina Crown and Root Rot of Pistachio in California. *Plants* **2020**, *9*, 134. [CrossRef]
239. Mircetich, S.M.; Rowhani, A. The relationship of cherry leaf roll virus and blackline disease of English walnut trees. *Phytopathology* **1984**, *74*, 423–428. [CrossRef]
240. McGranahan, G.H.; Leslie, C.A.; Woeste, K.E. Backcross breeding walnuts for resistance to the cherry leaf roll virus. *Acta Hortic.* **1997**, *442*, 121–128. [CrossRef]
241. Ferretti, L.; Corsi, B.; Luongo, L.; Dal Cortivo, C.; Belisario, A. A survey of cherry leaf roll virus in intensively managed grafted English (Persian) walnut trees in Italy. *J. Plant Pathol.* **2017**, *99*, 423–427. [CrossRef]
242. Woeste, K.; McGranahan, G.; Bernatzky, R. The identification and characterization of a genetic marker linked to hypersensitivity to the cherry leaf roll virus in walnut. *Mol. Breed.* **1996**, *2*, 261–266. [CrossRef]
243. Woeste, K.; McGranahan, G.H.; Bernatzky, R. Randomly amplified polymorphic DNA loci from a walnut backcross [(*Juglans hindsii* × *J. regia*) × *J. regia*]. *J. Am. Soc. Hortic. Sci.* **1996**, *121*, 358–361. [CrossRef]
244. McGranahan, G.; Leslie, C.; Bujazha, D.; Dandekar, A.; Beede, B.; Olson, B.; Reil, W.; Jamindar, S. Walnut improvement program. In *Annual Walnut Research Report*; California Walnut Board, University of California-Davis: Davis, CA, USA, 2003; pp. 1–46.
245. McGranahan, G.H.; Leslie, C.A. Advances in genetic improvement of walnut at the University of California, Davis. *Acta Hortic.* **2005**, *705*, 117–122. [CrossRef]
246. Kluepfel, D.; Leslie, C.; Aradhya, M.; Browne, G.; Hasey, J.; Pope, K.; McKenry, M.; Westphal, A.; Hackett, W.; Bostock, R.; et al. Development of disease-resistant walnut rootstocks: Integration of conventional and genomic approaches (SCRI- match Year 3). In *Annual Walnut Research Report*; California Walnut Board, University of California-Davis: Davis, CA, USA, 2015; pp. 1–5.
247. Escobar, M.A.; Civerolo, E.L.; Summerfelt, K.R.; Dandekar, A.M. RNAi-mediated oncogene silencing confers resistance to crown gall tumorigenesis. *Proc. Natl. Acad. Sci. USA* **2001**, *98*, 13437–13442. [CrossRef]
248. Escobar, M.A.; Leslie, C.A.; McGranahan, G.H.; Dandekar, A.M. Silencing crown gall disease in walnut (*Juglans regia* L.). *Plant Sci.* **2002**, *163*, 591–597. [CrossRef]
249. Walawage, S.L.; Britton, M.T.; Leslie, C.A.; Uratsu, S.L.; Li, Y.; Dandekar, A.M. Stacking resistance to crown gall and nematodes in walnut rootstocks. *BMC Genom.* **2013**, *14*, 668. [CrossRef]
250. Duval, H.; Hoerter, M.; Polidori, J.; Confolent, C.; Masse, M.; Moretti, A.; Ghelder, V.; Esmenjaud, D. High-resolution mapping of the RMia gene for resistance to root-knot nematodes in peach. *Tree Genet. Genomes* **2014**, *10*, 297–306. [CrossRef]
251. Claverie, M.; Dirlewanger, E.; Bosselut, N.; Van Ghelder, C.; Voisin, R.; Kleinhentz, M.; Lafargue, B.; Abad, P.; Rosso, M.-N.; Chalhoub, B.; et al. The *Ma* Gene for Complete-Spectrum Resistance to *Meloidogyne* Species in *Prunus* Is a TNL with a Huge Repeated C-Terminal Post-LRR Region. *Plant Physiol.* **2011**, *156*, 779–792. [CrossRef]
252. Van Ghelder, C.; Esmenjaud, D.; Callot, C.; Dubois, E.; Mazier, M.; Duval, H. *Ma* Orthologous Genes in *Prunus* spp. Shed Light on a Noteworthy NBS-LRR Cluster Conferring Differential Resistance to Root-Knot Nematodes. *Front. Plant Sci.* **2018**, *9*, 1269. [CrossRef]
253. Van Ghelder, C.; Lafargue, B.; Dirlewanger, E.; Ouassa, A.; Voisin, R.; Polidori, J.; Kleinhentz, M.; Esmenjaud, D. Characterization of the RMja gene for resistance to root-knot nematodes in almond: Spectrum, location, and interest for *Prunus* breeding. *Tree Genet. Genomes* **2010**, *6*, 503–511. [CrossRef]
254. Barreneche, T.; Botta, R.; Cecile, R. Advances in breeding of chestnuts. In *Achieving Sustainable Cultivation of Tree Nuts*; Serdar, Ü., Fulbright, D., Eds.; Burleigh Dodds Science Publishing: London, UK, 2019.
255. Vannini, A.; Morales-Rodriguez, C. Integrated disease management in tree nut cultivation. In *Achieving Sustainable Cultivation of Tree Nuts*; Serdar, Ü., Fulbright, D., Eds.; Burleigh Dodds Science Publishing: London, UK, 2019.
256. Pereira-Lorenzo, S.; Costa, R.; Anagnostakis, S.; Serdar, U.; Yamamoto, T.; Saito, T.; Ramos-Cabrer, A.M.; Ling, Q.; Barreneche, T.; Robin, C.; et al. Interspecific hybridization of chestnut. In *Polyploidy and Hybridization for Crop Improvement*; Mason, A.S., Ed.; Taylor & Francis Group, LLC.: Abingdon, UK, 2016; pp. 377–407. [CrossRef]
257. McKay, J.W. Results of a Chinese chestnut rootstock experiment. *Ann. Rep. North. Nut Grow. Assoc.* **1947**, *38*, 83–85.
258. McKay, J.W.; Jaynes, R.A. *Chestnuts. Handbook on North American Nut Trees*; Northern Nut Growers' Association: Knoxville, TN, USA, 1969; pp. 264–286.
259. Schad, C.; Solignat, G.; Grente, J.; Venot, P. Recherches sur le châtaignier à la Station de Brive. *Ann. De L'amélioration des Plantes* **1952**, *3*, 369–458.
260. Breisch, H. *Châtaignes et Marrons*; CTIFL Monographie: Paris, France, 1995; p. 240.
261. Breisch, H.; Hennion, B. Les porte-greffe du châtaignier, vers plus de résistance. *Infos CTIFL* **2004**, *662*, 35–38.
262. Pereira-Lorenzo, S.; Fernández-López, J. Propagation of chestnut cultivars by grafting: Methods, rootstocks and plant quality. *J. Hortic. Sci.* **1997**, *72*, 731–739. [CrossRef]

263. Costa, R.; Santos, C.; Tavares, F.; Machado, H.; Gomes-Laranjo, J.; Kubisiak, T.; Nelson, C.D. Mapping and transcriptomic approches implemented for understanding disease resistance to *Phytophthora cinammomi* in *Castanea* sp. *BMC Proc.* **2011**, *5*, 18. [CrossRef]
264. Wilkinson, J. *Nut Grower's Guide: The Complete Handbook for Producers and Hobbyists*; Landlinks Press: Collingwood, VIC, Australia, 2005.
265. Casey, J.; Casey, B. Australia. In *Following Chestnut Footprints (Castanea spp.) Cultivation and Culture, Folklore and History, Tradition and Uses*; ISHS: Leuven, Belgium, 2009; pp. 14–19.
266. Rutter, P.A.; Miller, G.; Payne, J.A. Chestnuts (*Castanea*). *Genet. Resour. Temp. Fruit Nut Crop.* **1991**, *290*, 761–790. [CrossRef]
267. Miller, G.; Miller, D.D.; Jaynes, R.A. Chestnuts. In *Fruit Breeding. Vol. III Nuts*; Janick, J., Moore, J.N., Eds.; Wiley: New York, NY, USA, 1996; pp. 99–123.
268. Pereira-Lorenzo, S.; Ballester, A.; Corredoira, E.; Viéitez, A.M.; Agnanostakis, S.; Costa, R.; Bounous, G.; Botta, R.; Beccaro, L.G.; Kubisiak, L.T.; et al. *Fruit Breed*; Springer: Boston, MA, USA, 2012; pp. 729–769.
269. Ding, B.; Itaya, A.; Qi, Y. Symplasmic protein and RNA traffic: Regulatory points and regulatory factors. *Curr. Opin. Plant Biol.* **2003**, *6*, 596–602. [CrossRef]
270. Mallory, A.C.; Mlotshwa, S.; Bowman, L.H.; Vance, V.B. The capacity of transgenic tobacco to send a systemic RNA silencing signal depends on the nature of the inducing transgene locus. *Plant J.* **2003**, *35*, 82–92. [CrossRef]
271. Dutt, M.; Li, Z.T.; Kelley, K.T.; Dhekney, S.A.; Van Aman, M.; Tattersall, J.; Gray, D.J. Transgenic rootstock protein transmission in grapevines. *Acta Hortic.* **2007**, *738*, 749–754. [CrossRef]
272. Agüero, C.B.; Uratsu, S.L.; Greve, C.; Powell, A.L.T.; Labavitch, J.M.; Meredith, C.P.; Dandekar, A.M. Evaluation of tolerance to Pierce's disease and *Botrytis* in transgenic plants of *Vitis vinifera* L. expressing the pear PGIP gene. *Mol. Plant Pathol.* **2005**, *6*, 43–51. [CrossRef] [PubMed]
273. Dandekar, A.M.; Jacobson, A.; Ibáñez, A.M.; Gouran, H.; Dolan, D.L.; Agüero, C.B.; Uratsu, S.L.; Just, R.; Zaini, P. Trans-graft protection against Pierce's disease mediated by transgenic grapevine rootstocks. *Front. Plant. Sci.* **2019**, *10*, 84. [CrossRef] [PubMed]
274. Escobar, M.A.; Civerolo, E.L.; Polito, V.S.; Pinney, K.A.; Dandekar, A.M. Characterization of oncogene-silenced transgenic plants: Implications for *Agrobacterium* biology and post-transcriptional gene silencing. *Mol. Plant Pathol.* **2003**, *4*, 57–65. [CrossRef] [PubMed]
275. Gouran, H.; Phu, M.; Britton, M.; Leslie, C.; Dandekar, A.M. *Crown Gall Resistant Walnut Rootstocks: Analysis of the Graft Union for Transmission of Genetic and Biological Materials in Walnut Research Reports*; California Walnut Board: Sacramento, CA, USA, 2012; pp. 67–77.
