# Supplementary material for: Provenance and family variations in early growth of Manchurian walnut (Juglans mandshurica Maxim.) and selection of superior families
Source: PLoS One. 2024 Mar 7;19(3):e0298918. doi: 10.1371/journal.pone.0298918 (PMC10919699; doi:10.1371/journal.pone.0298918)
Supplement: S1 File — (ZIP) [file pone.0298918.s004.zip › Agricultural uses of juglone opportunities and challenges.pdf]

agronomy

MDPI

Review

Agricultural Uses of Juglone: Opportunities and Challenges

A. K. M. Mominul Islam [1,2,*] and Joshua R. Widhalm [1,3,*]

1 Department of Horticulture and Landscape Architecture, Purdue University, 625 Agriculture Mall Dr, West Lafayette, IN 47907, USA
2 Department of Agronomy, Bangladesh Agricultural University, Mymensingh 2202, Bangladesh
3 Purdue Center for Plant Biology, Purdue University, West Lafayette, IN 47907, USA
* Correspondence: akmmominulislam@bau.edu.bd (A.K.M.M.I.); jwidhalm@purdue.edu (J.R.W.); Tel.: +1-765-496-3891 (J.R.W.)

Received: 4 September 2020; Accepted: 28 September 2020; Published: 1 October 2020

check for updates

Abstract: Application of conventional synthetic pesticides and agrochemicals has boosted the yield and productivity of crops by reducing pest infestation and promoting crop growth yet increasing reliance on many of these products poses serious environmental threats. This has led to growing interest in obtaining more environmentally friendly alternatives to conventional pesticides and agrochemicals. Allelochemicals produced by plants, fungi, and microbes offer options for developing novel natural product-based pesticides and agrochemicals that are effective but with lower environmental half-lives. Here, we review the current state of knowledge about the potential use of juglone (5-hydroxy-1,4-naphthoquinone), the allelochemical produced by black walnut trees (*Juglans nigra*), which has been investigated for applications across a range of different agricultural purposes. We then offer our perspective on what opportunities and challenges exist for harnessing juglone as a component of sustainable agriculture.

Keywords: allelochemical; agrochemical; biostimulant; *Juglans*; juglone; natural product; pesticide; sustainable agriculture; urease inhibitor

1. Introduction

Juglone, 5-hydroxy-1,4-napthoquinone (Figure 1), is a quinoid compound that functions as an allelochemical when released from trees in the walnut family (Juglandaceae) into the rhizosphere. Juglone has been isolated from several Juglandaceae members, including *Juglans nigra* L. (black walnut), *J. regia* L. (English walnut), *J. cinerea* L., *J. ailantifolia* Carr., *J. mandshurica* Maxim., *Carya tomentosa* Nutt., *C. ovata* (Mill.) K. Koch, *C. illinoensis* (Wangenh.) K. Koch (pecan), *Ptero-carya fraxinifolia* (Lam.) Spach, and *Platycarya strobilacea* Siebold & Zucc. [1,2]. Juglone is excreted from roots and exuded from litterfall in its reduced form, hydrojuglone [3–6]. Hydrojuglone is colorless, nontoxic, and is abundant in leaves, roots, husks, and bark [7]. Upon release, hydrojuglone is oxidized in air to its toxic form, juglone [8–10]. Juglone taken up by sensitive plants has general inhibitory effects on growth and development [4]. Moreover, juglone is the bioactive compound in certain plant parts used in traditional medicines for treating ailments including allergies, gastrointestinal abnormalities, cancer, and different fungal, bacterial, and viral infections [9]. Due to its general toxicity, juglone itself is unlikely to ever become a clinically significant drug. However, efforts are ongoing to synthesize juglone analogs with enhanced bioavailability, lower toxicity, and improved selectivity [11].

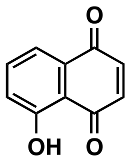

Figure 1. The chemical structure of juglone (5-hydroxy-1,4-naphthoquinone).

The allelopathic effects of black walnut on neighboring plants have been reported for centuries and remain a concern for home gardeners today [8,12–14]. Plinius Secundus (Pliny the Elder) is credited with being the first to report the phytotoxic effects of black walnut. It was not until the 1850s, however, that juglone, previously known as nucin, was first isolated [15]. By 1887, juglone could be synthesized [16], and in 1881 the first scientific article related to its allelopathy was published [17]. In 1928, juglone was determined to be the compound responsible for the phytotoxicity of walnut trees [18]. Today, natural products like juglone are touted as promising alternatives to synthetic agrochemicals due to their reported pesticidal properties and other biochemical activities of agricultural interest. Perhaps most notably, juglone exhibits inhibitory effects on several weed species [19], as characterized by effects like leaf wilting and yellowing or damaging the roots through induction of reactive oxygen species (ROS) together with calcium accumulation resulting to death [20,21]. Juglone is also reported as being lethal to larval development and insect flight muscle mitochondria, to have sedative effects on fish and other animals, to have mutagenic, carcinogenic, and lethal effects on animal cells, and to have repellent, antifeedant, antimicrobial, antifungal, and antiparasitic properties [19,22–26]. However, toxicity of juglone varies depending on several factors, such as the donor plant species, quantity released and amount accumulated, soil pH, texture, and organic matter content [27,28]. Therefore, it is worth investigating the use of juglone in agricultural applications and to study the factors precluding it from being implemented as a chemical tool. In this review, we provide an overview of the current state of knowledge on juglone, which encompasses nearly 100 years of research and observations. We also address the challenges and prospects to harnessing it as a natural product-based pesticide and/or agrochemical. Finally, we identify areas where further research is needed to implement juglone across areas of sustainable agriculture.

2. Prospects of Juglone as a Natural Product-Based Pesticide

Since the dawn of agriculture, pests have posed threats to crops, fish, livestock, and non-target insects. When it comes to food crops, for example, it is estimated that over 140,000 species of insects, weeds, nematodes, and microorganisms, including pathogenic fungi, viruses, and bacteria contribute to agricultural losses [29]. On average, 26–50% of crop losses have been reported in rice, potato, coffee, maize, cotton, wheat, soybean, barley, and sugar beet [30]. In total, around one-third of the total production of major crops is damaged due to pests worldwide [31]. More recently, the Food and Agriculture Organization (FAO) of the United Nations estimated that 19–30% of cereals, 33–60% of roots and tubers, and 37–55% of fruits and vegetables are damaged globally by crop pests [32].

To meet the demand for the agricultural output needed to sustain an increasing global population, 3.5 billion kg of active ingredient is used in the form of synthetic pesticides each year by farmers [33]. While pesticides are regulated in certain countries, farmers and their environments in developing countries are often exposed to toxic chemicals that are banned or restricted in other countries. This exposure is further exacerbated when inappropriate dosages, application techniques, equipment, and/or storage practices are employed [34]. Continued use of the same pesticides has also increased the rate at which pests develop resistances to available chemical active ingredients. Moving forward, developing new pest control products will be constrained by stringent environmental, toxicological, and regulatory requirements [35]. This opens the door for producing natural product-based pesticides. Natural products are compounds biosynthesized by living organisms and which have biological activities resulting from Darwinian selection. Thus, they offer a diverse source of new chemical

structures and modes of action. They are also likely to be met with more public and regulatory acceptance than synthetic chemicals, especially considering that they generally have lower environmental half-lives [36]. Juglone has emerged as a strong candidate for developing natural product-based alternatives to currently used pesticides for controlling insect pests, microorganisms, nuisance algae, and weeds. In the sections that follow, we highlight literature reporting on the effectiveness of juglone in controlling these pests.

2.1. Insecticidal Properties

Toxic effects, such as weight reduction, antifeedant activity, deterioration of morphology and sexual development, and reduction in egg hatching, of juglone and other naphthoquinones on certain phytophagous insects have been widely reported [25,37–40]. The melon or cotton aphid (*Aphis gossypii* Glover) is a major insect pest of cotton, melon, pumpkin, pepper, and tomato worldwide [41], causing damage to crops through direct feeding, photosynthesis inhibition, and acting as a viral vector [42,43]. As a result, plants become stunted and senesce [44]. To control the pest, large amounts of chemical insecticides are applied every year, which has led to broad-spectrum resistance to organophosphate, carbamate, and pyrethroid insecticides [45]. Using NMR-based "metabonomics," a subset of metabolomics in which metabolomes are measured and mathematically modelled, Lv et al. [25], found that juglone causes physiological disturbances in *A. gossypii* hemolymph that may underly its insecticidal properties.

Galleria mellonella, or the greater wax moth, is a grave threat to apiculture as its larvae feed on honeycombs, pollen, honey, and beeswax in hives [46], causing significant losses [47,48]. In Turkey, some beekeepers use *J. regia* leaves and/or walnut husks to control *G. mellonella* infestation in beehives. To identify the role of juglone in this control mechanism, Altuntaş et al. [26], and Erbaş and Altuntaş [48] incorporated the compound into the *G. mellonella* first-instar larvae diet and found that juglone prolonged larval developmental time, decreased pupal and adult weights, and lowered total egg numbers and egg hatchability. These findings are supported by similar results from other studies in which juglone was incorporated into insect diets [26,37,40,49,50]. Juglone's toxic effects have also been observed on *Aedes aegypti* (yellow fever mosquito), *Drosophila melanogaster*, *Manduca sexta* (tobacco hornworm) [49], *Lymantria dispar* (gypsy moth) [37] and its larvae [51], *Callosamia promethea* (saturniid moth) [38], *Trichoplusia ni* [52], *Tetranychus urticae* [53], and *Pieris rapae* and *Helicoverpa armigera* [40]. Therefore, it seems that there are opportunities for using both purified juglone as well as extracts or litter from walnut-producing species for controlling multiple types of insect pests.

2.2. Bactericidal Properties

Several articles have been published on the antibacterial activities of juglone against a number of bacteria ranging from those that affect animal guts to those that contaminate livestock products to others that represent plant pathogens [54–62]. Clark et al. [63], reported that juglone has moderate inhibitory activity against Gram-positive and acid-fast bacteria, and little activity against Gram-negative bacteria. In contrast, strong bactericidal activity of juglone was observed on *Erwinia amylovora*, a Gram-negative bacterium that is the causal agent of fire blight disease in pome fruit [58]. Juglone also inhibits key enzymes in the Gram-negative bacterium *Helicobacter pylori* [64]. Using a proteomics approach, Wang et al. found that in *Staphylococcus aureus*, proteins functioning in the tricarboxylic acid cycle and in DNA, RNA, and protein synthesis are inhibited by juglone [62]. In addition, juglone was found to increase oxidoreductase activity in cells and to create a peroxidative environment, significantly reducing cell wall formation and increasing membrane permeability [61]. Such changes to the bacterial cell biochemical environment likely have the capacity to affect antibiotic resistance activity [60].

Just as there are benefits to the bactericidal properties of juglone, there are examples where caution needs to be taken when introducing juglone into an environment or introducing beneficial bacteria into a juglone-rich environment. Nitrogen-fixing shrubs and trees are sometimes planted in walnut orchards to increase available nitrogen in the soil. This practice is cost effective and environmentally friendly as

it reduces nitrogen fertilizer requirements. However, Dawson and Seymour [65] reported that juglone slightly inhibits growth of *Rhizobium* and completely inhibits growth of *Frankia* species which form associations with actinorhizal plants [66]. In contrast, some bacteria can degrade juglone in soil [67], and certain *Pseudomonas* species can use juglone as their sole carbon source [68]. Thus, the presence of these organisms in the soil could undermine the efficacy of applying juglone or juglone-containing extracts in field or other uncontrolled settings.

2.3. Fungicidal Properties

Pathogenic fungi are responsible for damaging crops in both pre- and post-harvest stages, and also for the increased mortality of edible insects. Synthetic chemical fungicides are typically applied to control such pathogenic fungi, though a few natural product-based fungicides are available [69–72]. Clark et al. [63], compared the efficacy of juglone with several commercially available antifungal agents (clotrimazole, triacetin, tolnaftate, griseofulvin, zinc undecylenate, selenium sulfide, liriodenine, and liriodenine methiodide) and reported that the moderate antifungal activity of juglone was as effective as zinc undecylenate and selenium sulfide. In addition, the antifungal activity of juglone was observed against *Penicillium* spp., *Aspergilluus* spp., *Hansenula* spp., and *Saccharomyces carlsbergensis* [55,56].

Higher levels of juglone in pecan cultivars and other species in the Juglandaceae family correlate with increased resistance to pecan scab caused by *Fusicladium effusum* [73]. Foliar application of juglone to bean seedlings was found to confer better protection from rust compared to certain commercial fungicides [74]. On the other hand, Arasoglu et al. [75], compared the antifungal properties of free juglone with its poly (D,L-lactic-co-glycolic acid) [PLGA] nanoparticle formulation against *Aspergillus flavus*, *Candida albicans*, and *Fusarium* spp. Their results indicate that the juglone-encapsulated nanoparticle was more effective than free juglone. Hence, juglone or its PLGA nanoparticle formulations might be useful as a tool for the developing biofungicides.

Comparisons of the antifungal activity of green walnut husk extracts with pure juglone against the plant pathogenic fungi *Alternaria alternata*, *Rhizoctonia solani*, *Botrytis cinerea*, *Fusarium culmorum*, *Phytophthora infestans*, as well as *Ascosphaera apis* revealed that juglone is not the only component responsible for inhibiting mycelial growth [76]. Phenolic compounds were also found to synergistically contribute to the activity of the extracts possibly by modifying the antifungal activity of juglone [76]. In another study, Sytykiewicz et al. [77], examined the antifungal potential of methanolic, ethyl acetate, and acid-hydrolyzed methanolic extracts of *J. regia* leaves against pathogenic *Candida albicans* strains and observed that the methanolic extract produced the highest anticandidal activity. Methanolic extracts of *J. regia* leaves contain a range of phenolic acids (e.g., caffeic, chlorogenic, cinnamic, and coumaric acids), tannins, and flavonoids [77]. Interestingly, some fungi are proposed to play a role in the transfer of juglone from plants into the soil, such as the case with arbuscular mycorrhizal fungal hyphae transporting juglone from black walnut roots into the rhizosphere [78,79]. Thus, some fungi have evolved mechanisms to cope with high levels of juglone in the rhizosphere.

2.4. Algacidal Properties

Climate change and eutrophication caused by agricultural runoff has promoted the proliferation of "algal blooms" caused by cyanobacteria, diatoms, and green algae that threaten drinking water and aquatic ecosystems [80–82]. Bloom-forming cyanobacteria and nuisance algae shade light for other phytoplankton and reduce water quality by depleting oxygen, producing foul odors [83], and endanger aquatic food webs and human health through released toxins [84–87]. Juglone was shown to inhibit the growth and physiological performance of cyanobacteria including *Microcystis aeruginosa* [88], *Anabaena variabilis*, *A. flos-aquae*, and *Nostoc commune* [89,90]. Growth of Streptophytic (*Closterium acerosum*, *Micrasterias thomasiana*, and *Spirogyra grevilleana*) and chlorophytic (*Pandorina morum* and *Eudorina californica*) freshwater green algae is also inhibited by juglone at concentrations ranging from 0.1–1 mM [91]. Interestingly, lower concentrations, 0.1–1 mg L^{-1} (0.57–5.7 μM), were found

to stimulate growth of chlorophytic freshwater green algae *Chlorella vulgaris*, *Scenedesmus ecornis*, and *S. quadricauda* and the freshwater diatoms *Asterionella formosa*, *Fragilaria crotonensis*, and *Synedra acus* [92]. Park et al. [92] also found that juglone can inhibit the harmful bloom-forming nuisance cyanobacterium *Microcystis aeruginosa* and the freshwater diatom *Stephanodiscus hantzschii* by up to 93% and 75%, respectively, at 1 mg L^{-1} (5.7 µM). The same study found that even less juglone (0.1 mg L^{-1}; 0.57 µM) inhibits growth of the cyanobacterial species *A. flos–aquae*, *Oscillatoria curviceps*, and *Phormidium subfuscum*. Thus, cyanobacteria generally appear to be more susceptible to juglone than do green algae and diatoms. While cellular morphology and anatomy may play a role, given the propensity of juglone to induce ROS formation [93], this is consistent with studies showing that cyanobacteria are more susceptible to hydrogen peroxide than are green algae and diatoms [94,95]. It should be noted that in addition to algae, juglone also acts as a general toxicant to fish and other marine organisms [23,91] and must therefore be used with caution (also see Section 5 and cited references for more information).

2.5. Phytotoxic (Herbicidal) Properties

Several articles have been published reporting on the phytotoxic properties of juglone on the germination and growth of different herbaceous and woody crops species (Table 1). Like other 1,4-naphthoquinones, juglone's phytotoxicity comes from generation of ROS and glutathione (GSH) depletion [93], and from impairing plasma membrane H^{+}-ATPase [96], a mode of action distinct from existing synthetic commercial herbicides [97]. Rapid irreversible growth inhibition in maize coleoptile segments indicates that the impairment of plasma membrane H^{+}-ATPase is via alkylation with juglone [98]. To study juglone phytotoxicity, known concentrations of juglone, most often ranging from µM to mM concentrations, are applied either in hydroponic or soil culture and inhibition is observed [1,99,100]. Nonetheless, little remains known about actual juglone concentrations present in soil [27,28,101], or about the time required to build up sufficient amount of juglone to show toxicity to neighboring species [99]. Dana and Lerner [14] and Strugstad and Despotovski [23], have reported a number of vegetables, fruits, flowers, landscape, and field crop species that are susceptible and tolerant of juglone. Investigation of the effects of juglone on Norway spruce (*Picea abies*) revealed that it has more potent post-germination effects than it does on germination, perhaps suggesting that seeds are more biochemically equipped to neutralize ROS [102]. The seed coats of some species have been proposed to function as barriers to juglone, however, Kocaçaliskan et al. [103], observed no significant differences of juglone's inhibitory effect on seedling growth and protein content when comparing intact and coatless cucumber seeds. At the same time, differences in germination rate between species with relatively thick (cucumber) versus thin (cress and tomato) seed coats were observed [104]. Interestingly, a recent study found that black walnut extracts increased the height and number of leaves of rice (*Oryza sativa*) but had the opposite effect on height in wheat (*Triticum aestivum*) [105]. At the same time, Chi et al. [21], reported that juglone significantly reduces rice root growth at low concentrations (5–50 µM) and leads to changes in gene transcription associated with cell growth, cell wall formation, chemical detoxification, and abiotic stress responses with rapid induction of ROS. Juglone was also found to induce oxidative damage to the root apical meristem via ROS formation in lettuce [106]. It therefore seems that metabolic differences between species, and perhaps between above- and below-ground organs of the same species, contribute to determining susceptibility of plants to juglone. See Table 1 for additional examples of how juglone affects plant growth and development.

Table 1. Reported effects of juglone or juglone-containing materials on plants. Ref., reference.

Species	Growth Effect (Solution Tested)	Parts or Processes Affected	Ref.
Lonicera maackii, *Lespedeza cuneata*, *Trifolium incarnatum*, *Alnus glutinosa*, *Elaeagnus umbellata*	Decreased (0.01–1 mM juglone)	Shoot elongation and dry weight accumulation	[1]
Cucumis melo cv. Kiş Kavunu	Increased (1 mM juglone)	Elongation, fresh and dry weights, and polyphenol oxidase enzyme	[107]
Cucumis sativus cv. Beith Alpha	Decreased (1 mM juglone)	Elongation, fresh and dry weights, and protein content of cotyledons	[103]
	Increased (1 mM juglone)	Polyphenol oxidase enzyme activity	
Solanum lycopersicum cv. Rio Grande, *Cucumis sativus* cv. Çengelköy, *Lepidium sativum* cv. Bandirma, *Medicago sativa* cv. Yerli	Decreased (1 mM juglone; 10% (*w/v*) *J. regia* leaf aqueous extract)	Seed germination and seedling growth	[104]
Cucumis melo	Increased (1 mM juglone; 1/8 of 10% (*w/v*) *J. regia* leaf aqueous extract)	Seedling growth	
Cucumis sativus cv. Beith Alpha	Decreased (0.01–1 mM juglone)	Germination	[108]
Brassica rapa L.	Decreased (2% (*w/v*) ethyl acetate extract of *J. regia* rhizosphere and adjacent soil)	Seed germination, shoot and root length, peroxidase and malondialdehyde (MDA) activity	[109]
Day-neutral Strawberry (*Fragaria* × *ananassa* L.) cultivar Fern	Decreased (1 mM juglone; 10% (*w/v*) *J. regia* leaf aqueous extract)	Fruit yield per plant, number of fruits per plant, average fruit weight, crowns per plant, number of leaves, leaf area, fresh root weight, total soluble solid, vitamin C, and acidity	[110]
Nicotiana tabacum	Decreased (10–50 µM juglone)	Seedling growth	[111]
	Increased (10–50 µM juglone)	Reactive oxygen species and proline concentration	
Triticum aestivum	Decreased (*J. nigra* leaf aqueous extract)	Plant height and number of leaves	[105]
Oryza sativa	Increased (*J. nigra* leaf aqueous extract)		
Zea mays and *Glycine max*	Decreased (10–100 µM juglone)	Root shoot dry weight and length, and H+-ATPase activity	[96]
Zea mays and *Glycine max*	Decreased (100 µM juglone)	Shoot and root relative growth rates, leaf photosynthesis, transpiration, stomatal conductance, and leaf and root respiration	[112]
Raphanus sativus,	Decreased (*J. nigra* leaf aqueous extract)	Germination rate radical and plumule length, and seedling dry weight	[113]
Cucumis sativus cv. Beith Alpha, *C. melo* cv. Ananas	Decreased (1 mM juglone)	Seedling elongation, fresh and dry weights, catalase and superoxide dismutase activities	[114]
C. melo cv. Kis Kavunu	Increased (1 mM juglone)		
Cucumis sativus cv. Beith Alpha,	Increased (1 mM juglone)	Malondialdehyde (MDA) levels	[114]
C. melo cv. Ananas			
C. melo cv. Kis Kavunu	Decreased (1 mM juglone)		
Medicago polymorpha,	Increased (100 µM juglone)	Leaf chlorosis	[115]
Medicago polymorpha and *M. lupulina*		Reduce glutathione (GSH), GSH and oxidized glutathione ratios, and antioxidant activity	
Purshia tridentata (Pursh.) D.C.	Decreased (100 µM juglone)	Plant growth and total protein content	[116]
Lactuca sativa var. angustata	Decreased (180 g *J. regia* leaf litter per pot with 15 kg soil)	Growth and physiological processes	[117]

Differential regulation of abiotic stress responses also alters susceptibility to juglone. Increased levels of ROS and proline were found in the roots of juglone-inhibited tobacco seedlings [111], and lignification was induced in juglone-inhibited soybean roots [118,119]. Attenuated toxic effects in roots were observed in proline-pretreated tobacco seedlings when subjected to juglone, indicating that juglone toxicity may be countered by preventing ROS accumulation [111]. In another study, Terzi and Kocaçaliskan [120] observed that inhibition of seed germination and elongation, reduction of fresh and dry weights of cress (*Lepidium sativum* cv. Bandırma) seeds by juglone were alleviated when seeds were pretreated with gibberellic acid and kinetin. In addition, gibberellic acid was found to be more effective than kinetin in alleviation of juglone stress [120].

Juglone is an attractive allelochemical-based herbicide candidate because of its reported efficacy at inhibiting germination and repressing growth of many weeds [19]. In a laboratory study, Sytykiewicz et al. [121] observed higher germination suppression of weed species, *Papaver rhoeas* (corn poppy) and *Agrostemma githago* (corn cockle) by juglone application (1 μM–10 mM) than the crop species *Triticum aestivum* cv. Nawra (spring wheat) and *Avena sativa* cv. Maczo (spring oat). Similarly, Topal et al. [122], evaluated the herbicidal activity of juglone on the growth of four weed species, *Sinapis arvensis* (wild mustard), *Cirsium arvense* (creeping thistle), *Papaver rhoeas* (field poppy), and *Lamium amplexicaule* (henbit) along with two crop species, *Triticum vulgäre* Vill. cv. Gerek 79 (wheat) and *Hordeum vulgäre* L. cv. Kislik (barley) as control. Juglone at higher concentrations (1.15–5.74 mM) completely inhibited the growth of field poppy while a significant amount of reduction on the elongation and fresh weight of all weed species was found with no inhibition on the crops. Higher inhibitory activity on the growth of weeds than crops was also reported by Cachiţă-Cosma et al. [123]. From a laboratory, greenhouse, and field trial of a black walnut extract (NatureCur®, Redox Chemicals LLC, Burley, ID, USA) against horseweed (*Conyza canadensis*), hairy fleabane (*Conyza bonariensis*), purslane (*Portulaca oleraceae*), and tall annual morning glory (*Ipomoea purpurea*), NatureCur® shows pre- and post-emergent bioherbicide potential [124]. The possibility of juglone derivatives (*O*-acyl and *O*-alkyl) and the isomeric 1,4-naphthoquinone, lawsone, also offer promising leads for developing more potent and targeted herbicides [125].

3. Juglone as a Biostimulant

Most plant allelochemicals produce inhibitory effects at higher doses but can function as biostimulants at lower doses [126]. The biostimulatory activity of allelochemicals at lower doses is also known as hormesis [127–131] and may guide discovery of new growth hormones. The inhibitory potential of allelochemicals against target species is the focus of most phytotoxin studies [130], although for herbicides, lower doses have been investigated for hormetic responses [132,133]. This is important to consider for allelochemicals like juglone given that under natural settings soil concentrations may not be sufficient to achieve inhibitory effects observed in vitro or in other controlled conditions, and instead have a biostimulatory effect. Indeed, the presence of *J. nigra* has been reported to improve forage cover in pastures [134] and bioassays have indicated that at low concentrations (typically ≤ 1 μM) juglone exhibits biostimulatory activity e.g., [1,92,118,135]. Other studies (e.g., [136]) have indicated similar effects testing dilutions of extracts from juglone-containing plant materials.

The stimulatory activity of an allelochemical depends on several factors, including the type of allelochemical, the species affected, the trait being measured (e.g., biomass, height, leaf area, protein content, enzyme activity), surrounding environmental conditions (e.g., temperature, moisture, salinity or nutrient content, etc.), plant density, and time of exposure [128,130,131]. Stimulation of a particular trait in one species at a given concentration may not be the same in another species. For example, at 5.7 μM, juglone inhibited the growth of *Stephanodiscus hantzschii* by more than 60%, while at the same time exhibited hormetic activity on three other diatoms, *Asterionella formosa*, *Fragilaria crotonensis*, and *Synedra acus* [92]. Along the same lines, Table 1 summarizes a range of reported inhibitory and stimulatory effects of juglone, juglone-producing species, and juglone-containing materials on various plant species. Examination of the effects of treating cucumber (*Cucumis sativus* cv. Beith Alpha) seeds with juglone found that while elongation, seedling fresh and dry weights, and cotyledon protein content significantly decreased, polyphenol oxidase activity, which offers protection against pathogens, increased [103]. In another study, two different muskmelon cultivars, *Cucumis melo* cv. Ananas and *C. melo* cv. Kis Kavunu, were found to respond oppositely to juglone based on measurements of seedling elongation, fresh and dry weights, catalase and superoxide dismutase activities, and levels of the lipid peroxidation end product malonyldialdehyde (Table 1) [114].

Mechanisms underlying the hormetic activities of allelochemicals, including juglone, are not well understood. Chobot and Hadacek [137], reported that the antioxidant properties of juglone are responsible for the stimulatory activity on the germination of *Sinapis alba* under higher oxidative

stress. In addition to affecting redox cycling, juglone spontaneously forms adducts with the reduced form of the endogenous antioxidant GSH [6]. While at high concentrations juglone depletes GSH levels leading to deleterious effects, at low concentrations, juglone may stimulate expression of genes encoding glutathione *S*-transferases (GSTs), vacuolar transporters, and/or GSH biosynthetic enzymes. Compounds which induce such effects are called "safeners" [138] and are used to increase crop resistance to herbicides and may confer other types of defensive or xenobiotic detoxification effects. Thus, juglone may also hold potential as a natural product-based safener.

It is important to consider the involvement of several mechanisms in conferring hormetic activity of juglone, or its affect in combination with other compounds. For example, the herbicide glyphosate, which impedes the shikimate pathway (the source of aromatic amino acids and lignin precursors), when administered at low non-herbicidal doses was found to result in more elastic cell walls in soybean [129]. Interestingly, it was only observed in non-transgenic lines indicating the mechanism of hormesis is linked to the herbicide target site [139]. In contrast, soybean root growth is proposed to be inhibited by juglone in part due to the excessive production of lignin. Three-day-old seedlings exposed to 5 µM juglone for 24 h were found to have reduced root length but with increased phenylalanine ammonia-lyase (PAL) activity, which is responsible for the first step of the core phenylpropanoid pathway, and increased levels of lignin [119]. This example illustrates that even targeting the same cellular process can have inhibitory or stimulatory effects depending on the mechanism involved. Thus, more research is needed to identify the potential benefits and drawbacks of hormetic doses of juglone when used alone or in combination with other compounds on crop plants. It is also critical to consider other unintended effects on other types of organisms if it is to be further considered as a biostimulant in cropping systems.

4. Juglone as a Urease Inhibitor

Urea accounts for over half of the world's nitrogen fertilizer consumption due to its high nitrogen content (46%), its low relative cost per nitrogen unit, its availability in most markets, its high water solubility, its low corrosion capacity, its compatibility across fertilizer formulations, and its high foliar uptake [140,141]. Crops require a steady supply of nitrogen to sustain growth and development compared to other essential plant nutrients. Once nitrogen fertilizer is applied to fields, it is directly absorbed by plants, and the excess amount is lost in ionic or gaseous forms via leaching, volatilization, and the denitrification process, thereby becoming unavailable to plants [142–144].

Urease is an enzyme in plants, fungi, and bacteria that accelerates hydrolysis of urea to ammonia and carbon dioxide at a speed of one-hundred-trillion-fold [145]. Nearly, 79–89% of urease activity in soil derives from extracellular enzymes present in the soil matrix [146]. Depending on environmental conditions, 10–40% of nitrogen applied as urea can be lost as ammonia from the soil surface due to extracellular soil urease activity [147]. This loss of excess nitrogen from crop fields creates air and water (eutrophication) pollution that directly, or indirectly after conversion to other forms, contributes to biodiversity loss, human health hazards, stratospheric ozone depletion, acid rain, and climate change [148–151]. To combat nitrogen losses from soil and to enhance nitrogen use efficiency, various attempts have been made by fertilizer manufacturers to stabilize urea and control nitrogen release attributes [143]. These include surface coatings (e.g., sulfur, polymers etc.), N-(*n*-butyl) thiophosphoric triamide (BTPT/NBPT; Agrotain®, Koch Agronomic Services, Wichita, KS, USA), 25% N-(propyl) thiophosphoric triamide + 75% NBPT (Limus®, BASF USA), phenylphosphorodiamidate (PPD/PPDA), and hydroquinone [140,143,152]. Urease inhibitors are applied to the soil surface or directly to urea granules to slow the action of extracellular soil ureases. Activities of these inhibitors is affected by soil moisture content, pH, and temperature [153]. Delaying urea hydrolysis at the soil surface gives urea an opportunity to penetrate into the soil through precipitation or irrigation [145]. Once infiltrated to a depth of approximately 5–10 cm, released ammonia is trapped and mineralized into other plant usable forms.

A natural product-based ureases inhibitor should function efficiently in all types of soils under various moisture regimes, and at the same time be easy to produce, easy-to-use, less harmful to the crops, and be economical [154]. Kot et al. [155], reported that juglone is a strong, time and concentration-dependent inactivator of urease that irreversibly inhibits via covalent modifications of protein thiols. Conventional urease inhibitors hinder urea hydrolysis at the surface, but do not persist for long periods of time and are weakly mobile in soil. Indeed, juglone is hydrophobic and relatively immobile in soil [14], making it a strong candidate as a natural product-based urease inhibitor. At the same time, juglone is light sensitive and begins to photodegrade within few days [28]. It is also rapidly metabolized by certain soil bacteria that can utilize it as a carbon source [67]. But to date there is a lack of information related to the urease inhibitory activity of juglone under field conditions, though a recent study suggests that soil urease activity is reduced around *J. regia* [156]. This raises the prospect that juglone or walnut extracts applied through either surface drenches or coating of urea granules could be effective at managing urease activity in agricultural settings.

5. Knowledge Gaps and Future Prospects

Considering consumer demand for greener and more sustainable agricultural solutions and the growing number of pests developing resistances to conventional pesticides, natural product-based pesticide and agrochemical development is gaining interest. While natural products have been used for developing certain types of pesticides, they have been vastly underutilized for developing herbicides in particular [157]. Around 20% of conventional pesticides are either natural products or natural product-derived substances, including triketones (herbicides), spinosyn, neonicotinoids and pyrethroids (insecticides), and strobilurin (fungicides) [157]. Structural complexity, costly structure determination and synthesis, obtaining sufficient quantities, labile characteristics of some compounds, and general toxicity are among the obstacle hindering development of natural product-based pesticides and agrochemicals [158]. Below, we have detailed some of the specific limitations presenting barriers to developing juglone-based applications, either through application of pure compound or as part of integrating juglone-producing species or juglone-containing materials into cropping practices:

1. Juglone concentration may vary with species, age of the plant, seasons, and locations. For example, de Scisciolo et al. [27], recorded up to 10-fold variation in juglone concentrations in soils under different walnut trees, while Coder [159], recorded higher juglone levels in the lower parts of the leaf crown. The concentration of juglone appears to be highest during the leaf opening period of walnut and during fruit formation, but it may vary depending on walnut species [3].
2. Batch-to-batch variation and post-harvest effects on juglone content. Carnat et al. [160], found no juglone in extracted *J. regia* dried leaves, while Girzu et al. [161], extracted fresh leaves of the same species and determined juglone accounts for 0.5% of the fresh weight. Juglone concentration also varies across different parts of walnut trees [162], which can lead to unpredictable potencies of mulches derived from litter or unused portions of walnut industry byproducts, for example.
3. Juglone exerts off-target toxicity to beneficial organisms, for example, earthworms [163], N-fixing bacteria [65,66], and fish [91,164,165].
4. The general phytotoxicity of juglone to a variety of specialty crops, like asparagus, cabbage, eggplant, pepper, potato, tomato, apple, blackberry, blueberry, pear, and tobacco limits its use in horticultural production. Nonetheless, there are a number of species that appear to be more juglone tolerant [14].
5. Toxicity may vary depending on the soil types, for example, juglone activity is higher in poor quality soils [28,166] and in moist soil compared to dry soil [167].
6. Oxidized juglone is semi-volatile. While juglone was not detected in headspace collections from intact green husks, it could be detected in collections from blended husks [168]. Thus, while juglone may have low volatility when reduced or in aqueous solution, or be bound as a glycoside in intact tissues, free juglone in pure form or in disrupted tissues (e.g., mulches) has the potential

to volatilze which could lead to off-target movement and effects on nearby insects, vertebrates, plants, and microorganisms.

7. Juglone is light sensitive and begins to photodegrade within a few days [28]. Surface or foliar applications of juglone therefore may be subject to shortened environmental half-lives that reduce its efficacy.

If the limitations of juglone can be properly addressed, there is great potential to harness it as a natural product-based pesticide, biostimulant, urease inhibitor, and/or other potential agrochemicals. To further develop these potential applications, future research should be directed toward:

1. Investigating the biostimulatory (hormetic) and inhibitory activity of juglone on different crops and weeds at various stages of growth. While most research has focused on studying the phytotoxicity of juglone (Table 1), identifying application rates leading to hormetic doses of juglone at early stages in crops may also contribute to suppressing weed growth by enhancing crop growth [130,169].
2. Understanding the activity of juglone in natural field settings. Most research on juglone has been conducted under strictly controlled environmental conditions, either in laboratory or greenhouse settings [170]. The accumulation of any allelochemical, however, is heavily influenced by environmental conditions [171–174]. In the case of juglone, this further depends on the route by which juglone reaches the environment (Figure 2).
3. Identifying ways to reduce production costs. The cost of chemically synthesizing a natural product, producing it through metabolic engineering in a heterologous system, or of cultivating the producing species and extracting and purifying the target compound must be economically competitive in order to become a practical substitute to conventional synthetic pesticides and agrochemicals. While efficient methods for synthesizing juglone have been reported (e.g., [175]), identifying the remaining unknown genes in juglone biosynthesis [12] should be prioritized to enable biotechnological platforms for producing juglone in engineered biological systems in the field. Moreover, juglone is synthesized in most organs of black and English walnut trees, including the husks, hulls, and leaves [23], which become waste products of the hardwood and food industries. Millions of tons of English walnut shells from walnut kernel processing are generated worldwide but generally end up as waste [176]. Thus, together with other underutilized parts of walnut trees, there is an abundance of inexpensive juglone-containing source material that could be directly used in agricultural applications or for extraction of pure juglone.
4. Exploring the design of novel juglone derivatives [125] that balance alteration of lipophilicity with aqueous solubility [177] and which do not compromise Lipinski's "Rule of 5" set for physicochemical parameters of pharmaceuticals and fitted for agrochemicals by Tice [178]. Nanoparticle encapsulation is another revolutionary technique that has been shown to increase antimicrobial activity and duration of juglone [179]. Its application in agricultural settings could help reduce off-target movement and toxicity of juglone and improve water solubility of more lipophilic juglone derivatives.
5. Improving basic knowledge about juglone's mode(s) of action and molecular target sites in insects, vertebrates, plants, and microorganisms, the molecular mechanisms involved in deploying juglone into the environment, the uptake of juglone in target organisms, and the metabolism-based mechanisms that allow juglone-producing plants and other types of juglone-tolerant organisms to counter or resist the effects of juglone.

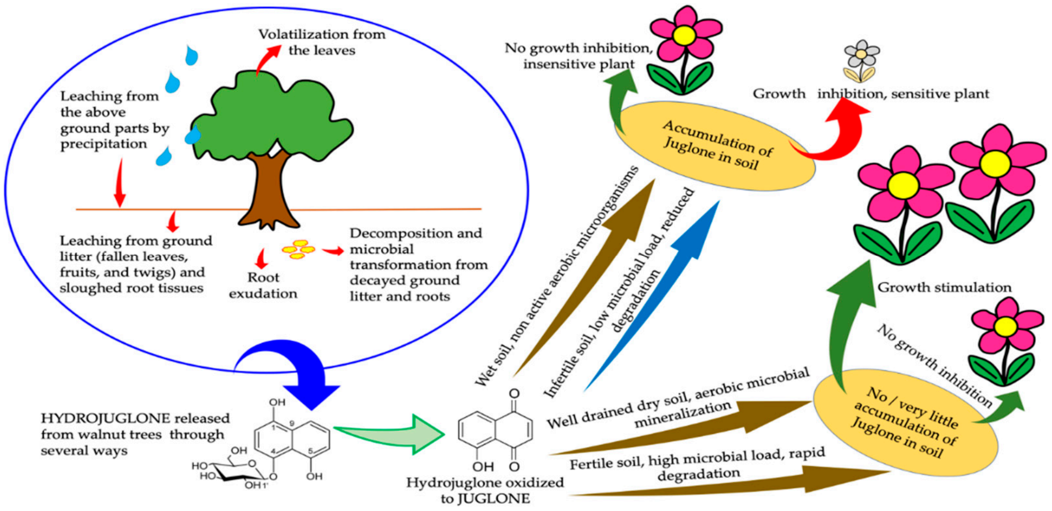

Figure 2. Routes of release, environmental fates, and phytotoxic and biostimulatory effects of juglone.

6. Conclusions

Natural product-based pesticide and agrochemical discovery offers great potential for developing alternatives to synthetic chemicals and for identifying compounds with new modes of action. Allelochemicals, like juglone, can play a pivotal role in this regard, although a number of limitations still exist. To date, most studies have focused on the inhibitory properties of juglone and have been conducted under controlled laboratory or greenhouse conditions. Hence, the transfer of laboratory and greenhouse trials into field conditions is crucial for gaining an understanding of environmental effects on juglone activity and persistence. In this review, we have highlighted significant research findings related to roles for juglone as a novel pesticide and its potential as a candidate for developing other agrochemicals. Despite the opportunities, challenges still exist to adopting juglone for applications related to pest management, to its use as a biostimulant, or for reducing nitrogen losses. It is our intention with this review to not only provide an overview on what has been done, but to identify areas in which research on juglone is still needed in order to integrate it, and perhaps other structurally similar quinones, as components of various sustainable agriculture strategies.

Author Contributions: A.K.M.M.I. & J.R.W. conceptualized the work; A.K.M.M.I. wrote the first draft with contributions from J.R.W.; A.K.M.M.I. and J.R.W. revised and edited the work. All authors have read and agreed to the published version of the manuscript.

Funding: This work was supported by start-up funds from Purdue University to J.R.W. and by the USDA National Institute of Food and Agriculture Hatch Project number 177845 to J.R.W.

Acknowledgments: We acknowledge Cliff Johnston at Purdue University for financial support of A.K.M.M.I.

Conflicts of Interest: The authors declare no conflict of interest. The funders had no role in the design of the study; in the collection, analyses, or interpretation of data; in the writing of the manuscript, or in the decision to publish the results.

References

1. Rietveld, W.J. Allelopathic effects of juglone on germination and growth of several herbaceous and woody species. *J. Chem. Ecol.* **1983**, *9*, 295–308. [CrossRef]
2. Zhou, J.; Xie, G.; Yan, X. *Encyclopedia of Traditional Chinese Medicines—Molecular Structures, Pharmacological Activities, Natural Sources and Applications*; Springer: Heidelberg, Germany; Dordrecht, The Netherlands; London, UK; New York, NY, USA, 2011.
3. Lee, K. Nature and Occurrence of Juglone in *Juglans nigra* L. Master's Thesis, Kansas State University, Manhattan, KS, USA, 1967; pp. 297–298.
4. Willis, R.J. *Juglans* spp., juglone and allelopathy. *Allelopath. J.* **2000**, *7*, 1–55.

5. Sytykiewicz, H. Expression patterns of glutathione transferase gene (GstI) in maize seedlings under juglone-induced oxidative stress. *Int. J. Mol. Sci.* **2011**, *12*, 7982–7995. [CrossRef]
6. Ahmad, T.; Suzuki, Y.J. Juglone in oxidative stress and cell signaling. *Antioxidants* **2019**, *8*, 91. [CrossRef]
7. Ercisli, S.; Turkkal, C. Allelopathic effects of juglone and walnut leaf extracts on growth, fruit yield and plant tissue composition in strawberry cvs. "Camarosa" and "Sweet Charlie". *J. Hortic. Sci. Biotechnol.* **2005**, *80*, 39–42. [CrossRef]
8. Jose, S. Black walnut allelopathy: Current state of the science. In *Chemical Ecology of Plants: Allelopathy in Aquatic and Terrestrial Ecosystems*; Inderjit, Mallik, A.U., Eds.; Birkhäuser: Basel, Switzerland, 2002; pp. 149–172.
9. Babula, P.; Adam, V.; Havel, L.; Kizek, R. Noteworthy secondary metabolites naphthoquinones—Their occurrence, pharmacological properties and analysis. *Curr. Pharm. Anal.* **2009**, *5*, 47–68. [CrossRef]
10. Matawska, I.; Bylka, W.; Widy-Tyszkiewicz, E.; Stanisz, B. Determination of the juglone content of *juglans regia* leaves by GC/MS. *Nat. Prod. Commun.* **2015**, *10*, 1239–1242. [CrossRef]
11. Zhang, X.B.; Zou, C.L.; Duan, Y.X.; Wu, F.; Li, G. Activity guided isolation and modification of juglone from *Juglans regia* as potent cytotoxic agent against lung cancer cell lines. *BMC Complement. Altern. Med.* **2015**, *15*, 1–8. [CrossRef]
12. McCoy, R.M.; Utturkar, S.M.; Crook, J.W.; Thimmapuram, J.; Widhalm, J.R. The origin and biosynthesis of the naphthalenoid moiety of juglone in black walnut. *Hortic. Res.* **2018**, *5*, 67. [CrossRef] [PubMed]
13. Willis, R.J. *The History of Allelopathy*; Springer: Dordrecht, The Netherlands, 2007; ISBN 9781402040931.
14. Dana, M.N.; Lerner, B.R. *Black Walnut Toxicity*; Department of Horticulture, Purdue University, Cooperative Extension Service: West Lafayette, IN, USA, 2001; pp. 1–2.
15. Vogel, A.; Reischauer, C. Buchner Neues. *Rep. Fur Pharm.* **1856**, *5*, 106.
16. Bernthsen, A.; Semper, A. Ueber die Constitution des Juglons und seine Synthese aus Naphtalin. *Berichte Dtsch. Chem. Gesellschaft* **1887**, *20*, 934–941. [CrossRef]
17. Hoy, P.R.; Stickney, J.S. Toxic action of black walnut. *Trans. Wis. State Hortic. Soc.* **1881**, *11*, 166–167.
18. Davis, E.F. The toxic principle of *Juglans nigra* as edentified with synthetic juglone, and its toxic effects on tomato and alfalfa plants. *Am. J. Bot.* **1928**, *15*, 620.
19. Dayan, F.E.; Duke, S.O. Biological activity of allelochemicals. In *Plant-derived Natural Products: Synthesis, Function, and Application*; Osbourn, A.E., Lanzotti, V., Eds.; Springer: New York, NY, USA, 2009; pp. 361–384. ISBN 9780387854984.
20. Massey, A.B. Antagonism of the walnuts (*Juglans nigra* L. and *J. cinerea* L.) in certain plant associations. *Phytopathology* **1925**, *15*, 773–784.
21. Chi, W.C.; Fu, S.F.; Huang, T.L.; Chen, Y.A.; Chen, C.C.; Huang, H.J. Identification of transcriptome profiles and signaling pathways for the allelochemical juglone in rice roots. *Plant Mol. Biol.* **2011**, *77*, 591–607. [CrossRef]
22. Nowicka, B.; Żądło, A.; Pluciński, B.; Kruk, J.; Kuczyńska, P. The oxidative stress in allelopathy: Participation of prenyllipid antioxidants in the response to juglone in *Chlamydomonas reinhardtii*. *Phytochemistry* **2017**, *144*, 171–179. [CrossRef]
23. Strugstad, M.P.; Despotovski, S. A summary of extraction, synthesis, properties and potential uses of juglone: A literature review. *J. Ecosyst. Manag.* **2012**, *13*, 1–16.
24. Cai, L.; Wei, G.X.; Van Der Bijl, P.; Wu, C.D. Namibian chewing stick, Diospyros lycioides, contains antibacterial compounds against oral pathogens. *J. Agric. Food Chem.* **2000**, *48*, 909–914. [CrossRef]
25. Lv, S.T.; Du, W.X.; Bai, S.M.; Chen, G. Insecticidal effect of juglone and its disturbance analysis in metabolic profiles of *Aphis gossypii* glover using 1H NMR-based metabonomics approach. *Phytoparasitica* **2018**, *46*, 521–531. [CrossRef]
26. Altuntaş, H.; Duman, E.; Kılıç, G. Juglone induced oxidative and genotoxic stress in the model insect *Galleria mellonella* L. (Pyralidae: *Lepidoptera*). *Int. J. Trop. Insect Sci.* **2020**. [CrossRef]
27. de Scisciolo, B.; Leopold, D.J.; Walton, D.C. Seasonal patterns of juglone in soil beneath *Juglans nigra* (black walnut) and influence of *J. nigra* on understory vegetation. *J. Chem. Ecol.* **1990**, *16*, 1111–1130. [CrossRef] [PubMed]
28. von Kiparski, G.R.; Lee, L.S.; Gillespie, A.R. Occurrence and fate of the phytotoxin juglone in alley soils under black walnut trees. *J. Environ. Qual.* **2007**, *36*, 709–717. [CrossRef] [PubMed]
29. Dhaliwal, G.S.; Jindal, V.; Dhawan, A.K. Lossess due to insect pests. *Indian J. Ecol.* **2010**, *37*, 1–7.

30. Oerke, E.C.; Dehne, H.W. Safeguarding production—Losses in major crops and the role of crop protection. *Crop Prot.* **2004**, *23*, 275–285. [CrossRef]
31. Oerke, E.C. Crop losses to pests. *J. Agric. Sci.* **2006**, *144*, 31–43. [CrossRef]
32. Gustavsson, J.; Cederberg, C.; Sonesson, U.; van Otterdijk, R.; Meybeck, A. *Global Food Losses and Food Waste: Extent, Causes and Prevention*; FAO: Rome, Italy, 2011; ISBN 9789251072059.
33. Pretty, J.; Bharucha, Z.P. Integrated pest management for sustainable intensification of agriculture in Asia and Africa. *Insects* **2015**, *6*, 152–182. [CrossRef]
34. Damalas, C.A.; Eleftherohorinos, I.G. Pesticide exposure, safety issues, and risk assessment indicators. *Int. J. Environ. Res. Public Health* **2011**, *8*, 1402–1419. [CrossRef]
35. Sparks, T.C.; Hahn, D.R.; Garizi, N.V. Natural products, their derivatives, mimics and synthetic equivalents: Role in agrochemical discovery. *Pest Manag. Sci.* **2017**, *73*, 700–715. [CrossRef]
36. Loiseleur, O. Natural products in the discovery of agrochemicals. *Chimia (Aarau)* **2017**, *71*, 810–822. [CrossRef]
37. Lindroth, R.L.; Anson, B.D.; Weisbrod, A.V. Effects of protein and juglone on gypsy moths: Growth performance and detoxification enzyme activity. *J. Chem. Ecol.* **1990**, *16*, 2533–2547. [CrossRef]
38. Thiboldeaux, R.L.; Lindroth, R.L.; Tracy, J.W. Differential toxicity of juglone (5-hydroxy-1,4-naphthoquinone) and related naphthoquinones to saturniid moths. *J. Chem. Ecol.* **1994**, *20*, 1631–1641. [CrossRef] [PubMed]
39. Sorokin, N.; Whitaker, J. The Impacts of Selected Natural Plant Chemicals on Terrestrial Invertebrates. In *Secondary Metabolites in Soil Ecology*; Karlovsky, P., Ed.; Springer: Berlin/Heidelberg, Germany, 2008; pp. 255–268.
40. Hu, W.; Du, W.; Bai, S.; Lv, S.; Chen, G. Phenoloxidase, an effective bioactivity target for botanical insecticide screening from green walnut husks. *Nat. Prod. Res.* **2018**, *32*, 2848–2851. [CrossRef] [PubMed]
41. Koo, H.N.; An, J.J.; Park, S.E.; Kim, J.I.; Kim, G.H. Regional susceptibilities to 12 insecticides of melon and cotton aphid, *Aphis gossypii* (Hemiptera: *Aphididae*) and a point mutation associated with imidacloprid resistance. *Crop Prot.* **2014**, *55*, 91–97. [CrossRef]
42. Dang, Q.L.; Lee, G.Y.; Choi, Y.H.; Choi, G.J.; Jang, K.S.; Park, M.S.; Soh, H.S.; Han, Y.H.; Lim, C.H.; Kim, J.C. Insecticidal activities of crude extracts and phospholipids from Chenopodium ficifolium against melon and cotton aphid, *Aphis gossypii*. *Crop Prot.* **2010**, *29*, 1124–1129. [CrossRef]
43. Pan, Y.; Peng, T.; Gao, X.; Zhang, L.; Yang, C.; Xi, J.; Xin, X.; Bi, R.; Shang, Q. Transcriptomic comparison of thiamethoxam-resistance adaptation in resistant and susceptible strains of Aphis gossypii Glover. *Comp. Biochem. Physiol. Part D Genomics Proteom.* **2015**, *13*, 10–15. [CrossRef]
44. Li, Z.Q.; Zhang, S.; Luo, J.Y.; Wang, C.Y.; Lv, L.M.; Dong, S.L.; Cui, J.J. Ecological adaption analysis of the cotton aphid (*Aphis gossypii*) in different phenotypes by transcriptome comparison. *PLoS ONE* **2013**, *8*. [CrossRef]
45. Koo, H.N.; Lee, S.W.; Yun, S.H.; Kim, H.K.; Kim, G.H. Feeding response of the cotton aphid, *Aphis gossypii*, to sublethal rates of flonicamid and imidacloprid. *Entomol. Exp. Appl.* **2015**, *154*, 110–119. [CrossRef]
46. Kwadha, C.A.; Ong'Amo, G.O.; Ndegwa, P.N.; Raina, S.K.; Fombong, A.T. The biology and control of the greater wax moth, *Galleria mellonella*. *Insects* **2017**, *8*, 61. [CrossRef]
47. Charrière, J.D.; Imdorf, A. Protection of honey combs from wax moth damage. *Am. Bee J.* **1999**, *139*, 627–630.
48. Erbaş, E.D.; Altuntaş, H. Lethal and sublethal effects of juglone on the life-history traits of *Galleria mellonella* L. (Lepidoptera: *Pyralidae*). *Acta Zool. Bulg.* **2020**, *72*, 43–48.
49. Mitchell, M.J.; Smith, S.L. Effects of the chitin synthetase inhibitor plumbagin and its 2-demethyl derivative juglone on insect ecdysone 20-monooxygenase activity. *Experientia* **1988**, *44*, 990–991. [CrossRef] [PubMed]
50. Piskorski, R.; Dorn, S. How the oligophage codling moth *Cydia pomonella* survives on walnut despite its secondary metabolite juglone. *J. Insect Physiol.* **2011**, *57*, 744–750. [CrossRef] [PubMed]
51. Sun, M.; Wang, Y.; Song, Z.; Fang, G. Insecticidal activities and active components of the alcohol extract from green peel of *Juglans mandshurica*. *J. For. Res.* **2007**, *18*, 62–64. [CrossRef]
52. Akhtar, Y.; Isman, M.B.; Niehaus, L.A.; Lee, C.H.; Lee, H.S. Antifeedant and toxic effects of naturally occurring and synthetic quinones to the cabbage looper, *Trichoplusia ni*. *Crop Prot.* **2012**, *31*, 8–14. [CrossRef]
53. Akhtar, Y.; Isman, M.B.; Lee, C.H.; Lee, S.G.; Lee, H.S. Toxicity of quinones against two-spotted spider mite and three species of aphids in laboratory and greenhouse conditions. *Ind. Crops Prod.* **2012**, *37*, 536–541. [CrossRef]

54. Pereira, J.A.; Oliveira, I.; Sousa, A.; Valentão, P.; Andrade, P.B.; Ferreira, I.C.F.R.; Ferreres, F.; Bento, A.; Seabra, R.; Estevinho, L. Walnut (*Juglans regia* L.) leaves: Phenolic compounds, antibacterial activity and antioxidant potential of different cultivars. *Food Chem. Toxicol.* **2007**, *45*, 2287–2295. [CrossRef]
55. Wu, Z.; Chen, G.; Wang, Y. Inhibition effect of juglong on several food deterioration microorganisms. *China Brew.* **2009**, *8*, 76–78.
56. Yakubovskaya, A.Y.; Pokhilo, N.D.; Anufriev, V.F.; Anisimov, M.M. Synthesis and antimicrobial and antifungal activities of compounds of the naphthazarin series. *Pharm. Chem. J.* **2009**, *43*, 396–398. [CrossRef]
57. Tan, D.T.C.; Osman, H.; Mohamad, S.; Kamaruddin, A.H. Synthesis and antibacterial activity of juglone derivatives. *J. Chem. Chem. Eng.* **2012**, *6*, 84–89.
58. Fischer, T.C.; Gosch, C.; Mirbeth, B.; Gselmann, M.; Thallmair, V.; Stich, K. Potent and specific bactericidal effect of juglone (5-Hydroxy-1,4- naphthoquinone) on the fire blight pathogen *Erwinia amylovora*. *J. Agric. Food Chem.* **2012**, *60*, 12074–12081. [CrossRef]
59. Zakavi, F.; Golpasand Hagh, L.; Daraeighadikolaei, A.; Farajzadeh Sheikh, A.; Daraeighadikolaei, A.; Leilavi Shooshtari, Z. Antibacterial effect of *Juglans regia* bark against oral pathologic bacteria. *Int. J. Dent.* **2013**, *2013*. [CrossRef]
60. Zmantar, T.; Miladi, H.; Kouidhi, B.; Chaabouni, Y.; Ben Slama, R.; Bakhrouf, A.; Mahdouani, K.; Chaieb, K. Use of juglone as antibacterial and potential efflux pump inhibitors in *Staphylococcus aureus* isolated from the oral cavity. *Microb. Pathog.* **2016**, *101*, 44–49. [CrossRef] [PubMed]
61. Wang, J.; Cheng, Y.; Wu, R.; Jiang, D.; Bai, B.; Tan, D.; Yan, T.; Sun, X.; Zhang, Q.; Wu, Z. Antibacterial activity of juglone against *Staphylococcus aureus*: From apparent to proteomic. *Int. J. Mol. Sci.* **2016**, *17*, 965. [CrossRef] [PubMed]
62. Wang, J.; Wang, Z.; Wu, R.; Jiang, D.; Bai, B.; Tan, D.; Yan, T.; Sun, X.; Zhang, Q.; Wu, Z. Proteomic analysis of the antibacterial mechanism of action of juglone against *Staphylococcus aureus*. *Nat. Prod. Commun.* **2016**, *11*, 825–827. [CrossRef] [PubMed]
63. Clark, A.M.; Jurgens, T.M.; Hufford, C.D. Antimicrobial activity of juglone. *Phyther. Res.* **1990**, *4*, 11–14. [CrossRef]
64. Kong, Y.H.; Zhang, L.; Yang, Z.Y.; Han, C.; Hu, L.H.; Jiang, H.L.; Shen, X. Natural product juglone targets three key enzymes from *Helicobacter pylori*: Inhibition assay with crystal structure characterization. *Acta Pharmacol. Sin.* **2008**, *29*, 870–876. [CrossRef] [PubMed]
65. Dawson, J.O.; Seymour, P.E. Effects of juglone concentration on growth in vitro of *Frankia* ArI3 and *Rhizobium japonicum* strain 71. *J. Chem. Ecol.* **1983**, *9*, 1175–1183. [CrossRef]
66. Neave, I.A.; Dawson, J.O. Juglone reduces growth, nitrogenase activity, and root respiration of actinorhizal black alder seedlings. *J. Chem. Ecol.* **1989**, *15*, 1823–1836. [CrossRef]
67. Williamson, G.B.; Weidenhamer, J.D. Bacterial degradation of juglone—Evidence against allelopathy? *J. Chem. Ecol.* **1990**, *16*, 1739–1742. [CrossRef]
68. Schmidt, S.K. Degradation of juglone by soil bacteria. *J. Chem. Ecol.* **1988**, *14*, 1561–1571. [CrossRef]
69. Bock, C.H.; Hotchkiss, M.W.; Shapiro-Ilan, D.I.; Brock, J.H.; Brenneman, T.B.; Wilkins, B.; Wells, D.E.; Wells, L.; Mizell, R.F. A comparison of organic fungicides: Alternatives for reducing scab on pecan. *Org. Agric.* **2019**, *9*, 305–314. [CrossRef]
70. Abbey, J.A.; Percival, D.; Abbey, L.; Asiedu, S.K.; Prithiviraj, B.; Schilder, A. Biofungicides as alternative to synthetic fungicide control of grey mould (*Botrytis cinerea*)—Prospects and challenges. *Biocontrol Sci. Technol.* **2019**, *29*, 241–262. [CrossRef]
71. Beckerman, J. Disease Management Strategies. Available online: https://extension.purdue.edu/extmedia/BP/BP-69-W.pdf (accessed on 5 June 2020).
72. Gopi, R.; Avasthe, R.K.; Kalita, H.; Yadav, A.; Das, S.K.; Dinisha, R.A.I. Eco-friendly management of tomato late blight using botanicals, bio-control agents, compost tea and copper fungicides. *Indian J. Agric. Sci.* **2020**, *90*, 35–39.
73. Hedin, P.A.; Langhans, V.E.; Graves, C.H. Identification of juglone in pecan as a possible factor of resistance to *Fusicladium effusum*. *J. Agric. Food Chem.* **1979**, *27*, 92–94. [CrossRef]
74. Duke, S.O. Natural pesticides from plants. In *Advances in New Crops, Proceedings of the First National Symposium "New Crops: Research, Development, Economics", Indianapolis, IN, USA, 23–26 October 1988*; Janick, J., Simon, J.E., Eds.; Timber Press: Portland, OR, USA, 1990; pp. 511–517. ISBN 0881921661.

75. Arasoglu, T.; Mansuroglu, B.; Derman, S.; Gumus, B.; Kocyigit, B.; Acar, T.; Kocacaliskan, I. Enhancement of antifungal activity of juglone (5-Hydroxy-1,4-naphthoquinone) using a poly(d, l -lactic-co-glycolic acid) (PLGA) nanoparticle system. *J. Agric. Food Chem.* **2016**, *64*, 7087–7094. [CrossRef]
76. Wianowska, D.; Garbaczewska, S.; Cieniecka-Roslonkiewicz, A.; Dawidowicz, A.L.; Jankowska, A. Comparison of antifungal activity of extracts from different *Juglans regia* cultivars and juglone. *Microb. Pathog.* **2016**, *100*, 263–267. [CrossRef]
77. Sytykiewicz, H.; Chrzanowski, G.; Czerniewicz, P.; Leszczyński, B.; Sprawka, I.; Krzyżanowski, R.; Matok, H. Antifungal activity of *Juglans regia* (L.) leaf extracts against candida albicans isolates. *Pol. J. Environ. Stud.* **2015**, *24*, 1339–1348. [CrossRef]
78. Achatz, M.; Rillig, M.C. Arbuscular mycorrhizal fungal hyphae enhance transport of the allelochemical juglone in the field. *Soil Biol. Biochem.* **2014**, *78*, 76–82. [CrossRef]
79. Achatz, M.; Morris, E.K.; Müller, F.; Hilker, M.; Rillig, M.C. Soil hypha-mediated movement of allelochemicals: Arbuscular mycorrhizae extend the bioactive zone of juglone. *Funct. Ecol.* **2014**, *28*, 1020–1029. [CrossRef]
80. Mantzouki, E.; Visser, P.M.; Bormans, M.; Ibelings, B.W. Understanding the key ecological traits of cyanobacteria as a basis for their management and control in changing lakes. *Aquat. Ecol.* **2016**, *50*, 333–350. [CrossRef]
81. O'Neil, J.M.; Davis, T.W.; Burford, M.A.; Gobler, C.J. The rise of harmful cyanobacteria blooms: The potential roles of eutrophication and climate change. *Harmful Algae* **2012**, *14*, 313–334. [CrossRef]
82. Monchamp, M.E.; Spaak, P.; Domaizon, I.; Dubois, N.; Bouffard, D.; Pomati, F. Homogenization of lake cyanobacterial communities over a century of climate change and eutrophication. *Nat. Ecol. Evol.* **2018**, *2*, 317–324. [CrossRef] [PubMed]
83. Brooks, B.W.; Lazorchak, J.M.; Howard, M.D.A.; Johnson, M.V.V.; Morton, S.L.; Perkins, D.A.K.; Reavie, E.D.; Scott, G.I.; Smith, S.A.; Steevens, J.A. Are harmful algal blooms becoming the greatest inland water quality threat to public health and aquatic ecosystems? *Environ. Toxicol. Chem.* **2016**, *35*, 6–13. [CrossRef] [PubMed]
84. Yang, Z.; Xiang, F.; Minter, E.J.A.; Lü, K.; Chen, Y.; Montagnes, D.J.S. The interactive effects of microcystin and nitrite on life-history parameters of the cladoceran *Daphnia obtusa*. *J. Hazard. Mater.* **2011**, *190*, 113–118. [CrossRef]
85. Sun, H.; Lü, K.; Minter, E.J.A.; Chen, Y.; Yang, Z.; Montagnes, D.J.S. Combined effects of ammonia and microcystin on survival, growth, antioxidant responses, and lipid peroxidation of bighead carp *Hypophthalmythys nobilis* larvae. *J. Hazard. Mater.* **2012**, *221–222*, 213–219. [CrossRef]
86. Otten, T.G.; Paerl, H.W. Health effects of toxic xyanobacteria in U.S. drinking and recreational waters: Our current understanding and proposed direction. *Curr. Environ. Health Rep.* **2015**, *2*, 75–84. [CrossRef]
87. Pham, T.L.; Utsumi, M. An overview of the accumulation of microcystins in aquatic ecosystems. *J. Environ. Manag.* **2018**, *213*, 520–529. [CrossRef]
88. Hou, X.; Huang, J.; Tang, J.; Wang, N.; Zhang, L.; Gu, L.; Sun, Y.; Yang, Z.; Huang, Y. Allelopathic inhibition of juglone (5-hydroxy-1,4-naphthoquinone) on the growth and physiological performance in *Microcystis aeruginosa*. *J. Environ. Manag.* **2019**, *232*, 382–386. [CrossRef]
89. Krajci, W.M.; Lynch, D.L. The inhibition of various micro-organisms by crude walnut hull extracts and juglone. *Microbios Lett.* **1977**, *4*, 175–181.
90. Bragg, J. Effects of Juglone (5′-Hydroxy-1, 4-Naphthoquinone) on the Algae *Anabaena flos-aquae*, *Nostoc commune*, and *Scenedesmus acuminatus*. *J. Ark. Acad. Sci.* **1986**, *40*, 52–55.
91. Kessler, C.T. Effect of juglone on freshwater algal growth. *J. Chem. Ecol.* **1989**, *15*, 2127–2134. [CrossRef] [PubMed]
92. Park, M.H.; Kim, K.; Hwang, S.J. Differential effects of the allelochemical juglone on growth of harmful and non-target freshwater algae. *Appl. Sci.* **2020**, *10*, 2873. [CrossRef]
93. Widhalm, J.R.; Rhodes, D. Biosynthesis and molecular actions of specialized 1,4-naphthoquinone natural products produced by horticultural plants. *Hortic. Res.* **2016**, *3*, 16046. [CrossRef] [PubMed]
94. Barrington, D.J.; Ghadouani, A. Application of hydrogen peroxide for the removal of toxic cyanobacteria and other phytoplankton from wastewater. *Environ. Sci. Technol.* **2008**, *42*, 8916–8921. [CrossRef] [PubMed]
95. Barrington, D.J.; Ghadouani, A.; Ivey, G.N. Environmental factors and the application of hydrogen peroxide for the removal of toxic cyanobacteria from waste stabilization ponds. *J. Environ. Eng.* **2011**, *137*, 952–960. [CrossRef]

96. Hejl, A.M.; Koster, K.L. Juglone disrupts root plasma membrane H+-ATPase activity and impairs water uptake, root respiration, and growth in soybean (*Glycine max*) and corn (*Zea mays*). *J. Chem. Ecol.* **2004**, *30*, 453–471. [CrossRef]
97. Dayan, F.E.; Duke, S.O. Natural compounds as next-generation herbicides. *Plant Physiol.* **2014**, *166*, 1090–1105. [CrossRef]
98. Rudnicka, M.; Polak, M.; Karcz, W. Cellular responses to naphthoquinones: Juglone as a case study. *Plant Growth Regul.* **2014**, *72*, 239–248. [CrossRef]
99. Rietveld, W.J. The significance of allelopathy in black walnut cultural systems. *North. Nut Grow. Assoc. Annu. Rep.* **1981**, *72*, 117–134.
100. Fisher, R.F. Juglone Inhibits Pine Growth Under Certain Moisture Regimes. *Soil Sci. Soc. Am. J.* **1978**, *42*, 801–803. [CrossRef]
101. Ponder, F.; Tadros, S.H. Juglone concentration in soil beneath black walnut interplanted with nitrogen-fixing species. *J. Chem. Ecol.* **1985**, *11*, 937–942. [CrossRef] [PubMed]
102. Segura-Aguilar, J.; Hakman, I.; Rydström, J. The effect of 5OH-1,4-naphthoquinone on Norway spruce seeds during germination. *Plant Physiol.* **1992**, *100*, 1955–1961. [CrossRef] [PubMed]
103. Kocaçalişkan, I.; Ceylan, M.; Terzi, I. Effects of juglone on seedling growth in intact and coatless seeds of cucumber (*Cucumis sativus* cv. Beith Alpha). *Sci. Res. Essays* **2008**, *4*, 39–41.
104. Kocaçaliskan, I.; Terzi, I. Allelopathic effects of walnut leaf extracts and juglone on seed germination and seedling growth. *J. Hortic. Sci. Biotechnol.* **2001**, *76*, 436–440. [CrossRef]
105. Ochekwu, E.B.; Uzoma, M.C. Allelopathic effects of *Juglans nigra* on wheat and rice. *Int. J. Innov. Agric. Biol. Res.* **2020**, *8*, 15–23.
106. Babula, P.; Vaverkova, V.; Poborilova, Z.; Ballova, L.; Masarik, M.; Provaznik, I. Phytotoxic action of naphthoquinone juglone demonstrated on lettuce seedling roots. *Plant Physiol. Biochem.* **2014**, *84*, 78–86. [CrossRef]
107. Kocaçalişkan, I.; Turan, E.; Terzi, I. Juglone effects on seedling growth in intact and coatless seeds of muskmelon. *Afr. J. Biotechnol.* **2008**, *7*, 4446–4449.
108. Terzi, I. Allelopathic effects of juglone and decomposed walnut leaf juice on muskmelon and cucumber seed germination and seedling growth. *Afr. J. Biotechnol.* **2008**, *7*, 1870–1874.
109. Cui, C.; Cai, J.; Zhang, S. Allelopathic effects of walnut (*Juglans regia* L.) rhizospheric soil extracts on germination and seedling growth of turnip (*Brassica rapa* L.). *Allelopath. J.* **2013**, *32*, 37–48.
110. Ercisli, S.; Esitken, A.; Turkkal, C.; Orhan, E. The allelopathic effects of juglone and walnut leaf extracts on yield, growth, chemical and PNE compositions of strawberry cv. Fern. *Plant Soil Environ.* **2005**, *51*, 283–287. [CrossRef]
111. Chen, S.Y.; Chi, W.C.; Trinh, N.N.; Cheng, K.T.; Chen, Y.A.; Lin, T.C.; Lin, Y.C.; Huang, L.Y.; Huang, H.J.; Chiang, T.Y. Alleviation of allelochemical juglone-induced phytotoxicity in tobacco plants by proline. *J. Plant Interact.* **2015**, *10*, 167–172. [CrossRef]
112. Jose, S.; Gillespie, A.R. Allelopathy in black walnut (Juglans nigra L.) alley cropping. II. Effects of juglone on hydroponically grown corn (*Zea mays* L.) and soybean (*Glycine max* L. Merr.) growth and physiology. *Plant Soil* **1998**, *203*, 199–205. [CrossRef]
113. Javanmard, H.R.; Karchegani, H.M. Determination of allelopathic effect of walnut (*Juglone regia* L.) on germination and initial development crops. *Ecol. Environ. Conserv.* **2016**, *22*, 1703–1708.
114. Altikat, S.; Terzi, I.; Kuru, H.I.; Kocacaliskan, I. Allelopathic effects of juglone on growth of cucumber and muskmelon seedlings with respect to antioxidant enzyme activities and lipid peroxidation. *J. Environ. Prot. Ecol.* **2013**, *14*, 1244–1253.
115. Torabi, Z.; Rafiei, F.; Shabani, L.; Danesh Shahraki, A. Physiological and molecular response of annual Medicago species to juglone. *Acta Physiol. Plant* **2015**, *37*. [CrossRef]
116. Vogel, C.S.; Dawson, J.O. Effect of juglone on growth in vitro of *Frankia* isolates and nodulation of *Alnus glutinosa* in soil. In *Frankia and Actinorhizal Plants*; Lalonde, M., Camiré, C., Dawson, J.O., Eds.; Springer: Dordrecht, The Netherlands, 1985; Volume 18, pp. 79–89. ISBN 978-94-009-5147-1.
117. Wang, Q.; Xu, Z.; Hu, T.; Ur Rehman, H.; Chen, H.; Li, Z.; Ding, B.; Hu, H. Allelopathic activity and chemical constituents of walnut (*Juglans regia*) leaf litter in walnut-winter vegetable agroforestry system. *Nat. Prod. Res.* **2014**, *28*, 2017–2020. [CrossRef]

118. Böhm, P.A.F.; Zanardo, F.M.L.; Ferrarese, M.L.L.; Ferrarese-Filho, O. Peroxidase activity and lignification in soybean root growth-inhibition by juglone. *Biol. Plant.* **2006**, *50*, 315–317. [CrossRef]
119. Böhm, P.A.F.; Böhm, F.M.L.Z.; Ferrarese, M.L.L.; Salvador, V.H.; Soares, A.R.; Ferrarese-Filho, O. Effects of juglone on soybean root growth and induction of lignification. *Allelopath. J.* **2010**, *25*, 465–474.
120. Terzi, I.; Kocaçalişkan, I. Alleviation of juglone stress by plant growth regulators in germination of cress seeds. *Sci. Res. Essays* **2009**, *4*, 436–439.
121. Sytykiewicz, H.; Kozak, A.; Łukasik, I.; Sempruch, C.; Goławska, S.; Mitrus, J.; Kurowska, M.; Kmieć, K.; Chrzanowski, G.; Leszczyński, B. Juglone-triggered oxidative responses in seeds of selected cereal agrosystem plant species. *Pol. J. Environ. Stud.* **2019**, *28*, 2389–2397. [CrossRef]
122. Topal, S.; Kocaçalişkan, I.; Arslan, O.; Tel, A.Z. Herbicidal effects of juglone as an allelochemical. *Phyt. Ann. Rei Bot.* **2007**, *46*, 259–269.
123. Cachiţă-Cosma, D.; Maior, C.; Corbu, S. Arguments for using the allelopathic compound juglone as a natural pesticide. *Environ. Eng. Manag. J.* **2015**, *14*, 1089–1095.
124. Shrestha, A. Potential of a black walnut (*Juglans nigra*) extract product (NatureCur®) as a pre- and post-emergence bioherbicide. *J. Sustain. Agric.* **2009**, *33*, 810–822. [CrossRef]
125. Durán, A.G.; Chinchilla, N.; Molinillo, J.M.G.; Macías, F.A. Influence of lipophilicity in O-acyl and O-alkyl derivatives of juglone and lawsone: A structure–activity relationship study in the search for natural herbicide models. *Pest Manag. Sci.* **2018**, *74*, 682–694. [CrossRef] [PubMed]
126. Liu, D.L.; An, M.; Johnson, I.R.; Lovett, J.V. Mathematical Modeling of Allelopathy. III. A Model for Curve-Fitting Allelochemical Dose Responses. *Nonlinearity Biol. Toxicol. Med.* **2003**, *1*, 154014203908444. [CrossRef]
127. An, M. Mathematical modelling of dose-response relationship (hormesis) in allelopathy and its application. *Nonlinearity Biol. Toxicol. Med.* **2005**, *3*. [CrossRef]
128. Belz, R.G.; Hurle, K.; Duke, S.O. Dose-response—A challenge for allelopathy? *Nonlinearity Biol. Toxicol. Med.* **2005**, *3*. [CrossRef]
129. Duke, S.; Cedergreen, N.; Belz, R.; Velini, E. Hormesis: Is it an important factor in herbicide use and allelopathy? *Outlooks Pest Manag.* **2006**, *17*, 29–33.
130. Abbas, T.; Nadeem, M.A.; Tanveer, A.; Chauhan, B.S. Can hormesis of plant-released phytotoxins be used to boost and sustain crop production? *Crop Prot.* **2017**, *93*, 69–76. [CrossRef]
131. Vargas-Hernandez, M.; Macias-Bobadilla, I.; Guevara-Gonzalez, R.G.; Romero-Gomez, S.d.J.; Rico-Garcia, E.; Ocampo-Velazquez, R.V.; Alvarez-Arquieta, L.d.L.; Torres-Pacheco, I. Plant hormesis management with biostimulants of biotic origin in agriculture. *Front. Plant Sci.* **2017**, *8*, 1–11. [CrossRef]
132. Belz, R.G.; Cedergreen, N.; Duke, S.O. Herbicide hormesis—Can it be useful in crop production? *Weed Res.* **2011**, *51*, 321–332. [CrossRef]
133. Abbas, T.; Nadeem, M.A.; Tanveer, A.; Zohaib, A.; Rasool, T. Glyphosate hormesis increases growth and yield of chickpea (*Cicer arietinum* L.). *Pak. J. Weed Sci. Res.* **2015**, *21*, 533–542.
134. Smith, R.M. Some effects of black locusts and black walnuts on southeastern ohio pastures. *Soil Sci.* **1942**, *53*, 385–398. [CrossRef]
135. Funk, D.; Case, P.; Rietveld, W.; Phares, R. Effects of juglone on the growth of coniferous seedlings. *For. Sci.* **1979**, *25*, 452–454.
136. Bahuguna, S.; Bahuguna, A.; Prasad, B.; Singh, N. Seed germination and seedling growth of wheat and barley influenced by the allelopathic effect of walnut (*Juglans regia* L.) leaf extracts under mid hills of Uttarakhand Agri-Silvi system. *Asian J. Agric. Res.* **2014**, *8*, 164–169. [CrossRef]
137. Chobot, V.; Hadacek, F. Milieu-dependent pro- and antioxidant activity of juglone may explain linear and nonlinear effects on seedling development. *J. Chem. Ecol.* **2009**, *35*, 383–390. [CrossRef] [PubMed]
138. Riechers, D.E.; Kreuz, K.; Zhang, Q. Detoxification without intoxication: Herbicide safeners activate plant defense gene expression. *Plant Physiol.* **2010**, *153*, 3–13. [CrossRef]
139. Velini, E.D.; Alves, E.; Godoy, M.C.; Meschede, D.K.; Souza, R.T.; Duke, S.O. Glyphosate applied at low doses can stimulate plant growth. *Pest Manag. Sci.* **2008**, *64*, 489–496. [CrossRef]
140. Modolo, L.V.; da-Silva, C.J.; Brandão, D.S.; Chaves, I.S. A minireview on what we have learned about urease inhibitors of agricultural interest since mid-2000s. *J. Adv. Res.* **2018**, *13*, 29–37. [CrossRef]
141. International Fertilizer Association. Fertilizer Outlook 2019—2023. In Proceedings of the IFA Annual Conference, Montreal, QC, Canada, 11–13 June 2019.

142. Liu, C.W.; Sung, Y.; Chen, B.C.; Lai, H.Y. Effects of nitrogen fertilizers on the growth and nitrate content of lettuce (*Lactuca sativa* L.). *Int. J. Environ. Res. Public Health* **2014**, *11*, 4427–4440. [CrossRef]
143. Zaman, M.; Nguyen, M.L.; Blennerhassett, J.D.; Quin, B.F. Reducing NH3, N2O and NO3- -N losses from a pasture soil with urease or nitrification inhibitors and elemental S-amended nitrogenous fertilizers. *Biol. Fertil. Soils* **2008**, *44*, 693–705. [CrossRef]
144. Cameron, K.C.; Di, H.J.; Moir, J.L. Nitrogen losses from the soil/plant system: A review. *Ann. Appl. Biol.* **2013**, *162*, 145–173. [CrossRef]
145. Modolo, L.V.; de Souza, A.X.; Horta, L.P.; Araujo, D.P.; de Fátima, Â. An overview on the potential of natural products as ureases inhibitors: A review. *J. Adv. Res.* **2015**, *6*, 35–44. [CrossRef] [PubMed]
146. Paulson, K.N.; Kurtz, L.T. Locus of urease activity in soil. *Soil Sci. Soc. Am. J.* **1969**, *33*, 897–901. [CrossRef]
147. Cantarella, H.; Otto, R.; Soares, J.R.; de Brito Silva, A.G. Agronomic efficiency of NBPT as a urease inhibitor: A review. *J. Adv. Res.* **2018**, *13*, 19–27. [CrossRef] [PubMed]
148. Ikemoto, Y.; Teraguchi, M.; Kobayashi, Y. Plasma levels of nitrate in congenital heart disease: Comparison with healthy children. *Pediatr. Cardiol.* **2002**, *23*, 132–136. [CrossRef]
149. Erisman, J.W.; Galloway, J.N.; Seitzinger, S.; Bleeker, A.; Dise, N.B.; Petrescu, R.; Leach, A.M.; de Vries, W. Consequences of human modification of the global nitrogen cycle. *Philos. Trans. R. Soc. B Biol. Sci.* **2013**, *368*, 1621. [CrossRef]
150. Peel, J.L.; Haeuber, R.; Garcia, V.; Russell, A.G.; Neas, L. Impact of nitrogen and climate change interactions on ambient air pollution and human health. *Biogeochemistry* **2013**, *114*, 121–134. [CrossRef]
151. Suddick, E.C.; Whitney, P.; Townsend, A.R.; Davidson, E.A. The role of nitrogen in climate change and the impacts of nitrogen-climate interactions in the United States: Foreword to thematic issue. *Biogeochemistry* **2013**, *114*, 1–10. [CrossRef]
152. Michel, H.J. Ureaseinhibitoren [Urease Inhibitors]. *Pharmazie* **1980**, *35*, 63–68.
153. Zanin, L.; Tomasi, N.; Zamboni, A.; Varanini, Z.; Pinton, R. The urease inhibitor NBPT negatively affects DUR3-mediated uptake and assimilation of urea in maize roots. *Front. Plant Sci.* **2015**, *6*, 1007. [CrossRef] [PubMed]
154. Mohanty, S.; Patra, A.K.; Chhonkar, P.K. Neem (*Azadirachta indica*) seed kernel powder retards urease and nitrification activities in different soils at contrasting moisture and temperature regimes. *Bioresour. Technol.* **2008**, *99*, 894–899. [CrossRef] [PubMed]
155. Kot, M.; Karcz, W.; Zaborska, W. 5-Hydroxy-1,4-naphthoquinone (juglone) and 2-hydroxy-1,4-naphthoquinone (lawsone) influence on jack bean urease activity: Elucidation of the difference in inhibition activity. *Bioorg. Chem.* **2010**, *38*, 132–137. [CrossRef] [PubMed]
156. Kot, M.; Bucki, M.; Olech, Z. Influence of walnut trees (*Juglans regia* L.) on soil urease activity. *Pol. J. Agron.* **2020**, *40*, 3–6.
157. Cantrell, C.L.; Dayan, F.E.; Duke, S.O. Natural products as sources for new pesticides. *J. Nat. Prod.* **2012**, *75*, 1231–1242. [CrossRef]
158. Duke, S.O.; Lydon, J. Natural Phytotoxins as Herbicides. In *Pest Control with Enhanced Environmental Safety*; Duke, S.O., Menn, J.J., Plimmer, J.R., Eds.; American Chemical Society: Washington, DC, USA, 1993; pp. 110–124. ISBN 780841213807.
159. Coder, K.D. Seasonal changes of juglone potential in leaves of black walnut (*Juglans nigra* L.). *J. Chem. Ecol.* **1983**, *9*, 1203–1212. [CrossRef]
160. Carnat, A.; Petitjean-Freytet, C.; Muller, D.; Lamaison, J.L. Percentage of principal components in the leaves of walnut *Juglan regia* L. *Plantes Med. Phyther.* **1993**, *26*, 332–339.
161. Gîrzu, M.; Fraisse, D.; Carnat, A.P.; Carnat, A.; Lamaison, J.L. High-performance liquid chromatographic method for the determination of juglone in fresh walnut leaves. *J. Chromatogr. A* **1998**, *805*, 315–318. [CrossRef]
162. Cosmulescu, S.; Trandafir, I.; Achim, G.; Baciu, A. Juglone content in leaf and green husk of five walnut (*Juglans regia* L.) cultivars. *Not. Bot. Horti Agrobot. Cluj-Napoca* **2011**, *39*, 237–240. [CrossRef]
163. Scott, R.; Sullivan, W.C. A review of suitable companion crops for black walnut. *Agrofor. Syst.* **2007**, *71*, 185–193. [CrossRef]
164. Marking, L.L. Juglone (5-hydroxy-1,4-naphthoquinone) as a fish toxicant. *Trans. Am. Fish. Soc.* **1970**, *99*, 510–514. [CrossRef]

165. Radix, P.; Seigle-Murandi, F.; Benoit-Guyod, J.L.; Krivobok, S. Toxicity of walnut husk washing waters. *Water Res.* **1992**, *26*, 1503–1506. [CrossRef]
166. Yang, L.; Wang, P.; Kong, C. Effect of larch (*Larix gmelini* Rupr.) root exudates on Manchurian walnut (*Juglans mandshurica* Maxim.) growth and soil juglone in a mixed-species plantation. *Plant Soil* **2010**, *329*, 249–258. [CrossRef]
167. Saha, D.; Marble, S.C.; Pearson, B.J. Allelopathic effects of common landscape and nursery mulch materials on weed control. *Front. Plant Sci.* **2018**, *9*, 733. [CrossRef] [PubMed]
168. Buttery, R.G.; Light, D.M.; Nam, Y.; Merrill, G.B.; Roitman, J.N. Volatile components of green walnut husks. *J. Agric. Food Chem.* **2000**, *48*, 2858–2861. [CrossRef] [PubMed]
169. Farooq, N.; Abbas, T.; Tanveer, A.; Jabran, K. Allelopathy for Weed Management. In *Co-Evolution of Secondary Metabolites*; Mérillon, J.M., Ramawat, K.G., Eds.; Springer Nature: Cham, Switzerland, 2020; pp. 505–519.
170. Cheng, F.; Cheng, Z. Research progress on the use of plant allelopathy in agriculture and the physiological and ecological mechanisms of allelopathy. *Front. Plant Sci.* **2015**, *6*, 1020. [CrossRef]
171. Vidal, R.A.; Bauman, T.T. Fate of allelochemicals in the soil. *Ciência Rural* **1997**, *27*, 351–357. [CrossRef]
172. Inderjit. Soil: Environmental effects on allelochemical activity. *Agron. J.* **2001**, *93*, 79–84.
173. Inderjit; Bhowmik, P.C. Sorption of benzoic acid onto soil colloids and its implications for allelopathy studies. *Biol. Fertil. Soils* **2004**, *40*, 345–348.
174. Trezzi, M.M.; Vidal, R.A.; Junior, A.A.B.; von Hertwig Bittencourt, H.; da Silva Souza Filho, A.P. Allelopathy: Driving mechanisms governing its activity in agriculture. *J. Plant Interact.* **2016**, *11*, 53–60. [CrossRef]
175. Barret, R.; Daudon, M. An efficient synthesis of juglone. *Synth. Commun.* **1990**, *20*, 2907–2912. [CrossRef]
176. Ayrilmis, N.; Kaymakci, A.; Ozdemir, F. Physical, mechanical, and thermal properties of polypropylene composites filled with walnut shell flour. *J. Ind. Eng. Chem.* **2013**, *19*, 908–914. [CrossRef]
177. Macías, F.A.; Mejías, F.J.R.; Molinillo, J.M.G. Recent Advances in Allelopathy for Weed Control: From Knowledge to Applications. *Pest Manag. Sci.* **2019**, *75*. [CrossRef] [PubMed]
178. Tice, C.M. Selecting the right compounds for screening: Does Lipinski's rule of 5 for pharmaceuticals apply to agrochemicals? *Pest Manag. Sci.* **2001**, *57*, 3–16. [CrossRef]
179. Arasoglu, T.; Derman, S.; Mansuroglu, B.; Yelkenci, G.; Kocyigit, B.; Gumus, B.; Acar, T.; Kocacaliskan, I. Synthesis, characterization and antibacterial activity of juglone encapsulated PLGA nanoparticles. *J. Appl. Microbiol.* **2017**, *123*, 1407–1419. [CrossRef] [PubMed]

© 2020 by the authors. Licensee MDPI, Basel, Switzerland. This article is an open access article distributed under the terms and conditions of the Creative Commons Attribution (CC BY) license (http://creativecommons.org/licenses/by/4.0/).
