# Supplementary material for: Provenance and family variations in early growth of Manchurian walnut (Juglans mandshurica Maxim.) and selection of superior families
Source: PLoS One. 2024 Mar 7;19(3):e0298918. doi: 10.1371/journal.pone.0298918 (PMC10919699; doi:10.1371/journal.pone.0298918)
Supplement: S1 File — (ZIP) [file pone.0298918.s004.zip › Allometric relationship between tree height and diameter of larch forests in China.pdf]

北京大学学报(自然科学版) 第 53 卷 第 6 期 2017 年 11 月
Acta Scientiarum Naturalium Universitatis Pekinensis, Vol. 53, No. 6 (Nov. 2017)
doi: 10.13209/j.0479-8023.2017.127

中国落叶松林胸径–树高相关关系的探讨

刘春云 方文静 蔡琼 马素辉 姜星星 吉成均† 方精云

北京大学地表过程分析与模拟教育部重点实验室,北京大学城市与环境学院生态学系, 北京 100871;
† 通信作者, E-mail: jicj@pku.edu.cn

摘要 基于幂函数$H = aD^b$, 对我国广泛分布的8种落叶松属(*Larix* Mill.)乔木184个样方胸径(D)与树高(H)的相关生长关系进行分析, 并探讨林分密度、林分总胸高断面积、年均温度和年降水量对相关生长系数(b值)的影响。结果表明, 相关生长系数在不同落叶松属间存在显著差异(P<0.05): 东北地区的兴安落叶松林(0.65±0.11)和黄花落叶松林(0.68±0.10)的相关生长系数显著高于西南地区的四川红杉林(0.51±0.17)和红杉林(0.54±0.15) (F=2.34, P=0.026)。林分密度和总胸高断面积不能解释b值的空间变异(P > 0.05), 但年均温与b值显著负相关(R^2=0.09, P<0.001)。研究结果说明落叶松属的不同种之间胸径–树高关系不同, 且温度可能是导致不同落叶松林分胸径–树高关系存在差异的主要原因。

关键词 胸径–树高; 相关生长; 落叶松
中图分类号 Q948

Allometric Relationship between Tree Height and Diameter of Larch Forests in China

LIU Chunyun, FANG Wenjing, CAI Qiong, MA Suhui, JIANG Xingxing, JI Chengjun†, FANG Jingyun

Key Laboratory for Earth Surface Process (MOE), Department of Ecology, College of Urban and Environmental Sciences, Peking University, Beijing 100871; † Corresponding author, E-mail: jicj@pku.edu.cn

Abstract 184 plots from larch (*Larix* Mill.) forests across China were sampled to characterize the height-diameter relationships and investigate the effects of structure and climate on them based on the power function $H = aD^b$. The results showed that the height-diameter relationship differed greatly among species: b-value of *Larix gmelinii* (0.65±0.11) and *L. olgensis* (0.68±0.10) were significantly higher than *L. mastersiana* (0.51±0.17) and *L. potaninii* (0.54±0.15) (F=2.34, P=0.026). These differences were significantly influenced by mean annual temperature, but no statistic relationships were detected between stem density, total basal area and mean annual prepetition against b-value. These results indicated that as mean annual temperature increased, a higher proportion of biomass was allocated into diameter growth than height growth (R^2=0.09, P<0.001).

Key words diameter-height; allometric scaling; *Larix*

胸径(diameter at breast height, DBH)和树高(tree height)的变化是树木生长的两个重要方面。前者为径向生长, 主要与树木的机械支持能力、水分吸收能力以及叶生物量有关; 后者为垂直生长, 反映垂直方向上林木截取光照的能力[1–2]。胸径与树高之间存在密切的相关生长关系(allometry, 又称异速生长), 反映树木对水平生长和垂直生长的权衡[1]。目前, 描述生物体器官之间数量关系的相关生长方程多以幂函数形式表示[3–5]。

由于树木胸径的测定方法简单、准确, 而测定树高相对困难且存在较大误差, 因此, 根据胸径测量数据和胸径–树高的相关生长方程推算树高的方

国家科技基础性专项《中国植被志》编研(2015FY210200)和国家自然科学基金(31321061)资助
收稿日期: 2016–05–22; 修回日期: 2016–05–30; 网络出版日期: 2017–08–06

法得到广泛使用，建立简单而准确的胸径–树高模型尤为重要[6–9]。

树木胸径和树高的相关生长关系受个体发育和外部环境的双重影响，物种的差异和光照、温度、水分等自然环境的改变都会影响树木的异速生长规律[2,10–15]。受光环境的显著影响[15]，安徽马尾松和杉木的胸径–树高关系不同。在我国东北地区，温度显著影响树木的胸径–树高关系，随着冬季低温胁迫的增强，更多的生物量资源用于胸径生长，树干更为粗壮[14]。在新疆地区，水分是针叶树种胸径–树高关系的限制因子，更多的年降水量导致更多的生物量资源分配于树木的垂直生长[16]。

对同一物种来说，气候是影响树木胸径和树高关系的重要因素。关于胸径–树高关系受哪种环境因子的影响，结论往往会因为研究对象、研究区域的尺度不同而存在差异。在研究的空间尺度和环境梯度较小时，可能会得出胸径–树高生长关系的幂指数与地形、土壤养分、林分密度和林龄等都没有显著关系的结论[17]；当环境梯度较大时，胸径–树高关系会随着气候、立地条件的不同而有规律地变化[14]。目前关于胸径–树高关系的研究多集中于区域尺度上不同物种、不同发育阶段个体的胸径–树高关系的差异，研究尺度较为狭窄[2]。由于物种分布的局限性和大尺度调查工作的缺乏，在更大地理尺度上探讨树木相关生长关系的研究较少见，对种以上水平(属)物种胸径–树高关系的环境影响机制的探讨十分不足。

落叶松属(*Larix* Mill.)植物均为落叶乔木，系裸子植物门松科，分布于北半球温带高山、寒温带和寒带地区[18]。我国有 10 个种和 1 变种，分布范围与我国东亚季风边缘带基本上重合，具有分布广泛的特点[19]。本文以我国落叶松属乔木为研究对象，通过野外大范围设立样地和每木调查的方法，实际测量落叶松的胸径和树高，研究不同落叶松种的胸径与树高相关关系及其对环境因子的响应。

1 材料和方法

1.1 样方调查

本文选择以 8 种落叶松属乔木为优势树种的 184 个森林样方，于 2000—2004 年和 2013—2015 年分两次进行调查，包括兴安落叶松(*Larix gmelinii*) (54 个样方)、黄花落叶松(*L. olgensis*) (13 个样方)、华北落叶松(*L. principis-rupprechtii*) (45 个样方)、新疆落叶松(*L. sibirica*) (14 个样方)、太白红杉(*L. chinensis*) (22 个样方)、西藏红杉(*L. griffithiana*) (4 个样方)、四川红杉(*L. mastersiana*) (18 个样方)和红杉(*L. potaninii*) (14 个样方) (图 1 和表 1)。除西藏红杉林 4 个样方(800 m^2)、华北落叶松林 2 个样方(400 m^2)和 1 个样方(1200 m^2)外，其余 177 个样方的面积均为 600 m^2 (20 m× 30 m)。本研究的所有样方中落叶松的株数均为 10 株以上，共调查落叶松个体 8338 株。

1.2 数据来源

对样方内所有胸径大于 3 cm 的乔木进行每木调查，即记录树种、测定所有乔木的胸径(1.3 m 树高处)及树高，同时记录调查样方所在位置的经度(°E)、纬度(°N)和海拔高度(m)。计算的林分指标包括林分密度、林分总胸高断面积以及落叶松乔木的平均胸径、最大胸径、平均树高和最大树高。

气候数据(1950—2000 年)来自 World Clim 数据库(www.worldclim.org/)，利用样方的经纬度信息获取所在样点的多年平均温度(mean annual temperature, MAT)和平均降水量(mean annual precipitation, MAP)[20]。

1.3 数据分析

1.3.1 胸径–树高关系的拟合

对每个落叶松林样方的胸径和树高采用方程(1)或(2)进行拟合[21–22]:

$$H = aD^b, \tag{1}$$

$$\log H = a + b\log D, \tag{2}$$

其中，H 为树高(m); D 为胸径(cm); a 为方程的截距; b 为斜率，即相关生长系数，表示生物量在胸径和树高中的分配关系[1,14]。

1.3.2 统计分析

对各样地拟合的胸径–树高方程的参数 a 和 b 进行单因素方差分析，检验其在不同落叶松林间的差异。采用一般线性模型(general linear models, GLM)探讨林分密度、总胸高断面积、年均温度、年降水量与 b 值的相关关系。应用广义混合线性模型(generalized linear mixed model, GLMM)，以林分密度、总胸高断面积、年均温度为固定因子，以样方及物种作为随机因子，探讨环境因子对 b 值的影响。统计分析使用 SPSS 11.0 (2001, SPSS Inc., USA), Matlab R2015a (The MathWork Inc., Natick, MA, USA)和 R 3.0.3 软件 (http://www.R-project.org/)。

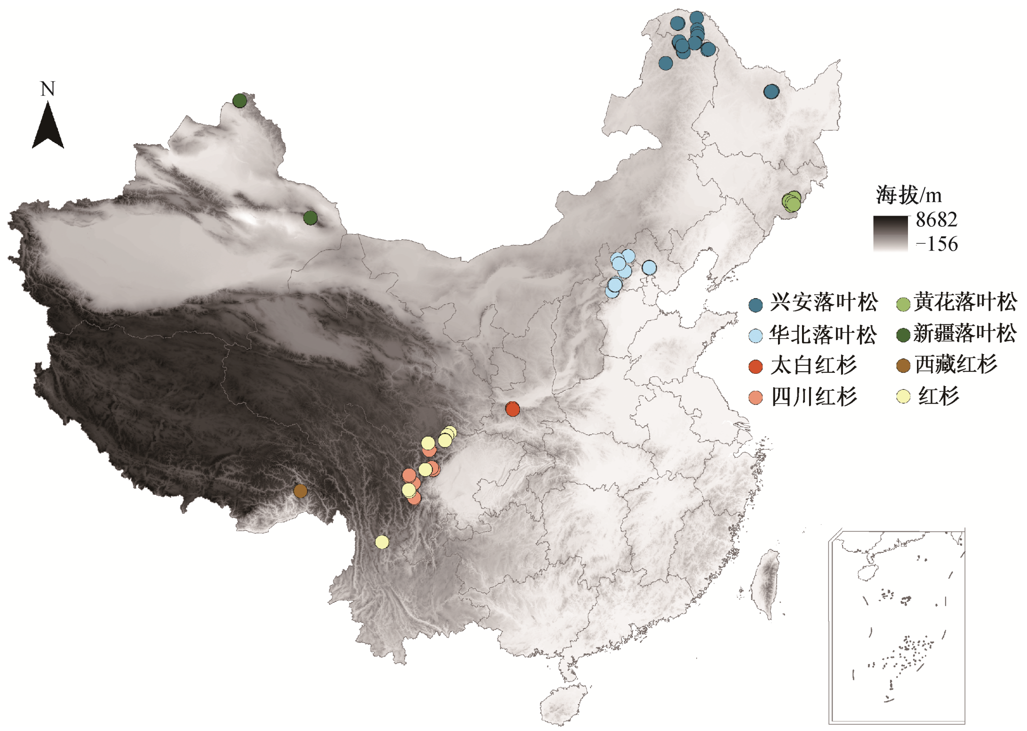

图 1　研究样方位置分布
Fig. 1　Location of study sites

表 1　184 个研究样方的基本信息
Table 1　Location and structural characteristics of the 184 larch forest plots

物种	纬度/(°N)	经度/(°E)	海拔/m	样方数量	林分密度/(株 · hm^{-2})	平均胸径/cm	平均树高/m	总胸高断面积/(m^{2}· hm^{-2})
兴安落叶松 *Larix gmelinii*	48.05~52.86	121.51~129.23	279~1114	54	1576.2±727.7	14.2±5.5	13.2±3.6	27.65±7.13
黄花落叶松 *L. olgensis*	42.06~42.41	127.96~128.43	652~1851	13	1314.1±646.7	19.2±8.0	14.2±5.5	46.16±15.77
华北落叶松 *L. principis-rupprechtii*	39.61~41.33	114.75~117.51	948~2650	45	1396.5±699.6	17.9±3.8	11.1±2.6	30.90±8.02
新疆落叶松 *L. sibirica*	43.31~48.68	86.79~93.68	1460~2680	14	1100.0±352.0	22.8±5.0	16.0±4.0	32.56±5.57
太白红杉 *L. chinensis*	33.92~33.99	107.76~107.79	3100~3300	22	1034.8±478.2	18.7±2.4	8.2±1.1	32.77±16.82
西藏红杉 *L. griffithiana*	29.29	95.35	3250~3750	4	1212.5±455.2	18.3±12.6	9.1±4.1	20.44±8.71
四川红杉 *L. mastersiana*	29.38~31.88	101.55~102.98	2730~4002	18	1417.6±755.1	24.6±9.8	12.2±3.9	27.96±16.51
红杉 *L. potaninii*	27.11~32.77	100.22~103.92	2790~3868	14	1373.8±847.7	25.2±15.2	12.3±3.8	26.80±17.74

说明：林分密度、平均胸径、平均树高和总胸高断面积的数据为平均值±标准差(SD)。

2　结果

2.1　落叶松林的胸径和树高生长

从表 2 可以看出, 184 个落叶松林调查样方的平均胸径和树高均存在较大差异(表 2)。从平均胸径看, 落叶松林分的平均胸径的范围为 6.3~53.4 cm。平均胸径的极大值出现在云南玉龙雪山的红杉林, 极小值出现在西藏红杉林。红杉林的平均胸径在 8 种落叶松林中最大(25.2±15.2 cm), 其次为四川红杉林(24.5±9.8 cm)和新疆落叶松林(22.7±5.0 cm), 平均胸径最小的是兴安落叶林(14.2±5.5 cm), 其余 4 种落叶松差别不大, 平均值在 17.5~19.5 cm 之间。从最大胸径的平均值看, 黄花落叶松林的最大胸径值最高(59.4±17.2 cm), 西藏红杉林(29.9±20 cm)最低, 其余 6 种落叶松的最大胸径平均值在 32.5~45.0 cm 之间, 从高到低依次为新疆落叶松林、四川红杉林、太白红杉林、红杉林、兴安落叶松林、华北落叶松林。

落叶松林的平均树高分布范围为 5.2~26.7 m。其中, 新疆落叶松林的平均树高均值最高(16.0±4.0 m), 黄花落叶松林(14.2±5.5 m)和兴安落叶松林(13.2±3.6 m)次之, 太白红杉林(8.2±1.1 m)和西藏红杉林(9.1±4.1 m)最低, 其余树种相差不大。落叶松属乔木的最大树高范围为 8.0~38.0 m。最大树高和平均树高的情况相似, 表现为黄花落叶松林(30.8±6.9 m)最高, 新疆落叶松林(24.4±5.2 m)和兴安落叶松林(24.0±3.9 m)次之, 太白红杉林(13.4±2.4 m)和西藏红杉林(15.3±5.7 m)最低, 其余类型无显著差异。

2.2　落叶松属植物的胸径–树高关系

如图 2 所示, 落叶松属林分的胸径–树高关系均表现为较好的幂函数关系(R^2 >0.6, P<0.001)。总体来说(不区分树种), 所有落叶松林分的胸径–树高函数为 H=2.8$D^{0.51}$ (R^2=0.21, P<0.001)。就不同树种而言, 华北落叶松的斜率(b 值)最高, 为 0.70; 黄花落叶松和新疆落叶松次之, 均为 0.67, 高于西藏红杉(0.49)和红杉(0.52); 太白红杉、四川红杉和兴安落叶松的 b 值无显著差异, 分别为 0.60, 0.61 和 0.61。

由于幂函数模型能够较好地描述不同落叶松林分的胸径–树高关系, 所以使用该模型分别模拟全部调查样方(n=184)落叶松胸径与树高的幂函数关系。结果显示, 其中 182 个样方的落叶松胸径–树高均呈显著的幂函数关系。单因素方差分析结果表明, 黄花落叶松(0.68±0.10)和兴安落叶松(0.65±0.11)的 b 值显著高于其余树种, 而华北落叶松、四

表 2　落叶松林调查样方的胸径树高特征

Table 2　DBH and height characteristics of eight larch species in China

森林类型	样方数	平均胸径/cm	最大胸径/cm	平均树高/m	最大树高/m
兴安落叶松林	54	14.2±5.5<br>6.5~43.4	38.3±11.7<br>15.4~66.8	13.2±3.6<br>7.2~26.7	24.0±3.9<br>15.8~34.0
黄花落叶松林	13	19.2±8.0<br>12.1~37.7	59.4±17.2<br>33.1~91.5	14.2±5.5<br>6.8~26.4	30.8±6.9<br>14.0~38.0
华北落叶松林	45	17.8±3.8<br>10.3~26.9	32.5±8.9<br>16.9~75.8	11.0±2.6<br>5.2~17.5	16.2±3.4<br>9.5~25.4
新疆落叶松林	14	22.7±5.0<br>12.6~28.9	44.9±11.7<br>27.1~68.8	16.0±4.0<br>9.9~24.8	24.4±5.2<br>17.7~31.7
太白红杉林	22	18.6±2.4<br>15.4~24.7	42.4±12.1<br>27.9~80.0	8.2±1.1<br>6.5~10.8	13.4±2.4<br>9.1~18.0
西藏红杉林	4	18.3±12.6<br>6.3~33.9	29.9±20.0<br>10.8~54.1	9.1±4.1<br>5.6~14.4	15.3±5.7<br>8.0~22.0
四川红杉林	18	24.5±9.8<br>11.5~43.8	43.3±15.7<br>22.2~72.6	12.2±3.9<br>7.1~19.7	16.9±3.4<br>11.8~23.0
红杉林	14	25.2±15.2<br>7.9~53.4	42.1±21.7<br>23.7~89.8	12.2±3.8<br>6.2~21.6	17.9±4.1<br>13.2~28.8

说明: 数据为平均值±标准差(SD), 最小值~最大值。

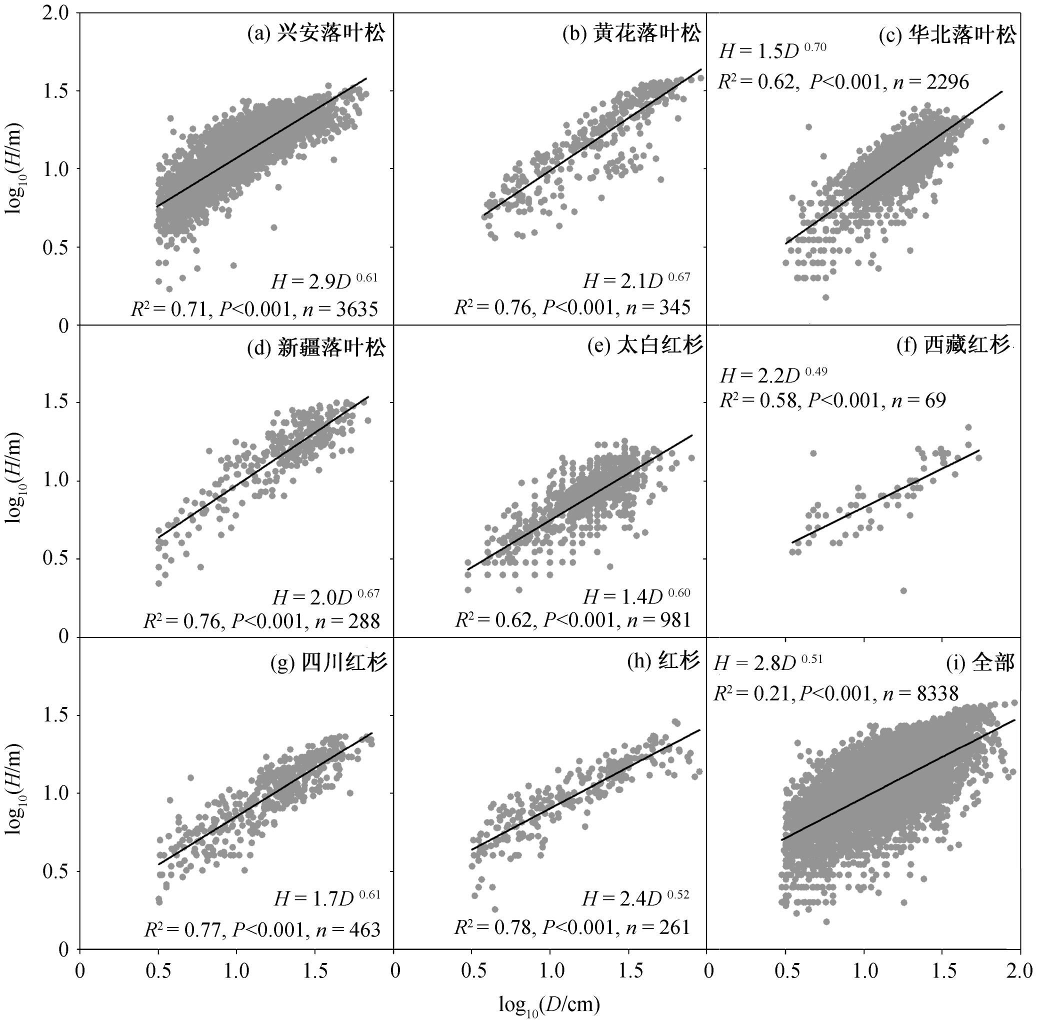

图 2　落叶松属植物的胸径–树高关系

Fig. 2　Allometric relationship between diameter and height for larch forests in China

川红杉和红杉的 *b* 值较低，分别为 0.56±0.23、0.51±0.17 和 0.54±0.15。其余 3 种落叶松的 *b* 值无显著差异：新疆落叶松为 0.61±0.15、太白红杉为 0.60±0.17，西藏红杉为 0.64±0.26 (F=2.34, P=0.026, 见表 3)。

2.3　环境因子对落叶松胸径–树高关系的影响

表 4 给出林分密度、总胸高断面积、年均温度、年降水量与胸径–树高关系的斜率 *b* 值的线性相关系数。结果显示，*b* 值与年均温呈显著的负相关关系(R^2=0.09, P<0.001)，其余环境因子不能解释落叶松胸径和树高关系的 *b* 值空间变异(P > 0.05)(图 3)。在进行线性混合模型分析之前，先对各环境因子进行皮尔森(Pearson)相关分析，结果发现年均温度与年降水量显著相关(r=0.51, P<0.001)，与其他因子无显著关系(表 5)，故剔除降水因子，以林分密度、林分断面积、年均温度为固定因子，以物种和样方为随机因子，建立混合线性模型对 *b* 值进行拟合。

线性混合模型分析结果显示，年均温度与胸径树高关系之间呈显著的负相关关系(P<0.001)，而其他因子对胸径–树高的相关生长无显著影响(表 6)。胸径–树高关系的 *b* 值与年均温度有显著的负相关关系，说明在研究区域内，年均温度越低，*b* 值越高，相对更多的生物量用于树高生长，树木形态趋于细高(如东北地区的兴安落叶松和黄花落叶松)。年均温度越高，*b* 值越低，相对更多的生物量用于胸径生长，树木形态趋于矮壮(比如西南地区的四川红杉和红杉)。在物种水平上，兴安落叶松和四川红杉的年均温度与 *b* 值呈显著的负相关关系，其他 6 种落叶松则关系不显著(图 4)。

表 3 不同落叶松树种胸径–树高关系方程参数的单因素方差分析结果

Table 3 Comparison of the DBH-height-relationship among forests dominated by eight larch species in China

落叶松种类	*a*	*b*	样本量
兴安落叶松	2.71(1.24)^{a}	0.65(0.11)^{a}	54
黄花落叶松	2.26(0.80)^{ab}	0.68(0.10)^{a}	13
华北落叶松	2.96(2.20)^{a}	0.56(0.23)^{b}	45
新疆落叶松	2.64(1.32)^{ab}	0.61(0.15)^{ab}	13
太白红杉	1.59(0.80)^{b}	0.60(0.17)^{ab}	22
西藏红杉	1.64(0.90)^{ab}	0.64(0.26)^{ab}	4
四川红杉	2.93(1.88)^{a}	0.51(0.17)^{b}	18
红杉	2.56(1.42)^{ab}	0.54(0.15)^{b}	13

注：上角标 a 和 b 表示差异显著性(P<0.05)，括号内数字为标准偏差(SD)。

表 4 不同环境因子对落叶松胸径–树高关系b值的解释

Table 4 Effects of environmental factors on the b-value in Equation of $\log H=a+b\log D$ for larch forests in China

环境因子	R^2	P
林分密度	<0.001	0.961
总胸高断面积	<0.001	0.989
年均温度	0.090	<0.001
年降水量	0.006	0.312

3 讨论

前人对我国东北湿润区山地森林的研究表明，树木的胸径–树高关系受到温度的显著影响，随着冬季低温胁迫的增强，更多的生物量资源分配于胸径生长，树干更为粗壮，但是没有对落叶松林进行具体分析，只是指出落叶松林的资源分配–气候关系与其他森林类型不同，兴安落叶松林尖削度(胸径 DBH 和树高 H 的比值)与最冷季均温无显著关系[14]。本研究的结果也表明，兴安落叶松的胸径–树高关系与其他森林类型不同，年均温度会对兴安落叶松的胸径–树高关系产生显著影响，且年均温越低，树木形态越趋于细高。Huang 等[23]对加拿大阿尔伯塔省(49—60°N)主要森林群落的胸径与树高的关系用 $H=aD^b$ 方程拟合，其中落叶松属植物的 b 值约为 0.80，而本研究中的 b 值为 0.54~0.68(表 3)。Huang 等[23]研究区的纬度和落叶松的 b 值均高于本文研究对象，即研究区更加寒冷，且树木形态细高，支持本研究结果。

部分研究表明，水分显著影响胸径–树高关系，随着水分条件的改善，更多的生物量资源分配于树高生长[10,13]。本文中年降水对 b 值无影响，可能是由于研究对象和区域环境的不同。Callaway 等[10]对比了山地和沙漠的黄松，两种生境的水分条件差异显著，在水分胁迫的逆境条件下，树木需要投入

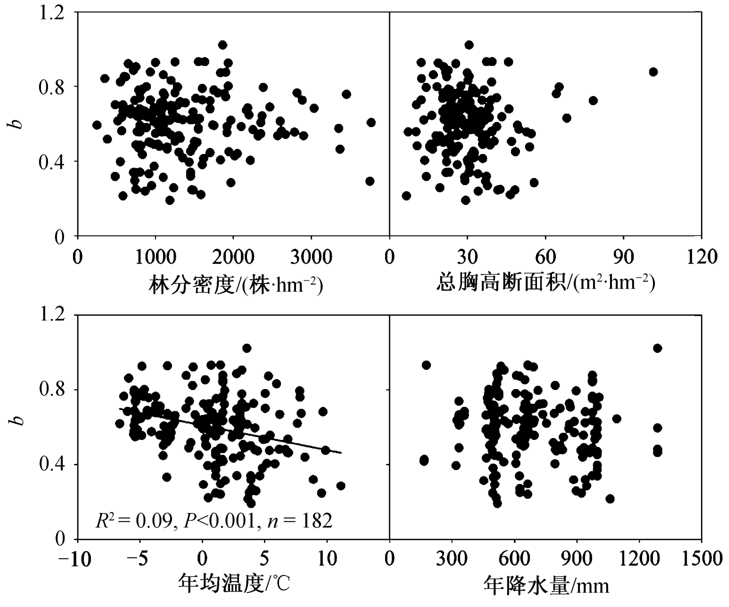

图 3 不同环境因子与斜率 b 值的关系

Fig. 3 Relationship between environmental factors and b-value for larch forests in China

表 5　各环境因子之间的皮尔森相关系数

Table 5　Pearson correlations among structural measurements and environmental factors for larch forests in China

环境因子	林分密度	总胸高断面积	年均温度	年降水量
林分密度	1.00	0.09	−0.08	−0.13
总胸高断面积	0.09	1.00	0.08	0
年均温度	−0.08	0.08	1.00	0.51***
年降水量	−0.13	0	0.51***	1.00

注: * 0.01<*P*<0.05; ** 0.001<*P*<0.01; *** *P*<0.001。

表 6　各环境因子线性混合模型分析结果

Table 6　Summary of generalized linear mixed model for effects of environmental factors and structural measurements for larch forests in China

环境因子	估计值	DF	*T*	*P*	显著性
截距	0.607782	178	6.52	<0.001	***
林分密度	−0.000006	178	−0.29	0.770	
胸高断面积	0.000347	178	0.35	0.720	
年均温度	−0.013421	178	−3.80	<0.001	***

注: * 0.01<*P*<0.05; ** 0.001<*P*<0.01; *** *P*<0.001。

更多资源用于边材生长，以保证水分运输。Martínez等[13]的研究地点为荒漠的林灌交错带，水分亦是限制该区域树木生长的重要因子。李利平等[16]对新疆山地针叶林的研究表明，虽然在整个新疆地区水分是胸径–树高关系的限制因子，但是北疆地区与南疆地区存在差异，北疆地区水分条件和生产力优于南疆，北疆针叶林的胸径–树高关系受年均温度的影响较大而受年降水量的影响较小，南疆则受年降水量影响较大。本研究中新疆落叶松林正是分布于北疆的阿尔泰山和东天山北坡，落叶松林的水分条件相对良好，对胸径–树高关系的影响不大。

虽然本文研究的是 8 种落叶松，且分布范围涵盖全国，但是落叶松属植物的生态位较为狭窄，主要分布于寒冷湿润的林线附近，且主要为阴坡，生境较为单一，气候梯度较窄，因此本研究中除年均温度外，其他环境因子都没有对胸径–树高关系产生显著影响。

4　结论

本文的研究结果表明：1) 我国落叶松属不同树种之间，胸径–树高的相关关系并非一致，且我国东北地区的兴安落叶松林和黄花落叶松林的相关生长系数(*b* 值)显著高于我国西南地区的四川红杉林和红杉林，前者树木形态趋于细高，后者树木形态趋于粗矮；2) 林分密度和总胸高断面积等生物因子不能解释落叶松不同种之间 *b* 值的空间变异；3) 落叶松生长地区年均温度的差异可能是导致不同落叶松种胸径–树高关系存在差异的主要原因。探讨我国

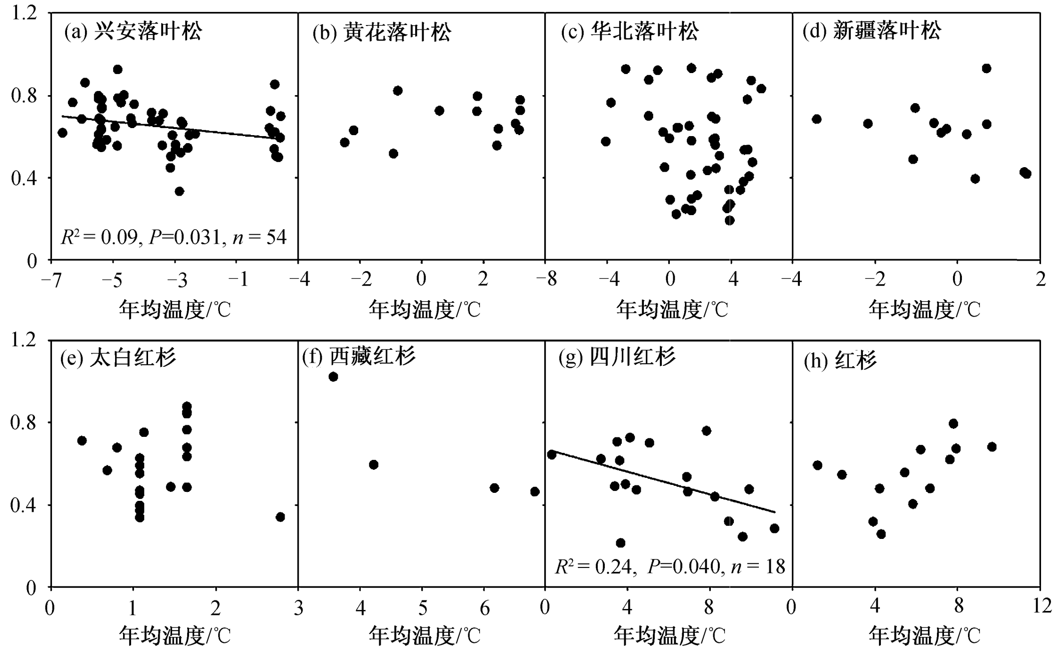

图 4　落叶松不同树种的年均温度与 *b* 值的相关关系

Fig. 4　Relationship between mean annual temperature and *b*-value for larch forests in China

落叶松属不同种胸径与树高的关系及其对环境因子的响应，有助于理解该物种资源径向和垂直分配的空间分异及驱动机制，亦可为森林经营实践中材积量的估算提供参考。

致谢 北京大学朱剑霄、李鹏、陶胜利、胡小康、姚辉、李超同学在野外调查和数据分析中给予帮助，长白山科学研究院宗占江研究员在物种鉴定方面提供宝贵意见，在此表示衷心感谢。

参考文献

[1] Aiba S I, Kohyama T. Tree species stratification in relation to allometry and demography in a warm-temperate rain forest. Journal of Ecology, 1996, 84 (2): 207–218

[2] King D A. Allometry and life history of tropical trees. Journal of Tropical Ecology, 1996, 12(1): 25–44

[3] Peters R H. The ecological implications of body size. Cambridge: Cambridge University Press, 1983: 197–198

[4] Niklas K J. Size-dependent variations in plant growth rates and the “3/4 power rule”. American Journal of Botany, 1994, 81(2): 134–144

[5] 韩文轩, 方精云. 幂指数异速生长机制模型综述. 植物生态学报, 2008, 32(4): 951–960

[6] Curtis R O. Height-diameter and height-diameter-age equations for second-growth Douglas-fir. Forest Science, 1967, 13(4): 365–375

[7] Huang S, Titus S J, Lakusta T W, et al. Ecologically based individual tree height-diameter models for major Alberta tree species. Alberta Environmental Protection, Land and Forest Services, Forest Management Division, 1994: 27–28

[8] Sharma M, Parton J. Height-diameter equations for boreal tree species in Ontario using a mixed-effects modeling approach. Forest Ecology and Management, 2007, 249(3): 187–198

[9] Azimeh M, Ahto K. Are allometric relationships between tree height and diameter dependent on environmental conditions and management?. Trees, 2016: doi 10.1007/s00468-016-1379-4

[10] Callaway R M, DeLucia E H, Schlesinger W H. Biomass allocation of montane and desert ponderosa pine: an analog for response to climate change. Ecology, 1994, 75(5): 1474–1481

[11] Aiba S I, Kitayama K. Structure, composition and species diversity in an altitude-substrate matrix of rain forest tree communities on Mount Kinabalu, Borneo. Plant Ecology, 1999, 140(2): 139–157

[12] Thornley J H M. Modelling stem height and diameter growth in plants. Annals of Botany, 1999, 84(2): 195–205

[13] Martínez A J, López-Portillo J. Allometry of *Prosopis glandulosa* var. *torreyana* along a topographic gradient in the Chihuahuan desert. Journal of Vegetation Science, 2003, 14(1): 111–120

[14] Wang X P, Fang J Y, Tang Z Y, et al. Climatic control of primary forest structure and DBH-height allometry in Northeast China. Forest Ecology and Management, 2006, 234(1): 264–274

[15] Cheng X P, Umeki K, Honjo T, et al. Height growth, diameter-height relationships and branching architecture of pinus massoniana and cunninghamia lanceolata in early regeneration stages in anhui province, eastern china, effects of light intensity and regeneration mode. Forestry Studies in China, 2011, 13(1): 1–12

[16] 李利平, 安尼瓦尔·买买提, 王襄平. 新疆山地针叶林乔木胸径–树高关系分析. 干旱区研究, 2011, 28(1): 47–53

[17] 樊艳文, 王襄平, 曾令兵, 等. 北京栓皮栎林胸径–树高相关生长关系的分析. 北京林业大学学报, 2011, 33(6): 146–150

[18] 中国科学院中国植物志编辑委员会. 中国植物志(第 7 卷). 北京: 科学出版社, 1978: 169–196

[19] 张兰生, 方修琦, 任国玉. 全球变化. 北京: 高等教育出版社, 2004: 88–109

[20] Hijmans R J, Cameron S E, Parra J L, et al. Very high resolution interpolated climate surfaces for global land areas. International Journal of Climatology, 2005, 25(15): 1965–1978

[21] Stoffels A, Soest J V. The main problems in sample plots. 3. Height regression. Ned Boschb Tijdschr, 1953, 25: 190–199

[22] Stage A R. Prediction of height increment for models of forest growth. USDA Forest Service Research Paper, 1975, INT-164: 20

[23] Huang S, Titus S J, Wiens D P. Comparison of nonlinear height-diameter functions for major Alberta tree species. Canadian Journal of Forest Research, 1992, 22(9): 1297–1304

word版下载：http://www.ixueshu.com
