# Supplementary material for: Provenance and family variations in early growth of Manchurian walnut (Juglans mandshurica Maxim.) and selection of superior families
Source: PLoS One. 2024 Mar 7;19(3):e0298918. doi: 10.1371/journal.pone.0298918 (PMC10919699; doi:10.1371/journal.pone.0298918)
Supplement: S1 File — (ZIP) [file pone.0298918.s004.zip › Analyses of genotypic variation in white poplar clones at four sites in China.pdf]

WHITE, T. L., G. R. HODGE and G. L. POWELL (1993): Advanced-generation breeding strategy for slash pine in the southeastern United States. Silvae Genet **42**: 359–371.

WILSON, A. J., D. MCDONALD, H. K. MOGHARDAM, C. M. HERBINGER and M. M. FERGUSON (2003): Marker-assisted estimation of quantitative genetic parameters in rainbow trout, *Oncorhynchus mykiss*. Genet Res **81**: 145–156.

YAMADA, Y. (1962): Genotype by environment interaction and genetic correlation of the same trait under different environments. Jpn J Genet **37**: 498–509.

YANCHUCK, A. D. (2001): A quantitative framework for breeding and conservation of forest tree genetic resources in British Columbia. Can J For Res **31**: 566–576.

YE, T. Z., K. J. S. JAYAWICKRAMA and G. R. JOHNSON (2007): Efficiency of including first-generation information in second-generation ranking and selection: results of computer simulation. Tree Genet Genomes **3**: 319–328.

YU, Q. and P. PULKKINEN (2003): Genotype-environment interaction and stability in growth of aspen hybrid clones. For Ecol Manage **173**: 25–35.

Analyses of Genotypic Variation in White Poplar Clones at Four Sites in China

By XIYANG ZHAO[1),*)], WEI HOU[2)], HUIQUAN ZHENG[3)] and ZHIYI ZHANG[4)]

(Received 19th November 2012)

Abstract

Growth characteristics have a complex inheritance pattern, and the gene-environment interaction makes predicting tree responses to environmental change difficult. The primary goals of this study are to evaluate the variation in growth traits of different white poplar clones and to measure the inter-site variation and ecological sensitivities. In the experiment, a total of 30 white poplar clones were planted and measured over 5 years for height (H) and diameter at breast height (DBH) at four different sites in North China. ANOVA results showed that there were significant differences in H and DBH between clones at each site ($P < 0.01$). Phenotypic and genotypic variation, and the repeatability of H and DBH, increased with the tree growth, which suggested that the inter-clone variation became gradually larger under the control of genetic factors. Under a selection ratio of 20%, the genetic gains of H and DBH also increased with the tree growth at the same site. Correlation analysis showed a significantly positive association between H and DBH both at the same age and sites, but the correlation coefficients decreased with increasing age. In conclusion, genetic gains were not equal between different sites, indicating differences in the influence of environment on the poplar genotype. Further investigations may be able to determine the role of environment for tree breeding programs and genetic selection.

Key words: poplar, variation, repeatability, genetic gain.

1. Introduction

Poplar naturally grows in temperate forests of the northern hemisphere, and is widely used for various solid wood and panel products (e.g. pulp, paper and fiber products) and as a source of energy (SEYED, 2011; BRADSHAW et al., 2000; FANG et al., 1999; GAMBLES et al., 1984). It is also an attractive and valuable forest resource because of its fast growing nature and can be easily propagated from both seed and vegetative reproduction (SCHREIBER et al., 2011; COOKE et al., 2007).

Growth characteristics are complex in inheritance and are greatly influenced by various environmental conditions. Different species and clones have various performances in different environments (FANG et al., 1999). The gene-environment (G×E) interaction describes the situation where a number of genotypes respond differently to various environments, so that the effects of genotypes and environments are not statistically additive (LYNCH et al., 1998). The existence of G×E interaction makes it impossible to interpret the main effects of genotype and environment and to predict the performance of genotypes in changing environments (MARRON et al., 2010). In general, the environment can act on clonal in three different ways: (1) the G×E interaction

1) State Key Laboratory of Tree Genetics and Breeding, Northeast Forestry University, Harbin, 150040, People's Republic of China. E-Mail: zhaoxyphd@163.com.

2) Department of Preventive Medicine, Stony Brook University. Stony Brook, NY 11794, USA. E-Mail: wei.hou@stonybrookmedicine.edu.

3) Guangdong Academy of Forestry, Guangzhou, 510520 People's Republic of China. E-Mail: zhenghq@sinogaf.cn.

4) National Engineering Laboratory for Tree Breeding, Beijing Forestry University, Beijing 100083, People's Republic of China. E-Mail: zhangzy.bjfu@gmail.com.

*) Corresponding author: XIYANG ZHAO. State Key Laboratory of Tree Genetics and Breeding, Northeast Forestry University, No.26 Hexing Road Xiangfang District, Harbin, People's Republic of China, 150040. Tel: +0086-451-82190607, Fax: +0086-451-82190607. E-Mail: zhaoxyphd@163.com.
The first two authors contributed equally to this work.

DOI:10.1515/sg-2013-0023
edited by Thünen Institute of Forest Genetics

is not significant; (2) the G×E interaction is significant due to changes in differences among genotypes but not due to changes in genotype ranking; and (3) the G×E interaction is significant due to changes in genotype ranking from one environment to another (NICOLAS et al., 2007). Only the last case will cause problems for the breeder because a genotype selected for its growth vigor may not necessarily be vigorous if it grows in a different environment.

In this experiment, a total of 30 white poplar clones were used to investigate the variation of height (H) and diameter at breast height (DBH) of different age at four sites. The primary objectives of this study are to: (1) compare growth traits of different poplar clones; (2) estimate genotypic inter-clonal variation and repeatability of H and DBH, and estimate inter-site variation and ecological sensitivity of poplar clones; (3) estimate genotypic and phenotypic parameters and their age trends; and (4) predict selection gains and correlated responses.

2. Materials and Methods

2.1 Sites description and material

Four sites were selected for plantation in North China: Guanxian (GX), Ningyang (NY), Weixian (WX) and Fengfeng (FF). Main characteristics of the four sites are presented in *Table 1*. Soils at the four sites represent in which white poplar clones are expected to be planted in North China. The NY site has sandy loam soil, whereas other sites which were originally abandoned agricultural land have rich soils.

Thirty white poplar clones were used in this study (P20, P22, P23, P26, P28, P30, P42, P46, P49, P50, P53, P63, P64, P67, P69, P76, P77, P78, P83, P85, P87, P88, P98, P99, P101, P103, P104, P105, P106, and P107), which were obtained by cross experiments in 2000, and the parents all belong to white poplar (*Populus tomentosa* × (*P. tomentosa* × *P. bolleana*)). The experimental plantations were established with 1-year-old seedlings in March 2006 by utilizing the randomized complete block design (MARRON et al., 2006), with four blocks at each site. In each block, clones were planted in row plots with four trees at 3×4 m spacing.

2.2 Statistical analyses

All common trees were measured for H and DBH at 3–5 years at the four sites. Abnormal data due to tree death or broken were excluded from the analyses. Individual trees were measured by using an unbalanced longitudinal schedule (*Table 2*). There were at least three time measures at GX and at most five time measures at NY. The others have four time measures (WX and FF). Statistical analyses were carried out by using SPSS version 13.0 (Chicago, SPSS Inc). The significance of fixed effects was evaluated using *F* tests. The linear mixed-effect models (1) were used for joint analysis of the four sites together (DHILLON, 2012):

$$y_{ijkl}=\mu + S_i + B_{j(i)} + C_k + CS_{ik} + BC_{j(i)k} + \varepsilon_{ijkl}, \quad (1)$$

where y_{ijkl} is the performance of the l_{th} ramet of the k_{th} clone growing in the j_{th} block of the i_{th} site; μ is the overall mean; S_i is the effect of the i_{th} site (i = 1,……4); $B_{j(i)}$ is the effect of the j_{th} block within the i_{th} site (j = 1,……4), C_k is the effect of the k_{th} clone (k = 1,……30); CS_{ik} is the interactive effect of K_{th} clone and It_h site, $BC_{j(i)k}$ is the interactive effect of K_{th} clone and J_{th} block (within i_{th} site) and ε_{ijkl} is the random error.

Variation among ramets of the sampled clones was analyzed by analysis of variance (ANOVA) within sites (HANSEN et al., 1996):

Table 1. – Main characteristics of poplar clonal trials.

Site	Longitude°E	Latitude° N	Altitude	Annual average Temperature	Annual rainfall(mm)
FF	114°14′	36.26	100	14.1℃	627 mm
WX	155.16	37.04	37	13.6℃	500mm
GX	115.22	36.62	32.00	13.0℃	584mm
NY	116.53	35.55	91	13.4℃	689mm

Table 2. – Measuring schedules for H and DBH in four sites during 2006–2010.

Sites	H					DBH				
	2006	2007	2008	2009	2010	2006	2007	2008	2009	2010
GX	-	-	X	X	X	-	-	X	X	X
WX	-	X	X	X	X	-	X	X	X	X
FF	-	X	X	X	X	-	X	X	X	X
NY	X	X	X	X	X	X	X	X	X	X

Note: X denotes that the trees were measured and – denotes that the trees were not measured.

DOI:10.1515/sg-2013-0023
edited by Thünen Institute of Forest Genetics

$$y_{ij} = \mu + \alpha_i + \beta_j + \alpha\beta_{i(j)} + \varepsilon_{ij}, \quad (2)$$

where y_{ij} is the performance of the ramet of ith clone within the jth block, μ is the overall mean; α_i is the effect of the clone (i = 1,……30); β_j is the effect of the block (i = 1,……3); $\alpha\beta_{i(j)}$ is the random effect of the ith clone with in jth block and ε_{ij} is the random error.

The genotypic coefficient of variation (GCV) was calculated using the following formula (HAI et al., 2008):

$$GCV = \frac{\sqrt{\sigma_c^2} \times 100}{\bar{X}}, \quad (3)$$

where $\bar{X}$ is the phenotypic mean of the trait H (DBH) and the σ^2_c is the genotypic variance component of H (DBH). The coefficient of phenotypic variation (PCV) was obtained from the phenotypic variance component as: $\sigma^2_p = \sigma^2_c + \sigma^2_e$.

The individual repeatability R was calculated as (HANSEN et al., 1996):

$$R = \frac{\sigma_c^2}{\sigma_c^2 + \sigma_b^2 + \sigma_e^2}, \quad (4)$$

where σ^2_c is the genetic variance components between clones, σ^2_b is the block variance and σ^2_e is the error variance component.

The phenotype correlation $r_A(xy)$ of traits x and y was calculated as (PLIURA et al., 2007):

$$r_A(xy) = \frac{\sigma_{a(xy)}}{\sqrt{\sigma^2_{a(x)} \cdot \sigma^2_{a(y)}}} \quad (5)$$

where $\sigma^2_{a(x)}$ is the clone variance component for trait x, $\sigma^2_{a(y)}$ is the clone variance component for trait y and $\sigma^2_{a(xy)}$ is the clone covariance component.

2.3 Stability parameter concepts

H or DBH during the fifth year may reflect the response of the clones to the environments of the experiment fields. Since H and DBH were significantly correlated during the fifth year, only DBH was used for the stability study (*Table 7*). FINLAY (1963) used the estimated regression coefficient bi of individual performance against site means to measure stability and relative adaptability. Regression coefficients (bi) were estimated using the following regression model (YU et. al.,2003):

Table 3. – Variance Analysis for H and DBH in the different variation source.

traits	year	Variation source	df	MS	F	P
	2006	clone	29	2.428	5.406	<0.01
	2007	site	2	116.586	163.867	<0.01
		clone	29	18.233	25.627	<0.01
		site × clone	58	3.174	4.462	<0.01
		Error	758	0.711		
	2008	sites	3	145.900	236.742	<0.01
		clone	29	40.499	65.715	<0.01
		site × clone	87	2.845	4.617	<0.01
H		Error	1119	0.616		
	2009	sites	3	221.076	248.283	<0.01
		clone	29	67.530	75.841	<0.01
		site × clone	87	3.526	3.960	<0.01
		Error	1119	0.890		
	2010	sites	3	232.504	210.277	<0.01
		clone	29	86.298	78.048	<0.01
		site × clone	87	5.658	5.117	<0.01
		Error	1119	1.106		
	2006	clone	29	1.611	5.401	<0.01
	2007	site	2	123.764	166.486	<0.01
		clone	29	21.295	28.645	<0.01
		site × clone	58	2.364	3.180	<0.01
		Error	758	0.743		
	2008	sites	3	110.659	118.149	<0.01
		clone	29	66.360	70.852	<0.01
		site × clone	87	4.077	4.353	<0.01
DBH		Error	1119	0.937		
	2009	sites	3	85.127	60.020	<0.01
		clone	29	122.692	86.505	<0.01
		site × clone	87	5.507	3.883	<0.01
		Error	1119	1.418		
	2010	sites	3	110.731	64.039	<0.01
		clone	29	195.212	112.898	<0.01
		site × clone	87	6.753	3.905	<0.01
		Error	1119	1.729		

DOI:10.1515/sg-2013-0023
edited by Thünen Institute of Forest Genetics

$$Y_i = a + bx_i + e_i, \quad (6)$$

where y is the clonal value at the site i, a is the intercept of the site, x_i is the mean of all clones at the ith site, and e_i is the unknown error. A variety with b_i value close to 1 indicates average stability, relatively speaking, which is equally adapted to good and poor sites; high values of b_i (>1) indicates low stability, and low values of bi (<1) indicates high stability.

3. Results

3.1 Variation among all variation sources

Results of ANOVA for H and DBH across four sites were presented in *Table 3*. All effects, including clone × site interactions were highly significant ($P<0.01$) based on overall F tests.

3.2 Average H and DBH for all trees at different sites

The average H and DBH of all trees at four sites were summarized in *Table 4*. The average H of four sites all increased greatly during growth years. Especially at FF, the average H changed from 6.44 m to 9.91 m from 2007 through 2010. At WX, the average H grew faster from 2007 to 2009 (5.46 m–8.86 m) than from 2009 to 2010 (8.86 m–9.62 m). The NY site showed the lowest growth velocity, and the average H changed from 4.32 m to 7.85 m from 2006 to 2010.

The average DBH of 30 clones at FF was also higher than other sites for different years (*Table 4*). It changed from 5.88 cm to 10.62 cm from 2007 to 2010. In 2007, the average DBH at NY (5.18 cm) was higher than that of WX (4.47 cm), but after two years, NY had the lowest DBH (9.19 cm).

There were significant diversities in H and DBH among the four sites in each year. In 2007, average Hs of FF, WX and NY were 6.44 m, 5.46 m and 5.21 m respectively. Average DBHs were 5.88 cm, 5.18 cm, and 4.47 cm for FF, NY, and WX respectively. In 2008, average H and DBH of FF were the highest, and those of NY were the lowest. In 2010, FF had the highest average H and DBH, and NY had the lowest H and DBH among all four sites.

3.3 Age trends in variation among poplar clones.

Ranges of H and DBH for all clones at each site were shown in *Table 4*. Average H of each clone at FF varied from 4.07 m to 8.79 m in 2007, while the maximum was 2.16 times the minimum. In 2010, the range was from 5.23 m to 13.22 m with a discrepancy of 2.53 times. Ranges of DBH among different clones at FF also changed significantly across years. The range was from 3.32 cm to 8.12 cm in 2007, but the range was from 4.14 cm to 16.32 cm in 2010. Other sites also presented the same trend that as the age went up; the width of range became larger. PCV and GCV of H and DBH at four sites in different years were also displayed in *Table 4*. The clonal PCV and GCV for DBH were higher than those for H, and GCV was lower than PCV. PCV and GCV of H and DBH at GX were smaller than those of other sites, while WX had higher variation. Taking one with another, PCV ranged from 15.34% to 23.40% for H and 19.42% to 31.88% for DBH, however GCV ranged from 14.43 to 22.20% for H and 18.43% to 29.83% for DBH. With the tree development and growth, PCV and GCV of H and DBH increased for all sites.

3.4 Age trends in repeatability

The clonal repeatability of H and DBH varied greatly year-by-year and reached the highest level in the last year at each site. The repeatability of H and DBH in FF

Table 4. – Variation parameter analysis of H and DBH of 30 poplar clones in different sites.

Site	Year	H					DBH				
		Average	Range	PCV	GCV	R	Average	Range	PCV	GCV	R
FF	2007	6.44	4.07-8.79	19.70	18.61	0.9629	5.88	3.32-8.12	23.64	22.32	0.9678
	2008	7.63	4.40-10.46	20.38	19.24	0.9745	7.98	3.58-11.86	26.68	25.20	0.9837
	2009	8.95	5.02-12.03	20.69	19.54	0.9759	9.59	3.93-14.50	27.82	26.27	0.9849
	2010	9.91	5.23-13.22	23.40	22.10	0.9809	10.62	4.14-16.32	29.18	27.55	0.9859
GX	2008	6.70	4.60-9.53	19.43	18.45	0.9694	6.99	4.15-8.95	19.42	18.43	0.9661
	2009	8.12	5.50-10.88	19.50	18.52	0.9724	8.67	4.49-11.87	22.95	21.78	0.9685
	2010	9.82	6.04-13.19	19.89	18.89	0.9730	10.54	5.10-15.99	25.04	23.77	0.9805
NY	2006	4.32	3.32-5.15	15.34	14.43	0.8150	3.01	1.7-4.03	21.48	20.20	0.8148
	2007	5.21	3.86-6.52	17.51	16.47	0.8959	5.18	3.52-6.98	19.82	18.64	0.9228
	2008	5.83	4.10-7.59	18.47	17.37	0.9237	6.56	4.24-9.00	21.45	20.18	0.9412
	2009	6.88	4.98-9.74	20.36	19.16	0.9545	8.32	5.10-11.93	23.96	22.54	0.9602
	2010	7.85	5.39-11.42	23.36	21.98	0.9661	9.19	5.67-14.00	26.75	25.17	0.9685
WX	2007	5.46	3.20-7.33	19.41	18.16	0.7977	4.47	2.20-7.13	24.11	22.56	0.8232
	2008	6.82	5.03-9.31	20.44	19.13	0.8652	6.79	3.95-9.86	25.13	23.51	0.8632
	2009	8.86	6.50-12.30	21.98	20.57	0.9030	8.61	5.31-12.41	27.69	25.90	0.8841
	2010	9.62	6.84-12.59	23.80	22.27	0.9048	10.02	5.70-14.84	31.88	29.83	0.9035

Note: the unit of H was m, DBH was cm, PCV and GCV were %.

DOI:10.1515/sg-2013-0023
edited by Thünen Institute of Forest Genetics

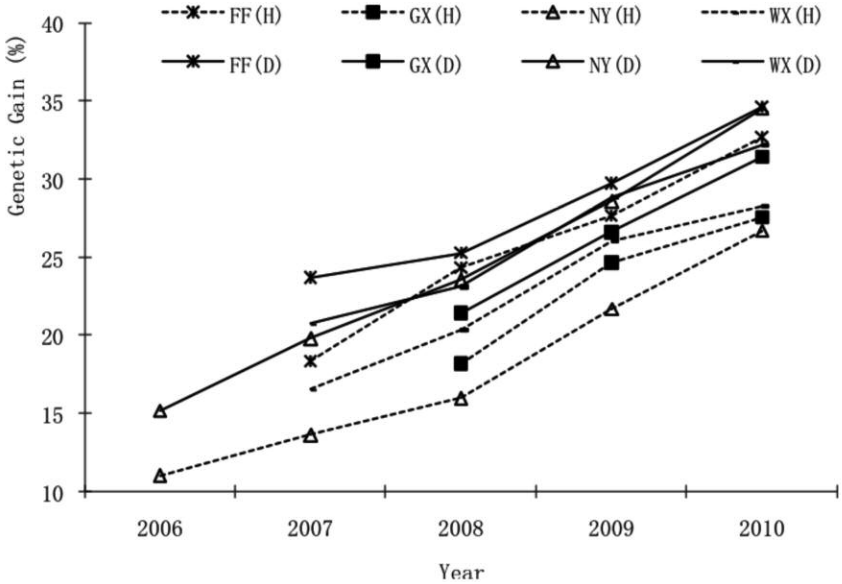

Figure 1. – Genetic gain of H and DBH of different sites in different years.

Table 5. – Correlation coefficient (r) between H and D in different years in GX and NY.

site	character	year	GX										NY				
			H		DBH			H					DBH				
			2009	2010	2008	2009	2010	2006	2007	2008	2009	2010	2006	2007	2008	2009	2010
GX	H	2008	0.971**	0.864**	0.779**	0.790**	0.787**	0.452*	0.776**	0.835**	0.884**	0.847**	0.603**	0.700**	0.721**	0.764**	0.756**
		2009		0.868**	0.804**	0.826**	0.818**	0.411*	0.748**	0.830**	0.846**	0.854**	0.606**	0.701**	0.731**	0.775**	0.774**
		2010			0.749**	0.779**	0.875**	0.370*	0.685**	0.783**	0.839**	0.894**	0.516**	0.689**	0.699**	0.731**	0.724**
	DBH	2008				0.992**	0.930**	0.332	0.622**	0.695**	0.770**	0.702**	0.755**	0.818**	0.858**	0.902**	0.876**
		2009					0.943**	0.351	0.649**	0.722**	0.803**	0.738**	0.745**	0.828**	0.872**	0.893**	0.896**
		2010						0.325	0.658**	0.762**	0.839**	0.814**	0.691**	0.845**	0.871**	0.906**	0.915**
NY	H	2006							0.762**	0.632**	0.465**	0.418*	0.713**	0.532**	0.445*	0.401*	0.365*
		2007								0.949**	0.847**	0.788**	0.781**	0.815**	0.790**	0.752**	0.737**
		2008									0.947**	0.908**	0.724**	0.859**	0.851**	0.826**	0.815**
		2009										0.972**	0.654**	0.864**	0.888**	0.856**	0.892**
		2010											0.561**	0.814**	0.836**	0.843**	0.890**
	DBH	2006												0.841**	0.803**	0.781**	0.736**
		2007													0.979**	0.945**	0.921**
		2008														0.983**	0.968**
		2009															0.993**

and GX all exceeded 0.95. At WX, the repeatability of H and DBH changed from 0.8150 to 0.9661 and from 0.8148 to 0.9685 respectively from 2007 to 2010. At NY, the repeatability of H and DBH in different years were all lower than other sites, but the numerical values were all higher than 0.7977. The repeatability of H and DBH varied from 0.7977 to 0.9048 and 0.8232 to 0.9035 respectively from 2007 to 2010.

3.5 Age trends in stability and genetic gain

DBH of the fifth year and estimated regression coefficients bi of 30 poplar clones were shown in *Table 8.* Clone P49 ($b_i = 0.94$) and P42 ($b_i = 1.09$) represented clones of average stability as defined by FINLAY (1963). Clone P22 represented a clone of high stability ($b_i = -0.14$). It performed relatively better on poor sites but its overall performance was poor (DBH5 = 8.82 cm). The unstable clone P98 ($b_i = 2.62$) performed relatively better on good sites.

The expected genetic gains for four sites combined were not shown because there was no repeatability for H and DBH due to non-significant difference among clones. *Fig. 1* presented the expected genetic gain resulting from different ages at each site. The genetic gain of DBH was higher than H at the same site. The genetic gain of DBH at FF was higher than other sites from 2007 to 2010, and the variation ranges from 23.69% to 34.63%. From 2008 to 2010, the genetic gain of DBH

DOI:10.1515/sg-2013-0023
edited by Thünen Institute of Forest Genetics

Table 6. – Correlation coefficient (r) between H and D in different years 4 sites.

site	character	year	WX								FF							
			H				DBH				H				DBH			
			2007	2008	2009	2010	2007	2008	2009	2010	2007	2008	2009	2010	2007	2008	2009	2010
GX	H	2008	0.745**	0.644**	0.785**	0.727**	0.485**	0.527**	0.534**	0.563**	0.771**	0.861**	0.875**	0.825**	0.630**	0.709**	0.739**	0.756**
		2009	0.734**	0.810**	0.778**	0.761**	0.511**	0.571**	0.580**	0.621**	0.748**	0.850**	0.865**	0.808**	0.665**	0.729**	0.758**	0.775**
		2010	0.679**	0.753**	0.725**	0.822**	0.469**	0.565**	0.587**	0.629**	0.862**	0.959**	0.963**	0.965**	0.775**	0.826**	0.847**	0.866**
	DBH	2008	0.707**	0.668**	0.680**	0.616**	0.796**	0.819**	0.829**	0.820**	0.673**	0.771**	0.764**	0.776**	0.878**	0.908**	0.914**	0.919**
		2009	0.710**	0.709**	0.729**	0.669**	0.783**	0.840**	0.835**	0.847**	0.679**	0.794**	0.786**	0.790**	0.880**	0.915**	0.927**	0.932**
		2010	0.727**	0.726**	0.707**	0.618**	0.725**	0.806**	0.815**	0.845**	0.788**	0.881**	0.862**	0.885**	0.928**	0.974**	0.983**	0.988**
NY	H	2006	0.458*	0.328	0.312	0.319	0.283	0.217	0.196	0.203	0.447*	0.393*	0.378*	0.324	0.267	0.289	0.302	0.303
		2007	0.706**	0.645**	0.582**	0.543**	0.511**	0.488**	0.450*	0.453*	0.713**	0.713**	0.681**	0.613**	0.546**	0.595**	0.607**	0.616**
		2008	0.700**	0.713**	0.695**	0.644**	0.516**	0.572**	0.556**	0.571**	0.755**	0.765**	0.768**	0.710**	0.634**	0.706**	0.716**	0.725**
		2009	0.708**	0.794**	0.718**	0.752**	0.544**	0.655**	0.659**	0.689**	0.743**	0.831**	0.796**	0.772**	0.700**	0.772**	0.790**	0.803**
		2010	0.646**	0.746**	0.772**	0.803**	0.444*	0.577**	0.599**	0.633**	0.711**	0.806**	0.793**	0.812**	0.642**	0.732**	0.758**	0.774**
	DBH	2006	0.673**	0.484**	0.464**	0.445*	0.684**	0.624**	0.567**	0.544**	0.592**	0.573**	0.570**	0.542**	0.679**	0.677**	0.674**	0.668**
		2007	0.654**	0.632**	0.637**	0.610**	0.696**	0.751**	0.740**	0.730**	0.715**	0.739**	0.692**	0.682**	0.809**	0.845**	0.836**	0.832**
		2008	0.656**	0.647**	0.658**	0.628**	0.715**	0.780**	0.769**	0.766**	0.678**	0.735**	0.692**	0.692**	0.805**	0.857**	0.855**	0.855**
		2009	0.699**	0.687**	0.711**	0.678**	0.738**	0.809**	0.801**	0.805**	0.673**	0.752**	0.725**	0.729**	0.819**	0.875**	0.873**	0.889**
		2010	0.688**	0.697**	0.727**	0.692**	0.710**	0.791**	0.793**	0.804**	0.642**	0.734**	0.712**	0.713**	0.785**	0.849**	0.863**	0.883**

Table 7. – Correlation coefficient (r) between H and D in different years in WX and FF.

site	character	year	WX							FF							
			H			DBH				H				DBH			
			2008	2009	2010	2007	2008	2009	2010	2007	2008	2009	2010	2007	2008	2009	2010
wx	H	2007	0.866**	0.728**	0.629**	0.724**	0.758**	0.694**	0.653**	0.690**	0.706**	0.720**	0.666**	0.651**	0.652**	0.670**	0.686**
		2008	1	0.920**	0.840**	0.678**	0.764**	0.757**	0.767**	0.641**	0.755**	0.782**	0.696**	0.627**	0.653**	0.685**	0.701**
		2009		1	0.971**	0.575**	0.711**	0.769**	0.813**	0.536**	0.680**	0.718**	0.645**	0.596**	0.641**	0.679**	0.698**
		2010			1	0.489**	0.644**	0.701**	0.813**	0.469**	0.592**	0.633**	0.556**	0.519**	0.561**	0.596**	0.614**
	DBH	2007				1	0.926**	0.873**	0.800**	0.509**	0.534**	0.506**	0.500**	0.767**	0.727**	0.713**	0.713**
		2008					1	0.972**	0.935**	0.531**	0.605**	0.588**	0.582**	0.819**	0.808**	0.803**	0.803**
		2009							0.983**	0.478**	0.597**	0.586**	0.586**	0.814**	0.818**	0.822**	0.824**
		2010								0.478**	0.609**	0.609**	0.603**	0.801**	0.811**	0.823**	0.829**
ff	H	2007									0.928**	0.867**	0.859**	0.790**	0.790**	0.765**	0.762**
		2008										0.971**	0.958**	0.825**	0.850**	0.866**	0.870**
		2009											0.975**	0.777**	0.824**	0.863**	0.861**
		2010												0.808**	0.862**	0.885**	0.896**
	DBH	2007													0.968**	0.940**	0.930**
		2008														0.991**	0.984**
		2009															0.998**

Note: In table 5 to 7, ** Correlation is significant at the 0.01 level (2-tailed). * Correlation is significant at the 0.05 level (2-tailed).

was the lowest, varying from 21.42% to 31.43%. The genetic gain of height at FF was higher than others from 2007 to 2010, and the range of variation was from 18.37% to 32.70%. The genetic gain of H at NY was lower than other sites from 2006 to 2010 and varied from 11.01% to 26.68%. The genetic gain increased with tree growth because the selection difference and repeatability increased year by year.

3.6 Age-age phenotype correlation

Correlation coefficients among all the combinations of traits and ages were shown in table 5–7. All coefficients were significant at each site, and the coefficients increased with the tree growth. The correlation coefficients between H and D varied from 0.779 to 0.875 for GX from 2008 to 2010. But at WX and FF, the coefficients varied from 0.724 to 0.813 and 0.790 to 0.896 respectively from 2007 to 2010. The data indicated the most times was NY form 2006 to 2010. The correlation coefficients between H and D were varied from 0.713 to 0.890. Among different sites, the correlation coefficients of H to H and D to D were also increased with age. It indicated that the environment influence was less and less significant and genetic impact was the dominating factor. But as the age difference increased, the coefficients decreased. At NY, the correlation coefficient between H in 2006 and H in 2010 was 0.418, and 0.778, 0.908 between H in 2007 and 2008 with H in 2010. The

DOI:10.1515/sg-2013-0023
edited by Thünen Institute of Forest Genetics

Table 8. – Stability and adaptability parameters of poplar clones in four sites.

Clone	Average DBH	b_i	Average DBH in FF	Average DBH in WX	Average DBH in GX	Average DBH in NY
P 107	15.13	2.12	16.48	14.89	16.18	13.31
P 106	14.78	1.93	16.52	12.90	15.99	13.56
P 46	13.62	-0.33	14.04	13.69	12.74	14.00
P 105	13.33	1.90	14.63	13.11	13.96	11.63
P 104	12.68	0.77	13.54	10.82	13.79	12.56
P 42	11.61	1.09	12.27	12.94	11.06	10.19
P 83	11.57	1.25	12.89	10.68	11.98	10.74
P 23	11.45	1.37	12.10	11.21	12.29	10.21
P 78	11.43	2.26	12.69	11.84	12.03	9.17
P 49	10.98	0.94	12.31	8.94	11.87	10.81
P 50	10.79	0.51	11.08	10.82	10.98	10.30
P 26	10.65	1.81	11.88	9.64	11.82	9.27
P 87	10.40	-0.19	10.86	9.16	10.56	11.03
P 85	10.26	-0.51	10.07	10.40	9.86	10.71
P 30	10.15	1.66	10.89	10.61	10.68	8.41
P 98	9.74	2.62	11.29	9.94	10.51	7.20
P 28	9.46	1.44	10.12	9.51	10.13	8.06
P 20	9.40	1.69	10.66	8.02	10.66	8.24
P 103	9.09	2.28	10.38	9.02	10.00	6.94
P 69	8.91	0.65	9.41	8.78	9.11	8.36
P 53	8.84	0.73	9.06	9.19	9.11	8.02
P 22	8.82	-0.14	8.58	9.20	8.69	8.81
P 101	8.80	1.89	9.56	9.50	9.40	6.76
P 88	8.74	1.74	10.23	8.39	9.08	7.27
P 64	8.10	0.58	8.09	8.47	8.46	7.39
P 99	7.58	0.46	6.92	9.21	7.66	6.53
P 76	7.10	0.35	6.72	7.47	7.63	6.58
P 67	7.04	0.33	6.66	8.50	6.80	6.21
P 77	6.27	-0.41	5.11	9.16	5.13	5.67
P 63	5.31	-0.80	4.23	5.74	5.41	5.86

correlation coefficient reached 0.972 especially between H of 2009 and 2010. The correlation coefficients between DBH also increased from the coefficients of 2006 with 2010 (0.736) to 2009 with 2010 (0.993). It all demonstrated that as the age went up, the measurement data were more accurate for prediction of the growth, and will be conducive to early selection.

4. Discussion

4.1 Variation among sites

Site effects reflect the response of tree to the combined effects of edaphic as well as local and regional climatic conditions (PLIURA et al., 2007). Significant site effects for growth rate had been reported previously for poplar clones (PLIURA, et al., 2007). The large differences in H and DBH growth in the first year may be due to the plants under planting shock at the start. The evaluation of vigor of aspen clones should be performed at 2 years after establishment in the field, then the data would be more reliable (YU et al., 2003). In this research, the poplar clones grew 5 years in different trials. From the repeatability we could conclude that the effects of environment were pimping in the last three years, so the estimation for clone was significant.

4.2 PCV, GCV and repeatability

The extent of variability in the breeding population was estimated by measuring different population parameters including phenotypic coefficient of variation (PCV) and genotypic coefficient of variation (GCV). The range for PCV for H and DBH in present study was in agreement with what observed in a previous study by PLIURA (PLIURA et al., 2007), in which PCV of H and DBH ranged from 10.05% to 16.40% and 20.00% to 31.60% respectively for poplar hybrid clones in four trials in Quebec. GCV is a more appropriate parameter than heritability for comparison of genetic variation and

DOI:10.1515/sg-2013-0023
edited by Thünen Institute of Forest Genetics

ability to respond to selection (HOULE, 1992). GCV was considerably lower than respective PCV. But all GCV estimates exceed 14.43%, which was higher than the results of DHILLOON et al. (2012). The observed high PCV and GCV were also showing little environment effect on the expression of H and DBH at different sites. Repeatability magnitude indicated the reliability with which the genotype will be recognized by its phenotype expression. In this research, the estimates of repeatability for growth traits at clone mean level ranged from 0.7977 to 0.9859, which is in general agreement with the studies of KIEN (KIEN et al., 2008) and LAMBETH (LAMBETH et al., 1994). High repeatability estimates indicated that the selection for these traits will be effective and less influenced by environmental effects (MANIEE, 2009).

4.3 Phenotypic correlations

Determining trends in age-age correlation is relatively simple for growth and yielding traits, requiring only patience and repeated assessment. Shortening the breeding cycles of tree through early selection can produce more genetic gain per unit year if there is a strong genetic correlation between early and mature traits (Goncalves, 2005). There were many reported age-age correlations for growth in conifers and *Hevea* species (GONCALVES, 2005; MATHESON et al., 1994; GONCALVES, 1998; KING, 1991). In poplar, KUMAR (2000) investigated the growth traits of 60 *P. deltoides* clones, and found out that the correlation coefficients increased with age and an early selection of poplar clones for rotation age of 6 years could be done effectively at age 4. The result provides a credible method for poplar tree early selection (KUMAR, 2000). In this study, there was a high correlation between H and DBH in the four different trials although the site means were very different (*Table 4*). The strong age-to-age correlations were also significant for H and DBH at different sites, especially after 2007. All coefficients appear positively significant, indicating that the selection for poplar clones was practical and predicting the yield was feasible.

4.4 Stability and genetic gain

It is particularly essential to include the gene × environment interaction in the analysis of variance over locations. In this study, the site × clone interaction was highly significant and each clone represented differentia among four trials. Similar results were found in researches of SARA (2006) and KARACIC (2006). Most breeding programs aimed at the development of widely adapted clones. However when genotype × environment interaction presents, material may be deployed to an environments which it is not best suited (ZOBEL and TALBERT, 1984). We found that clone P49 had average stability, but its DBH and H were not superior to others. So clone P49 may not be the best clone for breeding. Rational approach to selection of suitable clones is to choose which are not only superior in performance but also stable over a wide range of environments (YU et al., 2003). From *Table 8*, clones P107, P106, P46, P105, P104 and P42 had the best performances among the four sites. However, P107, P109 and P105 had low stability because of high values of b_i. Clones P107, P106, and P105 performed better at FF and GX than at NY and WX.

Genetic gains increased as the age went up, probably because the variation and repeatability among clones increased with the tree growth. High PCV and GCV may also cause higher genetic gain of DBH than that of H. Furthermore, the genetic gains were not equal among different sites. Perhaps the primary reason is that same clones have different performance in different environment, and ultimately significant genetic × environment interaction presented. In tree breeding and selection, we should better pay more attention to the environmental act on genotype, and then can comprehend the phenotype more clearly.

5. Conclusion

In conclusion, genetic gains in poplar clones differed between trees planted at different sites, which indicated differences in the impact of environment on the poplar genotype. Variation between clones may increase as a consequence of growth, with high PCV and GCV affecting the DBH compared to H. Further investigations are needed to determine the role of environment for tree breeding programs and genetic selection.

6. Acknowledgements

We acknowledge the Innovation Project of State Key Laboratory of Tree Genetics and Breeding (Northeast Forestry University). (No. 2013B09) support for the research, and we are also grateful to Professor RONGLING WU for critical reading of the manuscript.

7. References

BRADSHAW, H. D., R. CEULEMANS, J. DAVIS and R. STETTLER (2000): Emerging model systems in plant biology: poplar (*Populus*) as a model forest tree. Journal of Plant Growth Regulation. **19**: 306–313.

COOKE, J. E. and S. B. ROOD (2007): Trees of the people: the growing science of poplars in Canada and worldwide. Canadian Journal of Botany. **85**: 1103–1110.

DHILLON, G., A. SINGH, D. S. SIDHU and H. S. BRAR (2012): Variation among poplar clones for growth and crown traits under field conditions at two sites of northwestern India. Journal of forestry research. DOI: 10.1007/s11676-012-0269-x.

FANG, S. Z., X. Z. XU, S. X. LU and L. Z. TANG (1999): Growth dynamics and biomass production in short-rotation poplar plantations: 6-year results for three clones at four spacings. Biomass and Bioenergy. **17**: 415–425.

Finlay, W. K. and G. N. Wilkinson (1963): The analysis of adaptation in a plant breeding program. Aust. J. Agric. Res. **14**: 742–754.

GAMBLES, R. L. and L. ZSUFFA (1984): Conversion and use of poplar and willow biomass for food, forage and energy in North America. International Poplar Commission, October, Room, Fo: MISC/84/15.

GONCALVES, P., N. BORTOLETTO, A. CARDINAL, L. GOUVEA, R. COSTA and M. MORAES (2005): Age-age correlation for early selection of rubber tree genotypes in Sao Paulo State, Brazil. Genetics and Molecular Biology. **28**: 758–764.

DOI:10.1515/sg-2013-0023
edited by Thünen Institute of Forest Genetics

Goncalves, P., N. Bortoletto, F. Fonseca, O. Bataglia and A. Ortolani (1998): Early selection for growth vigour in rubber tree genotypes in Northwestern Sao Paulo State (Brazil). Genet Mol Biol. **21**: 515–521.

Hai, P. H., G. Jansson, C. Harwood, B. Hannrup and H. H. Thinh (2008): Genetic variation in growth, stem straightness and branch thickness in clonal trials of *Acacia auriculiformis* at three contrasting sites in Vietnam. For Ecol Manag **255**: 156–167.

Hansen, J. K. and H. Roulund (1996): Genetic parameters for spiral grain, stem form, Pilodyn and growth in 13 year old clones of Sitka Spruce (*Picea sitchensis* (Bong.) Carr.). Silvae Genet **46**: 107–113.

Houle, D. (1992): Comparing evolvability and variability of quantitative traits. Genetics. **130**: 195–204.

Karacic A. and M. Weih (2006): Variation in Growth and resource utilization among eitht poplar clones grown under different irrigation and fertilization fegimes in Sweden. Biomass and Bioenergy. **30**: 115–124.

Kien, N. D., G. Jansson, C. Harwood, C. Almqvist and H. H. Thinh (2008): Genetic variation in wood basic density and Pilodyn penetration and their relationships with growth, stem straightness and branch size for *Eucalyptus urophylla* S. T. Blake in Northern Vietnam. NZ J For Sci. **38**: 160–175.

King, J. N. and R. D. Burdon (1991): Time Trends in inheritance and projected efficiencies of early in a large 17-year old progeny test of *Pinus radiata*. Can J For Res. **21**: 1200–1207.

Kumar, D. and N. B. Singh (2001): Age-age correlation for early selection of clones of *Populus* in India. Silvae Genetica. **50**: 3–4.

Lambeth, C. C., M. Endo and J. Wright (1994): Genetic analysis of 16 clonal trials of *Eucalyptus* grandis and comparisons with seedlings checks. For. Sci. **40**: 397–411.

Li, J.Y., A. M. Brunner, R. Meilan and S. H. Strauss (2008): Stability of transgenes in trees: expression of two reporter genes in poplar over three field seasons. Tree Physiology. **29**: 299–312.

Liu, Z. J. and D. I. Dickmann (1996): Effects of water and nitrogen interaction on net photosynthesis, stomatal conductance, and water-use efficiency in two hybrid poplar clones. Physiologia Plantarum. **97**: 507–512.

Lynch, M. and B. Walsh (1998): Genetics and analysis of quantitative traits. Sinauer, Sunderland. Mass. pp: 105.

Matheson, A., D. J. Spencer and D. Magnussen (1994): Optimum age for selection in pinus radiata using basal area under bark for age:age correlations. Silvae Gen. **43**: 352–357.

Maniee, M., D. Kahrizi and R. Mohammadi (2009): Genetic variability of some morphophysiological traits in durum wheat (*Triticum turgidum* var. durum). Journal of Applied Sciences. **9**: 1383–1387.

Marron, N. and R. Ceulemans (2006): Genetic variation of leaf traits related to productivity in a *Populus deltoides* × *Populus nigra* family. Can. J. Forest Res. **36**: 390–400.

Marron, N., L. Ricciotti, C. Bastien, L. Beritognolo, M. Gaudet, I. Paolucci, F. Fabbrini, F. Salani, S. Y. Dillen, R. Ceulemans, M. Pinel, G. Taylor, G. S. Mugnozza and M. Sabatti (2010): Plasticity of growth and biomass production of an intraspecific *Populus alba* family grown at three sites across Europe during three growing seasons. Can. J. For. Res. **40**: 1887–1903.

Nicolas, M., Y. Sophie and C. Reinhart (2007): Evaluation of leaf traits for indirect selection of high yielding poplar hybrids. Environmental and Experimental Botany. **61**: 103–116.

Pliura, A., S. Y. Zhang, J. Mackay and J. Bousquet (2007): Genotypic variation in wood density and growth traits of poplar hybrids at four clonal trials. For Ecol Manag. **238**: 92–106.

Sara, O., P. Andrej, G. Zoran, I. Petar and R. Nenad (2006): Results of poplar Clone testing in field experiments. Genetika. **38**: 259–266.

Schreiber, S. G., U. G. Hacke, A. Hamann and B. T. Thomas (2011): Genetic variation of hydraulic and wood anatomical traits in hybrid poplar and trembling aspen. New phytologist. **190**: 150–160.

Seyed, M. S. (2011): Estimation of genetic parameters related to morphological traits in poplar clones. Americian Journal of Scientific Research. **27**: 105–110.

Yu, Q. B. and P. Pulkinen (2003): Genogype-environment interaction and stability in growth of aspen hybrid clones. Forest Ecology and Management. **173**: 25–35.

Zobel, B. J. and J. T. Talbert (1984): Applied forest tree ismprovement. John Wiley and Sons, Inc. pp: 505.

DOI:10.1515/sg-2013-0023
edited by Thünen Institute of Forest Genetics
