# Supplementary material for: Provenance and family variations in early growth of Manchurian walnut (Juglans mandshurica Maxim.) and selection of superior families
Source: PLoS One. 2024 Mar 7;19(3):e0298918. doi: 10.1371/journal.pone.0298918 (PMC10919699; doi:10.1371/journal.pone.0298918)
Supplement: S1 File — (ZIP) [file pone.0298918.s004.zip › Analysis of genetic effects on a complete diallel cross test of Pinus koraiensis.pdf]

# Euphytica


## Analysis of genetic effects on a complete diallel cross test of Pinus koraiensis

--Manuscript Draft--

| | |
|---|---|
| **Manuscript Number:** | EUPH-D-18-00847R1 |
| **Full Title:** | Analysis of genetic effects on a complete diallel cross test of Pinus koraiensis |
| **Article Type:** | Original Article |
| **Keywords:** | Pinus koraiensis; full-sib family; variation; heritability; genetic gain |
| **Corresponding Author:** | xiyang zhao, ph.D.<br>Northeast Forestry University<br>harbin, CHINA |
| **Corresponding Author Secondary Information:** | |
| **Corresponding Author's Institution:** | Northeast Forestry University |
| **Corresponding Author's Secondary Institution:** | |
| **First Author:** | Deyang Liang |
| **First Author Secondary Information:** | |
| **Order of Authors:** | Deyang Liang |
| | Biying Wang |
| | Shuanglin Song |
| | Jingyuan Wang |
| | Lianfu Wang |
| | Qi Wang |
| | Xuanbai Ren |
| | xiyang zhao, ph.D. |
| **Order of Authors Secondary Information:** | |
| **Funding Information:** | National Key Research and Development Program of China (2017YFD0600601) — Professor xiyang zhao |
| **Abstract:** | To evaluate elite Pinus koraiensis materials, 34 full-sib P. koraiensis families were taken as examples. Tree height and diameter at breast height were assessed. Variance analysis results showed that familial variance sources for different traits in different growth years were extremely significantly different. The average phenotypic variation coefficients of tree height, diameter at breast height and volume among families in different growth years ranged from 7.57 to 15.70%, 10.37 to 12.89% and 24.44 to 28.13%, respectively. The family heritabilities of all traits ranged from 0.910 to 0.990, which are high values. A significant and positive correlation was observed among all traits, with values ranging from 0.43 to 0.99. According to general and special combining ability analyses, female parents F4 and F2 and male parents M7 and M13 had high levels of general combining ability for all the traits. Families PK05 (F9 ×M14) and PK06 (F2×M14) showed the highest and the lowest special combining ability values in all the traits. Using the method of comprehensive multiple-traits to evaluate the families by traits in the 18th growth year at the rate of less than 10%, families PK40, PK05 and PK22 were selected as elite families, where the genetic gains in tree height, diameter at breast height and volume were 14.43%, 11.29% and 24.72%, respectively. This study provides the materials and theoretical basics for the improvement of seeds for orchard building. |
| **Response to Reviewers:** | Dear Editor<br>We are honored to resubmit our manuscript to your journal, Euphytica. Thank you very |


Powered by Editorial Manager® and ProduXion Manager® from Aries Systems Corporation

| | |
|---|---|
| | much for your nice suggestions and comments on the improvement of our manuscript. The manuscript entitled “Analysis of genetic effects on a complete diallel cross test of Pinus koraiensis” is the first revised version of our previous manuscript, and it has been revised according to thoughtful suggestions and comments from reviewers. We did our best to modify the manuscript and the modifications were as follows.<br>(1)The language should be polished.<br>First of all, thanks for your advice. The language had been polished.<br>(2) Due to the importance of provenance for mating evaluating in coniferous tree genetic improvement. In discussion, the provenances of the selected P.koraiensis parents should be analysed and discussed.<br>First of all, thanks for your advice. The materials of the study were only selected from the local P.koraiensis , so there was only one provenance. We have made modifications and explanations in the materials and experimental design.<br>(3) The height heritability results in Table 3 are greater than 0.9 even 0.99 which performed like qualitative traits' heritability approximately. While the height of trees is supposed to be quantitative trait generally.<br>First of all, thanks for your advice. After consideration, the reason for this may be due to the high planting density of the materials, the early growth is mainly based on tree height. Meanwhile, there were a mass of trees in the same family, the tree height difference within the family is small, all above leaded to the high tree heritability.<br>(4) In the Table 4 the correlation coefficients of height of each year was calculated by itself in every block which performed as 0.99. It's meaningless and known as 1.<br>First of all, thanks for your advice. We have deleted the correlation coefficients of height, DBH and V within each year in the table and made modifications in the manuscript.<br>(5) In the Table 6, the combination informations repeated in every rows, which are unnecessary.<br>First of all, thanks for your advice. The problem had been solved. |

Powered by Editorial Manager® and ProduXion Manager® from Aries Systems Corporation

Click here to access/download;Manuscript;manuscript 2019.3.29.docx
Click here to view linked References

# Analysis of genetic effects on a complete diallel cross test of *Pinus koraiensis*

Deyang Liang[1], Biying Wang[1], Shuanglin Song[2], Jingyuan Wang[2], Lianfu Wang[2], Qi Wang[2], Xuanbai Ren[3], Xiyang Zhao[1*]

1 State Key Laboratory of Tree Genetics and Breeding, School of Forestry, Northeast Forestry University, Harbin, 150040, P. R. China

2 Forest Cultivation Center, Linjiang Forestry Bureau of Jilin, Linjiang, 134600, P. R. China

3 Forestry Technical Advice Station of Jilin Province, Changchun, 130000, P. R. China

Corresponding author:

✉Xiyang Zhao: Tel: +0086-451-82192225; Fax: +0086-451-82192225;

Email: zhaoxyphd@163.com.

The first three authors contributed equally to this work.

**Abstract**: To evaluate elite *Pinus koraiensis* materials, 34 full-sib *P. koraiensis* families were taken as examples. Tree height and diameter at breast height were assessed. Variance analysis results showed that familial variance sources for different traits in different growth years were extremely significantly different. The average phenotypic variation coefficients of tree height, diameter at breast height and volume among families in different growth years ranged from 7.57 to 15.70 %, 10.37 to 12.89 % and 24.44 to 28.13 %, respectively. The family heritabilities of all traits ranged from 0.910 to 0.990, which are high values. A significant and positive correlation was observed among all traits, with values ranging from 0.43 to 0.99. According to general and special combining ability analyses, female parents F4 and F2 and male parents M7 and M13 had high levels of general combining ability for all the traits. Families PK05 (F9 ×M14) and PK06 (F2×M14) showed the highest and the lowest special combining ability values in all the traits. Using the method of comprehensive multiple-traits to evaluate the families by traits in the 18$^{th}$ growth year at the rate of less than 10 %, families PK40, PK05 and PK22 were selected as elite families, where the genetic gains in tree height, diameter at breast height and volume were 14.43 %, 11.29 % and 24.72 %, respectively. This study provides the materials and theoretical basics for the improvement of seeds for orchard building.

Keywords: *Pinus koraiensis*; full-sib family; variation; heritability; genetic gain

**Introduction:**

*Pinus koraiensis* is one of the most important native and economically valuable tree species in northeastern China, as well as in the far eastern region of Russia and the Korean peninsula (Barnes et al. 1992). *P. koraiensis* is fond of light but grows normally under poor light (Yang, 2013). For centuries, *P. koraiensis* has been extensively used as a major source of excellent timber and for natural remedies and edible pine nuts because of its excellent wood properties and the nutritional value of the pine nuts (Imbs et al. 1998; Nergiz et al. 2004). Because *P. koraiensis* serves as an important forest species, many studies have been conducted, analyzing its growth traits (Han et al. 2015; Wang et al. 2002), leaf traits (Liu et al. 2015), flowering characteristics (Wang et al. 2007), wood properties (Liang et al. 2016), photosynthetic index (Sun et al. 2016), sap flow (Moon et al. 2016), the nutritional components of the seed (Azad et al., 2009) and net primary productivity (Fang et al. 2016).

Because of the difficulty of cuttage, tissue culture and other asexual propagation methods in *P. koraiensis* (Zhao et al., 2018), seed orchards have been the principal method to improve *P. koraiensis*. In northeast China, several seed orchards were established in the 1960's, and a large number of seeds have been produced at each seed orchard since the beginning of the 21st century (Xia et al. 2016). Although several variance analysis studies were done on the selection of *P. koraiensis* families (Zhang et al., 2003; Zhao et al., 2018), all the materials were focused on half-sib families. The effects of the male parent were seldom investigated and analyzed. In this study, 34 *P. koraiensis* full-sib families were taken as materials and tree height and diameter at breast height in different growth years were investigated and evaluated. This research can provide the materials and theoretical basics to improve seed orchards and establish second-generation seed orchards.

## Materials and methods

### Experimental sites

The experiments were conducted at the Naozhi Forestry Seed Orchard (41°05'N, 126°06'E) located in the western hillsides of Changbai Mountain, Linjiang City, Jilin Province of northeastern China. The temperate monsoon climate of the region features an average frost-free season, mean annual precipitation, mean annual temperature and mean elevation of 128 days, 744 mm, 8.2°C and 510 m, respectively.

### Materials and experimental design

Thirty-four *P. koraiensis* full-sib families were taken as experimental materials (Table 1), all materials were selected from the local provenance. Control pollination was conducted in the spring of 1998. Seeds were collected in autumn 1999 and were sown in 2000. The experimental design consisted of 3 blocks planted in 2004 with four-year-old seedlings of each family using a completely randomized block design in row plots containing 12 trees at 2 ×3 m spacing.

### Method

Tree height (H) and diameter at breast height (DBH) of all living and unbroken plants were measured in autumn 2009 (the 10$^{th}$ growth year), 2011 (the 12$^{th}$ growth year), 2013 (the 14$^{th}$ growth year), 2015 (the 16$^{th}$ growth year) and 2017 (the 18$^{th}$ growth year). The volume (V) of a single tree was calculated using the method of Zhang (2016) as follows:

$$V=(H+3)g_{1.3}f \quad (1)$$

where $f$ is the experimental form factor of 0.33.

### Statistical analysis

Statistical analysis was carried out using SPSS 19.0 (Statistical Package for the Social Sciences) software. The significance of fixed effects was tested by analysis of

variance (ANOVA) *F*-tests. Variation among families in the same year was analyzed by ANOVA according to Hansen (1997) using Eq. (2):

$$y_{ij} = \mu + \alpha_i + \beta_j + \alpha\beta_{ij} + \varepsilon_{ij} \quad (2)$$

where $y_{ij}$ is the performance of an individual of family *i* within block *j*, $\mu$ is the overall mean, $\alpha_i$ is the family effect ($i$ = 1,…,34), $\beta_j$ is the block effect ($i$ = 1,…,3), $\alpha\beta_{ij}$ is the random effect of family *i* within block *j* and $\varepsilon_{ij}$ is the random error.

Genotypic ($\sigma_\gamma^2$) and phenotypic ($\sigma_p^2$) variances were calculated referring to ANOVA using the mean square and the following formulas (Metougui et al., 2017):

$$\sigma_\gamma^2 = \frac{A-B}{r}, \quad (3)$$

$$\sigma_p^2 = \sigma_\gamma^2 + \sigma_\varepsilon^2, \quad (4)$$

Where $\mathbf{A} = \sigma_\varepsilon^2 + r\sigma_\gamma^2$, is the Mean Square between families, $B = \sigma_\varepsilon^2$ is the Mean Square within families, and **r** is the number of replications.

The general combine ability (GCA) and special combine ability (SCA) were calculated using the following formulas according to Chen (2005):

$$GCA_i = \bar{X}_{i.} - \bar{X}_{..} \quad (5)$$

$$SCA_{ij} = X_{ij} - \bar{X}_{..} - GCA_i - GCA_j \quad (6)$$

The phenotypic Coefficient of Variation PCV (%) was estimated by the formula below (Jonah et al., 2013):

$$PCV = \frac{\sqrt{\sigma_p^2}}{\bar{X}} x100 \quad (7)$$

Where $\bar{X}$ is the mean value of a growth characteristic among all families.

The estimation of heritability of a character for the families was calculated using Eq. (8) (Hansen et al. 1997):

$$h^2 = \frac{\sigma_A^2}{\sigma_A^2 + \sigma_b^2 + \sigma_e^2} \quad (8)$$

where $\sigma_A^2$ is the additive genetic variance component between families, $\sigma_b^2$ is the block variance and $\sigma_e^2$ is the error variance component.

The phenotypic correlation coefficient was calculated using Eq. (9) (Fernando et al. 2016):

$$r_A(xy) = \frac{\sigma_{a(xy)}}{\sqrt{\sigma_{a(x)}^2 \cdot \sigma_{a(y)}^2}} \quad (9)$$

where $\sigma^2_{a(xy)}$ denotes the phenotypic covariance between traits of $x$ and $y$, and $\sigma^2_{a(xy)}$ and $\sigma^2_{a(y)}$ denote the phenotypic variance of trait $x$ and $y$, respectively.

The comprehensive evaluation was conducted with the method of $Q_i$ value evaluation where $Q_i$ was calculated using Eq. (10) (Liu et al. 2015):

$$Q_i = \sqrt{\sum_{j=1}^{n} a_i} \quad (10)$$

Where $a_i = X_{ij}/X_{j\max}$, $Q_i$ is the comprehensive valuation value of family $i$, $X_{ij}$ is the mean of a character, $X_{j\max}$ is maximum value of a character, and $n$ is the number of the evaluation index.

The estimated genetic gain was calculated using Eq. (11) (Silva et al. 2008):

$$\Delta G = (h^2 W / \bar{X}) \times 100\% \quad (11)$$

where $\Delta G$ is the genetic gain of a trait for the families, $h^2$ is the heritability of the trait, $W$ is the difference of the average values of the traits between the selected families and all families, namely, the selection difference, and $\bar{X}$ is the mean value of the growth characteristic among all families.

## Results

**ANOVA**

The ANOVA results are shown in Table 2. Extremely significant differences among families in different growth years are apparent. Therewasalso a significant difference among blocks in different traits at the $10^{th}$ growth year, but except for trait DBH at the $12^{th}$ growth year, there were no significant differences among blocks for any traits. Most family × block effects showed no significant differences.

**Genetic and variation parameters**

The genetic and variation parameters of H, DBH and V among different families are shown in Table 3. The average of H, DBH and V ranged from 5.31 to 9.92 m, from 2.87 to 5.11 cm and from 0.0018 to 0.0090 $m^3$ (from $10^{th}$ to $18^{th}$), respectively. PCVs of H, DBH and V ranged from 7.57 to 15.70 %, 10.37 to 12.89 % and 24.44 to 27.78 %, respectively, and the values showed decreased trends as age increased. The heritability values of all the traits in different growth years were all higher than 0.91.

**Correlation analysis**

Correlations among H, DBH and V in different growth years are shown in Table 4. In the same growth year, the correlation coefficients between H with DBH, H with V, and DBH with V ranged from 0.46 (the $16^{th}$ growth year) to 0.51 (the $12^{th}$ growth year), 0.65 (the $10^{th}$ growth year) to 0.73 (the $18^{th}$ growth year), and 0.85 (the $10^{th}$ growth year) to 0.93 (the $18^{th}$ growth year), respectively.In all, there were extremely significant positive correlations among all growth traits (0.43 to 0.94) in the same or different growth year.

**General combining ability**

The results of the general combining ability of different parents are shown in Table 5. Female parents F 4 and F 2 had a high level of general combining ability in all the traits, while female 9 had the lowest level of general combining ability in all the traits.

Interestingly, female parent F 3 had a negative GCA value for trait H (-0.069), but the highest value for DBH (0.229) and a higher value for V (7.241×E-04). For male parents, M 7 and M 13 had a higher level of general combining ability in all the traits, while male 12 had the lowest level of general combining ability in all the traits. Also, interestingly, male parent M 11 had the highest GCA value for trait H, but negative GCA values for trait DBH (-0.107) and V (-5.882×E-06).

**Special combining ability**

The results of the special combining ability of different parents are shown in Table 6. For H, PK05 (F9×M14) had the highest SCA value (2.999), followed by PK40 (F2×M10, 1.783) and PK34 (F2×M13, 1.350). PK25 (F4×M7), PK33 (F3×M10), PK01 (F9×M13) and PK06 (F2×M14) had lower SCA values, of -1.002, -1.178, -1.381 and -2.592, respectively. For DBH, PK05 also had the highest SCA value (0.963), followed by PK37 (F4×M9, 0.696), and PK31 (F7×M10, 0.519). PK33 (F3×M10), PK49 (F4×M11), PK34 (F2×M13) and PK02 (F7×M9) had lower SCA values, of -0.587, -0.596, -0.628 and -1.320, respectively. For V, PK05 had the highest SCA value (5.06E-03), followed by PK37 (2.66E-03) and PK22 (F3×M7, 2.53E-03). PK14 (F2×M7), PK06 (F2×M14), PK33 (F3×M10) and PK02 (F7×M9) had lower SCA values, of -2.34E-03, -2.35E-03, -2.71E-03 and -4.32E-03, respectively.

**Multitrait comprehensive evaluation and genetic gain**

By the method of multiple-traits comprehensive, when the families were evaluated by H, DBH and V in 18$^{th}$ growth year, with the selected rate of 10 %, families PK40, PK05 and PK22 were selected as elite families (Table 7). The average H, DBH and Vof elite families were 11.46 m, 5.82 cm and 0.277 m$^3$, which were higher than the

total average by 15.46 %, 12.34 % and 24.96 %, and the genetic gain was 14.43 %, 11.29 % and 24.72 %, respectively.

## Discussion

### Analysis of variance

Understanding genetic variation within populations, families or clones is most important to understand the structure of experimental materials (Safavi et al. 2010), and ANOVA is one of the most important methods to estimate the extent of variability in breeding research (Zhao et al. 2015). In this study, there are significant differences among families in all investigated traits in different growth years, which indicates that it is feasible to identify excellent families (Liang et al., 2018). The source of variation in the blocks effect showed no significance after the 14$^{th}$ growth year in different traits, suggesting that the edaphic conditions of different blocks were similar, and the variation was mainly caused by genotype.

### Genetic variance parameters

The coefficients of variation and heritability are the most important genetic and variation parameters in tree genetics and breeding (He et al., 2011). PCV, which denoted the discrete degree of groups, was the important index for the selection of breeding programs (Ren et al. 2010). In this study, PCVs of the different traits investigated ranged from 7.57 to 28.13 %, indicating a wide range of phenotypic performance in different growth years. PCVs of H (7.57 % to 15.70 %) were similar to PCVs of DBH (10.37 % to 12.89 %). However, they were lower than PCVs of V (24.44 % to 28.13 %) in different growth years, which indicated that evaluating and selecting families using V at young ages was reasonable. On the other hand, from the 10$^{th}$ growth year to the 18$^{th}$ growth year, PCVs of H, DBH and V show a downward trend, similar to studies in *P. koraiensis* (Zhang et al. 2016) and Masson Pine (Zhang

et al. 2013), but different to a study of poplar (Zhao et al. 2013). It is possible that different tree species, environments and ages have different growth trends and patterns, leading to different variation trends in different growth years (Xia, et al., 2016). The heritability can reflect the ability of a trait to be passed on to offspring (Jiang et al., 2018). Traits are more stable when the heritability is higher, and the environmental effect will be smaller (Seyed, 2011). In this study, the heritabilities of all traits ranged from 0.910 to 0.990, which indicates high heritability, in agreement with other studies of *P. koraiensis* (Wang et al. 2007) and *Larix olgensis* (Yin et al. 2016). High PCV and heritability values indicated that it would be less influenced by environmental effects, and the results were beneficial for the selection and evaluation of elite families (Maniee et al. 1998).

**Correlation analysis**

Phenotypic correlation reflects the relationship among different traits, and the age–age correlation of growth traits is an important parameter for early selection (Goncalves et al. 2005). In this study, an extremely significant positive correlation ($P$<0.01) was found among H, DBH and V at different growth years, similar to research in *Pinus radiata* (Matheson et al. 1994) and poplar (Zhao et al. 2013), which indicated that a strong correlation also existed between H, DBH and V with relatively little influence from environmental factors. In addition, the phenotypic correlation coefficients among H, DBH or V in different growth years ranged from 0.43to 0.93, similar to but higher than research in *Castanopsis fissa* (Zhong et al. 2015), Masson pine (Zeng et al. 2013) and larch (Sun et al. 2016). The results may suggest that the slow growth rate of *P. koraiensis* and the higher age-age correlation coefficient could provide an effect indication of anticipated mature growth, which would be benefit for early selection of

slow growing tree species and could also shorten the breeding cycle (Jesus et al. 1992; Kumar et al. 2002; Greaves et al. 1997; Hannrup et al. 1998; Osorio et al. 2003).

**Combining ability analysis**

For trees, especially coniferous tree species, it is difficult for breeders to improve even a single trait by crossing because of long life cycles. General combining ability and specific combining ability are critically important to estimate crossbreeding effects in tree breeding (Owusu et al., 2017). The selection of appropriate parents can generate elite families that can enhance breeding effects and shorten the breeding period (Sluder. 1996). GCA is determined by the additive gene action of the parental genotype that is passed to the offspring (Qi et al., 2010). In this study, females F4 and F2 showed higher GCA values in traits H, DBH and V, which indicated that with these two clones as female parents, the offspring will grow well in these traits. The male parents M7 and M13 showed higher GCA values in different growth traits, which indicated that these two clones should be considered as male parents in the next step (Youngkoo et al., 2000). The SCA effect primarily reflects differences in the gene frequencies between the parents (Viana et al. 2013). It is the deviation of the expected result based on the average performance of the hybrid combination and its parents that is affected by the external environmental conditions. Although it could not be stably inherited between the parents and offspring, the results can guide the utilization of heterosis and the breeding of hybrids (Rumpunen et al., 2003). In this study, PK05 (F 9× M14) had the highest SCA values of all the investigated traits, which indicated that strong heterosis appeared with a cross of these two parents. F 9 and M14 could provide excellent materials for establishing a special hybrid seed orchard (Chen et al., 2005)

**Multitrait comprehensive evaluation and genetic gain**

There are many different methods for selecting elite materials according to different breeding targets (Zhao et al., 2016). P. *koraiensis* is one of the most important commercial tree species. Growth traits are the most important characteristic for evaluating *Pinus* families. Comprehensive evaluation methods have been developed to breed multiple characteristics to cultivate materials with a strong integrated ability (Yin et al., 2017). However, in a study of *Populus deltoids*, Guan et al. (2005) found that too many characteristics lead to low genetic gains in individual characteristics and unclear selection targets. In this study, three traits were investigated to evaluate each family. At the selected rate of 10 %, families PK40, PK05 and PK22 were selected as elite families, and the genetic gains of H, DBH and V were 14.43 %, 11.29 % and 24.72 %, respectively. These findings were lower than in other studies of *P. koraiensis* (Wang et al. 2007) . This may be due to the experimental materials, which were obtained by controlled pollination, and all the parents were superior materials that were selected from the natural distribution (Harry et al. 2011). The elite families that were selected could be used for improved variety applied and the elite parents could be used to establish the improved seed orchard (Wang et al., 2000).

**Conclusion**

Due to long breeding and rotation cycles, the selection of superior families in *P. koraiensis* is very important for efforts designed to enhance genetic gain and improve breeding efficiency. In this study, 34 *P. koraiensis* full-sib families were taken as materials and H, DBH and V of different single trees were investigated. Three elite families and 4 elite parents were selected based on combining ability analysis and comprehensive evaluation according to three growth traits. The elite parents could be used to establish improved seed orchards and special hybrid seed orchards. The elite families could be used for improved variety applying. This study can provide the

materials, methods and theoretical basics for the improvement of seed orchards and special hybrid seed orchard development.

**Acknowledgments**

We acknowledge The National Key Research and Development Program of China (2017YFD0600601) support for this research.

**References**

Azad MA, Bae JH, Kim JS, Lim JK, Song KS, Shin BS, Kim HR (2009) Isolation and characterization of a novel thermostable α-amylase from korean pine seeds. New Biotechnology 26(3):143-149.

Barnes BV, Xu ZB, Zhao SD (1992) Forest ecosystems in an old-growth pine-mixed hardwood forest of Changbai mountains preserve in northeastern China. Can. J. For. Res. 22: 144-160.

Chen XY, Shen XH (2005) Forest tree breeding. Higher Education Press.

Fang Q, Wang Y, Shao XM (2016) The effect of climate on the net primary productivity (NPP) of *Pinus koraiensis* in the Changbai Mountains over the past 50 years. Trees 30(1): 281-294.

Fernando PG, James HR, Oliver F, Randi F, Brian JS, Richard S, Robert S, Mark FD, David BN (2016) Analysis of the genetic variation in growth, ecophysiology, and chemical and metabolomic composition of wood of *Populus trichocarpa* provenances. Tree Genetics & Genomes 15: 965-972.

Goncalves P, Bortoletto N, Cardinal A, Gouvea L, Costa R, Moraes M (2005) Age-age correlation for early selection of rubber tree genotypes in Sao Paulo State, Brazil. Genetics and Molecular Biology 28: 758-764.

Greaves BL, Borralho NM, Raymond CA, Evans R, Whiteman PH (1997) Age-age correlation in, and relationships between basic density and growth in *Eucalyptus nitens*. Silvae Genetica 46(50): 264-270.

Guan LH, Pan HX, Huang MR, Shi JS (2005) Research on growth and wood properties joint genetic improvement of new clones of *Poplus deltoides* (I-69) × *P. euramericana* (I-45). J Nanjing For Univ 29(2):6–10

Han S, Lee SJ, Yoon TK, Han SH, Lee J, Kim SH, Wang J, Cho MS, Son Y (2015) Species-specific growth and photosynthetic responses of first-year seedlings of four coniferous species to open-field experimental warming. Turkish Journal of Agriculture and Forestry 39(2): 342-349.

Hannrup B, Ekberg I (1998) Age-age correlations for tracheid length and wood density in *Pinus sylvestris*. Canadian Journal of Forest Research 28(9): 1373-1379.

Hansen J, Roulund H (1997) Genetic parameters for spiral grain, stem form, pilodyn and growth in 13 years old clones of sitka spruce (*Picea sitchensis* (Bong.) Carr.). Silvae Genet 46 : 107-113.

Harry XW, Leopoldo S (2011) Effect of selection method on genetic correlation and gain in a two-trait selection scheme. Australian Forestry 74(1): 36-42.

He GP, Xu YQ, Qi M, Shen FQ, Zhang JZ, Luo WJ (2011) Genetic variation and individual plant selection of main economic traits of progenies from the second - generation seed orchards of Chinese fir. Forest Research 24 (1): 123-126.

Imbs AB, Nevshupova NV, Pham LQ (1998) Triacylglycerol composition of *Pinus koraiensis* seed oil. Journal of American Oil Chemists Society 75(7): 865-870.

Jesus VH, Adams WT (1992) Age-age correlations and early selection for wood destiny in young coastal douglars-fir. Forest Science 38(2): 467-478.

Jiang GY, Jiang LP, Song SL, Wang JY, Wang Q, Wang LF, Zhang P, Zhao XY (2018) Genetic variance analysis and excellent fruit-timber families selection of half-sib *Pinus koraiensis*. Bulletin of Botanical Research, 38(5): 775-784

Jonah P, Aliyu B, Jibung G, Abimiku O (2011) Phenotypic and genotypic correlation in bambara groundnut (*Vigna subterranea* [L.] Verdc) in Mubi, Adamawa State, Nigeria[J]. World Journal of Agricultural Sciences 7 (3) : 298-303.

Kumar S, Lee J (2002) Age-age correlation and early selection for end of rotation wood density in radiata pine. Forest Genetics 9(4): 323-330.

Liang DY, Ding CJ, Zhao GH, Leng WW, Zhang M, Zhao XY, Qu GZ (2018) Variation and selection analysis of *Pinus Koraiensis* clones in northeast China. Journal of Forestry Research 29 (3): 611-622.

Liang DY, Jin YZ, Zhao GH, Dong YH, Leng WW, Chen CL, Wang H, Zhao XY (2016) Variance analyses of growth and wood characteristics of 50 *Pinus koraiensis* clones. Journal of Beijing Forestry University 38(6): 51-59.

Liu MR, Yin SP, Si DJ, Shao LT, Li Y, Zheng M, Wang FW, Li SC, Liu GF, Zhao XY (2015) Variation and genetic stability analyses of transgenic *TaLEA poplar* clones from four different sites in China. Euphytica 2: 331-342.

Liu ZL, Chen JM, Jin GZ, Qi YJ (2015) Estimating seasonal variations of leaf area index using litterfall collection and optical methods in four mixed evergreen-deciduous forests. Agricultural and Forest Meteorology 209: 36-48.

Maniee M, Kahrizi D, Mohammadi R (1998) Genetic variability of some morpho-physiological traits in durum wheat (var.). African Journal of Biotechnology 9(30): 4687-4691.

Matheson A, Spencer DJ, Magnussen D (1994) Optimum age for selection in *Pinus radiata* using basal area under bark for age-age correlations. Silvae Gen 43: 352-357.

Metougul ML, Mokhtari M, Maughan PJ, Jellen EN, Benlhabib O (2017) Morphological variability, heritability and correlation studies within an argan tree population (*Argania spinosa (L.)* Skeels) preserved in situ. International Journal of Agriculture and Forestry 7: 42-51.

Moon M, Kim T, Park J, Cho S, Ryu D, Suh S, Kim HS (2016) Changes in spatial variations of sap flow in korean pine trees due to environmental factors and their effects on estimates of stand transpiration. Journal of Mountain Science 13(6): 1024-1034.

Nergiz C, Donmez I (2004) Chemical composition and nutritive value of *Pinus pinea* L. seeds. Food Chemistry 86: 365-368.

Osorio LF, White TL, Huber DA (2003) Age-age and trait-trait correlation for *Eucalyptus grandis* hill ex maiden and their implications for optimal selection age and design of clonal trails. Theoretical & Applied Genetics 106(4): 735-743.

Owusu GA, Nyadanu D, Obeng-Antwi K, Amoah A, Danso FC, Amissah S (2017) Estimating gene action, combining ability and heterosis for grain yield and agronomic traits in extra-early maturing yellow maize single-crosses under three agro-ecologies of Ghana. Euphytica 213(12): 287.

Qi YZ (2010) Combining ability and cluster analysis of some maize inbred lines. Journal of Sourthwet University (Natural Science Edition) 32 (2): 19-25.

Ren HD, Yao XH, Kang WL, Li S, Wang KL, Duan FW (2010) Genetic variation and early selection or provenance and families of *Acacia mearnsii*. Scientia Silvae Sinicae 03: 153-160.

Rumpunen K, Kviklys D (2013) Combining ability and patterns of inheritance for plant and fruit traits in Japanese quince (*Chaenomeles japonica*). Euphytica 132(2):139-149.

Safavi SA, Pourdad SA, Mohmmad T, Mahoud K (2010) Assessment of genetic variation among safflower (*Carthamus tinctorius* L.) accessions using agro-morphological traits and molecular markers. Journal of Food Agriculture & Environment 8: 616-625.

Seyed MS (2011). Estimation of genetic parameters related to morphological traits in poplar clones. American Journal of Scientific Research 27: 105-110.

Silva F, Pereira MG, Ramos HC, Damasceno J, Pereira T, Gabriel A, Viana AP, Ferreguetti GA (2008) Selection and estimation of the genetic gain in segregating generations of papaya (*Carica papaya* L.). Crop Breeding & Applied Biotechnology, 8: 1-8.

Sluder ER (1996) Two-stage selection in slash pine produces good gains in fusiform rust resistance. South J Appl For 20(3):143-147.

Sun YR, Zhu JJ, Sun QJ, Yan QL (2016) Photosynthetic and growth responses of *Pinus koraiensis* seedlings to canopy openness: Implications for the restoration of mixed-broad leaved korean pine forests. Environmental & Experimental Botany 129: 118-126.

Viana J, DeLima R, Mundim G, Conde A, Vilarinhl A (2013) Relative efficiency of the genotypic value and combining ability effects on reciprocal recurrent selection. Theor Appl Genet 126: 889–899

Wang GY, Ning YP, Jin JH, Zhang SH, Yi HB (2000) Study on the establishment technique of seed orchard for korean pine improved generations[J]. Journal of Northeast Forestry University 3 (28): 68- 69.

Wang HM, Xia DA, Wang WJ, Yang SW (2002) Genetic variations of wood properties and growth characters of korean pines from different provenance. Journal of Forest Research 13(4): 277-280.

Wang W, Shi SL, Liu Y (2015) Present situation and prospect of artificial korean pine reproductive growth. Protection Forest Science and Technology 3: 96-98.

Wang YX, Dong YH, Wu PL, Han YX, Wu DH, Wu ZZ (2007) Effect analysis of establishing seedling seed orchard of *Pinus koraiensis*. Jilin Forestry Science and Technology 36(1): 1-5.

Xia H, Zhao GH, Si DJ, Yin SP, Li Y, Zheng M, Zhao XY (2016) Construction and management technology of tree seed orchard in China. Journal of West China Forestry Science 45(2): 46-51.

Yang Y (2013) Breeding technology of korean pine seedling in northern Liaoning province. The Friend of Farmer 24: 91.

Yin SP, Xiao ZH, Zhao GH, Zhao X, Sun XY, Zhang Y, Wang FW, Li SC, Zhao XY, Qu GZ (2017) Variation analyses of growth and wood properties of *Larix olgensis* clones in China. Journal of Forestry Research 28(4): 687-697.

Yin SP, Zhao GH, Xia H, Sun XY, Pan YY, Wang FW, Li SC, Zhao XY (2016) Progeny test of half-sibs families and excellent families selection in *Larix olgensis*. Journal of Southwest Forestry University 36(1): 64-69.

Youngkoo C, Scott R A (2011) Combining ability of seed vigor and seed yield in soybean.[J]. Euphytica 112(2): 145-150.

Zeng LH, Zhang Q, He BX, Lian HM, Cai YL, Wang YS, Luo M (2013) Age trends in genetic parameters for growth and resin-yielding capacity in masson pine. Silvae Genetica 62 : 7-18.

Zhang F, Wang G, Ning Y, Zhang S, Niu Z, Wang X (2003) The genetic test of fast-growing characters and heriditary selection for height growth of *Pinus koraiensis* filial generation. Journal of Northeast Forestry University 31**,** 68-69.

Zhang Q, Zeng LH, He BX, Lian HM, Cai YL (2013) Age changes and genetic analysis of resin-yield capacity of open-pollinated families of masson pine. Scientia Silvae Sinicae 01: 48-52.

Zhang Z Zhang HG, Zhang L (2016) Age variation in productivity and family selection of open-pollinated families of korean pine (*Pinus koraiensis*). Bulletin of Botanical Research 36(2): 305-309.

Zhao GH, Leng WW, Zhang T, Xu LS, Jin X, Feng L, Li HB, Zhao XY (2018) Variance analysis of growth traits of 51 *Pinus koraiensis* seedlings families. Bulletin of Botanical Research 38(4): 590-596.

Zhao XY, Li Y, Zhao L, Wu RL, Zhang ZY (2013) Analysis and evaluation of growth and adaptive performance of white poplar hybrid clones in different sites. Journal of Beijing Forestry University 35: 7-14.

Zhao XY, Xia H, Wan XW, Wang C, Liang DY, Li KL, Liu GF (2016) Variance and stability analyses of growth characters in half-sib *Betula platyphylla* families at three different sites in China. Euphytica 208: 173-186.

Zhao XY, Xia H, Wang XW, Wang C, Liang DY, Li KL, Liu GF (2015) Variance and stability analyses of growth characters in half-sib *Betula platyphylla* families at three different sites in China. Euphtyica 208 (1): 173-186.

Zhong ZK, Liang SH, Wang YL, Zhang Q (2015) Early growth performance of superior families's progenies of *Castanopsis fissa* in north Guangdong. Guangdong Forestry Science and Technology 31(6): 35-41.

table Click here to access/download;table;Tables.docx

Table 1 Name and parents of 34 *P. koraiensis* families

| Female \ Male | M7 | M9 | M10 | M11 | M12 | M13 | M14 |
|---|---|---|---|---|---|---|---|
| F2 | PK14 | PK29 | PK40 | PK19 | -- | PK34 | PK06 |
| F3 | PK22 | -- | PK33 | PK09 | PK17 | PK07 | -- |
| F4 | PK25 | PK37 | -- | PK49 | -- | -- | -- |
| F6 | PK23 | -- | PK50 | -- | -- | PK24 | PK20 |
| F7 | -- | PK02 | PK31 | PK16 | -- | PK30 | PK42 |
| F8 | -- | PK21 | PK12 | PK26 | PK08 | PK39 | PK46 |
| F9 | -- | -- | PK44 | PK15 | PK47 | PK01 | PK05 |

Table 2 ANOVAs of H, DBH and V of *P. koraiensis* in different growth years

| Age | Traits | Variance source | df | SS | Ms | F | sig |
|---|---|---|---|---|---|---|---|
| | | Family | 33 | 206.07 | 6.24 | 101.33 | 0.000 |
| | H | block | 2 | 2.14 | 1.07 | 17.54 | 0.000 |
| | | Family × block | 66 | 4.49 | 0.07 | 1.10 | 0.296 |
| | | Family | 33 | 40.37 | 1.22 | 45.18 | 0.000 |
| 10th | DBH | block | 2 | 1.40 | 0.70 | 25.85 | 0.000 |
| | | Family × block | 66 | 3.25 | 0.05 | 1.82 | 0.001 |
| | | Family | 33 | 8.72×E-05 | 2.64×E-06 | 23.67 | 0.000 |
| | V | block | 2 | 2.62×E-06 | 1.31×E-06 | 11.74 | 0.000 |
| | | Family × block | 66 | 9.57×E-06 | 1.40×E-07 | 1.30 | 0.086 |
| | | Family | 33 | 264.17 | 8.01 | 80.50 | 0.000 |
| | H | block | 2 | 0.17 | 0.08 | 0.85 | 0.429 |
| | | Family × block | 66 | 8.65 | 0.13 | 1.32 | 0.075 |
| | | Family | 33 | 52.34 | 1.59 | 39.95 | 0.000 |
| 12th | DBH | block | 2 | 0.78 | 0.39 | 9.82 | 0.000 |
| | | Family × block | 66 | 5.02 | 0.08 | 1.92 | 0.000 |
| | | Family | 33 | 2.37×E-04 | 7.18×E-06 | 18.42 | 0.000 |
| | V | block | 2 | 1.84×E-06 | 9.20×E-07 | 2.35 | 0.098 |
| | | Family × block | 66 | 3.33×E-05 | 5.10×E-07 | 1.30 | 0.088 |
| | | Family | 33 | 309.43 | 9.38 | 57.36 | 0.000 |
| | H | block | 2 | 0.81 | 0.41 | 2.48 | 0.086 |
| | | Family × block | 66 | 24.18 | 0.37 | 2.24 | 0.000 |
| | | Family | 33 | 61.44 | 1.86 | 32.47 | 0.000 |
| 14th | DBH | block | 2 | 0.12 | 0.06 | 1.05 | 0.350 |
| | | Family × block | 66 | 7.92 | 0.12 | 2.09 | 0.000 |
| | | Family | 33 | 4.90×E-04 | 1.48×E-05 | 12.66 | 0.000 |
| | V | block | 2 | 1.04×E-06 | 5.20×E-07 | 0.44 | 0.642 |
| | | Family × block | 66 | 1.01×E-04 | 1.53×E-06 | 1.31 | 0.079 |
| | | Family | 33 | 395.95 | 12.00 | 62.38 | 0.000 |
| | H | block | 2 | 0.10 | 0.05 | 0.27 | 0.766 |
| | | Family × block | 66 | 21.22 | 0.32 | 1.67 | 0.004 |
| | | Family | 33 | 77.54 | 2.35 | 31.70 | 0.000 |
| 16th | DBH | block | 2 | 0.45 | 0.22 | 3.03 | 0.051 |
| | | Family × block | 66 | 13.56 | 0.21 | 2.77 | 0.000 |
| | | Family | 33 | 1.05×E-03 | 3.20×E-05 | 11.32 | 0.000 |
| | V | block | 2 | 8.85×E-05 | 4.42×E-06 | 1.57 | 0.211 |
| | | Family × block | 66 | 2.63×E-04 | 3.98×E-06 | 1.41 | 0.036 |
| 18th | H | Family | 33 | 425.76 | 12.90 | 56.10 | 0.000 |

| | | | | | | |
|---|---|---|---|---|---|---|
| | block | 2 | 0.27 | 0.14 | 0.59 | 0.553 |
| | Family × block | 66 | 75.14 | 1.14 | 4.95 | 0.000 |
| | Family | 33 | 83.74 | 2.54 | 33.42 | 0.000 |
| DBH | block | 2 | 0.09 | 0.04 | 0.59 | 0.554 |
| | Family × block | 66 | 9.24 | 0.14 | 1.84 | 0.001 |
| | Family | 33 | 1.47×E-03 | 4.47×E-05 | 11.08 | 0.000 |
| V | block | 2 | 4.50×E-06 | 2.25×E-06 | 0.56 | 0.573 |
| | Family × block | 66 | 3.40×E-04 | 5.15×E-06 | 1.28 | 0.101 |

Table 3 Genetic and variation parameters of H, DBH and V in different years

| Age | Traits | Average | SD | Minimum | Maximum | PCV | $h^2$ |
|---|---|---|---|---|---|---|---|
| | H | 5.31 | 0.83 | 3.47 | 6.90 | 15.70 | 0.990 |
| 10 | DBH | 2.87 | 0.37 | 1.84 | 3.57 | 12.89 | 0.978 |
| | V | 0.0018 | 0.0005 | 0.0006 | 0.0029 | 27.78 | 0.958 |
| | H | 6.63 | 0.94 | 4.53 | 8.43 | 7.57 | 0.988 |
| 12 | DBH | 3.51 | 0.42 | 2.34 | 4.28 | 11.97 | 0.975 |
| | V | 0.0032 | 0.0009 | 0.0012 | 0.0049 | 28.13 | 0.946 |
| | H | 7.84 | 1.02 | 5.57 | 9.84 | 13.01 | 0.983 |
| 14 | DBH | 4.09 | 0.45 | 2.82 | 4.90 | 11.00 | 0.969 |
| | V | 0.0048 | 0.0013 | 0.0019 | 0.0072 | 27.08 | 0.921 |
| | H | 9.18 | 1.15 | 6.88 | 11.59 | 12.52 | 0.984 |
| 16 | DBH | 4.78 | 0.51 | 3.28 | 5.65 | 10.67 | 0.968 |
| | V | 0.0074 | 0.0019 | 0.0031 | 0.0106 | 25.68 | 0.912 |
| | H | 9.92 | 1.20 | 7.33 | 12.30 | 12.10 | 0.982 |
| 18 | DBH | 5.11 | 0.53 | 3.60 | 6.03 | 10.37 | 0.970 |
| | V | 0.0090 | 0.0022 | 0.0037 | 0.0129 | 24.44 | 0.910 |

Note: The units of H, DBH and V were m, cm, $m^3$, respectively.

Table 4 Correlation coefficients of H, DBH and V among different years

| Age and traits | | The 10th growth year | | | The 12th growth year | | | The 14th growth year | | | The 16th growth year | | | The 18th growth year | |
|---|---|---|---|---|---|---|---|---|---|---|---|---|---|---|---|
| | | H | DBH | V | H | DBH | V | H | DBH | V | H | DBH | V | H | DBH |
| The 10th growth year | DBH | 0.48$^{**}$ | | | | | | | | | | | | | |
| | V | 0.65$^{**}$ | 0.85$^{**}$ | | | | | | | | | | | | |
| | H | | 0.50$^{**}$ | 0.71$^{**}$ | | | | | | | | | | | |
| The 12th growth year | DBH | 0.50$^{**}$ | | 0.94$^{**}$ | 0.51$^{**}$ | | | | | | | | | | |
| | V | 0.71$^{**}$ | 0.93$^{**}$ | | 0.72$^{**}$ | 0.87$^{**}$ | | | | | | | | | |
| | H | | 0.50$^{**}$ | 0.71$^{**}$ | | 0.52$^{**}$ | 0.73$^{**}$ | | | | | | | | |
| The 14th growth year | DBH | 0.48$^{**}$ | | 0.94$^{**}$ | 0.50$^{**}$ | | 0.93$^{**}$ | 0.50$^{**}$ | | | | | | | |
| | V | 0.70$^{**}$ | 0.93$^{**}$ | | 0.71$^{**}$ | 0.94$^{**}$ | | 0.72$^{**}$ | 0.86$^{**}$ | | | | | | |
| | H | | 0.50$^{**}$ | 0.72$^{**}$ | | 0.51$^{**}$ | 0.73$^{**}$ | | 0.50$^{**}$ | 0.71$^{**}$ | | | | | |
| The 16th growth year | DBH | 0.45$^{**}$ | | 0.91$^{**}$ | 0.47$^{**}$ | | 0.91$^{**}$ | 0.47$^{**}$ | | 0.91$^{**}$ | 0.47$^{**}$ | | | | |
| | V | 0.68$^{**}$ | 0.93$^{**}$ | | 0.69$^{**}$ | 0.93$^{**}$ | | 0.70$^{**}$ | 0.93$^{**}$ | | 0.71$^{**}$ | 0.91$^{**}$ | | | |
| | H | | 0.52$^{**}$ | 0.73$^{**}$ | | 0.53$^{**}$ | 0.74$^{**}$ | | 0.51$^{**}$ | 0.72$^{**}$ | | 0.48$^{**}$ | 0.71$^{**}$ | | |
| The 18th growth year | DBH | 0.43$^{**}$ | | 0.92$^{**}$ | 0.45$^{**}$ | | 0.91$^{**}$ | 0.45$^{**}$ | | 0.92$^{**}$ | 0.45$^{**}$ | | 0.92$^{**}$ | 0.46$^{**}$ | |
| | V | 0.67$^{**}$ | 0.94$^{**}$ | | 0.68$^{**}$ | 0.94$^{**}$ | | 0.69$^{**}$ | 0.94$^{**}$ | | 0.69$^{**}$ | 0.92$^{**}$ | | 0.73$^{**}$ | 0.93$^{**}$ |

Table 5 Analysis of H, DBH and V general combining ability of different parents

| Traits | Female Parent | GCA | Male Parent | GCA |
|---|---|---|---|---|
| H | F 4 | 0.920 | M 11 | 0.697 |
| | F 2 | 0.570 | M 13 | 0.463 |
| | F 8 | 0.342 | M 7 | 0.006 |
| | F 3 | -0.069 | M 9 | -0.039 |
| | F 6 | -0.339 | M 10 | -0.190 |
| | F 7 | -0.427 | M 14 | -0.507 |
| | F 9 | -0.879 | M 12 | -1.050 |
| DBH | F 3 | 0.229 | M 7 | 0.233 |
| | F 2 | 0.186 | M 13 | 0.135 |
| | F 4 | 0.106 | M 14 | 0.057 |
| | F 8 | 0.095 | M 10 | 0.021 |
| | F 6 | -0.095 | M 9 | -0.040 |
| | F 7 | -0.147 | M 11 | -0.107 |
| | F 9 | -0.407 | M 12 | -0.450 |
| V | F 2 | 8.941×E-04 | M 7 | 6.941×E-04 |
| | F 4 | 8.441×E-04 | M 13 | 6.608×E-04 |
| | F 3 | 7.241×E-04 | M 14 | 1.041×E-04 |
| | F 8 | 4.108×E-04 | M 11 | -5.882×E-06 |
| | F 7 | -4.759×E-04 | M 9 | -5.588×E-05 |
| | F 6 | -7.559×E-04 | M 10 | -1.725×E-04 |
| | F 9 | -1.716×E-03 | M 12 | -1.989×E-03 |

Table 6 Analysis of H, DBH and V special combining ability of different parents

| Family | combination | SCA | | |
|---|---|---|---|---|
| | | H | DBH | V |
| PK01 | 9×13 | -1.381 | 0.055 | -1.00E-03 |
| PK02 | 7×9 | -0.151 | -1.320 | -4.32E-03 |
| PK05 | 9×14 | 2.999 | 0.963 | 5.06E-03 |
| PK06 | 2×14 | -2.592 | -0.210 | -2.35E-03 |
| PK07 | 3×13 | 0.409 | 0.289 | 1.46E-03 |
| PK08 | 8×12 | 0.332 | 0.179 | 5.23E-04 |
| PK09 | 3×11 | -0.905 | -0.419 | -2.07E-03 |
| PK12 | 8×10 | 0.212 | -0.143 | -2.94E-04 |
| PK14 | 2×7 | -0.982 | -0.486 | -2.34E-03 |
| PK15 | 9×11 | -0.365 | -0.203 | -1.03E-03 |
| PK16 | 7×11 | -0.557 | 0.397 | 5.26E-04 |
| PK17 | 3×12 | 0.932 | 0.424 | 1.61E-03 |
| PK19 | 2×11 | 0.177 | 0.474 | 2.06E-03 |
| PK20 | 6×14 | -0.221 | -0.149 | -9.04E-04 |
| PK21 | 8×9 | -0.459 | 0.048 | -4.11E-04 |
| PK22 | 3×7 | 0.816 | 0.461 | 2.53E-03 |
| PK23 | 6×7 | 0.087 | -0.215 | -5.94E-04 |
| PK24 | 6×13 | -0.271 | -0.167 | -6.61E-04 |
| PK25 | 4×7 | -1.002 | -0.186 | -1.29E-03 |
| PK26 | 8×11 | 0.965 | 0.285 | 1.84E-03 |
| PK29 | 2×9 | -0.907 | 0.336 | 4.06E-04 |
| PK30 | 7×13 | 0.667 | 0.225 | 8.59E-04 |
| PK31 | 7×10 | -0.640 | 0.519 | 7.93E-04 |
| PK33 | 3×10 | -1.178 | -0.587 | -2.71E-03 |
| PK34 | 2×13 | 1.350 | -0.628 | -1.31E-03 |
| PK37 | 4×9 | 0.113 | 0.696 | 2.66E-03 |
| PK39 | 8×13 | 0.028 | 0.364 | 1.57E-03 |
| PK40 | 2×10 | 1.783 | 0.215 | 2.32E-03 |
| PK42 | 7×14 | 0.257 | 0.113 | 1.62E-03 |
| PK44 | 9×10 | -0.008 | 0.049 | -6.75E-05 |
| PK46 | 8×14 | -0.452 | -0.349 | -1.77E-03 |
| PK47 | 9×12 | -0.658 | -0.520 | -1.55E-03 |
| PK49 | 4×11 | 0.227 | -0.596 | -1.99E-03 |
| PK50 | 6×10 | 0.633 | 0.086 | 8.73E-04 |

Table 7 Multicharacter comprehensive evaluation of *P. koraiensis* families

| Family | *Qi* | Family | *Qi* | Family | *Qi* |
|---|---|---|---|---|---|
| PK40 | 1.71 | PK17 | 1.58 | PK08 | 1.52 |
| PK05 | 1.70 | PK49 | 1.58 | PK20 | 1.50 |
| PK22 | 1.69 | PK21 | 1.58 | PK44 | 1.49 |
| PK37 | 1.68 | PK50 | 1.58 | PK42 | 1.49 |
| PK26 | 1.68 | PK25 | 1.57 | PK06 | 1.48 |
| PK19 | 1.68 | PK16 | 1.56 | PK15 | 1.47 |
| PK07 | 1.67 | PK24 | 1.55 | PK33 | 1.47 |
| PK39 | 1.66 | PK23 | 1.54 | PK01 | 1.46 |
| PK34 | 1.65 | PK31 | 1.54 | PK02 | 1.38 |
| PK30 | 1.64 | PK14 | 1.54 | PK47 | 1.31 |
| PK29 | 1.59 | PK09 | 1.53 | | |
| PK12 | 1.59 | PK46 | 1.52 | | |
