# Supplementary material for: Provenance and family variations in early growth of Manchurian walnut (Juglans mandshurica Maxim.) and selection of superior families
Source: PLoS One. 2024 Mar 7;19(3):e0298918. doi: 10.1371/journal.pone.0298918 (PMC10919699; doi:10.1371/journal.pone.0298918)
Supplement: S1 File — (ZIP) [file pone.0298918.s004.zip › Analysis of variation in growth traits of different species and families of Juglans mandshurica.pdf]

DOI:10.13759/j.cnki.dlxb.2023.08.006

胡桃楸不同种源及家系间生长性状的遗传变异[1)]

刘劲宏　李志新　张含国　　宋莉昕　　杨岳
（林木遗传育种国家重点实验室（东北林业大学），哈尔滨，150040）　（宾县万人欢林场）　（宾县林草局）

摘　要　在黑龙江省宾县万人欢林场试验地，选择胡桃楸（*Juglans mandshurica* Maxim.）种源试验林、家系试验林为研究对象，种源试验林试验种子来自黑龙江省15个种源地（以当地试验种子为对照）、家系试验林试验种子来自14个种源的133个胡桃楸半同胞家系（以当地试验种子为对照）；种源、家系试验林均于2014年秋季播种，2016年春季定植，试验采用完全随机区组设计，种源试验林为1个小区（小区9~10株，4次重复）、家系试验林为5个小区（小区8~12株，4次重复），株行距均为3 m×4 m。分别于2019、2020、2021年11月份，对5~7年生胡桃楸种源、半同胞家系试验林的树高、胸径等性状进行每木测量，利用测量的3 a树高、胸径，计算2020年连年生长量、2021年连年生长量；以树高连年生长量、胸径连年生长量、家系遗传力、变异系数、种源现实增益、家系遗传增益为评价指标，采用方差分析、布雷津多性状综合评定法，分析胡桃楸种源与家系间生长性状遗传变异规律、遴选优良种源家系。结果表明：种源各年度间大部分性状在各变异来源间差异极显著，家系各年度各性状在各变异来源间差异极显著。种源树高变异系数变化范围为21.97%~38.79%、胸径变异系数变化范围为27.99%~36.47%，家系树高变异系数变化范围为24.23%~41.54%、胸径变异系数变化范围为30.08%~45.33%。家系各年度各性状遗传力的变化范围为0.79~0.90，其中7年生树高超过了0.90。利用多性状综合评价法，以20%的入选率进行优良种源、家系选择，初步选出3个优良种源（宾县大泉子、富锦、宝清）、27个优良家系；入选的3个种源，树高的现实增益为9.75%、胸径的现实增益为13.80%、树高连年生长量的现实增益为9.54%、胸径连年生长量的现实增益为8.50%；入选的27个优良家系，树高的遗传增益为11.84%、胸径的遗传增益为14.32%、树高连年生长量的遗传增益为8.84%、胸径连年生长量的遗传增益为6.39%。对胡桃楸的生长性状综合分析表明，在胡桃楸种源和家系间均存在丰富的变异，可以进行优良种源、家系的初步筛选。

关键词　胡桃楸；种源；家系；生长性状；遗传变异

分类号　S718.46

Analysis of Variation in Growth Traits of Different Species and Families of *Juglans mandshurica*//Liu Jinhong, Li Zhixin, Zhang Hanguo (State Key Laboratory of Tree Genetics and Breeding, Northeast Forestry University, Harbin 150040, P. R. China); Song Lixin (Bin County Wanrenhuan Forestry); Yang Yue (Bin County Forestry and Grass Bureau)//Journal of Northeast Forestry University, 2023, 51(8): 9-17, 64.

In the experimental site of Wanhuan Forest Farm in Bin County, Heilongjiang Province, the experimental forest of *Juglans mandshurica* Maxim. seed source and family experimental forest were selected as the research objects, and the seed source experimental forest experimental forest was from 15 seed source areas in Heilongjiang Province (local experimental seeds were used as the control), and the experimental seeds of the family experimental forest were from 133 semi-sibling families of *J. mandshurica* from 14 seed sources (local experimental seeds were used as the control). The seed source and family experimental forest were sown in the fall of 2014 and planted in the spring of 2016, and the experiment adopted a completely random block design, the seed source experimental forest was 1 community (9-10 plants in the community, 4 repeats), and the family test forest was 5 communities (8-12 plants in the community, 4 repeats), and the plant row spacing was 3 m×4 m. In November 2019, 2020 and 2021, the tree height and breast diameter of the 5-7-year-old walnut seed source and semi-sibling family test forest were measured for each tree, and the measured 3-a tree height and breast diameter were used to calculate the annual growth in 2020 and 2021. Taking the annual growth of tree height, breast diameter annual growth, family heritability, coefficient of variation, seed source reality gain, and family genetic gain as the evaluation indexes, analysis of variance and comprehensive evaluation of Brezin multiple traits were used to analyze the genetic variation of growth traits between the seed source and the family lineage, and select excellent seed source lineages. The results showed that most of the traits in each year of the seed source were very different between the sources of variation, and the traits in each year of the family line were very different between the sources of variation. The variation range of high coefficient of variation of seed source trees was 21.97%-38.79%, the variation range of breast diameter coefficient variation coefficient was 27.99%-36.47%, the variation range of high coefficient of variation of family trees was 24.23%-41.54%, and the variation range of chest diameter coefficient of variation was 30.08%-45.33%. The heritability of each trait in each year of the family line ranged in 0.79-0.90, among which the height of the 7-year-old tree exceeded 0.90. Using the comprehensive evaluation method of multiple traits, excellent seed sources and lineages were selected with a 20% inclusion rate, and 3 excellent seed sources (Daquanzi, Fujin and Baoqing in Binxian County) and 27 excellent family lines were preliminarily selected. Among the three selected species, the realistic gain of tree height was 9.75%, the realistic gain of breast diameter was 13.80%, the realistic gain of continuous growth of tree height was 9.54%, and the realistic gain of continuous growth of breast diameter was 8.50%. The genetic gain of the 27 selected excellent family trees was 11.84%, the genetic gain of breast diameter was 14.32%, the genetic gain of tree height continuous growth was 8.84%, and the genetic gain of breast diameter continuous annual growth was 6.39%. The comprehensive analysis of the growth traits of walnut showed that there were abundant variations between the seed sources and families of Walnut Rowan, and the preliminary screening of excellent seed sources and families could be carried out.

Keywords　*Juglans mandshurica*; Seed source; Family; Growth traits; Genetic variation

---

1）黑龙江省应用技术研究与开发计划项目（GA19B201-7）。

第一作者简介：刘劲宏，男，1997年2月生，林木遗传育种全国重点实验室（东北林业大学），硕士研究生。E-mail：771710843@qq.com。

通信作者：张含国，林木遗传育种全国重点实验室（东北林业大学），教授。E-mail：hanguozhang1@sina.com。

收稿日期：2022年10月17日。
责任编辑：张　玉。

(C)1994-2023 China Academic Journal Electronic Publishing House. All rights reserved.　http://www.cnki.net

胡桃楸(*Juglans mandshurica* Maxim.),又称核桃楸、山核桃,是胡桃科(Juglandaceae)胡桃属(*Juglans*)落叶阔叶乔木,与水曲柳、黄波椤并称“东北三大硬阔”,是国家Ⅱ级珍稀树种和中国珍稀濒危树种的三级保护植物[1-2]。主要分布于我国东北地区的小兴安岭、完达山脉、长白山区和辽宁东部,华北地区也有零星分布[3-5],是东北阔叶红松林重要的伴生树种。

种源及优良家系的评价选择是林木育种的重要方法[6],也是林木遗传改良最基础的工作[7],其结果可为选育地区提供优质、高产、稳定的应用材料,对于实现适地适种源具有重要意义[8]。颜廷武等[9]对胡桃楸进行种源区划,并对少数种源、家系进行初步评价;卢贤博等[10]对4个胡桃楸种源内的28个家系进行变异分析和遗传评估,初步筛选出1个种源、3个家系以及10个优良单株;韩玉霞等[6]对39个4年生胡桃楸家系树高、地径进行分析,结果表明,树高、地径在家系间差异极显著,并选出4个优良家系。但目前选育良种较少,无法满足实际造林生产需求,并且由于胡桃楸的用途广、经济价值巨大,天然林破坏严重,资源储有量严重不足[11]。因此应开展种源家系变异分析及优良种源家系的选择研究,加快优良品系选育,繁育生长性状高、材性好的优良品种,为林业生产提供良种、提高胡桃楸林分的生长量和质量。

为此,本研究在黑龙江省宾县万人欢林场试验地,选择胡桃楸种源试验林、家系试验林为研究对象,种源试验林试验种子来自黑龙江省15个种源地(以当地试验种子为对照)、家系试验林试验种子来自14个种源的133个胡桃楸半同胞家系(以当地试验种子为对照);分别于2019、2020、2021年11月份,对5~7年生胡桃楸种源、半同胞家系试验林的树高、胸径等性状进行每木测量,利用测量的3 a树高、胸径,计算2020年连年生长量、2021年连年生长量;以树高连年生长量、胸径连年生长量、家系遗传力、变异系数、种源现实增益、家系遗传增益为评价指标,采用方差分析、布雷津多性状综合评定法,分析胡桃楸种源与家系间生长性状遗传变异规律、遴选优良种源家系。旨在为核桃楸的良种选育提供参考。

1 研究方法

试验地位于黑龙江省宾县万人欢林场(东经128°4′29″~128°19′13″、北纬45°44′20″~45°57′18″),地处张广才岭西坡,整体地势南高北低;最高海拔558.6 m,平均海拔340 m;最大坡度35°,平均坡度17°。气候条件属中温带大陆性季风气候,年平均气温2.4 ℃,年降水量600~800 mm,无霜期130 d左右,植物生长期140 d左右,年日照时间2 560 h。土壤环境为暗棕壤型。

试验包含种源试验林、家系试验林。其中,种源试验林试验种子来自黑龙江省15个种源地,包括宝清、宾县大泉子、东方红、东京城、富锦、虎林、珲春、金山屯、临江、牡丹江、七台河、三岔子、铁力、五常、亚布力和当地试验种子为对照;家系试验林试验种子包括来自14个种源(种源名称用字母命名,其中BQ为宝清、DQ为宾县大泉子、DFH为东方红、DJC为东京城、HL为虎林、HC为珲春、MDJ为牡丹江、QTH为七台河、QY为清原、SC为三岔子、TL为铁力、WC为五常、WRH为万人欢、YBL为亚布力)的133个胡桃楸半同胞家系和当地试验种子为对照。种源、家系试验林均于2014年秋季播种,2016年春季定植,试验采用完全随机区组设计,种源试验林为1个小区(4次重复,小区9~10株)、家系试验林为5个小区(4次重复,小区8~12株),株行距均为3 m×4 m。

分别于2019、2020、2021年11月份,对5~7年生胡桃楸种源、半同胞家系试验林的树高、胸径等性状进行每木测量。利用塔尺测量树高,树高测量精度为±0.01 m;胸径利用胸径尺测定,测量精度为±0.1 cm。利用测量的3 a树高、胸径计算生长量,其中2020年连年生长量为生长量Ⅰ、2021年连年生长量为生长量Ⅱ。

家系遗传力计算公式[12]:$h^2=1-1/F$,式中F为方差分析的F值。

变异系数计算公式[13]:$C_V=(S/\bar{x})\times100\%$,式中$S$为标准差、$\bar{x}$为某一性状群体平均值。

方差分析线性模型[14]:$Y_{ij}=\mu+B_i+P+P_{i,ji}+M_{eiij}$、$Y_{ij}=\mu+B_i+F+F_{i,ji}+M_{eiij}$,式中$\mu$为总体平均值、$B_j$为区组效应、$P$为种源效应、$F$为家系效应、$P_{i,ji}$为区组与种源的协同作用、$F_{i,ji}$为区组与家系的协同作用、$M_{eiij}$为机误。

种源现实增益估算公式:$\Delta G_r=W/\bar{x}$,式中W为选择差。

家系遗传增益估算公式[15]:$\Delta G=h^2W/\bar{x}$。

种源、家系比较,采用布雷津多性状综合评定法对种源、家系进行综合评定[16],计算公式:$Q_i=(\sum_{j=1}^{n}a_i)^{1/2}$、$a_i=X_{ij}/X_{j,\max}$,式中$Q_i$为综合评价值、$X_{ij}$为某一性状的平均值、$X_{j,\max}$为某一性状的最优值、$n$为评价指标的数量。

(C)1994-2023 China Academic Journal Electronic Publishing House. All rights reserved. http://www.cnki.net

2 结果与分析

2.1 胡桃楸种源的生长性状变异

对胡桃楸种源试验林 16 个种源各生长性状变异参数进行了分析(见表 1、表 2)。

5~7 年生胡桃楸种源，树高变异系数分别为 31.62%、25.70%、21.97%，树高连年生长量Ⅰ的变异系数为 29.27%、树高连年生长量Ⅱ的变异系数为 38.79%。5 年生胡桃楸，树高变异系数较大的 3 个种源为五常、亚布力、珲春，变异系数较小的 3 个种源为三岔子、牡丹江、宾县大泉子。6 年生胡桃楸，树高变异系数较大的 3 个种源为五常、亚布力、东京城，变异系数较小的 3 个种源为铁力、宾县大泉子、三岔子。7 年生胡桃楸，树高变异系数较大的 3 个种源为五常、宝清、亚布力，变异系数较小的 3 个种源为三岔子、东方红、宾县大泉子。树高连年生长量Ⅰ，变异系数较大的 3 个种源为亚布力、珲春、临江，变异较小的 3 个种源为铁力、宾县大泉子、三岔子；树高连年生长量Ⅱ，变异系数较大的 3 个种源为虎林、亚布力、临江，变异较小的 3 个种源为宾县大泉子、三岔子、宝清。

表 1　胡桃楸种源树高性状变异参数估计结果

5 年生树高		6 年生树高		7 年生树高		树高连年生长量Ⅰ		树高连年生长量Ⅱ	
种源	变异系数/%	种源	变异系数/%	种源	变异系数/%	种源	变异系数/%	种源	变异系数/%
五常	38.75	五常	31.87	五常	29.69	亚布力	38.54	虎林	59.36
亚布力	34.78	亚布力	30.31	宝清	26.45	珲春	33.12	亚布力	45.27
珲春	34.28	东京城	27.96	亚布力	23.84	临江	33.07	临江	43.45
东京城	33.76	对照	25.22	东京城	23.67	牡丹江	32.85	七台河	42.70
对照	32.70	东方红	25.15	铁力	23.23	五常	32.15	铁力	42.23
富锦	32.67	宝清	24.88	金山屯	22.90	宝清	30.39	珲春	40.89
铁力	31.56	七台河	24.84	虎林	20.89	金山屯	28.91	牡丹江	38.92
宝清	30.54	虎林	24.09	富锦	20.82	东京城	28.83	富锦	38.59
临江	29.64	牡丹江	23.68	对照	20.56	虎林	28.57	五常	38.03
金山屯	29.08	临江	23.51	珲春	19.76	七台河	28.12	金山屯	35.55
七台河	28.72	金山屯	23.40	牡丹江	19.20	对照	27.67	东京城	34.93
东方红	27.19	珲春	23.29	七台河	17.04	东方红	26.13	东方红	34.25
虎林	26.35	富锦	22.70	临江	16.75	富锦	23.10	对照	33.51
三岔子	25.94	铁力	22.00	三岔子	16.26	铁力	22.75	大泉子	30.32
牡丹江	25.33	大泉子	20.71	东方红	15.10	大泉子	21.91	三岔子	30.15
大泉子	25.25	三岔子	17.86	大泉子	14.43	三岔子	16.91	宝清	27.76

表 2　胡桃楸种源胸径性状变异参数估计结果

5 年生胸径		6 年生胸径		7 年生胸径		胸径连年生长量Ⅰ		胸径连年生长量Ⅱ	
种源	变异系数/%	种源	变异系数/%	种源	变异系数/%	种源	变异系数/%	种源	变异系数/%
东京城	39.05	亚布力	37.58	五常	32.53	亚布力	37.83	临江	46.92
三岔子	38.01	铁力	35.88	珲春	31.85	临江	35.25	珲春	42.87
金山屯	36.47	对照	34.77	宝清	31.45	东方红	32.61	铁力	41.14
铁力	36.20	五常	34.25	金山屯	30.84	珲春	32.54	牡丹江	40.58
虎林	36.11	东京城	33.19	亚布力	30.52	牡丹江	32.31	对照	39.50
亚布力	35.73	珲春	33.11	对照	28.26	七台河	30.96	虎林	38.53
对照	35.17	宝清	32.90	东京城	27.55	三岔子	30.45	富锦	36.61
富锦	34.78	七台河	32.22	富锦	26.40	东京城	29.24	东京城	35.68
大泉子	34.42	富锦	31.10	虎林	26.29	铁力	28.69	宝清	34.88
宝清	33.90	牡丹江	30.77	铁力	25.18	五常	28.12	亚布力	32.94
五常	33.13	临江	30.54	大泉子	24.92	宝清	27.57	五常	32.41
七台河	32.32	三岔子	30.53	临江	24.40	大泉子	25.63	金山屯	31.89
牡丹江	30.66	东方红	30.47	牡丹江	24.34	虎林	25.49	三岔子	31.57
珲春	29.40	大泉子	30.47	七台河	23.23	对照	23.94	东方红	31.28
东方红	28.56	金山屯	30.14	三岔子	23.05	金山屯	22.69	大泉子	29.52
临江	27.30	虎林	28.36	东方红	19.38	富锦	20.85	七台河	25.18

5~7 年生胡桃楸种源，胸径变异系数分别为 35.06%、33.40%、27.99%，胸径连年生长量Ⅰ的变异系数为 29.70%、胸径连年生长量Ⅱ的变异系数 36.47%。5 年生胡桃楸，胸径变异系数较大的 3 个种源为东京城、三岔子、金山屯，变异系数较小的 3 个种源为珲春、东方红、临江。6 年生胡桃楸，胸径

(C)1994-2023 China Academic Journal Electronic Publishing House. All rights reserved.　　http://www.cnki.net

变异系数较大的3个种源为亚布力、铁力、对照，变异系数较小的3个种源为宾县大泉子、金山屯、虎林。7年生胡桃楸，胸径变异系数较大的3个种源为五常、珲春、宝清，变异系数较小的3个种源为七台河、三岔子、东方红。胸径连年生长量Ⅰ，变异系数较大的3个种源为亚布力、临江、东方红，变异系数较小的3个种源为对照、金山屯、富锦；胸径连年生长量Ⅱ，变异系数较大的3个种源为临江、珲春、铁力，变异系数较小的3个种源为东方红、宾县大泉子、七台河。

2.2 胡桃楸家系的生长性状变异

对胡桃楸种源试验林133个家系进行分析，选取各性状变异系数排名靠前5名、居中5名、靠后5名的分析结果（见表3、表4）。

5~7年生胡桃楸家系，树高变异系数分别为29.98%、27.09%、24.23%，树高连年生长量Ⅰ的变异系数为34.32%、树高连年生长量Ⅱ的变异系数为41.54%。5年生胡桃楸，树高变异系数排名前5名、居中5名、后5名的家系，树高变异系数均值分别为39.75%、28.07%、18.76%；6年生胡桃楸，树高变异系数排名前5名、居中5名、后5名的家系，树高变异系数均值分别为34.23%、24.47%、16.52%；7年生胡桃楸，树高变异系数排名前5名、居中5名、后5名的家系，树高变异系数均值分别为29.46%、22.18%、13.05%。树高连年生长量Ⅰ，变异系数排名前5名、居中5名、后5名的家系，变异系数均值分别为49.74%、32.51%、21.13%；树高连年生长量Ⅱ，变异系数排名前5名、居中5名、后5名的家系，变异系数均值分别为63.10%、39.56%、22.94%。

5~7年生胡桃楸家系，胸径变异系数分别为32.28%、33.48%、30.08%，胸径连年生长量Ⅰ的变异系数为38.54%、胸径连年生长量Ⅱ的变异系数为45.33%。5年生胡桃楸，胸径变异系数排名前5名、居中5名、后5名的家系，胸径变异系数均值分别为43.60%、29.05%、15.98%；6年生胡桃楸，胸径变异系数排名前5名、居中5名、后5名的家系，胸径变异系数均值分别为46.49%、30.14%、20.37%；7年生胡桃楸，胸径变异系数排名前5名、居中5名、后5名的家系，胸径变异系数均值分别为38.30%、26.86%、18.19%。胸径连年生长量Ⅰ，变异系数排名前5名、居中5名、后5名的家系，变异系数均值分别为65.48%、37.16%、20.70%；胸径连年生长量Ⅱ，变异系数排名前5名、居中5名、后5名的家系，变异系数均值分别为66.22%、43.70%、27.27%。

对各种源、家系树高及胸径变异分析结果表明，除家系6年生比5年生的胸径变异系数略有升高外，其余各种源、家系树高及胸径变异系数均随着林龄的增大而呈现出递减的趋势，且种源与家系在各年份树高、胸径的变异系数相近。在种源、家系间，树高、胸径的后一年连年生长量的变异系数都大于前一年，与树高、胸径变异规律不同，且家系连年生长量变异系数大于种源。种源、家系间，树高的变异系数比较稳定，如五常、亚布力种源连续3 a都进入种源排名前3，宾县大泉子和三岔子种源连续3 a排名位于后3名。由于林龄较小，部分单株起初并未拥有胸径，随着时间增长开始拥有胸径，导致胸径变异系数在种源、家系中并不稳定，存在较大变动幅度。从上述结果中可以看出，无论种源、家系间均存在丰富变异，存在选择潜力，可以为今后选优提供材料基础。

表3 胡桃楸家系树高性状变异参数估计结果

5年生树高		6年生树高		7年生树高		树高连年生长量Ⅰ		树高连年生长量Ⅱ	
家系	变异系数/%	家系	变异系数/%	家系	变异系数/%	家系	变异系数/%	家系	变异系数/%
WC8	41.38	DJC15	35.10	TL16	32.13	TL3	52.78	TL30	70.66
YBL6	41.14	SC8	34.40	DJC20	31.45	HL4	50.94	SC18	65.51
DJC7	39.98	YBL6	34.26	YBL6	28.25	TL30	50.69	MDJ7	61.19
TL16	36.48	TL16	33.81	DQ23	27.84	HL12	48.77	MDJ14	60.22
DJC15	36.06	DQ6	33.58	HL12	27.63	WC4	45.53	DQ7	57.91
⋮	⋮	⋮	⋮	⋮	⋮	⋮	⋮	⋮	⋮
DJC6	28.18	QTH3	24.54	WC7	22.55	WC19	32.76	QTH5	39.87
DQ6	28.16	DQ7	24.54	MDJ4	22.26	WC29	32.47	HL1	39.74
HL22	28.05	DQ1	24.48	MDJ14	22.18	QTH1	32.46	HL4	39.72
QY1	28.00	WRH14	24.42	QY2	22.09	QY2	32.43	DJC24	39.47
WRH10	27.96	SC20	24.35	WRH10	21.82	WC8	32.42	WC19	38.98
⋮	⋮	⋮	⋮	⋮	⋮	⋮	⋮	⋮	⋮
YBL12	20.28	SC1	17.61	SC22	14.40	SC22	23.28	HL15	24.51
TL5	20.08	QTH8	17.27	SC27	14.13	DJC17	22.86	YBL6	24.37
DQ3	18.89	YBL12	16.09	TL5	13.73	SC27	22.08	YBL13	23.42
WRH16	18.13	DQ3	16.08	SC1	13.37	QTH6	21.42	SC11	23.24
SC1	16.40	SC27	15.55	HL15	10.98	DQ3	16.00	SC13	19.16

(C)1994-2023 China Academic Journal Electronic Publishing House. All rights reserved. http://www.cnki.net

表 4　胡桃楸家系胸径性状变异参数估计结果

5 年生胸径		6 年生胸径		7 年生胸径		胸径连年生长量Ⅰ		胸径连年生长量Ⅱ	
家系	变异系数/%	家系	变异系数/%	家系	变异系数/%	家系	变异系数/%	家系	变异系数/%
TL16	49.21	TL30	50.81	TL16	40.75	HL4	71.18	BQ1	69.22
SC11	46.41	DJC15	46.60	DJC20	38.50	HL23	66.25	HL8	67.50
MDJ3	42.83	HC11	45.88	MDJ3	38.37	DQ11	65.42	DJC5	65.24
HL6	40.18	WRH7	44.68	BQ1	36.97	SC18	62.47	HL4	64.98
HL8	39.35	DQ23	44.46	HL23	36.93	TL3	62.07	WRH6	64.15
⋮	⋮	⋮	⋮	⋮	⋮	⋮	⋮	⋮	⋮
QTH7	29.21	WRH2	30.32	QTH6	27.06	DQ19	37.50	DQ11	44.17
QTH9	29.04	DQ17	30.17	WRH10	26.92	SC17	37.35	YBL16	44.13
DJC17	29.03	WRH4	30.13	DJC6	26.87	WRH16	37.03	DQ14	43.99
DJC8	29.00	DJC6	30.10	CK	26.74	WRH8	36.99	MDJ10	43.66
DJC26	28.97	QTH4	29.97	DJC13	26.71	HL14	36.92	WRH7	42.52
⋮	⋮	⋮	⋮	⋮	⋮	⋮	⋮	⋮	⋮
DJC12	18.78	DJC2	21.68	SC5	19.25	QY7	23.02	QTH4	29.52
SC23	17.48	DQ3	20.38	DQ15	19.01	WRH3	21.68	DQ1	29.23
DJC13	16.25	HL15	20.17	DQ3	18.59	TL16	21.30	BQ6	27.73
YBL13	13.74	QTH9	20.02	QTH9	17.54	DQ3	19.13	WC14	27.28
DJC4	13.67	SC1	19.62	HL15	16.56	DJC12	18.37	WRH16	22.62

2.3　胡桃楸生长性状方差分析及种源家系的选择

家系处于不同设计，为克服环境对试验造成的影响，先对 5 个区分别进行方差分析，然后再标准化(除与小区对照)后合并进行分析(见表 5)。对种源试验林各性状方差分析表明，大部分性状在各变异来源间的差异均达极显著；树高连年生长量Ⅰ在种源间差异显著；7 年生的胸径在种源间呈差异显著，5 年生的胸径、6 年生的胸径在种源间差异不显著；胸径连年生长量Ⅰ在区组间差异不显著，胸径连年生长量Ⅱ在“种源×区组”间差异不显著。对家系试验林不同家系各性状方差分析表明，各年份各性状在其他变异来源间的差异均达极显著。因此，胡桃楸的生长性状在种源及家系间具有较大遗传差异，具备良种选择和改良的遗传基因基础，在选择适应性强、生长量高的优良种源及家系中具有较大改良潜力。对各家系胡桃楸遗传力进行分析，5 年生树高的遗传力为 0.87、6 年生树高的遗传力为 0.86、7 年生树高的遗传力为 0.90，树高连年生长量Ⅰ的遗传力为 0.87、树高连年生长量Ⅱ的遗传力为 0.82；5 年生胸径的遗传力为 0.79、6 年生胸径的遗传力为 0.84、7 年生胸径的遗传力为 0.88；胸径连年生长量Ⅰ的遗传力为 0.80、胸径连年生长量Ⅱ的遗传力为 0.84。树高与胸径遗传力随林龄的提升而增大，生长量遗传力随林龄提升而减少。家系所有性状遗传力均超过 0.70，属于较高水平。

表 5　胡桃楸种源、家系不同性状方差分析结果

性状		种源				家系				
		变异来源	自由度	均方	*F* 值	变异来源	自由度	均方	*F* 值	遗传力
树高	5 年生树高	种源	15	0.432	2.175 **	家系	132	0.478	7.803 **	0.87
		区组	3	4.813	24.207 **	区组	3	1.780	29.050 **	
		种源×区组	45	0.558	2.808 **	家系×区组	396	0.339	5.525 **	
	6 年生树高	种源	15	0.782	2.819 **	家系	132	0.337	7.119 **	0.86
		区组	3	5.152	18.577 **	区组	3	1.342	28.392 **	
		种源×区组	45	0.928	3.345 **	家系×区组	396	0.297	6.284 **	
	7 年生树高	种源	15	0.773	2.257 **	家系	132	0.326	9.646 **	0.90
		区组	3	5.461	15.936 *	区组	3	0.111	3.289 *	
		种源×区组	45	1.221	3.564 **	家系×区组	396	0.216	6.388 **	
	树高连年生长量Ⅰ	种源	15	0.142	2.91 **	家系	132	1.483	7.462 **	0.87
		区组	3	0.572	11.711 **	区组	3	10.583	53.263 **	
		种源×区组	45	0.159	3.249 **	家系×区组	396	1.254	6.312 **	
	树高连年生长量Ⅱ	种源	15	0.155	1.720 *	家系	132	1.328	5.562 **	0.82
		区组	3	0.495	5.502 **	区组	3	10.163	42.553 **	
		种源×区组	45	0.181	2.019 **	家系×区组	396	1.203	5.037 **	

(C)1994-2023 China Academic Journal Electronic Publishing House. All rights reserved.　http://www.cnki.net

续(表5)

性状		种源				家系				
		变异来源	自由度	均方	F值	变异来源	自由度	均方	F值	遗传力
胸径	5年生胸径	种源	15	0.553	1.464	家系	132	0.377	4.691**	0.79
		区组	3	5.475	14.498**	区组	3	3.890	48.436**	
		种源×区组	45	0.914	2.421**	家系×区组	396	0.400	4.983**	
	6年生胸径	种源	15	1.356	1.672	家系	132	0.579	6.308**	0.84
		区组	3	8.964	11.054**	区组	3	1.143	12.445**	
		种源×区组	45	1.490	1.838**	家系×区组	396	0.489	5.324**	
	7年生胸径	种源	15	1.917	1.823*	家系	132	0.505	8.447**	0.88
		区组	3	9.587	9.116**	区组	3	0.721	12.076**	
		种源×区组	45	2.348	2.233**	家系×区组	396	0.385	6.438**	
	胸径连年生长量Ⅰ	种源	15	0.353	2.576**	家系	132	1.457	4.924**	0.80
		区组	3	0.136	0.995	区组	3	6.280	21.224**	
		种源×区组	45	0.250	1.823**	家系×区组	396	1.151	3.888**	
	胸径连年生长量Ⅱ	种源	15	0.565	2.315**	家系	132	1.748	6.300**	0.84
		区组	3	0.402	1.646	区组	3	9.977	35.953**	
		种源×区组	45	0.230	0.943	家系×区组	396	1.897	6.836**	

注：* 表示差异显著($P<0.05$)；** 表示差异极显著($P<0.01$)。

对胡桃楸种源间生长性状均值进行分析比较(见表6、表7)，按照20%入选率对各性状进行筛选，结果表明：5年生树高入选种源为宾县大泉子、宝清、七台河，树高均值为1.84 m，高出当年群体均值13.58%，高出当年群体对照19.48%；6年生树高入选种源为宾县大泉子、富锦、虎林，树高均值为2.77 m，高出当年群体均值13.99%，高出当年群体对照17.37%；7年生树高入选种源为宾县大泉子、虎林、富锦，树高均值为3.53 m，高出当年群体均值11.36%，高出当年群体对照13.50%。5年生胸径入选种源为五常、亚布力、宾县大泉子，胸径均值为2.21 cm，高出当年群体均值11.06%，高出当年群体对照17.55%；6年生胸径入选种源为宾县大泉子、富锦、虎林，胸径均值为3.50 cm，高出当年群体均值18.24%，高出当年群体对照17.45%；7年生胸径入选种源为宾县大泉子、宝清、富锦，胸径均值为4.61 cm，高出当年群体均值13.83%，高出当年群体对照12.71%。树高连年生长量Ⅰ入选的种源为宾县大泉子、五常、富锦，树高连年生长量Ⅰ均值为0.92 m，高出当年群体均值21.05%，高出当年群体对照24.32%；树高连年生长量Ⅱ入选的种源为宝清、宾县大泉子、富锦，树高连年生长量Ⅱ均值为0.76 m，高出当年群体均值10.14%，高出当年群体对照5.56%。胸径连年生长量Ⅰ入选的种源为虎林、宾县大泉子、富锦，胸径连年生长量Ⅰ均值为1.35 cm，高出当年群体均值17.39%，高出当年群体对照8.00%；胸径连年生长量Ⅱ入选的种源为宝清、富锦、宾县大泉子，胸径连年生长量Ⅱ均值为1.41 cm，高出当年群体均值24.78%，高出当年群体对照23.68%。其中，个别种源各性状在5、6、7年生中均多次入选，如宾县大泉子、宝清、富锦、七台河、虎林、五常等种源，其中宾县大泉子种源各性状在所有年度中均入选前3名，虎林、富锦种源后2年各性状均值排名靠前，宝清种源树高、胸径连年增长量Ⅱ均值排名靠前。

表6　胡桃楸种源树高性状均值

5年生树高		6年生树高		7年生树高		树高连年生长量Ⅰ		树高连年生长量Ⅱ	
种源	均值/m	种源	均值/m	种源	均值/m	种源	均值/m	种源	均值/m
宾县大泉子	1.96	宾县大泉子	2.97	宾县大泉子	3.73	宾县大泉子	1.00	宝清	0.89
宝清	1.81	富锦	2.68	虎林	3.47	五常	0.88	宾县大泉子	0.78
七台河	1.75	虎林	2.67	富锦	3.38	富锦	0.87	富锦	0.78
虎林	1.71	五常	2.61	宝清	3.33	铁力	0.84	七台河	0.76
富锦	1.70	宝清	2.56	七台河	3.30	虎林	0.81	东京城	0.74
铁力	1.68	铁力	2.51	五常	3.26	三岔子	0.77	五常	0.73
东京城	1.66	三岔子	2.47	铁力	3.21	七台河	0.75	东方红	0.72
牡丹江	1.66	七台河	2.47	三岔子	3.17	对照	0.72	对照	0.72
三岔子	1.65	牡丹江	2.41	牡丹江	3.15	金山屯	0.71	三岔子	0.70
五常	1.63	对照	2.36	对照	3.11	宝清	0.71	虎林	0.69
亚布力	1.57	东京城	2.34	东京城	3.09	东京城	0.70	铁力	0.68
对照	1.54	亚布力	2.29	亚布力	3.00	临江	0.69	牡丹江	0.68
珲春	1.50	临江	2.22	东方红	2.99	东方红	0.69	珲春	0.66
临江	1.49	珲春	2.19	临江	2.92	牡丹江	0.68	亚布力	0.65
东方红	1.49	金山屯	2.17	珲春	2.84	珲春	0.68	临江	0.61
金山屯	1.45	东方红	2.15	金山屯	2.81	亚布力	0.67	金山屯	0.60

(C)1994-2023 China Academic Journal Electronic Publishing House. All rights reserved. http://www.cnki.net

表 7　胡桃楸种源胸径性状均值

5 年生胸径		6 年生胸径		7 年生胸径		胸径连年生长量Ⅰ		胸径连年生长量Ⅱ	
种源	均值/cm	种源	均值/cm	种源	均值/cm	种源	均值/cm	种源	均值/cm
五常	2.25	宾县大泉子	3.55	宾县大泉子	4.96	虎林	1.37	宝清	1.51
亚布力	2.19	富锦	3.50	宝清	4.45	宾县大泉子	1.36	富锦	1.38
宾县大泉子	2.19	虎林	3.45	富锦	4.41	富锦	1.33	宾县大泉子	1.35
虎林	2.18	亚布力	3.14	虎林	4.34	对照	1.28	七台河	1.33
宝清	2.17	五常	3.12	七台河	4.27	三岔子	1.19	东方红	1.27
富锦	2.16	七台河	2.98	五常	4.12	五常	1.19	亚布力	1.19
铁力	2.05	对照	2.98	对照	4.09	铁力	1.13	对照	1.18
七台河	2.01	铁力	2.95	牡丹江	3.98	宝清	1.12	五常	1.17
东京城	1.94	宝清	2.93	亚布力	3.96	东方红	1.11	东京城	1.15
牡丹江	1.89	三岔子	2.85	东京城	3.94	东京城	1.11	珲春	1.15
对照	1.88	牡丹江	2.82	铁力	3.92	七台河	1.10	牡丹江	1.13
三岔子	1.85	东京城	2.78	东方红	3.85	金山屯	1.04	金山屯	1.13
金山屯	1.80	金山屯	2.71	三岔子	3.85	牡丹江	1.01	三岔子	1.06
临江	1.79	珲春	2.68	珲春	3.80	亚布力	0.98	虎林	1.03
东方红	1.69	东方红	2.54	金山屯	3.60	珲春	0.97	铁力	0.99
珲春	1.69	临江	2.53	临江	3.45	临江	0.96	临江	0.95

对胡桃楸家系间生长性状进行多重分析比较(见表 8、表 9),按照 20%入选率对各性状进行筛选。结果表明:5 年生树高排名前 20%的家系,树高均值为 1.90 m,高出当年群体均值 17.28%,高出对照家系均值 15.85%;6 年生树高排名前 20%的家系,树高均值为 2.80 m,高出当年群体均值 16.67%,高出对照家系均值 12.00%;7 年生树高排名前 20%的家系,树高均值为 3.59 m,高出当年群体均值 14.70%,高出对照家系均值 9.79%;树高连年生长量Ⅰ排名前 20%的家系,树高连年生长量Ⅰ均值为 0.90 m,高出当年群体均值 16.88%,高出对照家系均值 12.50%;树高连年生长量Ⅱ排名前 20%的家系,树高连年生长量Ⅱ均值为 0.80 m,高出当年群体均值 19.40%,高出对照家系均值 12.68%。5 年生胸径排名前 20%的家系,胸径均值为 2.31 m,高出当年群体均值 16.67%,高出对照家系均值 12.14%;6 年生胸径排名前 20%的家系,胸径均值为 3.46 cm,高出当年群体均值 18.09%,高出对照家系均值 17.69%;7 年生胸径排名前 20%的家系,胸径均值为 4.54 cm,高出当年群体均值 17.62%,高出对照家系均值 12.38%;胸径连年生长量Ⅰ排名前 20%的家系,胸径连年生长量Ⅰ均值为 1.38 cm,高出当年群体均值 16.95%,高出对照家系均值 20.00%;胸径连年生长量Ⅱ排名前 20%的家系,胸径连年生长量Ⅱ均值为 1.36cm,高出当年群体均值 22.52%,高出对照家系均值 14.29%。入选家系,5 年生树高的遗传增益为 13.95%、6 年生树高的遗传增益为 14.23%、7 年生树高的遗传增益为 13.39%、树高连年生长量Ⅰ的遗传增益为 14.19%、树高连年生长量Ⅱ的遗传增益为 16.13%,5 年生胸径的遗传增益为 13.10%、6 年生胸径的遗传增益为 15.47%、7 年生胸径的遗传增益为 15.46%、胸径连年生长量Ⅰ的遗传增益为 13.88%、胸径连年生长量Ⅱ的遗传增益为 19.13%。

表 8　胡桃楸家系树高性状均值

5 年生树高		6 年生树高		7 年生树高		树高连年生长量Ⅰ		树高连年生长量Ⅱ	
家系	均值/m	家系	均值/m	家系	均值/m	家系	均值/m	家系	均值/m
QY10	2.13a	QY10	3.07a	QY10	3.96a	WRH2	0.97a	BQ2	0.93a
QTH2	2.01ab	WRH2	3.03ab	BQ2	3.82ab	QTH8	0.94ab	WRH10	0.92ab
WRH2	2.01ab	QTH2	3.03abc	QTH9	3.82ab	QTH2	0.94ab	DQ14	0.89abc
QY9	2.00abc	QTH8	2.94abcd	QTH2	3.80abc	DJC7	0.93abc	SC1	0.87abcd
QTH5	1.99abcd	QY9	2.92abcde	WRH2	3.77abcd	QY9	0.92abcd	HL22	0.86abcde
BQ3	1.99abcd	QTH5	2.91abcdef	BQ6	3.73abcde	WRH11	0.92abcd	QTH9	0.83abcdef
QY3	1.97abcde	QY2	2.90abcdef	QTH5	3.73abcde	QTH5	0.91abcd	WC9	0.83abcdef
QY2	1.94abcdef	DJC7	2.90abcdef	QY9	3.68abcdef	MDJ10	0.91abcd	WRH2	0.82abcdefg
QTH8	1.92abcdefg	BQ6	2.86abcdefg	HL22	3.67abcdefg	MDJ11	0.91abcd	QTH2	0.82abcdefgh
DFH19	1.91abcdefg	SC27	2.82abcdefgh	SC1	3.64abcdefgh	WRH14	0.91abcd	HL26	0.80abcdefghi
HL7	1.91abcdefgh	QY7	2.81abcdefghi	WRH16	3.64abcdeefghi	WRH16	0.91abcde	QY10	0.79abcdefghij
SC27	1.90abcdefghi	QTH9	2.79abcdefghij	QY2	3.60abcdeefghij	QY7	0.90abcde	BQ6	0.79abcdefghijk
WRH15	1.88abcdefghij	BQ3	2.79abcdefghij	QY3	3.57abcdefefghij	QTH9	0.89abcdef	WRH16	0.79abcdefghijkl
⋮	⋮	⋮	⋮	⋮	⋮	⋮	⋮	⋮	⋮

(C)1994-2023 China Academic Journal Electronic Publishing House. All rights reserved.　http://www.cnki.net

续(表 8)

5 年生树高		6 年生树高		7 年生树高		树高连年生长量Ⅰ		树高连年生长量Ⅱ	
家系	均值/m	家系	均值/m	家系	均值/m	家系	均值/m	家系	均值/m
DQ1	1.21ooppqq	YBL13	1.81ssttuuvv	DJC4	2.39nnoopp	HL15	0.54ccddeeff	MDJ7	0.50vwxy
DJC5	1.20ppqq	SC25	1.79ssttuuvv	SC18	2.39nnoopp	DQ11	0.53ddeeff	HL12	0.50vwxy
TL30	1.18ppqq	DJC4	1.75ttuuvv	TL30	2.37nnoopp	YBL6	0.52eeff	MDJ9	0.49wxy
DJC4	1.17ppqq	DQ1	1.68uuvv	TL3	2.32oopp	DQ1	0.47ff	SC18	0.49xy
YBL13	1.16qq	HL23	1.67vv	DQ1	2.26pp	HL23	0.46ff	MDJ2	0.47y

注:表中列出不同林龄时树高性状中排名前 13 与后 5 的家系;其中,a、b、c、…、z 为邓肯(Duncan)分析的等级前 26 名排序;aa、bb、cc、…、vv 为后续排序;$P<0.05$。

表 9 胡桃楸家系胸径性状均值

5 年生胸径		6 年生胸径		7 年生胸径		胸径连年生长量Ⅰ		胸径连年生长量Ⅱ	
家系	均值/cm	家系	均值/cm	家系	均值/cm	家系	均值/cm	家系	均值/cm
QTH2	2.63a	QY2	3.88a	QTH5	5.06a	DJC4	1.59a	WC3	1.53a
DJC7	2.61ab	SC27	3.77ab	QTH9	4.90ab	HL8	1.52ab	MDJ5	1.46ab
QY2	2.53abc	QTH5	3.75ab	QY10	4.87abc	TL7	1.45abc	WC1	1.44abc
QY10	2.52abcd	QTH2	3.73ab	QY2	4.84abcd	MDJ10	1.44abcd	BQ2	1.43abcd
WRH9	2.51abcd	QY10	3.71abc	QY9	4.73abcde	DJC6	1.44abcd	HL23	1.43abcde
WRH2	2.50abcde	BQ6	3.70abcd	HL7	4.70abcdef	YBL12	1.44abcd	QTH12	1.42abcdef
WRH15	2.40abcdef	QTH9	3.62abcde	BQ6	4.68abcdefg	QTH5	1.41abcde	WRH3	1.42abcdefg
QTH5	2.38abcdefg	DJC7	3.54abcdef	BQ2	4.64abcdefgh	BQ6	1.40abcdef	WRH16	1.39abcdefgh
WC9	2.36abcdefgh	WRH10	3.53abcdefg	WRH16	4.63abcdefghi	DJC7	1.40abcdef	WC14	1.39abcdefghi
QY7	2.34abcdefghi	MDJ10	3.50abcdefgh	QTH2	4.58abcdefghij	SC27	1.40abcdef	MDJ11	1.38abcdefghij
TL7	2.27abcdefghij	WRH2	3.48abcdefghi	WRH2	4.56abcdefghij	QTH9	1.39abcdefg	WC5	1.37abcdefghijk
QY9	2.26abcdefghij	BQ2	3.45abcdefghij	DJC7	4.56abcdefghij	SC29	1.39abcdefg	QTH4	1.37abcdefghijkl
WC8	2.26abcdefghij	YBL12	3.42abcdefghijk	HL1	4.50abcdefghijk	QY7	1.38abcdefgh	QTH9	1.36abcdefghijklm
⋮	⋮	⋮	⋮	⋮	⋮	⋮	⋮	⋮	⋮
HL15	1.44ffgghhiijj	SC20	2.21gghhiijjkk	TL16	2.85ooppqqrrss	SC8	0.89klmno	MDJ3	0.79xyzaabb
DQ1	1.44gghhiijj	DQ1	2.13hhiijjkk	TL30	2.66ppqqrrss	SC25	0.87lmno	DQ1	0.76yzaabb
SC20	1.43hhiijj	HL23	2.10iijjkk	TL3	2.64qqrrss	DJC15	0.84mno	DJC4	0.73zaabb
DJC4	1.35iijj	TL3	2.06jjkk	SC25	2.61rrss	DQ11	0.72no	SC25	0.70aabb
YBL13	1.33jj	SC25	2.02kk	DQ1	2.50ss	YBL6	0.59o	HL15	0.66bb

注:表中列出不同林龄时树高性状中排名前 13 与后 5 的家系;其中,a、b、c、…、z 为邓肯(Duncan)分析的等级前 26 名排序;aa、bb、cc、…、ss 为后续排序;$P<0.05$。

2.4 胡桃楸优良种源家系的综合评价

以树高、胸径、树高连年生长量、胸径连年生长量 4 个性状对胡桃楸种源进行综合评价,以 20%的入选率对 16 个种源进行评价选择。由表 10 可见:种源宾县大泉子、富锦、宝清入选,种源 Q_i 值均值为 1.59,入选的 3 个种源,种源宾县大泉子 Q_i 值为 1.71、种源富锦 Q_i 值为 1.65、种源宝清 Q_i 值 1.64。树高平均值为 3.48 m、胸径平均值为 4.61 cm、树高连年生长量平均值为 0.82 m、胸径连年生长量平均值为 1.26 cm,分别比种源群体平均值高 9.78%、13.83%、9.33%、8.62%,分别比对照高 11.90%、15.25%、12.33%、5.44%;树高现实增益平均值为 9.75%、胸径现实增益平均值为 13.80%、树高连年生长量现实增益平均值为 9.54%、胸径连年生长量现实增益平均值为 8.50%。单性状种源选择中,宾县大泉子、富锦、宝清、虎林、五常种源入选;其中宾县大泉子、富锦种源在各性状比较中均入选前 3 名,因此在综合对比中优势明显;宝清家系虽在树高、胸径分析中未入选前 3,但其连年生长量排名靠前,综合评定时排名位于第 3 名。

以树高、胸径、树高连年长量、胸径连年长量 4 个性状对胡桃楸家系进行综合评价,以 20%的入选率对 133 个家系进行评价选择(见表 11)。结果表明:27 个家系入选,入选家系 Q_i 值均值为 1.67,BQ2、BQ6、QTH5、QTH9、DQ3、MDJ10、WRH16 等入选家系,在单性状家系选择中性状均值排名均在前列。入选的 27 个家系,树高平均值为 3.54 m、胸径平均值为 4.51 cm、树高连年生长量平均值为 0.83 m、胸径连年生长量平均值 1.32 cm,分别比家系群体平均值高 13.10%、16.84%、10.67%、10.00%,分别比对照高 8.26%、11.63%、9.93%、12.82%;树高遗传增益均值为 11.84%、胸径遗传增益均值为 14.32%、树高连年生长量遗传增益均值为 8.84%、胸径连年生长量遗传增益均值为 6.39%。入选家系中,七台

(C)1994-2023 China Academic Journal Electronic Publishing House. All rights reserved. http://www.cnki.net

河、清原各占 14.81%,万人欢、虎林各占 11.11%,宝清、牡丹江、宾县大泉子、三岔子、五常各占 7.41%,东京城、东方红、铁力各占 3.70%。

表 10　胡桃楸种源综合评价(Q_i 值)结果

种源	Q_i 值	种源	Q_i 值
大泉子	1.71	三岔子	1.57
富锦	1.65	牡丹江	1.57
宝清	1.64	东方红	1.56
虎林	1.63	珲春	1.53
五常	1.62	亚布力	1.53
七台河	1.62	金山屯	1.51
铁力	1.59	临江	1.49
对照	1.59	平均值	1.59
东京城	1.57		

表 11　胡桃楸家系综合评价(Q_i 值)结果

家系	Q_i 值	家系	Q_i 值
BQ2	1.74	QY2	1.66
QTH9	1.73	SC1	1.66
QTH5	1.73	HL7	1.66
BQ6	1.70	WC9	1.65
QTH2	1.69	HL1	1.64
WRH16	1.69	QTH12	1.64
MDJ10	1.68	SC27	1.64
QY10	1.68	DFH19	1.64
QY9	1.68	DQ14	1.64
HL22	1.67	WRH4	1.63
DQ3	1.67	WC1	1.63
MDJ11	1.67	QY7	1.63
WRH2	1.67	TL7	1.63
DJC7	1.67	平均值	1.67

3　结论与讨论

在林木育种研究中,方差分析是评估变异幅度的重要方法[17]。本研究中,种源试验林中的 16 个种源,各性状虽然大部分性状在各变异来源间的差异达到极显著水平,但胸径相关性状在种源间显著水平较低,在 7 年生时才开始达到显著水平;家系各性状,在各变异来源间的差异均达到极显著水平。这种情况是由于种源间差异显著性小于种源内,造林初期环境条件对种源影响较大,导致胸径性状在种源间差异不显著;随着林龄增加,种源对当地环境适应,开始逐渐显示出自身差异性,差异显著水平开始提高;而家系,由于差异性较强,在前期各性状之间差异便能达到极显著水平。各种源、家系间,各性状存在较大差异,具有丰富的遗传变异,说明胡桃楸种源、家系存在较大改良潜力。

遗传和变异是林木育种研究的主要内容,是林木遗传改良的基础,变异系数可对群体的变异程度进行评价,能对某个性状的遗传变异情况进行评估[18-19]。本研究中,种源间,不同年份树高性状的变异系数变化范围为 21.97%~38.79%,胸径性状的变异系数变化范围为 27.99%~36.47%,无论是树高还是胸径,变异系数均随着林龄增大而减小;家系间,不同年份树高性状的变异系数变化范围为 24.23%~41.54%,胸径性状的变异系数变化范围为 30.08%~45.33%,除 6 年生胸径变异系数比 5 年生胸径变异系数略有增加外,树高、胸径性状变异系数在其余各年度间均随着林龄增加而呈现递减趋势;种源与家系变化趋势相同,说明胡桃楸种源、家系各性状间存在丰富的变异,同一种源、家系家族特征随林龄增大变得更明显。此外,本研究中,无论是种源还是家系,胸径的变异系数都高于树高的变异系数。本研究中,家系各性状遗传力均大于 0.70,且树高、胸径的遗传力大于 0.80,属于高遗传力,表明试验家系各性状能在子代中稳定遗传。一般认为,同一群体的各种性状的遗传力的估值存在一定规律性,往往表现为遗传力随林龄增加而增大的趋势[20],本研究所选家系材料存在丰富变异,更有利于家系间选择。

综合评价可以对多个性状同时进行评价,选育出的优良家系和单株更稳定[21]。本研究在对胡桃楸种源、家系的评价中,以 20%的入选率对胡桃楸种源、家系进行筛选,共筛选出 3 个种源、27 个家系。入选的 3 个种源,树高、胸径、树高连年生长量、胸径连年生长量,分别比种源群体平均值高 9.78%、13.83%、9. 33%、8. 62%;树高现实增益平均值为 9.75%、胸径现实增益平均值为 13.80%、树高连年生长量现实增益平均值为 9.54%、胸径连年生长量现实增益平均值为 8.50%。入选的 27 个家系,树高、胸径、树高连年生长量、胸径连年生长量,分别比家系群体平均值高 13.10%、16.84%、10.67%、10.00%;树高遗传增益均值为 11.84%、胸径遗传增益均值为 14.32%、树高连年生长量遗传增益均值为 8.84%、胸径连年生长量遗传增益均值为 6.39%。入选种源、家系表现出明显的生长优势,选择出的种源家系可尝试在生产中加以应用。

参　考　文　献

[1]　王东娜,牟长城,冯富娟.胡桃楸 ISSR-PCR 反应体系的建立及优化[J].实验室研究与探索,2010,29(11):18-22,37.
[2]　刘玉平.宽甸地区核桃楸高效培育技术[J].现代农业科技,2020(15):155-156.
[3]　朱红波,赵云,林士杰,等.核桃楸资源研究进展[J].中国农学通报,2011,27(25):1-4.
[4]　曹政.胡桃楸[J].农业科学实验,1982(11):43,44.
[5]　王宇.东北地区胡桃楸遗传多样性 SRAP 研究[D].哈尔滨:东北林业大学,2007.
[6]　韩玉霞,吴琳,于琪,等.39 个核桃楸家系苗期生长性状变异分析[J].吉林林业科技,2020,49(6):1-4.

(下转 64 页)

(C)1994-2023 China Academic Journal Electronic Publishing House. All rights reserved.　http://www.cnki.net

(4):755-762.
[16] 国家药典委员会.中华人民共和国药典[M].北京:中国医药科技出版社,2015.
[17] 林平,李吉跃,陈崇.银杏光合生理生态特性研究[J].北京林业大学学报,2008,30(6):22-29.
[18] 赵辉,吕良贺,路鑫,等.杂种金叶银杏叶片光合特性分析[J].南京林业大学学报(自然科学版),2020,44(1):193-199.
[19] 苑景淇,于忠亮,兰雪涵,等.遮阴对濒危植物朝鲜崖柏光合特性的影响[J].南京林业大学学报(自然科学版),2022,46(5):58-66.
[20] 张往祥,吴家胜,曹福亮.光强对银杏光合作用和光化学效率的影响[J].南京林业大学学报(自然科学版),2002,26(6):5-9.
[21] ZHANG L, WANG G P, WANG G B, et al. *Ginkgo biloba* L. responds to red and blue light: via phenylpropanoid and flavonoid biosynthesis pathway[J]. Forests, 2021, 12(8). doi: 10.3390/f12081079.
[22] PACHECO F V, ALVARENGA I C A, RIBEIRO JUNIOR P M, et al. Growth and production of secondary compounds in monkey-pepper (*Piper aduncum* L.) leaves cultivated under altered ambient light[J]. Australian Journal of Crop Science, 2014, 8(11): 1510-1516.
[23] 朱灿灿.银杏叶次生代谢产物的环境诱导机制及其调控[D].南京:南京林业大学,2010.
[24] 曹福亮,汪贵斌,郁万文.银杏叶用林定向培育技术体系的集成[J].南京林业大学学报(自然科学版),2014,38(6):146-152.
[25] 汪贵斌,郭旭琴,常丽,等.温度和土壤水分对银杏叶黄酮类化合物积累的影响[J].应用生态学报,2013,24(11):3077-3083.
[26] 钱龙梁,李佳佳,曹福亮,等.生物遮阴对银杏幼苗次生代谢的影响[J].南京林业大学学报(自然科学版),2019,43(3):189-194.
[27] 王华田,谢宝东,姜岳忠,等.光照强度对银杏叶片发育及黄酮和内脂含量的影响[J].江西农业大学学报(自然科学版),2002,24(5):617-622.
[28] 冷平生,苏淑钗,王天华,等.光强与光质对银杏光合作用及黄酮苷与萜类内酯含量的影响[J].植物资源与环境学报,2002,11(1):1-4.
[29] JUN S Y, SATTLER S A, CORTEZ G S, et al. Biochemical and structural analysis of substrate specificity of a phenylalanine ammonia-lyase[J]. Plant Physiology, 2018, 176(2): 1452-1468.
[30] TOHGE T, WATANABE M, HOEFGEN R, et al. The evolution of phenylpropanoid metabolism in the green lineage[J]. Critical Reviews in Biochemistry and Molecular Biology, 2013, 48(2): 123-152.
[31] 董理想,倪君.银杏中黄酮类化合物生物合成、调控机制及其影响因素的研究进展[J].中国中药杂志,2017,42(18):3497-3503.

(上接 17 页)

[7] ZAS R, MERLO E, FERNÁNDEZ LÓPEZ J. Genetic parameter estimates for Maritime pine in the Atlantic coast of North-west Spain[J]. International Journal of Forest Genetics, 2004, 11(1): 45-53.
[8] PINYOPUSARERK K, WILLIAMS E R. Range-wide provenance variation in growth and morphological characteristics of *Casuarina equisetifolia* grown in Northern Australia[J]. Forest Ecology and Management, 2000, 134(1/2/3): 219-232.
[9] 颜廷武,于世河,王骞春,等.辽宁地区核桃楸半同胞家系间苗期生长差异分析[J].辽宁林业科技,2020(6):13-15.
[10] 芦贤博,徐连峰,庞忠义,等.胡桃楸种源家系幼龄期生长变异及选择研究[J].林业科学研究,2022,35(1):20-30.
[11] 李佳娜,高瑞馨.我国胡桃楸的遗传育种研究进展[J].安徽农业科学,2020,48(17):4-7.
[12] 续九如.林木数量遗传学[M].北京:高等教育出版社,2006.
[13] WANG F, ZHANG Q H, TIAN Y G, et al. Comprehensive assessment of growth traits and wood properties in half-sib *Pinus koraiensis* families[J]. Euphytica, 2018, 214(11). doi: 10.1007/s10681-018-2290-4.
[14] 陈晓阳,沈熙环.林木育种学[M].北京:高等教育出版社,2005.
[15] 王虹,师尚礼,张旭业,等.紫花苜蓿多元杂交后代产量和品质一般配合力分析及遗传参数的估算[J].草业学报,2016,25(3):126-134.
[16] 朱之悌.林木遗传学基础[M].北京:中国林业出版社,1990.
[17] SAFAVI S A, POURDAD S S, TAEB M, et al. Assessment of genetic variation among safflower (*Carthamus tinctorius* L.) accessions using agro-morphological traits and molecular markers[J]. Journal of Food Agriculture & Environment, 2010, 8(3/4): 616-625.
[18] MWASE W F, SAVILL P S, HEMERY G E. Genetic parameter estimates for growth and form traits in common ash (*Fraxinus excelsior*, L.) in a breeding seedling orchard at Little Wittenham in England[J]. New Forest, 2008, 36(3): 225-238.
[19] METOUGUI M L, MOKHTARI M, MAUGHAN P J, et al. Morphological variability, heritability and correlation studies within an argan tree population (*Argania spinosa* (L.) Skeels) preserved in situ[J]. International Journal of Agriculture and Forestry, 2017, 7(2): 42-51.
[20] 曹汉洋.杉木第 2 代种子园半同胞子代测定及早期选择[J].南京林业大学学报(自然科学版),2011,35(1):19-23.
[21] 梁德洋.红松种子园亲本无性系及其子代变异选择研究[D].哈尔滨:东北林业大学,2021.

(上接 22 页)

[6] SUBRAMANIAN K N, NICODEMUS A, RADHAMANI A. Teak improvement in India[M]. Rome: Food and Agriculture Organization of the United Nations Forest Genetic Resources No.22, 1994.
[7] SUHAENDI H. Teak improvement in Indonesia[M]//KASHIO K, WHITE K. Teak for the future-proceedings of the second regional seminar on teak, Yangon, Myanmar. Bangkok: Food and Agriculture Organization of the United Nations Regional Office for Asia and the Pacific, 1998: 179-188.
[8] 邝炳朝.我国柚木栽培的回顾和展望[M]//中国林业科学研究院热带林业研究所.中国林业科学研究院热带林业研究所建所三十周年纪念文集.广州:中国林业科学研究院热带林业研究所,1992:69-72.
[9] 王宝生,曹月华,黄良胜.柚木茎尖的培养[J].植物学报,1980,22(2):200-201.
[10] 曹月华,王宝生.柚木(*Tectona grandis* L. f.)培养中诱导生根和移栽成活率的研究[J].植物学报,1981,23(6):434-440.
[11] 裘珍飞,曾炳山,刘英.柚木组培苗移植技术研究[J].广东林业科技,2001,17(4):1-5.
[12] 梁坤南,周再知,马华明,等.我国珍贵树种柚木人工林发展现状、对策与展望[J].福建林业科技,2011,38(4):173-178.
[13] 史富强,童清,杨华景,等.柚木优良无性系的早期选择[J].东北林业大学学报,2014,42(2):14-16.
[14] 黄桂华.柚木种质资源遗传变异和优良无性系早期选择的研究[D].北京:中国林业科学研究院,2015.
[15] 周强,黄桂华,张绍祥,等.肥料施肥对不同柚木无性系早期生长的影响[J].东北林业大学学报,2022,50(7):6-10.
[16] 邝炳朝,郑淑珍,罗明雄,等.柚木种源主要性状聚合遗传值的评价[J].林业科学研究,1996,9(1):7-14.
[17] PAYNE R W, LANE P W, AINSLEY A E, et al. The Genstat 5-reference manual[M]. Oxford: Oxford University Press, 1989.
[18] 林元震.R 与 ASReml-R 统计学[M].北京:中国林业出版社,2017.
[19] 何贵平,陈益泰,关志山,等.杉木无性系生长与分枝习性的遗传变异[J].林业科学研究,1997,10(5):556-559.
[20] 赵承开.杉木优良无性系早期选择年龄和增益[J].林业科学,2002,38(4):53-60.
[21] 徐化成.油松地理变异和种源选择[M].北京:中国林业出版社,1992.
[22] 袁嘉祖,冯晋臣.模糊数学及其在林业中的应用[M].北京:中国林业出版社,1988.
[23] 盛志廉,陈瑶生.数量遗传学[M].北京:科学出版社,1999.
[24] 卢翠香,邓紫宇,任世奇,等.11 个桉树无性系木材性状比较与选择[J].广西林业科学,2021,50(4):427-433.
[25] 严涵薇,程雅静,余彤彤,等.10 年生黑杨派无性系材性径向遗传变异分析与综合评价[J].林业科学研究,2021,34(6):28-37.
[26] 杨艳,唐洁,李永进,等.7 个南方适生杨树无性系生长和木材纤维性状分析与评价[J].浙江农林大学学报,2022,39(4):807-813.

(C)1994-2023 China Academic Journal Electronic Publishing House. All rights reserved. http://www.cnki.net
