# Supplementary material for: Provenance and family variations in early growth of Manchurian walnut (Juglans mandshurica Maxim.) and selection of superior families
Source: PLoS One. 2024 Mar 7;19(3):e0298918. doi: 10.1371/journal.pone.0298918 (PMC10919699; doi:10.1371/journal.pone.0298918)
Supplement: S1 File — (ZIP) [file pone.0298918.s004.zip › AnalysisofgeneticeffectsonacompletediallelcrosstestofBetulaplatyphylla.pdf]

ResearchGate

See discussions, stats, and author profiles for this publication at: https://www.researchgate.net/publication/267761714

Analysis of genetic effects on a complete diallel cross test of Betula platyphylla

Article *in* Euphytica · November 2014

DOI: 10.1007/s10681-014-1147-8

CITATIONS
17

READS
46

9 authors, including:

Xiyang Zhao
Northeast Forestry University

61 PUBLICATIONS **264** CITATIONS

SEE PROFILE

Guifeng Liu

81 PUBLICATIONS **1,373** CITATIONS

SEE PROFILE

Some of the authors of this publication are also working on these related projects:

Project National Key Research and Development Program of China (Grant No. 2016YFD0600404) View project

Project Transcriptome analysis of Pinus koraiensis in response to cold stress View project

All content following this page was uploaded by Xiyang Zhao on 26 October 2018.

The user has requested enhancement of the downloaded file.

Euphytica (2014) 200:221–229
DOI 10.1007/s10681-014-1147-8

Analysis of genetic effects on a complete diallel cross test of *Betula platyphylla*

Xiyang Zhao · Xiuyan Bian · Mengran Liu · Zhixin Li · Ying Li · Mi Zheng · Wenhua Teng · Jing Jiang · Guifeng Liu

Received: 3 November 2013 / Accepted: 8 May 2014 / Published online: 23 May 2014
© Springer Science+Business Media Dordrecht 2014

Abstract The study utilized the offspring of a 5 × 5 diallel cross of *Betula platyphylla* as the experimental material. Tree height (H), diameter at breast height (DBH) and stem straight degree (SSD) were measured at the fourth and eighth growth year (SSD were measured only in the eighth growth year). ANOVA analyses showed that growth traits in different growth years were significant among families ($P < 0.01$). There existed extremely significant correlations between H and DBH in the same year. Although the sequencing of average H and DBH of different families were vicissitudinous at the 4th and 8th year, there also existed significant correlations between H and DBH in different year. The general combining ability, special combining ability (SCA) and reciprocal effect (REC) of different hybridized combinations were also different remarkably. The mean square of SCA was significantly higher than GCA, implied that the non-additive effect played major roles. When B5, B8 and MB15 were selected as parents, the offspring performed perfectly in traits of H, DBH and SSD at the eighth growth year respectively. B5 × XB11, B8 × XB11 and B8 × MB15 had highest SCA values in traits of H, DBH, and SSD at the eighth growth year respectively. Consequently, in order to achieve optimal outcomes, seed orchard building and clonal propagation should be considered simultaneously in birch's genetic improvement process.

Keywords *Betula platyphylla* · Diallel cross · GCA · SCA · REC

Abbreviations

DBH	Diameter at breast height
H	Tree height
GCA	General combining ability
REC	Reciprocal effect
SSD	Stem straight degree
SCA	Specific combining ability

Xiyang Zhao and·Xiuyan Bian have contributed equally to this work.

X. Zhao · X. Bian · M. Liu · Z. Li · Y. Li · M. Zheng · W. Teng · J. Jiang · G. Liu (✉)
State Key Laboratory of Tree Genetics and Breeding, School of Forestry, Northeast Forestry University, Harbin 150040, People's Republic of China
e-mail: liuguifeng@126.com

X. Zhao
e-mail: zhaoxyphd@163.com

Introduction

Birch (*Betula platyphylla*) is the most extensively distributed broadleaf tree in northeastern, northern, northwestern and southwestern forest areas of China, and nearly one-third of the Daxing'an Mountains are mainly covered with it (Zeng et al. 2003). Because of rapid growth, strong adaptability, wide distribution and excellent wood quality, birch is one of the most important commercial tree species for papermaking, furniture making and plywood production (Li et al. 1995). Many

studies were conducted using birch, including intensive breeding (Yang et al. 2004), genetic and variation on provenance (Jiang et al. 2001), fiber length experiments (Wei et al. 2006), fungi in bark (Linnakoski et al. 2009; Liimatainen et al. 2012), hybridization breeding (Li et al. 2006), transgenic studies (Zhan et al. 2006) and molecular marker studies (Wei et al. 2010; Jiang et al. 2011). Due to low survival rate of cutting and grafting, provenances and families were the primary research materials in studies and crossbreeding was still the most important method for breeding new varieties.

Diallel cross was a set of possible combinations between lines, breeds or general populations (Jakubec et al. 1987). The system has been widely used in breeding either for the evaluation of combining ability and heterotic potential of varieties in crosses or for basic studies on the genetic structure of populations (Bahari et al. 2012; Sharma and Fanta 2010; Vuylsteke and Eeuwijk 2008; Lukonge et al. 2008; Moterle et al. 2012). Parameters including general combining ability (GCA), specific combining ability (SCA) and reciprocal effect (REC), were critically important to estimate crossbreeding effects for tree breeding. Those parameters help to identify the most desirable combiner that may be used to exploit hybrid vigor and heterosis became a routine tool for breeders to produce progeny that exhibit more desirable phenotype than those of their parental populations (Williams et al. 2002; Mekky et al. 2008). Generally, the degree of heterosis is significantly correlated with the degree of genetic resemblance between parental populations and is expected to be proportional to the degree of heterozgosity of the crosses (Sheridan 1981). Diallel analysis allows estimation of maternal effect needed to determine whether reciprocal crosses are likely to be equivalent (Waleed and Sajida 2011). Herein, an experiment was conducted in a 5 × 5 diallel mating system between different five birch parents to estimate crossbreeding effects (GCA, SCA and REC) for growth traits measured at the 4th and 8th growth years. Using offspring's growth traits to evaluate parental effects will help identify excellent parents for birch breeding.

Materials and methods

Experiment sites

The experimental forest land is located at the Maoer Mountain Experiment Forestry Center (45°20′N, 127°30′E), which has dark color and rich organic matter in soil. The climate was suitable for birch growth.

Materials and experiment design

Cross experiments were conducted in birch intensified seed orchard of the Northeast Forestry University in May of 2005. Five *B. platyphylla* (B5, B8, XB11, EB14, MB15) were selected as parents. The crossing design was shown in Table 1. Offspring were grown in the greenhouse for 2 years and then planted in the experimental field in the spring of 2007. The experiment utilized a randomized complete block design, with three blocks containing 20 families and 18 singles were planted in duplicate rows of each family. Seedlings were planted in rows with a 3 m × 3 m spacing for each block. H and DBH were measured at the 4th and 8th years and SSD were estimated by quantification based on (Zhao et al. 2012) at the 8th year after offsprings measured (Table 2).

Data analysis

Statistical analysis was carried out using the statistical package for the social science (SPSS) version 13.0 and the data processing system (DPS). SSD data were switched by SQRT in the calculation process. Significance of fixed effects were tested using *F* tests. Linear models (1) were used for joint analysis of the families by block together

$$Y_{ijkl} = \mu + x_{ij} + b_k + e_{ijkl,} \quad (1)$$

where y_{ijkl} is the performance of the ramet growing in the kth block; μ is the overall mean; x_{ij} is the of heterozygosis effect between i and j parent. B_k is the effect of the block. $(xb)_{ijk}$ is the effect of heterozygosis

Table 1 5 × 5 diallel cross design of cross parents in *B. platyphylla*

♀	♂				
	B 5	B 8	XB 11	EB 14	MB 15
B 5	–	×	×	×	×
B 8	×	–	×	×	×
XB 11	×	×	–	×	×
EB 14	×	×	×	–	×
MB 15	×	×	×	×	–

Table 2 Investigation criteria and scores of stem straightness degree

Traits	Scores				
	1	2	3	4	5
SSD	More than two obviously bend points in the stem	More than two slightly bend points or one obviously bend point in the stem	Two slightly bend points in the stem	One slightly bend point in the stem	Straight completely in the stem

Table 3 Variance analysis model of combining ability

Variation source	Degree of freedom (df)	Expected mean square (EMS)
GCA	$P-1$	$\sigma^2+2(P-2)\frac{1}{P-1}\sum_{i=1}^{P} g_i^2$
SCA	$P(P-3)/2$	$\sigma^2+2\frac{2}{P(P-3)}\sum_{i=1}^{P}\sum_{j=2}^{P} S_{ij}^2$
REC	$P(P-1)/2$	$\sigma^2+2\frac{2}{P(P-1)}\sum_{i=1}^{P}\sum_{j=2}^{P} r_{ij}^2$
Error	$ab(c-1)$	σ^2

by block and ε_{ijkl} is the random error. Model (1) can be decomposed to model (2) as the following:

$$X_{ij}=g_i+g_j+s_{ij}+r_{ij,} \tag{2}$$

where $g_{\rm i}$ and $g_{\rm j}$ were the GCA of parent i and j respectively. $s_{\rm ij}$ and $r_{\rm ij}$ was SCA effect and REC by heterozygosis effect between i and j parent respectively. Combining ability analysis were calculated as Table 3 (Griffing 1956).

The Penotypic correlation $r_A(xy)$ of traits x and y was calculated as the following (Pliura et al. 2007):

$$r_A(xy)=\frac{\sigma_{a(xy)}}{\sqrt{\sigma^2_{a(x)}\cdot\sigma^2_{a(y)}}}$$

where $\sigma^2_{a(x)}$ is the family variance component for the trait x, $\sigma^2_{a(y)}$ is the clone variance component for the trait y and $\sigma_{a(xy)}$ is the family covariance component.

Results

ANOVA analyses results

ANOVA analyses of H, DBH and SSD among different families and blocks were shown in Table 4. At the 4th year, variances among families and blocks were all significantly different in H and DBH. At the 8th year, variances among families were significant in traits of H, DBH and SSD, but among blocks, SSD traits were not significant. Because of significant variance among families, the evaluated for families and their parents were effective.

Average H, DBH and SSD of different parents

The average H, DBH and SSD of offspring of different hybridized combination at the 4th and 8th years were shown in Table 5. At the 4th year, the average H of offspring of B5 as the female parent was the highest (2.78 m), and the average H of offspring of B8 as the female parent was the lowest (2.57 m). The average DBH of the seedlings of different parents as female parents varied from 1.44 to 1.72 m. Offspring of B5 as the female parent indicated fast high growth. Offspring of EB14 as the male parent showed a higher average H and DBH than that of offspring of other parents, while offspring of XB11 had lowest averages. When trees grew up, the sorted order of average H and DBH of the seedlings belong to different parents were changed. For tree height, the seedlings of B5 and XB11 showed the height value of female and male parent respectively. The offspring of B8 showed the highest DBH whatever be male or female parents and MB15 were showed the highest value on SSD.

Average growth traits in different families

Average growth traits of different families were shown in Table 6. At the 4th year, average H and DBH of different hybridized combinations were 2.66 m and 1.60 cm respectively. H ranged from 2.34 m (B 8 × MB 15) to 3.02 m (B 5 × EB 14) and DBH varied between 1.29 cm (B 8 × MB 15) and 1.94 cm (B 5 × EB 14). At the 8th year, average H, DBH and SSD were 6.51 m, 6.34 cm and 1.73 respectively. The

Table 4 ANOVA analysis of H, DBH and S of *B. platyphylla*

Age	Variation source	H			DBH			SSD		
		df	*MS*	*F*	*df*	*MS*	*F*	*df*	*MS*	*F*
4	Family	19	1.79	7.21**	19	209.42	6.74**			
	Block	2	8.08	32.61**	2	332.50	10.70**			
8	Family	19	4.58	9.10**	19	6.85	5.92**	19	0.31	3.03**
	Block	2	1.87	3.72*	2	12.45	10.76**	2	0.12	1.15†

** *P* value < 0.01; * *P* value < 0.05; † *P* value > 0.05

Table 5 The average value of each trait of different parent as female or male

Parents	4 years old				8 years old					
	Female		Male		Female			Male		
	H (m)	DBH (cm)	H (m)	DBH (cm)	H (m)	DBH (cm)	SSD	H (m)	DBH (cm)	SSD
B 5	2.78	1.72	2.75	1.65	6.71	6.23	1.71	6.62	6.20	1.77
B 8	2.57	1.59	2.66	1.56	6.48	6.70	1.67	6.55	6.49	1.67
XB 11	2.65	1.55	2.53	1.50	6.42	6.16	1.68	6.71	6.41	1.69
EB 14	2.58	1.44	2.80	1.74	6.36	6.20	1.77	6.27	6.32	1.71
MB 15	2.74	1.68	2.59	1.54	6.61	6.44	1.79	6.41	6.31	1.78

Table 6 Significance of difference analysis of H, DBH and S in *B. platyphylla*

Hybridized combination	4 years old		8 years old		
	H (m)	DBH (cm)	H (m)	DBH (cm)	SSD
B 5 × B 8	2.60	1.54	6.47	6.08	1.61
B 5 × XB 11	2.55	1.49	7.01	6.16	1.68
B 5 × EB 14	3.02	1.94	6.41	6.00	1.75
B 5 × MB 15	2.94	1.92	6.96	6.69	1.80
B 8 × B 5	2.61	1.68	6.47	6.58	1.52
B 8 × XB 11	2.60	1.73	6.9	7.06	1.66
B 8 × EB 14	2.71	1.67	6.56	7.09	1.67
B 8 × MB 15	2.34	1.29	5.98	6.06	1.83
XB 11 × B 5	2.89	1.75	6.94	5.9	1.74
XB 11 × B 8	2.62	1.39	6.55	6.63	1.60
XB 11 × EB 14	2.59	1.49	5.88	5.91	1.62
XB 11 × MB 15	2.51	1.56	6.30	6.21	1.76
EB 14 × B 5	2.83	1.64	6.71	6.64	1.95
EB 14 × B 8	2.55	1.45	6.26	6.12	1.68
EB 14 × XB 11	2.39	1.31	6.05	5.74	1.74
EB 14 × MB 15	2.56	1.38	6.4	6.28	1.72
MB 15 × B 5	2.64	1.53	6.37	5.69	1.88
MB 15 × B 8	2.86	1.85	6.93	7.13	1.80
MB 15 × XB 11	2.59	1.49	6.89	6.67	1.69
MB 15 × EB 14	2.86	1.85	6.24	6.26	1.80
Average	2.66	1.60	6.51	6.34	1.73
Range	2.34–3.02	1.29–1.94	5.88–7.01	5.69–7.13	1.52–1.95

three traits ranged from 5.88 m to 7.01 m, 5.69 to 7.13 cm and 1.52 to 1.95 respectively. Family B 5 × XB 11 showed the highest average tree height, family MB 15 × B 8 showed the largest DBH and family EB 14 × B 5 had the most excellent stem straight degree.

ANOVA analyses of GCA, SCA and REC among different hybridized combinations

ANOVA analyses of GCA, SCA and REC were conducted for all hybridized combinations and results were shown in Table 7. There existed significant differences ($P < 0.01$) in GCA, SCA and REC among different hybridized combinations both at the 4th and 8th year.

GCA of different cross combinations

GCA was the most important gist of the cross parent selection. In this study, values of GCA effects were shown in Table 8. At the 4th year, B5 as the cross parents obtained higher GCA for H and DBH (0.1263 and 1.1367 for H and DBH respectively). Other parents showed lower or negative values. At the 8th year, B5 also showed positive GCA values for H and SSD, but showed a negative value for DBH. For DBH, B8 had the highest and positive value at the 8th year (0.3305). Parents B5, EB14, and MB15 all displayed positive GCA values for trait SSD, which indicated that these parents can promote the offspring's stem trait through crossing. Generally, B5, B8 and MB15 presented higher GCA values for traits H, DBH and SSD respectively. Therefore these three parents should be serious considered for the cross parent selection.

SCA and REC for each hybridized combination

If a certain parent has the ability to combine well with all other testers, it has higher GCA. But when the combination is only well in a certain cross, the SCA will be higher. REC was a very important gist for breeder to choose between orthogonal and reciprocal

Table 7 ANOVA analysis of GCA, SCA and REC of 5 × 5 complete diallel cross of *B. platyphylla*

Traits	Variation source	4 years old			8 years old	
		df	*MS*	*F*	*MS*	*F*
H	GCA	4	0.00	15.21**	0.178	19.07**
	SCA	5	0.00	6.54**	170.19	18,299.42**
	REC	10	0.00	11.57**	0.19	20.61**
DBH	GCA	4	3.44	9.28**	0.24	11.23**
	SCA	5	1.26	3.39**	161.37	7,533.02**
	REC	10	5.35	14.42**	0.42	19.59**
S	GCA	4			0.02	12.25**
	SCA	5			11.96	6,393.00**
	REC	10			0.01	4.66**

** *P* value<0.01

Table 8 GCA effect value of *B. platyphylla* parental

Parents	4 years old		8 years old		
	H	DBH	H	DBH	SSD
B 5	0.1263	1.1367	0.2025	−0.1709	0.0218
B 8	−0.0577	−0.2402	−0.0003	0.3305	−0.0724
XB 11	−0.0985	−0.9648	0.0701	−0.0786	−0.0515
EB 14	0.0374	−0.0467	−0.2691	−0.1198	0.0222
MB 15	−0.0075	0.115	−0.0032	0.0388	0.0800

Table 9 SCA and REC effect value of *B. platyphylla* parental

Combination	4 years old				8 years old					
	H		DBH		H		DBH		SSD	
	SCA	REC	SCA	REC	SCA	REC	SCA	REC	SCA	REC
B 5 × B 8	0.1200	−0.0206	0.8205	−0.6708	−0.2472	−0.0025	−0.1817	−0.2364	−0.1104	0.0406
B 5 × XB 11	0.0225	−0.1737	0.0398	−1.2932	0.1865	0.0366	−0.0638	0.1328	0.0158	−0.0307
B 5 × EB 14	0.0910	0.0955	0.8080	1.4948	0.1112	−0.1464	0.0495	−0.3186	0.0099	−0.0996
B 5 × MB 15	0.0065	0.1431	0.0272	1.9432	−0.0507	0.2913	−0.0203	0.4978	0.0847	−0.0420
B 8 × XB 11	0.1085	−0.005	0.8737	1.7484	0.1388	0.1709	0.2658	0.4846	0.0295	0.0314
B 8 × EB 14	0.0145	0.1084	0.1021	−1.2699	0.1638	0.1544	0.2512	0.2136	0.0008	−0.0055
B 8 × MB 15	0.0030	−0.258	0.1552	−2.7685	−0.0552	−0.4781	−0.119	−0.5347	0.0801	0.0117
XB 11 × EB 14	0.1165	0.0989	0.1003	0.9041	−0.3531	−0.0842	−0.321	0.0888	−0.0205	−0.0608
XB 11 × MB 15	0.0144	−0.0395	0.0895	0.3631	0.0278	−0.276	0.1336	−0.228	−0.0249	0.0349
EB 14 × MB 15	0.0109	−0.1543	0.0092	−0.2314	0.0781	0.0818	0.0057	0.0074	−0.0651	−0.038

combinations. The effects of REC include positive and negative effects, while positive effects indicated better evaluated traits of orthogonal combinations than those of reciprocal combinations and negative effects indicated the opposite. In this experiment, SCA and REC effects were shown in Table 9. At the 4th year, the hybridized combination B5 × B8 showed the highest SCA value (0.1200) in trait H, but the REC value was negative (−0.0206), which suggested that effects of the reciprocal cross were more outstanding. For DBH, the SCA value of B8 × XB11 (0.8737) were obviously higher than other combinations, and the REC value were 1.7484, which indicated that orthogonal effects were better than that of the reciprocal cross. Similarly, the SCA value of B8 × XB11 was the highest among the 10 cross combinations (0.2658) for DBH and the REC effect was also positive and higher (0.4846) at the 8th year. But for H, the SCA value of B5 × B8 was negative (−0.2472), and B5 × XB11 (0.1865) were the most excellent hybridized combination. The REC effect of B5 × XB11 was positive but the value was lower (0.0366) which indicated that there was no obvious difference between the effects of orthogonal and reciprocal. For SSD, combination B5 × MB15 presented higher SCA value (0.0847) which indicated that cross experiment using these two parents can improve the stem traits in offspring. However the negative REC value (−0.0420) suggested that MB15 × B5 combination effects were more excellent than the orthogonal combination.

Correlation analysis of H and DBH in different year

Correlation coefficients of H and DBH in different years were shown in Table 10. The correlations between H and DBH in the same year were significant (0.90 at the 4th year and 0.97 at the 8th year respectively) and the coefficients increased with tree growth. There also existed significant positive correlations between H and H, DBH and DBH, H and DBH in different years, which suggested that early evaluations were effective.

Discussion

Understanding genetic variation within individuals or populations is critical to efficient usage of genetic resources in breeding research (Safavi et al. 2010).

Table 10 Correlation coefficient of H and DBH of *B. platyphylla* in different year

Traits	DBH(4th year)	H(8th year)	DBH(8th year)
H (4th year)	0.90**	0.47*	0.46*
DBH (4th year)		0.48*	0.48*
H (8th year)			0.97**

** significant correlation at the 0.01 level (2-tailed); * significant correlation at the 0.05 level (2-tailed)

ANOVA analysis was the most important method to estimate the extent of variability in the breeding population. In this study, the variation of H, DBH, SSD, GCA, SCA and REC showed significant difference, indicating that estimations and selections of excellent families and parents were effective.

It was demonstrated that with the age went up, the measurements were more accurate for prediction of the growth, and will be conducive to early selection. But shortening the breeding cycles of tree through early selection can produce more genetic gain per unit year if there is a strong genetic correlation between early and mature traits (Goncalves et al. 2005). There were many reported age–age correlations for growth in conifers and *Hevea* species (Goncalves et al. 2005; Matheson et al. 1994; Goncalves et al. 1998; King and Burdon 1991). In poplar, (Kumar and Singh 2001) found out that the correlation coefficients increased with age and an early selection of poplar clones for rotation age of 6 years can be done effectively at age 4. The result provided a credible suggestion for other trees early selection (Kumar and Singh 2001). In this research, the correlations between H and DBH in the same year were higher and increased with the tree growth up (0.90–0.97). Although the the sequencings of average H and DBH of different families were vicissitudinous at the 4th and 8th year, there existed significant correlations between H and DBH in different year, which illustrated that early evaluation and selection were effective.

GCA and SCA effects were partitioned based on a proposed model to evaluate each parent when it is used as a female or a male in its hybrid combinations (Galal 2011). Selecting appropriate materials to be parents can generate excellent offspring which can enhance breeding effects and shorten breeding period (Sluder 1993, 1996). For trees, it was very difficult for breeders to improve excellent traits by crossing because of long life cycles. The combining ability was one piece of the most important information for developing breeding strategies. Especially for birch, the seed propagation was the primary method for propagation because of the difficulty to clone. With the help of combining ability we can obtain preeminent parents which can produce admirable offspring and furthermore can improve the birch population. In the previous research (Li et al. 2006), the results about genetic effects of seed and seeding traits of birch in 5 × 5 complete diallel cross design indicated that there were significant differences in the characteristics of seed and growth traits among crossing combinations. At the meantime, the differences of the SCA and REC of thousand-grain-weight, germination percentage of seed, seedling height and ground diameter also showed significant. In this present study, although the sequencing of GCA and SCA values were different in different years, the trends were same and correlation coefficients of different year were significant. So we take the 8th year parameters to illustrate the combining ability. Parent B5, B8 and MB15 had higher GCA values in H, DBH and SSD respectively and they should be considered as excellent parents to improve the homologous traits. The average H, DBH, and SSD in Table 5 also proved that average growth traits of offspring of these three parents were higher than others. SCA effect primarily reflects the differences in the gene frequencies between the parents (Viana et al. 2013). Hybridized combinations B 5 × XB 11, B 8 × XB 11 and B 8 × MB 15 showed the highest SCA values in the traits of H, DBH, and SSD at the 8th growth year respectively, which indicated that the offspring of this hybridized combination have higher average H, DBH and SSD. Reciprocal cross had different effects for different traits. For H and SSD, the REC effect values were 0.0366 and 0.0117, suggesting that with B 5 × XB 11and B 8 × MB 15 being parents, resulta of orthogonality and reciprocal cross were similar. But for DBH, the REC effect value was 0.4686, which indicated that the orthogonality cross effect were better than the reciprocal cross.

Combining ability analysis helps in the identification of parents with higher GCA and parental combinations with higher SCA. Based on combining ability analysis of different characters, higher SCA indicates non-additive gene effects and higher GCA effects refer to a greater role of additive gene effects controlling these characters (Romanus et al. 2008). The estimation of additive and non-additive gene action through combining ability analysis could be useful in determining the possibility of application of heterosis among progenies of good hybrids (Stuber 1994). If additive effects play the main role in the combining ability, it should be intensively considered and the seed orchard should be a better method in the process of genetic improvement. In case of non-additive effects were main functions, clone propagation and selection should be considered (Qi et al. 2008). In this study, the presence of highly significant

GCA and SCA variances for all the characters demonstrated the importance of both additive and non-additive genes in the expression of the traits. Results of the ANOVA analysis showed that the mean square of SCA were higher than GCA, which indicated that non-additive effect was the primary effect and the clone propagation method should be used for birch. But due to difficulty to clone, excellent provenance and families were the main methods for birch breeding. So in the process of genetic improvement of birch, the technology of clone propagation and seed orchard should be consider contemporarily, which will provide much more benefits.

Conclusion

Because of vegetative propagation was difficult to be realized, provenance and families selection were still the most important methods to propagate in birch. Especially in China, strengthening the seed orchard of birch at the Northeast Forestry University can produce a mass of excellent seeds. Excellent parents selection was the central of the seed orchard set up, so parental estimation and selection were the most important procedure in birch breeding. In this study, after eight years field trial, three parents (B5, B8, MB15) and hybridized combinations (B 5 $\times$ XB 11, B 8 $\times$ XB 11 and B 8 $\times$ MB 15) were selected which will improve H, DBH and SSD respectively. These parents and combinations should be emphasized in the seed orchard building at the next step. In addition, the technology of vegetative propagation should also be developed.

Acknowledgments This work was supported by a grant from the National Science & Technology Pillar Program of China (No. 2012BAD21B02). We also thank Drs. Wei Hou and Huiquan Zheng for critical reading of the manuscript.

References

Bahari M, Rafii MY, Saleh GB, Latif MA (2012) Combining ability analysis in complete diallel cross of watermelon (*Citrullus lanatus* (Thunb.) Matsum. & Nakai). Sci World. doi:10.1100/2012

Galal M (2011) Partitioning of general and specific combining ability effects for estimating maternal and reciprocal effects. J Agric Sci 3(2):213–222

Goncalves P, Bortoletto N, Fonseca F, Bataglia O, Ortolani A (1998) Early selection for growth vigour in rubber tree genotypes in northwestern Sao Paulo State (Brazil). Genet Mol Biol 21:515–521

Goncalves P, Bortoletto N, Cardinal A, Gouvea L, Costa R, Moraes M (2005) Age-age correlation for early selection of rubber tree genotypes in Sao Paulo State, Brazil. Genet Mol Biol 28:758–764

Griffing B (1956) Concept of general and specific combining ability in relation to diallel crossing systems. Aust J Biol Sci 9:463–493

Jakubec V, Komender P, Nitter G, Fewson D, Soukupova Z (1987) Crossbreeding in farm animals. 1. Analysis of complete diallel experiments by means of three models with application to poultry. J Anim Breed Genet 104:283–294

Jiang J, Yang CP, Liu GF, Liu YX, Ren XQ (2001) Analysis of genetic variation within and among *Betula platyphylla* provenance and provenance division using RAPD markers. Bull Bot Res 21:136–140

Jiang TB, Zhou BR, Gao FL, Guo BZ (2011) Genetic linkage maps of white birches (*Betula platyphylla* Suk. and *B. pendula* Roth) based on RAPD and AFLP markers. Mol Breeding 27:347–356

King JN, Burdon RD (1991) Time trends in inheritance and projected efficiencies of early in a large 17-year old progeny test of *Pinus radiata*. Can J For Res 21:1200–1207

Kumar D, Singh NB (2001) Age–age correlation for early selection of clones of *Populu*s in India. Silvae Genet 50:3–4

Li P, Fang G, Sun C (1995) Wood characteristics of pulpwood. Chem Ind For Prod 15:13–18

Li KL, Jiang J, Jiang Y, Xia DA, Yang CP, Liu GF (2006) Analysis of the genetic effects of seed and seedling traits of *Betula platyphylla* 5 $\times$ 5 complete diallel cross design. J Beijing For Univ 28:82–87

Liimatainen J, Karonen M, Sinkkonen J, Helander M, Salminen JP (2012) Phenolic compounds of the inner bark of *Betula pendula*: seasonal and genetic variation and induction by wounding. J Chem Ecol 38(11):1410–1418

Linnakoski R, Beer ZW, Rousi M, Solhein H, Wingfield MJ (2009) *Ophiostoma denticiliatum* sp. nov. and other Ophiostoma species associated with the birch bark beetle in southern Norway. Persoonia 23:9–15

Lukonge E, Labuschagne M, Herselman L (2008) Combining ability for yield and fibre characteristics in Tanzanian cotton germplasm. Euphytica 161:383–389

Matheson A, Spencer DJ, Magnussen D (1994) Optimum age for selection in pinus radiata using basal area under bark for age:age correlations. Silvae Genet 43:352–357

Mekky SS, Galal A, Zaky HI, Zein A (2008) Diallel crossing analysis for body weight and egg production traits of two native Egyptian and two exotic chicken breeds. Int J Poult Sci 7:64–71

Moterle LM, Braccini A, Scapim CA, Pinto R, Goncalves L, Rodrigues R, Junior A (2012) Combining ability of popcorn lines for seed quality and agronomic traits. Euphytica 185:337–347

Pliura A, Zhang SY, Mackay J, Bousquet J (2007) Genotypic variation in wood density and growth traits of poplar hybrids at four clonal trials. For Ecol Manage 238:92–106

Qi M, Peng JS, He GP (2008) Research on some issues about diallel cross test of Chinese fir. For Res 21(5):724–728

Romanus KG, Husseln S, Mashela WP (2008) Combining ability analysis and association of yield and yield components among selected cowpea lines. Euphytica 162:205–210

Safavi SA, Pourdad SA, Mohmmad T, Mahmoud K (2010) Assessment of genetic variation among safflower (*Carthamus tinctorius* L.) accessions using agro-morphological traits and molecular markers. J Food Agric Environ 8(1):616–625

Sharma MK, Fanta S (2010) Variance balanced designs for complete diallel cross. J Agric Biotechnol Sustain Dev 2(4):56–60

Sheridan AK (1981) Crossbreeding and heterosis. Anim Breed 49:131–144

Sluder ER (1993) Results at age 15 years from a half-diallel cross among 10 loblolly pines selected for resistance to fusiform rust (*Cronartium quercuum* f. sp. *fusiforme*). Silvae Genet 42(4–5):223–230

Sluder ER (1996) Two-stage selection in slash pine produces good gains in fusiform rust resistance. South J Appl For 20(3):143–147

Stuber CW (1994) Heterosis in plant breeding. Plant Breed Rev 12:227–251

Viana J, DeLima R, Mundim G, Conde A, Vilarinhl A (2013) Relative efficiency of the genotypic value and combining ability effects on reciprocal recurrent selection. Theor Appl Genet 126:889–899

Vuylsteke M, Eeuwijk F (2008) The use of general and specific combining abilities in a context of gene expression relevant to plant breeding. Euphytica 161:115–122

Waleed M, Sajida A (2011) Use of full diallel cross to estimate crossbreeding effects in laying chickens. Int J Poult Sci 10(3):197–204

Wei ZG, Yang CP, Pan H (2006) Identification of molecular markers associated with birch fiber length trait by multiple regression analysis. Mol Plant Breed 4:835–840

Wei ZG, Zhang KX, Yang CP, Liu GF, Liu GJ, Lian L, Zhang HG (2010) Genetic linkage maps of *Betula platyphylla* Suk based on ISSR and AFLP markers. Plant Mol Biol Report 28:169–175

Williams SM, Price SE, Siegel PB (2002) Heterosis of growth and reproductive traits in fowl. Poult Sci 81:1109–1112

Yang CP, Liu GF, Wei ZG, Wu YL, Zhou YM (2004) Study on intensive breeding technique of accelerating *Betula platyphylla* flowering and seeding early. Sci Silvae Sin 40:14–17

Zeng J, Zou YP, Bai JY, Zheng HS (2003) RAPD analysis of genetic variation in natural populations of *Betula alnoides* from Guangxi, China. Euphytica 134:33–41

Zhan YG, Su T, Han M, Sun D (2006) A multiplex polymerase chain reaction method for rapid detection of foreign genes in transgenic birch (*Betula platyphylla*). Bull Bot Res 26:480–485

Zhao XY, Ma KF, Shen YB, Zhang M, Li KY, Wu RL, Zhang ZY (2012) Characteristic variation and selection of forepart hybrid clones of Sect. *Populus*. J Beijing For Univ 34(2):45–51

View publication stats
