# Supplementary material for: Provenance and family variations in early growth of Manchurian walnut (Juglans mandshurica Maxim.) and selection of superior families
Source: PLoS One. 2024 Mar 7;19(3):e0298918. doi: 10.1371/journal.pone.0298918 (PMC10919699; doi:10.1371/journal.pone.0298918)
Supplement: S1 File — (ZIP) [file pone.0298918.s004.zip › Breeding of Persian walnut Aiming to introduce late-leafing and early-harvesting varieties by targeted hybridization.pdf]

Scientia Horticulturae 295 (2022) 110885

Contents lists available at ScienceDirect

Scientia Horticulturae

journal homepage: www.elsevier.com/locate/scihorti

Breeding of Persian walnut: Aiming to introduce late-leafing and early-harvesting varieties by targeted hybridization

Mehdi Fallah [a], Kourosh Vahdati [a,*], Darab Hasani [b], Mousa Rasouli [c], Saadat Sarikhani [a,*]

[a] Department of Horticulture, College of Aburaihan, University of Tehran, Tehran, Iran
[b] Temperate Fruits Research Center, Horticultural Science Research Institute (HSRI), Agricultural Research, Education and Extension Organization (AREEO), Karaj, Iran
[c] Department of Horticulture and Landscape Engineering, Faculty of Agriculture, University of Malayer, Malayer, Iran

ARTICLE INFO

Keywords:
Hybridization
Fruit set
Pollen tube
Walnut progeny
Late-leafing
Nut characteristics

ABSTRACT

Due to climate change, late-spring frost is considered as a main limiting factor for walnut production. The sustainable strategy to alleviate this challenge is to use late-leafing cultivars with desirable nut characteristics. In order to achieve late-leafing and early-harvesting cultivars, this breeding program including 3635 controlled crosses between 'Persia', 'Caspian', 'Chandler', 'Pedro' as female parents and 'Franquette', 'Lara' and superior genotype 35T as male parents was conducted during 2018–2019. The results showed that there is a significant difference between the cross combinations in terms of fruit set and pollen tube growth. So that, the highest final fruit set was observed in the cross ♀Chandler × ♂35T (67.90%). Also, the fruit growth period of different cross combinations varied between 134 and 157 days. The nut weight, kernel weight and kernel percentage of the obtained progenies ranged between 8.38–12.13 g, 4.19–8.24 g and 41.50–68.67%, respectively. The highest kernel percentage was observed in ♀Persia × ♂35T cross combination (68%). The hybrid seeds were planted in pots under greenhouse condition in the first year, and after initial growth, one-year-old seedlings were transferred to the nursery. Evaluation of the studied seedlings in 2020 showed that there was a significant variation (45 days) between seedlings in terms of leafing date. The early-and late-leafing genotypes were obtained when 35T and 'Franquette' used as male parents, respectively. Although, the studied seedlings have not yet reached the maturity phase for evaluating harvest date, but due to the great variation in leafing date, we hope to reach late-leafing and early-harvesting genotypes with desirable nut traits in the continuation of this breeding program.

1. Introduction

The fruit industry, with an export value of $ 161 billion, is one of the most economically important agricultural sectors in the world. Up to 21.4% of the world fruit export value is related to nut trees which their production and export value have respectively increased by ~35.6% and 107% fruits over the past 10 years (2010–2019). Persian walnut is the main nut trees in the world in term of production which accounts for 25.8% of the world's tree nuts production. After China and the United States, Iran as the third largest producer in the world, produce 7.1% of the world's walnuts (FAO, 2019).

Walnut production like other agricultural crops is largely depended on climate. In recent years, climate change has major negative impact on agriculture crops. So that, crops yield has reduced by 1, 2% over the past 100 years due to climate change (Wiebe et al., 2015). Climate change have highly negative affect on walnut production like other crops. Previous studies showed that climate changes will reduce the suitable lands for walnut cultivation in Iran by 6.2% during 2020–2050 (Vahdati et al., 2019b). Lack of available water, rising temperatures, the reduction of winter chill hours, and late-spring frost are some of the most important damages of climate change (Hassankhah et al., 2017; Hajinia et al., 2021).

Late-spring frost considers as a limiting factor for walnut production in many growing countries (Kazemi et al., 2018). So that, most of the walnut orchards in the north and northwest of Iran were damaged by late-spring frost in 2018. Using late-leafing varieties is the sustainable and efficient strategy to mitigate the severe damage caused by late-spring frost (Akca and Ozongun, 2004). In addition, late-leafing walnut verities are resistant to walnut blight (*Xanthomonas arboricola* pv. *juglandis*) (Bernard et al., 2018).

There are various breeding strategies to release late-leafing walnut cultivars. Utilizing genetic diversity and identifying superior and late-

* Corresponding authors.
E-mail addresses: kvahdati@ut.ac.ir (K. Vahdati), saadat.sarikhani@ut.ac.ir (S. Sarikhani).

https://doi.org/10.1016/j.scienta.2022.110885
Received 11 August 2021; Received in revised form 27 November 2021; Accepted 3 January 2022
Available online 12 January 2022
0304-4238/© 2022 Elsevier B.V. All rights reserved.

Table 1
Cross combination and number of controlled crosses of Persian walnut cultivars/genotypes.

	Male parents					
	Lara	35T		Franquette		Open pollination
	Cultivar	Main traits	Late-leafing<br>Late-harvest<br>Lateral bearing	Mid-leafing<br>Early-harvest<br>Lateral bearing	High late-leafing<br>Late-harvest<br>Terminal bearing	
**Female parents**	Persia	Late-leafing<br>Mid-harvest<br>Lateral bearing	150	150	150	150
	Caspian	Late-leafing<br>Mid-harvest<br>Lateral bearing	150	150	150	150
	Chandler	Late-leafing<br>Late-harvest<br>Lateral bearing	150	150	150	150
	Pedro	Late-leafing<br>Late-harvest<br>Lateral bearing	150	150	150	150

leaf genotypes is a common strategy to achieve late-leafing cultivars in walnut origin and diversity centers (Akca and Ozongun, 2004; Khorami et al., 2012; Ebrahimi et al., 2015; Mahmoodi et al., 2016; Sutyemez et al., 2021). In addition to germplasm evaluation, the natural or controlled cross is a widely used approaches for obtaining desirable cultivars (Vahdati et al., 2019a). In other words, by selecting superior cultivars/genotypes and implementing breeding programs such as hybridization to incorporate desirable traits into the desired cultivars, in addition to achieve the late leafing cultivars, the fruit and kernel characteristics of the genotypes as well as economic yield can be improved (Zeneli et al., 2005). The targeted hybridization is a common strategy in the USA and France walnut breeding programs (Bernard et al., 2018). The USA hybridization programs was started by Eugene F. Serr and Harold E. Forde (1948 to 1979) with the aim of increasing yield, kernel quality, late-leafing, and disease resistance (Tulecke and McGranahan, 1994). This breeding program led to introduce 10 commercial cultivars e.g. 'Chandler', 'Howard' and 'Sunland' (Vahdati et al., 2019a). The USA walnut breeding program based on hybridization was continued by Gale H. McGranahan from 1982 to 2009 and Charles A. Leslie since 2009 with the aim of resistance to blight and Cherry leaf roll virus (CLRV), early harvesting date, high yield, and kernel quality. Several important and commercial cultivars were introduced as a result of this hybridization program; which the last ones were 'Ivanhoe', 'Solano', and 'Durham' (Bernard et al., 2018, Vahdati et al., 2019a). In France, the first crossing program was carried out by Germain from 1977 to 1995 which led to releasing 'Ferjean', 'Fernette' and 'Fernor' cultivars (Germain, 1997). 'Feradam', 'Ferbel', 'Fertignac', and 'Ferouette' are new French walnut cultivars introduced as a result of the continuation of the cross-breeding program in 2010 (Bernard et al., 2018).

Considering high genetic diversity of Persian walnut in Iran, as an origin center of walnut, the walnut breeding programs in this country have been focused on the exploitation of genetic diversity (Khorami et al., 2018; Khadivi et al., 2019; Hassani et al., 2020; Kouhi et al., 2020; Sarikhani et al., 2021; Bujdoso and Cseke, 2021). Among other breeding strategies, molecular breeding has also been used in Iran's walnut breeding programs. In contrast to germplasm evaluation, less attention has been paid to hybridization in Iran's walnut breeding programs. In 1996, some hybridizations were conducted in Horticultural Science Research Institute (HSRI), Karaj, Iran for high yield and lateral bearing which is currently in the progeny evaluation stage (Hassani et al., 2020).

The success of breeding programs depends on different factors, such as the heritability of the studied traits. In this regard, the heritability of

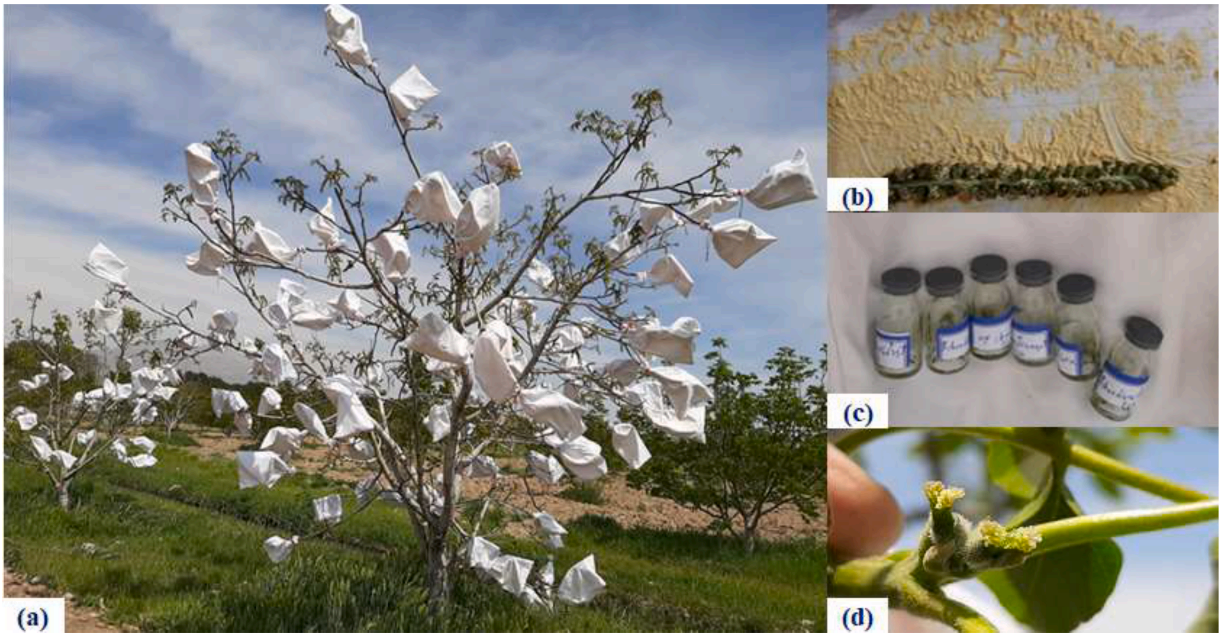

Fig. 1. (a) Isolation of flowers in female parents of the studied trees of Persian walnut; (b) Pollen collection; (c) Pollen storage in small glass vials; (d) The female flower stage is suitable for hybridization.

Table 2
The climate condition of the hybridization orchard site during hybridization period.

Year	Date	Temperature (°C) Min.	Max.	Wind speed (km.h^{-1})	Humidity (%)	Weather condition
2018	22-Apr	8	15	5	40	Sunny
	24-Apr	7	21	10	35	Sunny
	28-Apr	12	21	6	29	Sunny
	30-Apr	8	19	13	49	Sunny
2019	16-Apr	10	18	12	16	Sunny
	18-Apr	10	16	9	32	Sunny
	21-Apr	3	17	11	29	Sunny
	25-Apr	2	18	7	40	Sunny

Source: https://www.worldweatheronline.com

late-leafing trait was estimated 0.80 by Hansche et al. (1972). Also, a positive and strong correlation was reported between leafing and harvest date (Amiri et al., 2010; Sarikhani Khorami et al., 2014; Sutyemez et al., 2018). Late harvesting of fruits lead to increase the risk of damage causes by early-fall frost. Due to the importance of releasing late-leaf cultivars for reducing the late-spring frost damage, this study was conducted as the first targeted walnut hybridization program aiming to release commercial cultivars characterized by high yield and quality, late-leafing, and early harvesting.

2. Materials and Methods

2.1. Plant materials

This research was carried out at the walnut research orchard of Horticultural Science Research Institute (HSRI), Karaj-Iran during 2018–2020. The used plant materials were the progeny of the crosses between some commercial cultivars including 'Chandler', 'Pedro', 'Persia' and 'Caspian' as the female parent and the late-leafing cultivars/ genotypes including 'Franquette', 'Lara' and the superior genotype of 35T as the male parents (Table 1). 'Persia' and 'Caspian' are newly released Iranian cultivars characterized by late-leafing, mid-harvest date, and high yield with light to extra light kernel color (Hassani et al, 2020). Also, 35T is a superior genotype obtained from one of the Iranian's walnut germplasm evaluation programs which is characterized by 100% lateral fruit bearing.

2.2. Targeted hybridization

At the bud swelling stage, the branches of the female parent trees with sufficient flower buds were selected and covered by special isolation bags to prevent open pollination before blooming of female flowers as well as after controlled pollination (Fig. 1a). Also, the catkins of female parents were removed before covering the branches to prevent unwanted pollination. In order to collect pollen, mature catkins were harvested at the beginning of the anther shedding stage and placed on cellophane paper for 24 h at 22 °C (Fig. 1b). The collected pollens were stored in small glass vials at 4 °C until pollination (Fig. 1c) (Hassani et al., 2012). For targeted crosses, the branches in different directions of trees were selected and artificial pollination was performed using paint brush and pollination syringe when the angle between the two stigma lopes were at about 35°. (Fig. 1d) (McGranahan and Leslie, 2009). The exact dates of pollination and number of pollinated flowers were calculated. Also, climatic conditions were recorded during hybridization

Table 3
Analysis of variance of fruit set and number of flowers of the studied cross combinations.

Sources of variation	df	♀ Persia Number of flowers	Initial fruit set	Final fruit set	♀Caspian Number of flowers	Initial fruit set	Final fruit set	♀ Chandler Number of flowers	Initial fruit set	Final fruit set	♀ Pedro Number of flowers	Initial fruit set	Final fruit set
Year	1	477.04 ns	390.99 ns	108.80ns	10.66 ns	2.78 ns	110.21ns	495.04 ns	34.84 ns	72.38ns	57.04 ns	0.37 ns	1.76 ns
Year (Rep)	4	375.04	152.32	77.75	37.62	114.96	34.60	305.16	143.44	141.28	92.54 ns	72.64	32.83
Progeny	3	858.59 ns	738.11**	827.05**	577.05 ns	214.55ns	247.31*	315.59 ns	807.53**	975.16**	142.81 ns	136.90 ns	168.79ns
Year × Progeny	3	477.15 ns	42.90 ns	4.30 ns	249.11 ns	14.16 ns	2.18 ns	412.37 ns	0.66 ns	16.39 ns	239.48 ns	39.94 ns	14.70 ns
Error	12	194.20	113.81	86.16	123.12	84.46	71.83	203.86	66.67	53.99	92.86	87.86	91.58
CV (%)		29.80	19.67	22.02	30.59	18.02	24.87	11.70	13.66	14.88	25.90	16.85	20.84

ns, * and **: No significant, Significant at 5 and 1% probably levels, respectively.

Table 4
The initial and final fruit set of the studied cross combinations in 2018 and 2019.

Groups	Crosses	First year and second		
		Number of flowers	Initial fruit set (%)	Final fruit set (%)
I	♀ Persia × ♂ Lara	246	47.40 ^{b}	29.85 ^{b}
	♀ Persia × ♂ 35T	319	69.87 ^{a}	58.08 ^{a}
	♀ Persia × ♂ Franquette	153	54.21 ^{b}	41.28 ^{b}
	♀ Persia × ♂ OP	193	45.40 ^{b}	39.37 ^{b}
II	♀ Caspian × ♂ Lara	265	44.52 ^{ns}	25.24 ^{b}
	♀ Caspian × ♂ 35T	269	57.47 ^{ns}	40.47 ^{a}
	♀ Caspian × ♂ Franquette	151	54.41 ^{ns}	36.21 ^{ab}
	♀ Caspian × ♂ OP	185	47.57 ^{ns}	34.37 ^{ab}
III	♀ Chandler × ♂ Lara	189	57.45 ^{b}	41.12 ^{b}
	♀ Chandler × ♂ 35T	290	76.80 ^{a}	67.90 ^{a}
	♀ Chandler × ♂ Franquette	258	52.30 ^{b}	47.48 ^{b}
	♀ Chandler × ♂ OP	224	52.52 ^{b}	40.86 ^{b}
IV	♀ Pedro × ♂ Lara	188	53.94 ^{ns}	42.02 ^{ns}
	♀ Pedro × ♂ 35T	228	59.75 ^{ns}	53.34 ^{ns}
	♀ Pedro × ♂ Franquette	259	59.99 ^{ns}	46.06 ^{ns}
	♀ Pedro × ♂ OP	218	50.15 ^{ns}	42.15 ^{ns}

- The means in each group were separately compared. In each group, the means with the same letters were not significantly different from each other

(Table 2).

2.3. The studied traits

2.4.1. Percentage of fruit set and growth rate of pollen tube

In order to determine the percentage of fruit set at the studied cross combinations, the number of fruits formed on the branches were counted two times after pollination (30 days after hybridization and at the harvest day). The fruit set was calculated by the ratio of the number of fruits formed to the number of flowers pollinated on the branch (Bhat et al., 2016).

Fluorescent microscopy was used to detect pollen tube in the pistil and to study the effective pollination period (EPP) in each cross. For this purpose, pistils were cut at 24, 48, 72 and 96 h after pollination and placed in glass vials containing 10 ml of FAA solution (5% of 50% formaldehyde, 5% of glacial acetic acid, and 90% of 70% alcohol) (Pollegioni et al., 2011). Then, the samples were removed from the fixation solution and after washing with distilled water were kept in glass vials containing 10 ml of 5% sodium sulfite. Afterward, samples were placed in an autoclave at 110 °C in a pressure of 1.2 kg.cm^{-2} for 15 min to soften the pistil tissue. Eventually, the pollen tubes were stained with hematoxylin-aniline blue solution for 24 h (Jefferies, 1979). The growth of pollen tubes into pistils was investigated by fluorescent microscopy (MC300, Micros Austria) and the time of pollen tubes entered into the ovary was recorded (Luza and Polito, 1991).

Table 5
Percentage of pollen germination on the stigma and time required for the pollen tube to penetrate into the ovule.

Crosses	Pollen germination (%)		Pollen tube growth		
	6 h	24 h	48 h	60 h	72 h
♀ Persia × ♂ Lara	14 ^{hi}	72 ^{g}			
♀ Persia × ♂ 35T	28 ^{bcd}	95 ^{a}			
♀ Persia × ♂ Franquette	23 ^{e}	77 ^{f}			
♀ Persia × ♂ OP	25 ^{de}	85 ^{bcd}			
♀ Caspian × ♂ Lara	12 ^{i}	78 ^{ef}			
♀ Caspian × ♂ 35T	30 ^{bc}	89 ^{b}			
♀ Caspian × ♂ Franquette	27 ^{cd}	75 ^{fg}			
♀ Caspian × ♂ OP	22 ^{e}	82 ^{de}			
♀ Chandler × ♂ Lara	15 ^{gh}	75 ^{fg}			
♀ Chandler × ♂ 35T	34 ^{a}	94 ^{a}			
♀ Chandler × ♂ Franquette	31 ^{b}	79 ^{ef}			
♀ Chandler × ♂ OP	26 ^{d}	82 ^{de}			
♀ Pedro × ♂ Lara	17 ^{fg}	70 ^{gh}			
♀ Pedro × ♂ 35T	28 ^{bcd}	87 ^{bc}			
♀ Pedro × ♂ Franquette	18 ^{f}	79 ^{ef}			
♀ Pedro × ♂ OP	20 ^{e}	84 ^{cd}			

2.4.2. Pomological and phenological traits of progenies

The studied pomological and phenological characteristics including length of growth season, harvest date, nut weight, nut size (i.e. nut length, width, and diameter), shell thickness, packing tissue thickness, ease of removal of kernel, shell seal, kernel weight, kernel percentage, and kernel color were calculated based on IPGRI descriptors (IPGRI, 1994; Khadivi et al., 2019; Akca et al., 2020).

For evaluating progenies, the nuts harvested from each cross combination were planted in 10 kg pots in greenhouse conditions after stratification at 4 °C for 4 weeks. The obtained seedlings then were transferred to a nursery in the second year. The budbreak time of seedlings was recorded for two consecutive years.

2.6. Statistical analysis

The obtained data for fruit set, pollen germination and pomological traits were analyzed as a completely randomized design with three replications using SPSS software (ver. 22). Due to hybridization in two consecutive years, the combined analysis for fruit set was performed. Statistical analysis for pomological traits was performed separately for 2018 and 2019 data. Means were compared using the same software and Duncan's multiple range test. The Spearman's correlation coefficient was used to evaluate correlation between pomological and phenological traits of progenies using the relevant packages of R software.

3. Results and discussion

3.1. Fruit set percentage and pollen tube growth

No significant difference was observed between year, and year × progeny in term of fruit set and number of flowers. Despite the number of flowers, the fruit set percentage in different cross combinations was significantly different (Table 3). In the first group ('Persia' as the female parent), there was a significant difference between different male parents in terms of initial and final fruit set. The rate of final fruit set in the mean of first and second year ranged from 29.85% to 58.08%. The highest final fruit set was related to cross of ♀Persia × ♂35T (Table 4). In the second group ('Caspian' as the female parent), the significant difference was not observed between the male parents in terms of the initial fruit set. Furthermore, there was a significant difference between different crosses in terms of the final fruit set. The highest final fruit set was observed in ♀Caspian × ♂35T (40.47%) cross combination (Table 4). In the third group ('Chandler' as the female parent), the means of initial and final fruit set were significantly different between the cross combinations. So that, the highest and the lowest final fruit set were observed in ♀Chandler × ♂35T (67.90%) and ♀Chandler × ♂ Open pollination (40.86%) cross combinations, respectively. In the fourth groups ('Pedro' as the female parent), there was no significant difference between different crosses in terms of initial and final fruit set (Table 4).

Pollen deficiency reduces the fruit set (Polito and Li, 1985). This fact was confirmed by the findings of our study when the fruit set increased in controlled pollination compared to open pollination (Atefi, 1990). In contrast, the fruit set of ♀Persia × ♂Lara and ♀Caspian × ♂Lara cross combinations were lower than the open pollination, which is consistent with the results of Kumar (2017) who reported reducing of the fruit set in 'Netar Akhrot' walnut genotype in controlled pollination compared to open pollination. The lower fruit set may be attributed to younger age of plants, the genetic combination, and climatic and environmental factors (Bhat et al, 2016); In addition, lower fruit set in controlled pollination might be caused by the excessive pollen grain deposition on the stigma. So that, McGranahan et al. (1994) reported that excess pollen grains on

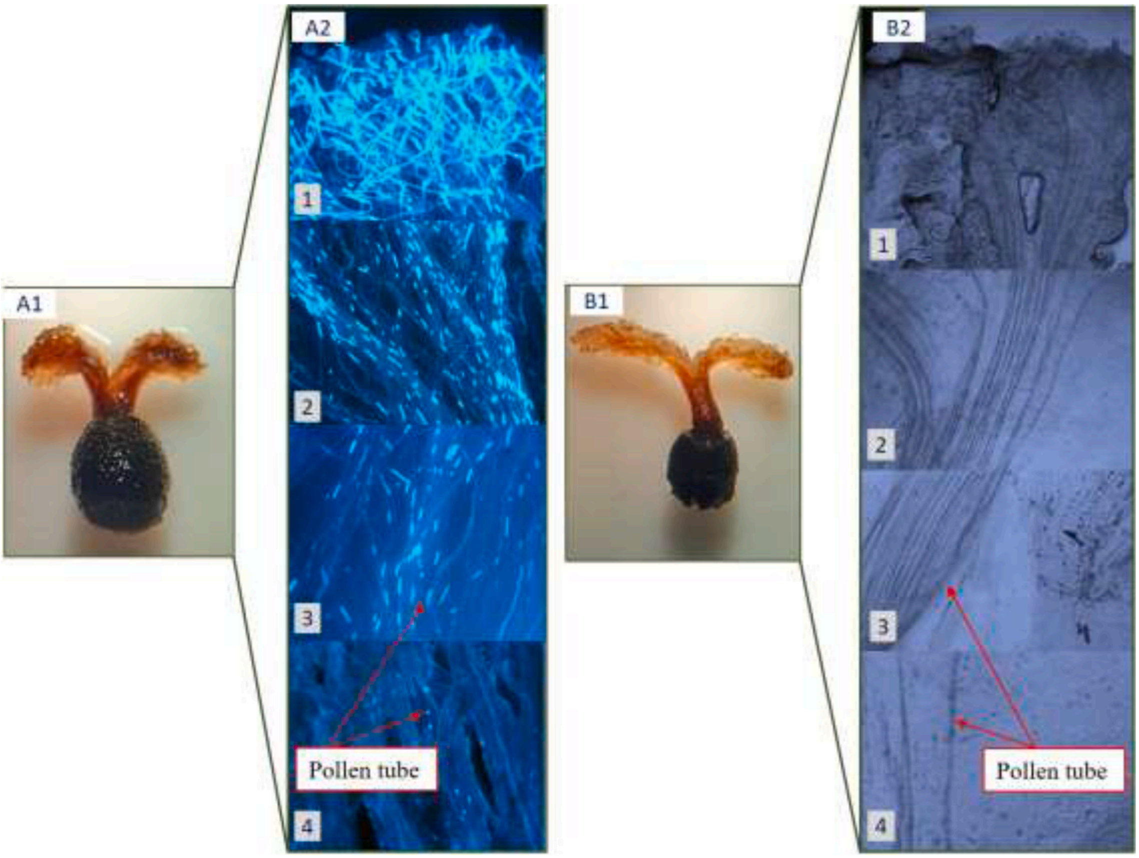

Fig. 2. A1 and B1: Pistil of 'Chandler' and 'Caspian' cultivars, respectively; A2 and B2: Pollen tube growth in 'Chandler' and 'Caspian' pistils, respectively (1: Stigma, 2: Upper part of style, 3: Middle part of style, 4: Ovary).

the flower stigma of 'Serr' walnut cultivar increased of pistillate flower abscission (PFA) and decreased fruit set.

Based on the obtained results, the fruit set percentage varied in the different cross combinations and the highest fruit set was obtained when 35T considered as male parent (43.4–70.9%). These results were consistent with the results of previous studies which reported 60–80% fruit set in different crosses (Kumar, 2017; Rovira and Aleta, 1997). Also, the obtained results of fruit drop are supported by McGranahan and Leslie (1990) who reported that there is sporadic fruit drop in walnut which may continue throughout the growing season.

The percentage of pollen germination and pollen tube growth in different parts of the walnut pistil (stigma, style, and ovary) controlled pollination were examined 6, 24, 48, 60, and 72 h after pollination. The results showed that the percentage of pollen germination on the stigma increased at the studied crosses from 6 to 24 h after pollination. In different cross combinations, the mean percentage of germination was 12–34% at 6 h after pollination and 72–95% at 24 h after pollination (Table 5).

The time required for the pollen tube to reach the ovary varied in different cross combinations. In ♀Persia × ♂35T, ♀Chandler × ♂35T, ♀Chandler × ♂Franquette and ♀Pedro × ♂35T cross combinations, the pollen tube growth reached the ovary 48 h after pollination and in other crosses, this happened 60 h after pollination. The penetration of the pollen tube into the ovary increased at 72 h after pollination (Table 5).

The level of stigma receptivity varies depending on the development stage of flowers' pistil. Luza and Polito (1991) reported that pollen germination fails in young flowers at stage 1 (1-2 days after opening the flowers). In contrast, in the flowers at stage 2 (2–6 days after opening the flowers), 35% and 92% of pollen germination occurred at 6th and 18th h after pollination, respectively (Luza and Polito, 1991). Therefore, the flowers at stage 2 were used for crossing in our study.

In the present study, at some crosses, the pollen tube reached the ovary 48 h after pollination. Our result is in consistent with the results of Luza and Polito (1991), who reported that the pollen tube reached to the ovary 48 h after pollination in 'Franquette' cultivar. In some of the crosses in this study, the pollen tube reached the ovary 60 h after pollination, which was not consistent with the results of Luza and Polito (1991). This inconsistency can be explained by the fact that they only used one cross combination with the same pistil size, whereas in the present study, several cross combinations were used which had different pistil size. The length of flower style varied in different female parents. The cultivar 'Caspian' had the longest and 'Chandler' cultivar had the shortest style length (Fig. 2A1 and B1). This phenomenon is due to genetic factors (Sarkissian and Harder, 2001). Pollegioni et al. (2011) reported that the pistil size of the flower (the length of the style to the end of the ovary) affects the time that pollen tube reaches the ovary. They stated that flowers with a longer pistil length (especially the length of the style) need more time. Considering the time taken pollen tube reaches to the ovary in all crosses in cultivar 'Caspian', which was 60 h, and the long style of this cultivar, the results of this study were consistent with the fact that the length of the style affects the time of pollen tube entry to the ovary. Finally, in addition to cultivar, the time of pollen tube growth depends on the nutritional status of the tree and environmental conditions including wind, rain and temperature (Sanzol and Herroero, 2001).

3.2. *Phenological and pomological characteristics of progenies*

Pomological and phenological characteristics were examined separately for 2018 and 2019. Based on the results, there was a significant difference between progeny in terms of pomological traits (Table S1). Phenological characteristics also differed in different parental groups.

Table 6
The pomological and phenological characteristics of walnut hybrid progenies in 2018 and 2019.

Crosses	Nut weight (g)		Kernel weight (g)		Kernel Percentage		Nut length (mm)		Nut width (mm)		Nut diameter (mm)	
	2018	2019	2018	2019	2018	2019	2018	2019	2018	2019	2018	2019
♀ Persia × ♂ Lara	11.90 ^{ab}	11.55 ^{a}	7.52 ^{a}	7.25 ^{abc}	63.25 ^{ab}	62.78 ^{ab}	38.38 ^{bc}	38.56^{bcd}	33.11^{abc}	33.50 ^{ab}	36.88 ^{a}	37.13 ^{a}
♀ Persia × ♂ 35T	12.13 ^{a}	11.99 ^{a}	8.13 ^{a}	8.24 ^{a}	67.05 ^{a}	68.67 ^{a}	43.80 ^{a}	45.54 ^{a}	32.78^{abc}	34.24 ^{ab}	36.76 ^{ab}	37.26 ^{a}
♀ Persia × ♂ Franquette	11.19 ^{abcd}	10.51 ^{a}	6.74 ^{bc}	6.60 ^{bcd}	60.24 ^{bc}	62.72 ^{ab}	37.63 ^{bcd}	37.67^{bcd}	33.78 ^{a}	34.08 ^{ab}	36.07 ^{ab}	36.23 ^{ab}
♀ Persia × ♂ OP	11.59 ^{abc}	11.68 ^{a}	7.43 ^{ab}	7.35 ^{ab}	64.13 ^{ab}	63.05 ^{ab}	38.08 ^{bc}	36.95^{cde}	32.89^{abc}	31.37^{cde}	36.89 ^{a}	36.53 ^{a}
♀ Caspian × ♂ Lara	9.95 ^{de}	11.19 ^{ab}	5.95 ^{de}	6.41^{bcde}	60.42 ^{bc}	58.63 ^{bc}	40.10 ^{b}	39.84 ^{bc}	34.05 ^{a}	33.75 ^{ab}	36.69 ^{ab}	36.76 ^{a}
♀ Caspian × ♂ 35T	11.04 ^{abcd}	10.08 ^{ab}	6.31^{cd}	5.76 ^{def}	57.07 ^{c}	57.18 ^{bc}	39.54 ^{bc}	37.66^{bcd}	33.69 ^{ab}	33.33^{abc}	36.19 ^{ab}	35.67^{abc}
♀ Caspian × ♂ Franquette	10.00 ^{de}	10.10 ^{ab}	5.71 ^{def}	5.45 ^{defg}	57.14 ^{c}	53.77 ^{cd}	34.80 ^{d}	34.00 ^{e}	32.17^{abc}	31.09 ^{e}	33.13^{d}	32.83 ^{d}
♀ Caspian × ♂ OP	9.18 ^{ef}	9.42 ^{ab}	5.27 ^{efg}	5.05 ^{efg}	57.44 ^{c}	53.78 ^{cd}	36.14 ^{cd}	35.87 ^{de}	31.12 ^{c}	30.74 ^{e}	33.68 ^{cd}	33.54^{cd}
♀ Chandler × ♂ Lara	10.49 ^{bcde}	10.88 ^{ab}	5.01 ^{fgh}	4.92 ^{fg}	47.78 ^{de}	45.18 ^{ef}	38.89 ^{bc}	38.41^{bcd}	31.80^{abc}	31.33^{cde}	36.69 ^{ab}	36.76 ^{a}
♀ Chandler × ♂ 35T	10.33 ^{cde}	10.38 ^{ab}	5.34 ^{efg}	5.25 ^{efg}	51.68 ^{d}	50.56 ^{de}	38.97 ^{bc}	37.75^{bcd}	32.88^{abc}	32.25^{bcd}	34.64^{bcd}	33.70 ^{cd}
♀ Chandler × ♂ Franquette	10.83 ^{abcd}	11.31 ^{a}	4.80 ^{ghi}	5.19 ^{efg}	44.33 ^{ef}	45.89 ^{ef}	38.63 ^{bc}	38.70^{bcd}	32.84^{abc}	32.63^{bcd}	32.74 ^{d}	32.75 ^{d}
♀ Chandler × ♂ OP	10.53 ^{bcde}	9.62 ^{ab}	4.39 ^{hi}	4.19 ^{g}	41.50 ^{f}	43.53 ^{f}	40.79 ^{b}	41.25 ^{b}	32.35^{abc}	32.46^{bcd}	35.33 ^{abc}	35.73^{abc}
♀ Pedro × ♂ Lara	8.38 ^{f}	8.64 ^{b}	4.23 ^{i}	4.32 ^{g}	50.57 ^{d}	50.04 ^{de}	36.13 ^{cd}	36.08 ^{de}	31.40 ^{bc}	31.30^{de}	33.94^{cd}	34.00^{bcd}
♀ Pedro × ♂ 35T	11.14 ^{bcde}	10.96 ^{ab}	5.64 ^{def}	5.99 ^{cdef}	50.80 ^{d}	54.53 ^{cd}	40.21 ^{b}	40.86 ^{b}	33.54 ^{ab}	34.92 ^{a}	36.05 ^{ab}	36.21 ^{ab}
♀ Pedro × ♂ Franquette	10.90 ^{bcde}	11.31^{a}	5.01 ^{fgh}	5.21 ^{efg}	45.94 ^{e}	46.09 ^{ef}	39.17 ^{bc}	38.87^{bcd}	33.71^{ab}	33.67 ^{ab}	36.36 ^{ab}	36.36 ^{ab}
♀ Pedro × ♂ OP	9.99 ^{de}	11.47 ^{a}	4.60 ^{ghi}	5.23 ^{efg}	46.06 ^{e}	45.58 ^{ef}	38.48 ^{bc}	37.68^{bcd}	33.50 ^{ab}	33.18^{abcd}	35.49 ^{abc}	35.38^{abc}

Means in each column, followed by similar letters are not significantly different at 5% probability level.

Continue Table 6. The pomological and phenological characteristics of walnut hybrid progenies in 2018 and 2019.

Crosses	Shell thickness (mm)	ERK	Packing tissue Thickness (mm)	Nut shape	Kernel fill		Kernel color		Hybridization Date		Fruit growth period (days)		Harvesting date	
	2018	2019	2018–19	2018	2019	2018–19	2018	2019	2018–19	2018	2019	2018–19	2018	2019
♀ Persia × ♂ Lara	0.78 ^{d}	0.84 ^{d}	1	0.05 ^{f}	0.07 ^{fg}	R	7	7	E.l	22-April	16-April	134	3-Sept.	28-Aug.
♀ Persia × ♂ 35T	0.60 ^{e}	0.64 ^{e}	1	0.05 ^{f}	0.05 ^{g}	R	7	7	E.l	22-April	16-April	134	3-Sept.	28-Aug.
♀ Persia × ♂ Franquette	0.86^{bcd}	0.85 ^{d}	1	0.09 ^{bc}	0.09 ^{de}	R	7	7	L	22-April	16-April	134	3-Sept.	28-Aug.
♀ Persia × ♂ OP	0.88^{bcd}	0.98 ^{cd}	2	0.10 ^{ab}	0.14 ^{a}	R	7	7	L	22-April	16-April	134	3-Sept.	28-Aug.
♀ Caspian × ♂ Lara	0.83^{bcd}	0.95 ^{cd}	1	0.05 ^{f}	0.05 ^{g}	R	7	7	L	24-April	18-April	136	7-Sept.	1-Sept.
♀ Caspian × ♂ 35T	0.91^{bcd}	0.91 ^{cd}	1	0.06 ^{de}	0.08 ^{ef}	R	7	7	L	24-April	18-April	136	7-Sept.	1-Sept.
♀ Caspian × Franquette	0.91^{bcd}	0.97 ^{cd}	1	0.07 ^{cd}	0.09 ^{de}	R	7	7	L	24-April	18-April	136	7-Sept.	1-Sept.
♀ Caspian × ♂ OP	0.97 ^{bc}	0.99 ^{cd}	2	0.13 ^{a}	0.13 ^{ab}	R	7	7	L	24-April	18-April	136	7-Sept.	1-Sept.
♀ Chandler × ♂ Lara	1.17 ^{a}	1.21 ^{a}	1	0.11 ^{ab}	0.13 ^{ab}	L.t	5	5	L	30-April	25-April	157	4-Oct.	29-Sept.
♀ Chandler × ♂ 35T	1.18 ^{a}	1.18 ^{a}	1	0.10 ^{ab}	0.12 ^{abc}	L.t	5	5	E.l	30-April	25-April	157	4-Oct.	29-Sept.
♀ Chandler ×♂ Franquette	1.02 ^{a}	1.03 ^{bc}	1	0.11 ^{ab}	0.13 ^{ab}	L.t	3	3	L	30-April	25-April	157	4-Oct.	29-Sept.
♀ Chandler × ♂ OP	1.16 ^{a}	1.16 ^{ab}	1	0.10 ^{ab}	0.10 ^{cd}	L.t	3	3	L	30-April	25-April	157	4-Oct.	29-Sept.
♀ Pedro × ♂ Lara	0.98 ^{b}	1.02 ^{bc}	1	0.11 ^{ab}	0.11 ^{bc}	B.o	5	5	E.l	28-April	21-April	147	22-Sept.	15-Sept.
♀ Pedro × ♂ 35T	1.21 ^{a}	1.19 ^{a}	1	0.11 ^{ab}	0.09 ^{de}	S.t	5	5	E.l	28-April	21-April	147	22-Sept.	15-Sept.
♀ Pedro × ♂ Franquette	1.20 ^{a}	1.22 ^{a}	1	0.10 ^{ab}	0.14 ^{a}	B.o	3	3	L	28-April	21-April	147	22-Sept.	15-Sept.
♀ Pedro × ♂ OP	1.19 ^{a}	1.31 ^{a}	1	0.11 ^{ab}	0.11 ^{bc}	S.t	3	3	L	28-April	21-April	147	22-Sept.	15-Sept.

Kernel fill: 3: Poor; 5: Moderate; 7; Well; Ease of removal of kernel (ERK): 1: Very easy; 5: Moderate; 9: Very difficult; Nut shape: Round (R), Long trapezoid (L.t), Broad ovate (B.o), Short trapezoid (S.t); Kernel color; Extra light (E.l), Light (L)

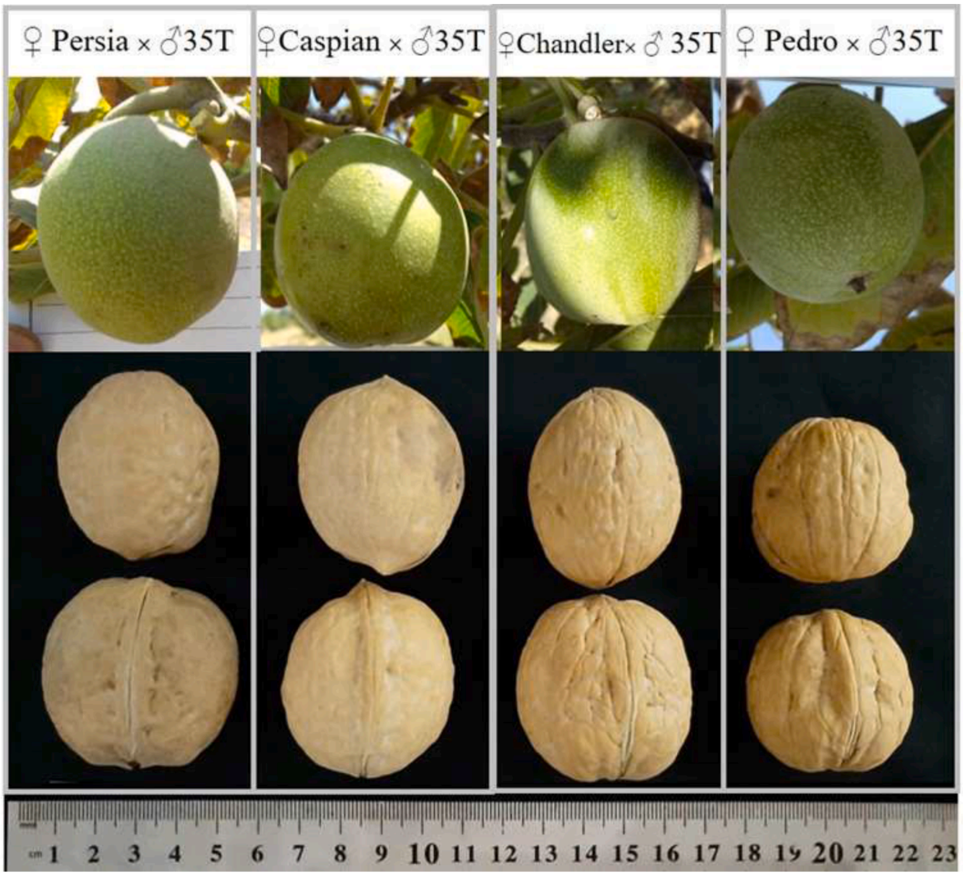

Fig. 3. The nuts of Persian walnut obtained from some crosses in this study.

The hybridization dates in 2018 and 2019 for 'Persia', 'Caspian', 'Pedro', and 'Chandler' cultivars were 22, 24, 28, 30 and 16, 18, 21, 25 April, respectively. Also, the harvest dates in 2018 and 2019 for 'Persia', 'Caspian', 'Pedro', and 'Chandler' cultivars were 3, 7, 22 September, 4 October, and 28 August, 1, 15, 29 September, respectively. In general, the fruit growth period for 'Persia', 'Caspian', 'Pedro', and 'Chandler' cultivars were 134, 136, 145, and 157 days, respectively (Table 6). However, we should consider that leafing and harvest date and ripening period depends on environmental conditions because of their low heritability (Bukucu et al., 2020; Ramos 1998).

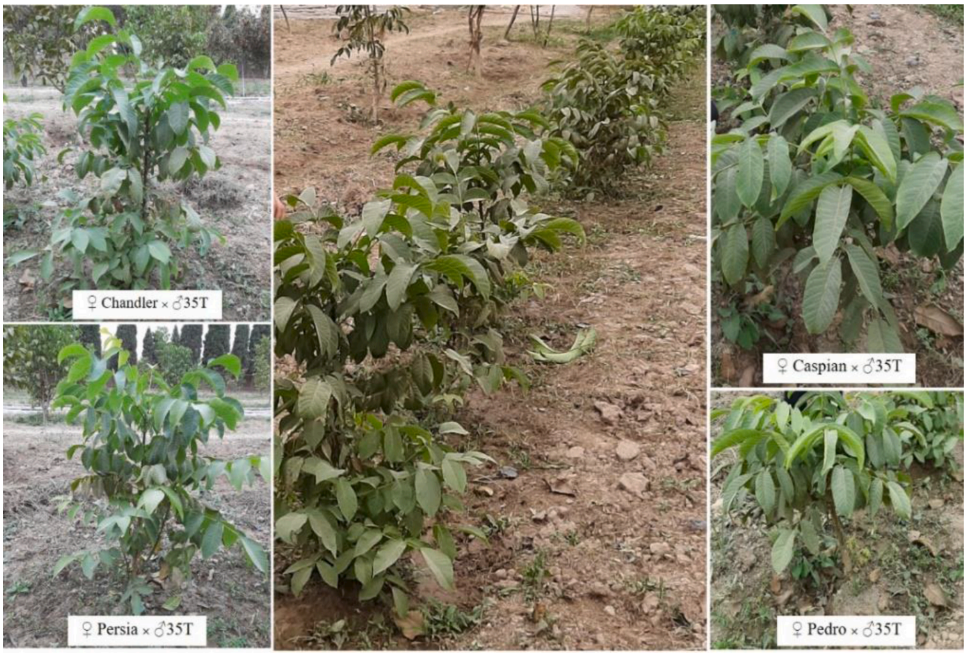

Fig. 4. The Persian walnut seedlings obtained from different crosses.

Table 7
The budbreak period in different seedlings obtained from crosses of Persian walnut cultivars.

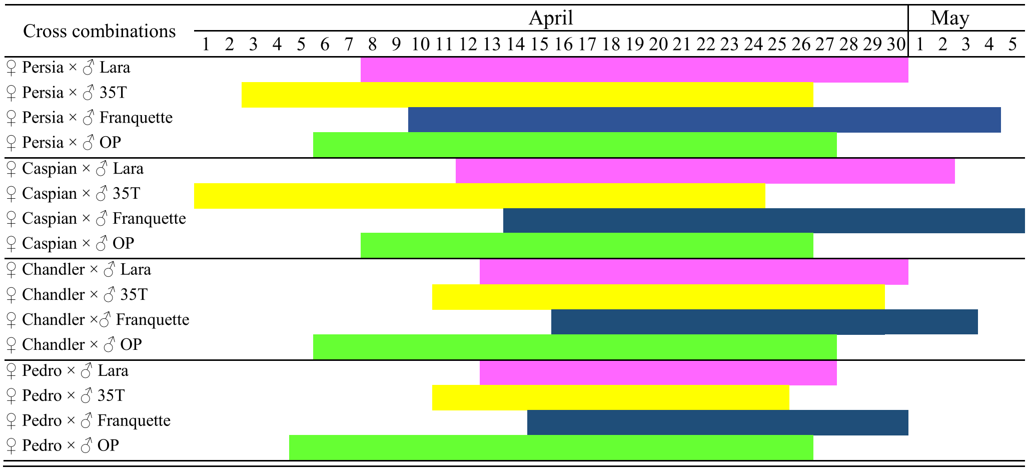

The results showed that ♀Persia × ♂35T (11.99, 12.3 g for 2018 and 2019, respectively) and ♀Pedro × ♂Lara (8.38, 8.64 g) cross combination had the highest and lowest nut weight, respectively. The kernel weight of progenies ranged between 4.19 and 8.24 g. The kernel percentage in the different cross groups was significantly different. So that, kernel percentage in 2018 and 2019 of 'Persia', 'Caspian', 'Chandler', and 'Pedro' groups varied between 60.24–68.67, 53.77–60.42, 41.50–51.68, and 45.58–54.53%, respectively. Also, the progenies obtained from ♀Persia × ♂35T cross showed the highest kernel percentage (67.05% and 68.67% in 2018 and 2019, respectively) (Table 6).

Since the studied parents were commercially superior cultivars/genotypes with favorable pomological characteristics, so the shell thickness (0.60–1.31 mm), packing tissue thickness (0.05–0.14 mm), ease of removal of kernel, and kernel color (light-extra light) of the obtained progenies were in desirable range which was consistent with the previous results on walnut superior genotypes Mahmoodi et al., 2019; Khadivi et al., 2019; Bujdoso et al., 2021). The size and shape of the nuts obtained from crosses were different. The nuts shape of some progenies is presented in Fig. 3.

The effect of pollen type on pomological characteristics was in agreement with the results previously reported in walnut (Golzari et al., 2016; Rasouli et al., 2019) and hazelnut (Balik and Beyhan, 2019). Golzari et al. (2016) evaluated xenia and metaxenia potentials in crosses of 'Jamal', 'Chandler', 'Hartley' and 'Pedro' (female parents) with 'Serr', 'Z60' and 'Damavand' (male parents) and reported that phenological characteristics including nut size (i.e. weight, length, width, diameter), kernel weight, shell-thickness and kernel percentage were affected by pollen grain type.

The high kernel percentage (more than 46%) is a main walnut breeding objective (Germain, 1997; Korac et al., 1997). Zeneli et al. (2005), Aslantas (2006), and Ebrahimi et al. (2015) evaluated walnut germplasms in Albania, Turkey and Iran, respectively, and reported that

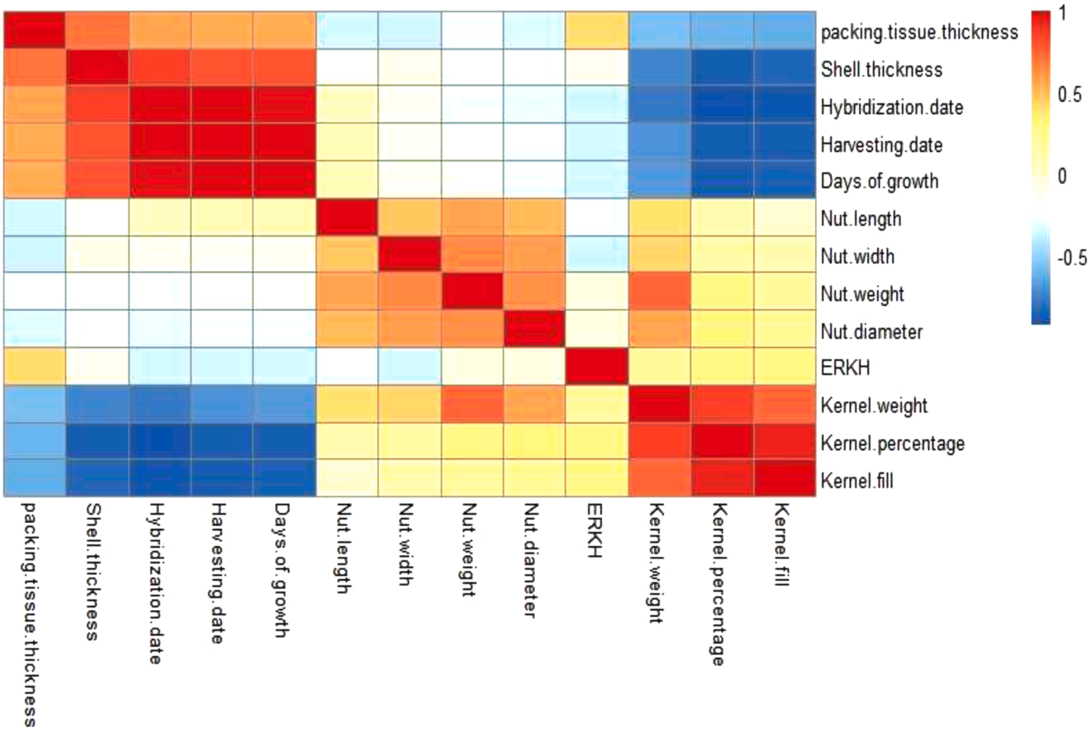

Fig. 5. Heatmap of Spearman's correlations between phenological and pomological traits of the studied walnut progenies.

the average kernel percentage of superior genotypes were 63.80% for Albania, 67.14% for Turkey and 62.18% for Iran. Hassani et al. (2020) reported that the kernel percentage of open-pollinated 'Persia', 'Caspian', and 'Chandler' cultivars were 63.1%, 56.0%, and 43.6 %, respectively, which was consistent with our results. In the current study, the high kernel percentage was observed in progenies which Iranian cultivars, 'Persia' and 'Caspian', were their female parents. Due to high heritability of this trait (Hansche et al., 1972), we hope to find promising offspring with the high kernel percentage and other desirable traits in the continuation of this program.

3.3. Evaluating seedlings obtained from crosses

As a result of two-year hybridization, a large population of progeny was obtained in 2018 (715 seeds) and 2019 (883 seeds). After evaluations of pomological characteristics, 651 and 819 hybrid seed were planted in pots under greenhouse condition in 2018 and 2019, respectively. After initial growth, one-year-old seedlings were transferred to the fruit tree nursery in Pakdasht, Tehran province (Fig. 4). Ombrothermic diagram of the nursery was presented in Fig. S1.

The most important strategy to overcome late-spring frost is to use genetic potential. Late-leafing genotypes with desirable nut characteristics can be a reliable solution for sustainable walnut production, especially in areas where there is late-spring frost damage (Akca and Ozongun, 2004; Vahdati et al., 2019). The results of this study showed that there is a significant variation (45 days) between the studied seedlings in terms of leafing date. The mid- and high late-leafing genotypes were obtained when 35T and 'Franquette' were used as male parents, respectively (Table 7). Bukucu et al. (2020) reported that the leafing date of the seedlings resulting from walnut hybridization varied from March 16 to May 21. Also, Hassankhah et al. (2017) reported that the budbreak of different walnut genotypes varied from March 16 to April 2. Although, the studied seedlings in the juvenile phase of growth and development have not yet reached the maturity stage to evaluate their harvest date, but due to the great variation in leafing date, we hope to reach late-leafing and early-harvesting genotypes with desirable nut traits in the continuation of this breeding program.

3.4. Correlation analysis

The two-year data from different crosses were used for correlation analysis. Spearman correlation analysis showed that a strong and positive correlation exist between the hybridization and harvesting date, the fruit growth period and shell thickness which was consistent with the results of Mahmoodi et al. (2019) and Khadivi et al. (2019). These traits had a negative correlation ($P < 0.01$) with kernel weight, kernel percentage, and kernel fill. The results are consistent with the results reported by Hassani et al. (2020). A strong positive correlation was observed between nut weight, nut length, nut width, nut diameter and kernel weigh, which was consistent with the reports of other researchers (Sharma and Sharma, 2001; Amiri et al., 2010; Sarikhani Khorami et al., 2014; Poggetti et al., 2017). Also, a slight positive correlation ($P<0.05$) was observed between nut weight and nut size with kernel percentage, ease of removal of kernel and kernel fill. Finally, a positive correlation was observed between packing tissue thickness and shell thickness (Fig. 5).

4. Conclusion

Late-spring frost is a challenge that made us eager to design a breeding program to overcome this problem. Due to the high heritability (0.80) of this trait and other related phenological traits, targeted hybridization between different cultivars is a reliable strategy to achieve late-leafing and early-harvest cultivars with desirable nut traits. Although, 'Chandler' cultivar is a relatively late-leafing variety with desirable nut traits, but due to long growth period and late fall of the leaves, it suffers from autumn frosts. Therefore, the main objective of this study was to alleviate late-spring and early-autumn frosts. In this regard, the present study was conducted by 3635 controlled crosses aiming to release late-leafing and early-harvest cultivars. A large breeding population with a variation of 45 days in leafing time was obtained. The studied progenies had favorable phenological and pomological traits which increases the chances of introducing late-leaf and early-harvesting cultivars with desirable nut traits in this breeding program.

CRediT authorship contribution statement

Mehdi Fallah: Methodology, Data curation, Writing – original draft. **Kourosh Vahdati:** Conceptualization, Supervision, Project administration, Writing – review & editing. **Darab Hasani:** Investigation, Writing – review & editing. **Mousa Rasouli:** Investigation, Writing – review & editing. **Saadat Sarikhani:** Investigation, Software, Validation, Writing – review & editing.

Declaration of Competing Interest

The authors declare that they have no known competing financial interests or personal relationships that could have appeared to influence the work reported in this paper.

Acknowledgments

This work has been supported by the Center of International Scientific Studies & Collaboration (CISSC), Ministry of Science Research and Technology. We also would like to thank Iran National Science Foundation (INSF), Center of Excellence for Walnut Improvement and Technology of Iran, and the University of Tehran for their supports.

Supplementary materials

Supplementary material associated with this article can be found, in the online version, at doi:10.1016/j.scienta.2022.110885.

References

Akca, Y., Ozongun, S., 2004. Selection of late leafing, late flowering, laterally fruitful walnut (*Juglans regia*) types in Turkey. N. Z. J. Crop Hortic. Sci. 32, 337–342.

Akca, Y., Yuldaşulu, Y.B., Murad, E., Vahdati, K., 2020. Exploring of walnut genetic resources in Kazakhstan and evaluation of promising selections. Int. J. Hortic. Sci. Technol. 7, 93–102.

Amiri, R., Vahdati, K., Mohsenipoor, S., Mozaffari, M.R., Leslie, C., 2010. Correlations between some horticultural traits in walnut. HortScience 45, 1690–1694.

Aslantas, R., 2006. Identification of superior walnut (*Juglans regia*) genotypes in north-eastern Anatolia, Turkey. N. Z. J. Crop Hortic. Sci. 34, 231–237.

Atefi, J., 1990. Preliminary research of Persian walnut and correlation between pair characters. Acta Hortic. 442, 101–108.

Balik, H.I., Beyhan, N., 2019. Xenia and metaxenia in hazelnuts: effects of pollinizer cultivars on nut set and nut characteristics of some hazelnut cultivars. Acad. J. Agric. 8, 9–18.

Bernard, A., Lheureux, F., Dirlewanger, E., 2018. Walnut: past and future of genetic improvement. Tree Genet. Genomes 14, 1.

Bhat, K.M., Hassan, S., Bhat, M.A., Mir, M.A., Kirmani, N., 2016. Pollination studies in walnut (*Juglans regia* L.). Int. Q. J. Life Sci. 11, 2683–2686.

Bujdoso, G., Cseke, K., 2021. The Persian (English) walnut (*Juglans regia* L.) assortment of Hungary: nut characteristics and origin. Sci. Hortic. 283, 110035, 2021.

Bujdoso, G., Illes, B., Varjas, V., Cseke, K., 2021. Is "Esterhazy II", an old walnut variety in the Hungarian gene bank, the original genotype? Plants 10, 854.

Bukucu, S.B., Ozcan, A., Sutyemez, M., Yildirim, E., 2020. Determination in the phenological difference levels of seedlings of some walnut genotypes (*Juglans regia* L.). J. Ecol. Environ. Res. 18, 4807–4815.

Ebrahimi, A., Khadivi-Khub, A., Nosrati, Z., Karimi, R., 2015. Identification of superior walnut (*Juglans regia*) genotypes with late leafing and high kernel quality in Iran. Sci. Hortic. 193, 195–201.

FAO, 2019. FAO Statistical Yearbook. Food and Agriculture Organization of the United Nations. Available at: http://www.fao.org/faostat/en/#data/QC.

Germain, E., 1997. Genetic improvement of the Persian walnut (*Juglans regia* L.). Acta Hortic. 442, 21–31.

Golzari, M., Hassani, D., Rahemi, M., Vahdati, K., 2016. Xenia and metaxenia in Persian walnut (*Juglans regia* L.). J. Nuts 7, 101–108.

Hajinia, Z., Sarikhani, S., Vahdati, K., 2021. Exploring low-chill genotypes of Persian walnut (*Juglans regia* L.) in west of Iran. Genet. Resour. Crop Evol. 3, 1–12.
Hansche, P.E., Beres, V., Forde, H.I., 1972. Estimates of quantitative genetic properties of walnut and their implications for cultivar improvement. J. Am. Soc. Hortic. Sci. 97, 279–285.
Hassani, D., Atefi, J., Haghjoian, R.A., Dastjerdi, R., Keshavarzi, M., Mozaffari, M.R., Soleimani, A., Rahmanian, A.R., Nematzadeh, F., Malmir, A.S., 2012. Damavand, a new walnut cultivar as a pollinizer for Iranian walnut cultivars and genotypes. Seed Plant Improv. J. 28, 529–531.
Hassani, D., Mozaffari, M.R., Soleimani, S., Dastjerdi, R., Rezaee, R., Keshavarzi, M., Vahdati, K., Fahadan, A., Atefi, J., 2020. Four new Persian walnut cultivars of Iran: Persia, Caspian, Chaldoran, and Alvand. HortScience 55, 1162–1163.
Hassankhah, A., Vahdati, K., Rahemi, M., Hassani, D., Sarikhani Khorami, S., 2017. Persian walnut phenology: effect of chilling and heat requirements on budbreak and flowering date. Int. J. Hortic. Sci. Technol. 4, 259–271.
IPGRI, 1994. Descriptors for Walnut (Juglans spp.). International Plant Genetic Resources Institute, Rome, Italy.
Jefferies, C.J., 1979. A hematoxylin/aniline blue stain for the study of embryo sacs and pollen tubes in a single section. Proc. R. Microbiol. Soc. 14, 229.
Kazemi, N., Sharifzadeh, M., Ahmadvand, M., 2018. Protecting walnut orchards against frost: a test of extended theory of planned behavior. Weather Clim. Soc. 10, 709–722.
Khadivi, A., Montazeran, A., Rezaei, M., Ebrahimi, A., 2019. The pomological characterization of walnut (*Juglans regia* L.) to select the superior genotypes – an opportunity for genetic improvement. Sci. Hortic. 248, 29–33.
Khorami, S.S., Arzani, K., Roozban, M.R., 2012. Identification and selection of twelve walnut superior and promising genotypes in Fars Province. Seed Plant Improv. J. 28, 277–296.
Khorami, S.S., Arzani, K., Karimzadeh, G., Shojaeiyan, A., Ligterink, W., 2018. Genome size; a novel predictor of nut weight and nut size of walnut trees. HortScience 53, 275–282.
Korac, M., Cerovic, S., Golosin, B., Miletic, R., 1997. Collecting, evaluation and utilization of walnut (*Juglans regia* L.) in Yugoslavia. Plant Genet. Res. Newsl. 111, 72–74.
Kouhi, M., Rezaei, A., Hassani, D., Sarikhani, S., Vahdati, K., 2020. Phenotypic evaluation and identification of superior Persian walnut (*Juglans regia* L.) genotypes in Mazandaran province, Iran. J. Nuts 11, 315–326.
Kumar, A., 2017. Extent of fruit set and retention under different modes of pollination in Persian walnut (*Juglans regia* L.). Acta Hortic. 696, 327–330.
Luza, J.G., Polito, V.S., 1991. Porogamy and chalozogamy in walnut (*Juglans regia* L.). The University of Chicago Bot.Gaz. 152, 100–106.
Mahmoodi, R., Dadpour, M.R., Hassani, D., Zeinalabedini, M., Vendramin, E., Micali, S., Zaare, F.N., 2019. Development of a core collection in Iranian walnut (*Juglans regia* L.) germplasm using the phenotypic diversity. Sci. Hortic. 249, 439–448.
Mahmoodi, R., Hassani, D., Amiri, M.E., Jaffaraghaei, M., 2016. Phenological and pomological characteristics of five promised walnut genotypes in Karaj. J. Nuts 7, 1–8.
McGranahan, G.H., Leslie, C, Jain, S.M., Pryadarshan, P.M., 2009. Breeding walnuts (*Juglans regia*). Breeding Plantation Tree Crop: Temperate Species. Springer, New York, pp. 249–273.
McGranahan, G.H., Leslie, C., Ballington, J.R., Moore, J.N., 1990. Walnuts (*Juglans*). In: Genetic Resources of Temperate Fruit and Nut Crops, II. ISHS, Secretariat, Netherlands, pp. 907–951.
McGranahan, G.H., Voyiatzis, D.G., Catlin, P.B., Polito, V.S., 1994. High pollen loads can cause pistillate flower abscission in walnut. J. Am. Soc. Hortic. Sci. 119, 505–509.
Poggetti, L., Ermacora, P., Cipriani, G., Pavan, F., Testolin, R., 2017. Morphological and carpological variability of walnut germplasm (*Juglans regia* L.) collected in North Eastern Italy and selection of superior genotypes. Sci. Hortic. 225, 615–619.
Polito, V.S., Li, N.Y., 1985. Pistil late flower differentiation in English walnut (*Juglans regia* L.): a developmental basis for heterodichogamy. Sci. Hortic. 26, 333–338.
Pollegioni, P., Van der Linden, G., Belisario, A., Gras, M., Anselmi, N., Olimpieri, I., Luongo, L., Santini, A., Turco, E., Scarascia Mugnozza, G., Malvolti, M.E., 2011. Mechanisms governing the responses to anthracnose pathogen in *Juglans* spp. J. Biotechnol. 159, 251–264.
Ramos, D., 1998. Walnut Production Manual, 3373. UCANR Publication, University of California.
Rasouli, M., Ershadi, B.Q., Karimi, R., 2019. The effect of pollen type of some walnut genotypes on fruit set, and fruit quantitative and qualitative characteristics of MSG15, MKG23 and MKG24 as seed parents. Plant Prod. 42, 307–320.
Rovira, M., Aleta, N., 1997. Pistillate flower abscission on four walnut cultivars. Acta Hortic. 442, 231–234.
Sanzol, J., Herroero, M., 2001. The effective pollination period in fruit trees. Sci. Hortic. 90, 1–17.
Sarikhani Khorami, S., Arzani, K., Roozban, M.R., 2014. Correlations of certain high-heritability horticultural traits in Persian walnut (*Juglans regia* L.). Acta Hortic. 1050, 61–68.
Sarikhani, S., Vahdati, K., Ligterink, W., 2021. Biochemical properties of superior Persian walnut genotypes originated from southwest of Iran. Int. J. Hortic. Sci. Technol. 8, 13–24.
Sarkissian, T.S., Harder, L.D., 2001. Direct and indirect responses to selection on pollen size in *Brassica napa* L. J. Evol. Biol. 14, 456–468.
Sharma, O.C., Sharma, S.D., 2001. Correlation between nut and kernel characters of Persian walnut seedlings trees of Garsa valley in Kullu district of Himachal Pradesh. Acta Hortic. 544, 129–132.
Sutyemez, M., Bukucu, S.B., Keles, O., Ozcan, A., Yıldırım, E., Boyacı, S., 2021. Phenological differences, genetic diversity, and population structure of genotypes obtained from seeds of Kaman-1 walnut cultivar. J. Food Qual. 2021. ID 5579875.
Sutyemez, M., Ozcan, A., Bukucu, B., 2018. Walnut cultivars through cross-breeding: 'Diriliş' and '15 Temmuz. J. Am. Pomol. Soc. 72, 173–180.
Tulecke, W., McGranahan, G., 1994. The Walnut Germplasm Collection of UC Davis. DANR, UC Davis.
Vahdati, K., Arab, M.M., Sarikhani, S., Sadat-Hosseini, M., Leslie, C.A., Brown, P.J., Al-Khayri, J., Jain, S., Johnson, D., 2019a. Advances in Persian walnut (*Juglans regia* L.) breeding strategies. Advances in Plant Breeding Strategies: Nut Beverage Crops. Springer, Cham.
Vahdati, K., Massah Bavani, A.R., Khosh-Khui, M., Fakour, P., Sarikhani, S., 2019b. Applying the AOGCM-AR5 models to the assessments of land suitability for walnut cultivation in response to climate change: a case study of Iran. PLOS One 14, e0218725.
Wiebe, K., Lotze-Campen, H., Sands, R., Tabeau, A., van der Mensbrugghe, D., Biewald, A., Bodirsky, B., Islam, S., Kavallari, A., Mason-D`Croz, D., Müller, C., 2015. Climate change impacts on agriculture in 2050 under a range of plausible socioeconomic and emissions scenarios. Environ. Res. Lett. 10, 085010.
Zeneli, G., Kola, H., Dida, M., 2005. Phenotypic variation in native walnut populations of Northern Albania. Sci. Hortic. 105, 91–100.
