# Supplementary material for: Provenance and family variations in early growth of Manchurian walnut (Juglans mandshurica Maxim.) and selection of superior families
Source: PLoS One. 2024 Mar 7;19(3):e0298918. doi: 10.1371/journal.pone.0298918 (PMC10919699; doi:10.1371/journal.pone.0298918)
Supplement: S1 File — (ZIP) [file pone.0298918.s004.zip › Combining ability analysis for nutritional quality and yield in maize (Zea mays L.).pdf]

The Bioscan 10(2): 785-788, 2015 (Supplement on Genetics and Plant Breeding)
AN INTERNATIONAL QUARTERLY JOURNAL OF LIFE SCIENCES
www.thebioscan.in

COMBINING ABILITY ANALYSIS FOR NUTRITIONAL QUALITY AND YIELD IN MAIZE (*ZEA MAYS* L.)

RUMANA KHAN* AND R. B. DUBEY
Department of Plant Breeding and Genetics, Rajasthan College of Agriculture,
Maharana Pratap University of Agriculture and Technology, Udaipur - 313 001, Rajasthan, INDIA
e-mail: khanrumana315@gmail.com

KEYWORDS
Zea mays L.
Heterosis
Starch
Protein

Received on :
14.03.2015

Accepted on :
17.06.2015

***Corresponding author**

ABSTRACT

A set of eight diverse inbred lines (P_1 to P_8) were crossed in diallel design to access their combining ability for yield and nutritional quality traits. The analysis of variance for combining ability revealed significant mean sum of squares due to GCA and SCA for all the traits. The ratio of σ^2SCA/σ^2GCA was greater than one for all the traits, thereby indicating the preponderance of non-additive gene effects in the expression of these traits. Inbred lines P_5, P_6 and P_8 for grain yield per plant, P_4, P_5, P_7 and P_8 for oil content, P_3 and P_4 for starch content and P_3, P_6, P_7 and P_8 for protein content were identified as most promising parents due to having good general combining ability. Hybrid P_6 x P_8 showed highest positive significant SCA effects along with positive significant economic heterosis (9.14 %) for grain yield per plant. Hybrid P_1 x P_6 exhibited highest positive significant SCA effects for oil content. The hybrid P_2 x P_5 showed highest significant SCA effects, economic heterosis (3.33 %) and maximum *per se* performance (66.61 %) for starch content. Hybrid P_2 x P_6 depicted highest positive significant SCA effects with good economic heterosis (6.37 %) for protein content. Hence these hybrids appear to be very promising combination for actual exploitation.

INTRODUCTION

Maize (*Zea mays* L.; 2N=20) is the third most widely distributed crop of the world, being grown in diverse seasons and ecologies with highest production and productivity among food cereals. The breeding strategy for exploitation of heterosis in maize (*Zea mays* L.) through the cultivation of single cross hybrids is primarily dependent on the development and identification of high *per se* performing diverse, vigorous and productive inbred lines and their subsequent evaluation for combining ability in cross combinations to identify single crosses with high heterotic effects. The two parent conventional single cross hybrids practically replaced double cross and three way cross hybrids in most of the developed countries (Mauria *et al.*, 1998). Single cross hybrids are considered most desirable as the breeding and seed production is much easier than the multi-parent hybrids (Vasal *et al.*, 1995). Maize crop serves as a source of basic raw material for a number of industries *viz.*, starch, protein, oil, alcoholic beverages, food, sweeteners, cosmetics, bio-fuels etc. In India, development of maize hybrids was aimed mainly to capitalize on the expression of heterosis for grain yield and other attributes of agronomic importance in spite of the fact that maize has unique distinction of a crop as a source of various industrial products. With available germplasm both exotic and indigenous, it is possible to develop maize hybrids which are nutritionally superior and industrially important with respect to high oil, protein and starch content.

Quality traits can impart very high economic importance to this crop, if exploited properly. Corn oil is considered most suitable for human nutrition as it possesses a very high proportion (about 80 %) of unsaturated fatty acids *viz.*, oleic acid and linoliec acid with a very low content of cholesterol (Singh *et al.*, 1998). In spite of this, limited breeding work has been done for exploiting the potentiality of maize as a source of edible oil in India. In general, oil content is negatively correlated with yield. Efforts are being made to keep balance in potential yield of maize and its oil content by selecting appropriate genotypes having high oil content. It is a by-product of starch industry. The high oil strains of USA have about 18-20 per cent oil on whole kernel basis (Jugenheimer, 1961). If this objective is realized, maize crop in India can attain very high industrial importance as a supplementary oil seed crop because of its high productivity vis-à-vis traditional oil seed crops (Joshi *et al.*, 1985) and as an important source of starch and quality protein. Maize is the major source of starch produced worldwide. In USA 95% starch manufactured is from maize. Efforts are needed to develop maize hybrids and composites having high amylase and amylopectin for use in the industry as specialized starch. Hybrids like "Hi starch" which was developed to take care for quality and amount of starch belong to full season maturity group which requires assured moisture conditions. Selection of parents on the basis of phenotypic performance alone is not a sound procedure since phenotypically superior lines may yield poor recombination. It is therefore, essential that parents should be chosen on the basis of their genetic value. The performance of parent may not necessarily reveal it to be a good or poor combiner. Therefore, gathering information on nature of gene effects and their expression in terms of combining ability is necessary. At the same time, it also elucidates the nature of gene action involved in the inheritance of characters. The

concept of good combining ability refers to the potential of a parental form of producing by its crossing with another parent superior offspring for the breeding process and it is widely used in the breeding of cross-pollinated plants. Information and exact study of combining ability can be useful in regard to selection of breeding methods and selection of lines for hybrid combination.

Present investigation was therefore, undertaken to access the nature of gene actions involved in the inheritance of quality traits along with grain yield and to identify best general combiner inbred lines and also the best hybrids with high SCA effects which can be exploited directly as single cross hybrid.

MATERIALS AND METHODS

A set of eight diverse inbred lines were crossed in diallel mating design (Griffing Model-I Method-II) during *Rabi* 2011 to generate 28 half-diallel crosses (reciprocals excluded). Thus a total of 40 entries *viz.*, 28 hybrids, 8 parents and 4 checks *viz.*, PHEM-2, DHM-117, SUPER-9681 and HM-5 were evaluated in randomized block design with three replications in a single row plot of 4 meters length having 60 cm x 25 cm (R x P) crop geometry during *Kharif* 2012.The observations were taken for seed yield, oil content, starch content and protein content on five randomly selected competitive plants of each entry in each replication. The Soxhlet method developed by A.O.A.C. (1970) was used for the estimation of oil content in percentage. Starch content was estimated by the Anthrone reagent method (Morris D. L. 1948) and nitrogen content by Micro Kjeldhal's method and the value of nitrogen content was multiplied by a factor of 6.25 and averaged and their mean values were subjected to various statistical and biometrical analyses. The analysis of variance was carried out for randomized block design separately for all the traits on plot mean basis as per standard statistical procedure described by Fisher 1918.The variances for general combining ability and specific combining ability were tested against their respective error variances derived from ANOVA reduced to mean level. Significance test for GCA and SCA effects were performed using t-test. The combining ability analysis for diallel mating design was performed according to Model-I (fixed effect) Method-II (parents and one set of F_1s without reciprocals) proposed by Griffing (1956).

RESULTS AND DISCUSSION

The analysis of variance for combining ability (Table 1) revealed significant mean sum of squares due to GCA and SCA for all the traits. The ratio of σ^2SCA/σ^2GCA was greater than one for all the traits, thereby indicating the preponderance of non-additive gene effects in the expression of these traits. These results are in accordance with the findings of Subramanian and Subbaraman (2006), Kumar (2008), Abdel-Moniam *et al.* (2009), Vivek *et al.* (2009), Kose and Turgut (2011), Afshar and Bahram (2012), Soni and Khanorkar (2013), Amiruzzaman *et al.* (2013) and Krupaker *et al.* (2013) .

General combining ability

The estimates of GCA effects revealed that good general combiner inbred lines for grain yield per plant were P_5, P_6 and P_8. As regard quality traits, inbred lines P_4, P_5, P_7 and P_8 were found good general combiners for oil content, P_3 and P_4 for starch content and P_3, P_6, P_7 and P_8 for protein content. In general inbred lines P_6 and P_8 were found good general combiners for quality and yield related traits (Amiruzzaman *et al.,* 2013) (Table 2). Thus these inbred lines with highest magnitude of GCA effects for grain yield per plant and nutritional traits could be used for the development of composite variety.

Specific combining ability

Grain yield per plant

Hybrid P_6 x P_8 showed highest positive significant SCA effects along good *per se* performance (147.33 g/plant) and positive significant economic heterosis (9.14 %) over the best check for grain yield per plant. This was a cross between good x good GCA effect parent for grain yield per plant (Kumar P. S. 2008). Another hybrid P_5 x P_6 showed good GCA effect along

Table 1: Analysis of variance for combining ability in maize

SN	Characters	Source			Var. Model I		
		GCA	SCA	Error	σ^2GCA	σ^2SCA	σ^2 SCA / σ^2GCA
1	Grain yield per plant	1344.84**	1293.64**	34.3184	917.368	35261.1	38.4372
2	Oil content	0.460657**	0.218988**	0.00130205	0.321549	6.09522	18.9558
3	Starch content	1.85126**	2.45502**	0.00882274	1.28971	68.4936	53.1081
4	Protein content	0.939009**	1.81865**	0.00371665	0.654705	50.818	77.3014

*,** Significant at 5 % and 1 % level of significance respectively

Table 2: GCA effects of inbred lines for grain yield and nutritional quality traits

Pedigree	Code	Starch content	Protein content	Oil content	Grain yield per plant
EI-718	P1	-0.08**	-0.43**	-0.15**	-10.44**
EI -561	P2	-0.21**	-0.10**	-0.43**	-6.98**
EI- 586	P3	0.51**	0.05*	-0.07**	-13.64**
EI -719	P4	0.79**	-0.22**	0.15**	-9.61**
EI – 721	P5	-0.13**	-0.29**	0.22**	15.46**
HKI – 193 -1	P6	-0.20**	0.24**	0.01	13.23**
EI-667	P7	-0.13**	0.34**	0.13**	2.86
DMRQPM-106	P8	-0.55**	0.40**	0.14**	9.13**

*,** Significant at 5% and 1% level of significance respectively

Table 3: Specific combining ability effects of hybrids for grain yield per plant

S.N.	Hybrids	SCA/GCA effects	Economic heterosis (%) over the best check DHM-117	Grain yield per plant (g)
1.	P_6 x P_8	37.42**	9.14*	147.33
2.	P_3 x P_8)	34.95**	-	118.00
3.	P_2 x P_7	34.55**	-	118.00
4.	P_4 x P_7	34.19**	-	115.00
5.	P_5 x P_6	32.75**	10.37**	149.00
6.	EI-561 (P_2)	-6.98**	-	17.67
7.	EI-586 (P_3)	-13.64**	-	25.33
8.	EI-719 (P_4)	-9.61**	-	20.67
9.	EI-721 (P_5)	15.46**	-	60.00
10.	HKI-193-1(P_6)	13.23**	-	47.33
11.	EI-667 (P_7)	2.86	-	58.33
12.	DMRQPM-106 (P_8)	9.13**	-	33.33
13.	DHM-117			135.00

*,** Significant at 5 % and 1 % level of significance respectively.

Table 4: Specific combining ability effects of hybrids for oil content

S.N.	Hybrids	SCA/GCA effects	Economic heterosis (%) over the best check PHEM-2	*Per se* Performance Oil content (%)	Grain yield per Plant (g)
1.	P_1 x P_6	0.75**	-	6.27	98.00
2.	P_2 x P_5	0.63**	-	6.08	106.67
3.	P_6 x P_8	0.50**	-	6.31	147.33
4.	P_4 x P_5	0.45**	-	6.48	121.67
5.	P_5 x P_8	0.45**	-	6.48	115.00
6.	EI-718 (P_1)	-0.15**	-	5.34	40.67
7.	EI-561 (P_2)	-0.43**	-	4.89	17.67
8.	EI-719 (P_4)	0.15**	-	5.60	20.67
9.	EI-721 (P_5)	0.22**	-	5.50	60.00
10.	HKI-193-1 (P_6)	0.01	-	5.07	47.33
11.	DMRQPM-106 (P_8)	0.14**	-	6.09	33.33
12.	PHEM-2			6.50	100.67

*,** Significant at 5 % and 1 % level of significance respectively

Table 5: Specific combining ability effects of hybrids for starch content

S.N.	Hybrids	SCA/GCA effects	Economic heterosis (%) over the best check PHEM-2	*Per se* performance Starch content (%)	Grain yield per plant(g)
1.	P_2 x P_5	3.47**	3.33**	66.61	106.67
2.	P_4 x P_5	2.38**	3.18**	66.52	121.67
3.	P_3 x P_8	2.33**	2.01**	65.76	118.00
4.	P_4 x P_6	2.22**	2.81**	66.28	108.33
5.	P_3 x P_7	1.55**	1.44**	65.40	77.00
6.	EI-561 (P_2)	-0.21**	-	61.87	17.67
7.	EI-586 (P_3)	0.51**	-	63.30	25.33
8.	EI-719 (P_4)	0.79**	-	63.48	20.67
9.	EI-721 (P_5)	-0.13**	-	61.09	60.00
10.	HKI-193-1(P_6)	-0.20**	-	61.13	47.33
11.	EI-667 (P_7)	-0.13**	-	62.58	58.33
12.	DMRQPM-106 (P_8)	-0.55**	-	62.78	33.33
13.	PHEM-2			64.47	100.67

*, ** Significant at 5 % and 1 % level of significance respectively

with highest economic heterosis (10.37 %) and maximum *per se* performance (149.00 g/ plant) for grain yield per plant. This was also a cross between good x good GCA effect parent for grain yield per plant (Vieira *et al.*, 2011). Out of total 28 hybrids, five best hybrids which exhibited highest significant positive SCA effects for grain yield per plant were *viz.*, P_6 x P_8, P_3 x P_8, P_2 x P_7, P_4 x P_7 and P_5 x P_6 (Table 3).

Oil content

The hybrid P_1 x P_6 exhibited highest positive significant SCA effects for oil content and it was a cross between poor x average GCA effect parent for oil content. It has 6.27 % oil content. None of the hybrid exhibited positive significant economic heterosis for oil content over the best check PHEM-2. Out of 28 hybrids, five best hybrids exhibiting highest magnitude of positive significant SCA effects for oil content are *viz.*, P_1 x P_6, P_2 x P_5, P_6 x P_8, P_4 x P_5 and P_5 x P_8 (Table 4).

Starch content

The hybrid P_2 x P_5 showed highest significant SCA effects,

Table 6: Specific combining ability effects of hybrids for protein content

S.N.	Hybrids	SCA/GCA effects	Economic heterosis (%) over the best check DHM-117	*Per se* performance Protein content (%)	Grain yield per Plant (g)
1.	$P_2 \times P_6$	1.80**	6.37**	11.41	116.33
2.	$P_6 \times P_7$	1.77**	10.23**	11.82	115.00
3.	$P_6 \times P_8$	1.69**	9.95**	11.79	147.33
4.	$P_1 \times P_4$	1.49**	_	10.31	88.33
5.	$P_4 \times P_8$	1.46*	3.57**	11.11	110.00
6.	EI-718 (P_1)	-0.43**	-	7.53	40.67
7.	EI-561 (P_2)	-0.10**	-	8.65	17.67
8.	EI-719 (P_4)	-0.22**	-	7.92	20.67
9.	HKI-193-1 (P_6)	0.24**	-	7.20	47.33
10.	EI-667 (P_7)	0.34**	-	7.86	58.33
11.	DMRQPM-106 (P_8)	0.40**	-	8.14	33.33
12.	DHM-117			10.72	135.00

*,** Significant at 5 % and 1 % level of significance respectively.

economic heterosis (3.33%) and *per se* performance (66.61 %) for starch content along with good *per se* performance for grain yield per plant. This was a cross between poor x poor GCA effect parent for starch content. Out of 28 hybrids, five best hybrids exhibiting highest positive significant SCA effects for starch content are *viz.*, $P_2 \times P_5$, $P_4 \times P_5$, $P_3 \times P_8$, $P_4 \times P_6$ and $P_3 \times P_7$. All these hybrids also exhibited positive significant economic heterosis for starch content (Table-5).

Protein content

Hybrid $P_2 \times P_6$ depicted highest positive significant SCA effects for protein content with good economic heterosis (6.37 %) and *per se* performance for protein content (11.41 %) and grain yield per plant (116.33 g/plant). This was a cross between poor x good GCA effect parents for protein content. Another hybrid $P_6 \times P_7$ exhibited good SCA effects along with highest economic heterosis (10.23 %) and maximum *per se* performance (11.82 %) for protein content. This was a cross between good x good GCA effect parents for protein content. The five best hybrids exhibiting highest magnitude of SCA effects for protein content are *viz.*, $P_2 \times P_6$, $P_6 \times P_7$, $P_6 \times P_8$, $P_1 \times P_4$ and $P_4 \times P_8$ (Table 6).

Hence these hybrids appear to be very promising combination for actual exploitation and could be recommended for testing in multi-location trials.

REFERENCES

Amiruzzaman, M., Islam, M. A., Lutful Hasan, Monjurul Kadir and Rohman, M. M. 2013. Heterosis and combining ability in a diallel among elite inbred lines of maize (*Zea mays* L.) *Emirates J. Food and Agriculture.* **25(2):** 132-137.

Abdel-Moneam, M. A., Attia, A., El-Emery, M. I. and Fayad, E. A. 2009. Combining ability and heterosis for some agronomic traits in crosses of maize. *Pakistan J. Biological Sciences.* **12(5):** 433-438.

Afshar, E. and Bahram, H. 2012. Combining ability and gene action for maturity and agronomic traits in different heterotic groups of maize inbred lines and their diallel crosses. *J. Crop Science and Biotechnology.* **15(3):** 219-229.

A. O. A. C. 1970. Official methods of analysis (11th edn.). *Associationof official Agricultural Chemists, Washington., D.C., USA.*

Fisher, R. A. 1918. The correlation between relatives on the supposition of Mendelian inheritance. *Tran. Roy. Soc. Edingrugh.* **52:** 399-433.

Griffing, B. 1956. Concept of general and specific combining ability in relation to diallel crossing systems. *Aust. J. Biol. Sci.* **9:** 463-493.

Joshi, V. N., Sharma, G. S. and Ranwah, B. R. 1985. Prospects of Maize (*Zea mays* L.) as a new oil seed crop. In oil seed production, constraints and opportunities (Edited by H.C. Shrivastava and S. Bhaskaran). IBH Publi. Co. pp. 529-533.

Jugenheimer, R. W. 1961. Breeding for oil and protein content in maize. *Euphytica.* **10:** 152-156.

Kose, A. and Turgut, I. 2011. Determination of general, specific combining ability and heterosis in maize inbred lines and their diallel crosses. *Ziraat Fakultesi Dergisi, Akdeniz Universitesi.* **24(1):** 39-46.

Krupakar, A., Kumar, B. and Marker, S. 2013. Combining ability for yield and quality traits in single cross hybrids of maize (*Zea mays* L.). *The Bioscan.* **8(4):** 1347-1355.

Kumar, P. S. 2008. Combining ability studies in maize (*Zea mays* L.). *Advances in Plant Sciences.* **21(1):** 167-169.

Mauria, S., Gupta, N. P., Zaidi, P. H. and Singh, N. N. 1988. Maize research in India- Progress and future challenges. Indian Farming (April, 1998). An ICAR publication.

Morris, D. L. 1948. Quantitative determination of carbohydrate with Derwood's anthrone reagent. *Science.* **107:** 254-255.

Singh, A. K., Shahi, J. P., Singh, J. K. and Singh, R. N. 1998. Combining ability for maturity and height traits in maize (*Zea mays* L.). *Annals of Agric. Res.* **19(2):** 162-166.

Soni, N. V. and Khanorkar, S. M., 2013. Association of geneticdivergence with heterosis, combining ability and mean value forquantitative traits in Popcorn (*Zea mays* var. Everta). *The Bioscan* **8(4):** 1363-1367.

Subramanian, A. and Subbaraman, N. 2006. Combining ability analysis of yield and its contributing traits in maize. *Indian J. Agric. Res.* **40(2):** 131-134.

Vasal, S. K., Dhillon, B. S. and Srinivasan, G. 1995. Changing scenario of hybrid maize breeding and research strategies to develop two-parent hybrids. In Hybrid Research and Development. M. Rai and S. Mauria (Ed.) PP. 20-36. Indian Society of Seed Technology. *IARI,* New Dehli-110012.

Vieira, R. A., SCApim, C. A., Tessmann, D. J. and Hata, F. T. 2011. Diallel analysis of yield, popping expansion, and southern rust resistance in popcorn lines. *Rev. Ciênc. Agron.* **42(3):** 774-780.

Vivek, B. S., Crossa, J. and Alvarado, G. 2009. Heterosis and combining ability among CIMMYT's mid-altitude early to intermediate maturing maize (*Zea mays* L.) populations. *Maydica.* **54(1):** 97-107.
