# Supplementary material for: Provenance and family variations in early growth of Manchurian walnut (Juglans mandshurica Maxim.) and selection of superior families
Source: PLoS One. 2024 Mar 7;19(3):e0298918. doi: 10.1371/journal.pone.0298918 (PMC10919699; doi:10.1371/journal.pone.0298918)
Supplement: S1 File — (ZIP) [file pone.0298918.s004.zip › Combining ability analysis of fruit yield and morphological traits in greenhouse cucumber (Cucumis sativus L).)]

Combining ability analysis of fruit yield and morphological traits in greenhouse cucumber (*Cucumis sativus* L.)

Maryam Golabadi[1], Pooran Golkar[2], and Abdolreza Eghtedary[3]

[1]Department of Agronomy and Plant Breeding, College of Agriculture, Isfahan (Khorasgan) Branch, Islamic Azad University, Isfahan, Iran (e-mail: m.golabadi@khuisf.ac.ir); [2]Institute of Biotechnology and Bioengineering, Isfahan University of Technology, 84156 83111 Isfahan, Iran; and [3]Department of Agronomy and Plant Breeding, College of Agriculture, Isfahan (Khorasgan) Branch, Islamic Azad University, Isfahan, Iran. *Received 10 December 2013, accepted 3 November 2014. Published on the web 7 April 2015.*

Golabadi, M., Golkar, P. and Eghtedary, A.-R. 2015. **Combining ability analysis of fruit yield and morphological traits in greenhouse cucumber (*Cucumis sativus* L.)**. Can. J. Plant Sci. **95**: 377–385. Knowledge about the genetic control of the different quantitative characters related to fruit yield and its components is still insufficient in greenhouse cucumbers. This information is useful in planning breeding programs in this economically important crop. In this study, the genetics of morphological traits and fruit yield was investigated using a 9 × 9 full diallel population of cucumbers for the greenhouse market. Data were collected on internode length, leaf length, leaf width, fruit length, fruit diameter, number of fruits per plant, yield per fruit and yield per plant. Variance components showed that both the additive and the dominant gene effects played significant roles in the genetic control of the traits studied. Genetic control of internode length, leaf width and number of fruits per plant was accomplished by additive effects. The significant mean squares of reciprocal crosses for all of the studied traits suggested that maternal inheritance also played an important role in the inheritance of these traits. Significant general combining ability for fruit yield revealed that both selection and hybridization methods would lead to desirable genetic improvements in cucumber through accumulation of desirable alleles from parents in the target genotype, but that hybridization would be preferred.

Key words: Action, additive, diallel, dominance

Golabadi, M., Golkar, P. et Eghtedary, A.-R. 2015. **Analyse de l'aptitude à la combinaison pour le rendement fruitier et les caractères morphologiques du concombre de serre (*Cucumis sativus* L.)**. Can. J. Plant Sci. **95**: 377–385. Nos connaissances sur le contrôle génétique des différents caractères quantitatifs associés au rendement fruitier et à ses éléments laissent toujours à désirer pour le concombre de serre. Or, de telles connaissances auraient leur utilité pour planifier les programmes d'hybridation de cette culture importante pour l'économie. Dans le cadre de cette étude, les chercheurs ont examiné la génétique des caractères morphologiques et du rendement fruitier en recourant à une population 9 × 9 totalement dialléle de concombres destinés au marché des légumes de serre. Les données recueillies portaient sur la longueur des entrenæuds, la longueur et la largeur des feuilles, le diamètre des fruits, le nombre de fruits par plant, le rendement par fruit et le rendement par plant. Les composants de la variance indiquent que les effets des gènes additifs et dominants jouent un rôle significatif dans le contrôle génétique des caractères examinés. La longueur des entrenæuds, la largeur des feuilles et le nombre de fruits par plant sont génétiquement contrôlés par des effets additifs. La variance significative des croisements réciproques pour l'ensemble des caractères étudiés laisse supposer que la transmission par la mère joue aussi un rôle important dans l'hérédité de ces caractères. Une aptitude à la combinaison générale significative pour le rendement fruitier révèle que les méthodes de sélection et d'hybridation aboutiraient à des améliorations génétiques intéressantes pour le concombre grâce à l'accumulation des allèles souhaitables des deux parents dans le génotype ciblé, mais qu'il faudrait privilégier l'hybridation.

Mots clés: Action, additif, diallèle, dominance

Can. J. Plant Sci. Downloaded from cdnsciencepub.com by 116.95.134.189 on 06/17/23
For personal use only.

Cucumber (*Cucumis sativus* L.) is a widely cultivated plant and one of the most important cucurbitaceous vegetables grown worldwide (Plader et al. 2007) with great economic importance (Harlan 1975). Greenhouse cucumbers are one of the most important vegetable crops grown in Iran. According to a 2012 Food and Agriculture Organization report (FAO 2012), Iran, with an annual production of 1 600 000 mt, ranks third in global cucumber production after China and Turkey. Although cultivation of imported raw materials is associated with high economic costs for Iranian farms, limited research seems to have been conducted on greenhouse cucumber breeding. Moreover, no adequate information is available on the lines, cultivars, gene action, or genotype × environment interactions for the different traits of cucumber under the farming conditions in Iran.

Based on the above considerations, it is obvious that seed production primarily warrants a sound and adequate knowledge of the gene actions of different traits

Abbreviations: **FD**, fruit diameter; **FL**, fruit length; **GCA**, general combining ability; **IL**, internode length; **LL**, leaf length; **LW**, leaf width; **NFP**, number of fruits per plant in every pickling; **PCA**, principal component analysis; **PF**, predictability factor; **SCA**, specific combining ability; **YF**, yield per fruit; **YP**, yield per plant in every pickling

Can. J. Plant Sci. Downloaded from cdnsciencepub.com by 116.95.134.189 on 06/17/23
For personal use only.

including morphological traits and fruit yield. Accordingly, it is essential to plan breeding experiments that shed more light on the recessive or dominant actions of traits. Using such data, breeders can select the best breeding procedure for different traits in cucumber.

In F_1 hybrid breeding, analysis of combining ability has been used in practical plant improvement programs to determine the relative importance of general combining ability (GCA), specific combining ability (SCA) of the parents in the performance of F_1 hybrids, and superior parents for crossing in hybridization programs (Yoshioka et al. 2010). General combining ability is the manifestation of the additive gene action for the selection of parents and SCA represents the non-additive gene action (Singh et al. 2011).

Breeding for fruit yield is an important objective in many cucumber breeding programs (Wehner et al. 2000). Information about gene action with regards to different traits will be helpful in selecting appropriate breeding procedures aimed at fruit yield improvement. There are different traits that are related to fruit weight and fruit number such as fruit length; fruit diameter; fruit length/diameter (L:D) ratio; vine length; leaf area that includes leaf length and leaf width (Hormuzdi and More 1989; Serce et al. 1999; Lopez-Sese and Staub 2002; Shetty and Wehner 2002; Blanco et al. 2005; Fan et al. 2006; Afangideh and Uyoh 2007; Cho et al. 2007; De Wilde and Duyfjes 2010); number of nodes per vine (Singh et al. 2011); plant height (Mule et al. 2011); and phenological traits including days to 50% flowering, anthesis, and first female flower formation (Singh et al. 2011). Leaf area is an important variable for most physiological and agronomic studies involving plant growth, light interception, photosynthetic efficiency, evapotranspiration, or response to fertilizers and irrigation (Blanco et al. 2005).

Different genetic analyses have been conducted to study the combining ability in cucumber for fruit yield and yield components (Lopez and Staub 2002; Xing Fang et al. 2004; Munshi et al. 2006; Mule et al. 2011; Sarkar and Sirohi 2011; Singh et al. 2011; Olfati et al. 2012). Genetic studies have also been reported on the number of female flower in cucumber (Wahid et al. 2003; Singh et al. 2011).

Selective mating designs such as diallel, which may allow inter-mating of the selects in different cycles and exploit both additive and non-additive gene effects, could be useful for the genetic improvement of fruit yield and nutritional purposes (Singh and Pawar 2005). Such studies have been conducted on some horticultural traits of cucumber including partenocarpy (Sun et al. 2006), fruit texture (Yoshioka et al. 2010), carotenoid content (Navazio and Simon 2001), fruit yield and its components (Dogra and Kanwar 2011; Jagesh et al. 2013), and lateral branching and number of flowering (both female and male) nodes (Lopez-Sese and Staub 2002). Improving fruit yield and its marketing requires adequate information regarding the nature of GCA and SCA of the parents available in a wide array of genetic material to be used in hybridization programs. The present study was carried out to estimate the combining ability, gene action, superior crosses for fruit yield, and the morphologic characters of greenhouse cucumbers.

MATERIAL AND METHODS

The genotypic materials selected from different geographical regions for use in this study are listed (Table 1).

Nine genotypes based on variation in different traits (Neda, Tornado, Amiral, Sco 4184, Storm, Karim, Atilgan, Vista and Raneem) were crossed in a complete-diallel design in 2011. The properties of these genotypes are available in a previous study (Golabadi et al. 2012).

This work was carried out at the Research Greenhouse of the Islamic Azad University of Isfahan (Khorasgan) Branch, Iran, (lat. 32°63′, long. 51°36′). Gibberellic acid (Merck Schuchardt OHG) at a concentration of 1500 ppm was used to induce androgenesis at the two- to four-leaf stage at intervals of 1–3 wk. The F_1 generation was planted in autumn 2012, in soil with an equal rate of peat moss, perlite and cocopeat (coir) bed. Treatments were arranged in a randomized complete block design (RCBD) with three replications. In each plot, the spaces between and within double rows were 90 (cm) and 50 (cm), respectively, while double rows were spaced at 180 (cm). Nutrient levels in the irrigation solution water were (mg L^{-1}) N 216, P 58, K 286, Ca 185, Mg 185, S 43, Fe 5.59, Mn 1.97, B 0.7, Zn 0.2, Cu 0.07 and Mo 0.05. Different fertilizers were used based on soil analysis that included: potassium nitrate, ammonium nitrate, magnesium nitrate, iron and other mineral elements such as sulphate dissolved in the irrigation water. Dichlorvos, Trigard, Abamectin and organic neem oil were applied for insect control. During the growing period, the diurnal greenhouse air temperature was kept at 25–30°C and the nocturnal temperature at 19–21°C with a relative humidity of about 60%. Drip irrigation was applied when needed. The source of water was urban water with electrical conductivity (EC) of 0.4 dSm^{-1}. The pH of the irrigation water was adjusted to 6.5 by nitric acid.

The agronomic and morphologic traits investigated included: internode length (IL) (cm), leaf length (LL) (cm), leaf width (LW) (cm), fruit length (FL) (cm), fruit

Table 1. Cucumber genotypes and origins

Entry	Parent	Origin
1	Neda	Turkey
2	Tornado	Spain
3	Amiral	Spain
4	Sco4184	Denmark
5	Storm	Netherland
6	Karim	Russia
7	Atilgan	France
8	Vista	France
9	Raneem	Netherland

diameter (FD) (cm), number of fruits per plant in every pickling (NFP), yield fruit^{-1} (YF) (g) and yield plant^{-1} in every pickling (YP) (g). Data were recorded from eight randomly selected plants in each genotype of the F_1 generation for horticultural traits. Each plot was harvested daily, if there was a fruit of marketable size on each plant. Then the numbers and the weights of all the fruits harvested in each plot were recorded. Yield per fruit was measured based on the mean weight harvested of every fruit in every pickling. The length of nodes per vine on the main stem was measured from node number 15 to node number 20. The lengths of 15 fruits at edible maturity and randomly harvested in 5 d (three fruits per day) were recorded from the base to the apex and reported as average values. The diameters of the same 15 fruits were measured at maximum thickness using a vernier caliper. Leaf length was measured from base to tip and leaf width was recorded at the widest points.

Combining abilities (general and specific) were estimated following Method 1, Model 1 of Griffing (1956) using SAS software version 9.1 (Zhang et al. 2005). Parental genotypes were selected from different sources, and the data were analyzed as a fixed model. The genotypic variance was partitioned into variance components, including GCA, SCA and reciprocal (REC).

The analysis of combining ability was based on the following model:

$$X_{ij} = \mu + g_i + g_j + S_{ij} + r_{ij} + 1/bc \leftarrow \sum_k \sum_l e_{ijkl},$$

where μ is the population mean; g_i is the GCA effects of ith parent; g_j is the GCA effects of jth parent; S_{ij} is SCA of the crosses between the ith and the jth parents; r_{ij} are the reciprocal effects between the ith and the jth parents; e_{ijkl} is the environmental effect that is associated with i_{jkl} observation; b is the the number of replications; and c is the the number of samples per replication.

The relative importance of variances due to GCA and SCA were compared via the predictability factor (PF) $[2\delta^2_{GCA}/(2\delta^2_{GCA}+2\delta^2_{SCA})]$. The closer this ratio is to unity, the greater the predictability based on the general combining ability alone (Baker 1978). The broad-sense and narrow sense heritability of traits were estimated using variance components and calculated as (Mahmud and Keramer 1951):

$$H_b = \frac{\sigma_A^2 + \sigma_D^2}{\sigma_A^2 + \sigma_D^2 + \sigma_e^2} \quad H_n = \frac{\sigma_A^2}{\sigma_A^2 + \sigma_D^2 + \sigma_e^2}$$

Principal component analysis (PCA) could convert a set of observations of possibly converted variables into a set of values of linearly uncorrelated variables that are less than the number of original variable. Principal component analysis and trait correlation carried out to analysis the interrelations between traits with SAS software version 9.1 (SAS Institute Inc. 2002).

RESULTS AND DISCUSSION

General and Specific combining Abilities of Studied Traits

Internode Length

The results of the present experiments revealed that, unlike SCA effects, GCA effects were significant for internode length (Table 2). Comparison of the variance components and predictability factor indicated the relative superiority of additive gene effects over dominant gene effects for the genetic control of IL. This finding is inconsistent with those reported by Dogra and Kanwar (2011) on cucumber; the discrepancy might be due to differences in the parental materials used and the environmental conditions of the two studies for making diallel crosses. Dogra and Kanwar (2011) demonstrated that SCA (σ^2s) was more important than GCA (σ^2g) for IL, indicating the preponderance of dominant gene action for internode length.

Table 2. Analysis of variance for eight agronomic traits in cucumber[z]

	D.F	IL	LL	LW	FL	FD	NFP	YF^{-1}	YP^{-1}
Replication	2	27.02NS	8.33NS	61.59NS	1.9NS	0.058NS	0.68NS	0.13NS	3284.2**
Crosses	80	119.97**	17**	47.67*	4.21**	0.34**	1.92**	188.15**	10823.75**
GCA	8	515.17**	31.48**	78.04**	7.4*	0.90**	3.05**	462.53**	14665.2*
SCA	36	51.08NS	12.22**	36.83NS	3.81**	0.20**	1.11NS	126.53**	8398.92**
Reciprocal	36	100.31**	18.75**	51.77*	3.91**	0.35**	2.49**	188.79**	12394.91**
Residual	160	37.76	3.08	34.85	1.78	0.05	0.85	40.81	428
PF		0.87	0.29	0.80	0.24	0.44	0.87	0.44	0.25
δ^2_{GCA}		8.58±4.75	0.35±0.27	0.76±0.67	0.066±0.06	0.012±0.008	0.036±0.027	116.99±122.1	6.24±4.1
δ^2_{SCA}		2.6±2.9	1.7±0.72	0.36±2.06	0.37±0.22	0.3±0.012	0.047±0.063	695.42±490.1	15.85±7.4
δ^2_A		17.16	0.7	1.52	0.12	0.024	0.072	233.9	12.48
δ^2_D		2.6	1.7	0.36	0.37	0.03	0.047	695.42	15.85
h^2_b		0.64	0.96	0.13	0.45	0.84	0.58	0.98	0.16
h^2_n		0.55	0.20	0.11	0.11	0.37	0.39	0.24	0.07

[z]IL, internode length; LL, leaf length; LW, leaf width; FL, fruit length; FD, fruit diameter; NFP, number of fruits plant^{-1}; YF^{-1}, yield fruit^{-1}; YP^{-1}, yield plant^{-1}; PF, predictability factor; h^2_b, broad-sense heritability, h^2_n, narrow-sense heritability.
Means of three replications. *, ** significant at $P<0.05$ and $P<0.01$, respectively. NS, not significant.

Can. J. Plant Sci. Downloaded from cdnsciencepub.com by 116.95.134.189 on 06/17/23
For personal use only.

Can. J. Plant Sci. Downloaded from cdnsciencepub.com by 116.95.134.189 on 06/17/23
For personal use only.

Plant height is a major characteristic for crop management and productivity (Liebig and Fricke 2002). In greenhouse cucumber production systems, where plants are vertically trained, the number of internodes and their individual lengths determine plant height. Identification of internode length inheritance could, therefore, initiate new breeding methods for improving plant height.

Leaf Size (Leaf Length and Leaf Width)

Analysis of variance in this study revealed that general combining ability effects were significant with respect to leaf length and leaf width; however, SCA was significant only for leaf length (Table 2). These findings imply the importance of both additive and dominant genetic controls of LL (Table 2). The higher value of δ^2_D, compared with that of δ^2_A, and the high deviation of the predictability factor from unity indicate the predominance of gene action in the genetic control of LL. Unlike the genetic control of leaf length, that of leaf width was found to be predominantly controlled by additive effects (Table 2), indicating that selection methods would be preferred for its enhancement. Since selection for leaf size (length and width) may indirectly influence photosynthesis, information on the genetic control of these traits might also be applicable in enhancing the photosynthetic capability of the leaf.

Fruit Size (Fruit Length and Fruit Diameter)

Fruit size is an important determinant of fruit yield, quality, and marketability. Analysis of variance in the present study showed that GCA and SCA were significant for fruit length and fruit diameter (Table 2). This finding is similar to the results reported by Lopez-Sese and Staub (2002), Singh et al. (2011), and Mule et al. (2011). However, different results have been reported with respect to combining ability by Fredrick and Staub (1989), who claimed a significant role for GCA effects on the genetic control of fruit length. The significance of GCA and SCA effects imply that both additive and dominant gene actions have significant effects on the genetic control of FL and FD. Variance component analysis and predictability factor showed that dominance had greater effects on the genetic control of FL and FD (Table 2). These results agree with those reported by Dogra and Kanwar (2011) and Sarkar and Sirohi (2011), but are inconsistent with those reported by Hormuzdi and More (1989).

Fruit Yield

Another result obtained from the analysis of variance in the present study is the significant effects of GCA and SCA in greenhouse cucumber on yield fruit^{-1} and yield plant^{-1} (Table 2). This is in agreement with the findings of Dogra and Kanwar (2011) and Jagesh et al. (2013). Olfati et al. (2012) claimed that GCA and SCA effects were significant for marketable yield, nonmarketable yield and, total yield in cucumber. The deviation of PF from unity and the higher magnitude of δ^2_D, compared with δ^2_A, imply the dominant genetic control of fruit yield, which is consistent with the findings of Sarkar and Sirohi (2011), Dogra and Kanwar (2011), and Jagesh et al. (2013). However, the predominant role of additive gene action in the genetic control of nonmarketable yield has also been reported (Olfati et al. 2012). It is likely that genetic factors are affected by different genotypic materials. Fruit yield has been reported as the most important breeding trait in cucumber (Singh et al. 2011; Olfati et al. 2012) and it maybe concluded that selection of those traits that are responsible for high heritability and that are correlated with yield could improve fruit yield in greenhouse cucumber.

Number of Fruits per Plant

Based on the results of the present experiment, general combining ability (GCA) was significant for the genetic control of number of fruits per plant (Table 2), which is an important yield component trait that affects cucumber yield, while SCA effects were not (Table 2). Our results indicating the significant effects of GCA and SCA on fruit yield per plant and yield per fruit are also confirmed by Abd El-Hafez et al. (1997).

The relatively small deviation of PF from unity observed in this study indicates the prime importance of additive gene action for the genetic control of number of fruits per plant suggesting that cyclic selection should be effective for improving number of fruits per plant. Lopez-Sese and Staub (2002) reported that fruit number was under the genetic control of both additive and dominant gene actions. Sarkar and Sirohi (2011) also reported the over-dominant gene action for the genetic control of fruit number per plant and suggested heterosis breeding for achieving higher yields in cucumber.

GCA mean square was found to be considerably larger than that of SCA for IL, NFP, and LW, indicating that additive genetic effects were of major importance in governing these traits. A close agreement was also observed between yield per fruit and yield per plant as affected by GCA.

Combining Ability Analysis of Reciprocal Crosses

The reciprocal effects in F_1 diallel crosses estimated by Griffing's analysis were significant for all the traits studied. These results indicate that extra-nuclear genes also have a significant contribution to the control of all the traits investigated. Therefore, the transfer of desirable traits could probably be easier with the production of cytoplasmic male sterility (CMS) lines. On the other hand, the use of reciprocal crosses could be used for different purposes in hybrid seed production.

As no report is yet available on cytoplasmic effects on the inheritance of these traits in cucumber, cytoplasmic breeding methods may be proposed for experiments aimed at improving the traits mentioned.

Can. J. Plant Sci. Downloaded from cdnsciencepub.com by 116.95.134.189 on 06/17/23
For personal use only.

Trait Heritability

The traits investigated here showed a wide range of 0.16 (yield fruit^{-1}) to 0.96 (leaf length) for broad-sense heritability (Table 2). This is suggestive of the fact that environmental factors have great effects on phenotypic variations of LL, FD, and YF (Table 2). Kumar et al. (2009) reported a high broad-sense heritability degrees of fruit length (100%), yield fruit^{-1} (99%), and fruit diameter (99%) that are almost similar to the results obtained in the present study. For the number of fruits per plant (98.66%), however, the same authors reported a high level of broad-sense heritability for number of fruits per plant (98.66%), which was different from ours (Kumar et al. 2009). The difference could be attributed to differences in environmental factors (especially temperature and humidity). Afangideh and Uyoh (2007) reported medium broad-sense heritability for fruit length (32%), which is to some extent similar to ours, whereas Serquen et al. (1997) reported very low broad-sense heritability (8%) for FL. In the case of number of fruits per plant, Afangideh and Uyoh (2007) reported a medium broad-sense heritability (58.26%) that is similar to our findings. Few reports, if any, seem to be available on the heritability of internode length, leaf length, and leaf width.

Estimates of heritability provide an index of transmissibility of characters to progenies. The narrow-sense heritability values were found to vary from 0.07 (yield plant^{-1}) to 0.55 (internode length) (Table 2). Narrow-sense heritability values below 70 for the studied traits were taken to imply moderate achievements to be expected if selections were made for these traits.

GCA Effects and Mean Performance of the Parents

GCA and SCA effects for the selected parents as combined with other genotypes were estimated in this study. Estimates of GCA effects varied from 4.56 (Neda) to −5.4 (Karim) for internode length (Table 3). Based on these estimates, the Karim cultivar with the least GCA value for internode length was considered to be an appropriate parent for hybridization as a negative combiner to reduce internode length. The highest GCA values for leaf length (1.22) and leaf width (2.21) were recorded for Neda and Tornado parents, respectively (Table 3). These parents could, therefore, be selected in hybridization programs for improving leaf size that would, in turn, enhance photosynthetic capacity. Leaf area is an important variable for most physiological and agronomic studies involving plant growth, light interception, photosynthetic efficiency, evapotranspiration, as well as responses to fertilizers and irrigation (Blanco and Folegatti 2005). General combining ability effects for fruit length varied from 0.7 (Sco4184) to −0.5 (Storm) (Table 3). GCA effects for fruit diameter ranged between 0.17 (Tornado) and −0.22 (Karim) (Table 3).

Fruit shape is a marketable character and fruit size is an indicator of yield improvement. The results of this study indicate that the Tornado and Sco4184 genotypes may be good combiners for breeding these traits. Mule et al. (2011) reported the best general combiners for fruit length and fruit diameter in a line × tester analysis. Greater GCA values for fruit length and fruit diameter indicate the capacity of the parent to produce superior progenies for internode length in the main stem when combined with another parent.

GCA effects for number of fruits per plant varied from 0.32 (Storm) to −0.32 (Sco4184). This implies that the Storm genotype could be selected as a best combiner in hybridization programs for improving NFP. However, the Sco4184 genotype exhibited the least value of

Table 3. General combining ability effects and mean values for nine cucumber parents estimated by diallel analyses[z]

Parent	GCA/Mean	IL	LL	LW	FL	FD	NFP	YF^{-1}	YP^{-1}
Neda	GCA	4.56**	1.22**	0.53	0.16	−0.04*	0.16	1.57*	23.77**
	Mean	54.47	27.42	29.4	14.48	2	4.91	63.19	315.08
Tornado	GCA	−1.17	1.13**	2.21**	0.009	0.17**	0.18	0.34	16.47
	Mean	48.72	27.33	31.06	14.32	2.22	4.96	61.96	307.78
Amiral	GCA	−0.39	−0.05	−0.24	0.31	−0.007	−0.09	0.25	−3.30
	Mean	49.51	26.14	28.16	14.63	2.03	4.66	61.87	288
Sco4184	GCA	2.37**	0.46**	0.36	0.70**	0.08**	−0.32**	6.10**	9.80
	Mean	52.77	26.66	29.21	15	2.13	4.43	67.72	301.12
Storm	GCA	−3.4**	−0.77**	−1.25	−0.5**	−0.016**	0.32**	−3.32**	4.17
	Mean	46.50	25.42	27.6	13.82	1.88	5.08	58.28	295.5
Karim	GCA	−5.4**	−0.43**	−0.09	0.06	−0.22**	0.11	−3.47**	−16.63
	Mean	44.51	25.76	28.75	14.38	1.82	4.87	58.14	274.68
Atilgan	GCA	0.42	−0.32	−1.06	−0.31	0.06**	−0.4*	0.72	−29.1**
	Mean	50.33	25.87	27.78	14	2.11	4.35	62.34	262.21
Vista	GCA	2.71**	−0.62**	1.07	−0.32	0.11**	0.01	0.03	3.51
	Mean	52.62	25.57	29.93	14	2.16	4.77	61.65	294.83
Raneem	GCA	0.28	−0.61**	−1.51*	−0.12	−0.008	0.004	−2.22**	−8.70
	Mean	50.19	25.5	27.34	14.19	2.03	4.76	59.4	282.61

[z]IL, internode length; LL, leaf length; LW, leaf width; FL, fruit length; FD, fruit diameter; NFP, number of fruits plant^{-1}; YF^{-1}, yield per fruit; YP^{-1}, yield per plant.

*, ** Significant at $P < 0.05$ and $P < 0.01$, respectively.

Can. J. Plant Sci. Downloaded from cdnsciencepub.com by 116.95.134.189 on 06/17/23
For personal use only.

GCA for NFP. Therefore, this genotype may reduce NFP in crossing programs, but increase YF^{-1} and FL. On the other hand, the Storm genotype exhibited a high value of GCA for NFP but low values for other traits. Therefore, the progenies of this parent may have high levels of NFP; however, fruits with low values of FL, FD are small. Finally, GCA effects for yield per fruit (YF^{-1}) varied between 6.10 (Sco4184) and –3.47 (Karim). Also, the Neda and Sco4184 genotypes were found to be good new combiners containing positive alleles for enhancing YF^{-1}.

General combining ability effects for yield per plant (YP^{-1}) varied from 23.77 (Neda) to −29.1 (Atilgan) (Table 3). Parents with significant GCA effects resulting from additive gene effects were found to be good combiners for enhancing fruit yield and number of fruit per plant.

Based on GCA effects, the parental genotype of Neda was found to be the best combiner containing positive alleles for improving IL, LL, and YP^{-1}.

Mean Comparison of Morphological Traits and Fruit Yield in F_1 Generation

The mean crosses for the different traits studied are shown in Table 4. The highest (63.5) and the lowest (33.22) mean values for IL were recorded for Neda × Vista and Karim × Raneem crosses, respectively. The results show that the Karim × Raneem cross is a good combination for reducing internode length. The mean values of crosses for leaf length varied from 22.37 (Vista × Raneem) to 29.93 (Tornado × vista). Therefore, 'Tornado × Vista' would be the most promising crossing for improving of leaf length. Leaf width mean varied between 'Karim × Vista' (31.45) to 'Storm × Raneem' (24.20). The highest mean values for fruit length (15.9) and fruit diameter (2.69) were recorded for 'Amiral × Raneem' and 'Tornado × Atilgan' crosses, respectively. Therefore these superior crosses could be selected as candidate genotypes in breeding programs aimed at fruit size enhancement. The highest mean value of number of fruit $plant^{-1}$ was recorded to 'Storm × Atilgan' (6.65);

Table 4. Mean comparison of agronomic traits of F_1 generation in 9 × 9 diallel cross of cucumber[z]

Crosses/traits	IL	LL	LW	FL	FD	NFP	YF^{-1}	YP^{-1}
Neda × Tornado	54.66	29.66	31	14.16	2.10	4.22	57.41	242
Neda × Amiral	56	29.04	30.25	14.66	2.11	4.85	61.27	298.28
Neda × Sco4184	51.33	28.20	29.45	15.20	2.20	5.11	61.19	319.68
Neda × Storm	56.16	26.87	30.16	13.37	1.75	5.74	68.55	395.22
Neda × Karim	55.33	28.41	30.7	13	1.70	4.63	70.75	327
Neda × Atilgan	59.16	27.66	29.83	13.33	1.82	5.13	61.51	316.14
Neda × Vista	63.5	29.12	30.87	12.66	1.85	6	67.36	401.3
Neda × Raneem	41.66	25.29	28.29	14.7	1.82	4.94	57.35	283.58
Tornado × Amiral	44.83	27.5	29.20	13.6	2.27	5.08	57.52	291.16
Tornado × Sco4184	49.50	28.83	30.37	15.57	2.08	4.71	62	305.71
Tornado × Storm	44.66	27.50	30.20	13.68	2.26	3.83	50.16	191.66
Tornado × Karim	39.33	22.81	27.75	14.08	2.20	4.05	57.5	233.7
Tornado × Atilgan	43.16	29.91	30.83	16.7	2.69	6.16	65.57	396.00
Tornado × Vista	48.66	29.93	31.66	13.66	2.29	5.14	63.28	325.95
Tornado × Raneem	43.33	28.20	30.25	14.25	2.64	5.19	68.78	356.09
Amiral × Sco4184	55.50	25.41	29.33	14.66	2.45	4.20	58.33	242.12
Amiral × Storm	42.16	25.83	28.87	14.45	2.02	5.36	68.85	365.73
Amiral × Karim	53.33	26.95	29.37	13.91	1.92	4.58	59.62	271.3
Amiral × Atilgan	49.33	26.29	28.50	15.20	2.04	4.9	60.6	295.8
Amiral × Vista	51.66	24.33	26.75	14.91	2.26	5.05	59.71	302.52
Amiral × Raneem	48.33	24.66	27.50	15.91	2.23	4.21	59.78	253.16
Sco4184 × Storm	49.16	26.91	29.41	14.75	2.58	4.60	72.16	333.65
Sco4184 × Karim	52.16	26.33	28.16	15	2.21	3.57	77.71	276.10
Sco4184 × Atilgan	53.33	25.58	28	15.22	2.20	4.25	85.33	362.70
Sco4184 × Vista	54.83	25.37	29.54	15.60	2.75	5.06	95.65	481.78
Sco4184 × Raneem	55.50	22.66	25	14.96	2.51	3.83	65	247.8
Storm × Karim	42.50	22.81	27.75	13.3	1.74	5.5	55.96	307.55
Storm × Atilgan	45.11	27.66	29.83	13.41	1.45	6.65	46.87	309
Storm × Vista	48.16	29.33	31.66	13.21	1.42	5.68	62.09	348.86
Storm × Raneem	41.94	23.45	24.20	12.25	1.21	6.38	55.87	353.14
Karim × Atilgan	43.05	25.62	26.08	13.91	2	6.39	60.36	245.17
Karim × Vista	44.72	26.04	31.45	13.16	1.44	5.88	57.92	337.76
Karim × Raneem	33.22	24.37	24.79	15.50	1.55	3.67	54.91	201.89
Atilgan × Vista	51.33	27.12	29.87	12.95	2.09	4.08	56.10	231.99
Atilgan × Raneem	51.16	26.25	27.83	13.41	2.14	3.25	62.76	201.15
Vista × Raneem	55.66	22.37	25.16	14.41	2.25	5.05	56.5	286.4
LSD (5%)	9.82	2.80	9.44	2.13	0.35	1.47	33.10	10.22

[z]IL, internode length; LL, leaf length; LW, leaf width; FL, fruit length; FD, fruit diameter; NFP, number of fruits $plant^{-1}$; YF^{-1}, yield per fruit; YP^{-1}, yield per plant.

Can. J. Plant Sci. Downloaded from cdnsciencepub.com by 116.95.134.189 on 06/17/23
For personal use only.

this superior cross could, therefore, be suggested for improving NFP. The highest values of YF^{-1} (95.65) and YP^{-1} (481.78) were recorded for the Sco4184 × vista cross (Table 4).

Trait Correlation Analysis

Based on the correlation coefficient analysis carried out, NFP and YP^{-1} revealed the highest positive and significant correlation ($r = 0.73$**) (Table 5), which is logical, as fruit weight per plant should naturally increase if the number of fruit per plant increases. Yield per plant and yield per fruit showed the highest positive and significant correlation with other traits. A negative and significant correlation was observed between NFP and FD. As expected, fruit diameter decreased with increasing number of fruits per plant. Controversial results have been reported in this regard for the different morphologic traits in cucumber (Cramer and Wehner 2000; Golabadi et al. 2013). These authors found a positive correlation between fruit yield and fruit number per pickling but a negative one between fruit yield per pickling and internode length. Therefore, yield per fruit may be improved by indirect selection of NFP.

Principle Component Analysis

In this research, PCA showed that only the first two components would account for a maximal value of total variance (98%) in the variables observed (Table 6). The relative importance of each trait can be characterized by the rank of the contribution (%) to the explanation of the observed phenotypic variation. In the first PC, yield per plant produced high loading values (Table 6). Therefore, this component was designated as the fruit weight. The leaf width trait produced high loading values for the second component, which was designated as the leaf area (Table 6). For future research under similar conditions, these two traits with high loading values may be regarded as the selection criteria for use in cucumber breeding programs aimed at improving fruit weight and leaf area in lines and hybrids. Cui et al. (1995) and Golabadi et al. (2013) used PCA on different traits in cucumber in order to accommodate many traits into a few comprehensive indices.

CONCLUSIONS

Cultivation of greenhouse cucumber has been considered as the main objective in producing off-season products in arid and semi-arid regions of Iran. Development of superior F_1 hybrids and good general combiner genotypes play important roles in the successful cultivation of greenhouse cucumber in the arid climate of Iran. Comparison of genetic components (δ^2_A and δ^2_D), predictability factor, and narrow-sense heritability showed the predominant role of dominance gene action for LL, FL, FD, YF^{-1}, and YP^{-1}, and the additive gene action for IL, NFP, and LW. Improving dominant and additive gene action traits, therefore, calls for the development of hybrid cultivars and breeding procedures, respectively, which are based on selection in segregating population. Moreover, selection of fruit yield based on fruit number may also be successful in breeding programs. Another finding of the present study was the fact that all the traits studied were mainly controlled by the nuclear and maternal effects (the mother plant). Since genetic improvement of fruit yield and its marketability are the major goals of greenhouse cucumber breeding in Iran, the superior genotypes in this study could be used in recombination breeding programs to accumulate suitable genes that are responsible for improving yield. The genetic control of other fruit quality traits such as skin color and skin diameter may be investigated in future studies.

The ideotype genotype of greenhouse cucumber seems to be the one with a high number of fruit per plant, wide leaves, short internodes, a long plant height, a high number of nodes, an indeterminate growth, and high fruit length and diameter. Of course, fruit length and diameter form a variable that depends on the product's marketability in each region. In Iran, greenhouse cucumbers of 15×2.5 (cm^2) are preferred.

Finally, the parents Neda and Sco4184, which possess favorable genes for fruit yield enhancement are suggested for use in new hybridization programs aimed at improving fruit yield of greenhouse cucumber. The Sco4184 genotype was found capable of increasing fruit yield by increasing FD and FL, while the Neda genotype was able to increase YP^{-1} by increasing vegetable growth and

Table 5. Correlation coefficient between different traits[z]

Traits	YP^{-1}	YF^{-1}	NFP	FD	FL	LW	LL	IL
YP^{-1}	1	0.59**	0.73**	0.02	0.01	0.11	0.34**	0.15
YF^{-1}		1	−0.1	0.31**	0.24*	0.06	0.1	0.32**
NFP			1	−0.26*	−0.19	0.1	0.35**	−0.08
FD				1	0.23*	−0.06	−0.14	0.27*
FL					1	0.07	0.01	0.06
LW						1	0.16	−0.01
LL							1	0.19
IL								1

[z]IL, internode length; LL, leaf length; LW, leaf width; FL, fruit length; FD, fruit diameter; NFP, number of fruits $plant^{-1}$; YF^{-1}, yield per fruit; YP^{-1}, yield per plant.
*, ** Significant at $P < 0.05$ and $P < 0.01$, respectively.

Can. J. Plant Sci. Downloaded from cdnsciencepub.com by 116.95.134.189 on 06/17/23
For personal use only.

Table 6. The percentage of genetic correlation matrix and the eigenvectors resulting from the principal component analysis in cucumber[z]

Traits	Vector 1	Vector 2
YF^{-1}	0.08	−0.01
IL	0.02	−0.02
LL	0.01	0.03
LW	0.02	0.99
FL	0.002	0.01
FD	0.001	−0.002
NFP	0.009	0.001
YP^{-1}	0.99	−0.02
Proportion variance	0.94	0.04
Cumulative variance	0.94	0.98

[z]IL, internode length; LL, leaf length; LW, leaf width; FL, fruit length; FD, fruit diameter; NFP, number of fruits plant^{-1}; YF^{-1}, yield per fruit; YP^{-1}, yield per plant.

photosynthetic surface by IL, LL, and LW. Also, the Storm genotype was found suitable for increasing FNP and decreasing FL. The Sco4184 × vista cross was found applicable in hybridization programs aimed at improving fruit yield.

Abd El-Hafez, A. A., El-Doweny, H. H. and Wadid, M. M. 1997. Estimation of combining ability for some new genetic resources of cucumber and their F_1 hybrids under high temperature conditions. J. Agric. Sci. Mansoura Univ. **22**: 427–439.

Afangideh, U. and Uyoh, E. A. 2007. Genetic variability and correlation studies in some varieties of cucumber (*Cucumis sativus* L.). Jordan J. Agric. Sci. **3**: 376–384.

Baker, C. M. 1978. Issues in diallel analysis. Crop Sci. **18**: 533–536.

Blanco, F. F. and Folegatti, M. V. 2005. Estimation of leaf area for greenhouse cucumber by linear measurements under salinity and grafting. Sci. Agric. (Piracicaba, Braz.) **62** (4): 305–309.

Cho, Y. Y., Oh, S., Oh, M. M. and Son, J. E. 2007. Estimation of individual leaf area, fresh weight, and dry weight of hydroponically grown cucumbers (*Cucumis sativus* L.) using leaf length, width, and SPAD value. Sci. Hortic. **111**: 330–334.

Cramer, C. S. and Wehner, T. C. 1998. Fruit yield and yield component means and correlations of four slicing cucumber populations improved through six to ten cycles of recurrent selection. Am. Soc. Hortic. Sci. **123**: 388–395.

Cramer, C. S. and Wehner, T. C. 2000. Fruit yield and yield component correlations of four pickling cucumber populations. Cucurbit. Genet. Coop. Rep. **23**: 12–15.

Cui, H., Zhang, M., Meng, H. and Deng, J. 1995. Principal component analysis for traits selection in cucumber breeding. Cucurbit Genet. Coop. Rep. **18**: 10–12.

De Wilde, W. J. J. O. and Duyfjes, B. E. E. 2010. *Cucumis sativus* L. forma hardwickii (Royle) W.J. de Wilde & Duyfjes and feral forma sativus. Thai For. Bull. (Bot) **38**: 98–107.

Dogra, B. S. and Kanwar, M. S. 2011. Exploitation of combining ability in cucumber (*Cucumis sativus* L.). Res. J. Agric. Sci. **2**: 55–59.

Fan, Z., Robbins, M. D. and Staub, J. E. 2006. Population development by phenotypic selection with subsequent marker-assisted selection for line extraction in cucumber (*Cucumis sativus* L.). Theor. Appl. Genet. **112**: 843–855.

FAO. **2012**. FAO STAT. [Online] Available: fao.org.

Fredrick, L. R. and Staub, J. E. 1989. Combining ability analyses of fruit yield and quality in near-homozygous lines derived from cucumber. J. Am. Soc. Hortic. Sci. **114**: 332–338.

Golabadi, M., Eghtedari, A. R. and Golkar, P. 2013. Determining relationships between different horticultural traits in (*Cucumis sativus* L.) genotypes with multivariate analysis. SABRAO J. Breed. Genet. **45**: 447–457.

Golabadi, M., Golkar, P. and Eghtedary, M. R. 2012. Assessment of genetic variation in cucumber (*Cucumis sativus* L.) genotypes. Euro. J. Exp. Bio. **2**: 826–831.

Griffing, B. 1956. Concept of general and specific combining ability in relation to diallel crossing systems. Aust. J. Biol. Sci. **9**: 463–493.

Harlan, J. R. 1975. Crops and man. ASA, CSSA, Madison, WI.

Hormuzdi, S. G. and More, T. A. 1989. Studies on combining ability in cucumber (*Cucumis Sativus* L.). Indian J. Genet. Plant Breed. **49**: 161–165.

Jagesh, K., Munshi, A. D., Kumar, R., Sureja, A. K. and Sharma, R. K. 2013. Combining ability and its relationship with gene action in slicing cucumber. Indian J. Hortic. **70**: 135–138.

Kanwar, M. S., Korla, B. N. and Sanjeev, K. 2003. Evaluation of cucumber genotypes for yield and qualitative traits. Himachal J. Agric. Res. **29** (1, 2): 43–47.

Kumar, S., Chandra, Y., Brijpal Bisen, Y. and Dixit, S. K. 2009. Genetic variability, heritability and genetic advance for some traits in cucumber (*Cucumis sativus* L.). Veg. Sci. **36**: 220–221.

Liebig, H. P. and Fricke, A. 2002. Cucurbitaceae (Kurbisgewachse). Pages 330–345 *in* H. Krug, H. P. Liebig, and H. Stutzel, eds. Gemuse production. Verlag Eugen. Ulmer, Stuttgart, Germany.

Lopez-Sese, A. I. and Staub, J. 2002. Combining ability analysis of yield components in cucumber. J. Am. Soc. Hortic. Sci. **127**: 931–937.

Mahmud, I. and Keramer, H. H. 1951. Segregation for yield height and maturity following a soybean cross. Agron. J. **43**: 605–609.

Mule, P. N., Khandelwel, V., Patil, A. B. and Chaudhary, B. R. 2011. Combining ability studies in cucumber (*Cucumis sativus* L.). Veg. Sci. **38**: 203–205.

Munshi, A. D., Kumar, R. and Panda, B. 2006. Combining ability in cucumber (*Cucumis sativus* L.). Indian J. Agric. Sci. **76**: 750–752.

Navazio, J. P. and Simon, P. W. 2001. Diallel analysis of high carotenoid content in cucumbers. J. Am. Soc. Hortic. Sci. **126**: 100–104.

Olfati, J. A., Samizadeh, H., Rabiei, B. and Peyvast, G. H. 2012. Griffing's methods comparison for general and specific combining ability in cucumber. Sci. World J. **2012**: 1–4.

Plader, W., Burza, W. and Malepszy, S. 2007. Cucumber. Biotechnology in agriculture and forestry 59: Transgenic Crops.

Sarkar, M. and Sirohi, P. S. 2011. Diallel analysis of quantitative characters in cucumber (*Cucumis sativus* L.). Veg. Sci. **38**: 73–75.

SAS Institute Inc. 2002. SAS/STAT 9 user's guide. SAS Institute Inc., Cary, NC.

Serce, S., Navazio, J. P., Gokce, A. F. and Staub, J. E. 1999. Nearly isogenic cucumber genotypes differing in leaf size and

plant habit exhibit differential response to water stress. JASHS **124**: 358–365.

Serquen, F. C., Bacher, J. and Staub, J. E. 1997. Genetic analysis of yield components in cucumber at low plant density. J. Am. Soc. Hortic. Sci. **122**: 522–528.

Shetty, N. V. and Wehner, T. C. 2002. Estimation of fruit grade weights based on fruit number and total fruit weight in cucumber. Hortic. Sci. **37**: 1117–1121.

Singh, R., Singh, A. K., Kumar, S., Singh, B. K. and Singh, S. P. 2011. Studies on combining ability in Cucumber (*Cucumis Sativus* L.). Veg. Sci. **38**: 49–52.

Singh, S. and Pawar, I. S. 2005. Theory and application of biometrical genetics. CBS Press, Frederiksberg, Denmark.

Sun, R., Lower, L. and Staub, J. E. 2006. Analysis of generation means and components of variance for parthenocarpy in cucumber (*Cucumis sativus L.*). Plant Breed. **125**: 277–280.

Wahid, M. M., Medany, M. A. and Abou-Hadid, A. F. 2003. Diallel analysis for yield and vegetative characteristics in cucumber (*Cucumis sativus* L.) under low temperature conditions. ISHS Acta Hortic. 598.

Wehner, T. C., Shetty, N. V. and Clark, R. L. 2000. Screening the cucumber germplasm collection for combining ability for yield. Hortic. Sci. **35**: 1141–1150.

Xing Fang, G., Ping, Z. S. and Qing, X. C. 2004. Analysis of combining ability of early yield and total yield character of cucumber cultivated in open field in spring. China Veg. **6**: 13–15.

Yoshioka, Y., Sugiyama, M. and Sakata, Y. 2010. Combining ability analysis of fruit texture in cucumber by mechanical measurement. Breed. Sci. **60**: 65–70.

Zhang, Y., Kang, M. S. and Lamkey, K. R. 2005. Diallel-SAS 05: A comprehensive program for Griffing's and Gardner-Eberhart analysis. Agron. J. **97**: 1097–1106.

Can. J. Plant Sci. Downloaded from cdnsciencepub.com by 116.95.134.189 on 06/17/23
For personal use only.
