# Supplementary material for: Provenance and family variations in early growth of Manchurian walnut (Juglans mandshurica Maxim.) and selection of superior families
Source: PLoS One. 2024 Mar 7;19(3):e0298918. doi: 10.1371/journal.pone.0298918 (PMC10919699; doi:10.1371/journal.pone.0298918)
Supplement: S1 File — (ZIP) [file pone.0298918.s004.zip › Combining phenotype, genotype, and environment to uncover genetic components underlying water use efficiency in Persian walnut.pdf]

Journal of Experimental Botany, Vol. 71, No. 3 pp. 1107–1127, 2020
doi:10.1093/jxb/erz467 Advance Access Publication October 23, 2019
This paper is available online free of all access charges (see https://academic.oup.com/jxb/pages/openaccess for further details)

RESEARCH PAPER

Combining phenotype, genotype, and environment to uncover genetic components underlying water use efficiency in Persian walnut

Mohammad M. Arab[1], Annarita Marrano[2], Rostam Abdollahi-Arpanahi[3], Charles A. Leslie[2], Hao Cheng[4], David B. Neale[2] and Kourosh Vahdati[1,*]

[1] Department of Horticulture, College of Aburaihan, University of Tehran, Tehran, Iran
[2] Department of Plant Sciences, University of California, Davis, CA 95616, USA
[3] Department of Animal and Poultry Science, College of Aburaihan, University of Tehran, Tehran, Iran
[4] Department of Animal Science, University of California, Davis, CA, USA

* Correspondence: kvahdati@ut.ac.ir

Received 19 July 2019; Editorial decision 7 October 2019; Accepted 8 October 2019

Editor: Fabrizio Costa, Fondazione Edmund Mach, Italy

Downloaded from https://academic.oup.com/jxb/article/71/3/1107/5602662 by guest on 31 January 2024

Abstract

Walnut production is challenged by climate change and abiotic stresses. Elucidating the genomic basis of adaptation to climate is essential to breeding drought-tolerant cultivars for enhanced productivity in arid and semi-arid regions. Here, we aimed to identify loci potentially involved in water use efficiency (WUE) and adaptation to drought in Persian walnut using a diverse panel of 95 walnut families (950 seedlings) from Iran, which show contrasting levels of water availability in their native habitats. We analyzed associations between phenotypic, genotypic, and environmental variables from data sets of 609 000 high-quality single nucleotide polymorphisms (SNPs), three categories of phenotypic traits [WUE-related traits under drought, their drought stress index, and principal components (PCs)], and 21 climate variables and their combination (first three PCs). Our genotype–phenotype analysis identified 22 significant and 266 suggestive associations, some of which were for multiple traits, suggesting their correlation and a possible common genetic control. Also, genotype–environment association analysis found 115 significant and 265 suggestive SNP loci that displayed potential signals of local adaptation. Several sets of stress-responsive genes were found in the genomic regions significantly associated with the aforementioned traits. Most of the candidate genes identified are involved in abscisic acid signaling, stomatal regulation, transduction of environmental signals, antioxidant defense system, osmotic adjustment, and leaf growth and development. Upon validation, the marker–trait associations identified for drought tolerance-related traits would allow the selection and development of new walnut rootstocks or scion cultivars with superior WUE.

Keywords: Abiotic stress, climate change, drought tolerance, genotyping array, genotype–environment analysis, genome-wide association study (GWAS), SNP markers.

Introduction

Persian walnut (*Juglans regia* L.) is one of the nut crops domesticated in Persia (Bayazit *et al.*, 2007) and today it is cultivated for nut and wood production in Asia, Europe, Oceana, and the Americas (Aradhya *et al.*, 2017; Bernard *et al.*, 2018). Walnut yield in many regions is reduced by biotic and abiotic stresses, such as drought, salt, and spring frost (Cochard *et al.*, 2002;

© The Author(s) 2019. Published by Oxford University Press on behalf of the Society for Experimental Biology.
This is an Open Access article distributed under the terms of the Creative Commons Attribution Non-Commercial License (http://creativecommons.org/licenses/by-nc/4.0/), which permits non-commercial re-use, distribution, and reproduction in any medium, provided the original work is properly cited. For commercial re-use, please contact journals.permissions@oup.com

Lotfi *et al.*, 2009*a*, *b*; Khodadadi *et al.*, 2016; Jinagool *et al.*, 2018; Knipfer *et al.*, 2018; Liu *et al.*, 2019). Water deficit is among the most prevalent environmental factors limiting walnut productivity in current areas of walnut cultivation, many of which are arid or semi-arid (Aleta *et al.*, 2009; Vahdati *et al.*, 2009; Famula *et al.*, 2019). In addition, global climate change is increasing the frequency of severe drought conditions in many walnut cultivation regions (Lotfi *et al.*, 2010; Karimi *et al.*, 2018). In particular during the juvenile phase, drought can severely compromise walnut development, thus eventually limiting yield potential (Vahdati *et al.*, 2009; Liu *et al.*, 2019). Therefore, a better understanding of the genetic make-up of water use efficiency (WUE) and leaf-related traits that underlie the adaptive response to drought stress is an essential prerequisite toward more effective breeding activities (Salekdeh *et al.*, 2009).

Stable isotope analysis using ^{13}C and ^{15}N as a surrogate measure of WUE offers the potential to assess large numbers of accessions under greenhouse or field conditions. The discrimination of stable carbon isotopes ($\Delta^{13}C$) has long been used in a wide range of plant species as an indirect and reliable measure of the ratio of photosynthetic activity to stomatal conductance for screening genotypes for potential WUE (Farquhar *et al.*, 1989; Rebetzke *et al.*, 2002). In contrast, the $\delta^{15}N$ has been used much less extensively (Robinson *et al.*, 2000). As $\Delta^{13}C$ is linearly related to the ratio of intercellular CO_2 concentration (C_i) to the atmospheric CO_2 partial pressure, a reduced $\Delta^{13}C$ indicates stomatal closure under drought stress (Farquhar *et al.*, 1989). There have been few comprehensive studies analyzing the effect of the inheritance of $\Delta^{13}C$ and $\delta^{15}N$ on WUE in walnut under normal conditions, including comparisons of carbon isotope discrimination among 22 *J. regia* families (Aletà *et al.*, 2009) and within the Walnut Improvement Program of University of California, Davis (UC Davis WIP) (Famula *et al.*, 2019). The difficulties in measuring WUE have limited the number of studies.

Leaves play key roles in photosynthesis and adaptation to climate. Some investigations revealed how morphological leaf traits, such as shape, size, area, color, and curvature, vary with geography and environment, but the correlation between leaf morphology and environmental factors is still undefined (Tian *et al.*, 2016). Plants also strive to balance carbon uptake and water loss under drought stress through changes in leaf morphology. Hence, assessing the diversity of leaf traits under both control and stress conditions, and their correlation with WUE traits could elucidate the relationships among these phenotypes as well as helping to select drought-tolerant cultivars.

Despite considerable progress in understanding the physiological mechanisms that walnut employs in response to drought stress (Cochard *et al.*, 2002; Lotfi *et al.*, 2010; Jerszurki *et al.*, 2017; Knipfer *et al.*, 2018), how physiology of drought tolerance differs in nature and the genomic regions involved in this variation are largely unknown in walnut. Walnut genome sequencing (Martínez-García *et al.*, 2016), subsequently developing a high-density 700K single nucleotide polymorphism (SNP) genotyping array (Marrano *et al.*, 2019), now allows walnut breeders to extensively and quickly mine natural variation and associated phenotypic variation with the underlying SNPs based on linkage disequilibrium (LD) (Arab *et al.*, 2019). Genomics enables the identification of candidate genes controlling drought-related traits (Tuberosa and Salvi, 2006). Recently, genome-wide association study (GWAS), as a newly developed genetic approach, has been employed to investigate the genetic architectures of complex traits in fruit trees (Cao *et al.*, 2016; Marrano *et al.*, 2018; Arab *et al.*, 2019). However, very few studies to date have used this approach to dissect the genetic basis of drought tolerance in fruit trees, due to the difficulties in assessment of large association panels under water deficit conditions. For the first time, Famula *et al.* (2019) have investigated in-depth the genomic and phenotypic variability in the WUE of walnut in the UC Davis walnut improvement program. They performed a GWAS for WUE-related traits under normal conditions with the Axiom *J. regia* 700K SNP array on diverse walnut genotypes (241 mature trees) from the UC Davis walnut breeding program and identified four markers associated with $\Delta^{13}C$. However, the complex genetic architecture of drought-related traits such as WUE makes it crucial to use a different walnut population of varied geographic origin in GWAS to validate markers identified by Famula *et al.* (2019) and for the discovery of new markers. To date, GWAS related to WUE have not been carried out in common-garden experiments using populations evolved under diverse climatic conditions, in particular walnut in relation to drought environments. This is partially due to the difficulty of deploying a water deficit experiment on an association panel consisting of genotypes with divergent phenology.

Walnut populations in Iran provide an unparalleled opportunity to explore variation in adaptive responses to drought stress and WUE. These native genetic resources have an extensive geographic distribution and have experienced a wide range of climates and soil compositions for thousands of generations (Vahdati *et al.*, 2009; Arab *et al.*, 2019). The variation in Iranian walnut genotypes (naturally open pollinated), grown across a wide variety of habitats that includes extremely dry conditions, suggests that, in nature, walnut has evolved to endure drought stress with an array of morphological and physiological adaptations. Thus, understanding the basis of walnut adaptation to climate is crucial for germplasm conservation and management under climate change. This natural variation should be exploited to improve WUE and, therefore, guarantee stable yield of cultivated walnut varieties under limited water conditions. Common-garden and gene mapping experiments can help to further explore the phenotypic diversity in drought adaptation segregating in local walnut populations in Iran. One of the newly emerged approaches to dissect adaptation in an explicit molecular framework is landscape genomics (genotype–environment association), which aims to identify candidate loci for adaptation through populations adapted to different environments (Vangestel *et al.*, 2018).

The aim of our study is to dissect adaptive genomic variation underlying drought- and WUE-related traits in Persian walnut using a diverse panel of 95 walnut families (950 seedlings) from Iran, which show contrasting levels of water availability in their native habitats. In particular, our main objectives were to: (i) explore the natural genetic variability of WUE and leaf-related traits in our collection under controlled and water deficit conditions; (ii) associate genetic variation in WUE and leaf-related traits with environmental variables; (iii) identify the

Downloaded from https://academic.oup.com/jxb/article/71/3/1107/5602662 by guest on 31 January 2024

markers related to WUE and leaf-related traits through GWAS; (iv) identify the candidate loci underlying adaptation to environmental variables; and (v) detect the most likely underlying candidate genes linked to identified markers. We performed association analysis by integrating genotype, phenotypes, and environment variables, thus providing new insights into the genetic basis of drought adaptations (alleles gained or lost) and WUE, in view of developing new cultivars with resilience in the face of climate change.

Materials and methods

Plant material

After an initial screening based on climate data, 95 walnut mother trees were selected from the native walnut gene pools across Iran. These local populations were located in distinct geographical regions (eight provinces) to capture variation in climate, geology, and topography. From 2015 to 2017, leaves and open-pollinated seeds (at least 60 per mother tree) were sampled, and geographical information for each mother tree was recorded (Fig. 1; Supplemenrtary Table S1 at *JXB* online). All sampled mother trees were originally open-pollinated seedlings (now 50–500 years old, and apparently healthy), growing in valleys located at 100–1000 m distance from each other, with an average interpopulation distance of 600 km, as described by Arab *et al.* (2019). Geographic information (GPS) was used to determine climatic parameters and to describe the edaphic conditions of sampled areas.

Experimental design

In tree crops, phenotypic traits related to growth, such as phenology, biotic and abiotic stress resistance, and WUE, are related to survival during the seedling and juvenile phases and are important elements of total lifetime fitness (Postma and Ågren, 2016; Lind *et al.*, 2017). Therefore, seeds collected from the 95 Iranian genotypes were established in a common garden (Arab *et al.*, 2019; Fig. 1; Supplementary Table S1) using a completely randomized design. Early lifetime phenotypes of 95 mother trees at natural sites were then estimated.

In detail, in 2015 open-pollinated seeds were obtained from 95 mother trees adapted to different climate condition across Iran. Prior to planting, seeds were soaked in running tap water for 10 d, treated with a fungicide (Captan 5%), and then placed in a refrigerator for 4–6 weeks at 4–6 °C (Vahdati *et al.*, 2009). Seeds were then planted in 7 liter polyethylene pots containing a soil, sand, and manure mixture. Twenty seeds per mother tree were planted directly into the soil and, in order to minimize the impact of water stress on growth, they were irrigated every 10 d from April to June, and 3–4 times per month in July and August during the 2 years of phenotyping. Seedlings were grown in a greenhouse at 25/20 °C (day/night), a 16 h photoperiod, and 40–50% relative humidity, and fertilized with macro- and microelements each month. After 1 year, seedlings were transferred to 15 liter pots containing a mixture of soil, sand, and manure (3:1:1, v/v/v). From these 95 half-sib families, a total of 950 saplings were produced (at least 10 plants per family), of which 6–8 uniform saplings from each family were selected for the drought stress experiment. In addition, morphological traits were recorded when saplings reached ~2 years of age. Two or three saplings per treatment in each half-sib family were phenotyped for leaf-related parameters and WUE under both normal and drought stress conditions.

The stress experiment was conducted in the Research Greenhouses of the Department of Horticulture, University of Tehran (Pakdasht, Tehran, Iran), using a factorial completely randomized design. Plants from each family were randomly assigned to either the control or the drought treatment group. Before the experiment began, in order to avoid stress shock, irrigation intervals were gradually increased in the drought treatment group. Finally, drought stress for 14 d was initiated at the primary leaf stage in 2-year-old saplings. Control plants received regular irrigation, while water was withheld from the drought-stressed plants for 14 d.

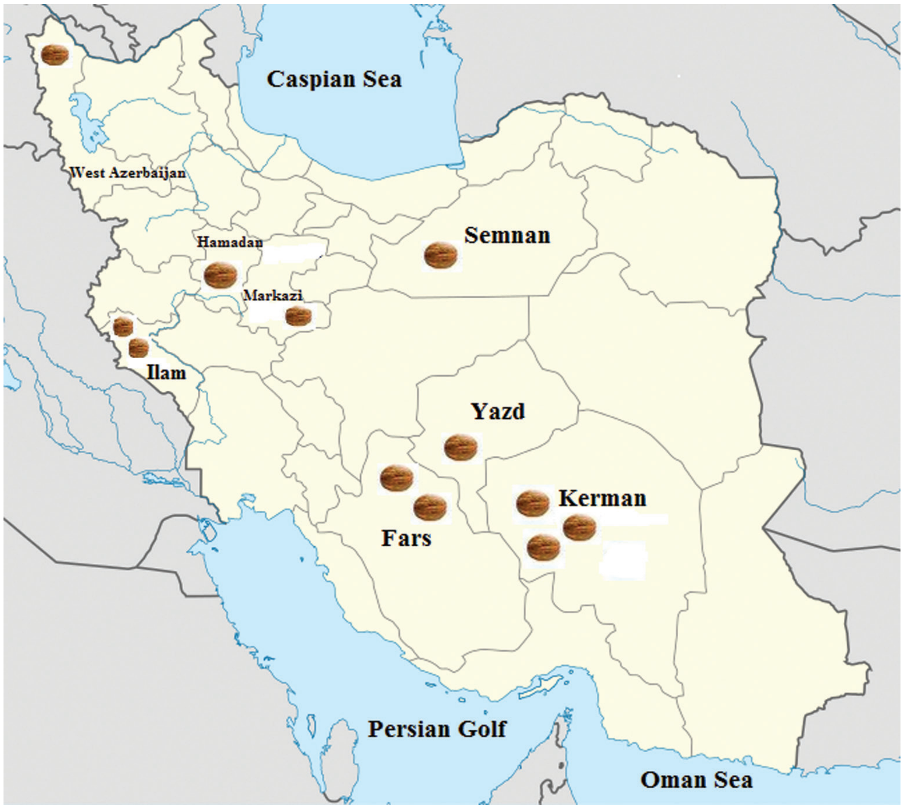

Fig. 1. Collection sites of the studied Iranian walnut genotypes. Collection areas are indicated with walnut seed (adapted from Arab *et al.*, 2019). (This figure is available in color at *JXB* online.)

Downloaded from https://academic.oup.com/jxb/article/71/3/1107/5602662 by guest on 31 January 2024

Phenotyping

Leaf image

Photos were taken from leaves 14 d after drought stress application, using an imaging box (45×45×45 cm) equipped with four white-colored fluorescent bulbs (32 W, 2630 lux) and a 10 megapixel camera (Canon, Powershot G12, USB connection, Japan) equipped with a 28–140 mm equivalent zoom lens. Camera settings, plant position, and light conditions were fixed, and three images were taken per sapling. Images were analyzed using the Matlab software package. Initial images were converted to binary images and leaf-related parameters were calculated using shape analysis. In brief, all extraneous leaves were first cropped from the picture. Then RGB color-space images of leaves were converted into gray-scale levels, a threshold technique was used to isolate each object from its background, and the pixel values of the gray-scaled images were then fixed to values of 1 or 0 to produce a binary image. Finally, the small objects of the binary images inside the image were recognized and then the image noise was filled. The final image was used to compute area (Ar), perimeter (Peri), major length (MAL), minor length (MIL), aspect ratio (AS), elongation (EL), and roundness (RO). Elongation (a) and roundness (b) values were calculated using the following equations:

$$\text{a) EL} = \frac{a}{b} \quad \text{b) RO} = \frac{4\pi \times A_C}{P_C^2}$$

where a and b are the major and minor axis lengths, and A_C and P_C are the area and perimeter of leaves.

Carbon and nitrogen isotope composition (δ13C, δ15N)

The WUE-related traits, including carbon isotope composition ($\delta^{13}C$), nitrogen isotope composition ($\delta^{15}N$), and carbon and nitrogen contents, were measured using leaf samples collected in mid-June 2017. Under both normal and stress conditions, two leaves, each from a different shoot, were collected from three saplings per family, pooled together, and sealed in aluminum foil. We will call each leaf pool a sample for clarity, even though each pool represents one family per treatment. Each sample was bulked, oven-dried at 60 °C for 72 h, and then coarsely ground. Finally, to determine $\delta^{13}C$ and $\delta^{15}N$, 2.5–4 mg of tissue per sample (control and treatment) were submitted to the Stable Isotope Facility at the University of California, Davis for isotope analysis (http://stableisotopefacility.ucdavis.edu/) as described by Famula *et al.* (2019).

To evaluate the drought tolerance of each family, a drought stress index (DSI) was estimated using the formula: DSI=(value of trait under drought condition)/(value of trait under well-watered condition)×100 (Wójcik-Jagła *et al.*, 2013). In addition, the principal component analysis (PCA) of all traits ranking value for each walnut family was calculated via the formula: ranking value=[PC1×contribution of PC1 (%)]+[PC2×contribution of PC2 (%)]+[PC3×contribution of PC3 (%)]+(PC4×contribution of PC4 (%)]+[PC5×contribution of PC5 (%)] (Liu *et al.*, 2015).

Statistical analysis of phenotypic data

Statistical analyses were performed using Minitab (Minitab, Inc., State College, PA, USA). Descriptive statistics and normality tests were run on data and their residuals. ANOVA was calculated using the general linear model (GLM) with families (G), drought stress treatment (T), and G×T as fixed effects. Multivariate statistical analyses including hierarchical cluster analysis (HCA), PCA, and correlation analysis were conducted using the R package factoextra (Kassambara and Mundt, 2017). A Bayesian statistical approach as implemented in the R package MCMCglmm (Hadfield, 2010) was also applied to estimate individual breeding values (EBVs) for the most important WUE-related traits (Supplementary Protocol S1). The EBV of each individual was used to identify the strong and weak individuals for $\Delta^{13}C$ and $\delta^{15}N$ traits.

DNA extraction, genotyping, and SNP allele calling

Sample genotyping was previously described in Arab *et al.* (2019). Briefly, total genomic DNA (gDNA) was extracted from ~40 mg of dry leaf tissue sampled from 95 mother trees across all eight Iranian walnut populations using the E-Z 96® Plant DNA Kit and according to the recommended protocol (Omega Bio-tek; Norcross, GA, USA) and 15 ng μl^{-1} of high-quality gDNA from each individual was sent to Affymetrix (now part of Thermo Fisher Scientific, Santa Clara, CA, USA; www.affymetrix.com) for genotyping with the new Axiom™ *J. regia* 700K SNP array (Marrano *et al.*, 2019) on the Affymetrix GenTitan platform. SNP allele calling was done by the Bioinformatics Core of Affymetrix using its three workflows, namely Best Practices, Sample QC, and Genotyping (http://www.bea.ki.se/documents/axiom_genotyping_solution_analysis_guide.pdf). The samples with dish QC value ≥0.82 and a QC call rate of ≥97% were considered for further analysis. The SNPs were then classified into six performance classes, and only *Poly High Resolution* (*PHR*) SNPs filtered for missing rate (>20%) and minor allele frequency (<5%) were used for the following genetic analysis. Genomic variation and population structure analyses of the present association panel are also completely described by Arab *et al.* (2019). PCA and fastSTRUCTURE approaches were applied, identifying four major subgroups resembling sample geographical origin. LD was estimated between all SNPs in the whole Iranian walnut population using PLINK v1.9 (Purcell *et al.*, 2007). The classical r^2 estimate of the correlation between genotypes was used. An LD decay plot of r^2 versus physical distance (kb) was created with the R package ggplot2 (Wickham, 2016).

Climate and geographic data

For each tree with a known collection location, climate data were gathered from the WorldClim database (Hijmans *et al.*, 2005). The data set contained a total of 22 variables, comprising latitude, longitude, altitude, and 19 bioclimatic variables (Supplementary Table S2). The bioclimatic variables are summary statistics of temperature and precipitation. We also included annual average potential evapotranspiration (PET), and a measure of aridity derived from precipitation and PET data (Zomer *et al.*, 2008). PCA was conducted on these environmental variables to reduce their high dimensionality. Then, the combinations of environmental variables in the form of PCs were also added to the genotype–environment association analysis.

Genome-wide association study

The GWAS was performed for 18 drought-related traits under both stress and normal conditions. For each trait, five different phenotypic inputs and various models were used in genotype–phenotype association analysis to: (i) identify single nucleotide variants (SNVs) for each condition; and (ii) ensure that SNVs were robust across models. Phenotypic data used as input included: (i) average performance of progenies for each trait under both normal and stress conditions; (ii) DSI; (iii) PC1–PC5 of phenotypic data; (iv) PCs of DSI values; and (v) climate variables and their combinations (PC1–PC3). The GWAS analysis was carried out by applying two different mixed models implemented in the R package GAPIT v3.0 (Lipka *et al.*, 2012): Settlement of Mixed Linear Model Under Exclusive Relationship (SUPER; Q. Wang *et al.*, 2014) and the Fixed and Random Model Circulating Probability Unification (FarmCPU; Liu *et al.*, 2016). Population structure (PCs) and familial relatedness (kinship matrix) were taken into account in all models to avoid spurious marker–trait associations (Yu *et al.*, 2006). The optimum number of PCs to include in the model was defined for each trait based on the forward model selection approach using the Bayesian information criterion, as implemented in the 'model.selection' function of GAPIT. Quantile–quantile (QQ) plots were used to select the best fitted model. Three common *P*-value adjustments were used for multiple testing correction: Bonferroni, false discovery rate (Benjamini and Hochberg, 1995), and suggestive thresholds based on the effective number of independent tests (Meff) following the method of Gao *et al.* (2008). The number of independent tests calculated for our study was 99 449, so that the significant *P*-value was 5×10^{-7} (0.05/99 449). The suggestive threshold was then set at $P=1.006\times10^{-5}$ (1/99 449). A lower suggestive *P*-value of $9.95.0\times10^{-5}$ ($-\log_{10} P=5$) was also used to identify the most significant marker–trait associations.

Downloaded from https://academic.oup.com/jxb/article/71/3/1107/5602662 by guest on 31 January 2024

Functional annotation of candidate SNPs

To identify putative candidate genes underlying drought tolerance in walnut, a BLASTNX was performed using the SNP probe sequences of the most significant and suggestive associations as queries against the NCBI protein databases (www.ncbi.nlm.nih.gov/). Based on the LD decay in our walnut panel, candidate genes were also searched in windows of ±10 kb around the most significant SNPs for each trait using the walnut gene annotation v1.0 aligned onto the new chromosome-level reference genome available at https://www.hardwoodgenomics.org/Genome-assembly/2539069.

Results

Genotypic variability and correlations between the geographic and environmental parameters and the evaluated phenotypic data

The panel of 95 diverse Iranian walnut families was exposed to water deficit, and the performances of stressed and well-watered seedlings were compared. Drought responses of well-watered and drought-stressed seedlings were measured using five physiological parameters related to WUE, seven leaf-related parameters, and two leaf color measurement systems (RGB and Lab). A normal distribution was observed for all traits except for %C, leaf area, and leaf color (Figs 2, 3; Supplementary Table S3). The variation in WUE-related traits differed significantly between genotypes and treatments (Supplementary Tables S3–S5). Our results showed great phenotypic variation among traits; the highest coefficient of variation was observed for δ^{15}N under normal conditions (28.51%), followed by δ^{15}N under stress conditions (27.49%); and the lowest coefficient of variation was observed for the b parameter (leaf color) under normal conditions (1.3%) (Supplementary Table S3). Reduced mean values for Δ^{13}C (higher WUE), %C, %N, and leaf growth were observed under water deficit, but δ^{15}N values were similar in both well-watered and stressed treatments. More descriptive statistics on the measured traits are summarized in Supplementary Table S3.

We estimated the Pearson correlation coefficient among the morphophysiological traits and geographical and environmental parameters of origin. Significant positive correlations were observed between annual precipitation at the location where seeds were collected and Δ^{13}C under either watering condition (r=0.42, P≤0.01; r=0.24, P≤0.05, respectively; Table 1). The Δ^{13}C of well-watered seedlings was negatively correlated with longitude (E) (r= –0.29, P≤0.001) and altitude (m) (r= –0.27, P≤0.001) of the collection site. These results suggest that the genotypes of arid regions, especially Yazd province, can be used as donor parents for breeding walnut cultivars for a changing climate. The %N of both normally watered and stressed seedlings was negatively correlated with collection site average annual temperature (r= –0.16, P≤0.05; r= –0.19, P≤0.05, respectively) and average temperature in the coldest and hottest months of the year (r= –0.20, P≤0.05; r= –0.20, P≤0.05, respectively; Table 1). There were also significant correlations for leaf growth-related traits and leaf color with the environmental conditions and geographical locations where the genotypes had originated (Table 1).

Under normal watering, we observed a positive correlation among leaf growth parameters, between %C and %N, and between Δ^{13}C and δ^{15}N. Under water deficit, correlations between Peri, Ar, MA, and MIL, as well as between RGB and the a and b color parameters, were significantly positive (Supplementary Fig. S1). Also, we observed a negative significant correlation between aspect ratio and RO and EL, as well as between all color parameters and L (Supplementary Fig. S1). In summary, correlation results show that morphophysiological traits, particularly leaf-related parameters, are important indicators of WUE under drought.

Physiological and leaf-related parameter assessment by heat map and PCA

Water deficit significantly affected Δ^{13}C, %C, %N, and the C:N ratio, but had no significant effect on δ^{15}N (Supplementary Table S4). ANOVA showed that the effects of water deficit and genotype were significant (P≤0.05) for all traits except red color (Supplementary Table S5). For AS, EL, and RO, the interaction between water deficit and families was also significant (P≤0.05) (Supplementary Table S5).

We observed considerable differences between families in their responses to water deficit (Fig. 4). Most of the seedlings exposed to water deficit clustered together (Group B in Fig. 4) and separately from plants grown under well-watered conditions (Group A in Fig. 4), indicating that water deficit treatment alters both the physiological and leaf-related parameters. Interestingly, some progeny collected from the driest regions and grown under water deficit clustered with the well-watered group (Group A in Fig. 4), suggesting that these genotypes are able to maintain their performance under water deficit treatment. HCA showed four distinct groups (Fig. 4). The four main leaf morphological measurements (Fig. 4, Group I) differed between the control (Group A) and stress treatment (Group B). Thus, leaf morphology appears to correlate closely with mid- or long-term water deficit in walnut. Also, δ^{15}N-related data clustered into group II, with most families showing a decrease under drought treatment (Fig. 4). Two color system parameters (RGB and Lab), aspect ratio, Δ^{13}C, and the C/N ratio clustered into group III (Fig. 4). All families showed a decrease in aspect ratio, a and b parameters, and green color under water deficit, and most exhibited decreased Δ^{13}C. Under both conditions, we observed a large variation in Δ^{13}C among families (Fig. 4; Table 2; Supplementary Table S6). Genotypes from dry regions, including Yazd, Kerman, and Fars, showed a higher WUE on average. Leaf elongation, roundness, %C, and %N clustered into group IV and exhibited lower values in most families under drought treatment (Fig. 4). Overall, most families demonstrated decreased leaf growth and increased WUE under water deficit treatment.

To assess the contributions of each parameter, we performed PCA on WUE and leaf-related phenotypes collected from seedlings after 2 weeks of water deficit treatment (Fig. 5). Most parameters were positively associated with the control group (Fig. 5), but the leaf color system (a and b) was negatively correlated with the water deficit treatment (Fig. 5). The three leaf color parameters (red, green, and blue) and the C/N ratio did not significantly contribute to family separation under either condition. In addition, PCA using the DSI values

Downloaded from https://academic.oup.com/jxb/article/71/3/1107/5602662 by guest on 31 January 2024

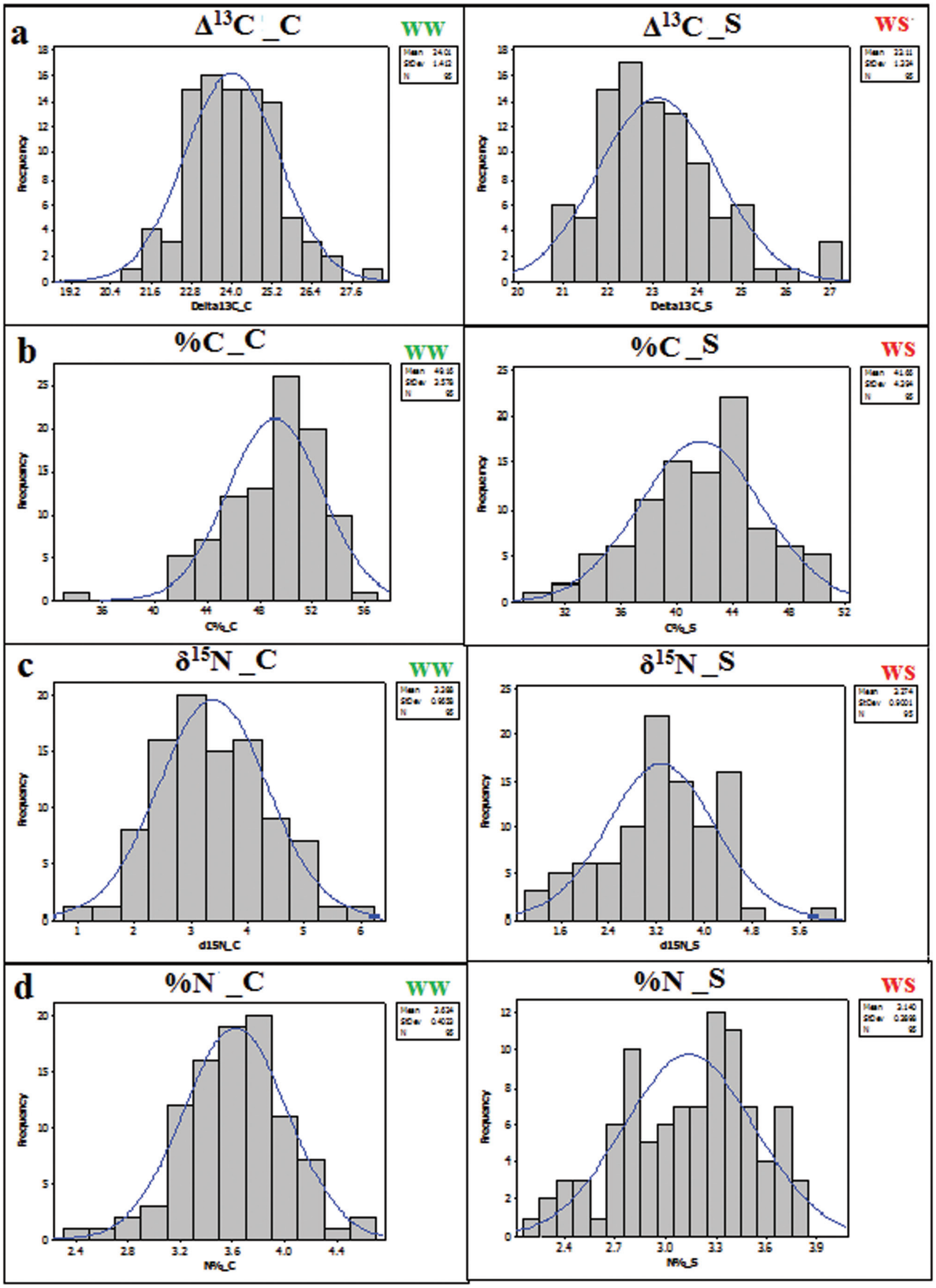

Fig. 2. Frequency distribution for water use efficiency-related traits of 95 walnut families. (a) Carbon isotope discrimination (Δ^{13}C), (b) carbon percentage (%C), (c) nitrogen isotope discrimination (δ^{15}N), (d) nitrogen percentage (%N). WW, well-watered; WS, water stressed.

of measurements clustered families with similar responses to water deficit into three major groups (Supplementary Fig. S2). The first five PCs explained 68.03% of the total phenotypic variance in DSI within our panel of 95 walnut families (Supplementary Fig. S2).

Ranking of walnut families using EBV and integrated PCA values

The 15 individuals with the best EBV for Δ^{13}C and δ^{15}N under the well-watered and stress conditions are listed in Table 2 and

Downloaded from https://academic.oup.com/jxb/article/71/3/1107/5602662 by guest on 31 January 2024

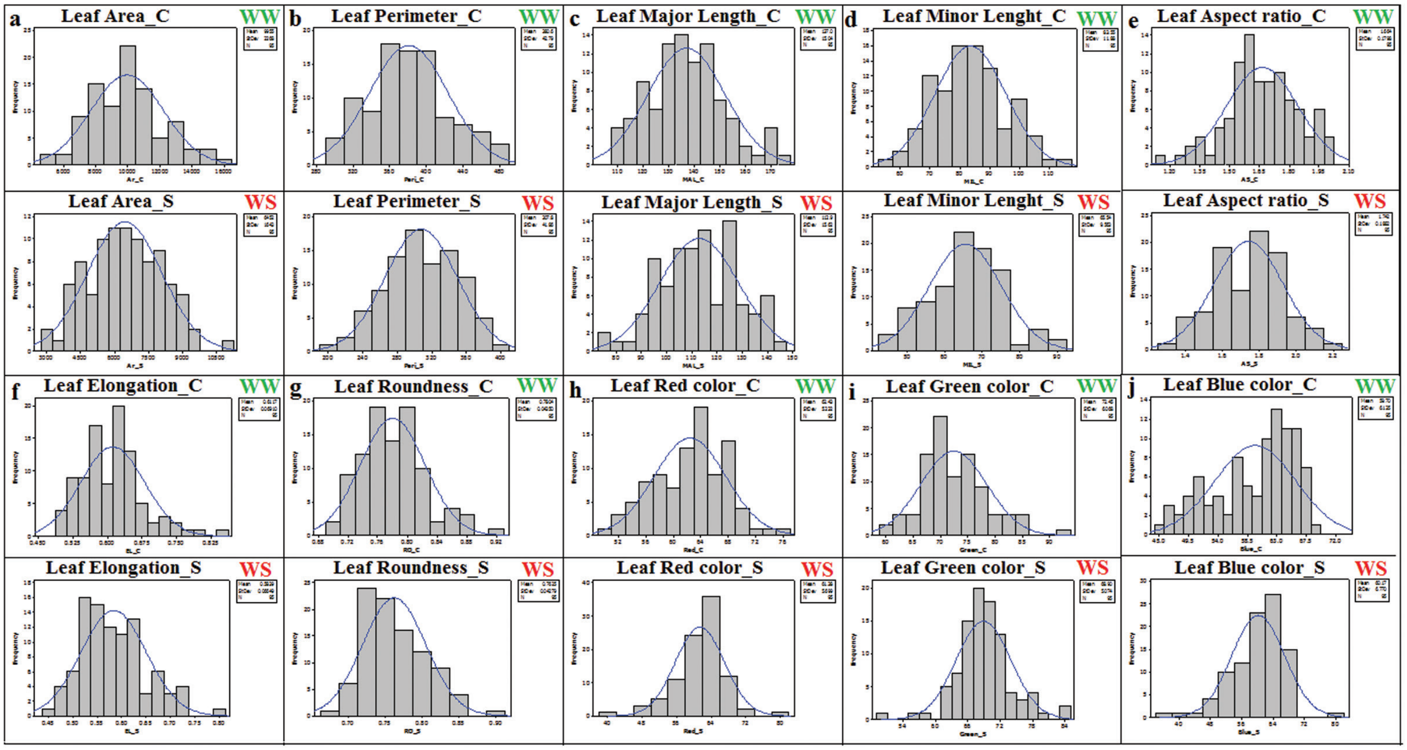

Fig. 3. Frequency distribution for leaf-related traits of 95 walnut families. (a) Area (Ar), (b) perimeter (Peri), (c) major length (MAL), (d) minor length (MIL), (e) aspect ratio (AS), (f) elongation (EL), and (g) roundness (RO) as well as RGB color system (h) red color, (i) green color, and (j) blue color. WW, well-watered; WS, water stressed. (This figure is available in color at *JXB* online.)

Supplementary Tables S6 and S7. A ranking value for each family was calculated using the following formula: ranking value=(22.04%×PC1)+(19.7%×PC2)+(15.9%×PC3)+(10.31%×PC4)+(0.08%×PC5) (Liu *et al.*, 2015). Families originating form Markazi, Fars, Kerman, and Semnan provinces had relatively higher ranking values, suggesting that they were more drought tolerant (Table 3). In general, ranking results showed that genotypes from dry or cold regions were more resistant to drought and had higher WUE.

Genotyping, population structure, and linkage disequilibrium

As previously reported (Arab *et al.*, 2019), all 95 samples analyzed with the Axiom *J. regia* 700K SNP array passed the quality check and were then used for the SNP clustering. Among the six SNP classes generated with the SNP clustering algorithm, we used only the *PHR* loci (323 273; 53.03%), which are polymorphisms with good cluster resolution and high-quality control measures (Supplementary Fig. S3). The population structure analysis, using both PCA and fastSTRUCTURE approaches, divided the Iranian walnut panel into four main groups, reflecting their geographic partitioning (group I, Kerman province; group II, Fars and Yazd provinces; group III, Semnan province; and group IV, other provinces) (Arab *et al.*, 2019). Also, we observed low levels of relatedness among the 95 Iranian walnut individuals, indicating low gene flow among the four subgroups (Supplementary Fig. S4). Our LD decay analysis showed that the LD (r^2) in our Iranian collection decayed below 0.2 within 10 kb, plateaued at ~10–50 kb, and then decayed to background levels (r^2<0.1) by 50–100 kb (Fig. 6). These findings indicated the possibility of performing GWAS with reasonable resolution using the 700K SNP array.

Genome-wide association analysis

We explored genotype–phenotype associations within our collection of 95 mother trees and the 18 traits related to WUE across treatments and of their DSI with 307 960 *PHR* SNPs using the SUPER and FarmCPU analysis in GAPIT. After Bonferroni multiple correction test [–log (P)≥7.0], a total of 15 SNP–trait associations (11 unique SNPs) were identified on chromosomes 2, 3, 5, 7, 9, 11, 12, and 15. Nine peaks with –log (P) values >8 in a Manhattan plot were strong signals of association between the WUE-related traits. As the Bonferroni correction test was too conservative, Bonferroni correction based on the simpleM method (Gao *et al.*, 2008) was performed, obtaining significant and suggestive *P*-value thresholds equal to $5×10^{-7}$ (0.05/99 449) and $1.006×10^{-5}$ (1/99 449), respectively. Based on these less conservative thresholds, 22 and 266 signals, respectively, were identified as significantly and suggestively associated with the traits of interest (Table 4; Supplementary Tables S8, S9). In addition, FarmCPU identified significant loci for some traits where SUPER failed to detect any loci, and the identified loci were mostly novel. In particular, out of the 22 most significant marker–trait associations (17 unique SNPs), 15 SNPs were found associated with trait mean of phenotypic performance and DSI, while two were associated with PCs from phenotypic data and DSI (Table 4). In detail, two SNPs on chromosomes 1 significantly associated with DSI of Δ^{13}C and each accounted for 21% of the phenotypic variation (Table 4; Fig. 7a). Six marker–trait associations on chromosomes 1,

Downloaded from https://academic.oup.com/jxb/article/71/3/1107/5602662 by guest on 31 January 2024

Table 1. Pearson correlations among ecological and geographical parameters with carbon isotope discrimination and leaf phenotypes in the studied walnut genotypes under normal and drought conditions.

Variable	Longi (E)	Latitu (N)	Altitu (m)	Precip[a]	Temp Ave[b]	Temp Min[c]	Temp Max[d]
Δ^{13}C_**C**	–0.29***	0.07^{NS}	–0.27***	0.42***	0.02^{NS}	0.05^{NS}	–0.03^{NS}
Δ^{13}C_**S**	–0.15^{NS}	0.05^{NS}	–0.15^{NS}	0.24*	0.09^{NS}	0.12^{NS}	0.06^{NS}
**%N_C**	–0.02^{NS}	0.01^{NS}	–0.02^{NS}	0.03^{NS}	–0.16*	–0.20	–0.15^{NS}*
**%N_S**	0.20*	–0.14^{NS}	0.17^{NS}	–0.08^{NS}	–0.19*	–0.14^{NS}	–0.20*
**Ar_C**	–0.13^{NS}	0.04^{NS}	–0.12^{NS}	0.10^{NS}	0.22*	0.19^{NS}	0.21*
**Ar_S**	–0.02^{NS}	–0.02^{NS}	–0.01^{NS}	–0.02^{NS}	0.21*	0.19*	0.20*
**Peri_C**	–0.12^{NS}	0.05^{NS}	–0.12^{NS}	0.13^{NS}	0.24*	0.22*	0.23*
**Peri_S**	0.003^{NS}	–0.06^{NS}	0.01^{NS}	0.02^{NS}	0.20*	0.23*	0.17^{NS}
**MAL_C**	–0.08^{NS}	0.05^{NS}	–0.10^{NS}	0.10^{NS}	0.21*	0.19*	0.21*
**MAL_S**	0.04^{NS}	–0.11^{NS}	0.05^{NS}	0.02^{NS}	0.19*	0.25**	0.14^{NS}
**MIL_C**	–0.15^{NS}	0.04^{NS}	–0.14^{NS}	0.10^{NS}	0.19^{NS}	0.16^{NS}	0.17^{NS}
**MIL_S**	–0.10^{NS}	0.06^{NS}	–0.08^{NS}	–0.02^{NS}	0.19*	0.12^{NS}	0.21*
**AS_C**	0.14^{NS}	–0.04^{NS}	0.11^{NS}	–0.03^{NS}	–0.03^{NS}	0.008^{NS}	–0.03^{NS}
**AS_S**	0.18^{NS}	–0.20*	0.15^{NS}	0.06^{NS}	0.009^{NS}	0.16^{NS}	–0.07^{NS}
**EL_C**	–0.13^{NS}	0.02^{NS}	–0.10^{NS}	0.04^{NS}	0.02^{NS}	–0.009^{NS}	0.01^{NS}
**EL_S**	–0.21*	0.23**	–0.18^{NS}	–0.06^{NS}	–0.003^{NS}	–0.17 ^{NS}	0.09^{NS}
**RO_C**	–0.13^{NS}	0.02^{NS}	–0.10^{NS}	0.03^{NS}	0.02^{NS}	–0.009^{NS}	0.02^{NS}
**RO_S**	–0.20*	0.22**	–0.17^{NS}	–0.06^{NS}	–0.005^{NS}	–0.17 ^{NS}	0.09^{NS}
**a_C**	–0.12^{NS}	0.16^{NS}	–0.14^{NS}	0.02^{NS}	–0.04^{NS}	–0.07^{NS}	0.004^{NS}
**a_S**	–0.22*	0.28**	–0.25**	0.1^{NS}	0.03^{NS}	–0.03^{NS}	0.11^{NS}
**b_C**	–0.09^{NS}	0.23*	–0.13^{NS}	–0.04^{NS}	–0.07^{NS}	–0.12^{NS}	0.01^{NS}
**b_S**	–0.20*	0.23*	–0.23*	0.11^{NS}	0.05^{NS}	0.01^{NS}	0.09^{NS}
**L_C**	0.11^{NS}	–0.23*	0.15^{NS}	0.03^{NS}	0.06^{NS}	0.11^{NS}	–0.01^{NS}
**L_S**	0.21*	–0.24*	0.24*	–0.12^{NS}	–0.05^{NS}	–0.007^{NS}	–0.10^{NS}

**Correlation is significant at the 0.01 level; *correlation is significant at the 0.05 level; ^{NS}, non-significant.
Trait abbreviations are explained in detail in the Materials and methods.
C and S indicate normal and stress conditions, respectively.
[a] Average annual precipitation.
[b] Average annual temperature.
[c] Average temperature in the coldest month of the year.
[d] Average temperature in the hottest month of the year.

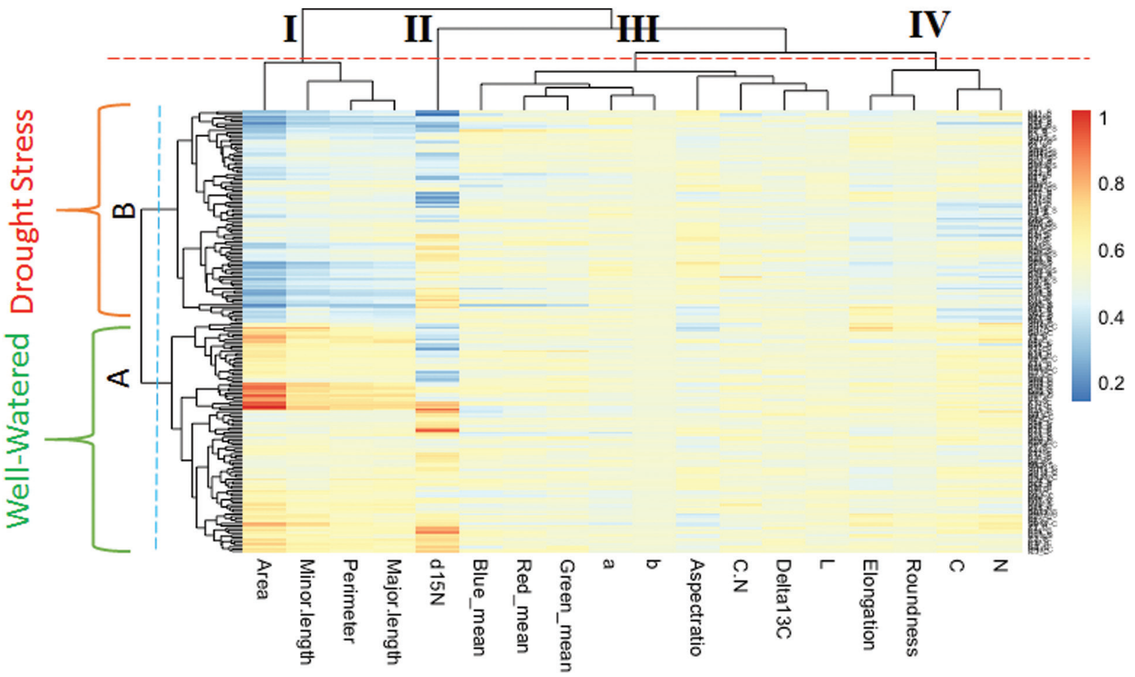

Fig. 4. Heatmap and hierarchical clustering analyses for morphological and physiological traits under well-watered and water deficit stress conditions in 95 walnut families after 14 d of treatment. (This figure is available in color at *JXB* online.)

Downloaded from https://academic.oup.com/jxb/article/71/3/1107/5602662 by guest on 31 January 2024

Table 2. Estimated breeding values for the top 15 and weakest five mother trees under drought for carbon isotope discrimination ($\Delta^{13}C\_S$)

Province	ID	Phenotype	Rank	Province	ID	EBV	Rank
**The top 15 mother trees**							
Yazd	YT2	20.86	1	Yazd	YT2	–0.57	1
Semnan	SeSh7	20.89	2	Semnan	SeSh7	–0.56	2
Kerman	KR13	20.95	3	Kerman	KR13	–0.54	3
Yazd	YT6	21.07	4	Fars	FaEq11	–0.52	4
Fars	FaEq11	21.08	5	Yazd	YT6	–0.52	5
Kerman	KRH4	21.09	6	Kerman	KRH4	–0.51	6
Kerman	KR7	21.34	7	Semnan	SeSh8	–0.43	7
Semnan	SeSh8	21.38	8	Kerman	KR7	–0.42	8
Ilam	IlEy2	21.4	9	Ilam	IlEy2	–0.42	9
Kerman	KBG5	21.63	10	Kerman	KBG5	–0.38	10
Markazi	MDJ2	21.74	11	Semnan	SeSh3	–0.34	11
Semnan	SeSh3	21.81	12	Fars	FaEq3	–0.34	12
Fars	FaEq3	21.81	13	Semnan	SeSh6	–0.33	13
Kerman	KR9	21.85	14	Markazi	MDJ2	–0.32	14
Semnan	SeSh6	21.9	15	Kerman	KBG7	–0.30	15
**The weakest five mother trees**							
Ilam	IlEy6	25.46	91	Ilam	IlEy6	0.60	91
Kerman	KR12	25.99	92	Kerman	KR12	0.72	92
Fars	FaEq2	26.75	93	Fars	FaEq2	0.91	93
Kerman	KR16	26.93	94	Semnan	SeSh4	0.95	94
Semnan	SeSh4	26.93	95	Kerman	KR16	0.97	95

2, 3, 9, 11, and 12 were identified for DSI of %C (Table 4; Fig. 7c). The percentage of phenotypic variation (R^2) that each significant locus associated with DSI of %C could explain ranged from 2% to 31%, and the strongest SNP peak accounted for 27% of the variation. For $\delta^{15}N$, one significant SNP–trait association was detected on chromosome 15 when using with average trait performance under normal conditions, explaining 26% of the phenotypic variation (Table 4; Fig. 7d). Two SNPs located on chromosomes 3 and 4 significantly associated with DSI of the C/N ratio and explained 21% and 22% of the phenotypic variation, respectively (Table 4; Fig. 7f). For DSI of both AS and RO, a common SNP–trait association on chromosome 5 was identified and accounted for 28% and 25% of the variation, respectively (Table 4; Fig. 8a–c). Also, we found a common marker–trait association on chromosome 7 for red, green, and blue color under normal conditions, in which R^2 was 27–29 % (Table 4). In addition, two SNPs for DSI of blue color were identified on chromosome 11 which explained 0.27% and 0.13% of the phenotypic variation, respectively (Table 4; Fig. 8e).

According to the suggestive threshold, 266 SNP–trait associations (164 unique SNPs) were identified. Of these, 131 SNPs were associated with mean of phenotypic performance and DSI of traits, and 33 SNPs were associated with PCs of phenotypic data and PCs of DSI of traits (Supplementary Tables S8, S9). In total, we identified a nearly equal number of associations in normal conditions (83) and drought stress (98) although the suggestive loci varied across and within traits and treatments. Furthermore, 27 out of 83 associations in normal conditions, and 43 out of 98 associations in drought stress conditions were related to more than one trait, possibly due to pleiotropic effects of genes. For the DSI of traits, we detected 85 associations, of which 27 were connected with more than one trait (Supplementary Tables S8, S9). Manhattan plots and QQ plots for most of the important traits under well-watered and stress conditions are shown in Supplementary Figs S5 and S6. Four SNPs on chromosome 11 and three SNPs on chromosomes 2 and 11 were detected for $\Delta^{13}C$ under normal and %C under stress conditions, respectively. Eight SNPs were identified for $\delta^{15}N$ under normal and drought conditions, of which seven SNPs were located on chromosome 10. In addition, for DSI of $\delta^{15}N$, four SNPs on chromosomes 5 and 6 were detected. Under normal conditions, five SNPs on chromosome 16 were identified for the C/N ratio. Two and six SNPs were detected for the DSI of Ar and Peri on chromosome 14, of which two SNPs were found associated with both traits. Overall, some significant markers are common among traits. For instance, several lead SNPs located on chromosomes 2, 5, and 6 were associated with AS, EL, and RO. Comparing the results of identified marker–trait associations showed that under the stress condition, some of the leaf-related trait associations were detected on the same chromosome and at the same positions; for example, eight, 14, and 14 SNPs on chromosome 2 were identified for AS, EL, and RO, respectively (Supplementary Fig. S5). Therefore, at this position, a putative major quantitative trait locus (QTL) for WUE and leaf growth may be located. Furthermore, for DSI of EL and RO, six common SNPs were found on chromosome 5. A lead SNP at chromosome 10 was associated with %C and %N traits. For leaf color-related traits, lead SNPs at several associated loci were located on chromosomes 7 and 11. By further lowering the P-values to $9.95.0\times10^{-4}$ ($-\log_{10} P$=4), we found the same marker–trait associations for WUE-related traits (i.e. $\Delta^{13}C$, $\delta^{15}N$, and C/N) on chromosomes 1, 7, and 16 as those

Downloaded from https://academic.oup.com/jxb/article/71/3/1107/5602662 by guest on 31 January 2024

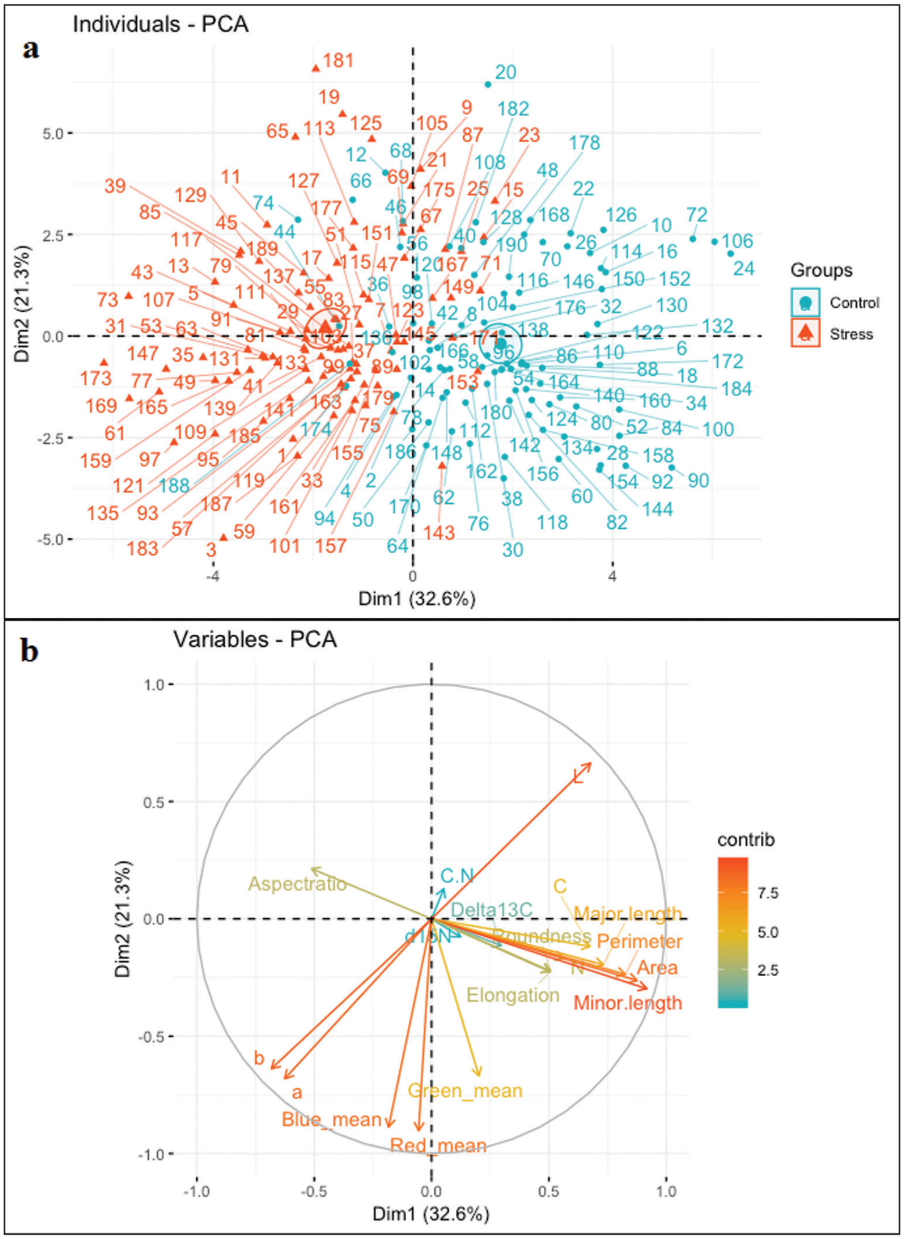

Fig. 5. Principal component analysis biplot of morphological and physiological traits of 95 walnut families under well-watered and drought stress conditions after 14 d of treatment. (This figure is available in color at *JXB* online.)

reported by Famula *et al.* (2019). In summary, our results show that several SNPs were associated with more than one trait. These finding are consistent with the quantitative nature of drought tolerance and the strong correlation among the different traits studied.

Genotype–environment association

We conducted a genotype–environment association analysis to identify candidate genomic regions that could contribute to local adaptation. After Bonferroni correction based on the simpleM method, with the significance threshold set (*P*-value 5×10^{-7}), the FarmCPU, and SUPER models identified 174 SNP–trait associations (115 unique SNPs); of these, 98 SNPs were associated with 21 climate variables and their combinations. (first three PCs), and 17 SNPs were related to three geographical variables (Table 5; Supplementary Table S10). Eighty-one peaks with –log (*P*) values >9 in a Manhattan plot indicated strong signals of associations with the climate variables. Manhattan plots and QQ plots for the most important traits are shown in Fig. 9 and Supplementary Fig. S6. In addition, multi-trait associations were identified for different climate variables. For instance, a lead SNP located on chromosome 8 was associated with aridity, BIO12, BIO13, BIO16, and longitude (Table 5; Fig. 9; Supplementary Fig. S6). For BIO2, BIO3, BIO15, BIO17, and latitude, a common SNP–trait association on chromosome 11 was also identified. In addition, we found a common SNP–trait association on chromosome 5 for BIO3, BIO14, BIO18, and latitude (Table 5; Fig. 9; Supplementary Fig. S6). A lead SNP associated with BIO4, BIO13, BIO19, PC1, PC2, and PET was identified on chromosome 15 (Table 5; Fig. 9; Supplementary Fig S6). The percentage of phenotypic

Downloaded from https://academic.oup.com/jxb/article/71/3/1107/5602662 by guest on 31 January 2024

Table 3. The five major components (PC1, PC2, PC3, PC4, and PC5) and PCA ranking values of the physiological parameters of 95 walnut families after 14 d of drought stress

Province	Genotype	PC1	PC2	PC3	PC4	PC5	Score	Rank
Markazi	MDJ3	26.15	72.12	167.90	0.16	15.10	281.43	1
Kerman	KR8	8.81	0.82	0.68	173.38	43.70	227.40	2
Fars	FaBa2	85.15	78.17	53.35	8.76	0.13	225.55	3
Kerman	KRH4	124.46	18.01	57.69	14.11	0.07	214.35	4
Ilam	IlIl6	16.95	0.08	128.79	2.16	35.62	183.60	5
Fars	FaEq15	54.78	46.85	3.98	30.45	40.39	176.44	6
Fars	FaEq11	19.39	19.95	129.17	5.13	0.01	173.65	7
West Azar	WAK2	161.06	6.28	3.12	0.31	0.01	170.77	8
Fars	FaEq14	6.32	69.57	4.25	46.39	31.58	158.11	9
Fars	FaEq5	81.02	15.76	0.43	33.25	23.70	154.17	10
Kerman	KRH3	6.75	21.89	39.17	1.35	80.68	149.84	11
Fars	FaEq12	91.01	24.69	5.65	25.09	1.29	147.73	12
Semnan	SeSh1	16.40	49.10	57.61	16.58	3.83	143.52	13
Fars	FaBa4	55.18	68.48	13.16	2.92	1.33	141.07	14
Semnan	SeSh5	2.49	19.37	13.44	85.64	17.99	138.93	15
Kerman	KBG2	33.58	0.07	60.01	6.68	37.62	137.95	16
Kerman	KR12	46.72	63.84	1.37	24.93	0.01	136.88	17
Kerman	KB1	23.58	89.61	9.20	10.96	0.57	133.93	18
Markazi	MDJ4	32.90	72.75	13.55	0.05	12.18	131.43	19
Ilam	Il12	3.04	99.38	5.51	2.48	19.86	130.26	20

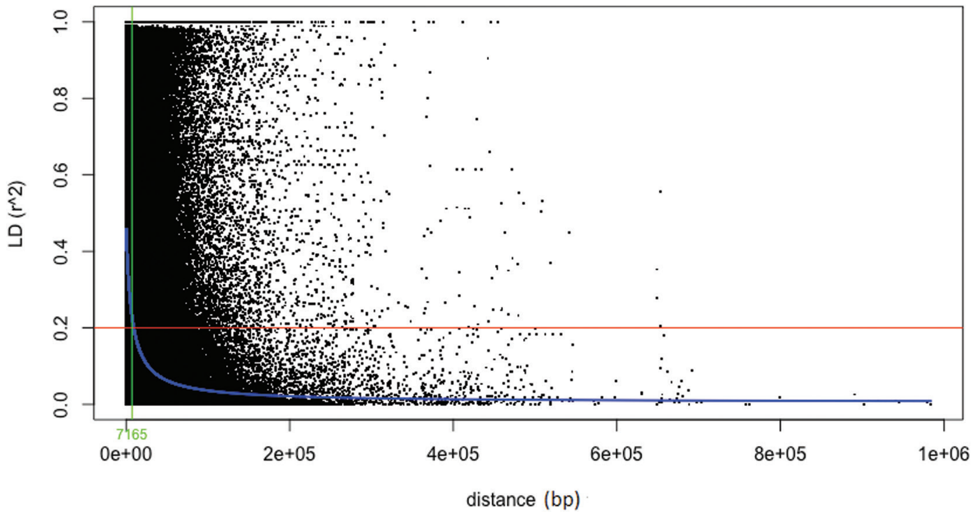

Fig. 6. Genome-wide linkage disequilibrium (LD) decay in the studied Iranian walnut collection. (This figure is available in color at *JXB* online.)

variation (R^2) that each significant SNP explained ranged from 2% to 16%. According to the suggestive threshold, 370 SNP–trait associations (265 unique SNPs) were identified; of these, 233 SNPs were related to 21 climate variables and their combinations, and 32 SNPs were associated with three geographical variables (Supplementary Table S10).

Functional annotation of candidate SNPs

By annotating the 17 most significant SNPs associated with WUE-related traits (Table 4), we found that the most significant SNPs associated with the DSI of Δ^{13}C on chromosome 1 fell in a gene encoding *S*-adenosylmethionine carrier 1 (Fig. 7). Four markers associated with the DSI of %C on chromosomes 1, 2, 9, and 12 were linked to genes encoding protein RFT1, alpha-glucosidase 2, geraniol 8-hydroxylase-like, and pleiotropic drug resistance protein 1-like, respectively (Table 4). Also, the SNP on chromosome 15 associated with δ^{15}N under normal watering is located within a gene encoding abscisic acid (ABA) 8'-hydroxylase 4-like (Fig. 7). On chromosome 4, a marker associated with the DSI of the C/N ratio was linked to a gene encoding an leucine repeat-rich (LRR) receptor-like serine/threonine-protein kinase. Common SNPs on chromosome 5 associated with the DSI of AS and RO fell in a gene coding for a 1-NAD-dependent malic enzyme. A significant marker associated with DSI of blue color is located within a gene encoding receptor-like protein kinase. Supplementary Table S8 provides the curation details of all 164 suggestive SNPs associated with drought-related traits that were annotated using BLASTx queries. Under water stress, eight markers associated with RO, AS, and EL on chromosome 2 were linked to genes encoding a cysteine-rich receptor-like protein kinase,

Downloaded from https://academic.oup.com/jxb/article/71/3/1107/5602662 by guest on 31 January 2024

Table 4. Functional annotations of the significantly associated SNPs for water use efficiency-related traits

Trait	Condition	SNP ID	CHR	Position	*P*-value	MAF	R^2	*J. regia* annotation
$\Delta^{13}C$	DSI	AX-170596078	1	13451392	2.30×10^{-7}	0.41	0.21	*S*-Adenosylmethionine carrier 1, chloroplastic/ mitochondrial, transcript variant X3
$\Delta^{13}C$	DSI	AX-170596083	1	13455420	2.30×10^{-7}	0.41	0.21	*S*-Adenosylmethionine carrier 1, chloroplastic/ mitochondrial, transcript variant X3
%C	DSI	AX-170954210	3	28588711	1.16×10^{-10}	0.1	0.27	Unknown
%C	DSI	AX-170815236	9	20690309	6.76×10^{-10}	0.05	0.31	Geraniol 8-hydroxylase-like
%C	DSI	AX-171133848	12	27686126	1.19×10^{-8}	0.44	0.19	Pleiotropic drug resistance protein 1-like
%C	DSI	AX-171480755	2	27191391	1.89×10^{-8}	0.35	0.02	α-Glucosidase 2
%C	DSI	AX-170835519	11	8665150	2.20×10^{-8}	0.06	0.03	Uncharacterized protein LOC108992038
%C	DSI	AX-171488640	1	2164492	5.33×10^{-7}	0.27	0.03	Protein RFT1 homolog, transcript variant X4
$\delta^{15}N$	Normal	AX-171076260	15	5413017	4.67×10^{-8}	0.1	0.26	Abscisic acid 8'-hydroxylase 4-like
%N	DSI	AX-170815236	9	20690309	5.75×10^{-7}	0.05	0.20	Geraniol 8-hydroxylase-like
C/N	DSI	AX-171507746	4	29059556	3.10×10^{-7}	0.27	0.22	Probable LRR receptor-like serine/threonine-protein kinase
C/N	DSI	AX-171113225	3	33413317	5.52×10^{-7}	0.05	0.21	Protein trichome birefringence-like 34
AS	DSI	AX-170978215	5	1540443	1.17×10^{-7}	0.15	0.28	1-NAD-dependent malic enzyme 62 kDa isoform, mitochondrial-like and 2-nucleolar GTP-binding protein
RO	DSI	AX-170978215	5	1540443	6.53×10^{-7}	0.15	0.25	1-NAD-dependent malic enzyme 62 kDa isoform, mitochondrial-like and 2- nucleolar GTP-binding protein
Red	Normal	AX-170739733	7	50573086	6.85×10^{-7}	0.35	0.27	Uncharacterized protein LOC108986320
Green	Normal	AX-171049033	7	36924913	1.54×10^{-7}	0.09	0.28	40S ribosomal protein S24-1-like, transcript variant X2
Blue	Normal	AX-170739733	7	50573086	1.09×10^{-7}	0.35	0.29	Uncharacterized protein LOC108986320
Blue	DSI	AX-170919261	11	11922980	9.80×10^{-9}	0.48	0.27	Putative receptor-like protein kinase At4g00960
Blue	DSI	AX-170634325	11	28707562	5.19×10^{-7}	0.44	0.13	AP-2 complex subunit sigma
PC1	Normal	AX-170739733	7	50573086	8.13×10^{-8}	0.35	0.26	Uncharacterized protein LOC108986320
PC1	Normal	AX-170739733	7	50573086	1.74×10^{-7}	0.35	0.26	Uncharacterized protein LOC108986320
PC3	DSI	AX-171091758	11	10312034	9.72×10^{-8}	0.19	0.24	Unknown

CHR, chromosome; MAF, minor allele frequency; R^2 (%), proportion of variation explained by an SNP.

a pentatricopeptide repeat (PPR)-containing protein, cytochrome P450, and a cyclin-dependent kinase (CDK). Since, marker–trait association signal can be influenced by the effect of size and LD, we considered clusters of SNPs with more moderate *P*-values. In doing so, several drought stress-responsive genes were found linked to the marker–trait associations identified, such as a PPR-containing protein, an F-box protein, a mitogen-activated protein kinase (MAPK), a wall-associated receptor kinase, transcription factors (WRKY, bHLH, and HSP), a putative peroxidase, a glutathione *S*-transferase-like, and an auxin transport protein (Supplementary Table S8).

The results of the genotype–environment association analysis revealed that the significant SNP on chromosome 15 with the greatest number of associated environmental variables (six) fell in a gene coding for transcription factor MYB1R1. Three lead SNP markers on chromosomes 8, 11, and 5, each associated with at least four climate variables, were linked to the genes calcium-dependent protein kinase 11 (CDPK11)-like, DAR GTPase 3, and omega-3 fatty acid desaturase, respectively (Table 5).

Discussion

To evaluate the extent of natural variation in walnut responses to drought in Iran, we phenotyped a collection of 95 Iranian walnut families for WUE and leaf-related traits. For the first time in walnut, we characterized drought tolerance using a common-garden experiment, therefore reducing environmental influence. By using >307 960 SNPs evenly distributed across the walnut genome, we increased the power and resolution of GWAS for detecting significant marker–trait associations for drought tolerance in walnut, despite the limited size of our association panel. In addition, the wide range of continuous and normally distributed drought-related phenotypes observed under both normal and water deficit conditions highlights the quantitative and complex nature of the traits studied.

Correlation between phenotypes and environmental characteristics

We found trait–climate relationships similar to a previous common-garden study (Aletà *et al.*, 2009). In general, our results showed a positive correlation between $\Delta^{13}C$ and precipitation at the geographical origin and a negative correlation between $\Delta^{13}C$ and altitude. We also found a positive association between leaf growth-related traits and temperature, and a negative association between leaf color and geographical parameters, as already found by Mitchell *et al.* (2018). Our results are in line with Körner (1999) who stated that plants tend to have larger leaves in warm areas. Our findings of associations between leaf size and temperature are consistent with Moles *et al.* (2014) who found that mean annual temperature is generally a better predictor of plant traits than mean annual precipitation. In combination, our findings suggest that

Downloaded from https://academic.oup.com/jxb/article/71/3/1107/5602662 by guest on 31 January 2024

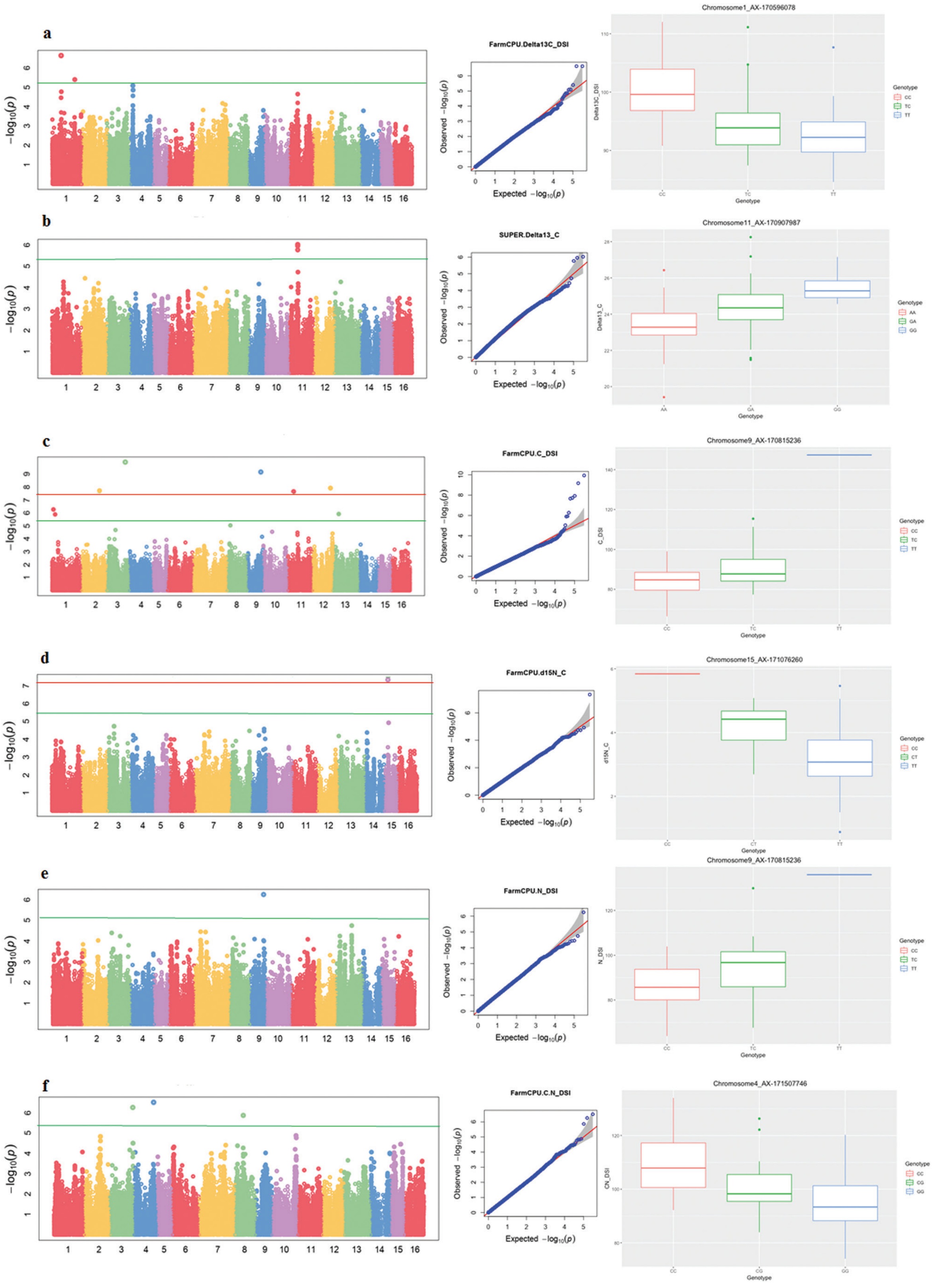

Fig. 7. Manhattan plots (left), quantile–quantile plots (middle), and box plot (right) of association analysis using the Q+K model for traits: (a) $\Delta^{13}C$ ($\Delta^{13}C$_DSI), (b) $\Delta^{13}C$ ($\Delta^{13}C$_C), (c) %C (C_DSI), (d) $\delta^{15}N$ ($\delta^{15}N$_C), (e) %N (N_DSI), and (f) C/N (C/N_DSI). The *y*-axis of the Manhattan plots shows the –log10 (*P*-values) of SNP association. Each dot represents an SNP. The upper horizontal line represents the Bonferroni-corrected significance threshold, while the lower line indicates the suggestive threshold. The threshold value was calculated by Bonferroni correction based on the tested number of SNP markers (P<0.05/99 449). (This figure is available in color at *JXB* online.)

interpopulation differences in $\Delta^{13}C$ as an indicator of WUE in Iranian walnut may evolve through positive selection at the local condition sites (drier and hotter), and precipitation may be a more important driver than temperature for WUE in walnut in Iran. Thus, we expect germplasm selection in Yazd and Kerman toward increased WUE. Our results are in

Downloaded from https://academic.oup.com/jxb/article/71/3/1107/5602662 by guest on 31 January 2024

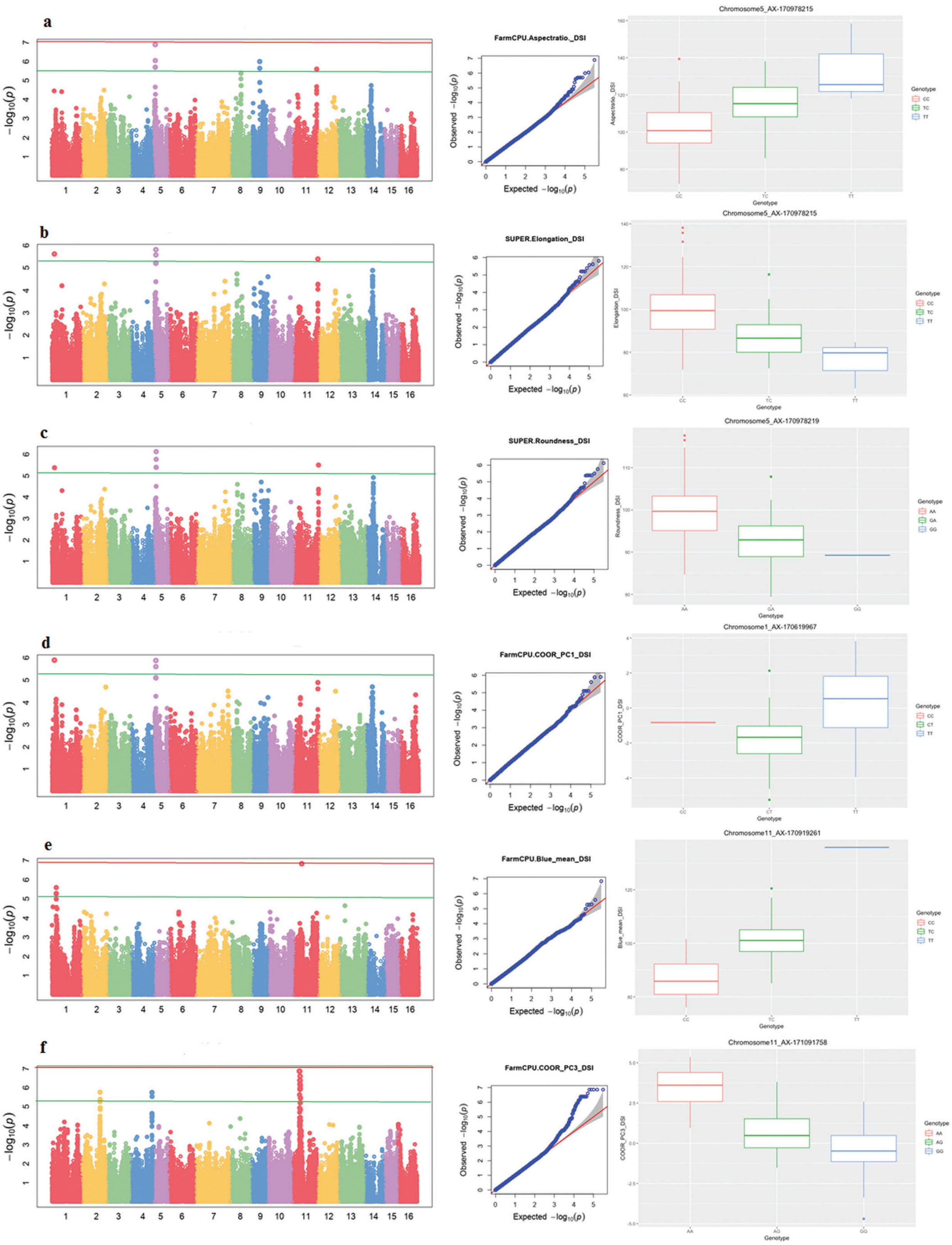

Fig. 8. Manhattan plots (left), quantile–quantile plots (middle), and box plot (right) of association analysis using the Q+K model for traits: (a) aspect ratio (AS_DSI), (b) elongation (EL_DSI), (c) roundness (RO_DSI), (d) principal component (PC1_DSI), (e) blue (Blue_DSI), and (f) principal component (PC3_DSI). The *y*-axis of the Manhattan plots shows the –log10 (*P*-values) of SNP association. Each dot represents an SNP. The upper horizontal line represents the Bonferroni-corrected significance threshold, while the lower line indicates the suggestive threshold. The threshold value was calculated by Bonferroni correction based on the tested number of SNP markers (P<0.05/99 449). (This figure is available in color at *JXB* online.)

agreement with Vahdati *et al.* (2009) and Lotfi *et al.* (2010) who stated that water availability is one of the most important abiotic determinants of the growth of walnut in Iran. Therefore, walnut trees that are adapted to hotter and drier regions, especially Yazd and Kerman, could be a valuable resource for future cross-breeding programs (cross with Chandler as a commercial

Downloaded from https://academic.oup.com/jxb/article/71/3/1107/5602662 by guest on 31 January 2024

Table 5. Functional annotations of the significantly associated SNPs for climate variables

Model	Trait	SNP ID	CHR	Position	*P*-value	MAF	R^2	*J. regia* annotation
FarmCPU	Altitude	AX-170937679	7	30111357	2.56×10^{-7}	0.06	0.04	Probable LRR receptor-like serine/threonine-protein kinase RFK1
SUPER	Aridity	AX-170663968	7	47984591	1.44×10^{-18}	0.07	0.08	Putative serine/threonine-protein kinase
SUPER	Aridity	AX-171503176	12	16945475	3.52×10^{-8}	0.14	0.06	Heat stress transcription factor A-5
SUPER	BIO2	AX-170704342	13	38138398	1.47×10^{-37}	0.15	0.11	GDSL esterase/lipase At4g10955-like
FarmCPU	BIO3	AX-170735664	10	33995671	2.27×10^{-10}	0.07	0.05	Synaptotagmin-5-like
FarmCPU	BIO3	AX-171515497	4	8503479	3.80×10^{-10}	0.17	0.05	Protein YLS9-like
FarmCPU	BIO13	AX-170641855	8	485984	2.94×10^{-8}	0.09	0.05	Protein NRT1/ PTR FAMILY 5.10-like
FarmCPU	BIO19	AX-170835389	11	8744062	8.78×10^{-10}	0.06	0.13	Cytochrome P450 84A1-like, transcript variant X1
FarmCPU	Longitude	AX-171071733	7	6080152	5.54×10^{-8}	0.06	0.03	Receptor-like protein 2
FarmCPU	PC2	AX-171528251	1	17400933	1.80×10^{-8}	0.50	0.02	S-Type anion channel SLAH1-like
FarmCPU	PET	AX-170836081	11	8280972	2.40×10^{-11}	0.1	0.1	Probable ubiquitin-conjugating enzyme E2, transcript variant X2
FarmCPU	Aridity	AX-171503554	8	6144078	4.65×10^{-14}	0.07	0.11	Calcium-dependent protein kinase 11-like
SUPER	BIO12				1.76×10^{-12}		0.14	
FarmCPU	BIO13				2.78×10^{-17}		0.14	
FarmCPU	BIO16				1.12×10^{-12}		0.13	
FarmCPU	Longitude				1.35×10^{-11}		0.07	
SUPER	BIO2	AX-170569995	11	22100214	3.49×10^{-18}	0.05	0.13	DAR GTPase 3, chloroplastic, transcript variant X2
FarmCPU	BIO3				6.09×10^{-14}		0.05	
FarmCPU	BIO15				3.81×10^{-15}		0.16	
FarmCPU	BIO17				8.86×10^{-10}		0.1	
FarmCPU	Latitude				9.58×10^{-10}		0.04	
FarmCPU	BIO3	AX-171526620	5	6786466	7.66×10^{-9}	0.06	0.04	Omega-3 fatty acid desaturase, endoplasmic reticulum-like, transcript variant X2
FarmCPU	BIO14				6.46×10^{-11}		0.11	
FarmCPU	BIO18				1.20×10^{-7}		0.14	
SUPER	Latitude				1.86×10^{-18}		0.06	
SUPER	BIO4	AX-170929810	15	9052875	2.37×10^{-7}	0.22	0.04	Transcription factor MYB1R1
FarmCPU	BIO13				5.05×10^{-8}		0.06	
FarmCPU	BIO19				1.79×10^{-9}		0.1	
FarmCPU	PC1				5.26×10^{-8}		0.05	
FarmCPU	PC2				2.19×10^{-16}		0.1	
FarmCPU	PET				2.50×10^{-8}		0.12	
SUPER	Aridity	AX-170891580	9	14032504	5.28×10^{-27}	0.08	0.06	Transcription termination factor MTERF2, chloroplastic-like, transcript variant X1
SUPER	Latitude				8.92×10^{-24}		0.07	
FarmCPU	Longitude				5.46×10^{-14}		0.05	
FarmCPU	BIO19	AX-170754261	2	1309667	3.04×10^{-13}	0.41	0.07	4-Coumarate-CoA ligase-like 9
FarmCPU	PC2				1.50×10^{-12}		0.04	

CHR, chromosome; MAF, minor allele frequency; R^2 (%), proportion of variation explained by an SNP.

cultivar) in order to release a new walnut cultivar with high yield and WUE.

Phenotypic variation among genotypes

Drought stress significantly altered the WUE-related traits and leaf-related parameters in all 95 walnut families (Fig. 4). Various approaches and statistical models, such as correlation analysis, PCA, and clustering, have been proposed for analyzing and interpreting large phenotypic data sets collected from plants grown under drought conditions (Vahdati *et al.*, 2009; Liu *et al.*, 2015). In this study, heatmap, PCA, and correlation analysis showed that family differences in drought tolerance and WUE were largely due to variation in leaf parameters, especially leaf growth (Fig. 5). This suggests that leaf characteristics may be affected more by drought and can be good indicators of water deficit in walnut. This is consistent with earlier reports of drought studies in walnut (Lotfi *et al.*, 2010). During drought conditions, photosynthesis may be inhibited due to leaf chlorosis and stomatal closure. Consequently, the Rubisco enzyme activity will be inhibited and transpiration will increase, ultimately leading to depleted carbohydrate reserves, reduced growth rate, and promotion of plant senescence (Rosati *et al.*, 2006). On the other hand, under drought conditions, most of the families exhibited decreased $\Delta^{13}C$. Our results are in agreement with those of Zhang *et al.* (2006), suggesting that the stomata may close in response to drought stress via ABA-dependent and independent pathways, decreasing water loss through transpiration. We also found that some genotypes that originated from dry regions, such as YT2, YT6, SeSh7, and KR13, have relatively good WUE (Table 2). Although we found that families from dry regions had comparatively greater WUE, WUE was highly variable (Table 2) and may not be a sufficient or appropriate index for assessing drought tolerance (Blum, 2009).

Downloaded from https://academic.oup.com/jxb/article/71/3/1107/5602662 by guest on 31 January 2024

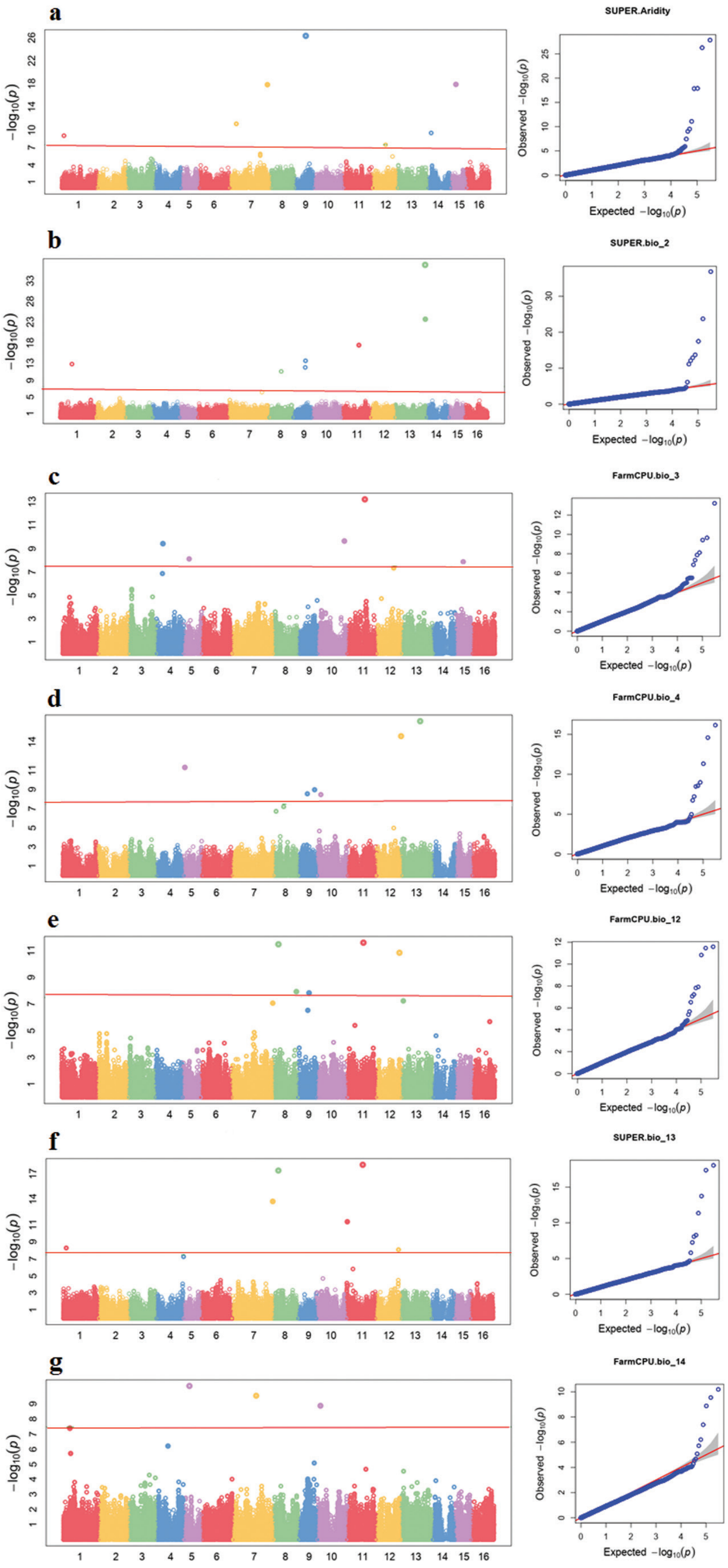

Fig. 9. Manhattan plots (left), quantile–quantile plots (middle), and box plot (right) of association analysis using the Q+K model for climate variables: (a) aridity, (b) BIO2, (c) BIO3, (d) BIO4, (e) BIO12, (f) BIO13, and (g) BIO14. The *y*-axis of the Manhattan plots shows the –log10 (*P*-values) of SNP association. Each dot represents an SNP. The horizontal line represents the Bonferroni-corrected significance threshold. The threshold value was calculated by Bonferroni correction based on the tested number of SNP markers (P<0.05/99 449). (This figure is available in color at *JXB* online.)

To comprehensively assess the relative drought tolerance of the 95 families, a ranking value was also calculated for each of the accessions analyzed (Table 3). Based on their ranking values, families MDJ3, KR8, FaBa2, KRH4, and IlIl6 were the most tolerant of drought. In contrast, when families were ranked according to Δ^{13}C, most genotypes from Yazd and some

Downloaded from https://academic.oup.com/jxb/article/71/3/1107/5602662 by guest on 31 January 2024

of the Kerman genotypes showed the best WUE. These regions are the driest parts of the country. Therefore, different mechanisms of walnut drought tolerance and adaptation are likely to be involved, and the correlation of Δ^{13}C with geographic and climate factors at the sites of origin demonstrates its potential involvement in drought adaptation. Our results are consistent with earlier studies in walnut (Aletà *et al.*, 2009) and other trees (Cregg and Zhang, 2001; Cumbie *et al.*, 2011), which found that the variation in Δ^{13}C was correlated with geographical origin and soil water availability. Therefore, water availability acted as a selective force and drove local adaptation of walnut trees across the eight Iranian provinces investigated in this study.

Population structure and linkage disequilibrium

Genetic structure analysis using different approaches (Arab *et al.*, 2019) classified our panel into four main groups, according to their geographical origin. The extent of LD in the genome is one of the most important factors influencing the possibility of LD-based association mapping (Lewis and Knight, 2012; Marrano *et al.*, 2018). Previous studies proposed that there was a very different range of LD extension in different crops and different chromosomal regions (Sonah *et al.*, 2015; Marrano *et al.*, 2018). Our results show that the observed percentage of SNP loci in LD in our Iranian collection is similar to that of other fruit trees (Myles *et al.*, 2011; Marrano *et al.*, 2018). These results fit with expectations for natural populations having high diversity and a large degree of outbreeding. As previous studies have pointed out, the causes of LD mainly include mutation, selection, migration, genetic drift, population bottlenecks, and admixture (Morrell *et al.*, 2005; Mackay and Powell, 2007). The rapid LD decay (r^2=0.2, 10 kb) observed in our collection is most probably due to the open-pollinated origin of our materials and the high levels of recombination in the genomes of local walnut populations. The rapid LD decay observed in our study is comparable with those found in other outcrossing woody crops such as grapevine (*Vitis vinifera* L.; 10 kb; Myles *et al.*, 2011) (Fig. 6). This value of LD decay indicates the high potential of Iranian collections for GWAS, as already demonstrated previously for nut quality-related traits (Arab *et al.*, 2019). However, the small sample size of our association panel limited the power of our GWAS.

Genotype and phenotype associations

To identify the genomic regions underlying WUE and drought tolerance, WUE traits and leaf-related parameters under normal watering and drought stress treatments were used separately for GWAS. We found different marker–trait associations for most of the traits in the two treatments. Our findings suggest that various mechanisms may control WUE and leaf-related traits under different watering conditions, in line with what was stated by Mickelbart *et al.* (2015). In addition, our GWAS results indicate the polygenic inheritance of drought tolerance in walnut, as already observed in walnut (Famula *et al.*, 2019) and other tree crops such as apple (H. Wang *et al.*, 2018) and loblolly pine (Cumbie *et al.*, 2011). In particular, Famula *et al.* (2019) identified four SNPs associated with Δ^{13}C in a collection of >60 genotypes (241 mature trees) from the UC Davis walnut breeding program, grown under normal field conditions. By using a less stringent *P*-value threshold, we found marker–trait associations in the same genomic regions identified by Famula *et al.* (2019). However, we identified marker–trait associations for Δ^{13}C in addition to those of Famula *et al.* (2019), most probably due to differences in the growing conditions between the two collections (greenhouse versus field) as well as plant material age and genetic background. These discrepancies between our study and that of Famula *et al.* (2019) confirm the complex architecture of drought tolerance (Dhanapal *et al.*, 2015; X. Wang *et al.*, 2018).

Proteins involved in WUE and leaf-related traits were identified across most of the walnut chromosomes (Table 4; Supplementary Table S9). On chromosome 1, *S*-adenosylmethionine carrier 1, which is involved in the *S*-adenosyl-l-methionine (SAM) cycle, was found to be associated with the DSI of Δ^{13}C. The metabolites in the SAM cycle are well known to play key roles in cell wall metabolism and biosynthesis of polyamine and ethylene for stress tolerance. Overexpression of SAM has been shown to increase drought tolerance in Arabidopsis. This indicates that increases in SAM could enhance the osmoregulant metabolism of plants dealing with drought stress (Wang *et al.*, 2016). On chromosome 12, a pleiotropic drug resistance protein that has been shown to be involved in abiotic stress response (Crouzet *et al.*, 2006) was found to be associated with the DSI of the %C. In addition, for the DSI of %C, we found an α-glucosidase on chromosome 2. In plants under drought stress, photosynthesis can be suppressed while sucrose is synthesized by starch degradation. In addition, in plants exposed to abiotic stresses (e.g. a combination of heat and drought) the expression of α-amylase, β-amylase, and α-glucosidase, which are required for starch degradation, significantly increase (Rizhsky *et al.*, 2004). On chromosome 15, an ABA 8'-hydroxylase, which is primarily a regulator of ABA catabolism in plants, was found to be associated with δ^{15}N under normal conditions. Results of previous studies have shown that ABA catabolic inhibitors that target ABA 8'-hydroxylase can regulate the ABA content in plants, resulting in drought tolerance (Kitahata *et al.*, 2005). On chromosome 4, LRR receptor-like serine/threonine-protein kinase, which is a key regulator of ABA signaling, is associated with the DSI of the C/N ratio (X. Wang *et al.*, 2018). The involvement of serine/threonine-protein kinases in the response to environmental stresses and ABA signaling has already been demonstrated previously.

On chromosome 5, an NAD-dependent malic enzyme and a nucleolar GTP-binding protein were found to be associated with DSI of both AS and RO. Previous studies have shown that this malic enzyme plays a role in the closure of stomata and the alteration of plant water use (Laporte *et al.*, 2002). Functional analysis in Arabidopsis showed that nucleolar GTP-binding protein regulates guard cell signaling in response to environmental stress through ABA-mediated pathways (Lee *et al.*, 2018). For the DSI of blue color, we identified a receptor-like protein kinase as one of the top candidates. The potential role of receptor-like protein kinase (RLKs) in

Downloaded from https://academic.oup.com/jxb/article/71/3/1107/5602662 by guest on 31 January 2024

abiotic stress response, including the ABA response and calcium signaling, has been reported in several studies (Ye *et al.*, 2017). Interestingly, on chromosome 2, we identified several genes correlated with three traits under stress (Supplementary Table S8). GWAS linked a cysteine-rich receptor-like protein kinase, a PPR-containing protein, a cytochrome P450, and a CDK with RO, AS, and EL under drought stress. RLKs have been reported to enhance ABA sensitivity in Arabidopsis (Lu *et al.*, 2016), conceivably conferring drought tolerance. PPR proteins are a large gene family in plants and play an important role in regulating plant responses to abiotic stress. Interestingly, overexpression of a cytosol–nucleus dual-localized PPR protein (SOAR1) in Arabidopsis confers tolerance to drought, salt, and cold stresses, by integrating ABA-dependent and independent signaling pathways (Jiang *et al.*, 2015). Recent investigation revealed that CDKs are involved in the signaling control of stress tolerance (Kitsios *et al.*, 2011).

In agreement with previous studies (Curtin *et al.*, 2017; Kalladan *et al.*, 2017), our results show that GWAS analyses contain noteworthy information beyond just a few significant signals (lowest *P*-value SNPs). We identified additional candidate genes involved in stress response by using less conservative *P*-values. For AS, RO, and %N under stress, we found a candidate gene encoding a wall-associated receptor kinase, which has been shown to be involved in cell elongation and plant development. Earlier studies in Arabidopsis have revealed that wall-associated kinases are expressed throughout plant development and are involved in cell expansion (Wagner and Kohorn, 2001). In addition, we found genes encoding an F-box protein and a PPR-containing protein as candidate loci for different traits. These proteins regulate ABA signaling and drought stress responses (Lyzenga and Stone, 2012). A MAPK gene, a member of a gene family important in the transduction of various abiotic stress and developmental signals, was found to be associated with $\Delta^{13}C$. It has been reported that plant hormones such as ABA and ethylene influence signaling through MAPK cascades (Jagodzik *et al.*, 2018). Another drought-responsive gene, the ethylene receptor protein gene, which is involved in ABA signaling, was found to be associated with the $\Delta^{13}C$ under drought condition. It has been proven that ABA influences plant development, as well as response to drought stress, through regulation of stomatal opening and closure (Wilkinson and Davies, 2002). Additionally, some annotations of WUE-related traits in the present study were also found in previous studies of WUE in walnut (Famula *et al.*, 2019) and other tree species (Cumbie *et al.*, 2011; Guerra *et al.*, 2016).

In summary, candidate genes involved in many processes related to the response to abiotic stresses, such as ABA signaling, regulation of stomatal function, photosynthesis, plant growth and development, and hormonal regulation, have been detected in this study. Most of the genes identified for WUE traits are involved in ABA signaling, the transduction of environmental signals, and, therefore, the response to drought stress (X. Wang *et al.*, 2018). Our findings indicate that the ABA signaling pathway and stomatal regulation underlie drought tolerance in walnut. Other candidate genes for WUE-related traits encode transcription factors, such as WRKY, bHLH, AP2-like ethylene-responsive protein, and heat shock 70 kDa protein, which have been reported to be involved in drought response (Yang *et al.*, 2017; Khan *et al.*, 2018), and their overexpression could increase tolerance to water deprivation (Zhai *et al.*, 2016). Moreover, peroxidase and glutathione *S*-transferase-like proteins, which are involved in anti-oxidative mechanisms, were found to be associated with the traits studied. Therefore, our results indicated that WUE is a complex quantitative trait, and diverse mechanism are involved.

Genetic bases of environmental stress adaptation

Persian walnut trees are exposed to different stressors in their environment, including drought, heat, and salinity, and have evolved specific physiological and molecular mechanisms to respond (Aleta *et al.*, 2009; Vahdati *et al.*, 2009; Famula *et al.*, 2019). Trees that have developed in Iranian or other Central Asian climates have been subjected to these abiotic stresses over time, which can be expected to result in localized adaptations (Arab *et al.*, 2019).

Our results revealed several interesting proteins involved in Persian walnut abiotic stress response. For instance, a CDPK encoded on chromosome 8 was associated with aridity, BIO12, BIO13, BIO16, and longitude (Table 5). CDPKs have been reported previously to be involved in diverse abiotic stress signaling pathways (Franz *et al.*, 2011). In particular, CDPKs act in the perception of abiotic stress stimuli to enhance the transcription of stress-related candidate genes (Franz *et al.*, 2011; Schulz *et al.*, 2013). A functional link between CDPKs and ABA signaling through the activation of ABA-inducible promotors has also been reported (Franz *et al.*, 2011). An omega-3 fatty acid desaturase (FAD) endoplasmic reticulum-like protein encoded on chromosome 5 was associated with BIO3, BIO14, BIO18, and latitude (Table 5). FADs contribute to the adaptability of plants to temperature fluctuation through membrane stabilization and reactive oxygen species scavenging, and are considered key factors in plant tolerance to various stresses (Román *et al.*, 2012; H.S. Wang *et al.*, 2014).

In addition, on chromosome 15, transcription factor MYB1R1, which is involved in the regulation of drought-responsive genes, was found to be associated with BIO4, BIO13, BIO19, PC1, PC2, and PET (Table 5). This transcription factor participates in stomatal closure in plants in response to ABA, and confers salt and drought tolerance (Shin *et al.*, 2011). Furthermore, a YLS9-like protein encoded on chromosome 4 was found to be associated with BIO3. YLS9-type proteins are implicated in leaf senescence, an important plant adaptation to fluctuations in environmental conditions (Müller *et al.*, 2017). Our results indicate that genotype–environment association could assist plant breeding by identification of genomic regions potentially adapted to environmental stress.

Conclusion

We have shown a detailed approach to uncover known and candidate genes potentially involved in drought adaptation and WUE in Persian walnut by integrating multiple disciplines. Combining the common-garden experiment, genotype–phenotype, and genotype–environment associations, making use of the phenotype–genotype–environment information

Downloaded from https://academic.oup.com/jxb/article/71/3/1107/5602662 by guest on 31 January 2024

from a walnut collection representing a diverse cross-section of the Iranian walnut gene pool and a high-density SNP array, allowed us to identify genomic regions involved in WUE and adaptation to drought. Under drought stress, the power to detect relevant loci for WUE-related traits and leaf-related parameters via GWAS increased, most probably because under water deficit conditions different WUE and leaf growth can be achieved by different families through different physiological mechanisms and corresponding gene networks, thus the number of marker–trait association increased. Although a number of identified regions have already been described in other populations, our study contributes to evaluating their relevance in local walnut germplasm and highlights the presence of additional QTLs related to WUE, which will be valuable information for establishing breeding populations. Consistent with previous studies, we confirmed the polygenic nature of WUE and drought adaptation in walnut. Most of the candidate genes controlling these traits are involved in response to abiotic stresses. For example, several candidate genes involved in ABA signaling and stomatal regulation were found close to our identified markers, confirming their role in WUE and drought adaptation. In the future, functional genomic characterization of these genes using transcriptome analysis (e.g. RNA-Seq or Tag-Seq analysis) or CRISPR/Cas9-based genome editing can provide additional checkpoints in walnut drought tolerance. In conclusion, our study provides insights into the genetic control of drought adaptation in walnut, enabling future marker-assisted selection and the development of new walnut rootstocks or cultivars with improved WUE. Our study based on 95 samples defines the scope of further research in terms of traits and SNPs that should be given special attention, in a larger population, combined with new phenotyping approaches under both field and greenhouse conditions.

Supplementary data

Supplementary data are available at *JXB* online.

Fig. S1. The correlation coefficient between physiological measurements in 95 walnut families under well-watered and drought stress conditions.

Fig. S2. Principal component analysis biplot of the DSI of morphological and physiological traits of 95 walnut families under well-watered (control) and drought stress conditions after 14 d of treatment.

Fig. S3. Proportion of the Axiom® Walnut 700K SNP array markers classified into each of the six classes.

Fig. S4. Principal component and kinship analyses of Persian walnut genetic data.

Fig. S5. Manhattan plots and quantile–quantile plots of association analysis using the Q+K model.

Fig. S6. Manhattan plots and quantile–quantile plots of association analysis using the Q+K model.

Table S1. The information of 95 walnut genotype analyzed under normal and drought stress conditions.

Table S2. All the environmental variables considered for genome–environment association analyses (FarmCPU and SUPER)

Table S3. Descriptive statistics for phenotypic variation in WUE-related traits of the walnut association panel under normal and drought stress conditions.

Table S4. Paired two sample *t*-test for means for the effect of drought stress on carbon isotope discrimination, nitrogen isotope discrimination, carbon percentage, nitrogen percentage, and the carbon to mnitrogen ratio with the data of 14 d.

Table S5. ANOVA for the effects of families, treatments, and the interaction between them.

Table S6. Breeding values for the top 15 and weakest five mother trees under normal conditions for carbon isotope discrimination.

Table S7. Breeding values for the top 15 and weakest five mother trees under normal and drought stress conditions for nitrogen isotope discrimination.

Table S8. Genes identified by a genome-wide association study underlying variation in water use efficiency in walnut (*Juglans regia* L.)

Table S9. The suggestive SNPs associated with WUE and leaf-related traits and candidate genes underlying different loci of these traits in normal and water deficit stress conditions based on 700K SNPs.

Table S10. The significant and suggestive SNPs associated with climate variable and candidate genes underlying different loci of these variables based on 700K SNPs.

Protocol S1. R code

Acknowledgements

We would like to thank the Iran National Science Foundation (INSF), the Center of Excellence for Walnut Improvement and Technology of Iran, University of Tehran, and the University of California-Davis for their support. We also thank the California Walnut Board for funding the genotyping assay. The authors are also grateful to Randi Famula and Gina Maria Sideli for their technical assistance, and Brian Allen and Omid Gholami for their lab support.

References

Aletà N, Vilanova A, Díaz R, Voltas J. 2009. Genetic variation for carbon isotope composition in *Juglans regia* L.: relationships with growth, phenology and climate of origin. Annals of Forest Science **66**, 413.

Arab MM, Marrano A, Abdollahi-Arpanahi R, Leslie CA, Askari H, Neale DB, Vahdati K. 2019. Genome-wide patterns of population structure and association mapping of nut-related traits in Persian walnut populations from Iran using the Axiom J. regia 700K SNP array. Scientific Reports **9**, 6376.

Aradhya M, Velasco D, Ibrahimov Z, Toktoraliev B, Maghradze D, Musayev M, Bobokashvili Z, Preece JE. 2017. Genetic and ecological insights into glacial refugia of walnut (*Juglans regia* L.). PLoS One **12**, e0185974.

Bayazit S, Kazan K, Gülbitti S, Cevik V, Ayanoğlu H, Ergül A. 2007. AFLP analysis of genetic diversity in low chill requiring walnut (*Juglans regia* L.) genotypes from Hatay, Turkey. Scientia Horticulturae **111**, 394–398.

Benjamini Y, Hochberg Y. 1995. Controlling the false discovery rate: a practical and powerful approach to multiple testing. Journal of the Royal Statistical Society: Series B (Methodological) **57**, 289–300.

Bernard A, Lheureux F, Dirlewanger E. 2018. Walnut: past and future of genetic improvement. Tree Genetics & Genomes **14**, 1.

Blum A. 2009. Effective use of water (EUW) and not water-use efficiency (WUE) is the target of crop yield improvement under drought stress. Field Crops Research **112**, 119–123.

Downloaded from https://academic.oup.com/jxb/article/71/3/1107/5602662 by guest on 31 January 2024

Cao K, Zhou Z, Wang Q, *et al*. 2016. Genome-wide association study of 12 agronomic traits in peach. Nature Communications **7**, 13246.

Cochard H, Coll L, Le Roux X, Améglio T. 2002. Unraveling the effects of plant hydraulics on stomatal closure during water stress in walnut. Plant Physiology **128**, 282–290.

Cregg BM, Zhang JW. 2001. Physiology and morphology of *Pinus sylvestris* seedlings from diverse sources under cyclic drought stress. Forest Ecology and Management **154**, 131–139.

Crouzet J, Trombik T, Fraysse AS, Boutry M. 2006. Organization and function of the plant pleiotropic drug resistance ABC transporter family. FEBS Letters **580**, 1123–1130.

Cumbie WP, Eckert A, Wegrzyn J, Whetten R, Neale D, Goldfarb B. 2011. Association genetics of carbon isotope discrimination, height and foliar nitrogen in a natural population of *Pinus taeda* L. Heredity **107**, 105–114.

Curtin SJ, Tiffin P, Guhlin J, *et al*. 2017. Validating genome-wide association candidates controlling quantitative variation in nodulation. Plant Physiology **173**, 921–931.

Dhanapal AP, Ray JD, Singh SK, Hoyos-Villegas V, Smith JR, Purcell LC, Andy King C, Cregan PB, Song Q, Fritschi FB. 2015. Genome-wide association study (GWAS) of carbon isotope ratio ($\delta^{13}C$) in diverse soybean [*Glycine max* (L.) Merr.] genotypes. Theoretical and Applied Genetics **128**, 73–91.

Famula RA, Richards JH, Famula TR, Neale DB. 2019. Association genetics of carbon isotope discrimination and leaf morphology in a breeding population of *Juglans regia* L. Tree Genetics & Genomes **15**, 6.

Farquhar GD, Ehleringer JR, Hubick KT. 1989. Carbon isotope discrimination and photosynthesis. Annual Review of Plant Biology **40**, 503–37.

Franz S, Ehlert B, Liese A, Kurth J, Cazalé AC, Romeis T. 2011. Calcium-dependent protein kinase CPK21 functions in abiotic stress response in *Arabidopsis thaliana*. Molecular Plant **4**, 83–96.

Gao X, Starmer J, Martin ER. 2008. A multiple testing correction method for genetic association studies using correlated single nucleotide polymorphisms. Genetic Epidemiology **32**, 361–369.

Guerra FP, Richards JH, Fiehn O, Famula R, Stanton BJ, Shuren R, Sykes R, Davis MF, Neale DB. 2016. Analysis of the genetic variation in growth, ecophysiology, and chemical and metabolomic composition of wood of *Populus trichocarpa* provenances. Tree Genetics & Genomes **12**, 6.

Hadfield JD. 2010. MCMC methods for multi-response generalized linear mixed models: the MCMCglmm R package. Journal of Statistical Software **33**, 1–22.

Hijmans RJ, Cameron SE, Parra JL, Jones PG, Jarvis A. 2005. Very high resolution interpolated climate surfaces for global land areas. International Journal of Climatology **25**, 1965–78.

Jagodzik P, Tajdel-Zielinska M, Ciesla A, Marczak M, Ludwikow A. 2018. Mitogen-activated protein kinase cascades in plant hormone signaling. Frontiers in Plant Science **9**, 1387.

Jerszurki D, Couvreur V, Maxwell T, Silva LD, Matsumoto N, Shackel K, de Souza JL, Hopmans J. 2017. Impact of root growth and hydraulic conductance on canopy carbon–water relations of young walnut trees (*Juglans regia* L.) under drought. Scientia Horticulturae **226**, 342–52.

Jiang SC, Mei C, Liang S, Yu YT, Lu K, Wu Z, Wang XF, Zhang DP. 2015. Crucial roles of the pentatricopeptide repeat protein SOAR1 in Arabidopsis response to drought, salt and cold stresses. Plant Molecular Biology **88**, 369–385.

Jinagool W, Lamacque L, Delmas M, Delzon S, Cochard H, Herbette S. 2018. Is there variability for xylem vulnerability to cavitation in walnut tree cultivars and species (*Juglans* spp)? HortScience **53**, 132–7.

Kalladan R, Lasky JR, Chang TZ, Sharma S, Juenger TE, Verslues PE. 2017. Natural variation identifies genes affecting drought-induced abscisic acid accumulation in *Arabidopsis thaliana*. Proceedings of the National Academy of Sciences, USA **114**, 11536–11541.

Karimi S, Karami H, Mokhtassi-Bidgoli A, Tavallali V, Vahdati K. 2018. Inducing drought tolerance in greenhouse grown *Juglans regia* by imposing controlled salt stress: the role of osmotic adjustment. Scientia horticulturae **239**, 181–92.

Kassambara A, Mundt F. 2017. Package 'factoextra'. Extract and visualize the results of multivariate data analyses. http://www.sthda.com/english/rpkgs/factoextra.

Khan SA, Li MZ, Wang SM, Yin HJ. 2018. Revisiting the role of plant transcription factors in the battle against abiotic stress. International Journal of Molecular Sciences **19**, 1634.

Khodadadi F, Tohidfar M, Mohayeji M, Dandekar AM, Leslie CA, Kluepfel DA, Butterfield T, Vahdati K. 2016. Induction of polyphenol oxidase in walnut and its relationship to the pathogenic response to bacterial blight. Journal of the American Society for Horticultural Science **141**, 119–124.

Kitahata N, Saito S, Miyazawa Y, *et al*. 2005. Chemical regulation of abscisic acid catabolism in plants by cytochrome P450 inhibitors. Bioorganic & Medicinal Chemistry **13**, 4491–4498.

Kitsios G, Doonan JH. 2011. Cyclin dependent protein kinases and stress responses in plants. Plant Signaling & Behavior **6**, 204–209.

Knipfer T, Barrios-Masias FH, Cuneo IF, Bouda M, Albuquerque CP, Brodersen CR, Kluepfel DA, McElrone AJ. 2018. Variations in xylem embolism susceptibility under drought between intact saplings of three walnut species. Tree Physiology **38**, 1180–1192.

Körner C. 1999. Alpine plant life. Functional ecology of high mountain ecosystems. Berlin Heidelberg: Springer.

Laporte MM, Shen B, Tarczynski MC. 2002. Engineering for drought avoidance: expression of maize NADP-malic enzyme in tobacco results in altered stomatal function. Journal of Experimental Botany **53**, 699–705.

Lee S, Rojas C, Oh S, Kang M, Choudhury S, Lee HK, Allen R, Pandey S, Mysore K. 2018. Nucleolar GTP-binding protein 1-2 (NOG1-2) interacts with jasmonate-ZIMDomain protein 9 (JAZ9) to regulate stomatal aperture during plant immunity. International Journal of Molecular Sciences **19**, 1922.

Lewis CM, Knight J. 2012. Introduction to genetic association studies. Cold Spring Harbor Protocols **2012**, 297–306.

Lind BM, Friedline CJ, Wegrzyn JL, Maloney PE, Vogler DR, Neale DB, Eckert AJ. 2017. Water availability drives signatures of local adaptation in whitebark pine (*Pinus albicaulis* Engelm.) across fine spatial scales of the Lake Tahoe Basin, USA. Molecular Ecology **26**, 3168–3185.

Lipka AE, Tian F, Wang Q, Peiffer J, Li M, Bradbury PJ, Gore MA, Buckler ES, Zhang Z. 2012. GAPIT: genome association and prediction integrated tool. Bioinformatics **28**, 2397–2399.

Liu B, Liang J, Tang G, Wang X, Liu F, Zhao D. 2019. Drought stress affects on growth, water use efficiency, gas exchange and chlorophyll fluorescence of *Juglans* rootstocks. Scientia Horticulturae **250**, 230–235.

Liu X, Huang M, Fan B, Buckler ES, Zhang Z. 2016. Iterative usage of fixed and random effect models for powerful and efficient genome-wide association studies. PLoS Genetics **12**, e1005767.

Liu Y, Zhang X, Tran H, Shan L, Kim J, Childs K, Ervin EH, Frazier T, Zhao B. 2015. Assessment of drought tolerance of 49 switchgrass (*Panicum virgatum*) genotypes using physiological and morphological parameters. Biotechnology for Biofuels **8**, 152.

Lotfi N, Vahdati K, Hassani D, Kholdebarin B, Amiri R. 2009*a*. Peroxidase, guaiacol peroxidase and ascorbate peroxidase activity accumulation in leaves and roots of walnut trees in response to drought stress. Acta Horticulturae **861**, 309–316.

Lotfi N, Vahdati K, Kholdebarin B, Amiri R. 2010. Soluble sugars and proline accumulation play a role as effective indices for drought tolerant screening in Persian walnut (*Juglans regia* L) during germination. Fruits **65**, 97–112.

Lotfi N, Vahdati K, Kholdebarin B, Ashrafi EN. 2009*b*. Germination, mineral composition, and ion uptake in walnut under salinity conditions. HortScience **44**, 1352–1357.

Lu K, Liang S, Wu Z, Bi C, Yu YT, Wang XF, Zhang DP. 2016. Overexpression of an Arabidopsis cysteine-rich receptor-like protein kinase, CRK5, enhances abscisic acid sensitivity and confers drought tolerance. Journal of Experimental Botany **67**, 5009–5027.

Lyzenga WJ, Stone SL. 2012. Abiotic stress tolerance mediated by protein ubiquitination. Journal of Experimental Botany **63**, 599–616.

Mackay I, Powell W. 2007. Methods for linkage disequilibrium mapping in crops. Trends in Plant Science **12**, 57–63.

Marrano A, Martínez-García PJ, Bianco L, *et al*. 2019. A new genomic tool for walnut (*Juglans regia* L.): development and validation of the high-density Axiom™ *J. regia* 700K SNP genotyping array. Plant Biotechnology Journal **17**, 1027–1036.

Downloaded from https://academic.oup.com/jxb/article/71/3/1107/5602662 by guest on 31 January 2024

Marrano A, Micheletti D, Lorenzi S, Neale D, Grando MS. 2018. Genomic signatures of different adaptations to environmental stimuli between wild and cultivated *Vitis vinifera* L. Horticulture Research **5**, 34.

Martínez-García PJ, Crepeau MW, Puiu D, *et al*. 2016. The walnut (*Juglans regia*) genome sequence reveals diversity in genes coding for the biosynthesis of non-structural polyphenols. The Plant Journal **87**, 507–532.

Mickelbart MV, Hasegawa PM, Bailey-Serres J. 2015. Genetic mechanisms of abiotic stress tolerance that translate to crop yield stability. Nature Reviews. Genetics **16**, 237–251.

Mitchell N, Carlson JE, Holsinger KE. 2018. Correlated evolution between climate and suites of traits along a fast–slow continuum in the radiation of Protea. Ecology and Evolution **8**, 1853–1866.

Moles AT, Perkins SE, Laffan SW, *et al*. 2014. Which is a better predictor of plant traits: temperature or precipitation?. Journal of Vegetation Science **25**, 1167–1180.

Morrell PL, Toleno DM, Lundy KE, Clegg MT. 2005. Low levels of linkage disequilibrium in wild barley (*Hordeum vulgare* ssp. spontaneum) despite high rates of self-fertilization. Proceedings of the National Academy of Sciences, USA **102**, 2442–2447.

Müller M, Seifert S, Lübbe T, Leuschner C, Finkeldey R. 2017. De novo transcriptome assembly and analysis of differential gene expression in response to drought in European beech. PLoS One **12**, e0184167.

Myles S, Boyko AR, Owens CL, *et al*. 2011. Genetic structure and domestication history of the grape. Proceedings of the National Academy of Sciences, USA **108**, 3530–3535.

Postma FM, Ågren J. 2016. Early life stages contribute strongly to local adaptation in *Arabidopsis thaliana*. Proceedings of the National Academy of Sciences, USA **113**, 7590–7595.

Purcell S, Neale B, Todd-Brown K, *et al*. 2007. PLINK: a tool set for whole-genome association and population-based linkage analyses. American Journal of Human Genetics **81**, 559–575.

Rebetzke GJ, Condon AG, Richards RA, Farquhar GD. 2002. Selection for reduced carbon isotope discrimination increases aerial biomass and grain yield of rainfed bread wheat. Crop Science **42**, 739–45.

Rizhsky L, Liang H, Shuman J, Shulaev V, Davletova S, Mittler R. 2004. When defense pathways collide. The response of Arabidopsis to a combination of drought and heat stress. Plant Physiology **134**, 1683–1696.

Robinson D, Handley LL, Scrimgeour CM, Gordon DC, Forster BP, Ellis RP. 2000. Using stable isotope natural abundances (δ^{15}N and δ^{13}C) to integrate the stress responses of wild barley (*Hordeum spontaneum* C. Koch.) genotypes. Journal of Experimental Botany **51**, 41–50.

Román Á, Andreu V, Hernández ML, Lagunas B, Picorel R, Martínez-Rivas JM, Alfonso M. 2012. Contribution of the different omega-3 fatty acid desaturase genes to the cold response in soybean. Journal of Experimental Botany **63**, 4973–4982.

Rosati A, Metcalf S, Buchner R, Fulton A, Lampinen B. 2006. Tree water status and gas exchange in walnut under drought, high temperature and vapour pressure deficit. The Journal of Horticultural Science and Biotechnology **81**, 415–420.

Salekdeh GH, Reynolds M, Bennett J, Boyer J. 2009. Conceptual framework for drought phenotyping during molecular breeding. Trends in Plant Science **14**, 488–496.

Schulz P, Herde M, Romeis T. 2013. Calcium-dependent protein kinases: hubs in plant stress signaling and development. Plant Physiology **163**, 523–530.

Shin D, Moon SJ, Han S, *et al*. 2011. Expression of StMYB1R-1, a novel potato single MYB-like domain transcription factor, increases drought tolerance. Plant Physiology **155**, 421–432.

Sonah H, O'Donoughue L, Cober E, Rajcan I, Belzile F. 2015. Identification of loci governing eight agronomic traits using a GBS–GWAS approach and validation by QTL mapping in soya bean. Plant Biotechnology Journal **13**, 211–221.

Tian M, Yu G, He N, Hou J. 2016. Leaf morphological and anatomical traits from tropical to temperate coniferous forests: mechanisms and influencing factors. Scientific Reports **6**, 19703.

Tuberosa R, Salvi S. 2006. Genomics-based approaches to improve drought tolerance of crops. Trends in Plant Science **11**, 405–412.

Vahdati K, Lotfi N, Kholdebarin B, Hassani D, Amiri R, Mozaffari MR, Leslie C. 2009. Screening for drought-tolerant genotypes of Persian walnuts (*Juglans regia* L.) during seed germination. HortScience **44**, 1815–9.

Vangestel C, Eckert AJ, Wegrzyn JL, Clair JB, Neale DB. 2018. Linking phenotype, genotype and environment to unravel genetic components underlying cold hardiness in coastal Douglas-fir (*Pseudotsuga menziesii* var. *menziesii*). Tree Genetics & Genomes **14**, 10.

Wagner TA, Kohorn BD. 2001. Wall-associated kinases are expressed throughout plant development and are required for cell expansion. The Plant Cell **13**, 303–318.

Wang H, Zhao S, Mao K, Dong Q, Liang B, Li C, Wei Z, Li M, Ma F. 2018. Mapping QTLs for water-use efficiency reveals the potential candidate genes involved in regulating the trait in apple under drought stress. BMC Plant Biology **18**, 136.

Wang HS, Yu C, Tang XF, Zhu ZJ, Ma NN, Meng QW. 2014. A tomato endoplasmic reticulum (ER)-type omega-3 fatty acid desaturase (LeFAD3) functions in early seedling tolerance to salinity stress. Plant Cell Reports **33**, 131–142.

Wang Q, Tian F, Pan Y, Buckler ES, Zhang Z. 2014. A SUPER powerful method for genome wide association study. PLoS One **9**, e107684.

Wang X, Cai X, Xu C, Wang Q, Dai S. 2016. Drought-responsive mechanisms in plant leaves revealed by proteomics. International Journal of Molecular Sciences **17**, 1706.

Wang X, Chen ZH, Yang C, *et al*. 2018. Genomic adaptation to drought in wild barley is driven by edaphic natural selection at the Tabigha evolution slope. Proceedings of the National Academy of Sciences, USA **115**, 5223–5228.

Wickham H. 2016. ggplot2: elegant graphics for data analysis. New York: Springer-Verlag.

Wilkinson S, Davies WJ. 2002. ABA-based chemical signalling: the co-ordination of responses to stress in plants. Plant, Cell & Environment **25**, 195–210.

Wójcik-Jagła M, Rapacz M, Tyrka M, Kościelniak J, Crissy K, Zmuda K. 2013. Comparative QTL analysis of early short-time drought tolerance in Polish fodder and malting spring barleys. Theoretical and Applied Genetics **126**, 3021–3034.

Yang G, Zhang W, Liu Z, Yi-Maer AY, Zhai M, Xu Z. 2017. Both JrWRKY2 and JrWRKY7 of *Juglans regia* mediate responses to abiotic stresses and abscisic acid through formation of homodimers and interaction. Plant Biology **19**, 268–278.

Ye Y, Ding Y, Jiang Q, Wang F, Sun J, Zhu C. 2017. The role of receptor-like protein kinases (RLKs) in abiotic stress response in plants. Plant Cell Reports **36**, 235–242.

Yu J, Pressoir G, Briggs WH, *et al*. 2006. A unified mixed-model method for association mapping that accounts for multiple levels of relatedness. Nature Genetics **38**, 203–208.

Zhai M, Sun Y, Jia C, Peng S, Liu Z, Yang G. 2016. Over-expression of *JrsHSP17.3* gene from *Juglans regia* confer the tolerance to abnormal temperature and NaCl stresses. Journal of Plant Biology **59**, 549–58.

Zhang J, Jia W, Yang J, Ismail AM. 2006. Role of ABA in integrating plant responses to drought and salt stresses. Field Crops Research **97**, 111–9.

Zomer RJ, Trabucco A, Bossio DA, Verchot LV. 2008. Climate change mitigation: a spatial analysis of global land suitability for clean development mechanism afforestation and reforestation. Agriculture, Ecosystems & Environment **126**, 67–80.

Downloaded from https://academic.oup.com/jxb/article/71/3/1107/5602662 by guest on 31 January 2024
