# Supplementary material for: Provenance and family variations in early growth of Manchurian walnut (Juglans mandshurica Maxim.) and selection of superior families
Source: PLoS One. 2024 Mar 7;19(3):e0298918. doi: 10.1371/journal.pone.0298918 (PMC10919699; doi:10.1371/journal.pone.0298918)
Supplement: S1 File — (ZIP) [file pone.0298918.s004.zip › Comparative physiology of allopatric Populus species geographic clines in photosynthesis, height growth, and carbon isotope discrimination in common gardens.pdf]

frontiers in Plant Science

ORIGINAL RESEARCH
published: 14 July 2015
doi: 10.3389/fpls.2015.00528

Comparative physiology of allopatric *Populus* species: geographic clines in photosynthesis, height growth, and carbon isotope discrimination in common gardens

Raju Y. Soolanayakanahally[1,2]*, Robert D. Guy[2], Nathaniel R. Street[3], Kathryn M. Robinson[3], Salim N. Silim[1], Benedicte R. Albrectsen[3] and Stefan Jansson[3]

[1] Agroforestry Development Centre, Agriculture and Agri-Food Canada, Indian Head, SK, Canada, [2] Department of Forest and Conservation Sciences, University of British Columbia, Vancouver, BC, Canada, [3] Department of Plant Physiology, Umeå Plant Science Centre, Umeå University, Umeå, Sweden

OPEN ACCESS

Edited by:
Glenn Thomas Howe,
Oregon State University, USA

Reviewed by:
Rebekka Bögelein,
University of Trier, Germany
Erwin Dreyer,
Institut National de la Recherche Agronomique, France

***Correspondence:**
Raju Y. Soolanayakanahally,
Agroforestry Development Centre,
Agriculture and Agri-Food Canada, #2 Government Road, Indian Head, SK, S0G 2K0, Canada
raju.soolanayakanahally@agr.gc.ca

Specialty section:
This article was submitted to Functional Plant Ecology, a section of the journal Frontiers in Plant Science

Received: 29 August 2014
Accepted: 29 June 2015
Published: 14 July 2015

Citation:
Soolanayakanahally RY, Guy RD, Street NR, Robinson KM, Silim SN, Albrectsen BR and Jansson S (2015) Comparative physiology of allopatric Populus species: geographic clines in photosynthesis, height growth, and carbon isotope discrimination in common gardens. Front. Plant Sci. 6:528. doi: 10.3389/fpls.2015.00528

Populus species with wide geographic ranges display strong adaptation to local environments. We studied the clinal patterns in phenology and ecophysiology in allopatric *Populus* species adapted to similar environments on different continents under common garden settings. As a result of climatic adaptation, both *Populus tremula* L. and *Populus balsamifera* L. display latitudinal clines in photosynthetic rates (*A*), whereby high-latitude trees of *P. tremula* had higher *A* compared to low-latitude trees and nearly so in *P. balsamifera* ($p = 0.06$). Stomatal conductance (g_s) and chlorophyll content index (CCI) follow similar latitudinal trends. However, foliar nitrogen was positively correlated with latitude in *P. balsamifera* and negatively correlated in *P. tremula*. No significant trends in carbon isotope composition of the leaf tissue ($\delta^{13}C$) were observed for both species; but, intrinsic water-use efficiency (WUE_i) was negatively correlated with the latitude of origin in *P. balsamifera*. In spite of intrinsically higher *A*, high-latitude trees in both common gardens accomplished less height gain as a result of early bud set. Thus, shoot biomass was determined by height elongation duration (HED), which was well approximated by the number of days available for free growth between bud flush and bud set. We highlight the shortcoming of unreplicated outdoor common gardens for tree improvement and the crucial role of photoperiod in limiting height growth, further complicating interpretation of other secondary effects.

Keywords: comparative physiology, poplar, common garden, latitude, bud set, photosynthesis, carbon isotope discrimination, water-use efficiency

Introduction

The "common garden" approach (Kawecki and Ebert, 2004), whereby samples of genotypes collected from different populations are directly compared under the same environmental conditions, either outdoors in a greenhouse, or in a growth chamber, is widely used to study local adaptation. Since the late 18th century, field "provenance trials" have been used in forestry to ensure that sources of seed produce well-adapted, productive trees for reforestation or afforestation

(Linhart and Grant, 1996). Common garden experiments or provenance trials also provide material for tree breeding and *ex-situ* conservation. Through association studies, common gardens that include accessions from a large number of populations have become extremely useful in uncovering the molecular-genetic basis for differences in physiological traits and their functional regulation (McKown et al., 2013a, 2014). They also yield invaluable data to model and predict tree responses to climate change (Wang et al., 2006), especially when replicated across the landscape (e.g., Rehfeldt et al., 1999).

Phenotypic selection studies, including those that describe the relationships of plant physiological traits across latitudes, can be used to examine the evolution of particular traits and to test adaptive hypotheses. Variations in growth rates and morphological characters have been well documented in latitudinally separated populations that have been sampled or collected over vast geographic ranges. In this context, latitude is really a proxy for environmental factors that drive selection, such as growing season length, photoperiod, temperature, and water availability. The general understanding is that high-latitude plants grow more slowly and are shorter in height compared to plants from low latitudes (Luquez et al., 2007; Moles et al., 2009). Differences in stature, however, may depend more on phenology and environment than on intrinsic growth potential. Indeed, high latitude populations of balsam poplar (*Populus balsamifera* L., section: *Tacamahaca*) are capable of higher rates of carbon assimilation (*A*) and height growth than their southern counterparts when freed from photoperiodic restrictions (Soolanayakanahally et al., 2009). The timing of height growth cessation is inversely correlated with the latitude of origin, and photoperiodic responses may be the only major adaptive mechanism responsible for this phenomenon (Schnekenburger and Farmer, 1989). Quite similarly, Howe et al. (1995) observed a longer critical photoperiod for bud set in *P. trichocarpa* ecotypes from 53°N compared to ecotypes from 34°N.

Ecophysiological attributes such as photosynthesis, respiration, leaf nitrogen (N), and growth phenology all vary with leaf lifespan (Wright et al., 2004a). Trait correlations across major plant functional groups strongly associate leaf lifespan and leaf mass per area (LMA) with climate variation (Wright et al., 2004b). Species with short-lived foliage generally have higher *A*, owing to trade-offs in carbon and nitrogen allocation between biochemistry and structure. A strong positive correlation between photosynthesis of a leaf and its nitrogen concentration is well recognized in plant species (Evans, 1989). Even in deciduous trees, where the green-cover period (GCP) is less than 1 year, there is a negative relationship between *A* and leaf lifespan among (Reich et al., 1995) and within (e.g., Gornall and Guy, 2007) species. Classic studies of adaptive variation in *Oxyria digyna* (Mooney and Billings, 1961; Billings et al., 1971) indicated a latitudinal cline in photosynthesis toward higher *A* in arctic ecotypes. Particularly in *Populus* species occupying large geographic areas, enhanced photosynthetic rates are driven by larger stomatal conductance (g_s) (Gornall and Guy, 2007; Soolanayakanahally et al., 2009; McKown et al., 2013a). The existence of such clines, especially when common to more than one species along the same or similar environmental gradient, constitutes strong evidence for adaptive trait selection (Endler, 1973).

Landscape patterns in ecophysiological traits of vegetation are beginning to be recognized at species and community levels, but rarely within species, which can be at odds with higher levels of organization. For example, Soolanayakanahally et al. (2009) reported that LMA increases with latitude of origin within balsam poplar, whereas in a meta-analysis of data for 2548 species, Wright et al. (2004b) found the opposite trend across biomes. In this study, we examine latitudinal patterns in photosynthesis as an adaptation to growing season length in allopatric species of *Populus*. The genus *Populus* presents many advantages to comparative physiologists seeking to understand the biological significance of physiological adaptation, including ease of propagation, access to large collections of native germplasm from temperate through sub-arctic habitats, and substantial genetic tools and resources.

Stomata partly determine intrinsic water-use efficiency (WUE_i), which is important to plant productivity, by mediating the diffusion of CO_2 into leaves and water vapor out. Assimilation-averaged water-use efficiency can be estimated *via* its correlation with the stable carbon isotopic composition of plant tissues (Farquhar et al., 1982). The carbon isotope ratio ($\delta^{13}C$) of plant tissue provides an integrated measure of internal plant physiological and external environmental properties influencing photosynthesis over the time when the carbon was fixed (Anderson et al., 1996). Variations in WUE_i and $\delta^{13}C$ have been well characterized along geographic gradients in moisture, temperature, and nutrients in many species. The variations in $\delta^{13}C$ have adaptive significance in *Pinus ponderosa* Dougl. *ex* Laws. (Zhang et al., 1997), *Chrysothamnus nauseosus* (Donovan and Ehleringer, 1994), *Pinus contorta* Dougl. *ex* Loud. (Guy and Holowachuk, 2001), and *Populus nigra* L. (Chamaillard et al., 2011).

Although species of different origin can display similar clines in phenotypic traits as a result of natural selection, a preferred phenotype may be achieved by different combinations of underlying traits. For example, latitudinal variation in *A* is associated with higher g_s among field-grown populations of black cottonwood (*Populus trichocarpa* Torr. & Gray, section: *Tacamahaca*) (Gornall and Guy, 2007), but not in greenhouse-grown balsam poplar (Soolanayakanahally et al., 2009), with consequent effects on respective trends in WUE_i and $\delta^{13}C$. This is somewhat surprising given that these two North American species are very closely related and hybridize extensively where their ranges overlap (Farrar, 1995). We wondered, therefore, whether similar patterns in *A*, g_s, and related ecophysiological traits would be found in a more disparate member of the genus *Populus*. In this study, we compared data of different *Populus* species, grown under natural conditions in common gardens in the field, to understand commonalities and differences within the genus. Relevant data were already available for *P. trichocarpa*, so we generated similar datasets for *P. tremula* and *P. balsamifera*. We then used the combined dataset to answer the following questions:

1. Do latitudinal clines in A exist in allopatric *Populus* species adapted to similar environments on different continents when measured during active growth in common gardens?
2. Are clinal patterns in height growth better explained by timing of bud set or by photosynthetic assimilation rates?

Material and Methods

Balsam Poplar Common Garden

Balsam poplar has a continuous natural range from Alaska to Newfoundland and south to Michigan. The plant material used here is a subset of the larger Agriculture Canada Balsam Poplar (AgCanBaP) collection consisting of 65 provenances (Soolanayakanahally et al., 2013). For the present purposes, five populations (**Table 1**) with 15 genotypes per population were planted into an outdoor common garden at Indian Head (50.33° N 103.39° W), Canada in mid-August 2005. This location is near the southern edge of the species range where photoperiod is always limiting during summer for most of the high-latitude populations. Stem cuttings (6–9 cm in length) with a minimum of two buds were rooted in Spencer-Lemaire RT 420A Rootrainer® containers (Beaver Plastics, Acheson, Canada) filled with a mixture of Sunshine-2 (Sun Gro Horticulture, Vancouver, Canada) growing mix (60%), peat (30%), and vermiculite (10%). The rooted cuttings were grown in a greenhouse with natural light supplemented by cool-white fluorescent lamps to provide a 19-h photoperiod and a minimum PPFD of 400 μmol m^{-2} s^{-1} at plant level. Maximum day and night temperatures were maintained close to 25 and 18°C, respectively. Upon flushing, the cuttings were kept well watered and fertilized weekly with ½-strength Hoagland's solution (Hoagland and Arnon, 1950). When the plants were approximately 45 cm tall, they were moved to a shade house for a period of 2 weeks before planting in a complete block design with three replicates per genotype spaced two meters apart. At the common garden site, maximal photoperiod is 16 h 12 min, with mean annual precipitation of 435 mm, and mean maximum and minimum temperatures during the growing season (May–August) of 22 and 8°C, respectively. The year in which measurements were taken, 2007, was relatively wet. The garden was weeded annually.

Aspen Common Garden

Eurasian aspen is distributed throughout Europe from the Mediterranean to northern Scandinavia and eastwards to Siberia and central Asia. We used the Swedish Aspen (SwAsp) collection planted at Sävar (63.80° N 20.30° E) in June 2004. The SwAsp collection contains 116 genotypes collected from 12 localities in Sweden (**Table 1**, 10 genotypes per population except for Luleå where only six genotypes were sampled). For more information on the collection and establishment of the common garden, refer to Luquez et al. (2007). In contrast to balsam poplar, the aspen common garden is located close to the northern edge of the species range, and photoperiod is not limiting during summer. At Sävar, the maximal photoperiod is 20 h 53 min, with mean annual precipitation of 482 mm, and mean maximum and minimum temperatures during the growing season (May–August) of 23 and 6°C, respectively. Spot weeding was done annually within a 0.5 m perimeter around each tree.

TABLE 1 | Geographic coordinates and mean elevation of origin for populations established in common gardens, by species.

Species	Population	Latitude	Longitude	Elevation
P. balsamifera (North America)	Fredericton (FRE)	46.40	67.25	147
	Rouyn Noranda (RNA)	48.60	78.67	310
	Love (LOV)	53.63	105.50	419
	Grand Prairie (GPR)	54.75	118.63	769
	White Horse (WHR)	60.70	135.33	770
P. tremula (Europe)	Ronneby	56.27	15.21	49
	Simlång	56.71	13.25	173
	Ydre	57.79	15.28	219
	Vårgårda	57.99	12.93	158
	Brunsberg	59.63	12.96	84
	Uppsala	59.81	17.91	17
	Älvdalen	61.22	13.97	354
	Delsbo	61.73	16.71	98
	Umeå	63.93	20.63	37
	Dorotea	64.36	16.44	382
	Luleå	65.62	22.19	13
	Arjeplog	66.20	18.43	445

Latitude (° N), longitude (° W, North America and ° E, Europe), elevation (m).

Gas Exchange Measurements

In both species, measurements were taken during active growth (i.e., well before bud set) in the absence of any water stress. For balsam poplar, measurements were made in the first 2 weeks of July 2007 on five populations (six trees per population; $n = 30$). For aspen, measurements were made on all 116 genotypes from June 24 to July 10 2008. Gas exchange was measured on all trees once, with the measurement tree randomized among populations and days of measurement.

Carbon assimilation (A), measured as light saturated photosynthesis, and stomatal conductance (g_s) were measured on clear, sunny days using a LC *Pro*$^+$ portable gas exchange system (Analytical Development Co., Ltd., Hoddesdon, UK). Measurements were made between 9:00 and 12:00 h on fully expanded sun leaves on each tree. CO_2 concentrations inside the cuvette were 360–370 μL L^{-1}. Air temperature was maintained at 25°C to yield a leaf temperature of approximately 26.5°C. Relative humidity was 60–70%, resulting in a leaf-to-air vapor pressure difference of 1.24–1.56 kPa. PPFD was 1500 μmol m^{-2} s^{-1} supplied by a mixed red/blue LED unit mounted on top of the cuvette. After stabilization of intercellular CO_2 concentration (C_i), three measurements were recorded over a period of 3 min and averaged to determine A (μmol CO_2 m^{-2} s^{-1}) and g_s (mol H_2O m^{-2} s^{-1}) following von Caemmerer and Farquhar (1981). The WUE_i was calculated as the ratio of A to g_s (i.e., μmol CO_2 mol^{-1} H_2O).

Following gas exchange measurements, the chlorophyll content index (CCI) was determined on five leaves per tree with an Opti-Sciences CCM-200 meter (Hudson, NH, USA). The CCM-200 uses calibrated light emitting diodes and receptors

to calculate the CCI, which is defined as the ratio of light transmission through the leaf at 931 nm to that at 653 nm. The efficacy of CCI for rapid and non-destructive estimation of relative total chlorophyll or foliar nitrogen content is well-established for many forest tree species (van den Berg and Perkins, 2004).

Leaf Tissue Sampling

Whole leaf samples were collected from 75 balsam poplar trees (i.e., 15 genotypes × 5 populations) and 348 aspen trees (i.e., 116 genotypes × 3 ramets/genotype) after the gas exchange measurements were completed. These were used for analysis of $\delta^{13}C$ and nitrogen content (Leaf N, µmol N cm^{-2}). Leaf tissue was dried to constant mass and then ground to fine powder. Homogenized subsamples of ~2.5 mg were packed in tin capsules and sent to the University of California at Davis Stable Isotope Facility for combustion and analysis by an online continuous flow dual analyzer coupled to an isotope ratio mass spectrometer (Europa Scientific Integra, Cheshire, England, UK). The $\delta^{13}C$ value of the leaf tissue is reported in per mil (‰) units relative to the arbitrary standard Vienna Pee Dee Belemnite (VPDB):

$$\delta^{13}C = [(^{13}C/^{12}C)_{\text{Sample}} - (^{13}C/^{12}C)_{\text{VPDB}}]/(^{13}C/^{12}C)_{\text{VPDB}} \times 1000$$

The overall, long-term sample preparation and analysis error between repeated analyses of the same ground tissue was less than ±0.11‰.

Growth Measurements

Final height of all genotypes was measured after bud set in 2007 for balsam poplar and 2008 for aspen. The 30 balsam poplar trees used for gas exchange measurements were harvested for shoot biomass determination at the end of the 2007 growing season. Numbers of leaves were counted for each tree and the leaf area was measured using a LI-3100 leaf area meter (LI-COR Biosciences, Lincoln, NE, USA). Dry mass was recorded after the stems and leaves were oven-dried to a constant weight. LMA was expressed as the leaf mass to area ratio.

Seasonal Phenology

In both species, spring and autumn phenology, characterized by dates of bud flush and bud set, respectively, were monitored twice weekly. The phenological stages and their characteristics are described in Soolanayakanahally et al. (2013). A scale of 0–10 was used to describe the different phenological stages, where 0 represented a spring stage where the buds were still closed and non-swollen, and 10 an autumn stage when there was complete leaf senescence. Height elongation duration (HED) was calculated as the number of days between bud flush and bud set. For balsam poplar, the length of the GCP, defined as the number of days from bud flush to when 80% of the leaves had abscised, was also recorded.

Statistical Analysis

Data analyses were conducted in SigmaStat version 2.03. Pearson's correlation coefficients (r) among physiology, growth, and geographic variables were calculated to determine the relationships among all variables across genotypes. Later, Bonferroni correction was applied to minimize the chances of making a Type I error. Multiple linear regressions were carried out on the data to select latitude as a common descriptive variable. The population means with standard errors are reported.

Results

For both of the collections used in this study, latitude, longitude, and elevation of origin are confounded, particularly in balsam poplar (**Table 1**). Although we report correlation coefficients between physiological variables and all three geographic descriptors in **Tables 2, 3**, relationships with longitude and/or elevation were in every case not significant after latitude was accounted for in multiple linear regressions (not presented). In balsam poplar, the number of frost-free days (which would tend to include the independent effects of latitude and elevation on temperature and growing season length) is actually a marginally better predictor than latitude in several cases (also not presented). However, for comparison to the other data sets generated or used in this study, we focus on latitude.

During active growth (i.e., before bud set) field gas exchange measurements showed significantly higher A in aspen populations coming from high latitudes compared with those from low latitudes ($p = 0.001$; **Figure 1B**). However, there was a non-significant correlation between A and latitude in balsam poplar ($p = 0.066$; **Figure 1A**). Mean photosynthetic rate was higher in balsam poplar compared to aspen, but differences between populations (as a function of latitude of origin) were more pronounced in aspen. Variation in stomatal conductance (g_s), as a function of latitude, paralleled variation in A in aspen (**Tables 3**) and the correlation between A and g_s was very

TABLE 2 | Pearson's correlation coefficients among physiology, growth, and geographic variables in *P. balsamifera* populations.

Variable	Latitude	Longitude	Elevation
A	0.853	0.865	0.825
g_s	**0.928**	**0.894**	0.845
WUE_i	**−0.944**	**−0.885**	−0.802
$\delta^{13}C$	−0.668	−0.590	−0.582
Leaf N	**0.968***	**0.964***	**0.884**
CCI	**0.940**	**0.944**	**0.924**
LMA	**0.999***	**0.985***	**0.911**
GCP	−0.852	−0.759	−0.645
HED	**−0.989***	**−0.962***	−0.860
Height	**−0.941**	**−0.879**	−0.782
Biomass	**−0.904**	**−0.895**	−0.802

Significant correlations are in bold ($p < 0.05$) or followed by an asterisk ($p < 0.01$). A, assimilation rate (µmol CO_2 m^{-2} s^{-1}); g_s, stomatal conductance (mol H_2O m^{-2} s^{-1}); WUE_i, intrinsic water use efficiency (µmol CO_2 mol^{-1} H_2O); $\delta^{13}C$, carbon isotope composition of leaf (‰); Leaf N, leaf nitrogen density (µmol N cm^{-2}); CCI, chlorophyll content index; LMA, leaf mass area (mg cm^{-2}); GCP, green-cover period (days); HED, height elongation duration (days); Height (cm) and Biomass (g).

strong for both *P. balsamifera* ($r = 0.816$) and *P. tremula* ($r = 0.717$).

WUE_i was negatively related with latitude in balsam poplar ($p < 0.05$, **Table 2**), however, $\delta^{13}C$ was non-significant with latitude ($p > 0.05$, **Table 2**). In aspen, both WUE_i and $\delta^{13}C$ were non-significant with latitude of origin ($p > 0.05$, **Table 3**). But, WUE_i and $\delta^{13}C$ positively correlated with each other in balsam poplar (Supplement 1). Changes in $\delta^{13}C$ as a function of latitude were similar in the two species (**Figures 1C,D**). In balsam poplar, WUE_i ranged from 57.9 to 69.2 μmol CO_2 mol^{-1} H_2O among populations, whereas in aspen it was from 43.3 to 59.1 μmol CO_2 mol^{-1} H_2O. The species difference in WUE_i was also reflected in $\delta^{13}C$ values, which ranged from −26.66 to −27.64‰ in balsam poplar and −28.51 to −29.29‰ in aspen. Trees with more negative $\delta^{13}C$ values (balsam poplar) or lower WUE_i (balsam poplar and aspen) had higher g_s (Supplement 1).

TABLE 3 | Pearson's correlation coefficients among physiology, growth, and geographic variables in *P. tremula* populations.

Variables	Latitude	Longitude	Elevation
A	**0.798***	**0.709***	0.158
g_s	**0.712***	0.575	0.264
WUE_i	−0.564	−0.402	−0.269
$\delta^{13}C$	−0.572	−0.262	−0.525
Leaf N	**−0.869***	**−0.609**	−0.224
CCI	**0.832***	**0.695**	0.365
LMA	−0.443	−0.346	−0.139
HED	**−0.945***	**−0.664**	−0.338
Height	**−0.905***	**−0.645**	−0.331

Significant correlations are in bold ($p < 0.05$) or followed by an asterisk ($p < 0.01$). A, assimilation rate ($\mu mol\ CO_2\ m^{-2}\ s^{-1}$); g_s, stomatal conductance ($mol\ H_2O\ m^{-2}\ s^{-1}$); WUE_i, intrinsic water use efficiency ($\mu mol\ CO_2\ mol^{-1}\ H_2O$); $\delta^{13}C$, carbon isotope composition of leaf (‰); Leaf N, leaf nitrogen density ($\mu mol\ N\ cm^{-2}$); CCI, chlorophyll content index; LMA, leaf mass area ($mg\ cm^{-2}$); HED, height elongation duration (days) and Height (cm).

Leaf N increased significantly with latitude, longitude, and elevation in balsam poplar (**Table 2** and **Figure 2C**). Both Leaf N and LMA were strongly positively correlated with all three geographic parameters. Nitrogen per unit mass was quite uniform (not presented). Therefore, enhanced *A* with latitude was associated with increased Leaf N more so than g_s. The overriding influence of g_s was even more apparent in aspen, where Leaf N decreased significantly with latitude (**Table 3**, **Figure 2D**), while *A* was enhanced, again consistent with a reduced water-use efficiency. In sharp contrast to balsam poplar (**Figure 2A**), however, LMA in aspen had no correlation with latitude when calculated across all 116 genotypes (not presented) or across the 12 population means (**Table 3**, **Figure 2B**). Luleå, a northern population in Sweden, seemed to have an anomalously low LMA. Leaf N density was positively correlated with $\delta^{13}C$ in aspen genotypes, but not with $\delta^{13}C$ in balsam poplar (Supplement 1).

Although higher rates of *A* were not associated with higher Leaf N in aspen (in contrast to balsam poplar), they

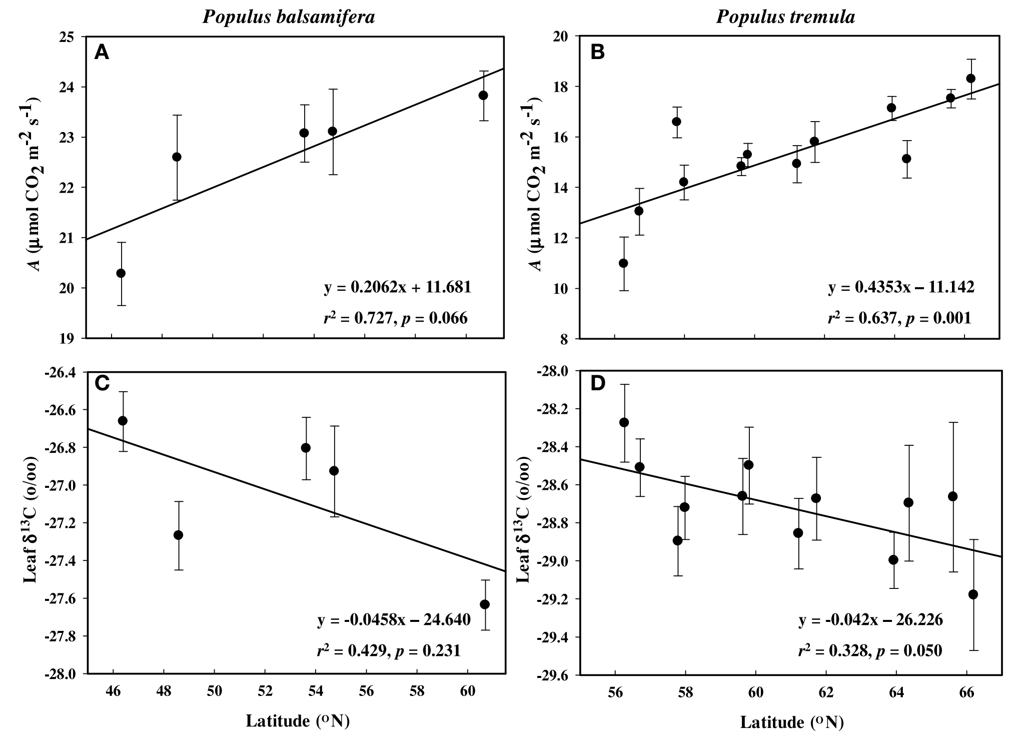

FIGURE 1 | Mean net assimilation rate (*A*) and leaf $\delta^{13}C$ (‰) across latitude measured during active growth in common gardens. (A,C) *P. balsamifera* and **(B,D)** *P. tremula*. Error bars are ±SE of the means.

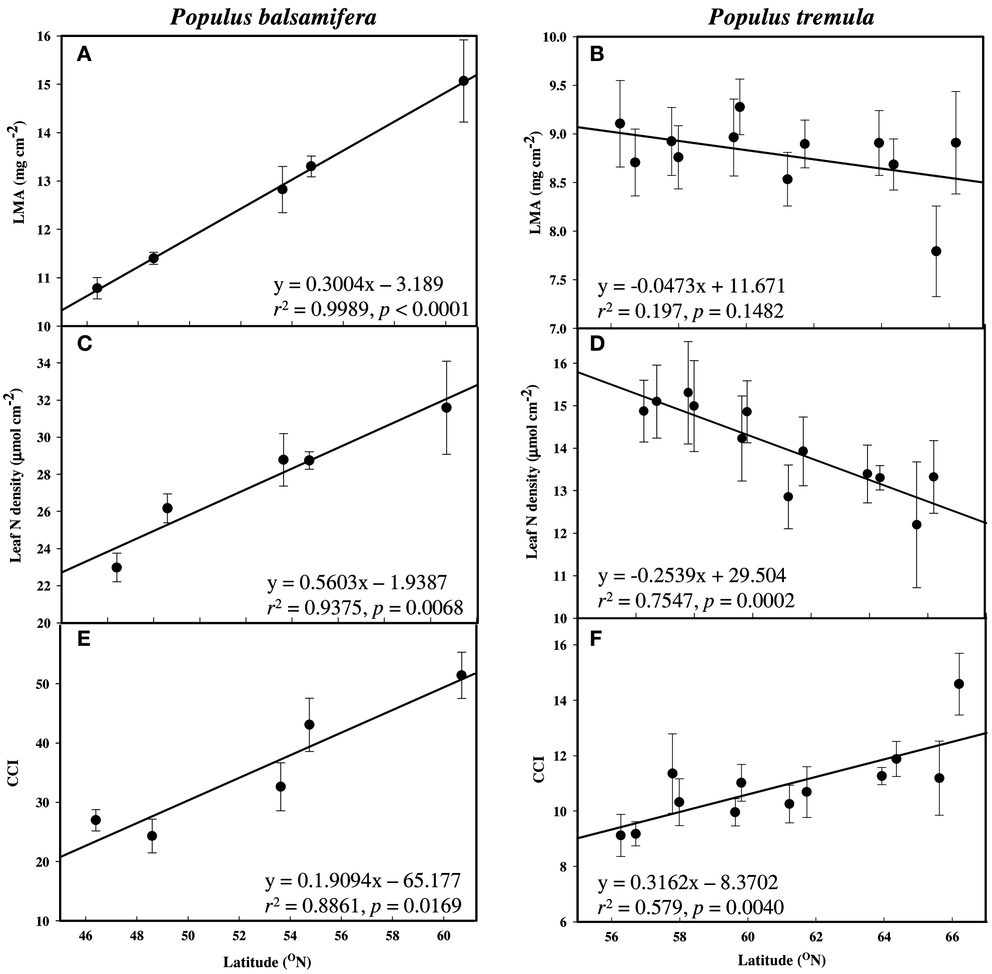

FIGURE 2 | Mean leaf mass area (LMA), leaf nitrogen density (Leaf N) and chlorophyll content index (CCI) across latitude measured during active growth in common gardens. (A,C,E) *P. balsamifera* and **(B,D,F)** *P. tremula*. Error bars are ±SE of the means.

were supported by higher chlorophyll per unit leaf area, as indicated by CCI. CCI was positively and significantly related to latitude (**Figures 2E,F**) and longitude in both species, and most particularly in balsam poplar (**Tables 2**, **3**).

In both balsam poplar and aspen, HED and height had strong inverse relationships with latitude (**Tables 2, 3**, **Figure 3**). Regardless of provenance, buds flushed within a few days of each other as soon as spring temperatures permitted, whereas dates of bud set spanned a much greater range. For example, in 2007, the White Horse balsam poplar population set bud in the first week of August ($\bar{X}$ = Julian day 217) whereas the Fredericton population set bud in the first week of September ($\bar{X}$ = Julian day 247). High-latitude populations set bud early compared to low-latitude populations and population rankings were consistent year-to-year in both common gardens (not shown).

Balsam poplar shoot biomass was determined at the end of the third growing season and HED was negatively correlated with latitude of origin (**Table 2**). HED ($r^2 = 0.958$; $p = 0.0037$) was a better predictor of shoot biomass than GCP (**Figure 4**, Supplement 2). Because *A* decreases with HED while biomass increases, biomass and *A* were negatively correlated ($r = -0.422$, $p < 0.05$). Mean shoot biomass was four-fold greater for the population from Fredericton (low-latitude) than for the population from White Horse (high-latitude) ($r = -0.904$, $p < 0.001$, **Table 2**). GCP exceeded HED by an average of 67 days, ranging from 50 to 78 days across populations with no clear latitudinal trend.

Discussion

Photosynthesis in Balsam Poplar and Aspen

In spite of relatively short photoperiods at the Indian Head common garden, and less so at Sävar, all genotypes went through a period of active growth before they set bud. Photosynthetic rates measured during this period increased with increasing latitude of origin in aspen but not in balsam poplar. The studies by Benowicz et al. (2000), Gornall and Guy (2007), and Soolanayakanahally et al. (2009) suggest that a greater rate of photosynthesis in high-latitude tree genotypes is an adaptation to short growing season length. A trend toward increasing rates of net photosynthesis in

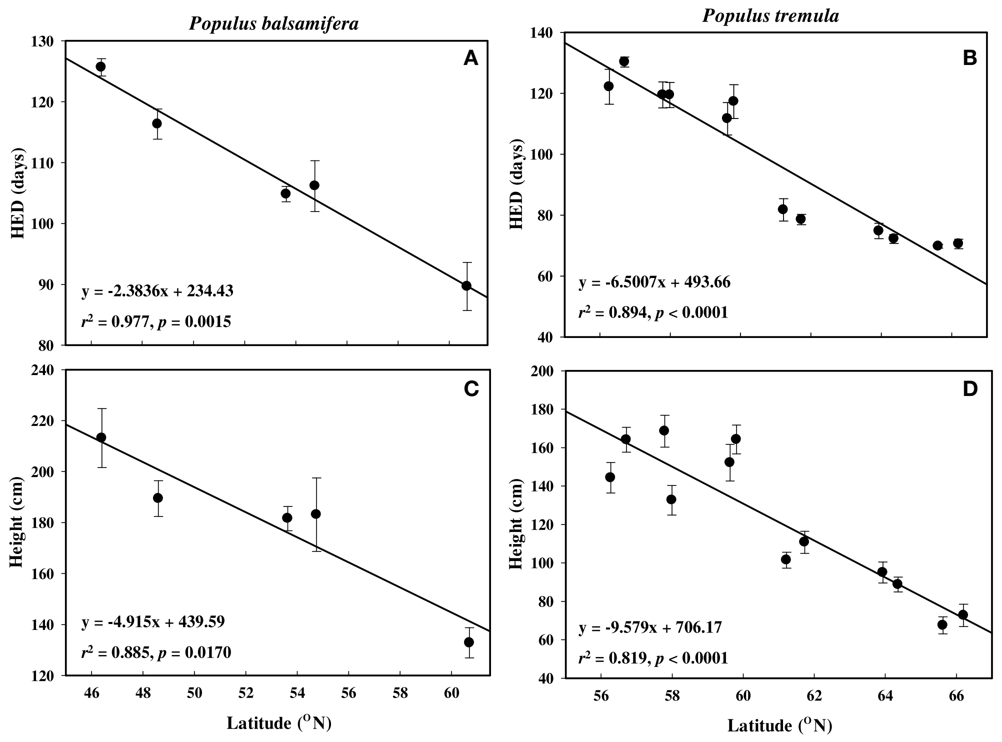

FIGURE 3 | Mean height elongation duration (HED) and height growth across latitude used in this study. (A,C) *P. balsamifera* and **(B,D)** *P. tremula*. Error bars are ±SE of the means.

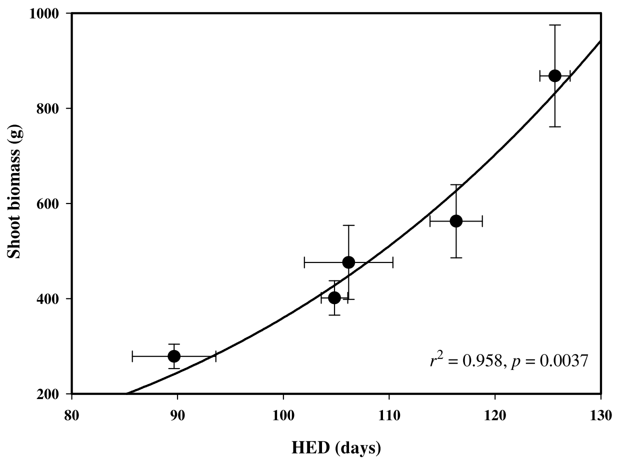

FIGURE 4 | Relationship between shoot biomass and height elongation duration (HED) among populations of *P. balsamifera* at the Indian Head common garden (50.33° N, 105.73° W). Non-linear regression Equation (2) ($f = y_0 + a * x$) is plotted. Error bars ± standard errors of the means.

relation to increasing latitude of origin has been seen in several plants (Reich et al., 1996), and even lichens (Schipperges et al., 1995). A similar relationship appears to exist with elevation. Ovaska (1988) found that mountain birch populations from high elevations had greater leaf photosynthetic rates per unit area than those from low elevation. Likewise, Oleksyn et al. (1998) reported that high elevation populations of Norway spruce [*Picea abies* (L.) Karst.] had higher photosynthetic rates than did populations from lower elevations. Higher *A* during active growth in short-growing season genotypes does not necessarily persist beyond budset. Johnsen et al. (1996) found that black spruce [*Picea mariana* (Mill) B.S.P.] trees from Yukon (63° N) had higher *A* than trees from Ontario (45° N) when measured in a 23-year-old common garden during the peak of summer (June–July), but this ranking was reversed when measurements were made later in the season (September–October; i.e., after bud set).

Higher photosynthetic rates in high elevation populations of Norway spruce were supported by a higher percentage of nitrogen in needles with no changes in LMA (Oleksyn et al., 1998). In contrast, in the present study, and under greenhouse conditions (Soolanayakanahally et al., 2009), high latitude populations of balsam poplar tended to have higher photosynthetic rates ($p < 0.06$), as well as significantly higher LMA and leaf N density, but not percent N. Aspen, on the other hand, followed neither of these patterns. Although LMA and Leaf N remained highly correlated across all genotypes, they did not correlate with trends in *A*. In aspen, higher photosynthetic rates in high latitude populations seemed almost entirely due to higher g_s. Variation in foliar nitrogen among different tree populations had no effect or was inconsistent with photosynthetic rate in *Pinus strobus* L. (Reich and Schoettle, 1988) and *Pinus radiata* D. Don. (Sheriff et al., 1986), which also appears to be the case in field-grown aspen. The leaf optical properties, light intensity, and leaf water content could influence CCI values (Biber, 2007),

and an intriguing result of greater CCI with latitude while leaf N declines remains largely unexplained in aspen. In addition, the trembling nature of aspen leaves with similar greenness on either side of lamina might scatter the light signals in a different way compared to balsam poplar leaves. Overall, physiological and leaf functional traits may account for variation in growth and biomass; however their expression is strongly controlled by daylength and seasonality (Yu et al., 2001; McKown et al., 2013b).

Seasonal Growth Phenology and Height

Photosynthetic rates were significantly higher in more northern populations of aspen, and a similar trend was observed in balsam poplar. Although photosynthetic rates tended to be higher in the populations adapted to shorter growing seasons, these populations grew less because of earlier bud set. However, stem height was the best predictor of biomass among fast growing hybrid poplars for use in poplar breeding and tree improvement (Rae et al., 2004). Populations of both species were not at all differentiated in terms of date of bud flush, but there was strong clinal variation in the timing of bud set (Luquez et al., 2007; Soolanayakanahally et al., 2013). In temperate climates, spring temperatures increase and cumulative heat sums have a strong effect on the timing of bud flush (Lechowicz, 1984; Olson et al., 2013). In addition, chilling requirements might also influence bud flush date (Campbell and Sugano, 1975). However, fall bud set is primarily controlled by day length. Pauley and Perry (1954) observed earlier height growth cessation (bud set) in latitudinally diverse populations of *Populus* species which continued to grow until the daylength fell below a critical threshold that varied with the latitude of origin. The initiation of bud formation is visually apparent within a few days of growth cessation. Our findings and other studies highlight the generality of adaptive clines in bud set (Howe et al., 1995; Li et al., 2003). The period of HED is, therefore, well approximated by the number of days between the recorded dates of bud flush and bud set. When grown outside, the high latitude populations of balsam poplar and aspen accomplished less growth during the summer (**Tables 2**, **3**). Plant height and shoot biomass (**Figure 4**) were tightly related to HED. In contrast, when height growth cessation was avoided under extended days in a greenhouse, height increment paralleled *A* in 21 latitudinally different provenances of balsam poplar (Soolanayakanahally et al., 2009). Indeed, short-season genotypes appear to have higher peak rates of stem elongation during free growth, even in outdoor common gardens (pers. comm. Salim Silim). Similarly, Mylecraine et al. (2005) and Mimura and Aitken (2007) found faster spring growth rates among northern populations of Atlantic white-cedar [*Chamaecyparis thyoides* (L.) B.S.P.] and Sitka spruce, respectively, in range-wide provenance tests encompassing their entire latitudinal ranges (29–44° N and 40–61° N, respectively).

Körner and Renhardt (1987) suggested that high latitude herbaceous perennials invest more in root growth to protect their productivity in case of shoot loss caused by growing season frost events or during winter. It is possible that trees and shrubs may do likewise, reducing potential shoot growth in northern provenances. Latitudinal clines in height growth cessation in response to photoperiod were first documented in European aspen by Sylvén (1940, in Pauley, 1949) and in North American cottonwoods by Pauley and Perry (1954). Photoperiodic responses among black cottonwood ecotypes were also observed by Howe et al. (1995). Cannell and Willett (1976) studied carbon partitioning in potted black cottonwood seedlings from 46 to 58° N, but they reported that all provenances had similar carbon partitioning between roots and shoots until height growth cessation. However, because carbon partitioning after bud set favors roots (Ledig et al., 1970), which continue to grow, black cottonwood from northern provenances finished the growing season with a higher root:shoot ratio. Enhanced late-season partitioning to root growth, compounded over years, might increase belowground respiratory costs to further impact relative rates of shoot growth. Cannell and Willett (1976), however, reported that differences in root:shoot ratio did not persist year-to-year because they were corrected each spring by proportionally greater shoot growth among northern genotypes.

Comparative Physiology of Populus

For comparative purposes, **Table 4** summarizes correlations reported here in outdoor common gardens for *P. balsamifera* and *P. tremula*, as well as for *P. trichocarpa* from Gornall and Guy (2007) and McKown et al. (2013a). The table also presents data for *P. balsamifera* grown under extended photoperiod in an indoor "common garden" as reported by Soolanayakanahally et al. (2009).

Across all studies, photosynthetic assimilation rates (*A*) were always measured during active growth irrespective of the growth conditions, and in all five studies, *A* increased with latitude of origin (field grown balsam poplar, $p > 0.05$). The consistency of this geographic cline in allopatric *Populus* species, as well as other woody plants, is strongly suggestive of its global adaptive significance. We have previously noted that higher *A* in populations from higher latitudes can be achieved in different ways. For example, Soolanayakanahally et al. (2009) found that higher internal (mesophyll) conductance, and not g_s, was associated with enhanced *A* in greenhouse-grown *P. balsamifera*, whereas g_s was correlated with *A* across field-grown *P. trichocarpa* populations ($r = 0.757$, $p < 0.05$, Gornall and Guy, 2007; $r = 0.70$, $p < 0.05$, McKown et al., 2013a). Mesophyll conductance was not estimated in both studies, but g_s increased with latitude in outdoor common gardens across all three species considered in **Table 4**, including *P. balsamifera*. Different trends in g_s between the field-grown and greenhouse-grown balsam poplars largely account for different trends in WUE_i, which decreased with latitude of origin in the former but increased in the latter. However, WUE_i was not significant with latitude of origin in field-grown *P. termula* (**Table 4**). No latitudinal pattern in WUE_i was observed in field-grown black cottonwood by Gornall and Guy (2007), but in a broader study involving more populations, WUE_i decreased with latitude also in this species (McKown et al., 2013a).

Trends in leaf $\delta^{13}C$ were in full concurrence with WUE_i. A strong correlation between $\delta^{13}C$ and WUE_i has been reported for numerous tree species (Zhang and Marshall, 1994; Zhang et al., 2004; Pointeau and Guy, 2014). Conversely, the relationship between $\delta^{13}C$ and WUE_i at the leaf level did not scale up to

TABLE 4 | Comparative physiology among *P. balsamifera*, *P. tremula*, and *P. trichocarpa*.

Variables	*P. balsamifera*		*P. tremula*	*P. trichocarpa*	
	Greenhouse (Soolanayakanahally et al., 2009) $n = 210$	Field (present study) $n = 30$	Field (present study) $n = 116$	Field (Gornall and Guy, 2007) $n = 30$	Field (McKown et al., 2013a) $n = 461$
A	$+^{***}$	ns	$+^{***}$	$+^{*}$	$+^{***}$
g_s	ns	$+^{*}$	$+^{***}$	$+^{*}$	$+^{*}$
WUE_i	$+^{***}$	$-^{*}$	ns	ns	$-^{*}$
$\delta^{13}C$	$+^{*}$	ns	ns	ns	ns
Leaf N	$+^{***}$	$+^{***}$	$-^{***}$	$+^{*}$	$+^{*}$
CCI	$+^{***}$	$+^{*}$	$+^{***}$	not available	$+^{*}$
Height	$+^{***}$	$-^{*}$	$-^{***}$	$-^{*}$	$-^{*}$
LMA	$+^{***}$	$+^{***}$	ns	ns	ns

*Summary of correlations for all genotypes between physiological and morphological variables with latitude. All results are from present study except where indicated. *$p < 0.05$; **$p < 0.01$; ***$p < 0.001$; ns, no significant change. The statistical tests for the present study are from **Tables 2**, **3**. A, assimilation rate (μmol CO_2 m^{-2} s^{-1}); g_s, stomatal conductance (mol H_2O m^{-2} s^{-1}); WUE_i, intrinsic water use efficiency (μmol CO_2 mol^{-1} H_2O); $\delta^{13}C$, carbon isotope composition of leaf (‰); Leaf N, leaf nitrogen density (μmol N cm^{-2}); CCI, chlorophyll content index; Height (cm); LMA, leaf mass area (mg cm^{-2}).*

harvestable stems in *Eucalyptus grandis* (Olbrich et al., 1993); hence, recurrent measurements of gas exchange across the growing season are crucial to better infer the outcomes (Wang et al., 2013). The differences in $\delta^{13}C$ values among the *P. balsamifera* and *P. tremula* populations were small and non-significant with latitude of origin (**Tables 2, 3**). In *P. balsamifera*, however, the cline was positive under greenhouse conditions (Soolanayakanahally et al., 2009), but trended in the opposite direction in the outdoor common garden (this study). Several authors have described *in situ* variation in $\delta^{13}C$ with latitude and elevation within species (Zhang et al., 1993). Such variation may be genetic or environmental or both. Clearly, however, growth in a common garden to assess the genetic component will not necessarily exclude the effects of environment. As noted above, earlier height growth cessation in high latitude genotypes may favor increased investment into root growth and a higher root:shoot ratio. Increased partitioning to roots might permit access to water from a greater relative volume of soil resulting in higher g_s, lower WUE_i, and increased isotope discrimination. When photoperiodic restrictions are removed under greenhouse conditions, high latitude genotypes can exhibit greater height growth than low-latitude genotypes. This reversal in height rankings among trees grown in the greenhouse (long photoperiod) vs. an outdoor common garden (short photoperiod) complicates the interpretation of the adaptive significance of water-use efficiency and the evaluation of growth rates for tree improvement. Hence, provenance trial results should be interpreted cautiously if not replicated at different latitudes.

Differences in LMA and Leaf N accounted for most of the variation in A reported by Soolanayakanahally et al. (2009) for balsam poplar populations under greenhouse conditions and, in the present study, an outdoor common garden. Leaf N density, but not LMA, was also associated with enhanced A in black cottonwood (Gornall and Guy, 2007; McKown et al., 2013a). However, aspen showed no association between A and Leaf N or between A and LMA (Supplement 1). CCI, on the other hand, paralleled A across all studies where it was assessed. This observed variance in leaf functional traits seems to be largely driven by the adaptation of a given population to latitude (daylength); however, common garden environmental factors might also influence trait-to-trait relationships. Other studies also found weak correlations between greenhouse juvenile and field mature trees (Cornelissen et al., 2003; Smith et al., 2011). As expected, final height was negatively correlated with latitude of origin across all three *Populus* species when grown outdoors under a natural photoperiodic regime.

Conclusions

Latitudinal clines in A exist among allopatric *Populus* species adapted to similar environments on different continents. We interpret this as a common and, therefore, an important adaptation to short growing seasons. In fact, this appears to be a convergent trait in that the underlying physiological mechanisms responsible for higher A are not consistent across species. In spite of intrinsically higher photosynthetic rates, high latitude populations consistently accomplish less height during the growing season as a result of earlier bud set leading to growth cessation. A shortcoming of unreplicated outdoor common gardens or provenance trails to assess growth rates for purposes of tree improvement is the crucial role of photoperiod in limiting plant height (Howe et al., 1995; Way and Montgomery, 2014). Because height growth is largely predetermined by phenological events, the effects of other important traits that determine performance are effectively obscured. Furthermore, subsequent effects on growth complicate the interpretation of other important adaptive traits such as root:shoot ratio, WUE_i, and $\delta^{13}C$ (McKown et al., 2013b).

Author Contributions

All authors designed and performed the experiments.

Acknowledgments

RS would like to thank Agriculture and Agri-Food Canada (AAFC) for providing financial support. This work was collectively supported by grants from AAFC to SS, a Natural Sciences and Engineering Research Council (Canada) Discovery Grant to RG and from the Swedish Research Council (VR) and the Swedish Research Council for Environment, Agricultural Sciences and Spatial Planning (Formas) to SJ. Thanks to Don Reynard for planting and maintaining the common garden at Indian Head and also Christiane Catellier for help with instrumentation.

Supplementary Material

The Supplementary Material for this article can be found online at: http://journal.frontiersin.org/article/10.3389/fpls.2015.00528

References

Anderson, J. E., Williams, J., Kriedemann, P. E., Austin, M. P., and Farquhar, G. D. (1996). Correlations between carbon isotope discrimination and climate of native habitats for diverse eucalypt taxa growing in a common garden. *Aust. J. Plant Physiol.* 23, 311–320. doi: 10.1071/PP9960311

Benowicz, A., Guy, R. D., and El-Kassaby, Y. A. (2000). Geographic pattern of genetic variation in photosynthetic capacity and growth in two hardwood species from British Columbia. *Oecologia* 123, 168–174. doi: 10.1007/s004420051002

Biber, P. D. (2007). Evaluating a chlorophyll content meter on three coastal wetland plant species. *J. Agri. Food Environ. Sci.* 1, 1–11.

Billings, W. D., Godfrey, P. J., Chabot, B. F., and Bourque, D. P. (1971). Metabolic acclimation to temperature in arctic and alpine ecotypes of *Oxyria digyna*. *Arctic Alpine Res.* 3, 277–289. doi: 10.2307/1550044

Campbell, R. K., and Sugano, A. I. (1975). Phenology of bud burst in Douglas-fir related to provenance, photoperiod, chilling and flushing temperature. *Bot. Gaz.* 136, 290–298. doi: 10.1086/336817

Cannell, M. G. R., and Willett, S. C. (1976). Shoot growth phenology, dry matter distribution and root:shoot ratios of provenances of *Populus trichocarpa*, *Picea sitchensis* and *Pinus contorta* growing in Scotland. *Silvae Genet.* 25, 49–59.

Chamaillard, S., Fichot, R., Vincent-Barbaroux, C., Bastien, C., Depierreux, C., Dreyer, E., et al. (2011). Variations in bulk leaf carbon isotope discrimination, growth and related leaf traits among three *Populus nigra* L. populations. *Tree Physiol.* 31, 1076–1087. doi: 10.1093/treephys/tpr089

Cornelissen, J. H. C., Cerabolini, B., Puyravaud, J. P., Maestro, M., Werger, M. J. A., Aerts, R., et al. (2003). Functional traits of woody plants: correspondence of species rankings between field adults and laboratory-grown seedlings? *J. Veg. Sci.* 14, 311–322. doi: 10.1111/j.1654-1103.2003.tb02157.x

Donovan, L. A., and Ehleringer, J. R. (1994). Carbon isotope discrimination, water-use efficiency, growth and mortality in a natural shrub population. *Oecologia* 100, 347–354. doi: 10.1007/BF00316964

Endler, J. A. (1973). Gene flow and population differentiation. *Science* 179, 243–250. doi: 10.1126/science.179.4070.243

Evans, J. R. (1989). Photosynthesis and nitrogen relationships in leaves of C_3 plants. *Oecologia* 78, 9–19. doi: 10.1007/BF00377192

Farquhar, G. D., O'Leary, M. H., and Berry, J. A. (1982). On the relationship between carbon isotope discrimination and the intercellular carbon dioxide concentration in leaves. *Aust. J. Plant Physiol.* 9, 121–137. doi: 10.1071/PP9820121

Farrar, J. L. (1995). *Trees in Canada*. Markham, ON: Fitz Henry and Whiteside Ltd.

Gornall, J. L., and Guy, R. D. (2007). Geographic variation in ecophysiological traits of black cottonwood (*Populus trichocarpa*). *Can. J. Bot.* 85, 1202–1213. doi: 10.1139/B07-079

Guy, R. D., and Holowachuk, D. L. (2001). Population differences in stable carbon isotope ratio of *Pinus contorta* Dougl. ex Loud.: relationship to environment, climate of origin, and growth potential. *Can. J. Bot.* 79, 274–283. doi: 10.1139/b01-001

Hoagland, D. R., and Arnon, D. J. (1950). The water culture method for growing plants without soil. *Calif. Agric. Exp. Stn.* 347, 1–32.

Howe, G. T., Hackett, W. P., Furnier, G. R., and Klevorn, R. E. (1995). Photoperiodic responses of a northern and southern ecotype of black cottonwood. *Physiol. Plant.* 93, 695–742. doi: 10.1111/j.1399-3054.1995.tb05119.x

Johnsen, K. H., Seiler, J. R., and Major, J. E. (1996). Growth, shoot phenology and physiology of diverse seed sources of black spruce.2. 23-year-old field trees. *Tree Physiol.* 16, 375–380. doi: 10.1093/treephys/16.3.375

Kawecki, T. J., and Ebert, D. (2004). Conceptual issues in local adaptation. *Ecol. Lett.* 7, 1225–1241. doi: 10.1111/j.1461-0248.2004.00684.x

Lechowicz, M. J. (1984). Why do temperate deciduous trees leaf out at different times? Adaptation and ecology of forest communities. *Am. Nat.* 124, 821–842. doi: 10.1086/284319

Ledig, F. T., Bormann, F. H., and Wenger, F. K. (1970). The distribution of dry matter growth between shoots and roots in loblolly pine. *Bot. Gaz.* 131, 349–359. doi: 10.1086/336552

Li, C., Junttila, O., Ernstsen, A., Heino, P., and Palva, E. T. (2003). Photoperiodic control of growth, cold acclimation and dormancy development in silver birch (*Betula pendula*) ecotypes. *Physiol. Plant.* 117, 206–212. doi: 10.1034/j.1399-3054.2003.00002.x

Linhart, Y. B., and Grant, M. C. (1996). Evolutionary significance of local genetic differentiation in plants. *Ann. Rev. Ecol. Syst.* 27, 237–277. doi: 10.1146/annurev.ecolsys.27.1.237

Luquez, V., Hall, D., Albrectsen, B. R., Karlsson, J., Ingvarsson, P., and Jansson, S. (2007). Natural phenological variation in aspen (*Populus tremula*): the SwAsp collection. *Tree Genet. Genomes* 4, 279–292. doi: 10.1007/s11295-007-0108-y

McKown, A. D., Guy, R. D., Azam, S., Drewes, E. C., and Quamme, L. K. (2013b). Seasonality and phenology alter leaf functional traits. *Oecologia* 172, 653–665. doi: 10.1007/s00442-012-2531-5

McKown, A. D., Guy, R. D., Klápste, J., Geraldes, A., Friedmann, M., Cronk, Q. C. B., et al. (2013a). Geographical and environmental gradients shape phenotypic trait variation and genetic structure in *Populus trichocarpa*. *New Phytol.* 201, 1263–1276. doi: 10.1111/nph.12601

McKown, A. D., Klápste, J., Guy, R. D., Geraldes, A., Porth, I., Hannemann, J., et al. (2014). Genome-wide association implicates numerous genes underlying ecological trait variation in natural populations of *Populus trichocarpa*. *New Phytol.* 203, 535–553. doi: 10.1111/nph.12815

Mimura, M., and Aitken, S. N. (2007). Adaptive gradients and isolation-by-distance with postglacial migration in *Picea sitchensis*. *Heredity* 99, 224–232. doi: 10.1038/sj.hdy.6800987

Moles, A. T., Warton, D. I., Warman, L., Swenson, N. G., Laffan, S. W., Zanne, A. E., et al. (2009). Global patterns in plant height. *J. Ecol.* 97, 923–932. doi: 10.1111/j.1365-2745.2009.01526.x

Mooney, H. A., and Billings, W. D. (1961). Comparative physiological ecology of arctic and alpine populations of *Oxyria digyna*. *Ecol. Monogr.* 31, 1–29. doi: 10.2307/1950744

Mylecraine, K. A., Kuser, J. E., Zimmermann, G. L., and Smouse, P. E. (2005). Rangewide provenance variation in Atlantic white-cedar (*Chamaecyparis thyoides*): early survival and growth in New Jersey and North Carolina plantations. *For. Ecol. Manag.* 216, 91–104. doi: 10.1016/j.foreco.2005.05.053

Olbrich, B. W., Le Roux, D., Poulter, A. G., Bond, W. J., and Stock, W. D. (1993). Variation in water use efficiency and $\delta^{13}C$ levels in *Eucalyptus grandis* clones. *J. Hydrol.* 150, 615–633. doi: 10.1016/0022-1694(93)90128-v

Oleksyn, J., Modrzynski, J., Tjoelker, M. G., Zytkowiak, R., Reich, P. B., and Karolewski, P. (1998). Growth and physiology of *Picea abies* populations from elevational transects: common garden evidence for altitudinal ecotypes and cold adaptation. *Funct. Ecol.* 12, 573–590. doi: 10.1046/j.1365-2435.1998.00236.x

Olson, M. S., Levsen, N., Soolanayakanahally, R. Y., Guy, R. D., Schroeder, W. R., Keller, S. R., and Tiffin, P. (2013). The adaptive potential of *Populus balsamifera* L. to phenology requirements in a warmer global climate. *Mol. Ecol.* 22, 1214-1230. doi: 10.1111/mec.12067

Ovaska, J. (1988). *Hieskoivun (Betula pubescens Ehrh.) Pohjoisten Provenienssien Fotosynteettinen Adaptaatio*. MSc Thesis. Department of Plant Physiology, University of Turku, Turku.

Pauley, S. S. (1949). Forest-tree genetics research: *Populus* L. *Econ. Bot.* 3, 299–330. doi: 10.1007/BF02859100

Pauley, S. S., and Perry, T. O. (1954). Ecotypic variation of the photoperiodic response in *Populus*. *J. Arnold Arbor.* 35, 167–188.

Pointeau, V. M., and Guy, R. D. (2014). Comparative resource-use efficiencies and growth of *Populus trichocarpa* and *Populus balsamifera* under glasshouse conditions. *Botany* 92, 443–451. doi: 10.1139/cjb-2013-0303

Rae, A. M., Robinson, K. M., Street, N. R., and Taylor, G. (2004). Morphological and physiological traits influencing biomass productivity in short rotation coppice poplar. *Can. J. For. Res.* 34, 1488–1498. doi: 10.1139/x04-033

Rehfeldt, G. E., Ying, C. C., Spittlehouse, D. L., and Hamilton, D. A. (1999). Genetic responses to climate in *Pinus contorta*: niche breadth, climate change, and reforestation. *Ecol. Monogr.* 69, 375–407. doi: 10.1890/0012-9615(1999)069[0375:GRTCIP]2.0.CO;2

Reich, P. B., Oleksyn, J., and Tjoelker, M. G. (1996). Needle respiration and nitrogen concentration in Scots Pine populations from a broad latitudinal range: a common garden test with field-grown trees. *Funct. Ecol.* 10**,** 768-776. doi: 10.2307/2390512

Reich, P. B., and Schoettle, A. W. (1988). Role of phosphorus and nitrogen in photosynthetic and whole plant carbon gain and nutrient use efficiency in eastern white pine. *Oecologia* 77, 25–33. doi: 10.1007/BF00380920

Reich, P. B., Walters, M. B., Kloeppel, B. D., and Ellsworth, D. S. (1995). Different photosynthesis-nitrogen relations in deciduous hardwood and evergreen coniferous tree species. *Oecologia* 104, 24–30. doi: 10.1007/BF00365558

Körner, C. H., and Renhardt, U. (1987). Dry matter partitioning and root length/leaf area ratios in herbaceous perennial plants with diverse altitudinal distribution. *Oecologia* 74, 411–418. doi: 10.1007/BF00378938

Schipperges, B., Kappen, L., and Sonesson, M. (1995). Intraspecific variations of morphology and physiology of temperate to arctic populations of *Cetraria nivalis*. *Lichenologist* 27, 517–529. doi: 10.1016/S0024-2829(05) 80011-8

Schnekenburger, F., and Farmer, R. E. Jr. (1989). Genetic variance in growth of balsam poplar under 16- and 8-hour photosynthetic periods. *Forest Sci.* 35, 903–919.

Sheriff, D. W., Nambiar, K. S., and Fife, D. N. (1986). Relationships between nutrient status, carbon assimilation and water use efficiency in *Pinus radiata* (D. Don) needles. *Tree Physiol.* 2, 73–88. doi: 10.1093/treephys/2.1-2-3.73

Smith, E. A., Collette, S. B., Boynton, T. A., Lillrose, T., Stevens, M. R., Bekker, M. F., et al. (2011). Developmental contributions to phenotypic variation in functional leaf traits within quaking aspen clones. *Tree Physiol.* 31, 68–77. doi: 10.1093/treephys/tpq100

Soolanayakanahally, R. Y., Guy, R. D., Silim, S. N., Drewes, E. C., and Schroeder, W. R. (2009). Enhanced assimilation rates and water use efficiency with latitude through increased photosynthetic capacity and internal conductance in balsam poplar (*Populus balsamifera* L.). *Plant Cell Environ.* 32, 1821–1832. doi: 10.1111/j.1365-3040.2009.02042.x

Soolanayakanahally, R. Y., Guy, R. D., Silim, S. N., and Song, M. (2013). Timing of photoperiodic competency causes phenological mismatch in balsam poplar (*Populus balsamifera* L.). *Plant Cell Environ.* 36, 116–127. doi: 10.1111/j.1365-3040.2012.02560.x

Sylvén, N. (1940). Lång-och kortdagstyper av de svenska skogsträden. *Svensk Papperstidning* 43, 317–324; 332–342; 350–351.

van den Berg, A. K., and Perkins, T. D. (2004). Evaluation of a portable chlorophyll meter to estimate chlorophyll and nitrogen contents in sugar maple (*Acer saccharum* Marsh.) leaves. *Forest Ecol. Manag.* 200, 113-117. doi: 10.1016/j.foreco.2004.06.005

von Caemmerer, S., and Farquhar, G. D. (1981). Some relationships between the biochemistry of photosynthesis and the gas exchange of leaves. *Planta* 153, 376–387. doi: 10.1007/bf00384257

Wang, D., LeBauer, D., Kling, G., Voigt, T., and Dietze, M. C. (2013). Ecophysiological screening of tree species for biomass production: trade-off between production and water use. *Ecosphere* 4, 138. doi: 10.1890/ES13-00156.1

Wang, T., Hamann, A., Yanchuk, A., O'neill, G. A., and Aitken, S. N. (2006). Use of response functions in selecting lodgepole pine populations for future climates. *Glob. Change Biol.* 12, 2404–2416. doi: 10.1111/j.1365-2486.2006.01271.x

Way, D. A., and Montgomery, R. A. (2014). Photoperiod constraints on tree phenology, performance and migration in a warming world. *Plant Cell Environ.* doi: 10.1111/pce.12431

Wright, I. J., Groom, P. K., Lamont, B. B., Poot, P., Prior, L. D., Reich, P. B., et al. (2004a). Leaf trait relationships in Australian plant species. *Funct. Plant Biol.* 31, 551–558. doi: 10.1071/FP03212

Wright, I. J., Reich, P. B., Westoby, M., Ackerly, D. D., Baruch, Z., Bongers, F., et al. (2004b). The worldwide leaf economics spectrum. *Nature* 428, 821–827. doi: 10.1038/nature02403

Yu, Q., Tigerstedt, P. M. A., and Haapanen, M. (2001). Growth and phenology of hybrid aspen clones (*Populus tremula* L. X *Populus tremuloides* Michx.). *Silva Fenn.* 35, 15–25. doi: 10.14214/sf.600

Zhang, J., and Marshall, J. D. (1994). Population differences in water-use efficiency of well-watered and water-stressed western larch seedlings. *Can. J. For. Res.* 24, 92–99. doi: 10.1139/x94-014

Zhang, J., Marshall, J. D., and Jaquish, B. C. (1993). Genetic differentiation in carbon isotope discrimination and gas exchange in *Pseudotsuga menziesii*. *Oecologia* 93, 80–87. doi: 10.1007/BF00321195

Zhang, W. J., Feng, Z., Cregg, M. B., and Schumann, M. C. (1997). Carbon isotopic composition, gas exchange, and growth of three populations of ponderosa pine differing in drought tolerance. *Tree Physiol.* 17, 461–466. doi: 10.1093/treephys/17.7.461

Zhang, X., Zang, R., and Li, C. (2004). Population differences in physiological and morphological adaptations of *Populus davidiana* seedlings in response to progressive drought stress. *Plant Sci.* 166, 791–797. doi: 10.1016/j.plantsci.2003.11.016

Conflict of Interest Statement: The authors declare that the research was conducted in the absence of any commercial or financial relationships that could be construed as a potential conflict of interest.

Copyright © 2015 Soolanayakanahally, Guy, Street, Robinson, Silim, Albrectsen and Jansson. This is an open-access article distributed under the terms of the Creative Commons Attribution License (CC BY). The use, distribution or reproduction in other forums is permitted, provided the original author(s) or licensor are credited and that the original publication in this journal is cited, in accordance with accepted academic practice. No use, distribution or reproduction is permitted which does not comply with these terms.
