# Supplementary material for: Provenance and family variations in early growth of Manchurian walnut (Juglans mandshurica Maxim.) and selection of superior families
Source: PLoS One. 2024 Mar 7;19(3):e0298918. doi: 10.1371/journal.pone.0298918 (PMC10919699; doi:10.1371/journal.pone.0298918)
Supplement: S1 File — (ZIP) [file pone.0298918.s004.zip › Correlation analysis of S1 families of maize for grain yield and its components.pdf]

ResearchGate

See discussions, stats, and author profiles for this publication at: https://www.researchgate.net/publication/262419694

Correlation Analysis of S1 Families of Maize for Grain Yield and its Components

Article *in* International Journal of Agriculture and Biology · November 2001

CITATIONS
43

READS
439

2 authors:

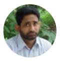

Muhammad Yousuf
Pakistan Agricultural Research Council

17 PUBLICATIONS **97** CITATIONS

SEE PROFILE

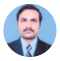

Muhammad Saleem
University of Management and Technology (Pakistan)

37 PUBLICATIONS **389** CITATIONS

SEE PROFILE

Some of the authors of this publication are also working on these related projects:

Coordination and development activities regarding Rice, Maize, Sorghum, Millet and Fodder Crops View project

Development of Rice Hybrids for Different Rice Growing Ecologies of Pakistan View project

All content following this page was uploaded by Muhammad Yousuf on 13 March 2014.

The user has requested enhancement of the downloaded file.

INTERNATIONAL JOURNAL OF AGRICULTURE & BIOLOGY
1560–8530/2001/03–4–387–388
http://www.ijab.org

Correlation Analysis of S1 Families of Maize for Grain Yield and its Components

MUHAMMAD YOUSUF AND MUHAMMAD SALEEM
Department of Plant Breeding and Genetics, University of Agriculture, Faisalabad–38040, Pakistan

ABSTRACT

Seventy-four S1 families obtained from maize population C-17 were grown in a triplicated randomized complete block design to estimate the genotypic and phenotypic correlation coefficients among various plant traits. Grain yield per plant showed significant genotypic correlation with plant height, number of kernel rows per ear and number of kernels per row. Correlation analysis showed that indirect selection for grain yield is possible through selection for number of kernel rows per ear and 100-grain weight.

Key Words: Maize; S1 families; Correlation

INTRODUCTION

Maize (*Zea mays* L.) is the third important cereal food crop of the world after wheat and rice (Poehlman, 1997). Grain yield in maize is a complex character and is the result of interrelationships of its various yield components (Grafius, 1960). Thus, information on genotypic and phenotypic correlation coefficients among various plant traits helps to ascertain the degree to which these are associated with economic productivity. The association between two characters can directly be observed as phenotypic correlation while genotypic correlation expresses the extent to which two traits are genetically associated. Both genotypic and phenotypic correlations among and between pairs of agronomic traits provide scope for indirect selection in a crop breeding programme. The S1 family selection in maize is considered as an efficient method of population improvement but has not been extensively exploited compared to other methods (Genter & Alexander, 1966; Hakim *et al.,* 1969). Altinibas and Algan (1993) found significant correlation of days to silking and number of kernel rows with grain yield. They also added that grain yield per plant is positively correlated with and is significantly affected by 100- grain weight. El-saad *et al.* (1994) selected 300 S1 families at Sids and evaluated at three locations. Highly significant genotypic correlation coefficients were obtained between grain yield per plant, days to 50% silking and plant height. Stojsin and Kannenberg (1994) studied five maize populations and found the highest correlation coefficients (0.95) between days to tasseling and silking. Similar correlation was found between anthesis and plant height. The correlation of grain yield with tasseling and silking was positive but non-significant.

MATERIALS AND METHODS

The studies were conducted in the experimental area of the Department of Plant Breeding and Genetics, University of Agriculture, Faisalabad during the year 1998-99. Maize population C-17, a composite developed at AARI, Faisalabad, was used as source material. Seventy-four S1 families developed from the population were assigned to two blocks, each containing 37 families in a triplicated randomized complete block design (RCBD). The experimental unit consisted of single row plot of 4.5 m length with plant to plant and row to row distance of 30 cm and 75 cm, respectively. The data were recorded for number of days taken to tasseling, number of days taken to silking, plant height, number of ears per plant, number of kernel rows per ear, number of kernels per row, 100-grain weight and grain yield per plant. The data were subjected to analysis of variance and covariance. Phenotypic and genotypic correlation coefficients were computed according to Kwon and Torrie (1964).

RESULTS AND DISCUSSION

The estimates of genotypic and phenotypic correlation coefficients between number of days taken to tasseling and grain yield per plant were positive and non-significant (Table I) as reported by Troyer (1990), and Stojsin and Kannenberg (1994). Correlation between number of days taken to silking and grain yield per plant was positive and non-significant both at the genotypic and phenotypic levels (El-Nagouli *et al.,* 1983; Troyer, 1990; Altinibas & Algan, 1993). Association of grain yield per plant and plant height was negative; significant at the genotypic and non-significant at the phenotypic level (Jasa-vega, 1985). Number of ears per plant and grain yield per plant showed positive and non-significant genotypic and phenotypic correlation, which is in accord with Shevardnadze and Goginashvili (1988). Number of kernel rows per ear was negatively associated with grain yield per plant, the correlation coefficient was significant at the genotypic level and non-significant at phenotypic level. The correlation between the number of kernels per row and grain yield per plant was positive both at genotypic and phenotypic levels but significant at genotypic level and non-significant at phenotypic level (Altinibas & Algan, 1993). Association of

Table I. Estimates of genotypic [r (g)] and phenotypic [r (p)] correlation coefficients for grain yield parameters

Characters		Number of days taken to tasseling	Number of days taken to silking	Plant height	Number of ears per plant	Number of kernel rows per ear	Number of kernels per row	100 – grain weight	Grain yield per plant
Number of days	r (g)		0.375**	-0.081*	0.190	-0.012	0.066	0.105	0.030
taken to tasseling	r (p)		- 0.283**	-0.075	0.004	-0.034	0.041	0.059	0.034
Number of days	r (g)			-0.016	0.332*	0.376*	0.002	0.066	0.044
taken to silking	r (p)			-0.015	0.065	0.275**	0.008	0.062	0.006
Plant height	r (g)				0.084	0.118*	-0.184*	0.132*	-0.113*
	r (p)				0.028	0.101	-0.130	0.111	-0.089
Number of ears per	r (g)					0.822*	-1.322	0.492	0.684
plant	r (p)					0.111	-0.292**	0.085	0.106
Number of kernel	r (g)						-0.075	0.259*	-0.083*
rows per ear	r (p)						-0.012	0.209**	-0.054
Number of kernels	r (g)							0.278*	0.109*
per row	r (p)							0..137	0.036
100 – grain weight	r (g)								
	r (p)								
Grain yield per	r (g)								-0.033
plant	r (p)								-0.028

* = Significant, ** = Highly Significant

100-grain weight and grain yield per plant was negative and non-significant at genotypic and phenotypic levels. 100-grain weight showed significant genotypic correlation with plant height, number of kernel rows per ear and number of kernels per row. Number of kernels per row showed significant correlation with plant height at the genotypic level and highly significant correlation at phenotypic level with number of ears per plant. Number of kernel rows per ear showed significant correlation at the genotypic level with number of days taken to silking, plant height and number of ears per plant. From the study it may be concluded that improvement in S1 families of maize can be done by simultaneous selection of number of kernel rows per ear and 100-grain weight.

REFERENCES

Altinibas, M. and N. Algan, 1993. Correlation among earliness, yield and yield components and quality traits in hybrid maize. *Anadolu*, 3: 40–62.

El-Nagouli, O.O., M.A. Abul Fadi, A.A. Ismail and M. Khamis, 1983. Genotypic and phenotypic correlations and path analysis in maize and their implications in selection. *Agron. Abst. Amer. Soc. Agron.,* 62–3.

El-Saad, S.F.A., M.M.A. Regheb and A.A.A. Aziz, 1994. Genetic variance and correlation studies in a yellow maize population. *Bull. Fac. Agri., Univ. Cairo,* 45: 811–6.

Genter, C.F. and M.W. Alexander, 1966. Development and selection of productive S1 inbred lines of corn. *Crop Sci.*, 13: 524–7.

Grafius, J.E., 1960. Does overdominance exist for yield in corn? *Agron. J.,* 52: 361.

Hakim, R.M., J.C. Sentz and V.R. Carangel, 1969. Mass and family selection for yield in a tropical variety of maize. *Agron. Absts. Amer. Soc. Agron.,* pp: 7–8, Madison. W.I. USA.

Jasa-vega, P.J., 1985. Divergent mass selection for line to line anthesis in a maize population. *Dissertation Absts. Int. B (Sci. and Engineering),* 45: 3422.

Kown, S.H. and J.H. Torrie, 1964. Heritability and inter-relationship among traits of two soybean populations. *Crop Sci.*, 4: 196–8.

Poehlman, J.M., 1997. *Breeding Field Crops,* 4th ed. Avi. Pub. Co., Inc. Westport, Connecticut, USA.

Shevardnadze, G.A. and K.A. Goginashvili, 1988. Correlations between some phenotypic characters in prolific maize. *Soc. Aka. Nauk. Gru. SSR,* 131: 597–9.

Stojsin, D. and L.W. Kannenberg, 1994. Genetic changes associated with different methods of recurrent selection in five maize populations.II. Indirectly selected traits. *Crop Sci.,* 23: 1466–72.

Troyer, F., 1990. Selection for early flowering in corn; Three adapted synthetics. *Crop Sci.*, 30: 896–900.

(Received 19 July 2001; Accepted 13 August 2001)

View publication stats
