# Supplementary material for: Provenance and family variations in early growth of Manchurian walnut (Juglans mandshurica Maxim.) and selection of superior families
Source: PLoS One. 2024 Mar 7;19(3):e0298918. doi: 10.1371/journal.pone.0298918 (PMC10919699; doi:10.1371/journal.pone.0298918)
Supplement: S1 File — (ZIP) [file pone.0298918.s004.zip › Determination in the phenological difference levels of seedlings of some walnut genotypes (Juglans regia L.)]

DETERMINATION IN THE PHENOLOGICAL DIFFERENCE LEVELS OF SEEDLINGS OF SOME WALNUT GENOTYPES (*JUGLANS REGIA* L.)

BÜKÜCÜ, Ş. B.[1*] – ÖZCAN, A.[2] – SÜTYEMEZ, M.[3] – YILDIRIM, E.[3]

[1]*Department of Plant and Animal Production, Silifke Taşucu Vocational School, Selçuk University, 33900 Silifke Taşucu, Mersin, Turkey*

[2]*Afsin Vocational School, University of Kahramanmaras Sutcu Imam, 46500 Afsin, Kahramanmaras, Turkey*

[3]*Department of Horticulture, Faculty of Agriculture, University of Sütçü İmam, 46040 Onikisubat, Kahramanmaraş, Turkey*

**Corresponding author*
e-mail: burakbukucu@gmail.com, phone: +90-553-638-4276

(Received 26th Feb 2020; accepted 25th May 2020)

Abstract. Walnut is a tree adversely affected by late spring and early autumn frosts. Therefore, it is one of the main goals to obtain late leafing and early defoliation genotypes in breeding studies on walnuts. The aim of this research is to determine the levels of phenological differences in walnut genotypes derived from the open-pollinated seeds of Bilecik, Chandler, Franquette, Howard, Kaman 1, Maraş 12, Pedro, Sütyemez 1 and Gimar (Gimnut). In this study, the bud burst, leafing, leaf yellowing and defoliation dates of seventy-five genotypes obtained from nine different walnut cultivars and their parents were determined. Cluster and PCA analyses were performed on a total of 684 genotypes to determine genetic diversity based on phenological markers. As a result of cluster analysis, all genotypes including their parents were clustered in three major groups. PCA analysis also confirmed the presence of genetic diversity-based phenological markers in our walnut seedling collection with PC1 50.226%, PC2 36.635%, PC3 12.413% and PC4 0.726% of the total variation. Pearson Correlation Coefficient among our walnut collection showed that there were very significant associations with budburst and leafing date (r = 0.97). As a result, we determined a wide variation in our walnut seedling collection, and these findings are potentially useful for future breeding studies in terms of late leafing and early defoliation.
Keywords: *cluster analyses, PCA, breeding, budbrust, leafing, defoliation*

Introduction

Walnut is among the widely cultivated nuts in the world due to its nutrient contains. English or Persian walnut (*Juglans regia* L.) is one of the 20 species of the genus *Juglans*, which belongs to the *Juglandaceae* family (Şen, 2011). *J. regia* L. is long-lived, deciduous, monoecious and heterodichogamous. Walnut tree domestication is thought to be carried out in Central Asia, and today it is distributed and grown commercially over a wide geographical range, including West-Central Asia, southern Europe, North and South America, Australia and New Zealand (Gunn et al., 2010).

Walnut is the most cultivated nuts in the world. Due to the important nutrients it contains, the demand for this species of fruit is increasing every day. Therefore, scientific studies and production fields of this fruit are increasing every day. Genetic and morphological markers have become indispensable parts of plant breeding studies. To date, genetic diversity studies have been carried out with molecular or morphological markers on various fruit species including walnut under different

http://www.aloki.hu ● ISSN 1589 1623 (Print) ● ISSN 1785 0037 (Online)
DOI: http://dx.doi.org/10.15666/aeer/1803_48074815
© 2020, ALÖKI Kft., Budapest, Hungary

ecological conditions (Hernández-Delgado et al., 2007; Hong et al., 2008; Ferreira et al., 2010; Torres-Calzada et al., 2013; Rana et al., 2015; Kabiri et al., 2018; Sütyemez et al., 2018). Although morphological characters are often stated to hinder a clear character distinction among genotypes due to environmental impacts (Wang et al., 2015), they are indispensable and essential for breeding studies. That is, morphological markers have an important role in the selection and classification of the promising genotypes in plant breeding studies. Flowering-related traits, growing habits, yield and fruit quality are very important parameters for walnut breeding. Late leafing and early defoliation also are important breeding characteristics in walnuts, especially to avoid late spring and early autumn frosts.

The objective of this study was to determine the levels of genetic differences in walnut genotypes obtained by open-pollination of 9 different cultivars (Bilecik, Chandler, Franquette, Howard, Kaman 1, Maraş 12, Pedro, Sütyemez 1 and Gimar) by using some important phenological markers. Results obtained from this characterization would provide useful information and contribute to future walnut breeding programs.

Materials and methods

Materials

This study was carried out in the vegetation period of 2017 and 2018 and, the data of these 2 consecutive years were used in the data analysis. In the study, nine important walnut genotypes including Bilecik, Chandler, Franquette, Howard, Kaman 1, Maraş 12, Pedro, Sütyemez 1, Gimar in Nuts Application and Research Center (SEKAMER), Kahramanmaraş, Turkey and 75 seedlings obtained from their open-pollinated seeds were used as material. According to the parents, the healthy seedlings were given codes between 1 and 75. The codes given to genotypes according to their parents are presented in *Table 1*. Bilecik, Kaman 1, Maras 12 and Sütyemez 1 walnut cultivars originated from Turkey and are grown considerably commercially in Turkey. Among these walnut cultivars, Maraş-12 has a cluster-bearing habit and Sütyemez-1 has very large nuts (Sütyemez, 2016; Sütyemez et al., 2019). Howard, Pedro and Chandler are walnut cultivars of USA origin. These cultivars have good nut quality and fruitfulness. Franquette is of French origin and its late leafing is an important trait of this cultivar (Ramos, 1997). In order to determine the genetic diversity, four phenological features were identified on a total of 684 seedlings including their parents, which are very important for breeding.

Table 1. *Descriptive statistics for phenological traits in our walnut collection*

Parent	Seedling code
Bilecik	BI1-75
Chandler	CH1-75
Franquette	FR1-75
Gimar	GI1-75
Howard	HO1-75
Kaman1	KA1-75
Maraş12	MA1-75
Pedro	PE1-75
Sutyemez1	SU1-75

http://www.aloki.hu ● ISSN 1589 1623 (Print) ● ISSN 1785 0037 (Online)
DOI: http://dx.doi.org/10.15666/aeer/1803_48074815
© 2020, ALÖKI Kft., Budapest, Hungary

Methods

Phenological traits

In the study, phenological traits such as budburst, leafing, leaf yellowing, and defoliation dates were examined to determine the levels of genetic difference. The walnut accessions were characterized based on the Descriptor for Walnut (IPGRI, 1994) and Sütyemez (1998). List of traits and their definitions are presented in *Table 2*.

Table 2. *Definitions used in the determination of phenological traits. (Source: IPGRI, 1994; Sütyemez, 1998)*

Traits	Description
Date of budburst	When over 50% of terminal buds have enlarged and the bud scales have split exposing the green of the leaves inside
Leafing date	Date when 50% of terminal buds have enlarged and the bud scales have split exposing the green leaves
Leaf yellowing date	The date when more than 50% of the green leaves on the plant turn yellow
Defoliation date	When all the leaves of the plant fall

Data analysis

The observation of phenotypic data as dates was recorded as the number of days from January 1st for statistical analyses. The data were analyzed statistically with descriptive statistics, cluster analyses, Principal Component Analyses (PCA) and correlation by using the JMP13 Statistical Package Program for genetic diversity based on phenological traits. Phenological pair-wise distances of the walnut genotypes were clustered using Ward's method (Anderberg, 1973).

Results and discussion

Phenological traits are very significant during the domestication and introduction of fruit species (Khadivi-Khub et al., 2015). We determined a very large phenological diversity in our walnut accessions. Genotypes were found to have quite different characteristics in terms of phenological traits. The highest coefficient of variation belonged to budburst (CV = 12.49%), while the lowest CV was given by leaf yellowing (1.86%) (*Table 3*). The summary findings of budburst, leafing, leaf yellowing and defoliation dates of genotypes are presented in *Table 3*. Khadivi-Khub et al. (2015) reported that CV for leafing date was 77.78% on 540 walnut tree accessions selected in Neiriz region, Iran.

Table 3. *Descriptive statistics for phenological traits in our walnut collection*

Traits	Unit	Min.	Max.	Mean	SD	CV (%)
Budburst	Day	64	134	86.84	10.84	12.49
Leafing	Day	74	140	95.76	10.37	10.83
Leaf-yellowing	Day	290	342	309.78	5.77	1.86
Defoliation	Day	306	363	333.20	7.39	2.22

SD standard deviation, CV coefficient of variation = (SD / mean) × 100

http://www.aloki.hu ● ISSN 1589 1623 (Print) ● ISSN 1785 0037 (Online)
DOI: http://dx.doi.org/10.15666/aeer/1803_48074815
© 2020, ALÖKI Kft., Budapest, Hungary

Late spring and early autumn frosts cause significant economic losses in walnut. The yield is significantly reduced due to spring frosts, especially in early leafing and flowering genotypes. Therefore, late leafing is an ideal character in walnut to escape the spring frost injury as shown by other walnut cultivars such as Chico, Serr, Ashley and Sunland (Khadivi et al., 2019). Early defoliation date is important for the ecological region with a short vegetation period. Because late spring frosts, as well as autumn frosts, can cause significant losses in walnut. In this study, it was aimed to determine the new genotypes having both of these important traits and to determine the relationships between them. The date of leafing ranged from 16 March to 21 May for all walnut genotypes. The date of defoliation was between 3 November and 30 December. Among all genotypes, the earliest budburst and leafing was observed in SU72 and the latest in FR70. Franquette is known as one of the most late-leafing walnut cultivars in the world. Among the walnut genotypes obtained, it is important that a large proportion of the latest leafing twenty genotypes were obtained from the seeds of the Franquette cultivar. Furthermore, as a result of the observations made about the defoliation date, it was determined that the earliest defoliation genotype was FR 72 and the latest was FR66. Hassankhah et al. (2017) reported that the budburst dates of 6 different walnut genotypes ranged from 16 March to 2 April. In a study conducted by Büküсü and Sütyemez (2016), it was reported that the defoliation dates for 8 important walnut genotypes changed between 8-29 November. Akca et al. (2018) reported that the dates of defoliation ranged from 17 November to 4 December in 12 different walnut genotypes.

Pearson Correlation Coefficient was used to determine the associations between the phenological traits. We determined a very significant correlation between budburst and leafing dates (r = 0.970). In addition, a significant positive correlation was found between leaf yellowing and defoliation date (r = 0.482). In addition, we found positive relationships between leafing and defoliation dates (r = 0.050). However, since this value was quite low, the not strong association was detected between them. Amiri et al. (2010) found similar relationships associated with these two traits (r = 0.298). Significant correlations between leafing date and some horticultural traits were determined on walnut by other researchers (Ebrahimi et al., 2015; Khadivi-Khub et al., 2015; Abedi and Parvaneh, 2016). However, studies investigating the relationships with the dates of the defoliation remains are limited (Amiri et al., 2010). Correlations of phenological traits are presented in *Figure 1*.

Multivariate Analysis can summarize the variability of a complex dataset and present it in a most interpretable form, such as principal components (Ribeiro et al., 2013). In other words, the aim of PCA is to determine the main factors and effective parameters to discriminate among accessions (Khadivi-Khub, 2015). Therefore, PCA analysis was carried out to make an overall view of the differences in our walnut accession. PCA showed that the first component (PC1) for phenological traits explained 50.226% of the total variance. The second component (PC2) represented a total variance of 36.635%, while the third (PC3) and fourth (PC4) components explained 12.413% and 0.726% of the total variance, respectively. The bi-plot segregated the walnut genotypes into groups based partially on their parents (*Table 4*; *Fig. 2*). Although PCA analysis has been performed on many horticultural traits in walnut, the findings of this phenological traits studied have remained limited (Cosmulescu and Trandafir, 2011; Ercisli et al., 2012; Pop et al., 2013; Bou Abdallah et al., 2016; Arab et al., 2019).

APPLIED ECOLOGY AND ENVIRONMENTAL RESEARCH 18(3):4807-4815.
http://www.aloki.hu ● ISSN 1589 1623 (Print) ● ISSN 1785 0037 (Online)
DOI: http://dx.doi.org/10.15666/aeer/1803_48074815
© 2020, ALÖKI Kft., Budapest, Hungary

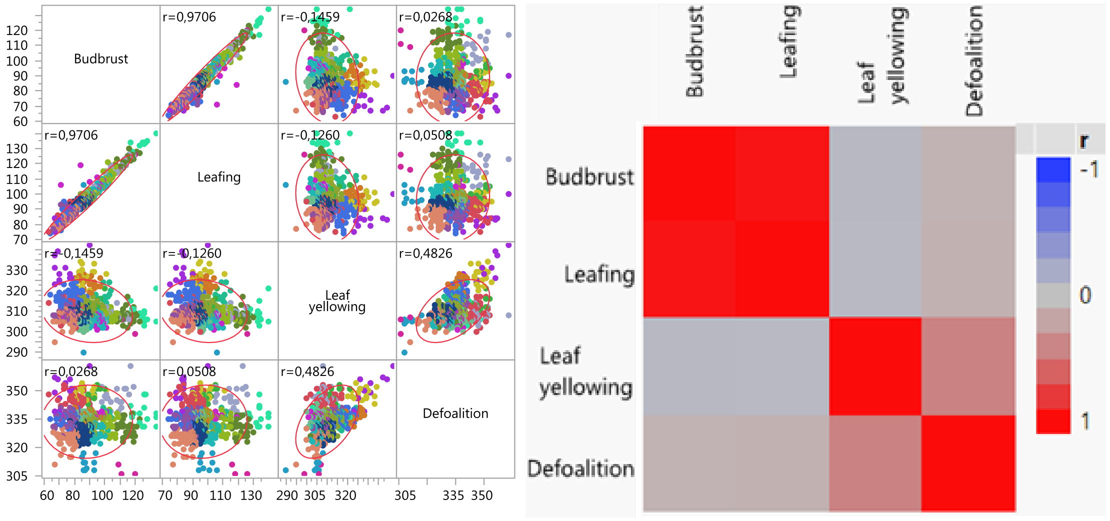

***Figure 1.** Scatterplot matrix and heatmap of correlations of phenological traits*

***Table 4.** Eigenvectors of the principal components (PC) for the studied walnut accessions*

Phenological traits	PC1	PC2	PC3	PC4
Budbrust	0.69**	0.11	0.09	0.70**
Leafing	0.69**	0.14	0.18	-0.71**
Leaf-yellowing	-0.21	0.67**	0.71**	0.01
Defoliation	-0.05	0.72**	-0.69**	0.01
% of variance	50.226	36.635	12.413	0.726
Cumulative variance	50.226	86.861	99.274	100

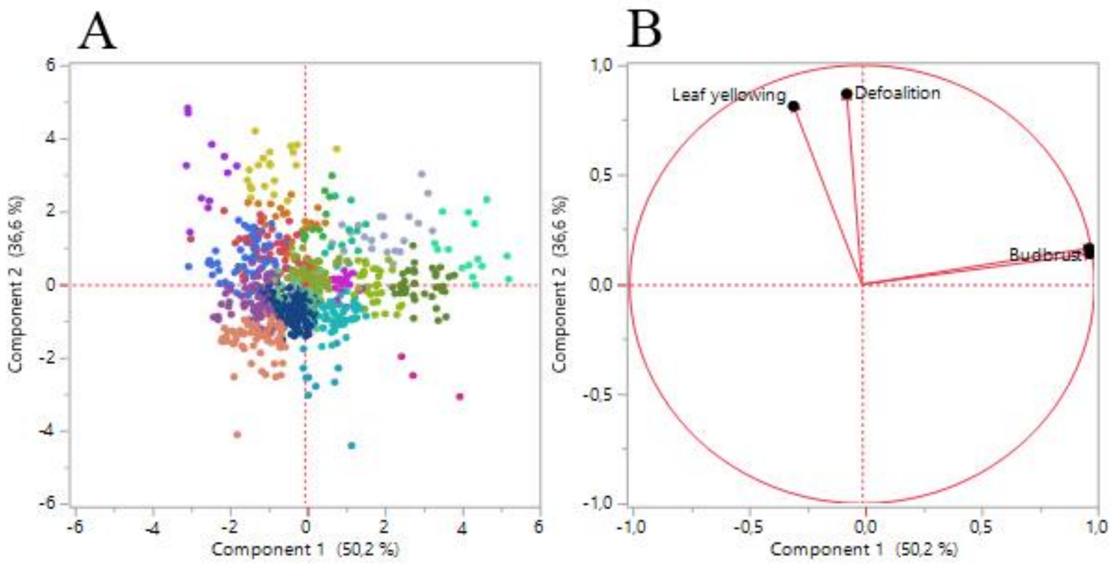

***Figure 2.** (A) Scatter plot for the first two principal components for the studied walnut accessions based on phenological traits. (B) Principal component analysis biplot of phenological traits of 684 walnut genotypes*

http://www.aloki.hu ● ISSN 1589 1623 (Print) ● ISSN 1785 0037 (Online)
DOI: http://dx.doi.org/10.15666/aeer/1803_48074815
© 2020, ALÖKI Kft., Budapest, Hungary

Cluster analysis of the 684 genotypes including seedling and their parents on the basis of four phenological traits was performed to estimate the relationships between the walnut genotypes in a dendrogram (*Fig. 3*). Based on this analysis, walnut accessions were classified into mainly three cluster groups. Genotypes obtained from the seeds of Bilecik and Kaman-1 cultivars, which were early leafing and deciduous compared to other genotypes examined, were generally included in Cluster 1. In Cluster 3, it has been determined that genotypes were generally derived from seeds of late-leaved cultivars such as Franquette, Chandler, and Pedro cluster together. Other genotypes examined in the study were found to be in Cluster 3 (*Fig. 3*). The results of the cluster analysis partially confirmed the results of PCA on the genotypes (*Fig. 2*). These findings show that although walnut has heterozygote, it has dominant genes especially in terms of traits like leafing and defoliation dates. Morphological traits have been used effectively in detecting genetic variation on walnut in various studies (Arzani et al., 2008; Ebrahimi et al., 2010, 2011; Ghasemi et al., 2012; Norouzi et al., 2013; Hussain et al., 2016; Cosmulescu and Stefanescu, 2018; Rezaei et al., 2018).

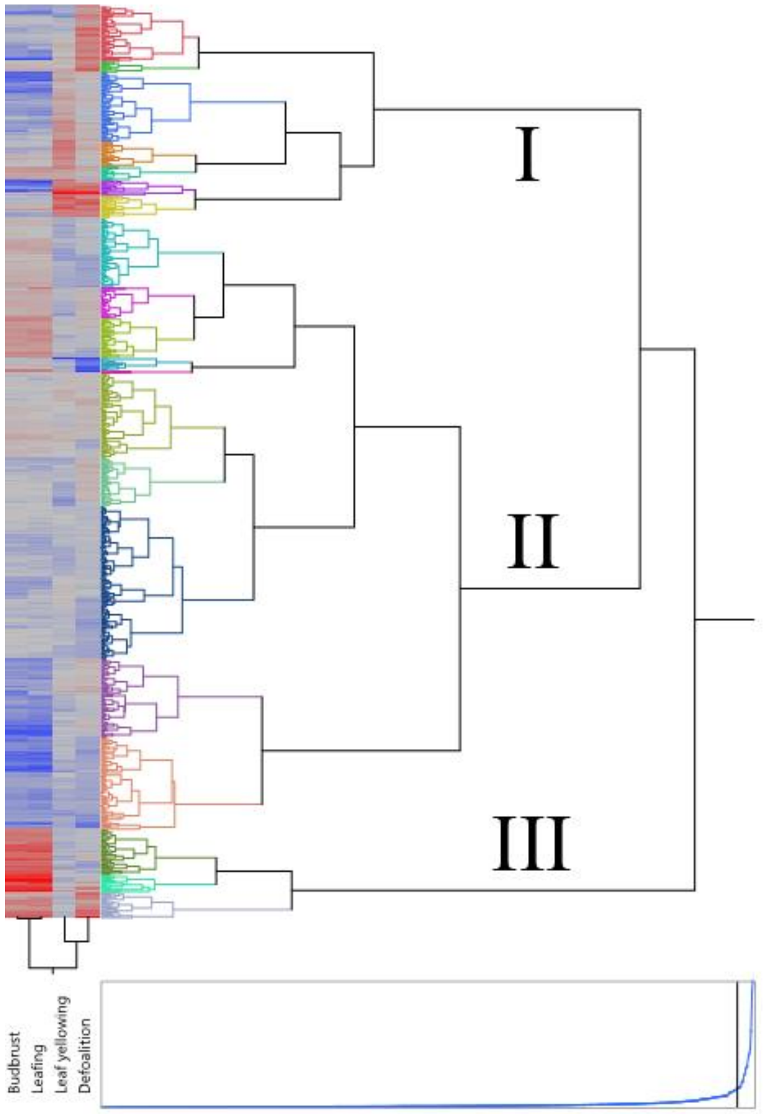

Figure 3. *Phenotypic clustering of 684 walnut genotypes based on Ward's phenological pair-wise distance and phenological heat map*

http://www.aloki.hu ● ISSN 1589 1623 (Print) ● ISSN 1785 0037 (Online)
DOI: http://dx.doi.org/10.15666/aeer/1803_48074815
© 2020, ALÖKI Kft., Budapest, Hungary

Conclusions

Walnut seedlings constitute the infrastructure of breeding programs both in terms of production and plant resources. Genotypes obtained from seeds in fruit species such as walnut are very important sources in terms of biodiversity. Walnut has a long juvenile period therefore breeding programs require a long time. Breeding programs carried out in the young periods of walnut contribute to shortening this period and reaching the early goal. Thus, identification of phenotypic differences provides an insight for future breeding studies. The results from the present study clearly showed that morphological markers are able to partially differentiate seedling obtained from open pollinated walnut cultivars seed. Based on our results, morphological markers should be used to describe genetic diversity and relationships among seedling walnut genotypes. Moreover, the findings provide an important source of information in the selection of walnut genotypes with new superior properties in the future.

REFERENCES

[1] Abedi, B., Parvaneh, T. (2016): Study of correlations between horticultural traits and variables affecting kernel percentage of walnut (*Juglans regia* L.). – Journal of Nuts 7(1): 35-44. DOI: 10.22034/JON.2016.522951.

[2] Amiri, R., Vahdati, K., Mohsenipoor, S., Mozaffari, M. R., Leslie, C. (2010): Correlations between some horticultural traits in walnut. – HortScience 45(11): 1690-1694. DOI: 10.21273/HORTSCI.45.11.1690.

[3] Anderberg, M. R. (1973): Cluster Analysis for Applications. – Academic Press, New York, pp. 2-18.

[4] Arab, M. M., Marrano, A., Abdollahi-Arpanahi, R., Leslie, C. A., Askari, H., Neale, D. B., Vahdati, K. (2019): Genome-wide patterns of population structure and association mapping of nut-related traits in Persian walnut populations from Iran using the Axiom *J. regia* 700K SNP array. – Scientific Reports 9(1): 6376. DOI: 10.1038/s41598-019-42940-1.

[5] Arzani, K., Mansouri-Ardakan, H., Vezvaei, A., Roozban, M. R. (2008): Morphological variation among Persian walnut (*Juglans regia*) genotypes from central Iran. – New Zealand Journal of Crop and Horticultural Science 36(3): 159-168. DOI: 10.1080/01140670809510232.

[6] Bou Abdallah, I., Baatour, O., Mechrgui, K., Herchi, W., Albouchi, A., Chalghoum, A., Boukhchina, S. (2016): Essential oil composition of walnut tree (*Juglans regia* L.) leaves from Tunisia. – Journal of Essential Oil Research 28(6): 545-550. DOI: 10.1080/10412905.2016.1166157.

[7] Bükücü, Ş. B., Sütyemez, M. (2016): The determination of the chilling requirements of some walnut (*Juglans regia* L.) cultivars and types. – Turkish Journal of Agricultural and Natural Science 3(4): 305-310.

[8] Cosmulescu, S., Stefanescu, D. (2018): Morphological variation among Persian walnut (*Juglans regia*) genotypes within the population and depending on climatic year. – Scientia Horticulturae 242: 20-24. DOI: 10.1016/j.scienta.2018.07.018.

[9] Cosmulescu, S., Trandafir, I. (2011): Variation of phenols content in walnut (*Juglans regia* L.). – South Western Journal of Horticulture, Biology and Environment 2(1): 25-33.

[10] Ebrahimi, A., Fatahi, M. M., Zamani, Z. A., Vahdati, K. (2010): An investigation on genetic diversity of 608 Persian walnut accessions for screening of some genotypes of superior traits. – Iranian Journal of Horticultural Sciences 40(4): 83-94.

http://www.aloki.hu ● ISSN 1589 1623 (Print) ● ISSN 1785 0037 (Online)
DOI: http://dx.doi.org/10.15666/aeer/1803_48074815
© 2020, ALÖKI Kft., Budapest, Hungary

[11] Ebrahimi, A., Fatahi, R., Zamani, Z. (2011): Analysis of genetic diversity among some Persian walnut genotypes (*Juglans regia* L.) using morphological traits and SSRs markers. – Scientia Horticulturae 130(1): 146-151. DOI: 10.1016/j.scienta.2011.06.028.
[12] Ebrahimi, A., Khadivi-Khub, A., Nosrati, Z., Karimi, R. (2015): Identification of superior walnut (*Juglans regia*) genotypes with late leafing and high kernel quality in Iran. – Scientia Horticulturae 193: 195-201. DOI: 10.1016/j.scienta.2015.06.049.
[13] Ercisli, S., Sayinci, B., Kara, M., Yildiz, C., Ozturk, I. (2012): Determination of size and shape features of walnut (*Juglans regia* L.) cultivars using image processing. – Scientia Horticulturae 133: 47-55. DOI: 10.1016/j.scienta.2011.10.014.
[14] Ferreira, J. J., Garcia-González, C., Tous, J., Rovira, M. (2010): Genetic diversity revealed by morphological traits and ISSR markers in hazelnut germplasm from northern Spain. – Plant Breeding 129(4): 435-441. DOI: 10.1111/j.1439-0523.2009.01702.x.
[15] Ghasemi, M., Arzani, K., Hassani, D. (2012): Evaluation and identification of walnut (*Juglans regia* L.) genotypes in Markazi province of Iran. – Crop Breeding Journal 2(2): 119-124. DOI: 10.22092/cbj.2012.100429.
[16] Gunn, B. F., Aradhya, M., Salick, J. M., Miller, A. J., Yongping, Y., Lin, L., Xian, H. (2010): Genetic variation in walnuts (*Juglans regia* and *J. sigillata*; *Juglandaceae*): species distinctions, human impacts, and the conservation of agrobiodiversity in Yunnan, China. – American Journal of Botany 97(4): 660-671. DOI: 10.3732/ajb.0900114.
[17] Hassankhah, A., Vahdati, K., Rahemi, M., Hassani, D., Sarikhani Khorami, S. (2017): Persian Walnut phenology: effect of chilling and heat requirements on budbreak and flowering date. – International Journal of Horticultural Science and Technology 4(2): 259-271. DOI: 10.22059/IJHST.2018.260944.249.
[18] Hernández-Delgado, S., Padilla-Ramírez, J. S., Nava-Cedillo, A., Mayek-Pérez, N. (2007): Morphological and genetic diversity of Mexican guava germplasm. – Plant Genetic Resources 5(3): 131-141. DOI: 10.1017/S1479262107827055.
[19] Hong, S. K., Kim, W. G., Yun, H. K., Choi, K. J. (2008): Morphological variations, genetic diversity and pathogenicity of Colletotrichum species causing grape ripe rot in Korea. – The Plant Pathology Journal 24(3): 269-278. DOI: 10.5423/PPJ.2008.24.3.269.
[20] Hussain, I., Sulatan, A., Shinwari, Z. K., Raza, G., Ahmed, K. (2016): Genetic diversity based on morphological traits in walnut (*Juglans regia* L.) landraces from Karakoram Region-I. – Pak. J. Bot. 48(2): 653-659.
[21] IPGRI (1994): Descriptors for Walnut (*Juglans* spp.). – International Plant Genetic Resources Institute, Rome.
[22] Kabiri, G., Bouda, S., Elhansali, M., Haddioui, A. (2018): Morphological and pomological variability analysis of walnut (*Juglans regia* L.) genetic resources from the middle and high Atlas of Morocco. – Atlas Journal of Biology 2018: 575-582. DOI: 10.5147/ajb.v0i0.179.
[23] Khadivi-Khub, A., Ebrahimi, A., Sheibani, F., Esmaeili, A. (2015): Phenological and pomological characterization of Persian walnut to select promising trees. – Euphytica 205(2): 557-567. DOI: 10.1007/s10681-015-1429-9.
[24] Khadivi, A., Montazeran, A., Yadegari, P. (2019): Superior spring frost resistant walnut (*Juglans regia* L.) genotypes identified among mature seedling origin trees. – Scientia Horticulturae 253: 147-153. DOI: 10.1016/j.scienta.2019.04.041.
[25] Norouzi, R., Heidari, S., Asgari-Sarcheshmeh, M. A., Shahi-Garahlar, A. (2013): Estimation of phenotypical and morphological differentiation among some selected Persian walnut (*Juglans regia* L.) accessions. – Intl. J. Agron. Plant Prod. 4(9): 2438-2445.
[26] Pop, I. F., Vicol, A. C., Botu, M., Raica, P. A., Vahdati, K., Pamfil, D. (2013): Relationships of walnut cultivars in a germplasm collection: comparative analysis of phenotypic and molecular data. – Scientia Horticulturae 153: 124-135. DOI: 10.1016/j.scienta.2013.02.013.

© 2020, ALÖKI Kft., Budapest, Hungary

[27] Ramos, D. E. (ed.) (1997): Walnut Production Manual (Vol. 3373). – UCANR Publications Oakland.
[28] Rana, J. C., Chahota, R. K., Sharma, V., Rana, M., Verma, N., Verma, B., Sharma, T. R. (2015): Genetic diversity and structure of Pyrus accessions of Indian Himalayan region based on morphological and SSR markers. – Tree Genetics & Genomes 11(1): 821. DOI: 10.1007/s11295-014-0821-2.
[29] Rezaei, Z., Khadivi, A., ValizadehKaji, B., Abbasifar, A. (2018): The selection of superior walnut (*Juglans regia* L.) genotypes as revealed by morphological characterization. – Euphytica 214(4): 69. DOI 10.1007/s10681-018-2153-z.
[30] Ribeiro, A. B., Bonafé, E. G., Silva, B. C., Montanher, P. F., Santos Júnior, O. O., Boeing, J. S., Visentainer, J. V. (2013): Antioxidant capacity, total phenolic content, fatty acids and correlation by principal component analysis of exotic and native fruits from Brazil. – Journal of the Brazilian Chemical Society 24(5): 797-804. DOI: 10.5935/0103-5053.20130105.
[31] Şen, S. M. (2011): Ceviz yetiştiriciliği, besin değeri, folklorü. 4th Ed. – ÜÇM Yayıncılık, Ankara.
[32] Sütyemez, M. (1998): Researches on walnut selection and fertilization biology of selected types in Kahramanmaraş region. (Kahramanmaraş bölgesinde ceviz seleksiyonu ve seçilmiş bazı tiplerin döllenme biyolojileri üzerine araştırmalar). – PhD Thesis. Çukurova Unıversity Institute of Natural and Applied Sciences.
[33] Sutyemez, M. (2016): New Walnut Cultivars: Maras 18, Sutyemez 1, and Kaman 1. – HortScience 51(10): 1301-1303. DOI: 10.21273/HORTSCI10972-16.
[34] Sütyemez, M., Özcan, A., Bükücü, Ş. B. (2018): Walnut cultivars through cross-breeding: 'DİRİLİŞ'and '15 TEMMUZ'. – The American Pomological Society 72(3): 173-180.
[35] Sütyemez, M., Bükücü, Ş. B., Özcan, A. (2019): Maraş 12: A walnut cultivar with cluster-bearing habit. – HortScience 54(8): 1437-1438. DOI: 10.21273/HORTSCI14226-19.
[36] Torres-Calzada, C., Tapia-Tussell, R., Higuera-Ciapara, I., Perez-Brito, D. (2013): Morphological, pathological and genetic diversity of Colletotrichum species responsible for anthracnose in papaya (*Carica papaya* L). – European Journal of Plant Pathology 135(1): 67-79. DOI: 10.1007/s10658-012-0065-7.
[37] Wang, H., Wu, W., Pan, G., Pei, D. (2015): Analysis of genetic diversity and relationships among 86 Persian walnut (*Juglans regia* L.) genotypes in Tibet using morphological traits and SSR markers. – The Journal of Horticultural Science and Biotechnology 90(5): 563-570. DOI: 10.1080/14620316.2015.11668715.

http://www.aloki.hu ● ISSN 1589 1623 (Print) ● ISSN 1785 0037 (Online)
DOI: http://dx.doi.org/10.15666/aeer/1803_48074815
© 2020, ALÖKI Kft., Budapest, Hungary
