# Supplementary material for: Provenance and family variations in early growth of Manchurian walnut (Juglans mandshurica Maxim.) and selection of superior families
Source: PLoS One. 2024 Mar 7;19(3):e0298918. doi: 10.1371/journal.pone.0298918 (PMC10919699; doi:10.1371/journal.pone.0298918)
Supplement: S1 File — (ZIP) [file pone.0298918.s004.zip › Developing individual tree crown width models for Larix principis-rupprechtii.pdf]

分 类 号：____________　　密 级：____________

学校代号：10538　　学 号：20141100422

中南林业科技大学

Central South University of Forestry & Technology

硕士学术学位论文

华北落叶松天然林单木冠幅模型研究

作者姓名：符亚健

导师姓名：吕 勇

培养学院：林学院

学科名称：森林经理学

研究方向：森林经营数表与森林可持续经营

提交日期：2017 年 5 月

中国知网 https://www.cnki.net

Developing individual tree crown width models for *Larix principis-rupprechtii*

By

FU Yajian

A thesis submitted in partial fulfillment of the

Requirements for the degree of

Master of Agronomy

Supervisor

Professor Lv Yong

Central South University of Forestry and Technology

498 Shaoshan South Road， Tianxin District

Changsha Hunan 410004， P.R.CHINA

May, 2017

中国知网 https://www.cnki.net

中国知网 https://www.cnki.net

摘 要

华北落叶松(*Larix principis-rupprechtii*)是我国华北地区山地的主要造林树种。其优点众多，生长速度快、木材材质好、用途广、腐朽耐力强，是营造经济生态林的良好树种，同时也是杰出的防护林树种。树冠是树木进行光合作用和积累能量的重要场所，它反映了树木的生长活力和竞争力，是树木一个重要的健康指标。冠幅是描述树冠生长的重要指标也是森林生长收获预估模型的重要预测变量。因此准确预估冠幅对森林可持续经营和森林生态研究极其重要。本文以多种树木林分因子为协变量构建了一般冠幅-胸径预测模型，并且分析了地域效应和区组效应以及嵌套在区组里的样地效应对华北落叶松冠幅影响，以此为基础使用了非线性混合效应模型构建了相应的冠幅模型，最后还考虑了冠幅与东西南北四个方向的冠径的相关性，并使用了四种可加性模型方法构建了相应的模型系统，四种模型系统都解决了冠幅与东西南北四个方向的冠径的相容性；最后通过综合比较确定了最优的可加性冠幅模型系统。主要研究结果如下：

（1）三参数的逻辑斯蒂模型：$CW=a/1+\exp[b+c\ln(D+1)]$能较好反映华北落叶松天然次生林冠幅和直径之间的非线性关系，与其它候选模型相比，该模型有较高的拟合精度，并且模型各参数都具有一定的生物学意义；对象木冠长（CL）、对象木树高（H）和每公顷株数（M）对冠幅影响较大，当这些因子作为预测变量时能明显改进模型的预测精度；得到的改进后的冠幅模型表达式为：

$$CW=1.4875-0.0311H+\exp[(-3.6416-0.0002M)+(2.4346+0.0045CL)\ln(D+1)]$$

通过大量实验数据验证，该模型具有较高的预测精度。

（2）研究发现区组效应和嵌套在区组里面的样地效应对华北落叶松的随机影响较大，当模型考虑这些随机影响时模型预测精度能进一步显著提高；指数方差函数且预测变量为胸高直径能有效剔除模型的异方差，表达式为：$\mathrm{var}(\varepsilon_{ijk})=\sigma^2\exp(2\gamma x_{ijk})$；利用所构建的嵌套两水平非线性混合效应模型预测冠幅时，利用随机抽取的 4 株样地计算随机效应参数效果较好。当地域效应作用在固定效应参数β_1和β_5上时，模型对应的 AIC（5425）最小而 LogLik（-2697）最大最终构建了华北落叶天然林非线性回归效应单木冠幅模型。

（3）以模型（4-3）为基础模型，使用非线性联立方程组（NSE）、 非线性似然不相关回归(NSUR)、比例平差法(AP）和最小二乘法独立回归(OLSSR)方法构建冠幅可加性模型系统，这几种方法都能有效的考虑总冠幅和各树冠半径

中国知网 https://www.cnki.net

之间的相关性。通过综合对比这几种可加性模型系统，对于总冠幅，分级联合控制平差法构建的冠幅模型系统对应的指标 δ，$RMSE$ 和 TRE 均要低于 NSUR、AP 和 OLSSR 模型系统；所以在可加性模型构建冠幅模型方法中，分级联合控制平差法构建的冠幅可加性模型系统拟合效果最好。

关键词：冠幅模型；非线性混合效应模型；非线性联立方程组；可加性；华北落叶松

中国知网 https://www.cnki.net

ABSTRACT

The *Larix principis-rupprechtii* forests are almost managed for the objectives of timber production, ecosystems or watershed protection, and habitats for specific animals. Larch forests in this region are usually characterized by a large amount of biomass and high primary productivity, which may be related to high adaptability of the tree species to the extreme low winter temperatures as well as to the efficient utilization of water from the melting zone of permafrost soil during the hot and dry summer season. This is why these larch species are widely used for timber production and afforestation in the temperature zone of China.Tree crown is an important place for photosynthesis and accumulation of energy. It reflects the growth vigor and competition ability of trees, and is an important health index of trees. The crown width is to describe the important variables to predict important index of canopy growth is also a harvest forest growth prediction model.This paper constructs a prediction model with a variety of crown diameter trees stand factors as covariates, and analyzes the influence of regional effect and group effect and nested in block in the kind of effect on the larch crown, on the basis of using the nonlinear mixed effect model constructed crown model, also consider the correlation the crown and the four corners of the world in four directions and crown diameter, and the use of four kinds of additive model method to build a model system, four models are solved compatibility the diameter of the crown and the four corners of the world four direction of the crown; finally through comprehensive comparison to determine the additivity of crown model system optimization. The main results are as follows:

(1) Logistic model with three parameters: CW=a/1+exp[b+cln (D+1)] can better reflect the nonlinear relationship between the amplitude and the diameter of the natural secondary forest of larch, compared with other candidate models, the model has high fitting precision, and have certain biological significance of the parameters of the model object; wood crown length (CL), object height (H) and per hectare (M) has great influence on the crown, when the forecast accuracy is significantly improved model of these factors as predictors of the crown; the expressions of the improved model

中国知网 https://www.cnki.net

for: $CW = 1.4875 - 0.0311H + \exp[(-3.6416 - 0.0002M) + (2.4346 + 0.0045CL)\ln(D+1)]$

Through a large number of experimental data, the model has higher prediction accuracy.

(2) The study found large random effects and nested block effects in block inside the plots of larch, when considering these random effects model prediction accuracy can be further increased significantly; the variance function index and predictive variables for breast height diameter can be effectively removed model heteroscedasticity, expression is: $\mathrm{var}(\varepsilon_{ijk}) = \sigma^2 \exp(2\gamma x_{ijk})$; the construction of the two level nested nonlinear mixed effect model to predict crown, using a random sample of 4 strains of samples calculated random effect parameter is better. When the regional effect in the fixed effect parameters and corresponding model, AIC (5425) and LogLik (-2697) nonlinear minimum maximum final construction of Larix natural forest crown models such as the regression effect shown in (5-19).

(3) Model (4-3) as the based model, using nonlinear simultaneous equations (NSE), nonlinear likelihood regression (NSUR), the ratio adjustment method (AP) and least squares regression (OLSSR) method to construct independent crown additive model system, these methods can reflect the correlation between the total crown and the effective radius of the crown. Through the comprehensive comparison of these kinds of additive model for the total system, crown width, crown model of joint control system construction adjustment method and corresponding grading index, and were lower than NSUR, AP and OLSSR model system; therefore, the joint control of construction grading adjustment method the crown additive model system the highest prediction accuracy, the final model the expression as shown in (5-7).

Key words: Crown width model, Nonlinear mixed-effects model, Nonlinear simultaneous equations, Additive, *Larix principis-rupprechtii*

中国知网 https://www.cnki.net

目 录

摘要......I
ABSTRACT......III
1 绪论......1
1.1 研究背景......1
1.2 研究目的与意义......2
1.3 课题来源......3
1.4 研究进展......3
1.4.1 非线性混合效应模型国内外研究进展及发展动态......3
1.4.2 可加性模型国内外研究现状及发展动态......6
1.4.3 冠幅模型研究进展......9
1.5 主要研究内容......10
2 研究地区概况和数据......12
2.1 研究区概况......12
2.1.1 关帝山国有林场概况......12
2.1.2 伯强国有林场概况......13
2.2 数据来源......14
2.3 样地主要林分因子统计......15
3 非线性回归冠幅模型建立与分析......17
3.1 材料与方法......17
3.1.1 实验数据......17
3.1.2 基础模型......17
3.1.3 预测变量确定......18
3.1.4 参数估计及改进模型评价......19
3.2 结果与分析......19
3.2.1 模型选择......19
3.2.2 模型改进......20
3.2.3 模型比较......20
3.3 小结......21

中国知网 https://www.cnki.net

4 非线性混合效应冠幅模型研建......22
4.1 材料与方法......22
4.1.1 实验数据......22
4.1.2 非线性混合效应模型......22
4.1.3 基础模型......23
4.1.4 形式参数构造......23
4.1.5 随机效应参数方差（ψ_1和ψ_2）结构......24
4.1.6 误差项方差协方差（**R**）结构......24
4.1.7 地域效应......24
4.1.8 参数估计......25
4.1.9 模型预测......25
4.1.10 PA 模型......25
4.1.11 区组水平......25
4.1.12 区组和嵌套在区组里面的样地效应（区组+区组*样地）......26
4.1.13 模型评价......26
4.2 结果与分析......27
4.2.1 基础模型......27
4.2.2 嵌套两水平非线性混合效应冠幅模型......28
4.2.3 误差项方差协方差（**R**）结构......29
4.2.4 地域效应......31
4.2.5 模型参数估计......32
4.2.6 模型预测和评价......32
4.3 小结......34
5 冠幅可加性模型研建......35
5.1 材料与方法......35
5.1.1 实验数据......35
5.1.2 基础模型......35
5.1.3 非线性联立方程组（NSE）......36
5.1.4 非线性似然无关回归方法(NSUR)......39
5.1.5 比例平差法（AP）......40
5.1.6 最小二乘法独立回归（OLSSR）......41

中国知网 https://www.cnki.net

5.1.7 模型评价......41
5.2 结果与分析......42
5.2.1 基础模型......42
5.2.2 总量直接控制平差法和分级联合控制平差法比较......43
5.2.3 冠幅可加性模型比较和评价......46
5.3 小结......48
6 结论与讨论......49
6.1 结论......49
6.2 讨论......49
6.3 创新点......50
参考文献......51
附录 A 非线性回归和非线性混合效应模型 R 程序......59
附录 B 似然不相关回归 R 程序......63
致谢......67

中国知网 https://www.cnki.net

1 绪论

1.1 研究背景

自从工业革命以来，人类社会高速发展，全球人口程爆炸式地增长，已经对环境产生了不可逆转的破坏，所导致的全球气候变暖、土地荒漠化、资源过度开发等问题日益严重。人类的可持续发展遭受到了严重的挑战，在生存环境日益遭到严重的破坏下，林木资源的总量逐渐下降，随着社会的发展人类对林木产品的需求也日益增多，由此与人类可持续发展这个当今社会大课题产生了重大的矛盾。在全球环境日益不断恶化、发展问题更加严重的情况下，联合国于 1992 年在巴西里约召开了联合国环境与发展会议（UNCED），该会议又称为“地球会议”，“里约宣言”指出：和平、发展和保护环境是互相依存、不可分割的，世界各国应在环境与发展领域加强国际合作，为建立一种新的、公平的全球伙伴关系而努力。这次会议提出了森林的可持续经营理念，该理念在过去几十年全球范围内得到了广泛的传播和一致的认可。恢复世界森林资源的重要举措和实现人类可持续发展的重要保证是实施森林的可持续经营、培育人工次生林[1]。

当前我国的森林资源发展十分地迅速，在取得一定的成就的同时，也存在着许许多多的问题。根据国务院新闻办公布的最新的第八次全国森林资源清查结果显示，全国森林覆盖率 21.63%，森林面积 2.08 亿公顷，森林蓄积 151.37 亿 m^3。人工林蓄积 24.83 亿 m^3' 面积 0.69 亿 hm。清查结果表明，我国林业资源进入了数量增长、质量提升的稳步发展时期。但是，我国森林覆盖率远低于全球 31% 的平均水平，人均森林面积仅为世界人均水平的 1/4，人均森林蓄积只有世界人均水平的 1/7，森林资源总量的相对不足、质量不高、分布不均的状况仍未得到根本改变，造林绿化改善生态的任务任重而道远；加强森林的可持续经营的要求非常迫切；森林有效供给与日益增长的社会需求的矛盾依然突出[2]。

准确预测树木和森林生长收获情况是森林资源可持续经营的重要基础，近些年来林分树种预测模型越来越受到国内外林业工作者的重视，作为研究森林预估林分生长量、收获量以及生长变化规律的林分树种预测模型被以各种方式建立和提出，其中已有相当一部分的树种预测模型被用于用于林业生产实践中。原因是林分树种预测模型主要具有下面几种用途，1、可以为采伐计划提供基础数据。2、模型的预估结果可以为森林管理决策提供林木资源方面的依据；3、对各种育林

中国知网　https://www.cnki.net

措施的效应和影响进行评价；4、通过经营模型和生长模型实现资源数据的更新。随着数学理论的进展，电子计算功能的日益强大，信息论、系统论、控制论等信息学科的奠定，森林可持续经营的思想对森林资源信息和利用的要求以及林木调查技术的变革，都促进森林生长模型的研究日渐深入，并呈现出许多新动向[3]。

1.2 研究目的与意义

单木生长模型是以林分中各单株林木与其相邻木之间的竞争关系为基础，描述单株木生长过程的模型。这类模型产生于 60 年代[4],近几十年来，由于单木生长模拟系统的算法优化和计算机技术快速发展，现实则以单木（非林分）作为预测单位的林分生长预测系统。这类模型与径阶分布模型或全林分模型的主要区别在于：径阶分布模型或全林分模型的预测变量是径阶或林分统计量，而单木模型中至少有些预测变量是单株林木的统计量。依据这类模型可以直接判定各单株木的生长潜力和生长状况，以及判定采用林分密度控制措施后的各保留木得生长状况，并且，这些信息对于林分的集约经营是非常有价值的，因此对于指导林分经营，单木生长模型具有其特殊的意义[5]。

非线性混合效应模型（Nonlinear Mixed Effects Models,简称 NLMEMs）是根据回归函数依赖随机效应参数和固定效应参数的非线性关系而建立的。它是分析多水平数据、重复调查数据以及纵向数据的近代统计学方法，既能刻画个体差异又能反映总体的平均变化趋势的统计分析工具。近些年，非线性混合效应模型受到越来越多的学者关注，而且被引入到很多门学科，如林学、农学、医学和工学等。经过许多年的不断发展，有学者已提出了单水平和嵌套多水平非线性混合效应模型的多种参数估计方法，典型的计算软件有 S-Plus 和 SAS[6]。

树冠是树木进行光合作用和积累能量的重要场所，它反映了树木的生长活力和竞争力，是树木一个重要的健康指标。冠幅是描述树冠生长的重要指标之一，它可以用来计算树木的竞争指数和作为协变量来预测树高或胸径生长量等；此外，冠幅也是林分可视化的重要参数。国内外对冠幅的研究主要集中在定性和图表研究，部分学者利用传统的回归方法建立冠幅与一些林分因子，例如林分密度、胸高断面积、树高、胸高直径等因子的线性关系，从而进一步分析这些因子对冠幅的影响。然而在实际应用中，由于气候条件、人为因素、立地条件以及经营措施对调查因子的随机干扰，林分调查因子与冠幅间可能呈现较复杂的非线性关系。同时，所调查的数据常为多水平数据或重复调查数据，如不同立地条件中对树木冠幅重复观察，这些数据的调查对象间可能存在有明显的异方差和自相关

中国知网　https://www.cnki.net

等。而传统的回归分析方法是假定数据间相互独立且非异质性，是反应林分总体变化情况，对于研究对象（林分类型）个体间的差异程度，传统的回归方法无法解决；然而，混合模型能有效地解决此类问题[7]。

华北落叶松(*Larix principis-rupprechtii*) 是松科（*Pinaceae*）落叶松属乔木（*Larix*）,高可达 30 米，胸径 1 米，中国特产，是我国华北地区高山针叶林带中的主要森林树种。其优点众多，主要优点有腐朽耐力强、木材材质好、生长速度快、用途广，是营造经济生态林的优质树种，同时也是良好的防护林树种。山西、河北是华北落叶松天然林分布的主要地区，随着气候的变化、人类活动的增加等诸多因素，如今的华北落叶松天然林呈片段分布，天然群体主要局限在各个山区顶部[8]。

1.3 课题来源

课题来源于国家林业局林业公益性行业科研专项项目：“太行山西侧生态公益林提质增效关键技术研究（201404417）”。目的是提高生态公益林的质量和效益。

项目主要研究的内容是：太行山西侧典型生态公益林提质增效评价指巧体系、不同尺度（单木、林分、景观和区域）目标结构、生长收获模型、提质增效关键技术。将为有效提升太行山西侧公益林的质效水平、服务功能以及可持续经营提供理论基础和技术支撑。研究重点围绕华北落叶松天然次生林单木冠幅模型开展。

1.4 研究进展

1.4.1 非线性混合效应模型国内外研究进展及发展动态

非线性混合效应模型（Nonlinear Mixed Effects Models,简称 NLMEMs）是根据回归函数依赖随机效应参数和固定效应参数的非线性关系而建立的。它是分析多水平数据、重复调查数据以及纵向数据的近代统计学方法，既能刻画个体差异又能反映总体的平均变化趋势的统计分析工具[9-11]。近些年，随着非线性混合效应模型的理论研究和计算软件不断深化和完善，非线性混合效应模型已被广泛应用到森林生长收获模型中，并成为现代林业模型上的一个核心内容[12-20]

为了含义确切，把已有文献的 Multilevel NLMEMs 译为嵌套多水平非线性混合效应模型。以后文中的多因素不仅包括嵌套多水平也包括多因素的其他类型）。

中国知网 https://www.cnki.net

为直观起见，用一个实际例子加以说明。假设树高 H 与树木直径 D 的关系是[21-22]：

$$H = 1.3 + \exp(\phi_1 + \phi_2 / D) + \varepsilon \qquad (1\text{-}1)$$

其中，ϕ_1和ϕ_2为形式参数（简称形参），ε为误差项。假设实验数据来自 5 个区组（Block），每个区组包含 4 个样地（Plot），分别测定每个样地的树高（H）和直径（D）。环境和林分因子考虑海拔（A）、林分密度（SD）和地位级指数（SI）。海拔按照≥700 m 和<700 m 分为两个等级（分别记为 1, 2）。林分密度按区间 500 株/ha 划分为 6 个等级（分别记为 1,2,…,6）。地位级指数从 15 m 开始，按间隔 2 m 划分 5 个等级（分别记为 1,2,…,5）。其中，海拔、区组和样地为逐级嵌套关系，其它变量间为相互交错关系。

如果只考虑单个因素对模型中因变量的随机影响，称为单水 NLMEMs[23]。以模型（1-1）为例，当考虑区组对树高的随机影响，并假定由区组产生的随机效应作用在形参ϕ_1上（以下实例中，除特别声明外，都假定随机效应参数作用在形参ϕ_1上），模型表达式为：

$$H = 1.3 + \exp\left[(\beta_1 + u_1) + \phi_2 / D\right] + \varepsilon \qquad (1\text{-}2)$$

其中，β_1为固定效应参数；u_1为区组产生的随机效应参数；ε为误差项。模型（1-2）就是单水平 NLMEMs。同样，可以考虑多个逐级嵌套因子对树高的影响，该类型模型称之为嵌套多水平 NLMEMs[24]。以嵌套两水平为例，假如考虑区组以及嵌套在区组中的样地对树高的随机影响，模型写为：

$$H = 1.3 + \exp\left[(\beta_1 + u_1 + v_1) + \phi_2 / D\right] + \varepsilon \qquad (1\text{-}3)$$

其中，v_1为样地产生的随机效应参数。类似的可以推广到逐级嵌套更多水平，例如三水平。对于嵌套多水平 NLMEMs 必须要求随机效应因子之间逐级嵌套。

Pinheiro 和 Bates（2000）定义的单水平和嵌套多水平 NLMEMs 已被广泛应用[25-27]，并得到领域内以及其它领域许多学者的一致认可[28-30]。典型的计算软件有 SAS（Nlinmix 宏和 Nlmixed 模块）[31-32]和 S-Plus/R（nlme 函数）[33]。

但近几年随着 NLMEMs 应用不断深化，单水平和嵌套多水平 NLMEMs 已经不能满足实际的需要。例如，在模型（1-1）中，当考虑林分密度（SD）和地

中国知网 https://www.cnki.net

位级指数（SI）以及它们之间交互作用（SD*SI）对树高生长的随机干扰时，由于 SD 和 SI 这两个因子不相互嵌套而是相互交错关系，利用嵌套多水平非线性混合效应模型无法分析此类问题。因此，Pinheiro 和 Bates（2000）把非线性混合效应模型仅分为单水平和嵌套多水平非线性混合效应模型在应用中受到局限。按照实际需要，对于模型中任意一个形式参数，相应的随机效应有以下几种可能情况：

$$\begin{cases}\text{单因素，例如 A, Block, Plot, SD, SI} \\ \text{多因素}\begin{cases}\text{只有主因素，例如 SD+SI+Block} \\ \text{只有交互因素，例如 SD*SI, SD*SI*Block} \\ \text{只有逐级嵌套因素，例如 Block(Plot), A(Block(Plot))} \\ \text{上述类型的混合，例如 SD+SI+SD*SI+Block(Plot)}\end{cases}\end{cases}$$

因此，不同形参对应的不同随机效应类型将会衍生出多种类型的非线性混合效应模型。单水平和嵌套多水平非线性混合效应模型只是其中的 2 种特殊类型。直到 2012 年，符利勇（2012）提出一种正态 NLMEMs 的标准表达式。NLMEMs 的一般标准形式是：

$$\begin{cases} \underset{1\times1}{y_{ij}} = f(\underset{p\times1}{\boldsymbol{\phi}_i}, \underset{1\times s}{\boldsymbol{\upsilon}_{ij}}) + \varepsilon_{ij}, i=1,\dots,M, j=1,\dots,n_i \\ \underset{p\times1}{\boldsymbol{\phi}_i} = \underset{p\times p_0}{\mathbf{A}_i}\underset{p_0\times1}{\boldsymbol{\beta}} + \sum_{E\in\Omega}\underset{p\times q^{(E)}}{\mathbf{B}_i^{(E)}}\underset{q^{(E)}\times1}{\mathbf{u}_i^{(E)}} \\ E(\mathbf{u}_i^{(E)})=0, \mathrm{var}(\mathbf{u}_i^{(E)}) = \underset{q^{(E)}\times q^{(E)}}{\boldsymbol{\psi}^{(E)}}, \mathrm{cov}(\mathbf{u}_i^{(E)}, \mathbf{u}_i^{(F)})=0, F\in\Omega, E\neq F \\ \boldsymbol{\varepsilon}_i \sim N(0, \underset{n_i\times n_i}{\mathbf{R}_i}), \boldsymbol{\varepsilon}_i = (\varepsilon_{i1},\dots,\varepsilon_{in_i})^T \end{cases} \quad (1\text{-}4)$$

其中，υ_{ij}和y_{ij}分别为第i个对象s维自变量值和第j次观测的 1 维因变量值。$\boldsymbol{\beta}$为$p_0\times1$维固定效应参数向量，$\boldsymbol{\phi}_i$是p维形式参数向量。$\mathbf{A}_i$和$\mathbf{B}_i^{(E)}$分别是$\boldsymbol{\beta}$和$\mathbf{u}_i^{(E)}$的设计矩阵。$\mathbf{u}_i^{(E)}$是构造变量E在第i个对象上产生的$q^{(E)}\times1$维随机效应参数向量，$\boldsymbol{\psi}^{(E)}$为$\mathbf{u}_i^{(E)}$的协方差矩阵，对于不同的$E\in\Omega$，$\mathbf{u}_i^{(E)}$相互独立。ε_{ij}为误差项，对于不同的j ε_{ij}相互独立，同时还假定ε_{ij}与所有$\mathbf{u}_i^{(E)}$相互独立。当$\mathbf{u}_i^{(E)}$（$E\in\Omega$）和ε_{ij}都服从正态分布时，模型（1-4）称为正态 NLMEMs （本项目所谈及的 NLMEMs 除特别声明外都默认为正态 NLMEMs）。

以模型（1-1）为例，当考虑 SD 和 SI 以及它们之间交互作用 SD*SI 对树高生长的随机干扰时，基于模型（1-4），模型写为：

中国知网 https://www.cnki.net

$$H=1.3+\exp\left[\left(\beta_1+u_1^{(SD)}+u_1^{(SI)}+u_1^{(SD*SI)}\right)+\phi_2/D\right]+\varepsilon \tag{1-5}$$

其中，$u_1^{(SD)}$，$u_1^{(SI)}$和$u_1^{(SD*SI)}$分别是由 SD、SI 以及 SD*SI 产生的随机效应参数。对应模型（1-4），$m=2$，$\Theta=\{(1,1),(1,2),...,(1,5),(2,1)...,(6,5)\}$，$M=30$，$\Omega=\{SD,SI,SD*SI\}$。

模型（1-4）包含多种类型的非线性混合效应模型，例如逐级嵌套多水平非线性混合效应模型、单水平非线性混合效应模型、包括主效应和交互效应的非线性混合效应模型、只含主效应的多因素非线性混合效应模型以及某几种类型组合的一般性非线性混合效应模型等。模型中固定效应参数和随机效应参数可以考虑分级（即数量化）。同时还把正态非线性混合效应模型的标准表达式推广到参数方差与某些因素（称为组变量）有关的非线性混合效应模型（考虑组变量的非线性混合效应模型）。因此该模型比传统的 NLMEMs 表达式更为一般化，具有更广的用途。同时，符利勇（2012）给出线性逼近—逐步 2 次规划算法计算模型（1-4），并已在 ForStat 2.2 正式版本上创建"非线性混合模型"模块。迄今，除 ForStat 外，我们尚未发现国内外拥有其它可以计算包括所有随机效应类型的 NLMEMs 通用计算软件。

混合效应模型在林业应用上历史较长，可以追溯到 1961 年，Clutter 在博士论文中指出在回归分析中相应的模型校正是很有必要的，而且通过对固定样地重复抽样可以避免假定模型中随机效应相互独立[34]。而考虑交互作用的 NLMEMs 的应用非常少见[35-44]。 对于度量误差模型，自从 20 世纪 90 年代才逐步在林业上应用。到目前为止，已有较多的报道[45-53]。

1.4.2 可加性模型国内外研究现状及发展动态

林业上，可加性模型最初起源于 20 世纪后期生物量建模中，它是基于总生物量模型研究基础上，进一步构建各分项（干材、干皮、树枝和树叶）生物量方程系统，使得该方程系统得到的各分项生物量之和等于总生物量。到目前为止，经过十多年的发展，国内外已提出了不同可加性建模方法，其中最常见的有非线性似然无关回归方法[54-57]、比例平差法[58-60]和线性或非线性联合估计方法等[61-66]。

比例平差法是解决单木生物量相容性问题的最简单且最直接的办法，它是基于各分量占总量的比例之和等于 1 提出[67]。各分量模型中参数是通过最小二乘法

中国知网 https://www.cnki.net

独立求解得到。根据各分量分配层次的不同，可得到总量直接控制平差法和分级联合控制平差法。以总量直接控制平差法为例，由总量（以地上生物量为例）直接平差分配给干材、干皮、树枝和树叶，从而保证各分量之和等于总量。具体各分量的模型表达式如下[68]：

$$
\begin{aligned}
&W_1 = f_1(\mathbf{x}) / \left[f_1(\mathbf{x}) + f_2(\mathbf{x}) + f_3(\mathbf{x}) + f_4(\mathbf{x})\right] W_5 + \varepsilon_1 \\
&W_2 = f_2(\mathbf{x}) / \left[f_1(\mathbf{x}) + f_2(\mathbf{x}) + f_3(\mathbf{x}) + f_4(\mathbf{x})\right] W_5 + \varepsilon_2 \\
&W_3 = f_3(\mathbf{x}) / \left[f_1(\mathbf{x}) + f_2(\mathbf{x}) + f_3(\mathbf{x}) + f_4(\mathbf{x})\right] W_5 + \varepsilon_3 \\
&W_4 = f_4(\mathbf{x}) / \left[f_1(\mathbf{x}) + f_2(\mathbf{x}) + f_3(\mathbf{x}) + f_4(\mathbf{x})\right] W_5 + \varepsilon_4 \\
&W_5 = f_5(\mathbf{x}) + \varepsilon_5
\end{aligned}
\qquad (1\text{-}6)
$$

其中，$f_1(\mathbf{x})$、$f_2(\mathbf{x})$、$f_3(\mathbf{x})$、$f_4(\mathbf{x})$和$f_5(\mathbf{x})$分别为干材、干皮、树枝和树叶生物量以及地上总生物量的基础模型（下面类同）。W_1、W_2、W_3、W_4和W_5分别表示干材、干皮、树枝和树叶生物量以及地上总生物量的估计值（下面类同），参数由传统最小二乘法计算得到。分级联合控制比例平差法，即首先把地上总生物量分配给树干和树冠，然后再把树干生物量分配给干材和干皮，树冠生物量分配给树枝和树叶。该方法不但实现、树枝、干皮、树叶和干材之和等于总量，而且还实现了树冠和树干之和等于总量。模型表达式与总量直接控制平差法相类似，此处不再重复阐述[69]。

非线性似然无关回归法（nonlinear seemingly unrelated regressions）最先由 Parresol（2001）引入生物量建模中。该方法是根据非线性似然无关理论保证了各分量生物量方程的可加性（即生物量相容），同时单木生物量各分量之间相互关系可通过误差项方差协方差矩阵描述，因此被广泛应用。模型表达式如下：

$$
\begin{aligned}
&W_1 = f_1(\mathbf{x}) + \varepsilon_1 \\
&W_2 = f_2(\mathbf{x}) + \varepsilon_2 \\
&W_3 = f_3(\mathbf{x}) + \varepsilon_3 \\
&W_4 = f_4(\mathbf{x}) + \varepsilon_4 \\
&W_5 = f_5(\mathbf{x}) = f_1(\mathbf{x}) + f_2(\mathbf{x}) + f_3(\mathbf{x}) + f_4(\mathbf{x}) + \varepsilon_5
\end{aligned}
\qquad (1\text{-}6)
$$

其中，各变量定义见公式（1-6），模型参数是由非线性似然无关回归方法估计得到，该方法的详细介绍见 Parresol（2001）[70]。

在比例平差法基础上，唐守正等（2000）提出了线性或非线性联合估计方法。该方法将各分量进行联合建模，联立求解，是近代统计分析方法之一[71]。它研究

中国知网　https://www.cnki.net

当模型的因变量是另外模型的自变量或模型之间有共同参数等参数估计问题。与非线性似然无关回归方法一样，该方法不仅保证各分量生物量相容，同时还可分析各分量之间的相关性。同样，非线性联立方程组也考虑总量直接控制和分级联合控制两种方案。同样以总量直接控制法为例，模型表达式如下：

$$\begin{cases} W_1 = f_1(\mathbf{x}) / \left[f_1(\mathbf{x}) + f_2(\mathbf{x}) + f_3(\mathbf{x}) + f_4(\mathbf{x}) \right] W_5 + \varepsilon_1 \\ W_2 = f_2(\mathbf{x}) / \left[f_1(\mathbf{x}) + f_2(\mathbf{x}) + f_3(\mathbf{x}) + f_4(\mathbf{x}) \right] W_5 + \varepsilon_2 \\ W_3 = f_3(\mathbf{x}) / \left[f_1(\mathbf{x}) + f_2(\mathbf{x}) + f_3(\mathbf{x}) + f_4(\mathbf{x}) \right] W_5 + \varepsilon_3 \\ W_4 = f_4(\mathbf{x}) / \left[f_1(\mathbf{x}) + f_2(\mathbf{x}) + f_3(\mathbf{x}) + f_4(\mathbf{x}) \right] W_5 + \varepsilon_4 \\ W_5 = f_5(\mathbf{x}) + \varepsilon_5 \end{cases} \tag{1-7}$$

各变量定义见公式（1-6）。与总量控制比例平差法相似，唯一不同是该方法对各分量进行联合建模，模型中参数是通过联立方程组法求解得到[72]。

对于三种方法，比例平差法中各分项生物量方程对应的参数是通过最小二乘法独立求解得到。显然，这些参数是在不考虑各分项生物量相容性的前提下给出的最优估计。当考虑相容性后可能不再是最优估计[73]。非线性似然无关回归法是通过对各分项生物量相加从而解决相容性问题，因此预测精度相对较高。但由于参数是基于非线性似然无关理论估计得到，计算时对参数初始值依赖较大，如果参数的初始值给定不合理时计算很难达到收敛[74]。而非线性联立方程组是将各分量进行联合建模，联立求解，既保证了各分项生物量之间的相容性，又能得到更优化的参数估计[75]。因此该方法最受学者青睐，迄今已在国内外被广泛应用。

总而言之，从应用层面，当前可加性模型的构建主要集中在生物量建模中，对于冠幅模型的构建，迄今尚未解决可加性问题，即东、西、南和北冠幅总和等于冠幅的两倍[76]。从理论层面，当前可加性模型还处于初步发展阶段，方程系统（1-5）—方程系统（1-7）需要同时满足以下三个假定条件才能应用：（1）参数不考虑受随机因子的干扰，即方程系统中每个方程局限于传统的回归模型而不能是混合效应模型；（2）方程系统除各分项生物量含有误差外，其他变量（林分或树木因子）都假定不含有度量误差；（3）方程系统（1-5）中各变量误差必须为独立等方差结构，方程系统（1-6）和（1-7）中各变量误差必须为独立等方差结构或无结构类型[77]。然而，在实际应用中，如果要考虑某些因子（例如地域）对各分项生物量的随机影响，或者方程系统中进一步考虑林分或树木因子含有度量误差，或者方程系统中误差结构为其他结构类型例如自相关情形，现有的生物量方程系统尚能解决。

中国知网 https://www.cnki.net

1.4.3 冠幅模型研究进展

冠幅模型是森林生长收获预估模型的核心内容。在 2005 年之前，对冠幅的研究主要集中在图表和定性层面[78-80]，利用传统回归方法建立冠幅与一些林分因子，例如林分密度、胸高断面积、胸高直径和树高等因子的线性关系[81-82]。通常情形下，由于所分析数据来源于多水平数据或重复调查数据，如对同一株树木不同时间段多次观察或不同立地条件下在相同样地中对树木冠幅重复观察等，因此数据之间可能存在明显的异相关和自相关等[83]。回归分析方法是假定数据间相互独立且非异质性[84]，反映林分总体变化情况，无法分析不同林分因子或水平对冠幅生长的随机影响。但是，混合模型能有效地解决此类问题[85-87]。

国内外有少数一部分学者利用混合模型来预测冠幅，例如，Sánchez-González et al 利用单水平 NLMEMs 构建西班牙栓皮栎冠幅模型，该研究中把样地作为随机效应因子，研究结果为混合模型的预测精度比普通回归模型高[88]。模型中只考虑了样地平方平均直径和对象木胸高直径对冠幅影响，同时如何利用所建模型建模之外的样地进行预测并没有报道。雷相东等把样地作为随机效应因子，利用线性混合模型对吉林省汪清林业局 9 个树种进行建模，并得到较好的效果[89]。该模型尽管分析多个林分变量对冠幅的线性影响，但在实际中这些因子可能与冠幅呈现较复杂的非线性关系。与此同时，除样地外，还有许多林分因子，如地位指数等，可能对冠幅有随机影响[90]。总之，大多已有冠幅模型是基于某特定林分或树种提出，模型中通常含有多个林分变量，实际应用中对数据要求很严，模型实用性差。对于考虑两个和两个以上相互嵌套的林分调查因子对冠幅影响到目前为止还尚未见。

基于上述问题，符利勇等以湖南省黄丰桥国有林场调查的杉木样本为例，把嵌套在立地指数中的样地和立地指数作为随机效应因子，详细介绍如何利用嵌套两水平 NLMEMs 构建冠幅模型。在构建杉木冠幅模型时建立了以样地和立地指数为嵌套 2 水平 NLMEMs 冠幅模型，并通过对数似然和指标 AIC 和确定最佳形式参数随机效应组合类型，用常数加幂函数、幂函数以及指数函数 3 种形式的残差方差模型消除异方差。最终选择了逻辑斯蒂形式的冠幅直径模型（1-8）作为基础模型[91]：

$$CW_{ijk} = \phi_1 / \left[1 + \phi_2 \exp\left(-\phi_3 D_{ijk}\right)\right] + \varepsilon_{ijk} \tag{1-8}$$

中国知网 https://www.cnki.net

其中，$\phi_1-\phi_3$为模型参数，CW_{ijk}和D_{ijk}分别为第i^{th}立地指数第j^{th}样地中第k^{th}株单木对应的冠幅和直径，ε_{ijk}为误差项。

为了提高模型预测精度，需进一步考虑除直径外的其它因子。为避免模型中过多参数和变量间共线性，选择了与冠幅相关性较大的样地优势木平均高（DH）、对象木枝下高（HCB）和对象木树高（H）作为新增加的林分变量，模型表达式为：

$$CW_{ijk}=\frac{\varphi_1+\varphi_4 DH_{ijk}}{1+(\varphi_2+\varphi_5 HCB_{ijk})\exp\left[-\left(\varphi_3+\varphi_6 H_{ijk}\right)D_{ijk}\right]}+\varepsilon_{ijk}$$

其中，DH_{ij}立地指数第i等级中第j个样地的优势木平均高（m）；HCB_{ijk}分别为第i等级中第j个样地第k株对象木枝下高；ϕ_4，ϕ_5和ϕ_6为形式参数。

在确定随机效应参数不同组合时，共分析了 63 种不同随机效应组合形式。在 12 种计算收敛的形式中，当立地指数效应和立地指数与样地的嵌套效应同时作用在ϕ_3和ϕ_5上时，AIC=2714.64 最小，Log lik=-1344.32 最大，模型表达式为：

$$CW_{ijk}=\frac{\beta_1+\beta_4 DH_{ijk}}{1+\left[\beta_2+\left(\beta_5+u_{5i}+u_{5ij}\right)HCB_{ijk}\right]\exp\left[-\left(\beta_3+u_{3i}+u_{3ij}+\beta_6 H_{ijk}\right)D_{IJK}\right]+\varepsilon_{ijk}}$$

式中：β_1-β_6为固定效应参数；u_{3i}和u_{5i}分别为第i^{th}个立地指数作用在ϕ_3和ϕ_5上的随机效应参数；u_{3ij}和u_{5ij}分别为第i^{th}个立地指数第j^{th}样地作用在ϕ_3和ϕ_5上的随机效应参数。

上式所构建的冠幅模型有效考虑了立地指数和嵌套在立地指数中的样地效应对冠幅的影响，明显提高了模型预测精度。然而，随着当前森林生态研究的不断深入，需要在之前传统的冠幅模型基础上进一步研究不同方向的冠幅模型，例如东、西、南和北冠幅模型，以及东、西、南和北冠幅和总冠幅之间的可加性模型，迄今为止，尚未存在相关的研究。为此，本研究将重点分析大尺度下地域随机效应对冠幅的影响以及冠幅可加性模型构建。

1.5 主要研究内容

以山西省关帝山自然保护区和山西省五台山伯强林场内华北落叶松天然次生林为研究对象，在无明显破坏和病虫害的林分中分别设置面积为 20m×20m 华北落叶松天然林固定标准地 67 块和 49 块。以单木冠幅数据为基础，重点构建冠

中国知网 https://www.cnki.net

幅模型。本研究 主要内容如下：

（1）、利用传统方法构建单木冠幅模型。思路为利用林业上常用的冠幅模型对天然落叶松单木冠幅进行拟合，选出最好的模型对冠幅进行预测。

（2）、在上一节的基础上，介绍如何利用近代统计模型方法，即非线性混合效应模型法构建天然落叶松单木冠幅模型并最终得到最优的模型。

（3）、分别构建冠幅各分项目（东、南、西、北冠径和总冠幅）模型，最后首次介绍如何利用非线性联立方程组构建冠幅可加性模型。

中国知网 https://www.cnki.net

2 研究地区概况和数据

2.1 研究区概况

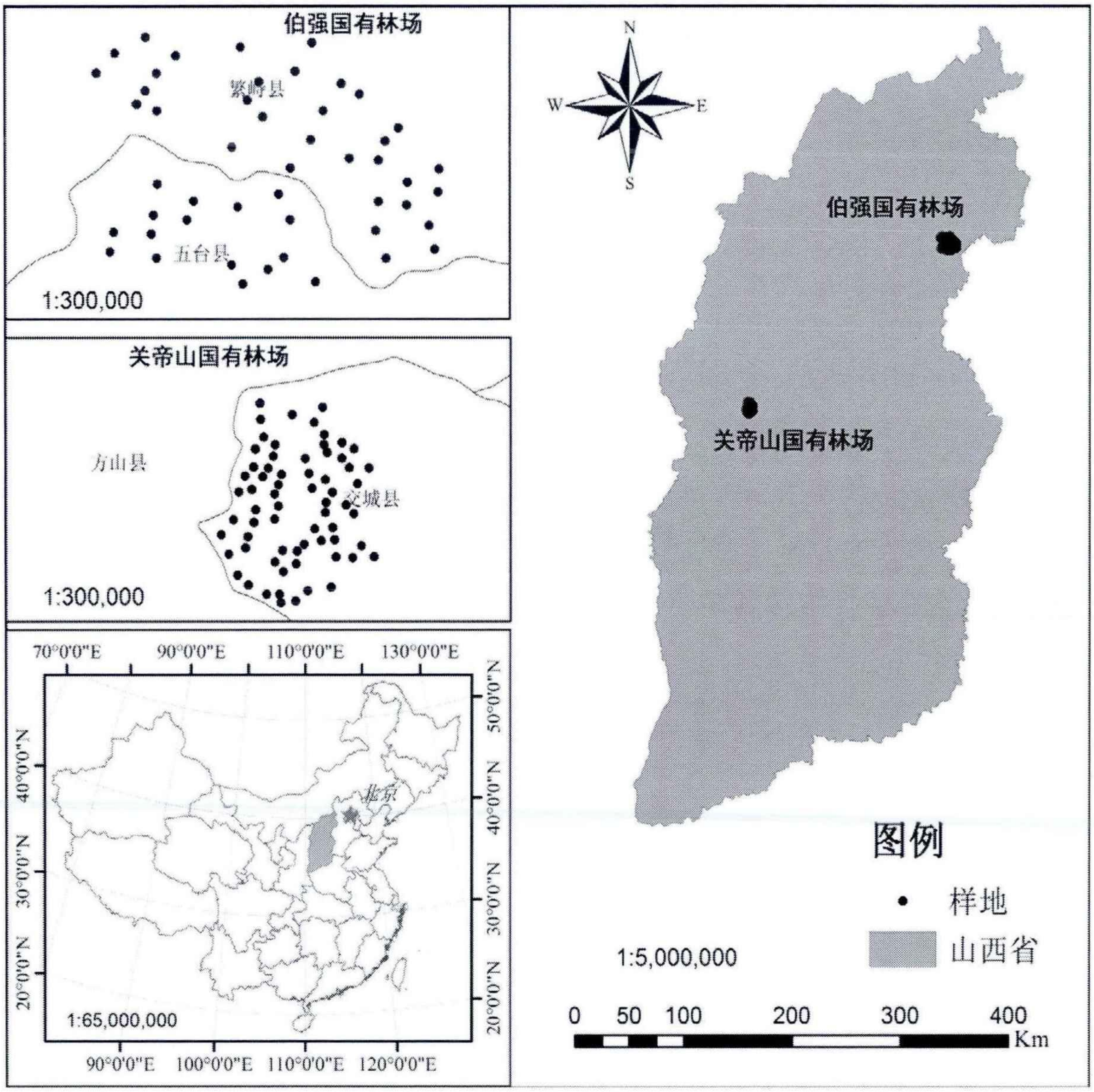

图 2-1 关帝山和五台山研究区位置图

2.1.1 关帝山国有林场概况

山西省关帝山国有林场地处吕梁山脉中段，位于方山县东北部和交城县西北部交界处见图（1）。地理坐标为：111°22′～111°33′E，37°45′～37°55′N，总面积 10443.5hm^2，海拔 1600～2831m. 是以保护华北落叶松（*Larix*

中国知网 https://www.cnki.net

principis-rupprechtii)、云杉（*Picea spp*)的天然林及世界珍禽褐马鸡（*Crossoptilon mantchuricum*)为主的森林和野生动物类型自然保护区，也是关帝山国家森林公园所在地。

保护区年平均气温 4.3℃，最冷月（1 月）均温-10.2℃，处于温暖带大陆性季风气候区；年均降水量 822.6*mm*,大部分地区≥10℃的积温 1800～2950*h*，无霜期 100～125*d*。最热月（7 月）均温 17.5℃，极端最高气温 32.0℃。

保护区森林覆盖率高达 85%，有林地面积 7709.7hm^2，占总面积的 73.8%，活立木蓄积 1272499m^3。由于海拔高差较大，植被垂直分布带非常明显,自上而下可以分为亚高山灌丛草甸带、寒温性针叶林带、山地落叶阔叶林带、山地温性针阔叶混交林带以及山地温性针叶林带。寒温性针叶林是关帝山国家森林公园的主要组成部分，由于云杉林和华北落叶松林的面积大且林相好，环境非常适合于国家级重点保护动物褐马鸡的生存繁衍，所以成为了褐马鸡的主要栖息地[92]。

2.1.2 伯强国有林场概况

五台山林业局伯强国有林场位于山西省东北部繁峙县中南部，属滹沱河支流、羊眼河的发源地，地处五台峰—北台北麓，位于东经 113°30′—113°53′，北纬 39°04′—39°16′之间。主要管辖太平沟、山幸庄、宫黄沟、车厂四个营林区，总经营面积 10307.3 公顷。东邻庄旺林场，西接臭冷杉自然保护区，南与五台山林场接壤，北与砂河镇相连。行政区域隶属于繁峙县，林场涉及繁峙县东山金山铺、神堂堡、横涧 4 个乡镇的 60 多个村庄。伯强林场属科级事业单位，隶属于山西省五台山国有林管理局。

林场境内属五台山剥蚀断块高中山区，地貌以土石质山为主，境内山脉走向由西南向东北逐渐降低，区内山地连绵，沟壑纵横，相对高差大。阴坡陡峭，阳坡土层薄。主要岩石为片麻岩、花岗岩和台山片岩。

林场境内气候属暖温带季风大陆性气候，四季分明，气候垂直变化明显。冬季漫长而严寒，春季干旱而多风，夏季温和且湿润，秋季凉爽又多雨。主要气象　因子为：年平均气温 6.3℃左右，极端最高气温 37.6℃，极端最低气温-24. 3 ℃，无霜期约 130 天左右，全年平均降水量为 400mm 下，且集中在 7、8、9 三个月内，整个地形西高东低，最高主峰北台顶叶斗峰海拔 3061.1 米，境内滤巧河、羊眼河横贯其中。

林场境内土壤垂直变化特别明显，海拔 2500 米以上为台山亚高山草甸土，2500-1800 米为山地森林踪壤土，1800-1600 米为山地淋溶褐土，1600 米以下为

中国知网　https://www.cnki.net

山地褐土。林内腐殖层厚，土壤厚度为30cm-50cm，有机质含量高，土壤结构好，PH值为中性微酸性。

伯强林场经营总面积10307.3公顷，其中林业用地面积10280.5公顷，占99.7%，非林地面积26.8公顷，占0.3%。伯强林场经营面积10275.8公顷，其中国家生态公益林面积为5700公顷，地方公益林面积为4575.8公顷。

林场经营范围属太行山系，区内植物种类较多。根据调查并参考有关资料统计，伯强林场范围内大约有植物82科，316属，447种。主要植物有：乔木：云杉、华北落叶松、臭冷杉、青杆、油松、红桦、白桦等；灌木；山亚绣球、虎榛子、绣线菊、沙棘、黄刺玫、山刺玫、忍冬、六道木、丁香、五台锦鸡儿、胡枝子、小叶鼠李等；草本主要有禾本科和嵩类等[93]。

2.2 数据来源

数据采集工作是在2015年7-9月完成。在山西省交城县西北部和方山县东北部交界处的关帝山国家森林公园和五台山伯强国有林场，按不同的海拔和坡向，在无明显破坏和病虫害的林分中总共设置了0.04公顷（20米×20米）的华北落叶松天然林固定样地116块；其中关帝山国家森林公园设置了67块固定样地，伯强林场设置了49块固定样地。实测了固定样地的相关林分结构特征指标，主要包括林分起源，郁闭度，林分年龄以及胸径大于5cm华北落叶松单木的树高，胸径，枝下高和东、西、南、北四个方向冠幅。共调查华北落叶松3369株。
其中树高用的是超声波测高仪来测量；东、南、西、北四个方向的冠幅测量是以人手持激光测距仪站在每个方向的树冠最大距离投影处测量到树干的垂直距离。究中的冠幅指的是四个方向冠幅直径的平均值，计算公式为:

$$CW=(CR_S+CR_N+CR_E+CR_W)/2$$

其中，CW 为总冠幅 CW、CR_E、CR_S、CR_W和 CR_N分别为东、南、西和北冠幅。每株优势木年龄是通过生长锥在树木胸高处钻孔所取得的年轮数来确定，并测得每块样地优势木的近五年生长量。冠幅CW、东西方向冠幅 CW_{EW}、南北方向冠幅 CW_{SN}、东西南北四个方向的树冠半径 CR_E、CR_W、CR_S 和 CR_N 与对应的5个树木因子胸径D、树高H、枝下高HCB、优势木树高DH、优势木胸径DD之间的关系分布见图2-2。

中国知网 https://www.cnki.net

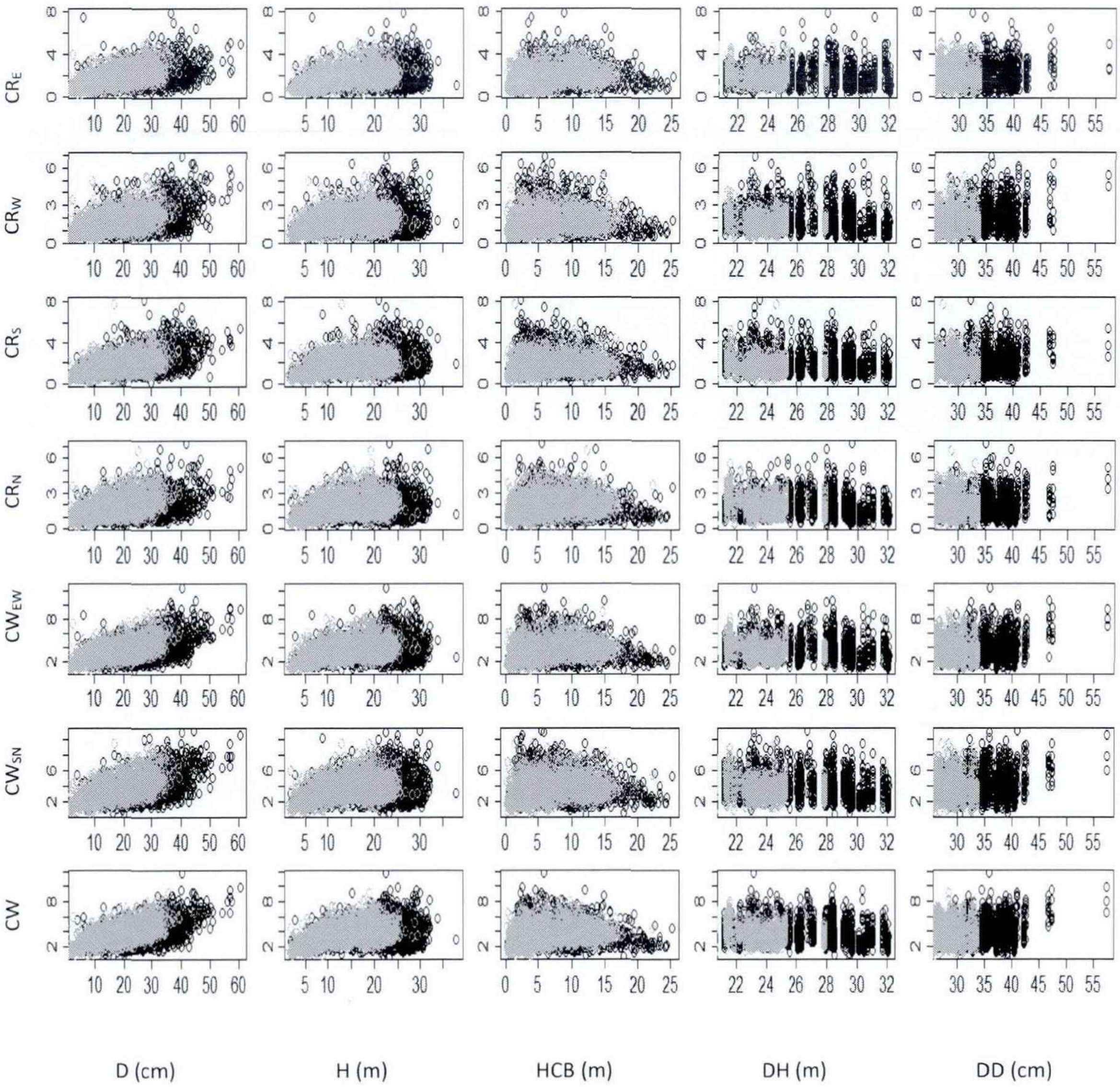

图 2-2 两个研究地区华北落叶松冠幅（CW）、南北冠幅（CW_{SN}）、东西冠幅（CW_{EW}）、北面冠幅（CR_N）、南面冠幅（CR_S）、西面冠幅（CR_W）和东面冠幅（CR_E）与胸径（D）、树高（H）、枝下高（HCB）、优势木树高（DH）、优势木胸径（DD）变化关系图；黑色点表示关帝山国有林场样地数据，灰色点表示伯强林场样地数据

2.3 样地主要林分因子统计

构建冠幅模型时，需把固定样地随机分为两组，一组作为建模数据，另一组作为检验数据。建模数据包含 84 块固定样地共 2250 组观测值，检验数据包含 35 块固定样地共 1119 观测值，数据统计信息见表 2-3。

中国知网　https://www.cnki.net

表 2-3 建模数据和检验数据统计信息

Table 2-3 Summary statistics for modeling and validation data

	建模数据				检验数据			
变量因子	Min	Max	Mean	SD	Min	Max	Mean	SD
D (cm)	5	60.5	22.2	10.1	5	57.0	21.8	9.8
H (m)	1.5	37.8	17.5	7.2	1.9	33.9	16.9	7.3
HCB (m)	0.1	25.3	7.1	4.5	0.2	22.1	7.1	4.4
DH (m)	11.5	32.1	23.0	5.1	10.7	31.9	22.4	5.4
CR_E(m)	0.0	7.4	1.9	0.9	0.4	7.9	1.8	0.9
CR_W(m)	0.1	6.9	1.9	0.9	0.3	6.3	1.8	0.9
CR_S(m)	0.5	8.2	2.0	1.0	0.1	7.8	1.9	0.9
CR_N (m)	0.1	7.2	2.0	0.9	0.1	6.8	1.9	0.9
CW_{EW} (m)	0.7	12.5	3.7	1.5	0.7	10.6	3.6	1.4
CW_{SN} (m)	0.6	11.1	4.0	1.6	0.5	9.4	3.8	1.5
CW (m)	0.7	11.7	3.9	1.4	0.6	9.5	3.7	1.3

注: Min = 最小值, Max = 最大值, Mean = 平均值, SD = 标准差, D= 胸径, H= 树高, HCB=枝下高, DH= 优势木树高, CR_E=东树冠半径, CR_W=西树冠半径, CR_S=南树冠半径, CW_{EW}=东西冠幅, CW_{SN}=南北冠幅, CR_N=北树冠半径, CW=冠幅

中国知网 https://www.cnki.net

3 非线性回归冠幅模型建立与分析

通过对现有研究得知，当前关于华北落叶松单木冠幅模型的研究非常少见。本章重点利用传统回归分析方法构建华北落叶松单木冠幅模型。首先利用建模数据，从 9 个常见的冠幅—直径模型形式中确定一个拟合效果较好的模型用于单木冠幅模型构建；为提高模型精度，需从所调查的林分和单木因子中筛选出与冠幅相关性较强的因子作为模型预测变量；最后对所构建的冠幅模型进行系统比较与评价。

3.1 材料与方法

3.1.1 实验数据

本章所有使用的数据（包括建模数据和检验数据）见第二章介绍。

3.1.2 基础模型

选用 9 个常用的冠幅-胸径模型（表 3-1）作为构建华北落叶松天然林单木冠幅模型的候选模型。首先利用建模数据求解各候选模型的参数，基于所估计的参数对检验数据进行预测。利用残差平方和（RSS）、剩余均方根误差（RMSE）和决定系数（R^2）3 个指标对模型进行评价，各指标计算公式如（3-1）—（3-3）式所示。其中，当 RSS 和 RMSE 的值越接近 0 而 R^2 的值越接近 1，说明模型的拟合精度越高。

$$RSS = \sum_{i=1}^{n} (\hat{y}_i - y_i)^2 \tag{3-1}$$

$$RMSE = \sqrt{\frac{\sum_{i=1}^{n} (\hat{y}_i - y_i)^2}{n - r}} \tag{3-2}$$

$$R^2 = 1 - \frac{\sum_{i=1}^{n} (y_i - \hat{y}_i)^2}{\sum_{i=1}^{n} (y_i - \bar{y})^2} \tag{3-3}$$

其中，（3-1）—（3-3）式中：y_i ，$\hat{y}$，$\bar{y}_i$分别为冠幅的实测值、预测值和实测值的平均值，n 为总观测样本数，r 为模型参数个数。

中国知网　https://www.cnki.net

表 3-1 候选冠幅模型

Table 3-1 Candidate model for crown width

模型编号	模型表达式	来源
1	$CW=1/(a+b/D)$	罗玲等[11]
2	$\ln CW=a+b\ln D$	卢昌泰等[12]
3	$CW=a+bD$	Sánchez-González et al.等[13]
4	$CW=aD^b$	Sönmez 等[14]
5	$CW=[D/(a+bD)]^2$	Sönmez 等[14]
6	$CW=a(b)^D$	Sánchez-González et al.等[13]
7	$CW=\exp(a+bD)$	Sönmez 等[14]
8	$CW=a+bD+cD^2$	Sönmez 等[14]
9	$CW=a/1+\exp[b+c\ln(D+1)]$	Sönmez 等[14]

注：CW 为冠幅（m），D 为胸径（cm），a、b、c 为模型参数。

通过残差平方和、剩余均方根误差和决定系数对各候选模型进行比较。对于二参数模型（模型 I）和三参数模型（模型 II），为了比较两类模型精度差异是否显著，本研究选用了似然比检验方法。似然比具体计算公式如（3-4）式所示。

$$LRT = 2\lg(L_1 / L_2) = 2(\lg L_1 - \lg L_2) \quad (3\text{-}4)$$

式中：L_1 和 L_2 分别为模型 I 和模型 II 的似然函数值，LRT 为似然比，服从自由度为 $k_1 - k_2$ 的 λ 分布。给定可靠性 $1-\alpha = 0.95$，当 $LRT \geq \lambda_\alpha(k_1 - k_2)$ 表明这模型 I 和模型 II 差异显著；反之，模型 I 和模型 II 差异不显著，故选择参数较少的模型 I。

3.1.3 预测变量确定

除与胸径相关外，冠幅还受其它林分变量的影响，如林分测树因子、立地因子以及林分竞争因子等。模型中增加一些林分变量能降低林分或单木差异对冠幅的影响[94-97]。

林木因子包括林分年龄（A)、林分密度（M）、树高（H）、冠长（RCL) 等；立地因子有立地指数（SI)；林分竞争因子包括样地平方平均直径（MDD)、样地优势木平均树高（Dt)、样地算数平均树高（MH）、样地优势木平均高（DH) 等。

中国知网 https://www.cnki.net

为了避免模型中过多参数和变量间的共线性，将选择与冠幅相关性较大的因子作为最终的林分或单木因子进行建模。

3.1.4 参数估计及改进模型评价

本研究所有计算都在 R 软件 nls 函数上实现，选用的方法为非线性最小二乘法。计算程序见附件 1。

基于建模数据和检验数据，选用平均偏差（Bias）（3-5）、剩余均方根误差（RMSE）（3-2）和决定系数（R^2）（3-3）对最优冠幅-直径（基础模型）和改进的冠幅模型进行比较和评价，最终确定用于对华北落叶松天然林单木冠幅进行预测的最优模型。

$$Bias=\sum_{i=1}^{n}(\hat{y}-y_i)/n \qquad (3\text{-}5)$$

3.2 结果与分析

3.2.1 模型选择

表 3-2 为模型 1 至模型 9 的评价指标，其中模型 8、模型 9 为三参数模型，余下的模型都为二参数模型。RSS 和 RMSE 的值越接近 0，R^2 的值越接近 1，说明模型的拟合能力越强。对各模型评价指标（表 3-2）进行综合分析，对于二参数模型，不管是建模数据还是检验数据，模型 6 对应的 *RSS 和 RMSE* 最小而 R^2 最大。对于三参数模型，模型 9 对应的效果最好。

对模型 6 和模型 9 做似然比检验，结果表明，两模型间差异显著（F=36.7,p＜0.0001），且模型 9 表现较好，因此，选择模型 9 作为构建建立华北落叶松天然林单木冠幅模型的基础模型，其结构形式为：

$$CW=a/1+\exp[b+c\ln(D+1)] \qquad (3\text{-}6)$$

其中，CW 为单木冠幅（m），D 为单木胸高直径(cm), ε 为误差项。

中国知网 https://www.cnki.net

表 3-2 各模型评价指标

Table 3 evaluation indexes for each model

模型	建模数据 Modelling data			检验数据 Validation data		
	RSS	*RMSE*	R^2	*Bias*	*RMSE*	R^2
1	523.708	14.587	0.283	0.445	6.718	0.302
2	506.059	14.095	0.307	0.425	6.477	0.327
3	489.264	13.628	0.330	0.412	6.286	0.347
4	506.059	14.093	0.307	0.349	6.475	0.327
5	541.665	15.087	0.259	0.457	6.971	0.275
6	476.654	13.276	0.348	0.395	6.023	0.374
7	477.614	13.296	0.338	0.374	6.019	0.364
8	474.315	13.211	0.351	0.405	6.177	0.358
9	414.341	12.216	0.391	0.262	5.483	0.381

3.2.2 模型改进

通过对所有候选林木因子进行分析得知对象木冠长（CL）、树高（H）和每公顷株数（M）与冠幅相关性较强，因此把这些因子作为最终的单木或林分因子用来构建冠幅模型。通过对各因子不同组合对模型精度影响进行分析，发现当对象木树高作用在参数 a 上，每公顷株数作用在参数 b 上，对象木冠长作用在参数 c 上模型拟合效果最好，最终改进模型的表达式如下：

$$CW = (a + a_1 H) + \exp[(b + b_1 M) + (c + c_1 CL)\ln(D+1)] \quad (3\text{-}7)$$

其中， a_1, b_1, c_1 分别为模型新增参数。

基于建模数据，利用非线性最小二乘法对模型进行拟合，得到模型参数估计值，把参数代入模型（3-7），模型表达式如下：

$$CW = 1.4875 - 0.0311H + \exp[(-3.6416 - 0.0002M) + (2.4346 + 0.0045CL)\ln(D+1)] \quad (3\text{-}8)$$

3.2.3 模型比较

利用基础模型（3-6）和改进的模型（3-8）对检验数据进行预测，得到的评价指标分别为：对于基础模型（3-6），Bias=0.262、剩余均方根误差 RMSE=5.483 和决定系数 R^2=0.381，对于改进的模型（3-8），Bias=0.203、剩余均方根误差

中国知网 https://www.cnki.net

RMSE=4.113 和决定系数 R^2=0.425.除此之外，对基础模型（3-6）和改进模型（3-8）进行似然比检验结果表明，两模型差异显著(F=46.46,p<0.001),因此表明对象木冠长（CL）、对象木树高（H）和每公顷株数（M）对冠幅影响较大，当模型中考虑这些变量时能明显改进模型预测精度。

3.3 小结

利用非线性最小二乘法对华北落叶松单木冠幅模型构建得知：

（1）三参数的逻辑斯蒂模型能较好反映冠幅和直径之见的非线性关系，与其它候选模型相比，该模型有较高的拟合精度。

（2）对象木冠长（CL）、对象木树高（H）和每公顷株数（M）对冠幅影响较大，当这些因子作为预测变量时能明显改进模型的预测精度。

（3）模型（3-7）有较高的预测精度，因此推荐使用模型估计华北落叶松单木冠幅。

中国知网 https://www.cnki.net

4 非线性混合效应冠幅模型研建

本研究主要对象是天然华北落叶松林，当前华北落叶松天然群体呈片段分布，因此不同地域相同直径或树高的华北落叶松的单木冠幅差异较大。而利用非线性混合效应模型能有效描述各区域（例如不同区组和样地）间的随机差异。然而迄今为止，利用该方法构建华北落叶松单木冠幅模型的研究较少，尤其针对华北落叶松天然次林几乎没有。为此，本研究以华北落叶松天然次生林单木数据为例，利用嵌套两水平非线性混合效应模型方法构建冠幅模型。分析区组以及嵌套在区组里的样地对冠幅的随机影响。为研究成果为今后华北落叶松天然次生林的科学经营提供一点理论依据。

4.1 材料与方法

4.1.1 实验数据

在庞泉沟自然保护区根据不同的海拔和坡向，在无明显人工破坏和自然灾害的林分中设置了华北落叶松天然次生林标准样地 69 块（20m×20m）。类似地，在五台山地区设置了华北落叶松天然次生林标准地 47 块。实测各样地的林分变量和立地因子，主要包括郁闭度、枝下高、海拔、坡向和坡度等。对于胸径大于 5cm 的活立木，实测其树高和胸径，其中树高是用超声波测高仪来测量。通过剔除异常值和非落叶松树种，最终 3369 株华北落叶松单木用于构建非线性混合效应冠幅模型。根据立地类型不同，把所有样地划分为 7 个区组，其中庞泉沟自然保护区 4 个区组共 1504 株，五台山地区 3 个区组共 1455 株。所有数据划分为两部分，建模数据和检验数据，详细介绍见第二章。

4.1.2 非线性混合效应模型

嵌套多水平 NLMEMs，以两水平为例，表达式为[98]：

$$y_{ijk} = f(\boldsymbol{\phi}_{ijk}, \boldsymbol{\upsilon}_{ijk}) + \varepsilon_{ijk}, i = 1,\ldots,M, j = 1,\ldots,M_i, k = 1,\ldots,n_{ij} \quad (4\text{-}1)$$

$$\boldsymbol{\phi}_{ijk} = \boldsymbol{A}_{ijk}\boldsymbol{\beta} + \boldsymbol{B}_{i,jk}\boldsymbol{u}_i + \boldsymbol{B}_{ijk}\boldsymbol{u}_{ij}, \boldsymbol{u}_i \sim N(0,\boldsymbol{\psi}_1), \mathbf{u}_{ij} \sim N(0,\boldsymbol{\psi}_2) \quad (4\text{-}2)$$

其中，M 为第一水平因子等级数；M_i 为第一水平因子第 i 等级对应的第二水平因子等级数；n_{ij} 为第一水平因子第 i 等级第二水平因子第 j 等级的重复观测次数；y_{ijk} 和 $\boldsymbol{\upsilon}_{ijk}$ 分别为第一水平因子第 i 等级第二水平因子第 j 等级对应的第 k 次重复调查时因变量和自变量的观测值；f 是关于参数向量 $\boldsymbol{\phi}$ 和 $\boldsymbol{\upsilon}_i$ 的非线性函数；$\boldsymbol{\beta}$ 为 $p\times1$ 维

中国知网　https://www.cnki.net

固定效应参数；$\mathbf{u}_i$ 为 $q_1\times1$ 维的第一水平随机效应参数，假定服从期望为0方差为 $\boldsymbol{\psi}_1$ 的正态分布；$\mathbf{u}_{ij}$ 为 $q_2\times1$ 维的第二水平随机效应参数，假定服从期望为0方差为 $\boldsymbol{\psi}_2$ 的正态分布；ϕ_{ij} 为形式参数（简称形参），它与 $\boldsymbol{\beta}$、$\mathbf{u}_i$ 及 $\mathbf{u}_{ij}$ 呈线性函数关系；$\mathbf{A}_{ijk}$、$\mathbf{B}_{i.jk}$ 和 $\mathbf{B}_{ijk}$ 分别为 $\boldsymbol{\beta}$、$\mathbf{u}_i$ 和 $\mathbf{u}_{ij}$ 的设计矩阵；ε_{ijk} 为随机误差项，假定服从期望为零，方差为 $\mathbf{R}$ 的正态分布，并假定 $\mathbf{u}_i$、$\mathbf{u}_{ij}$ 和误差项 ε_{ijk} 之间相互独立。

立地类型是影响林分生长的主要因子[99-100]，本研究中各区组是按照不同类型进行划分的，并且区组分布具有很大的随机性。除此之外，分布在各区组里的样地也是随机分布，因此为了定量描述区组以及嵌套在区组里面的样地对冠幅的影响，本研究将构建嵌套两水平的非线性混合效应冠幅模型，其中区组为第一水平随机效应因子，嵌套在区组里的样地为第二水平随机效应因子。

4.1.3 基础模型

符利勇等(2013)以胸径、树高、枝下高、优势木高作为协变量构建了以logistic形式的杉木单木冠幅模型，该模型预测精度比其他几种候选模型（例如，指数、幂函数和威布尔函数）预测精度明显要高。利用该模型对本研究中华北落叶松单木冠幅模型进行拟合同样发现该模型具有较高的预测精度，为此，本研究选择该冠幅模型作为基础模型用于构建非线性混合效应华北落叶松冠幅模型，模型表达式为：

$$CW_{ijk}=\frac{\phi_1+\phi_2 DH_{ij}}{1+(\phi_3+\phi_4 HCB_{ijk})\exp\left[-(\phi_5+\phi_6 H_{ijk})D_{ijk}\right]}+\varepsilon_{ijk} \tag{4-3}$$

其中，$CW_{ijk}, HCB_{ijk}, D_{ijk}$ 分别为第 i 区组中第 j 样地第 k 株对象木对应的冠幅（m）和枝下高（m）和胸高直径（cm）；DH_{ij} 为第 i 区组第 j 样地的平均优势高；ε_{ijk} 为误差项，ϕ_1-ϕ_6 为待估参数。

4.1.4 形式参数构造

模型（4-3）有6个形式参数 ϕ_1-ϕ_6，并且本研究分析区组和嵌套在区组中的样地对冠幅的影响，因此共衍生出63种非线性混合效应模型。通过对所有类型的非线性混合效应模型进行计算，利用评价指标AIC和对数似然Loglik选出一种最优随机效应组合构造类型[101]。为避免模型参数过多，本研究还利用似然比检验[102]：

$$LRT=2\log(L_1/L_2)=2[\log(L_1)-Log(L_2)]$$

其中，L_1 和 L_2 分别为随机类型1和随机类型2的似然函数值，LRT 服从自由度为

中国知网 https://www.cnki.net

k_1-k_2 的 λ 分布。给定可靠性 $\alpha=0.05$，当 $LRT \ge \lambda_\alpha(k_1-k_2)$ 拒绝原假设，说明这两个模型差异显著，反之，两个模型差异不显著，故选择含随机效应参数较少的模型。

4.1.5 随机效应参数方差（ψ_1 和 ψ_2）结构

研究中假定 ψ_1 和 ψ_2 为无结构类型，以 3×3 维矩阵为例，ψ_1 或 ψ_2 写为：

$$\begin{pmatrix} \sigma_1^2 & \rho_{12} & \rho_{13} \\ \rho_{21} & \sigma_2^2 & \rho_{23} \\ \rho_{31} & \rho_{32} & \sigma_3^2 \end{pmatrix}$$

其中，σ_i^2（$i=1,2,3$）为第 i 个随机效应参数方差，ρ_{ij}（$j=1,2,3$，$i\neq j$）为第 i 个随机效应与第 j 个随机效应的协方差，满足 $\rho_{ij}=\rho_{ji}$。

4.1.6 误差项方差协方差（R）结构

对于重复观测数据，误差项的方差协方差矩阵 $\mathbf{R}$ 中可能存在明显异方差和自相关，为解决该问题，本研究选用以下的方差-协方差矩阵类型：

$$\mathbf{R}_{ij}=\sigma^2\mathbf{G}_{ij}^{0.5}\mathbf{\Gamma}_{ij}\mathbf{G}_{ij}^{0.5}\quad, \tag{4-4}$$

其中，σ^2 为误差扩散的比例因子，由模型中残差方差值所给定；$\mathbf{\Gamma}_{ij}$ 是用来描叙对象内误差自相关性的 $n_{ij}\times n_{ij}$ 维矩阵。通过前期分析得知本实例中各样地内观测数据之间没有明显的相关性，故 $\mathbf{\Gamma}_{ij}$ 为单位矩阵；$\mathbf{G}_{ij}$ 是用来描叙对象内方差异质性的 $n_{ij}\times n_{ij}$ 维对角矩阵，对角元素为相应误差项的标准差。

本研究通过对残差方差增加权重消除异方差。选择的方差函数有：指数函数、幂函数和常数加幂函数。通过 AIC 和似然比检验确定一个最好的残差方差模型[103]。三种方差函数的表达式如下：

类型 1：指数函数

$$\mathrm{var}(\varepsilon_{ijk})=\sigma^2\exp(2\gamma x_{ijk})\quad, \tag{4-5}$$

类型 2：幂函数

$$\mathrm{var}(\varepsilon_{ijk})=\sigma^2 {x_{ijk}}^{2\gamma}\quad, \tag{4-6}$$

类型 3 常数加幂函数

$$\mathrm{var}(\varepsilon_{ijk})=\sigma^2(\gamma_1+x_{ijk}^{\gamma_2})^2\quad, \tag{4-7}$$

其中，x_{ijk} 为模型（4-3）中其中的一个预测变量，γ,γ_1，和 γ_2 分别为待估参数。

4.1.7 地域效应

本研究中由于试验数据来自两个区域，即庞泉沟自然保护区和五台山，由于立地条件，气候和环境因子的差异，使得两个研究区华北落叶松生长差异较大，

中国知网 https://www.cnki.net

为了定量描述之间的差异程度，本研究构建了一个基于区域效应的哑变量，即P=0 表示数据来自庞泉沟自然保护区，P=1 表示数据来自五台山地区。

4.1.8 参数估计

本研究计算是在 R 软件 nlme 函数上实现，该函数中参数估计方法为 LB 算法，主要包含两个步骤:惩罚最小二乘步（PNLS）和线性混合效应步（LME），模型中所有待估参数是通过这两个步骤相互交替运算得到，详细计算见符利勇和唐守正（2012），唐守正等（2015）。

参数估计时，由于 ML 通常能提供较好的拟合统计量，但所估计的参数量为有偏，而 REML 估计量为无偏，因此模型比较时选用 ML，最终混合模型的计算选用 REML。

4.1.9 模型预测

在利用考虑立地指数与样地交互效应和立地指数的嵌套两水平 NLMEMs 对冠幅预测时，需考虑两种情形[104]：

（1）固定效应响应，又称总体平均响应，即在预测冠幅时，需在预测样地中测量模型中所含的对象木胸高直径和林分变量。

（2）主体特定（Subject-specific）响应，简称 SS 响应，预测冠幅时，需在预测样地中测量一定数量对象木的林分因子、胸高直径以及冠幅来估计随机效应参数（立地指数与样地交互效应和立地指数效应）[105]。通常子样地中测量的对象木株树越多，模型预测精度越高。为考虑模型实用性和测量成本，Calama and Montero 认为随机测量 4 株对象木就能达到理想精度[106]，本研究也采用该方法。

4.1.10 PA 模型

PA 模型（Population-averaged model，又称总体平均模型）是指模型中所有随机效应参数值默认为 0，形式参数只含固定效应部分[107]即：

$$CW_{ijk}=f(\mathbf{x}_{ijk},\hat{\boldsymbol{\phi}}_{ij}),\tag{4-8}$$

其中，CW_{ijk} 和 $\mathbf{x}_{ijk}$ 分别为第 i 个区组第 j 个样地中第 k 株华北落叶松冠幅和自变量；$\hat{\boldsymbol{\phi}}_{ij}$ 为形式参数，在总体平均模型中，它只包含固定效应参数 $\hat{\boldsymbol{\beta}}$。

4.1.11 区组水平

假定最终模型中只考虑区组对冠幅影响，而不考虑其它效应（如嵌套在区组里面的样地效应），即公式（4-8）中 $\hat{\boldsymbol{\phi}}_{ij}$ 含有 $\hat{\boldsymbol{\beta}}$ 和 $\hat{\mathbf{u}}_i$。通常 $\hat{\mathbf{u}}_i$ 由下式计算得到（Vonesh

中国知网 https://www.cnki.net

and Chinchilli 1997）

$$\hat{\mathbf{b}}_i \approx \hat{\mathbf{G}}\hat{\mathbf{C}}_i^T(\hat{\mathbf{R}}_i + \hat{\mathbf{C}}_i\hat{\mathbf{G}}\hat{\mathbf{C}}_i^T)^{-1}\hat{\mathbf{e}}_i \tag{4-9}$$

其中，$\hat{\mathbf{b}}_i$=$\hat{\mathbf{u}}_i$为区组所产生的$q_1 \times 1$维随机效应；$\hat{\mathbf{G}}$=$\hat{\psi}_1$为$q_1 \times q_1$维方差协方差矩阵；

$\hat{\mathbf{C}}_i = \hat{\mathbf{Z}}_i$为$n_i \times q_1$维设计矩阵；$n_i = \sum_{i=1}^{M_i} n_{ij}$。

表 1 中，由于检验数据的区组与建模数据的区组完全相同，因此不需重新计算$\hat{\mathbf{u}}_i$，预测时可直接取自最终模型参数的估计结果。

4.1.12 区组和嵌套在区组里面的样地效应（区组+区组*样地）

当模型中同时考虑嵌套在区组里面的样地效应和区组效应对冠幅影响时，公式（4-8）的ϕ_{ij}中除$\hat{\boldsymbol{\beta}}$外还有$\mathbf{u}_i$和$\mathbf{u}_{ij}$。同样随机效应由公式（4-9）计算得出，此时，

$\hat{\mathbf{b}}_i = (\mathbf{u}_i^T, \mathbf{u}_{i1}^T, \mathbf{u}_{i2}^T, ..., \mathbf{u}_{iM_i}^T)^T$为$(q_1 + M_i q_2) \times 1$维增广随机效应参数向量，

$\hat{\mathbf{G}}$=$diag(\psi_1, \psi_2, ..., \psi_2)$为$(q_1 + M_i q_2) \times (q_1 + M_i q_2)$维对角块正定矩阵；

$$\hat{\mathbf{C}}_i = \begin{bmatrix} \mathbf{Z}_{i1} & \mathbf{E}_{i1} & 0 & 0 & 0 \\ \mathbf{Z}_{i2} & 0 & \mathbf{E}_{i2} & 0 & 0 \\ \vdots & 0 & 0 & \ddots & 0 \\ \mathbf{Z}_{iM_i} & 0 & 0 & 0 & \mathbf{E}_{iM_i} \end{bmatrix}_{n_i \times (q_1 + M_i q_2)}$$ 为设计矩阵。

与只考虑立地指数效应相类似，表 1 中检验数据与建模数据有相同的立地指数，因此不需重新计算$\hat{\mathbf{u}}_i$。

4.1.13 模型评价

本研究利用平均残差$\bar{e}$，残差方差δ，均方根误差$RMSE$，总相对误差TRE和修正决定系数R_α^2对模型进行评价，指标计算见公式（4-10）—公式（4-14）。

$$\text{平均残差}\ \bar{e} = \sum(CW_t - \hat{CW}_t)/n \tag{4-10}$$

$$\text{残差方差}\ \delta = \sum(CW_t - \hat{CW}_t)^2/(n-1) \tag{4-11}$$

$$\text{均方根误差}\ RMSE = \sqrt{\bar{e}^2 + \delta} \tag{4-12}$$

$$\text{总相对误差}\ TRE = 100\sum_{t=1}^{n}(CW_t - \hat{CW}_t)^2 / \sum_{t=1}^{n}\hat{CW}_t \tag{4-13}$$

中国知网 https://www.cnki.net

$$\text{修正决定系数}\ R_{\alpha}^{2}=1-\frac{(n-1)\sum_{t=1}^{n}(CW_{t}-\hat{CW}_{t})^{2}}{(n-L)\sum_{t=1}^{n}(CW_{t}-\overline{CW})^{2}} \tag{4-14}$$

其中，CW_t和$\hat{CW}_t$分别为第t个株树对应的冠幅实测值和估计值，

n为总观测数；$\overline{CW}$为平均冠幅。

4.2 结果与分析

4.2.1 基础模型

模型（4-3）的参数估计值见表 4-1。模型（4-3）的误差项假定了独立等方差，因此方差-协方差矩阵只有一个参数。所有参数都显著地不等于 0（$p<0.05$）。参数β_2为负值，表明随着 DH 的增加 CW 逐渐减少。基于拟合结果，基础模型（4-3）对应的评价指标$\bar{e}$、δ、$RMSE$、TRE和 R_{α}^{2} 分别为：0.0005、 0.8572、0.9259、 22.1684 和 0.5618。

表 4-1 各模型的参数估计值及评价指标

	参数	模型（4-3）	模型(4-15)	模型（4-17）	模型（4-18）
固定效应参数	β_1	17.5669	11.4986	10.8333	12.4305
	β_2	-0.2686	-0.1549	-0.1357	-0.1429
	β_3	5.6996	3.8790	3.8478	3.4042
	β_4	0.1917	0.1311	0.1308	0.1521
	β_5	0.0751	0.0913	0.0983	0.0703
	β_6	0.0009	-0.0010	-0.0012	-0.0007
随机效应参数	α_1				-2.7840
	α_5				0.0363
方差参数	$s_2^{(B)}$		0.0177	0.0181	0.0337
	$s_4^{(B)}$		0.0284	0.0333	0.0739
	$\sigma_{24}^{(B)}$		1	0.9990	1
	$s_2^{(B*P)}$		0.0326	0.0303	0.0269
	$s_4^{(B*P)}$		0.1121	0.1104	0.0790
	$\sigma_{24}^{(B*P)}$		-0.2510	-0.4020	-0.9980
	γ			0.0094	0.0098
	σ^2	0.9269	0.7726	0.6223	0.6150
统计指标	AIC	6051.61	5484.99	5448.62	5425.20
	-2LL	-3018.81	-2729.50	-2710.31	-2696.60

中国知网 https://www.cnki.net

4.2.2 嵌套两水平非线性混合效应冠幅模型

模型（4-3）中含有 6 个形式参数（ϕ_1—ϕ_6），当同时考虑区组效应和嵌套在区组里面的样地交互效应时，有 63 种不同组合形式。对所有组合形式计算，共有 15 种计算收敛，其中当区组效应和嵌套在区组里面的样地效应同时作用在ϕ_2和ϕ_4上时 $AIC = 5484$ 最小，$\text{Log}lik = -2729$ 最大，模型表达式为：

$$CW_{ijk} = \frac{\beta_1 + (\beta_2 + u_{2i} + u_{2ij})DH_{ij}}{1 + [\beta_3 + (\beta_4 + u_{4i} + u_{4ij})HCB_{ijk}]\exp[-(\beta_5 + \beta_6 H_{ijk})D_{ijk}]} + \varepsilon_{ijk} \quad (4\text{-}15)$$

其中，$\beta_1 - \beta_6$ 为固定效应参数；u_{2i} 和 u_{4i} 分别为第 i^{th} 区组作用在ϕ_2和ϕ_4上的随机效应参数；u_{2ij} 和 u_{4ij} 分别为第 i^{th} 个区组中第 j^{th} 个样地作用在ϕ_2和ϕ_4上的随机效应参数；值得注意的是 $\mathbf{u}_i = (u_{2i}, u_{4i})^T$ 与下标 i 有关，并且 $\mathbf{u}_i$ 假定为服从期望为零方差为 $\mathbf{\Psi}_1$ 的正态分布；$\mathbf{u}_{ij} = (u_{2ij}, u_{4ij})^T$ 与下标 i 和 j 有关，并且假定 $\mathbf{u}_{ij}$ 服从期望为零方差协方差矩阵为 $\mathbf{\Psi}_2$ 的正态分布。$\mathbf{u}_i, \mathbf{u}_{ij}$，和 ε_{ijk} 之间假定相互独立。

模型（4-15）的参数估计值见表 4-1。所有参数估计值都显著地不等于 0（$p < 0.05$）。结果表明即使考虑区组效应以及嵌套在区组中的样地效应对冠幅的影响，模型（4-15）中依然存在异方差情形，见图 4-1。

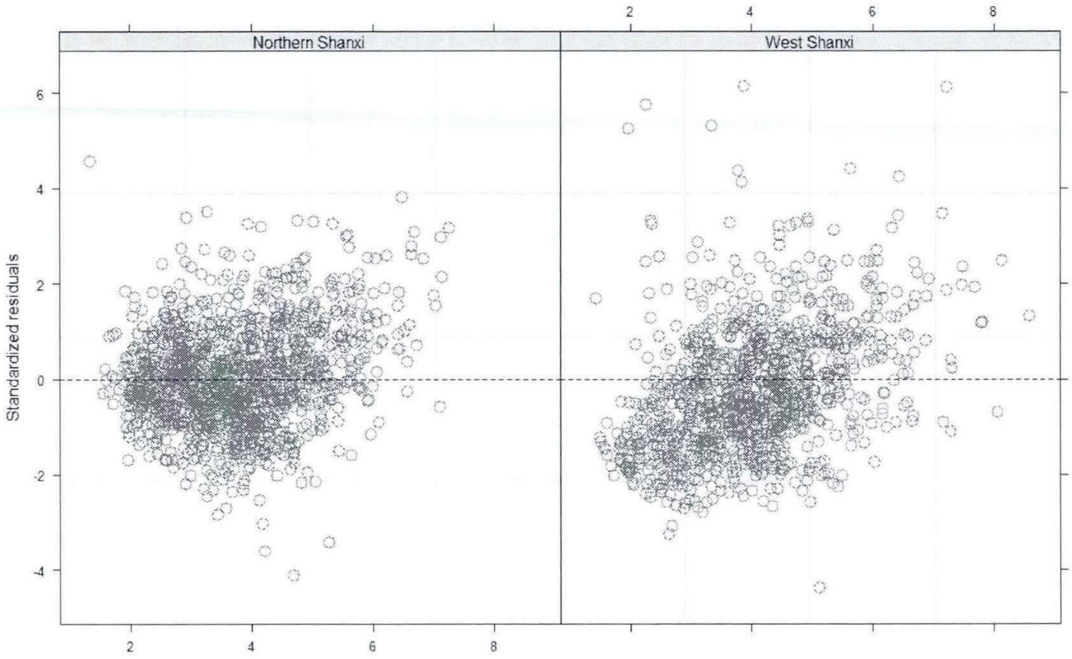

图 4-1 模型（4-15）的标准残差分布图

中国知网 https://www.cnki.net

4.2.3 误差项方差协方差（R）结构

表 4-3 为模型（4-15）对应 3 种加权残差方差模型在预测变量为 D，H，HCB 和 DH 以及不加权（独立等方差）时模型的评价指标。从表中可知，常数加幂函数且预测变量为 D 计算不收敛。对于各收敛的模型，考虑残差加权的 3 种模型对应的指标 AIC 和 Loglik 与不加权模型差异显著（P＜0.001），表明 3 种加权残差方差模型能明显解除异方差。在所有类型中，指数函数（4-16）且 D 作为预测变量对应的指标最小，故选它为模型（4-15）残差加权函数。表达式如下：

$$\mathrm{var}(\varepsilon_{ijk}) = \sigma^2 \exp(2\gamma D_{ijk}) \qquad (4\text{-}16)$$

因此，最终的嵌套两水平非线性混合效应冠幅模型为：

$$\begin{cases} CW_{ijk} = \dfrac{\beta_1 + (\beta_2 + u_{2i} + u_{2ij})DH_{ij}}{1 + [\beta_3 + (\beta_4 + u_{4i} + u_{4ij})HCB_{ijk}]\exp[-(\beta_5 + \beta_6 H_{ijk})D_{ijk}]} + \varepsilon_{ijk} \\ \mathrm{var}(\varepsilon_{ijk}) = \sigma^2 \exp(2\gamma D_{ijk}) \end{cases} \qquad (4\text{-}17)。$$

中国知网 https://www.cnki.net

表 4-2 各方差函数在胸高直径 D、树高 H、枝下高 HCB 和样地优势树高 DH 为预测变量时的评价指标，其中 1 表示模型的随机效应假定为独立等方差类型，EF 表示指数方差函数，PF 为幂方差函数，CPF 为常数加幂方差函数，F 表示计算不收敛，LL 为对数似然，LR 为似然比。

方差	D				H				HCB				DH			
函数	AIC	-2LL	LR	p value	AIC	-2LL	LR	p value	AIC	-2LL	LR	p value	AIC	-2LL	LR	p value
1	5485	-2730			5485	-2730			5485	-2730			5485	-2730		
EF	5449	-2710	38.33	<0.0001	5482	-2727	5.11	0.0238	5471	-2721	16.21	0.0001	5470	-2721	16.57	<0.0001
PF	5471	-2722	15.04	0.0001	5484	-2728	3	0.0831	5482	-2727	5.16	0.0231	5472	-2722	15.47	0.0001
CPF	F	F	F	F	5482	-2727	6.06	0.0484	5484	-2727	5.16	0.0757	5472	-2721	16.78	0.0002

中国知网 https://www.cnki.net

图 4-2 为嵌套两水平的非线性混合效应冠幅模型（4-17）的残差分布图。从图中得知，异方差明显减少，因此进一步表明指数函数（4-15）且 D 作为预测变量的方差函数能有效解释模型异方差。

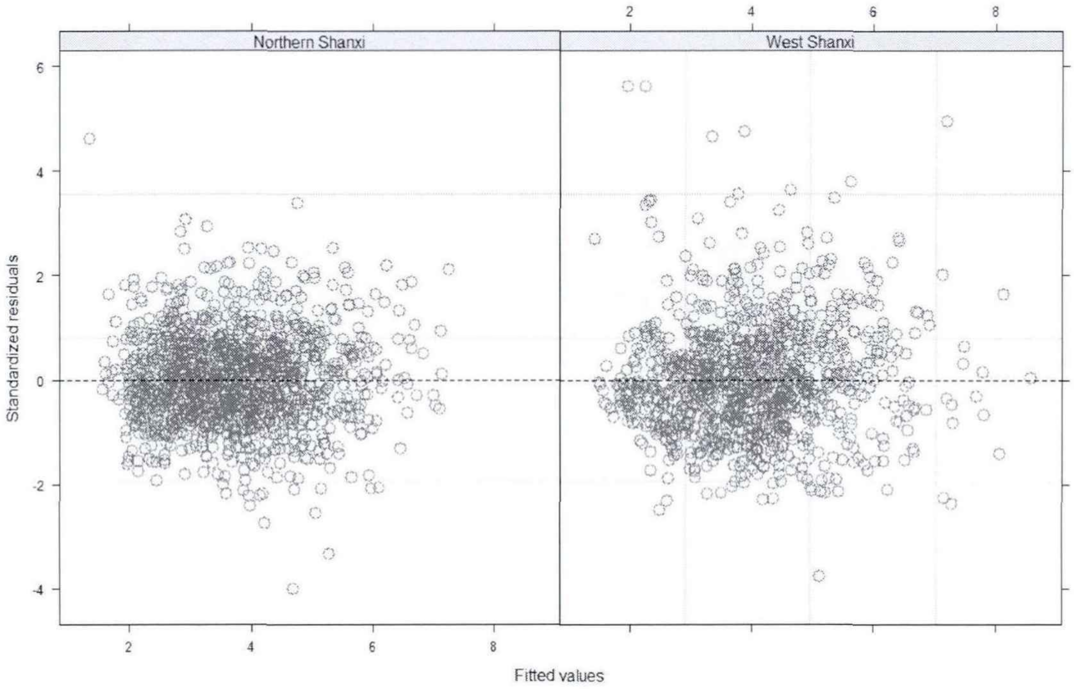

图 4-2 模型（4-17）的残差分布图

4.2.4 地域效应

与嵌套两水平随机效应类似，当模型考虑地域效应时，总共有 63 种不同地域效应和固定效应参数的组合形式。在这些考虑地域效应的嵌套两水平非线性混合效应模型时，只有 10 种类型计算收敛，当地域效应作用在固定效应参数 β_1 和 β_5 上时，模型对应的 AIC（5425）最小而 LogLik（-2697）最大。模型表达式如下：

$$\begin{cases} CW_{ijk} = \dfrac{\beta_1 + \alpha_1 P_{ijk} + (\beta_2 + u_{2i} + u_{2ij})DH_{ij}}{1 + [\beta_3 + (\beta_4 + u_{4i} + u_{4ij})HCB_{ijk}]\exp[-(\beta_5 + \alpha_5 P_{ijk} + \beta_6 H_{ijk})D_{ijk}]} + \varepsilon_{ijk} \\ \boldsymbol{\varepsilon}_{\mathbf{ij}} = (\varepsilon_{ij1}, \ldots, \varepsilon_{ijn})^T \sim N\left(0, \mathbf{R}_{\mathbf{ij}} = \sigma^2 \mathbf{G}_{\mathbf{ij}}^{0.5} \boldsymbol{\Gamma}_{\mathbf{ij}} \mathbf{G}_{\mathbf{ij}}^{0.5}\right) \\ \mathbf{G}_{\mathbf{ij}} = diag\left(\sigma^2 \exp(2\gamma D_{ij1}), \ldots, \sigma^2 \exp(2\gamma D_{ijn})\right) \\ \boldsymbol{\Gamma}_{\mathbf{ij}} = \mathbf{I}_n \end{cases} \qquad (4\text{-}18)$$

其中，α_1 和 α_5 分别为作用在 β_1 和 β_5 上的地域固定效应，P_{ijk} 为与地域有关的哑

中国知网 https://www.cnki.net

变量。

4.2.5 模型参数估计

表 4-1 给出了模型（4-17）和模型（4-18）的参数估计值以及评价指标（AIC 和 Loglik）。模型（4-18）对应的 AIC 最小而 LogLik 最大表明区组效应以及嵌套在区组里面的样地效应对冠幅的影响较大。把固定效应参数估计值代入到模型（4-18）中，得到落叶松冠幅的一般模型表达式为：

$$CW_{ijk}=\frac{12.4305-2.7840P_{ijk}+(-0.1429+u_{2i}+u_{2ij})DH_{ij}}{1+[3.4042+(0.1521+u_{4i}+u_{4ij})HCB_{ijk}]\exp[-(0.0703+0.0363P_{ijk}-0.0007H_{ijk})D_{ijk}]}+\varepsilon_{ijk}$$

（5-19）

其中

$$\mathbf{u}_i=\begin{bmatrix}u_{2i}\\u_{4i}\end{bmatrix}\sim N\left\{\begin{bmatrix}0\\0\end{bmatrix},\mathbf{\Psi}_1=\begin{pmatrix}0.0337 & 1\\1 & 0.0739\end{pmatrix}\right\},$$

$$\mathbf{u}_{ij}=\begin{bmatrix}u_{2ij}\\u_{4ij}\end{bmatrix}\sim N\left\{\begin{bmatrix}0\\0\end{bmatrix},\mathbf{\Psi}_2=\begin{pmatrix}0.0269 & -0.9980\\-0.9980 & 0.0790\end{pmatrix}\right\},$$

$$\varepsilon_{ij}\sim N\left(0,\mathbf{R}_{ij}=0.6150\mathbf{G}_{ij}^{0.5}\mathbf{I}_{n_{ij}}\mathbf{G}_{ij}^{0.5}\right),$$

$$\mathbf{G}_{\mathbf{ij}}=diag\left(0.6150\exp(0.0196D_{ij1}),\ldots,0.6150\exp(0.0196D_{ijn_{ij}})\right)。$$

4.2.6 模型预测和评价

模型（4-17）和模型（4-18）对应的$\bar{e}$、δ、$RMSE$和TRE具有相同变化趋势（见表 4-3）。不管多少株样木用于计算随机效应参数，模型（4-17）和模型（4-18）对应的$\bar{e}$、δ、$RMSE$和TRE都比其对应的 PA 水平和基础模型（4-3）要好。随着用于计算随机效应参数的样地株数增加，模型（4-17）和模型（4-18）对应的预测精度也随之增加。各统计指标的减少率随机样地株数的增加而增加，对于模型（4-17）当样木株数达到 5 株，对于模型（4-18）样木株数达到 4 株以后，各统计指标对应的减少率逐渐减少。对于模型（4-17），当利用 5 株样木计算随机效应参数时，与模型自身的 PA 水平相比，对应的评价指标值$\bar{e}$、δ、$RMSE$和TRE分别减少了 65%、31%、 19% 和 31 %。对于模型（4-18），当利用 4

中国知网 https://www.cnki.net

株样木计算随机效应参数时，与模型自身的PA水平相比，对应的评价指标值$\bar{e}$、δ、*RMSE*和*TRE*分别减少了69%、31%、 19% 和 30 %。

表4-3 模型（4-3）、模型（4-17）和模型（4-18）在总体平均水平（PA）、区组水平（Block）和区组和样地水平（Block +Block*Plot）对应的模型评价指标，其中对于区组和样地水平，共有8种情形（1-8株样木）计算随机效应参数，$\bar{e}$为平均残差，δ残差方差，*RMSE*为均方根误差，*TRE*为总相对误差

模型	$\bar{e}$	δ	*RMSE*	*TRE*
模型（4-3）	-0.2023	1.4003	1.2005	37.1015
模型（4-17）				
PA	-0.2882	1.3538	1.1987	36.1885
Block	-0.1943	1.2479	1.1339	34.4967
Block +Block*Plot (from 1 tree)	-0.1644	1.1095	1.0661	31.4866
Block +Block*Plot (from 2 trees)	-0.1461	1.0543	1.0371	29.6147
Block +Block*Plot (from 3 trees)	-0.1225	1.0027	1.0088	27.5016
Block +Block*Plot (from 4 trees)	-0.1001	0.9365	0.9729	25.1486
Block +Block*Plot (from 5 trees)	-0.0732	0.8244	0.9109	22.4961
Block +Block*Plot (from 6 trees)	-0.0572	0.7556	0.8711	20.9656
Block +Block*Plot (from 8 trees)	-0.0453	0.6934	0.8339	19.6341
Block +Block*Plot (from 10 trees)	-0.0421	0.6527	0.8090	18.5664
模型（4-18）				
PA	-0.2798	1.3321	1.1876	35.5987
Block	-0.1827	1.2239	1.1213	34.1435
Block +Block*Plot (from 1 tree)	-0.1529	1.0826	1.0517	31.2574
Block +Block*Plot (from 2 trees)	-0.1371	1.0325	1.0253	29.3040
Block +Block*Plot (from 3 trees)	-0.1142	0.9814	0.9972	27.1375
Block +Block*Plot (from 4 trees)	-0.0861	0.9178	0.9619	24.8156
Block +Block*Plot (from 5 trees)	-0.0694	0.8169	0.9065	22.2312
Block +Block*Plot (from 6 trees)	-0.0543	0.7487	0.8670	20.8381
Block +Block*Plot (from 8 trees)	-0.0429	0.6885	0.8309	19.5860
Block +Block*Plot (from 10 trees)	-0.0374	0.6470	0.8052	18.4993

根据T检验表明所有模型对应的平均残差都显著地等于0（$p < 0.05$）（见表4-3）。不管用多少株样木计算随机效应参数，模型（4-18）对应的$\bar{e}$、δ 、*RMSE*

中国知网 https://www.cnki.net

和TRE都显著小于相对应情形时的模型（4-17）。例如，当利用4株样木计算随机效应参数时，模型（4-18）对应的$\bar{e}$、δ、$RMSE$和TRE比模型（4-17）要少14%、2%、1%和1%。因此表明地域效应对冠幅的影响较大，当地域效应被考虑时明显改进模型预测精度。除此之外，与基础模型（4-3）相比，模型（4-17）对应的$RMSE$和TRE分别下降了19%和32%，因此进一步说明区组随机效应和嵌套在区组里面的样地效应对冠幅的影响较大。

综合以上分析把同时考虑区组随机效应、嵌套在区组里面的样地随机效应以及地域效应的模型（4-18）作为预测华北落叶松冠幅的最终模型。

4.3 小结

本研究首次利用嵌套两水平非线性混合效应模型构建华北落叶松单木冠幅模型。由于实验数据来自中国北方地区两个具有代表性的落叶松分布区域，因此模型考虑了地域效应。结果表明：

（1）地域效应对华北落叶松冠幅的影响较大，当考虑该效应时，模型预测精度明显提高；

（2）区组效应和嵌套在区组里面的样地效应对华北落叶松的随机影响较大，当模型考虑这些随机影响时模型预测精度能进一步显著提高；

（3）指数方差函数且预测变量为胸高直径能有效剔除模型的异方差；

（4）利用所构建的嵌套两水平非线性混合效应模型预测冠幅时，利用随机抽取的4株样地计算随机效应参数效果较好。

中国知网 https://www.cnki.net

5 冠幅可加性模型研建

树冠在树木的生长过程中具有重要的作用，它反映了树木的长期竞争水平。对树冠结构信息的描述也越来越引起森林经营者的重视。尤其近几年，随着计算机技术的发展和森林生态研究的深入，对冠幅的研究逐步从平均冠幅到不同方向的冠幅，其中最常见的是东、南、西和北冠幅。因此，构建各分项冠幅模型与总冠幅模型的相容性一直为世界生态学家和林学家所重视。到目前为止，针对冠幅可加性模型的研建较少，可加性模型主要集中为生物量建模领域。对于可加性建模，当前已提出了多种方法，最常见的有非线性似然无关回归方法、比例平差法和线性或非线性联合估计方法等。以生物量建模为例，非线性似然无关理论保证了树冠生物量、干皮和干材的可加性，同时通过误差项方差协方差矩阵描述单木生物量各分量之间相互关系。比例平差法是基于各分项占总量的比例之和等于1，它是解决单木生物量相容性问题的最简单且最直接的办法。通过最小二乘法独立求解得到各分量模型中参数。基于比例平差最优估计是在不考虑单木生物量相容性的情况下给出的，唐守正等提出了非线性或线性联合估计方法，该方法将各分项进行联立求解，联合建模。然而，利用上述研究方法对冠幅建模至今尚未报道。为此，本节重点介绍如何利用非线性似然无关回归方法、比例平差法和线性或非线性联合估计方法等构建冠幅可加性模型，研究成果将为森林经营和森林生态研究提供理论基础[108-112]。

5.1 材料与方法

5.1.1 实验数据

本研究需要构建总冠幅（CW），东冠幅半径（CR_E）、 西冠幅半径（CR_W）、东西冠幅（CW_{EW}）、南冠幅半径 （CR_S）、北冠幅半径（CR_N）和南北冠幅（CW_{SN}）与林木因子之间的关系模型，因此需要每个样地的单木信息和林分变量等，实验数据的详细介绍见第二章。

5.1.2 基础模型

继续选择以胸径、树高、枝下高、优势木高作为协变量构建了以 logistic 形式的杉木单木冠幅模型，该模型预测精度比其他几种候选模型（例如，指数、幂函数和威布尔函数）预测精度明显要高。利用该模型对本研究中华北落叶松单木

中国知网 https://www.cnki.net

总冠幅和各分项冠幅模型进行拟合同样发现该模型具有较高的预测精度，为此，选择该基础模型所构建冠幅模型作为基础模型用于构建华北落叶松总冠幅和各分项冠幅模型以及他们之间的相容性冠幅模型。所提出的冠幅模型表达式为：

$$CW = f(\mathbf{x},\boldsymbol{\beta}) = \frac{\beta_1 + \beta_2 DH}{1 + (\beta_3 + \beta_4 HCB)\exp\left[-(\beta_5 + \beta_6 H)D\right]} + \varepsilon$$

其中，$\mathbf{x}$为预测变量，包括 DH、HCB、H 和 D；$\boldsymbol{\beta} = (\beta_1, \beta_2, \beta_3, \beta_4, \beta_5, \beta_6)$为 6 维的参数向量；$\varepsilon$为误差项。$CW_{ijk}, HCB_{ijk}, D_{ijk}$分别为第$i$区组中第$j$样地第$k$株对象木对应的冠幅（m）和枝下高（m）和胸高直径（cm）；DH_{ij}为第i区组第j样地的平均优势高；ε_{ijk}为误差项，ϕ_1-ϕ_6为待估参数。

本研究为了考虑关帝山国家森林公园和伯强国有林场之间冠幅生长差异，因此构建了一个哑变量，即 P=0 时表示关帝山国家森林公园，P=1 时表示伯强国有林场。假定 P 作用在参数β_1和β_2上，模型表达式如下：

$$CW = f(\mathbf{x},\boldsymbol{\beta}) = \frac{\beta_1 + k_1 P + (\beta_2 + k_2 P)DH}{1 + (\beta_3 + \beta_4 HCB)\exp\left[-(\beta_5 + \beta_6 H)D\right]} + \varepsilon \quad (5\text{-}1)$$

模型（5-1）最终用于构建总冠幅和各分项冠幅模型的基础模型。

5.1.3 非线性联立方程组（NSE）

近代统计分析方法之一度量误差模型，它是研究当模型中的变量带有度量误差时的参数估计等问题，它的广义多元一般表达式为：

$$\begin{cases} \mathbf{f}(\mathbf{y}_i, \mathbf{x}_i, \boldsymbol{\beta}) = 0, i = 1, \ldots, n \\ \mathbf{Y}_i = \mathbf{y}_i + \mathbf{e}_i \\ E(\mathbf{e}_i) = 0, Var(\mathbf{e}_i) = \boldsymbol{\Sigma} \end{cases} \quad (5\text{-}2)$$

其中，$f = (f_1, f_2, \cdots, f_m)^T$为$m$维已知向量函数值，在本研究中由冠幅和冠幅半径所对应的基础模型来确定；n为总观测数；x_i是没有误差的$1 \times q$维观测向量；Y_i是真值y_i的$1 \times p$维观测向量，e_i是其观测误差；c是$k \times 1$维参数向量；Σ是已知或未知的$p \times p$维正定矩阵）；一般满足$p \ge m$；当f不为(y_i, x_i)和c的双线性函数时，就是非线性度量误差模型，否则称为线性度量误差模型。特别是$p = m$时，模型为非线性度量误差联立方程组。本研究是假定模型中总冠幅以及各分项冠幅含有度量误差，而所有林分变量（自变量）不含度量误差，即利用非线性联立方

中国知网 https://www.cnki.net

程组构建各分项生物量方程系统。非线性联立方程组的详细介绍见文献。

对于任意数据结构，模型（5-2）都保证了参数的相容性。不像最小二乘法需要对模型中部分或全部因变量和自变量做约束。模型（5-2）可以通过二步度量误差模型算法来求解参数，具体两个步骤如下：

第 1 步：首先令 $\boldsymbol{\psi}=\mathbf{I}$，通过极小化目标函数

$$Q_1(\mathbf{y}_i,\boldsymbol{\beta})=\sum_{i=1}^{n}(\mathbf{Y}_i-\mathbf{y}_i)\boldsymbol{\psi}^{-1}(\mathbf{Y}_i-\mathbf{y}_i)^T \tag{5-3}$$

得到参数 $\boldsymbol{\beta}$，根据观测值 $\mathbf{Y}_i$ 和 $\mathbf{x}_i$，利用模型（5-2）计算得到 $\hat{\mathbf{y}}_i$，根据 $\hat{\mathbf{y}}_i$ 计算得出方差-协方差矩阵估计值 $\hat{\boldsymbol{\psi}}$：

$$\hat{\boldsymbol{\psi}}=\frac{1}{n}\sum_{i=1}^{n}(\mathbf{Y}_i-\hat{\mathbf{y}}_i)^T(\mathbf{Y}_i-\hat{\mathbf{y}}_i) \tag{5-4}$$

第 2 步：在利用 $\hat{\boldsymbol{\psi}}$，通过极小化目标函数

$$Q_2(\mathbf{y}_i,\boldsymbol{\beta})=\sum_{i=1}^{n}(\mathbf{Y}_i-\mathbf{y}_i)\hat{\boldsymbol{\psi}}^{-1}(\mathbf{Y}_i-\mathbf{y}_i)^T \tag{5-5}$$

得到最终的参数 $\boldsymbol{\beta}$ 的估计值。

二步度量误差模型算法已在 ForStat 2.1 上实现非线性联立方程组法的参数估计和详细计算步骤见参考文献唐守正等（2015）[113]。

从相容性定义出发，要满足各分项之和等于总量，实际上就是满足各分项占总量的比例之和等于 1，唐守正等（2000）以此为基础提出了比例平差法。根据各分量分配层次的不同，可得到总量直接控制方案（Procedure Ⅰ）和分级联合控制方案（Procedure Ⅱ）[114]。

5.1.3.1 总量直接控制平差法

由总量直接平差分配给东冠幅半径 CR_E、西冠幅半径 CR_W、南冠幅半径 CR_S 和北冠幅半径 CR_N，从而保证各分量之和等于总量。具体各分项的模型表达式如下：

中国知网 https://www.cnki.net

$$\begin{cases} cw_{Ei} = \dfrac{f_E(\mathbf{x}_i, P_i, \boldsymbol{\beta}_E)}{f_E(\mathbf{x}_i, P_i, \boldsymbol{\beta}_E) + f_W(\mathbf{x}_i, P_i, \boldsymbol{\beta}_W) + f_S(\mathbf{x}_i, P_i, \boldsymbol{\beta}_S) + f_N(\mathbf{x}_i, P_i, \boldsymbol{\beta}_N)} cw_i \\ cw_{Wi} = \dfrac{f_W(\mathbf{x}_i, P_i, \boldsymbol{\beta}_W)}{f_E(\mathbf{x}_i, P_i, \boldsymbol{\beta}_E) + f_W(\mathbf{x}_i, P_i, \boldsymbol{\beta}_W) + f_S(\mathbf{x}_i, P_i, \boldsymbol{\beta}_S) + f_N(\mathbf{x}_i, P_i, \boldsymbol{\beta}_N)} cw_i \\ cw_{Si} = \dfrac{f_S(\mathbf{x}_i, P_i, \boldsymbol{\beta}_S)}{f_E(\mathbf{x}_i, P_i, \boldsymbol{\beta}_E) + f_W(\mathbf{x}_i, P_i, \boldsymbol{\beta}_W) + f_S(\mathbf{x}_i, P_i, \boldsymbol{\beta}_S) + f_N(\mathbf{x}_i, P_i, \boldsymbol{\beta}_N)} cw_i \\ cw_{Ni} = \dfrac{f_N(\mathbf{x}_i, P_i, \boldsymbol{\beta}_N)}{f_E(\mathbf{x}_i, P_i, \boldsymbol{\beta}_E) + f_W(\mathbf{x}_i, P_i, \boldsymbol{\beta}_W) + f_S(\mathbf{x}_i, P_i, \boldsymbol{\beta}_S) + f_N(\mathbf{x}_i, P_i, \boldsymbol{\beta}_N)} cw_i \\ cw = f_T(\mathbf{x}_i, P_i, \boldsymbol{\beta}_T) \\ \mathbf{Y}_i = \mathbf{y}_i + \mathbf{e}_i, \mathbf{Y}_i = (CR_{Ei}, CR_{Wi}, CR_{Si}, CR_{Ni}, CW)^T, \mathbf{y}_i = (cr_{Ei}, cr_{Wi}, cr_{Si}, cr_{Ni}, cw)^T, \\ E(\mathbf{e}_i) = 0, \operatorname{var}(\mathbf{e}_i) = \sigma^2 \boldsymbol{\psi}, i = 1, \dots, n \end{cases} \tag{5-6}$$

其中，$i = 1,\dots,n$，n为总观测数，CW_i、CR_{Ei}、CR_{Wi}、CR_{Si}和CR_{Ni}分别为第 i 株样木对应的东冠幅半径、西冠幅半径、南冠幅半径和北冠幅半径的观测值，含有度量误差；$cw_i, cr_{Ei}, cr_{Wi}, cr_{Si}$和 cr_{Ni}分别为东冠幅半径、西冠幅半径、南冠幅半径和北冠幅半径的真实值，不含有度量误差；$f_E(\mathbf{x}_i, P_i, \boldsymbol{\beta}_E)$，$f_W(\mathbf{x}_i, P_i, \boldsymbol{\beta}_W), f_S(\mathbf{x}_i, P_i, \boldsymbol{\beta}_S)$，$f_N(\mathbf{x}_i, P_i, \boldsymbol{\beta}_N)$ 和$f_T(\mathbf{x}_i, P_i, \boldsymbol{\beta}_T)$分别为$CR_E$、$CR_W$、$CR_S$、$CR_N$和 CW 对应的基础模型；$\boldsymbol{\beta}_E$，$\boldsymbol{\beta}_W$，$\boldsymbol{\beta}_S$，$\boldsymbol{\beta}_N$，和 $\boldsymbol{\beta}_T$分别为CR_E、CR_W、CR_S、CR_N和 CW 对应的参数；P_i为第i样木对应的哑变量值；方差-协方差矩阵$\boldsymbol{\psi}$ 大小为4×4，主要用于描述总冠幅以及各分项冠幅之间的相关性。

5.1.3.2 分级联合控制平差法

分级联合控制比例平差法，即首先把总冠幅分配给东西冠幅（CW_{EW}）和南北冠幅（CW_{SN}），然后再把东西冠幅分配给东冠径和西冠径，南北冠幅（CW_{SN}）分配给南冠径和北冠径。该方法不但实现了东、南、西、北冠径之和等于 2 倍总冠幅，而且还实现了东西冠幅和南北冠幅之和等于总冠幅的两倍。模型表达式如下：

中国知网 https://www.cnki.net

$$\begin{cases} cw_{Ei}=\dfrac{f_E(\mathbf{x}_i,P_i,\boldsymbol{\beta}_E)}{f_E(\mathbf{x}_i,P_i,\boldsymbol{\beta}_E)+f_W(\mathbf{x}_i,P_i,\boldsymbol{\beta}_W)}\dfrac{f_{EW}(\mathbf{x}_i,P_i,\boldsymbol{\beta}_{EW})}{f_{EW}(\mathbf{x}_i,P_i,\boldsymbol{\beta}_{EW})+f_{SN}(\mathbf{x}_i,P_i,\boldsymbol{\beta}_{SN})}cw \\ cw_{Wi}=\dfrac{f_W(\mathbf{x}_i,P_i,\boldsymbol{\beta}_W)}{f_E(\mathbf{x}_i,P_i,\boldsymbol{\beta}_E)+f_W(\mathbf{x}_i,P_i,\boldsymbol{\beta}_W)}\dfrac{f_{EW}(\mathbf{x}_i,P_i,\boldsymbol{\beta}_{EW})}{f_{EW}(\mathbf{x}_i,P_i,\boldsymbol{\beta}_{EW})+f_{SN}(\mathbf{x}_i,P_i,\boldsymbol{\beta}_{SN})}cw \\ cw_{Si}=\dfrac{f_S(\mathbf{x}_i,P_i,\boldsymbol{\beta}_S)}{f_S(\mathbf{x}_i,P_i,\boldsymbol{\beta}_S)+f_N(\mathbf{x}_i,P_i,\boldsymbol{\beta}_N)}\dfrac{f_{SN}(\mathbf{x}_i,P_i,\boldsymbol{\beta}_{SN})}{f_{EW}(\mathbf{x}_i,P_i,\boldsymbol{\beta}_{EW})+f_{SN}(\mathbf{x}_i,P_i,\boldsymbol{\beta}_{SN})}cw \\ cw_{Ni}=\dfrac{f_N(\mathbf{x}_i,P_i,\boldsymbol{\beta}_N)}{f_S(\mathbf{x}_i,P_i,\boldsymbol{\beta}_S)+f_N(\mathbf{x}_i,P_i,\boldsymbol{\beta}_N)}\dfrac{f_{SN}(\mathbf{x}_i,P_i,\boldsymbol{\beta}_{SN})}{f_{EW}(\mathbf{x}_i,P_i,\boldsymbol{\beta}_{EW})+f_{SN}(\mathbf{x}_i,P_i,\boldsymbol{\beta}_{SN})}cw \\ cw=f_T(\mathbf{x}_i,P_i,\boldsymbol{\beta}_T) \\ \mathbf{Y}_i=\mathbf{y}_i+\mathbf{e}_i,\mathbf{Y}_i=(CR_{Ei},CR_{Wi},CR_{Si},CR_{Ni},CW)^T,\mathbf{y}_i=(cr_{Ei},cr_{Wi},cr_{Si},cr_{Ni},cw)^T \\ E(e_i)=0,\mathrm{var}(e_i)=\sigma^2\boldsymbol{\psi},i=1,\ldots,n \end{cases} \tag{5-7}$$

其中，$f_{EW}(\mathbf{x}_i,P_i,\boldsymbol{\beta}_{EW})$ 和 $f_{SN}(\mathbf{x}_i,P_i,\boldsymbol{\beta}_{SN})$分别为东西冠幅（$CW_{EW}$）和南北冠幅（$CW_{SN}$）对应的基础模型；总冠幅和各分项冠幅之间的相关性通过方差-协方差矩阵$\boldsymbol{\psi}$中元素来解释。模型系统中其它参数和变量定义见模型系统（5-6）。

5.1.4 非线性似然无关回归方法(NSUR)

非线性似然无关回归方法（nonlinear seemingly unrelated regressions，NSUR）由 Parresol（2001）提出，该方法不仅保证了各分项树冠半径之和等于总冠幅两倍外，同时考虑了各树冠半径以及总冠幅之间的相关性，因此被广泛应用。模型表达式如下[115]：

$$\begin{aligned} CW_E &= f_E(\mathbf{x},P,\boldsymbol{\beta}_E)+\varepsilon_E \\ CW_W &= f_W(\mathbf{x},P,\boldsymbol{\beta}_W)+\varepsilon_W \\ CW_S &= f_S(\mathbf{x},P,\boldsymbol{\beta}_S)+\varepsilon_S \\ CW_N &= f_N(\mathbf{x},P,\boldsymbol{\beta}_N)+\varepsilon_N \\ CW &= (CW_E+CW_W+CW_S+CW_N)/2+\varepsilon_T \end{aligned} \tag{5-8}$$

其中，$y=(CW_E,CW_W,CW_S,CW_N,CW)^T$ 和 $\mathbf{x}$ 分别为 $n\times 5$ 和 $n\times q$ 大小矩阵；$e=(\varepsilon_E,\varepsilon_W,\varepsilon_S,\varepsilon_N,\varepsilon)^T$为$5\times N$矩阵；$\boldsymbol{\beta}^T=(\boldsymbol{\beta}_E,\boldsymbol{\beta}_W,\boldsymbol{\beta}_S,\boldsymbol{\beta}_N,\boldsymbol{\beta}_T)^T$为$5\times k$矩阵[$k=8$为基础模型（5-1）的参数个数]。

模型系统中假定了对于不同的单木，误差项ε_{ti}($t=E,W,S,N,T$； $i=1,\ldots,n$)之间相互独立，对于相同的单木，各误差项之间呈相关性。因此，本研究中，假定：

$$E(\varepsilon_{t_1 i_1},\varepsilon_{t_2 i_2}\mid X)=\begin{cases}0, & \text{if } i_1\neq i_2, i_1,i_2=1,\ldots,N; t_1,t_2=E,W,S,N,T \\ \sigma_{t_1 t_2}, & \text{if } i_1=i_2\end{cases} \tag{5-9}$$

中国知网 https://www.cnki.net

X 为 $\boldsymbol{\beta}$ 的 $n\times k$ 设计矩阵；令 $\Sigma=[\sigma_{t_1t_2}]$ 为每个观测点对应的 5×5 条件方差，误差项 e 的方差协方差矩阵等于：

$$\mathbf{R}=E(e^Te\,|\,X)=\Sigma\otimes\mathbf{I}_N$$

模型系统（5-8）通常是由广义最小二乘法计算得到，在第一步时，利用最小二乘法分别拟合总冠幅和各树冠半径得到残差 ε_t，然后利用 ε_t 计算矩阵 Σ 中的元素 $\hat{\sigma}_{t_1t_2}=(\hat{\varepsilon}_{t_1}^T\hat{\varepsilon}_{t_2})/N$。在第二步时，在已知方差-协方差矩阵 $\hat{\mathbf{R}}=\hat{\Sigma}\otimes\mathbf{I}_N$ 的条件下，利用广义最小二乘法求解 $\boldsymbol{\beta}$，计算公式如下：

$$\hat{\beta}=(\mathbf{x}^T(\hat{\Sigma}^{-1}\otimes\mathbf{I}_N)\mathbf{x})^{-1}\mathbf{x}^T(\hat{\Sigma}^{-1}\otimes\mathbf{I}_N)y$$

和

$$\mathrm{cov}(\hat{\beta})=\sigma^2(\mathbf{x}^T(\hat{\Sigma}^{-1})^{-1}\otimes\mathbf{I}_N)\mathbf{x})^{-1}$$

其中，$f_1(\mathbf{x})$、$f_3(\mathbf{x})$、$f_4(\mathbf{x})$、$f_6(\mathbf{x})$ 和 $f_7(\mathbf{x})$ 分别为地上总生物量以及各分项干材、干皮、树枝和树叶生物量的基础模型，模型参数是由非线性似然无关回归方法估计得到，该方法的详细介绍见 Parresol（2001）。总冠幅和各分项树冠半径之间的相关性通过方差协方差 Σ 来解释。模型系统（5-8）的参数估计 R 代码见附件 2。关于 NSUR 的详细介绍见参考文献 Parresol (1999, 2001)和唐守正等（2015）[116-118]。

5.1.5 比例平差法（AP ）

与总量直接控制平差法类似，比例平差法也是由总量直接平差分配给东、南、西和北树冠半径，从而保证各分量之和等于两倍总量。对于比例平差法，总冠幅和各树冠半径都是通过最小二乘法独立拟合得到。CR_E、CR_W、CR_S、CR_N 和 CW 对应的计算公式如下：

中国知网 https://www.cnki.net

$$
\begin{aligned}
\hat{CR}_E &= \frac{2f_E(\mathbf{x},P,\hat{\boldsymbol{\beta}}_E)}{f_E(\mathbf{x},P,\hat{\boldsymbol{\beta}}_E)+f_W(\mathbf{x},P,\hat{\boldsymbol{\beta}}_W)+f_S(\mathbf{x},P,\hat{\boldsymbol{\beta}}_S)+f_N(\mathbf{x},P,\hat{\boldsymbol{\beta}}_N)}\hat{CW}+\varepsilon_E \\
\hat{CR}_W &= \frac{2f_W(\mathbf{x},P,\hat{\boldsymbol{\beta}}_W)}{f_E(\mathbf{x},P,\hat{\boldsymbol{\beta}}_E)+f_W(\mathbf{x},P,\hat{\boldsymbol{\beta}}_W)+f_S(\mathbf{x},P,\hat{\boldsymbol{\beta}}_S)+f_N(\mathbf{x},P,\hat{\boldsymbol{\beta}}_N)}\hat{CW}+\varepsilon_W \\
\hat{CR}_S &= \frac{2f_S(\mathbf{x},P,\hat{\boldsymbol{\beta}}_S)}{f_E(\mathbf{x},P,\hat{\boldsymbol{\beta}}_E)+f_W(\mathbf{x},P,\hat{\boldsymbol{\beta}}_W)+f_S(\mathbf{x},P,\hat{\boldsymbol{\beta}}_S)+f_N(\mathbf{x},P,\hat{\boldsymbol{\beta}}_N)}\hat{CW}+\varepsilon_S \qquad (5\text{-}10) \\
\hat{CR}_N &= \frac{2f_N(\mathbf{x},P,\hat{\boldsymbol{\beta}}_N)}{f_E(\mathbf{x},P,\hat{\boldsymbol{\beta}}_E)+f_W(\mathbf{x},P,\hat{\boldsymbol{\beta}}_W)+f_S(\mathbf{x},P,\hat{\boldsymbol{\beta}}_S)+f_N(\mathbf{x},P,\hat{\boldsymbol{\beta}}_N)}\hat{CW}+\varepsilon_N \\
\hat{CW} &= f(\mathbf{x},P,\hat{\boldsymbol{\beta}})+\varepsilon
\end{aligned}
$$

其中，$\hat{CR}_E$，$\hat{CR}_W$，$\hat{CR}_S$，$\hat{CR}_N$和$\hat{CW}$分别为 CR_E、CR_W、CR_S、CR_N和 CW 对应的估计值；$\hat{\boldsymbol{\beta}}$，$\hat{\boldsymbol{\beta}}_E,\hat{\boldsymbol{\beta}}_W,\hat{\boldsymbol{\beta}}_S$ 和 $\hat{\boldsymbol{\beta}}_N$分别为 CR_E、CR_W、CR_S、CR_N和 CW 对应的模型参数估计值。

5.1.6 最小二乘法独立回归（OLSSR）

利用基础模型（5-1）分别对 CR_E、CR_W、CR_S和 CR_N进行建模，得到各自树冠半径的模型参数估计值，总冠幅估计量通过下式计算得到：

$$\hat{CW}=(\hat{CW}_E+\hat{CW}_W+\hat{CW}_S+\hat{CW}_N)/2$$

其中，$\hat{CR}_E$，$\hat{CR}_W$，$\hat{CR}_S$，$\hat{CR}_N$和$\hat{CW}$分别为 CR_E、CR_W、CR_S、CR_N和 CW 对应的估计值。

5.1.7 模型评价

首先利用建模数据和检验数据分别对 CR_E、CR_W、CR_S、CR_N和 CW 基础模型（5-1）进行比较和评价。选用的评价指标有：平均偏差（$\bar{e}$）、残差方差（δ）、均方根误差（$RMSE$）、总相对误差（TRE）和修正决定系数（R_α^2），计算公式见（5-11）—（5-15）。然后利用建模数据和检验数据对总量直接控制平差法和分级联合控制平差法进行比较，确定一个最优的非线性联立方程组冠幅可加性模型。最后基于$\bar{e}$，δ，$RMSE$和 TRE等评价指标对最优的冠幅模型与非线性似然无关回归方法、比例平差法和最小二乘法独立回归进行系统比较。

$$\bar{e}=\sum(y_i-\hat{y}_i)/n \qquad (5\text{-}11)$$

中国知网 https://www.cnki.net

$$\delta = \sum (y_i - \hat{y}_i)^2 / (n-1) \tag{5-12}$$

$$RMSE = \sqrt{\bar{e}^2 + \xi} \tag{5-13}$$

$$TRE = 100\sum_{i=1}^{n}(y_i - \hat{y}_i)^2 / \sum_{i=1}^{n}\hat{y}_i \tag{5-14}$$

$$R_\alpha^2 = 1 - \frac{(n-1)\sum_{i=1}^{n}(y_i - \hat{y}_i)^2}{(n-P)\sum_{i=1}^{n}(y_i - \bar{y})^2} \tag{5-15}$$

其中，y_i 和 $\hat{y}_i$ 分别为第i株样木对应的总冠幅和树冠半径的观测值和预测值，需指出的是$RMSE$包含了平均偏差和残差方差，因此作为模型的主要评价因子。

5.2 结果与分析

5.2.1 基础模型

利用模型（5-1）对 CR_E、CR_W、CR_S、CR_N和 CW 进行拟合，得到的模型参数估计值见表 5-1。除了β_6，基础模型（5-1）对应的总冠幅和各分项树冠半径的参数估计值都显著地不等于零。基础模型（5-1）对应的总冠幅和各分项树冠半径的拟合结果和评价指标见表 5-2。

表 5-1 模型（5-1）利用最小二乘法对东树冠半径（CR_E）、西树冠半径（CR_W）、南树冠半径（CR_S）、北树冠半径（CR_N）和总冠幅（CW）分开拟合时对应的参数估计值

参数	CR_E	CR_W	CR_S	CR_N	CW_{EW}	CW_{SN}	CW
β_1	5.9471	21.4953	10.1567	7.1215	20.6796	17.6719	18.5692
	(0.8818)	(9.2512)	(1.1120)	(1.1538)	(3.3754)	(1.7114)	(1.9196)
β_2	-0.0741	-0.3607	-0.2014	-0.0859	-0.3098	-0.2988	-0.2978
	(0.0219)	(0.1650)	(0.0285)	(0.0260)	(0.0646)	(0.0408)	(0.0416)
β_3	4.7128	12.6883	4.4821	5.6577	6.9529	5.0960	5.7345
	(0.6375)	(5.5075)	(0.5266)	(0.8085)	(1.1198)	(0.4761)	(0.5757)
β_4	0.1779	0.4744	0.2982	0.1935	0.2373	0.2394	0.2316
	(0.0370)	(0.1474)	(0.0464)	(0.0352)	(0.0287)	(0.0283)	(0.0227)
β_5	0.0750	0.0570	0.0759	0.0850	0.0649	0.0800	0.0724
	(0.0084)	(0.0061)	(0.0079)	(0.0087)	(0.0051)	(0.0059)	(0.0047)
β_6	-0.0006	-0.0007	-0.0005	-0.0012	-0.0007	-0.0009	-0.0008
	(0.0002)	(0.0001)	(0.0002)	(0.0002)	(0.0001)	(0.0001)	(0.0001)

中国知网 https://www.cnki.net

续表 5-1

参数	CR_E	CR_W	CR_S	CR_N	CW_{EW}	CW_{SN}	CW
k_1	0.4669	-3.2004	-2.7163	0.4205	-1.2661	-2.5405	-1.9687
	(0.5613)	(2.1219)	(0.6496)	(0.6288)	(1.2027)	(0.9105)	(0.8692)
k_2	0.0229	0.1230	0.0887	0.0216	0.1084	0.1164	0.1109
	(0.0232)	(0.0838)	(0.0258)	(0.0259)	(0.0517)	(0.0375)	(0.0364)

注：: $\beta_1-\beta_6$ 和 k_1,k_2 分别为模型参数。

表 5-2 模型（5-1）对应东树冠半径（CR_E）、西树冠半径（CR_W）、南树冠半径（CR_S）、北树冠半径（CR_N）和总冠幅（CW）拟合结果和评价指标

	建模数据					检验数据			
变量	$\bar{e}$	δ	$RMSE$	TRE	R_a^2	$\bar{e}$	δ	$RMSE$	TRE
CR_E	0.0020	0.5750	0.7583	30.4955	0.2851	-0.0943	0.5779	0.7660	31.0410
CR_W	-0.0006	0.5307	0.7285	28.5253	0.3531	-0.0953	0.5205	0.7277	28.3095
CR_S	0.0004	0.6228	0.7892	31.0224	0.3763	-0.0919	0.5622	0.7554	28.2862
CR_N	-0.0010	0.5558	0.7455	28.0731	0.3310	-0.1241	0.5894	0.7777	30.3775
CW_{EW}	0.0006	1.1059	1.0516	29.5135	0.4731	-0.1916	1.2734	1.1446	34.8162
CW_{SN}	-0.0008	1.2116	1.1007	30.3841	0.5038	-0.2173	1.2431	1.1359	32.1787
CW	-0.0001	0.8481	0.9209	21.9292	0.5664	-0.2043	0.9651	1.0034	25.9071

注：$\bar{e}$、δ、$RMSE$、TRE 和 R_a^2 分别为平均偏差、残差方差、均方根误差、总相对误差和修正决定系数。

通过 T 检验得知 CR_E、CR_W、CR_S、CR_N 和 CW 对应基础模型（5-1）的 $\bar{e}$ 都显著地等于零（($p>0.05$）。从表 5-2 中得知，对于不同树冠分项，基础模型（5-1）对应的预测精度差异较大。总体而言，CW 对应的基础模型（5-1）预测精度最高。例如，对于拟合数据，CW 对应的 R_a^2 为 0.57，分别比 CR_E、CR_W、CR_S，CR_N、CW_{EW} 和 CW_{SN} 大于 98.67%, 60.41%, 50.52%, 71.12%, 19.72% 和 12.43%。对于检验数据，CW 对应的 TRE 为 0.57，分别比 CR_E、CR_W、CR_S，CR_N、CW_{EW} 和 CW_{SN} 小于 16.54%、 8.49%、 8.41%、 14.72%、 25.59% 和 19.49%。

5.2.2 总量直接控制平差法和分级联合控制平差法比较

模型系统（5-6）和（5-7）的拟合结果见表 5-3。两个模型系统都满足 CR_E、CR_W、CR_S 和 CR_N 之和等总冠幅的两倍。通过 T 检验得知模型系统（5-6）和（5-7）对应 CR_E、CR_W、CR_S、CR_N 和 CW 的 $\bar{e}$ 都显著地等于零($p>0.05$）。模型系统（5-6）

中国知网 https://www.cnki.net

和（5-7）估计出的 CR_E, CR_W, CR_S, CR_N, 和 CW 稍大于观测值。模型系统（5-7）对应的所有评价指标比模型系统（5-6）要好。例如，对于建模数据，模型系统（5-7）对应的 CR_E, CR_W, CR_S, CR_N, 和 CW 的 *RMSE* 值分别比模型系统（5-6）要小 38.83%, 20.12%, 14.41%, 18.94% 和 15.91%。模型系统（5-7）对应的 CR_E, CR_W, CR_S, CR_N, 和 CW 的 R_α^2 值分别比模型系统(5-6)要大 13.03%, 8.87%, 2.90%, 6.03% 和 10.39%。对于检验数据，模型系统（5-7）对应的 CR_E, CR_W, CR_S, CR_N, 和 CW 的 *RMSE* 值分别比模型系统（5-6）要小 6.51%, 5.78%, 9.16%, 2.56% 和 4.70%。

模型系统（5-7）对应的参数估计值见表 5-4。模型系统（5-7）中的参数估计值 k_1 和 k_2 都显著不等于零表明在相同直径条件下关帝山和五台山两地的华北落叶松冠幅差异较大。模型系统（5-7）中的所有参数都显著地不等零，并且各自参数都有一定的生物学意义，因此本研究选用模型系统（5-7）对华北落叶松冠幅进行估计。

表 5-3 模型系统（5-6）和（5-7）评价指标

变量	建模数据 $\bar{e}$	建模数据 δ	建模数据 *RMSE*	建模数据 *TRE*	建模数据 R_α^2	检验数据 $\bar{e}$	检验数据 δ	检验数据 *RMSE*	检验数据 *TRE*
模型系统（5-6）									
CR_E	-0.0120	0.6935	0.8329	27.0650	0.2510	-0.0175	0.5088	0.7135	26.8541
CR_W	-0.0047	0.5149	0.7176	21.4326	0.3587	-0.0569	0.4037	0.6379	19.4227
CR_S	-0.0115	0.6044	0.7775	21.8867	0.3859	-0.0733	0.4152	0.6485	20.6815
CR_N	-0.0061	0.7325	0.8559	24.2362	0.2854	-0.0894	0.4550	0.6804	23.0218
CW	-0.0162	0.9543	0.9770	18.1091	0.5372	-0.1214	0.6946	0.8422	17.8430
模型系统（5-7）									
CR_E	-0.0017	0.2596	0.5095	24.4184	0.2837	-0.0120	0.4626	0.6803	25.1048
CR_W	-0.0021	0.3285	0.5732	17.426	0.3905	-0.0131	0.3649	0.6042	18.3002
CR_S	-0.0119	0.4427	0.6655	19.3670	0.3971	-0.0647	0.3568	0.6008	18.7875
CR_N	-0.0025	0.4813	0.6938	21.7485	0.3026	-0.0538	0.3715	0.6119	22.4323
CW	-0.0113	0.6749	0.8216	16.4427	0.5930	-0.1135	0.5530	0.7523	17.0049

注：CR_E、CR_W、CR_S、CR_N 和 CW 分别为东树冠半径、西树冠半径、南树冠半径、北树冠半径和总冠幅；$\bar{e}$、δ、*RMSE*、*TRE* 和 R_α^2 分别为平均偏差、残差方差、均方根误差、总相对误差和修正决定系数。

中国知网 https://www.cnki.net

中国知网 https://www.cnki.net

表 5-4 模型系统（5-7）和（5-8）的参数估计值

参数	模型系统 (5-7)							模型系统 (5-8)			
(SE)	CR_E	CR_W	CR_S	CR_N	CW_{EW}	CW_{SN}	CW	CR_E	CR_W	CR_S	CR_N
β_1	5.9024	19.0275	9.7025	6.3208	21.2372	18.0562	18.3077	6.0698	18.5313	9.6374	6.001
	(0.7431)	(6.8164)	(1.0849)	(1.0241)	(3.1059)	(1.4913)	(1.9012)	(1.0458)	(7.5485)	(1.0704)	(1.0576)
β_2	-0.0618	-0.2136	-0.1864	-0.0684	-0.2975	-0.2550	-0.2763	-0.0808	-0.268	-0.1802	-0.0556
	(0.0132)	(0.1221)	(0.0270)	(0.0249)	(0.0403)	(0.0417)	(0.0397)	(0.0249)	(0.1216)	(0.0271)	(0.0256)
β_3	4.5238	10.6839	4.1643	4.8531	6.4628	4.4823	5.8682	4.1899	11.5725	4.1046	4.9516
	(0.5174)	(4.7230)	(0.5064)	(0.7650)	(1.0874)	(0.4295)	(0.5784)	(0.691)	(4.7434)	(0.5035)	(0.749)
β_4	0.1745	0.5327	0.3548	0.2446	0.1905	0.2136	0.2149	0.1765	0.5343	0.3541	0.2521
	(0.0362)	(0.1481)	(0.0487)	(0.0415)	(0.0243)	(0.0210)	(0.0205)	(0.0392)	(0.1513)	(0.0485)	(0.0439)
β_5	0.0613	0.0602	0.0797	0.0908	0.0482	0.0640	0.0695	0.0671	0.0619	0.0792	0.0932
	(0.0085)	(0.0063)	(0.0084)	(0.0091)	(0.0047)	(0.0041)	(0.0042)	(0.0088)	(0.0067)	(0.0082)	(0.011)
β_6	-0.0005	-0.0008	-0.0006	-0.0014	-0.0005	-0.0007	-0.0007	-0.0004	-0.0009	-0.0007	-0.0015
	(0.0002)	(0.0001)	(0.0002)	(0.0002)	(0.0001)	(0.0001)	(0.0001)	(0.0002)	(0.0001)	(0.0002)	(0.0003)
k_1	-0.7937	-4.4671	-4.1821	-0.2245	-1.4340	-2.2105	-1.9144	-0.7589	-5.3737	-4.0833	-0.2328
	(0.5824)	(2.3045)	(0.6651)	(0.6345)	(1.0483)	(0.8449)	(0.8510)	(0.6012)	2.6251	0.6737	0.6396
k_2	0.0544	0.2219	0.1549	0.0563	0.9758	0.1078	0.1025	0.0836	0.2469	0.1616	0.0578
	(0.0249)	(0.1064)	(0.0261)	(0.0260)	(0.0464)	(0.0214)	(0.0328)	(0.0257)	(0.1146)	(0.0263)	(0.0261)

注：CR_E、CR_W、CR_S、CR_N、CW_{EW}、CW_{SN}和 CW 分别为东树冠半径、西树冠半径、南树冠半径、北树冠半径、东西冠幅、南北冠幅和总冠幅 $\beta_1-\beta_6$，k_1、k_2 分别模型参数。

5.2.3 冠幅可加性模型比较和评价

为了与模型系统（5-7）进行比较，本研究还利用了建模数据对 NSUR、AP 和 OLSSR 进行了拟合。利用检验数据，对模型系统（5-7）、NSUR、AP 和 OLSSR 进行评价。

图 5-1 为模型系统（5-7）、NSUR、AP 和 OLSSR 基于建模数据对应的 CR_E、CR_W、CR_S、CR_N 和 CW 各自的评价指标（$\bar{e}$、δ、*RMSE* 和 *TRE*）。所有模型结构都保证 CR_E、CR_W、CR_S 和 CR_N 之和等于 CW 的两倍。通过 T 检验得知所有模型系统对应 CR_E、CR_W、CR_S、CR_N 和 CW 的 $\bar{e}$ 都显著地等于零($p > 0.05$)。除了 OLSSR 估计的 CR_E、CR_S 和 CW 小于观测值外，其它所有模型系统估计的树冠分项都偏大。与 AP 相比，OLSSR 有较高的预测精度，但是这种差异不是非常显著。对于总冠幅，模型系统（5-7）的预测精度最高、其次是 NSUR、OLSSR 和 AP。例如，对于总冠幅，模型系统（5-7）对应的指标 δ、*RMSE* 和 *TRE* 比 NSUR 要低于 31.08%、16.99 % 和 13.43% ，比 OLSSR 要低于 45.96%、26.48% 和 25.09%，比 AP 低于 20.42%、10.78% 和 25.02%: 指标 R_a^2 比 NSUR 要高于 12.23%，比 OLSSR 高于 26.04%，比 AP 高于 4.70%。

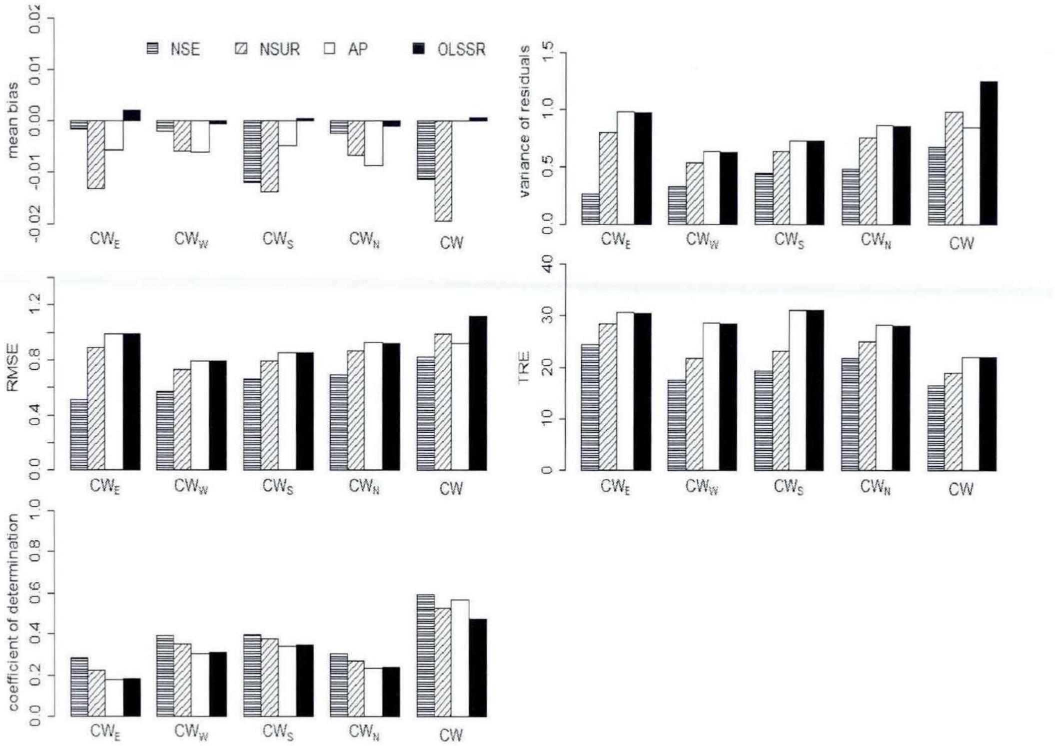

图 5-1 对于建模数据，四种模型结构包括模型系统（5-7）、非线性似然无关回归方法（NSUR）、比例平差法（AP）和最小二乘法独立回归（OLSSR）分别对应东树冠半径（CR_E）、西树冠半径（CR_W）、南树冠半径（CR_S）、北树冠半径（CR_N）和总冠幅（CW）的评价指标均方根误差（*RMSE*）、总相对误差（*TRE*）和分布图

中国知网 https://www.cnki.net

图 5-2 为模型系统（5-7）、NSUR、AP 和 OLSSR 基于检验数据对应的 CR_E、CR_W、CR_S、CR_N 和 CW 各自的评价指标（$\bar{e}$、δ、*RMSE* 和 *TRE*）。与建模数据计算结果相类似，所有模型结构都保证 CR_E、CR_W、CR_S 和 CR_N 之和等于 CW 的两倍。所有模型系统估计的树冠分项都偏大。通过 T 检验得知所有模型系统对应 CR_E、CR_W、CR_S、CR_N 和 CW 的 $\bar{e}$ 都显著地等于零($p > 0.05$）。与 OLSSR 相比，AP 有较高的预测精度，但是这种差异不是非常显著。对于总冠幅，模型系统（5-7）的预测精度最高、其次是 NSUR、AP 和 OLSSR。例如，对于总冠幅，模型系统（5-7）对应的指标 δ、*RMSE* 和 *TRE* 比 NSUR 要低于 12.89%、31.42 % 和 17.08%、比 AP 要低于 44.44%、42.70%、25.02% 和 34.36%，比 OLSSR 低于 42.74%、25.02%、和 34.39%；指标 R_a^2 比 NSUR 要高于 8.19% ，比 OLSSR 高于 34.36%，比 AP 高于 34.39%。因此模型系统（5-7）最终被推荐用于估计华北落叶松总冠幅和各分项树冠半径。

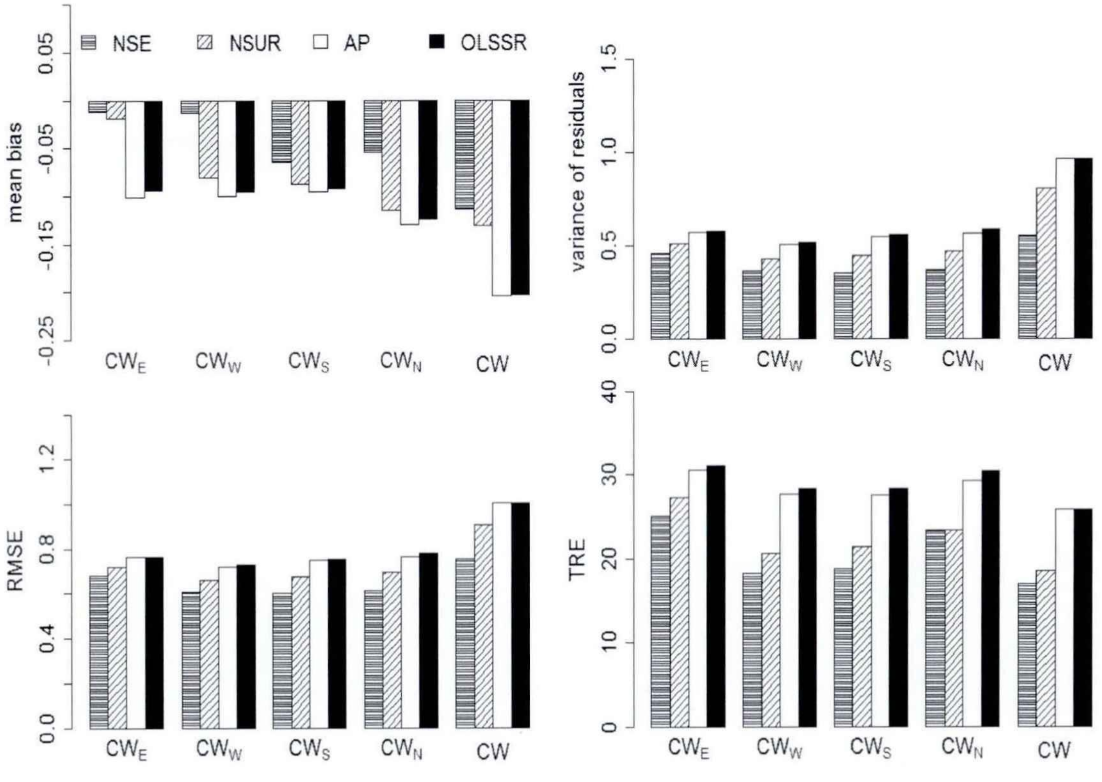

图 5-2 对于检验数据，四种模型结构包括模型系统（5-7）、非线性似然无关回归方法（NSUR）、比例平差法（AP）和最小二乘法独立回归（OLSSR）分别对应东树冠半径（CR_E）、西树冠半径（CR_W）、南树冠半径（CR_S）、北树冠半径（CR_N）和总冠幅（CW）的评价指标平均偏差（$\bar{e}$）、残差方差（δ）、均方根误差（*RMSE*）和总相对误差（*TRE*）分布图

中国知网 https://www.cnki.net

5.3 小结

选用了模型(5-1)作为基础模型并利用了 NSE, NSUR, AP 和 OLSSR 方法分别构建了华北落叶松冠幅模型系统，四种方法构建的冠幅模型系统均能有效考虑总冠幅和各树冠半径之间的相关性。通过比较非线性联立方程组中的两种方法发现，其中分级联合控制平差法构建的冠幅模型系统拟合效果更好。最后通过综合比较分析发现在所有可加性模型系统中，模型系统（5-7）构建的冠幅模型预测效果最好。

中国知网 https://www.cnki.net

6 结论与讨论

6.1 结论

(1)三参数的逻辑斯蒂模型：$CW=a/1+\exp[b+c\ln(D+1)]$能较好反映华北落叶松天然次生林冠幅和直径之间的非线性关系，与其它候选模型相比，该模型有较高的拟合精度，并且模型各参数都具有一定的生物学意义；对象木冠长（CL）、对象木树高（H）和每公顷株数（M）对冠幅影响较大，当这些因子作为预测变量时能明显改进模型的预测精度；得到的改进后的冠幅模型表达式为：

$$CW=1.4875-0.0311H+\exp[(-3.6416-0.0002M)+(2.4346+0.0045CL)\ln(D+1)]$$

通过大量实验数据验证，该模型具有较高的预测精度。

（2）研究发现区组效应和嵌套在区组里面的样地效应对华北落叶松的随机影响较大，当模型考虑这些随机影响时模型预测精度能进一步显著提高；指数方差函数且预测变量为胸高直径能有效剔除模型的异方差，表达式为：$\mathrm{var}(\varepsilon_{ijk})=\sigma^2\exp(2\gamma x_{ijk})$；利用所构建的嵌套两水平非线性混合效应模型预测冠幅时，利用随机抽取的 4 株样地计算随机效应参数效果较好。当地域效应作用在固定效应参数β_1和β_5上时，模型对应的 AIC（5425）最小而 LogLik（-2697）最大最终所构建华北落叶天然林的非线性回归效应冠幅模型如文中（5-19）所示。

（2）以模型（4-3）为基础模型，使用非线性联立方程组（NSE）、 非线性似然不相关回归(NSUR)、比例平差法(AP）和最小二乘法独立回归(OLSSR）方法构建冠幅可加性模型系统，这几种方法都能有效的考虑总冠幅和各树冠半径之间的相关性。通过综合对比这几种可加性模型系统，对于总冠幅，分级联合控制平差法构建的冠幅模型系统对应的指标δ，$RMSE$ 和TRE均要低于 NSUR、AP 和 OLSSR 模型系统；因此，分级联合控制平差法构建的冠幅可加性模型系统预测精度最高，最终模型表达式如文中（5-7）所示。

6.2 讨论

华北落叶松生长快，材质优良，用途大，对不良气候的抵抗力较强，并有保土、防风的效能，是我国黄河流域高山地区及辽河上游高山地区的森林更新和荒山造林的重要树，所以准确掌握华北落叶松的生长状态具有重要的意义；而树冠

中国知网 https://www.cnki.net

是树木进行光合作用和积累能量的重要场所，它反映了树木的生长活力和竞争力，是树木一个重要的健康指标。冠幅是描述树冠生长的重要指标之一，它可以用来计算树木的竞争指数和作为协变量来预测树高或胸径生长量等。研究构建了拟合度较好的一般华北落叶松冠幅预测模型以及考虑了地域效应、区组效应和嵌套在区组里的样地效的非线性混合效应冠幅模型，进一步提高了预测精度；最后还考虑到了不同方向冠径的相关性对冠幅的影响，以此构建了可加性冠幅模型系统，最终为华北落叶松冠幅预测提供了一整套比较全面可靠模型系统。但是在其它树种利用这套方法建立冠幅模型时，各项参数指标需要从新计算，非常耗时耗力，在今后的研究中，如果能消除树种差异的影响。建立其它树种都通用的冠幅模型系统，那将会非常有意义。

6.3 创新点

添加了其它林分单木因子作为协变量、考虑分析了地域效应、区组效应和嵌套在区组里的样地效应和不同方向冠径的相关性对冠幅的影响，最终为华北落叶松提供了一整套比较全面可靠的冠幅模型预测系统。此外所利用的多种数学模型来建立冠幅模型的方法，能为其它树种的冠幅模型的建立提供方法和经验。

中国知网 https://www.cnki.net

参考文献

[1] 联合国关于环境与发展问题重要会议.新华网 2012

[2] 第八次全国森林资源清查结果公布.中国林业网.2014

[3] 胡萍. 日本落叶松林分生长预测及收获预估[D].甘肃农业大学,2007.5

[4] Newham R.M The development of a stand model for douglas-fir Ph. D Thesis Univ.British Columbia Vancouver 1964.201

[5] 王雪梅. 陕西渭北刺槐林分生长收获模型研究[D].西北农林科技大学,2001.

[6] 符利勇. 非线性混合效应模型及其在林业上应用[D].中国林业科学研究院,2012.

[7] 符利勇,孙华. 基于混合效应模型的杉木单木冠幅预测模型[J]. 林业科学, 2013,08:65-74.

[8] 范英明. 华北落叶松天然群体 SSR 标记遗传多样性分析[D].北京林业大学, 2014.

[9] Sheiner, L.B., Beal, S.L. 1980. Evaluation of Methods for Estimating Population Pharmacokinetic Parameters. I. Michaelis-Menton Model Routine Clinical pharmacokinetic Data. J. Pharmacokinet. Biopharm, 8: 553-571.

[10] Bates, D.M., Watts, D.G. 1988. Nonlinear Regression Analysis and Its Applications. New York: Wiley.

[11] Pinheiro, J.C., Bates, D.M. 2000. Mixed-Effects Models in S and S-PLUS. Spring-Verlag, New York, NY.

[12] Zhang,Y.J., Borders, B.E.2004. Using a system mixed-effects modeling method to estimate tree compartment biomass for intensively managed loblolly pines-an allometric approach. For. Ecol. Manage, 194: 145- 157.

[13] Nanos, N., Rafael, C., Gregorio, M., Luis, G. 2004. Geostatistical Prediction of Height-Diameter Models . For. Ecol. Manage, 195: 221-235.

[14] Calama, R., Montero, G. 2004. Interregional Nonlinear Height-Diameter Model with Random Coefficients for Stone Pine in Spain. Can. J. For. Res, 34: 150-163.

[15] Hall, D.B., Clutter, M. 2004. Multivariate multilevel nonlinear mixed effects models for timber yield predictions. Biometrics, 60: 16-24.

[16] Fehrmann, L., Lehtonen, A., Kleinn, C., Tomppo, R. 2008. Comparison of linear and mixed -effect regression models and a k –nearest neighbor approach for

中国知网 https://www.cnki.net

estimation of single-tree biomass. Can. J. For. Res, 38: 1-9.

[17] Meng, S.X., Huang, S. 2009. Improved Calibration of Nonlinear Mixed-Effects Models Demonstrated on a Height Growth Function. For. Sci, 55(3): 239-248.

[18]Lindstrom, M.J., Bates, D.M. 1990. Nonlinear Mixed Effects Models for Repeated Measures Data. Biometrics, 46: 673-687.

[19] 符利勇，唐守正．基于非线性混合模型的杉木优势木平均高．林业科学，2012, 48 (7): 66-71.

[20] 符利勇，孙华，张会儒，雷相东，雷渊才，唐守正. 2013a. 不同郁闭度下胸高直径对杉木冠幅特征因子的影响．生态学报, 33(8): 2434-2443.

[21] Yang, Y., Huang, S. 2011. Comparison of different methods for fitting nonlinear mixed forest models and for making predictions. Can. J. For. Res, 41(8): 1671-1686

[22] Pinheiro, J.C., Bates, D.M. 2000. Mixed-Effects Models in S and S-PLUS. Spring-Verlag, New York, NY.

[23] 祖笑锋,李秋实,倪成才,覃先林,Nigh Gorden. 非线性混合效应生长模型的拟合、随机效应预测和应变量预测间对应关系[J]. 林业科学,2016,(10):72-79.

[24]符利勇．非线性混合效应模型及其在林业上应用[D].中国林业科学研究院,2012.

[25] Hall, D.B., Clutter, M. 2004. Multivariate multilevel nonlinear mixed effects models for timber yield predictions. Biometrics, 60: 16-24.

[26] Pandhard, X., Samson, A. 2009. Extension of the SAEM algorithm for nonlinear mixed models with 2 levels of random effects. Biostatistics, 10(1):121-135.

[27] Yang, Y., Huang, S. 2011. Comparison of different methods for fitting nonlinear mixed forest models and for making predictions. Can. J. For. Res, 41(8): 1671-1686

[28] Hall, D.B., Clutter, M. 2004. Multivariate multilevel nonlinear mixed effects models for timber yield predictions. Biometrics, 60: 16-24.

[29] Meng, S.X., Huang, S. 2009. Improved Calibration of Nonlinear Mixed-Effects Models Demonstrated on a Height Growth Function. For. Sci, 55(3): 239-248.

[30] Pandhard, X., Samson, A. 2009. Extension of the SAEM algorithm for nonlinear mixed models with 2 levels of random effects. Biostatistics, 10(1):121-135.

[31] Littell, R.C, Milliken, G.A., Stroup, W.W., Wolfinger, R.D. 1996. SAS System for Mixed Models. SAS Institute Inc., Cary, NC.

[32] Littell, R.C., Milliken, G.A., Stroup, W.W., Wolfinger, R.D., Schabenberber, O.

中国知网 https://www.cnki.net

2006. SAS for Mixed Models. 2nd ed., SAS Institute Inc, Cary, NC.

[33] Pinheiro, J.C., Bates, D.M. 2000. Mixed-Effects Models in S and S-PLUS. Spring-Verlag, New York, NY.

[34] Clutter, J. L. 1961. The development of compatible analytic models for growth and yield of Loblolly pine. Ph. D. dissertation, Duke University.

[35] Fu, L.Y., Zeng, W.S., Tang, S.Z., Sharma, R.P., Li, H.K. 2012. Using Linear Mixed Model and Dummy Variable Model Approaches to Construct Compatible Single-Tree Biomass Equations at Different Scales— A Case Study for Masson Pine in Southern China. J. For. Sci, 58(3):101-115.

[36] Meng, S.X., Huang, S. 2009. Improved Calibration of Nonlinear Mixed-Effects Models Demonstrated on a Height Growth Function. For. Sci, 55(3): 239-248.

[37] Nanos, N., Rafael, C., Gregorio, M., Luis, G. 2004. Geostatistical Prediction of Height-Diameter Models . For. Ecol. Manage, 195: 221-235.

[38] Strathe, A.B., Danfaer, A., Sørensen, H., Kebreab, E. 2010. A Multilevel Nonlinear Mixed-Effects Approach to Model Growth in Pigs. J. Anim. Sci, 88: 638-649.

[39] 罗玲,廖超英.榆林沙区樟子松冠幅与胸径的相关关系分析[J].安徽农学通报, 2007(24):92-97.

[40] Goelz, J.C.G., Burk,T.E. 1996. Measurement error causes bias in site index equations. Can. J. For. Res, 26:1585-1593.

[41] 张鹏,王新杰,高志雄,等.将乐地区马尾松最优冠幅模型研究[J].西北林学院学报,2015,30（4）:94-98.

[42] Williams, M.S., Schreuder, H.T. 2000. Guidelines for Choosing Volume Equations in the Presence of Measurement Error in Height.Can. J. For. Res, 30:306-310.

[43] Sharma, M., Parton, J. 2007. Height-Diameter Equations for Boreal Tree Species in Ontario Using a mixed-effects Modeling Approach. For. Ecol. Manage, 249: 187-198.

[44] 王璞,马履一,段劼,等.基于FVS的华北落叶松人工林树高生长量模型研究[J].西北林学院学报,2013,28(2):187-190.

[45] Gertner, G.Z. 1990. The Sensitivity of Measurement Error in Stand Volume Estimation. Can. J. For. Res, 20:800-804.

[46] 雷相东,张则路,陈晓光.长白落叶松等几个树种冠幅预测模型的研究[J].北京林业大学学报,2006（6）:75-79.

中国知网 https://www.cnki.net

[47] Kangas, A.S. 1998. Effect of Errors-in-Variables on Coefficients of a Growth Model and on Prediction of Growth. For. Ecol. Manage,102: 203-212.

[48] 吕飞舟,李新建,冯强,等.蒙古栎次生林林木竞争压力指数研究[J].林业资源管理,2015(2):71-76.

[49] 吴明山，胥辉. 2008. 度量误差对材积模型的影响及参数估计研究. 北京林业大学学报, 30(5): 83-86.

[50] 曾伟生，唐守正. 2010a. 利用混合模型方法建立全国和区域相容性立木生物量方程. 29(4):1-6.

[51] 曾伟生，唐守正. 2010b. 利用度量误差模型方法建立相容性立木生物量方程系统.林业科学研究, 23(6): 797-802.

[52] 李永慈，唐守正，李海奎，汤孟平. 2004. 用度量误差模型方法编制相容的生长过程表和材积表. 生物数学学报, 19 (2) : 199- 204

[53] 符利勇，孙华，张会儒，雷相东，雷渊才，唐守正. 2013a. 不同郁闭度下胸高直径对杉木冠幅特征因子的影响. 生态学报, 33(8): 2434-2443.

[54] Bi, H., Turner, J., Lambert, M.J. 2004. Additive biomass equations for native eucalypt forest trees of temperate Australia. Trees, 18: 467-479.

[55] Dong L., Zhang L., Li F. 2015. A three-step proportional weighting system of nonlinear biomass equations. Forest science, 61(1): 35-45.

[56] Parresol, B.R. 2001. Additivity of nonlinear biomass equations. Can. J. For. Res. 31: 865-878.

[57] 符利勇，雷渊才，曾伟生. 2014a. 几种相容性生物量模型及估计方法的比较. 林业科学, 50(6): 42-54.

[58] 唐守正，张会儒，胥 辉. 2000. 相容性生物量模型的建立及其估计方法研究. 林业科学, 36: 19 - 27

[59] 符利勇，雷渊才，孙伟，唐守正，曾伟生. 2014b.不同林分起源的相容性生物量模型构建. 生态学报, 34(6): 1-10.

[60] 符利勇，雷渊才，曾伟生. 2014a. 几种相容性生物量模型及估计方法的比较. 林业科学, 50(6): 42-54.

[61] Dong L., Zhang L., Li F. 2015. A three-step proportional weighting system of nonlinear biomass equations. Forest science, 61(1): 35-45.

[62] Fuller, W.A. (1987). Measurement Error Models. New York: John Wiley & Sons.Zou W., Zeng W., Zhang L., Zeng M. 2015. Modeling crown biomass for four pine species in China. Forests, 6(2): 433-449.

[63] 唐守正，张会儒，胥 辉. 2000. 相容性生物量模型的建立及其估计方法研究.

中国知网 https://www.cnki.net

林业科学, 36: 19 - 27

[64] Hall, D.B., Clutter, M. 2004. Multivariate multilevel nonlinear mixed effects models for timber yield predictions. Biometrics, 60: 16-24.

[65] 高红.塞罕坝林场华北落叶松人工林生长模型的研究[J].林业调查规划,2006(4):25-30.

[66] Yang, Y., Huang, S. 2011. Comparison of different methods for fitting nonlinear mixed forest models and for making predictions. Can. J. For. Res, 41(8): 1671-1686

[67] 卢昌泰,李吉跃,等.马尾松胸径与根径和冠径的关系研究[J].北京林业大学学报,2008(1):58-63.

[68] Jenkins, J.C., Chojnacky, D.C., Heath, L.S. 2003. Birdsey, R.A. National-scale biomass estimators for United States tree species. For. Sci, 49(1):12-35.

[69] 符利勇, 雷渊才, 曾伟生. 2014a. 几种相容性生物量模型及估计方法的比较. 林业科学, 50(6): 42-54.

[70] Parresol, B.R. 2001. Additivity of nonlinear biomass equations. Can. J. For. Res. 31: 865-878.

[71] 唐守正, 张会儒, 胥 辉. 2000. 相容性生物量模型的建立及其估计方法研究. 林业科学, 36: 19 - 27

[72] 符利勇, 雷渊才, 曾伟生. 2014a. 几种相容性生物量模型及估计方法的比较. 林业科学,

[73] Zeng, W.S., Tang, S.Z. 2012. Modeling Compatible Single-Tree Biomass Equations of Masson Pine (Pinus massoniana) in Southern China. J. For. Res, 23(4): 593-598.

[74] 李海奎,宁金魁.2012. 基于树木起源、立地分级和龄组的单木生物量模型. 生态学报, 32(3): 740-757.

[75] 符利勇，唐守正，张会儒，张则路，曾伟生. 2015. 东北地区两个主要树种地上生物量通用方程构建.　生态学报，35(1): 150-157.

[76] 薛立, 杨鹏. 2004. 森林生物量研究综述.福建林学院学报, 24(3): 283-288.

[77] 项文化, 田大伦, 闫文德. 2003. 森林生物量与生产力研究综述.中南林业调查规划, 22(3):57-60.

[78] Wykoff W R.A basal area increment model for individual conifers in the Northern Rock Mountains. Forest Science,1995.41(2):360-377

[79] Monserud R A,Sterba H. A basal area increment model for individual trees growing in even-and uneven-aged forest stands in Austria. Forest Ecology and

中国知网　https://www.cnki.net

Management,1996,80(1/3):57-80

[80] Gill SJ,Biging G S, Murphy A C.2000. Modeling conifer tree crown radius and est imating canopy cover. Forest Ecology and Management,126(3):405-416

[81] Bechtold W A.2004.Largest crown-width prediction models for 53 species in the western United States. Western Journal of Applied

[82] Uzoh, F.C.C., and Oliver, W.W. Individual tree diameter increment model for managed even-aged stands of ponderosa pine throughout the western United States using a multilevel linear mixed effects model. Forest Ecology and Management, 2008, 256,438-445.

[83] Garrett M F, Laird N M, Ware J H. Applied Longitudinal Analysis. Wiley-Interscience. John Wiley and Sons, Inc., Publication, New Jersey,2004.

[84] Keselman H J, Algina J, Kowalchuk R K, Wolfinger R D. A comparison of recent approaches to the analysis of repeated measurements. Br. J. Math. Stat. Psychol, 1999, 52, 63–78.

[85] Uzoh, F.C.C., and Oliver, W.W. Individual tree diameter increment model for managed even-aged stands of ponderosa pine throughout the western United States using a multilevel linear mixed effects model. Forest Ecology and Management, 2008, 256,438-445.

[86] 符利勇. 2012. 非线性混合效应模型及其在林业上应用.中国林业科学研究院, 博士论文.

[87] 符利勇, 张会儒, 唐守正. 2012a. 基于非线性混合模型的杉木林优势木平均高. 林业科学, 48(7): 66-71.

[88] Sánchez-González M,Caellas I,Montero G.Generalized height-diameter and crown diameter prediction models for cork oak forestsin Spain. Forest Systems . 2008

[89] 雷相东,张则路,陈晓光.长白落叶松等几个树种冠幅预测模型的研究[J].北京林业大学学报,2006（6）:75-79.

[90] Garber S M, Maguire D A. Modeling Stem Taper of Three Central Oregon Species Using Nonlinear Mixed Effects Models and Autoregressive Error Structures. Forest Ecology and Management, 2003,179: 507~522.

[91] 符利勇,孙华. 基于混合效应模型的杉木单木冠幅预测模型[J]. 林业科学, 2013,08:65-74.

[92] 冀卫荣,胡俊杰,孟翔,李友莲. 庞泉沟国家级自然保护区落叶松林地表甲虫的多样性[J]. 林业科学,2008,(09):95-100.

[93] 陈鹏飞. 生态公益林提质增效评价系统研究[D].中南林业科技大学,2016.

中国知网 https://www.cnki.net

[94] Uzoh, F.C.C., and Oliver, W.W. Individual tree diameter increment model for managed even-aged stands of ponderosa pine throughout the western United States using a multilevel linear mixed effects model. Forest Ecology and Management, 2008, 256,438-445.

[95] Yang, Y., Huang, S. 2011. Comparison of different methods for fitting nonlinear mixed forest models and for making predictions. Can. J. For. Res, 41(8): 1671-1686

[96] Fang Z, Bailey R L. Nonlinear Mixed Effects Modeling for Slash Pine Dominant Height Growth Following Intensive Silvicultural Treatments. Forest Science, 2001, 47:287-300.

[97] Pinheiro, J.C., Bates, D.M. 2000. Mixed-Effects Models in S and S-PLUS. Spring-Verlag, New York, NY.

[98] 符利勇, 孙华. 2013. 基于混合效应模型的杉木单木冠幅预测模型. 林业科学, 49(8): 65-74.

[99] Yang, Y., Huang, S. 2011. Comparison of different methods for fitting nonlinear
mixed forest models and for making predictions. Can. J. For. Res, 41(8): 1671-1686

[100] Keselman H J, Algina J, Kowalchuk R K, Wolfinger R D. A comparison of recent approaches to the analysis of repeated measurements. Br. J. Math. Stat. Psychol, 1999, 52, 63–78.

[101] Fang Z, Bailey R L. Nonlinear Mixed Effects Modeling for Slash Pine Dominant Height Growth Following Intensive Silvicultural Treatments. Forest Science, 2001, 47:287-300.

[102] Vonesh,E.F., and Chinchilli, V.M. Linear and nonlinear models for the analysis of repeated measurements. Marcel Dekker, New York, 1997.

[103] Calama,R., Montero,G. Multilevel linear mixed model for tree diameter increment in stone pine (pinus pinea): a calibrating approach. Sliva Fennica, 2005, 39(1),37-54.

[104] Leites L P, Robinson A P. Improving Taper Equations of Loblolly Pine with Crown Dimensions in a Mixed-Effects Modeling Framework. Forest Science, 2004, 50: 204~212.

[105] Yang, Y., Huang,S., Meng, S.X., Trincado, G., and VanderSchaaf, C.L. A multilevel individual tree basal area increment model for aspen in boreal mixedwood stands. Canadian journal of forest research, 2009, 39,2203-2214.

中国知网 https://www.cnki.net

[106] Calama,R., Montero,G. Multilevel linear mixed model for tree diameter increment in stone pine (pinus pinea): a calibrating approach. Sliva Fennica, 2005, 39(1),37-54.
[107] Yang, Y., Huang,S., Meng, S.X., Trincado, G., and VanderSchaaf, C.L. A multilevel individual tree basal area increment model for aspen in boreal mixedwood stands. Canadian journal of forest research, 2009, 39,2203-2214.
[108] Parresol, B.R. 2001. Additivity of nonlinear biomass equations. Can. J. For. Res. 31: 865-878.
[109] Bi, H., Turner, J., Lambert, M.J. 2004. Additive biomass equations for native eucalypt forest trees of temperate Australia. Trees, 18: 467-479.
[110] 唐守正, 张会儒, 胥 辉. 2000. 相容性生物量模型的建立及其估计方法研究. 林业科学, 36: 19 - 27
[111]骆期邦, 曾伟生, 贺东北, 包拓华, 林文端. 1999. 立木地上部分生物量模型的建立及其应用研究. 自然资源学报, 14(3): 271 – 277
[112] 唐守正, 张会儒, 胥 辉. 2000. 相容性生物量模型的建立及其估计方法研究.林业科学, 36: 19 - 27
[113] 唐守正, 李勇, 符利勇. 2015. 生物数学模型的统计学基础. 北京: 科学出版社, 第二版.
[114] 唐守正, 张会儒, 胥 辉. 2000. 相容性生物量模型的建立及其估计方法研究. 林业科学, 36: 19 - 27
[115] Parresol, B.R. 2001. Additivity of nonlinear biomass equations. Can. J. For. Res. 31: 865-878.
[116]Parresol B. R. 1999. Assessing Tree and Stand Biomass: A Review with Examples and, Critical Comparisons. Forest Science, 45(4): 573-593.
[117] Parresol, B.R. 2001. Additivity of nonlinear biomass equations. Can. J. For. Res. 31: 865-878.
[118] 唐守正, 李勇, 符利勇. 2015. 生物数学模型的统计学基础. 北京: 科学出版社, 第二版.

中国知网 https://www.cnki.net

附录 A 非线性回归和非线性混合效应模型 R 程序

```
setwd("C:/Users/Desktop/冠幅数据")
library(nlme)
ModellingData<-read.table("C:/Users/Desktop/CW/modelling
data.txt",sep="\t",header=T)
ValidationData<-read.table("C:/Users/Desktop/CW/validation    data.txt",    sep="\t",
header=T)
###base model
##w.eq1<-w~(b1+b2*DH)/(1+(b3+b4*HCB)*exp(-(b5+b6*H)*D))
fm0<-nls(w~(b1+b2*DH)/(1+(b3+b4*HCB)*exp(-(b5+b6*H)*D)),data=ModellingD
ata,start=c(b1=5.9466 ,b2=-0.0741,b3=4.7125,b4=0.1779,b5=0.0750,b6=-0.0006))
summary(fm0)
AIC(fm0)
logLik(fm0)
fm1<-nlme(w~(b1+b2*DH)/(1+(b3+b4*HCB)*exp(-(b5+b6*H)*D)),data=Modelling
Data,fixed=list(b1+b2+b3+b4+b5+b6~1),random=list(block=(b2+b4~1),plot=(b2+b4
~1)),start=c(10.8,-0.135,3.84,0.136796,0.091678,-0.001))
fm2.D<-update(fm1,weights=varPower(form=~D))
fm2.H<-update(fm1,weights=varPower(form=~H))
fm2.DH<-update(fm1,weights=varPower(form=~DH))
fm2.HCB<-update(fm1,weights=varPower(form=~HCB))
anova(fm1,fm2.D)
anova(fm1,fm2.H)
anova(fm1,fm2.DH)
anova(fm1,fm2.HCB)
fm3.D<-update(fm1,weights=varExp(form=~D))
fm3.H<-update(fm1,weights=varExp(form=~H))
fm3.DH<-update(fm1,weights=varExp(form=~DH))
fm3.HCB<-update(fm1,weights=varExp(form=~HCB))
anova(fm1,fm3.D)
```


中国知网 https://www.cnki.net

```
anova(fm1,fm3.H)
anova(fm1,fm3.DH)
anova(fm1,fm3.HCB)
fm4.D<-update(fm1,weights=varConstPower(form=~D))
fm4.H<-update(fm1,weights=varConstPower(form=~H))
fm4.DH<-update(fm1,weights=varConstPower(form=~DH))
fm4.HCB<-update(fm1,weights=varConstPower(form=~HCB))
anova(fm1,fm4.D)
anova(fm1,fm4.H)
anova(fm1,fm4.DH)
anova(fm1,fm4.HCB)
fm1<-nlme(w~(b1+b2*DH)/(1+(b3+b4*HCB)*exp(-(b5+b6*H)*D)),data=Modelling
Data,fixed=list(b1+b2+b3+b4+b5+b6~1),random=list(block=(b2+b4~1),plot=(b2+b4
~1)),start=c(10.8,-0.135,3.84,0.136796,0.091678,-0.001))
summary(fm1)
fm2.D<-update(fm1,weights=varExp(form=~D))
summary(fm2.D)
fm1<-nlme(w~(b1+k1*P+b2*DH)/(1+(b3+b4*HCB)*exp(-(b5+k5*P+b6*H)*D)),dat
a=ModellingData,fixed=list(b1+b2+b3+b4+b5+b6+k1+k5~1),random=list(block=(b2
+b4~1),plot=(b2+b4~1)),start=c(12.8016295473,-0.1531697813,3.4775576921,0.149
2122925,0.0660979971,-0.0005716994,-2.6517134864,0.0335453989))
fm3.D<-update(fm1,weights=varExp(form=~D))
summary(fm3.D)
w.eq1<-w~(b1+k1*P+(b2+k2*P)*DH)/(1+(b3+b4*HCB)*exp(-(b5+b6*H)*D))
fm1.we<-nls(we.eq1,data=ModellingData,start=c(b11=5.9466 ,b12=-0.0741,b13=4.71
25,b14=0.1779,b15=0.0750,b16=-0.0006,k11=0.4669,k12=0.0229))
summary(fm1.we)
fit.we<-predict(fm1.we)
pre.we<-predict(fm1.we,ValidationData)
fit.ww<-predict(fm1.ww)
pre.ww<-predict(fm1.ww,ValidationData)
fit.ws<-predict(fm1.ws)
```


中国知网 https://www.cnki.net

```
pre.ws<-predict(fm1.ws,ValidationData)
fit.wn<-predict(fm1.wn)
pre.wn<-predict(fm1.wn,ValidationData)
fit.wew<-predict(fm1.wew)
pre.wew<-predict(fm1.wew,ValidationData)
fit.wsn<-predict(fm1.wsn)
pre.wsn<-predict(fm1.wsn,ValidationData)
fit.w<-predict(fm1.w)
pre.w<-predict(fm1.w,ValidationData)
fwe<-Compute.Index(ModellingData$we,fit.we)
fww<-Compute.Index(ModellingData$ww,fit.ww)
fws<-Compute.Index(ModellingData$ws,fit.ws)
fwn<-Compute.Index(ModellingData$wn,fit.wn)
fwew<-Compute.Index(ModellingData$wew,fit.wew)
fwsn<-Compute.Index(ModellingData$wsn,fit.wsn)
fw<-Compute.Index(ModellingData$w,fit.w)
Indexes<-rbind(fwe,fww,fws,fwn,fwew,fwsn,fw)
pwe<-Compute.Index(validation$we,pre.we)
pww<-Compute.Index(validation$ww,pre.ww)
pws<-Compute.Index(validation$ws,pre.ws)
pwn<-Compute.Index(validation$wn,pre.wn)
pwew<-Compute.Index(validation$wew,pre.wew)
pwsn<-Compute.Index(validation$wsn,pre.wsn)
pw<-Compute.Index(validation$w,pre.w)
Indexes1<-rbind(pwe,pww,pws,pwn,pwew,pwsn,pw)

Compute.Index<-function(Obsv,Estv){
##mean bias pe
e<-Obsv-Estv
pe<-mean(e)
##variance of residuals s
s<-var(e)
```


中国知网 https://www.cnki.net

```
## root mean square error RMSE
RMSE<-sqrt(pe^2+s)
##total relative error TRE
TRE<-100*sum(e^2)/sum(Estv)
##adjusted coefficient of determination R2
R2<-1-(length(Obsv)-1)*sum(e^2)/((length(Obsv)-24)*sum((Obsv-mean(Obsv))^2))
return(list(pe=pe,s=s,RMSE=RMSE,TRE=TRE,R2=R2))
}
```


中国知网 https://www.cnki.net

附录 B 似然不相关回归 R 程序

```
setwd("C:/Users/Desktop/crown width")
install.packages("Rcpp_0.12.7.zip", repos=NULL,type="source")
install.packages("minqa_1.2.4.zip", repos=NULL,type="source")
install.packages("nloptr_1.0.4.zip", repos=NULL,type="source")
install.packages("lme4_1.1-12.zip", repos=NULL,type="source")
install.packages("pbkrtest_0.4-6.zip", repos=NULL,type="source")
install.packages("quantreg_5.29.zip", repos=NULL,type="source")
install.packages("SparseM_1.72.zip", repos=NULL,type="source")
install.packages("car_2.1-3.zip", repos=NULL,type="source")
install.packages("zoo_1.7-13.zip", repos=NULL,type="source")
install.packages("lmtest_0.9-34.zip", repos=NULL,type="source")
install.packages("sandwich_2.3-4.zip", repos=NULL,type="source")
install.packages("systemfit_1.1-18.zip", repos=NULL,type="source")
library(car)
library(zoo)
library(lmtest)
library(sandwich)
library(systemfit)
ModellingData<-read.table("modelling data.txt"， sep="\t",header=T)
FittingData<-read.table("validation data.txt", sep="\t",header=T)
we.eq4<-we~(b11+k11*P+(b12+k12*P)*DH)/(1+(b13+b14*HCB)*exp(-(b15+b16*
H)*D))
ww.eq4<-ww~(b21+k21*P+(b22+k22*P)*DH)/(1+(b23+b24*HCB)*exp(-(b25+b26*
H)*D))
ws.eq4<-ws~(b31+k31*P+(b32+k32*P)*DH)/(1+(b33+b34*HCB)*exp(-(b35+b36*H
)*D))
wn.eq4<-wn~(b41+k41*P+(b42+k42*P)*DH)/(1+(b43+b44*HCB)*exp(-(b45+b46*
H)*D))
w.eq4<-w~(we+ww+ws+wn)/2
```


中国知网 https://www.cnki.net

```
lables<-list("east crown width","west crown width","south crown width","north crown width","total crown width")
inst<-~DH+HCB+D+H+P
start.values<-c(b11=18.5698327,b12=-0.2978116,b13=5.73457,b14=0.231584,b15=0.0724258,b16=-0.0007919,k11=-1.9688543,k12=0.1109245,
b21=18.5698327,b22=-0.2978116,b23=5.73457,b24=0.231584,b25=0.0724258,b26=-0.0007919,k21=-1.9688543,k22=0.1109245,
b31=18.5698327,b32=-0.2978116,b33=5.73457,b34=0.231584,b35=0.0724258,b36=-0.0007919,k31=-1.9688543,k32=0.1109245,
b41=18.5698327,b42=-0.2978116,b43=5.73457,b44=0.231584,b45=0.0724258,b46=-0.0007919,k41=-1.9688543,k42=0.1109245)
model<-list(we.eq4,ww.eq4,ws.eq4,wn.eq4,w.eq4)
model.sur<-nlsystemfit("SUR",model,start.values,data=ModellingData,eqnlabels=labels)
print (model.sur)
DH<-validation$DH
HCB<-validation$HCB
H<-validation$H
D<-validation$D
P<-validation$P
pwe4<-(6.069835323-0.7588593812*P+(-0.0807860445+0.0835813567*P)*DH)/(1+(4.1899294757+0.1765473345*HCB)*exp(-(0.0671494552-0.000373947*H)*D))
pww4<-(18.5313403099-5.3736650207*P+(-0.2680203269+0.2469266131*P)*DH)/(1+(11.5725353029+0.5342635625*HCB)*exp(-(0.0618895323-0.0008615427*H)*D))
pws4<-(9.6373944256-4.0832646953*P+(-0.1801575672+0.1616050878*P)*DH)/(1+(4.1045740406+0.3541346827*HCB)*exp(-(0.0792082906-0.0006644498*H)*D))
pwn4<-(6.0010064254-0.2328388639*P+(-0.055571981+0.057825491*P)*DH)/(1+(4.9515517626+0.2520644788*HCB)*exp(-(0.0931662059-0.0014575102*H)*D))
pw4<-(pwe4+pww4+pws4+pwn4)/2
Compute.Index<-function(Obsv,Estv){
##mean bias pe
```


中国知网 https://www.cnki.net

```
e<-Obsv-Estv
pe<-mean(e)
##variance of residuals s
s<-var(e)
## root mean square error RMSE
RMSE<-sqrt(pe^2+s)
##total relative error TRE
TRE<-100*sum(e^2)/sum(Estv)
##adjusted coefficient of determination R2
R2<-1-(length(Obsv)-1)*sum(e^2)/((length(Obsv)-24)*sum((Obsv-mean(Obsv))^2))
return(list(pe=pe,s=s,RMSE=RMSE,TRE=TRE,R2=R2))
}
Iwe<-Compute.Index(validation$we,pwe4)
Iww<-Compute.Index(validation$ww,pww4)
Iws<-Compute.Index(validation$ws,pws4)
Iwn<-Compute.Index(validation$wn,pwn4)
Iw<-Compute.Index(validation$w,pw4)
Indexes<-rbind(Iwe,Iww,Iws,Iwn,Iw)
```


中国知网 https://www.cnki.net

中国知网 https://www.cnki.net

致 谢

首先衷心地感谢我的导师吕勇教授和朱光玉副教授。2014 年 9 月，我来到了长沙，师从于吕勇教授，吕老师从研究生培养计划、课程的选择以及毕业论文方面给予了认真的安排与指导，让我的科研能力得到了一定的提高。吕老师严谨的治学态度、勤奋的工作精神对我产生了很大的影响。2015 年 9 月，我来到了北京中国林业科学研究院资源信息研究所，师从于雷渊才研究员，雷老师关心学生的学习，在雷老师的指导下，我的的科研视野得到了进一步开阔，在雷老师的指导下，我参与了林业公益性科研项目，并参与了该项目的外业调查工作，雷老师从毕业论文的选题、构思、撰写等方面都提出了宝贵的意见。感谢朱光玉副教授、李健军教授对本论文提出了宝贵意见。

感谢参加为本次论文数据的外业调查人员。有中南林业科技大学程鹏飞，以及山西农业大学林学、森林经理学、森林生态学专业的曹慧、李东昌、黄逸鹏、余秋鹏等硕士研究生同学。在李健军教授的带领下，大家克服了重重困难，圆满完成了外业工作。同时感谢中国林业科学研究院资源信息研究所的沈剑波博士、段光爽博士以及五台山国有林管理局伯强林场的大力支持和帮助。

感谢中南林业科技大学 2014 级硕士研究生钱升平、康立、杨佳、龙时胜等同学平时对我的帮助。

最后，非常感谢我挚爱的父母，感谢他们一直以来给我极大的鼓励和支持。感谢在百忙之中参加论文审阅和答辩的专家和各位老师，同学。

作者： 符亚健

2017 年 5 月于中南林业科技大学

中国知网 https://www.cnki.net
