# Supplementary material for: Provenance and family variations in early growth of Manchurian walnut (Juglans mandshurica Maxim.) and selection of superior families
Source: PLoS One. 2024 Mar 7;19(3):e0298918. doi: 10.1371/journal.pone.0298918 (PMC10919699; doi:10.1371/journal.pone.0298918)
Supplement: S1 File — (ZIP) [file pone.0298918.s004.zip › Development and prospects of the walnut industry in China.pdf]

Development and Prospects of the Walnut Industry in China

J. Tian, Y. Wu, Y. Wang and F. Han
Pomology Institute
Shanxi Academy of Agricultural Sciences
Taigu 030815, Shanxi
China

Keywords: resource, cultivar, breeding, processing, export

Abstract

China is one of the world's centers of origin for walnut, with a cultural history of over 2,000 years. China possesses abundant walnut resources, including wild resources and breeding germplasm. Over the past fifty years, China has made some big achievements in improving walnut cultivation. These include new high quality varieties and advances in grafting techniques making the new varieties widely cultivated rapidly. Walnut exports started in 1921 from China. China was the biggest walnut export country in the world for a time. Nuts and kernels were the main export products. Presently, China is the global leader in walnut plantings. However, as a walnut export country China is No. 2, next to the United States. At present, the processing of walnut still focuses on rough processing, while little is done on further and fine processing. The main processed products are nutritional health foods such as walnut oil, walnut milk and walnut powder, as well as artworks. There are many resources and varieties of walnut in China, but most of these exist in decentralized operations, and levels of cultural management and further processing are relatively low. Requirements for walnut and its products increase year by year both in the domestic and oversea market. Therefore, the development potential for the walnut industry in China is very large. Further research on improved cultivation and processing techniques is needed in order to enhance market competitive ability.

INTRODUCTION

Walnut is a deciduous fruit tree of the Juglandaceae family and *Juglans* genus. It has a large production area with a long history in China. Walnut kernels have high nutritional value with a unique flavor and can be processed for the production of quality oil. The bark of the walnut tree, the green hull of walnut as well as the walnut wood all have their unique usages. Walnut is also an important economic forest species. China is one of the walnut centers of origin and the biggest cultivation country in the world. Walnut has spread all over China with a very long cultural history. People in China not only have a lot of experience in planting and management, but also have explored the edible and medical value of walnut and have created and mastered many methods to use it (Xi and Zhang, 1994).

WALNUT RESOURCES IN CHINA

There is abundant germplasm of walnut in China. According to Chinese and foreign reports, and combining the research of archaeology, cytology and zymology, Rongting Xi (1981, 1990) and Wenheng Yang (1984, 1987) suggested that China was one of the walnut centers of origin for the world. There are five species in *Juglans* native to China: walnut (*J. regia*), Manchurian walnut (*J. manshurica*), Chinese walnut (*J. cathayensis*), *Juglans sigillata* (*J. sigillata*) and Hebei walnut (*J. hopeiensis*) (Xi and Zhang, 1994).

Wild Walnut Resources in China

Different walnut resources possess diverse characteristics, so the large number of wild walnut resources provides a big advantage for walnut breeding in China.

1. Walnut. This is the main cultivated species in China. After a long period of

domestication, different cultivars adapted to various local conditions have formed the backbone of cultivation in different growing areas. Xinjiang wild walnut is an endangered species. It is the relic of the precious temperate deciduous broad-leaved forest of the tertiary period. Its distribution area is rather small being found only in the two counties located in Ili Valley of the Tianshan Mountains. It is distributed vertically in the lower hillside or the valley bottom at an altitude of 1,200 m to 1,600 m (Zeng, 2005).

2. Manchurian Walnut. This species is native to north and northeast of China, as well as to the Far East areas of Russia. It is mainly distributed on Changbai Mountain and Xiaoxinganling with an altitude of 500 m~1,000 m in the northeast of China. It is one of the three precious hardwood forest species, also a relic of the tertiary period. It is a very old fruit tree species with high economic value and wide adaptabilities. It has a straight-root system with strong resistance to drought and cold. It can tolerate temperatures of -50°C, so it can be used as a parent for the breeding of cold-resistant walnut. However, it is difficult to get kernels out of the nut because of the thick shell and small kernel (Zhou, 1994).

3. Chinese Walnut. This species is native to mountainous regions in subtropical areas of China, southward to Guangxi and Taiwan province. It is spread widely in native broadleaf forests. The nut is oval with a thick shell, apical apex, small kernel and 6−8 suture lines (Xi and Zhang, 1994). A variety in this species exists, named 'East China wild walnut'. The shell of this variety is relatively smooth, and there are only two suture lines. It is mainly spread in provinces which are located in the south of Yangtze River basin (Wang et al., 2007).

4. *Juglans Sigillata*. This is a special walnut species in China, originating from the southwest of China. It has a strong tolerance to high humidity and heat, yet is not so resistant to drought and cold. There are a great many quality cultivars with various nut sizes and shapes for this species, which can meet various demands of breeding targets (Han and He, 2004). It is mainly distributed in the southwest of China, along the valley of the Nu River, Lancang River, Jinsha River, Min River, and Bralunaputra River (Xi and Zhang, 1994).

5. Hebei Walnut. This is a rare and particular germplasm found in Hebei province. It was suggested that the species is a natural hybrid of walnut and Manchurian walnut. Under natural conditions, it always grows mixed with walnut and Manchurian walnut. The nut has a thick shell, small kernel and small yield. It is the species which has the smallest distribution area and the least quantity in the *Juglans* plants. It is spread naturally in north Taihang Mountains in Hebei Province (Pei et al., 2006). Hebei Walnut is famous for its large size, attractive appearance and beautiful veins, and it has a high ornamental and health-keeping value, so it can be used to create nice artworks, and it is also used to create amulets and health balls in China, all of which make it popular with people of various countries.

There are other less commercial walnut species in China, such as bunchy walnut (clustered walnut), red-flesh walnut, oversize walnut (36 g/nut), Baishui walnut, single-leaf walnut, shell-less walnut and so on (Duan and Zhang, 2004).

Walnut Resources for Breeding in China

Walnut breeding has a long history in China. Since ancient times, the Chinese people have used the method of seedling selection for making new cultivars. After improvement over many generations, people made the wild walnut varieties which have become the ones which are currently cultivated. Walnut breeding in China stayed with seedling selection for a very long time. This situation could not meet the needs of commercial production because of the big variation among offspring. In the 1970s, with the spreading of walnut propagation techniques in northern areas, walnut breeding started to be clonal selection (Feng et al., 2006). There are now a group of improved clonal cultivars in China. Large-scale productive walnut orchards were established by top grafting to the original seedlings trees, and numbers of new varieties suitable for different operation types were evaluated.

Presently, there are 7 walnut germplasm gene pools with more than 600 germplasms in the world. There are more than 200,000,000 walnut seedling trees in China. Each of them is a special genetic type generated by cross-pollination, which means that they are hybrid progenies of natural crossing (Feng et al., 2006). This enormous seedling population is a big treasure that supplies rich resources for walnut breeding research.

Walnut breeding research in China started in 1950s, but it did not get enough attention until the 1970s when the research entered a full-developing period. By the end of 2003, over 50 quality cultivars, 120 improved strains and 140 local cultivars had been selected and bred through resource investigation, introduction, seedling selection and cross breeding in China. Cultivars selected in China are mainly divided into two categories: early-fruiting ones such as 'Liaoning No. 1', 'Liaoning No.4' 'Wen 185', 'Zha 343', 'Luguang', 'Zhonglin No.1', 'Zhonglin No. 3', 'Zhonglin No.5', 'Xifu No. 1', 'Xifu No. 2' and 'Xiangling', 'inboxiang No. 1', etc. and late-fruiting ones such as 'Present No. 2', 'Jinlong No. 1', 'Jinlong No. 2', 'Qingxiang', and 'Xiluo No. 1', etc. (Table 1) (Xi and Liu, 2005).

HISTORY AND CURRENT SITUATION OF THE WALNUT INDUSTRY IN CHINA

After establishment of the new China, the walnut industry in China developed rapidly as a result of the efforts of excellent researchers and workers in the walnut industry. These changes include selection and breeding of new varieties, development of cultural techniques and storage and processing techniques.

History of Walnut Industry in China

According to the textual research, 'Fenzhou walnut' in Shanxi province had a cultural history of 2,000 years (Wang, 2003). Even so, walnut was always regarded as a small nut or sundry fruit and did not get enough attention (Wang and Hao, 2006). Thereafter, in a long historical process, walnut production was developed slowly until the foundation of the new China.

Recently, because of the breeding of new varieties, breakthroughs in propagation techniques and the urgent need of farmers to become economically rich, the walnut industry has developed rapidly with financial aid from the government in China. Walnut was regarded as a leading economic industry in more than 100 counties in a dozen provinces of north China. Before 1996, immature grafting techniques were responsible for limiting the popularization of the fine walnut varieties. Yet through the efforts of scientific researchers all over China, many grafting methods for walnut were created, such as square budding, twig grafting, seedling grafting on planted stock etc., which fundamentally solved the key problem of large-scale popularization of fine walnut varieties (Wang and Hao, 2006).

Present Situation of Walnut Cultivation in China

1. Cultural Distribution of Walnut in China. Walnut and *Juglans sigillata* are widely spread and cultivated making up of the main part of walnut cultivation in China, mainly in Provinces of Yunnan, Shanxi, Shaanxi, Hebei, Sichuan, Xinjiang, Henan, Liaoning, Gansu, Shandong, Ningxia, Qinghai, Anhui, Jiangsu, Hubei, Hunan, Guangxi, Tianjin, Beijing, Guizhou, and Tibet. Walnut spreads all over China, while *Juglans sigillata* is mainly spread in the southwest areas such as Yunnan, Guizhou, west of Sichuan and south of Tibet. Walnut cultivation is vertically distributed from the altitude of -154 m (Turpan basin of Xinjiang) to 4,200 m (Lakao county of Tibet).

2. General Information of Walnut Production in China. In 1991, the total cultivation area of walnut in China was 918,000 ha. Presently, it has increased to 1, 300,000 ha with about 200,000 ha coming into production, and the rest are young orchards planted in the past 10 years (Xi and Liu, 2005).

Before the establishment of the new China, walnut yield across the whole country was less than 50,000 t. In the 1950s, it increased to 100,000 t, but in the 1960s, it decreased to 40,000−50,000 t, while in the 1970s it increased to 70,000–80,000 t (Duan

and Zhang, 2004). In 1981, the total yield was 110,000 t, and in 1993 it was 192,000 t (Feng et al., 2006). Since 1996, walnut yield of China has increased steadily and continuously. In 2000, China became the number one walnut production country. At that time the yield was 300,000 t, which exceeded that of the United States. Walnut output of China in different years is presented in Figure 1. Annual walnut output of each of the main production provinces is more than 10,000 t and the total yield of the ten main production provinces accounts for 90% of the whole country production (Fig. 2). Among the provinces, Yunnan Province is the number one for walnut yield, which always accounts for around 20% of the total yield in the whole country (Wang et al., 2007).

3. The Main Characteristics of Walnut Cultivation in China. Walnut production in China mainly shows the following aspects: (1) it has a long history and rich resources in China. There are 216 fine clonal varieties, 164 improved seedling varieties in personal farmyards and 486 single plant lines in China (Han et al., 2004). (2) Mixed varieties and low energy input management are the two main characters of walnut cultivation in China. (3) China has the largest walnut cultural area in the world, but the unit yield is lower than the world average as a result of the less developed, small-scale peasant economy. (4) There is an enormous development potential for the walnut industry in China along with the consistent perfection of agricultural systems and the rapid development of agricultural productivity (Zhang, 2004).

WALNUT PROCESSING AND EXPORT IN CHINA

General Information for Walnut Processing in China

The amount of walnut kernel which people eat directly is very small. Presently, the main walnut processing products in Chinese markets are walnut oil, walnut protein, walnut powder, walnut milk, walnut products with different flavors and artworks made of walnut.

Besides being used in the food industry, walnut can also be used in fields of medicine, chemistry, industrial arts, etc. The bark of walnut tree contains ketone compounds, so it can be used as an industrial raw material and in pharmaceutical preparations. The leaves contain not only ketone compounds, but also phenols and several pigments, which can be used in antiphlogistic drugs and dye. In addition, walnut shell is a good material for creating activated carbon (Liu et al., 2004).

Traditional treatments for walnut in China are: eating directly, processing simply or exporting walnut materials abroad directly. These limitations on processing greatly decrease the economic value of walnut. In order to change our resource superiority into economic superiority, China should pay much more attention to the further processing of walnut, make comprehensive utilization and reasonable exploitation of walnut, and force industrialization.

Walnut Export in China

Walnut produced in China is mainly consumed domestically. The export amount is less than 10% of the total yield. Yet China had a traditional advantage in the trade of walnut. In 1921, walnut export in China was 6,710 t. It decreased to less than 1,000 t in the 1930s and 1940s. From the beginning of the new China to the 1970s, China was always the main walnut export country all over the world. Especially in the 1960s, walnut from China entered the markets of England and Germany. During this period, walnut exports from China occupied 40−50% of the whole world walnut market. In the 1970s, the walnut industry in the United States developed rapidly, which made the exports from China decrease greatly. At present, China just accounts for 20% of the total world trade amount (Han and He, 2004; Duan and Zhang, 2004). Walnut export from China is presented in Figure 3. The main reason for the decline was the selection and breeding of quality walnut cultivars were not paid enough attention in China, which caused irregular nut sizes, poor appearances and instable qualities, thus China lost competitive power in the international market.

On the other hand, walnut kernel from China is classified clearly, has complete specifications and an excellent taste, that's why walnut kernel export from China stays in the top position all over the world. Yet other countries are also making efforts in improving their export competitiveness for their walnut kernel, which puts much pressure on the trade of Chinese walnut kernels. Walnut kernel exports from China are around 8,000 t. Yet levels of food processing and commodity packaging are relatively low, which makes China stay in a low position of world trade in processed walnut products. So China should pay much attention to this aspect in order to change their unfavorable situation.

DEVELOPMENT POTENTIAL AND PROSPECT OF THE WALNUT INDUSTRY IN CHINA

Per capital consumption of walnut in China is just 0.16 kg per capita per year, which is much lower than some developed countries. However, people's requirement for walnut will increase constantly in the next few decades. Once per capita consumption rises to 0.5 kg, the yearly need of walnut in China will come to 650,000 t or more. At present, processed food made from walnut increases year by year, which requires over 50,000 t of walnut every year. With the progress of medical science, people's demands for nutrition and healthy foods are increasing, and the need for walnut and its by-products in various countries will also increase. Such favorable trend in both domestic and international market provides the pre-conditions for the development of the walnut industry in China.

A big advantage in walnut resources within China guarantees the breeding of quality cultivars, the development of new processed products, as well as the improvement of cultural and processing techniques. There is a big potential for the increase of yield of old walnut orchards and a big potential for increasing the breadth of soils which can be used to cultivate walnut, all of which give the walnut industry in China a promising prospect. So, during the next several decades, China should take corresponding measures for development of its walnut industry in order to enhance market competitive ability. These measures will include; breeding cultivars with traits of precociousness, high yield, strong resistance and nice nut quality, enhancing management of existing resources, regulating behavior of fruit growers, combining and matching yield increasing techniques, adopting new cultural techniques, energetically developing further processing, doing more research on new products, accelerating introduction of advanced technologies and equipment, improving walnut quality and yield and quality of processed products.

Literature Cited

Duan, H.X. and Zhang, Z.H. 2004. Generation situation, problems and develop method of Chinese walnut. Fruit Growers' Friend 1:4-5.

Editional Board of China Agricultural Yearbook. 1997-2007. China Agricultural Yearbook. China Agriculture Publishing, Peking.

Feng, L.F., Lv, F.D., Zhang, Y.P. et al. 2006. Research progress of China walnut breeding and cultivation technique. Economic Forest Researches 24(2):69-73.

Han, H.B. and He, F. 2004. Review and prospect of China walnut breeding. Economic Forest Researches 22(3):45-50.

Liu, W., Zhang, Z.H. and Ma, X.D. 2004. Present situation and prospect of China walnut further processing. Fruit Growers' Friend 1:8-9.

Pei, D., Liu, R.H. et al. 2006. Research on development and protection of Hebei walnut resource. Forest Resource Management 4:66-69.

Wang, H.X., Zhang, Z.H. and Xuan, L.C. 2007. Research progress of China walnut germplasm and breeding. Hebei Journal of Forest and Orchard Research 22(4): 387-391.

Wang, J.C. 2003. Attention problems of walnut economic forest development. Forests Economy 4:62.

Wang, K.J. and Hao, Y.B. 2006. Suggestions and problems in walnut industrialization of China. Rural Science and Technology 4:60.

Wu, Y., Cai, E., Yan, S.J., Kou, X.H. et al. 2001. Research on supercritical CO_2 fluid extraction technology of walnut oil. J. of Agricultural Engineering 17(6):135-138.
Xi, R.T. 1981. Discussion of China walnut origin. China Fruits 4:47-50.
Xi, R.T. 1990. Origin textual research of Chinese walnut. J. of Hebei Agricultural University 13(1):89-93.
Xi, R.T. and Liu, M.J. 2005. Chinese dry fruits. Chinese Forestry Publishing, Peking.
Xi, R.T. and Zhang, Y.P. 1994. Chinese fruit tree annals. Chinese Forestry Publishing, Peking.
Yang, W.H. 1984. Walnut of China. J. of Hebei Agricultural University 2:1-9.
Yang, W.H. and Cheng, S.Z. 1987. Isoenzyme change of walnut fruit and leaf during development process. J. of Hebei Agricultural University 10(4):164-166.
Zeng, B. 2005. Current situation and development of Xinjiang wild walnut. Northern Fruits 4:1-3.
Zhang, Z.R. 2004. International production and sales and countermeasures of Chongqing walnut industry development. J. of Western Chongqing College 3(3):71-72.
Zhou, Y.L. 1994. Vegetation of China Xiaoxing'an Mountains. Science Press. Peking.

Tables

Table 1. Main characters of walnut cultivars in China.

Varieties	Appearance traits of nut	Dimensions (cm)	Weight of nut (g)	Kernel weight (g)	Thickness of nut shell (mm)	Kernel proportion (%)	Stress resistance	Variety types
Liaoning No.1	round, smooth	3.5×3.4×3.5	9.4	5.6	0.9	59.6	cold, diseases	early fruiting Protandrous
Zha 343	ovoid, smooth	4.6×3.6×3.8	16.4	8.9	1.2	54	drought, diseases	early fruiting Protandrous
Zhonglin No.1	round, rough	4.0×3.7×3.9	14	7.5	1.0	54	drought	early fruiting Protogynous
Xifu No.1	elliptical, smooth	4.0×3.5×3.2	12.5	6.6	1.2	53	vigorous	early fruiting Protandrous
Xilin No.2	round, smooth	4.1×3.9×3.8	14.2	8.6	1.2	61	vigorous	early fruiting Protogynous
Xiangling	ovoid, smooth	4.1×3.5×3.8	12.2	7.8	1.09	64	diseases	early fruiting Protandrous
Jinlong No.1	round, smooth	3.7×3.8×4.0	14.8	9.1	1.16	61	drought, diseases	late fruiting Protandrous
Jinboxiang No.1	elliptical, smooth	4.5×3.8×3.6	15.2	9.2	1.15	61	cold, diseases	early fruiting Protandrous

Figures

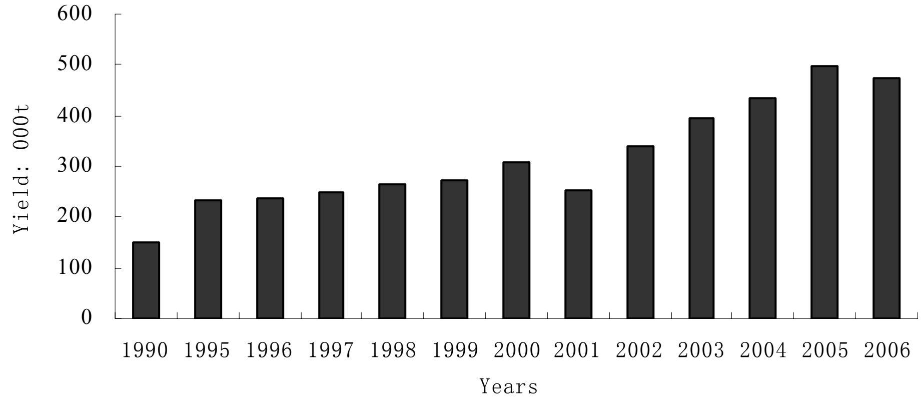

Fig. 1. Walnut yield of China in different years (from 'China agricultural yearbook', 1997-2007).

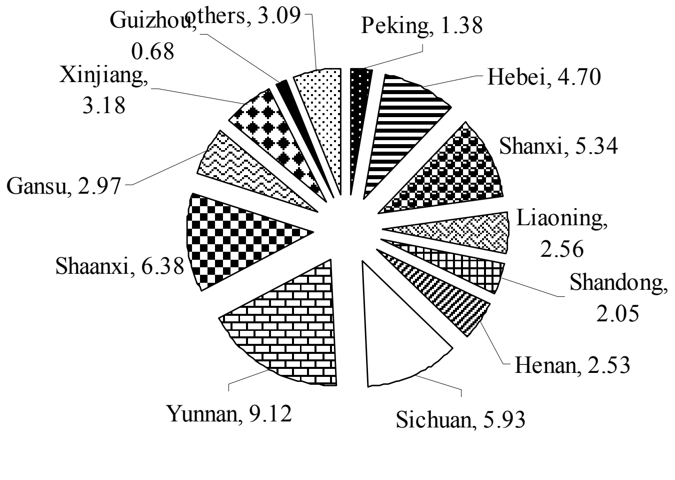

Fig. 2. Walnut output of the main walnut production provinces of China in 2006 (from 'China agricultural yearbook', 2007).

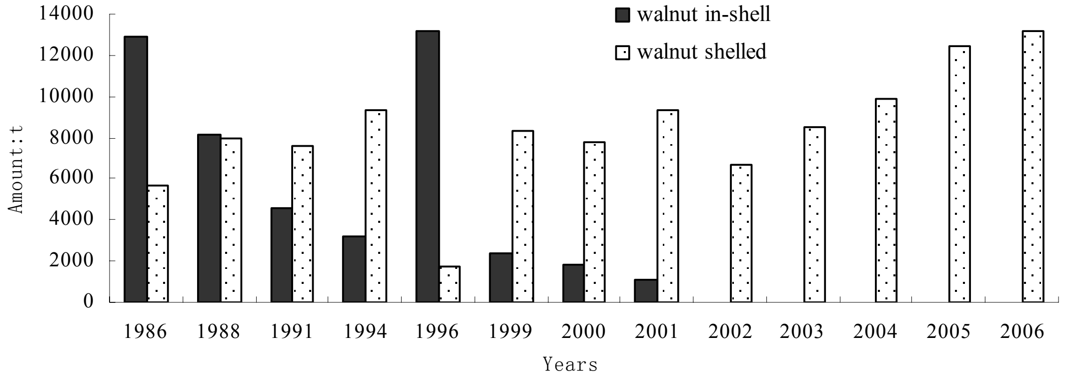

Fig. 3. Chinese walnut exports (from ‘China agricultural yearbook’, 2001-2007).
