# Supplementary material for: Provenance and family variations in early growth of Manchurian walnut (Juglans mandshurica Maxim.) and selection of superior families
Source: PLoS One. 2024 Mar 7;19(3):e0298918. doi: 10.1371/journal.pone.0298918 (PMC10919699; doi:10.1371/journal.pone.0298918)
Supplement: S1 File — (ZIP) [file pone.0298918.s004.zip › Differences in growth characters of Xanthoceras sorbifolium from different distribution areas and analysis on its correlation with geographical-climatic factors.pdf]

植物资源与环境学报，2019，**28**(3)：44-50，57
Journal of Plant Resources and Environment

不同分布区文冠果的生长性状差异及其与地理-气候因子的相关性分析

张　毅[1a,1b]**，敖　妍**[1a,1b,①]**，刘觉非**[2]**，赵磊磊**[3]**，张永明**[3]
(1. 北京林业大学：a. 省部共建森林培育与保护教育部重点实验室，b. 国家能源非粮生物质原料研发中心，北京 100083；
2. 赤峰市林业种苗站，内蒙古 赤峰 024000；3. 赤峰市翁牛特旗林业局，内蒙古 赤峰 024500)

摘要： 以内蒙古、河北和河南的 7 个文冠果(*Xanthoceras sorbifolium* Bunge)群体为研究对象，连续 3 a 对 11 个生长性状进行观测；在此基础上，采用相关性分析、通径分析和决策分析对其生长性状与地理-气候因子间的关系进行分析，并明确影响文冠果生长性状的主导因子。结果显示：供试 7 个文冠果群体间的生长性状存在极显著($P<0.01$)差异，其中，地径的平均变异系数最大(46.44%)，叶形指数和叶密度的平均变异系数最小(均为 11.80%)；而在群体内仅树高、地径、冠幅、第 1 轮活枝直径和叶面积存在极显著或显著($P<0.05$)差异，其他生长性状均无显著差异，表明文冠果的各生长性状在群体间存在广泛变异，而在群体内则变异程度不一致，且不同群体间的地径和树高等生长性状不稳定，但与叶片相关的生长性状相对稳定。相关性分析结果表明：纬度、经度和海拔与树高、地径、冠幅、叶长、叶宽、叶面积和叶密度总体上呈显著或极显著负相关；年均温、≥10 ℃年积温、年日照时数和年降水量与树高、地径和冠幅等多数生长性状呈显著或极显著正相关，说明纬度、经度和海拔增加不利于其树高和地径等 7 个生长性状的增长，而气温、日照时数和降水量的适度增加有利于文冠果的生长。通径分析和决策分析结果表明：≥10 ℃年积温是所有生长性状的直接作用因子，并且是树高、枝下高、分枝角度和叶形指数的最主要决策因子；冠幅和叶密度、地径和叶长、叶宽和叶面积、第 1 轮活枝直径的最主要决策因子分别为经度、年日照时数、年降水量、无霜期。综合分析结果表明：文冠果的生长性状呈现明显的地理变异规律，且不同地理-气候因子对其生长性状的影响程度存在明显差异；在文冠果的引种栽培过程中应综合考虑多种环境因子对其生长性状的影响，以提高优良种源选择的准确度。

关键词： 文冠果；生长性状；地理-气候因子；相关性；直接通径系数；决策系数

中图分类号： Q948.11；Q949.755.5　**文献标志码：** A　**文章编号：** 1674-7895(2019)03-0044-07
DOI：10.3969/j.issn.1674-7895.2019.03.06

Differences in growth characters of *Xanthoceras sorbifolium* from different distribution areas and analysis on its correlation with geographical-climatic factors　ZHANG Yi[1a,1b], AO Yan[1a,1b,①], LIU Juefei[2], ZHAO Leilei[3], ZHANG Yongming[3] (1. Beijing Forestry University: a. Key Laboratory for Silviculture and Conservation of Ministry of Education, b. National Energy R&D Center for Non-food Biomass, Beijing 100083, China; 2. Forestry Seedling Station of Chifeng City, Chifeng 024000, China; 3. Forestry Bureau of Ongniud Banner of Chifeng City, Chifeng 024500, China), *J. Plant Resour. & Environ.*, 2019, **28**(3): 44-50, 57

Abstract: Taking 7 populations of *Xanthoceras sorbifolium* Bunge from Inner Mongolia, Hebei, and He'nan as research objects, 11 growth characters were observed consecutively for 3 a; on the basis, the relationships between growth characters and geographical-climatic factors were analyzed by using correlation, path, and decision analyses, and the major factors impacting growth characters of *X.*

收稿日期： 2018-09-07
基金项目： 国家自然科学基金青年基金项目(31600241)；中央高校基本科研业务费专项资金项目(2015ZCQ-LX-02)
作者简介： 张　毅(1996—)，男，广东揭阳人，硕士研究生，主要从事能源植物培育理论与技术方面的研究。
①**通信作者** E-mail：aoyan316@163.com

sorbifolium were clarified. The results show that there are extremely significant ($P<0.01$) differences in growth characters among 7 populations of *X. sorbifolium* tested, in which, the average coefficient of variation of ground diameter is the largest (46.44%), while that of leaf shape index and leaf density is the smallest (both are 11.80%); there are extremely significant or significant ($P<0.05$) differences in tree height, ground diameter, crown width, diameter of the first round of living branch, and leaf area within population, but no significant difference in other growth characters, indicating that there are universal variations in each growth character of *X. sorbifolium* among populations, while the variation degree is inconsistent within population, and growth characters including ground diameter and tree height, etc. are not stable among different populations, but leaf-associated growth characters are relatively stable. The correlation analysis result shows that latitude, longitude, and altitude show significant or extremely significant negative correlations with tree height, ground diameter, crown width, leaf length, leaf width, leaf area, and leaf density in general; annual mean temperature, ≥10 ℃ annual accumulated temperature, annual sunshine hours, and annual precipitation all show significant or extremely significant positive correlations with most growth characters including tree height, ground diameter, and crown width, etc., indicating that the increase of latitude, longitude, and altitude is not good for the increase of 7 growth characters including tree height and ground diameter, etc., while proper increase of temperature, sunshine hours, and precipitation is beneficial for the growth of *X. sorbifolium*. Path and decision analyses results show that ≥10 ℃ annual accumulated temperature is the direct action factor of all growth characters, and is the major decision factor of tree height, clear bole height, branch angle, and leaf shape index; the major decision factors of crown width and leaf density, ground diameter and leaf length, leaf width and leaf area, and diameter of the first round of living branch are longitude, annual sunshine hours, annual precipitation, and frostless period, respectively. The comprehensive analysis result shows that the growth characters of *X. sorbifolium* show evident geographical variation law, and there are obvious differences in influence degree of different geographical-climatic factors on the growth characters; effects of multiple environmental factors on the growth characters should be comprehensively considered in introduction and cultivation process of *X. sorbifolium* to increase the accuracy of superior provenance selection.

Key words: *Xanthoceras sorbifolium* Bunge; growth characters; geographical-climatic factors; correlation; direct path coefficient; decision coefficient

文冠果(*Xanthoceras sorbifolium* Bunge)隶属于无患子科(Sapindaceae)文冠果属(*Xanthoceras* Bunge)，集中分布于内蒙古、河北、山西和陕西等地[1]。由于文冠果对环境适应性强，具有耐寒、耐旱和耐盐碱等特性，并且其结果期长、种子含油率高、综合利用价值大，已成为造林绿化、退耕还林以及防风固沙的首选树种，并被国家林业和草原局列为生物柴油生产的重点资源树种之一[2]。目前，研究者已对文冠果进行了较为系统的研究，主要集中在资源调查、种子品质、遗传变异、落花落果、栽培技术、化学成分和油脂提取等方面[3]；对其生长特性也有一定的研究，结果显示：不同环境条件下文冠果的树高、地径和冠幅等生长性状的差异较大[4-6]。

植物的生长发育和表型性状是基因与环境互作的结果，除与其自身的遗传特性有关外，在很大程度上易受地理、气候和土壤等外部因子的直接或间接影响[7]。由于分布范围广，生长环境条件差异大，在长期进化过程中，不同分布区域的文冠果形成了与当地环境相适应的生长性状，虽然其对不利的生长环境具有一定的抗逆性，但长势和产量均较差[8,4]。目前，对文冠果生长性状与地理-气候因子关系的研究多集中于某一特定生态区内的光照、水分和海拔等单一生态因子对其生长性状的影响[9]，而针对不同分布区多种环境因子对文冠果生长性状的综合影响进行研究，对文冠果适生立地的选择具有重要意义。

为明确不同分布区中影响文冠果生长性状的主导因子，作者以来源于内蒙古、河北和河南的 7 个文冠果实生群体为研究对象，连续 3 a 对 11 个生长性状进行观测，并采用 Duncan's 新复极差法和单因素方差分析法对供试文冠果群体的生长性状进行比较，采用相关性分析、通径分析和决策分析对各生长性状与多个地理-气候因子间的相关性进行分析，以期为文冠果适生立地和优良种质资源的选择及其标准化栽培管理措施的制定提供基础数据。

1 样地概况和研究方法

1.1 样地概况

在内蒙古坤都(P1)、内蒙古天山(P2)、内蒙古塞沁塔拉(P3)、内蒙古朱代沟(P4)、河北承德(P5)、河北蔚县(P6)和河南陕县(P7)分别选择1个文冠果实生群体,记录各群体的纬度、经度和海拔;根据每个采样点的地理坐标,从中国气象数据网(http://data.cma.cn/)获取2015年7月至2017年7月各群体的年均温、≥10 ℃年积温、年日照时数、年降水量、年蒸发量和无霜期的数据(表1)。

供试7个文冠果群体均位于温带草原或暖温带落叶阔叶林区域,主要伴生树种有榆树(*Ulmus pumila* Linn.)和刺槐(*Robinia pseudoacacia* Linn.)等;土壤为栗钙土或褐土,土层厚度150~160 cm。

表1 7个文冠果群体样地的自然概况
Table 1 Natural situation of plots of 7 populations of *Xanthoceras sorbifolium* Bunge

样地[1)]Plot[1)]	纬度 Latitude	经度 Longitude	海拔/m Altitude	年均温/℃ Annual mean temperature	≥10 ℃年积温/℃ ≥10 ℃ annual accumulated temperature	年日照时数/h Annual sunshine hours	年降水量/mm Annual precipitation	年蒸发量/mm Annual evaporation	无霜期/d Frostless period
P1	N44°13′	E119°18′	550	5.6	2 900	2 860	332	2 060	123
P2	N43°10′	E120°03′	420	5.7	2 850	3 020	346	2 055	149
P3	N42°57′	E119°00′	510	5.9	2 680	2 900	311	2 212	133
P4	N42°56′	E119°16′	500	5.8	2 690	2 925	343	2 023	121
P5	N40°58′	E118°50′	623	9.5	3 620	2 542	515	1 990	129
P6	N39°45′	E114°25′	850	6.6	3 010	2 900	407	1 580	128
P7	N34°38′	E111°37′	760	13.8	4 320	2 263	570	2 354	220

[1)] P1: 内蒙古坤都 Kundu of Inner Mongolia; P2: 内蒙古天山 Tianshan of Inner Mongolia; P3: 内蒙古塞沁塔拉 Segutala of Inner Mongolia; P4: 内蒙古朱代沟 Zhudaigou of Inner Mongolia; P5: 河北承德 Chengde of Hebei; P6: 河北蔚县 Yuxian of Hebei; P7: 河南陕县 Shanxian of He′nan.

1.2 生长性状测定方法

于2015年7月至2017年7月,在每个群体内选取长势和株龄(40~50 a)基本一致且处于盛果期的样株100株,各样株间距大于20 m。

采用布鲁莱斯测高器(精度0.01 m,哈尔滨光学仪器厂)测量树高;采用围尺(精度0.1 cm)测量地径和第1轮活枝直径;采用卷尺(精度0.01 m)测量冠幅和枝下高(地面至第1轮活枝的高度);采用量角器(精度0.01°)测量第1轮活枝的分枝角度。

在树冠的上、中、下3个部位分别随机采摘正常发育的小枝1支,统计从顶部向下10 cm枝段的成熟小叶数量,并按照公式"叶密度=成熟小叶数量/10"计算叶密度[8]47,结果取平均值;在每支小枝上随机选取3枚成熟叶片,用直尺(精度0.01 cm)测量叶片的长度和宽度,结果取平均值,并按照公式"叶形指数=叶长/叶宽"和"叶面积=叶长×叶宽×2/3"分别计算叶形指数和叶面积[8]47。

1.3 数据统计和分析

以连续3 a观测数据的平均值为基础,并采用EXCEL 2018和SPSS 20.0软件对数据进行统计和分析。采用Duncan′s新复极差法和单因素方差分析法研究各生长性状的差异。参照文献[10]计算各生长性状与地理-气候因子间的Pearson相关系数。采用通径分析研究地理-气候因子与文冠果生长性状的相互作用,并在此基础上利用决策系数[$R(i)^2$]分析地理-气候因子对各生长性状的决定程度;按照公式"$R(i)^2=2P_ir_{iy}-P_i^2$"计算决策系数,式中,P_i为直接通径系数,r_{iy}为单相关系数[11-12]。

2 结果和分析

2.1 不同文冠果群体生长性状的变异分析

不同文冠果群体生长性状的差异见表2,不同文冠果群体生长性状的变异系数见表3。

由表2可见:供试7个文冠果群体的生长性状在群体间存在极显著($P<0.01$)差异;但群体内个体间生长性状的差异水平略有不同,其中,个体间的树高、地径和冠幅存在极显著差异,第1轮活枝直径和叶面积存在显著($P<0.05$)差异,其他性状则无显著差异。表明文冠果生长性状在群体间存在广泛变异,而在群体内则变异程度不一致。在供试7个文冠果群体中,河北承德群体的树高、地径、冠幅、叶长、叶宽、叶面积

表2　不同文冠果群体生长性状的比较($\bar{X}$±*SD*) 1)

Table 2　Comparison on growth characters of *Xanthoceras sorbifolium* Bunge from different populations ($\bar{X}$±*SD*) 1)

群体 2) Population 2)	H/m	D_G/cm	H_{CB}/m	W_C/m	A_B/(°)	D_{LB}/cm
P1	2.88±1.22d	10.96±5.03d	0.91±0.18c	2.39±0.80d	36.61±4.83bc	11.75±4.29b
P2	2.04±0.68e	4.96±2.35f	0.57±0.10d	1.68±0.67e	39.77±4.81b	2.28±0.81e
P3	3.47±1.45c	8.95±3.88e	0.98±0.16bc	2.77±0.91c	36.73±6.58bc	7.53±2.31d
P4	4.05±1.75b	14.19±6.80b	1.20±0.32a	3.78±1.11b	45.95±8.34a	14.83±4.19a
P5	5.12±1.85a	19.71±8.83a	1.01±0.16bc	4.15±1.36a	34.17±6.16bc	9.74±3.68c
P6	1.84±0.55e	4.58±2.20f	0.47±0.05d	1.62±0.62e	32.07±4.58c	2.70±0.58e
P7	3.66±1.24c	12.97±6.20c	0.98±0.14bc	2.34±0.84d	35.96±6.49bc	8.61±2.85cd
均值 Average	3.56±1.44	11.65±5.20	0.95±0.38	2.86±1.02	36.44±14.25	10.53±5.03
群体间的 *F* 值 *F* value among populations	143.292**	252.900**	15.536**	136.854**	7.813**	68.291**
群体内的 *F* 值 *F* value within population	48.621**	96.534**	4.568	57.894**	2.115	23.476*

群体 2) Population 2)	L_L/cm	W_L/cm	LSI	A_L/cm^2	D_L/cm^{-1}
P1	4.61±0.61c	1.51±0.25c	3.10±0.43b	4.71±1.29d	1.17±0.15b
P2	5.09±0.68b	1.61±0.19b	3.24±0.41a	5.54±1.04b	1.00±0.11d
P3	4.59±0.62c	1.63±0.20b	2.85±0.36d	5.07±1.41cd	1.14±0.13bc
P4	4.65±0.60c	1.69±0.33b	2.80±0.39d	5.31±1.47bc	1.04±0.13c
P5	5.16±0.54a	1.92±0.45a	2.69±0.31e	6.60±2.31a	1.28±0.15a
P6	4.93±0.60bc	1.64±0.24b	3.02±0.28bc	5.47±1.42bc	1.12±0.13bc
P7	5.02±0.66b	1.68±0.25b	3.00±0.27c	5.73±1.55b	1.15±0.14b
均值 Average	4.79±0.67	1.68±0.34	2.91±0.39	5.44±1.72	1.13±0.15
群体间的 *F* 值 *F* value among populations	25.222**	33.977**	38.598**	51.017**	47.148**
群体内的 *F* 值 *F* value within population	5.346	10.745	12.332	28.489*	23.671

1) H：树高 Tree height；D_G：地径 Ground diameter；H_{CB}：枝下高 Clear bole height；W_C：冠幅 Crown width；A_B：分枝角度 Branch angle；D_{LB}：第1轮活枝直径 Diameter of the first round of living branch；L_L：叶长 Leaf length；W_L：叶宽 Leaf width；LSI：叶形指数 Leaf shape index；A_L：叶面积 Leaf area；D_L：叶密度 Leaf density. 同列中不同的小写字母表示差异显著(P<0.05) Different lowercases in the same column indicate the significant (P<0.05) difference. *：P<0.05；**：P<0.01.

2) P1：内蒙古坤都 Kundu of Inner Mongolia；P2：内蒙古天山 Tianshan of Inner Mongolia；P3：内蒙古塞沁塔拉 Segutala of Inner Mongolia；P4：内蒙古朱代沟 Zhudaigou of Inner Mongolia；P5：河北承德 Chengde of Hebei；P6：河北蔚县 Yuxian of Hebei；P7：河南陕县 Shanxian of He'nan.

表3　不同文冠果群体生长性状的变异系数

Table 3　Coefficient of variation of growth characters of *Xanthoceras sorbifolium* Bunge from different populations

群体 1) Population 1)	变异系数/% 2) Coefficient of variation 2)											
	H	D_G	H_{CB}	W_C	A_B	D_{LB}	L_L	W_L	LSI	A_L	D_L	均值 Average
P1	42.10	46.06	19.63	33.46	13.19	36.50	13.20	16.64	13.91	27.44	12.60	24.98
P2	32.28	46.97	18.39	39.81	18.15	35.67	10.53	12.08	12.73	18.79	11.14	23.32
P3	41.82	43.48	16.08	32.46	17.91	28.24	13.59	17.57	12.48	27.76	11.64	23.91
P4	43.74	47.92	16.75	29.10	14.26	30.62	12.97	19.56	13.81	27.68	11.35	24.34
P5	36.35	44.81	14.13	32.77	18.02	37.77	13.27	23.51	11.42	35.06	12.18	25.39
P6	29.82	48.04	9.88	38.22	12.09	34.03	12.27	14.52	9.39	25.94	11.95	22.38
P7	34.12	47.81	14.45	35.97	18.05	33.08	13.20	14.70	8.85	27.08	11.76	23.55
均值 Average	37.17	46.44	15.61	34.54	15.95	33.70	12.72	16.94	11.80	27.11	11.80	23.98

1) P1：内蒙古坤都 Kundu of Inner Mongolia；P2：内蒙古天山 Tianshan of Inner Mongolia；P3：内蒙古塞沁塔拉 Segutala of Inner Mongolia；P4：内蒙古朱代沟 Zhudaigou of Inner Mongolia；P5：河北承德 Chengde of Hebei；P6：河北蔚县 Yuxian of Hebei；P7：河南陕县 Shanxian of He'nan.

2) H：树高 Tree height；D_G：地径 Ground diameter；H_{CB}：枝下高 Clear bole height；W_C：冠幅 Crown width；A_B：分枝角度 Branch angle；D_{LB}：第1轮活枝直径 Diameter of the first round of living branch；L_L：叶长 Leaf length；W_L：叶宽 Leaf width；LSI：叶形指数 Leaf shape index；A_L：叶面积 Leaf area；D_L：叶密度 Leaf density.

和叶密度显著高于其他群体,内蒙古朱代沟群体的枝下高、分枝角度和第 1 轮活枝直径显著高于其他群体,内蒙古天山群体的叶形指数显著高于其他群体。

由表 3 可见: 不同文冠果群体生长性状的变异系数差异较大。其中,平均变异系数最大的生长性状为地径(46.44%) ,其次为树高(37.17%) ; 平均变异系数最小的生长性状为叶形指数和叶密度(均为 11.80%) ,叶长的平均变异系数也较小(12.72%) 。表明不同文冠果群体的地径和树高等生长性状不稳定,而与叶片相关的生长性状则相对稳定。

2.2 文冠果生长性状与地理-气候因子的相关性分析

供试文冠果生长性状与地理-气候因子的相关性分析结果见表 4。结果表明: 供试文冠果群体样地的地理位置、温度、光照和降水等因子与文冠果生长性状存在不同程度的相关性。

在地理因子方面,纬度、经度和海拔与树高、地径、冠幅、叶长、叶宽、叶面积和叶密度总体上呈显著($P<0.05$) 或极显著($P<0.01$) 负相关,表明纬度、经度和海拔增加对文冠果树高和地径等 7 个生长性状有抑制作用。在温度因子方面,年均温和≥10 ℃年积温与树高、地径、枝下高、冠幅、叶长、叶宽和叶面积均呈显著或极显著正相关,表明气温增加对文冠果树高和地径等 7 个生长性状有促进作用。在光照因子方面,年日照时数与所有生长性状均呈显著或极显著正相关,表明日照时数增加可促进文冠果的生长发育,其中,年日照时数与地径的相关性最密切(相关系数为 0.961) 。在降水因子方面,年降水量与树高、地径、枝下高、冠幅和第 1 轮活枝直径均呈显著或极显著正相关,与叶长、叶宽、叶面积和叶密度均呈显著或极显著负相关,表明年降水量增加对文冠果树高和地径等 5 个生长性状有促进作用,但对其叶片生长发育有一定的抑制作用。此外,年蒸发量和无霜期与叶长、叶宽、叶形指数、叶面积和叶密度均呈显著或极显著正相关,表明年蒸发量和无霜期增加可促进文冠果叶片的生长发育。

表 4 文冠果生长性状与地理-气候因子间的相关系数

Table 4 Correlation coefficient between growth characters of *Xanthoceras sorbifolium* Bunge and geographical-climatic factors

生长性状[1)] Growth character[1)]	相关系数[2)] Correlation coefficient[2)]								
	纬度 Latitude	经度 Longitude	海拔 Altitude	年均温 Annual average temperature	≥10 ℃年积温 ≥10 ℃ annual accumulated temperature	年日照时数 Annual sunshine hours	年降水量 Annual precipitation	年蒸发量 Annual evaporation	无霜期 Frostless period
H	−0.608**	−0.501*	−0.757**	0.785**	0.847**	0.842**	0.632**	0.021	0.785**
D_G	−0.662**	−0.564*	−0.821**	0.924**	0.945**	0.961**	0.564*	0.043	0.924**
H_{CB}	0.008	0.043	−0.617**	0.578*	0.588**	0.561*	0.830**	−0.004	0.578*
W_C	−0.555*	−0.645**	−0.847**	0.622**	0.676**	0.709**	0.617**	−0.663**	0.622**
A_B	0.016	0.511*	−0.566*	−0.044	0.571*	0.554*	−0.029	0.569*	−0.044
D_{LB}	0.696**	0.719**	−0.571*	−0.663**	0.573*	0.706**	0.832**	−0.604**	0.663**
L_L	−0.710**	−0.673**	−0.006	0.758**	0.718**	0.784**	−0.594**	0.655**	0.758**
W_L	−0.634**	−0.576**	−0.579**	0.709**	0.712**	0.768**	−0.566*	0.632**	0.709**
LSI	−0.009	−0.037	−0.573**	0.051	0.597**	0.507*	−0.013	0.584**	0.551*
A_L	−0.679**	−0.625**	−0.554*	0.752**	0.738**	0.802**	−0.584**	0.587**	0.752**
D_L	−0.607**	−0.641**	−0.585**	−0.796**	0.591**	0.623**	−0.597**	0.601**	0.796**

[1)] H: 树高 Tree height; D_G: 地径 Ground diameter; H_{CB}: 枝下高 Clear bole height; W_C: 冠幅 Crown width; A_B: 分枝角度 Branch angle; D_{LB}: 第 1 轮活枝直径 Diameter of the first round of living branch; L_L: 叶长 Leaf length; W_L: 叶宽 Leaf width; LSI: 叶形指数 Leaf shape index; A_L: 叶面积 Leaf area; D_L: 叶密度 Leaf density.

[2)] * : $P<0.05$; **: $P<0.01$.

2.3 文冠果生长性状与地理-气候因子的通径分析和决策分析

剔除通径系数不显著($P>0.05$) 的自变量,采用通径分析研究地理-气候因子与文冠果生长性状的相互作用,所得直接通径系数见表 5。为了进一步分析地理-气候因子对文冠果各生长性状的影响程度,以通径分析结果为基础计算地理-气候因子对文冠果生长性状的决策系数,结果见表 6。

从直接通径系数(表 5) 可见: 在地理因子方面,纬度和海拔均为树高和地径的直接作用因子,经度和海拔均为枝下高、冠幅、第 1 轮活枝直径和叶密度的直接作用因子。在气候因子方面,≥10 ℃年积温为

所有生长性状的直接作用因子; 年日照时数和年降水量均为树高、分枝角度、叶长、叶形指数和叶面积的直接作用因子; 年蒸发量为地径、冠幅、第 1 轮活枝直径、叶长、叶宽、叶形指数和叶面积的直接作用因子; 无霜期为树高、冠幅、第 1 轮活枝直径和叶密度的直接作用因子。

从决策系数(表 6) 可见: 经度是冠幅和叶密度的最主要决策因子，决策系数分别为 7. 664 和 5. 743; ≥10 ℃年积温是树高、枝下高、分枝角度和叶形指数的最主要决策因子，决策系数分别为 5. 667、5. 054、3. 251 和 16. 852; 年日照时数是地径和叶长的最主要决策因子，决策系数分别为 10. 395 和 2. 357; 年降水量是叶宽和叶面积的最主要决策因子，决策系数分别为 11. 151 和 3. 444; 无霜期是第 1 轮活枝直径的最主要决策因子，决策系数为 10. 838。

表 5　文冠果生长性状与地理-气候因子间的直接通径系数
Table 5　Direct path coefficient between growth characters of *Xanthoceras sorbifolium* Bunge and geographical-climatic factors

生长性状[1] Growth character[1]	直接通径系数[2] Direct path coefficient[2]							
	纬度 Latitude	经度 Longitude	海拔 Altitude	≥10 ℃年积温 ≥10 ℃ annual accumulated temperature	年日照时数 Annual sunshine hours	年降水量 Annual precipitation	年蒸发量 Annual evaporation	无霜期 Frostless period
H	0. 702	—	-0. 232	-0. 873	-0. 649	0. 771	—	0. 505
D_G	-0. 228	—	-0. 631	-1. 313	-1. 822	—	-0. 450	—
H_{CB}	—	-0. 295	-0. 326	-1. 286	-1. 207	—	—	
W_C	—	0. 947	-0. 122	-0. 518	—	-0. 166	-0. 078	-0. 236
A_B	—	—	—	1. 097	0. 809	-0. 359	—	—
D_{LB}	—	1. 968	1. 620	1. 776	—	—	2. 576	-2. 776
L_L	—	—	—	0. 463	1. 104	0. 877	0. 172	—
W_L	—	—	—	-1. 713	—	1. 942	0. 270	—
LSI	—	—	—	2. 819	0. 974	-1. 940	-0. 250	—
A_L	—	—	—	-0. 919	0. 487	1. 662	0. 254	—
D_L	—	1. 428	-0. 266	0. 338	—	—	—	0. 843

1) H: 树高 Tree height; D_G: 地径 Ground diameter; H_{CB}: 枝下高 Clear bole height; W_C: 冠幅 Crown width; A_B: 分枝角度 Branch angle; D_{LB}: 第 1 轮活枝直径 Diameter of the first round of living branch; L_L: 叶长 Leaf length; W_L: 叶宽 Leaf width; LSI: 叶形指数 Leaf shape index; A_L: 叶面积 Leaf area; D_L: 叶密度 Leaf density.
2) —: 无数据 No datum.

表 6　文冠果生长性状与地理-气候因子间的决策系数
Table 6　Decision coefficient between growth characters of *Xanthoceras sorbifolium* Bunge and geographical-climatic factors

生长性状[1] Growth character[1]	决策系数[2] Decision coefficient[2]							
	纬度 Latitude	经度 Longitude	海拔 Altitude	≥10 ℃年积温 ≥10 ℃ annual accumulated temperature	年日照时数 Annual sunshine hours	年降水量 Annual precipitation	年蒸发量 Annual evaporation	无霜期 Frostless period
H	4. 642	—	1. 631	5. 667	4. 221	5. 076	—	3. 456
D_G	1. 224	—	3. 116	8. 581	10. 395	—	2. 594	—
H_{CB}	—	1. 118	1. 007	5. 054	4. 601	—	—	—
W_C	—	7. 664	1. 736	2. 180	—	2. 091	1. 157	2. 435
A_B	—	—	—	3. 251	0. 801	0. 275	—	—
D_{LB}	—	0. 079	0. 542	1. 988	—	—	9. 768	10. 838
L_L	—	—	—	0. 238	2. 357	1. 888	0. 536	—
W_L	—	—	—	10. 224	—	11. 151	1. 595	—
LSI	—	—	—	16. 852	1. 567	8. 866	0. 725	—
A_L	—	—	—	2. 599	1. 140	3. 444	0. 747	—
D_L	—	5. 743	1. 231	1. 525	—	—	—	

1) H: 树高 Tree height; D_G: 地径 Ground diameter; H_{CB}: 枝下高 Clear bole height; W_C: 冠幅 Crown width; A_B: 分枝角度 Branch angle; D_{LB}: 第 1 轮活枝直径 Diameter of the first round of living branch; L_L: 叶长 Leaf length; W_L: 叶宽 Leaf width; LSI: 叶形指数 Leaf shape index; A_L: 叶面积 Leaf area; D_L: 叶密度 Leaf density.
2) —: 无数据 No datum.

3 讨论和结论

文冠果的自然分布范围广，生境条件复杂，通过长期的地理隔离和自然选择，使得文冠果群体间和群体内存在丰富的变异，这为文冠果良种选育和种子区划奠定了重要基础。本研究结果表明：在不同分布区，文冠果的生长性状存在极显著（$P<0.01$）差异，其中，地径、树高、冠幅和第1轮活枝直径的变异程度较大，变异系数分别为46.44%、37.17%、34.54%和33.70%，从这些群体中选择优良种源的潜力很大。河北承德群体的树高、地径、冠幅、叶长、叶宽、叶面积和叶密度显著（$P<0.05$）高于其他群体，且在供试7个群体中，河北承德群体的地理位置居中，纬度和经度均较低，热量、水分和光照条件较好，可作为文冠果优良种源选择的重点区域。

大量的研究结果表明：不同植物种类的生长对地理因子的响应程度不同，例如，毛红椿〔*Toona ciliata* var. *pubescens*（Franch.）Hand.-Mazz.〕的地径生长呈现纬度渐变模式[13]；花楸树〔*Sorbus pohuashanensis*（Hance）Hedl.〕[14]、乳源木莲（*Manglietia yuyuanensis* Law）[15]和野生玫瑰（*Rosa rugosa* Thunb.）[16]的生长性状呈现经纬度双重渐变模式，其中纬度起主要作用；而暴马丁香〔*Syringa reticulata* subsp. *amurensis*（Ruprecht）P. S. Green et M. C. Chang〕的株高和地径与纬度和经度均无显著相关性[17]，这与各种类分布区的地理位置差异有关[18]。本研究选取的7个文冠果群体的地理位置跨度较大，位于北纬34°~44°、东经111°~120°、海拔420~850 m的区域内，生境条件差异明显，导致文冠果的生长性状随纬度、经度和海拔的变化呈现并存的渐变模式。相关性分析结果显示：纬度和经度与文冠果的树高、地径、冠幅、叶长、叶宽、叶面积和叶密度均呈显著或极显著负相关，表明随纬度和经度增加，文冠果多数生长性状的增长幅度呈减小趋势；而海拔与文冠果所有生长性状总体上呈显著或极显著负相关，原因可能为海拔每上升100 m，气温降低0.6 ℃，直接影响文冠果的生长速率。

文冠果的生长性状受多种气候因子的综合影响，其中温度、光照和水分为主要影响因子[19]。大量研究结果表明：温度对不同植物的生长性状有不同程度的影响效应，例如，年均温与野生玫瑰[16]、暴马丁香[17]和香椿〔*Toona sinensis*（A. Juss.）Roem.〕[20]的树高和地径呈显著正相关，但与梭梭〔*Haloxylon ammodendron*（C. A. Mey.）Bunge〕[21]的株高和地径无显著相关性。本研究结果表明：年均温和≥10 ℃年积温与文冠果的大多数生长性状呈显著或极显著正相关，表明气温适度升高有利于文冠果的光合作用等生理活动，从而促进其生长发育。文冠果为喜光植物，年日照时数与其所有生长性状均呈显著或极显著正相关，表明日照时数增加可提高文冠果的光合效率，使其体内有机物质不断积累，有利于其生长发育。年降水量与文冠果的树高和地径等生长性状均呈显著或极显著正相关，但与叶片的相关生长性状均呈显著或极显著负相关，这可能是因为本研究供试的文冠果群体均位于中国北方地区，相对干旱，适宜程度的降水能够促进文冠果树高和地径等生长性状的增长，但由于文冠果不耐涝，因而在低洼水涝地区其叶片生长发育可能受到一定的限制。通径分析和决策分析结果表明：不同气候因子对文冠果生长性状的影响程度存在差异，其中，≥10 ℃年积温是文冠果所有生长性状的直接作用因子，并且是树高、枝下高、分枝角度和叶形指数的最主要决策因子；而年日照时数是地径和叶长的最主要决策因子，年降水量是叶宽和叶面积的最主要决策因子。因此，在引种栽培过程中应考虑多种气候因子对文冠果生长性状的综合作用，以提高其优良种源选择的准确度。

植物的表型性状是基因与环境互作的结果，除了地理-气候因子，文冠果生长发育还受其自身遗传特性的影响，因此，今后应关注环境因子和植物内因对文冠果生长性状的综合影响效应。另外，本研究仅就文冠果不同地理群体的生长性状与气候因子年均值的关系进行了探讨，对与生长性状相对应的气候资料（如日均温）等数据缺乏详细分析，且没有在对生长性状进行长期观测的基础上结合多年气候资料进行深度探讨，因而，针对文冠果生长性状对环境因子的响应规律有待进一步深入研究。

参考文献：

[1] 谢志玉，张文辉. 干旱和复水对文冠果生长及生理生态特性的影响[J]. 应用生态学报，2018，29(6)：1759-1767.

[2] 张 刚，魏典典，邬佳宝，等. 干旱胁迫下不同种源文冠果幼苗的生理反应及其抗旱性分析[J]. 西北林学院学报，2014，29(1)：1-7.

（下转第57页 Continued on page 57）

[20] 鲁　艳. 哈密盆地裸果木群落物种多样性及其优势种群生态位研究[D]. 乌鲁木齐: 新疆大学资源与环境学院，2007: 15-16.
[21] HIJMANS R J，CAMERON S，PARRA J L，et al. Very high resolution interpolated climate surfaces for global land areas[J]. International Journal of Climatology，2005，25(15): 1965-1978.
[22] KIEHL J T，HACK J J，BONAN G B，et al. The national center for atmospheric research community climate model: CCM3[J]. Journal of Climate，1998，11(6): 1131-1149.
[23] KNUTTI R，SEDLACEK J. Robustness and uncertainties in the new CMIP5 climate model projections[J]. Nature Climate Change，2013，3(4): 369-373.
[24] 周天军，邹立维. IPCC 第五次评估报告全球和区域气候预估图集评述[J]. 气候变化研究进展，2014，10(2): 149-152.
[25] 王运生，谢丙炎，万方浩，等. ROC 曲线分析在评价入侵物种分布模型中的应用[J]. 生物多样性，2007，15(4): 365-372.
[26] 郭彦龙，卫海燕，路春燕，等. 气候变化下桃儿七潜在地理分布的预测[J]. 植物生态学报，2014，38(3): 249-261.
[27] 高　蓓，卫海燕，郭彦龙，等. 应用 GIS 和最大熵模型分析秦岭冷杉潜在地理分布[J]. 生态学杂志，2015，34(3): 843-852.
[28] 马松梅，张明理，陈　曦. 沙冬青属植物在亚洲中部荒漠区的潜在地理分布及驱动因子分析[J]. 中国沙漠，2012，32(5): 1301-1307.
[29] 吕佳佳. 气候变化对我国主要珍稀濒危物种分布影响及其适应对策研究[D]. 北京: 中国环境科学研究院，2009: 5.
[30] 吴建国，吕佳佳. 气候变化对我国干旱区分布及其范围的潜在影响[J]. 环境科学研究，2009，22(2): 199-206.

（责任编辑: 佟金凤）

(上接第 50 页　Continued from page 50)

[3] 牟洪香. 木本能源植物文冠果(*Xanthoceras sorbifolium* Bunge)的调查与研究[D]. 北京: 中国林业科学研究院林业研究所，2006: 20-25.
[4] 敖　妍，韩树文，杨晓辉. 立地条件和栽植密度对文冠果生长结实的影响[J]. 中南林业科技大学学报，2016，36(6): 11-14.
[5] 柴春山，戚建莉，薛　睿，等. 甘肃省文冠果优良单株的选择及其性状特征[J]. 经济林研究，2017，35(4): 22-28.
[6] 刘五爱，王志贤. 立地条件及造林方式对文冠果造林效果的影响[J]. 园艺与种苗，2016(6): 44-46.
[7] 卜　静，李登武，王冬梅. 玉竹品质与主要生态因子的相关性[J]. 应用生态学报，2012，23(6): 1447-1454.
[8] 敖　妍. 木本能源植物文冠果类型划分、单株选择及相关研究[D]. 北京: 中国林业科学研究院林业研究所，2010.
[9] 张晓燕. 神东矿区不同种源地文冠果生长适宜性及耐盐性研究[D]. 呼和浩特: 内蒙古农业大学水土保持学院，2012: 32-37.
[10] 杨庆珍，王增绘，付　娟，等. 黄芪化学成分与生态因子的相关性[J]. 应用生态学报，2015，26(3): 732-738.
[11] 何忠俊，梁社往，丁　颖，等. 三七主根稳定碳同位素组成与生态因子的关系[J]. 生态环境学报，2015，24(4): 561-568.
[12] 刁松锋，邵文豪，董汝湘，等. 无患子光合生理日变化及其与生理生态因子的关系[J]. 西北植物学报，2014，34(4): 828-834.
[13] 刘　军，张海燕，姜景民，等. 毛红椿种实和苗期生长性状地理种源变异[J]. 南京林业大学学报(自然科学版)，2011，35(3): 55-59.
[14] 郑　健，胡增辉，郑勇奇，等. 花楸树种源间表型性状的地理变异分析[J]. 植物资源与环境学报，2012，21(3): 50-56.
[15] 李因刚，周志春，金国庆，等. 乳源木莲苗生长和形态的地理种源分化[J]. 林业科学研究，2007，20(1): 35-39.
[16] 童　冉，吴小龙，姜丽娜，等. 野生玫瑰种群表型变异[J]. 生态学报，2017，37(11): 3706-3715.
[17] 杨晓霞，冷平生，郑　健，等. 暴马丁香不同种源种子和幼苗的表型性状变异及其与地理-气候因子的相关性[J]. 植物资源与环境学报，2016，25(3): 80-89.
[18] 林　玲，王军辉，罗　建，等. 砂生槐天然群体种实性状的表型多样性[J]. 林业科学，2014，50(4): 137-143.
[19] 张　振，张含国，周　宇，等. 红松多无性系群体的种实性状变异研究[J]. 北京林业大学学报，2015，37(2): 67-78.
[20] 刘　军，陈益泰，姜景民，等. 香椿种源苗期性状变异与原产地生态因子典型相关分析[J]. 东北林业大学学报，2010，38(11): 27-29.
[21] 王葆芳，张景波，杨晓晖，等. 梭梭不同种源间种子性状和幼苗生长性状与地理和气候因子的关系[J]. 植物资源与环境学报，2009，18(1): 28-35.

（责任编辑: 郭严冬）
