# Supplementary material for: Provenance and family variations in early growth of Manchurian walnut (Juglans mandshurica Maxim.) and selection of superior families
Source: PLoS One. 2024 Mar 7;19(3):e0298918. doi: 10.1371/journal.pone.0298918 (PMC10919699; doi:10.1371/journal.pone.0298918)
Supplement: S1 File — (ZIP) [file pone.0298918.s004.zip › Diversity in Fruit Morphology and Nutritional Composition of Juglans mandshurica Maxim in Northeast China.pdf]

frontiers in Plant Science

ORIGINAL RESEARCH
published: 10 February 2022
doi: 10.3389/fpls.2022.820457

Diversity in Fruit Morphology and Nutritional Composition of *Juglans mandshurica* Maxim in Northeast China

Zhixin Li[1], Weihuai Wang[1], Haixiao Zhang[2], Jinhong Liu[1], Baoying Shi[3], Weizhao Dai[3], Kewu Liu[4] and Hanguo Zhang[1]**

[1] State Key Laboratory of Tree Genetics and Breeding, Northeast Forestry University, Harbin, China, [2] Jilin Provincial Academy of Forestry Sciences, Changchun, China, [3] Wuchang Baolongdian Seed Forest Farm in Heilongjiang, Wuchang, China, [4] Heilongjiang Academy of Forestry, Mudanjiang, China

OPEN ACCESS

Edited by:
Rebecca Grumet,
Michigan State University,
United States

Reviewed by:
Kourosh Vahdati,
University of Tehran, Iran
Sezai Ercisli,
Atatürk University, Turkey

***Correspondence:**
Kewu Liu
liukewu111@163.com
Hanguo Zhang
hanguozhang1@sina.com

Specialty section:
This article was submitted to
Plant Breeding,
a section of the journal
Frontiers in Plant Science

Received: 23 November 2021
Accepted: 17 January 2022
Published: 10 February 2022

Citation:
Li Z, Wang W, Zhang H, Liu J, Shi B, Dai W, Liu K and Zhang H (2022) Diversity in Fruit Morphology and Nutritional Composition of Juglans mandshurica Maxim in Northeast China. Front. Plant Sci. 13:820457. doi: 10.3389/fpls.2022.820457

Although Manchurian walnut (*Juglans mandshurica* Maxim) is widely distributed in northeast China, very few studies had been reported on its diversity among different populations. We surveyed 12 *J. mandshurica* populations in their native habitats across the northeast region of China and profiled 13 fruit morphological traits. We found a large degree of variations for these traits, especially for fruit weight (coefficient of variation, or CV of 22.00%), nut weight (CV of 19.42%), and kernel weight (CV of 19.89%). Statistical analysis showed that a large portion of the total variation can be attributed to within-population variation (66.64%), followed by random error (20.96%). We also comprehensively quantified the nutritional composition including fatty acids, amino acids, vitamins, and micronutrients. Similar to fruit morphological traits, we found large variation for most kernel components, which mostly can be explained by within-population variation. Further correlation analysis revealed the dependence of some morphological and nutritional traits on key geographical and ecological factors such as latitude, accumulated temperature, and day length. For instance, a significant positive correlation was found between fruit dimensions and equivalent latitude and precipitation, indicating that such factors should be considered for breeding. Taken together, our data provided a rich dataset for characterizing the variation among *J. mandshurica* populations and a foundation for selective breeding.

Keywords: *Juglans mandshurica* Maxim, fruit morphology, nutritional composition, diversity, environmental factors

INTRODUCTION

Manchurian walnut (*Juglans mandshurica* Maxim) is one of the 21 Juglans tree species in the family *Juglandaceae* (Lu, 1982). Along with other three species (*J. regia*, *J. ailantifolia*, and *J. cathayensis*), *J. mandshurica* is distributed in northeast Asia including China, Korea, and Russia. Due to its fast-growing property, it has also been extensively cultivated in the temperate climate regions. Traditionally, *J. mandshurica* had been used as food, medicine, and ornament for thousands of years (Luan et al., 2020). Increasing interest and usage of *J. mandshurica* recently are on its

medicinal potentials as it provides a rich source for various bioactive compounds that exhibit pharmacologically important properties (Vahdati, 2014; Wang et al., 2020; Cho et al., 2021). However, increased usage and loss of habitat by human activities also render *J. mandshurica* an endangered species (Wang et al., 2011).

Despite the high economic value and wild distribution of *J. mandshurica*, few studies had been performed to evaluate the diversity among different populations. Diversity in fruit has gained popularity recently because they may vary in a plethora of bioactive compounds, giving different medical values, flavor, and taste (Sahin and Anapali, 2002). Walnut fruits from six geographic provenances in China had been surveyed for morphological traits such as nut dimensions (longitudinal/lateral/transverse diameter), nut weight, shell thickness, and kernel weight (Zhang et al., 2021). Variations in these traits were found both within and among the six populations. However, this study is limited to only six locations in eastern Liaoning, a province in northeast China. Other efforts had been devoted to developing genetic makers to assess the genetic diversity (Woeste et al., 2002; Qu et al., 2011). For example, twenty microsatellite loci were identified in assessing the polymorphism and genetic structure in five native habitats including Xiaoxing'anling, Zhangguancailing, Changbai Mountain, Laoyeling, and Wada Mountain (Chen et al., 2013).

Nut characteristics and nutritional attributes also show a wide range of diversity among different walnut species (Pakrah et al., 2021). Rich in unsaturated fatty acids, proteins, vitamins, and other micronutrients, walnut provides high-quality foods (Pollegioni et al., 2017; Chatrabnous et al., 2018). By quantifying major components in the kernel of common walnut (*J. regia* L.), Geng et al. (2021) found distinct nutritional composition profiles for kernels from different geographical areas). For instance, much higher levels of flavonoids and vitamin E were found in nuts from Yunnan compared to other provinces. Recently, the genetic control of nut characteristics such as yield and pellicle color had been investigated by identifying major quantitative trait locus (QTL) for these traits (Arab et al., 2019; Kefayati et al., 2019; Marrano et al., 2019). However, the diversity of nut characteristics in *J. mandshurica* has not been reported yet.

Here, we comprehensively analyzed both fruit morphological traits and kernel nutritional composition in 12 populations across northeast China. The fruit phenotypical traits included fruit size (length/width), fruit shape, fruit weight, nut size (vertical/lateral/transverse diameter, and mean diameter), nut weight, nut roundness, shell thickness, kernel weight, and kernel rate. For nutritional composition, we quantified the content of crude fat, various fatty acids, vitamins, and other micronutrients. We found that both fruit morphological traits and nut nutritional composition show high levels of variability in 12 *J. mandshurica* populations. Interestingly, we found that within-population variation accounts for a significant portion of the total variation for most traits. Correlation analysis also showed the impact of geographical and ecological factors on these traits. Thus, our data not only revealed the diversity among *J. mandshurica* populations, but also provided a foundation for selective breeding of desired traits.

MATERIALS AND METHODS

Walnut Samples

Samples were collected from 12 native habitats of *J. mandshurica* (**Figure 1** and **Supplementary Table 1**) in northeast China. Nine of them were located in the Heilongjiang province including Daquanzi forest farm in Binxian (BX), Wulindong forestry farm of Dongfanghong Forestry Bureau in Raohe county (DFH), Dougouzi forest farm of Dongjing Forestry Bureau in Ning'an city (DJC), Qihu forest farm of Hulin city (HL), Jinshantun forestry Bureau of Yichun city (JST), Malian forest farm of Jiayin county (JY), Maolin forest farm of Tieli City (TL), Baolongdian forest farm in Wuchang city (WC), and the Fish Pond Management Office in Yabuli (YBL). The other three locations were in the Jilin province: Qinglongtai forest farm of Hunchun city (HC), Naozhi forest farm of Linjiang city (LJ), and Jingshan forest farm of Sanchazi Forestry Bureau in Baishan city (SC). Geographical and ecological conditions for each location were summarized in **Supplementary Table 1**. For each location, fruits were collected from 20 to 31 individual trees. The distance between two individual trees was above 30 m. For each tree, around 100 fruits were collected.

Quantification of Morphological Traits

To quantify morphological traits, 30 fruits were randomly selected from the 12 populations. In total, 13 traits were assessed. Vernier calipers (precision of 0.01 mm) were used to quantify the following six traits: fruit length (FL), fruit

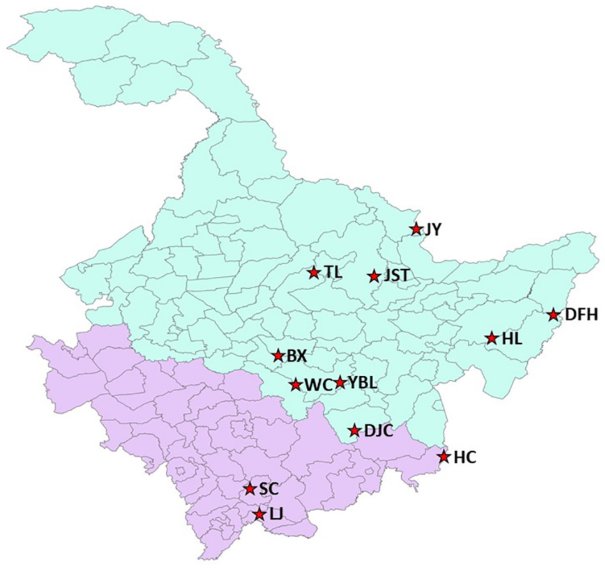

FIGURE 1 | Geographic locations of 12 *J. mandshurica* populations in this study. Light blue indicates Heilongjiang Province and light purple indicates Jilin Province. The geographic conditions for each site can be found in **Supplementary Table 1**. BX, Binxian, HLJ; DFH, Raohe, HLJ; DJC, Ning'an, HLJ; HC, Hunchun, JL; HL, Hulin, HLJ; JST, Yichun, HLJ; JY, Jiayin, HLJ; LJ, Linjiang, JL; SC, Baishan, JL; TL, Tieli, HLJ; WC, Wuchang, HLJ; YBL, Yabuli, HLJ. HLJ, Heilongjiang province; JL, Jilin province.

width (FW), nut longitudinal diameter (NV), nut transverse diameter (NT), nut lateral diameter (NL), and shell thickness (TS). A scale (precision of 0.01 g) was used to measure the following three traits: fruit weight (FT, average of 10 fruits), nut weight (NW), and kernel weight (KW). The remaining four traits, index of fruit shape (IF), mean diameter (DM), index of nut roundness (IR), and kernel rate (KR), were calculated using the following formulas: IF = FL/FW; MD = (NV + NT + NL)/3; IR = (NT + NL)/(2 × NV); and KR = KW/MW × 100%.

Kernel Nutritional Composition

Kernel nutritional composition was quantified in eight of the 12 populations (BX, DJC, HL, JST, SC, TL, WC, and YBL). For each individual tree of the selected populations, 15 kernels were randomly selected. The composition was assessed according to established national standard for water content (State Food and Drug Administration, 2016c), fatty acids (State Food and Drug Administration, 2016b), amino acids (State Food and Drug Administration, 2016a), vitamins (State Food and Drug Administration, 2016e,f), and trace elements (State Food and Drug Administration, 2016d). Three replicates were performed.

Statistical Analysis

A nested analysis of variance was used for statistical test with the following linear model:$Y_{ijk} = \mu + S_i + T_{(i)j} + \varepsilon_{ijk}$, in which Y_{ijk} denotes the k-th observation value of the j-th individual of the i-th population, μ denotes the average of all individuals from all populations, S_i denotes the fixed population effect, $T_{(i)j}$ denotes random effect of individuals within the same population, and ε_{ijk} denotes the random error. The degree of dispersion of the traits was quantified with coefficient of variation (CV) using the formula CV (%) = $S/\bar{X} \times 100$, in which $\bar{X}$ and S denote the mean and the standard deviation, respectively. In addition, the relative range was calculated according to $R_i^{'} = R^{'}/R_0$, in which $R^{'}$ and R_0 denote the range of a single population and all populations, respectively. The phenotypic differentiation coefficient was calculated using the formula V_{ST} (%) = $\left[\delta^2_{t/s}/(\delta^2_{t/s} + \delta^2_s)\right] \times 100$, in which $\delta^2_{t/s}$ and δ^2_s denote intra-population and inter-population variance component, respectively. Equivalent latitude was calculated according to the following equation: Equivalent latitud = latitude + (Altitude − 300) × a^{-1}, in which a = 140 when the altitude is ≥ 300 or a = 200 when the altitude is < 300. All statistical analysis were performed in SPASS or R.

RESULTS

Fruit Morphological Diversity of *Juglans mandshurica*

Statistics of the 13 traits of 12 populations were summarized in **Table 1**. Overall, we found a high degree of variation for most of the traits (**Figure 2** and **Supplementary Table 2**). Traits with the highest CVs were fruit weight (15.3–55.3 g with the mean of 31.1 g, CV of 22.00%), kernel weight (0.7–3.4 g with the mean of 1.8 g, CV of 19.89%), and nut weight (4.3–16.7 g with the mean of 9.4 g, CV of 19.42%). In addition, both kernel rate (6.26–28.90% with the mean of 19.07%, CV of 14.32%) and shell thickness (2.4–10.5 mm with the mean of 5.7 mm, CV of 13.14%) also showed a high degree of variation. Traits that showed a moderate variation (CV around 10%) included nut vertical diameter (27.5–58.3 mm with the mean of 42.9 mm, CV of 10.92%), the index of roundness (0.42–0.90 with the mean of 0.64, CV of 10.52%), nut lateral diameter (17.3–38.8 mm with the mean of 27.1 mm, CV of 10.06%), index of fruit shape (0.94–2.05 with the mean of 1.41, CV of 9.81%), fruit length (36.0–71.2 mm with the mean of 53.0 mm, CV of 9.79%), and nut transverse diameter (27.5–58.3 mm with the mean of 42.9 mm, CV of 9.51%). Finally, traits that showed the lowest variation were fruit width (25.1–51.8 mm with the mean of 38.0 mm, CV of 8.85%) and the mean diameter (24.4–40.7 mm with the mean of 32.1 mm, CV of 8.26%). To further appreciate the variation in fruit morphology, fruit length was selected as a representative (moderate level of CV) for visualization (**Figure 3**). A broad distribution was found in each population with a large degree of overlapping among groups. Similarly, other traits also showed a wide distribution (**Supplementary Figure 1**).

Inter-Group and Intra-Group Variations of Fruit Morphology

The overall variation among the 12 populations prompted us to investigate the source of the diversity. A nested ANOVA analysis revealed both inter-group and intra-group variations are significant (p < 0.01, **Supplementary Table 3**), indicating a high degree of diversity within and among the 12 populations. To discern the components contributing to the total phenotypic variance, we calculated the variance component and phenotypic differentiation coefficient (**Table 2**). We found that the total variation can be explained by inter-group variation (66.64%), random error (20.96%), and intra-group variation (12.40%), suggesting that within-population variation is the major contributor of fruit morphological diversity. In addition, phenotypic differentiation coefficient ranged 2.17% (index of roundness) to 31.17% (shell thickness) for the 13 traits with an average of 15.62%. We next evaluated the CVs of the 13 fruit morphological traits for each of the 12 populations (**Supplementary Table 4**). Consistent with the variance analysis, we found a high level of variation within individual populations (F = 49.98, p < 0.01). By contrast, similar CVs were observed among different populations for most of the 12 traits.

Analysis of the range of fruit morphology separated the traits into two groups (**Supplementary Table 5**). The first group included 11 out of 13 traits (expect nut weight and kernel rate) with a broad distribution of the relative range (e.g., R' of 83.60 and 82.60% for BX and DFH; and R' of 53.70 and 54.51% for HC and JST). The second group included nut weight and kernel rate, with the highest and smallest values for the relative range observed in the populations JY/JST ($R^{'}$ of 65.54%/65.01%) and HC (R' of 35.89%), respectively. Furthermore, variance analysis of the relative range revealed a significant intra-population difference (F = 8.992). In addition, multiple comparison analysis showed that the 12 populations can be classified into three groups

TABLE 1 | Summary of 13 fruit morphological traits of 12 *J. mandshurica* populations.

Fruit morphology	Max	Min	*R*	Mean	*SD*	CV (%)
Fruit length (mm)	71.2	36.0	35.2	53.0	5.2	9.79
Fruit width (mm)	51.8	25.1	26.7	38.0	3.4	8.85
Index of fruit shape	2.05	0.94	1.11	1.40	0.14	9.81
Fruit weight (g)	55.3	15.3	39.9	31.1	6.8	22.00
Nut vertical diameter (mm)	58.3	27.5	30.8	42.9	4.7	10.92
Nut transverse diameter (mm)	37.0	14.8	22.2	27.3	2.6	9.51
Nut lateral diameter (mm)	38.8	17.3	21.4	27.1	2.7	10.06
Mean diameter (mm)	40.7	24.4	16.3	32.1	2.7	8.26
Index of roundness	0.90	0.42	0.48	0.64	0.07	10.52
Shell thickness	10.5	2.4	8.1	5.7	0.8	13.14
Nut weight (g)	16.7	4.3	12.4	9.4	1.8	19.42
Kernel weight (g)	3.4	0.7	2.7	1.8	0.4	19.89
Kernel rate (%)	28.90	6.26	22.64	19.07	2.73	14.32

CV, covariance efficiency; Max, maximum; Min, minimum; R, range; SD, standard deviation.

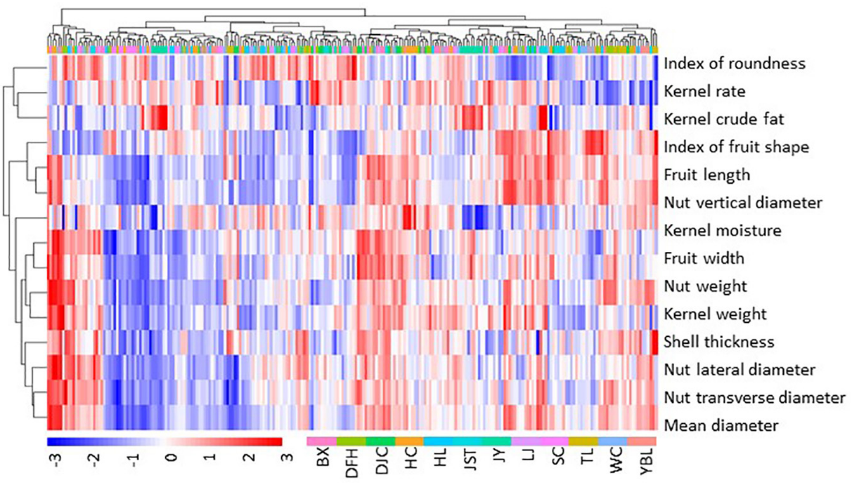

FIGURE 2 | Heatmap of fruit morphology. Fruit morphology data were scaled by subtracting the mean. Thus, positive values indicate above the mean (red), and negative values indicate below the mean (blue). Data of individual trees from each site, color coded as shown in the bottom of the figure, were used. BX, Binxian, HLJ; DFH, Raohe, HLJ; DJC, Ning'an, HLJ; HC, Hunchun, JL; HL, Hulin, HLJ; JST, Yichun, HLJ; JY, Jiayin, HLJ; LJ, Linjiang, JL; SC, Baishan, JL; TL, Tieli, HLJ; WC, Wuchang, HLJ; YBL, Yabuli, HLJ. HLJ, Heilongjiang province; JL, Jilin province.

based on the relative range (large to small): group 1 (BX, DFH, SC, and TL), group 2 (WC, DJC, YBL, HL, LJ, and JY6), and group 3 (JST and HC).

Nutritional Analysis of *Juglans mandshurica* Kernel

We performed a comprehensive analysis of the kernel components including crude fat, fatty acids (FAs), amino acids (AAs), vitamins, and micronutrients (**Figure 4** and **Table 3**). In line with the variations observed in fruit morphology, we also found a broad range of diversity in the kernel components. In addition, individuals within the same group did not show any grouping pattern, indicating a high degree of within-group variation. The water content was 1.72–10.97% (mean of 6.49%; CV of 20.52%), and the crude fat was 42.32–73.35% (mean of 56.05%; CV of 7.21%). Ten FAs were detected, with linoleic acid (mean of 64.02%, CV of 4.20%), oleic acid (mean of 19.35%, CV of 17.76%) and α-linolenic acid (mean of 12.24%, CV of 17.95%) accounting for 95.61% of the total FA amount. While the content of saturated FAs ranged from 3.09 to 5.05% (mean of 3.99%), unsaturated FAs were 94.95–96.91% (mean of 96.01%; monounsaturated FAs: 13.00–30.00% with the mean of 19.70%; polyunsaturated FAs: 66.00–83.00% with the mean of 76.31%). In addition, the ω-6/ω-3 ratio was between 3.29 and 7.54 (mean of 5.40), indicating that these unsaturated FAs can be easily be absorbed by human. Remarkably, human essential FAs accounted for 65.58–83.27% (mean of 76.26%) of total FAs in *J. mandshurica* kernel.

A total of 16 amino acids were quantified in the kernel (15.19–36.75 g, mean of 24.59 g, per 100 g of kernel). The content for essential amino acids was 4.10–13.39 g. The content of each

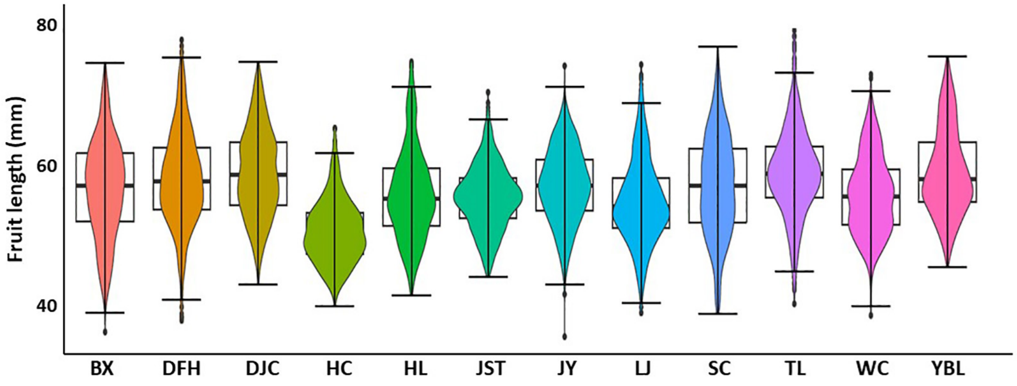

FIGURE 3 | Boxplot showing the distribution of fruit length in 12 *J. mandshurica* populations. BX, Binxian, HLJ; DFH, Raohe, HLJ; DJC, Ning'an, HLJ; HC, Hunchun, JL; HL, Hulin, HLJ; JST, Yichun, HLJ; JY, Jiayin, HLJ; LJ, Linjiang, JL; SC, Baishan, JL; TL, Tieli, HLJ; WC, Wuchang, HLJ;YBL, Yabuli, HLJ. HLJ, Heilongjiang province; JL, Jilin province.

TABLE 2 | Variance component and phenotypic differentiation coefficient by fruit morphology.

Fruit morphology	Variance component			Percentage variation (%)			Phenotypic differentiation coefficient (%)
	Inter-group	Intra-group	Random error	Inter-group	Intra-group	Random error	
Fruit length	3.2669	18.0335	6.3173	11.83	65.30	22.87	15.34
Fruit width	1.2608	7.1726	3.1015	10.93	62.18	26.89	14.95
Index of fruit shape	0.00029	0.01282	0.00593	1.52	67.32	31.15	2.21
Fruit weight	9.7818	35.8845	2.5556	20.28	74.42	5.30	21.42
Nut vertical diameter	2.6650	15.7618	3.9913	11.89	70.31	17.80	14.46
Nut transverse diameter	0.6472	4.9888	1.0927	9.62	74.14	16.24	11.48
Nut lateral diameter	1.0249	5.3737	1.1546	13.57	71.15	15.29	16.02
Mean diameter	1.2563	4.9906	0.9882	17.36	68.98	13.66	20.11
Index of roundness	0.00008	0.00356	0.00091	1.74	78.30	19.96	2.17
Shell thickness	0.1222	0.2698	0.1751	21.55	47.58	30.87	31.17
Nut weight	0.5751	2.2291	0.6765	16.52	64.04	19.44	20.51
Kernel weight	0.0154	0.0786	0.0356	11.86	60.67	27.47	16.35
Kernel rate	0.00010	0.00048	0.00020	12.56	61.93	25.51	16.86
Mean				12.40	66.64	20.96	15.62

amino acid was between 0.01 and 9.80 g with CVs ranging from 12.44% (Asp) to 126.56% (Pro). Three vitamins were measured (per 100 g of kernel) and showed variations in the following order: vitamin E (0.19–2.98 μg, mean of 0.96 μg, CV of 62.09%), vitamin A (95.38–445.32 μg, mean of 218.64 μg, CV of 37.76%), and vitamin B6 (1.96–9.66 mg, mean of 4.55 mg, CV of 37.23%). The content of micronutrients (per kilograms of kernel) showed moderate variations in the following order: Fe (mean of 33.02 mg, CV of 32.14%), Ca (mean of 693.00 mg, CV of 26.81%), Zn (mean of 30.32 mg, CV of 23.19%), P (mean of 5874.74 mg, CV of 16.13%), and Mg (mean of 2710.65 mg, CV of 15.01%).

Inter- and Intra-Population Variations of Kernel Nutritional Composition

Statistical analysis of various compounds in the kennel showed that both inter- and intra-population variations were significant ($p < 0.001$, **Supplementary Table 6**). For most nutritional compositions including fatty acids, amino acids, vitamin A and E, Fe, Zn, Ca, and Mg, we found the total variation can be largely attributed to intra-population variation (**Table 4**). For instance, a much larger variance component (%) was found for within-population variation compared to among-population variation for all fatty acids such as palmitic acid (93.95% vs. 4.02%). Similarly, the within-population variation accounted for 86.58 and 80.44% for vitamin A and E, respectively. By contrast, vitamin B6 was the only one that showed a significantly higher among-population variation than within-population variation (62.06% vs. 34.87%). In addition, the micronutrient P showed a similar distribution of the two (45.67 and 46.89% for inter- and intra-population variations, respectively).

Impact of Geographical and Ecological Factors on Fruit Morphology and Nutritional Composition

Correlation analysis of the association between geographical/ecological factors and various traits quantified

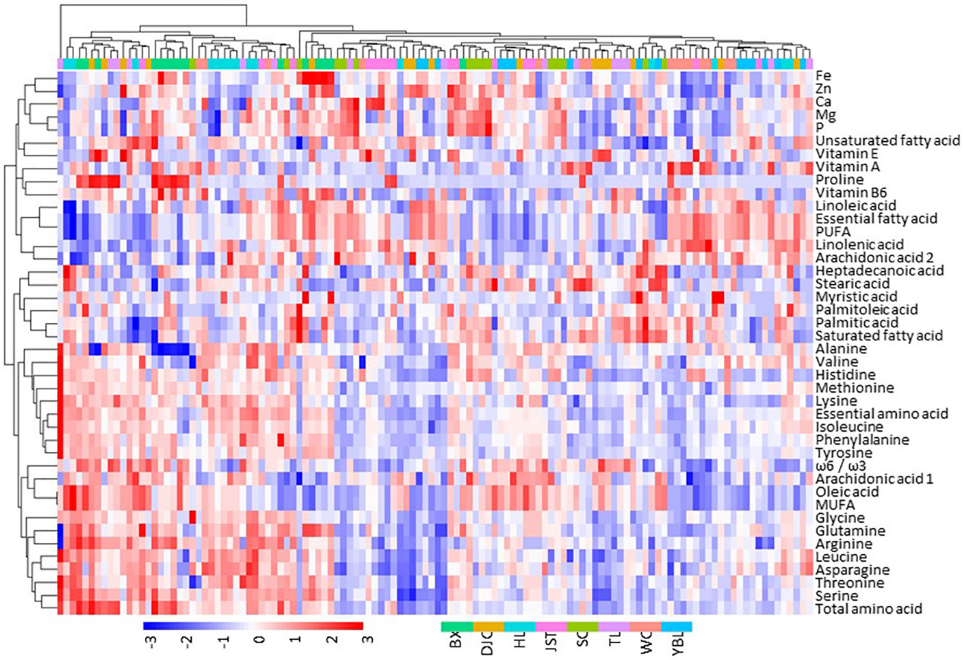

FIGURE 4 | Heatmap showing the distribution of major components in *J. mandshurica* kernel. Nutritional composition data were scaled by subtracting the mean. Thus, positive values indicate above the mean (red), and negative values indicate below the mean (blue). Data of individual trees from each site, color coded as shown in the bottom of the figure, were used. BX, Binxian, HLJ; DJC, Ning'an, HLJ; HL, Hulin, HLJ; JST, Yichun, HLJ; SC, Baishan, JL; TL, Tieli, HLJ; WC, Wuchang, HLJ;YBL, Yabuli, HLJ. HLJ, Heilongjiang province; JL, Jilin province.

here was summarized in **Supplementary Table 7**. We found that fruit morphology is strongly associated with geographical location and ecological conditions (temperature and humidity). First, the fruit length, width, weight, and mean diameter all showed a significant positive correlation with latitude, equivalent latitude, and precipitation. By contrast, the fruit weight, nut vertical diameter, mean diameter, nut weight, and shell thickness showed a significant negative correlation with longitude. Both nut vertical diameter and shell thickness also showed a positive correlation with altitude. In addition, a negative correlation was found between traits such as fruit size, fruit weight, nut size, nut weight, and nutshell thickness and temperature. For day length, no correlation was found for most traits except shell thickness (a negative correlation). Together, these data suggested a trend of change in fruit traits based on the geographic variation from southeast to northwest.

Similar analysis showed that the crude fat content is associated with equivalent latitude and temperature. Interestingly, we found different fatty acids are correlated with different geographical/ecological factors with the following association pairs: linoleic acid and longitude; α-linolenic acid and altitude and temperature; and oleic acid and humidity. For vitamins, while vitamin A showed no correlations with any geographical/ecological factor, many factors were potentially associated with vitamin B6 (longitude, equivalent latitude, temperature, humidity, and day length) and vitamin E (latitude, equivalent latitude, temperature, and humidity). Different factors were found to be associated with micronutrients such as Fe (day length), Zn (latitude, longitude, temperature, and day length), Ca (altitude and accumulated temperature), and P (temperature). Finally, there was no correlation between the content of amino acids and geographical/ecological factors. The correlation between Zn/vitamin B6 and longitude was shown in **Figure 5**.

Principle Component Analysis and Countersting of the 12 Populations

The data of both fruit morphology and kernel nutritional composition were used for PCA (**Supplementary Figures 2**, **3**). For fruit morphology, the first and second PCs explained 54.3 and 19.1% of the total variations, respectively. For kernel nutritional composition, the first and second PCs explained 36.8 and 19.3% of the total variations, respectively. No consistent pattern was found from the two PCA plots, indicating a strong influence of traits (morphology or kernel composition) on sample grouping. Consistent with this, clustering analysis also showed differential grouping patterns using different trait data (**Figure 6**). This indicates the orthogonality of different traits quantified in this study and variations within the same population.

DISCUSSION

Here, we found an overall high level of morphological diversity in *J. mandshurica*. This is consistent with a previous report in which large variations were found among six populations in the

TABLE 3 | Nutritional composition analysis of *J. mandshurica* kernel.

	Max	Min	*R*	Mean	*SD*	CV (%)
H_2O (%)	10.97	1.72	9.24	6.49	1.33	20.52
Crude fat (%)	73.35	42.32	31.03	56.05	4.04	7.21
Myristic acid (%)	0.14	0.00	0.14	0.03	0.02	73.67
Palmitic acid (%)	3.82	2.06	1.76	2.94	0.28	9.47
Heptadecanoic acid (%)	0.04	0.03	0.02	0.03	0.00	10.58
Stearic acid (%)	1.39	0.62	0.77	1.00	0.14	14.01
Saturated fatty acid (%)	5.05	3.09	1.96	3.99	0.33	8.31
Linoleic acid (%)	69.05	55.62	13.43	64.02	2.57	4.02
Alpha-linolenic acid (%)	18.82	8.42	10.40	12.24	2.20	17.95
Essential fatty acids (%)	83.27	65.58	17.69	76.26	3.47	4.55
Arachidonic acid (%)	0.07	0.03	0.04	0.05	0.01	14.95
Polyunsaturated fatty acid (%)	83.00	66.00	18.00	76.31	3.47	4.55
Palmitonoic acid (%)	0.15	0.04	0.11	0.06	0.01	22.43
Oleic acid (%)	29.70	12.42	17.28	19.35	3.44	17.76
Arachidonic acid (%)	0.37	0.21	0.17	0.29	0.03	11.00
Monounsaturated fatty acid (%)	30.00	13.00	17.00	19.70	3.46	17.54
Unsaturated fatty acid (%)	96.91	94.95	1.96	96.01	0.33	0.35
ω-6/ω-3	7.54	3.29	4.25	5.40	0.97	17.93
Thr (g/100 g)	1.47	0.39	1.09	0.78	0.12	15.76
Val (g/100 g)	2.11	0.14	1.97	1.03	0.20	19.05
Ile (g/100 g)	2.66	0.08	2.58	0.82	0.20	23.75
Leu (g/100 g)	2.92	0.75	2.17	1.78	0.24	13.78
Phe (g/100 g)	3.80	0.44	3.37	1.04	0.28	26.61
Lys (g/100 g)	1.39	0.12	1.26	0.54	0.12	22.14
Asp (g/100 g)	3.57	1.91	1.66	2.68	0.33	12.44
Ser (g/100 g)	1.88	0.68	1.20	1.31	0.20	15.58
Glu (g/100 g)	7.41	2.24	5.17	5.07	0.88	17.26
Gly (g/100 g)	3.25	0.60	2.65	1.20	0.25	21.21
Ala (g/100 g)	1.81	0.07	1.74	0.92	0.29	31.86
Met (g/100 g)	2.45	0.01	2.44	0.35	0.22	61.79
Tyr (g/100 g)	2.40	0.24	2.16	0.60	0.20	32.67
His (g/100 g)	2.11	0.38	1.73	0.73	0.18	25.20
Arg (g/100 g)	5.76	0.36	5.40	3.76	0.72	19.17
Pro (g/100 g)	9.80	0.06	9.74	1.99	2.52	126.56
Essential amino acid (g/100 g)	13.39	4.10	9.29	5.99	0.99	16.46
Amino acid (g/100 g)	36.75	15.19	21.57	24.59	4.32	17.58
Vitamin B6 (mg/100 g)	9.66	1.96	7.70	4.55	1.69	37.23
Vitamin E (μg/100 g)	2.98	0.19	2.79	0.96	0.59	62.09
Vitamin A (μg/100 g)	445.32	95.38	349.94	218.64	82.56	37.76
Fe (mg/kg)	81.82	11.69	70.13	33.02	10.61	32.14
Zn (mg/kg)	50.64	14.17	36.47	30.32	7.03	23.19
Ca (mg/kg)	1383.00	263.70	1119.30	693.00	185.81	26.81
Mg (mg/kg)	3748.00	1616.00	2132.00	2710.65	406.81	15.01
P (mg/kg)	8781.00	3403.00	5378.00	5874.74	947.43	16.13

CV, covariance efficiency; Max, maximum; Min, minimum; R, range; SD, standard deviation.

eastern Liaoning province (Zhang et al., 2021). For most traits, the level of variation is comparable between the two studies, as evidenced by similar CVs for traits such as nut weight (18.37 and 19.42% for the previous and this study, respectively, same order hereafter), kernel weight (19.42 and 19.89%), kernel rate (14.52 and 14.32%), and roundness index (9.92 and 10.52%). The only exception was shell thickness, which showed a CV of 25.11% in the previous report, but a moderate variation in our study (CV of 13.14%). Thus, a higher level of variation may exist in specific *J. mandshurica* populations. In addition, the variation observed here in *J. mandshurica* is also commensurate with findings in other Juglans species. For instance, significant variation was found for traits such as nut weight (8.0–23.0 g), kernel weight (4.0–14.0 g), kernel rate (40.0–72.2%), and shell

TABLE 4 | Variance component and phenotypic differentiation coefficient by kernel components.

Nutritional compositions	Variance component			Percentage variation (%)			Phenotypic differentiation coefficient (%)
	Inter-group	Intra-group	Random error	Inter-group	Intra-group	Random error	
H_2O	5.37E-05	9.50E-05	2.56E-05	30.79	54.51	14.70	36.10
Crude fat	2.50E-04	1.12E-03	1.87E-04	16.02	72.02	11.96	18.19
Myristic acid	3.74E-09	3.52E-08	5.07E-09	8.49	79.98	11.53	9.59
Palmitic acid	3.13E-07	7.33E-06	1.58E-07	4.02	93.95	2.03	4.10
Heptadecanoic acid	6.33E-11	7.24E-11	4.34E-10	11.12	12.72	76.16	46.65
Stearic acid	4.56E-07	1.57E-06	8.58E-09	22.45	77.12	0.42	22.55
Saturated fatty acid	2.70E-07	1.06E-05	1.89E-07	2.43	95.87	1.70	2.47
Linoleic acid	8.63E-05	5.71E-04	5.53E-06	13.02	86.14	0.83	13.13
Alpha-linolenic acid	1.05E-04	3.93E-04	4.27E-07	21.01	78.90	0.09	21.03
Essential fatty acids	5.08E-05	1.17E-03	3.34E-06	4.14	95.59	0.27	4.15
Arachidonic acid	2.67E-10	4.88E-10	2.30E-09	8.73	15.95	75.32	35.37
Polyunsaturated fatty acid	5.07E-05	1.18E-03	3.33E-06	4.12	95.61	0.27	4.13
Palmitonoic acid	7.65E-10	1.06E-08	3.35E-09	5.20	71.99	22.80	6.74
Oleic acid	5.95E-05	1.15E-03	2.67E-06	4.93	94.85	0.22	4.94
Arachidonic acid	1.09E-08	9.42E-08	2.76E-09	10.14	87.30	2.56	10.41
Monounsaturated fatty acid	6.23E-05	1.16E-03	2.75E-06	5.10	94.68	0.23	5.11
Unsaturated fatty acid	2.70E-07	1.07E-05	1.90E-07	2.43	95.86	1.71	2.47
ω-6/ω-3	2.87E-01	6.80E-01	2.16E-03	29.59	70.19	0.22	29.65
Thr	0.0029	0.0110	0.0019	18.10	69.88	12.02	20.57
Val	0.0001	0.0309	0.0073	0.19	80.79	19.02	0.23
Ile	0.0033	0.0328	0.0029	8.50	84.13	7.36	9.18
Leu	0.0062	0.0456	0.0088	10.30	75.16	14.55	12.05
Phe	0.0083	0.0610	0.0086	10.64	78.28	11.08	11.97
Lys	0.0014	0.0106	0.0026	9.58	72.94	17.48	11.61
Asp	0.0102	0.0812	0.0196	9.20	73.15	17.65	11.18
Ser	0.0111	0.0297	0.0032	25.11	67.58	7.31	27.09
Glu	0.1944	0.4916	0.1077	24.49	61.94	13.57	28.34
Gly	0.0044	0.0552	0.0058	6.68	84.47	8.85	7.32
Ala	0.0175	0.0552	0.0105	21.02	66.41	12.57	24.04
Met	0.0046	0.0407	0.0016	9.88	86.74	3.39	10.22
Tyr	0.0044	0.0318	0.0031	11.25	80.88	7.86	12.21
His	0.0073	0.0257	0.0030	20.33	71.47	8.20	22.15
Arg	0.0818	0.3639	0.0801	15.55	69.21	15.24	18.35
Pro	1.2834	4.6754	0.1314	21.07	76.77	2.16	21.54
Essential amino acid	0.1008	0.8350	0.0549	10.18	84.29	5.54	10.77
Amino acid	5.6496	12.8046	0.9626	29.10	65.95	4.96	30.61
Vitamin B6	1.92	1.08	0.10	62.06	34.87	3.07	64.03
Vitamin E	0.067	0.280	0.001	19.28	80.44	0.28	19.33
Vitamin A	890.32	5922.44	27.96	13.02	86.58	0.41	13.07
Fe	43.32	58.71	9.69	38.77	52.55	8.67	42.46
Zn	20.27	26.59	5.45	38.75	50.83	10.42	43.26
Ca	6479.8	24731.8	3649.9	18.59	70.94	10.47	20.76
Mg	69292.9	92539.9	14248.3	39.35	52.56	8.09	42.82
P	448347.0	436633.5	71144.1	46.89	45.67	7.44	50.66

thickness (0.1–3.0 mm) in six populations of *J. regia* L (Khadivi-Khub et al., 2015). Similar morphological variations were found in other *J. regia* L populations in the mountain regions of central Iran (Arzani et al., 2008), northern Iran (Alinia-Ahandani et al., 2014), Turkey (Balci et al., 2001), and the Himalayan region (Shah et al., 2021).

We also found that within-population variation can explain much of the total variation for most fruit traits, indicating a high level of variability among individuals. Consistent with this finding, a significant portion of the total variation in nutritional composition can be attributed to within-population variation. In fact, it accounted for > 80% for major kernel compounds

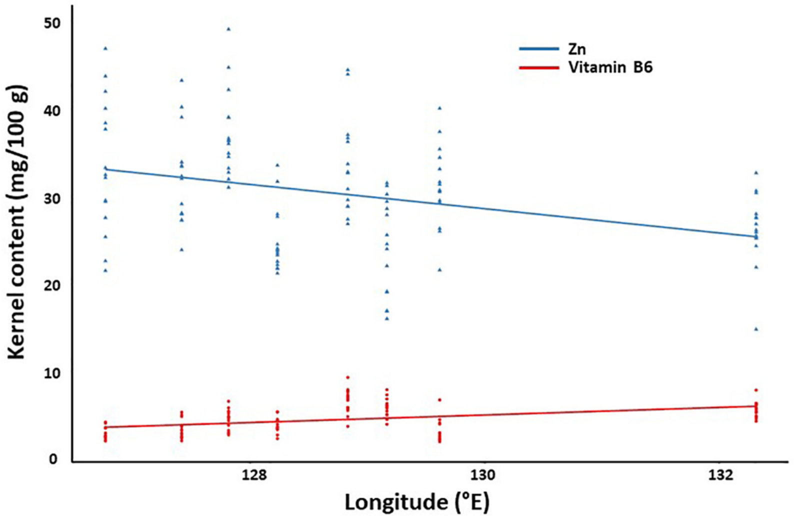

FIGURE 5 | Correlation between kernel components and geographical factors. The content of zinc and vitamin B6 were plotted against the longitude as a representative.

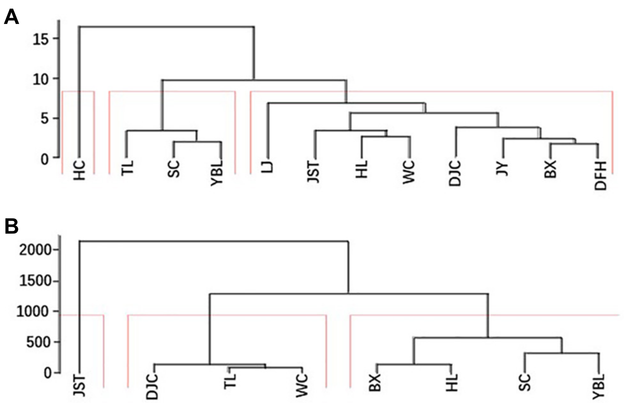

FIGURE 6 | Two clustering maps. For each site, the average of all individuals was sued for clustering. **(A)** Clustering using morphological data. **(B)** Clustering using kernel nutritional compositions. BX, Binxian, HLJ; DJC, Ning'an, HLJ; HL, Hulin, HLJ; JST, Yichun, HLJ; SC, Baishan, JL; TL, Tieli, HLJ; WC, Wuchang, HLJ;YBL, Yabuli, HLJ. HLJ, Heilongjiang.

including fatty acids (palmitic acid, linoleic acid, oleic acid, and arachidonic acid, amino acids (threonine, valine, and glycine), and vitamins (vitamin E and A). Thus, it is reasonable to expect a large degree of genetic diversity among induvial trees. Indeed, a recent genetic analysis using microsatellite markers showed a high level of individual-to-individual variation in *J. regia* L (Ebrahimi et al., 2017). The diversity among individuals within the same population was further supported by another previous study, which showed that genetic relatedness is not correlated to the geographic proximity in *J. regia* L. (Mohsenipoor et al., 2010). Future investigation is needed to validate the genetic source of variation as there is no large-scale genetic analysis in *J. mandshurica* yet.

Besides genetic control, geographical and ecological conditions are important factors in determining the growth and fruit quality of walnut (Diaz et al., 2005; Xu et al., 2016; Paź-Dyderska et al., 2021). It has been demonstrated that the interaction between genetic effects and environmental factors contribute to the heritability and thus the level of variation of many traits in walnut (Sarikhani et al., 2014). Thus, variation in the genetic background would lead to distinct interactions with the local environment, which could further result in diversity in adaption and morphological traits. In our study, we found significant correlations between environmental factors and various traits. The most significant correlation was between the content of vitamin B6 and the accumulated temperature (r = 0.507). Moreover, the overall variation trend based on the geological areas (from southeast to northeast) suggested a continuous variation, in line with previous studies that fruit

morphology variation along the latitude gradient (Zhang et al., 2017; Gao et al., 2021). However, the correlation coefficient was small for most of the statistically significant correlations (e.g., r = 0.188 for fruit length and equivalent latitude; r = 0.173 for fruit width and latitude). Thus, in addition to continuous variation, considerable regional variation also existed. This is consistent with the large degree of within-population variation for both fruit morphology and nutrient composition. Lastly, random variation also accounted for the diversity of some traits.

CONCLUSION

Our comprehensive analysis on both economically important phenotypic traits and nutritional composition revealed extensive diversity among 12 *J. mandshurica* populations. Notably, within-population variation was the main source of the total variation for most traits. Further analysis of the impact of environmental factors on multiple traits showed variation patterns along with the geographic areas and ecological gradient. Thus, the total variation by environmental factors can be dissected into continuous variation, regional variation, and random variation. Our dataset on the diversity of multiple fruit traits and kernel nutritional composition provides a foundation for future selective breeding. In addition, the large degree of variations observed here also reflects genetic diversity, which needs more investigation for further breeding programs.

DATA AVAILABILITY STATEMENT

The original contributions presented in the study are included in the article/**Supplementary Material**, further inquiries can be directed to the corresponding author/s.

AUTHOR CONTRIBUTIONS

ZL, HGZ, and KL designed the study. ZL, HXZ, BS, and WD collected samples. ZL, HXZ, WW, and JL quantified the traits and performed data analysis. ZL wrote the manuscript. HGZ revised the manuscript. All authors reviewed the manuscript.

FUNDING

This study was supported by the Heilongjiang Applied Technology Research and the Development Program Project (GA19B201). The Tree Genetics and Breeding Innovation Team of the Heilongjiang Touyan Innovation Program was thanked for generous support.

SUPPLEMENTARY MATERIAL

The Supplementary Material for this article can be found online at: https://www.frontiersin.org/articles/10.3389/fpls.2022.820457/full#supplementary-material

REFERENCES

Alinia-Ahandani, E., Ramandi, H. D., Sarmad, J., Asadi-Samani, M., Yavari, A., and Ahandani, R. A. (2014). Evaluation of morphological diversity among somepersian walnut accessions (Juglans regia L.) in Guilan, Northern Iran. *Int. J. Plant Biol. Res.* 2:8.

Arab, M. M., Marrano, A., Abdollahi-Arpanahi, R., Leslie, C. A., Askari, H., Neale, D. B., et al. (2019). Genome-wide patterns of population structure and association mapping of nut-related traits in Persian walnut populations from Iran using the Axiom J. regia 700K SNP array. *Sci. Rep.* 9:6376. doi: 10.1038/s41598-019-42940-1

Arzani, K., Mansouri-Ardakan, H., Vezvaei, A., and Roozban, M. R. (2008). Morphological variation among Persian walnut (Juglans regia) genotypes from central Iran. *N.Z. J. Crop Hortic. Sci.* 36, 159–168. doi: 10.1080/01140670809510232

Balci, I., Balta, F., Kazankaya, A., and Sen, S. (2001). Promising native walnut genotypes (Juglans regia L.) of the east black sea region of Turkey. *J. Am. Pomol. Soc.* 55:204.

Chatrabnous, N., Yazdani, N., and Vahdati, K. (2018). Determination of Nutritional Value and Oxidative Stability of Fresh Walnut. *J. Nuts* 09, 11–20.

Chen, C. M., Han, S. J., Yuan, S. S., Wang, C. J., and Yu, J. H. (2013). Isolation and characterization of 20 polymorphic microsatellite markers for Juglans mandshurica (Juglandaceae). *Appl. Plant Sci.* 1:1200009. doi: 10.3732/apps.1200009

Cho, S. H., Park, S., Jeong, H., Kim, S. R., Jeong, M. S., Choi, M., et al. (2021). Anti-Inflammatory Activity of 4-((1R,2R)-3-Hydroxy-1-(4-hydroxyphenyl)-1-methoxypropan-2-yl)-2-methoxyphenol Isolated from Juglans mandshurica Maxim. in LPS-Stimulated RAW 264.7 Macrophages and Zebrafish Larvae Model. *Pharmaceuticals* 14:771. doi: 10.3390/ph14080771

Diaz, R., Alonso, E., and Fernádez-López, J. (2005). Genetic and geographic variation in seed traits of common walnut (juglans regia l.) among twenty populations from the west of spain. *Acta Hortic.* 705, 137–141. doi: 10.17660/ActaHortic.2005.705.15

Ebrahimi, A., Zarei, A., Fardadonbeh, M. Z., and Lawson, S. (2017). Evaluation of genetic variability among "Early Mature" Juglans regia using microsatellite markers and morphological traits. *PeerJ* 5, e3834–e3834. doi: 10.7717/peerj.3834

Gao, S., Ren, Y., Masabni, J., Zou, F., Xiong, H., and Zhu, J. (2021). Influence of Geographical and Climatic Factors on Quercus variabilis Blume Fruit Phenotypic Diversity. *Diversity* 13:329. doi: 10.3390/d13070329

Geng, S., Ning, D., Ma, T., Chen, H., Zhang, Y., and Sun, X. (2021). Comprehensive Analysis of the Components of Walnut Kernel (Juglans regia L.) in China. *J. Food Qual.* 2021:9302181.

Kefayati, S., Ikhsan, A. S., Sutyemez, M., Paizila, A., Topçu, H., Bükücü, Ş. B., et al. (2019). First simple sequence repeat-based genetic linkage map reveals a major QTL for leafing time in walnut (Juglans regia L.). *Tree Genet. Genomes* 15:13. doi: 10.1007/s11295-019-1318-9

Khadivi-Khub, A., Ebrahimi, A., Mohammadi, A., and Kari, A. (2015). Characterization and selection of walnut (Juglans regia L.) genotypes from seedling origin trees. *Tree Genet. Genomes* 11:54. doi: 10.1007/s11295-015-0882-x

Lu, A. (1982). The geographical dispersal of Juglandaceae. *Acta Phytotaxon. Sin.* 20, 257–271.

Luan, F., Wang, Z., Yang, Y., Ji, Y., Lv, H., Han, K., et al. (2020). Juglans mandshurica Maxim.: a Review of Its Traditional Usages, Phytochemical Constituents, and Pharmacological Properties. *Front. Pharmacol* 11:569800. doi: 10.3389/fphar.2020.569800

Marrano, A., Sideli, G. M., Leslie, C. A., Cheng, H., and Neale, D. B. (2019). Deciphering of the Genetic Control of Phenology, Yield, and Pellicle Color in Persian Walnut (Juglans regia L.). *Front. Plant Sci.* 10:1140. doi: 10.3389/fpls.2019.01140

Mohsenipoor, S., Vahdati, K., Amiri, R., and Mozaffari, M. R. (2010). Study of the genetic structure and gene flow in Persian walnut (Juglans regia L.) using SSR markers. *Acta Hortic.* 861, 133–142. doi: 10.17660/actahortic.2010.861.17

Pakrah, S., Rahemi, M., Nabipour, A., Zahedzadeh, F., Kakavand, F., and Vahdati, K. (2021). Sensory and nutritional attributes of Persian walnut kernel influenced by maturity stage, drying method and cultivar. *J. Food Process Preserv.* 45:e15513. doi: 10.1111/jfpp.15513

Paź-Dyderska, S., Jagodziński, A. M., and Dyderski, M. K. (2021). Possible changes in spatial distribution of walnut (Juglans regia L.) in Europe under warming climate. *Reg. Environ. Change* 21:18. doi: 10.1007/s10113-020-01745-z

Pollegioni, P., Woeste, K., Chiocchini, F., Del Lungo, S., Ciolfi, M., Olimpieri, I., et al. (2017). Rethinking the history of common walnut (Juglans regia L.) in Europe: its origins and human interactions. *PloS One* 12:e0172541. doi: 10.1371/journal.pone.0172541

Qu, J., Hao, Y., Zhu, Y., Wu, C., Wang, W., and Leng, P. (2011). Studies on germplasm of Juglans by EST-SSR markers. *Acta Hortic. Sin.* 38, 441–448.

Sahin, U., and Anapali, O. (2002). Physico-Chemical and Physical Properties of some Substrates Used in Horticulture. *Eur. J. Hortic. Sci.* 67, 55–60.

Sarikhani, S., Arzani, K., and Roozban, M. R. (2014). Correlations of certain high-heritability horticultural traits in Persian walnut (Juglans regia L.). *Acta Hortic.* 1050, 61–68. doi: 10.17660/actahortic.2014.1050.6

Shah, R. A., Bakshi, P., Sharma, N., Jasrotia, A., Itoo, H., Gupta, R., et al. (2021). Diversity assessment and selection of superior Persian walnut (Juglans regia L.) trees of seedling origin from North-Western Himalayan region. *Resour. Environ. Sustain.* 3:100015. doi: 10.1016/j.resenv.2021.100015

State Food, and Drug Administration. (2016c). *Determination of Moisture in Foods*. Shenzhen: National food safety standard.

State Food, and Drug Administration. (2016b). *Determination of Fatty Acids in Foods*. Shenzhen: National food safety standard.

State Food, and Drug Administration. (2016a). *Determination of Amino Acids in Foods*. Shenzhen: National food safety standard.

State Food, and Drug Administration. (2016e). *Determination of Vitamin B6 in Foods*. Shenzhen: National food safety standard.

State Food, and Drug Administration. (2016f). *Determination of Vitamins A, D, and E in Foods*. Shenzhen: National food safety standard.

State Food, and Drug Administration. (2016d). *Determination of Trace Elements in Foods*. Shenzhen: National food safety standard.

Vahdati, K. (2014). Traditions and folks for walnut growing around the silk road. *Acta Hortic.* 1032, 19–24. doi: 10.17660/actahortic.2014.1032.1

Wang, D., Mu, C., Gao, Z., and Feng, F. (2011). ISSR analysis of genetic diversity of Juglans mandshurica Maxim. populations. *Nonwood For. Res.* 29, 22–29.

Wang, J., Wu, T., Fang, L., Liu, C., Liu, X., Li, H., et al. (2020). Peptides from walnut (*Juglans mandshurica Maxim.*) protect hepatic HepG2 cells from high glucose-induced insulin resistance and oxidative stress. *Food Funct.* 11, 8112–8121. doi: 10.1039/d0fo01753a

Woeste, K., Burns, R., Rhodes, O., and Michler, C. (2002). Thirty polymorphic nuclear microsatellite loci from black walnut. *J. Hered.* 93, 58–60. doi: 10.1093/jhered/93.1.58

Xu, Y., Han, H., Wang, H., Chen, L., Ma, Q., and Pei, D. (2016). Phenotypic and genetic diversities of nuts of walnut(Juglans regia) populations originated from seedlings in Daba mountains. *Lin Ye Ke Xue* 52, 111–119.

Zhang, H., Yang, X., Yu, M., Han, Y., and Wu, T. (2017). Variations in seed size and seed mass related to tree growth over 5 years for 23 provenances of Quercus acutissima from across China. *J. For. Res.* 28, 917–923. doi: 10.1007/s11676-017-0375-x

Zhang, L., Lu, X., Zhou, Q., and Deng, J. (2021). Morphological Variability between Geographical Provenances of Walnut Fruit (*Juglans mandshurica*) in the Eastern Liaoning Province, P.R. China. *Pol. J. Environ. Stud.* 30, 4353–4364. doi: 10.15244/pjoes/131806

Conflict of Interest: The authors declare that the research was conducted in the absence of any commercial or financial relationships that could be construed as a potential conflict of interest.

Publisher's Note: All claims expressed in this article are solely those of the authors and do not necessarily represent those of their affiliated organizations, or those of the publisher, the editors and the reviewers. Any product that may be evaluated in this article, or claim that may be made by its manufacturer, is not guaranteed or endorsed by the publisher.

Copyright © 2022 Li, Wang, Zhang, Liu, Shi, Dai, Liu and Zhang. This is an open-access article distributed under the terms of the Creative Commons Attribution License (CC BY). The use, distribution or reproduction in other forums is permitted, provided the original author(s) and the copyright owner(s) are credited and that the original publication in this journal is cited, in accordance with accepted academic practice. No use, distribution or reproduction is permitted which does not comply with these terms.
