# Supplementary material for: Provenance and family variations in early growth of Manchurian walnut (Juglans mandshurica Maxim.) and selection of superior families
Source: PLoS One. 2024 Mar 7;19(3):e0298918. doi: 10.1371/journal.pone.0298918 (PMC10919699; doi:10.1371/journal.pone.0298918)
Supplement: S1 File — (ZIP) [file pone.0298918.s004.zip › Drought stress affects on growth, water use efficiency, gas exchange and chlorophyll fluorescence of Juglans rootstocks.pdf]

Scientia Horticulturae 250 (2019) 230–235

Contents lists available at ScienceDirect

Scientia Horticulturae

journal homepage: www.elsevier.com/locate/scihorti

Drought stress affects on growth, water use efficiency, gas exchange and chlorophyll fluorescence of *Juglans* rootstocks

Binghua Liu[a,b], Jing Liang[a,b], Guimin Tang[c], Xiaofang Wang[a,b], Fangchun Liu[a], Dengchao Zhao[a,b,*]

[a] Shandong Academy of Forestry, 250014, Jinan, Shandong, China
[b] Economic Forest Products Quality Inspection Test Center of State Forestry Administration (Jinan), 250014, Jinan, Shandong, China
[c] Shandong Yingcai University, 251400, Jinan, Shandong, China

ARTICLE INFO

Keywords:
Growth rate
Photosynthesis
Photochemical efficiency
Walnut
Water deficit
Water use efficiency

ABSTRACT

Seedlings of three selected *Juglans* rootstocks (*Juglans mandshurica* Maxim, *Juglans nigra* L. and *Juglans regia* L. cv. Jizhaomian) were analyzed in terms of growth, water use efficiency (WUE), chlorophyll fluorescence and photosynthetic response to drought stress. Significant differences among seedlings of the three analyzed *Juglans* rootstocks were observed on performance in response to water deficit. *Juglans mandshurica* Maxim. and *Juglans regia* L. cv. Jizhaomian showed larger increase in WUE and smaller reduction in growth rate, chlorophyll fluorescence and gas exchange parameters than *Juglans nigra* L. under drought-stressed condition. It suggests that *Juglans mandshurica* Maxim. and *Juglans regia* L. cv. Jizhaomian are more tolerant to drought stress and more suitable as walnut rootstock in water deficit regions as compared with *Juglans nigra* L.

1. Introduction

In arid and semi-arid regions, increasing serious water deficits has become a major environmental factor adversely affecting plant growth, productivity, and survival (Shao et al., 2009). Drought triggers a wide variety of plant responses, including morphological, physiological, biochemical and molecular changes, that eventually affects plant growth and yields (Anjum et al., 2011; Zlatev and Lidon, 2012). It has been discovered that plants have developed various mechanisms for drought adaptation, including well-developed root systems, adjustments to growth rate, modifications to plant structure, more efficient water utilization, osmotic adjustment, and certain antioxidant enzyme activities (Chaves and Oliveira, 2004; McDowell et al., 2008).

Fruit trees are grown and used as food, medicine and other products to sustain and enhance human life (Ercisli et al., 2003; Oztemiz et al., 2017; Vijayan et al., 2008). Walnut (*Juglans regia* L.), one of the oldest cultivated fruit species originating from Central Asia, is commercially planted throughout the Southern Europe, Northern Africa, Eastern Asia, USA and Western South America. According to the FAO (2017), China ranked first globally in walnut production with 1.925 million tons of walnuts in shell and accounting for 47% of the world's total walnut production. Walnut tree needs large amount of water for optimum growth and productivity and is generally sensitive to drought stress (Gauthier and Jacobs, 2011; Jerszurki et al., 2017; Sun et al., 2011). In China, it is mainly cultivated in arid and semiarid regions, the increasing serious water deficit in those areas is one of the major limitations to walnut cultivation, and thus the capacity of walnut tree to withstand drought stress is of great economic importance. Grafting superior and commercial cultivars onto rootstocks capable of reducing the effect of water stress is considered to be a promising tool to reduce water losses in production, to enhance drought resistance and to improve water-use efficiency (WUE) under drought conditions (Schwarz et al., 2010). As reviewed by Warschefsky et al. (2016), rootstocks are widely used for vegetative propagation of woody perennial trees (e.g., *Juglans*, *Citrus*, *Malus*, *Prunus*, *Coffea*) in agriculture, horticulture, and silviculture.

Recently, great progress has been achieved in breeding for high stress-tolerance walnut rootstocks (Grauke and Thompson, 2003;

Abbreviations: BGR, relative basal diameter growth rate; C_i, intercellular CO_2 concentration; C_{chl}, chlorophyll content; F_m, maximum chlorophyll fluorescence yield of the dark-adapted state; F_v, variable fluorescence; F_s, fluorescence in stable status; F'_v, variable fluorescence of the light-adapted state; F'_m, maximum chlorophyll fluorescence yield of the light-adapted state; F'_0, minimal fluorescence of the light-adapted state; F_v/F_m, maximal photochemical efficiency; F_v'/F_m', excitation energy capture efficiency of PSII reaction centres; g_s, stomatal conductance; HGR, relative height growth rate; P_n, photosynthetic rate; qP, photochemical quenching; RGR, plant relative growth rate; T_r, transpiration rate; WUE, water-use efficiency; WUE_I, intrinsic water-use efficiency; ΦPSII, effective PSII quantum yield

* Corresponding author at: 42, East Wenhua Road, Shandong Academy of Forestry, 250014, Jinan, Shandong, China.
E-mail address: zdc-1@163.com (D. Zhao).

https://doi.org/10.1016/j.scienta.2019.02.056
Received 22 November 2018; Received in revised form 15 February 2019; Accepted 17 February 2019
Available online 23 February 2019
0304-4238/ © 2019 Published by Elsevier B.V.

Hackett et al., 2010; Leslie and McGranahan, 2014; Vahdati et al., 2009). Many different types of rootstocks are being used for walnut cultivation and each has a particular set of advantages and limitations for adaptation to different geographical regions. For example, black walnut (*Rhysocaryon,*) such as northern California black walnut (*J. hindsii*), 'Paradox' hybrids (*J. hindsii* × *J. regia*), and French hybrids (*J. regia* × *J. major* or *J. regia* × *J. nigra*) are often been used as rootstock to increase vigor, productivity and resistance to drought and soil-borne diseases (Baumgartner et al., 2013; Browne et al., 2015). Manchurian walnut (*Juglans mandshurica* Maxim.), one of the rare tree species in Northeast China, is often been used as vigorous rootstock to enhance drought and cold tolerance, and to improve plant growth (Tian et al., 2010). Chinese wingnut (*Pterocarya stenoptera*) is often been used as walnut rootstock to enhance resistant to *phytophthora* and waterlogging injury (Browne et al., 2011). *Juglans regia* L. cv. Jizhaomian is a unique wild resource of walnut best adapted to soil and climate of Shandong province China, and often been used as rootstock by local farmers. Gauthier and Jacobs (2011) proposed that the effects of *Juglans nigra* L. and *Juglans mandshurica* Maxim. on physiological responses to water deficit were mainly attribute to their deep root system, stomatal sensitivity to humidity, and drought-induced leaf abscission habit.

In the present study, we compared the performance of three *Juglans* rootstock (*Juglans nigra* L., *Juglans mandshurica* Maxim. and *Juglans regia* L. cv. Jizhaomian) seedlings under drought stress conditions. Our objective was to analyze their differences in growth, water use efficiency, chlorophyll fluorescence and photosynthetic response to drought stress. Such knowledge could assist growers in solving current practical problems in walnut tree management, and provide useful information about the selection of proper walnut rootstocks that are most appropriate for arid and semi-arid regions of China.

2. Materials and methods

2.1. Plant material and experimental design

One-year-old seedlings of three *Juglans* rootstocks, including *Juglans mandshurica* Maxim, *Juglans nigra* L. and *Juglans regia* L. cv. Jizhaomian, grown from seed were used as experimental materials. All plants were greenhouse-grown in plastic pots (38 cm × 23 cm, the pot volume was adequate for root growth) filled with a local topsoil : sand : grass peat mix (5 : 1 : 1, v : v : v) (pH = 7.46) at the plant nursery of Shandong Academy of Forestry, Jinan (36°40′ N, 117°00′ E), Shandong Province, China. Plants were grown without supplementary illumination with night and day temperatures at 20 to 25 ℃ and relative humidity at 65–80 %. Prior to the start of our experiments, all trees were irrigated daily and supplied weekly with 100% Hoagland's solution (pH = 6.5). After two months of growth under well-watered conditions, 30 uniform seedlings of each rootstock were chosen to initiate drought stress treatment on 12 May 2014. Two watering regimes were implemented over a 60-d period: 1) well-watered (control), in which 15 seedlings of each rootstock were irrigated every other day to 80% field capacity; and 2) drought stress, with 15 seedlings of each rootstock being maintained at 50% field capacity by irrigating every other day. All treatments were applied at 18:00 HR. Soil field capacities were determined with a digital moisture recorder (ZTS-II; Zhejiang, China) and the volume of water added to each pot was recorded for WUE calculations. To avoid edge effects, all pots were rotated weekly. These experiments were terminated on 12 July 2014.

Surface evaporation was minimized by covering the potting media surface with a 3-cm layer of sieved (2 mm) sand. As a control, six pots without plants per treatment were used to determine evaporative water loss from the soil surface throughout the experimental period. Transpiration water loss was evaluated gravimetrically by weighing all pots and calculating the changes in weight that occurred between watering events. The amount of water lost via transpiration was then added back to each pot during irrigation.

2.2. Measurement of physiological parameters

2.2.1. Growth

At the end of the experiment, the final plant height and basal diameter were recorded from six five-tree replicates of each treatment and rootstock. Plant height was measured from the base of the stem, at soil level, to the terminal bud of the main stem; Basal diameter was measured with a digital micrometer (0.001 mm) at the soil surface. After harvesting, plants materials were oven-dried at 70 ℃ to a constant weight and the final total dry biomass was then recorded. Relative height growth rate (HGR), relative basal diameter growth rate (BGR) and relative growth rate (RGR) were calculated by the standard formulas: $HGR = (lnH_F - lnH_I) / t$, $BGR = (lnB_F - lnB_I) / t$, $RGR = (lnW_F - lnW_I) / t$, where H_I, B_I and W_I are the initial height, basal diameter, and dry biomass, respectively; H_F, B_F and W_F are the final height, basal diameter, and dry biomass, respectively; and t is the time interval.

2.2.2. Long-term water-use efficiency

We defined long-term water-use efficiency (WUE_L) as the ratio of dry biomass produced to total water transpired during the experimental period. WUE_L was calculated as: $WUE_L = (W_F - W_I) / W_W$, where W_F, W_I and W_W are the final dry mass, the initial dry mass, and the total amount of water (weight) transpired during the period, respectively.

2.2.3. Chlorophyll content (C_{chl})

Chlorophyll was extracted from 0.5 g fresh leaf materials for 72 h in the dark using acetone (80%). Absorbances at 647 and 664 nm were determined with a Shimadzu UV-/vis spectrophotometer (Model UV2401PC, Shimadzu, Riverwood Drive, Columbia, MD, USA) and used to calculate leaf chlorophyll content according to Guerfel et al. (2009). Mean values for C_{chl} recorded at the six time points were used to demonstrate the effect of rootstock and soil water over the experimental period.

2.2.4. Gas exchange

Gas exchange parameters were measured every 10 days on the fifth leaf from the shoot apex using a Li-Cor 6400 portable photosynthesis system (Li-Cor Inc., Lincoln, NE, USA). Net photosynthetic rate (P_n), transpiration rate (T_r), stomatal conductance (g_s) and intercellular carbon dioxide concentration (C_i) were obtained from six plants per treatment. Measurements were made on sunny days (9:00 to 11:00 h) at 1500 μmol m^{-2} s^{-1} PPFD, as provided by a Q-Beam (blue and red diode) light source. Leaf temperature, ambient water vapor pressure, and CO_2 concentration were maintained at 28.7 ± 1.0 ℃, 1.30 ± 0.15 kPa, and 380 μmol m^{-2} s^{-1}, respectively. Instantaneous water use efficiency (WUE_I) was calculated as the ratio between net photosynthesis and transpiration ($WUE_I = P_n / T_r$).

2.2.5. Chlorophyll fluorescence

Chlorophyll fluorescence was measured with an integrating fluorescence fluorometer (LI-6400-40 leaf chamber fluorometer, Li-Cor). After the samples were adapted to darkness for 1 h, the minimum fluorescence (F_0) was measured with weak modulated irradiation (< 0.1 μmol m^{-2} s^{-1}). A 600 ms saturating flash (> 7000 μmol m^{-2} s^{-1}) was applied to determine the maximum chlorophyll fluorescence yield (F_m), variable fluorescence (F_v) and maximum photochemical efficiency (F_v/F_m). Immediately afterwards, the leaf was continuously irradiated with red–blue actinic beams (1400 μmol m^{-2} s^{-1}) and equilibrated for 30 min to record fluorescence in stable status (F_s). Following this irradiation, another saturation flash (> 6000 μmol m^{-2} s^{-1}) applied to determine variable fluorescence (F'_v) and maximum chlorophyll fluorescence yield (F'_m) in the light. After the flash, actinic irradiation was removed, far-red irradiation was given, and minimal fluorescence in the light (F'_0) was recorded. Effective PSII quantum yield (ΦPS_{II}), photochemical quenching (qP) and excitation energy capture efficiency of PSII reaction centers (F'_v/F'_m) were calculated as

$\Phi PS_{II} = (F'_m - F'_s) / F'_m$, $qP = (F'_m - F'_s) / (F'_m - F'_0)$ and $F'_v / F'_m = (F'_m - F'_0) / F'_m$), respectively.

2.3. Statistical analyses

The experiments were performed using a completely randomized design. All the measurements were conducted in sextuplicate. Data were presented as mean ± standard deviation (SD). Statistical analysis was carried out using the SPSS-13.0 for Windows statistical software package (Standard released version 13.0 for Windows; SPSS Inc., IL, USA). Analyses of two-way variance (ANOVA) were used to evaluate the effects of rootstock and drought treatment. Tukey's HSD (honestly significant difference) post hoc test ($P \leq 0.05$) was performed to test the existence of statistical differences between different walnut rootstocks under well-watered and drought-stressed conditions.

3. Results and discussion

3.1. Growth

Plant growth is one of the most fundamental processes of vegetable kingdom, being remarkably influenced by environmental variables including water changing factor. Growth rate of plant is always been used as an important indicator of plant vitality and of plant reaction to environmental stress (Dobbertin, 2005). Generally, drought stress exhibited an inhibitory effect on the growth rate of plants (Skirycz and Inzé, 2010; Tardieu et al., 2011; Zlatev and Lidon, 2012). In the present study, drought treatment had significant ($P \leq 0.001$) negative effects on RGR, HGR and BGR of the analyzed *Juglans* rootstock seedlings (Table 1), that is consistent with researches on *Malus* (Liu et al., 2012a, 2012b) and *Poplar* (Yin et al., 2005).

In addition, significant ($P \leq 0.05$) genotypic differences in growth rate for drought response were observed (Table 1). *Juglans regia* L. cv. Jizhaomian showed the smallest variation in RGR (30.30%), HGR (38.33%) and BGR (40.00%), followed by *Juglans mandshurica* Maxim. (the variation of RGR, HGR and BGR were 57.04%, 40.45% and 75.00%, respectively) and *Juglans nigra* L. (the variation of RGR, HGR and BGR were 65.26%, 54.37% and 80.00%, respectively) (Table 1). It suggested that *Juglans regia* L. cv. Jizhaomian was the most drought-tolerant genotype that can be used as rootstock to enhance walnut drought-tolerance in water deficit regions. The large variation of growth rate response to drought stress is mainly due to the genetic factors may be valuable for breeding programs or cultivation under certain conditions (Aletà et al., 2009; Vahdati et al., 2009).

3.2. Water-use efficiency

WUE is a functional characteristic related to plant growth, productivity, and performance and is often been used as an important index of plant adaptability to water limited conditions. It was reported that WUE can be improved by keeping plant under a certain degree of soil water deficit (Ma et al., 2010). Our research showed that a water deficit (i.e., 50% field capacity) caused values for WUE_L ($P \leq 0.001$) and WUE_I ($P \leq 0.05$) to rise significantly in all seedlings of the three *Juglans* rootstocks (Tables 1 and 2). It possibly due to a decrease in g_s (Table 2) that has been detected in our previous studies on *Malus* (Liu et al., 2012a, 2012b). The extent of this response in WUE_L was greatest in *Juglans mandshurica* Maxim. (16.20%), followed by *Juglans regia* L. (15.70%) and *Juglans nigra* L. (5.86%) (Table 1). Meanwhile, *Juglans regia* L. cv. Jizhaomian showed the largest variation of WUE_I (44.22%), followed by *Juglans mandshurica* Maxim (31.03%) and *Juglans nigra* L. (5.40%) (Table 2). The results suggested that *Juglans mandshurica* Maxim. and *Juglans regia* L. showed better adaptability to drought stress than *Juglans nigra* L. Aletà et al. (2009) and Sun et al. (2011) demonstrated that genetic variation for WUE, usually assessed by carbon isotope composition, in *Juglans regia* L. was related with growth, phenology and climate of origin. Genotypic differences in WUE for drought response also have been reported on many other tree species such as *Malus* (Liu et al., 2012a, 2012b; Ma et al., 2010) and *Populus* (Monclus et al., 2009).

3.3. Gas exchange

Gas exchange has been widely used to detect the effects of stress on functioning of the photosynthetic system. As shown in Table 2, drought stress significantly hampered the gas exchange parameters including P_n, ($P \leq 0.05$) T_r ($P \leq 0.01$), g_s ($P \leq 0.05$) and C_i ($P \leq 0.05$) of seedlings of the three *Juglans* rootstocks. It could be due to decrease in leaf expansion, impaired photosynthetic apparatus, premature leaf senescence, oxidation of chloroplast lipids and changes in structure of pigments and proteins (Anjum et al., 2011; Zlatev and Lidon, 2012). The decreased C_i may inhibit carbon uptake and, ultimately, growth (Earl, 2002). For all genotypes, g_s was affected more than P_n by water stress, and that WUE_I increased when less water was available. This indicated an optimization of carbon uptake versus water loss. Moreover, the greater reduction in g_s demonstrated that non-stomatal components could play an important role in limiting photosynthesis when plants undergo prolonged water deficit in the field (Earl, 2002). It suggests that g_s might be used as an indicator of WUE under water limited

Table 1
Influence of drought on growth rate and water-use efficiency of different *Juglans* rootstock seedlings.

Rootstocks		RGR (mg day^{-1})	HGR (cm day^{-1})	BGR (mm day^{-1})	WUE (g L^{-1})
Juglans mandshurica Maxim.	Control	1.12 ± 0.02^{ab}	0.89 ± 0.01^{b}	0.04 ± 0.01^{bc}	53.22 ± 3.42^{b}
	Drought	0.48 ± 0.01^{bc}	0.53 ± 0.01^{c}	0.01 ± 0.00^{c}	61.84 ± 5.71^{ab}
	Variation (%)	−57.04	−40.45	−75.00	16.20
Juglans regia L.	Control	1.32 ± 0.02^{a}	1.20 ± 0.02^{a}	0.10 ± 0.01^{a}	60.71 ± 3.90^{ab}
	Drought	0.92 ± 0.01^{b}	0.74 ± 0.01^{bc}	0.06 ± 0.01^{b}	70.24 ± 2.77^{a}
	Variation (%)	−30.30	−38.33	−40.00	15.70
Juglans nigra L.	Control	0.95 ± 0.01^{ab}	1.03 ± 0.01^{ab}	0.05 ± 0.01^{b}	39.74 ± 2.00^{c}
	Drought	0.33 ± 0.01^{c}	0.47 ± 0.00^{c}	0.01 ± 0.01^{c}	42.07 ± 4.50^{c}
	Variation (%)	−65.26	−54.37	−80.00	5.86
Two way ANOVA analyses					
F_R		45.18***	30.51***	12.33***	22.61***
F_D		61.83***	29.89***	11.90***	34.88***
$F_{R \times D}$		10.27*	12.33*	5.61	11.76**

Control: 80% soil field capacity, drought: 50% soil field capacity. RGR: plant relative growth rate, HGR: relative height growth rate, BGR: relative basal diameter growth rate, WUE: water−use efficiency. Values are means of six replicates ± standard deviation (SD). Small case superscript letters in the same column show statistically significant differences among different *Juglans* rootstocks for the same parameter under control and drought water regimes at $P \leq 0.05$ based on Duncan's means tests. F_R: rootstock effect, F_D: drought effect, $F_{R \times D}$: rootstock × drought interaction effect. *, **, and ***: significant at $P \leq 0.05$, 0.01, and 0.001, respectively.

Table 2
Influence of drought on chlorophyll content and gas exchanges of different *Juglans* rootstock seedlings.

Rootstock		C_{chl} (mg g^{-1})	P_n (μmol m^{-2} s^{-1})	T_r (mmol $m^{-2}s^{-1}$)	g_s (mmol $m^{-2}s^{-1}$)	C_i (μmol mol^{-1})	WUE_I (μmol $mmol^{-1}$)
Juglans mandshurica Maxim	Control	2.87 ± 0.25^{ab}	18.27 ± 1.98^{ab}	5.71 ± 1.23^{ab}	0.30 ± 0.13^{ab}	264.50 ± 11.39^{a}	3.19 ± 0.25^{bc}
	Drought	2.42 ± 0.01^{b}	12.04 ± 2.70^{bc}	2.88 ± 0.47^{c}	0.19 ± 0.08^{b}	230.12 ± 20.00^{b}	4.18 ± 0.32^{a}
	Variation (%)	−15.68	−34.10	−49.56	−36.67	−13.00	31.03
Juglans regia L.	Control	3.74 ± 0.18^{a}	19.71 ± 2.83^{a}	6.50 ± 1.19^{a}	0.34 ± 0.07^{a}	243.72 ± 9.42^{ab}	3.03 ± 0.07^{bc}
	Drought	2.82 ± 0.22^{ab}	12.81 ± 1.00^{bc}	2.93 ± 0.92^{c}	0.21 ± 0.01^{b}	204.65 ± 10.17^{bc}	4.37 ± 0.30^{a}
	Variation (%)	−24.59	−35.01	−54.92	−38.24	−16.03	44.22
Juglans nigra L.	Control	2.31 ± 0.27^{b}	16.37 ± 2.49^{ab}	5.50 ± 1.11^{ab}	0.27 ± 0.09^{ab}	190.30 ± 14.45^{bc}	2.78 ± 0.07^{c}
	Drought	1.05 ± 0.30^{c}	10.55 ± 2.13^{c}	3.60 ± 0.72^{bc}	0.13 ± 0.02^{c}	178.04 ± 6.19^{c}	2.93 ± 0.12^{c}
	Variation (%)	−54.55	−35.55	−34.55	−51.85	−6.44	5.40
Two way ANOVA analyses							
F_R		5.33^{*}	3.14^{**}	3.62^{**}	6.27^{**}	10.02^{*}	10.38^{**}
F_D		17.25^{***}	7.88^{*}	11.36^{**}	6.42^{*}	13.75^{*}	6.84^{*}
$F_{R \times D}$		6.74^{*}	10.03^{*}	2.22	9.56^{*}	12.17^{**}	3.28

Control: 80% soil field capacity, drought: 50% soil field capacity. C_{chl}: chlorophyll content, P_n: photosynthetic rate, T_r: transpiration rate, g_s: stomatal conductance, C_i: intercellular CO_2 concentration, WUE_I: intrinsic water − use efficiency. Values are means of six replicates ± standard deviation (SD). Small case superscript letters in the same column show statistically significant differences among different *Juglans* rootstocks for the same parameter under control and drought water regimes at $P \le 0.05$ based on Duncan's means tests. F_R: rootstock effect, F_D: drought effect, $F_{R \times D}$: rootstock × drought interaction effect. *, **, and ***: significant at $P \le 0.05$, 0.01, and 0.001, respectively.

conditions (Gulías et al., 2012). Differently, Rosati et al. (2006) and Cochard et al. (2002) reported that drought-induced photosynthesis inhibition in walnut (*Juglans regia* L.) was mainly due to the close of stomata which impairs gas exchange.

In addition, genotypic differences in the gas exchange parameters for drought response were observed (Table 2, $P \le 0.05$). The largest decline in values of P_n and g_s was observed on *Juglans nigra* L. (the variation of P_n and g_s was 35.55% and 51.85%, respectively) followed by *Juglans regia* L. cv. Jizhaomian (the variation of P_n and g_s was 35.01% and 38.24%, respectively) and *Juglans mandshurica* Maxim (the variation of P_n and g_s was 34.01% and 36.67%, respectively). Meanwhile, *Juglans regia* L. cv. Jizhaomian showed the largest decline in the value of T_r and C_i (the variation T_r and C_i was 54.92% and 16.03%, respectively), followed by *Juglans mandshurica* Maxim (the variation of T_r and C_i was 49.56% and 13.00%, respectively) and *Juglans nigra* L. (the variation of T_r and C_i was 34.55% and 6.44%, respectively). As a result, *Juglans mandshurica* Maxim. and *Juglans regia* L. cv. Jizhaomian showed better adaptability to drought stress than *Juglans nigra* L. as manifested by their drought response in growth and WUE analyzed above (Table 1). Substantial genotypic variations in gas exchanges under water deficit conditions also have been found on many other tree species including *Malus* (Liu et al., 2012a, 2012b; Ma et al., 2010), *Populus* (Monclus et al., 2009), *Olea* (Guerfel et al., 2009), etc.

3.4. Leaf chlorophyll content

Chlorophyll is the main component of photosynthetic pigments that are important to plants mainly for harvesting light and production of reducing powers, and the chlorophyll content of leaf (C_{chl}), an important indicator of photosynthetic capability, can directly affect photosynthetic potential and hence primary production (Gitelson et al., 2003). Reduction or no-change in leaf chlorophyll content under drought-stressed conditions has been reported in many species and the intensity depends on the duration and severity of drought (as reviewed by Anjum et al., 2011). In our study, water deficit significantly decreased leaf C_{chl} of *Juglans* seedlings (Table 2, $P \le 0.001$), that could be the main cause of inactivation of photosynthesis as manifested by the reduction of gas exchange parameters analyzed above. Correspond with the genotypic variation in P_n, significant ($P \le 0.05$) difference in C_{chl} was observed in different *Juglans* seedlings. *Juglans nigra* L. showed the largest decline in values of C_{chl} (54.55%), followed by *Juglans regia* L. cv. Jizhaomian (24.59%) and *Juglans mandshurica* Maxim (15.68%) (Table 2). Furthermore, water deficit induced reduction in C_{chl}, as a result of either slow synthesis or fast breakdown, has been considered as a typical symptom of oxidative stress (Smirnoff, 1993) and could be used as an indicators of drought tolerance for different genotypes (Guerfel et al., 2009).

3.5. Chlorophyll fluorescence

Chlorophyll fluorescence has been widely used as a fast, non-destructive and relatively simple technique to detect the effects of stress including water deficit on functioning of the photosynthetic system (Bolhar-Nordenkampf et al., 1989; Lichtenthaler and Rinderle, 1988). We observed that chlorophyll fluorescence parameters were significantly influenced by drought and *Juglans* rootstocks (Table 3, $P \le 0.01$). Under well-watered condition, *Juglans regia* L. cv. Jizhaomian showed higher values for F_v/F_m, F'_v/F'_m, qP and ΦPSII than *Juglans mandshurica* Maxim and *Juglans nigra* L. F_v/F_m of well-watered *Juglans* seedlings ranged from 0.79 to 0.85 that was in consistent with the typical value which was in the range of 0.75-0.85 for non-stressed plants (Bolhar-Nordenkampf et al., 1989). Sixty-day drought stress significantly decreased F_v/F_m, F'_v/F'_m, qP and ΦPSII; *Juglans nigra* L. induced a much greater reduction in F_v/F_m, F'_v/F'_m and qP than other two rootstock seedlings. It suggested that drought stress induced much more adverse photoinhibitory impairment in *Juglans nigra* L. than others. The decline of F_v/F_m in response to drought stress suggested that *Juglans mandshurica* Maxim more drought-tolerant than *Juglans regia* L. cv. Jizhaomian and *Juglans nigra* L. Still, the largest decline of ΦPSII was observed in *Juglans regia* L., cv. Jizhaomian followed by *Juglans mandshurica* Maxim and *Juglans nigra* L. Genotyical variarion in chlorophyll fluorescence parameters also have been studied in other species to evaluate the crop production under stress conditions (Araus et al., 1998; Baker and Rosenqvist, 2004).

4. Conclusions

Significant differences among seedlings of the three analyzed *Juglans* rootstocks were observed on growth, water use efficiency, chlorophyll fluorescence and photosynthetic response to drought stress. *Juglans mandshurica* Maxim. and *Juglans regia* L. cv. Jizhaomian showed better adaptability to drought stress than *Juglans nigra* L. and can be used as rootstock to enhance walnut drought-tolerance in water deficit regions.

However, researches on the influence of the analyzed *Juglans* rootstocks on stress response in grafted walnut (*Juglans regia* L.) tress should be carried out to further explain how rootstock affects plants' response to a variety of environmental stresses including drought. It could

Table 3
Influence of drought on chlorophyll fluorescence of different *Juglans* rootstock seedlings.

Rootstock		F_v/F_m	F_v'/F_m'	qP	ΦPSII
Juglans mandshurica Maxim	Control	0.84 ± 0.11^{a}	0.62 ± 0.01^{a}	0.75 ± 0.01^{a}	2.56 ± 0.08^{ab}
	Drought	0.79 ± 0.02^{ab}	0.57 ± 0.02^{ab}	0.74 ± 0.01^{a}	1.87 ± 0.10^{bc}
	Variation (%)	−5.95	−8.06	−1.33	−26.95
Juglans regia L.	Control	0.85 ± 0.07^{a}	0.62 ± 0.00^{a}	0.75 ± 0.03^{a}	2.73 ± 0.12^{a}
	Drought	0.78 ± 0.04^{ab}	0.59 ± 0.01^{ab}	0.73 ± 0.00^{ab}	1.99 ± 0.08^{bc}
	Variation (%)	−8.24	−4.84	−2.67	−27.11
Juglans nigra L.	Control	0.79 ± 0.01^{ab}	0.53 ± 0.01^{b}	0.67 ± 0.01^{bc}	2.07 ± 0.03^{b}
	Drought	0.65 ± 0.05^{c}	0.40 ± 0.01^{c}	0.65 ± 0.01^{c}	1.62 ± 0.01^{c}
	Variation (%)	−17.72	−43.33	−2.99	−21.74
Two way ANOVA analyses					
F_R		4.26***	9.73***	5.11***	12.53***
F_D		5.57**	16.28**	7.59***	16.42***
$F_{R\times D}$		9.73**	11.41**	9.06**	7.17**

Control: 80% soil field capacity, drought: 50% soil field capacity. F_v/F_m: maximal photochemical efficiency, F_v'/F_m': excitation energy capture efficiency of PSII reaction centers, qP: photochemical quenching, ΦPSII: effective PSII quantum yield. Values are means of six replicates ± standard deviation (SD). Small case superscript letters in the same column show statistically significant differences among different *Juglans* rootstocks for the same parameter under control and drought water regimes at $P \leq 0.05$ based on Duncan's means tests. F_R: rootstock effect, F_D: drought effect, $F_{R\times D}$: rootstock × drought interaction effect. *, **, and ***: significant at $P \leq 0.05$, 0.01, and 0.001, respectively.

provide better scientific evidence for future drought resistance breeding programs or selection of proper walnut rootstocks that are most appropriate for arid and semi-arid regions of China.

Acknowledgements

This work was supported by the Investigation Catalogue of Walnut Genetic Resources in Shandong Province (GR-2017-21) and the National Natural Science Foundation of China (3157030757). The authors are grateful to Yang Li for help in revising our English composition.

References

Aletà, N., Vilanova, A., Díaz, R., Voltas, J., 2009. Genetic variation for carbon isotope composition in *Juglans regia* L.: relationships with growth, phenology and climate of origin. Ann. For. Sci. 66, 413.
Anjum, S.A., Xie, X.Y., Wang, L.C., Saleem, M.F., Chen, M., Wang, L., 2011. Morphological, physiological and biochemical responses of plants to drought stress. Afr. J. Agr. Res. 6 (9), 2026–2032.
Araus, J.L., Amaro, T., Voltas, J., Nakkoul, H., Nachit, M.M., 1998. Chlorophyll fluorescence as a selection criterion for grain yield in durum wheat under Mediterranean conditions. Field Crop. Res. 55, 209–223.
Baker, N.R., Rosenqvist, E., 2004. Applications of chlorophyll fluorescence can improve crop production strategies: an examination of future possibilities. J. Exp. Bot. 55, 1607–1621.
Baumgartner, K., Fujiyoshi, P., Browne, G.T., Leslie, C., Kluepfel, D.A., 2013. Evaluating paradox walnut rootstocks for resistance to armillaria root disease. HortScience 48, 68–72.
Bolhar-Nordenkampf, H.R., Long, S.P., Baker, N.R., Oquist, G., Schreiber, U., Lechner, E.G., 1989. Chlorophyll fluorescence as a probe of the photosynthetic competence of leaves in the field: a review of current instrumentation. Funct. Ecol. 3 (4), 497–514.
Browne, G.T., Grant, J.A., Schmidt, L.S., Leslie, C.A., McGranahan, G.H., 2011. Resistance to *Phytophthora* and graft compatibility with Persian walnut among selections of Chinese wingnut. HortScience 46 (3), 371–376.
Browne, G.T., Leslie, C.A., Grant, J.A., Bhat, R.G., Schmit, L.S., Hacket, W.P., Kluepfel, D.A., Robinson, R., McGranahan, G.H., 2015. Resistance to species of *Phytophthora* identified among clones of *Juglans microcarpa* × *J. regia*. HortScience 50, 1136–1142.
Chaves, M.M., Oliveira, M.M., 2004. Mechanisms underlying plant resilience to water deficits: prospects for water saving agriculture. J. Exp. Bot. 55, 2365–2384.
Cochard, H., Coll, L., Le Roux, X., Améglio, T., 2002. Unraveling the effects of plant hydraulics on stomatal conductance during water stress in walnut. Plant Physiol. 128, 282–290.
Dobbertin, M., 2005. Tree growth as indicator of tree vitality and of tree reaction to environmental stress: a review. Eur. J. Forest Res. 124, 319–333.
Earl, H.J., 2002. Stomatal and non-stomatal restrictions to carbon assimilation in soybean (*Glycine max*) lines differing in water use efficiency. Environ. Exp. Bot. 48, 237–246.
Ercisli, S., Esitken, A., Cangi, R., Sahin, F., 2003. Adventitious root formation of kiwifruit in relation to sampling date, IBA and *Agrobacterium rubi* inoculation. Plant Growth Regul. 41, 133–137.
FAO, 2017. FAOSTAT Data. Food and Agriculture Organization of the United Nations, Rome.
Gauthier, M.M., Jacobs, D.F., 2011. Walnut (*Juglans spp.*) ecophysiology in response to environmental stresses and potential acclimation to climate change. Ann. For. Sci. 68, 1277–1290.
Gitelson, A.A., Gritz, Y., Merzlyak, M.N., 2003. Relationships between leaf chlorophyll content and spectral reflectance and algorithms for non-destructive chlorophyll assessment in higher plant leaves. J. Plant Physiol. 160, 271–282.
Grauke, L.J., Thompson, T.E., 2003. Rootstock development in temperate nut crops. Acta Hortic. 622, 553–556.
Guerfel, M., Baccouri, O., Boujnah, D., Chaibi, W., Zarrouk, M., 2009. Impacts of water stress on gas exchange, water relations, chlorophyll content and leaf structure in the two main Tunisian olive (*Olea europaea* L.) cultivars. Sci. Hortic. 119, 257–263.
Gulías, J., Seddaiu, G., Cifre, J., Salis, M., Ledda, L., 2012. Leaf and plant water use efficiency in cocksfoot and tall fescue accessions under differing soil water availability. Crop Sci. 52, 2321–2331.
Hackett, W., Leslie, C., Grant, J., Lampinen, B., McGranahan, G.H., Anderson, K., Bob, B., Buchner, R., Caprile, J., DeBuse, C., Hasey, J., Manterola, N., Robinson, R., Kluepfel, D., Browne, G., McKenry, M., 2010. Clonal Propagation of Walnut Rootstock Genotypes for Genetic Improvement, Walnut Research Reports. California Walnut Board. pp. 65–83.
Jerszurki, D., Couvreur, V., Maxwell, T., Silva, L.C.R., Matsumoto, N., Shackel, K., Souza, J.L.M., Hopmans, J., 2017. Impact of root growth and hydraulic conductance on canopy carbon-water relations of young walnut trees (*Juglans regia* L.) under drought. Sci. Hortic. 226, 342–352.
Leslie, C.A., McGranahan, G.H., 2014. The California walnut improvement program: Scion breeding and rootstock development. Tian, J.B. (Ed.), Proceedings of the 7th International Walnut Symposium 1050, 81–88.
Lichtenthaler, H.K., Rinderle, U., 1988. The role of chlorophyll fluorescence in the detection of stress conditions in plants. Crit. Rev. Anal. Chem. 19, 529–581.
Liu, B.H., Cheng, L., Ma, F.W., Zou, Y.J., Liang, D., 2012a. Growth, biomass allocation, and water use efficiency of 31 apple cultivars grown under two water regimes. Agrofor. Syst. 84, 117–129.
Liu, B.H., Cheng, L., Liang, D., Zou, Y.J., Ma, F.W., 2012b. Growth, gas exchange, water-use efficiency and carbon isotope composition of 'Gale Gala' apple trees grafted onto 9 wild Chinese rootstocks in response to drought stress. Photosynthetica 50, 401–410.
Ma, X.W., Ma, F.W., Li, C.Y., Mi, Y.F., Bai, T.H., Shu, H.R., 2010. Biomass accumulation, allocation, and water-use efficiency in 10 *Malus* rootstocks under two watering regimes. Agrofor. Syst. 80, 283–294.
McDowell, N., Pockman, W.T., Allen, C.D., Breshears, D.D., Cobb, N., Kolb, T., Plaut, J., Sperry, J., West, A., Williams, D.G., Yepez, E.A., 2008. Mechanisms of plant survival and mortality during drought: why do some plants survive while others succumb to drought? New Phytol. 178, 719–739.
Monclus, R., Villar, M., Barbaroux, C., Bastien, C., Fichot, R., Delmotte, F.M., Delay, D., Petit, J.M., Bréchet, C., Dreyer, E., Brignolas, F., 2009. Productivity, water-use efficiency and tolerance to moderate water deficit correlate in 33 poplar genotypes from a *Populus deltoides* × *Populus trichocarpa* F1 progeny. Tree Physiol. 29, 1329–1339.
Oztemiz, S., Kuden, A., Nas, S., Lavkor, I., 2017. Efficacy of *Trichogramma evanescens* and *Bacillus thuringiensis* var. kurstaki in control of *Cydia pomonella* (L.) in Turkey. Turk. J. Agric. For. 41, 201–207.
Rosati, A., Metcalf, S., Buchner, R., Fulton, A.N., Lampinen, B., 2006. Tree water status and gas exchange in walnut under drought, high temperature and vapour pressure deficit. J. Hortic. Sci. Biotechnol. 81, 415–420.
Schwarz, D., Rouphael, Y., Colla, G., Venema, J.H., 2010. Grafting as a tool to improve tolerance of vegetables to abiotic stresses: thermal stress, water stress and organic pollutants. Sci. Hortic. 127, 162–171.
Shao, H.B., Chu, L.Y., Jaleel, C.A., Manivannan, P., Panneerselvam, R., Shao, M.A., 2009. Understanding water deficit stress-induced changes in the basic metabolism of higher plants-biotechnologically and sustainably improving agriculture and the ecoenvironment in arid regions of the globe. Crit. Rev. Biotechnol. 29, 131–151.
Skirycz, A., Inzé, D., 2010. More from less: plant growth under limited water. Curr. Opin. Biotechnol. 21, 197–203.

Smirnoff, N., 1993. The role of active oxygen in the response of plants to water deficit and desiccation. New Phytol. 125, 27–58.
Sun, S.J., Meng, P., Zhang, J.S., Wan, X.C., 2011. Variation in soil water uptake and its effect on plant water status in *Juglans regia* L. during dry and wet seasons. Tree Physiol. 31, 1378–1389.
Tardieu, F., Granier, C., Muller, B., 2011. Water deficit and growth. Co-ordinating processes without an orchestrator? Curr. Opin. Plant Biol. 14, 283–289.
Tian, J., Wu, Y., Wang, Y., Han, F., 2010. Development and prospects of the walnut industry in China. Int. Soc. Hortic. Sci. 861, 31–38.
Vahdati, K., Lotfi, N., Kholdebarin, B., Hassani, D., Amiri, R., Mozaffari, M.R., Leslie, C., 2009. Screening for drought-tolerant genotypes of Persian walnuts (*Juglans regia* L.) during seed germination. HortScience 44, 1815–1819.
Vijayan, K., Chakraborti, S.P., Ercisli, S., Ghosh, P.D., 2008. NaCI induced morpho-biochemical and anatomical changes in mulberry (*Morus spp.*). Plant Growth Regul. 56, 61–69.
Warschefsky, E.J., Klein, L.L., Frank, M.H., Chitwood, D.H., Londo, J.P., von Wettberg, E.J.B., Miller, A.J., 2016. Rootstocks: diversity, domestication, and impacts on shoot phenotypes. Trends Plant Sci. 21, 418–437.
Yin, C.Y., Wang, X., Duan, B.L., Luo, J.X., Li, C.Y., 2005. Early growth, dry matter allocation and water use efficiency of two sympatric *Populus* species as affected by water stress. Environ. Exp. Bot. 53, 315–322.
Zlatev, Z., Lidon, F.C., 2012. An overview on drought induced changes in plant growth, water relations and photosynthesis. Emir. J. Food Agric. 24, 57–72.
