# Supplementary material for: Provenance and family variations in early growth of Manchurian walnut (Juglans mandshurica Maxim.) and selection of superior families
Source: PLoS One. 2024 Mar 7;19(3):e0298918. doi: 10.1371/journal.pone.0298918 (PMC10919699; doi:10.1371/journal.pone.0298918)
Supplement: S1 File — (ZIP) [file pone.0298918.s004.zip › Early growth of breadfruit in a variety× environment trial.pdf]

Early Growth of Breadfruit in a Variety × Environment Trial

Noa Kekuewa Lincoln,* Alyssa Cho, Graham Dow, and Theodore Radovich

ABSTRACT

Breadfruit [*Artocarpus altilis* (Parkinson) Fosberg] is growing in agronomic importance throughout the global tropics as a result of its potentially high yield and nutritious, carbohydrate-rich fruit. A bottleneck to expanded production of breadfruit is the lack of agronomic performance metrics and growth parameters, including an understanding of variety × environment interactions among commonly grown varieties. To address this knowledge gap, four varieties of breadfruit (ʻOtea, Fiti, Puaʻa, Maʻafala) available at the commercial scale were established in a randomized complete block design at five diverse sites in Hawaiʻi. Soil, architectural, physiological, and climate data were collected on 18-mo-old trees in 2018. Growth rates varied considerably for height (0.44–2.78 m yr^{-1}), diameter at breast height (0.85–5.19 cm yr^{-1}), and canopy width (0.14–1.35 m yr^{-1}). Diameter was significantly correlated to tree height (r^2 = 0.88), canopy width (r^2 = 0.83), and canopy volume (r^2 = 0.94). Growth was significantly different between sites, and some variety × site interactions were significant. Univariate and multivariate linear regressions were used to explore relationships between growth and climate, soils, and architecture. Future work will focus on the continued evaluation of variety × environmental influence on fruit production and fruit quality and on promoting this network of sites as a valuable resource for the study of an underutilized crop species with substantial potential to be a major crop in the tropics.

Core Ideas

- Breadfruit, while rapidly growing in importance globally, suffers from a lack of agronomic study.
- To our knowledge, the first variety × environment trial network of breadfruit was established in Hawaiʻi.
- Examination of initial growth rates show considerably higher rates than represented in the literature.
- Multiple climate and soil parameters significantly correlate to early growth.

Published in Agron. J. 111:3020–3027 (2019)
doi:10.2134/agronj2019.02.0068
Supplemental material available online

Copyright © 2019 The author(s). Re-use requires permission from the publisher.

Breadfruit [*Artocarpus altilis* (Parkinson) Fosberg] is a large tropical tree that has served as a staple crop for many Pacific peoples for millennia. In the past centuries, it has been introduced to new tropical regions and has become a prominent local food in several countries, with significant growth in its global distribution, processing, and marketing over the last 30 yr (Lincoln et al., 2018). Most recently, the enormous potential of breadfruit to affect global food security and climate-smart agriculture in the tropics has been recognized and promoted (Lucas and Ragone 2012; McGregor et al., 2016), including being listed as one of the 35 priority crops listed in the International Treaty on Plant Genetic Resources for Food and Agriculture (FAO, 2009). As one of the few staple crops that grow on long-lived perennial trees, breadfruit has the potential to dramatically shift contemporary agricultural practices away from the cultivation of annually harvested crops in the global tropics beyond the Pacific. As a tree crop, breadfruit provides a myriad of environmental benefits, including carbon sequestration, improved soil health, water and nutrient use efficiency, and a reduced need for chemical inputs (Kang, 1997; Kang and Akinnifesi, 2000; Ragone, 1997).

Breadfruit is highly productive, with annual yields of 6 t ha^{-1} (fruit dry weight) reported and yields of 10 t ha^{-1} in orchard settings theorized (Lincoln et al., 2018). The fruit is highly nutritious compared with other staple carbohydrates regarding protein, fiber, vitamin, and mineral content (Jones et al., 2011; Turi et al., 2015). Furthermore, breadfruit has extensive resource applications and has shown potential for use in latex, insect repellent, and beauty products (Jones et al., 2012; Meilleur et al., 2004; Navarro et al., 2007; Whitnell and Murch, 2018). Recent advances in technology (e.g., commercial-scale propagation and value-added products) and efforts in promotion (e.g., public education and tree giveaways) have resulted in a substantial increase in the awareness and commercial viability of breadfruit (Langston and Lincoln, 2018; Lysak, 2018; Ragone et al., 2016; Taylor et al., 2011; Thompson and Abraham, 2016).

Despite the increase in breadfruit cultivation, the species is underutilized (Ragone, 2007) and suffers from significant lack of research investment (Lincoln et al., 2018). Neglected and underutilized species are seen as marginalized agricultural species and are generally ignored by researchers, policymakers, and breeders (Padulosi et al., 2002, 2013). Such crops have the

N.K. Lincoln, A. Cho, T. Radovich, Dep. of Tropical Plant and Soil Sciences, College of Tropical Agriculture and Human Resources, Univ. of Hawaiʻi at Mānoa, Honolulu, HI 96822; G. Dow, Dep. of Biology, Boston Univ., Boston, MA 02215. Received 10 Feb. 2019. Accepted 8 July 2019. *Corresponding author (nlincoln@hawaii.edu).

Abbreviations: DBH, diameter at breast height; LAI, leaf area index; PAR, photosynthetic active radiation; SPAD, single-photon avalanche diode.

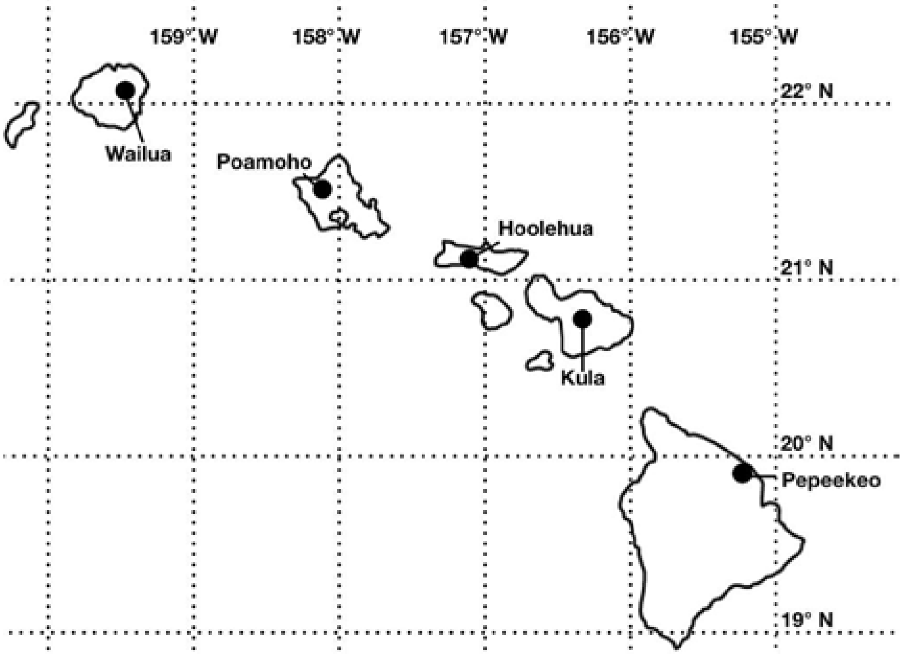

Fig. 1. Location of the five breadfruit trial locations reported on in this paper on five islands in Hawai'i.

potential to contribute to food and nutrition security, enhance resilience of agricultural systems, and preserve traditional food systems and cultural identities (Padulosi et al., 2013). Although considerable ethnobotanical information has been documented for breadfruit, there is a general lack of empirical agronomic studies, including variety × environment interactions. Some previous research has focused on the effects of solar radiation on flower set, of planting arrangement and row orientation on yield (Roberts-Nkrumah, 2002; Gloster and Roberts-Nkrumah 2016), of canopy dimensions on fruit yield (Fownes and Raynor, 1993; Gloster and Roberts-Nkrumah 2013), and of pruning on growth form (Gloster and Roberts-Nkrumah 2016; Roberts-Nkrumah, 2016).

In Hawai'i, breadfruit served as an important staple crop prior to European arrival (Winter et al., 2018), playing an important role in agroecosystems and socio-political development (Lincoln and Ladefoged, 2014). Following contact, Hawai'i experienced a rapid decline in diversified traditional agricultural systems and crops, replaced by large-scale monoculture for export markets akin to many colonized tropical societies (Levin, 2015; Kagawa-Viviani et al., 2018). Hawai'i is experiencing a rapid redevelopment of breadfruit production, with an exponential increase in commercial tree plantings over the last 20 yr (Langston and Lincoln, 2018) that has stemmed, at least in part, from a very strong public education campaign (Ragone et al., 2016) and cultural connection to the crop (Meilleur et al., 2004). For the first time, establishment of orchard-style cultivation of breadfruit is occurring, with at least 12 substantial orchards (100+ trees) and over 50 smaller orchards (20+ trees) representing over 6000 trees now planted in Hawai'i established for commercial production of the fruit (Langston and Lincoln, 2018). At least in part, the growth in plantings is related to the commercial propagation of breadfruit through tissue culture, whereas traditional cloning techniques, such as root cuttings or maracotting, were a hinderance to orchard establishment.

Due to a historical lack of research and investment into its growing conditions, agronomy, physiology, and cropping systems, it is likely that substantial gains in breadfruit yield could be made with relatively little effort into agronomic research and breeding (Sraffa, 2005; Willcox, 1954). Therefore, we have established multiple variety trials in diverse environments and soil types across Hawai'i to begin a formal assessment of common commercially available varieties to enhance our knowledge of breadfruit cultivation. To our knowledge, this is the first variety × environment trial conducted for breadfruit. In assessing growth and development after 18 mo, our objective was to describe the dominant abiotic environmental factors associated with differences in initial growth of varieties among diverse sites.

MATERIALS AND METHODS

Location

Variety trials were established at five sites on five islands in Hawai'i: Wailua (Kauai), Poamoho (O'ahu), Ho'olehua (Molokai), Kula (Maui), and Pepeekeo (Hawai'i) (Fig. 1; Table 1). The sites represent a range of environmental and soil conditions. Average annual climate data for each site was extracted via electronic maps from the Rainfall and Evapotranspiration Atlases of Hawaii (Giambelluca et al., 2013, 2014).

Varieties

The varieties evaluated (Table 2) consisted of four varieties that are available at commercial scale via tissue culture: 'Otea, Pua'a, Ma'afala, and Fiti. These varieties have been widely distributed globally, including to Hawai'i (Lincoln et al., 2018). They were obtained as tissue cultured plugs by mail order (www.globalbreadfruit.com).

Establishment and Management

All material was grown in a common greenhouse with bimonthly fertilization using Sustane 4N-2.6P-3.3K (organic fertilizer from compost and turkey manure; Sustane Natural

Table 1. Brief overview of trial site locations in Hawai‘i.

Location	Island	Affiliation	Unique attributes
Ho‘olehua	Moloka‘i	Demonstration Agricultural Farm, CTAHR†	High winds
Kula	Maui	Agricultural park, CTAHR	Cold temperatures
Pepeekeo	Hawai‘i	Private partner	Within sea spray zone
Poamoho	O‘ahu	Agricultural research station, CTAHR	Acidic soils
Wailua	Kauai	Agricultural research center, CTAHR	High rain/cloud cover

† College of Tropical Agriculture and Human Resources at the University of Hawaii at Mānoa.

Fertilizers Inc., Cannon Falls, MN) and daily irrigation, initially in 1-gal pots and transplanted into 3-gal pots at 3 mo. At initial establishment, trees were highly uniform, and all measured between 74 and 80 cm tall. The planting sites were standardized for experimental design, installation, and management. Sites were prepared by removing grass, laying weed mat, softening 1-m^3 holes, mixing 250 mL Sustane 4N-2.6P-3.3K organic fertilizer into the hole, and applying a second 250-mL application as a top dressing immediately after planting. Irrigation was established, and weed mat was installed to prevent the regrowth of weeds and grasses. Sites are fertilized bi-annually with standardized surface application as applied during establishment.

Experimental Design

Installation design was identical across the five sites, with each site serving as a replicate. Each site included 20 trees (five trees each of the four test varieties) in a randomized block design so that each of the five rows served as a block and contained one of each of the varieties in a random position. Trees were established in November 2016 at a 30-ft spacing.

Data Collection

Soil samples were taken from each site immediately prior to planting. Three composite samples (consisting of three cores each) were randomly collected 2 m from an outplant position to a depth of 30 cm. Samples were passed through a 2-mm sieve. Subsets were oven dried for moisture content, pH was determined using a 2:1 ratio with deionized water, organic matter was determined using a standard loss on ignition method, nitrate and ammonium were determined with a 2 M KCl extraction, extractable nutrients were assessed with Mehlich 3 extraction, and exchangeable cations were determined using ammonium acetate extraction buffered at pH 7. Determination of pH, organic matter, and inorganic nitrogen were conducted at the Indigenous Cropping Systems Laboratory at the University of Hawai‘i at Mānoa. Determination of extractable nutrients was conducted at Brookside Laboratories Inc. (New Bremen, OH).

At the time of planting, tree height, base diameter, and branching were documented. In March 2018, measurements of tree architecture, nutrient content, and physiology were recorded. Tree architecture included diameter at breast height (DBH), height, height to the first branch, total primary and secondary branches, and canopy width (averaged width measured facing the four cardinal directions). An estimated canopy volume was calculated assuming a pyramidal crown shape, applying the average canopy radius, and calculating canopy height as the total height less the height of the first branch. Three measurements of canopy density were taken: (i) a leaf-intersection measurement in which a vertical pole was threaded midway between the trunk and canopy edge at each the cardinal directions, (ii) a photogrammetry method that used tree profile pictures taken from the four cardinal directions and calculated the percentage area of light gaps using randomly generated points, and (iii) a photogrammetry method that used a curved mirror to image the canopy from beneath the tree from the four cardinal directions and calculated the percentage area of lights gaps using randomly generated points. A relative chlorophyll index was recorded using a single-photon avalanche diode (SPAD) meter (502 Plus Chlorophyll; Spectrum Technologies, Inc., Aurora, IL), in which 10 measurements on three separate canopy leaves in the third position were recorded. Photosynthetic yield, or light use efficiency, was measured using the Plant Photosynthetic Meter 300 (Environmental Analysis & Remote Sensing Earth Environmental Monitoring B.V., Delft, The Netherlands) on the same leaves measured for chlorophyll after artificially shading the leaves for 10 min (see www.ears.nl for description of instrument technology for measuring photosynthetic yield). Leaf punches of known area (117.75 cm^2 total) were extracted from three separate canopy leaves in the third position, dried, and weighed to determine leaf-mass area.

Table 2. Passport data for the four varieties of breadfruit established in five replicated planting trials in Hawai‘i. Information from National Tropical Botanical Gardens (2017).

	Ma'afala	Ulu Fiti	Otea	Pua'a
Alternative names		Ulu Falaoa		
Geographic origin	Samoa	Fiji	French Polynesia	French Polynesia
Distribution	Samoa, Tonga, Fiji, Cook Islands, Vanuatu, Micronesia	Rotuma, Samoa	Society Islands	Society Islands, Marquesas
Seediness	mostly seedless	few seeds	seedless (sterile)	seedless (sterile)
Fruit shape	oval	round to heart	broad ovoid	oval to heart
Weight, kg	0.6–1.1	1.1–2.8	1.4–2.5	1.0–3.1
Average leaf size, cm	36 × 30	39 × 27	43 × 36	44 × 34
Average number of lobes	8	6	8	7
Lobe depth	moderate/shallow	moderate	moderate/deep	moderate/deep
Male flower size, cm	11 × 2	17 × 3	21 × 4	29 × 4

Table 3. Average annual climate parameters for site locations.

Site	Soil type	Elevation	Cloud frequency	Evaporative potential	Net radiation	Rainfall	Relative humidity	Temperature		
								Average	Maximum	Minimum
		m	%	mm	w m^{-2}	mm	%	°C		
Hoolehua	Oxisol	57.1	0.44	2891.5	129.8	473.3	72.0	23.4	28.1	19.6
Kula	Aridisol	471.3	0.47	1917.6	118.9	560.1	82.8	20.8	25.6	16.7
Pepeekeo	Andisol	6.7	0.59	2508.6	144.8	3376.0	71.3	22.6	26.4	19.0
Poamoho	Oxisol	208.9	0.53	2807.4	130.1	910.9	76.7	22.3	26.9	18.4
Wailua	Inceptisol	149.0	0.64	3362.0	126.6	2018.9	74.8	22.2	26.7	18.6

Statistical Analysis

Data were analyzed in JMP Pro 13.1.0. For site and variety comparisons, ANOVA was applied to parameters of interest, and significant group differences were determined by Tukey–Kramer HSD tests. For consideration of important parameters with respect to growth, we first conducted linear regressions between parameters of interest and growth as measured by diameter, height, and canopy width. Multivariate regression models were conducted using parameters in each of three categories: site climate, tree architecture and physiology, and soil nutrients. For multivariate regressions, we removed variables when high (>0.9) cross-correlations occurred and removed variables that did not meet a linear correlation standard (<0.1). A generalized linear model using standard least squares was conducted with 75% of the data set, with 25% withheld for validation. A forward selection method was used; we stopped introduction of parameters when the effect level of the new variable was insignificant ($P > 0.05$).

RESULTS

Summary data for all measurements is provided by site (Supplementary Table 1) and variety (Supplementary Table 2).

Site Characterization

The sites represent a range of settings (Table 1) and a range of climatic (Table 3) and soil conditions (Table 4).

Growth

Measurements of height, DBH, canopy width, and canopy volume showed normal distribution and a substantial range with strong, significant relationships between the different growth parameters (Table 5). Across all sites and varieties differences in growth rates were expressed, with over a 600% difference in growth rates between the highest and lowest extremes.

Differences in growth by site were apparent (Fig. 2), with some divergence by variety and minimal interactive (site × variety) effects. An ANOVA verified significant differences in growth rate between sites. Tukey–Kramer analysis indicated four significantly different groups: Pepeekeo (A), Wailua (B), Hoolehua (B, C), Kula (C, D), and Poamoho (D). There were no significant differences between varieties across sites. However, interactions between site and variety indicate site-specific differences in early varietal growth. For instance, the rank order of varieties' growth rates differed for each site, and some significant divergence of varieties within each site could be seen using Tukey–Kramer analysis, although these effects were sparse at this time.

Growth and Climate

We examined a suite of climate data that included elevation, cloud frequency, net radiation, Penman–Monteith potential evaporation, rainfall, relative humidity, vapor pressure differential, and air temperature (minimum, average, and maximum). Some parameters were removed due to high cross correlation (Körner, 2007). Most of the remaining parameters demonstrated highly significant relationships to growth, with the strongest relationships being with annual rainfall, net radiation, and elevation (Table 6). A multivariate linear regression model between DBH and all climate parameters, with an adjusted r^2 of 0.66 ($P < 0.001$), indicated that the climate parameters of importance were, in descending order, annual rainfall, cloud frequency, minimum temperature, and net radiation. The inclusion of variety in the model was insignificant.

Growth and Soil

Sites represented strong differences in natural soil parameters and fertility driven by substrate age and climate (Table 2). Using linear regression, soil parameters that correlated highly with growth were extractable sulfur, percent organic matter, total exchange capacity, and extractable phosphorus (Table 6). Multivariate regression that exhibited an adjusted r^2 of 0.67 ($P < 0.001$) indicated that the parameters of importance, in descending order, were organic matter, total exchange capacity, and extractable phosphorus. These soil parameters, however, are highly correlated to the climate parameters of interest and may simply represent cross correlation. The inclusion of variety into the model was insignificant.

Table 4. Measured soil parameters for site locations in Hawai'i established with replicated breadfruit and breadnut plantings.

Site location	Soil pH	TEC†	Organic matter	NO_3–N	NH_4–N	P‡	Ca	Mg	K	Fe	Mn	Cu	Zn
		cmol kg^{-1}	%	ppm		mg kg^{-1}							
Hoolehua	7.1	13.14	4.42	91.10	6.17	26.67	1428	389	515	43	249	1.02	2.82
Kula	7.4	19.40	4.14	11.90	10.33	118.33	2353	540	841	87	189	2.78	4.79
Pepeekeo	5.8	4.61	22.39	3.43	11.30	5.00	524	51	69	87	10	3.11	1.89
Poamoho	5.5	8.65	7.89	1.13	12.27	7.67	865	104	93	45	15	1.66	0.52
Wailua	5.3	3.85	18.04	7.23	19.00	8.00	299	64	29	113	3	1.52	1.15

† Total exchange capacity of the soil.

‡ Phosphorus as phosphate.

Table 5. Growth parameter summary statistics and relationships to diameter at breast height (DBH) for breadfruit trees across all varieties and sites.

Parameter	Range	Mean (SD)	Equation relating to DBH	r^2
Height, m	0.44–2.78	1.43 (0.51)	= –0.092 + 0.55 × DBH	0.88***
Canopy width, m	0.14–0.27	0.74	= –122.1 + 117.6 × Sqrt(DBH)	0.83***
Canopy volume, m^3	0.01–2.32	2.31	= $(–0.99 + 0.64 × DBH)^2$	0.94***

*** Significant at the 0.001 probability level.

Architecture and Physiology

Measurements of architecture included total number of branches, height to first branch, average branch spacing, average leaf intersection, vertical light penetration, and horizontal light penetration. These measurements represent the stature of the tree (i.e., how dense or compact the growth of the tree is). Much of the variation in these parameters was driven by variety, with Ma'afala and Fiti being considerably denser and more compact than Otea and Pua'a, having significantly more branches per unit height and less light penetration both vertically and horizontally. However, there are clear site differences in architecture in addition to differences in growth. For instance, the density of branches (i.e., the number of branches per unit height) falls into two significantly different groups when controlling for variety. Similar patterns occur for most of the architectural measurements, indicating a clear response in tree stature and density based on site environment.

Two physiological measurements were made for chlorophyll and photosynthetic yield. The SPAD results showed no significant differences between sites or varieties or interactions between the two, indicating fairly consistent chlorophyll content across all treatments. Photosynthetic yield showed no differences between varieties but showed significant differences between sites, with most sites being significantly different from each other (using a Tukey–Kramer difference test, four significantly different groups defined the five sites). The rank order of photosynthetic yield mirrored the difference in growth rates.

Architecture/Physiology and Growth

Of the architectural and physiological measurements made, a measure of leaf intersection as a proxy for leaf area index (LAI) had the strongest correlation (Table 6). The multivariate model, with an adjusted r^2 of 0.44 ($P < 0.001$), indicates that the parameters of importance, in descending order, were leaf intersection, horizontal light penetration, and the number of

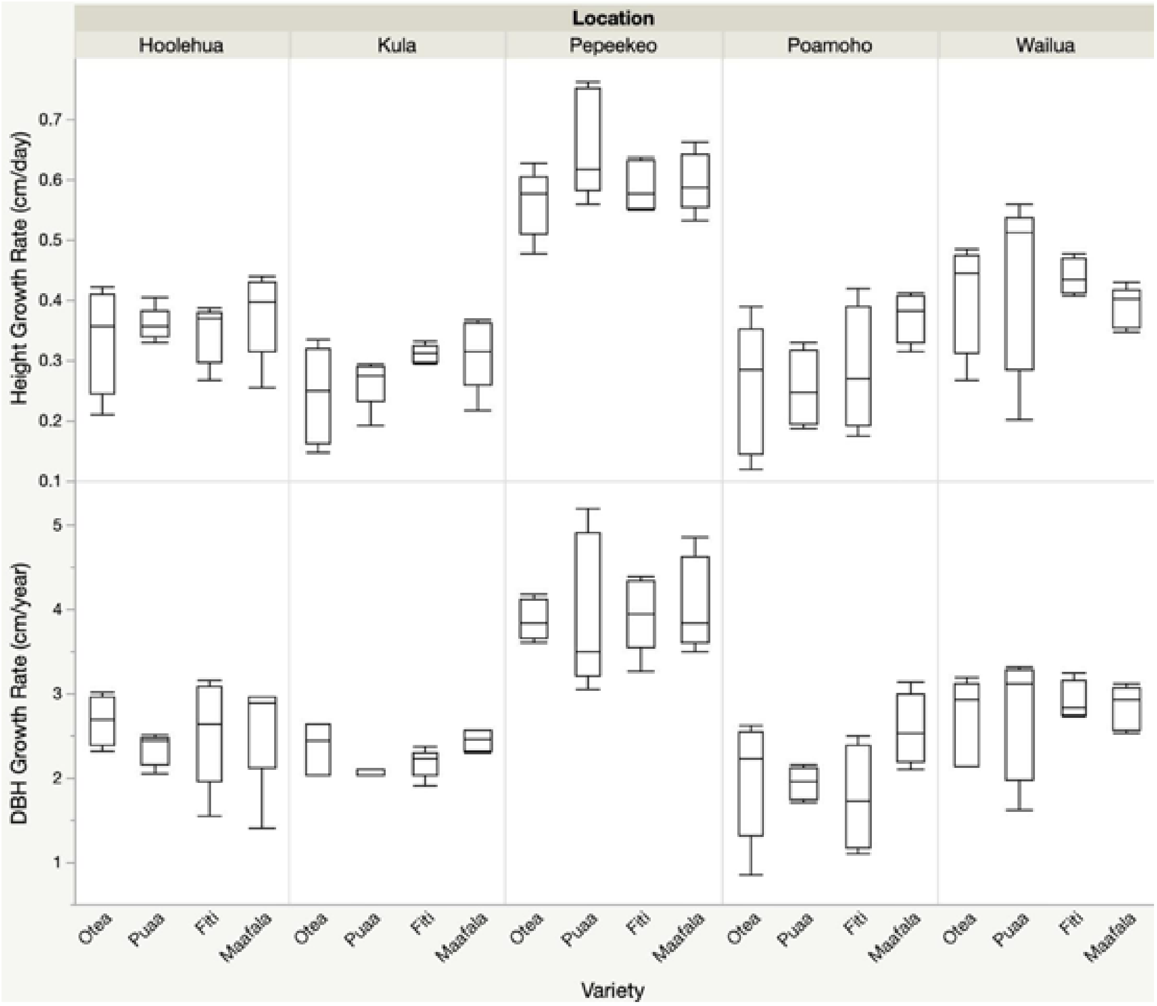

Fig. 2. Breadfruit height and diameter at breast height (DBH) growth by site and variety.

Table 6. Linear regression r^2 values examining the relationship between growth and climate, soil, and tree parameters.

Parameter	Height	DBH†	Canopy width
Climate			
Elevation	0.45*	0.39*	0.36*
Cloud frequency	0.25*	0.17*	0.29*
Net radiation	0.56*	0.48*	0.41*
Annual rainfall	0.65*	0.57*	0.57*
Relative humidity	0.36*	0.27*	0.32*
Minimum air temperature	0.17*	0.10*	0.16*
Vapor pressure differential	0.27*	0.19*	0.24*
Soils			
Total exchange capacity, meq/100 g	0.38*	0.27*	0.41*
pH	0.14*		0.16*
Organic matter, %	0.58*	0.48*	0.55*
Estimated N release, kg N acre^{-1}	0.38*	0.28*	0.38*
S, ppm	0.67*	0.58*	0.59*
P, mg kg^{-1}	0.31*	0.20*	0.28*
Olsen P, mg kg^{-1}	0.16*	0.08*	0.17*
Ca, mg kg^{-1}	0.29*	0.18*	0.33*
Mg, mg kg^{-1}	0.27*	0.25*	0.35*
K, mg kg^{-1}	0.24*	0.13*	0.25*
Na, mg kg^{-1}	0.20*	0.18*	0.21*
B, mg kg^{-1}	0.17*	0.09*	0.19*
Fe, mg kg^{-1}	0.11*	0.15*	0.17*
Mn, mg kg^{-1}	0.19*	0.11*	0.22*
Cu, mg kg^{-1}	0.14*	0.23*	0.08**
Tree architecture and physiology			
SPAD results		0.08**	0.11*
Photosynthetic yield	0.17*	0.11**	0.19*
Number of secondary branches	0.18*	0.19*	0.24*
Number of branches	0.14*	0.09**	0.25*
Leaf internode length	0.27*	0.23*	0.30*
Leaf intersection	0.46*	0.42*	0.40*
Vertical light penetration, %	0.10*	0.18*	

* Significant at the 0.05 probability level.

** Significant at the 0.01 probability level.

† Diameter at breast height.

branches, all of which are indicative of the density of growth of the tree. The inclusion of variety into the model was significant and improved the adjusted r^2 to 0.48 ($P < 0.001$).

DISCUSSION

Breadfruit is reportedly fast growing in favorable conditions, growing in height 0.5 to 1.5 m yr^{-1} and in trunk diameter to 0.5 to 1 m in the first 10 to 12 yr (Ragone, 2008). The growth rates observed in this study tended to be higher than previously reported values. Growth in height ranged from 0.4 to 2.8 m yr^{-1} (average, 1.4 m yr^{-1}), and change in trunk diameter ranged from 0.9 to 5.2 cm yr^{-1} (mean, 2.8 cm yr^{-1}). Due to the fertilization and weed management used in these trials, we suggest that the growth rates represented by these trials are likely in line with what commercial growers might observe. Across all varieties and sites, strong relationships between different growth measurements, including height, trunk diameter, and canopy width, were recorded. That these relationships hold well may indicate that they could broadly be applied to *Artocarpus altilis*, but it is likely that these relationships breakdown for the interspecific hybrids of *A. altilis x mariannensis* that grow much larger trunk diameters. Broader usage of the derived equations could provide validation or refutation.

Significant differences in growth between sites occurred, suggesting that climatic and environmental drivers are manifest in growth across sites. The climate and range of breadfruit are well summarized in Ragone (1997), with some additional information found in Jones et al. (2011). There have been no systematic trials of the climate tolerance of breadfruit, with existing information being anecdotal and based on observations of breadfruit distribution. However, at an early stage we saw some variety × environment interaction effects that suggest site-specific usage of varieties may maximize productivity and expand the range of breadfruit cultivation.

Highly important climate factors were rainfall, solar radiation, and cloud frequency. Cloud frequency is a positive model parameter; that is, increased cloud cover increases growth. We believe this relates to breadfruit's relatively low photosynthetic saturation, which in Hawai'i occurs at ~1000 μmol m^{-2} s^{-1} of photosynthetic active radiation (PAR) (G. Dow and N.K. Lincoln, unpublished data, 2018). The two sites that exhibited the highest growth rates had relatively high levels of cloud frequency, and broadly breadfruit appears to function best in environments where PAR is moderated. Indeed, during measurements, we noticed extreme "slumps" in photosynthetic activity at sites with full sun that occurred as early as 10:00 AM and exhibited a near complete shutdown of activity on canopy leaves (G. Dow and N.K. Lincoln, unpublished data, 2018). Although not a climate parameter, leaf intersection was an important model parameter. In addition to serving as a proxy for LAI and increasing total photosynthetic capacity of the tree, we suggest that a high leaf intersection also creates self-shading of the lower leaves, lowering PAR exposure throughout the canopy and helping to avoid photosynthetic shutdown. Breadfruit leaves on most varieties are highly segregated and, although they are large, tend to have high levels of light penetration. Further studies should investigate what could be a complex interaction between leaf segregation, light and wind penetration, and photosynthetic rates in breadfruit.

Despite fertilization, soil parameters showed high correlation to growth, in particular total exchange capacity, organic matter concentration, and extractable sulfur and phosphorus. We found a negative relationship between total exchange capacity and growth. In a separate project that examined trees from 61 breadfruit production sites in Hawai'i (Lincoln and Langston, 2018), a strong negative relationship was observed between tree health (as measured by SPAD, photosynthetic capacity, and fruit size) and soil pH. High-pH (alkaline) soils drive higher total exchange capacity calculations by definition and could explain the observed relationship between exchange capacity and growth in this study; however, this leads to a question of why higher soil pH negatively affects breadfruit tree health and production.

Our initial results further support previous research into breadfruit architecture. For instance, Gloster and Roberts-Nkrumah (2013) found strong differences in LAI between varieties but not between light penetration rates. Similarly, we observed significant differences between varieties for leaf intersection and a weak correlation between leaf intersection and vertical light penetration. Overall, divergence in architecture of

the varieties was stronger than the divergence in growth. Further emphasis on how the varieties respond to climatic factors in terms of their architecture and how those differences manifest in yields is an important future direction of this study. This may be important to understand differences in yields based on site. Previous research has shown that, within a site, specific tree measurements can help to predict yields. For instance, Roberts-Nkrumah (2002) found that canopy width was the only tree canopy parameter that significantly correlated with fruit set, whereas Fownes and Raynor (1993) found that canopy volume was the best predictor of yield, followed closely by DBH. However, these studies were focused in localized plantings within a similar environment. Differences in architecture in our study were minimal within each site, meaning they would not be useful in predicting differences between trees at a single site but were very divergent across sites and are likely important in predicting potential yields across environments. Because the fruit develop at branch tips only, the variability in density of branches across sites, we suspect, will correlate to the number of fruit set at each site.

We anticipate that initial differences seen across sites and varieties will translate to variety-specific responses of yield to environment. A broad range of yields are reported for breadfruit, from 25 to over 2000 kg per tree (Lincoln et al., 2018), but the majority of studies are anecdotal and based on broad estimations using fruit counts over a short period, with few systematic yield analyses. Liu et al. (2014) demonstrate that, in Hawai'i, most varieties produced fruit 4 yr after planting, with an average of 47 ± 8 fruit produced by the young trees, increasing to 130 ± 22 fruit per tree after 7 yr and continuing to increase throughout the study. Measurements indicate differences in net primary productivity potential, such as SPAD and leaf intersection, which has been shown to correlate to productivity in other crop species (e.g., Charbonnier et al., 2017; Madugundu et al., 2017) and in breadfruit (Lincoln et al., 2019).

CONCLUSION

We have established a network of five sites with replicated breadfruit trials consisting of four commercially available varieties. Sites in this statewide trial represent a broad range of natural climate and soil parameters and will provide a resource for future research and observation. To our knowledge, this is the first systematic evaluation of breadfruit varieties performance in a series of environments. Initial results show a significant divergence of growth rates by site and some divergence between varieties at the site level. Growth characteristics of height, canopy width, and canopy volume all correlated strongly. Several site characteristics showed a correlation to initial growth rates.

We hope to promote the existence of this network of sites as a significant resource for the study of an undervalued crop species with tremendous potential to be a major crop in the tropics. We welcome collaboration and use of the network by other researchers interested in breadfruit, tropical fruit, or tree crops in general. Future work will focus on the continued evaluation of the breadfruit varieties for production and fruit quality, but we have also laid the groundwork to use these sites to examine breadfruit variety × environment interactions, developmental physiology, and impacts on soil.

ACKNOWLEDGMENTS

This work was supported in part by the USDA National Institute of Food and Agriculture HATCH (project 8035-H) and McIntire-Stennis (project 8038M-MS) programs managed by the College of Tropical Agriculture and Human Resources, the Western SARE Research and Education program (grant SW17-050), and the Maui Country Office of Economic Development.

REFERENCES

Charbonnier, F., O. Roupsard, G. Le Maire, J. Guillemot, F. Casanoves, A. Lacointe, and A. Clément-Vidal. 2017. Increased light-use efficiency sustains net primary productivity of shaded coffee plants in agroforestry system. Plant Cell Environ. 40(8):1592–1608. doi:10.1111/pce.12964

FAO. 2009. International treaty on plant genetic resources for food and agriculture, 56. FAO, Rome, Italy. http://www.fao.org/3/a-i0510e.pdf (accessed 12 Feb. 2018).

Fownes, J.H., and W.C. Raynor. 1993. Seasonality and yield of breadfruit cultivars in the indigenous agroforestry system of Pohnpei, Federated States of Micronesia. Trop. Agric. 70:103–109.

Giambelluca, T.W., Q. Chen, A.G. Frazier, J.P. Price, Y.L. Chen, P.S. Chu, et al. 2013. Online rainfall atlas of Hawai'i. Bull. Am. Meteorol. Soc. 94(3):313–316. doi:10.1175/BAMS-D-11-00228.1

Giambelluca, T.W., X. Shuai, M.L. Barnes, R.J. Alliss, R.J. Longman, T. Miura, et al. 2014. Evapotranspiration of Hawaii. Final report submitted to the U.S. Army Corps of Engineers-Honolulu District and the Commission on Water Resource Management, Honolulu, HI.

Gloster, M.C., and L.B. Roberts-Nkrumah. 2013. Effect of cultivar on light interception in breadfruit (*Artocarpus altilis*). Presented at: Caribbean Food Crops Society 49th Annual Meeting, Port of Spain, Trinidad and Tobago. 13 June–6 July 2013. Caribbean Food Crops Society, Mayagüez, Puerto Rico.

Gloster, M.C., and L. Roberts-Nkrumah. 2016. Comparative evaluation of early tree growth and development of two breadfruit cultivars in response to tree arrangement and pruning at two locations on Trinidad. In: L.B. Roberts-Nkrumah and E.J. Duncan, editors, Proceedings of the International Breadfruit Conference– Commercialising breadfruit for food and nutrition security. Univ. of the West Indies, Republic of Trinidad and Tobago. p. 126.

Jones, A.M.P., D. Ragone, D.W. Bernotas, and S.J. Murch. 2011. Beyond the bounty: Breadfruit (Artocarpus altilis) for food security and novel foods in the 21st century. Ethnobot. Res. Appl. 9:129–149. doi:10.17348/era.9.0.129-149

Jones, A.M.P., J.A. Klun, C.L. Cantrell, D. Ragone, K.R. Chauhan, P.N. Brown, and S.J. Murch. 2012. Isolation and identification of mosquito (*Aedes aegypti*) biting deterrent fatty acids from male inflorescences of breadfruit (*Artocarpus altilis* (Parkinson) Fosberg). J. Agric. Food Chem. 60(15):3867–3873. doi:10.1021/jf300101w

Kagawa-Viviani, A., P. Levin, E. Johnston, J. Ooka, J. Baker, M. Kantar, and N.K. Lincoln. 2018. I ke ēwe 'āina o ke kupuna: Hawaiian ancestral crops in perspective. Sustainability 10(12):4607. doi:10.3390/su10124607

Kang, B.T. 1997. Alley cropping: Soil productivity and nutrient recycling. For. Ecol. Manage. 91(1):75–82. doi:10.1016/S0378-1127(96)03886-8

Kang, B.T., and F.K. Akinnifesi. 2000. Agroforestry as alternative land-use production systems for the tropics. Nat. Resour. Forum 24(2):137–151. doi:10.1111/j.1477-8947.2000.tb00938.x

Körner, C. 2007. The use of 'altitude' in ecological research. Trends Ecol. Evol. 22(11):569–574. doi:10.1016/j.tree.2007.09.006

Langston, B., and N. Lincoln. 2018. The role of breadfruit in biocultural restoration and sustainability in Hawai'i. Sustainability 10(9):3965.

Levin, P. 2015. Searching for sustainable agriculture in Hawai'i. In: J. Chirico and G.S. Farley, editors, Thinking like an island: Navigating a sustainable future in Hawaii. Univ. of Hawai'i Press, Honolulu. p. 46–78. doi:10.21313/hawaii/9780824847616.003.0004

Lincoln, N.K., and T.N. Ladefoged. 2014. Agroecology of pre-contact Hawaiian dryland farming: The spatial extent, yield and social impact of Hawaiian breadfruit groves in Kona. Hawai'i. J. Arch. Sci. 49:192–202. doi:10.1016/j.jas.2014.05.008

Lincoln, N.K., and B. Langston. 2018. Assessment of soil, foliar, and fruit nutrient concentrations for breadfruit producers in Hawai'i. Coll. of Trop. Agr. Human Resour., Univ. of Hawai'i, Honolulu.

Lincoln, N.K., T. Radovich, K. Acosta, E. Isele, and A. Cho. 2019. Towards standardized leaf sampling for foliar nutrient concentrations in breadfruit. HortTechnology 29(4):2019.

Lincoln, N.K., D. Ragone, N. Zerega, L.B. Roberts-Nkrumah, M. Merlin, and A.M. Jones. 2018. Grow us our daily bread: A review of breadfruit cultivation in traditional and contemporary systems. Hortic. Rev. (Am. Soc. Hortic. Sci.) 46:299–384.

Liu, Y., A.M.P. Jones, S.J. Murch, and D. Ragone. 2014. Crop productivity, yield and seasonality of breadfruit (*Artocarpus* spp., Moraceae). Fruits 69(5):345–361. doi:10.1051/fruits/2014023

Lucas, M.P. and D. Ragone. 2012. Will breadfruit solve the world hunger crisis? New developments in an innovative food crop. ArcNews, Summer:6–7.

Lysak, M. 2018. Improving food and nutrition security through exploration of market potentials by revitalizing breadfruit as a strategy for adapting to climate change. Doctoral dissertation. Dep. of Plant and Environmental Science, Univ. Copenhagen, Copenhagen, Denmark.

Madugundu, R., K.A. Al-Gaadi, E. Tola, A.G. Kayad, and C.S. Jha. 2017. Estimation of gross primary production of irrigated maize using Landsat-8 imagery and Eddy Covariance data. Saudi J. Biol. Sci. 24(2):410–420. doi:10.1016/j.sjbs.2016.10.003

McGregor, A.M., L.D. Tora, and V. Lebot. 2016. Planting breadfruit orchards as a climate change adaptation strategy for the Pacific islands. Acta Hortic. 1128:55–66. doi:10.17660/ActaHortic.2016.1128.8

Meilleur, B.A., R.R. Jones, C.A. Titchenal, and A.S. Huang. 2004. Hawaiian breadfruit: Ethnobotany, nutrition, and human ecology. CTAHR, Honolulu, HI.

National Tropical Botanical Gardens. 2017. ntbg.org/breadfruit/database (accessed 19 Mar. 2017).

Navarro, M., S. Malres, J.P. Labouisse, and O. Roupsard. 2007. Vanuatu breadfruit project: Survey on botanical diversity and traditional uses of Artocarpus altilis. Acta Hortic. 757:81–88. doi:10.17660/ActaHortic.2007.757.9

Padulosi, S., T. Hodgkin, J.T. Williams, and N. Haq. 2002. Underutilized crops: Trends, challenges, and opportunities. In: V.R. Rao, A.H.D. Brown, and M. Jackson, editors, Managing plant genetic diversity. CABI, Oxfordshire, UK. p. 323–338. doi:10.1079/9780851995229.0323

Padulosi, S., J. Thompson, and P. Rudebjer. 2013. Fighting poverty, hunger and malnutrition with neglected and underutilized species: Needs, challenges and the way forward. Bioversity International, Rome, Italy. http://www.bioversityinternational.org/ (accessed 10 Jan 2017).

Ragone, D. 1997. Breadfruit: *Artocarpus altilis* (Parkinson) Fosberg. Promoting the conservation and use of underutilized and neglected crops. International Plant Genetic Resources Institute, Rome, Italy.

Ragone, D. 2007. Breadfruit: Diversity, conservation and potential. Acta Hortic. 757:19–30. doi:10.17660/ActaHortic.2007.757.1

Ragone, D. 2008. Farm and forestry production and marketing profile for breadfruit (*Artocarpus altilis*). In: C.R. Elevitch, editor, Specialty crops for Pacific Island agroforestry. Permanent Agriculture Resources, Holualoa, HI.

Ragone, D., C.R. Elevitch, and A. Dean. 2016. Revitalizing breadfruit in Hawaii: A model for encouraging the cultivation and use of breadfruit in the tropics. Trop. Agric. 93:213–224.

Roberts-Nkrumah, L.B. 2002. Management implications of the distribution of fruit set on breadfruit (*Artocarpus altilis*) trees. Acta Hortic. 632:201–207.

Roberts-Nkrumah, L. 2016. Evaluation of regrowth responses of breadfruit (*Artocarpus altilis*) cultivars 'Yellow' and 'Ma'afala' to different levels of pruning. Trop. Agric. (Trinidad) 93(Special Issue 1):127–139.

Sraffa, P. 2005. The laws of returns under competitive conditions. In: G.Z. Sun, editor, Readings in the economics of the division of labor: The classical tradition. Vol. 2. World Sci. Pub., Singapore. p. 216–233. doi:10.1142/9789812701275_0022

Taylor, M., R. Paroda, H. Jaenicke, and P. Mathur. 2011. Implementing the strategy for the conservation through use of underutilised crops in the Pacific. Acta Hortic. 979:533–539.

Thompson, J., and N. Abraham. 2016. Breadfruit in St. Vincent and the Grenadines: The breadfruit festival and prospects for commercialisation. Trop. Agric. (St Augustine) (Special Issue):107–110.

Turi, C.E., Y. Liu, D. Ragone, and S.J. Murch. 2015. Breadfruit (*Artocarpus altilis* and hybrids): A traditional crop with the potential to prevent hunger and mitigate diabetes in Oceania. Trends Food Sci. Tech. 45(2):264–272.

Whitnell, K.L., and S.J. Murch. 2018. From student research to commercialization: A case study. Planta Medica International Open 5(1). doi:10.1055/s-0038-1644977

Willcox, O.W. 1954. Quantitative agrobiology: III. The Mitscherlich equation and its constants. Agron. J. 46:323–326. doi:10.2134/agronj1954.00021962004600070008x

Winter, K.B., N.K. Lincoln, and F. Berkes. 2018. The socio-ecological keystone concept: A quantifyable metaphor for understanding the structure, function, and resilience of a biocultural system. Sustainability 10:3294. doi:10.3390/su10093294
