# Supplementary material for: Provenance and family variations in early growth of Manchurian walnut (Juglans mandshurica Maxim.) and selection of superior families
Source: PLoS One. 2024 Mar 7;19(3):e0298918. doi: 10.1371/journal.pone.0298918 (PMC10919699; doi:10.1371/journal.pone.0298918)
Supplement: S1 File — (ZIP) [file pone.0298918.s004.zip › Effect of soil preparation of slope seperated level ditch on the growth and development of walnut in Loess hills.pdf]

黄土区退耕地隔坡水平沟整地对核桃生长发育的影响

赵雨明[1], 侯兴琼[2]

(1. 山西省林业科学研究院, 山西 太原 030012; 2. 晋中市林业局, 山西 榆次 030600)

摘 要: 隔坡水平沟整地技术显著地促进了旱坡地核桃幼树的生长发育, 6年生核桃地径平均达 6.12 cm, 树高 221 cm, 冠幅 245.5 cm× 238.3 cm, 新梢长 85 cm, 新梢粗 1.82 cm, 核桃生长迅速的特性得到了充分体现。剖根试验表明, 1年生核桃幼树根系水平分布达 119 cm~126 cm, 垂直分布达 60 cm~65 cm, 水平根条数达 14条 ~15条; 3年生时, 根系水平分布已达到 143 cm~156 cm, 根系最大分布范围已经交接; 6年生时, 根系已经向下延伸至 4 m 以下的坡面。幼树期产量迅速上升, 7年生时平均产量达 2.56 kg/株, 1 728 kg/hm², 达到了盛果期产量的 57.6%.

关键词: 黄土区; 隔坡水平沟; 核桃; 生长发育
中图分类号: S664.1 文献标识码: A 文章编号: 1007-726X(2010)04-0013-05

Effect of Soil Preparation of Slope_seperated Level Ditch on the Growth and Development of Walnut in Loess Hills

Zhao Yuming[1], Hou Xingqiong[2]

(1. Shanxi Academy of Forestry Sciences, 030012 Taiyuan, China; 2. Jinzhong Forestry Bureau, 030600 Yuci, China)

Abstract: The technique of soil preparation of slope_separated level ditch effectively promote young plant growth and development of walnut. At the age of 6 years, the diameter at ground has reached 6.12 cm averagely, and height 221 cm, crown diameter 245.5 cm× 238.3 cm, shoot length 85 cm, shoot diameter 1.82 cm. Walnut trees showed the rapid growth property in the area. The root test shows that, the root level distribution has reached 119 cm to 126 cm, and vertical distribution 60 cm to 65 cm at the age of 1 year, the number of root has reached 14 to 15. At the age of 3 years in 5 m× 3 m close planting garden, the root largest distribution ranges have been joined. The yields increased rapidly at young growth stage. At the age of 7 years, the yields of average single_plant reached 2.56 kg, and that of per hm² 1 728 kg, reaching 57.6 percent in full fruit period.

Key words: Loess hills; slope_separated level ditch; walnut; growth and development

20世纪 90年代初, 针对山西省西部黄土丘陵区干旱缺水, 枣树幼树不能适龄结果和大树不能丰产、稳产的难题, 山西省林科院在石楼县进行了专题研究, 先后开展了 "旱坡地枣树幼树丰产栽培技术研究" 和 "黄河中游旱坡地枣树密植栽培丰产技术研究" 等研究, 在把现代径流林业抗旱新技术和枣树栽培丰产技术有机结合的基础上, 成功地建立起集水密植丰产枣园, 后被完善为 "郝家山模式"。这一模式在水土保持方面的突破主要体现在集水工程技术——隔坡水平沟整地上。新技术在黄土丘陵区枣树栽培中得到了广泛应用, 取得了巨大成效, 改写了阳坡山地不能进行枣树密植栽培的历史, 大大提高了旱坡地枣树生产力和水土保持的生态效益。近年来, 在退耕还林生态经济林建设中也进行了推广应用。核桃是黄土区生态经济林建设的先锋树种之一, 旱坡地密植栽培后, 栽培模式和栽培技术都发生了重大改变, 借鉴枣树研究成果, 同步研究隔坡水平沟整地方法对核桃生长发育的影响, 是研究山地核桃肥水管理、病虫害防治、整形修剪、适宜树形和密度等密植丰产栽培技术的前提和基础。

收稿日期: 2010-12-01
基金项目: 林业科学技术推广项目 [2006] 24号资助
作者简介: 赵雨明 (1959—), 男, 山西兴县人, 1988年毕业于山西农业大学, 教授级高级工程师。

1 材料与方法

1.1 试验地与试材

试验地设在石楼县小蒜镇下山村晋林山退耕还林地。石楼县位于吕梁山西麓,黄河东岸,辖4镇,5乡,总面积1 742 km^2,人口9.91×10^4人。地势东高西低,群山连绵,地表覆盖着深厚的黄土,因受降水侵蚀、冲刷,沟壑纵横,地形破碎。全境分为山地和丘陵地两部分,西部和北部及城关一带均为黄土丘陵区,属温带大陆性气候。年均气温为9.2 ℃,1月份平均温度为-7 ℃,7月份平均温度为22.7 ℃;年均降水量为550 mm;霜冻期为9月下旬至次年4月下旬,无霜期180 d~190 d。

小蒜镇位于石楼县西北部、黄河东岸,2002年被规划为生态重建重点乡。晋林山方圆约666.7 hm^2耕地全部规划为退耕还林地,树种有核桃、杏树、五角枫、侧伯、刺槐、柠条、紫穗槐等,其中核桃66.67 hm^2,目前已经成林,石楼县也因此成为全省退耕还林重点县样板。

试验用地0.667 hm^2,均为隔坡水平沟整地,隔坡间距5 m,株距3 m。试材为核桃园中1年生(2006年秋季造林),3年生(2004年秋季造林)和6年生(2002年秋季造林)核桃树,供试品种为中林3号。试验地土层深厚,肥力较高,林地经营管理好,道路交通也较为便利。

1.2 研究方法

1.2.1 地上部分生长发育

每个处理设3次重复,每重复选定10株,调查记载植株干高、树高、地径、冠幅、新梢生长量等。进入结果期后,调查统计逐年平均单株产量和每公顷产量,均以鱼鳞坑整地作为对照。

1.2.2 根系生长发育

1.2.2.1 1年生与3年生核桃根系

每个处理分3次重复,每重复内随机抽取1株,采用整株剖挖方法,记载根系最大分布范围和集中分布范围,记录水平根数量、根长、根粗、毛细根数量等,记载垂直根长度和粗度,研究植株根系在土壤中的分布情况。

1.2.2.2 6年生核桃根系

每个处理分2次重复,每重复内随机抽取1株,在距主干4 m,3 m,2 m,1 m处挖4个剖面,面积为50 cm×50 cm,同时剖开主干周围,记载各剖面和主干周围主侧根数量、粗度,研究植株根系在土壤中的分布情况。

2 结果与分析

2.1 隔坡水平沟整地对核桃地上部分的影响

隔坡间距5 m,株距3 m的1年生,3年生和6年生核桃树地上部分生长发育情况见表1。

表1 隔坡水平沟整地核桃树地上部分生长发育情况

树龄	整地方式	地径/cm	干高/cm	树高/cm	冠幅/cm	新梢生长量/cm	
						长度	粗度
1年生	隔坡水平沟	1.16	—	24.2	38.4×30.6	14.2	0.73
	鱼鳞坑	1.03	—	21.1	32.3×30.1	10.5	0.62
3年生	隔坡水平沟	2.56	52	134.0	85.2×53.1	57.1	1.57
	鱼鳞坑	2.17	54	108.0	60.1×40.5	50.3	1.25
6年生	隔坡水平沟	6.12	53	221.0	245.5×238.3	85.2	1.82
	鱼鳞坑	4.83	57	175.0	165.2×178.0	64.7	1.65

由表1可知,隔坡水平沟整地较好地解决了退耕地土壤水分、养分平衡和耕作层以下土质坚硬的难题,同样可以在旱坡地上成功地建立核桃密植丰产园。1年生,3年生和6年生核桃植株均表现出了速生习性,在地径、树高、冠幅和新梢生长量等各项生长指标方面均极显著地高于对照、鱼鳞坑整地。1年生核桃地径为1.16 cm,树高24.2 cm,冠幅38.4 cm×30.6 cm,新梢生长量长为14.2 cm,粗为0.73 cm,显著高于对照。3年生时差异加大,地径为2.56 cm,树高134.0 cm,冠幅85.2 cm×53.1 cm,新梢生长量长为57.1 cm,粗为1.57 cm,各项指标均显著高于对照。到6年生时优势更加明显,地径平均达到6.12 cm,比对照提高了26.70%;树高221.0 cm,比对照提高了26.29%;冠幅245.5 cm×238.3 cm,比对照提高了98.95%;新梢生长量长为85.2 cm,比对照提高了31.68%,粗为1.82 cm,比对照提高了10.30%。核桃生长迅速的特性得到了充分体现,表明隔坡水平沟整地技术在集水、节水和活土层加厚等关键环节发挥了作用。试验表明,核桃到6年生时,工程整地效应仍然十分明显。核桃喜光喜温,在

土壤肥沃、土层深厚、水分充足的立地条件下生长发育良好, 黄河中游石楼县的气候、土壤条件正好满足了这种要求。经集水工程整地后, 较好地解决了土壤水分、养分和土质疏松透气等问题, 显著地促进了核桃幼树的生长发育。

2.2 隔坡水平沟整地对核桃根系生长的影响

2.2.1 1年生核桃根系生长发育

1年生核桃根系生长发育见表 2.

表 2　1年生核桃根系调查

项目 重复	整地 方式	根系最大分布范围/cm		根系集中分布范围/cm		不同根粗范围的水平根系(侧根)								
						0.18 cm~0.26 cm			0.27 cm~0.50 cm			0.51 cm~1.00 cm		
		水平	垂直	水平	垂直	数量/条	长度范围/cm	平均长度/cm	数量/条	长度范围/cm	平均长度/cm	数量/条	长度范围/cm	平均长度/cm
1	隔坡水平沟	120	62	62	45	5	22~45	37.00	5	61~87	72.42	4	70~82	75.50
	鱼鳞坑	45	30	30	16	6	10~45	20.67	4	45~52	48.25			
2	隔坡水平沟	126	65	63	44	6	30~49	43.42	5	64~92	76.43	4	79~98	88.54
	鱼鳞坑	52	34	40	20	7	15~54	23.45	5	48~54	50.34			
3	隔坡水平沟	119	60	66	48	6	26~47	39.51	4	59~87	72.23	4	71~84	78.21
	鱼鳞坑	48	32	32	18	5	14~46	22.24	6	42~58	49.87	1	72	72.00

由表 2可知, 核桃幼树期间前 1 a~2 a地上部分生长相对较慢, 而根系却进行着旺盛的生长。隔坡水平沟整地显著地促进了 1年生核桃幼树根系的生长发育。根系最大分布范围: 水平分布达 119 cm~126 cm, 垂直分布为 60 cm~65 cm; 而对照水平分布为 45 cm~52 cm, 垂直分布为 30 cm~34 cm. 根系集中分布范围: 水平分布达 62 cm~66 cm, 垂直分布为 44 cm~48 cm; 而对照水平分布为 30 cm~40 cm, 垂直分布为 16 cm~20 cm.

水平根分布处理间有 3个级别的根, 而对照有 2个 ~3个级别的根。进一步分析可知, 不同级别水平根分布数量和生长量差异十分显著: 隔坡水平沟整地重复间水平根数量达 14条 ~15条, 而对照仅有 10条 ~12条。其中根粗在 0.18 cm~0.26 cm范围内, 隔坡水平沟整地根数为 5条 ~6条, 平均长度在 37.00 cm~43.42 cm之间; 对照为 5条 ~7条, 平均长度在 20.67 cm~23.45 cm之间。根粗在 0.27 cm~0.50 cm范围内, 隔坡水平沟整地根数为 4条 ~5条, 平均长度在 72.23 cm~76.43 cm之间; 对照为 4条 ~6条, 平均长度在 48.25 cm~50.34 cm之间。根粗在 0.51 cm~1.00 cm范围内, 隔坡水平沟整地根数为 4条, 平均长度在 75.50 cm~88.54 cm之间; 对照仅有 1个重复有此级别的根, 数量为 1条, 长度为 72.00 cm.

2.2.2 3年生核桃根系生长发育

3年生核桃根系生长发育见表 3.

表 3　3年生核桃根系调查

项目 重复	整地 方式	根系最大分布范围/cm		根系集中分布范围/cm		不同根粗范围的水平根系(侧根)								
						0.10 cm~0.50 cm			0.51 cm~1.00 cm			1.01 cm~1.50 cm		
		水平	垂直	水平	垂直	数量/条	长度范围/cm	平均长度/cm	数量/条	长度范围/cm	平均长度/cm	数量/条	长度范围/cm	平均长度/cm
1	隔坡水平沟	143	76	69	34	13	17~70	58.34	13	74~157	91.00	4	119~146	129.57
	鱼鳞坑	84	58	53	25	9	15~64	42.36	5	60~113	74.14	2	88~92	90.00
2	隔坡水平沟	156	80	74	32	15	19~82	62.53	14	82~152	95.42	5	128~154	136.34
	鱼鳞坑	86	54	56	28	11	18~74	44.57	6	68~121	77.33	1	89	89.00
3	隔坡水平沟	148	74	73	37	14	19~73	60.86	11	75~150	92.37	4	124~150	131.47
	鱼鳞坑	89	51	59	30	14	20~81	47.31	8	70~118	81.52	3	91~97	95.00

由表 3可知, 隔坡水平沟 3年生核桃根系最大分布范围: 水平分布已经达到 143 cm~156 cm, 垂直分布为 74 cm~80 cm; 而对照水平分布为 84 cm~89 cm, 垂直分布为 51 cm~58 cm. 根系集中分布范围: 水平分布达 69 cm~74 cm, 垂直分布为 32 cm~37 cm; 对照水平分布为 53 cm~59 cm, 垂直

分布为 25 cm~30 cm。经隔坡水平沟工程整地后，隔坡间距 5 m，株距 3 m的核桃密植园，3年生时根系最大分布范围已经交接，生长发育得到了充分展示。如果栽植密度过大，幼树期间株间存在肥水竞争。旱坡地肥水有限，3 m×5 m低密度的密植园较好地调节了肥水有限和生长发育的矛盾。

3年生核桃和 1年生核桃相比，根数和生长量显著增加。水平根数量达 29条 ~34条，而对照仅有 16条 ~25条，差异十分显著。重复间粗度在 0.1 cm~0.5 cm范围内的水平根数量为 13条 ~15条，平均长度在 58.34 cm~62.53 cm范围内；而对照水平根数量为 9条 ~14条，平均长度在 42.36 cm~47.31 cm范围内。粗度在 0.51 cm~1.00 cm范围内的水平根数量为 11条 ~14条，平均长度在 91.00 cm~95.42 cm范围内；而对照为 5条 ~8条，平均长度在 74.14 cm~81.52 cm范围内。粗度在 1.01 cm~1.50 cm范围内的水平根数量为 4条 ~5条，平均长度在 129.57 cm~136.34 cm范围内，而对照为 1条 ~3条，平均长度在 89 cm~95 cm范围内。以上数据说明栽培环境的改变显著地促进了核桃根系的生长发育。

2.2.3 6年生核桃根系生长发育

6年生核桃根系生长发育见表 4.

表 4 6年生核桃根系在不同坡面的剖面分布情况

剖面与主干的距离 /m	整地方式	重复	垂直分布 / cm	不同根粗范围内根的数量 /条					
				2.51~3.00 cm	2.01~2.50 cm	1.51~2.00 cm	1.01~1.50 cm	0.51~1.00 cm	0.11~0.50 cm
4	隔坡水平沟	1	5~35	—	—	—	—	1	3
		2	5~40	—	—	—	—	1	4
	鱼鳞坑	1	—	—	—	—	—	—	—
		2	—	—	—	—	—	—	—
3	隔坡水平沟	1	5~31	—	—	—	1	1	6
		2	5~35	—	—	—	1	2	5
	鱼鳞坑	1	5~33	—	—	—	—	1	7
		2	5~34	—	—	—	—	2	8
2	隔坡水平沟	1	5~45	—	—	1	4	2	6
		2	5~50	—	—	1	3	3	5
	鱼鳞坑	1	5~45	—	—	—	2	7	6
		2	5~47	—	—	—	3	8	5
1	隔坡水平沟	1	5~60	1	1	1	8	6	9
		2	5~58	1	1	2	9	5	9
	鱼鳞坑	1	5~47	—	1	1	5	4	7
		2	5~43	—	—	2	6	5	8
主干四周	隔坡水平沟	1	5~65	1	1	2	11	7	10
		2	5~63	1	1	3	13	8	9
	鱼鳞坑	1	5~45	—	1	2	8	7	10
		2	5~48	—	—	3	9	8	13

由表 4可知，不同整地方式的 6年生核桃植株根系在坡面上的分布数量和大小仍差异很大。

坡面距主干 4 m的剖面，隔坡水平沟植株已经有 0.51 cm~1.00 cm级以下 2个级别的根系分布，重复间根的数量为 4条 ~5条，而对照还没有分布。坡面距主干 3 m的剖面，隔坡水平沟植株有 1.01 cm~1.50 cm级以下 3个级别的根系，根数为 8条；对照有 0.51 cm~1.00 cm级以下 2个级别的根系，根数为 8条 ~10条。坡面距主干 2 m的剖面，隔坡水平沟植株有 1.51 cm~2.00 cm级以下 4个级别的根系，根数为 12条 ~13条；对照有 1.01 cm~1.50 cm级以下 3个级别的根系，根数为 15条 ~16条。坡面距主干 1 m的剖面，隔坡水平沟植株有 2.51 cm~3.00 cm级以下 6个级别的根系，根数为 26条 ~27条；对照有 2.01 cm~2.50 cm级以下 4个 ~5个级别的根系，根数为 18条 ~21条。主干四周，隔坡水平沟植株有全部 6个级别的根系，根数为 32条 ~35条；而对照有 4个 ~5个级别的根系，根数为 28条 ~33条。

核桃 6年生时，隔坡水平沟植株根系已经向下延伸至 4 m以外的隔坡坡面，而对照根系尚未伸至 4 m以下的坡面。隔坡水平沟整地加速了核桃幼树

的生长发育, 地上部分树形选择和栽培技术要和根系承受力相对应。树形选择应以开心形为主, 以控制树冠, 达到快速成形、提早结果的目的; 栽培技术要以集约经营技术为主, 精耕细作, 加大肥水管理力度, 维持营养生长和生殖生长的平衡。随着年龄的增加, 树冠逐渐成形, 隔坡坡面单位土体内根的密度加大, 水肥竞争更加激烈。可见, 在肥水有限的旱坡地上建立密植园, 隔坡间距 5 m, 株距 3 m的密度也是旱坡地核桃栽培的最佳密度。

2.3 隔坡水平沟整地对核桃产量的影响

隔坡水平沟整地对核桃产量的影响见表 5.

表 **5** 幼树期间核桃密植园产量

整地方式	4年生(05年)		6年生(07年)		7年生(08年)	
	/(kg株$^{-1}$)	/(kg hm^{-2})	/(kg株$^{-1}$)	/(kg hm^{-2})	/(kg株$^{-1}$)	/(kg hm^{-2})
隔坡水平沟	0.98	661.5	2.04	1 377.00	2.56	1 728.0
鱼鳞坑	0.32	216.0	1.01	681.75	1.46	985.5

注: 5年生(06年)时遭特殊自然灾害(4月上旬冰雹)袭击, 核桃几近绝产。

由表 5可知, 隔坡水平沟整地显著地影响了核桃产量的形成, 幼树期产量迅速上升, 7年生时已经进入了盛果初期。

4年生核桃, 平均单株产量为 0.98 kg,每公顷产量为 661.5 kg,而对照平均单株产量仅为 0.32 kg,每公顷产量为 216.0 kg. 6年生时单株产量上升到 2.04 kg,每公顷产量为 1377 kg,对照平均单株产量为 1.01 kg,每公顷产量为 681.75 kg. 7年生时, 单株产量已经达到 2.56 kg,每公顷产量为 1 728 kg,对照平均单株产量为 1.46 kg,每公顷产量为 985.5 kg.

由此可见, 隔坡水平沟整地改变了水平沟内深层土壤的理化性状和水肥气热, 结合密植栽培, 有效地促进了核桃幼树快速成形和提早结果, 7年生的核桃每公顷产量已上升到 1728 kg.

3 结论

黄土丘陵区退耕地采用隔坡水平沟整地, 进行核桃密植丰产栽培是完全可行的。

隔坡水平沟整地技术显著地促进了核桃幼树地上部分的生长发育, 6年生核桃地径平均达 6.12 cm,树高 221 cm,冠幅 245.5 cm×238.3 cm,新梢生长量长为 85.2 cm,粗为 1.82 cm,核桃生长迅速的特性得到了充分体现。

剖根试验表明, 1年生核桃幼树根系水平分布达 119 cm~126 cm,垂直分布为 60 cm~65 cm;水平根达 14条 ~15条。3年生时根系水平分布已经达到 143 cm~156 cm,株间水平根系已经交接; 垂直分布为 74 cm~80 cm,水平根数量达 29条 ~34条。

6年生核桃根系已经向下延伸至 4 m以外的坡面, 隔坡水平沟整地加速了核桃幼树的生长发育, 地上部分树形选择和栽培技术要和根系承受力相对应。同时表明, 在肥水有限的旱坡地上建立密植园, 隔坡间距 5 m,株距 3 m的密度也是核桃栽培的最佳密度。

隔坡水平沟显著地促进了核桃产量的形成, 幼树期产量迅速上升, 3年生时开始挂果, 4年生时形成产量, 7年生时已经进入了盛果初期, 平均单株坚果产量达 2.56 kg,每公顷产量为 1728 kg,已经达到了盛果期产量的 57.6%.

参考文献:

[1] 赵雨明. 旱坡地枣树密植园施肥试验[J]. 东北林业大学学报, 2001(4): 120-124.

[2] 赵雨明, 卢桂宾, 史敏华, 等. 旱坡地枣树密植园密度与根系关系的研究[J]. 山西林业科技, 1998(4): 10-14, 42.

[3] 邝立刚. 黄土丘陵旱坡地枣树密植丰产技术[G]//西部大开发科教现行与可持续发展. 中国科协 2000年学术年会文集. 北京: 中国科技出版社, 2000.

[4] 卢桂宾, 杨海青. 旱坡地密植枣园试验简报[J]. 山西果树, 1995(1): 40.

[5] 卢桂宾. 旱坡地枣树密植园的丰产树形[J]. 东北林业大学学报, 2001(4): 134-138.
