# Supplementary material for: Provenance and family variations in early growth of Manchurian walnut (Juglans mandshurica Maxim.) and selection of superior families
Source: PLoS One. 2024 Mar 7;19(3):e0298918. doi: 10.1371/journal.pone.0298918 (PMC10919699; doi:10.1371/journal.pone.0298918)
Supplement: S1 File — (ZIP) [file pone.0298918.s004.zip › Effects of branch drawing on the leaf growth, fruit quality and yield of Gala apple.pdf]

西北林学院学报 2009, 24(2): 71～74
Journal of Northwest Forestry University

不同拉枝角度对嘎拉苹果叶片及果实产量和品质的影响[①]

杜 荣[1], 曲俊贤[1], 赵增强[2], 韩明玉[1], 范崇辉[1*]

(1. 西北农林科技大学 园艺学院,陕西 杨陵 712100; 2. 丹凤县林果站,陕西 丹凤 726200)

摘 要:为探讨嘎拉苹果细长纺锤形小主枝的适宜拉枝角度,分别进行了 40°～50°、60°～70°、80°～90°、100°～110° 4 个水平的拉枝处理,测定了叶片质量、果实品质和单枝产量相关指标。结果表明,拉枝角度 80°以上比 70°以下果实品质好,单枝产量高。拉枝角度 80°～90°比 100°～110°时单叶干重、果实单果重、可溶性蛋白质含量高,而果实着色指数和 Vc 含量低,且差异显著;单叶鲜重、果实光洁指数、果皮叶绿素含量、果形指数、果锈指数、硬度、可溶性固形物、可溶性总糖、可滴定酸含量等指标值及单枝产量差异不显著。在陕西渭北南部苹果产区,嘎拉苹果纺锤形小主枝的拉枝角度以 90°～100°为宜。

关键词:不同拉枝角度;嘎拉;叶片质量;果实品质;单枝产量

中图分类号:S661.1 **文献标识码**:A **文章编号**:1001-7461(2009)02-0071-04

Effects of Branch Drawing on the Leaf Growth, Fruit Quality and Yield of Gala Apple

DU Rong[1], QV Jun-xian[1], ZHAO Zeng-qiang[2], HAN Ming-yu[1], FAN Chong-hui[1*]

(1. *College of Horticulture, Northwest A&F University, Yangling, Shaanxi* 712100, *China*;
2. *Danfeng County Forest-Fruit Station, Danfeng, Shaanxi* 712100, *China*)

Abstract: In order to investigate the proper angle of branch drawing to form slender spindle-shaped like sub-main, branches of Gala, a main cultivar of apple. Four treatments were designed: 40°～50°, 60°～70°, 80°～90°, 100°～110° and some indices were measured, such as leaf and fruit quality, and fruit yield of a branch. The results showed that the qualities of fruit with the branch angle over 80° were better than those with the branch angles below 70°. When the branch angle were 80°～90°, the leaf dry weight, fruit weight and soluble protein were higher than those with the angle of 100°～110°, but lower in color index and Vc content and with significant differences. No significant differences were found between the indices such as fresh weight, smoothing and cleaning index, chlorophyll, fruit shape index, fruit rust index, hardness, soluble solids, total soluble sugar, titratable acid and the yield of unit branch. Considered comprehensivly, the suitable branch drawing angles of Gala cultivar were between 90°～100°.

Key words: different angle of branch; Gala apple; leaf quality; fruit quality; yield of a branch

2005 年我国苹果面积 189 万 hm^2,产量 2 401 万 t,分别占世界总面积和总产量的 36.2%和 37.8%,在世界苹果产业中占有举足轻重的地位。但我国和陕西的苹果产业仍然存在着产量低、品质差等问题。拉枝是现代果树修剪的常用方法之一,对幼树拉枝可促进树冠扩大,早成形和结果;大树拉枝可改善树体的通风透光条件,提高光能利用率,促进果树内膛照光和果实着色,从而提高果实的产量与品质。关于苹果拉枝技术前人已做过较多研究[1-6],但生产中仍存在一些问题,如苹果主要品种和树形拉枝的适宜角度未进行深入研究。因此,主要对生产主栽品种嘎拉细长纺锤形小主枝的适宜角度进行了研究,旨在探讨不同拉枝角度对嘎拉果实产量和品质的影响,为生产应用提供依据。

① **收稿日期**:2008-07-31 **修回日期**:2008-10-09
基金项目:农业部项目(2006G-28,nyhyzx07-024);现代农业产业技术体系项目(MATS);国家支撑计划(2007BAK31B01-04)。
作者简介:杜荣,女,在读硕士,研究方向:果树生理。
* **通讯作者**:范崇辉,男,教授,主要从事果树生理生态的教学与研究。

(C)1994-2023 China Academic Journal Electronic Publishing House. All rights reserved. http://www.cnki.net

1 材料与方法

1.1 试验园概况

试验设在宝鸡市农业科技苹果专家大院进行。该果园海拔850 m,年平均气温12.0～12.5℃,年降雨量601 mm,无霜期209 d,昼夜温差大,苹果树1998年冬季栽植,主栽品种富士,授粉品种嘎拉,以M26为矮化中间砧,树形采用细长纺锤形,株行距2.5 m×4.2 m,树势健壮,有灌溉条件,砂壤土,管理水平较高。

1.2 试验设计与方法

1.2.1 拉枝处理 2006年3月选生长势、高度、方位基本一致的枝条作为单位枝分别进行40°～50°(Ⅰ),60°～70°(Ⅱ),80°～90°(Ⅲ),100°～110°(Ⅳ)4个水平的拉枝处理,每个处理6株,共24株。

1.2.2 叶片处理 2007年秋季新梢停止生长后随机采取处理枝基部粗度、长度基本相同的新梢中部第6、7、8完整无损叶片,每处理取100片。取叶后立即外裹湿毛巾,装入塑料袋带回实验室,冲洗干净,用电子天平测定单叶鲜重、比叶鲜重、叶片厚度。然后于105℃烘箱烘15 min杀青,再置80℃烘箱烘24 h至恒重,测单叶干重、比叶干重。以上指标均重复3次。

1.2.3 果实处理 2007年8月中旬果实成熟期,每个处理随机采30个果实,带回实验室测定品质指标。

每个处理取10个果实,用电子天平称量果实单果重,用游标卡尺测定果实纵横径后计算果形指数。

果实着色指数分5级:0级果面不着色,1级果面着色1%～30%,2级果面着色30.1%～60%,3级果面着色60.1%～90%,4级果面着色90.1%以上;果面光洁指数分为4级:1级果面粗糙,2级果面较粗糙,色较暗,3级果面较光滑,4级果面光洁细腻。果面果锈指数分5级:0级果面无锈斑,1级果面0.5 cm^2以下锈斑,2级果面0.5～1.0 cm^2锈斑,3级果面1.0～2.0 cm^2锈斑,4级果面2.0 cm^2以上锈斑。其中着色指数=Σ(各级果数×级数)/总果数;果面光洁指数=Σ(各级果数×级数)/总果数;果锈指数=Σ(各级果数×级数)/总果数。

果实硬度为去皮硬度,用手持硬度计测量,每个果实在胴部不同部位取5个点。每处理测5个果实,取平均值。

用手持糖量计测定可溶性固形物;用比色法测定叶绿素[7];用NaOH酸碱中和滴定法测定可滴定酸;用蒽酮法测定可溶性总糖;用考马斯亮蓝G-250测定蛋白质;用钼蓝比色法测定Vc。以上指标各处理重复4次,取平均值。

1.2.4 单枝产量 在试验树上选高度、方位、生长势基本一致的不同拉枝角度处理枝条作为单位枝,分别测定每个枝条上的所有果实的总重量,每个处理选5个枝条,取平均值。

1.3 数据分析

用DPS软件对各指标数据进行方差和显著性分析。

2 结果与分析

2.1 不同拉枝角度对叶片质量的影响

从表1可以看出,叶片的单叶鲜重和单叶干重随着拉枝角度的增大呈增加的趋势,在处理Ⅲ时,达到最大值,随后下降,而且不同角度间的差异不同。单叶鲜重处理Ⅲ与处理Ⅳ差异不显著,处理Ⅲ、处理Ⅳ与处理Ⅱ、处理Ⅰ存在极显著差异($P<0.01$),处理Ⅱ与处理Ⅰ存在极显著差异。单叶干重处理Ⅲ与处理Ⅳ存在显著差异,处理Ⅲ、处理Ⅳ与处理Ⅱ、处理Ⅰ存在极显著差异,处理Ⅱ与处理Ⅰ存在极显著差异。比叶鲜重、比叶干重和叶片厚度均随着拉枝角度的增大而增大,在处理Ⅲ时达到最大值,随后又随着拉枝角度的增大而呈下降趋势,各拉枝角度间差异不显著($P>0.05$)。

表1 不同拉枝角度对叶片质量的影响

Table 1 Effect of branch drawing angle on leaf quality

处理	单叶鲜重/g	单叶干重/g	比叶鲜重/(g·cm^{-2})	比叶干重/(g·cm^{-2})	叶片厚度/μm
Ⅰ	0.91cC	0.33dC	0.009 4	0.003 4	288.98
Ⅱ	1.07bB	0.42cB	0.009 7	0.003 8	319.01
Ⅲ	1.53aA	0.67aA	0.011 7	0.005 1	320.41
Ⅳ	1.47aA	0.59bA	0.010 6	0.004 3	318.61

注:同行数据后不同字母表示差异显著(小写表示 $P<0.05$,大写表示 $P<0.01$)。表2、表3同。

2.2 不同拉枝角度对果实品质的影响

由表2可以看出在拉枝角度较小时,单果重与拉枝角度呈正相关,拉枝角度达到处理Ⅲ时单果重最大,后随拉枝角度的增大单果重呈下降趋势,不同拉枝角度处理间存在极显著差异($P<0.01$);果实着色指数随着拉枝角度的增大而增加,在处理Ⅳ值

(C)1994-2023 China Academic Journal Electronic Publishing House. All rights reserved. http://www.cnki.net

最大，与其他处理间均存在极显著差异，处理Ⅲ与处理Ⅱ存在显著差异，与处理Ⅰ存在极显著差异，处理Ⅱ与处理Ⅰ存在极显著差异；在拉枝角度较小时，果面光洁指数与拉枝角度呈正相关，在处理Ⅲ时值最大，与处理Ⅱ、处理Ⅰ存在极显著差异($P<0.01$)，与处理Ⅳ差异不显著。处理Ⅳ与处理Ⅱ不存在显著差异，与处理Ⅰ存在极显著差异，处理Ⅱ与处理Ⅰ存在显著差异；果皮叶绿素含量与拉枝角度呈现负相关，处理Ⅲ与处理Ⅳ差异不显著，处理Ⅲ、处理Ⅳ与其他2个处理间存在极显著差异，处理Ⅱ与处理Ⅰ也存在极显著差异；角度较小时果形指数与拉枝角度呈正相关，处理Ⅲ果形指数值最大，随后减小，但不同拉枝角度处理对果形指数的影响差异不显著；果锈指数与拉枝角度呈现负相关，处理Ⅲ与处理Ⅳ差异不显著，处理Ⅲ、处理Ⅳ与其他2个处理存在极显著差异，处理Ⅱ与处理Ⅰ存在极显著差异。

表2　不同拉枝角度对嘎拉果实外观品质的影响

Table 2　Effect of branch drawing angle on fruit external quality

处理	单果重/g	着色指数	光洁指数	果皮叶绿素	果形指数	果锈指数
Ⅰ	184.8dD	2.81dC	1.80cC	0.0661aA	0.894	4.07aA
Ⅱ	220.5cC	3.08cB	2.41bBC	0.0464bB	0.896	3.51bB
Ⅲ	291.3aA	3.18bB	3.32aA	0.0195cC	0.901	2.39cC
Ⅳ	245.4bB	3.56aA	2.91abAB	0.0148cC	0.899	2.45cC

由表3可以看出角度较小时果实硬度随拉枝角度的增大而增加，在处理Ⅲ时值最大，随后开始减小，处理Ⅲ与Ⅳ差异不显著，但与Ⅱ差异显著，与处理Ⅰ差异极显著；处理Ⅳ与Ⅱ差异不显著，与处理Ⅰ差异显著；处理Ⅱ与Ⅰ差异不显著。可溶性固形物在角度小时随拉枝角度的增大而增加，在处理Ⅲ值最大，与处理Ⅳ差异不显著，与处理Ⅱ、Ⅰ差异极显著；处理Ⅳ与处理Ⅱ差异不显著，与处理Ⅰ差异显著；处理Ⅱ与处理Ⅰ差异不显著。可溶性总糖在角度小时与拉枝角度呈正相关，在处理Ⅲ值最大，随后开始减小，处理Ⅲ与处理Ⅳ差异不显著，均与处理Ⅱ、处理Ⅰ差异极显著；处理Ⅱ与处理Ⅰ差异极显著。可滴定酸在角度较小时，与拉枝角度呈负相关，在处理Ⅲ时值最小，与处理Ⅳ差异不显著，与处理Ⅱ、处理Ⅰ差异极显著；拉枝角度处理Ⅳ与处理Ⅱ差异显著，与处理Ⅰ差异极显著；处理Ⅱ与处理Ⅰ差异不显著。在角度较小时可溶性蛋白质与拉枝角度呈正相关，在处理Ⅲ值最大，随后又减小，处理Ⅲ与其他各处理均存在极显著差异；处理Ⅳ与处理Ⅱ差异不显著，与处理Ⅰ差异极显著；处理Ⅱ与处理Ⅰ存在显著差异；Vc含量在处理Ⅳ值最大，各处理间均存在显著差异。

表3　不同拉枝角度对嘎拉苹果内在品质的影响

Table 3　Effect of branch drawing angle on fruit internal quality

处理	硬度	可溶性固形物	可溶性总糖	可滴定酸	可溶性蛋白质	Vc含量
Ⅰ	8.15cB	118.55cB	83.19cC	3.70aA	7.06cC	4.57dD
Ⅱ	8.44bcAB	132.27bcB	101.83bB	3.48aAB	8.20bBC	6.03bB
Ⅲ	9.46aA	136.85aA	120.75aA	2.63bC	10.67aA	5.52cC
Ⅳ	9.16abAB	130.05abAB	115.63aA	2.89bBC	9.11bB	7.21aA

2.3　不同拉枝角度对单枝产量的影响

由表4可以看出在枝条的生长势基本相近的情况下，拉枝角度在处理Ⅲ和处理Ⅳ时单枝产量明显比处理Ⅰ和Ⅱ高。枝条长度60～80 cm时处理Ⅳ的单枝产量比处理Ⅲ高0.4倍，而处理Ⅲ比处理Ⅰ和Ⅱ高0.9～2.4倍。枝条长度在80～100 cm时，处理Ⅲ的产量最高，比处理Ⅰ和Ⅱ高1.7～2.8倍，和处理Ⅳ差异不明显。在枝条长度100～120 cm时，处理Ⅳ的产量比处理Ⅰ和Ⅱ高1.4～1.6倍，与处理Ⅲ产量相当。在枝条长度120～140 cm时，处理Ⅲ的值高于处理Ⅰ和Ⅱ2.0～2.1倍，比处理Ⅳ高0.27倍。因此在枝条生长势基本一致的情况下，拉大枝条角度有利于提高产量。

表4　不同拉枝角度对单枝产量的影响

Table 4　Effect of branch drawing angle an fruit yield in a single banch

g

处理	枝条长度/cm			
	60～80	80～100	100～120	120～140
Ⅰ	295.2	463.5	729.3	867.8
Ⅱ	522.7	657.3	788.2	909.0
Ⅲ	1 000.9	1 781.1	1 903.6	2 734.5
Ⅳ	1 438.0	1 707.4	1 924.3	2 154.8

3　结论与讨论

叶片是果树的光合器官，拉枝对果树叶片质量及光合效能有一定影响。许利军[8]指出苹果拉枝后树体通风透光状况改善，特别是下部及内膛叶片光

(C)1994-2023 China Academic Journal Electronic Publishing House. All rights reserved.　http://www.cnki.net

合效能提高。吴鲜亮[9]等对苹果梨幼树研究得出，拉枝后叶片的叶绿素含量增加了，使光合效率提高，光合产物增加。本试验研究表明，嘎拉苹果拉枝后单叶鲜重和单叶干重增加，但比叶重和叶片厚度增加不显著，拉枝 80°以上时，叶片重量大，叶片质量有所改善，对促进叶片光合作用有积极意义。

不同拉枝角度对果树的产量与品质有重要影响。高建国[10]认为红富士苹果拉枝可抑制枝条生长势，增加枝条受光面积，有利于养分的积累，促进果实增大。文解华[11]对黑宝石李进行拉枝试验结果表明，拉枝明显增加产量和提高果实品质。李勇武[12]研究认为，富士苹果拉枝 110°、嘎拉苹果拉枝 90°左右时果实品质最好。本试验研究结果表明嘎拉苹果在拉枝角度为 80°～110°比 40°～70°时果实品质优，单枝产量高。拉枝角度为 80°～90°时单叶干重、单果重和可溶性蛋白质含量最高，拉枝角度为 100°～110°时果实着色指数和 Vc 含量最大。

参考文献：

[1] 张继义，赵国生，胡约军，等.苹果梨幼树拉枝试验[J].中国果树，2001(1)：19.

[2] 朴松树.扭拉枝整形使小苹果幼树早产早丰[J].北方园艺，1990(10)：33-34.

[3] 何世珑，周军马，恩 明，等.苹果幼树拉枝效应观察初报[J].宁夏农林科技，1994(1)：21-23.

[4] 郭宝林.苹果幼旺树增枝促花处理的效应[J].中国果树，1999(3)：16-17.

[5] 戴文圣.拉枝对不同品种幼龄梨树生长结果的影响[J].浙江林学院学报，1996(2)：123-129.

[6] 江政俊，郃微森.夏季拉枝成形改造放任旺长梨树[J].中国南方果树，2001(3)：55.

[7] 高俊风.植物生理研究技术[M].北京：高等教育出版社，2001：101-103.

[8] 许利军.苹果“强拉枝”及拉后的管理[J].西北园艺，2004(4)：13-14.

[9] 吴鲜亮，何志爱，杨勇.拉枝对苹果梨幼树生长发育的影响[J].内蒙古农业科技，2008(1)：54-55.

[10] 高建国.红富士苹果树生长季修剪关键技术[J].西北园艺，2005(4)：44.

[11] 文解华.黑宝石李不同时间拉枝促花试验[J].广西园艺，2005，16(5)：36-37.

[12] 李勇武，韩明玉，范崇辉，等.不同拉枝角度对苹果果实品质的影响[J].西北农林科技大学学报：自然科学版，2006，34(11)：157-159.

[13] 薛兴军，刘春林.香梨开心小树拉枝时间及角度的反应试验[J].园艺特产，2005(6)：19.

(上接第 62 页)

3 结论与讨论

松树的针叶束实质就是茎极度缩短的短枝，只是在通常条件下，因受顶端优势的抑制，而呈休眠状态。一旦打破休眠，其芽就会恢复分生能力，并发育成枝条。研究表明，适宜的营养液能提高根系的质量，利于针叶束更快、更好地适应移栽的环境。

在陕西地区，柴松针叶束水培较适宜的采叶时间在 12～1 月份。

采叶时以采当年生枝条中下部的粗壮针叶为好；采叶后冷藏 30～35 d，有利于生根。

切基对柴松针叶束生根有促进作用。

单独使用生长调节剂而不用营养液处理可以生根，但生根时间长，并且根系质量差；而只用营养液培养很难生根。

采自 2 a 生柴松的针叶束可作为水培生根的材料，最佳的生长调节剂及其浓度为 NAA 80 $mg \cdot L^{-1}$ 处理 24 h；最佳的营养液配方为硼酸 70 $mg \cdot L^{-1}$、硝酸铵 50 $mg \cdot L^{-1}$、Vb_1 30 $mg \cdot L^{-1}$、磷酸二氢钾 20 $mg \cdot L^{-1}$。

参考文献：

[1] 陕西省林业科学研究所，陕西省防护林建设工作队.陕西主要树种造林技术[M].西安：陕西科学技术出版社，1992：14-15.

[2] 乐海波，张成高，范柏林.油松针叶束繁殖研究[J].湖北林业科技，2007 (1)：26-31.

[3] 王瑞勤，李凤兰，谭丽婷.油松针叶束离体培养下器官建成的解剖学研究[J].北京林业大学学报，1994，16 (4)：28-32.

[4] 周心铁，杨承桂.松树针叶束育苗[M].北京：中国林业出版社，1988.

[5] 杨承桂，周全国，赵文平，等.湿地松无性系针叶束育苗生根的研究[J].湖北林业科技，1990 (3)：21-24.

[6] 陶民保，王友玉，徐郭俊，等.湿地松火炬松针叶束水培育苗试验初报[J].江苏林业科技，1998，15 (4)：13-15.

[7] 李福秀，张晓明，周体林，等.思茅松针叶束水培生根的研究[J].西南林学院学报，2005，25(1)：25-27.

[8] 罗延恂.松属针叶束水培育苗[J].吉林林业科技，1985 (3)：8-9.

[9] 王金祥，严小龙，潘瑞炽.不定根形成与植物激素的关系[J].植物生理学通讯，2005，41(2)：133-142.

[10] 郑彩霞，李凤兰，高荣孚，等.松树针叶束内源激素与单宁的变化[J].北京林业大学学报，1996，5(2)：55-62.

[11] 高甲荣.油松针叶营养元素含量与叶龄及采叶部位的关系[J].陕西林业科技，1998(1)：1-9.

(C)1994-2023 China Academic Journal Electronic Publishing House. All rights reserved. http://www.cnki.net
