# Supplementary material for: Provenance and family variations in early growth of Manchurian walnut (Juglans mandshurica Maxim.) and selection of superior families
Source: PLoS One. 2024 Mar 7;19(3):e0298918. doi: 10.1371/journal.pone.0298918 (PMC10919699; doi:10.1371/journal.pone.0298918)
Supplement: S1 File — (ZIP) [file pone.0298918.s004.zip › Effects of mixing proportion on the growth, stem form quality and spatial utilizataion ability of Manglietia yuyuanensis in mixed forests with Cunninghamia lanceolata and M. yuyuanensis.pdf]

第 44 卷 第 1 期
2020 年 1 月

南京林业大学学报(自然科学版)
Journal of Nanjing Forestry University (Natural Sciences Edition)

Vol.44, No.1
Jan., 2020

DOI:10.3969/j.issn.1000-2006.201810003

杉莲混交林中乳源木莲生长形质、空间利用能力的混交比例效应

欧建德[1],吴志庄[2*],康永武[3]

(1.福建省明溪县林业局,福建 明溪 365200;2.国家林业和草原局竹子研究开发中心,浙江 杭州 310012;3.福建省沙县林业局,福建 沙县 365500)

摘要:【目的】针阔混交林以其有效改善针叶纯林树种结构简单、生态功能低效、生产力低下局面的特点而被广泛推广。杉木乳源木莲混交林(简称为"杉莲混交林")是中国南方林区重要的混交林类型,系统分析各混交比例类型下乳源木莲生长性状、干形形质以及空间利用能力和综合表现的效应与规律,为科学构建杉莲混交林提供理论依据。【方法】以福建省沙县 12 年生不同混交比例杉莲混交林和纯林中的乳源木莲为研究对象,调查乳源木莲的生长形质和空间利用能力等系列性状表现,分析乳源木莲在各混交类型中生长、干形形质、空间利用能力等系列性状的差异变化;应用主成分分析法综合有显著混交比例类型效应的乳源木莲生长形质和空间利用能力表现,揭示乳源木莲生长、干形形质、空间利用能力综合表现的混交比例类型效应和变化规律,并基于总体表现进行混交比例类型的评价与优选。【结果】乳源木莲各项生长指标均有显著的混交比例效应,胸径、树高、单株材积随混交比例减少而逐渐增加;乳源木莲尖削度和胸高形数随混交比例减少而逐渐减少,枝下高则随混交比例减少而逐渐增加;乳源木莲冠高、树冠体积、树冠表面积及生长空间指数随混交比例减少而逐渐增大,但树冠圆满度则随混交比例减少而逐渐减小,乳源木莲在生长形质及空间利用能力的综合得分值呈现随混交比例减少而逐渐增大;混交比例对乳源木莲的冠幅和枝下高比例无显著影响。【结论】混交比例类型显著改变杉莲混交林中乳源木莲生长、干形形质和空间利用能力及其综合表现,合理混交比例类型可显著提高其生长、干形形质和空间利用能力,乳源木莲生长、干形形质和空间利用能力等性状有着不同的混交比例效应,开展综合评价十分必要。综上,杉莲混交林在密度为 2 500 株/hm^2时,以 2 杉 1 莲混交模式下乳源木莲生长形质及空间利用能力表现最好。

关键词:乳源木莲;杉木;生长性状;干形形质;空间利用能力;主成分分析;混交比例效应

中图分类号:S758 **文献标志码**:A **开放科学(资源服务)标识码(OSID)**:

文章编号:1000-2006(2020)01-0089-08

Effects of mixing proportion on the growth, stem form quality and spatial utilizataion ability of *Manglietia yuyuanensis* in mixed forests with *Cunninghamia lanceolata* and *M. yuyuanensis*

OU Jiande[1], WU Zhizhuang[2*], KANG Yongwu[3]

(1. Mingxi County Forestry Bureau of Fujian Province, Mingxi 365200, China; 2. China National Bamboo Research Center, Hangzhou 310012, China; 3. Shaxian County Forestry Bureau of Fujian Province, Shaxian 365500, China)

Abstract: 【Objective】Coniferous-broad-leaved mixed forests have been widely promoted for their functions in improving the simple structure, low ecological function, and low productivity of pure coniferous stands. *Cunninghamia lanceolata* and *Manglietia yuyuanensis* mixed forest is an important type of mixed forest in southern China. The effects and laws of growth, stem form as well as quality, spatial utilization ability, and comprehensive performance of *C. lanceolata* and *M.*

收稿日期:2018-10-03 **修回日期**:2019-03-26

基金项目:中央财政林业科技推广示范资金项目(标准化示范[2014]2 号);三明市双创城市示范项目((2017)72 号)。

第一作者:欧建德(smmxojd@163.com),教授级高级工程师,ORCID(0000-0001-5023-1809)。*通信作者:吴志庄(wzzcaf@126.com),副研究员,ORCID(0000-0003-3469-498X)。

引文格式:欧建德,吴志庄,康永武,等. 杉莲混交林中乳源木莲生长形质、空间利用能力的混交比例效应[J]. 南京林业大学学报(自然科学版),2020,44(1):89-96. OU J D, WU Z Z, KANG Y W. Effects of mixing proportion on the growth, stem form quality and spatial utilizataion ability of *Manglietia yuyuanensis* in mixed forests of *Cunninghamia lanceolata* with *M. yuyuanensis*[J]. Journal of Nanjing Forestry University (Natural Sciences Edition), 2020, 44(1): 89-96. DOI: 10.3969/j.issn.1000-2006.201810003.

yuyuanensis mixed forest under different mixing proportions were systematically analyzed, to provide a theoretical basis for the scientific construction of *C. lanceolata* and *M. yuyuanensis* mixed forests.【Method】*M. yuyuanensis* trees from 12-year-old *C. lanceolata* and *M. yuyuanensis* mixed forests under different mixing proportions and *M. yuyuanensis* pure stands in Shaxian County of Fujian Province were selected as the research objects. The growth quality, stem form and space utilization ability of *M. yuyuanensis* were investigated, and differences as well as changes in growth, stem form quality, and spatial utilization ability of *M. yuyuanensis* under different types were analyzed. Based on the characteristics of growth, stem form quality, and spatial utilization ability, a principal component analysis was applied to the performance of growth form and spatial utilization ability of *M. yuyuanensis*, revealing the mixed proportion effect and change rule of comprehensive performance of growth, stem form quality, and spatial utilization ability of *M. yuyuanensis* and evaluating and optimizing the mixed proportion type based on the overall performance of *M. yuyuanensis*.【Result】The height, DBH(breast-hight diameter) and individual volume of *M. yuyuanensis* presented a significant mixed proportion effect. The DBH, height and volume of individual trees increased with the decrease in mixed proportion. Mixed proportion significantly affected tip sharpness, under-branch height, and shape of breast height. The sharpness and form factor of breast height decreased with the decrease in mixing proportion, while the under-branch height increased with a decrease in the mixing proportion. The spatial utilization ability, such as crown length, crown fullness, crown volume, crown surface area, and growth spatial index of *M. yuyuanensis* were significantly affected by the mixing ratio. The crown length, crown volume, crown surface area, and growth spatial index of *M. yuyuanensis* increased as mixing ratio decreased, while its crown fullness increased. The mixing proportion significantly affected the comprehensive performance of growth and form quality and spatial utilization ability of *M. yuyuanensis*. The comprehensive score value increased gradually with a decrease in the mixed proportion. Mixing proportion had no significant effect on crown width and under-branch height of *M. yuyuanensis*.【Conclusion】Mixing proportions can significantly change the growth, stem form quality, spatial utilization ability and comprehensive performance of *M. yuyuanensis* in *C. lanceolata-M. yuyuanensis* mixed forests. Rational mixed proportion types can significantly improve the growth, stem form quality, and spatial utilization ability of *M. yuyuanensis*, which is very important. In the mixed forest of *C. lanceolata-M. yuyuanensis*, the growth, stem form quality, and spatial utilization ability of *M. yuyuanensis* present different mixed proportion effects; therefore, comprehensive studies are needed. At a density below 2 500 plants/hm^2 in *C. lanceolata-M. yuyuanensis* mixed forests, the growth quality and spatial utilization ability of *M. yuyuanensis* performed the best under the model of two *C. lanceolata* to one *M. yuyuanensis*.

Keywords: *Manglietia yuyuanensis*; *Cunninghamia lanceolate*; growth trait; stem form quality; spatial utilization; principal component analysis; mixed-proportion effect

针阔混交林以其能有效改善针叶纯林树种结构简单、生态功能低效、生产力低下的局面而被广泛推广。树木干形形质关系着经济价值，提升林木干材品质是用材林培育质量的控制内容[1-9]。森林的空间结构与空间利用能力往往决定了林分结构的稳定性、森林生产力及发展潜力，成为当前的研究热点[1-2,10-12]。探明树种混交后的生长形质和空间利用能力效应，是合理混交树种组成与比例的基础。当前有关混交林效应的研究大多围绕生长[13-14]、生态功能[15-17]等方面进行，未见干形形质及空间利用能力方面的报道。

乳源木莲（*Manglietia yuyuanensis*）是中国南方混交林阔叶树种，经济价值不低于杉木[18]，当前研究主要集中在其生长效应[18-21]、竞争关系[21-22]、生态效应[23-26]等方面，尚未见乳源木莲生长形质与空间利用能力方面的报道。杉木（*Cunninghamia lanceolata*）乳源木莲混交林（以下简称“杉莲混交林”）是南方林区重要的混交林类型，笔者以福建省沙县12年生不同乳源木莲混交比例类型的杉莲混交林和乳源木莲纯林为研究对象，应用主成分分析法系统分析各混交比例下乳源木莲生长、干形形质及其空间利用能力性状和综合表现的效应和规律，以期为科学构建杉莲混交林提供依据。

1 材料与方法

1.1 研究区概况及试验材料

试验地设在福建省沙县际口（117°48′E，26°26′N），试验区为丘陵地带，属中亚热带东南季风型气候，年平均气温19.3 ℃，极端最高气温41.0 ℃，极端最低气温-6.5 ℃，无霜期290~320 d，年降水量约1 780 mm，年平均相对湿度80%以上。

研究材料为12年生杉木-乳源木莲混交林，造林材料为1年生乳源木莲与杉木裸根苗，挖明穴造林，造林密度为2 500株/hm^2，营建的乳源木莲

纯林,及 1 杉 1 莲、2 杉 1 莲比例的杉木乳源木莲混交林,采用行间混交方式。造林后连续抚育 3 a,每年 4—5 月、8—9 月全面除草,造林后次年进行扩穴培土。试验林分地处低山丘陵,海拔 150~250 m,坡度平均 20°,阴坡的中、下坡位,土壤为由砂岩、花岗岩发育形成的山地红壤,土层厚度 0.8 m,地力中等,土壤 pH 为 5.2。林下植被主要为亚热带常绿阔叶树种和蕨类植物,盖度为 80%~90%。林分生长状况见表 1。

表 1　不同杉莲混交模式下林分生长情况

Table 1　Stand growth of *C. lanceolata-M. yuyuanensis* forest under different mixed forest patterns

混交模式 mixed pattern	代号 code	树种 tree species	林分密度/(株·hm^{-2}) stand density	平均胸径/cm mean DBH	平均树高/m mean tree height	平均单株材积/m^3 average individual volume	树种蓄积/(m^3·hm^{-2}) volume of tree species	林分总蓄积/(m^3·hm^{-2}) total stand volume
乳源木莲纯林 *M. yuyuanensis* pure forest	Ⅰ	乳源木莲 *M. yuyuanensis*	2 325	11.51	8.33	0.052 6	122.262 4	122.262 4
1 杉 1 莲 mixed forest of 1 *C. lanceolata* and 1 *M. yuyuanensis*	Ⅱ	杉木 *C. lanceolata*	1 142	13.45	10.77	0.085 7	97.768 2	
		乳源木莲 *M. yuyuanensis*	1 158	12.21	9.07	0.062 0	71.746 1	
		合计 total	2 300					169.514 3
2 杉 1 莲 mixed forest of 2 *C. lanceolata* and 1 *M. yuyuanensis*	Ⅲ	杉木 *C. lanceolata*	1 558	13.17	10.69	0.081 8	127.514 8	
		乳源木莲 *M. yuyuanensis*	733	12.60	9.81	0.070 3	51.517 5	
		合计 total	2 291					179.032 2

1.2　试验设计与数据调查

采用 3 处理 3 重复的完全随机区组设计:处理Ⅰ为乳源木莲纯林,处理Ⅱ为中等比例乳源木莲混交林类型(1 杉 1 莲),处理Ⅲ为低比例乳源木莲混交林类型(2 杉 1 莲)。2017 年 12 月分别在 12 年生的不同人工混交林类型林分下坡位,随机布设 3 个规格为 20 m×20 m 临时标准地,作为 3 个重复。采用每木调查法,分别测定乳源木莲树高、枝下高、地径、胸径、东西与南北冠幅。

1.3　指标测定与计算

(1) 平均冠幅[1-2]:以树木东西、南北方向冠幅长度的平均值表示树木的平均冠幅。

$C_D = (C_{D,ew}+C_{D,sn})/2$。

其中:C_D 为平均冠幅,m;$C_{D,ew}$ 为树木东西冠幅长,m;$C_{D,sn}$ 为树木南北冠幅长,m。

(2) 冠高[1-2]:指树干第 1 个活枝到树梢的高度,其计算公式为

冠高(C_L)= 树高(H)-枝下高(H_B)。

其中:C_L 为树冠高度,m;H 为树高,m;H_B 为枝下高,m;枝下高是指树干基部至树干第 1 个活枝高度。

(3) 树冠圆满度[1-2]:以冠幅与冠长的比值表示,即 $C_{FR} = C_D/C_L$。其中:C_{FR} 为树冠圆满度;C_D 为树木平均冠幅,m;C_L 为树冠高度,m。

(4) 单株材积[18]:

$V = 0.000\ 052\ 76 D_{DBH}^{1.882\ 161} H^{1.009\ 317}$。

其中:V 为单株材积,m^3;D_{DBH} 为胸径,cm。

(5) 尖削度[2]:以地径与树高的比值表示,即尖削度=地径/树高。

其中:尖削度,cm/m;地径,cm;树高,m。

(6) 胸高形数:

$f_{1.3} = V/(g_{1.3} \times H)$。

其中:$f_{1.3}$ 为胸高形数;$g_{1.3}$ 为胸高断面积,m^2[5,9]。

(7) 枝下高比例:

枝下高比例=枝下高/树高。

其中:枝下高,m;树高,m。

(8) 树冠体积[1-2]:

$C_V = \prod/12 \times C_{D,ew} \times C_{D,sn} \times C_L$。

其中:C_V 为树冠体积,m^3。

(9) 树冠表面积[1-2]:

$C_{SA} = \prod/4 \times C_D \times (4C_L^2 + C_D^2)^{1/2}$。

其中:C_{SA} 为树冠表面积,m^2。

(10) 生长空间指数[1-2]:

$G_{SI} = C_V/D_{DBH}$。

其中:G_{SI} 为生长空间指数,m^3/cm。

1.4　乳源木莲生长形质及空间利用能力综合评价

主成分分析法适用于多性状的综合评价[1,5,27-28]。选择有显著混交比例效应的乳源木莲生长形质与空间利用能力系列性状进行主成分分析;构建相应主成分性状指标体系,并建立主成分得分值评价数学模型[1,5,27-28],计算综合得分[1,5]。

1.5 数据分析

采用Excel 2003和SPSS 21.0软件对数据进行统计分析,数据用平均值±标准误表示。采用单因素方差分析法(One-way ANOVA)和Duncan法进行方差分析和多重比较($\alpha=0.05$),采用SPSS 21.0软件进行主成分分析。

2 结果与分析

2.1 混交比例对乳源木莲生长及干形形质的影响

研究发现(表2),混交比例显著影响12年生乳源木莲胸径($F_{(2,6)}=12.10$)、树高($F_{(2,6)}=99.97$)、单株材积($F_{(2,6)}=28.24$)等生长性状。乳源木莲胸径、树高、单株材积均呈现随混交比例减少而逐渐增加的趋势;乳源木莲比例减少至1杉1莲时其胸径显著增大,且1杉1莲与2杉1莲混交林间无显著差异;乳源木莲比例减少至1杉1莲时其树高与单株材积显著增大,而2杉1莲时表现最好,纯林的表现最差。

对干形形质的研究发现,除枝下高比例外,混交比例类型显著影响12年生乳源木莲的尖削度($F_{(2,6)}=78.35$)、枝下高($F_{(2,6)}=66.33$)、胸高形数的($F_{(2,6)}=23.32$)等形质性状(表2)。乳源木莲尖削度呈现随其混交比例减少而逐渐减少的变化,乳源木莲比例减少至1杉1莲时其尖削度显著减少,纯林时最大、干材尖削度也大,2杉1莲混交林最小、干材尖削度也小。乳源木莲枝下高随其混交比例减少呈现逐渐增加的趋势,乳源木莲比例减少至1杉1莲时枝下高显著增大,纯林时枝下高最小,2杉1莲时枝下高最大。乳源木莲胸高形数随其混交比例减少而呈现逐渐减小的趋势,乳源木莲比例减少至1杉1莲时胸高形数显著减少,纯林胸高形数最大、干材也较圆满,1杉1莲与2杉1莲混交林间其胸高形数较小且无显著性差异、干材欠圆满。

表2 不同混交模式中乳源木莲生长形质性状表现

Table 2 Performance of growth characteristics of *M.yuyuanensis* in different mixed patterns

混交模式代号 code of mixed pattern	胸径/cm DBH	树高/m tree height	单株材积/m^3 individual volume	尖削度/($cm\cdot m^{-1}$) taperingness	枝下高/m branch height	枝下高比例 branch height ratio	胸高形数 form factor at breast height
Ⅰ	11.51±0.15 b	8.33±0.10 c	0.052 6±0.001 1 c	1.90±0.04 a	3.57 ±0.04 c	0.429±0.000 a	0.606±0.001 a
Ⅱ	12.21±0.28 a	9.07±0.15 b	0.062 0±0.002 9 b	1.65±0.01 b	3.86±0.05 b	0.425±0.006 a	0.584±0.009 b
Ⅲ	12.60±0.35 a	9.81±0.13 a	0.070 3±0.003 9 a	1.56±0.04 c	4.05±0.07 a	0.413±0.010 a	0.574±0.008 b
F	12.10	99.97	28.24	78.35	66.33	4.24	23.32

注:表中数据为平均值±标准误。同列不同字母表示数值间差异显著($P<0.05$)。下同。The data in the table are the mean±SE. The different lowercase letters in a list indicated significant difference($P<0.05$). The same below.

2.2 混交比例对乳源木莲地上空间利用能力的影响

不同混交模式中乳源木莲空间利用能力性状及综合得分表现见表3。由表3可以看出,除平均冠幅外,乳源木莲混交比例类型显著影响其12年生冠高($F_{(2,6)}=45.70$)、树冠圆满度($F_{(2,6)}=11.56$)以及树冠体积($F_{(2,6)}=14.51$)、树冠表面积($F_{(2,6)}=22.27$)、生长空间指数($F_{(2,6)}=7.77$)等空间利用能力指标,改变着乳源木莲的空间利用能力。乳源木莲的冠高、树冠体积和树冠表面积均呈现随混交比例减少而逐渐增大的趋势,乳源木莲比例减少至1杉1莲时其冠高、树冠体积显著增大,纯林的冠高、树冠体积最小,2杉1莲时最大;乳源木莲比例减少至1杉1莲时其树冠表面积增加,纯林的最小,1杉1莲与2杉1莲混交林间树冠表面积较大且显著差异。乳源木莲生长空间指数呈现随其混交比例减少而逐渐增大的趋势,乳源木莲比例减少增加至2杉1莲混交时其生长空间指数显著增大,2杉1莲的最大,纯林与1杉1莲混交林间的较小且无显著性差异。综合以上分析可知,乳源木莲混交后的地上生长空间利用能力更好,预示着后期生长过程中有着最好的生长潜力与可能。结果显示(表3),乳源木莲混交比例类型显著影响其树冠圆满度,树冠圆满度随其比例减少而减少,比例减少至1杉1莲时树冠圆满度显著减小,纯林时树冠圆满最大、冠形最宽阔,1杉1莲与2杉1莲混交林间树冠圆满度较小、冠形较狭长且无显著性差异。乳源木莲地上空间利用能力随其比例减少的变化趋势与树高、胸径和单株材积的变化趋势一致,验证了前人认为树种的地上空间利用能力影响其生长的结论[1-2,29]。本研究结果还显示,混交比例类型对12年生乳源木莲冠幅生长无显著影响,这与12年生试验林分充分郁闭,位于林分下层的乳源木莲树冠水平方向生长(冠幅生长)主要受林分密度的影响有关。

表 3　不同混交模式中乳源木莲空间利用能力性状及综合得分表现

Table 3　Performance of spatial utilization ability and comprehensive score of *M.yuyuanensis* in different mixed patterns

混交模式代号 code of mixed pattern	平均冠幅/m crown mean width	冠高/m crown length	树冠圆满度 crown fullness ratio	树冠体积/m^3 crown volume	树冠表面积/m^2 crown surface area	生长空间指数/($m^3\cdot cm^{-1}$) growth spatial index	综合得分 comprehensive score
Ⅰ	2.34±0.08 a	4.76±0.06 c	0.55±0.02 a	9.41±0.54 b	20.21±0.57 b	0.70 ±0.03 b	7.54±0.17 c
Ⅱ	2.39±0.07 a	5.21±0.13 b	0.49 ±0.03 b	9.96±0.34 b	21.84±0.20 b	0.70±0.04 b	8.14±0.03 b
Ⅲ	2.48±0.08 a	5.76±0.17 a	0.47±0.00 b	12.46±1.11 a	25.20±1.50 a	0.85±0.08 a	9.26±0.43 a
F	2.72	45.70	11.56	14.51	22.27	7.77	19.18

2.3　混交比例对乳源木莲生长形质及空间利用能力综合表现的影响

研究表明，乳源木莲生长形质及空间利用能力性状间的混交比例类型间差异变化以及显著性水平不尽相同；乳源木莲平均胸径、树高、单株材积、尖削度、枝下高、胸高形数、冠高、树冠圆满度、树冠体积、树冠表面积、生长空间指数等 11 个生长形质、空间利用能力性状指标有着显著的混交比例效应，为此选择 11 个性状指标进行主成分分析。经分析，第 1～7 主成分的方差特征值分别为 9.508、1. 090、0.247、0.100、0.051、0.004、0. 000，贡献率依次为 86. 436%、9. 911%、2. 245%、0. 911%、0. 460%、0. 036%、0. 001%。各主成分表达式为：

$z_1 = -0.097X_1 + 0.105X_2 + 0.103X_3 - 0.098X_4 + 0.100X_5 - 0.100X_6 + 0.104X_7 - 0.095X_8 + 0.092X_9 + 0.099X_{10} + 0.079X_{11}$；

$z_2 = -0.226X_1 - 0.032X_2 - 0.116X_3 + 0.121X_4 - 0.181X_5 + 0.234X_6 + 0.040X_7 + 0.325X_8 + 0.434X_9 + 0.294X_{10} + 0.606X_{11}$；

$z_3 = 1.201X_1 - 0.214X_2 + 0.665X_3 + 1.159X_4 - 0.301X_5 - 0.390X_6 - 0.168X_7 + 0.635X_8 + 0.251X_9 + 0.101X_{10} - 0.164X_{11}$；

$z_4 = 0.429X_1 - 0.051X_2 + 0.334X_3 - 1.122X_4 + 1.870X_5 + 0.622X_6 - 0.966X_7 + 1.890X_8 - 0.020X_9 - 0.167X_{10} - 0.094X_{11}$；

$z_5 = -0.612X_1 + 0.918X_2 + 0.860X_3 + 2.436X_4 + 2.170X_5 + 2.199X_6 + 0.299X_7 - 1.308X_8 + 0.123X_9 - 0.667X_{10} + 0.216X_{11}$；

$z_6 = 2.800X_1 - 1.857X_2 + 5.816X_3 - 4.814X_4 - 5.539X_5 + 10.200X_6 - 0.055X_7 - 2.350X_8 + 2.271X_9 + 3.840X_{10} - 4.799X_{11}$；

$z_7 = 30.516X_1 + 23.874X_2 - 37.382X_3 + 7.299X_4 + 1.324X_5 + 23.707X_6 + 34.048X_7 + 16.131X_8 - 54.066X_9 + 40.573X_{10} + 3.397X_{11}$。

综合得分值 $Q = \sum$ 各主成分值×贡献率。式中：z_1 为第 1 主成分；Q 为综合得分值；X_1 为胸径，cm；X_2 为树高，m；X_3 为单株材积，m^3；X_4 为尖削度，cm/m；X_5 为枝下高，m；X_6 为胸高形数；X_7 为冠高，m；X_8 为树冠圆满度；X_9 为树冠体积，m^3；X_{10} 为树冠表面积，m^2；X_{11} 为生长空间指数，m^3/cm。

根据生长形质及空间利用能力综合得分计算与方差分析结果（表 3），可以发现乳源木莲混交比例类型显著影响 12 年生乳源木莲生长形质及空间利用能力的综合表现（$F_{(2,6)} = 19.18$），混交比例改变其生长形质表现和空间利用能力；乳源木莲的生长形质、空间利用能力综合得分呈现随其比例减小而逐渐增大的变化趋势；乳源木莲比例减少至 1 杉 1 莲时综合得分显著增大，2 杉 1 莲时综合得分最大、综合表现最好，1 杉 1 莲时次之，纯林时综合得分最小、综合表现最差。

3　讨　论

以福建省沙县 12 年生乳源木莲纯林以及不同混交比例类型的杉莲混交林为研究对象，调查分析乳源木莲生长、干形形质、空间利用能力等系列性状的混交比例效应，揭示其生长、干材品质、空间利用能力以及综合表现的混交比例响应规律。研究发现，乳源木莲生长形质、空间利用能力以及综合表现有着显著的混交比例类型效应，合理的混交比例类型可提高其生长形质及空间利用能力。研究认为，在 2 500 株/hm^2 的林分密度，以低乳源木莲混交比例类型的 2 杉 1 莲模式中的生长形质及空间利用能力的总体表现最好。鉴于本试验林的乳源木莲枝下高比例均在 0. 4 以上，建议及时开展间伐，调整并保持低杉莲混交比例。

混交比例显著影响杉莲混交林中乳源木莲的生长表现，这与前人对于红锥（*Castanopsis hystrix*）[13]、桤木（*Alnus cremastogyne*）[14] 树种的研究结论一致。导致乳源木莲胸径、树高、单株材积随混交比例减小而逐渐增加的变化，与乳源木莲幼龄

阶段喜阴的生物学特性[19-20]有关；随乳源木莲占比减少，位居混交林分冠层的杉木能够为其提供愈加荫蔽的生长环境，是不同混交比例类型形成的差异性林分结构，以及由此产生差异性生长环境和种内与种间竞争差异共同作用的结果。

混交比例显著影响杉莲混交林中乳源木莲的干形形质，与前人研究混交比例影响乳源木莲形质的结论[25,30]一致，并与前人林分结构改变干形形质的研究结论[1,6]相验证。导致乳源木莲树干尖削度随混交比例减小而逐渐减小的趋势，是因位于混交林冠层的杉木比例随着乳源木莲比例减小而增大，从而形成更加荫蔽的乳源木莲生长环境；为获得充足光照乳源木莲采取相对于径向生长优先树高生长的策略所致，这与前人对南方红豆杉（*Taxus wallichina* var. *mairei*）[6]、马尾松（*Pinus massoniana*）[31]树种的研究结论相一致。乳源木莲枝下高随混交比例减小逐渐增加的变化原因，可能是位于混交林冠层的杉木的比例增大，加剧乳源木莲自然整枝所致，与前人混交比例改变竞争强度[21]的研究结论相验证。混交比例类型间的乳源木莲干材品质的变化，是因混交比例差异改变并形成的差异性林分结构，并由此形成差异性的生境及林木竞争共同作用的结果。同时发现，混交比例对乳源木莲枝下高比例无显著性影响。

混交比例显著影响杉莲混交林中乳源木莲空间利用能力，与前人关于林分结构改变生长空间利用能力的研究结论[1,25]相印证。出现乳源木莲冠高、树冠体积和表面积随其混交比例减小而逐渐增大的变化，可能是因混交比例减小，对冠幅生长无显著影响、而树冠长度生长显著增大综合作用的结果。导致乳源木莲生长空间指数随其混交比例减小而逐渐增大的变化，可能是因树冠体积与胸径生长随混交比例的增长幅度差异综合作用的结果。树冠圆满度随混交比例减小而减小，这与乳源木莲为获取充足的光照而采用狭长的树冠形状以响应其混交比例减小，从而形成更加荫蔽的生境，验证了前人关于光强影响树冠圆满度的研究结论[32-33]。混交比例对乳源木莲冠幅生长无显著性影响，这与位于混交林分下层乳源木莲的树冠水平方向生长（冠幅生长）主要受到林分密度的影响有关。

混交比例类型间的乳源木莲生长形质和地上生长空间利用能力的综合表现是因不同混交比例形成的差异性林分结构，以及由此形成差异性的生境与林木竞争共同作用的结果。低混交比例（木莲比例低于50%）类型的乳源木莲生长形质及空间利用能力综合表现最好，有着良好的生长与干形形质基础，以及强劲的后续生长潜力。为此，建议采用低混交比例（木莲占比）模式培育乳源木莲用材林。

参考文献（reference）：

[1] 欧建德. 造林密度对峦大杉生长形质及林分分化的影响[J]. 东北林业大学学报，2018，46（1）：7-11. OU J D. Effect of planting density on growth, form quality and stand differentiation of *Cunninghamia konishii* plantation[J]. Journal of Northeast Forestry University, 2018, 46(1): 7-11. DOI:10.13759/j.cnki.dlxb.2018.01.002.

[2] 欧建德，吴志庄，康永武. 峦大杉与杉木人工林的生长形质、林分分化和空间利用比较[J]. 东北林业大学学报，2018，46（7）：7-11. OU J D, WU Z Z, KANG Y W. Comparison of growth, stand differentiation, form quality and space utilization of *Cunninghamia konishii* and *C. lanceolata* plantation[J]. Journal of Northeast Forestry University, 2018, 46(7): 7-11. DOI:10.13759/j.cnki.dlxb.2018.07.002.

[3] 欧建德，吴志庄. 南方红豆杉人工林树干干形异常的诊断及处置[J]. 南京林业大学学报（自然科学版），2017，41（3）：95-99. OU J D, WU Z Z. Early judgment and disposal technology for stem form abnormity of *Taxus chinensis* var. *mairei* plantation[J]. Journal of Nanjing Forestry University (Natural Sciences Edition), 2017, 41(3): 95-99. DOI:10.3969/j.issn.1000-2006.201603024.

[4] 欧建德，吴志庄. 南方红豆杉修枝后生长与干形动态表现[J]. 浙江农林大学学报，2017，34（1）：104-111. OU J D, WU Z Z. Growth and stem form quality with pruning in *Taxus wallichiana* var. *mairei*[J]. Journal of Zhejiang A&F University, 2017, 34(1): 104-111. DOI: 10.11833/j.issn.2095-0756.2017.01.015.

[5] 欧建德，吴志庄. 南方红豆杉修枝经营措施优化及评价[J]. 南京林业大学学报（自然科学版），2017，41（1）：117-122. OU J D, WU Z Z. Optimization of pruning operation method for *Taxus wallichina* var. *mairei*[J]. Journal of Nanjing Forestry University (Natural Sciences Edition), 2017, 41(1): 117-122. DOI:10.3969/j.issn.1000-2006.2017.01.018.

[6] 欧建德，吴志庄.林下套种对南方红豆杉树冠形态结构及干形变化的影响[J].西南林业大学学报，2016，36（5）：106-110. OU J D, WU Z Z. The Effect of interplant under the canopy on *Taxus chinensis* var. *mairei* crown structure and stem form changes[J]. Journal of Southwest Forestry College, 2016, 36(5): 106-110. DOI:10.11929/j.issn.2095-1914.2016.05.018.

[7] 欧建德，吴志庄，罗宁. 林窗大小对杉木林内南方红豆杉生长与形质的影响[J]. 应用生态学报，2016，27（10）：3098-3104. OU J D, WU Z Z, LUO N. Effects of forest gap size on the growth and form quality of *Taxus wallichina* var. *mairei* in *Cunninghamia lanceolata* forests[J]. Chinese Journal of Applied Ecology, 2016, 27(10): 3098-3104. DOI: 10.13287/j.1001-

9332.201610.018.

[8] 欧建德,吴志庄.经营措施及地形因子与南方红豆杉杈干关系[J].东北林业大学学报,2016,44(9):24-28. OU J D, WU Z Z. Relationship between management measures and terrain factors of *Taxus wallichina* var. *mairei* artificial pure forest and its forking [J]. Journal of Northeast Forestry University,2016,44(9):24-28. DOI:10.3969/j.issn.1000-5382.2016.09.006.

[9] 欧建德. 基于多元统计分析的南方红豆杉幼林修枝技术[J]. 南京林业大学学报(自然科学版), 2016, 40(3): 183-187. OU J D. Pruning techniques of *Taxus chinensis* var. *mairei* young plantations based on multivariate statistical analysis[J]. Journal of Nanjing Forestry University(Natural Sciences Edition), 2016, 40(3): 183-187. DOI: 10.3969/j. issn. 1000-2006. 2016. 03.030.

[10] 柏广新, 孙志虎, 高波, 等. 长白山林区天然次生林胡桃楸的适宜生长空间[J]. 林业科学, 2009, 45(12): 8-15. BAI G X, SUN Z H, GAO B, et al. Optimal growing space for *Juglans mandshurica* in second growth forests in Changbai Mountains[J]. Scientia Silvae Sinicae, 2009, 45(12): 8-15. DOI:10.3321/j. issn:1001-7488.2009.12.002.

[11] 张成程, 李凤日, 赵颖慧. 落叶松人工林空间结构优化的探讨[J]. 植物研究, 2008, 28(5): 632-636,640. ZHANG C C, LI F R, ZHAO Y H. Discussion on optimization of forest spatial structure of *Larix olgensis* plantation[J].Bulletin of Botanical Research, 2008, 28(5): 632-636,640.

[12] 贾亚运, 何宗明, 周丽丽, 等. 造林密度对杉木幼林生长及空间利用的影响[J]. 生态学杂志, 2016, 35(5): 1177-1181. JIA Y Y, HE Z M, ZHOU L L, et al. Effects of planting densities on the growth and space utilization of young *Cunninghamia lanceolata* plantation[J]. Chinese Journal of Ecology, 2016, 35(5): 1177-1181. DOI: 10. 13292/j. 1000-4890. 201605.035.

[13] 黄云鹏. 杉木与红锥混交林生长量及混交比例的研究[J]. 福建林学院学报, 2008, 28(3):271-275. HUANG Y P. Study on growth increment and mixed ratio of a mixed forest of *Cunninghamia lanceolata* and *Castanopsis hystrix*[J]. Journal of Fujian College of Forestry, 2008, 28(3):271-275. DOI:10.3969/j. issn.1001-389X.2008.03.018.

[14] 谢福荣. 杉木桤木混交林生长量与土壤肥力的研究[J]. 福建林学院学报, 2006, 26(2):161-164. XIE F R. Growth and soil fertility of a mixed plantation of Chinese fir with *Alnus cremastogyne* [J]. Journal of Fujian College of Forestry, 2006, 26(2): 161-164. DOI:10.13324/j.cnki.jfcf.2006.02.015.

[15] 殷沙, 赵芳, 欧阳勋志. 马尾松木荷不同比例混交林枯落物和土壤持水性能比较分析[J]. 江西农业大学学报, 2015, 37(3): 454-460. YIN S, ZHAO F, OUYANG X Z. A comparison on water-holding capacity of forest litter and soil of mixed forests of *Pinus massoniana* and *Schima superba* in different proportions [J]. Acta Agriculturae Universitatis Jiangxiensis, 2015, 37(3): 454-460. DOI:10.13836/j.jjau.2015071.

[16] 赖国桢, 曹梦, 潘萍, 等. 马尾松木荷不同比例混交林植被碳密度特征[J]. 中南林业科技大学学报, 2018, 38(2): 108-113. LAI G Z, CAO M, PAN P, et al. Carbon density of vegetation of mixed plantation of *Pinus massoniana* and *Schima superba* in different proportions[J]. Journal of Central South University of Forestry & Technology, 2018, 38(2): 108-113. DOI: 10. 14067/j.cnki.1673-923x.2018.02.017.

[17] 杨玉盛, 陈光水, 谢锦升, 等. 杉木-观光木混交林群落 N、P 养分循环的研究[J]. 植物生态学报, 2002, 26(4): 473-480. YANG Y S, CHEN G S, XIE J S, et al. Nutrient cycling of N and P by a mixed forest of *Cunninghamia lanceolata* and *Tsoongiodendron odorum* in subtropical China[J]. Acta Phytoecologica Sinica, 2002, 26(4): 473-480.

[18] 周东雄. 杉木乳源木莲混交林林分生产力研究[J]. 林业科技开发, 2004, 18(5):23-25. ZHOU D X. Productive forces of mixed forest stand of *Cunninghamia lanceolata* with *Manglietia yuyuanensis* [J]. China Forestry Science and Technology, 2004, 18(5):23-25. DOI:10.3969/j.issn.1000-8101.2004.05.007.

[19] 潘世华. 坡向和坡位对杉木乳源木莲混交林生长的影响[J]. 华东森林经理, 2016, 30(2):32-35. PAN S H. Influences of slope directions and positions on growth of mixed forests between *Cuninghamia lanceolata* and *Manglietia yuyuanensis* [J]. East China Forest Management, 2016, 30(2):32-35. DOI:10.3969/ j.issn.1004-7743.2016.02.010.

[20] 廖国华. 杉木乳源木莲混交林间伐效应研究[J]. 福建农业学报, 2006, 21(4): 411-414. LIAO G H. The effect of thinning on mixed forest of Chinese fir and *Manglietia yuyuanensis*[J]. Fujian Journal of Agricultural Sciences, 2006, 21(4): 411-414. DOI:10.3969/j.issn.1008-0384.2006.04.026.

[21] 曹永慧, 李生, 陈存及, 等. 乳源木莲杉木混交林生长及其竞争关系分析[J]. 林业科学, 2005, 41(5):201-206. CAO Y H, LI S, CHEN C J, et al. The growth of *Manglietia yuyuanensis* stand mixed with *Cunninghamia lanceolata* and the interspecific competition between the tree species [J]. Scientia Silvae Sinicae, 2005, 41(5):201-206. DOI:10.3321/j.issn:1001-7488.2005. 05.037.

[22] 詹步清. 乳源木莲混交林种内及种间竞争研究[J]. 福建林学院学报, 2002, 22(3):274-277. ZHAN B Q. Study on the inner-species competition and inter-species competition in mixed forest of *Manglietia yuyuanensis* [J]. Journal of Fujian College of Forestry, 2002, 22(3):274-277. DOI: 10.3969/j. issn. 1001-389X.2002.03.020.

[23] 周东雄. 杉木乳源木莲混交林凋落物研究[J]. 生态学杂志, 2005, 24(6):595-598. ZHOU D X. Litter of mixed forest of *Cunninghamia lanceolata* and *Manglietia yuyuanensis* [J]. Chinese Journal of Ecology, 2005, 24(6):595-598. DOI:10. 13292/j.1000-4890.2005.0002.

[24] 陈善治, 周东雄. 杉木乳源木莲混交对地力影响的初步研究[J]. 福建林业科技, 1995(2):44-47. CHEN S Z, ZHOU D X. Preliminary research on effects of the mixture of Chinese fir and *Manglietia yuyuanensis* on the soil fertiity [J]. Journal of Fujian Forestry Science and Technology, 1995(2):44-47.

[25] 欧建德,康永武,吴志庄.乳源木莲生长形质、林分分化及空间利用能力的混交效应[J].东北林业大学学报,2019,47(3):

12-16. OU J D, KANG Y W, WU Z Z. Mixed effect on the growth, form quality, stand differentiation and spatial utilization ability of *Manglietia yuyuanensis* in *Cunninghamia lanceolata-Manglietia. yuyuanensis* mixed forest[J]. Journal of Northeast Forestry University, 2019, 47(3): 12-16. DOI: 10.13759/j.cnki.dlxb.2019.03.003.

[26] 周东雄. 杉木乳源木莲混交林分特征与固土保水功能[J]. 福建林学院学报, 2004, 24(1): 68-71. ZHOU D X. The feature and function for fixing of soil and conservation of water of mixed forests of *Cunninghamia lanceolata* and *Manglietia yuyuanensis* [J]. Journal of Fujian College of Forestry, 2004, 24(1): 68-71. DOI: 10.3969/j.issn.1001-389X.2004.01.017.

[27] 康永武, 罗宁, 欧建德. 造林密度对南方红豆杉人工林生长性状的影响[J]. 西南林业大学学报(自然科学), 2017, 37(3): 47-52. KANG Y W, LUO N, OU J D. Effects of planting density on the growth traits of *Taxus wallichina* plantation[J]. Journal of Southwest Forestry University (Natural Sciences), 2017, 37(3): 47-52.

[28] 欧建德, 吴志庄. 南方红豆杉盆栽轻型基质配方优化[J]. 东北林业大学学报, 2015, 43(9): 52-55, 89. OU J D, WU Z Z. Optimized medium ingredient with a light medium for *Taxus chinensis* var. *mairei* potted plants [J]. Journal of Northeast Forestry University, 2015, 43(9): 52-55, 89. DOI: 10.3969/j.issn.1000-5382.2015.09.011.

[29] 欧建德, 吴志庄, 康永武. 峦大杉树冠特征与生长形质通径分析[J]. 东北林业大学学报, 2018, 46(11): 8-11, 40. OU J D, WU Z Z, KANG Y W. Path analysis between canopy characteristics and growth, form quality of *Cunninghamia konishii* [J]. Journal of Northeast Forestry University, 2018, 46(11): 8-11, 40. DOI: 10.13759/j.cnki.dlxb.2018.11.002.

[30] 谢芳. 乳源木莲优质干材混交培育的研究[J]. 林业科学, 2003, 29(2): 84-90. XIE F. Cultivating high-quality stemwood of *Manglietia yuyuanensis* by mixing with *Cunninghamia lanceolata*[J]. Scientia Silvae Sinicae, 2003, 29(2): 84-90. DOI: 10.3321/j.issn: 1001-7488.2003.02.014.

[31] 程希平, 王妍方, 杨晓军, 等. 马尾松幼树干形生长模式的研究[J]. 中南林业科技大学学报, 2014, 34(4): 20-24. CHENG X P, WANG Y F, YANG X J, et al. Study on stem form growth pattern of *Pinus massoniana* saplings [J]. Journal of Central South University of Forestry & Technology, 2014, 34(4): 20-24. DOI: 10.3969/j.issn.1673-923X.2014.04.005.

[32] 李肇锋, 潘军, 王金盾, 等. 光环境对闽楠幼树生长及其表型可塑性的影响[J]. 西南林业大学学报, 2014, 34(6): 65-69. LI Z F, PAN J, WANG J D, et al. The effects of light environment on the growth and the phonotypic plasticity of the seedlings of *Phoebe bournei*[J]. Journal of Southwest Forestry University, 2014, 34(6): 65-69. DOI: 10.3969/j.issn.2095-1914.2014.06.011.

[33] 欧建德. 不同人工光环境对南方红豆杉幼树观赏性状及树冠表型可塑研究[J]. 农学学报, 2013, 3(1): 45-49. OU J D. Study on morphological plasticity and ornamental traits of landscape type *Taxus chinensis* var. *mairei* under different artificial light condition [J]. Journal of Agriculture, 2013, 3(1): 45-49. DOI: 10.3969/j.issn.1007-7774.2013.01.010.

(责任编辑　王国栋)

word版下载：http://www.ixueshu.com
