# Supplementary material for: Provenance and family variations in early growth of Manchurian walnut (Juglans mandshurica Maxim.) and selection of superior families
Source: PLoS One. 2024 Mar 7;19(3):e0298918. doi: 10.1371/journal.pone.0298918 (PMC10919699; doi:10.1371/journal.pone.0298918)
Supplement: S1 File — (ZIP) [file pone.0298918.s004.zip › Evaluation of the tree form quality of middle-aged Pinus tabuliformis plantation under different canopy densities in Huanglong Mountains, Northwest China.pdf]

应 用 生 态 学 报 2015 年 7 月 第 26 卷 第 7 期
Chinese Journal of Applied Ecology, Jul. 2015, 26(7): 1945-1953

黄龙山不同郁闭度油松中龄林林木形质评价*

尤健健 张文辉** 邓 磊
(西北农林科技大学西部环境与生态教育部重点实验室, 陕西杨凌 712100)

摘 要 以黄土高原南部黄龙山林区油松人工中龄林为研究对象,按郁闭度 CD<0.65(类型1)、0.65≤CD<0.75(类型2)、0.75≤CD<0.85(类型3)、CD≥0.85(类型4)将样地划分4个等级.综合林木生长、干形和分枝情况,运用层次分析法建立林木形质评价层次结构模型和指标体系,对4种郁闭度类型的油松林林木的形质水平进行了综合评价.结果表明:林木生长、干形和分枝3大类形质评价因素及其包括的胸径、树高、径高比、尖削度、通直度、分杈率、活枝下高、侧枝数、最大侧枝基径和侧枝平均基径10项指标,可以全面地反映油松林林木形质水平.其中,通直度、分杈率和胸径3个指标的总权重达0.7382,对林木形质水平影响最大,是油松林木形质的主要决定因素.随着林分郁闭度的减小,林木形质综合得分表现为先升高后下降,类型2郁闭度下达到最高的90.28分,该类郁闭度下林木形质水平最优.本评价方法操作简单,可解决林木形质评价的量化问题,评价模型体系也可在黄土高原其他林木形质评价中借鉴和应用.

关键词 油松中龄林; 郁闭度; 林木形质; 层次分析法; 黄土高原

文章编号 1001-9332(2015)07-1945-09 **中图分类号** S753.3 **文献标识码** A
DOI:10.13287/j.1001-9332.20150506.019

Evaluation of the tree form quality of middle-aged *Pinus tabuliformis* plantation under different canopy densities in Huanglong Mountains, Northwest China. YOU Jian-jian, ZHANG Wen-hui, DENG Lei (*Education of Ministry Key Laboratory of Environment and Ecology in West China, Northwest A&F University, Yangling 712100, Shaanxi, China*). -*Chin. J. Appl. Ecol.*, 2015, **26**(7): 1945-1953.

Abstract: To clarify the effects of different canopy densities on tree form quality of *Pinus tabuliformis*, a hierarchical indicator system was structured, which brought about a set of grading criteria to evaluate tree form quality of the middle-aged *P. tabuliformis* plantation under four canopy densities in Huanglong Mountains by using the analytic hierarchy process (AHP). Plots were divided into four classes according to the stand canopy density (CD): CD<0.65(type 1), 0.65≤CD<0.75 (type 2), 0.75≤CD<0.85 (type 3) and CD≥0.85 (type 4). The results indicated that, by comprehensive analysis of ten related indicators, *i.e.*, diameter at breast height (DBH), tree height, diameter height ratio, taperingness, stem straightness, forking ratio, height under living branch, number of branches, max-branch base diameter and average base diameter of branches, the tree form quality could be evaluated. Among these factors, stem straightness, forking ratio and DBH were the most important ones influencing the tree form quality with a total weight of 0.7382. So, these three indicators were the major determinants of tree form quality. The comprehensive scores of the tree form quality evaluations fluctuated as the canopy density decreased and topped at 90.28 when CD was valued at 0.75 (type 2). The indicators and evaluation system developed in this study were easy to operate, and quite fit for solving the quantity problem of the tree form quality evaluation. Our system would be capable for applications in evaluating the tree form quality of other tree species on the Loess Plateau.

Key words: middle-aged *Pinus tabuliformis* plantation; canopy density; tree form quality; AHP; the Loess Plateau.

* 陕西省科技统筹创新工程计划项目(2014KTCL02-04)和国家"十二五"科技支撑计划项目(2012BAD22B0302)资助.
** 通讯作者. E-mail: zwhckh@163.com
2014-09-17 收稿, 2015-03-23 接受.

林木形质是树木在生长过程中受自身生长特性和环境条件的影响而形成的，它不仅影响木材加工工艺和使用质量，也对树木出材率、经济价值产生影响[1-2].无论是公益林还是用材林，培育高质量、大径级木材与保持生态功能是相得益彰的，而林木形质评价能为培育高质量大径级木材提供重要依据.近年来，对林木形质研究多集中在华东、华中及华南地区，树种包括马尾松(*Pinus massoniana*)、日本落叶松(*Larix kaempferi*)、樟树(*Cinnamomum camphora*)等[3-7].主要涉及林木形质指标或木材性质对造林密度或间伐强度的响应以及优良形质选育等方面，关于林木形质综合评价的研究较少，尤其是在黄土高原地区等生态脆弱区，林木形质方面的研究更加缺乏.

油松(*Pinus tabuliformis*)是我国北方地区重要的用材树种，由于其对干旱瘠薄的立地条件具有较强的耐受力和适应性，多年来在黄土高原植被恢复建设过程中一直被用作主要的造林树种[8-10].油松主干通直、高大，木材轻软，强重比大，在建筑、家具、造纸等领域都有广泛应用，属优良用材[11].近年来，随着生态平衡失调问题日益突出以及人们对环境问题的日渐重视，受国际上可持续发展林业理论的影响，我国林业也从单纯的公益防护林或经济林型向生态经济综合型转变[12].因此，运用科学的森林经营技术，在黄土高原南部林区森林发挥生态功能的基础上培育高质量、大径级木材是该地区林业发展的方向.森林近自然经营主要目标是在生态、防护功能提升的基础上培育更多的优质木材和林产品，通过抚育间伐控制林地郁闭度条件，并对林木形质进行综合评价是优化森林经营措施的有效途径.

综合评价中各个因素标度数值的确定是一个难点，对于大多数工作者来说很难准确打分并确定权重.层次分析法(analytic hierarchy process, AHP)可以把定性问题定量化，从而避免主观性，减少人为失误。判断矩阵和一致性度量检验的应用可以确保评价结果更加客观合理[13].AHP 模型已在农业、林业及生态环境评价等领域得到了广泛的应用[14-16].

本研究以黄土高原南部黄龙山林区油松人工林为对象，采用层次分析法对不同郁闭度下林木形质进行综合评价，旨在建立林木形质综合评价的层次结构模型，为各地制定林木形质评价指标体系提供参考.

1 研究地区与研究方法

1.1 研究区概况

研究区位于陕西省黄龙山林区界头庙林场(35°28′49″—36°02′01″ N，109°38′49″—110°12′47″ E)，海拔 1100~1500 m.该地区为典型的黄土高原丘陵沟壑地貌，属大陆性暖温带半湿润气候类型.年均气温 8.6 ℃，≥10 ℃的年积温为 2953.7 ℃，全年无霜期 126~186 d，年均降雨量 606.2 mm[17].森林植被位于暖温带针阔叶混交地带，植被乔木层除油松外还有辽东栎(*Quercus liaotungensis*)、茶条槭(*Acer ginnala*)、白桦(*Betula platyphylla*)、山杨(*Populus davidiana*)、榆树(*Ulmus pumila*)等；灌木种类丰富，主要有绣线菊(*Spiraea thunbergii*)、黄蔷薇(*Rosa hugonis*)、悬钩子(*Rubus corchorifolius*)、胡枝子(*Lespedeza bicolor*)、丁香(*Syring apekinensis*)、卫矛(*Euony musalatus*)、陕西荚蒾(*Viburnum schensianum*)等；草本层主要有苔草(*Carexl anceolata*)、茜草(*Rubia cordifolia*)和披碱草(*Elymus dahuricus*)等.

1.2 样地设置与调查

调查样地设置在 1988 年营造的油松人工纯林内，造林地原为撂荒地，初植密度 3900 株·hm^{-2}. 1998 年进行了强度基本一致的定株抚育，2006 年进行了不同强度(保留郁闭度)的间伐试验，此后林地处于自然恢复和保护状态，未经受过较大的外界干扰.于间伐当年在对试验区全面踏查的基础上，选择立地条件相似的林分，设置 12 块固定监测样地，面积为 20 m×20 m，调查林分保留郁闭度，并按保留郁闭度(CD)大小将样地分为 4 种类型：CD<0.65 为类型 1；0.65≤CD<0.75 为类型 2；0.75≤CD<0.85 为类型 3；CD≥0.85 为类型 4.样地基本林分特征见表 1.

调查林分郁闭度采用样点统计法[18]，在每块样地内随机设置50个样点，判断样点是否被树冠遮

表 1 样地基本林分特征

Table 1 Stand characteristics of the plots

郁闭度类型 Canopy density type	样地编号 Plot No.	海拔 Altitude (m)	坡向 Aspect (°)	坡度 Slope (°)	坡位 Location	郁闭度 Canopy density
类型 1	1	1482.05	半阴坡	16	上部	0.60
Type 1	2	1486.05	阴坡	12	上部	0.61
	3	1480.88	阴坡	10	上部	0.61
类型 2	1	1466.50	半阴坡	15	中上部	0.71
Type 2	2	1474.38	阴坡	11	上部	0.68
	3	1470.21	阴坡	13	上部	0.71
类型 3	1	1466.30	阴坡	11	上部	0.82
Type 3	2	1450.45	阴坡	14	中上部	0.79
	3	1452.85	阴坡	17	中上部	0.81
类型 4	1	1481.56	阴坡	15	中上部	0.88
Type 4	2	1483.76	阴坡	12	上部	0.87
	3	1475.63	半阴坡	14	上部	0.90

盖,统计被遮盖样点数,计算出郁闭度:

郁闭度=被树冠遮盖的样点数/50　(1)

2013 年 8 月按保留郁闭度类型依次在每块样地内随机抽取油松 18、19、22 和 24 株进行林木形质指标调查,共调查 12 块样地 249 株油松.调查树高、胸径、基径、树干中央直径、通直度、活枝下高、侧枝数(基径≥1 cm)、侧枝基径和分杈木数量.其中,树干通直度按通直、较通直、一般、弯曲、严重弯曲分为 5 级,分别记为 5、4、3、2、1 分.

计算径高比、尖削度和分杈率:

径高比=胸径/树高　(2)

尖削度指 $T_{0.5}$ 平均尖削度[19]:

$$T_{0.5}=(D_0-D_{0.5})/L \quad (3)$$

式中: D_0 为树干基部直径; $D_{0.5}$ 为树干中央直径; L 为 1/2 树高.

分杈率为样地内有分杈的林木占该样地林木总数的比率:

分杈率=分杈木株数/样地林木总株数　(4)

1.3　形质评价方法

基于层次分析法的系统性原则,将评价体系分为有隶属关系的 3 层递阶层次结构,从上到下依次为:目标层——林木形质;约束层——生长、干形和分枝情况;指标层——影响约束层评价因素的各项具体指标,以此建立层次结构模型.结合专家咨询法,按照构建各层评价因子的判断矩阵并进行一致性检验,若判断矩阵的随机一致性比率 CR<0.1,表明具有满意的一致性,可用于权重的计算;反之,需调整判断矩阵,直至满足要求.运用高斯迭代法求解各层次上评价指标判断矩阵的最大特征根及其对应的特征向量,得到各评价指标对目标层的权重,最后再结合野外实地调查数据,计算林木形质综合评分.油松林木形质的综合得分根据公式(5)计算:

$$V_i=\sum_{j=1}^{10} X_{ij}W_j \quad (5)$$

式中: V_i 为第 i 类样地的林木形质综合评分; X_{ij} 为第 i 类样地第 j 个指标所对应的分值; W_j 为第 j 个指标的权重.

1.4　数据处理

利用 SPSS 18.0 软件对不同郁闭度下林木形质指标进行单因素方差分析(one-way ANOVA),结合 Duncan 检验比较参数间差异性($\alpha=0.05$).采用 SigmaPlot 12.0 软件作图.

2　结果与分析

2.1　林木形质评价层次模型的构建

在综合考虑影响林木形质的多种因素的基础上,运用层次分析法,通过约束层 3 个方面(生长情况、干形情况、分枝情况)和指标层 10 个评价指标(树高、胸径、径高比、树干尖削度、树干通直度、分杈率、活枝下高、侧枝数、侧枝最大基径、侧枝平均基径)构建油松林木形质评价层次模型(图 1).

2.2　判断矩阵的构建

针对判断矩阵的准则,每 2 个评价指标按 A 与 B 同等重要、A 比 B 稍重要、A 比 B 重要、A 比 B 明显重要和 A 比 B 极端重要分别以 1、3、5、7、9 作为标度,并以 2、4、6、8 表示相邻判断的中间值,用这些值的倒数表示 2 个指标的反比较.对同一层次中的各因素间相对于上一层次某项因子的相对重要性给予判断,从而得出各评价因子的判断矩阵.求解判断矩阵的最大特征根及其对应的特征向量,经过多次

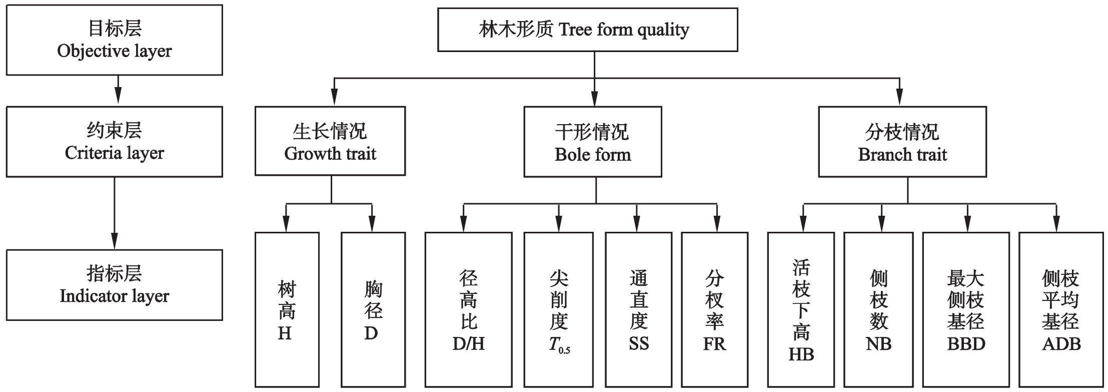

图 1　油松林木形质评价层次结构

Fig.1　Hierarchical structure of tree form quality evaluation of *Pinus tabuliformis*.

H: Tree height; D: DBH; D/H: Diameter height ratio; $T_{0.5}$: Taperingness; SS: Stem straightness; FR: Forking ratio; HB: Height under living branch; NB: Number of branches; BBD: Max-branch base diameter; ADB: Average base diameter of branches.

表 2 林木形质评价判断矩阵

Table 2 Determining matrix of tree form quality evaluation

项目 Item	生长情况 Growth trait	干形情况 Bole form	分枝情况 Branch trait	贡献率 Relative contribution rate
生长情况 Growth trait	1	1/5	3	0.1884
干形情况 Bole form	5	1	7	0.7306
分枝情况 Branch trait	1/3	1/7	1	0.0810

表 3 生长情况判断矩阵

Table 3 Determining matrix of growth traits

项目 Item	胸径 DBH	树高 Height	贡献率 Relative contribution rate
胸径 DBH	1	2	0.6667
树高 Height	1/2	1	0.3333

表 4 干形情况判断矩阵

Table 4 Determining matrix of bole form

项目 Item	径高比 Diameter height ratio	尖削度 Taperingness	通直度 Stem straightness	分杈率 Forking ratio	贡献率 Relative contribution rate
径高比 Diameter height ratio	1	1	1/5	1/5	0.0808
尖削度 Taperingness	1	1	1/5	1/5	0.0808
通直度 Stem straightness	5	5	1	3	0.5316
分杈率 Forking ratio	5	5	1/3	1	0.3069

一致性检验和调整，使判断矩阵符合要求.油松林木形质评价目标层和约束层判断矩阵见表 2，生长情况、干形情况及分枝情况的判断矩阵分别见表 3、表 4 和表 5.

经一致性检验，得出目标层和约束层评价因子判断矩阵的 CR 值为 0.062，干形指标判断矩阵和分枝指标判断矩阵的 CR 值分别为 0.057 和 0.063，均<0.1，所以它们都满足层次分析的一致性检验要求，说明具有满意的一致性，可以用来进行指标权重的计算.由于生长情况判断矩阵为二阶判断矩阵，其 RI 值(平均随机一致性指标)为 0，因此不做判断一致性检验.

表 5 分枝情况判断矩阵

Table 5 Determining matrix of branch traits

项目 Item	活枝下高 Height under living branch	侧枝数 Number of branches	最大侧枝基径 Max-branch base diameter	侧枝平均基径 Average base diameter of branches	贡献率 Relative contribution rate
活枝下高 Height under living branch	1	1/3	1/5	1/5	0.0639
侧枝数 Number of branches	3	1	1/5	1/5	0.1107
最大侧枝基径 Max-branch base diameter	5	4	1	1/3	0.3021
侧枝平均基径 Average base diameter of branches	5	4	3	1	0.5233

2.3 评价指标权重的计算

用高斯迭代法求解各层次上评价指标判断矩阵的最大特征根及其对应的特征向量，得到林木形质评价指标权重(表 6).10 个评价指标对林木形质影响的权重表现为：通直度(0.3884)>分杈率(0.2242)>胸径(0.1256)>树高(0.0628)>径高比(0.0590)=尖削度(0.0590)>侧枝平均基径(0.0424)>最大侧枝基径(0.0245)>侧枝数(0.0090)>活枝下高(0.0052).通直度、分杈率和胸径的总权重达 0.7382，可见，这 3 个形质指标是评价林木形质的关键因素，在很大程度上决定了林木形质的优劣.

2.4 评价指标分级和林木形质分级

根据样地实时调查数据的波动范围，将各评价指标实测值划分成 5 个区间，代表 5 个质量等级[14]，分别按照各形质指标所表达的生物学意义，给出质量状况等级并按百分制赋值.质量状况等级依次用优、良、中、较差和差表示，对应的得分值依次为 100、80、60、40 和 20(表 7).本研究中各形质指标分级表示不同郁闭度下油松林木形质的相对水平，不具有绝对性.

2.5 林木形质评价

由图 2 可以看出，保留郁闭度除对油松树高影响不显著外，对其余形质评价指标都有显著影响.随

表 6 评价指标权重

Table 6 Relative weight of evaluating indicators

约束层 Criteria layer	指标层 Indicator layer	权重 Weight
生长情况 Growth trait	胸径 DBH	0.1256
	树高 Tree height	0.0628
干形情况 Bole form	径高比 Diameter height ratio	0.0590
	尖削度 Taperingness	0.0590
	通直度 Stem straightness	0.3884
	分杈率 Forking ratio	0.2242
分枝情况 Branch trait	活枝下高 Height under living branch	0.0052
	侧枝数 Number of branches	0.0090
	最大侧枝基径 Max-branch base diameter	0.0245
	侧枝平均基径 Average base diameter of branches	0.0424

表 7　林木形质评价指标等级及评分标准

Table 7　Criteria and indicator interval partition, quality-class and scores for tree form quality evaluation

约束层 Criteria layer	指标层 Indicator layer	区间划分 Interval partition	质量状况 Quality-class	得分 Score
生长情况 Growth trait	胸径 DBH (cm)	<9.00	差 Worst	20
		[9.00,10.00)	较差 Worse	40
		[10.00,11.00)	中 Medium	60
		[11.00,12.00)	良 Good	80
		≥12.00	优 Best	100
	树高 Tree height (m)	<5.00	差 Worst	20
		[5.00,5.50)	较差 Worse	40
		[5.50,6.00)	中 Medium	60
		[6.00,6.50)	良 Good	80
		≥6.50	优 Best	100
干形情况 Bole form	径高比 Diameter height ratio	<1.50	差 Worst	20
		[1.50,1.60)	较差 Worse	40
		[1.60,1.70)	中 Medium	60
		[1.70,1.80)	良 Good	80
		≥1.80	优 Best	100
	尖削度 Taperingness	<1.00	优 Best	100
		[1.00,1.50)	良 Good	80
		[1.50,2.00)	中 Medium	60
		[2.00,2.50)	较差 Worse	40
		≥2.50	差 Worst	20
	通直度 Stem straightness	<3.00	差 Worst	20
		[3.00,3.50)	较差 Worse	40
		[3.50,4.00)	中 Medium	60
		[4.00,4.50)	良 Good	80
		≥4.50	优 Best	100
	分杈率 Forking ratio (%)	<5.00	优 Best	100
		[5.00,6.00)	良 Good	80
		[6.00,7.00)	中 Medium	60
		[7.00,8.00)	较差 Worse	40
		≥8.00	差 Worst	20
分枝情况 Branch trait	活枝下高 Height under living branch (m)	<3.00	差 Worst	20
		[3.00,3.50)	较差 Worse	40
		[3.50,4.00)	中 Medium	60
		[4.00,4.50)	良 Good	80
		≥4.50	优 Best	100
	侧枝数 Number of branches	<30.00	优 Best	100
		[30.00,35.00)	良 Good	80
		[35.00,40.00)	中 Medium	60
		[40.00,45.00)	较差 Worse	40
		≥45.00	差 Worst	20
	最大侧枝基径 Max-branch base diameter (cm)	<2.00	优 Best	100
		[2.00,2.50)	良 Good	80
		[2.50,3.00)	中 Medium	60
		[3.00,3.50)	较差 Worse	40
		≥3.50	差 Worst	20
	侧枝平均基径 Average base diameter of branches (cm)	<1.50	优 Best	100
		[1.50,2.00)	良 Good	80
		[2.00,2.50)	中 Medium	60
		[2.50,3.00)	较差 Worse	40
		≥3.00	差 Worst	20

表 8　样地林木形质综合得分及形质分级

Table 8　Tree form quality evaluation and grade of the plots

郁闭度类型 Canopy density type	综合评分 Synthesis value	形质等级 Tree form quality grade
类型 1 Type 1	77.51	Ⅲ
类型 2 Type 2	90.28	Ⅰ
类型 3 Type 3	88.80	Ⅱ
类型 4 Type 4	75.32	Ⅲ

着保留郁闭度的减小,胸径不断增大,并在郁闭度降低到类型 2 以下时胸径显著增大.同时,林木径高比和尖削度也显著增大,侧枝数显著增多,活枝下高显著降低,侧枝基径显著增大.类型 1 郁闭度下油松分杈率显著高于其他郁闭度林分.林木通直度随郁闭度的增大而增大,但在类型 4 郁闭度下有所降低.

由表 8 可以看出,在进行油松林木形质等级划分时,综合评分≥90 分的为优等形质,记为Ⅰ级; 综合得分在 80~90 的形质水平为良好,记为Ⅱ级; 综合得分在 70~80 的为中等形质水平,记为Ⅲ级,综合得分在 60~70 的形质水平较差,记为Ⅳ级; 综合得分<60 分的形质水平差,记为Ⅴ级.类型 2 郁闭度下林分林木形质最优,综合评分达 90.28,类型 3 郁闭度下林分林木形质综合评分稍低于前者,林木形质水平良好,类型 1 和类型 4 郁闭度下样地林木形质水平均处于中等水平.总体看,油松林木形质水平随林分保留郁闭度的降低而升高,但当郁闭度降低至类型 1 时,林木形质水平反而降低.

3　讨　　论

林木形质的形成是个长期的过程,除了受自身遗传因素的影响外,也受到外界环境因素的共同作用[20-21].森林郁闭度影响着林分内各个生态因子的变化,如风、光、温度的时空分布以及降水的再分配等[22],林木生长及树干形质也会随着林地生境条件的改变而改变.对黄龙山林区油松中龄林林木形质的层次分析表明: 不同保留郁闭度下林分林木形质水平有明显差异,且随着保留郁闭度的减小,林木形质水平先升高后降低,当保留郁闭度在类型 2 时,林木形质水平达到最优.林木形质直接影响木材加工利用和木材的品质.干形圆满通直,不仅出材率高,而且木材品质较好; 而树干弯曲,一方面影响木材加工利用,使出材率明显下降[23],另一方面因树干弯曲而产生的应力木,会使木材品质降低,树干上的节

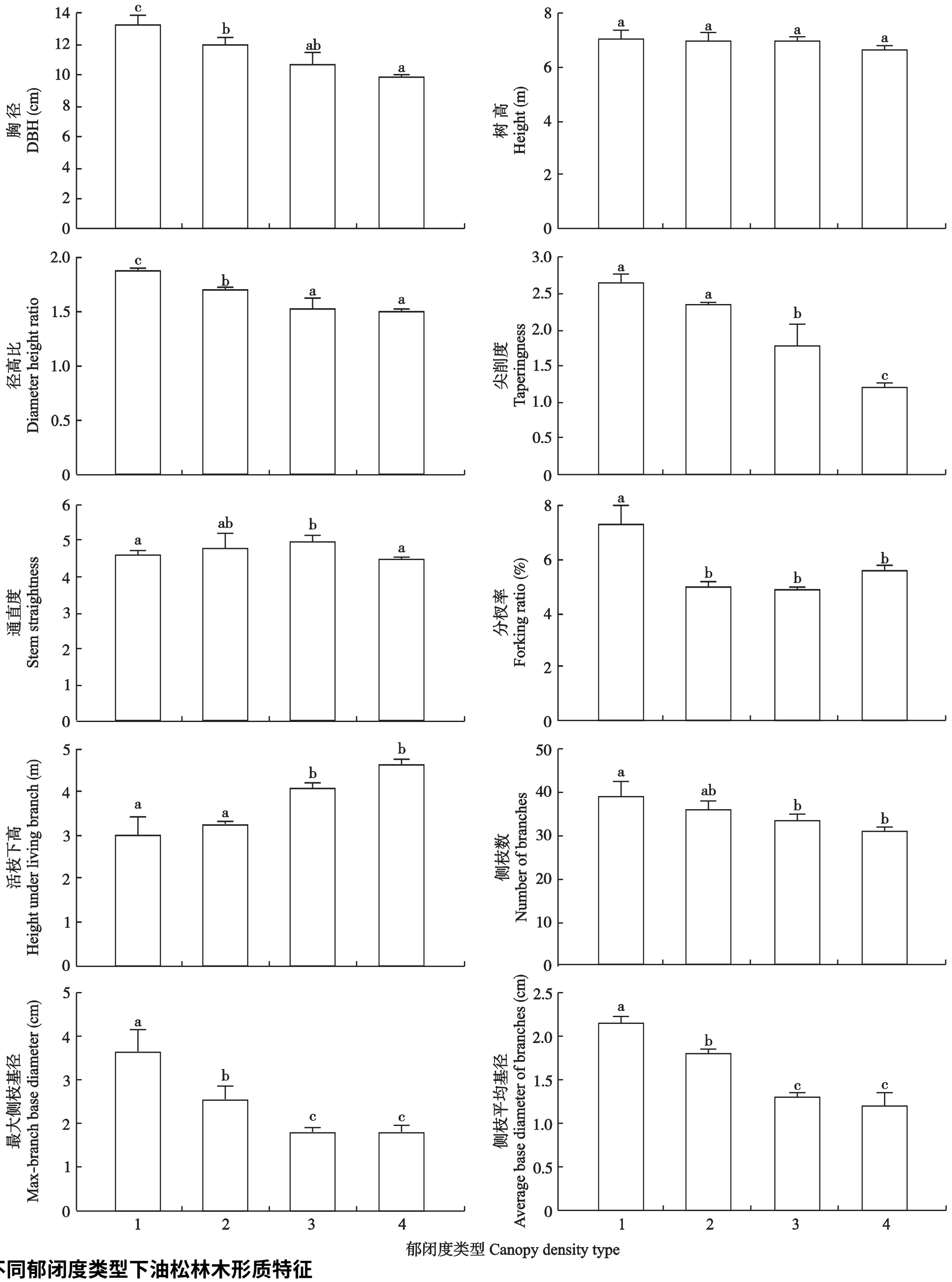

图 2　不同郁闭度类型下油松林木形质特征

Fig.2　Characteristics of tree form quality of *Pinus tabuliformis* under different canopy density types.

不同字母表示郁闭度间差异显著(P<0.05) Different letters meant significant difference among different canopy densities at 0.05 level.

子也会严重降低胶合板等板材的强度，使产品降级[24].分杈干形成的双心(包括三心)材增加了木材构造的不均匀性和加工的困难性，且分杈出现在树干较低位置也会极大地影响原木出材率[25].从评价指标权重来看，林木通直度、分杈率和胸径对林木形质的影响最大，表明提高通直度、促进林木胸径增长并降低分杈率是林木形成优良形质的关键.

由于植物体是固着生长，当外部环境和生态因子变化时，它们会通过形态可塑性来适应环境，从而形成不同的形态和构型，提高其生存适宜度和竞争能力[26-27].郁闭度的减小对应着林窗的增大和增多，改变了光照在林分中的分布，进而改变了林木各

部位对光资源的利用和再分配,提高了树冠对光能的利用效率.同时,林内光照增强使土壤温度升高,加快了土壤中有机质的分解,使土壤速效养分增加,从而提高了林地土壤肥力,进而促进林木的生长[28].然而,随着树木生长空间的增大,林木树冠和胸径显著增大,光合产物向上部树干分配的比例减少,从而导致树干尖削度增大,并且树冠的增大对应着分枝的增粗和增长,从而导致更多、更大节子的产生,甚至形成分杈干,因此,单纯追逐生长量的快速增大,会导致木材质量下降[29].

对各形质评价指标的方差分析表明,保留郁闭度除对油松树高影响不显著外,对其他形质评价指标都有显著影响.随着保留郁闭度的减小,胸径、径高比都显著增大,对形成优良形质有利,但同时林木尖削度也显著增大,侧枝数显著增多,活枝下高显著降低,侧枝基径也显著增大,这对培育优良形质都是不利的变化.本研究中,4 种郁闭度下林木通直度均处于较高水平,但类型 1 郁闭度下油松分杈率显著高于其他郁闭度林分,这在更大程度上降低了林木形质水平.当郁闭度降低到类型 1 水平时,林地内分杈干木数量有所增加,这可能是由于缺少相邻木树冠的遮挡和束缚,侧枝生长点长期接受光照,从而使侧枝快速发育,形成分杈.这也与刘英杰等[30]和陈永亮等[31]对红松人工林林木分杈及分杈与林分密度的关系的研究结果一致.大量研究表明,造林时适当增大初植密度可提高林木通直度水平,促进自然整枝,减小林木尖削度[3-4,32],而促进胸径生长、减小分杈率则需要通过抚育间伐等森林经营措施控制合理的林分密度结构[10,33-34].本研究中,当保留郁闭度为类型 2 时,对林木形质影响最大的通直度、分杈率和胸径指标都达到了良好以上水平,且树高、径高比、尖削度、侧枝数和侧枝基径等指标均达到了中等以上水平,林木形质相对最优,这与木材生产实际[19,23]相符合,体现出形质评价体系的科学性和合理性.但在该郁闭度下林木的活枝下高指标仍处于较差水平,所以建议在森林经营过程中及时进行修枝,以避免在树干的较低位置形成节子,从而影响出材质量.

本评价层次结构模型所选评价指标均为国内外林木形质研究[34-36]中高频出现的指标,且指标数据易于获取,有可比性,该模型体系也可在黄土高原其他林木形质评价中应用和借鉴.林木的生长与树冠紧密相关,树冠作为林木进行光合作用的主要场所,对林分的太阳能分配、养分循环、降雨量分配等起着重要作用[37].冠层特性(冠幅、冠长、冠体积等)影响着光合产物的分配[38-39],对木材结构形成起重要作用,最终影响木材形质和性质[40-41].然而,由于树冠特征对树干形质的影响最终会表现在胸径、树高、尖削度以及分枝等指标上[41-44],所以在进行综合评价时,未将其作为指标纳入评价体系.本研究中,油松林木形质综合评价得分和分级结果只反映了该研究区油松中龄林在不同郁闭度下林木形质的相对水平,不具有绝对性,只能用于优化森林经营措施.若要指导生产实践,还需根据木材形质的相关分级标准进行进一步研究.

参考文献

[1] Kellogg RM, Warren WG. Evaluating western hemlock stem characteristics in terms of lumber value. *Wood and Fiber Science*, 1984, **16**: 583-597

[2] Tara LK, Peter MB. Long-term response of yellow-poplar to thinning in the southern Appalachian Mountains. *Forest Ecology and Management*, 2014, **312**: 148-153

[3] Shen H-H (谌红辉), Ding G-J (丁贵杰), Wen H-H (温恒辉), *et al*. Effects of planting density on growth and economic benefit of Masson pine plantation. *Forest Research* (林业科学研究), 2011, **24**(4): 470-475 (in Chinese)

[4] Liu Q-H (刘青华), Zhou Z-C (周志春), Zhang K-M (张开明), *et al*. Initial stand density and provenance effects on the growth and wood basic density of Masson pine. *Scientia Silvae Sinicae* (林业科学), 2010, **46**(9): 58-64 (in Chinese)

[5] Sun X-M (孙晓梅), Zhang S-G (张守攻), Li S-Y (李时元), *et al*. Multi-traits selection of open-pollinated *Larix kaempferi* families for pulpwood purpose. *Scientia Silvae Sinicae* (林业科学), 2005, **41**(4): 48-54 (in Chinese)

[6] Wu J-Y (吴际友), Long Y-Z (龙应忠), Yu G-F (余格非), *et al*. Genetic analysis and combined selection of main economic characters of half-sib families for Slash pine. *Scientia Silvae Sinicae* (林业科学), 2000, **36**(suppl.): 56-61 (in Chinese)

[7] Zhang Q (张 谦), Zeng L-H (曾令海), Cai Y-L (蔡燕灵), *et al*. Genetic analyses on growth and form traits of open-pollinated families of *Cinnamomum camphora*. *Journal of Central South University of Forestry & Technology* (中南林业科技大学学报), 2014, **34**(1): 1-6 (in Chinese)

[8] Han W-J (韩文娟), Yuan X-Q (袁晓青), Zhang W-H (张文辉). Effects of gap size on seeding natural regeneration in artificial *Pinus tabulaeformis* plantation. *Chinese Journal of Applied Ecology* (应用生态学报), 2012, **23**(11): 2940-2948 (in Chinese)

[9] Han W-J (韩文娟), Cao X-P (曹旭平), Zhang W-H (张文辉), *et al*. Effect of ground cover on early rege-

neration of *Pinus tabulaeformis* plantation. *Scientia Silvae Sinicae* (林业科学), 2014, **50**(1): 49-54 (in Chinese)

[10] Gao Y-C (高云昌), Zhang W-H (张文辉), He J-F (何景峰), *et al*. Effects of thinning intensity on *Pinus tabulaeformis* plantation in Huanglong Mountain, northwest China: A comprehensive evaluation. *Chinese Journal of Applied Ecology* (应用生态学报), 2013, **24**(5): 1313-1319 (in Chinese)

[11] Xu H-C (徐化成). *Pinus tabulaeformis*. Beijing: Chinese Forestry Publishing House, 1993 (in Chinese)

[12] Sun S-C (孙书存), Gao X-M (高贤明), Bao W-K (包维楷), *et al*. Density effects on tree growth and community structure of Chinese pine plantations in the upper reaches of the Min River, China. *Chinese Journal of Applied & Environmental Biology* (应用与环境生物学学报), 2005, **11**(1): 8-13 (in Chinese)

[13] Yang B (杨 斌), Yang G-Z (杨国州), Zhang Y-D (张延东), *et al*. Selecting optimum tree species of farmland protection forest in Linxia Beiyuan by analytical hierarchy process. *Scientia Silvae Sinicae* (林业科学), 2006, **42**(6): 49-55 (in Chinese)

[14] Wang N-J (王乃江), Zhang W-H (张文辉), Tong J-X (同金侠), *et al*. Forest quality evaluation in Caijiachuan state forest station on Loess Plateau. *Scientia Silvae Sinicae* (林业科学), 2010, **46**(9): 7-13 (in Chinese)

[15] Cao Y-G (曹银贵), Zhou W (周 伟), Wang J (王静), *et al*. Comparative on regional cultivated land intensive use based on principal component analysis and analytic hierarchy process in Three Gorges Reservoir Area. *Transactions of the Chinese Society of Agricultural Engineering* (农业工程学报), 2010, **26**(4): 291-296 (in Chinese)

[16] Zhang X-P (张晓萍), Yang Q-K (杨勤科), Li R (李锐). Diagnostic indicators of catchment health: A new method of evaluation of ecological environment. *Bulletin of Soil and Water Conservation* (水土保持通报), 1998, **18**(4): 57-62 (in Chinese)

[17] Deng L (邓 磊), Zhang W-H (张文辉), He J-F (何景峰). Effects of different management measures of *Quercus liaotungensis* in the Huanglong Mountain. *Acta Botanica Boreali-Occidentalia Sinica* (西北植物学报), 2011, **31**(1): 159-166 (in Chinese)

[18] Li Y-N (李永宁), Zhang B-L (张宾兰), Qin S-Y (秦淑英), *et al*. Review of research and application of forest canopy closure and its measuring methods. *World Forestry Research* (世界林业研究), 2008, **21**(1): 40-46 (in Chinese)

[19] Liu X-E (刘杏娥), Wang X-Q (王小青), Jiang Z-H (江泽慧), *et al*. Effects of planting density on tree growth and wood quality and modeling the wood quality of *Populus* ×*xiaohei*. *Journal of Beijing Forestry University* (北京林业大学学报), 2007, **29**(6): 161-166 (in Chinese)

[20] Yu B-Y (余碧云), Zhang W-H (张文辉), He T (何婷), *et al*. Effects of forest gap size on the architecture of *Quercus variablis* seedlings on the south slope of Qinling Mountains, West China. *Chinese Journal of Applied Ecology* (应用生态学报), 2014, **25**(12): 3399-3406 (in Chinese)

[21] Adolfo R, Andrea P, Silvia C, *et al*. The role of tree architecture in super high density olive orchards. *Scientia Horticulturae*, 2013, **161**: 24-29

[22] Zhu J-J (朱教君), Kang H-Z (康宏樟), Hu L-L (胡理乐). Estimation on optical porosity or canopy closure for a forest stand with hemispherical images. *Chinese Journal of Ecology* (生态学杂志), 2005, **24**(10): 1234-1240 (in Chinese)

[23] Barbara L, Fernando D, Claudio B, *et al*. Stem form and compression wood formation in young *Pinus radiate* trees. *Canadian Journal of Forest Research*, 2010, **40**: 26-36

[24] Pan H-X (潘惠新), Huang M-R (黄敏仁), Li H-G (李火根), *et al*. Research on the genetic variation of new clonal stem form traits in *Populus deltoides*. *Journal of Nanjing Forestry University* (南京林业大学学报), 1999, **23**(5): 1-6 (in Chinese)

[25] Xu Y-M (徐有明). Wood Technology. Beijing: Chinese Forestry Publishing House, 2006 (in Chinese)

[26] Wu G-L (武高林), Du G-Z (杜国祯). Advances in plant morphological growth strategy. *World Science and Technology Research and Development* (世界科技研究与发展), 2007, **29**(4): 47-51 (in Chinese)

[27] Li J-Q (李俊清), Zang R-G (臧润国), Jiang Y-X (蒋有绪). Review on studies of architecture and morphological diversity for *Fagus sylvatica* L. *Acta Ecologica Sinica* (生态学报), 2001, **21**(1): 151-155 (in Chinese)

[28] Ran R (冉 然), Zhang W-H (张文辉), He J-F (何景峰), *et al*. Effects of thinning intensities on population regeneration of natural *Quercus variabilis* forest on the south slope of Qinling Mountains. *Chinese Journal of Applied Ecology* (应用生态学报), 2014, **25**(3): 695-701 (in Chinese)

[29] Han F (韩 飞), Li F-R (李凤日), Ling M (梁明). Influence of stand density on knot and stem form of larch. *Journal of Northeast Forestry University* (东北林业大学学报), 2010, **38**(6): 4-8 (in Chinese)

[30] Liu Y-J (刘英杰), Chen L-X (陈立新), Zhang Z-R (张兆荣). Preliminary study on the reason of stem devergence of Korean pine plantation. *Journal of Northeast Forestry University* (东北林业大学学报), 1998, **26**(2): 11-14 (in Chinese)

[31] Chen Y-L (陈永亮), Geng X-W (耿叙武), Li G-Q (李桂秋), *et al*. The relationship between the plantation density of *Pinus koraiensis* and its forking. *Journal of Northeast Forestry University* (东北林业大学学报), 2000, **28**(3): 32-35 (in Chinese)

[32] Shen H-H (谌红辉), Ding G-J (丁贵杰). Study on planting density effects for Masson pine plantation. *Scientia Silvae Sinicae* (林业科学), 2004, **40**(1): 92-98 (in Chinese)

[33] Cao Y (曹 云), Yang J (杨 劼), Song B-Y (宋炳

煜), *et al*. Effects of artificial tending on *Pinus tabulaeformis* forest growth and its structural characteristics. *Chinese Journal of Applied Ecology* (应用生态学报), 2005, **16**(3): 397-402 (in Chinese)

[34] Shen H-H (谌红辉), Fang S-Z (方升佐), Ding G-J (丁贵杰), *et al*. Thinning density effects on Masson pine plantation. *Scientia Silvae Sinicae* (林业科学), 2010, **46**(5): 84-91 (in Chinese)

[35] Julia D, Suzanne WS, Jürgen B, *et al*. Trade-offs among establishment success, stem morphology and productivity of under planted *Toona ciliata*: Effects of nurse-species and thinning density. *Forest Ecology and Management*, 2009, **259**: 1846-1855

[36] Christian K, Edgar K, Patrick P, *et al*. Growth and form of *Quercus robur* and *Fraxinus excelsior* respond distinctly different to initial growing space: Results from 24-year-old Nelder experiments. *Journal of Forestry Research*, 2013, **24**: 1-14

[37] Gong N-N (公宁宁), Ma L-Y (马履一), Jia L-M (贾黎明), *et al*. Effects of different stand densities and site conditions on crown of *Pinus tabulaeformis* plantations in Beijing mountain area. *Journal of Northeast Forestry University* (东北林业大学学报), 2010, **38**(5): 9-12 (in Chinese)

[38] Timo K. Crown architecture and stemwood production in Norway spruce (*Picea abies* (L.) Kant.). *Tree Physiology*, 1988, **4**: 337-346

[39] Glencross K, Lencross, JD, Nichols JC, *et al*. Spacing affects stem form, early growth and branching in young whitewood (*Endospermum medullosum*) plantations in Vanuatu. *International Forestry Review*, 2012, **14**: 442-451

[40] Wang X-Q (王小青), Liu X-E (刘杏娥), Ren H-Q (任海青). Effects of crown attributes on wood characteristics and increments of *Populus* ×*xiaohei*. *Forest Research* (林业科学研究), 2007, **20**(6): 801-806 (in Chinese)

[41] Larson PR. Stem form development of forest trees. *Forest Science Monograph*, 1963, **5**: 42

[42] Jiang L-C (姜立春), Jiang Y-H (蒋雨航). Modeling effects of crown characteristics on stem taper of Dahurian larch using mixed model. *Journal of Beijing Forestry University* (北京林业大学学报), 2014, **36**(2): 10-14 (in Chinese)

[43] Fu L-Y (符利勇), Sun H (孙 华), Zhang H-R (张会儒), *et al*. Effects of diameter at breast height on crown characteristics of Chinese fir under different canopy density conditions. *Acta Ecologica Sinica* (生态学报), 2013, **33**(8): 2434-2443 (in Chinese)

[44] Dong Y-F (董玉峰), Jiang Y-Z (姜岳忠), Zhang M-Z (张明哲), *et al*. Correlation between branch characteristics and growth traits, stem form for varieties in poplar plantation. *Journal of Central South University of Forestry & Technology* (中南林业科技大学学报), 2014, **34**(2): 34-38 (in Chinese)

作者简介 尤健健,男,1988 年生,硕士研究生. 主要从事天然林保护及森林经营研究. E-mail: silviculture@163.com

责任编辑 孙 菊
