# Supplementary material for: Provenance and family variations in early growth of Manchurian walnut (Juglans mandshurica Maxim.) and selection of superior families
Source: PLoS One. 2024 Mar 7;19(3):e0298918. doi: 10.1371/journal.pone.0298918 (PMC10919699; doi:10.1371/journal.pone.0298918)
Supplement: S1 File — (ZIP) [file pone.0298918.s004.zip › Exploring the antiviral activity of juglone by computational method.pdf]

Journal of Receptors and Signal Transduction

http://informahealthcare.com/rst
ISSN: 1079-9893 (print), 1532-4281 (electronic)

J Recept Signal Transduct Res, 2014; 34(6): 456–457
© 2014 Informa Healthcare USA, Inc.
DOI: 10.3109/10799893.2014.917325

informa healthcare

RESEARCH ARTICLE

Exploring the antiviral activity of juglone by computational method

Shailima R. D. Vardhini[1,2]

[1]St. Mary's College, Yousufguda, Hyderabad, Andhra Pradesh, India and [2]Celesta Research Lab, Mahabubnagar, Andhra Pradesh, India

Abstract

Nature has been the best source of medicines for a long time. Many plant extracts have been used as drugs. Juglone occurs in all parts of the Juglandaceae family and is found extensively in the black walnut plants. It possesses antifungal, antimalarial, antibacterial and antiviral properties besides exhibiting cytotoxic effects. Juglone has gained interest by the researchers for its anticancer properties. This article elucidates the antiviral activity of the Juglone by the computational method.

Keywords

1a8g, antiviral, drug design, Juglone, natural drugs

History

Received 24 February 2014
Revised 1 April 2014
Accepted 4 April 2014
Published online 20 May 2014

Journal of Receptors and Signal Transduction Downloaded from informahealthcare.com by Technische Universiteit Eindhoven on 01/09/15
For personal use only.

Introduction

Juglone is a naturally occurring plant component (1). It has a naphthoquinone and is seen in the leaves, bark and wood of butternut (*Juglans Cinerea*), walnut (*Juglans regia*) and Manchurian walnut (*Juglans mandshurica*) (2). In China, the bark, branches and the exocarp of the immature fruits are used in treating many types of cancers (3). The components obtained from the black walnut trees are used against the acne and in hair dying (4). The plant species *J. regia* possess this aromatic phytochemical in all parts (5) and is reported to have the oldest known allelopathic compound (6). Juglone has attracted the researchers for its anticancer properties (7–10). Juglone (natural compounds) is also known for its cytotoxic properties (11–13), antifungal, antibacterial and antiviral properties (4,14).

Though juglone is reported to possess the antiviral property, the available study is minimal. The objective of this investigation is to establish the antiviral activity of juglone *in silico*. To accomplish this, the protein 1a8g was selected.

1a8g, HIV protease is an enzyme present in the HIV, a retro virus, which is essential for its life cycle (15,16). This acts by cleaving the newly formed polyproteins to create an active protein component of HIV virion without which the virion remains uninfectious (17). This ability of the homodimer protein makes it a prime drug target.

Materials and methods

The protein of interest, 1a8g, was downloaded from the Protein Data Bank (USCD, San Diego, CA; Rutgers, New Jersey) and was imported onto the Molegro. The ligands for the study were selected from PubChem (Bethesda, MD) and were imported onto the Molegro. Both the protein and the ligand were prepared by assigning ''always''. The active sites on the protein were then detected (Figure 1). On detection, it showed one cavity with the volume 281.088.

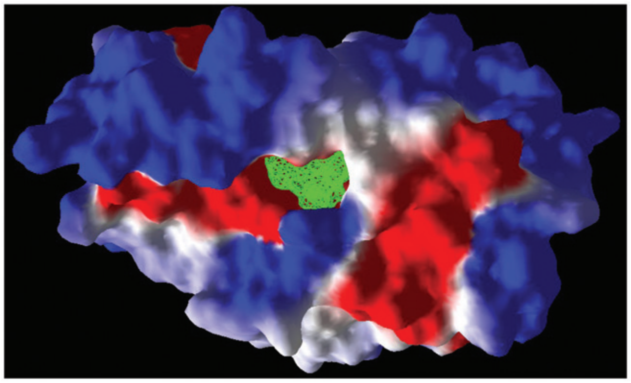

Figure 1. Protein with active site.

Results

The Molegro Virtual Docker (Cambridge, MA) was used to run the docking. The four selected ligands bound with the protein successfully generating 20 poses (Table 1 and Figure 2).

The fourth ligand showed the highest binding affinity with 114.967 dock score. The hydrogen bonds that were present between the ligand and the protein are ASP 29, ASP 30 and ASP 30 (Table 2).

Conclusion

Juglone is a plant extract present abundantly in the walnut plants (18). It is widely known to have the anticancer

Address for correspondence: Dr. Shailima R. D. Vardhini, St. Mary's College, Yousufguda, Hyderabad, Andhra Pradesh, India, and Celesta Research Lab, Mahabubnagar, Andhra Pradesh, India. E-mail: shailima.rampogu@gmail.com

RIGHTSLINK

Table 1. Dock results.

Name	Ligand	MolDock Scone	Rerank Scone	HBond
☐ [03]3806	3806	−91.501	−76.2276	−2.5
☐ [00]3806	3806	−91.2673	−73.471	−4.04672
☐ [04]3806	3806	−91.124	−76.3343	−3.10932
☐ [02]3806	3806	−90.3145	−73.1725	−2.45776
☐ [01]3806	3806	−89.6244	−74.8575	−5.42399
☐ [00]6011...	60116266	−96.0727	−79.7716	−3.75737
☐ [01]6011...	60116266	−94.006	−78.4725	−2.14087
☐ [02]6011...	60116266	−92.3643	−76.6894	−4.16963
☐ [03]6011...	60116266	−90.1145	−74.8902	−4.29193
☐ [04]6011...	60116266	−87.666	−71.8214	−6.35603
☐ [00]6669...	66695376	−92.3996	−76.6707	−4.19206
☐ [03]6669...	66695376	−91.4926	−76.2205	−2.5
☐ [01]6669...	66695376	−90.3487	−72.2455	−4.43407
☐ [02]6669...	66695376	−89.6389	−74.8617	−5.42524
☐ [04]6669...	66695376	−89.3862	−73.9742	−2.43836
☐ [01]7054...	70541023	−115.189	−92.2813	0
☑ [00]7054...	70541023	−114.967	−86.325	−4.63145
☐ [02]7054...	70541023	−114.657	−90.6893	−2.03821
☐ [04]7054...	70541023	−111.49	−87.703	−4.01169
☐ [03]7054...	70541023	−110.743	−89.4703	−2.69013

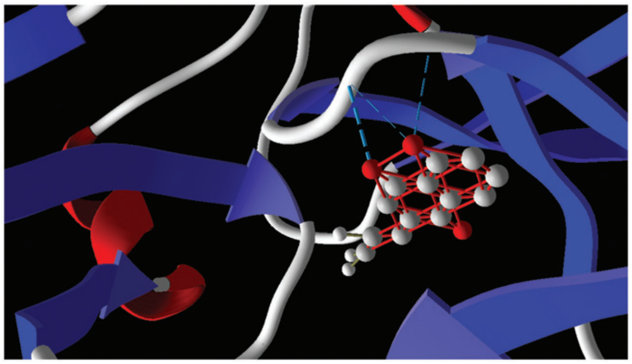

Figure 2. Protein–ligand docking.

Table 2. Hydrogen bond value.

Interactions [3]
☑ Interaction: Hydrogen Bond. Value = −0.568547
☑ Interaction: Hydrogen Bond. Value = −1.16634
☑ Interaction: Hydrogen Bond. Value = −2.27662

activities. There are a very few reports (11) on the antiviral property of juglone. This article could successfully evaluate this property in silico, hence proving juglone to be one of the potential drugs against viruses.

Journal of Receptors and Signal Transduction Downloaded from informahealthcare.com by Technische Universiteit Eindhoven on 01/09/15
For personal use only.

Declaration of interest

The author reports no declaration of interest.

References

1. Paulsena MT, Ljungman M. The natural toxin juglone causes degradation of p53 and induces rapid H2AX phosphorylation and cell death in human fibroblasts. Toxicol Appl Pharmacol 2005;209: 1–9.
2. Funt RC, Martin J. Black walnut toxicity to plants, humans and horses. Ohio State University Extension Fact sheet, HYG-1148-93; 1993. Available from: http://ohioline.osu.edu/hyg-fact/1000/1148.html [last accessed 10 Jan 2014].
3. Liu L, Li W, Koike K, et al. New alpha-tetralonylglucosides from the fruit of *Juglans mandshurica*. Chem Pharm Bull 2004;52: 566–9.
4. Inbaraj JJ, Chignell CF. Cytotoxic action of juglone and plumbagin: a mechanistic study using HaCaT keratinocytes. Chem Res Toxicol 2004;17:55–62. PMID: 14727919.
5. Harborne JB. Introduction to ecological biochemistry. 4th ed. London, New York: Academic Press.; 1988.
6. Erdemoglu N, Küpeli E, Yesilada E. Anti-inflammatory and antinociceptive activity assessment of plants used as remedy in Turkish folk medicine. J Ethnopharmacol 2003;89:123–9.
7. Gatti L, Perego P. Cellular resistance to oxaliplatin and drug accumulation defects. In: Bonetti A, Leone R, Muggia FM, Howell SB, eds. Cellular resistance to oxaliplatin and drug accumulation defects platinum and other heavy metal compounds in cancer chemotherapy. USA: Humana Press; 2009:115–117.
8. Johnson J, Syed D, Heren C, et al. Carnosol, a dietary diterpene, displays growth inhibitory effects in human prostate cancer PC3 cells leading to G2-phase cell cycle arrest and targets the 5′-AMP-activated protein kinase (AMPK) pathway. Pharm Res 2008;25: 2125–34.
9. Barathi S, Vardhini SRD, Chitra P, Arulselvi P. Cytotoxic effect of juglone on human peripheral blood lymphocytes. Asian J Pharm Clin Res 2013;6:178–86.
10. Babula P, Adam V, Havel L, Kizek R. Naphthoquinones and their pharmacological properties. Ceska Slov Farm 2007;56:114–20.
11. Kamei H, Koide T, Kojima T, et al. Inhibition of cell growth in culture by quinones. Cancer Biother Radiopharm 1998;13:185–8.
12. Aithal BK, Kumar MR, Rao BN, et al. Juglone, a naphthoquinone from walnut, exerts cytotoxic and genotoxic effects against cultured melanoma tumor cells. Cell Biol Int 2009;33:1039–49.
13. Rippmann JF, Hobbie S, Daiber C, et al. Phosphorylation-dependent proline isomerization catalyzed by Pin1 is essential for tumor cell survival and entry into mitosis. Cell Growth Differ 2000;11: 409–16.
14. Clark AM, Jurgens TM, Hufford CD. Antimicrobial activity of juglone. Phytother Res 2006;4:11–14.
15. Brik A, Wong C-H. HIV-1 protease: mechanism and drug discovery. Org Biomol Chem 2003;1:5–14.
16. Balakrishnan M, Srivastava RC, Mayank P. Homology modeling and docking studies between HIV protease and carbamic acid. Indian J Biotechnol 2010;9:96–100.
17. Kohl NE, Emini EA, Schleif WA, et al. Active human immunodeficiency virus protease is required for viral infectivity. Proc Natl Acad Sci USA 1988;85:4686–90.
18. Gîrzu M, Carnat A, Privat A.-M, et al. Sedative effect of walnut leaf extract and juglone, an isolated constituent. Pharm Biol 1998;36: 280–6.

RIGHTSLINK
