# Supplementary material for: Provenance and family variations in early growth of Manchurian walnut (Juglans mandshurica Maxim.) and selection of superior families
Source: PLoS One. 2024 Mar 7;19(3):e0298918. doi: 10.1371/journal.pone.0298918 (PMC10919699; doi:10.1371/journal.pone.0298918)
Supplement: S1 File — (ZIP) [file pone.0298918.s004.zip › Genetic Diversity of Juglans mandshurica Populations in Northeast China Based on SSR Markers.pdf]

frontiers | Frontiers in Plant Science

ORIGINAL RESEARCH
published: 30 June 2022
doi: 10.3389/fpls.2022.931578

Check for updates

Genetic Diversity of *Juglans mandshurica* Populations in Northeast China Based on SSR Markers

Qinhui Zhang[1,2], Xinxin Zhang[1,2], Yuchun Yang[3], Lianfeng Xu[4], Jian Feng[5], Jingyuan Wang[6], Yongsheng Tang[6], Xiaona Pei[2*] and Xiyang Zhao[2*]

[1] State Key Laboratory of Tree Genetics and Breeding, Northeast Forestry University, Harbin, China, [2] College of Forestry and Grassland, Jilin Agricultural University, Changchun, China, [3] Jilin Provincial Academy of Forestry Sciences, Changchun, China, [4] Qiqihar Branch of Heilongjiang Academy of Forestry, Qiqihar, China, [5] Liaoning Academy of Forest Science, Shenyang, China, [6] Linjiang Forestry Bureau of Jilin Province, Lijiang, China

OPEN ACCESS

Edited by:
Daniel Pinero,
National Autonomous University of Mexico, Mexico

Reviewed by:
Xiaoming Pang,
Beijing Forestry University, China
Asit Ray,
Siksha O Anusandhan University, India

***Correspondence:**
Xiaona Pei
xiaonapei2020@163.com
Xiyang Zhao
zhaoxyphd@163.com

Specialty section:
This article was submitted to Functional Plant Ecology, a section of the journal Frontiers in Plant Science

Received: 29 April 2022
Accepted: 03 June 2022
Published: 30 June 2022

Citation:
Zhang Q, Zhang X, Yang Y, Xu L, Feng J, Wang J, Tang Y, Pei X and Zhao X (2022) Genetic Diversity of Juglans mandshurica Populations in Northeast China Based on SSR Markers. Front. Plant Sci. 13:931578. doi: 10.3389/fpls.2022.931578

Juglans mandshurica is a native tree species in Northeast China. Due to habitat destruction and human disturbance, its population size has sharply decreased. Currently, information on molecular markers of *J. mandshurica* is limited and cannot meet the needs of germplasm resource evaluation and molecular marker-assisted breeding of *J. mandshurica*. Based on transcriptomic data from three tissues (leaves, bark, and fruit pericarp), we developed expressed sequence tag-simple sequence repeats (EST-SSRs) for *J. mandshurica,* and 15 polymorphic EST-SSR primers were initially selected. The average number of alleles (*Na*), expected heterozygosity (*He*), and the polymorphic information content (*PIC*) at different loci were 18.27, 0.670, and 0.797, respectively. Population genetic diversity analysis revealed that the average *Na*, *He,* and Shannon information indices (*I*) for 15 *J. mandshurica* populations were 6.993, 0.670, and 1.455, respectively. Among them, population Hunchun exhibited the highest genetic diversity ($Na = 7.933$, $He = 0.723$, and $I = 1.617$), while population Heihe exhibited the lowest genetic diversity ($Na = 4.200$, $He = 0.605$, and $I = 1.158$). STRUCTURE analysis, neighbor-joining method cluster analysis, and principal coordinate analysis showed that the 343 individuals of *J. mandshurica* from 15 populations were clustered into three categories. Category 1 (green) had 147 individuals from eight populations in Qingyuan, Caohekou, Jian, Ningan, Yongji, Baishishan, Helong, and Maoershan; category 2 (blue) had 81 individuals from three populations in Hulin, Boli, and Sanchazi; and category 3 (red) had 115 individuals from four populations in Heihe, Hunchun, Fangzheng, and Liangshui. Analysis of molecular variance (AMOVA) showed that genetic variations among and within individuals accounted for 16.22% and 21.10% of the total genetic variation, respectively, indicating that genetic variations within populations were greater than genetic variations among populations. The average genetic differentiation coefficient (*Fst*) and gene flow (*Nm*) between different populations were 0.109 and 4.063, respectively, implying moderate levels of genetic differentiation and gene flow. Based on

the genetic diversity characteristics of different populations, we proposed various genetic conservation strategies for *J. mandshurica*.

Keywords: *Juglans mandshurica*, EST-SSR, genetic diversity, genetic structure, genetic differentiation, conservation strategies

INTRODUCTION

Juglans mandshurica Maxim., a deciduous tree of the Juglandaceae family, is native to northern and northeastern China and is mainly distributed in the Changbai Mountains and Lesser Khingan Mountains at elevations of 500–1,000 m. It is also distributed in Russia, Japan, and Korea and can withstand low temperatures of −50°C. As an ancient fruit tree species, it is economically valuable (Tian et al., 2009; Zhou et al., 2015). It is composed of a hard, dense, elastic, corrosion-resistant, and easy-to-process elite material for military joins and furniture (Zhu et al., 2011). Its fruits are of high quality with high oil content. Moreover, it is an excellent dried fruit species with a promising future in Northeast China (Pang et al., 2019). *J. mandshurica* also has high medicinal value. Its green husk, bark, roots, and leaves contain juglone, which exhibits good antitumor (Mallavadhani et al., 2014), antifungal (Sharma et al., 2009), antiviral (Vardhini, 2014), antioxidant (Chobot and Hadacek, 2009), anthelmintic (Islam and Widhalm, 2020), and hypoglycemic activities (Hosseini et al., 2014). It has potential applications in the pharmaceutical, agroforestry, food, and cosmetic industries. Collection and evaluation of plant germplasm resources is a prerequisite for the utilization of wild cultivated genes, which enhances plant genetic improvement and further assists in breeding programs (El-Esawi, 2017; Mohammed et al., 2020). In China, the northeast forest area is the largest natural forest area, with a rich abundance of *J. mandshurica* and rich variation in the growth, wood, and seed properties of different resources (Song et al., 2017). However, driven by financial gain, *J. mandshurica* in Northeast China has been illegally and excessively cut, leading to the loss of a large number of good genes of *J. mandshurica* and the destruction of natural forests. The frequent occurrence of forest fires has also constrained the development and utilization of germplasm resources, leading to a sharp decline in local economic and ecological benefits. Studies on *J. mandshurica* have been conducted in China from the 1980s to 1990s (Yang et al., 1990); however, the long growth cycle and low survival rate of asexual propagation, such as cutting and grafting, have led to slow progress in research on *J. mandshurica*. Most studies have focused on medicinal value development (Jin et al., 2015), pharmacological effects (Yao et al., 2017), clinical effects (Park et al., 2012), and toxicity tests (Sun et al., 2007), with a limited number of studies focusing on germplasm collection, conservation, development, and utilization.

In recent years, with advances in molecular biology and high-throughput sequencing technologies, molecular markers, transcriptome sequencing, and genome sequencing have been gradually applied to study the genetic structure, genetic diversity, and relatedness among or within species, which have allowed the exploration of the evolutionary origin of species at the individual and population levels. Molecular marker technologies, such as SRAP (Wang, 2007; Zhang et al., 2020), ISSR (Wang et al., 2011), and SSR (Liu, 2015; Wang et al., 2016; Dang et al., 2021), have been used to study the genetic diversity of *J. mandshurica*; however, a limited number of studies have used EST-SSRs as markers. We collected germplasm resources for *J. mandshurica* in the forest area of northeastern China and studied the intraspecific genetic diversity based on transcriptome sequencing and EST-SSR molecular marker technologies. We aimed to: (1) use Illumina HiSeq 2000 to sequence the RNA of *J. mandshurica* and to develop polymorphic EST-SSR primers using the obtained transcriptome data; (2) analyze the genetic diversity of different *J. mandshurica* populations in northeastern China using the developed EST-SSR primers; and (3) propose genetic conservation strategies for *J. mandshurica* in northeastern China.

MATERIALS AND METHODS

Plant Materials and DNA Extraction

A total of 343 samples from 15 representative *J. mandshurica* populations were collected from the main distribution areas of Heilongjiang, Jilin, and Liaoning Provinces in Northeast China (**Figure 1**, **Table 1**). Sampling was spaced at least 100 m apart. Fresh, pest-free leaves of *J. mandshurica* were collected and snap-frozen in liquid nitrogen for DNA extraction, which was performed by the CTAB method, as described by Zhao and Woeste (2011), with some modifications. Assessment of DNA quality was performed by 1% agarose gel electrophoresis, while DNA purity and concentration were evaluated by a Micro-Spectrophotometer (KAIAO K5600, Beijing KAIAO Technology Development Co., Ltd., China). Sample DNA solutions were diluted to a working concentration of 35 ng/μL and stored at −20°C.

SSR Primer Development

Three tissue samples (leaves, bark, and fruit pericarp) were collected from a healthy *J. mandshurica* plant in Harbin, Heilongjiang Province, China. RNA extraction and sequencing were performed by Annaroad Gene Technology (Beijing) Co., Ltd., Beijing, China and Biomarker Technologies Co., Ltd., Beijing, China. Based on transcriptome data, SSRs were detected and localized by the MIcroSAtellite identification tool (MISA) (http://pgrc.ipk-gatersleben.de/misa/) (Patil et al., 2020). Minimum SSR motif repeats were identified by 10 mononucleotides, six dinucleotides, five trinucleotides, tetranucleotide, pentanucleotide, and hexanucleotide motif repeats. Primer 3 version 4.1.0 (https://bioinfo.ut.ee/primer3/) (Preethi et al., 2020) was used to design the primers. Primer length ranged from 18 to 24 bp, GC contents ranged from 40 to

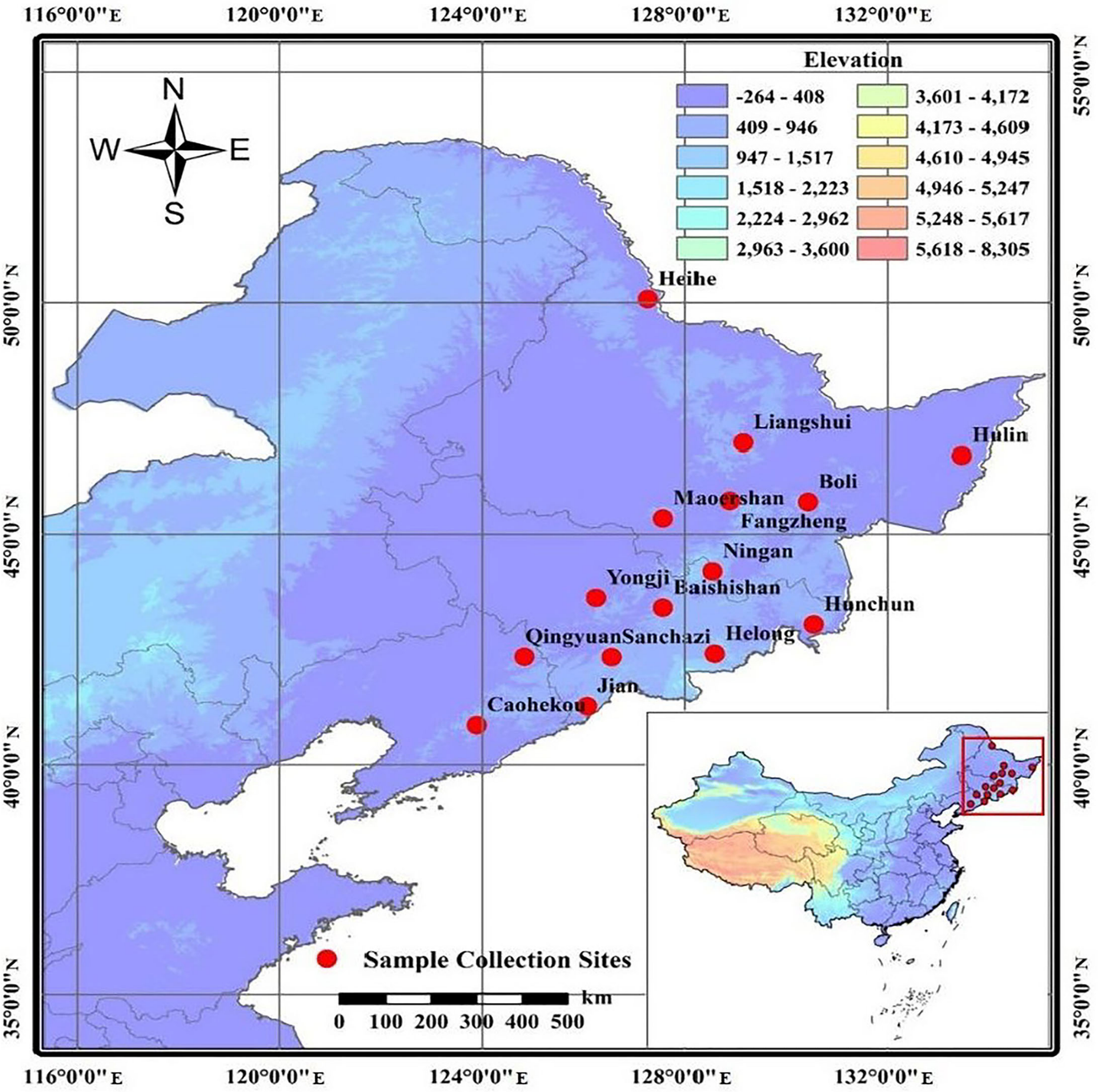

FIGURE 1 | Geographical distribution of *Juglans mandshurica* collected in Northeast China.

60%, annealing temperatures ranged from 55 to 60°C, and the size of PCR products ranged from 100 to 350 bp.

Primer Screening and PCR Amplification

A total of 240 primer pair sequences were randomly selected and synthesized (Tsingke Biotechnology Co., Ltd., Herbin, China) (**Supplementary Table S1**), and a universal M13 sequence (5′-TGTAAAACGACGGGCCAGT-3′) labeled with four fluorescent dyes (HEX, ROX, FAM, and TAMRA) was added in front of the 5′ end of the forward primers (Li et al., 2020). To screen the primers, 16 samples were randomly selected from eight populations for PCR amplification. The reaction system (20 μL) contained 10 μL of 2× Super PCR Mix (Beijing Genomics Institution, Beijing, China), 0.2 μL of forward primer (10 μM), 0.8 μL of reverse primer (10 μM), 0.5 μL of M13 primer with a fluorescent label (Sangon Biotech (Shanghai) Co., Ltd., Shanghai, China), 2 μL of DNA, and 2.0 μL of ddH_2O. The PCR conditions were as follows: predenaturation at 94°C for 4 min; 30 cycles of denaturation at 94°C for 30 s, annealing at 60°C for 30 s, and extension at 72°C for 30 s; and a final extension step at 72°C for 10 min.

TABLE 1 | Summary of *J. mandshurica* sampling locations in Northeast China.

Provenance	Longitude (E)	Latitude (N)	Elevation (m)	Annual temperature (°C)	Annual rainfall (mm)	Forest-free days (d)	Annual sunshine (h)
Heihe	127°16′	50°04′	160	−1.9	572	153	2,655
Hunchun	130°32′	43°02′	300	5.7	618	150	2,322
Fangzheng	128°52′	45°43′	227	2.6	611	125	2,500
Liangshui	129°08′	46°58′	416	0.3	676	110	2,375
Ningan	128°31′	44°11′	267	4.0	514	137	2,655
Caohekou	123°53′	40°51′	233	6.1	930	128	2,394
Jian	126°04′	41°16′	500	0.4	631	148	2,375
Qingyuan	124°49′	42°20′	511	4.9	935	127	2,433
Yongji	126°14′	43°37′	220	5.3	677	142	2,390
Sanchazi	126°33′	42°19′	520	2.5	726	110	2,300
Boli	130°25′	45°41′	405	3.9	526	155	2,467
Hulin	133°28′	46°41′	222	2.7	690	135	2,422
Helong	128°34′	42°24′	442	4.8	536	120	2,413
Baishishan	127°34′	43°24′	600	3.8	720	115	2,477
Maoershan	127°34′	45°20′	400	2.4	700	125	2,522

TABLE 2 | EST-SSR primer information of *J. mandshurica*.

Locus	Forward Primer	Reverse Primer	Motif	Tm (°C)	Size (bp)
NEJM-1	GGTTTTCTTGCATTTTGCTG	GCTTTGGTTTCAATTTGGCT	(AG)15	58.81	277
NEJM-2	TTCGCTGCTTCCTCTCTCTC	TCGTGTTGTCCGTTCTCATC	(CT)8	59.83	250
NEJM-3	CAACTCGTACGTGCGATCAT	AGTGAGAGCTTGGAGCTTGG	(CT)15	59.75	228
NEJM-4	GAGATGAGTGGGAGCTTTGC	GGCTTGTGATCCGAAATCAT	(TC)8	59.93	273
NEJM-5	GAGCGAAAACTGTGCCTTTC	TTGGCTAGAGGTTGAAGGGA	(CT)10	59.90	248
NEJM-6	TCTCTTGCGCTTTGTTTTCA	ACTGATTCGTTTCACTCGCA	(GA)15	59.59	221
NEJM-7	AGACTCGAAGGCTCTGCTTG	TATGGCACCTTTGTGGTCAA	(TC)15	59.93	206
NEJM-8	TGGGGTGTTGATACACAAAGA	CCTCAGCAACAGTCCCTTTC	(TG)8	59.36	272
NEJM-9	CTGAGCAAGTCAACATCCGA	CCCATTGGCGAAACTTGTAT	(AC)7	59.90	239
NEJM-10	CTGACCCGACCCACTAATGT	TCCAAATCGGACTCTGATACC	(TA)11	59.42	220
NEJM-11	AGAAGTGATGAACCGCTCGT	GGTGCGTGACAGTATTCTCG	(CT)11	59.60	185
NEJM-12	GACCTCAAACCAGATCTTCCA	CCAGTTCCCAGGTGATGTCT	(TC)15	59.54	264
NEJM-13	CAGGAATGAACACGGCAGTA	CCTGGATTTTGTTCCTCTGC	(AT)8	59.69	236
NEJM-14	TAACGCAATCCCAACACAAA	GTATTCGTCTGCTCCGCTTC	(AG)12	59.98	219
NEJM-15	CTAATGGTTGGTGTGCCAGA	GAACCACAAGGATTCCCAGA	(CT)12	59.73	182

All PCR products were sent to Sangon Biotech (Shanghai) Co., Ltd., Shanghai, China for capillary electrophoresis sequencing. Ultimately, 15 pairs of polymorphic EST-SSRs were selected and characterized (**Table 2**), and 15 primer pairs were used to analyze the genetic diversity of 343 samples from 15 natural *J. mandshurica* populations. The different Mantel tests were performed with the R package "vegan" and were based on 1,000 permutations.

Statistical Analysis

For subsequent analyses, the primary microsatellite data obtained by capillary electrophoresis were converted into GenePOP format using the MS-Tools program (Li et al., 2015). The total number of alleles (*Na*), effective number of alleles (*Ne*), Shannon information index (*I*), observed heterozygosity (*Ho*), expected heterozygosity (*He*), overall fixation index (*Fit*), inbreeding coefficient (*Fis*), genetic differentiation coefficient (*Fst*), gene flow (*Nm*), number of private alleles (*NPA*), and Hardy–Weinberg equilibrium (*HWE*) were calculated by the GenAlEx 6.502 program (Gultyaeva et al., 2019). PIC calc software was used to calculate the polymorphic information content (*PIC*) (Nagy et al., 2012), while the CONVERT 1.31 program (Marta et al., 2012) was used to convert the GenePOP data format to STRUCTURE data format. The Bayesian clustering method in STRUCTURE 2.3.4 was used to analyze the genetic structure of the *J. mandshurica* population (Mohammed et al., 2020), while the *K* value was tested from 2 to 10, with three replications at each *K*, 100,000 generations burnin period, and 100,000 Markov Chain Monte Carlo (MCMC) repetitions. The LnP (*K*) and ΔK plots were generated by the online tool Structure Harvester (http://taylor0.

biology.ucla.edu/structureHarvester/) (Ravelombola et al., 2018), and the best K value was selected using the ΔK maximum value principle. Nei's (1983) genetic distances between populations were calculated by PowerMarker 3.25 program (Poudel et al., 2019), and the computed distances were used for the construction of phylogenetic trees by using the neighbor-joining method (NJ). Annotation and visualization of phylogenetic trees were performed using Mega 7.0.26 software and the Interactive Tree of Life online tool (https://itol.embl.de) (Letunic and Bork, 2021). Analysis of molecular variance (AMOVA) and principal coordinate analysis (PCoA) were performed using GeneAlEx 6.502 software (Gultyaeva et al., 2019).

RESULTS

EST-SSR Loci Detection

A total of 63,552 EST-SSR loci were detected based on the transcriptome data of *J. mandshurica*. Among them, mononucleotide repeat units were the most abundant, accounting for 55.80% of all SSRs, followed by dinucleotides and trinucleotides with 32.14 and 10.43%, respectively (**Figure 2A**). The lowest number of tandem repeats was 5 (4,292, 6.75%), with 10 tandem repeats accounting for the highest percentage (13,382, 21.06%), followed by 11 (7,384, 11.62%), 6 (6,600, 10.39%), and 12 (5,949, 9.36%) repeats (**Figure 2D**). In addition, there were 40 sequence types of 63,552 EST-SSRs, among which the A/T mononucleotide motif (34,503, 5.29%) was the most dominant, followed by AG/CT (7,529, 11.85%) and GA/TC (7,294, 11.48%) motifs (**Figures 2B,C**).

Polymorphic EST-SSR Primer Development

A total of 240 EST-SSRs were randomly selected and synthesized for polymorphism investigation (**Table 3**). A total of 274 alleles were detected at the 15 EST-SSR loci. The *Na* per locus varied from 10 (NEJM-8) to 33 (NEJM-6), with a mean of 18.27, whereas the *Ne* ranged from 1.728 (NEJM-4) to 6.170 (NEJM-6) with a mean of 3.937. The *I* ranged from 0.635 (NEJM-4) to 2.005 (NEJM-6) with a mean of 1.455. The *Ho* ranged from 0.132 (NEJM-4) to 0.775 (NEJM-9) with a mean of 0.564; *He* ranged from 0.322 (NEJM-4) to 0.831 (NEJM-6) with a mean of 0.670. The *PIC* ranged from 0.662 (NEJM-5) to 0.912 (NEJM-6) with a mean of 0.797. The *Fit* ranged from 0.141 (NEJM-9) to 0.812 (NEJM-4) with a mean of 0.317; *Fis* ranged from 0.025 (NEJM-8) to 0.590 (NEJM-4) with a mean of 0.177; *Fst* ranged from 0.039 (NEJM-7) to 0.541 (NEJM-4) with a mean of 0.184; *Nm* ranged from 0.212 (NEJM-4) to 6.191 (NEJM-7) with a mean of 1.967; and *NPA* ranged from 1 (NEJM-9) to 9 (NEJM-15) with a mean of 3.867, accounting for 21.17% of *Na*.

Genetic Diversity of the *J. mandshurica* Population

Genetic diversity analysis was performed on 343 individuals from 15 populations of *J. mandshurica* using the primers shown in **Table 4**. The range of variations in *Na* among the different populations was from 4.200 (Heihe) to 8.133 (Fangzheng and Maoershan), and the mean value was 6.933. For *Ne*, the range was from 2.894 (Heihe) to 4.651 (Helong), with an average of 3.937. For *I,* variations ranged from 1.158 (Heihe) to 1.617 (Hunchun), with a mean value of 1.455. Variations in *Ho* ranged from 0.477 (Jian) to 0.665 (Fangzheng), with a mean of 0.564, whereas variations in *He* ranged from 0.605 (Heihe) to 0.723 (Hunchun), with a mean value of 0.670. *NPA* varied from 1 (Qingyuan, Boli and Baishishan) to 10 (Hulin) with a mean value of 3.867. The fixation index (*F*) varied from 0.026 (Heihe) to 0.326 (Maoershan) with a mean value of 0.177. In this study, $F > 0$ indicates the absence of heterozygotes and excess of pure heterozygotes in the *J. mandshurica* population and the presence of inbreeding. Furthermore, the Hunchun population showed high genetic diversity ($Na = 7.933$, $He = 0.723$, and $I = 1.617$), whereas the Heihe population exhibited a relatively low level of genetic diversity ($Na = 4.200$, $He = 0.605$, and $I = 1.158$).

Genetic Structure of the *J. mandshurica* Population

The genetic structure of the *J. mandshurica* population in Northeast China was analyzed using the STRUCTURE program. The optimal number of clusters (K) was determined by comparing the LnP (D) and ΔK based on the rate of change in LnP (D). The LnP (D) value increased continuously with the number of clusters (K) from 2 to 10 based on estimated posterior probability of genotype data, and the inflection point appears when $K = 3$ and $K = 5$ (**Figure 3A**). Two pronounced peaks of ΔK were observed (**Figure 3B**). Because the structure analysis cannot clearly classify the test samples into five categories when $K = 5$, $K = 3$ was used as the optimal K value. Based on this value, 343 individuals of *J. mandshurica* were grouped into three genetic categories (category 1 in green, category 2 in blue, and category 3 in yellow) (**Figure 3C**). Category 1 consisted of 147 individuals from eight populations for Qingyuan, Caohekou, Jian, Ningan, Yongji, Baishishan, Helong, and Maoershan; category 2 consisted of 81 individuals from three populations for Hulin, Boli, and Sanchazi; and category 3 consisted of 115 individuals from four populations for Heihe, Hunchun, Fangzheng, and Liangshui.

Principal Coordinates and Evolutionary Tree Analysis of the *J. mandshurica* Population

To further analyze the genetic relationships among natural populations of *J. mandshurica*, principal coordinate analysis (PCoA) was performed using Nei's genetic distances of 343 individuals from 15 populations (**Figure 4**). The PC1 horizontal axis explained 13.90% of the total variations, while the PC2 vertical axis explained 5.86% of the total variations. The two principal axes explained 19.76% of the total genetic variation. The 15 natural populations were divided into three categories, which were generally consistent with the results obtained in the population structure analysis: category 1 included the Qingyuan, Caohekou, Jian, Ningan, Yongji, Baishishan, Helong, and Maoershan populations; category 2 included the Hulin, Boli, and Sanchazi populations; and category 3 included the Heihe, Fangzheng, and Liangshui populations. Nei's genetic distance was used to construct a neighbor-joining dendrogram (**Figures 5**, **6**) in which all samples were clustered into three categories.

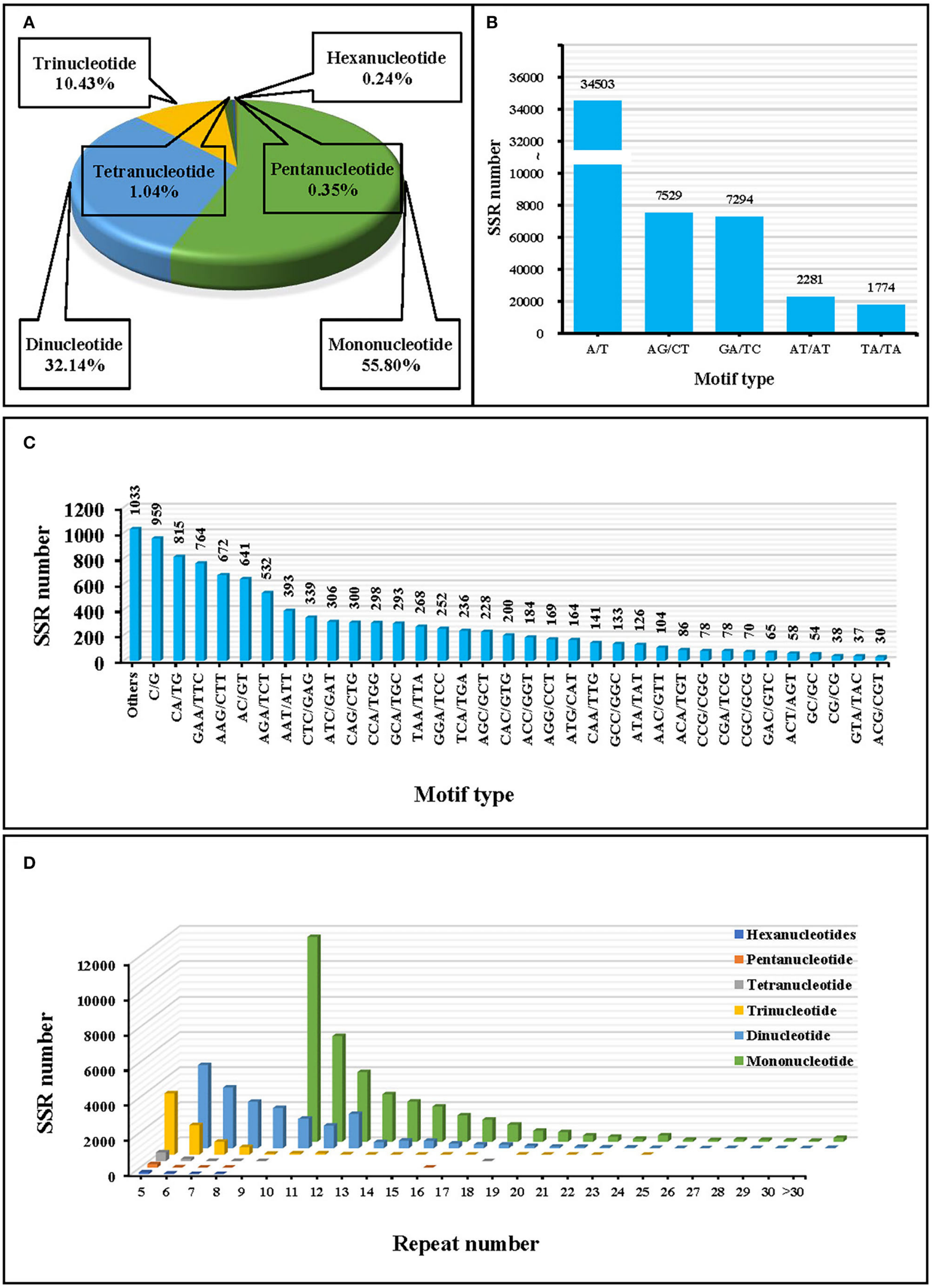

FIGURE 2 | Statistics of simple sequence repeats (SSRs) in *J. mandshurica*. **(A)** Statistics of the proportion of different motif types. **(B,C)** Statistics of the SSR numbers of different motif types. **(D)** Statistics of the number of repeat types for different motifs.

TABLE 3 | EST-SSR primer polymorphisms of *J. mandshurica*.

Locus	Na	Ne	I	Ho	He	PIC	Fit	Fis	Fst	Nm	NPA	HWE
NEJM-1	19	2.393	1.031	0.450	0.475	0.832	0.470	0.052	0.440	0.318	4	NS
NEJM-2	20	4.556	1.674	0.673	0.770	0.891	0.251	0.126	0.143	1.494	3	NS
NEJM-3	18	5.138	1.749	0.646	0.772	0.866	0.265	0.163	0.121	1.813	4	NS
NEJM-4	13	1.728	0.635	0.132	0.322	0.668	0.812	0.590	0.541	0.212	2	*
NEJM-5	12	3.192	1.348	0.510	0.671	0.662	0.272	0.240	0.043	5.627	3	NS
NEJM-6	33	6.170	2.005	0.701	0.831	0.912	0.236	0.157	0.094	2.414	4	NS
NEJM-7	17	4.183	1.565	0.667	0.747	0.747	0.142	0.107	0.039	6.191	6	NS
NEJM-8	10	2.797	1.191	0.609	0.625	0.696	0.178	0.025	0.157	1.343	3	NS
NEJM-9	17	5.920	1.925	0.775	0.820	0.894	0.141	0.055	0.091	2.511	1	NS
NEJM-10	22	3.995	1.625	0.707	0.735	0.828	0.159	0.039	0.125	1.744	5	NS
NEJM-11	17	4.651	1.496	0.580	0.646	0.853	0.331	0.102	0.255	0.730	5	NS
NEJM-12	18	4.646	1.722	0.683	0.763	0.851	0.209	0.105	0.116	1.897	2	NS
NEJM-13	16	3.536	1.281	0.319	0.631	0.791	0.606	0.495	0.221	0.881	2	NS
NEJM-14	21	3.119	1.374	0.601	0.664	0.752	0.225	0.095	0.144	1.490	5	NS
NEJM-15	21	3.025	1.209	0.406	0.581	0.719	0.461	0.301	0.229	0.842	9	*
Mean	18.27	3.937	1.455	0.564	0.670	0.797	0.317	0.177	0.184	1.967	3.867	

**Denotes significant departure from Hardy Weinberg equilibrium at $P < 0.05$.*

TABLE 4 | Genetic diversity parameters of different populations of *J. mandshurica*.

Provenance	Sample	Na	Ne	I	Ho	He	NPA	F
Heihe	6	4.200	2.894	1.158	0.573	0.605	3	0.026
Hunchun	27	7.933	4.505	1.617	0.657	0.723	4	0.094
Fangzheng	29	8.133	4.359	1.588	0.665	0.716	2	0.080
Liangshui	27	7.333	4.132	1.562	0.614	0.714	3	0.136
Ningan	11	5.867	3.776	1.388	0.581	0.655	3	0.164
Caohekou	10	5.533	3.475	1.319	0.573	0.639	2	0.121
Jian	17	6.467	3.841	1.438	0.477	0.670	6	0.286
Qingyuan	20	6.733	3.967	1.436	0.529	0.666	1	0.266
Yongji	27	7.467	4.025	1.525	0.557	0.687	3	0.243
Sanchazi	27	6.800	3.824	1.395	0.564	0.642	5	0.112
Boli	27	6.267	3.669	1.363	0.572	0.648	1	0.133
Hulin	34	7.600	3.957	1.445	0.553	0.642	10	0.166
Helong	26	8.067	4.651	1.589	0.539	0.704	8	0.256
Baishishan	26	7.467	3.888	1.475	0.516	0.663	1	0.248
Maoershan	29	8.133	4.086	1.531	0.487	0.677	6	0.326
Mean		6.933	3.937	1.455	0.564	0.670	3.867	0.177

This was inconsistent with the aforementioned results, and some individuals were assigned to different categories (**Figure 6**).

Genetic Differentiation and Gene Flow of the *J. mandshurica* Population

Genetic variation among different populations of *J. mandshurica* was investigated using the AMOVA tool (**Table 5**). The obtained results revealed significant genetic differentiation at the 0.01 level among the populations ($Fst = 0.162$, $P < 0.001$). Moreover, 21.10 and 62.68% of the genetic variations were attributed to variations among individuals and within individuals, respectively, whereas variations among populations accounted for the total genetic variance of 16.22%, indicating that genetic variations within populations were much higher compared to genetic differentiation among populations. The *Fst* value was 0.162 ($0.15 < Fst < 0.25$), indicating a moderate level of genetic differentiation among populations. The *Fis* value was 0.252 ($Fis > 0$), indicating the loss of heterozygotes and the presence of inbreeding between populations. The *Fst* (**Figure 7**, lower left) and *Nm* (**Figure 7**, upper right) values among 15 populations of *J. mandshurica* were calculated. The data revealed genetic differences and gene flow among different populations of *J. mandshurica*. The *Fst* value varied from 0.011 to 0.208 with a mean value of

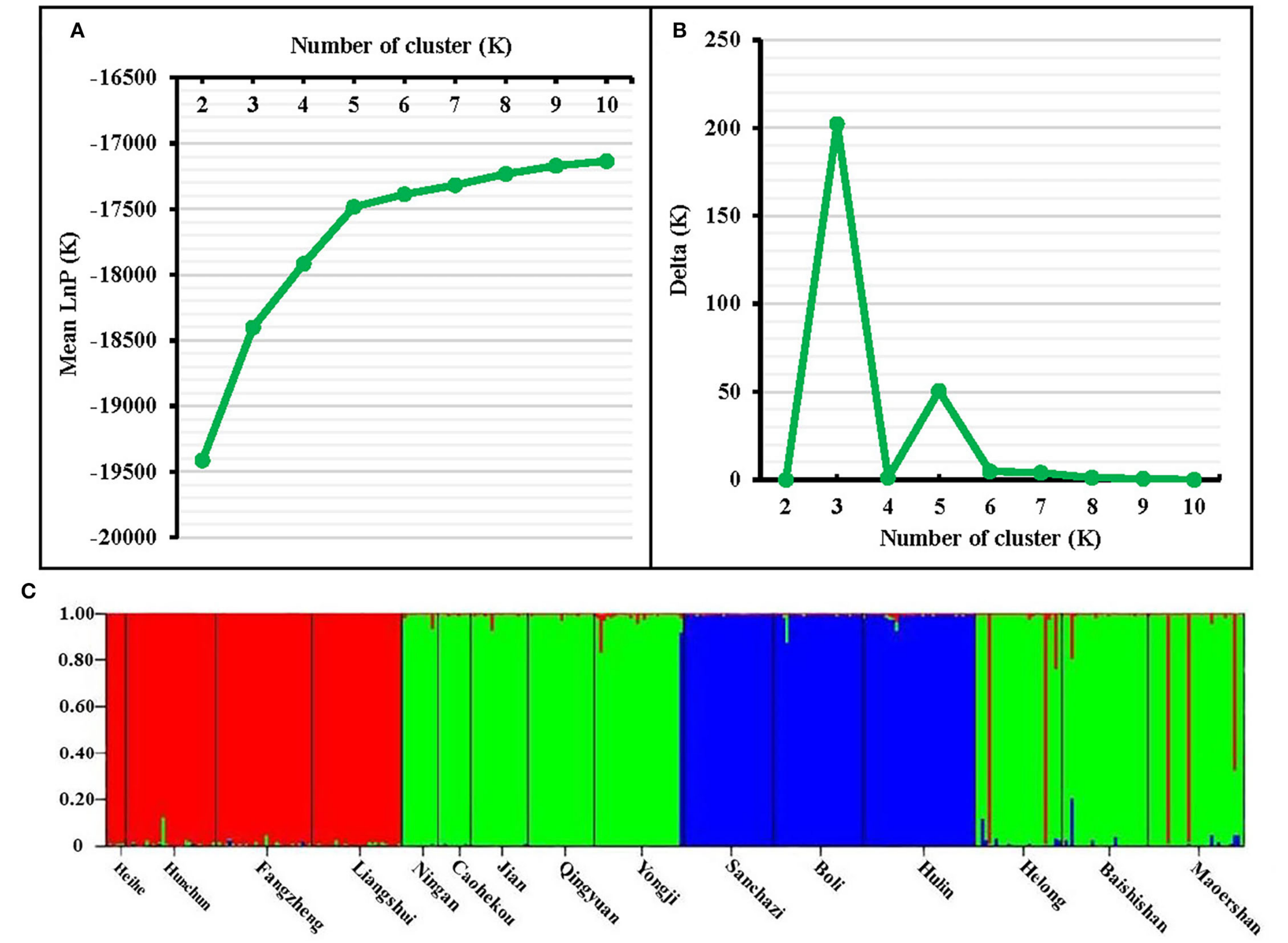

FIGURE 3 | STRUCTURE analysis of the natural population of *J. mandshurica* based on 15 EST-SSRs. **(A)** Calculation of population structure using Mean LnP (K). **(B)** Relations between the optional number of cluster K and Delta K. **(C)** Genetic structure map of 15 natural populations of *J. mandshurica* based on STRUCTURE analysis ($K = 3$).

0.109, and the *Nm* value varied from 0.950 to 22.422 with a mean value of 4.063 among the different populations. The highest *Fst* and the lowest *Nm* values were found between the Sanchazi and Caohekou populations, followed by the Hulin and Caohekou populations. The lowest *Fst* and the highest *Nm* values were found between the Sanchazi and Hulin populations.

Genetic Distance and Genetic Uniformity of the *J. mandshurica* Population

Nei's genetic distances were used to depict the genetic relationships among the 15 *J. mandshurica* populations, and a smaller genetic distance and larger genetic uniformity indicate a close genetic relationship. In this study, pairwise population genetic distance ranged from 0.073 to 0.743 (**Figure 8**, lower left), and the genetic uniformity between populations ranged from 0.156 to 0.958 (**Figure 8**, upper right). The maximum genetic distance appeared between the Sanchazi and Heihe populations, suggesting that they are not closely related. This was followed by the Heihe and Boli populations, and the minimum genetic distance was observed between the Sanchazi and Hulin populations, suggesting that the two populations are closely related.

The Correlations Between the Molecular Traits and Environmental Factors

Mantel tests were performed to explore the correlations between molecular traits and environmental factors. The results showed that except for *Na*, no significant correlation was observed between genetic diversity parameters and environmental factors (**Table 6**). *Na* had a significantly positive correlation with latitude ($r = 0.2974$) and annual sunshine ($r = 0.2592$), but with weak correlation coefficients. These results indicate that the molecular traits of *J. mandshurica* populations were less influenced by environmental factors.

DISCUSSION

Due to the challenges associated with asexual reproduction and with most of the *J. mandshurica* resources basically existing in a

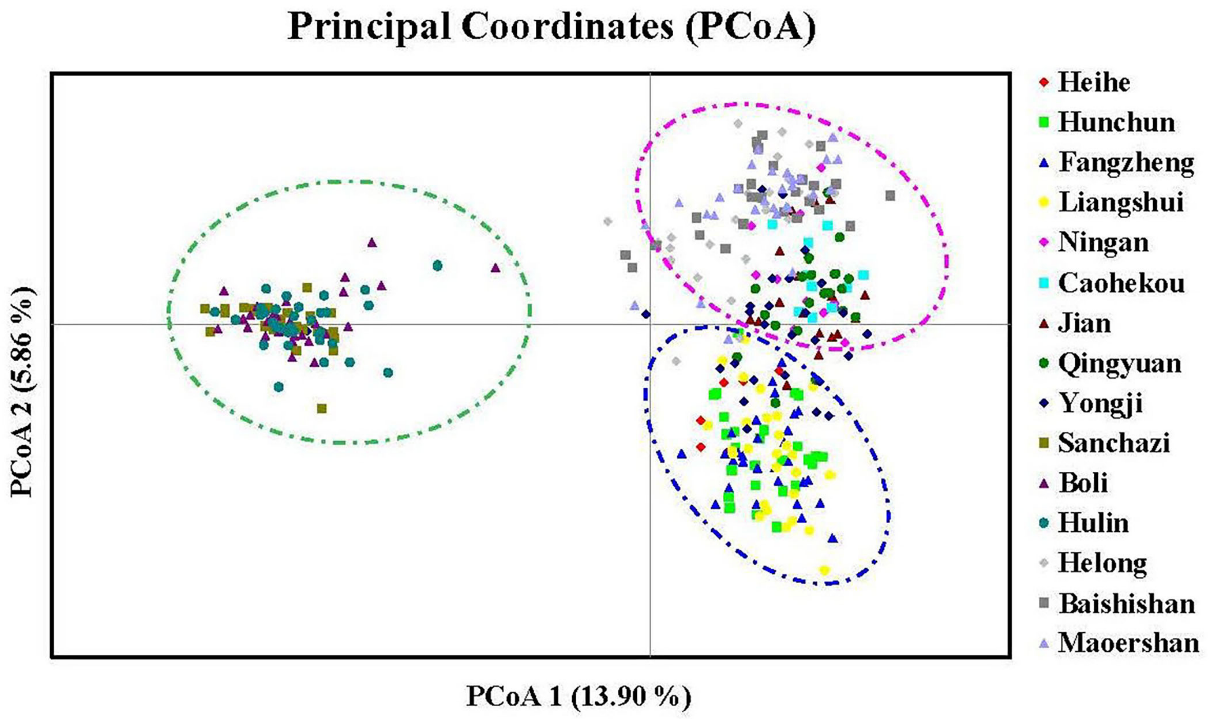

FIGURE 4 | Principal coordinate analysis (PCoA) based on the genetic distance of 343 individuals for *J. mandshurica*.

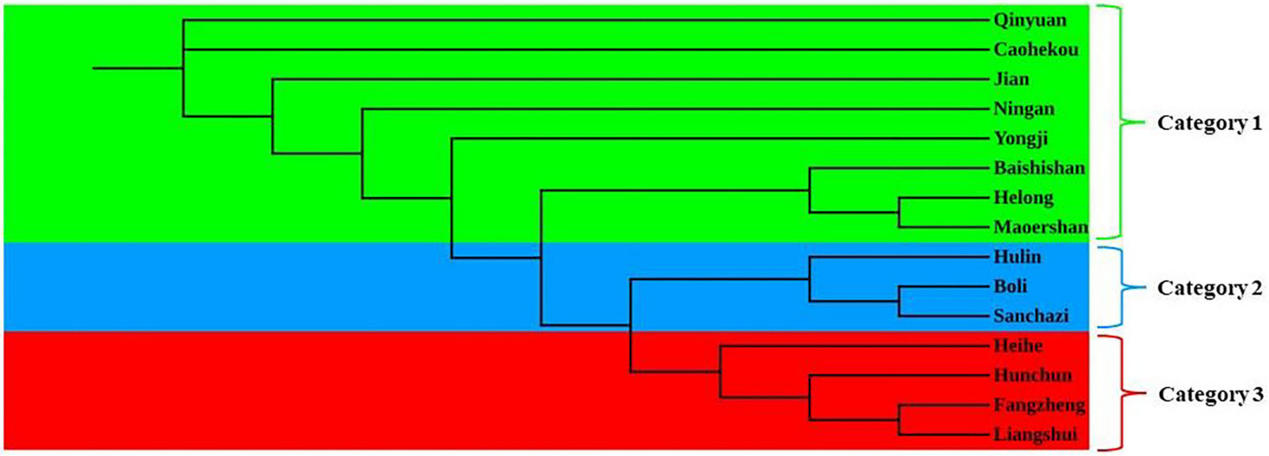

FIGURE 5 | Neighbor-joining tree of 15 *J. mandshurica* populations.

wild or semi-wild state, genetic improvement of *J. mandshurica* is lagging (Zhang et al., 2021). To develop a reasonable, effective breeding strategy and accelerate genetic improvements of *J. mandshurica*, a comprehensive evaluation of *J. mandshurica* resources in natural populations is important. We used the EST-SSR molecular marker to analyze the genetic diversity of *J. mandshurica* populations in Northeast China. Compared to other molecular marker technologies, this molecular marker is characterized by codominance, stable amplification, and good repeatability. It is a commonly used genetic diversity analysis method (Rahemi et al., 2012). Our findings have positive implications for conservation and genetic improvements of *J. mandshurica* resources in Northeast China.

EST-SSR Primer Development

The EST-SSRs have been extensively studied in the plant, animal, and microbial species. Compared to genomic SSRs, EST-SSRs have many advantages, including low sequencing

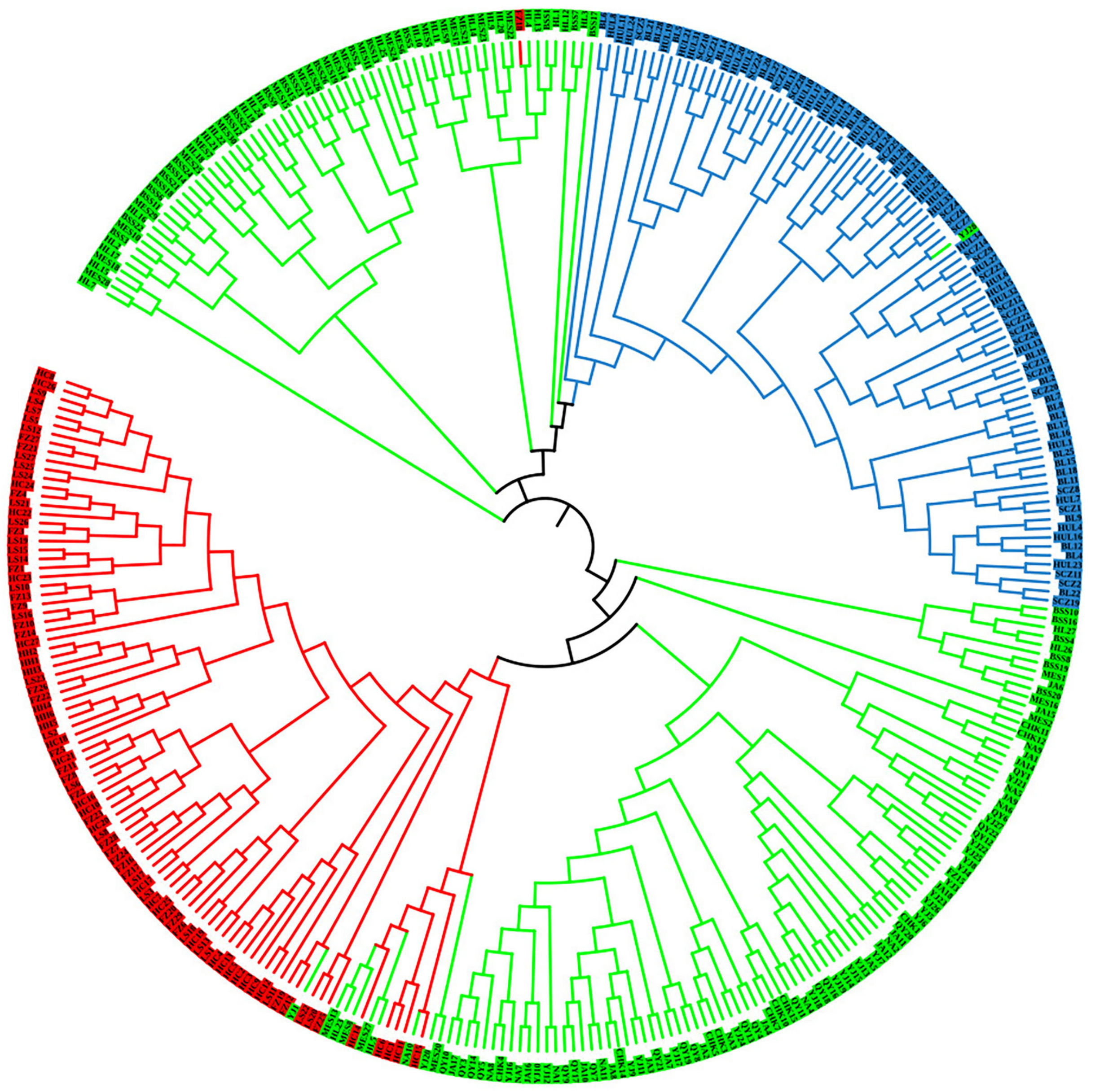

FIGURE 6 | Neighbor-joining tree of 343 *J. mandshurica* individuals (HH: Heihe; HC: Hunchun; FZ: Fangzheng; LS: Liangshui; NA: Ningan; CHK: Caohekou; JA: Jian; QY: Qingyuan; YJ: Yongji; SCZ: Sanchazi; BL: Boli; HUL: Hulin; HL: Helong; BSS: Baishishan; and MES: Maoershan).

costs, identification of the genetic relationship between similar species, and analysis of population genetics (Durand et al., 2010). We found 63,552 EST-SSR loci in the transcriptome sequence of *J. mandshurica*. Then, 240 loci were randomly selected to synthesize primers, and 15 pairs of highly polymorphic EST-SSR primers were finally obtained after screening. In the transcriptome of *J. mandshurica*, among the six nucleotide repeat types, mononucleotide and dinucleotide repeat motifs were the most abundant, accounting for 55.80 and 32.14%, respectively. Distributions of EST-SSR repeat motifs for *J. mandshurica* are comparable to those of *Ziziphus jujuba* (Xiao et al., 2015), *Sesamum indicum* (Uncu et al., 2015), and *Tectona grandis* (Yasodha et al., 2018). SSR repeat motifs are associated with the level of species evolution, and the content of short repeat motifs increases with the time of species evolution or variation frequency (Liu et al., 2020). In this study, the proportion of short repeat motifs was relatively high, indicating a high species variation frequency of *J. mandshurica*. Mononucleotide repeats (A/T) were the most abundant motif class, followed by dinucleotide repeats (AG/CT), while the most common trinucleotide repeats were GAA/TTC and AAG/CTT. Comparable findings have been reported for *Vigna angularis*

TABLE 5 | Analysis of molecular variance for the *J. mandshurica* population.

Variance source	df	SS	MS	VC	PV (%)	Fit	Fis	Fst
Among populations	14	748.284	53.449	1.032	16.22%			
Among individuals	328	2188.162	6.671	1.342	21.10%			
Within individuals	343	1367.500	3.987	3.987	62.68%			
Total	685	4303.946		6.361	100%	0.373***	0.252***	0.162***

****Denotes significant differences at P < 0.001.*

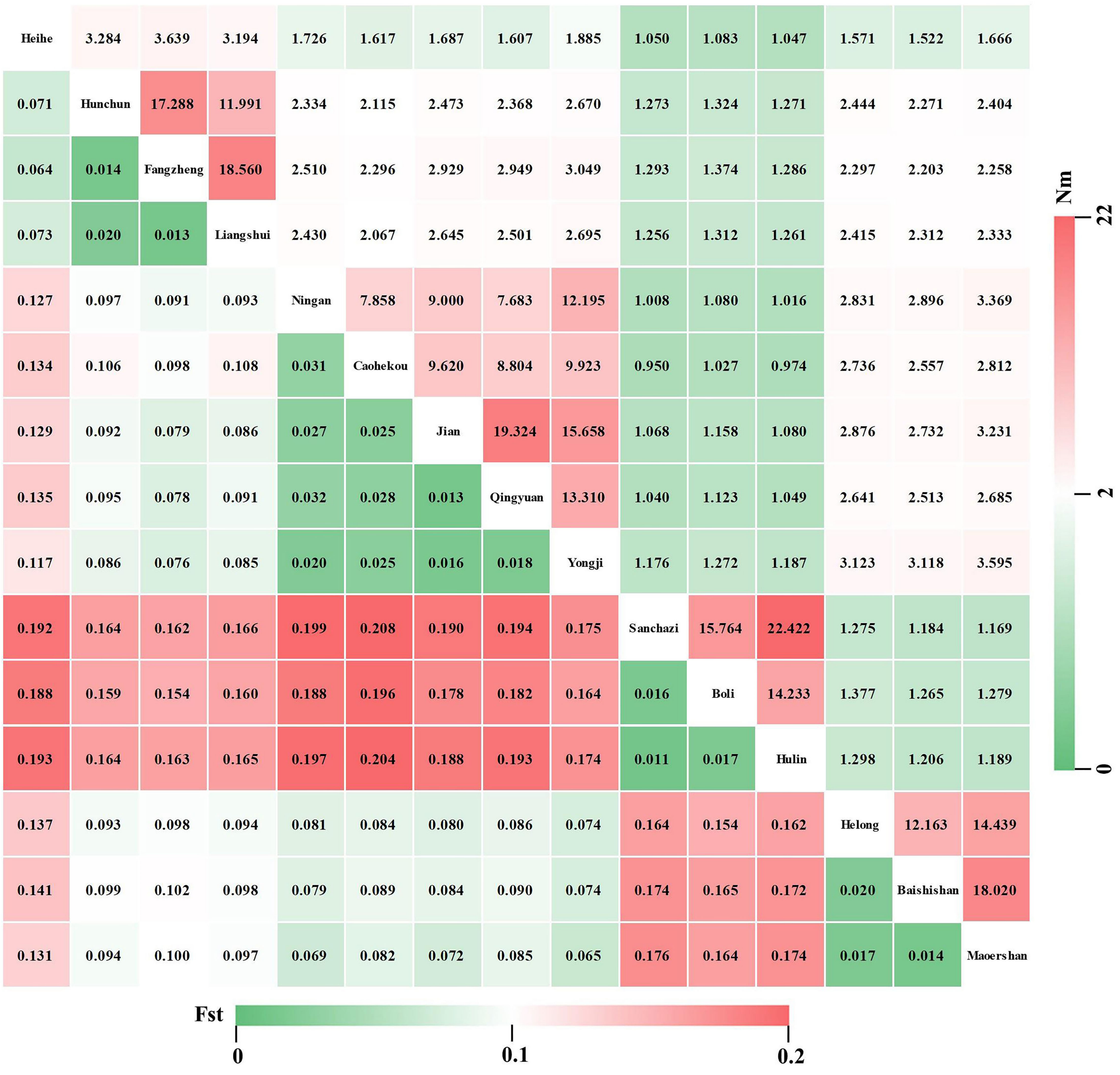

FIGURE 7 | Genetic differentiation coefficient (lower left) and gene flow (upper right) among different populations of *J. mandshurica*.

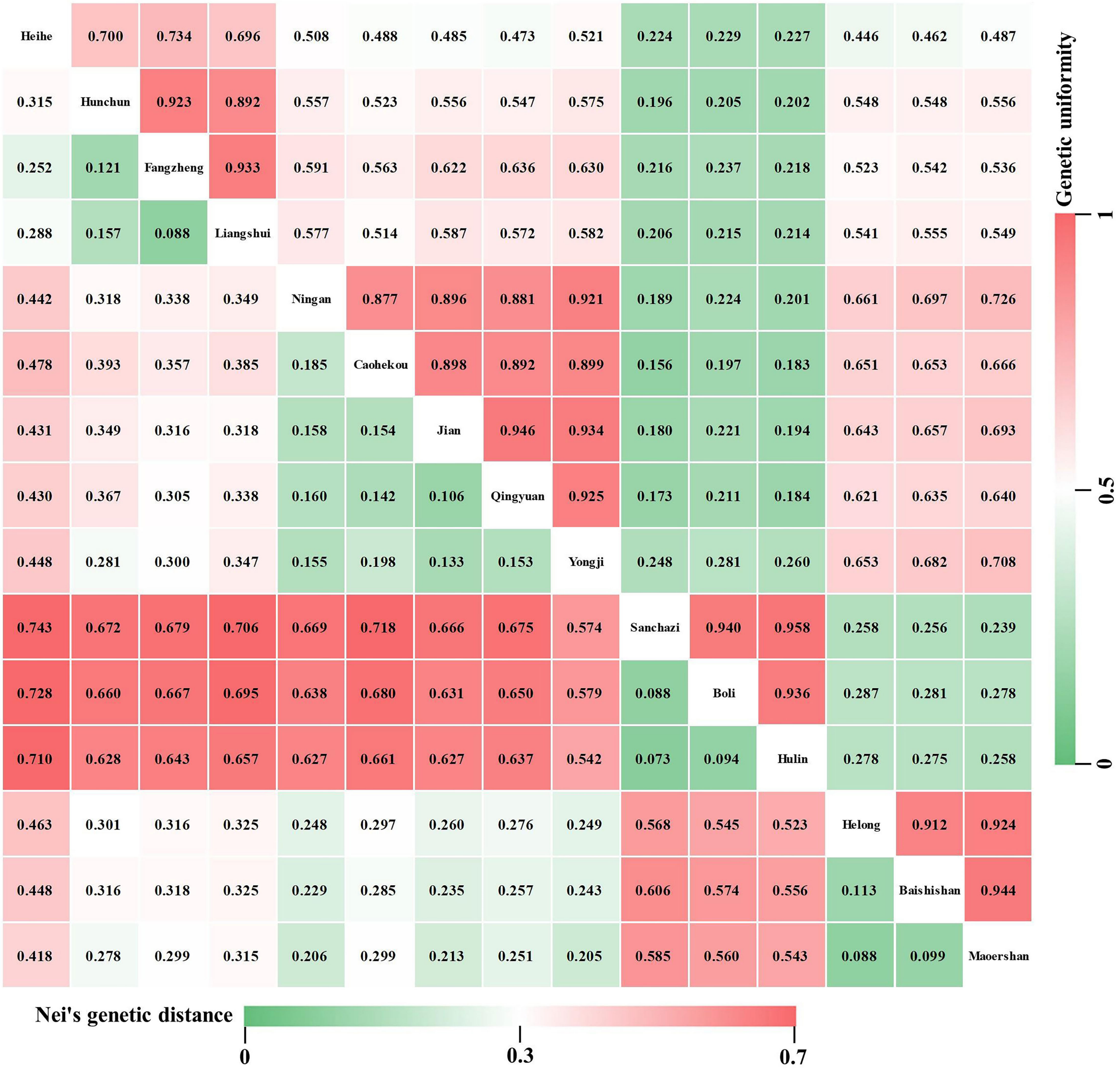

FIGURE 8 | Nei's genetic distance (lower left) and genetic uniformity (upper right) among different populations of *J. mandshurica*.

TABLE 6 | Relationship between molecular traits and environmental factors revealed by Mantel test.

Environment factors	*Na*	*Ne*	*I*	*Ho*	*He*	*NPA*	*F*
Longitude (E)	−0.0554	−0.0584	−0.0068	−0.0724	0.0263	0.1369	−0.0993
Latitude (N)	0.2974*	0.2548	0.2457	−0.1246	0.1516	−0.1166	0.1358
Elevation (m)	0.1673	0.1101	0.1153	−0.0032	0.0730	−0.0424	0.1577
Annual temperature (°C)	0.2603	0.2672	0.2553	−0.0080	0.2125	−0.1356	0.1407
Annual rainfall (mm)	0.2207	0.1309	0.1162	−0.1695	−0.0739	−0.1037	−0.1374
Forest-free days (d)	−0.0008	−0.0243	−0.0075	−0.0968	−0.0271	−0.1257	−0.0825
Annual sunshine (h)	0.2592*	0.1155	0.1658	−0.0405	0.0858	−0.1102	0.0970

Correlation is significant at 5% level.

(Chen et al., 2015) and *J. sigillata* (Feng et al., 2018). In most species, dinucleotides and trinucleotides are the most common types of transcriptomic SSR repeat motifs (Kantety et al., 2002; Varshney et al., 2002), which is confirmed by the results of this study.

Initially, we screened 15 EST-SSR loci, and the mean values for *Na*, *He,* and *PIC* for the 15 loci were 18.27, 0.670, and 0.797, respectively. The polymorphism level of the loci for *J. mandshurica* was higher than that reported by Hu et al., 2016 for the same plant and higher than that of *J. regia* (Torokeldiev et al., 2019), *J. cathayensis* (Dang et al., 2015), *J. hopeiensis* (Hu et al., 2015), and *J. sigillata* (Feng et al., 2018). *PIC* is an important parameter that expresses the degree of genetic diversity among plants, and its evaluation is beneficial for the establishment of plant gene pools and accelerating the breeding process (Avval, 2017). The *PIC*s for the EST-SSR loci screened in this study were all >0.5, implying highly polymorphic loci (Botstein et al., 1980). Therefore, the EST-SSR primers developed in this study are highly polymorphic and can be used for genetic diversity evaluation of *J. mandshurica* breeding resources.

Population Genetic Diversity

Genetic diversity is the product of the long-term evolution of species and is a key factor in population survival and evolution (Barrett and Kidwell, 1998; González et al., 2020). Genetic diversity studies have important implications for forest breeding and the management of endangered tree species (Millar and Westfall, 1992). Heterozygosity is often used to measure the degree of genetic variation and can provide useful information for the protection of endangered species (Schmidt et al., 2021). In this study, the results based on EST-SSR markers showed that the *He* and *Ho* of 15 *J. mandshurica* populations were 0.670 and 0.564, respectively, which was similar to previous research results (Liu, 2015) and higher than those of *J. sigillata* (Chen et al., 2020) and *Amygdalus Mira* (Bao et al., 2018), and the level of genetic diversity was relatively high but lower than that of *J. regia* (Shamlu et al., 2018; Guney et al., 2021). Many factors, including mating systems, evolutionary history, and the level of environmental heterogeneity, influence the level of genetic diversity (Booy et al., 2000). As a tertiary relict plant, *J. mandshurica* is mostly distributed in the eastern mountains of Northeast China (Tang et al., 2019), and the mutations accumulated from a longer evolutionary history have led to a rich genetic basis for the peccary. Phylogeographical studies have shown that *J. mandshurica* had two separate refugia during the Quaternary ice age and that there was asymmetric gene flow (Bai et al., 2010). The existing surviving populations may have retained and inherited an overall rich genetic base of ancestors, giving them a high genetic diversity. The results of Wang et al. (2016) on the phylogeography of *J. mandshurica* also prove this point. Biologically, *J. mandshurica* is a hermaphroditic heterozygous species (Zhang et al., 2019), which is mainly outcrossing. It can produce new genotypes through sexual recombination. The heterodichogamy flowering mechanism effectively prevents self-pollination in *J. mandshurica*. These are probably the main reasons why the current population of *J. mandshurica* still maintains a high level of genetic diversity (Nybom and Bartish, 2000). Additionally, the *Ho* value was found to be lower than the *He* value in all populations, indicating heterozygous deletion. Inbreeding, non-random mating, or population structure destruction may lead to the lack of heterozygotes (Liao et al., 2019). Thus, further analysis is warranted.

Genetic diversity analysis of the 15 *J. mandshurica* populations revealed various differences. The Hunchun population had the highest genetic diversity level ($Na = 7.933$, $He = 0.723$, and $I = 1.158$), while the Heihe population had the lowest ($Na = 4.200$, $He = 0.605$, and $I = 1.617$). Bai et al. (2010) reported that there may be two refugia in the distribution area of *J. mandshurica*, one of which is in the northeast and is mainly distributed in Changbai Mountain. Changbai Mountain is a scenic tourist site with frequent human activities, which may affect the population of *J. mandshurica*, leading to plant habitat fragmentation (Aguilar et al., 2008). Fragmented plant habitats inhibit pollen dispersal and gene flow between populations, thereby affecting genetic diversity in the population (Vranckx et al., 2012). The Hunchun population is located near the Northeast refuge, which is not a tourist city. The existence of refuge and less human disturbance may be the reason for the high genetic diversity of the Hunchun population. The Heihe population is located in the northernmost part of China and is distant from other *J. mandshurica* populations. During field investigations, few *J. mandshurica* were found in this population, yet *J. mandshurica* is a tall tree that is suitable for wind-induced cross-pollination. Marked environmental differences and population isolation may destroy the coadaptive allele combination of parents and reduce the adaptability of distant offspring (Hufford et al., 2012). The fixed index (*F*) for both populations was small but >0, indicating an excess of pure heterozygotes in both populations and a slight inbreeding phenomenon (Wright, 1950).

Environmental factors, such as temperature, elevation, and rainfall, may influence the genetic variation of forests. We used the Mantel test to study the relationship between molecular traits of *J. mandshurica* populations and environmental factors. However, no clear geographical variation pattern was observed in molecular diversity. This is different from what was found in the studies of other species (Hu et al., 2010; Li et al., 2013; Zhang et al., 2017). This finding indicates that the geographic variation at the gene level in the natural populations of *J. mandshurica* may be located with some randomness and is less influenced by the environment, the specific reasons for which need to be further investigated.

Population Genetic Structure

The genetic structure reveals the distribution pattern of genetic diversity among and within populations, reflecting the adaptive potential of various species to the environment (Melo et al., 2014). The 15 natural populations of *J. mandshurica* in this study can be divided into three categories: category 1 denotes the Changbai Mountain area, category 2 denotes the Wanda Mountain area, and category 3 denotes the Lesser Khingan Mountain area. Comparable findings were obtained by neighbor-joining method cluster analysis and

principal coordinate analysis. The Sanchazi population, which is located in the Changbai Mountain area, is not included in category 1. The reason may be that the Sanchazi population is located in Jingyu County, Jilin Province, which was affected by the war. Human factors lead to fragmentation of the population's habitat and inhibit gene exchange (Haag et al., 2010). In addition, relatively isolated or dispersed populations may also reduce the success rate of pollination (Ghazoul, 2005), thereby affecting the genetic structure.

Population Genetic Differentiation

To elucidate the genetic variations among *J. mandshurica* populations, AMOVA was conducted. Genetic variations in *J. mandshurica* were mainly found at the individual level, accounting for 63% of the total genetic variation. Genetic variations among populations accounted for only 16%, indicating that within-population genetic differentiation is greater than among populations, which is consistent with the findings of Wang (2007), Wang et al. (2011), Dang et al. (2021), and Zhang et al. (2021). This may be attributed to the life history and habitat of *J. mandshurica*. *J. mandshurica* is a heterodichogamy deciduous species whose cross-pollination feature results in a high gene flow within the population (Hamrick and Godt, 1996) and a high degree of heterozygosity, thereby improving genetic diversity within the population and reducing the degree of differentiation among populations (Hao et al., 2017). Therefore, when selecting *J. mandshurica* populations with high genetic diversity, the focus should be on the selection of individuals within the population, which is beneficial for the genetic improvement of *J. mandshurica*.

The detection of genetic differentiation is a key to promote the genetic improvement of forests (Li et al., 2021). *Fst* measures the degree of differentiation among populations and is a widely used descriptive statistical indicator in population and evolutionary genetics (Holsinger and Weir, 2009). In this study, the mean *Fst* for the *J. mandshurica* population was 0.109, implying a moderate degree of genetic differentiation (Wright, 1950). This is higher than that reported by Wang et al. (2016). Differences may be attributed to varying research materials and different selection pressures of gene fragments where the selected SSRs were located (Hu et al., 2016). Gene flow (*Nm*) refers to the movement of genes within and among populations, which in the plant kingdom is mainly achieved through the migration or movement of genetic material carriers, such as pollen, seeds, spores, and vegetative bodies. It affects the degree of genetic variation in the population (Zhang et al., 2003). The effects of genetic drift and genetic differentiation can be effectively eliminated when gene flow is >1 (Slatkin, 1985). In this study, the average *Nm* of the *J. mandshurica* population was 4.063, which is relatively high when compared to the results of Wang et al. (2011). *J. mandshurica* is pollinated by the wind; therefore, in this study, *J. mandshurica* may have achieved gene exchange in different geographical locations through long-distance pollen flow (Victory et al., 2006), thereby eliminating geographic isolation and reducing genetic differentiation among populations. Genetic distance and genetic uniformity are indicators of genetic relationships among populations or individuals, which can be further used to analyze the degree of genetic differentiation among the 15 populations. In this study, the variation range of genetic uniformity among populations was 0.156–0.958, with an average of 0.524, which is lower than those reported by Wang (0.83 and 0.9217 in 2007 and 2011, respectively). The average genetic distance was 0.393, which is higher than that of Zhang (0.0049) (2021). The results show great differences, indicating that genetic relationships among *J. mandshurica* populations in this study are relatively distantly related. The differences in genetic uniformity and genetic distance among different populations indicate great fluctuations in genetic variations.

Genetic Conservation Strategy

Studies on genetic diversity and genetic structure are prerequisites for formulating species protection measures (Newton et al., 1999; Wuyun et al., 2015). *J. mandshurica* is a native tree species in Northeast China. It has been listed as a local protected wild plant in Jilin Province, Hebei Province, and Beijing; however, only a nature reserve has been established in Changbai Mountain, and no measures have been undertaken in other distribution areas where it is still the main cut tree species (http://www.iplant.cn/rep/). We show that the *J. mandshurica* population in Northeast China has high genetic diversity, indicating that the endangerment of *J. mandshurica* is not the cause of genetic variation but is mainly affected by human disturbance and habitat destruction and degradation (Wang et al., 2011). In this study, the genetic diversity of the Heihe population was relatively low, and the number of individuals was small. Botanical gardens or arboretums can be used for *ex situ* protection of living *J. mandshurica* in this area. The genetic diversity of the Hunchun population is relatively high and can be protected by establishing a nature reserve. In addition, the germplasm resources of *J. mandshurica* should be systematically collected. Given that there are challenges in asexual propagation strategies, such as cuttings, grafting, and tissue cultures of *J. mandshurica*, the Germplasm Resources Bank of *J. mandshurica* can be established by collecting seeds. On this basis, combined with whole-genome information of *J. mandshurica* (Bai et al., 2018; Yan et al., 2021), SNP and other molecular marker technologies should be used to perform molecular marker-assisted breeding and whole-genome selection breeding to accelerate the progress of genetic improvement for *J. mandshurica*.

CONCLUSION

We developed 15 EST-SSR primers using the transcriptome data of *J. mandshurica*, and 15 loci exhibited high levels of genetic polymorphism, which can be used for genetic diversity evaluation of *J. mandshurica* populations. Genetic diversity analysis was performed on 343 individuals from 15 natural populations of *J. mandshurica* in Northeast China using 15 EST-SSR primers. The *J. mandshurica* populations exhibited high levels of genetic diversity and moderate genetic differentiation. Based on STRUCTURE analysis, neighbor-joining method cluster analysis, and principal coordinate analysis, the 15 populations can be divided into three categories: Changbai Mountain area, Wanda Mountain area, and Lesser Khingan Mountain area. With regard

to the characteristics of the *J. mandshurica* population, there is a need to formulate corresponding genetic conservation strategies to maintain the genetic diversity of the *J. mandshurica* population in Northeast China to the greatest extent possible and to provide a basis for the subsequent development and utilization of important genetic resources of *J. mandshurica*.

DATA AVAILABILITY STATEMENT

Publicly available datasets were analyzed in this study. This data can be found here: https://www.ncbi.nlm.nih.gov/. Transcriptome data of different tissues for *Juglans mandshurica* PRJNA816294.

AUTHOR CONTRIBUTIONS

Conceptualization: QZ and XZhao. Methodology: XZhan and XP. Validation: YY. Resources: LX, JF, JW, and YT. Writing—original draft preparation: QZ. Writing—review and editing: XZ. All authors have read and agreed to the published version of the manuscript.

FUNDING

This work was supported by the Fundamental Research Funds for the Central Universities (No. 2572021AW31) and the Scientific Research Start-up Funds of Jilin Agricultural University (No. 2021002).

SUPPLEMENTARY MATERIAL

The Supplementary Material for this article can be found online at: https://www.frontiersin.org/articles/10.3389/fpls.2022.931578/full#supplementary-material

REFERENCES

Aguilar, R., Quesada, M., Ashworth, L., Herreriasdiego, Y., and Lobo, J. (2008). Genetic consequences of habitat fragmentation in plant populations: susceptible signals in plant traits and methodological approaches. *Mol. Ecol.* 17, 5177–5188. doi: 10.1111/j.1365-294X.2008.03971.x

Avval, S. E. (2017). Assessing polymorphism information content (PIC) using SSR molecular markers on local species of *Citrullus colocynthis*. Case study: Iran, Sistan-Balouchestan province. *J. Mol. Biol. Res.* 7, 42–49. doi: 10.5539/jmbr.v7n1p42

Bai, W. N., Liao, W. J., and Zhang, D. Y. (2010). Nuclear and chloroplast DNA phylogeography reveal two refuge areas with asymmetrical gene flow in a temperate walnut tree from East Asia. *New Phytol.* 188, 892–901. doi: 10.1111/j.1469-8137.2010.03407.x

Bai, W. N., Yan, P. C., Zhang, B. W., Woeste, K. E., Lin, K., and Zhang, D. Y. (2018). Demographically idiosyncratic responses to climate change and rapid Pleistocene diversification of the walnut genus Juglans (Juglandaceae) revealed by whole-genome sequences. *New Phytol.* 217, 1726–1736.

Bao, W. Q., Wuyun, T. N., Du, H. Y., Li, T. Z., Liu, H. M., Wang, L., et al. (2018). Genetic diversity and population structure of *Amygdalus mira* in the Tibet plateau in China based on SSR markers. *Sci. Silvae Sin.* 54, 30–41. doi: 10.11707/j.1001-7488.20180204

Barrett, B. A., and Kidwell, K. K. (1998). AFLP-based genetic diversity assessment among wheat cultivars from the pacific northwest. *Crop Sci.* 38, 1261–1271. doi: 10.2135/cropsci1998.0011183X003800050025x

Booy, G., Hendriks, R. J. J., Smulders, M. J. M., Groenendael, J. M. V., and Vosman, B. (2000). Genetic diversity and the survival of populations. *Plant Biol.* 2, 379–395. doi: 10.1055/s-2000-5958

Botstein, D., White, R. L., Skolnick, M., and Davis, R. W. (1980). Construction of a genetic linkage map in man using restriction fragment length polymorphisms. *Am. J. Hum. Genet.* 32, 314.

Chen, H. L., Liu, L. P., Wang, L. X., Wang, S. H., Somta, P., and Chen, X. Z. (2015). Development and validation of EST-SSR markers from the transcriptome of adzuki bean (*Vigna angularis*). *PLoS ONE* 10:e0131939. doi: 10.1371/journal.pone.0131939

Chen, S. Y., Wu, T., Xiao, L. J., Ning, D. L., and Pan, L. (2020). Genetic diversity of *Juglans sigillata* Dode germplasm in Yunnan Province, China, as revealed by SSRs. *Plant Genet. Resour.* 18, 417–426. doi: 10.1017/S1479262120000441

Chobot, V., and Hadacek, F. (2009). Milieu-dependent pro-and antioxidant activity of juglone may explain linear and nonlinear effects on seedling development. *J. Chem. Ecol.* 35, 383–390. doi: 10.1007/s10886-009-9609-5

Dang, M., Liu, Z. X., Chen, X., Zhang, T., Zhou, H. J., Hu, Y. H., et al. (2015). Identification, development, and application of 12 polymorphic EST-SSR markers for an endemic Chinese walnut (*Juglans cathayensis* L.) using next-generation sequencing technology. *Biochem. Syst. Ecol.* 60, 74–80. doi: 10.1016/j.bse.2015.04.004

Dang, M., Zhou, H. J., Woeste, K. E., Yue, M., Zhang, Y., Zhao, G. F., et al. (2021). Comparative phylogeography of *Juglans regia* and *J. mandshurica* combining organellar and nuclear DNA markers to assess genetic diversity and introgression in regions of sympatry. *Trees* 35, 1993–2007. doi: 10.1007/s00468-021-02167-y

Durand, J., Bodénès, C., Chancerel, E., Frigerio, J. M., Vendramin, G., Sebastiani, F., et al. (2010). A fast and cost-effective approach to develop and map EST-SSR markers: oak as a case study. *BMC Genom.* 11:570. doi: 10.1186/1471-2164-11-570

El-Esawi, M. A. (2017). SSR analysis of genetic diversity and structure of the germplasm of faba bean (*Vicia faba* L.). *C. R. Biol.* 340, 474–480. doi: 10.1016/j.crvi.2017.09.008

Feng, X. J., Yuan, X. Y., Sun, Y. W., Hu, Y. H., Zulfiqar, S., Ouyang, X. H., et al. (2018). Resources for studies of iron walnut (*Juglans sigillata*) gene expression, genetic diversity, and evolution. *Tree Genet. Genom.* 14, 1–15. doi: 10.1007/s11295-018-1263-z

Ghazoul, J. (2005). Pollen and seed dispersal among dispersed plants. *Biol. Rev.* 80, 413–443. doi: 10.1017/S1464793105006731

González, A. V., Gómez-Silva, V., Ram'irez, M. J., and Font'urbel, F. E. (2020). Meta-analysis of the differential effects of habitat fragmentation and degradation on plant genetic diversity. *Conserv. Biol.* 34, 711–720. doi: 10.1111/cobi.13422

Gultyaeva, E. I., Kazartsev, I. A., and Shaydayuk, E. L. (2019). Molecular-genetic polymorphism of *Puccinia triticina* in southern Dagestan relating to the center of the common evolution between agent causing leaf rust and wheat. *Russ. J. Genet.* 55, 418–425. doi: 10.1134/S1022795419040045

Guney, M., Kafkas, S., Keles, H., Zarifikhosroshahi, M., Gundesli, M. A., Ercisli, S., et al. (2021). Genetic diversity among some walnut (*Juglans regia* L.) genotypes by SSR markers. *Sustainability* 13, 6830. doi: 10.3390/su1312 6830

Haag, T., Santos, A. S., Sana, D. A., Morato, R. G., Cullenjr, L., Crawshawjr, P. G., et al. (2010). The effect of habitat fragmentation on the genetic structure of a top predator: loss of diversity and high differentiation among remnant populations of Atlantic Forest jaguars (*Panthera onca*). *Mol. Ecol.* 19, 4906–4921. doi: 10.1111/j.1365-294X.2010.04856.x

Hamrick, J. L., and Godt, M. J. W. (1996). Conservation genetics of endemic plant species. *Conserv. Genet.* 1996, 281–304. doi: 10.1007/978-1-4757-2 504-9_9

Hao, L., Zhang, L., Zhang, G. S., Wang, Y., Han, S. L., and Bai, Y. R. (2017). Genetic diversity and population genetic structure of

Salix psammophila. *Acta Bot. Boreal-Occident. Sin.* 37, 1507–1516. doi: 10.7606/j.issn.1000-4025.2017.08.1507

Holsinger, K. E., and Weir, B. S. (2009). Genetics in geographically structured populations: defining, estimating and interpreting F_{ST}. *Nat. Rev. Genet.* 10, 639–650. doi: 10.1038/nrg2611

Hosseini, S., Huseini, H. F., Larijani, B., Mohammad, K., Najmizadeh, A., Nourijelyani, K., et al. (2014). The hypoglycemic effect of *Juglans regia* leaves aqueous extract in diabetic patients: a first human trial. *DARU, J. Pharm. Sci.* 22, 1–5. doi: 10.1186/2008-2231-22-19

Hu, Y. H., Zhao, P., Zhang, Q., Wang, Y., Gao, X. X., Zhang, T., et al. (2015). De novo assembly and characterization of transcriptome using Illumina sequencing and development of twenty five microsatellite markers for an endemic tree *Juglans hopeiensis* Hu in China. *Biochem. Syst. Ecol.* 63, 201–211. doi: 10.1016/j.bse.2015.10.011

Hu, Y. P., Wang, L., Xie, X. L., Yang, J., Li, Y., and Zhang, H. G. (2010). Genetic diversity of wild populations of *Rheum tanguticum* endemic to China as revealed by ISSR analysis. *Biochem. Syst. Ecol.* 38, 264–274. doi: 10.1016/j.bse.2010.01.006

Hu, Z., Zhang, T., Gao, X. X., Wang, Y., Zhang, Q., Zhou, H. J., et al. (2016). De novo assembly and characterization of the leaf, bud, and fruit transcriptome from the vulnerable tree *Juglans mandshurica* for the development of 20 new microsatellite markers using Illumina sequencing. *Mol. Genet. Genom.* 291, 849–862. doi: 10.1007/s00438-015-1147-y

Hufford, K. M., Krauss, S. L., and Veneklaas, E. J. (2012). Inbreeding and outbreeding depression in *Stylidium hispidum*: implications for mixing seed sources for ecological restoration. *Ecol. Evol.* 2, 2262–2273. doi: 10.1002/ece3.302

Islam, A. K. M. M., and Widhalm, J. R. (2020). Agricultural uses of juglone: opportunities and challenges. *Agronomy* 10, 1500. doi: 10.3390/agronomy10101500

Jin, M., Diao, S. B., Zhang, C. H., Cao, S., Sun, J. F., Li, R., et al. (2015). Two new diarylheptanoids isolated from the roots of *Juglans mandshurica*. *Nat. Prod. Res.* 29, 1839–1844. doi: 10.1080/14786419.2015.1009063

Kantety, R. V., Rota, M. L., Matthews, D. E., and Sorrells, M. E. (2002). Data mining for simple sequence repeats in expressed sequence tags from barley, maize, rice, sorghum and wheat. *Plant Mol. Biol.* 48, 501–510. doi: 10.1023/A:1014875206165

Letunic, I., and Bork, P. (2021). Interactive Tree Of Life (iTOL) v5: an online tool for phylogenetic tree display and annotation. *Nucleic Acids Res.* 49, W293–W296. doi: 10.1093/nar/gkab301

Li, D. Y., Long, C., Pang, X. M., Ning, D. L., Wu, T., Dong, M. L., et al. (2020). The newly developed genomic-SSR markers uncover the genetic characteristics and relationships of olive accessions. *PeerJ.* 8, e8573. doi: 10.7717/peerj.8573

Li, J., Zhao, M., Wei, S. C., Luo, Z. H., and Wu, H. (2015). Geologic events coupled with Pleistocene climatic oscillations drove genetic variation of Omei treefrog (*Rhacophorus omeimontis*) in southern China. *BMC Evol. Biol.* 15:289. doi: 10.1186/s12862-015-0572-1

Li, M., Wang, S. X., Gao, B. J. (2013). Analysis of genetic diversity of chinese pine (*Pinus tabulaeformis*) natural secondary forest populations and correlation with theirs habitat ecological factors. *Acta Ecol. Sin.* 33, 3602–3610. doi: 10.5846/stxb201211271680

Li, X., Zhao, M. H., Xu, Y. J., Li, Y., Tigabu, M., and Zhao, X. Y. (2021). Genetic diversity and population differentiation of *Pinus koraiensis* in China. *Horticulturae* 7, 104. doi: 10.3390/horticulturae7050104

Liao, R. Y., Luo, Y. X., Yisilam, G., Lu, R. S., Wang, Y. G., and Li, P. (2019). Development and characterization of SSR markers for *Sanguinaria canadensis* based on genome skimming. *Appl. Plant Sci.* 7, e11289. doi: 10.1002/aps3.11289

Liu, J. Q., Fang, Y., and Chen, Y. X. (2020). Analysis of SSR distribution characteristics and primer development of *Cornus florida* transcriptome. *Mol. Plant Breed.* 18, 6769–6775. doi: 10.13271/j.mpb.018.006769

Liu, J. Z. (2015). *Based on the analysis of species distribution model of Juglans mandshurica genetic diversity and virtual screening of flavonoids compounds on Juglans mandshurica* (Master). Northeast Forestry University, Harbin.

Mallavadhani, U. V., Prasad, C. V., Shrivastava, S., and Naidu, V. G. M. (2014). Synthesis and anticancer activity of some novel 5, 6-fused hybrids of juglone based 1, 4-naphthoquinones. *Eur. J. Med. Chem.* 83, 84–91. doi: 10.1016/j.ejmech.2014.06.012

Marta, B., Lenka, P., Petra, B., Katerina, K., and Tomáš, U. (2012). Genetic differences between wild and captive populations of the peregrine falcon (*Falco peregrinus*) and the saker falcon (*Falco cherrug*) living in the czech republic. *J. Agric. Sci. Technol.* 2, 642–651.

Melo, A. T. D. O., Coelho, A. S. G., Pereira, M. F., Blanco, A. J. V., and Franceschinelli, E. V. (2014). High genetic diversity and strong spatial genetic structure in *Cabralea canjerana* (Vell.) Mart. (Meliaceae): implications to Brazilian Atlantic Forest tree conservation. *Nat. Conserv.* 12, 129–133. doi: 10.1016/j.ncon.2014.08.001

Millar, C. I., and Westfall, R. D. (1992). Allozyme markers in forest genetic conservation. *New For.* 6, 347–371. doi: 10.1007/BF00120652

Mohammed, H., Jaiswal, S. K., Mohammed, M., Mbah, G. C., and Dakora, F. D. (2020). Insights into nitrogen fixing traits and population structure analyses in cowpea (*Vigna unguiculata* L. Walp) accessions grown in Ghana. *Physiol. Mol. Biol. Plants* 26, 1263–1280. doi: 10.1007/s12298-020-00811-4

Nagy, S., Poczai, P., Cernák, I., Gorji, A. M., Hegedus, G., and Taller, J. (2012). PICcalc: an online program to calculate polymorphic information content for molecular genetic studies. *Biochem. Genet.* 50, 670–672. doi: 10.1007/s10528-012-9509-1

Newton, A. C., Allnutt, T. R., Gillies, A. C. M., Lowe, A. J., and Ennos, R. A. (1999). Molecular phylogeography, intraspecific variation and the conservation of tree species. *Trends Ecol. Evol.* 14, 140–145. doi: 10.1016/S0169-5347(98)01555-9

Nybom, H., and Bartish, I. V. (2000). Effects of life history traits and sampling strategies on genetic diversity estimates obtained with RAPD markers in plants. *Perspect. Plant Ecol. Evol. Syst.* 3, 93–114. doi: 10.1078/1433-8319-00006

Pang, H. Y., Li, H. L., Long, Z. Y., and Qi, Y. H. (2019). Study on seed traits and seedling growth characteristics of *Juglans mandshurica* Maxim. from different provenances. *Seed* 38, 89–92. doi: 10.16590/j.cnki.1001-4705.2019.09.089

Park, G., Jang, D. S., and Oh, M. S. (2012). *Juglans mandshurica* leaf extract protects skin fibroblasts from damage by regulating the oxidative defense system. *Biochem. Biophys. Res. Commun.* 421, 343–348. doi: 10.1016/j.bbrc.2012.04.013

Patil, P. G., Singh, N. V., Parashuram, S., Bohra, A., Mundewadikar, D. M., Sangnure, V. R., et al. (2020). Genome wide identification, characterization and validation of novel miRNA-based SSR markers in pomegranate (*Punica granatum* L.). *Physiol. Mol. Biol. Plants.* 26, 683–696. doi: 10.1007/s12298-020-00790-6

Poudel, M. R., Ghimire, S. K., Pandey, M. P., Dhakal, K. H., Thapa, D. B., and Khadka, D. K. (2019). Assessing genetic diversity for drought and heat stress tolerance of Nepalese wheat genotypes by SSR markers. *EurAsian J. BioSci.* 13, 941.

Preethi, P., Rahman, S., Naganeeswaran, S., Sabana, A. A., Gangaraj, K. P., Jerard, B. A., et al. (2020). Development of EST-SSR markers for genetic diversity analysis in coconut (*Cocos nucifera* L.). *Mol. Biol. Rep.* 47, 9385–9397. doi: 10.1007/s11033-020-05981-8

Rahemi, A., Fatahi, R., Ebadi, A., Taghavi, T., Hassani, D., Gradziel, T., et al. (2012). Genetic diversity of some wild almonds and related *Prunus species* revealed by SSR and EST-SSR molecular markers. *Plant Syst. Evol.* 298, 173–192. doi: 10.1007/s00606-011-0536-x

Ravelombola, W., Shi, A. N., Weng, Y. J., Mou, B. Q., Motes, D., Clark, K., et al. (2018). Association analysis of salt tolerance in cowpea (*Vigna unguiculata* (L.) Walp) at germination and seedling stages. *Theor. Appl. Genet.* 131, 79–91. doi: 10.1007/s00122-017-2987-0

Schmidt, T. L., Jasper, M. E., Weeks, A. R., and Hoffmann, A. A. (2021). Unbiased population heterozygosity estimates from genome-wide sequence data. *Methods Ecol. Evol.* 12, 1888–1898. doi: 10.1111/2041-210X.13659

Shamlu, F., Rezaei, M., Lawson, S., Ebrahimi, A., Biabani, A., and Khan-Ahmadi, A. (2018). Genetic diversity of superior Persian walnut genotypes in Azadshahr, Iran. *Physiol. Mol. Biol. Plants* 24, 939–949. doi: 10.1007/s12298-018-0573-9

Sharma, N., Ghosh, P., Sharma, U. K., Sood, S., Sinha, A. K., and Gulati, A. (2009). Microwave-assisted efficient extraction and stability of juglone in different solvents from *Juglans regia*: quantification of six phenolic constituents by validated RP-HPLC and evaluation of antimicrobial activity. *Anal. Lett.* 42, 2592–2609. doi: 10.1080/00032710903202055

Slatkin, M. (1985). Gene flow in natural populations. *Annu. Rev. Ecol. Syst.* 16, 393–430. doi: 10.1146/annurev.es.16.110185.002141

Song, J. X., Li, J., Guo, C., Zhou, Y. C., Wang, H., and Zhang, L. J. (2017). Selection of fruit variation type of *Juglans mandshurica* germplasm resources

in the eastern Liaoning Mountain region. *Mol. Plant Breed.* 15, 3798–3802. doi: 10.13271/j.mpb.015.003798

Sun, M. L., Wang, Y. M., Song, Z. Q., and Fang, G. Z. (2007). Insecticidal activities and active components of the alcohol extract from green peel of *Juglans mandshurica*. *J. For. Res.* 18, 62–64. doi: 10.1007/s11676-007-0011-2

Tang, L. L., Zhang, M., Zhao, X. L., Kang, M. Y., Liu, H. Y., and Gao, X. M., et al. (2019). Species distribution and community assembly rules of *Juglans mandshurica* in North China. *Chin. J. Plant Ecol.* 43, 753–761. doi: 10.17521/cjpe.2018.0161

Tian, J., Wu, Y., Wang, Y., and Han, F. (2009). Development and prospects of the walnut industry in China. *VI Int. Walnut Symp.* 861, 31–38. doi: 10.17660/ActaHortic.2010.861.2

Torokeldiev, N., Ziehe, M., Gailing, O., and Finkeldey, R. (2019). Genetic diversity and structure of natural *Juglans regia* L. populations in the southern Kyrgyz Republic revealed by nuclear SSR and EST-SSR markers. *Tree Genet. Genom.* 15, 1–12. doi: 10.1007/s11295-018-1311-8

Uncu, A. Ö., Gültekin, V., Allmer, J., Allmer, J., Frary, A., and Doganlar, S. (2015). Genomic simple sequence repeat markers reveal patterns of genetic relatedness and diversity in sesame. *Plant Genome* 8, 1–12. doi: 10.3835/plantgenome2014.11.0087

Vardhini, S. R. D. (2014). Exploring the antiviral activity of juglone by computational method. *J. Recept. Signal Transduct.* 34, 456–457. doi: 10.3109/10799893.2014.917325

Varshney, R. K., Thiel, T., Stein, N., Langridge, P., and Graner, A. (2002). In silico analysis on frequency and distribution of microsatellites in ESTs of some cereal species. *Cell. Mol. Biol. Lett.* 7, 537–546.

Victory, E. R., Glaubitz, J. C., Rhodes, O. E., and Woeste, K. (2006). Genetic homogeneity in *Juglans nigra* (Juglandaceae) at nuclear microsatellites. *Am. J. Bot.* 93, 118–126. doi: 10.3732/ajb.93.1.118

Vranckx, G. U. Y., Jacquemyn, H., Muys, B., and Honnay, O. (2012). Meta-analysis of susceptibility of woody plants to loss of genetic diversity through habitat fragmentation. *Conserv. Biol.* 26, 228–237. doi: 10.1111/j.1523-1739.2011.01778.x

Wang, D. N., Mu, C. C., Gao, Z., and Feng, F. J. (2011). ISSR analysis of genetic diversity of *Juglans mandshurica* Maxim populations. *Nonwood For. Res.* 29, 22–29. doi: 10.14067/j.cnki.1003-8981.2011.02.017

Wang, W. T., Xu, B., and Zhang,., D. Y., Bai, W. N. (2016). Phylogeography of postglacial range expansion in *Juglans mandshurica* (Juglandaceae) reveals no evidence of bottleneck, loss of genetic diversity, or isolation by distance in the leading-edge populations. *Mol. Phylogenet. Evol.* 102, 255–264. doi: 10.1016/j.ympev.2016.06.005

Wang, Y. (2007). *The study on the genetic diversity of Juglans mandshurica Maxim in the northeast of China* (Master). Northeast Forestry University, Harbin.

Wright, S. (1950). The genetical structure of populations. *Annal. Eugen.* 15, 323–354. doi: 10.1038/166247a0

Wuyun, T., Amo, H., Xu, J. S., Ma, T., Uematsu, C., and Katayama, H. (2015). Population structure of and conservation strategies for wild Pyrus ussuriensis Maxim. in China. *PLoS ONE* 100: e0133686. doi: 10.1371/journal.pone.0133686

Xiao, J., Zhao, J., Liu, M. J., Liu, P., Dai, L., and Zhao, Z. H. (2015). Genome-wide characterization of simple sequence repeat (SSR) loci in Chinese jujube and jujube SSR primer transferability. *PLoS ONE* 10:e0127812. doi: 10.1371/journal.pone.0127812

Yan, F., Xi, R. M., She, R. X., Chen, P. P., Yan, Y. J., Yang, G., et al. (2021). Improved de novo chromosome-level genome assembly of the vulnerable walnut tree *Juglans mandshurica* reveals gene family evolution and possible genome basis of resistance to lesion nematode. *Mol. Ecol. Resour.* 21, 2063–2076. doi: 10.1111/1755-0998.13394

Yang, S. W., Liu, G. F., Zhang, S. Y., Wang, H. R., Peng, H. M., Yang, C. P., et al. (1990). A study on the geographic variation and preliminary selection for the best provenances of *Juglans mandshurica*. *J. Northeast For. Univ.* 18(S2), 72–76.

Yao, G. D., Cheng, Z. Y., Shang, X. Y., Gao, P. Y., Huang, X. X., and Song, S. J. (2017). Coumarins from the bark of *Juglans mandshurica* exhibited anti-hepatoma activities via inducing apoptosis. *J. Asian Nat. Prod. Res.* 19, 1134–1142. doi: 10.1080/10286020.2017.1292256

Yasodha, R., Vasudeva, R., Balakrishnan, S., Sakthi, A. R., Abel, N., Binai, N., et al. (2018). Draft genome of a high value tropical timber tree, Teak (*Tectona grandis* L. f): insights into SSR diversity, phylogeny and conservation. *DNA Res.* 25, 409–419. doi: 10.1093/dnares/dsy013

Zhang, D. M., Shen, X. H., Zhang, H. X., and Shen, J. M. (2003). Advances in gene flow and paternity analysis of forest population. *For. Res.* 16, 488–494. doi: 10.13275/j.cnki.lykxyj.2003.04.019

Zhang, H., Hu, D. C., Xie, X. M., Liu, D., Zhang, P., and Wang, Z. L. (2020). Analyses on population structure and genetic diversity of *Juglans mandshurica* in Queshan provincial nature reserve of Shandong. *J. Plant Resour. Environ.* 29, 34–42. doi: 10.3969/j.issn.1674-7895.2020.03.05

Zhang, L. J., Guo, C., Qin, B. T., Lu, X. J., Yang, Y. C., Qi, Y. H., et al. (2019). Phonological characteristics of flowering and pollen viability of *Juglans mandshurica*. *J. Northeast For. Univ.* 47, 4–8. doi: 10.13759/j.cnki.dlxb.2019.05.002

Zhang, Q. H., Yu, S. H., Pei, X. N., Wang, Q. C., Lu, A. J., Cao, Y., et al. (2021). Within-and between-population variations in seed and seedling traits of *Juglans mandshurica*. *J. For. Res.* 2021, 1–12. doi: 10.1007/s11676-021-01381-1

Zhang, S., Gu, J. T., Wang, J. M., Zuo, L. H., and Yang, M. S. (2017). Analysis on introduction trial and genetic diversity of black locust populations. *Acta Hortic. Sin.* 44, 1609–1618. 10.16420/j.issn.0513-353x.2016-0964

Zhao, P., and Woeste, K. E. (2011). DNA markers identify hybrids between butternut (*Juglans cinerea* L.) and Japanese walnut (*Juglans ailantifolia* Carr.). *Tree Genet. Genom.* 7, 511–533. doi: 10.1007/s11295-010-0352-4

Zhou, Y. Y., Yang, B. Y., Jiang, Y. Q., Liu, Z. X., Liu, Y. X., Wang, X. L., et al. (2015). Studies on cytotoxic activity against HepG-2 cells of naphthoquinones from green walnut husks of *Juglans mandshurica* Maxim. *Molecules* 20, 15572–15588. doi: 10.3390/molecules200915572

Zhu, H. B., Zhao, Y., Lin, S. J., Yang, Y. C., Zhao, S. S., Sun, L. G., et al. (2011). The progress of *Juglans mandshurica* Maxim. *Chin. Agric. Sci. Bull.* 27, 1–4.

Conflict of Interest: The authors declare that the research was conducted in the absence of any commercial or financial relationships that could be construed as a potential conflict of interest.

Publisher's Note: All claims expressed in this article are solely those of the authors and do not necessarily represent those of their affiliated organizations, or those of the publisher, the editors and the reviewers. Any product that may be evaluated in this article, or claim that may be made by its manufacturer, is not guaranteed or endorsed by the publisher.

Copyright © 2022 Zhang, Zhang, Yang, Xu, Feng, Wang, Tang, Pei and Zhao. This is an open-access article distributed under the terms of the Creative Commons Attribution License (CC BY). The use, distribution or reproduction in other forums is permitted, provided the original author(s) and the copyright owner(s) are credited and that the original publication in this journal is cited, in accordance with accepted academic practice. No use, distribution or reproduction is permitted which does not comply with these terms.
