# Supplementary material for: Provenance and family variations in early growth of Manchurian walnut (Juglans mandshurica Maxim.) and selection of superior families
Source: PLoS One. 2024 Mar 7;19(3):e0298918. doi: 10.1371/journal.pone.0298918 (PMC10919699; doi:10.1371/journal.pone.0298918)
Supplement: S1 File — (ZIP) [file pone.0298918.s004.zip › Genetic stability analysis of introduced Betula pendula, Betula kirghisorum, and Betula pubescens families in saline-alkali soil of northeastern China.pdf]

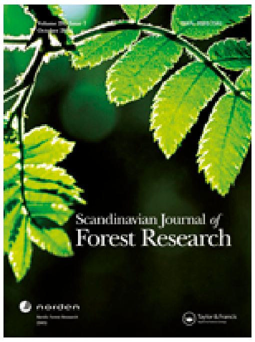

Taylor & Francis
Taylor & Francis Group

Scandinavian Journal of Forest Research

ISSN: 0282-7581 (Print) 1651-1891 (Online) Journal homepage: http://www.tandfonline.com/loi/sfor20

Genetic stability analysis of introduced Betula pendula, Betula kirghisorum, and Betula pubescens families in saline-alkali soil of northeastern China

Xi-yang Zhao, Xiu-yan Bian, Zhi-xin Li, Xiu-wei Wang, Cheng-jun Yang, Gui-feng Liu, Jing Jiang, Y. Kentbayev, B. Kentbayeva & Chuan-ping Yang

To cite this article: Xi-yang Zhao, Xiu-yan Bian, Zhi-xin Li, Xiu-wei Wang, Cheng-jun Yang, Gui-feng Liu, Jing Jiang, Y. Kentbayev, B. Kentbayeva & Chuan-ping Yang (2014) Genetic stability analysis of introduced Betula pendula, Betula kirghisorum, and Betula pubescens families in saline-alkali soil of northeastern China, Scandinavian Journal of Forest Research, 29:7, 639-649, DOI: 10.1080/02827581.2014.960892

To link to this article: http://dx.doi.org/10.1080/02827581.2014.960892

Accepted author version posted online: 03 Sep 2014.
Published online: 03 Oct 2014.

Submit your article to this journal

Article views: 71

View related articles

View Crossmark data

Full Terms & Conditions of access and use can be found at
http://www.tandfonline.com/action/journalInformation?journalCode=sfor20

Download by: [Northeastern Forestry University] **Date:** 10 December 2015, At: 16:24

Scandinavian Journal of Forest Research, 2014
Vol. 29, No. 7, 639–649, http://dx.doi.org/10.1080/02827581.2014.960892

Taylor & Francis
Taylor & Francis Group

Downloaded by [Northeastern Forestry University] at 16:24 10 December 2015

RESEARCH ARTICLE

Genetic stability analysis of introduced *Betula pendula, Betula kirghisorum*, and *Betula pubescens* families in saline-alkali soil of northeastern China

Xi-yang Zhao[a], Xiu-yan Bian[a], Zhi-xin Li[a], Xiu-wei Wang[a], Cheng-jun Yang[a], Gui-feng Liu[a], Jing Jiang[a], Y. Kentbayev[b], B. Kentbayeva[b] and Chuan-ping Yang[a*]

[a]*State Key Laboratory of Tree Genetics and Breeding, Northeast Forestry University, Harbin 150040, China;* [b]*Department of Forest Resources and Game Management, Kazakh National Agrarian University, Almaty 050010, Kazakhstan*

(*Received 26 July 2013; accepted 29 August 2014*)

Approximately, 20% of arable land worldwide, as well as nearly half of irrigated land, is subjected to salt stress. Osmotic stress and ion toxicity due to saline soils cause low crop yields. In this study, we introduced 18 families of salt-tolerant birch (*Betula pendula* Roth., *Betula kirghisorum* Sav.-Ryczg., and *Betula pubescens* Ehrh) into five high-salinity locations in northeastern China and evaluated their survival abilities. We also analyzed variation and stability of genotype–environment interactions of the different families under an additive main effect and multiplicative interaction model. Survival rate analysis indicated that the introduced families were well adapted to the high-salinity environments, whereas native families died. Variation analysis revealed significant differences between location × family interaction mean values for height and basic stem diameter (BSD), suggesting that most genotypes responded differently to different sites. The heritability of tree height and BSD at different sites varied from 0.416 to 0.940, with the coefficient of phenotypic variation ranging from 9.88% to 35.53%. Stability analysis indicated that some families had high tree heights but were sensitive to environmental conditions, whereas others were resistant but had average tree heights. These results suggest that families should be bred in various habitats to assess growth under favorable and unfavorable environments.

Keywords: *Betula pendula*; half-sib families; salt-tolerant; stability; introduction; variation

Introduction

Soil salinity, a major abiotic stress reducing plant productivity, affects large terrestrial areas of the world (Toshio & Eduardo 2005). Currently, approximately 20% of arable land, and nearly half of irrigated land worldwide, is subjected to salt stress. In China, the total area of saline-alkali soil is approximately 8.11×10^7 ha, which comprises 8–9% of the total land area. China also contains nearly 700 million hectares of soil suffering from secondary salinization, which represents approximately 10% of arable land (Xu 2004). Osmotic stress and ion toxicity due to saline soils lead to low crop yields and negatively affect growth of agricultural and forestry industries (Alarcon et al. 1993). Soil salinization is thus considered to be an ecological disaster. Transformation of saline soil and improvement of saline soil productivity are needed to increase economic development. The productivity of saline areas can be increased in two ways. First, high-salinity soil can be improved using rational irrigation and drainage methods, by washing soils with fresh water and by chemically modifying saline-alkali soil to improve growing conditions. Second, biological methods can be used to improve plant growth in saline-alkali soil, for example, by breeding plants that are tolerant to high-salt conditions.

Many drawbacks are associated with the improvement of saline-alkali soil. For example, the application of fresh water causes a decline in soil fertility. In addition, in some regions, irrigation with highly saline water due to a lack of fresh water can cause secondary salinization. Indeed, improvement of high-salinity soil requires a great deal of manpower, materials, and financial resources and is also quite difficult to perform over extensive areas (Liu et al. 2007). The second approach, the improvement of plant growth in high-salinity soil, is therefore more desirable. Using plant varieties well adapted to saline areas is the most effective, economical way to utilize high-salinity soils, but only shrubs and a few tree species are adapted to saline areas in China.

Betula platyphylla Suk is the most extensively distributed broadleaf tree in northern and southwestern forest areas of China. The largest birch stand volume is found in the northeastern Chinese forest region, where nearly one-third of the Daxing′an Mountains are dominated by birch (Zeng et al. 2003). Because birch exhibits rapid growth, strong adaptability, wide distribution (Li et al. 1995), and excellent wood quality, this tree is one of the most

*Corresponding author. Email: yangcp@nefu.edu.cn
Xi-yang Zhao and Xiu-yan Bian contributed equally to this work.

© 2014 Taylor & Francis

Downloaded by [Northeastern Forestry University] at 16:24 10 December 2015

important commercial tree species for paper, furniture, and plywood production in China. In addition, birch is a pioneer species of secondary broad-leaved forests of northern China (Zhang et al. 2012). Many investigations have been performed with birch, including intensive breeding (Yang et al. 2004) and molecular marker studies (Wei et al. 2010; Jiang et al. 2011), surveys of provenance division and genetic variation (Jiang et al. 2001), and hybridization breeding experiments (Li et al. 2006).

In the central Asian country of Kazakhstan, salt-tolerant populations of birch species *Betula pendula* Roth., *Betula kirghisorum* Sav.-Ryczg., and *Betula pubescens* Ehrh, which belong to sect. *Betula*, are mainly distributed in the northwestern inland arid zone in the Kostanay State Southern Nawu Er Rent Farm Protected Area. This desert steppe region has a mean annual precipitation of 233 mm. The area is dotted with various salt lakes and exhibits heavy soil salinization. *B. kirghisorum*, the most representative salt-tolerant species, is closely related to *B. pendula*, and is considered to have evolved from populations of that species growing in saline soil habitats. The other two types of salt-tolerant birch, *B. pendula* and *B. pubescens*, are also distributed in eastern Kazakhstan and in northern and western Siberian steppe regions (Sha & Han 1958).

In this experiment, we focused on growth traits of 18 introduced families of *B. pendula*, *B. kirghisorum*, and *B. pubescens*. Our study objectives were to: (1) compare the survival rates of each family and species; (2) examine the effects of genotype × environment (G × E) interactions on plant growth; and (3) compare the growth traits of different birch families and determine which families exhibit the best growth parameters under high-salinity conditions.

Materials and methods

Site description and materials

The materials used in this study included two native Chinese families of *B. pubescens* obtained in 2009 from the Northeast Forestry University seed orchard and 18 introduced birch half-sib families from Uhl's Grandmother Protection Zone in Kazakhstan, i.e. 10 *B. pendula* (families 1, 2, 3, 12, 13, 14, 15, 16, 19, and 22), 4 *B. kirghisorum* (4, 5, 17, and 20), and 4 *B. pubescens* (7, 8, 9, and 18) families. Experimental plantations were established in April 2011 at five sites in China: (A) Cai You Jiu Chang, Daqing, Heilongjinag Province, (B) Mao′er Mountains, Shangzhi, Heilongjiang Province, (C) Sha Er Qin, Inner Mongolia, (D) Xin Hua Dian Chang, Daqing, Heilongjinag Province, and (E) Black Mountains, Liaoning Province. Characteristics of the five sites are shown in Table 1. The experimental design consisted of five blocks per site. One-year-old seedlings of half-sib families were planted using a randomized complete block design (Marron & Ceulemans 2006) in row plots containing 20 trees at a spacing of 3 m × 4 m.

Statistical analysis

Tree height (H) and basic stem diameter (BSD) of all living, unbroken plants in the five sites were measured after leaf fall in 2012. Survival rates of the different families were calculated from the number of surviving vs. planted trees. Statistical analysis was carried out using SPSS and DPS software. The significance of fixed effects was tested by analysis of variance (ANOVA) F-tests. The linear model (Equation 1) used for joint analysis of the five sites (Dhillon et al. 2012) was as follows:

$$y_{ijkl} = \mu + S_i + B_{j(i)} + C_k + CS_{ik} + BC_{j(i)k} + \varepsilon_{ijkl}, \quad (1)$$

where y_{ijkl} is the performance of individual l of family k growing in block j of site i, μ is the overall mean, S_i is the effect of site i ($i = 1, \ldots, 5$), $B_{j(i)}$ is the effect of block j within site i ($j = 1, \ldots, 5$), C_k is the effect of family k ($k = 1, \ldots, 18$), CS_{ik} is the interactive effect of family k and site i, $BC_{j(i)k}$ is the interactive effect of clone k and block j (within site i) and ε_{ijkl} is the random error.

Variation among families was analyzed by ANOVA within sites according to Hansen and Roulund (1997) using the following equation:

$$y_{ij} = \mu + \alpha_i + \beta_j + \alpha\beta_{i(j)} + \varepsilon_{ij}, \quad (2)$$

where y_{ij} is the performance of an individual of family i within block j, μ is the overall mean, α_i is the family

Table 1. Main geographical and environmental characteristics of different sites.

Site	Longitude °E	Latitude °N	Altitude	Annual average temperature (°C)	Annual rainfall (mm)	pH value	Conductivity (μs/cm)	Soil type
A	125°04′	46°33′	148	4.2	550	8.22	0.20	Sandy soil
B	127°31′	45°16′	291	2.4	650	8.29	0.20	Sylvogenic soil
C	111°44′	40°33′	1064	6.3	535	8.10	0.16	Sandy soil
D	124°47′	46°14′	135	4.2	550	8.33	0.17	Sandy soil
E	122°07′	41°41′	38	8.0	586	7.22	0.05	Sand clay

Note: Sites A, B, C, D, and E, which represent Cai You Jiu Chang in Daqing, Heilongjinag province, Mao er mountains in Shangzhi, Heilongjiang province, Sha Er Qin in Inner Mongolia, Xin Hua Dian Chang in Daqing, Heilongjinag province, and Black Mountains in Liaoning Province, respectively. The same below.

Downloaded by [Northeastern Forestry University] at 16:24 10 December 2015

effect (i = 1, …, 18), β_j is the block effect (i = 1, …, 5), $\alpha\beta_{i(j)}$ is the random effect of family i within block j, and ε_{ij} is the random error.

The coefficient of phenotypic variation (PCV) was calculated using the formula (Hai et al. 2008):

$$\mathrm{PCV} = \frac{\mathrm{SD}}{\overline{X}}, \quad (3)$$

where $\overline{X}$ and SD are, respectively, the phenotypic mean and standard deviation of the trait (H or BSD).

Heritability (H^2) was calculated following Hansen and Roulund (1997) as:

$$H^2 = \frac{\sigma_A^2}{\sigma_A^2 + \sigma_b^2 + \sigma_e^2}, \quad (4)$$

where σ_A^2 is the additive genetic variance component between families, σ_b^2 is the block variance, and σ_e^2 is the error variance component.

The phenotypic correlation $r_A(xy)$ of traits x and y in the same sites was calculated according to Bi et al. (2000) as:

$$r_A(xy) = \frac{\mathrm{COV}_{p(x,y)}}{\sigma_p(x)\sigma_p(y)}, \quad (5)$$

where $\mathrm{COV}_{p(x,y)}$ is the covariance between families, $\sigma_p(x)$ is the variance component for trait x, and $\sigma_p(y)$ is the variance component for trait y.

B-type correlations of traits between environments x and y, $r_g(xy)$ was calculated (Burdon 1977) as follows:

$$r_g(xy) = \frac{\mathrm{COV}_{g(x,y)}}{\sigma_{gx}^2 \bullet \sigma_{gy}^2}, \quad (6)$$

where $\mathrm{COV}_{g(x,y)}$ is covariance for groups between the trait as it is expressed in environments x and y, respectively, and σ_{gx}^2 and σ_{gy}^2 are the variances between groups in environments x and y, respectively.

An additive main effects and multiplicative interaction (AMMI) model of tree height (H) was calculated following Zobel et al. (1988) as:

$$Y_{jtr} = \mu + \alpha_i + \beta_j + \sum_{k=1}^{n} \lambda_k \psi_{ik} \delta_{jk} + \varepsilon_{ijr}, \quad (7)$$

where μ is the grand mean, α_i is the provenance mean deviation (or provenance effect) with i = 1, … 5, β_j is the site effect with j = 1, … 5, λ_k is the singular value for axis k with k = 1, …, n, ψ_{ik} is the provenance eigenvector value for axis k, δ_{jk} is the site eigenvector for axis k, ρ_{ij} is the residual, ε_{ijr} is the error with r = 1, …, 5 replications, ε_{ijr} = Y_{ijr} minus the mean μ_{ij}, and ε_{ijr} is distributed normally with mean zero and standard deviation σ.

Results

Survival rates of different families

Survival rates of different families are shown in Table 2. Average survival rates at site E (Black Mountains) were obviously higher than at the other sites, while those at site D were much lower. Among species, survival rates of *B. kirghisorum* were higher than those of *B. pendula* and *B. pubescens* families. The average survival rate of the introduced families was 0.60, whereas no native birch trees survived.

Variation among all sources of variation

Results of ANOVA for H and BSD across the five sites are presented in Table 3. All effects, including family × site interactions, were highly significant ($P < 0.01$) in the overall F-tests.

Variation parameter analysis of H *and BSD at the five different sites*

ANOVA results from different families at the same sites are shown in Table 4. Significant differences ($P < 0.01$) were revealed among families. Heritability (H^2) of the different traits varied from 0.416 to 0.940, with the highest and lowest hereditabilities of BSD observed at sites C and D, respectively. The parameter H^2 in site C was the highest. However, H^2 of BSD in site D was lower, which was 0.416. The PCV of H and BSD ranged from 9.88% to 35.53%. For both traits, this parameter was lowest at site C and highest at site A.

Average H *and BSD of all trees at different sites*

Mean H and BSD of all families at each site are shown in Table 5. Average H across the five sites stabilized over the course of the experiment. Average H of all plants was highest at site E, with a mean value of 1.81 ± 0.36 m and a variation range of 1.53 ± 0.35 m to 1.98 ± 0.31 m. The next highest H value was found at site C, with a mean value of 1.33 ± 0.16 m and a variation range of 1.16 ± 0.19 m to 1.49 ± 0.06 m. The lowest mean value was 0.99 ± 0.45 m at site D, with values ranging from 0.74 ± 0.48 m to 1.18 ± 0.33 m.

Average BSD of the 18 families was also highest at site E (Table 5), with a mean BSD of 2.68 ± 0.53 cm and a variation range of 2.27 ± 0.49 m to 2.96 ± 0.52 m. The next highest BSD was observed at site A, with an average of 1.64 ± 0.81 cm and a range of 1.04 ± 0.83 to 2.12 ± 0.95 cm. The lowest average BSD, 1.22 ± 0.18 cm, was observed at site C, where it ranged from 1.02 ± 0.21 to 1.44 ± 0.07 cm.

Table 2. Survival rates of different half-sib families of birch at different sites.

Species	Families	Sites A	B	C	D	E	Average of different families
B. pendula	1	0.48	0.59	0.41	0.05	0.90	0.49
	2	0.88	0.70	0.45	0.08	0.90	0.60
	3	0.58	0.63	0.63	0.17	0.90	0.58
	12	0.88	0.65	0.43	0.22	0.83	0.60
	13	0.80	0.70	0.48	0.37	0.81	0.63
	14	0.63	0.62	0.54	0.22	0.80	0.56
	15	0.50	0.77	0.60	0.22	0.73	0.56
	16	0.85	0.55	0.46	0.37	0.83	0.61
	19	0.80	0.58	0.60	0.10	0.78	0.57
	22	0.90	0.67	0.38	0.35	0.83	0.62
Average of 10 *B. pendula* families		0.73	0.65	0.50	0.21	0.83	0.58
B. kirghisorum	4	0.85	0.78	0.50	0.37	0.96	0.69
	5	0.90	0.51	0.49	0.47	0.88	0.65
	17	0.93	0.76	0.55	0.4	0.83	0.69
	20	0.83	0.70	0.49	0.25	0.74	0.60
Average of 4 *B. kirghisorum* families		0.88	0.69	0.51	0.37	0.85	0.66
B. pubescens	7	0.60	0.53	0.50	0.13	0.89	0.53
	8	0.75	0.74	0.49	0.17	0.86	0.60
	9	0.70	0.67	0.58	0.37	0.89	0.64
	18	0.85	0.68	0.45	0.13	0.83	0.59
Average of 4 *B. pubescens* families		0.73	0.66	0.50	0.20	0.87	0.59
Average of all the families		0.76	0.66	0.50	0.25	0.84	0.60

Note: The unit of survival rates was %.

Correlation analysis of H *and BSD between different sites*

Correlations of *H* and BSD at the five sites are shown in Table 6. Correlation coefficients of *H* and BSD at the same site were significant at the $P < 0.01$ level. Within-site correlation coefficients of *H* and BSD ranged from 0.790 to 0.947. Site C showed the highest correlation coefficient, while site B had the lowest value.

The B-type correlation between *H* at different sites ranged from 0.376 to 0.694; the correlation coefficient between sites A and D was the highest, and that between sites C and E was the lowest. In addition, correlation coefficients of *H* and BSD between site C and the other sites were the lowest. Moreover, the correlation coefficient between BSD at different sites ranged from 0.313 to 0.727; the coefficient between sites B and E was the largest, and that between sites C and E was the smallest. The correlation coefficient of *H* and BSD at different sites varied from 0.334 to 0.767. The smallest correlation coefficient (0.334) was found between sites D and C and also between sites E and C; the largest was that between sites A and D.

Table 3. Variation analysis of tree height (*H*) and BSD of 18 half-sib families of birch (*B. kirghisorum*, *B. pendula*, and *B. pubescens*) at five different sites. $P < 0.01$ indicated that there was significant difference between the homologous variation source.

Traits	Source	SS	df	MS	*F*	*P*
H	Family	3.434	17	0.202	2.912	<0.01
	Site	419.362	4	104.840	1511.487	<0.01
	Family × site	22.457	68	0.330	4.761	<0.01
	Error	68.669	990	0.069		
	Total	513.922	1079			
BSD	Family	3.797	17	0.223	2.408	<0.01
	Site	54.817	4	13.704	147.725	<0.01
	Family × site	21.449	68	0.315	3.400	<0.01
	Error	91.841	990	0.093		
	Total	171.905	1079			

Downloaded by [Northeastern Forestry University] at 16:24 10 December 2015

Table 4. Results from ANOVA within each site: significance of family effect (MS, *F*, and Sig), heritability coefficient (H^2), PCV of tree height (*H*), and BSD for birch at five different sites.

Site	Trait	Df	MS	*F*	Sig.	H^2	PCV (%)
A	*H*	17	0.646	4.129	$P < 0.01$	0.758	35.19
	BSD	17	0.859	2.933	$P < 0.01$	0.659	35.53
B	*H*	17	0.087	3.766	$P < 0.01$	0.734	15.98
	BSD	17	0.099	2.858	$P < 0.01$	0.650	15.18
C	*H*	17	0.107	11.012	$P < 0.01$	0.909	9.88
	BSD	17	0.173	16.687	$P < 0.01$	0.940	12.44
D	*H*	17	0.155	3.801	$P < 0.01$	0.737	22.6
	BSD	17	0.157	1.712	$P < 0.01$	0.416	23.7
E	*H*	17	0.197	5.769	$P < 0.01$	0.827	11.97
	BSD	17	0.528	4.525	$P < 0.01$	0.779	14.41

Note: There was significant difference for *H* and BSD in different sites ($P < 0.01$).

Downloaded by [Northeastern Forestry University] at 16:24 10 December 2015

AMMI analysis of different birch families at the five sites

Results of the AMMI ANOVA for the 18 birch families examined under the five environmental conditions are shown in Table 7. All three components, i.e. genotype (G), environment (E), and G × E interaction, were highly significant ($P < 0.001$). The main effects of G and E accounted for 0.77% and 94.19% of the total variation, respectively, while the G × E interaction accounted for 5.04% of the total variation in *H*. The G × E interaction was further partitioned into improved principal component analysis 1 (IPCA1) and IPCA2 components. The IPCA1 component was significant and accounted for 54.08% of the total G × E interaction sum of squares (Table 7). The main effects (G and E) accounted for 94.96% and IPCA1 accounted for 2.73% of the total variation in the G × E data. The AMMI model (Figure 1) thus jointly gave a model fit of 97.69%.

Biplot of genotype–environment interaction

A biplot generated from the AMMI analysis captured 97.69% of the treatment sum of squares. Because IPCA1 scores of genotypes close to zero had little interaction across environments, the overall mean rankings were extraordinarily reliable. By contrast, the ranking of genotypes with large IPCA1 scores (either positive or negative overall rankings) was less reliable. Genotypes and locations combined with IPCA1 scores of the same sign produced positive specific interaction effects, whereas combinations of positive signs had negative specific interactions. From the AMMI model (Figures 1 and 2), we determined that E_A, E_C, and E_D had positive values for families 4, 5, 7, 8, 9, 14, 15, 17, 19, and 22. Similarly, E_B and E_E had positive interactions with families 1, 2, 3, 12, 13, 16, 18, and 20. The mean *H* at site E was obviously the highest, and its G × E interaction was better than at site A as well. Although site C still had a low G × E interaction, its mean *H* was inferior to that of site E. Site D values were close to the horizontal ordinate, with the lowest mean *H*. The mean output at site B was less than at site A but better than at site D.

AMMI analysis allowed us to determine the total G × E interaction effect of each genotype, which was then further divided into interaction effects of individual environments. A genotype showing a low G × E interaction is stable over a range of environments. A genotype showing high positive interactions in an environment obviously has the ability to exploit the natural conditions of a specific environment and is therefore the genotype most adapted to that environment. When examining values above the horizontal ordinate on the biplot (Figure 1), we observed that the IPCA1 value of family 5, 0.72, was distant from the horizontal ordinate, suggesting that it was the most unstable of all studied families. The mean *H* value of this family was 1.50, which was greater than the total mean of all families. IPCA1 values of the other families were close to the horizontal ordinate. Family 19 had the highest mean family *H* (1.57) and was closest to the horizontal ordinate, with an IPCA1 value of 0.0797. The data suggested that family 15 was the most stable, although its mean family *H* was the lowest. The IPCA1 scores of the other families were located around the horizontal ordinate. Below the horizontal ordinate, the most unstable family was family 20, with a distance from the horizontal ordinate of 0.3457 and a mean *H* of 1.47 m. The most stable family was family 12, which had an IPCA1 value of 0.12 and a mean *H* of 1.49 m. The values of all other families approached the horizontal ordinate. By observing the overall biplot, we determined that family 19 was the most stable family and family 5 the least.

Discussion

Improvement of plant growth in saline-alkali soil, a widespread type, is urgently needed. Many techniques have been used to improve plant growth in saline-alkali

Downloaded by [Northeastern Forestry University] at 16:24 10 December 2015

Table 5. Mean and multiple comparisons of tree height (*H*) and BSD of 18 families at five different sites.

	H					BSD				
Family	Site A	Site B	Site C	Site D	Site E	Site A	Site B	Site C	Site D	Site E
1	0.88 ± 0.33c	0.94 ± 0.19abcd	1.49 ± 0.06a	0.84 ± 0.48bc	1.66 ± 0.27bcd	1.57 ± 0.77abc	1.27 ± 0.28bc	1.44 ± 0.07a	1.27 ± 0.35ab	2.54 ± 0.40abc
2	1.02 ± 0.44c	1.07 ± 0.26abc	1.45 ± 0.08a	1.12 ± 0.42ab	1.89 ± 0.38ab	1.04 ± 0.83c	1.22 ± 0.25bc	1.36 ± 0.07abc	1.51 ± 0.37a	2.65 ± 0.54abc
3	1.00 ± 0.38c	0.87 ± 0.25d	1.37 ± 0.10abcd	0.88 ± 0.32bc	1.74 ± 0.31abc	1.29 ± 0.76bc	1.15 ± 0.33c	1.26 ± 0.12cdef	1.19 ± 0.38ab	2.70 ± 0.36abc
4	1.26 ± 0.41bc	0.94 ± 0.22bcd	1.37 ± 0.10abcd	1.11 ± 0.44ab	1.77 ± 0.29ab	1.69 ± 0.77abc	1.23 ± 0.25bc	1.28 ± 0.12cde	1.51 ± 0.68a	2.49 ± 0.52abc
5	1.88 ± 0.38a	0.91 ± 0.18cd	1.39 ± 0.12abcd	0.99 ± 0.44abc	1.56 ± 0.39cd	1.31 ± 0.68bc	1.33 ± 0.30abc	1.26 ± 0.19cdef	1.46 ± 0.77a	2.32 ± 0.53bc
7	1.33 ± 0.36bc	1.16 ± 0.24a	1.46 ± 0.12a	0.98 ± 0.39abc	1.78 ± 0.36ab	1.78 ± 0.77abc	1.43 ± 0.29ab	1.40 ± 0.12ab	1.30 ± 0.56ab	2.57 ± 0.57abc
8	1.26 ± 0.41bc	1.13 ± 0.27ab	1.40 ± 0.18abcd	0.74 ± 0.48c	1.83 ± 0.35ab	1.91 ± 0.73ab	1.40 ± 0.35abc	1.31 ± 0.22bcd	1.02 ± 0.68b	2.57 ± 0.45abc
9	1.32 ± 0.41bc	1.14 ± 0.24ab	1.44 ± 0.17abcd	0.87 ± 0.44bc	1.88 ± 0.31ab	1.77 ± 0.74abc	1.30 ± 0.31bc	1.36 ± 0.20abc	1.26 ± 0.70ab	2.68 ± 0.37abc
12	1.28 ± 0.50bc	1.08 ± 0.29abc	1.16 ± 0.19f	1.06 ± 0.51ab	1.98 ± 0.31a	1.70 ± 0.91abc	1.27 ± 0.32bc	1.02 ± 0.21h	1.29 ± 0.68ab	2.88 ± 0.53a
13	1.19 ± 0.47bc	1.10 ± 0.26abc	1.30 ± 0.18cde	1.05 ± 0.38ab	1.95 ± 0.39a	1.56 ± 0.89abc	1.33 ± 0.38abc	1.24 ± 0.19cdef	1.33 ± 0.66ab	2.92 ± 0.54a
14	1.42 ± 0.43bc	1.09 ± 0.28abc	1.30 ± 0.14cde	0.94 ± 0.44abc	1.88 ± 0.35ab	1.84 ± 0.81ab	1.28 ± 0.33bc	1.20 ± 0.16defg	1.32 ± 0.72ab	2.73 ± 0.47ab
15	1.19 ± 0.40bc	1.10 ± 0.25abc	1.29 ± 0.16cde	0.88 ± 0.55bc	1.75 ± 0.34abc	1.68 ± 0.72abc	1.30 ± 0.28bc	1.15 ± 0.13fg	1.24 ± 0.77ab	2.54 ± 0.48abc
16	1.16 ± 0.44bc	1.10 ± 0.23abc	1.32 ± 0.14bcde	0.97 ± 0.46abc	1.82 ± 0.32ab	1.66 ± 0.93abc	1.33 ± 0.33abc	1.14 ± 0.09fg	1.35 ± 0.63ab	2.93 ± 0.62a
17	1.23 ± 0.34bc	1.07 ± 0.21abc	1.26 ± 0.13def	1.18 ± 0.33a	1.53 ± 0.35d	1.63 ± 0.64abc	1.20 ± 0.30bc	1.14 ± 0.10fg	1.36 ± 0.73ab	2.27 ± 0.49c
18	1.11 ± 0.46bc	1.01 ± 0.25abcd	1.28 ± 0.11cde	1.01 ± 0.61ab	1.92 ± 0.28a	1.66 ± 0.90abc	1.35 ± 0.33abc	1.14 ± 0.08fg	1.32 ± 0.90ab	2.84 ± 0.45a
19	1.60 ± 0.39ab	1.00 ± 0.25abcd	1.32 ± 0.10cde	0.98 ± 0.39abc	1.85 ± 0.30ab	2.12 ± 0.95a	1.31 ± 0.32abc	1.17 ± 0.09efg	1.35 ± 0.71ab	2.96 ± 0.52a
20	1.03 ± 0.71c	1.13 ± 0.24ab	1.23 ± 0.13ef	1.02 ± 0.53ab	1.97 ± 0.40a	1.28 ± 91bc	1.55 ± 0.89a	1.09 ± 0.13gh	1.24 ± 0.70ab	2.94 ± 0.55a
22	1.43 ± 0.42bc	1.06 ± 0.26abc	1.17 ± 0.16f	1.12 ± 0.48ab	1.85 ± 0.32ab	1.96 ± 0.86ab	1.36 ± 0.224abc	1.07 ± 0.11gh	1.33 ± 0.70ab	2.75 ± 0.48ab
Average	1.26 ± 0.44	1.05 ± 0.25	1.33 ± 0.16	0.99 ± 0.45	1.81 ± 0.36	1.64 ± 0.81	1.31 ± 0.38	1.22 ± 0.18	1.31 ± 0.69	2.68 ± 0.53

Note: The letters a, b, c, d, and e represent multiple comparisons results according to least-significant difference test.

Table 6. Correlation coefficients of tree height (*H*) and BSD at different sites.

		H					BSD				
Traits		A	B	C	D	E	A	B	C	D	E
H	A	1	0.642**	0.504**	0.694**	0.553**	0.791**	0.650**	0.425**	0.767**	0.599**
	B		1	0.483**	0.667**	0.756**	0.732**	0.790**	0.443**	0.661**	0.731**
	C			1	0.399**	0.376**	0.494**	0.468**	0.947**	0.573**	0.386**
	D				1	0.636**	0.644**	0.645**	0.334**	0.875**	0.670**
	E					1	0.649**	0.689**	0.334**	0.642**	0.915**
BSD	A						1	0.669**	0.420**	0.664**	0.700**
	B							1	0.411**	0.638**	0.727**
	C								1	0.511**	0.313**
	D									1	0.677**
	E										1

Note: All the coefficients were significant positive correlation. A, B, C, D, and E replace the different sites and **correlation is significant at the 0.01 level (two-tailed).

soils, the most effective of which are biological methods. Nevertheless, plants seldom survive under high-salt conditions. In this study, we evaluated the abilities of salt-tolerant birch families from Kazakhstan to adapt to high-salinity areas in northeastern China. When assessing the suitability of a plant for a particular environment, it is important to examine not only plant survival but also the interactions between the plant's genotype and the environment. These interactions may be quite complex. G × E interaction effects can be reduced by selecting stable plant families (Yu & Pulkkien 2003). A genotype is considered to be stable if its response to the environment is small. By selecting stable families adapted to multiple high-salinity environments, we should be able to identify birch families having potential productivity in saline-alkali soils.

In this study, we first examined the survival rates of introduced vs. native birch families in high-salinity regions. We determined that introduced birch families (*B. kirghisorum*, *B. pendula*, and *B. pubescens*) had higher survival rates than birch families native to China; this outcome indicates that the introduction of these families was successful, at least in the early growth years. We also found that the trees performed differently in different locations. For example, low survival rates were observed at site D, which has a high soil pH and conductivity, whereas high survival rates were recorded at site E, which is characterized by a low soil pH and conductivity. Other environmental factors, such as photoperiod, temperature, and rainfall, were undoubtedly also acting on the trees. Our results suggest that the different birch families were strongly affected by such environmental factors.

Variation between sites and families

To examine family adaptability and stability, we examined the variance in *H* and BSD among the families at various sites. Burdon (1977) has suggested that genotypes superior in one environment may not be superior elsewhere. In this study, we carefully noted the specific characteristics of each environment, a vital detail for performing an informative experiment. Average *H* and BSD values were highest at site E, suggesting that site salinity levels were probably the most critical environmental factor for the 18 families. The average *H* of the 18 families was lowest at site D, while the average BSD was lowest at site C. This result suggests that the environment has an important influence on *H*.

To further explore the influence of environment on the variability of average *H* and BSD values among families, we employed ANOVA, which can be used to

Table 7. AMMI ANOVA of 18 birch genotypes for tree height (*H*) under five different environmental conditions.

Source	df	SS	% of G-E SS	MS	*F*	*P*	% of G × E interaction SS
G	17	3.4388	0.77	0.2023	2.9142	< 0.0001	–
E	4	418.98	94.19	104.745	1509.057	< 0.0001	–
G × E	68	22.4226	5.04	0.3297	4.7506	< 0.0001	–
IPCA1	20	12.1258	2.73	0.6063	1.9477	0.0076	54.08
IPCA2	18	5.9389	1.34	0.3299	1.06	0.3888	26.49
IPCA3	16	0	0	0	0	0.9999	0
Error	990	68.7168	–	0.0694	–	–	–

Note: The IPCA1 component was significant and accounted for 54.08% of the total G × E interaction sum of squares.

Downloaded by [Northeastern Forestry University] at 16:24 10 December 2015

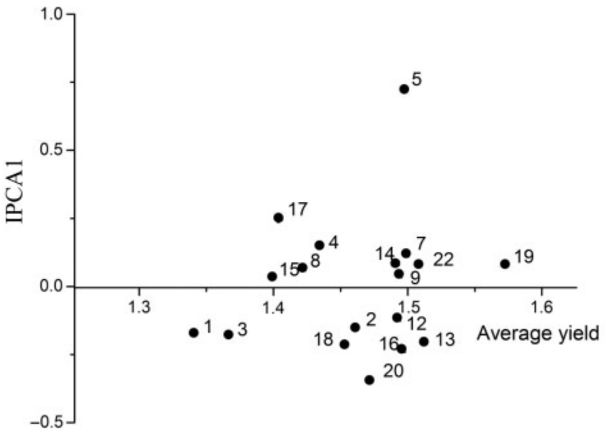

Figure 1. H biplot of genotype by environment interactions of different birch families. Abscissa indicated tree height value and the ordinate showed IPCA1 value of different families, respectively. Family 19 was higher than other families in different sites. Most of the families were stable in different sites and family 5 was low stability than other families.

determine whether G × E interactions are a crucial source of variation (Zobel et al. 1988). Variance is primarily caused by three factors – environment, genotype, and G × E interactions, which leads to different yields when genotypes are grown in several different environments. Understanding these factors is vital for breeding. When breeders develop improved plant varieties, they need to implement a plan to deal with the effects of the interaction between these factors (Annicchiarico 2002). Such information aids in parental selection from a base population and contributes to the evaluation of adaptation (Aina et al. 2007). Variance analysis of *H* and BSD site × family effects (Table 2) produced highly significant values ($P < 0.01$), indicating that different sites had different degrees of influence on different families. Likewise, results from ANOVA of *H* and BSD among families in identical sites (Table 3) illustrate that different families performed differently in this study, and that diverse genotypes performed differently within the same site.

ANOVA demonstrated that trait *H* was significantly affected by E ($P < 0.0001$), which accounted for 94.19% of the sum of squares component, thereby indicating that environmental effects clearly dominated. Genotypes with IPCA1 scores far from zero (12.1258) had a perfect interaction across environments (Crossa et al. 1991), demonstrating that these families were dramatically influenced by the environment.

PCV and H^2 *of* H *and BSD for the 18 birch families*

Understanding genetic variation within populations is critical for efficient use of genetic resources in breeding research (Safavi et al. 2010). In this study, we examined the extent of variability in a breeding population by measuring different population parameters such as PCV. The PCV of *H* and BSD at different sites ranged from 9.88% to 35.53%, reflecting a wide range of genotype performance across different environments (Frew 2003). These results are in agreement with those of Pliura et al. (2007), who found that the PCV of *H* and diameter at breast height (DBH) ranged from 10.05% to 16.40% and from 20.00% to 31.60%, respectively, for poplar hybrid clones in four trials in Quebec, Canada. The low PCV uncovered indicates that *H* and BSD variation between individuals in the same family was low, indicating a high degree of family stability. Nevertheless, low PCV among families indicates insignificant to excellent family selection, as little difference between families leads to low genetic gain. Yield stability data are consequently useful for concurrently selecting high-yielding and stable genotypes (Kang & Pham 1991).

Correlation between H *and BSD at different sites*

Although *H* and BSD are clearly related, the relationship between these parameters is rather complex (Sumida et al. 2013). Estimates of correlations are necessary to assess whether selection based on tree height would significantly affect BSD and whether such a correlation will be observed at different sites. Owing to phenotypic plasticity, variation always exists between plants of the same family grown at different sites. It is therefore important for breeders to consider correlations of the same trait between different sites (Burdon 1977; Johnson 1997), and also of different traits in the same site. In this study, we observed significant variation in *H*, BSD, and *H* and BSD in the same family at different sites and within the same site. Selecting for one trait can therefore affect the selection of another trait at the same site or other sites. Within the same site, the highest correlation coefficient was 0.947 (site C), which indicates that selecting trees with excellent *H* will probably lead to the selection of trees with excellent BSD.

AMMI analysis

Different experimental sites represent different environments that vary with respect to such factors as longitude, latitude, altitude, annual average temperature, annual average rainfall, and soil composition (Ortiz et al. 2001; Yu & Pulkkien 2003). In our study, each birch family experienced a wide range of environmental conditions among different sites. AMMI model analysis, which combines ANOVA and principal components analysis, is a powerful tool for describing relationships among sites and among genotypes. Using this technique, yield can be more accurately estimated than with traditional methods. The AMMI model provides adjusted yield estimates, allowing the performance of each genotype to be rated under each environmental condition. The model also provides a comprehensive analysis of complex interactions, which improves the accuracy of breeding and

Downloaded by [Northeastern Forestry University] at 16:24 10 December 2015

Downloaded by [Northeastern Forestry University] at 16:24 10 December 2015

increases experimental efficiency (Gauch & Zobel 1996). AMMI model analysis generates a graphic representation (or biplot) of the major effects of the interactions (PCA1) of both genotypes and environments concurrently (Kempton 1984). The biplot graph, which clearly displays complex yield patterns (Gauch 1992; Krualee et al. 2012), can capture 90% of treatment variation (Krualee et al. 2012). Although AMMI model analysis results are based simply on yield statistics (not environmental data), Ebdon and Gauch (2002) have reported that AMMI environmental statistics correlate with environmental factors. An ideal plant variety exhibits both high yields and stability of performance (Eberhart & Russell 1966) over a wide range of environments (Allard & Bradshaw 1964). In an AMMI biplot, genotypes that are distributed near the origin have minimal interactions with the environment, while genotypes located away from the origin are more sensitive to environmental interactive forces (Misra et al. 2009). The data distribution of our AMMI biplot (Figure 2) suggests that site E was the most favorable site. Site E is located in Liaoning Province, with a higher annual average temperature, a higher annual average rainfall, and more fertile soil than the other sites. These factors may explain why site E was the best site in this experiment. Furthermore, the climate in site E is quite similar to the native climate of the introduced species. This observation is consistent with the suggestion of Rehfeldt and Gallo (2001) that introduction of tree species into foreign environments is most successful when the foreign climate is similar to the native one.

Site C lies in Inner Mongolia, which is located at a high altitude with low annual rainfall and sandy soils. Tree heights were therefore low at site C. Sites A and D both lie in DaQing, but their outputs were quite different because saline soils (and nutrients) were distributed differently

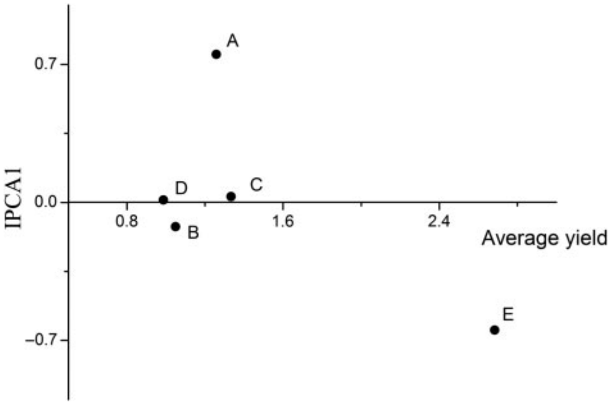

Figure 2. *H* biplot of genotype by environment interactions of different birch families growth in different sites. Abscissa indicated tree height value and the ordinate showed IPCA1 value of different sites, respectively. The average tree height in site E is higher than the other sites, but sites D, B, and C were much more stable.

between these two sites. In addition, sites A and D had lower annual average temperatures than all other sites except site B. Site B is located in the Mao′er Mountains, which has a characteristic mountain climate with low annual average temperatures and a wide range of annual rainfall totals; the altitude is the highest in Heilongjiang Province. AMMI biplots are often used to identify appropriate cultivars for various environmental conditions. Yan (2001) defined an ideal genotype on the basis of both mean yield and stability and determined that genotypes can be ranked based on their biplot distance from the ideal genotype. In the current study, we determined that family 19 exhibited the best performance, indicating that this introduced variety of *B. pendula* exhibited excellent characteristics regardless of environment. Nevertheless, plants in this family grew best at site E, which was similar to their native habitat. The growth of family-5 plants was strongly influenced by the environment, displaying ideal growth under appropriate conditions but poor growth in other habitats. The height of trees in the other families did not vary among sites, indicating that the ability of these plants to adapt to different environments was weak. Indeed, plants exhibiting wide adaptation perform well in nearly all environments (Annicchiarico 2002). Identifying introduced varieties with wide adaptation and high yields provides valuable information for future plant introduction.

Variety introduction

The introduction of plant varieties has historically been followed by selection for those that are able to survive and produce high yields under local conditions. In China, many species, including poplar and pecan, have been successfully introduced in the past several years. Because growth characteristics are inherited in a complex manner and are greatly influenced by various environmental conditions, the successful introduction of a species requires planting the species in different environments to test its growth characteristics and genetic stability (Seyed 2011). Different species perform differently in different environments (Fang et al. 1999). Examining G × E interactions enables interpretation of the main effects of genotype and environment as well as prediction of the performance of a genotype in changing environments (Marron et al. 2010). In the current study, different families performed differently at different sites. To gain further insights into the potential of these introduced birch families to become established in high-salinity environments, the growth of these trees under saline conditions should be reexamined in several years.

Conclusions

The high PCV and H^2 values observed in this study indicate that the evaluation and selection of introduced

Downloaded by [Northeastern Forestry University] at 16:24 10 December 2015

birch families were a fruitful approach. Of the introduced families, family 19 exhibited the best performance, while site E provided the most favorable environment among the five experimental habitats. Although survival rates were high for all introduced birch families, our results are preliminary pending the flowering and fruiting of these families. Future efforts will include the annual monitoring of survival rate, investigation of growth traits, and calculation of variation and stability parameters. After trees begin flowering, cross breeding should be applied to continually improve this excellent germplasm resource.

Funding

This work was supported by the Breeding Technology Research of Valuable Timber Species [grant number 2012BAD01B05]; Excellent Tree Selected and Seed Orchard Technology in Birch [grant number 2012BAD21B02].

References

Aina OO, Dixon AGO, Akinrinde EA. 2007. Additive main effects and multiplicative interaction (AMMI) analysis for yield of Cassava in Nigeria. J Biol Sci. 7:796–800.

Alarcon J, Sanchez-Blanco M, Bolarin M, Torrecillas A. 1993. Water relations and osmotic adjustment in *Lycopersicon esculentum* an *L. pennellii* during short-term salt exposure and recovery. Physiol Plant. 89:441–447.

Allard RW, Bradshaw A. 1964. Implications of genotype-environmental interactions in applied plant breeding. Crop Sci. 4:503–508.

Annicchiarico P. 2002. Genotype × environment interactions: challenges and opportunities for plant breeding and cultivar recommendations. Washington (DC): Food & Agriculture Org.

Bi CX, Guo JZ, Wang HW, Shu QY. 2000. The correlation and path analysis on the quantitative characters of Chinese pine. J Northwest For Univ. 15:7–12.

Burdon R. 1977. Genetic correlation as concept for studying genotype-environment interactions in forest breeding. Silvae Genet. 26:168–175.

Crossa J, Fox P, Pfeiffer W, Rajaram S, Gauch HG. 1991. AMMI adjustment for statistical analysis of an international wheat yield trial. Theor Appl Genet. 81:27–37.

Dhillon G, Singh A, Sidhu DS, Brar HS. 2012. Variation among poplar clones for growth and crown traits under field conditions at two sites of north-western India. J For Res. 24:61–67.

Ebdon J, Gauch H. 2002. Additive main effect and multiplicative interaction analysis of national turfgrass performance trials. Crop Sci. 42:489–496.

Eberhart S, Russell W. 1966. Stability parameters for comparing varieties. Crop Sci. 6:36–40.

Fang S, Xu X, Lu S, Tang L. 1999. Growth dynamics and biomass production in short-rotation poplar plantations: 6-year results for three clones at four spacings. Biomass Bioenergy. 17: 415–425.

Frew M. 2003. Yield stability in common bean (*Phaseolus vulgaris* L.) genotypes. Euphytica. 130:147–153.

Gauch H Jr. 1992. Statistical analysis of regional yield trials: AMMI analysis of factorial designs. Amsterdam (Netherlands): Elsevier.

Gauch HG Jr, Zobel RW. 1996. AMMI analysis of yield trials. In: Kang MS, Gauch HG, editors. Genotype–by–environment interaction. Boca Raton (FL): CRC Press.

Hai PH, Jansson G, Harwood C, Hannrup B, Thinh HH. 2008. Genetic variation in growth, stem straightness and branch thickness in clonal trials of *Acacia auriculiformis* at three contrasting sites in Vietnam. For Ecol Manage. 255: 156–167.

Hansen J, Roulund H. 1997. Genetic parameters for spiral grain, stem form, pilodyn and growth in 13 years old clones of Sitka spruce (*Picea sitchensis* (Bong.) Carr.). Silvae Genet. 46:107–113.

Jiang J, Yang CP, Liu GF, Liu YX, Ren XQ. 2001. Analysis of genetic variation within and among *Betula platyphylla* provenance and provenance division using RAPD markers. J Northeast For Univ. 29:30–34.

Jiang TB, Zhou BR, Gao FL, Guo BZ. 2011. Genetic linkage maps of white birches (*Betula platyphylla* Suk. and *B. pendula* Roth) based on RAPD and AFLP markers. Mol Breeding. 27:347–356.

Johnson GR. 1997. Site-to-site genetic correlations and their implications on breeding zone size and optimum number of progeny test sites for coastal Douglas-fir. Silvae Genet. 46:280–285.

Kang M, Pham H. 1991. Simultaneous selection for high yielding and stable crop genotypes. Agron J. 83:161–165.

Kempton RA. 1984. The use of biplots in interpreting variety by environment interactions. J Agric Sci. 103:123–135.

Krualee S, Sdoodee S, Eksomtramage T, Sereeprasert V. 2012. Stability of fresh fruit bunch of oil palm cross (*Elaeis Guineensis* Jacq.) in southern Thailand. Sabrao J Breed Genet. 44:1–8.

Li KL, Jiang J, Jiang Y, Xia DA, Yang CP, Liu GF. 2006. Analysis of the genetic effects of seed and seedling traits of *Betula platyphylla* 5×5 complete diallel cross design. J Beijing For Univ. 28:82–87.

Li P, Fang G, Sun C. 1995. Wood characteristics of pulpwood. Chem Ind For Prod. 15:13–18.

Liu ZH, Shi LR, Bai LR, Zhao KF. 2007. Effects of salt stress on the contents of chlorophyll and organic solutes in *Aeluropus littoralis* var. *sinensis* Dbeaux. J Plant Physiol Mol Biol. 33:165–172.

Marron N, Ceulemans R. 2006. Genetic variation of leaf traits related to productivity in a *Populus deltoides* × *Populus nigra* family. Can J For Res. 36:390–400.

Marron N, Ricciotti L, Bastien C, Beritognolo I, Gaudet M, Paolucci I, Fabbrini F, Salani F, Dillen SY, Ceulemans R, et al. 2010. Plasticity of growth and biomass production of an intraspecific *Populus alba* family grown at three sites across Europe during three growing seasons. Can J For Res. 40:1887–1903.

Misra RC, Das S, Patnaik MC. 2009. AMMI model analysis of stability and adaptability of late duration finger millet (*Eleusine coracana*) genotypes. World Appl Sci J. 6: 1650–1654.

Ortiz R, Wagoire WW, Hill J, Chandra S, Madsen S, Stolen O. 2001. Heritability of and correlations among genotype-by-environment stability statistics for grain yield in bread wheat. Theor Appl Genet. 103:469–474.

Pliura A, Zhang SY, Mackay J, Bousquet J. 2007. Genotypic variation in wood density and growth traits of poplar hybrids at four clonal trials. For Ecol Manage. 238:92–106.

Rehfeldt GE, Gallo LA. 2001. Introduction of ponderosa pine and Douglas-fir to Argentina. New For. 21:35–44.

Safavi SA, Pourdad SA, Mohmmad T, Mahmoud K. 2010. Assessment of genetic variation among safflower (*Carthamus tinctorius* L.) accessions using agro-morphological traits and molecular markers. J Food Agric Environ. 8:616–625.

Sha HF, Han GY. 1958. Salt resistance of plants. Beijing: Science Press; p. 15– 17.

Seyed MS. 2011. Estimation of genetic parameters related to morphological traits in poplar clones. Am J Sci Res. 27: 105–110.

Sumida A, Miyaura T, Torii H. 2013**.** Relationships of tree height and diameter at breast height revisited: analyses of stem growth using 20-year data of an even-aged *Chamaecyparis obtusa* stand. Tree Physiol. 33:106–118.

Toshio Y, Eduardo B. 2005. Developing salt-tolerant crop plants: challenges and opportunities. Trends Plant Sci. 10: 615–620.

Wei ZG, Zhang KX, Yang CC, Liu GF, Liu GJ, Zhang HG. 2010. Genetic linkage maps of *Betula platyphylla* Suk based on ISSR and AFLP markers. Plant Mol Biol Rep. 28:169–175.

Xu HG. 2004. The halophyte and salinization ecological governance. Beijing: China Agricultural Science and Technology Press.

Yan W. 2001. GGEbiplot—a windows application for graphical analysis of multienvironment trial data and other types of two-way data. Agron J. 93:1111–1118.

Yang CP, Liu GF, Wei ZG, Wu YL, Zhou YM. 2004. Study on intensive breeding technique of accelerating *Betula platyphylla* flowering and seeding early. Sci Silvae Sin. 40:14–17.

Yu Q, Pulkkien P. 2003. Genotype–environment interaction and stability in growth of aspen hybrid clones. For Ecol Manage. 173:25–35.

Zhang BK, Wang D, Yang CP, Liu GF, Liu G, Zhang H, Lian L, Wei ZG. 2012. Linkage map construction and QTL analysis for *Betula platyphylla* Suk using RAPD, AFLP, ISSR and SSR. Silvae Genet. 61:1–9.

Zeng J, Zou YP, Bai JY, Zheng HS. 2003. RAPD analysis of genetic variation in natural populations of *Betula alnoides* from Guangxi, China. Euphytica. 134:33–41.

Zobel RW, Madison JW, Gauch HG. 1988. Statistical analysis of a yield trial. Agron J. 80:388–393.

Downloaded by [Northeastern Forestry University] at 16:24 10 December 2015
