# Supplementary material for: Provenance and family variations in early growth of Manchurian walnut (Juglans mandshurica Maxim.) and selection of superior families
Source: PLoS One. 2024 Mar 7;19(3):e0298918. doi: 10.1371/journal.pone.0298918 (PMC10919699; doi:10.1371/journal.pone.0298918)
Supplement: S1 File — (ZIP) [file pone.0298918.s004.zip › Genetic variability and divergence studies in pod and seed traits of Pongamia pinnata (L.) Pierre., accessions in Bay Islands.pdf]

Journal of Forestry Research (2014) 25(2): 351−358
DOI 10.1007/s11676-013-0422-1

ORIGINAL PAPER

Genetic variability and divergence studies in pod and seed traits of *Pongamia pinnata* (L.) Pierre., accessions in Bay Islands

I. Jaisankar • M. Sankaran • D.R. Singh • V. Damodaran

Received: 2012-04-20; Accepted: 2012-07-15
© Northeast Forestry University and Springer-Verlag Berlin Heidelberg 2013

Abstract: In this study, we assessed the variability in pod, seed traits and oil content of 24 accessions of *Pongamia pinnata* collected from different parts of the Andaman and Nicobar Islands, India. The experiment was conducted at the Central Agricultural Research Institute, Port Blair during 2010 to 2011. The variability studies for pod and seed traits revealed that, the accession CPT-6 collected from New Wandoor in South Andaman recorded the maximum values for eight traits viz. pod length (75.51 mm), pod width (34.62 mm), pod thickness (17.55 mm), 100 pod weight (770.33 g), seed width (21.23 mm), 100 seed weight (377.00 g), oil percentage (43.92%) and germination percentage (94.7%). However, the maximum seed length (26.46 mm) was recorded from CPT 2 and the maximum pod-seed ratio was obtained from CPT 13 and CPT 4 (2.50 and 2.44 respectively). The phenotypic and genotypic coefficients of variations were also close to each other for all traits, but the 100 pod and seed weight exhibited higher phenotypic and genotypic coefficient of variation than the other traits. Estimates of broad sense heritability ranged from 0.30 (for seed length) to 0.95 (pod length) and genetic advance as percent of the mean ranged between 11.66% and 57.40% with seed length giving the lowest value and 100 pod weight giving the highest value. Both the phenotypic and genotypic correlation between pod length, pod width, pod thickness, 100 pod weight, seed width and 100 seed weight and between pod thickness, 100 pod weight, seed width, 100 seed weight and germination percentage were found to be strong. On the basis of non hierarchical Euclidian cluster analysis, 24 accessions were grouped into six clusters. The maximum numbers of seven accessions were included in cluster VI. The maximum intra cluster distance (10.238) shown by cluster VI and the maximum inter cluster distance (17.021) between V and III followed by III and II (15.942). Among the six clusters formed the cluster III recorded maximum oil percentage, 100 pod weight, 100 seed weight, pod length, pod thickness, pod width, seed width and germination percentage, while cluster V recorded maximum pod seed ratio and germination percentage. The present findings suggest that the crossing between accessions of cluster V and cluster III will result in a wide spectrum of variability in subsequent generations.

Keywords: *Pongamia pinnata*, correlation, heritability, genetic gain, genetic divergence, oil percentage

Fund project: This work was financially supported by the National Oil Seeds and Vegetable Oils Development Board, Gurgaon, Ministry of Agriculture, Government of India.

The online version is available at http://www.springerlink.com

I. Jaisankar (✉), M. Sankaran, D.R. Singh, V. Damodaran
Central Agricultural Research Institute, Indian Council of Agricultural Research, Port Blair, Andaman and Nicobar Islands, India. E-mail: ijaisankar@yahoo.com

Corresponding editor: Hu Yanbo

Introduction

Pongamia pinnata (Linn.) Pierre is an arboreal legume tree, commonly known as Indian-beech, poonga-oil-tree, pongam tree, karanja tree, karum and kanji. It is part of the Leguminosae family with high potential for oil production and the ability to grow on marginal land. This tree has potential for the biodiesel industry (Scott et al. 2008). This medium-sized tree is indigenous to the Indian subcontinent and South-East Asia, and has been successfully introduced to humid tropical regions of the world and to parts of Australia, New Zealand, China, and the United States. The mature tree can withstand water logging and slight frost and is highly tolerant of salinity. It is common along seashores with its roots in fresh or saltwater. *P. pinnata* is a multipurpose tree with every part of the tree having specific use. Its root, bark, leaves, sap, and flowers have medicinal properties and have an effect on wide array of organisms including insect pests, nematodes, and molluscs (Srinivasan et al. 2003; Baswa et al. 2001). Leaves are used as lactagogue fodder, especially in arid regions and also as green manure. Dried leaves are used in stored grains to repel insects. Leaves are active against *Micrococcus*; their juice is used for cold, cough, diarrhea, dyspepsia, flatulence, gonorrhea, and leprosy (Muthu et al. 2006). Flowers are used to treat diabetes; roots for cleaning gums, teeth, ulcers and bark for bleeding piles (Duke 1983). The wood is not durable and hence limited to cabinetmaking, cartwheels, posts, and fuel. The ash of the wood is used in dyeing (Allen and Allen 1981). The seedcake

is used as cattle and poultry feed and biogas production. Furthermore, the waste pulp is used as an organic fertilizer (Shrinivasa 2001). It is mainly valued for its seeds consisting of 30–40% oil rich in triglycerides. The oil is also valued in folk medicine to enhance the pigmentation of skin affected by leucoderma and used as a liniment to treat scabies, herpes, and rheumatism (Burkill 1966). Besides these advantages, pongamia seed oil has similar properties to that of diesel (Heller 1996) and has gained the importance as bio-diesel and is fast emerging as a viable alternative to fossil fuel. In meeting the future demands for bio-diesel it will be important to establish extensive commercial-scale pongamia plantations. However, the progress will be hampered by several factors viz. shortage of elite planting material, low viability of the seeds, and insufficient seed germination due to fungal contamination during their storage, seedling susceptibility to *Rhizoctonia hiemalis* leading to premature defoliation, blight and retarded growth, and the presence of a hard seed coat that reduces germination capability (Edwards and Naithani 1999). Moreover, the constraint of *Pongamia* plants established by vegetative propagation through stump cuttings are not deep rooted, and are easily uprooted (Azam et al. 2005). Today the challenging task is to screen the naturally available *P. pinnata* genetic resources to select the best planting material for higher productivity. Seeds from proven source or plus trees form the backbone of any successful tree improvement and afforestation programme. Seed parameter and germination behaviour are the most important for afforestation programme and these characters are interdependent and polygenically controlled. For a successful promotion of large-scale plantations, there is a need for carefully planned and well directed seed source research. The most successful tree improvement programme is that where proper seed sources were used. The loss from using the wrong sources can be great and even disastrous (Zobel and Talbert 1984). To exploit the potential of available resource base, variability and genetic analysis of 24 Pongamia accessions selected from various locations in the Andaman and Nicobar Islands were assessed for pod and seed traits as a scope for a further breeding program. Inter relationship among direct and indirect influence of component traits of seed/seedlings is important in predicting the correlated response to directional selection and in the detection of traits as useful markers. The knowledge of genetic variability and association between pod and seed traits can provide considerable help in genetic improvement of the species. Keeping all this in view, an effort has been made to evaluate the extent of variation and relationship of pod and seed traits behaviour of Candidate Plus trees (CPTs) collected from various parts of the Andaman and Nicobar Islands, India.

Materials and methods

An extensive wild germplasm exploration survey was conducted to select the high yielding CPTs of *Pongamia pinnata* at the fruiting stage (Table 1). The selection was made on phenotypic assessments of desirable economic characters viz., yield potential, crown diameter, total height, diameter at breast height level, and free from pests and disease. A total of 24 CPTs (morphologically superior trees) were selected from latitude ranging N 09º12′44.2"–N 13º16′52.34" and longitude E 092º32′30.6" – E 093º04′26.76". The morphological observations of the selected trees were recorded (Table 2). The total height of the standing tree was recorded with the help of Blume- Leiss Altimeter. Diameter at breast height level was recorded with the use of tree caliper and the crown diameter was recorded with the measuring tape. From each CPT, 3 kg of mature pods were collected during December 2010–July 2011. The observations for 11 quantitative characters (four pods and seven seeds) were recorded at the Central Agricultural Research Institute, Port Blair (11°36" N latitude and 92°42" E longitude; elevation 60 m above MSL) during 2010–2011.

Table 1: Details of candidate plus trees *Pongamia pinnata* accessions (CPTs) collected from the various parts of the Andaman and Nicobar Islands

S. No.	CPTs	Site data		
		Latitude	Longitude	Altitude (m)
1.	Marine hill (CPT 1)	N11º40′27.7"	E092º44′14.8"	16
2.	Marine dockyard(CPT 2)	N11º38′18.4"	E092º43′33.0"	3
3.	Jirkatang (CPT3)	N11º50′12.2"	E092º39′15.8"	5
4.	Badmaashpahad (CPT 4)	N11º36′21.6"	E092º42′08.3"	10
5.	Tirur (CPT 5)	N11º43′05.1"	E092º36′47.2"	8
6.	New Wandoor (CPT 6)	N11º35′54.5"	E092º37′21.6"	2
7.	Burmanallah (CPT 7)	N11º32′20.6"	E092º43′51.4"	16
8.	Wandoor (CPT 8)	N11º35′33.3"	E092º36′41.2"	10
9.	Chidiyatapu (CPT 9)	N11º33′43.6"	E092º43′16.09"	4
10.	Radha nagar beach (CPT 10)	N11º59′04.2"	E092º37′13.4"	3
11.	Chouldari (CPT 11)	N11º38′03.8"	E092º40′05.5"	6
12.	Neill Kendra (CPT 12)	N11º50′32.82"	E093º02′47.79"	5
13.	Shoalbay (CPT 13)	N11º 42′53"	E092º 43′00"	18
14.	Temple Myo (CPT 14)	N11º 41′30.5"	E092º 36′36.1"	18
15.	Smith Island (CPT 15)	N13º 18.09′40"	E093º 04′26.76"	13
16.	Sand pocket (CPT 16)	N10º37′24.9"	E092º32′30.6"	2
17.	Teetop-1(CPT 17)	N09º12′44.4"	E092º44′58.7"	10
18.	Teetop-2 (CPT 18)	N09º12′44.2"	E092º44′57.5"	9
19.	Diglipur Jetty (CPT 19)	N13º16′52.34"	E093º01′36.50"	27
20.	Saawai (CPT 20)	N09º12′58.1"	E092º43′38.5"	10
21.	Herbertabad (CPT 21)	N11º42′43.5"	E092º36′53.8"	17
22.	Panighat (CPT 22)	N11º 39′28.7"	E092º 44′08.2"	54
23.	Nimbudera (CPT 23)	N12º 43′12.3"	E092º 53′11.2"	16
24.	Shaitankari (CPT 24)	N11º 43′14.8"	E092º 40′05.0"	63

Pod characteristics

The pods were cleaned and stored in muslin bags at ambient conditions. All lots were dried under similar temperature and humidity conditions to reach constant weight. A total of 300

healthy pods were collected from each CPT to make three replications containing 100 pods per replication. Observation on four pod characters viz. length, width, thickness and 100-pod weight were recorded. With the use of a digital vernier caliper, the pod length was measured from the tip of the pod to the point of attachment of the pod to the stalk; pod width and pod thickness were measured and expressed in mm. The weight of the 100-pods was recorded by weighing in electrical balance; average value was calculated and expressed in grams.

Table 2: Characteristics of candidate plus trees of *Pongamia pinnata* accessions

CPTs	Candidate Plus Tree Characteristics					Pod Characteristics			
	Height (m)	DBH* (cm)	No. of branches	Crown dia. (m)	Seed yield (kg ·tree^{-1}·a^{-1})	Texture	Tip	Size	Shape
CPT 1	14.8	90	9	14.5	45	smooth	beaked	Small	bulged
CPT 2	18	162	20	15.8	104	smooth	beaked	Small	bulged
CPT 3	9.8	126.5	14	16	75	rough	mucronate	Medium	bulged
CPT 4	11.8	121.6	19	15.8	216	smooth	beaked	Medium	flat
CPT 5	17	181.5	21	17.6	129	rough	mucronate	Large	bulged
CPT 6	19.4	164.5	27	15	309	rough	beaked	Large	bulged
CPT 7	12.5	43.4	16	8.6	85	smooth	mucronate	Small	flat
CPT 8	10.6	51.9	18	9.5	211	rough	mucronate	Large	bulged
CPT 9	12.4	55.9	22	13.4	70	rough	mucronate	Medium	flat
CPT 0	12.3	61.2	21	12.7	131	rough	mucronate	Large	bulged
CPT 11	11.6	65.4	20	10.3	109	rough	mucronate	Medium	flat
CPT 12	12.5	146.5	22	12.5	104	smooth	mucronate	Large	bulged
CPT 13	18.6	95.4	21	10.8	244	rough	mucronate	Medium	bulged
CPT 14	15.4	106.5	19	10.7	152	rough	mucronate	Medium	bulged
CPT 15	17.4	99.6	16	8.9	59	smooth	beaked	Small	bulged
CPT 16	13	152	11	15.6	46	rough	beaked	Small	bulged
CPT 17	14.5	97.6	22	13.7	101	smooth	beaked	Small	bulged
CPT 18	15.6	112.5	19	12.8	52	rough	mucronate	Small	flat
CPT 19	17.3	96.5	18	10.7	98	rough	beaked	Small	flat
CPT 20	16.5	87.6	19	9.6	64	rough	mucronate	Small	flat
CPT 21	11.2	83.4	21	9.8	52	rough	beaked	Medium	flat
CPT 22	10.7	98.6	23	12.6	135	rough	mucronate	Medium	bulged
CPT 23	11.5	110.7	15	14.6	97	smooth	beaked	Medium	flat
CPT 24	12.8	106.5	19	17.5	100	rough	beaked	Large	bulged

*Diameter at breast height level

Seed characteristics

Samples of 300 seeds were collected from each CPT to make three replications containing 100 seeds per replication. Measurement of morphometric characters such as seed length and seed width were taken using a digital vernier caliper and expressed in mm. Seed length : seed width ratio was the seed length divided by seed width. The 100-seed weight was obtained by weighting 100 pure seed and was expressed in grams. The pod-seed ratio was obtained as ratio of 100-pod weight divided by 100-seed weight. Oil content of seeds was estimated by the solvent extraction method using soxhlet apparatus with three replicates for each seed lot. Petroleum ether (boiling point 60ºC–80ºC) was used as solvent. Germination percentage (GP) was computed as the portion of number of germinated seeds to that of sown seeds and expressed in percentage.

Statistical analysis

The pod and seed parameters were analysed using analysis of variance (ANOVA) and the Duncan Multiple Range Test (DMRT) to understand the significance of differences between the pods and seeds of CPTs (Panse and Sukhatme 1976). Prior to ANOVA, the percentage data set (GP) was arcsine-transformed to meet the normality assumption (Zar 1996). The phenotypic variation for each trait was partitioned into components due to genetic (hereditary) and non-genetic (environmental) factors and estimated using the following formula (Johanson et al. 1955):

$$V_p = MSG / r;$$
$$V_g = (MSG - MSE) / r;$$
$$V_e = MSE$$

where MSG, MSE and r are the mean squares of CPTs, mean squares of error and number of replications, respectively. The phenotypic variance (V_p) is the total variance among phenotypes when grown over the range of environments of interest, the genotypic variance (V_g) is the part of the phenotypic variance that can be attributed to genotypic differences among the phenotypes, and the error variance (V_e) is part of the phenotypic variance due to environmental effects.

To be able to compare the variation among traits, phenotypic coefficients of variation (PCV) and genotypic coefficients of variation (GCV) were computed according to the method suggested by Burton (1952):

$$PCV = (\sqrt{V_p} / X) \times 100$$

$$GCV = (\sqrt{V_g} / X) \times 100$$

where, V_p, V_g and X are the phenotypic variance, genotypic variance and grand mean for each pod and seed-related trait, respectively.

Broad sense heritability (h^2B) was calculated according to Allard (1999) as the ratio of the genotypic variance (V_g) to the phenotypic variance (V_p). Genetic advance (GA) expected and GA as per cent of the mean assuming selection of the superior 5% of the genotypes were estimated in accordance with Johanson et al. (1955) as:

$$GA = K \cdot h^2 B \cdot \sqrt{V_p}$$

$$GA(as\ \%\ of\ the\ mean) = (GA/X) \times 100$$

K is the selection differential (2.06 for selecting 5% of the genotypes).

Euclidean distance cluster analysis captures the kinetic styles of patterns of variation in the parameters observed and recorded. Intra- and inter-cluster distances were calculated to identify accessions that can be used as parents for desired traits in future breeding programmes. The broad genetic divergence was calculated by using non-hierarchical Euclidian cluster analysis (Sachan et al. 2004)

Results

Pod and seed traits

The mean performance of pod traits (pod length, pod width, pod thickness and 100-pod weight) and seed traits (seed length, seed width, seed length- width ratio, 100-seed weight, pod-seed ratio, total oil percentage, and germination percentage) from 24 CPTs of *Pongamia pinnata* revealed significant difference among the CPTs (Table 3). Variability studies for pod and seed traits revealed that the accession CPT-6 recorded maximum values for eight traits viz. pod length (75.51 mm), pod width (34.62 mm), pod thickness (17.55 mm), 100 pod weight (770.33 g), seed width (21.23 mm), 100 seed weight (377.00 g), oil percentage (43.92%) and germination percentage (94.7%). However maximum seed length (26.46 mm) was recorded from CPT 2 and the maximum pod seed ratio was obtained from CPT 13 and CPT 4 (2.50 and 2.44 respectively). The accession CPT 20 exhibited the lowest recorded values for five traits viz. seed length (16.45 mm), seed length width ratio (1.02), 100 seed weight (148 g) and total oil percentage (31.20 %). However, the lowest pod length (38.81 mm), pod width (21.76 mm), pod thickness (10.48 mm), 100 pod weight (275.33 g), seed length (16.83 mm), seed width (14.62 mm), pod seed ratio (1.40) and germination percentage (52.7%) was recorded by accessions CPT 18, CPT 15, CPT 7, CPT 1, CPT 12, CPT 12, CPT 1 and CPT 23, respectively.

Genetic variability and association studies

The amount of genetic variation expressed by different pod and seed traits could be judged through the study of phenotypic coefficient of variation (PCV) and genotypic coefficient of variation (GCV). The phenotypic and genotypic coefficient of variations were also close to each other for all traits, but the 100 pod weight and 100 seed weight exhibited higher PCV and GCV than the other traits (Table 4). The magnitude of the error variance was relatively lower than the genotypic variance for all traits. Estimates of broad sense heritability ranged from 0.30 (for seed length) to 0.95 (pod length), genetic advance as percent of the mean ranged between 11.66% and 57.40% with the seed length giving the lowest value and 100 pod weight giving the highest value. The magnitude of genotypic variance was higher than the error variance in the one hand, which the phenotypic and genotypic variances were close to each other on the other hand. It indicates that the phenotypic component was the major contributor to the total variance for these traits (100 pod weight, 100 seed weight and total oil percentage). This variability due to genetic variance further indicates considerable scope for selection.

In the present study the genotypic coefficient of variation and the genetic advances as percent of the mean were found to be high for the 100 pod weight. Higher GCV indicates that worthwhile improvement could be achieved for this trait through simple selection while higher genetic advance value suggests that population means for 100 pod weight may be changed considerably by selecting the superior 5% of the genotypes. Genotypic and phenotypic association of pod and seed traits was in the same direction and that the genotypic estimates were higher than the phenotypic ones (Table 5). Germination percentage exhibited positive significant correlation with pod thickness, seed width and 100 seed weight both at the genotypic and phenotypic level. However, the pod width, 100 pod weight and total oil percentage expressed positive significant correlation only at genotypic level. 100-pod weight trait showed strong correlation with 100 seed weight. In general, the genotypic correlation coefficient values were higher than corresponding phenotypic values. The genotypic correlation is an estimated value, whereas the phenotypic correlation is a derived value from the genotypic and environmental interaction. Both the phenotypic and genotypic correla-

tion between pod length, pod width, pod thickness, 100 pod weight, seed width and 100 seed weight and between pod thickness, 100 pod weight, seed width, 100 seed weight and germination percentage were strong. This offers an opportunity to select phenotypes based on these traits.

Table 3: Mean performance of selected genotypes for pod and seed traits *Pongamia pinnata* accessions

CPTs	Pod traits				Seed traits						
	Length (mm)	Width (mm)	Thickness (mm)	100 pod Weight (g)	Length (mm)	Width (mm)	Length width ratio	100 seed weight (g)	Pod seed ratio	Total oil percentage (%)	Germination percentage (%)
CPT 1	43.39^{kl}	23.53^{ijk}	14.81^{bcd}	275.33^{k}	23.39^{abcd}	15.85^{fghij}	1.48^{abcde}	196.83^{hijk}	1.40^{e}	34.48^{efgh}	85.3^{bcde}
CPT 2	47.63^{j}	23.33^{ijkl}	12.36^{ghi}	346.33^{hijk}	26.46^{a}	16.26^{defgh}	1.63^{ab}	247.50^{defgh}	1.40^{e}	34.43^{efgh}	78.0^{efgh}
CPT 3	65.28^{cde}	30.55^{cd}	14.40^{bcdef}	531.67^{cd}	20.34^{cdef}	18.00^{bcdef}	1.13^{fg}	349.33^{ab}	1.71^{de}	32.23^{hij}	76.3^{fghi}
CPT 4	62.82^{def}	24.20^{hijk}	13.71^{cdefg}	486.33^{de}	26.23^{ab}	17.40^{bcdef}	1.50^{abcd}	295.67^{bcd}	2.44^{a}	34.07^{fghij}	79.0^{defg}
CPT 5	69.47^{b}	31.80^{c}	15.23^{bcd}	591.00^{c}	25.02^{abc}	18.18^{bcde}	1.38^{bcdef}	298.17^{bc}	1.71^{de}	31.32^{ij}	74.7^{ghi}
CPT 6	75.51^{a}	34.62^{a}	17.55^{a}	770.33^{a}	26.12^{ab}	21.23^{a}	1.23^{defg}	377.00^{a}	2.07^{abcd}	43.92^{a}	94.7^{a}
CPT 7	40.77^{lm}	28.73^{de}	10.48^{i}	343.67^{hijk}	20.74^{bcdef}	17.24^{efghi}	1.20^{efg}	201.83^{ghij}	1.71^{de}	35.60^{cdefg}	68.7^{ijk}
CPT 8	69.48^{b}	32.08^{bc}	15.39^{bc}	674.67^{b}	21.84^{bcdef}	18.84^{bc}	1.15^{fg}	284.00^{cd}	2.11^{abcd}	31.40^{ij}	84.3^{cdef}
CPT 9	56.15^{gh}	24.90^{ghi}	11.24^{hi}	425.00^{efg}	23.81^{abc}	18.27^{cde}	1.30^{cdef}	216.67^{fghi}	1.98^{abcd}	31.43^{ij}	65.0^{jk}
CPT10	70.03^{b}	33.88^{ab}	16.13^{ab}	719.33^{ab}	19.47^{cdef}	17.59^{cdefg}	1.11^{fg}	253.17^{cdefg}	2.36^{ab}	40.77^{b}	89.7^{abc}
CPT 11	55.06^{h}	30.43^{cd}	13.36^{defg}	477.67^{de}	22.62^{bcde}	18.39^{bcd}	1.24^{defg}	225.83^{efghi}	2.16^{abcd}	37.50^{bcd}	64.3^{jk}
CPT 12	65.63^{cd}	29.26^{d}	14.65^{bcde}	595.67^{c}	16.83^{f}	14.62^{k}	1.15^{fg}	226.83^{efghi}	2.32^{abc}	33.53^{fghij}	60.7^{kl}
CPT 13	61.66^{ef}	30.42^{cd}	14.55^{bcdef}	498.67^{de}	23.72^{abc}	19.07^{ab}	1.24^{defg}	273.67^{cde}	2.50^{a}	38.13^{bc}	81.3^{defg}
CPT 14	49.79^{ij}	22.83^{jkl}	13.54^{cdefg}	484.33^{de}	24.88^{abc}	16.90^{fghij}	1.47^{abcde}	289.50^{cd}	1.75^{de}	37.27^{bcde}	64.0^{jk}
CPT 15	48.84^{j}	21.76^{l}	12.73^{fgh}	338.67^{hijk}	20.36^{cdef}	14.72^{jk}	1.38^{bcdef}	198.83^{hijk}	1.72^{de}	34.10^{fghi}	63.3^{jk}
CPT 16	47.94^{j}	23.76^{ijk}	14.85^{bcd}	394.67^{fgh}	24.99^{abc}	16.67^{ghijk}	1.50^{abc}	242.50^{defgh}	1.64^{de}	33.17^{ghij}	81.0^{defg}
CPT 17	46.12^{jk}	22.43^{kl}	14.85^{bcd}	385.00^{fgh}	23.92^{abc}	15.30^{hijk}	1.56^{abc}	213.33^{fghi}	1.80^{cde}	33.16^{ghij}	62.3^{jk}
CPT 18	38.81^{m}	23.87^{ijk}	12.82^{efgh}	291.67^{jk}	22.77^{abcde}	16.46^{hijk}	1.37^{bcdef}	159.00^{jk}	1.99^{abcd}	36.20^{bcdef}	78.3^{defg}
CPT 19	52.75^{hi}	24.47^{hijk}	13.52^{cdefg}	374.33^{fghi}	24.18^{abc}	14.65^{jk}	1.66^{a}	175.00^{ijk}	2.13^{abcd}	38.27^{bc}	69.7^{hij}
CPT 20	42.02^{lm}	27.01^{ef}	11.98^{ghi}	302.33^{ijk}	16.45^{f}	16.07^{ghijk}	1.02^{g}	148.00^{k}	2.13^{abcd}	31.20^{j}	86.7^{abcd}
CPT 21	56.11^{gh}	25.96^{fgh}	12.78^{efgh}	365.33^{ghij}	20.95^{cdef}	15.11^{ijk}	1.38^{bcdef}	157.33^{jk}	2.32^{abc}	37.57^{bcd}	69.7^{hij}
CPT 22	60.84^{f}	24.68^{hi}	14.60^{bcdef}	446.67^{ef}	18.05^{def}	15.49^{ghijk}	1.17^{fg}	262.00^{cdef}	1.71^{de}	37.83^{bcd}	92.3^{ab}
CPT 23	59.11^{fg}	26.57^{fg}	12.33^{ghi}	339.33^{hijk}	17.55^{ef}	15.32^{ghijk}	1.14^{fg}	181.67^{ijk}	1.87^{bcde}	31.30^{ij}	52.7^{l}
CPT 24	68.43^{bc}	31.20^{c}	14.81^{bcd}	593.33^{c}	20.02^{cdef}	15.40^{ghijk}	1.29^{cdefg}	285.83^{cd}	1.70^{de}	34.70^{efgh}	82.7^{defg}
Mean	56.6	27.18	13.86	460.47	22.11	16.79	1.32	240.01	1.94	35.07	75.19
SEd	1.85	0.91	0.95	38.65	2.81	1.13	0.14	26.77	0.28	1.44	4.25
CD	3.72	1.84	1.92	77.81	5.66	2.28	0.28	53.88	0.55	2.9	8.55

Trait means not followed by the same superscript letter and significantly different at p=0.05.

Table 4: Genetic estimates of parent tree pod and seed traits of *Pongamia pinnata* accessions

	GV	PV	EV	GCV	PCV	ECV	Heritability	GA (%) of mean
Pod length	120.23	125.34	5.11	19.37	19.78	4	95.92	39.08
Pod width	14.89	16.14	1.25	14.2	14.78	4.12	92.23	28.09
Pod thickness	2.12	3.48	1.36	10.5	13.46	8.42	60.85	16.87
100 pod weight	18463.37	20704.39	2241.01	29.51	31.25	10.28	89.18	57.4
Seed length	5.17	17.03	11.86	10.28	18.66	15.57	30.34	11.66
Seed width	2.17	4.1	1.93	8.77	12.05	8.27	52.98	13.16
Seed length width ratio	0.02	0.05	0.03	11.05	17.11	13.06	41.75	14.71
100 seed weight	3192.72	4267.27	1074.55	23.54	27.22	13.66	74.82	41.95
Pod seed ratio	0.06	0.17	0.11	12.69	21.48	17.33	34.92	15.45
Total oil percentage	8.94	12.05	3.11	8.52	9.9	5.03	74.17	15.12
Germination percentage	114.33	141.36	27.03	14.22	15.81	6.91	80.88	26.34

GV= Genotypic variance, PV= Phenotypic variance, EV= Environmental variance, GCV= Genotypic coefficient of variation, PCV= Phenotypic coefficient of variation, ECV= Environmental coefficient of variation

Table 5: Correlation matrix of pod and seed traits of *Pongamia pinnata* accessions

		Pod width	Pod thickness	100 pod weight	Seed length	Seed width	Seed length width ratio	100 seed wt	Pod- seed ratio	Total oil percentage	Germination percentage
Pod length	G	0.764**	0.758**	0.921**	-0.025	0.551**	-0.459*	0.764**	0.470*	0.252	0.303
	P	0.733**	0.568**	0.872**	-0.003	0.396*	-0.28	0.673**	0.271	0.211	0.274
Pod width	G	1	0.574**	0.820**	-0.241	0.691**	-0.764**	0.559**	0.457*	0.258	0.384*
	P	1	0.397*	0.780**	-0.118	0.539**	-0.508**	0.464*	0.29	0.215	0.322
Pod thickness	G		1	0.838**	0.285	0.478*	-0.089	0.790**	0.114	0.445*	0.600**
	P		1	0.600**	0.104	0.225	-0.024	0.448*	0.134	0.277	0.438*
100 pod	G			1	0.093	0.670**	-0.430*	0.808**	0.430*	0.321	0.396*
weight	P			1	0.031	0.492*	-0.308	0.711**	0.296	0.302	0.301
Seed length	G				1	0.383*	0.671**	0.489*	-0.213	0.331	0.159
	P				1	0.489*	0.792**	0.256	-0.096	0.16	0.057
Seed width	G					1	-0.425*	0.787**	0.326	0.347	0.446*
	P					1	-0.138	0.507**	0.137	0.21	0.354
Seed length	G						1	-0.154	-0.457*	0.083	-0.206
width ratio	P						1	-0.069	-0.203	0.059	-0.18
100 seed	G							1	0.137	0.227	0.422*
weight	P							1	-0.225	0.237	0.354
Pod seed ratio	G								1	0.436*	0.092
	P								1	0.138	-0.065
Total oil	G									1	0.405*
percentage	P									1	0.282

** Significant at 1% level; * Significant at 5% level, G= Genotypic association, P= Phenotypic association

Divergence studies

On the basis of non hierarchical Euclidian cluster analysis, 24 accessions were grouped in to six clusters (Table 6). The maximum number of seven accessions were included in cluster VI followed by cluster I (6 accessions). In contrast, clusters II and V included only two accessions. The cluster pattern exhibited that geographical diversity need not to necessarily be correlated with genetic diversity. The inter- and intra-cluster distances are presented in Table 7. The intra cluster distances ranged from 3.532 to 10.238 with maximum value in cluster VI followed by I and cluster III. Minimum intra cluster distance was found in cluster II. The highest inter cluster distance was found between clusters V and III (17.021) followed by clusters III and II (15.942). The minimum inter cluster distance was observed between clusters IV and II (7.020).

Cluster mean indicated significant variation among clusters particularly for oil content and seed weight (Table 8). The cluster III recorded maximum oil percentage, 100 pod weight, 100 seed weight, pod length, pod thickness, pod width, seed width and germination percentage, while cluster V recorded maximum pod seed ratio and germination percentage. Thus it may be suggested that crosses involving under cluster III and V may result in substantial segregates and further selection for overall improvement of species.

Table 6: Composition of Euclidean clusters for pod and seed traits in *Pongamia pinnata*

Cluster	No. of CPTs	Name of the CPTs
I	6	CPT 1, CPT 2, CPT 3, CPT 4, CPT 5, CPT 16
II	2	CPT 15, CPT 17
III	3	CPT 6, CPT 8, CPT 24
IV	4	CPT7, CPT 9, CPT 19, CPT 21
V	2	CPT 18, CPT 20
VI	7	CPT 10, CPT 11, CPT 12, CPT 13, CPT 14, CPT 22, CPT 23

Table 7: Estimates of inter and intra- cluster distances for pod and seed traits in *Pongamia pinnata* accessions

Clusters	I	II	III	IV	V	VI
I	**9.72**	9.275	12.62	9.465	10.518	9.989
II		**3.532**	15.942	7.02	8.292	10.615
III			**9.316**	14.328	17.021	11.397
IV				**7.957**	8.447	9.837
V					**4.847**	12.24
VI						**10.238**

Table 8: Cluster mean value for pod and seed traits in *Pongamia pinnata* accessions

Clusters	Pod length	Pod width	Pod thickness	100 Pod weight	Seed length	Seed width	Seed length width ratio	100 seed weight	Pod seed ratio	Oil percentage	Germination percentage
I	56.09	26.19	14.23	437.56	24.41	17.06	1.43	271.75	1.72	33.28	79.06
II	47.48	22.1	13.8	361.83	22.14	15.01	1.47	206.08	1.76	33.63	62.83
III	72.75	32.63	15.92	679.44	22.66	18.5	1.22	315.61	1.96	36.67	87.22
IV	51.45	26.02	12.01	377.08	22.42	16.32	1.39	187.75	2.04	35.72	68.25
V	40.42	25.44	12.4	297	19.62	16.27	1.19	153.5	2.06	33.7	82.5
VI	60.3	28.3	14.17	508.81	20.45	16.77	1.22	244.67	2.1	36.36	72.14

Discussion

The study of pod and seed morphological characters of the natural populations is often considered to be useful step in the study of the genetic variability. Seed weight depends on reserve food material that is produced as a result of endosperm and is dominated by the maternal traits; it is also influenced by nutrient availability at the time of seed setting and environmental factors (Allen 1960; Johnsen et al.1989). The seeds from various CPTs exhibited significant variability in pod and seed traits that could be attributed to isolations, which in turn influence gene flow. Significant variability of seed characters like seed size and weight was observed in selected plus trees (Bagchi and Sharma 1989). Similar observations with reference to the relationship and variation in pod and seed characteristics were also reported by Divakara et al. 2010; Sunil et al. 2010; Mukta et al. 2009 and Rao et al. 2011. In the case of seed oil percentage, significant variation was observed in the seeds collected from the different sources and the value ranged from 31.20–43.93%. Genetic control of seed size traits has been observed in several tree species like *Tectona grandis* (Jayasankar et al. 1999), *Strychnos cocculoides* (Mkonda et al. 2003) and *Juniperus procera* (Mamo et al. 2006).

The characters such as 100 pod weight and germination percentage exhibited significant variation among CPTs of *P. pinnata* similar variation has also been reported in *Azadirachta indica* (Jindal et al. 1999; Kaura et al. 1998), *Jatropha curcas* (Kumar et al. 2003). In most plant species, seeds vary in their degree of germinability between and within populations and between and within individuals (Gera et al. 2000; Sivakumar et al. 2002). Correlated quantitative traits are major interest in an improvement program, as the improvement of one character may cause simultaneous changes in the other characters. Here the 100 pod weight and 100 seed weight are under strong genetic control with high GCV, PCV, heritability and GA (as % of the mean). Heritability has an important place in tree breeding as it provides an index of the relative role of heredity and environment in the expression of various traits. Dorman (1976) reported that heritability estimate is important in tree improvement programme. It is also useful for ranking importance of each trait in cross breeding programmes. Gains from tree breeding programmes depend on the type and extent of genetic variability. Hence, improvement in these characters can lead to improve germination percentage and total oil percentage respectively. The results agree with the findings in *Grewia optiva* (Chauhan 1989) and *Jatropha curcas* (Kaushik et al. 2007), which also exhibited positive correlation between 100 seed weight and germination percentage.

The D^2 statistic, which is based on several characters, is one of the powerful tools to assess the relative contribution of different component traits to the total diversity. This makes it possible to quantify the degree of divergence between populations to understand the trend of evaluation and to choose genetically diverse parents for obtaining desirable recombination. The clustering pattern in this study revealed that trees from different islands and regions were grouped together in a cluster vise- versa and suggested that the geographical diversity did not necessarily represent genetic diversity. This was in line with the results obtained earlier through D^2 analysis by Kaushik[a] et al. 2007; Saini et al. 2004 and Kaushik 2005. The trees that originated in one region had been distributed in different clusters, indicating that the trees with the same geographic origin could have undergone changes for different characters under selection.

Maximum intra-cluster distance (10.238) shown by cluster VI is an indicator of selection of parents for hybridization within the cluster. The maximum inter cluster distance (17.021) between clusters V and III followed by clusters III and II (15.942), indicating wider genetic diversity between the trees in these groups and selection of parents from such clusters for hybridization programs would help to achieve novel hybrids. The minimum inter-cluster distance indicated that trees in these clusters were of close relationship. Hence, selection of parents from these two clusters is to be avoided. The clusters, having high inter-cluster distance and high mean value, would produce divergent trees and therefore should be selected. Clusters V and III have high inter cluster distance, indicating that trees belonging to these clusters should be selected as parents for breeding programme.

Our findings can help identify better genotypes of *P. pinnata* with better yield and high oil content, which will benefit energy plantations in the island coastal areas.

Reference

Allard RW. 1999. *Principles of plant breeding, 2nd edt*. New York: John Wiley & Sons, Inc, p 485.

Allen GS. 1960. Factors affecting the viability and germination behaviour of coniferous seed. IV. Stratification period and incubation temperature, *Pseudostuga menziesii* (Mirb.) Franco. *For Chron*, **36**: 18−19.

Allen ON, Allen EK. 1981. *The Leguminosae*. USA: The University of Wis-

consin Press.

Azam MM, Waris A, Nahar NM. 2005. Prospects and potential of fatty acid methyl esters of some nontraditional seed oils for use as biodiesel in India. *Biomass and Bioenergy*, **29**: 293–302.

Bagchi SK, Sharma VP. 1989. Biometrical studies on seed characters of *Santalum album* L. *Silvae Genet*, **38**: 152–153.

Baswa M, Rath CC, Dash SK, Mishra RK. 2001. Antibacterial activity of Karanja (*Pongamia pinnata*) and neem (*Azadirachta indica*) seed oil: a preliminary report. *Microbios*, **105**:183–189

Burkill JH. 1966. *A dictionary of economic products of Malay peninsula L. 2 vols*. Kuala Lumpur, Malaysia: Art Printing Works Publisher.

Burton GW. 1952. Quantitative inheritance in grass. *In*: Proceedings of the Sixth International Grassland Congress, Pennsylvania State College, USA, 17, 23 August 1952, pp. 277–283.

Chauhan V. 1989. Germination ecology of seeds of *Grewia optiva* Drummond I. Seed polymorphism and germination response. *J Tree Sci*, **8**: 42–44.

Divakara BN, Alur AS, Tripati S. 2010. Genetic variability and relationship of pod and seed traits in *Pongamia Pinnata* (L.) Pierre., a potential agroforestry tree. *International Journal of Plant Production*, **4** (2): 129–141.

Dorman KW. 1976. The genetics and biodiversity of southern Pines. *In*: *Agriculture Handbook*. Washington, D.C.: USDA, US Forest Service, p. 471,

Duke JA. 1983. Handbook of Energy Crops, unpublished, Excerpt from the Internet. Available at: <http://www.hort.purdue.edu/newcrop/duke_energy/*Pongamia_pinnata*.html>.

Edwards DGW, Naithani SC. 1999. *Seed and Nursery Technology of Forest Trees*. New Delhi: New Age International.

Gera M, Gera N, Ginwal HS. 2000. Seed trait variations in *Dalbergia sissoo* Roxb. *Seed Sci Technol*, **28**: 467–475.

Heller J. 1996. Physic nut. *Jatropha curcas* L. Promoting the conservation and use of underutilized and neglected crops. 1. Institute of Plant Genetics and Crop Plant Research, Gatersleben/ International Plant Genetic Resources Institute, Rome.

Jayasankar S, Babu LC, Sudhakar K, Unnithan VKG. 1999. Provenance variation in seed and germination characteristics of teak (*Tectona grandis* L.F.). *Seed Sci Technol*. **27**: 131–139.

Jindal SK, Satyavir, Pancholy A. 1999. Variability and associations for seed yield, oil content and tree morphological traits in neem (*Azadirachta indica*). *Journal of Tropical Forest Science*, **11**: 320–2 [special 10th year anniversary issue].

Johnsen O, Dietrichson J, Skaret G. 1989. Phenotypic changes in progenies of northern clones of *Picea abies* (L.) Karst. grown in a southern seed orchard. III. Climate changes and growth in a progeny trial. *Scand J For Res*, **4**: 343–350.

Johnson HW, Robinson HF, Comstock RE. 1955. Genotypic and phenotypic correlations on soyabean and their implications in selection. *Agronomy Journal*, **47**: 477–83.

Kaura SK, Gupta SK, Chowdhury J B. 1998. Morphological and oil content variation in seeds of *Azadirachta indica* A. Juss. (Neem) from northern and western provenances of India. *Journal of Plant Foods and Human Nutrition* (Formerly Qualitas Plantarum), **52**: 132–136.

Kaushik N. 2005. Inter- and intra-provenance variation in neem (*Azadirachta indica* A. Juss) from different agroclimatic zones of Haryana. PhD thesis, FRI Deemed University, DehraDun.

Kaushik N[a], Kumar S, Kumar K, Beniwal RS, Kaushik N, Roy S. 2007. Genetic variability and association studies in pod and seed traits of *Pongamia pinnata* (L.) Pierre in Haryana, India. *Genet Resour Crop Evol,* **54**: 1827–1832

Kaushik NK, Kumar S, Kumar, Roy S. 2007. Genetic variability and divergence studies in seed traits and oil content of Jatropha (*Jatropha curcas* L.) accessions. *Biomass and Bioenergy,* **31**: 497–502.

Kumar S, Parimallam R, Arjunan MC, Vijayachandran SN. 2003. Variation in *Jatropha curcas* seed characteristics and germination. *In*: Hegde NG, Daniel JN, Dhar S (eds), *Proceeding of the national workshop on Jatropha and other perennial oilseed species*. Pune, India, pp. 63–6.

Mamo N, Mihretu M, Fekadu M, Tigabu M, Teketay D. 2006. Variation in seed and germination characteristics among *Juniperus procera* populations in Ethiopia. *For Ecol Manage*, **225**: 320–327.

Mkonda A, Lungu S, Maghembe JA, Mafongoya PL. 2003. Fruit-and seed-germination characteristics of *Strychnos cocculoides* an indigenous fruit tree from natural populations in Zambia. *Agrofor Sys*, **58**: 25–31.

Mukta N, Murthy IYLN, Sripal P. 2009. Variability assessment in *Pongamia pinnata* (L.) Pierre germplasm for biodiesel traits. *J Industrial Crops and Products*, **29** (2-3): 536–540.

Muthu C, Ayyanar M, Raja N, Ignacimuthu S. 2006. Medicinal plants used by traditional healers in Kancheepuram District of Tamil Nadu, India. *J. Ethnobiol Ethnomed*, **2**: 43.

Panse VG, Sukhatme PV. 1976. *Statistical methods for agricultural workers*. New Delhi: ICAR, p.361.

Rao GR, Arun K Shanker, Srinivas I, Korwar GR, Venkateswarlu B. 2011. Diversity and variability in seed characters and growth of *Pongamia pinnata* (L.) Pierre accessions. *Trees.* **25**:725–734

Sachan JN, Sial P, Singh B, Pattnaik RK (2004) Analysis of genetic divergence for quality improvement in toria (*Brassica rapa* L.spp. toria). *Environ Ecol,* **22**: 283–286

Saini ML, Jain P, Singh JV. 2004. Genetic diversity in a germplasm collection of guar [*Cyamopsis tetragonaloba* (L.) Taub.]. *Forage Research*, **30**: 92–5.

Scott PT, Pregelj L, Chen N, Hadler JS, Djordjevic MA, Gresshoff PM. 2008. *Pongamia pinnata*: Anuntapped resource for the biofuels industry of the future. *Bioenerg Res*, **1**: 2–11.

Shrinivasa U. 2001. A viable substitute for diesel in rural India. *Curr Sci*, **80**: 1483–1484.

Sivakumar V, Parthiban KT, Singh BG, Gnanambal VS, Anandalakshmi R, Geetha S. 2002. Variability in drupe characters and their relationship on seed germination in teak (*Tectona grandis* L.F.). *Silvae Genet*, **51**: 232–237.

Srinivasan K, Muruganandan S, Lal J, Chandra S, Tandan SK, Raviprakash V, Kumar D. 2003. Antinociceptive and antipyreticactivities of *Pongamia pinnata* leaves. *Phytol Res,* **17**: 259–264

Sunil N, Kumar V, Sivaraj N, Lavanya C, Prasad RBN, Rao BVSK, Varaprasad KS. 2010. Variability and divergence in *P. Pinnata* (L.) Pierre germplasm- a candidate tree for biodiesel. *J Bioenergy*, **1** (6): 382–391.

Zar J. 1996. *Biostatistical Analysis*. New Jersey: Prentice-Hall Inc., p.662.

Zobel B, Talbert JJ. 1984. *Applied Forest Tree Improvement*. New York: John Wiley and Sons, pp. 75–116.
