# Supplementary material for: Provenance and family variations in early growth of Manchurian walnut (Juglans mandshurica Maxim.) and selection of superior families
Source: PLoS One. 2024 Mar 7;19(3):e0298918. doi: 10.1371/journal.pone.0298918 (PMC10919699; doi:10.1371/journal.pone.0298918)
Supplement: S1 File — (ZIP) [file pone.0298918.s004.zip › Genetic variation among and within provenances of Adansonia digitata L.(Baobab) in seed germination and seedling growth from selected natural populations in Malawi.pdf]

Agroforest Syst (2012) 86:419–431
DOI 10.1007/s10457-011-9472-6

Genetic variation among and within provenances of *Adansonia digitata* L. (Baobab) in seed germination and seedling growth from selected natural populations in Malawi

C. R. Y. Munthali · P. W. Chirwa · F. K. Akinnifesi

Received: 7 June 2011 / Accepted: 30 December 2011 / Published online: 15 January 2012
© Springer Science+Business Media B.V. 2012

Abstract Baobab (*Adansonia digitata* L.) trees have a wide variety of subsistence and economic values across sub-Saharan Africa. Knowledge of the genetic variation within and between the species is essential for management and designing domestication, improvement and conservation strategies. Fifty-nine half-sib families were evaluated in the nursery to determine the genetic variation and control within and between the populations from four silvicultural zones. Seed germination and seven seedling growth parameters were assessed. Total phenotypic variance, family variance, within family variance, individual narrow sense heritability, and additive genetic coefficient of variation (AGCV) were derived from observed and expected mean squares. There were highly significant differences in seed germination, plant height, root collar diameter, number of leaves, shoot height and diameter, tuber diameter and weight. Provenance variation in germination ranged from 46.7 ± 3.3 to 68.7 ± 3.3% while tree-to-tree variation ranged from 6.3 ± 8.6 to 95.5 ± 8.6%. Coefficient of variation in seedling growth variables ranged from 18.6 to 43.6%. Individual narrow sense heritabilities (h^2) ranged from 0.07 to 0.71. AGCV ranged from 3.21 to 14.67%. Morphological traits showed that mainland populations were genetically distant from the island one. High and moderate additive genetic control of traits and AGCV show the potential that Baobab can also respond well to tree improvement. High phenotypic variation found in the study offers an opportunity to effect selection of superior attributes at both provenance and individual tree-to-tree level.

Keywords Indigenous · Breeding · Selection · Diversity

C. R. Y. Munthali (✉)
Mzuzu University, Private Bag 1, Luwinga, Mzuzu, Malawi
e-mail: chimuleke.munthali@gmail.com

C. R. Y. Munthali
Department of Forestry, Stellenbosch University, Stellenbosch, South Africa

P. W. Chirwa
Pretoria University, Lynwood Road, Hatfield, Pretoria 0002, South Africa

F. K. Akinnifesi
World Agroforestry Centre (ICRAF), Southern Africa Regional Programme, Chitedze Agricultural Research Station, P.O. Box 30798, Lilongwe 3, Malawi

Introduction

The miombo woodland contains over 200 tree species that bear edible fruits (Akinnifesi et al. 2008). More than 80% of the rural population in sub-Saharan Africa is poor and traditionally depend on forest for most of their livelihood such as use of fruits (Kalaba et al. 2010). Harvesting of the indigenous tree fruits from the wild can increase rural annual income by US$300 to US$2000 per household (Kalaba et al. 2010).

Economic impact analysis of indigenous fruit trees have shown that fruit collection, consumption and sale reduce income poverty by 33% (Akinnifesi et al. 2008). The contribution of indigenous fruits of the miombo woodlands is especially important in the Southern African Development Community (SADC) mainly for the marginalized groups in the society, such as women, children and the landless (Akinnifesi et al. 2006; Shackleton et al. 2007). For instance, indigenous fruits contribute on average about 42% of the annual food basket and a majority of households rely on indigenous fruits during famine (Campbell et al. 1997). In a study conducted in Zimbabwe, Mithöfer et al. (2006) reported that indigenous fruits reduced the probability of falling below the poverty threshold, thereby vulnerability to severe famine, during the critical food shortage period by 30%. In the case of *A. digitata,* farmers sell fruits to buy food. Shackleton and Shackleton (2004) have reported that non timber food products (NTFPs) assist households in times of adversity such as death or retrenchment of the head of the household or breadwinner, droughts, floods, frosts or diseases leading to crop failure or death of livestock. Furthermore, collection of indigenous fruits contributes between 5.5 and 6.5% of the total household income in the rural communities of southern Africa (Akinnifesi et al. 2008; Kalaba et al. 2010.

About 85% of rural households in South Africa rely on edible fruits and wild spinaches (Shackleton and Shackleton 2004) and collect about 104 kg of edible fruits and 58 kg of wild spinaches. In addition, some indigenous fruit trees are important sources of medicine for the rural communities with about two-thirds of households utilizing indigenous fruit trees for medicinal purposes (Kalaba et al. 2010). In southern Africa, farmers traditionally integrate crops and fruit trees such as baobabs on their farm land either as wild, semi-domesticates or cultivated (Akinnifesi et al. 2008). A global study by Ruiz-Pérez et al. (2004) indicated that harvesting of non-timber forest products from wild and semi-wild or lightly managed forests, is a viable subsistence strategy of households, providing extra income to household who depend on agriculture or off-farm income sources. *Adansonia digitata* L. (Baobab) tree is a characteristic species for drier areas, especially sites with annual rainfall between 500 and 800 m (Shivcharn and Gunnar 2004). The baobab is at its best on deep well-drained soil at altitudes of between 450 to 600 m above sea level with a rainfall between 300 and 500 mm per annum (Wickens 1982; Wilson 1988). However, it also occurs from sea-level to at least 1500 m and from areas receiving as little as 90 mm of rainfall to as much as 1400 mm annually, with extension to higher rainfall influenced by man (Wilson 1988). It extends from northern Transvaal and Namibia to Ethiopia, Sudan and fringes of the Sahara (Gebauer et al. 2002; Sidibe and Williams 2002; Wickens 1982; Wilson 1988). Baobab has many socio-economic uses with nearly every part of the tree being used by human beings (Sidibe and Williams, 2002). According to ICUC (2002), baobab oil from seed is for cooking and is sold in local markets as well as international markets for use in cosmetics industries. Furthermore, its pulp is used for synthesis of beverages at local scale and processed industrially. Presently, pulp powder is an important export commodity to EU markets (Gruenwald and Galiza 2005). In Australia, a Baobab Fruit Company is conducting trials on the use of tubers as carrots with an initial cultivation project of 4,000 plants (Gruenwald and Galiza 2005). The leaves are sold as well-sought important vegetables in parts of West Africa. Apart from manufacturing juice, baobab fruit has huge potential for making jam, oil and wine (Akinnifesi et al. 2008; Ham and Akinnifesi 2006).

Past research and extension efforts towards indigenous fruits were hampered due to biasness towards cultivation of exotic fruit tree species (Akinnifesi et al. 2008). However, for two decades, the World Agroforestry Centre (ICRAF) and its partner institutions have conducted research especially on domestication of indigenous fruit trees in southern Africa (Akinnifesi et al. 2008). For example, field trials on provenance and family variation have been done in southern Africa for *Uapaka kirkiana* and *Sclerocarya birrea* (Akinnifesi et al. 2008; Chirwa et al. 2007). In recent market studies (Akinnifesi et al. 2008), baobab has emerged as one of the priority species for domestication in southern Africa. Indeed, nutritional studies on *A. digitata* in Malawi have shown that baobab nutritional value is high for the supply of ascorbic acid, fat, carbohydrates, phosphorus, calcium, magnesium, iron, potassium and sodium all important for mankind (Saka et al. 1992; Saka and Msonthi 1994; Saka et al. 2008).

The supply of *A. digitata* in Malawi continues to rely on wild populations which are unfortunately dwindling due to various factors (Chirwa et al. 2006). However, sustainable supply of baobab products can

only be achieved through planting (Shivcharn and Gunnar 2004). In order to succeed in baobab domestication, an understanding of genetic variation is important (Zobel and Talbert, 1984).

Genetic diversity is the basis of all biodiversity and is a key requirement for the long term survival of a species especially in environments subject to climatic change or introduction of new pest, pathogens and competitors (Pakkad et al. 2008). Knowledge of the genetic variation within the species is essential to design a strategy to promote the use and conservation of indigenous fruit trees meant for on-farm cultivation (Haq et al. 2008). Genetic divergence information is also required in identifying parents for hybridization programmes intended to identify more heterotic recombinants (Sandhu et al. 2006). With large geographical distribution of baobab, morphotypes have developed (Sidibe and Williams 2002). Within the species, there is evidence showing the occurrence of a number of local forms differing in habit, size, quality of the fruit and leaf vitamin content (Assogbadjo et al. 2009). In addition, in Mali and Sudan variation in tree bark colour, pulp taste, tree height and diameter and leaf vitamin C content have been reported. Sidibe and Williams (2002) have proposed assessment of patterns of genetic diversity of baobab in relation to local distribution. Genetic variation among and within provenances of different species have been evaluated through seedlings in several studies (Mwase et al. 2006; Mwitwa et al. 2007, 2008; Ngulube et al. 1997). This study was undertaken to characterize, quantify the genetic variation and estimate the genetic control of *Adansonia digitata* L. seed germination and seedling growth characteristics in five natural populations existing in four diverse ecological conditions using a nursery study.

Materials and methods

Materials

A total of 59 half-sib families from Karonga, Likoma Island, Salima, Mwanza and Chikwawa were selected on the basis of differences in silvicultural classification of Malawi Hardcastle (1978) (Table 1). Likoma Island was included because of its isolation. According to Hardcastle (1978), silviculture zone L has mean annual rainfall (MAR) >1600 mm with predominantly weathered ferralitic soils. Silviculture zone Ba has MAR ranging between 710 and 850 mm and characterized by calcimorphic soils overlaying vertisols. Silviculture zone J has MAR ranging between 1200 and 1600 mm and characterized by ferrallitic soils whilst silviculture zone A has MAR ranging between 710 and 840 mm with vertisols. Seed was collected from randomly selected parent trees located at distances of approximately 100 m apart.

Nursery evaluation

The nursery experiment was carried out at Mzuzu University green house from 1st October, 2008 consisting of 59 families nested in five provenances. The experiment was laid out as a complete randomized block design with four replications consisting of twenty-five black polythene tubes as a treatment unit. The rooting medium was dark-grey miombo soil mixed with sand in the ratio of 2:1 respectively. Prior to sowing, seed pre-treatment involved nicking by removing a small part of seed coat. Two seeds were sown per tube at a depth of about 4 cm. After 30 days, seedlings were thinned to one per tube. Water was applied when necessary to keep the medium moist.

Data collection

Seed was considered germinated when two true leaves appeared on the surface of the growing medium. Germinated seed were recorded daily for 30 days. At the age of three months all the seedlings were assessed for the number of leaves, root collar diameter, length and area of third leaf, total leaf area, and seedling height. At five months, only five seedlings were systematically selected from each treatment by sampling the first five seedlings in a row. To determine root parameters, seedlings were removed from the polythene tubes and soil washed carefully off the root system and left to dry before weighing. The root (tuber) and shoot were separated at the root collar. The seedlings were assessed for fresh tuber weight, tuber diameter, shoot diameter and height.

Data analysis

Data were analysed as randomized complete block using the nested effects model. Arcsine transformation

Table 1 Physical description of sites (population/provenance) and number of trees (families) sampled

Population	Number of sampled families	Population code	Silvicultural zone after Hardcastle (1978)	Average annual Stress Period (weeks)*	Altitude (m)	Mean annual rainfall (mm)	Mean annual temperature (^{0}C)	Soil
Karonga	10	K	L	7″ Field capacity:15<br>12″ Field capacity″:10	475–1,000	>1600	23–25	Ferrisols dominant; regosols
Salima	15	S	Ba	7″ Field capacity:28<br>12″ Field capacity:26	200–1,200	710–850	20–25	Alluvial calcimorphic soils above the vertisols
Mwanza	15	M	J	7″ Field capacity:16<br>12″ Field capacity:11	900–1,500	1,200–1,600	19–21	Sandy ferrallitic
Chikwawa	15	C	A	7″ Field capacity:35<br>12″ Field capacity:35	<200	710–840	>25	Vertisols
Likoma Island	10	L	L	7″ Field capacity:15<br>12″ Field capacity″:10	475–1,000	>1,600	23–25	Ferrisols, alluvial calcimorph, regosols, lithosols

Source: Hardcastle (1978)

* Information on average annual stress calculations are reported by Hardcastle (1978)

was done for germination percentage (Fowler et al. 1998) prior to analysis.

Least significant difference (LSD) was used to identify significant differences between provenances and families. Estimates of narrow-sense heritability (h^2) was calculated from the variance components, obtained from the decomposition of the mean squares obtained from analysis of variance procedure. Narrow-sense heritability was calculated according to the formula given by Zobel and Talbert (1984) as follows:

$$h^2 = \frac{\sigma_A^2}{\sigma_p^2} = \frac{4\sigma_f^2}{(\sigma_w^2 + \sigma_f^2)}$$

where coefficient of relationship was taken as 0.25 for half-sib families, σ_f^2 the variance between families, σ_w^2 the variance within families and σ_p^2 total phenotypic variation.

The additive genetic coefficient of variation (AGCV) values were calculated using the formula given by Cornelius (1993) as follows:

$$\text{AGCV} = 100(\sigma_A/\mu)$$

where σ_A is the additive genetic standard deviation and μ the phenotypic (and genotypic) mean.

Results

Provenance variation in germination percentage

In the nursery, seed germination commenced within nine days after sowing for all populations. At 14 days, almost all viable seed had germinated in all populations. Seed germination was significantly different ($P \geq 0.001$) among provenances, with Chikwawa having the highest germination (68.7 ± 3.3%) and

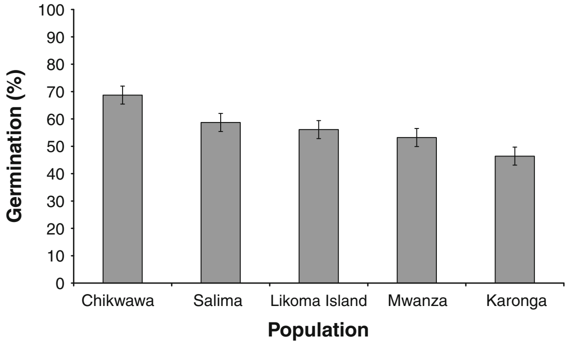

Fig. 1 Variation in mean germination percentage among five populations of *Adansonia digitata* L.

Karonga having the lowest germination (46.4 ± 3.3%) at 23 days after sowing (Fig. 1).

Family variation in germination percentage

At 30 days after sowing, germination percentage was significantly different ($P \geq 0.001$) among families within provenance (Fig. 2). Across all the provenances, mean germination percentage ranged from 6.3 ± 8.6% in one tree from Mwanza (M8) to 95.6 ± 8.6% in another tree in the same population (M6) (Fig. 2). Within Chikwawa C3 (85.4 ± 8.6%) and C5 (85.6 ± 8.6%) were superior to the rest in germination whilst the least germination was observed in C15 (36.5 ± 8.6%). Similarly, M6, M13, M15 had superior germination percentage (ranging from 84.8 ± 8.6 to 95.6 ± 8.6%). M8 was the most inferior with germination of 6.3 ± 8.6%, but not significantly different from M5 and M11 both at 16.4 ± 8.6%. In Salima, S2, S6 and S3 had superior germination ranging from 85.4 ± 8.6 to 91.7 ± 8.6% whereas the least germination was found in S4 (11.4 ± 8.6%) and S7 (18.9 ± 8.6%). In Likoma, L3 (95.2 ± 8.6%) had the most outstanding germination with L9 (32.1 ± 8.6%) having the least. In Karonga K12 (84.2 ± 8.6%) was the best performer whilst K14 (13.9 ± 8.6) was the least. However, K7, K13, K2 and K11 had poor germination as well ranging from 28 ± 8.6 to 45.3 ± 8.6%.

Provenance variation in seedling growth traits

After germination, most of the seedlings developed vigorously; although a few seedlings were nibbled by grass hoppers soon after germination. At three months

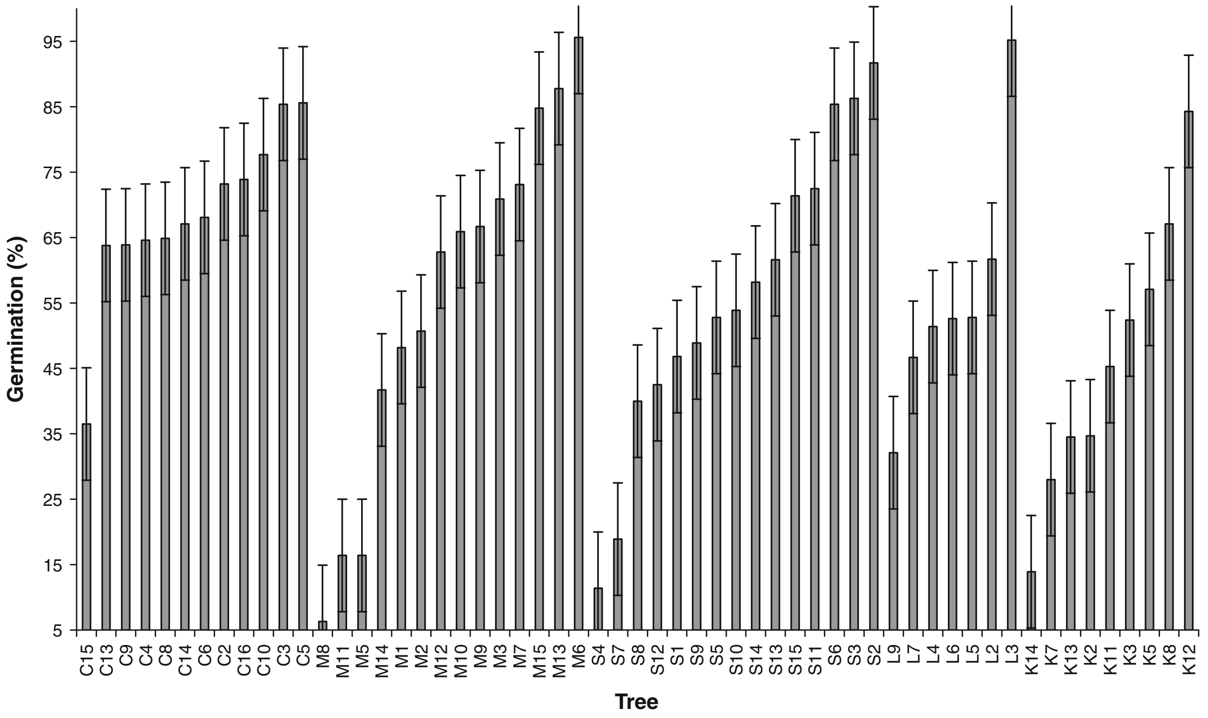

Fig. 2 Variation in mean germination percentage between trees within family and among five provenances of *Adansonia digitata* L. at 30 days after sowing (*C* Chikwawa, *M* Mwanza, *S* Salima, *L* Likoma Island and *K* Karonga; label of individual trees range from 1 to 15)

Table 2 Plant growth attributes at 3 and 5 months after sowing

Provenance	Plant height (cm)	Plant rcd (mm)	Number of leaves	Shoot height (cm)	Tuber weight (g)	Tuber diameter (mm)	Shoot diameter (mm)
	3 months after sowing			5 months after sowing			
Karonga	16.7 ± 0.246a	5.1 ± 0.060a	8.7 ± 0.124a	17.9 ± 0.325a	20.7 ± 0.724b	20.1 ± 0.334b	5.4 ± 0.140a
Salima	16.1 ± 0.195a	5.1 ± 0.048a	8.3 ± 0.099b	17.9 ± 0.258a	20.7 ± 0.575b	20.3 ± 0.266b	5.6 ± 0.111a
Chikwawa	14.8 ± 0.182b	5.1 ± 0.045a	7.6 ± 0.092c	16.5 ± 0.258b	23.9 ± 0.574a	21.8 ± 0.265a	5.4 ± 0.111a
Mwanza	14.5 ± 0.187b	5.1 ± 0.046a	7.5 ± 0.094c	15.8 ± 0.263b	21.5 ± 0.586b	20.4 ± 0.271b	5.3 ± 0.113a
Likoma	13.9 ± 0.256c	4.5 ± 0.063b	7.8 ± 0.129c	16.0 ± 0.357b	16.2 ± 0.797c	20.0 ± 0.271b	4.8 ± 0.154b
LSD	0.616	0.063	0.311	0.864	1.93	0.890	0.307
CV%	25	18.6	24.1	24.5	43.6	20.6	33.3

Means followed by the same letter are not significantly different, *rcd* root collar diameter, *LSD* Least significant difference

after sowing, there were significant differences ($P \geq 0.001$) in plant height among provenances, with Karonga (16.7 ± 0.246 cm) having the tallest plants whilst the shortest were from Likoma Island (13.9 ± 0.256 cm), the rest of the populations were intermediate and were not significantly different (Table 2). Root collar diameter was significantly different ($P \geq 0.001$) among provenances, with Karonga (5.1 ± 0.060 mm), Salima (5.1 ± 0.048 mm), Chikwawa (5.1 ± 0.045 mm) and Mwanza (5.1 ± 0.046 mm) having the largest diameter whilst the smallest diameter were from Likoma Island (4.5 ± 0.063 mm). The number of leaves on the whole seedling was significantly different ($P \geq 0.001$) among provenances with Karonga having the largest number of leaves (8.7 ± 0.124) whilst Chikwawa (7.6 ± 0.092), Mwanza (7.5 ± 0.095) and Likoma Island (7.8 ± 0.124) had the least. At five months after sowing, shoot height was significantly different ($P \geq 0.001$) among provenance, with Karonga (17.9 ± 0.325 cm) and Salima (17.9 ± 0.258 cm) having the tallest seedlings whilst the shortest were from Chikwawa (16.5 ± 0.258 cm), Mwanza (15.8 ± 0.263 cm) and Likoma Island (16.0 ± 0.357 cm). Tuber weight was significantly different ($P \geq 0.001$) among provenances, with Chikwawa (21.8 ± 0.574 g) having the heaviest whilst Likoma Island had the lightest (16.2 ± 0.724 g). Tuber diameter was significantly different ($P \geq 0.001$) among provenances, with Chikwawa having the largest diameter (21.8 ± 0.265 mm), whilst Karonga, Salima, Mwanza and Likoma were intermediate (ranging from 20.0 ± 0.271 to 20.4 ± 0.270 mm). Shoot diameter was significantly different ($P \geq 0.001$) among provenances with Karonga, Salima, Chikwawa and Mwanza having the largest diameter ranging from 5.3 ± 0.113 to 5.6 ± 0.111 mm whilst Likoma Island had the smallest (4.8 ± 0.154 mm).

Family variation in seedling traits

Variation in seedling height within all provenances was significantly different ($P \geq 0.001$) among the families (Fig. 3). Across all provenances, seedling height ranged from 10.9 ± 0.52 cm in one tree in Mwanza (M8) to 19.4 ± 0.52 cm in another tree in Salima (S5). Superior growth in Chikwawa was observed in C7, C16 and C2 ranging from 17 ± 0.52 to 17.9 ± 0.52 cm with C15 (11.9 ± 0.52 cm) being the most inferior. The most outstanding growth in Mwanza was found in M15, M7, M3 and M12 ranging from 15.2 ± 0.52 to 16.1 ± 0.52 cm whilst M8 (10.9 ± 0.52 cm) was the most inferior. In Salima the best performer was S5 (19.4 ± 0.52 cm) while S15 (12.7 ± 0.52) and S14 (13.3 ± 0.52 cm) were the worst. In Likoma the most outstanding growth was found in L7 (15.6 ± 0.52 cm) with L2 and L4 being the poor performers at 12.5 ± 0.52 cm. In Karonga superior growth was observed in K3 (18.7 ± 0.52 cm) and K5 (18.2 ± 0.52 cm) whilst K14 (13.4 ± 0.52 cm) was the most inferior.

Variation in tuber weight within all provenances was significantly different ($P \geq 0.001$) among the families (Fig. 4). Across all the provenances, tuber weight ranged from 14.4 ± 1.5 g in one tree in Likoma Island (L5) to 30.9 ± 1.5 g in another tree in Chikwawa (C7). Within Chikwawa, outstanding growth was observed in C7 (30.9 ± 1.5 g) and C8 (30.8 ± 1.5 g) whilst the

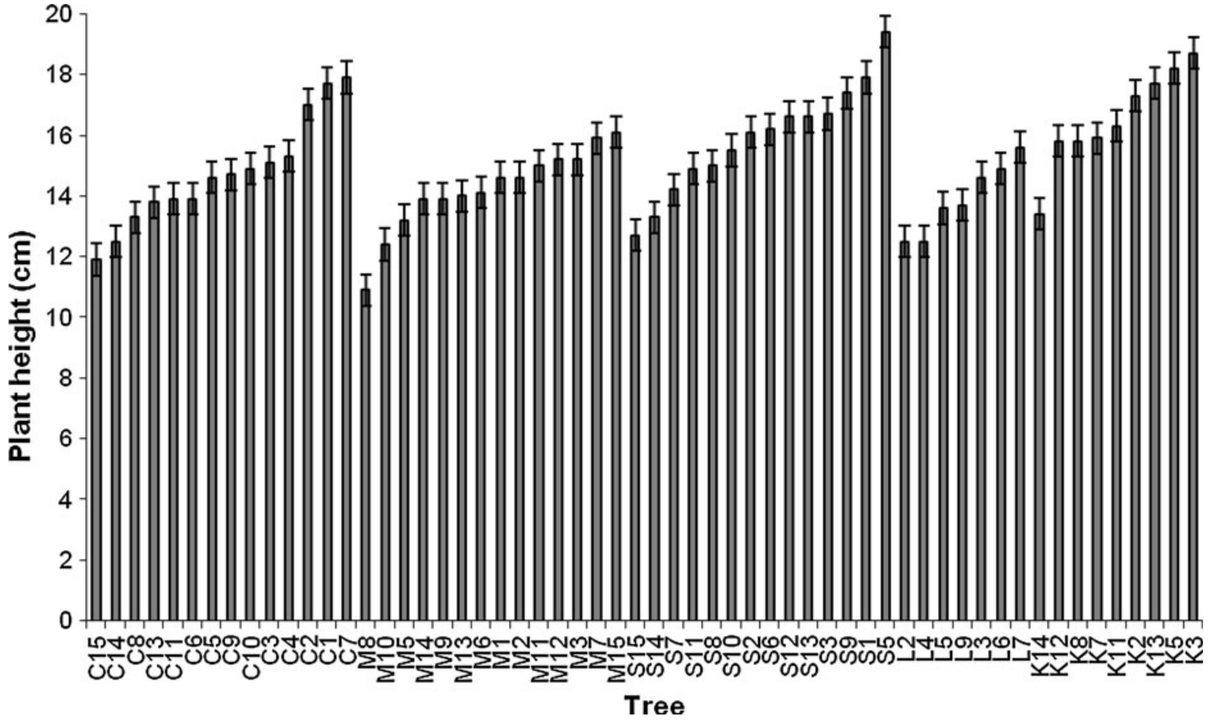

Fig. 3 Family variation in plant height within five provenances in *Adansonia digitata* L. three months after sowing. (*C* Chikwawa, *M* Mwanza, *S* Salima, *L* Likoma Island and *K* Karonga; label of individual trees range from 1 to 15)

inferior growth was found in C2, C3 and C16 ranging from 17.9 ± 1.5 to 19.4 ± 1.5 g. Within Mwanza, superior growth was observed in M2, M5, M13, M1 and M11 ranging from 23.3 ± 1.5 to 26.2 ± 1.5 g whereas the inferior growth was found in M6, M3, M10 and M14, ranging from 16.2 ± 1.5 to 18.6 ± 1.5 g. In Salima, superior growth was found in S15 (25.9 ± 1.5 g) whereas S5, S10 and S2 were inferior ranging from 14.2 ± 1.5 to 16.5 ± 1.5 g. Within Likoma, the best growth was observed in L6 (19.3 ± 1.5 g) whereas the least was in L5 (14.4 ± 1.5 g) and L9 (14.7 ± 1.5). In Karonga the outstanding growth was found in K14 (21.4 ± 1.5 g) whilst the worst was in K11 (15.1 ± 1.5 g).

Phenotypic and genetic variation and control of seedling growth parameters

Results in Table 3 show genetic control and variance components of seedling traits. Strong additive gene effects was found for germination percentage ($h^2 = 0.53$), plant height ($h^2 = 0.71$) total leaf area ($h^2 = 0.65$) and number of leaves ($h^2 = 0.66$) 36 days after sowing but decreased as time elapsed. Moderate additive gene effects was found in height to first leaf ($h^2 = 0.44$), tuber weight ($h^2 = 0.34$), third leaf area ($h^2 = 0.32$), tuber diameter ($h^2 = 0.20$) and root collar diameter ($h^2 = 0.15$). Shoot diameter showed the lowest genetic control ($h^2 = 0.07$). Within population variation (σ_w^2) ranged from 87.2 to 96.4% of the total variation (σ_p^2) for all the traits. AGCV was moderate for germination percentage (11.2%), tuber weight (12.8%), total leaf area (12%), and third leaf area (14.7%) whilst that for the other traits could be considered low (<10).

Discussion

Germination percentage

The prevailing differences in provenance and families in germination in the nursery investigation were essentially genetic in origin (Sniezko and Stewart 1989). Germination for all populations and families started 9–11 days after sowing and within 14 days

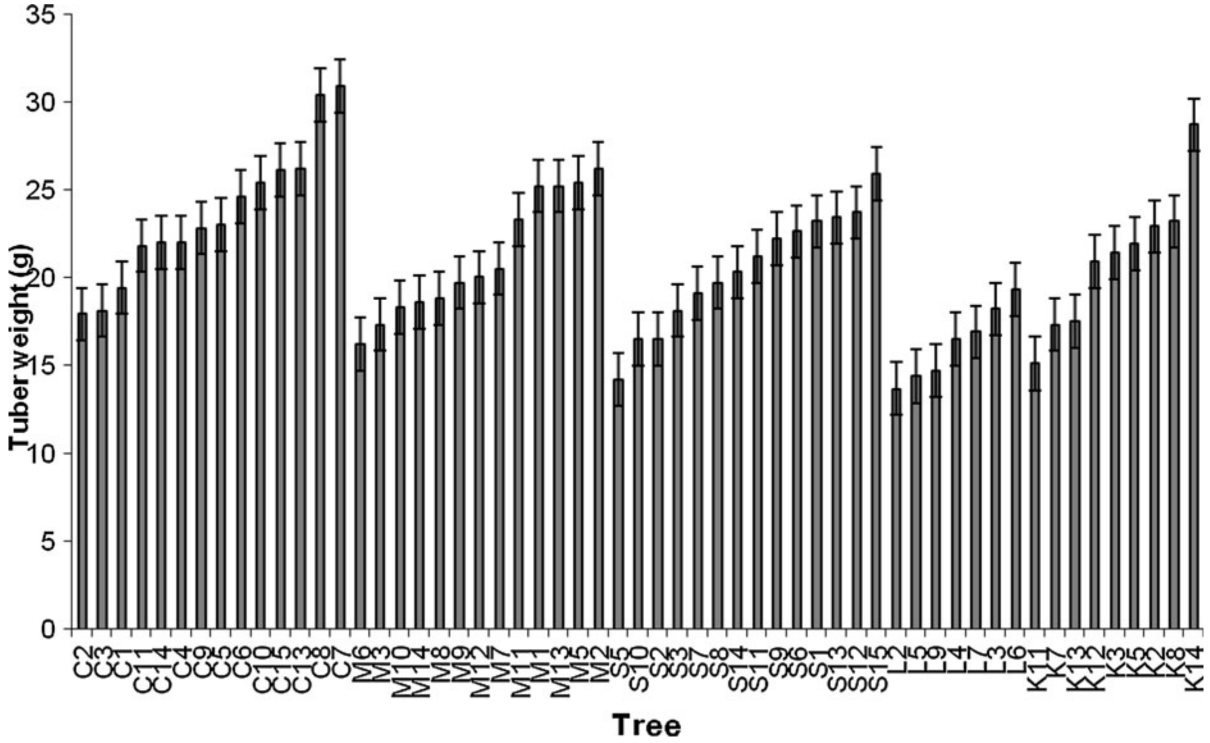

Fig. 4 Family variation in tuber weight within five provenances in *Adansonia digitata* L. five months after sowing. (*C* Chikwawa, *M* Mwanza, *S* Salima, *L* Likoma Island and *K* Karonga; label of individual trees range from 1 to 15)

Table 3 Values of total phenotypic variance (σ_p^2), family variance ($\sigma_{f(p)}^2$), within family variance (σ_w^2), individual-plant- narrow sense heritability (h^2) and additive genetic coefficient of variation (AVCV) for 13 variables in *Adansonia digitata*L. in Malawi

Variable	σ_p^2	σ_w^2	$\sigma_{f(p)}^2$	h^2	AGCV
Germination percentage	0.031	0.027	0.004	0.53	11.16
Five months after sowing					
Shoot diameter	3.150	3.097	0.054	0.07	4.36
Shoot height	17.409	15.735	1.674	0.38	7.71
Tuber weight	86.233	78.943	7.290	0.34	12.80
Tuber diameter	17.366	16.507	0.859	0.20	4.49
Number of leaves	3.718	3.480	0.238	0.26	6.16
Plant height	14.658	13.508	1.150	0.31	3.21
3 months after sowing					
Third leaf length	0.555	0.5180	0.036	0.26	8.30
Third leaf area	70.998	63.526	6.472	0.32	14.67
Total leaf area	968.209	811.374	156.835	0.65	12.04
Number of leaves	2.438	2.035	0.4025	0.66	10.07
Plant height	700.870	576.579	124.295	0.71	9.0
Root collar diameter	0.877	0.845	0.032	0.15	3.77
Height to first leaf	70.203	62.470	7.733	0.44	9.51

most of the viable seed had germinated. In this case seed origin seems to have had no influence on the initiation of germination. Since days to germination were the same, this may imply that the population origin is the same and that these populations have so far not evolved differently for this trait (Andersen et al.

2008). Thus days taken to start germination have failed to characterize the population into different ecological (silvicultural) zones. It is anticipated that high-vigour seeds germinate faster than low-vigour seed under any condition (Schmidt 2000). In the current study, germination capacity was poor mostly for Karonga population which is mainly attributed to fruit handling. However, mean germination was generally high for the other populations and most of the families within populations (Figs. 1 and 2). The results indicate need for careful post handling of fruits to retain high seed physiological quality (viability). However, some families such as M8, M11, M5, S4 and S7 (Fig. 2) had poor germination despite proper fruit and seed handling. It may be inferred that the origin of the problem was maternal. Poor germination may be a sign of inbreeding Zobel and Talbert (1984). According to Andersen et al. (2008) most of the variation in germination in *Abies guatemalensis* was due to proportion of seeds with viable embryos. Effectiveness of pollinators and breeding system has an impact on viability of embryos Zobel and Talbert (1984). *Adansonia digitata* L. is outcrossing (Assogbadjo et al. 2006) but tree density in Malawi is dwindling such that in most populations there may be only two (2) adult trees per hectare (Chirwa et al. 2006) increasing chances of related mating. In this study, albinism occurred in some families at low frequency. Albino plants are a sign of inbreeding (Zobel and Talbert 1984). Variation studies in germination between and within populations in miombo species including baobabs are still scanty. Chithila et al. (2008) found variation in germination between four baobab provenances in Malawi ranging from 0 to 60% depending on method of pretreatment. Munthali (2007) reported commencement of germination in Malawian baobabs at 7 days after sowing and germination percentage ranging from 23 to 75%, depending on pretreatment method. ICUC (2002) reported that one is unlikely to have 100% viability in baobab but that healthy seeds should give 70–85% germination. In this study, some families achieved up to 95% germination showing high physiological quality. Published work in other miombo species such as in *Uapaka kirkiana* (Mwase et al. 2006; Ngulube et al. 1997) have reported significant variation in seed germination between indigenous populations in Malawi. The variation has been attributed to genetic as well as environmental factors. The presence of significant population and family variation in germination percentage shows need for proper selection of seed source. The differences in germination in the present study likewise could be linked to family variation (genetic), fruit maturity at collection, fruit and seed handling and original ecological factors (Schmidt 2000). Sustainable supply of indigenous fruits can only be attained through planting (Akinnifesi et al. 2006). Highest quality transplants are required in domestication programmes; hence high germination capacity is essential (Ngulube et al. 1997).

Seedling growth

Understanding variation existing in the species is prerequisite before starting any tree breeding programme Zobel and Talbert (1984). A number of population genetics studies through nursery studies have been done (Andersen et al. 2008; Marcar et al. 2002; Masamba 1999; Mwase et al. 2006; Mwitwa et al. 2008; Munthali 1999; Ngulube et al. 1997; Sniezko and Stewart 1989). Variation in seedling traits during the nursery phase is usually considered genetic in origin (Ngulube et al. 1997). The study showed that there were substantial variation in shoot and root traits between provenances and families in baobabs. The clusters of tuber weight, seedling diameter and plant height vividly showed that main land populations are similar genotypes while the one from the Likoma Island is different (Table 2). The clusters also appeared to show that seed sources from drier sites (with long duration of stress period) (Table 1) had generally vigorous growth, for instance Chikwawa was mostly superior in most growth traits while Likoma Island was mostly underperforming in majority of the traits. According to Ngulube et al. (1997), variation in plant biomass discloses overall differences in growth ability among populations. Generally, Chikwawa from the lowest altitude had the most outstanding growth in most traits. Similarly, Andersen et al. (2008) have reported superior growth in populations from low altitude areas compared to high altitude area. The results show that farmers who would like to establish nurseries for production of baobab root tubers (carrots) should choose the Chikwawa population as a first choice followed by the other mainland populations and avoid the Likoma Island population due to its inferiority. Marcar et al. (2002) have shown that better performing *Eucalyptus grandis* provenances in glasshouse trials also performed equally well in the field

trials. It may also follow therefore that if the biomass at seedling stage is maintained up to later stages, as expressed through the differences in biomass and its partitioning in this study, then this might already be an indication of populations that will have superior growth later (Ngulube et al. 1997). The baobab populations may also be having different net assimilation capacity leading to variation in seedling parameters. The differences found could be attributed to strong natural selection and ecological barriers to gene flow (Sorensen et al. 1990). However, provenance and family trials are still required to assess juvenile and mature correlations and genotype x environmental interaction (Chirwa et al. 2007; Zobel and Talbert 1984). Notwithstanding, present results might project variation in fruit traits in future as well. These nursery results depict possibility of identifying population variation easily and cheaply useful for countries that can not afford molecular studies. The current study compares well with studies done on baobab in Benin (Assogbadjo et al. 2006) where morphometric analysis showed significant differences within and among populations across the climatic zones largely influenced by abiotic factors (temperature and rainfall). Variation in seedling growth traits have been associated with distance between locations, elevation and latitude as well which are linked with adaptive variation of species (Sorensen et al. 1990). Subsequently, clusters found in present results in different traits might be an indication that populations in baobabs exists as ecotypes partitioned by both climatic and geomorphological factors. This study has been done at national level but *A. digiata* L occurs at extremely wide geographical scale. Hence, it will be important to study the pattern of genetic variation at regional scale (southern Africa) to design domestication and tree improvement activities. High genetic variation found between and within populations in all traits as shown by high coefficient of variation means that for tree improvement to succeed, selection should be at both population and family level (within population).

Heritability

Narrow-sense heritabilities provide a basis for predicting the genetic gain that could be achieved from selection on a particular trait (Marcar et al. 2002). The estimates in Table 3 have shown that there were considerable differences in genetic control of traits. For instance, germination percentage, total leaf area, number of leaves, plant height and height to first leaf were more strongly genetically influenced. Only shoot diameter and root collar diameter showed weak additive genetic effects (0.07–0.15) but the rest of the traits have moderate genetic control. Low heritability values mean that phenotype is a weak predictor of genotype (Marcar et al. 2002). There were fluctuations in value of additive genetic effect as seedlings were approaching dormant stage in growth. Apical dormancy in seedlings of *Pterocarpus angolensis* has been attributed to seasonal changes in climate that has effects on the physiological response of plants (Mwitwa et al. 2007). High heritability values indicate that much of the variation for a given trait observed in a population had a genetic origin (Zobel and Talbert, 1984). Strong and intermediate genetic control is favourable for selection in breeding programmes as it shows breeding programmes need not use large numbers of families to attain high genetic gains. The variance components have shown that much of the variation in most traits was explained by the within-family variation meaning that selection intensity will be high at this level. The results are supported by Zobel and Talbert (1984) who have indicated that more than 90% of the variation in forest trees resides within population. AGCV values have indicated existence of moderate genetic diversity in germination percentage, tuber weight, total leaf area, third leaf area, and number of leaves. The high values could mean preponderance of heterozygosity in the population which is favourable for evolution since it favours high genetic diversity (Zobel and Talbert, 1984). There is generally scanty information on genetic information in most tropical tree species. Mwitwa et al. (2007), reported narrow sense heritability for seventeen shoot and root variables ranging from low to moderate (0.09–0.35); and also in shoot die back in *Pterocarpus angolensis* of 0.07 in the first growing season and 0.42 in the second season (Mwitwa et al. 2008). These values are similar to those from this study; only that some of the traits in this study have high heritability. Furthermore, heritability has been shown to change with season or time (Mwitwa et al. 2007); which has also been depicted in this study. This may imply that selection should be done at the time plants are vigorously growing. These results also compare well with reported work by Cornelius (1993) who analysed trends of heritabilities and AGCV for several tree species as follows: for heritability, mean

ranged from 0.21 to 0.50 and median 0.19 to 0.48 while for AGCV the mean ranged from 5.34 to 23.10 and median 5.10 to 20.30. Sirisena and Senayayake (2000)reported similar values of heritability in banana clones ranging from 4 to 46% and genetic coefficient of variation ranging from 5 to 12%. Hence, it can be implied that genetic control of traits in *Adansonia digitata* L. may be similar to other tree species. High and moderate heritabilities may be due to wide genetic variability of the germplasm belonging to different genomic groups (Sirisena and Senayayake 2000). AGCV of greater than 10% indicate some promise for genetic improvement (Sirisena and Senayayake 2000). The high estimates of narrow-sense heritabilities coupled with considerable morphometric variation observed may indicate that superior gains would be attained in baobab from selection and breeding. However the appearance of the albino seedlings in some families is a matter of concern since it is sign of inbreeding and increase of homozygosity in the population (Zobel and Talbert 1984) which reduces heterosis.

Conclusion

The initiation of seed germination has shown that baobab populations are genetically close. Even though, germination percentage showed significant differences between and within the populations, the trait can not be easily used to characterize the populations according to silvicultural zones. However, while seedling variations cannot be easily used to specifically delineate populations into definite ecotypes, the general trend emerging was that mainland trees were genetically distant to Likoma Island population and that the severity of stress period of the locations seem to have further genetically diverged populations into ecotypes even on the mainland. These results are also important to tree breeders as they may detect variation at an early stage and guide in breeding; and the use of phenotypic variation may also provide a faster method of detecting variation between and within the populations particularly useful for countries that can not afford use of biotechnological methods in showing population diversity. The high genetic variation and genetic control of traits are encouraging results showing great potential of improving baobab through selection and breeding. The low germination capacity in some families despite good fruit and seed handling may be linked to inbreeding as a result of very low *Adansonia* tree densities in Malawi thereby promoting mating of close relatives. This is an area that should be critically researched since inbreeding has numerous harmful effects on fruit trees.

Acknowledgments The first author would like to thank Universities of Mzuzu and Stellenbosch for the financial support. University of Pretoria is thanked for facilitating the writing up of the article. We also extend our thanks to all those that were involved in the caring of the experiment and data collection.

References

Akinnifesi FK, Kwesiga F, Mhango J, Chilanga T, Mkonda A, Kadu CAC, Kadzere I, Mithofer D, Saka JDK, Silesh G, Ramadhani T, Dhliwayo P (2006) Towards the development of Miombo fruit trees as commercial tree crops in southern Africa. For Trees Livelihoods 16:103–121

Akinnifesi FK, Sileshi G, Ajayi OC, Chirwa PW, Kwisiga FR, Harawa R (2008) Contributions of agroforestry research and development to livelihood of smallholder farmers in southern Africa: 2. fruit, medicicinal, fuelwood and fodder trees systems. Agric J 3(1):76–88

Andersen JP, Cordova JPP, Nielsen UB, Kollmann J (2008) Provenance variation in germination and seedling growth of *Abies guatamalensis* Rehder. For Ecol Manag 255: 1831–1840

Assogbadjo AE, Kyndt T, Sinsin B, Gheysen G, Van Damme P (2006) Patterns of genetic and morphometric diversity in Baobab (*Adansonia digitata*) populations across different climatic zones of Benin (West Africa). Ann Bot 97: 819–830

Assogbadjo AE, Kyndt T, Chadare FJ, Sinsin B, Gheysen G, Eyog-Matig O, Van Damme P (2009) Genetic fingerprinting using AFLP cannot distinguish traditionally classified baobab morphotypes. Agroforest Syst 75:157–165

Campbell B, Luckert M, Scoones I (1997) Local level valuation of savanna resources: a case study from Zimbabwe. Econ Bot 51:59–77

Chirwa M, Chithila V, Kayambazinthu D, Dohse C (2006) Distribution and population structures of *Adansonia digitata* in some parts of Ntcheu, Dedza and Mangochi districts, Malawi. FRIM, Zomba

Chirwa PW, Bwanali RJ, Meke, Sagona W, Munthali CRY, Mwabumba L (2007) Growth and phenology of a three-to four-year-old *Sclerocarya birrea* international provenance trial in Malawi. South hemisphere For J 69(1):49–54

Chithila V, Tembo C, Namoto M (2008) The germination of baobab (*Adansonia digitata* L.) seeds 90 days after storage. FRIM Report No. 08001. ODC: 181.525

Cornelius J (1993) Heritabilities and additive genetic coefficients of variation in forest trees. Can J For Res 24:372–379

Fowler J, Cohen P, Jarvis P (1998) Practical statistics for field biology. Wiley, New York

Gebauer J, El-Siddig K, Ebert G (2002) Baobab (*Adansonia digitata* L.): a review on a multipurpose tree with promising future in the Sudan. Gartenbauwissenschaft 67(64): 155–160

Gruenwald J, Galiza M (2005) Market brief in the European Union for selected natural ingredients derived from native species: *Adansonia digitata* L. Baobab. UNCTAD/BioTrade facilitation programme. Nertherlands and Switzerland. www.biotrade.org

Ham C, Akinnifesi FK (2006) Zimbabwe, Zambia, Malawi and Tanzania: priority fruit species and products for tree domestication and commercialization. IK Notes No. 94

Haq C, Bowe C, Dunsiger (2008) Challenges to stimulating the adoption and impact of indigenous fruit trees in tropical agriculture. In: AKinnifesi FK, Leak RRB, Ajayi OC, Silesh G, Tchoundjeu Z, Matakala P, Kwesiga FR (eds) Indigenous fruit trees in the tropics domestication utilisation, and commercialization, Columns Design Ltd, Reading, UK

Hardcastle PD (1978) A preliminary silvicultural classification of Malawi. Forestry Research Institute of Malawi. Fore Res Record No. 57

ICUC (2002) Fruits for the future, Baobab. International center for underutilized crops. Factsheet No.4, Southampton, UK

Kalaba FK, Chirwa PW, Syampungani S, Ajayi OC (2010) Contribution of agroforestry to biodiversity and livelihoods improvement in rural communities of southern African regions. In: Teja Tscharntke, Christoph Leuschner, Edzo Veldkamp, Heiko Faust, Edi Guhardja, Arifuddin Bidin (eds) Tropical rainforests and agroforests under global change, Springer Berlin

Marcar NE, Crawford DF, Saunders A, Matheson AC, Arnold RA (2002) Genetic variation among and within provenances and families of *Eucalyptus grandis* W. Hill and E. globules Labill. Subsp. Globules seedlings in response to salinity and waterlogging. For Ecol Manag 162:231–249

Masamba CRL (1999) Factors influencing accelerated seedling tree growth in Malawian provenances of *Faildherbia albida* Del. Dissertation, Wye College, University of London

Mithöfer D, Waibel H, Akinnifesi FK (2006). The role of food from natural resources in reducing vulnerability to poverty: a case study from Zimbabwe. Working Paper 2006 No. 1, 13 pp. Development and agricultural economics. Faculty of economics and management, University of Hannover, Germany

Munthali CRY (1999) Seed and seedling variation of *Pterocarpus angolensis* DC from selected natural populations of Malawi. Dissertation, University of Stellenbosch

Munthali AM (2007) Seed preatment and germination of *Adansonia digitata* L. Dissertation, Mzuzu University

Mwase WF, Bjornstad A, Ntupanyama YM, Kwapata MB, Bokosi JM (2006) Phenotypic variation in fruit, seed and seedling traits of nine *Uapaka kirkiana* provenances found in Malawi. South Afr For J No.208, 15–21

Mwitwa JP, Munthali CRY, van Wyk G (2007) Half-sib family variation in shoot and root traits of seedlings of *Pterocarpus angolensis* (Family: Fabaceae; syn. Papilionaceae). South Hemisphere For J 69(2):91–94

Mwitwa JP, Munthali CRY, van Wyk G (2008) Heritability of shoot die-back and root biomass in sixteen *Pterocarpus angolensis* (Fabaceae) half-sib families from Malawi, Namibia and Zambia. South Hemisphere For J 70(3): 221–226

Ngulube MR, Hall JB, Maghembe JA (1997) Fruit, seed, and seedling variation in *Uapaka kirkiana* from natural populations in Malawi. For Ecol Manag 98:209–219

Pakkad G, Ueno S, Yoshimaru H (2008) Genetic diversity and differentiation of *Quercus semiserrata* Roxb. in northern Thailand revealed by nuclear and chloroplast microsatellite markers. For Ecol Manag 255:1067–1077

Ruiz-Pérez M, Belcher B, Youn Y, Achdiawan R, Alexiades M, Aubertin C, Caballero J, Campbell B, Clement C, Cunningham T, Fantini A, de Foresta H, Fernández CG, Gautam KH, Martínez PH, Jong W, Kusters K, Kutty MG, López C, Fu M, Alfaro MAM, Nair TK R, Ndoye O, Ocampo R, Rai N, Ricker M, Schreckenberg K, Shackleton S, Shanley P, Sunderland T (2004) Markets drive the specialization strategies of forest peoples. Ecol soc vol.1–23

Saka JDK, Msonthi JD (1994) Nutritional value of edible fruits of indigenous wild trees in Malawi. For Ecol Manag 64:245–248

Saka JDK, Msonthi JD, Sambo EY (1992) Dry matter, acidity and ascorbic acid contents of edible wild fruits growing in Malawi. Trop Sci 32:217–221

Saka JDK, Kadzere I, Ndabikunze BK, Akinnifesi FK, Tiisekwa BPM (2008) Product development: nutritional value, processing and utilization of indigenous fruits from the Miombo ecosystem. In: AKinnifesi FK, Leak RRB, Ajayi OC, Silesh G, Tchoundjeu Z, Matakala P, Kwesiga FR. (eds) Indigenous fruit trees in the tropics domestication utilisation, and commercialization. Columns Design Ltd, Reading, UK

Sandhu JS, Singh P, Singh A (2006) Genetic divergence in chickpea in different environments. Trop Sci 46(1):23–30

Schmidt L (2000) Guide to handling of tropical and subtropical forest seed. Danida Forest Seed Centre. Krogerupvej 21 DK-3050 Humlebaek, Denmark

Shackleton C, Shackleton S (2004) The importance of non-timber forest products in rural livelihood security and as safety nets: a review of evidence from South Africa. South Afr J Sci 100:658–664

Shackleton S, Shanley P, Ndoye O (2007) Invisible but viable: recognizing local markets for non-timer forest products. Int For Rev 9(3):697–712

Shivcharn SD, Gunnar G (2004) Local management practices influence the viability of the baobab (*Adansonia digitata* Linn.) in different land use types, Cinzana, Mali. Agric Ecosyst Environ 101:85–103

Sidibe M, Williams JT (2002) Baobab. *Adansonia digitata* L.. International Centre for Underutilised Crops, Southhampton

Sirisena JA, Senayayake SGJN (2000) Estimation of variability parameters within 'Mysore' banana clones and their implication for crop improvement. Sci Hortic 84:49–66

Sniezko RA, Stewart HTL (1989) Range-wide provenance variation in growth and nutrition of *Acacia albida* seedlings propagated in Zimbabwe. For Ecol Manag 27: 179–197

Sorensen FC, Robert KC, Jerry FF (1990) Geographic variation in growth and phenology of seedlings of *Abies procera*/*A. mannifica* complex. For Ecol Manag 36:205–232

Wickens GE (1982) The Baobab-Africa's upside-down tree. Kew Bulletin 37(2):173–209

Wilson RT (1988) Vital statistics of the baobab (*Adansonia digitata*). Afr J Ecol 26:197–206

Zobel B, Talbert J (1984) Applied forest tree improvement. John Wiley Sons, New York
