# Supplementary material for: Provenance and family variations in early growth of Manchurian walnut (Juglans mandshurica Maxim.) and selection of superior families
Source: PLoS One. 2024 Mar 7;19(3):e0298918. doi: 10.1371/journal.pone.0298918 (PMC10919699; doi:10.1371/journal.pone.0298918)
Supplement: S1 File — (ZIP) [file pone.0298918.s004.zip › Genetic variation analysis of growth and form traits of Castanopsis hystrix in the second generation seed orchard.pdf]

http://xuebao.scau.edu.cn
doi: 10.7671/j.issn.1001-411X.2017.05.014

杨会肖, 廖焕琴, 杨晓慧, 等. 2 代种子园红锥生长和形质性状遗传变异分析[J]. 华南农业大学学报, 2017, 38(5): 81-85.

2 代种子园红锥生长和形质性状遗传变异分析

杨会肖，廖焕琴，杨晓慧，张卫华，潘　文
(广东省森林培育与保护利用重点实验室/广东省林业科学研究院,广东 广州 510520)

摘要:【目的】有效评价和利用红锥 *Castanopsis hystrix* 2 代种子园资源、挖掘其优良性状。【方法】对广东省龙眼洞林场 2 代种子园内 56 个红锥无性系生长和形质性状进行调查,分析各性状的变异系数、不同性状间的相关关系,并进行主成分分析和聚类分析。【结果】56 个红锥无性系的 10 个生长和形质性状的变异系数介于 11%~47%,平均变异系数为 28.2%。树高与胸径、冠幅与树高、胸径间呈极显著正相关,胸径与分枝数、分枝大小与叶片疏密间也存在极显著正相关,冠幅与分枝角度、尖削度与分枝大小存在显著的负相关。10 个性状可以综合为 3 个主成分,前 3 个主成分累计贡献率达 85%。根据系统聚类将 56 个红锥无性系划分为 4 个组。【结论】不同红锥无性系的生长性状和形质性状间变异系数较大,遗传多样性较丰富,且性状间存在不同程度的相关性,为开展红锥种内杂交育种的亲本选择奠定基础。

关键词: 红锥; 生长性状; 形质性状; 重复力; 遗传相关; 主成分分析; 聚类分析
中图分类号: S722.3　　**文献标识码:** A　　**文章编号:** 1001-411X(2017)05-0081-05

Genetic variation analysis of growth and form traits of *Castanopsis hystrix* in the second generation seed orchard

YANG Huixiao, LIAO Huanqin, YANG Xiaohui, ZHANG Weihua, PAN Wen
(Guangdong Provincial Key Laboratory of Silviculture, Protection and Utilization/
Guangdong Academy of Forestry, Guangzhou 510520, China)

Abstract:【Objective】To evaluate and utilize the resources in the second generation seed orchard of *Castanopsis hystrix,* and develop elite genotypes with genetic merit.【Method】We investigated the growth and form traits of 56 *C. hystrix* clones in the second generation seed orchard at Longyandong forest farm, Guangdong Province. The variation coefficients and correlations of different traits were analyzed. Principal component analysis and cluster analysis were performed.【Result】The variation coefficients of ten growth and form traits of 56 *C. hystrix* clones ranged from 11% to 47%, and the average was 28.2%. Height and diameter at breast height, crown width and height, crown width and diameter at breast height, diameter at breast height and branch number, branch size and leaf density all had significant positive correlations($P<0.01$). Crown width and branch angle, taper degree and branch size had significant negative correlations($P<0.05$). Principal component analysis indicated that ten traits could be explained by the first three principal components, accounting for 85% of cumulative proportion of variations. Based on hierarchical cluster analysis, 56 clones were divided into four groups.【Conclusion】Growth and form traits among *C. hystrix* clones had extensive variation with rich genetic diversity. Different degrees of correlations are found among traits. This study provides a basis for parent selection in breeding intraspecific hybrid of *C. hystrix*.

收稿日期:2016-12-12　　**优先出版时间:**2017-07-14
优先出版网址:http://kns.cnki.net/kcms/detail/44.1110.s.20170714.0859.028.html
作者简介:杨会肖(1981—),女,助理研究员,博士,E-mail: hxyang@sinogaf.cn; 通信作者: 张卫华(1977—),女,教授级高工,博士,E-mail: zwh523@sinogaf.cn
基金项目:广东省科技计划项目(2014B020202002, 2016B070701008);广东省科技创新专项(2014KJCX003)

http://xuebao.scau.edu.cn

Key words: *Castanopsis hystrix*; growth trait; form trait; repeatability; genetic correlation; principal component analysis; cluster analysis

红锥 *Castanopsis hystrix* 又名红黎、赤黎、黎木、刺栲、红栲、红橡栲，为壳斗科栲属常绿乔木，是华南地区重要的乡土阔叶珍贵用材和高效多用途树种[1-3]。自20世纪70年代末以来，广西、广东、福建等省区已开展红锥天然林资源的调查、材性试验、良种选育、栽培试验、混交林技术、生物学特性、木材利用技术以及病虫害防治等方面的研究[4-6]。我国阔叶树种子园各项技术研究虽起步较晚，但可借鉴杉木 *Cunninghamia* spp.和松树 *Pinus* spp.等的种子园营建及经营技术上的研究成果[7]，包括园址选择、亲本开花结实特性、控制授粉、树体管理、土壤管理等方面[8]。本文以广东省龙眼洞林场2代红锥种子园为研究对象，对种子园内的56个红锥无性系的生长性状树高、胸径和冠幅，以及形质性状尖削度、通直度、分枝角度、分枝数、分枝大小、叶片疏密和树形进行调查，比较红锥生长性状和形质性状的差异性及其相关性，采用主成分分析法和聚类分析法对不同无性系的生长性状和形质性状进行综合评价，旨在为红锥常规杂交育种研究和优异基因的挖掘提供参考。

1 材料与方法

1.1 调查材料

试验地设在广东省龙眼洞林场墓园工业区内(东经113°21′~113°27′，北纬23°10′~23°18′)。该地年降雨量1 694 mm，年均温21.8 ℃，海拔120 m。种子园林地为深厚的壤质赤红壤，土层厚40~60 cm，pH4.8~5.2。该园为2008年嫁接的2代种子园，56个无性系均为广西、广东和福建种源的种子经过子代测定选择的优良个体。种子园试验采用完全随机区组设计，7次重复，重复内的家系采用不相邻小区种植，各重复内家系植株数量为3~20株，株行距5.0 m×5.0 m。2015年12月对红锥56个无性系1 030个单株进行了树高、胸径、冠幅、通直度、分枝角度和分枝数的测量，具体测定方法见文献[9]。此外，还有4个指标：尖削度=树干直径(1/2树高位置)/树干直径(1.3 m树高位置)；分枝大小，总体侧枝粗<1.5 cm为1级，总体侧枝粗[1.5, 2.0) cm为2级，总体侧枝粗≥2.0 cm为3级；叶片疏密，疏松计为1，紧密计为2；树形，分为圆满型、三角形和伞形3种类型，依次计为1、2和3。

1.2 数据统计分析

利用Excel对原始数据进行整理，采用R软件“psych”包进行主成分分析和样品聚类分析，其中聚类分析采用系统聚类法。应用SAS软件进行不同红锥无性系间各生长性状和形质指标的方差分析、相关性分析。其中，调用GLM过程进行方差分析并选择TYPEⅢ的平方和类型[10]。红锥种子园生长和形质性状观测数据按下列模型进行方差分析：

$$Y_{ijk} = u + B_i + C_j + e_{ijk},$$

式中，Y_{ijk}为无性系的性状观测值，u为总体平均值，B_i为区组效应，C_j为无性系效应，e_{ijk}为剩余误差，并计算参数：无性系重复力(R^2)，$R^2=1-1/F$，其中，F为方差分析TYPEⅢ的F值；变异系数(CV)，CV=SD/M，其中，SD和M分别表示表型标准差和均值；遗传相关系数(r)，$r = \mathrm{cov}XY/\sqrt{\sigma_X^2\sigma_Y^2}$，其中cov为协方差，$\sigma^2$为方差。

2 结果与分析

2.1 2代红锥种子园生长和形质性状变异分析

对56个红锥无性系生长和形质的10个性状指标的变异分析结果见表1。红锥平均树高7.8 m、胸径9.1 cm、尖削度0.80、冠幅3.90 m、通直度3.9、分枝角度56°、分枝数5.8个、分枝大小为2.20级、叶片疏密为1.4、树形为1.60。除尖削度的变异系数较小外，其他均高于15%，说明这些性状个体间的差异较大，性状表现不稳定，因此在建立红锥高世代种子园时有必要对建园材料进行再选择。红锥的生长和形质性状变异系数排序为：分枝

表1 2代种子园红锥生长和形质性状变异分析

Tab. 1 Variation analysis for growth and form traits of *Castanopsis hystrix* in the second generation seed orchard

项目	树高/m	胸径/cm	尖削度	冠幅/m	通直度	分枝角度/(°)	分枝数/个	分枝大小/级	叶片疏密	树形
最小值	5.0	5.0	0.37	1.50	1.0	30	2.0	1.00	1.0	1.00
最大值	16.0	17.0	0.97	6.60	5.0	100	19.0	3.00	2.0	3.00
平均值	7.8	9.1	0.80	3.90	3.9	56	5.8	2.20	1.4	1.60
标准差	1.4	2.2	0.09	0.86	1.0	15	2.7	0.73	0.5	0.62
变异系数(CV)/%	18	24	11	22	27	26	47	34	35	38

http://xuebao.scau.edu.cn

数>树形>叶片疏密>分枝大小>通直度>分枝角度>胸径>冠幅>树高>尖削度。

2.2 2 代红锥种子园生长和形质性状方差分析

按单株小区试验设计进行方差分析，分别对 56 个红锥无性系生长和形质性状的差异显著性进行检测。由表 2 可知，除尖削度的区组效应未能达到显著水平外，其他性状的区组效应均达显著或极显著水平。不同性状的无性系效应检测 F 值介于 1.27~2.14，除分枝角度外，其他性状的 F 值显著性水平均达到显著或极显著。不同性状的重复力为 0.21~0.53，表明这些性状在单株水平上受到中等水平的遗传控制，进一步开展无性系选择潜力较大。

表 2 2 代种子园红锥生长和形质性状方差分析

Tab. 2 Variance analysis for growth and form traits of *Castanopsis hystrix* in the second generation seed orchard

性状	变异来源	自由度	平方和	均方	F	P	重复力
树高	无性系	55	173.10	3.16	1.71	<0.001	0.42
	区组	6	164.20	16.55	8.96	<0.001	
	误差	330	1 624.30	1.85			
胸径	无性系	55	365.00	6.56	1.50	0.010	0.33
	区组	6	193.00	19.10	4.38	<0.001	
	误差	330	3 848.00	4.37			
尖削度	无性系	55	0.68	0.01	1.50	0.010	0.33
	区组	6	0.10	0.01	1.28	0.240	
	误差	330	6.90	0.01			
冠幅	无性系	55	75.00	1.34	1.98	<0.001	0.50
	区组	6	31.40	3.14	4.64	<0.001	
	误差	330	595.00	0.68			
通直度	无性系	55	114.80	2.05	2.14	<0.001	0.53
	区组	6	58.70	5.88	6.13	<0.001	
	误差	330	845.10	0.96			
分枝角度	无性系	55	14 469.00	258.40	1.27	0.090	0.21
	区组	6	16 304.00	1 630.40	8.03	<0.001	
	误差	330	178 829.00	203.00			
分枝数	无性系	55	711.00	12.70	1.92	<0.001	0.48
	区组	6	294.00	29.37	4.45	<0.001	
	误差	330	5 628.00	6.61			
分枝大小	无性系	55	48.60	0.87	1.74	<0.001	0.42
	区组	6	12.30	1.23	2.46	0.010	
	误差	330	436.50	0.50			
叶片疏密	无性系	55	23.51	0.42	1.86	<0.001	0.46
	区组	6	11.01	1.10	4.88	<0.001	
	误差	330	198.68	0.23			
树形	无性系	55	32.60	0.57	1.35	0.070	0.32
	区组	6	13.30	1.03	1.26	0.060	
	误差	30	236.50	0.60			

2.3 2 代红锥种子园生长和形质性状的相关性分析

对不同红锥无性系生长和形质性状的相关性分析结果(表 3)表明，不同红锥无性系树高与胸径、冠幅、分枝数之间存在极显著正相关性(P<0.001)；胸径与冠幅、分枝数、分枝大小和叶片疏密之间存在极显著正相关性(P<0.001)；叶片疏密与分枝角度、分枝数和分枝大小间存在明显的正相关性(P<0.05 或 P<0.01)。冠幅与分枝角度，尖削度与分枝大小间存在显著的负相关(P<0.001 或 P<0.05)，但相关值较低，介于–0.13~ –0.28。可见红锥无性系生长量对分枝数、分枝大小和叶片疏密具有较大的影响，但对尖削度、通直度、分枝角度和树形影响不大。

2.4 2 代红锥种子园生长和形质性状的主成分分析

主成分分析结果(表 4)表明：第 1 主成分的特征值为 3.18，方差贡献率为 44%，代表了全部信息的 44%。第 2 主成分的特征值为 1.77，方差贡献率为 28%，代表了全部信息的 28%。第 3 主成分的特征值为 1.29，方差贡献率为 13%，代表了全部信息的

http://xuebao.scau.edu.cn

表3 2代种子园红锥生长和形质性状间的相关性分析 1)

Tab. 3 Correlation analysis of different growth and form traits of *Castanopsis hystrix* in the second generation seed orchard

性状	树高	胸径	尖削度	冠幅	通直度	分枝角度	分枝数	分枝大小	叶片疏密
胸径	0.51***								
尖削度	0.20	0.04							
冠幅	0.60***	0.63***	0.19						
通直度	0.06	0.01	0.39**	0.07					
分枝角度	0.06	0.04	–0.14	–0.13***	–0.22				
分枝数	0.50***	0.51***	–0.06	0.44*	0.22	0.08			
分枝大小	0.11	0.60***	–0.28*	0.32	–0.07	–0.16	0.31*		
叶片疏密	0.22	0.55***	0.20	0.22	–0.09	0.29*	0.40**	0.35**	
树形	0.22	0.17	0.18	0.11	0.17	0.00	0.21	–0.16	–0.02

1)*、**和***分别表示0.05、0.01和0.001水平显著相关(简单相关系数法)。

表4 2代种子园红锥生长和形质性状间的主成分分析

Tab. 4 Principal component analysis of growth and form traits of *Castanopsis hystrix* in the second generation seed orchard

特征向量	PC1	PC2	PC3
树高	0.71	0.23	0.15
胸径	0.88	–0.16	–0.06
尖削度	0.15	0.73	0.16
冠幅	0.77	0.14	–0.19
通直度	0.12	0.68	–0.22
分枝角度	0.04	–0.34	0.84
分枝数	0.74	0.02	0.07
分枝大小	0.55	–0.50	–0.50
叶片疏密	0.61	–0.26	0.35
树形	0.23	0.49	0.28
特征值	3.18	1.77	1.29
解释的方差贡献率/%	44	28	13
累积贡献率/%	44	72	85

13%。前3个主成分的特征值均大于1，且累积方差贡献率为85%，包含了全部指标的大部分信息，因此可以选取前3个主成分作为红锥生长和形质性状选择的综合指标。

在第1主成分中，胸径、树高、冠幅占有较高载荷，说明第1主成分反映的是与生长量有关的指标信息，可称为生长量因子。在第2主成分中，尖削度、通直度占有较高的载荷，说明第2主成分反映了与树形有关的信息，可称为树形因子。在第3主成分中，分枝角度占有较高的载荷，反映的是与侧枝有关的指标信息，可称为侧枝因子。由此可知，红锥无性系之间的差异主要表现在生长量、树形和侧枝。

2.5 2代红锥种子园生长和形质性状的聚类分析

以所测的10个生长和形质性状的平均值为参数，采用系统聚类法对56个红锥无性系进行聚类分析，结果见图1。10个生长和形质性状采用系统聚类能够将56个无性系完全区分开，说明所研究的生长和形质性状能够反映各个样品间的差异。取阈值为15时，56份红锥无性系可分为4组：第1组包含8份材料，约占总样本数的14.3%；第2组包含

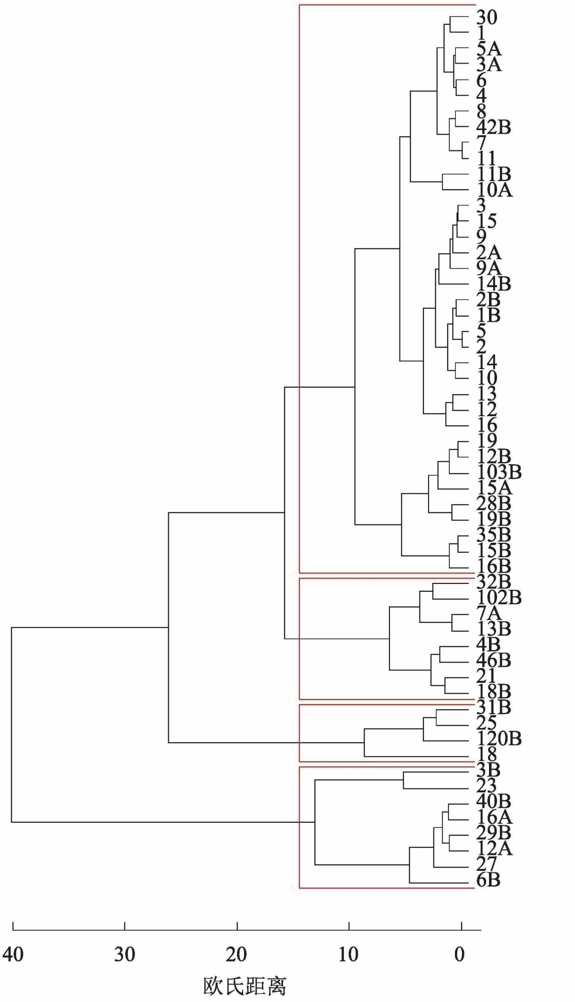

图1 2代种子园红锥56个无性系的生长和形质性状的聚类

Fig. 1 Cluster dendrogram based on growth and form traits of 56 clones in the second generation seed orchard of *Castanopsis hystrix*

http://xuebao.scau.edu.cn

4 份材料，约占总样本数的 7.1%；第 3 组包含 8 份材料，约占总样本数的 14.3%；第 4 组包含 36 份材料，分为 3 个亚组，约占总样本数的 64.3%。由此可见，56 个红锥无性系具有各自的生长和形质表型特征，这为开展红锥种内杂交育种的亲本选择奠定基础。

3 讨论与结论

通过对 2 代红锥种子园 56 份无性系单株生长和形质性状调查表明，10 个生长和形质性状的变异系数介于 11%~47%，方差分析也表明各无性系不同性状间差异达显著或极显著水平，不同性状的重复力为 0.21~0.53。大量研究表明林木种子园不同无性系间的生长性状和形质性状间存在显著差异[11-12]。廖焕琴等[9]发现红锥 1.5 代种子园无性系间的不同性状表现出显著或极显著差异。陈苏英等[13]的研究发现 1.5 代杉木种子园不同无性系的生长性状(树高、胸径、单株材积)间存在显著差异。孙文生[14]的研究发现红松 *Pinus koraiensis* 种子园各无性系间的结实量存在极显著差异，立地条件的不同、管理方式差异、树龄及不同无性系对种子园结实量具有显著影响。因此，有必要对红锥种子园内各无性系植株进行调查与清理，淘汰不适合本地生长的无性系植株。

各生长和形质性状间存在不同程度的相关性。树高与胸径、冠幅与树高、胸径间极显著正相关，相关系数分别为 0.51、0.60 和 0.63。胸径与分枝数、分枝大小和叶片疏密间也存在极显著正相关，相关系数介于 0.51~0.60。这与廖焕琴等[9]的研究结果一致。冠幅与分枝角度、尖削度与分枝大小存在显著的负相关，相关值较低。可见，对相关性系数较高的性状进行选择时，对一个性状的改良，可能同时影响另一个表型性状。因此，在对生长和形质等综合性状进行改良时，应关注性状间的正负相关性并充分利用这些相关性提高林木育种效率。

对 56 份红锥无性系生长和形质性状的主成分分析结果表明，前 3 个主成分累积贡献率达 85%，因此所选的前 3 个性状具有较强的代表性。大量研究表明主成分分析结果与参试资源和性状指标的选择有关系[15]。从对 56 份红锥无性系的主成分分析可以看出，不同性状在 3 个主成分中具有明显不同的载荷值，载荷值的绝对值越大，说明该因子对当前变量的影响程度越大[16]，因此可以将 9 个生长和形质性状归类为：第 1 主成分以胸径性状为主，第 2 主成分以尖削度为主，第 3 主成分以分枝角度性状为主。由此可知，红锥无性系之间的差异主要表现在生长量、尖削度和分枝角度。

参考文献：

[1] 中国树木志编委会. 中国要树种造林技术[M]. 北京: 中国林业出版社, 1981: 520-524.
[2] 郑万均. 中国树木志: 第 1 卷[M]. 北京: 中国林业出版社, 1983.
[3] 《广东森林》编辑委员会. 广东森林[M]. 广州: 广东科技出版社, 1990.
[4] 王明怀, 陈建新. 红锥等 8 个阔叶树种抗旱生理指标比较及光合作用特征[J]. 广东林业科技, 2005, 21(2): 1-5.
[5] 黄永权, 梁东成, 张方秋. 广东省红锥遗传改良进展及改良策略初探[J]. 广东林业科技, 2004, 20(4): 58-60.
[6] 王明庥. 林木育种学概论[M]. 北京: 中国林业出版社, 1989.
[7] 周邦社,杨新兵.植被和坡向对土壤温度与土壤热通量变化的影响[J].河北农业大学学报,2011,34(2):80-85.
[8] 潘勇军, 王兵, 陈步峰, 等. 短轮伐期桉树人工林土壤温度特征分析[J].林业科学研究,2011,24(3):404-409.
[9] 廖焕琴, 张卫华, 张方秋, 等. 红锥 1.5 代改良种子园无性系生长和形质性状变异分析[J]. 林业与环境科学, 2016, 32(4): 23-27.
[10] 黄少伟, 谢维辉. 实用 SAS 编程与林业试验数据分析[M]. 广州: 华南理工大学出版社, 2001.
[11] 崔宝禄, 杨俊明, 郑辉, 等. 我国针叶树种子园结实量的研究进展[J]. 河北林果研究, 2005, 20(2): 120-123.
[12] 曹汉洋. 杉木第 2 代种子园半同胞子代测定及早期选择[J]. 南京林业大学学报(自然科学版), 2011, 35(1): 19-23.
[13] 陈苏英, 马祥庆, 吴鹏飞, 等. 1.5 代杉木种子园不同无性系生长和结实性状的评价[J]. 热带亚热带植物学报, 2014, 22(3): 281-291.
[14] 孙文生.红松种子园优质高产经营技术研究[D].北京: 北京林业大学, 2006: 1-100.
[15] 刘玉皎, 宗绪晓. 青海蚕豆种质资源形态多样性分析[J]. 植物遗传资源学报, 2008, 9(1): 79-83.
[16] 王明明, 王建华, 宋振巧, 等. 木瓜属品种资源的数量分类研究[J]. 园艺学报, 2009, 36(5): 701-710.

【责任编辑　李晓卉】

http://xuebao.scau.edu.cn

word版下载：http://www.ixueshu.com
