# Supplementary material for: Provenance and family variations in early growth of Manchurian walnut (Juglans mandshurica Maxim.) and selection of superior families
Source: PLoS One. 2024 Mar 7;19(3):e0298918. doi: 10.1371/journal.pone.0298918 (PMC10919699; doi:10.1371/journal.pone.0298918)
Supplement: S1 File — (ZIP) [file pone.0298918.s004.zip › Genetic Variation in Growth Traits in a Quercus robur L. Open-Pollinated Progeny Test of the Slavonian Provenance.pdf]

Genetic Variation in Growth Traits in a *Quercus robur* L. Open-Pollinated Progeny Test of the Slavonian Provenance

By S. BOGDAN, I. KATIČIĆ-TRUPČEVIĆ and D. KAJBA*

University of Zagreb, Faculty of Forestry, Department of Forest Genetics and Dendrology
Svetošimunska 25, HR-10000 Zagreb, Croatia

(Received 4th July 2003)

Abstract

The study presents evaluation of an open-pollinated progeny test of 21 selected plus trees from Slavonian pedunculate oak provenance in Croatia (in the central part of Drava river valley). The test was established in 1992 with two-year-old seedlings. Heights were measured at 5 to 13 years and diameters at breast height (DBH) at 10 to 13 years after sowing.

Variances caused by the population within provenance effect were not significant during the studied period. In contrast, variance components caused by family effect were statistically significant, and ranged from 11.1 to 18.6% and from 2.2 to 10.6% for height and DBH respectively. Statistically significant variances caused by the family effect indicate that most of genetic variation of productive traits was within the studied populations.

The estimated family mean narrow sense heritabilities varied from 0.62 to 0.78 and from 0.28 to 0.65, while individual heritabilities ranged from 0.48 to 0.80 and from 0.09 to 0.46 for height and DBH, respectively.

Realised gain from the test and expected genetic gains by two possible methods of selection for the measured traits have been calculated for: *i.*) realised gain i.e. superiority of selected plus trees progenies over control plants (bulks from unselected trees within the provenance) *ii.*) individual within provenance mass selection of first generation plus trees at the same ages as those represented in studied trial and *iii.*) backward selection among first generation plus trees after open-pollinated testing. Estimated genetic gains indicate that the highest gain could be expected by backward selection among first generation plus trees after open-pollinated progeny testing (9.7 to 22.3% and 6.8 to 17.3% over control means).

These results indicate that due to significant within population variation and high additive variances, improvement for productive traits in the studied oak populations could be achieved by use of seeds and plants from selected plus trees (especially from already established clonal seed orchard), at least in younger ages.

Key words: Quercus robur, open-pollinated progeny test, genetic variation, genetic parameters, selection.

Introduction

Pedunculate oak (*Quercus robur* L.) is, along with *Q. petraea* and *Q. suber*, the most important European oak species. Despite its importance, it has a relatively low representation in breeding programmes. The reasons for this include its long rotation length, irregular seed production, difficulties in storing seeds over a long period and difficulties with vegetative propagation. Knowledge of the amount and pattern of the genetic variability of pedunculate oak quantitative traits is limited, although progeny tests have been established relatively recently (SAVILL and KANOWSKI, 1993; VIDAKOVIĆ et al., 2000).

The most valuable pedunculate oak stands in Croatia are situated in Slavonia, between the rivers Sava, Drava and Danube. In contrast to other oak forests in Europe, this region has not been under forestry management until the second half of the 19th century because it had been part of the Military Frontier District i.e. buffer zone between the Austro-Hungarian and the Turkish Empires (KLEPAC, 1996). Major exploitation of oak wood in the area began in 1871 and from the beginning natural regeneration has been applied using local seed sources. Therefore genetic resource and structure of these stands uniquely reflects the original conditions of this lowland oak populations in Europe. In Central Europe, especially in Hungary, pedunculate oak of Slavonian origin has been extensively planted since the end of the 19th century. It has proven its genetic superiority based on the exceptionally straight stem, relatively regular crown and ascending branches. Although some Hungarian botanists considered this provenance as a subspecies, recent genetic studies do not confirm this hypothesis. The superiority of Slavonian oak provenance is probably due to fact that large seed quantities imported in Hungary originated from untouched forests of primeval character, where selective cutting did not have an effect on the genetic composition of the stands (MÁTYÁS, 1997).

In the 1950s natural oak stands in Croatia were evaluated in terms of wood productivity and quality. The results and additional ecological studies delineated three seed zones and seven seed regions (GRAČAN et al., 1995). Over the past decades, the periodicity of seed crops has not been regular, which has limited natural regeneration and artificial planting in partial naturally regenerated stands. In order to increase genetic quality and reduce seed periodicity, it was decided to establish a clonal seed orchard (VIDAKOVIĆ, 1996).

Forty plus trees were selected in the seed region of Central Drava valley (aprox. 19,500 ha) based on 10 evaluated traits (height, DBH, volume, trunk length, branching habit, branch thickness, occurrence of spiral grain, stem straightness, forking and health). For growth traits, plus trees had to be at least one standard deviation superior to stand means. Quality traits were evaluated by an expert team. The first clonal seed orchard was established near town of Nasice in the same seed zone.

Research results concerning genetic parameters during tree development and the success of early selection in different forest tree species are described in numerous papers. Most research has been done on conifers (among recent publications see e.g. LEE et al., 2002; LEE et al., 2002a; MATHESON et al., 2002). However, the knowledge of genetic parameters evaluated in pedunculate oak sib and/or clonal tests is limited (NEPVEU, 1984; JENSEN et al., 1997). The goal of this study is to investigate within/among population genetic variation in this

*) phone: 00385 1 235 2537, fax: 00385 1 235 2505
e-mail: sasa.bogdan@zg.htnet.hr
katicicida@hotmail.com
davorin.kajba@zg.htnet.hr

DOI:10.1515/sg-2004-0036
edited by Thünen Institute of Forest Genetics

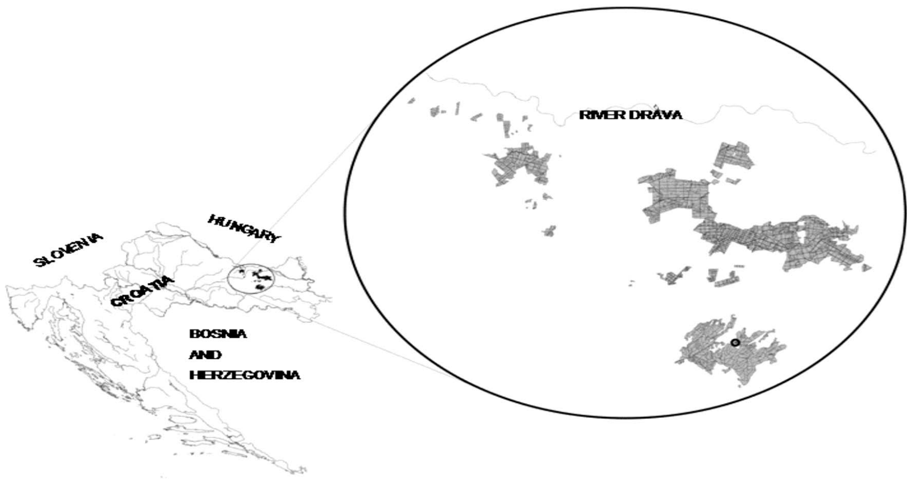

Figure 1. – Map of the Central Drava Valley seed region where plus trees were selected.

valuable part of the species range, to identify trends in genetic parameters and heritabilites of growth traits as well as possibilities for improvement of these traits based on different selection strategies.

Material and Methods

Material

Of a total of 40 selected plus trees from the seed region of Central Drava Valley (*Figure 1*), acorns were collected from 21 trees that yielded abundant seed quantities in 1989. An open-pollinated progeny test was planted in the spring of 1992 with 2 years old plants in a spacing of 2.0 x 2.0 m with 4 plants per square plot. The experimental design was a randomised complete block system with 5 replications. The test also included bulk seed lots from three natural populations from the same seed region (populations Koska, Slatina and Donji Miholjac), which served as a control.

Measurements

Heights of each plant were measured every second year from 5 to 9 years after sowing (H5, H7, H9), and annually from 10 to 13 yrs after sowing (H10, H11, H12, H13). Diameters at breast height were measured annually from year 11 after sowing (DBH11, DBH12 and DBH13).

Statistical analysis

The analysis of variance was done with the GLM procedure using the linear model:

$$Y_{ijk} = \mu + f_i + R_j + I_{ij} + \varepsilon_{ijk}; \qquad (1)$$

where: Y_{ijk} – individual tree observation; μ – overall mean; F_i – effect of the *i-th* open pollinated family; R_j – the effect of the *j-th* replication I_{ij} – the family x replication interaction; ε_{ijk} – the random error effect. All studied effects were considered random and normally distributed.

Estimation of genetic parameters

The REML method of VARCOMP procedure was used to estimate the variance components of all effects. All statistical analyses were done with the SAS software for Windows release 8.2. (SAS INSTITUTE INC., 1989). Additive and phenotypic variances were estimated with the usual formulae (WRIGHT, 1976; BECKER, 1992; FALCONER and MACKAY, 1996):

$$\sigma_f^2 = \tfrac{1}{4} V_A; \qquad (2)$$

$$V_{PH} = \sigma_f^2 + \sigma_I^2 + \sigma_e^2; \qquad (3)$$

where σ_f^2 is family variance component; V_A is additive genetic variance; V_{PH} is phenotypic variance; σ_I^2 is variance component due to the family x replication interaction and σ_e^2 is variance due to the random error.

Narrow sense heritability was calculated on the basis of individual tree values (h_i^2) and family means (h_f^2) in the manner described in more detail in COTTERILL and ZED (1980) and elsewhere:

$$h_i^2 = \frac{4\sigma_f^2}{\sigma_f^2 + \sigma_I^2 + \sigma_e^2}; \qquad (4)$$

$$h_f^2 = \frac{\sigma_f^2}{\sigma_f^2 + \frac{k_2\sigma_I^2}{k_1} + \frac{\sigma_e^2}{k_1}}; \qquad (5)$$

where: k_1, k_2 are coefficients for σ_f^2 and σ_I^2, respectively, in the expected mean square for the family effect (proc GLM).

Response to selection

The realised gain from the test and expected genetic gains by two possible methods of selection for the measured traits were calculated by three methods which included:

i) realised gain was calculated as the difference between means of selected plus tree progenies and means of unselected controls (bulk seed lots from three natural populations within the provenance).

ii) expected genetic gain by individual within provenance mass selection of first generation plus trees at the same ages as those represented in studied trial. The applied formula is (JENSEN et al., 1997):

$$\Delta G_1 = i\,\sigma_P\, h_i^2; \qquad (6)$$

Where σ_P and h_i^2 refers to individuals in sample plots of size and environment comparable to analysed field trial. *i* is standardised selection intensity of one.

iii) expected genetic gain by backward selection among first generation plus trees. It is assumed that plus trees are put into clonal seed orchard and genetic thinning will be performed

DOI:10.1515/sg-2004-0036
edited by Thünen Institute of Forest Genetics

Table 1. – Height (H) and diameter at breast height (DBH) of open-pollinated progenies as well as control plants from natural populations (DM = population Donji Miholjac; K = population Koska; PS = population Podravska Slatina) at various ages from sowing.

family	H5 (cm)	H7 (cm)	H9 (cm)	H10 (cm)	H11 (cm)	H12 (cm)	H13 (cm)	DBH11 (cm)	DBH12 (cm)	DBH13 (cm)
4	121.0	246.7	375.4	461.0	472.0	540.5	586.5	6.1	7.3	8.7
6	113.4	218.8	321.9	406.8	413.2	498.3	537.9	5.0	6.3	7.6
22	95.3	200.6	302.5	380.5	387.1	477.4	528.4	5.1	6.5	7.6
21	106.5	214.9	319.5	386.8	400.0	471.5	526.0	5.0	6.4	7.6
10	108.3	221.9	303.0	384.0	398.8	482.8	511.0	5.4	6.5	7.8
2	101.9	203.1	303.5	373.8	387.3	473.9	503.7	5.1	5.9	7.5
1	101.7	197.2	290.3	363.2	380.5	455.6	498.9	4.7	5.5	7.0
17	108.2	216.6	305.3	369.8	377.0	458.2	495.3	4.8	5.7	7.1
26	101.4	207.8	316.4	361.3	379.5	462.6	487.5	4.9	5.7	6.8
11	92.4	193.0	276.5	346.3	355.0	437.5	484.5	4.3	5.1	6.3
32	107.8	213.6	311.3	373.3	381.8	463.5	481.0	5.1	6.1	7.2
25	116.2	211.1	307.0	377.8	380.5	459.5	481.0	5.0	6.2	6.7
13	99.5	185.9	275.8	348.9	351.7	436.9	456.1	4.6	5.3	6.3
19	100.5	195.4	274.7	333.0	338.8	427.4	449.5	4.5	5.6	6.6
24	101.6	190.3	273.9	319.5	327.9	436.3	432.6	4.7	4.9	6.1
20	93.7	190.2	268.9	327.8	333.0	398.9	424.0	4.4	5.1	5.9
39	109.4	190.6	277.0	335.0	327.4	416.5	406.3	4.3	4.9	5.6
38	86.4	178.8	246.4	298.0	315.5	400.6	403.7	3.9	4.6	6.2
7	63.1	130.3	164.3	215.6	230.3	352.2	379.3	3.2	4.0	4.7
18	73.1	161.0	221.4	278.6	309.2	385.9	371.2	3.3	4.1	4.9
40	56.5	135.1	193.4	296.5	275.9	342.7	365.6	3.0	3.6	4.8
**mean**	**98.0**	**195.4**	**282.3**	**349.4**	**358.2**	**441.8**	**467.1**	**4.8**	**5.5**	**6.6**
DM	97.9	181.1	256.2	325.5	335.3	427.2	462.1	4.3	5.1	6.6
K	100.3	197.8	285.7	350.8	357.0	447.8	469.0	4.9	5.4	6.6
PS	91.5	183.4	281.3	342.2	356.7	424.7	460.5	4.7	5.4	6.4
**mean**	**96.6**	**187.4**	**274.4**	**339.5**	**349.7**	**433.2**	**463.9**	**4.6**	**5.3**	**6.5**

after open-pollinated testing of clones. The applied formula is (JENSEN et al., 1997):

$$\Delta G_2 = 2(i\,\sigma_P\,h_f^2); \qquad (7)$$

Where σ_P and h_f^2 refers to families in sample plots of size and environment comparable to analysed field trial. i is standardised selection intensity of one.

Results and Discussion

Growth measurements

The mean height values of the studied open-pollinated families are shown in *Table 1.* The individual plant heights in the test varied from 97.4 cm at age 5 years after sowing (H5) to 465.5 cm at age 13 years (H13). The ranking of the studied families according to height did not change significantly during the investigated period. The same was observed in the diameter at breast height.

Families "4" and "6", displayed the best results during the entire study period, likewise the families "38", "18", "40" and "7" showed the poorest growth values. Competition has not occurred because survival rates were around 90 %.

Estimates of variance components and genetic parameters

The variance components due to population and family effects are shown as a percent of the total variability in *Table 2.* It can be seen that variances caused by population effect were not significant during investigated period. KRAHL-URBAN (1959) found high heritabilities in *Quercus robur* progeny tests i. e. significant individual variation of forking, multiple leader and stem straightness. Moreover, molecular studies on *Q. petraea* and *Q. robur* showed that most of the nuclear gene diversity (74 %) is distributed within populations (KREMER and PETIT, 1993). Statistically significant variances caused by family effect show that most of genetic variation is within populations of Slavonian oak. ERIKSSON and EKBERG (2001) presented a graph on within/among population genetic variation for random mating populations with varying strength of natural selection and population gene flow. Climax species such as *Q. robur* with large stands and with strong gene flow under random mating would be expected to have a large within population variation. Despite a constant decrease and fragmentation of the oak forests since the end of 19th century, results of this study suggest a good genetic condition of Slavonian provenance. Molecular analysis should confirm this indication.

Family (h_f^2) as well as individual (h_i^2) narrow sense heritabilities at various ages are shown in *Table 3.* The estimated heritabilities for height remained relatively high over the entire study period. Heritabilities for diameter at breast height were somewhat lower. Height is under stronger genetic control which is in accordance with many other studies on forest species.

Table 2. – The percentage of variance components for plant height and DBH at population and family levels at various ages. (^{ns} = not significant. **, *** = significant at 1% and 0.1% levels, respectively).

Trait	Population	Family
H5	0.0^{ns}	14.4^{***}
H7	0.0^{ns}	13.5^{***}
H9	0.0^{ns}	18.6^{***}
H10	0.0^{ns}	16.2^{***}
H11	0.2^{ns}	15.1^{***}
H12	1.6^{ns}	11.1^{***}
H13	2.2^{ns}	12.1^{***}
DBH11	0.9^{ns}	2.2^{ns}
DBH12	0.0^{ns}	10.6^{***}
DBH13	0.9^{ns}	8.7^{**}

DOI:10.1515/sg-2004-0036
edited by Thünen Institute of Forest Genetics

Table 3. – Heritabilities, realised response and expected genetic gains for measured quantitative traits at various ages.

	H5	H7	H9	H10	H11	H12	H13	DBH11	DBH12	DBH13
h_i^2	0.62	0.59	0.80	0.72	0.66	0.48	0.52	0.09	0.46	0.37
h_f^2	0.70	0.74	0.78	0.76	0.74	0.62	0.67	0.28	0.65	0.58
R (%)	1.5	4.2	2.9	2.9	2.4	2.0	0.7	3.2	3.6	1.3
ΔG_1 (%)	17.5	16.4	21.4	17.9	16.6	9.2	11.7	7.2	16.1	12.0
ΔG_2 (%)	18.8	17.7	22.3	18.8	17.6	9.7	12.6	6.8	17.3	12.9

Realised gains (R) measured as the difference between plus trees progeny means and mean of control plots are presented as a percentage of the control in *Table 3.* It showed a positive but quite low values (0.7 to 4.2% for height and 1.3 to 3.2% for DBH). Although the studied traits are under relatively strong genetic control, plus tree progeny means are not much higher than the control plants from natural populations, probably because of mating with inferior individuals. Another reason could be the small number of plants in family or control plots, which may not represent a true sample of original populations.

Significantly higher genetic gains could be expected using individual mass selection of first generation plus trees (ΔG_1) in environments comparable to the field trial. The estimated expected genetic gains by backward selection among first generation plus trees after open-pollinated progeny testing (ΔG_2) showed the highest values. Estimated genetic gains are presented as percentages of control plots means (*Table 3*). Because oaks are grown for long rotations the problem of early selection for late expressed traits is serious and therefore breeding oaks for production traits alone is not recommended by JENSEN et al. (1997). However, the results suggest that owing to significant within population variation and additive variances, improvement for productive traits in oak populations could be expected by the use of seeds and plants from selected plus trees (especially from already established clonal seed orchards).

Conclusions

Genetic variability of pedunculate oak assessed by analysis of quantitative traits in an open-pollinated progeny test did not show differentiation between sampled populations in part of its distribution range in Croatia (Central Drava Valley seed region). Most of the genetic variation has been observed within populations. These as well as the estimated genetic gains indicate possibilities for improvement by individual mass selection of plus trees and establishment of a clonal seed orchard.

Acknowledgement

We thank to professor CSABA MÁTYÁS and two anonymous referees for helpful suggestions.

References

BECKER, W. A. (1992): Manual of Quantitative Genetics. Academic Enterprises. Pullman, USA. pp. 191.

COTTERILL, P. P. and P. G. ZED (1980): Estimates of genetic parameters for growth and form traits in four *Pinus radiata* D. Don progeny tests in South Australia. Aust. For. Res. **10**: 155–167.

ERIKSSON, G. and I. EKBERG (2001): An Introduction to Forest Genetics. SLU, Uppsala. Chapter **6**: 60–61.

FALCONER, D. S. and T. F. C. MACKAY (1996): Introduction to Quantitative Genetics. Longman Group London. 464 p.

GRAČAN, J., A. KRSTINIĆ, S. MATIĆ, Đ. RAUS and Z. SELETKOVIĆ (1995): Šumski sjemenski rajoni u Hrvatskoj. "Hrvatske šume" Zagreb, p. 111, (In Croatian).

JENSEN, J. S., H. WELLENDORF, K. JAGER, S. M. G. DE VRIES, V. JENSEN (1997): Analysis of a 17-year Old Dutch Open-Pollinated Progeny Trial With *Quercus robur* L. Forest Genetics **4**(3): 139–147.

KLEPAC, D. (1996): Old pedunculate oak forests and their contribution in the development of Croatia. *In:* KLEPAC, D. (ed.) Pedunculate oak in Croatia. Hrvatska Akademija Znanosti i Umjetnosti, 'Hrvatske šume' d.o.o. Vinkovci-Zagreb. 377–384.

KRAHL-URBAN, J. (1959): Die Eichen. Paul Parey Verlag, Hamburg, 288.

KREMER, A. and R. J. PETIT (1993): Gene diversity in natural populations of oak species. Ann. Sci. For. 50, Suppl 1, 186–202.

LEE, S. J., J. WOOLLIAMS, C. J. A. SAMUEL and D. C. MALCOLM (2002): A study of population variation and inheritance in Sitka spruce II. Age trends in genetic parameters for vigour trends and optimum selection ages. Silvae Genetica **51**(2–3), 55-72.

LEE, S. J., J. WOOLLIAMS, C. J. A. SAMUEL and D. C. MALCOLM (2002a): A study of population variation and inheritance in Sitka spruce III. Age trends in genetic parameters and optimum selection ages for wood density, and genetic correlations with vigour traits. Silvae Genetica **51**(4), 143–151.

MÁTYÁS, C. (1997): Homage to the oaks of Slavonia: the last relics of the Central European lowland primeval forests. Forest Genetics **4**: 2, 58 p.

MATHESON, A. C., H. X. WU, D. J. Spencer, C. A. RAYMOND and A. R. GRIFFIN (2002): Inbreeding in *Pinus radiata.* III. The effect of inbreeding on age-age correlation and early selection efficiency. Silvae Genetica **51**(2–3), 115–127.

NEPVEU, G. (1984): Contrôle héréditaire de la densité et de la rétractibilité du bois de trois espèces de Chêne (*Quercus petraea*, *Quercus robur* et *Quercus rubra*). Silvae Genetica **33**(4–5), 110–115.

SAS INSTITUTE INC. (1989): SAS User's guide: Statistics 1989 Edition. Cary, NC, USA.

SAVILL, P. S. and P. J. KANOWSKI (1993): Tree improvement programs for European oaks: goals and strategies. Ann. Sci. For. **50**, Suppl 1, 368–383.

VIDAKOVIĆ, M., D. KAJBA, S. BOGDAN, V. PODNAR and J. BEĆAREVIĆ (2000): Estimation of genetic gain in a progeny trial of pedunculate oak (*Quercus robur* L.). Glas. Šum. Pokuse, Vol. **37**: 375–381.

VIDAKOVIĆ, M. (1996): Establishment of clone seed orchard of pedunculate oak. *In:* KLEPAC, D. (ed.) Pedunculate Oak (*Quercus robur* L.) in Croatia. HAZU, Centar za znanstveni rad Vinkovci i "Hrvatske šume" d.o.o. Vinkovci – Zagreb 1996, 127–138.

WRIGHT, J. W. (1976): Introduction to Forest Genetics. Academic Press, New York. 463 p.

DOI:10.1515/sg-2004-0036
edited by Thünen Institute of Forest Genetics
