# Supplementary material for: Provenance and family variations in early growth of Manchurian walnut (Juglans mandshurica Maxim.) and selection of superior families
Source: PLoS One. 2024 Mar 7;19(3):e0298918. doi: 10.1371/journal.pone.0298918 (PMC10919699; doi:10.1371/journal.pone.0298918)
Supplement: S1 File — (ZIP) [file pone.0298918.s004.zip › Genetic variation of Fraxinus angustifolia natural populations in Greece based on nuclear and chloroplast microsatellite markers.pdf]

Eur J Forest Res (2012) 131:1151–1161
DOI 10.1007/s10342-011-0586-1

ORIGINAL PAPER

Genetic variation of *Fraxinus angustifolia* natural populations in Greece based on nuclear and chloroplast microsatellite markers

R. M. Papi · K. A. Spanos · D. A. Kyriakidis

Received: 15 March 2011 / Revised: 24 September 2011 / Accepted: 2 November 2011 / Published online: 18 December 2011
© Springer-Verlag 2011

Abstract Assessment of genetic variation within and among populations is an essential parameter for the effective conservation of forest genetic resources. In this work, the genetic diversity within and among natural populations of the forest tree species *Fraxinus angustifolia* Vahl in Greece was studied using selected nuclear and chloroplast microsatellite DNA loci. Eight natural populations of *F. angustifolia* were identified in different locations of the mainland of Greece, and a total of 230 individuals were studied. High polymorphism was observed within populations, while the genetic differentiation among populations was moderate. Intra-population diversity was correlated with geographical coordinates, but no isolation by distance was observed. Of the three haplotypes identified, only one was dominant. Putative ancestral haplotypes were found at small spatial scales suggesting that population expansions could have originated in the region. This study located in sympatry haplotypes that in other parts of Europe are in allopatry, reinforcing the notion of population expansions from the south of Balkans including Greece. Suggestions for conservation and management of *F. angustifolia* are also reported.

Keywords *Fraxinus angustifolia* · Genetic diversity · Population differentiation · Nuclear microsatellite loci · Chloroplast microsatellite loci

Communicated by R. Matyssek.

R. M. Papi · D. A. Kyriakidis (✉)
Laboratory of Biochemistry, Department of Chemistry, Aristotle University of Thessaloniki, 54124 Thessaloniki, Greece
e-mail: kyr@chem.auth.gr

R. M. Papi · K. A. Spanos
Forestry Institute, National Agricultural Research Foundation, Loutra Thermis, Greece

Introduction

The Mediterranean basin has been characterized as a "hot spot" wealth of genetic diversity and the major area for the conservation of the European gene resources (Petit et al. 2003). Forest biodiversity is also very high in this region, where more than 100 tree species can be found compared to <30 species located in European temperate forests (Fady-Welterlen 2005). Moreover, the anticipated rapid environmental changes are likely to force Mediterranean tree species to migrate to higher latitudes (Petit et al. 2005), although diverse forest tree populations seem to adapt well to rapid environmental changes (Mueller-Starck 1989). Thus, conservation of Mediterranean species and their genetic resources is a very important issue, and the assessment of their genetic structure and diversity for conservation and restoration is needed. In addition to genetic variation, information on mating system and pollen and seed dispersal are very important.

Microsatellite markers have been widely used to study population genetics, evolutionary processes and conservation or management of biological resources (Jarne and Lagoda 1996). Nuclear microsatellites (nSSRs) are multi-allelic within and among populations and inherited in a codominant fashion (Morgante and Olivieri 1993). In contrast, chloroplast microsatellites (cpSSRs) are usually maternally inherited, and therefore, they are useful in phylogenetic and evolutionary studies (Newton et al. 1999; FRAXIGEN 2005).

Fraxinus angustifolia Vahl, the narrow-leaved ash, belongs to the *Oleaceae* family (Wallander and Albert 2000; FRAXIGEN 2005). Two other *Fraxinus* species are native in Europe, *Fraxinus excelsior* L., the common ash, and *Fraxinus ornus* L., the flowering manna ash.

F. angustifolia is closely related to *F. excelsior,* and sometimes the two species are morphologically confused. *F. angustifolia* is hermaphrodite with variable morphology that enabled the establishment of several taxa based on differences in the samara and leaf morphology. It is reproduced by seed, stump sprouts, stem sprouts and often by root sprouts in flooding soils. It has a preference for humid or temporary flooding sites, and it occurs throughout most of the Iberian Peninsula, on the Mediterranean coasts of France, Italy and Greece, in the coastal areas of Morocco, Algeria and Tunisia and in the Caspian Sea (Wallander 2001; Tutin et al. 1972; FRAXIGEN 2005). *Fraxinus* species can be used in reforestation, restoration of degraded riparian ecosystems and temporary flooding areas, and therefore, selection of genetic material with adaptability and good growth in different ecological environments is of highly importance (FRAXIGEN 2005; Spanos et al. 2004).

In the past, deciduous riparian and lowland water friendly mixed forests of *F. angustifolia* in Greece (e.g. *Ulmus minor, U. laevis, Fraxinus angustifolia*, *Salix* spp., *Populus alba*, *Populus nigra, Populus canescens, Quercus robur*) covered large areas (Spanos et al. 2004). These forests were found on fertile soils with plenty of soil water. Some decays ago, most of these forests have been deforested and converted into rich agricultural or grazing lands. This is the main reason for today scattered and limited distribution of *F. angustifolia* in S. Europe and particularly in Greece (FRAXIGEN 2005). The species today is considered as a threatened noble hardwood and needs urgent conservation according to the National Forest Law with emphasis to in situ conservation (Spanos et al. 2004; FRAXIGEN 2005).

The present study aimed to assess the genetic variation of *F. angustifolia* Vahl, in Greece. We have selected eight populations, the most known as remnant, mature and old grown, distributed from Northern to Southern Greece. These selected populations are the least disturbed and show high naturalness and biodiversity value. The number of populations chosen is adequate to make genetic studies and to draw valid conclusions due to the scattered and fragmented distribution of *F. angustifolia* and the small geographical area of Greece. Furthermore, some of these populations (e.g. Nestos, Doirani) are under the NATURA protection status, while the rest of the populations are under strict protection (National Forest Law). Selected nSSR and cpSSR markers (Lefort et al. 1999; Brachet et al. 1999; Weising and Gardner 1999) were used for the identification of genetic diversity of *F. angustifolia* and for the definition of population structure. These data provided useful knowledge for the implementation of a conservation strategy of *F. angustifolia* genetic resources.

Materials and methods

Plant material

Samples from eight *F. angustifolia* natural populations located all over continental Greece (Table 1) were collected between June and July 2003. Populations were properly selected to fulfil the following criteria. All populations used in this study are found on alluvial plains, as shown in Table 1. In the mixed forests of *F. angustifolia,* ash in general covers 30–50% in the broader area (habitat), but in each sampled stand, ash covers >50% of all tree species, oaks, elms, white and black poplars. Therefore, ash is the dominant tree species in the sampled stand, while in the whole habitat/forest type it may be a dominant or codominant tree species. It should be detailed that 50 m is the minimal distance between sampled individuals. Data on the soil composition, the general climate, forest vegetation zone and stand type of the studied populations are presented in Table 1. Young leaves from 30 non-adjacent trees were sampled in each population, except for the population from Kalavrita, where only 20 trees were sampled due to limited access because of the water ponds created by the local river. In total, 230 individuals were sampled. After collection, leaves were immediately dried using silica gel (10–15 g/g plant material) and kept at 0–4°C prior to DNA extraction.

DNA isolation and microsatellite analysis

Total DNA was extracted from approximately 100 mg dry leaves using the DNeasy Plant Mini Kit (QIAGEN). Samples were frozen in liquid nitrogen and hand ground. To improve extraction, cell lysis was performed at 65°C for 30 min.

Five primer pairs of nSSR loci (FEMSATL4, FEMSATL11, FEMSATL16, FEMSATL19 and M2-30) that had been established previously for *F. excelsior* (Brachet et al. 1999; Lefort et al. 1999) were used in the amplification reactions. Amplification reactions were carried out as described in Heuertz et al. (2001) and performed on a MJ Research P200 thermal cycler. The forward primer of each pair was labelled with a fluorescent dye (LI-COR, IR-Dye800 and IR-Dye700) to enable the detection of PCR products in LI-COR IR^2 DNA analyser. Labelling with different dye allowed the pooling of two PCR products together in one well.

Six universal primer pairs of cpSSR loci (ccmp2, ccmp3, ccmp4, ccmp6, ccmp7 and ccmp10) that had previously been developed for angiosperms were also used (Weising and Gardner 1999). Amplification reactions were carried out as described in Heuertz et al. (2004a) with the only modifications that annealing temperature was 50°C instead of 55°C, and the final elongation was performed for 8 min. The forward primer of each pair was also labelled with a fluorescent dye (LI-COR, IR-Dye800 and IR-Dye700).

Table 1 *F. angustifolia* natural populations sampled in Greece

Code	Population name	Longitude (°)	Latitude (°)	Altitude (m)	(Forest vegetation zone)/stand type	Climate/soil	Sample size
09A	Komotini	25.23.3E	40.59.3N	10–15	(Ostryo carpinion)	Sub-Mediterranean	30
					Mixed ash, oak, elm, white poplar, oriental plane	Alluvial deposits (sandy–loamy,	
					Ash cover in the broader area: 30%	pH range: 6.0–7.0)	
10A	Nestos	24.46.5E	40.53.7N	18–20	(Ostryo carpinion)	Sub-Mediterranean	30
					Mixed, oak, ash, elm, white poplar, black poplar, oriental plane	Alluvial deposits (sandy–loamy, pH range: 6.0–7.0)	
					Ash cover in the broader area: 25–30%		
11A	Doirani	22.46.4E	41.14.7N	50	(Quercion fraineto)	Mediterranean (Thermophilous subcontinental broadleaves)	30
					Mixed, oak, ash, elm, oriental plane, alder	Alluvial deposits (sandy–clayey, pH range: 6.0–7.0)	
					Ash cover in the broader area: 30–40%		
12A	Ierissos	23.49.0E	40.27.6N	10–20	(Quercion ilicis)	Meso-Mediterranean	30
					Mixed, oak, ash, elm, white poplar, oriental plane, maple	Alluvial deposits (sandy–loamy, pH range: 6.0–7.0)	
					Ash cover in the broader area: 30%		
13A	Omolio	22.37.2E	39.53.6N	16–20	(Quercion ilicis)	Meso-Mediterranean	30
					Mixed, oak, ash, elm, white poplar, oriental plane	Alluvial deposits (silty–sandy, pH range: 6.0–7.0)	
					Ash cover in the broader area: 40–50%		
14A	Evia	23.25.3E	38.49.1N	120	(Quercion ilicis)	Meso-Mediterranean	30
					Mixed, oak, ash, elm, white poplar, oriental plane	Alluvial deposits (clayey –sandy, pH range: 6.0–7.0)	
					Ash cover in the broader area: 30%		
15A	Louros	20.45.6E	39.09.2N	50	(Quercion ilicis)	Meso-Mediterranean	30
					Mixed, oak, ash, elm, white poplar, oriental plane	Alluvial deposits (sandy–loamy, pH range: 6.0–7.0)	
					Ash cover in the broader area: 30%		
16A	Kalavrita	22.04.1E	37.56.3N	870	(Quercion fraineto)	Sub-Mediterranean	20
					Mixed, oak, ash, elm, oriental plane, white poplar, maple	Alluvial deposits (sandy–loamy, pH range: 6.0–7.0)	
					Ash cover in the broader area: 30%		

Spanos et al. (2004), FRAXIGEN (2005)

Electrophoresis and detection of PCR products were carried out on denaturing polyacrylamide gels (6.5% v/v acrylamide–bisacrylamide 38:2, 25 cm) using LI-COR IR^2 DNA analyser. Gels run for 1.5 h at 1,500 V in TBE buffer. The resulting electrophorograms were analysed with SAGA software from LI-COR.

Data analysis

Genetic diversity within populations

The statistical analysis of the nSSR data was performed with FSTAT V2.9.3.2 software (Goudet 2001). The total number of detected variants (designated here as "alleles") (N_A), the allelic richness (A_S) based on the minimum population size of 20 diploid individuals, the average observed and expected heterozygosity within populations (H_O and H_S), unbiased estimator of Nei (1987), the total gene diversity (H_T) and Wright's inbreeding coefficient (F_{IS}) were calculated for all loci and individuals in the studied populations. Alleles were characterized as private (A_p) if they presented a frequency of more than 5% in one population and did not occur in any other population (Payn et al. 2008). F_{IS} was estimated according to Weir and Cockerham (1984). Jackknifing over loci was performed to estimate standard errors. For F_{IS} and F_{IT} (Wright's inbreeding coefficient of an individual relative

to the total population), alleles were permuted among individuals within samples (1,000 permutations).

MICRO-CHECKER software (van Oosterhout et al. 2004) was used to check for genotyping errors and estimation of null alleles. The program constructs random genotypes by randomizing the observed alleles for each locus within samples and then compares the observed genotypes with the distribution of randomized genotypes. Deviations from Hardy–Weinberg (HW) equilibrium were determined for each locus in each population using GENEPOP 4.0.7 (Rousset 2008). A Markov chain algorithm, implemented in GENEPOP 4.0.7, estimates the exact P value associated with the null hypothesis of HW equilibrium and the standard error of this estimate. The number of dememorizations and batches was 1,000 and 20, respectively. All pairs of loci in each population were tested for genotypic linkage disequilibrium based on Markov chain algorithm using GENEPOP 4.0.7 and 1,000 dememorizations.

Pearson's correlation analysis was performed between intra-population parameters and latitude or longitude for each population to estimate the correlation of genetic variation within populations with geographical coordinates.

For the cpSSR markers, the length variants at each locus were combined into haplotypes based on the results of Heuertz et al. (2006). The statistical analysis of the cpSSR data, the haplotypic richness (A_r), the number of private haplotypes (K_p) and the gene diversity (H) was performed with Contrib software (Petit et al. 1998). Contributions of each population to total diversity (CT) and total allelic richness (CTR) were calculated for nSSR according to Petit et al. (1998).

Differentiation between populations

Genetic differentiation between populations (F_{ST}) was determined using FSTAT software (Weir and Cockerham 1984). The significance of F_{ST} was tested for the 95 and 99% confidence intervals based on 1,000 permutations. Population differentiation was tested by randomizing genotypes among samples (1,000 randomizations) assuming random mating within samples. The above tests were performed using FSTAT. Pairwise F_{ST} between populations was estimated using GENEPOP software version 4.0.7.

Population genetic structure

The correlation between $F_{ST}/(1 - F_{ST})$ (Rousset 1997) and the natural logarithm of geographical distance between populations was estimated using SPAGeDI 1.2 software (Hardy and Vekemans 2002) to test for isolation by distance. Significance of the correlation was assessed by 1,000 random permutations. Genetic distances between populations were estimated using Nei's genetic distance Ds (Nei 1978) as implemented in SPAGeDI 1.2. The resulting distance matrix was used to construct a consensus midpoint-rooted neighbour-joining dendrogram, after testing 59 possible dendrograms, using Phylip V3.67 software (Felsenstein 2007). Population's genetic structure was illustrated using Treeview32 software.

Estimation of mating system

The estimation of the mating system parameters in *F. angustifolia* was performed using a multilocus mating system program-MLTR (Ritland 2002). In the mixed-mating model, plants self-fertilize at rate s and the remaining fraction of progeny were derived from outcrosses. The multilocus and the average single-locus outcrossing rates (t_m and t_s, respectively) were obtained under the mixed-mating model (MMM). A positive difference between t_m and t_s indicated the occurrence of inbreeding between relatives (biparental inbreeding) (Ritland 1990). An approximate measure of the fraction of apparent selfing due to biparental inbreeding was obtained from the difference $1 - r_s$, whereas r_s was the correlation of selfing between two members of a family, or the normalized variance of selfing rate among families. For all the estimates, the Newton–Raphson method was chosen. Standard errors of estimates were obtained based upon 1,000 bootstraps, while resampling was performed in the family.

Detection of recent bottleneck

Populations were tested for a recent reduction of their effective population size (Cornuet and Luikart 1996) using the BOTTLENECK software (Piry et al. 1999). The mutation-drift equilibrium was assumed and the software calculated the deviation in observed gene diversity from the expected equilibrium value that was computed from the observed number of alleles. If a population is suffering from a recent bottleneck, a larger reduction of its number of alleles at neutral loci is observed compared to the gene diversity. As suggested by Piry et al. (1999) in the case of microsatellite loci, the two-phased model of mutation (TPM) was used with 95% stepwise mutation model (SMM) and 5% multistep mutations. The TPM is intermediate to the SMM and IAM (infinite alleles model). The Wilcoxon signed-rank test was used for the estimation of the significance of heterozygote excess because it provides relatively high power and it can be used with as few as four polymorphic loci and any number of individuals.

Results

Genetic variation at cpSSR loci

For the determination of *F. angustifolia* haplotypes presented in Greece, 15 samples from the 7 populations

(except 16A) were analysed with the same cpSSR markers used previously in a wide European study (Heuertz et al. 2006). Samples of *F. excelsior* and *F. angustifolia* from Great Britain and Spain (supplied under FRAXIGEN research program, EVK2-CT-2001-00180) with known haplotypes were analysed and used as a standard for haplotype's definition. The length variants at each of the six cpSSR loci were combined according to the results of Heuertz et al. (2006). Ccmp2 and ccmp4 loci were monomorphic resulting only in fragments of 194 and 140 bp, respectively, while the other four loci (ccmp3, ccmp6, ccmp7 and ccmp10) presented low levels of polymorphism. The small number of sample size might be one of the reasons for the low polymorphism that was assessed. Another possible explanation is the fact that cpSSRs are not as polymorphic as the nSSRs, especially when the studied area is regional and not continental. The combination of these loci (Table 2) revealed that three different haplotypes are present in *F. angustifolia* populations in Greece. Figure 1 shows their geographical distribution. Haplotype H01 is the most dominant in Greece, existed in four populations (09A, 11A, 13A and 14A), while H03 is the second, found in two populations (10A and 15A). Haplotype H05 was also found in population 12A. The total haplotypic diversity h_T was estimated to be 0.8095 using Contrib software.

Genetic variation at nSSR loci

The five nSSR loci used previously (Brachet et al. 1999; Lefort et al. 1999) for *F. excelsior* were also applied to this study of genetic variation of *F. angustifolia*. The number of

Table 2 Haplotype distribution and frequency in Greek populations of *F. angustifolia*

Haplotype	Population	D_{HT}	Haplotype frequency (%)	Size of amplification product (bp) for each loci					
				ccmp2	ccmp3	ccmp4	ccmp6	ccmp7	ccmp10
H01	09A	0.667	55.32	194	97	140	97	118	103
	11A	1.000							
	13A	0.667							
	14A	0.667							
H03	10A	0.833	29.79	194	97	140	99	117	103
	15A	0.833							
H05	12A	1.000	14.89	194	97	140	98	117	103

The frequency of each haplotype was estimated for the number of individual genotyped; D_{HT} divergence from other population, total $h_T = 0.8095$

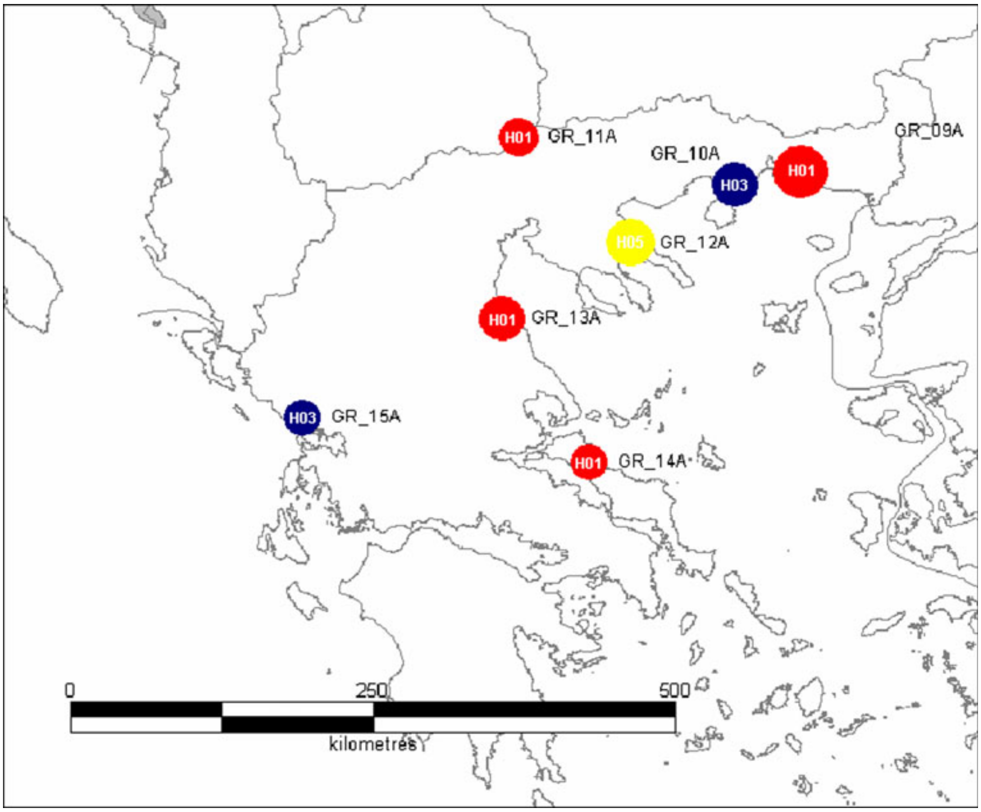

Fig. 1 *F. angustifolia* chloroplast microsatellite haplotypes detected in Greece

alleles detected over the eight populations revealed that all loci are highly polymorphic. In a total of 230 individuals, 131 alleles were detected with an average number of 26.2 alleles per locus, while the average number of alleles over the five nSSR loci ranged from 8.2 (population 16A) to 15.4 (population 09A). The allelic richness, A_S, per locus and over all populations was estimated based on the minimum population size of 20 individuals. It presented a minimum value of 6.344 for the locus FEMSATL16, while the maximum value was 18.169 for FEMSATL11 (Table 3). No evidence of null alleles was found as estimated using MICRO-CHECKER (data not shown) (Chakraborty et al. 1992; Brookfield 1996). Two private alleles were identified, one in population 15A and the other in population 16A. Across all loci, the linkage disequilibrium test revealed statistical independence/dependence of our loci.

The average expected heterozygosity, H_S, for each locus over the studied populations ranged from 0.391 (FEMSATL19) to 0.918 (FEMSATL11), while the values of the observed heterozygosity, H_O, for the same loci were 0.385 and 0.909, respectively (Table 3).

Gene diversity, H_T, was very high for locus FEMSATL11 and M2-30 (0.947 and 0.933, respectively), slightly lower for FEMSATL4 (0.846) and even lower for FEMSATL19 (0.422). Over all nSSR loci H_T was high and ranged from 0.5812 (population 14A) to 0.839 (population 09A). Figure 2 shows the contribution of each population to the total gene diversity and allelic richness, with population 09A being the most contributory. The inbreeding coefficient, F_{IS}, as an indicator of heterozygosity excess showed a positive value for all loci, ranged from $F_{IS} = 0.014$ (FEMSATL19) to $F_{IS} = 0.208$ (FEMSATL16), and for all populations (except 16A), indicating a heterozygosity deficit (Table 3). Over all loci and populations, the mean inbreeding coefficient was 0.085. An overall significant departure from Hardy–Weinberg equilibrium (Fisher's exact test) was observed in 5 out of 8 populations (Table 3). Total genetic diversity recorded for all loci and populations of *F. angustifolia* in Greece was high (0.766), while the differentiation between populations was low (0.056), revealing that only 5.6% of the differentiation among populations contributed to the total diversity.

The results of the BOTTLENECK statistics are also presented in Table 3. The estimation was based on 2,000 replications. All populations presented almost the same probability for heterozygosity excess and suggested that populations have not experienced a recent bottleneck. The

Table 3 Genetic variation parameters of *F. angustifolia* for the studied loci (A) and populations (B)

Locus	N_A	A_S	H_O	H_S	H_T	F_{IT} (*SE*)	F_{IS} (*SE*)	F_{ST} (*SE*)
(A)								
FEMSATL4	32	13.430	0.703	0.803	0.846	0.175** (0.037)	0.124** (0.039)	0.058 (0.015)
FEMSATL11	33	18.169	0.909	0.918	0.947	0.045** (0.022)	0.014^{ns} (0.026)	0.032 (0.007)
FEMSATL16	17	6.344	0.492	0.603	0.682	0.307** (0.089)	0.208** (0.090)	0.126 (0.073)
FEMSATL19	17	6.549	0.385	0.391	0.422	0.114** (0.066)	0.044^{ns} (0.099)	0.077 (0.036)
M2-30	32	16.347	0.860	0.913	0.933	0.087** (0.025)	0.064** (0.027)	0.025 (0.012)
All loci	26.2	12.168	0.670	0.726	0.766	0.137** (0.046)	0.085** (0.033)	0.056 (0.018)

Population	N	A	A_R	A_p	H_E	var(H_E)		F_{IS}	Probability**** (one tail for H excess)
						intra	inter		
(B)									
09A	30	15.4	13.246	–	0.8390	0.0011	0.0114	0.010^{ns}	0.95313
10A	30	12.0	10.726	–	0.7368	0.0026	0.0222	0.090*	1.00000
11A	30	10.6	9.545	–	0.7500	0.0016	0.0393	0.193***	0.98438
12A	30	13.2	11.667	–	0.7658	0.0022	0.0357	0.144***	0.98438
13A	30	12.0	10.662	–	0.7228	0.0019	0.0613	0.162**	0.96875
14A	30	10.4	8.926	–	0.5812	0.0021	0.1217	0.041^{ns}	0.98438
15A	30	10.0	8.916	1	0.6534	0.0019	0.0750	0.122^{ns}	0.95313
16A	20	8.2	8.200	1	0.7554	0.0016	0.0183	−0.138***	0.95313

N_A total number of alleles, N sample size, A average number of alleles per locus, A_S allelic richness per locus and overall population, A_R mean allelic richness per population, A_p number of private alleles, H_O average observed heterozygosity, H_S average expected heterozygosity within populations, H_T total gene diversity, H_E mean expected heterozygosity, var(H_E) intra-locus and inter-locus variance of H_E, F_{IT} mean Wright's inbreeding coefficient in the total population, F_{IS} mean Wright's inbreeding coefficient within populations, F_{ST} mean genetic differentiation based on allele identity, *SE* standard error (Jackknife over loci), *P* values: ns, $P > 0.05$; *, $P < 0.05$; **, $P < 0.01$; ***, $P < 0.001$; **** probability using Wilcoxon's signed-rank test as determined by BOTTLENECK software

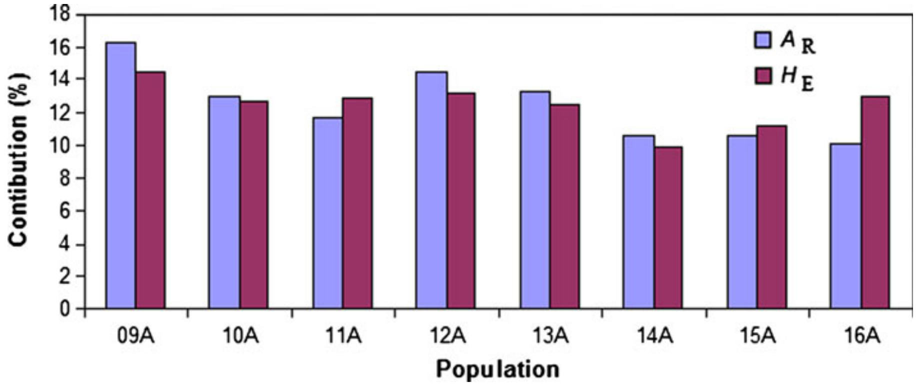

Fig. 2 Contribution of each population to the total allelic richness and total gene diversity. A_R, allelic richness; H_E, expected heterozygosity

Wilcoxon signed-rank test revealed a normal L-shaped distribution for all populations as expected under mutation-drift equilibrium. The Pearson correlation analysis revealed that the genetic diversity, A_R and H_E, within *F. angustifolia* populations in Greece is significantly positive correlated with both latitude and longitude (Fig. 3). The correlation coefficient of the allelic richness versus latitude and longitude was $r = 0.7213$ ($P < 0.001$) and $r = 0.\ 7771$ ($P < 0.001$), respectively, while the correlation coefficient of heterozygosity versus latitude and longitude was $r = 0.5298$ ($P < 0.001$) and $r = 0.4861$ ($P < 0.001$) (Fig. 3a and b). When Wright's inbreeding coefficient was correlated with the geographical variables, a positive correlation was observed only versus latitude ($r = 0.6321$, $P < 0.001$), while the correlation with longitude was close to zero ($r = -0.0524$, $P < 0.001$). Therefore, higher gene diversity and allelic richness was found within populations in north-eastern Greece, while lower values were observed in south-western country. Values of inbreeding coefficient were also higher in northern Greece.

The positive values of inbreeding coefficient could be attributed in part to the presence of null alleles, or the inbreeding between relatives (biparental inbreeding), restricted seed and moderate pollen dispersal, or the Wahlund effect due to the presence of two or more breeding subunits inside a given population. Since the use of MICRO-CHECKER software demonstrated that none of the loci contain null alleles, the biparental inbreeding in *F. angustifolia* was determined. The estimation of the multilocus and the single-locus population outcrossing rates was performed using the MLTR program, for the analysis of the mating system. When the entire family was resampled, the multilocus outcrossing rate was found to be $t_m = 0.961 \pm 0.111$, and the single-locus outcrossing rate was $t_s = 0.947 \pm 0.106$, while their difference was 0.014 ± 0.072. The positive value of their difference indicates that biparental inbreeding occurs in *F. angustifolia.* Ignoring the effects of biparental inbreeding, an expected value of the selfing rate could be estimated according to the equation $s = 2F_{IS}/(1 + F_{IS})$ (Hartl and

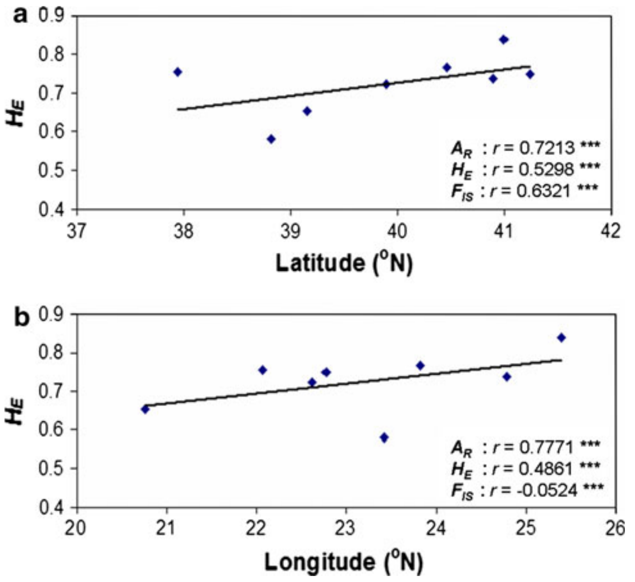

Fig. 3 Pearson's correlation analysis between genetic diversity parameters of *F. angustifolia* populations and geographical coordinates **a** H_E versus latitude and **b** H_E versus longitude. A_R allelic richness; F_{IS} Wright's inbreeding coefficient per population; *P* values: ***, $P < 0.001$

Clark 1989). The expected value of the selfing rate was estimated to be 0.157. The heterozygosity deficit could be also attributed to a spatial Wahlund effect since the individuals in our populations were far from each other, thus each population may consist of spatial breeding subpopulations. Sometimes, Wahlund effect is observed when highly polymorphic molecular markers are used (Morand et al. 2002; Bottin et al. 2005), and the nSSRs that have been used in our study were found polymorphic.

The pairwise F_{ST} values of the eight *F. angustifolia* populations ranged from 0.0170 (11A vs. 13A) to 0.1742 (14A vs. 16A) indicating a moderate population genetic differentiation (data not shown). Highly significant ($P <$ 0.001) genetic differentiation was found in 26 out of 28 pairs of populations. A Mantel test was performed to estimate the correlation between the variable $F_{ST}/(1 - F_{ST})$ for each pair of population and the natural logarithm of the

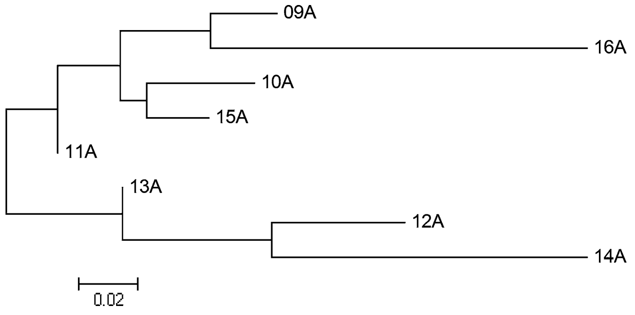

Fig. 4 Genetic distance between pairs of populations of *F. angustifolia* studied in Greece. The midpoint-rooted dendrogram was constructed using the neighbour-joining cluster algorithm and Nei's (1978) genetic distance among pairs of populations

geographical distances between them. A positive but not significant correlation between $F_{ST}/(1-F_{ST})$ and the natural logarithm of distance ($r = 0.0078$, $P > 0.05$) was observed among the eight natural populations of *F. angustifolia* in Greece.

The relationship between the natural populations of *F. angustifolia* in Greece, based on Nei's genetic distances, is summarized in a midpoint-rooted dendrogram (Fig. 4) according to the neighbour-joining clustering algorithm. Two geographically distant populations 09A and 16A were clustered together and were close to another cluster of distant populations 10A and 15A. Closer to these two clusters were populations 11A and 13A, while populations 12A and 14A formed another cluster that is separated from the others with a long branch.

Discussion

The genetic diversity and differentiation of eight natural populations of *F. angustifolia* in Greece was studied based on nSSR and cpSSR loci. Since a wide genetic study of *F. angustifolia* all over Europe has been performed based on cpSSR loci (Heuertz et al. 2006), and no other study has been done in Greece, it is interesting to know the genetic structure of populations in Greece in terms of the evolutionary history of the species. Furthermore, *F. angustifolia* and *F. ornus* are highly important hardwood species in Greece for both, wood quality and nature conservation, while *F. excelsior* is of minor importance since it is rarely found in the country, possibly in some isolated groups in the northern borders (Spanos et al. 2004; FRAXIGEN 2005).

Our study using nSSR loci revealed high levels of within-population variation and low genetic differentiation between them. The loci were highly polymorphic in *F. angustifolia* and displayed a large number of alleles ($H_T = 0.766$ and $A_S = 12.168$) in a relatively small number of individuals. The existence of a significant heterozygosity deficit in *F. angustifolia* Greek populations and a moderate genetic differentiation amongst them indicates that gene flow has not been able to erase the population structure in *F. angustifolia*. This is supported by the pairwise F_{ST} values between the eight *F. angustifolia* populations indicating that, despite the small contribution of differentiation among populations to the total genetic diversity, the populations could be consider genetically distinct. Our result demonstrate that narrow-leaf ash populations still harbour most of their genetic diversity despite the small sizes in many cases (e.g. populations of 300–500 trees) and could be explained by the fact that *F. angustifolia* in Greece has been fragmented and remained in small scattered populations and metapopulations only recently. Another reason is that the species has developed high root and stem sprouting ability and thus is able to maintain viable population sizes, compete with environmental stress and compete with other tree species. The high intra-population genetic diversity demonstrates that both sexual reproduction (wind pollination) and clonal propagation (stem sprouting and root suckering—easy process in wet environments and fertile soils) contribute to the maintenance of species genetic diversity (FRAXIGEN 2005). The high intra-population variation and low genetic differentiation between populations seem to be the general pattern for temperate wind pollinated forest tree species (Lowe et al. 2004; Hartl and Clark 1989).

Since no data on genetic variation of *F. angustifolia* based on nSSR markers are available, our results could be compared to those obtained on *F. excelsior*, a closely related species. In European studies of *F. excelsior* based on nSSR loci (Morand et al. 2002; Heuertz et al. 2001; FRAXIGEN 2005; Ferrazzini et al. 2007), it was found that genetic diversity within populations and allelic richness were high. The same observation was reported for *F. mandshurica* in northeast China (Hu et al. 2008). Moreover, two contrasting patterns of within and among population diversity between western and southern European populations of common ash have been observed (Heuertz et al. 2004b; Ferrazzini et al. 2007). The first pattern consisted of high allelic and genetic diversity within populations, low differentiation among populations and occurred mainly in western and central Europe, Italy, as well as in Lithuania. The second pattern consisted of relatively low allelic and genetic diversity within populations, relatively strong differentiation among populations and occurred in Sweden and southeast Europe. Besides Europe, the former pattern has been observed for *Fraxinus* spp. in S. Korea, where the use of molecular marker I-SSR proved that the highest proportion of genetic diversity (>90%) was distributed within populations, while only a relatively small part (4–10%) being evident among populations (Cho et al. 2002).

Fraxinus angustifolia is closely related to *F. excelsior,* and evidence of hybridization between them has been reported by various groups based on observations of

intermediate morphologies (Rameau et al. 1989), the genotyping of natural populations with RAPD markers (Jeandroz et al. 1996) and the assessment of the morphological and genetic structure of putative hybrid populations (Fernandez-Manjarres et al. 2006). In the *F. angustifolia* populations that were studied, the morphological characteristics of the individuals indicated that no intermediates/hybrids are present in these populations.

Since MICRO-CHECKER analysis revealed that no null alleles were present in *F. angustifolia* populations, other common causes of positive inbreeding coefficient values were examined. Evidence for the occurrence of biparental inbreeding came from the within populations analysis of the genetic structure. An association between genetic relatedness and spatial position of individuals was detected. Apart from the biparental inbreeding, self-pollination is possible in *F. angustifolia,* and the selfing rate in the populations studied was estimated to be 0.157. This result is in accordance with the reproductive system of *F. angustifolia*. As already mentioned, *F. angustifolia* is hermaphrodite, and although the start of the stigma receptivity precedes the start of the opening of anthers and the release of pollen, there is an overlap between the male and female phases making the self-pollination possible. Another reason for the heterozygosity deficit is the Wahlund effect which may be due to distinct breeding subpopulations or to the highly polymorphic molecular markers that were used.

As mentioned previously, the distribution of the deciduous forests of *F. angustifolia* in Greece is rapidly limited. Therefore, the populations were tested for a possible recent reduction of their effective size (Piry et al. 1999; Cornuet and Luikart 1996). No evidence of a recent bottleneck in any of *F. angustifolia* populations in Greece was revealed.

Correlation analysis between population genetic diversity and geographical coordinates showed a positive link for both latitude and longitude, with populations from NE Greece showing higher gene diversity compared to populations from lower latitudes. Negative correlations of the genetic diversity with geographical gradients have been observed in *F. mandshurica* populations of NE China (Hu et al. 2008). No evidence of correlation between geographical distance and genetic differentiation of the population studied was observed. Since Greece is a mountainous country interrupted by agricultural land in the plains and river valleys, it is possible that closed populations are isolated and consequently more genetically differentiated due to the presence of mountain chains or vast agricultural land while more distant populations could be connected because of a regular presence of the species in between. This was further confirmed by the population structure of *F. angustifolia* in Greece, as obtained from the midpointed-rooted neighbour-joined dendrogram. It indicated that geographically distant populations were clustered together and this is in accordance with the finding that no significant isolation by distance was presented.

The use of chloroplast microsatellite loci revealed three cpDNA haplotypes in Greece, H01, H03 and H05. Haplotype H01 was the most dominant in Greece, with the next most common being H03. Finally, haplotype H05, which is present in several populations from the eastern Alps, was found in one population in Greece. Heuertz's studies revealed a specific geographical organization of the four most common chloroplast haplotypes (H01, H02, H03 and H04) that are widespread and showed little overlap in their distribution regions (Heuertz et al. 2006). Our data are in accordance with the fact that higher haplotype diversity is recorded in southern European populations compared with those from further north. A decline in the number of haplotypes with increasing distance from a refugium is consistent with fossil pollen data and with theoretical models of postglacial migration suggesting that postglacial expansion occurred from several glacial refugia located in all three southern European peninsulas and the Alps.

Fraxinus. angustifolia is an important constituent of European mixed broadleaved woodlands, and in recent years, there is an increased interest of planting *F. angustifolia* not only for timber production, but also for long-term sustainability, conservation of biodiversity and ecological restoration (FRAXIGEN 2005; Spanos et al. 2004). For the conservation and restoration programmes, it is crucial to maintain the genetic variation of *F. angustifolia* natural populations and especially the adaptive variation that improves the fitting of the individuals and defines their adaptation/survival under various environmental conditions. Adaptive variation is measured in specialized field trials such as reciprocal transplant experiments (RTEs), which is difficult and time-consuming compared to the estimation of neutral variation using microsatellite markers or other molecular markers. Since high neutral variation is believed to reflect high adaptive variation, molecular markers are widely used in conservation and restoration studies even if they are not directly acknowledged as indicative markers (Gebremedhin et al. 2009; Holderegger et al. 2006; Vendramin and Morgante 2005; Boshier and Young 2000).

Our results showed that the genetic diversity of *F. angustifolia* in Greece is high within populations and moderate differences between populations exist, thus sampling as many individuals as possible from a few populations is recommended to maintain the high levels of genetic variation. In areas where ash populations are very small and/or highly fragmented, the recommendation to use local seed must be interpreted with caution because there may be a risk of significant inbreeding leading to reduced genetic quality of the seed. In general, when collecting clonal material, sampling 30 trees in a species with random

outcrossing and 60 trees in a completely selfing species, allows us to conserve 95% of alleles with a frequency of >5% (FAO 1995). Clonal material could be collected via grafts from mature trees to be grafted on ash seedlings for clonal seed orchard establishment. Alternative, collection of cuttings from selected genotypes (<10 years age) could be performed to reproduce (vegetatively) the desired characteristics for economic or ornamental plantations. For ex situ conservation of the species, grafts and cuttings are used. When seeds are collected, sampling depends on the number of seed collected per tree and the mating/pollination system of the tree species. It has been shown (FRAXIGEN 2005) that wind pollination (*Fraxinus excelsior*, *F. angustifolia*) is likely to result in trees producing seeds from many fathers. Therefore, in *Fraxinus excelsior* and *F. angustifolia,* as much as many seeds (e.g. 500) are collected from the mother tree (all over the crown), the higher genetic diversity is conserved (FRAXIGEN 2005). In addition, collecting seed from at least 10 trees will conserve 95% of the alleles with frequency >5%, while a collection from 20 trees will include additional rare alleles (95% of alleles with frequency >2.5%) (FRAXIGEN 2005). These calculations assume collection of at least 500 viable seeds per tree. Spacing between seed trees is important in ensuring a diverse genetic collection. Collecting at distances greater than seed dispersal will ensure that the sampled trees are unrelated, although the pollen pools siring seed may be still related. Thus, in *F. angustifolia,* collection of seeds from trees which are 100 m apart (where 90% of the seed and pollen is dispersed) is likely to ensure the seed trees are unrelated, but there is still possibility to be mating with a similar selection of fathers. Therefore, in *F. angustifolia,* seed trees need to be at least 150 m apart to ensure collection from both unrelated mother trees and different pollen pools. Thus, the distance of 150 m between seed trees is a good recommendation (Spanos et al. 2004). Additionally, seed can be collected in mast and non-mast years, since genetic variation is high in both years.

In conclusion, genetic variation of *F. angustifolia* was assessed presenting high within-population diversity and a moderate population differentiation. Significant correlation with the geographical variables was observed, while populations were not isolated by distance. No indications for the existence of hybrids of *F. excelsior* and *F. angustifolia* were found in the populations studied. Since most populations of *F. angustifolia* in Greece are fragmented and scattered, in some cases isolated, and found in remnant old grown stands, in situ conservation is more important than ex situ. *F. angustifolia* has high ability in population expansion and survival under optimum environmental conditions (in the previously mentioned habitats). Therefore, for an integrated conservation of species, genetic resources (ecological aspects, conservation, breeding) not only the population but the whole habitat should be maintained. In conclusion, in situ conservation of narrow-leaf ash populations is of high priority to maintain inter-population and intra-population species genetic diversity, particularly under the face of climate change.

Acknowledgments This work was financially supported by FRAXIGEN (EVK2-CT-2001-00180), funded by FP5 of EU. The authors would like to thank Dionysios Gaitanis and Dimosthenis Mylonas for collecting plant material and field work.

References

Boshier DH, Young AG (2000) Forest conservation genetics: limitations and future directions. In: Young AG, Boshier DH, Boyle T (eds) Forest conservation genetics: principles and practice. CSIRO Publishing, Australia

Bottin L, Verhaegen D, Tassin J, Olivier I, Vaillant A, Bouvet MJ (2005) Genetic diversity and population structure of an insular tree, *Santalum austrocaledonicum* in New Caledonian archipelago. Mol Ecol 14:1979–1989

Brachet S, Jubier MF, Richard M, Junc-Muller B, Frascaria-Lacoste N (1999) Rapid identification of microsatellite loci using 5′ anchored PCR in the common ash *Fraxinus excelsior*. Mol Ecol 8:160–163

Brookfield JFY (1996) A simple new method for estimating null allele frequency from heterozygote deficiency. Mol Ecol 5:453–455

Chakraborty R, De Andrade M, Daiger SP, Budowle B (1992) Apparent heterozygote deficiencies observed in DNA typing data and their implications in forensic applications. Ann Hum Genet 56:45–57

Cho KJ, Chung JM, Kim WW, Hong YP (2002) Genetic structure analysis of three *Fraxinus* species populations in Korea. Paper presented at the 'Genetics' Symposium, Stará Lesná, Slovakia

Cornuet JM, Luikart G (1996) Description and power analysis of two tests for detecting recent population bottlenecks from allele frequency data. Genetics 144:2001–2014

Fady-Welterlen B (2005) Is there really more biodiversity in Mediterranean forest ecosystems? Taxon 54:905–910

FAO (1995) Collecting woody perennials. In: Guarino L, Rao R, Reid R (eds) Collecting plant genetic diversity: technical quidelines. CAB International, Wallingford, pp 485–509

Felsenstein J (2007) PHYLIP: Phylogeny inference package, version 3.67c, Department of Genome Sciences and the Department of Biology, University of Washington, Seattle

Fernandez-Manjarres JF, Gerard PR, Dufour J, Raquin C, Frascaria-Lacoste N (2006) Differential patterns of morphological and molecular hybridization between *Fraxinus excelsior* L. and *Fraxinus angustifolia* Vahl (Oleaceae) in eastern and western France. Mol Ecol 15:3245–3257

Ferrazzini D, Monteleone I, Belletti P (2007) Genetic variability and divergence among Italian populations of common ash (*Fraxinus excelsior* L.). Ann For Sci 64:159–168

FRAXIGEN (2005) Ash species in Europe: biological characteristics and practical guidelines for sustainable use. Oxford Forestry Institute, Department of Plant Sciences, University of Oxford, Oxford

Gebremedhin B, Ficetola GF, Naderi S, Rezaei HR, Maudet C, Rioux D, Luikart G, Flagstad O, Thuiller W, Taberlet P (2009) Frontiers in identifying conservation units: from neutral markers to adaptive genetic variation. Anim Conserv 12:107–109

Goudet J (2001) FSTAT, a program to estimate and test gene diversities and fixation indices (version 2.9.3). Available from http://www.unil.ch/izea/softwares/fstat.html

Hardy OJ, Vekemans X (2002) SPAGeDi: a versatile computer program to analyse spatial genetic structure at the individual or population levels. Mol Ecol Notes 2:618–620

Hartl DL, Clark AG (1989) Principles of population genetics. Sinauer Associates, Sunderland

Heuertz M, Hausman J, Tsvetkov I, Frascaria-Lacoste N, Vekemans X (2001) Assessment of genetic structure within and among Bulgarian populations of the common ash (*Fraxinus excelsior* L.). Mol Ecol 10:1615–1623

Heuertz M, Fineschi S, Anzidei M, Pastorelli R, Salvini D, Paule L, Frascaria-Lacoste N, Hardy OJ, Vekemans X, Vendramin GG (2004a) Chloroplast DNA variation and postglacial recolonization of common ash (*Fraxinus excelsior* L.) in Europe. Mol Ecol 13:3437–3452

Heuertz M, Hausman JF, Hardy OJ, Vendramin GG, Frascaria-Lacoste N, Vekemans X (2004b) Nuclear microsatellites reveal contrasting patterns of genetic structure between western and southern European populations of the common ash (*Fraxinus excelsior* L.). Evolution 58:976–988

Heuertz M, Carnevale S, Fineschi S, Sebastiani F, Hausman F, Paule L, Vendramin GG (2006) Chloroplast DNA phylogeography of European ashes, *Fraxinus* sp. (*Oleaceae*): roles of hybridization and life history traits. Mol Ecol 15:2131–2140

Holderegger R, Kamm U, Gugerli F (2006) Adaptive vs. neutral genetic diversity: implications for landscape genetics. Landscape Ecol 21:797–807

Hu LJ, Uchiyama K, Shen HL, Saito Y, Tsuda Y, Ide Y (2008) Nuclear DNA microsatellites reveal genetic variation but a lack of phylogeographical structure in an endangered species, *Fraxinus mandshurica*, across north-east China. Ann Bot 102:195–205

Jarne P, Lagoda PJL (1996) Microsatellites, from molecules to populations and back. Trends Ecol Evol 11:424–429

Jeandroz S, Frascaria-Lacoste N, Bousquet J (1996) Molecular recognition of the closely related *Fraxinus excelsior* and *F. oxyphylla* (*Oleaceae*) by RAPD markers. Forest Genet 3:237–242

Lefort F, Brachet S, Frascaria-Lacoste N, Edwards KJ, Douglas GC (1999) Identification and characterization of microsatellite loci in ash (*Fraxinus excelsior* L.) and their conservation in the olive family (*Oleaceae*). Mol Ecol 8:1088–1089

Lowe A, Harris S, Ashton P (2004) Ecological genetics—design, analysis, and application. Blackwell, UK/USA/Australia

Morand ME, Brachet S, Rossignol P, Dufour J, Frascaria-Lacoste N (2002) A generalized heterozygote deficiency assessed with microsatellites in French common ash populations. Mol Ecol 11:377–385

Morgante M, Olivieri AM (1993) PCR-amplified microsatellites as markers in plant genetics. Plant J 3:175–182

Mueller-Starck G (1989) Genetic implications of environmental stress in adult forest stands of *Fagus sylvatica* L. In: Scholz F, Gregorius H, Rudin D (eds) Genetic effects of air pollutants in forest tree populations. Springer, Berlin, pp 127–142

Nei M (1978) Estimation of average heterozygosity and genetic distance for small number of individuals. Genetics 89:583–590

Nei M (1987) Molecular evolutionary genetics. Columbia University Press, New York

Newton AC, Allnutt TR, Gillies ACM, Lowe AJ, Ennos RA (1999) Molecular phylogeography, intraspecific variation and the conservation of tree species. Trends Ecol Evol 14:140–145

Payn KG, Dvorak WS, Janse BJH, Myburg AA (2008) Microsatellite diversity and genetic structure of the commercially important tropical tree species *Eucalyptus urophylla*, endemic to seven islands in eastern Indonesia. Tree Genet Genome 4:519–530

Petit R, El Mousadik A, Pons O (1998) Identifying populations for conservation on the basis of genetic markers. Conserv Biol 12:844–855

Petit RJ, Aguinagalde I, de Beaulieu JL, Bittkau C, Brewer S, Cheddada R, Ennos R, Fineschi S, Grivet D, Lascoux M, Mohanty A, Müller-Starck GM, Demesure-Musch B, Palmé A, Martin JP, Rendell S, Vendramin GG (2003) Glacial refugia: hotspots but not melting pots of genetic diversity. Science 300:1563–1565

Petit R, Hampe A, Cheddadi R (2005) Climate changes and tree phylogeography in the Mediterranean. Taxon 54:877–885

Piry S, Luikart G, Cornuet JM (1999) BOTTLENECK: a computer programme for detecting recent reductions in the effective population size using allele frequency data. J Hered 90:502–503

Rameau JC, Mansion D, Dume G (1989) Flore forestiere Francaise, guide ecologique illustre. Tome I Plaines et Collines Institute pour le Developpement Forestier, Paris

Ritland K (1990) A series of FORTRAN computer programs for estimating plant mating systems. J Heredity 81:235–237

Ritland K (2002) Extensions of models for the estimation of mating systems using *n* independent loci. Heredity 88:221–228

Rousset F (1997) Genetic differentiation and estimation of gene flow from *F*-statistics under isolation by distance. Genetics 145: 1219–1228

Rousset F (2008) GENEPOP'007: a complete reimplementation of the GENEPOP software for Windows and Linux. Mol Ecol Notes 8:103–106

Spanos K, Papi R, Mylonas D, Gaitanis D, Kyriakidis D (2004) The European research programme FRAXIGEN: ash for the future, defining ash populations for conservation, regeneration and ecological adaptation—works in Greece. In: Forest genetics and tree breeding in the age of genomics: progress and future. Proceedings of a joint conference of IUFRO Division 2 Charleston, South Carolina, USA, pp 421–429

Tutin TG, Heywood VH, Burges NA (1972) Flora Europaea. Cambridge University Press, Cambridge

van Oosterhout C, Hutchinson WF, Wills DPM, Shipley P (2004) MICRO-CHECKER: software for identifying and correcting genotyping errors in microsatellite data. Mol Ecol Notes 4:535–538

Vendramin GG, Morgante M (2005) Genetic diversity in forest tree populations and conservation: analysis of neutral and adaptive variation. In: The role of biotechnology, Villa Gualino, Turin, Italy—5–7 March, 2005, pp 129–130

Wallander E (2001) Evolution of wind-pollination in *Fraxinus* (*Oleaceae*)—an ecophylogenetic approach. Göteborg University, Göteborg

Wallander E, Albert VA (2000) Phylogeny and classification of *Oleaceae* based on rps16 and trnl-F sequence data. Am J Bot 87:1827–1841

Weir BS, Cockerham CC (1984) Estimating F-statistics for the analysis of population structure. Evolution 38:1358–1370

Weising K, Gardner RC (1999) A set of conserved PCR primers for the analysis of single sequence repeat polymorphisms in chloroplast genomes of dicotyledonous angiosperms. Genome 42:9–19
