# Supplementary material for: Provenance and family variations in early growth of Manchurian walnut (Juglans mandshurica Maxim.) and selection of superior families
Source: PLoS One. 2024 Mar 7;19(3):e0298918. doi: 10.1371/journal.pone.0298918 (PMC10919699; doi:10.1371/journal.pone.0298918)
Supplement: S1 File — (ZIP) [file pone.0298918.s004.zip › Genome-wide association analysis and pathway enrichment provide insights into the genetic basis of photosynthetic responses to drought stress in Persian walnut.pdf]

Horticulture Research

Horticulture Research, 2022, **9**: uhac124
https://doi.org/10.1093/hr/uhac124

Downloaded from https://academic.oup.com/hr/article/doi/10.1093/hr/uhac124/6603865 by guest on 14 January 2023

Article

Genome-wide association analysis and pathway enrichment provide insights into the genetic basis of photosynthetic responses to drought stress in Persian walnut

Mohammad M. Arab[1], Patrick J. Brown[2], Rostam Abdollahi-Arpanahi[3], Seyed Sajad Sohrabi[4], Hossein Askari[5], Sasan Aliniaeifard[6], Ali Mokhtassi-Bidgoli[7], Mohsen B. Mesgaran[2], Charles A. Leslie[2], Annarita Marrano[2], David B. Neale[2] and Kourosh Vahdati[1,*]

[1]Department of Horticulture, College of Aburaihan, University of Tehran, Tehran, Iran
[2]Department of Plant Sciences, University of California, Davis, CA 95616
[3]Department of Animal and Dairy Science, University of Georgia, Athens, GA, USA
[4]Department of Plant Production and Genetic Engineering, Faculty of Agriculture, Lorestan University, Khorramabad, Iran
[5]Department of Plant Sciences and Biotechnology, Faculty of Life Sciences and Biotechnology, Shahid Beheshti University, Tehran, Iran
[6]Photosynthesis laboratory, Department of Horticulture, College of Aburaihan, University of Tehran, Tehran, Iran
[7]Department of Agronomy, Faculty of Agriculture, Tarbiat Modares University, Tehran, Iran
*Corresponding author. E-mail: kvahdati@ut.ac.ir

Abstract

Uncovering the genetic basis of photosynthetic trait variation under drought stress is essential for breeding climate-resilient walnut cultivars. To this end, we examined photosynthetic capacity in a diverse panel of 150 walnut families (1500 seedlings) from various agro-climatic zones in their habitats and grown in a common garden experiment. Photosynthetic traits were measured under well-watered (WW), water-stressed (WS) and recovery (WR) conditions. We performed genome-wide association studies (GWAS) using three genomic datasets: genotyping by sequencing data (∼43 K SNPs) on both mother trees (MGBS) and progeny (PGBS) and the Axiom™ *Juglans regia* 700 K SNP array data (∼295 K SNPs) on mother trees (MArray). We identified 578 unique genomic regions linked with at least one trait in a specific treatment, 874 predicted genes that fell within 20 kb of a significant or suggestive SNP in at least two of the three GWAS datasets (MArray, MGBS, and PGBS), and 67 genes that fell within 20 kb of a significant SNP in all three GWAS datasets. Functional annotation identified several candidate pathways and genes that play crucial roles in photosynthesis, amino acid and carbohydrate metabolism, and signal transduction. Further network analysis identified 15 hub genes under WW, WS and WR conditions including *GAPB, PSAN, CRR1, NTRC, DGD1*, *CYP38*, and *PETC* which are involved in the photosynthetic responses. These findings shed light on possible strategies for improving walnut productivity under drought stress.

Introduction

Walnut (*Juglans regia* L.) was domesticated in ancient Persia and is today cultivated for its edible nuts throughout temperate and semi-arid regions from Asia to Europe and the Americas [1]. Iran is a prominent centre of diversity for walnut, and ranks third in in-shell walnut production after China and the United States [2]. Walnut production in Central Asia and across the globe is constrained by abiotic stresses, particularly drought, heat, and salinity [3,4]. Drought is likely the most challenging abiotic stress for walnut, with ever-increasing global water scarcity driving large production losses [5–8]. Therefore, understanding the physiological and molecular mechanisms of drought tolerance in walnuts has become more important worldwide, with more extended drought periods expected in the coming decades. Walnut populations adapted to their native habitats around Iran offer an opportunity to better understand walnut responses to drought stress. So far, most studies conducted in walnut have focused on physiological aspects of drought stress, whereas molecular mechanisms are less well documented [3]. Therefore, a priority task for accelerating walnut improvement is deciphering the molecular genetic basis of drought related traits.

Photosynthesis is a key physiological mechanism involved in adaption to abiotic stresses and regulation of plant development [9]. Under drought conditions, photosynthesis decreases due to stomatal closure, decreased

Received: 9 November 2021; **Accepted:** 17 May 2022; **Published:** 8 June 2022; **Corrected and Typeset:** 1 August 2022
© The Author(s) 2022. Published by Oxford University Press on behalf of Nanjing Agricultural University. This is an Open Access article distributed under the terms of the Creative Commons Attribution License (https://creativecommons.org/licenses/by/4.0/), which permits unrestricted reuse, distribution, and reproduction in any medium, provided the original work is properly cited.

CO_2 availability in the chloroplast, and decreased carboxylation efficiency [10]. Excess light energy during drought-induced stomatal closure can cause serious damage to the plant through generation of reactive oxygen species (ROS) [11]. Plants have evolved diverse protective strategies to cope with excessive light, including non-photochemical quenching (NPQ), antioxidant production, and the regulation of electron transport to moderate ROS formation or detoxify ROS after they form [12]. Because excess light energy absorbed by chlorophyll can either be dissipated through NPQ or re-emitted as fluorescence [11], chlorophyll fluorescence analysis is a powerful tool for tracking fluxes of light absorption by chlorophyll through the electron transport chain [13]. The "OJIP" test represents a fast and non-destructive analysis of polyphasic chlorophyll fluorescence kinetics that has been employed for quick and precise assessment of biophysical aspects of photosynthesis under abiotic stress [14]. The OJIP test, together with gas exchange measurements, have been used successfully to study the photosynthetic apparatus of various tree crops, under abiotic stress conditions, including walnut [7,8]. Despite extensive physiological studies, the genetic basis underlying variation in walnut photosynthesis under both water-stress and re-watering conditions remains largely unknown.

A first-draft reference genome of Persian walnut was released in 2016 (Chandler v1.0 [15]), followed by the development of a high-density Axiom™ *J. regia* 700 K SNP genotyping array [16] that facilitated advanced genomic studies, including QTL mapping and genome wide association studies (GWAS). These new genomic tools have been extensively used for investigating genomic diversity and association mapping in walnut [17–21]. However, there are few studies on the genetic basis of physiological traits in walnut [3,5]. Recently, two annotated, chromosome-level assemblies of the walnut genome [22,23] have been released, enabling SNP identification at chromosome scale and the application of genomic tools in plant breeding programs. SNP arrays provide reliable and robust markers for a multitude of applications in breeding programs and population genomic studies. However, SNP arrays are species- and population specific and ascertainment bias is one of their main drawbacks [24,25]. SNP array development also requires prior knowledge, and has a relatively high cost [24,25]. On the other hand, genotyping-by-sequencing (GBS) and restriction site associated DNA sequencing (RADseq) approaches have the potential to generate large marker datasets at low cost with minimal ascertainment bias, although large amounts of missing data and heterozygote undercalling are significant drawbacks that can only be partially addressed by imputation [24,25]. Therefore, GBS offers a cost-efficient alternative or complement to high-throughput genotyping arrays for gaining genomic information [25].

GWAS successfully identified many SNPs underlying a wide range of traits in plants. However, for complex or polygenic traits, the statistical power of GWAS for identifying variants of small effect is restricted by the stringent levels set for significance threshold and by insufficient numbers of high-frequency polymorphisms identified in most panels [26,27]. So, many small effect SNP markers are always ignored and most of the genetic variants contributing to the trait remains hidden [28]. Further, since many associated SNPs are noncoding it can be problematic to identify the molecular mechanisms by which they may act. Pathway or gene set enrichment analysis as a complementary method to GWAS can help tackle the aforesaid problems through assessing modules of functionally related genes instead of focusing only on one or a few markers that are most significantly associated [26,27]. Therefore, this approach by pooling information across many genetic variants (SNPs) can identify potentially relevant biological pathways or molecular mechanisms even when individual SNPs fail to reach a stringent significance threshold.

In this study, we take advantage of natural variation in local walnut populations of Iran to investigate the genetic control of photosynthetic-related traits under well-watered (WW), water-stressed (WS), and water-recovery (WR) conditions, combining GWAS with network and pathway enrichment analyses. Given that the expression of physiological and photosynthetic traits in Persian walnut is under strong genetic control, we hypothesized that locally adapted Persian walnut populations would express different levels of trait plasticity under water-stress (mild and severe) and re-watering conditions. Our main objectives are: (1) to assess natural genetic variation and phenotypic plasticity in photosynthetic traits in a diverse collection of walnut trees under water-stress and re-watering conditions; (2)to uncover genomic regions contributing to photosynthetic trait variation through GWAS; and finally (3) to identify key pathways and hub genes related to photosynthesis under well-watered, water-stressed and recovery conditions through pathway and network analysis.

Results

Natural variation in photosynthetic traits of Persian walnut populations

From the 150 walnut families collected from major walnut-growing regions of Iran (Table S1), 30 photosynthetic-related traits, largely classified into two main categories (gas exchange and chlorophyll fluorescence measurements), were evaluated in walnut plants grown under control, water-stress, and recovery conditions (Figure S1). A brief description of calculations for each measured phenotype is provided in Table 1.

High phenotypic variation was observed among families for all the traits measured. Most traits revealed a near normal distribution (Figure 1; Figures S2-3). There was significant ($P \leq 0.001$) genotypic variation in both

Downloaded from https://academic.oup.com/hr/article/doi/10.1093/hr/uhac124/6603865 by guest on 14 January 2023

Table 1. Calculations and definitions of water relations, gas-exchange and fluorescence (Strasser et al., 2000, 2004) parameters measured in the study with their broad classification

Phenotype category	Phenotype	Calculation	Definition
		**Drought score and water relations**	
Drought score	DS		Drought scoring system based on the appearance characteristics of seedlings
	RWC	(fresh weight (FW) – dry weight (DW)) / (turgor weight (TW) – DW)	Leaf relative water content (RWC)
		**Gas-exchange parameters**	
Gas-exchange parameters	P_n		Net photosynthetic rate (μmol CO_2 m^{-2} s^{-1})
	T_r		Transpiration rate (mmol H_2O m^{-2} s^{-1})
	g_s		Stomatal conductance (mol H_2O m^{-2} s^{-1})
	Ci		Intercellular CO_2 concentration (μmol CO_2 mol^{-1} air)
	Ca		Atmospheric CO_2 concentration
	WUEintri	P_n/g_s	WUEintri in μmol CO_2 mmol H_2O^{-1}
	WUEinst	P_n/T_r	WUEinst in μmol CO_2 mmol H_2O^{-1}
	CE	P_n/C_i	An estimate of carboxylation efficiency of Rubisco
		**Fluorescence parameters**	
Primary fluorescence measurements	F_0	$F_0 = F_{50\mu s}$	Minimum Fluorescence, when all PSII RCs are open
	F_J	$F_J = F_{2m}$	Fluorescence intensity at the J-step (2 ms)
	F_I	$F_I = F_{60ms}$	Fluorescence intensity at the I-step (60 ms)
	F_M	$F_M = F_{1s}$	Maximum fluorescence, when all PSII RCs are closed
	F_V	$F_V = F_M - F_0$	Variable fluorescence
	V_J	$V_J = (F_J-F_0)/(F_M-F_0)$	Variable Fluorescence at the J-step
	V_I	$V_I = (F_I-F_0)/(F_M-F_0)$	Variable fluorescence at the I-step
Fluorescence ratios	F_M/F_0		
	F_V/F_0		Maximum efficiency of photochemistry
	F_V/F_M	$(F_M - F_0)/F_M$	Maximum yield of primary photochemistry
Derived parameters	M_0	$4(F_{300}\mu s - F_0)/(F_M - F_0)$	Rate of reaction center closure
	N	$(Area/(F_M - F_0)) \times M_0 \times (1/V_J)$	Turn-over number Q_A reduction events between time 0 and F_M
	Ψ_0	$\Psi_o = ET_0/TR_0 = 1- V_J$	Likelihood that a trapped exciton can move an electron further than $Q_A{}^-$
	φ_{Eo}	$\Phi_{Eo} = ET_0/ABS = (1-(F_0/F_M)) \times \Psi$	Quantum yield of electron transport to intersystem electron acceptors (between photosystem II and I)
	φ_{Do}	$\Phi_{Do} = 1- \Phi p_0 = (F_0/F_M)$	Quantum yield at time 0 for energy dissipation
	Φ_{pav}	$\Phi_{pav} = \Phi p_0(1-V_{av})$	Average quantum yield of primary photochemistry
	PI_{ABS}	$(RC/ABS) \times (\varphi_{Po}/1- \varphi_{Po})$	Performance Index of PSII normalized for equal absorption
Energy flux parameters	ABS/RC	$ABS/RC = M_0 \times (1/V_J) \times (1/\Phi p_0)$	Energy absorption by antenna per reaction center (indicator of antenna size for PSII)
	TR_0/RC	$TR_0/RC = M_0 \times (1/V_J)$	Flux of excitons trapped per reaction center: reduction of Pheophytin and Q_A
	ET_0/RC	$ET_0/RC = M_0 \times (1/V_J) \times \Psi_0$	Energy flux for electron transport per reaction center: beyond $Q_A{}^-$
	DI_0/RC	$DI_0/RC = (ABS/RC)-(TR_0/RC)$	Flux ratio of energy dissipation per reaction center

categories of photosynthetic traits across treatments, except for some gas exchange parameters (C_i and C_i/C_a under drought recovery) and chlorophyll fluorescence measurements (V_I under drought, as well as, F_I and F_M under recovery) (Tables S2-S5). Significant treatment effects ($P \leq 0.001$) on all traits pointed out the expression of phenotypic plasticity under drought stress. Most of the photosynthetic related traits showed substantial reductions. However, φ_{Do}, ABS/RC, DI_0/RC, WUE, and WUEi were increased for plants grown under drought stress compared to the control plants.

We observed several significant correlations among photosynthetic traits under drought stress (Figure 2A; Figures S4). For instance, drought stress tolerance index (DS) was negatively correlated with the gas exchange parameters g_s, C_i, T_r, and P_n but positively correlated with the F_V/F_M, RWC, and WUE (Figure 2A). Our results also showed a negative correlation between WUE and C_i, g_s, and T_r. The photosynthetic trait correlations under recovery conditions were almost in line with the DSI of the studied traits. Furthermore, we observed significant regional differentiation in gas exchange

Downloaded from https://academic.oup.com/hr/article/doi/10.1093/hr/uhac124/6603865 by guest on 14 January 2023

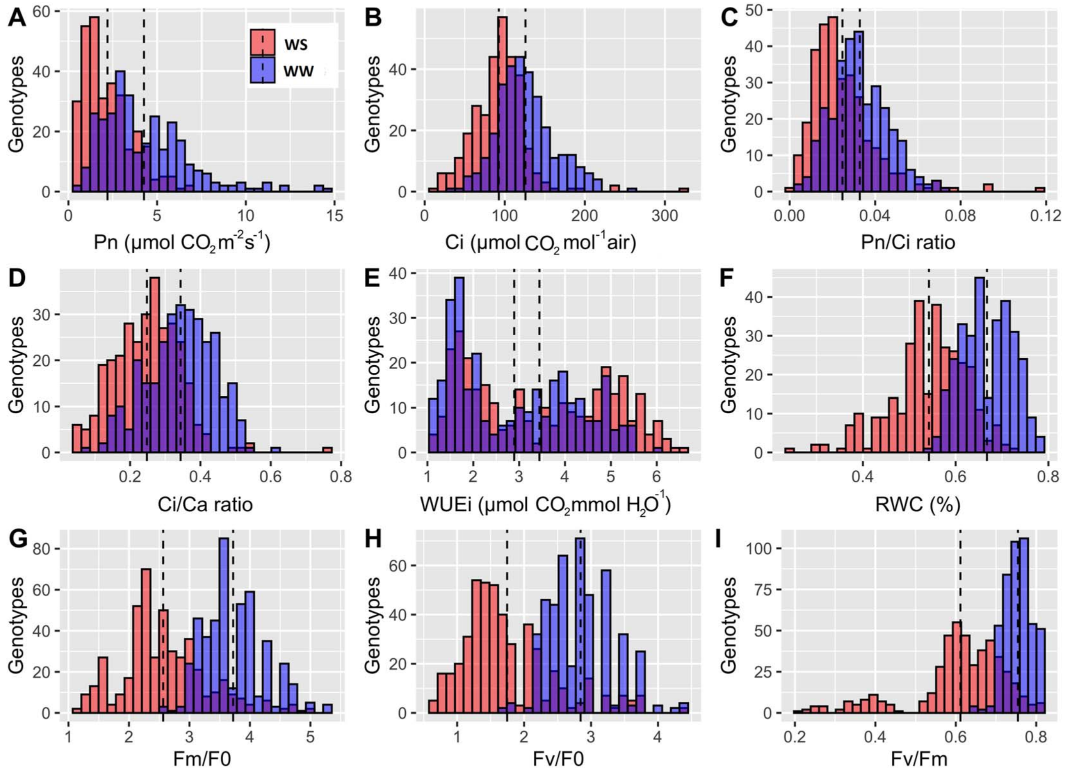

Figure 1. Distribution of the photosynthetic related traits of 6-month-old plants of walnut under well-watered (blue), severe stress (red) conditions and overlap between them (purple) in the first-year experiment. (a) P_n, (b) C_i, (c) P_n/C_i, (d) C_i/C_a, (e) WUE_i, (f) RWC, (g) F_M/F_0, (h) F_V/F_0, and (i) F_V/F_M in 140 Iranian walnut families. Traits are indicated on the x-axis and number of families on the y-axis. Dashed vertical lines indicate the mean of each distribution.

measurements (Figure 3; Figure S5). The highest WUE under severe drought stress was found in Markazi and Isfahan populations.

We performed principal component analysis (PCA) on all photosynthetic traits of the 140 families during stress to further explore the key parameters and provide an integrated view of the relationships among traits within populations (Figure 2B; Figures S4). PCA using DSI (value of trait under WS / value of trait under WW) of the studied traits showed that the first five components (PC1–5) cumulatively explained more than 90% of the total variation for the photosynthetic traits across the panel under severe drought stress (Figure 2B). The first principal component (PC1), explaining more than 39% of the total variation, was associated with photosynthetic traits; positively with F_V/F_M (78%), WUE (58%) and RWC (57%), and negatively with Ci (66%), g_s (66%), T_r (65%), and P_n (54%) (Figure 2B). Since PC1 has a positive and high correlation with water relation parameters and F_V/F_M index it could be viewed as a quality of plant water status and photosynthetic efficiency under severe drought stress. As a result, PC1 can be considered as a drought tolerant component in our studied walnut panel. On the other hand, since previous studies in plants have shown that positively correlated traits with PC1 (F_V/F_M, WUE and RWC) have higher heritability than other studied traits, they can be used as biomarkers for selection of drought tolerant genotypes in future studies. The second principal component (PC2), explaining more than 15% of the total variation, was positively associated with phenotypic variation of WUE (57%) (Figure 2B).

SNP calling and population structure

From the 95 mother trees genotyped using both the SNP array (MArray) and GBS (MGBS), and the 150 families genotyped through GBS (PGBS), 94, 87, and 136 gave good quality data, respectively, and were used as three separate panels for further genomic analysis. The Array-scored SNPs were categorized into six default groups of Affymetrix Power Tools (APT) according to clustering performance as follows; 1) Poly High Resolution (PHR), 2) Mono High Resolution (MHR), 3) No Minor Homozygote (NMH), 4) Call Rate Below Threshold (CRBT), 5) Off-Target Variant (OTV) and 6) Other as described by Arab et al (2019) [17]. PCA and population structure estimates for each set of panels genotyped through GBS (PGBS and MGBS; Figure 2C and D) divided our panels into four main clusters based on their geographical locations. Data from the Axiom *J. regia* 700 K SNP array (Arab et al., 2020) also classified mother trees into four main groups. These results confirm that our walnut panels (MArray, MGBS and PGBS) comprise mainly four genetic clusters. Therefore, the optimal number of genetic groups was chosen as four for association mapping studies to control the family structure. LD decayed (shown by r [2] < 0.2) within 10 kb across the genome as described by Arab et al. (2020) [3].

Downloaded from https://academic.oup.com/hr/article/doi/10.1093/hr/uhac124/6603865 by guest on 14 January 2023

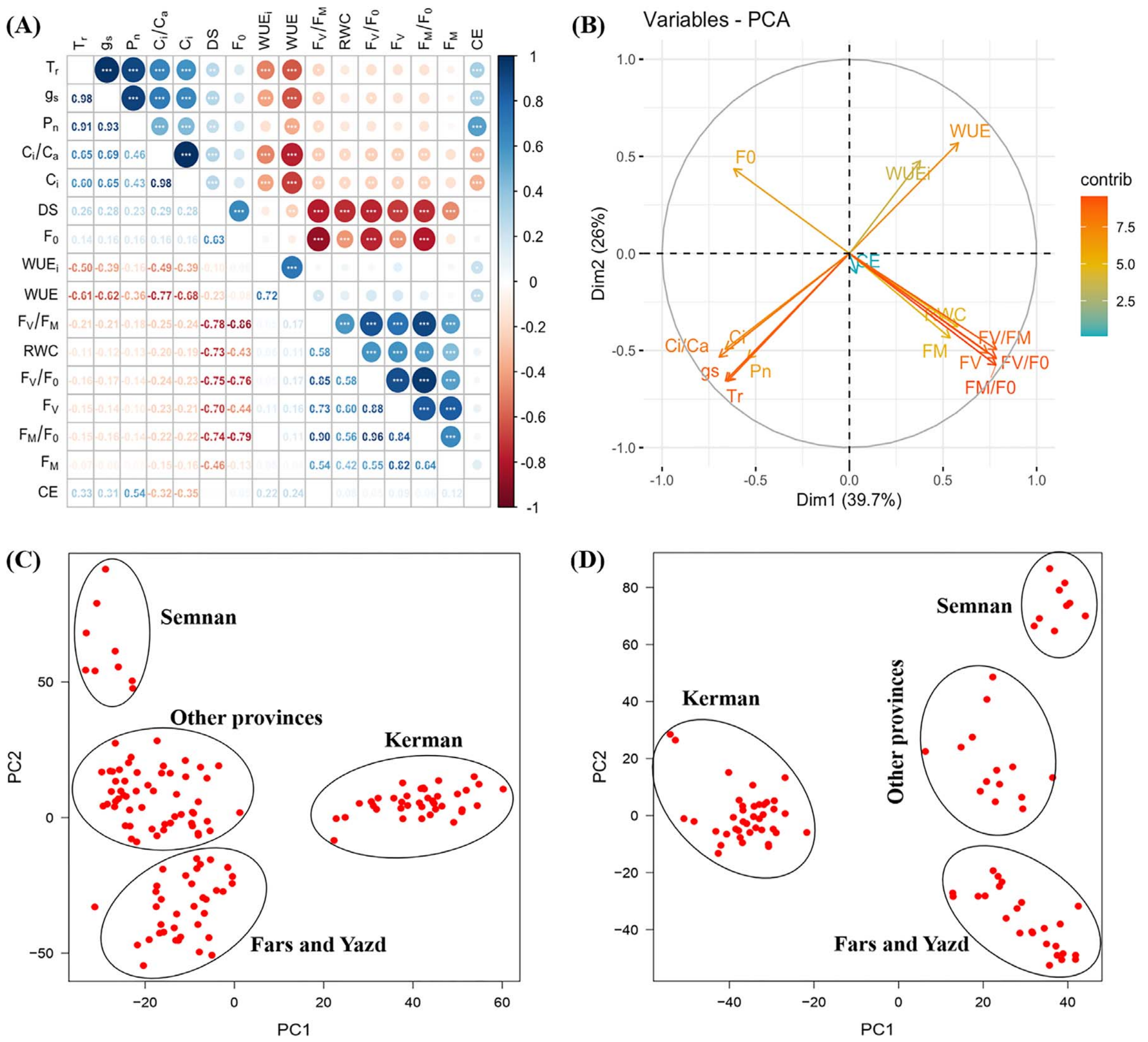

Figure 2. Correlation plot of photosynthetic traits and principal component analysis (PCA) plots of phenotypic data and SNP markers. (A) Correlation coefficient of plasticity in trait value (DSI), between all gas exchange and chlorophyll fluorescence phenotypes in the 140 walnut families grown in a common garden under severe water stress condition in the first-year experiment. The colour spectrum, bright blue to bright red represents highly positive to highly negative correlations. Stars in circle indicate the significance of correlations (*$P \leq 0.05$, **$P \leq 0.01$, and ***$P \leq 0.001$). (B) Principal component analysis of photosynthetic traits with the first two components showing variation in plasticity in trait value in the 140 walnut families grown in a common garden under severe water stress condition in the first-year experiment. The traits coloured by red contributed more to the variation explained by PC1 and PC2, than those coloured by blue. (C) PCA of the 140 Persian walnut families using 44 207 GBS-derived SNPs (PGBS). (D) PCA of the 87 Persian walnut mother trees using 40 828 GBS-derived SNPs (MGBS). See Table 1 for the definition of measured traits.

Genome wide association study

After filtering for minor allele frequency (MAF > 5%) and missing rate (< 10%), we obtained three SNP panels including 295 685 polymorphisms (MArray), 40 828 SNPs (MGBS), and 43 607 SNPs (PGBS). For ease of reading and understanding, we divided the GWAS results into six categories, based on the correspondence of the genotyping approach and the studied trait results. Also, for each of the categories, we classified the results into 5 distinct groups based on the experimental conditions (WW, WS and WR) and phenotypic plasticity of traits (DSI = WS/WW∗100; DRI = WR/WW∗100; see Methods) (Table 2; Table S6).

Given the significant (α/n) and suggestive (1/n) thresholds (where n is marker number; see Methods), we identified 578 and 1543 unique SNPs, respectively, for the studied phenotypic traits (Table 2, Table S6, Supplementary data sets S1-S3). Our results found a total of 198 (34%), 228 (40%) and 152 (26%) significant associations for at least one photosynthetic-related trait under all conditions using the MArray, MGBS and PGBS datasets, respectively (Table 2; Figure 4). We also identified a total of 544 (35%), 524 (34%) and 481 (31%) suggestive SNPs associated with all photosynthetic traits under all conditions through the MArray, MGBS and PGBS datasets, respectively (Table S6; Figure S6). One and five of the suggestive SNPs identified were in common between the MArray and MGBS, and the MGBS and PGBS datasets, respectively. These results indicate that the strong significance of these marker-trait associations identified using two different genotyping methods.

Downloaded from https://academic.oup.com/hr/article/doi/10.1093/hr/uhac124/6603865 by guest on 14 January 2023

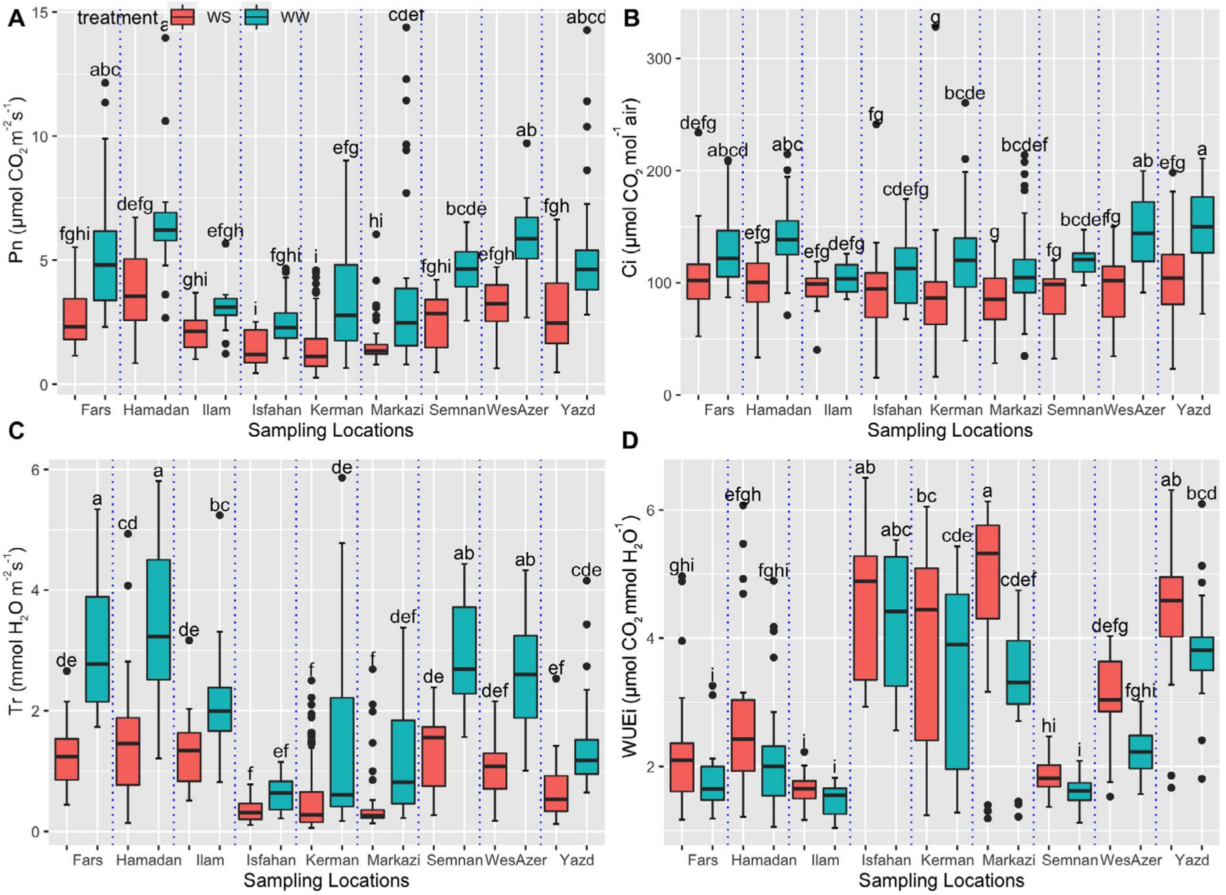

Figure 3. Box plots of the regional differentiation of the gas exchange parameters in the 140 walnut families grown in a common garden under well-water and severe water stress conditions in the first-year experiment. (A) P_n, (B) C_i, (C) T_r and (D) WUE_i. WW: well-water; WS: water-stress. Different letters indicate statistically significant differences at the level of $p < 0.01$ (Tukey's test).

Table 2. Summary of significant marker-trait associations identified by GWAS analysis using two approaches (FarmCPU and MLMM) for all the photosynthetic traits across six categories (A–F) in well-water (WW), water-stress (WS), water-recovery (WR) conditions and for drought stress index (DSI) and drought recovery index (DRI) of traits as a relative measure

Trait Classification	Marker-Trait	WW	WS	WR	DSI	DRI
(A) Chl fluorescence (Mother trees- Array)	Associations	63	69	10	41	23
	Unique SNPs	50	41	9	35	18
(B) Chl fluorescence (Mother trees- GBS)	Associations	59	64	32	31	9
	Unique SNPs	43	45	26	23	5
(C) Chl fluorescence (Progeny-GBS)	Associations	51	14	26	14	68
	Unique SNPs	38	10	23	13	55
(D) Gas exchange (Mother trees- Array)	Associations	28	15	2	11	1
	Unique SNPs	25	14	2	10	1
(E) Gas exchange (Mother trees- GBS)	Associations	56	38	13	14	24
	Unique SNPs	32	29	10	14	21
(F) Gas exchange (Progeny-GBS)	Associations	2	4	5	-	8
	Unique SNPs	2	4	4	-	8
Total associations	Associations	259	204	88	111	133
	Unique SNPs	190	143	74	95	108
Associations detected by the FarmCPU approach	Associations	71	47	14	24	32
	Unique SNPs	53	40	14	24	27
Associations detected by the MLMM approach	Associations	188	157	74	87	101
	Unique SNPs	146	115	65	75	88
SNPs detected by both approaches	Total SNPs	259	204	88	111	133
	Unique SNPs	199	155	79	99	115

We identified 434 and 176 SNPs associated with chlorophyll fluorescence and gas exchange phenotypes, respectively. Of those, 131, 96, 58, 71, and 78 SNPs linked to chlorophyll fluorescence phenotypes were identified under WW, WS, and WR conditions and phenotypic plasticity of traits (DSI and DRI), respectively (Table 2;

Downloaded from https://academic.oup.com/hr/article/doi/10.1093/hr/uhac124/6603865 by guest on 14 January 2023

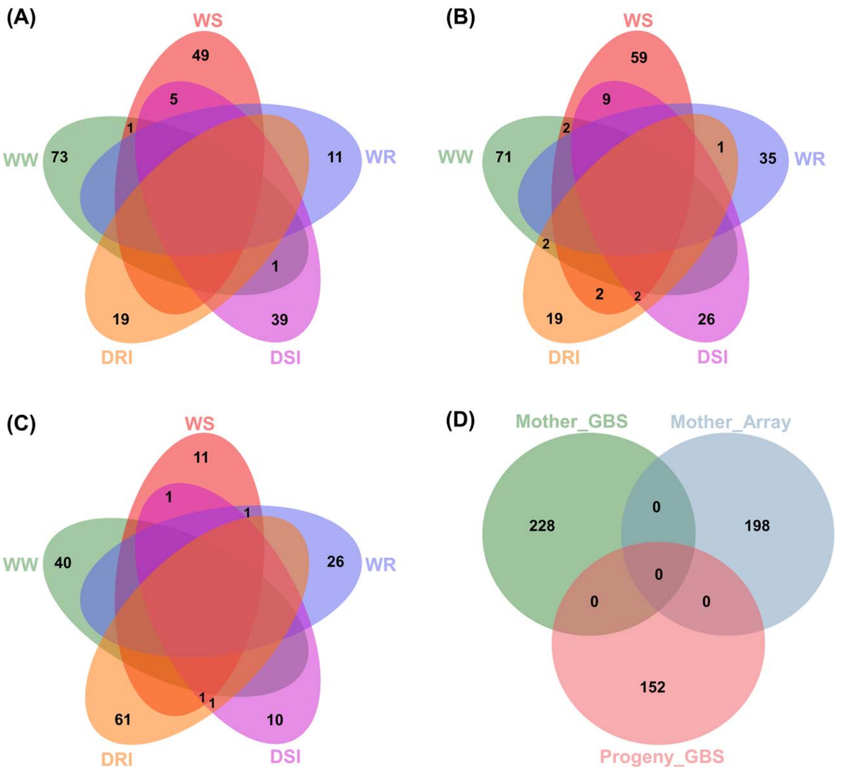

Figure 4. Venn diagrams depicting the significant SNPs identified for all the photosynthetic traits across experiments (First and second years) and different conditions through GWAS using different datasets MArray (A), MGBS (B) and PGBS (C) and highlight the concordance between different datasets (D). Well-water: WW, water-stress: WS, water-recovery: WR, drought stress index: DSI, drought recovery index: DRI.

Supplementary data set S4). We also found 59, 47, 16, 24, and 30 SNPs associated with gas exchange parameters under WW, WS, and WR conditions and phenotypic plasticity of traits (DSI and DRI), respectively. We observed only a few significant trait-locus associations across experiments, indicating different responses of walnut to drought stress and re-watering. In addition, we detected 1699 suggestive SNPs associated with gas exchange (n = 1104) and chlorophyll fluorescence (n = 565) phenotypes (Supplementary data set S4). The highest number of photosynthetic-associated SNPs was identified under WW (508), followed by WS (407), DSI (221), DRI (300), and WR (263). More details are summarized in Tables S6–S8, and Supplementary data set S5.

Significant associations were identified on all the walnut chromosomes. The greatest number of associations was found on chromosome 7 (60 SNPs) and the lowest number of associations was found on chromosome 14 (17 SNPs). We also found SNPs that were associated with multiple traits. A total of 11, 28, and 113 significant ($p < 0.05/n$) SNPs were associated with more than three, two, and one trait, respectively. Among these, the marker AX-170754326 on chromosome 2 was simultaneously associated with thirteen traits related to the chlorophyll fluorescence. We also identified a significant SNP (S11_15758875) located on chromosome 11 associated with seven traits related to chlorophyll fluorescence. Using the suggestive threshold ($p < 1/n$), 33, 47 and 95 SNPs were associated with more than five, four and three traits, respectively. In particular, the SNP AX-170754326 was simultaneously associated with twenty traits related to chlorophyll fluorescence. Also, the locus S4_5195821 on chromosome 4 was associated with fourteen chlorophyll fluorescence related traits. Our results are in line with the quantitative (multigenic) nature of drought tolerance and the strong correlation among the studied photosynthetic traits.

By further lowering the *P*-values threshold to $9.95.0 \times 10^{-5}$ ($-\log_{10}P = 5$), we also observed clusters of linked SNPs associated to a single trait. For example, twenty-three, twenty-three, twenty-two, nineteen and twelve suggestive SNPs on chromosome 4 were associated with DRI of F_V, PC2, φ_{Do}, F_V/F_M and F_M/F_0, respectively. Also, under normal condition, thirty and twenty-nine suggestive SNPs on chromosome 4 were identified for the CE and g_s traits. For DSI of PC1, F_V/F_M, F_M/F_0, φ_{Do}, C_i and RWC were identified twenty-six, twenty-four, twenty-three, seventeen, fifteen and fifteen suggestive SNPs on chromosomes 1, 2, 7, 2, 5 and 8 respectively. Under water stress condition, eighteen, fifteen, fourteen, eleven, and ten suggestive SNPs on chromosome 2 were identified for the F_M/F_0, ABS/RC, PC1, $F_{i,}$ and F_M respectively. On the other hand, forty-eight, forty-seven, thirty-nine suggestive SNPs on chromosome 8 were linked to Ψ_0, φ_{Eo} and V_J under normal respectively. Therefore, at these positions, a putative major quantitative trait locus (QTL) for studied traits may be located. More

Downloaded from https://academic.oup.com/hr/article/doi/10.1093/hr/uhac124/6603865 by guest on 14 January 2023

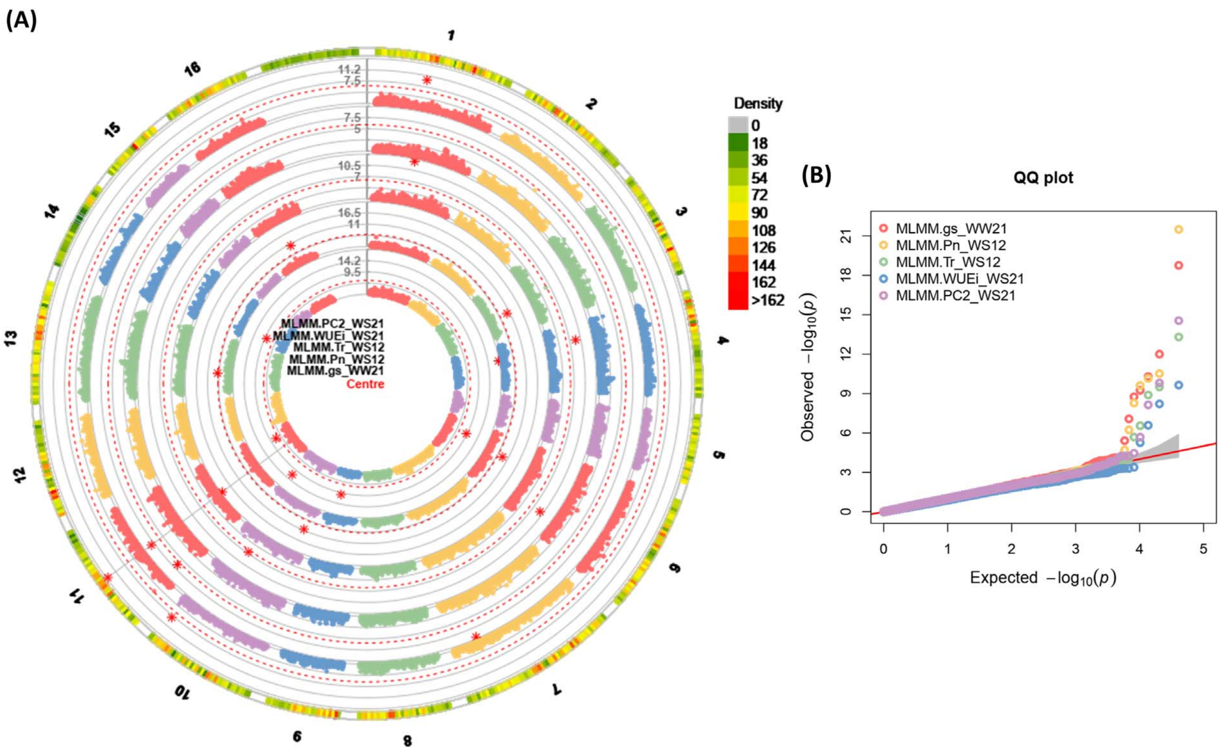

Figure 5. (A) and (B) Circular *Manhattan plots (left), and* quantile-quantile *plots (right) of association analysis using the MGBS dataset and MLMM (Q + K) model for* gas-exchange related *traits from centre to the outside of plot including; (a)* gs_WW21, *(b)* Pn_WS12, *(c)* T_r_WS12, *(d)* WUE_i_WS21, *and (e)* PC2_WS21. The outermost circle shows SNP density in 1 Mb windows for each chromosome *where green to red indicates low to high marker density*. Black bold line (Y-axis) represents –Log10 P-value. The circles of red dashed lines *represent the Bonferroni-corrected significance threshold. Red stars indicate genome-wide significantly associated SNPs. Vertical grey dashed lines are drawn through GWAS findings to indicate multi-trait associations.* For QQ plots, X-axis represents expected $-\log_{10}$ (p-value) and Y-axis is observed $-\log_{10}$ (p-value) of each SNPs. Well-water: WW, water-stress: WS.12: severe drought stress in the first year, 21: severe drought stress in the second year.

information regarding the suggestive SNPs is summarized in Supplementary data sets S6-S7. Manhattan plots and QQ plots for most of the important studied traits under different conditions are shown in Figures 5-8 and Figures S7-S12.

Candidate gene identification for significant SNPs

Candidate genes underlying each measured trait were selected, based primarily on the significant and suggestive SNPs within the gene or in the flanking regions (Tables 3-4). A comprehensive list of identified genes is provided in Supplemental data sets S6-S7. Our BLASTX results showed out of 578 and 1543 significant and suggestive SNPs identified by GWAS, 67 (11%) and 204 (13%) SNPs were located inside the gene, respectively (Supplementary data set S6). When we searched 20 kb windows around the significant and suggestive SNPs associated with photosynthetic traits, 382 (66%) and 1043(68%) SNPs were functionally annotated based on the best/top BLAST alignments for each SNP (Supplementary data set S7). Most of the significant or suggestive SNPs located within or nearby the genes which were involved in the regulation of photosynthesis and drought tolerance. More details are described in supplementary file.

Gene-set enrichment and network analysis

We extracted all genes within 20 kb around the significant and suggestive SNPs. Given a *P*-value of 9.95×10^{-5} ($-\log_{10}P = 5$), we detected 5907 (MArray), 1588 (MGBS) and 1497 (PGBS) SNPs associated with all the photosynthetic traits under all conditions (Figure 9A). Of these, 61 and SNPs were common between MGBS and PGBS, and 9 SNPs between MArray and MGBS (Figure 9A). We identified 6254 candidate genes adjacent to the SNPs of Array dataset, while 1621 and 1615 candidate genes were detected near the MGBS and PGBS SNPs, respectively (Figure 9B). Of these, 874 candidate genes were identified by more than one GWAS dataset (MArray, MGBS, and PGBS). We also found that 67 genes were common between the three datasets (Figure 9B). On the other hand, 3123, 4098 and 2268 SNPs associated with studied traits under WW, WS and WR conditions were located within or 10 kb upstream or downstream of 3376, 4468 and 2582 genes in the walnut gene annotation v2.0, respectively (Figure 9C and D). The identified genes were characterized, and various KEGG pathways and GO terms were found to be particularly relevant to drought tolerance and photosynthesis.

We identified 96, 104 and 96 KEGG pathways using identified genes associated with photosynthetic traits under WW, WS and WR conditions, respectively, of which

Downloaded from https://academic.oup.com/hr/article/doi/10.1093/hr/uhac124/6603865 by guest on 14 January 2023

Table 3. Functional annotations of the associated SNPs located within the candidate genes for gas exchange parameters measured under drought stress and recovery in the first and second year experiments

Method	Model	SNP	Position	Chrom	Trait	P.value	maf	R2	Sequence Description	Blast Top Hit E-Value	Blast Top Hit Accession
MGBS	MLMM	S10_5 181 343	5 181 343	Chr10	Pn_WS12	3.06E-11	0.07	0.08	chaperone protein ClpB3, chloroplastic-like	6.00E-09	KAF5457676
MArray	FarmCPU	AX-171161268	25 869 736	Chr11	CE_DSI12	2.19E-09	0.1	0.05	transcription factor UPBEAT1	6.70E-09	XP_018842308
MGBS	MLMM	S4_20 095 205	20 095 205	Chr4	gs_WR22	2.19E-08	0.07	0.16	probable LRR receptor-like serine/threonine-protein kinase At3g47570	2.90E-09	XP_018815270
MGBS	MLMM	S10_14 590 765	14 590 765	Chr10	RWC_DSI12	8.37E-08	0.37	0.07	ethylene-responsive transcription factor ERF054-like	3.70E-09	XP_018838445
MArray	FarmCPU<br>MLMM	AX-171161782	6 654 073	Chr16	CE_WW21<br>gs_WW21<br>PC1_WW21<br>Tr_WW21	6.48E-07<br>2.14E-06<br>1.31E-05<br>5.11E-05<br>3.69E-06<br>1.56E-05<br>5.16E-05	0.17	0.23<br>0.20<br>0.17<br>0.14	thylakoid lumenal 17.4 kDa protein, chloroplastic	2.10E-11	XP_018842827
MArray	FarmCPU<br>MLMM	AX-171195950	1 291 896	Chr13	DS_WR22<br>DS_WS21<br>DS_WS12	9.21E-06<br>1.13E-05<br>1.91E-05<br>2.11E-05<br>3.58E-05	0.38	0.22<br>0.21<br>0.2	zinc finger protein 4-like	2.20E-10	XP_018827763
MArray	FarmCPU<br>MLMM	AX-171203906	5 028 296	Chr2	gs_WS12<br>CE_WS21	1.83E-06<br>5.65E-06<br>2.80E-05	0.12	0.19<br>0.18	ABC transporter A family member 2-like isoform X2	1.40E-10	XP_035543787
PGBS	MLMM<br>FarmCPU	S1_3 218 672	3 218 672	Chr1	PC3_DSI12<br>CE_DSI12	3.82E-06<br>5.01E-06<br>5.32E-06<br>2.77E-05	0.18	0.11<br>0.13	serine/threonine-protein phosphatase 4 regulatory subunit 3-like isoform X3	1.80E-8	KAF5464745
MArray	MLMM<br>FarmCPU	AX-171223024	3 113 160	Chr6	PC2_WW21	9.09E-06<br>1.12E-05	0.43	0.12	wall-associated receptor kinase 2-like	4.40E-07	XP_035546447
MArray	FarmCPU<br>MLMM	AX-170962930	15 525 358	Chr5	Tr_DSI12<br>gs_DSI12	9.34E-06<br>2.99E-05<br>1.14E-05<br>3.92E-05	0.11	0.19<br>0.15	transcription factor bHLH155-like isoform X2	3.70E-06	KAF5482376
MArray	FarmCPU<br>MLMM	AX-171159263	15 485 089	Chr5	Tr_DSI12<br>gs_DSI12	9.34E-06<br>2.99E-05<br>1.14E-05	0.11	0.18<br>0.16	DEAD-box ATP-dependent RNA helicase 41 isoform X1	4.90E-09	XP_018840854
MGBS	FarmCPU	S11_4 360 438	4 360 438	Chr11	CE_DRI22	1.03E-05	0.34	0.20	protein FAR1-RELATED SEQUENCE 5-like	5.10E-10	KAF5454297
MArray	FarmCPU<br>MLMM	AX-171210671	39 073 355	Chr1	gs_DSI12<br>Tr_DSI12	1.23E-05<br>1.74E-05<br>3.12E-05	0.05	0.18<br>0.17	probable chlorophyll(ide) b reductase NYC1, chloroplastic isoform X1	1.10E-09	XP_035539329
MArray	FarmCPU<br>MLMM	AX-171595118	761 867	Chr3	Tr_DSI21<br>Pn_DSI21<br>PC1_DSI21<br>gs_DSI21	1.54E-05<br>1.99E-05<br>2.53E-05<br>5.35E-05<br>1.67E-05<br>2.41E-05<br>4.15E-05<br>7.01E-05	0.23	0.17<br>0.16<br>0.04<br>0.15	pentatricopeptide repeat-containing protein At5g12100, mitochondrial	4.70E-12	KAF5473779

(Continued)

Downloaded from https://academic.oup.com/hr/article/doi/10.1093/hr/uhac124/6603865 by guest on 14 January 2023

Table 3. Continued

Method	Model	SNP	Position	Chrom	Trait	P.value	maf	R2	Sequence Description	Blast Top Hit E-Value	Blast Top Hit Accession
PGBS	FarmCPU<br>MLMM	S8_12 601 147	12 601 147	Chr8	Tr_WR22	2.53E-05<br>5.12E-05	0.16	0.15	protein FAR1-RELATED SEQUENCE 5-like	3.80E-12	XP_035550680
MArray	FarmCPU	AX-171210670	39 073 346	Chr1	Tr_DSI12	2.58E-05	0.05	0.17	probable chlorophyll(ide) b reductase NYC1, chloroplastic isoform X1	1.50E-10	XP_035539329
MArray	FarmCPU<br>MLMM	AX-171200931	1 158 989	Chr5	PC1_WS21<br>Ci_DSI21	2.74E-05<br>3.15E-05<br>3.81E-05<br>4.05E-05	0.20	0.14<br>0.14	E3 ubiquitin-protein ligase KEG isoform X1	3.70E-12	XP_018818212
MArray	MLMM<br>FarmCPU	AX-170590700	22 447 120	Chr16	gs_DRI22<br>Pn_DRI22<br>Tr_DRI22	2.98E-05<br>4.61E-05<br>6.67E-05<br>4.24E-05<br>6.31E-05<br>7.95E-05	0.25	0.12<br>0.15<br>0.12	serine/threonine-protein kinase PBS1-like	1.70E-10	XP_018838998
MArray	FarmCPU<br>MLMM	AX-171201119	2 039 053	Chr5	PC2_WS21	3.01E-05<br>4.78E-05	0.11	0.18	ATPase 11, plasma membrane-type-like isoform X2	2.30E-09	XP_018816318
MArray	FarmCPU	AX-171163808	562 727	Chr8	PC3_DSI12	3.05E-05	0.14	0.17	F-box protein CPR1-like isoform X1	1.90E-11	KAF5461318
MArray	FarmCPU	AX-170562520	18 034 619	Chr4	WUE_WS21<br>WUEi_WS12	3.08E-05<br>3.12E-05	0.14	0.11<br>0.14	galactan beta-1,4-galactosyltransferase GALS3-like	2.90E-10	KAF5472203
PGBS	MLMM	S14_4 949 136	4 949 136	Chr14	PC2_WS12	3.46E-05	0.08	0.11	cytochrome b561 and DOMON domain-containing protein At3g25290-like	1.40E-10	KAF5446463
MArray	FarmCPU	AX-171166866	36 403 551	Chr7	PC3_WS12	3.87E-05	0.23	0.15	mitogen-activated protein kinase kinase kinase NPK1	2.20E-09	XP_018840497
MArray	FarmCPU<br>MLMM	AX-171160624	3 197 511	Chr4	CE_DSI21	5.61E-05<br>8.85E-05	0.3	0.17	light-harvesting complex-like protein OHP1, chloroplastic	1.00E-10	XP_018841680
MArray	FarmCPU	AX-171206253	15 247 129	Chr1	WUE_DSI21	6.38E-05	0.3	0.15	transcription factor MYB93-like	2.70E-10	KAF5480862
PGBS	MLMM	S5_2 941 282	2 941 282	Chr5	PC1_WS21<br>Ci.Ca_WS21	6.98E-05<br>2.29E-05	0.14	0.07<br>0.09	phosphatidylinositol-3-phosphatase myotubularin-1-like isoform X1	1.20E-10	KAF5470068
PGBS	FarmCPU	S16_22 311 907	22 311 907	Chr16	Pn_DRI22	7.20E-05	0.38	0.12	glucose-6-phosphate 1-dehydrogenase, chloroplastic-like	8.30E-08	KAF5443584
PGBS	MLMM	S15_531 589	531 589	Chr15	CE_WR22	7.90E-05	0.23	0.12	protein EPIDERMAL PATTERNING FACTOR 2	0.0000016	KAF5444497

CHR: chromosome; MAF: minor allele frequency; R2 (%): proportion of variation explained by an SNP. WW: well-water; WS: water-stress; DSI: drought stress index; WR: water-recovery; DRI: drought recovery index. 12: severe drought stress in the first year, 21: severe drought stress in the second year and 22: recovery condition in the second year.

Downloaded from https://academic.oup.com/hr/article/doi/10.1093/hr/uhac124/6603865 by guest on 14 January 2023

Table 4. Functional annotations of the associated SNPs located within the candidate genes for chlorophyll fluorescence parameters measured under drought stress and recovery in the first and second year experiments

Method	Model	SNP	Position	Chrom	Trait	P.value	maf	R2	Sequence Description	Blast Top Hit E-Value	Blast Top Hit Accession
MGBS	MLMM<br>FarmCPU	S1_3 020 891	3 020 891	Chr1	F_V_WR22<br>F_M_WR22<br>F_I_WR22<br>F_J_WR22	9.05E-25<br>1.21E-12<br>7.61E-11<br>5.66E-06<br>9.40E-05<br>6.79E-05<br>1.29E-05<br>2.56E-05	0.09	0.25<br>0.29<br>0.30<br>0.26	probable receptor-like protein kinase At1g30570	2.90E-09	XP_018822983
MGBS	MLMM<br>FarmCPU	S7_4 088 661	4 088 661	Chr7	F_M/F_0_DSI21<br>F_V/F_0_DSI21<br>φ_{Pav}_WS21<br>PI_{ABS}_WS21	1.07E-19<br>3.89E-19<br>1.18E-08<br>4.25E-10	0.08	0.29<br>0.28<br>0.32<br>0.25	vacuolar sorting protein 39	9.30E-10	KAF5463988
PGBS	MLMM	S8_15 239 273	15 239 273	Chr8	PI_{ABS}_WW21	1.00E-13	0.42	0.16	protein CELLULOSE SYNTHASE INTERACTIVE 3-like	8.90E-08	XP_018830259
MArray	MLMM	AX-171212226	25 226 489	Chr16	F_V/F_0_WS21<br>F_M/F_0_WS21	1.04E-13<br>1.04E-13	0.32	0.16<br>0.16	cyclase-associated protein 1-like	1.20E-08	XP_018807064
PGBS	MLMM	S13_30 285 764	30 285 764	Chr13	DI_0/RC_WW21	1.58E-09	0.07	0.09	glucan endo-1,3-beta-glucosidase	1.10E-09	XP_018824600
MArray	MLMM	AX-171174023	10 769 441	Chr13	F_I_DSI21	1.85E-07	0.45	0.24	ubiquinone biosynthesis protein COQ4 homolog, mitochondrial-like	8.20E-11	KAF5449503
MArray	FarmCPU	AX-171179454	7 717 597	Chr1	F_J_WS21<br>TR_0/RC_WS21<br>F_I_WS21<br>F_M_WS21<br>F_V_WS21<br>F_V/F_M_DSI21<br>F_M/F_0_WS21<br>F_V/F_0_WS21	1.98E-05<br>3.24E-05<br>3.55E-05<br>3.81E-05<br>4.77E-05<br>5.46E-05<br>9.81E-05<br>9.81E-05	0.23	0.27<br>0.26<br>0.25<br>0.24<br>0.25<br>0.25<br>0.23<br>0.23	LRR receptor-like serine/threonine-protein kinase RPK2	5.00E-08	XP_018816677
PGBS	MLMM	S4_1 477 560	1 477 560	Chr4	F_M_WS21<br>F_I_WS21	5.78E-07<br>6.45E-07	0.39	0.08<br>0.07	BAG family molecular chaperone regulator 4	1.70E-08	KAF5471506
MGBS	FarmCPU	S12_21 754 474	21 754 474	Chr12	DI_0/RC_DSI21	1.62E-06	0.28	0.01	pentatricopeptide repeat-containing protein At5g19020, mitochondrial	1.00E-08	XP_018840533
MArray	FarmCPU	AX-171177765	1 333 358	Chr2	DI_0/RC_WS21<br>ABS/RC_WS21<br>ABS/RC_DSI21<br>φ_{D0}_WS21<br>F_V/F_M_WS21<br>F_V/F_M_DSI21<br>DI_0/RC_DSI21<br>N_DSI21<br>φ_{Pav}_WS21<br>PC1_WS21	2.32E-06<br>2.39E-06<br>5.31E-06<br>8.26E-06<br>8.26E-06<br>9.33E-06<br>9.34E-06<br>2.46E-05<br>2.97E-05<br>3.57E-05	0.48	0.34<br>0.35<br>0.32<br>0.3<br>0.3<br>0.29<br>0.29<br>0.26<br>0.27<br>0.26	cyclin-dependent kinase G-2-like isoform X1	6.90E-09	XP_018822176

(Continued)

Downloaded from https://academic.oup.com/hr/article/doi/10.1093/hr/uhac124/6603865 by guest on 14 January 2023

Table 4. Functional annotations of the associated SNPs located within the candidate genes for chlorophyll fluorescence parameters measured under drought stress and recovery in the first and second year experiments

Method	Model	SNP	Position	Chrom	Trait	P.value	maf	R2	Sequence Description	Blast Top Hit E-Value	Blast Top Hit Accession
MArray	FarmCPU	AX-171176298	17 931 021	Chr4	ET_0/RC_WR22<br>V_I_WS21<br>φ_{D0}_DRI22<br>F_V/F_M_DRI22<br>N_DSI21<br>F_V_DRI22	3.29E-06<br>1.63E-05<br>2.42E-05<br>3.05E-05<br>3.10E-05<br>4.87E-05	0.05	0.3<br>0.24<br>0.25<br>0.26<br>0.26<br>0.26	leucine-rich repeat receptor-like serine/threonine-protein kinase BAM3	7.50E-10	XP_018814580
MArray	FarmCPU<br>MLMM	AX-171200020	13 088 532	Chr2	V_I_DRI22	3.34E-06<br>7.97E-06	0.23	0.28	glucan endo-1,3-beta-glucosidase-like	1.20E-08	KAF5447740
MArray	FarmCPU<br>MLMM	AX-171218271	3 371 396	Chr6	F_0_DSI11	7.35E-06<br>1.17E-05	0.23	0.19	wall-associated receptor kinase-like 1	4.00E-06	XP_018812976
PGBS	FarmCPU	S6_3 429 326	3 429 326	Chr6	TR_0/RC_DRI22	6.15E-06	0.16	0.28	wall-associated receptor kinase 2-like	1.40E-09	XP_035546447
MArray	FarmCPU	AX-171217577	28 076 649	Chr11	F_0_DSI21	1.56E-05	0.44	0.29	glutathione S-transferase-like	3.30E-10	KAF5455705
MArray	MLMM	AX-171180361	19 366 994	Chr7	ABS/RC_WS21	1.91E-05	0.14	0.01	9-cis-epoxycarotenoid dioxygenase NCED6, chloroplastic	3.50E-11	XP_018817253
MArray	FarmCPU	AX-171192601	9 713 679	Chr13	TR_0/RC_DSI21<br>F_0_DSI21	1.934E-05<br>5.72E-05	0.36	0.30<br>0.26	pentatricopeptide repeat-containing protein At3g04130, mitochondrial	4.40E-10	XP_018857296
MArray	FarmCPU<br>MLMM	AX-171166172	18 425 461	Chr1	ψ_0_DRI22	2.13E-05<br>3.19E-05	0.49	0.27	beta-amylase-like	6.10E-09	XP_018846417
MGBS	FarmCPU	S11_4 360 438	4 360 438	Chr11	φ_{E0}_WW21<br>V_J_WW21<br>ψ_0_WW21<br>ET_0/RC_WW21	2.66E-05<br>2.69E-05<br>2.69E-05<br>4.34E-05	0.35	0.25<br>0.26<br>0.26<br>0.26	protein FAR1-RELATED SEQUENCE 5-like	5.10E-10	KAF5454297
PGBS	MLMM<br>FarmCPU	S11_10 333 165	10 333 165	Chr11	F_0_WS21	3.13E-05<br>3.33E-05	0.1	0.24	zinc finger BED domain-containing protein DAYSLEEPER-like	1.60E-07	XP_035547471
MArray	MLMM<br>FarmCPU	AX-171195135	42 508 998	Chr1	V_J_WR22<br>ψ_0_WR22	3.20E-05<br>3.20E-05<br>3.27E-05<br>3.27E-05	0.2	0.26<br>0.24	cytochrome P450 94A2-like	1.10E-09	KAF5482357
MArray	FarmCPU<br>MLMM	AX-171206801	1 322 756	Chr15	Fv_WS11	5.06E-06<br>3.81E-05	0.17	0.19	calcium-dependent protein kinase 32-like	7.30E-11	XP_018834447
MArray	MLMM	AX-171141014	11 272 317	Chr11	TR_0/RC_WW21	3.67E-05	0.18	0.01	protein FAR-RED IMPAIRED RESPONSE 1-like	6.10E-07	XP_035546226
MArray	FarmCPU	AX-171159752	37 390 204	Chr10	DI_0/RC_WW21	4.37E-05	0.11	0.2	glucose-6-phosphate 1-dehydrogenase, chloroplastic-like isoform X3	6.60E-12	XP_035550548
MArray	FarmCPU	AX-171199574	16 297 096	Chr8	φ_{Pav}_DRI22	6.96E-05	0.43	0.21	2-alkenal reductase (NADP(+)-dependent)-like	3.30E-09	KAF5462701
PGBS	FarmCPU<br>MLMM	S1_20 893 964	20 893 964	Chr1	F_V/F_M_WS12<br>F_0_WS12	5.06E-06<br>1.21E-05<br>9.81E-06<br>3.45E-05	0.11	0.14<br>0.12	G-type lectin S-receptor-like serine/threonine-protein kinase LECRK1	3.30E-11	XP_018859482
MArray	MLMM	AX-170695720	2 303 269	Chr11	F_V_WS12	1.82E-05	0.29	0.14	acyl-CoA-binding domain-containing protein 4	4.70E-09	XP_018850062
MArray	MLMM<br>FarmCPU	AX-171197482	2 063 855	Chr10	F_V/F_0_WS12	1.95E-05<br>4.49E-05	0.28	0.16	protein KINESIN LIGHT CHAIN-RELATED 2-like	2.00E-08	KAF5457244

CHR: chromosome; MAF: minor allele frequency; *R2* (%): proportion of variation explained by an SNP. WW: well-water; WS: water-stress; DSI: drought stress index; and DRI: drought recovery index. 11: moderate drought stress in the first year; 12: severe drought stress in the first year; 21: severe drought stress in the second year; and 22: recovery condition in the second year.

Downloaded from https://academic.oup.com/hr/article/doi/10.1093/hr/uhac124/6603865 by guest on 14 January 2023

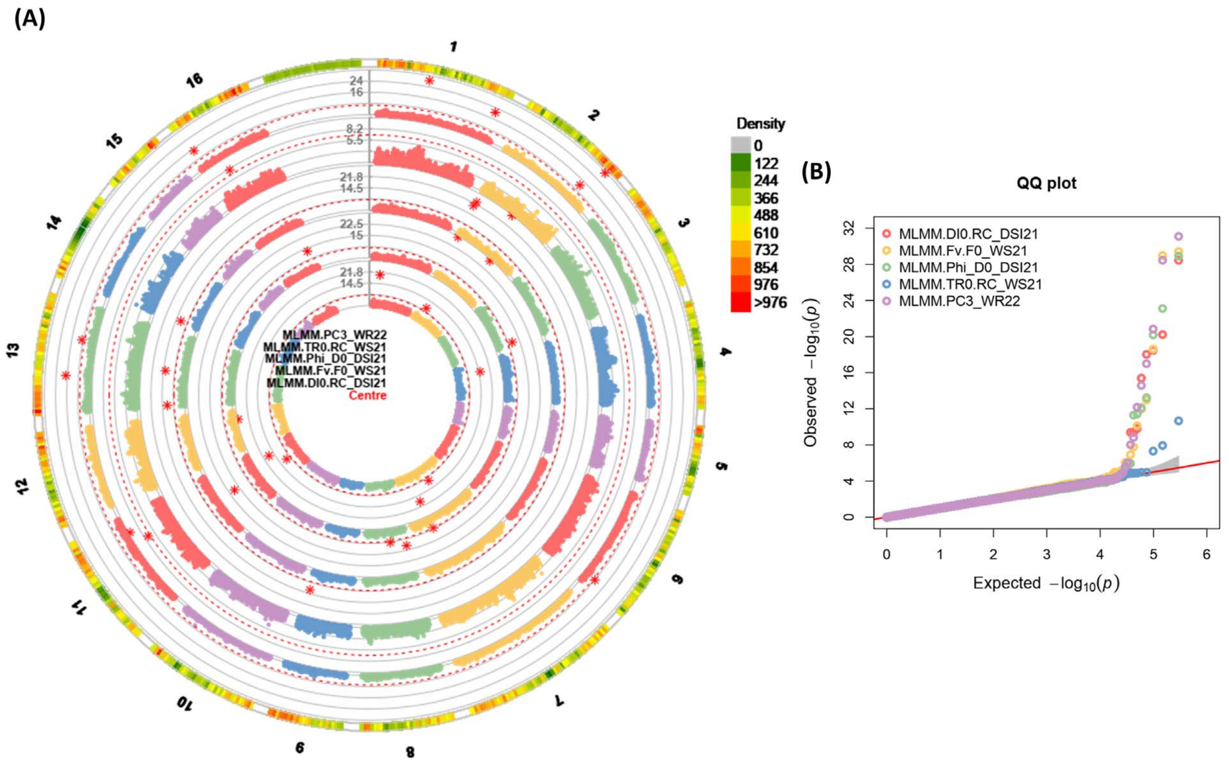

Figure 6. (A) Circular *Manhattan plots (left)*, *and* (B) quantile-quantile *plots (right) of association analysis using the MArray dataset and MLMM* (Q + K) *model for chlorophyll* fluorescence *related traits from centre to the outside of plot including; (a)* DI_0/RC_DSI21, *(b)* F_V/F_0_WS21, *(c)* φ_{Do}_DSI21, *(d)* TR_0/RC_WS21, *and (e)* PC3_WR22. The outermost circle shows SNP density in 1 Mb windows for each chromosome *where green to red indicates low to high marker density*. Black bold line (Y-axis) represents –Log10 P-value. The circles of red dashed lines *represent the Bonferroni-corrected significance threshold. Red stars indicate genome-wide significantly associated SNPs*. For QQ plots, X-axis represents expected $-\log_{10}$ (p-value) and Y-axis is observed $-\log_{10}$ (p-value) of each SNPs. Water-stress: WS, water-recovery: WR, drought stress index: DSI. 21: severe drought stress in the second year and 22: recovery condition in the second year.

15, 26 and 20 were significantly enriched (Figure 10A). Enriched pathways were related to metabolic processes including carbohydrate metabolism, amino acid metabolism, lipid metabolism, energy metabolism and signal transduction. We found that few pathways were shared by genes associated with photosynthetic traits in WW, WS and WR conditions, and several unique pathways were identified for genes associated with photosynthetic traits in each condition (Figure 10A). For instance, a few KEGG pathways related to carbohydrate metabolism including pentose and glucuronate interconversions (KEGG:00040), C5-Branched dibasic acid metabolism (KEGG:00660), and N-Glycan biosynthesis (KEGG:00510), were shared by genes associated with photosynthetic traits in all conditions. We found several pathways, including Ribosome (KEGG:03010), inositol phosphate metabolism (KEGG:00562), tyrosine metabolism (KEGG:00350), pentose phosphate pathway (KEGG:00030), Cysteine and methionine metabolism (KEGG:00270), starch and sucrose metabolism (KEGG: 00500), and glycolysis/gluconeogenesis (KEGG:00010) showed an overrepresentation of significant genes associated with photosynthetic traits under WS condition. On the other hand, sphingolipid metabolism (KEGG:00600), sulfur relay system (KEGG:04122), Phosphonate and phosphinate metabolism (KEGG:00440), carotenoid biosynthesis (KEGG:00906), and cyanoamino acid metabolism (KEGG:00460) pathways were unique to genes detected for photosynthetic traits under WR condition. We also identified several others pathways related to lipid and amino acid metabolisms, such as fatty acid biosynthesis (KEGG:00061), nitrogen metabolism (KEGG:00910), tryptophan metabolism (KEGG:00380), and biotin metabolism (KEGG:00780), were specific to genes associated with photosynthetic traits under WW condition (Figure 10A).

As a complementary approach to the KEGG survey, we found 25, 29 and 19 significant GO terms using identified genes, which were associated with photosynthetic traits under WW, WS and WR conditions, respectively (Figure S13). GO terms of the protein metabolic process (GO:0019538), lipid phosphorylation (GO:0046834), phosphatidylinositol phosphate kinase activity (GO:0016307), and carbohydrate biosynthetic process (GO:0016051) under WS condition, as well as, response to photooxidative stress (GO:0080183), membrane lipid metabolic process (GO:0006643), response to oxidative stress (GO:0006979) and antioxidant activity (GO:0016209) under WR condition were the important enriched terms (Figure S13). These results indicated different genetic controls of photosynthetic traits under drought and recovery conditions in Persian walnut.

To provide further insight into the interaction in the pathways related to drought-response related genes,

Downloaded from https://academic.oup.com/hr/article/doi/10.1093/hr/uhac124/6603865 by guest on 14 January 2023

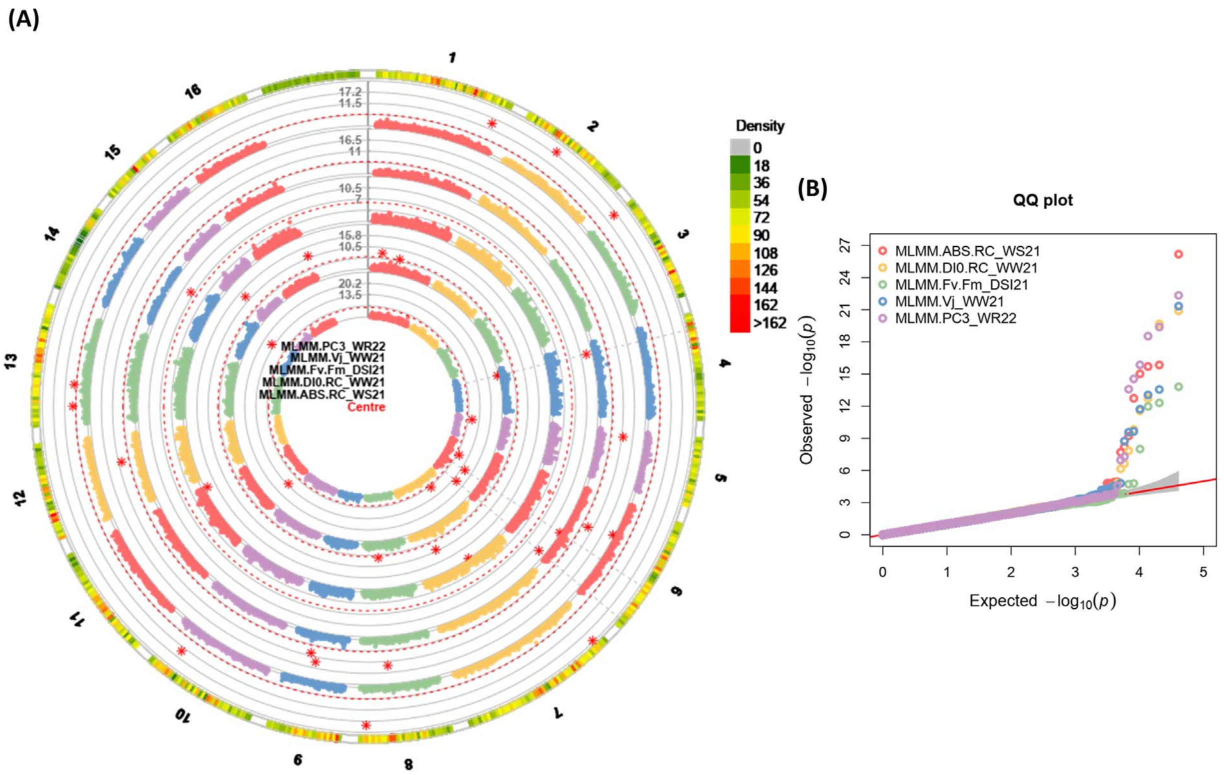

Figure 7. (A) and (B) Circular *Manhattan plots (left), and* quantile-quantile *plots (right) of association analysis using the MGBS dataset and MLMM (Q + K) model for chlorophyll* fluorescence *related traits from centre to the outside of plot including; (a) ABS/RC_WS21, (b)* DI_0/RC_WW21, *(c)* F_V/F_M_DSI21, *(d)* V_J_WW21, *and (e) PC3_WR22*. The outermost circle shows SNP density in 1 Mb windows for each chromosome *where green to red indicates low to high marker density*. Black bold line (Y-axis) represents –Log10 P-value. The circles of red dashed lines *represent the Bonferroni-corrected significance threshold. Red stars indicate genome-wide significantly associated SNPs. Vertical grey dashed lines are drawn through GWAS findings to indicate multi-trait associations*. For QQ plots, X-axis represents expected $-\log_{10}$ (p-value) and Y-axis is observed $-\log_{10}$ (p-value) of each SNPs. Well-water: WW, Water-stress: WS, water-recovery: WR, drought stress index: DSI. 21: severe drought stress in the second year and 22: recovery condition in the second year.

we proceeded beyond enriching pathways to identify highly modulated drought and photosynthesis specific sub-networks. The protein–protein interactions (PPI) were identified using the STRING database based on the genes associated with the photosynthetic traits in each condition separately (WW, WS and WR) and the network was subsequently constructed using Cytoscape (Figure 10B-D). The most important identified hub genes were directly or indirectly involved in the photosynthesis and drought stress responses (Figure 10B-D).

Discussion

Given the challenges of climate change and consequent water shortage in Persian walnut production areas, understanding the complex physiological and genetic basis of drought tolerance and adaptation in walnut is becoming increasingly important [3]. Natural variation in photosynthetic traits in Persian walnut is a largely unexploited resource that can provide useful information for breeding or engineering photosynthetic efficiency. In the current study, we explored natural variation in photosynthetic parameters in Persian walnut by combining a common garden approach with GWAS and pathway enrichment analysis. We have identified both genomic regions and pathways that suggest important adaptive mechanisms exist within the walnut populations sampled in this study. Here, we discuss our main findings and their implications for walnut breeding.

Natural variation in photosynthetic traits of Persian walnut populations

Photosynthesis is highly susceptible to drought stress and can be studied by measuring gas exchange parameters or analysing chlorophyll fluorescence [29]. In present study, photosynthetic traits varied widely under WW, WS, and WR conditions, suggesting that genetic improvement of these traits is feasible in walnut. Water stress (WS) and subsequent recovery (WR) treatments significantly affected all traits compared to the well-watered control (WW), in agreement with previous reports in walnut [6,8]. Under water stress conditions most families exhibited decreased net photosynthetic rate (P_n), stomatal conductance (g_s), transpiration rate (T_r), and intercellular CO_2 concentration (C_i), whilst both intrinsic and instantaneous water use efficiency (WUE) increased substantially. These findings are consistent with those of Zhang et al. (2006) [30] and Arab et al. (2020) [3], suggesting that stomatal closure via the ABA-dependent pathway resulted in decreasing water loss and increasing WUE. We also observed high correlation between P_n, T_r, and g_s implying regulation of stomatal aperture affected P_n

Downloaded from https://academic.oup.com/hr/article/doi/10.1093/hr/uhac124/6603865 by guest on 14 January 2023

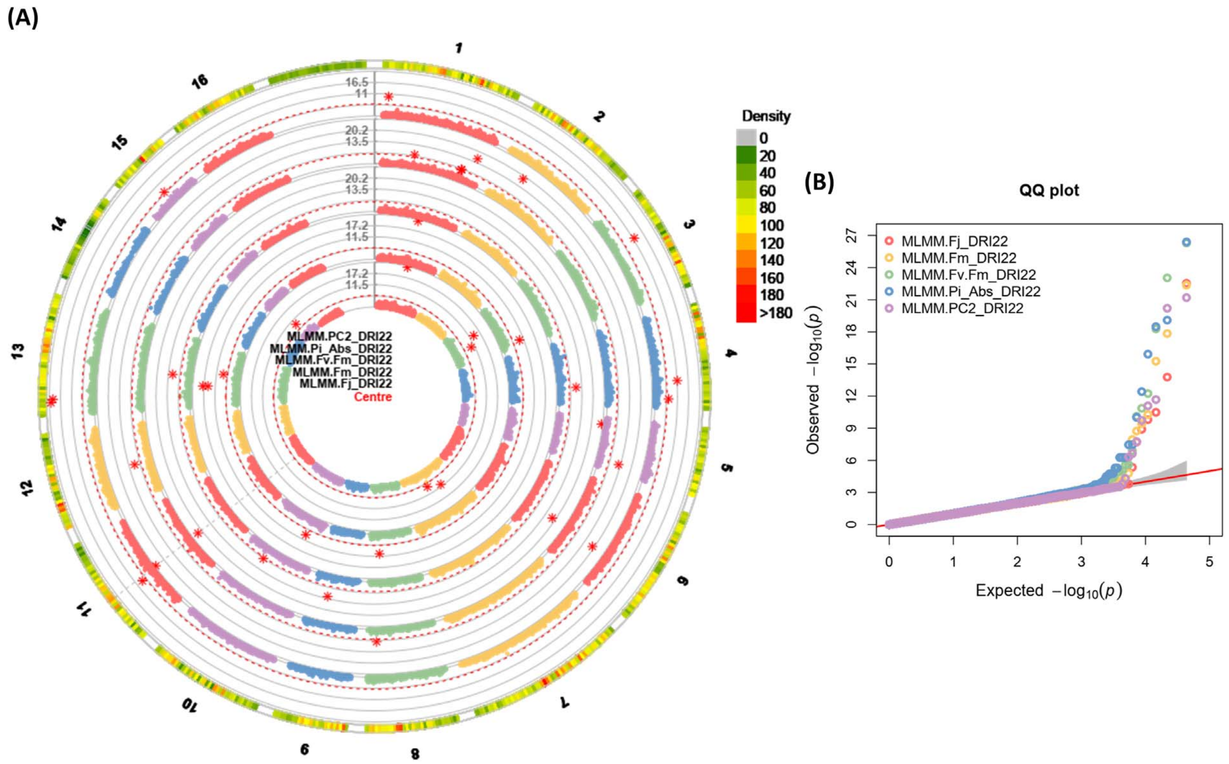

Figure 8. (A) and (B) Circular *Manhattan plots (left), and* quantile-quantile *plots (right) of association analysis using the PGBS dataset and MLMM* (Q + K) *model for chlorophyll* fluorescence *related traits from centre to the outside of plot including; (a)* F_J_DRI22, *(b)* F_M_DRI22, *(c)* F_V/F_M_DRI22, *(d)* PI_{ABS}_DRI22, *and (e) PC2_DRI22.* The outermost circle shows SNP density in 1 Mb windows for each chromosome *where green to red indicates low to high marker density.* Black bold line (Y-axis) represents –Log10 P-value. The circles of red dashed lines *represent the Bonferroni-corrected significance threshold. Red stars indicate genome-wide significantly associated SNPs. Vertical grey dashed lines are drawn through GWAS findings to indicate multi-trait associations.* For QQ plots, X-axis represents expected $-\log_{10}$ (p-value) and Y-axis is observed $-\log_{10}$ (p-value) of each SNPs. Drought recovery index: DRI. 22: recovery condition in the second year.

under normal and stress conditions. Our results are consistent with the results of Rosati et al. (2006) [31] who reported that reduced photosynthesis is mainly associated with the stomatal closure, which consequently influences leaf biochemical processes. Furthermore, Our OJIP-test results showed that drought stress caused a significant inhibition of both PSII and beyond PSII electron transport activities. Our finding demonstrated a decrease in the maximal quantum yield of PSII (F_V/F_M), and decline of Ψ_0 and φ_{Eo} under water stress, especially in drought sensitive genotypes, reflects the accumulation of Q_A, indicating blockage of electron transfer from Q_A to Q_B on the PSII acceptor side, which was reported by Kalaji et al. (2016) [32]. These results are consistent with Liu et al. (2019) [8] who showed that drought stress affects photosynthetic electron transport of walnut plants.

SNP-array and GBS are complementary for understanding genetic basis of photosynthetic response to drought and re-watering in Persian walnut

GWAS identified at least one SNP with a significant or suggestive association with the majority of photosynthetic traits either in the WW, WS and WR conditions or plasticity of the trait across treatments.

There was little overlap between MArray, MGBS, and PGBS results (Figure 9A). The first reason is that there is very little SNP overlap between array and GBS datasets (~5 K SNPs). A second reason is that there are different numbers of individuals (94, 87, and 136) for each analysis. In line with Elbasyoni et al. (2018) [24], the lack of agreement between the GWAS analyses with array and GBS data is likely due to ascertainment bias inherent in the Array-scored SNPs because markers were discovered in a diverse panel independent from our walnut genotypes, as well as low coverage data in GBS. In agreement with Negro et al. (2019) [25], we identified more significant or suggestive SNPs associated with photosynthetic traits using the SNP-array compared to GBS. This result can be due to the higher genome coverage of the SNP-array. Based on our small dataset, we conclude that combining both datasets for GWAS may be expected to boost the likelihood of identifying trait-SNP associations. Our results showed the infrequent occurrence of genomic loci with a significant association for a trait under different treatments (WW, WS and WR conditions). This could be explained by substantial genetic-environment interaction for the majority of the traits. These highlight distinct mechanisms of photosynthetic response to drought and re-watering in Persian walnut. Interestingly, we identified far fewer significant and suggestive loci overlapping with plasticity in studied traits. This suggests independent genetic control of the expression of a trait and its plasticity [3].

Downloaded from https://academic.oup.com/hr/article/doi/10.1093/hr/uhac124/6603865 by guest on 14 January 2023

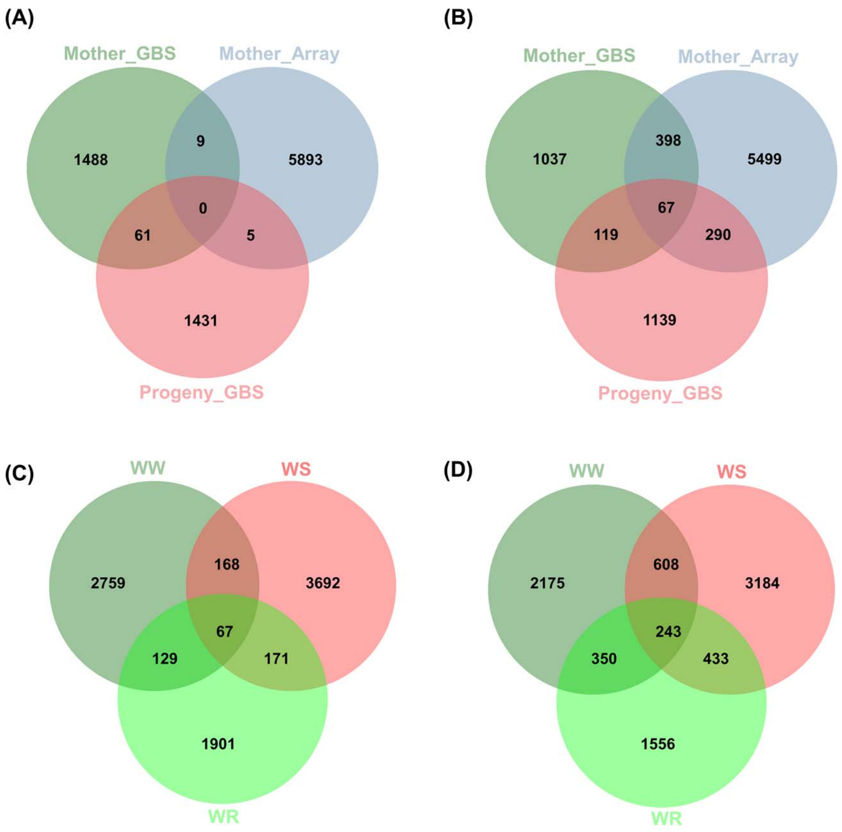

Figure 9. Complementarity of the three data sets to detect SNP-trait associations and candidate genes. (A) and (B); the number of specific genomic regions (A) and candidate genes (B) detected by each data set for all the photosynthetic traits across experiments (First and second years) and different conditions. (C) and (D); the number of genomic regions (C) and candidate genes (D) detected by both Array and GBS data sets across experiments (First and second years) under well-watered (WW), water-stressed (WS), and water-recovery (WR) conditions.

Functional annotation revealed the complex and distinct mechanisms of photosynthetic response to drought and re-watering in Persian walnut

The photosynthetic traits characterized here are all quantitative traits, so multiple genomic regions with small effects are expected. We first searched for genes containing significant and suggestive GWAS SNPs (Table 2 and S6), and identified several potential candidates underlying photosynthetic trait variation. Here, we highlight some of the most important (Tables 3-4).

A peak SNP on chromosome 10 found to be associated with P_n under water stress condition was located within a *CLPB3* gene encoding a chaperone protein ClpB3. Previous study in *Arabid*opsis has demonstrated that ClpB/Hsp100 family of proteins is involved in chloroplast development [33]. On chromosome 10, a significant SNP located within a gene encoding an ethylene-responsive transcription factor (ERF), was associated with DSI of RWC. Many studies demonstrated that several ERFs bind to both GCC box and dehydration-responsive elements (DRE) and act as a key regulatory hub in plant responses to biotic and abiotic stresses [34]. On chromosome 16, we found a significant SNP associated with CE, gs, Tr and PC1 under well-water condition fell in a gene (*TL17*) encoding thylakoid lumenal protein. Recently lumen proteins shown to play important roles in regulating thylakoid biosynthesis and the activity of photosynthetic protein complexes, especially photosystem II [35]. On chromosome 1, a significant SNP which has been linked with F_V, F_M, F_I, and F_J under recovery condition, we found to be located inside a gene encoding a receptor-like protein kinase (RLK) which is involved in abiotic stress responses, including calcium signaling and antioxidant defense system [36]. In addition, on chromosome 2, a SNP associated with DI_0/RC, ABS/RC, φ_{D0}, F_V/F_M, φ_{Pav}, and PC1 under drought stress fell in a gene encoding cyclin-dependent kinase (CDK). Previous studies have shown that CDKs as core cell cycle regulators play key role in diverse aspects of plant responses to abiotic stress [36]. Our results also showed that several other SNP markers associated with the studied traits were located within the genes encoding different protein kinases, especially receptor-like kinases (RLKs), calcium-dependent protein kinases (CDPKs), and mitogen-activated protein kinase (MAPK) cascades. As a result, it can be concluded that protein kinases play crucial roles in Persian walnut responses to the both of drought stress and recovery conditions through various signal transduction pathways.

On chromosome 4, a significant SNP located within a gene *BAG4* encoding protein BAG family molecular chaperone regulator 4, was found to be associated with the F_M and F_I under water-stress. Among the plant Bcl-2 associated athanogene (*BAG*) genes, *BAG4* has been

Downloaded from https://academic.oup.com/hr/article/doi/10.1093/hr/uhac124/6603865 by guest on 14 January 2023

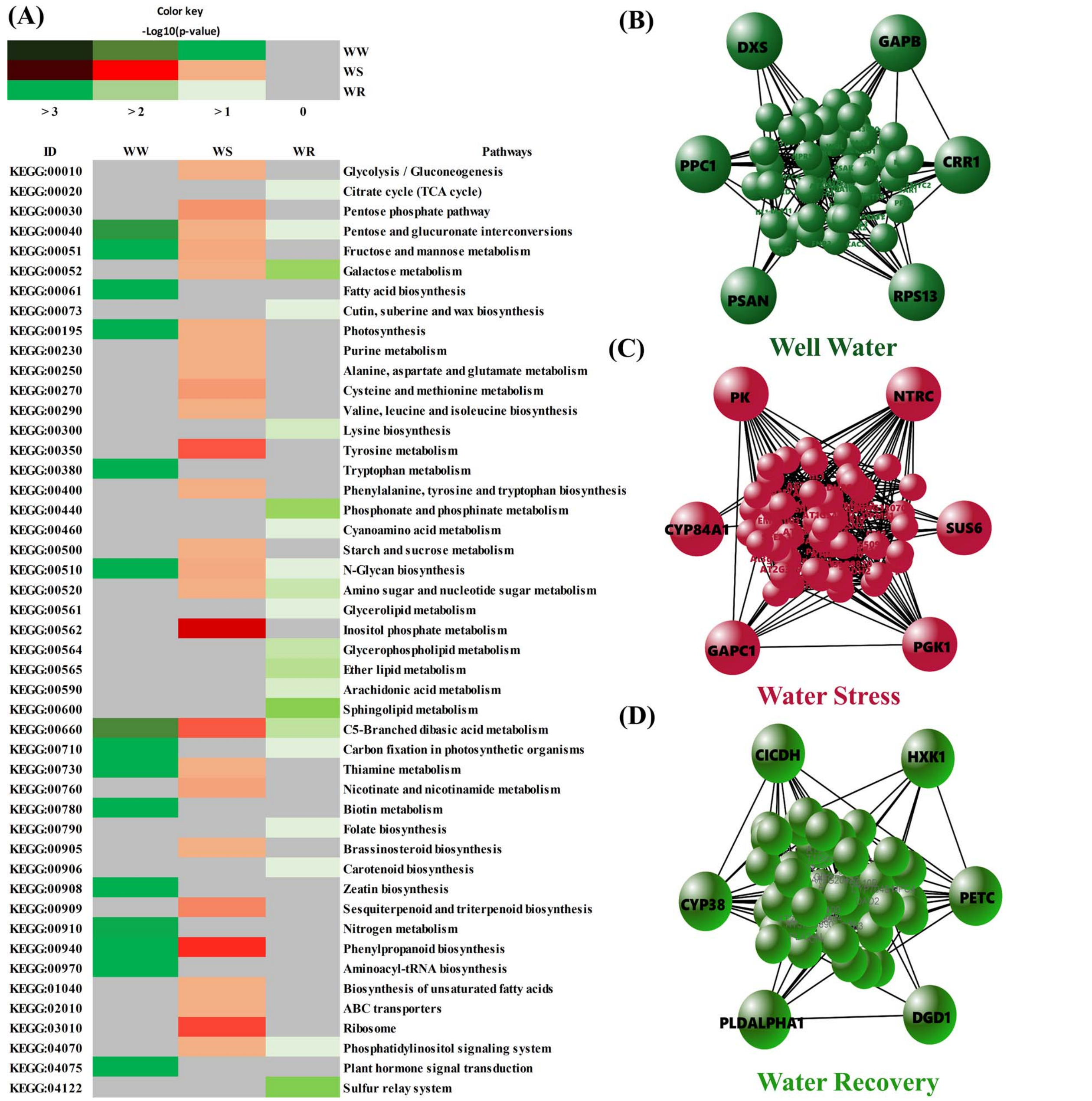

Figure 10. Enrichment and network analysis highlighted key pathways and hub genes involved in photosynthetic responses in walnut under drought and recovery conditions. (A) Top KEGG descriptions significantly enriched using genes associated with photosynthetic traits under WW, WS and WR conditions. Different colours in X-axis represent different significant levels of the KEGG pathways. The y-axis represents the KEGG pathways. Sub-networks of the most important hub genes from all the genes associated with photosynthetic traits under (B) well-watered (WW), (C) water-stressed (WS), and (D) water-recovery (WR) conditions are shown.

extensively studied and its overexpression in tobacco plants confers tolerance to abiotic stresses [37]. Three peak SNPs on chromosomes 12, 13 and 3 respectively associated with DSI of DI_0/RC, DSI of TR_0/RC and F_0 and DSI of P_n, T_r, g_s and PC1 were located in a gene encoding pentatricopeptide repeat-containing protein, thought to play crucial roles in plant responses to abiotic stresses. Jiang et al. (2015) [38] showed that upregulation of the pentatricopeptide repeat *SOAR1* expression in *Arabidopsis* enhances stomatal closure and plant tolerance to multiple abiotic stresses (drought, salinity and cold). On chromosome 11, a SNP associated with F_V under drought stress falls within an *ACBP4* gene encoding an Acyl-CoA-binding domain-containing protein that may function as an intracellular carrier of acyl-CoA esters. Du et al. (2013) [39] revealed that overexpression of *ACBP2* (*ACBP2-OXs*) in Arabidopsis confers tolerance to drought by promoting ABA signalling and stomatal closure. On chromosome 6, a wall-associated receptor kinase that may function as a signalling receptor of an extracellular matrix component was found to be linked with the DSI of F_0, DRI of TR_0/RC and PC2 under well-water condition. Hou et al. (2005) [40] have shown that various cell wall-associated receptor kinase (*WAK*) gene family members are involved

Downloaded from https://academic.oup.com/hr/article/doi/10.1093/hr/uhac124/6603865 by guest on 14 January 2023

in abiotic stress responses as well they are required for cell elongation and development.

Interestingly, we found two SNPs on chromosomes 8 and 11 located within the *FRS5* gene that encodes the protein FAR1-RELATED SEQUENCE, which has been linked with multiple traits, including T_r under re-watering condition as well as φ_{E0}, V_J, ψ_0 ET_0/RC, and TR_0/RC under well-water condition. On the other hand, of particular interest to us are SNPs/or genes correlated with more than one trait. We searched in windows of ±10 kb around the SNPs associated with multi-traits (Supplementary data set S7). For example, GWAS identified two SNPs on chromosomes 2 linked to the protein FAR1-RELATED SEQUENCE, which individually associated with 10 and 8 traits (some in multiple environments). Recently, Ma and Li (2018) [41] have reported that this protein plays multiple roles in an extensive range of biological processes, including oxidative stress responses, chlorophyll biosynthesis, and starch synthesis.

Taken altogether, our functional annotation revealed a high number of identified loci that were either close or within the known genes that play crucial roles in photosynthetic processes, including ABA signalling, regulation of stomatal function, chlorophyll biosynthesis, antioxidant biosynthesis, starch synthesis, lipid metabolism, and transduction of environmental signals. Other candidate genes identified in our study encode transcription factors, such as MYB, WRKY, bHLH, AP2-like ethylene-responsive protein, and HSP70, which are involved in drought responses in plant. Our results show that drought tolerance and recovery involve distinct and diverse mechanisms. Their polygenic nature represents a constraint on development of new trait combinations and needs to be considered when attempting to breed drought tolerance walnut rootstocks and cultivars.

Gene-set enrichment analysis identified key pathways involved in photosynthetic responses to drought and re-watering in Persian walnut

We complement our BLASTx results with gene-set enrichment analysis using KEGG and GO databases to identify molecular mechanisms underlying photosynthetic responses and drought tolerance in walnut. Pathway analysis revealed significantly enriched pathways that were linked to photosynthesis and drought stress responses. Our KEGG results indicate that photosynthesis pathway under both WW and WS conditions as well as carbon fixation in photosynthetic organisms pathway under WW and WR conditions were enriched with identified genes associated with photosynthetic traits. Previous studies showed that photosynthesis rate declined under WS in walnut [8], however, photosynthesis activity is essential for plant acclimation to WS [42]. Also, KEGG results showed that several amino acid and carbohydrate metabolism-related pathways such as tyrosine metabolism, cysteine and methionine metabolism and starch and sucrose metabolism were enriched under WS. In addition, GO terms related to signaling pathways such as phosphatidylinositol phosphate kinase activity and carbohydrate biosynthetic process were the important enriched terms under WS condition. It is well-documented that drought stress causes the accumulation of reactive oxygen species (ROS) in the cells, which in turns mediates multiple biological processes such as signal transduction pathways activation, oxidoreductase activities, and carbohydrate metabolic processes that are implicated in regulating the response to drought stress [43]. Additionally, high ROS doses have negative effects on cell protection [4].

Likewise, our KEGG results showed that ribosome pathway was significantly enriched by genes affecting photosynthetic efficiency under WS. In agreement with these finding, previous study in *Arabidopsis thaliana* suggest that ribosome biosynthesis highly increased in response to drought and recovery [44]. Plant have evolved several protective adaptations to respond to drought by up regulation of a considerable number of transcripts, therefore, they need a high number of ribosomes to translate these transcripts to proteins [45]. Also, it was suggested that plants need a significant number of ribosomes during the recovery phase to renew or repair their proteins. Moreover, biological functional analysis revealed that genes associated with photosynthetic traits under WS mapped to carbohydrate metabolism GO terms.

KEGG finding revealed that sphingolipid metabolism, sulfur relay system, carotenoid biosynthesis, and cyanoamino acid metabolism were enriched by identified genes associated with photosynthetic traits under WR condition. Also, our results showed that the membrane lipid metabolic process, response to oxidative stress and antioxidant activity GO terms were enriched with these genes under WR condition. These findings suggest that plants have begun to grow and repair damaged tissues under WR condition. On the other hand, we found a number of enriched pathways shared between the two experimental conditions including; Galactose metabolism and Amino sugar and nucleotide sugar metabolism between WS and WR conditions, and Fructose and mannose metabolism, photosynthesis, and phenylpropanoid biosynthesis between WW and WS conditions, as well as carbon fixation in photosynthetic organisms between WW and WR conditions. Therefore, in line with previous studies [3,4,6] can be concluded that various metabolic processes are involved in Persian walnut adaptation to drought stress. In agreements with our results it has been suggested that amino acid (i.e. proline) and carbohydrates metabolisms play important roles during the drought stress response in Persian walnut [4,6]. Many studies have found that amino acid metabolism is closely related to drought tolerance [46]. Moreover, several studies have shown that carbohydrate metabolism as one of the key plant processes for absorbing the energy generated during photosynthesis occupies a vital function in drought stress responses

Downloaded from https://academic.oup.com/hr/article/doi/10.1093/hr/uhac124/6603865 by guest on 14 January 2023

in addition to acting as energy sources [47,48]. It was reported that that the increasing of sugars and other compatible solutes, contributes to osmotic adjustment under drought stress [4]. Hence, our results suggest that maybe osmotic adjustment is one of the important mechanisms of response to drought in Persian walnut. Overall, these results suggest that multiple biological processes are involved in drought stress responses as well as amino acid and carbohydrate metabolisms may play important roles in Persian walnut seedling responses to drought stress.

Network analysis highlighted hub genes involved in photosynthetic responses to drought and re-watering in Persian walnut

Among many biological pathways activated in plants under environmental stresses, the photosynthesis and cell growth processes are the most sensitive to drought and recovery. Our network analysis further identified the most important hub genes that are directly or indirectly involved in the complex interaction network linked to photosynthetic and drought-related stress responses. Among these, 15 associated with photosynthetic processes (*GAPB, PSAN,* and *CRR1* under WW condition; *PGK1* and *NTRC* under WS condition; *DGD1*, *CYP38*, and *PETC* under recovery condition), carbon and nitrogen metabolism (*PPC1* under WR condition), carbohydrate metabolism (*SUS6* and *GAPC1* under WS condition),regulation of stomatal movement (*PLDALPHA1* under WR condition), and cell growth and development (*DXS, RPS13A,* and *HXK1* under WW and WR conditions) were the most important drought-responsive genes. The *GAPA* and *GAPB* genes encode one of the two subunits forming respectively the photosynthetic glyceraldehyde-3-phosphate dehydrogenase (GAPDH) and chloroplast localized glyceraldehyde-3-phosphate dehydrogenase, which play crucial roles in plant metabolism and are involved in abiotic stress response [49]. The *CRR1* gene is required for both formation and activity of the chloroplast NAD(P)H dehydrogenase (NDH) complex of the photosynthetic electron transport chain [50]. Naranjo et al. (2016) [51] showed *NTRC* plays an important role in the control of photosynthetic electron transport in *Arabidopsis*. The *RPS1* gene is required for optimal plastid performance and plays an important role in biosynthesis of thylakoid membrane proteins [52]. The *PETC* gene is a component of the cytochrome b6-f complex, which mediates electron transfer between PSII and PSI, cyclic electron flow around PSI, and state transitions [53]. Overall, these findings (promising candidate genes) will accelerate future efforts aimed at improving walnut drought tolerance.

Conclusion and perspectives

We characterized photosynthetic traits in diverse walnut families (n = 150) grown in a controlled greenhouse under well-watered, water-stressed, and re-watered conditions. GWAS analysis was performed using over 295 K array-scored and 43 K GBS-scored SNPs. Our main conclusions are the following: (1) Combining two genotyping technologies with different SNP distributions and densities allowed the identification of more marker-trait associations. (2) Identification of different genomic regions under drought stress and drought recovery conditions in walnut suggests that their genetic control is different. (3) Multiple candidate genes previously reported to be associated with photosynthesis and drought tolerance were identified. For the traits obtained from analysis of chlorophyll fluorescence, because of the time and/or labour intensity of data collection, a relatively small number of selected families (60) were evaluated and hence the power of GWAS was reduced. Therefore, a larger walnut population and more SNPs are clearly required to obtain more accurate GWAS results. On the other hand, GWAS of other drought-related traits such as water relations and biochemical parameters in future studies will complement the results of our study. In addition, because of the complex multigenic nature of drought tolerance, the most significant SNPs associated with photosynthetic traits are probably not the true causative loci. (4) The integration of GWAS and enrichment analysis was helpful for identifying promising candidate genes and pathways for further study. Together these findings provide new insight into possible drought tolerance mechanisms in walnut.

Materials and methods

Plant material and experimental design:

Plant materials used for this study consisted of a diverse panel of Persian walnut composed of 150 mother trees (local populations; 50- to 500-year-old open pollinated seedlings) from different geographical regions in Iran. This panel is expected to capture most of the genomic variation within locally-adapted populations (Table S1). Each of the sampled populations were located in a distinct habitat with very diverse environmental conditions (e.g. climate, geology, and topography). GPS coordinates and elevation were used to determine climatic parameters of the sampled areas (WorldClim [54]). At least 60 seeds along with leaf samples were collected from each of the 150 mother trees in 2015. A detailed list of mother trees is presented in Table S1.

To evaluation early-lifetime phenotypes of mother trees, and progeny photosynthetic performance under drought condition, collected seeds were established in a common garden. Seeds were stratified to break dormancy and 20 seeds per mother tree (half-sib family) were subsequently planted on 7-liter polyethylene pots (15 cm × 15 cm × 50 cm deep) in a potting mix (2:1:1 (v/v/v), soil:sand:leaf manure). More details are described in the supplementary file and by Arab et al. (2020) [3]. Ten uniform seedlings from each of the 150 families were then selected to initiate a common garden experiment. Two water stress experiments were

Downloaded from https://academic.oup.com/hr/article/doi/10.1093/hr/uhac124/6603865 by guest on 14 January 2023

carried out under greenhouse conditions (25 (±5) °C, 45 (±10) % RH, and photoperiod of ~16 h) over two years at the Research Greenhouses of the Department of Horticulture, University of Tehran, Pakdasht, Tehran, Iran. In the first experiment, 10 uniform seedlings (6-month-old) from each family were randomly assigned to either the well-watered or the water-stressed group. Water stress was applied by withholding and setting three levels of moisture treatment; (i) well-watered (above 75% FC), (ii), mild water-stressed (~40–50% FC), and (iii) severely water-stressed (~25–35% FC) (see Figure S1). In the fall when the buds were dormant, 9-month-old sapling were transplanted into 15-liter polyethylene pots (25 cm × 25 cm × 70 cm deep) containing a mix soil as previous. In the second experiment, 15-month-old sapling were arranged in two groups as first experiment. They then were subjected to water stress by withholding and setting three levels of moisture treatment; (i) well-watered (above 75% FC), (ii), severely water-stressed (~25–35% FC), and (iii) re-watered following severe stress (see Figure S1). Experiments were laid out in a factorial completely randomized design with two factors (family and water treatment) and 2–3 replications.

Drought score index and relative water content (RWC)

Score index was visually graded on a range of 1 to 9 according to the appearance characteristics of the plant (1 to 9 indicates perfectly healthy plants to damaged and dying plants). Fresh leaves samples (ten uniform leaf discs) were collected from each plant, weighed [fresh weight (FW)], and placed in a petri dish filled with distilled deionized water for 24 h. Surface water on the leaves was removed through tissue paper and the leaves were weighed [turgor weight (TW)] and dried at 70°C. After 24 h, dry weight (DW) of samples was recorded and RWC was calculated as follow: (FW-DW)/(TW-DW) × 100 (Table 1).

Photosynthesis measurements

Gas-exchange photosynthetic parameters were measured three times on the 150 families using an infrared gas analysing system, IRGA (LI-6400, Li-Cor, Inc., Lincoln, NE, USA) under severe water-stress level in the first and second experiments, and at the end of recovery period in the second experiment. Measurements were performed on two fully-expanded, upper-canopy leaflets (fifth leaf in basipetal order) per plant, with controlled atmosphere (~400 μmol CO_2 mol^{-1}; 25 ± 2°C and ~ 50–60% relative humidity) and photosynthetic active radiation of 1200–1500 μmol photons m^{-2} s^{-1}. The infra-red gas analyser system (IRGA) was manually adjusted and the levels of CO_2 and H_2O references were fixed before measurements. For each time-point, measurements were taken on only undamaged leaves during two consecutive days from 9 a.m. till 3 p.m. Net photosynthetic rate (P_n in μmol CO_2 m^{-2} s^{-1}), transpiration rate (T_r in mmol H_2O m^{-2} s^{-1}), stomatal conductance (g_s in mol H_2O m^{-2} s^{-1}), and intercellular CO_2 concentration (C_i in μmol CO_2 mol^{-1} air) were recorded from two plants per treatment. P_n, T_r and g_s were used to calculate instantaneous and intrinsic WUE (WUEinst = P_n/T_r in μmol CO_2 mmol H_2O^{-1}; WUEintri = P_n/g_s in μmol CO_2 mol H_2O^{-1}, respectively). P_n/C_i ratio (CE) was taken as an estimate of carboxylation efficiency of Rubisco [55].

Chlorophyll fluorescence measurements in the first experiment

In the first experiment, parameters obtained for the 150 families from the analysis of chlorophyll fluorescence were recorded under mild (3 weeks after drought) and severe (5 weeks after drought) stress. For measuring chlorophyll *a* fluorescence, we used the same leaf immediately after the gas exchange analysis. The measurements were performed in the greenhouse during two consecutive days from 9 AM until 3 PM, using a portable chlorophyll fluorometer (PAM-2500, Walz, Effeltrich, Germany). After a dark-adapted period (30 min) with dark leaf clips, the minimum fluorescence (F_0) was measured using weak modulated irradiation light [<0.1 μmol (photons) m^{-2} s^{-1}]. Afterwards, a 800 ms saturating flash at 6000 μmol (photons) m^{-2} s^{-1} was applied to determine the maximum chlorophyll fluorescence (F_M), variable fluorescence ($F_V = F_M - F_0$) and maximum quantum yield of PSII (F_V/F_M) using the equation in Table 1.

Chlorophyll fluorescence measurements in the second experiment

In the second experiment, chlorophyll fluorescence parameters of 60 selected extreme families (very drought tolerant to very sensitive) were measured under severe stress (24 days after drought) and recovery (two weeks after re-irrigation). Polyphasic Chl a fluorescence transients (OJIP-test) were measured using a portable fluorometer (Fluorpen FP 100-MAX, Photon Systems Instruments, Drasov, Czech Republic) in the middle part of the sapling in young fully-expanded walnut leaflets with 3 replicates for each treatment (control or drought) after 20 min dark adaptation. To fully ensure that all PSII centers are open, plants were allowed to dark-adapt overnight, and the lights were extinguished in the greenhouse until measurements were concluded pre-dawn (between 1 and 5 a.m.). The fluorescence measurements were taken by a saturating light of ~3000 μmol m^{-2} s^{-1}. Fluorescence intensities were recorded at four time points: 50 μs (O), 2 ms (J), 60 ms (I), and maximum fluorescence at around 1 s (P). Measurements related to the OJIP test were calculated based on the approaches described by Strasser et al. (2000, 2004) [13,29]. The definition of the measured parameters and detailed calculation formulas are listed in Table 1. More details of the OJIP-test are given in the supplementary file.

Downloaded from https://academic.oup.com/hr/article/doi/10.1093/hr/uhac124/6603865 by guest on 14 January 2023

Calculation of phenotypic plasticity

The response of genotypes to drought stress for all measured traits was expressed as a relative change in water-stress compared with well-water conditions using the drought stress index (DSI) described in Wójcik-Jagła et al. (2013) [56] and calculated as follows: DSI = (value of trait under water-stressed condition) / (value of trait under well-watered condition) × 100. The drought recovery index (DRI) was calculated using the same equation but substituting the value of trait under recovery condition in place of the value of trait under drought condition.

Statistical analysis

Statistical analyses were performed using Minitab software (Minitab, Inc., State College, PA, USA) and R environment (R Development Core Team, 2017) and the related R packages. Descriptive statistics and normality tests were run on both of the data and their residuals, respectively. The results of descriptive statistics were plotted using ggplot2 R package [57]. Analysis of variance of each experiment was performed separately. We applied general linear models (GLM) to test the effect of families (F), water stress or re-watering treatment (T), and their interaction (F × T) on each photosynthetic-related trait under mild and severe drought stress, and subsequently re-watering. Pearson correlation analysis was conducted via factoextra package [58] in R.

DNA extraction, GBS library construction, and sequencing

In this study, 95 out of 150 mother trees were chosen for retrospective tissue sampling and mother-tree genotyping. In other words, genotype data are only available for 95 of the 150 mother trees. Mature fresh leaves were sampled from each family and mother tree (150 + 95 = 245 in total), immediately frozen in liquid nitrogen, and lyophilized prior to DNA extractions. Genomic DNAs of 95 mother trees and 150 families (pooled leaf tissue from 10 six-month-old individuals per each family) were isolated from young leaflets from 40 mg of dry leaves using the E-Z 96 Plant DNA Kit (Omega Bio-tek; Norcross, GA) according to the manufacturer's instructions. The DNA concentrations were determined using Qubit dsDNA High Sensitivity (HS) Assay Kits (InVitrogen, Life Technologies), after adjusting to 50 ng/μl 10 μl aliquots (500 ng in total) were used for the library preparation. GBS was performed on 245 walnut samples using a two-enzyme GBS protocol previously described [59] with a few modifications. Briefly, individual DNA samples were digested using HindIII-HF (3 U) and MseI (1.5 U) followed by ligation of individual-specific barcode and common adapters using T4 DNA ligase. Ligated samples were pooled together (96-plex libraries) then amplified by polymerase chain reaction (PCR). Purification of final libraries was performed using magnetic beads (Omega Bio-Tek, GA, USA) for fragment selection in the range of 150-500 bp. Library size distribution was checked using a 2100 BioAnalyzer (Agilent Technologies, CA, USA). Three 96-plex libraries were pooled to generate a single lane (288-plex) sequencing libraries. Libraries were sequenced through single end 90 bp sequencing read length using Illumina HiSeq4000 of the University of California Davis Genome Center (https://dnatech.genomecenter.ucdavis.edu/illumina-library-sequencing/).

SNPs calling, filtering, and imputation

SNP calling was performed using the TASSEL 5 GBS v2 SNP-calling pipeline [60] and the walnut cv. Chandler v2.0 reference genome [22]. 64 bp tags that occurred at least ten times were mapped using Burrows-Wheeler Aligner (BWA) with default parameters [61]. SNPs with average coverage below 1 (n = 22 328) or low coverage across individuals (n = 7885; data in <20% of individuals) were discarded before SNP calling, as were SNPs with extremely high coverage (n = 14 462; log(average coverage) > 2.75; likely repetitive DNA) and SNPs with an excess of heterozygous genotypes (n = 7067; inbreeding coefficient F < −0.05 in the mother trees only; likely paralogous SNPs). The latter two thresholds were established by comparing the proportion of SNPs matching the Axiom array at different thresholds (Figure S14). Since some of our samples represent pooled tissue from multiple individuals, we relaxed the last threshold to discard only the few SNPs that showed the largest excess of heterozygous calls. After SNP calling, the resulting vcf file was filtered to exclude taxa and SNPs with >90% missingness and SNPs with minor allele count <20. Missing genotype calls were then imputed using Beagle 5.0 [62] using default parameters. After imputation, we further filtered the datasets to exclude SNPs with minor allele frequency less than 5% in both mother trees and their offspring (MGBS and PGBS) and SNPs with severe departures from the Hardy–Weinberg equilibrium only in mother trees (MGBS) using PLINK v1.9 software [63].

Genotyping with the Axiom *J. regia* 700 K SNPs array, and quality control

The 95 mother trees were genotyped at 609658 SNPs evenly distributed throughout the walnut genome using the high-density walnut array from Affymetrix [16] as described by Arab et al (2019) [17].

Single and multi-trait genome-wide association mapping

GWAS was performed for 30 photosynthetic traits under well-water (WW), water-stress (WS) and recovery (WR) conditions. Specifically, the phenotypic data included: (i) average performance of families for each trait under WW, WS and WR conditions; (ii) DSI and DRI; (iii) PC1–PC5 of phenotypic data; and (iv) PCs of DSI and DRI values. Three SNP panels including the array data on 94 mother trees (n = 295 685 SNPs), GBS data on 87 mother trees (n = 40 828 SNPs) and GBS data on 136 families (n = 43 607 SNPs) were used separately for GWAS. A subset of traits was measured on 60 selected families, in which

Downloaded from https://academic.oup.com/hr/article/doi/10.1093/hr/uhac124/6603865 by guest on 14 January 2023

case GWAS was performed using just 60 individuals. Single trait GWAS was conducted by applying two different models implemented in the R package Genome Association and Prediction Integrated Tools (GAPIT v3.0 [64]): (*i*) the Fixed and Random Model Circulating Probability Unification (FarmCPU [65]), and (*ii*) the Multiple Loci Linear Mixed Model (MLMM [66]). Population structure (PCA) and genetic relatedness (kinship matrix) were calculated in GAPIT and included in the GWAS models as a random effect to control spurious associations [67]. Population structure analysis is described in detail in the supplementary file. The best number of PCs to include in the GWAS models was determined based on the Bayesian Information Criterion (BIC), as implemented in the "model selection" function of GAPIT and scree-plot of PCA results. Quantile–quantile (QQ) plots were applied to check if the model was correctly fitted. Bonferroni genome-wide thresholds were set to define significant associations in each dataset. The significant Bonferroni *P*-value thresholds were 1.7E-7, 1.22E-6 and 1.15E-6 for the MArray, MGBS, and PGBS datasets, respectively. The suggestive *P*-value thresholds (1/number of SNP markers) were 3.38E-6, 2.44E-5, and 2.29E-5 for the MArray, MGBS, and PGBS datasets, respectively. A lower suggestive *P*-value of 9.99×10^{-5} ($-\log_{10}P = 5$) was also used to detect the suggestive SNP–trait associations for gene set enrichment and network analysis.

Gene annotation and pathway enrichment analysis

Significant and suggestive SNPs identified in the GWAS were used to search for putative candidate genes controlling photosynthetic traits in walnut. Flanking sequences of the significant and suggestive SNPs from both the SNP Array and GBS method were annotated using the National Centre for Biotechnology Information (NCBI; https://www.ncbi.nlm.nih.gov) BLASTX function. A SNP was considered to be located within a gene if it falls within a gene sequence. Then, based on the LD decay in our walnut panel [3], 20-kb windows were drawn around the identified marker-trait associations (10 kb upstream and downstream the SNP position) to search for candidate genes using the annotation v2.0 [22]. Enriched functional annotation clusters of the associated walnut candidate genes were defined using Blast2GO V5.0 tool [68] (E-value $\geq 1 \times 10^{-5}$) implemented in Gene Ontology (GO [69]) and Kyoto Encyclopedia of Genes and Genomes (KEGG [70]) databases. The protein–protein interactions (PPI) were identified using the STRING database [71] based on the candidate genes and the network was subsequently constructed using Cytoscape [72]. Furthermore, hub genes were identified in the PPI networks using cytoHubb [73].

Acknowledgments

This work has been supported by the Center of International Scientific Studies & Collaboration (CISSC), Ministry of Science Research and Technology of Iran. We would like to thank Iran National Science Foundation (INSF), Iran's National Elite Foundation (INEF), Center of Excellence for Walnut Improvement and Technology of Iran, University of Tehran, and the University of California, Davis for their support. We also gratitude the California Walnut Board for funding the genotyping assay.

Author Contributions

MMA: designed, performed the project, data analysis and wrote the manuscript. PJB, RAA and HA: directed the project, contributed to the data analysis, interpretation of the results and revised the manuscript. SA, AMB and SSS: designed some experiments, analysed the data and revised manuscript. CAL and AM: designed some parts of the project, and revised manuscript. MBM: helped with environmental data collation and respective analysis. DBN and KV: designed, directed the project and revised manuscript. All authors listed, have made direct and intellectual contribution to the work, and approved it for publication.

Data availability

All data supporting the findings of the present study are available within the paper and its supplementary material files.

Conflict of Interests

The authors declare no conflicts of interest.

Supplementary data

Supplementary data is available at *Horticulture Research* online.

References

1. Vahdati K, Arab MM, Sarikhani S et al. Advances in Persian walnut (*Juglans regia L.*) breeding strategies. In: Al-Khayri JM, Jain SM, Johnson DV. (eds.), *Advances in Plant Breeding Strategies: Nut and Beverage Crops: Volume 4*. New York: Springer, 2019, 401–72.
2. Vahdati K, Arab MM, Sarikhani S. Advances in biotechnology and propagation of nut trees in Iran. In: Egorov E, Ilina I, Zaporozhets N. (eds.), *BIO Web of Conferences*. Krasnodar, Russia: EDP Sciences, 2020;**25**:01003.
3. Arab MM, Morrano A, Abdollahi-Arpanahi R et al. Combining phenotype, genotype, and environment to uncover genetic components underlying water use efficiency in Persian walnut. *Comparative Study*. 2020;**71**:1107–27.
4. Vahdati K, Lofti N, Kholdebarin B et al. Screening for drought-tolerant genotypes of Persian walnuts (*Juglans regia L*) during seed germination. *Hort Science*. 2009;**44**:1815–9.
5. Famula RA, Richards JH, Famula TR et al. Association genetics of carbon isotope discrimination and leaf morphology in a breeding population of *Juglans regia L. Tree Genet Genomes*. 2019;**15**:1–13.
6. Karimi S, Karami H, Mokhtassi-Bidgoli A et al. Inducing drought tolerance in greenhouse grown *Juglans regia* by imposing con-

Downloaded from https://academic.oup.com/hr/article/doi/10.1093/hr/uhac124/6603865 by guest on 14 January 2023

trolled salt stress: the role of osmotic adjustment. *Sci Hortic*. 2018;**239**:181–92.

7. Knipfer T, Reyecs C, Momayyezi M et al. A comparative study on physiological responses to drought in walnut genotypes (RX1, Vlach, VX211) commercially available as rootstocks. *Trees*. 2020;**34**:665–78.
8. Liu B, Liang J, Tang G et al. Drought stress affects on growth, water use efficiency, gas exchange and chlorophyll fluorescence of Juglans rootstocks. *Sci Hortic*. 2019;**250**:230–5.
9. Oakley CG, Savage L, Lotz S et al. Genetic basis of photosynthetic responses to cold in two locally adapted populations of Arabidopsis thaliana. *J Exp Bot*. 2018;**69**: 699–709.
10. Nikinmaa E, Holtta T, Hari P et al. *Assimilate transport in phloem sets conditions for leaf gas exchange*, *Plant Cell Environ*. 2013;**36**: 655–69.
11. Herritt M, Dhanapal AP, Purcell LC et al. Identification of genomic loci associated with 21chlorophyll fluorescence phenotypes by genome-wide association analysis in soybean. *BMC Plant Biol*. 2018;**18**:1–19.
12. van Bezouw RF, Keurentjes JJ, Harbinson J et al. Converging phenomics and genomics to study natural variation in plant photosynthetic efficiency. *Plant J*. 2019;**97**:112–33.
13. Strasser RJ, Srivastava A, Tsimilli-Michael M. The fluorescence transient as a tool to characterize and screen photosynthetic samples. In: Yunus M, Pathre U, Mohanty P. (eds.), *Probing Photosynthesis: Mechanism, Regulation & Adaptation*. Boca Raton, Florida: CRC Press, 2000, 445–83.
14. Mathur S, Mehta P, Jajoo A. Effects of dual stress (high salt and high temperature) on the photochemical efficiency of wheat leaves (Triticum aestivum). *Physiol Mol Biol Plants*. 2013;**19**: 179–88.
15. Martínez-García PJ, Crepeau MW, Puiu D et al. The walnut (*Juglans regia*) genome sequence reveals diversity in genes coding for the biosynthesis of non-structural polyphenols. *Plant J*. 2016;**87**:507–32.
16. Marrano A, Martinez-Garcia PJ, Bianco L et al. A new genomic tool for walnut (*Juglans regia* L.): development and validation of the high-density axiom™ *J. regia* 700K SNP genotyping array. *Plant Biotechnol J*. 2019;**17**:1027–36.
17. Arab MM, Marrano A, Abdollahi-Arpanahi R et al. Genome-wide patterns of population structure and association mapping of nut-related traits in Persian walnut populations from Iran using the axiom J regia 700K SNP array. *Sci Rep*. 2019;**9**:1–14.
18. Bernard A, Marrano A, Donkpegan A et al. Association and linkage mapping to unravel genetic architecture of phenological traits and lateral bearing in Persian walnut (*Juglans regia* L.). *BMC Genomics*. 2020;**21**:1–25.
19. Bükücü ŞB, Sutyemez M, Kefayati S et al. Major QTL with pleiotropic effects controlling time of leaf budburst and flowering-related traits in walnut (*Juglans regia L*). *Sci Rep*. 2020;**10**:1–10.
20. Sideli GM, Marrano A, Montanari S et al. Quantitative phenotyping of shell suture strength in walnut (*Juglans regia L*) enhances precision for detection of QTL and genome-wide association mapping. *PLoS One*. 2020;**15**:e0231144.
21. Marrano A, Sideli GM, Leslie CA et al. Deciphering of the genetic control of phenology, yield, and pellicle color in Persian walnut (*Juglans regia L.*). *Front Plant Sci*. 2019;**10**:1140.
22. Marrano A, Britton M, Zaini PA et al. High-quality chromosome-scale assembly of the walnut (*Juglans regia L.*) reference genome. *Gigascience*. 2020;**9**:giaa050.
23. Zhu T, Wang L, You FM et al. Sequencing a Juglans regia× J microcarpa hybrid yields high-quality genome assemblies of parental species. *Hortic Res*. 2019;**6**:1–16.
24. Elbasyoni IS, Lorenz AJ, Guttieri M et al. A comparison between genotyping-by-sequencing and array-based scoring of SNPs for genomic prediction accuracy in winter wheat. *Plant Sci*. 2018;**270**: 123–30.
25. Negro SS, Millet EJ, Madur D et al. Genotyping-by-sequencing and SNP-arrays are complementary for detecting quantitative trait loci by tagging different haplotypes in association studies. *BMC Plant Biol*. 2019;**19**:1–22.
26. Li H, Thrash A, Tang JD et al. Leveraging GWAS data to identify metabolic pathways and networks involved in maize lipid biosynthesis. *Plant J*. 2019;**98**:853–63.
27. Thrash A, Tang JD, DeOrnellis M et al. PAST: the pathway association studies tool to infer biological meaning from GWAS datasets. *Plants (Basel)*. 2020;**9**:58.
28. Esmaeili-Fard SM, Gholizadeh M, Hafezian SH et al. Genes and pathways affecting sheep productivity traits: genetic parameters, genome-wide association mapping, and pathway enrichment analysis. *Front Genet*. 2021;**12**.
29. Strasser RJ, Tsimilli-Michael M, Srivastava A. Analysis of the chlorophyll a fluorescence transient in Chlorophyll a fluorescence. In: Papageorgiou GC, Govindjee S. (eds.), *Chlorophyll a Fluorescence: A Signature of Photosynthesis*. Dordrecht: Springer Dordrecht, 2004, 321–62.
30. Zhang J, Jia W, Yang J et al. Role of ABA in integrating plant responses to drought and salt stresses. *Field Crop Res*. 2006;**97**: 111–9.
31. Rosati A, Metcalf S, Buchner R et al. Tree water status and gas exchange in walnut under drought, high temperature and vapour pressure deficit. *J Hortic Sci Biotechnol*. 2006;**81**:415–20.
32. Kalaji HM, Jajoo A, Oukarroum A et al. Chlorophyll a fluorescence as a tool to monitor physiological status of plants under abiotic stress conditions. *Acta Physiol Plant*. 2016;**38**:102.
33. Lee U, Rioflorido I, Hong SW et al. The Arabidopsis ClpB/Hsp100 family of proteins: chaperones for stress and chloroplast development. *Plant J*. 2007;**49**:115–27.
34. Müller M, Munné-Bosch S. Ethylene response factors: a key regulatory hub in hormone and stress signaling. *Plant Physiol*. 2015;**169**:32–41.
35. Järvi S, Gollan PJ, Aro EM. Understanding the roles of the thylakoid lumen in photosynthesis regulation. *Front Plant Sci*. 2013;**4**:434.
36. Chen X, Ding Y, Yang Y et al. Protein kinases in plant responses to drought, salt, and cold stress. *J Integr Plant Biol*. 2021;**63**:53–78.
37. Doukhanina EV, Chen S, van der Zalm E et al. Identification and functional characterization of the BAG protein family in Arabidopsis thaliana. *J Biol Chem*. 2006;**281**:18793–801.
38. Jiang SC, Mei C, Liang S et al. Crucial roles of the pentatricopeptide repeat protein SOAR1 in Arabidopsis response to drought, salt and cold stresses. *Plant Mol Biol*. 2015;**88**:369–85.
39. Du ZY, Chen MX, Chen QF et al. Overexpression of Arabidopsis acyl-CoA-binding protein ACBP2 enhances drought tolerance. *Plant Cell Environ*. 2013;**36**:300–14.
40. Hou X, Tong H, Selby J et al. Involvement of a cell wall-associated kinase, WAKL4, in Arabidopsis mineral responses. *Plant Physiol*. 2005;**139**:1704–16.
41. Ma L, Li G. FAR1-related sequence (FRS) and FRS-related factor (FRF) family proteins in Arabidopsis growth and development. *Front Plant Sci*. 2018;**9**:692.
42. Flexas J, Barón M, Bota J et al. Photosynthesis limitations during water stress acclimation and recovery in the drought-adapted

Downloaded from https://academic.oup.com/hr/article/doi/10.1093/hr/uhac124/6603865 by guest on 14 January 2023

Vitis hybrid Richter-110 (V. berlandieri× V rupestris). *J Exp Bot*. 2009;**60**:2361–77.
43. Apel K, Hirt H. Reactive oxygen species: metabolism, oxidative stress, and signal transduction. *Annu Rev Plant Biol*. 2004;**55**: 373–99.
44. Georgii E, Jin M, Zhao J et al. Relationships between drought, heat and air humidity responses revealed by transcriptome-metabolome co-analysis. *BMC Plant Biol*. 2017;**17**:1–23.
45. Hosseini SZ, Ismaili A, Nazarian-Firouzabadi F et al. Dissecting the molecular responses of lentil to individual and combined drought and heat stresses by comparative transcriptomic analysis. *Genomics*. 2021;**113**:693–705.
46. Shi H, Ye T, Chen F et al. Manipulation of arginase expression modulates abiotic stress tolerance in Arabidopsis: effect on arginine metabolism and ROS accumulation. *J Exp Bot*. 2013;**64**: 1367–79.
47. Pedroso FK, Prudente DA, Bueno ACR et al. Drought tolerance in citrus trees is enhanced by rootstock-dependent changes in root growth and carbohydrate availability. *Environ Exp Bot*. 2014;**101**: 26–35.
48. Woldesemayat AA, Ntwasa M. Pathways and network based analysis of candidate genes to reveal cross-talk and specificity in the sorghum (Sorghum bicolor (L) Moench) responses to drought and it's co-occurring stresses. *Environ Exp Bot*. 2018;**9**:557.
49. Li X, Wei W, Li F et al. The plastidial glyceraldehyde-3-phosphate dehydrogenase is critical for abiotic stress response in wheat. *Int J Mol Sci*. 2019;**20**:1104.
50. Shimizu H, Shikanai T. Dihydrodipicolinate reductase-like protein, CRR1, is essential for chloroplast NAD (P) H dehydrogenase in Arabidopsis. *Plant J*. 2007;**52**:539–47.
51. Naranjo B, Mignée C, Krieger-Liszkay A et al. The chloroplast NADPH thioredoxin reductase C, NTRC, controls non-photochemical quenching of light energy and photosynthetic electron transport in Arabidopsis. *Plant Cell Environ*. 2016;**39**: 804–22.
52. Yu H-D, Yang XF, Chen ST et al. Downregulation of chloroplast RPS1 negatively modulates nuclear heat-responsive expression of HsfA2 and its target genes in Arabidopsis. *PLoS Genet*. 2012;**8**:e1002669.
53. Munekage Y, Takeda S, Endo T et al. Cytochrome b6f mutation specifically affects thermal dissipation of absorbed light energy in Arabidopsis. *Plant J*. 2001;**28**:351–9.
54. Hijmans RJ, Cameron SE, Parra JL et al. Very high resolution interpolated climate surfaces for global land areas. *Climatology*. 2005;**25**:1965–78.
55. Farquhar GD, Sharkey TD. Stomatal conductance and photosynthesis. *Annu Rev Plant Physiol*. 1982;**33**:317–45.
56. Wójcik-Jagła M, Rapacz M, Tyrka M et al. Comparative QTL analysis of early short-time drought tolerance in polish fodder and malting spring barleys. *Theor Appl Genet*. 2013;**126**:3021–34.
57. Wickham HJC. ggplot2-Elegant Graphics for Data Analysis. In: Gentleman R, Hornik K, Parmigiani G. (eds.), *Use R!*. Switzerland: Springer International Publishing, 2016, 89–107.
58. Kassambara A. Factor Analysis of Mixed Data In: Kassambara A. (ed.), *Practical Guide to Cluster Analysis in R: Unsupervised Machine Learning (Multivariate Analysis)*. South Carolina: CreateSpace Independent Publishing Platform, 2017, 149–64.
59. Poland JA, Brown PJ, Sorrells ME et al. Development of high-density genetic maps for barley and wheat using a novel two-enzyme genotyping-by-sequencing approach. *PLoS One*. 2012;**7**:e32253.
60. Glaubitz JC, Casstevens TM, Lu F et al. TASSEL-GBS: a high capacity genotyping by sequencing analysis pipeline. *PLoS One*. 2014;**9**:e90346.
61. Li H, Durbin R. Fast and accurate long-read alignment with burrows–wheeler transform. *Bioinformatics*. 2010;**26**:589–95.
62. Browning BL, Zhou Y, Browning SR. A one-penny imputed genome from next-generation reference panels. *Am J Hum Genet*. 2018;**103**:338–48.
63. Purcell S, Neale B, Todd-Brown K et al. PLINK: a tool set for whole-genome association and population-based linkage analyses. *Am J Hum Genet*. 2007;**81**:559–75.
64. Lipka AE, Tian F, Wang Q et al. GAPIT: genome association and prediction integrated tool. *Bioinformatics*. 2012;**28**:2397–9.
65. Liu X, Huang M, Fan B et al. Iterative usage of fixed and random effect models for powerful and efficient genome-wide association studies. *PLoS Genet*. 2016;**12**:e1005767.
66. Segura V, Vilhjálmsson BJ, Platt A et al. An efficient multi-locus mixed-model approach for genome-wide association studies in structured populations. *Nat Genet*. 2012;**44**:825–30.
67. Yu J, Pressoir G, Briggs WH et al. A unified mixed-model method for association mapping that accounts for multiple levels of relatedness. *Nat Genet*. 2006;**38**:203–8.
68. Götz S, Garcia-Gomez JM, Terol J et al. High-throughput functional annotation and data mining with the Blast2GO suite. *Nucleic Acids Res*. 2008;**36**:3420–35.
69. The Gene Ontology Consortium. The gene ontology resource: 20 years and still GOing strong. *Nucleic Acids Res*. 2019;**47**: D330–D338.
70. Kanehisa M, Goto S, Kawashima S et al. The KEGG resource for deciphering the genome. *Nucleic Acids Res*. 2004;**32**:277D–280.
71. Mering CV, Huynen M, Jaeggi D et al. STRING: a database of predicted functional associations between proteins. *Nucleic Acids Res*. 2003;**31**:258–61.
72. Shannon P, Markiel A, Ozier O et al. Cytoscape: a software environment for integrated models of biomolecular interaction networks. *Genome Res*. 2003;**13**:2498–504.
73. Chin C-H, Chen SH, Wu HH et al. cytoHubba: identifying hub objects and sub-networks from complex interactome. *BMC Syst Biol*. 2014;**8**:1–7.

Downloaded from https://academic.oup.com/hr/article/doi/10.1093/hr/uhac124/6603865 by guest on 14 January 2023
