# Supplementary material for: Provenance and family variations in early growth of Manchurian walnut (Juglans mandshurica Maxim.) and selection of superior families
Source: PLoS One. 2024 Mar 7;19(3):e0298918. doi: 10.1371/journal.pone.0298918 (PMC10919699; doi:10.1371/journal.pone.0298918)
Supplement: S1 File — (ZIP) [file pone.0298918.s004.zip › Genome-wide patterns of population structure and association mapping of nut-related traits in Persian walnut populations from Iran using the Axiom J. regia 700K SNP array.pdf]

SCIENTIFIC REPORTS

OPEN

Genome-wide patterns of population structure and association mapping of nut-related traits in Persian walnut populations from Iran using the Axiom *J. regia* 700K SNP array

Received: 7 November 2018
Accepted: 12 April 2019
Published online: 23 April 2019

Mohammad Mehdi Arab[1], Annarita Marrano[2], Rostam Abdollahi-Arpanahi[3], Charles A. Leslie[2], Hossein Askari[4], David B. Neale[2] & Kourosh Vahdati[1]

Persian plateau (including Iran) is considered as one of the primary centers of origin of walnut. Sampling walnut trees originating from this arena and exploiting the capabilities of next-generation sequencing (NGS) can provide new insights into the degree of genetic variation across the walnut genome. The present study aimed to explore the population structure and genomic variation of an Iranian collection of Persian walnut (*Juglans regia* L.) and identify loci underlying the variation in nut and kernel related traits using the new Axiom *J. regia* 700K SNP genotyping array. We genotyped a diversity panel including 95 walnut genotypes from eight Iranian provinces with a variety of climate zones. A majority of the SNPs (323,273, 53.03%) fell into the "Poly High Resolution" class of polymorphisms, which includes the highest quality variants. Genetic structure assessment, using several approaches, divided the Iranian walnut panel into four principal clusters, reflecting their geographic partitioning. We observed high genetic variation across all of the populations ($H_O = 0.34$ and $H_E = 0.38$). The overall level of genetic differentiation among populations was moderate ($F_{ST} = 0.07$). However, the Semnan population showed high divergence from the other Iranian populations (on average $F_{ST} = 0.12$), most likely due to its geographical isolation. Based on parentage analysis, the level of relatedness was very low among the Iranian walnuts examined, reflecting the geographical distance between the Iranian provinces considered in our study. Finally, we performed a genome-wide association study (GWAS), identifying 55 SNPs significantly associated with nut and kernel-related traits. In conclusion, by applying the novel Axiom *J. regia* 700K SNP array we uncovered new unexplored genetic diversity and identified significant marker-trait associations for nut-related traits in Persian walnut that will be useful for future breeding programs in Iran and other countries.

The genus *Juglans*, one of the most important genera of the *Juglandaceae* family, contains approximately 21 species, of which Persian walnut (*Juglans regia* L.) is the most economically important for nut production. Persian walnut is a long-lived, monoecious, open-pollinated and dichogamous tree, widely cultivated across temperate and subtropical regions[1–4]. Persian walnut trees are native to the mountainous regions of central Asia[5–7], and today are distributed and grown commercially over a wide geographical range, including west-central Asia, southern Europe, North and South America, Australia and New Zealand[1,8–10].

[1]Department of Horticulture, College of Aburaihan, University of Tehran, Tehran, Iran. [2]Department of Plant Sciences, University of California, Davis, CA, 95616, USA. [3]Department of Animal and Poultry Science, College of Aburaihan, University of Tehran, Tehran, Iran. [4]Department of Plant Sciences and Biotechnology, Faculty of Life Sciences and Biotechnology, Shahid Beheshti University, Tehran, Iran. Correspondence and requests for materials should be addressed to K.V. (email: kvahdati@ut.ac.ir)

Iran and Afghanistan are thought to be among the main centers of origin and domestication of walnut[5,11]. In Iran, walnut has been planted widely for both nut and wood production, grows in extensive naturalized populations, and plays a key role in the country's economy and culture[2,3,11]. Iranian walnut populations inhabit areas of widely varying precipitation, temperature, altitude and latitude[12]. Walnut trees have been seed-propagated in this area for thousands of years and dichogamy has promoted considerable genetic variation within existing natural or planted seedling populations[13,14]. Due to propagation primarily by seed, considerable phenotypic variability can be observed for different traits in the natural walnut populations of Iran[2,13,14]. Therefore, this valuable native walnut gene pool, located throughout the country and containing widely varying alleles, could be a valuable resource for the development of walnut breeding programs in Iran, either by direct selection or by use of superior genotypes in cross-breeding programs[2,12–14].

Information regarding the amount of genetic divergence between geographically separated walnut populations in Iran can be used to enhance the effectiveness of breeding programs[2,15,16]. During recent decades the genetic diversity and structure of Persian walnut populations in Iran has been studied using both morphological and molecular markers, and some promising genotypes (e.g. for high yield, good kernel color and late leafing) have been selected and introduced[2,3,11,14]. However, there are wide areas populated by walnut trees in different parts of Iran that are still genetically unexplored[15]. These genetic materials are most likely adapted to the local environmental conditions and may have high levels of tolerance to biotic and abiotic stresses[16,17]. Hence, these native populations potentially could provide resources for sustainable high yields under the climate change scenario[16]. Therefore, assessing the genomic variation and differentiation of Iranian walnut genotypes will facilitate the effective use of these valuable genetic resources in future breeding programs, not only in Iran but also in other countries.

Patterns of walnut population genetic diversity and structures have been studied using several molecular marker systems, including randomly amplified polymorphic DNA (RAPDs)[15], and simple sequence repeat (SSRs)[18–24]. However, there is no information in the literature on analysis of walnut genetic resources in Iran using high throughput genotyping platforms. Advances in next-generation sequencing (NGS) and the continuous decrease in cost have facilitated the discovery of whole genome single nucleotide polymorphisms (SNPs)[25–27]. SNP markers are the most abundant type of sequence variations. Distributed throughout the genome, they are the best candidates for performing advanced genetic analyses such as genome-wide association studies (GWAS) and genomic selection (GS)[25–28]. Ciarmiello *et al.*[29] developed a simple technique for genetic characterization of walnut through exploiting SNPs in internal transcribed spacers (ITS). You *et al.*[30] released a 6K Infinium SNP array based on 'Chandler' for use in walnut genetic improvement. A full walnut genome sequence ('Chandler' reference genome) has recently been published[31]. In addition, Marrano *et al.*[25] released a high density Axiom *J. regia* 700K SNP array after high depth re-sequencing of 27 founders of the Walnut Improvement Program of University of California, Davis.

GWAS takes advantage of natural genetic variation, which is found extensively in many fruit species[26,28]. This approach makes it possible to simultaneously screen a large number of individuals for genomic variation, allowing identification of novel alleles underlying various traits[26,28]. However, few GWAS studies in nut tree crops have been reported so far, due to complex population structures that arose during a long domestication process[26,28]. Understanding the genetics of characters related to nut and kernel quality is crucial for walnut breeding programs worldwide and no study has identified regions (quantitative trait loci, QTLs) in the walnut genome associated with these traits. Therefore, it may be beneficial to use a diverse panel of walnuts of varied geographic origin in GWAS studies to identify useful new genetic variation for nut and kernel-related traits.

The walnut improvement program of the Horticulture Science Research Institute (HSRI) in Iran was launched in the early 1980's with the aim of developing new cultivars. As a first step, in 1983 selected superior genotypes from the Iranian walnut gene pool were established in Karaj, Shahrood, Mashhad and Uremia. In 1994, seven genotypes from the Karaj collection were propagated by grafting and planted, along with eight French/Californian commercial cultivars, at Karaj. This resulted in the release of the first Iranian walnut cultivars, Jamal (Z63) and Damavad (Z30) in 2009–2010[32]. Although the fourth phase of the walnut improvement program at HSRI based on traditional breeding continues, climate change and increasing environmental stresses (especially spring frost, drought and salinity) make it vital to initiate a new scion and rootstock breeding program using genomic-based approaches. Thus, we characterized a representative collection of the natural genetic and phenotypic variation present in Iran as first step towards future walnut sampling and the introduction of molecular breeding for Persian walnut in Iran.

The main objectives of the present study were to: (i) assess population structure, genomic variation and differentiation among Iranian walnut populations, (ii) evaluate the performance of the newly designed Axiom *J. regia* 700K SNP array on Iranian walnut genetic resources, and (iii) identify SNPs significantly associated with nut and kernel-related traits using GWAS. The present study is the first to directly assess the population diversity of Iranian walnut genotypes using high-density SNP markers. Although some of these materials have low agronomic value compared to commercial walnut cultivars, they may contain useful resilience alleles that have been lost in the modern genetic resources of walnut.

Results

Phenotypic variation and multivariate analysis. We measured 22 seed-related traits in a walnut collection comprising 95 genotypes, assembled from different parts of Iran (Table 1; Supplementary Table S1; Supplementary Fig. S1). These plant materials were collected from eight provinces ranging from the northwest of Iran (West Azerbaijan) with cold weather to the southeast of Iran (Kerman) with very arid regions. These were all old walnut trees from open pollinated seedlings grown in the valleys among mountainous areas. Twelve nuts were evaluated from each tree. As shown in Table 2, we observed a great phenotypic variation within our walnut collection. The ease of kernel removal from nuts, which varied on a scale from 1 to 7.67, with an average of 2.75,

Country	Province	Region	Sample size	Altitude (m)	Longitude (E)	Latitude (N)	Annual rainfall (mm)	Annual avg. temp. (C)
Iran	Kerman	Baft-Gugher	13	2763	56°23′	29°31′	247.55	15.33
Iran	Kerman	Rabor	16	2730	56°57′	29°24′	267	15
Iran	Kerman	Rabor-Hanza	6	2850	57°12′	29°19′	267	15
Iran	Kerman	Bardsir	6	2823	56°29′	29°37′	72.5	14.6
Iran	Fars	Eqlid	15	2167	52°47′	30°54′	305.35	12.98
Iran	Fars	Bavanat	5	2407	53°31′	30°26′	209.5	15.85
Iran	Semnan	Shahmirzad	9	1999	53°33′	35°55′	206.24	12.91
Iran	Ilam	Ilam	6	1387	46°30′	33°40′	560.54	16.93
Iran	Ilam	Eyvan	6	1140	46°15′	33°52′	691	17.1
Iran	Yazd	Taft	6	2450	53°48′	31°45′	57.05	19.48
Iran	Markazi	Delijan-Jasb	4	1998	50°48′	34°06′	171.66	17.59
Iran	West Azerbaijan	Khoy	2	1215	44°58′	38°35′	285	12.57
Iran	Hamadan	Nahavand	1	1644	48°25′	34°15′	375	14.58
**Reference cultivar in genotyping array**								
USA	California	Davis	1	9	121°28′ W	38°33′	508	16.1

Table 1. Geographical and ecological data of the walnut populations studied.

No.	Trait	Abbr.	Unit	Min.	Max.	Mean	SD	CV (%)
1	Nut length	NuLe	mm	27.69	50.38	38.68	5.29	13.67
2	Nut width	NuWi	mm	26.008	40.71	32.24	2.68	8.30
3	Nut thickness	NuTh	mm	25.54	40.48	31.68	2.77	8.75
4	Nut weight	NuWe	g	7.71	20.11	12.99	2.39	18.35
5	Kernel percentage	KePe	%	38.31	67.36	50.46	5.23	10.36
6	Shape index	ShIn		91.73	161.84	121.34	14.87	12.25
7	Size index	SiIn		27.32	41.91	34.20	3.06	8.94
8	Round index	RoIn		0.62	1.09	0.84	0.10	12.07
9	Nut shape	NuSh	Code (1–9)	1.33	7.92	4.65	1.94	41.81
10	Shell thickness	SheTh	mm	0.98	2.63	1.70	0.34	19.84
11	Shell color	SheCo	Code (1–9)	1.17	8.25	4.19	1.52	36.30
12	Shell texture	SheTe	Code (1–9)	1.08	8.42	4.93	1.81	36.58
13	Shell seal	SheSe	Code (1–9)	1.67	7.67	5.32	1.28	24.11
14	Shell strength	SheSt	Code (1–9)	1.33	8.08	4.93	1.30	26.44
15	Packing tissue thickness	PaTiTh	Code (1–7)	1.08	5.92	2.38	1.01	42.56
16	Kernel weight	KeWe	g	3.83	8.97	6.50	1.04	16.09
17	Kernel color	KeCo	Code (1–9)	1	8.17	3.60	1.41	39.34
18	Kernel plumpnes	KePl	Code (1–7)	2.25	6.42	4.03	1.09	27.04
19	Kernel shrivel	KeSh	Code (1–7)	1.17	6.33	2.73	1.00	36.61
20	Kernel vein	KeVe	Code (1–7)	1.08	6.42	3.35	1.35	40.23
21	Kernel filled	KeFi	Code (1–7)	2.75	6.42	4.20	0.98	23.32
22	Ease of kernel removal from nuts	EKeNu	Code (1–9)	1	7.67	2.75	1.29	46.88

Table 2. Fruit traits utilized in the studied walnut genotypes. [a]SD is an abbreviation of standard deviation, which was calculated based on the measured values of twelve seeds. [b]CV is an abbreviation of coefficient of variation, which was estimated as the ratio of the standard deviation to the mean of all genotypes.

had the maximum coefficient of variation (46.88%), whereas nut width, which varied from 26.01 to 40.72 mm with an average of 32.24, had the lowest coefficient of variation (8.30%) (Table 2).

Correlation coefficients were used to determine the relationships between seed-related traits (Table 3). Significant positive correlations were observed between a size index and nut-related traits including nut length (r = 0.85**), nut width (r = 0.89**), nut thickness (r = 0.81**), and nut weight (r = 0.77**) (Table 3). A roundness index was negatively correlated with nut length (r = −0.80**), nut weight (r = −0.38**) and the size Index (r = −0.39**) (Table 3). Kernel weight was positively correlated with nut length (r = 0.45**), nut width (r = 0.46** nut thickness (r = 0.47**), nut weight (r = 0.83**), and size index (0.54**) (Table 3). Furthermore, kernel percentage was positively correlated with kernel fill and kernel plumpness, and negatively correlated with nut length, and nut width (Table 3).

Principal component analysis (PCA) was used to identify patterns of diversity among genotypes, based on phenotypic traits (Supplementary Figs S2 and S3). PCA showed that the first eight components explained 82.1%

Character	NuLe	NuWi	NuTh	NuWe	KePe	SiIn	RoIn	SheTh	SheSe	KeWe	KeCo	KePl	KeVe	KeFi
NuLe	1.00													
NuWi	0.54**	1.00												
NuTh	0.41**	0.94**	1.00											
NuWe	0.72**	0.65**	0.56**	1.00										
KePe	−0.56**	−0.42**	−0.27**	−0.46**	1.00									
SiIn	0.85**	0.89**	0.81**	0.77**	−0.53**	1.00								
RoIn	−0.80**	0.04 ^{ns}	0.21*	−0.38**	0.42**	−0.39**	1.00							
SheTh	0.11 ^{ns}	0.20*	0.22*	0.30**	0.12 ^{ns}	0.19 ^{ns}	0.05 ^{ns}	1.00						
SheSe	0.29**	0.16 ^{ns}	0.09 ^{ns}	0.25*	−0.14 ^{ns}	0.24*	−0.26*	0.08 ^{ns}	1.00					
KeWe	0.45**	0.46**	0.47**	0.83**	0.1 ^{ns}	0.54**	−0.18 ^{ns}	0.40**	0.20*	1.00				
KeCo	−0.06 ^{ns}	−0.005 ^{ns}	0.03 ^{ns}	−0.06 ^{ns}	0.02 ^{ns}	−0.03 ^{ns}	0.07 ^{ns}	0.19 ^{ns}	−0.07 ^{ns}	−0.05 ^{ns}	1.00			
KePl	−0.18 ^{ns}	−0.24*	−0.18 ^{ns}	−0.15 ^{ns}	0.52**	−0.23*	0.07 ^{ns}	−0.03 ^{ns}	−0.006 ^{ns}	0.16 ^{ns}	−0.12 ^{ns}	1.00		
KeVe	0.29**	0.13 ^{ns}	0.01 ^{ns}	−0.02 ^{ns}	−0.29**	0.21*	−0.32**	0.02 ^{ns}	0.18*	−0.20*	0.33**	−0.36**	1.00	
KeFi	−0.34**	−0.25*	−0.20*	−0.25*	0.54**	−0.33**	0.23*	−0.08 ^{ns}	−0.015 ^{ns}	0.06 ^{ns}	−0.25*	0.66**	−0.45**	1.00

Table 3. Correlations among the fruits traits in the studied genotypes of walnut. **Correlation is significant at the 0.01 level. *Correlation is significant at the 0.05 level. ^{ns}Correlation is no significant. Traits abbreviations are explained in detail in the material and method section.

of the total variance among the 95 Iranian walnut genotypes (Supplementary Table S2). The first principal component accounted for 25.9% of the total phenotypic variation and showed the highest positive correlation with nut length (0.37), size index (0.35), nut weight (0.32), nut width (0.27), and nut thickness (0.22) (Supplementary Table S2; Fig. S3). The second principal component accounted for 15. 1% of the phenotypic diversity and positively correlated with kernel weight (0.35), nut thickness (0.28), nut weight (0.27), and nut width (0.26) as well as negatively depends on shell color (−0.34), kernel vein (−0.28), and shell texture (−0.26) (Supplementary Table S2; Fig. S3). The bi-plot segregated the genotypes into groups based mainly on their geographical origin (Supplementary Figs S2 and S3).

Genotyping and data quality control. We genotyped the whole Iranian collection using the latest the Applied Biosystem Axiom *J. regia* 700K SNP array (Table 1; Supplementary Table S1; Fig. S1). By applying default thresholds (dish quality control – dQC < 0.82 and quality control call rate < 0.97), all of the 95 samples passed the quality standards. The average cluster call rate and average reproducibility were 99.75% and 99.95%, indicating high quality genotyping results.

The SNPs were categorized into the six default classes using Affymetrix Power Tools (APT): 1) *Poly High Resolution (PHR*), which comprises polymorphic SNPs with three high-resolution genotypic clusters; 2) *No Minor Homozygote* (*NMH*), SNPs with no samples of the minor homozygous genotypes; 3) *Mono High Resolution* (*MHR*), SNPs which are monomorphic across the genotypes studied; 4) *Call Rate Below Threshold* (*CRBT*), SNPs with genotype call rate below threshold (97%); 5) *Off-Target Variant* (*OTV*) polymorphisms, where the genotyping data with low-intensity cluster resulted from dissimilarity between the probe and the target sequences and; (6) *Other*, which includes SNPs with no clear cluster pattern of the genotypic data.

A summary of the distribution of all SNPs in the different categories is shown in Table 4. Overall, the vast majority of the SNPs (323,273; 53.03%) fell into the PHR class of polymorphisms, which represent the highest quality variants. These were filtered for missing rate (<20%) and minor allele frequency (MAF > 0.05), obtaining a final subset of 313,657 PHR SNPs that were used in the subsequent analyses.

Population structure analysis. To study the structure of Persian walnut populations and the genetic relationship among samples, three different analyses were performed. Principal Component Analysis (PCA) and cluster analysis (CA) were used to assess the genetic distances among Iranian walnut genotypes by using a linkage disequilibrium (LD)-pruned SNP subset of 33,336 PHR polymorphisms. Figure 1a shows the first two principal components, which explained 8.05 and 7.33% of the total variation, respectively. PC1 clearly distinguishes Kerman individuals from the other provinces, while along PC2, genotypes from Fars and Yazd provinces clustered separately from Semnan (Fig. 1a). The cultivar Chandler, which was used as standard during the genotyping process, grouped with individuals from Ilam (Fig. 1a). Overall, we identified a clear separation of the Iranian walnut genotypes into four main genetic clusters centered in (1) Kerman, (2) Fars and Yazd, (3) Semnan, and (4) Ilam, Markazi, Hamedan, and West Azerbaijan provinces (Fig. 1a). By overlaying a map of Iran, we can observe a coincidence between these four walnut subpopulations and potential barriers to gene flow, such as mountains and deserts.

In contrast, the cluster analysis on a genome-wide Identical-By-State (IBS) matrix grouped the Iranian walnut genotypes into five groups (Supplementary Fig. S4): (1) Yazd, Fars and West Azerbaijan, (2) Kerman, (3) Ilam, Markazi and Hamedan, (4) Semnan and (5) Ilam and Chandler (Fig. 2b). As with PCA, the cluster analysis assigned all genotypes to their geographical regions (Supplementary Fig. S4), showing the Fars-Yazd and Semnan groups as the two most distant and the genotypes from Ilam and Markazi as genetically closest.

Category	Number of Markers	% of Markers
PolyHighResolution	323,273	53.03%
NoMinorHom	78,476	12.87%
MonoHighResolution	43,904	7.20%
CallRateBelowThreshold	37,869	6.21%
OffTargetVariant	33,468	5.49%
Other	67,721	11.11%
AAvarianceX	2,587	0.42%
AAvarianceY	2,944	0.48%
ABvarianceX	4,471	0.73%
ABvarianceY	7,024	1.15%
BBvarianceX	3,178	0.52%
BBvarianceY	4,727	0.78%
HomHomResolution	16	0.00%
Total	609,658	100.00%

Table 4. Summary of SNP data generated in walnut populations using Axiom *J. regia* 700K SNP array. The SNPs classes are explained in detail in the results section.

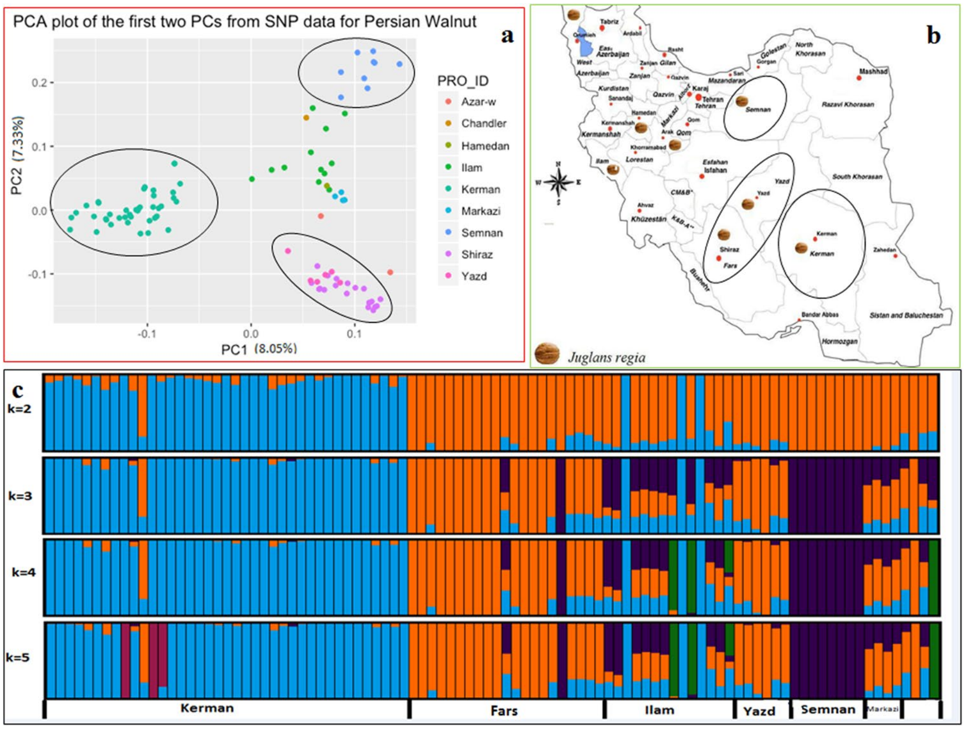

Figure 1. (**a**) Relationships among Persian walnut populations samples as represented by principal component analysis (PCA) using 33,336 genome-wide SNPs profiles (PRO_ID: different provinces of Iran); (**b**) Geographical distribution of the studied walnut samples across Iran; and (**c**) Admixture proportions of 95 Iranian walnut accessions as assigned using fastSTRUCTURE and the admixture option for K = 2 to K = 5. Each vertical bar exemplifies a sample (95 Iranian walnut genotypes sampled in eight provinces of Iran).

We then applied the model-based clustering approach implemented in fastSTRUCTURE software to determine the most likely number of genetic groups (K) within our Iranian walnut collection. According to the best choice algorithm function of fastSTRUCTURE, the most likely K ranged from 2 to 5 (Fig. 1c). At K2 the two main groups comprised (1) Kerman individuals (n = 41), (2) all other genotypes (Fig. 1c). At K3 the three major groups encompassed (1) Kerman individuals (n = 41), (2) Semnan individuals (n = 9), and (3) the genotypes from other provinces (Fig. 1c). At K4, in addition to the clusters identified at K3, we observed a fourth group including the cultivar Chandler and two individuals from Ilam (Fig. 1c). At K5, we identified a further substructure with the Kerman population dividing it into two groups, (1) thirty-eight Kerman individuals, and (2) three very old Kerman individuals (Fig. 1c). As with the cluster analysis, most of the Ilam population (n = 10) and all of the Markazi (n = 4) individuals were not clearly assigned to a defined group by fastSTRUCTURE, showing

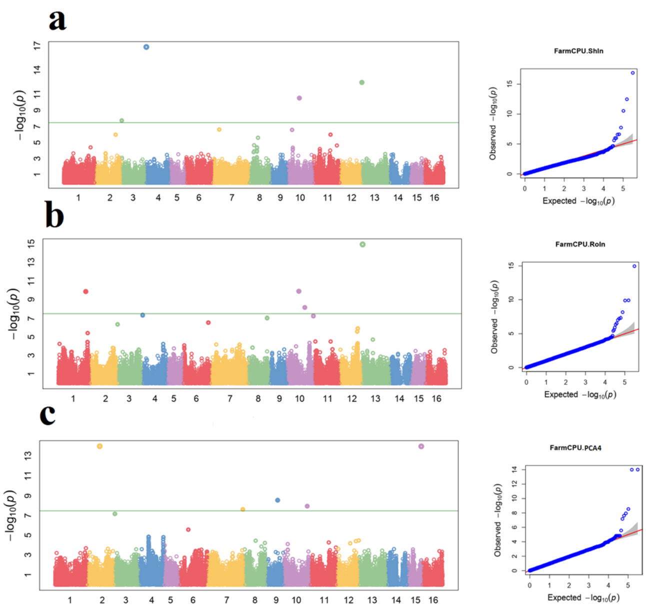

Figure 2. Manhattan plots (left) and quantile-quantile plots (right) of association analysis using the Q + K model for traits including; (**a**) Shape Index (ShIn); (**b**) Round index (RoIn) and (**c**) principal component analysis (PC). The y-axis of Manhattan plots shows the −log10 (P values) of SNP association. Each dot represents a SNP. The horizontal dashed green line represents the Bonferroni-corrected significance threshold. The threshold value was calculated by Bonferroni correction based on the tested number of SNP markers ($P < 0.05/99449$).

admixture with the Kerman, Semnan and Fars groups (Fig. 1c, K6, Supplementary Table S3). According to the marginal likelihood graph, the most likely number of subgroups in our Iranian walnut diversity panel was K3 (Supplementary Fig. S5). The three- methods of population structure analysis each identified different degrees of substructure, but overall we can conclude that the Iranian walnut population panel comprises mainly four genetic clusters (Fig. 1a–c; Supplementary Figs S4, 5).

Genetic variation and differentiation. The average values of observed heterozygosity (H_o) and expected heterozygosity (H_e) were 0.34 and 0.38, respectively but the level of genetic diversity varied among the Iranian walnut populations (Table 5).

The lowest value of allelic richness (A_r) was found in the West Azerbaijan population (1.44), while the Ilam and Kerman populations both showed the highest value of A_r (1.62) (Table 5). The observed heterozygosity (H_o) ranged from 0.30 in the Markazi population to 0.34 in the Ilam and West Azerbaijan populations (0.33) (Table 5). The expected heterozygosity (Nei's gene diversity, H_e) varied from 0.26 (West Azerbaijan) to 0.34 (Kerman). The lowest (0.31) and highest (0.35) unbiased expected heterozygosity values (UH_E) were identified in the Semnan and Ilam populations respectively (Table 5). The fixation index (F_{IS}) ranged from −0.29 in the West Azerbaijan population to 0.03 in Kerman and Fars populations, but on average, we observed low values of F_{IS} in all Iranian walnut populations, indicating a deficit of homozygotes (Table 5).

To study the amount of genetic differentiation among the Iranian walnut populations, we evaluated genetic differentiation parameters (including G'_{ST}, D_{Jost} and F_{ST}) for each pairwise comparison between the Iranian walnut populations (Table 6 and Supplementary Tables S4, 5).

The global F_{ST}, F_{IT} and F_{IS} values (0.07, 0.11 and 0.04 respectively) among our Iranian walnut populations indicate moderate genetic differentiation (Supplementary Table S4). A clear differentiation was observed between the Semnan population and those of Yazd, Fars, West Azerbaijan and Kerman provinces (Table 6). The same differentiation was also observed when estimating the D_{Jost} (Supplementary Table S5).These findings are in agreement with the above analysis of population structure. Comparison to the Iranian map (Fig. 1b), indicates that all of the genomic results for our population correlate with geographical and ecological location.

Parental analysis. We investigated the level of relatedness within our walnut collection, including 'Chandler' as a commercial walnut cultivar. A pair of Kerman genotypes had a proportion of IBD alleles (PI-HAT) value > 99% and were therefore considered to be genetically identical. The first and second-degree relationships, the most informative, are shown in Table 7. In first-degree relationships, the probability of parent-offspring pairs sharing zero, one, and two IBD alleles (Z0, Z1, and Z2) is expected to be closer to 0, 1 and 0 respectively, while for second-degree pairs, Z0, Z1, and Z2 are expected to be roughly 0.5, 0.5 and 0 respectively. Nine pairs of individuals showed PI_HAT equal to or higher than 0.5, and were then considered to be first-degree relatives (Table 7). Eight pairs showing PI-HAT ≈ 0.25–0.35 and relatively high Z0 and Z1 (≈0.5) values were considered second-degree pairs (Table 7). Interestingly, we identified a second-degree relationship between two individuals

Province	Sample Size	A_r	H_o	H_e	UH_E	F_{IS}
Kerman	41	1.61	0.33	0.34	0.34	0.03
Fars	20	1.58	0.31	0.32	0.33	0.03
Ilam	12	1.62	0.34	0.34	0.35	0.00
Semnan	9	1.51	0.30	0.29	0.31	−0.04
Yazd	6	1.54	0.32	0.30	0.32	−0.09
Markazi	4	1.51	0.30	0.29	0.33	−0.04
West Azerbaijan	2	1.44	0.33	0.26	0.35	−0.29

Table 5. Basic descriptive population genetic parameters for each Iranian walnut population across all loci (313,657 SNPs). The parameters calculated are; N = Number of individuals per population sample genotyped per locus, A_r = Allelic richness per population, H_o = observed heterozygosity per locus, H_e = expected heterozygosity per locus, UH_E = unbiased expected heterozygosity, F_{IS} = inbreeding coefficient per population.

	Kerman	Fars	Ilam	Semnan	Yazd	Markazi	West Azerbaijan
Kerman	—						
Fars	0.06	—					
Ilam	0.05	0.06	—				
Semnan	0.10	0.12	0.07	—			
Yazd	0.07	0.04	0.07	0.14	—		
Markazi	0.06	0.05	0.03	0.09	0.07	—	
West Azerbaijan	0.04	0.00	0.02	0.11	0.05	0.01	—

Table 6. Pairwise values of Wright's fixation index (F_{ST}) between populations of Iranian walnut across all loci (313,657 SNPs).

from Ilam population and the cv. 'Chandler'. Overall, we identified first and second-degree relationships only between individuals of the same province.

Association mapping for nut-related traits. Due to the great phenotypic variability observed in our collection for nut-related traits, we ran GWAS to dissect, for the first time in walnut, the genetic basis of these traits. GWAS was performed using as phenotypic entrance, both the average of 12 kernels per tree and the individual best linear unbiased predictors (BLUPs), as well as the first five PCs of the phenotypic data. We applied the SUPER and FarmCPU algorithms, accounting for both population structure and kinship. A total of 55 loci on 11 chromosomes were significantly associated with six nut and kernel-related traits and the first five PCs of the phenotypic data (Fig. 2; Supplementary Table S6). Three SNPs on chromosome 7 were identified for nut weight. Six SNPs on chromosome 3 were identified for kernel percentage. For both nut width and nut thickness a common SNP on chromosome 1 was identified. Six SNPs, two on chromosome 10 and one each on chromosomes 3, 4, 7, and 13, were identified for shape index. For roundness index, nine SNPs were identified - three on chromosome 10 and one each on chromosomes 1, 3, 4, 6, 8, and 13. We also found 19 SNPs significantly associated with the first five PCs of the phenotypic data. The allelic effect and minor allele frequency (MAF) for these ranged from −5.72 to 12.24 and 0.05 to 0.50 respectively. No significant associations were found for other traits but at a suggestive threshold we found association for all of the traits studied except kernel shrivel, kernel veins, and packing tissue thickness.

Considering the suggestive threshold, we identified 56 loci associated with 13 nut and kernel related traits (Supplementary Table S6). Seven SNPs, six on chromosome 7, and one on chromosome 13 were identified for shell texture. For shell seal, six were on chromosome 13, and one on chromosome 14. Six SNPs on chromosomes 8 (n = 3) and 13 (n = 3) were detected for kernel filled trait. We found 5 SNPs each associated with kernel weight, kernel plumpness, and ease of kernel removal from nuts. Four SNPs each were associated with nut length, size index, shell color, and kernel color and located across chromosomes 1, 2, 4, 6, 7, 10, 11, 12, 13, and 16. Two SNP on chromosome 4 were detected for nut shapes and one SNP each on chromosomes 3 and 4 were identified for shell strength. A single SNP on chromosome 13 was identified for shell thickness. The allelic effect and MAF for these suggestive SNPs ranged from −1.94 to 3.04 and 0.07 to 0.49 respectively.

The 55 significant (41 unique sequence) and 56 suggestive SNPs associated with nut and kernel-related traits were annotated using BLASTx queries (Supplementary Table S6). We found that the most significant SNPs associated with both shape index and round index, fell in genes coding for the WRKY transcription factor 70 isoform X1 (E value = 1E-12; (Fig. 2) and WD repeat-containing protein 3 isoform X2 (E value = 7E-09). Also, the SNP on chromosome 13 associated to kernel fill is located within a gene encoding for the cyclic DOF factor 2-like (*E* value = 1E-08). Other genes identified as involved in the studied traits included were the WD repeat-containing protein RUP2, the acidic endochitinase-like, the LRR receptor-like serine/threonine-protein kinase GSO1, the classical arabinogalactan protein 9-like, the cysteine-rich receptor-like protein kinase 25, and the NAC domain-containing protein 43 genes (Supplementary Table S6). However, no genes were identified for some marker-trait associations ("None" in the Supplementary Table S6).

FID1	IID1	FID2	IID2	Z0[a]	Z1[b]	Z2[c]	PI_HAT[d]
Identical individuals or clone							
Kerman	KBG9	Kerman	KBG12	0	0.0045	0.9955	0.9977
RCA: Parent-Offspring							
Semnan	SeSh4	Semnan	SeSh7	0.1243	0.6722	0.2036	0.5397
Fars	FaEq13	Fars	FaEq15	0.3379	0.2866	0.3756	0.5188
Kerman	KBG3	Kerman	KBG6	0.1294	0.7442	0.1264	0.4985
Yazd	YT3	Yazd	YT4	0.1298	0.7485	0.1217	0.496
Kerman	KBG12	Kerman	KBG13	0.1344	0.7559	0.1097	0.4876
Kerman	KBG9	Kerman	KBG13	0.1336	0.7582	0.1082	0.4873
Kerman	KR10	Kerman	KB6	0.1407	0.748	0.1112	0.4853
Kerman	KR11	Kerman	KBG11	0.1327	0.78	0.0873	0.4773
Kerman	KR9	Kerman	KB5	0.1414	0.7736	0.085	0.4718
RCA: 2nd degree							
Ilam	IlEy3	USA	Chandler	0.2026	0.7449	0.0525	0.425
Ilam	IlEy2	USA	Chandler	0.1966	0.7573	0.046	0.4247
Ilam	IlIl2	Ilam	IlIl3	0.5151	0.2654	0.2195	0.3522
Fars	FaEq3	Fars	FaEq7	0.512	0.3239	0.1641	0.326
Fars	FaEq6	Fars	FaEq14	0.5607	0.2846	0.1546	0.2969
Fars	FaEq5	Fars	FaEq7	0.5596	0.2999	0.1405	0.2905
Kerman	KBG2	Kerman	KBG8	0.5493	0.3593	0.0915	0.2711
Fars	FaEq3	Fars	FaEq5	0.6523	0.2024	0.1453	0.2465
Ilam	IlEy2	Ilam	IlEy3	0.6236	0.2753	0.1012	0.2388
Kerman	KBG1	Kerman	KBG6	0.6501	0.2468	0.1031	0.2265
Kerman	KBG2	Kerman	KBG6	0.7058	0.1462	0.148	0.2211

Table 7. Parentage analysis and relationship categories assignment (RCA) for Iranian walnut genotyps obtained by SNP allelic profiles using PLINK. FID1 = Family ID for the first sample; IID1 = Individual ID for the first sample; FID2 = Family ID for the second sample, IID2 = Individual ID for the second sample; [a]probability to share zero IBD allele; [b]probability to share one IBD allele; [c]probability to share two IBD allele; [d]relatedness measure.

Discussion

Iran has a long history of walnut production and is the third leading country in walnut production worldwide (445,829 in-shell tons[33]). Most walnut trees grown in Iran originated from seed, and exhibit considerable diversity in yield, quality, and resistance to abiotic and biotic stresses[2,11,12]. Environmental stresses and climate change are reducing walnut yield[17]. To face these challenges, native genotypes with interesting phenotypic traits need to be explored and preserved for future development of improved scion and rootstock varieties[16]. In this regard, as a pre-selection step, we evaluated various Iranian walnut populations in their native habitats for traits including climatic adaptations, precocity, yield, nut quality and resistance to biotic and abiotic stresses. Based on the profiles of all these traits, we selected the most interesting and variable 95 genotypes for the assessment of walnut genomic variation as a first step towards future walnut sampling and the introduction of molecular breeding for Persian walnut in Iran. These individuals originated from eight Iranian provinces (Table 1), rich in native walnut trees adapted to local conditions[2,15,16,23]. Many of the sampled trees are estimated to be at least 100, and some up to 500, years old. In particular, this collection of walnut trees exhibited especially high levels of phenotypic variation for nut-related traits, which were high correlated to each other, as reported in previous work[17,34–38].

The study of genomic variation and genetic differentiation in domesticated or natural populations is important for understanding patterns of local adaptation and dissecting the genetic basis of traits of interest. Access to the new Axiom *J. regia* 700K SNP array[25] allowed us to explore in-depth the genome-wide allelic variation within our Iranian walnut genetic resources.

Compared to previous surveys of Iranian walnut[23,36–38], we characterized a gene pool covering most of the Iranian walnut distribution at higher genetic resolution. Although the latest Axiom *J. regia* 700K SNP array was designed using the deep re-sequencing of 27 California walnut accessions, the conversion rate for Iranian samples (53.03%) was similar to that observed for the California material[25], confirming this array's value in assessing the population structure and genomic variation of Iranian walnut populations.

Using the genetic profiles of high-quality SNPs evenly distributed across the genome, we identified four distinct genetic groups in our walnut collection (Fig. 1a,b). Such population structure can be explained by the geographical proveniences of our genotypes as well as the various climate conditions to which they adapted. For instance, the walnut genotypes from Kerman clearly separated from the others (Fig. 1a,b). The Kerman population includes many individuals located at high altitude and a set of three very old trees that formed a separate group at K5. In addition, most of the individuals from Ilam and Markazi were admixed with the genotypes from Kerman, Semnan, and Fars. This level of genetic similarity could be the result of human-mediated exchanges

among these provinces. Overall, our population structure results are in line with previous genetic analysis of Persian walnut[7,23,39,40].

The average heterozygosity (Ho = 0.34) was similar to that of UC Davis WIP accessions (Ho = 0.3) examined by Marrano *et al.*[25] using the same SNP array. In contrast, our Iranian collection was more heterozygous than six *J. regia* populations from Kerman province studied by Vahdati *et al.*[16] (0.23), but less heterozygous than other Persian walnut germplasm described in previous genetic surveys[23,40,41]. This discrepancy can be explained by the use in this study of SNP markers, which are bi-allelic and therefore detect polymorphisms differently from the multi-allelic SSRs[42]. In the Kerman, Fars, and Ilam populations, H_e was slightly higher than H_o, probably due to the Wahlund effect or inbreeding. On the other hand, the values of Ho and He in the West Azerbaijan population suggest low inbreeding and large genetic variation. However, these findings could also be due to the small sample size of our walnut panel. Collecting additional walnut material from Iran will be essential in providing further support for our conclusions.

As in Marrano *et al.*[25], overall most of the Iranian populations exhibited little inbreeding and considerable genetic variation, as expected due to the dichogamous nature of walnut which promotes outcrossing. The high inbreeding coefficient found by Vahdati *et al.*[16] suggests significant heterozygote deficiency, likely due to crossing between closely related individuals. We observed greater genetic variation than Vahdati *et al.*[16], probably because we assessed more diverse populations from eight provinces and using genome-wide markers. In addition, although walnut is a mixed-mating tree species, negative F_{IS} values are expected in adult trees because breeding is usually purged at an early age, thus age of the tree and sampling is an important indicator in determining F_{IS} values[41]. Absence of inbreeding may also be due to an "isolate breaking effect" that occurs when previously isolated populations interbreed[43,44].

F_{ST} values for most wind-pollinated tree species tend to be lower than 0.10[41], indicating that more than 90% of the neutral genetic variation is maintained within populations. Our global F_{ST} value of 0.07, indicates that 93% of the genetic diversity of our walnut collection occurs within the seven Iranian populations (Supplementary Table S2). Our results agree with previous studies reporting overall F_{ST} values for *J. regia* populations in Europe, Africa and Asia[40], China[10,45] and six from Kerman province[16]. These findings possibly reflect the human-mediated dispersal of *J. regia* in space and time, resulting in a reassembly and homogenization of walnut genetic diversity.

The pairwise F_{ST} analysis suggests the Semnan population is the most genetically differentiated from the other provinces. Semnan province is far from the other studied provinces so neither pollen nor natural seed dispersal from them is feasible. Thus, it will be interesting to explore more in-depth the Semnan population for crossing in future Iranian walnut breeding. The Ilam-West Azerbaijan, and Ilam-Markazi populations showed lower pairwise F_{ST} values, suggesting gene flow among these populations. Due to their geographical location, there is a possibility of wind-driven cross-pollination, seed dispersal by animal movement, and seed movement of superior genotypes between orchards by walnut growers.

To date, there is no published parentage analysis of Iranian walnut populations. Although walnut can be propagated by several methods[46], propagation of valuable trees from Iranian landraces or natural populations has been almost exclusively by seed, via humans or birds. However, we found a clonal relationship between one pair of very old individuals from Kerman province (Gugher) that were genetically identical and likely propagated by natural layering. Based on our observation at the site, natural layering occurred when a branch of the older tree (more than 500 years old) touched the ground, producing adventitious roots and eventually a new tree. Vahdati and Khalighi[47] have previously reported vegetative propagation by layering in walnut. This is the first report of vegetative propagation in an Iranian walnut population.

Parentage analysis identified seventeen pairwise relationships among the Iranian genotypes, nine classified as first and eight as second-degree relationships. All of these were between individuals within given populations, probably due to open pollination or seed exchange between local walnut growers. Interestingly, we identified a second-degree relationship between the cultivar Chandler and two individuals from the Ilam population, possibly indicating one of the ancestors of 'Chandler' could have originated from Iran. Our results are consistent with Tulecke[48] that stated the parents of Chandler might have originated from Iran. These parentage results, together with the genomic variation and differentiation analysis, are of interest both for clarifying the relationships between walnut accessions in view of GWAS analysis, and for accurate planning of future breeding programs in Iran.

Due to the limited number of individuals per population, our conclusions about genetic differentiation among populations have to be considered preliminary. The results of this study can be improved by increasing the sample size from the populations that we had a limited number of samples of them in our survey (Hamadan, West Azerbaijan, Markazi, and Yazd), and from the Semnan population as the most genetically differentiated from the other populations. In addition, in future studies, sampling of young and old trees from the same locations might reveal if local regeneration methods tend to preserve local genetic diversity or obscure it by the importation of new genetic types. Additional collections from other Iranian provinces and further genomic analysis will provide additional evidences to our results.

As further proof of the value of our walnut collection, we performed association mapping for nut and kernel-related traits, identifying marker-trait associations for 19 of the 22 traits studied. Our GWAS results revealed 55 significant SNPs (41 of unique sequence) associated with the variation of six traits, and 56 suggestive SNPs associated with 13 traits. None of the markers identified for the studied traits were previously mapped. In some cases, the same SNP was associated with different traits, which could be explained by the high correlation observed among them, or pleiotropic effects. Limited information exists in walnut regarding the genetic based of nut and kernel-related traits. The annotation of the significant and suggestive SNPs revealed genetic mechanisms for the studied traits also identified for other plant species. In particular, it has been already reported that the genes encoding for the WRKY transcription factor, the LRR receptor-like serine/threonine-protein kinase GSO1, the NAC domain-containing protein 43, and cyclic DOF factor 2-like are likely involved in embryo development[49]

and, therefore, the determination of seed size[50], as well as in the development of the epidermal surface in embryos and cotyledons[51], seed size/weight[52], and seed maturation[53]. The SNPs identified in this study, if appropriately validated, could be used as potential markers for marker-assisted breeding in walnut.

Conclusion

Genome-wide markers offer new opportunities for better understanding genomic variation and architecture of horticulturally important traits in walnut. We used the Axiom *J. regia* 700K SNP array to characterize Iranian walnut genotypes and to verify that the genetic variation available in our panel was suitable to perform an informative association mapping study. We observed a conversion rate similar to that obtained using the same SNP array in a Californian walnut collection. This indicates the Axiom *J. regia* 700K SNP array is a robust and valid genomic tool for further exploring the genetic variation and differentiation of walnut worldwide.

Population structure analysis of this Iranian walnut collection showed four main groups. Total differentiation among the populations was moderate, reflecting the occurrence of cross-hybridization events between native populations. Pairwise F_{ST} analysis found the Semnan population to be the most genetically differentiated and further in-depth examination of it should be prioritized in view of its value for future breeding programs. Overall, we observed consistency among the different genetic analyses employed and results were in accord with geographical and ecological information. Our findings demonstrate that large genetic variation still exists within Iranian walnut populations located in one of the main centers of origin and domestication of Persian walnut. Also, the potential of our population for future GWAS studies was confirmed through the results of association mapping for nut and kernel-related traits. The information generated in this study will be useful for better understanding the genetic basis of adaptation in walnut and identifying resilience alleles to be used by future breeding programs in addressing the challenges of climate change. Our invaluable collection of walnut genotypes adapted to diverse climates and altitudes across Iran were maintained at the Nut Crops collection orchard of Aburaihan Campus, University of Tehran. All the seven populations investigated in present study, along with additional material collected from other parts of Iran, have been established in a common garden to investigate in the future the genetic architecture of local adaptation and the correlation among genotypes and both environmental variables and drought-related traits using GWAS approaches.

Materials and Methods

Sample collection and phenotypic measurements. In the first step, pre-selection of Iranian walnut genotypes based on phenotypic records from the Iranian Ministry of Agriculture and local growers was performed with the aim of selecting superior genotypes to be used in future scion and rootstock breeding programs. We selected 95 genotypes to characterize at both phenotypic and genomic levels. In particular, the selected 95 individuals inhabit disjunctive mountainous areas, and are old walnut trees from open pollinated seedlings (50- to 500-year-old) with trunk diameters greater than 50 cm. They represent local populations (seedlings) that were randomly planted by humans or birds (mostly crows), and grow across wide areas in different parts (valleys or mountains) of Iran. Seeds of the studied genotypes collected from walnut populations located in eight main walnut producers provinces in Iran. The walnut trees investigated in the present research were separated from each other by 366–1768 km (approximately 890 km on average). We sampled four locations from Kerman (Baft-Gugher, Rabor, Rabor-Hanza and Bardsir), two each from Fars (Eqlid and Bavanat) and Ilam (Ilam and Eyvan) and one each from Semnan, Yazd, Markazi, West Azerbaijan and Hamadan (Table 1; Supplementary Table S1; Fig. S1). We planned to collect a minimum of ten samples per location; however, the number of samples collected per region was not equal because of different plant density found in each regions. These native genotypes are considered diverse on a regional scale since each region has gradually selected individuals adapted to environmental, horticultural, cultural and traditional features of the location. Therefore, the 95 selected genotypes likely represent a large part of the full genetic diversity found in Iranian walnut populations. A summary of their profiles for nut and kernel related traits are shown in Table 2.

Leaf tissue and open-pollinated seeds (at least 60 nuts per mother tree) were sampled. Twelve nuts of each selected genotype were used to evaluate 22 fruit-related traits, based on International Plant Genetic Resources Institute (IPGRI) or BI descriptors[54]. These included nut length (NuLe), nut width (NuWi), nut thickness (NuTh), nut weight (NuWe), kernel percentage (KePe), shape index (ShIn), size index (SiIn), round index (RoIn), nut shape (NuSh), shell thickness (SheTh), shell color (SheCo), shell texture (SheTe), shell seal (SheSe), shell strength (SheSt), packing tissue thickness (PaTiTh), kernel weight (KeWe), kernel color (KeCo), kernel plumpness (KePl), kernel shrivel (KeSh), kernel vein (KeVe), kernel fill (KeFi), and ease of kernel removal from nuts (EKeNu). Statistical analyses including descriptive statistics and normality testing on data and their residuals were performed in Minitab 18 statistical software (Minitab, Inc., State College, PA, USA). Multivariate statistical analyses, including principal component analysis (PCA), and correlation analysis were conducted using R[55]. Pearson and Spearman's rank correlation coefficients were used to determine the relationships between two continuous variables and two continuous or continuous-ordinal variables respectively.

Plant materials and DNA extraction. This study examined 95 adult walnut trees grown locally in diverse parts of Iran with various climates. The plant material was collected from eight Iranian provinces: Kerman, Fars, Ilam, Semnan, Yad, Markazi, West Azerbaijan and Hamedan. A detailed list is presented in Table 1. During the summer of 2017 mature fresh leaves were collected, immediately frozen in liquid nitrogen, and lyophilized. Geographical information for each tree was recorded along with detailed climate and population data (Table 1, Fig. 1b).

Total genomic DNA was extracted from 40 mg of dry leaves using the E-Z 96 Plant DNA Kit (Omega Bio-tek; Norcross, GA) according to the manufacturer's instructions. DNA was quantified using Qubit dsDNA High Sensitivity (HS) Assay Kits (InVitrogen, Life Technologies). The quality of DNA samples was checked by agarose gel electrophoresis.

Genotyping with the Axiom *J. regia* 700K SNPs array. DNA samples were adjusted to the recommended concentration of 15 ng/μL in 50 μL aliquots and sent to Affymetrix (now part of Thermo Fisher Scientific, Santa Clara, CA; www.affymetrix.com) for genotyping using the new Axiom *J. regia* 700K SNPs array[25] on the Affymetrix GenTitan platform. Genomic DNA of the cultivar Chandler was used as a control.

SNP allele calling and data analysis. SNP allele calling was performed by the Bioinformatics Core of Affymetrix as described in the Axiom Genotyping Solution Data Analysis Guide (http://www.bea.ki.se/documents/axiom_genotyping_solution_analysis_guide.pdf). The samples with a dQC value ≥ 0.82 and a QC call rate $\geq 97\%$ were considered for further analysis. SNPs were then classified into six major classes: PHR, NMH, OTV, MHR, CRBT and Other.

Analysis of population structure. PHR SNPs filtered for missing rate (>20%) and MAF (<5%), and LD-pruned (plink commands: indep-pairwise 50 5 0.25) were used to perform the population structure analysis. Two approaches were used: (i) Principal Component Analysis (PCA) and (ii) fastSTRUCTURE analysis. The PCA analysis was performed using the R package 'SNPRelate'[56]. The PCA plot was constructed using the R package ggplot2. A Bayesian clustering approach using fastSTRUCTURE software v1.0[57] was then applied. A number of clusters (K values) ranging from 2 to 10 were tested, with ten replicates each using the default convergence criterion and priors. The most likely K value was chosen by plotting the marginal likelihood of the data, and with the best choice function implemented in fastSTRUCTURE. The results of all replicates for each K cluster were summarized using CLUMPAK (http://clumpak.tau.ac.il/)[58].

To confirm further the subgroups identified by the above analysis, individual dissimilarities for each pair of individuals were calculated and used for hierarchical cluster analysis by the R package 'SNPRelate'[56].

Genomic diversity and differentiation among Iranian walnut populations. The genomic diversity of populations was estimated using the 'diveRsity' package[59], which is available in R[55]. Mean number of alleles per locus (A), mean observed heterozygosity (H_o), expected heterozygosity (H_e), unbiased expected heterozygosity (UH_e), allelic richness per population (Ar), and inbreeding coefficient (F_{IS}) were calculated for SNPs with a missing rate < 20% and MAF > 5% across the different sub-populations of Iranian walnut accessions. Populations with only one individual were excluded from the analysis.

Two important measurements of within-population genomic variation at a marker locus are the expected heterozygosity (H_e or gene diversity) and the observed heterozygosity[60]. These are computed as:

$$H_e = \sum\nolimits_{i=1}^{k}\sum\nolimits_{j=i+1}^{k} 2p_ip_j = 1 - \sum\nolimits_{i=1}^{k} p_i^2 \text{ for k alleles} \tag{1}$$

Where k is the number of distinct alleles at a locus, and *pi* $(i = 1, 2, \ldots k)$ is the frequency of allele i in the population.

And

$$H_o = H_e(1 - \mathrm{F}) \tag{2}$$

Where F ranges from 0 (no inbreeding) to 1 (completely inbred population)

Genetic differentiation statistics between subpopulations for each locus and across all loci were computed using the R package 'diveRsity'. These included: G'_{ST} = Hedrick's standardized "differentiation" per locus[61], D_{Jost} = Jost's true allelic differentiation per locus[62], and the three unbiased estimators of Wright's F-statistics[63] within-population inbreeding coefficient (F_{IS}), total-population inbreeding coefficient F (F_{IT}), and the among-population genetic differentiation coefficient $\theta(F_{ST})$. The index F_{ST} was computed as:

$$F_{ST}(\theta) = \frac{\sigma_w}{\sigma_a + \sigma_b + \sigma_w} \tag{3}$$

where σ_w is the variance of the allele frequencies between populations, σ_b is the variance of the allele frequencies between individuals within populations, and σ_a is the variance of the allele frequencies between gametes within individuals.

PGDSpider version 2.1.1.5[64] was used to convert PLINK files (PED and MAP) to Genepop format as data input format for 'diveRsity' R package.

Relatedness Analysis. The PLINK 1.9 software[65] was employed on each pair of the Iranian walnut genotypes to infer relatedness for all pairwise comparisons among the 95 Iranian walnut accessions. Pairwise IBD analysis was used to explore the first-degree and second-degree relationships among individuals as the proportion of the SNPs at which there were zero, one, or two shared IBD alleles represented by Z0, Z1, and Z2 respectively. Relatedness was then measured using the PLINK PI_HAT parameter, which indicates the proportion of SNPs in IBD between individual pairs. Pairs of accessions with PI_HAT value > 95% were considered to be genetically identical. We considered indvidual pairs to be first and second-degree relatives if they had PI_HAT values ≥ 0.47 and 0.22 respectively.

Association mapping for nut-related traits. Association mapping was performed for 22 seed-related traits using the average performance of each genotype, BLUPs and the first five PCs of the phenotypic data. GWAS was carried out by applying three models: MLMM[66], SUPER[67] and Fixed and random model Circulating Probability Unification (FarmCPU)[68] method, as implemented in GAPIT[69]. Population structure and familial relatedness were taken into account in all models. We determined suggestive thresholds to correct the p-value for

multiple testing using the approach described by Gao *et al.*[70]. The significant and suggestive P values were 5e^{-07} and 1.006e^{-05} respectively. Manhattan plots were constructed accordingly using the GAPIT. For each trait, the significant SNPs were compared and annotated using the walnut gene annotation v1.0 (taxid: 51240)[31].

References

1. McGranahan, G. H. & Leslie, C. A. Walnuts (Juglans). *Acta Hortic.* **290**, 905–951 (1991).
2. Arzani, K., Mansouri-Ardakan, H., Vezvaei, A. & Roozban, M. R. Morphological variation among Persian walnut (*Juglans regia*) genotypes from central Iran. *New Zeal J Crop Hort.* **36**, 159–168 (2008).
3. Karimi, R., Ershadi, A., Vahdati, K. & Woeste, K. Molecular characterization of Persian walnut populations in Iran with microsatellite markers. *Hortscience.* **45**, 1403–1406 (2010).
4. Vischi, M. *et al*. Genetic Diversity of Walnut (*Juglans regia* L.) in the Eastern Italian Alps. *Forests.* **8**, 81 (2017).
5. Bayazit, S. *et al*. AFLP analysis of genetic diversity in low chill requiring walnut (*Juglans regia* L.) genotypes from Hatay, Turkey. *Sci Hort.* **111**, 394–398 (2007).
6. Chen, X., Xu, L., Zhang, S. L. & Liu, Z. Q. Walnut Genebank in China National Clonal Plant Germplasm Repository. *Acta Hortic.* **1050**, 89–94 (2014).
7. Pollegioni, P. *et al*. Rethinking the history of common walnut (*Juglans regia* L.) in Europe: Its origins and human interactions. *PloS One.* **12**, 0172541 (2017).
8. Krussman, G. Manual of cultivated broad-leaved trees and shrubs, Vol. II. Timber Press, Portland, Ore (1986).
9. Hemery, G. E., Savill, P. S. & Thakur, A. Height growth and flushing in common walnut (*Juglans regia* L.): 5-year results from provenance trials in Great Britain. *Forestry* **78**(2), 121–133 (2005).
10. Gunn, B. F. *et al*. Genetic variation in walnuts (*Juglans regia* and *J. sigillata*; Juglandaceae): species distinctions, human impacts, and the conservation of agrobiodiversity in Yunnan, China. *Am J Bot.* **97**, 660–671 (2010).
11. Atefi, J. Study on phonological and pomological characters on walnut promising clones in Iran. *Acta Hortic* **442**, 101–108 (1997).
12. Atefi, J. Comparison of some promising Iranian walnut clones and foreign varieties. *Acta Hortic.* **544**, 51–59 (2001).
13. Eskandari, S., Hassani, D. & Abdi, A. Investigation on genetic diversity of Persian walnut and evaluation of promising genotypes. *Acta Hortic.* **705**, 159–163 (2005).
14. Ebrahimi, A., Fatahi, R. & Zamani, Z. Analysis of genetic diversity among some Persian walnut genotypes (*Juglans regia* L.) using morphological traits and SSRs markers. *Sci Hort.* **130**(1), 146–151 (2011).
15. Fatahi, R., Ebrahimi, A. & Zamani, Z. Characterization of some Iranians and foreign walnut genotypes using morphological traits and RAPD markers. *Hortic Environ Biotechnol.* **51**, 51–60 (2010).
16. Vahdati, K. *et al*. Genetic diversity and gene flow of some Persian walnut populations in southeast of Iran revealed by SSR markers. *Plant Syst Evol.* **301**, 691–699 (2015).
17. Khorami, S. S., Arzani, K., Karimzadeh, G., Shojaeiyan, A. & Ligterink, W. Genome Size: A Novel Predictor of Nut Weight and Nut Size of Walnut Trees. *Hort Science* **53**, 275–282 (2018).
18. Pollegioni, P. *et al*. Long-term human impacts on genetic structure of Italian walnut inferred by SSR markers. *Tree Genet Genomes* **7**, 707–723 (2011).
19. Mahmoodi, R., Rahmani, F. & Rezaee, R. Genetic diversity among *Juglans regia* L. genotypes assessed by morphological traits and microsatellite markers. *Span J Agric Res* **11**, 431–437 (2013).
20. Pop, I. F. *et al*. Relationships of walnut cultivars in a germplasm collection: comparative analysis of phenotypic and molecular data. *Sci Hort* **153**, 124–135 (2013).
21. Najafi, F. *et al*. Isolation and characterization of novel microsatellite markers in walnut (*Juglans regia* L.). *Am J Plant Sci.* **5**, 409–415 (2014).
22. Dang, M. *et al*. Identification, development, and application of 12 polymorphic EST-SSR markers for an endemic Chinese walnut (*Juglans cathayensis* L.) using next-generation sequencing technology. *Biochem Syst Ecol.* **60**, 74–80 (2015).
23. Ebrahimi, A., Zarei, A., McKenna, J. R., Bujdoso, G. & Woeste, K. E. Genetic diversity of Persian walnut (*Juglans regia*) in the cold temperate zone of the United States and Europe. *Sci Hort.* **220**, 36–41 (2017).
24. Roor, W., Konrad, H., Mamadjanov, D. & Geburek, T. Population differentiation in common walnut (*Juglans regia* L.) across major parts of its native range—insights from molecular and morphometric data. *J Hered.* **108**, 391–404 (2017).
25. Marrano, A. *et al*. A new genomic tool for walnut (*Juglans regia* L.): development and validation of the high-density Axiom™ *J. regia* 700K SNP genotyping array. *Plant Biotechnol J.* 1–10 (2019).
26. Laurens, F. *et al*. An integrated approach for increasing breeding efficiency in apple and peach in Europe. *Hortic Res* **5**, 11 (2018).
27. Shah, U. N. *et al*. Bio-techniques for improvement of qualitative & quantitative traits in walnut (*Juglans regia*). *Advances in Horticultural Science* **32**, 113 (2018).
28. Van Nocker, S. & Gardiner, S. E. Breeding better cultivars, faster: applications of new technologies for the rapid deployment of superior horticultural tree crops. *Hortic Res* **1**, 14022 (2014).
29. Ciarmiello, L. F. *et al*. A PCR based SNPs marker for specific characterization of English walnut (*Juglans regia* L.) cultivars. *Mol Biol Rep* **38**, 1237–1249 (2011).
30. You, F. M. *et al*. Genome-wide SNP discovery in walnut with an AGSNP pipeline updated for SNP discovery in allogamous organisms. *BMC genomics* **13**, 1–16 (2012).
31. Martínez-García, P. J. *et al*. The walnut (*Juglans regia*) genome sequence reveals diversity in genes coding for the biosynthesis of non-structural polyphenols. *Plant J.* **87**, 507–532 (2016).
32. Hassani, D. *et al*. Genetic improvement of Persian walnut (*Juglans regia* L.) in Iran. In *VII International Walnut Symposium* **1050**, pp. 95–102 (2013).
33. FAO. FAOSTAT Production Crops, http://faostat.fao.org/site/567/default.aspx# ancor (2014).
34. Sharma, O. C. & Sharma, S. D. Genetic divergence in seedling trees of Persian walnut (*Juglans regia* L.) for various metric nut and kernel characters in Himachal Pradesh. *Sci Hort* **88**, 163–171 (2001).
35. Amiri, R., Vahdati, K., Mohsenipoor, S., Mozaffari, M. R. & Leslie, C. Correlations between some horticultural traits in walnut. *HortScience* **45**, 1690–1694 (2010).
36. Rezaei, Z., Khadivi, A., ValizadehKaji, B. & Abbasifar, A. The selection of superior walnut (*Juglans regia* L.) genotypes as revealed by morphological characterization. *Euphytica* **214**, 69 (2018).
37. Ebrahimi, A., Khadivi-Khub, A., Nosrati, Z. & Karimi, R. Identification of superior walnut (*Juglans regia*) genotypes with late leafing and high kernel quality in Iran. *Sci Hort.* **193**, 195–201 (2015).
38. Khadivi-Khub, A. & Ebrahimi, A. The variability in walnut (*Juglans regia* L.) germplasm from different regions in Iran. *Acta physiol plant.* **37**, 57 (2015).
39. Aradhya, M. *et al*. Genetic and ecological insights into glacial refugia of walnut (*Juglans regia* L.). *PloS one.* **12**, e0185974 (2017).
40. Ebrahimi, A., Zarei, A., Lawson, S., Woeste, K. E. & Smulders, M. J. M. Genetic diversity and genetic structure of Persian walnut (*Juglans regia*) accessions from 14 European, African, and Asian countries using SSR markers. *Tree Genet Genomes.* **12**, 114 (2016).
41. Malvolti, M. E., Fineschi, S. & Pigliucci, M. Morphological Integration and Genetic Variability in *Juglans regia* L. *J Hered* **85**, 389–94 (1994).

42. Emanuelli, F. *et al*. Genetic diversity and population structure assessed by SSR and SNP markers in a large germplasm collection of grape. *BMC plant biol* **13**, 39 (2013).
43. Karimi, R. *et al*. Morphological and molecular evaluation of Persian walnut populations in northern and western regions of Iran. *J Nuts* **2**, 21–31 (2014).
44. Hamrick, J. L., Godt, M. J. W., Murawski, D. A. & Loveless, M. D. Correlations between species traits and allozyme diversity: implications for conservation biology. Genetics and conservation of rare plants, 75–86 (1991).
45. Wang, H., Pei, D., Gu, R. S. & Wang, B. Q. Genetic diversity and structure of walnut populations in central and southwestern China revealed by microsatellite markers. *J Am Soc Hortic Sci.* **133**, 197–203 (2008).
46. Vahdati, K., Razaee, R. & Mirmasoomi, M. Micropropagation of some dwarf and early mature walnut genotypes. *Biotechnology.* **8**, 171–175 (2009).
47. Vahdati, K. & Khalighi, A. Persian walnut stooling in Iran. In *IV International Walnut Symposium* **544**, pp. 527–530 (1999).
48. Tulecke, W. The Walnut Germplasm Collection of the University of California, Davis: A Description of the Collection and a History of the Breeding Program of Eugene F. Serr and Harold I. Forde. Genetic Resources Conservation Program. Division of Agriculture and Natural Resources, University of California (1994).
49. Grunewald, W. *et al*. Transcription factor WRKY23 assists auxin distribution patterns during Arabidopsis root development through local control on flavonol biosynthesis. *Proc Natl Acad Sci USA* **109**, 1554–1559 (2012).
50. Johnson, C. S., Kolevski, B. & Smyth, D. R. TRANSPARENT TESTA GLABRA2, a trichome and seed coat development gene of Arabidopsis, encodes a WRKY transcription factor. *The Plant Cell* **14**, 1359–1375 (2002).
51. Tsuwamoto, R., Fukuoka, H. & Takahata, Y. GASSHO1 and GASSHO2 encoding a putative leucine-rich repeat transmembrane-type receptor kinase are essential for the normal development of the epidermal surface in Arabidopsis embryos. *The Plant Journal* **54**, 30–42 (2008).
52. Mathew, I. E., Das, S., Mahto, A. & Agarwal, P. Three rice NAC transcription factors heteromerize and are associated with seed size. *Front Plant Sci* **7**, 1638 (2016).
53. Corrales, A. R. *et al*. Characterization of tomato Cycling Dof Factors reveals conserved and new functions in the control of flowering time and abiotic stress responses. *J. Exp. Bot* **65**, 995–1012 (2014).
54. IPGRI. Descriptors for walnut (*Juglans spp*.). Rome, Italy, International Plant Genetic Resources Institute: 51 pp (1994).
55. R Core Team. R: A language and environment for statistical computing. R Foundation for Statistical Computing, Vienna, Austria, http://www.R-project.org/ (2014).
56. Zheng, X. *et al*. A high-performance computing toolset for relatedness and principal component analysis of SNP data. *Bioinformatics.* **28**, 3326–3328 (2012).
57. Raj, A., Stephens, M. & Pritchard, J. K. fastSTRUCTURE: variational inference of population structure in large SNP data sets. *Genetics* **197**, 573–589 (2014).
58. Kopelman, N. M., Mayzel, J., Jakobsson, M., Rosenberg, N. A. & Mayrose, I. Clumpak: a program for identifying clustering modes and packaging population structure inferences across K. *Mol Ecol Resour.* **15**, 1179–1191 (2015).
59. Keenan, K., McGinnity, P., Cross, T. F., Crozier, W. W. & Prodöhl, P. A. diveRsity: An R package for the estimation and exploration of population genetics parameters and their associated errors. *Methods Ecol Evol.* **4**, 782–788 (2013).
60. Nei, M. Analysis of gene diversity in subdivided populations. *Proc Natl Acad Sci USA* **70**, 3321–3323 (1973).
61. Hedrick, P. W. A standardized genetic differentiation measure. *Evolution* **59**, 1633–1638 (2005).
62. Jost, L. O. U. G_{ST} and its relatives do not measure differentiation. *Mol Ecol* **17**, 4015–4026 (2008).
63. Weir, B. S. & Cockerham, C. C. Estimating F-statistics for the analysis of population structure. *Evolution.* **38**, 1358–1370 (1984).
64. Lischer, H. E. & Excoffier, L. PGDSpider: an automated data conversion tool for connecting population genetics and genomics programs. *Bioinformatics* **28**, 298–299 (2011).
65. Purcell, S. *et al*. PLINK: a tool set for whole-genome association and population-based linkage analyses. *Am J Hum Genet.* **81**, 559–575 (2007).
66. Segura, V. *et al*. An efficient multi-locus mixed-model approach for genome-wide association studies in structured populations. *Nature genet.* **44**, 825 (2012).
67. Wang, Q., Tian, F., Pan, Y., Buckler, E. S. & Zhang, Z. A SUPER powerful method for genome wide association study. *PloS one.* **9**, e107684 (2014).
68. Liu, X., Huang, M., Fan, B., Buckler, E. S. & Zhang, Z. Iterative usage of fixed and random effect models for powerful and efficient genome-wide association studies. *PLoS genet.* **12**, e1005767 (2016).
69. Lipka, A. E. *et al*. GAPIT: genome association and prediction integrated tool. *Bioinformatics* **28**, 2397–2399 (2012).
70. Gao, X., Starmer, J. & Martin, E. R. A multiple testing correction method for genetic association studies using correlated single nucleotide polymorphisms. *Genet Epidemiol.* **32**, 361–369 (2008).

Acknowledgements

We would like to thank the Iran National Science Foundation (INSF), the Center of Excellence for Walnut Improvement and Technology of Iran, University of Tehran, and the University of California-Davis for their support. We also thank the California Walnut Board for funding the genotyping assay.

Author Contributions

M.M.A.: designed, performed the project, and wrote the manuscript. A.M.: directed the project, contributed to the interpretation of the results and revised manuscript. R.A.A.: contributed to the interpretation of the results and revised the manuscript. C.A.L.: designed the project, and revised manuscript. H.A.: designed the project. D.B.N.: designed, directed the project and revised manuscript. K.V.: designed, directed the project and revised manuscript. All authors listed, have made direct and intellectual contribution to the work, and approved it for publication.

Additional Information

Supplementary information accompanies this paper at https://doi.org/10.1038/s41598-019-42940-1.

Competing Interests: The authors declare no competing interests.

Publisher's note: Springer Nature remains neutral with regard to jurisdictional claims in published maps and institutional affiliations.

Open Access This article is licensed under a Creative Commons Attribution 4.0 International License, which permits use, sharing, adaptation, distribution and reproduction in any medium or format, as long as you give appropriate credit to the original author(s) and the source, provide a link to the Creative Commons license, and indicate if changes were made. The images or other third party material in this article are included in the article's Creative Commons license, unless indicated otherwise in a credit line to the material. If material is not included in the article's Creative Commons license and your intended use is not permitted by statutory regulation or exceeds the permitted use, you will need to obtain permission directly from the copyright holder. To view a copy of this license, visit http://creativecommons.org/licenses/by/4.0/.

© The Author(s) 2019
