# Supplementary material for: Provenance and family variations in early growth of Manchurian walnut (Juglans mandshurica Maxim.) and selection of superior families
Source: PLoS One. 2024 Mar 7;19(3):e0298918. doi: 10.1371/journal.pone.0298918 (PMC10919699; doi:10.1371/journal.pone.0298918)
Supplement: S1 File — (ZIP) [file pone.0298918.s004.zip › Geographical variation and preliminary selection of the best provenance of Juglans mandshurica.pdf]

DOI:10.13759/j.cnki.dlxb.1990.s2.011

第18卷 育种专刊 东北林业大学学报 Vol.18 Sp.Iss.
1990年8月 JOURNAL OF NORTHEAST FORESTRY UNIVERSITY Aug. 1990

胡桃楸地理变异规律及最佳种源的初步选择

杨书文 刘桂丰 张世英 王会仁
彭宏梅 杨传平 夏德安 张 靖
(东北林业大学)

【摘 要】

通过对帽儿山试验林场胡桃楸种源试验幼树资料的研究表明：胡桃楸的生长性状，适应性状和形态性状分别与经度、纬度呈显著负相关，表现出以经向变异为主，纬向变异为辅，经纬双向渐变的趋势。同时为帽儿山及毗邻地区造林用种初步选出了最佳种源。

主题词：胡桃楸；种源试验；地理变异；种源选择

胡桃楸（*Juglans manclshumca*）主要分布于我国东北的小兴安岭、张广才岭、老爷岭、长白山区和辽宁省东部山区[1]。它材质坚硬、致密、纹理通直美观，是珍贵用材树种和经济树种。目前，胡桃楸已由原来的野生状态到造林试验初见成效，不远的将来，大面积造林势必在东北广大地区展开。为今后造林用种有据可依，本课题首次研究了胡桃楸地理变异规律，并进行最佳种源的初步选择，得到了初步结果。

1 试验材料和研究方法

1.1 种源布点及试验设计

在胡桃楸自然分布区内均匀设置8个采种点，它们是：辽宁省的宽甸，吉林省的临江、汪清和舒兰，黑龙江省的东方红、桦南、帽儿山和带岭。1985年秋分别从各点采集种子，翌春在帽儿山林场直播造林。采用完全随机区组设计，5次重复；双行小区，每小区50穴，每穴播5粒种子，试验地周围有保护行。

1.2 性状观测

造林后每年对胡桃楸进行生长性状（树高、地径）、适应性状（保存率）、形态性状（冠幅、侧枝数、侧芽数）等调查。在播种前进行了千粒重、种子长与宽的形态测量。

1.3 研究方法

首先对各调查性状进行方差分析，在差异显著的基础上将有变异的性状与经纬度及

?1994-2015 China Academic Journal Electronic Publishing House. All rights reserved. http://www.cnki.net

生态因子进行相关分析，了解各性状的地理变异规律及与生态因子的关系。最后选出帽儿山及毗邻地区的最佳种源。

2 结果与分析

2.1 生长性状的地理变异趋势

对胡桃楸3个年度的生长分别进行方差分析得出F值为3.16*（1 a 生）、3.70**（2 a 生）和4.32**（3 a 生），其结果均达到差异显著或极显著，随着年龄的增长，高生长分化越来越大。

树高和地径与经纬度及生态条件的相关分析得出（见表1），树高和地径与经度呈显著的负相关，与纬度呈较大程度的负相关，地径与年降水量呈显著正相关，即经度偏西，纬度偏南，降水量越大的地区，树高、地径生长量越大，呈现经纬双向渐变的趋势。

表1　种源各性状与经纬度和生态因子相关系数

性状	地理坐标和生态因子					
	纬度	经度	年均温	年积温（≥60℃）	年平均降水量	蒸发量
千粒重	−0.2146	−0.0471	0.2443	0.4435	0.1560	0.3746
种子长	0.6173	0.6763	−0.4456	−0.4456	0.1909	0.5444
种子宽	0.6172	0.1057	−0.4007	−0.2303	−0.0995	0.0101
树高	−0.4587	−0.7016*	0.2296	0.2855	0.6941	0.3130
地径	−0.5997	−0.7524*	0.1548	0.1626	0.7511*	0.1553
保存率	−0.7142*	−0.5024	0.8350**	0.7807*	0.5630	0.7669*
冠幅	−0.3881	−0.7906*	0.1349	0.2059	0.6308	0.2338
侧枝数	−0.3035	−0.6563	0.0984	0.1909	0.5444	0.0716
侧芽数	−0.4292	−0.6068	−0.2050	0.1963	0.6901	0.1092

注：* 代表相关显著，** 代表相关极显著

从以上分析可见，经度越小，降雨量越大，这似乎与我国降雨量分布特点，从东向西，降雨越少相矛盾。但就核桃楸分布区的局部范围内降雨有其独特一点。在分布区最东边的种源东方红经度为133.17°，年降水为650.94 mm，而分布区最西边的宽甸经度为104.78°，年降水高达为1 158.00 mm，与我国大范围降雨特点出现相反的结果，这是胡桃楸分布区内几大山脉对局部范围降雨影响不同造成的。

2.2 适应性状的地理变异趋势

胡桃楸的适应性状主要观测了造林保存率，经方差分析得出各种源间F值为9.527**，差异达到了极显著水平。相关分析得出（见表1），保存率与纬度呈显著的负相关，与年均温、≥10℃的积温及蒸发量分别呈极显著和显著的正相关，即低纬度（偏

?1994-2015 China Academic Journal Electronic Publishing House. All rights reserved.　http://www.cnki.net

南）、温度高、蒸发量大的地区种源幼树保存率高。胡桃楸适应性的这种变异趋势也是其对环境长期适应的结果。低纬度种源的胡桃楸为了适应高温、蒸发量大的生态条件，生存能力必然要强，保存率高。否则幼树在剧烈蒸腾，大量失水情况下而枯死。

2.3　形态性状的地理变异趋势

根据主要形态性状中冠幅、侧枝数，侧芽数与经纬度及生态因子的相关（见表1）可见，三个性状都与经纬度呈负相关，其中经度对三个性状的影响高于纬度。冠幅与经度呈显著负相关。其它生态因子与形态性状有一定的相关性，但均未达到相关显著水平。综合评定，胡桃楸的形态性状主要受经纬度双重控制，经度影响较大，在分布区内，随种源地理位置偏向西南，冠幅大，侧枝粗，侧芽多。表现出与生长性状、适应性状同样的变异趋势。

除以上3个方面的性状外，还进行了种子性状的变异趋势分析，但规律性不强。

2.4　优良种源的初步选择

为了选出帽儿山及毗邻地区造林的最佳种源，在高生长和适应性状方差分析的基础上进行了8个种源树高及保存率（转换的）平均数间的差异显著性测验（L. S. R 测验），结果见表2。

表2　胡桃楸3年生树高和保存率 L.S.R 测验结果

种源	舒兰	宽甸	带岭	帽儿山	临江	汪清	东方红	桦南
三年树高	92.6	92.4	78.3	74.9	71.9	69.8	68.5	64.9
5%	——	——	——					
			——	——	——	——	——	——
1%	——	——	——	——				
				——	——	——	——	——

种源	舒兰	宽甸	汪清	帽儿山	临江	东方红	桦南	带岭
成活率%	96.2	89.0	78.0	77.3	67.3	63.3	48.6	17.8
5%	——	——	——	——				
				——	——	——	——	——
1%	——	——	——	——	——			
					——	——	——	——

从表2可见，舒兰、宽甸两个种源无论在生长性状，还是在适应性状上都表现优良。从高生长看，舒兰、宽甸之间无差异，与帽儿山（当地）差异显著，与临江、汪清、东方红、桦南等差异极显著。各种源高生长相比较，舒兰、宽甸分别高出当地对照的23.6%和23.4%，高出最差种源（桦南）的42.7%和42.4%。

从各种源生长性状与适应性状的排列顺序可见，除带岭这一个种源的变化较大外，其它种源在两组排列基本吻合。带岭种源这一特殊情况有待进一步研究分析。

综上所述，帽儿山及毗邻地区胡桃楸造林用适应选择辽宁省的宽甸和吉林省舒兰两种源。

?1994-2015 China Academic Journal Electronic Publishing House. All rights reserved.　http://www.cnki.net

2.5 种源高生长的稳定性及早期选择的可行性

2.5.1 将胡桃楸各种源历年高生长按年度绘点，并连成三条曲线[2]（见图1）。

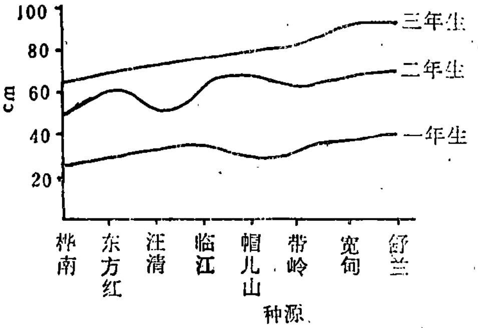

图1 各种源历年高生长曲线

从图可见，种源从桦南至宽甸的高生长在不同年度内均呈平缓上升趋势。除汪清、东方红二种源有个别波动现象外，其它种源均呈稳定生长，尤其舒兰、宽甸二种源在高生长上处于曲线的最高峰，因此可见各种源高生长在各年度排序较稳定的。

2.5.2 根据不同种源的1至3 a 高生长进行秩次相关分析[3]，结果分别为 $r_{(1-2年)}=0.744$，$r_{(1-3年)}=0.928\,5^{**}$，$r_{(2-3年)}=0.839\,0^{*}$，1 a 与 3 a，2 a 与 3 a 高生长相关紧密，因此可以认为种源的早期选择是可行的。

2.6 优良种源高生长性状遗传增益的估算

按照上述所选的优良种源（宽甸、上营）占所有种源的百分率（入选率）和种源高生长方差分析的种源遗传力$\left(1-\frac{1}{F}\right.$，$F$ 为种源方差除以机误方差$\left.\right)$为据，进行种源遗传增益的估算，其结果为 10.18 %。若在帽儿山及毗邻的生态条件相似地区采用最佳种源造林，按目前高生长计算可望得到 10.18 %遗传增益。

3 结论与建议

3.1 胡桃楸的生长性状、适应性状和形态性状分别与经度和纬度相关显著，呈现出以经度为主，纬度为辅的双重负向渐变趋势，即越靠近分布区西南方向的种源生长量越大，适应性愈强。

3.2 帽儿山及毗邻地区最佳种源初选为宽甸和舒兰。其中宽甸种源高生长分别高出当地对照种源和最差种源 23.4% 和 42.4%，舒兰种源高生长分别高出上述对比者 23.6% 和 42.7%。若采用宽甸、舒兰二种源造林可望得到 10.18 %的遗传增益。

3.3 高生长的稳定性分析和秩次相关分析证明上述最佳种源的选择是可靠的。

3.4 根据初选结果在该林场可进一步进行中间试验或进行优良种源的推广工作。

参　考　文　献

1 周以良等．黑龙江省树木志．哈尔滨：黑龙江科学技术出版社，1986，173—175
2 于秉君等．5年生长白落叶松种源试验研究．东北林业大学学报，1988，16（3）：27—33
3 杨书文．长白落叶松种源选择的研究．东北林业大学学报，1984，12（4）：20—29

?1994-2015 China Academic Journal Electronic Publishing House. All rights reserved.　http://www.cnki.net

A STUDY ON THE GEOGRAPHIC VARIATION AND PRELIMINARY SELECTION FOR THE BEST PROVENANCES OF JUGLANS MANDSHURICA

Yang Shuwen Liu Guifeng Zhang Shiying Wang Huiren
Peng Hongmei Yang Chuanping Xia Dean Zhang Jing
(Northeast Forestry University)

ABSTRACT

The analysis of the data from young *Juglans mandshurica* plantations on the Maoershan Experimental Forest Farm indicates that variations in growth, adaptional and morphological traits are negatively correlated with longitude and latitude strongly. It is a dual cline rely mainly on longitude. The best provenances have been determined for the reforestation of Maoershan and its neighbouring regions.

Descriptors: *Juglans mandshurica*; Provenance test; Geographic variation; Provenance selection

?1994-2015 China Academic Journal Electronic Publishing House. All rights reserved. http://www.cnki.net
