# Supplementary material for: Provenance and family variations in early growth of Manchurian walnut (Juglans mandshurica Maxim.) and selection of superior families
Source: PLoS One. 2024 Mar 7;19(3):e0298918. doi: 10.1371/journal.pone.0298918 (PMC10919699; doi:10.1371/journal.pone.0298918)
Supplement: S1 File — (ZIP) [file pone.0298918.s004.zip › Growth and wood density of 25-year-old Masson's pine inter-family variation and selection.pdf]

林业科学研究 2010, 23(6): 804～808
Forest Research

文章编号: 1001-1498(2010)06-0804-05

25年生马尾松生长和木材基本密度家系变异与选择

林思京

(福建省华安县西陂国有林场, 福建 华安 363803)

摘要: 以福建省华安县西陂林场的25年生来自省内不同产地的46个马尾松自由授粉家系试验林为试材, 研究了达主伐年龄时马尾松生长、干形和木材基本密度的家系差异以及性状间的相关性, 并依据不同培育目标选择优良家系。结果表明, 马尾松生长、树干圆满度和木材基本密度皆存在显著的家系差异, 其中生长和树干通直度变异相对较大, 而树干圆满度和木材基本密度变异较小, 变异系数在10%以下。生长和干形性状受到弱至中度的家系遗传控制, 家系遗传力为0.23～0.46, 木材密度受到的遗传控制相对较强, 家系遗传力为0.46。性状遗传相关分析结果显示, 选择速生种源可同时改良树干圆满度, 但对树干通直度和木材基本密度影响不大。根据不同用材对材质材性要求, 选出24个纸浆纤维材优良家系和6个锯材优良家系, 平均木材干物质量和胸径生长皆为对照的1.16倍。

关键词: 马尾松; 家系; 生长; 干形; 木材基本密度; 选择
中图分类号: S791.248 文献标识码: A

Growth and Wood Density of 25-year-old Masson's Pine: Inter-family Variation and Selection

LIN Si-jing

(Xipi Forest Farm of Hua'an County, Fujian Province, Hua'an 363803, Fujian, China)

Abstract: 25-year-old Masson's pine (*Pinus massoniana*) trials including 46 open-pollinated families located at Hua'an county in Fujian province were used to reveal the variation among families in growth, stem-form qualities and wood basic density, and select superior families suitable for producing pulpwood and building timber, respectively. The results indicated that there existed significant differences among families in growth, stem fullness and wood basic density. The variation was relative larger for growth and stem straightness than the other traits. The coefficients of variation of stem fullness and wood basic density were below 10%. It was found that growth, stem form and wood basic density were under moderate to weak family genetic control, and family heritability ranged from 0.23 to 0.46. Among test traits, the family heritability of wood basic density was the largest which reached 0.46. Genetic correlation suggested that the stem fullness could be concurrently improved by selecting the fast-growing families. However, the wood basic density and stem straightness were not influenced by growth. 24 and 6 superior families were selected for pulpwood and sawn timber, respectively. Both of the mean wood dry matter accumulation and DBH growth were 1.16 times that of the control.

Key words: *Pinus massoniana*; family; growth; stem form; wood basic density; selection

马尾松(*Pinus massoniana* Lamb.)是我国松属(*Pinus* L.)中分布最广的树种, 生长快, 适应性强, 是南方山地主要的针叶商品用材树种之一, 广泛用于制浆造纸、建筑、松香原料等。由于分布区气候、土壤条件以及栽培措施的不同, 已有研究认为马尾松种源生长呈典型的纬向连续渐变地理变异模式,

收稿日期: 2010-03-15
基金项目: "十一五"国家科技支撑专题"高产优质多抗马尾松新品种选育"(2006BAD01A1403)
作者简介: 林思京(1964—), 男, 福建武平人, 高级工程师, 主要从事林木良种和森林培育技术研究与生产.

?1994-2018 China Academic Journal Electronic Publishing House. All rights reserved. http://www.cnki.net

并在南方各主产区建立了较大规模的良种繁育基地，选育出一批优良种源和家系在生产上广泛应用[1-3]。但马尾松性状的遗传变异不仅存在于不同地理种源间，而且也广泛存在于家系和个体等层次。季孔庶等[4]对 8年生无性系种子园自由授粉子代半同胞家系生长、干形和材性指标进行了分析，认为家系间存在较为丰富的变异，具选育潜力。基于家系间遗传变异丰富，很多研究根据不同培育目标已为当地生产单位筛选出一批优良家系，木材密度遗传增益为 8.7%，材积平均遗传增益 8.4%～40%，最高遗传增益可达 108%，增产效果显著，具有良好的推广应用前景[4-8]，但已有研究多是幼龄阶段的研究结果，无法预测成熟材材性遗传变异规律和遗传改良效果。

在林木改良过程中最终希望综合性状得到改良，因此性状间的相关性成为关心的问题之一。但目前对性状之间的相关性研究结果并不一致，木材密度、生长性状及它们之间的关系受遗传因子和环境因子的影响[9]。如 A. D. Yanchuk等[10]研究 36年生加拿大白杨（Populus canadensis Moench）时发现木材基本密度与生长速度之间负相关不显著。D. Po等[11]在对海岸松（P. pinaster Ait）的研究中指出不考虑生长而单单进行材性育种无意义，因为密度与生长呈负相关，故应在已经过生长改良的群体中选择材性优良的群体。也有研究显示木材密度与生长不相关，可进行独立选择[12]。施季森[13]则认为杉木（Cunninghamia lanceolata（Lamb.）Hook.）木材密度与树高存在正相关，与胸径和材积存在负相关。

20世纪 80年代初，福建省开展了系统的马尾松选育工作，于 1980年和 1982年在全省进行了优树选择，并于 1983年春将优树自由授粉种子进行育苗，1984年春在三明官庄林场、华安西陂林场、永春碧卿林场同时建立了子代测定林。本研究利用福建华安西陂林场的 46个马尾松优良家系的测定林，研究已达主伐年龄时马尾松生长、干形和木材基本密度性状的家系差异以及性状之间的关系，并以纸浆纤维材和锯材培育目标为生产上筛选一批速生优质的马尾松优良家系。

1 材料与方法

1.1 材料来源

试验材料为设置在福建省华安县西陂林场的 25年生马尾松优树自由授粉家系测定林，计有 46个家系参试，其中 27个家系来自福建泉州产地，18个家系来自南平产地，1个家系来自当地作为对照。该试验林位于 117°30′E，25°06′N，年均气温 20.9 ℃，年降水量 1 618 mm，无霜期 330 d左右，年日照时数 2 000 h以上，土壤为山地黄壤，肥力中等，土层厚度在 80 cm以上。试验采用完全随机设计，30次重复，2株小区，株行距 2 m×2 m，块状整地，穴规 40 cm×40 cm×30 cm。试验林于 1984年春利用 1年生裸根苗造林，造林成活率在 95%以上。造林后连续抚育 3年至幼林郁闭。

1.2 试验林调查和材性测定

由于部分重复保存不完整，这里只利用 12～16个重复的材料。2008年 12月在每个重复的试验区中随机选择 1株，测量树高、胸径、树干通直度、树干圆满度（用树干下部 4 m高处直径与胸径之比表示）等指标。树干通直度按通直、较通直、一般、弯曲、严重弯曲 5级，分别记分为 5、4、3、2和 1，分数越高越通直。同时在植株胸高上坡方位用 6 mm直径的生长锥钻取 1根髓心至树皮的完整无疵木芯。对所取木芯用最大含水量法测定木材基本密度[14]。

1.3 数据分析

单株材积（V）按公式 $V=0.000\,062\,341\,803\times D_{1.3}^{1.856\,149\,7}\times H^{0.956\,849\,2}$ 估算[2]，其中 $D_{1.3}$ 为胸径，H 为树高。以小区单株测定值为单元，采用 SAS/STAT 软件中的 GLM程序进行性状方差分析，以验证家系的效应。性状遗传相关分析则以单株测定值为单元。方差分析和遗传相关分析时树干通直度经 $\sqrt{X}$ 数据转换。家系遗传力 $H^2=(V_1-V_2)/V_1$，单株遗传力 $h^2=4(V_1-V_2)/(V_1+2V_2)$，其中 V_1 为家系间方差，V_2 为机误方差。

2 结果与分析

2.1 生长、干形和木材基本密度的家系遗传变异

生长性状方差分析结果（表 1）显示，马尾松生长、木材基本密度和树干圆满度皆存在极显著的家系差异。25年生时家系平均胸径变幅为 12.46～19.54 cm，平均值为 16.03 cm，胸径生长量最大的家系是最小家系的 1.57倍。相对于胸径生长，家系间平均单株材积生长变异最大，变幅范围为 0.077 9～0.200 3 m³，材积生长量最大家系是最小家系的 2.57倍。家系间树高生长差异相对较小，变异系数

?1994-2018 China Academic Journal Electronic Publishing House. All rights reserved. http://www.cnki.net

仅为胸径变异系数的 59.98%。45个家系间木材基本密度变幅为 0.446 4～0.535 7 g·cm^{-3}，最大和最小家系间相差 20%。对于两个干形指标，树干通直度在家系间差异不显著，平均得分为 3.10，而树干圆满度变异系数尽管较小，但在家系间仍差异极显著。

表 1 马尾松家系生长、干形和木材基本密度的差异分析

性状	变异来源		平均值	变幅	变异系数/%
	家系	机误			
胸径/cm	28.066 6*	18.666 3	16.03	12.46～19.54	26.94
树高/m	6.225 2**	3.808 8	13.04	11.82～14.16	14.96
材积/m³	0.010 2*	0.006 6	0.139 4	0.077 9～0.200 3	58.05
木材基本密度/(g·cm^{-3})	0.004 0**	0.002 2	0.491 1	0.446 4～0.535 7	9.44
树干通直度	0.906 3	0.695 5	3.10	2.57～3.69	26.79
树干圆满度	0.008 8**	0.005 1	0.843 3	0.792～0.893	8.22
木材干物质量/kg	2 390.469 6**	1 789.230 0	69.025	36.181～98.295	61.28

2.2 生长、干形和木材基本密度的遗传控制及性状相关

2.2.1 家系各性状所受遗传控制 从表 2可以看出，参试性状基本上受到中度家系遗传控制，家系遗传力在 0.23～0.46之间，其中木材基本密度受家系遗传控制相对较强，家系遗传力为 0.46。生长、干形和木材基本密度受弱度的加性遗传控制，单株遗传力较小，变化在 0.08～0.22之间，尤其是树干通直度受环境影响较大，单株遗传力不到 0.1。

表 2 马尾松生长、干形和木材基本密度的家系和单株遗传力估算值

遗传力	胸径	树高	材积	木材基本密度	树干通直度	树干圆满度
家系遗传力	0.33	0.39	0.36	0.46	0.23	0.42
单株遗传力	0.14	0.17	0.15	0.22	0.08	0.20

2.2.2 性状相关 性状相关分析(表 3)表明，25年生时马尾松家系木材基本密度虽有随胸径、树高和单株材积生长量的增加而降低的趋势，但相关性未达到 0.1显著性概率水平。生长性状与树干圆满度呈高度正遗传相关（ξ=0.665 5～0.742 6），而与树干通直度相关性不显著。由此可推断，在选择速生家系时可同时改良其树干圆满度，但对树干通直度和木材基本密度影响较小，在遗传上相互独立，选择速生家系不会导致木材基本密度下降。

表 3 马尾松家系生长与干形和木材基本密度的遗传相关

性状	树干通直度	树干圆满度	木材基本密度
胸径	0.128 6	0.666 8	−0.196 6
	(0.394 2)	(<0.000 1)	(0.190 5)
树高	0.219 1	0.742 6	−0.199 3
	(0.143 5)	(<0.000 1)	(0.184 1)
材积	0.165 9	0.665 5	−0.181 5
	(0.270 5)	(<0.000 1)	(<0.227 4)

2.3 纸浆纤维材和锯材目标的优良家系选择

马尾松主要用作纸浆纤维材和锯材，两种用材对材质材性要求不同。纸浆纤维材选育目标要求速生丰产，单位时间单位面积的纸浆材收获量最大。这里以单株木材干物质量(单株材积与木材基本密度的乘积)为选择指标，共筛选出 24个木材干物质量大于对照的马尾松纸浆纤维材优良家系(表 4)，包括 8个来自建阳产地的家系和 16个来自晋江产地的家系，25年生时平均单株木材干物质量在 67.58～98.30 kg，较对照高出 0.4%～46.16%。

表 4 纸浆纤维材优良家系各性状均值

家系	平均单株材积/m³	木材基本密度/(g·cm^{-3})	木材干物质量/kg
2/4	0.147 9	0.485 6	72.153
7/28	0.155 8	0.507 6	79.461
15/81	0.129 7	0.516 3	68.445
16/90	0.135 8	0.521 7	70.333
23/121	0.151 8	0.477 4	72.608
23/123	0.160 4	0.474 2	75.529
23/125	0.170 0	0.495 0	84.450
23/131	0.184 9	0.508 2	93.000
77/559	0.169 6	0.467 3	78.124
78/565	0.200 3	0.491 0	98.295
79/573	0.168 0	0.446 4	76.514
80/580	0.163 7	0.463 9	76.294
80/581	0.162 9	0.504 6	83.081
80/582	0.172 0	0.489 6	84.190
80/583	0.174 0	0.481 2	85.395
82/589	0.153 8	0.494 1	76.230
82/591	0.179 5	0.477 5	86.729
82/592	0.156 8	0.491 8	80.831
82/594	0.175 8	0.498 7	90.115
83/600	0.155 0	0.460 7	75.002
83/601	0.145 3	0.465 3	69.313
103/819	0.142 6	0.481 1	70.335
105/824	0.147 2	0.518 2	76.308
105/829	0.128 3	0.527 2	67.583

?1994-2018 China Academic Journal Electronic Publishing House. All rights reserved. http://www.cnki.net

锯材优良家系要求径生长量大、木材密度高、干形好。以胸径大于对照的 20%，木材基本密度不低于对照，树干通直度得分不低于 3分，树干圆满度在 0.8以上作为选择条件，共筛选出 6个锯材优良家系 (表 5)。

表 5　锯材优良家系各性状均值

家系	胸径 / cm	树干圆满度	树干通直度
82/592	16.55	0.827	3.13
80/581	16.74	0.844	3.08
23/125	17.70	0.868	3.33
82/594	17.71	0.858	2.92
23/131	18.55	0.873	3.53
78/565	19.54	0.886	3.00

以纸浆纤维材和锯材为培育目标，分别选出 24个纸浆纤维材优良家系：2/4、7/28、15/81、16/90、23/121、23/123、23/125、23/131、77/559、78/565、79/573、80/580、80/581、80/582、80/583、82/589、82/591、82/592、82/594、83/600、83/601、103/819、105/824、105/829；6个锯材优良家系：82/592、80/581、23/125、82/594、23/131、78/565。其中 78/565家系表现最好，木材干物质量比对照高 46.16%，其次为 23/131、82/594、82/591、80/583、23/125、80/582、80/581家系，木材干物质量较对照高 23.30% ～ 38.20%。对于入选的优良家系，既可利用其亲本材料作为进一步开展定向育种的改良代种子园的建园材料和杂交亲本，也可从优良家系中进一步选择优良个体进行无性化推广应用。

3　结论与讨论

马尾松遗传改良包括种源、家系、个体等不同层次水平的选择和利用。对马尾松的遗传改良的报道开始大多集中在林分和种源水平，而在家系水平上的研究较少，为此各省区相继开展了家系试验研究[16-20]。福建省位于我国南部，为马尾松优良种源的主产区[2]，故在试验区选择福建产地的马尾松品种，在适应性上也比外来产地的马尾松品种有更强的优势。本文利用已达主伐年龄的马尾松家系试验林，研究了 25年生的来自福建南平和泉州的马尾松家系生长、木材基本密度和树干圆满度的变异情况，结果表明除树干通直度外，其它性状在家系间差异显著，它们在家系水平上存在着很大的遗传改良潜力。在各性状中，生长性状家系变异相对较大，而木材基本密度和树干圆满度变异较小，因此在遗传育种改良中应以生长量为主要选择指标，其它指标为辅；鉴于更多变异来源于个体间，在选择优良家系的基础上进行优良个体的选择能取得显著的效果。

了解生长与材性的遗传相关有助于速生优质林木新品种的选育，但生长与材性的相关性因树种、年龄、材料来源等而异。如季孔庶等[4]对福建漳平五一林场 8年生马尾松子代测定林进行研究，提出生长性状与木材基本密度存在显著正相关关系，而郑仁华等[21]利用福建三明市中村采育场 9年生马尾松家系测定林材料，研究认为木材基本密度与胸径、材积等生长性状呈极显著负相关。本文则认为 25年生马尾松家系胸径、树高和单株材积与木材基本密度不相关，生长和木材基本密度可独立选择，进一步证实了其在 12年生时的研究结果[15]。

参考文献：

[1] 全国马尾松地理种源试验协作组. 马尾松种源变异及种源区划分的研究[J]. 亚热带林业科技, 1987, 15(2): 81－89
[2] 周志春, 傅玉狮, 吴天林. 马尾松生长和材性的地理遗传变异及最优种源区的划定[J]. 林业科学研究, 1993, 6(5): 556－564
[3] 刘青华, 金国庆, 张 蕊, 等. 24年生马尾松生长、形质和木材基本密度种源变异与种源区划[J]. 林业科学, 2009, 45(10): 55－61
[4] 季孔庶, 樊民亮, 徐立安. 马尾松无性系种子园半同胞子代变异分析和家系选择[J]. 林业科学, 2005, 41(6): 43－49
[5] 杨章旗. 马尾松种子园优良家系生长性状选择[J]. 福建林学院学报, 2006, 26(1): 45－48
[6] 郑天汉, 张柏松. 马尾松优良家系材积生长性状研究[J]. 林业勘察设计, 2002(2): 15－18
[7] 范林元, 赖焕林, 季孔庶, 等. 马尾松实生种子园建园家系遗传值估算与优良家系评选[J]. 东北林业大学学报, 2004, 32(4): 4－5
[8] 胡集瑞. 马尾松种子园建园亲本性状遗传变异及优质速生无性系选育[J]. 福建林业科技, 2008, 35(2): 21－24
[9] Zobel B J, van Buijtenen J P. Wood Variation: Its Causes and Control[M]. Berlin: Springer-Verlag, 1989: 7－421
[10] Yanchuk A D, Dancik B P. Variation and heritability of wood density and fiber length of trembling aspen in Alberta, Canada[J]. Silvae Genetica, 1984, 33(1): 11－16
[11] Pot D, Chantre G, Rozenberg P, et al. Genetic control of pulp and timber properties in maritime pine (Pinus pinaster Ait)[J]. Annals of Forest Science, 2002, 59(5-6): 1286－560
[12] Beaulieu J. Genetic variation in tracheid length and relationships with growth and wood traits in eastern white spruce (Picea glauca)[J]. Wood and Fiber Science, 2003, 35(4): 609－616
[13] 施季森. 杉木林木材性的遗传和变异的研究[J]. 南京林业大学学报, 1987(4): 15－23
[14] Smith D M. Maximum moisture content method for determining spe-

?1994-2018 China Academic Journal Electronic Publishing House. All rights reserved.　http://www.cnki.net

cific gravity of small wood samples[R]. US Forest Service, Forest Products Laboratory, 1954, Report No. 2014

[15] 林思京. 福建省马尾松优树子代初步评定[J]. 福建林学院学报, 1997, 17(3): 255－258

[16] 杨宗武, 郑仁华, 傅忠华, 等. 马尾松工业用材优良家系选择的研究[J]. 林业科学, 2003, 39(SP 1): 74－80

[17] 郑仁华, 杨宗武, 梁松庆, 等. 马尾松建筑材优良家系的选择[J]. 福建林学院学报, 2002, 22(1): 1－3

[18] 郑仁华, 施季森, 杨宗武, 等. 马尾松纸浆材优良家系的选择[J]. 南京林业大学学报, 2002, 26(5): 1－6

[19] 徐立安, 陈天华, 王章荣, 等. 马尾松种源子代材性变异与制浆造纸材优良种源选择[J]. 南京林业大学学报, 1997, 21(2): 1－6

[20] 周志春, 金国庆, 周世水. 马尾松自由授粉家系生长和材质的遗传分析及联合选择[J]. 林业科学研究, 1994, 7(3): 263－268

[21] 郑仁华, 陈国金. 马尾松家系木材基本密度遗传变异的研究[J]. 西北林学院学报, 2010, 25(2): 6－9

[22] 周志春, 金国庆, 秦国峰. 马尾松幼龄材密度、管胞长度的地理遗传变异及性状相关[J]. 林业科学研究, 1990, 3(4): 393－397

?1994-2018 China Academic Journal Electronic Publishing House. All rights reserved. http://www.cnki.net
