# Supplementary material for: Provenance and family variations in early growth of Manchurian walnut (Juglans mandshurica Maxim.) and selection of superior families
Source: PLoS One. 2024 Mar 7;19(3):e0298918. doi: 10.1371/journal.pone.0298918 (PMC10919699; doi:10.1371/journal.pone.0298918)
Supplement: S1 File — (ZIP) [file pone.0298918.s004.zip › Heritability of morphological traits in apple early-ripening full-sib and half-sib offspring and its potential use for assisted selection.pdf]

HortScience 47(3):328–333. 2012.

Heritability of Morphological Traits in Apple Early-ripening Full-sib and Half-sib Offspring and Its Potential Use for Assisted Selection

Hassan Hajnajari[1]
Department of Horticulture, Seed and Plant Improvement Research Institute, Mahdasht Road, Shahrake Nahalo Bazr, Alborz Province-(Karaj) 3183964653, Iran

Bahaeddin Chashnidel and Kourosh Vahdati
Department of Horticulture, College of Abouraihan, University of Tehran, Tehran, Iran

Mohsen Ebrahimi
Department of Agronomy and Crop Breeding, College of Abouraihan, University of Tehran, Tehran, Iran

Alireza Nabipour
Department of Oil Seeds, Seed and Plant Improvement Research Institute, Karaj, Iran

Esmaeil Fallahi
University of Idaho, Parma Research and Extension Center, 29603 University of Idaho Lane, Parma, ID 83660

Additional index words. early screening, full-sib offspring, heritability, juvenility, morphological traits

Abstract*. To select the superior parents for the breeding program we oriented to produce new early-ripening cultivars, we evaluated more than 60 productive and vegetative characters in 108 native and imported cultivars grown in national collection for commercial apples located in Karaj, Iran, using an apple descriptor. The germination percentage of 100,000 hybrid seeds was 7% in the first year. In the second year, only 3000 from 7000 seedlings had to pass the final screening. Use of morphological markers as an early selection technique showed high efficiency in the apple breeding program. This study was conducted to determine heritability of certain morphological traits potentially used for selection of fruit early-ripening progenies in apple (*Malus ×domestica* Borkh.). Morphological traits in 3- and 4-year-old seedlings from 28 half-sib and 16 full-sib families were studied in 2007 and 2008, respectively. Parent–progeny correlations were analyzed using true-to-type progenies and their relative parents, all in the juvenile phase. Significant variations were observed between parental cultivars and half-sib and full-sib progenies for most of the studied traits. Positive correlations were found between seedling height and other morphological characters. A highly significant correlation ($r = 0.98$) was observed between branch distance along seedling stem and number of branches. The results showed that the heritability varied among the traits, ranging from moderate to high values. A high level of heritability was found in leaf chlorophyll concentration (LCC) and seedling heights in both half-sib and full-sib families. Heritability of half-sib progenies derived from the early-ripening parents was higher than the late-ripening parents. The relatively high heritability estimates for morphological traits, particularly in early-ripening cross combinations, suggested narrow-sense heritability as a criterion for early screening. The progenies of early cultivars exhibited the maximum and minimum heritability for seedling height (0.91) and leaf length (0.56), respectively. Oppositely, in the late-ripening progenies, the maximum and minimum heritability were found in LW and TD traits (0.79 and 0.42), respectively.

Received for publication 1 Nov. 2011. Accepted for publication 20 Jan. 2012.
We thank the Seed and Plant Improvement Institute for providing support for this research.
[1]To whom reprint requests should be addressed; e-mail hassanhajnajari@yahoo.com.

Length of the juvenile phase seems to be the main obstacle of the fruit tree breeding program because of the land area required for breeding plots, the fact that it is time-consuming, and costly nursery management, all restricting breeding advancement. A large variation in tree architecture and other morphological characters was found in different fruit species, for example, apple (Lespinasse, 1977), pear (Sansavini and Musacchi, 1994), and peach (Scorza, 1984). Implementation of these traits for progeny selection in the breeding program demands genetic studies to distinguish the environmental and the genetic variances from the phenotypic variances, to calculate the expected genetic contribution in the form of heritability, and to estimate their combining ability (Gallais, 1989; Hill et al., 1998). Efficient early selection techniques were applied on seedlings younger than 2 years old (Alston and Battle, 1992; Kazlovskaya, 2005; Koc et al., 2009; Larsen et al., 2006; Segura et al., 2006). Traits of blooming and ripening dates (Hansche et al., 1966; Kester, 1965; Tancred et al., 1995) and fruit quality (Brown, 1960; Dicenta et al., 1993; Kester et al., 1977) have been widely used for study of their inheritance in fruit tree species. In most cases, data collected from the successive years were as replications to estimate the genetic and non-genetic variance. Morphological characteristics regarding tree growth habit (Sampson and Cameron, 1965), the compact habit (Lapins, 1974), dwarfism (Alston, 1976; Decourtye, 1967), and spur types (Alston and Watkins, 1974; Decourtye and Lantin, 1969) were used for genetic analysis. As a result of annual shoot development, tree size is an always increasing variable. Several studies showed that shoot length decreases during consecutive annual biological cycles in several species and in different cultural and climatic conditions. In apricot and apple trees, the mean number of nodes per annual shoot decreased over successive years (Costes et al., 2003) as a result of a decrease in the number of newly formed shoots. In addition, the decrease in the mean number of nodes was similar for all shoots of similar ages within the trees, whichever their branching orders (Costes et al., 2003). However, the final size cannot be predicted from a single-year measurement but from successive years. The phenotypic variance of annual shoot length also decreases on time (Costes et al., 2004). Large variation in time of ripening has been noted in apple cultivars, which facilitates classifying them into nine classes of earliness from extremely early to very late ripening (Watkins and Smith, 1982). Earliness has been found to have relatedness with several morphological traits such as leaf length, seedling height, trunk diameter, leaf chlorophyll concentration, and number of branches (Kazlovskaya, 2005). Tancred et al. (1995) investigated the inheritance of ripening date in apples and obtained high heritability and additive genetic components of variance. Heritability of phenotypic variance in a selection would present in the next generation (Falconer, 1981; Hanson, 1963). However, accurate heritability estimates may be obtainable if phenotypes will present in many trees of successive years (Hardner et al., 2002; Liebhard et al., 2003; Yao and Mehlenbacher, 2000). Heritability has been estimated using several full-sib progenies (Durel et al., 1998; Oraguzie et al., 2001; Tancred et al., 1995). Recently, Liebhard et al. (2003) estimated genetic and environmental variance and identified some emphasized qualitative

trait loci for tree height, collar diameter, and phenological traits in apple offspring. Most genetic studies on the inheritance of the columnar tree habit suggested that a single dominant gene, *Co,* was implicated (Lapins, 1974, 1976). Brown (1960) found the progeny mean for fruit shape to be approximately equal to the midparent value, but the progeny mean for fruit size was less than the midparent value. Isozymic genes showed promise as markers for a limited number of specific characters but there was insufficient polymorphism among cultivated apples limiting their use in a breeding program (Alston and Battle, 1992). Correspondence between phenotypic and genotypic indices of Danish populations of European crab apple (*M. sylvestris*) confirmed that relying exclusively on either morphological or molecular characters as diagnostic markers in studies of hybridization between *M.* ×*domestica* and *M. sylvestris* might lead to fallible results (Larsen et al., 2006). The main aims of the current study were: 1) to evaluate the effect of maternal and paternal parent on morphological characteristics of offspring; 2) to compare the performance of 3- and 4-year-old seedlings obtained from different cross combinations between early and late ripening cultivars; 3) to estimate the heritability of morphological traits using progenies from both open and controlled pollinations of apple cultivars; and 4) to analyze correlations between traits and develop an efficient method for selecting desirable progenies in early growth stages of the juvenile phase. The parents were selected based on the previous evaluations made on 108 cultivars and genotypes existing in the national commercial apple cultivar collection using ab apple descriptor (Watkins and Smith, 1982), revealing high genetic variations in phenological, pomological, morphological traits regardless of ripening time (Hajnajari, 2008).

Materials and Methods

Plant materials. In 2005, controlled and uncontrolled crosses (open pollination) were made between 32 different Iranian native apple and introduced parents belonging to eight different classes, from early- to very late-ripening classes (Hajnajari et al., 2008). These cultivars demonstrated various ripening times including early, mid, late, and very late ripening. The list of cultivars based on ripening time included very early ripening: 'Golab-e Sahne'; early: 'Golab-e Kohanz', 'Kompouti' syn. 'Sharbati', 'Haji-e karaj', 'Mashhad Nouri'; mid-early: 'Koli Mohalat' syn. 'Gol Bahar', 'Sheikh Ahmad', 'Ardabil1', 'Sultani-e Shabestar', 'Heidarzade', 'Jonathan'; mid: 'Stayman'; Mid-Late: 'Red Delicious', 'Yellow Spur', 'Early Red One', 'Starking', 'Glockenapfel', 'Idared', 'Golden Smoothee', 'Red Chief', 'Khorsijan'; late: 'Top Red Delicious', 'Golden Delicious', 'Prime Gold'; late to very late: 'Akhlemad-e Mashhad', 'Granny Smith', 'Red Rome Beauty', 'Winesap' (Hajnajari, 2008, 2010). Parent–progeny correlations were relieved also by use of true-to-type propagated plants of the relative parents all in the juvenile phase. In creating full-sib families (FS), hybridizations between parents were oriented on the basis of their predetermined class of earliness, yield per tree, and fruit quality (Hajnajari, 2008). Half-sib (HS) families were open-pollinated offspring from 28 parents. By sowing almost 100,000 hybrid seeds obtained from 44 predefined cross combinations, a total of 7,000 offspring was produced. In total, 2620 studied progenies were from HS families and 180 from FS families. All hybrid seeds were directly sown in rows spaced at 100 cm × 15 cm in the nursery under a mist system to facilitate the growth of young seedlings, protecting them against abiotic stresses of high temperatures and low relative humidity. Nursery management practices included furrow irrigation, mechanical weed control, and wooly aphid attacks during late spring and summer. In the final season of investigation, the number of seedlings per full-sib and half-sib families varied from five to 500, and a total of 2700 seedlings was evaluated for data collection. In 2007 and 2008, measurements were made on 2700 apple 3- to 4-year-old offspring including 16 full-sib and 28 half-sib families (Table 1) grown in Kamal Abad Research Station, Seed and Plant Improvement Institute, located at Karaj, Iran (lat. 35°48′ N, long. 51°2′ E).

Data collection and analysis. Ten morphological traits and architectural characteristics were evaluated, which were previously reported to have a significant correlation with early ripening in apple cultivars (Kazlovskaya, 2005). These traits included seedling height (SH), leaf length (LL), leaf width (LW), peduncle length (PL), LCC, all with 10 replicates per progeny; trunk diameter (TD); branch's distance along seedling trunk (BD); number of branches (NB); number of branches with suitable angle on the trunk (NBA); approximate angle of 75 grade, judged accurately by visual scrutiny; and number of branches longer than 20 cm (NBL) excluding shorter branches. Total chlorophyll concentration was measured by a chlorophyll meter (Model CCM-200; Opti-sciences Inc.). Ten healthy leaves of offspring were collected methodically from the midpart of branches distributing around the middle belt of tree height at the late stage of vegetative growth. Progenies were scored by mean values of LL and LW. All other traits were similarly scored giving them equal weight. Likewise, each progeny was consequently ranked in a certain position of the predefined scaled chart based on expression intensities of studied markers. Only the progenies ranked in the first and second classes were selected by morphological traits and the remaining was discarded. Analysis of variance and mean comparisons for all of the seedling families were performed by using Duncan's multiple range tests at $P \leq 0.01$ by SAS 9.0 software (SAS Institute Inc., 2000). Narrow-sense heritability in half-sib progenies was estimated according to Hill et al (1998): $h_n^2 = 4\sigma_{BF}^2 / \left(\sigma_{BF}^2 + \sigma_{WF}^2\right)$, where σ_{BF}^2 and σ_{WF}^2 refer to inter- and intrafamily variances, respectively. The variance component was calculated by dividing mean squares to the variance of components. Narrow-sense heritability in the

Table 1. Narrow-sense heritability in the full-sib and half-sib apple crosses.

Full-sib crosses	Full-sib crosses	Half-sib crosses	Half-sib crosses	Half-sib crosses	Half-sib crosses
Winesap × Starking (FS1)	Stayman × Golden Delicious (FS9)	Akhlemad-e Mashhad (HS1)	Golden Smoothee (HS8)	Red Delicious (HS15)	Kompouti (HS22)
Golden Delicious × Winesap (FS2)	Heidarzade × Granny Smith (FS10)	Golab-e Kohanz (HS2)	Gravenstein (HS9)	Red Rome Beauty (HS16)	Heidarzade (HS23)
Early Red One× Winesap (FS3)	Heidarzade × Ardabil (FS11)	Starking (HS3)	Haji-e karaj (HS10)	Shafei (HS17)	Sheikh Ahmad (HS24)
Golden Delicious × Shafei (FS4)	Heidarzade × Khorsijan (FS12)	Early Red One (HS4)	Jonathan (HS11)	Sultani-e Shabestar (HS18)	Glockenapfel (HS25)
Golden Delicious × Jonathan (FS5)	Heidarzade × Early Red One (FS13)	Golab-e Kohanz Damavand (HS5)	Mashhad Nouri (HS12)	Stayman (HS19)	Golden Delicious (HS26)
Jonathan × Starking (FS6)	Heidarzade × Idared (FS14)	Golab-e Sahne (HS6)	Red Chief (HS13)	winesap (HS20)	Koli Mohalat (HS27)
Golab-e sahne × Top Red Delicious (FS7)	Stayman × Early Red One (FS15)	Granny Smith (HS7)	Prime Gold (HS14)	Yellow Spur (HS21)	DST (HS28)
Stayman × Khorsijan (FS8)	Stayman × Scarlet wilson (FS16)				

half-sib families can be intended by correlation estimate among the family's components and similarity level among the families is equal to 25% of additive genetic variance. The Pearson correlation coefficient test was applied to estimate phenotypic correlations between the measured traits ($P \leq 0.01$). Narrow-sense heritability (h_n^2) in full-sib progenies was calculated according to the following equation (Falconer, 1981): $h_n^2 = \text{bo}\bar{p}$, where $\text{bo}\bar{p}$ refers to the regression values between progenies and the parental mean values. It is known that the covariance between the value of progenies and the mean parental value $(\text{Covo}\bar{p})$ is equal to 50% of the additive genetic variance (V_A) in a full-sib progeny test, according to the following equation:

$$\text{Covo}\bar{p} = 1/2\,V_A.$$

Results

Relatedness among morphological traits. Results showed significant variations for morphological traits were found between parents within half-sib progenies and between full-sib offspring (data not shown). Mean comparisons of morphological traits, estimated for parents, half-sib, and full-sib progenies, are displayed in Tables 2, 3, and 4. Early cultivars attained maximum mean values for LL, LCC, NB, and SH 9.9, 45.3, 13, and 199.5, respectively (Table 2). Contrarily, very late parents exhibited maximum values for LW (4.3) and PL (2.96), whereas maximum rates for BD were registered in the Parents 11, 11.3, and 13.1, respectively, belonging to three different ripening classes as very early–early, early, and early–mid.

Heritability of morphological traits in full-sib families. In the full-sib families, maximum (6.3) and minimum (4.67) LL were found, respectively, in the progenies of 'Heidarzade' × 'Ardabil' (FS_{11}) and 'Winsap' × 'Starking' (FS_1) cross combinations (Table 3), whereas the progenies of the two following cross combinations 'Heidarzade' × 'Granny Smith' (FS_{10}) and 'Winsap' × 'Starking' (FS_1) presented accordingly maximum (3.65) and minimum LW (2.03) (Table 3). In addition, high levels of LCC were found in progenies of the 'Heidarzade' × 'Ardabil' (FS_{11}) family. These results are in accordance with those obtained by Kenis and Keulemans (2007) calculated for mean comparisons of architectural traits in full-sib progenies derived from crossing 'Telamon' and 'Braeburn'.

Heritability of morphological traits in half-sib families. Mean values for all morphological traits showed significant variations in the half-sib progenies (Table 4), which was confirmed by the results demonstrated through maximum mean values for LL (9.65), TD (3.1), and SH (317.6) for the progenies of 'Sheikh Ahmad' (HS_{24}). In addition, offspring of 'Golab-e Sahne' (HS_6) exhibited high variation for all morphological traits. Maximum grade of LCC (34.39) was observed in 'Stayman' (HS_{19}) families, and its minimum amount (13.55) was registered in 'Golden Delicious' (HS_{26}) families. Maximum LW (3.65) was found in 'Granny Smith' (HS_7) progenies. Results showed that progenies of early cultivars Golab-e Sahne (HS_6), Sheikh Ahmad (HS_{24}), Golab-e Kohanz (HS_2), Heidarzade (HS_{23}), and Stayman (HS_{19}) had maximum mean values for all morphological traits, except PL. Mean comparison results regarding morphological characteristics revealed that the early parent cultivars and their progenies were significantly different from the offspring derived from the rest of cross combinations. The progenies of early parents presented the highest values for most of the studied traits. These results were in agreement with those by Kazlovskaya (2005) and Segura et al. (2006) in apple cultivars.

Relatedness between morphological traits and architectural traits. Significant positive correlations (r = 0.8112*, 0.701*, 0.902**, 0.836**, and 0.9803*) existed between SH and architectural traits NBL, NBA, TD, BD, and NB correspondingly (Table 5). Maximum correlation (r = 0.9803) was observed between SH and NB traits. Likewise, among leaf characters, a strong correlation was found between LL and LW (r = 0.941**). Interestingly, LCC was positively correlated with LL (r = 0.764*). Narrow-sense heritability (hn2) of morphological traits was independently estimated for progenies of half-sib (Table 6) and full-sib (Table 7) families within two growth seasons (2007–2008). Besides, heritability of the morphological traits was calculated separately for the half-sib offspring obtained from early-to-mid ripening

Table 2. Mean values of morphological and architectural traits of the apple parent cultivars in the juvenile period.

Traits[z]	Very early–early ripening	Early ripening	Early–mid ripening	Midripening	Mid–late ripening	Late ripening	Late–very late ripening	Very late ripening
LL	9.9 a+	9.8 a	9.2 ab	8.3 ab	8.25 ab	7.75 bc	7.5 bc	6.8 c
LW	3.4 bc	3.9 abc	3.6 abc	4.3 a	3.3 c	3.8 abc	3.5 bc	4 ab
PL	2.6 ab	2.7 ab	2.65 ab	2.8 a	2.4 b	2.2 bc	2.1 bc	2.96 a
LCC	45.3 a	38 ab	35.5 ab	42.4 a	37.8 ab	36.3 ab	30.7 b	26.1 b
NBL	10 a	8.6 ab	9 a	8.2 ab	7.4 b	6.5 b	8.3 ab	6.2 b
SH	199.5 a	186.8 b	167.8 ab	176.6 ab	172.8 ab	160.7 b	178.1 ab	142.4 b
TD	8.8 a	9.7 a	7.7 ab	7.5 ab	8.1 ab	6.9 b	7.9 ab	6.4 b
NBA	8 ab	9 a	7.1 ab	8.3 ab	7.6 ab	9.2 a	6.1 b	5.3 b
NB	13 a	11 ab	9 bc	11 ab	8.7 bc	8.7 bc	7.2 c	7.5 c
BD	11 ab	11.3 ab	13.1 a	7.3 b	10.2 ab	9.1 ab	8.1 ab	6.8 b

Means separated using Duncan's multiple range test (alpha = 0.01). Means that have common letters are not significantly different.

[z]LL = leaf length; LW = leaf width; PL = pedicle length; LCC = leaf chlorophyll concentration; NBL = number of branches longer than 20cm; NBA = number of branches with suitable angle on base trunk; SH = seedling height; TD = trunk diameter; NB = number of branches and BD = branches distance along the stem.

Table 3. Means of morphological and architectural traits in apple progenies of the full-sib crosses.

	FS_1	FS_2	FS_3	FS_4	FS_5	FS_6	FS_7	FS_8	FS_9	FS_{10}	FS_{11}	FS_{12}	FS_{13}	FS_{14}	FS_{15}	FS_{16}
NBA	10.75 ab	12.17 a	8.64 abc	2.80 c	6.13 abc	11.80 a	10.33 ab	8.33 abc	7.30 abc	4.79 bc	9.63 abc	7.50 abc	4.55 bc	4.64 bc	9 abc	7 abc
NB	37.2 bcd	39 b	29 bcdef	16.8 f	24.13 cdef	37.6 bc	39.83 b	23.93 cdef	21.11 ef	26.6 bcdef	31.5 bcde	29.5 bcdef	16.21 f	17.1 f	23.43 def	57 a
NBL	14.13 ab	15.5 a	12.18 ab	4.2 b	10.53 ab	15.2 a	14.17 ab	13.29 ab	10.33 ab	13.22 ab	17.56 a	12.75 ab	6.8 b	7.46 ab	12.72 ab	14.5 ab
BD	5.31 bc	5.93 bc	7.03 abc	3 c	6.5 4abc	7.07 abc	8.7 ab	7.64 ab	10.45 a	6.49 abc	9.16 ab	7.67 ab	7.61 ab	6.53 abc	8.91 ab	9.17 ab
TD	2.45 abc	2.52 abc	2.36 abc	1.88 c	2.3 abc	2.66 ab	2.2 bc	2.53 abc	2.55 abc	1.9 c	2.87 ab	2.38 abc	2.14 bc	2.28 abc	2.5 abc	2.95 a
LCC	20.95 e	20.15 cde	23.79 de	27.74 cde	24.07 de	21.98 de	29.16 bcde	34.1 abc	26.91 cde	28.38 cde	37.77 a	30.18 abcd	28.67 cde	26.48 cde	37.27 ab	27.55 cde
SH	278.75 abc	267 bc	297.82 abc	250. bc	299 abc	310.2 ab	268.5 bc	293.64 abc	271.2 abc	238.22 c	339 a	273 bc	260.8 bc	305 ab	295 abc	312.13 ab
LL	4.67e	5.14 cde	5.28 bcde	5.46 bcde	5.03 cde	4.83 de	5.35 bcde	5.54 bcd	5.84 abc	6.04 ab	6.3 a	5.1 cde	5.28 bcde	5.1 Cde	5.27 bcde	5.34 bcde
LW	2.03 d	3.05 bc	3.04 bc	3.04 bc	2.76 C	2.6 c	3.02 bc	3.03 bc	3.31 ab	2.11 d	2.92 bc	2.76 c	2.74 c	2.95 Bc	3.02 bc	3.65 a
PL	1.76 cdef	1.77 cdef	1.84 cdef	1.76 cdef	1.63 F	1.67 f	1.98 bcde	1.96 Bcde	2.34 a	1.7 def	1.76 cdef	1.8 cdef	2.05 bc	2 Bcd	2.21 ab	1.8 cdef

Means separated using Duncan's multiple range test (alpha = 0.01). Means with common letters are not significantly different.

LL = leaf length; LW = leaf width; PL = pedicle length; LCC = leaf chlorophyll concentration; NBL = number of branches longer than 20 cm; NBA = number of branches with suitable angle on base trunk; SH = seedling height; TD = trunk diameter; NB = number of total branches; BD = branches distance along the stem; FS = full-sib.

Table 4. Means of morphological and architectural traits in apple progenies of the half-sib crosses.

	HS_1	HS_2	HS_3	HS_4	HS_5	HS_6	HS_7	HS_8	HS_9	HS_{10}	HS_{11}	HS_{12}	HS_{13}	HS_{14}
NBA	3.53 defg	3.5 defg	3.44 defg	3.27 efg	7.42 Abcde	7.72 abcd	3.56 defg	3.06 fg	2.33 fg	4.38 cdefg	2.89 fg	10.29 a	5.11 cdefg	3.93 cdefg
NB	15.8 fghi	19.27 defghi	15.73 fghi	19.7 defghi	32.45 Abc	27.5 abcde	19.89 defghi	22.75 cdefgh	21 defghi	22.86 cdefgh	24.91 bcdefg	37 a	26 bcdef	11.6 I
NBL	6.2 def	6.9 cdef	4.59 def	5.31 def	8.76 Cde	12.64 bc	8.45 cde	5.35 def	4.82 def	9.13 cde	5.63 def	17.05 ab	8.3 cdef	4.6 def
BD	7.81 abcdefg	6.1 cdefg	7.48 abcdefg	9.33 abcd	7.79 abcdefg	8.6 abcde	6.64 bcdefg	7.6 abcdefg	4.67 g	8.8 abcde	9.64 abc	6.38 bcdefg	4.83 fg	9.15 abcde
TD	2 defg	1.83 fgh	1.89 fg	2.13 bcdefg	2.23 Bcdefg	2.68 ab	1.99 defg	1.97 defg	1.72 gh	2.47 bcde	2.16 bcdefg	2.49 bcde	1.93 efg	1.97 defg
LCC	23.23 efgh	25.49 bcdefg	22.14 efgh	25.75 bcdefg	22.53 Efgh	31.83 ab	19.56 ghi	20.04 ghi	21.18 fgh	27.78 abcdef	26.55 bcdefg	30.43 abcd	25.71 bcdefg	21.68 fgh
SH	247.1 defghi	206.6 ijk	300.9 abc	241.3 fghi	296.4 Abc	311.8 a	191.3 k	271.5 abcdefgh	225.1 hij	279.7 abcdef	239 fghi	315 a	207.6 ijk	245.5 efjhi
LL	5.25 c	5.98 bc	5.53 bc	5.35 bc	5.65 Bc	8.66 a	4.87 c	6.5 abc	6.25 abc	6.81 b	7.35 ab	7.73 ab	4.78 c	7.05 ab
LW	2.75 cdefg	3.59 a	2.94 bcdef	2.62 efg	3.02 Bcdef	2.6 fg	3.65 a	2.87 bcdef	2.75 cdefg	3.14 bc	3.05 bcde	2.98 bcdef	2.63 defg	2.93 bcdef
PL	1.75 fghij	2.53 a	1.92 defgh	2.06 cdefg	2.13 Bcdef	1.72 ghij	1.47 ij	1.95 defgh	1.85 defgh	1.9 defgh	1.99 defg	2.2 abcd	1.72 ghij	2 defg

	HS_{15}	HS_{16}	HS_{17}	HS_{18}	HS_{19}	HS_{20}	HS_{21}	HS_{22}	HS_{23}	HS_{24}	HS_{25}	HS_{26}	HS_{27}	HS_{28}
NBA	5 cdefg	5.25 cdefg	5.6 cdefg	6.39 abcdef	5.5 cdefg	3.8 cdefg	5.65 cdefg	6.18 bcdef	7.86 abc	10 ab	2.6 fg	1.5 g	3.56 defg	3.18 fg
NB	29.14 abcd	19.7 defghi	21.57 defghi	20.59 defghi	20.6 defghi	15.28 fghi	13.38 hi	22.26 defghi	25.8 bcdef	33.91 ab	16.92 efghi	14 ghi	23.86 cdefgh	14.4 ghi
NBL	7.36 cdef	8.25 cedf	6.57 def	10.28 cd	8.87 cde	5 def	7.42 cdef	8.88 cde	12.67 bc	17.95 a	3.6 ef	2.5 f	6.9 cdef	4.73 def
BD	7.63 abcdefg	9.18 abcde	5.7 efg	6.73 bcdefg	8.68 abcde	7.43 abcdefg	10.39 a	9.8 ab	8.33 abcdef	9.78 ab	10.5 a	5 fg	7.01 abcdefg	5.95 defg
TD	1.99 defg	2.17 bcdefg	2.53 bcd	2.63 abc	2.09 cdefg	1.69 gh	2.01 defg	1.75 gh	2.36 bcdef	3.1 a	1.32 h	1.65 gh	1.99 defg	2.16 bcdefg
LCC	22.71 efgh	23.14 efgh	17.95 hi	24.67 cdefgh	34.39 a	23.5 defgh	25.51 bcdefg	27.75 abcde	29.25 abcde	31.5 abc	21.56 fgh	13.55 i	17.75 hi	27.94 abcdef
SH	245.9 efghi	246.9 defghi	307.8 ab	293 abcd	241.1 fghi	299.8 ghi	264.8 bcdefgh	200.2 ijk	276.3 abcdefg	317.6 a	175.7 k	258 cdefgh	200.2 ijk	290.1 abcde
LL	5.91 bc	6.78 b	6.13 bc	5.71 bc	7.62 ab	5.45 bc	6.33 abc	5.82 abc	5.74 bc	9.65 a	7.02 ab	6.1 bc	5.08 c	5.8 bc
LW	2.38 gh	2.94 bcdef	3.17 bc	3.06 bcd	3.01 bcdef	2.9 bcdef	3.04 bcde	3.07 bc	2.98 bcdef	3.6 a	3.15 bc	3.3 ab	2.2 h	3.05 bcde
PL	1.38 j	1.88 defgh	2.13 bcde	2.07 cdefg	2.2 abcd	1.85 defgh	1.99 defg	2.08 cdefg	1.59 hij	1.77 efghi	2.4 abc	2.45 ab	1.72 ghij	1.95 defgh

Means separated using Duncan's multiple range test (alpha = 0.01). Means with the same letters are not significantly different.
LL = leaf length; LW = leaf width; PL = pedicle length; LCC = leaf chlorophyll concentration; NBL = number of branches longer than 20 cm; NBA = number of branches with suitable angle on base trunk; SH = seedling height; TD = trunk diameter; NB = total number of branches; BD = branches distance along the stem; HS = half-sib.

and late-to-very late open-pollinated parents (Table 8). In half-sib families, moderate to high heritability (0.45 to 0.85) was estimated for all traits in 2 successive years of the juvenile phase (Table 6). Maximum heritability was calculated for LCC (0.85) and SH (0.84) in 4-year-old seedlings. Architectural characters of both SH and NB presented the highest heritability (0.80 and 0.84 and 0.80 and 0.79) in the two consecutive growths seasons. Heritability estimates for leaf traits confirmed that LL had high heritability, 0.68 and 0.71, in 2007–2008, regularly. Lowest heritability was estimated for LW (0.45 and 0.48). In full-sib progenies, highest heritability was estimated for LCC (0.79) and SH (0.71) in 4-year-old hybrids and the minimum was obtained for PL (0.45) in 3-year-old seedlings (Table 7).

Narrow-sense heritability for architectural characters. Narrow-sense heritability for architectural characters in full-sib and half-sib progenies ranged between 0.47 to 0.75 and 0.65 to 0.79, respectively, in two growing seasons (Tables 6 and 7). Heritability estimates obtained for full-sib progenies were lower than those of half-sib progenies for most of morphological traits in both years. Likewise, narrow-sense heritability was calculated for half-sib progenies in early- and late-ripening cultivars for morphological traits separately. Early families had higher heritability than the late-ripening families for morphological traits (Table 8). The progenies of early cultivars exhibited the maximum and minimum heritability for SH (0.91) and LW (0.56), respectively. It was also noted that the descendants of early and mid–early cultivars had a high rate of heritability for all vegetative characters related to earliness. In opposition, in the late-ripening progenies, maximum and minimum heritability were attributed to LW and TD traits (0.79 and 0.42, respectively) (Table 8). Tree architectural traits in early families had higher heritability than late-ripening families. Among architectural characters, BD had the highest heritability (0.90) (Table 8). Heritability estimates illustrated the existence of high genetic variability for all of the studied traits in both families. The high heritability of morphological characters observed in half-sib progenies and even in full-sib offspring in both years could be the reason for the strong ability in transmission of the earliness from early parents to progenies. These results were in concordance with the report of Tancred et al. (1995), confirming that ripening time benefited high heritability in apple cultivars. In addition, these results could be supplementary to the positive correlation coefficients between morphological and tree architectural characters in the juvenile phase.

Discussion

Use of morphological markers may accelerate the time-consuming procedure of progeny screening resulting from the offspring juvenility phase as a biological barrier and decreased the high expenses caused by a long period of time for nursery field occupation and relative laborious management (Rweyongeza

Table 5. Correlations among phenotypic traits of leaf length (LL), leaf width (LW), length peduncle (LP), leaf chlorophyll concentration (LCC), number of branches longer than 20 cm (NBL), number of branches with suitable angle on along trunk (NBA), seedling height (SH), trunk diameter (TD), branches distance (BD). and number of branches total (NB) in apple seedlings during the 2008 growth season.

	LL	LW	LP	LCC	NBL	NBA	SH	TD	BD	NB
LL	1	0.941**	0.612	0.764*	0.532	0.458	0.812*	0.762*	0.552	0.458
LW		1	0.7712*	0.6115	0.6717	0.5432	0.8314*	0.811*	–0.3324	–0.324
LP			1	0.5561	0.2914	0.3645	0.2231	0.292	0.066	0.135
LCC				1	0.3524	0.4715	0.5462	0.393	0.122	–0.154
NBL					1	0.7656*	0.8112*	0.8356*	0.6751	0.885**
NBA						1	0.701*	0.1514	0.286	0.791**
SH							1	0.902**	0.836**	0.9803**
TD								1	–0.616	0.584
BD									1	0.668*
NB										1

*, **Significant at 5% and 1% level of probability, respectively.

Table 6. Estimation of additive genetic variance (V_A), phenotypic variance (V_P), and narrow-sense heritability (h_n^2) for morphological traits in half-sib progenies obtained from imported apple cultivars and Iranian native genotypes (parents) based on the data collected 2007 and 2008.

	3 years old (2007)			4 years old (2008)		
	V_A	V_P	h_n^2	V_A	V_P	h_n^2
SH	78,326.42	98,076.35	0.80	114,580.80	136,394.21	0.84
LL	4.8	6.39	0.67	5.42	8.41	0.65
LW	1.87	4.15	0.45	2.09	4.36	0.48
TD	8.62	12.86	0.68	9.81	13.77	0.71
LP	1.93	3.94	0.49	2.17	4.71	0.46
LCC	z	z	z	1,442.35	1,694.62	0.85
NB	3,658.62	4,583.68	0.80	3,539.84	4,499.92	0.79
BD	142.51	192.58	0.74	153.04	134.02	0.65
NBL	693.27	949.68	0.73	765.4	1,109.28	0.69
NBA	286.25	420.96	0.68	307.8	462.34	0.67

^{z}Data not collected for LCC in 3-year-old trees.
LL = leaf length; LW = leaf width; LP = length of peduncle; LCC = leaf chlorophyll concentration; NBL = number of branches longer than 20 cm; NBA = number of branches with suitable angle on along trunk; SH = seedling height; TD = trunk diameter; BD = branches distance; NB = total number of branches.

Table 7. Estimates of additive genetic variance (V_A), phenotypic variance (V_P), and narrow-sense heritability (h_n^2) for morphological traits in full-sib progenies obtained from crossing apple cultivars and Iranian native genotypes (parents) based on data collected during two growing seasons 2007 and 2008.

	3 years old (2007)			4 years old (2008)		
	V_A	V_P	h_n^2	V_A	V_P	h_n^2
SH	768.42	1130.03	0.68	868.32	1222.99	0.71
LL	0.92	1.51	0.61	1.14	2.07	0.55
LW	0.68	1.07	0.63	0.72	1.06	0.68
TD	0.54	1.15	0.47	0.93	1.79	0.52
LP	1.59	1.31	0.45	1.2	2.48	0.48
LCC	z	z	z	39.03	49.41	0.79
NB	138.71	198.16	0.70	194.86	259.81	0.75
BD	12.81	22.9	0.58	17.04	32.77	0.52
NBL	53.23	98.57	0.54	61.12	120.43	0.51
NBA	42.6	69.84	0.61	49.96	90.84	0.55

^{z}Not collected data for LLC in 3 year olds.
Traits morphological include leaf length (LL), leaf width (LW), length peduncle (LP), leaf chlorophyll concentration (LCC), number of branches longer than 20cm (NBL), number of branches with suitable angle on along trunk (NBA), seedling height (SH), trunk diameter (TD), branches distance (BD), and number of branches total (NB).

et al., 2004), still to separate the genetic and environmental variations (Dierig et al., 2001). Nevertheless, high costs resulting from nursery construction, energy, and qualified technicians will make a real obstacle lifting up the number of seedlings to 7000 yearly. At the end of data registration of morphologies, there was an identity card of each genotype in which the marker traits were scored. Based on the comments by Sato et al. (2008), the proportion of obtaining superior offspring in fruit breeding program depends not only on the family mean, but also on the variance within the family. All genetic components are under control of gene frequency; as the result, they may differ from a population to another to the genealogy of population (Bohren et al., 1961; Falconer, 1963). Our results are agreed with these reports (Tables 7 and 8). If the offspring grown in the single parcel belong to a defined half-sib or full-sib family, the estimated hereditability in different periods of time are considered to fit in with the differences between families (Kennedy, 1981). Hereditability differences between half-sib and full-sib families are practically attributed to the genetic component (Falconer, 1963). It was demonstrated that time of ripening in early cultivars was positively correlated with architectural traits such as SH, TD, BD, and NB and morphological traits like LL (Hajnajari et al., 2009; Kazlovskaya, 2005; Visser et al., 1976). Therefore, these traits can be used as valid and efficient criteria for screening of apple progenies for earliness during the juvenile phase in the breeding program. Consistent with our observations, Gallais (1989) reported when correlations between target traits and easy-to-measure characters explain a sufficient part of variance, genetic correlations provide a means to predict the target traits based on the values of selected variables. Based on our results, SH and other architectural characters adding up to leaf traits in the juvenile phase can be used for selection of earliness. Kazlovskaya (2005) and Visser et al. (1970) proposed that these variables could be applied efficiently for pre-screening of early apple genotypes in the juvenile phase. High correlations between certain morphological markers and fruit early-ripening progenies strengthen the existence of genetic linkage among the relative traits and/or preponderant dominance of responsible genes. The present results regarding SH narrow-sense heritability are in accordance with the outcomes reported by Rezaee et al. (2009) in walnut half-sib families for the same trait. The different results between the full-sib and half-sib crosses were owing to the unknown genetic origin of male parents in half-sib families. In this case the efficacy of the screening method will be improved if the morphological markers enjoy also cytoplasmic heredity. Others reported that ≈60% of the selected low chilling candidates were of the columnar type when the Brazilian apple 'Primicia' was used as a reference variety (Jacob, 2010). Besides, ripening time itself in apple cultivars has a high heritability as well (Tancred et al., 1995). The range of heritability estimates for architectural traits in 1-year-old seedlings within full-sib families, for example 'Starkrimson' × 'Granny Smith', had higher evaluations than appraisals reported by Segura et al. (2006). Rather being the result of different age groups of progenies (3–4 years old), this difference could be caused by the elevated number of offspring (2700) derived from numerous cross combinations (44) in this research compared with the parallel work made by Segura et al. (2006). There are similar reports regarding high heritability of morphological traits in apple trees, which have given emphasis to the same concept including tree habit (Lapins, 1974; Sampson and Cameron, 1965) and spur-type bearing habit (Alston and Watkins, 1974; Decourtye and Lantin, 1969). Furthermore, for most of the studied morphological traits, the estimated narrow-sense heritability was found to be high, so this suggests that the screening method based on morphological traits might be efficiently used in screening of progenies for earliness trait during the juvenile period. It is in concordance with the conclusion made by Jules (2010). Others found the highest competency in combined application of genetic and morphological markers (Koc et al., 2009; Larsen et al., 2006). Based on the latest worldwide results, early screening of progenies is more confidential with, where possible, the combined use of morphological and molecular markers. Sato et al. (2008) have used positively morphological markers for selection of dwarfing pears and deduced that the plant height of seedlings in Japanese pear was greatly reduced by inbreeding, even in 1-year-old seedlings. Recent data confirmed the relative efficacy of morphological markers technique. Time of ripening in early cultivars was positively correlated with architectural and morphological traits (Hajnajari et al., 2009; Kazlovskaya., 2005; Rezaee et al., 2009; Tancred et al., 1995). Therefore, these traits

Table 8. Estimates of additive genetic variance (V_A), phenotypic variance (V_P), and narrow-sense heritability (h_n^2) for morphological traits in half-sib progenies obtained from early–mid, late–very late-ripening apple cultivars, and Iranian native genotypes as parents.

	Early–midripening apple cultivars			Late–very-late ripening apple cultivars		
Traits	V_A	V_P	h_n^2	V_A	V_P	h_n^2
SH	175,793.04	193,179.16	0.91	71,094.72	94,017.8	0.76
LL	5.64	7.42	0.76	2.83	5.98	0.48
LW	2.37	4.23	0.56	1.53	1.93	0.79
TD	10.96	12.83	0.85	2.84	6.76	0.42
LP	2.39	4.19	0.57	1.93	3.86	0.50
LCC	1,160.88	1,319.18	0.88	659.12	988.12	0.67
NB	3,022.56	3,875.08	0.78	1,628.72	2,273.01	0.72
BD	141.6	157.33	0.90	101.04	170.52	0.59
NBL	947.62	1,538.31	0.77	134.21	298.24	0.45
NBA	358.23	477.64	0.75	51.04	116	0.44

Traits morphological include leaf length (LL), leaf width (LW), length peduncle (LP), leaf chlorophyll concentration (LCC), number of branches longer than 20cm (NBL), number of branches with suitable angle on along trunk (NBA), seedling height (SH), trunk diameter (TD), branches distance (BD), and number of branches total (NB).

can be used as valid and efficient criteria for screening of progenies for earliness during the juvenile phase in breeding programs.

Literature Cited

Alston, F. 1976. Dwarfing and lethal genes in apple progenies. Euphytica 25:505–514.

Alston, F.H. and I. Battle. 1992. Genetic markers in apple breeding. Phytoparasitica 20, 89S-92S. Hort. Res. International, East Malling, West Malling, Kent, UK.

Alston, F.H. and R. Watkins. 1974. Apple breeding at East Malling. Proc. Eucarpia Fruit Breeding Symp. 1973:14–29.

Bohren, B.B., H.E. McKean, and Y. Yamada. 1961. Relative efficiencies of heritability estimated based on regression of offspring on parent. Biometrics 17:481–491.

Brown, A.G. 1960. The inheritance of shape, size and season of ripening in progenies of the cultivated apple. Euphytica 9:327–337.

Costes, E., A. Belouin, L. Brouard, and M. Le Lezec. 2004. Development of young pear tree architecture and occurrence of first flowering: A varietal comparison. J. Hort. Sci. Biotechnol. 79:67–74.

Costes, E., H. Sinoquet, J.J. Kelner, and C. Godin. 2003. Exploring within tree architectural development of two apple tree cultivars over 6 years. Ann. Bot. (Lond.) 91:91–104.

Decourtye, L. 1967. Etude de quelques caracteres a controle genetique simple chez le pommier (Malus sp.) et le poirier (Pyrus communis). Annales de l'Amelioration des Plantes 17:243–265.

Decourtye, L. and B. Lantin. 1969. Contribution a la connaissance des mutants spurs de pommier; heredite de caractere. Annales de l'Amelioration des Plantes 19:227–238.

Dicenta, F., G. Garcia, and E.A. Carbonnel. 1993. Heritability of fruit characters in almond. J. Hort. Sci. 68:121–126.

Dierig, D.A., D.T. Ray, T.A. Coffelt, F.S. Nakayama, G.S. Leake, and G. Lorenz. 2001. Heritability of height, width, rubber, and latex in guayule (Parthenium argentatum). Ind. Crops Prod. 13:229–238.

Durel, C.E., F. Laurens, A. Fouillet, and Y. Lespinasse. 1998. Utilization of pedigree information to estimate genetic parameters from large unbalanced data sets in apple. Theor. Appl. Genet. 96:1077–1085.

Falconer, D.S. 1963. Quantitative inheritance, p. 193–216. In: Burdette, W.J. (ed.). Methodology in mammalian genetics. Holden-Day, San Francisco, CA.

Falconer, D.S. 1981. Introduction to quantitative genetics. Longman, London, UK.

Gallais, A. 1989. Theorie de la selection en amelioration des plantes. John Libbey Eurotext, Paris, France.

Hajnajari, H. 2008. National fruit collections of Iran, germplasm and pomology. Agriculture Education Publication, Karaj, Iran.

Hajnajari, H. 2010. Cultivar evaluation program of the national Iranian apple collection in the last decade. Proceedings of the International Scientific Conference of Fruit Growing Intensification in Belarus: Traditions, Progress, Prospects. p. 33–39.

Hajnajari, H., B. Chashnidel, K. Vahdati, and O. Agheli. 2009. Cluster analyses of early and late apple cultivars in juvenile phase. Short papers of 6th Iranian Hort Congress, 13–16 July. Gilan University. Rasht, Iran.

Hajnajari, H., I. Dehghani Shouraki, A. Khandan, and M.L. Fakhraei. 2008. National guideline for the conduct of tests for distinctness, uniformity and stability in Apple. Plant Variety Registration Department, Seed and Plant Certification and Registration Institute, Karaj, Iran.

Hansche, P.E., V. Beres, and R.M. Brooks. 1966. Heritability and genetic correlation in the sweet cherry. Proc. Amer. Soc. Hort. Sci. 88:173–183.

Hanson, W.D. 1963. Heritability, p. 125–129. In: Hanson, W.D. and H.F. Robinson (eds.). Statistical genetics and breeding. Publ. 982. Natl. Acad. Aci.-Natl. Res. Counc., Washington, DC.

Hardner, C.M., C.W. Winks, R.A. Stephenson, E.G. Gallagher, and C.A. McConchie. 2002. Genetic parameters for yield in macadamia. Euphytica 125:255–264.

Hill, J., H.C. Becker, and P.M.A. Tigerstedt. 1998. Quantitative and ecological aspects of plant breeding. H. Chapman and A. J. Hall, London, UK.

Jacob, H.B. 2010. Breeding experiments of apple varieties with columnar growth and low chilling requirements. Acta Hort. 872:159–164.

Jules, J. 2010. Biochemical and molecular markers in plant breeding, p. 37. Vol. 9. In: Stuber, C.W. (ed.). Plant breeding reviews.

Kazlovskaya, Z. 2005. National workshop on fruit breeding. Horticulture Department, Seed and Plant Improvement Institute, Karaj, Iran.

Kenis, K. and J. Keulemans. 2007. Study of tree architecture of apple (Malus domestica Borkh.) by QTL analysis of growth traits. Mol. Breed. 19:193–208.

Kennedy, B.W. 1981. Variance component estimation and prediction of breeding values. Can. J. Genet. Cytol. 23:565–578.

Kester, D.E. 1965. Inheritance of time of bloom in certain progenies of almond. J. Amer. Soc. Hort. Sci. 87:214–221.

Kester, D.E., P. Hansen, and V.R.N. Beres Asay. 1977. Variance components and heritability of nut and kernel in almond. J. Amer. Soc. Hort. Sci. 102:264–266.

Koc, A., M. Akbulut, E. Orhan, Z. Celik, S. Bilgener, and S. Ercisli. 2009. Identification of Turkish and standard apple rootstocks. Genet. Mol. Res. 8:420–425.

Lapins, K.O. 1974. Spur type growth habit in 60 apple progenies. J. Amer. Soc. Hort. Sci. 99:568–572.

Lapins, K.O. 1976. Inheritance of compact growth type in apple. J. Amer. Soc. Hort. Sci. 101:133–135.

Larsen, A.S., C.B. Asmussen, E. Coart, D.C. Olrik, and E.D. Kjær. 2006. Hybridization and genetic variation in Danish populations of European crab apple (Malus sylvestris). Tree Genet. Genomes 2:86–97.

Lespinasse, J.M. 1977. La conduite du pommier: Types de fructification, incidence sur la conduite de l'arbre. INVUFLEC, Paris, France.

Liebhard, R., M. Kellerhals, W. Pfammatter, M. Jertmini, and C. Gessler. 2003. Mapping quantitative physiological traits in apple (Malus domestica Borkh.). Plant Mol. Biol. 52:511–526.

Oraguzie, N.C., M.E. Hofstee, L.R. Brewer, and C. Howard. 2001. Estimation of genetic parameters in a recurrent selection program in apple. Euphytica 118:29–37.

Rezaee, R., K. Vahdati, and M. Valizadeh. 2009. Variability of seedling vigor in Persian walnut as influenced by the vigor and bearing habit of the mother tree. J. Hortic. Sci. Biotechnol. 84:228–232.

Rweyongeza, D.M., F.C. Yeh, and N.K. Dhir. 2004. Genetic parameters for seasonal height and height growth curves of white spruce seedlings and their implications to early selection. For. Ecol. Mgt. 187:159–172.

Sampson, D.R. and D.F. Cameron. 1965. Inheritance of bronze foliage, extra petals and pendulous habit in ornamental crabapples. Proc. Amer. Soc. Hort. Sci. 86:717–722.

Sansavini, S. and S. Musacchi. 1994. Canopy architecture, training and pruning in the modern European pear orchards: An overview. Acta Hort. 367:152–153.

SAS Institute Inc. 2000. SAS user's guide. SAS Institute, Cary, NC.

Sato, A., Y. Sawamura, N. Takada, and T. Hirabayashi. 2008. Relationship between inbreeding coefficients and plant height of 1-year-old seedlings in crosses among Japanese pear (*Pyrus pyrifolia* Nakai) cultivars/selections. Sci. Hort. 117:85–88.

Scorza, R. 1984. Characterization of four distinct peach tree growth types. J. Amer. Soc. Hort. Sci. 109:455–457.

Segura, V., C. Cilas, F. Laurens, and E. Costes. 2006. Phenotyping progenies for complex architectural traits: A strategy for 1-year-old apple trees (Malus domestica Borkh.). Tree Genet. Genomes 2:140–151.

Tancred, S.J., A.G. Zeppa, M. Cooper, and J.K. Stringer. 1995. Heritability and patterns of inheritance of the ripening date of apples. HortScience 30:325–328.

Visser, T. 1970. The relation between growth, juvenile period and fruiting of apple seedlings and its use to improve breeding efficiency. Euphytica 19:293–302.

Visser, T., G.H. Verhaegh, and D.P. Vries. 1976. A comparison of apple and pear seedlings with reference to the juvenile period. I. Seedling growth and yield. Euphytica 25:343–351.

Watkins, R. and R.A. Smith. 1982. Descriptors list for apple (Malus). IBPGR, Rome, Italy.

Yao, Q. and S.A. Mehlenbacher. 2000. Heritability, variance components and correlation of morphological and phenological traits in hazelnut. Plant Breed. 119:369.
