# Supplementary material for: Provenance and family variations in early growth of Manchurian walnut (Juglans mandshurica Maxim.) and selection of superior families
Source: PLoS One. 2024 Mar 7;19(3):e0298918. doi: 10.1371/journal.pone.0298918 (PMC10919699; doi:10.1371/journal.pone.0298918)
Supplement: S1 File — (ZIP) [file pone.0298918.s004.zip › Improving disease resistance of butternut (Juglans cinerea), a threatened fine hardwood a case for single-tree selection through genetic improvement and deployment.pdf]

Tree Physiology 26, 121–128
© 2005 Heron Publishing—Victoria, Canada

Improving disease resistance of butternut (*Juglans cinerea*), a threatened fine hardwood: a case for single-tree selection through genetic improvement and deployment†

CHARLES H. MICHLER,[1,2] PAULA M. PIJUT,[1] DOUGLASS F. JACOBS,[3] RICHARD MEILAN,[3] KEITH E. WOESTE[1] and MICHAEL E. OSTRY[4]

[1] *USDA Forest Service, North Central Research Station, Hardwood Tree Improvement and Regeneration Center, West Lafayette, IN 47907-2061, USA*

[2] *Corresponding author (cmichler@fs.fed.us)*

[3] *Department of Forestry and Natural Resources, Purdue University, West Lafayette, IN 47907-2061, USA*

[4] *USDA Forest Service, North Central Research Station, St. Paul, MN 55108, USA*

Received October 29, 2004; accepted April 15, 2005; published online October 3, 2005

Summary Approaches for the development of disease-resistant butternut (*Juglans cinerea* L.) are reviewed. Butternut is a threatened fine hardwood throughout its natural range in eastern North America because of the invasion of the exotic fungus, *Sirococcus clavigignenti-juglandacearum* Nair, Kostichka and Kuntz, which causes butternut canker. Early efforts were made to identify and collect putatively resistant germ plasm, identify vectors and to characterize the disease. More recently, molecular techniques have been employed to genetically characterize both the pathogen and the resistant germ plasm. Much of the host resistance may originate from hybridization with a close Asian relative, Japanese walnut (*Juglans ailanthifolia* Carr.), and from a few natural phenotypic variants. Further genetic characterization is needed before classical breeding or genetic modification can be used to produce canker-resistant trees.

Keywords: hybridization, polymorphism, regeneration, Sirococcus clavigignenti-juglandacearum, tree breeding, vegetative propagation.

Natural history of butternut

This review focuses on approaches for the development of canker-resistant butternut (*Juglans cinerea* L.). Butternut, also known as white walnut or oilnut, is a fine hardwood species in the family Juglandaceae, section *Trachycaryon* (Manning 1978), or more appropriately, section *Cardiocaryon* (Fjellstrom and Parfitt 1994). Butternut hybridizes with *J. regia* L. (*Dioscaryon*) and species in the section *Cardiocaryon*, but not with *J. nigra* L. (*Rhysocaryon*). Two hybrids of *J. cinerea* are recognized: *J. cinerea* × *J. regia* = *J.* × *quadrangulata* (Carr.) Rehd. and *J. cinerea* × *J. ailantifolia* Carriére = *J.* × *bixbyi* Rehd. (USDA NRCS 2004*b*). Native to North America, from New Brunswick to Georgia, and west to Minnesota and Arkansas, butternut is not an abundant species (Schultz 2003) and is seldom found in pure stands, but rather in association with four northern and central mixed mesophytic forest cover types: sugar maple (*Acer saccharum* Marsh.)–basswood (*Tilia americana* L.), yellow-poplar (*Liriodendron tulipifera* L.)–white oak (*Quercus alba* L.)–northern red oak (*Quercus rubra* L.), beech (*Fagus sylvatica* L.)–sugar maple, and river birch (*Betula nigra* L.)–sycamore (*Acer pseudoplatanus* L.) (Rink 1990). A relatively slow-growing tree, butternut attains a mean height of 12 to 18 m, a mean diameter of 30–61 cm and seldom exceeds 75 years of age (Rink 1990). Butternut is shade-intolerant and is considered to be one of the most winter hardy of the *Juglans* species (USDA Hardiness Zone range of 3 to 7). Butternut grows best on moist, rich, well-drained loamy soils, though it also grows quite well in drier, rocky soils, especially of limestone origin (Rink 1990, Cogliastro et al. 1997). On deep soils, butternut forms a taproot and wide-spreading lateral roots.

Butternut is monoecious, with male and female flowers maturing at different times. Staminate catkins (6–14 cm) preformed on the previous year's wood appear as small, scaly, cone-like buds and the female flowers occur in two-to eight-flowered spikes borne on the current year's shoots. The fruit is a drupe-like, furrowed nut enclosed in a thick, indehiscent husk that develops from a floral involucre. Fruit occur singly or in clusters of from two to five, and are edible, sweet and oily. The nut varies from ellipsoid to subcylindric or ovoid, is 3–6 cm in length, two-celled, and has a hard pericarp. Leaves are alternate, pinnately compound, 30–60 cm long, with seven to 19 leaflets. Butternut stems are stout, reddish buff to greenish gray, pubescent or smooth and have a chambered chocolate-brown pith. *Juglans cinerea* has a ridged and furrowed bark; the ridges are whitish and the furrows are grayish black and upon exposure to air, the inner bark turns yellow. The sapwood of butternut is nearly white and the heartwood is light

† *This paper was among those presented at the 18th North American Forest Biology Workshop, which was sponsored by the Society of American Foresters and the USDA Forest Service, and hosted by the Michigan Technological University, Houghton, MI, July 11–15, 2004.*

Downloaded from https://academic.oup.com/treephys/article/26/1/121/1676171 by guest on 31 January 2024

brown. The wood is moderately light in weight, rather coarse textured, moderately weak in bending and endwise compression, relatively low in stiffness, moderately soft and moderately high in shock resistance (Forest Products Laboratory 1999). The chromosome number of butternut is 2n = 32. Butternut trees produce seed at about 20 years of age, with good seed crops occurring every 2 to 3 years (Rink 1990). Seeds of butternut, like most *Juglans* spp., have a dormant embryo, but dormancy can be broken by fall sowing or by moist, prechilling of seeds at 3–5 °C for 3–4 months (Dirr 1998).

Historically, the inner bark of butternut and its nut husks were used to produce an orange or yellow dye for Confederate troop uniforms and the inner bark has mild cathartic properties (Peattie 1950, Dirr 1998). Native Americans extracted oil from crushed butternuts and the sap of butternut has been used to make syrup (Goodell 1984). The roots and fruit husks of butternut exude juglone, a naphthoquinone, which is allelopathic (Rink 1990). Butternut is an underused hardwood species valued economically and ecologically for its wood and edible nuts (Ostry and Pijut 2000). Quality butternut wood commands a high market price for many uses, including furniture, veneer, cabinets, paneling and fine woodworking, because it machines easily, finishes well and resembles black walnut (*Juglans nigra*) when stained. In areas where quality butternut wood is available, it ranks eighth out of the top 28 species for prime veneer and saw logs (Peterson 1990). There has been limited selection of butternuts for superior genotypes, other than for nut quality and production (McDaniel 1981, Goodell 1984, Milikan and Stefan 1989, Milikan et al. 1990, Ostry and Pijut 2000).

Butternut canker: disease distribution and impact

In 1967, it was reported that stem cankers were present on all but two butternut trees in a woodlot in southwestern Wisconsin (Wisconsin Conservation Department 1967). This was the first reported observation of what is now referred to as butternut canker. Butternut dieback and decline throughout the northeastern USA had previously been attributed to *Melanconis juglandis* Ellis & Everhart Graves (Graves 1923), its perfect state, and is now commonly found in its imperfect state, *Melanconium oblongum* Berk., on branches killed by butternut canker and on senescing branches.

A survey of butternut in Wisconsin in 1976 revealed that 31 and 9% of the trees were diseased and dead, respectively. In a follow-up survey in 1992, 92 and 27% of the trees were diseased and dead, respectively (Carlson and Guthmiller 1993). A survey in the eastern USA showed that butternut canker was present in at least 14 of the 16 states surveyed and the disease had already eliminated inventoried butternut populations in North and South Carolina (Anderson and LaMadeleine 1978). The most recent USDA Forest Service Forest Inventory and Analysis survey data revealed that overall, in seven Midwestern states, the number of butternut trees in all size classes decreased by 23% (USDA NCRS 2004*a*).

In 1992, Minnesota enacted a moratorium on the harvest of healthy butternut on state lands. In many states, butternut is listed as a “species of concern” or a “sensitive species” and it is a Regional Forester Sensitive Species in the Eastern Region in 13 of the 16 National Forests. In Canada, the disease was first detected in Ontario and Quebec in 1991 (Davis et al. 1992) and in New Brunswick in 1997 (Harrison et al. 1998). Butternut was listed as endangered by the Committee on the Status of Endangered Wildlife in Canada in November 2003.

Disease description

The fungus responsible for butternut canker disease, *Sirococcus clavigignenti-juglandacearum* Nair, Kostichka and Kuntz, was described as a new species in 1979 (Nair et al. 1979). Though there have been no reports of this fungus outside of North America, it is thought to be an exotic pathogen (Furnier et al. 1999).

Conidia of *S. clavigignenti-juglandacearum* develop in pycnidia under the bark of diseased trees. The conidia are exposed when hyphal pegs, arising from a stroma, break open the bark. Rainsplash and probably insects (Katovich and Ostry 1998, Halik and Bergdahl 2002), birds and other animals disseminate the conidia that are produced in sticky masses throughout the growing season. The fungus can be seed-borne in black walnut as well as in butternut (Innes 1997).

Elongated annual cankers commonly originate at leaf scars and buds in the crowns of trees. The sunken cankers often have inky black centers with whitish margins. Perennial branch and stem cankers eventually develop throughout trees as a result of conidia washing down from sporulating cankers in the upper crowns (Tisserat and Kuntz 1983). Trees are girdled and killed by coalescing stem cankers.

Host range

Butternut is the only species killed by butternut canker; however, black walnut (Ostry et al. 1997) and a cultivated selection of Japanese walnut known as heartnut (*J. ailanthifolia* var. *cordiformis* (Makino.) Rehder) (Ostry 1997*a*) are occasional hosts. Experimentally inoculated plants of several grafted *Juglans* spp. and hybrids, including some of the leading cultivars of Persian walnut grown commercially in California also demonstrated susceptibility to the fungus (Ostry and Moore, unpublished data).

Seedlings of several other hardwood species are susceptible in greenhouse experiments and may harbor the fungus (Ostry 1997*b*). Species in *Carya*, a genus in the walnut family (Juglandaceae), that were found to be susceptible include pecan (*C. illinoensis* (Wangenh.) K. Koch) and shagbark hickory (*C. ovata* (Mill.) K. Koch). The fungus has been recovered from northern red oak (*Quercus rubra*), black oak (*Q. velutina* Lam.), white oak (*Q. alba*) and black cherry (*Prunus serotina* Ehrh.). Bitternut hickory (*C. cordiformis* (Wangenh.) K. Koch) has also exhibited susceptibility in greenhouse tests (Ostry and Moore, unpublished data). These preliminary re-

Downloaded from https://academic.oup.com/treephys/article/26/1/121/1676171 by guest on 31 January 2024

sults indicate that species of genera other than *Juglans* may serve as a reservoir of the pathogen within forests and raise the possibility that the original source of the pathogen was a species other than *Juglans*.

Breeding for resistance: the end user imperative

Before discussing how a canker-resistant butternut might be bred, an overarching consideration should be made clear: end users or clients determine the goals, products and, ultimately perhaps, the methods of a breeding program. Without a clearly identified client group or an understanding of the original genetic diversity of butternut, it is difficult to know how many target environments should be considered for breeding. These unknowns affect all the scenarios described below.

Assessment of natural disease resistance

Healthy butternut has been found growing adjacent to trees infected and killed by the disease in areas throughout its range. Many of these putatively resistant trees or their progeny have been propagated for further assessment (Ostry et al. 2003). Preliminary data in repeated, controlled inoculations indicate wide phenotypic variation in susceptibility to canker among butternuts (Ostry and Woeste 2004).

Although some candidate trees may prove to be resistant, they may not be pure butternuts. Butternut hybridizes with *J. regia* to produce *J.* × *quadrangulata* and with Japanese walnut (*J. ailanthifolia*) to produce *J.* × *bixbyi*. Additionally, *J. ailanthifolia* var. *cordiformis* Maxim. hybridizes with butternut to produce buartnut (*J.* × *bixbyi*). Buartnuts are often phenotypically indistinguishable from butternuts and are common feral trees in portions of the butternut range. Japanese walnuts and their hybrids appear to be less susceptible to butternut canker than most butternuts. Whether there are canker-resistance genes in Japanese walnut is unclear. The striking hybrid vigor of buartnut can sometimes be used to distinguish it from butternut, and the apparent canker resistance of the hybrids may be associated with their vigor. Preliminary results indicate all known buartnuts have *J. ailanthifolia* as the female parent (Woeste and Ostry, unpublished data). If this observation withstands further scrutiny, we should be able to identify hybrids, even after many generations of backcrossing, by analysis of chloroplast polymorphisms. At present, the best tool for discriminating *Juglans* spp. hybrids is the sequence polymorphism found within the internal transcribed spacer (ITS) regions of the nuclear ribosomal DNA (Baldwin et al. 1995). Our preliminary data indicate that we can use the ITS region to determine if a tree is pure butternut or a hybrid. Comparative studies of microsatellite alleles from butternut, black walnut and Japanese walnut might also be useful for identifying hybrids.

We have identified two bark phenotypes among butternut trees of the same size and age (Ostry and Woeste 2004). The rarer phenotype has dark-colored bark with deep bark fissures resembling the bark of black walnut. Butternut bark is usually light gray with shallow bark fissures. These bark types, and various intermediate types, have been found on adjacent trees in scattered woodlots in Indiana, Minnesota and Wisconsin. Often the dark/deep bark phenotype is associated with healthy trees and the light/shallow bark with diseased trees (Ostry et al. 2003). The unusual appearance of the dark-barked trees led to speculation that they might be hybrids with black walnut. Our preliminary data (K.E. Woeste and Li, unpublished data), based on analysis of the sequence of the ribosomal ITS, indicate that dark-barked butternuts are not hybrids, although it is possible there has been gene flow from black walnut to butternut through Japanese walnut (Funk 1970). We have no data on the origins of the dark-barked phenotype. Similarly, we have yet to determine if the relationship between this phenotype and greater canker resistance is coincidental, based on genetic linkage, or the result of pleiotropic gene action.

Breeding options

To estimate the time frame and cost for a canker resistance breeding program, data are needed on the heritability of the dark-barked phenotype and the heritability of resistance seen in butternut or their hybrids. We must also determine if there is one or many resistance genes in butternut and Japanese walnut, and under what circumstances they function. Moreover, information concerning the etiology of butternut canker is needed to determine if what appears to be resistance is influenced by dynamic genotype × environment interactions. For example, one or more of the disease vectors may show preference for certain host phenotypes.

American chestnut (*Castanea dentata* (Marsh.) Borkh.) and American elm (*Ulmus americana* L.), two hardwoods threatened by exotic fungi, are instructive in this context. Breeders for both species have struggled with the advantages and disadvantages of using hybrids (or some type of hybrid introgression) as opposed to native germ plasm (Townsend and Douglass 2001; Sandra Anagnostakis, Connecticut Agricultural Experiment Station, New Haven, CT, personal communication).

If only butternuts are used for breeding, there are three questions to answer. (1) How much resistance is present in the germ plasm? (2) What is the heritability of the resistance phenotype(s) in the target environments? (3) How many resistance genes are likely to be important? Disadvantages to pursuing a pure butternut approach include the low seed yield and long juvenility of existing germ plasm. These factors dramatically increase the time required for breeding. Advantages to the pure butternut approach include the possible rapid release of resistant clones and high client acceptance. Although few end users may wish to deploy the current clonal butternut material available, the pure butternut strategy avoids the serious drawbacks of the two alternative approaches described below.

An approach based on hybridization, with or without subsequent generations of backcrossing, is feasible, but it may not appeal to prospective clients. As in the pure butternut approach, a hybrid approach requires research into how much re-

Downloaded from https://academic.oup.com/treephys/article/26/1/121/1676171 by guest on 31 January 2024

sistance is present in sexually compatible *Juglans* spp. and into the heritability of the resistant phenotypes in interspecific crosses and backcrosses. Though hybrids are vigorous, precocious trees, they could catalyze the evolution of a *Juglans* hybrid swarm and lead to weediness (Haysom and Murphy 2003). A distinct advantage, however, is that canker-resistant hybrids could be developed quickly, and the germ plasm distributed as seeds. As with the previously described pure butternut strategy, hybrids could incorporate the remaining local genetic diversity of butternut, but the early generation hybrids may be unable to fill the ecological and economic roles of pure butternuts.

Genetic engineering is also a viable option for developing a canker-resistant butternut. However, public acceptance, the potential for transgene spread and other regulatory hurdles are important considerations that must be considered. Opportunities for introducing resistance through genetic engineering and the challenges to be overcome are discussed in detail in the following sections.

Biotechnology

Genetic engineering provides an alternative means of introducing genes conferring disease resistance. Transgenes for fungal resistance have been categorized in several ways. Plants use a wide range of passive (expressed all the time) and active (inducible) mechanisms to defend against invasion, colonization and reproduction by pathogens. In general, an active response to pathogen attack involves the interaction between the product of a single, dominant resistance gene (*R*) in the plant host and that of a corresponding dominant avirulence gene (*AVR*) in the pathogen. This gene-for-gene model, originally proposed by Flor (1956), has recently been validated through the molecular analysis of various *R* and *AVR* genes (reviewed by Baker et al. 1997, Hammond-Kosack and Jones 1997, Stirling 2001).

Though much attention has been paid to inducible systems, there is abundant evidence that preformed defenses play a critical role in disease resistance. Preexisting structural or chemical barriers, or both, are assumed to provide a first line of defense against pathogens. These include thick cuticles (Dickman et al. 1989), narrow stomata (Jones 1987) and the presence of secondary metabolites such as flavonoids, phenolics, saponins and lactones (Stirling 2001).

Small, preformed proteins also play an important role in defending against pathogen attack. Many of these antimicrobial peptides are found predominantly in the outer cell layers of organs, but they are not unique to plants (Stirling 2001). For example, cecropins are peptides produced in the circulatory fluids of invertebrates in response to infection. Similarly, magainins are peptides that accumulate in the skin of amphibians, where they are thought to protect against microorganisms. Defensins are a family of antimicrobial peptides that have been found in mammals, insects and plants (Broekaert et al. 1995, 1997).

Septoria musiva Peck is a widespread fungal pathogen that limits the utility of hybrid poplars throughout the eastern USA. ESF12 is a small, synthetic peptide (18 amino acids in length) that structurally mimics magainins (Zasloff 1987) and, in a leaf disc assay, inhibits *S. musiva* (Powell et al. 1995). A small chitin-binding protein, Ac-AMP1 (29 amino acids), which was isolated from the seed coat of *Amaranthus caudatus* L., inhibits the growth of several plant-pathogenic fungi at much lower concentrations than other known antifungal, chitin-binding peptides (Broekaert et al. 1997). Hybrid poplar trees containing genes that encode both ESF12 and Ac-AMP1 have significantly greater resistance to *S. musiva* compared with non-transgenic control plants (Liang et al. 2002).

Finally, certain enzymes impart resistance to plant disease. Oxylate oxidases (OxO) catalyze the conversion of oxalic acid to hydrogen peroxide (H_2O_2) and carbon dioxide and accumulate in cereal embryos during germination (Lane 1994). The production of these enzymes is stimulated in response to fungal attack (Hurkman and Tanaka 1996). The mechanism by which OxO imparts protection to the plant is not completely understood, although it is known that the H_2O_2 liberated by OxO activity can be utilized in the cross-linking reactions involved in lignification (Olson and Varner 1993). Hybrid poplars transformed with the *OxO* gene from wheat were shown to have significantly higher resistance to *S. musiva* than untransformed plants (Liang et al. 2001).

Risk management of genetically modified trees

Though numerous transgenic herbaceous annual species are now being grown commercially in the USA (see: http://www.aphis.usda.gov/brs/), only one transgenic woody perennial, papaya (*Carica papaya* L.), has been approved for commercial distribution. Its release resulted from a concerted effort to save an entire industry from destruction by the ubiquitous ringspot virus in Hawaii (Gonsalves 1998). This exceptional case involves virtually no environmental risk because papaya was originally introduced into Hawaii (i.e., it is reproductively isolated) and because the Pacific Ocean is an effective physical barrier to transgene escape.

Presently, other transgenic tree species in the USA are grown only for research. There are three reasons for this limitation: (1) existing regulations were written for agronomic row crops, which are highly domesticated and have few, if any, wild relatives; (2) long-lived trees pose ecological concerns that differ from those of annual row crops; and (3) biotechnological techniques for use with trees have developed more slowly than biotechnological techniques for use with annual crops.

The U.S. Animal and Plant Health Inspection Service (APHIS), which has over-arching regulatory authority over all transgenic plants, requires that an effort be made to mitigate the risk of transgene spread to related species in the wild. Several transgene confinement strategies are currently being tested; most involve some form of flowering control (Meilan et al. 2001).

Downloaded from https://academic.oup.com/treephys/article/26/1/121/1676171 by guest on 31 January 2024

In general, herbaceous annual crops are heavily domesticated and have virtually no wild relatives with which they are sexually compatible. In addition, the products harvested (e.g., seed, fruit and pollen) are usually derived from the flowers. Thus, there is neither a need nor a desire to prevent flowering in these species. In contrast, forest trees are not domesticated and are grown primarily for their wood; reproductive growth is usually an impediment. This is largely true for butternut, although it may be grown for its nut crop.

To minimize the risk of transgene spread yet preserve the nut crop, it may be necessary to develop a late-acting sterility mechanism (i.e., mechanisms that prevent seed germination). A class of genes known as late embryogenesis abundant (*LEA*) genes are expressed in the final stages of embryo development (Thomas 1993). Some members of this gene family are expressed after the seed has reached its maximum size and its storage oils and proteins have accumulated. By transforming plants with a cytotoxin gene fused to the promoter from one of these *LEA* genes, the host would produce a fully formed but non-viable seed.

Because the nuts would be consumed by humans, it would be necessary to use a gene encoding a toxin that has no adverse effects on mammals. One candidate is a ribosome inhibiting protein (RIP) from the plant *Saponaria officinalis* L. This toxin prevents protein synthesis and is effective at very low concentrations. It is reported to have no effect if ingested by animals (M.L. Crouch, Indiana University, Bloomington, USA, personal communication).

There may be circumstances when it is desirable to have transgene spread in the environment. With an increase in international trade, there has been a proportionate increase in the number of foreign insects and diseases that have invaded the USA. Because our native trees did not coevolve with these introduced pests, they often lack innate resistance. If development of resistance through conventional breeding is unsuccessful, the spread of a disease resistance gene into wild populations of butternut may be the only effective means of saving the species from drastic reduction as suffered by American elm as a result of Dutch elm disease or the American chestnut as a result of chestnut blight.

Development of butternut propagation and tissue culture technologies

Butternut is easily propagated from seed by fall sowing or after cold stratification at 3–5 °C for 3–4 months, but the canker fungus is also seed-borne (Orchard 1984, Andre et al. 2001). Therefore, vegetative or clonal reproduction methods must be developed to conserve or produce clones of elite genotypes selected or genetically improved for canker resistance. As a conservation strategy, a program to graft scion wood from putatively canker-resistant trees and to establish germ plasm repositories within the natural range of butternut was initiated in 1992 (Ostry et al. 2003). Grafting selected butternut to black walnut rootstock has been successful, but it is time consuming and yields variable results depending on time of grafting, compatibility between scion and rootstock, overwintering of grafted plants, time of outplanting and winter injury (Millikan 1971, Van Sambeek et al. 2003). Midsummer field planting of in-leaf grafts with overhead irrigation was not a good alternative method to traditional overwintering of grafted plants in a controlled cold-storage environment and subsequent spring planting (Van Sambeek et al. 2003).

Juglans spp. are normally recalcitrant to routine, commercial-scale vegetative propagation by rooted cuttings. However, propagation of butternut can be achieved if the type of cutting, date of collection, auxin concentration and greenhouse parameters are carefully controlled. Propagation of 5- and 6-year-old butternut trees by hardwood and softwood cuttings has been successful (Pijut and Moore 2002). A low percentage (10.5 to 27.8%) of rooting of hardwood cuttings collected in mid-May was achieved. The greatest rooting success of 22.2 and 27.8% was achieved when hardwood cuttings were treated with 62 mM indole-3-butyric acid-potassium salt (K-IBA) or 74 mM indole-3-butyric acid (IBA), respectively. Better success was achieved with softwood cuttings than hardwood cuttings (87.5 versus 3.6%). Rooting success ranged from 15.8 to 87.5% when June softwood cuttings were treated with 62 or 74 mM IBA. Rooted hardwood and softwood cuttings were successfully overwintered in cold storage and had high field survival and good growth when planted the following year (Pijut 2004).

Tissue culture techniques such as micropropagation, in vitro regeneration and rooting, somatic embryogenesis, cryopreservation and genetic transformation systems must be developed to genetically modify and propagate butternut for specific characteristics, such as improved wood quality and insect pest or canker disease resistance. Somatic embryogenesis of butternut has been induced by culturing immature zygotic cotyledonary tissue (Pijut 1993, 1999). Best results, across genotypes tested, were obtained when cotyledon explants collected 8 to 9 weeks post-anthesis were induced to form embryogenic callus in darkness on Driver and Kuniyuki walnut (DKW) medium supplemented with 1.1 μM 6-benzylaminopurine (BA), 9.1 μM 2,4-dichlorophenoxyacetic acid (2,4-D) and 0.25 g l^{-1} L-glutamine, or on a Murashige and Skoog (MS) medium containing 1.1 μM BA, 9.1 μM 2,4-D and 1 g l^{-1} casein hydrolysate (CH). Somatic embryo development occurred after removal of plant growth regulators and glutamine, and a reduction in CH concentration. Globular to mature somatic embryos were differentiated, and conversion of somatic embryos into whole plants was complete, though at low frequency (2%). Plantlets survived for 3 to 4 months after acclimatization from the in vitro environment. The potential exists for genetic improvement of butternut if refinement of this somatic embryogenesis protocol can be achieved to increase the frequency of somatic embryos able to develop into whole plants and survive acclimatization.

Micropropagation of butternut by axillary bud culture has been developed (Pijut 1997). Nodal segments are cultured on MS medium supplemented with 0.2 g l^{-1} CH and 8.9 μM BA. Roots are initiated on microshoots by pulsing for 7 days in darkness on half-strength MS medium containing 0.1 g l^{-1} CH and 2.5 μM IBA. Rooted plantlets can then be successfully ac-

Downloaded from https://academic.oup.com/treephys/article/26/1/121/1676171 by guest on 31 January 2024

climatized ex vitro. The inability to store recalcitrant seeds, such as those of butternut, for long periods of time could create a serious challenge for germ plasm conservation, especially if butternut canker disease threatens to eliminate the species. Development of cryopreservation methods could alleviate this difficulty. Pence (1990) reported that only one embryonic axis of butternut survived desiccation and cryostorage, as determined by the greening and swelling of the shoot and callusing at the shoot base. Beardmore and Vong (1998) examined low (0 to –40 °C) and ultra-low (–196 °C) temperature tolerance of butternut embryonic axes. Embryonic axes with approximately 3 mm of cotyledonary tissue attached to the hypocotyl area germinated after exposure to low temperatures for 4 h and to ultra-low cryostorage for 24 h. Reducing the water content of the embryonic axes, by slow desiccation to 4.8% or less, resulted in an increased tolerance to –196 °C. These results suggest that butternut is amenable to low and ultra-low storage as a means of ex situ conservation.

Production and deployment of genetically improved trees

Nurseries producing butternut seedlings currently follow similar guidelines and cultural regimes to those used for black walnut production (Ostry and Pijut 2000). Seedlings are generally produced by bareroot nursery culture (Jacobs 2003). This method of seedling establishment often leads to a period of transplant stress during which seedlings must reestablish root–soil contact following the loss of structural and fine roots.

Seedling propagation in containers may provide a better means of rapidly testing grafted material, rooted cuttings, or in vitro culture for canker resistance. The preservation of the root system within the rooting medium of the container helps alleviate transplant stress. Container production also promotes a more uniform crop for germ plasm testing because rooting is confined to a fixed container volume. Rooted cuttings of butternut have been propagated in 655 cm^3 (6.4 cm diameter × 25.4 cm depth) containers (Pijut and Moore 2002), transplanted to 2835 cm^3 pots, and then successfully established in the field (Pijut 2004). Propagation protocols established for container production of other *Juglans* spp. can likely be effectively adapted for butternut.

Silvicultural guidelines specific to butternut have not been developed, but butternut's close relationship with black walnut suggests that recommendations should be similar for both species (Ostry et al. 2003). Deployment of resistant germ plasm will involve both enrichment plantings in forested areas and afforestation plantings.

Classification of butternut as intolerant to shading (Rink 1990) suggests that significant canopy openings are needed to facilitate seedling establishment and growth. Openings approximately 2–3 times the height of surrounding dominant trees may be the minimum size needed to establish butternut seedlings (Ostry et al. 1994). Growth of direct-seeded and 2-0 stock of butternut was more vigorous in clearcuts (0.4–2.0 ha) or heavy thinnings (2.8 m^2 basal area retention) than in light thinnings (5.6 m^2 basal area retention) or uncut controls (Ostry et al. 2003). Growth of planted butternut seedlings may be relatively slow compared with co-occurring hardwood species (Cogliastro et al. 1997). Slow initial growth and characteristic shade intolerance implies that effective control of weeds, deer browsing and rodent damage is needed during establishment (Cogliastro et al. 1993, Jacobs et al. 2004). Field survival of transplanted butternut rooted cuttings was 91% after 2 years on a site where mesh cages and effective chemical weed control were applied, versus only 67% in their absence (Pijut 2004).

Conclusions

Butternut, an ecologically and economically important forest tree of eastern North America, faces possible extinction as a result of the recent rapid advancement of infection from canker disease caused by *Sirococcus clavigignenti-juglandacearum*. Evidence of disease resistance within natural butternut populations suggests that the most promising path for conservation and deployment of resistant germ plasm is through single-tree selection and breeding. Advances in molecular techniques allow for rapid and accurate genetic characterization of individuals that exhibit resistance. Development of a formal butternut canker resistance program to breed selected trees will allow identification of multiple genotypes that exhibit resistance and help maintain genetic diversity of reintroduced populations. Genetic engineering offers an alternative means of introducing canker resistance into butternut, but faces uncertain application. Continued refinement of tissue culture, vegetative propagation, nursery production systems, and silvicultural guidelines for reintroduction will help ensure successful restoration of the next generation of canker-resistant butternut.

References

Anderson, R.L. and L.A. LaMadeleine. 1978. The distribution of butternut decline in the Eastern United States. USDA For. Serv. For. Survey Rep. S-3-78, 5 p.

Andre, R., L. Innes, F. Colas, M. Bettez and S. Mercier. 2001. Butternut canker in Québec: a 5-year history that led to seed treatments. *In* Canadian Tree Improvement Association: Tree Seed Work. Group News Bulletin, No. 34. Ed. R. Smith. Natural Resources Canada, Canadian Forest Service, Fredericton, NB, Canada, pp 14–16.

Baker, B., P. Zambryski, B. Staskawicz and S.P. Dinesh-Kumar. 1997. Signaling in plant–microbe interactions. Science 276:726–733.

Baldwin, B.G., M.J. Sanderson, J.M. Porter, M.F. Wojciechowski, C.S. Campbell and M.J. Donoghue. 1995. The ITS region of nuclear ribosomal DNA: a valuable source of evidence on angiosperm phylogeny. Ann. Mo. Bot. Gard. 82:247–277.

Beardmore, T. and W. Vong. 1998. Role of the cotyledonary tissue in improving low and ultra-low temperature tolerance of butternut (*Juglans cinerea*) embryonic axes. Can. J. For. Res. 28:903–910.

Broekaert, W.F., F.R. Terras, B.P. Cammue and R.W. Osborn. 1995. Plant defensins: novel antimicrobial peptides as components of the host defense system. Plant Physiol. 108:1353–1358.

Broekaert, W.F., B.P.A. Cammue, M.F.C. De Bolle, K. Thevissen, G.W. De Samblanx and R.W. Osborn. 1997. Antimicrobial peptides from plants. Crit. Rev. Plant Sci. 16:297–323.

Downloaded from https://academic.oup.com/treephys/article/26/1/121/1676171 by guest on 31 January 2024

Carlson, J.C. and M. Guthmiller. 1993. Incidence and severity of butternut canker in Wisconsin in 1976 and 1992. Phytopathology 83: 1352. Abstract only.

Cogliastro, A., D. Gagnon and A. Bouchard. 1993. Effect of site and silvicultural treatment on growth, biomass allocation and nitrogen utilization of seedlings of four broadleaf species in plantations in southwest Québec. Can. J. For. Res. 23:199–209.

Cogliastro, A., D. Gagnon and A. Bouchard. 1997. Experimental determination of soil characteristics optimal for the growth of ten hardwoods planted on abandoned farmland. For. Ecol. Manage. 96: 49–63.

Davis, C.N., D.T. Myren and E.J. Czerwinski. 1992. First report of butternut canker in Ontario. Plant Dis. 76:972.

Dickman, M.B., G.K. Podila and P.E. Kolattukudy. 1989. Insertion of cutinase gene into a wound pathogen enables it to infect intact host. Nature 342:446–448.

Dirr, M.A. 1998. Manual of woody landscape plants: their identification, ornamental characteristics, culture, propagation and uses. 5th Edn. Stipes Publishing, Champaign, IL, 1187 p.

Fjellstrom, R.G. and D.E. Parfitt. 1994. Walnut (*Juglans* spp.) genetic diversity determined by restriction fragment length polymorphisms. Genome 37:690–700.

Flor, H.H. 1956. The complementary genic systems in flax and flax rust. Adv. Genet. 8:29–54.

Forest Products Laboratory. 1999. Wood handbook: wood as an engineering material. USDA For. Serv. Gen. Tech. Rep. FPL-GTR-113, Madison, WI, 463 p.

Funk, D.T. 1970. Genetics of black walnut. USDA For. Serv. Res. Paper WO-10, Washington, DC, 13 p.

Furnier, G.R., A.M. Stolz, M.M. Raka and M.E. Ostry. 1999. Genetic evidence that butternut canker was recently introduced into North America. Can. J. Bot. 77:783–785.

Goodell, E. 1984. Walnuts for the northeast. Arnoldia 44:3–19.

Gonsalves, D. 1998. Control of papaya ringspot virus in papaya: a case study. Annu. Rev. Phytopathol. 36:415–437.

Graves, A.H. 1923. The Melanconis disease of the butternut *Juglans cinerea* L. Phytopathology 13:411–435.

Halik, S. and D.R. Bergdahl. 2002. Potential beetle vectors of *Sirococcus clavigignenti-juglandacearum* on butternut. Plant Dis. 86: 521–527.

Hammond-Kosack, K.E. and J.D.G. Jones. 1997. Plant disease resistance genes. Annu. Rev. Plant Physiol. 48:575–607.

Harrison, K.J., J.E. Hurley and M.E. Ostry. 1998. First report of butternut canker caused by *Sirococcus clavigignenti-juglandacearum* in New Brunswick, Canada. Plant Dis. 82:1282.

Haysom, K.A. and S.T. Murphy. 2003. The status of invasiveness of forest tree species outside their natural habitat: a global review and discussion paper. *In* Forest Health and Biosecurity Working Papers. CABI BioScience, FAO, Rome, Italy, pp 1–76.

Hurkman, W.J. and C.K. Tanaka. 1996. Germin gene expression is induced in wheat leaves by powdery mildew infection. Plant Physiol. 111:735–739.

Innes, L. 1997. *Sirococcus clavigignenti-juglandacearum* on butternut and black walnut fruit. *In* Foliage, Shoot and Stem Diseases of Trees: Proceedings of the IUFRO WP 7.02.02 Meeting, May 25–31, 1997, Québec City. Inf. Rep. LAU-X-122. Eds. G. Laflamme, J.A. Bérubé and R.C. Hamelin. Natl. Res. Canada, Can. For. Serv., Laurentian Forestry Centre, Sainte-Foy, Québec, QC, Canada, pp 129–132.

Jacobs, D.F. 2003. Nursery production of hardwood seedlings. Dept. For. Nat. Resources, Purdue University Cooperative Extension Service, FNR-212, West Lafayette, IN, 8 p.

Jacobs, D.F., A.L. Ross-Davis and A.S. Davis. 2004. Establishment success of conservation tree plantations in relation to silvicultural practices in Indiana, USA. New For. 28:23–36.

Jones, D.G. 1987. Disease resistance. *In* Plant Pathology, Principles and Practice. Open University Press, Milton Keynes, U.K., pp 52–72.

Katovich, S.A. and M.E. Ostry. 1998. Insects associated with butternut and butternut canker in Minnesota and Wisconsin. Great Lakes Entomol. 31:97–108.

Lane, B.G. 1994. Oxalate, germin and the extracellular matrix of higher plants. FASEB J. 8:294–301.

Liang, H., C.A. Maynard, R.D. Allen and W.A. Powell. 2001. Increased *Septoria musiva* resistance in transgenic hybrid poplar leaves expressing a wheat oxalate oxidase gene. Plant Mol. Biol. 45:619–629.

Liang, H., C.M. Catranis, C.A. Maynard and W.A. Powell. 2002. Enhanced resistance to the poplar fungal pathogen, *Septoria musiva*, in hybrid poplar clones transformed with genes encoding antimicrobial peptides. Biotechnol. Lett. 24:383–389.

Manning, W.E. 1978. The classification within the Juglandaceae. Ann. Mo. Bot. Gard. 65:1058–1087.

McDaniel, J.C. 1956. The pollination of Juglandaceae varieties: Illinois observations and review of earlier studies. Annu. Rep. North. Nut Grow. Assoc. 47:118–132.

McDaniel, J.C. 1981. Other walnuts including butternut, heartnut and hybrids. *In* Nut Tree Culture in North America. Ed. R.A. Jaynes. North. Nut Grow. Assoc., Hamden, NJ, pp 98–110.

Meilan, R., A. Brunner, J. Skinner and S. Strauss. 2001. Modification of flowering in transgenic trees. *In* Molecular Breeding of Woody Plants. Progress in Biotechnology Series. Eds. A. Komamine and N. Morohoshi. Elsevier Science, Amsterdam, pp 247–256.

Millikan, D.F. 1971. Propagation of *Juglans* species by fall grafting. Annu. Rep. North. Nut Grow. Assoc. 62:41–44.

Milikan, D.F. and S.J. Stefan. 1989. Current status of the butternut, *Juglans cinerea* L. Annu. Rep. North. Nut Grow. Assoc. 80:52–54.

Milikan, D.F., S.J. Stefan and K.S. Rigert. 1990. Selection and preservation of butternut, *Juglans cinerea* L. Annu. Rep. North. Nut Grow. Assoc. 81:22–25.

Nair, V.M.G., C.J. Kostichka and J.E. Kuntz. 1979. *Sirococcus clavigignenti-juglandacearum*: an undescribed species causing canker on butternut. Mycologia 71:641–646.

Olson, P.D. and J.E. Varner. 1993. Hydrogen peroxide and lignification. Plant J. 4:887–892.

Orchard, L.P. 1984. Butternut canker: host range, disease resistance, seedling-disease reactions and seed-borne transmission. Ph.D. Diss., Univ. Wisconsin, Madison, WI, 145 p.

Ostry, M.E. 1997*a*. *Sirococcus clavigignenti-juglandacearum* on heartnut (*Juglans ailanthifolia* var. *cordiformis*). Plant Dis. 81: 1461.

Ostry, M.E. 1997*b*. Butternut canker in North America 1967–1997. *In* Foliage, Shoot and Stem Diseases of Trees: Proc. IUFRO WP 7.02.02 Meeting, Inf. Rep. LAU-X-122. Eds. G. Laflamme, J.A. Bérubé and R.C. Hamelin. Natl. Res. Canada, Can. For. Serv., Laurentian Forestry Centre, Sainte-Foy, Québec, QC, Canada, pp 121–128.

Ostry, M.E. and P.M. Pijut. 2000. Butternut: an underused resource in North America. HortTechnology 10:302–306.

Ostry, M.E. and K. Woeste. 2004. Spread of butternut canker in North America, host range, evidence of resistance within butternut populations and conservation genetics. *In* Black Walnut in a New Century: Proc. 6th Walnut Council Research Symposium. Eds. C.H. Michler, P.M. Pijut, J. Van Sambeek, M. Coggeshall, J. Seifert, K.

Downloaded from https://academic.oup.com/treephys/article/26/1/121/1676171 by guest on 31 January 2024

Woeste and R. Overton. USDA For. Serv. North Central Res. Stn. Gen. Tech. Rep. NC-243, St. Paul, MN, pp 114–120.

Ostry, M.E., M.E. Mielke and D.D. Skilling. 1994. Butternut: strategies for managing a threatened tree. USDA For. Serv. North Central Res. Stn. Gen. Tech. Rep. NC-165, St. Paul, MN, 7 p.

Ostry, M.E., S. Katovich and R.L. Anderson. 1997. First report of *Sirococcus clavigignenti-juglandacearum* on black walnut. Plant Dis. 81:830.

Ostry, M.E., B. Ellingson, D. Seekins and W. Ruckheim. 2003. The need for silvicultural practices and collection of butternut germ plasm for species conservation. *In* Proc. 13th Cent. Hardwood For. Conf., Eds. J.W. Van Sambeek, J.O. Dawson, F. Ponder, Jr., E.F. Lowenstein and J.S. Fralish. USDA For. Serv. North Central Res. Stn. Gen. Tech. Rep. NC-234, St. Paul, MN, pp 551–555.

Peattie, D.C. 1950. A natural history of trees of Eastern and Central North America. Houghton Mifflin, Boston, MA, 606 p.

Pence, V.C. 1990. Cryostorage of embryo axes of several large-seeded temperate tree species. Cryobiology 27:212–218.

Peterson, T.A. 1990. Wisconsin forest products price review, timber edition. USDA Coop. Ext. Serv., Univ. Wisconsin, Madison, WI, 5 p.

Pijut, P.M. 1993. Somatic embryogenesis in butternut, *Juglans cinerea*. Can. J. For. Res. 23:835–838.

Pijut, P.M. 1997. Micropropagation of *Juglans cinerea* L. (Butternut). *In* Biotechnology in Agriculture and Forestry. Vol. 39. High-Tech and Micropropagation V. Ed. Y.P.S. Bajaj. Springer-Verlag, Berlin, pp 345–357.

Pijut, P.M. 1999. Somatic embryogenesis from immature fruit of *Juglans cinerea*. *In* Somatic Embryogenesis in Woody Plants. Vol. 4. Eds. S. Mohan Jain, P.K. Gupta and R.J. Newton. Kluwer Academic Publishers, Dordrecht, pp 415–429.

Pijut, P.M. 2004. Vegetative propagation of butternut (*Juglans cinerea*): field results. *In* Black Walnut in a New Century: Proc. 6th Walnut Council Research Symposium. Eds. C.H. Michler, P.M. Pijut, J. Van Sambeek, M. Coggeshall, J. Seifert, K. Woeste and R. Overton. USDA For. Serv. North Central Res. Stn. Gen. Tech. Rep. NC-243, St. Paul, MN, pp 37–44.

Pijut, P.M. and M.J. Moore. 2002. Early season softwood cuttings effective for vegetative propagation of *Juglans cinerea*. HortScience 37:697–700.

Powell, W.A., C.M. Catranis and C.A. Maynard. 1995. Synthetic antimicrobial peptide design. Mol. Plant–Microb. Interact. 8: 792–794.

Raikhel, N.V. and H.-L. Lee. 1993. Structure and function of chitin-binding proteins. Annu. Rev. Plant Physiol. 44:591–615.

Rink, G. 1990. *Juglans cinerea* L., butternut. *In* Silvics of North America. Vol. 2. Hardwoods. Tech. Coords. R.M. Burns and B.H. Honkala. USDA For. Serv. Agric. Handbook 654, Washington, DC, pp 386–390.

Schultz, J. 2003. Conservation assessment for butternut or white walnut (*Juglans cinerea* L.). USDA For. Serv., Eastern Region. http://www.fs.fed.us/r9/wildlife/tes/ca-overview/docs/plant_juglans_cinera-Butternut2003.pdf, Milwaukee, WI, pp 1–76.

Stirling, B.V. 2001. Molecular characterization of major gene resistance in a *Populus*-leaf rust pathosystem. Ph.D. Diss., Univ. Washington, Seattle, WA, 139 p.

Thomas, T.L. 1993. Gene expression during plant embryogenesis and germination: an overview. Plant Cell 5:1401–1410.

Tisserat, N. and J.E. Kuntz. 1983. Dispersal gradients of conidia of the butternut canker fungus in a forest during rain. Can. J. For. Res. 13:1139–1144.

Townsend, A.M. and L.W. Douglass. 2001. Variation among American elm clones in long-term dieback, growth and survival following *Ophiostoma* inoculation. J. Environ. Hortic. 19:100–103.

USDA NCRS. 2004*a*. Forest inventory and analysis. USDA For. Serv. North Central Res. Stn., St. Paul. http://www.ncrs.fs.fed.us/4801/

USDA NRCS. 2004*b*. The PLANTS Database, Version 3.5. USDA National Resources Conservation Service, National Plant Data Center, Baton Rouge, LA. http://plants.usda.gov.

Van Sambeek, J.W., M.E. Ostry and J.J. Zaczek. 2003. Survival and growth of deep-planted, in-leaf grafts in a germ plasm repository of canker-resistant butternut. *In* Proc. 13th Central Hardwood Forest Conference. Eds. J.W. Van Sambeek, J.O. Dawson, F. Ponder, Jr., E.F. Lowenstein and J.S. Fralish. USDA For. Serv. North Central Res. Stn. Gen. Tech. Rep. NC-234, St. Paul, MN, pp 476–479.

Wisconsin Conservation Department. 1967. Annual report on forest pest conditions in Wisconsin. Forest Management Division, Madison, WI, 67 p.

Zasloff, M. 1987. Magainins, a class of antimicrobial peptides from Xenopus skin: isolation, characterization of two active forms and partial cDNA sequence of a precursor. Proc. Natl. Acad. Sci. USA 84:5449–5453.

Downloaded from https://academic.oup.com/treephys/article/26/1/121/1676171 by guest on 31 January 2024
