# Supplementary material for: Provenance and family variations in early growth of Manchurian walnut (Juglans mandshurica Maxim.) and selection of superior families
Source: PLoS One. 2024 Mar 7;19(3):e0298918. doi: 10.1371/journal.pone.0298918 (PMC10919699; doi:10.1371/journal.pone.0298918)
Supplement: S1 File — (ZIP) [file pone.0298918.s004.zip › Juglans mandshurica fruits and seed variation and plus tree selection.pdf]

核桃楸种实性状变异规律及优良单株选择[1)]

张海啸
(林木遗传育种国家重点实验室(东北林业大学),哈尔滨,150040)

李爱清
(黑龙江省铁力林业局)

张含国　张磊
(林木遗传育种国家重点实验室(东北林业大学))

张剑斌
(黑龙江省森林与环境科学研究院)

摘　要　对12个地点天然林中核桃楸果实超过100个的单株种实形态性状(包括种子质量、种仁质量、种壳厚、出仁率、三径均值等)进行测定与分析,结果表明:种子质量和种仁质量变异系数(分别为19.9%和20.4%)较大,种壳厚和出仁率变异系数(分别为13.58%和14.42%)中等,三径均值变异系数(8.42%)稍小。对各种源内的种仁质量与种子形态性状拟合,多数种源结果较理想。同时对种仁质量和出仁率分别与环境因子拟合,结果较理想(R^2 分别为90.71%和76.61%)。通过聚类分析和综合坐标法选出优良种源为三岔子、铁力、金山屯、亚布力,并通过综合坐标法选出各种源优良单株为BX1、BX3、BX13、BX30、BX25,DFH2、DFH9、DFH13、DFH26,DJC2、DJC5、DJC8、DJC13,JY7、JY9、JY14、JY21,LJ2、LJ7、LJ30、LJ8,WC2、WC3、WC4、WC7,HC1、HC7、HC16,HL1、HL10、HL15、HL26、HL14,JST1、JST4、JST24、JST27,YBL1、YBL5、YBL14、YBL16、YBL9,TL5、TL7、TL20、TL30、TL10,SC16、SC17、SC23、SC3、SC18。选择主要提高了出仁率、种仁质量和种壳厚这三个直接影响核桃楸品质的指标,各种源入选率在15%~25%,出仁率增益在5.81%~24.28%,种仁质量增益在5.19%~25.77%,种壳厚除东京城、临江、金山屯和亚布力外,其他种源增益不明显。

关键词　核桃楸;种实性状;变异规律;单株选择;综合坐标法
分类号　S722.3

***Juglans mandshurica* Fruits and Seeds Variation and Plus Tree selection**//Zhang Haixiao(National Key Laboratory of Forest Tree Genetics and Breeding, Northeast Forestry University, Harbin 150040, P. R. China); Li Aiqing(Tieli Forestry Bureau); Zhang Hanguo, Zhang Lei(National Key Laboratory of Forest Tree Genetics and Breeding, Northeast Forestry University); Zhang Jianbin(Academy of Forest and Environment of Heilongjiang Province)//Journal of Northeast Forestry University,2017,45(3):-.

We studied the seed traits of northeast walnut (*Juglans mandshurica*) collected from 12 sites in natural forests. The coefficient of variation in seed weight (19.9%) and kernel weight (20.4%) were larger than others, shell thickness (13.58%) and kernel rate (14.42%) were medium, three-diameter mean (8.42%) was smaller. Fitting kernel weight and phenotypic traits of seed in different provenances showed good results. The results of fitting kernel weight and kernel rate with environmental factors were good. Selected superior provenances were Sanchazi, Tieli, Jinshantun, Yabuli by cluster analysis and comprehensive coordinate methods. Through the latter method, the selection of Plus Trees from provenances were BX1, BX3, BX13, BX30, BX25; DFH2, DFH9, DFH13, DFH26; DJC2, DJC5, DJC8, DJC13; JY7, JY9, JY14, JY21; LJ2, LJ7, LJ30, LJ8; WC2, WC3, WC4, WC7; HC1, HC7, HC16; HL1, HL10, HL15, HL26, HL14; JST1, JST4, JST24, JST27; YBL1, YBL5, YBL14, YBL16, YBL9; TL5, TL7, TL20, TL30, TL10; SC16, SC17, SC23, SC3, and SC18. The selection improved kernel rate, kernel weight and shell thickness that were three direct quality indicators. The inclusion rates were in 15%-25%, and Kernel rate and kernel weight gain were in 5.81%-24.28% and 5.19%-25.77%, respectively. The shell thickness in other sources gain were not obvious except in Dogjingcheng, Linjiang, Jinshantun and Yabuli.

Keywords　*Juglans mandshurica*; Cone and seed traits; Variation; Individual selection; Comprehensive coordinates

DOI:10.13759/j.cnki.dlxb.2017.03.001

核桃楸(*Juglans mandshurica*)属于胡桃科胡桃属的落叶乔木,又称胡桃楸、楸子。主要分布于中国小兴安岭、大兴安岭东南部、完达山脉、长白山、辽宁东部,苏联远东地区及朝鲜和日本。核桃楸材质坚硬、致密、耐冲击、易加工、耐腐蚀、刨面光滑、有光泽、花纹美观、油漆性能好,是军工、家具、建筑、船舰、木模、车辆、乐器及运动器械的优良用材[1-5]。核桃楸种仁营养丰富,含有8种人体所必需的氨基酸及多种微量元素,可直接食用;种仁含油率40%~63%,其中超过90%为不饱和脂肪酸,是高级食用油,具有一定的降血脂功效;果壳可制活性炭树皮和外果皮可提制褐色染料及单宁;叶、果皮、树皮、果肉可制农药;中药青龙衣(未成熟的青果皮)具有镇痛作用和抗肿瘤作用;核桃楸叶提取液、树皮具有抑制肿瘤细胞作用[6-9]。目前,对于核桃楸的选育作为用材林的研究较多,作为坚果林的选育研究较少[10-17]。文中通过对12个地点天然林中核桃楸果实的收集及观察测量,分析核桃楸种实性状变异规

1) 林业公益性行业科研专项(201304704)。
第一作者简介:张海啸,男,1989年8月生,林木遗传育种国家重点实验室(东北林业大学),硕士研究生。E-mail:245335490@qq.com。
通信作者:张含国,林木遗传育种国家重点实验室(东北林业大学),教授。E-mail:hanguozhang1@sina.com。
收稿日期:2016年11月7日。
责任编辑:任　俐。

(C)1994-2019 China Academic Journal Electronic Publishing House. All rights reserved.　http://www.cnki.net

律，初步选择优良种源及单株，为日后核桃楸坚果林良种选育工作提供一定参考。

1 材料与方法

材料于 2014 年 8 月末采自核桃楸分布区内 12 个地点的天然林，分别为宾县、东方红、东京城、虎林、珲春、嘉荫、金山屯、临江、铁力、三岔子、五常、亚布力，其地理位置及环境条件如表 1。各地点内均挑选结实量较高（单株果实超过 100）的 20 个左右单株进行果实采集，单株采集间隔大于 30 m。采种单株用地名缩写和单株序号进行编号，如宾县单株为 BX1～BX31。采集后每个单株随机挑选 30 个果实，测其果质量（称总质量后求均值）；每 10 个果实分为一组，分别测其果长、果径（每组内果实长、果实直径取均值，共 3 组）；进行去除果皮处理并在自然条件下风干后，测量种长、种宽（缝合线）、种厚（垂直缝合线）、三径均值（种长、宽、厚三者均值）、种质量（单粒种子质量）；然后对种子进行去壳处理并测量仁质量（单粒种仁质量），在种壳碎片中挑选出三片较大碎片测量其壳厚（取 3 次测量均值）；果长径比为果长与果径之比，长宽比为种长与种宽之比，长厚比为种长与种厚之比，宽厚比为种宽与种厚之比，果皮质量为果质量减去种子质量。

统计软件采用 MINITAB、DPS 等。种源间差异分析采用巢式设计，线性模型为 $X_{ijk}=\mu+S_i+T_{j(i)}+\varepsilon_{ijk}$，其中，$X_{ijk}$ 为第 i 个种源第 j 个单株第 k 个观测值；μ 为总体平均值；S_i 为种源间固定效应；$T_{j(i)}$ 为种源内单株间随机效应；ε_{ijk} 为随机误差。优良单株选择采用综合坐标法[18-19]，按 $P_i=\sqrt{\sum_i W_i(1-a_{ij})^2}$ 试计算坐标得分进行排序（P_i 越小越优），其中，$a_{ij}=X_{ij}/X_{j\max(\min)}$；$P_i$ 为第 i 个候选单株到标准点的距离；X_{ij} 为性状 j 的第 i 个观测值；$X_{j\max(\min)}$ 为性状 j 的最大（小）值；W_i 为性状 j 的权重。出仁率、种质量、仁质量、壳厚、三径均值权重分别为方式一（0.5、0.05、0.2、0.2、0.05）和方式二（0.35、0.05、0.4、0.15、0.05）两种，方式一的目的是为工业用仁选种，方式二的目的是为食用药用仁选种，为避免极大值（仁质量和出仁率）的优良基因损失，故将极值单株也选入优良单株（若综合坐标法未选入极大值单株）。

表 1 各地点地理位置与环境因子

种 源	纬度	经度	海拔/m	年均温/℃	≥5 ℃积温/℃	≥10 ℃积温/℃	年均降水量/mm
宾县（BX）	45°34′N	127°35′E	391	3.50	3 299	3 110	700
东方红（DFH）	46°30′N	133°44′E	144	1.00	2 867	2 577	600
东京城（DJC）	43°51′N	129°10′E	514	3.50	3 085	2 734	500
虎林（HL）	45°58′N	132°19′E	222	2.70	2 968	2 703	690
珲春（HC）	43°15′N	131°13′E	300	5.65	3 167	2 756	618
嘉荫（JY）	48°28′N	130°35′E	119	−1.10	2 500	2 000	550
金山屯（JST）	47°23′N	129°37′E	302	0.40	2 732	2 399	630
临江（LJ）	41°56′N	126°59′E	652	4.26	3 092	2 680	831
铁力（TI）	47°28′N	128°14′E	362	2.20	2 894	2 678	645
三岔子（SC）	42°31′N	126°46′E	583	4.00	2 923	2 558	759
五常（WC）	44°54′N	127°49′E	242	2.30	3 260	3 003	625
亚布力（YBL）	44°57′N	128°50′E	383	2.30	3 071	2 812	666

2 结果与分析

2.1 种源间及种源内差异分析

2.1.1 种源间差异分析

对核桃楸各性状进行方差分析结果如表 2 所示，各主要性状在种源间及种源内均存在丰富的变异，主要性状在种源间均存在极显著差异，且种源内的各单株间也存在极显著的差异（单果质量在单株间有不同程度的腐烂，所以未做种源内单株间方差分析）。描述果实及种子大小的性状变异系数较小，包括三径均值、果长、果径、果长径比，分别为 8.42%、8.69%、7.80%、8.27%，其他都高于 10.00%。

2.1.2 种源内变异分析

对各种源主要性状进行变异分析（表 3），各种源间性状差异较大，五常种质量均值最大（10.420 9 g），珲春种质量均值最小（7.461 9 g），五常均值超过珲春 28.39%；亚布力仁质量均值最大（2.004 4 g），珲春仁质量均值最小（1.503 6 g），亚布力均值超过珲春 24.99%；东京城壳厚均值最大（6.49 mm），珲春壳厚均值最小（5.14 mm），亚布力均值超过珲春 16.83%；铁力出仁率均值最高（20.46%），东京城出仁率均值最低（17.23%），铁力均值超过东京城 15.79%；亚布力三径均值最大（33.41 mm），珲春三径均值最小（29.15 mm），亚布力均值超过珲春 12.75%。各个性状在各种源内变异系数均较大。种质量性状金山屯变异系数最小（14.51%），三岔子变异系数最大（22.50%），平均变异系数为 17.74%；仁质量性状金山屯变异系数最小（15.45%），东方红变异系数最大（23.19%），平均变异系数为 19.04%；壳厚性状珲春变异系数最小（9.37%），金山屯变异

(C)1994-2019 China Academic Journal Electronic Publishing House. All rights reserved. http://www.cnki.net

系数最大(14.78%),平均变异系数为11.75%;出仁率珲春变异系数最小(8.22%),五常变异系数最大(20.30%),平均变异系数为13.13%;三径均值金山屯变异系数最小(6.22%),东京城变异系数最大(9.42%),平均变异系数为7.58%;果长金山屯变异系数最小(5.77%),三岔子变异系数最大(10.27%),平均变异系数为7.91%;果径铁力变异系数最小(5.73%),三岔子变异系数最大(8.80%),平均变异系数为7.21%;果长径比嘉荫变异系数最小(6.29%),东方红变异系数最大(9.86%),平均变异系数为8.07%。总体来看,种质量和仁质量变异系数较大,壳厚和出仁率变异系数中等,三径均值、果长、果径、果长径比变异系数较小。

表2　核桃楸主要性状方差分析

性状	均值	标准差	变异系数/%	均方(自由度)			F值	
				种源间	种源内	误差	种源间	种源内
出仁率	19.050 0	2.747 0	14.42	0.063 40(11)	0.011 3(237)	0.000 2(6 021)	6.15**	62.38**
仁质量	1.768 4	0.360 8	20.40	9.144 69(11)	1.878 8(237)	0.044 9(6 021)	11.35**	30.84**
种质量	9.373 2	1.864 9	19.90	338.072 00(11)	54.219 0(237)	0.869 0(6 021)	4.80**	41.87**
壳厚	5.730 0	0.778 4	13.58	78.203 10(11)	6.795 9(237)	0.220 4(6 021)	5.51**	50.82**
三径均值	32.051 0	2.697 0	8.42	801.648 00(11)	144.383 0(228)	1.503 0(6 930)	5.55**	96.05**
果长	52.922 0	4.597 0	8.69	268.7300 0(11)	54.290 0(324)	1.100 0(672)	4.95**	49.36**
果径	37.977 0	2.961 0	7.80	116.000 00(11)	21.740 0(324)	0.750 0(672)	5.34**	28.88**
果长径比	1.397 1	0.115 5	8.27	0.058 00(11)	0.038 0(324)	0.000 8(672)	1.52	44.95**
单果质量	31.357 0	6.740 0	21.49	269.010 00(11)		37.830 0(324)	7.11**	

注:**表示差异极显著($P<0.01$)。

表3　各种源内主要性状变异分析

种源	种质量			仁质量			壳厚			出仁率		
	均值/g	标准误/g	变异系数/%	均值/g	标准误/g	变异系数/%	均值/mm	标准误/mm	变异系数/%	均值/%	标准误	变异系数/%
宾县	9.937 1**	0.083 0	19.74	1.802 2**	0.015 2	19.99	5.64**	0.03	10.94	18.30**	0.001 0	13.08
东方红	9.690 3**	0.084 7	19.95	1.749 3**	0.017 8	23.19	5.67**	0.03	10.60	18.15**	0.001 3	15.73
东京城	9.873 3**	0.065 0	15.30	1.692 8**	0.012 4	17.08	6.49**	0.04	13.68	17.23**	0.001 0	13.73
虎林	9.010 4**	0.067 3	17.16	1.727 3**	0.015 7	20.85	5.24**	0.03	13.72	19.24**	0.001 2	14.83
珲春	7.461 9**	0.048 2	14.79	1.503 6**	0.010 9	16.57	5.14**	0.02	9.37	20.17**	0.000 7	8.22
嘉荫	8.971 2**	0.068 1	15.65	1.728 3**	0.018 5	22.13	5.84**	0.03	11.18	19.20**	0.001 3	13.46
金山屯	8.606 0**	0.050 6	14.51	1.739 5**	0.010 9	15.45	5.40**	0.03	14.78	20.31**	0.000 9	11.09
临江	9.161 5**	0.077 4	19.02	1.660 7**	0.013 5	18.29	5.81**	0.03	11.38	18.27**	0.000 9	11.70
铁力	9.395 7**	0.078 1	18.56	1.9017**	0.014 1	16.50	5.91**	0.03	11.00	20.46**	0.001 1	11.61
三岔子	9.817 3**	0.097 3	22.50	1.910 5**	0.016 5	19.60	5.59**	0.03	12.57	19.68**	0.001 0	11.18
五常	10.420 9**	0.091 6	18.73	1.806 9**	0.019 5	23.04	5.89**	0.03	10.16	17.57**	0.001 7	20.30
亚布力	10.169 6**	0.073 1	16.97	2.004 4**	0.013 4	15.74	6.18**	0.03	11.62	19.91**	0.001 1	12.63
平均值	9.376 3	0.073 7	17.74	1.768 9	0.014 9	19.04	5.73	0.03	11.75	19.04	0.001 1	13.13

种源	三径均值			果长			果径			果长径比		
	均值/mm	标准误/mm	变异系数/%	均值/mm	标准误/mm	变异系数/%	均值/mm	标准误/mm	变异系数/%	均值	标准误	变异系数/%
宾县	32.28**	0.11	8.16	52.88**	0.52	9.57	37.16**	0.29	7.62	1.42**	0.01	8.25
东方红	31.92**	0.10	7.81	54.12**	0.54	9.19	38.23**	0.31	7.54	1.42**	0.02	9.86
东京城	31.69**	0.12	9.42	54.78**	0.50	8.07	38.15**	0.33	7.55	1.44**	0.01	7.13
虎林	32.02**	0.10	7.71	52.20**	0.55	9.38	38.64**	0.34	7.76	1.36**	0.01	9.48
珲春	29.15**	0.08	6.29	47.96**	0.38	6.18	34.94**	0.35	7.73	1.38**	0.02	9.12
嘉荫	33.23**	0.09	6.29	53.25**	0.40	6.44	37.64**	0.26	5.93	1.42**	0.01	6.29
金山屯	31.01**	0.08	6.22	51.87**	0.32	5.77	38.37**	0.26	6.46	1.36**	0.01	6.51
临江	31.61**	0.10	7.85	51.41**	0.45	8.30	36.54**	0.25	6.43	1.41**	0.01	8.42
铁力	32.87**	0.11	8.18	54.89**	0.41	7.11	39.58**	0.24	5.73	1.39**	0.01	7.69
三岔子	32.88**	0.12	9.24	53.32**	0.58	10.27	38.56**	0.36	8.80	1.39**	0.01	8.53
五常	32.58**	0.09	6.83	52.18**	0.38	6.90	37.75**	0.30	7.61	1.39**	0.01	8.29
亚布力	33.41**	0.10	6.98	54.83**	0.45	7.71	39.26**	0.30	7.34	1.40**	0.01	7.25
平均值	32.05	0.10	7.58	52.81	0.46	7.91	37.90	0.30	7.21	1.40	0.01	8.07

注:**表示差异极显著($P<0.01$)。

2.2　各性状间相关分析

对核桃楸的各性状进行相关分析(表4),出仁率与种质量、壳厚、种长、种厚和三径均值均呈极显著负相关,与种宽、果长、果长径比呈显著负相关,与

(C)1994-2019 China Academic Journal Electronic Publishing House. All rights reserved.　http://www.cnki.net

仁质量呈极显著正相关,与宽厚比呈显著正相关;仁质量与种质量、壳厚、种长、种宽、种厚、三径均值、果长、果径、果皮质量、单果质量均呈极显著正相关;种质量与壳厚、种长、种宽、种厚、三径均值、果长、果径、果皮质量、单果质量均呈极显著正相关;壳厚与种长、种宽、种厚、三径均值、果长、果径、果皮质量、单果质量均呈极显著正相关,与宽厚比呈显著负相关;三径均值与长宽比、果长、果径、果长径比、果皮质量、单果质量呈极显著正相关,与长厚比呈显著正相关,与宽厚比呈显著负相关。

表4 性状间相关性分析

相关因子	仁质量(Y)	种质量(X_1)	壳厚	种长(X_2)	种宽(X_3)	种厚(X_4)	三径均值(X_5)	长宽比(X_6)	长厚比(X_7)	宽厚比(X_8)	果长(X_9)	果径(X_{10})	果长径比(X_{11})	果皮质量(X_{12})	单果质量
种质量(X_1)	0.707**														
壳厚	0.352**	0.596**													
种长(X_2)	0.496**	0.630**	0.379**												
种宽(X_3)	0.613**	0.704**	0.503**	0.468**											
种厚(X_4)	0.501**	0.644**	0.511**	0.418**	0.780**										
三径均值(X_5)	0.631**	0.783**	0.534**	0.859**	0.815**	0.798**									
长宽比(X_6)	0.047	0.123	0.013	0.700**	−0.300**	−0.181**	0.269**								
长厚比(X_7)	0.060	0.071	−0.059	0.620**	−0.217**	−0.448**	0.155*	0.850**							
宽厚比(X_8)	0.048	−0.066	−0.134*	−0.029	0.114	−0.529**	−0.162*	−0.114	0.422**						
果长(X_9)	0.472**	0.561**	0.314**	0.875**	0.313**	0.288**	0.699**	0.694**	0.615**	−0.029					
果径(X_{10})	0.565**	0.562**	0.393**	0.329**	0.552**	0.577**	0.541**	−0.081	−0.161*	−0.154*	0.496**				
果长径比(X_{11})	−0.001	0.094	−0.013	0.635**	−0.162*	−0.207**	0.26**	0.811**	0.797**	0.103	0.61**	−0.381**			
果皮质量(X_{12})	0.507**	0.481**	0.281**	0.507**	0.394**	0.355**	0.527**	0.235**	0.203**	−0.013	0.644**	0.788**	−0.032		
单果质量	0.603**	0.647**	0.381**	0.585**	0.503**	0.456**	0.637**	0.233**	0.193**	−0.027	0.688**	0.814**	−0.006	0.98**	
出仁率	0.319**	−0.434**	−0.358**	−0.216**	−0.155*	−0.218**	−0.244**	−0.118	−0.027	0.152*	−0.158*	−0.038	−0.129*	0.003	−0.097

注: **表示差异极显著($P<0.01$) ;相关系数为 Pearson 相关系数。

2.3 性状的回归分析

2.3.1 对仁质量性状的回归分析

由于核桃楸种子壳较厚且非常坚硬,仁质量的测量工作量很大,因此,分别以12个种源内以及总体的仁质量和出仁率为因变量,以核桃楸种实性状为自变量进行多元回归,以期为优良单株的间接选择提供理论基础。每个种源均删除系数不显著的自变量,结果表明,同一地点出仁率的决定系数均小于仁质量的决定系数,故表5只列出预测的 R^2 最大的仁质量回归方程及决定系数以供参考。对于不在12个种源内的其他种源,种仁质量的预测可用总的回归方程。

表5 各种源内仁质量与形态性状的拟合

种源	拟合的模型	R^2/%	调整的 R^2/%	预测的 R^2/%	P 值
宾县	$Y_{BX}=-1.285+0.122\,4X_5-0.515X_7$	83.66	81.62	76.44	0
东方红	$Y_{DFH}^{0}=1.477+18.27X_7^{-3}-5.75X_8^{-3}-3.04X_1^{0}\times X_7^{-3}+1.632X_1^{0}\times X_8^{-3}-15.81X_6^{-2}\times X_7^{-3}$	88.01	83.01	74.22	0
东京城	$Y_{DJC}^{0.5}=2.097-6.76X_7^{-3}-1.318X_8^{1.63}+0.0649(X_3/20)^5+0.191\,4X_1\times X_7^{-3}-0.62X_6^{-5}\times(X_3/20)^5+6.95X_7^{-3}\times X_8^{1.63}$	75.04	62.55	56.55	0.004
虎林	$Y_{HL}^{0}=-40.25+0.895X_1+97.2(X_2/20)^{-3}-45.48X_6^{-1}-1.656X_1\times(X_5/20)^{-2}-141.9(X_2/20)^{-3}\times(X_5/20)^{-2}+349.0X_4^{-1}\times X_8^{0.36}+62.89X_7^{-0.5}\times X_8^{0.36}$	93.65	89.60	84.85	0
珲春	$Y_{HC}=-0.630+0.201X_1+0.415X_7$	83.59	81.65	78.63	0
嘉荫	$Y_{JY}^{-0.5}=1.468-0.344\,6X_1^{0}+91\,273X_5^{-4.13}\times X_8^{-4}$	77.06	74.20	68.59	0
金山屯	$Y_{JST}=0.501-0.914X_3+0.002\,037X_3\times X_5+0.896X_4\times X_8$	80.33	76.40	70.71	0
临江	$Y_{LJ}=-114.0-6.506X_2+12.56X_5+57.49X_6+20.76X_7-2.668X_1\times X_6+2.860X_1\times X_7+0.176\,6X_2\times X_4-0.220\,1X_4\times X_5-3.505X_4\times X_7$	97.44	95.13	90.18	0
铁力	$Y_{TL}=11.96+0.166\,6X_1+0.630X_2+0.353X_4$	85.69	80.09	71.81	0
三岔子	$Y_{SC}=-0.127+0.077\,0X_1+0.045\,9X_3$	78.20	75.63	70.70	0
五常	$Y_{WC}=-24.14+0.142\,2X_5-29.64X_6-14.05X_7+24.12X_8-1.390X_9+48.57X_{11}+1.177X_6\times X_{10}+0.041\,4X_{11}\times X_{12}$	88.72	80.51	68.72	0.001
亚布力	$Y_{YBL}=17.73+0.088\,4X_5-12.25X_6+12.36X_7-18.81X_8$	87.27	83.64	77.08	0
总体	$Y_{总}=-0.420+0.094\,3X_1+0.040\,29X_3+0.140\,9X_7$	56.27	55.71	54.70	0

注: 回归方程中 X_1~X_8 和 Y 所代表的性状与表4中代表的相同; X^{-3}、Y^0 等分别表示 X 或 Y 经过 BOX−COX 变换(即 $X^*=\begin{cases}X^{\lambda},\lambda\neq0\\ \ln X,\lambda=0\end{cases}$ 或 $Y^*=\begin{cases}Y^{\lambda},\lambda\neq0\\ \ln Y,\lambda=0\end{cases}$) ,$\lambda$ 取值为−3或0。

(C)1994-2019 China Academic Journal Electronic Publishing House. All rights reserved. http://www.cnki.net

除东京城（56.55%）、嘉荫（68.59%）和五常（68.72%）三个地点的方程中预测的 R^2 较小外，其他地点的方程预测的 R^2 均大于 70%，达到了较好的预测效果。

2.3.2　性状与环境因子回归分析

以 12 个地点的仁质量与出仁率为因变量，以环境因子为自变量进行多元回归，删除不显著的自变量，得到其结果如下，预测的 R^2 均较大，预测效果较为理想，为优良种源的选择提供了理论指导。

仁质量 = 9.92 − 0.037 83 经度 − 0.061 1 纬度 − 0.015 24 海拔$^{0.5}$ − 0.022 72（≥5 ℃积温/1 000）^{4} + 0.083 13（≥10 ℃积温/1 000）^{3}；

R^2 为 97.27%，调整的 R^2 为 95.00%，预测的 R^2 为 90.71%，$P=0$。

出仁率（%）= 83.6 − 0.445 2 经度 + 0.394 纬度 − 1.454（≥10 ℃积温/1 000）^{3} + 2.310 年均温 − 0.031 1 年均降水量 − 0.083 9海拔$^{0.5}$×年均温 + 0.001 752（≥10 ℃积温/1 000）^{3}×年均降水量；

R^2 为 97.78%，调整的 R^2 为 93.89%，预测的 R^2 为 76.61%，$P=0.004$。

2.4　种源间聚类分析

对 12 个种源核桃楸的主要性状（出仁率、仁质量、种质量、壳厚、三径均值）进行聚类分析（标准化数据、欧氏距离、离差平方和法），聚类结果如图 1 所示，12 个种源可在 3.75 阈值处可分成三组，第一组为金山屯、虎林、珲春三个种源，特点为种子小、仁质量小、壳厚小、出仁率高；第二组为铁力、三岔子、亚布力三个种源，特点为种子大、仁质量大、壳厚大、出仁率高；第三组为东京城、临江、嘉荫、五常、东方红、宾县，特点为种子大小中等、仁质量中等、壳厚中等、出仁率低。

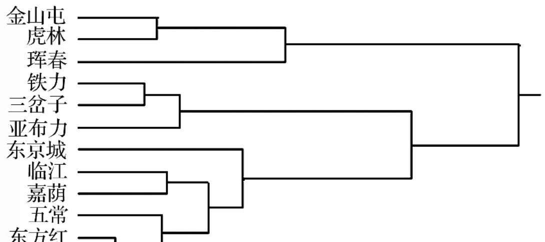

图 1　种源间主要性状聚类分析

从环境因子间聚类分析（图 2）可以看出，其分类基本与地理位置的远近相一致。而图 1、图 2 两种分类之间没有明显的一致性，间接说明环境因子对性状间的差异有影响。

2.5　优良种源选择与优良单株选择

2.5.1　优良种源选择：聚类分析和综合指数法

通过图 1 的聚类分析，可初步获得优良种源为第二组（出仁率高和仁质量大），即为铁力、三岔子、亚布力。

通过综合坐标法（表 6）（两种加权方法）选择的优良种源（前三名）分别为三岔子、铁力、金山屯和三岔子、铁力、亚布力。综合来看，优良种源为三岔子、铁力、金山屯、亚布力，入选率为 30%。

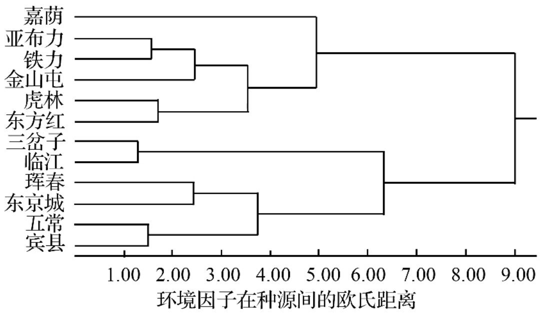

图 2　种源间环境因子聚类分析

2.5.2　优良单株选择：综合性状指数法选优

通过两种加权方式的综合坐标法对各个种源内进行综合选择，种源内每种加权方式分别选择前 3～4 个单株作为优良单株，两种方式选择后的优良单株加上极值（仁质量和出仁率）单株，总体作为种源内的优良单株。如表 6 所示，各种源内优良单株入选率在 15%～25%，改良的性状主要是出仁率、仁质量和壳厚。与各种源均值对比选出的优良单株的出仁率高出 5.81%～24.28%；仁质量性状高出均值 5.19%～25.77%；种质量的增益除嘉荫（13.19%）和珲春（5.68%）外其他种源不明显；壳厚性状东京城、临江、金山屯和亚布力分别低于均值 7.03%、9.47%、7.12%和 5.7%，增益效果明显，五常、珲春和铁力种源略低于均值，增益不明显，其他种源均略高于均值。

2.5.3　优良种源、优良单株选择：单一性状分别选择

通过对仁质量及出仁率的种源间方差分析并多重比较结果表明，以仁质量性状选择的优良种源为亚布力和三岔子（高于（均值±标准差）），以出仁率性状选择的优良种源为铁力和金山屯（高于（均值±标准差）单株很多，所以选择多重比较第一组）。综合来看，优良种源为亚布力、三岔子、铁力、金山屯，与 2.5.1 结果一致。

对各种源内仁质量及出仁率进行选择（优良单株为高于均值加 1 倍标准差）后综合整理，结果如表 7 所示，因为改良的性状为仁质量和出仁率，所以种质量和壳厚性状改良效果较弱，入选率为 5%～30%。与各种源均值对比选出的优良单株的出仁率高出 7.21%～29.65%；仁质量性状高出均值 0.47%～38.31%；种质量增益除宾县（−0.77%）、五常（−6.91%）和铁力（−10.37%）为负增益外，其他种源选择的优

(C)1994-2019 China Academic Journal Electronic Publishing House. All rights reserved.　http://www.cnki.net

良单株增益在0.54%~22.68%；壳厚性状除嘉荫、亚布力和铁力增益效果明显外（分别低于均值6.01%、7.14%和6.69%），其他种源增益不明显或负增益。

表6 综合坐标法选优及选择增益

种源	种源间		种源内				选择增益				入选
	方式一 P_i	方式二 P_i	方式一优良单株	方式二优良单株	综合后优良单株	增加极值单株	出仁率/%	种质量/%	仁质量/%	壳厚/%	率/%
宾县	0.096 4	0.096 1	3、30、10	13、3、1	1、3、13、30	25	7.83	2.02	9.94	0.53	25
东方红	0.110 3	0.113 7	26、9、2	13、26、9	2、9、13、26		20.38	0.42	19.39	1.81	20
东京城	0.177 5	0.170 9	13、8、5	13、8、2	2、5、8、13		13.98	-1.01	12.96	-7.03	20
嘉荫	0.109 2	0.119 7	14、7、9	14、7、21	7、9、14、21		8.97	13.19	23.97	0.58	20
临江	0.125 8	0.138 3	30、7、2	7、30、2	2、7、30	8	14.00	-5.20	7.67	-9.47	20
五常	0.127 6	0.118 9	7、2、4	7、2、3	2、3、4、7		24.28	2.05	25.77	-2.24	20
珲春	0.132 1	0.172 9	1、7、16	16、7、1	1、7、16		5.81	5.68	11.74	-2.63	15
虎林	0.081 8	0.099 8	26、10、15	26、10、1	1、10、15、26	14	17.54	-0.79	16.61	0.92	25
金山屯	0.076 2	0.095 7	4、24、1	4、24、27	1、4、24、27		8.54	3.44	12.69	-7.12	20
亚布力	0.093 1	0.080 6	1、16、14	1、5、16	1、5、14、16	9	9.78	-4.51	5.19	-5.70	25
铁力	0.074 4	0.070 2	20、30、5	20、30、7	5、7、20、30	10	8.06	-1.16	7.04	-0.05	25
三岔子	0.053 7	0.052 1	16、23、17	16、23、17	16、17、23	3、18	11.23	1.37	13.26	0.42	25

表7 单一性状分别选优及选择增益

种源	种源内选择			选择增益/%				入选
	按仁质量选择	按出仁率选择	两次综合	出仁率	种质量	仁质量	壳厚	率/%
宾县	12、13	25、9、16	9、12、13、16、25	10.44	-0.77	9.23	5.70	25
东方红	13、16	26、2	2、13、16、26	18.26	6.54	23.92	2.15	20
东京城	13、23	8、13	8、13、23	12.50	5.61	16.55	-0.92	15
嘉荫	14	无	14	12.28	22.68	38.31	-6.01	5
临江	7、12、11	8、2、30	2、7、8、11、12、30	8.44	5.39	13.10	-3.16	30
五常	7、2	4、2、15、7	2、4、7、15	29.65	-6.91	20.06	-1.39	20
珲春	16	1、20	1、16、20	8.19	5.70	14.20	3.88	15
虎林	14、24、1、26	10、26、14、15	1、10、14、15、24、26	15.12	3.18	18.27	2.91	30
金山屯	27、22	24	22、24、27	11.14	5.63	17.56	6.81	15
亚布力	5、22、1	9、25、1	1、5、9、22、25	7.21	0.54	6.17	-7.14	25
三岔子	18	3、16	3、16、18	13.33	4.16	17.85	2.59	15
铁力	20	10	10、20	12.77	-10.37	0.47	-6.69	10

综合性状选择和单一性状选择对比看来，由于单一性状选择的入选率较低，所以选择增益较综合选择法高，但选择出的优良单株数量不稳定（1~6个），且选择的单株不能兼顾两个或以上性状，尽管选择增益较高，但种源内选择出的优良单株间同一性状的差异较大，需进一步通过杂交等方法选育才能得到较稳定的结果。与单一性状选择相比，综合性状选择法兼顾了各个性状，有较强的优势，如果在选育过程中兼顾到极大值单株，在科研与生产中都会起到较好的效果。

3 结论与讨论

通过种源间差异分析与种源内变异分析发现，核桃楸种源间与种源内均存在丰富的变异，为良种选择提供了理论基础。且仁质量与种质量的变异系数高于出仁率、壳厚和三径均值，说明对仁质量与种质量的选择效果更好。

仁质量除与长宽比、长厚比和宽厚比无显著相关外，与其他性状均呈极显著相关；出仁率除与长宽比和长厚比无显著相关外，与其他性状均存在显著或极显著相关。值得注意的是，刘宏伟等[20]的研究表明，圆形的种子比长形种子的出仁率更高，李永荣等[18]、吴文龙[21]的研究表明，薄壳山核桃出仁率与果形指数（长宽比）相关性不显著，文中总体结果中能看到出仁率分别与长宽比和长厚比呈弱的负相关，但也没有达到显著水平（P 值分别为0.18和0.93）。种源内单独的相关分析发现，三岔子和亚布力各自种源内出仁率与宽厚比呈极显著正相关（相关系数分别为0.565和0.577）；宾县出仁率与长厚比在10%显著水平内达到显著负相关（相关系数为-0.385），表明宾县圆形种子比长形种子出仁率高；珲春出仁率与长厚比达到显著正相关（相关系数为0.537），出仁率与长宽比在10%显著水平内达到显著正相关（相关系数为0.416），表明圆形种子比长形种子的出仁率低；其他种源内无显著相关。综合来看，出仁率与种子长还是圆关系不稳定，每个地点

(C)1994-2019 China Academic Journal Electronic Publishing House. All rights reserved. http://www.cnki.net

关系不同,不同物种间关系也有不同[21-22],有待于进一步研究讨论。

由于核桃楸种子壳较厚且非常坚硬,仁质量的测量工作量很大,故文中分别对各种源的仁质量进行拟合,以期为优良单株的间接选择提供理论基础,可根据拟合结果初选优良单株后再进行仁质量测量。拟合结果表明,种源内出仁率的决定系数均小于仁质量的决定系数,其原因可能为出仁率与种实性状的相关性没有仁质量与种实性状的相关性强。包含所有种源总体的方程决定系数不大,进一步说明种源间存在差异,且对方程的拟合产生影响。但大部分种源内的拟合结果比较理想,在不破坏种子的情况下可以对种子仁质量进行初步估计,而出仁率则可以直接用估计的仁质量除以种质量得到,为仁质量与出仁率的估计提供参考。同时,对仁质量和出仁率分别与环境因子进行拟合,所得到的结果均比较理想,为今后的优良种源选择提供理论基础。

因单一性状独立选择法每选择一个性状,就会有将其他优良性状淘汰的风险,以致最终可供选择的优良单株较少[18,23],因此,文中对比了单一性状分别选择和综合选择的选择效果,最终通过综合坐标法与聚类分析法选出优良种源为三岔子、铁力、金山屯、亚布力,并通过综合坐标法对种源内单株进行选择,各种源选出的优良单株分别为BX1、BX3、BX13、BX30、BX25,DFH2、DFH9、DFH13、DFH26,DJC2、DJC5、DJC8、DJC13,JY7、JY9、JY14、JY21,LJ2、LJ7、LJ30、LJ8,WC2、WC3、WC4、WC7,HC1、HC7、HC16,HL1、HL10、HL15、HL26、HL14,JST1、JST4、JST24、JST27,YBL1、YBL5、YBL14、YBL16、YBL9,TL5、TL7、TL20、TL30、TL10,SC16、SC17、SC23、SC3、SC18。选择主要提高了出仁率、仁质量和壳厚这三个直接影响核桃楸品质的指标。每个种源均选择出相应的优良单株,入选率为15%~25%,选出的单株综合性状改良结果较为理想,各种源出仁率增益在5.81%~24.28%,仁质量增益在5.19%~25.77%,壳厚除东京城、临江、金山屯和亚布力外,其他种源增益不明显,种质量增益除嘉荫(8.81%)与珲春(5.68%)外,其他种源增益不明显。

参 考 文 献

[1] 中国农业百科全书编辑部.中国农业百科全书(林业卷上)[M].北京:农业出版社,1989:167-168.

[2] 袁润章.中国土木建筑百科辞典(工程材料上)[M].北京:中国建筑工业出版社,2008.

[3] 甄玉林.核桃楸的开发与利用[J].国土绿化,2009(7):52.

[4] 崔哲.胡桃楸的研究进展[J].现代医药卫生,2008,24(20):3061-3062.

[5] 刘广平,田立军,赵宝军.胡桃楸的综合利用与开发[J].中国林业,2007(15):27.

[6] XU H L, YU X F, QU S C, et al. Juglone, isolated from *Juglans mandshurica* Maxim, induces apoptosis via down-regulation of AR expression in human prostate cancer LNCaP cells[J]. Bioorganic & Medicinal Chemistry Letters,2013,23(12):3631-3634.

[7] 汪向升,张咏莉.核桃楸的化学成分分析及其抗肿瘤活性概述[J].热带医学杂志,2013,13(1):122-125.

[8] CARVALHO M, FERREIRA P L, MENDES V S, et al. Human cancer cell antiproliferative and antioxidant activities of *Juglans regia* L.[J]. Food and Chemical Toxicology,2010,48(1):441-447.

[9] 于阳阳.东北核桃楸种仁蛋白提取及降血压肽制备的研究[D].哈尔滨:东北林业大学,2012.7.

[10] 杨书文,刘桂丰,张世英,等.胡桃楸地理变异规律及最佳种源的初步选择[J].东北林业大学学报,1990,18(S2):72-76.

[11] 刘桂丰,杨书文.胡桃楸种源的初步区划及最佳种源选择[J].东北林业大学学报,1991,19(S2):189-195.

[12] 李广玉,张学政,张含国,等.胡桃楸苗木高生长种源家系变异规律初报[J].林业科技,2004,29(4):1-3.

[13] 张含国,邓继峰,张磊,等.胡桃楸种源家系变异规律及家系选择研究[J].西北林学院学报,2011,26(2):91-95.

[14] 褚宪丽,朱航勇,张含国,等.胡桃楸种源家系变异与选择[J].东北林业大学学报,2010,38(11):5-6.

[15] 苏喜廷,姚盛智,陈志诚,等.胡桃楸遗传改良中几项技术的试验研究[J].林业科技,2008,33(1):8-9.

[16] 袁显磊,祁永会,刘忠玲,等.核桃楸种源选择试验及其环境因子的影响[J].植物研究,2013,33(4):468-476.

[17] 朱红波,赵云,林士杰,等.核桃楸资源研究进展[J].中国农学通报,2011,27(25):1-4.

[18] 李永荣,李晓储,吴文龙,等.66个薄壳山核桃实生单株果实性状变异选择研究[J].林业科学研究,2013,26(4):438-446.

[19] 张守仁.坐标综合评定法的改进及应用[J].浙江林业科技,1992,12(5):51-53.

[20] 刘宏伟,王国义,孙美欧.核桃楸坚果型优树选择[J].中国林副特产,2015(6):41-43.

[21] 吴文龙,李永荣,方亮,等.薄壳山核桃果实性状的遗传变异与相关性研究[J].经济林研究,2010,28(3):25-30.

[22] 张罡,崔建国,邸爽,等.不同品种杂交榛果实表型性状的变异研究[J].西北林学院学报,2010,25(6):75-78.

[23] 陈晓阳,沈熙环.林木育种学[M].北京:高等教育出版社,2005:12.

(C)1994-2019 China Academic Journal Electronic Publishing House. All rights reserved. http://www.cnki.net
