# Supplementary material for: Provenance and family variations in early growth of Manchurian walnut (Juglans mandshurica Maxim.) and selection of superior families
Source: PLoS One. 2024 Mar 7;19(3):e0298918. doi: 10.1371/journal.pone.0298918 (PMC10919699; doi:10.1371/journal.pone.0298918)
Supplement: S1 File — (ZIP) [file pone.0298918.s004.zip › Juglans mandshurica Maxim. A Review of Its Traditional Usages, Phytochemical Constituents, and Pharmacological Properties.pdf]

frontiers in Pharmacology

REVIEW
published: 21 January 2021
doi: 10.3389/fphar.2020.569800

Juglans mandshurica Maxim.: A Review of Its Traditional Usages, Phytochemical Constituents, and Pharmacological Properties

Fei Luan[1,2†], Ziyan Wang[2†], Yan Yang[3], Yafei Ji[1], Haizhen Lv[2], Keqing Han[2], Daoheng Liu[2], Xiaofei Shang[4], Xirui He[3] and Nan Zeng[1]**

[1]*Department of Pharmacology, College of Pharmacy, Chengdu University of Traditional Chinese Medicine, Chengdu, China,* [2]*Department of Clinical Pharmacy, Shaanxi Provincial Hospital of Tuberculosis Prevention and Treatment, Xi'an, China,* [3]*Department of Bioengineering, Zhuhai Campus of Zunyi Medical University, Zhuhai, China,* [4]*Key Laboratory of Veterinary Pharmaceutical Development of Ministry of Agriculture, Key Laboratory of New Animal Drug Project, Lanzhou Institute of Husbandry and Pharmaceutical Sciences, Chinese Academy of Agricultural Sciences, Lanzhou, China*

OPEN ACCESS

Edited by:
Bey Hing Goh,
Monash University Malaysia, Malaysia

Reviewed by:
Javad Sharifi-Rad,
Shahid Beheshti University of Medical Sciences, Iran
Jayanta Kumar Patra,
Dongguk University Seoul, South Korea

***Correspondence:**
Xirui He
xiruihe6105194@163.com
Nan Zeng
19932015@cdutcm.edu.cn

†These authors have contributed equally to this work

Specialty section:
This article was submitted to Ethnopharmacology, a section of the journal Frontiers in Pharmacology

Received: *05 June 2020*
Accepted: *04 December 2020*
Published: *21 January 2021*

Citation:
Luan F, Wang Z, Yang Y, Ji Y, Lv H, Han K, Liu D, Shang X, He X and Zeng N (2021) Juglans mandshurica *Maxim.: A Review of Its Traditional Usages, Phytochemical Constituents, and Pharmacological Properties. Front. Pharmacol. 11:569800. doi: 10.3389/fphar.2020.569800*

Juglans mandshurica Maxim., also known as "Manchurian walnut" (Chinese) and "Onigurumi" (Japanese), is a medicinal plant widely distributed in Western and Central Asia, especially in China. It has been traditionally used to treat cancer, gastric ulcers, diarrhea, dysentery, dermatosis, uterine prolapse, and leukopenia. To date, more than 400 constituents including quinones (e.g. naphthoquinones, anthraquinones, naphthalenones, tetralones), phenolics, flavonoids, triterpenoids, coumarins, lignans, phenylpropanoids, diarylheptanoids, and steroids, were isolated and structurally identified from different plant parts of *J. mandshurica*. Among them, quinones, phenolics, triterpenoids, and diarylheptanoids, as the major bioactive substances, have been extensively studied and displayed significant bioactivity. Previous studies have demonstrated that *J. mandshurica* and a few of its active components exhibit a wide range of pharmacologically important properties, such as antitumor, immunomodulatory, anti-inflammatory, neuroprotective, anti-diabetic, antiviral, antimicrobial, and anti-melanogenesis activities. However, many investigations on biological activities were mainly based on crude extracts of this plant, and the major bioactive ingredients responsible for these bioactivities have not been well identified. Further *in vitro* and *in vivo* studies on the mechanisms of action of the pure bioactive compounds, and more elaborate toxicity studies as well as clinical studies are needed to ensure safety and effectiveness of the plant for human use. Taken together, the present review will provide some specific useful suggestions guide to further investigations and applications of this plant in the preparation of medicines and functional foods.

Keywords: *Juglans mandshurica*, traditional uses, phytochemistry, pharmacology, antitumor activities

INTRODUCTION

Juglans mandshurica Maxim, known as *Manchurian walnut* and *Onigurumi*, is a perennial and fast-growing deciduous broad-leaf tree reaching up to 20 m in the family Juglandaceae. It is extensively cultivated and distributed on a large scale throughout China, India, Japan, Siberia, Russia, and Korean Peninsula, *etc.* (Son, 1995; Machida et al., 2005; Bai et al., 2010; Wang et al., 2015; Hu et al., 2016; Li et al., 2018; Zhao et al., 2018; Zhao et al., 2019). In China, as hardwood tree species together with *Fraxinus mandshurica* Rupr. and *Phellodendron amurense* Rupr., it is mainly distributed in temperate to warm-temperate zones, and thus itgrown throughout many regions of northeast China, such as Heilongjiang and Liaoning provinces (Editorial Committee of Flora of China, 1979; Wang et al., 2020a). Now, it is officially listed as a national level II rare tree species and is also ranked as a rare and endangered tree species in China (Zhu et al., 2018). More importantly, every plant parts of *J. mandshurica*, including roots, stems, barks, branches, leaves, green husks, and immature fruits have important medical and health protection values, and have been used to prevent or treat multiple diseases for hundreds of years (see **Figure 1**; Zhao et al., 2019). As an example, "Bei-Qing–Long–Yi" (BQLY), the epicarp of immature fruits of *J. mandshurica*, has been used as traditional medicine for the treatment of cancer, gastric ulcers, diarrhea, dysentery, dermatosis, uterine prolapse, and leukopenia in northern China and Korea (Park et al., 2012; Liu et al., 2017; Park et al., 2017; Zhang et al., 2017; Huo et al., 2018; Zhou et al., 2019b). Currently, it is attracting increasing interest worldwide due to its various health-promoting effects. Nevertheless, overdosage or unreasonable use of BQLY can lead to some adverse reaction, such as nausea, vomiting, dizziness, dyspnea, palpitation, and even shock and death (Huo et al., 2017).

Phytochemical investigations on the different medicinal parts (roots, stems, barks, branches, leaves, and immature fruits) led to the isolation and identification of more than 400 compounds, including quinones, phenolics, flavonoids, lignans, coumarins, phenylpropanoids, triterpenoids, diarylheptanoids, and steroids. Among these compounds, quinones, phenolics, triterpenoids, and diarylheptanoids have been extensively studied and displayed the best bioactivity. As an example, naphthoquinone compounds obtained from green walnut husks of *J. mandshurica* were recognized as major active component that is mainly responsible for the anticancer activity, and the study on the bioactivity of these components has become a hotspot and attracted widespread attention from domestic and foreign researchers (Zhang et al., 2019). The kernels of the nuts of *J. mandshurica* also have high nutritional value, containing lipids (60–66%), proteins (15–20%), carbohydrates (1–15%), vitamins, and minerals (Wang et al., 2017b; Fang et al., 2018; Wang et al., 2020a). The lipids are also considered to be the main source for bioactivities owing to their abundant polyunsaturated fatty acids (Carey et al., 2020). Recent pharmacological studies have revealed that the active components and/or crude extracts of *J. mandshurica* display various biological activities, such as antitumor, immunoregulatory, anti-inflammatory, neuroprotective, anti-diabetic, antiviral, antimicrobial and anti-melanogenesis activities. More importantly, most of these claimed effects are consistent with those observed therapeutic actions of *J. mandshurica* in folk medicine.

Until recently, scientists have made a great contribution to report the chemical constituents and biological properties of *J. mandshurica*. However, no systematic review covering all-important aspects on this plant is available. In order to provide new insights for the in-depth exploration and comprehensive utilization of this plant, we systematically and critically summarize the current findings on botanical description, traditional usages, phytochemistry, pharmacology, and toxicology as well as the potential molecular mechanisms of *J. mandshurica*. Available information on this plant in this review enables people to explore their therapeutic potential, to highlight the gaps as well as provide the scientific basis for future study of this plant.

BOTANICAL DESCRIPTION AND TRADITIONAL USAGES

Botanical Description

J. mandshurica is a tree with gray bark that can grow up to a height of approximately 20 m. The odd-pinnate compound leaves can grow up to 80 cm on the sprout, the petiole is 9–14 cm in length, the leaflets are 6–17 cm in length and 2–7 cm in width. The shape of the leaflets is elliptical, oblong, ovate-elliptic or oblong-lanceolate, serrated, first sparsely pubescent on top, the underside is flat pilose with stellate hairs, the lateral leaflets are sessile, the apex is acuminate, and the base is truncated or heart-shaped. The male catkin inflorescence is 9–20 cm long, the inflorescence rachis is pubescent and usually has 12 stamens, the drug septum is gray-black pilose, the female spike is 5–6 mm in length and usually has 4–10 flowers, and the rachis is pubescent. The infructescence is approximately 10–15 cm in length, and infructescence pendulous with up to 5–7 fruits. The fruit is globular, ovate or elliptical with a sharp tip, and it is densely covered with glandular pubescence. Generally, it is approximately 3.5–7.5 cm in length and 3–5 cm in diameter. The fruit nucleus is 2.5–5 cm long with 8 longitudinal ridges on the surface, two of which are more prominent. The flowering period is in May and the fruit period from August to September (http://ppbc.iplant.cn/sp/10792).

Traditional Usages

Local and traditional usages of *J. mandshurica* in China can be traced back to the Han dynasty over 2000 years ago. Available literature shows that *J. mandshurica* has been used as popular herbal medicine and food by ethnic groups in many regions of the world, especially in Asian countries, such as China, Japan, and Korea to treat the various diseases like leucorrhoea, diarrhea, gastritis, leukopenia, dermatosis, and uterine prolapse (Liu et al., 2004a; Li et al., 2005; Xu et al., 2010; Park et al., 2012; Park and Oh, 2014; Yao et al., 2015b; Li et al., 2017b; Park et al., 2017; Chaudhary et al., 2019).

In China, *J. mandshurica*, bitter and pungent in taste, was firstly listed and recorded as the "highest-grade" medicine in the

TABLE 1 | Chemical constituents isolated and structurally identified from *J. mandshurica*.

NO.	Chemical constituents	Extracts	Parts	References
Quinones				
Naphthoquinones				
**1**	Juglone	EtOH	Green walnut husks	Zhou et al. (2019c)
		EtOH	Roots	Jin et al. (2016)
		MeOH	Leaves	Yao et al. (2015b)
		EtOH	Pericarps	Zhou et al. (2015e)
**2**	5-Methoxy-1,4-naphthoquinone	EtOH	Green walnut husks	Zhou et al. (2015b)
**3**	2-Hydroxy-1,4-naphthoquinone	EtOH	Green walnut husks	Zhou et al. (2019c)
**4**	3-Methoxy-juglone	EtOH	Green walnut husks	Zhou et al. (2015b)
		EtOH	Pericarps	Zhou et al. (2014a)
**5**	2-Ethoxy-juglone	EtOH	Roots	Zhao et al. (2019)
		EtOH	Green walnut husks	Zhou et al. (2015b)
**6**	3-Ethoxy juglone	EtOH	Green walnut husks	Zhou et al. (2019c)
		EtOH	Roots	Zhao et al. (2019)
**7**	5,8-Dihydroxy-1,4-naphthoquinone	EtOH	Green walnut husks	Zhou et al. (2015b)
**8**	3,5-Dihydroxy-1,4-naphthoquinone	EtOH	Green walnut husks	Zhou et al. (2019c)
**9**	2,5-Dihydroxy-1,4-naphthoquinone	EtOH	Green walnut husks	Zhou et al. (2019c)
**10**	1,4,8-Trihydroxy-3-naphthalene-carboxylic acid-1-O-β-D-glucopyranoside ethyl ester	EtOH	Green walnut husks	Zhou et al. (2019c)
**11**	(S)-(-)-3-(8-hydroxy-1,4-dioxo-1,4-dihydro-naphthalen-2-yl)-3-(4-hydroxy-3-methoxyphenyl)-propionic acid methyl ester	EtOH	Roots	Jiang et al. (2018)
**12**	4-(5-Hydroxy-1,4-dioxo-1,4-dihydro-naphthalen-2-ylamino)-butyric acid methyl ester	EtOH	Roots	Zhao et al. (2019)
**13**	5-Hydroxy-2-[(2-hydroxyethyl)-amino]-1,4-naphthalenedione	EtOH	Roots	Zhao et al. (2019)
**14**	(S)-(-)-3-(8-hydroxy-1,4-dioxo-1,4-dihydro-naphthalen-2-yl)-3-(4-hydroxy-3-methoxyphenyl)-propionic acid methyl ester	EtOH	Roots	Zhao et al. (2019)
**15**	1,4,8-Trihydroxynaphthalene-1-O-β-D-glucopyranoside	EtOH	Epicarp	Yang et al. (2015)
		EtOH	Green walnut husks	Zhou et al. (2015b)

(Continued in next column)

TABLE 1 | (*Continued*) Chemical constituents isolated and structurally identified from *J. mandshurica*.

NO.	Chemical constituents	Extracts	Parts	References
**16**	1,4,5-Trihydroxynaphthalene-1,4-di-O-β-D-glucopyranoside	EtOH	Epicarp	Yang et al. (2015)
		EtOH	Green walnut husks	Zhou et al. (2015b)
**17**	5-Hydroxy-2-(2-hydroxy-ethylamino)-1,4-naphthoquinone	EtOH	Roots	Jin et al. (2016)
**18**	Isosclerone	EtOH	Green walnut husks	Qiu et al. (2017)
**19**	2-Methoxy-juglone	EtOH	Green walnut husks	Zhou et al. (2015b)
		EtOH	Pericarps	Zhou et al. (2015d)
**20**	Engelharquinone	EtOH	Green walnut husks	Zhou et al. (2015b)
		EtOH	Pericarps	Zhou et al. (2015d)
**21**	1,4,5-Trihydroxynaphthalene-1,5-di-O-β-D-glucopyranoside	EtOH	Green walnut husks	Zhou et al. (2015b)
**22**	1,4,8-Trihydroxynaphthalene-1-O-β-D-[6′-O-(3″,4″,5″-trihydroxybenzoyl)]-glucopyranoside	EtOH	Green walnut husks	Zhou et al. (2015b)
**23**	3,6-Dihydroxy-4,5-dimethoxy-1,8-naphalic anhydride	EtOH	Stem barks	Lin et al. (2014)
**24**	3,4,5,6-Tetrahydroxy-1,8-naphalic anhydride	EtOH	Stem barks	Lin et al. (2014)
**25**	5-Hydroxy-2-methoxy-1,4-naphthoquinone	MeOH	Stem barks	Yao et al. (2014)
**26**	3,5-Dihydroxy-1,4-naphthoquinone	EtOH	Green walnut husks	Zhou et al. (2018b)
**27**	2-Ethoxy-5-hydroxynaphthalene-1,4-dione	EtOH	Pericarps	Zhou et al. (2015d)
**28**	Juglanperylenone A	EtOH	Stem barks	Lin et al. (2013)
**29**	Juglanperylenone B	EtOH	Stem barks	Lin et al. (2013)
Anthraquinones				
**30**	Juglanthraquinone C	EtOH	Roots	Zhao et al. (2019)
		EtOH	Roots	Jin et al. (2016)
**31**	1-Hydroxy-anthraquinone	EtOH	Roots	Zhao et al. (2019)
**32**	8-Hydroxyl-anthraquinone-1-carboxylic acid	EtOH	Epicarps	Zhou et al. (2016)
**33**	1,8-Dihydroxy-anthraquinone	EtOH	Pericarps	Zhou et al. (2014a)
**34**	1,3-Dihydroxy-2-methyl-anthraquinone	EtOH	Pericarps	Zhou et al. (2015e)
**35**	1-Hydroxy-2methyl-4-methoxy-anthraquinone	EtOH	Pericarps	Zhou et al. (2015e)
**36**	1-Methyl-3,8-dihydroxy-6-methoxy-anthraquinone	EtOH	Pericarps	Zhou et al. (2015e)
**37**	Xanthopurpurin	EtOH	Pericarps	Zhou et al. (2015e)
**38**	2-Hydroxy-3-methyl-anthraquinone	EtOH	Pericarps	Zhou et al. (2015e)

(Continued on following page)

TABLE 1 | (*Continued*) Chemical constituents isolated and structurally identified from *J. mandshurica*.

**39**	1-Hydroxy-5-pentyl-anthraquinone	EtOH	Stem barks	Jin et al. (2016)
**40**	1,5-Dihydroxy-9,10-anthraquinone-2-carboxylic acid methyl ester	EtOH	Stem barks	Lin et al. (2013)
Naphthalenones				
**41**	1,4,8-Trihydroxy-3-naphthalene-carboxylic acid-1-O-β-D-glucopyranoside ethyl ester	EtOH	Roots	Zhao et al. (2019)
**42**	1,4,8-Trihydroxy-naphthalene-1-O-β-D-glucopyanoside	EtOH	Green walnut husks	Zhou et al. (2018a)
**43**	5-Hydroxy-1,4-dioxo-1,4-dihydronaphthalen-2-ylamino)-butyric acid methyl ester	EtOH	Roots	Jin et al. (2016)
**44**	Juglanstetralone A	EtOH	Green walnut husks	Guo et al. (2015)
**45**	Juglanstetralone B	EtOH	Green walnut husks	Guo et al. (2015)
**46**	(4R)-3,4-dihydro-4-butoxy-5-hydroxy-naphthalen-1(2H)-one	EtOH	Green walnut husks	Chen et al. (2015)
**47**	1,4,8-Trihydroxynaphthalene-1-O-β-D-[6′-O-(4″-hydroxy-3″,5″-dimethyoxybenzoyl)]-glucopyranoside	MeOH	Stem barks	Min et al. (2002)
**48**	1,4,8-Trihydroxynaphthalene-1-O-β-D-[6′-O-(3″,4″,5″-trihydroxybenzoyl)]-glucopyranoside	MeOH	Stem barks	Min et al. (2002)
**49**	1,4,8-Trihydroxynaphthalene-1-O-D-glucopyranosyl-(1→6)-β-D-xylopyranoside	MeOH	Roots	Lee et al. (2000)
**50**	1,4,8-Trihydroxynaphthalene-1-O-β-D-glucopyranosyl-(1→6)-α-L-arabino-pyranoside	MeOH	Roots	Lee et al. (2000)
**51**	1-Hydroxy-4-methoxynaphthalene-1-O-β-D-glucopyranosyl-(1→6)-α-L-rhamnopyranoside	MeOH	Roots	Lee et al. (2000)
**52**	1,4,8-Trihydroxynaphthalene-1-O-[α-L-arabinofuranosyl-(1→6)-β-D-glucopyanoside]	MeOH	Stem barks	Min et al. (2000)
**53**	1,4,8-Trihydroxynaphthalene-1-O-β-D-[6′-O-(3″,5″-dihydroxy-4″-methoxybenzoyl)]-glucopyanoside]	MeOH	Stem barks	Min et al. (2000)
**54**	1,4,8-Trihydroxy-3-naphthalene-carboxylic acid-1-O-β-D-glucopyranoside methyl ester	MeOH	Roots	Kim et al. (1998)
Tetralones				
**55**	(4S)-4,5,8-trihydroxy-α-tetralone-5-O-β-D-glucopyranosyl-(1→6)-β-D-glucopyranosie	EtOH	Green walnut husks	Wang et al. (2019a)
**56**	(4S)-4,8-dihydroxy-α-tetralone-4-O-β-D-glucopyranosyl-(1→6)-β-D-glucopyranoside	EtOH	Green walnut husks	Wang et al. (2019a)

(Continued in next column)

TABLE 1 | (*Continued*) Chemical constituents isolated and structurally identified from *J. mandshurica*.

**57**	Juglanoside E	MeOH	Green walnut husks	Wang et al. (2019a)
		EtOH	Epicarp	Yang et al. (2015)
		EtOH	Roots	Zhao et al. (2019)
		MeOH	Fruits	Liu et al. (2004a)
**58**	Berchemiaside A	EtOH	Green walnut husks	Wang et al. (2019a)
		EtOH	Roots	Zhao et al. (2019)
**59**	Regiolone **(5)**	EtOH	Green walnut husks	Wang et al. (2019a)
		EtOH	Immature exocarps	Yang et al. (2019)
		EtOH	Pericarps	Zhou et al. (2014b)
		EtOH	Exocarps	Zhou et al. (2016)
**60**	Berchemiaside B	EtOH	Green walnut husks	Wang et al. (2019a)
**61**	Juglanbioside A	EtOH	Green walnut husks	Zhou et al. (2019b)
**62**	Juglanbioside B	EtOH	Green walnut husks	Zhou et al. (2019b)
**63**	Juglanbioside C	EtOH	Green walnut husks	Zhou et al. (2019b)
**64**	Juglanbioside D	EtOH	Green walnut husks	Zhou et al. (2019b)
**65**	Juglanbioside E	EtOH	Green walnut husks	Zhou et al. (2019b)
**66**	Juglanoside A	EtOH	Roots	Zhao et al. (2019)
		EtOH	Green walnut husks	Zhou et al. (2017)
		MeOH	Fruits	Liu et al. (2004a)
**67**	4(S)-5-methoxy-juglanoside A	EtOH	Green walnut husks	Zhou et al. (2019c)
**68**	4(S)-5-methoxy-juglanoside D	EtOH	Green walnut husks	Zhou et al. (2019c)
**69**	Juglanoside B	EtOH	Green walnut husks	Zhou et al. (2019c)
		MeOH	Fruits	Liu et al. (2004a)

(Continued on following page)

TABLE 1 | (*Continued*) Chemical constituents isolated and structurally identified from *J. mandshurica*.

**70**	4(S)-4,5,8-trihydroxy-α-tetralone-5-O-β-D-[6′-O-(3″,4″,5″-trihydroxybenzoyl)]-glucopyranoside	EtOH	Green walnut husks	Zhou et al. (2019c)
**71**	Juglonol A	EtOH	Immature exocarps	Yang et al. (2019)
**72**	Juglonol B	EtOH	Immature exocarps	Yang et al. (2019)
**73**	Juglonol C	EtOH	Immature exocarps	Yang et al. (2019)
**74**	Botrytone	EtOH	Immature exocarps	Yang et al. (2019)
**75**	(4R)-5,8-dihydroxy-4-methoxy-α-tetralone	EtOH	Immature exocarps	Yang et al. (2019)
		MeOH	Fruits	Machida et al. (2005)
**76**	Sclerone	EtOH	Immature exocarps	Yang et al. (2019)
**77**	(4S)-4-hydroxy-1-tetralone	EtOH	Immature exocarps	Yang et al. (2019)
		EtOH	Pericarps	Zhou et al. (2014b)
**78**	(4S)-45-dihydroxy-α-tetralone-4-O-β-D-glucopyranoside	EtOH	Green walnut husks	Zhou et al. (2017)
**79**	(4S)-4-hydroxy-α-tetralone-4-O-β-D-(6′-O-4″-hydroxylbenzoyl)-glucopyranoside	EtOH	Green walnut husks	Zhou et al. (2017)
**80**	(4S)-45-dihydroxy-α-tetralone-4-O-β-D-(6′-O-4″-hydroxylbenzoyl)-glucopyranoside	EtOH	Green walnut husks	Zhou et al. (2017)
**81**	(4S)-458-thihydroxy-α-tetralone-5-O-β-D-(6′-O-4″-hydroxylbenzoyl)-glucopyranoside	EtOH	Green walnut husks	Zhou et al. (2017)
**82**	4,5,8-Trihydroxy-α-tetralone-5-O-β-D-[6′-O-(4″-hydroxy-3″,5″-dimethoxybenzoyl)]-glucopyranoside	EtOH	Roots	Zhao et al. (2019)
**83**	4(S)-4,5,8-trihydroxy-α-tetralone-4-O-β-D-glucopyranoside	EtOH	Green walnut husks	Zhou et al. (2018a)
**84**	(4S)-4,5,8-dihydroxy-α-tetralone-5-O-β-D-[6′-O-(3″,4″,5″-trihydroxylbenzoyl)]-glucopyranoside	EtOH	Green walnut husks	Zhou et al. (2018a)
**85**	(4S)-4-hydroxy-α-tetralone-4-O-β-D-[6′-O-4″-hydroxylbenzoyl)]-glucopyranoside	EtOH	Green walnut husks	Zhou et al. (2018a)
**86**	(4S)-4,5-dihydroxy-α-tetralone-4-O-β-D-(6′-O-4″-hydroxylbenzoyl)-glucopyranoside	EtOH	Green walnut husks	Zhou et al. (2018a)
**87**	4,5-O-isopropylidene-α-tetralone	EtOH	Green walnut husks	Zhang et al. (2009)
**88**	4-Methoxy-α-tetralone-5-O-α-glucopyranoside	EtOH	Green walnut husks	Zhang et al. (2009)
**89**	4-Ethoxy-8-hydroxy-α-tetralone	EtOH	Green walnut husks	Zhang et al. (2009)
**90**	4(R)-ethoxy-8-hydroxy-α-tetralone	EtOH	Exocarps	Zhou et al. (2016)
**91**	(4R),5-dihydroxy-α-tetralone	EtOH	Epicarps	Zhou et al. (2016)

(Continued in next column)

TABLE 1 | (*Continued*) Chemical constituents isolated and structurally identified from *J. mandshurica*.

**92**	4-Butoxy-5,8-dihydroxy-3,4-dihydronaphthalen-1-one	EtOH	Green walnut husks	Qiu et al. (2017)
**93**	4-Ethoxy-5,8-dihydroxy-3,4-dihydronaphthalen-1-one	EtOH	Green walnut husks	Qiu et al. (2017)
**94**	5,8-Dihydroxy-4S-methoxy-β-tethalone	EtOH	Green walnut husks	Qiu et al. (2017)
**95**	5-Hydroxy-4-methoxy-α-naphthalen-1-one	EtOH	Green walnut husks	Qiu et al. (2017)
**96**	4,5,8-Trihydroxy-1,2,3,4-tetrahydronaphthalene-1-one	EtOH	Green walnut husks	Qiu et al. (2017)
**97**	1α,2α,4β-trihydroxy-1,2,3,4-tetrahydronaphthalene	EtOH	Green walnut husks	Qiu et al. (2017)
**98**	(4S)-4-hydroxy-α-tetralone	EtOH	Green walnut husks	Zhou et al. (2015b)
**99**	(4S)-5-hydroxy-4-methoxy-α-tetralone	EtOH	Green walnut husks	Zhou et al. (2015b)
		MeOH	Fruits	Machida et al. (2005)
**100**	Juglanoside C	MeOH	Fruits	Liu et al. (2004a)
**101**	Juglanoside D	MeOH	Fruits	Liu et al. (2004a)
**102**	(4S)-4,5,8-trihydroxy-α-tetralone-5-O-β-D-[6′-O-(3″,4″,5″-trihydroxybenzoyl)]-glucopyranoside	EtOH	Green walnut husks	Zhou et al. (2015b)
**103**	(4S)-4-hydroxy-α-tetralone-4-O-β-D-(6′-O-4″-hydroxylbenzoyl)-glucopyranoside	EtOH	Green walnut husks	Zhou et al. (2015b)
**104**	(4S)-4,5-dihydroxy-α-tetralone-4-O-β-D-(6′-O-4″-hydroxylbenzoyl)-glucopyranoside	EtOH	Green walnut husks	Zhou et al. (2015b)
**105**	(4S)-4,5,8-thihydroxy-α-tetralone-5-O-β-D-(6′-O-4″-hydroxylbenzoyl)-glucopyranoside	EtOH	Green walnut husks	Zhou et al. (2015b)
**106**	4,5-Dihydroxy-α-tetralone	EtOH	Green walnut husks	Chen et al. (2015)
**107**	4,8-Dihydroxy-1-tetralone	MeOH	Stem barks	Yao et al. (2014)
**108**	4′α,5′,8′-trihydroxy-α-tetralone-5′-O-β-D-[6-O-(4″-hydroxy-3″,5″-dimethoxybenzoyl)]-glucopyranose	MeOH	Stem barks	Yao et al. (2014)
**109**	4(R)-5-hydroxy-4-ethox-β-tetralone	EtOH	Green walnut husks	Zhou et al. (2018b)
**110**	4(S)-4,5-dihydroxy-α-tetralone	EtOH	Green walnut husks	Zhou et al. (2018b)
**111**	5-Hydroxy-4-methoxy-α-tetralone	EtOH	Pericarps	Zhou et al. (2015d)
**112**	Juglanone	MeOH	Fruits	Liu et al. (2010)
**113**	(4S)-4,8-dihydroxy-α-tetralone	MeOH	Fruits	Machida et al. (2005)

(Continued on following page)

TABLE 1 | (*Continued*) Chemical constituents isolated and structurally identified from *J. mandshurica*.

**114**	(4R)-4,8-dihydroxy-α-tetralone	MeOH	Fruits	Machida et al. (2005)
**115**	(4R)-5-hydroxy-4-methoxy-α-tetralone	MeOH	Fruits	Machida et al. (2005)
**116**	(4S)-5,8-dihydroxy-4-methoxy-α-tetralone	MeOH	Fruits	Machida et al. (2005)
**117**	(4S)-4,8-dihydroxy-5-methoxy-α-tetralone	MeOH	Fruits	Machida et al. (2005)
**118**	(4R)-4-hydroxy-α-tetralone	MeOH	Fruits	Machida et al. (2005)
**119**	(S)-(+)-4-hydroxytetralone	MeOH	Roots	Li et al. (2002)
**120**	4,5,8-Trihydroxy-α-tetralone-5-O-β-D-[6′-O-(4″-hydroxy-3″,5″dimethoxybenzoyl)]-glucopyanoside]	MeOH	Stem barks	Min et al. (2000)
**121**	4α,5,8-trihydroxy-α-tetralone-5-O-β-D-[6′-O-(3″,5″-dihydroxy-4″-methoxybenzoyl)]-glucopyanoside]	MeOH	Stem barks	Min et al. (2000)
**122**	4α,5,8-trihydroxy-α-tetralone-5-O-β-D-[6′-O-(3″,4″,5″-trihydroxybenzoyl)]-glucopyanoside]	MeOH	Stem barks	Min et al. (2000)
**123**	4,5,8-Trihydroxy-α-tetralone-5-O-β-D-[6′-O-(3″,5″-dimethoxy-4″-hydroxybenzoyl)]-glucopyranoside	MeOH	Roots	Kim et al. (1998)
**124**	2,6-Dimethoxy-1,4-benzoquinone	EtOH	Pericarps	Zhou et al. (2015e)
**125**	p-hydroxymethoxybenzobijuglone	EtOH	Leaves	Li et al. (2007b)
	Phenolics			
**126**	2-[4-(3-hydroxypropyl)-2-methoxyphenoxy]-1,3-propanediol	MeOH	Fruits	Kim et al. (2019)
**127**	(-)-Evofolin B	MeOH	Fruits	Kim et al. (2019)
**128**	(2S)-Schweinfurthinol	MeOH	Fruits	Kim et al. (2019)
**129**	Hydroxypropiophenone-4-O-β-D-glucopyranosyl-(1→6)-β-D-glucopyranoside	EtOH	Green husks	Zhou et al. (2017)
**130**	2-(4-Formyl-2-methoxyphenoxy)-propan-1,3-diol **(1)**	MeOH	Fruits	Park et al. (2017)
**131**	2-(4-Hydroxymethyl-2-methoxyphenoxy)-propan-1,3-diol	MeOH	Fruits	Park et al. (2017)
**132**	(+)-3-hydroxy-2-(4-hydroxy-3-methoxyphenyl)-1-(4-hydroxyphenyl)-propan-1-one	MeOH	Fruits	Park et al. (2017)
**133**	Threo-2-(4-hydroxy-3-methoxyphenyl)-1-(4-hydroxyphenyl)-propan-1,3-diol	MeOH	Fruits	Park et al. (2017)
**134**	2-(4-Hydroxy-3-methoxyphenyl)-1-(4-hydroxyphenyl)-1-methoxy-propan-3-ol	MeOH	Fruits	Park et al. (2017)
**135**	(2-glyceryl)-O-coniferaldehyde	MeOH	Fruits	Park et al. (2017)
**136**	1,2-Bis-(4-hydroxy-3-methoxyphenyl)-propane-1,3-diol	MeOH	Fruits	Park et al. (2017)
**137**	Salidroside	EtOH	Roots	Zhao et al. (2019)
**138**	6-O-(4′-hydroxy-3′,5′-dimethoxybenzoyl)-D-glucopyranose	EtOH	Roots	Zhao et al. (2019)
	6-O-(4′-hydroxy-3′,5′-dimethoxybenzoyl)-D-glucopyranose	MeOH	Stem barks	Yao et al. (2014)
**139**	4′-hydroxy-2′,6′-dimethoxyphenol-1-O-β-D-(6-O-syringoyl)-glucopyranoside	EtOH	Roots	Zhao et al. (2019)
**140**	5-O-cafffeoyl-quinic acid butyl ester	EtOH	Epicarps	Yang et al. (2015)
**141**	3,5-di-O-caffeoyl-quinic acid butyl ester	EtOH	Epicarps	Yang et al. (2015)
**142**	Vanillic acid-4-O-β-D-(6′-O-galloyl)-glucopyranoside	EtOH	Epicarps	Yang et al. (2015)
**143**	4-Hydroxy-2,6-dimethoxyphenol-1-O-β-D-glucopyranoside	EtOH	Epicarp	Yang et al. (2015)
**144**	4-Hydroxy-4-(3′-hydroxyphenol)-butanoic acid-4-O-β-D-glucopyranoside ethyl ester	EtOH	Husks	Zhou et al. (2018a)
**145**	4-Hydroxy-4-(3′-hydroxyphenol)-butyric acid-4-O-β-D-glucopyranoside methyl ester	EtOH	Husks	Zhou et al. (2018a)
**146**	1,4,8-Trihydroxy-3-naphthoic acid ethyl ester-1-O-β-D-glucopyanoside	EtOH	Husks	Zhou et al. (2018a)
**147**	Chrysophanol	EtOH	Pericarps	Zhou et al. (2014b)
**148**	Chlorogenic acid	EtOH	Pericarps	Zhou et al. (2014b)
**149**	p-hydroxybenzonic acid	EtOH	Pericarps	Zhou et al. (2014b)
		EtOH	Green walnut husks	Fu et al. (2020)
**150**	*p*-methoxyphenylacetic acid	EtOH	Pericarps	Zhou et al. (2014b)
**151**	1,4-Dihydroxybenzene	EtOH	Pericarps	Zhou et al. (2014b)
		EtOH	Green walnut husks	Fu et al. (2020)
**152**	Ethyl gallate	EtOH	Epicarps	Zhou et al. (2016)
		EtOH	Green walnut husks	Fu et al. (2020)
**153**	Methy 4-hydroxyphenylacetate	EtOH	Epicarps	Zhou et al. (2016)
**154**	5-Hydroxyl-1-(4′-hydroxphenyl)-7-(4-′′-hydroxy-3″-methoxyphenyl)-3-heptanone	EtOH	Epicarps	Zhou et al. (2016)
**155**	2,5-Dimethyl-1,3-benzenediol	EtOH	Green walnut husks	Fu et al. (2020)
**156**	Caffeic acid	EtOH	Green walnut husks	Fu et al. (2020)
**157**	Vanillic acid	EtOH	Green walnut husks	Fu et al. (2020)
		EtOH	Pericarps	Zhou et al. (2015d)
**158**	Syringic acid	EtOH	Green walnut husks	Fu et al. (2020)
		EtOH	Pericarps	Zhou et al. (2015c)

(Continued on following page)

TABLE 1 | (*Continued*) Chemical constituents isolated and structurally identified from *J. mandshurica*.

**159**	Protocatechuic acid	EtOH	Green walnut husks	Fu et al. (2020)
		EtOH	Pericarps	Zhou et al. (2015c)
**160**	2-Hydroxy-4-methoxy-3,6-dimethyl benzoic acid	EtOH	Green walnut husks	Fu et al. (2020)
**161**	3′-O-(E-4-coumaroyl)-quinic acid	EtOH	Green walnut husks	Fu et al. (2020)
**162**	5′-O-(E-4-coumaroyl)-quinic acid	EtOH	Green walnut husks	Fu et al. (2020)
**163**	3,3′-dimethoxylellagic acid	EtOH	Green walnut husks	Fu et al. (2020)
**164**	Dimethyl feruloyl-lactate	EtOH	Green walnut husks	Fu et al. (2020)
**165**	(S)-3-hydroxy-1,5-diphenyl-1-pentanone	EtOH	Green walnut husks	Fu et al. (2020)
**166**	Z-P-coumaryl-hexacosanoate	EtOH	Green walnut husks	Fu et al. (2020)
**167**	4-Hydroxybenzoic acid methyl ester	MeOH	Leaves	Yao et al. (2015b)
**168**	Methyl isoferulate	EtOH	Green walnut husks	Zhou et al. (2018b)
**169**	Mesodihydroguaiaretic acid	EtOH	Pericarps	Zhou et al. (2015c)
**170**	Protocatechuic acid methyl ester	EtOH	Pericarps	Zhou et al. (2015c)
**171**	4-Hydroxymethyl-2-methoxy phenol	EtOH	Pericarps	Zhou et al. (2015c)
**172**	Methyl gallate	EtOH	Pericarps	Zhou et al. (2014a)
**173**	Gallic acid	EtOH	Pericarps	Zhou et al. (2015d)
**174**	Vanillin	EtOH	Pericarps	Zhou et al. (2015d)
**175**	2,5-Dihydroxy-methyl-phenylacetate	EtOH	Pericarps	Zhou et al. (2015d)
**176**	p-hydroxy-benzaldehyde	EtOH	Pericarps	Zhou et al. (2015d)
**177**	4′-hydroxy-2′,6′-dimethoxyphenol-1-O-β-D-(6-O-syringoyl)-glucopyranoside	MeOH	Barks	Machida et al. (2009)
**178**	1-O-β-D-(6-O-syringoyl)-glucopyranoside	MeOH	Barks	Machida et al. (2009)
**179**	4′-hydroxy-2′-methoxyphenol-1-O-β-D-(6-O-syringoyl)-glucopyranoside	MeOH	Barks	Machida et al. (2009)
**180**	10-Hydrogenmyricananadiol	EtOH	Green peel	Li et al. (2017a)
**181**	Myricatomentogenin	EtOH	Green peel	Li et al. (2017a)
		EtOH	Green walnut husks	Qiu et al. (2017)
**182**	Myricanol	EtOH	Epicarps	Zhou et al. (2016)
**183**	5-Deoxymyricanone	EtOH	Epicarps	Zhou et al. (2016)
**184**	L-2-O-methyl-chiroinosicol	EtOH	Green walnut husks	Qiu et al. (2017)
**185**	Ethyl 3-methoxy-4-hydroxybenzoate	EtOH	Green walnut husks	Li et al. (2013)
**186**	Ethyl 3,4-dihydroxybenzoate	EtOH	Green walnut husks	Li et al. (2013)
**187**	Massonianoside D	EtOH	Pericarps	Zhou et al. (2015c)
**188**	Pterocarine	EtOH	Pericarps	Zhou et al. (2014a)
**189**	3,4-Dihydroxybenzoic acid	EtOH	Green walnut husks	Chen et al. (2015)
**190**	6-O-galloyl-D-glucopyranose	MeOH	Stem barks	Yao et al. (2014)
**191**	1-O-galloyl-β-D-glucopyranose	MeOH	Stem barks	Yao et al. (2014)
**192**	1,2,6-Trigalloylglucose	MeOH	Stem barks	Ngoc et al. (2008)
**193**	1,2,3,6-Tetragalloylglucose	MeOH	Stem barks	Ngoc et al. (2008)
**194**	1,2,3,4,6-penta-O-galloyl-β-D-glucose	Acetone	Barks	Ju et al. (2009)
Triterpenoids				
**195**	Klodorol B	EtOH	Green walnut husks	Zhou et al. (2019a)
**196**	1α,3β-dihydroxy-olean-18-ene	MeOH	Green walnut husks	Zhou et al. (2019a)
		EtOH	Pericarps	Zhou et al. (2014a)
**197**	Ursolic acid acetate	MeOH	Green walnut husks	Zhou et al. (2019a)
**198**	2α,3α,19α-trihydroxyurs-12-en-28-oic acid	MeOH	Green walnut husks	Zhou et al. (2019a)
**199**	20(R)-24β-hydroxy-20,25-epoxy-dammar-3-one	MeOH	Green walnut husks	Zhou et al. (2019a)
**200**	20β-hydroxydammara-23(24)-en-3-one	MeOH	Green walnut husks	Zhou et al. (2019a)
**201**	Dammara-20,24-dien-3β-ol	MeOH	Green walnut husks	Zhou et al. (2019a)
		EtOH	Pericarps	Zhou et al. (2010)
**202**	24-Methylenecycloartenone	EtOH	Roots	Zhao et al. (2019)
**203**	Sigmoiside B	EtOH	Roots	Zhao et al. (2019)
**204**	Oleanolic acid	EtOH	Green walnut husks	Zhou et al. (2015a)
		EtOH	Pericarps	Zhou et al. (2010)

(Continued on following page)

TABLE 1 | (*Continued*) Chemical constituents isolated and structurally identified from *J. mandshurica*.

**205**	Betulinic acid	EtOH	Green walnut husks	Zhang et al. (2009)
**206**	20(S)-hydroxydammar-24-en-3-on	EtOH	Green walnut husks	Zhou et al. (2015a)
**207**	20(S)-protopanaxadiol-3-one	EtOH	Green walnut husks	Zhou et al. (2015a)
		EtOH	Pericarps	Zhou et al. (2010)
**208**	20(S),24(R)-dihydroxydammaran-25-en-3-one	EtOH	Green walnut husks	Zhou et al. (2015a)
**209**	20(S),24(S)-dihydroxydammaran-25-en-3-one	EtOH	Green walnut husks	Zhou et al. (2015a)
**210**	1β,12β,20(S)-trihydroxydammar-24-en-3-one	EtOH	Green walnut husks	Zhou et al. (2015a)
**211**	12β,20(R),24(R)-trihydroxydammar-25-en-3-one	EtOH	Green walnut husks	Zhou et al. (2015a)
**212**	20(S)-protopanaxadiol	EtOH	Green walnut husks	Zhou et al. (2015a)
**213**	1β,3α,12β,20(S)-tetrol-24-ene-dammar	EtOH	Green walnut husks	Zhou et al. (2015a)
**214**	3-Epikatonic acid	EtOH	Green walnut husks	Zhou et al. (2015a)
**215**	2α-hydroxyoleanolic acid	EtOH	Green walnut husks	Zhou et al. (2015a)
**216**	2α,3β,23-trihydroxy-12-en-28-oleanolic acid	EtOH	Green walnut husks	Zhou et al. (2015a)
		EtOH	Pericarps	Zhou et al. (2010)
**217**	Ursolic acid	EtOH	Green walnut husks	Zhou et al. (2015a)
		EtOH	Root	Liu et al. (2009)
		EtOH	Pericarps	Zhou et al. (2015d)
**218**	3β-hydroxyurs-20-en-28-oic acid	EtOH	Green walnut husks	Zhou et al. (2015a)
**219**	2α-hydroxyursolic acid	EtOH	Green walnut husks	Zhou et al. (2015a)
**220**	3-Oxo-23-hydroxyurs-12-en-28-oic acid	EtOH	Green walnut husks	Zhou et al. (2015a)
**221**	2α,3β,23-trihydroxyurs-12-en-28-oic acid	EtOH	Green walnut husks	Zhou et al. (2015a)
**222**	2α,3β,23-trihydroxy-12-en-28-ursolic acid	EtOH	Pericarps	Zhou et al. (2010)
**223**	Corosolic acid	EtOH	Green walnut husks	Chen et al. (2015)

(Continued in next column)

TABLE 1 | (*Continued*) Chemical constituents isolated and structurally identified from *J. mandshurica*.

**224**	Arjunolic acid	EtOH	Green walnut husks	Chen et al. (2015)
**225**	3β,23-dihydroxy-olean-12-en-28-oic acid	EtOH	Green walnut husks	Chen et al. (2015)
**226**	3β,23-dihydroxy-urs-12-en-28-oic acid	EtOH	Green walnut husks	Chen et al. (2015)
**227**	3β,24-dihydroxy-12-en-28-ursolic acid	MeOH	Stem barks	Yao et al. (2014)
**228**	2α,3α,19α-trihydroxy-ursolic acid	EtOH	Pericarps	Zhou et al. (2014a)
**229**	3β,19β,28-trihydroxylupane 3-O-trans-caffeate	EtOH	Roots	Li et al. (2017b)
**230**	3β,19β,28-trihydroxylupane 3-O-cis-caffeate	EtOH	Roots	Li et al. (2017b)
**231**	Maslinic acid	EtOH	Stem barks	Lin et al. (2013)
**232**	Corosolic acid	EtOH	Stem barks	Lin et al. (2013)
**233**	3β-hydroxy-olean-11,13(18)-dien-28-oic acid	EtOH	Stem barks	Lin et al. (2013)
**234**	3β-acetoxy-olean-11,13(18)-dien-28-oic acid	EtOH	Stem barks	Lin et al. (2013)
**235**	Juglangenin A	EtOH	Stem barks	Zhang et al. (2012b)
Diarylheptanoids				
**236**	2-Oxatrycyclo-[13.2.2.13,7]-eicosa-3,5,7-(20),15,17,18-hexaen-10-one	EtOH	Green walnut husks	Wang et al. (2019a)
**237**	Juglanin A	EtOH	Green walnut husks	Wang et al. (2019a)
		EtOH	Green peel	Li et al. (2017a)
		EtOH	Roots	Zhao et al. (2019)
		EtOH	Pericarps	Zhou et al. (2010)
**238**	2-Oxatrycyclo-[13.2.2.13,7]-eicosa-3,5,7(20),15,17, 18-hexaen-10–16-diol	EtOH	Green walnut husks	Wang et al. (2019a)
**239**	(11S)-11,17-dihydroxy-3,4-dimethoxy-[7,0]-metacyclophane	EtOH	Green walnut husks	Wang et al. (2019a)
		MeOH	Leaves	Yao et al. (2015b)
**240**	(2S,3S,5S)-2,3,5-trihydroxy-1,7-bis-(4-hydroxy-3-methoxyphenyl)-heptane	EtOH	Roots	Diao et al. (2017)
**241**	(2S,3S,5S)-2,3-dihydroxy-5-O-β-D-xylopyranosyl-7-(4-hydroxy-3-methoxyphenyl)-1-(4-hydroxyphenyl)-heptane	EtOH	Roots	Diao et al. (2017)
**242**	Rhoiptelol C	EtOH	Roots	Zhao et al. (2019)
**243**	Rhoiptelol B	EtOH	Roots	Zhao et al. (2019)
**244**	3′,4″-epoxy-2-O-β-D-glucopyranosyl-1-hydroxyphenyl)-7-(3-methoxy-phenyl)-heptan-3-one	EtOH	Roots	Diao et al. (2017)

(Continued on following page)

TABLE 1 | (*Continued*) Chemical constituents isolated and structurally identified from *J. mandshurica*.

**245**	Juglanin D	EtOH	Green peel	Li et al. (2017a)
**246**	(-)-threo-3′,4″-epoxy-1-(4-hydroxyphenyl)-7-(3-methoxyphenyl)-heptan-2,3-diol	EtOH	Roots	Zhao et al. (2019)
**247**	(11R)-3,11,17-trihydroxy-2-methoxy-1,16-oxo-7,13-diphenyl-11-heptanol	EtOH	Roots	Zhao et al. (2019)
		EtOH	Green walnut husks	Zhou et al. (2020)
		MeOH	Leaves	Yao et al. (2015b)
**248**	(3R)-3′,4″-epoxy-1-(4-hydro-xyphenyl)-7-(3-methoxyphenyl)-heptan-3-ol	EtOH	Roots	Zhao et al. (2019)
**249**	Juglaside A	EtOH	Roots	Zhao et al. (2019)
**250**	(1α,3β,5α,6α)-1,5-epoxy-3,6-dihydroxy-1,7-bis-(3-methoxy-4-hydroxy-phenyl)-heptane	EtOH	Roots	Zhao et al. (2019)
**251**	Engelheptanoxide A	EtOH	Roots	Zhao et al. (2019)
**252**	(R)-4-(5-hydroxy-7-(4-hydro-xyphenyl)-heptyl)-2-methoxy-phenol	EtOH	Roots	Zhao et al. (2019)
**253**	(2S,3S,5S)-2,3,5-tri-hydroxy-1,7-bis-(4-hydroxy-3-methoxyphenyl)-heptane	EtOH	Roots	Zhao et al. (2019)
**254**	(2S,3S,5S)-2,3-dihydroxy-5-β-D-xylopyranosyl-7-(4-hydroxy-3-methoxyphenyl)-1-(4-hydroxyphenyl)-heptane	EtOH	Roots	Zhao et al. (2019)
**255**	1-(4-Hydro-xyphenyl)-7-(4-hydroxy-3-methoxyphenyl)-4-hepten-3-one	EtOH	Roots	Zhao et al. (2019)
**256**	Jugcathayenoside	EtOH	Green walnut husks	Zhou et al. (2020)
**257**	(1α,3β,5α,6α)-1,5-epoxy-3,6-dihydroxy-1-(3-methoxy-4-hydroxy-phenyl)-7-(4-hydroxyphenyl) -heptane	EtOH	Green walnut husks	Zhou et al. (2020)
**258**	(1α,3β,5α,6α)-1,5-epoxy-3,6-dihydroxy-1,7-bis-(3-methoxy-4-hydroxylphenyl)-heptane	EtOH	Green walnut husks	Zhou et al. (2020)
**259**	(1α,3β,5α,6α)-1,5-epoxy-3,6-dihydroxy-1,7-bis-(3-methoxy-4-hydroxylphenyl)-heptane	EtOH	Roots	Jin et al. (2015)
**260**	5(S)-5-hydroxy-1-(4-hydroxy-3-methoxyphenyl)-7-(4-hydroxyphenyl)-3-heptanone	EtOH	Green walnut husks	Zhou et al. (2020)
**261**	5-Hydroxy-1-(4′-hydroxyphenyl)-7-(4″-hydroxy-3″-methoxy)-3-heptanone	EtOH	Green walnut husks	Zhou et al. (2020)
**262**	Hexahydrocurcumin	EtOH	Green walnut husks	Zhou et al. (2020)
**263**	Juglanin C	EtOH	Green walnut husks	Zhou et al. (2020)
		MeOH	Leaves	Yao et al. (2015b)
**264**	1-(4′-hydroxyphenyl)-7-(3″-methylphenyl-4″-hydroxyphenyl)-4-ene-3-heptanone	EtOH	Green walnut husks	Zhou et al. (2020)
**265**	(11S,12R)-11,12,17-trihydroxy-2-methoxy-1,16-oxo-7,13-diphenyl-11,12-heptanol	EtOH	Green walnut husks	Zhou et al. (2020)
**266**	(12R)-12,17-dihydroxy-2-methoxy-1,16-oxo-7,13-diphenyl-3-heptanone	EtOH	Green walnut husks	Zhou et al. (2020)
**267**	1-(4′-hydroxyphenyl)-7-(3″-methylphenyl)-2-hydroxy-3′,4″-epoxy-3-heptanone	EtOH	Green walnut husks	Zhou et al. (2020)
**268**	(-)-threo-3′,4″-epoxy-1-(4-hydroxyphenyl)-7-(3-methoxyphenyl)-heptan-2,3-diol	EtOH	Roots	Jin et al. (2015)
**269**	Myricananin F	EtOH	Green walnut husks	Chen et al. (2015)
**270**	Myricatomentogenin	MeOH	Leaves	Yao et al. (2015b)
**271**	Rhein	EtOH	Stem barks	Lin et al. (2013)
**272**	Emodin	EtOH	Stem barks	Lin et al. (2013)
**273**	Anthrarufin	EtOH	Stem barks	Lin et al. (2013)
**274**	(5S)-5-hydroxy-7-(4-hydroxy-3methoxyphenyl)-1(4-hydroxyphenyl)-3-heptanone	MeOH	Roots	Li et al. (2002)
**275**	Diarylheptanone glucoside	MeOH	Roots	Kim et al. (1998)
	Flavonoids			
**276**	Rhamnetin-3-O-β-D-xylopyranoside	EtOH	Green peel	Li et al. (2017a)
**277**	Quercetin-3-O-α-L-arabinofuranoside	EtOH	Green peel	Li et al. (2017a)
**278**	Quercetin-3-O-β-D-xylopyranoside	EtOH	Green peel	Li et al. (2017a)
**279**	Apigenin	EtOH	Roots	Zhao et al. (2019)
**280**	Quercitrin	EtOH	Green peel	Li et al. (2017a)
		EtOH	Epicarp	Yang et al. (2015)
		MeOH	Stem barks	Min et al. (2003)
**281**	Kaempferol-3-O-β-D-glucopyranoside	EtOH	Epicarp	Yang et al. (2015)
		EtOH	Green walnut husks	Zhou et al. (2019d)
**282**	Quercetin-3-O-β-D-glucopyranoside	EtOH	Epicarp	Yang et al. (2015)
		EtOH	Green walnut husks	Zhou et al. (2019d)
**283**	Myricitrin	EtOH	Epicarp	Yang et al. (2015)
		MeOH	Stem barks	Min et al. (2003)
**284**	Afzelin	EtOH	Epicarp	Yang et al. (2015)
		MeOH	Stem barks	Min et al. (2003)
**285**	Hyperin	EtOH	Epicarp	Yang et al. (2015)

(Continued in next column)

(Continued on following page)

TABLE 1 | (*Continued*) Chemical constituents isolated and structurally identified from *J. mandshurica*.

**286**	Kaempferol	EtOH	Pericarps	Zhou et al. (2014b)
		MeOH	Stem barks	Min et al. (2003)
**287**	Pinostrobin	EtOH	Pericarps	Zhou et al. (2014b)
		EtOH	Green walnut husks	Li et al. (2013)
**288**	Onysilin	EtOH	Pericarps	Zhou et al. (2014b)
		EtOH	Green walnut husks	Li et al. (2013)
**289**	Juglanin B	EtOH	Pericarps	Zhou et al. (2014b)
		EtOH	Epicarps	Zhou et al. (2016)
		EtOH	Roots	Liu et al. (2009)
**290**	5-Hydroxy-3,7,3′,4′-tetramethoxyflavone	EtOH	Pericarps	Zhou et al. (2014b)
**291**	(2S)-5,7,4′-trihydroxy-dihydroflavonol	EtOH	Pericarps	Zhou et al. (2014b)
**292**	Apigenin	EtOH	Green walnut husks	Zhou et al. (2019d)
**293**	Tricin	EtOH	Green walnut husks	Zhou et al. (2019d)
**294**	Eupatilin	EtOH	Green walnut husks	Zhou et al. (2019d)
**295**	3,7,8,3′-tetrahydroxy-4′-methoxyflavone	EtOH	Green walnut husks	Zhou et al. (2019d)
**296**	3,5-Dihydroxy-7-methoxy-3′,4′-methylenedioxyflavone	EtOH	Green walnut husks	Zhou et al. (2019d)
**297**	Taxifolin	EtOH	Green walnut husks	Zhou et al. (2019d)
		MeOH	Stem barks	Min et al. (2003)
**298**	Quercetin-3-O-(6″-galloyl)-β-D-gllactopyranoside	EtOH	Green walnut husks	Zhou et al. (2019d)
**299**	Quercetin-3-O-(4″-O-acetyl)-α-L-rhamnopyranoside	EtOH	Green walnut husks	Zhou et al. (2019d)
**300**	Engeletin	EtOH	Green walnut husks	Zhou et al. (2019d)
**301**	Isoengeletin	EtOH	Green walnut husks	Zhou et al. (2019d)
**302**	Quercetin-3-O-β-D-glucuronide	EtOH	Green walnut husks	Zhou et al. (2019d)
**303**	Myricetin-3-O-β-D-glucuronide	EtOH	Green walnut husks	Zhou et al. (2019d)
**304**	Broussonol E	EtOH	Epicarps	Zhou et al. (2016)
**305**	Kaempferol-3-O-α-L-rhamnoside	EtOH	Epicarps	Zhou et al. (2016)
**306**	Quercetin-3-O-α-L-rhamnoside	EtOH	Epicarps	Zhou et al. (2016)
**307**	Wogonin	EtOH	Green walnut husks	Li et al. (2013)
**308**	Alpinetin	EtOH	Green walnut husks	Li et al. (2013)
**309**	5-Hydroxy-7,8-dimethoxyflavanone	EtOH	Green walnut husks	Li et al. (2013)
**310**	Quercetin	EtOH	Pericarps	Zhou et al. (2014a)
		MeOH	Stem barks	Min et al. (2000)
**311**	Juglbiflavone A	EtOH	Roots	Li et al. (2017b)
**312**	Myricetin	MeOH	Stem barks	Min et al. (2003)
**313**	1,3,5,8-Tetrahydroxy-xanthone	EtOH	Root	Liu et al. (2009)
**314**	1,3,8-Trihydroxy-5-methoxy-xanthone	EtOH	Root	Liu et al. (2009)
Lignans				
**315**	(+)-Sesamin	EtOH	Barks	Wang et al. (2019b)
**316**	(-)-Sesamin	EtOH	Barks	Wang et al. (2019b)
**317**	Juglansol A	EtOH	Barks	Zhang et al. (2017)
**318**	Balanophonin	EtOH	Barks	Zhang et al. (2017)
**319**	(+)-Epinoresinol	EtOH	Barks	Zhang et al. (2017)
**320**	(+)-Medioresinol	EtOH	Barks	Zhang et al. (2017)
**321**	(+)-Pinoresinol	EtOH	Barks	Zhang et al. (2017)
**322**	Erythro-(7S,8R)-guaiacyl-glycerol-β-O-4′-dihydroconiferyl ether	EtOH	Barks	Zhang et al. (2017)
**323**	Erythro-(7R,8S)-guaiacylglycerol-β-O-4′-dihydroconiferyl ether	EtOH	Barks	Zhang et al. (2017)
**324**	Threo-(7R,8R)-guaiacyl-glycerol-β-O-4′-dihydroconiferyl ether	EtOH	Barks	Zhang et al. (2017)
**325**	Erythro-guaiacylglycerol-β-O-4′-sinapyl ether	EtOH	Barks	Zhang et al. (2017)
**326**	(rel-(3R,3′S,4R,4′S)-3,3′,4,4′-tetrahydro-6,6′-dimethoxy-[3,3′-bi-2H-benzopyran]-4,4′-diol	EtOH	Barks	Zhang et al. (2017)
**327**	(7S,8R)-4,9,7′-trihydroxy-3′-methoxy-8′,9′-dinor-7,4′-epoxy-8,5′-neolignan	MeOH	Fruits	Park et al. (2017)
**328**	Threo-(7S,8S,7′E)-1′-formyl-4,7,9-trihydroxy-8-O-4′-neolignan	MeOH	Fruits	Park et al. (2017)
**329**	Erythro-(7R,8S,7′E)-1′-formyl-4,7,9-trihydroxy-8-O-4′-neolignan	MeOH	Fruits	Park et al. (2017)
**330**	Threo-(7S,8S)-3′-methoxy-4,7,9,9′-tetrahydroxy-8-O-4′-neolignan	MeOH	Fruits	Park et al. (2017)
**331**	Erythro-(7R,8S)-3′-methoxy-4,7,9,9′-tetrahydroxy-8-O-4′-neolignane	MeOH	Fruits	Park et al. (2017)

(Continued in next column)

(Continued on following page)

TABLE 1 | (*Continued*) Chemical constituents isolated and structurally identified from *J. mandshurica*.

**332**	(+)-Lyoniresinol	EtOH	Roots	Zhao et al. (2019)
**333**	(+)-Lyoniresinol-3α-O-β-D-glucopyranoside	EtOH	Roots	Zhao et al. (2019)
**334**	(7S,8R)-dihydrodehydrodiconiferyl alcohol	EtOH	Roots	Zhao et al. (2019)
Coumarins				
**335**	Juglansoside C	EtOH	Barks	Lou et al. (2019a)
**336**	Juglansin A	EtOH	Barks	Yao et al. (2017)
**337**	Xanthyoxylin	EtOH	Barks	Yao et al. (2017)
**338**	Braylin	EtOH	Barks	Yao et al. (2017)
**339**	6,7-Dimethoxyl-coumarin	EtOH	Barks	Yao et al. (2017)
**340**	6,7,8-Trimethoxyl-coumarin	EtOH	Barks	Yao et al. (2017)
**341**	Xanthyletin	EtOH	Barks	Yao et al. (2017)
**342**	Luvangetin	EtOH	Barks	Yao et al. (2017)
**343**	Norbraylin	EtOH	Barks	Yao et al. (2017)
**344**	5,6,7-Trimethoxyl-coumarin	EtOH	Barks	Yao et al. (2017)
**345**	Juglansoside A	EtOH	Barks	Lou et al. (2018)
**346**	Juglansoside B	EtOH	Barks	Lou et al. (2018)
**347**	5-Methoxyseselin	EtOH	Barks	Lou et al. (2018)
**348**	Apigravin	EtOH	Barks	Lou et al. (2018)
**349**	Alloxanthoxyletin	EtOH	Barks	Lou et al. (2018)
**350**	Isoschinilenol	EtOH	Barks	Lou et al. (2018)
**351**	7-Geranyloxy-6-methoxycoumarin	EtOH	Barks	Lou et al. (2018)
**352**	Fraxinol	EtOH	Stem barks	Lin et al. (2013)
**353**	Fraxetin	EtOH	Stem barks	Lin et al. (2013)
Phenylpropanoids				
**354**	Juglansnoid A	EtOH	Barks	Cheng et al. (2016)
**355**	Juglansnoid B	EtOH	Barks	Cheng et al. (2016)
**356**	Juglansnoid C	EtOH	Barks	Cheng et al. (2016)
**357**	(2E)-3-[4-(4-hydroxy-3-methylbutoxy)-phenyl]-2-propenal	EtOH	Barks	Cheng et al. (2016)
**358**	Boninenal	EtOH	Barks	Cheng et al. (2016)
**359**	(4′-hydroxy-3′-methylbutoxy)-benzaldehyde	EtOH	Barks	Cheng et al. (2016)
**360**	(E)-4-[4′-hydroxy-3′-methylbut-(E)-2′-enyloxy]-cinnamate	EtOH	Barks	Cheng et al. (2016)
**361**	Ailanthoidiol	EtOH	Barks	Cheng et al. (2016)
**362**	Methyl nitinoate	EtOH	Barks	Cheng et al. (2016)
**363**	Caffeic acid methyl ester	MeOH	Leaves	Yao et al. (2015b)
**364**	Trans-coumaric acid methyl ester	MeOH	Leaves	Yao et al. (2015b)
**365**	Ferulic acid	MeOH	Leaves	Yao et al. (2015b)
**366**	Cinnamic acid	MeOH	Leaves	Yao et al. (2015b)
		EtOH	Pericarps	Zhou et al. (2015d)
**367**	Trans-3-hydroxy-4-methoxy-cinnamic acid	EtOH	Green walnut husks	Zhou et al. (2018b)
**368**	4-(1-Hydroxy-1-methylethyl)-benzoic acid	EtOH	Green walnut husks	Zhou et al. (2018b)
**369**	(-)-Dihydrode-hydrodiconiferyl alcohol	EtOH	Pericarps	Zhou et al. (2015c)
Steroids				
**370**	Daucosterol	EtOH	Pericarps	Zhou et al. (2015d)
		MeOH	Green walnut husks	Chen et al. (2015)
**371**	Daucosterin	EtOH	Green walnut husks	Zhang et al. (2009)
**372**	24(R)-5α-stigmasterol	EtOH	Green walnut husks	Zhou et al. (2020)
**373**	β-sitosterol	EtOH	Green walnut husks	Chen et al. (2015)
		EtOH	Pericarps	Zhou et al. (2014a)
**374**	Stigmast-5-en-3β,7α-diol	EtOH	Green walnut husks	Chen et al. (2015)
**375**	Stigmast-5-en-3β,7β-diol	EtOH	Green walnut husks	Chen et al. (2015)
**376**	Stigmast-5-en-3β-ol	EtOH	Pericarps	Zhou et al. (2015c)
**377**	Stigmast-4-en-3-one	EtOH	Pericarps	Zhou et al. (2015c)
**378**	24(R)-5α-stigmastane-3,6-dione	EtOH	Pericarps	Zhou et al. (2015c)
**379**	Ligstroside	EtOH	Roots	Zhao et al. (2019)
**380**	Oleuropein	EtOH	Roots	Zhao et al. (2019)
Alkaloids				
**381**	N-methylflindersine	EtOH	Barks	Lou et al. (2019b)
**382**	Orixalone D	EtOH	Barks	Lou et al. (2019b)
**383**	Flindersine	EtOH	Barks	Lou et al. (2019b)
**384**	Dectamine	EtOH	Barks	Lou et al. (2019b)

(Continued in next column)

(Continued on following page)

TABLE 1 | (*Continued*) Chemical constituents isolated and structurally identified from *J. mandshurica*.

**385**	4-methoxy-N-methyl-2-quinolone	EtOH	Barks	Lou et al. (2019b)
**386**	Juglanaloid A	EtOH	Barks	Cheng et al. (2018a)
**387**	Juglanaloid B	EtOH	Barks	Cheng et al. (2018a)
Other compounds				
**388**	Galleon	EtOH	Green peel	Li et al. (2017a)
		EtOH	Pericarps	Zhou et al. (2010)
**389**	Hexyl-1-O-α-D-arabinofuranosyl-(1→6)-β-D-glucopyranoside	EtOH	Green husks	Zhou et al. (2017)
**390**	(4S,5S,7R,8R,14R)-8,11-dihydroxy-2,4-cyclo-eudesmane	EtOH	Pericarps	Zhou et al. (2014b)
**391**	Siaresinolic acid	EtOH	Green walnut husks	Zhang et al. (2009)
**392**	Dihydrophaseic acid	EtOH	Green walnut husks	Zhang et al. (2009)
**393**	Epi-dihydrophaseic acid	EtOH	Green walnut husks	Qiu et al. (2017)
**394**	Nodulisporone	EtOH	Green walnut husks	Qiu et al. (2017)
**395**	1-Ethyl malate	EtOH	Green walnut husks	Qiu et al. (2017)
**396**	1-Buthyl malate	EtOH	Green walnut husks	Qiu et al. (2017)
**397**	Succinic acid	EtOH	Green walnut husks	Qiu et al. (2017)
**398**	Ethyl-O-β-D-glucopyranoside	EtOH	Green walnut husks	Qiu et al. (2017)
**399**	3β,20-dihydroxy-5β-pregnant	EtOH	Green walnut husks	Zhou et al. (2020)
**400**	Octadecane	EtOH	Green husks	Chen et al. (2015)
**401**	2-Hydroxy-tetracosanoic acid-(2,3-dihydroxy-1- hydroxymethyl-heptadec-7-enyl)-amide	EtOH	Green husks	Chen et al. (2015)
**402**	Coniferylalcohol-9-O-β-D-glucopyranoside	EtOH	Pericarps	Zhou et al. (2015c)
**403**	Phenylethyl acid	EtOH	Pericarps	Zhou et al. (2015d)
**404**	(S)-(8E,10E)-12-hydroxy-7-oxo-8,10-octadecadienoic acid	MeOH	Stem barks	Yao et al. (2015a)
**405**	(S)-(8E,10E)-12-hydroxy-7-oxo-8,10-octadecadienoic acid methyl ester	MeOH	Stem barks	Yao et al. (2015a)
**406**	Methyl (7E,9E)-6,11-dioxononadeca-7,9-dienoate	EtOH	Stem barks	Lin et al. (2014)
**407**	Di-(2-ethylexyl)-phthalate	EtOH	Green walnut husks	Zhou et al. (2018b)

famous Chinese ancient classical book "*Compendium of Materia Medica*" (Simplified Chinese: 本草纲目) compiled by pharmacologist Shizhen Li (1518–1593 CE) in the Ming Dynasty (Zhang et al., 2018). According to another TCM monograph of "*Kaibao Bencao*" (Simplified Chinese: 开宝本草) in the Song Dynasty, BQLY has the functions of nourishing lungs and relieving asthma. Moreover, the decoction of kernels, barks, roots, and immature pericarps of *J. mandshurica* has been used as folk remedy for treating cancer, which was consistent with their heat clearing and detoxification effects (Lee et al., 2002; Li et al., 2003; Park et al., 2012; Yao et al., 2012; Xu et al., 2013; Gao et al., 2016; Wang et al., 2017a; Zhang et al., 2019). Interestingly, *J. mandshurica* is traditionally decocted together with chicken eggs to effectively prevent and treat multiple tumors in Chinese folk medicine (Wang et al., 2017a; Wang et al., 2017c).

It is important that various parts of this plant, including the green walnut husks, green peels, roots, stems, barks, branches, leaves and immature fruits have a great medicinal value in indigenous medicine. The green peels were extensively used as folk remedy for removing heat and detoxication, relieving dysentery, and improving eyesight (Li et al., 2017a). The barks were commonly used to treat urinary stones, lichen planus circumscriptus, chronic bronchitis, blurred vision, shigellosis, and HIV (Xin et al., 2014; Yao et al., 2017). Its fresh rejuvenated fruit has been used traditionally as a medicine for treatment of cancer and dermatosis, and as an anodyne to relieve aches in China (Liu et al., 2004a). The nuts are extensively used as food because of its considerable nutritional value (Wang et al., 2017b; Mu et al., 2017). In Japan, several parts of this plant have been used in folk medicines and the fruits have been commonly used for the treatment of chilblains and athlete's foot (Machida et al., 2005).

PHYTOCHEMICAL CONSTITUENTS

Currently, more than 400 comounds including quinones, phenolics, triterpenoids, diarylheptanoids, flavonoids, coumarins, lignans, phenylpropanoids, and steroids, *etc*. have been isolated and identified from different organs of *J. mandshurica* Among them, quinones, phenolics, triterpenoids, and diarylheptanoids are the most important and abundant bioactive constituents, which have been considered as the promising ingredients for future evaluation. Many ingredients with significant biological activities such as juglone, juglanthraquinone C, juglonol A, juglanin B, and juglansoside C might be used as markers for quantitative validatio and quality control of the plant in the future. The chemical compounds isolated and identified from *J. mandshurica* are summarized in **Table 1**, and structures of major bioactive compounds are presented in **Figure 2**.

Quinones

Until now, approximately **125** quinones and their derivatives have been identified from the different plant organs of *J. mandshurica*. Quinones found in this plant can be

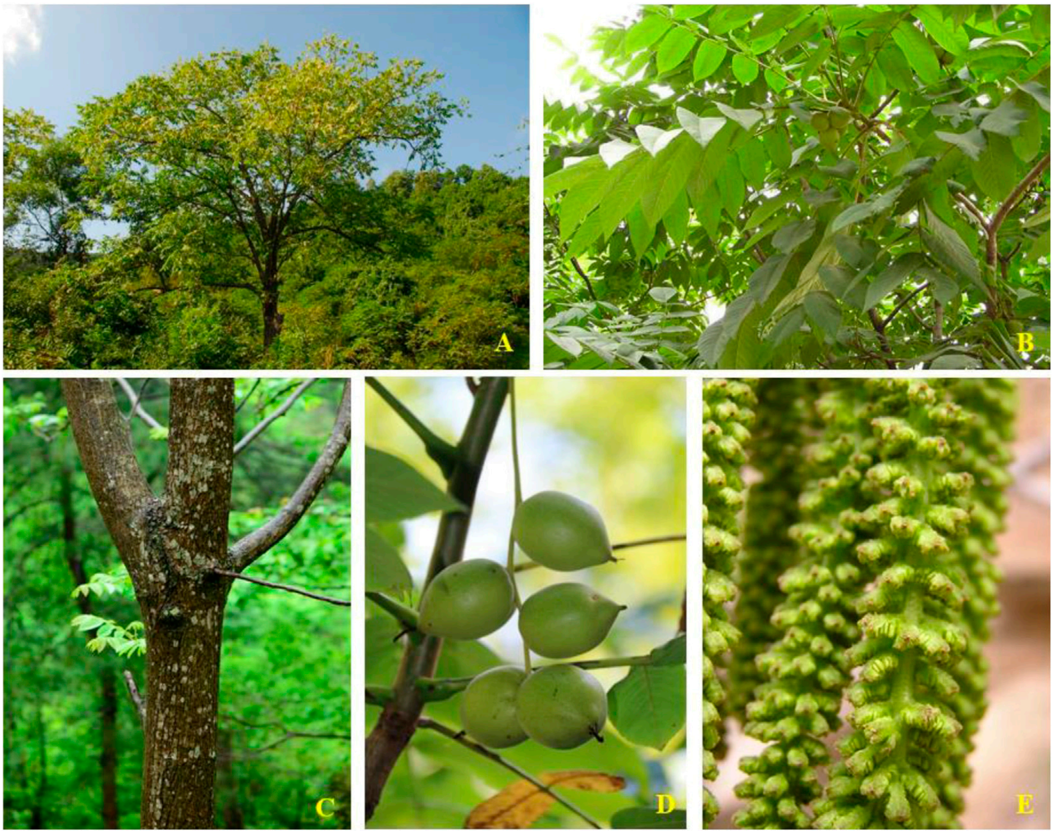

FIGURE 1 | *J. mandshurica* Maxim: **(A)** Whole plant; **(B)** Leaves; **(C)** Stembark; **(D)** Fruits; **(E)** Flowers.

structurally divided into naphthoquinones (**1–29**), anthraquinones (**30–40**), naphthalenones (**41–54**), tetralones (**55–123**), and benzoquinones (**124–125**) based on the structural characteristics. In recent years, the study on the bioactivity of naphthoquinone compounds obtained from *J. mandshurica* has become a hotspot, which was recognized as major active components for the anticancer activity (Zhang et al., 2019). However, few *in vivo* pharmacological activity evaluation and even clinical trials of these ingredients were still reported recently.

Phenolics

Nowadays, a total of **69** phenolics constituents (**126–194**) have been isolated and structurally characterized from the different parts of *J. mandshurica*. Nevertheless, only few bioactive phenolic compounds of this plant have been reported in recent years. To fully utilize the phenolics constituents of *J. mandshurica* in the development and application of cosmetic, functional foods and pharmaceutical products, more in-depth research on chemical ingredients and bioactivities are urgently needed.

Triterpenoids

To date, approximately forty-one triterpenoids (**195–235**) have been isolated and identified from the different parts of *J. mandshurica*. Among of them, dammarane-type triterpenoids isolated and identified from different medicinal parts of *J. mandshurica*, have captured more and more attention around the world due to their potent pharmacological activities, especially in antitumor properties (Salehi et al., 2019).

Diarylheptanoids

Diarylheptanoids own multiple pharmacological activities, raising ncreasingly attention over the last few decades (Sun et al., 2020). Currently, a total of 40 diarylheptanoids (**236–275**) were identified from the different parts of *J. mandshurica*. Among of them, compound **237–239**, showed outstanding cytotoxicity against the A549 and HeLa cells (Wang et al., 2019a).

Flavonoids

Flavonoids are widespread in the plant kingdom in free form or as glycosides, and many of them are natural drugs with various medical functions (Luan et al., 2019). Up to date, a total of **39** flavonoids (**276–314**) have been obtained and purified from the green peel, epicarp, stem barks, roots, green walnut husks, and pericarps of *J. mandshurica*. Amongst the isolated compounds, taxifolin (**297**) exhibited the strongest anti-HIV-1 activity against MT-4 cells (Min et al., 2002). However, pharmacological investigations on other flavonoids from *J. mandshurica* are

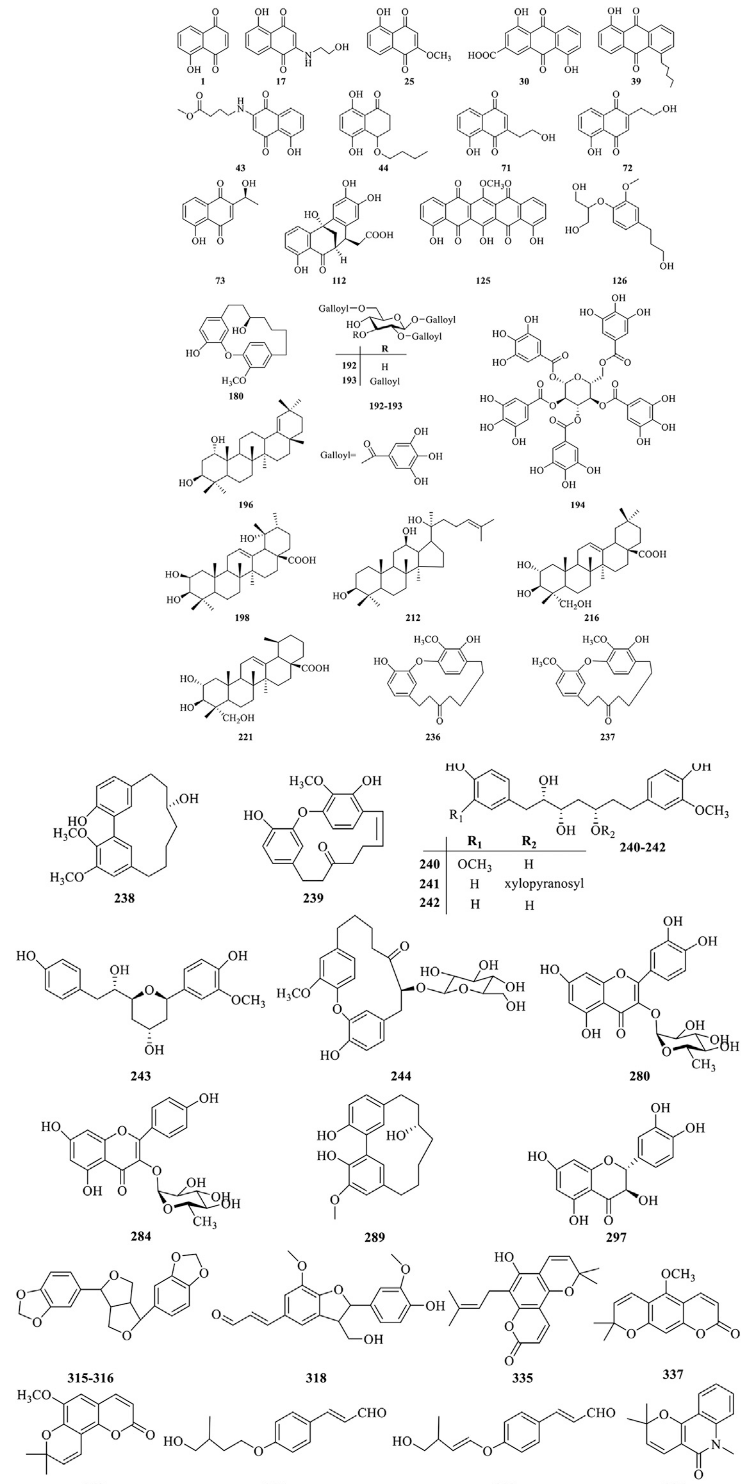

FIGURE 2 | Chemical structures of the major bioactive compounds from *J. mandshurica*.

TABLE 2 | The pharmacological activities of bioactive compounds and extracts of *J. mandshurica* ("↓", decrease; "↑", increase).

Biological activities	Tested substance	Types	Testing Subjects	Doses/duration of treatment	Mechanisms/effects	References
Antitumor activity						
	Juglone **(1)**	*In vitro*	Human hepatocellular carcinoma HepG2 cells	10, 20, and 30 μM for 24 h	Bcl-2 protein level ↓; cleaved-PARP, cleaved-caspase 3, LC3-II, and Beclin-1 proteins levels ↑	Wang et al. (2018a)
	Juglone **(1)**	*In vitro*	Human gastric cancer BGC-823, colon cancer HCT-15, and leukemia K562 cells	0.04, 0.2, 1.0, 5, 25, and 125 μM for 48 h	IC_{50} = 9.6, 27.8, and 35.5 μM, respectively	Zhou et al. (2019b)
	Juglone **(1)**	*In vitro*	Human cervical carcinoma HeLa cells	12.5, 25, 50, and 100 μmol/L for 24 h	IC_{50} = 33 μM, Bcl-2 expression ↓; Bax, caspase-3/-8/-9, and PARP expressions ↑	Zhang et al. (2012a)
	Juglone **(1)**	*In vitro*	Leukemia HL-60 cells	0, 0.5, 1.0, and 1.5 μg/ml for 48 h	Caspase-3, caspase-9, PARP, Smac, AIF, cytochrome c, and Bax/Bcl-2 expressions ↑	Xu et al. (2010)
	Juglone **(1)**	*In vitro*	Colon cancer CCL-228-SW 480 cells	20 μM for 24 h	Cleavage-caspase-3 expression↑; AIF activity↑	Bayram et al. (2019)
	Juglone **(1)**	*In vitro*	Human breast cancer MDA-MB231, HepG2, and gastric cancer SNU638 cells	0–100 μM for 24 h	IC_{50} = 4.46, 9.16, and 56.38 μM, respectively	Jin et al. (2016)
	Juglone **(1)**	*In vitro*	Human gastric cancer MGC-803, lung cancer A549, leukemia K562, and cervical cancer HeLa cells	0–100 μM for 24 h	IC_{50} = 25.90, 28.60, 39.06, and 44.90 μM, respectively	Yao et al. (2015b)
	Juglone **(1)**	*In vitro*	Prostate cancer LNCaP cells	5, 10, and 15 μM for 24 h	Caspase-3/9 ↑; androgen receptor (AR) and prostate-specific antigen (PSA) expressions ↓	Xu et al. (2013)
	Juglone **(1)**	*In vitro*	Cervical cancer Hela cells	10, 20, and 40 μM for 24 h	Bax, CytC, Fas, FasL, Caspase-3, p-JNK and p-c-Jun expressions ↑	Lu et al. (2017)
	Juglone **(1)**	*In vitro*	Pancreatic cancer BxPC-3 and PANC-1 cells	5, 10, 15, 20, 30, 40 and 50 μM for 24 h	IC_{50} = 21.05 μM and 21.25 μM, severally. Adhesion and invasion and MMP-2, MMP-9 and Phactr-1 expressions ↓	Avcı et al. (2016)
	5-Hydroxy-2-(2-hydroxy-ethylamino)-1,4-naphthoquinone **(17)**	*In vitro*	MDA-MB231, HepG2, and SNU638 cells	0–100 μM for 24 h	IC_{50} = 28.23, 12.17, and 51.71 μM, respectively	Jin et al. (2016)
	5-Hydroxy-2-methoxy-1,4-naphthoquinone **(25)**	*In vitro*	MGC-803, K562, cervical cancer SiHa, HeLa, A549, CaSKi and placental choriocarcinoma JAR cells	NM	IC_{50} = 2.0, 2.3, 2.7, 4.0, 5.3, 6.6, and 6.9 μM, severally	Yao et al. (2014)
	Juglanthraquinone C **(30)**	*In vitro*	HepG2 and BEL-7402 cells	1.25–20 μg/ml for 48 h	IC_{50} = 10.5 μg/ml. Akt and Foxo3a expressions ↑ and ROS level ↑	Hou et al. (2016)
	Juglanthraquinone C **(30)**	*In vitro*	HepG2 cells	2.5–10 μg/ml for 48 h	IC_{50} = 9.0 μg/ml. Ki67, cyclin A, CDK proteins expressions ↓; cyclin E, Cip1/p21, caspase-3/9 proteins expressions ↑; Bax/Bcl2 ratio ↑	Yao et al. (2012)
	1-Hydroxy-5-pentyl-anthraquinone **(39)**	*In vitro*	MDA-MB231, HepG2, and SNU638 cells	0–100 μM for 24 h	IC_{50} = 78.18, 64.01, and 88.47 μM, respectively	Jin et al. (2016)
	5-Hydroxy-1,4-dioxo-1,4-dihydronaphthalen-2-ylamino)-butyric acid methyl ester **(43)**	*In vitro*	MDA-MB231, HepG2, and SNU638 cells	0–100 μM for 24 h	IC_{50} = 21.15, 9.34, and 54.86 μM, severally	Jin et al. (2016)
	Juglanstetralone A **(44)**	*In vitro*	BGC-823 cells	104.81, 112.18, 121.18, 130.3, 140.11, 150.66, 162 and 174.19 μg/ml	IC_{50} = 125.89 μg/ml	Guo et al. (2015)

(Continued on following page)

TABLE 2 | (*Continued*) The pharmacological activities of bioactive compounds and extracts of *J. mandshurica* ("↓", decrease; "↑", increase).

Juglonol A **(71)**	*In vitro*	Human lung cancer NCI-H1975, HCC827, HepG2, breast cancer MD-AMB-231, leukemia HL-60, colon cancer CT26, and glioma C6	NM	IC_{50} in ranges of 9.5–31.6 μg/ml	Yang et al. (2019)
Juglonol C **(73)**	*In vitro*	NCI-H1975, HCC827, HepG2, MD-AMB-231, HL-60, CT26, and C6	NM	IC_{50} in ranges of 6.4–19.5 μg/ml	Yang et al. (2019)
p-hydroxy-methoxybenzobijuglone **(125)**	*In vitro*	BGC823 cells	0–25 μM for 24 h, 48 h, 72 h	IC_{50} = 10.6, 8.2, and 7.5 μM, respectively	Li et al. (2009)
p-hydroxy-methoxybenzobijuglone **(125)**	*In vitro*	HeLa cells	0–30 μM for 24 h, 48 h, 72 h	IC_{50} = 15.9, 12.2, and 10.7 μM, respectively	Li et al. (2007a)
10-Hydrogenmyricananadiol **(180)**	*In vitro*	NCI-H460 and K562 cells	1, 3, 10, 30, and 100 μmol/L	IC_{50} = 48.06 and 43.94 μmol/L, respectively	Li et al. (2017a)
1α,3β-dihydroxy-olean-18-ene **(196)**	*In vitro*	HepG-2 cells	0.5–200 μM for 48 h	IC_{50} = 18.22 μM	Zhou et al. (2019a)
2α,3α,19α-trihydroxyurs-12-en-28-oic acid **(198)**	*In vitro*	HepG-2 cells	0.5–200 μM for 48 h	IC_{50} = 17.32 μM	Zhou et al. (2019a)
20(S)-protopanaxadiol **(212)**	*In vitro*	HepG-2 cells	0.5–300 μM for 24 h	IC_{50} = 10.32 μM	Zhou et al. (2015a)
2α,3β,23-trihydroxy-12-en-28-oleanolic acid **(216)**	*In vitro*	HepG-2 cells	0.5–300 μM for 24 h	IC_{50} = 16.13 μM	Zhou et al. (2015a)
2α,3β,23-trihydroxyurs-12-en-28-oic acid **(221)**	*In vitro*	HepG-2 cells	0.5–300 μM for 24 h	IC_{50} = 15.97 μM	Zhou et al. (2015a)
2-Oxatrycyclo-[13.2.2.13,7]-eicosa-3,5,7-(20),15,17,18-hexaen-10-one **(236)**	*In vitro*	Human lung cancer A549 and cervical cancer HeLa cells	0.01, 0.1, 1, 10, and 100 μM	GI_{50} = 1.6 and 2.1 μM, respectively	Wang et al. (2019a)
Juglanin A **(237)**	*In vitro*	Human lung cancer A549 and cervical cancer HeLa cells	0.01, 0.1, 1, 10, and 100 μM	GI_{50} = 5.8 and 3.3 μM, respectively	Wang et al. (2019a)
2-Oxatrycyclo-[13.2.2.13,7]-eicosa-3,5,7(20),15,17, 18-hexaen-10–16-diol **(238)**	*In vitro*	Human lung cancer A549 and cervical cancer HeLa cells	0.01, 0.1, 1, 10, and 100 μM	GI_{50} = 2.4 and 1.9 μM, respectively	Wang et al. (2019a)
(11S)-11,17-dihydroxy-3,4-dimethoxy-[7,0]-metacyclophane **(239)**	*In vitro*	Human lung cancer A549 and cervical cancer HeLa cells	0.01, 0.1, 1, 10, and 100 μM	GI_{50} = 1.3 and 2.7 μM, respectively	Wang et al. (2019a)
Juglanin B **(289)**	*In vitro*	Human breast cancer SKBR3, BT474, MCF-7, MDA-MB-231 cells	0–40 μM for 24 and 48 h	IC_{50} = 20.07, 24.17, 26.35, 29.13 μM for 24 h, and 17.69, 19.85, 14.38, 23.25 μM for 48 h, respectively	Sun et al. (2017)
Juglanin B **(289)**	*In vitro*	SKBR3, BT474, MCF-7, MDA-MB-231 cells	2.5, 5.0 and 10 μM	Chk2, Cdc25C, Cdc2, Chk2, p27, cyclin D, Bad, Bax, cleaved caspase-3/-8/-9, and LC3B-II expressions↑; Cdc25C, Cdc2, Bcl-2 expressions ↓	Sun et al. (2017)
Juglanin B **(289)**	*In vivo*	Human breast cancer MCF-7 tumor-bearing mice	5 and 10 mg/kg for 7 days	Tumor volume↓; Cleaved caspase-3/-9, LC3BI, LC3BII and phosphorylated JNK expressions ↑;	Sun et al. (2017)
Balanophonin **(318)**	*In vitro*	Hep3B, A549, MCF-7, HepG2, and breast cancer Bcap-37 cells	6.25, 12.5, 25, 50, and 100 μM for 48 h	IC_{50} = 14.02, 23.42, 25.41, 40.68, and 66.07 μM, respectively	Zhang et al. (2018)
Juglansoside C **(335)**	*In vitro*	Hep3B cells	Log [1.0, 1.5, and 2.0] μM	IC_{50} = 70.9 μM	Lou et al. (2019a)
Xanthyoxylin **(337)**	*In vitro*	HepG2 cells	6.25, 12.5, 25, 50, and 100 μM for 48 h	IC_{50} = 62.30 μM. Cleaved-caspase 7 protein level ↑; PARP and pro-caspase 7 proteins levels ↓	Yao et al. (2017)
6,7,8-Trimethoxyl-coumarin **(340)**	*In vitro*	Hep3B cells	6.25, 12.5, 25, 50, and 100 μM for 48 h	IC_{50} = 76.12 μM. Cleaved-caspase 7 expression↑; PARP and pro-caspase 7 expressions ↓	Yao et al. (2017)

(Continued on following page)

TABLE 2 | (*Continued*) The pharmacological activities of bioactive compounds and extracts of *J. mandshurica* ("↓", decrease; "↑", increase).

	(2E)-3-[4-(4-hydroxy-3-methylbutoxy)-phenyl]-2-propenal **(357)**	*In vitro*	HepG2 and Hep3B cells	100 μM	IC_{50} = 58.58 and 69.87 μM, respectively	Cheng et al. (2017)
	Boninenal **(358)**	*In vitro*	HepG2 and Hep3B cells	100 μM	IC_{50} = 63.70 and 46.45 μM, respectively	Cheng et al. (2017)
	N-methylflindersine **(381)**	*In vitro*	Hep3B and HepG2 cells	100 μM	IC_{50} = 61.80 and 56.24 μM, respectively	Lou et al. (2019b)
	JME	*In vitro*	HeLa cells	25–1,000 μg/ml for 24 and 48 h	IC_{50} = 413.50 μg/ml for 24 h and 391.30 μg/ml for 48 h, respectively	Xin et al. (2014)
	JMM6	*In vitro*	BEL-7402 cells	30, 60 and 120 μg/ml	IC_{50} = 83.0 μg/ml	Zhang et al. (2013)
	JRP1	*In vitro*	S180 cells	25, 50 and 100 g/ml for 48 h	Cell growth ↓	Wang et al. (2015)
	JRP1	*In vivo*	S180 tumor-bearing mice	25, 50, and 100 mg/kg, i.p., for 21 days	Tumor growth ↓; IL-2, TNF-α and IFN-γ levels ↓; inhibition rates = 35.3%, 40.6% and 48.1%, severally	Wang et al. (2015)
	JMCE	*In vivo*	S180 tumor-bearing mice	100, 200, and 500 mg/kg, i.g., for 8 days	Tumor growth ↓; SOD activity↑; MDA content ↓; inhibition rates = 48.37%, 40.81%, and 36.52%, severally	Yao et al. (2009)
	EDJB	*In vivo*	H22 tumor-bearing mouse	0.64, 1.28, and 2.56 g/kg/d, i.p., 10 days	Tumor growth ↓; thymus index and spleen index↑; peripheral red blood cells and hemoglobin numbers ↑; white blood cells numbers ↓	Wang et al. (2017c)
	TT	*In vivo*	H22 tumor-bearing mouse	0.09 and 0.18 g/kg/d, i.p., for 10 days	Tumor growth ↓; inhibition rates = 34.22% and 36.92%, severally	Wang et al. (2017d)
	JA	*In vitro*	HepG2, MDA-MB-231, SGC-7901, A549 and Huh7 cells	0–80 μM for 48 h	IC_{50} = 24.94, 26.92, 36.27, 37.59, and 38.25 μM, respectively	Gao et al. (2016)
	JA	*In vitro*	HepG2 cells	23 μM	Caspase-3, PARP-1, cleaved-caspase-9, Apaf-1, HtrA2/Omi, Bax, XBP-1s, GRP78, cleaved Caspase-7, cleaved-caspase-12, and p21 expressions ↑; CyclinB1 and phosphorylated- CDK1 expressions ↓	Gao et al. (2016)
Anti-inflammatory activity						
	Juglone **(1)**	*In vitro*	Primary astrocytes induced by LPS	5, 10, 15, and 20 μM	TNF-α, IL-1β and IL-6 levels ↓; TLR4, MyD88, TAK1, p-IκBα, NF-κB, and p-NF-κB levels ↓	Peng et al. (2015)
	Juglone **(1)**	*In vivo*	High-fat diet-induced neuroinflammation in rats	0.25 and 1.0 mg/kg, i.g., for 70 days	TNF-α, IL-1β and IL-6 levels ↓; TLR4, MyD88, TAK1, p-IκBα, NF-κB, and p-NF-κB levels ↓	Peng et al. (2015)
	1,2,3,4,6-penta-O-galloyl-β-D-glucose **(194)**	*In vitro*	HaCaT cells	1.0, 5.0, and 10 μM	CCL17, CXCL-9, CXCL-10, and CXCL-11 expressions ↓; NF-κB and STAT1 ↓	Ju et al. (2009)
	(2S,3S,5S)-2,3,5-trihydroxy-1,7-bis-(4-hydroxy-3-methoxyphenyl)-heptane **(240)**, Rhoiptelol C **(242)**	*In vitro*	LPS-stimulated RAW264.7 cells	10, 30, and 100 μM	NO, TNF-α and IL-6 generation ↓	Diao et al. (2019)
	(2S,3S,5S)-2,3-dihydroxy-5-O-β-D-xylopyranosyl-7-(4-hydroxy-3-methoxyphenyl)-1-(4-hydroxyphenyl)-heptane **(241)**	*In vitro*	LPS-stimulated RAW264.7 cells	3, 10, 30 and 100 μM	NO and TNF-α generation ↓	Diao et al. (2019)

(Continued on following page)

TABLE 2 | (*Continued*) The pharmacological activities of bioactive compounds and extracts of *J. mandshurica* ("↓", decrease; "↑", increase).

	Rhoiptelol B **(243)**, 3′,4″-epoxy-2-O-β-D-glucopyanosyl-1-hydroxyphenyl)-7-(3-methoxyphenyl)-heptan-3-one **(244)**	*In vitro*	LPS-stimulated RAW264.7 cells	3, 10, 30 and 100 μM	NO, TNF-α and IL-6 generation ↓	Diao et al. (2019)
	Juglanin B **(289)**	*In vivo*	LPS-induced acute lung injury in mice	10 and 20 mg/kg, i.g., for 21 days	α-SMA, collagen type I, collagen type III, and TGF-β1 mRNA and protein expressions↓; IL-4, IL-6, IL-17, IL-18, TNF-α and IL-1β levels↓	Dong and Yuan (2018)
	JMLE	*In vivo*	DNCB-induced allergic dermatitis-like skin lesions of mice	0.5% JMLE	Skin severity and scratching scores↓; TNF-α, IgE, IL-1, and IL-13 levels ↓	Park and Oh (2014)
Neuroprotective activity						
	HP	*In vitro*	H_2O_2-induced PC12 cells	1.0, 1,5, 2.0, 2.5 mg/ml for 24 h	ROS ↓; GSH-Px activity ↑	Ren et al. (2018)
	HP	*in vivo*	Scopolamine-induced memory impairment in mice	200, 400, and 800 mg/kg, i.g., for 30 days	ACh, ChAT, AChE, 5-HT, DA, and NE contents ↑; SOD and GSH-Px activities↑; p-CaMK II expression ↑	Ren et al. (2018)
	EVSGPGLSPN	*In vitro*	H_2O_2-induced PC12 cells	12.5, 25, 50, and 100 μM	ROS ↓; CAT, GSH-px, SOD activities ↑; IKKβ, NF-κB p65, IL-1β, TNF-α, cytochrome C, caspase-3/9, and PARP expressions↓; p-CREB and synaptophysin expressions ↑	Liu et al. (2019)
	TWLPLPRYVLLPSPK, and KVPPLLY	*In vitro*	$A\beta_{25-35}$-induced PC12 cells	50 μM for 24 h	ROS ↓; GSH-Px activity and ATP contents↑; Beclin-1, LC3-I, LC3-II, and p-Akt/Akt expressions ↑; p62 and p-mTOR/mTOR expressions ↓	Zhao et al. (2020)
	WLPLPR, YVLLPSPK, and KVPPLLY	*In vitro*	$A\beta_{25-35}$-induced PC12 cells	100 μM for 24 h	LAMP1, LAMP2, and Cathepsin D expressions ↑	Zhao et al. (2020)
Anti-diabetic activity						
	JMEE	*In vitro*	α-glucosidase and α-amylase inhibitory activity	0.025 mg/ml	IC_{50} = 0.014 mg/ml for α-glucosidase and IC_{50} = 0.13 mg/ml for α-amylase	Wang et al. (2019c)
	LPLLR	*In vitro*	Insulin resistant (IR) hepatic HepG2 cells	100, 500, 1,000, 1,500, and 2000 μM	Inhibited the α-glucosidase (50.12%) and α-amylase (39.08%) at 2000 μM	Wang et al. (2020a)
	LPLLR	*In vitro*	Insulin resistant (IR) hepatic HepG2 cells	100 and 200 μM	IRS-1, PI3K, Akt, AMPK, GSK3β levels ↑; GS, GLUT4 ↑; G-6-Pase, PEPCK ↓	Wang et al. (2020a)
	LVRL, LRYL, VLLALVLLR	*In vitro*	High glucose-induced IR HepG2 cells model	12.5, 25, 50, and 100 μM for 24 h	IRS-1, PI3K, Akt, GSH-Px, CAT, SOD, Nrf2, HO-1 ↑; ROS, ERK, JNK, p38 ↓	Wang et al. (2020b)
Immunoregulatory activity						
	PH	*in vivo*	On the immune system of mice	200, 400, and 800 mg/kg, i.g., for 35 days	Thymus and spleen indexes, lymphocyte proliferation, macrophage activity ↑; $CD4^+$ and $CD8^+$ T cells numbers, IgA and sIgA levels ↑; IFN-α and IL-6 expressions ↑	Li et al. (2018)
	HP	*in vivo*	Mice stimulated by exhaustion swimming experiment	800 mg/kg, i.g., for 28 days	Spleen and thymus indexes ↑; T-lymphocyte proliferation and sIgA generation ↑	Fang et al. (2018)
Antiviral activity						
	1,2,6-Trigalloylglucose **(192)**	*In vitro*	Reverse transcriptase (RT) activity	NM	IC_{50} = 0.067 μM	Min et al. (2000)

(Continued on following page)

TABLE 2 | (*Continued*) The pharmacological activities of bioactive compounds and extracts of *J. mandshurica* ("↓", decrease; "↑", increase).

	1,2,3,6-Tetragalloylglucose **(193)**	*In vitro*	Reverse transcriptase (RT) and ribonuclease H inhibitory activities	NM	IC_{50} = 0.04 μM for RT and IC_{50} = 39.0 μM for ribonuclease H	Min et al. (2000)
	Taxifolin **(297)**	*In vitro*	HIV-1 virus MT-4 cells	NM	IC_{100} = 25 μg/ml and CC_{100} > 100 μg/ml	Min et al. (2002)
Anti-melanogenesis activity						
	2-[4-(3-hydroxypropyl)-2-methoxyphenoxy]-1,3-propanediol **(126)**	*In vitro*	B16F10 melanoma cells	0.5 and 1.0 μM for 48 h	Melanin content ↓; p-ERK protein expression ↑; MITF and tyrosinase protein expressions ↓	Kim et al. (2019)
Antimicrobial activity						
	Juglonol A **(71)**	*In vitro*	*S. aureus, E. faeculis, K. pneumonia, C. albicans, F. oxysporum, F. oxysporium, C. lagenarium, and P. asparagi*	NM	MIC values ranging 8–64 μg/ml, IC_{50} was 9.5–31.6 μg/ml to 7 cell lines	Yang et al. (2019)
	Juglonol B **(72)**	*In vitro*	*S. aureus*	NM	MIC = 8 μg/ml	Yang et al. (2019)
Hepatoprotective activity						
	Juglone **(1)**	*in vivo*	High-fat diet-induced liver injury of rats	0.25 and 1.0 mg/kg, i.g., for 70 days	AST, ALT, TG, TC, HDL and MDA levels ↓; SOD and LDL activities ↑	Peng et al. (2015)
Other activities						
	1,2,6-Trigalloylglucose **(192)**	*In vitro*	Complement system	50, 100, 200, and 400 μM for 0.5 h	IC_{50} = 136 μM	Min et al. (2003)
	1,2,3,6-Tetragalloylglucose **(193)**	*In vitro*	Complement system	20, 40, 80, 160, and 360 μM for 0.5 h	IC_{50} = 34 μM	Min et al. (2003)
	Apigenin **(279)**	*In vitro*	Complement system	NM	IC_{50} = 440 μM	Min et al. (2003)
	Afzelin **(284)**	*In vitro*	Complement system	NM	IC_{50} = 258 μM	Min et al. (2003)
	(+)-Sesamin **(315)**	*In vitro*	$A\beta_{1-42}$ aggregation inhibition activity by ThT assay	20 μM	Exhibited significant inhibition of $A\beta_{1-42}$ aggregation with the inhibition rate of 80.6%	Wang et al. (2019b)
	(-)-Sesamin **(316)**	*In vitro*	$A\beta_{1-42}$ aggregation inhibition activity by ThT assay	20 μM	Exhibited inhibition of $A\beta_{1-42}$ aggregation with the inhibition rate of 67.7%	Wang et al. (2019b)
	HP	*In vivo*	Mice stimulated by exhaustion swimming	200, 400, and 800 mg/kg, i.g., for 28 days	Swimming time ↑; liver glycogen contents ↑; lactic acid contents ↓	Fang et al. (2018)

NM, not mentioned; JMLE, *J. mandshurica* leaf extract; PH, protein hydrolyzates; HP, hydrolyzed peptide; JMEE, ethanol extract of the leaves of *J. mandshurica*; LPLLR, a novel pentapeptide (Leu-Pro-Leu-Leu-Arg) from the protein hydrolysates of *J. mandshurica*; JRP1, a water-soluble polysaccharide; JME, *J. mandshurica* extracts; JMM6, fractions; JMCE, chloroform extracts of *J. mandshurica* roots; EDJB, eggs decocted with *J. mandshurica* branches; TT, total tannins; JA, A ω-9 polyunsaturated fatty acid; TWLPLPR, YVLLPSPK, and KVPPLLY, three novel peptides; EVSGPGLSPN, peptide; LVRL, LRYL, and VLLALVLLR, three novel peptides.

very limited in the existing literature, and need to urgently conduct in future study.

Lignans

Lignans with chiral carbon atoms are usually consisted of a pair of enantiomers or several pairs of stereoisomers with different amount in nature, and the biological activities of enantiomers are not identical due to the chiral nature of the biological receptors (Pereira et al., 2011). Until now, **20** lignans (**315–334**) have been structurally identified from the barks, roots, and fruits of *J. mandshurica*.

Coumarins

Coumarins refer to the general term of o-hydroxycinnamic acid lactones with the basic skeleton of benzoben-α-pyranone parent nucleus, which is one of the main components of TCM (Jiang et al., 2020). At present, **19** coumarins (**335–353**) have been isolated and characterized from the stem barks of *J. mandshurica*, and mainly include simple coumarins and pyranocoumarins.

Phenylpropanoids

Phenylpropanoids displayed various biological effects including defending against herbivores, microbial attack, or other sources of injury. Nowadays, a total of **16** phenylpropanoids (**354–369**) have been isolated and structurally identified from the barks, leaves, pericarps, and green walnut husks of *J. mandshurica*. However, studies on biological effects of phenylpropanoids from *J. mandshurica* are very limited.

Steroids

So far, phytochemical investigations from the green walnut husks, roots, and epicarp of *J. mandshurica* have shown the presence of **11** steroids **(370–380)** including daucosterol **(370)**, daucosterin **(371)**,

24(R)-5α-stigmasterol **(372)**, β-sitosterol **(373)**, stigmast-5-en-3β,7α-diol **(374)**, stigmast-5-en-3β,7β-diol **(375)**, stigmast-5-en-3β-ol **(376)**, stigmast-4-en-3-one **(377)**, 24(R)-5α-stigmastane-3,6- dione **(378)**, ligstroside **(379)**, and oleuropein **(380)**. However, few bioactive steroids have been reported recently.

Alkaloids

Alkaloids is an important secondary metabolite and represent a relatively small class of compounds from this plant and possess remarkable antitumor activity. Until now, 7 alkaloids **(381–387)** have been isolated and structurally elucidated from the barks of *J. mandshurica*. However, there are not many studies on the biological activity of these alkaloids and therefore further research need to be explored.

Other Compounds

A few other classes of compounds (**388–407**) have been isolated from *J. mandshurica*. Among them, siaresinolic acid **(391)**, dihydrophaseic acid **(392)**, epi-dihydrophaseic acid **(393)**, nodulisporone **(394)**, 1-ethyl malate **(395)**, 1-buthyl malate **(396)**, succinic acid **(397)**, ethyl-O-β-D-glucopyranoside, 3β,20-dihydroxy- 5β-pregnant **(398)** were first isolated from green walnut husks of this plant (Zhang et al., 2009; Qiu et al., 2017).

PHARMACOLOGICAL PROPERTIES

To date, *J. mandshurica* have been explored for multiple pharmacological activities, such as antitumor, immunoregulatory, anti-inflammatory, neuroprotective, antidiabetic, antiviral, antimicrobial, and anti-melanogenesis activities. Next, these biological activities were discussed one by one in the following paragraphs, and the recapitulative summary was also presented in **Table 2**. The mechanism of the typical and representative pharmacological activities like antitumor, immune immunoregulation, antioxidant and

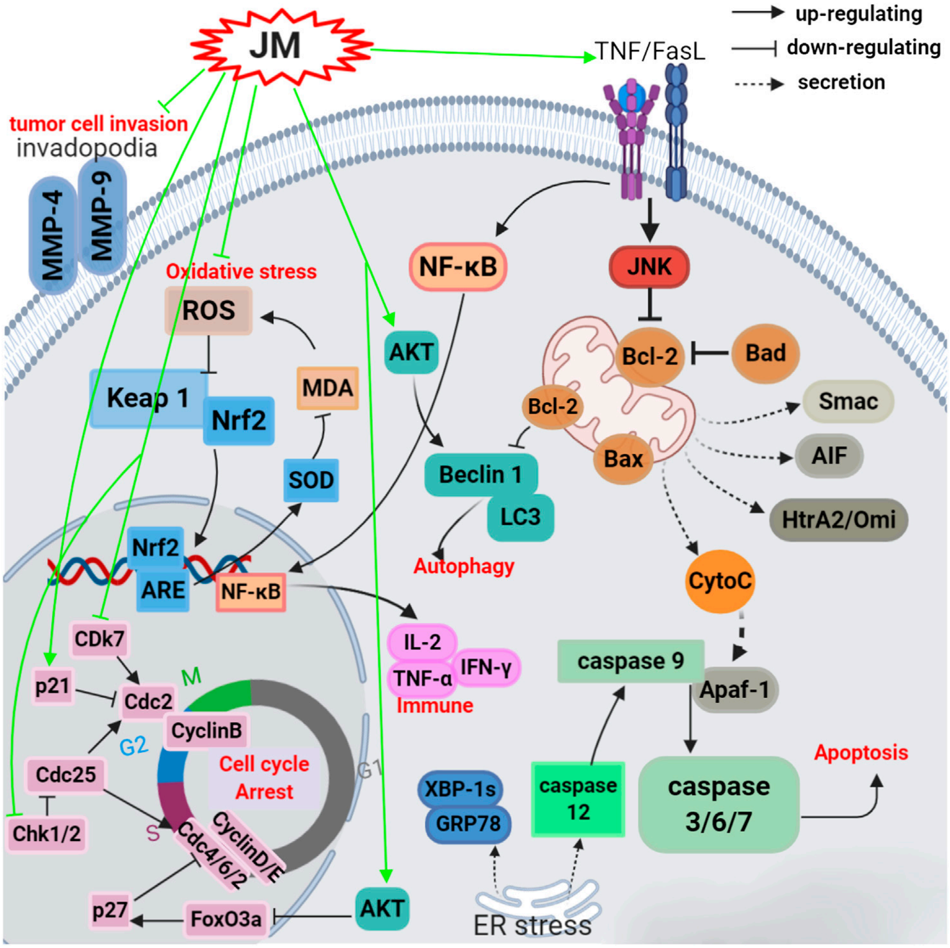

FIGURE 3 | Schematic representation of the possible mechanism of antitumor activity of *J. mandshurica*.

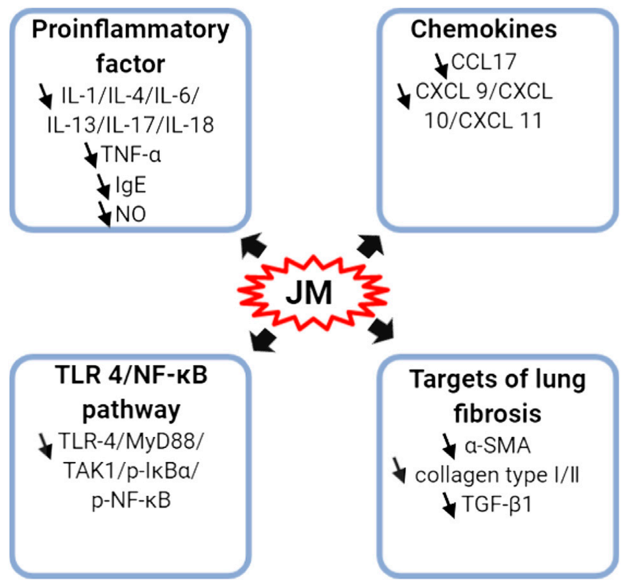

FIGURE 4 | Schematic representation of the molecular mechanism of anti-inflammatory of *J. mandshurica*.

neuroprotective activities of *J. mandshurica* are summarized and presented in the following **Figures 3–6**, respectively.

Antitumor Activity

A variety of the crude extracts, isolated compounds, and polysaccharides from *J. mandshurica* displayed significant antitumor activity both *in vitro* and *in vivo*. The underlying mechanisms of action of these components included induction of cell apoptosis and autophagy, cell cycle arrest, promotion of cell differentiation and inhibition of cell adhesion and invasion. Effects on telomerase activity and regulation of mRNA and protein expression levels of tumor-related factors were observed (see **Table 2** and **Figure 3**). In general, the antitumor activity of *J. mandshurica* has been effectively demonstrated in various human cancer cell lines, such as hepatocellular carcinoma HepG2, Hep3B, Huh7, and BEL-7402 cells (Yao et al., 2012; Zhang et al., 2013; Zhou et al., 2015a; Gao et al., 2016; Hou et al., 2016; Jin et al., 2016; Cheng et al., 2017; Yao et al., 2017; Wang et al., 2018a; Zhang et al., 2018; Lou et al., 2019a; Zhou et al., 2019a; Lou et al., 2019b), lung cancer A549, NCI-H460, and NCI-H1975 cells (Yao et al., 2014; Yao et al., 2015b; Gao et al., 2016; Jin et al., 2016; Li et al., 2017a; Zhang et al., 2018; Yang et al., 2019), breast cancer SKBR3, BT474, MCF-7, Bcap-37, and MDA-MB-231 cells (Gao et al., 2016; Jin et al., 2016; Sun et al., 2017; Zhang et al., 2018), cervical cancer Hela, SiHa, and CaSKi cells (Li et al., 2007a; Zhang et al., 2012a; Xin et al., 2014; Yao et al., 2014; Yao et al., 2015b; Lu et al., 2017; Wang et al., 2019a), gastric cancer SNU638, BGC-803, SGC-7901, and BGC-823 cells (Li et al., 2009; Yao et al., 2014; Yao et al., 2015b; Guo et al., 2015; Gao et al., 2016; Jin et al., 2016; Zhou et al., 2019b), prostate cancer LNCaP cells (Xu et al., 2013), pancreatic cancer BxPC-3 and PANC-1 cells (Avcı et al., 2016), colon cancer HCT 15 and CCL-228-SW 480 cells (Bayram et al., 2019; Zhou et al., 2019b), leukemia K562 and HL-60 cells (Xu et al., 2010; Yao et al., 2014; Yao et al., 2015b; Li et al., 2017a; Zhou et al., 2019b), placental choriocarcinoma JAR cells (Yao et al., 2014), and glioma C6 cells (Yang et al., 2019). It is worth noting that the isolated compounds 1, 17, 25, 30, 39, 43, 44, 71, 72, 73, 125, 180, 196, 198, 212, 216, 221, 236, 237, 238, 239, 289, 318, 335, 337, 340, 357, 358, and 381 displayed significant antitumor activity against on HepG2, A549, MCF-7, Hela, SiHa, MDA-MB-231, BGC-803, SGC-7901, BGC-823, LNCaP, BxPC-3, and PANC-1 *in vitro*. Besides, the antitumor activity of the compounds with mother nucleus of 1, 4-naphthoquinone substituted by hydroxy is stronger than that of methoxy substitution at the same position, and the compounds with 5-and 8-hydroxy groups have the strongest antitumor activity. The anti-tumor activity of naphthoquinone

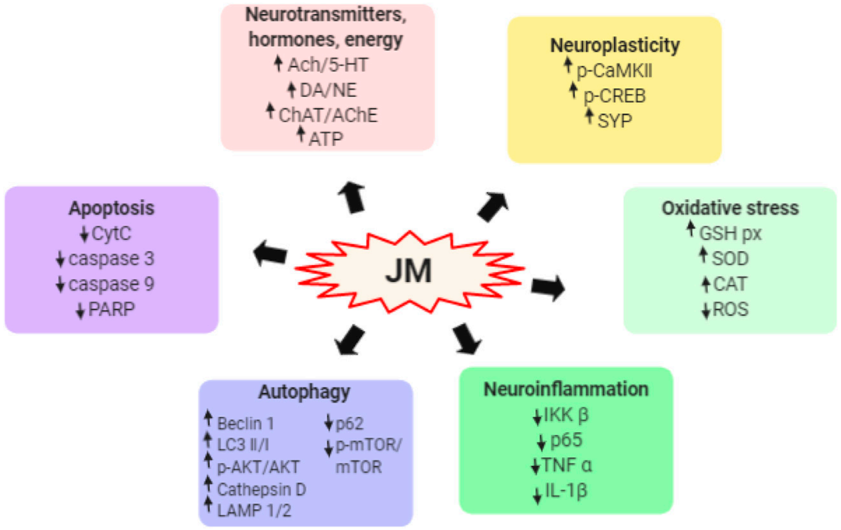

FIGURE 5 | Schematic representation the underlying mechanism of neuroprotective activity of *J. mandshurica*.

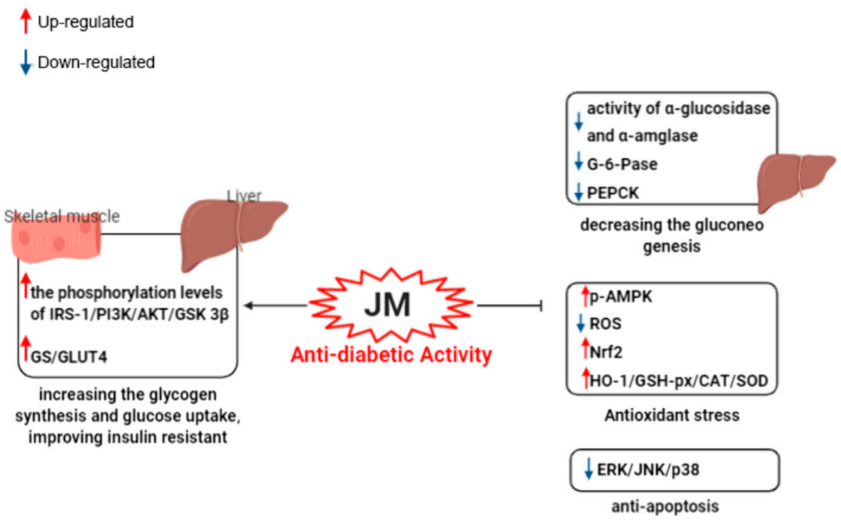

FIGURE 6 | Schematic representation of underlying the mechanism of anti-dabite activity of *J. mandshurica*.

type compounds is generally stronger than that of naphthone, naphthol and thier glycosides, and the naphthone glycosides showed the weakest antitumor activity (Zhang et al., 2019).

In vivo in mouse models, it has been demonstrated that *J. mandshurica* and its secondary products showed protective activity on MCF-7 tumor-bearing mice (Sun et al., 2017), S180 tumor-bearing mice (Yao et al., 2009; Wang et al., 2015), and H22 tumor-bearing mouse (Wang et al., 2017c; Wang et al., 2017d). A polysaccharide, namely JRP1, purified form the fruits, at doses of 25, 50 and 100 mg/kg, i.p., for 21 days, inhibited the tumor growth with inhibition rates of 35.3%, 40.6% and 48.1%, respectively, and decreased the index of spleen and thymus and increased the serum levels of immune regulatory markers such as IL-2, TNF-α and IFN-γ with a dose-dependent manner in S180 tumor-bearing mice (Wang et al., 2015). Orally administration with JMCE (at doses of 100, 200, and 500 mg/kg) to S180 tumor-bearing mice once a day for 8 days significantly elevated the indexes thymus and spleen, inhibited the growth of tumor with inhibition rates of 48.37%, 40.81%, and 36.52%, respectively. JMCE also increased the activity of SOD and decreased the content of MDA in the serum of tumor-bearing mice (Yao et al., 2009).

In traditional Chinese medicine as described by "Zhongguo Minjian Liaofa", branches of *J. mandshurica* are decocted together with chicken eggs. The eggs should be initially administered and the decoction should be administered when there are no obvious side effects. Eggs decocted with *J. mandshurica* branches **(EDJB)**, at doses of 0.64, 1.28, and 2.56 g/kg i.p. once a day for 10 days, suppressed the growth of tumor tissues and increased the body weights in H22 tumor-bearing mouse in a dose- and time-dependent manner. Moreover, EDJB dramatically elevated the thymus index and spleen index of tumor mice, improved the peripheral red blood cells and hemoglobin numbers as well as reduced the white blood cells numbers (Wang et al., 2017c), suggested EDJB has good anti-tumor effect against H22 cell. In addition, total tannins (TT) obtained from *J. mandshurica*, at doses of 0.09 and 0.18 g/kg once a day for 10 days, prominently inhibited the growth of tumor tissues in H22 tumor bearing mouse with an inhibition rate of 34.22% and 36.92%, respectively (Wang et al., 2017d).

Multidrug resistance (MDR) is a major obstacle that hinders the treatment of cancer. Wen et al. (2017) developed a self-assembled polyjuglanin nanoparticle, namely DOX/PJAD-PEG-siRNA, and evaluated its anticancer activity both *in vitro* and *in vivo*. *In vitro* results showed that it improved the cytotoxicity of doxorubicin (DOX) to A549/DOX and H69/CIS cell lines with MDR. Meanwhile, at concentrations of 2, 4, and 8 μg/ml, it significantly down-regulated the mRNA expressions of Kras, P-gp, and c-Myc in a dose-dependent manner (Wen et al., 2017). Moreover, DOX/PJAD-PEG-siRNA at 2 mg/kg for 21 days, significantly suppressed the growth of tumor, decreased the volume and weight of tumor, KI-67 positive levels and expressions of RAS and c-Myc, and increased the TUNEL positive levels and protein levels of p-JNK and p53 in drug-resistant xenografted nude mice when compared to the free DOX at same dose (Wen et al., 2017). These antitumor activities reported are consistent with the traditional usage such as the treatment of liver cancer, lung cancer, breast cancer, cervical cancer, and gastric cancer, *etc.*

Overall, *J. mandshurica* has prominent antitumor potential and has a good health benefit for human. Nevertheless, it is worth noting that most of the research conducted to study antitumor activity stay in the primary stage, and has employed *in vitro*-based methods and further more in-depth *in vivo* and mechanism of action investigations as well as clinical studies should therefore be encouraged and strengthened.

Immunoregulatory Activity

Li et al. (2018) first evaluated the immunoregulatory functions of the three protein hydrolyzates (PH), namely albumin, glutelin, and globin (molecular weights: 11–35 kDa) obtained from *J. mandshurica* in mice. The three compounds, glutelin, albumin, and globin at doses of 200, 400, and 800 mg/kg/d, for 35 days significantly increased the thymus and spleen indexes, lymphocyte

proliferation, macrophage activity, $CD4^+$ and $CD8^+$ T cells numbers, IgA and sIgA levels, and dose-dependently up-regulated mRNA and protein expression levels of IFN-α and IL-6 relative to that of the control group (Li et al., 2018). Simultaneously, a hydrolysate peptide (HP) isolated from *J. mandshurica* (molecular weight <3 kDa), at dose of 800 mg/kg/d for 28 days, obviously elevated the spleen and thymus indexes and promoting the spleen T-lymphocyte proliferation and sIgA generation in the intestinal tract of mice stimulated by exhaustion swimming experiment (Li et al., 2018).

Anti-Inflammatory Activity

A variety of isolated compounds and crude extracts from *J. mandshurica* displayed anti-inflammatory activity in various inflammatory related models, and the possible mechanism of action of active compounds were showed in **Figure 4**. In HaCaT cells induced by IFN-γ, 1.0, 5.0, and 10 μM 1,2,3,4,6-penta-O-galloyl-β- D-glucose (PGG, 194) notably inhibited the protein and mRNA expression levels of CCL17, reduced the protein expression of CXCL-9, CXCL-10, and CXCL-11, and prominently repressed the NF-κB activation as well as STAT1 activation (Ju et al., 2009). Furthermore, PGG obviously reduced the protein expression of CXCL-9, CXCL-10, and CXCL-11 (Ju et al., 2009). Peng et al. (2015) revealed that juglone **(1)**, at doses of 0.25 and 1.0 mg/kg, i.g., daily, for 70 days, significantly decreased the levels of TNF-α, IL-1β and IL-6 both in serum and hypothalamus tissues in rats with high-fat diet-induced neuroinflammation. Further investigations demonstrated that juglone suppressed the inflammatory responses *via* inhibition of TLR4/NF-κB signaling pathway by reducing the protein expressions of TLR4, MyD88, TAK1, p-IκBα, NF-κB, and p-NF-κB (Peng et al., 2015). In LPS-induced primary astrocytes, juglone at doses of 5, 10, 15, and 20 μM, could prominently down-regulate the expressions of these indicators involved in TLR4/NF-κB signaling pathway (Peng et al., 2015). Similarly, in LPS-stimulated acute lung injury mice model, juglanin B **(289)**, at dosages of 10 and 20 mg/kg, i.g., daily, for 21 days, significantly alleviated the lung fibrosis and inflammation cell infiltration via decreasing the mRNA and protein expressions of α-SMA, collagen type I, collagen type III, and TGF-β1 (Dong and Yuan, 2018). Moreover, juglanin B **(289)** notably decreased the levels of IL-4, IL-6, IL-17, IL-18, TNF-α and IL-1β as well as down-regulated the expression of phosphorylated NF-κB via suppressing the IKKα/IκBα signaling pathway (Dong and Yuan, 2018). In addition, five diarylheptanoids and their glycosides, (2S,3S,5S)- 2,3,5-trihydroxy-1,7-bis-(4-hydroxy-3-methoxyphenyl)-heptane **(240)**, (2S,3S,5S)- 2,3-dihydroxy-5-O-β-D-xylopyranosyl-7-(4-hydroxy-3-methoxyphenyl)-1-(4-hydroxyphenyl)-heptane **(241)**, rhoiptelol C **(242)**, rhoiptelol B **(243)**, and 3′,4″-epoxy -2-O-β-D-glucopyanosyl-1-hydroxyphenyl)-7-(3-methoxy-phenyl)-heptan-3-one **(244)** significantly and dose-dependently repressed the NO, IL-6 and TNF-α generation in LPS-stimulated RAW264.7 cells (Diao et al., 2019).

Besides, *J. mandshurica* leaves ethanol extract (JMLE) is particularly effective against allergic dermatitis. After treatment with 0.5% JMLE, the clinical skin severity scores (1.50%) were significantly decreased relative to that of the control group (3.83%), and scratching scores (96.33%) also remarkedly reduced relative to that of the control group (325.01%) in DNCB-induced allergic dermatitis-like skin lesions of mice (Park and Oh, 2014). Further study showed that JMLE obviously decreased the serum levels of TNF-α, IgE, IL-1, and IL-13 (Park and Oh, 2014), suggesting that JMLE might provide the theoretical basis for the further study of active ingredients against allergic dermatitis.

Neuroprotective Activity

Neurodegenerative diseases are characterized by a severe and progressive loss of neurons in the central nervous system, leading to cognitive, behavioral, and motor dysfunctions (Liu et al., 2019). Natural-derived peptides are effective substances in alleviating the oxidative stress and preventing neurotoxicity (Zhao et al., 2020). The hydrolyzed peptide (HP) obtained from *J. mandshurica* displayed important neuroprotective activity both *in vitro* and *in vivo*, and the underlying mechanism was displayed in **Figure 5**.

Three different molecular-weight HP (<3 kDa; 3–10 kDa; >10 kDa) obtained from *J. mandshurica*, and their antioxidant capacity were evaluated *in vitro* after treated with different concentrations (1.0, 1.5, 2.0, and 2.5 mg/ml). Results found that the lower molecular-weight HP (<3 kDa) exhibit higher and significant antioxidant activities via repressing the production of ROS and increasing the activity of glutathione peroxidase (GSH-Px) in the H_2O_2-induced PC12 cells, which than those of higher molecular-weight HP, suggesting that the antioxidant capacity of HP might be relate to molecular-weight (Ren et al., 2018). Similarly, *in vivo*, orally administrated with HP at doses of 200, 400, and 800 mg/kg daily for 30 days in scopolamine-induced memory impairment in mice, the total path for searching the platform was significantly shortened, the escape latency was significantly decreased, and the dwelling distance and time in the coverage zone were notably increased in the Morris water maze test. HP also extended the latency and lessened errors in the passive avoidance response tests (Ren et al., 2018). Mechanically, HP increased the contents of ACh, ChAT, AChE, 5-HT, DA, and NE, elevated the activities of the SOD and GSH-Px as well as up-regulated the protein expression of p-CaMK II in brain tissues of mice (Ren et al., 2018). Subsequently, another antioxidant peptide obtained from *J. mandshurica*, namely EVSGPGLSPN, at concentrations of 12.5, 25, 50, and 100 μM, dose-dependently decreased the production of ROS, and enhanced the activities of CAT, GSH-px, and SOD in H_2O_2-induced PC12 cells (Liu et al., 2019). Simultaneously, EVSGPGLSPN inhibited the IKKβ and p65 expressions to repress the NF-κB pathway activation, alleviated the neurotoxic cascade by overexpression of IL-1β and TNF-α. Furthermore, EVSGPGLSPN significantly inhibited the apoptosis of PC12 cells by down-regulating the expression of cytochrome C, caspase-3/9, and PARP as well as up-regulating the expression of p-CREB and synaptophysin in oxidatively damaged PC12 cells (Liu et al., 2019). These results indicated that EVSGPGLSPN may protect against H_2O_2-induced neurotoxicity by increasing the activity of antioxidant enzymes and blocking the NF-κB/caspase pathways.

In a recent study, three peptides, namely YVLLPSPK, TWLPLPR, and KVPPLLY, obtained from *J. mandshurica*, at a concentration of 50 μM for 24 h, prominently inhibited the generation of ROS, increased the activity of GSH-Px and contents of ATP, and alleviated apoptosis in $A\beta_{25-35}$-induced PC12 cells. It also promoted autophagy and affected the Akt/mTOR signaling pathway through up-regulating the protein expression levels of Beclin-1, LC3-I, LC3-II, LAMP1, LAMP2, Cathepsin and p-Akt/Akt as well as down-regulating the protein expression level of p62 and p-mTOR/mTOR at molecule levels (Zhao et al., 2020). Results from above studies indicated that *J. mandshurica* may serves as sustainable dietary supplement to further develop novel functional food to prevent or defer oxidation-incurred memory impairment damage and ageing/or age-related neurodegenerative diseases, such as Alzheimer's disease (AD) and Parkinson's disease (PD).

Antidiabetic Activity

Recent findings have demonstrated that *J. mandshurica* possess significant hypoglycemic activity *in vitro* and the possible mechanism of this action was showed in **Figure 6**. The ethyl acetate fractions extracted from ethanol extract of *J. mandshurica* leaves (JMEE) showed significant α-glucosidase and α-amylase inhibitory activity *in vitro* with IC_{50} of 14 and 130 μg/ml, which were stronger than that of the positive drug acarbose with IC_{50} of 44 and 158 μg/ml, respectively (Wang et al., 2019c). In insulin resistant (IR) hepatic HepG2 cells, LPLLR (Leu-Pro-Leu-Leu-Arg), a novel pentapeptide from the protein hydrolysates of *J. mandshurica*, at concentrations of 100 and 200 μM, increased the phosphorylation levels of insulin receptor substrate 1 (IRS-1), phosphatidylinositol 3-kinase (PI3K), protein kinase B (Akt), AMPK and GSK3β, and up-regulated the expression levels of GS and glucose transporter type 4 (GLUT4), while down-regulated the expression levels of G-6-Pase and PEPCK in IR hepatic HepG2 cells (Wang et al., 2020a). These findings suggested that LPLLR exerts anti-diabetic effect through increasing the glycogen synthesis and glucose uptake, as well as decreasing the gluconeogenesis. In addition, the peptide LPLLR possesses good stability under *in vitro* simulated gastrointestinal digestion, and the low molecular weight (610.4 Da) of LPLLR may be beneficial for its intestinal absorption. Nevertheless, more in-depth *in vivo* investigation is needed to explore the stability and absorption of LPLLR. Subsequently, in high glucose-induced IR and oxidative stress in HepG2 cells, three novel peptides, namely Leu-Val-Arg-Leu (LVRL), Leu-Arg-Tyr-Leu (LRYL), and Val-LeuLeu-Ala-Leu-Val-Leu-Leu-Arg (VLLALVLLR) from *J. mandshurica* at 12.5–100 μM, significantly improve glucose consumption, glucose uptake, GLUT4 translocation, and elevated the phosphorylation of IRS-1, PI3K, and Akt. The activities of GSH-Px, CAT, and SOD, the nuclear transport of Nrf2, and the protein expression of HO-1 were also increased. Furthermore, these peptides reduced high glucose-induced ROS overproduction and the phosphorylation of ERK, JNK, and p38 (Wang et al., 2020b). These results suggested that peptides from *J. mandshurica* could protect HepG2 cells from high glucose-induced IR and oxidative stress by activating IRS-1/PI3K/Akt and Nrf2/HO-1 signaling pathways.

Antimicrobial Activity

Three new juglone derivatives, namely juglonol A **(71)**, B **(72)**, and C **(73)**, isolated from the immature exocarps of *J. mandshurica* by Yang and his colleagues (2019) and their antimicrobial activity against Gram-positive (*S. aureus* and *E. faeculis*) and Gram-negative (*E. coli* and *K. pneumonia*) bacteria, yeast (*C. albicans*), and fungi (*F. oxysporum*, *F. oxysporium*, *C. lagenarium*, and *P. asparagi*) were evaluated. The results showed that juglonol A **(71)** obviously suppressed all tested strains except for *E. coli*. with the MIC values ranging 8 from 64 μg/ml However, juglonol B **(72)** only significantly inhibited the *S. aureus* with MIC value of 8 μg/ml (Yang et al., 2019). Juglonol A have also been demonstrated to exhibit modestly inhibitory activity against the non-small-cell lung carcinoma (NCI-H1975), lung adenocarcinoma (HCC827), hepatocellular carcinoma (HepG2), triple-negative breast cancer (MD-AMB-231), leukemia (HL-60), mouse colon cancer (CT26) and rat glioma (C6), and IC_{50} values were ranging from 9.5 to 31.6 μg/ml (Yang et al., 2019). These results suggested that the presence of juglone core or hydroxyethyl side chain is essential to the molecules' biological activity and that the position of substitution has a marked impact on the potency. Hence, juglonol A, as pan-inhibitors, might be cytotoxic.

Antiviral Activity

Min et al. (2000) found that 1,2,6-trigalloylglucose **(192)** and 1,2,3,6- tetragalloylglucose **(193)** isolated from barks of *J. mandshurica* showed the most potent anti-reverse transcriptase (RT) activity of HIV-1 with the IC_{50} values of 67 and 40 nM, respectively. In addition, compound **192** notably suppressed the ribonuclease H (RNase H) activity with IC_{50} values of 39 μM when used illimaquinone as a positive control (Min et al., 2000). Simultaneously, Min and his colleagues further found that taxifolin **(297)** displayed the most potent anti-HIV-1 activity against MT-4 cells with the IC_{100} value of 25 μg/ml and CC_{100} value of above 100 μg/ml (Min et al., 2002). However, the certain mechanism of anti-HIV-1 activity should be performed at molecule level in the future.

Anti-Melanogenesis Activity

Recently, Kim et al. (2019) obtained three phenolic ingredients from fruit of *J. mandshurica* and evaluated their anti-melanogenesis activity in B16F10 melanoma cells and primary human epidermal melanocytes. It was found that compound 2-[4-(3-hydroxypropyl)-2-methoxyphenoxy]-1,3-propanediol **(126)** at concentrations of 0.5 and 1.0 μM, showed the highest inhibitory effect through reducing the melanin content, increasing the p-ERK protein expression and decreasing MITF and tyrosinase protein expressions. These effects also could immediately reverse by PD98059, which a potent ERK inhibitor, indicated compound **126** effectively curbed melanogenesis mainly through p-ERK-associated MITF degradation (Kim et al., 2019). Therefore, *J. mandshurica* has the potential to suppress melanogenesis and can become a useful resource for developing novel skin-whitening agents to cure hyperpigmentation disorders.

PHARMACOKINETICS

Neither systemic evidences regarding the pharmacokinetics extracts from this plant nor evaluations of its target-organ toxicity have been performed. Few investigations have studied the pharmacokinetics parameters of *J. mandshurica* and its bioactive compounds in animal experiments. Chen et al. (2018) first measured the gallic acid and syringic acid concentrations in rat plasma after the intragastric administration of the aqueous extracts of *J. mandshurica* at dose of 12 g/kg using high performance liquid chromatography (HPLC). The maximum plasma concentration (C_{max}) was 0.64 μg/ml, while the time to reach peak concentration (T_{max}) and elimination half-life ($T_{1/2}$) were 61.80 and 184.21 min, respectively. The area under the plasma concentration-time curve ($AUC_{0\text{-}t}$) and $AUC_{0\text{-}\infty}$ of gallic acid was 96.37 μg min/mL, and 121.59 μg min/mL. Additionally, the C_{max}, T_{max}, $T_{1/2}$, $AUC_{0\text{-}t}$, and $AUC_{0\text{-}\infty}$ of syringic acid was 0.43 μg/ml, 30.67 min, 99.63 min, 40.33 μg min/mL, 47.02 μg min/mL, respectively (Chen et al., 2018).

Additionally, Chen et al. (2018b) studied the chemical ingredients distribution of the ethanol extracts of exocarp from *J. mandshurica* after orally administrated at concentration of 1.35 g/ml to rats. The results showed that a total of 54 ingredients have been identified, including 41 archetypes and 13 metabolites. The archetypes included 17 naphthoquinones, 9 diarylheptanoids, 7 flavonoids, 5 triterpenoids, and 3 polyphenols. The metabolites comprised 4 naphthoquinones, 3 diarylheptanoids, and 1 flavonoid, *etc*, were detected in rats' gastric tissues by UPLC-Q-TOF/MS technology for the first time (Cheng et al., 2018b). Similarly, 24 chemical components including 12 naphthoquinones, 5 flavonoids, 3 diarylheptanoids, and 4 triterpenoids were also detected in rats' kidney tissues by UPLC-Q-TOF/MS technology after orally administration of the ethanol extract of *J. mandshurica* at a dose of 1.35 g/ml to rats (Wang et al., 2018b).

Overall, these results might be contributed to explain the body's metabolic process and relative mechanism of action of various components from *J. mandshurica*, and provide a methodological reference for the evaluation of the safety and effectiveness of compounds in the accumulation in gastric and kidney tissues and relational adverse reactions as well as composition and tissue distribution. It also provides more comprehensive information for clarifying the substance basis of anti-tumor effects in *J. mandshurica*. Further investigations are required to explore the pharmacokinetics, metabolic stability, and the drug delivery system of *J. mandshurica* and its active components.

TOXICOLOGICAL INFORMATION

When evaluating the efficacy of drugs, toxicity and safety should be firstly taken into account. Although *J. mandshurica* as a popular Chinese herbal medicine is frequently used in TCM, information on the side effects and safety evaluations for this plant are seldom reported and insufficient to support their safety. Liu et al. (2004a) reported the acute toxicity of total extracts (TE), petroleum ether extracts (PEE), *n*-butanol extracts (*n*BE), aqueous extracts (AE), chloroform extracts (CE), and acetic ether extracts (AEE) from BQLY in mice by administering the increasing doses orally and intraperitoneal injection (TE, PEE, *n*BE, and AE at doses of 3.62, 4.25, 5.00, 5.88, and 6.29 g/kg, respectively; CE at doses of 400.2, 470.6, 553.6, 651.3, and 766.3 mg/kg; AEE at doses of 930.2, 1,094.4, 1,287.4, 1,514.7, and 1781.9 mg/kg) for 14 days. The results found that the treatment by gavage did not cause any deaths or side effects. However, the intraperitoneal injection with CE and AEE resulted in dose-dependent mortality with signs of toxicity, and the median lethal dose (LD_{50}) of CE and AEE were 575.38 mg/kg and 1,303.59 mg/kg, respectively. Simultaneously, the LD_{50} of TE, PEE, *n*BE, and AE were more than 5 g/kg both in intragastrical and intraperitoneal administration (Liu et al., 2004b). These findings suggested that intraperitoneally injected with chloroform extracts and acetic ether extracts from BQLY were toxic to mice. Recently, Ju et al. (2019) investigated the acute toxicity of aqueous extracts from the stem-barks of *J. mandshurica* in mice by orally administering the at maximum dose of 227.27 g/kg daily for continuous 14 days. They found that the treatment by aqueous extracts did not cause any deaths or side effects (Ju et al., 2019). Therefore, these results further confirmed that the aqueous extracts of *J. mandshurica* did not present the apparent toxicity, and might be relatively safe for human.

Additionally, studies showed that BQLY contain many toxic compounds, such as juglone (Huo et al., 2017). In previous study, Westfall et al. (1961) reported that the LD_{50} of juglone in mice was 2.5 mg/kg by gavage, the LD_{50} of intraperitoneal injection was 25 mg/kg, and the LD_{50} of rats was 112 mg/kg by gavage (Westfall et al., 1961). Chen et al. (2005) speculated that the reason for the toxicity of juglone was that it combines with blood components after entering the blood, causing a high concentration of juglone in the blood. Moreover, juglone can react with the sulfhydryl compounds in the gastrointestinal contents, resulting in low absorption of juglone during intragastric administration, which accumulates in the cardia antrum, causing toxicity. In addition, juglone and its metabolites can covalently bind to cytosolic proteins in the kidney, causing renal toxicity (Chen et al., 2005).

The toxicity studies regarding the *J. mandshurica* and its active components are still in the exploratory stage and mainly focused on acute toxicity study. Therefore, apart from the classical toxicological evaluation, research on chronic toxicity, toxicity mechanism, and toxicokinetics should be further conducted in several animal models and provide scientific explanations for its toxicity and safety applications in the future.

CONCLUSION AND FUTURE PERSPECTIVES

The present review systematically summarizes the findings of the latest research on the traditional usages, phytochemical constituents, pharmacological properties, and toxicities of different extracts and ingredients of *J. mandshurica*. As a historical herbal medicine, it has been traditionally and

popularly used in indigenous populations to treat cancer in China, Japan, Korea, and India more than 2000 years. Recent investigations have focused primarily on evaluating the anticancer activities of the extracts or isolated compounds of this plant. Until now, more than 400 chemical constituents have been isolated and identified from the different parts of *J. mandshurica*. Through a comprehensive analysis, we found that the quinones, phenolics, triterpenoids, and diarylheptanoids are major and important active compounds of *J. mandshurica* with numerous pharmacological activities shown *in vivo* and *in vitro* investigations.

However, there are also some points and aspects that need to be noted and researched further: **(1)** The quinones from *J. mandshurica* with prominent antitumor activity have captured researcher's attention increasingly, and further study on these compounds should be a priority. Until recently, however, *J. mandshurica* was still considered as folk medicine for the treatment of cancer and the related preclinical experiments results are questioned and unpersuasive, future studies are necessary to address issues regarding composition of the extract, explicability of preclinical experiments, and lack of transformation of the preclinical results to clinical efficacy. Hence, the clinical trial evaluations of *J. mandshurica*, including animal models and should be conducted urgently. **(2)** Although a great number of chemical ingredients had been isolated and identified from this plant, pharmacological evaluations on these compounds are limited to few compounds such as juglone, juglanstetralone A, p-hydroxymethoxybenzobijuglone, juglanthraquinone C, and juglanin. Therefore, deep phytochemical studies of *J. mandshurica* and its pharmacological properties, especially the mechanism of action of its bioactive constituents to illustrate the correlation between ethnomedicinal uses and biological activities will undoubtedly be the focus of further research. **(3)** Toxicological investigations are crucial to understand the safety of herbal drugs, but data on toxicological aspects of *J. mandshurica* were still rarely. Although research confirmed that many medicinal parts of *J. mandshurica* have little or no toxicity, BQLY has some adverse reactions, which may cause harm to human health. Thence, toxicity and safety assessment studies on BQLY extract and other effective extracts are necessary to ensure the full use of medicinal resources, to meet the Western evidence-based medicine standards, and to provide accurate evidence for clinical applications. Besides, the crude drugs should be strictly in accordance with traditional processing theories and subjected to ancient processing techniques (*Pao Zhi*), including cleaning, cutting, drying, and digesting, which can reduce their toxicity and exert maximal therapeutic efficacy by transforming the secondary plant metabolites. **(4)** Pharmacokinetics is an indispensable part of new drug development and rational clinical drug use. However, data on the pharmacokinetics of active compounds and crude extracts of *J. mandshurica* remain unclear.

Overall, *J. mandshurica* is a source for nutritional and medical compounds and is worthy of further studty owing to its health-promoting properties and its potential for further development in food industry. However, the existing health-related evidence on *J. mandshurica* is insufficient, and its clinical value has not been adequately studied. Therefore, comprehensive investigations on biological properties, especially the underlying mechanism of bioactiveties of *J. mandshurica* and its isolated compounds, should be conducted in order to support its ethnomedicinal uses. Besides, the development of healthcare products of *J. mandshurica* will undoubtedly be the focus of further research. Lastly, this study will help scientists to created additional potential health-promoting pharmaceuticals and functional foods based on *J. mandshurica*.

AUTHOR CONTRIBUTIONS

HL, KH, DL, and XS obtained and analyzed the literatures. FL, ZW, YJ, and YY wrote the manuscript. XH and NZ gave ideas and edited the manuscript. All authors read and approved the final version of the manuscript for publication.

FUNDING

This work was supported by the National Natural Science Foundation of China (Grant No. 82074094), the Open Research Fund of Chengdu University of Traditional Chinese Medicine Key Laboratory of Systematic Research of Distinctive Chinese Medicine Resources in Southwest China (Grant No. 2020XSGG002), the Xinglin Scholar Research Promotion Project of Chengdu University of Traditional Chinese Medicine (Grant No. CDTD2018014) and the Science and Technology Project of Zunyi (Grant No. ZSKH-HZ-(2020)-78).

REFERENCES

Avcı, E., Arıkoğlu, H., and Kaya, D. E. (2016). Investigation of juglone effects on metastasis and angiogenesis in pancreatic cancer cells. *Gene*. 588, 74–78. doi:10.1016/j.gene.2016.05.001

Bai, W. N., Liao, W. J., and Zhang, D. Y. (2010). Nuclear and chloroplast DNA phylogeography reveal two refuge areas with asymmetrical gene flow in a temperate walnut tree from East Asia. *New Phytol*. 188, 892–901. doi:10.1111/j.1469-8137.2010.03407.x

Bayram, D., Özgöçmen1, M., Armagan, I., Sevimli, M., Türel, G. Y., and Şenol, N. (2019). Investigation of apoptotic effect of juglone on CCL-228-SW 480 colon cancer cell line. *J. Cancer Res. Therapeut*. 15, 68–74. doi:10.4103/jcrt.JCRT_880_17

Carey, A. N., Fisher, D. R., Bielinski, D. F., Cahoon, D. S., and Shukitt-Hale, B. (2020). Walnut-associated fatty acids inhibit LPS-induced activation of BV-2 microglia. *Inflammation*. 43, 241–250. doi:10.1007/s10753-019-01113-y

Chaudhary, N., Sasaki, R., Shuto, T., Watanabe, M., Kawahara, T., Suico, M. A., et al. (2019). Transthyretin amyloid fibril disrupting activities of extracts and fractions from *Juglans mandshurica* Maxim. var. cordiformis (Makino) Kitam. *Molecules*. 24, 500. doi:10.3390/molecules24030500

Chen, C., Wang, T. M., Di, X., and Kang, T. G. (2018). A pharmacokinetic study of gallic acid and syringic acid in the water extraction of *Juglans mandshurica* Maxim. in rat plasma. *Chin. J. Ethnomed. Ethnopharmacy*. 27, 28–33

Chen, G., Pi, X. M., and Yu, C. Y. (2015). A new naphthalenone isolated from the green walnut husks of *Juglans mandshurica* Maxim. *Nat. Prod. Res*. 29, 174–179. doi:10.1080/14786419.2014.971789

Chen, L. J., Lebetkin, E. H., and Burka, L. T. (2005). Metabolism and disposition of juglone in male F344 rats. *Xenobiotica*. 35, 1019–1034. doi:10.1080/00498250500356621

Cheng, T., Wang, G. L., Huo, J. H., and Wang, W. M. (2018b). Distribution of exocarp components of *Juglans mandshurica* in rats' gastric tissues based on UPLC-Q-TOF/MS. *Chin. Tradit. Herb. Drugs*. 49, 2527–2539. doi:10.7501/j.issn.0253-2670.2018.11.007

Cheng, Z. Y., Du, Y. Q., Zhang, Q., Lin, B., Gao, P. Y., Huang, X. X., et al. (2018a). Two pairs of new alkaloid enantiomers with a spiro [benzofuranonebenzazepine] skeleton from the bark of *Juglans mandshurica*. *Tetrahedron Lett*. 59, 2050–2053. doi:10.1016/j.tetlet.2018.04.037

Cheng, Z. Y., Yao, G. D., Guo, R., Huang, X. X., and Song, S. J. (2017). Phenylpropanoids from *Juglans mandshurica* exhibit cytotoxicities on liver cancer cell lines through apoptosis induction. *Bioorg. Med. Chem. Lett*. 27, 597–601. doi:10.1016/j.bmcl.2016.12.005

Diao, S. B., Jin, M., Sun, J. F., Zhou, Y., Ye, C., Jin, Y., et al. (2019). A new diarylheptanoid and a new diarylheptanoid glycoside isolated from the roots of *Juglans mandshurica* and their anti-inflammatory activities. *Nat. Prod. Res*. 33, 701–707. doi:10.1080/14786419.2017.1408100

Dong, Z. W., and Yuan, Y. F. (2018). Juglanin suppresses fibrosis and inflammation response caused by LPS in acute lung injury. *Int. J. Mol. Med*. 41, 3353–3365. doi:10.3892/ijmm.2018.3554

Editorial Committee of Flora of China (1979). *Chinese Academy of Sciences. Flora of China*., Vol. 21. (Beijing: Science Press), 31–33

Fang, L., Ren, D. Y., Cui, L. Y., Liu, C. L., Wang, J., Liu, W., et al. (2018). Antifatigue, antioxidant and immunoregulatory effects of peptides hydrolyzed from Manchurian Walnut (*Juglans mandshurica* Maxim.) on mice. *Grain Oil Sci. Technol*. 1, 44–52. doi:10.3724/SP.J.1447.GOST.2018.18028

Fu, Q. F., Song, H. J., Zhu, L., Gao, H. R., Ma, D. N., Zhang, X. J., et al. (2020). Study on phenolic acid compounds of *Juglans mandshurica*. *Inf. Trad. Chin. Med*. 37, 44–47. doi:10.19656/j.cnki.1002-2406.200009

Gao, X. L., Lin, H., Zhao, W., Hou, Y. Q., Bao, Y. L., Song, Z. B., et al. (2016). JA, a new type of polyunsaturated fatty acid isolated from *Juglans mandshurica* Maxim, limits the survival and induces apoptosis of heptocarcinoma cells. *Apoptosis*. 21, 340–350. doi:10.1007/s10495-015-1202-5

Guo, L. N., Zhang, R., Guo, X. Y., Cui, T., Dong, W., Huo, J. H., et al. (2015). Identification of new naphthalenones from *Juglans mandshurica* and evaluation of their anticancer activities. *Chin. J. Nat. Med*. 13–0710. doi:10.1016/S1875-5364(15)30070-4

Hou, Y. Q., Yao, Y., Bao, Y. L., Song, Z. B., Yang, C., Gao, X. L., et al. (2016). Juglanthraquinone C induces intracellular ROS increase and apoptosis by activating the Akt/Foxo signal pathway in HCC cells. *Oxid. Med. Cell. Longev*., 494, 1623. doi:10.1155/2016/4941623

Hu, Z., Zhang, T., Gao, X. X., Wang, Y., Zhang, Q., Zhou, H. J., et al. (2016). De n*ovo* assembly and characterization of the leaf, bud, and fruit transcriptome from the vulnerable tree *Juglans mandshurica* for the development of 20 new microsatellite markers using Illumina sequencing. *Mol. Genet. Genom*. 291, 849–862. doi:10.1007/s00438-015-1147-y

Huo, J. H., Du, X. W., Sun, G. D., Dong, W. T., and Wang, W. M. (2018). Identification and characterization of major constituents in *Juglans mandshurica* using ultra performance liquid chromatography coupled with time-of-flight mass spectrometry (UPLC-ESI-Q-TOF/MS). *Chin. J. Nat. Med*. 16–0545. doi:10.1016/S1875-5364(18)30089-X

Huo, J. H., Du, X. W., Sun, G. D., Meng, Y. L., and Wang, W. M. (2017). Comparison of the chemical profiles of fresh-raw and dry-processed *Juglans mandshurica*. *J. Separ. Sci*. 40, 646–662. doi:10.1002/jssc.201600877

Jiang, H., Yang, L., Hou, A., Zhang, J., Wang, S., Man, W., et al. (2020). Botany, traditional uses, phytochemistry, analytical methods, processing, pharmacology and pharmacokinetics of Bupleuri Radix: a systematic review. *Biomed. Pharmacother*. 131, 110679. doi:10.1016/j.biopha.2020.110679

Jiang, Z., Diao, S. B., Li, R., Zhou, W., Sun, J. F., Zhou, Y., et al. (2018). One new 1, 4-napthoquinone derivative from the roots of *Juglans mandshurica*. *Nat. Prod. Res*. 32 (9), 1017–1021. doi:10.1080/14786419.2017.1375921

Jin, M., Diao, S. B., Zhang, C. H., Cao, S., Sun, J. F., Li, R., et al. (2015). Two new diarylheptanoids isolated from the roots of *Juglans mandshurica*. *Nat. Prod. Res*. 29, 1839–1844. doi:10.1080/14786419.2015.1009063

Jin, M., Sun, J. F., Li, R., Diao, S. B., Zhang, C. H., Cui, J. M., et al. (2016). Two new quinones from the roots of *Juglans mandshurica*. *Arch. Pharm. Res. (Seoul)*. 39, 1237–1241. doi:10.1007/s12272-016-0781-1

Ju, S. M., Song, H. Y., Lee, S. J., Seo, W. Y., Sin, D. H., Goh, A. R., et al. (2009). Suppression of thymus- and activation-regulated chemokine (TARC/CCL17) production by 1,2,3,4,6-penta- O-galloyl-β-D-glucose via blockade of NF-κB and STAT1 activation in the HaCaT cells. *Biochem. Biophys. Res. Commun*. 387, 115–120. doi:10.1016/j.bbrc.2009.06.137

Ju, X. C., Chen, C., Di, X., Xiao, H. H., Zhang, H., Zhai, Y. J., et al. (2019). Acute toxicity and *in vitro* anti-tumor activity of *Juglans mandshurica*. *Central South Pharm*. 17, 360–364. doi:10.7539/j.issn.1672-2981.2019.03.008

Kim, J. Y., Lee, E. J., Ahn, Y., Park, S., Kim, S. H., and Oh, S. H. (2019). A chemical compound from fruit extract of *Juglans mandshurica* inhibits melanogenesis through p-ERK-associated MITF degradation. *Phytomedicine*. 57, 57–64. doi:10.1016/j.phymed.2018.12.007

Kim, S. H., Lee, K. S., Son, J. K., Je, G. H., Lee, J. S., Lee, C. H., et al. (1998). Cytotoxic compounds from the roots of *Juglans mandshurica*. *J. Nat. Prod*. 61, 643–645. doi:10.1021/np970413m

Lee, K. S., Li, G., Kim, S. H., Lee, C. S., Woo, M. H., Lee, S. H., et al. (2002). Cytotoxic diarylheptanoids from the roots of Juglans mandshurica. *J. Nat. Prod*. 65, 1707–1708. doi:10.1021/np0201063

Lee, S. W., Lee, K. S., and Son, J. K. (2000). New naphthalenyl glycosides from the roots of *Juglans mandshurica*. *Planta. Med*. 66, 184–186. doi:10.1055/s-0029-1243129

Li, G., Cui, J. M., Kwon, Y., Seo, C. S., Lee, C. S., Woo, M. H., et al. (2005). Two new diarylheptanoids from Juglans *mandshurica*. *Bull. Kor. Chem. Soc*. 26, 1878–1880. doi:10.1002/chin.200616203

Li, G., Xu, M. L., Choi, H. G., Lee, S. H., Jahng, Y. D., Lee, C. S., et al. (2003). Four new diarylheptanoids from the roots of *Juglans mandshurica*. *Chem. Pharm. Bull*. 51, 262–264. doi:10.1248/cpb.51.262

Li, J., Wang, J., Liu, C. L., Fang, L., and Min, W. H. (2018). Protein hydrolyzates from Changbai mountain Walnut (*Juglans mandshurica* Maxim.) boost mouse immune system and exhibit immunoregulatory activities. *Evid. Based Complement. Alternat. Med*., 457, 6561. doi:10.1155/2018/4576561

Li, J., Xu, K. P., Zou, H., Long, H. P., Zou, Z. X., Kuang, J. W., et al. (2013). Chemical constituents in the green peel of *Juglans mandshurica* maxim. *Central South Pharm*. 11, 1–3. doi:10.7539/j.issn.1672-2981.2013.01.001

Li, J., Xu, K. P., Zou, Z. X., Zou, H., Long, H. P., Tan, L. H., et al. (2017a). Two new compounds from the green peel of *Juglans mandshurica*. *J. Asian. Nat. Prod. Res*. 19, 1087–1092. doi:10.1080/10286020.2017.1295228

Li, J., Xu, P. S., Zou, Z. X., Zou, H., Long, H. P., Tan, L. H., et al. (2017b). Three new compounds from the roots of *Juglans mandshurica* Maxim. *Phytochem. Lett*. 20, 40–44. doi:10.1016/j.phytol.2017.03.014

Li, Z. B., Bao, Y. M., Chen, H. B., Wang, J. Y., Hu, J. H., and An, L. J. (2007b). A cytotoxic compound from the leaves of *Juglans mandshurica*. *Chin. Chem. Lett*. 18, 846–848. doi:10.1016/j.cclet.2007.05.043

Li, Z. B., Wang, J. Y., Jiang, B., Zhang, X. L., An, L. J., and Bao, Y. M. (2007a). Benzobijuglone, a novel cytotoxic compound from Juglans mandshurica, induced apoptosis in HeLa cervical cancer cells. *Phytomedicine*. 14, 846–852. doi:10.1016/j.phymed.2007.09.004

Li, Z. B., Wang, J. Y., Yang, J., Zhang, X. L., An, L. J., and Bao, Y. M. (2009). Apoptosis of BGC823 cell line induced by p-hydroxymethoxybenzobijuglone, a novel compound from *Juglans mandshurica*. *Phytother. Res*. 23, 551–557. doi:10.1002/ptr.2685

Lin, H., Zhang, Y. W., Bao, Y. L., Wu, Y., Sun, L. G., Yu, C. L., et al. (2013). Secondary metabolites from the stem bark of *Juglans mandshurica*. *Biochem. Systemat. Ecol*. 51, 184–188. doi:10.1016/j.bse.2013.08.010

Lin, H., Zhang, Y. W., Hua, Y., Bao, Y. L., Wu, Y., Sun, L. G., et al. (2014). Three new compounds from the stem bark of *Juglans mandshurica*. *J. Asian. Nat. Prod. Res*. 16, 819–824. doi:10.1080/10286020.2014.923406

Liu, C. G., Wang, Y., Guo, S., Liu, Y. X., Sun, Y. P., Fu, L., et al. (2017). Study on chemical constituents from pericarps of Juglans mandshurica. *Inf. Trad. Chin. Med*. 34, 4–6. doi:10.19656/j.cnki.1002-2406.2017.04.002

Liu, C. L., Guo, Y., Zhao, F. R., Qin, H. X., Lu, H. Y., Fang, L., et al. (2019). Potential mechanisms mediating the protective effects of a peptide from walnut (*Juglans mandshurica* Maxim.) against hydrogen peroxide induced neurotoxicity in PC12 cells. *Food Funct*. 10, 3491–3501. doi:10.1039/c8fo02557f

Liu, G. R., Xu, K. P., Shen, J., Yang, F., Zou, H., and Tan, G. S. (2009). Antitumor chemical constituents from the roots of *Juglans mandshurica* Maxim. *Central South Pharm*. 7, 644–646

Liu, L. J., Li, W., Loike, K. Z., Zhang, S. J., and Nikaido, T. (2004a). New α-Tetralonyl glucosides from the fruit of *Juglans mandshurica*. *Chem. Pharm. Bull.* 52, 566–569. doi:10.1248/cpb.52.566

Liu, W., Lin, W. H., and Ji, Y. B. (2004b). Study on the acute toxicity experiment of mice and anti-tumor function *in vitro* of the qinglongyi. *China J. Chin. Mater. Med.* 29, 887–890.

Liu, L. J., Li, W., Sasaki, T., Asada, Y., and Koike, K. (2010). Juglanone, a novel α-tetralonyl derivative with potent antioxidant activity from *Juglans mandshurica*. *J. Nat. Med.* 64, 496–499. doi:10.1007/s11418-010-0435-4

Lou, L. L., Guo, R., Cheng, Z. Y., Zhao, P., Yao, G. D., Wang, X. B., et al. (2018). Coumarins from *Juglans Mandshurica* Maxim and their apoptosis-inducing activities in hepatocarcinoma cells. *Phytochem. Lett.* 15–20. doi:10.1016/j.phytol.2018.01.005

Lou, L. L., Zhao, P., Cheng, Z. Y., Guo, R., Yao, G. D., Wang, X. B., et al. (2019a). A new coumarin from *Juglans mandshurica* Maxim induce apoptosis in hepatocarcinoma cells. *Nat. Prod. Res.* 33, 1791–1793. doi:10.1080/14786419.2018.1434646

Lou, L. L., Cheng, Z. Y., Guo, R., Yao, G. D., and Song, S. J. (2019b). Alkaloids from *Juglans mandshurica* maxim induce distinctive cell death in hepatocellular carcinoma cells. *Nat. Prod. Res.* 33, 911–914. doi:10.1080/14786419.2017.1413571

Lu, Z., Chen, H., Zheng, X. M., and Chen, M. L. (2017). Experimental study on the apoptosis of cervical cancer Hela cells induced by juglone through c-Jun N-terminal kinase/c-Jun pathway. *Asian Pac. J. Trop. Med.* 10, 572–575. doi:10.1016/j.apjtm.2017.06.005

Luan, F., Han, K. Q., Li, M. X., Zhang, T., Liu, D. H., Yu, L. H., et al. (2019). Ethnomedicinal uses, phytochemistry, pharmacology, and toxicology of species from the genus *Ajuga* L.: a systematic review. *Am. J. Chin. Med.* 47, 959–1003. doi:10.1142/S0192415X19500502

Machida, K., Matsuoka, E., Kasahara, T., and Kikuchi, M. (2005). Studies on the constituents of *Juglans* species. I. Structural determination of (4*S*)- and (4*R*)-4-hydroxy-alpha-tetralone derivatives from the fruit of *Juglans mandshurica* MAXIM. var. sieboldiana. *MAKINO. Chem. Pharm. Bull.* 53, 934–937. doi:10.1248/cpb.53.934

Machida, K., Yogiashi, Y., Matsuda, S., Suzuki, A., and Kikuchi, M. (2009). A new phenolic glycoside syringate from the bark of *Juglans mandshurica* MAXIM. var. sieboldiana MAKINO. *J. Nat. Med.* 63, 220–222. doi:10.1007/s11418-009-0312-1

Min, B. S., Lee, H. K., Lee, S. M., Kim, Y. H., Bae, K. H., Otake, T., et al. (2002). Anti-human immunodeficiency virus-type 1 activity of constituents from *Juglans mandshurica*. *Arch. Pharm. Res. (Seoul)*. 25, 441–445. doi:10.1007/bf02976598

Min, B. S., Lee, S. Y., Kim, J. H., Lee, J. K., Kim, T. J., Kim, D. H., et al. (2003). Anti-complement activity of constituents from the stem-bark of *Juglans mandshurica*. *Biol. Pharm. Bull.* 26, 1042–1044. doi:10.1248/bpb.26.1042

Min, B. S., Nakamura, N., Miyashiro, H., Kim, Y. H., and Hattori, M. (2000). Inhibition of human immunodeficiency virus type 1 reverse transcriptase and ribonuclease H activities by constituents of *Juglans mandshurica*. *Chem. Pharm. Bull.* 48, 194–200. doi:10.1248/cpb.48.194

Mu, X. Y., Sun, M., Yang, P. F., and Lin, Q. W. (2017). Unveiling the identity of Wenwan walnuts and phylogenetic relationships of Asian Juglans species using restriction site-associated DNA-sequencing. *Front. Plant Sci.* 8, 1708. doi:10.3389/fpls.2017.01708

Ngoc, T. M., Hung, T. M., Thuong, P. T., Kim, J. C., Choi, J. S., Bae, K., et al. (2008). Antioxidative activities of galloyl glucopyranosides from the stem-bark of *Juglans mandshurica*. *Biosci. Biotechnol. Biochem.* 72, 2158–2163. doi:10.1271/bbb.80222

Park, G., Jang, D. S., and Oh, M. S. (2012). Juglans mandshurica leaf extract protects skin fibroblasts from damage by regulating the oxidative defense system. *Biochem. Biophys. Res. Commun.* 421, 343–348. doi:10.1016/j.bbrc.2012.04.013

Park, G., and Oh, M. S. (2014). Inhibitory effects of Juglans mandshurica leaf on allergic dermatitis-like skin lesions-induced by 2,4-dinitrochlorobenzene in mice. *Exp. Toxicol. Pathol.* 66, 97–101. doi:10.1016/j.etp.2013.10.001

Park, S., Kim, N., Yoo, G., Kim, S. N., Kwon, H. J., Jung, K., et al. (2017). Phenolics and neolignans isolated from the fruits of *Juglans mandshurica* Maxim. and their effects on lipolysis in adipocytes. *Phytochemistry*. 137, 87–93. doi:10.1016/j.phytochem.2017.01.019

Peng, X. H., Nie, Y., Wu, J. J., Huang, Q., and Cheng, Y. Q. (2015). Juglone prevents metabolic endotoxemia-induced hepatitis and neuroinflammation via suppressing TLR4/NF-κB signaling pathway in high-fat diet rats. *Biochem. Biophys. Res. Commun.* 462, 245–250. doi:10.1016/j.bbrc.2015.04.124

Pereira, A. C., Magalhães, L. G., Gonçalves, U. O., Luz, P. P., Moraes, A. C., Rodrigues, V., et al. (2011). Schistosomicidal and trypanocidal structure-activity relationships for (±)-licarin A and its (-)- and (+)-enantiomers. *Phytochemistry*. 72, 1424–1430. doi:10.1016/j.phytochem.2011.04.007

Qiu, J. Y., Wang, W. M., Li, J., Zhao, M., Wang, J. L., and Zhang, S. J. (2017). Chemical constituents in green walnut husks of *Juglans regia*. *Chin. Trad. Herb. Drugs*. 48, 2385–2389. doi:10.7501/j.issn.0253-2670.2017.12.005

Ren, D. Y., Zhao, F. R., Liu, C. L., Wang, J., Guo, Y., Liu, J. S., et al. (2018). Antioxidant hydrolyzed peptides from Manchurian walnut (*Juglans mandshurica* Maxim.) attenuate scopolamine-induced memory impairment in mice. *J. Sci. Food. Agric.* 98, 5142–5152. doi:10.1002/jsfa.9060

Salehi, B., Sener, B., Kilic, M., Sharif-Rad, J., Naz, R., Yousaf, Z., et al. (2019). *Dioscorea* plants: a genus rich in vital nutrapharmaceuticals-A review. *Iran. J. Pharm. Res.* 18, 68–89. doi:10.22037/ijpr.2019.112501.13795

Son, J. K. (1995). Isolation and structure determination of a new tetralone glucoside from the roots of *Juglans mandshurica*. *Arch Pharm. Res. (Seoul)*. 18, 203–205.

Sun, D. J., Zhu, L. J., Zhao, Y. Q., Zhen, Y. Q., Zhang, L., Lin, C. C., et al. (2020). Diarylheptanoid: a privileged structure in drug discovery. *Fitoterapia*. 142, 104490. doi:10.1016/j.fitote.2020.104490

Sun, Z. L., Dong, J. L., and Wu, J. (2017). Juglanin induces apoptosis and autophagy in human breast cancer progression via ROS/JNK promotion. *Biomed. Pharmacother.* 85, 303–312. doi:10.1016/j.biopha.2016.11.030

Wang, R. J., Wang, S., Xia, Y. J., Tu, M., Zhang, L. J., and Wang, Y. M. (2015). Antitumor effects and immune regulation activities of a purified polysaccharide extracted from *Juglan regia*. *Int. J. Biol. Macromol.* 72, 771–775. doi:10.1016/j.ijbiomac.2014.09.026

Wang, T. M., Fu, Y., Yu, W. J., Chen, C., Di, X., Zhang, H., et al. (2017a). Identification of polar constituents in the decoction of Juglans mandshurica and in the medicated egg prepared with the decoction by HPLC-Q-TOF MS^2. *Molecules*. 22, 1452. doi:10.3390/molecules22091452

Wang, T. M., Liu, J., Yi, T., Zhai, Y. J., Zhang, H., Chen, H. B., et al. (2017b). Multiconstituent identification in root, branch, and leaf extracts of *Juglans mandshurica* using ultra high-performance liquid chromatography with quadrupole time-of-flight mass spectrometry. *J. Separ. Sci.* 40, 3440–3452. doi:10.1002/jssc.201700521

Wang, T. M., Yu, W. J., Fu, Y., Di, X., Zhai, Y. J., Zhang, H., et al. (2017c). Inhibitory effect of the eggs decocted with branches of *Juglans mandshurica* on solid tumor of murine H22 hepatocarcinoma cell in mice. *Drugs Clin.* 32, 365–369. doi:10.7501/j.issn.1674-5515.2017.03.002

Wang, T. M., Yu, W. J., Fu, Y., Di, X., Zhai, Y. J., Kang, T. G., et al. (2017d). Vivo anti-tumor activity of total tannins from Juglans mandshurica, *China Medical Herald*. 14, 16–19.

Wang, P., Gao, C., Wang, W., Yao, L. P., Zhang, J., Zhang, S. D., et al. (2018a). Juglone induces apoptosis and autophagy via modulation of mitogen-activated protein kinase pathways in human hepatocellular carcinoma cells. *Food Chem. Toxicol.* 116, 40–50. doi:10.1016/j.fct.2018.04.004

Wang, G. L., Cheng, T., Huo, J. H., and Wang, W. M. (2018b). Analysis on chemical constituents from rat kidney tissues of *Juglans mandshurica* based on UPLC-Q-TOF/MS. *Chin. Tradit. Herb. Drugs*. 49, 3763–3769. doi:10.7501/j.issn.0253-2670.2018.16.006

Wang, A. D., Xie, C. J., Zhang, Y. Q., Li, M. C., Wang, X., Liu, J. Y., et al. (2019a). α-Tetralonyl glucosides from the green walnut husks of *Juglans mandshurica* and their antiproliferative effects. *Planta. Med.* 85, 335–339. doi:10.1055/a-0832-2328

Wang, J., Zhou, L., Cheng, Z. Y., Wang, Y. X., Yan, Z. Y., Huang, X. X., et al. (2019b). Chiral resolution and bioactivity of enantiomeric furofuran lignans from *Juglans mandshurica* Maxim. *Nat. Prod. Res.* 1-4. doi:10.1080/14786419.2019.1577839

Wang, H., Liu, H., Zhang, N. X., Zhang, H., Gao, W. Y., and Sun, J. M. (2019c). Screening and chemical analysis of hypoglycemic and antioxidant effective fractions from leaves of *Juglans mandshurica*. *Mod. Chin. Med.* 21, 312–315. doi:10.13313/j.issn.1673-4890.20180926005

Wang, J., Wu, T., Fang, L., Liu, C. L., Liu, X. T., Li, H. M., et al. (2020a). Anti-diabetic effect by walnut (*Juglans mandshurica* Maxim.)-derived peptide LPLLR through inhibiting α-glucosidase and α-amylase, and alleviating insulin resistance of hepatic HepG2 cells. *J. Funct. Foods*. 69, 103944. doi:10.1016/j.jff.2020.103944

Wang, J., Wu, T., Fang, L., Liu, C. L., Liu, X. T., Li, H. M., et al. (2020b). Peptides from walnut (*Juglans mandshurica* Maxim.) protect hepatic HepG2 cells from high glucose-induced insulin resistance and oxidative stress. *Food Funct*. 11, 8112–8121. doi:10.1039/d0fo01753a

Wen, Z. M., Jie, J., Zhang, Y., Liu, H., and Peng, L. P. (2017). A self-assembled polyjuglanin nanoparticle loaded with doxorubicin and anti-Kras siRNA for attenuating multidrug resistance in human lung cancer. *Biochem. Biophys. Res. Commun*. 493, 1430–1437. doi:10.1016/j.bbrc.2017.09.132

Westfall, B. A., Russell, R. L., and Auyong, T. K. (1961). Depressant agent from walnut hulls. *Science*. 134, 1617. doi:10.1126/science.134.3490.1617

Xin, N., Hasan, M., Li, W., and Li, Y. (2014). Juglans mandshurica Maxim extracts exhibit antitumor activity on HeLa cells *in vitro*. *Mol. Med. Rep*. 9, 1313–1318. doi:10.3892/mmr.2014.1979

Xu, H. L., Yu, X. F., Qu, S. C., and Sui, D. Y. (2013). Juglone, isolated from *Juglans mandshurica* Maxim, induces apoptosis via down-regulation of AR expression in human prostate cancer LNCaP cells. *Bioorg. Med. Chem. Lett*. 23, 3631–3634. doi:10.1016/j.bmcl.2013.04.007

Xu, H. L., Yu, X. F., Qu, S. C., Zhang, R., Qu, X. R., Chen, Y. P., et al. (2010). Anti-proliferative effect of Juglone from *Juglans mandshurica* Maxim on human leukemia cell HL-60 by inducing apoptosis through the mitochondria-dependent pathway. *Eur. J. Pharmacol*. 645, 14–22. doi:10.1016/j.ejphar.2010.06.072

Yang, B. Y., Jiang, Y. Q., Meng, Y., Liu, Y. X., Liu, Z. X., Xiao, H. B., et al. (2015). Studies on chemical constituents in n-butanol extracts from epicarp of green fruit of *Juglans mandshurica*. *Chin. Tradit. Herb. Drugs*. 46, 481–485. doi:10.7501/j.issn.0253-2670.2015.04.004

Yang, Q., Yao, Q. S., Kuang, Y., Zhang, Y. Z., Feng, L. L., Zhang, L., et al. (2019). Antimicrobial and cytotoxic juglones from the immature exocarps of *Juglans mandshurica*. *Nat. Prod. Res*. 33, 3203–3209. doi:10.1080/14786419.2018.1468326

Yao, D. L., Jiang, L. J., Zhou, W., Xu, T., Pen, L., and Li, G. (2009). Study on the inhibitory effect of chloroform extract of *Juglans mandshurica* root on mouse S180 sarcoma. *J. Chin. Med. Mater*. 32, 595–596. doi:10.13863/j.issn1001-4454.2009.04.042

Yao, D. L., Jin, M., Zhang, C. H., Luo, J., Li, R., Zheng, M. S., et al. (2014). A new phenolic glycoside from *Juglans mandshurica*. *Nat. Prod. Res*. 28, 998–1002. doi:10.1080/14786419.2014.902946

Yao, D. L., Zhang, C. H., Li, R., Luo, J., Jin, M., Piao, J. H., et al. (2015a). Two new conjugated ketonic fatty acids from the stem bark of *Juglans mandshurica*. *Chin. J. Nat. Med*. 13, 0299–0302. doi:10.1016/S1875-5364(15)30018-2

Yao, D. L., Zhang, C. H., Luo, J., Jin, M., Zheng, M. S., Cui, J. M., et al. (2015b). Chemical constituents from the leaves of *Juglans mandshurica*. *Arch Pharm. Res. (Seoul)*. 38, 480–484. doi:10.1007/s12272-014-0398-1

Yao, G. D., Cheng, Z. Y., Shang, X. Y., Gao, P. Y., Huang, X. X., and Song, S. J. (2017). Coumarins from the bark of *Juglans mandshurica* exhibited anti-hepatoma activities via inducing apoptosis. *J. Asian. Nat. Prod. Res*. 19, 1134–1142. doi:10.1080/10286020.2017.1292256

Yao, Y., Zhang, Y. W., Sun, L. G., Liu, B., Bao, Y. L., Lin, H., et al. (2012). Juglanthraquinone C, a novel natural compound derived from *Juglans mandshurica* Maxim, induces S phase arrest and apoptosis in HepG2 cells. *Apoptosis*. 17, 832–841. doi:10.1007/s10495-012-0722-5

Zhang, J. B., Liu, J. X., Zha, F., and Di, L. D. (2009). Chemical constituents in green walnut husks of *Juglans regia*. *Chin. Tradit. Herb. Drugs*. 40, 847–849

Zhang, W., Liu, A. H., Li, Y., Zhao, X. Y., Lv, S. J., Zhu, W. H., et al. (2012a). Anticancer activity and mechanism of juglone on human cervical carcinoma HeLa cells. *Can. J. Physiol. Pharmacol*. 90, 1553–1558. doi:10.1139/y2012-134

Zhang, Y. W., Lin, H., Bao, Y. L., Wu, Y., Yu, C. L., Huang, Y. X., et al. (2012b). A new triterpenoid and other constituents from the stem bark of *Juglans mandshurica*. *Biochem. Systemat. Ecol*. 44, 136–140. doi:10.1016/j.bse.2012.04.015

Zhang, Y. L., Cui, Y. Q., Zhu, J. Y., Li, H. Z., Mao, J. W., Jin, X. B., et al. (2013). The anti-tumor effect and biological activities of the extract JMM6 from the stem-barks of the Chinese *Juglans mandshurica* Maxim on human hepatoma cell line BEL-7402. *Afr. J. Tradit. Complement. Altern. Med*. 10, 258–269. doi:10.4314/ajtcam.v10i2.10

Zhang, X. N., Bai, M., Cheng, Z. Y., Yu, Z. G., and Huang, X. X. (2018). Cytotoxic lignans from the barks of *Juglans mandshurica*. *J. Asian. Nat. Prod. Res*. 20, 494–499. doi:10.1080/10286020.2017.1374256

Zhang, Y. C., Ge, P. L., Chen, J. H., Zhou, Y. Y., and Liu, Y. X. (2019). Advances in studies on naphthoquinones from green walnut husks of *Juglans mandshurica* and their anticancer activities. *Chin. Tradit. Herb. Drugs*. 50, 2251–2256. doi:10.7501/j.issn.0253-2670.2019.09.035

Zhang, Y. W., Lin, H., Li, S. S., Chen, J. B., Sun, Y. S., and Li, Y. X. (2017). High-speed counter-current chromatography assisted preparative isolation of bioactive compounds from stem bark of *Juglans mandshurica*. *J. Separ. Sci*. 40, 767–778. doi:10.1002/jssc.201601043

Zhao, P., Zhou, H. J., Potter, D., Hu, Y. H., Feng, X. J., Dang, M., et al. (2018). Population genetics, phylogenomics and hybrid speciation of *Juglans* in China determined from whole chloroplast genomes, transcriptomes, and genotyping-bysequencing (GBS). *Mol. Phylogenet. Evol*. 126, 250–265. doi:10.1016/j.ympev.2018.04.014

Zhao, Y., Zhou, W., Diao, S. B., Jiang, Z., Jin, M., and Li, G. (2019). Phytochemical investigation on the roots of Juglans mandshurica and their chemotaxonomic significance. *Biochem. Systemat. Ecol*. 87, 103957. doi:10.1016/j.bse.2019.103957

Zhao, F. R., Wang, J., Lu, H. Y., Fang, L., Qin, H. X., Liu, C. L., et al. (2020). Neuroprotection by walnut-derived peptides through autophagy promotion via Akt/mTOR signaling pathway against oxidative stress in PC12 cells. *J. Agric. Food Chem*. 68, 3638–3648. doi:10.1021/acs.jafc.9b08252

Zhou, Y. Y., Meng, Y., Jiang, Y. Q., Liu, Z. X., and Yang, B. Y. (2014a). Study on anti-tumor chemical constituents from pericarps of *Juglans mandshurica*. *J. Chin. Med. Mater*. 37, 1998–2001. doi:10.13863/j.issn1001-4454.2014.11.022

Zhou, Y. Y., Liu, Z. X., Meng, Y., Jiang, Y. Q., and Yang, B. Y. (2014b). Chemical constituents from active fraction in pericarps of *Juglans mandshurica*. *Chin. Tradit. Herb. Drugs*. 45, 2303–2306. doi:10.7501/j.issn.0253-2670.2014.16.004

Zhou, Y. Y., Yang, B. Y., Liu, Z. X., Jiang, Y. Q., Liu, Y. X., Fu, L., et al. (2015a). Cytotoxicity of triterpenes from green walnut husks of *Juglans mandshurica* Maxim in HepG-2 cancer cells. *Molecules*. 20, 19252–19262. doi:10.3390/molecules201019252

Zhou, Y. Y., Yang, B. Y., Jiang, Y. Q., Liu, Z. X., Liu, Y. X., Wang, X. L., et al. (2015b). Studies on cytotoxic activity against HepG-2 cells of naphthoquinones from green walnut husks of *Juglans mandshurica* Maxim. *Molecules*. 20, 15572–15588. doi:10.3390/molecules200915572

Zhou, Y. Y., Liu, Y. X., Jiang, Y. Q., Liu, Z. X., Niu, F., Yang, B. Y., et al. (2015c). Chemical constituents from pericarp of Juglans mandshurica Maxim. *Chin. Tradit. Pat. Med*. 37, 2669–2673. doi:10.3969/j.issn.1001-1528.2015.12.021

Zhou, Y. Y., Jiang, Y. Q., Meng, Y., Liu, Z. X., and Yang, B. Y. (2015d). Active parts constituents from the pericarps of *Jugland mandshurica* Maxim. *Chin. Tradit. Pat. Med*. 37, 332–335. doi:10.3969/j.issn.1001-1528.2015. 02.024

Zhou, Y. Y., Liu, Y. X., Meng, Y., Jiang, Y. Q., Liu, Z. X., Yang, B. Y., et al. (2015e). Quinones of *Juglans mandshurica* maxim. *Acta Chinese Medicine and Pharmacology*. 43, 8–10. doi:10.19664/j.cnki.1002-2392.2015.03.004

Zhou, Y. Y., Liu, Y. X., Jiang, Y. Q., Liu, Z. X., Yang, B. Y., and Xiao, H. B. (2016). Studies on anti-tumor chemical constituents in exocarps of *Juglans mandshurica*. *Chin. Tradit. Herb. Drugs*. 47, 2979–2983. doi:10.7501/j.issn.0253-2670.2016.17.004

Zhou, Y. Y., Liu, Q. Y., Yang, B. Y., Jiang, Y. Q., Liu, Y. X., Wang, Y., et al. (2017). Two new cytotoxic glycosides isolated from the green walnut husks of *Juglans mandshurica* Maxim. *Nat. Prod. Res*. 31, 1237–1244. doi:10.1080/14786419.2016.1233412

Zhou, Y. Y., Wang, Y., Song, H. J., Guo, S., Liu, Y., Liu, Y. X., et al. (2018a). Chemical constituents of *n*-butanol fraction from green walnut husks of *Juglans mandshurica*. *Chin. Tradit. Herb. Drugs*. 49, 4220–4225. doi:10.7501/j.issn.0253-2670.2018.18.004

Zhou, Y. Y., Song, H. J., Guo, S., Zhang, X. J., Fu, L., and Fu, Q. F. (2018b). Chemical constituents from EtOAc extract of Juglans mandshurica maxim. *Information on Traditional Chinese Medicine*. 35, 46–48. doi:10.19656/j.cnki.1002-2406.180146

Zhou, Y. Y., Song, H. J., Guo, S., Wang, Y., Gao, H. R., Zhang, X. J., et al. (2019a). A new triterpene from the green walnut husks of *Juglans mandshurica* Maxim. *J. Nat. Med.* 73, 800–804. doi:10.1007/s11418-019-01309-4

Zhou, Y. Y., Guo, S., Wang, Y., Song, H. J., Gao, H. R., Zhang, X. J., et al. (2019b). α-Tetralone glycosides from the green walnut husks of *Juglans mandshurica* Maxim. and their cytotoxic activities. *Nat. Prod. Res.* 1-9. doi:10.1080/14786419.2018.1561681

Zhou, Y. Y., Wang, Y., Guo, S., Song, H. J., Zhang, X. J., Liu, Y., et al. (2019c). Two new tetralone glycosides from the green walnut husks of *Juglans mandshurica* Maxim. *Nat. Prod. Res*. 33, 2932–2938. doi:10.1080/14786419.2018.1510397

Zhou, Y. Y., Song, H. J., Chen, H., Guo, S., Wang, Y., Gao, H. R., et al. (2019d). Study on flavonoids from green walnut husks of *Juglans mandshurica*. *Chin. Tradit. Herb. Drugs*. 50, 3588–3592. doi:10.7501/j.issn.0253-2670.2019.15.011

Zhou, Y. Y., Song, H. J., Li, J., Guo, S., Wang, Y., Gao, H. R., et al. (2020). Chemical constituents from the green walnut husks of *Juglans mandshurica*. *Chin. Tradit. Pat. Med*. 42, 375–381. doi:10.3969/j.issn.1001-1528.2020.02.020

Zhou, Y. Y., Wang, D., and Niu, F. (2010). Studies on constituents from pericarps of *Juglans mandshurica* with anti-tumor activity. *Chin. Tradit. Herb. Drugs*. 41, 11–14

Zhu, F. Q., Liang, Y. M., Xu, L., Wang, Y. F., Ding, J. L., and Kang, T. G. (2018). Study on Manchu medicine *Juglans mandshurica* herbal Textual research and DNA barcoding identification. *J. Chin. Med. Mater*. 41, 2073–2078. doi:10.13863/j.issn1001-4454.2018. 09. 011

Conflict of Interest: The authors declare that the research was conducted in the absence of any commercial or financial relationships that could be construed as a potential conflict of interest.

Copyright © 2021 Luan, Wang, Yang, Ji, Lv, Han, Liu, Shang, He and Zeng. This is an open-access article distributed under the terms of the Creative Commons Attribution License (CC BY). The use, distribution or reproduction in other forums is permitted, provided the original author(s) and the copyright owner(s) are credited and that the original publication in this journal is cited, in accordance with accepted academic practice. No use, distribution or reproduction is permitted which does not comply with these terms.

GLOSSARY

ABTS 2,2′-azino-bis-(3-ethylbenzenthiazoline-6-sulphonic) acids

Ach acetylcholine

AIF apoptosis-inducing factor

ATP adenosine 5′-triphosphate

A549/DOX DOX-resistant A549

AChE acetylcholinesterase

BQLY the epicarp of immature fruits

CXCL-9/10/11 chemokines

CCL-17 activation-regulated chemokine

ChAT choline acetyltransferase

CC100 maximum cytotoxic concentration

CDK-2 cyclin-dependent kinase 2

CAT catalase

DOX doxorubicin

DOX/PJAD-PEG-siRNA amphiphilic poly(juglanin (Jug) dithiodipropionic acid (DA))-b-poly(ethylene glycol) (PEG)-siRNA Kras with DOX

DNCB 2,4-dinitrochlorobenzene

DA dopamine

DPPH 1,1′-diphenyl-1-picrylhydrazyl

EDJB eggs decocted with J. mandshurica branches

ERK extracellular signal-regulated kinase

GSH glutathione

GSH-px glutathione peroxidase

5-HT 5-hydroxytryptamine

HIV human immunodeficiency virus

HO-1 heme oxygenase-1

H96/CIS Cisplatin-resistant H96

HP hydrolyzed peptide

IC50 50% inhibitory concentrations

IC100 complete inhibitory concentration

JNK c-Jun N-terminal kinase

JMEE J. mandshurica ethanol extracts

JMM6 a separated fraction of ethanol extract from J. mandshurica

JRP1 a water-soluble polysaccharide

JA ω-9 polyunsaturated fatty acid

JMCE chloroform extracts of J. mandshurica roots;

JMLE J. mandshurica leaves ethanol extract

KVPPLLY Lys-Val-Pro-Pro-Leu-Leu-Tyr

LPS lipopolysaccharide

IgA immunoglobulin A

IL-2 interleukin-2

IL-1β interleukin-1β

IL-4 interleukin-4

IL-6 interleukin-6

IL-13 interleukin-13

IL-17 interleukin-17

IL-18 interleukin-18

IFN-α interferon-α

IFN-γ interferon-γ

LAMP1/2 lysosome-associated membrane protein 1/2

mTOR mammalian target of serine/ threonine protein kinase rapamycin

MDR multidrug resistance

Nrf2 nuclear factor E2-related factor 2

NF-κB nuclear factor-κB

NE noradrenaline

p62 sequestosome 1

p-CaMK II phosphorylation of CaM-dependent protein kinase II

ROS reactive oxygen species

sIgA secretory IgA

SOD superoxide dismutase

α-SMA α-smooth muscle-actin

TCM Traditional Chinese Medicine

TWLPLPR Thr-Trp-Leu-Pro-Leu-Pro-Arg

TNF-α tumor necrosis factor-α

TGF-β1 transforming growth factor-β1

TLR-4 Toll like receptor-4

YVLLPSPK Tyr-Val-Leu-Leu-Pro-Ser-Pro-Lys

△Ψm mitochondrial membrane potential
