# Supplementary material for: Provenance and family variations in early growth of Manchurian walnut (Juglans mandshurica Maxim.) and selection of superior families
Source: PLoS One. 2024 Mar 7;19(3):e0298918. doi: 10.1371/journal.pone.0298918 (PMC10919699; doi:10.1371/journal.pone.0298918)
Supplement: S1 File — (ZIP) [file pone.0298918.s004.zip › Microwave-assisted efficient extraction and stability of juglone in different solvents from Juglans regia quantification of six phenolic constituents by validated RP-HPLC and evaluation of antimicrobial activity.pdf]

Analytical Letters

ISSN: 0003-2719 (Print) 1532-236X (Online) Journal homepage: http://www.tandfonline.com/loi/lanl20

Microwave-Assisted Efficient Extraction and Stability of Juglone in Different Solvents from Juglans regia: Quantification of Six Phenolic Constituents by Validated RP-HPLC and Evaluation of Antimicrobial Activity

Nandini Sharma , Partha Ghosh , Upendra K. Sharma , Swati Sood , Arun K. Sinha & Arvind Gulati

To cite this article: Nandini Sharma , Partha Ghosh , Upendra K. Sharma , Swati Sood , Arun K. Sinha & Arvind Gulati (2009) Microwave-Assisted Efficient Extraction and Stability of Juglone in Different Solvents from Juglans regia: Quantification of Six Phenolic Constituents by Validated RP-HPLC and Evaluation of Antimicrobial Activity, Analytical Letters, 42:16, 2592-2609, DOI: 10.1080/00032710903202055

To link to this article: http://dx.doi.org/10.1080/00032710903202055

Published online: 29 Oct 2009.

Submit your article to this journal

Article views: 109

View related articles

Citing articles: 10 View citing articles

Full Terms & Conditions of access and use can be found at
http://www.tandfonline.com/action/journalInformation?journalCode=lanl20

Download by: [KU Leuven University Library] **Date:** 22 April 2016, At: 02:32

Analytical Letters, 42: 2592–2609, 2009
Copyright © Taylor & Francis Group, LLC
ISSN: 0003-2719 print/1532-236X online
DOI: 10.1080/00032710903202055

Downloaded by [KU Leuven University Library] at 02:32 22 April 2016

CHROMATOGRAPHY

Microwave-Assisted Efficient Extraction and Stability of Juglone in Different Solvents from *Juglans regia*: Quantification of Six Phenolic Constituents by Validated RP-HPLC and Evaluation of Antimicrobial Activity

Nandini Sharma,[1] Partha Ghosh,[1] Upendra K. Sharma,[1] Swati Sood,[2] Arun K. Sinha,[1] and Arvind Gulati[2]
[1]Natural Plant Products Division, Institute of Himalayan Bioresource Technology (CSIR), Palampur, India
[2]Hill Area Tea Science Division, Institute of Himalayan Bioresource Technology (CSIR), Palampur, India

Abstract: In the present study, microwave-assisted extraction was compared with conventional approaches for the efficient extraction of juglone and other phenolics from *Juglans regia* bark. The effect of different solvents was also studied and ethyl acetate was found to be a better solvent in terms of juglone yield and stability. Further, a simple and fast RP-HPLC method was developed and validated for the determination of juglone and other bioactive phenolics like gallic acid, caffeic acid, quercetin, myricetin, and quercitrin in these extracts. In addition, the extracts were tested for antimicrobial activity against 16 microorganisms where all the extracts showed broad spectrum activity.

Received 16 April 2009; accepted 17 July 2009.

The authors are grateful to the Council of Scientific & Industrial Research, New Delhi, India, for its financial support during the course of this project. The authors are also thankful to the Director, IHBT, for providing necessary facilities during the course of the work. N. Sharma and U. K. Sharma are thankful to CSIR, Delhi, for the award of the senior research fellowship.

Address correspondence to Arun K. Sinha, Natural Plant Products Division, Institute of Himalayan Bioresource Technology (CSIR), Palampur-176061, HP, India. E-mail: aksinha08@rediffmail.com

Keywords: Antimicrobial activity, juglone, *Juglans regia*, microwave assisted extraction, phenolic compounds, RP-HPLC, stability

INTRODUCTION

Common walnut (*Juglans regia* L.) belongs to the family Juglandaceae and is extensively used in the Indian (Ayurvedic) and Greco-Arab traditional systems of medicine for the treatment of various common ailments and cancer. It is reported to be astringent, antifungal, diuretic, laxative, tonic, blood purifier and detoxifier (Haque et al. 2003; Stamper et al. 2006; Bhatia et al. 2006). *J. regia* bark is used in some countries as a toothbrush and as a dye for coloring the lips (Alkhawajah 1997). It is also used as an anthelmintic and is applied to skin eruptions and ulcers (Kirtikar and Basu 1975).

In common walnut, naphthoquinones and flavonoids are considered the major phenolic compounds (Wichtl and Anton 1999). Among the various naphthoquinones, juglone (5-hydroxy-1,4-naphthoquinone) is of great interest due to its chemical reactivity and bioactivity. Juglone is reported as active ingredient for hair color (Ghosh and Sinha 2008). It is found in fresh walnut leaves (Bruneton 1993; Girzu et al. 1998; Solar et al. 2006), husks (Binder, Benson, and Flath 1989; Buttery et al. 2000; Fukuda, Ito, and Yoshida 2003; Stamper et al. 2006), inner root bark (Hedin, Langhans, and Graves 1979) and also in the stem bark (Mouhajir et al. 2001). Juglone is known to undergo reversible oxido-reduction reactions with the simultaneous formation of free radicals (Anderson et al. 2001). Further, Girzu et al. (1998) reported that juglone is not stable in methanolic solution (with a loss of about 20% after 24 hours) whereas chloroform extract could not be preserved for a longer time. Therefore, a crucial task is the choice of an efficient extraction technique, including appropriate solvent, extraction time, and temperature in order to provide the highest quantity of the desired analyte.

In order to reduce or eliminate the use of organic solvent and to improve the extraction process, newer sample preparation methods, such as microwave-assisted extraction (MAE), ultrasound-assisted extraction (UAE), accelerated solvent extraction, etc., have been introduced for the efficient extraction of bioactive compounds from plants to increase their therapeutic functionality (Proestos and Komaitis 2006; Sharma et al. 2006). Among these, MAE is the simplest and most economical technique in terms of lesser solvent consumption and considerable reduction in extraction time (Pan, Niu, and Liu 2003; Sharma et al. 2006; Martino et al. 2006; Proestos and Komaitis 2006; Cos et al. 2006). In recent years, many papers have been published on the applicability of MAE for the extraction of

Downloaded by [KU Leuven University Library] at 02:32 22 April 2016

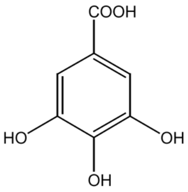

1. Gallic acid

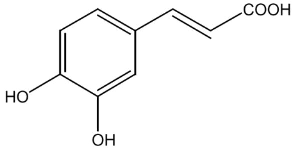

2. Caffeic acid

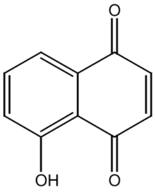

6. Juglone

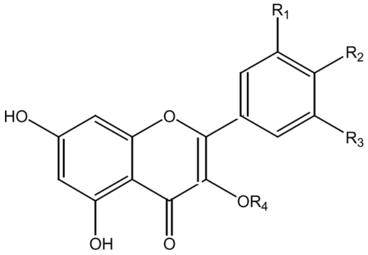

Compound	R_1	R_2	R_3	R_4
3. Quercitrin	OH	OH	H	rhamnose
4. Myricetin	OH	OH	OH	H
5. Quercetin	OH	OH	H	H

Figure 1. Chemical structures of the quantified phenolic compounds in different extracts of *J. regia.*.

bioactive compounds from plants (Sharma et al. 2008). Nevertheless, to the best of our knowledge, there is no report available that could illustrate the feasibility of MAE as a rapid and efficient extraction tool for the extraction of juglone and other phenolic compounds from *J. regia.*

Thus, the present study investigates the feasibility of MAE as an alternate and effective approach for the extraction of juglone from *J. regia*, along with its comparison with other reported techniques, i.e., maceration and ultrasound-assisted extraction (UAE). Several solvents of different polarity were examined to find the best extraction conditions and the extracts were analyzed with the help of HPLC. Further, some other phenolics like quercetin, myricetin, quercetin rhamnoside, caffeic acid and gallic acid, in addition to juglone (Fig. 1), were also quantified in the extracts by a validated RP-HPLC method. Simultaneously, the obtained extracts were subjected to antimicrobial activity against certain bacteria and fungi.

EXPERIMENTAL

Plant Material and Chemicals

J. regia bark and leaves were obtained from Kashmir and Palampur region of India and plant material was confirmed at our biodiversity division. The bark was air-dried at room temp (25 ± 5°C) and relative humidity of 50 ± 5%, powdered, and stored in air-tight plastic bags.

Downloaded by [KU Leuven University Library] at 02:32 22 April 2016

HPLC-grade acetonitrile (MeCN), trifloroacetic acid (TFA), and methanol were purchased from E. Merck (Merck, Darmstadt, Germany). HPLC-grade water was purchased from J. T. Baker (USA). Gallic acid, quercetin, quercitrin, caffeic acid, and myricetin standards were purchased from Sigma (USA). Juglone was from Acros Organics (USA). All of the samples and solvents were filtered through a 0.45 µm membrane filter (Millipore, Germany) and degassed prior to use.

Extraction of Plant Material

Different solvent systems (chloroform, ethyl acetate, methanol, water) were used to determine the effectiveness of solvent type on the extraction of juglone and other phenolic compounds from the bark of *J. regia*.

Microwave-Assisted Extraction (MAE)

About 2 g of powdered plant material was extracted with 20 mL of chloroform, ethyl acetate, methanol, and water in a focused microwave (CEM Discover) for 10–40 min. On mass yield basis, an extraction time of 20 min at 150 W microwave power and 50°C temperature was taken as optimum. The extracts were filtered and concentrated to dryness under vacuum (temperature, 45–50°C) and then subjected to lyophilization until a constant weight was obtained.

Ultrasound-Assisted Extraction (UAE)

About 2 g each of powdered plant material was sonicated with 20 mL of chloroform, ethyl acetate, methanol, and water in an ultrasonicator bath (Elma Ultrasonic, Germany) at a controlled temperature 40 ± 5°C for 30–60 min. An extraction time of 40 min was taken as optimum on mass yield basis. The extracts were filtered and concentrated to dryness under vacuum (temperature 45–50°C) and then subjected to lyophilization until a constant weight was obtained.

Maceration

About 2 g each of powdered plant material was macerated overnight in 20 mL of chloroform, ethyl acetate, methanol, and water at room temperature. The extract obtained was filtered and concentrated fully under vacuum (temperature 45–50°C) and lyophilized until a constant weight was obtained.

Downloaded by [KU Leuven University Library] at 02:32 22 April 2016

All of the extractions were performed in triplicate. All samples were kept in a nitrogen atmosphere and 4°C until further use. For the quantitative determination of compounds by HPLC, concentrated extracts were dissolved in methanol (analytical grade) to obtain a sample solution of 2 mg/mL. The extracts were filtered through a 0.45 μm membrane filters prior to use.

Instrumentation and Chromatographic Conditions

In order to develop an analytical method with shorter retention time and a good resolution of peaks for juglone and other phenolics, HPLC was performed on Waters Model 600 pump system controlled by a Waters 600 automated gradient controller, a Waters 717 plus auto injector, and a Waters 2996 photodiode array detector (Waters Associates, Milford, MA, USA). Chromatographic separations were performed on a LiChrospher RP-18 column (250 mm × 4.6 mm, 5 mm) (Merck, Darmstadt, Germany). The mobile phase was a mixture of A: 0.05% trifloro acetic acid in water and B: acetonitrile–methanol (70:30, v/v), with a gradient programed as follows: A: 80–0% in 0–10 min and back to 80% in 20 min with a flow-rate of 1 mL/min. The injected volume was 20 μL in each assay. The spectra of compounds were recorded from 200 to 500 nm, and the detection wavelengths were 254 nm for gallic acid, myricetin, quercitrin, quercetin, and juglone while caffeic acid was detected at 320 nm.

Stability Study

The degradation rate (decrease of juglone concentration over time) was determined in methanol and ethyl acetate. Juglone solutions were prepared in HPLC grade methanol and AR grade ethyl acetate, followed by storage in the dark at 4°C in amber glass bottles. Initial concentrations were 2 mg/ml. Actual concentrations were determined by the developed reversed phase HPLC in triplicate. Samples were drawn at 24 h intervals respectively from each of methanol and ethyl acetate solution. Aliquots of 20 μL were injected and monitored at 254 nm.

HPLC Validation Studies

Identification of Constituents and Peak Purity

Peaks were identified on the basis of retention times and by comparison with those of the reference standard compounds (Fig. 2). A peak purity

Downloaded by [KU Leuven University Library] at 02:32 22 April 2016

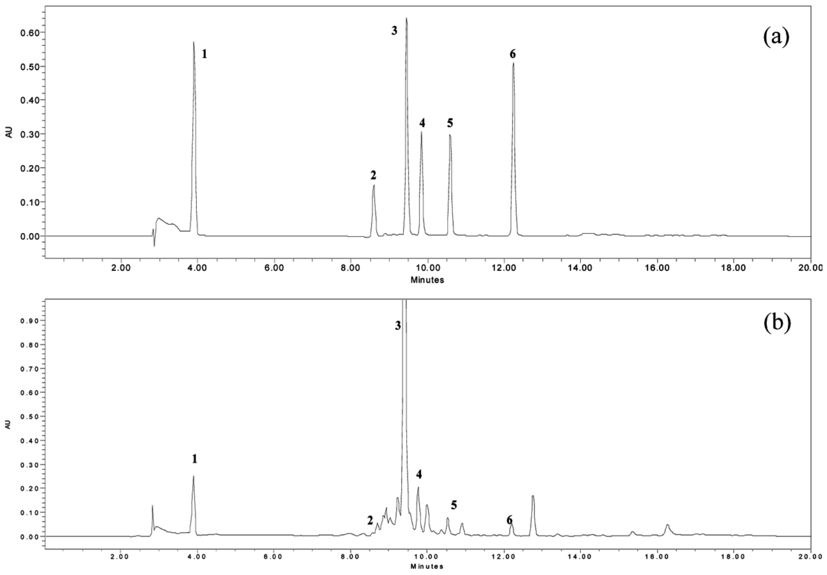

Figure 2. (a) HPLC chromatogram of standards: gallic acid (**1**), caffeic acid (**2**), quercitrin (**3**), myricetin (**4**), quercetin (**5**) and juglone (**6**); (b) HPLC chromatogram of ethyl acetate extract.

test was performed using a photo diode array detector coupled to the HPLC system and comparing the UV spectrum of each peak with those of reference standards at the start, middle, and end of the peak.

Calibration Curve

The stock solution (1 mg/mL) of each standard compound (**1–6**) was freshly prepared in methanol and desired concentrations were obtained by serial dilution for standard curve preparation (Table 1). The calibration graphs were plotted after linear regression of the peak area vs. concentration and both detection limits (LODs) and quantification limits (LOQs) were measured following the standard methods.

Repeatability

The precision of the chromatographic determination for the proposed method, expressed as a relative standard deviation (RSD %), was

Downloaded by [KU Leuven University Library] at 02:32 22 April 2016

Table 1. Parameters of the linearity, detection limit, and quantification limit for chemical compounds present in the bark of *J. regia*

Compound	Linearity range (µg/mL)	Linear equation	r^2	LOD (µg/mL)	LOQ (µg/mL)
Gallic acid (**1**)	1–60	$Y = 31998.65 \times -37025$	0.997	0.16	0.50
Caffeic acid (**2**)	1–60	$Y = 22267.58 \times -6887$	0.998	0.08	0.25
Quercitrin (**3**)	30–900	$Y = 46825 \times -539923$	0.999	0.08	0.25
Myricetin (**4**)	0.312–28	$Y = 58325 \times +11358$	0.997	0.05	0.15
Quercetin (**5**)	0.25–16	$Y = 112824 \times -23172$	0.998	0.04	0.12
Juglone (**6**)	1.6–50	$Y = 63623 \times -30697$	0.997	0.13	0.40

calculated by six replicate injections (n = 6) of each compound (intra-day and inter-day). The standard solutions used for repeatability experiments were the same as used in the calibration curve experiment (Table 2).

Table 2. Repeatability of intra-day and inter-day analysis

Compound	Concentration	RSD%	
		Intraday (n = 6)	Interday (n = 6)
Gallic acid (**1**)	60	0.36	0.44
	30	0.60	0.08
	15	0.28	0.70
Caffeic acid (**2**)	60	0.44	0.19
	30	0.83	0.34
	15	0.98	0.93
Quercitrin (**3**)	64	0.76	0.55
	32	0.50	0.74
	16	0.80	0.80
Myricetin (**4**)	28	0.76	0.56
	14	0.50	0.92
	7	0.38	0.97
Quercetin (**5**)	16	0.38	0.80
	8	0.39	0.92
	4	0.78	0.75
Juglone (**6**)	52	0.25	0.35
	26	0.43	0.22
	13	0.93	0.73

Downloaded by [KU Leuven University Library] at 02:32 22 April 2016

Downloaded by [KU Leuven University Library] at 02:32 22 April 2016

Recovery

For percent recovery, three sets of ethyl acetate extract of barks of *J. regia* were prepared (conc. 2 mg/mL). Three different concentrations of standard compounds **1** (60, 30, and 15 μg/mL); **2** (60, 30, and 15 μg/mL); **3** (64, 32, and 16 μg/mL); **4** (28, 14, and 7 μg/mL); **5** (16, 8, and 4 μg/mL) and **6** (52, 26, and 13 μg/mL) were prepared. The three sets of extracts were then individually spiked with 1 mL of each standard compounds (**1–6**) from all the three spiking concentrations. The samples, to which standards were added, were pretreated and analyzed using the developed HPLC method for measuring the percentage recovery.

Antimicrobial Activity

The antimicrobial activity was screened against 16 microbial strains including *Staphylococcus aureus* (MTCC 3160), *Bacillus subtilis* (MTCC 121), *Micrococcus luteus* (MTCC 2470), *Burkholderia cepacia* (MTCC 438), *Escherichia coli* (MTCC 43), *Klebseilla pneumoniae* (MTCC 109), *Pseudomonas aeruginosa* (MTCC 424), *Enterobacter cloacae* (MTCC 509), *Candida albicans* (MTCC 3017), *Issatchenkia orientalis* (MTCC 231), *Aspergillus flavus* (MTCC 277), *Aspergillus niger* (MTCC 404), *Aspergillus ochraceous* (MTCC 4893), *Aspergillus parasiticus* (MTCC 2797), *Aspergillus sydowii* (MTCC 4335), and *Trichophyton rubrum* (MTCC 296) procured from the Institute of Microbial Technology, India. The broth dilution method was employed for the determination of minimum inhibitory concentration (MIC) and minimum microcidal concentration (MMC) (Cos et al. 2006). Stock solution was prepared by dissolving the extracts in DMSO. Two-fold dilution series of the extracts were prepared for the dose range 2000–2 μg/ml in sterilized Mueller-Hinton broth for bacteria and Sabouraud dextrose broth for fungi in 96-well microtiter plates. The microtiter plates were inoculated with freshly grown bacterial cultures in Mueller-Hinton broth and yeast cultures in Sabouraud dextrose broth. The inoculation of filamentous fungal strains was done by preparing spore and cell suspension in Sabouraud dextrose broth. The uninoculated sterilized medium with DMSO and without DMSO served as the control. Standard antibiotics ampicillin served as the positive controls for bacteria and nystatin for fungi. The microtiter plates were incubated at 37°C for 24 hours for bacteria, 28°C for 24 hours for *Candida albicans* and *Issatchenkia orientalis*, 28°C for 7 days for *Trichophyton rubrum*, and 28°C for 5 days for rest of the fungi. After incubation, 5 μL of resazurin indicator solution (5 mg/mL) was added to each well and the plates were again incubated

for 12 hours. MIC was recorded as the lowest extract concentration which prevented a color change from purple to pink. MMC was determined by plating the samples of MIC dilution and assessing no growth on Mueller-Hinton agar and Sabouraud dextrose agar after incubation at the desired temperatures. The lowest concentration which completely killed the inoculated microorganism was recorded as the MMC. All experiments were performed in triplicate.

Downloaded by [KU Leuven University Library] at 02:32 22 April 2016

RESULTS AND DISCUSSION

Extraction Optimization

In the recent years, application of microwave for the extraction of secondary metabolites from plants has been increasing (Pan, Niu, and Liu 2003; Sharma et al. 2006; Martino et al. 2006) whereby comparison of MAE has been made with other extraction systems by investigating the influence of duration of microwave irradiation, solvent to material ratio, extraction temperature, and types of solvents (Pan, Niu, and Liu 2003). In this study too, MAE has been compared with other techniques for the extraction of juglone from *J. regia* bark.

Further, the efficient extraction of analytes depends upon the solvent choice which, in turn, is determined by the solubility of the analytes of interest, by the interaction between solvent and matrix, and finally by the microwave absorbing properties of the solvent determined by its dielectric constant (Jassie et al. 1997). For the optimization of conditions for the efficient extraction of juglone from *J. regia* bark, maceration, MAE and UAE was performed in solvents ranging from non-polar to polar such as chloroform, ethyl acetate, methanol, and water. In case of maceration, maximum yield of extract was obtained in methanol (12.21%) followed by ethyl acetate (5.53%), water (4.40%), and chloroform (2.12%); however, analysis by HPLC showed that the content of juglone was more in chloroform extract (0.0146%) in comparison to methanol (0.006%) and ethyl acetate extract (0.009%) while it was not detected in water extract. Based upon these observations, chloroform seems to be the solvent of choice for extraction of juglone. This result is in concurrence with the study of Hadjmohammadi and Kamel (2006) where it is reported that chloroform is the best solvent for extraction of juglone (2.69%) from *Pterocarya fraxinifolia* leaves by refluxing. In another study, Girzu et al. 1998 extracted juglone (0.498%) from fresh walnut leaves using chloroform as solvent by maceration. Nevertheless with MAE and UAE, content of juglone was found higher in ethyl acetate extract (0.0147% with MAE and 0.0105% with UAE) instead of

Downloaded by [KU Leuven University Library] at 02:32 22 April 2016

chloroform (0.002% with MAE and 0.003% with UAE) though yield of the extract was again higher in methanol (10.8% and 9.87%, respectively). This anomalism with chloroform in microwave irradiation may be due to its low dielectric constant ultimately leading to poor yields of analytes (Hayes 2002); whereas, lower content of juglone in methanol may be attributed to its decomposition in it, as evident from the earlier report (Girzu et al. 1998). Even though water has the highest dielectric constant, it did not yield any amount of juglone under microwave irradiation as it may not be able to sufficiently solubilize juglone. Thus, ethyl acetate was optimized as the best solvent for the extraction of juglone from the bark of *J. regia* (collected from Kashmir) because of its good heating capacity under microwave and its ability to solubilize juglone and, as a result, inevitably used in further studies. The aforementioned extraction technique was also applied to *J. regia* bark/leaves collected from the Palampur region of India; however, they did not yield any impressing results in terms of juglone content.

Stability of Juglone

Juglone is reported to undergo degradation in solvents such as acetonitrile, methanol, or in acidic medium as well as saline water (Girzu et al. 1998; Hadjmohammadi and Kamel 2006; Wright et al. 2007). In this study, we have examined the decomposition of juglone in methanol and ethyl acetate solution at dark and 4°C over a period of a week. The concentration of juglone in these solutions was calculated from a standard curve derived from a freshly prepared solution and degradation % is summarized in Table 3. From the studies, it is evident that after 24 h juglone does not undergo decomposition in ethyl acetate solution whereas a 6% loss was observed in methanol. Thus, ethyl acetate was optimized as a better solvent in terms of stability and higher extraction of juglone. Further, to avoid the problems of preservation, the samples were extracted within an hour and the extract was analyzed on the same day.

Table 3. Degradation (%) of juglone in methanol and ethyl acetate at 4°C

	24 h	48 h	72 h	96 h	120 h	144 h	168 h
Methanol	6	12	17	20	21	25	33
Ethyl acetate	–[a]	4	8	12	13	15	17

[a]No degradation.

Downloaded by [KU Leuven University Library] at 02:32 22 April 2016

HPLC Method Development, Validation, and Quantification Studies

HPLC is a preferred technique for its high sensitivity, precision, and specificity in comparison to other techniques such as TLC, GC/GC-MS, spectrophotometric, and fluorescence methods. There have been few HPLC methods for the determination of napthaquinones including juglone (Stensen and Jensen 1994, Girzu et al. 1998, Hadjmohammadi and Kamel 2006; Colaric et al. 2005; Babula et al. 2006). However, to the best of our knowledge, no RP-HPLC method is available for the determination of phenolic compounds in walnut bark. In this regard, we have proposed a simple, rapid, and specific RP-HPLC method for the determination of juglone along with other important five phenolic compounds in *J. regia* bark.

The proposed chromatographic method was validated to determine the linearity, LOD, LOQ, accuracy, and precision of each compound. The linearity, LOD, and LOQ for six compounds (**1–6**) were investigated and results are presented in Table 1. Linear equation between the concentration of the standards injected and the peak area can be expressed as $y = mx + c$, where y is the concentration and x is the peak area of the standard, and m and c are constants. A good linearity was achieved in the range 0.997–0.999 for all the compounds. Detection limit is the lowest amount of analyte in a sample that can be detected, but not necessarily quantified. LOD for all the compounds (**1–6**) was in the range 0.04–0.16 µg/mL (Table 1). The LOQ, which is defined as the lowest concentration that can be determined with acceptable accuracy and precision for all the compounds (**1–6**), was experimentally verified by six injections and found in the range 0.12–0.50 µg/mL (Table 1).

The intra-day and inter-day precision (repeatability) of the method was calculated by six replicate injections of three different concentrations of each compound (**1–6**), respectively. Precision was expressed as %RSD. The intraday and interday %RSD of chromatographic determination was observed in the range of 0.25–0.98 and 0.08–0.97%, respectively (Table 2). Hence, the results showed good precision of the developed RP-HPLC method.

Recoveries of the experiment were performed in order to study the accuracy of the method. The optimized RP-HPLC conditions were applied for determination of percentage recovery of compounds (**1–6**) in *J. regia* (collected from Kashmir). Recoveries ranged between 94.53 and 105.09% (Table 4) which testifies the accuracy of the proposed method for determination of the compounds. Overall, the validated method was found to be suitable for quantification of juglone and other phenolic compounds.

Table 4. Recovery study of compounds analyzed

Compounds	Amount present (μg/mL)	Amount added (μg/mL)	Observed amount (μg/mL)	% recovery
**1**	45.60	30.0	74.50	98.50
		15.0	61.80	101.90
		7.5	55.60	104.80
**2**	4.69	30.0	34.60	99.85
		15.0	18.77	95.32
		7.5	11.80	96.80
**3**	367.30	32.0	405.80	101.62
		16.0	387.50	101.09
		8.0	369.40	98.42
**4**	21.04	14.0	34.40	98.17
		7.0	29.25	104.31
		3.5	25.40	103.50
**5**	4.18	8.0	12.80	105.09
		4.0	7.80	95.35
		2.0	6.30	101.94
**6**	5.22	26.0	32.10	102.80
		13.0	18.15	99.61
		6.5	11.08	94.53

Quantitative Determination of Compounds (1–6) in Bark Extracts of *J. regia*

Juglone and other phenolic compounds (**1–6**) were quantitated using the developed and validated RP-HPLC method in the microwave-assisted extracts of bark of *J. regia*. The calculated amount of each compound (**1–6**) is as shown in Table 5. Further quantification and identification

Table 5. Contents of compounds present in the microwave-assisted extracts of *J. regia* bark

S. no.	Ethyl acetate (%)	Methanol (%)	Water (%)
Gallic acid **(1)**	0.1250	0.2030	0.3440
Caffeic acid **(2)**	0.0048	0.0398	0.0050
Quercitrin **(3)**	0.8620	0.7398	0.6144
Myricetin **(4)**	0.0531	0.0515	0.0310
Quercetin **(5)**	0.0208	0.0202	0.0111
Juglone **(6)**	0.0147	0.0029	–

Downloaded by [KU Leuven University Library] at 02:32 22 April 2016

of the remaining secondary metabolites present in *J. regia* bark by the developed method is currently under progress.

Antimicrobial Activity

The antimicrobial activity of synthetic and natural compounds including plant extracts has been recognized for many years and has formed the basis of many applications including food preservation, pharmaceuticals, and medicine (Narad et al. 1995; Tzoris et al. 2003; Murthy et al. 2006). *J. regia* contains napthaquinones as major phenolic compounds (Wichtl and Anton 1999) which are reported to possess very interesting spectrum of antimicrobial activities (Babula et al. 2009). In this direction, extracts of *J. regia* bark and marker compound juglone were tested for antimicrobial activity against 16 microorganisms and the results are given in Table 6. The extracts showed antimicrobial activity against most of the microorganisms whereas juglone was active only against *Pseudomonas aeruginosa* and *Burkholderia cepacia*. Enhanced activity of extracts may be due to the synergistic effect of other compounds present in them. Most of the fungal test organisms were resistant to the aforementioned plant extracts, except *Trichophyton rubrum*. Methanolic extract showed broad spectrum antimicrobial activity against bacteria and filamentous fungi under study while *Bacillus subtilis* appears to be most susceptible among the test organisms. Earlier, leaf extracts of *J. regia* have been reported to inhibit the growth of Gram positive bacteria but not gram negative bacteria and fungi (Preira et al. 2007). Moreover, MIC and MMC values of standard antibiotics used (Ampicillin and Nystatin) were found to be relatively higher than the extracts against *Pseudomonas aeruginosa* and *Burkholderia cepacia* (Table 6). Further fractionation of methanolic and ethyl acetate extracts did not lead to any improvement in antimicrobial activity.

CONCLUSIONS

In this report, we demonstrated the feasibility and ease of microwave for the extraction of juglone from *J. regia* bark. Our results showed that while ethyl acetate is a better extracting solvent in terms of juglone yield and stability, methanolic extract showed higher antimicrobial activity against the tested organisms. Simultaneously, a simple and fast RP-HPLC method was developed and validated for the identification and quantification of six phenolic compounds (**1**–**6**) present in the extracts of *J. regia* bark. The results are promising and demonstrate

Downloaded by [KU Leuven University Library] at 02:32 22 April 2016

Downloaded by [KU Leuven University Library] at 02:32 22 April 2016

Table 6. Antimicrobial activity of the various *J. regia* bark extracts (μg/mL) by Broth micro dilution method

	Methanolic extract		Ethyl acetate extract		Water extract		Juglone		Ampicillin[a]/Nystatin[b]	
Microorganism	MIC	MMC	MIC	MMC	MIC	MMC	MIC	MMC	MIC	MMC
Candida albicans (3017)	–	–	–	–	–	–	–	–	7.8	7.8
Issatchenkia orientalis (231)	–	–	–	–	–	–	–	–	31.3	31.3
Aspergillus flavus (277)	1000	1000	–	–	–	–	–	–	62.5	62.5
Aspergillus niger (404)	500	500	–	–	–	–	–	–	15.6	62.5
Aspergillus ochraceous (4893)	1000	1000	–	–	–	–	–	–	62.5	125
Aspergillus parasiticus (2797)	–	–	–	–	–	–	–	–	62.5	125
Aspergillus sydowii (4335)	500	500	–	–	–	–	–	–	3.9	3.9
Trichophyton rubrum (296)	1000	2000	250	500	2000	2000	–	–	31.3	62.5

(*Continued*)

Downloaded by [KU Leuven University Library] at 02:32 22 April 2016

Table 6. Continued

	Methanolic extract		Ethyl acetate extract		Water extract		Juglone		Ampicillin[a]/Nystatin[b]	
Microorganism	MIC	MMC	MIC	MMC	MIC	MMC	MIC	MMC	MIC	MMC
Staphylococcus aureus (3160)	500	1000	500	1000	1000	2000	–	–	2.0	3.9
Bacillus subtilis (121)	250	500	125	250	–	–	–	–	3.9	7.8
Micrococcus luteus (2470)	1000	2000	1000	2000	2000	–	–	–	2.0	3.9
Burkholderia cepacia (438)	500	500	1000	1000	500	500	250	500	2000.0	2000.0
Escherichia coli (43)	–	–	–	–	–	–	–	–	31.3	62.5
Enterobacter cloacae (509)	–	–	–	–	–	–	–	–	2000.0	2000.0
Klebseilla pneumoniae (109)	–	–	–	–	–	–	–	–	2000.0	2000.0
Pseudomonas aeruginosa(424)	250	500	250	500	250	500	125	250	1000.0	1000.0

[a]antibiotics for bacteria.
[b]antibiotics for fungi. MTCC No. in parenthesis.

the potential for using MAE for the efficient extraction of natural products. This would help in reduction of requisite time for the chemical analysis and characterization of such compounds.

REFERENCES

Alkhawajah, A. M. 1997. Studies on the antimicrobial activity of *Juglans regia*. *Am. J. Chin. Med.* 25: 175–180.

Anderson, K. J., S. S. Teuber, A. Gobeille, P. Cremin, A. L. Waterhouse, and F. M. Steinberg. 2001. Walnut polyphenolics inhibit in vitro human plasma and LDL oxidation. *J. Nutr.* 131: 2837–2842.

Babula, P., V. Adam, L. Havel, and R. Kizek. 2009. Noteworthy secondary metabolites naphthoquinones-their occurrence, pharmacological properties and analysis. *Curr. Parmaceut. Anal.* 5: 47–68.

Babula, P., R. Mikelova, V. Adamb, R. Kizek, L. Havel, and Z. Sladky. 2006. Using of liquid chromatography coupled with diode array detector for determination of naphthoquinones in plants and for investigation of influence of pH of cultivation medium on content of plumbagin in *Dionaea muscipula*. *J. Chromatogr. B* 842: 28–35.

Bhatia, K., S. Rahman, M. Ali, and S. Raisuddin. 2006. *In vitro* antioxidant activity of *Juglans regia* L. bark extract and its protective effect on cyclophosphamide-induced urotoxicity in mice. *Redox Rep.* 11: 273–279.

Binder, R. G., M. E. Benson, and R. A. Flath. 1989. Eight 1,4-naphtoquinones from *Juglans regia*. *Phytochem.* 28: 2799–2801.

Bruneton, J. 1993. In *Pharmacognosie, Phitochimie Plantes Medicinales*, 556–558. Paris: Tec. & Doc.

Buttery, R. G., D. M. Light, Y. Nam, G. B. Merrill, and J. N. Roitman. 2000. Volatile components of green walnut husks. *J. Agric. Food Chem.* 48: 2858–2861.

Colaric, M., R. Veberic, A. Solar, M. Hudina, and F. Stampar. 2005. Phenolic acids, syringaldehyde, and juglone in fruits of different cultivars of juglans regia L. *J. Agric. Food Chem.* 53: 6390–6396.

Cos, P., A. J. Vlietinck, B. V. Berghe, and L. Maes. 2006. Anti-infective potential of natural products: how to develop a stronger *in vitro* proof-of-concept. *Ethanopharmacol.* 106: 290–302.

Fukuda, T., H. Ito and T. Yoshida. 2003. Antioxidative polyphenols from walnuts (*Juglans regia* L.). *Phytochem.* 63: 795–801.

Ghosh, P., and A. K. Sinha. 2008. Hair colors: Classification, chemistry and a review of chromatographic and electrophoretic methods for analysis. *Anal. Lett.* 41: 2291–2312.

Girzu, M., D. Fraisse, A. P. Carnat, A. Carnat, and J. L. Lamaison. 1998. High performance liquid chromatographic method for the determination of juglone in fresh walnut leaves. *J. Chromatogr. A* 805: 315–318.

Hadjmohammadi, M. R., and K. Kamel. 2006. Determination of juglone (5-hydroxy 1,4,-naphthoquinone) in *Pterocarya flaxinifolia* by RP-HPLC. *Iran J. Chem. Chem. Eng.* 25: 73–76.

Downloaded by [KU Leuven University Library] at 02:32 22 April 2016

Haque, R., B. Bin-Hafeez, S. Parvez, S. Pandey, I. Sayeed, M. Ali, and S. Raisuddin. 2003. Aqueous extract of walnut (*Juglans regia* L.) protects mice against cyclophosphamideinduced biochemical toxicity. *Human Experiment. Toxicol.* 22: 473–480.

Hayes, B. L. 2002. *Microwave Synthesis: Chemistry at the Speed of Light*. Matthews, NC: CEM publishing.

Hedin, P. A., V. E. Langhans, and C. H. Graves. 1979. Identification of juglone in pecan as a possible factor of resistance to Fusicladium effusum. *J. Agric. Food Chem.* 27: 92–94.

Jassie, L., R. Revesz, T. Kierstead, E. Hasty, and S. Matz. 1997. In *Microwave enhanced chemistry: Fundamentals, sample preparation and Applications*, eds. H. M. S. Kingston and S. J. Haswell, 569. Washington, DC: American Chemical Society.

Kirtikar, S., and B. D. Basu. 1975. Juglandaceae. In *Indian medicinal plants*, eds. E. Blatter, J. F. Cauis, and K. S. Mhaskar, 2347–2349. Dehradun: Bishen Singh, Mahendra Pal Singh.

Martino, E., H. Ramaiola, M. Urbano, F. Bracco, and S. Collina. 2006. Microwave-assisted extraction of coumarin and related compounds from *Melilotus officinalis* (L.) Pallas as an alternative to Soxhlet and ultrasound-assisted extraction. *J. Chromatogr. A* 1125: 147–151.

Mouhajir, F., J. A. Pedersen, M. Rejdali, G. H. N. Towers. 2001. Phenolics in Moroccan medicinal plant species as studied by electron spin resonance spectroscopy. *Pharmaceut. Biol.* 39: 391–391.

Murthy, M. M., M. Subramanyam, K. V. Giridhar, and A. Jetty. 2006. Antimicrobial activities of bharangin from *Premna herbaceae* Roxb. and bharangin monoacetate. *J. ethnopharmacol.* 104: 290–292.

Narad, S., N. N. Mishra, P. Pandey, A. Kumar, and K. S. Pitre. 1995. Electroanalytical and bacterial effect on N-(5-nitro-2-furfurylidine) 3-amino-2-oxazolidone (Furazolidone) and its metal complexes. *Anal. Lett.* 28: 2005–2016.

Pan, X., G. Niu, and H. Liu. 2003. Microwave-assisted extraction of tea polyphenols and tea caffeine from green tea leaves. *Chem. Eng. Process.* 42: 129–133.

Preira, J. A., I. Oliviera, A. Sousa, P. Valentao, P. B. Andrade, I. C. F. R. Ferreira, F. Ferreres, A. Bento, R. Seabra, and L. Estevinho. 2007. Walnut (*Juglans regia* L.) leaves: Phenolic compounds, antibacterial activity and antioxidant potential of different cultivars. *Food Chem. Toxicol.* 45: 2287–2295.

Proestos, C., and M. Komaitis. 2006. Ultrasonically assisted extraction of phenolic compounds from aromatic plants: Comparison with conventional extraction techniques. *J. Food Qual.* 29: 567–582.

Sharma, A., S. C. Verma, N. Saxena, N. Chadda, N. P. Singh, and A. K. Sinha. 2006. Microwave- and ultrasound-assisted extraction of Vanillin and its quantification by high-performance liquid chromatography in Vanilla planifolia. *J. Sep. Sci.* 29: 613–619.

Sharma, U. K., K. Sharma, N. Sharma, A. Sharma, H. P. Singh, and A. K. Sinha. 2008. Microwave-assisted efficient extraction of different parts of hippophae rhamnoides for the comparative evaluation of antioxidant activity and

Downloaded by [KU Leuven University Library] at 02:32 22 April 2016

quantification of its phenolic constituents by reverse-phase high-performance liquid chromatography (RP-HPLC). *J. Agric. Food Chem.* 56: 374–379.

Solar, A., M. Colaric, V. Usenik, and F. Stampar. 2006. Seasonal variations of selected flavonoids, phenolic acids and quinones in annual shoots of common walnut (*Juglans regia* L.). *Plant Sci.* 170: 453–461.

Stampar, F., A. Solar, M. Hudina, R. Veberic, and M. Colaric. 2006. Traditional walnut liquor-cocktail of phenolics. *Food Chem.* 95: 627–631.

Stensen, W., and E. Jensen. 1994. High performance liquid chromatographic separation of naphthoquinones and their derivatives: Effect of hydrogen bonding on retention. *J Chromatgr. A* 659: 87–93.

Tzoris, A., E. A. H. Hall, G. A. J. Besselink, and P. Bergvelt. 2003. Testing the durability of polymyxin B immobilization on a polymer showing antimicrobial activity: a novel approach with the ion step method. *Anal. Lett.* 36: 1781–1803.

Wichtl, M., and R. Anton. 1999. In *Plantes therapeutiques*, 291–293. Paris: Tec. & Doc.

Wright, D. A., C. L. Mitchelmore, R. Dawson, and H. G. Cutler. 2007. The influence of water quality on the toxicity and degradation of juglone (5-hydroxy 1,4-naphthoquinone). *Environmental Technol.* 28: 1091–1101.

Downloaded by [KU Leuven University Library] at 02:32 22 April 2016
