# Supplementary material for: Provenance and family variations in early growth of Manchurian walnut (Juglans mandshurica Maxim.) and selection of superior families
Source: PLoS One. 2024 Mar 7;19(3):e0298918. doi: 10.1371/journal.pone.0298918 (PMC10919699; doi:10.1371/journal.pone.0298918)
Supplement: S1 File — (ZIP) [file pone.0298918.s004.zip › ‘Helete Güneşi’, a new walnut cultivar with late leafing, early harvest date, and superior nut traits.pdf]

agriculture

MDPI

Article

'Helete Güneşi', a New Walnut Cultivar with Late Leafing, Early Harvest Date, and Superior Nut Traits

Mehmet Sütyemez [1,*], Şakir Burak Büküçü [2] and Akide Özcan [3]

[1] Department of Horticulture, Agricultural Faculty, Kahramanmaras Sutcu Imam University, Kahramanmaras 46040, Turkey
[2] Department of Plant and Animal Production, Silifke Tasucu Vocational School, Selcuk University, Silifke Tasucu, Mersin 33900, Turkey; burak.bukucu@selcuk.edu.tr
[3] Department of Organic Farming, Goksun Vocational School, Kahramanmaras Sutcu Imam University, Kahramanmaras 46650, Turkey; akideozcan@ksu.edu.tr
* Correspondence: sutyemez@ksu.edu.tr

Abstract: 'Helete Güneşi' was selected among different genotypes obtained from crossing 'Maraş 18' × 'Chandler' in Turkey. The present study compares phenological and pomological traits of 'Helete Güneşi' with those of its parents so as to scale their performances. 'Helete Güneşi' staged leaf out on 22 April, whereas its parents, 'Chandler' and 'Maraş 18', did on 20 and 12 April, respectively. The harvest date of 'Helete Güneşi' was as early as 17 September, whereas 'Chandler' and 'Maraş 18' began to be harvested on 5 October and 15 September, respectively. Defoliation in 'Helete Güneşi' occurred about 1 month earlier than 'Chandler'. The nut weight and kernel percentage of 'Helete Güneşi' were 13.41 g and 53.39%, respectively, whereas in 'Chandler' the values were 12.73 g and 48.23%, respectively, but were 14.62 g and 53.76% in 'Maraş 18'. 'Helete Güneşi' had a higher yield value compared to its parents. The results demonstrated that 'Helete Güneşi' has superior traits in being selected for late leafing date, early harvest date, high yield, and good nut quality. Therefore, it can be considered as a valuable genetic resource in future breeding programs around the world.

Keywords: walnut; *Juglans regia* L.; cultivar; crossbreeding; late leafing; lateral bud flowering; yield; early harvest

check for updates

Citation: Sütyemez, M.; Büküçü, Ş.B.; Özcan, A. 'Helete Güneşi', a New Walnut Cultivar with Late Leafing, Early Harvest Date, and Superior Nut Traits. *Agriculture* **2021**, *11*, 991. https://doi.org/10.3390/agriculture11100991

Academic Editors: Kourosh Vahdati, Anita Solar and Neus Aletà

Received: 7 September 2021
Accepted: 9 October 2021
Published: 12 October 2021

Publisher's Note: MDPI stays neutral with regard to jurisdictional claims in published maps and institutional affiliations.

Copyright: © 2021 by the authors. Licensee MDPI, Basel, Switzerland. This article is an open access article distributed under the terms and conditions of the Creative Commons Attribution (CC BY) license (https://creativecommons.org/licenses/by/4.0/).

1. Introduction

Walnut (*Juglans regia* L.) is a very important source of nutrition for the human body and a valuable plant for the ecological cycle as it usually has a long lifespan. It is a hard-shelled fruit species and is cultivated in various regions of the world. Walnut production in the world has increased significantly since 1985 and has reached nearly 5 million tons a year [1]. Among various factors affecting walnut production is the increase in public awareness about the positive effects of nutritional elements in walnut, along with a gradual increase in demand for walnut as a result of population growth [2–7].

Global climate change, diseases, and pests, as well as a decrease in the area of agricultural lands have affected plant cultivation. In addition, increasing demand for walnut and other negative factors make it necessary for walnut producers to grow productive and high-quality walnut cultivars that can tolerate different biotic and abiotic factors, thereby indicating the significance of walnut breeding programs. In today's world, walnut-growing countries such as the US, China, France, Turkey, and Iran have recently focused on walnut crossbreeding programs to develop new walnut cultivars with superior traits.

One of the most critical ecological factors that limit walnut cultivation is the late-spring frost. High yield, early harvest date, and tolerance to plant diseases/pests are favorable traits in a good walnut cultivar. In addition, high nut quality directly boosts the market value of walnuts [8–14]. The main objective of walnut breeding programs is to obtain new walnut cultivars with these traits. Thanks to walnut breeding studies in

different countries, several walnut cultivars such as 'Chandler', 'Franquette', 'Fernor', 'Howard', 'Serr', and 'Pedro' are currently entrenched in the global market [15,16]. Similar to current trends in the world, walnut breeding programs have become popular in Turkey over the past recent years. For instance, two patents were obtained for two important walnut cultivars, i.e., 'Maraş 18' and 'Sütyemez-1' [17]. The first walnut crossbreeding program was initiated by Prof. Mehmet Sütyemez in 2005 [18]. Within the scope of this crossbreeding program, the first crossbred walnut cultivar ('Diriliş') in Turkey was released in 2016 [19]. Likewise, 'Helete Güneşi' is now a crossbred walnut cultivar with superior traits after being selected among different genotypes resulting from the cross combinations of 'Maraş 18' × 'Chandler'. A patent was obtained for 'Helete Güneşi' from the Ministry of Agriculture and Forestry in 2021 [20].

The present study aimed to compare 'Helete Güneşi' with its parents ('Maraş 18' and 'Chandler') in terms of their phenological and pomological traits.

2. Materials and Methods

2.1. Materials

The present study was conducted in crossbreeding plots affiliated with the University of Kahramanmaras Sutcu Imam, Faculty of Agriculture, Nut Application and Research Center (SEKAMER). The first crossbreeding attempt that would lead to different combinations was initiated by Sütyemez in 2005 [18]. The aim of this breeding program is to obtain productive and high-quality genotypes with late leafing, early harvest, and early leaf defoliation. 'Helete Güneşi' is one of the cultivars obtained from this breeding program. Within the framework of this program, the present study focused on 'Helete Güneşi', which was selected among different genotypes obtained from 'Maraş 18' × 'Chandler' combinations. In order to minimize the effects of rootstock on plant performance, 'Helete Güneşi' and its parents were grafted onto seedlings of the same seed source in the same year and then were transplanted into the field in 2012. Phenological traits were observed on 10 plants per cultivar.

The study area (SEKAMER) was located in Kahramanmaraş province, Turkey (37°35′27″ N and 37°03′28″ E), which is 930 m above sea level and has a mild climate. Annual precipitation level and average temperature are 727 mm and 16.9 °C, respectively. The soil structure is generally suitable for walnut cultivation.

2.2. Method

Mean values for phenological and pomological traits were recorded in three consecutive years (2018, 2019, and 2020). IPGRI [21] and UPOV [22] criteria were used in characterizing these traits. A total of 26 different traits, i.e., 14 phenological and 12 pomological traits, were analyzed. The walnut cultivars were observed every other day so as to document the phenological traits. The nuts were collected during their harvest maturity and represented each tree for pomological analysis. After removing the green shells, the nuts were dried until the moisture content fell below 8%. The dried nuts were kept in the laboratory for 24 h, and a total of 75 nuts from each cultivar were used for pomological analysis. Shell thickness was measured using a digital caliper. Nut weight and kernel weight were calculated using an electronic scale with an accuracy of 0.01 g. The mean values of data described phenological and pomological traits (Tables 1 and 2).

Table 1. Description of the phenological traits.

Traits	Description
Leafing date	Date when 50% of terminal buds had enlarged and the bud scales had split, exposing the green leaves
First male bloom date	When first pollen shedding occurred
Last male bloom date	When last pollen shedding occurred
First female bloom date	Date of initial pistillate flower receptivity
Last female bloom date	Date of last pistillate flower receptivity
Male flowering times	Duration of catkins receptiveness
Female flowering times	Duration of female flower receptiveness
Female abundance	Female flower abundance: 3: Light; 5: Intermediate; 7: High
Catkin abundance	Male flower abundance: 3: Light; 5: Intermediate; 7: High
Lateral bud flowering	Percentage of lateral buds with female flowers
Dichogamy	Overlapping duration of female flowers and catkins receptiveness, status 1 Protandrous; 2 Protogynous; 3 Unknown
Yield	Yield per tree of 7-, 8-, and 9-year-old trees, determined as kg
Harvest date	The date when the green husk begins to crack on the trees
Defoliation date	The date when 75% of the leaves on the trees fall

Table 2. Description of the pomological traits.

Traits	Description
Nut shape	1: Round; 2: Triangular; 3: Broad ovate; 4; Ovate; 5: Short trapezoid; 6: Long trapezoid; 7: Broad eliptic; 8: Eliptic; 9: Cordate
Shell texture	1: Very smooth; 3: Smooth; 5: Medium; 7: Rough; 9: Very rough
Shell colour	1: Very light; 3: Light; 5: Medium; 7: Dark; 9: Very dark
Shell strength	1: Paper; 3: Weak; 5: Intermediate; 7: Strong
Shell thickness (mm)	Near the center of a halved shell was measured with a digital caliper.
Nut weight (g)	Average of total 75 nuts
Kernel weight (g)	Average of total 75 nuts
Kernel percentage (%)	Kernel weight/nut weight × 100
Kernel color	1: Extra light; 2: Light; 3: Light amber; 4: Amber
Kernel fill	3: Poor; 5: Moderate; 7: Well
Kernel flavor	1: Satisfactory; 2: Unsatisfactory
Ease of removal of kernel halves	1: Very easy; 3: Easy; 5: Moderate; 7: Difficult; 9: Very difficult

The data were analyzed using variance analysis to calculate lateral bud flowering, yield per tree, nut weight, kernel weight, kernel percentage, and shell thickness values. Mean values of each cultivar were compared using a multiple range test with the least significant difference of 5% (LSD). JMP 13 package program was used for statistical data analysis.

3. Results and Discussions

The present study mainly aimed to offer insights into the details of 'Helete Güneşi' and its performance. Comparisons were made with previous crossbreeding research and also with the parents of 'Helete Güneşi'. Phenological and pomological characteristics of 'Helete Güneşi' were evaluated in contrast to the features of its parents (Tables 3 and 4). As can be seen in Table 3, the first leafing date in 'Helete Güneşi' was 22 April, which was 2 days later than 'Chandler' and 10 days later than 'Maraş 18' (Figure 1). A later leafing date in 'Helete Güneşi', compared to 'Chandler', is a promising phenological trait.

Table 3. Phenological traits of 'Helete Güneşi' and its parents.

Phenological Traits	Cultivars		
	**Helete Güneşi**	**Chandler**	**Maraş 18**
Leafing date	22 April	20 April	12 April
First male bloom date	23 April	21 April	13 April
Last male bloom date	2 May	30 April	20 April
First female bloom date	29 April	1 May	21 April
Last female bloom date	9 May	8 May	28 April
Male flowering times	9 days	9 days	7 days
Female flowering times	10 days	7 days	7 days
Female abundance	High (7)	High (7)	Intermediate (5)
Catkin abundance	Intermediate (5)	High (7)	Intermediate (5)
Lateral bud flowering	98.8% ± 1.42	90.7% ± 3.10	70.2% ± 3.93
Dichogamy	Protandrous	Protandrous	Protandrous
Yield (kg/tree) (7th-8th-9th year on tree)	9.36 kg ± 1.11	7.03 kg ± 0.89	4.14 kg ± 0.86
Harvest date	17 September	5 October	15 September
Defoliation date	7 November	6 December	3 November

Table 4. Pomological traits of 'Helete Güneşi' and its parents.

Pomological Traits	Cultivars		
	**Helete Güneşi**	**Chandler**	**Maraş 18**
Nut shape	Broad elliptic (8)	Ovate (5)	Ovate (5)
Shell texture	Very smooth (1)	Medium (5)	Smooth (3)
Shell color	Light (3)	Light (3)	Very light (1)
Shell strength	Weak (3)	Weak (3)	Intermediate (5)
Shell thickness	1.10 mm ± 0.12	1.19 mm ± 0.10	1.37 mm ± 0.14
Nut weight	13.41 g ± 1.25	12.73 g ± 1.36	14.62 g ± 1.41
Kernel weight	7.16 g ± 0.71	6.13 g ± 0.69	7.86 g ± 0.82
Kernel percentage	53.39% ± 1.70	48.23% ± 1.61	53.76% ± 1.86
Kernel color	Light (2)	Light (2)	Light (2)
Kernel fill	Well (7)	Well (7)	Well (7)
Ease of removal of kernel halves	Easy (3)	Very easy (1)	Easy (3)
Kernel flavor	Satisfactory (1)	Satisfactory (1)	Satisfactory (1)

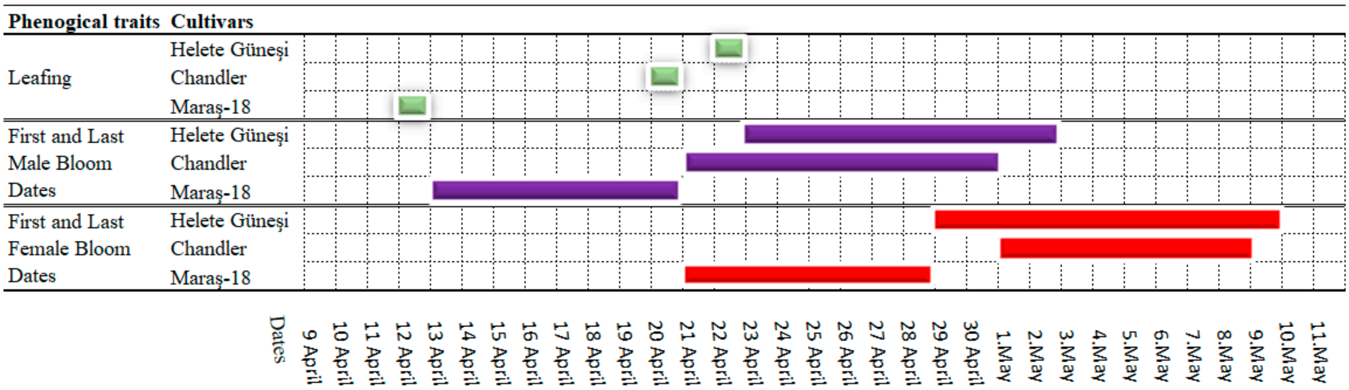

Figure 1. The comparison of some phenological traits in different walnut cultivars.

Many studies in the existing literature have listed 'Chandler' having a later leafing date then many walnut genotypes [16,23–27]. Various studies have underlined the importance of a late leafing date that can help protect the tree from late-spring frost [11,15,17,28–30].

'Helete Güneşi' can be seen as an important genetic resource for walnut cultivation because of the said trait.

Walnut is a monoic fruit species and, because of its hereditary nature, is characterized by a high dichogamy tendency. Therefore, the determination of active periods in male and female flowering times is of vital importance in terms of fertilization biology.

The mean values of phenological traits in walnut cultivars through the three consecutive years are given in Table 3. In 'Helete Güneşi', the male bloom period lasted for 9 days between 23 April and 2 May, while the female bloom period lasted for 10 days between 29 April and 9 May. In 'Chandler', however, the male bloom period lasted for 9 days between 21 and 30 April, and the female bloom period lasted for 7 days between 1 and 8 May. Meanwhile, regarding 'Maraş 18', the male bloom period lasted for 7 days between 13 and 20 April, whereas the female bloom period lasted for 7 days between 21 and 28 April (Figure 1, Table 3).

The dichogamy status of a cultivar is determined as the overlap between male and female bloom dates. All walnut cultivars analyzed in the present study displayed a protandrous flowering trait (Table 3). This was not a surprising result because previous studies in the existing literature pointed to the fact that 'Chandler' has a protandrous flowering trait [12,27,31]. Another study by Sütyemez [17] demonstrated that 'Maraş 18' is also protandrous in flowering. It can be thus stated that the findings in the present study are in agreement with previous research in the available literature. Pollinators of 'Helete Güneşi' can benefit walnut orchards if they are planted in the right combination of male–female flowering overlap.

Walnut breeding programs usually aim at higher rates of lateral bud flowering and a high abundance of female flowers, which can be considered as two important parameters in assessing yield potential in a given cultivar [12,16,28,32].

Phenological observations in the present study demonstrated that 'Helete Güneşi' had a fairly high lateral bud flowering rate (98.8%). Furthermore, this cultivar had a high level of female flower abundance. Therefore, the suitability of these traits has made 'Helete Güneşi' superior to 'Chandler' in some respects, since the latter is also known to have a high percentage of lateral bud flowering (90.7%). On the other hand, lateral bud flowering and female flower abundance in 'Maraş 18' were calculated as 70.2% and intermediate, respectively. In sum, the level of male flower abundance in 'Helete Güneşi', 'Chandler', and 'Maraş 18' were intermediate, high, and intermediate, respectively.

In 10 same-age plants (mean values of 7th, 8th, and 9th years), the mean values of yield among the three cultivars under the same ecological conditions were calculated as 9.36 kg/per tree in 'Helete Güneşi', 7.03 kg/per tree in 'Chandler', and 4.14 kg/per tree in 'Maraş 18' (Table 3). It was evident that 'Helete Güneşi' displayed a higher yield value compared to the credible yield of 'Chandler'. Statistical differences between lateral bud flowering and yield per tree in each walnut cultivar are shown in Figure 2. Thus, it can be suggested that 'Helete Güneşi' is superior to 'Chandler' and 'Maraş 18' cultivars in terms of lateral bud flowering and total yield.

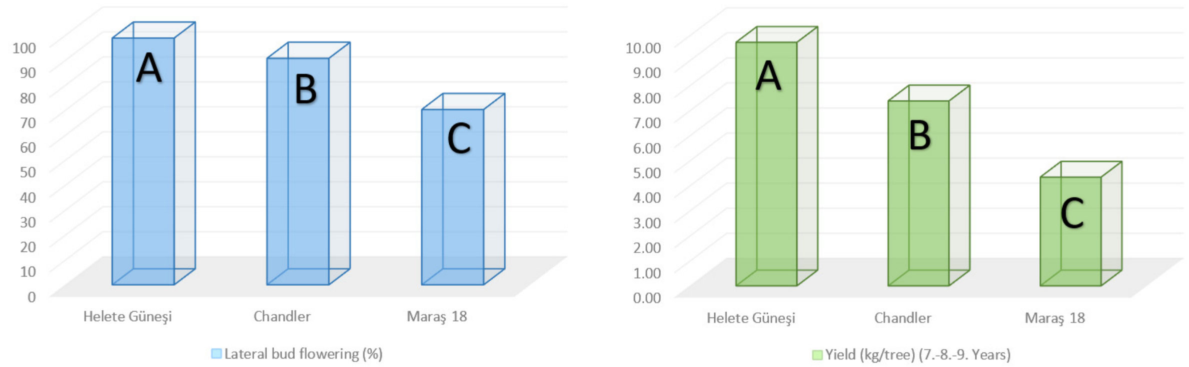

Figure 2. A comparison of three cultivars in terms of yield per tree and lateral bud flowering. Different letters over the columns represent significant differences based on the LSD multiple range test ($p = 0.05$).

Another important phenological parameter that determines the economic potential of a cultivar is "harvest date", which directly affects the spot value of marketed products. While it applies to walnut as well, an early harvest date can be seen as a protective measure against early-autumn frost events. Sütyemez [17] reported that the harvest date for 'Maraş 18' was earlier compared to 'Chandler'. In the current research, the harvest dates of 'Helete Güneşi' and 'Maraş 18' cultivars were 17 and 15 September, respectively, whereas the harvest date of 'Chandler' was 5 October, i.e., 18 days later than 'Helete Güneşi' (Table 3). Therefore, an early harvest date in 'Helete Güneşi' can be seen as another promising trait in the phenological features of this cultivar.

Despite its lesser importance than late-spring frost, early-autumn frost can affect the success rate of walnut cultivation and the sustainability of orchard management. Due to its late defoliation date, 'Chandler' is not wholly suitable for agricultural lands exposed to frequent, early-autumn frost. In contrast, 'Maraş 18' is a walnut cultivar with an early defoliation date. Specifically, defoliation dates in 'Helete Güneşi', 'Maraş 18', and 'Chandler' cultivars were 7 November, 3 November, and 6 December, respectively. In other words, the defoliation date in 'Helete Güneşi' occurred 30 days earlier than 'Chandler' and 4 days later than 'Maraş 18' (Table 3).

Pomological analysis helps determine fruit quality and has been widely used in many breeding studies to identify fruit cultivars with superior traits. The most important traits in a high-quality walnut cultivar are nut weight, kernel weight, kernel percentage, kernel color, kernel fill, and ease of removal of kernel halves [12,16,23,28,33,34].

Twelve different traits were analyzed for the purpose of characterizing pomological traits among the three different walnut cultivars in the present study. The findings indicated that 'Helete Güneşi' had a broad elliptic nut shape, a very smooth shell texture, and a light shell color, thereby indicating remarkable differences in comparison with its parents in terms of nut shape. Shell thickness in 'Helete Güneşi', 'Chandler', and 'Maraş 18' cultivars were 1.10 mm, 1.19 mm, and 1.37 mm, respectively. In other words, the shell strength of 'Helete Güneşi' was comparatively weak (Table 4).

Nut weight is not the sole determiner of walnut quality since kernel weight is also a crucial criterion. In the available literature, there are various reports on kernel weight in different walnut genotypes, ranging from 3.62 to 27.00 g [17,33–38]. In addition, it is reported that a high-quality cultivar should have a kernel percentage of over 50% [39].

In the present study, the nut weight and kernel weight of 'Helete Güneşi' were 13.41 g and 7.16 g, respectively, while its kernel percentage was 53.39%. According to standards on walnut quality, thus 'Helete Güneşi' can be seen as a high-quality cultivar in terms of kernel percentage. Nut weight, kernel weight, and kernel percentage were 14.62 g, 7.86 g, and 53.76% in 'Maraş 18', whereas in 'Chandler' they were 12.73 g, 6.13 g, and 48.23%, respectively. With these descriptions, the 'Helete Güneşi' cultivar struck a position between the two parent cultivars in terms of kernel weight, although it was closer to 'Maraş 18' in terms of kernel percentage (Table 4).

The statistical analysis of nut weight, kernel weights, kernel percentage, and shell thickness of 'Helete Güneşi' and its parents indicated statistically significant differences among these three cultivars (Figure 3). Previous studies on the 'Chandler' cultivar showed that its nut weight and kernel weight ranged from 7.7 g to 14.0 g and from 3.32 g to 7.00 g, respectively [17,27,31,40]. Similarly, Sütyemez [17] reported that the kernel weight and kernel percentage of 'Maraş 18' ranged from 13 to 15 g and from 52% to 57%, respectively.

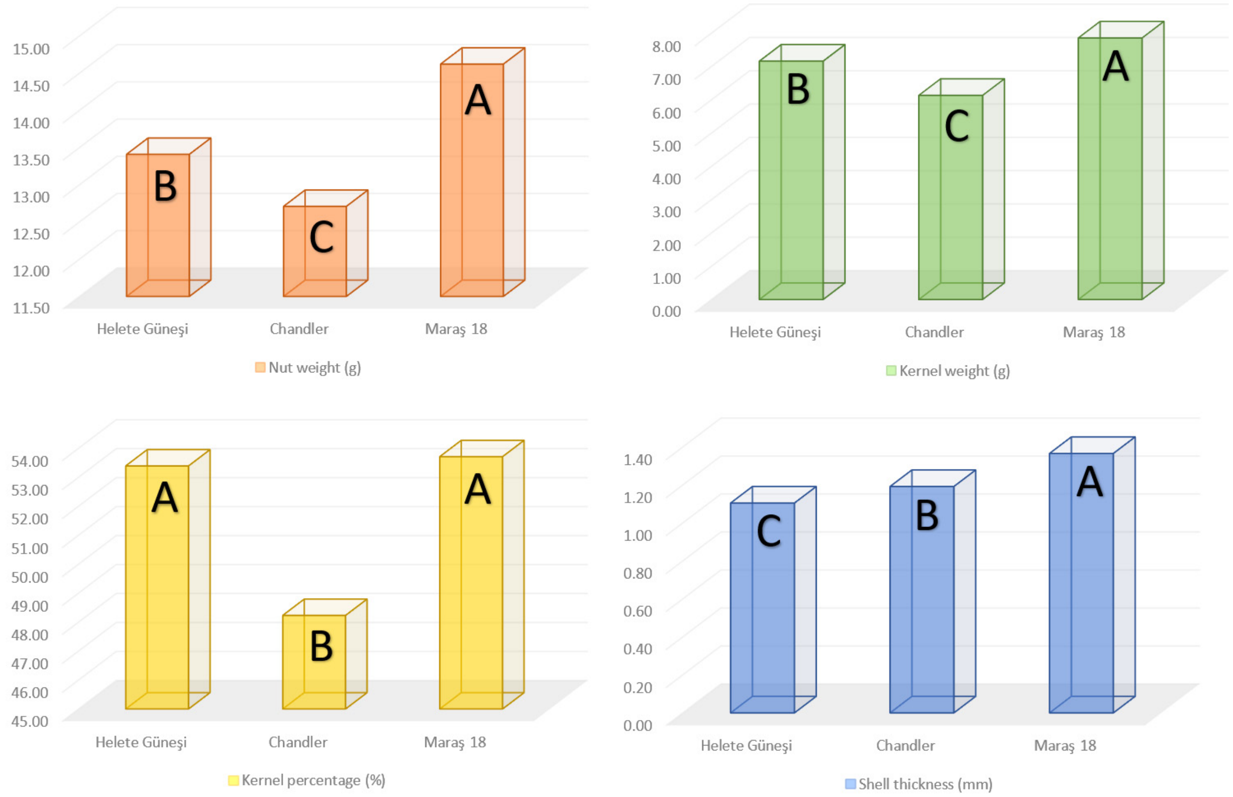

Figure 3. A comparison of three cultivars in terms of kernel weight, shell thickness, shelled nut weight, and kernel percentage. Different letters over the columns represent significant differences based on the LSD multiple range test ($p = 0.05$).

A suitable degree of kernel fill was observed in the three cultivars of this study. Meanwhile, kernel color is another important indicator of market value for a walnut cultivar. It was found that the three cultivars in the present study were similar to each other in this respect since they had a light kernel color. The current findings describe the kernel color of 'Chandler' and 'Maraş 18' as the same as those in the available literature [17,19,27]. Finally, ease of removal of kernel halves was easy in the case of 'Helete Güneşi' and 'Maraş 18' cultivars, while it was very easy in the 'Chandler' cultivar. Kernel flavor in the three cultivars was tested by a group of 20 people, as a result of which, all three cultivars had kernels with satisfactory flavors. For a more tangible description of 'Helete Güneşi', a pictorial overview of its kernels and the tree is depicted in Figure 4.

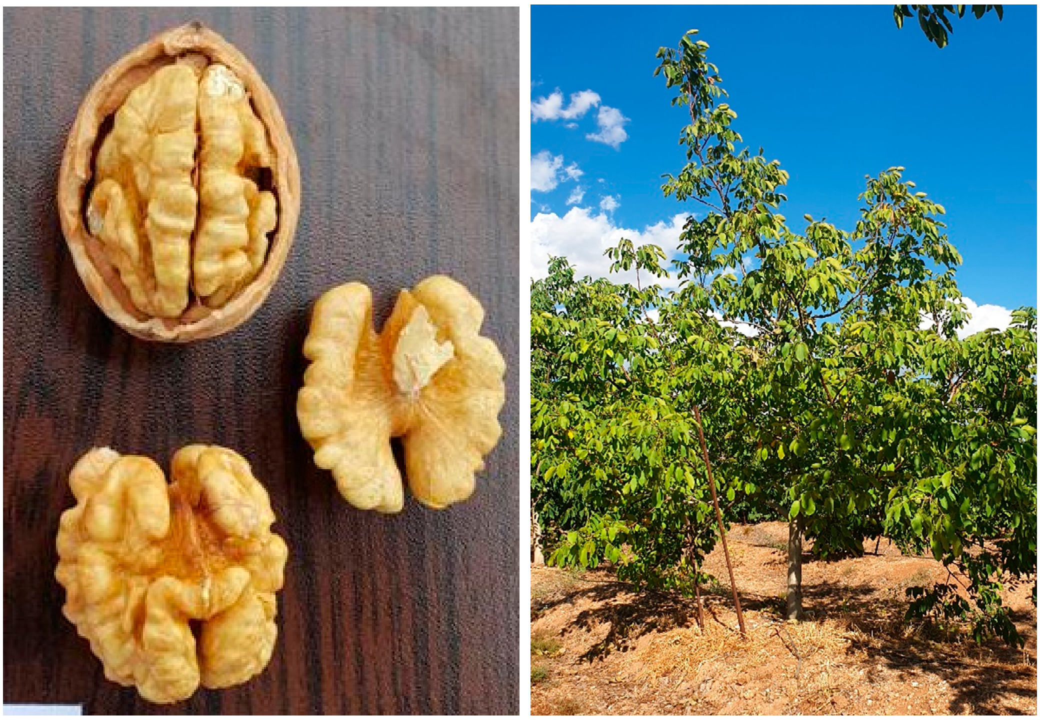

Figure 4. An overview of nuts, kernels, and the tree of 'Helete Güneşi'.

4. Conclusions

Plant breeding is increasingly becoming important in light of opportunities for economic investment and by motives to maintain genetic diversity. While numerous walnut cultivars have been registered around the world so far, more breeding programs are needed to produce higher quality and productive walnut cultivars for solving existing problems, such as spring and autumn frosts, in walnut cultivation.

Here, a thorough comparison with established walnut cultivars showed that 'Helete Güneşi' is rather superior because of its late-leafing ability, early harvest date, high rate of lateral bud flowering, total yield per tree, and high-quality nut traits such as high kernel weight and percentage.

The superiority of 'Helete Güneşi' over 'Chandler' can be claimed in terms of several phenological and pomological values, which bear a vital importance for walnut cultivation. 'Helete Güneşi' can serve as a contribution to walnut cultivation in the world, while offering a genetic resource for walnut crossbreeding in the future.

5. Patent

'Helete Güneşi' was registered by the Ministry of Agriculture and Forestry in Turkey in 2021.

Author Contributions: Conceptualization, M.S., Ş.B.B. and A.Ö.; Methodology, M.S., Ş.B.B. and A.Ö.; Software, M.S., Ş.B.B. and A.Ö.; Validation, M.S., A.Ö. and Ş.B.B.; Investigation, M.S., Ş.B.B. and A.Ö.; Resources, M.S., Ş.B.B. and A.Ö.; Data Curation, M.S., Ş.B.B. and A.Ö.; Writing—Original Draft Preparation, M.S., Ş.B.B. and A.Ö.; Writing—Review and Editing, M.S., Ş.B.B. and A.Ö.; Visualization, M.S., Ş.B.B. and A.Ö. All authors have read and agreed to the published version of the manuscript.

Funding: This research received no external funding.

Institutional Review Board Statement: Not applicable.

Informed Consent Statement: Not applicable.

Data Availability Statement: All data.

Conflicts of Interest: The authors declare no conflict of interest.

References

1. FAO. Available online: http://www.fao.org/faostat/en/#data/QC/visualize (accessed on 5 August 2021).
2. Vahdati, K.; Bavani, A.R.; Khosh-Khui, M.; Fakour, P.; Sarikhani, S. Applying the AOGCM-AR5 models to the assessments of land suitability for walnut cultivation in response to climate change: A case study of Iran. *PLoS ONE* **2019**, *14*, e0218725. [CrossRef]
3. Jahanbani, R.; Ghaffari, S.M.; Vahdati, K.; Salami, M.; Khalesi, M.R.; Sheibani, N.; Moosavi-Movahedi, A.A. Kinetics study of protein hydrolysis and inhibition of angiotensin converting enzyme by peptides hydrolysate extracted from walnut. *Int. J. Peptide Res. Ther.* **2018**, *24*, 77–85. [CrossRef]
4. Arranz, S.; Pérez-Jiménez, J.; Saura-Calixto, F. Antioxidant capacity of walnut (*Juglans regia* L.): Contribution of oil and defatted matter. *Eur. Food Res. Tech.* **2008**, *227*, 425–431. [CrossRef]
5. Gharibzahedi, S.M.T.; Mousavi, S.M.; Hamedi, M.; Rezaei, K.; Khodaiyan, F. Evaluation of physicochemical properties and antioxidant activities of Persian walnut oil obtained by several extraction methods. *Indust. Crop Prod.* **2013**, *45*, 133–140. [CrossRef]
6. Zibaeenezhad, M.; Aghasadeghi, K.; Hakimi, H.; Yarmohammadi, H.; Nikaein, F. The effect of walnut oil consumption on blood sugar in patients with diabetes mellitus type 2. *Int. J. Endocrinol. Metab.* **2016**, *14*, e34889.
7. Gorji, N.; Moeini, R.; Memariani, Z. Almond, hazelnut and walnut, three nuts for neuroprotection in Alzheimer's disease: A neuropharmacological review of their bioactive constituents. *Pharmacol. Res.* **2018**, *129*, 115–127. [CrossRef] [PubMed]
8. Aslantas, R. Identification of superior walnut (*Juglans regia*) genotypes in north-eastern Anatolia, Turkey. *N. Z. J. Crop Horti. Sci.* **2006**, *34*, 231–237. [CrossRef]
9. Rezaee, R.; Vahdati, K.; Grigoorian, W.; Valizadeh, M. Walnut grafting success and bleeding rate as affected by different grafting methods and seedling vigor. *J. Hortic. Sci. Biotech.* **2008**, *83*, 94–99. [CrossRef]
10. McGranahan, G.; Leslie, C. Walnut. In *Fruit Breeding*; Springer: Boston, MA, USA, 2012; pp. 827–846.
11. Ebrahimi, A.; Khadivi-Khub, A.; Nosrati, Z.; Karimi, R. Identification of superior walnut (*Juglans regia*) genotypes with late leafing and high kernel quality in Iran. *Sci. Hortic.* **2015**, *193*, 195–201. [CrossRef]
12. Sütyemez, M.; Bükücü, Ş.B.; Özcan, A. Maraş 12: A walnut cultivar with cluster-bearing habit. *HortScience* **2019**, *54*, 1437–1438. [CrossRef]
13. Chatrabnous, N.; Yazdani, N.; Tavallali, V.; Vahdati, K. Preserving quality of fresh walnuts using plant extracts. *LWT* **2018**, *91*, 1–7. [CrossRef]
14. Mahmoudian, M.; Rahemi, M.; Karimi, S.; Yazdani, N.; Tajdini, Z.; Sarikahni, S.; Vahdati, K. Role of kaolin on drought tolerance and nut quality of Persian walnut. *J. Saudi Soc. Agri. Sci.* **2021**, *20*, 409–416.
15. Germain, E. Production and Economics of Nut Crops. In Proceedings of the II International Course on Production and Economics of Nut Tree Crops, Adana, Turkey, 18–29 May 1998.
16. Ramos, D.E. (Ed.) *Walnut Production Manual*; UCANR Publications: Davis, CA, USA, 1997; Volume 3373.
17. Sütyemez, M. New walnut cultivars: Maras 18, Sutyemez 1, and Kaman 1. *HortScience* **2016**, *51*, 1301–1303. [CrossRef]
18. Sütyemez, M.; Baymiș, M. Kalite ve Verim Bakımından Üstün Özelliklere Sahip Bazı Ceviz (Juglans regia L.) Tip ve Çeşitlerinin Karşılıklı Melezlenmesi Suretiyle Yeni Çeşit Eldesi Üzerine Araştırmalar. Available online: https://app.trdizin.gov.tr/proje/T0RjNU5EUT0/kalite-ve-verim-bakimindan-ustun-ozelliklere-sahip-bazi-ceviz-juglans-regia-l-tip-ve-cesitlerinin-karsilikli-melezlenmesi-suretiyle-yeni-cesit-eldesi-uzerine-arastirmalar (accessed on 8 October 2021).
19. Sütyemez, M.; Özcan, A.; Bükücü, Ş.B. Walnut cultivars through cross-breeding: 'Dirilis' and '15 Temmuz'. *J. Am. Pomol. Soc.* **2018**, *73*, 46.
20. Republic of Turkey Ministry of Agriculture and Forestry. Available online: https://www.tarimorman.gov.tr/BUGEM/TTSM/Sayfalar/Detay.aspx?SayfaId=87 (accessed on 2 July 2021). (In Turkey)
21. IPGRI. *Descriptors for walnut (Juglans spp.)*; International Plant Genetic Resources Institute: Rome, Italy, 1994; p. 54.
22. UPOV (Union Internationale Pour le Protection des Obtentions Vegetales). *Draft Guidelines for the Conduct of Tests for Distinctness Homogeneity and Stability Walnut (Juglans regia L.)*; UPOV: Geneva, Switzerland, 2014; Volume TG/125/7, p. 28.
23. Sütyemez, M.; Kaska, N. Comparison of the behaviours of some locally selected genotypes and local and foreign walnut cultivars under ecological conditions of K. Maras. *Acta Hortic.* **2004**, *705*, 151–157. [CrossRef]
24. Luedeling, E.; Gassner, A. Partial least squares regression for analyzing walnut phenology in California. *Agric. For. Meteorol.* **2012**, *158*, 43–52. [CrossRef]
25. Hassankhah, A.; Vahdati, K.; Rahemi, M.; Hassani, D.; Sarikhani Khorami, S. Persian walnut phenology: Effect of chilling and heat requirements on budbreak and flowering date. *Int. J. Hortic. Sci. Technol.* **2017**, *4*, 259–271.
26. Yıldız, E.; Okatan, V.; Çolak, A.M.; Türk, M.U. Vegetative and phenological characteristics of promising walnut (*Juglans regia* L.) Genotypes in Uşak (Turkey) Province. Kyrgyzstan, 20-23 September 2017. *Eurasian Agric. Nat. Sci. Congr.* **2017**, *1*, 17.
27. Hassani, D.; Mozaffari, M.R.; Soleimani, A.; Dastjerdi, R.; Rezaee, R.; Keshavarzi, M.; Vahdati, K.; Fahadan, A.; Atefi, J. Four new persian walnut cultivars of Iran: Persia, Caspian, Chaldoran, and Alvand. *HortScience* **2020**, *55*, 1162–1163. [CrossRef]
28. McGranahan, G.; Leslie, C. Breeding walnuts (*Juglans regia*). In *Breeding Plantation Tree Crops: Temperate Species*; Springer: New York, NY, USA, 2009; pp. 249–273.
29. Botu, M.; Tudor, M.; Botu, I.; Cosmulescu, S.; Papachatzis, A. Evaluation of walnut cultivars in the conditions of the Oltenia's hill area regarding functioning potential. *An. Univ. Din Craiova Biol. Hortic. Tehnol. Prelucr. Prod. Agric. Ing. Mediu.* **2010**, *15*, 94–103.
30. Khadivi, A.; Montazeran, A.; Yadegari, P. Superior spring frost resistant walnut (*Juglans regia* L.) genotypes identified among mature seedling origin trees. *Sci. Hortic.* **2019**, *253*, 147–153. [CrossRef]

31. Manthos, I.; Rouskas, D. 'Ourania' walnut. *HortScience* **2021**, *56*, 521–522. [CrossRef]
32. Arzani, K.; Mansouri-Ardakan, H.; Vezvaei, A.; Roozban, M.R. Morphological variation among Persian walnut (*Juglans regia*) genotypes from central Iran. *N. Z. J. Crop Hortic. Sci.* **2008**, *36*, 159–168. [CrossRef]
33. Akça, Y.; Yuldaşulu, Y.B.; Murad, E.; Vahdati, K. Exploring of walnut genetic resources in Kazakhstan and evaluation of promising selections. *Int. J. Hortic. Sci. Technol.* **2020**, *7*, 93–102.
34. Cosmulescu, S.; Ionescu, M. Phenological and pomological properties of promising walnut (*Juglans regia* L.) genotype with cluster fruiting from selected native population in Oltenia, Romania. *Gen. Res. Crop Evol.* **2021**, *68*, 2289–2297. [CrossRef]
35. Sharma, O.C.; Sharma, S.D. Genetic divergence in seedling trees of Persian walnut (*Juglans regia* L.) for various metric nut and kernel characters in Himachal Pradesh. *Sci. Hortic.* **2001**, *88*, 163–171. [CrossRef]
36. Mosivand, M.; Hassani, D.; Payamnour, V.; Jafar Aghaei, M. Comparison of tree, nut, and kernel characteristics in several walnut species and inter-specific hybrids. *Crop Breed. J.* **2013**, *3*, 25–30.
37. Ghasemi, M.; Arzani, K.; Hassani, D. Evaluation and identification of walnut (*Juglans regia* L.) genotypes in Markazi province of Iran. *Crop Breed. J.* **2012**, *2*, 119–124.
38. Gülsoy, E.; Kuş, E.; Altıkat, S. Determination of physico-mechanical properties of some domestic and foreign walnut (*Juglans regia* L.) varieties. *Acta Sci. Pol. Hortorum Cultus* **2019**, *18*, 67–74. [CrossRef]
39. Vahdati, K. Traditions and folks for walnut growing around the silk road. *Acta Hortic.* **2014**, *1032*, 19–24. [CrossRef]
40. Mahmoodi, R.; Hassani, D.; Amiri, M.E.; Jaffaraghaei, M. Phenological and pomological characteristics of five promised walnut genotypes in Karaj. *J. Nuts* **2016**, *7*, 1–8.
