# Supplementary material for: Provenance and family variations in early growth of Manchurian walnut (Juglans mandshurica Maxim.) and selection of superior families
Source: PLoS One. 2024 Mar 7;19(3):e0298918. doi: 10.1371/journal.pone.0298918 (PMC10919699; doi:10.1371/journal.pone.0298918)
Supplement: S2 File — (ZIP) [file pone.0298918.s005.zip › Morphological Variability between Geographical Provenances of Walnut Fruit (Juglans mandshurica) in the Eastern Liaoning Province, P.R. China.pdf]

Pol. J. Environ. Stud. Vol. 30, No. 5 (2021), 4353-4364
DOI: *10.15244/pjoes/131806* ONLINE PUBLICATION DATE: 2021-05-21

Original Research

Morphological Variability between Geographical Provenances of Walnut Fruit (*Juglans mandshurica*) in the Eastern Liaoning Province, P.R. China

Lijie Zhang[1,2], Xiujun Lu[1,2], Qiang Zhou[1], Jifeng Deng[1,2]*

[1]College of Forestry, Shenyang Agricultural University, Shenyang, Liaoning Province, People's Republic of China
[2]Key Laboratory of Forest Tree genetics and Breeding of Liaoning Province, Shenyang, Liaoning Province, People's Republic of China

Received: 5 October 2020
Accepted: 18 December 2020

Abstract

The eastern Liaoning Province of China has rich morphological diversity in walnut fruit, which is beneficial for selecting promising characters for marketability purposes. However, only a few reports have addressed morphological diversity in this region. In this study, *J. mandshurica* nuts and kernels from six geographical provenances were assessed for morphological traits, such as nut longitudinal diameter, nut lateral diameter, nut transverse diameter, mean diameter, nut weight, kernel weight, shell thickness, nut sutural thickness, kernel percentage, and index of roundness. Morphological traits proved to be quite variable and showed differences both within and among the geographical provenances. The frequency distribution of the traits had single peaks and followed a normal distribution. Principal component analysis revealed that 81.062% of the total variance was explained by the first three components. An unweighted PGM with averaging cluster analysis divided the geographical provenances into two groups; cluster I, containing five geographical provenances, and cluster II, containing only one. The study highlighted that the traits related to nut weight were of importance for discrimination, and Fushun is the optimal geographical provenance for breeding and selection. This study could provide a basis and reference for further understanding of the genetic resources of *Juglans mandshurica* fruit types.

Keywords: Phenotypic diversity, *Juglans mandshurica*, Eastern Liaoning Province, variation, principal component analysis

*e-mail: jifeng-deng@syau.edu.cn

Introduction

Geographical provenance (a distinct geographical place to obtain seeds or propagating materials, which originated from the same tree species) variation of forest trees plays an important role in tree breeding studies and is of great significance for efficient utilization of their germplasm resources [1-2]. The fruit facilitates species reproduction and has relatively stable reproduction characteristics, which show strong adaptability in complex and changeable environments [3]. Morphological traits are specific and genetically stable external reproductive characteristics, which are the result of interactions between genetic variation and environmental factors [3]. Variations in morphological traits reflect the variability of genotypes, populations, ecotypes, and geographical provenance [4-5]. Over the last few decades, scientists have found that there are considerable variabilities in morphological traits within and among geographical provenances under the influence of eco-environmental stress, and fruit morphological traits may vary greatly among individuals, even within the same geographical provenance [6-8]. Some fruits show strong adaptability and have more flexible survival strategies [9-10]. Therefore, studying the phenotypic variations of fruit is helpful to understand the effects of environmental and genetic factors on species and how well plants adapt to environmental changes in distributed areas through long-term selection and evolution. Moreover, it provides the basis for forest improvement and breeding, *ex situ* conservation, seed selection, seed production, and genetic diversity protection [11-16].

Juglans mandshurica is commonly known as “hickory” and is included in the “three hard and broad tree species” along with *Fraxinus mandshurica* and *Phellodendron amurense*, which are tree species distributed in the northeastern forest region of China [17-18]. *J. mandshurica* is a tertiary relict species and is a national class II and class III protected rare plant and endangered tree species in China [19]. Natural *J. mandshurica* forests are mainly distributed in the Xiaoxinganling Mountains, Wanda Mountains, Changbai Mountains, and the eastern mountainous areas of the Liaoning province in China, and partly in far eastern Russia, North Korea, and Japan [20-21]. *J. mandshurica* has substantial economic value, because of its nutritional, medicinal, and collection values. In general, the nut kernels contain 57%-65% fat, of which 3%-6% are saturated fatty acids; 15%-29% protein; and 5%-7% fiber, of which 25% is soluble. The kernels also contain small amounts of plant sterols and other phytochemicals. Moreover, walnuts are the only nut that is rich in ALA Omega-3s, which are essential fatty acids to various body processes [20, 22-25]. However, the existing *J. mandshurica* resources are distributed mostly as natural forests, and due to excessive logging, the imbalance between harvesting and breeding has led to imminent danger for the natural *J. mandshurica* resources in recent years [23]. At present, the yield of *J. mandshurica* walnut fruits has declined sharply, and the timber yield accounts for less than 1% of the total timber output in the eastern mountainous areas of Liaoning, China [26]. Therefore, there is an urgent need for development and sustainable utilization of *J. mandshurica*. Previous studies on germplasm resources of *J. mandshurica* from northeastern China revealed that *J. mandshurica* is a monoecious plant with different flowers and has two mating types, which are randomly distributed in the population. The reproductive characteristics of *J. mandshurica* during its dioecious maturation lead to the rare flowering of male and female flowers, which seriously affects the pollination and fruit setting rate, thus affecting its yield and quality [26-27]. Though *J. mandshurica* has been extensively studied, most studies have focused on its pharmacological properties [28-33] and population dynamics [34]. Few reports, to our knowledge, have addressed the morphological variability of walnut fruits in the eastern Liaoning Province, P.R. China, which has the greatest natural distribution of *J. mandshurica*. Therefore, the aim of the present study was to utilize morphological characteristics to assess the differences in geographical provenances of *J. mandshurica* naturally grown in the eastern Liaoning region. This is the first report, to our knowledge, on the morphological variability of walnut fruits grown throughout this area and would provide a basis for effective management and sustainable utilization of *J. mandshurica*.

Materials and Methods

Field Survey

Based on geographical distribution, climate differences, and local heterogeneity in the eastern Liaodong region, six representative natural geographical provenances of *J. mandshurica* were selected along the Changbai mountain ranges: Benxi, Dandong, Xinbin, Qingyuan, Huanren, and Fushun (Table 1). The plots were determined in the spring of 2017; for each provenance, three 100×100 m plots were set up as replicates, and for each plot, the topography was flat and without human interference. No major natural disasters such as fire, pests, or disease occurred in the selected regions over the last decade. In the early 1950s, a large-scale fire almost destroyed the original forest, resulting in the natural regeneration of the site to a secondary forest, which was composed of *Fraxinus mandshurica*, *J. mandshurica*, and *Quercus mongolica* in the tree layer. In the early 1960s, patches of the *F. mandshurica* and *Q. mongolica* forests were partially cleared. The selected plots were located on slopes between 18° and 23° and at elevations ranging from 400 to 550 m asl. The soil in all stands was typical brown forest soil. The forest stands were all half-mature,

Table 1. Topographical and climatological data of the geographical provenances.

Geographical Provenance	Latitude	Longitude	Average annual temperature (ºC)	Average annual precipitation (mm)	Maximum air temperature (ºC)	Lowest air temperature (ºC)	Frost-free period (d)
Benxi	40°49′–41°35′	123°34′–125°46′	5.85	876.25	33.0	–32.0	127
Dandong	39°43′–41°09′	123°22′–125°42′	7.43	931.20	36.0	–17.0	173
Xinbin	41°14′–41°58′	124°15′–125°57′	5.47	898.60	29.0	–12.0	150
Qingyuan	41°47′–42°28′	124°20′–125°28′	4.97	775.59	37.0	–38.0	120
Huanren	40°25′–41°34′	124°43′–125°47′	5.66	946.55	28.0	–19.0	153
Fushun	41°14′–42°28′	123°39′–125°28′	6.04	800.64	38.0	–33.0	145

stand canopy density was above 0.6, and the height of the secondary forests was 15-20 m. Superior trees (based on the diameter at breast height, tree height, height under living branches, crown width, and shape) were simultaneously investigated from late August to early September (surveyed 6 times per month) for morphological diversity analysis based on their mature period.

Morphological Data

For each provenance, 10 sample trees were selected in every plot; in total, 180 sample trees were selected from six geographical provenances in the eastern Liaoning mountainous area. Nuts were then obtained from the sample trees. The selection criteria were diameter at breast height, tree height, height under living branches, crown width, and shape, as well as growth conditions of the sample trees that were superior to those of the average trees in the plot. During this study, the different locations (west, northeast, and southeast) and slope positions (upper, middle, and lower) of the plots were also considered for the selection of the sample trees, but this was not strictly due to the actual situations of the plots. A total of 10 morphological characteristics (nut longitudinal diameter, nut lateral diameter, nut transverse diameter, mean diameter (Equation 1), nut weight, kernel weight, shell thickness, nut sutural thickness, kernel percentage, and index of roundness (Equation 2)) were recorded during the present study. There were 30 replicates for the nut and kernel data of each geographical provenance, and the average values and coefficients of variation were then calculated. Nut weight and kernel weight were measured by a precision electronic autobalance (0.001 g). Nut longitude diameter, nut transverse diameter, nut lateral diameter, shell thickness, and nut sutural thickness were measured with Vernier calipers.

$$MD = (LonD + TD + LatD)/3 \quad (1)$$

...where, LonD is nut longitudinal diameter, TD is nut transverse diameter, LatD is nut lateral diameter, and MD is mean diameter.

$$IR = (TD + LatD)/(2 \quad LonD) \quad (2)$$

...where, IR represents index of roundness.

Fruit Appearance

Nuts from our sample trees were preliminarily classified as 12 shape forms: spherical, spherical with one-side saddle-backing, spherical with two-side saddle-backing, shuttle, nucleus with one-side saddle-backing, nucleus with on-side long saddle-backing, nucleus with two-side saddle-backing, cone, deep wrinkle, bended longitudinal ridge, saddle-backing with hooked beak, and others (Fig. 1). The shape forms were judged based on the fruit appearance, including the apex, longitudinal edge, and corrugation depression between kernel edges. Various walnut shapes have both commercial and aesthetic values [3-4]. Different fruit appearances also illustrate the variations within and among provenances.

Statistical Analysis

A Pearson's correlation coefficient and one-way analysis of variance (ANOVA) were used to distinguish the correlation (significantly correlated at $p<0.05$ and $p<0.01$) and significant differences (at the 0.05 level and 0.01 level) between nut longitudinal diameter, nut lateral diameter, nut transverse diameter, mean diameter, nut weight, kernel weight, shell thickness, nut sutural thickness, kernel percentage, and index of roundness. Principal component analysis (PCA) and UPGMA (unweighted PGM with averaging) cluster analysis of geographical provenances based on morphological traits were all completed using SPSS 22.0 software (IBM, Armonk, NY, USA). Plotting was completed using OriginPro 9.0 software (OriginLab Corp., Northampton, MA, USA).

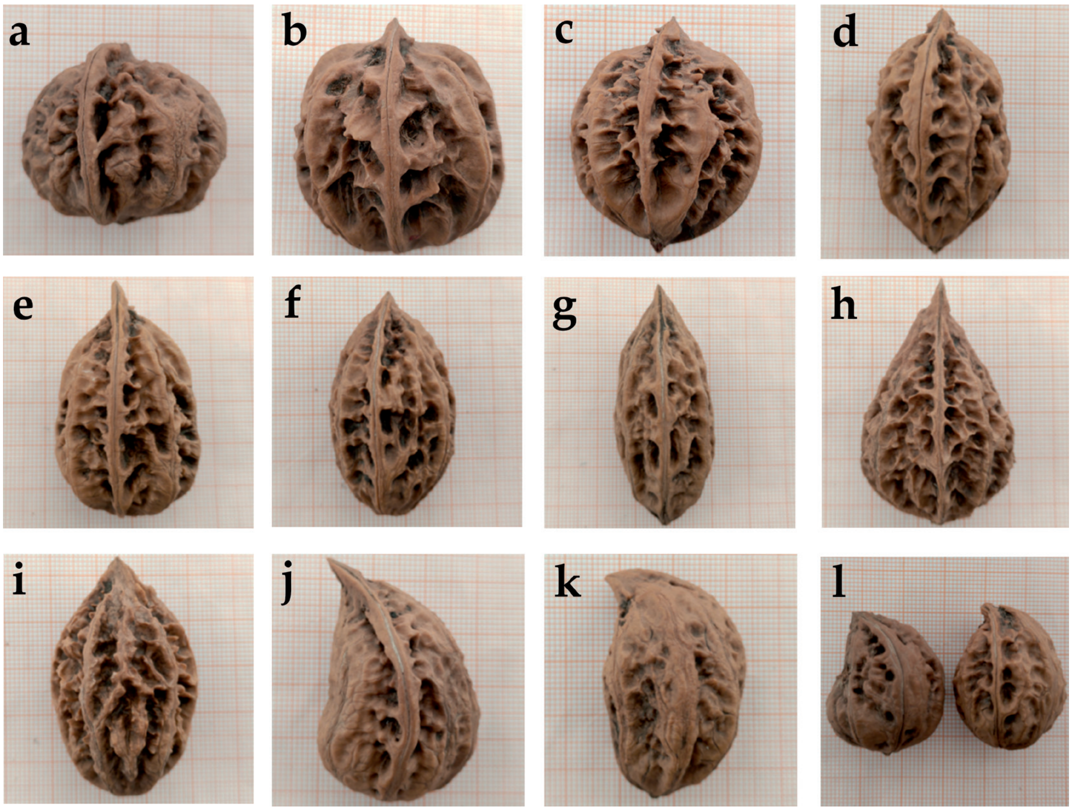

Fig. 1. Appearance of *Juglans mandshurica* fruits among geographical provenances. a represents spherical shape, b represents spherical with one-side saddle-backing shape, c represents spherical with two-side saddle-backing shape, d represents shuttle shape, e represents nucleus with one-side saddle-backing shape, f represents nucleus with on-side long saddle-backing shape, g represents nucleus with two-side saddle-backing shape, h represents cone shape, i represents deep wrinkle shape, j represents bended longitudinal ridge shape, k represents saddle-backing with hooked beak shape, and l represents other shape forms.

Results

Morphological Variability Assessment

The variance analysis of the nut and kernel morphological traits of *J. mandshurica* among geographical provenances showed that great variations occurred in most of the traits. The maximum, 99%, minimum, 1%, mean values, raw data distribution, and coefficient of variation (Cv) measured for each trait are presented in Fig. 2. A high variation was found in important quantitative features related to the fruit. If we divided the coefficients of variation into high level (>20%), medium level (10%–20%), and low level (<10%), shell thickness and nut sutural thickness showed a huge variation (25.105% and 28.824%, respectively); nut longitudinal diameter, nut weight, kernel weight, and kernel percentage exhibited a medium level of variation (11.040%, 18.365%, 19.417%, and 14.520%, respectively); and nut transverse diameter, nut lateral diameter, mean diameter, and index of roundness had the smallest coefficients of variation (9.93%, 9.41%, 7.80%, and 9.92%, respectively). The average nut longitudinal diameter, nut lateral diameter, nut transverse diameter, mean diameter, nut weight, kernel weight, shell thickness, nut sutural thickness, kernel percentage, and index of roundness were 41.21 mm, 27.85 mm, 27.37 mm, 31.68 mm, 10.03 g, 1.69 g, 0.98 mm, 5.29 mm, 17.02%, and 0.70, respectively. Data distribution histograms plotted relative to box plots of the studied traits are shown in Fig. 2; nut longitudinal diameter, nut weight, and kernel percentage have more uniform distribution than that of the other traits.

Distribution Frequency of Morphological Traits

The results of the distribution frequency of the morphological characteristics of *J. mandshurica* fruit are shown in Fig. 3. The distribution frequency of the nut longitudinal diameter had obvious double peaks, while the other morphological traits had a single peak and followed a normal distribution. Compared to the nut longitudinal diameter and kernel percentage, the distribution frequency of the nut weight was more even.

Analysis of Variance of Morphological Traits Among Geographical Provenances

The differences between the morphological traits among geographical provenances were further

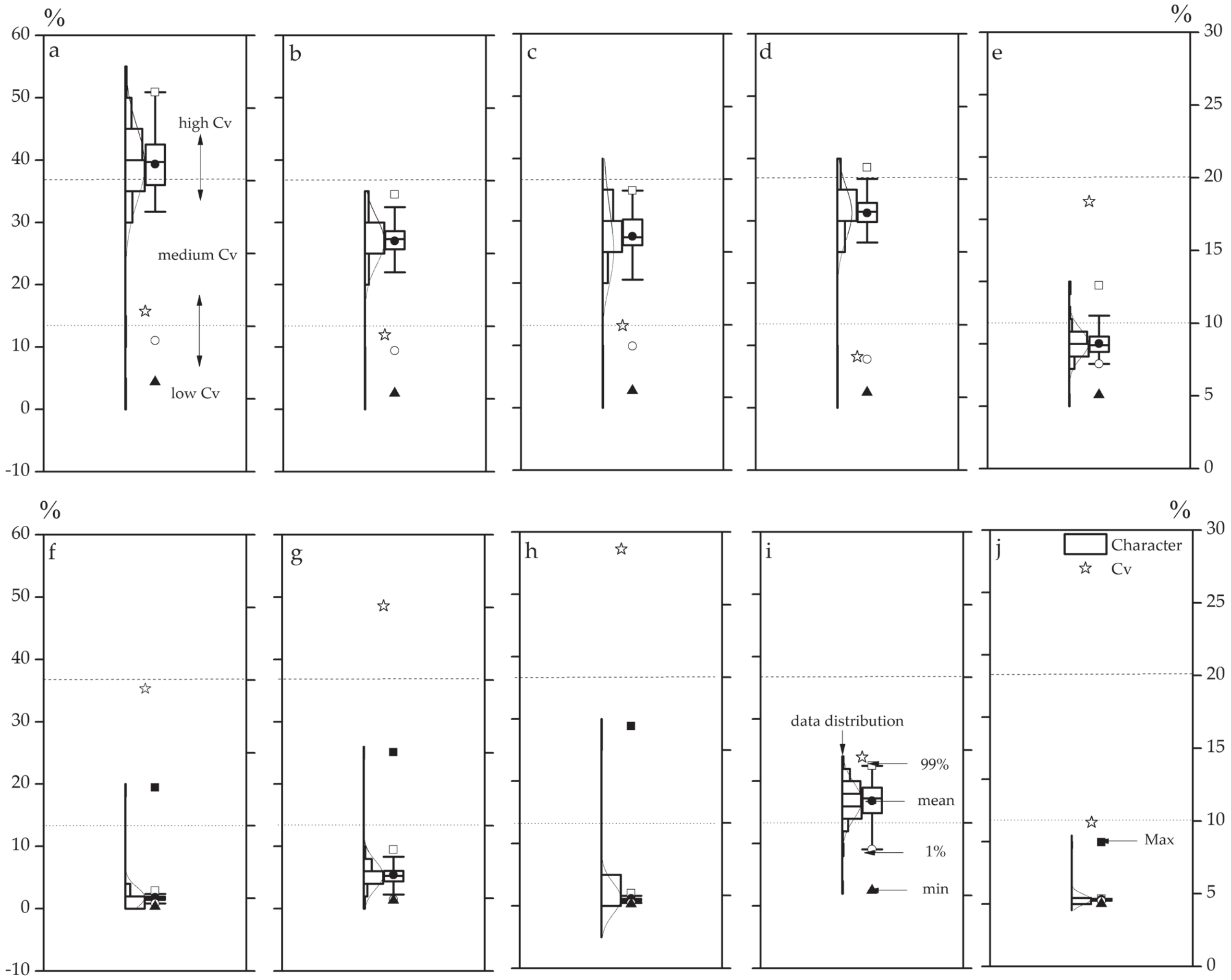

Fig. 2. Descriptive statistics related to the ten morphological traits of *Juglans mandshurica* nuts and kernels in six provenances (maximum, 99%, minimum, 1%, mean values, raw data distribution, and coefficient of variation measured for each trait). Max represents Maximal value, Min represents minimal value, mean represents average value, and Cv represents coefficient of variation. a, b, c, d, e, f, g, h, i, and j represent LonD, TD, LatD, MD, NW, KW, SheT, SutT, KP, and IR, respectively. LonD represents nut longitudinal diameter, TD represents nut transverse diameter, LatD represents nut lateral diameter, MD represents mean diameter, NW represents nut weight, KW represents kernel weight, SheT represents shell thickness, SutT nut sutural thickness, KP represents kernel percentage, and IR represents index of roundness.

analyzed. Fig. 4 shows that the maximum Cv values of the nut longitudinal diameter, mean diameter, and index of roundness in the Dandong geographical provenance; the nut lateral diameter, nut weight, nut sutural thickness, and kernel percentage in the Xinbin geographical provenance; the nut transverse diameter and shell thickness in the Huanren geographical provenance; and the nut weight and kernel weight in the Fushun geographical provenance were 12.78%, 9.05%, 11.11%, 10.26%, 11.41%, 34.90%, 25.65%, 19.21%, 11.11%, 20.24%, and 22.09%, respectively. In addition, the minimum Cv values of the mean diameter, kernel percentage, and index of roundness in the Benxi geographical provenance; nut transverse diameter, nut weight, kernel weight, and shell thickness in the Qingyuan geographical provenance; the nut lateral diameter in the Huanren geographical provenance; and the nut longitudinal diameter, nut transverse diameter, and nut sutural thickness in the Fushun geographical provenance were 6.22%, 8.24%, 7.20%, 7.55%, 12.66%, 14.67%, 15.84%, 6.04%, 8.06%, 8.55%, and 13.76%, respectively. The maximum values of the kernel weight and kernel percentage in the Dandong geographical provenance were 1.913 g and 19.14%, respectively; the nut longitudinal diameter and mean diameter in the Xinbin geographical provenance were 39.296 mm and 31.547 mm; and the nut transverse diameter, nut lateral diameter, nut weight, shell thickness, nut sutural thickness, and index of roundness in the Fushun geographical provenance were 26.908 mm, 29.272 mm, 11.182 g, 6.765 mm, 0.935 mm, and 0.754, respectively.

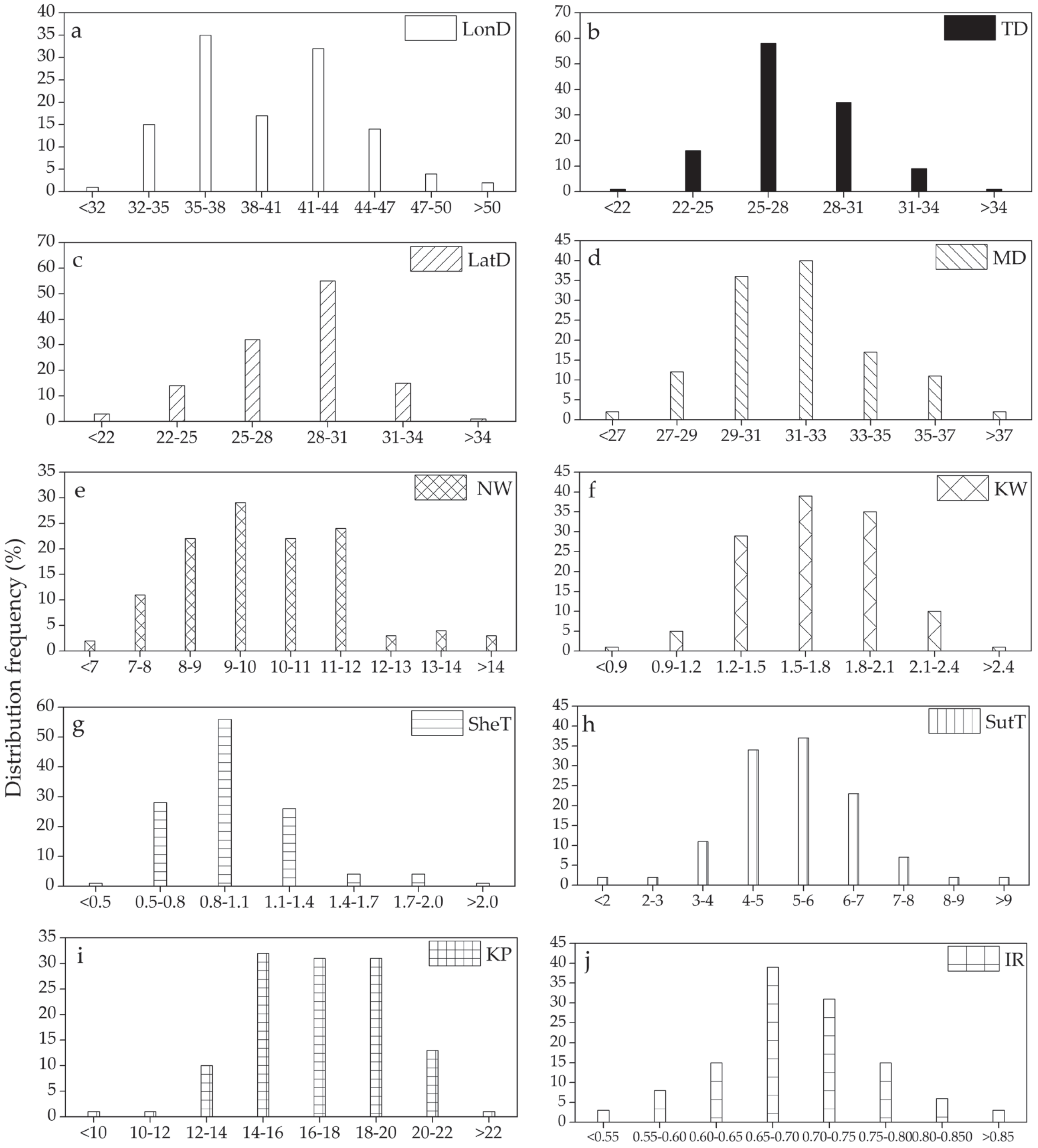

Fig 3. Distribution frequency histograms based on the morphological traits.

In Table 2, except the mean diameter ($p>0.05$), there were significant differences in the nut longitudinal diameter, nut lateral diameter, kernel weight, and shell thickness among the geographical provenances ($p<0.05$). There were extremely significant differences in the nut transverse diameter, nut weight, nut sutural thickness, kernel percentage, and index of roundness among the geographical provenances ($p<0.01$).

Correlations between Morphological Traits

Ten morphological traits of nuts and kernels were evaluated using a Pearson correlation analysis (Table 3). The results showed that the mean diameter was positively correlated with the nut longitudinal diameter, nut transverse diameter, nut lateral diameter, nut weight ($p<0.01$), kernel weight ($p<0.01$), nut sutural thickness ($p<0.05$), shell thickness, and index of roundness. The nut lateral diameter was positively correlated with the nut longitudinal diameter and nut transverse diameter ($p<0.01$). The nut transverse diameter was positively correlated with the nut longitudinal diameter ($p<0.01$). The index of roundness was positively correlated with the nut transverse diameter ($p<0.01$), nut lateral diameter ($p<0.01$), and nut weight ($p<0.05$), and negatively correlated with the nut longitudinal diameter

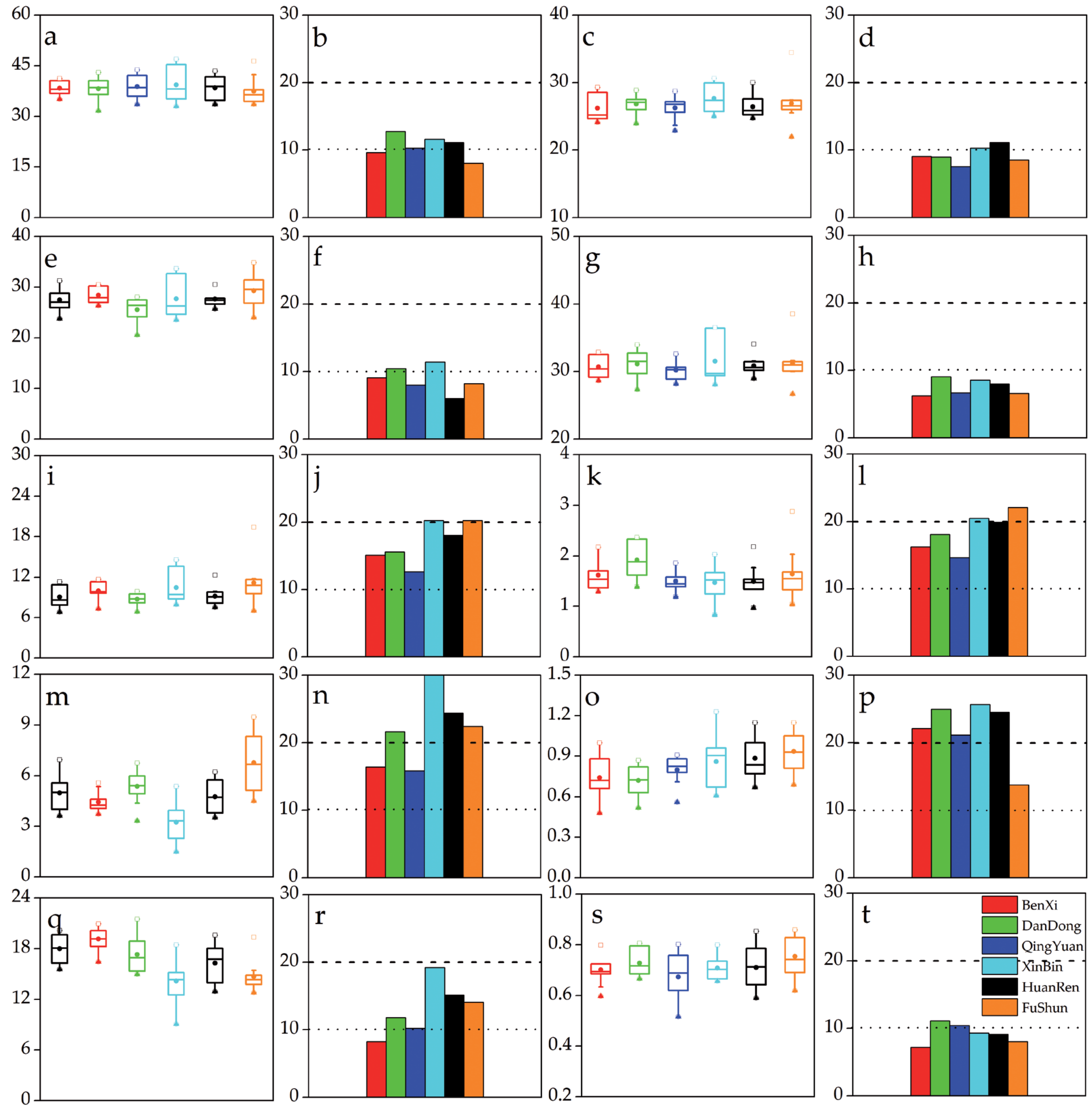

Fig. 4. Box plots of descriptive statistics related to the morphological traits among the geographical provenances. a, c, e, g, i, k, m, o, q, and s represent the maximum, 99%, minimum, 1%, and mean values for LonD, TD, LatD, MD, NW, KW, SheT, SutT, KP, and IR, respectively. b, d, f, h, j, l, n, p, r, and t represent the coefficient of variation measured for LonD, TD, LatD, MD, NW, KW, SheT, SutT, KP, and IR, respectively.

and kernel percentage. The kernel percentage was positively correlated with the longitudinal diameter and kernel weight, and negatively correlated with the nut weight ($p<0.05$). Shell thickness was positively correlated with the longitudinal diameter, nut weight, nut transverse diameter, and nut sutural thickness. Nut sutural thickness was positively correlated with the nut longitudinal diameter, nut transverse diameter ($p<0.05$), nut lateral diameter, mean diameter ($p<0.05$), nut weight ($p<0.05$), and kernel weight ($p<0.05$). Kernel weight was positively correlated with the nut longitudinal, transverse, lateral, mean, diameters, and nut weight ($p<0.01$ for all correlated parameters). The nut weight was positively correlated with the nut longitudinal, transverse, lateral, and mean diameters, and the kernel weight ($p<0.01$ for all correlated parameters). Generally, kernel weight is in some way related to the nut weight; the results of this study were consistent with those of previous studies. Table 3 displays cases of heavier nuts with low kernel percentage; however, the relationship was not overly strict.

Principal Component Analysis of Morphological Traits

PCA was performed to identify the principal distinguishing traits of the variability. About 81.062% of the total variance was explained by the first three

Table 2. Results of the analysis of variance for quantitative traits.

Variable	Sum of Squares	Mean Square	**Fisher**	***p***
LonD	27.791	5.558	2.343	0.046
TD	9.471	1.894	6.314	0.0001
LatD	46.423	9.285	2.903	0.017
MD	4.038	0.808	2.058	0.076
NW	22.529	4.506	3.470	0.006
KW	1.027	0.205	2.700	0.024
SheT	49.670	9.934	2.374	0.043
SutT	0.445	0.0890	5.664	0.00010
KP	0.0120	0.00200	5.838	0.00010
IR	0.0340	0.00700	4.494	0.0010

[1] *p* represents statistical significance; the difference was considered significant at $p<0.05$ and extremely significant at $p<0.01$.

Table 3. Correlations between the morphological traits of *Juglans mandshurica* fruit.

Character	LonD	TD	LatD	MD	NW	KW	SheT	SutT	KP	IR
LonD	1.000									
TD	0.341*	1.000								
LatD	0.184	0.643**	1.000							
MD	0.780**	0.789**	0.706**	1.000						
NW	0.455*	0.723**	0.775**	0.810**	1.000					
KW	0.536**	0.558**	0.585**	0.730**	0.705**	1.000				
SheT	0.203	0.253	0.130	0.255	0.188	0.100	1.000			
SutW	0.170	0.337*	0.276	0.318*	0.370*	0.365*	0.371*	1.000		
KP	0.222	–0.130	–0.150	0.0300	–0.248	0.492*	–0.0900	0.0600	1.000	
IR	–0.284	0.536**	0.633**	0.254	0.376*	0.170	0.0100	0.150	–0.251	1.000

[1] Pearson analysis of morphological traits. * Correlation is significant at the $p<0.05$ level (2-tailed). ** Correlation is significant at the $p<0.01$ level (2-tailed).

components (Table 4). PC1, PC2 and PC3 accounted for about 42.329%, 24.476% and 14.257% of the total variance.

The principal morphological attributes that identified the PCA factors were nut weight, kernel weight (Component 1), index of roundness (Component 2), and kernel percentage (Component 3) (Table 5).

Cluster Analysis of Geographical Provenances Based on Morphological Traits

A dendrogram based on the UPGMA analysis revealed two separate clusters: I and II (Fig. 5). Cluster I consisted of five geographical provenances (gp.), while cluster II only included one. Cluster I was further split into the groups IA and IB. Group IA consisted of four geographical provenances (gp. Benxi, gp. Dandong, gp. Qingyuan, and gp. Xinbin), while groups IB and II contained a single geographical provenance (gp. Huanren and gp. Fushun, respectively). Group IA was further split into the IA1 and IA2 subgroups. The subgroup IA1 included three geographical provenances (gp. Benxi, gp. Dandong, and gp. Qingyuan). Subgroup 1 contained two geographical provenances (gp. Benxi and gp. Dandong), while subgroup 2 contained a single geographical provenance (gp. Qingyuan).

Discussion

Juglans mandshurica is widely distributed in northeastern China and with high economic value of tree species providing timber, woody grain, and oil in

Table 4. Eigen values, variance, and cumulative values of the first three factors contributing to 81.062% of the total variance.

Component	Eigen value	Variance (%)	Cumulative variance (%)
1	4.233	42.329	42.329
2	2.448	24.476	66.805
3	1.426	14.257	81.062
4	0.999	9.985	91.048
5	0.375	3.746	94.794
6	0.358	3.580	98.374
7	0.154	1.544	99.918
8	0.00600	0.0590	99.977
9	0.00200	0.0230	100.000
10	1.058E-8	1.058E-7	100.000

The principal morphological attributes that identified the PCA factors were nut weight, kernel weight (Component 1), index of roundness (Component 2), and kernel percentage (Component 3) (Table 5).

this region, the survival of *J. mandshurica* is under unprecedented threat, in addition to a lack of proper characterization and management [35]. Studying the relevant morphological traits of fruit is an important prerequisite for further selection of plant genetic resources, which are the foundation of breeding and classification [36-37]. In this study, a certain amount of variability in different phenotypic traits was revealed among *J. mandshurica* nuts and kernels from six geographical provenances in eastern Liaoning, China because of the distance-span and geographical distribution.

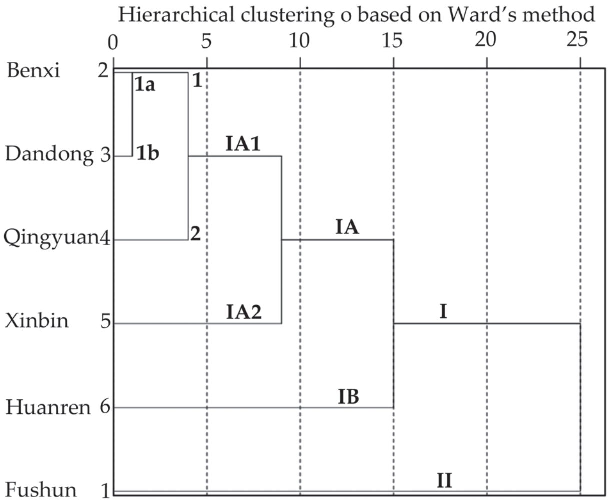

Fig. 5. Hierarchical clustering of *Juglans mandshurica* fruits based on Ward's method using Euclidian distance with standard.

Table 5. Component scores of the first three factors of the total variance.

Variance	Component		
	1	2	3
LonD	0.540	–0.821	0.072
TD	0.804	0.194	–0.0990
LatD	0.766	0.261	–0.0770
MD	0.854	–0.308	–0.0150
NW	0.942	0.0740	–0.161
KW	0.936	0.0360	0.610
SheT	0.187	0.361	–0.00400
SutW	0.389	0.399	–0.614
KP	–0.0300	–0.0140	0.961
IR	0.106	0.957	–0.130

Fruit size and shape determine market value and have great physical influence on grading and sorting due to both utilization and aesthetic appeal [38,39]. Based on appearance, shape, longitudinal ridge, apical, and wrinkle of *J. mandshurica* fruits from different geographical provenances, 12 typical morphological features, including spherical, spherical with one-side saddle-backing, spherical with two-side saddle-backing, shuttle, nucleus with one-side saddle-backing, nucleus with on-side long saddle-backing, nucleus with two-side saddle-backing, cone, deep wrinkle, bended longitudinal ridge, saddle-backing with hooked beak, and others, were screened. All these phenotypic features were only based on the appearance of *J. mandshurica* nuts, but they reflected a high level of variation in morphological traits among the geographical provenances. Therefore, nut longitudinal diameter, nut transverse diameter, nut lateral diameter, mean diameter, shell thickness, nut sutural thickness, and index of roundness were selected based on the above analysis. Furthermore, nut weight, kernel weight, and kernel percentage are the most outstanding properties to determine yield as well fruit quality, and hence are of consumer preference. The Cv indicates the degree of dispersion of each trait and can accurately understand its genetic characteristics. In the present study, shell thickness and nut sutural thickness showed a huge variation (25.105% and 28.824%, respectively) compared to nut transverse diameter, nut lateral diameter, mean diameter, and index of roundness (9.93%, 9.41%, 7.80%, and 9.92%, respectively), as well as nut longitudinal diameter, nut weight, kernel weight, and kernel percentage (11.04%, 18.365%, 19.417%, and 14.520%, respectively), which showed medium variation change. Similar variation in fruit morphology has been reported in previous studies [40, 41]. There was a significant difference between all these morphological traits and they were also closely correlated with each other; in addition, the frequency

distribution of morphological traits followed a normal distribution, which proved that the selected variables were representative and suited for further statistical analysis.

Morphological traits varied between the geographical provenances. Nut weight, kernel weight, and kernel percentage make up the main edible portion of fruits, and therefore are highly valuable pomological attributes for consumer preference and industrial applications. Positive values of nut weight, kernel weight, and kernel percentage are desirable. In this study, the highest nut weight value (11.18 g) was recorded for the Fushun geographical provenance with a Cv of 20.22%, while the lowest value (8.67 g) was observed for the Qingyuan geographical provenance with a Cv of 12.66%. The highest kernel weight value (1.91 g) was recorded for the Dandong geographical provenance with a Cv of 18.10%, while the lowest value (1.47 g) was observed for the Xinbin geographical provenance with a Cv of 20.48%. The highest kernel percentage value (19.1%) was recorded for the Dandong geographical provenance with a Cv of 11.79%, while the lowest value (14.14%) was observed for the Xinbin geographical provenance with a Cv of 19.21%. The predominant fruit shape found in this study was round, but the mean diameter and index of roundness had low Cvs. Compared to shell thickness and nut sutural thickness, the distribution frequency of nut weight and kernel weight were more even. Thus, nut weight and kernel weight were the important morphological traits owing to their high values and variabilities. The nut weight and kernel weight play a vital role in pomological attributes that can be combined with other traits through breeding. In addition, significant correlations were found between the morphological characteristics, especially with nut weight. The positive correlations between nut weight and most of the characteristics ranged from 0.188 to 0.810; nut weight is positively related to nut longitudinal diameter, nut transverse diameter, nut lateral diameter, mean diameter, shell thickness, nut sutural thickness, kernel weight, and index of roundness. Since larger fruits harbor larger stones, there was no higher kernel percentage with larger nut weight, and a negative correlation was obtained between nut weight and kernel percentage (−0.248); however, it was not significant. PCA allows for the identification of patterns in data as well as highlighting the similarities and differences [43−45]. PC1, PC2, and PC3 accounted for about 42.329%, 24.476%, and 14.257% of the total variance, respectively. The principal morphological attributes that identified the PCA factors were nut weight, kernel weight, index of roundness, and kernel percentage. Such similarities and differences have also been reported in previous studies [46−48]. Based on the above results, nut weight is the most important phenotypic characteristic because of its suitability and ease of identification, and can be used to grade and sort the fruit.

Multivariate analysis is an important statistical technique used to analyze data from more than one variable and reveal the relationship between the subjects based on a set of shared attributes [41, 42]. Data generated from the *J. mandshurica* nuts and kernels was used for the construction of a dendrogram to assess the relatedness among the geographical provenances. The cluster analysis based on morphological traits such as nut longitudinal diameter, nut transverse diameter, nut lateral diameter, mean diameter, shell thickness, nut sutural thickness, nut weight, kernel weight, kernel percentage, and index of roundness generated two main clusters. Cluster I consisted of five geographical provenances, while cluster II only included one geographical provenance (gp. Fushun). The nut and kernel characteristics of this geographical provenance had the largest values for nut transverse diameter (26.908 mm), nut lateral diameter (29.272 mm), nut weight (11.18 g), shell thickness (6.756 mm), nut sutural thickness (0.935 mm), and index of roundness (0.754), as well as high Cvs for nut weight (20.24%) and kernel weight (22.09%), which were quite distinct from the other geographical provenances. Cluster I was further split into groups IA and IB. In addition, group IA was further split into subgroups IA1 and IA2. Subgroup IA1 included three geographical provenances (gp. Benxi, gp. Dandong, and gp. Qingyuan), which had relatively low values for nut transverse diameter, nut lateral diameter, mean diameter, nut weight, and nut sutural thickness, and low Cv values for nut transverse diameter, mean diameter, nut weight, kernel weight, shell thickness, kernel percentage, and index of roundness. In summary, *J. mandshurica* fruits in the Fushun geographical provenance are relatively well adapted to the local climate (average annual temperature of 6.04ºC, average annual precipitation of 800.64 mm, and frost-free period of 145 d) and thus have the potential for use in future breeding programs.

The distribution of *J. mandshurica* is mainly concentrated in northeastern China. This species is mostly scattered along the banks of valleys and foothills at an altitude of 300-800 m and mixed into Korean pine broad-leaved mixed forests; small natural forests can be formed along the valleys in secondary broad-leaved forests. Its special habitat led to phenotypic variation in *J. mandshurica* fruits [4]. Comprehensive research is needed in the future due to the complex correlations between its phenotypic characters and environmental conditions such as temperature, humidity, rainfall, frost-free period, and topographic factors affecting the fruiting characteristics of *J. mandshurica* [35, 49-52]. More thorough studies could be carried out regarding morphological characteristics like kernel skin color, which can be used as a criterion for germplasm resource collection, preservation, and utilization.

Conclusions

To our knowledge, this study is the first of its kind in northeastern China to evaluate the proper utilization

of *J. mandshurica* nuts and kernels from different geographical provenances. The study highlighted that morphological traits such as nut longitudinal diameter, nut transverse diameter, nut lateral diameter, mean diameter, nut weight, kernel weight, shell thickness, nut sutural thickness, kernel percentage, and index of roundness can be utilized for fast and easy discrimination based on fruit appearance. The morphological diversity of *J. mandshurica* has been used to describe and identify germplasm sources and collect suitable genotypes for cultivation, preservation, and utilization, as well as to set quality standards and sort fruit cultivars. Our results revealed important morphological characteristics that can be selected during breeding and cultivation. The relatively large variation and value in nut weight indicated that it is a particularly important trait for breeding of large fruit sized for better marketability and returns to the growers. Based on this evaluation, *J. mandshurica* nuts and kernels from the Fushun geographical provenance with excellent fruit traits represent a highly precious variety.

Acknowledgements

This work is supported by funds from the National Key Research and Development Plan of China 2017YFD0600600, Foundation for State Major Research Project of China 2017YFD0600605 and National Natural Science Foundation of China 31800609.

References

1. O´BRIEN E.K., MAZANEC R.A., KRAUSS S.L. Provenance variation of ecologically important traits of forest trees implications for restoration. Journal of Applied Ecology, **44**, 583, **2007**.
2. KLEEMANN F., FRAGSTEIN M.V., VORNAM B., MÜLLER A., LEUSCHNER C., HOLZSCHUH A., TSCHARNTKE T., FINKELDEY R., POLLE A.Relating genetic variation of ecologically important tree traits to associated organisms in full-sib aspen families. European Journal of Forest Research,, **130**, 707, **2011**.
3. GAO Z.Y, ZHANG H.F., CHEN G.P., FENG X.M., ZHAO T.J., GAO X., SHI F.C. Fruit stone morphology and geographic variation in *Juglans mandshurica* populations. Chinese Journal of Applied and Environmental Biology, **23**, 609, **2017**.
4. MÜLLER A., HORNA V., KLEEMANN F., VORNAM B., LEUSCHNER C. Physiological vs. morphological traits controlling the productivity of six aspen full-sib families. Biomass Bioenergy, **56**, 274, **2013**.
5. ANEGBEH P.O., UKAFOR V., USORO C., TCHOUNDJEU Z., LEAKEY R.R.B., SCHRECKENBERG K. Domestication of Dacryodes edulis: 1. Phenotypic variation of fruit traits from 100 trees in southeast Nigeria. New Forests, **29**, 149, **2005**.
6. YANG L., SHEN H.L., LIANG L.D., CUI X.T., WANG A.Z. Phenotypic diversity of fruit and seed collected from six wild *Sorbus pohuashanensis* Provenances. Journal of Northeast Forestry University, **37**, 8, **2009**.
7. CHEN L.J., DENG X.M., DING M.M., LIU M.Q., LI J.C., HUI W.K., LIAO B.Y., CHEN X.Y. Geographic variation in traits of fruit stones and seeds of *Melia azedarach*. Journal of Beijing Forestry University, **36**, 15, **2014**.
8. YAO D., JIN M., ZHANG C., LUO J., JIANG Z., ZHENG M., CUI J., LI G. Chemical constituents of the leaves of *Juglans mandshurica*. Chemistry of Natural Compounds, **52**, 93, **2016**.
9. HUANG J., YU H., GUAN X., WANG G., GUO R. Accelerated dryland expansion under climate change. *Nat.* Nature Climate Change, **6**, 166, **2016**.
10. HUANG J., JI M., XIE Y., WANG S., HE Y., RAN J. Global semi-arid climate change over last 60 years. Climate Dynamics, **46**, 1131, **2015**.
11. WARUHIU A.N., KENGUE J., ATANGANA A.R., TCHOUNDJEU Z., LEAKEY, R.R.B. Domestication of *Dacryodes edulis*. 2. Phenotypic variation of fruit traits in 200 trees from four populations in the humid lowlands of Cameroon. Journal of Food Agriculture and Environment, **2**, 340, **2004**.
12. LI Y.R., LI X.C., WU W. L., ZHAI M.G., GUO Z.R. Study on Nut characteristics variation and superior tree selection of *Carya illinoensis*. Forest Research, **26**, 438, **2013**.
13. LEAKEY R., SHACKLETON S. Characterization of phenotypic variation in Marula (*Sclerocarya birrea*) fruits, nuts and kernels in South Africa and Namibia. International Migration Review, **39**, 381, **2002**.
14. SHEN L., XU R., LIU S., JUN C., XU C.Q., XIE C.X., LIU T.N. Phenotypic variation of seed traits of *Haloxylon Ammodendron* and its affecting factors. Biochemical Systematics and Ecology, **60**, 81, **2015**.
15. GIL L., CLIMENT J., NANOS N., MUTKE S., ORTIZ I., SCHILLER G. Cone morphology variation in *Pinus canariensis* Sm. Plant Systematics and Evolution, **235**, 35, **2002**.
16. GARCIA R., SIEPIELSKI A.M., BENKMAN C.W. Cone and seed trait variation in whitebark pine *Pinus albicaulis* (Pinaceae) and the potential for phenotypic selection. American Journal of Botany, **96**, 1050, **2018**.
17. LUO J.X., LI X.Q., SUN P., WANG L.H., HU G.R., WANG F.L., ZHENG W. Variation pattern of tracheid and wood density in natural populations of *Picea asperata*. Journal of Beijing Forestry University, **26**, 80, **2004**.
18. ZHANG Y.W., LIN H., BAO Y.L., WUA Y., YU C.L., HUANG Y.X., LI Y.X. A new triterpenoid and other constituents from the stem bark of *Juglans mandshurica*. Biochemical Systematics and Ecology, **44**, 136, **2012**.
19. WANG D.N., MOU C.C., GAO Z., FENG F.J. ISSR analysis of genetic diversity of *Juglans mandshurica* Maxim. populations. Nonwood Forest Research, **29**, 22, **2011**.
20. JIN M., SUN J., LI R., DIAO S., ZHANG C., CUI J., SON J.K., ZHOU W., LI G. Two new quinones from the roots of *Juglans mandshurica*. Archives of Pharmacal Research, **39**, 1237, **2016**.
21. ZHAO M.H., JIANG Z.T., LIU T., LI R. Flavonoids in *Juglans regia* L. leaves and evaluation of in vitro antioxidant activity via intracellular and chemical methods. The Scientific World Journal, **2014**, 1, **2014**.
22. ZHAO Y., ZHOU W., DIAO S., JIANG Z., JIN M., LI G. Phytochemical investigation on the roots of *Juglans mandshurica* and their chemotaxonomic significance. Biochemical Systematics and Ecology, 103957, **2019**.

23. ZHOU Y.Y., SONG H.J., GUO S., WANG Y. A new triterpene from the green walnut husks of *Juglans mandshurica* Maxim. Journal of Natural Medicines, **73**, 800, **2019**.
24. SALIMI M., MAJD A., SEPAHDAR Z., AZADMANESH K., IRIAN S. Cytotoxicity efects of various *Juglans regia* (walnut) leaf extracts in human cancer cell lines. Pharmaceutical Biology, **50**, 1416, **2012**.
25. HASAN T.N., GRACE B.L., SHAF G., HAZZANI A.A., ALSHATWI A.A. Anti-proliferative efects of organic extracts from root bark of *Juglans regia* L. (RBJR) on MDA-MB-231 human breast cancer cells: role of Bcl-2/Bax, caspases and Tp53. Asian Pacific Journal of Cancer Prevention, **12**, 525, **2011**.
26. LI Y.F., JIANG S., CHEN X.H. Advances in Study of *Juglandaceae* Plant. Journal of Inner Mongolia Agricultural University, **27**: 148, **2006**.
27. MA W.L., LUO J.C., JIN T., JONI K. Study on dynamics of *Juglans mandshurica* population from Changbai Mountain. Bulletin of Botanical Research, **27**, 249, **2007**.
28. GUO L.N., ZHANG R. Identification of new naphthalenones from *Juglans mandshurica* and evaluation of their anticancer activities. Chinese Journal of Natural Medicines, **13**, 707, **2015**.
29. CARVALHO M., FERREIRA P.L., MENDES V.S., SILVA R., PEREIRA J., JERÓNIMO C., SILVA B. Human cancer cell antiproliferative and antioxidant activities of *Juglans regia* L. Food and Chemical Toxicology, **48**, 441, **2010**.
30. XU H.L., YU X.F., QU S.C., SUI D.Y. Juglone, isolated from *Juglans mandshurica* Maxim, induces apoptosis via down-regulation of AR expression in human prostate cancer LNCaP cells. Bioorganic and Medicinal Chemistry Letters, **23**, 3631, **2013**.
31. MACHIDA K., YOGIASHI Y., MATSUDA S., SUZUKI A., KIKUCHI M. A new phenolic glycoside syringate from the bark of *Juglans mandshurica* MAXIM. var. *sieboldiana* MAKINO. Journal of Natural Medicines, **63**, 220, **2009**.
32. WANG R.J., SHI W., XIA Y.J., TU M.W.L.J., ZHANG L.J., WANG Y.M. Antitumor effects and immune regulation activities of a purifed polysaccharide extracted from *Juglan regia*. International Journal of Biological Macromolecules, **72**, 771, **2015**.
33. SHARMA P., RAVIKUMARG G., KALAISELVI M., GOMATHI D., UMA C. In vitro antibacterial and free radical scavenging activity of green hull of *Juglans regia*. Journal of Pharmaceutical and Biomedical analysis, **3,** 298, **2013**.
34. HU Z., ZHANG T., GAO X.X., WANG Y., ZHANG Q., ZHOU H.J., ZHAO G.F., WANG M.L., WOESTE K.E., ZHAO P. De novo assembly and characterization of the leaf, bud, and fruit transcriptome from the vulnerable tree *Juglans mandshurica* for the development of 20 new microsatellite markers using Illumina sequencing. Molecular Genetics and Genomics, 1, **2015**.
35. LI X., WEN Y., ZHANG J., LIU L., JIN L., YAN T., WANG Y. The effect of low-temperature event on the survival and growth of *Juglans mandshurica* seedlings within forest gaps. Journal of Forest Research, **29**, 943, **2018**.
36. ASMA B.M., OZTURK K. Analysis of morphological, pomological and yield char acteristics of some apricot germplasm in Turkey. Genetic Resources and Crop Evolution, **52**, 305, **2005**.
37. BADENES M.L., MARTINEZ-CALVO J., LLACER G. Analysis of apricot germplasm from the European ecogeographical group. Euphytica, **102**, 93, **1998**.
38. SHARMA D.P., SHARMA N., BAWA R., RAJESH K. Potential of apricot growing in the arid-cold desert region of North-Western Himalayas. Acta Horticulturae, **696**, 61, **2005**.
39. ERDOGAN D., GUNER M., DURSUN E., GEZER I. Mechanical harvesting apricots. Biosystems Engineering, **85**, 19, **2003**.
40. KRICHEN L., AUDERGON J.M., TRIFI-FARAH N. Variability of morphological characters among Tunisian apricot germplasm. Hortscience, **179**, 328, **2014**.
41. WANI A.A., ZARGAR S.A., MALIK A.H., KASHTWARI M., NAZIR M., KHUROO A.A., AHMAD F., DAR T.A. Assessment of variability in morphological characters of apricot germplasm of Kashmir, India. Hortscience, **225**, 630, **2017**.
42. POGGETTI L., ERMACORA P., CIPRIANI G., PAVAN F., TESTOLIN R. MorPhological and carpological variability of walnut germplasm (*Juglans regia* L.) collected in North-Eastern Italy and selection of superior genotypes. Hortscience, **225**, 615, **2017**.
43. MILOŠEVIĆ T., MILOŠEVIĆ N., GLIŠIĆ I., KRŠKA B. Characteristics of promising apricot (*Prunus armeniaca* L.) genetic resources in Central Serbia based on blossoming period and fruit quality. Hortscience, **37**, 46, **2010**.
44. MILOSEVIC T., MILOSEVIC N. Genetic variability and selection in natural populations of vineyard peach (*Prunus persica* ssp. *vulgaris* Mill.) in the krusevac region (Central Serbia). Agrociencia, **44**, 297, **2010**.
45. AZODANLOU R., DARBELLAY C., LUISIER J.L., VILLETTAZ J.C., AMADÒ R. Development of a model for quality assessment of tomatoes and apricots. Food Science and Technology, **36**, 223, **2003**.
46. CHEN G.P., CHENG S.S., CONG M.Y., LIU J., GAO X., WANG H., SHI F.C. Effects of litter decomposition on soil nutrients in three broad-leaved forests. Chinese Journal of Plant Ecology, **33**, 874, **2014**.
47. SHE C.Q., YANG W.X., FANG S.Z., SHANG X.L. Phenotypic Diversity of Seed Traits in *Cyclocarya paliurus* Natural Population. Acta Application Ecology, **20**, 2351, **2009**.
48. CHEN S.Y., YAND H., HAN J., ZHANG D.W., ZHAO S.S., ZHANG Z.H., GUO Z.L., YANG Y.C. Provenance variation of seed traits of *Juglans mandshurica* in Changbai mountains, northeastern China. Journal of Beijing Forestry University, **37**, 32, **2015**.
49. BLENNOW K., LINDKVIST L. Models of low temperature and high irradiance and their application to explaining the risk of seedling mortality. Forest Ecology and Management, **135**, 289, **2000**.
50. CLARK J.S. Why trees migrate so fast: confronting theory with dispersal biology and the paleorecord. American Naturalist, **152**, 204, **1998**.
51. GRAY A.N., SPIES T.A., PABST R.J. Canopy gaps affect long-term patterns of tree growth and mortality in mature and old-growth forests in the Pacific Northwest. Forest Ecology and Management, **281**, 111, **2012**.
52. GUO R., ZENG R., ZHANG J.X., ZHANG X.L., SUN G.Y. Effects of different light intensities on reactive oxygen species metabolism and chlorophyll fluorescence parameters in leaves of flue-curing tobacco seedlings after chilling stress. Acta Agriculturae Boreali-Sinica, **30**, 225, **2015**.
