# Supplementary material for: Provenance and family variations in early growth of Manchurian walnut (Juglans mandshurica Maxim.) and selection of superior families
Source: PLoS One. 2024 Mar 7;19(3):e0298918. doi: 10.1371/journal.pone.0298918 (PMC10919699; doi:10.1371/journal.pone.0298918)
Supplement: S2 File — (ZIP) [file pone.0298918.s005.zip › Morphological Variability, Heritability and Correlation Studies within an Argan Tree Population (Argania spinosa (L.) Skeels) Preserved in situ.pdf]

International Journal of Agriculture and Forestry 2017, 7(2): 42-51
DOI: 10.5923/j.ijaf.20170702.02

Morphological Variability, Heritability and Correlation Studies within an Argan Tree Population (*Argania spinosa* (L.) Skeels) Preserved *in situ*

Mohamed Louay Metougui[1,*], Mimoun Mokhtari[2], Peter J. Maughan[3], Eric N. Jellen[3], Ouafae Benlhabib[1]

[1]Department of Production, Protection & Plant Biotechnology, Hassan II Institute of Agronomy and Veterinary Medicine, Rabat, Morocco
[2]Department of Horticulture, Horticultural Complex of Agadir, Hassan II Institute of Agronomy and Veterinary Medicine, Ait Melloul, Morocco
[3]Department of Plant & Wildlife Sciences, Brigham Young University, Provo, UT, USA

Abstract *Argania spinosa* (L.) Skeels is an endemic tree to Morocco, that produce a high-quality oil used for both cosmetic and culinary purposes. In this study, the morphological diversity of argan trees was investigated in an *in situ* preserved population, at Admine reserve in southwestern Morocco over three years. A total of 122 trees were characterized using 30 quantitative traits. Significant differences among genotypes and among years were seen for all traits. Correlation analysis between traits showed that vigor traits (leaf and shoot sizes) were positively correlated with fruit traits. Broad-sense heritability estimates were high for clustered leaf traits and thorn numbers (H^2>0.90) and for most of the fruit, stone and almond traits (H^2>0.70). Cluster analysis using Euclidian distances was used to establish the relationships among the argan germplasm and grouped the genotypes into five relatively homogenous clusters. The relationship among the traits was also analyzed by principal component analysis. Highly discriminating traits were those related to fruit, stone and almond morphologies, as well as clustered leaf size, shoot length, and glomeruli density.

Keywords *Argania spinosa*, Argan, Diversity, Morphological characterization, Genetic conservation

1. Introduction

Argan tree (*Argania spinosa* L. skeels) belongs to the monospecific genus *Argania* and is the only representative of the tropical family Sapotaceae in Morocco [1]. This tree is endemic to Southwest Morocco, where it occupies an area of around 952,200 ha [2]. Outside this major settlement, significant outlying populations are present in the valley of Oued Grou in southeastern Rabat, on the Mediterranean slope of the Beni-Snassen mountains in northern Oujda [3], and in Tindouf region in eastern Algeria [4].

Argan is a multi-purpose tree, with leaves and fruit used for livestock feed, and the wood for carpentry and fuel [5]. The primary product of this tree, however, is the highly prized oil derived from its almonds, which has culinary, pharmaceutical, and cosmetic uses [6]. Argan oil contains about 80% unsaturated fatty acids (mainly oleic and linoleic acid) and important levels of γ-tocopherols – a strong antioxidant. Multiple studies have shown the argan oil effect on lowering cholesterol, triacylglycerol, and cardiovascular risks [7, 8]. It has also been shown that argan oil has hypotensive and antiatherogenic properties [7-9].

Argan oil is one of the most expensive edible oils in the world, reaching a retail price of 300$/L in the US and European markets where it's the subject of several cosmetic patents [6]. According to the Moroccan independent institution for export control and coordination, the export of 1000 tons of argan oil in 2015 was worth nearly 20 million US dollars. For the local population in the arid zones where argan grows, the tree plays an important socio-economic role. Indeed, it is estimated that nearly 19% of the local population's income depends on this tree, with major processing activities, including oil extraction, being traditionally accomplished by women. Thus the argan tree represents an invaluable resource which merits intensive study and preservation [10].

Several studies described the genetic diversity seen within argan tree. The first studies dating from the 1990s, showed a strong geographical structure of argan populations using isozymes and chloroplast DNA polymorphisms [11, 12]. Based on fruit and stone morphological variability, Bani-Aameur and Ferradous [13] reported the existence of large variances within populations in contrast with low inter-population diversity. Ait Aabd et *al.* confirmed the high within population diversity using fruit traits and oil

* Corresponding author:
metougui@gmail.com (Mohamed Louay Metougui)
Published online at http://journal.sapub.org/ijaf
Copyright © 2017 Scientific & Academic Publishing. All Rights Reserved

content and reported for the first time the heritability of those characters [14]. These studies were limited to fruit related traits and didn't consider the existing variability between genotypes in the vegetative part (leaf and shoot traits).

Understanding the true genetic diversity within a species is critical for developing *in situ* and *ex situ* conservation strategies, which are desperately needed to protect the variability and fecundity of this species. Such studies also provide the basis necessary for developing core breeding germplasm collections used to initiate plant improvement programs. Here we present a study of genetic diversity within the Admine reserve in upper Southwestern Morocco based on the morphological characterization of over 100 argan genotypes using 30 quantitative fruit and vegetative characteristics. The aim of the present study was to determine the amount of genetic diversity within this *in situ* preserved population, quantify the variability levels of each trait, and identify the most significant discriminating traits in the species.

2. Material and Methods

2.1. Study Area

The study was conducted at the Admine Natural Reserve of the Horticultural Complex in Agadir, in upper southwestern Morocco. The reserve extends over 28 ha and is located at 9°28'35"W, 30°21'58"N (Figure 1) at an average elevation of 30 m above sea level. The study area is a natural argan forest which has been protected from human pressure for more than 30 years. The area features the subtropical-semiarid climate with warm summers and mild winters which are optimal for argan growth. The average annual rainfall in this region is 300 mm (2000-2016). During the study period, rainfall varied from a minimum of 10 mm (2013) to a maximum of 431 mm (2014).

In area, the annual average high temperature varies between 19°C – 37°C, while the average low temperature varies from 6 to 21°C. Occasionally, the area experiences Saharan winds in summer, which increase the temperature above 40°C, and lowers the relative humidity to under 10%.

2.2. Plant Material

The study was carried out during three years from 2013 to 2015. The Admine Natural Reserve area was divided into 2500 m² plots, from which one tree was randomly selected. A total of 122 trees were studied, covering all the area, and representing nearly 10% of the total number of trees of the reserve. All the studied trees were mature (over 20 years old) and vigorous, with no apparent disease symptoms.

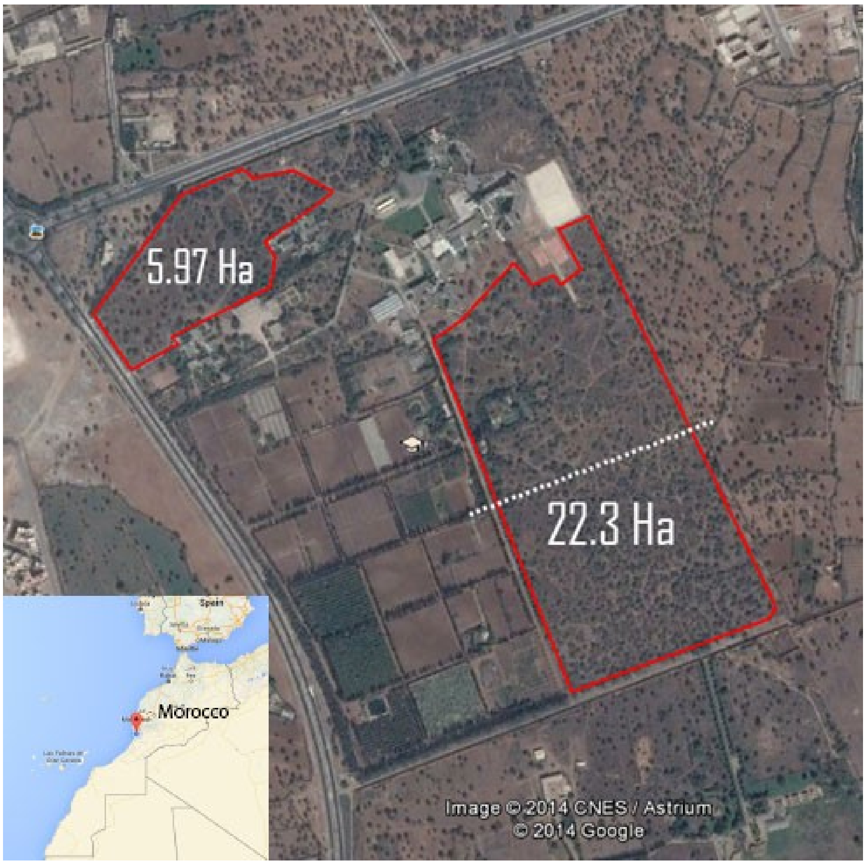

Figure 1. Map showing the study area

2.3. Morphological Descriptors and Statistical Analysis

To assess diversity within the studied population, we used a total number of 30 quantitatively scored morphological traits (Table 1). The characterization included tree, leaf, shoot, fruit, stone, and almond traits. Leaf and shoot traits were recorded for all three years of the study; fruit, stone and almond traits were recorded in 2014 and 2015; while tree height and diameter were recorded only once in 2013.

Minimum, maximum, mean and coefficient of variation (CV) were computed for each trait. The CV was determined as a variability indicator. To compare the heterogeneity of the measured traits between genotypes, across years and estimate the genotype by year interaction effect, a two-way Analysis of Variance (ANOVA) was performed for each trait. To identify the correlations between traits, a Pearson coefficient, and p-value correlation matrix was computed.

The distances between genotypes were computed using the Euclidean norm based on similarity levels, using standardized data. Clustering of the genotypes was performed using Ward's hierarchical clustering method [15]. To find the patterns of the main variations and associations between the morphological traits in the studied population and the most discriminant traits, a principal component analysis (PCA) was performed using data from all the years of the study.

The analysis of variance, clustering, and dendrogram were computed using Minitab 17.1.0 software, while the correlation matrix, PCA and PCA biplot were computed using PAST 3.10 software.

Table 1. List of Traits Used for Argan Characterization, with the Used Units, Sample Size and Measurement Methods

Organ	Trait	Abv	Ut	Spl	Measurement Method
**Tree**	Height	**TH**	m	**1**	Height and diameters were estimated from pictures using the comparison with a measurable control object method
	Average crown spread	**ACS**	m	**1**	
**Leaves**	Simple leaves length	**SLL**	cm	**10**	Length and width at the largest point, and the surface of a fully expanded simple leaf including the petiole taken from the year's shoot
	Simple leaves width	**SLL**	cm	**10**	
	Simple leaves surface	**SLS**	cm²	**10**	
	SL width-to-length ratio	**SLR**	-	**10**	Simple leaf width divided by its length
	Clustered leaves length	**CLL**	cm	**10**	Length and width at the largest point, and the surface of a fully expanded leaf including the petiole taken from a leaves cluster on the last year's shoot
	Clustered leaves width	**CLW**	cm	**10**	
	Clustered leaves surface	**CLS**	cm²	**10**	
	CL width-to-length ratio	**CLR**	-	**10**	Clustered leaf width divided by its length
	Leaves per Cluster	**LpC**	-	**10**	Number of leaves per leaf cluster on a last year's shoot
**Shoot**	Length	**StL**	cm	**10**	Length of a fully elongated year's shoot
	Glomeruli number	**GN**	-	**10**	Number of flower glomeruli on a fully elongated year's shoot
	Glomeruli density	**GD**	-/cm	**10**	Glomeruli number divided by the shoot length
	Thorn Density	**ThN**	-/cm	**10**	Number of thorns on the last year's lignified shoot divided by it length
**Fruit**	Length	**FL**	cm	**10**	Length of a fruit measured from the proximal to the distal end
	Diameter	**FD**	cm	**10**	Diameter of a mature fruit at its widest point
	Diameter-to-Length Ratio	**FDLr**	-	**10**	Fruit diameter divided by its length
	Weight	**FW**	g	**10**	Weight of a whole fruit
**Stone**	Length	**SL**	cm	**10**	Length of a stone measured from the proximal to the distal end
	Diameter	**SD**	cm	**10**	Diameter of a stone fruit at its widest point
	Diameter-to-Length Ratio	**SDLr**	-	**10**	Stone diameter divided by its length
	Weight	**SW**	g	**10**	Weight of a whole stone
	Carpel Number	**CaN**	-	**10**	Number of chambers in a stone
**Almond**	Length	**AL**	cm	**10**	Length of an almond at its longest point
	Width	**AWi**	mm	**10**	Width of an almond at its widest point
	Width-to-Length Ratio	**AWLr**	-	**10**	Almond width divided by its length
	Thickness	**ATh**	mm	**10**	Thickness of a whole almond
	Weight	**AWe**	mg	**10**	Weight of a whole almond
	Almonds per Stone	**ApS**	-	**10**	Number of almonds present in a stone

Ut: Unit; Abv: Abbreviation; Spl: Number of measured sample per genotype.

2.4. Genetic Estimates

Genotypic ($\sigma^2 g$) and Phenotypic variances ($\sigma^2 p$) were calculated using the mean square from the ANOVA tables as formulated by Comstock and Robinson [16]:

$$\sigma^2{}_g = \frac{MS_g - MS_{gy}}{r \times y} \quad (1)$$

$$\sigma^2{}_p = \frac{MS_g}{r \times y} \quad (2)$$

where MS_g is the Mean Square for genotype, MS_{gy} the Mean Square for genotype to year interaction, r the number of replication and y the number of years.

Heritability in the broad sense (H^2) was estimated according to Allard [17]:

$$H^2 = \frac{\sigma^2{}_g}{\sigma^2{}_p} \quad (3)$$

Phenotypic Coefficient of Variation (PCV) and Genotypic Coefficient of Variation (GCV) were computed as described by Singh and Chaudhary [18].

$$PCV(\%) = \frac{\sqrt{\sigma^2{}_p}}{X} \times 100 \quad (4)$$

$$GCV(\%) = \frac{\sqrt{\sigma^2{}_g}}{X} \times 100 \quad (5)$$

where X is the sample's mean.

3. Results

Mean, minimum, maximum and coefficient of variation (CV) values for the studied characters are given in Table 2. The highest CV identified was for thorn density (78.01%) due to the skewness of its distribution. The next highest CV was that of the simple leaf surface (36.44%), followed by tree height, simple leaf width/length ratio, and almond weight. The lowest CV was that of the stone's diameter (10.23%). Tree height and crown spread varied widely with CVs of 34.88% and 29.85%, respectively.

The average tree height was 3.5 meters and ranged from a maximum of 8.76 m to a minimum of 1.89 m. The crown spread varied between 3.04 and 12.8 m with an average of 3.04 m. Both tree dimension parameters had a highly significant positive linear correlation (r=0.86).

3.1. Leaf Traits

Argan trees usually present two types of leaves: simple leaves that grow on newly formed shoots and clustered leaves that grow on older woody shoots. Simple leaf area ranged between 0.79 to 4.38 cm², with an average length of 3.51 cm, width of 0.78 cm, and a surface of 2.4 cm². Clustered leaves were grouped in 2 to 9 leaves per cluster with a leaf area ranging from 0.65 cm² to 3.07 cm² and an average area of 1.62 cm². The CVs of simple leaf dimensions were consistently higher than those of clustered leaves (Table 2). Clustered leaf width/length ratios were equal to 0.18, which were lower than the simple leaf ratio (0.25).

Leaf size varied over years. Specifically, there was a better growth of simple leaves in 2015 than in 2013 as illustrated for leaf length (4.11 vs. 3.02 cm), leaf width (0.96 vs. 0.67 cm) and leaf area (2.85 vs. 1.8 cm²) (Table 3). The clustered leaf size variation was lower, varying respectively in its length, width and area from 3.24 to 3.32 cm, 0.57 to 0.59 cm and 1.57 to 1.68 cm² over the three years.

Table 2. Minimum, Maximum, Mean Values and Coefficient of Variation of the Studied Morphological Traits

Organ	Trait	Min	Max	Mean	CV (%)
**Tree**	**TH**	1.89	8.76	3.5	34.88
	**ACS**	3.04	12.79	5.56	29.85
**Leaves**	**SLL**	1.77	5.52	3.51	19.95
	**SLW**	0.36	1.36	0.78	28.53
	**SLS**	0.79	4.38	2.39	36.44
	**SLr**	0.07	0.43	0.23	32.11
	**CLL**	2.02	4.72	3.28	15.89
	**CLW**	0.36	0.99	0.58	22.29
	**CLS**	0.65	3.07	1.62	29.53
	**CLr**	0.11	0.34	0.18	25.53
	**LpC**	2.28	7.39	4.70	20.05
**Shoot**	**StL**	4.03	17.33	9.08	28.43
	**GN**	5.92	21.88	12.27	24.55
	**GD**	0.82	2.02	1.39	13.35
	**ThN**	0.00	1.15	0.45	78.01
**Fruit**	**FL**	1.46	4.68	2.93	19.28
	**FD**	1.29	2.49	2.01	11.18
	**FDLr**	0.40	0.99	0.71	14.82
	**FW**	0.87	5.12	2.97	27.73
**Stone**	**SL**	0.97	3.31	2.12	18.24
	**SD**	0.89	1.65	1.35	10.23
	**SDLr**	0.38	0.91	0.66	15.31
	**SW**	0.47	2.89	1.81	28.03
	**CaN**	1.90	3.70	2.38	14.06
**Almond**	**AL**	0.78	2.48	1.61	17.45
	**AWi**	0.57	1.05	0.82	12.11
	**AWLr**	0.31	0.75	0.52	14.35
	**ATh**	1.85	4.65	2.99	16.74
	**AWe**	55.29	376.00	184.76	31.44
	**ApS**	1.00	2.30	1.37	17.61

3.2. Shoot Characterization

The length of new shoots varied widely between genotypes, ranging from 4.03 to 17.33 cm. Glomeruli number ranged between 5.92 and 21.88 per shoot, and from 0.82 to 2.02 per cm (Table 2). Over the years evaluated, 2013 presented the best growth in term of stem length and number of glomeruli/stem, with 9.61 cm and 12.77 glomeruli, respectively, while 2015 had the highest glomeruli density (1.45 per cm).

Thorn density on woody shoots ranged between 0 (spineless trees) to 1.15 thorns/cm. The density was slightly

higher in 2014 (0.48 thorn/cm) than 2013 and 2015 (0.44 thorn/cm; Table 3).

Table 3. Mean Values over Years and F-Values from Two-Way ANOVA of the Studied Traits

	Mean per year			Source of variation		
**Traits**	**2013**	**2014**	**2015**	**G**	**Y**	**GYI**
**SLL**	3.02	3.41	4.11	26.2***	669.2***	1.3**
**SLW**	0.67	0.76	0.92	46.7***	561.7***	1.5***
**SLS**	1.80	2.54	2.85	15.6**	233.9***	**0.7NS**
**SLr**	0.23	0.23	0.23	-	-	-
**CLL**	3.24	3.32	3.30	15.4***	3.7*	4.0***
**CLW**	0.57	0.59	0.57	28.3***	3.5*	4.2***
**CLS**	1.68	1.57	1.61	13.0***	9.3***	3.8***
**CLr**	0.18	0.18	0.18	-	-	-
**LpC**	4.52	4.59	4.99	17.3***	52.6***	1.5***
**StL**	9.61	9.13	8.91	33.6***	17.3***	7.0***
**GN**	12.77	11.64	12.67	21.6***	24.2***	8.3***
**GD**	1.37	1.33	1.45	-	-	-
**ThN**	0.44	0.48	0.44	227.5***	39.4***	2.0***
**FL**	-	2.74	3.24	96.9***	1646.3***	17.5***
**FD**	-	1.98	2.07	16.5***	55.4***	6.7***
**FDLr**	-	0.74	0.65	-	-	-
**FW**	-	2.79	3.34	74.5***	723.7***	17.4***
**SL**	-	1.98	2.34	91.6***	2003***	17.4***
**SD**	-	1.33	1.39	14.5***	51.0***	3.9***
**SDLr**	-	0.69	0.61	-	-	-
**SW**	-	1.61	2.13	68.1***	1888***	19.0***
**CaN**	-	2.38	2.39	14.0***	*0.0NS*	3.7***
**AL**	-	1.51	1.77	58.8***	1316.7***	13.8***
**AWi**	-	8.14	8.49	39.6***	79.3***	6.3***
**AWLr**	-	0.55	0.49	-	-	-
**ATh**	-	3.03	2.97	18.2***	*1.3NS*	2.1***
**AWe**	-	177.7	197.7	53.0***	262.9***	21.2***
**ApS**	-	1.27	1.49	3.9***	78.9***	2.4***

G: Genotype; Y: Year; GYI: Genotype Year Interaction; ***, **, * significant at 0.1%, 1% & 5% respectively; NS: Non-significant

3.3. Fruit Characterization

Fruit, stone, and almond sizes were measured during two seasons, 2014 and 2015. The fruit weight varied extensively from 0.8 to 5.12 g. Fruit size varied among genotypes from 1.46 to 4.68 cm in length and 1.29 to 2.49 cm in diameter. Stone weight ranged between 0.47 to 2.89 g, and almond weight ranged between 55.29 to 376.00 mg. Stone and almond size showed comparable growth as the fruit and ranged for stones from 0.97 to 3.31 cm in length and 0.89 to 1.65 cm in diameter. The highest almond size was 2.48 cm in length, 1.05 cm in width and 4.65 mm in thickness. The smallest almond size was 0.78 cm, 0.57 cm, and 1.85 mm in length, width, and thickness respectively (Table 2).

The fruit diameter-to-length ratio averaged 0.71 and varied between 0.4 and 0.99, respectively for round and apiculate/fusiform type fruits. Thus, this argan population is made up of 79% oval and drop shaped fruits (ratio of 0.60-0.85), 7% round fruits, and 14% apiculate/fusiform fruits.

Stone diameter and length were positively correlated (r=0.86), with the diameter-to-length ratio following a similar distribution as the fruits diameter-to-length ratio. Almond width-to-length ratio average 0.52, ranging from 0.31 to 0.75 and showed a positive linear correlation with the fruit ratio (r=0.72). CVs were similar across all fruit characteristics, with the highest CV value for the fruit weight and the lowest for the diameter-to-length ratio. Fruit, stone, and almond size, as well as stone weight were higher in 2015 than 2014. Almond thickness did not show a significant difference between the years (Table 3). Lastly, the number of chambers per fruit stone ranged from one to five and contained one to four almonds, with an average of 2.38 chambers and 1.37 almonds per stone.

3.4. Analysis of Variance and Correlation

The ANOVA showed highly significant ($p<0.01$) differences between genotypes (G) for the 22 tested morphological traits, as well as statistically relevant differences between years (Y) except for the number of chambers and almond thickness. The GY interaction was significant for most of the traits except for the simple leaf surface (Table 3).

Variance components, which represent the factors' individual contribution to the total variation, revealed that the genotype was the dominant factor. Its contribution to the total variation ranged between 14.18% for the number of almonds/seed and 89.79% for thorns density (Figure 2). The number of almonds per seed differed slightly between trees, but with considerable variation among trees (the residual represents 71.87% of ApS total variance).

The bivariate Pearson correlations revealed a significant correlation between fruit, stone, and almond size and weight. A significant correlation ($p<0.01$) between tree height and the number of leaves per cluster (r=0.27), stone length (r=0.26), and almond length (r=0.26). Similarly, a significant correlation was also found between shoot length and simple leaf surface (r=0.42), clustered leaf surface (r=0.41), number of glomeruli per shoot (r=0.85), fruit length (r=0.36) and weight (r=0.34), seed length (r=0.37) and weight (r=0.32), almond length (r=0.42), width (r=0.25) and weight (r=0.36) and glomeruli density (r=-0.50). The number of glomeruli per shoot and clustered leaf length were positively correlated with fruit, seed and almond length, and weight.

3.5. Genetic Estimates

Genotypic and phenotypic variances, broad-sense heritability, genotypic and phenotypic coefficients of variation of 22 traits are reported in Table 4. The results revealed substantial phenotypic and genotypic variances among trees for all the traits. For most characters, a large proportion of the phenotypic variation was accounted for by

the genetic component, highlighting the importance of genetics on the expression of these characters. Heritability was greater than 0.59 for all traits except for ApS, which showed a heritability value of 0.38.

Table 4. Estimate of Genetic Variables for the Studied Traits

Traits	σ^2g	σ^2p	H^2	GCV (%)	PCV (%)
**SLL**	0.47	0.49	0.95	19.5	35.4
**SLW**	0.05	0.05	0.97	28.4	51.2
**SLS**	0.74	0.78	0.96	36.1	65.3
**CLL**	0.19	0.26	0.74	13.3	27.3
**CLW**	0.01	0.02	0.85	21.0	40.3
**CLS**	0.17	0.24	0.71	25.3	53.2
**LpC**	0.81	0.89	0.92	19.2	35.6
**FL**	0.23	0.28	0.82	16.2	31.8
**FD**	0.02	0.04	0.59	7.2	16.6
**FW**	0.43	0.56	0.77	22.1	44.7
**SL**	0.10	0.12	0.81	14.9	29.3
**SD**	0.01	0.01	0.73	7.5	15.5
**SW**	0.14	0.20	0.72	21.0	43.7
**CaN**	0.09	0.12	0.74	12.5	25.9
**AL**	0.05	0.07	0.77	13.9	28.1
**AWi**	0.75	0.89	0.84	10.5	20.3
**ATh**	0.23	0.26	0.88	16.0	30.2
**Awe**	1447	2413	0.60	20.6	47.1
**ApS**	0.02	0.05	0.38	9.9	28.4
**StL**	5.30	6.69	0.79	25.4	50.5
**GN**	4.97	8.10	0.61	18.2	41.1
**ThN**	1.24	1.26	0.99	77.6	138.1

The phenotypic coefficient of variation (PCV) values ranged from 15.5% (SD) to 169% (ThN) and were higher than those of genotypic coefficient of variation (GCV) for all the traits, reflecting the influence of environment on the expression of traits. The maximum GCV estimates were of ThN (77.6%) followed by SLS (36.1%), while fruit diameter (7.2%), stone diameter (7.5%), and almond per stone (9.9%) recorded the lowest GCV values (Table 4).

3.6. Cluster Analysis

The genetic variability among genotypes was studied to know the extent of divergence between the genotypes and gather them into relatively homogeneous clusters. The dendrogram summarizes the phylogenic relationship among 112 genotypes (Figure 3).

Based on Euclidian distance, the argan collection was grouped into five major clusters. Cluster II, the largest, included 29 genotypes, while cluster I, the smallest, included only 14 genotypes.

Average distance values among (average distance from centroid) and between clusters (distances between cluster centroids) are presented in Table 5. Such measures provide an insight into the nature of genetic divergence at intra and inter-cluster levels. Intra-cluster distances were in most cases smaller than inter-cluster distances. The distance values ranged between 3.22 and 8.11. Among the five clusters, the maximal intra-cluster distance was found within cluster I (4.77), while the minimal distance was within the cluster IV (4.25). Regarding the inter-cluster distance, the maximal value was found between clusters I and V (8.11) followed by cluster I and IV (5.96), while the smallest distance was identified between clusters II and III (3.22).

Table 5. Inter and Intra (bold) Cluster Distance Matrix

	n	C.I	C.II	C.III	C.IV	C.V
C.I	14	**4.7669**	4.2244	5.6793	5.9592	8.1128
C.II	29	4.2244	**4.2892**	3.2204	4.2072	5.1920
C.III	28	5.6793	3.2204	**4.3282**	4.6390	4.0234
C.IV	19	5.9592	4.2071	4.6390	**4.2479**	4.8630
C.V	22	8.1128	5.1920	4.0234	4.8630	**4.5409**

C.I, C.II, C.III, C.IV and C.V: Cluster I, II, III, IV and V respectively

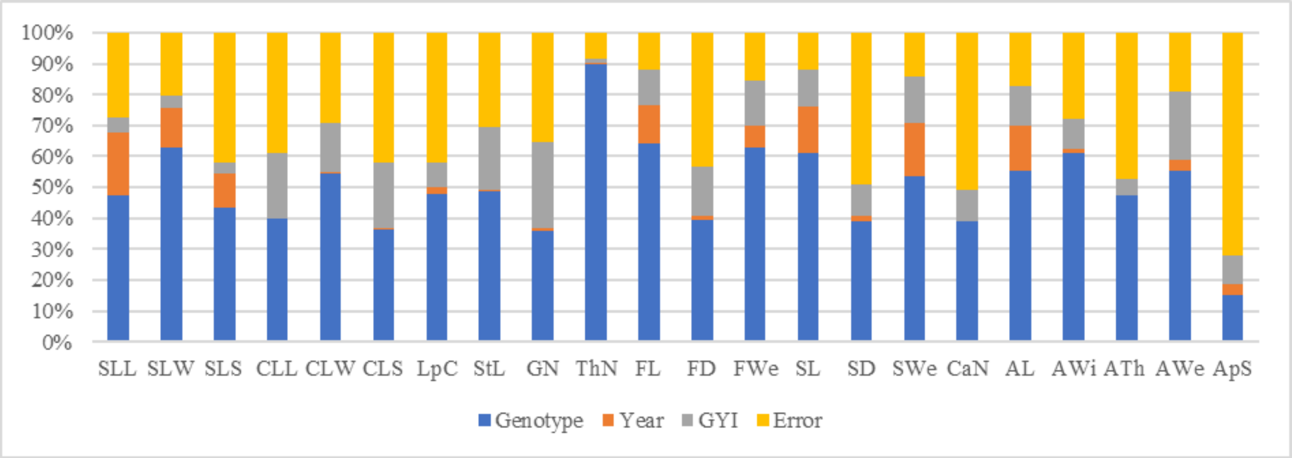

Figure 2. Contribution of sources to total variance

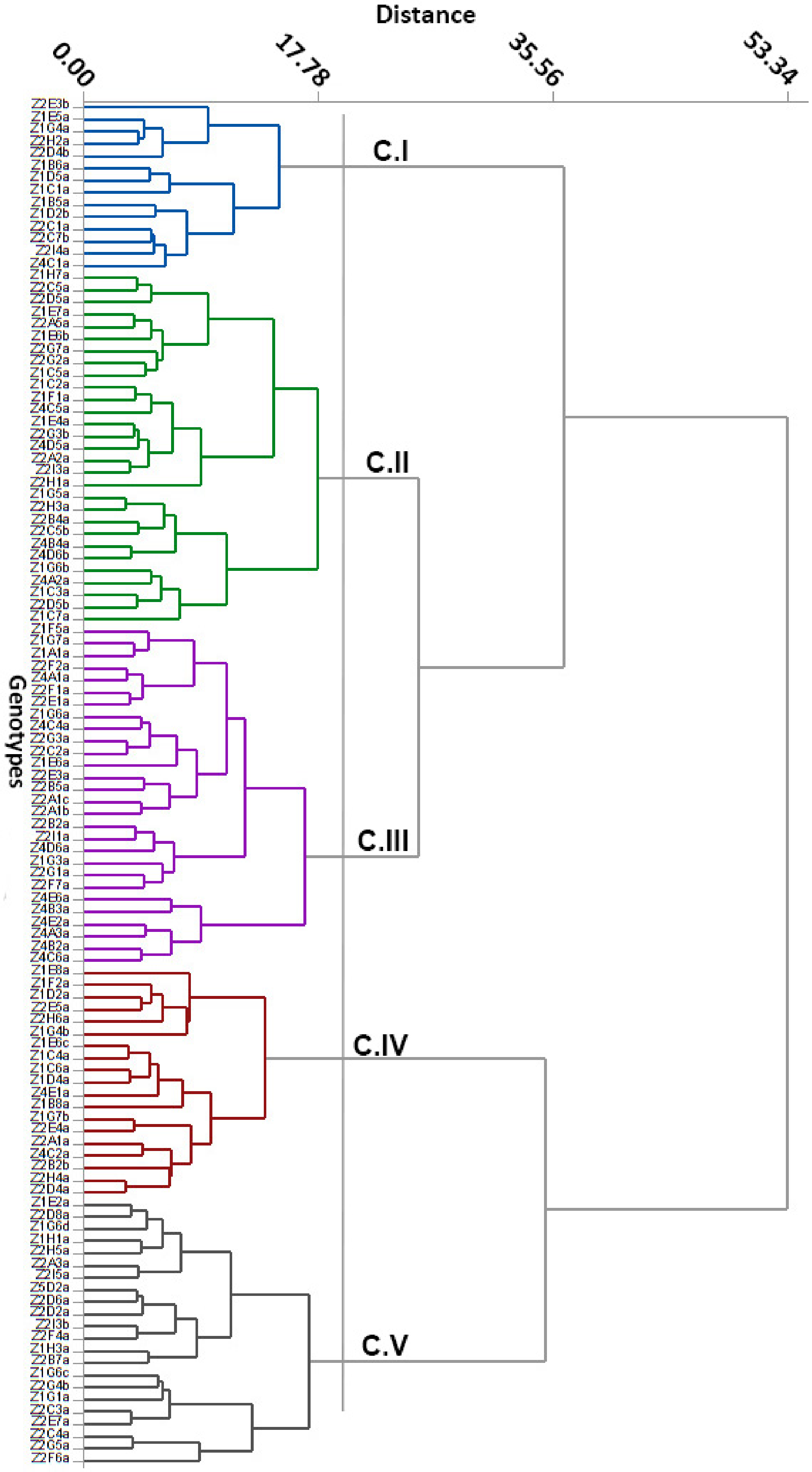

Figure 3. Genotypes' clustering using ward linkage and Euclidean distance

The clusters mean values showed significant differences between clusters for all of the traits. Cluster II grouped tall trees with a wide crown, long and narrow leaves, intermediate length shoots, and medium sized ellipsoid fruits. The first cluster's trees have the same leaves characteristics as cluster II, with long shoots, high glomeruli number, long and heavy fruits, stones and almonds. Cluster III trees have small size leaves, high glomeruli density, and medium fruit size. Cluster IV grouped trees with the wider leaves and shorter fruits. Cluster V trees are smaller, have short shoots, a small glomeruli number, small sized and light fruits, stones, and almonds.

Principal component analysis (PCA) computes the variable contribution to the total variation among the principle differentiation axes. The eigenvalues are often used to select factors that discriminate the best between the entries. The sum of the eigenvalues is usually equal to the number of variables. Therefore, in this analysis, the first principal component retained 8.16 of the original variables (Table 6). The eight principal components, with an eigenvalue greater than one, accounted for 81.55% of the total variation; which suggests they are the principal components and should be used to summarize the original 30 variables. Out of the eight principal components, the first three contributed significantly to the total variation with respective values of 27.19, 14.70 and 10.54%.

The plot of the first two principal components showed a high dispersion of the genotypes but was able to fairly differentiate 4 of the 5 previously defined clusters (Figure 4).

Accordingly, the first principal component (PC1) had a high positive component loading from fruit, stone, and almond length and weight, shoot length, and glomeruli number; and high negative loading from fruit and stone diameter-to-length ratio. The second principal component (PC2) featured both simple and clustered leaf dimension traits (length, width, and surface) and simple leaf width/length ratio. The main contributing traits to the third principal component (PC3) were fruit and stone diameter, diameter/length ratio, and almond width, weight, and width/length ratio. The above traits contributed most to diversity and were the most effective at differentiating the genotypes.

Table 6. Eigenvalues, Variance and Cumulative Variance for the Eight Major Principal Components (PC) of the Principal Component Analysis

PC	Eigenvalue	Variance (%)	Cumulative Variance (%)
1	8.16	27.19	27.19
2	4,41	14.70	41.89
3	3,16	10.54	52.43
4	2,46	8.19	60.62
5	2,17	7.24	67.85
6	1,88	6.25	74.10
7	1,18	3.93	78.03
8	1,06	3.52	81.55

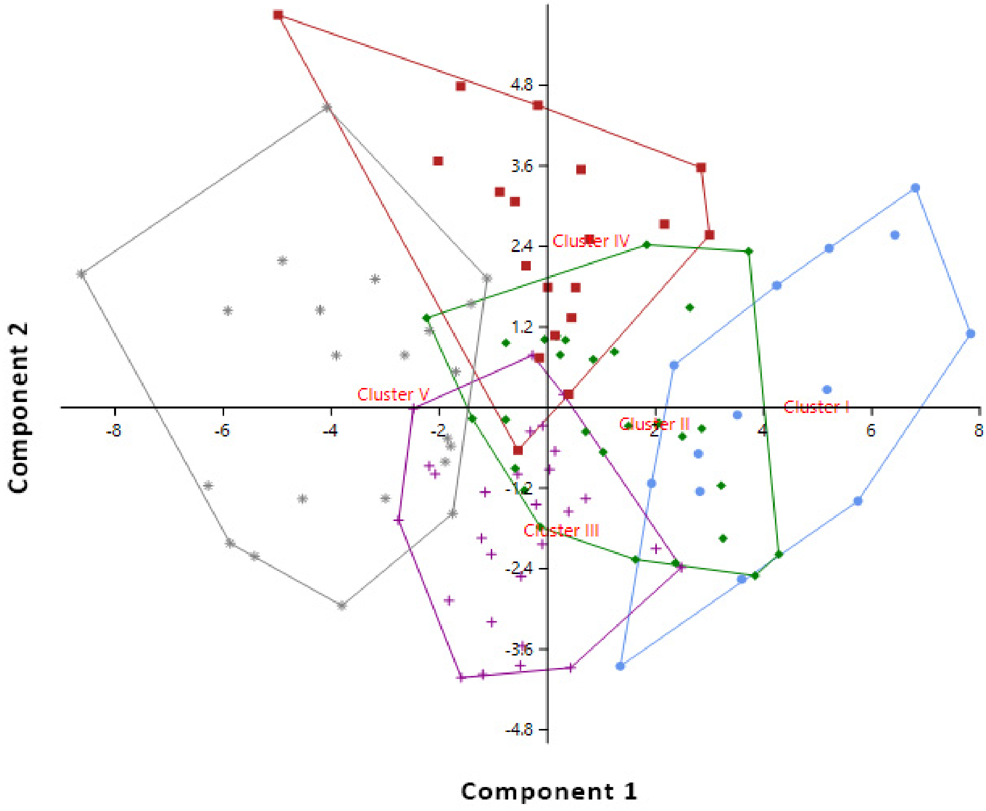

Figure 4. Two-dimensional PCA plot based on the 2 first components of the studied genotypes with their corresponding cluster (clusters are distinguished with the same colors as in the dendrogram)

4. Discussion

The understanding of the genetic structure of populations and the study of genetic parameters is essential for effective management of genetic resources and is the first step toward any domestication process. Here we study an *in situ* protected Argan population growing in the Admine natural preserve of the Horticultural Complex of Agadir. The collected data suggests that a significant phenotypic variability exists within the collection. This high within-population variability is promoted in argan because of its allogamy and entomophily [19, 20].

Variability within core germplasm collections is essential for any domestication work. Our data showed significant differences ($p<0.05$ to $p<0.001$) between genotypes for all traits, and between the years for most traits. These results are in agreement with those found within other argan populations [13, 14, 21].

Leaf size values were higher than those reported by Zahidi and Bani-Aameur for both simple and clustered leaves, except for width-to-length ratios which showed the same values [22]. A higher variability was also recorded within our population for simple and clustered leaf width and width-to-length ratio.

Fruit, stone and almond weights and dimensions were similar to those reported in previous studies [13, 21, 23]; but our CVs were higher than those reported by Bani-Aameur and Ferradous [13]. The collected data illustrate the high diversity of the Admine reserve population. Such considerable diversity may be related to the protected status of the study area, which helps the trees to develop without any human or animal pressure.

Significant variation between years was seen for most traits and could be related to the climatic difference between years, particularly rainfall. In fact, leaf, fruit, stone, and almond traits were greater in the cropping year 2015 when rainfall reached 350 mm, which was higher than the previous years (246 and 151 mm respectively for 2013 and 2014). Similar conclusions were made by Bani-Aameur and Ferradous, where they found an important year effect over fruit traits [24]. Interesting, the climatic difference did not seem to affect the qualitative traits (i.e., general shapes of fruit, stone, and leaves).

The correlation matrix between characters indicated that vigor related traits, including shoot length, simple leaf area, number of glomeruli, fruits, kernel and almond sizes were positively correlated. Such correlations between the vegetative organ of the tree and the size of the fruits were reported for several woody species and underscore the role photosynthetic organ play in increasing fruit size and weight [25-27].

The year's effect on the morphological traits allowed the estimation of heritability. Most of the traits had a broad-sense heritability coefficient greater than 0.60. The magnitude of this coefficient indicates the reliability with which a genotype can be evaluated through its phenotype. Globally, leaf traits had higher heritability than fruits related traits. This is mainly due to the time needed to develop fully expanded leaves and mature fruits [28, 29]. The last one being longer is consequently more affected by the climate.

The heritability was maximal ($H^2> 0.90$) for simple leaf characteristics, leaf number per cluster, and spine density which indicate the stability of those characters over disparate years. For, fruit, kernel, almond and clustered leaf traits, they had moderate to high heritability (H^2 between 0.59-0.89); this suggests that even with a significant environmental effect these traits should respond positively to selection. The number of almonds per stone presents a low H^2 value and thus is likely strongly influenced by the environment, especially the relative humidity and its effect on the pollination success. The relatively low heritability level of this trait in comparison with the rest of fruit traits is in agreement with previous studies [21, 30].

The genetic divergence, based on Euclidean distances, has split the argan collection into five homogeneous clusters; two of the five, Cluster I and IV, present a promising agronomic potential with essentially a high kernel weight. In the principal components analysis, the two first principal components explained only 41.89% of the total variance but they were able to differentiate four clusters over the five ones established through the genetic divergence study; that is because of the relatively high contribution of few traits in the genotype differentiation. The two first PC also showed that fruit and kernel traits are highly discriminating, while leaf traits were of less importance in classifying genotypes.

5. Conclusions

The main outcome of this agro-morphological characterization is the high variability that exists within the argan collection of Admine reserve. The broad phenotypic diversity detected within this gene pool should be used in selection/breeding programs and to establish a core collection. In fact, the argan conservation, selection, and commercial production are necessary to reduce human pressure on the forest and to prevent the genetic erosion of the species.

The morphological characterization revealed significant correlations between the trees' vegetative traits and the fruits' size; this will facilitate the early selection of productive genotypes. Most studied traits had a high heritability values and could be efficiently used for argan descriptors establishment. The study also showed the relevance of the fruit traits in differentiating between genotypes.

This investigation should be complemented by molecular characterization to fully elucidate the genetic structure of the species. The molecular tools in argan can be also used in marker-assisted selection to accelerate the emerging breeding programs.

REFERENCES

[1] Msanda, F., A. El Aboudi, and J.-P. Peltier, 2005, Biodiversité et biogéographie de l'arganeraie marocaine. Cahiers Agricultures. 14(4), 357-364.

[2] Lefhaili, A., Global Forest Resources Assessment 2015, Country Report Morocco. 2014, FAO: Rome. p. 10.

[3] Emberger, L., 1925, Le domaine naturel de l'arganier. Bulletin de la Societe Botanique de France. 72(4), 770-774.

[4] Kaabèche, M., A. Benkheira, and B. de Foucault, 2010, L'arganeraie d'Algérie: structure, écologie, syntaxonomie, dynamique. Acta Botanica Gallica. 157(3), 563-572.

[5] M'hirit, O., 1989, L'arganier: une espèce fruitière-forestière a usages multiples. in Les espèces ligneuses à usages multiples des zones arides et méditerranéennes. Zaragoza, Spain: Commission des Communautés européennes, Luxembourg. 141-168.

[6] Lybbert, T.J., A. Aboudrare, D. Chaloud, N. Magnan, and M. Nash, 2011, Booming markets for Moroccan argan oil appear to benefit some rural households while threatening the endemic argan forest. Proceedings of the National Academy of Sciences. 108 13963-13968.

[7] El Abbassi, A., N. Khalid, H. Zbakh, and A. Ahmad, 2014, Physicochemical characteristics, nutritional properties, and health benefits of argan oil: A review. Critical reviews in food science and nutrition. 54(11), 1401-1414.

[8] Charrouf, Z. and D. Guillaume, 2008, Argan oil: Occurrence, composition and impact on human health. European Journal of Lipid Science and Technology. 110(7), 632-636.

[9] Ould Mohamedou, M., et al., 2011, Argan oil exerts an antiatherogenic effect by improving lipids and susceptibility of LDL to oxidation in type 2 diabetes patients. International journal of endocrinology. 2011, 1-8.

[10] Ben Chakroun, F., 1990, Un systeme typique d'agroforesterie au Maroc: l'arganeraie. in Séminaire maghrébin d'agroforesterie. Jebel Oust, Tunisie. 19-26.

[11] El Mousadik, A. and R. Petit, 1996, Chloroplast DNA phylogeography of the argan tree of Morocco. Molecular Ecology. 5(4), 547-555.

[12] El Mousadik, A. and R. Petit, 1996, High level of genetic differentiation for allelic richness among populations of the argan tree [Argania spinosa (L.) Skeels] endemic to Morocco. Theoretical and Applied Genetics. 92(7), 832-839.

[13] Bani-Aameur, F. and A. Ferradous, 2001, Fruits and stone variability in three argan (Argania spinosa (L.) Skeels) populations. Forest Genetics. 8(1), 39-43.

[14] Ait Aabd, N., F. El Ayadi, F. Msanda, and A. El Mousadik, 2010, Genetic variability of argan tree and preselection of the candidate plus trees. Notulae Botanicae Horti Agrobotanici Cluj-Napoca. 38(3), 293-301.

[15] Ward, J.H., 1963, Hierarchical grouping to optimize an objective function. Journal of the American Statistical Association. 58, 236–244.

[16] Comstock, R.R. and H.F. Robinson, 1952, Genetic parameters, their estimation and significance. in 6th international Grassland Congress. Nat. publ. Co.Wash., D.C., U.S.A. 248-291.

[17] Allard, R.W., Principles of plant breeding. 1960, New York: John Wiley and Sons. 89-98.

[18] Singh, R.K. and B.D. Chaudhary, Biometrical Methods in Quantitative Analysis. 1985, New Delhi: kalayani Publishers. 253-260.

[19] Benlahbil, S., A. Zahidi, F. Bani-Aameur, and A. El Mousadik, 2015, Duration of Blossoming, Longevity of Argan Flower and Assessment of Pollen Fertility by Staining. International Journal of Agriculture and Forestry. 5(6), 291-304.

[20] Nerd, A., V. Irijimovich, and Y. Mizrahi, 1998, Phenology, breeding system and fruit development of Argan [Argania spinosa, Sapotaceae] cultivated in Israel. Economic botany. 52(2), 161-167.

[21] Ait Aabd, N., F. Msanda, and A. El Mousadik, 2012, Univariate and multivariate analysis of agronomical traits of preselected argan trees. Notulae Botanicae Horti Agrobotanici Cluj-Napoca. 40(2), 308-316.

[22] Zahidi, A., F. Bani-Aameur, and A. El Mousadik, 2013, Variability in leaf size and shape in three natural populations of Argania spinosa (L.) Skeels. International Journal of Current Research and Academic Review. 1, 13-25.

[23] Bani-Aameur, F., 2004, Morphological diversity of argan (Argania spinosa (L.) Skeels) populations in Morocco. Forest Genetics. 11(3-4), 311-316.

[24] Bani-Aameur, F., A. Ferradous, and P. Dupuis, 1999, Typology of fruits and stones of Argania spinosa (Sapotaceae). Forest Genetics. 6(4), 213-219.

[25] Cornelissen, J., 1999, A triangular relationship between leaf size and seed size among woody species: allometry, ontogeny, ecology and taxonomy. Oecologia. 118(2), 248-255.

[26] Primack, R.B., 1987, Relationships among flowers, fruits, and seeds. Annual Review of Ecology and Systematics. 18, 409-430.

[27] Roper, T. and W. Loescher, 1987, Relationships between leaf area per fruit and fruit quality in Bing sweet cherry. HortScience. 22(6), 1273-1276.

[28] Benaouf, Z., A. Miloudi, Z. Souidi, and Z. Arabi, 2015, Reproductivity and phenology of argan (argania spinosa (L.) skeels) a rare tree endemic to the west of algeria. Pakistan Journal of Botany. 47(6), 2181-2188.

[29] Bani-Aameur, F., L. Louali, and P. Dupuis, 1998, Maturation et chute des fruits de l'arganier. Revue Marocaine des Sciences Agronomiques et Vétérinaires. 18(3), 151-158.

[30] Ait Aabd, N., F. El Ayadi, F. Msanda, and A. El mousadik, 2011, Evaluation of agromorphological variability of argan tree under different environmental conditions in Morocco: implication for selection. International Journal of Biodiversity and Conservation. 3(3), 73-82.
