# Supplementary material for: Provenance and family variations in early growth of Manchurian walnut (Juglans mandshurica Maxim.) and selection of superior families
Source: PLoS One. 2024 Mar 7;19(3):e0298918. doi: 10.1371/journal.pone.0298918 (PMC10919699; doi:10.1371/journal.pone.0298918)
Supplement: S2 File — (ZIP) [file pone.0298918.s005.zip › Multi-trait variation analysis of Juglans mandshurica grafting seedlings.pdf]

学校代码：10225

学　　号：S22686

学 位 论 文

核桃楸无性系多性状变异规律分析

王玮槐

指导教师姓名：	张含国　教授　东北林业大学<br>李志新　讲师　东北林业大学		
申请学位级别：	硕　士	学 科 专 业 ：	林学
论文提交日期：	2022 年 3 月	论文答辩日期：	2022 年 5 月
授予学位单位：	东北林业大学	授予学位日期：	2022 年 6 月

答辩委员会主席：郭长虹 教授

论 文 评 阅 人：

東北林業大學

中国知网　https://www.cnki.net

University Code：10225
Register Code ：S22686

Dissertation for the Degree of Master

Multi-trait variation analysis of *Juglans mandshurica* grafting seedlings

**Candidate:**	**Weihuai Wang**
**Supervisor:**	**Prof.Hanguo Zhang**
**Associate Supervisor:**	**Zhixin Li**
**Academic Degree Applied for:**	**Master**
**Speciality:**	**Forestry**
**Date of Oral Examination:**	**May, 2022**
**University:**	**Northeast Forestry University**

中国知网 https://www.cnki.net

摘要

影响核桃楸嫁接成活的因素较多，技术要求也比较高，成活较困难，致使品种选育后繁育工作进程缓慢，严重影响了核桃楸的良种推广与生产。目前核桃楸的研究多数都是用于用材林和种仁营养成分及生物活性的测定方面，核桃楸开花晚，始花年龄一般为5-8 年，开展核桃楸性状相关及早期与晚期相关性研究，对于缩短核桃楸育种周期十分必要。本试验以核桃楸无性系为研究材料，对其生理生化、光合指标、激素种类、嫁接成活率以及子代生长性状进行测定，利用变异分析、方差分析、极差分析、多重比较及相关分析等方法，研究各性状在嫁接当年及进入结实初期（嫁接 5 年）无性系的变化规律，为核桃楸早期与晚期相关及优良无性系选择奠定基础。结果如下：

性状变异分析。1 年生核桃楸无性系生理与光合性状变异分析。性状的变异系数为14.70～52.09%（平均值 29.31%），变异最小的是叶绿素相对吸光值，最大的是气孔导度。核桃楸无性系 5 年生各性状的变异系数为 6.30～35.83%（平均为 21.66%），相较 1 年生变异减少，变异最小的是胞间 CO_2 摩尔分数，最大的是气孔导度。两个年度除胞间 CO_2 摩尔分数和蒸腾速率不同外，可溶性蛋白含量、可溶性糖含量、叶绿素相对吸光值、净光合速率和气孔导度 3 个群体（结实群体、未结实群体以及整个群体）一致。2 个年度可溶性蛋白含量和可溶性糖含量无性系间差异不显著，叶绿素相对吸光值、净光合速率和气孔导度 2 个年度无性系间差异显著（$p<0.05$）。两个年度的未结实群体叶绿素相对吸光值平均值高于结实群体 9.68%，净光合速率平均值低于结实群体 7.80%。叶绿素相对吸光值与胞间 CO_2 摩尔分数早晚一致性好，是早期与晚期相关和早期选择的重要性状。

内源激素变异分析。5 年生与 1 年生无性系群体中内源激素均为 6-BA 含量变异系数最高，6-BA 含量、GA_3 含量和 IAA 含量均表现为结实群体数值较大。5 年生群体的6-BA 含量比 1 年生的高，5 年生群体的 GA_3 含量和 IAA 含量比 1 年生的低。1 年生整体群体，三种激素含量无性系间差异显著，GA_3 含量在结实群体与未结实群体差异不显著，结实群体数值较大；6-BA 含量和 IAA 含量在 2 个群体差异显著，结实群体均值大。二个年度无性系 3 种激素进行配对 T 检验发现，三个群体差异均不显著。

性状及早晚相关分析。在结实群体中，GA_3 含量 1 年生与 5 年生群体正相关显著。1 年生群体相关分析中，在结实群体中各性状相关不显著。除胞间 CO_2 摩尔分数与 GA_3 含量、6-BA 含量、IAA 含量及嫁接成活率呈正相关，其他性状与 GA_3 含量、6-BA 含量、IAA 含量及嫁接成活率均呈负相关。在未结实群体中，气孔导度与 6-BA 含量正相关显著，GA_3 含量与嫁接成活率正相关显著。可溶性蛋白含量，可溶性糖含量，净光合速率，气孔导度和胞间 CO_2 摩尔分数与 GA_3 含量、6-BA 含量、IAA 含量和嫁接成活率呈正相关。在整体群体中，叶绿素相对吸光值与 GA_3 含量负相关显著，净光合速率与6-BA 含量正相关极显著，与 IAA 含量正相关显著，6-BA 含量与气孔导度，蒸腾速率正相关显著，6-BA 含量与净光合速率，IAA 含量正相关极显著。5 年生群体相关分析中，

中国知网 https://www.cnki.net

在结实群体中叶绿素相对吸光值与三种激素正相关。四个光合指标与 GA_3 含量和结实量正相关，与 6-BA 含量负相关。在未结实群体中四个光合指标与 GA_3 含量和 6-BA 含量负相关。在整体群体中，净光合速率与三种激素负相关，其中与 6-BA 含量达到了显著水平。胞间 CO_2 摩尔分数、蒸腾速率与三种激素正相关。

无性系结实量与 2019、2020 年树高及胸径呈正相关，即结实量较多系号的早期生长也较快。无性系嫁接成活率与 2019、2020 年树高及胸径呈负相关，其中除 2019 年胸径外均达到了显著水平。

关键词：核桃楸：无性系；生理生化指标；光合指标；激素；变异分析；早晚相关

中国知网 https://www.cnki.net

Abstract

There are many factors affecting the survival of walnut catalpa grafting, the technical requirements are relatively high, and it is more difficult to survive, resulting in a slow process of breeding after breeding varieties, which seriously affects the promotion and production of fine varieties of walnut catalpa. At present, most of the research on walnut catalpa is used for the determination of nutrient composition and biological activity of timber forests and seed kernels, walnut catalpa flowering late, the age of first flowering is generally 5-8 years, and the study of the correlation between walnut catalpa traits and early and late is very necessary to shorten the breeding cycle of walnut catalpa.In this study, with clone of *Juglans mandshurica* for research material, the physiological and biochemical, photosynthesis, hormone, graft survival rate and child growth properties are measured, using mutation analysis, variance analysis, such as poor analysis and correlation analysis method, To study the changes of each character in the clone at the year of grafting and at the beginning of fruiting (grafting 5 years), and to lay a foundation for the morning and evening correlation and selection of excellent clones.

Analysis on the variation of physiological and photosynthetic traits in clones of Annual juglans. The variation coefficient of the traits ranged from 14.70 to 52.09% (mean 29.31%), with the minimum variation in chlorophyll content (Chlorophyll relative light absorption value) and the maximum variation in stomatal conductance. The coefficient of variation of each character at 5 years was 6.30-35.83% (average 21.66%), and the variation was less than that at 1 year. The smallest variation was intercellular CO_2 concentration, and the largest was stomatal conductance. Except intercellular CO_2 concentration and transpiration rate, soluble protein content, soluble sugar content, chlorophyll content (Chlorophyll relative light absorption value), net photosynthetic rate and stomatal conductance were the same between the two years. There were no significant differences in soluble protein content and soluble sugar content between the two years, but significant differences in chlorophyll content (Chlorophyll relative light absorption value), net photosynthetic rate and stomatal conductance between the two years($p<0.05$). In two years, the average Chlorophyll relative light absorption value of non-fruity group was 9.68% higher than that of fruity group, and the average net photosynthetic rate was 7.80% lower than that of fruity group. Chlorophyll relative light absorption value is consistent with intercellular CO_2 concentration in the morning and evening, which is an important trait of morning and evening correlation and early selection.

The variation coefficient of endogenous hormone 6-BA content was the highest in both five-year and one-year groups, and 6-BA content, GA_3 content and IAA content were higher in fruiting groups. The content of 6-BA in the five-year group was higher than that in the one-year

中国知网 https://www.cnki.net

group, while the content of GA_3 and IAA in the five-year group was lower than that in the one-year group. In the whole group, there were significant differences in the content of three kinds of hormones among clones, but there was no significant difference in the content of GA_3 between fruiting group and non-fruiting group, and the value of GA_3 in fruiting group was larger. The contents of 6-BA and IAA were significantly different between the two groups, and the mean value of fruiting group was larger. Paired T tests of three hormones of the two annual clones found that the differences between the three groups were not significant.

In fruiting group, there was a significant positive correlation between annual and five-year group GA_3 content. Correlation analysis of annual group showed that there was no significant correlation among characters in fruity group. Except that intercellular CO_2 concentration was positively correlated with GA_3 content, 6-BA content, IAA content and grafting survival rate, other traits were negatively correlated with GA_3 content, 6-BA content, IAA content and grafting survival rate. There was a significant positive correlation between stomatal conductance and 6-BA content and between GA_3 content and grafting survival rate in unfruited groups. Soluble protein content, soluble sugar content, net photosynthetic rate, stomatal conductance and intercellular CO_2 concentration were positively correlated with GA_3 content, 6-BA content, IAA content and grafting survival rate. In the whole group, chlorophyll content (Chlorophyll relative light absorption value) was significantly negatively correlated with GA_3 content, and net photosynthetic rate was significantly positively correlated with 6-BA content, but significantly positively correlated with IAA content. 6-BA content was significantly positively correlated with stomatal conductance and transpiration rate, and 6-BA content was significantly positively correlated with net photosynthetic rate and IAA content. Chlorophyll content (Chlorophyll relative light absorption value) was positively correlated with three kinds of hormones in the fruited group. The four photosynthetic indexes were positively correlated with GA_3 content and seed setting, but negatively correlated with 6-BA content. The four photosynthetic indexes were negatively correlated with GA_3 content and 6-BA content in the non-fruiting group. In the whole group, the net photosynthetic rate was negatively correlated with the three hormones, among which the 6-BA content reached a significant level. Intercellular CO_2 concentration and transpiration rate were positively correlated with three hormones.

The yield of clones was positively correlated with tree height and DBH in 2019 and 2020, that is, the early growth of lines with higher yield was faster. The survival rate of clonal grafting was negatively correlated with tree height and DBH in 2019 and 2020, and reached a significant level except DBH in 2019.

Keywords： *Juglans mandshurica*; clone; Physiological biochemical indicators; photosynthetic indexess; hormone; mutation analysis

中国知网 https://www.cnki.net

目录

摘要 .... **I**
Abstract .... **III**
目录 .... **V**
1. 绪论 **1**
1.1. 引言 1
1.2. 核桃楸研究进展 1
1.3. 研究目的意义 4
1.4. 技术路线图 5
2. 影响核桃楸嫁接成活率因素分析 **6**
2.1. 材料与方法 6
2.1.1. 试验地点 6
2.1.2. 材料来源 6
2.1.3. 嫁接方法及嫁接成活率的调查 6
2.1.4. 数据处理和分析 7
2.2. 结果与分析 7
2.2.1. 核桃楸种源嫁接成活率分析 7
2.2.2. 不同因素嫁接成活率分析 8
2.2.3. 最佳处理组合分析 9
2.3. 本章小结 9
3. 核桃楸无性系生理生化及光合性状变异分析 **11**
3.1. 材料与方法 11
3.1.1. 试验地点 11
3.1.2. 材料来源 11
3.1.3. 光合及生理生化指标的测定 11
3.1.4. 数据处理和分析 11
3.2. 结果与分析 12
3.2.1. 各性状变异分析 12
3.2.2. 无性系各性状差异显著性分析 13
3.2.3. 早晚及性状相关分析 20
3.3. 本章小结 23
4. 核桃楸无性系内源激素变异分析 **25**
4.1. 材料与方法 25
4.1.1. 地点与材料 25

中国知网 https://www.cnki.net

4.1.2. 内源激素含量的测定 25
4.1.3. 数据处理与分析 25
4.2. 结果与分析 25
4.2.1. 无性系激素变异分析 25
4.2.2. 无性系间激素显著性分析 26
4.2.3. 核桃楸早晚及性状相关分析 30
4.3. 本章小结 31
5. 核桃楸无性系子代生长变异分析 32
5.1. 材料与方法 32
5.1.1. 材料 32
5.1.2. 调查方法 32
5.1.3. 数据处理与分析 32
5.2. 结果与分析 32
5.2.1. 无性系子代整体生长变异分析 32
5.2.2. 48 个无性系对应子代林生长变异分析 33
5.2.3. 19 个无性系对应子代林生长变异分析 34
5.3. 本章小结 36
讨论 37
结论 39
参考文献 40
攻读学位期间发表的学术论文 46
致谢 48

中国知网 https://www.cnki.net

1. 绪论

1.1. 引言

核桃楸（*Juglans mandshurica Maxim.*）属胡桃科（*Juglandaceae*）胡桃属（*Juglans*）落叶阔叶乔木，与水曲柳（*Fraxinus mandshurica Rupr.*）和黄波罗（*Phellodendron amurense Rupr.*）并称“东北三大硬阔”，被列为国家 II 级珍稀树种和珍稀濒危树种的三级保护植物[1]。主要分布于我国东北地区的小兴安岭、完达山脉、长白山区和辽宁东部，华北地区也有零星分布[2-5]，是东北阔叶红松林重要的伴生树种。但是存在良种繁育困难[6-7]，很难满足东北林区造林需求。由于核桃楸用途广泛，导致人为的大量采伐，野生核桃楸资源遭到严重破坏，因此核桃楸选育及定向培育研究意义重大。

1.2. 核桃楸研究进展

核桃楸因其具有丰富的材用及果用价值，越来越多的人对种源及无性系生长、结实特性及种实性状进行研究。国内外学者早在 20 世纪 70 年代就已经开始对林木早期选择的研究[8-11]。颜廷武等[12]的研究表明，幼龄期的核桃楸已具有代表性，对核桃楸进行早期选择是可行且有效的。Zhang 等[13]采用综合评价法,选择种子性状较好的 5 个家系和幼苗性状较好的 5 个家系作为优良材料。颜廷武等[12]从 121 个核桃楸半同胞家系中早期选择出 5 个优良半同胞家系。尤海舟等[14]研究核桃楸不同种源种子质量及苗期生长变异规律，认为核桃楸种子质量差异明显且存在广泛变异。陈思羽等[15]分析了空间地理变异和地形因子对不同种源核桃楸结实性状的影响。袁显磊等[16]在黑龙江省林口和兴隆试验点进行核桃楸种源试验并选择了适合林口地区的优良种源为绥阳、和龙、帽儿山和辉南种源；适合兴隆地区的优良种源为抚松、和龙、兴隆和绥阳种源。褚宪丽等[2]对 15 年生核桃楸种源(3 个)试验林进行了研究，牡丹峰种源的变异最小，生长性状与其他种源相比差异显著且保存率最高，为优良种源。芦贤博等[17]对万人欢林场核桃楸 4 个种源内的 28 个家系（6a）生长性状，初步选出 1 个优良种源（万人欢）与 3 个优良家系（WRH3、WRH5、QTH2）及 10 个优良单株。韩玉霞等[18]对 39 个核桃楸家系进行评价选择，选出 4 个优良家系。张含国等[19]在林口县青山林场对迎春、铁力、穆棱 3 个种源的 122 个家系 7 年生核桃楸子代林材料进行了分析，初选出 13 个家系。常君等[20]通过对比分析薄壳山核桃蛋白含量的变异特性和差异性，将 14 号、64 号和 45 号品种初选为高蛋白薄壳山核桃品种。张海啸[21]通过聚类分析和综合坐标法选出了 17 个核桃楸优良单株。张振[22]从 4 个种源的 60 个无性系中选择出 12 个优良无性系。S.D. Sharma 等[23]对 229 个单株的 25 个性状进行比较，选出 38 棵单株作为优树。李嘉琪[24]以吉林省白城市林木种子园的 304 个樟子松（*Pinus sylvestris var.*）无性系为材料，对其结实性状（2015、2016 和 2017 年的结实量）进行调查分析。发现 2015、2016 及 2017 年结实量之间均为正相关，2016 年与 2017 年结实量极显著正相关(r=0.150)。方乐金等[25]在安徽

中国知网 https://www.cnki.net

西田林场杉木种子园对从福建洋口林场引进的 25 个无性系，每系固定 6 株样株，分系分株连续进行 6a 的结实量观测与分析。结果表明，以无性系为计算单位的球果产量估算的相关系数，在各年度间均达到极显著水平。证明虽然无性系在不同年份的结实量有很大差异，而各无性系的相对产量却基本稳定。梁一池[26]在福建省对锥栗（*Castanea henryi (Skan) Rehd.*）嫁接苗进行品种园结合无性系测定，采用完全随机(CR)设计，每个无性系定植 50~80 株不等。开始结实后，每个无性系随机选择 8-10 株，进行定株观测 1987~1993 年单株坚果产量。多年方差分析结果表明：锥栗基因型×年份互作效应不显著，无性系产量年度间相关系数达到显著或极显著水平。

嫁接繁殖方面，Habibi Fariborz[27]和 Rasool Aatifa[28]叙述了子代与砧木之间果树嫁接的生理、生化和分子方面的基础。Aziz Ebrahimi[29]发现与在田间嫁接相比，在温室中可大大提高了所有嫁接方法的嫁接成功率。肖玉璞等人[30]通过在温室内进行核桃楸嫁接试验，得到不同无性系嫁接成活率存在显著差异的结论。翁春余等[31]对薄壳山核桃 17 个无性系进行了同一无性系 2 年生播种苗砧木嫁接试验发现各无性系间嫁接成活率差异显著。王红娟等[32]2007 年，从 12 个元宝枫（*Acer truncatum Bunge*）优良无性系上采穗，在晋宁县嫁接苗木 2400 株，设 5 次重复，随机区组排列，对其嫁接成活率及苗木的株高、基径、生长节数的年生长节律进行了调查与分析，结果表明：引种的 12 个无性系苗木中，92 号嫁接成活率最高，为 94%，88 号最低，为 62.5%。嫁接成活率在无性系内部变异不大，在无性系间达到了极显著差异。黄佳聪等[33]以 1 年生、地径大于 4mm 的滇橄榄（*Phyllanthus emblica Linn.*）留床实生苗为砧木。以 8 个滇橄榄优良无性系木质化枝条为接穗，开展滇橄榄嫁接试验。对不同无性系嫁接成活率差异性进行研究。结果表明：滇橄榄不同无性系间嫁接成活率差异达显著水平。戴承喜[34]研究大叶榉不同无性系嫁接苗的嫁接成活率及其生长情况。结果表明：大叶榉嫁接成活率平均达 64.90%，且不同无性系间嫁接成活率差异明显。乔谦[35]为探讨元宝枫各无性系嫁接成活能力方面的差异，以 14 个元宝枫优良无性系为试验材料，春季采用双舌接方式进行嫁接试验，对各无性系的嫁接成活率及其春梢性状的差异进行了分析比较。结果表明：嫁接后 35 天左右可以作为确定成活与否的适宜时间；各无性系之间成活率差异极显著。何芳婷[36]为比较油茶（*Camellia oleifera Abel*）芽苗砧嫁接后的生长指标变化情况，以 3 个油茶优良无性系岑软 2 号、岑软 3 号和长林 4 号作为对象进行嫁接试验，结果表明：3 个无性系嫁接后的成活率差异不显著。王瑞等[37]为继续优化油茶芽苗砧嫁接技术，以‘湘林’系列油茶优良无性系为材料，研究了 5 种不同的基质类型、2 种不同规格的容器杯、2 种嫁接部位、2 种接穗类型、砧木所保留根系的长度以及接穗叶片的保留程度对油茶芽苗砧嫁接苗成活率与苗木长势的影响。研究结果表明：基质以黄心土∶泥炭土∶河沙=2∶1∶1 最适宜；容器杯规格以 10 cm×12 cm 最适宜；嫁接位置以胚芽最适宜；接穗类型以腋芽最适宜;叶片保留程度以留叶 1/2 最适宜。初步建立了油茶芽苗砧嫁接技术体系，为提高芽苗砧嫁接成活率及新梢长势提供了理论依据。李正银[38]以云南省昭通市 21 个核桃优树无性系为试验材料，对其 1 年生嫁接苗苗高、地径进行遗传变异分

中国知网 https://www.cnki.net

析、方差分析及 Pearson 相关性检验。各核桃优树无性系间达到极显著水平。孙铭浩等[39]为加快青檀（*Pteroceltis tatarinowii Maxim.*）优良无性系的繁殖速度，对选育的青檀优良无性系 TX01 进行了嫁接育苗试验。结果表明：使用蜡封接穗进行切腹接繁育青檀良种苗木，嫁接成活率达到 92.8%，当年年底平均苗高为 230.5cm，苗木平均地面直径 1.46cm，嫁接口愈合良好，苗木生长健壮且整齐。是一种行之有效的快速繁育青檀良种苗木的好方法, 具有较高的经济收益和推广前景。谭飞燕等[40]以 15 个中国马褂木（*Liriodendron chinense*）无性系为材料进行嫁接试验。结果表明：不同马褂木无性系间的嫁接成活率存在显著差异，不同无性系间嫁接苗高的生长存在显著差异。樊光辉[41]为了实现杜仲（*Eucommia ulmoides Oliv.*）优良无性系，对 40 个杜仲优良无性系进行了嫁接试验研究。结果表明，40 个杜仲无性系嫁接成活率有差异。

周恩强等[42]对核桃子苗嫁接后测定和分析生理生化指标，得出单宁含量和砧木含水量对核桃嫁接成活影响较小，而可溶性蛋白质含量和可溶性糖含量是影响嫁接存活的主要因素。郑炳松[43]等研究表明可溶性蛋白质含量与可溶性糖含量等内部生理生化因子对山核桃嫁接成活有显著的影响。汤睿等[44]在阐述了 7 种不同核桃砧木苗和 4 种核桃嫁接方法的基础上，进一步分析了嫁接时期等因素对核桃嫁接成活率的影响。蒲光兰等[45]以 4 个嫁接时期核桃接穗内含物的变化特征为对象，研究内含物对核桃嫁接成活的影响。结果表明接穗蛋白质含量是影响核桃嫁接成活的重要因子。Anket Sharma[46]认为生长素，细胞分裂素和赤霉素可以影响核桃嫁接。Saravana Kumar R M[47]认为生长素在砧木和接穗上的应用增加了山核桃嫁接的成功率。Zhai，L. [48] ，李伟[49]认为通过增加砧木中的生长素水平，可以提高嫁接成功率。赵金秀[50]为加快培养大规格观赏牡丹（*Paeonia suffruticosa subsp.*）速度，在大规格凤丹牡丹上进行高枝嫁接观赏牡丹试验。结果表明：接穗用 500 ppm 萘乙酸或 500 ppm 吲哚丁酸激素处理，浸泡 5 小时，晾干后嫁接，成活率提高约 20%。罗兰芳[51]以 1 年生沉水樟（*Cinnamomum micranthum*）砧木和当年生或 1～2 年生的沉水樟穗条为材料，通过不同的激素处理、不同的嫁接方式和不同的嫁接处理的方式，分析了不同的嫁接方式对沉水樟的成活率的影响。结果表明：不同的激素处理、不同的嫁接方式和不同的嫁接处理的对照组与试验组均存在显著性差异或极显著性差异，其中不同的激素处理的处理组 5(1.5×10^{-6} 2,4-D+0.4×10^{-6} IAA)、劈接法和沙藏处理的沉水樟嫁接成活率分别达到 80.33%、69.67%和 87.66%。季树泉[52]为提高油橄榄（*Olea europaea L.*）嫁接成活率，开展了用吲哚丁酸，萘乙酸等激素处理油橄榄接穗切面，然后进行嫁接试验。几年来试验证明，嫁接中激素处理接穗效果良好。刘剑斌[53]在建阳市林木种苗站进行不同芽数与激素种类的嫁接育苗试验发现在 200 ppm NAA 处理下，千年桐（*Vernicia montana Lour.*）苗木存活率最高。宫永红[54]认为核桃接穗催醒是将接穗散开单根平放埋没在基质内 1~3 d。金丽丽[55]提出接穗的预催醒在嫁接前的 1~3 d 进行，1 月份前，未蜡封的接穗预催醒 2 d，蜡封的接穗预催醒 3 d；2 月以后，未蜡封的接穗预催醒 1 d，蜡封的接穗预催醒 2 d。王仕海等[56]和赵宝军等[57]对辽宁省冬季核桃室内嫁接技术进行综述，认为接穗催醒是嫁接试验所必需的过程。金丽

中国知网 https://www.cnki.net

丽等[55]和赵亚辉等[58]均认为采用催醒温床的基质须采用新鲜、干净、无霉烂和无杂质的粗锯末，其厚度为 40~50 cm。为增加透气性，较细的锯末要掺入一些刨花。基质入床前要进行消毒、温水加湿，将湿度调到锯末含水量 55%左右 (手握成团状，一触即散，手不湿为宜) 。

在内源激素含量影响方面，核桃楸果实发育过程中激素逐渐升高，尤其是赤霉素含量，较之前相比增长幅度很大，对人工调控核桃楸果实成熟有重要的指导意义[59]。Nanda Amrit K[60]介绍了 8 种激素在嫁接形成过程中的作用。宋福南[61]对七年生白桦（*Betula platyphylla Suk.*）嫁接幼树雌花发育过程中的激素含量进行研究分析高浓度ZR分别与低浓度的IAA、GA协同作用可促进白桦雌花分化。张旭等[62]以中间型无花果（*Ficus carica L.*）优选系M105 为试验材料，研究不同浓度赤霉素（GA_3）处理无授粉条件的秋果，实现单性结实的赤霉素响应窗口期内对果实激素水平的影响，结果表明：GAs含量、IAA含量和ZR含量在赤霉素处理后果实各部位均有提高，ABA含量在赤霉素处理后各部位均有明显下降。GA_3 处理后，提高座果率分别 30.89%、46.67%和 45.78%。

在生长性状与结实量相关方面，王芳等[63]以红松（*Pinus koraiensis*）半同胞子代家系为材料，对其生长性状(树高和胸径)、结实性状(连续 7 个结实年份的球果总数)进行调查，分析发现结实量与树高和胸径均呈极显著正相关，并利用多性状综合评价的方法，结合生长和结实等性状，优选出优良家系和单株。王庆娜[64]以红松亲本无性系及其子代为材料，对子代生长与结实量进行测定分析，认为树高和胸径与结实量正相关但未达显著水平。杨俊明[65]认为无性系再选择的正确途径是“结实能力--子代生长”联合选择，依据造林 5 年后的子代生长表现，无性系结实量与子代生长性状正相关。

1.3. 研究目的意义

近年来，核桃楸已选育出许多优良材料，但是由于核桃楸树种自身一些原因，如单宁含量高、髓心大及易发生伤流等，使得目前的核桃楸扦插繁殖较为困难，成活率也极低，因此国内外的核桃楸繁育研究多集中在嫁接和组培繁育方面。而且影响核桃楸嫁接成活的因素较多，技术要求也比较高，成活较困难，致使品种选育后繁育工作进程缓慢，严重影响了核桃楸的良种推广与生产。

目前核桃楸在用材林和种仁营养成分[66-68]及生物活性的测定方面研究也较多，而性状早期与晚期相关及早期选择研究较少。核桃楸较核桃（*Juglans regia L.*）开花晚，始花年龄一般为 5-8 年，开展早期与晚期相关性、树种各性状相关性研究，缩短育种周期十分必要。

本试验以 2020、2021 年嫁接的群体、嫁接当年与结实初期的 11 个无性系及子代林相关群体为研究目标，调查生理生化指标、光合指标、内源激素、嫁接成活率以及子代生长性状，利用变异分析、方差分析、极差分析及相关分析等方法，研究各性状在嫁接当年及进入结实初期（嫁接 5 年）无性系的变化规律，为核桃楸早期与晚期相关性及优

中国知网 https://www.cnki.net

良无性系选择提供参考。

1.4. 技术路线图

为找到影响核桃楸嫁接成活率因素，对 2020 年 7 个种源的 137 个无性系及 2021 年 2 个种源的 32 个无性系的嫁接成活率进行调查分析。为了解各性状间及性状前期与晚期相关关系，对 2021 年在林场苗圃嫁接培育的无性系及 2017 年建立的无性系收集区的无性系进行生理生化及光合性状的测定分析。为了解内源激素对嫁接成活率和结实量的影响，对 2021 年在林场苗圃嫁接培育的无性系及 2017 年建立的无性系收集区的无性系进行内源激素含量测定分析。对 2016 年定植的子代林中与 2020、2021 年嫁接系号对应的 48 个无性系及与 2016 年嫁接系号对应的 19 个无性系的生长性状进行调查分析。

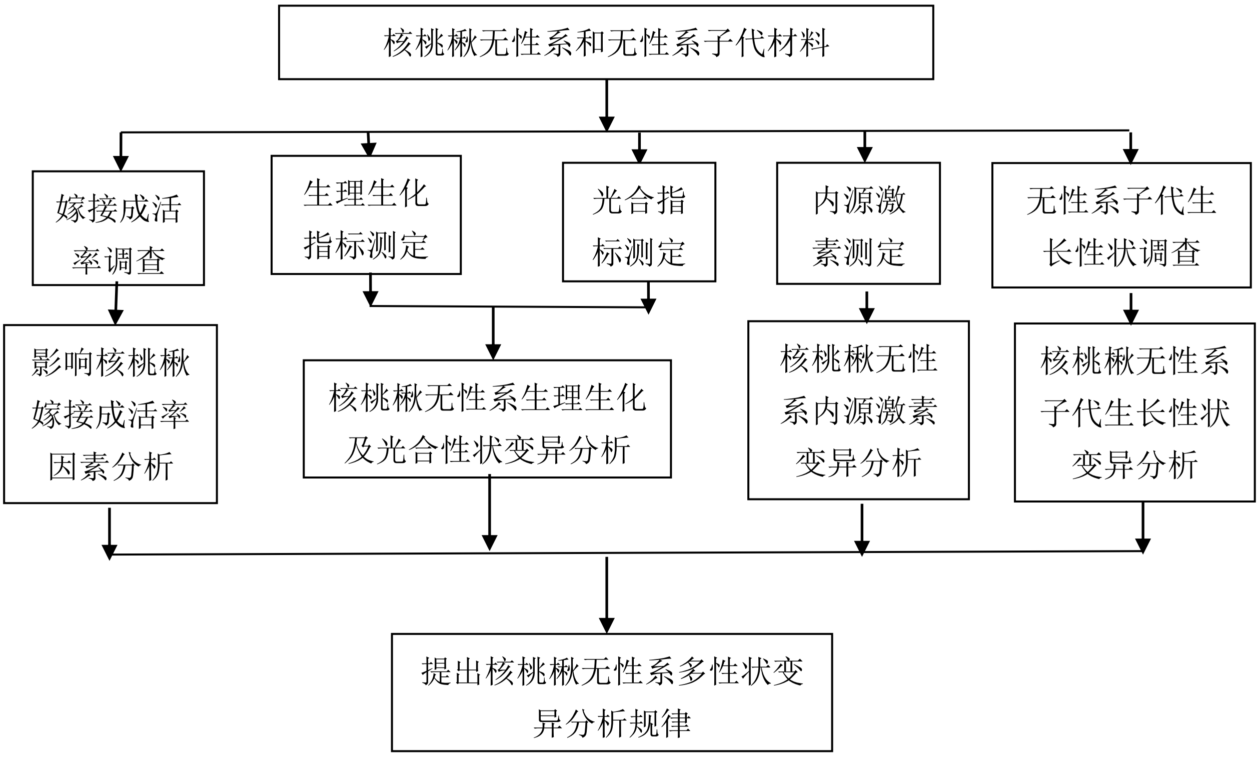

中国知网 https://www.cnki.net

2. 影响核桃楸嫁接成活率因素分析

核桃楸已选育出许多优良材料，但是又因其嫁接易存在伤流，使得目前的核桃楸无性繁殖较为困难，成活率也极低，而且影响核桃楸嫁接成活的因素较多，致使品种选育后规模化繁殖工作进程缓慢，严重影响了核桃楸的良种推广与生产。黄坚钦等[69]通过田间试验及数学分析方法，分析了山核桃嫁接成活的影响因子。结果表明：外源激素可以提高嫁接成活率。王白坡等[70]在 2002 年对山核桃研究进行综述认为外源激素可以提高嫁接成活率。开展影响核桃楸嫁接成活因素的研究十分必要。

2.1. 材料与方法

2.1.1. 试验地点

五常市宝龙店林场位于五常市东部，属于长白山系，张广才岭西坡，低山丘陵地带，海拔 197—621 米。地理坐标为东经 127°38′—127°55′，北纬 44°50′—44°58′。寒温带大陆性季风气候，年降水量 620.9 毫米，最低气温-40.9℃，最高气温 35.6℃，年平均气温 3.4℃，年无霜期 120 天左右。土壤以典型暗棕壤分布最广，其次为潜育暗棕壤。

2.1.2. 材料来源

2020 年嫁接材料来自 7 个种源（虎林、东京城、铁力、嘉荫、金山屯、大泉子、三岔子）的 137 个无性系，2021 年嫁接材料来自亚布力（YBL）和五常（WC）种源的 32 个无性系。

2.1.3. 嫁接方法及嫁接成活率的调查

2020 年每个无性系嫁接约 30 株，每个种源均大于 600 株，采用双舌接方法（催醒：接穗在嫁接 1 天前取出，放于的水中浸泡 12h。砧木在嫁接前 1 天晚上取出，埋在湿锯末内。嫁接前适当修剪过根，先将砧木距离根部 8cm 左右处剪断，用手拿着砧木，距离根部 4cm 处放在拉刀上往怀里拉，拉到断面成一斜面，断面保留厚度为 0.5mm 左右；再拿着砧木，在斜面上部占斜面总长的 1/3 处在劈刀上推一劈口，劈口长约 2~3cm。在接穗顶芽芽下 4-5cm 处用铲削成 4cm 左右长的斜面，用手拿着接穗在斜面上部占斜面总长的 1/3 处劈刀上推，劈口长约 2~3cm，接穗与砧木的劈口及斜面长度相对应。砧木、接穗的劈口与斜面呈“舌”形，将接穗的“舌”插入砧木劈口，使得接穗和砧木的插口部位两“舌”相贴，接穗削面上端和砧木削面下端要露出约 0.2cm 的木质部。形成层要对齐，如果接穗细，必须保证一边形成层对齐，使之紧密贴合。用 1.5mm 绳自上而下螺纹状绑 4-5 圈绑往，上下露白处不绑扎，保护愈伤组织生长位置。绑扎时，不要移动砧穗形成层对准的位置。）未加激素处理，用阔叶树锯末作为室内苗木培养基质。2021 年采取除正交设计外 32 个无性系嫁接均接穗催醒 12 小时、采用双舌接方法、未加激素处理并用阔叶树锯末作为室内苗木培养基质。正交设计中激素处理采取浸

中国知网 https://www.cnki.net

泡 4 小时。

正交设计用混合系号进行，选定拟水平法 $L_9 3^4$ 的正交表对嫁接试验的 4 个因素及 3 个水平进行设定，第一个因素是接穗催醒时间(1 天、4 天和 7 天)，第二个因素是激素种类(NAA、IBA 和 GA_3)，第三个因素是激素质量浓度(50 mg/g、150 mg/g 和 250 mg/g)，第四个因素是基质种类（针叶树锯末和阔叶树锯末），正交设计进行了三次重复，每个重复 300 株，另加一组对照（未加激素，接穗未催醒）。设计如表 2-1：

表 2-1 嫁接试验正交试验设计表

Tab. 2-1 Orthogonal experimental design table of grafting test

设计	接穗催醒时间/天	激素质量浓度 mg/g	基质种类	激素种类
1	1	50	针叶树	NAA
2	1	150	阔叶树	IBA
3	1	250	阔叶树	GA_3
4	4	50	阔叶树	GA_3
5	4	150	阔叶树	NAA
6	4	250	针叶树	IBA
7	7	50	阔叶树	IBA
8	7	150	针叶树	GA_3
9	7	250	阔叶树	NAA

嫁接成活率的调查：对 2020 年 137 个无性系、2021 年嫁接群体 32 个无性系及正交设计的嫁接成活率进行调查。

2.1.4. 数据处理和分析

数据分析主要包括方差分析、多重比较（SNK）、极差分析及相关分析（皮尔逊与斯皮尔曼），采用 Excel2016 和 SPSS18.0 处理与分析。嫁接成活率等百分率性状经过反正弦转换后进行分析。

2.2. 结果与分析

2.2.1. 核桃楸种源嫁接成活率分析

2020、2021 年核桃楸种源嫁接成活率进行分析（表 2-2、2-3），2020 年不同种源之间差异显著，不同种源嫁接成活率为 31.50%（虎林）～75.57%（大泉子）平均值为 55.17%，与多重比较结果一致，大泉子、三岔子及金山屯种源嫁接成活率分别为 75.59%、65.34%及 64.91%较种源平均值分别高 37.00%、18.43%及 17.45%，大泉子 12、三岔子 18 及大泉子 31 无性系嫁接成活率分别为 100%、100%及 100%较种源平均值高 81.26%、81.26%及 81.26%。虎林 04、虎林 24 及虎林 14 无性系嫁接成活率分别为 8%、8.70%及 9.33%较无性系平均值低 589.63%、534.46%及 491.11%。2021 不同种源之间差异不显著，不同种源嫁接成活率为 74.10%（亚布力）～78.82%（五常）平均值为 73.95%，五常种源嫁接成活率为 78.82%较种源平均值高 3.4%，五常 28、五常 17 及亚布力 8 无性系嫁接成活率分别为 95%、94.44%及 93.22%较种源平均值高 32.09%、31.31%及 29.61%。亚布力 2、五常 29 及亚布力 15 无性系嫁接成活率分别为 16%、

中国知网 https://www.cnki.net

35.42 及 51.67%较种源平均值低 362.19%、108.8%及 43.13%。

表 2-2 核桃楸种源嫁接成活率方差分析

Tab. 2-2 Analysis of variance of graft survival rate of Juglans provenances

		平方和	df	均方	F	Sig
2020 年嫁接成活率	组间	4.042	6	0.674	7.403	0.000
	组内	11.647	128	0.091		
	总数	15.688	134			
2021 年嫁接成活率	组间	0.039	1	0.039	0.628	0.434
	组内	1.883	30	0.063		
	总数	1.922	31			

表 2-3 2020 年核桃楸种源嫁接成活率多重比较

Tab. 2-3 Multiple comparison of grafting survival rate of juglans provenance in 2020

种源	N	均值/%	分组
虎林	21	31.50	c
东京城	18	36.62	bc
铁力	16	49.97	abc
嘉荫	16	53.41	ab
大泉子	27	64.07	a
金山屯	19	64.91	a
三岔子	20	65.34	a

2.2.2. 不同因素嫁接成活率分析

对2021年正交设计结果进行分析，不同激素种类嫁接成活率差异显著，不同激素浓度与不同接穗催醒时间嫁接成活率差异不显著（表2-4）。不同激素种类嫁接成活率为56.79%（GA_3）～80.44%（IBA），平均为72.45%。NAA、IBA较对照分别提高5.3%、5.7%，较GA_3提高41.1%、41.6%。不同激素浓度嫁接成活率为63.19%（150 mg/g）～78.58%（50 mg/g），50 mg/g较对照提高3.2%。不同接穗催醒时间嫁接成活率为67.83%（4天）～77.09%（1天），1天催醒时间较对照提高1.3%。

表2-4 不同因素嫁接成活率方差分析

Tab.2-4 Variance analysis of grafting survival rate under different influencing factors

	平方和	df	均方	F	Sig
校正模型	1.217a	7	0.174	2.265	0.074
截距	17.825	1	17.825	232.143	0.000
接穗催醒时间/天	0.097	2	0.049	0.633	0.542
激素质量浓度 mg/g	0.291	2	0.146	1.897	0.177
基质种类	0.003	1	0.003	0.042	0.839
激素种类	0.826	2	0.413	5.375	0.014
误差	1.459	19	0.077		
总计	22.911	27			
校正的总计	2.676	26			

a. R^2 =0 .455（调整 R^2 =0 .254）

中国知网 https://www.cnki.net

表 2-5 不同因素对嫁接成活率多重比较

Tab. 2-5 Multiple comparison of grafting survival rate under different influencing factors

接穗催醒时间	均值/%	分组	激素浓度	均值/%	分组	激素种类	均值/%	分组
4	67.83	a	150	63.19	a	GA_3	56.79	b
7	72.44	a	对照	76.12	a	对照	76.12	ab
对照	76.12	a	250	75.58	a	NAA	80.13	a
1	77.09	a	50	78.58	a	IBA	80.44	a

2.2.3. 最佳处理组合分析

极差分析结果可知：从 4 个因素并结合 R 值（因素极差值）的大小对比可知，激素种类是成活率最优因素，其次是激素质量浓度，再次是接穗催醒时间，最后是基质种类。因而 4 个因素的优劣排序为：因子 4（激素种类）>因子 2（激素质量浓度）>因子 1（接穗催醒时间）>因子 3（基质种类）。

具体结合各因子的最佳水平可知，激素种类以第 3 个水平即 IBA 最优，嫁接成活率达到了 80.44%，较处理平均值提高 11.02% 。激素质量浓度以第 1 个水平即 50mg/g 最优，嫁接成活率达到了 78.58%，较处理平均值提高 8.45% 。接穗催醒时间是第 1 个水平即 1 天是最优，嫁接成活率达到了 77.09%，较处理平均值提高 6.40% 。基质种类以第 2 个水平即阔叶树锯末基质是最优，嫁接成活率达到了 73.14%，较处理平均值提高 0.95% 。综合上述分析可知：最优因子为激素种类。最优组合为“激素种类 IBA、激素质量浓度 50mg/g、接穗催醒时间 1 天及基质种类阔叶树锯末基质”。

表 2-6 嫁接成活率极差分析

Tab.2-6 Range analysis table of grafting survival rate

	水平	接穗催醒时间	激素质量浓度 mg/g	基质种类	激素种类
嫁接成活率总和	1	231.27	235.75	213.24	240.4
	2	203.48	189.58	438.85	170.38
	3	217.33	226.75	-	241.31
嫁接成活率平均值	1	77.09	78.58	71.08	80.13
	2	67.83	63.19	73.14	56.79
	3	72.44	75.58	-	80.44
最佳水平		1	1	2	3
极差		9.26	15.39	2.06	23.64
水平数量		3	3	2	3
每水平重复数 r		3	3	4	3
折算系数 d		0.52	0.52	0.71	0.52
R'		8.34	13.86	2.93	21.29

2.3. 本章小结

2020 年嫁接成活率在不同种源之间差异显著，三岔子、金山屯和大泉子成活率较高，分别为 75.59%、65.34%及 64.91%较平均值高 37.00%、18.43%及 17.45%，大泉子 12、三岔子 18 及大泉子 31 无性系嫁接成活率分别为 100%、100%及 100%较无性系平均值高 81.26%、81.26%及 81.26%。虎林 04、虎林 24 及虎林 14 无性系嫁接成活率分别为 8%、8.70%及 9.33%较无性系平均值低 589.63%、534.46%及 491.11%。2021 年嫁接成活率在不同种源之间差异不显著，五常种源嫁接成活率为 78.82%较无性系平均值高

中国知网 https://www.cnki.net

3.4%，五常 28、五常 17 及亚布力 8 无性系嫁接成活率分别为 95%、94.44%及 93.22%较无性系平均值高 32.09%、31.31%及 29.61%。亚布力 2、五常 29 及亚布力 15 无性系嫁接成活率分别为 16%、35.42 及 51.67%较种源平均值低 362.19%、108.8%及 43.13%。不同激素种类嫁接成活率差异显著，不同激素浓度与不同接穗催醒时间的嫁接成活率差异不显著。NAA 和 IBA 激素处理较对照嫁接成活率提高 5.3%和 5.7%。GA_3 激素处理会降低嫁接成活率。用阔叶树锯末当基质可以提高嫁接成活率但未达显著水平。多因素处理后得出的最佳组合为激素种类 IBA、激素质量浓度 50 mg/g、催醒时间 1 天及基质种类阔叶树锯末基质。

中国知网 https://www.cnki.net

3. 核桃楸无性系生理生化及光合性状变异分析

生理生化活动是生命体基本的过程，通过测定植物的生理生化及光合性状来反映其生命活动规律，认识植物的生长发育等的规律，可以为核桃楸无性系选择提供参考。张露荷等[71]以枣为研究对象，测定叶片光合生理指标，认为净光合速率与气孔导度及蒸腾速率之间极显著正相关。刘博等[72]、王力刚等[73]和吴孝红等[74]也得到与张露荷等一致的结论。王云鹏等[75]探究木荷优树生长性状的早期与晚期相关性，为木荷改良效率的提高提供理论依据。苗清丽[76]以杂种落叶松为研究对象，通过性状相关及早期与晚期相关分析，为间接选择及早期选择做准备。

3.1. 材料与方法

3.1.1. 试验地点

同 2.1.1

3.1.2. 材料来源

接穗来源于黑龙江省的金山屯林业局、东京城林业局、虎林县林业局和吉林省三岔子林业局，砧木来源于五常市宝龙店林场。材料分 2 组：第一组为 2021 年在林场苗圃嫁接培育的无性系（1 年生），第二组为 2017 年建立的无性系收集区（5 年生，已部分开花结实），其中 5 个无性系开花结实（结实群体），6 个无性系未开花结实（未结实群体）。与 5 年生收集区相同的 1 年生无性系有 11 个，为便于比较分析也视为结实无性系（结实群体）和未结实无性系（未结实群体）。

3.1.3. 光合及生理生化指标的测定

光合指标的测定：2021 年 7 月底采用 Li-6400 便携式光合作用测定仪（Li-COR Inc. USA）测定每个无性系的 3 株树上健康、完整且朝南向阳方位的 3 片树叶的净光合速率（P_n）、气孔导度（G_s）、胞间 CO_2 摩尔分数（C_i）和蒸腾速率（T_r）。测定时间 8:00—11:30，测定过程中光合有效辐射约为 1800 μmol/（m^2 • s）。

生理生化指标的测定：2021 年 7 月底，采集每个无性系 3 个单株健康且完整的叶片，应用 BCA 法蛋白含量测定试剂盒（苏州格锐思生物科技有限公司生产）测定每株混合样可溶性蛋白质量分数，应用可溶性糖含量测定试剂盒（苏州格锐思生物科技有限公司生产）测定每株混合样可溶性糖质量分数，使用叶绿素相对吸光值-502 便携式叶绿素测定仪测定叶绿素相对吸光值，每个无性系测 3 株，每株选健康、完整且朝南向阳方位 3 片叶，每片叶测 3 次。

3.1.4. 数据处理和分析

数据分析主要包括变异分析、方差分析、独立样本 T 检验、配对样本 T 检验及相关分析（皮尔逊与斯皮尔曼），采用 Excel2016、SPSS18.0 处理与分析。可溶性蛋白含量及可溶性糖含量等百分率性状经过反正弦转换后进行分析。

中国知网 https://www.cnki.net

3.2. 结果与分析

3.2.1. 各性状变异分析

遗传变异是基因型与环境互作的结果，遗传变异大说明可供选择范围大，从中选择优良个体进行嫁接繁殖，用无性繁殖还可缩短生殖周期，达到早开花结实的目的。对1年生核桃楸11个无性系7个生理生化及光合性状进行分析（表3-1），核桃楸无性系的可溶性蛋白含量、可溶性糖含量、叶绿素相对吸光值、净光合速率、气孔导度、胞间CO_2摩尔分数及蒸腾速率等指标的变异幅度（平均值）分别为2.53%～7.08%（4.94%）、1.96%～4.12%（2.90%）、24.7～49.8（36.81）、1.57 μmol CO_2 m^{-2} s^{-1}～18.9 μmol CO_2 m^{-2} s^{-1}（10.97 μmol CO_2 m^{-2} s^{-1}）、0.05 mol H_2O m^{-2} s^{-1}～0.68 mol H_2O m^{-2} s^{-1}（0.35 mol H_2O m^{-2} s^{-1}）、235 μmol CO_2 mol^{-1}～923 μmol CO_2 mol^{-1}（314.66 μmol CO_2 mol^{-1}）和2.1 mmol H_2O m^{-2} s^{-1}～12.9 mmol H_2O m^{-2} s^{-1}（7.40 mmol H_2O m^{-2} s^{-1}）。各项单个指标中气孔导度变异系数最高为52.09%，叶绿素相对吸光值的变异系数最低为14.70%，各项指标平均变异系数为29.31%。

表3-1 核桃楸无性系1年生与5年生性状对比分析

Tab. 3-1 Comparative analysis of one-year and five-year clones of Juglans

性状	群体	极小值	极大值	均值	标准差	变异系数/%
可溶性蛋白含量/%	1 年生群体	2.53	7.08	4.94	1.24	25.1
	5 年生群体	3.52	7.08	5.72	1.27	22.18
	1 年生/5 年生	0.72	1	0.86	0.98	1.13
可溶性糖含量/%	1 年生群体	1.96	4.12	2.9	0.77	26.67
	5 年生群体	1.53	3.42	2.2	0.63	28.84
	1 年生/5 年生	1.28	1.2	1.32	1.22	0.92
叶绿素相对吸光值	1 年生群体	24.7	49.8	36.81	5.41	14.7
	5 年生群体	26.5	52	41.25	4.85	11.76
	1 年生/5 年生	0.93	0.96	0.89	1.12	1.25
净光合速率/μmol CO_2 m^{-2} s^{-1}	1 年生群体	1.57	18.9	10.97	3.79	34.54
	5 年生群体	4.93	18.9	11.65	2.65	22.73
	1 年生/5 年生	0.32	1	0.94	1.43	1.52
气孔导度/mol H_2O m^{-2} s^{-1}	1 年生群体	0.05	0.68	0.35	0.18	52.09
	5 年生群体	0.14	0.81	0.44	0.16	35.83
	1 年生/5 年生	0.36	0.84	0.8	1.13	1.45
胞间 CO_2 摩尔分数/μmol CO_2 mol^{-1}	1 年生群体	235	923	314.66	65.25	20.74
	5 年生群体	234	352	318.81	20.09	6.3
	1 年生/5 年生	1	2.62	0.99	3.25	3.29
蒸腾速率/mmol H_2O m^{-2} s^{-1}	1 年生群体	2.1	12.9	7.4	2.32	31.36
	5 年生群体	4.13	13.4	9.14	2.19	23.95
	1 年生/5 年生	0.51	0.96	0.81	1.06	1.31

对核桃楸 5 年生 11 个无性系 7 个生理生化及光合性状进行研究（表 3-1），核桃楸无性系的可溶性蛋白含量、可溶性糖含量、叶绿素相对吸光值、净光合速率、气孔导度、胞间 CO_2 摩尔分数及蒸腾速率等指标的幅度（平均值）分别为 3.52%～7.08%（5.72%）、1.53%～3.42%（2.20%）、26.50～52.00（41.25）、4.93 μmol CO_2 m^{-2} s^{-1}～18.90 μmol CO_2 m^{-2} s^{-1}（11.65 μmol CO_2 m^{-2} s^{-1}）、0.14 mol H_2O m^{-2} s^{-1}～0.81 mol H_2O m^{-2} s^{-1}（0.44 mol H_2O m^{-2} s^{-1}）、234 μmol CO_2 mol^{-1}～352 μmol CO_2 mol^{-1}（318.81 μmol CO_2 mol^{-1}）和 4.13 mmol H_2O m^{-2} s^{-1}～13.40 mmol H_2O m^{-2} s^{-1}（9.14 mmol H_2O m^{-2} s^{-1}）。各项单个指标中气孔导度变异系数最高为 35.83%，胞间 CO_2 摩尔分数的变异系数最低为

中国知网 https://www.cnki.net

6.30%，各项指标平均变异系数为 21.66%。

对核桃楸 11 个无性系 1 年生与 5 年生 7 个生理生化及光合性状进行对比分析，5 年生比 1 年生核桃楸无性系可溶性蛋白含量、叶绿素相对吸光值、净光合速率、气孔导度、胞间 CO_2 摩尔分数和蒸腾速率分别高 7.89%、6.03%、3.10%、11.43%、0.66%和 11.76%，1 年生比 5 年生核桃楸无性系可溶性糖含量高 15.91%。2 个年龄阶段都是气孔导度变异较大，胞间 CO_2 摩尔分数和叶绿素含量变异相对较小。

3.2.2. 无性系各性状差异显著性分析

3.2.2.1. 1 年生群体分析

（1）1 年生结实群体分析

对1年生结实群体进行显著性分析，可溶性蛋白含量及可溶性糖含量无性系间差异不显著（表3-2），可溶性蛋白含量为2.50%（HL12）～5.97%（DJC25），可溶性糖含量为1.96%（HL12）～4.12%（SC10）。叶绿素含量、净光合速率、气孔导度、胞间CO_2摩尔分数及蒸腾速率无性系间差异显著，叶绿素相对吸光值为32.15（SC21）～37.56（HL12）、净光合速率为9.38 μmol CO_2 m^{-2} s^{-1}（DJC25）～15.28 μmol CO_2 m^{-2} s^{-1}（SC15）、气孔导度为0.29 mol H_2O m^{-2} s^{-1}（SC10）～0.57 mol H_2O m^{-2} s^{-1}（SC15）、

表3-2 1年生结实群体不同性状方差分析

Tab. 3-2 Variance analysis of annual fruit group

性状		平方和	df	均方	F	Sig
可溶性蛋白含量	组间	0.002	4	0.0006	1.654	0.236
	组内	0.003	10	0.0003		
	总数	0.006	14			
可溶性糖含量	组间	0.001	4	0.0002	1.406	0.301
	组内	0.002	10	0.0002		
	总数	0.003	14			
叶绿素相对吸光值	组间	517.043	4	129.261	12.09	0.000
	组内	1389.857	130	10.691		
	总数	1906.9	134			
净光合速率	组间	194.824	4	48.706	10.492	0.000
	组内	185.693	40	4.642		
	总数	380.517	44			
气孔导度	组间	0.451	4	0.113	7.402	0.000
	组内	0.609	40	0.015		
	总数	1.06	44			
胞间 CO_2 摩尔分数	组间	5322.089	4	1330.522	5.112	0.002
	组内	10410.222	40	260.256		
	总数	15732.311	44			
蒸腾速率	组间	48.585	4	12.146	5.186	0.002
	组内	93.683	40	2.342		
	总数	142.267	44			

胞间 CO_2 摩尔分数为 293.67 μmol CO_2 mol^{-1}（SC10）～325.11 μmol CO_2 mol^{-1}（SC21）及蒸腾速率为 6.77 mmol H_2O m^{-2} s^{-1}（DJC25）～9.62 mmol H_2O m^{-2} s^{-1}（SC15）。HL12 无性系可溶性蛋白含量及可溶性糖含量较低，叶绿素含量较高；SC21 无性系叶绿素含

中国知网 https://www.cnki.net

量较低而胞间 CO_2 摩尔分数较高； SC15 无性系净光合速率、气孔导度及蒸腾速率较高。

(2)1 年生未结实群体分析

对 1 年生未结实群体进行性状分析，可溶性蛋白含量、可溶性糖含量及胞间 CO_2 摩尔分数无性系间差异不显著（表 3-3），可溶性蛋白含量为 3.92%（JST13）～7.08%（JST6）、可溶性糖含量为 2.15%（JST13）～3.99%（JST8）和胞间 CO_2 摩尔分数为 282 μmol CO_2 mol^{-1}（JST7）～374.78 μmol CO_2 mol^{-1}（JST8）。叶绿素相对吸光值、净光合速率、气孔导度及蒸腾速率无性系间差异显著，叶绿素相对吸光值为 32.15（JST6）～37.56（JST11）、净光合速率为 5.99 μmol CO_2 m^{-2} s^{-1}（JST7）～15.43 μmol CO_2 m^{-2} s^{-1}（JST13）、气孔导度为 0.11 mol H_2O m^{-2} s^{-1}（JST7）～0.53 mol H_2O m^{-2} s^{-1}（JST19）和蒸腾速率为 3.88 mmol H_2O m^{-2} s^{-1}（JST7）～9.52 mmol H_2O m^{-2} s^{-1}（JST19）。JST13 无性系可溶性蛋白含量和可溶性糖含量较低，而净光合速率较高；JST8 无性系可溶性糖含量和胞间 CO_2 摩尔分数较高；JST6 可溶性蛋白含量高，而叶绿素含量低；JST7 无性系胞间 CO_2 摩尔分数、净光合速率、气孔导度及蒸腾速率较低；JST19 无性系气孔导度和蒸腾速率较高。

表3-3 1年生未结实群体不同性状方差分析

Tab.3-3 Variance analysis of annual unfruit group

性状		平方和	df	均方	F	Sig
可溶性蛋白含量	组间	0.002	5	0.0004	1.58	0.239
	组内	0.003	12	0.0003		
	总数	0.005	17			
可溶性糖含量	组间	0.001	5	0.0001	0.312	0.897
	组内	0.005	12	0.0004		
	总数	0.006	17			
叶绿素相对吸光值	组间	1260.506	5	252.101	9.974	0.000
	组内	3943.194	156	25.277		
	总数	5203.7	161			
净光合速率	组间	581.898	5	116.38	14.904	0.000
	组内	374.804	48	7.808		
	总数	956.702	53			
气孔导度	组间	1.248	5	0.25	15.909	0.000
	组内	0.753	48	0.016		
	总数	2.002	53			
胞间 CO_2 摩尔分数	组间	46103.333	5	9220.667	1.247	0.302
	组内	355054.667	48	7396.972		
	总数	401158	53			
蒸腾速率	组间	193.466	5	38.693	11.196	0.000
	组内	165.89	48	3.456		
	总数	359.356	53			

(3)1 年生整体分析

对所有无性系进行分析，11 个无性系可溶性蛋白含量、可溶性糖含量及胞间 CO_2 摩尔分数无性系间差异不显著（表 3-4），可溶性蛋白含量为 2.50%（HL12）～7.08%（JST6）、可溶性糖含量为 1.96%（HL12）～4.12%（SC10）和胞间 CO_2 摩尔分数为

中国知网 https://www.cnki.net

235 μmol CO_2 mol^{-1}（JST7）～923 μmol CO_2 mol^{-1}（JST8）。叶绿素相对吸光值、净光合速率、气孔导度及蒸腾速率无性系间差异显著，叶绿素相对吸光值为 24.70（SC21）～49.80（JST11）、净光合速率为 1.57 μmol CO_2 m^{-2} s^{-1}（JST7）～18.90 μmol CO_2 m^{-2} s^{-1}（JST13）、气孔导度为 0.05 mol H_2O m^{-2} s^{-1}（JST7）～0.68 mol H_2O m^{-2} s^{-1}（SC15）及蒸腾速率为 2.10mmol H_2O m^{-2} s^{-1}（JST7）～12.90mmol H_2O m^{-2} s^{-1}（SC15）。HL12 无性系可溶性蛋白含量和可溶性糖含量较低，SC15 无性系气孔导度和蒸腾速率较高，JST7 无性系胞间 CO_2 摩尔分数、净光合速率、气孔导度及蒸腾速率较低。

除结实群体胞间CO_2摩尔分数无性系间差异显著，而整体与未结实群体不显著外，其余性状3个群体表现一致，可溶性蛋白含量和可溶性糖含量无性系间差异不显著，叶绿素相对吸光值、净光合速率、气孔导度及蒸腾速率无性系间差异显著。

表 3-4 1 年生整体群体性状方差分析

Tab.3-4 Variance analysis of annual group

性状		平方和	df	均方	F	Sig
可溶性蛋白含量	组间	0.005	10	0.0005	1.567	0.182
	组内	0.007	22	0.0003		
	总数	0.011	32			
可溶性糖含量	组间	0.002	10	0.0002	0.583	0.811
	组内	0.007	22	0.0003		
	总数	0.009	32			
叶绿素相对吸光值	组间	3336.531	10	333.653	17.893	0.000
	组内	5333.051	286	18.647		
	总数	8669.582	296			
净光合速率	组间	847.144	10	84.714	13.3	0.000
	组内	560.496	88	6.369		
	总数	1407.64	98			
气孔导度	组间	1.855	10	0.185	11.984	0.000
	组内	1.362	88	0.015		
	总数	3.217	98			
胞间 CO_2 摩尔分数	组间	51759.434	10	5175.943	1.246	0.274
	组内	365464.889	88	4153.01		
	总数	417224.323	98			
蒸腾速率	组间	267.99	10	26.799	9.085	0.000
	组内	259.572	88	2.95		
	总数	527.563	98			

结实与未结实群体T检验表明（表3-5），可溶性蛋白含量、可溶性糖含量及胞间CO_2摩尔分数在结实群体与未结实群体之间差异不显著，未结实群体均值较大。叶绿素相对吸光值2个群体之间差异显著，未结实群体均值较大。净光合速率、气孔导度及蒸腾速率2个群体之间差异显著且结实群体均值较大。该结论与具体无性系性状表现一致。

中国知网 https://www.cnki.net

表 3-5 不同性状 1 年生结实群体与未结实群体独立样本 T 检验
Tab.3-5 Independent sample t-test of annual fruit group and unfruit group

性状	均值差值	差分的 95%置信区间		t	df	Sig.(双侧)
		极小值	极大值			
可溶性蛋白含量	-0.00643	-0.01974	0.00688	-0.986	31	0.332
可溶性糖含量	-0.00501	-0.01673	0.00671	-0.872	31	0.390
叶绿素相对吸光值	-4.60123	-5.6882	-3.51427	-8.333	282	0.000
净光合速率	-0.58444	-1.50146	0.33257	-1.284	44	0.206
气孔导度	-0.11249	-0.18927	-0.0357	-2.952	44	0.005
胞间 CO_2 摩尔分数	-10.55556	-15.70395	-5.40716	-4.132	44	0.000
蒸腾速率	2.41911	1.47452	3.3637	5.161	44	0.000

3.2.2.2. 5 年生群体分析

(1)5 年生结实群体分析

对 5 年生结实群体进行性状分析，可溶性蛋白含量、可溶性糖含量、胞间 CO_2 摩尔分数及蒸腾速率无性系间差异不显著（表 3-6），可溶性蛋白含量为 3.96%（DJC25）～7.07%（SC10）、可溶性糖含量为 1.66%（SC15）～2.86%（DJC25）、胞间 CO_2 摩尔分数为 308.11 μmol CO_2 mol^{-1}（HL12）～325.56 μmol CO_2 mol^{-1}（SC21）及蒸腾速率为 7.52 mmol H_2O m^{-2} s^{-1}（SC10）～9.49 mmol H_2O m^{-2} s^{-1}（SC15）。

表 3-6 5 年生结实群体不同性状方差分析
Tab. 3-6 Variance analysis of five year old fruit group

性状		平方和	df	均方	F	Sig
可溶性蛋白含量	组间	0.002	4	0.0006	1.387	0.306
	组内	0.004	10	0.0004		
	总数	0.007	14			
可溶性糖含量	组间	0	4	0.0001	0.302	0.870
	组内	0.003	10	0.0003		
	总数	0.003	14			
叶绿素相对吸光值	组间	581.16	4	145.29	5.526	0.000
	组内	3418.054	130	26.293		
	总数	3999.214	134			
净光合速率	组间	431.843	4	107.961	33.868	0.000
	组内	127.509	40	3.188		
	总数	559.352	44			
气孔导度	组间	0.229	4	0.057	3.611	0.013
	组内	0.633	40	0.016		
	总数	0.862	44			
胞间 CO_2 摩尔分数	组间	1920.311	4	480.078	1.32	0.279
	组内	14546	40	363.65		
	总数	16466.311	44			
蒸腾速率	组间	22.615	4	5.654	2.048	0.106
	组内	110.433	40	2.761		
	总数	133.047	44			

叶绿素相对吸光值、净光合速率及气孔导度无性系间差异显著，叶绿素相对吸光值为 35.92（SC10）～41.64（HL12）、净光合速率为 6.49 μmol CO_2 m^{-2} s^{-1}（SC21）～15.7 μmol CO_2 m^{-2} s^{-1}（HL12）及气孔导度为 0.28 mol H_2O m^{-2} s^{-1}（SC21）～0.49 mol H_2O m^{-2} s^{-1}（SC10）。DJC25 无性系可溶性糖含量较高，而可溶性蛋白含量较低；SC15 无性系可溶性糖含量较低，蒸腾速率较高；SC10 无性系可溶性蛋白含量和气孔导度较

中国知网 https://www.cnki.net

高，而蒸腾速率及叶绿素含量较低；SC21 无性系净光合速率和气孔导度较低，HL12 胞间 CO_2 摩尔分数较低，而叶绿素含量和净光合速率较高。

(2)5 年生未结实群体分析

对 5 年生未结实群体进行性状分析，可溶性糖含量无性系间差异不显著（表 3-7），可溶性蛋白含量为 2.50%（JST19）～5.97%（JST6）、可溶性糖含量为 1.53%（JST11）～3.42%（JST6）。叶绿素相对吸光值、净光合速率、气孔导度、胞间 CO_2 摩尔分数及蒸腾速率无性系间差异显著，叶绿素相对吸光值为 40.03（JST6）～44.79（JST8）、净光合速率为 10.21 μmol CO_2 m^{-2} s^{-1}（JST19）～13.49 μmol CO_2 m^{-2} s^{-1}（JST11）、气孔导度为 0.25 mol H_2O m^{-2} s^{-1}（JST6）～0.59 mol H_2O m^{-2} s^{-1}（JST13）、胞间 CO_2 摩尔分数为 291.00 μmol CO_2 mol^{-1}（JST6）～336.44 μmol CO_2 mol^{-1}（JST8）及蒸腾速率为 7.07 mmol H_2O m^{-2} s^{-1}（JST6）～12.54 mmol H_2O m^{-2} s^{-1}（JST8）。JST6 无性系可溶性蛋白含量及可溶性糖含量较高，而叶绿素含量、气孔导度、胞间 CO_2 摩尔分数及蒸腾速率均较低。

表 3-7 5 年生未结实群体性状方差分析

Tab.3-7 Variance analysis of five year old unfruit group

性状		平方和	df	均方	F	Sig
可溶性蛋白含量	组间	0.002	5	0.0005	2.621	0.08
	组内	0.002	12	0.0002		
	总数	0.005	17			
可溶性糖含量	组间	0.001	5	0.0002	1.854	0.177
	组内	0.001	12	0.0001		
	总数	0.002	17			
叶绿素相对吸光值	组间	667.592	5	133.518	11.508	0.000
	组内	1809.889	156	11.602		
	总数	2477.481	161			
净光合速率	组间	58.282	5	11.656	8.119	0.000
	组内	68.913	48	1.436		
	总数	127.195	53			
气孔导度	组间	0.829	5	0.166	12.155	0.000
	组内	0.655	48	0.014		
	总数	1.485	53			
胞间 CO_2 摩尔分数	组间	14715.722	5	2943.144	16.892	0.000
	组内	8363.111	48	174.231		
	总数	23078.833	53			
蒸腾速率	组间	197.6	5	39.52	25.186	0.000
	组内	75.318	48	1.569		
	总数	272.918	53			

JST19 无性系可溶性蛋白含量和净光合速率较低，JST8 无性系叶绿素含量、胞间 CO_2 摩尔分数及蒸腾速率较高；JST11 可溶性糖含量较低，而净光合速率较高。

(3)5 年生整体群体分析

对 5 年生核桃楸 11 个无性系的 7 个生理生化及光合性状进行研究（表 3-8），可溶性蛋白含量和可溶性糖含量无性系间差异不显著，可溶性蛋白含量为 3.52%（JST19）～7.08%（SC10），可溶性糖含量为 1.53%（JST11）～3.42%（JST6）。叶

中国知网 https://www.cnki.net

绿素相对吸光值、净光合速率、气孔导度、胞间 CO_2 摩尔分数及蒸腾速率无性系间差异显著，叶绿素相对吸光值为 26.50（SC10）～52.00（JST8）、净光合速率为 4.93 μmol CO_2 m^{-2} s^{-1}（SC21）～18.9 μmol CO_2 m^{-2} s^{-1}（HL12）、气孔导度为 0.14 mol H_2O m^{-2} s^{-1}（JST6）～0.81 mol H_2O m^{-2} s^{-1}（JST13）、胞间 CO_2 摩尔分数为 234 μmol CO_2 mol^{-1}（JST6）～352 μmol CO_2 mol^{-1}（JST8）和蒸腾速率为 4.13 mmol H_2O m^{-2} s^{-1}（JST6）～13.40 mmol H_2O m^{-2} s^{-1}（JST8）。SC10 可溶性糖蛋白较高，而叶绿素含量较低；JST6 无性系可溶性糖含量较高，而气孔导度、胞间 CO_2 摩尔分数和蒸腾速率较低；JST8 叶绿素含量、胞间 CO_2 摩尔分数和蒸腾速率较高。

表 3-8 5 年生整体群体不同性状方差分析

Tab.3-8 Variance analysis of five year old group

性状		平方和	df	均方	F	Sig
可溶性蛋白含量	组间	0.005	10	0.0005	1.641	0.16
	组内	0.006	22	0.0003		
	总数	0.011	32			
可溶性糖含量	组间	0.001	10	0.0001	0.644	0.761
	组内	0.004	22	0.0002		
	总数	0.005	32			
叶绿素相对吸光值	组间	1738.718	10	173.872	9.512	0.000
	组内	5227.943	286	18.280		
	总数	6966.661	296			
净光合速率	组间	490.666	10	49.067	21.983	0.000
	组内	196.422	88	2.232		
	总数	687.088	98			
气孔导度	组间	1.114	10	0.111	7.607	0.000
	组内	1.288	88	0.015		
	总数	2.402	98			
胞间 CO_2 摩尔分数	组间	16662.242	10	1666.224	6.400	0.000
	组内	22909.111	88	260.331		
	总数	39571.354	98			
蒸腾速率	组间	284.02	10	28.402	13.456	0.000
	组内	185.751	88	2.111		
	总数	469.771	98			

除结实群体胞间 CO_2 摩尔分数和蒸腾速率不同外，其余性状 3 个群体一致。可溶性蛋白含量和可溶性糖含量无性系间差异不显著，叶绿素相对吸光值，净光合速率和气孔导度无性系间差异显著。

结实与未结实群体 T 检验表明，可溶性蛋白含量、气孔导度、胞间 CO_2 摩尔分数在结实群体与未结实群体之间差异不显著（表 3-9），未结实群体均值大。可溶性糖含量、净光合速率 2 个群体之间差异不显著，结实群体均值大。叶绿素相对吸光值、蒸腾速率 2 个群体之间差异显著，未结实群体均值大。

从 2 个年龄性状独立样本 T 检验来看，叶绿素相对吸光值在结实与未结实群体之间差异显著且均为未结实群体想对较高，胞间 CO_2 摩尔分数 2 个群体之间差异不显著且均为未结实群体想对较高。蒸腾速率 2 个群体差异显著，1 年生群体结实群体均值大，5

中国知网 https://www.cnki.net

年生群体未结实群体均值大。

表 3-9 不同性状 5 年生结实群体与未结实群体独立样本 T 检验

Tab.3-9 Independent sample t-test of five year old fruit group and unfruit group

性状	均值差值	差分的 95%置信区间		t	df	Sig.(双侧)
		极小值	极大值			
可溶性蛋白含量	-0.00228	-0.01589	0.01133	-0.342	31	0.735
可溶性糖含量	0.00101	-0.00834	0.01036	0.220	31	0.827
叶绿素相对吸光值	-2.57951	-3.68703	-1.47198	-4.588	237	0.000
净光合	0.12667	-1.08622	1.33955	0.210	44	0.834
气孔导度	0.05398	-0.0014	0.10935	1.965	44	0.056
胞间 CO_2 摩尔分数	-0.02222	-9.46851	9.42407	-0.005	44	0.996
蒸腾速率	1.69378	0.68547	2.70208	3.385	44	0.002

(4)二个年度无性系配对 T 检验分析

对未结实群体进行配对 T 检验发现（表 3-10），叶绿素相对吸光值、气孔导度及蒸腾速率在 1 年生和 5 年生群体之间差异显著，5 年生群体叶绿素相对吸光值、气孔导度及蒸腾速率想对较高。

表 3-10 不同性状 1 年生和 5 年生群体配对 T 检验

Tab.3-10 Paired t-test of annual group and five year old group

	性状	均值	标准差	差分的 95%置信区间		t	df	Sig.(双侧)
				极小值	极大值			
未结实群体	可溶性蛋白含量	-0.0059	0.02004	-0.01587	0.00406	-1.249	17	0.228
	可溶性糖含量	0.00973	0.0184	0.00058	0.01889	2.244	17	0.038
	叶绿素相对吸光值	-3.52346	5.11117	-4.31648	-2.73043	-8.774	161	0.000
	净光合速率	1.37889	4.9639	0.024	2.73377	2.041	53	0.046
	气孔导度	0.147	0.22025	0.08688	0.20712	4.905	53	0.000
	胞间 CO_2 摩尔分数	2.94444	85.54298	-20.40429	26.29318	0.253	53	0.801
	蒸腾速率	2.94185	3.06404	2.10553	3.77817	7.055	53	0.000
结实群体	可溶性蛋白含量	-0.01005	0.02931	-0.02628	0.00618	-1.329	14	0.205
	可溶性糖含量	0.00372	0.02234	-0.00865	0.01609	0.645	14	0.530
	叶绿素相对吸光值	-5.54519	6.7589	-6.69571	-4.39466	-9.532	134	0.000
	净光合速率	-0.16644	4.36106	-1.47665	1.14376	-0.256	44	0.799
	气孔导度	0.01976	0.21483	-0.04479	0.0843	0.617	44	0.540
	胞间 CO_2 摩尔分数	5.6	27.30002	-2.60183	13.80183	1.376	44	0.176
	蒸腾速率	0.30156	2.19812	-0.35883	0.96195	0.92	44	0.362
整体	可溶性蛋白含量	-0.00779	0.02436	-0.01643	0.00085	-1.837	32	0.076
	可溶性糖含量	0.007	0.02019	-0.00016	0.01416	1.992	32	0.055
	叶绿素相对吸光值	4.44242	5.99224	3.75814	5.12671	12.776	296	0.000
	净光合速率	0.67646	4.73952	-0.26882	1.62175	1.42	98	0.159
	气孔导度	0.08916	0.22586	0.04412	0.13421	3.928	98	0.000
	胞间 CO_2 摩尔分数	4.15152	65.52759	-8.91773	17.22076	0.63	98	0.530
	蒸腾速率	1.74172	2.99879	1.14362	2.33982	5.779	98	0.000

对结实群体进行配对 T 检验发现，叶绿素相对吸光值在 1 年生和 5 年生群体之间差异显著，5 年生群体叶绿素相对吸光值想对较高。对整个群体进行配对 T 检验发现，叶绿素相对吸光值、气孔导度及蒸腾速率在 1 年生和 5 年生群体之间差异显著，5 年生群体叶绿素相对吸光值、气孔导度及蒸腾速率想对较高。表明随着树龄增大，3 个性状平

中国知网 https://www.cnki.net

均值均有增大趋势。

3.2.3. 早晚及性状相关分析

3.2.3.1. 早期与晚期相关分析

对 1 年生和 5 年生群体各性状进行皮尔逊相关分析（表 3-11），叶绿素相对吸光值在整体群体内无性系之间、未结实群体内无性系之间均呈极显著正相关，在结实群体内无性系之间显著正相关，相关系数分别为 0.322、0.484 和 0.149。叶绿素相对吸光值在早期与晚期一致性较好，有利于早期选择。胞间 CO_2 摩尔分数在结实群体内无性系之间、未结实群体内无性系之间均呈极显著正相关，在整体群体内无性系之间显著正相关，相关系数分别为 0.296、0.512 及 0.442。蒸腾速率在结实群体内无性系之间、未结实群体内无性系之间显著正相关。净光合速率在未结实群体内无性系之间显著正相关，气孔导度在未结实群体内无性系之间显著负相关。

叶绿素相对吸光值和胞间 CO_2 摩尔分数无性系排名斯皮尔曼相关结果表明(表 3-12)，1 年生与 5 年生群体都达到了相关显著水平，相关系数分别为 0.326 和 0.227。从 2 个性状排名也证明其相关性较高（表 3-12），1 年生群体胞间 CO_2 摩尔分数由高到低前三名为 JST8、SC21 及 JST19，5 年生群体胞间 CO_2 摩尔分数前三名为 JST8、JST13 及 SC21；1 年生群体叶绿素相对吸光值前三名为 JST11、JST8 及 JST7，5 年生群体叶绿素相对吸光值前三名为 JST8、JST11 及 JST19。

表 3-11 不同性状 1 年生与 5 年生群体早期与晚期相关分析

Tab.3-11 Correlation analysis of annual group and five year old group

性状	结实群体	未结实群体	整体
可溶性蛋白含量	0.016	0.31	0.204
可溶性糖含量	-0.168	0.294	0.138
叶绿素相对吸光值	0.149*	0.484**	0.322**
净光合速率	0.114	0.445**	-0.14
气孔导度	0.287	-0.341*	0.156
胞间CO_2摩尔分数	0.442**	0.512**	0.296*
蒸腾速率	0.319*	0.333*	-0.106

**在 0.01 水平（双侧）上显著相关。*在 0.05 水平（双侧）上显著相关。

表 3-12 不同性状 1 年生与 5 年生群体性状排名

Tab.3-12 Character ranking of annual group and five year old group

胞间 CO_2 摩尔分数	1 年生群体排名	5 年生群体排名	叶绿素相对吸光值	1 年生群体排名	5 年生群体排名
DJC25	7	8	DJC25	7	10
HL12	6	9	HL12	5	4
JST11	9	6	JST11	1	2
JST13	5	2	JST13	6	6
JST19	3	4	JST19	4	3
JST6	8	11	JST6	8	9
JST7	11	10	JST7	3	8
JST8	1	1	JST8	2	1
SC10	10	5	SC10	9	11
SC15	4	7	SC15	10	7
SC21	2	3	SC21	11	5

3.2.3.2. 性状相关分析

中国知网 https://www.cnki.net

可溶性蛋白，可溶性糖都是重要的渗透调节物质和营养物质，它们的增加和积累能提高细胞的保水能力。植物体中生理生化指标越大植物抗逆性越高。光合指标可以间接代表植物生长速度，净光合速率越高说明植物生长越快，越有利于营养物质积累。王丽云等[77]对乌桕优株 1 年生子代幼苗的生长特性及光合指标进行研究，发现幼苗生长速率与净光合速率呈正显著相关。

（1）1 年生群体分析

为了解核桃楸各性状间的相关关系，对核桃楸 1 年生结实群体各性状进行相关分析（表 3-13），在结实群体中胞间 CO_2 摩尔分数与气孔导度、与蒸腾速率极显著正相关。净光合速率与气孔导度、与蒸腾速率极显著正相关。气孔导度与蒸腾速率极显著正相关。

在未结实群体中（表 3-14），叶绿素相对吸光值与净光合速率极显著正相关，与气孔导度显著正相关。净光合速率与气孔导度、与蒸腾速率极显著正相关。气孔导度与蒸腾速率极显著正相关。

表 3-13 1 年生结实群体性状相关分析

Tab.3-13 Correlation analysis of annual fruit group

性状	可溶性糖含量	叶绿素相对吸光值	净光合速率	气孔导度	胞间 CO_2 摩尔分数	蒸腾速率	GA_3 含量	6-BA 含量	IAA 含量	嫁接成活率
可溶性蛋白含量	-0.347	0.195	-0.072	0.267	0.27	0.137	-0.744	-0.338	-0.27	-0.545
可溶性糖含量		0.32	0.259	-0.028	-0.398	-0.034	-0.625	-0.614	-0.624	-0.539
叶绿素相对吸光值			0.113	0.102	-0.015	0.075	-0.741	-0.569	-0.47	-0.511
净光合速率				0.653**	0.047	0.657**	-0.573	-0.559	-0.385	-0.342
气孔导度					0.733**	0.963**	-0.353	-0.302	-0.172	-0.228
胞间 CO_2 摩尔分数						0.754**	0.09	0.162	0.375	0.256
蒸腾速率							-0.425	-0.313	-0.081	-0.158
GA_3 含量								0.572	-0.188	-0.105
6-BA 含量									0.367	-0.237
IAA 含量										0.662

**在 0.01 水平（双侧）上显著相关。

表 3-14 1 年生未结实群体性状相关分析

Tab.3-14 Correlation analysis of annual unfruit group

性状	可溶性糖含量	叶绿素相对吸光值	净光合速率	气孔导度	胞间 CO_2 摩尔分数	蒸腾速率	GA_3 含量	6-BA 含量	IAA 含量	嫁接成活率
可溶性蛋白含量	0.208	-0.159	0.07	-0.059	-0.018	-0.087	0.803	0.447	0.221	0.669
可溶性糖含量		0.108	-0.22	-0.129	-0.192	-0.212	0.014	0.55	0.525	0.29
叶绿素相对吸光值			0.353**	0.316*	-0.104	0.257	0.469	-0.164	0.648	0.201
净光合速率				0.858**	0.107	0.833**	0.038	0.773	0.048	0.397
气孔导度					0.218	0.946**	0.074	0.837*	0.323	0.42
胞间 CO_2 摩尔分数						0.188	0.397	0.792	0.751	0.637
蒸腾速率							-0.019	0.786	0.172	0.361
GA_3 含量								0.257	0.499	0.869*
6-BA 含量									0.502	0.437
IAA 含量										0.411

**在 0.01 水平（双侧）上显著相关。
*在 0.05 水平（双侧）上显著相关。

在整体群体中（表 3-15），叶绿素相对吸光值与净光合速率显著正相关，与胞间 CO_2 摩尔分数显著负相关。胞间 CO_2 摩尔分数与气孔导度、与蒸腾速率极显著正相关。

中国知网 https://www.cnki.net

气孔导度与净光合速率、与蒸腾速率极显著正相关。气孔导度与蒸腾速率极显著正相关。

表 3-15 1 年生整体群体性状相关分析

Tab.3-15 Correlation analysis of annual group

性状	可溶性糖含量	叶绿素相对吸光值	净光合速率	气孔导度	胞间 CO_2 摩尔分数	蒸腾速率	GA_3 含量	6-BA 含量	IAA 含量	嫁接成活率
可溶性蛋白含量	0.009	0.002	0.102	0.229	0.194	0.161	0.001	0.29	-0.053	0.007
可溶性糖含量		0.172	0.044	-0.022	-0.126	0.013	0.081	0.294	0.161	0.06
叶绿素相对吸光值			-0.039	-0.015	0.077	0.04	-0.346*	0.078	0.056	0.620*
净光合速率				0.802**	0.085	0.794**	0.341	0.465**	0.416*	-0.034
气孔导度					0.244*	0.952**	0.22	0.436*	0.233	-0.38
胞间 CO_2 摩尔分数						0.222*	0.052	0.215	-0.003	-0.618*
蒸腾速率							0.252	0.432*	0.235	-0.488
GA_3 含量								0.335	0.292	-0.023
6-BA 含量									0.510**	0.369
IAA 含量										0.338

**在 0.01 水平（双侧）上显著相关。

*在 0.05 水平（双侧）上显著相关。

（2） 5 年生群体分析

为了解核桃楸各性状间的相关关系，对核桃楸 1 年生群体各性状进行相关分析（表 3-16），在结实群体中，叶绿素相对吸光值与净光合速率显著负相关，与胞间 CO_2 摩尔分数显著正相关。胞间 CO_2 摩尔分数与气孔导度、与蒸腾速率极显著正相关，与净光合速率显著负相关。气孔导度与净光合速率及蒸腾速率极显著正相关。

表 3-16 5 年生结实群体性状相关分析

Tab.3-16 Correlation analysis of five year old fruit group

性状	可溶性糖含量	叶绿素相对吸光值	净光合速率	气孔导度	胞间 CO_2 摩尔分数	蒸腾速率	GA_3 含量	6-BA 含量	IAA 含量	结实量
可溶性蛋白含量	-0.132	0.145	0.121	0.056	0.01	0.144	-0.165	0.608	0.438	-0.872
可溶性糖含量		-0.042	-0.293	-0.203	0.017	-0.017	0.047	-0.508	0.485	-0.574
叶绿素相对吸光值			-0.312*	-0.043	0.326*	-0.063	0.312	0.528	0.759	-0.458
净光合速率				0.509**	-0.336*	0.008	0.55	-0.699	0.034	0.824
气孔导度					0.540**	0.709**	0.19	-0.401	-0.596	0.855
胞间 CO_2 摩尔分数						0.691**	0.045	-0.318	-0.732	0.786
蒸腾速率							0.109	-0.365	-0.662	0.836
GA_3 含量								-0.483	0.557	0.17
6-BA 含量									-0.082	-0.236
IAA 含量										-0.3

**在 0.01 水平（双侧）上显著相关。

*在 0.05 水平（双侧）上显著相关。

在未结实群体中（表 3-17），胞间 CO_2 摩尔分数与气孔导度、与蒸腾速率极显著正相关。蒸腾速率与气孔导度极显著正相关。

中国知网 https://www.cnki.net

表 3-17 5 年生未结实群体性状相关分析

Tab.3-17 Correlation analysis of five year old unfruit group

性状	可溶性糖含量	叶绿素相对吸光值	净光合速率	气孔导度	胞间 CO_2 摩尔分数	蒸腾速率	GA_3 含量	6-BA 含量	IAA 含量
可溶性蛋白含量	-0.21	-0.153	0.148	0.16	0.09	0.105	0.122	-0.478	0.24
可溶性糖含量		-0.146	-0.045	0.127	0.148	0.211	-0.183	0.332	-0.836*
叶绿素相对吸光值			-0.052	-0.046	-0.036	0.023	-0.441	-0.114	0.342
净光合速率				0.13	-0.205	-0.043	-0.255	-0.215	-0.243
气孔导度					0.891**	0.930**	-0.435	0.213	-0.686
胞间 CO_2 摩尔分数						0.897**	-0.216	0.257	-0.685
蒸腾速率							-0.373	0.172	-0.727
GA_3 含量								0.037	0.108
6-BA 含量									-0.19

**在 0.01 水平（双侧）上显著相关。*在 0.05 水平（双侧）上显著相关。

在整体群体中（表 3-18），结实量和生理生化指标负相关，与光合指标正相关。叶绿素相对吸光值与结实量显著负相关，气孔导度与蒸腾速率极显著正相关，与结实量显著正相关。胞间 CO_2 摩尔分数与净光合速率极显著负相关，与气孔导度及蒸腾速率极显著正相关。

表 3-18 5 年生整体群体性状相关分析

Tab.3-18 Correlation analysis of five year old group

性状	可溶性糖含量	叶绿素相对吸光值	净光合速率	气孔导度	胞间 CO_2 摩尔分数	蒸腾速率	GA_3 含量	6-BA 含量	IAA 含量
可溶性蛋白含量	-0.164	0.145	-0.021	0.221	0.173	0.227	0.417*	0.258	-0.031
可溶性糖含量		0.042	-0.02	-0.001	-0.029	0.07	0.018	-0.119	0.08
叶绿素相对吸光值			-0.172	0.038	0.161	0.084	-0.099	0.245	-0.016
净光合速率				0.315**	-0.263**	-0.021	-0.125	-0.349*	-0.238
气孔导度					0.747**	0.840**	0.059	-0.113	0.005
胞间 CO_2 摩尔分数						0.769**	0.194	0.194	0.144
蒸腾速率							0.249	0.071	0.19
GA_3 含量								0.152	0.243
6-BA 含量									0.049

**在 0.01 水平（双侧）上显著相关。
*在 0.05 水平（双侧）上显著相关。

在 1 年生和 5 年生所有群体中，胞间 CO_2 摩尔分数与气孔导度及蒸腾速率均极显著正相关，气孔导度与净光合速率及蒸腾速率极显著正相关。除 1 年生结实群体和 5 年生整体外，蛋白含量与净光合速率正相关。除 1 年生结实群体外，蛋白含量与气孔导度、与胞间 CO_2 摩尔分数、与蒸腾速率正相关。除 1 年生结实群体外，糖含量与净光合速率负相关。叶绿素含量在 1 年生三个群体中与净光合速率正相关，在 5 年生三个群体中与净光合速率负相关，叶绿素含量与净光合速率无显著相关关系。

3.3. 本章小结

核桃楸无性系 1 年生各性状的变异系数为 14.70～52.09%（平均值 29.31%），变异最小的是叶绿素相对吸光值，最大的是气孔导度。核桃楸无性系 5 年生各性状的变异系数为 6.30～35.83%（平均为 21.66%），相较 1 年生变异减少，变异最小的是胞间 CO_2 摩尔分数，最大的是气孔导度。两个年度的未结实群体叶绿素相对吸光值平均值显著高

中国知网 https://www.cnki.net

于结实群体 9.68%，净光合速率平均值显著低于结实群体 7.80%。叶绿素相对吸光值与胞间 CO_2 摩尔分数 1 年生与 5 年生群体早期与晚期一致性较好。叶绿素相对吸光值与结实量显著负相关，气孔导度与蒸腾速率、与结实量显著正相关；胞间 CO_2 摩尔分数与气孔导度、与蒸腾速率极显著正相关。

中国知网 https://www.cnki.net

4. 核桃楸无性系内源激素变异分析

已知的植物激素主要有以下 6 类：生长素、赤霉素、细胞分裂素、脱落酸、乙烯和油菜素甾醇。脱落酸是一种抑制生长的植物激素，乙烯是抑制生长的气体。油菜素甾醇的生长促进作用主要表现为加速细胞伸长[78]。不同的赤霉素生物活性不同，GA_3 的活性最高。大部分细胞分裂素在体内合成的部位是根尖，而 6-BA 合成部位是叶片。生长素（IAA）、GA_3 和 6-BA 均可以促进细胞分裂。王赵民等[79]应用 GA_3 对杉木进行喷布试验。结果表明：与对照相比，结果枝数、单果鲜重、出籽率及千粒重均有较大幅度的提高。李培旺等[80]发现叶片中 GA_3 对蓖麻主花序结实均有重要影响。郭东强等[81]发现低浓度的 IAA 有利于促进开花结实。因此开展核桃楸植物内源激素含量对无性系结实量影响的研究十分必要。

4.1. 材料与方法

4.1.1. 地点与材料

同 3.1.1、3.1.2

4.1.2. 内源激素含量的测定

2021 年 7 月底每个无性系采集南向 3 个单株健康且完整的叶片，由苏州梦熙生物公司采用高效液相色谱法检测 GA_3、6-BA 和 IAA 含量。

4.1.3. 数据处理与分析

数据分析主要包括变异分析、方差分析、独立样本 T 检验、配对样本 T 检验及相关分析（皮尔逊和斯皮尔曼），采用 Excel2016、SPSS18.0 处理与分析。

4.2. 结果与分析

4.2.1. 无性系激素变异分析

对宝龙店核桃楸 1 年生 11 个无性系 3 个内源激素含量进行分析（表 4-1），核桃楸无性系的 GA_3 含量、6-BA 含量和 IAA 含量等指标的变异幅度（平均值）分别为 0.38 μg/g FW～2.80 μg/g FW（1.14 μg/g FW）、0.17 μg/g FW～2.28 μg/g FW（0.91 μg/g FW）和 16.69 μg/g FW～213.34 μg/g FW（114.08 μg/g FW）。各项单个指标中 6-BA 含量变异系数最高为 60.48%，IAA 含量的变异系数最低为 48.84%。

对核桃楸 5 年生 11 个无性系 3 个激素含量进行研究，核桃楸无性系的 GA_3 含量、6-BA 含量和 IAA 含量等指标的变异幅度（平均值）分别为 0.41 μg/g FW～2.70 μg/g FW（1.12 μg/g FW）、0.11 μg/g FW～6.02 μg/g FW（1.07 μg/g FW）和 11.80 μg/g FW～251.95 μg/g FW（100.45 μg/g FW）。各项单个指标中 6-BA 含量变异系数最高为 99.85%，GA_3 含量的变异系数最低为 51.85%。

对核桃楸 11 个无性系 1 年生与 5 年生 3 个激素含量进行对比分析，5 年生比 1 年生

中国知网 https://www.cnki.net

核桃楸无性系 6-BA 含量高 16.86%，1 年生比 5 年生核桃楸无性系 GA_3 含量和 IAA 含量分别高 2.00%，13.56%。

表 4-1 核桃楸无性系激素变异分析

Tab.4-1 Analysis of hormone variation of *Juglans mandshurica* clones

性状	群体	极小值	极大值	均值	标准差	变异系数/%
GA_3 含量	1 年生群体	0.38	2.80	1.14	0.61	53.81
	5 年生群体	0.41	2.70	1.12	0.58	51.85
	1 年生/5 年生	0.93	1.04	1.02	1.06	1.04
6-BA 含量	1 年生群体	0.17	2.28	0.91	0.55	60.48
	5 年生群体	0.11	6.02	1.07	1.07	99.85
	1 年生/5 年生	1.55	0.38	0.86	0.52	0.61
IAA 含量	1 年生群体	16.69	213.34	114.08	55.72	48.84
	5 年生群体	11.80	251.95	100.45	64.27	63.98
	1 年生/5 年生	1.41	0.85	1.14	0.87	0.76

4.2.2. 无性系间激素显著性分析

4.2.2.1. 1 年生群体分析

（1）1 年生结实群体分析

对 1 年生结实群体进行激素性状分析，GA_3 含量无性系间差异不显著（表 4-2），GA_3 含量为 0.67 μg/g FW（DJC25）～1.73 μg/g FW（SC21），平均值为 1.25 μg/g FW，最高无性系大于平均值 38.05%。6-BA 含量无性系间差异显著，6-BA 含量为 0.77 μg/g FW（SC10）～2.08 μg/g FW（SC21），平均值为 1.31μg/g FW，最高无性系大于平均值 59.19%。IAA 含量无性系间差异不显著，IAA 含量为 108.00 μg/g FW（HL12）～161.95 μg/g FW（SC15），平均值为 136.82 μg/g FW，最高无性系大于平均值 18.37%。

表 4-2 1 年生结实群体无性系激素方差分析

Tab. 4-2 Variance analysis of annual fruit group

性状		平方和	df	均方	F	Sig
GA_3 含量	组间	2.154	4	0.538	1.479	0.280
	组内	3.641	10	0.364		
	总数	5.795	14			
6-BA 含量	组间	2.795	4	0.699	6.287	0.009
	组内	1.112	10	0.111		
	总数	3.907	14			
IAA 含量	组间	6766.797	4	1691.699	1.357	0.316
	组内	12468.775	10	1246.877		
	总数	19235.572	14			

（2）1 年生未结实群体分析

对 1 年生未结实群体进行激素性状分析，GA_3 含量无性系间差异显著（表 4-3），GA_3 含量为 0.44 μg/g FW（JST11）～1.87 μg/g FW（JST7），平均值为 1.04 μg/g FW，最高无性系大于平均值 78.81%。6-BA 含量无性系间差异显著，6-BA 含量为 0.23μg/g FW（JST8）～0.97 μg/g FW（JST6），平均值为 0.58 μg/g FW，最高无性系大于平均值 66.47%。IAA 含量无性系间差异显著，IAA 含量为 20.20 μg/g FW（JST8）～181.65 μg/g FW（JST19），平均值为 95.13 μg/g FW，最高无性系大于平均值 90.95%。

中国知网 https://www.cnki.net

表 4-3 1 年生未结实群体无性系激素方差分析
Tab. 4-3 Variance analysis of annual unfruit group

性状		平方和	df	均方	F	Sig
GA_3 含量	组间	4.249	5	0.850	6.313	0.004
	组内	1.615	12	0.135		
	总数	5.865	17			
6-BA 含量	组间	1.201	5	0.240	8.282	0.001
	组内	0.348	12	0.029		
	总数	1.549	17			
IAA 含量	组间	61409.916	5	12281.983	32.876	0.000
	组内	4483.069	12	373.589		
	总数	65892.985	17			

（3）1 年生整体群体分析

对所有无性系进行激素性状分析，11 个无性系 GA_3 含量无性系间差异显著（表 4-4），GA_3 含量为 0.44 μg/g FW（JST11）～1.87 μg/g FW（JST7），平均值为 1.14μg/g FW，最高无性系大于平均值 69.93%。6-BA 含量无性系间差异显著，6-BA 含量为 0.23 μg/g FW（JST8）～2.08 μg/g FW（SC21），平均值为 0.91 μg/g FW，最高无性系大于平均值 128.17%。IAA 含量无性系间差异显著，IAA 含量为 20.20 μg/g FW（JST8）～181.65 μg/g FW（JST19），平均值为 114.08 μg/g FW，最高无性系大于平均值 59.24%。

表 4-4 1 年生整体群体无性系激素方差分析
Tab. 4-4 Variance analysis of annual group

性状		平方和	df	均方	F	Sig
GA_3 含量	组间	6.758	10	0.676	2.829	0.02
	组内	5.256	22	0.239		
	总数	12.015	32			
6-BA 含量	组间	8.301	10	0.83	12.511	0.00
	组内	1.46	22	0.066		
	总数	9.76	32			
IAA 含量	组间	82393.988	10	8239.399	10.693	0.00
	组内	16951.844	22	770.538		
	总数	99345.832	32			

结实与未结实群体 T 检验表明，GA_3 含量在结实群体与未结实群体差异不显著（表 4-5），结实群体数值较大。6-BA 含量和 IAA 含量在 2 个群体差异显著，结实群体均值大。

表 4-5 激素 1 年生结实群体与未结实群体独立样本 T 检验
Tab.4-5 Independent sample t-test of annual fruit group and unfruit group

性状	均值差值	差分的 95%置信区间		t	df	Sig.(双侧)
		极小值	极大值			
GA_3 含量	-0.20839	-0.6508	0.23403	-0.964	29	0.34
6-BA 含量	-0.72532	-1.02444	-0.42619	-4.945	31	0.00
IAA 含量	-41.68533	-79.04977	-4.3209	-2.275	31	0.03

1 年生结实群体中，6-BA 含量无性系间差异显著，GA_3 和 IAA 含量无性系间差异

中国知网 https://www.cnki.net

不显著。未结实群体中，三种激素含量无性系间差异显著。1 年生整体群体中，三种激素含量无性系间差异显著。GA_3 含量、6-BA 含量和 IAA 含量，均为结实群体均值大。

4.2.2.2. 5 年生群体分析

（1）5 年生结实群体分析

对 5 年生结实群体进行激素性状变异分析，GA_3 含量无性系间差异不显著（表 4-6），GA_3 含量为 0.90 μg/g FW（DJC25）～1.63 μg/g FW（HL12），平均值为 1.24 μg/g FW，最高无性系大于平均值 31.60%。6-BA 含量无性系间差异不显著，6-BA 含量为 1.09 μg/g FW（SC10）～2.46 μg/g FW（DJC25），平均值为 1.47 μg/g FW，最高无性系大于平均值 66.94%。IAA 含量无性系间差异显著，IAA 含量为 18.77 μg/g FW（SC15）～184.10 μg/g FW（HL12），平均值为 110.21 μg/g FW，最高无性系大于平均值 67.05%。

表 4-6 5 年生结实群体无性系激素方差分析

Tab. 4-6 Variance analysis of five year old fruit group

性状		平方和	df	均方	F	Sig
GA_3 含量	组间	1.456	4	0.364	1.601	0.249
	组内	2.274	10	0.227		
	总数	3.73	14			
6-BA 含量	组间	3.782	4	0.946	0.418	0.792
	组内	22.637	10	2.264		
	总数	26.42	14			
IAA 含量	组间	43316.106	4	10829.027	5.466	0.013
	组内	19811.43	10	1981.143		
	总数	63127.536	14			

（2）5 年生未结实群体分析

表 4-7 5 年生未结实群体无性系激素方差分析

Tab. 4-7 Variance analysis of five year old unfruit group

性状		平方和	df	均方	F	Sig
GA_3 含量	组间	2.861	5	0.572	1.835	0.18
	组内	3.743	12	0.312		
	总数	6.604	17			
6-BA 含量	组间	1.399	5	0.28	0.857	0.537
	组内	3.918	12	0.326		
	总数	5.316	17			
IAA 含量	组间	57071.33	5	11414.266	14.594	0
	组内	9385.482	12	782.123		
	总数	66456.811	17			

对 5 年生未结实群体进行激素性状分析，GA_3 含量无性系间差异不显著（表 4-7），GA_3 含量为 0.57 μg/g FW（JST8）～1.60 μg/g FW（JST6），平均值为 1.02 μg/g FW，最高无性系大于平均值 57.29%。6-BA 含量无性系间差异不显著，6-BA 含量为 0.35 μg/g FW（JST7）～1.17 μg/g FW（JST6），平均值为 0.73 μg/g FW，最高无性系大于平均值 60.66%。IAA 含量无性系间差异显著，IAA 含量为 36.18 μg/g FW（JST8）～204.15 μg/g FW（JST13），平均值为 92.33 μg/g FW，最高无性系大于平均值

中国知网 https://www.cnki.net

121.12%。

（3）5 年生群体分析

对所有无性系进行激素变异分析，11 个无性系 GA_3 含量无性系间差异不显著（表 4-8），GA_3 含量为 0.57 μg/g FW（JST8）～1.63 μg/g FW（HL12），平均值为 1.12 μg/g FW，最高无性系大于平均值 45.61%。6-BA 含量无性系间差异不显著，6-BA 含量为 0.35 μg/g FW（JST7）～2.46 μg/g FW（DJC25），平均值为 1.07 μg/g FW，最高无性系大于平均值 130.82%。IAA 含量无性系间差异显著，IAA 含量为 18.77 μg/g FW（SC15）～204.15 μg/g FW（JST13），平均值为 100.45 μg/g FW，最高无性系大于平均值 103.23%。

表 4-8 5 年生群体无性系激素方差分析

Tab. 4-8 Variance analysis of five year old group

性状		平方和	df	均方	F	Sig
GA_3 含量	组间	4.705	10	0.471	1.721	0.139
	组内	6.016	22	0.273		
	总数	10.722	32			
6-BA 含量	组间	9.765	10	0.976	0.809	0.623
	组内	26.555	22	1.207		
	总数	36.32	32			
IAA 含量	组间	103003.865	10	10300.387	7.761	0
	组内	29196.911	22	1327.132		
	总数	132200.776	32			

结实与未结实群体 T 检验表明，GA_3 含量、6-BA 含量和 IAA 含量在结实群体与未结实群体差异不显著（表 4-9），6-BA 含量达到 6.4%的显著水平，结实群体数值较大。

表 4-9 激素处理 5 年生结实群体与未结实群体独立样本 T 检验

Tab.4-9 Independent sample t-test of five year old fruit group and unfruit group

性状	均值差值	差分的 95%置信区间		t	df	Sig.(双侧)
		极小值	极大值			
GA_3 含量	-0.21782	-0.62235	0.18671	-1.098	31	0.281
6-BA 含量	-0.7485	-1.54394	0.04694	-1.978	17.853	0.064
IAA 含量	-17.88256	-64.41814	28.65303	-0.786	29.05	0.438

对二个年度无性系 3 种激素进行配对 T 检验发现（表 4-10），三个群体差异都不显著。

5 年生结实群体中，三种激素含量无性系间差异显著。5 年生未结实群体中，GA_3 和 6-BA 含量无性系间差异不显著，IAA 含量无性系间差异显。5 年生整体群体中，GA_3 和 6-BA 含量无性系间差异不显著，IAA 含量无性系间差异显著。GA_3 含量、6-BA 含量和 IAA 含量，均为结实群体均值大。

中国知网 https://www.cnki.net

表 4-10 激素处理 1 年生和 5 年生群体配对 T 检验

Tab.4-10 Paired t-test of annual group and five year old group

	性状	均值	标准差	差分的 95%置信区间		t	df	Sig.(双侧)
				极小值	极大值			
整体	GA_3	0.02	0.79	-0.26	0.30	0.16	32	0.87
	6-BA	-0.15	1.13	-0.55	0.25	-0.79	32	0.44
	IAA	13.62	83.75	-16.07	43.32	0.93	32	0.36
未结实群体	GA_3	0.03	0.96	-0.45	0.50	0.12	17	0.91
	6-BA	-0.14	0.62	-0.45	0.16	-0.98	17	0.34
	IAA	2.80	87.13	-40.53	46.13	0.14	17	0.89
结实群体	GA_3	0.02	0.56	-0.29	0.33	0.12	14	0.91
	6-BA	-0.17	1.56	-1.03	0.70	-0.41	14	0.69
	IAA	26.61	80.52	-17.98	71.19	1.28	14	0.22

4.2.3. 核桃楸早晚及性状相关分析

4.2.3.1. 早期与晚期相关分析

对 1 年生与 5 年生群体各性状进行皮尔逊相关分析（表 4-11），GA_3 含量在结实群体正相关显著，相关系数为 0.55。斯皮尔曼相关结果表明，6-BA 含量在整体水平早晚正相关极显著，相关系数为 0.745，GA_3 含量在结实群体正相关显著，相关系数为 0.9。

表 4-11 1 年生与 5 年生群体激素早期与晚期相关分析

Tab.4-11 Hormone correlation analysis of annual group and five year old group

性状	整体群体		未结实群体		结实群体	
	皮尔逊	斯皮尔曼	皮尔逊	斯皮尔曼	皮尔逊	斯皮尔曼
GA_3 含量	0.117	0.2	-0.258	-0.143	0.55*	0.9*
6-BA 含量	0.145	0.745**	0.062	0.029	-0.187	0.4
IAA 含量	0.031	-0.218	0.025	0.2	-0.12	-0.7

**在 0.01 水平（双侧）上显著相关。

*在 0.05 水平（双侧）上显著相关。

4.2.3.2. 性状相关分析

（1）核桃楸 1 年生群体分析

为了解核桃楸激素与其他性状间的相关关系，对核桃楸 **1 年生**结实群体各性状进行相关分析（表 3-13)，除胞间 CO_2 摩尔分数与 GA_3 含量、6-BA 含量、IAA 含量和嫁接成活率呈正相关，其他性状与 GA_3 含量、6-BA 含量、IAA 含量和嫁接成活率均呈负相关。

在未结实群体中（表 3-14)，气孔导度与 6-BA 含量正相关显著，GA_3 含量与嫁接成活率正相关显著可溶性蛋白含量，可溶性糖含量，净光合速率，气孔导度和胞间 CO_2 摩尔分数与 GA_3 含量、6-BA 含量、IAA 含量和嫁接成活率呈正相关。

在整体群体中（表 3-15)，叶绿素相对吸光值与 GA_3 含量负相关显著，净光合速率与 6-BA 含量正相关极显著，与 IAA 含量正相关显著，6-BA 含量与气孔导度，蒸腾速率正相关显著，6-BA 含量与净光合速率，IAA 含量正相关极显著。

（2）核桃楸 5 年生群体分析

中国知网 https://www.cnki.net

为了解核桃楸激素与其他性状间的相关关系，对核桃楸**1 年生**结实群体各性状进行相关分析（表 3-16)，四个光合指标与 GA_3 含量和结实量正相关，与 6-BA 含量负相关

在未结实群体中（表 3-17)，可溶性蛋白含量与 IAA 含量负相关显著（表 33）。四个光合指标与 GA_3 含量和与 IAA 含量负相关。

在整体群体中（表 3-18)，可溶性蛋白含量与 GA_3 含量正相关显著（表 34）。净光合速率与三种激素负相关，其中与 6-BA 含量达到了显著水平。胞间 CO_2 摩尔分数，蒸腾速率与三种激素正相关。

4.3. 本章小结

5 年生群体与 1 年生群体均为 6-BA 含量变异系数最高，5 年生比 1 年生核桃楸无性系的 6-BA 含量高 16.86%，5 年生比 1 年生核桃楸无性系的 GA_3 含量和 IAA 含量低 1.96%和 11.94%。1 年生群体和 5 年生群体中三种激素含量均表现为结实群体数值较大。GA_3 含量在结实群体早晚正相关显著，GA_3 含量与嫁接成活率负相关，既 GA_3 含量越高的无性系嫁接成活率越低。1 年生群体中，除结实群体外，其他群体激素含量与气孔导度、胞间 CO_2 摩尔分数和蒸腾速率均为正相关；IAA 含量与可溶性蛋白含量、叶绿素相对吸光值和净光合速率在三个群体中均为负相关，除结实群体 IAA 含量与 GA_3 含量负相关外，三种激素之间均为正相关；5 年生三个群体中，三种激素与叶绿素相对吸光值和净光合速率均为负相关。

中国知网 https://www.cnki.net

5. 核桃楸无性系子代生长变异分析

了解亲本与子代间性状相关性有助于提高选择效率、缩短育种周期。王娟娟[82]对油松优树子代苗进行相关分析发现结实量与树高与胸径均呈正相关。赵祜[8]以青海云杉一代无性系种子园中筛选出的 18 个优良无性系 90 个分株为材料进行相关分析，青海云杉单株结实量与树冠高呈极显著正相关。

5.1. 材料与方法

5.1.1. 材料

2016 年定植的子代林中找到与 2020、2021 年嫁接系号相关的六个子代林区有 130 个无性系子代，其中与 2020、2021 年嫁接系号对应的有 48 个无性系：BX1、BX2、BX4、BX13、BX16、BX20、BX22、BX25、BX27、BX28、DJC1、DJC8、DJC11、DJC18、DJC21、DJC25、HL1、HL3、HL18、HL25、JST9、JST14、JST18、JST19、JY24、ML2、ML5、ML13、ML22、ML23、ML24、ML25、SC12、SC15、SC21、SC23、WC7、WC8、WC17、WC23、WC24、WC30、YBL1、YBL5、YBL7、YBL11、YBL19 和 YBL20。与 2016 年嫁接系号对应的有 19 个无性系：DJC8、DJC11、DJC12、DJC18、DJC25、HL18、HL19、HL22、HL25、JST9、JST19、SC19、SC21、WC7、WC8、WC9、WC10、WC17 和 WC30。

5.1.2. 调查方法

嫁接成活率的调查：对 2020 年 137 个无性系和 2021 年 32 个无性系的嫁接成活率进行调查。

树高与胸径的调查：对核桃楸子代林 2019 与 2020 年各区无性系子代树高及胸径进行每木调查。为了使每个小区调查数据具有可比性，2019 与 2020 年无性系子代树高及胸径都是除以该小区对照值后的比值。

结实量的调查：对结实初期（嫁接 5 年）无性系 2020 年结实量进行每株调查。

5.1.3. 数据处理与分析

数据分析主要包括变异分析、方差分析及皮尔逊相关分析，采用 Excel2016 和 SPSS18.0 处理与分析。嫁接成活率等百分率性状经过反正弦转换后进行分析。

5.2. 结果与分析

5.2.1. 无性系子代整体生长变异分析

5.2.1.1. 无性系子代树高、胸径变异分析

对 6 个子代林区 130 个无性系子代进行分析，2019、2020 年树高及胸径的变异幅度（平均值）分别为 1.06m～1.09m（1.08m）、1.07m～1.10m（1.09m）、0.57cm～0.61cm（0.59cm）和 0.87cm～0.91cm（0.89cm）。变异系数最大的是 2019 年胸径

中国知网 https://www.cnki.net

（91.31%），最小的是 2020 年树高（32.43%）。

表 5-1 树高与胸径变异分析

Tab.5-1 Variation analysis of tree height and DBH in 2019 and 2020

性状	N	差分的 95%置信区间		均值	标准差	变异系数/%
		极小值	极大值			
2019 年树高/m	2233	1.06	1.09	1.08	0.37	34.35
2020 年树高/m	2233	1.07	1.10	1.09	0.35	32.43
2019 年胸径/cm	2227	0.57	0.61	0.59	0.54	91.31
2020 年胸径/cm	2232	0.87	0.91	0.89	0.48	54.53

5.2.1.2. 无性系子代树高及胸径差异显著性分析

对 2019、2020 年树高及胸径进行分析，2019、2020 年年树高及胸径无性系子代间差异显著（表 5-2），2019、2020 年树高与胸径分别为为 0.69m（ML13）～1.62m（QY9）、0.71cm（ML13）～1.61cm（YBL7）、0.64m（五常 10）～1.46m（YBL7）和 0.49cm（JST29）～1.54cm（YBL7）。其中 ML13 的 2019、2020 年树高与胸径较小，YBL7 的 2019 和 2020 年树高与胸径较大。

表 5-2 树高与胸径方差分析

Tab.5-2 Variation analysis of tree height and DBH in 2019 and 2020

性状		平方和	df	均方	F	Sig
2019 年树高/m	组间	69.559	129	0.539	4.804	0.000
	组内	236.023	2103	0.112		
	总数	305.582	2232			
2020 年树高/m	组间	63.364	129	0.491	4.837	0.000
	组内	213.549	2103	0.102		
	总数	276.913	2232			
2019 年胸径/cm	组间	162.148	129	1.257	5.401	0.000
	组内	488.02	2097	0.233		
	总数	650.168	2226			
2020 年胸径/cm	组间	108.721	129	0.843	4.277	0.000
	组内	414.244	2102	0.197		
	总数	522.965	2231			

5.2.2. 48 个无性系对应子代林生长变异分析

5.2.2.1. 无性系子代生长变异分析

表 5-3 相关群体树高与胸径变异分析

Tab.5-3 Variation analysis of tree height and DBH of relevant group in 2019 and 2020

性状	N	差分的 95%置信区间		均值	标准差 deviation	变异系数/% variation
		极小值	极大值			
2019 年树高/m	879	1.04	1.09	1.06	0.38	35.36
2020 年树高/m	879	1.05	1.09	1.07	0.36	33.62
2019 年胸径/cm	879	0.52	0.59	0.56	0.54	98.03
2020 年胸径/cm	879	0.82	0.88	0.85	0.50	58.25

2019、2020 年树高及胸径的变异幅度（平均值）分别为 1.04m～1.09m（1.06m）、1.05m～1.09m（1.07m）、0.52cm～0.59cm（0.56cm）和 0.82cm～0.88cm（0.85cm）。变

中国知网 https://www.cnki.net

异系数最大的是 2019 年胸径（98.03%），最小的是 2020 年树高（33.62%）。

5.2.2.2. 无性系子代生长差异显著性分析

对 2019、2020 年树高及胸径进行分析，2019、2020 年树高与胸径无性系子代间差异显著（表 5-4），2019、2020 年树高与胸径分别为为 0.69m（ML13）～1.62m（YBL7）、0.71m（ML13）～1.61m（YBL7）、0.66cm（ML22）～1.46cm（YBL7）和 0.69cm（ML13）～1.54cm（YBL7）。其中 HL18 无性系嫁接成活率低，2019、2020 年树高及胸径高，五常 17 无性系嫁接成活率高，2019、2020 年树高及胸径高低。

表 5-4 相关群体树高与胸径方差分析

Tab.5-4 Variation analysis of tree height and DBH of relevant group in 2019 and 2020

性状		平方和	df	均方	F	Sig
2019 年树高/m	组间	29.93	47	0.637	5.614	0
	组内	94.256	831	0.113		
	总数	124.185	878			
2020 年树高/m	组间	26.821	47	0.571	5.469	0
	组内	86.713	831	0.104		
	总数	113.534	878			
2019 年胸径/cm	组间	55.403	47	1.179	4.773	0
	组内	205.234	831	0.247		
	总数	260.636	878			
2020 年胸径/cm	组间	47.296	47	1.006	4.955	0
	组内	168.772	831	0.203		
	总数	216.068	878			

5.2.2.3. 无性系成活率与无性系子代生长相关分析

嫁接成活率与 2019、2020 年树高及胸径呈负相关，其中除 2019 年树高与胸径外达到了显著水平。即嫁接成活率较高系号的早期生长较慢。

表 5-5 生长性状相关分析

Tab.5-5 Trait correlation analysis

性状	2020 年树高/m	2019 年胸径/cm	2020 年胸径/cm	嫁接成活率
2019 年树高/m	0.927**	0.828**	0.842**	-0.154*
2020 年树高/m		0.786**	0.886**	-0.155*
2019 年胸径/cm			0.818**	-0.141
2020 年胸径/cm				-0.157*

**在 0.01 水平（双侧）上显著相关。

*在 0.05 水平（双侧）上显著相关。

5.2.3. 19 个无性系对应子代林生长变异分析

5.2.3.1. 无性系子代林变异分析

2019、2020 年树高与胸径的变异幅度（平均值）分别为 1.06m～1.14m（1.10m）、1.05m～1.13m（1.09m）、0.54cm～0.66cm（0.60cm）和 0.84cm～0.94cm（0.89cm）。变异系数最大的是 2019 年胸径（91.99%），最小的是 2020 年树高（33.44%）。

中国知网 https://www.cnki.net

表 5-6 2 无性系子代树高与胸径变异分析

Tab.5-6 Variation analysis of tree height and DBH of families in 2019 and 2020

性状	差分的 95%置信区间		均值	标准差	变异系数/%
	极小值	极大值			
2019 年树高/m	1.06	1.14	1.10	0.39	35.54
2020 年树高/m	1.05	1.13	1.09	0.37	33.44
2019 年胸径/cm	0.54	0.66	0.60	0.55	91.99
2020 年胸径/cm	0.84	0.94	0.89	0.49	54.91

5.2.3.2. 无性系子代林差异显著性分析

对 2019、2020 年树高与胸径进行分析，2019、2020 年树高与胸径无性系子代间差异显著（表 5-7），2019、2020 年树高与胸径分别为为 0.77m（JST9）～1.46m（WC10）、0.76m（JST9）～1.40m（WC10）、0.33cm（JST9）～0.93cm（WC10）和 0.56cm（DJC18）～1.26cm（WC30）。其中 DJC25 无性系结实量最高，除 2019 年胸径第二高，其他生长性状均最高。

表 5-7 无性系子代树高与胸径方差分析

Tab.5-7 Variation analysis of tree height and DBH of families in 2019 and 2020

性状		平方和	df	均方	F	Sig
2019 年树高/m	组间	12.343	18	0.686	5.620	0.000
	组内	39.163	321	0.122		
	总数	51.506	339			
2020 年树高/m	组间	11.707	18	0.650	6.201	0.000
	组内	33.665	321	0.105		
	总数	45.371	339			
2019 年胸径/cm	组间	21.486	18	1.194	4.733	0.000
	组内	80.952	321	0.252		
	总数	102.438	339			
2020 年胸径/cm	组间	14.326	18	0.796	3.821	0.000
	组内	66.855	321	0.208		
	总数	81.181	339			

对 WC10、SC21、HL22、HL18 和 DJC25 共 5 个结实无性系与对应无性系子代进行相关分析。结实量与 2019、2020 年树高与胸径呈正相关，其中与 2019 年树高及胸径正相关显著，与 2020 年胸径达到了极显著水平。即结实量较多系号的早期生长也较快。

表 5-8 家系树高与胸径相关分析

Tab.5-8 Correlation analysis of family tree height and DBH in 2019 and 2020

性状	2020 年树高/m	2019 年胸径/cm	2020 年胸径/cm	结实量
19 年树高	0.951**	0.733**	0.834**	0.949*
19 年胸径		0.729**	0.849**	0.922*
20 年树高			0.850**	0.736
20 年胸径				0.970**

**在 0.01 水平（双侧）上显著相关

*在 0.05 水平（双侧）上显著相关

中国知网 https://www.cnki.net

5.3. 本章小结

6 个子代林区 130 个家系 2019、2020 年树高与胸径家系间差异显著，其中 ML13 的 2019、2020 年树高与胸径较小，YBL7 的 2019、2020 年树高与胸径较大，嫁接成活率为 92.73%。变异系数最大的是 2019 年胸径（91.31%），最小的是 2020 年树高（32.43%）。48 个无性系 2019、2020 年树高与胸径家系间差异显著，变异系数最大的是 2019 年胸径（98.03%），最小的是 2020 年树高（33.62%）。其中 HL18 无性系嫁接成活率低，2019、2020 年树高及胸径高，五常 17 无性系嫁接成活率高，2019、2020 年树高及胸径高低。19 个无性系 2019、2020 年树高与胸径家系间差异显著，变异系数最大的是 2019 年胸径（91.99%），最小的是 2020 年树高（33.44%）。其中 DJC25 无性系结实量最高，除 2019 年胸径第二高，其他生长性状均最高。相关分析中，嫁接成活率与 2019、2020 年树高与胸径呈负相关，其中除 2019 年胸径外均达到了显著水平。即嫁接成活率较高系号的早期生长较慢。对 5 个结实系号进行相关分析的，结实量与 2019、2020 年树高与胸径呈正相关。即结实量较多系号的早期生长也较快。

中国知网 https://www.cnki.net

讨论

在嫁接期间添加 NAA 激素可以提高嫁接成活率，这与张健等[84]2018 年在海南做的油茶嫁接试验及赵琳等[85]2010 年在云南做的白花油茶嫁接试验得到的结论一致。添加 IBA 激素可以提高嫁接成活率，这与 1999 年董高峰等[86]在广东对沙田柚进行的嫁接试验的结论一致。袁婷婷等[87]2014 年采用正交试验设计，研究了激素对油茶芽苗砧嫁接愈合及嫁接苗成活的影响，结果表明：在嫁接期间施加 IBA 激素提高嫁接成活率，这与本试验结论一致。邓朝佐等[88]在 1986 年对金银花进行嫁接试验也得到了相同的结论。郑颖等[89]2020 年对蒙古栎进行嫁接试验后认为 IBA 是一种激素,能促进伸长生长、诱导和促进植物细胞分化,促进切口愈伤组织的形成,从而有利于接口的愈合,提高嫁接成活率。滕贵波等[90]2012 年为提高美国红枫嫁接成活率，进行了以萘乙酸(NAA)和吲哚丁酸(IBA)处理接穗的嫁接试验后得到的结论相同。赵翔[91] 在广东对灰木莲进行嫁接繁殖技术的研究后，认为在嫁接期间添加 NAA 和 IBA 激素后可以提高嫁接成活率，与本试验结论相同。而添加 GA_3 激素会降低嫁接成活率，与唐辉等[92]2007 年做的银杏嫁接成活率试验得出的结论一致。黄坚钦等[69]通过田间试验及数学分析方法，分析了山核桃嫁接成活的影响因子。结果表明：激素可以提高嫁接成活率。王白坡等[70]在 2002 年对山核桃研究进行综述认为激素可以提高嫁接成活率。

从性状相关性分析中可以发现，1 年生群体中可溶性蛋白含量与嫁接成活率均负相关，既可溶性蛋白含量越高，嫁接成活率越低。周恩强等[42]于 2008 年对核桃子苗嫁接后测定和分析生理生化指标进行研究，得出可溶性蛋白质含量是降低嫁接成活率的主要因素之一。

本文试验地点都是在宝龙店林场，环境影响较小，主要还是无性系的遗传因素，本文旨在比较两组材料之间的差异。这与贾庆彬等[93]对不同林龄（18、23、27 a）时期的红松进行优良家系选择的方法相近。叶绿素相对吸光值和胞间 CO_2 摩尔分数早期与晚期相关均到达显著极显著水平，高红霞等[94]2011 年对红砂家系进行叶绿素含量测定，通过早期选择筛选 12 个家系为抗旱性优良家系。柴文敏等[95]2011 年对唐古特白刺以叶绿素含量为标准做早期选择，筛选出 5 个家系作为抗旱优良家系。2008 年李森[96]在四川通过对光合生理的研究，认为叶绿素含量可作为核桃品种选育的参考指标。叶绿素含量早期与晚期相关显著，胞间 CO_2 摩尔分数与净光合速率负相关，这与林兵等[97]2018 年对天然胡杨林的研究结果一致。

在 5 年生与 1 年生群体三种内源激素均表现为结实群体高于未结实群体。GA_3 含量与嫁接成活率正相关。早期 GA_3 含量高的无性系嫁接成活率高。秦柏婷等[59]研究发现，核桃楸果实发育过程中赤霉素含量逐渐升高，较之前相比增长幅度很大，张旭等人[62]发现赤霉素处理可以提高了无花果座果率。除结实群体外，其他群体中三种激素之间均为正相关；IAA 与嫁接成活率正相关，这与蒋丽娟等[98] 2008 年对光皮树研究后得出

中国知网 https://www.cnki.net

的结论一致。

无性系嫁接成活率与 2019、2020 年家系树高及胸径呈负相关，其中除 2019 年胸径外达到了显著水平，即嫁接成活率较高系号的早期生长较慢。这与辛建华等[99]和王华荣等[100]对楸树的结论一致。对 5 个结实系号进行相关分析的，结实量与 2019、2020 年树高及胸径呈正相关。即结实量较多系号的早期生长也较快。这与张鑫鑫等[101]2016 年对长白落叶松研究后得出生长性状之间及结实量与生长性状间的高相关系数的结论一致。李嘉琪等[24]2017 年对樟子松进行研究也得到了生长性状与结实量正相关的结论，其中胸径和结实量到达了显著水平。黄宇[102]2015 年对木荷进行生长、结实量及果实特征调查发现生长性状与结实量正相关。陈苏英等[103] 2012 年通过对杉木生长和球果特征的调查发现树高胸径与结实量呈正相关。林能庆[104] 2012 年对马尾松调查分析得出生长性状与当年结实量呈正相关的结论。

中国知网 https://www.cnki.net

结论

（1）不同激素种类嫁接成活率差异显著，不同激素浓度与不同接穗催醒时间嫁接成活率差异不显著。NAA、IBA 处理接穗会提高嫁接成活率，GA_3 处理会降低嫁接成活率。用阔叶树锯末当基质可以提高嫁接成活率但于针叶树锯末差异不显著。提高嫁接成活率的最佳组合为激素种类 IBA、激素质量浓度 50mg/g、接穗催醒时间 1 天、基质种类阔叶树锯末基质。

（2）叶绿素相对吸光值与胞间 CO_2 摩尔分数早晚一致性好，是早期与晚期相关和早期选择的重要性状。结实量与叶绿素相对吸光值负相关，与可溶性蛋白含量、可溶性糖含量、净光合速率、气孔导度、胞间 CO_2 摩尔分数、蒸腾速率、GA_3 含量及 6-BA 含量正相关；叶绿素相对吸光值与可溶性蛋白含量、可溶性糖含量、净光合速率、气孔导度、胞间 CO_2 摩尔分数、蒸腾速率及 6-BA 含量正相关，能够进行间接选择。

（3）1 年生群体和 5 年生群体中三种激素含量均表现为结实群体数值较大。GA_3 含量在结实群体早晚正相关显著，GA_3 含量与嫁接成活率正相关。早期 GA_3 含量高的无性系嫁接成活率高，未来的结实量也高。

（4）无性系嫁接成活率与 2019、2020 年家系树高及胸径呈负相关，其中除 2019 年胸径外达到了显著水平，即嫁接成活率较高系号的早期生长较慢。对 5 个结实系号进行相关分析的，结实量与 2019、2020 年树高及胸径呈正相关。即结实量较多系号的早期生长也较快。

中国知网 https://www.cnki.net

参考文献

[1] 王东娜，牟长城，冯富娟．核桃楸 ISSR-PCR 反应体系的建立及优化[J].实验室研究与探索, 2010, 29(11): 18-22.

[2] 褚宪丽，朱航勇，张含国等．核桃楸种源家系变异与选择[J].东北林业大学学报，2010, 38(11): 5-7.

[3] 朱红波，赵云，林士杰等．核桃楸资源研究进展 [J].中国农学通报, 2011, 27(25)；1-4.

[4] 曹政．核桃楸[J]．农业科学实验, 1982, 44-45.

[5] 王宇．东北地区核桃楸遗传多样性 SRAP 研究[D]．哈尔滨：东北林业大学, 2007.

[6] Shikai Zhang *et al*. Genetic Improvement in *Juglans mandshurica* and Its Uses in China: Current Status and Future Prospects[J]. Phyton (Buenos Aires), 2022, 91(3) : 489-505.

[7] Zhang, Qinhui *et al*. Correction to: Within- and between-population variations in seed and seedling traits of *Juglans mandshurica*[J]. Journal of Forestry Research, 2021: 1-1.

[8] Growth and Management of Black Walnut (*Juglans nigra L.*) on Strip-Mined Lands in Southeastern Kansas[J]. Transactions of the Kansas Academy of Science (1903-), 1970, 73(4):

[9] Zhang Heng *et al*. Climate response of radial growth and early selection of *Larix olgensis* at four trials in northeast China[J]. Dendrochronologia, 2022, 73

[10]马常耕，林静芳，李明鹤等．池杉速生无性系早期选择的研究[J]．林业科学, 1979(03): 194-198.

[11]Jean Marc Kaumbu Kyalamakasa *et al*. Early Selection of Tree Species for Regeneration in Degraded Woodland of Southeastern Congo Basin[J]. Forests, 2021, 12(2) : 117-117.

[12]颜廷武，于世河，王骞春等．辽宁地区核桃楸半同胞家系间苗期生长差异分析[J].辽宁林业科技, 2020(06): 13-15.

[13]Zhang, Qinhui *et al.* Within- and between-population variations in seed and seedling traits of *Juglans mandshurica*[J]. Journal of Forestry Research, 2021, : 1-12.

[14]尤海舟，郭福忠，金长谦等．核桃楸不同种源种子形态、质量及苗期生长研究[J]．江苏农业科学, 2020, 48(02): 155-158.

[15]陈思羽，杨辉，韩姣等．长白山区核桃楸结实性状种源变异分析[J]．北京林业大学学报, 2015, 37(12): 32-40.

[16]袁显磊，祁永会，刘忠玲等．核桃楸种源选择试验及其环境因子的影响[J].植物研究, 2013, 33(04): 468-476.

[17]芦贤博，徐连峰，庞忠义等．核桃楸种源家系幼龄期生长变异及选择研究[J].林业科学研究, 2022, 35(01): 20-30.

中国知网 https://www.cnki.net

[18]韩玉霞，吴琳，于琪等. 39 个核桃楸家系苗期生长性状变异分析[J].吉林林业科技, 2020, 49(06): 1-4.
[19]张含国，邓继，张磊等. 核桃楸种源家系变异规律及家系选择研究[J].西北林学院学报, 2011, 26(2): 91-95.
[20]常君，任华东，姚小华等. 41 个薄壳山核桃品种果实营养成分与脂肪酸组成的比较分析[J].西南大学学报(自然科学版), 2021, 43(02): 20-30.
[21]张海啸，李爱清，张含国等. 核桃楸种实性状变异规律及优良单株选择[J].东北林业大学学报, 2017, 45(03): 1-7.
[22]Zhen Zhang *et al*. Clonal variations in nutritional components of Pinus koreansis seeds collected from seed orchards in Northeastern China[J]. Journal of Forestry Research, 2016, 27(2) : 295-311.
[23]S.D. Sharma, O.C. Sharma. Selection of superior Persian walnut (*Juglans regia L*.) from a seedling population in Himachal Pradesh[J]. Advances in Horticultural Science, 2000, 14(4):
[24]李嘉琪，韩喜东，马盈慧等. 樟子松无性系生长性状与结实量变异研究[J].植物研究, 2020, 40(02): 217-223.
[25]方乐金，施季森. 杉木种子园无性系结实稳定性的遗传变异[J].南京林业大学学报(自然科学版), 2004(01): 17-20.
[26]梁一池，黄铭利. 锥栗优良无性系结实稳定性的研究[J]. 福建林学院学报，1998(03): 1-5.
[27]Habibi Fariborz, Liu Tie, Folta Kevin, Sarkhosh Ali. Physiological, biochemical, and molecular aspects of grafting in fruit trees. [J]. Horticulture research, 2022.
[28]Rasool Aatifa, Mansoor Sheikh, Bhat K. M. *et al*. Mechanisms Underlying Graft Union Formation and Rootstock Scion Interaction in Horticultural Plants[J]. Frontiers in Plant Science, 2020, 11.
[29]Aziz Ebrahimi and Kourosh Vahdati and Esmaeil Fallahi. Improved Success of Persian Walnut Grafting Under Environmentally Controlled Conditions[J]. International Journal of Fruit Science, 2007, 6(4) : 3-12.
[30]肖玉璞，刘宏伟，张淑华. 核桃楸嫁接试验初报[J] . 中国林副特产, 2015, No.139, 51-52.
[31]翁春余，邵慰忠，叶浩然等. 薄壳山核桃 17 个无性系嫁接试验[J]. 浙江林业科技, 2012, v. 32, 38-41.
[32]王红娟，段安安，蒋艳. 晋宁县引种元宝枫优良无性系嫁接苗木的年生长节律研究[J]. 西部林业科学, 2013, 42(02): 73-77.
[33]黄佳聪，吴建花，尹光顺等. 滇橄榄不同无性系嫁接试验研究[J]. 西部林业科学, 2017, 46(05): 68-72.

中国知网 https://www.cnki.net

[34]戴承喜，王旭军，余亚玲等. 大叶榉不同无性系嫁接效果比较[J]. 湖南林业科技, 2016, 43(06): 18-21.

[35]乔谦，丰震，李承水. 元宝枫无性系嫁接成活率及其春梢性状分析[J]. 山东农业科学, 2016, 48(05): 45-48.

[36]何芳婷，唐炜祁，陈顺秀等. 优良无性系油茶芽苗砧嫁接比较试验[J]. 现代农业科技, 2016(12): 165+178.

[37]王瑞，陈永忠，陈隆升等. 油茶优良无性系芽苗砧嫁接技术体系的研究[J]. 中南林业科技大学学报, 2013, 33(07): 77-80.

[38]李正银，蒋德惠，丁永平等. 昭通市核桃优树无性系 1 年生嫁接苗生长及变异研究[J]. 林业调查规划, 2020, 45(03): 158-164.

[39]孙铭浩，王加彬，王芬. 青檀优良无性系 TX01 嫁接育苗试验[J]. 林业科技通讯,2016(06) : 29-31.

[40]谭飞燕，蒋华，黄寿先等. 中国马褂木无性系嫁接繁殖性状变异[J]. 广东农业科学, 2013, 40(05): 45-47.

[41]樊光辉. 40 个杜仲无性系嫁接成活率与接芽萌发枝高生长量的比较试验[J]. 西北林学院学报, 2008(02) : 91-94.

[42]周恩强，王俊明，樊金拴等. 影响核桃嫁接成活因子分析[J]. 陕西林业科技, 2012, (2): 28.

[43]郑炳松，刘力，黄坚钦等. 山核桃嫁接成活的生理生化特性分析[J]. 福建林学院学报, 2002, 22(4): 320-324.

[44]汤睿，刘静波，刘劲等. 中国核桃嫁接繁殖技术研究进展[J]. 农学学报，2017，7(08): 60-65.

[45]蒲光兰，肖千文，赖腾跃等. 核桃砧木、穗条内含物及其与嫁接成活率的关系[J]. 陕西师范大学学报(自然科学版), 2016, 44(02): 79-84.

[46]Anket Sharma, Bingsong Zheng. Molecular Responses during Plant Grafting and Its Regulation by Auxins, Cytokinins, and Gibberellins[J]. Biomolecules, 2019, 9(9).

[47]Saravana Kumar R M, Gao Liu Xiao, Yuan Hu Wei, Xu Dong Bin, Liang Zhao, Tao Shen Chen, Guo Wen Bin, Yan Dao Liang, Zheng Bing Song, Edqvist Johan. Auxin enhances grafting success in Carya cathayensis (Chinese hickory). [J]. Planta, 2018, 247(3).

[48]Zhai, L., Wang, X., Tang, D. *et al*. Molecular and physiological characterization of the effects of auxin-enriched rootstock on grafting. Hortic Res 8, 74 (2021).

[49]Li Wei *et al*. Elevated auxin and reduced cytokinin contents in rootstocks improve their performance and grafting success. [J]. Plant biotechnology journal, 2017, 15(12) : 1556-1565.

中国知网 https://www.cnki.net

[50]赵金秀. 大规格牡丹高枝嫁接试验研究[J]. 山东林业科技, 2021, 51(03): 46-47+45.
[51]罗兰芳. 沉水樟嫁接技术研究[J]. 绿色科技, 2020(17): 90-91+95.
[52]季树泉. 激素处理接穗切面提高油橄榄嫁接成活降[J]. 四川林业科技通讯, 1979(03): 11-12.
[53]刘剑斌. 千年桐嫁接育苗技术的初步研究[J]. 林业勘察设计, 2012(01): 161-163.
[54]宫永红, 赵宝军, 刘枫. 北方地区核桃良种室内嫁接繁育技术[J]. 北方园艺, 2015(10): 65-67.
[55]金丽丽, 孙龙生, 刘万生. 核桃冬季室内嫁接育苗技术[J]. 北方园艺, 2009(02): 165-166.
[56]王仕海, 陈琦, 赵宝军等. 核桃冬季室内嫁接育苗技术[C]. 中国园艺学会干果分会成立大会暨第二届全国干果生产与科研进展学术研讨会论文集, 2001: 180-183.
[57]赵宝军, 宫永红. 核桃冬季室内嫁接技术[J]. 林业科技通讯, 1997(01): 34.
[58]赵亚辉, 裴更生. 优质核桃室内嫁接繁育技术[J]. 现代农业科技, 2010(24): 123-124+127.
[59]秦柏婷, 蔡佳友, 傅靖棋等. 核桃楸不同发育器官内源激素的动态变化[J/OL]. 分子植物种: 1-9[2021-12-30].
[60]Nanda Amrit K, Melnyk Charles W. The role of plant hormones during grafting. [J]. Journal of plant research, 2018, 131(1).
[61]宋福南, 杨传平, 刘雪梅. 白桦雌花发育过程中内源激素动态变化[J]. 植物生理学通讯, 2006(03): 465-466.
[62]张旭, 柴丽娟, 李艳美等. GA_3 对无花果秋果内源激素水平的影响[J]. 中国农业大学报, 2015, 20(03): 65-72.
[63]王芳, 王元兴, 王成录等. 红松优树半同胞子代家系生长、结实及抗病虫能力的变异特征[J]. 应用生态学报, 2019, 30(05): 1679-1686.
[64]王庆娜. 红松种子园优良亲本及子代评价选择[D]. 东北林业大学, 2017.
[65]杨俊明, 李盼威. 华北落叶松无性系结实能力变异与无性系再选择[J]. 河北科技师范学院学报, 2004(02): 32-35.
[66]Li Zhixin, Wang Weihuai, Zhang Haixiao, Liu Jinhong, Shi Baoying, Dai Weizhao, Liu Kewu, Zhang Hanguo. Diversity in Fruit Morphology and Nutritional Composition of *Juglans mandshurica Maxim* in Northeast China [J]. Frontiers in Plant Science, 2022, 13.
[67]YAŞAR AKÇA. Determination of Fruit Growth and Development in Walnut[J]. TURKISH JOURNAL OF AGRICULTURE AND FORESTRY, 2000, 24(3) : 349-354.

中国知网 https://www.cnki.net

[68]Kavosi Hojatollah and Khadivi Ali. The selection of superior late-leafing genotypes of Persian walnut (*Juglans regia L.*) among seedling originated trees based on pomological characterizations[J]. Scientia Horticulturae, 2021, 288.
[69]黄坚钦，方伟，丁雨龙等. 影响山核桃嫁接成活的因子分析[J]. 浙江林学院学报, 2002(03): 3-6.
[70]王白坡，程晓建，喻卫武. 山核桃嫁接育苗成活率探讨[J]. 浙江林学院学报, 2002(03): 7-10.
[71]张露荷，黄华梨，张广忠等. 甘肃沿黄灌区 30 个枣树品种光合及水分利用特性[J]. 经济林研究, 2018, 36(04): 160-164.
[72]刘博，黄华梨，王多锋等. 覆盖方式对干热河谷区枣园土壤温度和树体光合特性及果实品质的影响[J]. 西北农业学报, 2021, 30(03): 377-385.
[73]王力刚，韩海成，赵岭等. 黑龙江省西部半干旱区主要造林树种光合参数比较及相关关系研究[J]. 防护林科技, 2010(05): 14-18.
[74]吴孝红，胡鑫，汪贵斌等. 两种盐胁迫对苦楝幼苗光合特性的影响[J]. 北方园艺, 2019(20): 75-81.
[75]王云鹏，张蕊，周志春等. 木荷优树自由授粉家系早期生长性状遗传变异动态规律[J]. 林业科学, 2020, 56(09): 77-86.
[76]苗清丽. 杂种落叶松不同年龄化学成份变异研究及早期选择[D]. 东北林业大学, 2017.
[77]王丽云，蒋丽娟，张冬林等. 不同乌桕幼苗生长特性及光合指标日变化规律研究[J]. 湖南林业科技, 2011, 38(06): 50-53.
[78]李根，张然，王雨藤等. 油菜素甾醇调控植物生长发育及非生物胁迫的研究进展[J/OL]. 分子植物育种: 1-22[2022-03-26].
[79]王赵民，吴隆高，王嫩良等. GA_3 等 3 种植物生长调节剂对杉木结实和种子品质的影响[J]. 林业科技通讯, 1993(09): 27-29.
[80]李培旺，陈景震，张良波等. 蓖麻叶片内源激素变化对主花序开花结实的影响[J]. 经济林研究, 2016, 34(04): 113-116.
[81]郭东强，卢陆峰，卢晨升等. 邓恩桉开花结实期间内源激素含量变化[J]. 桉树科技, 2019, 36(04): 22-26.
[82]王娟娟. 油松优树子代测定及综合选择研究[D]. 西北农林科技大学, 2008.
[83]赵祐，王立，吕东. 青海云杉种子园无性系结实性状遗传变异研究[J]. 安徽农业科学, 2017, 45(35): 155-157.
[84]张健，叶天文，陈雅等. 外源激素处理接穗对海南油茶高接换冠的影响[J]. 经济林研究, 2020, 38(02): 53-59.
[85]赵琳，黄钰，李甜江等. 云油茶 5 个品种芽苗砧嫁接技术研究[J]. 西部林业科学, 2012, 41(05): 19-25.

中国知网 https://www.cnki.net

[86]董高峰，黄涛，李耿光等．外源激素对沙田柚茎尖微嫁接成活率的影响[J]．生态科学，2001(03): 26-30.

[87]袁婷婷，钟秋平，丁少净等．植物生长调节剂对油茶芽苗砧嫁接愈合的影响[J]．林业科学研究, 2015, 28(04): 457-463.

[88]邓朝佐，李富福，董学军．IBA 在金花茶扦插和嫁接繁殖中应用初探[J]．广西林业科技, 1987(04): 22-24.

[89]郑颖，于世河，冯健等．不同处理对蒙古栎嫁接成活率和苗木生长的影响[J]．林业科技通讯, 2020(05): 52-54.

[90]滕贵波，颜廷武，赵博文．美国红枫嫁接育苗技术研究[J]．辽宁林业科技，2016(06): 32-33.

[91]赵翔．灰木莲扦插和嫁接繁殖技术的研究[D]．东北林业大学, 2017.

[92]唐辉，韦霄，梁惠凌等．外源激素对银杏高位嫁接及其枝梢生长的影响[J]．中南林业科技大学学报, 2007(01): 85-87.

[93]贾庆彬，刘庚，赵佳丽等．红松半同胞家系生长性状变异分析与优良家系选择[J/OL]．南京林业大学学报(自然科学版): 1-12[2022-05-21].

[94]高红霞，苏世平，李毅等．基于渗透调解物质及叶绿素分析红砂抗旱优良家系的早期选择[J]．应用生态学报, 2016, 27(01): 40-48.

[95]柴文敏，李毅，苏世平等．唐古特白刺(Nitraria tangutorum)抗旱优良家系的生理特性[J]．中国沙漠, 2017, 37(06): 1158-1170.

[96]李森．早实核桃川早 1 号、蜀玲光合生理研究[D]．四川农业大学, 2010.

[97]林兵，武胜利，管文轲等．胡杨叶片的胞间 CO_2 浓度及气孔和非气孔限制的探究[J]．湖北农业科学, 2021, 60(13): 87-92.

[98]蒋丽娟，马倩，佟金权等．光皮树砧木生长指标及内源激素对嫁接成活率的影响[J]．中南林业科技大学学报, 2011, 31(03): 26-29.

[99]辛建华，许庆标，刘建军等．不同树龄楸树种质资源的嫁接成活影响因素[J]．山东林业科技, 2021, 51(04): 71-74.

[100] 王华荣，王海亭，张国立等．楸树不同嫁接方法对苗木成活率和生长量的影响[J]．安徽农业科学, 2007(30): 9505.

[101] 张鑫鑫，夏辉，赵昕等．长白落叶松种子园亲本生长与结实性状综合评价[J]．植物研究, 2017, 37(06): 933-940.

[102] 黄宇．木荷无性系种子园生长与结实性状的初步研究[J]．热带作物学报，2017, 38(02): 213-217.

[103] 陈苏英，马祥庆，吴鹏飞等．1.5 代杉木种子园不同无性系生长和结实性状的评价[J]．热带亚热带植物学报, 2014, 22(03): 281-291.

[104] 林能庆．马尾松无性系种子园结实量与生长性状的相关分析[J]．防护林科技，2013(10): 10-12.

中国知网 https://www.cnki.net

攻读学位期间发表的学术论文

[1]王玮槐, 李志新, 张含国, 刘劲宏, 代伟昭, 石宝英. 核桃楸无性系生理生化及光合指标的遗传变异及相关性[J]. 东北林业大学学报，2022，50(04)：1-7.

中国知网 https://www.cnki.net

致谢

本课题承蒙黑龙江省应用技术研究与开发计划项目“核桃楸良种选育、高效培育技术示范与推广”GA19B201-7 基金资助，特致殷切谢意。

衷心感谢导师张含国教授和李志新讲师对本人的精心指导。为我能按时地完成毕业论文提供了许多指导性的建议，借此论文完成之际，谨向导师致以最诚挚的感谢和敬意！还要感谢我的师兄师姐们，在论文的一些相关问题中，他们以自己的经验给我介绍了许多知识，同时，在这中间我也学到了不少与人沟通的能力。

还要感谢实验室提供的试验设备，使我能够方便及时地做实验。在此表示由衷的感谢！感谢林学院老师和同窗们的关心和支持！感谢所有帮助过我的人！

中国知网 https://www.cnki.net

中国知网 https://www.cnki.net
