# Supplementary material for: Provenance and family variations in early growth of Manchurian walnut (Juglans mandshurica Maxim.) and selection of superior families
Source: PLoS One. 2024 Mar 7;19(3):e0298918. doi: 10.1371/journal.pone.0298918 (PMC10919699; doi:10.1371/journal.pone.0298918)
Supplement: S2 File — (ZIP) [file pone.0298918.s005.zip › Non-Native Forest Tree Species in Europe The Question of Seed Origin in Afforestation.pdf]

forests MDPI

*Review*

# Non-Native Forest Tree Species in Europe: The Question of Seed Origin in Afforestation

**Paraskevi Alizoti [1,*], Jean-Charles Bastien [2], Debojyoti Chakraborty [3], Marcin Miroslav Klisz [4], Johan Kroon [5], Charalambos Neophytou [6], Silvio Schueler [3], Marcela van Loo [3], Marjana Westergren [7], Monika Konnert [8], Vlatko Andonovski [9], Kjell Andreassen [10], Peter Brang [11], Robert Brus [12], Branislav Cvjetković [13], Martina Đodan [14], Manuel Fernández [15], Josef Frýdl [16], Bo Karlsson [5], Zsolt Keserű [17], Andrej Kormutak [18], Vasyl Lavnyy [19], Tiit Maaten [20], Bill Mason [21], Georgeta Mihai [22], Cristina Monteverdi [23], Sanja Perić [14], Krasimira Petkova [24], Emil Borissov Popov [25], Matti Rousi [26], Srđan Milenko Stojnić [27] and Ivaylo Tsvetkov [26]**

1 School of Forestry and Natural Environment, Aristotle University of Thessaloniki, 54124 Thessaloniki, Greece
2 INRAE Centre Val de Loire–UMR BioForA, INRAE, 2163 Avenue de la Pomme de Pin, CS 40001 Ardon, CEDEX 2, 45075 Orleans, France; jean-charles.bastien@inrae.fr
3 Department of Forest Growth, Silviculture and Genetics, Austrian Federal Research Centre for Forests, Natural Hazards and Landscape (BFW), Seckendorff-Gudent-Weg 8, A-1131 Vienna, Austria; debojyoti.chakraborty@bfw.gv.at (D.C.); silvio.schueler@bfw.gv.at (S.S.); marcela.vanloo@bfw.gv.at (M.v.L.)
4 Department of Silviculture and Forest Tree Genetics, Forest Research Institute, 05-090 Raszyn, Poland; M.Klisz@ibles.waw.pl
5 Skogforsk-Forestry Research Institute of Sweden, 2250 Ekebo, SE-268 90 Svalöv, Sweden; johan.kroon@skogforsk.se (J.K.); curly.birch@gmail.com (B.K.)
6 Department of Forest and Soil Sciences, Institute of Silviculture, University of Natural Resources and Life Sciences (BOKU), Peter-Jordan-Straβe 82, 1190 Vienna, Austria; charalambos.neophytou@boku.ac.at
7 Slovenian Forestry Institute, Vecna pot 2, 1000 Ljubljana, Slovenia; marjana.westergren@gozdis.si
8 Bavarian Institute for Forest Genetics, 83317 Teisendorf, Germany; monika.konnert@gmx.de
9 Faculty of Forest Sciences, Landscape Architecture and Environmental Engineering, University Ss. Cyril and Methodius, P.O. Box 235, Skopje 1000, North Macedonia; makmontana1@t-hom.mk
10 Norwegian Institute of Bioeconomy Research (NIBIO), P.O. Box 115, 1431 Ås, Norway; kjell.Andreassen@Skogoglandskap.no
11 Swiss Federal Institute for Forest, Snow and Landscape Research (WSL), 8903 Birmensdorf, Switzerland; peter.brang@wsl.ch
12 Biotechnical Faculty, University of Ljubljana, Jamnikarjeva 101, 1000 Ljubljana, Slovenia; robert.brus@bf.uni-lj.si
13 Department for Forest Genetics and Afforestation/Reforestation, University of Banja Luka, 78000 Banja Luka, Republic of Srpska, Bosnia and Herzegovina; branislav.cvjetkovic@sf.unibl.org
14 Division for Silviculture, Croatian Forest Research Institute, 10450 Jastrebarsko, Croatia; martinat@sumins.hr (M.Đ.); sanjap@sumins.hr (S.P.)
15 Department of Agroforestry Sciences, School of Engineering, ETSI, Campus de El Carmen, Huelva University, 21071 Huelva, Spain; manuel.fernandez@dcaf.uhu.es
16 Department of Forest Tree Species Biology and Breeding, Forestry and Game Management Research Institute, 252 02 Jíloviště, Czech Republic; JosefFrydl@seznam.cz
17 Forest Research Institute, 4150 Püspokladany, Hungary; keseruzs@erti.hu
18 Plant Biology and Biodiversity Center, Institute of Plant Genetics and Biotechnology, Slovak Academy of Sciences, 950 07 Nitra, Slovakia; nrgrkorm@savba.sk
19 Department of Silviculture, Ukrainian National Forestry University, 79057 Lviv, Ukraine; lavnyy@gmail.com
20 Institute of Forestry and Rural Engineering, Estonian University of Life Sciences, 51014 Tartu, Estonia; tiit.maaten@emu.ee
21 Forest Research, Northern Research Station, Roslin, Midlothian EH25 95Y, UK; bill.mason@forestresearch.gov.uk
22 'Marin Dracea' National Institute for Reserach and Development in Forestry, 077190 Voluntari, Romania; gmihai_2008@yahoo.com
23 CREA Research Centre for Forestry and Wood, 52100 Arezzo, Italy; monteverdicris@libero.it
24 Department of Silviculture, Faculty of Forestry, University of Forestry, 10 Kliment Ohridski Blvd, 1797 Sofia, Bulgaria; kpet@abv.bg
25 Forest Research Institute, Bulgarian Academy of Sciences, 1756 Sofia, Bulgaria; emilpopov99@hotmail.com
26 Natural Resources Institute Finland (Luke), Latokartanonkaari 9, 00790 Helsinki, Finland; Matti.Rousi@luke.fi (M.R.); tsvet_i@yahoo.com (I.T.)

check for updates

**Citation:** Alizoti, P.; Bastien, J.-C.; Chakraborty, D.; Klisz, M.M.; Kroon, J.; Neophytou, C.; Schueler, S.; Loo, M.v.; Westergren, M.; Konnert, M.; et al. Non-Native Forest Tree Species in Europe: The Question of Seed Origin in Afforestation. *Forests* **2022**, *13*, 273. https://doi.org/10.3390/f13020273

Academic Editor: Dušan Gömöry

Received: 5 January 2022
Accepted: 3 February 2022
Published: 8 February 2022

**Publisher's Note:** MDPI stays neutral with regard to jurisdictional claims in published maps and institutional affiliations.

**Copyright:** © 2022 by the authors. Licensee MDPI, Basel, Switzerland. This article is an open access article distributed under the terms and conditions of the Creative Commons Attribution (CC BY) license (https://creativecommons.org/licenses/by/4.0/).

[27] Institute of Lowland Forestry and Environment, University of Novi Sad, Antona Čehova 13d, 21000 Novi Sad, Serbia; srdjan_stojnic@yahoo.com
* Correspondence: alizotp@for.auth.gr; Tel.: +30-231-0992-769

**Abstract:** Non-native forest tree species have been introduced in Europe since the 16th century, but only in the second half of the 20th century the significance of the seed source origin for their economic use was recognized, resulting in the establishment of numerous provenance trials at a national, regional, European and International level, as those led by IUFRO. Breeding programs have also been launched in the continent for the most economically important species. Aim of this work is the formulation of provenance recommendations for planting of five non-native tree species in Europe (Douglas fir, grand fir, Sitka spruce, lodgepole pine and black locust), based on the information obtained from twenty countries, in the frame of the EU FP-1403 NNEXT Cost Action. The survey revealed that official and non-official national recommendations, based on provenance research results, have been elaborated and followed at a different level and extend for the above five species, but only for Douglas fir recommendations exist in almost all the participating to the survey countries. The compilation of provenance recommendations across Europe for each species is presented in the current work. Besides the recommended introduced seed sources, European seed sources are also preferred for planting, due to ease of access and high availability of forest reproductive material. European breeding programs yielding genetic material of high productivity and quality constitute currently the seed source of choice for several species and countries. Consolidation of trial data obtained across countries will allow the joint analysis that is urgently needed to draw solid conclusions, and will facilitate the development of 'Universal-Response-Functions' for the species of interest, rendering possible the identification of the genetic material suitable for global change. New provenance trial series that will test seed sources from the entire climatic range of the species, established in sites falling within and outside the environmental envelopes of their natural ranges, are urgently needed to pinpoint and understand the species-specific climate constraints, as well as to correlate functional traits to the seed origin and the environmental conditions of the test sites, so that the selection of suitable forest reproductive material of non-native tree species in the face of climate change can be feasible.

**Keywords:** provenance recommendations; provenance testing; breeding programs; adaptation; exotic tree species; Douglas fir; Sitka spruce; grand fir; lodgepole pine; black locust

## 1. Introduction

Following the CBD [1] definition, the term 'non-native species' refers to "a species, subspecies or lower taxon, introduced (i.e., by human action) outside its natural past or present distribution; the term includes any part, gametes, seeds, eggs, or propagules of such species that might survive and subsequently reproduce", while according to FAO Global Forest Resources Assessment [2], and as accepted by Forest Europe (Indicator 4.4), the above definition is extended also to those species having a potential to spread (i.e., outside the range they occupy naturally or being able to occupy sites without direct or indirect introduction or care by humans).

In Europe, the first introductions of non-native tree species from other continents were carried out between the 16th and 18th century, mainly by botanists motivated by curiosity and botanical interest [3], in a time when knowledge on genetics and local adaptation was lacking. Some species, like northern red oak (*Quercus rubra* L.), black locust (*Robinia pseudoacacia* L.), black cherry (*Prunus serotina* Ehrh.) and boxelder maple (*Acer negundo* L.), introduced during the above period were used for both ornamental and forestry purposes and are still of economic value [4]. In the late 18th and the 19th century, the introduction of fast-growing non-native tree species was promoted in the frame of an extensive reforestation effort to counterbalance the overexploitation of autochthonous timber resources that largely powered industrialization [3]. During the same period, only

small trial plantations were established, testing introduced species like *Pinus strobus* L. [5]. Only at the beginning of the 20th century was it recognized that variation among trees may be partly inherited [6]. The underlying processes or mechanisms that caused the observed variation were still unknown at that time and the introduction of non-native tree species reproductive material was not based on gene-ecological knowledge and genetic criteria. Consequently, the use of reproductive material maladapted to the planting sites often resulted in extensive failures, as for example that of the interior variety of Douglas-fir in Central Europe [7]. Following such failures, the significant role of the seed origin for a successful introduction was increasingly recognized, resulting in the introduction of non-native species in a more organized way which considered also the existing ecological and genetic knowledge (e.g., [8–10]). Ever since, non-native tree species have been utilized in Europe as an important timber [11], pulp [12] and biomass/energy [13] resource, as well as a source of non-timber wood products [14].

From a genetic point of view, and according to [15], the term 'provenance' refers to the geographic location of the native population where the plant material originated, while the term 'seed source' to the geographic location from which the seed was obtained regardless of whether or not the parent trees were located in their native and autochthonous habitat. Most of the non-native forest tree species introduced in Europe display vast geographic variation within their native range. Hence, the performance and survival of different provenances may differ significantly when they are planted outside their natural distribution area [10,16–18]. The assessment though of the genetic differences among provenances and the characterization of the genetic patterns of adaptive geographic variation can only be succeeded following planting/testing of provenances in common gardens, i.e., provenance tests, which are established across the environmental range of the intended planting regions [15]. In this way, the most suitable provenances can be identified for each region. Provenance tests are of the utmost importance for species of high economic or ecological value, as they provide information on the plasticity, adaptive/growth potential and tolerance to insects, diseases and cumulative environmental stress [6].

In the second half of the 20th century, numerous provenance trials were established in Europe for economically important non-native tree species, which included provenances covering almost the entire natural range of those species, e.g., the International Union of Forest Research Organizations (IUFRO) trial series, initiated in 1966 for Douglas fir [19,20], and in 1968 for Sitka spruce [13,21]. Such trials provide, even today, valuable insights into adaptive and growth traits of different provenances. Studied traits included bud phenology [10,22–24], frost hardiness [10,25], drought tolerance [16,26,27], height and volume growth [28,29], wood density [30], as well as branching patterns [28,31]. In addition to the provenances originating from the native range, European seed sources/local land races have also been tested in provenance trials. A 'local land race' is formed when a species is introduced into an exotic environment and adapts through natural, and sometimes artificial, selection to the environmental conditions of the new planting zone [15]. In several cases, the European land races outperformed the provenances introduced from the places of origin, e.g., Sitka spruce [21] and Douglas fir [32,33] land races. However, not all species with potential economic value were equally represented in such field trials. For instance, while different cultivated varieties are available for black locust [34], no extensive provenance trials including native origins have been established so far in Europe.

The most widely used non-native species in Europe are fast growing ones, like Sitka spruce (*Picea sitchensis* (Bong.) Carr.), Douglas-fir (*Pseudotsuga menziesii* (Mirb). Franco), grand fir (*Abies grandis* (Douglas ex D. Don) Lindley), black locust (*Robinia pseudoacacia*), lodgepole pine (*Pinus contorta* Douglas), eucalypts (*Eucalyptus* spp.), northern red oak (*Quercus rubra* L.) and tree of heaven (*Ailanthus altissima* (Mill.) Swingle) [3].

The results from the provenance tests established in the continent for non-native tree species (i.e., the Douglas fir trials initiated in 1966 by IUFRO [35]) are already being used to formulate recommendations regarding the most suitable provenances for planting in operational plantations across European ecoregions. In addition to provenance testing,

breeding programs have also been launched for several economically important species (i.e., Sitka spruce in Britain and Ireland, Douglas fir in France [36]. In the framework of the breeding effort, seed orchards and progeny tests have been established for the production of improved Forest Reproductive Material (FRM), i.e., the case of Douglas fir [20]. Such sources have already been included into the FRM recommendations proposed by several countries (e.g., [20,37]).

Aim of the current paper is to present, for the first time, provenance recommendations for planting of five selected non-native tree species in Europe, based on the results of a survey that was carried out in the frame of the FP-1403 NNEXT Cost Action (Non-native tree species for European forests: experiences, risks and opportunities) and its Working Group 2—'Pathways'. Twenty participating countries responded to the survey. From the obtained responses, it was revealed that national recommendations and results from experimental trials established at a pan-European, regional or national level exist for only five non-native tree species, used in five or more European countries. The five selected species of interest in the current work are: (1) Douglas fir (*Pseudotsuga menziesii* (Mirb). Franco); the species with the broader network of provenance trials, being established in twenty countries and with already formulated provenance recommendations at the national scale in several countries-for this reason it has been selected as a 'model species' for European provenance recommendations in this work, (2) grand fir (*Abies grandis* (Douglas ex D. Don) Lindley); a species reported by seven countries, with limited occurrence in Central Europe, but with existing information on provenance performance; (3) Sitka spruce (*Picea sitchensis* (Bong.) Carr.); a species reported by five countries, extensively bred and with high economic importance for North-Western Europe; (4) lodgepole pine (*Pinus contorta* Douglas); a species reported by eight countries, mainly introduced into the Nordic countries, and (5) black locust (*Robinia pseudoacacia* L.); a species reported by thirteen countries that is distributed all over Europe and for which only European local land races or selected clonal lines are planted.

## 2. Methods

For the current review, a survey was carried out in the framework of the FP-1403 NNEXT Cost Action (Non-native tree species for European forests: experiences, risks and opportunities) activities, aiming towards the collection of information on the: (i) the status-quo of non-native tree species provenance research and of the research carried out on other genetic tests, and (ii) the formulated provenance recommendations and the existing guidelines on transfer of non-native tree species FRM in Europe. For this purpose, an extensive questionnaire was disseminated to the representatives of the FP 1403- NNEXT Cost Action partner countries, addressing the following topics (Supplementary Table S1): (i) Information on existing genetic tests: type of genetic test (provenance/clonal/progeny test), location, type of trial (IUFRO international/regional/national), number and origin of provenances or genetic material tested, important results; (ii) Information on provenance recommendations: existence or not, status of recommendations (official/unofficial, mandatory or not) at the national level, scientific connection of recommendations to genetic tests or not, and specific recommendations formulated at the national level for each one of the non-native tree species planted in each country.

The responses obtained from twenty-six partner countries indicated that five non-native tree species were the ones that attracted an extensive interest for testing and planting and were reported from five or more countries, namely Douglas fir, grand fir, Sitka spruce, lodgepole pine and black locust. Species that were reported by only one, two or three countries (i.e., eastern white pine, Japanese larch) were not considered as being of pan-European interest and were not included in the current study.

Information on the geographic coordinates (latitude and longitude and in some cases altitude) of the genetic trials established in Europe, as well as on the origin of the genetic materials tested within those genetic trials was provided by the representatives of the NNEXT Cost Action partner countries. The coordinates were checked for their consistency

and were converted to the World Geodetic System (WGS 84), which is the reference coordinate system used by the Global Positioning System (GPS). The coordinates were used to extract values of mean annual temperature (MAT) and annual precipitation (MAP) for the period 1970–2000 from the Worldclim database, version2 [38]. The extracted MAT and MAP were plotted to visualize the bioclimatic envelope of genetic trials and the origin of the genetic materials of five non-native species with respect to their native distribution and introduced range in Europe (Figure 1).

The information obtained from the NNEXT Cost Action partner countries on the genetic tests, tested genetic material, existing recommendations for planting and available literature at the national level were compiled to provide a pan-European overview on the research activities, the provenance/genetic testing carried out, and the results obtained, in order to formulate recommendations for planting provenances/seed sources (including European land races and material from breeding programs) of the five above mentioned species in different regions of the continent.

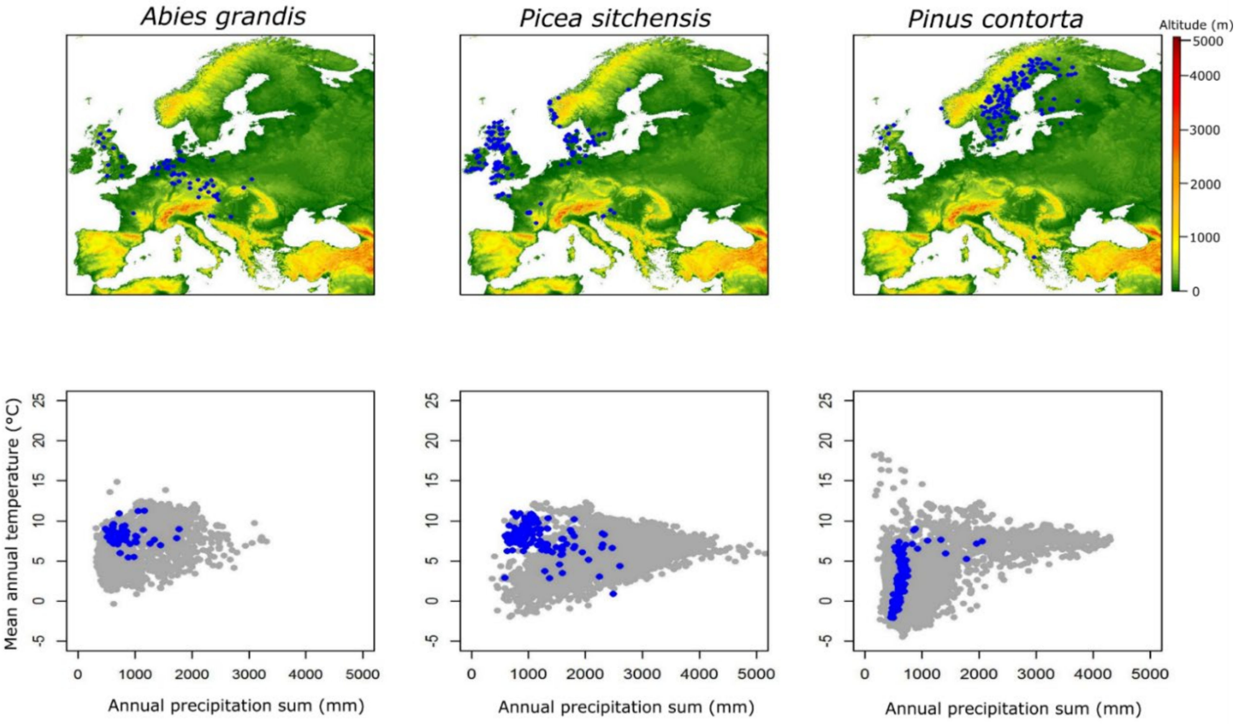


**Figure 1.** *Cont.*

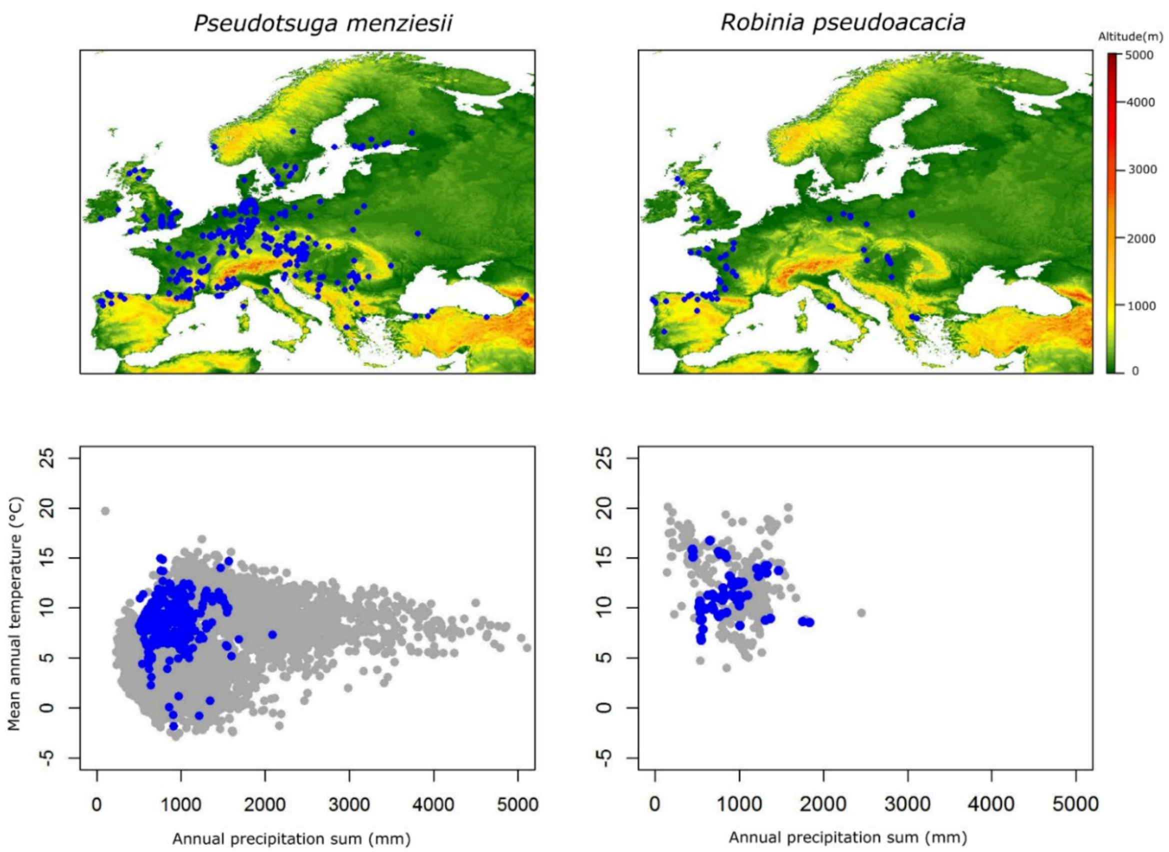


**Figure 1.** Geographic location of established genetic trials in Europe for the five non-native tree species of interest is indicated (map), together with their display within the bioclimatic parameter space represented by mean annual temperature and mean annual precipitation of the regions where each non-native tree species grows naturally (blue dots: bioclimatic plot of the establishment sites of genetic trials in Europe, grey dots: bioclimatic space of each species in its natural distribution).

## 3. Results

### *3.1. Provenance Testing in Europe*

As mentioned earlier, the necessity of establishing genetic tests for assessing the adaptive and growth potential of FRM of non-native species was realized during the second half of the 20th century. Since then, the provenance testing efforts for the most important, from a reforestation and production point of view non-native tree species, were mostly initiated and coordinated by the IUFRO Working Groups and Networks and took place in two periods for the species of interest in the current work. The first period (1966–1968) focused on Douglas fir, Sitka spruce and lodgepole pine, while the later one focused on grand fir.

Douglas fir is the species that attracted a lot of interest in Europe since the late 19th, due to the success of its first plantations, which originated most probably from the region close to mouth of Columbia River (between Washington and Oregon). The first provenance trial has been established in Chorin (east of Berlin) in 1910, triggered by the failures that followed the first period of the successful planting of the species and were due to imports from the interior part of its natural distribution [20]. In 1923, another provenance test involving 35 seed lots from British Columbia, Washington and Oregon was established in the Netherlands, followed by a multi-environment provenance trial across nine locations planted in 1932 in Germany [20]. In 1966, the IUFRO initiated a trial series for Douglas fir [19,20] by performing, during the period 1966–1968, a systematic collection of 182 provenances covering the whole natural range of the species, the so-called 'IUFRO Douglas fir collection'. The seed of the 124 provenances was distributed to 59 Institutions in 36 countries, among which 16 were European; namely Austria, Belgium, former Czechoslovakia,

Denmark, Finland, France, former East and West Germany, Hungary, Ireland, Italy, Norway, Spain, the Netherlands, the United Kingdom and the former Yugoslavia [19,20,39,40].

IUFRO initiated also the seed collection from 41 grand fir seed lots, originating from British Columbia, Washington, Oregon, Idaho and Montana, and elevations ranging from 0 to 1500 m., during the period 1974 to 1976 (Fletcher 1986). The above genetic material was used in 1980/1981 for the establishment of an IUFRO network of provenance trials in several European countries; i.e., France [41], Austria [42,43], Germany [44–46], the Netherlands [47], the United Kingdom [48], the Czech Republic [49] and Poland (e.g., [50,51]). Another extensive provenance trial was also established at a national scale in Germany with seed collected in 1976 along a west-east transect in Oregon, from the Pacific to the Cascades crest.

Sitka spruce also attracted an interest for forestry in Europe quite early, due to its high yield potential and high tolerance to adverse site conditions. For this reason, it has been tested in field trials since the first half of the 20th century. The first provenance trials though were established in Norway during the period 1915–1928, the United Kingdom in 1929, Germany in 1930, and Denmark [13,37,52,53], but were based mainly on commercial seed collections that covered only a small part of the species natural range. In 1968, and due to the species interest, IUFRO (International Union of Forest Research Organizations) initiated the activities for the establishment of a series of provenance tests for the species. During the period 1968 to 1970, the IUFRO teams collected FRM (seed) from 81 origins covering the entire natural distribution of the species [54]. The collection was carried out in two phases: the Washington-Oregon distribution in 1968 (by J. Turnbull and B. Hansen), and the British Columbia-Alaska distribution in 1970 (by A. Fletcher and N. Danby). The FRM of the 81 provenances collected (19 from Alaska, 41 from British Columbia, 11 from Washington, 8 from Oregon and 2 from California) was then distributed in an unbalanced pattern to 18 Institutes in 14 countries, out of which 11 were European (Belgium, Bulgaria, Denmark, France, Germany, Ireland, Latvia, the Netherlands, Norway, the United Kingdom and the former Yugoslavia) [55]. Due to the different number (3 to 81) of provenances obtained by the 18 Institutes of the 14 countries and the different requirements of each Institute's plan, it was decided from IUFRO in 1973 to organize an international provenance testing experiment involving ten common provenances, the so called 'IUFRO Sitka Spruce International Ten Provenance Experiments' [54]. The seed material from the ten provenances (two from Washington, one from Oregon, two from Alaska, four from British Columbia and one from Queen Charlotte Island) was sown in all 18 institutes in 1973, while the nursery phase was completed by during the autumn 1974 or 1975, allowing the establishment of the trials the following year [37,54,56].

Lodgepole pine captured an interest for introduction to Europe due to its wide range of environmental tolerance (growing in maritime, continental, subalpine conditions and in any type of site, including wet and dry soils, bearing characteristics of a pioneer species), and its high growth potential [57]. The species has been introduced in 1910 in Finland and its success was the reason behind the interest of the whole Fennoscandia in it [57]. The first plantations in Sweden were established in 1928, while in 1967 six provenance tests were established following an expedition and seed collection in 1963 [57]. Following the high interest of the northern European countries in the species, IUFRO coordinated in 1966–1968 the collection of 158 seed lots from the natural distribution of the species, which were then distributed to more than 20 (mostly European) countries, resulting to the establishment of a network of trials known as 'the IUFRO 70/71 experiments' [58–60].

Provenance testing on black locust in Europe is quite recent, following the also recent breeding efforts on the species. The recorded high presence of the species in Europe though is not analogous to the level of its provenance testing. In Hungary, the leading country in Europe in selection and breeding of black locust, only eight genetic trials exist focusing either on provenance or clone testing (e.g., [61–63]). In France a clonal test was planted in 2015 to compare Hungarian versus 250 French clones for their form and superior growth. Moreover, France together with Portugal, Spain, the United Kingdom

and Ireland launched in 2009 a transnational project and set up a network of 38 arboreta (known as REINFFORCE Arboretum & Demonstration Sites Network). Within this network, provenances of black locust growing in Bulgaria, Romania, Slovakia, North Macedonia and Turkey, along with other tree species were planted in 32 environmentally different sites (i.e., arboreta), distributed across all the countries participating in the REINFORCE Project [64], except for Ireland. Genetic trials (provenance or clonal tests) were established in many European countries; i.e., Italy [65], Bulgaria [66], Poland [67] and Austria [68].

Figure 1 indicates the geographic location of established genetic trials in Europe for the five non-native tree species of interest and their display within the bioclimatic parameter space, represented by mean annual temperature and mean annual precipitation of the natural distribution regions. The findings presented in Figure 1, suggest that genetic testing effort took place in specific European regions for most of the species, except for Douglas fir which is the only species tested across the whole continent in numerous trials, indicating the pan-European interest for the species, triggered by its high productivity, great genetic variation, adaptive plasticity and lack of potential to hybridize with other tree species growing in Europe and especially the autochthonous ones.

### 3.2. Provenance Recommendations for Planting in Europe

Results from field trials (provenance and progeny tests) often provide a good basis for the formulation of recommendations on FRM transfer, and this was attempted in the current work for the five non-native tree species of interest. However, as mentioned above, the distribution of provenance and progeny trials varies strongly across the continent, reflecting the importance of the five species for European forestry. Therefore, the recommended seed sources and the criteria used to formulate those recommendations may vary strongly in different cases and from country to country. For example, in some cases, there is a good knowledge on the growth performance of the seed sources (based on provenance research results) and detailed recommendations for the use of seed originating from specific seed zones in the native range are given in certain European countries. In other cases, European (non-native) seed sources are preferred, due to lack of information on the performance of native provenances, the ease of access, and their high availability. Finally, breeding programs may also comprise an important source of high-quality reproductive material and constitute the source of choice for some species and countries (e.g., lodgepole pine in Sweden). In Figure 2 the different options concerning the origin, selection criteria and type of recommended basic material are presented.

The scheme of FRM transfer rules is not common across European countries, while it may or may not be compulsory to follow those rules depending upon the country. Laws and regulations may require traceability and identifiability of FRM, but use of certain seed sources is not obligatory by law. However, compliance with FRM transfer guidelines is, in some cases, a criterion for receiving funding for afforestation (e.g., Germany, United Kingdom; [69]). In other cases, afforestation must be performed according to such guidelines in state forests (e.g., Baden-Württemberg in Germany; [70]). Also, the form of such recommendations can vary greatly from country to country. In the current work, we distinguish the official from the non-official recommendations. Official recommendations are defined as those issued by official authorities and can be mandatory or optional. If they are included in legal acts they are mandatory, but if they are part of official advices they are optional. Non-official recommendations refer to those stemming from research results included in scientific publications and reports from provenance trials. Based on the information collected from the Cost Action FP 1403-WP2 questionnaire responses and the above mentioned criteria, the availability and the type of provenance recommendations per species and country are presented in Table 1.

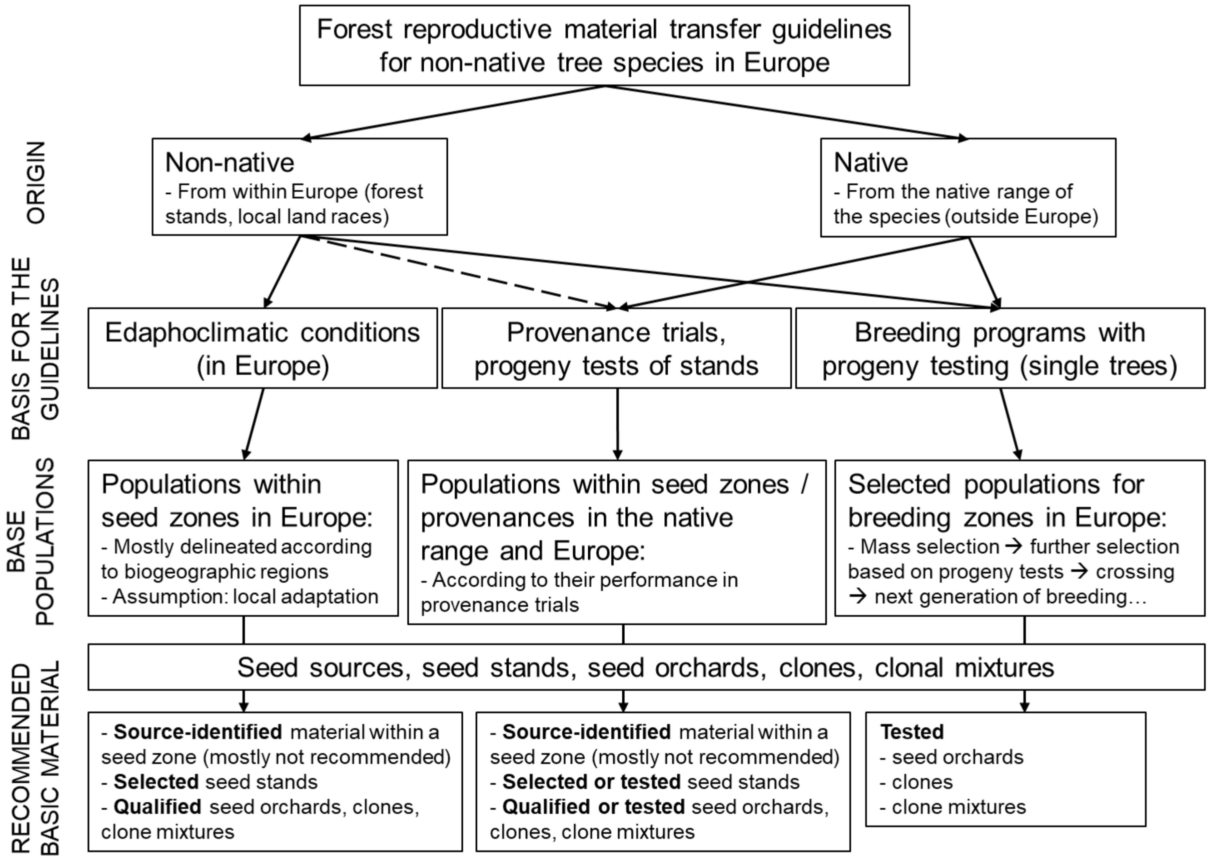


**Figure 2.** Forest reproductive material (FRM) transfer guidelines for non-native tree species in Europe. The flow chart presents different options of origin, basis for the guidelines, base populations and categories of recommended basic material.

**Table 1.** Availability and type of provenance recommendations in different European countries based on the questionnaire responses.

| | **Non Native Tree Species** | | | | |
|---|---|---|---|---|---|
| **Country** | ***Douglas fir* (*Pseudotsuga menziesii*)** | ***Grand fir¥* (*Abies grandis*)** | ***Sitka spruce* (*Picea sitchensis*)** | ***Lodgepole pine¥* (*Pinus contorta*)** | ***Black locust* (*Robinia pseudoacacia*)** |
| Austria | O | U | n | n | - |
| Bosnia | U | - | - | - | - |
| Bulgaria | U | - | - | - | - |
| Croatia | - | - | - | - | - |
| Estonia | - | - | - | U | - |
| Finland | - | - | - | - | - |
| France | - | - | - | - | - |
| Germany | - | - | - | - | - |
| Greece | U | - | - | - | - |
| Hungary | - | - | - | - | O |
| Italy | U | n [1] | n [1] | n [1] | U |
| North Macedonia | U | O | - | - | - |
| Norway | - | - | O | O | - |
| Poland | U | U | n | n | - |
| Romania | U | - | - | - | - |
| Slovenia | - | - | - | - | - |
| Spain | O | n | n | n | - |
| Sweden | - | n | n | O | - |
| Ukraine | - | - | - | - | - |
| United Kingdom | O | O | O | O | - |

(O): official recommendations available, (U): unofficial recommendations, (n): no recommendations, (n [1]): no specific recommendations, but general FRM legal rules apply, (-): no response.

### 3.2.1. *Douglas fir*

For *Douglas fir* (*Pseudotsuga menziesii*), and until the last quarter of the 20th century, seed transfers to Europe were based on an empirical assessment of ecological similarities between the native range and the plantation sites in Europe. Seed transfer rules for Europe took a new perspective given the numerous provenance tests, planted under the aegis of IUFRO in more 30 countries of the northern and southern hemispheres [71]. The IUFRO collection was planted in more than 110 sites in Europe and results of those provenance tests manifested the good adaptability and growth of the coastal variety and the poor performance of the interior variety [72]. Due to its much slower growth, when compared to the coastal variety, and its strong sensitivity to *Rhabdocline* needle cast, the interior type was found unsuitable for reforestation in Europe [20].

All the Douglas-fir seed sources recommended for reforestation in Europe, originate from the part of the range between 40° and 50° latitude, west of the Cascade Range and below 600 m of elevation [20,40]. Recommendations are based on a compromise between growth and survival.

In Central and Eastern Europe, tolerance to fall and winter frosts should be considered. Therefore, in this area, provenances from the middle elevation zone of the Cascade range in Washington and from northern Oregon seem to be best suited [29,73–76]. Provenances from this area that are widely recommended in many European countries include, among others, Newhalem (seed zone 402) and Darrington (seed zone 403) from the Northwest Washington Cascades, Bremerton (seed zone 222) from the East Slope of the Olympic Peninsula and Idanha (seed zone 452) from the Northwest Oregon Cascades [74,76–78] (Seed zone numbers and their relative location in the United States and Canada for the five non-native tree species reported here can be as found in the webpages http://www.forestseedlingnetwork.com/resources.aspx, last accessed 10 December 2021 and https://www2.gov.bc.ca/gov/content/industry/forestry, last accessed 10 December 2021).

In Oceanic Europe, where frost is less likely, coastal provenances from Washington and Vancouver Island are recommended mainly because of their excellent branching architecture and superior growth [40,79]. For example, in Ireland and Great Britain, coastal Washington provenances are the first choice for general use [40,80]. Seed sources from South West Cascades are recommended only for southern and eastern areas of Great Britain with a less pronounced oceanic climate [40].

In northern and high elevation areas of the Mediterranean Europe, similar provenances as the ones recommended above could be used, as well as provenances originating from Northern Oregon (Cascades and coastal) [77,81,82]. In southern or low elevation areas provenances from coastal southern Oregon and northern California (coastal and northern Sierra Nevada) could be recommended due to their better survival in comparatively harsher and drier sites of the Mediterranean region [39,83–87].

Provenance tests planted at high elevation (above 900m) are rare in Europe. Nevertheless, these few tests indicate that seed sources originating from a higher elevation in the natural range (above 600 m) and from a higher latitude (British Columbia) may have an advantage in growth and survival [88]. Thus, the altitude and latitude of FRM origin should be considered when reforestation is planned and carried out in mountainous sites of Europe.

Besides native provenances, basic material from Europe is also an important seed source for Douglas fir. A large number of selected and tested seed stands in Europe, as well as qualified and tested seed orchards and clones are available for harvesting across the continent [89]. Often, selected stands or qualified seed orchards are recommended as sources of FRM for use within a European seed zone [76,78,80]. European land races have also been recommended for use outside their area of origin, based on their good performance in provenance tests; as for example the land race of South West Baden (Germany) which is recommended as seed source in Austria, based on its performance in field trials [76]. Breeding programs offer an additional source of high quality FRM. The European Douglas-fir Improvement Research Cooperative (EUDIREC) established by collaborating research

organizations across Europe focuses mainly on building common breeding populations, improving seed production methods, ex-situ conservation and development of a database for European genetic resources [71]. In France, Douglas fir seed orchards established with native (North American) genetic material consist an important seed source. Backward selection of parents already took place in these seed orchards, based on results of progeny testing. Target traits for selection were height growth, stem straightness and forking [20,90]. The rogued seed orchards are recommended as FRM source also outside France [78].

#### 3.2.2. *Grand fir*

Concerning *Grand fir* (*Abies grandis*), guidelines for FRM transfer have been mainly issued by countries with established provenance trials, i.e., most of the central European countries, the United Kingdom to the West, Norway to the North and North Macedonia in south-eastern Europe. In most cases, recommendations are based on the results of provenance research, whereas, sometimes, use of European seed sources is also advised. In general, the most recommended provenances are located within the coastal part of the native range.

In particular, low and medium elevation provenances from the native range, beginning at the north-eastern tip of the Olympic Peninsula and stretching south to Southern Puget Trough and the eastern slope of the Coastal Range in Washington are highly recommended in most of the countries which reported guidelines (seed zones number 221, 222, 231, 232 and 241, but also seed zone 030 from the western slope of the Olympic Peninsula). This area includes the best performing provenances, e.g., Bear Mountain (Louella-Blyn), which uniformly displays superior growth across sites in Europe [91–94] and in the native range [95]. The most recommended seed zone 221, is included in the provenance recommendations of Ireland, the United Kingdom, the Netherlands, Germany, the Czech Republic, Poland, as well as North Macedonia.

Other areas within the coastal part of the native range that are recommended in two or more countries are: (i) The western slope of the Cascades: In particular, seed zone 403 including is recommended in Germany and Czechia. Provenance Buck Creek (Darrington), from this area, showed superior height growth and lower frost sensitivity across many test sites in both countries [46,94], (ii) Eastern Vancouver Island in British Columbia: Seed zone 1020 (Courtenay) is recommended for low to medium elevation sites in central and (north-)western (sub-atlantic) Europe (Ireland, Great Britain, the Netherlands, Germany, Czechia and Poland), as well as for warmer regions in the Southeast of the continent (North Macedonia). This provenance is characterized by early flushing and good growth [22,48,51,77,91,93], (iii) The Crest of the Cascades stretching south to Northern Oregon: Seed zones from this region (e.g., seed zones 251, 621 and 622) are recommended in the Netherlands and in Czechia under some restrictions [49]. Based on field trials, these provenances might be suitable for dry climates of Europe [93,96], (iv) The eastern slope of the Washington Cascades: Provenances from there are included in unofficial recommendations in Austria and North Macedonia since they perform better under warmer and drier conditions of central-eastern and south-eastern Europe [43,77], (v) The northernmost parts of the Vancouver Island and the Western Cascades of Washington include suitable provenances for Scandinavian countries [97].

Due to their higher frost resistance, the interior provenances from northern Idaho might be an option for Scandinavian countries [97] and areas of high elevation with a more continental climate [49,92]. However, it should be kept in mind that they display an overall inferior growth performance (e.g., [43,93]).

In addition to native provenances, basic material from Europe constitutes also an important source of FRM for grand fir. A number of identified seed sources, selected stands, as well as qualified seed orchards and clones are available in Ireland, Great Britain, Belgium, the Netherlands, Sweden, Germany, the Czech Republic, Poland and Slovakia [89]. These sources are included in the FRM transfer guidelines of several countries, including Ireland and Germany (e.g., Baden-Württemberg-Germany, [70]). In particular, a series of

"special provenances", which are assumed to represent local land races are recommended in north-western Germany [98]. In other cases, selected stands within a European seed zone are recommended (e.g., in Bavaria-Germany, [78]). To our knowledge, European seed sources have never been field tested, fact that increases uncertainty when FRM from these sources is used, while the existing breeding efforts are at the level of qualified seed orchards.

#### 3.2.3. *Sitka spruce*

Regarding *Sitka spruce* (*Picea sitchensis*), the experience of European countries in breeding programs is very diverse. Ireland, Great Britain, Denmark and the Scandinavian countries though are beyond any doubt the ones who developed the most advanced breeding programs for the species. The different intensity of provenance research and breeding activities on *Sitka spruce* among European countries resulted in various forms of FRM transfer guidelines. Current guidelines span from general suggestions about the most suitable indigenous material (seed zones or provenances), to recommendations on native origins, local land races and improved genetic material produced through intensive selection and controlled crossings in breeding programs. Undoubtedly, the significance of inter-provenance variation for the adaptation of *Sitka spruce* FRM to European conditions is a well-studied and known topic, in the context of using *Sitka spruce* seed sources for planting in Europe.

According to official and unofficial national recommendations, the main provenances recommended in selected European countries are originating from British Columbia; namely Haida Gwaii (seed zone 1110), Vancouver Island (seed zones 1010, 1020), Oregon (seed zones 041, 051, 052, 053, 061, 062, 071, 072, 081, 082, 090) and Washington (seed zones 012, 030). Depending on the harshness of site conditions, Haida Gwaii provenances are suggested as more cold hardy, whereas provenances from Oregon and Washington are considered to be more appropriate for milder conditions, however, the southern provenances when compared to the Haida Gwaii and Vancouver Island ones, provide significantly higher productivity [13,99–101]. For the extremely harsh conditions of western Norway, material originating from Seward, Alaska is recommended [13,53]. Still, Haida Gwaii provenances can be planted even in Scandinavia, and specifically in southwestern Norway and southern Sweden [52,53]. Nevertheless, results obtained from plus tree progeny testing indicate that the use of genetically improved FRM may be preferred over the indigenous seed sources for future use in Europe.

In the case of countries with advanced breeding programs such as the United Kingdom, Ireland or Denmark, the use of improved genetic material can be strongly recommended. Tested progenies, obtained from controlled crosses among selected parents, are vegetatively propagated and are the first choice for deployment in commercial plantations [102]. According to Samuel et al. [37], the use of improved forest reproductive material, on average, allows for 15–20% additional profit compared to the one obtained from unimproved material originating from Haida Gwaii. However, due to relatively rare mast seed years, the use of improved seed from seed orchards may not be sufficient to cover the needs, requiring also the use of seed from non-improved local stands of the species.

#### 3.2.4. *Lodgepole pine*

For *lodgepole pine* (*Pinus contorta*) the afforestation interest is currently limited to the northern European countries–especially Sweden–and, to a lesser extent in Great Britain. This is due to the good growth performance of the species in the Nordic countries [57,103] and to its lower susceptibility to insects (especially *Rhyacionia buoliana*; [104–106], compared to that recorded in more southern latitudes. Therefore, countries that issued FRM transfer guidelines and responded to our questionnaire are limited to the North-Northwest of the continent. In particular, official guidelines are available in Ireland, the United Kingdom, Norway and Sweden, while non official ones exist in Estonia.

In the above mentioned European regions and countries, provenance research and practical experience led to the recommendation of different varieties and provenances depending on the climatic conditions. In general, the *Pinus contorta* var. *latifolia* variety is better suited to regions with more continental climate, like Sweden and the inland sites of Norway, while the *Pinus contorta* var. *contorta* variety displays superior performance in areas under strong oceanic influence, like the British Isles and coastal areas of Norway.

In particular, coastal Alaskan provenances of the *contorta* variety have been recommended for growing in nursing mixtures with *Sitka spruce*, while north coastal origins of the same variety, including the Vancouver Island and Haida Gwaii seed sources, are considered suitable, as being hardy in general, in Great Britain [106] and in Ireland, especially for the infertile upland sites of the country [107]. South coastal provenances should be preferred in less exposed mineral sites of Ireland [107], whereas Skeena River seed source, which represents a transition zone between the varieties *contorta* and *latifolia*, has been recommended for less exposed mineral soils in the United Kingdom [106]. In Norway, coastal Alaskan provenances are also recommended for the coastal areas of the country, with the coastal provenance Skagway being recommended for the harsher coastal sites [108–110]. Along the Norwegian coast, an increase of survival was observed when the latitude of the seed origin was increasing [111]. Contrariwise, moving the *latifolia* variety provenances to the North, by two to five degrees (compared to the latitude of the seed origin), has been suggested in Sweden. Such a transfer enhances frost hardiness and, in contrast to an altitudinal transfer, is not connected with significant height losses [112–115]. Apart from Sweden, the *latifolia* variety, and particularly the provenances originating from the northern half of British Columbia and southern Yukon are recommended for south-eastern Norway [108–110]. Finally, provenances of the same variety from British Columbia and Montana are unofficially recommended in Estonia [116].

In addition to provenance research, breeding programs have been launched in order to provide forest reproductive material of high quality in two of the aforementioned countries. In Sweden, breeding has mainly focused on improving growth, survival and form traits [117,118]. Base material for breeding was initially collected for the *latifolia* variety from more than 100 native stands growing in Yukon, British Columbia and partly in Alberta, Canada. The current collection of plus trees mainly consists of young individuals selected from the best open pollinated families, that were OP progenies of the initially selected plus trees in the 100 native stands of the species (see [118] and citations therein). Seed orchards of the above mentioned breeding program are the preferred lodgpole pine seed source for the Swedish Forestry [119]. In Great Britain, a particular focus of a former breeding program was the identification of inter-provenance hybrids, combining the good vigor of the south coastal material with the good form of the interior provenances. However, due to changing trends in afforestation [120], environmental concerns and insect outbreaks there has been a major decline in afforestations with lodgepole pine and the breeding program has been terminated.

#### 3.2.5. *Black locust*

Regarding *Black locust* (*Robinia pseudoacacia*), and given the recent extensive experience obtained from the breeding programs carried out in specific European countries, the recommendations on FRM mainly refer to European basic material (seed sources, stands, orchards, clone/clonal mixtures). The species naturalization in Europe's conditions after its introduction led to the testing of local land races, and not of indigenous FRM material, in eight European countries (i.e., Hungary, Croatia, Germany, Austria, Belgium, Poland, France and Spain).

In Western and Central Europe, the use of FRM from seed sources, seed stands and orchards is country-specific. Mostly seed from Hungarian, Romanian and Bulgarian qualified seed orchards and selected seed stands is recommended in Western European countries, although according to the official national recommendations also local genetic material (collected from qualified seed orchards, or selected seed stands) can be planted.

Concerning FRM of clones and clonal mixtures, only specific clones are recommended in more than two countries. While Hungary is the leading country in selection and breeding of black locust, the majority of cultivars included in the national recommendations were field tested but not produced in the country. For Central and Western Europe the three tested clones Appalachia (Eppelecsi), Jáseed zonskiséri and Nyireségi are recommended, however four other clones (i.e., Kiskunsági, Üllôi, Zalai and Rózsaseed zonesìn) seem to be also appropriate. For countries with advanced black locust breeding programs, locally tested clones of good performance may be a promising alternative to the commonly recommended ones [63,66,68,121,122].

## 4. Discussion

The present review on non-native tree species provenance recommendations across Europe summarizes the huge efforts of several generations of forest scientists to assess the quantitative and adaptive genetic variation of non-native trees in Europe and to utilize this knowledge for successful, stable and productive reforestation programs. Although many trial series were established under the aegis of IUFRO in transnational cooperation, which included common seed harvests in the species' native range, common experimental designs, common standards and measurement periods, our analysis shows that little efforts were made to consolidate the measured trial data across countries and to provide results from joint data analysis, publications and provenance recommendations for the whole continent.

Instead, the majority of trials were analyzed at a national level providing field guidelines for nurseries and practitioners. So far, this "nationalizing" of international trials made it very difficult to draw common conclusions and to establish European breeding programs. Moreover, the lack of transnational analysis concealed the fact that many trials across Europe showed very comparable results and recommendations. For example, the most well-adapted and productive provenances of Douglas-fir for many European countries originate from the western Cascade Range in Oregon, Washington and British Columbia, and the exact order of ranks within a given trial rather depends on which provenances were tested at a certain test site and not necessarily on the origin of the provenances. Thus, our review confirmed North American studies on the adaptive landscape of these conifers in relation to traits such as growth, bud burst or cold resistant (e.g., [123–126]). These adaptive landscapes can be considered a result of population history, demography (e.g., [127]) and local adaptation [128], where population differentiation can mostly be linked to climate variables (e.g., mean temperatures, coldest month temperatures, etc.) along gradients from South-to-North and West-to-East. The amount of variation explained by climate variables differs among species and subspecies [126]. Across species, the European recommendations widely fit to these findings and suggest that plantations in Northern Europe or at higher elevation should rather include provenances that originate from higher altitudes or more northern areas of the species natural ranges, whereas plantations with lower frost risk should rather use genetic materials originating from coastal regions (not to confuse with the coastal subspecies) and lower elevations. The comparable recommendations among European countries and the high congruence to range-wide North American studies indicate that future experiments might not need to cover many different countries, but the most important ecoregions and bioclimatic zones. Moreover, existing models for quantitative and adaptive traits developed in North America [124,126] could be transferred to the European landscape to preselect the most promising seed sources. Recently, a similar transfer, but in the opposite direction, demonstrated the applicability of provenance-specific growth models of Douglas-fir from Central Europe to the species native range in Western North America [129]. For Sitka spruce and lodgepole pine such models are already available and should be applied for the European landscape for provenance preselection [123,130–132]. Similar accurate data are missing for grand fir, however similar geographic variation of traits was identified in European [133] and North American trials [134]. For black locust, so far no geographic variation among provenances was detected [135]. Overall, our review

highlights the need for better transnational (within Europe) and transcontinental exchange of data, models and seed sources, to select appropriate seed material for future plantations. In particular, such data exchange will be needed if new and so far not used or tested tree species need to be transferred in the light of climate change to Europe or vice versa. For example, North American conifers such as ponderosa pine (*Pinus ponderosa*), interior spruce (*Picea glauca*, *Picea engelmannii*, and their hybrids) are well studied species within their native range, fact that could potentially allow easy model transfers for provenance preselection to Europe [125,126,136–138].

Present provenance recommendations of non-native tree species in Europe are mainly based on seed collections within the 1960ties and 1970ties and on trials established within the 1970ties and 1980ties. These decades can be described as the most significant era of provenance research, with wide-ranging cooperation among countries and continents, which even allowed cooperation among political systems (e.g., see [139] for results on IUFRO-trials in Eastern Germany). By the end of the 1980ties, the most important international and national provenance trial series on Douglas-fir, grand fir, Sitka spruce and lodgepole pine were already established and the interest in new trial series was limited. And although non-native deciduous trees, such a black locust or red oak, are of similar importance as conifers, the provenance research community in most European countries lacked interest until the end of the 1990ties to establish single trials or trial series across several countries. Only recently, a number of European countries started transnational activities to establish arboreta with different black locust varieties.

Given that present provenance recommendations are based on measurements made in past climate on trees that were grown out of seed harvested more than 50 years ago, a growing number of scientists questioned the role of old provenance experiments for recommending future reforestation stock [140,141]. And indeed, comparisons of current temperature change to paleoclimatic variations suggest that new climates, substantially different from current conditions might evolve in the future and give rise to new ecosystems [142,143]. On the other hand, provenance trials were identified to be most valuable tools to identify the breadth of species climatic niches and to develop seed transfer and assisted migration schemes (e.g., [123,138,144]). For example, Chakraborty et al. [17,145] used a comprehensive dataset of provenance trials located in Austria and southern Germany to model tree height and basal area of Douglas-fir in relation to the climate of the plantation trial site and the climate of provenance origin. This model, a so-called 'Universal-Response-Function' Wang et al. [131] allowed to identify the most important climatic drivers for tree growth and to calculate the response of individual provenances to climate change. For Douglas-fir, Chakraborty et al. [17,145] found that populations originating from regions with average annual temperatures ranging among 6–8 °C perform best in the current climate, while future reforestations should make use of seed material from slightly warmer climatic origins whose mean temperature is between 7–9 °C. Such new seed sources might be either obtained from lower altitudes or from more southerly located seed zones in Northwest America. However, climate change might not only require adapted seed material, but will enable additional plantations on further forest sites: in particular high elevation sites (above 1000 m a.s.l.) will become more suitable for Douglas-fir [17]. 'Universal-Response-Functions', as done for Douglas-fir [145] or lodgepole pine [131], should also be developed for other non-native species to better utilize existing provenance data and to allow predictions for global change. But even existing models have limitations when applied for climate change because most trials series miss provenances from climatically extreme locations in the native range, as such provenances were not considered in previous trials due to practical reasons. For example, Douglas-fir trials in Europe rarely contain southern provenances from California or Mexico and northern provenances from northern British Columbia. Moreover, past common garden series miss trials at climatically extreme sites, and did not tested non-native species and their provenances consistently towards the species range limits [144,146]. Instead, past trials were established under optimal climate conditions to select the most productive planting stock for foresters. This constrains the

applicability of provenance recommendations and climate response models to climate change scenarios beyond the limit of the past trial climate. For example, Chakraborty et al. [17] estimated an increasing model uncertainty for trial locations with mean annual temperatures above 9 °C, a temperature which is predicted to occur frequently in many parts of Europe in the face of climate change. Thus, new trial series are urgently needed either within the species native or non-native range, which should rather aim at achieving a better understanding of the species-specific climate constraints and the correlations between functional trait variation and the environmental conditions of trial sites and origins of the tested seed sources. Carefully designed with provenances from the entire climatic range, and trial sites established within and outside of the species climatic range, such trial series would push our understanding of the species local adaptations and would allow identifying suitable future reforestation stock. Due to the availability of gridded climate data and multivariate climate response models such as the universal response functions or multivariate regression tree approach [126], such trial series can be achieved with fewer provenances planted on fewer trial sites without affecting the prediction accuracy of the models [131]. Thus, in 15–20 years new data for improved models allowing predictions for wider climate spaces could become available.

While the scientific interest in provenance research across Europe decreased in the late 1980ties, several countries increased their tree breeding activities which were ongoing since the 1970ties (e.g., [37]). Such activities were either based on the results of the previous provenance experiments or on selecting superior plus trees growing in commercial plantations, by subjecting them to intensive selection. For example, within the British tree breeding program for Sitka spruce, selection intensity among plus trees of commercial plantations was around 1:75,000 for the first ten years [147]. Another example is the Douglas-fir breeding program in France, where more than 500 provenances were tested in 62 trials to identify the best seed sources. This provenance testing was accompanied by progeny testing on 500 plus trees from France and IUFRO collections [20]. Similar programs exist for lodgepole pine in Sweden [57] and to a smaller extent for black locus in Hungary [121]. Successful breeding programs with significant improvements in productivity and quality already contribute to the national reforestation activities by providing seed materials from seed orchards and vegetative propagation [13]. Given (i) the regulatory frameworks on forest reproductive material in the EU (European Union) and OECD (Organisation for Economic Co-operation and Development), (ii) the decline of old forests for seed harvest, and (iii) the ongoing tree improvement programs in the species' native ranges, it can be expected that seed imports into the EU might further decline in the long-term. Thus, the European breeding programs are the promising perspective for future seed procurement. However, the various national programs need to be strongly promoted across the country borders and develop further into European breeding programs for transnational breeding zones [36].

## 5. Conclusions

Consolidation of trial data obtained across countries will render possible the joint analysis that is urgently needed, and will facilitate the development of 'Universal-Response-Functions' for the species of interest, rendering possible the identification the genetic material suitable for global change. New provenance trial series testing seed sources from the entire climatic range of the species, established in sites falling within and outside their ranges, are urgently needed for understanding the species-specific climate constraints and for correlating functional traits to the seed origin and the environmental conditions of the test sites, so that the selection of suitable forest reproductive material of non-native tree species in the face of climate change can be feasible.

**Supplementary Materials:** The following supporting information can be downloaded at: https://www.mdpi.com/article/10.3390/f13020273/s1, Table S1. Questionnaire of the survey carried out in the frame of the FP-1403 NNEXT Cost Action (Non-native tree species for European forests: experiences, risks and opportunities) and its Working Group 2-'Pathways' on a) non-native tree species established field trials and b) the existing official or non-official recommendations for planting of their FRM in the participating countries.

**Author Contributions:** Conceptualization, P.A. and M.K.; methodology, P.A., M.K., D.C., M.v.L., S.S., C.N., M.M.K., J.-C.B., M.W. and J.K.; construction of maps, D.C. and S.S.; information contributors, P.A., M.K., J.-C.B., D.C., M.M.K., J.K., C.N., S.S., M.v.L., M.W., V.A., K.A., P.B., R.B., M.Ð., M.F., J.F., B.K., B.C., Z.K., A.K., V.L., T.M., M.R., B.M., G.M., C.M., S.P., K.P., E.B.P., S.M.S. and I.T.; writing—original draft preparation, P.A., M.K., J.-C.B., C.N., M.M.K., S.S. and M.v.L.; writing—review and editing, P.A., C.N., S.S., M.v.L., M.K. and M.M.K. All authors have read and agreed to the published version of the manuscript.

**Funding:** This research received no external funding.

**Institutional Review Board Statement:** Not applicable.

**Informed Consent Statement:** Not applicable.

**Data Availability Statement:** Not applicable.

**Acknowledgments:** This paper is an output from the European COST Action FP1403 'Non-native tree species for European forests—experiences, risks and opportunities' (NNEXT), and specifically the Working group "Pathways", in which all authors participated and contributed information in the relevant questionnaire.

**Conflicts of Interest:** The authors declare no conflict of interest.

## References

1. CBD (Convention on Biological Diversity). Descisions adopted by the Conference of the Parties to the Convention on Biological Diversity at its sixth meeting. In Proceedings of the Sixth Meeting of the Conference of the Parties to the Convention on Biological Diversity, The Hague, The Netherlands, 7–19 April 2002.
2. FRA 2015: Forest Resources Assessment Working Paper 180—Terms and Definitions. Rome, Italy, 2015. Available online: https://www.fao.org/3/ap862e/ap862e00.pdf (accessed on 16 December 2021).
3. Nyssen, B.; Schmidt, U.E.; Muys, B.; van der Lei, P.B.; Pyttel, P. The history of introduced tree species in Europe in a nutshell. In *Introduced Tree Species in European Forests: Opportunities and Challenges*; Krumm, F., Vítková, L., Eds.; European Forest Institute (EFI): Freiburg, Germany, 2016; pp. 44–54.
4. Camenen, E.; Porté, A.J.; Benito Garzón, M. American trees shift their niches when invading Western Europe: Evaluating invasion risks in a changing climate. *Ecol. Evol.* **2016**, *6*, 7263–7275. [CrossRef] [PubMed]
5. Radu, S. *Pinus strobus*: Past and future in Europe. A page of silvicultural history and international scientific cooperation. *Ann. For. Res.* **2008**, *51*, 133–140.
6. Morgenstern, M. *Geographic Variation in Forest Trees: Genetic Basis and Application of Knowledge in Silviculture*; UBC Press: Vancouver, BC, Canada, 2011.
7. Kay, J.; Anderson, M.L. Douglas fir at home and abroad. *Emp. For. J.* **1928**, *7*, 22–40.
8. Kanzow, H. Die Douglasie: Aufstellung einer Ertragstafel auf Grund der Ergebnisse der preußischen Probeflächen und Auswertungen von Provenienzversuchen (Establishment of a yield table based on the results from Prussian field trials and evaluation of provenance tests). *Z. Forst-Jagdwes.* **1937**, *69*, 65–93, 113–139, 242–271.
9. Larsen, J.B.; Ruetz, W.F. Frostresistenz verschiedener Herkünfte der Douglasie (*Pseudotsuga menziesii*) und der Küstentanne (*Abies grandis*) entlang des 44. Breitengrades in Mittel Oregon (Frost resistance of different Douglas fir (*Pseudotsuga menziesii*) and grand fir (*Abies grandis*) provenances along the 44th latitude parallel in Mid Oregon). *Forstwiss. Cent.* **1980**, *99*, 222–233.
10. Cannell, M.G.R.; Rothery, P.; Ford, E.D. Competition within stands of *Picea sitchensis* and *Pinus contorta*. *Ann. Bot.* **1984**, *53*, 349–362. [CrossRef]
11. Kjær, E.D.; Lobo, A.; Myking, T. The role of exotic tree species in Nordic forestry. *Scand. J. For. Res.* **2014**, *29*, 323–332. [CrossRef]
12. Carneiro, M.; Fabião, A.; Madeira, M. Effects of site preparation and slash management on growth and understory vegetation of Eucalyptus globulus plantations along a rotation time span in Portugal. *Eur. J. For. Res.* **2014**, *133*, 941–955. [CrossRef]
13. Lee, S.; Thompson, D.; Hansen, J.K. Sitka Spruce (*Picea sitchensis* (Bong.) Carr). In *Forest Tree Breeding in Europe*; Pâques, L.E., Ed.; Springer: Dodrecht, The Netherlands, 2013; pp. 177–227.
14. Fotiadis, G.; Kyriazopoulos, A.P.; Fraggakis, I. The behaviour of *Ailanthus altissima* weed and its effects on natural ecosystems. *J. Environ. Biol.* **2011**, *32*, 801.
15. White, T.L.; Adams, W.T.; Neale, D.B. *Forest Genetics*; CABI: Oxford, UK, 2007; pp. 187–230.

16. Eilmann, B.; de Vries, S.M.G.; den Ouden, J.; Mohren, G.M.J.; Sauren, P.; Sass-Klaassen, U. Origin matters! Difference in drought tolerance and productivity of coastal Douglas-fir (*Pseudotsuga menziesii* (Mirb.)) provenances. *For. Ecol. Manag.* **2013**, *302*, 133–143. [CrossRef]
17. Chakraborty, D.; Wang, T.; Andre, K.; Konnert, M.; Lexer, M.J.; Matulla, C.; Weissenbacher, L.; Schueler, S. Adapting Douglas-fir forestry in Central Europe: Evaluation, application, and uncertainty analysis of a genetically based model. *Eur. J. For. Res.* **2016**, *135*, 919–936. [CrossRef]
18. Merceron, N. Processus Écologiques et Évolutifs Impliqués dans le Succès de L'introduction de *Quercus rubra* L. en Europe. Ph.D. Thesis, University of Liège, Liege, Belgium, 28 November 2016.
19. Kleinschmit, J.; Bastien, J.-C. IUFRO's role in Douglas-fir (*Pseudotsuga menziesii* (Mirb.) Franco) tree improvement. *Silvae Genet.* **1992**, *41*, 161–173.
20. Bastien, J.-C.; Sanchez, L.; Michaud, D. Douglas-Fir (*Pseudotsuga menziesii* (Mirb.) Franco). In *Forest Tree Breeding in Europe*; Pâques, L.E., Ed.; Springer: Dodrecht, The Netherlands, 2013; pp. 325–369.
21. Ying, C.; McKnight, L. *Proceedings of the IUFRO International Sitka Spruce Provenance Experiment*; Ministry of Forests of British Columbia: Victoria, BC, Canada; The Irish Forestry Board Co.: Wicklow, Ireland, 1993.
22. Lines, R. *Choice of Seed Origins for the Main Forest Species in Britain, Bulletin No. 66*; Forestry Commission: London, UK, 1987.
23. Daubree, J.; Kremer, A. Genetic and phenological differentiation between introduced and natural populations of *Quercus rubra* L. *Ann. Sci. For.* **1993**, *50*, 271s–280s. [CrossRef]
24. Eckhart, T.; Walcher, S.; Hasenauer, H.; van Loo, M. Genetic diversity and adaptive traits of European versus American Douglas-fir seedlings. *Eur. J. For. Res.* **2017**, *136*, 811–825. [CrossRef]
25. Braun, H.; Wolf, H. Untersuchungen zu Wachstum und Frosthärte von Douglasien-Populationen in Ostdeutschland (Study of growth and frost tolerance of Douglas fir populations in East Germany). *Beiträge Forstwirtsch. Landsch.* **2001**, *35*, 211–214.
26. Jansen, K.; Sohrt, J.; Kohnle, U.; Ensminger, I.; Gessler, A. Tree ring isotopic composition, radial increment and height growth reveal provenance-specific reactions of Douglas-fir towards environmental parameters. *Trees-Struct. Funct.* **2013**, *27*, 37–52. [CrossRef]
27. Huang, W.; Fonti, P.; Larsen, J.B.; Ræbild, A.; Callesen, I.; Pedersen, N.B.; Hansen, J.K. Projecting tree-growth responses into future climate: A study case from a Danish-wide common garden. *Agric. For. Meteorol.* **2017**, *247*, 240–251. [CrossRef]
28. Nielsen, U. Genetisk Variation i Sitkagran (*Picea sitchensis* (Bong.) Carr.) i Højdevækst, Stammeform og Frosthærdighed—Vurderet ud fra Danske Proveniens-, Afkoms- og Klonforsøg [Genetic Variation in Sitka Spruce (*Picea sithensis* (Bong.) Carr.) Regarding Height Growth, Stem Form and Frost Hardiness at the Provenance, Progeny, and Clonal Level, Based on Danish Field Trials]. Ph.D. Thesis, The Royal Veterinary and Agricultural University, Copenhagen, Denmark, 1994.
29. Moise, M. Studiul Variabilităţii Genetice Interpopulaţionale la Larice (*Larix decidua* Mill.) şi duglas (*Pseudotsuga menziesii* Mirb. Franco) în Culturi Comparative Multistaţionale din România (Study of Intrapopulation Genetic Variability for Larch and Douglas Fir In Multisite Comparative Trials). Ph.D. Thesis, Academy of Agricultural and Sylvicultural Sciences, Bucharest, Romania, 1998.
30. Murphy, P.G.; Pfeifer, A.R. Wood density and branching characteristics of Sitka spruce provenances grown in Ireland. In Proceedings of the Joint Meeting of Western Forest Genetics Association & IUFRO Working Parties S 2.02–05, 00, 12 and 14, Olympia, WA, USA, 20–24 August 1990; Volume 2, pp. 2–5.
31. Cannell, M.G.R. Production of branches and foliage by young trees of *Pinus contorta* and *Picea sitchensis*: Provenance differences and their simulation. *J. Appl. Ecol.* **1974**, *11*, 1091–1115. [CrossRef]
32. Larsen, J.B.; Kromann, H.K. Douglas-fir provenances in Denmark. *Forstl. Forsøgsvaesen I Danmark.* **1983**, *38*, 346–375.
33. Kjær, E.D.; Barner, H.; Andersen, A.M.D. Undersøgelse af afkom fra tre danske douglasgranfrøplantager. *Dan. Skovforen. Tidsskr.* **1996**, *81*, 1–17.
34. Straker, K.C.; Quinn, L.D.; Voigt, T.B.; Lee, D.K.; Kling, G.J. Black locust as a bioenergy feedstock: A review. *Bioenergy Res.* **2015**, *8*, 1117–1135. [CrossRef]
35. OECD. *Consensus Ducument on the Environmental Directorate*; Series on Harmonization of Regulatory Oversight in Biotechnology No. 43; OECD Environmental Health and Safety Publications: Paris, France, 2008.
36. Pâques, L.E. Introduction. In *Forest Tree Breeding in Europe*; Springer: Dodrecht, The Netherlands, 2013.
37. Samuel, S.; Fletcher, A.M.; Lines, R. *Choice of Sitka Spruce Seed Origins for Use in British Forests*; Bulletin No. 127; Forestry Commission: Edinburgh, UK, 2007.
38. Fick, S.E.; Hijmans, R.J. WorldClim 2: New 1-km spatial resolution climate surfaces for global land areas. *Int. J. Climatol.* **2017**, *37*, 4302–4315. [CrossRef]
39. Ducci, F.; Heois, B.; de Rogatis, A.; Proietti, R. *Pseudotsuga menziesii* (Mirb.) Franco, 1969–1970 IUFRO field experiment results in Europe and Italy. In Proceedings of the Atti IV Congress SISEF—Meridiani e Foreste, Rifreddo, Italy, 7–10 October 2005; pp. 1001–1010.
40. Fletcher, A.M.; Samuel, C.J.A. *Choice of Douglas Fir Seed Sources for Use in British Forests*; No. 129; Forestry Commission: Edinburgh, UK, 2010.
41. Bastien, J.C. Grand fir provenance experiment—Forêt Domaniale de Chaud. In Proceedings of the Joint Meeting of the IUFRO Working Parties S2.02.05, 06, 12 and 14, Limoges, France, 1–4 August 1995.
42. Liesebach, M.; Weißenbacher, L. Experience with *Abies grandis* in the summer dry regions of Austria. *Forst Holz* **2007**, *62*, 19–20.

43. Liesebach, M.; Schueler, S.; Weissenbacher, L. Provenance trials with Grand fir (*Abies grandis* D. (Don) Lindl.) in Austria—Suitability, growth performance and variation. *Austrian J. For. Sci.* **2008**, *125*, 183–200.
44. Rau, H.M.; Weisgerber, H.; Kleinschmit, J. Vorläufige Erfahrungen mit Küsten-Tannen-Provenienzen in West-Deutschland (Preliminary experience with grand fir provenances in West Germany). *Forst Holz* **1991**, *46*, 245–249.
45. Rau, H.-M.; Kleinschmit, J.; König, A.; Ruetz, W.; Svolba, J. Provenienzversuche mit Küstentanne (*Abies grandis* LINDL.) in Westdeutschland [Provenance trials with grand fir (*Abies grandis* LINDL.) in West Germany]. *Allg. Forst Jagdztg.* **1998**, *169*, 109–115.
46. Rau, H.M.; Schönfelder, E. Anbauerfahrungen mit Herkünften der Großen Küstentanne (*Abies grandis* LINDL.) in Westdeutschland—Ergebnisse der aufnahme von 18 Flächen im Alter 27 (Experience with grand fir (*Abies grandis* LINDL.) cultivation in West Germany—Results from 19 plots in the age of 27 years). *Austrian J. For. Sci.* **2008**, *125*, 201–216.
47. Kranenborg, K. *Abies grandis* provenance research in The Netherlands. In Proceedings of the Joint Meeting of the IUFRO Working Parties S2.02.05, 06, 12 and 14, Limoges, France, 1–4 August 1995.
48. Samuel, C.J.A. *The Influence of Seed Origin on the Growth of Grand Fir in Britain*; Forestry Commission: Edinburgh, UK, 1996.
49. Vančura, K.; Beran, B. Results on North American firs IUFRO tests in the Czech Republic. In Proceedings of the Joint Meeting of the IUFRO Working Parties S2.02.05, 06, 12 and 14, Limoges, France, 1–4 August 1995.
50. Burzyński, G.; Górczyński, J. *Odporność na Mróz Oraz Wstępna Ocena Przyfatności Hodowlanej Jodły Olbrzymiej Różnego Pochodzenia*; Technical Report; Forest Research Institute: Raszyn, Poland, 1990.
51. Dolnicki, A.; Kraj, W. Dynamics of frost resistance in various provenances of *Abies grandis* Lindl. *Acta Soc. Bot. Pol.* **1998**, *67*, 51–58. [CrossRef]
52. Magnesen, S. *Det Internasjonale Sitkagran-Proveniensforsøket pa Vestlandet [The International Sitka Spruce Provenance Trial in Western Norway]*; Norwegian Forest Research Institute: Aas, Norway, 1986.
53. Magnesen, S. *Forsøk med Nordlige Sitkagran-Provenienser på Vestlandet [Experiments with Northern Sitka Spruce Provenances in Western Norway]*; Report; Norwegian Forest Research Institute: Aas, Norway, 1999.
54. O'Driscoll, J. Sitka spruce international ten provenance experiment: Results to end of nursery stage. *For. Genet. Resour. Inf.* **1978**, *7*, 35–46.
55. Rollins, L.A.; Moles, A.T.; Lam, S.; Buitenwerf, R.; Buswell, J.M.; Brandenburger, C.R.; Flores-Moreno, H.; Nielsen, K.B.; Couchman, E.; Brown, G.S.; et al. High genetic diversity is not essential for successful introduction. *Ecol. Evol.* **2013**, *3*, 4501–4517. [CrossRef]
56. Orlić, S. International test of Sitka spruce (*Picea sitchensis* (Bong.) Carr.) provenances in Croatia. *Sumar. List.* **1998**, *122*, 213–220.
57. Elfving, B.; Ericsson, T.; Rosvall, O. The introduction of lodgepole pine for wood production in Sweden—A review. *For. Ecol. Manag.* **2001**, *141*, 15–29. [CrossRef]
58. Şimşek, Y. Untersuchungen über das Wachstum der *Pinus contorta* (Dougl.) von verschiedenen Herkunften im Osten des Schwarzmeergebietes der Turkei [Growth study of different *Pinus contorta* (Dougl.) provenances in the eastern Black Sea area of Turkey]. *Silvae Genet.* **1989**, *38*, 157–168.
59. Lindgren, D.; Fries, A.; Lindgren, K.; Löfmark, S. Lodgepole pine cuttings. In *Massproduction Technology for Genetically Improved Fast Growing Forest Tree Species*; AFOCEL/IUFRO: Bordeaux, France, 1992; Volume 1, pp. 105–111.
60. Lindgren, K. IUFRO Pinus contorta seed collections—Distributions and publications. In *Pinus contorta—From Untamed Forest to Domesticated Crop, Proceedings of the Meeting of IUFRO WP 2.02.02 and Frans Kempe Symposium, Umeå, Sweden, 24–28 August 1992*; Lindgren, D., Ed.; Department of Forest Genetics and Plant Physiology, Swedish University of Agricultural Sciences: Uppsala, Sweden, 1992; pp. 232–237.
61. Rédei, K.; Osváth-Bujtás, Z.; Balla, I. Clonal approaches to growing black locust (*Robinia pseudoacacia*) in Hungary: A review. *Forestry* **2002**, *75*, 547–552. [CrossRef]
62. Rédei, K.; Veperdi, I.; Meilby, H. Stand structure and growth of mixed white poplar (*Populus alba* L.) and black locust (*Robinia pseudoacacia* L.) plantations in Hungary. *Acta Silv. Lignaria Hung.* **2006**, *2*, 23–32.
63. Rédei, K. *Black Locust (Robinia pseudoacacia L.) Growing in Hungary*; Hungarian Forest Research Institute, Agroinform Kiadó: Budapest, Hungary, 2013; p. 78.
64. Orazio, C.; Debets, R.C.; Cantero, L.D.L.A.; Casero, J.D.; Recio, C.P.; Bravo, F.; Bengoetxea, N.G.; Gonzalez, A.A.; Jinks, R.; Paillassa, E.; et al. *Arboretum and Demonstration Site Catalogue REINFFORCE (REsource INFrastructures for Monitoring, Adapting and Protecting European Atlantic FORests under Changing Climate)*; IEFC-EFIATLANTIC: Cestas, France, 2013.
65. De Natale, F.; Floris, A.; Gasparini, P.; Scrinzi, G.; Tabacchi, G.; Tosi, V. *Inventario Nazionale delle Foreste e dei Serbatoi Forestali di Carbonio (National Inventory of Forests and Forest Carbon Sinks)*; MiPAAF—Ispettorato Generale del Corpo Forestale dello Stato, CRA-ISAFA: Trento, Italy, 2005.
66. Kalmukov, K. Improvement of the black locust stands in Bulgaria. In Proceedings of the Biennial International Symposium, Forest and Sustainable Development, Brașov, Romania, 15–16 October 2010; Małek, S., Jasik, M., Eds.; Transilvania University Press: Brașov, Romania, 2011; pp. 41–46.
67. Hasenauer, H.; Gazda, A.; Konnert, M.; Lapin, K.; Mohren, G.M.J.; Spiecker, H.; van Loo, M.; Pötzelsberger, E. *Non-Native Tree Species for European Forests: Experiences, Risks and Opportunities. COST Action FP1403 NNEXT Country Reports, Joint Volume*, 3rd ed.; University of Natural Resources and Life Sciences: Vienna, Austria, 2017.

68. Schueler, S.; Weißenbacher, L.; Sieberer, K. Robinien für Energie- oder Wertholz- die Sorte macht's! (Black locust for energy or valuable wood, the sort makes the difference). *Forstztg* **2006**, *117*, 8–9.
69. Konnert, M.; Fady, B.; Gömöry, D.; A'Hara, S.; Wolter, F.; Ducci, F.; Koskela, J.; Bozzano, M.; Maaten, T.; Kowalczyk, J. *Use and Transfer of Forest Reproductive Material in Europe in the Context of Climate Change*; European Forest Genetic Resources Program (EUFORGEN), Bioversity International: Rome, Italy, 2015; Volume xvi, 75p.
70. Anonymous. *Herkunftsempfehlungen für Forstliches Vermehrungsgut in Baden-Württemberg [Provenance Recommendations for Forest Reproductive Material]*; Forstliche Versuchs- und Forschungsanstalt Baden-Württemberg (FVA): Freiburg, Germany, 2018.
71. Lavender, D.P.; Hermann, R.K. *Douglas-Fir: The Genus Pseudotsuga*; Forest Research Publications Office, Oregon State University: Corvallis, OR, USA, 2014.
72. Spiecker, H.; Lindner, M.; Schuler, J.K. *Douglas-Fir—An Option for Europe. What Science Can Tell Us 9*; European Forest Institute (EFI): Joensuu, Finland, 2019.
73. Enescu, M.C. *Cercetari de Provenienta la Duglas si Larice (Provenance Research on Douglas Fir and European Larch)*; Seria A II-A; Centrul de Propaganda Tehnica Agricola, ICAS: Bucharest, Romania, 1984.
74. Petkova, K. Investigation of Douglas-fir provenance test in North-Western Bulgaria at age 20. *For. Ideas* **2011**, *17*, 131–140.
75. Ruetz, W. Applying the results of Douglas-fir Provenance research to practical forestry—An Example from Bavaria. In Proceedings of the IUFRO Working Party on Breeding Strategy for Douglas-Fir as an Introduced Species (W.P. S 2.02.05), Vienna, Austria; 1987; pp. 251–263.
76. Weißenbacher, L. Herkunftswahl bei Douglasie—Der Schlüssel für einen erfolgreichen Anbau (Provenance selection in Douglas Fir—The key for a successful cultivation). *BFW Prax.* **2008**, *16*, 3–5.
77. Andonovski, V. Adaptive Capability of Some Introduced Forest Tree Species. Master's Thesis, University Ss. Cyril and Methodius, Skopje, North Macedonia, 1995.
78. Anonymous. *Herkunftsempfehlungen für Forstliches Vermehrungsgut in Bayern [Provenance Recommendations for Forest Reproductive Material in Bavaria]*; Amt für forstliche Saat- und Pflanzenzucht (ASP), Bayerisches Staatsministerium für Ernährung, Landwirtschaft und Forsten: Munich, Germany, 2016; p. 168.
79. Michaud, D.; Permingeat, J.; Bouvet, A. Douglas fir provenances from natural areas; results after 15 years in France. In Proceedings of the Joint Meeting of the IUFRO Working Parties. S2.02.05, 06, 12 and 14, Limoges, France, 1–4 August 1995.
80. Anonymous. *Afforestation Scheme 2014-2020 Rates. Douglas Fir. Forestry*; Department of Agriculture, Food and the Marine: Dublin, Ireland, 2018. Available online: https://www.agriculture.gov.ie/forestservice/publications/ (accessed on 10 December 2018).
81. Zas Arregui, R.; Merlo, E.; Diaz, R.; Fernández López, J. Stability across sites of Douglas-fir provenances in northern Spain. *For. Genet.* **2003**, *10*, 71–82.
82. Lavadinović, V.; Isajev, V.; Rakonjac, L.; Marković, N. Effect of altitude and continentality of Douglas fir provenances on height increment in test plantations in Serbia. *Lesn. Časopis* **2008**, *54*, 53–59.
83. Lupi, E. Performance of Douglas-Fir (*Pseudotsuga menziesii* (Mirb.) Fanco) Provenances from California in France and Other Atlantic Europe Regions; Opportunities and Risks in a Changing Climate Context. Master's Thesis, Albert-Ludwigs University of Freiburg, Freiburg, Germany, 2016.
84. Ducci, A.; Tocci, A. Primi resultati della sperimentazione IUFRO 1969-70 su *Pseudotsuga menziesii* (Mirb.) Franco nel'appennino centro settentrionale (First results of the 1969–70 IUFRO experiment on *Pseudotsuga menziesii* (Mirb.) Franco in the central-northern Apennines). *Ann. Ist. Sper. Selvic.* **1987**, *XVIII*, 213–289.
85. Ducci, F.; de Rogatis, A. Risorse Genetiche Forestali in Italia—I. In *Situ*; CRA SEL: Arezzo, Italy, 2009; pp. 14–23.
86. Popov, E. Results of 20 year old Douglas-fir Provenance Experiment Established on the Northern Slopes of Rila Mountain in Bulgaria. *J. For. Sci.* **2014**, *60*, 394–399. [CrossRef]
87. Ducci, F.; Proietti, R.; de Rogatis, A.; Monteverdi, M.C.; Germani, A.; Bresciani, A.; Teani, A.; Lauteri, M.; de Dato, G.; Cutino, I. Gestione delle risorse genetiche della Douglasia in Italia in relazione agli scenari dei cambiamenti globali. *I Georg. Quad.* **2016**, 137–157.
88. Rosette, C. *Contribution à L'exploration de la Variabilité Infraspécifique du Douglas, Mémoire EN*; INRA: Orléans, France, 1986; 136p.
89. FOREMATIS. "Forest Reproductive Material Information System" European Commission. 2021. Available online: https://ec.europa.eu/forematis/ (accessed on 14 November 2021).
90. Pseudotsuga Menziesii, Vilmorain, France. 2021. Available online: https://www.vilmorin-tree-seeds.com/seeds/recherchepep-1223-pseudotsuga-menziesii.html (accessed on 14 November 2021).
91. Bennuah, S. Provenance Variation in Grand Fir (*Abies grandis* Lindley). Master's Thesis, University of Edinburgh, Edinburgh, UK, 1998.
92. Kulej, M.; Socha, J. Effect of provenance on the volume increment of grand fir (*Abies grandis* Lindl.) under mountain conditions of Poland. *J. For. Sci.* **2008**, *54*, 1–8. [CrossRef]
93. Rau, H.-M. *Ergebnisse des Westdeutschen IUFRO-Küstentannen-Provenienzversuches im Alter 27, Beiträge aus der Nordwestdeutschen Forstlichen Versuchsanstalt Band 4*; Universitätsverlag Göttingen: Göttingen, Germany, 2008.
94. Frýdl, J.; Dostál, J.; Beran, F.; Čap, J.; Fulín, M.; Frampton, J.; Božič, G.; Mátyás, C. Exotic *Abies* Species in Czech Provenance Trials: Assessment after Four Decades. *Acta Silv. Lignaria Hung.* **2018**, *14*, 9–34. [CrossRef]
95. Clark, J.; Murphy, G. Estimating forest biomass components with hemispherical photography for Douglas-fir stants in northwest Oregon. *Can. J. For. Res.* **2011**, *41*, 1060–1074. [CrossRef]

96. König, A. Herkunftsdifferenzierung von *Abies grandis* im norddeutschen Tiefland und Mittelgebirgsraum sowie Empfehlungen zur Proveninezwahl. *Forst Holz* **2007**, *62*, 14–17.
97. Magnesen, S. IUFRO *Abies grandis* provenances in Southern Norway. In Proceedings of the Joint Meeting of the IUFRO Working Parties S2.02.05, 06, 12 and 14, Limoges, France, 1–4 August 1995.
98. Anonymous. *Herkunftsempfehlungen Abies Grandis (Douglas ex. D. Don) Lindl. (Große Küstentanne). Niedersachsen—Übriges Bundesgebiet (HKG 830 02)*; NW-FVA, Abteilung C—Waldgenressourcen: Hann. Münden, Germany, 2018; Available online: https://www.nw-fva.de/HKE/getData?request=getPdfSpecies&sid=830&hid=83002&cid=3 (accessed on 14 November 2021).
99. Thompson, D.; Lally, M.; Pfeifer, A. Washington, Oregon or Queen Charlotte Islands? Which is the best provenance of Sitka spruce (*Picea sitchensis*) for Ireland? *Ir. For.* **2005**, *62*, 19–34.
100. Thompson, D. Development of improved Sitka spruce for Ireland. *Ir. For.* **2013**, *70*, 104–118.
101. Goeckede, J.; Grotehusmann, H.; Rau, H.M. Suitability of different Sitka spruce provenances for planting in north-west Germany. *Forstarchiv* **1974**, *85*, 75–83.
102. Mason, W.L.; Gill, J. Vegetative propagation of conifers as a means of intensifying wood production in Britain. *Forestry* **1986**, *59*, 155–172. [CrossRef]
103. Fries, A.; Elfving, B.; Ukrainetz, N. Growth and survival of lodgepole pine and Scots pine after 25 years in a reciprocal transplant experiment in Canada and Sweden. *Scand. J. For. Res.* **2017**, *32*, 287–296. [CrossRef]
104. Kriek, W. IUFRO provenances of *Pinus contorta* in The Netherlands. In Proceedings of the IUFRO Joint Meeting of Working Parties, Vancouver, BC, Canada; 1980; pp. 97–99.
105. Stephan, B.; Liesebach, M. Growth performance and wood characteristics of five *Pinus contorta* progenies. *Silvae Genet.* **1995**, *44*, 243–248.
106. Lines, R. *Experiments on Lodgepole Pine Seed Origins in Britain*; Forestry Commission Technical Paper 10; Forestry Commission: Edinburgh, UK, 1996.
107. Anonymous. *Afforestation Scheme 2014–2020 Rates. "Lodgepole Pine," Forestry*; Department of Agriculture, Food and the Marine: Dublin, Ireland, 2018. Available online: https://www.agriculture.gov.ie/forestservice/publications/ (accessed on 4 December 2018).
108. Dietrichson, J. *Contorta i Norsk Skogbruk*; Norsk Forstmannsforenings Temakonferanse: Oslo, Norway, 1985.
109. Magnesen, S. *Forsøk med Contortafuru i Utsatte Strøk på Vestlandet [Experiments with Contorta Pine in Exposed Areas in Western Norway]*; Norwegian Forest Research Institute: Aas, Norway, 1998.
110. Skrøppa, T. *Pinus contorta i Gravberget—Revisjon 1988/99 [Pinus Contorta in Gravberget—Revision 1988/99]*; Norwegian Forest Research Institute: Ås, Norway, 1992.
111. Magnesen, S. IUFRO Pinus contorta provenance experiments in west Norway and Vest Agder. In *Pinus contorta from Untamed Forest to Domesticated Crop, Proceedings of the IUFRO Working Party Meeting 1992 on Pinus contorta Provenances and Breeding and the Frans Kempe Symposium, Umeå, Sweden, 24–28 August 1992*; Swedish University of Agricultural Sciences: Uppsala, Sweden, 1992; pp. 183–192.
112. Ericsson, T. *Provenance Qualities of the Pinus contorta Breeding Base in Sweden*; Skogforsk: Uppsala, Sweden, 1993.
113. Lindgren, K.; Lindgren, D.; Rosvall, O. *Förflyttningsrekommendationer för Provenienser av Contortatall i Sverige. Arbetsrapport Nr. 27 (Seed Transfer Guidelines for Pinus contorta in Sweden. Working Report No. 27)*; Swedish University of Agricultural Sciences: Umeå, Sweden, 1988.
114. Jansson, G.; Danell, Ö.; Wilhelmsson, L. *Tillväxt, Överlevnad och Skador hos Provenienser av Contortatall i Svealand och Södra Norrland. Rapport Nr 10 (Growth, Survival and Damage of Provenances of Contorta Pine in Svealand and Southern Norrland)*; Skogforsk: Uppsala, Sweden, 1989.
115. Rosvall, O.; Andersson, B.; Ericsson, T. *Beslutsunderlag för Val av Skogsodlingsmaterial i Norra Sverige med Trädslagsvisa Guider [Species Specific Guidelines for Choosing Forest Regeneration Material for North Ern Sweden]*; Skogforsk: Uppsala, Sweden, 1998.
116. Kasesalu, H. Cultivation of the Lodgepole Pine (*Pinus contorta* DOUGL. Ex Loud.) at Järvselja (County Tartu). *Dendrol. Res. Est.* **2000**, *II*, 113–123.
117. Hayatgheibi, H.; Fries, A.; Kroon, J.; Wu, H.X. Genetic analysis of lodgepole pine (*Pinus contorta*) solid-wood quality traits. *Can. J. For. Res.* **2017**, *47*, 1303–1313. [CrossRef]
118. Hayatgheibi, H.; Fries, A.; Kroon, J.; Wu, H.X. Estimation of genetic parameters, provenance performances, and genotype by environment interactions for growth and stiffness in lodgepole pine (*Pinus contorta*). *Scand. J. For. Res.* **2019**, *34*, 1–11. [CrossRef]
119. Karlsson, B.; Kroon, J. Pinus contorta. In *Short Reviews on the Genetics and Breeding of Introduced to Europe Forest Tree Species*; Silva Slovenica Publishing Centre, Slovenian Forestry Institute: Ljubljana, Slovenia, 2016; pp. 20–24.
120. Mason, W.; Connolly, T. Nursing mixtures can enhance long term productivity of Sitka spruce (Picea sitchensis (Bong.) Carr.) stands on nutrient-poor soils. *Forestry* **2018**, *91*, 165–196. [CrossRef]
121. Rédei, K.; Osvath-Bujtas, Z.; Veperdi, I. Black locust (*Robinia pseudoacacia* L.) improvement in Hungary: A review. *Acta Silv. Lignaria Hung.* **2008**, *4*, 127–132.
122. Broshtilov, K. Growth of white acacia (*Robinia pseudoacacia* L.) vegetative generations on habitat of average productivity where chromic luvisoil is available. *For. Ideas* **2009**, *37*, 166–176.
123. Rehfeldt, G.E.; Ying, C.C.; Spittlehouse, D.L.; Hamilton, D.A., Jr. Genetic responses to climate in *Pinus contorta*: Niche breadth, climate change, and reforestation. *Ecol. Monogr.* **1999**, *69*, 375–378. [CrossRef]

124. St. Clair, J.B.; Mandel, N.; Vance-Borland, K.W. Genecology of Douglas-fir in Western Oregon and Washington. *Ann. Bot.* **2005**, *96*, 1199–1214. [CrossRef]
125. Rehfeldt, G.E.; Jaquish, B.C.; López-Upton, J.; Sáenz-Romero, C.; St. Clair, J.B.; Leites, L.P.; Joyce, D.G. Comparative genetic responses to climate for the varieties of *Pinus ponderosa* and *Pseudotsuga menziesii*: Realized climate niches. *For. Ecol. Manag.* **2014**, *324*, 126–137. [CrossRef]
126. Liepe, K.; Hamann, A.; Smets, P.; Fitzpatrick, C.; Aitken, S.N. Adaptation of lodgepole pine and interior spruce to climate: Implications for reforestation in a warming world. *Evol. Appl.* **2016**, *9*, 409–419. [CrossRef] [PubMed]
127. Van Loo, M.; Hintsteiner, W.; Pötzelsberger, E.; Schüler, S.; Hasenauer, H. Intervarietal and intravarietal genetic structure in Douglas-fir: Nuclear SSRs bring novel insights into past population demographic processes, phylogeography, and intervarietal hybridization. *Ecol. Evol.* **2015**, *5*, 1802–1817. [PubMed]
128. St. Clair, J.B. Genetic variation in fall cold hardiness in coastal Douglas-fir in western Oregon and Washington. *Can. J. For. Res.* **2006**, *84*, 1110–1121. [CrossRef]
129. Chakraborty, D.; Schueler, S.; Lexer, M.J.; Wang, T. Genetic trials improve the transfer of Douglas-fir distribution models across continents. *Ecography* **2019**, *42*, 88–101. [CrossRef]
130. Wang, T.; Hamann, A.; Yanchuk, A.; O'neill, G.A.; Aitken, S.N. Use of response functions in selecting lodgepole pine populations for future climates. *Glob. Chang. Biol.* **2006**, *12*, 2404–2416. [CrossRef]
131. Wang, T.; O'Neill, G.; Aitken, S.N. Integrating environmental and genetic effects to predict responses of tree populations to climate. *Ecol. Appl.* **2010**, *20*, 153–163. [CrossRef]
132. Xu, P.; Ying, C.C.; El-Kassaby, Y.A. Multivariate analyses of causal correlation between growth and climate in Sitka spruce. *Silvae Genet.* **2000**, *49*, 257–263.
133. Scholz, F.; Stephan, B. Growth and reaction to drought of 43 *Abies grandis* provenances in a greenhouse study. *Silvae Genet.* **1983**, *31*, 27–35.
134. Xie, Y.; Ying, C. Geographic variation of grand fir (Abies grandis) in the Pacific coast region: 10-year results from a provenance trial. *Can. J. For. Res.* **1993**, *23*, 1065–1072. [CrossRef]
135. Mebrahtu, T.; Hanover, J. Heritability and expected gain estimates for traits of black locust in Michigan. *Silvae Genet.* **1989**, *38*, 125–130.
136. Rehfeldt, G.E.; Jaquish, B.C.; Sáenz-Romero, C.; Joyce, D.G.; Leites, L.P.; St. Clair, J.B.; López-Upton, J. Comparative genetic responses to climate in the varieties of *Pinus ponderosa* and *Pseudotsuga menziesii*: Reforestation. *For. Ecol. Manag.* **2014**, *324*, 147–157. [CrossRef]
137. Rehfeldt, G.E.; Leites, L.P.; St. Clair, J.B.; Jaquish, B.C.; Sáenz-Romero, C.; López-Upton, J.; Joyce, D.G. Comparative genetic responses to climate in the varieties of *Pinus ponderosa* and *Pseudotsuga menziesii*: Clines in growth potential. *For. Ecol. Manag.* **2014**, *324*, 138–146. [CrossRef]
138. Gray, L.; Hamann, A.; John, S.; Rweyongeza, D.; Barnhardt, L.; Thomas, B. Climate change risk management in tree improvement programs: Selection and movement of genotypes. *Tree Genet. Genomes* **2016**, *12*, 23. [CrossRef]
139. Braun, H. Die Nutzung der Klonselektion als Züchtungsmethode am Beispiel des internationalen IUFRO—Douglasien-Herkunftsversuchs 1970 auf der Versuchsfläche Stralsund [The use of clone selection as a breeding method in the example of the international IUFRO Douglas fir origin trial in 1970 on the Stralsund trial area]. *Beiträge Forstwirtsch.* **1985**, *19*, 133–137.
140. Kölling, C. Die Douglasie im Klimawandel: Gegenwärtige und zukünftige Anbaubedingungen in Bayern. *LWF Wissen* **2008**, *59*, 12–21.
141. Isaac-Renton, M.G.; Roberts, D.R.; Hamann, A.; Spiecker, H. Douglas-fir plantations in Europe: A retrospective test of assisted migration to address climate change. *Glob. Chang. Biol.* **2014**, *20*, 2607–2617. [CrossRef]
142. Williams, S.; Jackson, S. Novel climates, no-analog communities, and ecological surprises. *Front. Ecol. Environ.* **2007**, *5*, 475–482. [CrossRef]
143. Ohlemüller, R.; Grittie, E.; Sykes, M.; Thomas, C. Quantifying components of risk for European woody species under climate change. *Glob. Chang. Biol.* **2006**, *12*, 1788–1799. [CrossRef]
144. Mátyás, C. Modelling climate change effects with provenance test data. *Tree Physiol.* **1994**, *14*, 797–804. [CrossRef] [PubMed]
145. Chakraborty, D.; Wang, T.; Andre, K.; Konnert, M.; Lexer, M.J.; Matulla, C.; Schueler, S. Selecting populations for non-analogous climate conditions using universal response functions: The case of Douglas-fir in central Europe. *PLoS ONE* **2015**, *10*, e0136357. [CrossRef] [PubMed]
146. Leites, L.; Robinson, A.; Rehfeldt, G.; Marshall, J.; Crookston, N. Height-growth response to climatic changes differs among populations of Douglas-fir: A novel analysis of historic data. *Ecol. Appl.* **2012**, *22*, 154–165. [CrossRef] [PubMed]
147. Fletcher, M. Breeding improved Sitka spruce. In *Super Sitka for the 90's. Forestry Commission Bulletin No 103*; Rook, D., Ed.; HMSO: London, UK, 1992; pp. 11–24.
