# Supplementary material for: Provenance and family variations in early growth of Manchurian walnut (Juglans mandshurica Maxim.) and selection of superior families
Source: PLoS One. 2024 Mar 7;19(3):e0298918. doi: 10.1371/journal.pone.0298918 (PMC10919699; doi:10.1371/journal.pone.0298918)
Supplement: S2 File — (ZIP) [file pone.0298918.s005.zip › Nonparametric Interval Estimators for the Coefficient of Variation.pdf]

Dongliang Wang[1] / Margaret K. Formica[2] / Song Liu[3]

Nonparametric Interval Estimators for the Coefficient of Variation

[1] Department of Public Health and Preventive Medicine, SUNY Upstate Medical University, Syracuse, NY, USA, E-mail: wangd@upstate.edu
[2] Department of Public Health and Preventive Medicine, SUNY Upstate Medical University, Syracuse, NY USA
[3] Department of Biostatistics and Bioinformatics, Roswell Park Cancer Institute, Buffalo, NY USA

Abstract:
The coefficient of variation (CV) is a widely used scaleless measure of variability in many disciplines. However the inference for the CV is limited to parametric methods or standard bootstrap. In this paper we propose two nonparametric methods aiming to construct confidence intervals for the coefficient of variation. The first one is to apply the empirical likelihood after transforming the original data. The second one is a modified jackknife empirical likelihood method. We also propose bootstrap procedures for calibrating the test statistics. Results from our simulation studies suggest that the proposed methods, particularly the empirical likelihood method with bootstrap calibration, are comparable to existing methods for normal data and yield better coverage probabilities for nonnormal data. We illustrate our methods by applying them to two real-life datasets.

Keywords: coefficient of variation, empirical likelihood, Jackknife empirical likelihood, bootstrap, Wilks' theorem
DOI: 10.1515/ijb-2017-0041
Received: June 5, 2017; **Revised:** February 15, 2018; **Accepted:** March 16, 2018

1 Introduction

Since being introduced by [1], the coefficient of variation (CV) has been widely used as a scaleless measure of variability in many disciplines, including renewal theory, reliability theory, medical and biological science, and public health. For a variable X with mean μ and variance σ^2, the population CV, also known as as the noise to signal ratio, is defined as $\tau = \sigma/\mu$ to measure the relative dispersion of X.

Given a real dataset, the population CV is estimated by the ratio of the sample standard deviation over the sample mean. A reliable interval estimate for the population CV oftentimes is more desirable than the point estimate. For instance, [2] investigated the use of CV in assessing variability of quantitative chemical and biological assays and suggested that "since the CV is estimated and has a distribution of its own, it may be prudent in some applications to employ not the point estimate but rather a more conservative estimate such as an upper percentile of the observed distribution of the CV". For sample size justifications in pharmaceutical studies where log-transformation over the clinical response is often necessary [3], the upper bound of the confidence interval for the CV from preliminary data should be considered over the sample CV in order to make conservative study planning. In general, an interval estimate is more informative than the sample CV with respects to determining whether the population CV exceeds a certain point.

Many statistical inference methods have been developed for constructing a reliable confidence interval of the CV. Under an assumption of Gaussianity, the exact confidence interval was derived by [4] and approximate intervals were derived by [5–12]. For non-normal distributions, [9] developed confidence intervals for Gamma and Weibull distributions. [13] proposed an interval estimator for the CV via deriving the sampling distribution of the inverse of the CV without making any assumption of the underlying distribution. Banik and Kibria [14] evaluated the performance of several bootstrap interval estimators given different underlying distributions. Monika et al. [15] provided a comprehensive review of interval estimators developed thus far for the CV, and suggested replacing the sample mean with the sample median for skewed data. The authors also compared the performances of the available methods via a relatively thorough simulation study. The readers are referred to [15] for more details about the above mentioned interval estimators for the CV. More recently, [16] extended the method proposed by [13]. Albatineh et al. [17] investigated the performance of many methods under ranked set sampling.

The goal of this paper is to propose novel non-parametric interval estimators for the CV, under the framework of the empirical likelihood (EL) methodologies. The EL method, formally proposed by Owen [18], has

Dongliang Wang is the corresponding author.
© 2018 Walter de Gruyter GmbH, Berlin/Boston.

Automatically generated rough PDF by *ProofCheck* from River Valley Technologies Ltd

Brought to you by | UCL - University College London
Authenticated
Download Date | 5/1/18 9:54 PM

been extensively employed in a wide variety of statistical methodology development; see a comprehensive review in Owen [19]. In general, the EL method is limited to making inferences for M-estimators defined by estimating equations and may encounter computational difficulty when applied to more complex non-linear statistics, such as U-statistics. To overcome this, the jackknife empirical likelihood (JEL) method, proposed by Jing and Zhou [20], applies the empirical likelihood method over the sample mean of jackknife pseudo-values instead of a U-statistic of the original data points, taking advantage of the fact that the jackknife pesudo-values are asymptotically independent. JEL has rapidly found many applications, including the inferences for copulas [21], ROC curves [22–24], and Gini index [25, 26], to name a few. In this paper we apply the EL and JEL methods to derive interval estimators for the population coefficient of variation.

The remainder of the paper is organized as follows. In Section 2 the novel EL and JEL methods are proposed for the inference of the CV. Results of simulation studies carried out to evaluate the empirical performance of the proposed methods are described in Section 3. Two examples of applying the proposed methods to real data sets are provided in Section 4, followed by the conclusions and discussions of future work in Section 5.

2 Methodologies

Throughout this section, let $X_1 = x_1, \cdots, X_n = x_n$ denote an independent and identically distributed sample of size n from a continuous distribution $F(X)$ with support over the positive real line, a non-zero expected value μ and existence of the variance σ^2. The coefficient of variation (CV) is defined as $\tau = \frac{\sigma}{\mu}$ and the sample CV is given as

$$\hat{\tau} = \frac{\hat{\sigma}}{\bar{X}} = \frac{\left(\frac{1}{n-1}\sum_{i=1}^{n}(X_i - \bar{X})^2\right)^{1/2}}{\bar{X}}, \tag{1}$$

where $\bar{X} = \frac{1}{n}\sum_{i=1}^{n} X_i$. It is not straightforward from eq. (1) that either EL or JEL method can be readily applied for interval estimation. In the rest of this section, we propose the novel EL and JEL methods after some adjustments.

2.1 Empirical likelihood confidence interval for a single CV

We firstly propose new interval estimators for a single CV via the empirical likelihood method. Let $m = \lfloor n/2 \rfloor$ denote the integer part of $n/2$. Define $Y_i = \frac{1}{2}(X_i - X_{m+i})^2$ and $Z_i = \frac{1}{2}(X_i^2 + X_{m+i}^2)$ for $i = 1, 2, \dots, m$. It is ready to show that

$$\tau = \left(\frac{\mathrm{E}Y_i}{\mathrm{E}Z_i - \mathrm{E}Y_i}\right)^{\frac{1}{2}},$$

or equivalently,

$$\mathrm{E}(Y_i - \tau^2(Z_i - Y_i)) = 0. \tag{2}$$

The estimating eq. (2) for τ allows us to readily apply the empirical likelihood method for inference. Towards this end, let $(\pi_1, \pi_2, \dots, \pi_m)$ be the probability vector associated with the derived data points $(\boldsymbol{Y}, \boldsymbol{Z}) = \{(Y_1, Z_1), \cdots, (Y_m, Z_m)\}$ such that $\sum_{i=1}^{n} \pi_i = 1$ and $\pi_i \geq 0 \;\forall\; 1 \leq i \leq m$. The empirical likelihood ratio function, evaluated at τ, is given by

$$L(\tau) = \max\left\{\prod_{i=1}^{m} m\pi_i : \pi_i \geq 0, \sum_{i=1}^{m}\pi_i = 1, \sum_{i=1}^{m}\pi_i(Y_i - \tau^2(Z_i - Y_i)) = 0\right\}. \tag{3}$$

Following standard Lagrange multiplier procedures, we have the log-empirical likelihood ratio as

$$-2\log l(\tau) = -2\log L(\tau) = 2\sum_{i=1}^{m}\log\Big(1 + \lambda(Y_i - \tau^2(Z_i - Y_i))\Big),$$

where λ satisfies

$$\frac{1}{m}\sum_{i=1}^{m}\frac{Y_i - \tau^2(Z_i - Y_i)}{1 + \lambda(Y_i - \tau^2(Z_i - Y_i))} = 0.$$

We establish the Wilks' theorem for $l(\tau)$ as follows:

Automatically generated rough PDF by ProofCheck from River Valley Technologies Ltd

Brought to you by | UCL - University College London
Authenticated
Download Date | 5/1/18 9:54 PM

Theorem 2.1
Let τ_0 denote the true value of the CV and assume that $EX_1^4 < \infty$, then

$$-2\log l(\tau_0) \xrightarrow{d} \chi_1^2, \text{ as } n \to \infty.$$

The proof of Theorem 2.1 follows from Theorem 3.4 in Owen (2001), by noting that $\text{Var}(Y_i - \tau^2(Z_i - Y_i))$ is bounded by cEX_1^4 and thus is finite, given $EX_1^4 < \infty$, where c is a finite constant.

Theorem 2.1 can be utilized to test $H_0 : \tau = \tau_0$ against $H_a : \tau \neq \tau_0$ for a given constant τ_0. It can alternatively be utilized to construct an (1-α)-level interval estimator for τ as

$$\tau : \mathfrak{R}_c = \{\tau : -2\log l(\tau) \leq c\}, \tag{4}$$

where c is the $1-\alpha$ quantile of χ_1^2 distribution.

As an alternative to calibrate the EL statistic using χ_1^2 distribution, one can use the bootstrap calibration to overcome the potential undercoverage problem, which the EL method occasionally encounters, particularly when the sample size is small. We propose the following procedure to implement the bootstrap calibration method.

1. Calculate $\hat{\tau}$ as in eq. (1).
2. For $b = 1, \cdots, B$,
   - Generate a bootstrap sample $(\boldsymbol{Y^*}, \boldsymbol{Z^*}) = \{(Y_1^*, Z_1^*), \cdots, (Y_m^*, Z_m^*)\}$ of size m from the original data $(\boldsymbol{Y}, \boldsymbol{Z}) = \{(Y_1, Z_1), \cdots, (Y_m, Z_m)\}$ with replacement;
   - Calculate the bootstrapped log-empirical likelihood ratio at $\hat{\tau}$ based on the bootstrapped sample $(\boldsymbol{Y^*}, \boldsymbol{Z^*})$ as

$$-2\log l^*(\hat{\tau}) = 2\sum_{i=1}^{m}\log\left(1 + \lambda(Y_i^* - \hat{\tau}^2(Z_i^* - Y_i^*))\right),$$

   where λ^* satisfies

$$\frac{1}{m}\sum_{i=1}^{m}\frac{Y_i^* - \hat{\tau}^2(Z_i^* - Y_i^*)}{1 + \lambda(Y_i^* - \hat{\tau}^2(Z_i^* - Y_i^*))} = 0.$$

3. Calculate the bootstrap calibrated $100(1-\alpha)\%$ EL confidence interval for τ as

$$\mathbb{R}_\alpha = \{\tau : -2\log l(\tau) \leq c_\alpha^*\}, \tag{5}$$

   where c_α^* is the $100(1-\alpha)$ percentile of the B bootstrapped log-empirical likelihood ratios $l^*(\hat{\tau})$.

2.2 Jackknife empirical likelihood confidence interval for a single CV

In this section, we propose new interval estimators for a single CV via the jackknife empirical likelihood method. We start up by introducing an estimating equation for τ

$$U_n(\tau) = \frac{\tau^2}{n}\sum_{i=1}^{n}X_i^2 - (\tau^2 + 1)s^2, \tag{6}$$

where s^2 is the sample variance $s^2 = \frac{1}{n-1}\sum_{i=1}^{n}(X_i - \bar{X})^2$ and τ can be estimated by solving $U_n(\tau) = 0$. By the JEL method in Jing *et al.* (2009), the jackknife pseudo-values from $U_n(\tau)$ can be expressed as

$$\hat{V}_i(\tau) = nU_n(\tau) - (n-1)U_{n-1}^{(-i)}(\tau), \tag{7}$$

where $U_{n-1}^{(-i)}(\tau)$ is the quantity at eq. (6) with the ith observation X_i deleted. The jackknife estimator of the CV follows up as

$$U_n(\tau) = \frac{1}{n}\sum_{i=1}^{n}\hat{V}_i(\tau).$$

Automatically generated rough PDF by ProofCheck from River Valley Technologies Ltd

Brought to you by | UCL - University College London
Authenticated
Download Date | 5/1/18 9:54 PM

We can then follow the standard empirical likelihood method for a univariate mean over the jackknife pseudo-values. Towards this end, let $\boldsymbol{\pi} = (\pi_1, \dots, \pi_n)$ be the probability vector. The jackknife empirical likelihood ratio at τ is given by

$$L(\tau) = \max\left\{\prod_{i=1}^{n} n\pi_i : \pi_i \geq 0, \sum_{i=1}^{n} \pi_i = 1, \sum_{i=1}^{n} \pi_i \hat{V}_i(\tau) = 0\right\}.$$

By the standard Lagrange multiplier technique, the log-transformed jackknife empirical likelihood ratio is

$$-2\log l(\tau) = -2\log L(\tau) = 2\sum_{i=1}^{n} \log\left(1 + \lambda \hat{V}_i(\tau)\right),$$

where $\lambda = \lambda(\tau)$ satisfies

$$\frac{1}{n}\sum_{i=1}^{n} \frac{\hat{V}_i(\tau)}{1 + \lambda \hat{V}_i(\tau)} = 0. \quad (8)$$

Let $h(X_1, X_2; \tau) = \frac{1}{2}(X_1^2 + X_2^2) - \frac{1}{2}(\tau^2 + 1)(X_1 - X_2)^2$ and $g(x) = \mathrm{E}h(x, X_2; \tau)$ be a function of $X_1 = x$ with the variance as $\sigma_g^2 = \mathrm{var}(g(X_1))$. We establish the Wilks' theorem for $l(\tau)$ as follows.

Theorem 2.2
Let τ_0 denote the true value of the CV and assume that $\mathrm{E}h^2(X_1, X_2) < \infty$ and $\sigma_g^2 > 0$, then

$$-2\log l(\tau_0) \xrightarrow{d} \chi_1^2, \text{ as } n \to \infty.$$

The proof of Theorem 2.2 follows directly from Theorem 2.1 in Jing *et al.* (2009) by noting that the quantity $U_n(\tau_0)$ is a U-statistics with a symmetric kernel $h(X_1, X_2; \tau_0)$ of degree $m = 2$. More details are provided in the appendix.

A $100(1-\alpha)\%$ JEL confidence interval for τ can be constructed using Theorem 2.2 as

$$\mathbb{R}_\alpha = \{\tau : -2\log l(\tau) \leq c\}, \quad (9)$$

where c denotes the $(1-\alpha)$ quantile of χ_1^2.
The following bootstrap calibration procedure is also proposed for the JEL method.

1. Calculate $\hat{\tau}$ as in eq. (1).
2. For $b = 1, \cdots, B$,
   - Generate a bootstrap sample $\mathbf{X^*} = \{X_1^*, X_2^*, \dots, X_n^*\}$ of size n from the original data $\mathbf{X} = \{X_1, X_2, \dots, X_n\}$ with replacement;
   - Calculate the jackknife pseudo-values $\hat{V}_i^*(\hat{\tau})$, $i = 1, \dots, n$ at $\tau = \hat{\tau}$ by eq. (7);
   - Calculate the bootstrapped jackknife empirical likelihood ratio after log-transformation at $\hat{\tau}$

   $$l^*(\hat{\tau}) = 2\sum_{i=1}^{n} \log\left(1 + \lambda^* \hat{V}_i^*(\hat{\tau})\right),$$

   where λ^* satisfies

   $$\frac{1}{n}\sum_{i=1}^{n} \frac{\hat{V}_i^*(\hat{\tau})}{1 + \lambda^* \hat{V}_i^*(\hat{\tau})} = 0.$$

3. Calculate the bootstrap calibrated $100(1-\alpha)\%$ JEL confidence interval for τ as

   $$\mathbb{R}_\alpha = \{\tau : -2\log R(\tau) \leq c_\alpha^*\}, \quad (10)$$

   where c_α^* is the $100(1-\alpha)$ percentile of the B bootstrapped jackknife empirical likelihood ratios $l^*(\hat{\tau})$.

Automatically generated rough PDF by *ProofCheck* from River Valley Technologies Ltd

Brought to you by | UCL - University College London
Authenticated
Download Date | 5/1/18 9:54 PM

3 Simulations and computations

For the purpose of our simulation studies we consider the CV value of 0.1, 0.3, 0.5 and 0.8 from a series of normal distributions, lognormal distributions and exponential distributions. To achieve it, we set the normal(μ, σ) distribution with the location parameter $\mu = 10$ and the scale parameter $\mu = 1, 3, 5$, and 8, respectively; we set the Laplace (μ, λ) distribution with the location parameter $\mu = 1$ and the scale parameter $\lambda = 0.0707$, 0.2121, 0.3536, and 0.5657, respectively. we set the shifted exponential distribution $\exp(\mu, \lambda)$ with the scale parameter $\lambda = 1$ and the location parameter $\mu = 9, 7/3, 1$, and $1/4$, respectively. The exponential distribution is considered since it is commonly used statistical model for real-life data analysis with heavily tails and mildly right-skewed with a skewness of 2.

We generate 2,000 Monte Carlo simulations for sample sizes of $n = 18, 30, 50, 100$ and 500 from normal, lognormal or exponential distribution and then calculate the 95% confidence intervals for τ utilizing the EL method at eq. (4), the EL-boot method at eq. (5), the JEL method at eq. (9) and the JEL-boot method at eq. (10). For the purpose of the comparison, we also calculate the confidence intervals for τ using the following methods.

- The Miller interval is calculated as

$$\left\{\tau : \hat{\tau} - Z_{\alpha/2}\Big(\frac{\hat{\tau}^2}{n-1}(\frac{1}{2} + \hat{\tau}^2)\Big)^{\frac{1}{2}} \le \tau \le \hat{\tau} + Z_{\alpha/2}\Big(\frac{\hat{\tau}^2}{n-1}(\frac{1}{2} + \hat{\tau}^2)\Big)^{\frac{1}{2}}\right\}$$

  where $Z_{\alpha/2}$ is the upper $\alpha/2$ quantile of the standard normal distribution. Miller (1991) originally proposed the method for normal distribution, approximating the asymptotic distribution of the sample CV with a normal distribution.

- The McKay interval, proposed by [5] for normal distribution, is derived via approximating the asymptotic distribution of the CV by χ^2 distribution and can be calculated as

$$\left\{\tau : \hat{\tau}\Big((\frac{\chi^2_{n-1,1-\alpha/2}}{n} - 1)\hat{\tau}^2 + \frac{\chi^2_{n-1,1-\alpha/2}}{n-1}\Big)^{-\frac{1}{2}} \le \tau \le \hat{\tau}\Big((\frac{\chi^2_{n-1,\alpha/2}}{n} - 1)\hat{\tau}^2 + \frac{\chi^2_{n-1,\alpha/2}}{n-1}\Big)^{-\frac{1}{2}}\right\},$$

  where $\chi^2_{n-1,\alpha/2}$ and $\chi^2_{n-1,1-\alpha/2}$ are the $\alpha/2$ and $1-\alpha/2$ quantiles of χ^2_{n-1} distribution.

- The Vangel interval is a modification of the McKay interval for normal distribution as in [8]

$$\left\{\tau : \hat{\tau}\Big((\frac{\chi^2_{n-1,1-\alpha/2} + 2}{n} - 1)\hat{\tau}^2 + \frac{\chi^2_{n-1,1-\alpha/2}}{n-1}\Big)^{-\frac{1}{2}} \le \tau \le \hat{\tau}\Big((\frac{\chi^2_{n-1,\alpha/2} + 2}{n} - 1)\hat{\tau}^2 + \frac{\chi^2_{n-1,\alpha/2}}{n-1}\Big)^{-\frac{1}{2}}\right\};$$

- The Panich interval, another modification of the McKay inteval by Panichkitkosolkul (2009) for normal distribution, is calculated by replacing $\hat{\tau}$ with $\tilde{\tau} = \hat{\tau}\sqrt{(n-1)/n}$, which is the maximum likelihood estimate of τ for normal distribution.
- The bootstrap intervals, including the basic (Boot), percentile (Boot-p) intervals and the interval from the bias-corrected and accelerated (BCa) bootstrap, are calculated with the sample CV as the statistic, using the *R* package *boot*; more details can be found in [24] and [25].
- The Boot-Miller interval is obtained by replacing the constants $\pm Z_{\alpha/2}$ in the Miller interval with the $\alpha/2$ and $1-\alpha/2$ quantiles of bootstrap samples. From the simulation results in [15], the Boot-Miller method seemingly performs better than the standard bootstrap t method.

The above listed methods for comparison are chosen since their superior performance has been shown in previous simulation studies in [15]., The parametric intervals, such as Miller, McKay, Vangel and Panich methods, were particularly developed for underlying normal distributions. For skewed lognormal and exponential distributions, modified Miller (m-Miller), McKay (m-McKay) and Vangel (m-Vangel) intervals are also calculated utilizing the sample median instead of the sample mean. For all bootstrap related methods, including EL-boot and JEL-boot, $B = 1000$ pseudo-samples were generated.

Tables 1–3 show the empirical coverage probabilities and average lengths of the 95% confidence intervals form different methods for normal, lognormal and exponential distributions, respectively. Major observations from our simulation results can be summarized as follows.

Automatically generated rough PDF by ProofCheck from River Valley Technologies Ltd

Brought to you by | UCL - University College London
Authenticated
Download Date | 5/1/18 9:54 PM

– In Tables 1–3, the EL and JEL methods universally encounter a undercoverage problem, which can be addressed by bootstrap calibration. The intervals from the EL and EL-boot methods are usually shorter than those from the JEL and JEL-boot methods, respectively. No matter whether they are calibrated by chi-square distribution or bootstrap, the interval estimators from the JEL methods are usually shorter than the counterparts from the EL methods, with coverage probabilities closer to the nominal level; and both EL and JEL methods in general perform better than standard bootstrap methods.

– For the normal distributions as shown in Table 1, it is no wonder that the parametric methods relying on the normality assumption enjoy the the advantage over their competitors, in terms of better coverage and shorter interval width. The performance of the JEL-boot interval is slightly worse for small sample size with $n < 50$. For large sample size with $n = 500$, EL and JEL are comparable to the parametric methods and no bootstrap calibration is necessary. Standard bootstrap procedures, such as basic, percentile and BCa bootstraps, are only comparable for large sample size and perform abruptly worse for small to medium sample size with $n \leq 100$.

– For the Laplace distributions as shown in Table 2, only the EL-boot and JEL-boot intervals indicate satisfactory coverage probabilities when $n \geq 50$. The performance of the EL and JEL methods is uniformly better than parametric methods and bootstrap. The intervals from parametric methods are obviously shorter than expected, most likely because the normality assumption is not met. Standard bootstrap methods, particularly the BCa method, may provide satisfactory coverage for large enough sample size ($n \geq 100$).

– For the exponential distributions as shown in Table 3, EL-boot and JEL-boot methods demonstrate a satisfactory performance given large sample size with $n = 500$. When n is small or medium, both EL-boot and JEL-boot still perform obviously better than standard bootstrap and parametric methods with an acceptable accuracy of coverage. The performance of parametric methods is generally very poor except for $\tau = 0.8$. It is also worthy noting that the coverage probabilities of parametric intervals become worse as sample size increases. There is room for improving the standard bootstrap methods unless sample size is large.

– The modified Miller (m-Miller), McKay (m-McKay) and Vangel (m-Vangel) methods and the boot-Miller method do not demonstrate significant improvement over their counterparts in the simulations and thus their results are not included in Tables 1–3 for the sake of simplicity.

Overall, the EL and JEL methods are comparable to or slightly worse than parametric methods for normal distribution and perform universally better than parametric and bootstrap methods for Laplace and exponential distributions. The bootstrap calibration can improve the coverage accuracy of the EL and JEL methods with a slightly wider interval estimators. We observe from our simulation studies that the JEL-boot method always performs well, no matter whether the data are sampled from normal, Laplace or exponential distribution.

Table 1: Coverage probabilities (average lengths) of the interval estimators for normal distributions at the nominal level of 95%.

		$n = 18$	$n = 30$	$n = 50$	$n = 100$	$n = 500$
$\tau = 0.1$	Miller	0.926(0.067)	0.926(0.051)	0.940(0.040)	0.935(0.028)	0.948(0.013)
	McKay	0.957(0.075)	0.948(0.055)	0.952(0.041)	0.940(0.029)	0.948(0.013)
	Vangel	0.957(0.075)	0.948(0.055)	0.952(0.041)	0.940(0.029)	0.948(0.013)
	Panich	0.959(0.073)	0.953(0.054)	0.949(0.041)	0.939(0.028)	0.946(0.013)
	Boot	0.732(0.073)	0.776(0.063)	0.824(0.063)	0.884(0.063)	0.932(0.055)
	Boot-p	0.680(0.073)	0.737(0.063)	0.790(0.063)	0.872(0.063)	0.932(0.055)
	BCa	0.744(0.073)	0.798(0.065)	0.847(0.065)	0.893(0.065)	0.936(0.059)
	EL	0.832(0.072)	0.870(0.063)	0.906(0.051)	0.921(0.038)	0.952(0.018)
	EL-boot	0.910(0.135)	0.924(0.100)	0.933(0.063)	0.936(0.042)	0.952(0.018)
	JEL	0.902(0.063)	0.914(0.050)	0.934(0.039)	0.936(0.028)	0.947(0.013)
	JEL-boot	0.938(0.097)	0.940(0.061)	0.945(0.043)	0.940(0.029)	0.948(0.013)
$\tau = 0.3$	Miller	0.913(0.218)	0.934(0.167)	0.948(0.129)	0.954(0.091)	0.952(0.040)
	McKay	0.952(0.258)	0.954(0.184)	0.955(0.136)	0.948(0.094)	0.958(0.041)
	Vangel	0.952(0.252)	0.958(0.182)	0.954(0.135)	0.948(0.093)	0.957(0.041)
	Panich	0.950(0.243)	0.954(0.178)	0.956(0.134)	0.950(0.093)	0.956(0.041)
	Boot	0.728(0.187)	0.780(0.171)	0.833(0.171)	0.878(0.171)	0.924(0.145)
	Boot-p	0.656(0.187)	0.744(0.171)	0.794(0.171)	0.852(0.171)	0.924(0.145)
	BCa	0.758(0.183)	0.808(0.174)	0.856(0.174)	0.888(0.174)	0.926(0.151)
	EL	0.820(0.244)	0.875(0.210)	0.903(0.169)	0.936(0.125)	0.950(0.057)
	EL-boot	0.917(0.500)	0.926(0.322)	0.930(0.210)	0.947(0.136)	0.952(0.058)
	JEL	0.897(0.222)	0.918(0.171)	0.938(0.131)	0.948(0.092)	0.951(0.041)

Automatically generated rough PDF by ProofCheck from River Valley Technologies Ltd

Brought to you by | UCL - University College London
Authenticated
Download Date | 5/1/18 9:54 PM

	JEL-boot	0.940(0.357)	0.941(0.214)	0.952(0.145)	0.947(0.096)	0.952(0.041)
**τ = 0.5**	Miller	0.900(0.416)	0.942(0.319)	0.944(0.245)	0.938(0.170)	0.952(0.076)
	McKay	0.946(0.618)	0.955(0.385)	0.958(0.274)	0.948(0.182)	0.955(0.078)
	Vangel	0.947(0.567)	0.956(0.371)	0.960(0.269)	0.948(0.180)	0.955(0.078)
	Panich	0.947(0.523)	0.958(0.361)	0.958(0.265)	0.946(0.179)	0.954(0.078)
	Boot	0.722(0.293)	0.789(0.253)	0.828(0.253)	0.848(0.253)	0.918(0.220)
	Boot-p	0.672(0.293)	0.745(0.253)	0.798(0.253)	0.828(0.253)	0.912(0.220)
	BCa	0.756(0.286)	0.816(0.255)	0.847(0.255)	0.861(0.255)	0.920(0.227)
	EL	0.835(0.532)	0.877(0.424)	0.913(0.336)	0.927(0.242)	0.944(0.109)
	EL-boot	0.920(1.039)	0.922(0.650)	0.943(0.419)	0.940(0.263)	0.947(0.110)
	JEL	0.899(0.517)	0.934(0.356)	0.938(0.263)	0.941(0.178)	0.950(0.077)
	JEL-boot	0.930(0.884)	0.951(0.450)	0.947(0.290)	0.945(0.183)	0.950(0.077)
**τ = 0.8**	Miller	0.914(0.899)	0.930(0.665)	0.940(0.494)	0.953(0.340)	0.950(0.150)
	McKay	0.950(2.006)	0.962(1.353)	0.976(0.715)	0.978(0.412)	0.966(0.166)
	Vangel	0.956(1.776)	0.965(1.174)	0.976(0.671)	0.978(0.402)	0.965(0.166)
	Panich	0.954(1.718)	0.962(1.382)	0.975(0.652)	0.978(0.398)	0.964(0.165)
	Boot	0.764(0.464)	0.800(0.387)	0.827(0.387)	0.865(0.387)	0.912(0.329)
	Boot-p	0.747(0.464)	0.786(0.387)	0.816(0.387)	0.855(0.387)	0.910(0.329)
	BCa	0.828(0.473)	0.854(0.401)	0.862(0.401)	0.887(0.401)	0.920(0.342)
	EL	0.874(1.688)	0.906(1.408)	0.920(0.919)	0.941(0.540)	0.950(0.226)
	EL-boot	0.935(2.328)	0.940(2.087)	0.938(1.234)	0.952(0.596)	0.950(0.227)
	JEL	0.947(1.769)	0.935(1.125)	0.932(0.612)	0.947(0.374)	0.943(0.153)
	JEL-boot	0.966(2.301)	0.947(1.596)	0.945(0.702)	0.952(0.389)	0.943(0.154)

Table 2: Coverage probabilities (average lengths) of the interval estimators for Laplace distributions at the nominal level of 95%.

		$n = 18$	$n = 30$	$n = 50$	$n = 100$	$n = 500$
**τ = 0.1**	Miller	0.795(0.065)	0.767(0.051)	0.802(0.040)	0.801(0.028)	0.772(0.013)
	McKay	0.834(0.073)	0.798(0.054)	0.813(0.041)	0.807(0.029)	0.770(0.013)
	Vangel	0.834(0.073)	0.798(0.054)	0.813(0.041)	0.806(0.029)	0.770(0.013)
	Panich	0.835(0.071)	0.798(0.053)	0.806(0.041)	0.806(0.028)	0.770(0.013)
	Basic	0.826(0.073)	0.842(0.063)	0.88(0.063)	0.916(0.063)	0.94(0.053)
	Percentile	0.767(0.073)	0.809(0.063)	0.858(0.063)	0.904(0.063)	0.934(0.053)
	BCa	0.832(0.078)	0.858(0.067)	0.894(0.067)	0.922(0.067)	0.938(0.057)
	EL	0.779(0.080)	0.823(0.074)	0.866(0.061)	0.916(0.048)	0.944(0.023)
	EL-boot	0.880(0.167)	0.896(0.152)	0.916(0.087)	0.932(0.058)	0.954(0.024)
	JEL	0.829(0.081)	0.854(0.066)	0.890(0.055)	0.920(0.042)	0.940(0.020)
	JEL-boot	0.902(0.234)	0.907(0.109)	0.922(0.073)	0.941(0.049)	0.950(0.020)
**τ = 0.3**	Miller	0.811(0.217)	0.824(0.167)	0.793(0.128)	0.807(0.091)	0.802(0.040)
	McKay	0.847(0.260)	0.840(0.184)	0.815(0.135)	0.818(0.093)	0.804(0.041)
	Vangel	0.85(0.253)	0.840(0.182)	0.812(0.134)	0.818(0.093)	0.804(0.041)
	Panich	0.848(0.244)	0.838(0.178)	0.803(0.133)	0.816(0.093)	0.801(0.041)
	Boot	0.831(0.242)	0.876(0.203)	0.872(0.203)	0.91(0.203)	0.93(0.166)
	Boot-p	0.783(0.242)	0.835(0.203)	0.855(0.203)	0.905(0.203)	0.928(0.166)
	BCa	0.846(0.277)	0.888(0.229)	0.892(0.229)	0.926(0.229)	0.932(0.183)
	EL	0.786(0.280)	0.842(0.239)	0.864(0.199)	0.908(0.154)	0.933(0.073)
	EL-boot	0.882(0.659)	0.907(0.440)	0.911(0.300)	0.936(0.192)	0.937(0.076)
	JEL	0.846(0.282)	0.877(0.223)	0.892(0.177)	0.925(0.133)	0.932(0.061)
	JEL-Boot	0.908(0.544)	0.926(0.369)	0.920(0.247)	0.940(0.162)	0.936(0.063)
**τ = 0.5**	Miller	0.829(0.419)	0.836(0.319)	0.837(0.243)	0.838(0.171)	0.841(0.076)
	McKay	0.869(0.665)	0.852(0.398)	0.858(0.273)	0.849(0.182)	0.842(0.078)
	Vangel	0.868(0.588)	0.849(0.378)	0.856(0.268)	0.850(0.181)	0.842(0.078)
	Panich	0.863(0.541)	0.844(0.368)	0.853(0.264)	0.850(0.180)	0.841(0.078)
	Boot	0.832(0.477)	0.862(0.379)	0.889(0.379)	0.913(0.379)	0.941(0.305)
	Boot-p	0.788(0.477)	0.836(0.379)	0.873(0.379)	0.906(0.379)	0.945(0.305)
	BCa	0.85(0.73)	0.88(0.464)	0.906(0.464)	0.928(0.464)	0.942(0.351)
	EL	0.790(0.691)	0.838(0.518)	0.884(0.397)	0.915(0.289)	0.934(0.133)
	EL-boot	0.884(1.174)	0.887(0.872)	0.922(0.634)	0.936(0.365)	0.940(0.139)
	JEL	0.858(0.682)	0.873(0.468)	0.904(0.345)	0.928(0.243)	0.944(0.108)
	JEL-Boot	0.906(1.063)	0.912(0.789)	0.930(0.503)	0.940(0.292)	0.950(0.112)
**τ = 0.8**	Miller	0.831(0.920)	0.850(0.666)	0.868(0.488)	0.894(0.341)	0.882(0.150)
	McKay	0.872(1.734)	0.898(1.240)	0.918(0.729)	0.922(0.416)	0.914(0.167)

Automatically generated rough PDF by *ProofCheck* from River Valley Technologies Ltd

Brought to you by | UCL - University College London
Authenticated
Download Date | 5/1/18 9:54 PM

Vangel	0.886(1.594)	0.902(1.132)	0.918(0.674)	0.924(0.405)	0.914(0.166)
Panich	0.878(1.471)	0.896(1.035)	0.915(0.666)	0.922(0.401)	0.914(0.166)
Boot	0.822(1.443)	0.856(0.842)	0.875(0.842)	0.921(0.842)	0.941(0.593)
Boot-p	0.804(1.443)	0.858(0.842)	0.882(0.842)	0.924(0.842)	0.944(0.593)
BCa	0.867(3.887)	0.902(1.426)	0.922(1.426)	0.94(1.426)	0.945(0.744)
EL	0.833(1.560)	0.879(1.389)	0.887(1.072)	0.927(0.645)	0.939(0.261)
EL-boot	0.904(2.224)	0.924(2.059)	0.922(1.493)	0.940(0.830)	0.943(0.272)
JEL	0.862(1.498)	0.916(1.388)	0.922(0.798)	0.94(0.471)	0.948(0.198)
JEL-Boot	0.918(2.051)	0.938(1.901)	0.941(1.129)	0.949(0.553)	0.947(0.204)

Table 3: Coverage probabilities (average lengths) of the interval estimators for exponential distributions at the nominal level of 95%.

		$n = 18$	$n = 30$	$n = 50$	$n = 100$	$n = 500$
$\tau = 0.1$	Miller	0.712(0.064)	0.713(0.050)	0.708(0.039)	0.708(0.028)	0.708(0.013)
	McKay	0.764(0.072)	0.749(0.053)	0.728(0.041)	0.725(0.028)	0.716(0.013)
	Vangel	0.764(0.072)	0.749(0.053)	0.727(0.041)	0.724(0.028)	0.716(0.013)
	Panich	0.752(0.070)	0.747(0.052)	0.725(0.040)	0.723(0.028)	0.711(0.013)
	Boot	0.732(0.073)	0.776(0.063)	0.824(0.063)	0.884(0.063)	0.932(0.055)
	Boot-p	0.680(0.073)	0.737(0.063)	0.790(0.063)	0.872(0.063)	0.932(0.055)
	BCa	0.744(0.073)	0.798(0.065)	0.847(0.065)	0.893(0.065)	0.936(0.059)
	EL	0.716(0.078)	0.788(0.077)	0.834(0.064)	0.898(0.052)	0.944(0.026)
	EL-boot	0.838(0.172)	0.868(0.170)	0.885(0.098)	0.924(0.068)	0.953(0.029)
	JEL	0.735(0.079)	0.786(0.064)	0.834(0.056)	0.888(0.045)	0.936(0.023)
	JEL-boot	0.843(0.243)	0.880(0.140)	0.896(0.090)	0.929(0.061)	0.948(0.025)
$\tau = 0.3$	Miller	0.796(0.206)	0.799(0.164)	0.806(0.126)	0.805(0.090)	0.795(0.040)
	McKay	0.864(0.243)	0.818(0.181)	0.828(0.133)	0.813(0.093)	0.795(0.041)
	Vangel	0.864(0.238)	0.822(0.179)	0.829(0.133)	0.814(0.092)	0.796(0.041)
	Panich	0.852(0.229)	0.818(0.175)	0.828(0.131)	0.816(0.092)	0.796(0.041)
	Boot	0.728(0.187)	0.780(0.171)	0.833(0.171)	0.878(0.171)	0.924(0.145)
	Boot-p	0.656(0.187)	0.744(0.171)	0.794(0.171)	0.852(0.171)	0.924(0.145)
	BCa	0.758(0.183)	0.808(0.174)	0.856(0.174)	0.888(0.174)	0.926(0.151)
	EL	0.733(0.211)	0.797(0.201)	0.848(0.177)	0.878(0.143)	0.936(0.074)
	EL-boot	0.844(0.409)	0.888(0.416)	0.902(0.268)	0.915(0.190)	0.946(0.081)
	JEL	0.738(0.179)	0.794(0.168)	0.838(0.145)	0.884(0.119)	0.932(0.062)
	JEL-boot	0.830(0.486)	0.866(0.357)	0.892(0.234)	0.914(0.166)	0.941(0.070)
$\tau = 0.5$	Miller	0.841(0.386)	0.868(0.302)	0.887(0.237)	0.862(0.168)	0.865(0.076)
	McKay	0.932(0.555)	0.928(0.362)	0.912(0.265)	0.886(0.179)	0.878(0.078)
	Vangel	0.932(0.507)	0.924(0.349)	0.912(0.260)	0.884(0.177)	0.878(0.078)
	Panich	0.922(0.478)	0.921(0.340)	0.910(0.256)	0.879(0.176)	0.877(0.078)
	Boot	0.722(0.293)	0.789(0.253)	0.828(0.253)	0.848(0.253)	0.918(0.220)
	Boot-p	0.672(0.293)	0.745(0.253)	0.798(0.253)	0.828(0.253)	0.912(0.220)
	BCa	0.756(0.286)	0.816(0.255)	0.847(0.255)	0.861(0.255)	0.920(0.227)
	EL	0.715(0.346)	0.801(0.322)	0.845(0.291)	0.878(0.234)	0.932(0.121)
	EL-boot	0.851(0.645)	0.888(0.603)	0.904(0.432)	0.916(0.305)	0.944(0.131)
	JEL	0.740(0.279)	0.797(0.244)	0.830(0.217)	0.857(0.174)	0.922(0.094)
	JEL-boot	0.810(0.756)	0.864(0.416)	0.879(0.334)	0.892(0.242)	0.932(0.106)
$\tau = 0.8$	Miller	0.907(0.766)	0.927(0.599)	0.938(0.470)	0.950(0.332)	0.963(0.149)
	McKay	0.989(1.883)	0.980(1.073)	0.982(0.645)	0.980(0.399)	0.976(0.165)
	Vangel	0.989(1.608)	0.978(0.928)	0.984(0.614)	0.980(0.390)	0.976(0.165)
	Panich	0.984(1.477)	0.974(0.896)	0.982(0.610)	0.977(0.386)	0.976(0.164)
	Boot	0.764(0.464)	0.800(0.387)	0.827(0.387)	0.865(0.387)	0.912(0.329)
	Boot-p	0.747(0.464)	0.786(0.387)	0.816(0.387)	0.855(0.387)	0.910(0.329)
	BCa	0.828(0.473)	0.854(0.401)	0.862(0.401)	0.887(0.401)	0.920(0.342)
	EL	0.754(0.638)	0.813(0.592)	0.847(0.516)	0.889(0.416)	0.936(0.211)
	EL-boot	0.908(1.156)	0.899(0.988)	0.922(0.739)	0.923(0.523)	0.942(0.226)
	JEL	0.872(0.522)	0.863(0.400)	0.865(0.330)	0.876(0.257)	0.920(0.137)
	JEL-boot	0.891(0.765)	0.886(0.531)	0.882(0.423)	0.906(0.326)	0.938(0.155)

It is worthy noting that both EL-boot and JEL-boot may suffer from the convex dual problem inherently arising in the computation of the profile empirical likelihood for a vector mean. For small sample size with $n \leq 30$ or large CV with $\tau \geq 0.5$, there is a possibility that a few very influential data points exist. If that is the

Automatically generated rough PDF by *ProofCheck* from River Valley Technologies Ltd

Brought to you by | UCL - University College London
Authenticated
Download Date | 5/1/18 9:54 PM

case, there might be no finite EL-boot or JEL-boot interval estimates because the empirical likelihood function could be bounded to a value too low to reach the critical value calibrated by bootstrap, which is usually high as the sample CV estimate might lie outside of the convex hull of transformed data points or the jackknife pseudo-values for a large portion of resampled data sets which do not contain the whole or partial set of the influential point. A simple soultion to the convex dual problem is to replace the EL-boot or JEL-boot interval with the one calibrated by chi-square distribution if the empirical likelihood function is bounded. The slightly modified EL-boot and JEL-boot methods perform very well as demonstrated in our extensive simulation studies.

In terms of computational cost, there is no doubt that the proposed methods are more expensive than their counterparts, due to the inclusion of the Lagrange and jackknife procedures. However, the propose methods do not pose any computational burden given the current personal computer configurations and can be easily implemented using commonly used statistical packages, such as the *emplik* package in *R*, developed by [26]. For instance, the amount of time needed to calculate both EL and EL-boot intervals for a data set of $n = 50, 100$, or 500 randomly generated from a normal distribution could be less than 3 seconds on a standard MacBook Pro with OS 10 and 2.6 GhZ Intel i5 processor. The major *R* functions to run the proposed EL and JEL methods are provided in the Appendix.

4 Examples

For the purpose of illustration, we apply our methods to two real datasets: one from clinical microbiology and the other from engineering.

4.1 An example from clinical microbiology

The CV has been widely used to measure the reproducibility of serological tests. We consider a dataset from [27], which consists of the measurements of the antibody titers on 30 distinct days of a single serum specimen from a serological test. The data are provided in Table 4. The Q–Q plot against the standard normal distribution in Figure 1 and the Shapiro-Wilk test for normality (p=0.414) suggest that the data may come from a normal distribution. The sample mean and standard deviation are 28.4 and 9.29, respectively. The sample CV is 0.327. The 90% and 95% confidence intervals from a variety of methods are provided in Table 5. Consistent with the observations from our simulation study, the confidence intervals from all methods are comparable except the EL-boot method, which gives obviously wider confidence intervals.

Table 4: The measurements of the antibody titers on 30 distinct days of a single serum specimen from a serological test, originally from [27].

10	14	14	19	21	22	22	22	23	23	25	25
26	27	28	28	29	29	31	32	32	33	34	36
36	37	39	39	40	56						

Automatically generated rough PDF by *ProofCheck* from River Valley Technologies Ltd

Brought to you by | UCL - University College London
Authenticated
Download Date | 5/1/18 9:54 PM

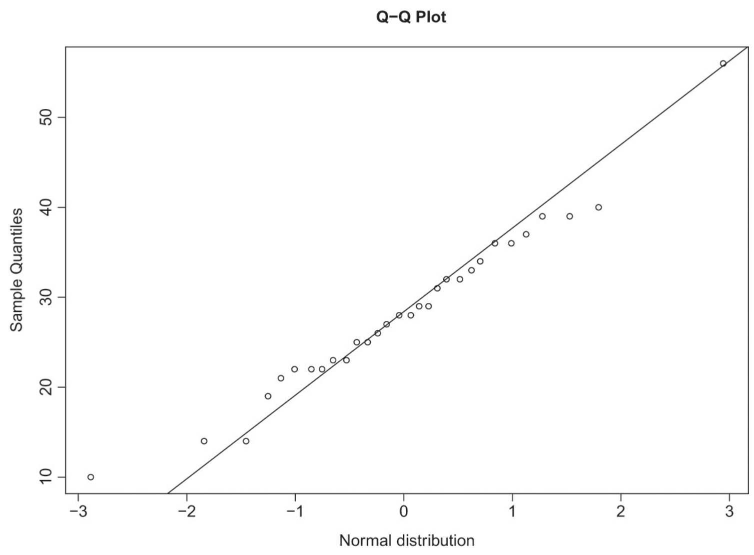

Figure 1 QQ plot for the measurements of the antibody titers on 30 distinct days of a single serum specimen from a serological test, from the clinical microbiology example.

Table 5: 90% and 95% confidence interval estimates from a variety of methods for the EISA example data.

		$\alpha = 0.10$		$\alpha = 0.05$
Miller	0.249	0.405	0.234	0.420
McKay	0.266	0.435	0.256	0.461
Vangel	0.265	0.432	0.256	0.458
Panich	0.261	0.424	0.251	0.450
Boot	0.261	0.419	0.238	0.436
Boot-p	0.235	0.394	0.218	0.417
BCa	0.259	0.432	0.245	0.466
Boot-Miller	0.252	0.407	0.240	0.421
EL	0.247	0.465	0.231	0.487
EL-boot	0.204	0.525	0.173	0.569
JEL	0.254	0.422	0.242	0.441
JEL-boot	0.251	0.426	0.236	0.452

Table 6: Times between successive air conditioner failures in a Boeing 720 aircraft, originally from [28].

90	10	60	186	61	49	14	24	56	20	79	84
44	59	29	118	25	156	310	76	26	44	23	62
130	208	70	101	208							

Table 7: 90% and 95% confidence interval estimates from a variety of methods for the Boeing 720 aircraft example data.

		$\alpha = 0.10$		$\alpha = 0.05$
Miller	0.557	1.139	0.501	1.194
McKay	0.635	1.534	0.604	1.862
Vangel	0.626	1.423	0.596	1.673
Panich	0.618	1.382	0.588	1.612
m-Miller	0.550	1.116	0.496	1.170
m-McKay	0.626	1.483	0.595	1.780
m-Vangel	0.618	1.382	0.588	1.612
Boot	0.712	1.057	0.682	1.107
Boot-p	0.638	0.983	0.589	1.014
BCa	0.714	1.097	0.684	1.125
Boot-Miller	0.576	1.140	0.512	1.204

Automatically generated rough PDF by *ProofCheck* from River Valley Technologies Ltd

Brought to you by | UCL - University College London
Authenticated
Download Date | 5/1/18 9:54 PM

EL	0.451	1.032	0.411	1.076
EL-boot	0.341	1.145	0.268	1.210
JEL	0.689	1.058	0.658	1.534
JEL-boot	0.677	1.077	0.643	1.610

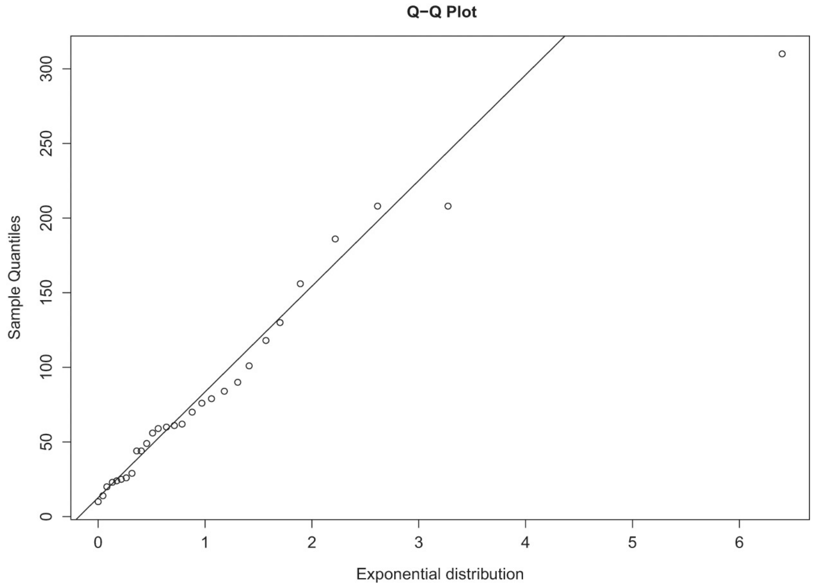

Figure 2 QQ plot for the times between successive air conditioner failures in a Boeing 720 aircraft, from the engineering example.

4.2 An example from engineering

To illustrate the difference of our methods with existing methods, we utilize a well-known engineering dataset, which was suggested from an exponential distribution. The dataset was originally from [28], consisting 29 records of the time intervals between successive failures of the air condition equipment in a Boeing 720 aircraft. The data are provided in Table 6 with the Q–Q plot against the exponential distribution provided in Figure 2. The p-value from the test of exponentiality proposed by [29] is 0.280. The sample mean and standard deviation are 83.5 and 70.81, respectively. The sample CV is 0.848. The 90% and 95% confidence intervals from a variety of methods are provided in Table 7. Consistent with the previous observations, the confidence intervals from all methods are comparable except those the EL-boot method, which are evidently wider. It is worthy noting the fact that the lower bounds of the confidence intervals from the EL and EL-boot methods are considerably smaller than those from other methods, which suggests that the EL and EL-boot methods are robust to the potential outliers from the data. All other methods yield similar interval estimates.

5 Discussions

In this paper we propose interval estimators for the coefficient of variation utilizing empirical likelihood and jackknife empirical likelihood methods. We also developed bootstrap procedures to calibrate the likelihood ratio statistics. Results from our simulation studies suggest that the coverage probabilities of our proposed methods are comparable to existing methods for normal data and are much closer to the nominal level for non-normal data. The JEL interval estimators perform slightly better than the EL ones and the bootstrap calibrated empirical likelihood method performs uniformly better than other methods in our simulations. We thus recommend the bootstrap JEL interval estimator in practice for the coefficient of variation, unless there is a strong belief that the data are from a Gaussian distribution.

Even though the proposed methods can be directly applied when both positive and negative values are possible, our definition of the CV is limited to ratio variables, which are continuous variables with natural zero potins indicating none of the variable being measured. The limitation is caused by the inherent drawbacks of the CV itself. The interpretation of the CV in presence of negative measurementscould be very misleading with a close-to-zero mean value and the comparisons of CVs could be invalid if the measurement variables do not correspond in their zero points; see more details in [30].

Automatically generated rough PDF by *ProofCheck* from River Valley Technologies Ltd

Brought to you by | UCL - University College London
Authenticated
Download Date | 5/1/18 9:54 PM

The proposed methods may be further extended to compare the difference of the CVs from two populations. The extension may involve an additional nuisance parameter, which needs to be profiled out for the empirical likelihood ratio statistic. The proposed methods could also be extended for the data subject to right censoring or limit of detection.

Acknowledgements

We are grateful to the constructive comments from the Associated Editor and the three anonymous Referees, which notably improved the quality of our manuscript.

Appendix

5.1 Proof of Theorem 2.2

Proof

Firstly we show that at $\tau = \tau_0$, $U_n(\tau)$ can be written as a U-statistic

$$U_n(\tau) = \binom{n}{2}^{-1} \sum_{1 \le i_1 < i_2 \le n} h(X_{i_1}, X_{i_2}; \tau),$$

where $h(X_1, X_2; \tau) = \frac{1}{2}\tau^2(X_1^2 + X_2^2) - \frac{1}{2}(\tau^2 + 1)(X_1 - X_2)^2$, since

$$s^2 = \frac{1}{n-1}\sum_{i=1}^{n}(X_i - \bar{X})^2 = \binom{n}{2}^{-1} \sum_{1 \le i_1 < i_2 \le n} \frac{1}{2}(X_{i1} - X_{i2})^2.$$

We then show that $\mathrm{E}h(X_1, X_2; \tau) = 0$, which can be easily derived by noting the facts that

$$\begin{aligned} \tau^2 &= \frac{\sigma^2}{\mu^2} = \frac{\sigma^2}{\mathrm{E}X^2 - \sigma^2}, \\ \sigma^2 &= \frac{1}{2}\mathrm{E}(X_1 - X_2)^2 \text{ and} \end{aligned}$$

$$\mathrm{E}X^2 = \frac{1}{2}\mathrm{E}(X_1^2 + X_2^2)$$

Thus Theorem 2.2 follows directly from Theorem 2.1 in Jing et al. (2009).

5.2 R script for the proposed methods

```
[baselinestretch=0.75]
if (F){
 install.packages("emplik")
}
library(emplik)
######################
## EL functions
######################
cvhat4el.f = function(x){## CV estimate for EL
 n = length(x)
 m = floor(n/2)
 y = z = rep(NA,m)
 for (i in 1:m){
 y[i] = (x[i]-x[m+i])^2/2
 z[i] = (x[i]^2+x[m+i]^2)/2
```

Automatically generated rough PDF by ProofCheck from River Valley Technologies Ltd

Brought to you by | UCL - University College London
Authenticated
Download Date | 5/1/18 9:54 PM

```
 }
 cvhat = sqrt(mean(y)/mean(z-y))
 out = list(cvhat=cvhat,x=x, y=y, z=z)
 return(out)
}
el.f = function(y,z,tau){
 zvals = y-tau^2*(z-y)
 tt = el.test(x=zvals,mu=0)
 ll = tt$"-2LLR"
 if(abs(sum(tt$wts)-length(y))>1) ll = 300
 out = list("-2LLR" = ll,zvals = zvals,tau = tau,n = n)
 return(out)
}
ci.el.f = function(x,avals = c(0.10,0.05),B = 1000,step = 0.01){
 nalpha = length(avals)
 ci.alpha = array(NA,dim = c(nalpha,3,2))
 cx.alpha = round(qchisq(1-avals,1),2) ## cut-off for chi-square
 n = length(x)
 m = floor(n/2)
 ttt = cvhat$el.f(x)
 cvhat = ttt$cvhat
 y = ttt$y
 z = ttt$z
 llboot = rep(NA,B)
 for (i in 1:B){
 idx = sample(1:m,replace=T)
 ystar = y[idx]
 zstar = z[idx]
 llboot[i] = el.f(tau=cvhat, y=ystar, z=zstar)$"-2LLR"
 }
 cboot.alpha = round(quantile(llboot,prob=1-avals,na.rm=T),2)
 for (i in 1:nalpha){
 if (el.f(tau = 1000,y = y, z = z)$"-2LLR" >= cx.alpha[i]){
 ci = findUL(step = step, fun = el.f, MLE = cvhat, y = y, z = z, level
 = cx.alpha[i])
 ci.alpha[i,1,] = c(ci$Low,ci$Up)
 }
 ci.alpha[i,3,] = ci.alpha[i,1,]
 if (el.f(tau = 1000,y = y, z = z)$"-2LLR" >=cboot.alpha[i]){
 ci = findUL(step = step, fun = el.f, MLE = cvhat, y = y, z = z,level
 = cboot.alpha[i])
 ci.alpha[i,3,] = c(ci$Low,ci$Up)
 }
 }
 out = list(ci.alpha = ci.alpha,x = x,avals = avals, B = B,cx.alpha = cx.alpha,
 cboot.alpha = cboot.alpha)
 return(out)
}
######################
## JEL
######################
cvhat4jel.f=function(x){## CV estimate for JEL
 n=length(x)
 y=x^2
 tt1 = mean(y)
 tt2 = sd(x)^2
 cvhat = sqrt(tt2/(tt1-tt2))
 out=list(cvhat=cvhat,x=x)
 return(out)
```

Automatically generated rough PDF by ProofCheck from River Valley Technologies Ltd

Brought to you by | UCL - University College London
Authenticated
Download Date | 5/1/18 9:54 PM

```
}
U_n.f=function(x,tau){
 n=length(x)
 y=x^2
 tt1 = mean(y)
 tt2 = sd(x)^2
 tt=tt1*tau^2-(tau^2+1)*tt2
 out=list(U=tt, tt1=tt1, tt2=tt2,x=x)
 return(out)
}
jkkf.f=function(x,tau){
 n=length(x)
 Un=U_n.f(x,tau)$U
 Un1=vv=rep(NA,n)
 for (i in 1:n){
 Un1[i]=U_n.f(x[-i],tau)$U
 }
 vjack=n*Un-(n-1)*Un1
 out=list(vjack=vjack,n=n,tau=tau,x=x,Un=Un)
 return(out)
}
jel.f=function(tau,x){
 n=length(x)
 vjack=jkkf.f(x=x,tau=tau)$vjack
 tt = el.test(x=vjack,mu=0)
 ll=tt$"-2LLR"
 if(abs(sum(tt$wts)-length(x))>1) ll=300
 out=list("-2LLR"=ll,vjack=vjack,tau=tau,n=n)
 return(out)
}
ci.jel.f=function(x,avals=c(0.10,0.05),B=1000,step=0.01){
 n=length(x)
 cvhat=cvhat4jel.f(x)$cvhat
 nalpha=length(avals)
 ci.alpha=array(NA,dim=c(nalpha,3,2))
 llboot=rep(NA,B)
 for (i in 1:B){
 xstar=sample(x,n,replace=T)
 llboot[i]=jel.f(tau=cvhat,x=xstar)$"-2LLR"
 }
 cx.alpha=qchisq(1-avals,1) ## cut-off for chi-square
 cboot.alpha=round(quantile(llboot,prob=1-avals,na.rm=T),2) ## bootstrap cutoff
 for (i in 1:nalpha){
 ci.alpha[i,1,]=rep(NA,2)
 if (jel.f(tau=1000,x=x)$"-2LLR">=cx.alpha[i]){
 ci=findUL(step=step, fun=jel.f, MLE=cvhat, x=x,level=cx.alpha[i])
 ci.alpha[i,1,]=c(ci$Low,ci$Up)
 }
 ci.alpha[i,3,] = ci.alpha[i,1,]
 if (jel.f(tau=1000,x=x)$"-2LLR">=cboot.alpha[i]){
 ci=findUL(step=step, fun=jel.f, MLE=cvhat, x=x,level=cboot.alpha[i])
 ci.alpha[i,3,]=c(ci$Low,ci$Up)
 }
 }
 out=list(ci.alpha=ci.alpha,x=x,avals=avals,B=B,cx.alpha=cx.alpha,
 cboot.alpha=cboot.alpha)
 return(out)
}
```

Automatically generated rough PDF by ProofCheck from River Valley Technologies Ltd

Brought to you by | UCL - University College London
Authenticated
Download Date | 5/1/18 9:54 PM

References

[1] Pearson K. Mathematical contributions to the theory of evolution? III. Regression, heredity and panmixia. Philos Trans R Soc A. 1896;187:253–318.
[2] Reed GF, Lynn F, Meade BD. Use of coefficient of variation in assessing variability of quantitative Assays. Clin Diagn Lab Immunol. 2002;9(6);1235–1239.
[3] Chow SC, Wang H. On sample size calculation in bioequivalence trials. J Pharmacok Pharmacod. 2001;28:155–169.
[4] Lehmann EL. Testing statistical hypothesis, 2nd ed. New York: Wiley, 1996.
[5] McKay AT. Distribution of the coefficient of variation and the extended t distribution. J Roy Statist Soc B. 1932;95:695–698.
[6] David FN. Note on the application of Fisher's k-statistics. Biometrika. 1949;36:383–393.
[7] Reh W, Scheffler B. Significance tests and confidence intervals for coefficients of variation. Comput Stat Data Anal. 1996;22(4):449–452.
[8] Vangel MG. Confidence interval for a normal coefficient of variation. The Am Stat. 1996;50:21–26.
[9] Wong ACM, Wu J. Small sample asymptotic inference for the coefficient of variation: normal and nonnormal models. J Stat Plann Inference. 2002;104:73–82.
[10] Verrill S, Johnson RA. Confidence bounds and hypothesis tests for normal distribution coefficients of variation. Commun Stat Theory Methods. 2007;36(12):2187–2206.
[11] Mahmoudvand R, Hassani H. Two new confidence intervals for the coefficient of variation in a normal distribution. J Appl Stat. 2009;36(4):429–442.
[12] Panichkitkosolkul W. Confidence intervals for the coefficient of variation in a normal distribution with a known population mean. Probab Stat J. 2013;Article ID 324940. DOI: 10.1155/2013/324940.
[13] Sharma KK, Krishna H. Asymptotic sampling distribution of inverse coefficient of variation and its applications. IEEE Trans Reliab. 1994;43(4):630–633.
[14] Banik S, Kibria BMG. Estimating the population coefficient of variation by confidence intervals. Commun Stat Simul Comput. 2011;40:1236–1261.
[15] Monika G, Kibria BMG, Albatineh AN, Ahmed NU. A comparison of some confidence intervals for estimating the population coefficient of variation: a simulation study. SORT 2012;36:45–68.
[16] Albatineh AN, Boubakari I, Kibria BMG. New confidence interval estimator of the signal-to-noise ratio based on asymptotic sampling distribution. Commun Stat Theory Meth. 2015. DOI: 10.1080/03610926.2014.1000498.
[17] Albatineh AN, Kibria BMG, Wilcox ML, Zogheib B. Confidence interval estimation for the population coefficient of variation using ranked set sampling: a simulation study. J Appl Stat. 2014;41:733–751.
[18] Owen A. Empirical likelihood ratio confidences for single functional. Biometrika. 1988;75:237–249.
[19] Owen AB. Empirical likelihood. Boca Raton, FL: Chapman and Hall/CRC Press, 2001.
[20] Jing B, Yuan, J, Zhou W. Jackknife empirical likelihood. Journal of the American Statistical Association. 2009;104(487):1224–1232.
[21] Peng L, Qi Y. Smoothed jackknife empirical likelihood method for tail copulas. TEST. 2010;19(3):514–536.
[22] Adimari G, Chiogna M. Jackknife empirical likelihood based confidence intervals for partial areas under ROC curves. Stat Sin. 2012;22:1457–1477.
[23] Yang H, Zhao Y. Smoothed jackknife empirical likelihood inference for the difference of ROC curves. J Multivariate Anal. 2013;115:270–284.
[24] Yang H, Zhao Y. Smoothed jackknife empirical likelihood inference for ROC curves with missing data. J Multivariate Anal. 2015;140:123–138.
[25] Wang D, Zhao Y, Gilmore DW. Jackknife empirical likelihood confidence interval for the Gini index. Stat Probab Lett. 2016;110:289–295.
[26] Wang D, Zhao Y. Jackknife empirical likelihood for comparing two Gini indices. Canadian J Stat. 2016;44(1):102–119.
[27] Canty A, Ripley B. Boot: Bootstrap R (S-Plus) Functions. R package version 1.3-18, 2016.
[28] Davison AC, Hinkley DV. Bootstrap methods and their applications. Cambridge: Cambridge University Press, 1997.
[29] Zhou M. emplik: Empirical likelihood ratio for censored/truncated data. R package version 1.0-3, 2016.
[30] Wood RJ, Durham TM. Reproducibility of serological titers. J Clin Microbiol 1980;11:541–545.
[31] Proschan F. Theoretical explanation of observed decreasing failure rate. Technometrics. 1963;5:375–383.
[32] Gail MH, Gastwirth JL. A scale-free goodness-of-fit test for the exponential distribution based on the Gini statistic. J R Stat Soc Ser B. 1978;40:350–357.
[33] Eisenberg DT. Telomere length measurement validity: the coefficient of variation is invalid and cannot be used to compare quantitative polymerase chain reaction and Southern blot telomere length measurement techniques. Int J Epidemiol. 2016;45:1295–1298.

Automatically generated rough PDF by *ProofCheck* from River Valley Technologies Ltd

Brought to you by | UCL - University College London
Authenticated
Download Date | 5/1/18 9:54 PM
