# Supplementary material for: Provenance and family variations in early growth of Manchurian walnut (Juglans mandshurica Maxim.) and selection of superior families
Source: PLoS One. 2024 Mar 7;19(3):e0298918. doi: 10.1371/journal.pone.0298918 (PMC10919699; doi:10.1371/journal.pone.0298918)
Supplement: S2 File — (ZIP) [file pone.0298918.s005.zip › Phenotypic variation of Syringa oblata Lindl.pdf]

DOI: [illegible] cnki lykxyj [illegible]
林业科学研究 2006, 19(2): 199～204
Forest Research

文章编号：1001-1498(2006)02-0199-06

紫丁香表型多样性研究

明 军[1]，顾万春[2*]

(1. 中国农业科学研究院蔬菜花卉研究所，北京 100081；2. 中国林业科学研究院林业研究所，北京 100091)

摘要：以紫丁香天然林分布区抽取的 4个天然群体和 1个栽培群体为拟似群体，进行了其表型多样性分析。结果表明：紫丁香表型性状存在着丰富的群体间、群体内变异，5个群体的果、种子、叶以及子代幼苗等 14个表型性状差异显著，变异系数为 14.25% ～29.13%，表型性状分化系数为 1.85% ～86.67%，平均值为 43.93%；群体间和群体内的差异均极显著，群体内的方差分量为 56.06%，其中有 10个种实性状与生态梯度值呈显著或极显著的相关，采用 Dist平均分类距离系数的聚类分析得到的群体间的遗传距离关系基本上与群体采种点的生态地理位置关系一致，其中山西五老峰群体独立为一组，北部三个群体聚为一组，栽培群体聚入了北部群体组，并且与辽宁北票群体较近。依据研究结果，紫丁香种质资源收集、保护策略应当是：基于表型分化系数群体间平均值达到较高水平，而群体内保留了种质的大部分变异，所以在分布区内应首先保护天然群体的存在，即尽可能多的群体数量，同时，要很好保护天然群体的完整性。育种亲本的选择策略应当是：在群体内收集、选择特异变异的同时，加强多群体的收集和选择。

关键词：紫丁香；天然群体；表型标记；遗传多样性
中图分类号：S722.3 文献标识码：A

Phenotypic Variation of *Syringa oblata* Lindl

MING Jun[1], *GU Wan-chun*[2]

(1. Institute of Vegetables and Flowers, Chinese Academy of Agricultural Sciences, Beijing 100081, China;
2. Research Institute of Forestry, CAF, Beijing 100091, China)

Abstract: *Syringa oblata* Lindl. is a native deciduous shrub species in China's temperate regions. Genetic resources of the species has been seriously degraded, remaining few scattered distributions. 4 natural populations and 1 cultivated population of *Syringa oblata*. were analyzed using morphological markers. Morphological diversity among and within populations were analyzed based on 14 phenotypic traits, including three capsule characters, two foliage traits, five seed and four seedling traits, of 30 sample trees from each population. Variance analysis showed that there were significant variation among/within populations. The coefficient of variation (*CV*) was 14.25% to 29.13%, and phenotypic differentiation coefficient (*Vst*) ranged from 1.85% to 86.67%, the mean value of 5 populations was 43.93%. The variation within population was the main part of the phenotypic variation, variance component of within population variance was 56.06%. There is close relationship between the 10 phenotypic traits and ecological gradient variation in spatial distribution. Cluster analysis based on average taxonomic distance (Dist) of morphological traits showed that the 5 populations of *Syringa oblata* were divided into two groups and that the cultivated population was clustered into northern group. According to the research result, the lilac germplasm resources collections and protection strategy should be focused on protecting more populations as many as

收稿日期：2005-06-03
基金项目：“十五”国家攻关“林木种质资源保存技术创新与利用研究”(2001BA511B10)，国家重大基础性工作项目“林木种质资源收集、保存、编目”(2001DEA10002)
作者简介：明 军(1963—)，男，湖北武汉人，副教授，博士；*通讯作者.
(C)1994-2019 China Academic Journal Electronic Publishing House. All rights reserved. http://www.cnki.net

possible. On the other hand, integrality is also important. A selective breeding strategy should be employed not only selecting genetic variation within but also among populations.

Key words: *Syringa oblata* Lindl.; natural population; morphological traits; genetic diversity

表型多样性主要研究群体在分布区内各种环境下的表型变异，是遗传多样性与环境多样性的综合体现。利用表型性状差异研究群体的遗传多样性具有简便、快速和节省费用等优点，在马尾松（*Pinus massoniana* Lamb.）、悬钩子（*Rubus* L.）、蜘蛛抱蛋（*Aspidistra elatior* Bl.）[1,2]、白皮松（*P. bungeana* Zucc. ex Endl.）[2,3]、云杉（*Picea asperata* Mast.）[4]、蒙古栎（*Quercus mongolicas* Fisch）等植物效果较好[5]。通过有效的采样、合理的数学统计方法，可以揭示群体的遗传规律、变异大小，可客观评价其遗传多样性。紫丁香（*Syringa oblata* Lind.）是丁香属分布最广、应用最多的中国原产种，欧美以紫丁香为亲本选育的品种近1 000个[6,7]。历史上紫丁香在其分布区内的农林业生产中常被视为杂灌木而遭受清除。目前，文献记载的许多分布点已经找不到紫丁香天然群落，显然部分遗传资源已经丢失；因此，对其进行科学合理评价，弄清其群体种质遗传多样性，对抢救和保护现有所剩不多的紫丁香天然群体和种质的创新利用，均具有重要的理论意义和参考价值。

1　材料和方法

1.1　群体与个体抽样及试验材料采集

在紫丁香自然分布区内抽样采集了4个天然群体和一个栽培群体（北京地区）。采样地的地理生态因子、林分情况见表1。每群体选取30～40个单株（丛）进行调查和采样，株（丛）间距离5倍株高以上，保证取样均匀性，最大限度降低母树间的亲缘关系。选择生长正常，无严重缺陷，无明显病虫害的单株（丛），在每个单株树冠中上部，采集成熟果，同时采集20～30个叶片。

1.2　性状测定方法

调查记录采种点的生态环境因子：经纬度、海拔、立地条件以及采种母株的植株高、地径、冠幅、结实情况等汇入采集表1。

从每个采集群体中，分别取30个采样母树，每个母树分别抽取20个叶片测定其叶长和叶宽；从每个采样母树上采集的果实中随机抽取30个果实，分别测量果长、果宽、果厚；每个采样单株分别2种种型，各随机抽取30粒种子，测量种长、种宽。使用游标卡尺共测量2个母树叶片和7个种实性状指标，精度为0.1 mm。

种子千粒质量：采用GB/T 3543.7-1995的百粒法，从每采样母树随机抽取100粒种子称质量，重复10次，测量精度0.001 g，取平均数换算成千粒质量。

子代幼苗表型测定，每个采样母树所采集的种子为1个家系，分家系在同一条件下播种、育苗，每个家系随机选30个单株，苗龄120 d，按群体家系分别测量记录真叶片数、苗高、第2对真叶的长和宽，共测240片。使用游标卡尺测量，测量精度为0.1 mm。

表1　紫丁香采样群体的地理位置及调查母树的生长情况

项目	天然群体				栽培群体
	内蒙大黑山	辽宁北票	辽宁喀左	山西五老峰	
经度/(°)E	118.97	120.40	120	110.30	116.20
纬度/(°)N	42.27	41.55	41.35	34.8	39.93
海拔/m	571	171～700	171～700	100～1 900	
年均气温/℃	6.8	8.3	8.3	10.7	11.8
年降水量/mm	358.1	507.1	507.1	453.5	635.3
坡度/(°)	30～45	30～45	30～40	45～65	
坡向	N	S、EN	SW	N	
地貌类型	低山区	低山区	低山区	中高山区	平原
土壤厚度/cm	5～20	5～50	5～50	5～20	
采样母树/株	34	36	34	38	30
(树高±SD)/m	1.30±0.41	1.54±0.17	1.04±0.24	4.08±0.79	1.77±0.14
(平均地径±SD)/cm	1.34±0.41	1.55±0.37	0.87±0.33	14.9±1.94	1.6±0.20
(冠幅±SD)/m	EW 1.91±0.37	3.38±0.78	1.30±0.31	4.25±0.85	2.86±0.64
	SN 1.80±0.53	3.30±0.52	1.40±0.24	4.25±0.85	3.16±0.44

注：SD为标准差。

(C)1994-2019 China Academic Journal Electronic Publishing House. All rights reserved.　http://www.cnki.net

1.3 统计分析方法及参数

(1)种实表型性状方差分析采用巢式设计方差分析, 线形模型为:

$$Y_{ijk}=\mu+S_i+T_{(i)j}+\varepsilon_{(ij)k} \quad (1)$$

式(1)中: Y_{ijk}为第 i个群体第 j个家系第 k个观测值; μ为总均值; S_i为群体效应(固定); $T_{(i)j}$为群体内家系效应(随机); $\varepsilon_{(ij)k}$为试验误差。

(2)表型分化系数(V_{st})

$$V_{st}=\left(\sigma^2_{t/s}/(\sigma^2_{t/s}+\sigma^2_s)\right)\times 100\% \quad (2)$$

式(2)中: $\sigma^2_{t/s}$为群体间方差分量, σ^2_s为群体内方差分量, V_{st}为表型分化系数, 表示群体间变异占遗传总变异的百分比[2]。

(3)表型性状极差。

(4)表型性状变异特征系数(CV)　CV表示表型性状离散程度, 用相对极差 R'_i表示极端差异程度。$R'_i=R_i/R_o\times 100\%$, R_i为群体内的极差; R_o为性状总极差。

(5)群体表型性状相似性聚类分析　采用 Dist 平均分类距离系数(E_{ij}):

$$E_{ij}=\sqrt{\sum_k \frac{1}{x_k}\left(\frac{x_{ki}}{x_i}-\frac{x_{kj}}{x_j}\right)} \quad (3)$$

i, j, k分别为第 i, j群体的第 k个性状。

利用 NTSYSpc2.1软件对表型数据进行非加权配对算术平均法(UPGMA)进行聚类分析[8]。

(6)使用 SAS8.0进行表型性状与生态因子的偏相关关系分析。

2 结果与分析

2.1 紫丁香群体表型性状的形态变异特征

采样母树的 10个表型性状均值及均值的标准差见表 2。

表 2　紫丁香 4个天然群体和 1个栽培群体表型性状的平均值及标准差(SD)

性状	天然群体				栽培群体
	内蒙大黑山	辽宁北票	辽宁喀左	山西五老峰	
果长 /mm	1.434±0.18	1.296±0.24	1.293±0.16	1.549±0.20	1.225±0.25
果宽 /mm	0.557±0.08	0.537±0.07	0.530±0.07	0.651±0.10	0.582±0.09
果厚 /mm	0.357±0.04	0.364±0.05	0.356±0.04	0.384±0.05	0.371±0.05
种子长 1 /mm	0.881±0.099	0.819±0.108	0.854±0.134	0.981±0.117	0.886±0.147
种子宽 1 /mm	0.331±0.053	0.218±0.028	0.221±0.035	0.369±0.044	0.263±0.062
种子长 2 /mm	0.912±0.111	0.831±0.103	0.855±0.101	1.044±0.116	0.881±0.132
种子宽 2 /mm	0.239±0.042	0.345±0.046	0.334±0.043	0.237±0.034	0.317±0.072
千粒质量 /g	5.909±1.707	4.873±1.410	4.618±1.334	4.082±1.181	3.320±0.959
叶长 /cm	6.373±2.25	4.383±0.68	5.208±1.31	4.524±1.04	5.990±1.00
叶宽 /cm	6.085±1.75	4.415±0.66	4.888±1.38	4.453±1.17	6.002±0.98

注: 种子长(宽)1为 1室宽种子; 种子长(宽)2为同侧 2室种子, 下同。

从表 2中可以看出: 紫丁香果的形态特征在群体间存在差异, 果宽、果厚的变异一致, 均以山西五老峰群体果长均值最大, 辽宁喀左为最小; 2种类型种子长变化与果长变化一致; 内蒙大黑山群体叶长、叶宽最大, 辽宁北票最小。

从表 3可以看出: 10个表型性状的平均变异系数有一定差异, 叶长的平均变异系数最大, 果厚性状的变异系数最小。变异系数表示性状值离散性特征, 变异系数越大, 则形状值离散程度越大, 说明果实厚度性状较其它性状的稳定性高, 果厚性状在一定程度上反映果实饱满程度, 叶长宽变化较大。

表 3　紫丁香 4个天然群体和 1个栽培群体表型性状的相对极差和变异系数(CV)

性状	天然群体								栽培群体		平均变异系数	群体间变异系数
	内蒙大黑山		辽宁北票		辽宁喀左		山西五老峰					
	相对极差	CV	相对极差	CV	相对极差	CV	相对极差	CV	相对极差	CV		
果长 /mm	0.67	12.57	0.60	11.32	0.73	12.87	0.81	13.21	0.98	19.11	15.27	9.4183
果宽 /mm	0.60	14.51	0.58	14.07	0.70	14.30	0.86	15.98	0.52	15.86	17.10	12.0557
果厚 /mm	0.82	13.08	1.00	15.16	0.58	13.37	0.64	13.80	0.54	14.37	14.25	12.3526
种子长 1 /mm	0.38	11.21	0.83	13.13	0.75	13.86	0.84	11.97	0.80	16.60	14.51	9.7109
种子宽 1 /mm	0.77	16.10	0.49	13.02	0.74	15.64	0.85	11.87	0.77	23.68	27.50	11.6145
种子长 2 /mm	0.49	12.23	0.73	12.40	0.72	11.84	0.79	11.08	0.77	14.95	15.03	9.2137
种子宽 2 /mm	0.73	17.44	0.79	13.25	0.88	12.95	0.73	14.45	0.97	22.77	22.98	11.8642
千粒质量 /g	0.54	12.05	0.95	20.35	0.18	9.22	0.72	21.62	0.21	8.38	24.00	6.2483
叶长 /cm	1.00	35.38	0.25	15.50	0.39	25.21	0.43	22.97	0.31	16.71	29.13	18.4285
叶宽 /cm	1.00	28.72	0.32	14.95	0.44	28.18	0.54	26.18	0.39	16.39	27.48	20.8348

(C)1994-2019 China Academic Journal Electronic Publishing House. All rights reserved. http://www.cnki.net

实地调查采集的栽培群体果实败育率较高，对应的果实、种子表型性状的变异也较大；而种子千粒质量和叶的变异小于天然群体，反映了栽培群体具有相对较好且相对稳定、一致度较高的生境条件。

2.2 子代幼苗表型特征

从 5个群体中抽取检测了 3个群体子代生长 120 d幼苗的苗高、真叶对数以及第 2对真叶的长宽(表 4)。表 4显示：苗期性状变异系数大于母树果实、种子和叶的表型变异值，这种变异表明子代个体之间存在的遗传差异的变化幅度大于其现存的经过环境选择的亲代的变异。

表 4 紫丁香天然群体子代幼苗 4个表型性状的平均值和变异系数

性状		群体			平均	群体间变异系数
		内蒙大黑山	辽宁北票	山西五老峰		
苗高 /cm	平均值	3.101	3.801	3.526	3.489	
	变异系数 /%	55.724 5	64.276 7	39.196 5		42.224 8
真叶数 /片	平均值	3.114	3.2	3.804	3.351	
	变异系数 /%	32.013	32.197 5	29.468 3		29.440 2
真叶长 /cm	平均值	1.922	1.559	1.373	1.623	
	变异系数 /%	95.460 4	44.916 9	32.730 6		67.977 3
真叶宽 /cm	平均值	1.727	1.394	1.354	1.492	
	变异系数 /%	69.856 3	45.422 7	32.513 5		51.757 9

2.3 紫丁香群体表型分化差异的显著性检验

10个表型性状在群体间和群体内均存在极显著差异。

按巢式设计方差分量比组成了各方差分量与群体间的表型分化系数(表 5)。

表 5 紫丁香表型性状的方差分量及群体间表型分化系数

性状	方差分量			多样性贡献百分比 /%		表型分化系数 /%
	群体间	群体内	随机误差	群体间	群体内	
果长	0.016 8	0.024 8	0.017 9	40.38	59.62	40.38
果宽	0.002 7	0.002 7	0.004 7	50.00	50.00	50.00
果厚	0.000 1	0.000 6	0.002 0	14.29	85.71	14.29
种子长 1	0.004 0	0.005 9	0.008 7	40.40	59.60	40.40
种子宽 1	0.005 2	0.000 8	0.001 1	86.67	13.33	86.67
种子长 2	0.007 8	0.005 3	0.007 0	59.54	40.46	59.54
种子宽 2	0.003 1	0.000 8	0.001 2	79.49	20.51	79.49
叶长	0.501 7	1.020 3	0.916 6	32.96	67.04	32.96
叶宽	0.566 9	0.401 7	0.401 7	58.53	41.47	58.53
苗高	0.032 2	1.703 7	2.170 5	1.85	98.15	1.85
真叶数	0.123 6	0.130 8	0.973 3	48.58	51.42	48.58
真叶长	0.064 7	0.140 2	1.217 0	31.58	68.42	31.58
真叶宽	0.035 0	0.095 1	0.596 0	26.90	73.10	26.90
平均	0.10	0.27	0.49	43.94	56.06	43.93

表 5显示：亲代表型性状和子代表型性状变异系数为 1.85% ~86.67%，这说明紫丁香天然群体表型变异变化较大，其中 2型种子宽的表型变异系数的均值分别达到了 86.67%、79.49%；果宽、种子长 2和叶宽的表型变异系数的均值达到了 50%，其中种子宽 1、种子宽 2、叶宽、种子长 2这 4个性状的群体间方差分量均超过了群体内方差分量。

2.4 紫丁香群体表型聚类分析

采用 Dist平均分类距离系数的 UPGMA聚类方法对 5个群体的 13个表型值数据进行聚类分析，结果见图 1。

(C)1994-2019 China Academic Journal Electronic Publishing House. All rights reserved. http://www.cnki.net

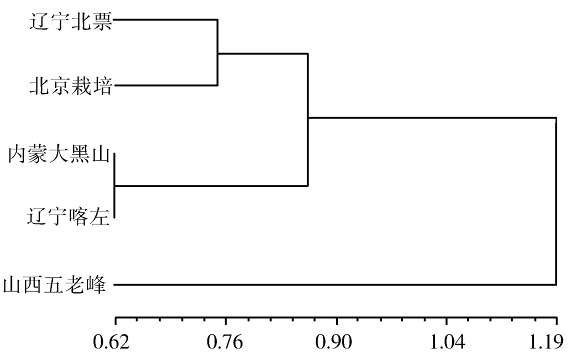

图 1　紫丁香表型性状 Dist平均分类距离聚类树型图

图1显示：群体间的 Dist平均分类距离基本上与群体采种点的生态梯度及地理距离关系一致，其中山西五老峰群体为一组，北部3个群体聚为另一组。值得注意的是栽培群体聚入了北部组，并且与北票群体较近。由此可以推断：栽培群体的亲本可能来自辽宁中部到河北北部地区。

2.5　紫丁香表型性状与采集点地理生态因子及生态梯度值的偏相关关系

对紫丁香采种母树的10个性状与采样地的地理气候因子及生态梯度的相关分析结果见表6。

表 6　紫丁香表型性状与地理生态因子间偏相关系数矩阵

性状	经度(E)	纬度(N)	海拔	年均气温	年降水量	生态梯度 EGA
果长	-0.6545	-0.6306	0.9524**	-0.1232	-0.7362	-0.1015
果宽	-0.9937**	-0.9446**	0.7050	0.6339	-0.0352	-0.7827*
果厚	-0.9239**	-0.9352**	0.5589	0.7742*	0.2304	-0.8865**
种子长 1	-0.9612**	-0.8965**	0.7637*	0.5001	-0.1829	-0.6634
种子宽 1	-0.7733*	-0.6417	0.7492	0.1386	-0.5369	-0.3115
种子长 2	-0.9312**	-0.8811**	0.8665**	0.3572	-0.3439	-0.5489
种子宽 2	0.6245	0.4778	-0.7075	0.0588	0.6756	0.1091
千粒质量	0.5043	0.5349	0.1431	-0.9664**	-0.8662**	0.9067**
叶长	0.2299	0.4605	-0.4537	-0.1461	-0.0856	0.2462
叶宽	0.1843	0.4236	-0.5106	-0.0265	0.0259	0.1413

紫丁香果宽、果厚、种子长1、种子长2这4个性状与经度、纬度呈极显著的负相关；千粒质量与年均气温、年降水量呈极显著的负相关，海拔高度与果长、种子长2呈极显著的正相关。千粒质量与生态梯度值呈极显著的正相关，果宽和果厚与生态梯度值分别呈显著的负相关和极显著负相关。

3　讨论与结论

3.1　紫丁香的表型变异丰富

紫丁香种内存在丰富的群体间、群体内变异，其5个群体的果、种子、叶及其子代幼苗等13个表型性状差异显著，变异系数较大，变幅为14.25%～29.13%。群体间表型性状的变异数据显示：果长、种子长和千粒质量3项表型指标的变异系数均比叶长、叶宽的小，表明在表型多样性评价研究中，繁殖器官性状指标具有较营养器官稳定的特性，尤其在木本等多年生植物的评价中应优先选用果实、种子等性状。对观赏植物，花部的性状也很重要，本研究由于采样季节的限制没有列入。

10个母树表型性状和4个子代表型性状分化系数的变异幅度为1.85%～86.67%，平均值达到了43.93%，高于白皮松(22.86%)[3]、泸定百合(*Lilium sargentiae* Wilson)(28.03%)[9]、云杉(30.99%)[4]，低于蒙古栎(56.09%)[7]，这表明紫丁香天然群体间表型分化系数较大，该物种可能存在较高的群体内自交率[10,11]。丁香开花规律的观察研究表明：其具有较高异花授粉结实率的同时，自交结实率也可高达70.9%[12,13]。

紫丁香栽培群体果实、种子表型性状的变异均大于天然群体的对应性状变异，与实地调查栽培群体种子败育率高的现象一致，当果实存在较高败育率时，必然导致果实和种子发育的饱满度和一致度下降，统计中采用随机抽样，这样造成统计数据中包含较多的不同阶段败育的、饱满度不同的果实种子；而种子千粒质量和叶的变异小于天然群体，这恰好反映了栽培群体具有相对较好且相对稳定的生境条件。

群体间子代变异系数均大于其亲代，而群体间分化系数小于其亲代，其中苗高的群体间的表型分化系数仅有1.85%，显然是由于栽培环境的一致性减小了群体所处环境因素造成的差异。

紫丁香在群体内和群体间果实、种子及其子代

(C)1994-2019 China Academic Journal Electronic Publishing House. All rights reserved.　http://www.cnki.net

幼苗等 14个表型性状上均存在变异,即不同群体的环境异质性导致了群体表型变异的差异,这些变异受到生境因素影响的同时也反映出种质的遗传多样性。

3.2 紫丁香的表型变异呈现地理气候因子梯度变化趋势

生态梯度值[14]与紫丁香果、种子、叶等表型性状相关关系显示了与东经经度、北纬纬度的变化基本一致的规律,即果实、种子、树体大小随着经度、纬度的增加而逐渐减小。显示辽宁东北部地区可能处于其分布区的东北部边缘,而位于山西东条山脉可能处于其自然分布的中部区域。

综上所述,紫丁香的表型性状在群体内和群体间均存在真实明显的遗传变异。因此,依据研究结果,紫丁香种质资源收集、保护策略应当是:基于表型分化系数群体间平均值达到较高水平,而群体内保留了种质的大部分变异,所以在分布区内应首先保护天然群体的存在,即尽可能多的群体数量,同时,要很好保护天然群体的完整性。育种亲本的选择策略应当是:在群体内收集、选择特异变异的同时,加强多群体的收集和选择。

参考文献:

[1] 葛颂,洪德元.遗传多样性及其检测方法[A].见:钱迎倩、马克平.生物多样性研究的原理和方法[M].北京:中国科学技术出版社,1994

[2] 顾万春,王棋,游应天,等.森林遗传资源学概论[M].北京:中国科学技术出版社,1998

[3] 李斌,顾万春,卢宝明.白皮松天然群体种实性状表型多样性研究[J].生物多样性,2002,10(2):181~188

[4] 罗建勋,李晓清,孙鹏,等.云杉天然群体的表型变异[J].东北林业大学学报,2003,31(1):9~11

[5] 李文英.蒙古栎天然群体遗传多样性研究[D].北京:北京林业大学,2003

[6] 臧淑英,崔洪霞.丁香花[M].上海:上海科学技术出版社,2000

[7] http://www.arboretum.harvard.edu/

[8] Rohlf F J. Numerical Taxonomy and Multivariate Analysis System Version 2.1[M]. Applied Biostatistics Inc, 2000

[9] 毛钧,张明宇,虞泓.泸定百合普洱居群遗传与变异研究[J].云南大学学报(自然科学版),2003,25(增刊):91~96

[10] Hamrick J L, Godt M J W, Sherman-Broyes S L. Factors influencing levels diversity in woody plant species[J]. New Forest, 1992,6:95~124

[11] 周世良,张方,王中仁,等.杭州石荠苧和石香薷的遗传多样性研究[J].遗传学报,1998,25(2):173~180

[12] 刘彦琴,焦英俊,吕立峰.紫丁香开花规律的观察研究[J].内蒙古农牧学院学报,1996,17(1):59~62

[13] 吴国良,杨志红,刘群龙.丁香开花授粉生物学特性研究[J].北京林业大学学报,1998,20(2):118~120

[14] 顾万春,李斌,游应天,等.生态梯度轴(EGA)区划林木育种区的研究[J].生态学报,1997,17(2):159~169

(C)1994-2019 China Academic Journal Electronic Publishing House. All rights reserved. http://www.cnki.net
