# Supplementary material for: Provenance and family variations in early growth of Manchurian walnut (Juglans mandshurica Maxim.) and selection of superior families
Source: PLoS One. 2024 Mar 7;19(3):e0298918. doi: 10.1371/journal.pone.0298918 (PMC10919699; doi:10.1371/journal.pone.0298918)
Supplement: S2 File — (ZIP) [file pone.0298918.s005.zip › Photosynthetic Physiological Characteristics of Naturally Regenerated Juglans mandshurica Saplings at Different Ages.pdf]

doi:10.3969/j.issn.1006-8023.2023.04.004

不同年龄阶段胡桃楸天然更新幼树的光合生理特性

袁钰晨,谢旭强,徐立清,于均屹,蔡智,王庆成*
(东北林业大学 林学院,哈尔滨 150040)

摘　要:探究不同树龄胡桃楸(*Juglans mandshurica*)幼树适宜的光环境条件,为制定林下胡桃楸幼树的人工抚育措施提供理论依据。以哈尔滨市方正县转山实验林场的树龄为 1~3 a(Ⅰ年龄阶段)、4~6 a(Ⅱ年龄阶段)和 7~9 a(Ⅲ年龄阶段)的胡桃楸天然更新幼树为研究对象,测定其在不同光环境(林缘、林隙和林下)的生长形态和光合指标。结果表明,随着光照强度的减弱,各年龄阶段的幼树生物量、苗高和地径降低,比叶面积增大;叶片叶绿素 a/b 值、光补偿点(Light Compensation Point, LCP)、最大净光合速率(maximum net photosynthetic rate, P_{max})、暗呼吸速率(Dark respiration rate, R_d)下降,叶绿素含量升高。在林下和林隙中,Ⅱ年龄阶段的幼树叶片最大净光合速率和叶绿素含量最高,叶片光补偿点、暗呼吸速率和叶绿素 a/b 最低;在林缘,随着树木年龄的增长叶片最大净光合速率和光补偿点逐渐增大。综上所述,林缘更适宜幼树的生长。此外,对 4~6 a 的胡桃楸幼树进行抚育时,可适当降低抚育强度;而对 1~3 a 和 7~9 a 的胡桃楸幼树进行人工抚育时,要加强抚育力度,及时对上层林木透光。

关键词:胡桃楸;幼树;树龄;生境;天然更新;光合生理
中图分类号:S754.5　　**文献标识码**:A　　**文章编号**:1006-8023(2023)04-0029-09

Photosynthetic Physiological Characteristics of Naturally Regenerated *Juglans mandshurica* Saplings at Different Ages

YUAN Yuchen, XIE Xuqiang, XU Liqing, YU Junyi, CAI Zhi, WANG Qingcheng*
(School of Forestry, Northeast Forestry University, Harbin 150040, China)

Abstract: This study was aimed to investigate the appropriate light conditions for *Juglans mandshurica* saplings of different ages, and provide a theoretical basis for the development of artificial tending measures for *J. mandshurica* saplings in the understory. In this study, natural regeneration of *J. mandshurica* saplings aged 1-3 a (age stage Ⅰ), 4-6 a (age stage Ⅱ) and 7-9 a (age stage Ⅲ) in the experimental forestry farm of Zhuan Shan, Fangzheng County, Harbin City were used as experimental subjects, determining their growth patterns and photosynthetic indicators in different light environments (forest edge, forest gap and understory). The results showed that: as light intensity diminished, the biomass, saplings height and ground diameter decreased, and the specific leaf area increased; chlorophyll a/b, light compensation point (LCP), the highest maximum net photosynthetic rate (P_{max}) and dark respiration rate (R_d) decreased, chlorophyll content increased in saplings of all ages. In the understory and the forest gap, saplings of age Ⅱ had the highest maximum net photosynthetic rate and chlorophyll content and the lowest values of light compensation point, dark respiration rate and chlorophyll a/b; the highest maximum net photosynthetic rate and light compensation point of saplings increases as saplings age in the forest edge. In summary, the forest edge is more suitable for the growth of saplings. In addition, when artificially tending *J. mandshurica* saplings between 4 and 6 years old, tending intensity can be reduced appropriately. In contrast, when artificially tending *J. mandshurica* saplings of 1-3 and 7-9 years old, and light transmittance of the upper forest layer should be strengthened timely.

Keywords: *Juglans mandshurica*; saplings; tree-ages; habitat; naturally regenerated; photosynthetic physiological

收稿日期:2022-11-29
基金项目:中央高校基本科研业务费专项资金项目(2572020DR04)
第一作者简介:袁钰晨,硕士研究生。研究方向为森林培育。E-mail: 1064365757@qq.com
***通信作者**:王庆成,博士,教授,博士生导师。研究方向为森林培育。E-mail: wqcnefu@163.com
引文格式:袁钰晨, 谢旭强, 徐立清, 等. 不同年龄阶段胡桃楸天然更新幼树的光合生理特性[J]. 森林工程,2023,39(4):29-37.
YUAN Y C, XIE X Q, XU L Q, et al. Photosynthetic physiological characteristics of naturally regenerated *Juglans mandshurica* saplings at different ages[J]. Forest Engineering, 2023,39(4):29-37.

(C)1994-2023 China Academic Journal Electronic Publishing House. All rights reserved. http://www.cnki.net

0 引言

天然林资源保护工程实施以来,东北国有林区全面禁止天然林的商业性采伐[1],森林资源的培育方式主要转为林冠下更新[2]。充分了解树种的生态学特性,特别是对光照的需求才能保证其在林冠下成功更新[3]。光照是重要的环境因子[4],森林中的光环境在空间和时间上均存在异质性,影响树木的生长发育[5]。研究表明,植物为适应异质光环境,在生长形态和生理上会产生不同响应,其可塑性变化是植物在不同光环境下的重要策略[6-7]。在形态上,随着光照条件的减弱,植物的光合能力降低,单位面积叶片生产的有机质减少,使叶片的比叶面积增大[8];同时植物增加地上部分的投入并减少根的分配,降低根冠比;在生理上,植物通过增加叶绿素含量并降低叶绿素 a/b 值、降低光饱和点和光补偿点等措施来适应弱光条件[9-12]。

与环境因子相比,植物自身的特性对光合作用的影响更为复杂和重要[13]。在不同的光环境下,不同年龄的植物为了自身生长和形态建成的需要会采取不同的响应策略,植物耐阴性也会随年龄增长发生改变,影响幼树保存率和森林更新。研究表明,红松(*Pinus koraiensis*)随着年龄的增长需光量逐渐增大,对强光的耐受性也增加[14]。也有研究发现随年龄的增长,不同年龄的植物光合能力也不同,2个品种的“早酥”梨叶片净光合速率随着年龄的增长而增加[15],光合能力增大;而火炬松(*Pinus taeda*)针叶净光合速率随着年龄的增长而下降,光合能力不断降低[16]。

胡桃楸(*Juglans mandshurica*)是东北地区珍贵的硬阔树种,具有较高观赏价值和经济价值[17]。由于过度采伐和忽视后备资源的培育,胡桃楸数量下降[18]。因此,开展胡桃楸后备资源的培育是林业生产的迫切任务。目前已有对胡桃楸光合生理的研究,表明胡桃楸幼苗有一定的耐阴性,具有在林下更新的能力。但以往研究大多采用同一年龄段的苗木[3,19-21],不同年龄阶段的苗木在不同光环境下是否具有不同的适应对策尚不明确;且在野外,除了林冠层分布模式不同导致的光抑制性外,还有土壤、水分和竞争等因子的影响,长期生长在野外的植物会出现与控制条件下光合特性不一致的情况[22],但目前对于胡桃楸的光合生理研究均采用人为控制试验,无法还原在天然林中胡桃楸对不同光环境的响应。因此,本研究通过对天然林中不同生境下不同年龄阶段的胡桃楸幼树进行研究,分析天然林中同一生境不同年龄阶段的幼树和同一年龄阶段不同生境的幼树生长形态和光合生理特点,旨在揭示随着树龄的增长幼树的光合生理变化规律,为胡桃楸幼树人工抚育措施提供理论支撑。

1 材料与方法

1.1 研究地概况

研究地位于哈尔滨市方正县转山实验林场(128°52′30″~129°06′40″ E ,45°46′01″~45°54′38″ N,)。林场地处长白山脉、张广才岭西坡、老爷岭北麓的低山丘陵地带,属中温带大陆性季风气候,年均气温 2.7 ℃,年均降水量 577 mm,无霜期 125 d[23]。

本研究选择的林分主要为天然起源的珍贵硬阔混交林,林分平均树高 16.2 m,平均胸径 22.0 cm,平均年龄 66 a。立地类型为阴缓坡中层暗棕壤类型,林分的平均郁闭度为 0.7。

1.2 研究方法

2021 年 7 月,在林场胡桃楸分布较多的天然林分中,选择 3 种生境。1)林下。距道边 30 m 以上,郁闭度 0.8 左右的林下;2)林隙。距道边 30 m 以上,面积为 50~100 m^2 的林隙;3)林缘。道边 10 m 范围内,全光照。选择生长正常的 1~9 年生胡桃楸幼树(根据主干芽鳞痕判断幼树树龄),将 3 种光环境下的胡桃楸幼树分为 3 个年龄阶段(1~3 a 为Ⅰ年龄阶段,4~6 a 为Ⅱ年龄阶段,7~9 a 为Ⅲ年龄阶段)。

1.3 生长形态和生理指标测定

1.3.1 生长和形态指标测定

3 种生境下的 3 个年龄阶段分别选取 5 株生长正常的幼树作为研究对象。用卷尺(精度 0.1 cm)和游标卡尺(精度 0.1 mm)测量胡桃楸幼树的树高、地径; 2021 年 9 月,每个处理随机选取 3 株胡桃楸幼树,将其整株挖出,分为根、茎、叶 3 部分,洗净阴干,带回实验室;置 65 ℃的烘箱中烘至恒重,用天平(精度 0.001 g)测定其干质量[24],记录根、茎、叶和总生物量。同时计算出根冠比(地下部分/地上部分)。

每株胡桃楸幼树上随机选 20 片叶子,测定其叶面积,然后在 105 ℃处理 10 min,在 65 ℃烘干到恒重,用电子天平(精度 0.001 g)称其干质量。同时计算比叶面积(叶面积/叶片干质量)。

(C)1994-2023 China Academic Journal Electronic Publishing House. All rights reserved. http://www.cnki.net

1.3.2　光合生理指标测定

1)叶片光响应曲线的绘制

于 2021 年 7—8 月选择几个连续的晴天,时间为 8:00—11:30 和 14:00—17:00。每株胡桃楸幼树上,选择颜色正常、无病虫害、无机械损伤的 3 片完全展开的成熟叶片,为避免水分亏缺,将所选成熟叶片所在的枝条剪下并立即插入水中,保持离体叶片的光合活力[25]。按照王凯等[20]的方法测定。用 LI-6400 便捷式光合仪(Li-Cor,Inc,USA)测定完成后,以光照强度(Photosynthetie photon flax density, PPFD)为横轴,P_n 为纵轴,绘制光响应曲线(P_n-PPFD 曲线)。采用非直角双曲线模型拟合出光补偿点(light compensation point,LCP)、最大光合速率(P_{max})、暗呼吸速率(R_d)和表观量子效率(apparent photosynthetic quantum efficiency,AQY)。

2)叶绿素含量的测定

将所采叶片避开叶脉后剪碎,采用丙酮、无水乙醇混合液浸提,直到叶片完全变为白色,在紫外分光光度计 663、645 nm 波长下比色,测定叶绿素 a、叶绿素 b 含量,并计算出叶绿素 a/b 和叶绿素总量[26]。

1.4　**数据处理**

利用 SPSS 20.0 完成采用单因素方差分析(one-way ANOVA)进行方差分析和差异显著性检验(P<0.01 和 P<0.05)。使用双因素方差分析(two-way ANOVA)对树龄和光照处理下的生理指标差异显著性进行检验。采用 SigmaPlot12.5(Systat, United States, California)制图。

2　结果与分析

2.1　不同生境和不同树龄胡桃楸幼树生长差异

各年龄阶段的幼树林缘的树高和地径均极显著大于林隙和林下(P<0.01),林隙也大于林下但差异不显著,如图 1 所示。幼树的根、茎、叶和总生物量都随着光照强度的增强而增加,各年龄阶段的生物量均是林缘极显著大于林隙和林下(P<0.01),林隙和林下没有显著差异,见表 1。各年龄阶段的幼树根冠比由大到小排序均为林缘、林隙、林下,其中Ⅰ年龄阶段差异显著(P<0.05),其他年龄阶段差异不显著,见表 1。Ⅰ年龄阶段幼树叶片的比叶面积由大到小顺序为林下、林隙、林缘,但均无显著性差异,其他年龄阶段林下均显著大于林缘(P<0.05),如图 2 所示。

2.2　生境和树龄对胡桃楸幼树叶片光合生理的影响

表 2 为 3 个年龄阶段的胡桃楸幼树在不同生境下的光合参数。由表 2 可知,对胡桃楸幼树的光合指标进行双因素方差分析,树龄对幼树的 4 个光合指标均有极显著的影响(P<0.01);生境极显著影响幼树光补偿点、净光合速率、暗呼吸速率(P<0.01),但对表观量子效率没有显著影响;树龄和生境的综合影响对 4 个光合指标均是极显著的。Ⅰ年龄阶段幼树在林缘的光补偿点和暗呼吸速率大于林隙和林

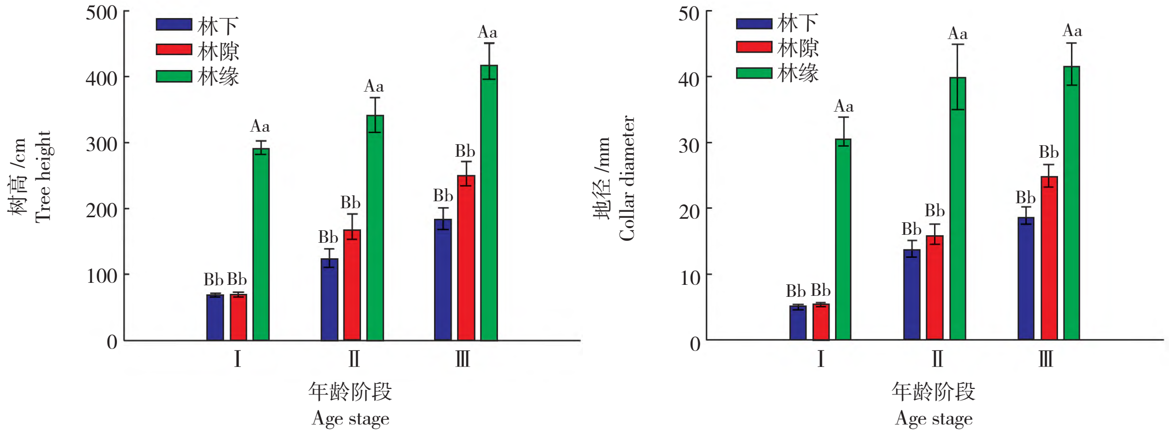

不同大写字母表示同一年龄阶段在不同生境之间具有显著差异(P<0.05),不同小写字母表示同一年龄阶段在不同生境之间具有极显著差异(P<0.01),下同。

Different capital letters indicate significant differences between habitats at the same age (P<0.05), different lowercase letters indicate extremely significant differences between habitats at the same age (P<0.01). The same below.

图 1　3 个年龄阶段的胡桃楸幼树在不同生境下的生长

Fig. 1 Growth of *Juglans mandshurica* saplings of three age stages in different habitats

(C)1994-2023 China Academic Journal Electronic Publishing House. All rights reserved.　http://www.cnki.net

表 1 3 个年龄阶段的胡桃楸幼树在不同生境下的生物量分配

Tab. 1 Specific leaf area of *Juglans mandshurica* saplings of three age stages in different habitats

树龄 Tree-age	生境 Habitat	根/g Root	茎/g Stem	叶/g Leaf	全株/g Whole plant	根冠比 Root/shoot ratio
Ⅰ	林下	1.463±0.410Bb	21.083±0.380Bb	0.613±0.070Bb	23.150±0.690Bb	0.067±0.020Cb
	林隙	10.700±7.280Bb	21.783±16.830Bb	2.507±0.850Bb	35.000±24.930Bb	0.510±0.100Ba
	林缘	375.480±56.690Aa	458.170±79.820Aa	65.875±15.910Aa	899.525±152.410Aa	0.721±0.020Aa
Ⅱ	林下	16.510±7.290Bb	40.173±7.430Bb	3.417±1.420Ba	60.100±15.920Bb	0.331±0.120Aa
	林隙	28.893±2.240Bb	52.533±4.680Bb	8.270±0.980Bb	89.697±6.760Bb	0.481±0.050Aa
	林缘	401.367±43.600Aa	623.513±6.530Aa	79.513±13.500Aa	1104.393±35.500Aa	0.573±0.070Aa
Ⅲ	林下	36.493±16.900Bb	97.937±36.590Bb	2.996±1.710Bb	137.427±54.930Bb	0.327±0.060Aa
	林隙	89.903±16.410Bb	190.277±24.810Bb	12.720±2.790Bb	292.900±41.670Bb	0.447±0.020Aa
	林缘	471.943±58.510Aa	903.853±59.570Aa	83.267±10.620Aa	1 459.063±118.470Aa	0.486±0.040Aa

注:不同大写字母表示同一年龄阶段在不同生境之间具有显著差异($P<0.05$),不同小写字母表示同一年龄阶段在不同生境之间具有极显著差异($P<0.01$)。

Note: Different capital letters indicate significant differences between habitats at the same age ($P<0.05$), different lowercase letters indicate extremely significant differences between habitats at the same age ($P<0.01$).

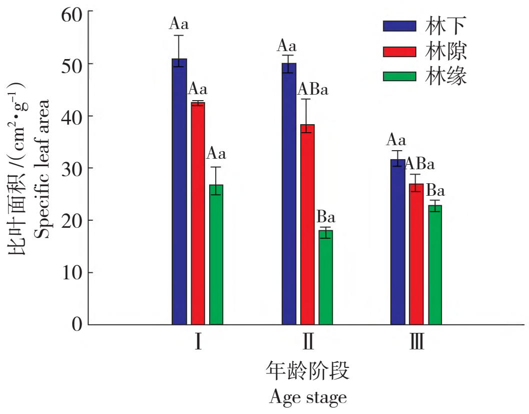

图 2 3 个年龄阶段的胡桃楸幼树在不同生境下的比叶面积

Fig. 2 Specific leaf area of *Juglans mandshurica* saplings of three age stages in different habitats

下,且均差异极显著,林缘的最大净光合速率极显著大于林下和林隙($P<0.01$)。Ⅱ年龄阶段幼树的光补偿点、最大净光合速率和暗呼吸速率由大到小顺序均为林缘、林隙、林下,且均差异极显著($P<0.01$)。Ⅲ年龄阶段的幼树光补偿点和最大净光合速率由大到小排序为在林缘、林隙、林下,且均差异极显著,暗呼吸速率在林下极显著小于林隙和林缘($P<0.01$),林隙显著小于林缘($P<0.05$),表观量子效率没有显著性差异。在林下,仅Ⅱ年龄阶段幼树的光补偿点显著低于Ⅰ、Ⅲ年龄阶段($P<0.05$),其余均无明显差异,但Ⅱ年龄阶段幼树的最大净光合速率最大,暗呼吸速率最小。在林隙,Ⅱ年龄阶段幼树的光补偿点极显著低于Ⅰ、Ⅲ年龄阶段,最大净光合速率极显著大于Ⅰ和Ⅲ年龄阶段($P<0.01$),暗呼吸速率也低于Ⅰ、Ⅲ年龄阶段,但差异不显著,表观量子效率差别不大。在林缘,Ⅲ年龄阶段幼树的光补偿点极显著小于Ⅰ、Ⅱ年龄阶段,最大净光合速率极显著大于Ⅰ、Ⅱ年龄阶段($P<0.01$);Ⅰ年龄阶段幼树的暗呼吸速率和表观量子效率极显著大于Ⅱ和Ⅲ年龄阶段($P<0.01$)。

表 3 为 3 个年龄阶段的胡桃楸幼树在不同生境下的叶绿素质量分数和 a/b 值。由表 3 可知,对胡桃楸幼树叶绿素含量和比值进行双因素方差分析,树龄对幼树的叶绿素 b 和总量具有显著的影响($P<0.05$);生境对 4 个指标均有显著影响($P<0.05$);树龄和生境综合影响对 4 个光合指标均不显著。各年龄阶段的幼树叶绿素 a、b 和叶绿素总量总体随着光照强度的减弱而增加,除Ⅰ、Ⅱ年龄阶段幼树叶绿素 b 含量和叶绿素总量在林缘显著低于林下和林隙外($P<0.05$),其他均差异不显著;各年龄阶段的叶绿素 a/b 在林缘略大于林隙和林下,差异均不显著。在林下和林隙,Ⅱ年龄阶段幼树的叶绿素 a、叶绿素 b 含量和叶绿素总量大于Ⅰ和Ⅲ年龄阶段,均没有显著性差异;在林缘随年龄增大叶绿素 a、叶绿素 b 含量和叶绿素总量增大,除林缘Ⅲ年龄阶段幼树叶绿素 b 显著大于Ⅰ年龄阶段和Ⅲ年龄阶段叶绿素 a 和叶绿素总量显著大于Ⅰ年龄阶段外($P<0.05$),其他均没有显著性差异。在林下和林隙,Ⅱ年龄阶段幼树叶绿素 a/b 小于Ⅰ和Ⅱ年龄阶段,均没有显著性差异;在林缘叶绿素 a/b 值差异不大。

(C)1994-2023 China Academic Journal Electronic Publishing House. All rights reserved. http://www.cnki.net

表 2 3 个年龄阶段的胡桃楸幼树在不同生境下的光合参数

Tab. 2 Photosynthetic parameters of *Juglans mandshurica* saplings of three age stages in different habitats

树龄 Tree-age	生境 Habitat	光补偿点/(μmol · m^{-2} · s^{-1}) Light compensation point	最大净光合速/(μmol · m^{-2} · s^{-1}) Maximum net photosynthetic rate	暗呼吸速率/(μmol · m^{-2} · s^{-1}) Dark respiration rate	表观量子效率/(μmol · m^{-2} · s^{-1}) Apparent photosynthetic quantum efficiency
Ⅰ	林下	7.16±0.11Ff	7.80±0.17Ee	0.54±0.05De	0.080±0.002ABab
	林隙	15.17±0.09Dd	8.48±0.29Ee	1.08±0.08Cc	0.078±0.001Bab
	林缘	28.84±0.52Aa	11.34±0.12Dd	2.38±0.20Aa	0.091±0.002Aa
Ⅱ	林下	6.33±0.17Ggf	8.48±0.23Ee	0.50±0.02De	0.082±0.010ABab
	林隙	13.84±0.05Ee	13.54±0.54Cc	0.97±0.12Ccd	0.075±0.001Bab
	林缘	27.52±0.23Bb	16.43±0.35Bb	1.52±0.15Bb	0.059±0.002Cc
Ⅲ	林下	7.60±0.17Ff	7.78±0.34Ee	0.62±0.05Dde	0.076±0.050Bab
	林隙	15.12±0.12Dd	11.63±0.05Dd	1.14±0.06Cbc	0.073±0.001Bbc
	林缘	20.92±0.40Cc	20.50±0.26Aa	1.49±0.30Bb	0.075±0.006Bab
树龄 Tree-age		***	***	**	**
生境 Habitat		***	***	**	NS
树龄×生境 Tree-age×habitat		***	***	**	**

注:不同小写字母表示在 0.01 水平上具有显著性差异($P<0.01$),不同大写字母表示在 0.05 水平具有显著性差异($P<0.05$);* 表示差异显著性;* 为 $P<0.05$,** 为 $P<0.01$,*** 为 $P<0.001$;NS 为不显著,下同。

Note: Different lowercase letters indicate significant differences at the 0.01 level, different capital letters indicate significant differences at the 0.05 level; * indicates significant difference; * indicates $P<0.05$, ** indicates $P<0.01$, *** indicates $P<0.001$; NS indicates not significant. The same below.

表 3 3 个年龄阶段的胡桃楸幼树在不同生境下的叶绿素含量和 a/b 值

Tab. 3 Chlorophyll (Chl) content and chlorophyll a/b of *Juglans mandshurica* saplings of three age stages in different habitats

树龄 Tree-age	生境 Habitat	叶绿素 a/(mg · g^{-1}) Chl a	叶绿素 b/(mg · g^{-1}) Chl b	叶绿素总量/(mg · g^{-1}) Chl	叶绿素 a/b Chl a/b
Ⅰ	林下	3.93±0.55Aab	1.32±0.18Aab	5.25±0.74ABa	2.95±0.02Aa
	林隙	3.42±0.13ABab	1.21±0.01ABabc	4.63±0.13ABab	2.81±0.11Aa
	林缘	2.53±0.46Bb	0.79±0.07Cc	3.32±0.53Cb	3.21±0.25Aa
Ⅱ	林下	4.29±0.37Aa	1.51±0.13Aa	5.80±0.50Aa	2.85±0.06Aa
	林隙	4.17±0.22Aa	1.52±0.11Aa	5.69±0.32Aa	2.76±0.12Aa
	林缘	3.28±0.39ABab	0.98±0.06Cbc	4.26±0.46BCab	3.29±0.22Aa
Ⅲ	林下	3.95±0.20Aab	1.23±0.01ABabc	5.18±0.21ABa	3.18±0.13Aa
	林隙	3.77±0.15Aab	1.21±0.04ABabc	4.98±0.19ABab	3.11±0.20Aa
	林缘	3.85±0.21Aab	1.21±0.10ABabc	5.06±0.25ABab	3.25±0.35Aa
树龄 Tree-age		NS	*	*	NS
生境 Habitat		*	***	**	*
树龄×生境 Tree-age×habitat		NS	NS	NS	NS

3 讨论

3.1 不同生境对胡桃楸幼树的影响

在一定的范围内,光照增强能够促进植物幼苗的生长,光照减弱会导致其生长减缓[27-28]。研究表明,林隙和林下幼树的树高和地径均极显著小于林缘(图 1);幼树的根、茎、叶生物量也均在林隙和林下生境极显著小于林缘,随着光照条件的减弱,各年龄阶段幼树的根、茎和叶生物量都降低(表 1),说明高光有利于幼树生长和生物量的积累,这与前人研究一致[29]。尽管植物适应光环境变化有多种途径,但生物量分配格局是苗木适应不同光环境的重

(C)1994-2023 China Academic Journal Electronic Publishing House. All rights reserved. http://www.cnki.net

要方式[30]。绝大多数研究结果显示,随着光照强度增加,生物量在根的分配增加,在地上部分投资减少,增加根冠比[3,5,12],这与本研究结果相似。

弱光环境下,叶片的形态变化是最直接反映其生长状况的指标之一。叶片的比叶面积体现了植物对环境因子的利用率、生存和生长措施[31],是植物用于投资单位质量干物质而得到的叶面积,能有效地反映植物的光捕获能力[32]。本研究中各年龄阶段的幼树比叶面积均随着光照强度的减弱表现出增大的趋势,与多数研究一致[8,33],这与叶片在低光环境下同化组织对输导和结构组织的相对比例提高有关[34],使植物用相同的干物质可以制造出更大的叶面积。

光响应曲线参数是衡量植物环境适应性的基本指标[35]。叶片最大净光合速率体现了植物光合能力的大小[36],光补偿点可以判断植物利用弱光能力的强弱[28],其值越大利用弱光的能力越强。本研究发现各年龄阶段幼树的光补偿点、最大净光合速率和暗呼吸速率由小到大顺序均为林下、林隙、林缘(表 2),说明幼树在弱光下净光合速率较低,光合产物的呼吸消耗较小,利用弱光的能力较强,在较弱的光照下就可以达到最大净光合速率,这与黄一鑫等[37]、靳甜甜等[38]研究结果相似;而林缘的幼树有更高的净光合速率,呼吸消耗更大,有更强的光合利用能力。

叶绿素是光合作用的光敏催化剂,其含量和比例是植物利用和适应环境因子的重要指标[39],提高叶绿素含量对叶片吸收和转化光能有利[40]。研究表明,植物会增加叶绿素含量,同时为了适应弱光环境会降低叶绿素 a/b 值[11,41]。本研究中,各年龄阶段的幼树叶绿素 a、叶绿素 b 和叶绿素总量由小到大顺序均为林缘、林隙、林下(表 3),在弱光环境中,植物的光氧化伤害减少、叶绿体内基粒变大,以及基粒片层的垛叠程度变高使得叶绿素含量升高[42]。各年龄阶段的叶绿素 a/b 均为林缘大于林隙和林下,且均没有显著差异(表 3);强光充足光照下,幼树可直接吸收冠层直射光,减少了叶绿素 a 向叶绿素 b 的转换[43],导致叶绿素 b 含量较低,致使叶绿素 a/b 值较大;而在林下和林隙弱光环境下,幼树加快了叶绿素 a 向 b 的转换,通过增加叶绿素 b 的含量来提高光能捕获能力,提高对弱光的适应性。

3.2 不同年龄阶段胡桃楸幼树的光合生理

随林木年龄的增长,各生长时期的林木环境适应性和生理特性也会随之改变[41]。林下和林隙在Ⅱ年龄阶段的幼树有最高的最大净光合速率、叶绿素含量,最低的暗呼吸速率、光补偿点和叶绿素 a/b,较高的表观量子效率(表 2)。较低的光补偿点使Ⅱ年龄阶段的幼树在弱光环境中可以最大限度地利用低光量子密度,并在较低的光环境下最大效率地进行光合作用,进而增加有机物积累,为幼树的生存和生长提供能量[44];较低的暗呼吸速率可以降低由于呼吸作用引起的碳消耗,保持碳代谢平衡[45]。可以说明Ⅱ年龄阶段的幼树利用弱光的能力最强,对弱光有较高的适应性,光合速率最高,在黑暗条件下呼吸消耗的光合产物较少,有利于有机物的积累,在弱光下有积极的措施来应对光胁迫,可以更好地生长。造成这个结果的原因可能是Ⅰ年龄阶段幼树叶片的生理机制发育还不完善,所以受到光胁迫的影响较大,光利用能力较弱,这与马文涛等[46]对胡杨的研究结果一致;而Ⅲ年龄阶段的胡桃楸从幼树到成年树转变,需光性增强,耐阴性减弱,这与红松的生长发育过程一致[47]。在林缘,随着年龄的增长幼树的最大净光合速率和叶绿素含量增加,暗呼吸速率、光补偿点和叶绿素 a/b 降低,说明在充足光照情况下,幼树随着树龄增长光合速率和需光性逐渐增加,耐阴性降低,有利于有机物的积累。在没有光照胁迫时,与美国山核桃(*Carya cathayensis*)的研究结果一致,年龄大的比年龄小的有更高的光合速率,光能利用能力更强[48]。

本研究发现,胡桃楸幼树的叶绿素相关指标不受其树龄和生境的显著综合影响(表 3),但幼树的光合指标均受到其树龄和生境极显著的综合影响(表 2),因此,在对幼树进行人工抚育时要充分考虑到树龄和光照 2 个方面。

4 结论

在林缘,胡桃楸幼树的净光合速率和生物量积累最高,生长最优;在林隙和林下,幼树的净光合速率和生物量积累降低,生长受限。因此,林缘最适宜胡桃楸幼树的生长。在林下和林隙生境下,4~6 a 的胡桃楸幼树的耐阴能力最强,对光需求较低;1~3 a 和 7~9 a 胡桃楸幼树的耐阴性较弱,对光需求较强。而在林缘生境下,随着年龄增长,胡桃楸幼树的耐阴性减弱,对光需求增高。因此,在对 4~6 a 的胡桃楸幼树进行抚育时,可适当降低抚育强度;而对 1~3 a 和 7~9 a 的胡桃楸幼树进行人工抚

(C)1994-2023 China Academic Journal Electronic Publishing House. All rights reserved. http://www.cnki.net

育时,要加强抚育力度,及时对上层林木进行透光,以满足胡桃楸幼树的生长需求,从而提高更新效果。

【参 考 文 献】

[1] 李朝洪,韦唯,刘舒欣. 国有林区天然林资源保护工程绩效评价—以黑龙江省重点国有林区为例[J]. 林业科学,2021,57(4):153-162.

LI C H, WEI W, LIU S X. Performance evaluation of natural forest resources protection project in state-owned forest region-a case study of key state-owned forest region in Heilongjiang province[J]. Scientia Silvae Sinicae, 2021, 57(4): 153-162.

[2] 张象君,王庆成,郝龙飞,等. 长白落叶松人工林林隙间伐对林下更新及植物多样性的影响[J]. 林业科学,2011,47(8):7-13.

ZHANG X J, WANG Q C, HAO L F, et al. Effect of gap thinning on the regeneration and plant species diversity in *Larix olgensis* plantation[J]. Scientia Silvae Sinicae, 2011, 47(8): 7-13.

[3] 孙欣欣. 遮荫对胡桃楸和紫椴苗木形态和生理的影响[D]. 哈尔滨:东北林业大学,2013.

SUN X X. Effect of shade on morphology and physiology in *Juglans mandshurica* and *Tilia amurensis* seedlings[D]. Harbin: Northeast Forestry University, 2013.

[4] 宋新章,张智婷,肖文发,等. 长白山杨桦次生林采伐林隙幼苗更新动态[J]. 林业科学,2008,44(3):13-20.

SONG X Z, ZHANG Z T, XIAO W F, et al. Regeneration dynamics of logging gaps in *Populus davidiana*-*Betula platyphylla* secondary forests in Changbai mountain[J]. Scientia Silvae Sinicae, 2008, 44(3): 13-20.

[5] 程晶,刘济明,王灯,等. 喀斯特特有植物罗甸小米核桃幼苗对光照强度的可塑性响应[J]. 应用与环境生物学报,2021,27(1):23-30.

CHENG J, LIU J M, WANG D, et al. Plasticity response of the karst endemic plant *Juglans regia* L. f. *luodianense* seedings to light intensity[J]. Chinese Journal of Applied and Environmental Biology, 2021, 27(1): 23-30.

[6] MCCONNAUGHAY K D M, COLEMAN J S. Biomass allocation in plants: ontogeny or optimality? A test along three resource gradients[J]. Ecology, 1999, 80(8): 2581-2593.

[7] 陈志成,刘晓静,刘畅,等. 锐齿栎幼苗生长、光合作用和非结构性碳对遮阴和模拟光斑的响应[J]. 生态学杂志,2017,36(4):935-943.

CHEN Z C, LIU X J, LIU C, et al. Responses of growth, photosynthesis and nonstructural carbohydrate of *Quercus aliena* var. *acuterrata* seedlings to shade and simulated sunfleck[J]. Chinese Journal of Ecology, 2017, 36(4): 935-943.

[8] 刘青青,马祥庆,李艳娟,等. 杉木种子萌发及幼苗生长对光强的响应[J]. 应用生态学报,2016,27(12):3845-3852.

LIU Q Q, MA X Q, LI Y J, et al. Response of seed germination and seedling growth to Chinese fir to difference light intensities[J]. Chinese Journal of Applied Ecology, 2016, 27(12): 3845-3852.

[9] 王满莲,韦霄,唐辉,等. 光强对三种喀斯特植物幼苗生长和光合特性的影响[J]. 生态学杂志,2015,34(3):604-610.

WANG M L, WEI X, TANG H, et al. Effect of light intensity on growth and photosynthesis of three Karst plants seedlings[J]. Chinese Journal of Ecology, 2015, 34(3): 604-610.

[10] 冯强,刘宁,王暾. 不同自然光环境下土庄绣线菊的生理生态响应[J]. 中南林业科技大学学报,2010,30(9):27-33.

FENG Q, LIU N, WANG T. Ecophysiological responses of *Spiraea pubescens* to different natural light regimes[J]. Journal of Central South University of Forestry & Technology, 2010, 30(9): 27-33.

[11] 徐飞,郭卫华,徐伟红,等. 不同光环境对麻栎和刺槐幼苗生长和光合特征的影响[J]. 生态学报,2010,30(12):3098-3107.

XU F, GUO W H, XU W H, et al. Effect of light intensity on the growth and photosynthesis seedings of *Quercus acutissima* and *Robinia pseudoacacia*[J]. Acta Ecologica Sinica, 2010, 30(12): 3098-3107.

[12] PORTSMUTH A, NIINEMETS U. Structural and physiological plasticity in response to light and nutrients in five temperate deciduous woody species of contrasting shade tolerance[J]. Functional Ecology, 2007, 21(1): 61-77.

[13] 胡乘风. 阔叶红松林冠层树种光合特性及光合生产潜力研究[D]. 北京:北京林业大学,2020.

HU C F. Study on photosynthetic characteristics and potential productivity of five canopy tree species in the broad-leaved Korean pine forest[D]. Beijing: Beijing Forestry University, 2020.

[14] 孙一荣,朱教君,于立忠,等. 不同光环境对红松幼苗光合生理特征的影响[J]. 生态学杂志,2009,28(5):850-857.

SUN Y R, ZHU J J, YU L Z, et al. Photosynthetic characteristics of *Pinus Kora* seedlings under different light regimes[J]. Chinese Journal of Ecology, 2009, 28(5): 850-857.

[15] 韩多红,周德峰,孟好军. 干旱荒漠区 2 个‘早酥’梨品种光合特性与树龄的相关性研究[J]. 果树资源学报,2020,1(3):1-5.

HAN D H, ZHOU D F, MENG H J. Correlation between photosynthetic characteristics and tree age of two ‘Zaosu’

(C)1994-2023 China Academic Journal Electronic Publishing House. All rights reserved. http://www.cnki.net

pear cultivars in arid desert[J]. Shanxi Fruits, 2020, 1(3): 1-5.

[16] DRAKE J E, RAETZ L M, DAVIS S C, et al. Hydraulic limitation not declining nitrogen availability causes the age-related photosynthetic decline in loblolly pine (*Pinus taeda* L.)[J]. Plant, Cell and Environment, 2010, 33(10): 1756-1766.

[17] 隋元杰,宋卫东,杨辉,等.加强保护与培育珍贵树种资源的建议[J].吉林林业科技,2009,38(1):59-61.
SUI Y J, SONG W D, YANG H, et al. Proposals to enhance the protection and cultivation of precious tree resources[J]. Journal of Jilin Forestry Science and Technology, 2009, 38(1): 59-61.

[18] 刘月,王君,杨雨春,等.不同林分密度胡桃楸胸径、树高、材积与冠幅关系[J].森林工程,2021,37(3):28-35.
LIU Y, WANG J, YANG Y C, et al. Relationships between crown width and DBH, tree height or volume of *Juglans mandshurica* in stands of different densities[J]. Forest Engineering, 2021, 37(3): 28-35.

[19] 孙一荣,朱教君,于立忠,等.不同光强下核桃楸、水曲柳和黄菠萝的光合生理特征[J].林业科学,2009,45(9):29-35.
SUN Y R, ZHU J J, YU L Z, et al. Photosynthetic characteristics of *Juglans mandshurica*, *Fraxinus mandshruica* and *Phellodendron amurense* under different light regimes[J]. Scientia Silvae Sinicae, 2009, 45(9): 29-35.

[20] 王凯,朱教君,于立忠,等.光环境对胡桃楸幼苗生长与光合作用的影响[J].应用生态学报,2010,21(4):821-826.
WANG K, ZHU J J, YU L Z, et al. Effects of light environment on *Juglans mandshuria* seedings growth and photosynthesis[J]. Chinese Journal of Applied Ecology, 2010, 21(4): 821-826.

[21] 张东来,葛文志,张玲.遮阴处理对胡桃楸幼苗形态特征及生理特性的影响[J].林业科技,2017,55(6):1-4.
ZAHNG D L, GE W Z, ZHANG L. Effect of light environment on *Juglans mandshurica* seedings growth and photosynthesis[J]. Forestry Technology, 2017, 55(6): 1-4.

[22] 王暾,郭晋平,刘宁,等.森林光环境对4种天然灌木的光合作用和形态的影响[J].林业科学,2011,47(6):56-63.
WANG T, GUO J P, LIU N, et al. Photosynthetic and morphological responses and plasticity of four naturally-regenerated shrubs under forest light environments[J]. Scientia Silvae Sinicae, 2011, 47(6): 56-63.

[23] 徐立清,崔东海,王庆成,等.张广才岭西坡次生林不同生境胡桃楸幼树根系构型及细根特征[J].应用生态学报,2020,31(2):373-380.
XU L Q, CUI D H, WANG Q C, et al. Root architecture and fine root characteristics of *Juglans mandshurica* saplings in different habitats in the secondary forest on the west slope of Zhangguangcailing, China[J]. Chinese Journal of Applied Ecology, 2020, 31(2): 373-380.

[24] 薛思雷.遮荫对水曲柳和蒙古栎苗木生长形态和光合生理的影响[D].哈尔滨:东北林业大学,2012.
XUE S L. Effects of shading on growth morphology and photosynthetic physiology in *Fraxinus mandshurica* and *Quercus mongolica* seedings[D]. Harbin: Northeast Forestry University, 2012.

[25] 唐艳,王传宽.东北主要树种光合作用可行的离体测定方法[J].植物生态学报,2011,35(4):452-462.
TANG Y, WANG C K. A feasible method for measuring photosynthesis in vitro for major tree species in northeastern China[J]. Chinese Journal of Plant Ecology, 2011, 35(4): 452-462.

[26] 石凯,李泽,张伟建,等.不同光照对油桐幼苗生长、光合日变化及叶绿素荧光参数的影响[J].中南林业科技大学学报,2018,38(8):35-42.
SHI K, LI Z, ZHANG W J, et al. Influence of different light intensity on the growth, diurnal change of photosynthesis and chlorophyll fluorescence of Tung tree seedling[J]. Journal of Central South University of Forestry & Tec, 2018, 38(8): 35-42.

[27] GONZALEZ-RIVAS B, TIGABU M, CASTRO-MARIN G, et al. Seed germination and seedling establishment of Neotropical dry forest species in response to temperature and light conditions[J]. Journal of Forestry Research, 2009, 20(2): 99-104.

[28] 孙中元,马艳军,刘杏娥,等.弱光环境对高地省藤幼苗生长与光合作用的影响[J].西南林业大学学报,2013,33(1):16-21.
SUN Z Y, MA Y J, LIU X E, et al. Effects of low light environment on the growth and photosynthesis of *Calamus nambariensis* seedlings[J]. Journal of Southwest Forestry University, 2013, 33(1): 16-21.

[29] VERDU M, GARCIA-FAYOS P. Nucleation processes in a Mediterranean bird-dispersed plant[J]. Functional Ecology, 1996,10(2): 275-280.

[30] 薛思雷,王庆成,孙欣欣,等.遮荫对水曲柳和蒙古栎光合、生长和生物量分配的影响[J].植物研究,2012,32(3):354-359.
XUE S L, WANG Q C, SUN X X, et al. Effects of shading on the photosynthetic characteristics, growth, and biomass allocation in *Fraxinus mandshurica* and *Quercus mongolica*[J]. Bulletin of Botanical Research, 2012, 32(3): 354-359.

[31] 张业.油松天然林林内主要植物种的叶功能性状研究[D].北京:北京林业大学,2012.

(C)1994-2023 China Academic Journal Electronic Publishing House. All rights reserved. http://www.cnki.net

ZHANG Y. The research of leaf functional trait of *Pinus Tabulaeformis* Carr. in China[D]. Beijing: Beijing Forestry University, 2012.

[32] CHEN H Y H, KLINKA K, KAYAHARA G J. Effects of light on growth, crown architecture, and specific leaf area for naturally established *Pinus contorta* var. *latifolia* and *Pseudotsuga menziesii* var. *glauca* saplings[J]. Canadian Journal of Forest Research, 1996, 26(7): 1149-1157.

[33] 康红梅,宋卓琴,曹冬梅,等.遮荫对两种灌木光合特性及叶绿素荧光参数的影响[J].北方园艺,2021(6):59-65.
KANG H M, SONG Z Q, CAO D M, et al. Effect of shade on photosynthetic properties and chlorophyll fluorescence parameters of two shrubs[J]. Northern Horticulture, 2021(6): 59-65.

[34] LAMBERS H, POORTER H. Inherent variation in growth rate between higher plants: a search for physiological causes and ecological consequences[J]. Advanced in Ecological Research, 1992, 22(1): 187-261.

[35] 鲍婧婷,王进,苏洁琼.不同林龄柠条(*Caragana korshinskii*)的光合特性和水分利用特征[J].中国沙漠,2016,36(1):199-205.
BAO J T, WANG J, SU J Q. Photosynthetic properties and water use characteristics in *Caragana korshinskii* in different ages[J]. Journal of Desert Research, 2016, 36(1): 199-205.

[36] 李勇,韩海荣,康峰峰,等.油松人工林冠层光合生理特性的空间异质性[J].东北林业大学学报,2013,41(4):32-35.
LI Y, HAN H R, KANG F F, et al. Spatial heterogeneity of photosynthetic characterisitics of *Pinus tabulaeformis* canopy[J]. Journal of Northeast Forestry University, 2013, 41(4): 32-35.

[37] 黄一鑫,程艳霞.森林光环境对4种乔木幼树光合和光谱反射特性的影响[J].生态学报,2022,42(22):1-9.
HUANG Y X, CHENG Y X. Photosynthetic characterisitics and spectral reflectance characteristics of four natural tree saplings under forest light environment[J]. Acta Ecologica Sinica, 2022, 42(22): 1-9.

[38] 靳甜甜,刘国华,胡婵娟,等.黄土高原常见造林树种光合蒸腾特征[J].生态学报,2008,28(11):5758-5765.
JIN T T, LIU G H, HU C J, et al. Characterisitics of photosynthetic and transpiration of tree common afforestation species in the loess plateau[J]. Acta Ecologica Sinica, 2008, 28(11): 5758-5765.

[39] LIU Y Q, SUN X Y, WANG Y, et al. Effects of shades on the photosynthetic characteristics and chlorophyll fluorescence parameters of *Urtica dioica*[J]. Acta Ecologica Sinica, 2007, 27(8): 3457-3464.

[40] VON CAEMMERER S, FARQUHAR G D. Effects of partial defoliation, changes of irradiance during growth, short-term water stress and growth at enhanced p(CO_2) on the photosynthetic capacity of leaves of *Phaseolus vulgaris* L[J]. Planta, 1984, 160(4): 320-329.

[41] 夏婵,李何,王佩兰,等.不同光照强度对赤皮青冈幼苗光合特性的影响[J].中南林业科技大学学报,2021,41(7):72-79.
XIA C, LI H, WANG P L, et al. Effects of shading on the photosynthetic characteristics of *Cyclobalanopsis gilva* seedlings[J]. Journal of Central South University of Forestry & Technology, 2021, 41(7): 72-79.

[42] ATANASOVA L, STEFANOV D, YORDANOV I, et al. Comparative characteristics of growth and photosynthesis of sun and shade leaves from normal and pendulum walnut (*Juglans regia* L.) trees[J]. Photosynthetica, 2003, 41(2): 289-292.

[43] TANAKA R, TANAKA A. Effects of chlorophyllide a oxygenase overexpression on light acclimation in *Arabidopsis thaliana*[J]. Photosynthesis Research, 2005, 85(3): 327-340.

[44] MAXWELL K, JOHNSON G N. Chlorophyll fluorescence—a practical guide[J]. Journal of Experimental Botany, 2000, 51(345): 659-668.

[45] BALIBREA M E, Dell´Amico J, Bolarín M C, et al. Carbon partitioning and sucrose metabolism in tomato plants growing under salinity[J]. Physiologia Plantarum, 2000, 110(4): 503-511.

[46] 马文涛,武胜利.不同林龄胡杨净光合速率对生态因子和生理因子的响应[J].云南大学学报(自然科学版),2020,42(5):1004-1013.
MA W T, WU S L. Net photosynthetic rate of *Populus euphratica* at different stand ages in response to ecological and physiological factors[J]. Journal of Yunnan University(Natural Sciences Edition), 2020, 42(5): 1004-1013.

[47] 姜树忠.红松光合特性及其更新对光照响应研究进展[J].辽宁林业科技,2022,(3):57-61.
JIANG S Z. Photosynthetic characteristics of pine and its renewal in response to light[J]. Liaoning Forestry Science and Technology, 2022, (3): 57-61.

[48] 吕芳德,黄菁,和红晓.不同树龄美国山核桃光合作用的试验与分析[J].中南林业科技大学学报,2011,31(3):44-46.
LYU F D, HUANG J, HE H X. Experimental analysis on photosynthesis of different ages *Carya illinoensis*[J]. Journal of Central South University of Forestry &Technology, 2011, 31(3): 44-46.

(C)1994-2023 China Academic Journal Electronic Publishing House. All rights reserved. http://www.cnki.net
