# Supplementary material for: Provenance and family variations in early growth of Manchurian walnut (Juglans mandshurica Maxim.) and selection of superior families
Source: PLoS One. 2024 Mar 7;19(3):e0298918. doi: 10.1371/journal.pone.0298918 (PMC10919699; doi:10.1371/journal.pone.0298918)
Supplement: S2 File — (ZIP) [file pone.0298918.s005.zip › Plant phenotypic plasticity in a changing climate.pdf]

Plant phenotypic plasticity in a changing climate

A.B. Nicotra[1], O.K. Atkin[1], S.P. Bonser[2], A.M. Davidson[1], E.J. Finnegan[3], U. Mathesius[1], P. Poot[4], M.D. Purugganan[5], C.L. Richards[6], F. Valladares[7] and M. van Kleunen[8]

[1] Research School of Biology, The Australian National University, Canberra, ACT, Australia
[2] Evolution and Ecology Research Centre & School of Biological, Earth and Environmental Sciences University of New South Wales Sydney, Australia
[3] CSIRO Plant Industry, Canberra, ACT, Australia
[4] School of Plant Biology, Faculty of Natural and Agricultural Sciences, University of Western Australia, Crawley, WA, Australia, and Science Division, Department of Environment and Conservation, Locked Bag 104, Bentley Delivery Centre, WA, Australia
[5] Department of Biology, Center for Genomics and Systems Biology, New York University, New York, NY, USA
[6] Department of Integrative Biology, University of South Florida, Tampa, FL, USA
[7] Instituto de Recursos Naturales. CSIC, Madrid, Spain
[8] Institute of Plant Sciences and Oeschger Centre, University of Bern, Bern, Switzerland

Climate change is altering the availability of resources and the conditions that are crucial to plant performance. One way plants will respond to these changes is through environmentally induced shifts in phenotype (phenotypic plasticity). Understanding plastic responses is crucial for predicting and managing the effects of climate change on native species as well as crop plants. Here, we provide a toolbox with definitions of key theoretical elements and a synthesis of the current understanding of the molecular and genetic mechanisms underlying plasticity relevant to climate change. By bringing ecological, evolutionary, physiological and molecular perspectives together, we hope to provide clear directives for future research and stimulate cross-disciplinary dialogue on the relevance of phenotypic plasticity under climate change.

Climate change and plant adaption

Climate change is altering the environments in which all organisms develop. Plant species can adjust to these novel conditions through phenotypic plasticity (see Glossary), adapt through natural selection or migrate to follow conditions to which they are adapted; these options are not mutually exclusive. For any given plant species or population, determining responses to environmental changes will require an understanding of the environmentally induced variation in the phenotype of individual plants. Once regarded as noise, phenotypic plasticity is now understood to be genetically controlled, heritable and of potential importance to species' evolution [1,2]. With mounting evidence from molecular and developmental biology, we are now at the threshold of gaining a sophisticated understanding of the mechanisms of plasticity, which will be crucial for predicting changes in species distributions, community composition and crop productivity under climate change [3,4].

Some authors have argued that plastic responses to rapid climate change are less important than adaptation

Corresponding author: Nicotra, A.B. (adrienne.nicotra@anu.edu.au).

Glossary

Adaptive plasticity: Phenotypic plasticity that increases the global fitness of a genotype (Figure 2).
Environmental sensing loci: Genes or gene regions that encode sensors, or receptors, for environmental signals, e.g. genes encoding photoreceptors or receptors detecting microbial signals.
Epialleles: Different forms or alleles of a gene that are identical in DNA sequence but differ in epigenetic markers. These epigenetic differences are usually associated with differing expressions of the epialleles. The causes of their formation are as yet poorly understood.
Epigenetic: Includes the mechanisms of gene regulation that lead to heritable, but potentially reversible, changes in gene expression without changing the DNA sequence of the gene (Box 1).
Fitness: The fitness of an individual is taken as the relative abundance and success of its genes (often measured as the number of surviving offspring) over multiple generations. In many cases, especially with large or long-lived species, direct estimates of fitness are not feasible and total biomass, seed number or biomass, survivorship or growth rates of a single generation are used as proxies.
Genome plasticity: A change in genome structure or organization associated with environmental signals, leading to the evolution of new phenotypes, might result from mutational hotspots, genome expansion, transposable elements or somatic recombination.
Genotype: When we refer to a genotype we do so in a population genetic sense, not in reference to a molecular sequence of a single gene, but to the complete genome.
Phenotype: The appearance or characteristics of an organism resulting from both genetic and environmental influences. In our terms, all organisms have a phenotype not just those expressing a mutation in a given gene of interest.
Phenotypic plasticity: The range of phenotypes a single genotype can express as a function of its environment.
Plant functional traits: Quantitative traits related to the fitness and success of individuals in a given environment, they provide good indicators about species' ecologies (e.g. what growth rates they are likely to exhibit, what recruitment strategy they rely on) and are often related to competitive status, commonness/rarity or dominance in the community (Box 2).
Plant functional types: Categorical assessments enabling plant species to be grouped according to functional position in a community or ecosystem. For example, classifications can be based on growth form (e.g. herb, grass, shrub), nitrogen fixing status, photosynthetic pathway or leaf longevity.
Post-transcriptional and post-translational modifications: Chemical modifications to mRNA or proteins that are made after an mRNA or protein is transcribed or translated, respectively (e.g. the phosphorylation of proteins).
Regulatory gene transcription: The process of making mRNA of a regulatory gene. The RNA is subsequently translated to form a protein, the product of the gene.
Signaling cascades: These are cascades of events that mediate cellular responses to external signals, for example the cascades of protein phosphorylation and second messenger generation following the perception of a signal by a receptor kinase.

 1360-1385/$ – see front matter © 2010 Elsevier Ltd. All rights reserved. doi:10.1016/j.tplants.2010.09.008 Trends in Plant Science, December 2010, Vol. 15, No. 12

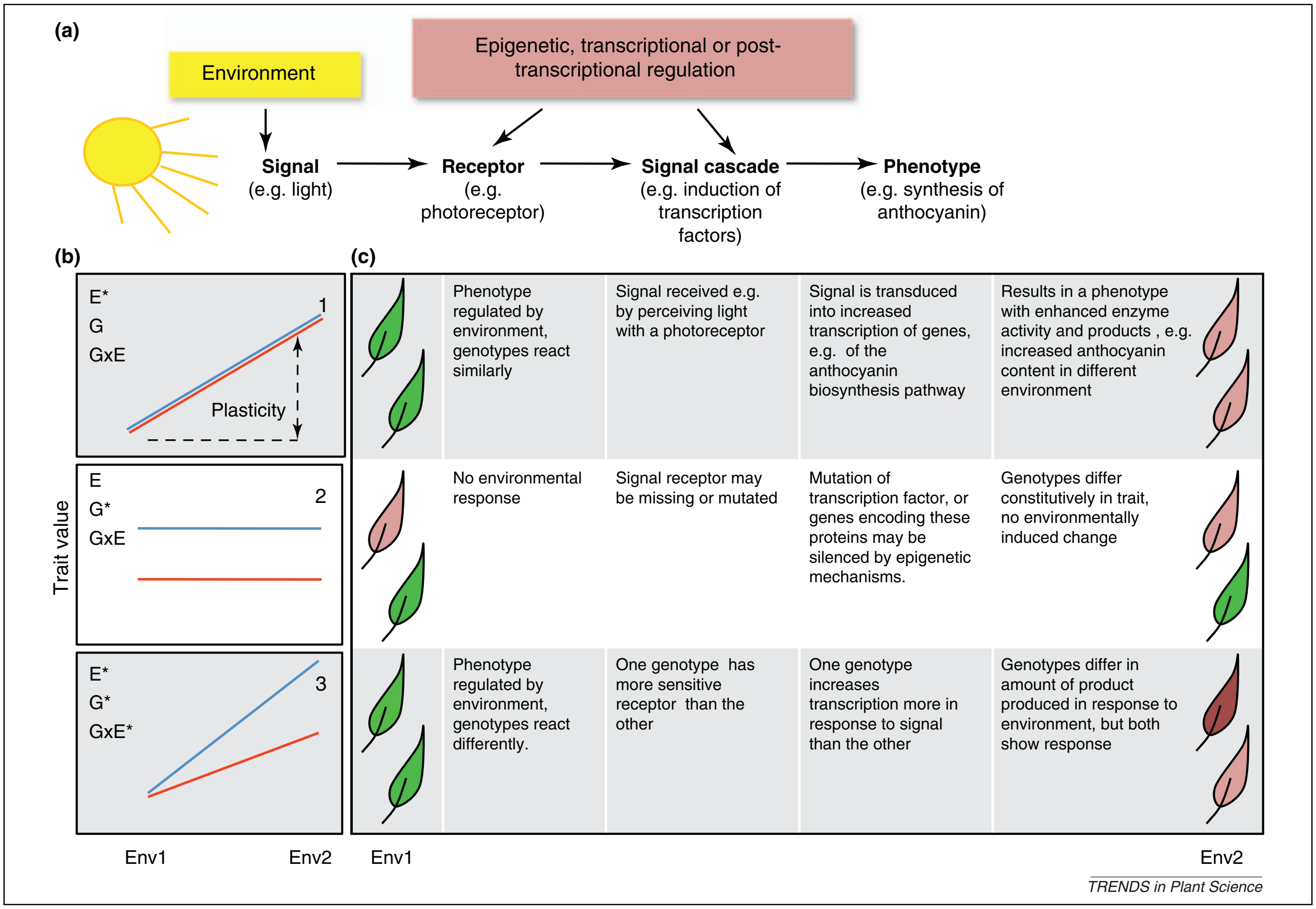

Figure 1. Anthocyanins are produced in leaves in response to excess light and temperature and osmotic extremes, and serve as a reversible plastic mechanism for the protection of photosynthetic machinery [86–88]. Here, we use an anthocyanin example to illustrate **(a)** the points in the molecular machinery, which translate an environmental signal (excess light in this case) into a phenotype. **(b)** In the evolutionary and ecological literature, these responses are commonly presented as reaction norms. Here, the blue and red lines indicate the reaction norms of two different genotypes responding to a change from a low light environment (Env1) to a high light one (Env2). The extent of phenotypic change in response to a signal is its phenotypic plasticity. Asterisks in the panels denote whether there is a significant effect of environment (E) or genotype (G), and whether there is a significant genotype by environment interaction ($G \times E$). **(c)** Likely examples of the mechanisms underlying the cases depicted in panels 1–3 are given separately for each point in the signal pathway. The leaves on the left and right represent the phenotypes in Env1 and Env2, respectively.

or shifts in the geographic range of distribution [5,6]. These studies argue that the failure to expand beyond current limits demonstrates that a species' adaptive potential has been largely exhausted, or argue that plasticity will be an unimportant factor because the cues that signaled the plastic responses in the first place might no longer be 'reliable' in changed climates [7]. However, as we show below, plastic changes in seed longevity, phenology, leaf lifespan and the temperature responses of metabolic processes are all well documented in response to elevated CO_2 and climate change factors.

There is general acceptance that high levels of genetic variation within natural populations improve the potential to withstand and adapt to novel biotic and abiotic environmental changes including the tolerance of climatic change [8]. A portion of this genetic variation determines the ability of plants to sense changes in the environment and produce a plastic response. For example, genetic variation in genes encoding temperature sensors and transcription factors regulating vernalization (see below) could help plant populations adapt to changes in temperature. Plasticity, therefore, can both provide a buffer against rapid climate changes and assist rapid adaption [2,9]. Thus, we argue that, in the context of rapid climate change, phenotypic plasticity can be a crucial determinant of plant responses, both short- and long-term.

Here, we provide a conceptual toolbox with definitions of the key theoretical elements and a synthesis of the current understanding of the molecular and genetic mechanisms underlying phenotypic plasticity, as relevant to climate change. We discuss how new developments in our understanding of signaling cascades and epigenetics in particular hold promise for interdisciplinary approaches to understanding the evolution of plasticity and for predicting how plasticity will influence the responses of native plants and agricultural systems to climate change. We aim to provide background on the ecological and evolutionary literature on phenotypic plasticity and outline emerging techniques in molecular biology. By bringing these perspectives together, we hope to stimulate crucial cross-disciplinary dialogues on the topic of plasticity and plant responses to climate change [2,9] (Box 1).

Molecular basis of plastic responses in key traits

The ability of an organism to express plasticity in a given trait must be mediated at the molecular level [10] (See

Box 1. Outstanding questions

Modern techniques and the potential for cross-disciplinary approaches mean that we are now in a position to address the following questions effectively.

Q1: Molecular basis of plasticity:
- What is the genetic control of plasticity and how is it linked to epigenetics?
- Can we identify 'plasticity genes'?
- Does identifying such plasticity genes improve our ability to predict the longer term responses of traits and species to climate change?

Q2: Adaptive plasticity:
- What traits are likely to show adaptive plasticity?
- Will species with differing ecologies (i.e. differing functional types) exhibit adaptive plasticity in different traits?
- Will the incidence of adaptive plasticity vary among types of traits (e.g. those related to anatomy versus allocation versus physiology)?

Q3: Functional traits:
- Are the traits most commonly identified as plant functional traits also those that show adaptive plasticity?
- Is plasticity in functional traits important in determining response to climate change under future climates, regardless of current adaptive value?

Q4: Plasticity and evolution:
- How has plasticity contributed to the diversification of lineages and can the evidence of this contribution be found by comparing the distribution of adaptive plasticity or relevant plasticity genes with population or species phylogenies?
- How will plasticity contribute to rapid evolution in response to climate change?
- How much variation is there for plasticity and how does it respond to selection?

Q5: Plasticity in crop species:
- Has breeding led to reductions in adaptive plasticity in contemporary crop varieties relative to older ones or wild ancestors?
- Can we breed for plasticity in key traits in agricultural systems to improve yield stability under climate change?

Figure 1 and Figure I in Box 2). For example, developmental transformations have been shown to be controlled by environmental signaling pathways that sense abiotic cues such as light and nitrogen [11] and drought [12], as well as biotic signals such as Nod factors that cause nodulation in legumes under low nitrogen conditions [13]. For many other environmentally induced phenotypic responses, the mechanisms of how environmental signals are sensed and processed are still largely unknown [e.g. 14,15]. An improved understanding of the molecular basis of environmentally induced changes in plant traits will yield insight into possible ecological and evolutionary responses in wild species and will be useful for engineering plasticity in crop species (Box 1, Q1).

Flowering time is a good example of a crucial trait that has been shown to be both under genetic control and plastic (see below). Under climate change, the temperature cues triggering the chain of events leading to flowering might cease to be reliable if they occur at the wrong time with respect to the lifecycle and ecology of the species. Such changes in cue, signal or response schemes might thereby elicit maladaptive responses [7]. Alternatively, they can lead to the expression of phenotypic responses that are currently hidden [16]. Current techniques in molecular biology and genetics allow for studies of plastic trait responses that scale from a description of molecular mechanism to the assessment of adaptive value under current or simulated future climates [17]. Thus far the genetic basis of plasticity has been examined in greatest depth in model and crop species. As new tools become available, the extension of these studies to more non-model species becomes increasingly possible and will help us determine the extent to which there are genetic homologs in other species (Box 2).

Plasticity in key plant functional traits in response to climate change

Plasticity is a characteristic of a given trait in response to a given environmental stimulus, rather than a characteristic of an organism as a whole. Likewise, some responses are examples of adaptive plasticity, providing a fitness benefit, whereas others are inevitable responses to physical processes or resource limitations [18,19] (Figure 2). Both adaptive and non-adaptive plasticity will play a role in the context of plant responses to climate change. Differentiating between the two is important to our understanding of both the current value and the evolution of plasticity (Box 1, Q2). The consensus from the theoretical literature is that adaptive phenotypic plasticity should evolve in heterogeneous environments where signals of environmental conditions are reliable [19,20]. Hypotheses about what sort of species will be most plastic also abound in the literature [21–26], yet our ability to predict patterns of plasticity in key traits in response to climate change remains limited.

Given that it is not feasible to assess plastic responses to current or future environments on all species, it is important to identify which traits are likely to show important plastic responses to particular changing environmental conditions and to develop predictors to enable us to generalize about the sorts of species likely to exhibit these plastic responses [9]. Those traits can then be examined in current or projected climate conditions to determine the extent of plasticity and assess the extent to which the underlying molecular and genetic pathways are shared (Box 2).

Plasticity in plant functional traits

In recent years, ecologists have categorized species according to plant functional types and have also identified several continuous plant functional traits that vary in predictable ways along environmental gradients. Functional types are widely used in global climate models to group species according to their function in the ecosystem or community (e.g. C3 or C4 grasses, herbs, shrubs, deciduous trees, N-fixing legumes, etc.). Functional traits are those that help describe the ecology of species using a few, easily quantified variables (e.g. seed size, plant height, leaf lifespan, leaf mass per area, etc.) [27]. Functional traits are relevant to both global climate models and mechanistic models of plant distributions (see below). Considering their probable importance, we advocate that plant functional traits should have priority for the investigation of (adaptive) phenotypic plasticity and identification of molecular and genetic mechanisms across species (Box 3).

Adaptive plasticity in functional traits is likely to assist rapid adaptation to new conditions. Thus, a natural ques-

Box 2. Bridging evolution, ecology and molecular biology

Plastic molecular responses to environmental signals can occur in many ways. An external stimulus must first be perceived at the cell surface by a receptor (Figure I, 1) that then initiates a signaling cascade. Responses to the environmental challenge (lightning bolts) can include the post-translational modifications of the components of signaling pathways [69] (2). Alternatively, regulatory gene transcription can occur in many ways and in response to a broad range of stimuli (3,4,6,7). Epigenetic processes, including DNA methylation (4,5), histone modification (4) and transposable element activation (5,6), can also alter gene expression (5,6) and thereby mediate plasticity [70]. Changes to the population of small RNAs can lead to post-transcriptional control (RNAi) as well as changes in chromatin modification (4,8). Lastly, the expansion of short repeat sequences can affect gene expression (7).

New developments in our understanding of signaling cascades and epigenetics in particular hold promise for understanding the evolution of plasticity in natural systems and for predicting how plasticity will influence the responses of native plants and agricultural systems to climate change. For example, mutant or gene expression studies are useful for discovering genes underlying specific responses [12,71,72]. Techniques, such as quantitative trait loci [72,73] and linkage disequilibrium mapping [71,74], have also been used to identify natural variants in plasticity genes [19]. Plasticity genes might also evolve by the diversification of gene families in which the promoters of different family members perceive specific environmental cues. Once specific genes that lead to genetic variation in phenotypic plasticity have been isolated, one can employ molecular population genetic analyses of natural 'plasticity alleles' to infer the evolutionary histories of plastic phenotypes and the evolutionary forces that shape variation in these key loci. On a genome-wide scale, approaches to characterizing gene expression and epigenetic changes, including high-throughput sequencing, microarrays and proteomic approaches, offer the possibility to characterize patterns of plasticity at the scale of the genome rather than gene by gene [75,76].

This discussion demonstrates how phenotypic responses to environmental signals can be correlated with molecular signals at single genes and across the entire genome. Further investigation to identify the genome architecture that confers the responsiveness of key traits to particular stimuli might enable the prediction of plastic responses to novel environments posed by climate change.

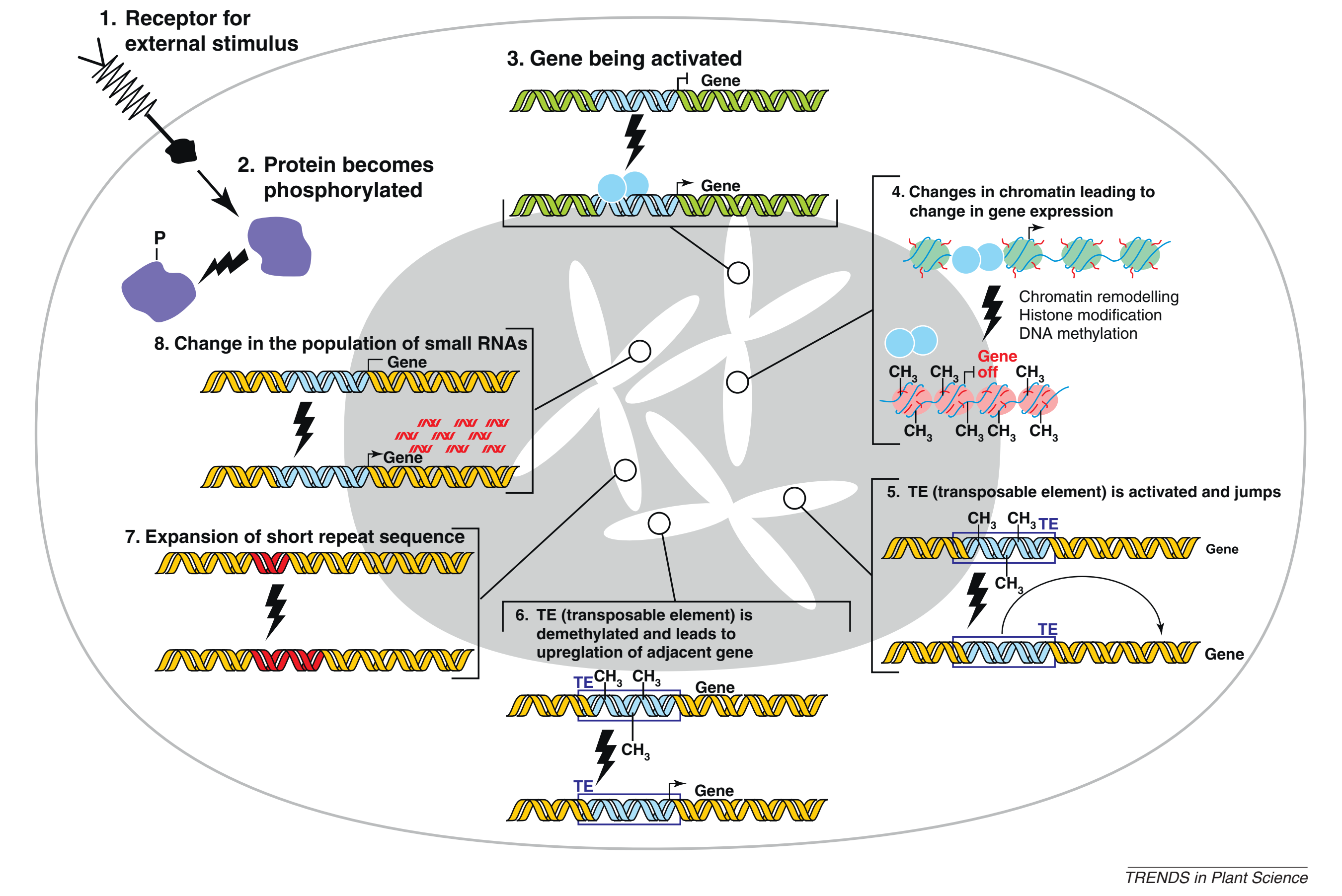

Figure I. A variety of signaling cascades can be triggered in response to environmental signals. The subsequent genetic and epigenetic changes can occur in different cells/tissues, but are here presented in a single cell.

tion to ask is whether we can predict patterns of plasticity in functional traits based on the means of those traits themselves or based on other aspects of a species' ecology (Box 1, Q3). Although many studies have compared patterns of phenotypic plasticity in small numbers of species of contrasting ecologies, little consensus has emerged. As such, this question might be best addressed using a meta-analysis approach [28].

Plasticity in leaf phenology, flowering time and seed or seedling traits

Some of the best-documented effects of climate change have been shifts in leaf phenology [29] and flowering time [30]. Among plant species included in a meta-analysis, 87% show shifts in phenology to earlier spring times [31]. These changes might reflect both genetic (i.e. rapid evolution) and plastic changes [30].

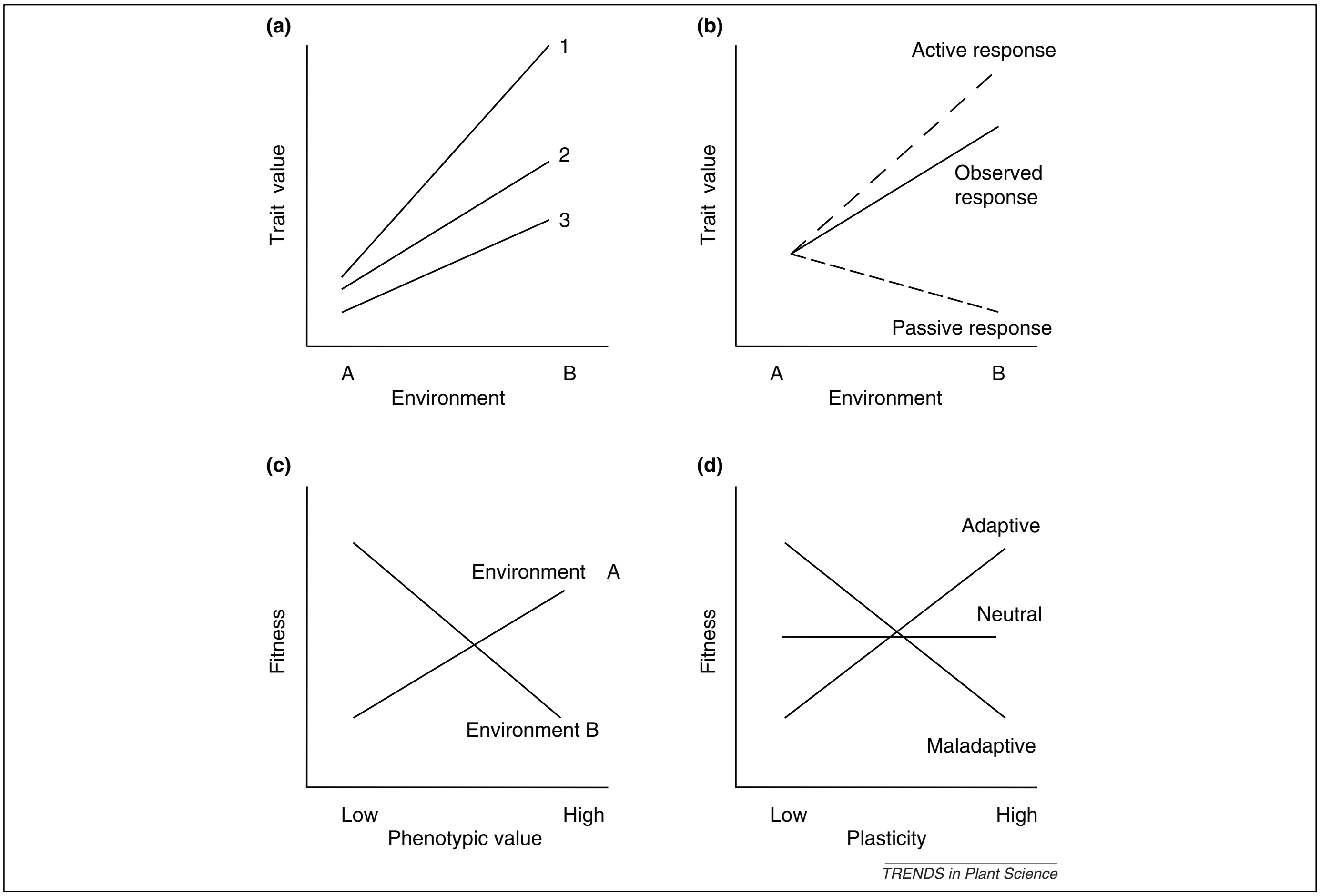

Figure 2. Generally, plasticity studies use factorial designs to assess genotype (or alternatively population or line) and environmental effects and their interactions (G × E). The interaction term is used to determine whether contrasting genotypes differ in their ability to alter phenotype in response to environmental signals (their reaction norms). **(a)** A reaction norm plot showing the response of three 'lines' (1–3) to two environments (A and B). The lines could be independent clonal genotypes [19], recombinant inbred lines [89], varieties or even populations and species. Line 1 shows the greatest phenotypic plasticity, line 3 the least. **(b)** An illustration of how an observed plastic response can be the result of active and passive responses occurring at the same time. For example, the passive response can reflect resource limitation, whereas the active response changes allocation to offset loss in fitness in environment B. Adaptive plastic responses are generally, but not necessarily, those that are active and that require a specific signal perception-transduction system allowing plants to change their development (adapted from [19]). **(c)** and **(d)** show tests of adaptive plasticity; such data are often analyzed using selection-gradient analyses [3,4,90,91]. In **(c)**, fitness is maximized at a high value of the phenotypic trait in environment A and at a low value in environment B, so that the ability of the genotype to alter its phenotype depending on the environment will itself be adaptive. **(d)** presents a different approach to assessing adaptive plasticity in which a measure of plasticity (absolute or an index) is regressed against average fitness; the relationship could be adaptive, neutral or even maladaptive (after) [19].

One example of an environmentally induced mechanism of regulating flowering time has been studied in detail in *Arabidopsis thaliana*. Flowering in *Arabidopsis* depends in part on the plastic downregulation of the transcription factor gene *FLC* (*FLOWERING LOCUS C*), which is regulated by epigenetic changes in histone modification in response to vernalization (prolonged exposure to cold) [30]. Here, the vernalization treatment acts via the transcriptional induction of a gene (*VIN3*), which then controls the recruitment or activity of protein complexes that modify chromatin and thereby silence the *FLC* locus [30]. Because FLC acts as a repressor of several flowering genes, its epigenetic silencing allows flowering to occur. The epigenetic silencing of *FLC* can only be reversed in the next generation. This pathway has been largely conserved in Brassicaceae, but with some variation [32]. The most extreme example being in *Arabis alpina* where *PERPETUAL FLOWERING* 1, an *FLC* ortholog, regulates flowering in response to vernalization and conditions a perennial growth habit [33].

The modifications of these regulatory pathways have been observed in several other plant species as well. For example, putative *FLC* homologs have been identified from different eudicot taxa including chicory (*Cichorium intybus*) [34] and sugar beet (*Beta vulgaris*) [35]. The sugar beet *FLC*-like gene is transiently downregulated by cold and delays flowering when expressed in *Arabidopsis* [35]. The response of *FLC* homologs in other species to cold and their roles in regulating flowering have not been elucidated. Phylogenetic analyses in four legumes found no close *FLC* homolog, but identified several homologs of genes that regulate FLC expression, e.g. VIN3 [36].

Despite the similarity in the physiology of the vernalization response between species, it is clear that this response has evolved independently in dicots and monocots. Cereals including rice, wheat and barley do not encode any homologs of *FLC*, but an unrelated transcription factor VRN2 plays a similar role as a flowering repressor as FLC [37,38], and a positive regulator of flowering VRN1, which is activated by cold temperatures to repress VRN2, is

Box 3. Key functional traits for assessment of plastic responses to climate change

The list in Table I suggests key traits for the investigation of adaptive phenotypic plasticity across a broad range of species. The choice of phenotypic traits of interest will vary with growth form and development stage, and also depends on whether plasticity is investigated in a controlled environment or in the field. However, developing a database of plasticity data is dependent on commonly measured traits, particularly in comparative work. These traits hold potential for incorporation into both mechanistic models of species distributions and models of vegetation distributions. This list also represents those traits for which molecular genetic mechanisms are of particular interest from ecological or evolutionary perspectives.

Table I. Key functional traits for the investigation of adaptive phenotypic plasticity

Priority	Trait	Biological significance	Refs
A	Leaf mass per unit area (LMA, the inverse of SLA, specific leaf area)	An easily measured correlate of relative growth rate, photosynthetic capacity, leaf lifespan and leaf nitrogen content.	[27,77–79]
A	Stomatal size, density	Stomata control water loss and uptake of CO_2.	[80,81]
A	Height at maturity	Indication of competitive position in a stand, relevant in herbaceous and woody species, harder to measure in long-lived species.	[78]
A	Flowering time, size at reproduction, phenology	Plasticity in these traits will determine the ability of many species to respond to a changing climate.	[82]
A	Seed size, number	Indicators of fitness; these can also be plastic in their own right.	[27,78]
B	Water use efficiency	Carbon gain as a function of water loss. Can be measured as an integrated measure using isotopes, but instantaneous measures are also of interest.	[83,84]
B	Leaf size, shape, thickness	Leaf form, as the site of photosynthesis, is crucial to growth and carbon balance.	[27]
B	Root-to-shoot ratio	The relative allocation of total plant mass to roots and shoots (i.e. leaves and stem)	[27]
B	Specific root length	Root length per unit mass, a belowground analog to SLA or LMA. Of interest from a global change perspective in particular as precipitation patterns shift.	[27,85]
B	Plant chemical defenses	Presence, absence and concentration of secondary metabolites employed in defense vary in many species depending on growth conditions and herbivore pressure.	[27,78]
B	Leaf pigmentation	Pigmentation changes (e.g. anthocyanin) is associated with the ability to protect the photosynthetic apparatus from excess light and could contribute to leaf longevity during senescence, as well as freezing-, drought- and osmotic-tolerance.	[86–88]

under epigenetic control [39]. The extension of these approaches to non-model or crop species holds exciting potential.

Plasticity in seed traits has also been documented in several species. For example, both warmer developmental temperatures [40,41] and maternal drought stress [42] can decrease seed dormancy. Elevated CO_2 can alter seed provisioning and slow seedling growth rates [43]. Dormancy prevents germination when the environment is unlikely to sustain subsequent plant growth; thus, seeds with reduced dormancy status might be more likely to germinate in inappropriate conditions. Seed longevity can also be plastic; for example, changes in temperature and rainfall experienced during seed development have the potential to halve seed longevity [44]. These examples demonstrate that the effects of climate change on plastic regeneration traits could be substantial. As yet, the mechanisms underlying these plastic responses and whether they could be adaptive under current conditions remain unknown.

Plasticity and shifts in the distribution of species and vegetation types under climate change

Future changes in climate could result in extinctions, range shifts, changes in major vegetation types and alterations in feedbacks between vegetation and the atmosphere. Indeed, the distribution of many plant species has already altered in response to climate change; some species have shown up to 6 km pole-ward migration each year over the past 16–132 years [31]. Recent years have seen tremendous progress in species distribution and vegetation models but as yet most of these models do not consider the phenotypic plasticity of existing genotypes or the evolution of either traits or plasticity itself [9].

Box 3 identifies plant functional traits in which plasticity is likely to be important to species responses to climate change, and which we therefore suggest have priority for research on plasticity and its underlying mechanisms. Below we consider how a better understanding of plasticity in these traits will contribute to predicting species distribution changes and shifts in vegetation types and how it can alter our approach to crop breeding.

Species distribution models

Niche-based models, in their simplest forms, take the climatic conditions of a species' current distribution and use modeled future climatic scenarios to project future distributions [45]. They generally assume that distributions reveal the ecological potential of the current gene pool and that the niche does not change over time [46]. However, the environmental conditions currently occupied by a species can fail to reveal the full extent of its potential range (fundamental niche) for reasons such as dispersal limitation, the effects of species interactions and the likelihood that portions of that potential niche are currently unexpressed because they do not correspond to any contemporary environment [46].

Phenotypic plasticity will be particularly important in predicting dynamics at population boundaries. At the trailing edge, plasticity can buffer population declines and influence the potential of the species to adapt to novel conditions [47]. At the leading edge, shifting species interactions might lead to unanticipated plastic responses. Recently, mechanistic models that incorporate physiological knowledge about variation within a species in response to environment have offered an alternative to purely correlative models [48,49]. For example, population declines

on the trailing edge of the distribution of 16 tree species were examined using a mechanistic model that incorporates plasticity in phenology [50]. Declines were generally attributable to a reduction in fruit maturation success resulting from maladaptive plastic responses to temperature changes that led to delays in early-season dormancy break [51].

Mechanistic models that combine evolutionary genetics, demography and the plasticity of key plant traits (Box 3) will improve our potential to model future species distributions [52]. These models are more time and labor demanding to parameterize than correlative niche-based models [53] and thereby we suggest that integrated mechanistic/correlational models [54] be strategically directed. For example, they could be useful to predict outcomes of non-equilibrium situations (e.g. species invasions).

Plasticity and predicting shifts in vegetation types

Climate change is also predicted to affect the global distribution patterns of vegetation types and their feedback on atmospheric CO_2 levels and temperatures. Dynamic global vegetation models (DGVMs) coupled to general circulation models are used to predict what plant functional types will dominate at particular locations [51]. Crucial to these predictions of increased CO_2 concentrations and temperatures are feedbacks from the climate-induced conversion of vegetation types, for example Amazonian tropical rainforests to savanna/grasslands [55]. Whether abrupt changes in vegetation types will actually occur, however, depends on the extent to which the existing vegetation can tolerate environmental change. Plastic changes in response to temperature or drought are commonly observed in leaf chemistry, biomass allocation and metabolic rates. Incorporating real values for the acclimation of respiration in response to growth temperature into DGVMs can decrease modeled rates of respiration and increase rates of net primary productivity by up to 20% in the tropics [56]. Plastic changes of this magnitude are likely to substantially alter the predicted rates of ecosystem net carbon exchange, with important but largely unknown consequences for future atmospheric CO_2 concentrations and global temperatures.

Some of the tools to incorporate phenotypic plasticity are already available for DGVMs [56,57]. Most of these models work on a functional type basis, where types might reflect growth form or photosynthetic pathway rather than incorporating detailed trait data for specific species. Thus, studies of whether species can be classified into functional types for plasticity are also needed to make use of these tools. Strategic data collection to answer these questions will require effective dialogue between modelers and biologists to identify both traits and key species or functional type definitions on which to focus efforts (Box 1, Q3).

Plasticity, phenotypic evolution and breeding in response to rapid changes

In the short-term, the plastic responses of existing genotypes will be of particular importance in determining plants' persistence under climate change. These plastic responses might, however, also have important consequences for longer term evolutionary pathways [58] (Box 1, Q4). Adaptive plasticity is likely to facilitate persistence and, therefore, reduce the chances of extinction in a novel environment, setting the stage for subsequent adaptive evolution by natural selection [58]. But even plasticity that is not currently adaptive (Figure 2) can provide sources of novel phenotypes important in phenotypic evolution [2,9].

As yet, studies of climate change-induced evolution under simulated and natural climatic conditions have rarely integrated plastic and genetic evolutionary responses [17]. Nevertheless, both abrupt and gradual climate changes will impose selection on plant populations. Abrupt climate changes will result in rapid hard selection for more stress-tolerant genotypes, whereas gradual climate changes are expected to impose soft selection mediated by intraspecific interactions [17]. There is also the possibility that genome-wide changes, including the random formation of epialleles, can be environmentally triggered [59] (Box 1). This genome plasticity is distinct from phenotypic plasticity but can provide a mechanism that generates phenotypically plastic responses [60]. Because epigenetic changes can happen much more rapidly than DNA sequence-based changes [60,61] and because they have been shown to respond to environmental stress [62], they could be particularly important in the face of a rapid change in climate. Experimental studies using classic plasticity designs with epigenetic markers or epi-RILs will be important tools to allow us to link genomic processes with the evolution of plastic responses [61,63–65].

Plasticity and crop breeding in a drier or more variable climate

Lastly, amid growing fears of food crises, we are particularly keen to motivate cross-disciplinary research that synthesizes applied research in crop systems with ecological and evolutionary theory. Crop scientists have traditionally focused on directional selection on plant traits to obtain higher yields in particular environments, or on breeding for homeostasis under a range of conditions [66]. Selection for increased phenotypic plasticity *per se* has not been directly addressed. Because selection is often conducted on trait values under a single productive condition, we suggest that it is currently unclear whether domestication and breeding have led to increased or decreased plasticity in traits indirectly associated with yield. Genetic lines selected for relative yield stability could have high phenotypic plasticity because relatively large morphological and physiological changes can underlie yield stability [66].

Breeding for phenotypic plasticity in traits other than yield will potentially afford resilience in an increasingly unpredictable environment [67]. For example, breeding for plasticity in water use traits could lead to better survival and higher average yields [68]. Likewise, novel approaches to identify key environmental sensing genes in crop and model systems can lead to an opportunity to breed for phenotypic plasticity to build resilience in an increasingly variable environment [68] (Box 1, Q5).

Concluding remarks

There is increasing evidence of the importance of plasticity in plants under climate change in both natural and agri-

cultural systems. Our aim has been to discuss the potential roles of plasticity in determining plant response to and effects of climate change in a way that is accessible and relevant to ecologists, physiologists and molecular biologists alike. We see progress in this field as being very much dependent on multidisciplinary approaches and the application of emerging techniques. We have identified outstanding questions in the field as directions for future research (Box 1). Many of these are extensions of long-standing questions such as how common and important is adaptive plasticity, what is the molecular genetic basis of plasticity and what is the relevance of plasticity in determining species distributions and vegetation processes? Answers to these tantalizing questions are now relevant in an applied context and are closer to our grasp thanks to exciting new technical progress and the potential for integrative multidisciplinary approaches.

Acknowledgements
We thank the ARC/NZ Research Network for Vegetation Function for supporting the plasticity working group from which this paper arose. Thanks to Carl Davies for the design of Figure I. We also thank three anonymous reviewers who provided insightful comments on an earlier version of this manuscript.

References
1 Bradshaw, A.D. (2006) Unraveling phenotypic plasticity - why should we bother? *New Phytologist* 170, 644–648
2 Lande, R. (2009) Adaptation to an extraordinary environment by evolution of phenotypic plasticity and genetic assimilation. *J. Evol. Biol.* 22, 1435–1446
3 van Kleunen, M. *et al.* (2007) Selection on phenotypic plasticity of morphological traits in response to flooding and competition in the clonal shore plant *Ranunculus reptans*. *J. Evol. Biol.* 20, 2126–2137
4 van Kleunen, M. and Fischer, M. (2001) Adaptive evolution of plastic foraging responses in a clonal plant. *Ecology* 82, 3309–3319
5 IPCC (2007) Climate Change 2007: Synthesis Report. http://www.ipcc.ch/publications_and_data/publications_and_data_reports.htm#1
6 Jump, A.S. and Penuelas, J. (2005) Running to stand still: adaptation and the response of plants to rapid climate change. *Ecol. Lett.* 8, 1010–1020
7 Visser, M.E. (2008) Keeping up with a warming world; assessing the rate of adaptation to climate change. *Proc. R. Soc. Lond. B. Biol. Sci.* 275, 649–659
8 Jump, A.S. *et al.* (2009) Environmental change and the option value of genetic diversity. *Trends Plant Sci.* 14, 51–58
9 Chevin, L-M. *et al.* (2010) Adaptation, plasticity, and extinction in a changing environment: towards a predictive theory. *PLoS Biol.* 8, e1000357
10 Schlichting, C.D. and Smith, H. (2002) Phenotypic plasticity: linking molecular mechanisms with evolutionary outcomes. *Evol. Ecol.* 16, 189–211
11 Krouk, G. *et al.* (2009) A systems approach uncovers restrictions for signal interactions regulating genome-wide responses to nutritional cues in *Arabidopsis*. *Plos Comput. Biol.* 5, DOI: 10.1371/journal.pcbi.1000326
12 Nilson, S.E. and Assmann, S.M. (2010) Heterotrimeric G proteins regulate reproductive trait plasticity in response to water availability. *New Phytologist* 185, 734–746
13 Madsen, E.B. *et al.* (2003) A receptor kinase gene of the LysM type is involved in legume perception of rhizobial signals. *Nature* 425, 637–640
14 Stitt, M. and Hurry, V. (2002) A plant for all seasons: alterations in photosynthetic carbon metabolism during cold acclimation in *Arabidopsis*. *Curr. Opin. Plant Biol.* 5, 199–206
15 Coupe, S.A. *et al.* (2006) Systemic signalling of environmental cues in *Arabidopsis* leaves. *J. Exp. Bot.* 57, 329–341
16 Schlichting, C.D. (2008) Hidden reaction norms, cryptic genetic variation, and evolvability. *Ann. N. Y. Acad. Sci.* 1133, 187–203
17 Reusch, T.B.H. and Wood, T.E. (2007) Molecular ecology of global change. *Mol. Ecol.* 16, 3973–3992
18 Weiner, J. (2004) Allocation, plasticity and allometry in plants. *Pers. Plant Ecol. Evol. And Syst.* 6, 207–215
19 van Kleunen, M. and Fischer, M. (2005) Constraints on the evolution of adaptive phenotypic plasticity in plants. *New Phytologist* 166, 49–60
20 Schmitt, J. *et al.* (2003) The adaptive evolution of plasticity: Phytochrome-mediated shade avoidance responses. *Integr. Comp. Biol.* 43, 459–469
21 Funk, J.L. (2008) Differences in plasticity between invasive and native plants from a low resource environment. *J. Ecol.* 96, 1162–1173
22 Muth, N.Z. and Pigliucci, M. (2007) Implementation of a novel framework for assessing species plasticity in biological invasions: responses of *Centaurea* and *Crepis* to phosphorus and water availability. *J. Ecol.* 95, 1001–1013
23 Burns, J.H. (2006) Relatedness and environment affect traits associated with invasive and non-invasive introduced Commelinaceae. *Ecol. Appl.* 16, 1367–1376
24 Murray, B.R. *et al.* (2002) How plant life-history and ecological traits relate to species rarity and commonness at varying spatial scales. *Aust. Ecol.* 27, 291–310
25 Poot, P. and Lambers, H. (2008) Shallow-soil endemics: adaptive advantages and constraints of a specialized root-system morphology. *New Phytologist* 178, 371–381
26 Pohlman, C.L. *et al.* (2005) Geographic range size, seedling ecophysiology and phenotypic plasticity in Australian *Acacia* species. *J. Biogeography* 32, 341–351
27 Cornelissen, J.H.C. *et al.* (2003) A handbook of protocols for standardised and easy measurement of plant functional traits worldwide. *Aust. J. Bot.* 51, 335–380
28 van Kleunen, M. *et al.* (2010) A meta-analysis of trait differences between invasive and non-invasive plant species. *Ecol. Lett.* 13, 235–245
29 Morin, X. *et al.* (2009) Leaf phenology in 22 North American tree species during the 21st century. *Global Change Biol.* 15, 961–975
30 Franks, S.J. and Weis, A.E. (2008) A change in climate causes rapid evolution of multiple life-history traits and their interactions in an annual plant. *J. Evol. Biol.* 21, 1321–1334
31 Parmesan, C. and Yohe, G. (2003) A globally coherent fingerprint of climate change impacts across natural systems. *Nature* 421, 37–42
32 Finnegan, E.J. *et al.* (2010) Vernalisation. In *Encyclopedia of Life Sciences*, http://www.els.net/, DOI: 10.1002/9780470015902.a0002048.pub3, John Wiley & Sons
33 Wang, Y. *et al.* (2003) The *Arabidopsis* homeobox gene, ATHB16, regulates leaf development and the sensitivity to photoperiod in Arabidopsis. *Dev. Biol.* 264, 228–239
34 Locascio, A. *et al.* (2009) Characterization of a MADS FLOWERING LOCUS C-LIKE (MFL) sequence in *Cichorium intybus*: a comparative study of CiMFL and AtFLC reveals homologies and divergences in gene function. *New Phytologist* 182, 630–643
35 Reeves, P.A. *et al.* (2007) Evolutionary conservation of the FLOWERING LOCUS C-mediated vernalization response: evidence from the sugar beet (*Beta vulgaris*). *Genetics* 176, 295–307
36 Hecht, V. *et al.* (2005) Conservation of *Arabidopsis* flowering genes in model legumes. *Plant Physiol.* 137, 1420–1434
37 Yan, L.L. *et al.* (2004) The wheat VRN2 gene is a flowering repressor down-regulated by vernalization. *Science* 303, 1640–1644
38 Greenup, A. *et al.* (2009) The molecular biology of seasonal flowering-responses in Arabidopsis and the cereals. *Ann. Bot.* 103, 1165–1172
39 Oliver, S.N. *et al.* (2009) Vernalization-induced flowering in cereals is associated with changes in histone methylation at the VERNALIZATION1 gene. *Proc. Natl. Acad. Sci. U. S. A.* 106, 8386–8391
40 Steadman, K.J. *et al.* (2004) Maturation temperature and rainfall influence seed dormancy characteristics of annual ryegrass (*Lolium rigidum*). *Aust. J. Agric. Res.* 55, 1047–1057
41 Qaderi, M.M. *et al.* (2006) Maturation temperature regulates germinability and chemical constituents of Scotch thistle (*Onopordum acanthium*) cypselas. *Can. J. of Botany-Revue Canadienne De Botanique* 84, 28–38
42 Meyer, S.E. and Allen, P.S. (1999) Ecological genetics of seed germination regulation in Bromus tectorum L. II. Reaction norms in response to a water stress gradient imposed during seed maturation. *Oecologia* 120, 35–43

43 Huxman, T.E. *et al.* (1998) The effects of parental CO2 environment on seed quality and subsequent seedling performance in *Bromus rubens*. *Oecologia* 114, 202–208
44 Kochanek, J. *et al.* (2010) Pre-zygotic parental environment modulates seed longevity. *Austral Ecology* early view April 2010
45 Thuiller, W. *et al.* (2005) Climate change threats to plant diversity in Europe. *Proc. Natl. Acad. Sci. U. S. A.* 102, 8245–8250
46 Colwell, R.K. and Rangel, T.F. (2009) Hutchinson's duality: the once and future niche. *Proc. Natl. Acad. Sci. U. S. A.* 106, 19651–19658
47 Thuiller, W. *et al.* (2008) Predicting global change impacts on plant species' distributions: Future challenges. *Perspect. Plant Ecol. Evol. Syst.* 9, 137–152
48 Kearney, M. and Porter, W. (2009) Mechanistic niche modelling: combining physiological and spatial data to predict species' ranges. *Ecol. Lett.* 12, 334–350
49 Wiens, J.A. *et al.* (2009) Niches, models, and climate change: Assessing the assumptions and uncertainties. *Proc. Natl. Acad. Sci. U. S. A.* 106, 19729–19736
50 Chuine, I. and Beaubien, E.G. (2001) Phenology is a major determinant of tree species range. *Ecol. Lett.* 4, 500–510
51 Morin, X. *et al.* (2008) Tree species range shifts at a continental scale: new predictive insights from a process-based model. *J. Ecol.* 96, 784–794
52 Chevin, L.M. and Lande, R. (2010) When do adaptive plasticity and genetic evolution prevent extinction of a density-regulated population? *Evolution* 64, 1143–1150
53 Pearson, R.G. and Dawson, T.P. (2003) Predicting the impacts of climate change on the distribution of species: are bioclimate envelope models useful? *Global Ecol. Biogeography* 12, 361–371
54 Jackson, S.T. *et al.* (2009) Ecology and the ratchet of events: climate variability, niche dimensions, and species distributions. *Proc. Natl. Acad. Sci. U. S. A.* 106, 19685–19692
55 Cox, P. (2001) Description of the "TRIFFID" Dynamic Global Vegetation Model In Met Office Hadley Centre Technical Note, 1–16
56 Atkin, O.K. *et al.* (2008) Using temperature-dependent changes in leaf scaling relationships to quantitatively account for thermal acclimation of respiration in a coupled global climate-vegetation model. *Global Change Biol.* 14, 2709–2726
57 Kattge, J. and Knorr, W. (2007) Temperature acclimation in a biochemical model of photosynthesis: a reanalysis of data from 36 species. *Plant Cell Environ.* 30, 1176–1190
58 Ghalambor, C.K. *et al.* (2007) Adaptive versus non-adaptive phenotypic plasticity and the potential for contemporary adaptation in new environments. *Funct. Ecol.* 21, 394–407
59 Finnegan, E.J. (2002) Epialleles – a source of random variation in times of stress. *Curr. Opin. Plant Biol.* 5, 101–106
60 Richards, C.L. *et al.* (2010) What role does heritable epigenetic variation play in phenotypic evolution? *Bioscience* 60, 232–237
61 Bossdorf, O. *et al.* (2008) Epigenetics for ecologists. *Ecol. Lett.* 11, 106–115
62 Verhoeven, A. *et al.* (2009) Seasonal changes in abundance and phosphorylation status of photosynthetic proteins in eastern white pine and balsam fir. *Tree Physiol.* 29, 361–374
63 Jablonka, E. and Raz, G. (2009) Transgenerational epigenetic inheritance: prevalence, mechanisms, and implications for the study of heredity and evolution. *Q. Rev. Biol.* 84, 131–176
64 Reinders, J. *et al.* (2009) Compromised stability of DNA methylation and transposon immobilization in mosaic *Arabidopsis* epigenomes. *Genes Dev.* 23, 939–950
65 Johannes, F. *et al.* (2009) Assessing the impact of transgenerational epigenetic variation on complex traits. *PLoS Genet.* 5, e1000530. doi:1000510.1001371/journal.pgen.1000530
66 Sadras, V.O. *et al.* (2009) Phenotypic plasticity of yield and phenology in wheat, sunflower and grapevine. *Field Crops Res.* 110, 242–250
67 Sambatti, J.B.M. and Caylor, K.K. (2007) When is breeding for drought tolerance optimal if drought is random? *New Phytologist* 175, 70–80
68 Nicotra, A.B. and Davidson, A. (2010) Adaptive plasticity in water use traits. *Funct. Plant Biol.* 37, 117–127
69 Nuhse, T.S. *et al.* (2007) Quantitative phosphoproteomic analysis of plasma membrane proteins reveals regulatory mechanisms of plant innate immune responses. *Plant J.* 51, 931–940
70 Chinnusamy, V. and Zhu, J.K. (2009) Epigenetic regulation of stress responses in plants. *Curr. Opin. Plant Biol.* 12, 133–139
71 Zhao, K.Y. *et al.* (2007) An *Arabidopsis* example of association mapping in structured samples. *Plos Genet.* 3, 71–82
72 Ungerer, M.C. *et al.* (2002) Quantitative trait loci for inflorescence development in *Arabidopsis thaliana*. *Genetics* 160, 1133–1151
73 Weinig, C. *et al.* (2002) Novel loci control variation in reproductive timing in *Arabidopsis thaliana* in natural environments. *Genetics* 162, 1875–1884
74 Ehrenreich, I.M. *et al.* (2009) Candidate Gene Association Mapping of *Arabidopsis* Flowering Time. *Genetics* 183, 325–335
75 Richards, C.L. *et al.* (2009) Perspectives on ecological and evolutionary systems biology. In *Plant Systems Biology* (Gutierrez, R.A. and Coruzzi, G.M., eds), pp. 331–351, Blackwell Publishing
76 Travers, S.E. *et al.* (2007) Ecological genomics: making the leap from model systems in the lab to native populations in the field. *Front. Ecol. Environ.* 5, 19–24
77 Wright, I.J. *et al.* (2004) The worldwide leaf economics spectrum. *Nature* 428, 821–827
78 Westoby, M. (1998) A leaf-height-seed (LHS) plant ecology strategy scheme. *Plant and Soil* 199, 213–227
79 Poorter, H. *et al.* (2009) Causes and consequences of variation in leaf mass per area (LMA): a meta-analysis. *New Phytologist* 182, 565–588
80 Hetherington, A.M. and Woodward, F.I. (2003) The role of stomata in sensing and driving environmental change. *Nature* 424, 901–908
81 Sack, L. *et al.* (2006) How strong is intracanopy leaf plasticity in temperate deciduous trees? *Am. J. Bot.* 93, 829–839
82 Metcalf, J.C. *et al.* (2003) Evolutionary demography of monocarpic perennials. *Trends Ecol. Evol.* 18, 471–480
83 Seibt, U. *et al.* (2008) Carbon isotopes and water use efficiency: sense and sensitivity. *Oecologia* 155, 441–454
84 Farquhar, G.D. *et al.* (1989) Carbon isotope discrimination and photosynthesis. *Annu. Rev. Plant Biol.* 40, 503–537
85 Hodge, A. (2004) The plastic plant: root responses to heterogeneous supplies of nutrients. *New Phytologist* 162, 9–24
86 Steyn, W.J. *et al.* (2002) Anthocyanins in vegetative tissues: a proposed unified function in photoprotection. *New Phytologist* 155, 349–361
87 Chalker-Scott, L. (1999) Environmental significance of anthocyanins in plant stress responses. *Photochem. Photobiol.* 70, 1–9
88 Tallis, M.J. *et al.* (2010) The transcriptome of *Populus* in elevated CO2 reveals increased anthocyanin biosynthesis during delayed autumnal senescence. *New Phytologist* 186, 415–428
89 Callahan, H.S. *et al.* (2005) Plasticity genes and plasticity costs: a new approach using an *Arabidopsis* recombinant inbred population. *New Phytologist* 166, 129–140
90 Stinchcombe, J.R. *et al.* (2004) Flowering time plasticity in Arabidopsis thaliana: a reanalysis of Westerman & Lawrence (1970). *J. Evol. Biol.* 17, 197–207
91 Callahan, H.S. and Pigliucci, M. (2002) Shade-induced plasticity and its ecological significance in wild populations of *Arabidopsis thaliana*. *Ecology* 83, 1965–1980
