# Supplementary material for: Provenance and family variations in early growth of Manchurian walnut (Juglans mandshurica Maxim.) and selection of superior families
Source: PLoS One. 2024 Mar 7;19(3):e0298918. doi: 10.1371/journal.pone.0298918 (PMC10919699; doi:10.1371/journal.pone.0298918)
Supplement: S2 File — (ZIP) [file pone.0298918.s005.zip › Pomological and Phenological Description of the New ‘Bahri Koz’Cultivar of Walnut (Juglans regia L.) and Its Nutritional Value.pdf]

Journal of Nuts 13(2) (2022) 131-139

Journal of Nuts
Journal homepage: ijnrs.damghaniau.ac.ir

ORIGINAL ARTICLE

Pomological and Phenological Description of the New 'Bahri Koz' Cultivar of Walnut (*Juglans regia* L.) and Its Nutritional Value

Mehmet Sütyemez[*1], Şakir Burak Bükücü[2], Akide Özcan[3]

[1]Department of Horticulture, Agricultural Faculty, Kahramanmaraş Sütçü İmam University, Kahramanmaraş 46040, Turkey

[2]Department of Plant and Animal Production, Silifke Taşucu Vocational School, Selçuk University, 33900 Silifke, Mersin, Turkey

[3]Department of Organic Farming, Göksun Vocational School, Kahramanmaraş Sütçü İmam University, Göksun 46650, Kahramanmaraş, Turkey

A R T I C L E I N F O

Keywords:

Cultivar;
Fatty acid;
Juglans regia L.;
Phenology;
Pomology

A B S T R A C T

'Bahri Koz' is a walnut cultivar that reaches a mid-early harvest maturity and has some superior fruit traits. In 2019, the cultivar was registered and patented by the Variety Registration and Seed Certification Center of the Republic of Türkiye Ministry of Agriculture and Forestry. To determine the pomological and phenological traits of the 'Bahri Koz', 27 different traits were examined. The pomological analysis determined that the in-shell nut weight of 'Bahri Koz' was 12-14g, the kernel weight was 6.5-7.0g, and the kernel ratio was 51-54%. The shell and kernel of the cultivar were determined to be light in color and the surface of the shell to have a smooth structure. 'Bahri Koz' had superior traits compared to the 'Chandler' cultivar in terms of some of the pomological parameters such as in-shell nut weight, kernel weight, and kernel percentage. The new cultivar was seen to reach leafing, harvest maturity, and defoliation at an earlier date than 'Chandler'. The dichogamy status of the cultivar was determined to be protandrous and in this respect, a pollinator cultivar was recommended for the establishment of orchards with 'Bahri Koz'. This cultivar was also shown to be rich in lipid and unsaturated fatty acid content.

Introduction

Walnut (*Juglans regia* L.) is an important fruit species of the genus *Juglans* that has been consumed by humanity since ancient times. Some of the characteristics of walnut include being long-lived, deciduous, monoecious, open-pollinated, and generally dichogamous. It is widely cultivated in temperate regions such as Asia, Europe, North and South America, South Africa, Australia, and New Zealand (McGranahan and Leslie, 2012). Walnut trees are commonly found in almost every corner of Anatolia as one of its regions of origin and among Turkish people there is a great traditional interest in walnut trees and their cultivation. This situation is very significant as it provides a wide variety for walnut breeders.

Walnut is an important delicious and high-energy food rich in healthy fatty acids, vitamins, and minerals. Walnut kernels have a high-fat content consisting mainly of unsaturated fatty acids (Chatrabnous *et al.*, 2018). Linoleic acid is the major

[*]**Corresponding author**: Email address: sutyemezmehmet@gmail.com
Received: 14 December 2021; Received in revised form: 25 February 2022; Accepted: 12 April 2022
DOI: 10.22034/jon.2022.1952690.1154

fatty acid type, followed by oleic acid and linolenic acid. Walnuts are also rich in vitamins A and E (Kafkas *et al*., 2017; Özcan and Sutyemez, 2019). Because of this healthy nutrient content, the consumption of walnuts is steadily increasing, as well as the scientific studies carried out on them. Some studies conducted in recent years have revealed that the consumption of walnuts reduces the risk of cardiovascular disease by increasing high-density lipoprotein (HDL) cholesterol and lowering low-density lipoprotein (LDL) cholesterol. Moreover, their consumption has also been found to reduce the risk of Alzheimer's disease. The positive effects of walnuts on human health are attributed to the high rate of unsaturated fatty acids (Spaccarotella *et al*., 2008; Orhan *et al*., 2011; Jahanbani *et al.*, 2016; Sánchez-González *et al*., 2017).

Initially, walnut-breeding studies began in the form of a method based on the selective breeding of walnut genotypes having superior traits grown from one open-pollinated seedling population (Sarikhani *et al.*, 2021). In subsequent years, cross-breeding studies began to be carried out in various countries such as the USA, France, China, and Türkiye. However, selection, which is a part of all breeding studies, is still used to choose and preserve naturally existing genotypes that have superior traits. These studies constitute an important genetic resource in terms of conserving genetic diversity and providing an infrastructure for transferring some important traits to future breeding studies.

Important considerations in walnut breeding programs include improvement of lateral bud flowering, nut quality, late leafing, early defoliation, and resistance to certain diseases and pests. Since the walnut is especially susceptible to damage by late spring frosts, late leafing has been the cornerstone of many breeding studies. In this respect, some important walnut cultivars such as 'Payne' and 'Franquette', which were identified as late-leafed in the 1800s, were selected. In the following years, both high lateral flowering and late leafing cultivars such as 'Chandler' and 'Howard' were developed in the USA, whereas France developed important cultivars such as 'Fernor' and 'Fernette', which also exhibited late leafing (Bernard *et al*., 2018). Türkiye's first walnut cultivars were registered in 1990 (Anonymous, 2021), followed by the development of the first cross-bred walnut cultivar named 'Diriliş' in 2016 (Sütyemez *et al*., 2018).

'Bahri Koz' is a new walnut cultivar from the Kahramanmaraş Sütçü İmam University walnut-breeding program in Türkiye. This cultivar was selected from seedling populations of Kahramanmaraş Province (Türkiye) and registered in the Turkish list of fruit varieties in 2019. In this study, carried out between 2019 and 2020, a number of phenological and pomological traits of the 'Bahri Koz' walnut cultivar were determined and compared with those of the 'Chandler' walnut cultivar.

Material and Methods

The 'Bahri Koz' walnut cultivar was selected from Kahramanmaraş Province in 1994 (Sütyemez, 1998) and subsequently registered and patented by the Republic of Türkiye as the 'Bahri Koz' cultivar in 2019 (Anonymous, 2021). To determine the performances of the 'Bahri Koz' and 'Chandler' cultivars, ten replicates were grafted over *Juglans regia*, cv. Maraş 18 rootstocks during the 2002 vegetation period. These walnut cultivars were planted in the same year at the Kahramanmaraş Sütçü İmam University Faculty of Agriculture Nut Application and Research Center (SEKAMER), located at 37° 35' 27" N latitude, 37° 03' 28" E longitude, and 930 m above sea level. As an average between Mediterranean and Continental climates, the climate of the region is mild, with annual precipitation of 727 mm and an average annual temperature of 16.9°C. The soil structure in general is suitable for walnut cultivation. In this study, we examined data for the years 2019 and 2020 and compared them with the data we obtained for the 'Chandler'. Some significant pomological and phenological traits were evaluated according to the Walnut Descriptor (IPGRI, 1994). These parameters

are presented in Tables 1 and 2. In this study, a fatty acid analysis was also conducted to determine the nutritional content of the 'Bahri Koz'. Oil extraction and fatty acid analysis of the nuts were carried out according to the methods of Bligh and Dyer (1959) and Kafkas *et al*. (2017) using gas chromatography.

Table 1. Descriptors of the phenological traits.

Traits	Description
**Leafing date**	Date when 50% of terminal buds have enlarged and the bud scales have split exposing the green leaves
**First male bloom date**	When first pollen shedding occurs
**Last male bloom date**	When last pollen shedding occurs
**First female bloom date**	Date of initial pistillate flower receptivity
**Last female bloom date**	Date of last pistillate flower receptivity
**Male flowering period**	Catkin receptive duration
**Female flowering period**	Female flower receptive duration
**Female abundance**	Female flower abundance: 3 (Light); 5 (Intermediate); 7 (High)
**Catkin abundance**	Male flower abundance: 3 (Light); 5 (Intermediate); 7 (High)
**Lateral bud flowering**	Percent of lateral buds with female flowers
**Dichogamy**	Female flowers and catkins receptive duration overlap status: 1 (Protandrous); 2 (Protogynous); 3 (Unknown)
**Estimated yield**	Rate in relation to age and volume of tree: 3 (Low); 5 (Intermediate); 7 (High)
**Harvest date**	Date when nuts are harvestable
**Defoliation date**	Date of defoliation

Table 2. Descriptors of the pomological traits.

Traits	Description
**Nut shape**	1: Round; 2: Triangular; 3: Broad ovate; 4; Ovate; 5: Short trapezoid; 6: Long trapezoid; 7: Broad eliptic; 8: Eliptic; 9: Cordate
**Shell texture**	1: Very smooth; 3: Smooth; 5: Medium; 7: Rough; 9: Very rough
**Shell color**	1: Very light; 3: Light; 5: Medium; 7: Dark; 9: Very dark
**Shell strength**	1: Paper; 3: Weak; 5: Intermediate; 7: Strong
**Shell thickness (mm)**	Measured with a digital caliper near center of half-shell
**In-shell weight (g)**	Average of total 50 nuts
**Kernel weight (g)**	Average of total 50 nuts
**Kernel veins (%)**	Percent of kernels with conspicuous veins
**Kernel percentage (%)**	Kernel weight/nut weight × 100
**Kernel color**	1: Extra light; 2: Light; 3: Light amber; 4: Amber
**Kernel fill**	3: Poor; 5: Moderate; 7: Well
**Kernel flavor**	1: Satisfactory; 2: Unsatisfactory
**Ease of removal of kernel halves**	1: Very easy; 3: Easy; 5: Moderate; 7: Difficult; 9: Very difficult

Results

Phenological traits

Traits such as leafing, flowering habits, harvest times, and defoliation date are used as the selection criteria in walnut-breeding programs. Leafing and defoliation dates in particular are among the main objectives of many walnut-breeding studies (Solar *et al*., 2014; Ebrahimi *et al*., 2015; Khadivi *et al*., 2019; Vahdati *et al.*, 2019; Bükücü *et al*., 2020; Hassani *et al.*, 2020; Kouhi *et al.*, 2020; Fallah *et al.*, 2022). Because the walnut is sensitive to spring and autumn frosts, significant economic losses occur when walnuts

are damaged by these two negative ecological conditions. The average leafing date of ‘Bahri Koz’ was observed as 28 March. In terms of this trait, it was emerging from dormancy 22 days earlier than ‘Chandler’. The average defoliation date of ‘Bahri Koz’ was determined as October 27, about nine days earlier than the ‘Chandler’. The calculations performed using these two phenological traits revealed that the average vegetation periods of the ‘Bahri Koz’ and ‘Chandler’ cultivars were 213 and 200 days, respectively.

Determining the blooming dates of the male and female flowers is very important in fruit species having a dichogamy tendency such as walnut. In this study, the first and last male bloom dates of ‘Bahri Koz’ were determined as 29 March and 5 April, respectively. Moreover, the female flower bloom date occurred between 7 April and 14 April. In light of these data, it was determined that the ‘Bahri Koz’ had a protandrous flowering trait that was similar to that of ‘Chandler’. Potential pollinizers of ‘Bahri Koz’ include ‘Sütyemez 1’ and ‘Howard’. Data for the ‘Chandler’ cultivar are presented in Table 3.

Although the catkin abundance was determined as high in ‘Chandler’, it was observed as intermediate in ‘Bahri Koz’. Lateral bud flowering is an important parameter that indicates the productivity of walnuts and it is among the goals of many walnut-breeding programs (Sütyemez *et al*., 2018; Khadivi *et al*., 2019). In this study, we examined this trait separately for the ‘Bahri Koz’ and ‘Chandler’ cultivars and found that the values were 70% and 91%, respectively. It was determined that ‘Bahri Koz’ had an intermediate estimated yield (Table 3).

Table 3. Phenological traits of ‘Bahri Koz’ and ‘Chandler’ walnut cultivars.

Phenological Traits	Cultivars	
	Bahri Koz	Chandler
Leafing date	28 March	19 April
First male bloom date	29 March	20 April
Last male bloom date	5 April	30 April
First female bloom date	7 April	28 April
Last female bloom date	14 April	5 May
Male flowering period	7 days	10 days
Female flowering period	7 days	7 days
Female abundance	Light (3)	High (7)
Catkin abundance	Intermediate (5)	High (7)
Lateral bud flowering	70%	91%
Dichogamy	Protandrous	Protandrous
Estimated yield	Intermediate (5)	High (7)
Harvest date	11 September	6 October
Defoliation date	27 October	5 December

Pomological traits

The study examined 13 different parameters to determine the pomological traits, presented as the average values between 2019 and 2020. The ‘Bahri Koz’ shell surface structure was determined as smooth and that of the ‘Chandler’ shell as medium. In addition, the in-shell nuts of ‘Bahri Koz’ were light-colored, large, and round. The shell thickness of the cultivar was 1.41 mm and its shell strength was intermediate. The average ‘Bahri Koz’ in-shell nut weight varied between 12 and 14 g, whereas the average kernel weight of the cultivar was 6.5-7.0 g and the kernel percentage was around 51-54%. The in-shell nut weight of ‘Chandler’ varied between 11 and 13 g, and its kernel weight between 5 and 6 g with 45-49% kernel percentage. ‘Bahri Koz’ yielded a superior performance compared to ‘Chandler’ in terms of in-

shell nut weight, kernel weight, and kernel percentage traits (Table 4). Some important pomological trait results of the 'Bahri Koz' are presented in Fig. 1.

The nuts were harvested under Kahramanmaraş ecological conditions around the second week of September, which was about three weeks earlier than the 'Chandler' harvest. Determining the harvest time of the walnut is very important for its subsequent storage, marketable life, and quality.

The kernel of 'Bahri Koz' was easily removed from the shell as a whole or as two halves, resulting in a very high ratio of intact kernels, which is an important quality trait for walnuts. The kernel color was light with vein values of 28%. The walnut kernel is edible even before full maturation, which some consumers prefer, and in this case, it is classified as fresh walnut. According to consumer opinions, 'Bahri Koz' had a more pleasant aroma and taste compared to 'Chandler'. 'Bahri Koz' nuts and kernels are illustrated in Fig. 2.

Table 4. Pomological traits of 'Bahri Koz' and 'Chandler' walnut cultivars.

Pomological Traits	Cultivars	
	**Bahri Koz**	**Chandler**
**Nut shape**	Round (1)	Ovate (5)
**Shell texture**	Smooth (2)	Medium (5)
**Shell color**	Light (3)	Light (3)
**Shell strength**	Intermediate (5)	Weak (3)
**Shell thickness**	1.41 mm	1.22 mm
**In-shell weight**	12-14 g	11-13 g
**Kernel weight**	6.5-7.0 g	5.0-6.0 g
**Kernel veins**	28%	17%
**Kernel percentage**	51-54%	45-49%
**Kernel color**	Light (2)	Light (2)
**Kernel fill**	Well (7)	Well (7)
**Ease of removal of kernel halves**	Moderate (5)	Very easy (1)
**Kernel flavor**	Satisfactory (1)	Satisfactory (1)

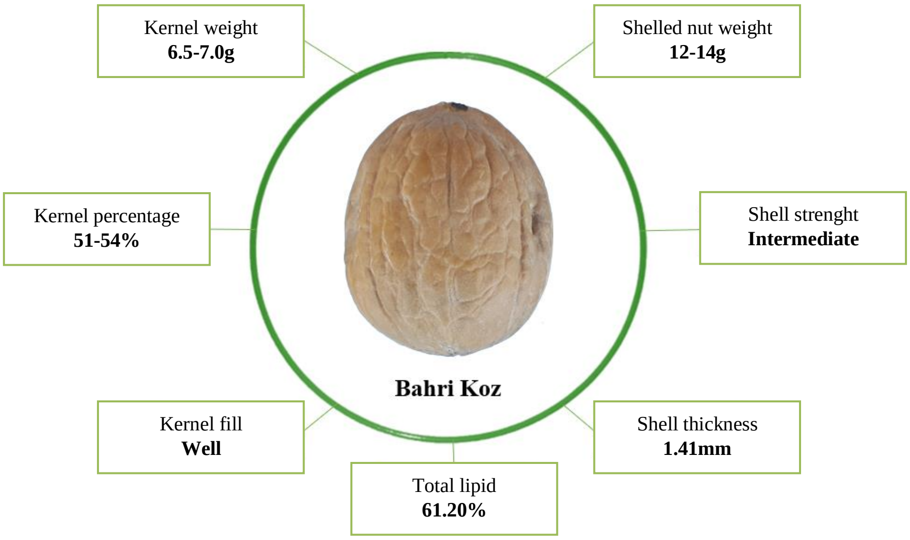

Fig. 1. Some significant trait values of 'Bahri Koz'.

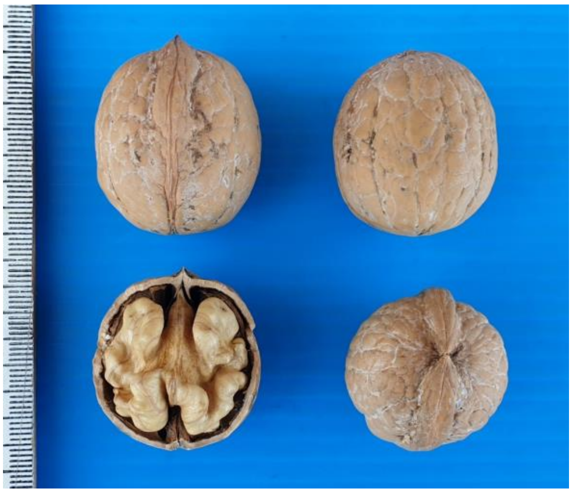

Fig. 2. Fruits of 'Bahri Koz' walnut cultivar.

Fatty acid content

The most important reason for the recent increase in the consumption of walnuts is that they are rich in unsaturated fatty acids, which are especially beneficial for human health. Many studies have reported that walnut consumption partially reduces the risk of cardiovascular problems and Alzheimer's disease (Lavedrine *et al*., 1999; Orhan *et al*., 2011; Sánchez-González *et al*., 2017). In this study, we evaluated the total lipids, saturated fatty acids (SFAs), and unsaturated fatty acids including monounsaturated fatty acids (MUFAs) and polyunsaturated fatty acids (PUFAs). These values in the cultivars are presented in Table 5. The total lipid content of 'Bahri Koz' was found to be 61.20%, which was lower than that of 'Chandler' (63.14%). The highest fatty acid value was 57.56% in linoleic acid, which is a significant unsaturated fatty acid. The total SFA value of the cultivar was determined as 7.59%, whereas the total MUFA value was found as 69.08% (Bayındır, 2021).

Table 5. Total lipid, saturated fatty acid and unsaturated fatty acid values of 'Bahri Koz' and 'Chandler' cultivars.

Cultivars	Total lipid	Myristic acid	Palmitic acid	Arachidic acid	SFAs
**Bahri Koz**	61.20±6.87	0.16±0.21	6.93±0.04	0.55±0.64	7.64±0.90
**Chandler**	63.14±11.14	0.00±0.00	7.52±0.03	0.07±0.09	7.59±0.12
**Cultivars**	**Oleic acid**	**MUFAs**	**Linoleic acid**	**α-linolenic acid**	**PUFAs**
**Bahri Koz**	23.33±0.50	23.33±0.50	57.56±0.59	11.52±0.22	69.08±0.81
**Chandler**	16.39±0.12	16.39±0.12	62.27±0.32	13.70±1.05	75.97±1.37

Discussion

'Bahri Koz' is an important walnut cultivar selected in Kahramanmaraş, Turkey. In this study, 14 phenological, 13 pomological, and fatty acid contents of 'Bahri Koz' and 'Chandler' were revealed. 'Chandler' is widely cultivated in the world so we compared 'Bahri Koz' with 'Chandler'.

As a result of the phenological examinations, the leafing dates of 'Bahri Koz' and 'Chandler' were 28 March and 19 April, respectively. Some studies have reported 'Chandler' leafing date was in the first week of April under the ecological conditions in Iran. (Mahmoodi *et al*., 2016; Hassankhah *et al*., 2017). The difference could be due to the different ecologies in which the experiment was conducted. It was determined that Chandler's defoliation date was later than Bahri Koz's. In the studies conducted in Turkey, it was reported that the defoliation date of 'Chandler' usually occurred later than in the local genotypes (Akça *et al*., 2013; Sutyemez, 2016). It was determined that both walnut cultivars had the

protandrous flowering trait. This finding was consistent with those of other studies investigating the flowering traits of Chandler (Ertürk *et al*., 2013; Sutyemez, 2016). Lateral bud flowering is the most important trait which is a sign of a high yield for a walnut cultivar. We determined Chandler's lateral bud flowering as 91% and Bahri Koz's as 70%. Manthos and Rouskas (2021) reported that Chandler's lateral bud flowering was 90%, as similar as in our study.

'Bahri Koz' outperformed 'Chandler' for most of the pomological traits studied. While the in-shell nut weight of 'Bahri Koz' was determined as 12-14 g, this value was determined as 11-13 g for 'Chandler'. Kernel percentages were determined as 51-54% and 45-49% for 'Bahri Koz' and 'Chandler', respectively. McGranahan and Leslie (2015) reported that the in-shell nut weight of 'Chandler' was 13.4 g, its kernel weight 6.6 g, and kernel percentage 49.5%. The partial differences obtained within the scope of the study could be attributed to varying cultivation, ecological, and maintenance conditions. Mahmoodi *et al*. (2016) determined the shell thickness of 'Chandler' as 1.28 mm. In this study, the shell thickness of this cultivar was determined as 1.22 mm. Findings for other pomological traits, such as nut shape, nut color, kernel fill and kernel color, examined for 'Chandler' were similar to the study conducted by Manthos and Rouskas (2021).

The fatty acid contents of 'Bahri Koz' and 'Chandler' were also presented in the study. Kafkas *et al*. (2017) reported that in 'Chandler', the total lipid content was 62.30%, total saturated fatty acid 9.44%, and total unsaturated fatty acid 80.33%, which were consistent with the values we obtained in our study.

Conclusions

The 'Bahri Koz' walnut cultivar has been registered and preserved within the framework of walnut-breeding studies of the Kahramanmaras Sutcu Imam University (KSU). This cultivar possesses a number of important nut traits including intermediate yields, superior fruit quality, and earlier harvesting periods. Moreover, the cultivar has a high nutritional content in terms of beneficial fatty acids. 'Bahri Koz' is especially suitable for walnut growing areas where late frost events in spring are not likely.

Conflict of interests

The authors declare that they do not have any conflict of interest.

References

Akça Y, Ünal B, Çelik M, Okay Y (2013) Comparison of some promising Turkish and foreign walnut cultivars. VII International Walnut Symposium. 1050, 143-150. doi: 10.17660/ActaHortic.2014.1050.17

Anonymous (2021) Republic of Turkey Ministry of Agriculture and ForestryVariety Registration and Seed Certifıcation Center, https: // www. tarimorman.gov.tr/BUGEM/TTSM/Sayfalar/ Detay.aspx?SayfaId=87.

Bayındır F (2021) Detection of fatty acid and tocopherol contents of some walnut types and genotypes, MSc thesis, University of Cukurova, Adana, Turkey. pp. 101.

Bernard A, Lheureux F, Dirlewanger E (2018) Walnut: past and future of genetic improvement. Tree Genetics & Genomes 14(1), 1-28. doi: 10.1007/s11295-017-1214-0.

Bligh EG, Dyer WJ (1959) A rapid method of total lipid extraction and purification. Canadian Journal of Biochemistry and Physiology. 37(8), 911-917. doi: 10.1139/o59-099

Bükücü ŞB, Özcan A, Sütyemez M, Yildirim E (2020) Determination in the phenological difference levels of seedlings of some walnut genotypes (*Juglans regia* L.). Applied Ecology and Environmental Research. 18(3), 4807-4815. doi: 10.15666 /aeer/ 1803 _48074815

Chatrabnous N, Yazdani N, Tavallali V, Vahdati K (2018) Preserving quality of fresh walnuts

using plant extracts. LWT 91:1-7. doi: 10.1016/j.lwt.2018.01.026

Ebrahimi A, Khadivi-Khub A, Nosrati Z, Karimi R (2015) Identification of superior walnut (*Juglans regia*) genotypes with late leafing and high kernel quality in Iran. Scientia Horticulturae. 193, 195-201. doi: 10.1016 /j. scienta.2015.06.049

Ertürk U, Mert C, Soylu A, Akça Y, Okay Y (2013) Evaluation of some domestic and foreign walnut cultivars in the conditions of Bursa, Turkey. VII International Walnut Symposium. 1050_14, 123-129. doi: ActaHorticulture. 2014.1050.14

Fallah M, Vahdati K, Hasani D, Rasouli M, Sarikhani S (2022) Breeding of Persian walnut: Aiming to introduce late-leafing and early-harvesting varieties by targeted hybridization. Scientia Horticulturae. 295. doi: 10.1016/j.scienta.2022.110885

Hassani D, Mozaffari MR, Soleimani A, Dastjerdi R, Rezaee R, Keshavarzi M, Vahdati K, Fahadan A, Atefi J (2020a) Four new persian walnut cultivars of Iran: Persia, Caspian, Chaldoran, and Alvand. HortScience. 55(7), 1162-1163. doi: 10.21273/HORTSCI15044-20

Hassankhah A, Vahdati K, Rahemi M, Hassani D, Sarikhani Khorami S (2017) Persian walnut phenology: effect of chilling and heat requirements on budbreak and flowering date. International Journal of Horticultural. Science and Technology. 4(2), 259-271. doi: 10.22059/IJHST.2018.260944.249

IPGRI (1994) Descriptors for walnut (*Juglans* spp.). Rome: International Plant Genetic Resources Institute. 1-54.

Jahanbani R, Ghaffari SM, Salami M, Vahdati K, Sepehri H, Namazi Sarvestani N, Sheibani N, Moosavi-Movahedi AA (2016) Antioxidant and anticancer activities of walnut (*Juglans regia* L.) protein hydrolysates using different proteases. Plant Foods and Human Nutrition. 71, 402-409. doi: 10.1007/s11130-016-0576-z

Kafkas E, Burgut A, Ozcan H, Ozcan A, Sutyemez M, Kafkas S, Türemis N (2017) Fatty acid, total phenol and tocopherol profiles of some walnut cultivars: a comparative study. Food and Nutrition Sciences. 8(12), 1074. doi: 10.4236/fns.2017.812079

Khadivi A, Montazeran A, Yadegari P (2019) Superior spring frost resistant walnut (*Juglans regia* L.) genotypes identified among mature seedling origin trees. Scientia Horticulturae. 253, 147-153. doi: j.scienta. 2019.04.041

Kouhi M, Rezaei A, Hassani D, Sarikhani S, Vahdati K (2020) Phenotypic evaluation and identification of superior Persian walnut (*Juglans regia* L.) Genotypes in Mazandaran Province, Iran. Journal of Nuts. 111(4), 315-326. doi: 10.22034/JON.2021.1919541.1104

Lavedrine F, Zmirou D, Ravel A, Balducci F, Alary J (1999) Blood Cholesterol and Walnut Consumption: A Cross-Sectional Survey in France. Preventive Medicine. 28, 333-339. doi: 10.1006/pmed.1999.0460

Mahmoodi R, Hassani D, Amiri ME, Jaffaraghaei M (2016) Phenological and pomological characteristics of five promised walnut genotypes in Karaj, Iran. Journal of Nuts. 7(1), 1-8.

Manthos I, Rouskas D (2021) Introduction of a New Interesting Walnut Cultivar "Leto". Plants. 10(12), 2738. doi: 10.3390/plants10122738

McGranahan G, Leslie C (2012) Walnut. In: Badenes M., Byrne D. (eds) Fruit Breeding. Handbook of Plant Breeding. Vol 8, Springer Boston, MA, pp. 827-846. doi: 10.1007/978-1-4419-0763-9_22

McGranahan G, Leslie C (2015) Walnut tree named 'Solano', U.S. Patent Application, No. 13/694,337.

Orhan IE, Suntar IP, Akkol EK (2011) *In vitro* neuroprotective effects of the leaf and fruit

extracts of *Juglans regia* L. (walnut) through enzymes linked to Alzheimer's disease and antioxidant activity. International Journal of Food Sciences and Nutrition. 62(8), 781-786. doi: 10.3109/09637486.2011.585964

Özcan A, Sütyemez M (2019) Cevizde Depolama Süresinin Protein ve Yağ Kompozisyonuna Olan Etkisinin Belirlenmesi," *Yüzüncü Yıl Üniv.Tarım Bilimleri Der.* vol. 29, no. 4, pp. 628-633. doi.org/10.29133/yyutbd.590308

Sánchez-González C, Ciudad CJ, Noe V, Izquierdo-Pulido M (2017) Health benefits of walnut polyphenols: An exploration beyond their lipid profile. Critical Reviews in Food Science and Nutrition. 57(16), 3373-3383. doi: 10.1080/10408398.2015.1126218

Sarikhani S, Vahdati K, Ligterink W (2021) Biochemical properties of superior Persian walnut genotypes originated from southwest of Iran. International Journal of Horticultural Science and Technology. 8(1), 13-24. doi: 10.22059/ijhst.2020.309363.392

Solar A, Veberic R, Stampar F (2014) 'Sava' and 'Krka' Walnut Cultivars. HortScience. 49(8), 1081-1082. doi: 10.21273/ HORTSCI. 49.8.1081

Spaccarotella KJ, Kris-Etherton PM, Stone WL, Bagshaw DM, Fishell VK, West SG, Lawrence FR, Hartman TJ (2008) The effect of walnut intake on factors related to prostate and vascular health in older men. Nutrition Journal. 7(1), 13. doi: 10.1186/1475-2891-7-13.

Sütyemez M (1998) Studies on walnut (*Juglans regia* L.) selection and fertilization biology of some selected types in Kahramanmaraş region. PhD thesis, University of Cukurova, Adana, Turkey.

Sutyemez M (2016) New walnut cultivars: Maras 18, Sutyemez 1, and Kaman 1. HortScience. 51(10), 1301-1303. doi: 10.21273/ HORTSCI10972-16

Sütyemez M, Özcan A, Bükücü ŞB (2018) Walnut cultivars through cross-breeding: 'Diriliş' and '15 Temmuz'. The American Pomological Society. 72(3), 173-180.

Vahdati K, Arab MM, Sarikhani S, Sadat Hosseini M, Leslie CA, Brown PJ (2019) Advances in Persian walnut (*Juglans regia* L.) breeding strategies. In: JM Al-Khayri, SM Jain and DV Johnson (eds.) Advances in plant breeding strategies, Vol 4. pp. 401-472. Nut and Beverage Crops. Springer, Switzerland. doi: 10.1007/978-3-030-23112-5_11.
