# Supplementary material for: Provenance and family variations in early growth of Manchurian walnut (Juglans mandshurica Maxim.) and selection of superior families
Source: PLoS One. 2024 Mar 7;19(3):e0298918. doi: 10.1371/journal.pone.0298918 (PMC10919699; doi:10.1371/journal.pone.0298918)
Supplement: S2 File — (ZIP) [file pone.0298918.s005.zip › Preliminary division and selection of the best provenance of Juglans mandshurica.pdf]

DOI:10.13759/j.cnki.dlxb.1991.s2.030

第19卷 育种专刊 东北林业大学学报 Vol.19 Sp.Iss.
1991年 5月 JOURNAL OF NORTHEAST FORESTRY UNIVERSITY May 1991

胡桃楸种源的初步区划及最佳种源选择

刘桂丰 杨书文 李俊涛 刘 强 王会仁 彭宏梅

（东北林业大学）

【摘要】

根据两次胡桃楸种源试验的各类性状，采用典型相关分析和主成分分析，将东北地区的胡桃楸划分为四个种源区。即长白山完达山种源区；吉林中部浅山种源区；辽宁东部种源区；小兴安岭松花江地区种源区。两次试验选出的最佳种源均为舒兰，但本溪和宽甸种源也很好。如果采用舒兰、本溪两种源在帽儿山造林，可得到8.6%的遗传增益。

主题词：胡桃楸；种源试验；种源区划；最佳种源选择

胡桃楸地理种源试验始于1985年。其目的是了解胡桃楸地理变异规律及模式，影响胡桃楸变异的主要生态因子、遗传与环境的交互作用。在此基础上划分种源区，选择优良种源。从而为胡桃楸的种子区划，良种基地布局，制订树木良种策略等提供科学依据。有关胡桃楸的地理变异规律及模式，笔者曾报道过[1]。对于种源区划或种子区区划还未曾有人报道。本文是在研究地理变异规律的基础上，利用先后两次种源试验材料，对胡桃楸进行种源区的初步区划和最佳种源的选择。有关试验材料与方法参见胡桃楸地理变异规律研究[1—5]。

1 胡桃楸的分类地位

胡桃楸（*Juglans mandshurica* Maxim.）又名核桃楸子（东北）、核桃楸（中国树木志，黑龙江树木志），属胡桃科（Juglandanceae）胡桃属（*Juglans* Linn.）。该属在我国分布有5个种，东北有3种（包括栽培种），胡桃楸是自然分布于东北地区面积最广的一种。因为胡桃楸的果核的形状及腔隙的变化很大。俄国植物学家司克沃尔错夫（B. W. Skvortzov，1927、1929年）根据分布于黑龙江、吉林两省的胡桃楸形态变化，先后发表了10个变种。即圆核核桃楸（Var. *rotunda* Skv.）；三角核核桃楸（Var. *triguetra* Skv.）；小核核桃楸（Var. *gracilis* Skv.）；齿核核桃楸（Var. *oblong* Skv.）；长卵核核桃楸（Var. *oblong* Skv.）；卵圆形核桃楸（Var. *mifunensis* Skv.）；大核核桃楸（Var. *komarovi* Skv.）；棱核核桃楸（Var. *girinensis*

?1994-2016 China Academic Journal Electronic Publishing House. All rights reserved. http://www.cnki.net

Shv)；倒卵核核桃楸（Var. *dode* Skv.)；扁核核桃楸（Var. *depressa* Skv.)。周以良认为：胡桃楸果核的形态虽然变化较大，但在同一株树上果核形态以及腔隙的多少也有变异，没有一定规律性，不能作为划分变种的依据，应属于胡桃楸的种内变化。国内有关分类书籍中，均未将胡桃楸分为变种。胡桃楸种源试验的种子采集，是依据《中国植物志》第二十一卷，《中国树木志》等书藉的分类结果，按一个种采集种子的。

2　胡桃楸的地理分布[3－4]

胡桃楸的适应性较强，分布范围广，以植物区系看，在温带针叶落叶阔叶林带和暖温带落叶阔叶林带均有胡桃楸的分布，从地理和行政区看，主要分布于东北小兴安岭海拔 500 m 以下，长白山海拔 1 000 m 以下。内蒙旅哲里木盟大青沟、山西、河北、河南、山东有散生胡桃楸。苏联远东地区、朝鲜、日本也有分布。

在东北胡桃楸多单株混生在红松阔叶混交林内，在次生阔叶林内可沿河谷形成小片天然林，但常与春榆、黄波罗、水曲柳、山杨、椴树等混生。胡桃楸在东北分布面积最为辽阔。因此，胡桃楸的种源试验主要在东北地区进行。

3　胡桃楸种源区的划分原则

以种源试验中具有显著性差异的生长性状，适应性状和生理生化性状为主要指标，以阻碍基因交流的山脉、河流、分布区的断裂带以及植被区划、气候区划、行政区划等作为种源区的划界参考。划分的同一种源区是由遗传表型相似的一个或多个种源组成，组成种源区的种源应该是相临近的，划分成的每个种源区在地域上应该是连续的。

4　种源区的划分[6]

种源间（第二次试验）各类生物因子与地理因子的典型相关分析表明（见表 1），第一对典型变量的相关系数达到显著水平，第一对典型变量反映的信息量也最多，故采用第一典型变量得分值作二维排序图（见图 1），并根据各种源在图中的分布，划分出 4 个种源区。即长白山完达山种源区（编号Ⅰ）；吉林中部浅山种源区（编号Ⅱ）；辽宁省东部种源区（编号Ⅲ）；小兴安岭松花江地区种源区（编号Ⅳ）。

4.1　种源区划的合理性验证

首先用第二次种源试验的各种源的地理因子与气候因子进行典型相关分析，结果第一对典型相关系数（$\lambda_1=0.973\,1^{**}$）达到相关极显著，第二对典型相关系（$\lambda_2=0.844\,6$）接近相关显著水平。用代表性较强的第一对典型变量得分值作二维排序图，并根据各种源在图中的位置区划，其结果与种源区划结果相似（见图 2）。

另外，根据第一次种源试验中，历年的生长性状与第 6 年观测到的形态性状、适应性状进行主成分分析，前两个主分量的累计贡献率达到 76.65%。用前两个主分量作二

?1994-2016 China Academic Journal Electronic Publishing House. All rights reserved.　http://www.cnki.net

表 1　种源间各类生物因子与地理因子典型相关分析

相关系数<br>性　状	第一典型变量<br>λ_1=0.862 1*<br>载　荷	第二典型变量<br>λ_2=0.741 6<br>载　荷
生物因子	U_1	U_2
1年生树高	−0.370 86	−1.308 40
2年生树高	1.096 52	0.574 34
2年生地径	0.186 94	−0.122 77
保　存　率	0.567 97	0.193 93
晚霜危害率	1.137 45	−0.316 59
叶 绿 素 a	0.787 05	−0.440 75
叶 绿 素 b	−0.371 66	−0.304 15
呼 吸 速 率	−0.366 34	0.347 77
地 理 因 子	V_1	V_2
纬　　度	1.115 07	0.466 80
经　　度	−1.012 64	0.660 17

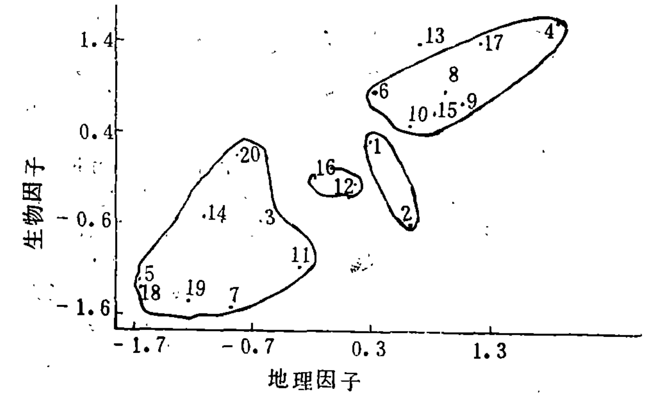

图 1　生物因子与地理因子第一典型变量得分值二维排序

1. 舒兰；2. 五常；3. 桦南；4. 绥棱；5. 八家子；6. 清河；7. 临江；8. 实县；9. 美溪；10. 帽儿山；11. 白石山，12　新宾；13. 本溪；14. 露水河；15. 凉水；16. 宽甸；17. 铁力；18. 汪清；19. 穆棱；20. 东京城。

维排序图，也可将参试种源划分4个种源区，且与上述划分结果相吻合（见图3）。

4.2　各种源区的范围及调种说明[7－8]

各种源区的范围见表2，种源区划见图4。

各地用种说明：①凡进行过种源试验的地区，可根据本地种源试验的结果选择最佳种源造林。②尚未进行过种源试验的地区，可根据临近地区的试验结果或选用本区的种源。

4.3　胡桃楸各种源区概况

各种源区的遗传、气候特点见表3。

?1994-2016 China Academic Journal Electronic Publishing House. All rights reserved.　http://www.cnki.net

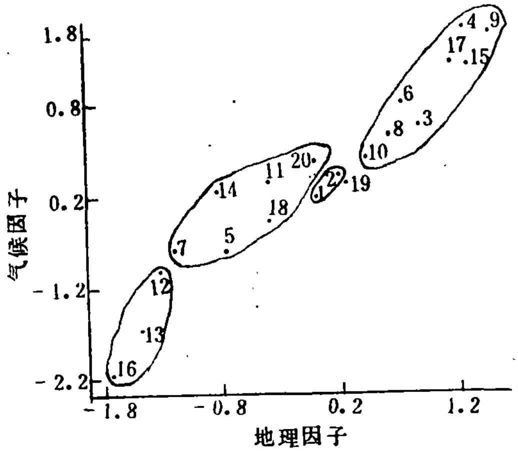

图 2 地理因子与气候因子典型变量得分值二维排序
1. 舒兰; 2. 五常; 3. 桦南; 4. 绥棱; 5. 八家子; 6. 清河; 7. 临江; 8. 宾县; 9. 苇溪; 10. 帽儿山; 11. 白石山; 12. 新宾; 13. 本溪; 14. 露水河; 15. 凉水; 16. 宽甸、17. 铁力; 18. 汪清; 19. 穆棱; 20. 东京城。

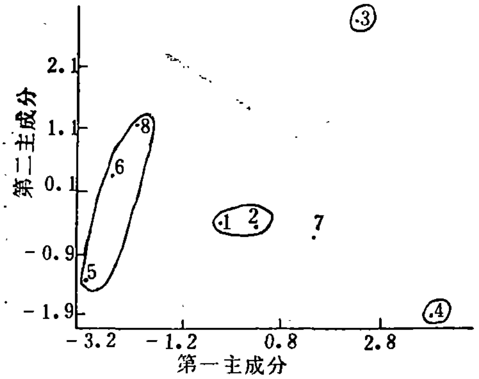

图 3 第一次种源试调各性状主成分分析排序
1. 带岭; 2. 帽儿山; 3. 宽甸; 4. 舒兰; 5. 桦南; 6. 东方红; 7. 临江; 8. 汪清。

表 2 种 源 区 的 范 围

种源区名称	编号	所含种源	范围(以市县为单位)
长白山完达山种源区	Ⅰ	穆棱东京城 桦南、露水河 白石山、临江市八家子汪清	佳木斯市、双鸭山市、鸡西市、牡丹江市绥化市、绥纷河市、浑江市、通化市、桦南县、勃利县、密山县、林口县、海林县、宁安县、穆棱县、东宁县、汪清县、珲春县和龙县、龙井县、抚顺县、长白县、集安县、桦甸县、靖宇县、蛟河县
吉林中部浅山区种源区	Ⅱ	舒兰 五常	吉林市、辽源市、开源市、铁岭市、口图县、舒兰县、五常县、伊通县、磐石县。
辽宁东部种源区	Ⅲ	本溪、宽甸 新宾	本溪市、丹东市、鞍山市、劳口市、恒仁县、宽甸县、庄河县、盖县、风城县复县、东钩县、新宾县。
小兴安岭松花江地区种源区	Ⅵ	清河 帽儿山 凉水、美溪 铁力、宾县 绥棱	伊春市、北安市、绥化市 黑河市、海伦县、孙克县 德都县、铁力县、依兰县 通河县、宾县、木兰县、方正县 尚志县、克山县、绥棱县、庆安县、延寿县

5 最佳种源的选择

对于最佳种源的选择，曾经根据第一次种源试验的3年生树高和保存率进行了初

?1994-2016 China Academic Journal Electronic Publishing House. All rights reserved. http://www.cnki.net

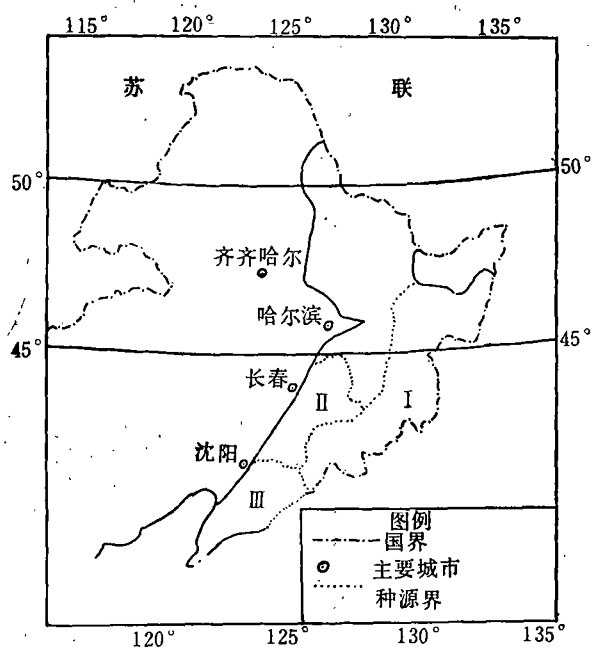

图 4　胡桃楸种源区划

选。如今，试验已达 6 年生，年但最佳种源未变，仍然为舒兰和宽甸两种源。

第二次种源试验，在增加了种源数量的情况下，用各种源高生长，冻害率和叶绿素 a 含量进行差异显著性测验（LSR 测验），重新选择最佳种源见表（4）。

表 3　胡桃楸各种源区基本特征

种源区	编号	遗传特点	气候特征 年均温（℃）	1月均温（℃）	7月均温（℃）	≥10℃ 积温	年降水（mm）
长白山完达山种源区	Ⅰ	生长中等适应性中等生长期中等	2.3<br>5.5	−17.1<br>−11.9	20.2<br>20.3	2 517.5<br>3 169.8	500<br>990
吉林中部种源区	Ⅱ	生长快适应性强生长期长叶绿素含量高，光合速率高	4.4<br>｜<br>6.4	−18.4<br>｜<br>−15.3	22.6<br>｜<br>23.7	2 861.6<br>｜<br>3 218.2	620<br>｜<br>650
辽宁东部种源区	Ⅲ	生长快适应性强叶绿素含量高光合速率高	6.4<br>｜<br>8	−15.3<br>｜<br>−12.3	23.7<br>｜<br>24.3	338.5<br>｜<br>347.7	800<br>｜<br>1 158
小兴安岭牡丹江地区种源区	Ⅳ	生长慢适应性差，叶绿素含量中等，呼吸速率高	−2.5<br>｜<br>−2.5	−23.9<br>｜<br>−20.5	20.0<br>｜<br>21.4	2 148<br>｜<br>2 454	500<br>｜<br>660

?1994-2016 China Academic Journal Electronic Publishing House. All rights reserved.　http://www.cnki.net

表 4 胡桃楸代表性状 LSR 测验结果

种源	树高	显著 0.05	标准 0.01	种源	叶绿素 a 含量	显著 0.05	标准 0.01	种源	冻害率	显著 0.05	标准 0.01
舒兰	37.2			舒兰	1.482			舒兰	0.57		
本溪	36.6			白石山	1.337			穆棱	0.66		
穆棱	33.7			本溪	1.312			白石山	0.68		
宽甸	32.7			新宾	1.308			本溪	0.74		
宾县	32.7			美溪	1.288			宽甸	0.75		
白石山	32.6			铁力	1.264			桦南	0.77		
清河	32.5			露水河	1.236			宾县	0.77		
绥棱	31.2			东京城	1.233			清河	0.80		
桦南	30.9			凉水	1.217			八家子	0.81		
五常	29.2			八家子	1.209			新宾	0.83		
临江	28.7			穆棱	1.193			汪清	0.83		
东京城	28.5			临江	1.180			帽儿山	0.83		
八家子	28.3			宽甸	1.175			五常	0.87		
帽儿山	28.2			五常	1.172			露水河	0.89		
铁力	28.1			帽儿山	1.167			临江	0.99		
凉水	28.0			汪清	1.093			凉水	0.90		
新宾	25.9			清河	1.092			绥棱	0.91		
露水河	25.6			桦南	1.050			东京城	0.91		
汪清	23.7			宾县	1.031			铁力	0.92		
美溪	23.1			绥棱	0.995			美溪	0.92		

从表 4 可见，高生长最大的仍然为舒兰种源，在0.05的显著性标准下，本溪和穆棱种源与舒兰种源间无显著差异，与其它所有种源差异显著，宽甸种源在所有参试的20个种源中，居第 4 位，表现也较好。各种源高生长相比较，舒兰和本溪种源分别高出当地对照种源（帽儿山）的 31.9%和 29.8%。高出最差种源（美溪）的 61.0%和 58.4%。

舒兰和本溪种源不但生长量最大，适应性也较强，舒兰种源的冻害率最低。本溪种源的冻率虽然高于舒兰种源，但它们之间差异不显著。舒兰和本溪种源的叶绿素 a 含量也较高。因此，可根据叶绿素含量作为种源早期选择的一个指标。

如果按照上述所选的优良种源（舒兰、本溪）占所有种源的百分率（入选率）和种源高生长方差分析的种源遗传力（$h^2=1-1/F$，F 为种源方差除以机误方差）为依据，进行种源的遗传增益估算，其结果为 8.6%。

6 胡桃楸种源试验早期选择的可靠性

根据第一次种源试验各年份树高间的相关分析表明（见表 5）：各年份树高间的相关系数均达相关显著或极显著水平，说明幼龄期的胡桃楸就有代表性，1 年生就可预测 6 年生的情况。

另外，根据胡桃楸22年生人工林早期选择的研究，如果以树高作为早期选择性状，最

?1994-2016 China Academic Journal Electronic Publishing House. All rights reserved. http://www.cnki.net

佳年龄定为6a。因此，本次研究所用1—2年生和6年生的材料是可靠的。

表5　1—6a树高相关系数表

年龄	1	2	3	4	5
2	0.9626**				
3	0.8535**	0.9029**			
4	0.8817**	0.9565**	0.9694**		
5	0.9198**	0.8943**	0.9297**	0.9052**	
6	0.7734	0.7308*	0.7975**	0.7324*	0.2773*

7 结　　论

7.1　分布在东北地区的胡桃楸可区划为4个种源区。即长白山完达山种源区（编号为Ⅰ）；吉林中部浅山区种源区（编号为Ⅱ）；辽宁东部种源区（编号为Ⅲ），小兴安岭松花江地区种源区（编号为Ⅳ）。其中第Ⅱ、第Ⅲ种源区为优良基因资源富集区。

7.2　两次种源试验，舒兰种源均为最佳。第二次试验中较好的还有本溪种源。第一次种源试验选出的宽甸种源。在第二次试验也较好。

7.3　如果选择舒兰和本溪种源在帽儿山造林，树高的遗传增益为8.6%。

参　考　文　献

1　杨书文，刘桂丰等．胡桃楸地理变异规律及最佳种源的初步选择．东北林业大学学报，1990，18（育种专刊）：83—76

2　杨书文，刘桂丰等．胡桃楸地理变异规律的再研究．东北林业大学学报，1991，19（育种专刊）

3　郑万钧．中国树木志．北京：中国林业出版社

4　中国科学院中国植物志编委会．中国植物志（第二十一卷）．北京：科学出版社，1978

5　周以良等．黑龙江树木志．哈尔滨：黑龙江科技出版社，1986

6　陈晓阳．典型相关分析在树种地理变异研究中的应用．北京林业大学学报，1990，12（3）：43—60

7　中国植被编辑委员会编著．中国植被．北京：科学出版社，1980

8　中华人民共和国林业部林业区划办公室主编．中国林业区划．北京：中国林业出版社，1987

?1994-2016 China Academic Journal Electronic Publishing House. All rights reserved.　http://www.cnki.net

THE PRIMARY DIVISION AND SELECTION FOR THE OPTIMAL PROVENANCE OF *JUGLANS MANDSHURICA*

Liu Guifeg Yang Shuwen Li Juntao
Liu Qiang Wang Huiren Peng Hongmei
(Northeast Forestry University)

ABSTRACT

On the basis of each characters of twice provenance tests for *Juglans mandshurica* by using canonical correlation analysis and principal component analysis, the provenances of *Juglans mandshurica* in north-eastern region are divided into four provenance districts: Changbaishan-wandashan provenance district; the low mountain provenance district in the middle of Jilin province; the provenance district of eastern Liaoning province; Lesser Xing'an mountain-Songhuajiang provenance district. The best provenance is regarded as the provenance in Shulan by twice provenance tests. In addition, the provenances of Benxi and Kuandian are also very good. If afforesting in Maoershan with the two provenances of shulan and Benxi, 8.6% of genetic gains can be obtained.

Descriptors: *Juglans mandshurica*; Provenance test; Provenance division; Selection for the optimal provenances

?1994-2016 China Academic Journal Electronic Publishing House. All rights reserved. http://www.cnki.net
