# Supplementary material for: Provenance and family variations in early growth of Manchurian walnut (Juglans mandshurica Maxim.) and selection of superior families
Source: PLoS One. 2024 Mar 7;19(3):e0298918. doi: 10.1371/journal.pone.0298918 (PMC10919699; doi:10.1371/journal.pone.0298918)
Supplement: S2 File — (ZIP) [file pone.0298918.s005.zip › Production area division of secondary forest of Juglans mandshurica in the central and eastern regions of Northeast China.pdf]

罗也,杨雨春,赵珊珊,任生,许长有,朱爱玲. 2022. 东北中东部胡桃楸天然次生林产区区划. 生态学杂志,**41**(12):2299-2305.

Luo Y, Yang YC, Zhao SS, Ren S, Xu CY, Zhu AL. 2022. Production area division of secondary forest of *Juglans mandshurica* in the central and eastern regions of Northeast China. *Chinese Journal of Ecology*, **41**(12):2299-2305.

东北中东部胡桃楸天然次生林产区区划

罗 也[1,2] 杨雨春[1,2*] 赵珊珊[3] 任 生[3] 许长有[4] 朱爱玲[4]

([1]吉林省林业科学研究院,长春 130033;[2]吉林省退化森林生态系统恢复与重建跨区域合作科技创新中心,长春 130033;[3]吉林省林业调查规划院,长春 130022;[4]柳河县林业局,吉林通化 135300)

摘 要 为准确了解东北中东部地区胡桃楸天然次生林产区分布情况,选取年均温、极端高温、极端低温、年降水量、年均日照,无霜期、积温等作为影响胡桃楸材积生长的气候因子,采用逐步回归分析法,确定影响胡桃楸材积的主导因子,通过数学模型模拟,筛选出最优模型并进行精度检验,进而划分胡桃楸天然次生林产区。结果表明:(1)年降水量和年均温为影响胡桃楸材积生长的主导因子,且材积与主导因子的最优数学模型为德林科所模型,$R^2=0.89$,赤池信息量标准 $AIC=640.07$,对数似然函数 $LL=-67.16$;(2)通过理论材积值与实际材积值相对误差比较分析,数学模型精度满足要求;(3)胡桃楸天然次生林最终划分为最适宜分布区、适宜分布区和一般分布区3个产区;(4)对胡桃楸3类产区的理论材积值进行单因素方差分析,3类产区之间差异达到极显著水平($P<0.01$),表明胡桃楸3类产区区划合理,结果准确可靠,研究结果可为胡桃楸天然次生林的合理经营和布局提供科学依据。

关键词 胡桃楸;材积;主导因子;逐步回归分析;产区区划

Production area division of secondary forest of *Juglans mandshurica* in the central and eastern regions of Northeast China. LUO Ye[1,2], YANG Yu-chun[1,2*], ZHAO Shan-shan[3], REN Sheng[3], XU Chang-you[4], ZHU Ai-ling[4] ([1] *Forestry Academy of Jilin Province*, *Changchun* 130033, *China*; [2] *Interregional Cooperation Science and Technology Innovation Center for Restoration and Reconstruction of Degraded Forest Ecosystem in Jilin Province*, *Changchun* 130033, *China*; [3] *Jilin Provincial Forestry Investigation and Planning Institute*, *Changchun* 130022, *China*; [4] *Forestry Bureau of Liuhe County*, *Tonghua* 135300, *Jilin*, *China*).

Abstract: Division of production areas of the secondary *Juglans mandshurica* forests is important for sustainable forest management in the central and eastern regions of Northeast China. Based on the data of annual mean temperature, extremely high temperature, extremely low temperature, mean annual precipitation, mean annual sunshine, frost-free period, and accumulated temperature, we examined the impacts of climate on the volume growth of *J. mandshurica* forests. The dominant factors affecting the volume of *Juglans mandshurica* were determined using stepwise regression analysis. Through mathematical model simulation, the optimal model was selected and its precision was tested, and the secondary forest area of *J. mandshurica* was divided. The results showed that: (1) Mean annual precipitation and mean annual temperature were the dominant factors affecting the volume growth of *J. mandshurica*, while the Delinco model was the best for describing the relationship between timber volume and the dominant factors ($R^2=0.89$, $AIC=640.07$, $LL=-67.16$). (2) The accuracy of the mathematical model met the requirements by comparing and analyzing the relative error between the theoretical and actual volume values. (3) Based on the best-predicted model, the secondary *J. mandshurica* forests were divided into three production areas: the most suitable distribution area, the suitable distribution area, and the general distribution area. (4) Furthermore, a one-way analysis of variance revealed that the theoretical volume values of the three production areas were significantly different from each other ($P<0.01$), suggesting that the divisions of the three production areas was accurate and reliable. These results provide insights for rational management and distribution of secondary *J. mandshurica* forests.

Key words: *Juglans mandshurica*; timber volume; dominant factor; stepwise regression analysis; production area division.

国家"十三五"重点研发计划项目(2017YFD0600605)、吉林省科技厅项目(20160203010NY,20190303072SF)和吉林省林业科技项目(2014-006)资助。

收稿日期:2021-08-10 接受日期:2022-04-08

*通讯作者 E-mail:yang-yu-chun@163.com

(C)1994-2023 China Academic Journal Electronic Publishing House. All rights reserved. http://www.cnki.net

产区区划是按照树种的生物学特性、产量及地域进行的划分，既从客观上反映不同地区的水热条件对林分生长的影响，又将各地区实际的和潜在的生长情况加以分析比较，从而通过不同的生长特点对各区域进行划分。我国早在 20 世纪 80 年代开始产区区划研究，主要包括农作物、中药材、果树和林业等。农作物产区划分研究目前主要以小麦（*Triticum aestivum*）、水稻（*Oryza sativa*）和棉花（*Gossypium*）为主，其中黑龙江地区小麦产区以综合指标编码值为基础方法，结合当地气候情况进行划分（祖世亨等，2001），北方小麦（管伟豆等，2021）和长江中下游水稻（程菁靓等，2019）以自然条件和产地进行产区划分。鄱阳湖地区棉花（林春等，2010）的产区则在自然条件的基础上，结合遥感影像数据进行划分，划分精确度更加可靠。中药材产区划分的方法主要以地理信息系统为平台，多采用空间聚类进行划分，方法相对单一，如芍药（*Paeonia lactiflora*）（魏志华等，2011a）、冬凌草（*Rabdosia rubescens*）（王新民等，2008）、杜仲（*Eucommia ulmoides*）（何方等，2010）、拓首乌（*Polygonum multiflorum*）（魏志华等，2011b）和裕丹参（*Salvia miltiorrhiza*）（魏志华等，2011c）等。果树产区划分与农作物的区划方法基本一致，如板栗（*Castanea mollissima*）（板栗丰产林标准化协作组，1989），而葡萄（*Vitis vinifera*）区划，需考虑不同品种葡萄的适宜产区，以便为葡萄酒的制造提供较好的原材料（Winkler *et al.*，1974；Coombe *et al.*，1987；翟衡等，2018）。对林业树种进行产区划分意义非常重要，从产区划分的提出至今，为合理经营各类树种，应用不同方法进行了大量树种的产区划分，在初级阶段，主要以自然条件为基础，通过气候因素和产地分布信息对安徽地区刺槐（*Robinia pseudoacacia*）（刘桂华等，1996）、河南杉木（*Cunninghamia lanceolata*）（魏岚等，2009）和黄连木（*Pistacia chinensis*）（任银玲等，2012）进行划分，随后为准确划分产区，采用了不同的划分方法。

数量化分类结合聚类分析是常用方法之一，应用该方法对湖北马尾松（*Pinus massoniana*）（庄尔奇等，1997）、浙江马尾松（高智慧等，1991）、西南地区葰竹（*Phyllostachys nidularia*）（黄甫昭等，2012）和苦楝（*Melia azedarach*）（程诗明等，2005）进行了产区划分，划分结果相较于只考虑产地分布而言，其精确度较高；逐步回归分析法也是常用方法之一，通过逐步回归分析，确定众多影响因子中主导因子，既准确又简便地对产区进行了划分，如四川粗枝云杉（*Picea asperata*）（四川省粗枝云杉纸浆材协作组，2001）、福建柳杉（*Cryptomeria japonica*）（庄晨辉等，1998）和东北地区落叶松（*Larix gmelinii*）、红松（*Pinus koraiensis*）、樟子松（*Pinus sylvestris* var. *mongolica*）（陈效群等，1990）的产区划分，其结果准确可靠。因此，本研究结合胡桃楸自然条件分布情况，采用逐步回归分析法。

胡桃楸（*Juglans mandshurica*）为胡桃科（Juglandaceae）胡桃属（*Juglans*）落叶乔木，是东北“三大硬阔”树种之一，主要分布于我国黑龙江、吉林、辽宁等东北中东部地区。胡桃楸材质坚硬，纹理通直，果实营养丰富，含油率高，是珍贵的用材和经济林树种之一（Chen *et al.*，2003；Hu *et al.*，2016）。由于过度的利用与采伐，现存胡桃楸林主要以天然次生混交林为主（张丽鹏等，2014；唐丽丽等，2019），其林地生产力低下。为更好地利用胡桃楸价值，相关学者进行了大量实验，包括胡桃楸遗传育种、栽培技术、资源开发、经济价值、立地指数、生物多样性和群落结构等方面的相关研究（陈思羽等，2015；Song *et al.*，2017；Salahuddin *et al.*，2018；及利等，2019；唐丽丽等，2019；罗也等，2019，2020a）。但对于东北中东部胡桃楸主要分布区划分方面研究未见报道。因此，本研究通过对东北中东部 21 个地区胡桃楸天然次生林材积和气候因子的相关性分析，采用逐步回归分析法，确定影响胡桃楸材积的主导因子，通过多数学模型模拟，筛选出最优模型并进行精度检验，进而划分胡桃楸天然次生林产区，为胡桃楸天然次生林合理经营与布局提供科学的理论依据。

1 研究地区与研究方法

1.1 研究区概况

研究区设在东北中东部地区胡桃楸天然次生林分布区域，主要分布在黑龙江省、吉林省和辽宁省，地理坐标为 123°58′13″E—130°24′10″E，40°52′25″N—46°48′50″N，该研究区域主要为大陆性季风气候，冬季寒冷干燥，夏季湿润多雨。该地区植被类型复杂，多以天然次生林分布为主，主要乔木树种有胡桃楸、水曲柳（*Fraxinus mandshurica*）、蒙古栎（*Quercus mongolica*）、紫椴（*Tilia amurensis*）、花曲柳（*Fraxinus rhynchophylla*）、春榆（*Ulmus pumila*）、色木槭（*Acer mono*）、白桦（*Betula platyphylla*）等。

1.2 数据调查

本研究在东北中东部地区胡桃楸主要分布区进行调查，共 21 个县市，布设 197 块样地，样地采用样

(C)1994-2023 China Academic Journal Electronic Publishing House. All rights reserved. http://www.cnki.net

圆法进行调查,半径为 17.85 m,对样圆内所有 DBH ≥5 cm 的活立木进行调查、挂号并定位,调查树种名称、胸径、树高等,记录每块样地的经纬度、海拔等相关信息,在每个县市布设样地时,充分考虑立地因素、林龄、林分密度和干扰情况等因子,布设上述因子的重复样地,消除非气候因素影响。林木材积根据各省立木材积表查得,各气候因子采用各县局 40 年内气象因子统计数据平均值(中国地面气候资料),并根据胡桃楸自身生物学特性,选择出年均温、极端高温、极端低温、年降水量、年均日照,无霜期、积温等 7 个气候因子进行分析,基本情况如表 1。

1.3 回归方程的建立

气候是影响林木生长的环境因子之一,虽然各气候因子对林木生长均起作用,但所起的作用不完全相同,其中包含一些主导因子,因此为准确了解影响胡桃楸生长的主导因子,首先需要建立以材积为因变量(Y_i),以各气候因子为自变量(X_i)的回归方程:

$$Y = b_0 + \sum_{i=1}^{n} b_i X_i$$

其次建立材积与主导因子之间的最优模型,通过最优模型的选择,进行精度检验与产区划分。

1.4 模型精度检验

将得到的主导因子带入到材积理论最优方程中,计算得到胡桃楸理论材积值,同时将理论材积值与实测材积值进行比较,并计算相对误差值。

1.5 产区划分

由于胡桃楸的标准年龄(A_0)为 50 龄(罗也等,2019),将每个县市的材积与年龄进行拟合(模型略去),计算出 50 龄的材积,并结合气候因子-材积数学回归模型进行产区划分。

1.6 区划原则

1.6.1 行政界线完整性原则 为了便于调查研究和经营管理,合理完成胡桃楸生长生产任务,使得区划成果得以稳步实施,因此在符合自然规律和遵守自然界限的前提下,产区区划力求保持县市行政界线的相对完整性。

1.6.2 综合因子和主导因子原则 胡桃楸材积生长受到综合因子的影响,区划时应考虑综合生境状况,但是各因子之间对于胡桃楸材积生长的影响不一,在诸多水、热等因子中,某些因子起到主导作用,与胡桃楸材积生长密切相关,因此区划时既要全面考虑综合因子效应,又要突出主导因子的作用。

1.6.3 区划依据 胡桃楸的生长因不同生境和气候环境的差异而有所不同,在不同条件下,胡桃楸材

表 1 不同地区胡桃楸材积与气候因子关系

Table 1 Relationship between volume of *Juglans mandshurica* and climate factors in different regions

地点 Location	材积 Volume ($m^3 \cdot hm^{-2}$)	海拔(x_1) Altitude (m)	年均温(x_2) Annual average temperature (℃)	极端高温(x_3) Extreme high temperature (℃)	极端低温(x_4) Extreme low temperature (℃)	年降水量(x_5) Annual precipitation (mm)	年均日照(x_6) Annual average sunshine (h)	无霜期(x_7) Frost-free period (d)	积温(x_8) Accumulated temperature (≥10 ℃)
铁力	73.04	353	2.4	36.3	-42.6	530.0	2420.0	128	2249.0
林口	60.21	406	4.5	37.8	-35.2	530.3	2400.0	123	2300.0
尚志	108.69	317	2.3	35.4	-41.0	666.1	2446.9	120	2400.0
穆棱	57.43	559	5.1	38.0	-35.0	540.0	2305.0	126	2600.0
海林	65.04	438	4.2	37.6	-38.8	536.3	2388.9	131	2416.2
五常	90.87	298	3.5	35.6	-45.5	625.0	2629.0	130	2650.0
蛟河	87.86	463	3.4	35.8	-40.5	708.8	2300.0	125	2525.0
汪清	60.04	491	4.9	37.5	-37.5	574.9	2234.0	126	2233.0
桦甸	91.28	493	3.9	36.3	-45.0	748.1	2379.0	125	2731.0
珲春	59.89	110	5.6	36.3	-32.5	617.9	2246.8	150	2220.0
辉南	89.35	637	5.0	35.0	-37.0	737.4	2296.0	138	2500.0
靖宇	93.39	624	3.7	36.0	-42.2	767.3	2259.0	104	2224.2
抚松	107.43	770	4.0	44.1	-34.8	800.0	2352.5	115	2274.9
和龙	53.81	604	5.6	36.2	-31.5	573.6	2387.2	138	2300.0
临江	110.64	594	3.0	36.0	-33.7	880.0	2600.0	148	2400.0
集安	94.89	719	6.0	36.0	-33.0	788.0	2319.0	129	2550.0
清源	79.20	646	5.4	37.2	-37.3	781.0	2433.0	133	2720.0
抚顺	70.38	446	7.0	38.1	-40.5	753.2	2485.0	145	2950.0
新宾	82.73	659	4.7	36.0	-34.0	769.6	2450.0	130	2800.0
本溪	82.98	401	6.9	37.5	-33.6	850.0	2589.0	140	2820.0
恒仁	77.47	553	6.4	36.3	-25.0	710.6	2685.6	156	2700.0

(C)1994-2023 China Academic Journal Electronic Publishing House. All rights reserved. http://www.cnki.net

积能够直接反映其生长适宜状况,因此,根据胡桃楸材积大小差异并结合实际生长情况,确定胡桃楸产区分布情况。

1.7 数据分析

实验数据和表格建立均采用 Excel 2007 进行整理,采用 SPSS 19.0 和 SigmaPlot 12.5 对数据进行逐步回归分析、单因素方差分析和模型建立与选择。

2 结果与分析

2.1 不同地区胡桃楸材积与各因子之间关系

通过多元逐步回归分析,剔除对因变量影响不显著的自变量,保留影响显著的自变量,从而建立简单、实用的材积与气候因子之间的数学模型。通过相关性分析发现(表 2),年均温和年降水量对胡桃楸材积影响显著($P<0.05$),其他因子影响不显著。且年降水量对胡桃楸材积的影响相较于年均温更显著,其中年均温与材积呈显著负相关关系,年降水量与材积呈显著正相关。

为建立材积与年降水量和年均温的最优模型,列举了拟合效果较好的 6 个模型(表 3),由表可知,德林科所式模型拟合效果最好,R^2 值最大,达到 0.89,赤池信息量标准(AIC)值最小,为 640.07,对数似然函数(LL)值最大,为−67.16,其方程式为:$y=-3012.7136-32.8227\ln x_2-0.4334\ln^2 x_2+880.5408\ln x_5-61.0765\ln^2 x_5$,式中 y 为材积,x_2 为年均温,x_5 为年降水量。

2.2 模型精度检验

通过模型精度检验发现(表 4),各地区胡桃楸材积理论值与实测值之间相对误差,均在 10.0%以下,计算结果表明该方程的精度能够满足要求。

2.3 胡桃楸天然次生林产区划分

根据气候因子-材积数学回归模型,结合胡桃楸的标准年龄(A_0)为 50 龄时的材积,得出胡桃楸天然次生林分布区内各林分每公顷材积。现将东北中东部地区胡桃楸天然次生林划分为三大产区(表 5,图 1),产区 I 为胡桃楸次生林生长最适宜分布区域,每公顷林木材积主要在 85 m^3 以上,年降水为 600~900 mm,年均温 1.0~5.5 ℃,该区域主要包括黑龙江省的尚志市、五常市,吉林省的舒兰市、蛟河市、桦甸市、辉南县、靖宇县、梅河口市、抚松县、柳河县、通化县、集安市、临江市、长白县、江源区。产区 II 为胡桃楸次生林生长适宜分布区域,每公顷林木材积在 70~85 m^3,年降水为 550~850 mm,年均温 3.0~7.0 ℃,该区域主要包括黑龙江省的铁力市、通河县、依兰县、方正县、宾县、阿城、延寿县,吉林省的敦化市、安图县,辽宁省的清源县、新宾县、本溪县、抚顺市、恒仁县。产区 III 为胡桃楸次生林生长一般分布区域,每公顷林木材积在 70 m^3 以下,年降水为 500~650 mm,年均温 4.0~6.0 ℃,该区域主要包括黑龙江省的林口县、海林市、穆棱市、宁安市、绥芬河

表 2 胡桃楸材积与气候因子相关性

Table 2 Correlation between volume of *Juglans mandshurica* and climate factors

	海拔(x_1) Altitude	年均温(x_2) Average annual temperature	极端高温(x_3) Extreme high temperature	极端低温(x_4) Extreme low temperature	年降水量(x_5) Annual precipitation	年均日照(x_6) Average annual sunshine	无霜期(x_7) Frost-free period	积温(x_8) Accumulated temperature (≥10 ℃)
样本数量 Samples number	21	21	21	21	21	21	21	21
相关性 Pearson	0.034	−0.447	0.019	−0.226	0.718	0.255	−0.246	0.089
Sig. P	0.090	0.021*	0.467	0.162	0.000*	0.132	0.142	0.351

注:* 表示在 0.05 水平上差异显著。

Note: * Indicates a significant difference at the 0.05 level.

表 3 胡桃楸材积与年降水量和年均温拟合模型

Table 3 Fitting model of volume of *Juglans mandshurica* with annual precipitation and average annual temperature

方程式名称 Equation name	表达式 Expression	参数 Parameter determination a	b	c	d	e	R^2	AIC	LL
迈耶式	$y=a+bx_2+cx_2x_5+dx_2^2x_5+ex_5$	49.5006	−12.4759	0.0004	0.0007	0.1117	0.8370	740.2987	−77.6814
孟宪宇式	$y=a+bx_2^2x_5+cx_2^3x_5+dx_2^2x_5\ln x_5$	18.0319	0.0390	0.0061	−0.0410	-	0.6479	1764.0744	−185.1878
山本和藏式	$y=ax_2^bx_5^c$	0.1671	−0.3883	1.0336	-	-	0.8430	802.1657	−84.1974
德林科所式	$y=a+b\ln x_2+c\ln^2x_2+d\ln x_5+e\ln^2x_5$	−3012.7136	−32.8227	−0.4334	880.5408	−61.0765	0.8861	640.0665	−67.1570
赵克升式	$y=ax_2^2x_5+bx_2^3x_5+cx_2^2+dx_2^2x_5\ln x_2$	0.0466	0.0068	−0.1556	−0.0471	-	0.6701	1950.2589	−204.7372
逐步回归式	$y=a+bx_2+cx_5$	27.3120	−7.7020	0.1290	-	-	0.8610	788.8105	−82.7951

注:AIC:赤池信息量标准;LL:对数似然函数。

Note: AIC: Akaike's information criterion; LL: Log-likelihood.

(C)1994-2023 China Academic Journal Electronic Publishing House. All rights reserved. http://www.cnki.net

表 4　胡桃楸理论材积与实际材积精度分析

Table 4　Accuracy analysis between theoretical and actual volume of *Juglans mandshurica*

地点 Location	理论材积值 Theoretical volume ($m^3 \cdot hm^{-2}$)	实测材积值 Actual volume ($m^3 \cdot hm^{-2}$)	相对误差 Relative error (%)
铁力	78.44	73.04	7.40
林口	57.23	60.21	4.95
尚志	102.80	108.69	5.41
穆棱	55.00	57.43	4.23
海林	60.86	65.04	6.43
五常	82.90	90.87	8.77
蛟河	94.76	87.86	7.85
汪清	63.14	60.04	5.17
桦甸	94.17	91.28	3.17
珲春	65.78	59.89	9.83
辉南	84.65	89.35	5.26
靖宇	97.75	93.39	4.67
抚松	97.88	107.43	8.89
和龙	58.33	53.81	8.41
临江	113.18	110.64	2.29
集安	88.03	94.89	7.23
清源	86.06	79.20	8.66
抚顺	74.62	70.38	6.03
新宾	89.81	82.73	8.56
本溪	82.86	82.98	0.15
恒仁	73.35	77.47	5.32

市、东宁县，吉林省的汪清县、珲春市、图们市、龙井市、和龙市、延吉市。

2.4　产区差异显著性检验

对以上划分的胡桃楸 3 类产区的理论材积值进行单因素方差分析，结果见表 6，由表可知，F 值为 43.59，通过查 0.01 水平上 F 界值表可得 $F_{0.01}=6.01$（$F>F_{0.01}$），3 类产区之间差异达到极显著水平（$P<0.01$），表明胡桃楸 3 类产区区划合理，结果可靠。

3　讨论与结论

产区区划是林业生产与经营的基础，研究产区区划可以为林分的合理布局提供理论依据，因此，许多学者研究了不同树种在不同地区的区划分布，其中多数学者主要以材积生长量、生产力、胸径年轮宽、平均优势高和立地类型等为基础进行划分，划分结果适用于特定树种。而胡桃楸为主要用材和经济树种，其生长状况以材积体现最为明显（孙楠等，2015；罗也等，2019），因此根据以往相关研究方法和结果，结合胡桃楸树种实际生长情况，本研究以胡桃楸天然次生林每公顷林分材积为基础，同时结合年均温、极端高温、极端低温、年降水量、年均日照，无霜期、积温等气候因子，对东北中东部地区胡桃楸天然次生林进行产区区划。

表 5　胡桃楸天然次生林产区划分

Table 5　Production area division natural secondary forest of *Juglans mandshurica*

产区 Production area	材积 volume ($m^3 \cdot hm^{-2}$)	地点 Site	水热条件 Hydrothermal conditions 年降水 Annual precipitation (mm)	年均温 Average annual temperature (℃)
Ⅰ	>85	黑龙江省尚志市、五常市；吉林省舒兰市、蛟河市、桦甸市、辉南县、靖宇县、梅河口市、抚松县、柳河县、通化县、集安市、临江市、长白县、江源区	600~900	1.0~5.5
Ⅱ	70~85	黑龙江省铁力市、通河县、依兰县、方正县、宾县、阿城、延寿县；吉林省敦化市、安图县；辽宁省清源县、新宾县、本溪县、抚顺市、恒仁县	550~850	3.0~7.0
Ⅲ	<70	黑龙江省林口县、海林市、穆棱市、宁安市、绥芬河市、东宁县；吉林省汪清县、珲春市、图们市、龙井市、和龙市、延吉市	500~650	4.0~6.0

研究发现，气候因子是影响树种材积生长的主要因素，年均温和年降水量显著影响材积生长，对于不同树种，年均温和年降水量存在促进或抑制作用（康洪梅等，2018；丁晓娟等，2016），极端高温和极端低温对树种普遍存在抑制作用（董立民，1988；丁晓娟等，2016），积温在树种不同年龄发育阶段影响程度不同（胡继文等，2019），年均日照和无霜期较其他气候因子影响较小，但同样起到非常重要的作用（董立民，1988；胡继文等，2019）。因此，气候因子对于不同树种的生长至关重要，且不同地区不同树种的主导影响因子不同。

表 6　各产区间材积方差分析

Table 6　Variance analysis of volume in each production area

误差来源 Source of error	平方和 Sum of square	自由度 Degree of freedom	均方 Mean square	F	P
组间 Between groups	4318.56	2	2159.28	43.59	0.00
组内 Inside group	891.71	18	49.54		
总数 Total	5210.27	20			

本研究通过逐步回归分析、数学模型选择和误差检验，得到年降水量和年均温是影响胡桃楸天然

(C)1994-2023 China Academic Journal Electronic Publishing House. All rights reserved.　http://www.cnki.net

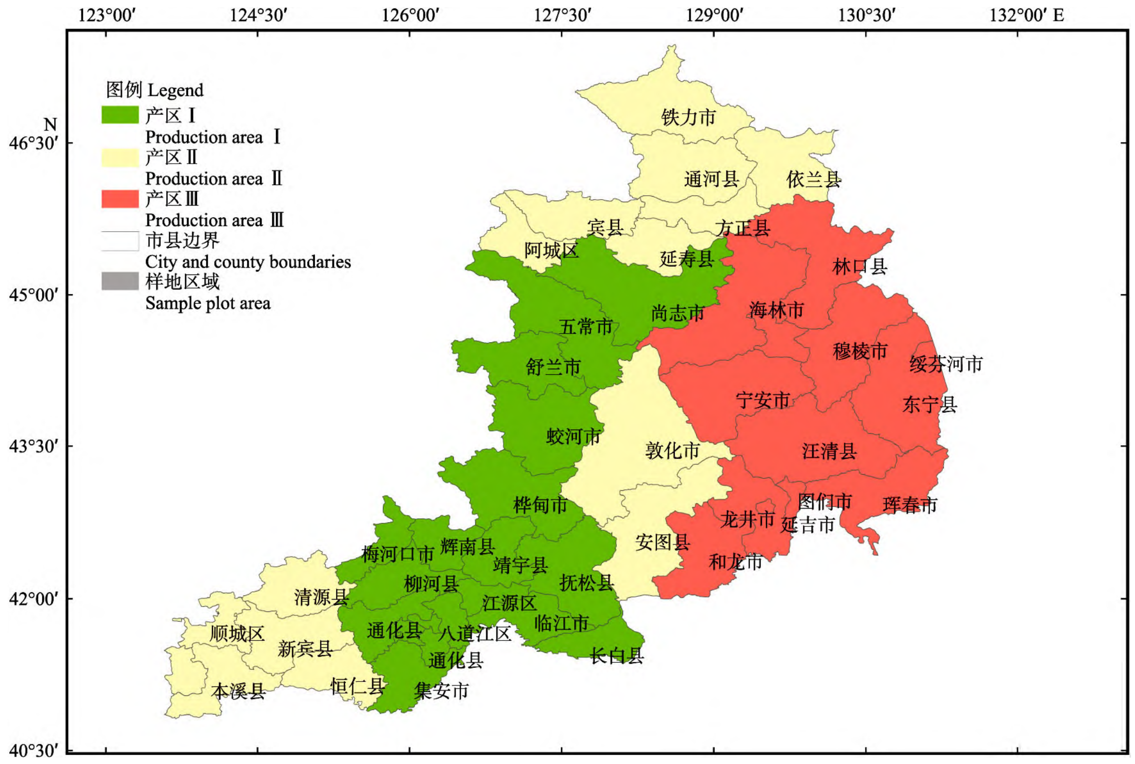

图 1 产区区划示意图

Fig.1 Schematic diagram of production area division

次生林林分材积的主导因子。同时,年降水量和年均温对各树种材积影响不一。影响毛白杨(董立民,1988)、香椿(胡继文等,2019)、樟子松(沈海龙等,1995)等材积生长量的主要气候因子均为年降水量,且香椿和樟子松材积生长量与年降水量呈显著正相关关系;影响云杉(刘铮等,2014)、湿地松(徐有明等,2001)等材积生长量的主要气候因子为年均温,且主要呈负相关关系。贾宏炎等(2019)研究发现,年降水量和年均气温是影响大青山柚木材积生长量的两个主要气象因子;丁晓娟等(2016)研究发现,西伯利亚落叶松材积与年降水量呈极显著正相关,与年均温呈显著负相关。本研究同样发现,胡桃楸材积与年降水量呈极显著正相关关系,与年均温呈显著负相关关系。而康洪梅等(2018)研究发现,云南蓝果树材积生长量与年均温呈正相关,与降水量呈负相关,这与本研究所得结果正好相反,出现以上结果原因可能与纬度、立地和树种有关,需进一步研究。

本研究通过年降水量、年均温与材积关系的分析比较,最终共得到 3 个产区(产区Ⅰ、Ⅱ、Ⅲ),产区Ⅰ为胡桃楸次生林生长最适宜分布区域,产区Ⅱ为胡桃楸次生林生长适宜分布区域,产区Ⅲ为胡桃楸次生林生长一般分布区域,通过对胡桃楸 3 类产区的理论材积值进行单因素方差分析表明,3 类产区之间理论材积达到极显著水平,证明胡桃楸 3 类产区区划合理。该产区区划结果可靠,能够代表东北地区胡桃楸天然次生林产区分布情况,该结果可为东北中东部地区胡桃楸天然次生林合理经营与布局提供科学依据。

参考文献

板栗丰产林标准化协作组. 1989. 我国板栗产区的划分及丰产栽培技术要点. 经济林研究, **7**(1): 102-104.

陈思羽, 杨 辉, 韩 姣, 等. 2015. 长白山区核桃楸结实性状种源变异分析. 北京林业大学学报, **37**(12): 32-40.

陈效群, 仲崇祺, 杨 凯. 1990. 落叶松、红松、樟子松产区区划的研究. 林业科技, **69**(3): 3-6+9.

程菁靓, 赵 龙, 杨 彦, 等. 2019. 我国长江中下游水稻产区铅污染分区划分方法研究. 农业环境科学学报, **38**(1): 70-78.

程诗明, 顾万春. 2005. 苦楝中国分布区的物候区划. 林业科学, **41**(3): 186-191.

丁晓娟, 陈蜀江, 黄铁成, 等. 2016. 阿尔泰山南坡西伯利亚落叶松生长量与气候变化的关系. 干旱区资源与环境, **30**(2): 98-103.

董立民. 1988. 杨树生长量与气候条件的关联度分析. 西北林学院学报, **3**(2): 73-79.

高智慧, 柴锡周, 周 琪, 等. 1991. 浙江省马尾松产区区划研究. 浙江林业科技, **11**(4): 1-9.

管伟豆, 肖 然, 李荣华, 等. 2021. 土壤镉污染北方小麦生

(C)1994-2023 China Academic Journal Electronic Publishing House. All rights reserved. http://www.cnki.net

产阈值及产区划分初探. 农业环境科学学报, **40**(5): 969-977.
何　方, 张康健, 王承南, 等. 2010. 杜仲产区的划分. 经济林研究, **28**(2): 86-87.
胡继文, 麻文俊, 沈元勤, 等. 2019. 香椿无性系苗期生长及早期选择研究. 林业科学研究, **32**(4): 165-170.
黄甫昭, 吕大勇, 王福升, 等. 2012. 西南地区篌竹主产区立地类型划分. 南京林业大学学报(自然科学版), **36**(2): 142-146.
贾宏炎. 2019. 广西大青山柚木人工林生长过程研究. 林业科学研究, **32**(1): 97-105.
及　利, 韩　姣, 王　芳, 等. 2019. 干旱胁迫对不同土壤基质下核桃楸幼苗的生理特性的影响. 植物研究, **39**(5): 722-732.
康洪梅, 张珊珊, 史富强, 等. 2018. 主要气候因子对极小种群野生植物云南蓝果树生长的影响. 东北林业大学学报, **46**(7): 23-27.
林　春, 辜晓青, 祝必琴. 2010. 鄱阳湖区棉花种植气候区划. 气象与减灾研究, **33**(1): 58-62.
刘桂华, 李宏开. 1996. 安徽省长江以北刺槐产区区划. 安徽农业大学学报, **23**(4): 536-541.
刘　铮, 白　英, 赵传燕, 等. 2014. 气候影响下祁连山自然保护区青海云杉林材积生长量差异性研究. 干旱区资源与环境, **28**(7): 171-176.
罗　也, 及　利, 杨雨春, 等. 2020a. 东北地区胡桃楸次生混交林乔木物种组成和多样性. 生态学杂志, **39**(9): 2887-2895.
罗　也, 王　君, 杨雨春, 等. 2020b. 利用随机效应模型模拟东北三省胡桃楸地位指数. 应用生态学报, **31**(8): 2549-2557.
罗　也, 杨雨春, 王　君, 等. 2019. 吉林省长白山区胡桃楸天然次生混交林立地指数模型. 应用生态学报, **30**(12): 4049-4058.
任银玲, 李中方, 张　翔, 等. 2012. 河南省生物质能源树种黄连木的种植区划. 贵州农业科学, **40**(6): 164-168.
沈海龙, 李世文, 胡详一, 等. 1995. 东北东部山地樟子松生长与气候因子的相关分析. 东北林业大学学报, **23**(3): 33-39.
四川省粗枝云杉纸浆材协作组. 2001. 粗枝云杉人工林产区区划研究. 四川林业科技, **22**(2): 73-78.
孙　楠, 李兴安, 张怡春. 2015. 采伐强度对水胡黄硬阔叶混交林主要树种及林下更新生长的影响. 林业科技, **40**(1): 30-33.
唐丽丽, 张　梅, 赵香林, 等. 2019. 华北地区胡桃楸林分布规律及群落构建机制分析. 植物生态学报, **43**(9): 753-761.
王新民, 谢彩香, 陈士林, 等. 2008. 冬凌草适宜产区区划研究. 安徽农业科学, **36**(31): 13677-13680.
魏　岚, 赵丰华. 2009. 河南杉木产区区划. 赤峰学院学报(自然科学版), **25**(5): 80-81.
魏志华, 王新民, 乔卿梅, 等. 2011a. 亳芍药材适宜产区区划研究. 北方园艺, (11): 170-172.
魏志华, 王新民, 乔卿梅, 等. 2011b. 柘首乌药材适宜产区区划研究. 特产研究, **33**(2): 25-27.
魏志华, 王新民, 乔卿梅, 等. 2011c. 裕丹参药材适宜产区区划研究. 广东农业科学, **38**(17): 24-25+237.
徐有明, 鲍春红, 周志翔, 等. 2001. 湿地松种源生长量、材性的变异与优良种源综合选择. 东北林业大学学报, **29**(5): 18-21.
翟　衡, 王赵盼, 杜远鹏. 2018. 我国葡萄气候区划及酿酒葡萄优势产区评价. 中国果树, (3): 5-12.
张丽鹏, 杨雨春, 赵珊珊, 等. 2014. 环境因子对长白山区天然核桃楸林生长的影响. 中国农学通报, **30**(4): 34-41.
庄晨辉, 严思钟, 李闽丽. 1998. 柳杉产区区划研究. 华东森林经理, **12**(1): 44-48.
庄尔奇, 刘　强, 高方彬, 等. 1997. 湖北省马尾松产区区划研究. 华中农业大学学报, **16**(1): 80-86.
祖世亨, 曲成军, 高英姿, 等. 2001. 黑龙江省冬小麦气候区划研究. 中国生态农业学报, **9**(4): 85-87.
Chen XW, Zhang XS, Zhou GS. 2003. Spatial characteristics and change for tree species along the North East China Transect (NECT). *Plant Ecology*, **164**: 65-74.
Coombe BG. 1987. Influence of temperature on composition and quality of grapes. *Acta Horticulture*, **206**: 23-33.
Hu Z, Zhang T, Gao XX, *et al*. 2016. De novo assembly and characterization of the leaf, bud, and fruit transcriptome from the vulnerable tree *Juglans mandshurica* for the development of 20 new microsatellite markers using illumina sequencing. *Molecular Genetics and Genomics*, **291**: 849-862.
Salahuddin, Boris R, Muhammad R, *et al*. 2018. Root order-based traits of Manchurian walnut & larch and their plasticity under interspecific competition. *Scientific Reports*, **8**: 9815.
Song NQ, Zhang JT, Zhao FG. 2017. The PCA index for measuring functional diversity and its application to *Juglans mandshurica* communities in the Beijing mountains, China. *International Journal of Biomathematics*, **10**: 127-139.
Winkler AJ, Cook JA, Kliever WM, *et al*. 1974. General Viticulture. Berkeley: University of California Press.

作者简介　罗　也,男,1991 年生,硕士,主要研究方向为森林培育。E-mail: 1549348929@ qq.com
责任编辑　张　敏

(C)1994-2023 China Academic Journal Electronic Publishing House. All rights reserved.　http://www.cnki.net
