# Supplementary material for: Provenance and family variations in early growth of Manchurian walnut (Juglans mandshurica Maxim.) and selection of superior families
Source: PLoS One. 2024 Mar 7;19(3):e0298918. doi: 10.1371/journal.pone.0298918 (PMC10919699; doi:10.1371/journal.pone.0298918)
Supplement: S2 File — (ZIP) [file pone.0298918.s005.zip › Progeny performance and selection of superior trees within families in Larix olgensis.pdf]

Euphytica (2020)216:60
https://doi.org/10.1007/s10681-020-02596-9

# Progeny performance and selection of superior trees within families in *Larix olgensis*

**Heng Zhang · Yuhangyi Zhang · Dawei Zhang · Lihu Dong · Kejian Liu · Ying Wang · Chuanping Yang · Vincent L. Chiang · Mulualem Tigabu · Xiyang Zhao**

Received: 1 August 2019 / Accepted: 10 March 2020
© Springer Nature B.V. 2020

**Abstract** *Larix olgensis* is one of the most important timber species in Northeast China. Although *L. olgensis* seed orchards have been established for many years and some progeny tests have been conducted, studies of progeny performance and the selection of superior families are still lacking. These tests are important for upgrading and improvement of seed orchards. Here, we estimate the genetic variation for major economic traits between families to provide selection materials for the establishment of a second-generation seed orchard. This study examined 71 half-sib families and 2 control families of *L. olgensis* in the Xiyang forest seed orchard in Yongji County, Northeast China. We measured tree height, diameter at breast height, volume, branch angle, stem straightness degree and crown width of 4-year-old progeny. Genetic parameters for the studied traits were determined, and comprehensive analyses of multiple traits and breeding values were used for selection of superior families and individual trees within families. The results show that all these traits are significantly different ($P < 0.01$) among families where family variance was greater than environmental variance. The phenotypic and genotypic coefficients of variation ranged from 9.01% to 78.22% and from 1.81% to 39.20%, respectively. The heritability values of families and individual trees ranged from 0.663 to 0.959 and 0.034 to 0.983, respectively. There was a significant positive correlation between investigated traits. Using the multiple-traits comprehensive evaluation method, seven families and 56 superior individual trees were selected. Selection of superior parents using breeding values for timber yield, stem quality and crown width as a proxy for planting density again resulted in the selection of seven superior families. We determined that the comprehensive multiple-traits evaluation approach is effective for selecting superior families and individual trees for the establishment of advanced second generation seed orchards. Families selected based on breeding value may be used as preferred parents for future hybridization breeding.

**Keywords** Seed orchard · Genetic variation · Family heritability · Genetic gain

The authors Heng Zhang, Yuhangyi Zhang, Dawei Zhang have contributed equally to this work.

H. Zhang · D. Zhang · L. Dong · C. Yang · V. L. Chiang · X. Zhao (✉)
State Key Laboratory of Tree Genetics and Breeding, Northeast Forestry University, Harbin 150040, People's Republic of China
e-mail: zhaoxyphd@163.com

Y. Zhang · K. Liu · Y. Wang
National Larch Breeding Base in Yongji County, Yongji 132201, Jilin, People's Republic of China

M. Tigabu (✉)
Southern Swedish Forest Research Centre, Swedish University of Agricultural Sciences, Alnarp, Sweden
e-mail: Mulualem.tigabu@slu.se
