# Supplementary material for: Provenance and family variations in early growth of Manchurian walnut (Juglans mandshurica Maxim.) and selection of superior families
Source: PLoS One. 2024 Mar 7;19(3):e0298918. doi: 10.1371/journal.pone.0298918 (PMC10919699; doi:10.1371/journal.pone.0298918)
Supplement: S2 File — (ZIP) [file pone.0298918.s005.zip › Provenance selection of Juglans mandshurica Maxim. and the effects of environmental factors.pdf]

植物研究 2013,33(4):468~476
Bulletin of Botanical Research

核桃楸种源选择试验及其环境因子的影响

袁显磊[1,2] 祁永会[2] 刘忠玲[2] 周志军[2] 毛子军[1]*

(1. 东北林业大学,哈尔滨 150040; 2. 黑龙江省林业科学研究所,哈尔滨 150081)

摘 要 收集13个核桃楸种源,在黑龙江省林口和兴隆两个试验点进行播种育苗,调查1年生苗木生长量,进行核桃楸种源选择试验。结果表明,不同种源核桃楸在两地播种后,苗高、基径生长量差异显著,适合林口地区的优良种源为绥阳、和龙、帽儿山和辉南种源;适合兴隆地区的优良种源为抚松、和龙、兴隆和绥阳种源。林口试验点核桃楸种子直径与1年生苗木基径生长量的相关系数达到了显著水平,可以作为反映基径生长量的指标,用线性函数模型来模拟核桃楸种子与苗木的生长情况的相关系数和拟合度相对较高。通过对13个种源地和2个试验点的主要环境因子差异与其1年生苗生长性状差异相关性分析,核桃楸不同种源1年生苗高与纬度和年平均气温等环境因子有一定相关性,且呈负相关,核桃楸种源可适当向北移栽。

关键词 核桃楸;种源选择;生长性状差异

中图分类号:S718.53 **文献标志码**:A **doi**:10.7525/j.issn.1673-5102.2013.04.014

Provenance Selection of *Juglans mandshurica* Maxim. and the Effects of Environmental Factors

YUAN Xian-Lei[1,2] QI Yong-Hui[2] LIU Zhong-Ling[2] ZHOU Zhi-Jun[2] MAO Zi-Jun[1]*

(1. Northeast Forestry University, Harbin 150040; 2. Heilongjiang Forest Research Institute, Harbin 150081)

Abstract A selective experiment on 13 *Juglans mandshurica* provenances was conducted in Linkou and Xinglong of Heilongjiang province by investigating the growth of one-year-old seedlings. The results showed significant differences in height and basal diameter growth of the provenances in the two test-sites. The excellent provenances adapted to Linkou area was Suiyang, Helong, Maoershan and Huinan. The excellent provenances were Fusong, Helong, Xinglong and Suiyang in Xinglong area. There was a significant correlation between the seed diameter and basal diameter growth of *J. mandshurica* in Linkou area. It could be regarded as the index of reflecting the basal diameter growth. The relationship between the growths of seed and seeding could be best described by the linear model. The correlations between the one-year-old seedlings growth of *J. mandshurica* and the main environmental factors of 13 provenances as well as two test-sites were analyzed. The results showed the height of one-year-old seedlings has a significantly negative correlation with the latitude and the annual temperature of locations. The provenances of *J. mandshurica* may be appropriately planting northwards.

Key words *Juglans mandshurica* Maxim.; provenance selection; variation of growth characters

核桃楸(*Juglans mandshurica* Maxim.)又名胡桃楸,为胡桃科(Uglandaceae)胡桃属(*Juglans*)阔叶落叶乔木,属第三纪孑遗植物,已列为国家三级保护植物[1~2],是东北阔叶红松混交林的主要伴生树种,与水曲柳、黄菠萝并称三大硬阔。核桃楸材质坚硬致密,弹性好,易加工,是优良的军用细木工和家具用材;果皮和树皮含有鞣质,种子富含脂肪,可食用或工业用;树皮及叶可药用,具有消暑解毒抗癌等作用[3]。由于过量采伐,核桃楸天然林大树接近枯竭。近些年,进行了一系列的人工更新试验。关于胡桃属苗木繁育及培育技术研究已有报道[4~6]。

核桃楸在东北分布面积最为辽阔,最适生长区

基金项目:林业公益性行业科研专项经费子项目(20110400105);973国家重点基础研究发展计划项目(2010CB951301)
第一作者简介:袁显磊(1979—),男,助理研究员,硕士研究生,主要研究方向为植物生理生态、森林培育。
* 通信作者:E-mail:zijunm@yahoo.com.cn
收稿日期:2012-11-25

(C)1994-2019 China Academic Journal Electronic Publishing House. All rights reserved. http://www.cnki.net

域为长白山地区和小兴安岭,因此,核桃楸的种源试验主要在东北地区进行[7~8]。根据核桃楸苗期地理种源变异规律、种源选择、早晚相关进行研究表明,生长性状、适应性状等种源间存在显著差异[9]。核桃楸种内的地理变异总趋势受经纬度双重控制,以纬度影响略大,呈现东北到西南的变化趋势[10]。种源可以适度地北移,其在移栽地的的生长优于当地的种源[11]。刘桂丰、杨书文等将东北地区的核桃楸种源划分为4个种源区,即长白山完达山种源区、吉林中部浅山种源区、辽宁东部种源区、小兴安岭松花江种源区。已有研究表明,虽然对核桃楸进行了一些种源试验,但种源地的和试验地都比较少,而不同地区引种的适宜种源可能各不相同,因此,扩大种源试验地点及增加参试种源对于核桃楸的种质资源保存及核桃楸优质林的发展是非常迫切的任务之一。

本文通过收集核桃楸自然分布区内不同种源种子进行播种育苗,对1年生幼苗生长进行观测,研究核桃楸不同种源苗期生长变异规律,选择核桃楸苗期生长优良种源,以期为核桃楸种源选择、培育优质苗木和遗传改良提供理论依据。了解核桃楸种源和家系变异规律,对收集与保存其种质资源,保护生物多样性,维护生态平衡及发展核桃楸经济林,具有较大的理论指导意义和生产实践意义。

1　试验地自然概况

试验地有两处,分别设在林口林业局湖水经营所苗圃和兴隆林业局元宝山苗圃。湖水经营所苗圃位于张广才岭东麓,海拔高度230 m,地理位置为130°14′E、45°30′N,土壤为轻粘土,黑土层厚度50 cm,pH为7.3,无霜期110~130 d,年平均降水量580 mm,年平均气温2.5℃。元宝山苗圃位于小兴安岭南麓,海拔高度160 m,地理位置为127°59′E、46°16′N,土壤为草甸土,土质、水质良好,pH6.5,无霜期115 d,年平均降水量602 mm,年平均气温1.7℃。该两处试验地分别为核桃楸的长白山完达山种源区及小兴安岭松花江种源区,代表了核桃楸在黑龙江省的适生地和天然分布区。

2　材料和方法

2.1　采种

供试材料为来自辽宁、吉林和黑龙江3个省的13个种源,其中在黑龙省江选择1#(美溪)、2#(帽儿山)、3#(五常)、4#(桃山)、5#(绥阳)、6#(兴隆)、7#(东京城)共7个种源,辽宁省选择8#(凤城)、9#(新宾)2个种源,在吉林省选择10#(辉南)、11#(桦甸)、12#(抚松)、13#(和龙)共4个种源。各种源均为2010年秋季采种,冬季沙藏,翌年春采用变温处理种子后进行播种育苗。核桃楸分布区图及采种地点见图1,13个种源地主要环境因子见表1。

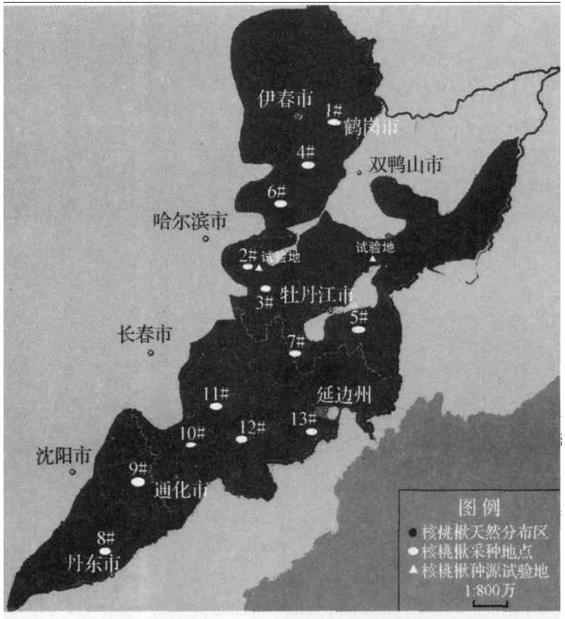

图1　东北三省(辽宁、吉林、黑龙江)核桃楸天然分布及其2个种源试验地

Fig. 1　Natural distributions of *J. mandshurica* in Liaoning, Jilin and Heilongjiang provinces, and two provenance experimental fields

2.2　育苗

播种前对13个种源进行种子千粒重、种子长度与种子直径的形态测量。于2011年春季在林口湖水和兴隆元宝山两个试验点进行垄式、分种源播种育苗。待出苗较整齐后,按苗圃常规管理进行适时浇水、除草、松土,促进苗木生长。

2011年10月,每个种源内随机选取30株苗木,用钢卷尺和游标卡尺测定1年生播种苗苗高和基径。

2.3　数据处理

对13个种源的核桃楸种子的千粒重、种子长度、种子直径分别进行单因素方差分析(one-way ANOVA)和多重比较,显著性水平设定为$p=0.05$。千粒重、种子长度方差齐性,采用最小显著差数法(LSD)检验,种子直径方差齐性不齐,采用Dunnett T3检验。变异系数的计算公式为:

$$变异系数\ C \cdot V=(标准偏差\ SD/平均值\ MN)\times 100\% \qquad (1)$$

(C)1994-2019 China Academic Journal Electronic Publishing House. All rights reserved.　http://www.cnki.net

表1 核桃楸种源地主要环境因子

Table 1 Main information on the provenances of *J. mandshurica*

编号<br>No.	种源地<br>Provenance	经度<br>Long.	纬度<br>Lat.	年平均气温<br>Annual average temperature (℃)	年平均降水量<br>Average annual precipitation(mm)	无霜期<br>Frost-free period (d)
1#	黑龙江伊春市美溪<br>Heilongjiang Yichun City Meixi	129°07′	47° 38′	0.4	640	100
2#	黑龙江尚志市帽儿山<br>Heilongjiang Shangzhi City Maoershan	127°30′	45°20′	2.8	723.8	125
3#	黑龙江五常市宝龙店<br>Hlongjiang Wuchang City Baolongdian	127°44	44°56′	3.4	620.9	130
4#	黑龙江伊春市桃山<br>Heilongjiang Yichun City Taoshan	128°21′	46°47′	2.8	665	113
5#	黑龙江绥阳<br>Heilongjiang Suiyang	129°41′	43°08′	3.0	550	122
6#	黑龙江兴隆<br>Heilongjiang Xinglong	127°59′	46°16′	1.2	602	115
7#	黑龙江东京城<br>Heilongjiang Dongjingcheng	128°07′	43°30′	4.2	550	125
8#	辽宁丹东市凤城<br>Ljaoning Dandong City Fengcheng	124°03′	40°27′	8.2	979.2	156
9#	辽宁抚顺市新宾<br>Liaoning Fushun City Xinbin	124°05′	41°55′	4.6	700	127
10#	吉林通化市辉南<br>Jilin Tonghua City Huinan	126°03′	42°18′	5.0	736.5	125
11#	吉林吉林市桦甸<br>Jilin Jilin City Huadian	126°44′	42°58′	3.9	748.1	110
12#	吉林白山市抚松<br>Jilin Baishan City Fusong	127°16′	42°20′	3.3	817.9	110
13#	吉林延边州和龙<br>Jilin Yanbian State Helong	129°00′	42°32′	4.9	520	125

对湖水、元宝山苗圃2个试点各种源1年生苗木苗高、基径分别进行单因素方差分析和多重检验。湖水不同种源苗木高生长方差齐性，采用LSD检验。湖水种源苗木基径生长，元宝山种源苗高和基径生长方差齐性不齐，采用Dunnett T3检验。

对不同种源水曲柳种子形态、千粒重和子代播种苗生长指标进行相关性分析，根据相关分析结果选择相关度较高的指标进行回归分析。将不同种源的核桃楸种子性状，在湖水、元宝山地区苗期生长性状进行聚类分析。

2.4 核桃楸种源早期选择的可靠性

根据刘桂丰等的研究，幼龄期的核桃楸就有代表性，1年生就可预测6年生的情况，另外根据核桃楸22年生人工林早期选择的研究，如果以树高作为早期选择性状，最佳年龄为6年。因此，本试验研究采用1年生苗进行种源早期评价具有可靠性。

3 试验结果与分析

3.1 不同种源核桃楸种子的形态、重量差异

对不同种源核桃楸种子形态的方差分析结果表明，13个种源间在千粒重、种子长度和种子直径均存在极显著差异($P<0.01$)。东京城种源种子长度最长，显著高于其他种源($P<0.05$)，种子直径较大，千粒重最重，显著高于其他种源($P<0.05$)。抚松种源种子直径最宽，种子长度较长，千粒重仅低于东京城种源。凤城种源种子长度和直径均最小，千粒重稍高于美溪种源。美溪种源千粒重最轻，说明饱满度较差。绥阳和帽儿山种源种子也较大，千粒重较重。

3.2 不同种源核桃楸1年生苗木生长表现

(1)湖水试验点的苗期生长表现

对不同种源核桃楸种子在湖水播种繁殖后1年生苗木高、径生长量方差分析13个种源子代的苗高、基径生长差异极显著($P<0.01$)。辉南种源苗高最高，显著高于其他种源($P<0.05$，表3)，高于平均值26%。美溪种源苗高显著低于其他种源($P<0.05$)。绥阳种源基径最大，显著高于其他种源($P<0.05$)，高于平均值29%。五常种源基径显著低于其他种源($P<0.05$)。

(C)1994-2019 China Academic Journal Electronic Publishing House. All rights reserved. http://www.cnki.net

表 2　不同种源核桃楸种子形态、重量

Table 2　Shape and weight of seeds of *J. mandshurica* from different provenances

种源编号 Provenance No.	千粒重 1 000-grain weight(g)	种子长度 Seed length(mm)	种子直径 Seed diameter(mm)
1#	8 616.67 ±269.50 d	39.97 ±3.19 g	27.30 ±2.85 ab
2#	10 010.00 ±104.40 bcd	46.27 ±4.44 b	30.37 ±3.18 a
3#	10 276.67 ±664.55 bc	42.98 ±4.41 ef	28.98 ±2.39 a
4#	9 666.67 ±299.55 bcd	44.05 ±4.12 cde	28.61 ±2.72 a
5#	10 066.67 ±208.16 bcd	46.70 ±5.14 ab	29.71 ±3.10 a
6#	9 480.00 ±227.15 bcd	45.60 ±5.31 bc	29.42 ±2.76 a
7#	12 870.00 ±3 398.13 a	48.45 ±6.48 a	30.10 ±3.08 a
8#	8 733.33 ±56.86 cd	38.17 ±3.52 h	26.74 ±1.99 b
9#	9 400.00 ±232.59 bcd	42.18 ±3.85 f	28.45 ±2.04 a
10#	9 853.33 ±227.44 bcd	43.30 ±4.75 def	30.66 ±8.74 a
11#	9 570.00 ±406.32 bcd	43.18 ±4.43 ef	27.99 ±3.21 ab
12#	10 446.67 ±205.50 b	45.22 ±4.02 bc	31.05 ±8.23 a
13#	8 983.333 ±126.62 bcd	45.82 ±4.14 bc	27.56 ±2.36 ab

注:表中数据为平均值 ± 标准差;千粒重 n =3;种子长度,种子直径 n =50

Note: Data in the table were mean ± standard deviation; The 1000-grain weight n =3; Seed length, Seed diameter n =50

种源间的平均高变异系数为 22.90%,变异系数范围 15.21% ~23.38%,其中美溪种源内变异系数最大,绥阳种源内变异系数最小。种源间的平均基径变异系数为 23.11%,大于种源内的变异系数范围 15.35% ~21.22%,其中美溪种源内变异系数最大,桦甸种源内变异系数最小(表 3)。

苗高前 5 名的分别是 10#、12#、5#、13#、2# 种源,基径前 5 名的分别是 5#、13#、10#、2#、11# 种源。综合苗高、基径来看,辉南、绥阳种源 1 年生苗木生长好,和龙、帽儿山种源 1 年生苗木生长也较好。

(2)元宝山的苗期生长表现

对不同种源核桃楸种子在元宝山播种繁殖后 1 年生苗木高、径生长量方差分析结果表明,13 个种源子代在高、径生长方面差异极显著($P <0.01$)。经多重比较发现,抚松种源最好,苗高和基径均最大,分别高出 13 个种源的平均值 21% 和 22%。兴隆、绥阳、和龙种源 1 年生苗木生长也较好(表 4)。东京城种源高径生长量均较低。

表 3　湖水试验点不同种源 1 年生苗木苗高及基径生长量

Table 3　Growths of height and basal diameter of one-year-old seedlings in Hushui test-site from different provenances

种源编号 Provenance No.	平均苗高 Average seedling height(cm)	变异系数 Coefficient of variation(%)	平均基径 Average basal diameter(mm)	变异系数 Coefficient of variation(%)
1#	17.83 ±4.16 i	23.38	9.94 ±2.10 bcde	21.22
2#	22.13 ±4.96 cde	22.44	10.72 ±2.25 abc	20.99
3#	20.10 ±3.85 efg	19.17	7.31 ±1.27 f	17.46
4#	18.37 ±3.39 ghi	18.50	9.65 ±1.65 cde	17.16
5#	23.47 ±3.56 bc	15.21	12.76 ±2.41 a	18.96
6#	17.87 ±3.32 hi	18.63	8.81 ±1.55 de	17.70
7#	19.97 ±3.61 fgh	18.10	9.38 ±1.44 cde	15.37
8#	20.55 ±3.69 def	17.99	8.66 ±1.56 de	18.05
9#	21.40 ±4.51 cdef	21.09	8.46 ±1.51 ef	17.93
10#	26.73 ±4.86 a	18.19	10.78 ±2.20 abc	20.40
11#	19.80 ±4.26 fghi	21.52	10.33 ±1.58 bc	15.35
12#	25.17 ±5.07 ab	20.15	10.11 ±1.84 bcd	18.22
13#	22.47 ±4.13 cd	18.39	11.83 ±2.15 ab	18.25
平均值 Mean	21.22 ±4.86	22.90	9.90 ±2.28	23.11

注:表中数据为平均值 ± 标准差,n =30　下同

Note: Data in the table were mean ± standard deviation, n = 30. The same as below.

种源间的平均高变异系数为 28.88%(表 4),种源内的变异系数范围 16.05% ~33.31%,其中美溪种源内变异系数最大,兴隆种源内变异系数最小。种源间的平均基径变异系数为 33.76%,种源内的变异系数范围 20.06% ~44.75%,其中凤城种源内变异系数最大,五常种源内变异系数最小。

苗高前 5 名的分别是 12#、6#、13#、5#、10# 种源,基径前 5 名的分别是 12#、13#、5#、6#、4# 种源。综合苗高、基径来看,抚松、兴隆、绥阳及和龙种源 1 年生苗木生长较好。

(C)1994-2019 China Academic Journal Electronic Publishing House. All rights reserved.　http://www.cnki.net

3.3 不同种源核桃楸种子形态、千粒重与苗木生长指标的相关性

从表5可以看出,两个试验点的种子长度与苗高的相关系数较接近,说明这种相关性较为稳定,可作为反映核桃楸评价幼苗高生长情况的一项指标。千粒重与苗高呈负相关关系,在控制了种子长度和种子直径的情况下,偏相关系数增加。湖水试验点种子直径与基径显著相关($P<0.05$),在控制了种子长度和千粒重的情况下,偏相关系数为0.616,在湖水试验点可选择种子直径作为反映基径生长量的指标。

3.4 苗期生长与种子性状相关指标的回归分析

选择与苗高相关性稳定的种子长度作为回归分析的自变量,选择与基径相关性较高的种子直径作为回归分析的自变量,同时引入多种模型,进行回归分析,根据拟合度进行比较,直至生成最佳模型。结果表明,两个试验点均以线性函数模型来估测核桃楸1年生苗苗木生长相对较准确(表6)。

表4 元宝山试验点不同种源1年生苗木高径生长量

Table 4 Growths of height and basal diameter of one-year-old seedlings in Yuanbaoshan test-site from different provenances

种源编号 Provenance No.	平均苗高 Average seedling height(cm)	变异系数 Coefficient of variation(%)	平均基径 Average base diamete(mm)	变异系数 Coefficient of variation(%)
1#	22.48±7.48 b	33.31	5.20±1.61 b	31.11
2#	22.37±5.49 b	24.57	7.29±1.62 a	22.31
3#	24.86±6.01 ab	24.17	7.13±1.43 a	20.06
4#	23.43±5.26 b	22.46	7.31±1.92 a	26.28
5#	26.87±5.98 ab	22.28	7.66±2.29 a	29.90
6#	30.37±4.87 a	16.05	7.59±1.94 a	25.63
7#	22.26±5.60 b	25.18	6.57±1.50 ab	22.96
8#	23.41±6.93 b	29.62	7.15±3.19 a	44.75
9#	25.69±7.42 ab	28.90	7.01±2.37 ab	33.89
10#	25.39±8.26 ab	32.55	6.91±3.00 ab	43.52
11#	22.45±7.79 b	34.72	7.01±2.83 ab	40.37
12#	30.60±8.84 a	28.90	8.82±3.19 a	36.26
13#	27.00±7.66 ab	28.39	8.03±2.49 a	31.06
平均值 Mean	25.23±7.28	28.88	7.23±2.44	33.76

表5 种子形态、千粒重与1年生苗木苗高、基径相关性分析

Table 5 Correlation analysis of seed shape and 1 000-seed weight with height and basal diameter of one-year-old seedlings

试验点 Test site	性状 Character	苗高 Seedling height		基径 Base diameter	
		相关系数 Correlation coefficient	偏相关系数 Partial correlation coefficient	相关系数 Correlation coefficient	偏相关系数 Partial correlation coefficient
湖水 Hushui	种子长度 Seed length	0.372	0.498	0.243	-0.121
	种子直径 Seed diameter	0.189	0.055	0.564*	0.616
	千粒重 The 1 000-grain weight	-0.062	-0.447	0.133	-0.295
元宝山 Yuanbaoshan	种子长度 Seed length	0.325	0.359	0.417	0.354
	种子直径 Seed diameter	0.357	0.396	0.394	0.281
	千粒重 The 1 000-grain weight	-0.085	-0.537	0.087	-0.364

* $P\leqslant0.05$

(C)1994-2019 China Academic Journal Electronic Publishing House. All rights reserved. http://www.cnki.net

表 6　核桃楸 1 年生苗木苗高与千粒重、基径与种子长度相关关系的数学模型

Table 6　Mathematical model of height and 1 000-seed weight, basal diameter and seed length of one-year-old seedlings of *J. mandshurica* Maxim.

数学模型 Mathematical mode	试验点 Test site	测量因子 The measuring factor	方程 Equation	R^2	F	P
线性回归 Linear regression	湖水 Hushui	苗高 Seedling height	$y = 199.383 + 18.07x$	0.138	1.760	0.212
		基径 Basal diameter	$y = -11.578 + 1.131x$	0.318	5.137	0.045
	元宝山 Yuanbaoshan	苗高 Seedling height	$y = 1141.46 + 31.417x$	0.106	1.299	0.279
		基径 Basal diameter	$y = 0.257 + 0.24x$	0.155	2.020	0.183
指数方程 Exponential equation	湖水 Hushui	苗高 Seedling height	$\ln Y = \ln(451.93) + 0.018X$	0.128	1.616	0.230
		基径 Basal diameter	$\ln Y = \ln(4.846) + 0.051X$	0.301	4.739	0.052
	元宝山 Yuanbaoshan	苗高 Seedling height	$\ln Y = \ln(1488.927) + 0.012X$	0.102	1.247	0.288
		基径 Basal diameter	$\ln Y = \ln(2.599) + 0.035X$	0.154	1.999	0.185
S 曲线 S curve	湖水 Hushui	苗高 Seedling height	$Y = e^{7.628 - 32.229X}$	0.125	1.564	0.237
		基径 Basal diameter	$Y = e^{4.458 - 40.821X}$	0.282	4.310	0.062
	元宝山 Yuanbaoshan	苗高 Seedling height	$Y = e^{1141.469 + 31.417X}$	0.106	1.299	0.279
		基径 Basal diameter	$Y = e^{2.968 - 28.917X}$	0.152	1.967	0.188
Powper	湖水 Hushui	苗高 Seedling height	$Y = 56.101X^{0.757}$	0.127	1.593	0.233
		基径 Basal diameter	$Y = 0.165X^{1.440}$	0.291	4.522	0.057
	元宝山 Yuanbaoshan	苗高 Seedling height	$Y = 336.488X^{0.531}$	0.11	1.362	0.268
		基径 Basal diameter	$Y = 0.257X^{0.240}$	0.155	2.020	0.183

3.5 不同种源聚类分析

将不同种源的核桃楸按照种子性状,在湖水、元宝山地区苗期生长量进行聚类分析,结果如图 2～4。从图 2 中可以看出,不同种源按照种子形态和重量可聚为 3 类:1#、8#、13# 为一类,种子各项指标较低;7# 单独为一类,种子各指标较高;其他各种源为一类。

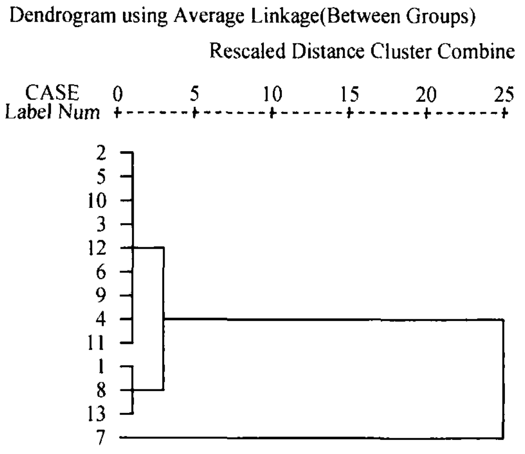

图 2　不同种源核桃楸种子性状聚类分析

Fig. 2　Cluster analysis for the seeds of *J. mandshurica* of different provenances

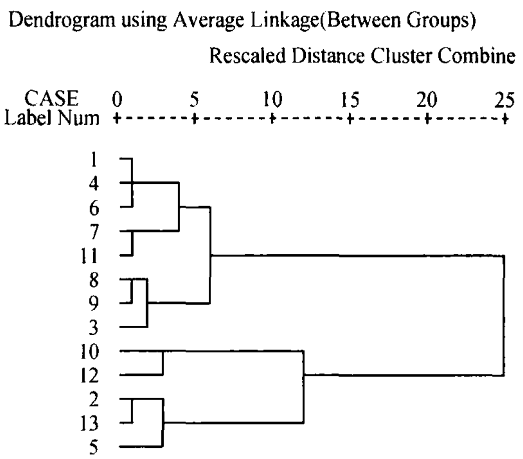

图 3　湖水试验点不同种源核桃楸高径生长的聚类分析

Fig. 3　Cluster analysis for the height growth of the *J. mandshurica* of different provenances in Hushui test-sites

从湖水试验点的生长情况来看(图 3),可分为 4 类:1#、4#、6#、7#、11# 聚为一类,高径生长量较低;2#、13#、5# 聚为一类,高径生长量较高;3#、8#、9# 聚为一类,苗高生长量高,但是基径生长量低;10#、12# 聚为一类,高径生长量居中。

从元宝山试验点的生长情况来看(图 4),4#、8#、1#、11#、2#、7# 聚为一类,高径生长量较低;5#、13#、9#、10#、3# 聚为一类,高径生长量较高;6# 和 12# 聚为一类高径生长量最高。

(C)1994-2019 China Academic Journal Electronic Publishing House. All rights reserved.　http://www.cnki.net

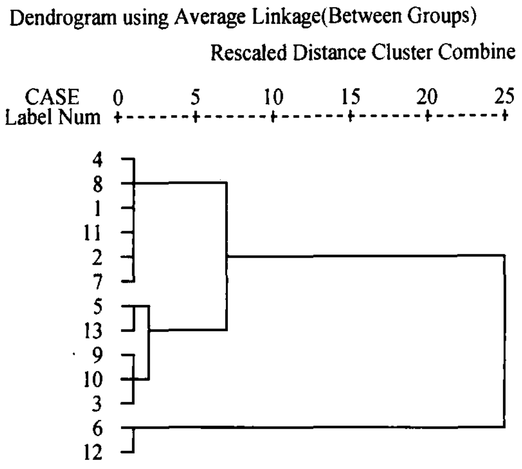

图4 元宝山试验点不同种源核桃楸高径生长的聚类分析

Fig. 4 Cluster analysis for the height growth of the *J. mandshurica* of different provenances in Yuanbaoshan test-sites

3.6 不同种源核桃楸苗期生长与主要环境因子关系

将种源地的经度、纬度、年平均气温、年平均降水量、无霜期、苗高和基径分别与2个试验地的对应环境因子及苗高和基径求差，建立苗木生长差异与各环境因子差关系（表7）。

（1）1年生苗木生长差异与经度差关系分析

各种源地与试验地1年生苗木生长差异与经度差异的相关分析表明（表7），林口湖水苗圃与兴隆元宝山苗圃2处试验点的苗高差与种源地经度差的 R^2 均趋于0，且表现出相反的正负相关，表明苗高差与经度差异的相关性不强。

在林口湖水苗圃苗木基径差与种源地经度差具有一定相关性，且呈正相关；在兴隆元宝山苗圃苗木基径差与经度差呈负相关，且相关性不强。

以上分析表明，1年生苗木生长差异与核桃楸种源地的经度差异相关性不强，不具有普遍规律性。

表7 1年生苗木生长差异与主要环境因子差异的回归模型

Table 7 Regression model between one-year-old seedling growth differences and main environmental factors

试验点<br>Test site	自变量<br>Argument	因变量<br>Dependent variable	方程<br>Equation	R^2
湖水<br>Hushui	经度差<br>Difference in longitude	苗高差<br>Difference in seedling height	$y=-0.863x^2-0.877\,7x-3.812\,8$	0.038 8
元宝山<br>Yuanbaoshan	经度差<br>Difference in longitude	基径差<br>Difference in basal diameter	$y=0.066x^2+0.238\,1x-5.707\,2$	0.009 3
湖水<br>Hushui	经度差<br>Difference in longitude	苗高差<br>Difference in seedling height	$y=0.108\,1x^2+1.172\,6x-0.689\,4$	0.239 4
元宝山<br>Yuanbaoshan	经度差<br>Difference in longitude	基径差<br>Difference in basal diameter	$y=-0.043\,6x^2-0.116x-0.331\,8$	0.024 6
湖水<br>Hushui	纬度差<br>Difference in latitude	苗高差<br>Difference in seedling height	$y=-0.197x^2-1.289\,5x-3.003\,5$	0.473 1
元宝山<br>Yuanbaoshan	纬度差<br>Difference in latitude	基径差<br>Difference in basal diameter	$y=-0.109\,8x^2-0.953\,2x-6.932\,4$	0.211 7
湖水<br>Hushui	纬度差<br>Difference in latitude	苗高差<br>Difference in seedling height	$y=-0.084\,1x^2-0.293\,3x-2.754\,8$	0.073 2
元宝山<br>Yuanbaoshan	纬度差<br>Difference in latitude	基径差<br>Difference in basal diameter	$y=-0.072\,9x^2-0.498x-0.923\,3$	0.382 2
湖水<br>Hushui	年平均气温差<br>Difference in annual average temperature	苗高差<br>Difference in seedling height	$y=-0.219\,8x^2+1.310\,1x-2.806$	0.319 6
元宝山<br>Yuanbaoshan	年平均气温差<br>Difference in annual average temperature	基径差<br>Difference in basal diameter	$y=-0.152\,8x^2+0.919\,1x-6.436\,4$	0.119
湖水<br>Hushui	年平均气温差<br>Difference in annual average temperature	苗高差<br>Difference in seedling height	$y=-0.057\,2x^2+0.124\,8x-2.740\,7$	0.056 8
元宝山<br>Yuanbaoshan	年平均气温差<br>Difference in annual average temperature	基径差<br>Difference in basal diameter	$y=-0.081\,5x^2+0.582\,7x-1.043\,9$	0.380 3
湖水<br>Hushui	年平均降水量差<br>Difference in average annual precipitation	苗高差<br>Difference in seedling height	$y=1E-06x^2+0.002\,6x-2.56$	0.018 6
元宝山<br>Yuanbaoshan	年平均降水量差<br>Difference in average annual precipitation	基径差<br>Difference in basal diameter	$y=6E-06x^2-0.000\,9x-5.719\,7$	0.002 7
湖水<br>Hushui	年平均降水量差<br>Difference in average annual precipitation	苗高差<br>Difference in seedling height	$y=1E-05x^2-0.007\,4x-2.363$	0.130 9
元宝山<br>Yuanbaoshan	年平均降水量差<br>Difference in average annual precipitation	基径差<br>Difference in basal diameter	$y=6E-06x^2+8E-05x-0.508\,8$	0.036
湖水<br>Hushui	无霜期差<br>Difference in frost-free period	苗高差<br>Difference in seedling height	$y=-0.003\,5x^2+0.092\,6x-1.81$	0.168 5
元宝山<br>Yuanbaoshan	无霜期差<br>Difference in frost-free period	基径差<br>Difference in basal diameter	$y=-0.002x^2+0.037\,6x-5.456\,7$	0.057 1
湖水<br>Hushui	无霜期差<br>Difference in frost-free period	苗高差<br>Difference in seedling height	$y=-0.000\,9x^2-0.012\,9x-2.677\,2$	0.095 6
元宝山<br>Yuanbaoshan	无霜期差<br>Difference in frost-free period	基径差<br>Difference in basal diameter	$y=-0.001\,1x^2+0.037\,8x-0.446\,5$	0.165 1

(C)1994-2019 China Academic Journal Electronic Publishing House. All rights reserved. http://www.cnki.net

(2)1 年生苗木生长差异与纬度差关系分析

2 处试验地的苗高差与种源地纬度差异均表现出一定相关性,且为负相关;苗木基径差与种源地纬度差虽呈负相关,但其相关性不强(表7)。

(3)1 年生苗木生长差异与年平均气温差关系分析

2 处试验地的苗高差与种源地年平均气温差均表现出一定相关性,且为负相关;苗木基径差与年平均气温差虽呈负相关,但在林口湖水苗圃其相关性表现不强(表7)。

(4)1 年生苗木长差异与年平均降水量差关系分析

2 处试验地的苗高差及苗木基径差均与种源地年平均降水量差呈正相关,但其相关性不强。

(5)1 年生苗木生长差异与无霜期差关系分析

林口湖水苗圃的苗高差异与种源地无霜期差异表现出一定相关性,且呈负相关;兴隆元宝山苗圃的苗木基径差异与种源地无霜期差异表现出一定相关性,且呈负相关;而兴隆元宝山苗圃的苗木基径差异及林口湖水苗圃的苗高差异与种源地无霜期差异虽呈负相关,但相关性较弱(表7)。

4　讨论

4.1　不同种源核桃楸种子的形态、重量与苗木生长指标的相关性

树木种子的形态具有较高的稳定性,是植物分类学上的一种重要性状,在林木遗传育种研究上也具有重要价值[12],可以在种质资源的收集、遗传评价基础之上开展种源试验,进一步选择优良种源和家系[13]。种子的大小和重量影响种子发芽,进而影响苗木的早期生长[14]。种子的某些性状与子代苗木生长有关[15],据对杉木、火炬松、巨桉等树种的研究表明,在苗圃选择超级苗木是有遗传增益的,早期选择具有一定的可靠性[16],对不同种源核桃楸种子形态和重量的测量表明,13 个种源间在千粒重、种子长度和种子直径 3 个方面存在极显著差异($P<0.01$)。播种试验表明两个试验点的种子长度与苗高的相关系数较高、林口试验点种子直径与基径生长量的相关系数达到了显著水平,可以作为反映基径生长量的指标。经回归分析,两个试验点均以线性函数模型来估测核桃楸 1 年生苗苗木生长相对准确。

4.2　不同种源核桃楸 1 年生苗生长表现分析

苗期试验是早期选择的重要途径之一,可了解各种源苗期的差异,从而培育适合造林试验用的苗木,1 年生苗的生长量是决定苗木质量的重要依据,苗高和基径是苗木生长量的直接表现,苗木的质量反映了苗木吸收、同化养分能力的大小,是衡量苗木生产力高低的重要指标之一[16]。通过对林口湖水试验点 1a 生播种苗苗木生长情况分析,辉南和绥阳种源最好,和龙和帽儿山种源较好,兴隆元宝山试验点则为抚松种源最好,高径生长量分别高出 13 个种源平均值的 21% 和 22%,绥阳、和龙、兴隆种源较好。张含国等对的 3 个不同种源 127 个家系的核桃楸研究试验表明,不同种源的核桃楸在林口青山林场播种造林,7 年后不同种源的苗高变异平均系数较 1 年生苗木时增加,种源内分化更加明显,但种源间差异有所缩小,变异幅度下降。种源内分化明显,其原因可能为不同家系的遗传性状不同,而随着树龄的增加,差异明显;种源间差异缩小,其原因可能为不同种源受相同生长环境影响,随树龄增加,其平均差异逐渐缩小。本试验也将继续关注 13 个种源苗木的生长情况。

4.3　不同种源聚类分析

通过对不同种源的种子重量和形态指标、两地播种苗苗期生长的聚类分析比较,可以看出,1#(美溪)种源种子小,千粒重低,在两个试验点的苗木生长量也低,但该种源内苗木生长变异系数大,表明该种源不同家系的遗传性状差异较大,如能在该地收集到外形较大的种子,可以考虑做家系选择。7#(东京城)种源种子大、千粒重重,但是在两个试验点的苗木生长量却也较低,这可能与东京城种源采集时间早(9 月中旬)有关,造成其种子成熟度不够,影响了出芽后苗木生长。2#(帽儿山)和 6#(兴隆)种源在两个试验点的生长表现不同,帽儿山种源在元宝山试验地的生长状况远不如在林口试验地,着可能与具体生境及环境因子的影响关系较大,帽儿山和林口试验点同属长白山系。兴隆种源在元宝山试验地的生长表现良好而在林口的生长表现较差,其原因是该种源地与元宝山试验地相距最近,均位于小兴安岭南坡,而距离林口试验点则很远。表明不同种源地和试验地之间复杂的地理、气候等因子对于种源移植具有很大影响,造成很大的种源差异与变异差异。种源与栽植地之间有很强的交互作用,表明种源的相对表现依赖于移植的地点[17]。5#(绥阳)、13#(和龙)种源在两个试验点的生长表现均较好,13#(和龙)种源,种子形态和重量不占优势,但表现出很好的生长潜力,反映出

(C)1994-2019 China Academic Journal Electronic Publishing House. All rights reserved.　http://www.cnki.net

这2个种源遗传性状优良,适合做进一步的试验点播种繁殖及优良家系选择研究。综合苗高与基径的生长情况,适合林口地区的优良种源为绥阳、和龙、帽儿山、辉南;适合兴隆地区的优良种源为抚松、和龙、兴隆、绥阳。

4.4 核桃楸不同种源苗木生长指标与主要环境因子的相关性

通过对13个种源地与2个试验点的主要环境因子差异与其1年生苗生长性状(苗高、基径)差异建立的回归方程可以看出,种源地与试验地的苗高差与其经度差、年平均降水量差相关性不明显,而与无霜期差2试验点表现不同,不具有规律性;与纬度差、年平均气温差具有一定相关性,且呈现负相关。很多实验观察表明,种源可以适度地北移,其在移栽地的的生长优于当地的种源[11]。但是如果北移太远,则将会遭受冻害而不如当地种源生长的好。如果向南移植,它们的生长也不如当地种源好。这表明种源地与移植地的气候差异和植物生长之间是一个曲线关系[17]。研究发现预测火炬松种源移植的最重要的因子之一是两地之间的温差和年平均最低温差[17],这与我们的试验结果基本吻合。综合上述分析,核桃楸不同种源1年生苗高与纬度和年平均气温等环境因子有一定相关性,且呈负相关,核桃楸种源可适当北移。另外,由建立的回归方程可见(表7),种源地与试验地的苗木基径差与主要环境因子差异相关性不明显或者不具有规律性,这种种源间的差别与试验地气候因子的不相关性表明一些种源地的选择压力较低或遗传因子占主导地位[18]。

参 考 文 献

1. 吴征镒. 植物地理(上册)[M]. 北京:科学技术出版社,1983:113-114.
2. 张含国,邓继峰,张磊,等. 核桃楸种源家系变异规律及家系选择研究[J]. 西北林学院学报,2011,26(2):91-95.
3. 朱红波,赵云,林士杰,等. 核桃楸资源研究进展[J]. 中国农学通报,2011,27(25):1-4.
4. 杜香莉,郭军战,冯汀. 我国核桃资源的综合利用研究[J]. 西北林学院学报,2003,18(3):82-85.
5. 范成民,董丽芬,朱帜,等. 核桃芽苗砧嫁接方法研究[J]. 西北林学院学报,2008,23(4):109-111.
6. 李保国,齐国辉,郭素平,等. 河北省太行山中南部核桃栽植时期及栽植技术研究[J]. 西北林学院学报,2006,21(4):83-84.
7. 刘桂丰,杨书文,李俊涛,等. 胡桃楸种源的初步区划及最佳种源选择[J]. 东北林业大学学报,1991,19(S):189-195.
8. 赵光仪,田兴军. 黄波罗、胡桃楸、水曲柳分布北限论析[J]. 东北林业大学学报,1991,19(4):290-294.
9. 杨书文,刘桂丰,赵克尊. 胡桃楸早期选择的初步研究[J]. 东北林业大学学报,1990,19(S):76-81.
10. 杨书文,刘桂丰,王会仁,等. 胡桃楸地理变异规律的再研究[J]. 东北林业大学学报,1991,19(S):183-188.
11. Wells O O, Wakeley P C. Geographic variation in survival, growth, and fusiform rust infection of planted loblolly pine [J]. For Sci Monogr, 1966, 11:40.
12. 魏志刚,高玉池,杨传平,等. 引种盐松不同种源种子表型性状和发芽特性[J]. 东北林业大学学报,2009,37(11):7-10.
13. 邵文豪,姜景民,栾启福,等. 乐昌含笑不同种源幼林生长性状变异和种源选择[J]. 江西农业大学学报,2011,33(4):701-706.
14. 姜景民,虞沫奎,胡世才,等. 湿地松种子分级育苗的研究[J]. 林业科学研究,1996,9(3):290-295.
15. 唐庆兰,黎海利,黄寿先,等. 大叶栎优树种子性状变异研究[J]. 广西林业科学,2006,35(1):12-13,33.
16. 佘诚棋,杨万霞,方升佐,等. 青钱柳种源间苗期性状变异分析[J]. 南京林业大学学报:自然科学版,2010,34(1):34-38.
17. Schmidtling R C. Use of provenance tests to predict response to climatic change: loblolly pine and *Norway spruce* [J]. Tree Physiology, 1994, 14:805-817.
18. Arend M, Kuster T, Günthardt-Goerg M S, et al. Provenance-specific growth responses to drought and air warming in three European oak species (*Quercus robur*, *Q. petraea* and *Q. pubescens*) [J]. Tree Physiology, 2011, 00, 1-11, doi:10.1093/tree phys/tpr004.

(C)1994-2019 China Academic Journal Electronic Publishing House. All rights reserved. http://www.cnki.net
