# Supplementary material for: Provenance and family variations in early growth of Manchurian walnut (Juglans mandshurica Maxim.) and selection of superior families
Source: PLoS One. 2024 Mar 7;19(3):e0298918. doi: 10.1371/journal.pone.0298918 (PMC10919699; doi:10.1371/journal.pone.0298918)
Supplement: S2 File — (ZIP) [file pone.0298918.s005.zip › Provenance study of Scots pine (Pinus sylvestris L.) in Belgium. I. Evaluation of phenoptypical traits.pdf]

Provenance study of Scots pine (*Pinus Sylvestris* l.) in Belgium. I. Evaluation of phenoptypical traits

ZHELEV, P.[1)], LUST, N.[2)]

[1)]*Department of Dendrology, University of Forestry, 1756 Sofia, Bulgaria*
[2)]*Laboratory of Forestry, Ghent University, Geraardsbergsesteenweg 267, B-9090 Melle-Gontrode, Tel +32-9-2522113, Fax +32-9-2525466, E-mail Noël.Lust@rug.ac.be*

1. Introduction

The provenance tests are among the classical investigation methods in forest genetics. They provide knowledge about the reaction of different origins of the species to a new environment. This information is both of scientific and practical importance. Of particular interest are some provenance experiments, established many decades ago. Despite of the fact, that their designs are not completely according to the modern statistical requirements, they can provide valuable information concerning the adaptation of different races of the species to the new environment (Giertych, 1986).

The Scots pine (*Pinus sylvestris* L.) is among the most important forest tree species because of its economical value and wide geographic range of distribution - from Iberian peninsula and Scotland to the Far East and from Scandinavia to Asia minor (Boratinsky, 1991). Because of its importance, this species is among the most studied forest tree species in Europe (Giertych & Matyas (eds.), 1991).

The provenance experiments chosen for this study are among the oldest in Europe (Nanson, 1974). They were established in 1908 and 1909, respectively, within the first IUFRO series on the provenance studies of Scots pine. The previous results were summarised by Wiedemann (1930), Delevoy (1937), Nanson (1968, 1974), Giertych (1979) and Giertych & Oleksyn (1992). The objective of this work was to summarise the results now, 85 years later. We decided to include as many characters as possible in the analysis. In the present work the results of the general phenotypic characteristics are reported. The data about some biochemical characteristics, needle morphology and anatomy and tree ring growth will be presented and discussed in subsequent papers.

2. Material et methods

2.1. The experiment

The object of present study are two provenance experiments of Scots pine (*Pinus sylvestris* L.) in Belgium. The first experiment is situated in Groenendal Forest Experimental Station, in "Foret de Soigne", near Brussels and the second one is in Pijnven, in the Northern part of Belgium.

After the works of Wiedemann (1930) and Delevoy (1937), no reports concerning Pijnven experiments have been published. Giertych and Oleksyn (1992) stated the hypothesis that probably Pijnven experiment could be reactivated, which is confirmed by the present study.
The provenances, included in the experiments are listed in table 1 and their geographic origin is presented in fig. 1. The order is according to the IUFRO numbering. The data about the geographic coordinates and altitude were presented by Giertych and Oleksyn (1992). Some information about the exact present names of the provenances was kindly provided by Prof. Dr. Maciej Giertych - Institute of Dendrology - Polish Academy of Sciences. However, the names from the original Experimental Station's documents are also presented in table 1. For historical details concerning the establishment of the experiments see also Giertych and Oleksyn (1992).

2.2. Characters assessed

The following characteristics were included in the analysis (following Oleksyn and Giertych, 1984):

1. Diameter (cm) - obtained by measuring all trees in provenances at breast height (1.3 m).
2. Height (m) measured on ten trees having diameter about the mean for the provenance.
3. Stem form - estimated on a 5 point scale where 1 indicates very crooked trees and 5 ideally straight ones.
4. Cleaning - estimated on a 5 point scale where 1 indicates trees with many dead branches and 5 - trees free of any dead branches.
5. Branchiness - estimated on a 5 point scale where 1 indicates very heavy branches and 5 very thin ones.
6. Branch angle - estimated on a three point scale where 1 indicates below 30°, 2 - an angle close to 45° and 3 - an angle close to 90°.

2.3. Data analysis

For estimation of the effect of provenance, site, replication within site and provenance x site interactions for the characteristics height and diameter, an analysis of variance was applied using the following model:

$$Y_{ijk} = \mu + S_i + P_j + R_k + SP_{ij} + e_{ijkl}$$

where Y_{ijk} is the value of the character of l-th individual in k-th replication in j-th provenance in i-th site;

μ is the overall mean;

S_i is the effect of the i-th site (i=1,2)

P_j is the effect of j-th provenance (j=1..8)

R_k is the effect of the k-th replication (k= 1..4)

SP_{ij} is the interaction term, specified by i-th site and j-th provenance

e_{ijkl} is the residual error

The provenances represented without replications and included in one site only (see table 1) were omitted from the ANOVA test. Linear regression analysis was performed for revealing the impact of latitude and longitude on the quantitative traits.

Table 1. *General information about the provenances and test sites studies*

PROVENANCES			Sites No of replications		Latitude (N)	Londitude	Altitude [m]
IUFRO No	Actual name	Name listed in the documents					
			Groenendaal	Pijnven			
1	Scotland		2	2	57° 14'	3° 42' W	200
2	France (Haute de la Loire)		2	2	44° 58'	3° 07' E	1140
3	Poland (Olsztyn)	Eastern Prussia	2	2	53° 40'	21° 30'	130
4	Belgium		2	2	50° 54'	5° 40'	104
5	Germany (Kaiserslautern)	Bavaria*	2	2	49° 25'	7° 45'	300
	Latvia (Riga)	Russia-Riga					
6	first collection		2	2	56° 45'	25° 45'	10
6a	second collection		2	2	56° 45'	25° 45'	10
7	Germany (Brandenburg)	Prussia-Brandenburg	2	2	52° 50'	14° 10'	40
8	Russia (Perm)		1	1	57° 00'	64° 00'	300
9	Bulgaria (Plovdiv)		1	-	42° 10'	23° 50'	1550
11	Slovakia (Spis)	Hungary-Nord	1	-	48° 45'	20° 45'	550
12	Slovakia (Bratislava)	Hungary-West	2	-	48° 30'	17° 00'	210

* Despite the name "Bavaria", written in the documents of the Forest Experimental Station in Groenendaal, the oppinion of Prof. Dr. M. Giertych (pers. comm.), based also on the data about the geographic longitude of Wiedemann (1930), is that the actual place of this provenance is Kaiserslautern (Rheipfalz).

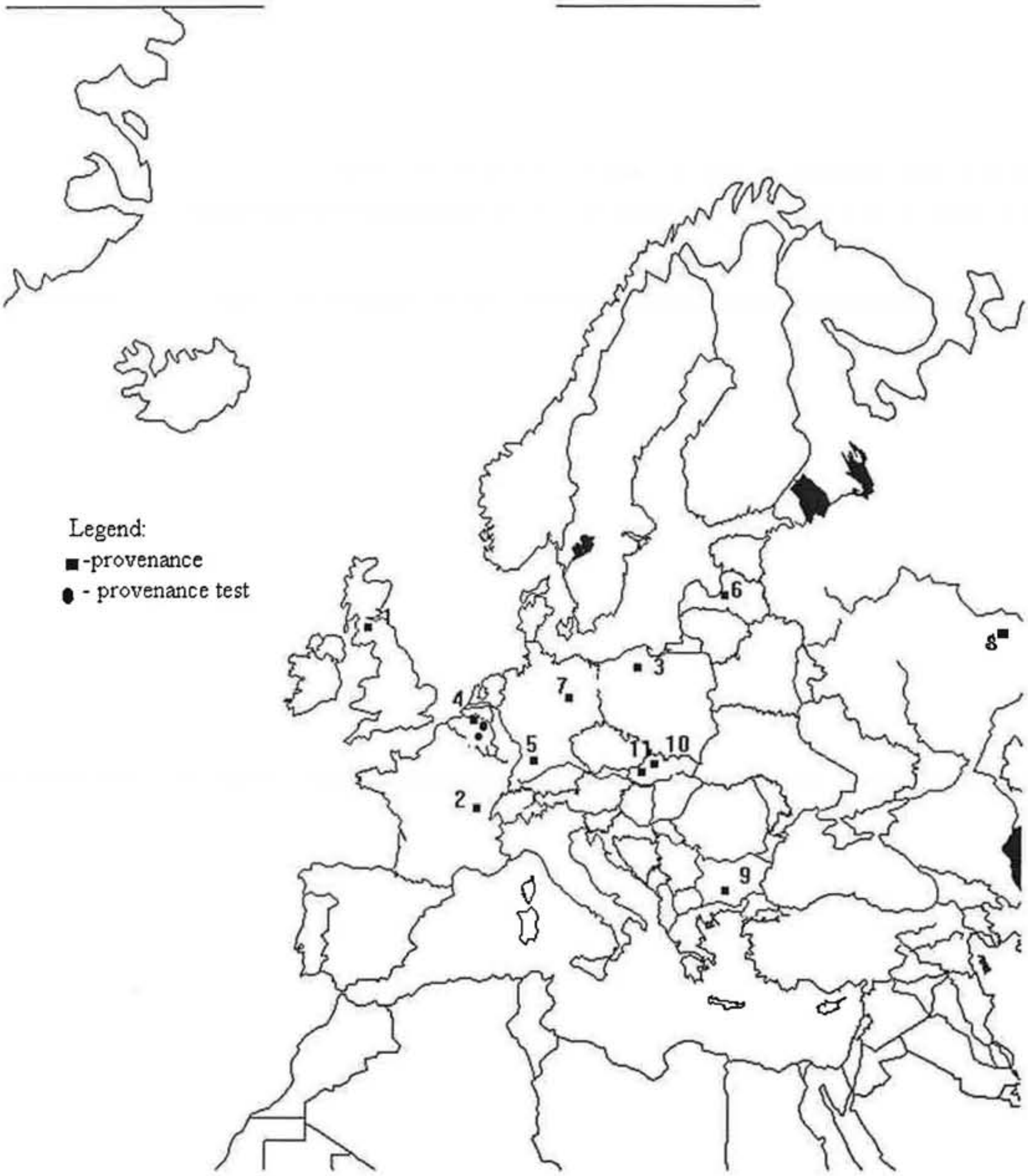

Figure 1. *Map of the provenances*

We used the true values of the latitude and longitude, where the minutes were converted into real numbers, i.e. 60 minutes were taken as equal to 1.00 and one minute to 0.0167. For example, if the latitude is 52°24', the value assigned is 52.40. Also correlation analysis was applied to detect the relationships among different traits (phenotypical correlations).

3. Results

3.1. Analysis of variance

The ANOVA test (tables 2 and 3) performed on the height and diameter growth revealed significant effects of the site conditions on the provenance performance. Also the provenance effects were statistically significant. The provenance x site interaction effect was significant only for the height growth.

Table 2. *Summary of the analysis of variance for the diameter (7 provenances included)*

Source of variation	D.F.	Mean square	F-ratio	Significance level
Site	1	945.39	31.13	***
Replication	6	521.76	17.18	***
Provenance	1	0.322	0.011	ns
Site x Provenance	6	50.56	1.665	ns
Residual	258	30.37		

Legend:
**** significant at confidence level $p \leq 0.001$*
ns - not significant

Table 3. *Summary of the analysis of variance for the height growth (8 provenances included)*

Source of variation	D.F.	Mean square	F-ratio	Significance level
Site	1	1785.6864	451.415	***
Replication	1	1.1770	0.298	ns
Provenance	7	105.3795	26.640	***
Site x Provenance	7	29.68038	7.503	***
Residual	131	3.9557554		

Legend:
**** $p \leq 0.0001$*
ns - non significant

3.2. Height growth

The measurements in both sites revealed the following trends in relation to the height growth of the provenances. The largest mean value for the height growth in the experiment Belle Etoile possess Riga - second collection (IUFRO No 6a), followed by Olsztyn and Bulgaria (table 4). The lowest mean value possess France (Haute de la Loire). The remaining provenances have intermediate values regarding this trait (Fig. 2).

For the site conditions of Pijnven first rank has again the provenance from Riga (second collection), followed by Kaiserslautern, and here the provenance from Perm expresses considerably lower values in comparison to the others. The provenance from France possess again low value for mean height, but higher than Perm (table 5, fig. 3).

Table 4. *Mean values of the traits studied at the site conditions in Groenendaal*

Provenance	CHARACTERISTICS					
	D	H	SF	CL	BR	BA
Scotland	42.53	26.15	3.09	1.62	2.37	2.87
France (Haute de la Loire)	32.43	24.25	2.59	2.22	2.95	2.78
Poland (Olsztyn)	30.61	29.50	3.00	2.79	2.16	2.36
Belgium	36.20	27.15	3.32	2.82	2.75	2.87
Germany (Kaiserslautern)	41.36	26.50	2.20	2.48	2.16	2.92
Latvia (Riga)						
first collection	33.65	27.92	3.65	3.43	3.05	2.85
second collection	34.36	30.75	3.55	2.89	2.92	2.73
Germany (Brandenburg)	36.29	26.4	3.31	2.78	2.66	2.87
Russia-Perm	32.22	25.7	4.00	2.89	3.22	3.00
Bulgaria (Plovdiv)	39.71	28.85	3.21	2.57	2.21	3.00
Slovakia (Spis)	37.50	26.8	3.25	2.40	2.55	2.90
Slovakia (Bratislava)	34.33	25.75	3.40	2.67	2.80	2.95

Legend:
D - diameter [cm]
H - height [m]
SF - stem form
CL - cleaning
BR - branchiness
BA - branch angle

Table 5. *Mean values of the traits studied for the provenances in the Pijnven site conditions (see table 4 for the abbreviations of traits)*

Provenance	Characteristics (Rank)					
	D	H	SF	CL	BR	BA
Scotland	37.72	19.5	3.04	2.77	2.54	2.72
France (Haute de la Loire)	28.22	16.8	2.00	2.22	2.33	3.00
Poland (Olsztyn)	33.40	22.25	3.25	2.91	2.94	2.84
Belgium	32.88	21.3	3.27	3.00	3.08	2.88
Germany (Kaiserslautern)	37.33	22.45	2.33	2.93	2.53	3.0
Latvia (Riga)						
first collection	31.76	24.9	3.58	3.11	3.00	2.96
second collection	29.68	21.4	3.87	3.52	3.74	2.93
Germany (Brandenburg)	36.39	21.6	3.13	3.08	2.30	2.91
Russia-Perm	17.44	13.6	1.92	2.28	1.84	2.92

		D [cm]	H [m]	SF	CL	BR	BA
	SCOT	37,72	19,5	3,04	2,77	2,54	2,72
	FR	28,22	16,8	2	2,22	2,33	3
	PL	33,4	22,25	3,25	2,91	2,94	2,84
	BEL	32,88	21,3	3,27	3	3,08	2,88
	KA	37,33	22,45	2,33	2,93	2,53	3
Latvia (Rig	R1	31,76	24,9	3,58	3,11	3	2,96
	R2	29,68	21,4	3,87	3,52	3,74	2,93
	BR	36,39	21,6	3,13	3,08	2,3	2,91
	PE	17,44	13,6	1,92	2,28	1,84	2,92

Figure 2. *Mean values for height and diameter for the provenances at the Groenendaal site conditions*

Abbreviations: SC-Scotland; FR-France; PL-Poland; BEL-Belgium; KA-Kaiserslautern; R1 and R2 —Riga 1 and Riga 2 respectively; BR-Brandenburg; PE-Perm (Russia); SL1 and S12-Slovakia 1 and Slovakia 2 respectively.

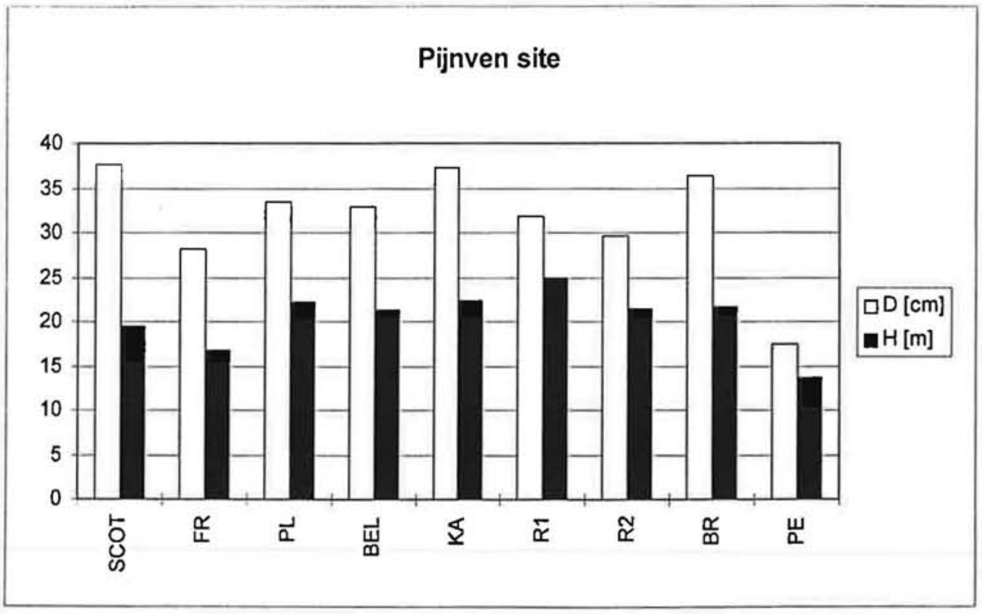

Figure 3. *Mean values for heigt and diameter for the provenances at the Groenendaal site conditions (For abbreviations see fig. 2).*

3.3. Diameter growth

For the site in Groenendaal (Belle etoile) the largest mean value in diameter was estimated for the provenance of Scotland (table 4). The second position keeps Germany (Kaiserslautern) and the third is Bulgaria. The lowest value of the diameter growth now can be attributed to Olsztyn, while the remaining provenances are between these values (fig. 2).

For the provenance test in Pijnven the very low values for diameter for the Perm provenance could be pointed out - 17.44 cm in comparison to the others. For example, the first positions keep Scotland (37.72 cm), followed immediately by Kaiserslautern (37.33 cm) and the other provenances, while the remaining provenances have intermediate values, but considerably higher than Perm (table 5, fig. 3).

3.4. Qualitative characters

Concerning the stem form, in Groenendaal site the straightest stems has the provenance from Perm (Russia). This is in contrast to its very low survival rate - only 9 trees remained. Since the stem form itself is not the most important characteristic, but all the characters have to be evaluated in a complex manner, it couldn't be estimated that Perm is a very suitable provenance for the site conditions there. The differences, however, are too small among the provenances. Kaiserslautern provenance possess the lowest mean value - 2.00, which indicates very crooked trees (table 4).

To the contrary, Perm provenance is the worst performer, concerning the same trait, in the site of Pijnven. Here the straightest stems are estimated for Riga (table 5).

The cleaning and branchiness are important characteristics, regarding the wood quality, since the heavy dead branches decreases the possibilities of using the wood. In both sites Riga provenance possess higher values for cleaning than the remaining ones. Scotland in Groenendaall and France and Perm in Pijnven are the worst performers regarding the cleaning.

Similar trend is estimated concerning the branchiness, where Riga posses highest values, and only Perm provenance values exceed those for Riga for the site in Groenendaal.

The branch angle values don't differ significantly among the provenances (Tables 4 and 5). Most of them are between 2.5 and 3, which means nearly horizontal, with an exception - Poland for the site in Groenendaal.

3.5. Phenotypic correlations

The correlation coefficients among the characters assessed (table 6) are significant in five cases. They are namely for the couples of characters: stem form - branchiness, stem form - cleaning, cleaning-branchiness, height-diameter and height-stem form. All the remaining correlation coefficients are not significant at 5 % probability level.

3.6. Regression analysis

For testing the effect of the geographic coordinates we plotted the values for diameter and height against the geographic latitude and longitude. This is of particular importance regarding the seed transfer. The only one significant regression was estimated for the effect of the longitude on the diameter and height growth for the site in Pijnven (fig. 6-9).

Table 6. *Matrix of the phenotypical correlation coefficients among the traits studied*

	D	H	SF	CL	BR	BA
D	1.0000					
H	0.5892*	1.0000				
SF	0.2206	0.5255*	1.0000			
CL	-0.1216	0.1127	0.5686**	1.0000		
BR	-0.0214	0.1609	0.7273***	0.6019**	1.0000	
BA	0.0514	-0.3127	-0.0411	-0.0196	0.1397	1.0000

Legend: signiificant at
* $p \leq 0.05$
** $p \leq 0.01$
*** $p \leq 0.001$

Abbreviation of traits: D- diameter, H - height, SF - stem form, CL - cleaning, BR - branchiness, BA - branch angle.

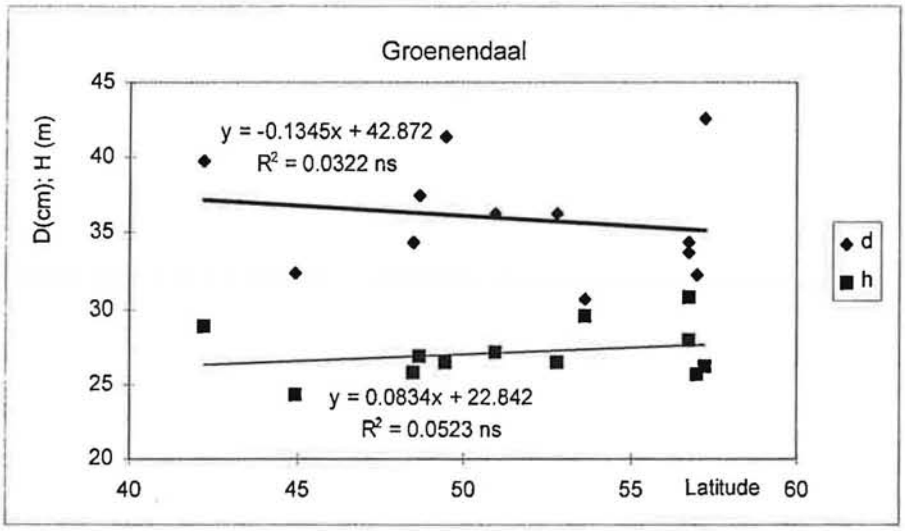

Figure 4. *Relationship between the latitude and height and diameter performance at Groenendaal site experiment.*

Legend: ns -not significant at 5 % probability level

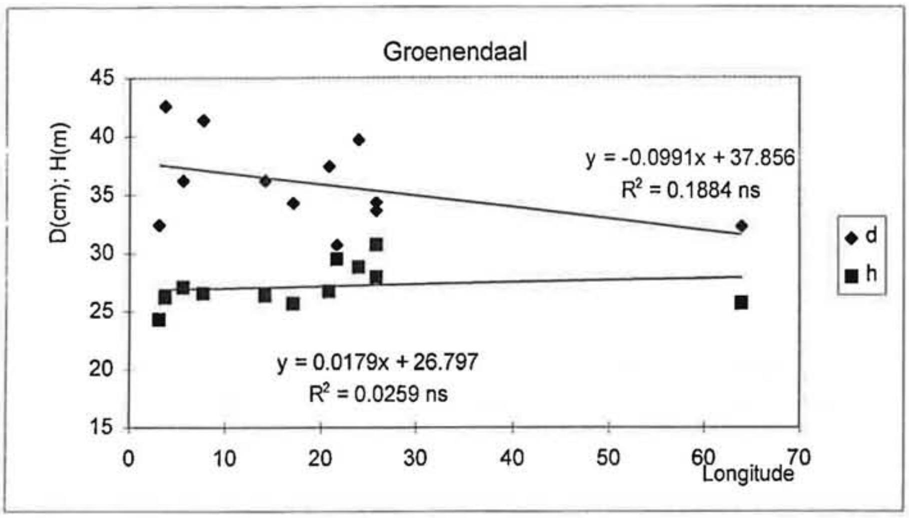

Figure 5. *Relationship between the longitude and height and diameter performance at Groenendaal site experiment.*

Legend: ns -not significant at 5 % probability level

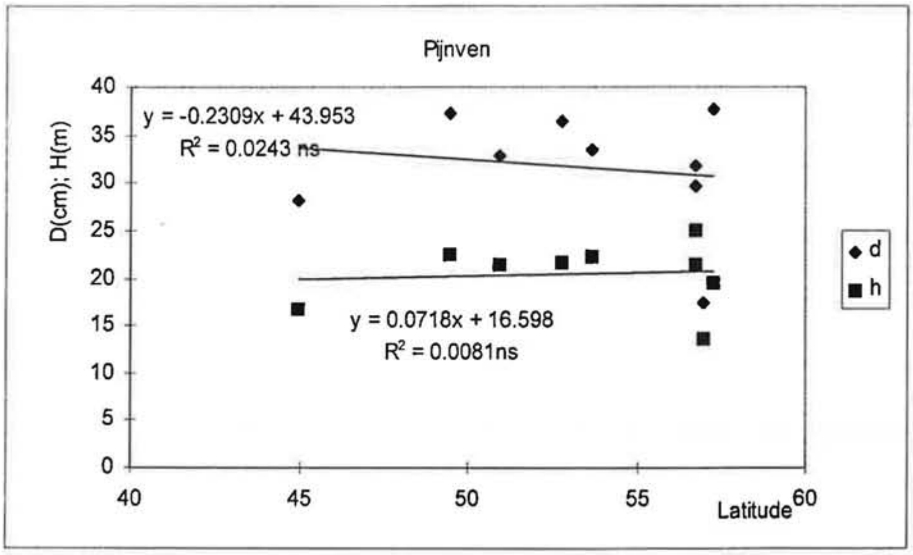

Figure 6. Relationship between the latitude and height and diameter performance at Pijnven site experiment.

Legend: ns - not significant at 5 % probability level

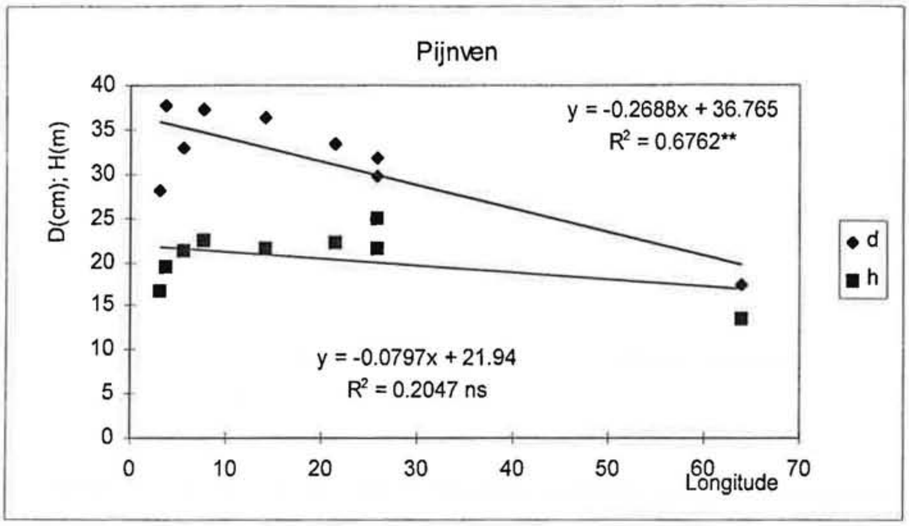

Figure 7. Relationship between the longitude and height and diameter performance at Pijnven site experiment.

Legend: ns - not significant at 5 % probability level
*** - significant at 1 % probability level.*

4. Discussion

There is a large amount of data and experience concerning the Scots pine provenances' performance. It is often pointed out that the marginal provenances regarding the area of distribution possess lower performance in comparison to the central ones (Giertych, 1991a,b). Giertych and Oleksyn (1992) presented a map, showing the most appropriate provenances for transfer. It is confirmed by this study, that the Baltic provenances are among the best performers. The local provenance from Belgium possess intermediate values. The relatively low values of the French provenance contrast the previous studies. The only one provenance from Balkan peninsula (i.e. Bulgaria) keeps the third rank both for height and diameter growth at the site conditions in Gronendaal. This is somewhat controversial to the statement, that usually these pronenances (tested quite seldom, however) are among the worst ones. Even though this provenance, together with the Scottish one, show relatively good values for diameter and height, their quality characteristics are lower, because of presence of strong branches, poor cleaning etc. These provenances and particularly Scotland represent the marginal Scots pine populations (Ennos, 1991).

It is confirmed once again the low performance of the Perm provenance. It could be pointed out also the contrast behaviour of this provenance regarding the survival rate in the two sites. While in Pijnven most of the trees survived, there was only nine trees alive in Groenendaal site. The values for height and diameter for Perm provenance in Pijnven were much lower, than the others, and therefore during our second visit in Pijnven, in March 1995, we recognized that all the trees were removed from the experiment. As it was pointed out by Giertych and Oleksyn (1992), this provenance could serve only as a link between the Eastern and Western provenances.

The ranking of Delevoy (1937) and Nanson (1968) were confirmed in most cases by the present experiment. They also outlined the provenances from Riga as best performers. It is expected, however, that provenances originating from similar climatic conditions (mild and wet climate) will be more adaptive than the others, originating from typical continental climate. Since the mortality affect the number of trees per unit area, we did not count the volume production. It could be more precious if the mean single tree volume is used as a character for comparison of the provenances.

Additional difficulties came from the several unreplicated provenances, that were omitted from the ANOVA test. There is some approaches of using analysis of variance even in unreplicated provenance tests, but it requires much more provenances, included in the experiment (Abraitis & Eriksson, 1996).

Nanson (1968) proved the high degree of correlation of a same trait at different age (age-age correlations), which were in most cases significant for the quantitative traits. Here we could not propose such comparison because of different manner of measurement.

Concerning the quality traits, such as stem form, branchiness, cleaning and branch angle, the differences are not as large, as in the growth characters. It is influenced also by subjective quantification of these traits, where the metric values assigned do not allow to process them statistically with the same success as the quantitative characters.

The provenance by site interactions was of importance only for height growth. This phenomenon is of interest because of the contrast site conditions. It is believed that the provenance by site interactions in forest trees are often detectable, but of small importance (see Matheson & Raymond, 1984, 1986 and Skroppa, 1984 for reviews). In the present case no special conclusions could be drawn because of the limited number of test sites.

The longitude correlated with the growth characters better than latitude. This finding confirm the trends estimated (e.g. Giertych & Oleksyn, 1992) regarding the seed transfer. Generally, our results correspond to the estimated most adaptive provenances in the IUFRO experimental series. We used the true values of the latitude and longitude in the regression analysis.

Finally it appear that the results of the two experiments studied confirm the general trends established from the wide range IUFRO provenance series.

5. Acknowledgements

P. Zhelev thanks many persons, involved in the study. Special thanks are due to Dr. Bart Muys (now in the University of Leuven), ir. Wim Buysse, ir. Johan Neirynck, ir. Danny Maddelein, ir. Geert Sioen, ir. Bruno de Vos - all from the Laboratory of Forestry, Univ. of Gent, Belgium, for help and courtesy during the stay in Belgium. Also warm thanks to ir. Bart de Cuyper - Forest Experimental Station in Groenendaal and Mr. Van Oost - Regional Forestry Service - Zonienwoud, and Mr. Van Geebelen - Regional Forestry Service - Pijnven. Special thanks to Prof. Dr. Maciej Giertych from the Institute of Dendrology - Kornik (Poland) for the information provided about the provenances. The financial support, provided by the Project No CIPA 3510 PL- 924604 of the European Community's Action for Cooperation in Sciences and Technology with Central and Eastern European Countries is also very much appreciated.

6. References

Abraitis, R. & Eriksson, G. (1996). *Pinus sylvestris* L. East European populations: Growth and behaviour in one Lithuanian field trial. Baltic Forestry, 2, 28-35.

Boratinsky, A. (1991). Range of natural distribution. In: M. Giertych & C. Matyas (eds.) Genetics of Scots pine. Akademiai Kiado, Budapest, 19-30.

Delevoy, G. (1937). Experience internationale sur l'origine du Pin-sylvestre. Bulletin de la Societe Centrale Forestiere de Belgique., 3, 97-124.

Ennos, R.A. (1991). Genetic variation in Caledonian pine populations: origins, exploitation and conservation. In: G. Muller-Starck and M. Ziehe (eds.). Genetic Variation in European Populations of Forest Trees. Sauerländer's Verlag, Frankfurt am Main, 235-249.

Giertych, M. (1979). Summary of results on Scots pine (*Pinus sylvestris* L.) height growth in IUFRO provenance experiments. Silvae genetica, 28 (4), 136-152.

Giertych, M. (1986). L'amélioration génétique des arbres forestiers - aujourd'huit et demain. Revue Forestiere Francaise, Special issue, 204-207.

Giertych, M. (1991a). Provenance variation in growth and phenology. In: M. Giertych and C. Matyas (eds.) Genetics of Scots pine. Elsevier : 87-101.

Giertych, M. (1991b). Inheritance of tree form. In: M. Giertych and C. Matyas (eds.) Genetics of Scots pine. Elsevier : 243-254.

Giertych, M. & Matyas, C. (eds.) (1991). Genetics of Scots pine. Series Developments in plant genetics and breeding No 3, Elsevier, 280 pp.

Giertych, M. & Oleksyn, J. (1992). Studies on genetic variation in Scots pine (*Pinus sylvestris* L.) coordinated by IUFRO. Silvae genetica, 41 (3), 133-143.

Matheson, A. C. & Raymond, C.A. (1984). Provenance x environment interaction: its detection, practical importance and use with particular reference to tropical forestry. In: Barnes R.D. and Gibson G.L. (eds). Commonwealth Forestry Institute, Oxford, and Forest Research Center, Harare: 81-117.

Matheson, A. C. & Raymond, C.A. (1986). A review of provenance x environment interaction: its practical use with particular reference to the tropics. Commonwealth Forestry Review, 65, 283-302.

Nanson, A. (1968). La valeur des tests precocées dans la selection des arbres forestiers en particulier au point de vue de la croissance. These du doctorat, Station des recherches des Eaux et Forets, Groenendaal-Hoeilaart. Faculte des sciences agronomiques, Gembloux.

Nanson, A. (1974). Outlines of forest tree breeding in Belgium. In: R. Toda (ed.): Forest tree breeding in the world: 40-45.

Oleksyn, J. (1988). Report on the IUFRO 1982 provenance experiment on Scots pine (*Pinus sylvestris* L.). Arboretum Kornickie, 33, 211-229.

Oleksyn, J. & Giertych, M. (1984). Results of a 70-years old Scots pine (*Pinus sylvestris* L.) provenance experiment in Pulawy, Poland. Silvae genetica, 33(1), 22-27.

Skroppa, T. (1984). A critical evaluation of methods available to estimate the genotype x environment interaction. Studia Forestalia Suecica, No 166, 3-14.

Wiedemann, G. (1930). Die Versuche über den Einfluss der Herkünft des Kiefernsamens. Zeitschrift für Forst und Jagdwesen, 62, 498-522.
