# Supplementary material for: Provenance and family variations in early growth of Manchurian walnut (Juglans mandshurica Maxim.) and selection of superior families
Source: PLoS One. 2024 Mar 7;19(3):e0298918. doi: 10.1371/journal.pone.0298918 (PMC10919699; doi:10.1371/journal.pone.0298918)
Supplement: S2 File — (ZIP) [file pone.0298918.s005.zip › Provenance variation and within-provenance genetic parameters in Eucalyptus urophylla across 125 test sites in Brazil, Colombia, Mexico, South Africa and Venezuela.pdf]

Tree Genetics & Genomes (2015) 11: 57
DOI 10.1007/s11295-015-0889-3

ORIGINAL PAPER

Provenance variation and within-provenance genetic parameters in *Eucalyptus urophylla* across 125 test sites in Brazil, Colombia, Mexico, South Africa and Venezuela

G. R. Hodge[1] · W. S. Dvorak[1]

Received: 13 November 2014 / Revised: 5 May 2015 / Accepted: 7 May 2015 / Published online: 21 May 2015
© Springer-Verlag Berlin Heidelberg 2015

Abstract Provenance and within-provenance genetic variation for stem volume growth in *Eucalyptus urophylla* was examined in a large trial series of 125 provenance/progeny tests in five countries. In general, heritability (within-provenance) for age-3 volume was around 0.15 for Brazil, Colombia, Mexico, and South Africa and was slightly lower in Venezuela, where tree volume was lower. Provenances from all seven islands in Indonesia where the species occurs were included in the trials. Substantial provenance variation was observed in all countries, with the best provenances showing from 25 to 30 % more volume than the mean. The average of between-country genetic correlation estimates for growth traits was 0.72, both among and within provenances. Provenance and family performance between Mexico and Venezuela were very similar, with estimated provenance and within-provenance genetic correlations of 1.00 and 0.90, respectively. There was some tendency for lower-elevation provenances to show better volume growth, but there were also very large differences observed between provenances located very close to one another. The results underscore the value of extensive provenance collections throughout the range of a species in order to ensure that the very best provenances are being captured.

Communicated by: R. Burdon

This article is part of the Topical Collection on *Breeding*

✉ G. R. Hodge
grh@ncsu.edu

1 Camcore, Department of Forestry and Environmental Resources, North Carolina State University, Raleigh, NC, USA

Keywords Genetic parameters · Provenance · Genotype× environment interaction · Conservation status · Type B correlations · Meta-analysis · *Eucalyptus urophylla*

Introduction

Eucalyptus urophylla is one of the most important species in tropical plantation forestry. It is occasionally used commercially as a pure species in regions of Southeast Asia, tropical Brazil, and along the western Gulf Coast in Mexico. However, it is better known as an important hybrid partner and is commonly crossed with other eucalypt species to produce progeny that often exhibit remarkable hybrid vigor for growth. Historically, *E. urophylla* has most often been hybridized with *Eucalyptus grandis* to produce fast-growing clonal plantations for the pulp and solid-wood industries in the Southern Hemisphere. Generally, the *E. grandis*×*E. urophylla* hybrid has better disease tolerance for some leaf and stem diseases, and higher wood density than pure *E. grandis*. More recently, *E. urophylla* has been tested as a hybrid partner with *Eucalyptus pellita* in the Republic of the Congo (Vigneron et al. 2000) and in Indonesia, and with *E. pellita* and *E. brassiana* in north-eastern Brazil, where the hybrids there are commercially planted as a source of high-density wood for bio-energy. Crosses between *E. urophylla* and cold-tolerant species like *E. benthamii* and *E. dunnii* are also now being tested in seasonally cool areas in southern Latin America. The *E. urophylla* parent brings increased growth and better rooting ability to the hybrid compared to the cold-tolerant eucalypt parent. In many ways, *E. urophylla* is a universal hybrid donor, since it can easily be crossed with a number of different eucalypt species and often produces reasonable crops of viable hybrid seeds.

E. urophylla is native to seven islands in the eastern Lesser Sunda Archipelago: Flores, Adonora, Lembata, Pantar, Alor, Timor, and Wetar (see Fig. 1). The species occurs from 70-m elevation (Wetar) to over 2500 m (Timor) (Gunn and McDonald 1991) across a myriad of soil types that are usually well-drained. Black and dark-grey volcanic soils typify the sites at Mt. Egon (Flores), Mt. Boleng (Adonara), and Mt. Lewotolo (Lembata). More ancient and weathered yellow-brown soils can be found on some sites on Mt. Mutis in western Timor and reddish-yellow soils derived from uplifted ocean crust typify the soil profiles on Wetar. Estimates of annual precipitation on the islands vary depending on the climatic models used. The FloraMap™ model (Jones and Gladkov, 1999) indicates that mean annual precipitation ranges from 820 to 1250 mm across the islands (Dvorak et al. 2008). Calculations using WorldClim™ (Hijmans et al. 2005, 2012) suggest higher yearly precipitation averages from 1130 to 1580 mm (Camcore, unpublished). Both models show Wetar to receive the least precipitation and the Timor highlands to be the wettest, with the WorldClim model indicating that some provenances on the latter receive approximately 2000 mm of rain annually. The length of the dry season varies by island. The island of Wetar and some provenances in the highlands of Timor have only a 3-month dry season (<50 mm of rain per month), while most of the other islands have more pronounced dry periods of 5 months between June and October. Of the seven islands, the climate on Wetar is most distinct from the other six islands, with lower, more evenly distributed rainfall (Dvorak et al. 2008).

Australian and French forest researchers were the first to make seed collections of *E. urophylla* in Indonesia for provenance testing in the 1960s and 1970s (see Martin and Cossalter 1975–1976; Turnbull and Brooker 1978; Jacobs 1981; FAO 1981; Moura 1981; Gunn and McDonald 1991). There have been a number of papers published in the last 2 decades on the results of provenance and family testing of *E. urophylla* from these early seed collections, including estimation of genetic parameters (see Vercoe and Clarke 1994; Wei and Borralho 1998; Rocha et al. 2006; Kien et al. 2009; Maid and Bhumibhamon 2009; Souza et al. 2011; and others). Results generally indicated that Mt. Egon and Mt. Lewotobi, on Flores Island, were the best in productivity and that the

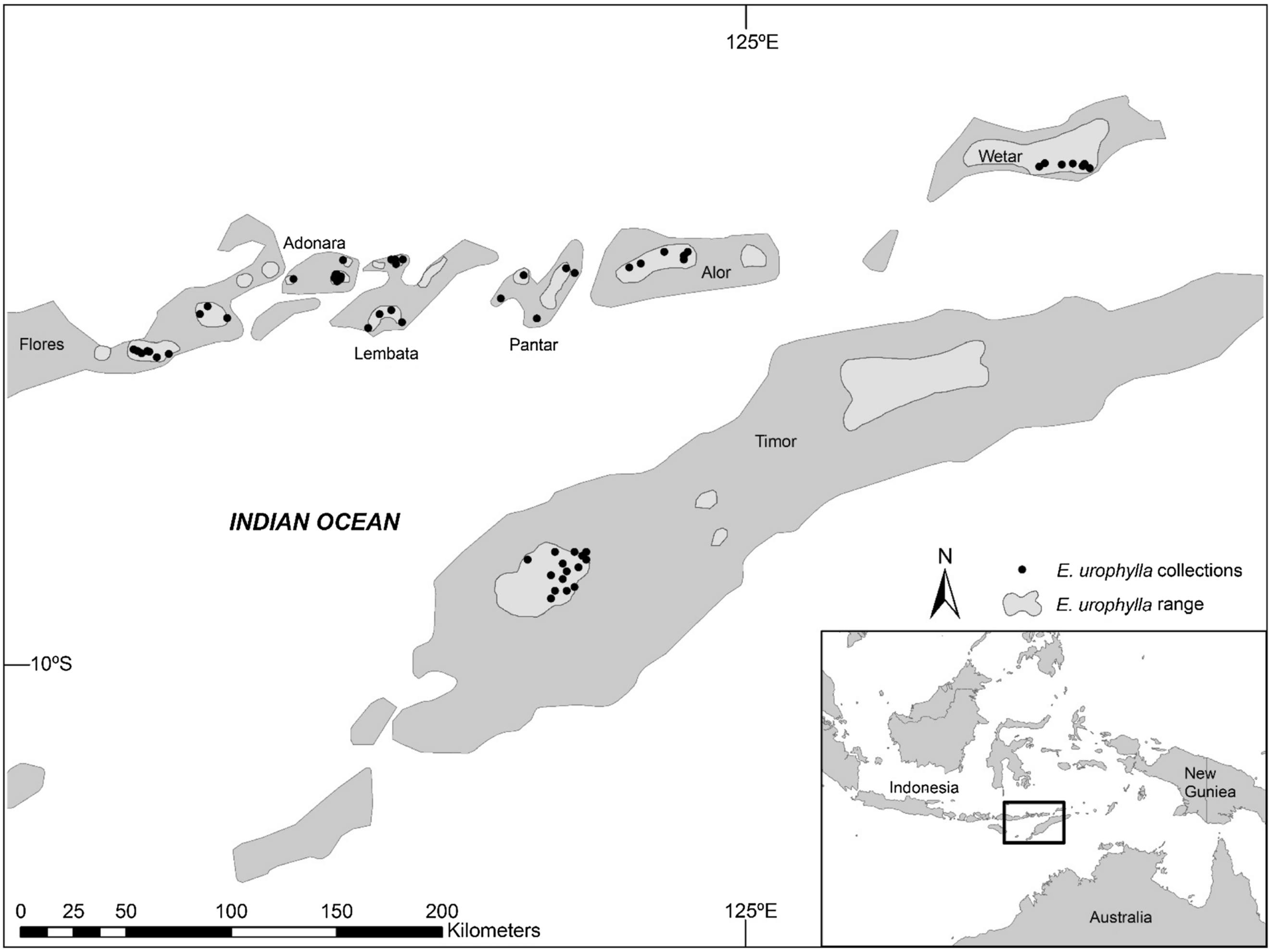

Fig. 1 Natural range of *E. urophylla* and provenances where family seed collections were made on seven islands in Indonesia

high-elevation Timor sources were the poorest in growth. However, Vercoe and Clarke (1994) concluded in their summary of international test results of *E. urophylla* trials in the tropics and subtropics that there was no one single superior provenance or island of origin, with the exception of the poor performance of high-elevation sources on Timor. The main limitation of many of these early *E. urophylla* studies was that only one to a few trials were established per country and sometimes the studies included populations represented by only a few open-pollinated families. In some cases, provenance/progeny trials were selectively thinned prior to assessment, which would bias differences among provenances and families, and affect the precision of estimates of genetic parameters.

As *E. urophylla* has grown in importance as a plantation species and a hybrid partner, governments, universities, and private sector organizations have made additional seed collections on the islands to resample known populations of proven quality and to locate new ones. One of the most recent comprehensive seed collections of *E. urophylla* were made by Camcore (International Tree Breeding and Conservation Program), North Carolina State University, and PT Sumalindo Hutani Jaya, a local Indonesian forest industry, from 1996 to 2003 (Hodge et al. 2001; Pepe et al. 2004; Dvorak et al. 2008). In the 7 years of seed collections, 62 populations and 1104 mother trees were sampled on the seven islands where *E. urophylla* naturally occurs. Camcore collections were different than prior ones in several respects. First, the collection efforts sampled a larger number of locations on most islands than previous collections. Second, there was an attempt to make multiple seed collections of *E. urophylla* on different aspects of the slopes of several volcanoes with a recent history of eruptions (Mt. Egon, Mt. Boleng, Mt. Lewotolo). This was to determine if different levels of natural and/or man-made disturbance influenced provenance performance in field trials dependent on the life history of the particular stands. Third, there was a mandate from the Camcore membership to assess the conservation status of each natural population sampled on the islands using standards of the International Union for the Conservation of Nature and Natural Resources (IUCN) (Pepe et al. 2004). The ultimate goal was to establish ex situ conservation parks of the *E. urophylla* in various countries (Mitchell 2010; Dvorak 2012). Fourth, all Camcore members participating in the field testing of the genetic material collected in Indonesia in the various countries were required to use the same test design, establish trees at approximately the same spacing, and make assessments at the same age to facilitate meaningful comparisons across sites and years.

In this paper, we examine provenance and family performance across two continents and five countries in 125 Camcore *E. urophylla* trials. Preliminary assessments of a subset of this trial series were made by Hodge et al. (2001) and Dvorak et al. (2008); the current data set is large and is essentially complete, as very few further measurements from these trials are expected. The large data set allows for precise results of provenance and family mean growth and survival at different locations, and good estimates of genetic parameters for breeding purposes, including genotype × environment interaction of provenances and families across a range of climatic and edaphic effects over large geographic areas. The continuing Camcore efforts on ex situ conservation of *E. urophylla* populations are summarized. Having good information on the geographical limits of transfer of genetic material in *E. urophylla* is important, especially during a period of global climatic fluctuations, for the development of pure-species and hybrid breeding programs, and the genetic composition and placement of ex situ conservation plantings in the tropics and subtropics.

Even though *E. urophylla* naturally occurs in the Republic of Indonesia and the Democratic Republic of Timor-Leste (formerly East Timor, Indonesia), we refer to *E. urophylla* occurring only in Indonesia in this paper. None of the Camcore seed collections made between 1996 and 2003 came from the Democratic Republic of Timor Leste because of political difficulties in entering the region as the local populace pushed for independence.

Materials and methods

Seed collections

Seed collections in natural populations of *E. urophylla* occurred from mid-June to early September over the 7 years from 1996 to 2003, depending on the seasonality of capsule ripening and elevation of the collection site. Seeds were collected from 10 to 30 trees per provenance whenever possible. Observations were made at each site to classify the stand as old-growth forests or secondary regeneration, and to describe soil characteristics (color and drainage) and conservation status. Also, it was noted if there was any *E. alba* present at the collection sites on the lower slopes (<500-m elevation) to explain observations of natural introgression with *E. urophylla* in progeny trials. There were multiple trips to some islands over years to sample different provenances. Seeds were kept separate by individual tree and then sent to Camcore, North Carolina State University, for repackaging and distribution. Seed distribution for tests occurred after each year of seed collection, so all families are not present at all test sites. In general, there were common control lots, typically multiprovenance bulks, used across years to maintain connectedness among the test data sets. Details of the collection sites and year of collection are presented in Pepe et al. (2004). Some latitude and longitude coordinates of various provenances sampled during the collections have been amended from earlier publications because of improved GPS and mapping

Table 1 Details for provenances of *Eucalyptus urophylla*

Code	Provenance	Island	Year collected	Cons. status[a]	Latitude	Longitude	Elevation		Ann. precip.[b]	Trees	$\widehat{G}_{prov}$				
							Min	Max			BRZ	COL	MEX	SAF	VEN
1	Doken	Adonara	1996	V	08° 20′ S	123° 15′ E	600	1000	1452	20	−12.0	−9.7	−21.1	−5.8	−19.8
3	Muda	Adonara	1996	V	08° 21′ S	123° 16′ E	600	900	1452	20	32.1	16.3	17.7	0.4	17.1
4	Lamalota	Adonara	1996	V	08° 16′ S	123° 16′ E	650	820	1292	20	3.1	3.9	−7.4	−0.7	−6.8
53	Lamalota Barat	Adonara	2002	V	08° 21′ S	123° 15′ E	866	866	1376	6	4.4	3.6	0.8	0.9	0.8
54	Dua Muda	Adonara	2002	V	08° 20′ S	123° 15′ E	733	733	1348	16	−7.9	−8.0	−4.2	−2.1	−3.8
55	Watololong	Adonara	2002	V	08° 20′ S	123° 15′ E	630	800	1292	14	−27.5	−26.5	−11.5	−6.7	−10.6
56	Gonehama	Adonara	2002	V	08° 20′ S	123° 16′ E	687	687	1452	15	−20.2	−23.8	0.4	−5.3	−0.1
57	Lamahela	Adonara	2002	V	08° 22′ S	123° 15′ E	856	856	1452	11	−4.7	−14.6	18.6	−2.1	16.4
58	Kawela	Adonara	2003	CR	08° 21′ S	123° 04′ E	600	600	1398	20	15.9	7.9	20.6	3.2	18.6
13	Mainang	Alor	1997	V	08° 14′ S	124° 39′ E	1100	1250	1577	20	11.8	11.6	14.7	5.9	12.9
14	Apui	Alor	1997	V	08° 16′ S	124° 44′ E	1100	1300	1571	20	−13.9	−0.8	−9.6	3.7	−9.5
15	Pintu Mas	Alor	1997	E	08° 17′ S	124° 33′ E	320	450	1301	20	26.7	11.6	14.4	−0.9	13.0
16	Watakika	Alor	1997	CR	08° 18′ S	124° 30′ E	350	600	1434	20	19.8	11.9	3.2	−0.5	2.6
27	Manabai	Alor	1999	E	08° 14′ S	124° 45′ E	400	400	1436	5	−6.6	−3.5	−10.1	−1.4	−8.1
28	Molpui	Alor	1999	E	08° 15′ S	124° 44′ E	400	400	1436	15	−3.8	−2.3	−6.8	−0.9	−5.3
17	Ille Nggele	Flores	1998	E	08° 39′ S	122° 27′ E	570	800	1381	23	16.0	11.5	20.3	4.0	18.1
18	Lere-Baukrenget	Flores	1998	E	08° 39′ S	122° 23′ E	700	750	1415	20	2.7	4.6	5.0	1.8	4.7
19	Kilawair	Flores	1998	E	08° 41′ S	122° 29′ E	225	530	1615	21	10.0	−3.3	32.7	1.9	28.6
20	Hokeng	Flores	1998	E	08° 31′ S	122° 47′ E	350	800	1502	27	1.3	−13.6	7.9	−6.2	6.4
24	Ille Meak	Flores	1999	E	08° 39′ S	122° 27′ E	680	680	1381	15	15.9	7.5	20.6	3.0	18.4
26	Hikong	Flores	1999	E	08° 30′ S	122° 40′ E	680	680	1375	26	6.4	6.1	5.4	2.6	6.2
45	Koangao	Flores	2001	E	08° 40′ S	122° 32′ E	220	220	1421	24	15.6	8.0	32.5	5.8	29.4
46	Kolibuluk/ Waibeller	Flores	2001	E	08° 40′ S	122° 25′ E	648	648	1723	18	0.8	0.4	14.9	0.4	13.5
47	Natakoli	Flores	2001	E	08° 37′ S	122° 24′ E	820	980	1415	16	−21.2	−18.4	−9.1	−4.7	−8.4
48	Palueh	Flores	2001	CR	08° 28′ S	122° 42′ E	540	600	1532	21	22.5	25.6	28.1	7.5	25.1
2	Padekluwa	Lembata	1996	E	08° 30′ S	123° 26′ E	700	900	1492	20	2.2	7.1	7.5	5.5	6.7
5	Jontona / Ille Ape 1	Lembata	1996	V	08° 16′ S	123° 29′ E	675	900	1312	20	−4.0	1.8	6.9	4.9	5.9
6	Labalekan	Lembata	1996	V	08° 32′ S	123° 31′ E	620	920	1346	20	8.1	5.6	16.3	4.2	14.9
31	Ille Kerbau	Lembata	2000	V	08° 29′ S	123° 29′ E	730	750	1421	16	24.0	20.2	6.8	5.1	10.2
32	Puor	Lembata	2000	E	08° 34′ S	123° 24′ E	900	980	1413	14	23.8	12.9	11.8	2.4	11.0
33	Jontona / Ille Ape 2	Lembata	2000	E	08° 17′ S	123° 30′ E	840	880	1440	25	24.6	7.2	11.6	1.2	9.8
34	Bunga Muda	Lembata	2000	V	08° 16′ S	123° 32′ E	600	700	1440	14	15.7	6.5	20.8	2.8	18.3
35	Lowokukung	Lembata	2000	V	08° 16′ S	123° 30′ E	540	650	1312	8	−12.0	−17.5	1.0	−7.0	2.5
30	Wasbila	Pantar	1999	E	08° 20′ S	124° 03′ E	380	380	1230	4	−1.8	−4.8	−1.8	−2.1	−0.3
36	Mauta	Pantar	2000	E	08° 26′ S	124° 10′ E	600	640	1398	31	−8.9	−23.2	4.8	−6.9	3.0
37	Lalapang	Pantar	2000	E	08° 19′ S	124° 16′ E	500	650	1429	17	−8.2	−11.4	14.4	−1.6	14.2
38	Delaki	Pantar	2000	E	08° 31′ S	124° 06′ E	780	840	1390	20	−1.9	−11.3	10.8	−1.7	12.6
39	Beangonong	Pantar	2000	E	08° 25′ S	123° 57′ E	450	680	1201	25	−9.1	−19.7	8.6	−4.0	5.4
7	Naususu	Timor	1997	LR	09° 38′ S	124° 13′ E	1200	1450	1759	20	−26.1	−19.8	−29.2	−7.4	−26.5
8	Mollo	Timor	1997	LR	09° 41′ S	124° 11′ E	1200	1600	1489	20	−27.3	−17.1	−30.8	−6.0	−29.5
9	Tutem	Timor	1997	LR	09° 35′ S	124° 17′ E	1200	1400	1563	20	−7.3	−0.3	−17.6	−0.6	−15.6
10	Tune	Timor	1997	LR	09° 33′ S	124° 19′ E	1100	1400	1481	20	−6.9	0.2	−23.6	−2.2	−20.8
11	Lelobatang	Timor	1997	LR	09° 41′ S	124° 14′ E	1200	1400	1578	13	−20.2	−14.4	−23.6	−5.4	−21.7
12	Fatumnase	Timor	1997	LR	09° 34′ S	124° 13′ E	1700	2000	1912	4	−14.1	−5.1	−20.6	−1.9	−19.2
21	Lelobatan	Timor	1998	LR	09° 43′ S	124° 10′ E	1400	1650	1424	34	−22.9	−9.8	−24.3	−0.7	−22.7
22	Leloboko	Timor	1998	LR	09° 37′ S	124° 10′ E	1400	1600	1467	23	−24.8	−10.2	−32.6	−2.8	−29.4
23	A'esrael	Timor	1998	LR	09° 36′ S	124° 14′ E	1655	1655	1909	11	−34.9	−20.0	−50.4	−6.9	−46.3
25	Fatuneno	Timor	1999	LR	09° 32′ S	124° 18′ E	1400	1400	1503	14	−5.0	−2.8	−7.6	−1.1	−7.1
29	Nenas	Timor	1999	LR	09° 35′ S	124° 12′ E	1605	1605	1550	20					

Table 1 (continued)

Code	Provenance	Island	Year collected	Cons. status[a]	Latitude	Longitude	Elevation		Ann. precip.[b]	Trees	$\widehat{G}_{prov}$				
							Min	Max			BRZ	COL	MEX	SAF	VEN
40	Bonmuti	Timor	2001	LR	09° 31′ S	124° 16′ E	1370	1370	1692	14	8.6	20.4	−19.0	3.4	−17.1
41	Nuapin	Timor	2001	LR	09° 31′ S	124° 11′ E	1800	2000	1555	22	−4.9	7.7	−13.5	0.8	−12.9
42	Oepopo	Timor	2001	LR	09° 31′ S	124° 19′ E	1340	1340	1503	12	−11.2	3.4	−20.9	−0.6	−19.7
43	Bonleu	Timor	2001	LR	09° 33′ S	124° 04′ E	1600	1800	1392	25	−18.1	1.2	−19.0	−1.0	−17.9
44	Nunbena	Timor	2001	LR	09° 40′ S	124° 16′ E	1200	1400	1479	27	−27.0	−6.4	−19.4	−2.7	−18.1
49	Ketur	Wetar	2002	LR	07° 52′ S	126° 27′ E	432	498	1116	16	23.2	18.6	14.0	5.1	12.6
50	Puaanan	Wetar	2002	LR	07° 51′ S	126° 27′ E	453	516	1116	15	29.0	23.0	18.5	6.3	16.6
51	Remamea	Wetar	2002	LR	07° 52′ S	126° 26′ E	409	542	1116	16	21.2	17.2	12.0	4.6	10.8
52	Talianan	Wetar	2002	LR	07° 53′ S	126° 28′ E	483	559	1018	11	28.1	25.2	11.8	6.4	10.8
59	Alasannaru	Wetar	2003	LR	07° 51′ S	126° 24′ E	580	612	1172	15	1.6	3.7	0.3	1.6	−0.2
60	Elun Kripas	Wetar	2003	LR	07° 51′ S	126° 17′ E	715	750	1136	15	4.3	0.6	6.2	0.2	5.4
61	Nesunhuhun	Wetar	2003	LR	07° 52′ S	126° 15′ E	600	642	1136	15	−1.8	1.2	−3.9	0.6	−3.9
62	Nakana Ulam	Wetar	2003	LR	07° 52′ S	126° 21′ E	680	750	1232	15	−1.4	2.3	−3.8	1.1	−3.9

Variables include latitude, longitude, minimum and maximum elevation, and annual precipitation. $\widehat{G}_{prov}$ for BRZ, COL, MEX, and VEN are the predicted provenance effect for volume gain (%) in Brazil, Colombia, Mexico, and Venezuela, respectively. $\widehat{G}_{prov}$ for SAF is the predicted provenance effect for diameter at breast height (DBH) gain (%) in South Africa

[a] Conservation status: *LR* low risk, *V* vulnerable, *E* endangered, *CR* critically endangered, using standards of the International Union for Conservation of Nature and Natural Resources (IUCN) (Farjon and Page 1999)

[b] Precipitation estimates generated from the WorldClim Global Climate Database using the Extract Climate Data by Points feature of DIVA-GIS (Hijmans et al. 2005, 2012)

capabilities. The list of all provenances sampled over the 7-year period with amended coordinates is provided in Table 1.

Test design and measurements

The provenance/progeny tests generally contained a subset of four to seven provenances and/or sources, with each provenance being represented by 8–15 open-pollinated families. The trial design was the same at all locations, a randomized complete block design, with provenances randomized in each replicate, and families randomized within the provenance sub-blocks. Families were planted in 6-tree row plots, with from 6–9 replicates per trial. Spacing was usually 3×3 m (1111 stems per hectare) in all countries except Venezuela, where spacing was 3.0×3.5 m (952 stems per ha). Test measurements were generally done at age 3 years, although some tests were measured at ages 2 or 5 and some tests were measured at multiple ages. Measurements were not available at all ages for all tests. In most tests, the growth traits *height* in meters and *DBH* in centimeters (diameter at breast height, outside bark, i.e., 1.3 m) were taken, and a generic index developed by Ladrach (1986) for juvenile trees was used to estimate volume as follows:

$$\text{Volume} = 0.00003\left(\text{DBH}^{2*}\text{height}\right)$$

In a number of the tests in South Africa, heights were not measured, only DBH. Table 2 lists the number of *E. urophylla* tests in Brazil, Colombia, Mexico, South Africa, and Venezuela, along with summary statistics for the latitude, longitude, elevation, and precipitation where tests were established. Figure 2 shows the general locations of the tests in each country, and Appendix 1 provides more detail on the number of tests at each age, the number of provenances, and number of families tested in each country. In total, measurements were available for 125 provenance/progeny tests and 190,388 trees. Calculations of growth and survival means for each test, and across all tests for a given country were done using SAS® (SAS Institute Inc. 2011).

Standardization of data

Forest tree growth traits often display a strong relationship between the mean of the trait and its phenotypic and genetic variances, such that field tests with bigger trees will have larger phenotypic and genetic variances than field tests with smaller trees (even if the tests are of the same age). To deal with this situation of heterogeneous variances (sometimes called scale effects), White et al. (2007) (pp. 398–400) recommend data standardization prior to ANOVA, variance component analysis, or multiple-site mixed model analysis. The goal is primarily to homogenize variances that will be pooled together in the linear model and to eliminate "spurious" genotype×environment interaction (Burdon 1977; Eisen and Saxton 1983; Hill 1984). For the analyses in the current paper,

Table 2 Summary statistics for latitude, elevation, and precipitation for provenance/progeny tests of *E. urophylla* established in various countries.

Country	Tests	Latitude			Elevation (m)			Precipitation (mm)		
		Median	Min	Max	Median	Min	Max	Median	Min	Max
Brazil	6	21° 39′ S	0° 38′ S	22° 15′ S	690	50	750	1250	1200	2000
Colombia	29	3° 34′ N	2° 29′ N	4° 49′ N	1571	947	2034	2078	981	2761
Mexico	14	17° 47′ N	17° 40′ N	17° 50′ N	40	25	50	2328	2172	2560
South Africa	39	28° 33′ S	24° 57′ S	28° 38′ S	160	60	978	1084	954	1538
Venezuela	37	8° 25′ N	7° 48′ N	9° 40′ N	150	75	245	1369	900	1639

standardization was done as follows: The individual-tree phenotypic coefficient of variation was calculated for each replicate for each growth trait, and a mean coefficient of variation (CV_y) was calculated for each species-country-trait combination. Phenotypic observations were then standardized in each replicate to a mean=100 and standard deviation=100 CV_y using PROC STANDARD in SAS®. Effectively, this is equivalent to dividing all observations by the phenotypic standard deviation, as recommended by White et al. (2007) (pp 399–400), followed by multiplying by a constant (100 CV_y) and adding a constant (100 %). The advantage of this standardization is that the population mean for the growth trait can be interpreted as 100 %, and the associated variances and standard deviations are the same size relative to the mean as in the raw data, thus all variance components and predicted breeding values can be directly interpreted in terms of percent gain (above or below 100 %) without any further need for back-transformation or rescaling.

Variance components and genetic parameters, by country

All variance component analyses were conducted using ASREML version 3.0 (Gilmour et al. 2006). Several variance component analyses were run.

First, an examination of the age–age genetic correlations in Venezuela was done. There were 28 tests measured at age 3 years in Venezuela. Among the 22 tests measured at age 2 years, 16 tests were also measured at age 3. Among the 13 tests measured at age 5, nine were also measured at age 3. Some of the age-2 and age-5 trials were also measured at age 3, so age–age correlations could be estimated. In the other countries, there were very few, if any tests measured at more than one age. For Venezuela, the age–age correlations (among ages 2, 3, and 5 years) were examined using a multitrait analysis in ASREML. This program is well-suited for multitrait analyses, and it allows the user to specify the structure or form of the variance–covariance (or correlation) matrix for each random effect in the linear model. Next, based on age–age correlation estimates near +1 (results discussed below), it was deemed reasonable to aggregate standardized growth trait data across the different ages, i.e., when data were not available for age 3, data from age 2 or age 5 years were used as though they were age-3 data. For each country, multisite, multitrait analyses for the three growth traits (height, DBH, volume) were conducted.

The linear model for all of the analyses was as follows:

$$y_{ijklm} = \mu + E_i + B(E)_{ij} + P_k + PE_{ik} + F(P)_{kl} + F(P)E_{ikl} + e_{ijklm}$$

where y_{ijklm}=phenotypic observation for the $ijklm^{th}$ tree,

μ=overall mean,

E_i=fixed effect of the ith test,

$B(E)_{ij}$=fixed effect of the jth block nested in the ith test,

P_k=random effect of the kth provenance, $E[P_k]=0$, $Var[P_k]=\sigma^2_{prov}$,

PE_{ik}=random interaction of the kth provenance and the ith test, $E[PE_{ik}]=0$, $Var[PE_{ik}]=\sigma^2_{pe}$,

$F(P)_{kl}$=random effect across sites of the lth family in the kth provenance, $E[F(P)_{kl}]=0$, $Var[F(P)_{kl}]=\sigma^2_f$,

$F(P)E_{ikl}$=random interaction of the lth family in the kth provenance and the ith test, $E[F(P)E_{ikl}]=0$, $Var[F(P)E_{ikl}]=\sigma^2_{fe}$

e_{ijklm}=random error term associated with the $ijklm^{th}$ tree, $E[e_{ijklm}]=0$, $Var[e_{ijklm}]=\sigma^2_e$.

Phenotypic variance within-provenance (σ^2_{phen}) was estimated as

$$\hat{\sigma}^2_{phen} = \hat{\sigma}^2_f + \hat{\sigma}^2_{fe} + \hat{\sigma}^2_e$$

Narrow-sense heritability within provenance (h^2) was estimated as

$$\hat{h}^2 = \hat{\sigma}^2_a / \hat{\sigma}^2_{phen}$$

where $\hat{\sigma}^2_a$ is the estimated additive variance, calculated as $\hat{\sigma}^2_a = 3\hat{\sigma}^2_f$. The covariance among open-pollinated families would typically be higher than one fourth of additive genetic variance; this could result from inbreeding and/or from a small number of effective male pollinators leading to the presence of some percentage of full-sibs (and possibly selfs) within the open-pollinated family (Squillace 1974). Thus, an estimated family variance was multiplied

by a coefficient of 3 instead of 4 to estimate additive variance and heritability. This has been found to give better agreement between parameter estimates from open-pollinated and control-pollinated data sets for the same genetic material (Dieters et al. 1995).

The amount of provenance variation was estimated as

$$\widehat{P} = \widehat{\sigma}^2_{prov} / \widehat{\sigma}^2_{phen}$$

This allows a direct comparison of estimates of provenance variance ($\widehat{P}^2$) and additive genetic variance ($\widehat{\sigma}^2_A$). Standard errors of $\widehat{P}^2$ and $\widehat{h}^2$ were estimated using the standard errors of $\widehat{\sigma}^2_{prov}$ and $\widehat{\sigma}^2_f$, respectively, as calculated by ASREML, and treating $\widehat{\sigma}^2_{phen}$ as a constant according to Dickerson's approximation (Dickerson 1969).

Type B genetic and provenance correlations (r_{Bg} and r_{Bprov}, respectively) were estimated as

$$r_{Bg} = \widehat{\sigma}^2_f \,/\, \left(\widehat{\sigma}^2_f + \widehat{\sigma}^2_{fe}\right)$$

$$r_{Bprov} = \widehat{\sigma}^2_{prov} \,/\, \left(\widehat{\sigma}^2_{prov} + \widehat{\sigma}^2_{pe}\right)$$

Type B correlations estimated as above measure the genetic or provenance correlation between the same trait expressed on two different sites (Burdon 1977). Assuming homogeneous variances, the type B correlation will measure the type of G×E interaction which is due to rank changes across environments. It is this type of interaction that is of most interest to tree breeders selecting provenances or families which will perform well across the range of sites. Type B correlations over multiple sites range between zero and one; $r_{Bg} \approx 1$ indicates a near-perfect correlation between performance in different environments, or in other words, an absence of genotype (or provenance)×environment interaction. Standard errors of r_{Bg} were estimated using the standard errors of $\widehat{\sigma}^2_f$ and $\widehat{\sigma}^2_{fe}$ and $Cov(\widehat{\sigma}^2_f, \widehat{\sigma}^2_{fe})$ from ASREML and a first-order approximation of a Taylor-series expansion (Lee and Forthofer 2006) and standard errors of r_{Bprov} estimated in a similar manner. It is important to note that, using this approximation, the standard error estimates will approach zero as the type B correlation estimate approaches a theoretical bound (i.e., zero or one).

The additive genetic coefficient of variation (GCV) was estimated as

$$\widehat{GCV} = 100^* \,\widehat{\sigma}_a / \,\bar{x}$$

where $\bar{x}$ is the trait mean. The *GCV* expresses the additive genetic standard deviation in percentage and gives a breeder an estimate of how much genetic improvement could be made in a trait.

Finally, ASREML was used to directly estimate genetic correlations among traits (height–DBH, height–volume, DBH–volume) and age–age genetic correlations for volume (VOL2–VOL3, VOL2–VOL5, and VOL3–VOL5) at the provenance (r_{prov}) and within-provenance (r_g) levels. For each random effect, ASREML can calculate the variance component for each trait and correlations among the traits, along with associated standard errors (Gilmour et al. 2006).

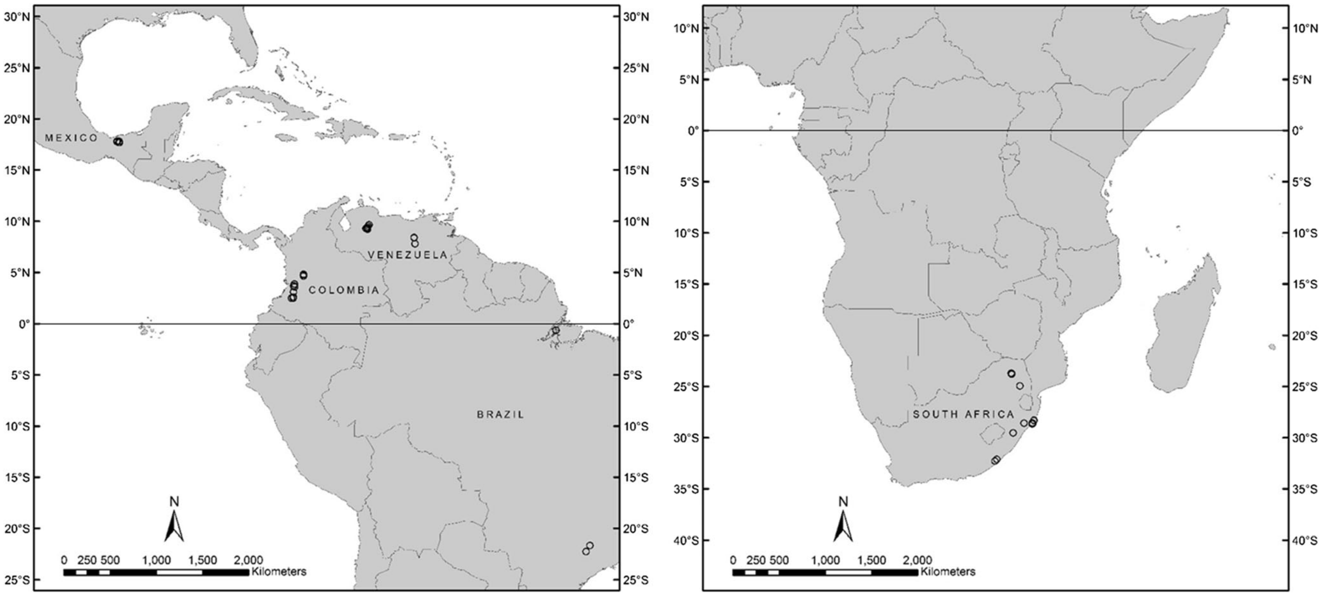

Fig. 2 General locations of 125 provenance / progeny tests of *E. urophylla* in the Camcore trial series. Test locations in Brazil (6 tests), Colombia (29 tests), Mexico (14 tests), and Venezuela (37 tests) (*left*). Test locations in South Africa (39 tests) (*right*). *Each circle* may indicate several tests within 50 km of each other

Across-country correlations and provenance BLUPs

When the individual-country analyses were completed, an across-country analysis was done using individual-tree standardized age-3 volume (VOL3) as the growth trait for Brazil, Colombia, Mexico, and Venezuela, and standardized age-3 DBH (DBH3) as the growth trait for South Africa. ASREML was used to conduct the multitrait analyses, with the growth trait in each country being treated as a distinct trait. Fixed and random effects in the models were the same as defined above. Country–country genetic correlations at the provenance (r_{prov}) and within-provenance (r_g) levels (and standard errors) were estimated directly from the ASREML output. These analyses were also used to produce best linear unbiased predictions (BLUPs) of provenance effects (G_{prov}) for VOL3 for each country. To examine if there were any impacts of differential survival, plot–volume sums were also analyzed, and BLUPs of provenance effects for this trait were also calculated.

Results

Growth results across countries

Mean survival, height, DBH, and volume/tree for *E. urophylla* in each country are presented in Table 3. Mean survival at age 3 years ranged from 68.6 % in Mexico to 90.4 % in Colombia. Growth rates were quite good in all countries. Even in Venezuela, which had the slowest growth, average height growth exceeded 3 m per year, with mean height of 9.8 m and mean DBH of 9.8 cm. In the other four countries, mean heights were generally around 12 m and mean DBH from 11 to 12 cm (Table 3).

Age–age correlations, Venezuela

Estimates of age–age provenance and within-provenance genetic correlations and other genetic parameters for volume growth in Venezuela are presented in Table 4. Most importantly, the age–age genetic correlations at the provenance and within-provenance levels across ages 2, 3, and 5 years are uniformly high, with all estimates above 0.94. All correlations with age-3 volume are 0.99 or above (with some estimates bounded at 1.00 by the ASREML program). The heritability estimates at ages 2 and 5 were slightly lower than at age 3 years ($\hat{h}^2$=0.05 for ages 2 and 5 years vs $\hat{h}^2$=0.09 for age 3). Similarly, ages 2 and 5 had slightly lower estimated type B genetic correlations than at age 3 (r_{Bg}=0.30 at age 2 and r_{Bg}= 0.35 at age 5 vs r_{Bg}=0.45 at age 3). The measurements do not overlap completely, for example, of the 22 tests with age-2 data, and the 28 tests with age-3 data, only 16 tests had both age-2 and age-3 data. Similarly, only nine tests had both age-3 and age-5 data. Since the different ages had similar genetic parameter estimates and estimated genetic correlations near + 1, it was deemed acceptable to aggregate age-3 standardized data with age-2 and age-5 standardized data; this resulted in a total of 39 tests with growth data in Venezuela.

Genetic parameters and growth trait correlations, by country

Provenance variation for growth traits was substantial in all five of the countries, with $\widehat{P}^2$ =0.04 to 0.08 in Brazil, Colombia, South Africa, and Venezuela. In Mexico, provenance variance was very important, with $\widehat{P}^2$ =0.16, 0.14, and 0.20 for height, DBH, and volume, respectively. Provenance×environment interaction was also lower in Mexico than in the other four countries. For example, for Mexico, r_{Bprov} for volume was 0.94, indicating almost zero provenance×environment interaction variance, while in the other four countries, r_{Bprov} was moderate, ranging from 0.68 to 0.83 (Table 5).

In every case, heritability estimates for the three growth traits in a given country were all very similar, differing by no more than 0.02. In general, $\hat{h}^2$ for growth traits was around 0.15, ranging from 0.14 to 0.18 for Brazil, Colombia, Mexico, and South Africa. In Venezuela, estimated heritabilities for growth traits were substantially lower, with $\hat{h}^2$=0.10, 0.08, and 0.09 for height, DBH, and volume, respectively. These lower heritabilities might be expected, given the slower growth rates observed in the Venezuelan trials, mentioned

Table 3 Growth and survival in progeny tests of *E. urophylla* averaged across all available progeny tests in various countries

Country	Age 2					Age 3					Age 5				
	Tests	Surv (%)	Height (m)	DBH (cm)	Volume (m^3)	Tests	Surv (%)	Height (m)	DBH (cm)	Volume (m^3)	Tests	Surv (%)	Height (m)	DBH (cm)	Volume (m^3)
Brazil	.	.	.	.	.	2	76.9	14.7	11.3	0.0640	4	73.2	15.0	10.3	0.0617
Colombia	4	95.3	11.7	10.3	0.0419	29	90.4	11.9	11.3	0.0585	.	.	.	.	.
Mexico	.	.	.	.	.	14	68.6	12.1	12.0	0.0617	.	.	.	.	.
South Africa	5	92.8	10.2	9.2	0.0312097993	35	90.0	12.4	12.4	0.0693	.	.	.	.	.
Venezuela	22	82.7	6.9	7.1	0.0147861759	28	80.0	9.8	9.8	0.0430	13	69.3	16.5	15.1	0.1442

Table 4 Age–age genetic correlation estimates within provenance (r_g, above diagonal) and at the provenance level (r_{prov} below diagonal), and other genetic parameter estimates for volume growth for *E. urophylla* at ages 2, 3, and 5 years in Venezuela

Country	Trait	Age-age genetic correlations			$\widehat{P}^2$	r_{Bprov}	$\widehat{h}^2$	r_{Bg}	$\widehat{GCV}$	$\widehat{\sigma}^2_{phen}$
		With vol2	With vol3	With vol5						
Venezuela	vol2		1.00 *na*	0.94 *± 0.07*	0.04 *± 0.01*	0.61 *± 0.11*	0.05 *± 0.01*	0.35 *± 0.07*	15.9	4677.7
Venezuela	vol3	1.00 *± 0.02*		1.00 *na*	0.08 *± 0.00*	0.79 *± 0.07*	0.09 *± 0.01*	0.45 *± 0.06*	20.8	4893.8
Venezuela	vol5	0.97 *± 0.03*	0.99 *± 0.01*		0.08 *± 0.00*	0.84 *± 0.08*	0.05 *± 0.01*	0.30 *± 0.08*	16.0	4982.8

±Standard errors listed in italics

above. Also contributing to a lower heritability in Venezuela is a high level of genotype×environment interaction; r_{Bg} for age-3 volume in Venezuela was 0.52. Colombia had moderate levels of genotype×environment interaction, with r_{Bg}=0.65 for volume, while the other countries had fairly low levels (or near zero) genotype×environment interaction (r_{Bg}=0.76, 0.79, and 1.00 for Mexico, South Africa, and Brazil, respectively) (Table 5).

Genetic correlations among the three different growth traits (height, DBH, and volume) for each country are also presented in Table 5. Regardless of trait pair or country, these correlation estimates are quite high. Estimates of height–volume genetic correlations (within provenance) range from r_g=0.89 to 0.95, and DBH–volume correlations range from r_g=0.97 to 1.00. Similarly, height–volume provenance correlation estimates range from r_{prov}=0.88 to 0.95, and DBH–volume provenance correlation estimates range from r_{prov}=0.96 to 1.00. In particular, it was of interest to examine the relationship between the genetic parameters for DBH and volume in South Africa, since DBH was the only trait measured in many of the South African tests. Comparing the estimated parameters for DBH and volume, provenance variation ($\widehat{P}^2$ =0.04 vs 0.06), heritability ($\hat{h}^2$=0.17 vs 0.18), and type B correlations (r_{Bprov}=0.72 vs 0.73 and r_{Bg}=0.82 vs 0.79) were all very similar (Table 5). The only parameter estimates that were substantially different were the variance components themselves, e.g., the phenotypic variance ($\widehat{h}^2_{phen}$) or the genetic coefficient of variation ($\widehat{GCV}$) (Table 5). But in terms of ranking families and provenance in a BLUP analysis, one would produce nearly identical rankings whether analyzing DBH or volume. It was therefore deemed acceptable in a multiple country analysis to analyze volume data for Brazil, Colombia, Mexico, and Venezuela, together with DBH data from South Africa.

Growth trait correlations between countries

A multiple-country analysis for growth traits was done both to examine between-country genetic correlation estimates and to

Table 5 Genetic correlation estimates within provenance (r_g, above diagonal) and at the provenance level (r_{prov} below diagonal), and other genetic parameter estimates for growth traits for *E. urophylla* at age 3 years in five countries

Country	Trait	Genetic Correlations of growth traits			$\widehat{P}^2$	r_{Bprov}	$\widehat{h}^2$	r_{Bg}	$\widehat{GCV}$	$\widehat{\sigma}^2_{phen}$
		With ht3	With dbh3	With vol3						
Brazil	ht3		0.87 *± 0.02*	0.89 *± 0.02*	0.05 *± 0.01*	0.82 *± 0.21*	0.17 *± 0.02*	1.00 *± 0.00*	9.3	506.5
Brazil	dbh3	0.85 *± 0.08*		0.97 *± 0.01*	0.04 *± 0.01*	0.81 *± 0.20*	0.15 *± 0.02*	1.00 *± 0.00*	11.8	912.6
Brazil	vol3	0.88 *± 0.06*	1.00 *± 0.01*		0.06 *± 0.01*	0.83 *± 0.17*	0.15 *± 0.02*	1.00 *± 0.00*	26.3	4586.0
Colombia	ht3		0.78 *± 0.03*	0.85 *± 0.02*	0.08 *± 0.01*	0.70 *± 0.08*	0.14 *± 0.01*	0.62 *± 0.06*	8.3	482.8
Colombia	dbh3	0.83 *± 0.06*		0.97 *± 0.00*	0.05 *± 0.00*	0.73 *± 0.08*	0.16 *± 0.01*	0.70 *± 0.06*	11.0	744.3
Colombia	vol3	0.91 *± 0.03*	0.98 *± 0.01*		0.06 *± 0.01*	0.68 *± 0.08*	0.16 *± 0.01*	0.65 *± 0.06*	24.7	3948.3
Mexico	ht3		0.89 *± 0.02*	0.92 *± 0.02*	0.16 *± 0.01*	0.89 *± 0.05*	0.17 *± 0.02*	0.77 *± 0.09*	8.8	471.5
Mexico	dbh3	0.97 *± 0.02*		0.98 *± 0.00*	0.14 *± 0.00*	0.91 *± 0.05*	0.16 *± 0.02*	0.74 *± 0.09*	10.4	675.0
Mexico	vol3	0.95 *± 0.02*	1.00 *± 0.00*		0.20 *± 0.00*	0.94 *± 0.04*	0.17 *± 0.02*	0.76 *± 0.09*	25.0	3699.6
South Africa	ht3		0.95 *± 0.01*	0.95 *± 0.01*	0.06 *± 0.01*	0.63 *± 0.09*	0.16 *± 0.01*	0.77 *± 0.04*	8.1	417.6
South Africa	dbh3	0.78 *± 0.08*		1.00 *na*	0.04 *± 0.00*	0.72 *± 0.08*	0.17 *± 0.01*	0.82 *± 0.03*	11.5	763.8
South Africa	vol3	0.85 *± 0.06*	0.96 *± 0.02*		0.06 *± 0.00*	0.73 *± 0.08*	0.18 *± 0.01*	0.79 *± 0.03*	25.0	3472.7
Venezuela	ht3		0.85 *± 0.02*	0.94 *± 0.01*	0.08 *± 0.01*	0.71 *± 0.07*	0.10 *± 0.01*	0.51 *± 0.06*	8.9	788.1
Venezuela	dbh3	0.90 *± 0.04*		0.98 *± 0.01*	0.06 *± 0.00*	0.69 *± 0.08*	0.08 *± 0.01*	0.58 *± 0.06*	8.0	749.5
Venezuela	vol3	0.94 *± 0.02*	0.98 *± 0.01*		0.08 *± 0.00*	0.73 *± 0.07*	0.09 *± 0.01*	0.52 *± 0.06*	20.9	4899.3

±Standard errors listed in italics

predict provenance effects. For this analysis, the traits of interest were volume for Brazil, Colombia, Mexico, and Venezuela, and DBH for South Africa. Across all country-pairs, the average of both types of genetic correlation estimates was 0.72. The between-country provenance correlation estimates range from r_{prov}=0.54 for Mexico–Colombia to r_{prov}= 1.00 for Mexico–Venezuela (Table 6). The between-country within-provenance genetic correlation estimates range from r_g=0.34 for Brazil–South Africa to r_g=0.92 for Colombia–Venezuela. The country pair that has the highest correlations of provenance and family performance is Mexico–Venezuela, with r_{prov}=1.00 and r_g=0.90. The country pair with the lowest correlations is probably South Africa–Brazil, with r_{prov}=0.63 and r_g=0.34.

Provenance BLUPs

Best linear unbiased predictions (BLUPs) were made for G_{prov} (provenance effect for volume, expressed in units of % gain above the unimproved population mean) for each country. Provenance rankings and BLUPs from the analysis using standardized individual-tree volume and from the analysis using plot volume were compared. Rankings and BLUPs were very similar, with correlations between provenance BLUPs from the two approaches ranging from 0.91 to 0.98 across the five countries. All results presented here are from the analysis using standardized individual-tree volume as the unit of observation.

Predictions of G_{prov} were made for Brazil, Colombia, Mexico, South Africa, and Venezuela and are listed in Table 1. It is important to recall that for Brazil, Colombia, Mexico, and Venezuela, the growth trait was VOL3 and, for South Africa, the growth trait was DBH3. Thus, the BLUPs for South Africa predict the percent gain in DBH, while the BLUPs for all other countries predict the percent gain in volume.

There was very large provenance variation for growth in all countries. The largest variation was in Mexico, where the best provenance had $\widehat{G}_{prov}$ =+32.7 % (#19 Kilawair, Flores) and the worst provenance had $\widehat{G}_{prov}$ =−50.0 % (#23 A'esrael, Timor), a range of 82.7 %. Less provenance variation was observed in Colombia, where the best provenance had $\widehat{G}_{prov}$ =+25.6 % (#48 Palueh, Flores) and the worst provenance had $\widehat{G}_{prov}$ = −26.2 % (#55 Watololong, Adonara), a range of 51.8 %. As would be expected, the range of variation in $\widehat{G}_{prov}$ corresponds to the estimated provenance variation ($\widehat{P}^2$) for volume in Table 5, where Mexico had $\widehat{P}^2$ =0.20 and Colombia had $\widehat{P}^2$ =0.06. But even in Colombia, there were very important differences among provenances for volume growth.

Provenance differences for DBH3 in South Africa ranged from $\widehat{G}_{prov}$ =+7.5 % (#48 Palueh, Flores) to −7.4 % (#7 Naususu, Timor). It was interesting that Palueh was the best provenance for growth in both Colombia and South Africa. To estimate $\widehat{G}_{prov}$ for VOL3 in South Africa, one could make a rough conversion from $\widehat{G}_{prov}$ for DBH3 as follows: $\widehat{G}_{prov(VOL3)}$ = $100\times[1-(1+\widehat{G}_{prov(DBH\,3)}\ /100)^3]$. This approximation would assume that the percent gain in height is equivalent to the gain in DBH, a fairly reasonable assumption since age–age correlations are near unity, the heritabilities and type B genetic correlations for the two traits are nearly identical, and the estimated *GCV* for height is only slightly lower than for DBH (Table 5). Using this approximation, the best provenance in South Africa, Palueh has an approximate provenance effect for volume of $\widehat{G}_{prov(VOL3)}$ =+24.2 %, quite comparable to the value of +25.6 % for the Palueh provenance in Colombia.

Table 6 Between-country genetic correlation estimates for 3-year volume growth of *E. urophylla* in five countries

r_g (above diag.) r_{prov} (below diag.)	Brazil	Colombia	Mexico	South Africa	Venezuela
**Brazil**		0.67 *± 0.07*	0.47 *± 0.09*	0.34 *± 0.10*	0.64 *± 0.11*
**Colombia**	0.89 *± 0.06*		0.90 *± 0.06*	0.89 *± 0.04*	0.92 *± 0.05*
**Mexico**	0.80 *± 0.07*	0.54 *± 0.13*		0.75 *± 0.06*	0.90 *± 0.10*
**South Africa**	0.63 *± 0.14*	0.75 *± 0.10*	0.63 *± 0.13*		0.75 *± 0.04*
**Venezuela**	0.80 *± 0.07*	0.54 *± 0.13*	1.00 *na*	0.63 *± 0.13*	

In South Africa, DBH was measured in 39 tests, while height and volume were measured in only 22 tests. For estimates involving South Africa, the genetic and provenance correlations are for DBH in South Africa and volume in the other country

Values above diagonal are within-provenance (r_g) and values below diagonal are at the provenance level (r_{prov}) (± standard errors listed in italics)

General performance of provenances from the seven islands

Across all sites, the performance of the 15 provenances from Timor was generally slightly below to greatly below the average of all provenances (Tables 1 and 7). Timor provenances performed somewhat better in Colombia and South Africa than in the other countries. Provenances from Pantar showed variable performance, generally poor in Brazil, Colombia, and South Africa, but above average in Mexico and Venezuela. Provenances from Lembata were generally good, holding second place for growth among the seven islands in all countries. Provenances from Flores were very good in Mexico and Venezuela, and provenances from Wetar were generally very good in Brazil, Colombia, and South Africa (Table 7). More specifically, the four collections in the eastern Ilwaki region of southern Wetar (#49, #50, #51 # 52), several populations on Mt. Egon and low-elevation sources close to the coast on Flores (#17, #19, #24, #26, #45, #48)), seven of the provenances on Lembata (exception # 33), and the three provenances in central and western Alor (#13, #15, #16) were generally better than average at most of the locations (especially in the tropical lowlands of Mexico and Venezuela). The seven collection sites on Mt. Boleng, Adonara (discussed below), were generally average to poor performers, except for one population on the southeastern slope of the volcano (#3 Muda) which was very good, and one other (#57 Lamahela) which was very good in Mexico and Venezuela (see discussion below).

Impact of conservation status

There is a strong impact of elevation on conservation status, with the lower-elevation provenances being at higher risk (Dvorak et al. 2008) (Table 8). Mean elevation for *Low Risk* provenances was 1180 m, and for *Critically Endangered* provenances was 548 m, with *Vulnerable* and *Endangered* provenances found at intermediate elevations. This makes sense, as the lower-elevation provenances are generally closer to towns and roads and are much easier for access for timber harvest or agricultural expansion.

In Brazil, Mexico, and Venezuela, there is a very clear pattern of lower-risk (and higher-elevation) provenances to be slower growing than higher-risk provenances. For example, for Mexico, moving from a conservation status of *Low Risk* to *Vulnerable* to *Endangered* to *Critically Endangered*, the mean predicted provenance effects go from –12.9 to +3.3 to +9.8 to +17.3 %. The same pattern is observed for Brazil and Venezuela and is similar, although less pronounced, in Colombia and South Africa.

Discussion

Effect of elevation and island

There appears to be tendency for low-elevation provenances to show faster growth than high-elevation provenances, and this has been reported previously (e.g., Ngulube 1989; Vercoe and Clarke 1994; Maid and Bhumibhamon 2009). In the current study, there is a clear relationship between elevation of the original provenance and volume growth, with lower-elevation provenances having larger provenance effects for volume. For Mexico, Brazil, and Venezuela, the correlation between $\widehat{G}_{prov}$ and elevation is moderate to strong, with $r=-0.74$ ($p<0.0001$) for both Mexico and Venezuela, and $r=-0.56$ ($p<0.0001$) for Brazil. For Colombia and South Africa, this correlation is statistically significant, but it is not strong ($r=-0.27$, $p<0.04$ for Colombia, and $r=-0.21$, $p<0.09$ for South Africa). However, the correlation between elevation and growth is confounded somewhat by island. Timor has much higher elevations than all of the other islands (except eastern Alor), and

Table 7 Mean predicted provenance effects of the seven islands for 3-year volume growth of *E. urophylla* in five countries

Island	Number	Elevation	Ann. precip.	$\widehat{G}_{prov}$				
				BRZ	COL	MEX	SAF	VEN
Adonara	9	749	1390	−1.8	−5.7	1.5	−2.0	1.3
Alor	6	673	1459	5.7	4.8	1.0	1.0	0.9
Flores	10	606	1476	7.0	2.8	15.8	1.6	14.2
Lembata	8	768	1397	10.3	5.5	10.3	2.4	9.9
Pantar	5	590	1330	−6.0	−14.1	7.4	−3.3	7.0
Timor	16	1483	1580	−16.1	−4.9	−23.5	−2.3	−21.6
Wetar	8	576	1130	13.0	11.5	6.9	3.2	6.0

BRZ, COL, MEX, and VEN are the predicted provenance effect ($\widehat{G}_{prov}$) for volume gain (%) in Brazil, Colombia, Mexico, and Venezuela, respectively. SAF is the predicted provenance effect ($\widehat{G}_{prov}$) for diameter at breast height (DBH) gain (%) in South Africa

Table 8 Mean predicted provenance effects for 3-year volume growth of *E. urophylla* for four different conservation status groups

Conservation status	Number	Elevation	Ann. precip.	$\widehat{G}_{prov}$				
				BRZ	COL	MEX	SAF	VEN
Low risk	23	1180	1424	−6.0	0.8	−12.9	−0.4	−12.0
Vulnerable	15	804	1406	−0.2	−2.1	3.3	−0.1	3.2
Endangered	20	611	1420	4.2	−1.7	9.8	−0.1	8.9
Critically endangered	3	548	1455	19.4	15.1	17.3	3.4	15.4

BRZ, COL, MEX, and VEN are the predicted provenance effect ($\widehat{G}_{prov}$) for volume gain (%) in Brazil, Colombia, Mexico, and Venezuela, respectively. SAF is the predicted provenance effect ($\widehat{G}_{prov}$) for diameter at breast height (DBH) gain (%) in South Africa. Conservation status using standards of the International Union for Conservation of Nature and Natural Resources (IUCN) (Farjon and Page 19)

when Timor provenances are removed from the data set, the correlations between $\widehat{G}_{prov}$ and elevation for Mexico and Venezuela drop to r=−0.26 and −0.27, with nonsignificant p values (p<0.08 and 0.07, respectively). In general, one can probably say that the higher-elevation provenances from Timor do not grow as fast as lower-elevation provenances from other islands, most likely because they originate in areas with shorter growing seasons.

Provenance variation within island

There was substantial provenance variation within island. For example, consider the provenances from Adonara Island, which had $\widehat{G}_{prov}$ for Mexico ranging from +20.6 % (#58 Kawela) to −21.1 % (#1 Doken), a range of 41.7 %. From Alor Island, $\widehat{G}_{prov}$ for Mexico went from +14.7 % (#13 Mainang) to −6.8 % (#28 Molpui), a range of 21.5 %. Across all seven islands, the mean range from best to worst provenance for $\widehat{G}_{prov}$ Mexico was 30.0 %. Patterns for $\widehat{G}_{prov}$ in Brazil, Colombia, Venezuela, and South Africa were very similar.

This level of provenance variation within an island is large, but perhaps more surprising is that large differences were found even among near-neighbor provenances. For example, on the island of Lembata, we had collections from four provenances on the Lewotolo volcano: #5 Jontona/Ille Ape1, #33 Jontona/Ille Ape2, #34 Bunga Muda, and #35 Lowokukung. All four of these provenances are within 3–5 km distance from one another and occupy a narrow elevational range from about 600 to 860 m (mid-elevation, see Table 1). One might expect there to be relatively little provenance variation for growth among these four provenances, but in fact, rather large differences were observed. Bunga Muda and Jontona/Ille Ape2 were very good growers (e.g., +20.8 and +11.6 % for $\widehat{G}_{prov}$ Mexico, respectively), and Jontona/Ille Ape1 and Lowokukung were substantially worse (e.g., +6.9 and + 1.0 % for $\widehat{G}_{prov}$ Mexico, respectively). The range in $\widehat{G}_{prov}$ Mexico for these four near-neighbor provenances was 19.8 %.

Another example of large provenance effects observed in near-neighbor provenances was seen on Adonara Island. As mentioned earlier, a group of seven provenances were collected on Mt. Boleng, on the eastern side of the island, all within 10 km of one another, and all from 687- to 866-m elevation (mid-elevation, see Table 1). To illustrate the provenance variation, consider the $\widehat{G}_{prov}$ Mexico values for the seven provenances circling the volcano, beginning with provenance #1, Dokeng, and moving in a northwesterly direction (Fig. 3): #1 Doken=−21.8 %, #55 Watolong=−11.5 %, #54 Dua Muda=−4.3 %, #53 Lamolota Barat=+0.8 %, #57 Lamahela=+ 18.6 %, #3 Muda=+17.7 %, and # 56 Gonehama=+0.4 %. This is a range of 39.7 % in $\widehat{G}_{prov}$ Mexico, in the distance of just few kilometers. The exact numbers and patterns for $\widehat{G}_{prov}$ in the other countries vary, but in all cases, the range among these seven provenances is large, considering how near they are to one another. For example, the range for $\widehat{G}_{prov(VOL3)}$ for Colombia is 42.8 %, for Brazil is 59.6 %, and for Venezuela is 27.7 %. The range for $\widehat{G}_{prov(DBH3)}$ for South Africa is −6.7 to 0.4 %, and converting those to approximate values for $\widehat{G}_{prov(VOL3)}$ would be −18.8 and +1.2 %, a range of 20.0 %.

Numerous studies have reported on the good performance of the Egon and/or Lewotobi sources from Flores Island (Vercoe and Clarke 1994; Wei and Borralho 1998; Maid and Bhumibhamon 2009; Kien et al. 2009). Even though a number of collectors have denoted Egon and Lewotobi with provenance designations, they are both massive volcanoes with potentially many different collection sites on their slopes, some of which probably have different geologic and genetic life histories. Therefore, comparisons of provenances results of collections made on the same volcano by various researchers over years need to be viewed cautiously unless GPS coordinates are provided.

In the current study, there were six provenances collected on Mt. Egon,: (#17 Ille Ngele, #18 Lere-Bukrenget, #19 Kilawair, #24 Ille Meak, #46 Kolibuluk, and #47 Natakoli). The most accessible of all of these provenances

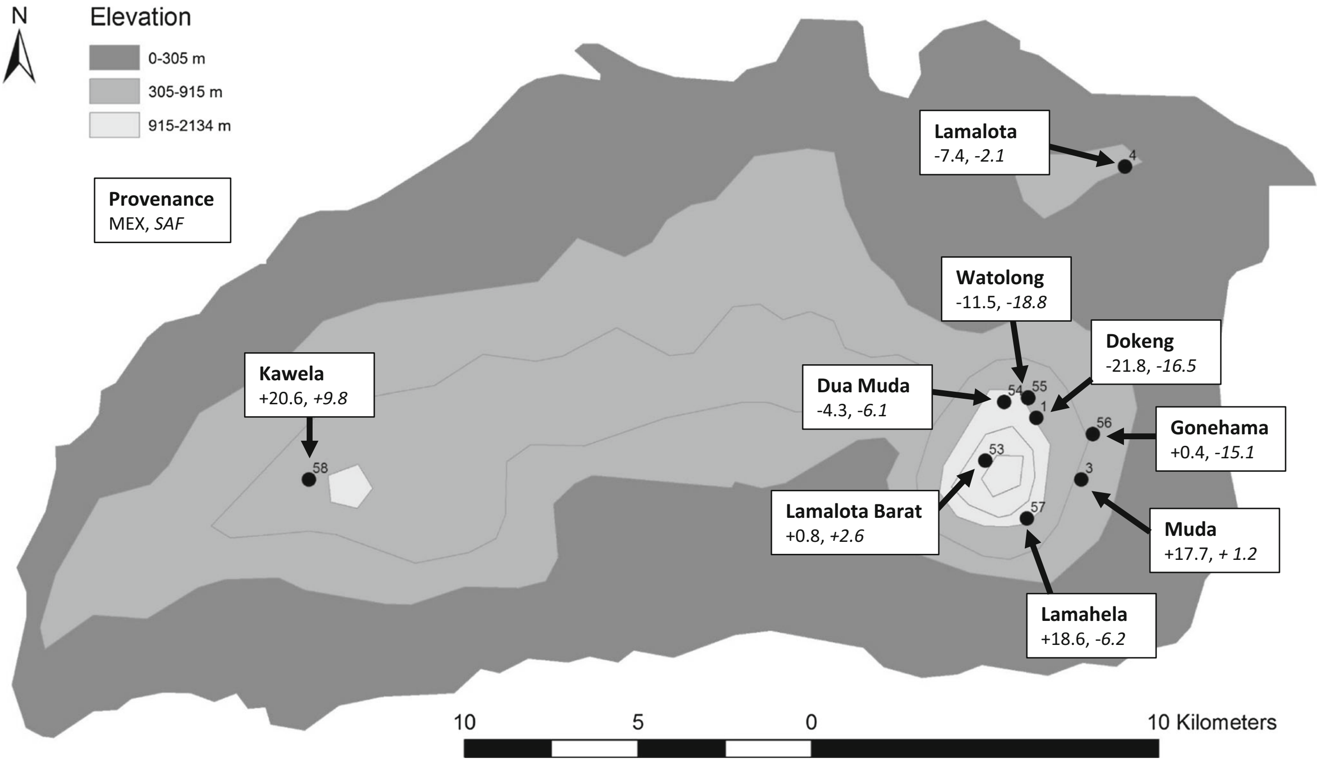

Fig. 3 Provenance effects for volume growth at age 3 years for *E. urophylla* provenances from Adonara Island, Indonesia. Values shown are the predicted provenance effects ($\hat{G}_{prov}$) for volume gain (%) in Mexico and South Africa, MEX and *SAF,* in normal font and italics, respectively. Values for South Africa are approximate, converted from the predicted provenance effect for diameter at breast height (DBH) gain (%)

by road is Ille Ngele, which probably served as the source of seed labeled as "Egon" by many collectors in the past. The reputation of Egon being a good source of seed probably comes from this location, and in the Camcore trials, this source is also very productive (unfortunately, the natural stands at this site are being destroyed by annual fires and agricultural encroachment). It is important to note, however, that there were some collection sites on Egon that were poor in performance across most sites (# 18 Lere-Baukrenget, #47 Natokoli) and others that were generally good for volume growth in Mexico and Venezuela but were just slightly above average in Brazil, Colombia, and Venezuela (Table 1).

In the current study, only one provenance was collected near, but not on, the slopes of Mt. Lewotobi on Flores Island, #20 Hokeng which possibly is the same site spelled "Hoken" by Vercoe and Clarke (1994). It may well be that, in general, "Lewotobi" is a good source, as indicated by numerous authors. However, we would expect that complete sampling of Lewotobi would produce some excellent provenances and some others that are very average or even poor, as is the case on as on Lewotolo, Boleng, and Egon. In our study, Hokeng was above average for volume growth in Mexico and Venezuela, but average or below average in Brazil, Colombia, and South Africa (Table 1). Progeny from this provenance exhibited the highest percentage of white-bark trees in trials in Zululand, South Africa (51 % of the trees) suggesting high levels of introgression with *E. alba* in natural stands (Dvorak et al. 2008). The low-elevation sources of Koangao and Palueh on Flores Island, which as far as we know had never been sampled in any collections before, showed excellent promise across all sites but probably do not exist anymore because of local human pressure. Their future rests in our ability to conserve these populations ex situ (discussed later).

Interesting trends in provenance variation for volume were also noted for provenances from Alor and Wetar. On Alor, six provenances were sampled, three from the eastern part of the island, #14 Apui, #27 Manabai, and #28 Molpui, and three from the central to western side of the island #13 Mainang, #15 Pintu Mas, and #16 Watakiki. The distance between the two groups is approximately 8 to 15 km. The provenances from the eastern side performed poorly across all locations where they were planted. However, provenances from the central to western side of the island performed above to greatly above average wherever established except in South Africa. We can find no reason in our study for this geographical pattern based on year of collection, forest type (old-growth or secondary), or elevation of the collection site. Vercoe and Clarke (1994), in their

summary of international provenance trial results from early collections, also note the relatively different performance of Alor provenances dependent on their geographic location on the island. They found that on one site in Brazil, the two groups from Alor grew as described above, but eventually, the western and central provenances fell in rank over time, while the rank of the eastern provenance improved. On a second site in Brazil and one in Colombia, the western and central provenances of *E. urophylla* maintained their excellent performance through ages 5 to 7 years (Vercoe and Clarke 1994).

On Wetar, eight provenances were collected from the southern part of the island in 2002 and 2003. The 2002 collection sampled four populations in the Ilwaki area: #49 Ketur, #50 Puaanan, #51 Remamea, and #52 Talianan. The 2003 collection was located 30 to 45 km west of Ilwaki where the 2002 collections occurred and included the provenances of #59 Alasannarua, #60 Elun Kripas, #61 Nesunhuhun, and #62 Nakana Ulam. Results showed that the four provenances from the 2002 collection were much superior to those collected further west on Wetar in 2003. The 2002 collections were geographically quite close to each other and were approximately 160 to 200 m lower in elevation than those sources collected 30 km further west in 2003. The 2002 and 2003 collections might thus represent genetically different groups. Another possibility is that the difference is simply due to flowering differences in the year of collection, discussed in more detail in the section below.

Possible causes of provenance effects

The presence of large differences in provenance effects among near-neighbor provenances is rather surprising. Eucalypts are known to exhibit inbreeding depression (e.g., Griffin and Cotterill 1988; Hardner and Potts 1995), and this factor might account for some of the observed differences. If only a small number of founders originally colonized an area, possibly after a volcanic eruption and subsequent lava flow and fire on a specific side of the cone, the resulting population of trees may be relatively inbred; this would result in some provenances showing substantial growth loss due to inbreeding, while neighboring stands in the vicinity colonized by a larger number of founders might produce much more vigorous progeny. Another possible explanation of some of the provenance differences could be differences in flowering percentage due to different years of collection, or different local climatic conditions among provenances. Differences in flowering percentage have been shown to have a large impact on the genetic quality of seed orchard crops in *E. nitens*. Specifically, seedlots from the same seed orchard, but collected in different years, were compared, and a 15 to 17 % volume advantage was observed for seedlots collected in years with prolific flowering (40 to 47 % flowering) versus years with limited flowering (15 % flowering) (Swain et al. 2013). For the current study, it seems possible that low levels of flowering in some provenances in the specific collection year would lead to less outcrossing, more inbreeding, and some growth loss.

However, it is also possible that the observed differences in provenance effects reflect true differences in the genetic quality of the provenances, unrelated to differences in inbreeding or life history. These could also be related to founder effects, that is to say, by random chance, that the genetic quality of the original genotypes to colonize an area was much better or much worse than the species mean. If so, this underscores the value of extensive provenance collections throughout the range of a species in order to ensure that the very best provenances are being captured.

Growth and yield

Provenance/progeny tests are designed to compare and rank genetic entries such as families and in order to produce precise rankings are generally planted in row plots, as were the vast majority of trials in this study. These data are also not full-rotation age but are somewhere on the order of one third of rotation age. As such, the data were not intended to be used to project growth and yield as in a formal mensurational or silvicultural study, but nevertheless, the data may have some value to at least obtain an estimate of MAI through 3 years. Assuming a spacing of 3×3 m (1111 stems per hectare) in all countries except Venezuela (where spacing was 3.0×3.5 m= 952 stems per ha) and multiplying by individual-tree volume and species survival (Table 6), unimproved *E. urophylla* was estimated to be growing about 19.6 m^3/year in Colombia, 15.7 m^3/year in Mexico, 23.1 m^3/year in South Africa, and 10.9 m^3/year in Venezuela. Our observations in the field trials suggest that good growth of *E. urophylla* is dependent on adequate soil depth, excellent water drainage, and establishment in frost-free environments. The growth rates in the highlands of southern Colombia might have been somewhat negatively influenced by the lack of a well-defined dry season and associated excessive soil moisture, and furthermore, at latitudes 2–4°N, the test sites were probably too high in elevation (>1500 m) for the lowland sources from Indonesia. In Mexico, growth was affected by the high water tables, and in some cases poor weed control, resulting in poor survival and poor MAI in coastal Veracruz/Tabasco States. Had survival been maintained at 90 % rather than 69 %, MAI for unimproved *E. urophylla* on these fertile soils would have been closer to 20.5 m^3/ha/year at age 3 years. The limiting factor for growth in Venezuela is an extended dry season of 7–8 months; in addition, many of Venezuelan tests were established on heavy clays, although site preparation on these sites included ripping to improve water penetration and site quality. One might

expect the MAI to increase as the plantations continue to grow and put on diameter through pulp rotation age of 7–8 years. For example, in Venezuela, a substantial number of tests were measured at age 5 years, providing a reasonable estimate of growth potential. These data indicate an increase in MAI from 10.9 m^3/year at age 3 years to 19.0 m^3/year at age 5 years.

Genetic parameters

In the current study, heritability estimates (narrow-sense within-provenance) were generally around 0.15 for height and DBH (as well as volume), with $\widehat{GCV}$ for height and diameter around 8 to 12 %. These correspond nicely with genetic parameter estimates from Kien et al. (2009) from two provenance–progeny trials in Vietnam, who estimated heritabilities of 0.19 to 0.22 for age 3 height and DBH, respectively, and $\widehat{GCV}$ around 7 to 12 %. Wei and Borralho (1998) also report narrow-sense within-provenance heritabilities for 3-year height and diameter of 0.23 and 0.18, respectively.

In this study, the ratio of provenance variance relative to phenotypic variance ($\widehat{P}^2$) ranged from 0.04 to 0.08 for the three growth traits in Brazil, Colombia, South Africa, and Venezuela (Table 5). These values are similar to those observed in four species of subtropical pines; for example, mean $\widehat{P}^2$ for age 8 volume was 0.04 (Hodge and Dvorak 2012). In the current study, provenance variance for *E. urophylla* growth traits in Mexico was very high, $\widehat{P}^2$ ranging from 0.14 to 0.20 (Table 5). However, even the lower levels of provenance variation observed in Brazil, Colombia, South Africa, and Venezuela are clearly of biological and economic importance, with substantial gains possible from selection of the best provenances. Selection of the top six provenances (out of 61 reported in this study) for each of the five countries would produce expected volume gains of 27.4 % in Brazil, 22.2 % in Colombia, 25.9 % in Mexico, 20.5 % in South Africa, and 23.1 % in Venezuela.

In the current study, there were limited data to estimate age–age correlations, with the only good estimates available for Venezuela, but their provenance and within-provenance genetic correlations among age 2, 3, and 5-year volume were near unity ($0.94 \leq r \leq 1.00$). These values are probably representative of the age–age correlations in other countries, despite the fact that these tests had lower growth rates than observed in other countries (Table 3). For example, in a study examining 319 tests of four pine species, age–age correlations at the provenance and genetic level were very high and near unity for age 3, 5, and 8-year volume for all species (Hodge and Dvorak 2012). No important differences in age–age correlations were observed between the two very fast-growing species (*P. tecunumanii* and *P. maximinoi*) and the two slower-growing species (*P. patula* and *P. greggii*).

Potential for genetic gain

For almost every species–country combination, there was important provenance variation for volume growth. Generally, the best provenance was 10 to 20 % better than the species mean, and there was a 20 to 35 % range from the best to worst provenance (Table 1). In addition to gains from provenance selection, breeders could make genetic gain from selection of good progeny from the better families within provenances. The genetic coefficient of variation (*GCV*) provides an estimate of the amount of within-provenance additive genetic variance, expressing the genetic standard deviation relative to the trait mean. For *E. urophylla* in these five countries, $\widehat{GCV}$ for 3-year volume averaged 24.4 and ranged from 20.9 (Venezuela) to 26.3 (Brazil). In other words, if selection among and with families resulted in an improvement of one genetic standard deviation for volume, this would be approximately 25 % volume gain above the provenance mean. Clearly, tree breeders have tremendous opportunity to make genetic gain even if making selections among families and progeny from the very best provenances.

In situ conservation status and Ex situ conservation efforts

All of the provenances on Timor and Wetar are classified as *Low Risk,* and there are no *Low Risk* provenances on any of the other islands (Pepe et al. 2004; Dvorak et al. 2008). All but one of the provenances on Adonara are *Vulnerable,* and all but one of the provenances on Flores are *Endangered*. All five provenances collected on Pantar are *Endangered,* while Lembata and Alor have a mix of conservation status.

The three highest-risk provenances, with *Critically Endangered* status, are #58 Kawela (Adonara) found at 600-m elevation, #16 Watakika (Alor) between 350- and 600-m elevation, and #48 Palueh (Flores) between 540- and 600-m elevation (Table 1). Most probably, the conservation status of #45 Koangau (Flores), the lowest elevation source sampled on Flores Island (220 m), should now be changed from *Endangered* to *Critically Endangered* because of the imminent threat from agriculture first reported more than a decade ago. Kawela is the only population sampled on Adonora that is not on Mt. Boleng (see Fig. 3). It is about 50 ha in size and consists of old-growth trees that are continually being harvested by locals to plant rice and vegetables. This provenance exhibits good productivity when planted in tropical climates. Watatika, Alor Island, is predominantly an old-growth forest, but encroachment into the stand increases every year. As mentioned above, Palueh is an excellent growing provenance in all five countries but was scheduled to be harvested by the local community for agricultural expansion in 2003. Most likely the

old trees no longer exist. Koangau, a unique coastal provenance, was an outstanding performer in Brazil, Mexico, and Venezuela, and a good performer in the other two countries also probably no longer exists on Flores. It is interesting to note that the populations of Kawela, Watakika, and Palueh were predominantly remnants of old-growth forests. It leads one to ask the question if inbreeding levels in these large old-growth forests are lower than in younger natural stands with presumably a different history of disturbance.

The seed collection efforts and the establishment of 125 trials represent a tremendous financial investment by the members of the Camcore program over the years. There is important variation for growth among provenances, both across and within islands, and genetic variation within provenances. Even though molecular assessment of a subset of populations across all seven islands shows that Wetar and Timor are the most genetically diverse (Payn et al. 2007, 2008), patterns in growth and adaptability would indicate that samples from entire range (all seven islands) should be included in an ex situ conservation program (Dvorak et al. 2008).

Camcore member organizations are making provenance, family and within-family selections in this trial series for use in their own breeding programs. Some organizations use *E. urophylla* primarily as a hybrid partner, often with *E. grandis*. In this case, pollen will be collected from selected trees and used to make hybrid crosses, and the selections will be conserved in clone banks. Other organizations are already using *E. urophylla* as a commercial species or have concluded that some of the better provenances, families, and clones from the Camcore trial series can be competitive with their current commercial species, such as *E. grandis* or *E. grandis* × *E. urophylla* hybrids. In this case, the *E. urophylla* selections will be propagated and tested in single-tree plot and block-plot clonal trials. Regardless of whether the interest in *E. urophylla* is as a hybrid partner or pure species, there is broad general interest in refining the population selected for growth and adaptability with further selection focused on important wood traits, such as density, pulp yield or lignin composition, or solid-wood characteristics such as minimal splitting. Finally, all members are conserving some of their *E. urophylla* genetic base in breeding orchards and clone banks in order to ensure the ability to make long-term genetic gain in the species. A source of ongoing discussion is whether we can keep *E. urophylla* free of contamination from other eucalypt pollen sources (if we choose to use open-pollinated breeding strategies) and whether this is necessary for the future. In addition to the clone banks used to archive selections in the breeding programs, the South African Camcore members have committed to long-term maintenance of genetic material from most of 61 *E. urophylla* provenances in the Camcore collections in permanent conservation parks. A conservation park is an area of land established in a specific climatic zone and ranges in size from approximately 20 to 35 ha. Each provenance plot is ¼ha in size and contains at least 50 genotypes from a minimum of 10 open-pollinated families established in at least two locations. The objective is to maintain genetic diversity of this important species, ensuring ability to adapt to new opportunities, new disease, and insect threats, and changing climatic and economic conditions (Dvorak 2012).

Conclusions

We concur with Vercoe and Clarke (1994) that there is not one provenance or one island that is always superior over the others in *E. urophylla* provenance testing. However, our international trial series has identified populations on the island of Wetar and Lembata that are very productive and highlights the good performance of coastal lowland provenances on Flores. Our results indicate that the general superiority of Mt. Egon and Mt. Lewotobi, Flores, might be overstated; there are good and poor sources on these volcanoes, and some variation should be expected. This points to the need for intensive provenance sampling and testing in *E. urophylla* to locate productive sources. For the same reasons, it is probably an overstatement to say that the Mt. Boleng, Adonara, source is always poor. The large provenance and family variation in *E. urophylla* set the stage for impressive gains through selection and breeding; the amount of additive variation found for height and DBH in our study is similar to that found in other commercial tree species. The future prospects of in situ conservation of populations on Flores, Adonara, Lembata, Pantar, and Alor are poor because of human population pressure and the lack of any financial value for the native eucalypt forests in local markets. We have conserved representative samples of most populations in our collections on the seven islands ex situ in the Camcore program.

Acknowledgments We would like to recognize the contributions of the Sumalindo researchers who supervised the various seed collections of *E. urophylla* over the 7-year period from 1996 to 2003. These include Arif Purwanto, Bambang Pepe, Maurits Sipayung, Suhartono Wijoyo, Suyadi, Teddy Sahelangi, Tribowo Suwartono, and Widiyatno. Collection trips would sometimes last 3 weeks and require hiking over difficult terrain for as much as 12 h/day to reach isolated stands. Without the help of these dedicated foresters, none of the genetic material from the Camcore seed collections described in this manuscript would ever have been made available for others to test in field trials across the various countries.

Many thanks also to all of the Camcore membership and particularly those research teams in Argentina, Brazil, Colombia, Mexico, South Africa, and Venezuela for establishing, measuring, and protecting these field trials so that results could be shared with others in the forest community.

Finally, we want to recognize the contributions of Andy Whittier and Robert Jetton (Camcore) for their assistance in map making and extracting precipitation data from prediction models.

Appendix

Table 9 Numbers of tests, provenances, and families of *E. urophylla* included in Camcore provenance/progeny tests in various countries

Country	Age 2			Age 3			Age 5			All ages combined			
	Tests	Provs	Fams	Tests	Provs	Fams	Tests	Provs	Fams	Tests	Provs	Fams	Trees
Brazil				2	12	136	5	31	387	6	40	523	18,965
Colombia	4	6	118	29	49	876				29	49	876	43,540
Mexico				14	42	442				14	42	442	23,053
South Africa	5	31	268	35	23	448				39	40	586	60,097
Venezuela	22	40	562	28	53	752	13	44	617	37	53	834	46,839

References

Burdon RD (1977) Genetic correlation as a concept for studying genotype-environment interaction in forest tree breeding. Silvae Genet 26:168–175

Dickerson GE (1969) Techniques for research in quantitative animal genetics. In: *Techniques and procedures in animal science research.* Am Soc Anim Sci, Albany, N.Y., pp 36–79

Dieters MJ, White TL, Hodge GR (1995) Genetic parameter estimates for volume from full-sib tests of slash pine (*Pinus elliottii*). Can J For Res 25:1397–1408

Dvorak WS (2012) The Strategic Importance of Applied Tree Conservation Programs to the Forest Industry in South Africa. South For 73(1):1–6, **74**

Dvorak WS, Hodge GR, Payn KG (2008) The conservation and breeding of *Eucalyptus urophylla*: a case study to better protect important populations and improve productivity. South For 70(2):77–85

Eisen EJ, Saxton AM (1983) Genotype x environment interactions and genetic correlations involving two environment factors. Theor Appl Genet 67:75–86

FAO (1981) International provenance trials of *Eucalyptus urophylla*. For Genet Resour Inf 10:40

Gilmour AR, Gogel BJ, Cullis BR, Thompson R (2006) *ASReml User Guide Release 2.0*. VSN International Ltd, Hemel Hempstead, HP1 1ES, UK. ISBN 1-904375-23-5

Griffin AR, Cotterill PP (1988) Genetic variation in growth of outcrossed, selfed and open-pollinated progenies of *Eucalyptus regnans* and some implications for breeding strategy. Silvae Genet 37:124–131

Gunn BV, McDonald MW (1991) *Eucalyptus urophylla* seed collections. FAO For Genet Res Inf 19:34–37

Hardner CM, Potts BM (1995) Inbreeding depression and changes in variation after selfing in *Eucalyptus globulus* ssp. *globulus*. Silvae Genet 44:46–54

Hijmans RJ, Cameron SE, Parra JL, Jones PG, Jarvis A (2005) Very high resolution interpolated climate surfaces for global land areas. Int J Climatol 25:1965–1978

Hijmans RJ, Guarino L, Mathur P (2012) DIVA-GIS, version 5. Manual. International Potato Center, Lima, Peru

Hill WG (1984) On selection among groups with heterogeneous variance. Anim Prod 39:473–477

Hodge GR, Pepe B, Wijoyo FS, Dvorak WS (2001) Early results of *Eucalyptus urophylla* provenance/progeny trials in Colombia and Venezuela. In: Proc. IUFRO Developing the Eucalypt of the Future. Working Party 2.08.03. Sept. 9–13 Valdivia, Chile

Hodge GR, Dvorak WS (2012) Growth potential and genetic parameters of four Mesoamerican pines planted in the Southern Hemisphere. Southern For 74:27–49

Jacobs MR (1981) Eucalypts for planting. FAO Forestry Series No. 11. FAO, Rome

Jones PG, Gladkov A (1999) FloraMapTM . Version 1. A computer tool for predicting the distribution of plants and other organisms in the wild. Centro Internaciónal de Agricultura Tropical (CIAT), Cali, Colombia

Kien ND, Jansson G, Harwood C, Thinh HH (2009) Genetic control of growth and form in *Eucalyptus urophylla* in Northern Vietnam. Journal of Trop Sci 21(1):50–56

Ladrach WE (1986) Thinning of *Pinus patula* by mechanical and selective method: results at 10 years. In: Whitmore JL, de Barros NF, Salazar R (eds) *Plantation Forests for Wood Production in the Neotropics*, p 17. Abstracts of Three IUFRO/MAB Symposia, Costa Rica

Lee ES, Forthofer RN (2006) Analyzing complex survey data, 2nd edn. Sage Publications Inc., Thousand Oaks, California, **91 pp**

Maid M, Bhumibhamon S (2009) Timor mountain gum improvement program in eastern Thailand. J Sust Dev 2(1):176–191

Martin B, Cossalter C (1975–1976) The Eucalypts of the Sunda Islands. Bois et Forêts des Tropiques. 163,3-25, 164, 3–14, 166, 3–22, 167, 3–24, 168, 3–17, 169,3-13. Centre Technique Forestier Tropical. CTFT, France

Mitchell G (2010) Camcore protecting endangered populations of important forest species. Wood SA & Timber Times, South Africa, pp 8–9

Moura VPG (1981) Provenance trials of *Eucalyptus urophylla* in east-central Brazil. Boletim de Pesquisa, Centro de Pesquisa Agropecuária dos Cerrados, EMBRAPA, Brazil (3) 22 pp

Ngulube MR (1989) Provenance variation in Eucalyptus urophylla in Malawi. For Ecol Manag 26(4):265–273

Payn K, Dvorak W, Janse B, Myburg A (2008) Microsatellite diversity and genetic structure of the commercially important tropical tree species *Eucalyptus urophylla*, endemic to seven islands in eastern Indonesia. Tree Genet Genomes 4(3):519–530

Payn KG, Dvorak WS, Myburg AA (2007) Chloroplast DNA phylogeography reveals the island colonisation route of Eucalyptus urophylla (Myrtaceae). Aust J Bot 55:673–683

Pepe B, Surata K, Suhartono R, Sipayung M, Purwanto A, Dvorak W (2004) Conservation status of natural populations of *Eucalyptus urophylla* in Indonesia & international efforts to protect dwindling gene pools. *FAO Forest Genetic Resources* No.31:61–63

Rocha M, Pires IE, Xavier A, Cruz CD, Rocha RB (2006) Genetic evaluation of half-sib *Eucalyptus urophylla* by the REML/BLUP and minimum squares procedure. Ciencia Florestal 16(3/4):369–379

SAS Institute Inc (2011) SAS/STAT® 9.3 User's Guide. SAS Institute Inc., Cary, NC

Souza C, Freitas MLM, de Moraes MLT, Sebben AM (2011) Estimates of genetic parameters for quantitative traits in open-pollinate families of *Eucalyptus urophylla*. Florestas 41(4):847–856

Squillace AE (1974) Average genetic correlations among offspring from open-pollinated forest trees. Silvae Genet 23:149–156

Swain TL, Verryn SD, Laing MD (2013) A comparison of the effect of genetic improvement, seed source and seedling seed orchard variables on progeny growth in *Eucalyptus nitens* in South Africa. Tree Genet Genomes 9:767–778

Turnbull JW, Brooker MIH (1978) Timor mountain gum *Eucalyptus urophylla* S. T. Blake. Forest Tree Leaflet No. 214. CSIRO, Melbourne

Vercoe T, Clarke B (1994) Trial growth performance of *Eucalyptus urophylla* S. T. Blake. Report to FAO Forestry Division on the growth of *Eucalyptus urophylla* in international provenance trials and other growth trials. CSIRO Division of Forestry

Vigneron, P, Bouvet J-M, Gouma R, Saya A, Gion J-M, Verhaegen D (2000) Eucalypt hybrids breeding in Congo. In: "Hybrid Breeding and Genetics of Forest Trees" Proceedings of QFRI/CRC-SPF Symposium, 9–14 April 2000, Noosa, Queensland, Australia. Eds. H.S. Dungey, M.J. Dieters, and D.G. Nikles, p.14-26

Wei X, Borralho NMG (1998) Genetic control of growth traits of *Eucalyptus urophylla* S. T. Blake in South East China. Silvae Genetica 47:158–165

White TL, Adams WT, Neale DA. 2007. *Forest Genetics*. CAB International. 704 pp.

Data Archiving Statement

Data used for this study are available on the public database Dryad (datadryad.org)
