# Supplementary material for: Provenance and family variations in early growth of Manchurian walnut (Juglans mandshurica Maxim.) and selection of superior families
Source: PLoS One. 2024 Mar 7;19(3):e0298918. doi: 10.1371/journal.pone.0298918 (PMC10919699; doi:10.1371/journal.pone.0298918)
Supplement: S2 File — (ZIP) [file pone.0298918.s005.zip › Provenance variation in growth characters of four subtropical pine species planted in Mexico.pdf]

New Forests (2005) 29:1–13
DOI 10.1007/s11056-004-0243-3 © Springer 2005

Provenance variation in growth characters of four subtropical pine species planted in Mexico

J. LOPEZ-UPTON[1,*], J.K. DONAHUE[2], F.O. PLASCENCIA-ESCALANTE[1] and C. RAMIREZ-HERRERA[1]
*[1]Colegio de Postgraduados, Programa Forestal, Km 36.5 Carr. Méx-Tex., Montecillo, Edo. Mex. 56230, Mexico; [2]Boise Cascade Corporation, P.O. Box 1060, De Ridder, Louisiana 70634, USA; *Author for correspondence (e-mail: uptonj@colpos.mx)*

Received 24 January 2003; accepted in revised form 16 August 2003

Key words: *Pinus greggii* var. *australis*, *P. maximinoi*, *P. patula*, *P. tecunumanii*, Species and provenance testing

Abstract. In 1995 a species/provenance test was established in the state of Puebla, Mexico to assess and compare the growth of *Pinus greggii* var. *australis*, *P. maximinoi*, *P. patula* and *P. tecunumanii*, including 10, 5, 11 and 4 provenances of each species, respectively. Each provenance test lot consisted of seed from 7 to 10 individual trees collected in natural stands. Range-wide samples were included in the test for *P. greggii* var. *australis* and *P. patula*. Material of *P. maximinoi* and *P. tecunumanii* from Guatemala, Honduras and Nicaragua, as well as Mexico was established in the test. The test site is in a mountainous area at 1440 m elevation, near 20 °N latitude. At 5 years of age significant differences were seen at both the species and provenance levels in survival, total height, diameter and volume. The overall test survival was 93%. Variance among species was three time greater than among provenances, comprising 25–33% of the total variation. *P. maximinoi* had the greatest height, diameter and volume (62 dm^3) followed by *P. tecunumanii* (53 dm^3). *P. greggii* var. *australis* had an average volume of 43 dm^3 and *P. patula* produced 30 dm^3. Although *P. maximinoi* had the lowest survival rate, the San Jeronimo provenance was the most productive of all 30 provenances tested; 98% survival and 73 dm^3 volume. This was equivalent to 2.8 cm annual diameter increment and 2 m in height growth per year. Significant growth differences among provenances within species indicate that potential gain exists using provenance selection.

Introduction

Mexico has a large number of forest tree species with good potential for commercial plantations, especially in zones with minimal environmental limitations. While a large number of Mexican pines have been tested world-wide, very few systematic species and provenance tests have been established in natural pine habitats in Mexico, to determine the best material for plantations. Determining the best species for a site is important, but finding the most productive provenance of a species can be critical to the financial success of a plantation project. The trial described in this report is the most comprehensive range-wide test of *Pinus greggii* var. *australis* Donahue and Lopez and *Pinus patula* Schiede ex Schlect. and Cham. planted within their natural distributions. It also includes provenances of *Pinus maximinoi* H.E. Moore and *Pinus tecunumanii* Eguiluz and J.P. Perry that have been included in international testing programs (Birks and Barnes 1990; Dvorak et al. 2000a,b).

Several subtropical pine species from Mexico and central America have demonstrated good growth in plantations outside of Mexico (Kietzka 1988; Dvorak et al. 1996; Dvorak et al. 2000a,b,c,d). While *P. patula* is currently the most utilized species for reforestation in Mexico (PRONARE 2000), it is a principal commercial forestry species in South Africa (van Wyk 1998). *P. greggii* Engelm. ex Parl. is the fourth most important species for production of seedlings for reforestation by the National Reforestation Program (PRONARE) in Mexico. Its use for plantations is increasing due to its favorable growth rates, abundance of seed and tolerance to moderate drought levels compared to other species. The species is prolific and precocious in its native habitat, making it an ideal candidate for seed orchard production (López 1986). It is also tolerant to moderate drought relative to other Mexican pines found on similar sites (Vargas and Muñoz 1991). Two varieties of *P. greggii* have been identified: var. *greggii* and var. *australis* (Donahue and López-Upton 1999). The variety *australis*, which grows in more subtropical environments has shown significantly greater growth rates than var. *greggii* in most field trials (Azamar et al. 2000; Dvorak et al. 2000a; Velasco 2001). *P. tecunumanii* and *P. maximinoi* are two species that have demonstrated great potential in subtropical environments in Brazil, Colombia, South Africa, as well as other countries (Crockford et al. 1990; Wright et al. 1993; Hodge and Dvorak 1999).

Large areas exist in Mexico that have ideal environments for fast-growing forest tree plantations but are not being utilized as such. Due to low financial returns for forestry, these areas have been converted to other activities such as grazing and farming that promote soil erosion and the silting in of water reservoirs, such as the case at the Necaxa Dam, near the test site. The objective of this study was to provide information on which pine species and provenances may be more productive for forestry in the mountains around Huauchinango, Puebla, so that the establishment of seed orchards can be initiated. But also to direct future research on the area.

Materials and methods

The trial is located on the 'Los Ayacahuites' farm, near the village of Patoltecoya, Huauchinango, state of Puebla, in Mexico, at 20°13′N latitude and 98°03′W longitude. The site is at 1440 m elevation, has a 60% slope with a southern aspect. It has a rainy, subtemperate climate, with an average temperature of 18.5 °C and an average annual precipitation of 2000 mm. Winters in this zone are practically frost-free. The topsoils in the immediate test area are predominately silty-clays.

A total of 30 provenances from Mexico and central America were included of the four species in the test (Table 1). Range-wide samples of *P. greggii* var. *australis* and *P. patula* were obtained, along with a good sampling of the north–south gradient of *P. tecunumanii*, and the southern distribution of *P. maximinoi* (Figure 1). Seeds of the latter two species were provided by the CAMCORE Cooperative at North Carolina State University.

Table 1. Details of the provenances used in the study

Site	State/Department, Country	Latitude	Longitude	Elevation (m)	Precipitation (mm)	Temperature (°C)
P. greggii var. *australis*						
Valle Verde	Querétaro, Mexico	21°29′	99°13′	1490	1350	18
El Madroño	Querétaro, Mexico	21°17′	99°10′	1740	1200	17
Laguna Seca	Hidalgo, Mexico	21°04′	99°10′	1720	850	18
San Joaquín	Querétaro, Mexico	20°56′	99°34′	2350	1100	16
El Piñón	Hidalgo, Mexico	20°56′	99°12′	1830	850	17
Pemuxtitla	Hidalgo, Mexico	20°49′	98°46′	1370	1640	18
Xochicoatlán	Hidalgo, Mexico	20°47′	98°42′	1840	1750	17
Xodhé	Hidalgo, Mexico	20°45′	99°22′	1845	700	17
Cieneguilla	Hidalgo, Mexico	20°45′	99°02′	1860	750	17
Patoltecoya	Puebla, Mexico	20°13′	98°03′	1440	2000	19
P. maximinoi						
Altamirano	Chiapas, Mexico	16°44′	92°03′	1315	1750	20*
San Jerónimo	Oaxaca, Mexico	16°10′	97°00′	1350	1950	20
San Juan Sacatepéquez	Guatemala, Guatemala	14°41′	90°38′	1790	1140	21
Tatumbla	Fco.Morazán, Honduras	14°01′	87°07′	1500	908	26
San José Bayuncún	Nueva Segovia, Nicaragua	13°45′	86°20′	1100	1180	23
P. patula						
Pinal de Amoles	Querétaro, Mexico	21°07′	99°39′	2400	1050	14
Encarnación	Hidalgo, Mexico	20°52′	99°13′	2400	1070	14
Zacualtipan	Hidalgo, Mexico	20°39′	98°40′	2220	1550	14
Tlahuelompan	Hidalgo, Mexico	20°38′	98°35′	2020	1600	15
Huayacocotla	Veracruz, Mexico	20°32′	98°29′	2030	1350	15
Estación Apulco	Hidalgo, Mexico	20°24′	98°22′	2190	1050	14
Acaxochitlán	Hidalgo, Mexico	20°10′	98°12′	2290	1250	14
Ahuazotepec	Puebla, Mexico	20°01′	98°12′	2460	1200	13
Zacatlán-norte	Puebla, Mexico	19°58′	97°59′	2000	1170	15

Table 1. (continued)

Site	State/Department, Country	Latitude	Longitude	Elevation (m)	Precipitation (mm)	Temperature (°C)
Zacatlán-este	Puebla, Mexico	19°56′	97°58′	2360	1200	15
Tlaxco	Tlaxcala, Mexico	19°39′	98°04′	2800	860	13
P. tecunumanii						
Chanal	Chiapas, Mexico	16°42′	92°25′	2180	1238	17
Rancho Nuevo	Chiapas, Mexico	16°41′	92°35′	2310	1238	15
San Esteban	Olancho, Honduras	15°15′	85°38′	950	1071	25*
Yucul	Matagalpa, Nicaragua	12°56′	85°46′	1000	1394	22

* Estimated by the authors. Climate information for *P. greggii* and *P. patula* from INEGI (1980). All other information from Greaves (1979), Dvorak and Donahue (1988), Wright et al. (1993) and Dvorak et al. (2000b,d).

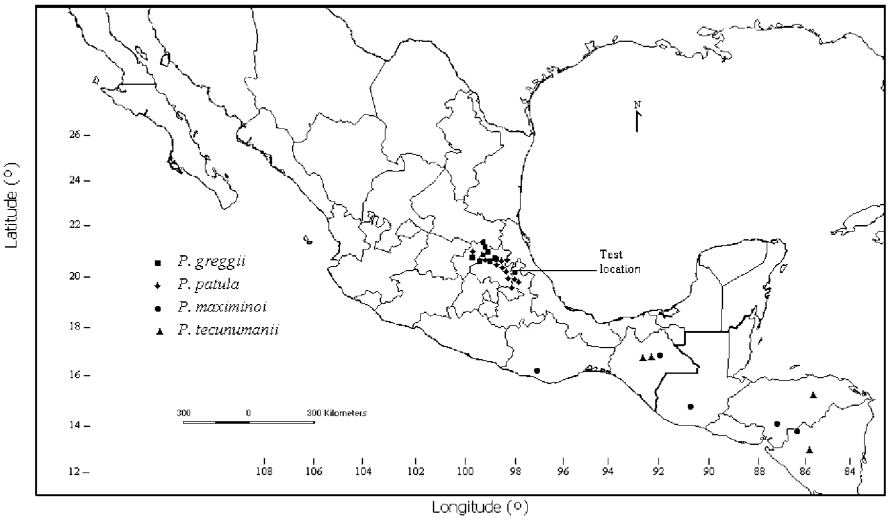

Figure 1. Distribution of species and provenances tested in Huauchinango, Puebla State, Mexico.

Each provenance test lot of *P. greggii* var. *australis* and *P. patula* were comprised of seeds from 10 families, while those of *P. maximinoi* and *P. tecunumanii* were made up of seven families. Due to a lack of sufficient seed of some provenances, bulk lots were used to provide a balance test. The best trees found at each location were chosen to represent the provenance. These were the largest, in term of volume, and those that had the best stem form. Even though some populations are quite small, distance between selections was a minimum of 100 m to reduce the probability of inbreeding in future generations should they be included in a seed orchard.

The test was planted in July 1995, at a 3 m × 3 m spacing, using nursery stock 6–7 months old. The experimental design was a randomized complete blocks with split plots and 10 replications. Species comprised the main plots, with provenances nested within species as subplots. Individual plot size was five trees for a total of 1500 test trees (30 provenances × 5 trees × 10 replications).

Survival, total height and diameter at 1.37 m were measured at 5 years of age (e.g., Hodge and Dvorak 1999). Individual tree volume, outside bark was calculated using a formula developed by Romero (1993) for *P. patula* at age 5: volume = 0.000060208 (diameter)$^{1.78610033}$ (height)$^{1.0268489}$.

The following model was used for the analyses of variance:

$$Y_{ijkm} = \mu + R_i + S_i + \mathrm{RS}_{ij} + P(\mathrm{S})_{kj} + \mathrm{RP}(\mathrm{S})_{ijk} + \eta_{ijkm} \quad (1)$$

where $i = 1 \ldots 10$; $j = 1 \ldots 4$; $k = 1 \ldots 30$; $m = 1 \ldots 5$; R = replication effect; S = species effect; RS = replication × species interaction; P(S) = provenance within species effect; RP(S) = replication × provenance within species interaction; η = sampling error.

For survival, plot means were calculated and the replication × provenance within species interaction is the residual error. Prior to analysis the data was transformed using the arcsine of the square root of percentage. All statistical analyses were done with the Statistical Analysis System (SAS Institute Inc. 1999). To determine whether there was greater variation among provenances than among species variance components were derived considering species and provenance effects as random (Littell et al. 1996). The PROC MIXED procedure and Restricted Maximum Likelihood Method were used to produce unbiased estimators due to unbalance number of provenances among species and mortality.

To compare differences among species and provenances within species, these effects were considered fixed, while all others were considered random. The Satterthwaite and LSMEANS options of the PROC MIXED procedure were used to determine the correct degrees of freedom and adjusted means for the comparisons, due to the unbalanced number of provenances among species (Littell et al. 1996). To test the random effects (replication effects and their interactions with others) the PROC GLM procedure was used (SAS Institute Inc. 1999).

Results and discussion

The analysis of variance showed significant differences ($p \leq 0.001$) among species for all variables; among provenances, all were significant except for survival (Table 2). Overall test survival was good (93.8%) at 5 years. Commercial plantations of *P. greggii* and *P. patula* near the test site generally have at least 85% survival at 5 years.

Despite the amount of variation among species and provenances, the greatest amount of variation was seen within provenances; 50–63% of the estimated total (Table 2). Since each provenance test lot was created by mixing seeds together of several individual trees, variation due to families (between and within) is confounded within the provenance variation (σ^2_e). Although the amount of variation seen in this test was less than some progeny tests, it is similar to levels seen in other forest tree species (e.g., Riemenschneider 1988; Magnussen and Yeatman 1990). The variation evident in the test indicates that gain is possible should selections be made among provenances.

Variation between (σ^2_s) and within species ($\sigma^2_{p(s)}$) comprised a significant portion of the total phenotypic variation (34–42%) and was greater than the estimated variance between main plots (σ^2_{rs}) and subplots ($\sigma^2_{rp(s)}$). This suggests that a good improvement strategy for this site and situation would be to select the best provenances within the best species.

P. maximinoi produced the greatest volume of all species, double the amount of *P. patula*, the poorest performer (Table 3). *P. tecunumanii* had 80% more volume than *P. patula*. *P. greggii* and *P. tecunumanii* had the best survival (97%), while on average, *P. maximinoi* had the lowest survival (85%). Individual provenance survival for *P. maximinoi* ranged from 76 to 98%.

Despite the large difference in latitude, the *P. maximinoi* and *P. tecunumanii* provenances had greater growth than the native provenances of *P. greggii* and *P.*

Table 2. Means, variance components (%) and total variance for the variables assessed in the test

Variable	Overall mean	Variance components in % (degrees of freedom)					σ^2_{TOT}
		$\sigma^2_s(3)$	$\sigma^2_{rs}(27)$	$\sigma^2_{p(s)}(26)$	$\sigma^2_{rp(s)}(234)$	$\sigma^2_e(1107)$	
Survival	93.8%	11.07**	6.92*	2.48 n.s.	79.53	–	0.0594
Height	8.05 m	31.59**	1.71*	8.16**	7.79**	50.75	2.4394
Diameter	11.25 cm	25.73**	2.47*	8.18**	0.55 n.s.	63.07	7.3228
Volume	42.40 dm^3	33.09**	3.12*	9.13**	0.30 n.s.	54.36	562.88

σ^2_s = Variance among species; σ^2_{rs} = replications × site variance; $\sigma^2_{p(s)}$ = variance among provenances within species; $\sigma^2_{rp(s)}$ = replication × provenance variance; σ^2_e = variance within provenances (sampling error); σ^2_{TOT} = total variance of the other sources of variance. *$p \leq 0.05$. **$p \leq 0.01$. n.s. = not significant. For survival $\sigma^2_{rp(s)}$ is the error term.

Table 3. Survival, height, diameter and volume at 5 years of age

Species	Mean (range among provenances)			
	Survival (%)	Height (m)	Diameter (cm)	Volume (dm^3)
P. greggii var. *australis*	96.6a (92–100)	8.05c (7.28–8.73)	11.43b (10.2–12.4)	42.67c (31.9–52.3)
P. maximinoi	85.2b (76–98)	9.34a (8.73–9.88)	13.02a (11.2–14.1)	61.95a (46.9–73.3)
P. patula	94.2a (84–100)	7.25d (6.19–7.92)	9.84c (8.3–11.0)	29.60d (19.0–39.7)
P. tecunumanii	97.0a (96–98)	8.72b (8.16–9.28)	12.51a (11.9–13.2)	53.27b (45.6–60.4)

Means followed by the same letter are not significantly different ($p \leq 0.05$).

patula. Altamirano, the best provenance of *P. maximinoi* produced 130% more volume than the local source of *P. greggii* (Patoltecoya), and produced 151% more volume than the local source of *P. patula* (Acaxochitlán). These results, as shown in Figure 2, demonstrate the potential importance of species, as well as provenance selection to production forestry in Mexico. Volume of the best provenance of each species tested and the local commercially used provenances of *P. greggii* and *P. patula*.

Similar growth differences were seen at 18 months of age in this same test, *P. maximinoi* and *P. greggii* had fewer growth flushes than *P. patula* and *P. tecunumanii*. However the average length of each flush (space between branch whorls) was greater in *P. maximinoi* and *P. greggii* than the latter two, particularly in *P. maximinoi* (Salazar et al. 1999), a difference so significant that it resulted in this species having the maximum growth. These differences would cause *P. patula* and *P. tecunumanii* to produce wood with more knots. Salazar et al. (1999) also found that while *P. greggii* and *P. tecunumanii* both have a similar growth rate during most of the year, *P. tecunumanii* continues growing later into the fall season. This probably accounts for the differences in growth between the two species on this site.

P. patula was the principal plantation species on the farm for wood production from 1985 to 1990. The seed was collected locally at Acaxochitlán, state of Hidalgo

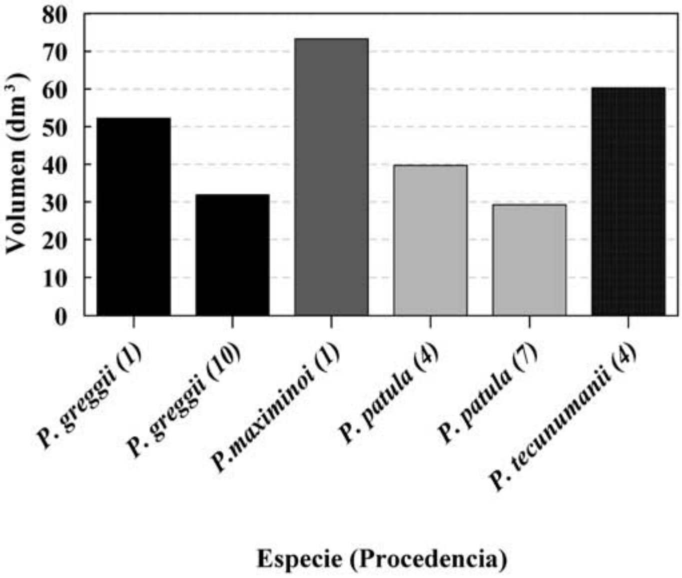

Figure 2. Volume of the best provenance of each species tested and the local commercially used provenances of *P. greggii* and *P. patula*. The provenance code coincides with Table 1.

(Fernández Alonso† personal communication). Subsequently a local source of *P. greggii* var. *australis* (Patoltecoya) was planted and showed superior growth at 7 years of age. *P. greggii* var. *australis* also grew better than *P. patula* in this test (Table 3). Moreover, the best *P. greggii* provenance (Valle Verde) produced 32% more volume than the best *P. patula*, Tlahuelompan (Figure 2). This advantage of *P. greggii* over *P. patula* was somewhat unexpected as it was believed (based on rainfall data) that the *P. greggii* provenances were adapted to sites with less precipitation than those of *P. patula*, and thus would grow less. It is possible that *P. greggii* var. *australis* provenances such as Laguna Seca and El Piñón take advantage of the additional moisture provided by the frequent fogs which occur on those sites resulting in adaptation to higher levels of moisture than rainfall data indicate. Also *P. greggii* var. *australis* occurs naturally on warmer sites than *P. patula*, giving it an advantage at this frost-free test site. The elevation of the test site is lower than most of the *P. greggii* var. *australis* provenances, and significantly lower (600–1400 m) than all of the *P. patula* material (Table 1). International field trials have shown that *P. patula* is more appropriate for planting on more temperate, higher elevation sites than this test site (Evans 1992). In central Mexico good *P. patula* sites would be at elevations of 2000 m and above.

In general, the more productive *P. patula* provenances were those closer to the test site geographically, while the better *P. greggii* were more distant, to the north in the states of Querétaro and Hidalgo, and those with greater rainfall. It should be noted that had the test included provenances of *P. greggii* var. *greggii* from the

Table 4. Survival, height, diameter and volume by provenance at 5 years of age

Provenance	Survival (%)	Height (m)	Diameter (cm)	Volume (dm^3)
P. greggii var. *australis*				
Valle Verde	100a	8.64a	12.35a	52.26a
El Madroño	96a	8.22ab	11.19bc	41.61bcd
Laguna Seca	98a	8.37ab	11.91ab	48.07ab
San Joaquín	98a	7.55bcd	11.21bc	38.68de
El Piñón	94ab	8.25ab	11.96ab	46.14abc
Pemuxtitla	98a	8.72a	11.76ab	46.82abc
Xochicoatlán	92b	8.14abc	12.05ab	48.05ab
Xodhé	92b	7.47cd	10.21d	32.85e
Cieneguilla	98a	7.85bcd	11.20bc	40.30cd
Patoltecoya	100a	7.28d	10.36cd	31.91e
P. maximinoi				
Altamirano	86ab	9.88a	14.05a	73.33a
San Jerónimo	98a	9.70a	14.12a	72.72a
San Juan Jacatépequez	84b	9.50ab	13.48ab	65.52a
Tatumbla	76b	8.73b	12.22bc	51.25b
San Jose Bayuncún	82b	8.91b	11.26c	46.93b
P. patula				
Pinal de Amoles	92ab	7.92a	10.81ab	36.62ab
Encarnación	100a	7.15bcd	9.55cd	27.71cde
Zacualtipan	94a	7.67ab	10.19abc	32.75bcd
Tlahuelompan	96a	7.87a	11.04a	39.72a
Huayacocotla	94a	7.20bcd	9.37d	26.44de
Estación Apulco	94a	7.08cd	9.71cd	27.87cde
Acaxochitlán	84b	7.30bcd	9.96bcd	29.25cde
Ahuazotepec	96a	7.06cd	9.53cd	27.11cde
Zacatlán-norte	94a	7.54abc	10.40abc	33.13abc
Zacatlán-este	96a	6.81d	9.34d	25.89e
Tlaxco	96a	6.19e	8.31e	19.05f
P. tecunumanii				
Chanal	96a	8.16c	11.93b	45.61b
Rancho Nuevo	98a	8.54bc	11.91b	47.33b
San Esteban	98a	8.89ab	13.22a	59.75a
Yucul	96a	9.28a	12.96a	60.38a

Means followed by the same letter are not significantly different ($p \leq 0.05$), comparisons are made of provenances within species.

northern Mexico states of Coahuila and Nuevo León, instead of var. *australis*, the results could have been very different. *P. greggii* var. *greggii* occurs on sites with minimal precipitation, at higher elevations with occasional frosts (Donahue and Lopez Upton, 1996) and grows considerably slower than var. *australis* (Azamar et al. 2000; Dvorak et al. 2000a; Velasco 2001).

Of the *P. greggii* provenances, Valle Verde grew the best, producing 64% more volume than the poorest *P. greggii*, Patoltecoya, which was the locally occurring provenance, and used as a comparison (Table 4). Patoltecoya has also shown inferior growth among *P. greggii* var. *australis* provenances in a previous study done

on this site by López et al. (1999). This local source was comprised of very few individual trees and it is possible that inbreeding had occurred, reducing its performance (Ramírez et al. 1997). At the same time the more productive trees may have been removed in previous selective cutting (high-grading) leaving the poor performers to dominate the population. A third factor that may affect this populations' performance is that it is located at a geographical and elevational extreme in the species' distribution (Table 1). Poor performance of extreme range populations is commonly seen in forest tree species (Rehfeldt 2000). Similarly Xodhé, an inferior source, is a small, reduced population growing on a very dry site. While it may have problems with inbreeding (Sorensen 1997), it also may be material adapted to slow growth in a dry environment (López et al. 2000).

P. tecunumanii as a species averaged 1.74 m annual height increment, with a range of 1.63–1.86 among provenances. Eight-years-old CAMCORE Cooperative trials of *P. tecunumanii* in South Africa gave similar results (1.65–1.78 m) and had superior growth in Colombia (1.87–2.11 m) (Vázquez and Dvorak 1996). As this test site in Mexico has few limiting factors for growth like the Colombian site, it remains to be seen if annual height increments will reach 2 m a year.

Of the *P. tecunumanii* material, Yucul was the best, producing 32% more volume than Chanal, the poorest (Table 4). The two Mexican provenances, which are considered high-elevation sources (2100+ m), grew less than those from central America, which are from low elevations (950–1,000 m). The test site is located at 1440 m above sea level, which is more comparable to the central American provenances. Significant differences in growth were not seen within the two subgroups of *P. tecunumanii* provenances. However these same provenances in trials in other countries have grown significantly different from each other (Dvorak et al. 2000d).

P. tecunumanii could outperform *P. maximinoi* on sites above 1600 m elevation or where light frosts occur (Dvorak et al. 2000b). In central and southern Mexico frosts occur at 2000–2100 m elevation. Of all the test material, the Mexican *P. maximinoi* sources, Altamirano and San Jeronimo grew the best (Table 4). Both sources had annual increments of 2.8 cm in diameter, 2.0 m in height and 73 dm^3 in volume at 5 years of age. In CAMCORE trials in Brazil and Colombia the Tatumbla provenance outgrew San Jeronimo at 3, 5 and 8 years of age. Internodal length in this test was measured by Salazar et al. (1999) at 18 months of age and no significant differences were found among the species. Internodal length can be an indicator of disposition to foxtail, which may be related to the broken top problem. Five percent of the *P. maximinoi* trees in our trial were found to have broken tops, apparently due to wind. In general, within species, the better provenances in volume production were closer to the test site elevation and precipitation than poorer performers. In fact the two best provenances in the test, which were *P. maximinoi*, had the most similar elevation and precipitation to the test site (Figure 3). Interestingly it is the two species considered native to the area *P. greggii* and *P. patula* that deviate somewhat from this pattern. Although one of these provenances may have a similar environment to the test site, reduced population size and deleterious effects of inbreeding could cause it to perform poorer than another provenance with a more dissimilar environment.

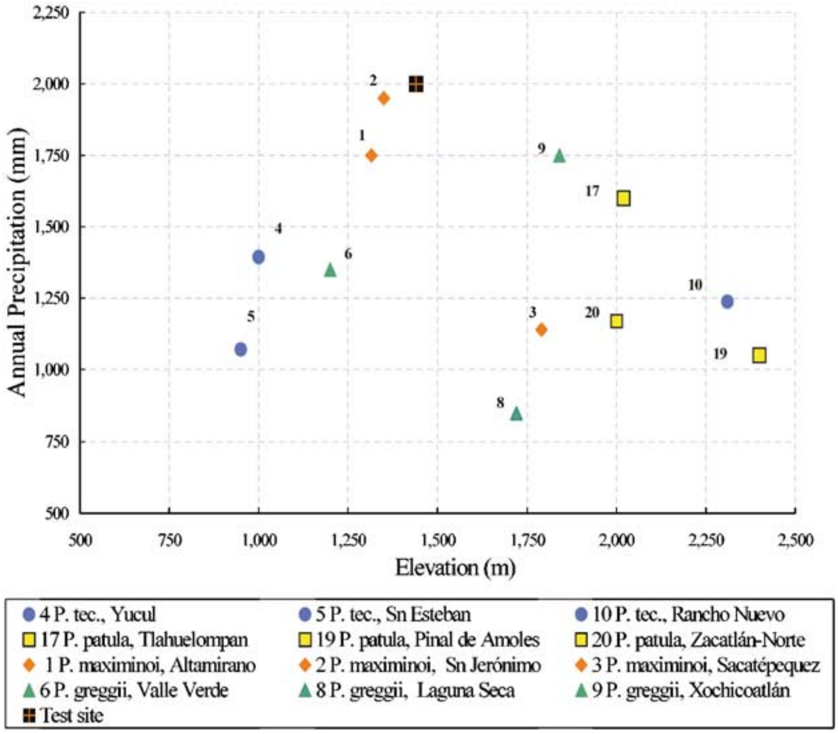

Figure 3. Elevation and precipitation of the best three provenances of each species, in relation to the test site. The number beside each provenance is its ranking in volume production of all 30 provenances.

Conclusions

In the area near Huauchinango, state of Puebla, Mexico, trees of *P. maximinoi* and *P. tecunumanii* had greater growth at 5 years of age than the local commercial species, *P. greggii* var. *australis* and *P. patula*. Within each species tested differences were found among provenances, and in some cases correlations were seen between volume production and latitude, elevation and precipitation. *P. maximinoi* had the best volume production, and the Mexican provenances were superior. Given the potential the species has shown here, trials with provenances from central Mexico should be established, along with a larger sampling of *P. tecunumanii*. Further study of potential foxtailing problems in *P. maximinoi* need to be assessed at the family level. The low-elevation provenances of *P. tecunumanii* were second overall to *P. maximinoi* in growth. The *P. greggii* var. *australis* provenances from northern Querétaro and Hidalgo grew best, while *P. patula* provenances of lower elevation, and those near to the Huauchinango area did the best among this species' material.

Acknowledgements

This research was partially supported by the Consejo Nacional de Ciencia y Tecnología (CONACYT) México P220-CCOR-904153. The CAMCORE Cooperative at North Carolina State University provided seed of *P. maximinoi* and *P. tecunumanii* that helped expand the scope of this test.

References

Azamar O.M., López U.J., Vargas H.J.J. and Plancarte B.A. 2000. Evaluación de un ensayo de procedencias-progenies de *Pinus greggii* y su conversión a huerto semillero. In: 1er Congreso Nacional de Reforestación. Montecillo, México, 7 p.

Birks J.S. and Barnes R.D. 1990. Provenance variation in *Pinus caribaea*, *P. oocarpa* and *P. patula* ssp. *tecunumanii*. Tropical Forestry Papers, Oxford Forestry Institute, University of Oxford, UK, 40 p.

Crockford K.J., Dunsdon A.J., Baylis W.H., Birks J.S. and Barnes R.D. 1990. Evaluation of tropical pine provenances and progeny tests. ODA Research Scheme R. 4346. Final Report. Oxford Forestry Institute, University of Oxford, UK 136 p.

Donahue J.K. and López-Upton J. 1996. Geographic variation in leaf, cone and seed morphology of *Pinus greggii* in native forests. For. Ecol. Manage. 82: 145–157.

Donahue J.K. and López-Upton J. 1999. A new variety of *Pinus greggii* (Pinaceae) in Mexico. Sida 18: 1103–1113.

Dvorak W.S. and Donahue J.K. 1988. *Pinus maximinoi* seed collections in Mexico and central America. CAMCORE Bulletin on Tropical Forestry No. 4. Raleigh, NC, 47 p.

Dvorak W.S., Kietzka J.E. and Donahue J.K. 1996. Three-year survival and growth of provenances of *Pinus greggii* in the tropics and subtropics. For. Ecol. Manage. 83: 123–131.

Dvorak W.S., Kietzka J.E., Donahue J.K., Hodge G.R. and Stanger T.K. 2000a. *Pinus greggii*. In: Conservation and Testing of Tropical and Subtropical Forest Tree Species by the CAMCORE Cooperative. NCSU, Raleigh, NC, pp. 52–73.

Dvorak W.S., Gutiérrez E.A., Gaspare W.J., Hodge G.R., Osorio L.F., Bester C. and Kikuti P. 2000b. *Pinus maximinoi*. In: Conservation and Testing of Tropical and Subtropical Forest Tree Species by the CAMCORE Cooperative. NCSU, Raleigh, NC, pp. 106–127.

Dvorak W.S., Hodge G.R., Kietzka J.E., Malan F.S., Osorio L.F. and Stanger T.K. 2000c. *Pinus patula*. In: Conservation and Testing of Tropical and Subtropical Forest Tree Species by the CAMCORE Cooperative. NCSU, Raleigh, NC, pp. 148–173.

Dvorak W.S., Hodge G.R., Gutiérrez E.A., Osorio L.F., Malán F.S. and Stanger T.K. 2000d. *Pinus tecunumanii*. In: Conservation and Testing of Tropical and Subtropical Forest Tree Species by the CAMCORE Cooperative. NCSU, Raleigh, NC, pp. 188–209.

Evans J. 1992. Plantation Forestry in the Tropics; Tree Planting for Industrial, Social, Environmental, and Agroforestry Purposes. 2nd edn. Claredon Press, Oxford, 403 p.

Greaves A. 1979. Descriptions of seed sources and collections for provenances of *Pinus oocarpa*. Tropical Forestry Papers, Commonwealth Forestry Institute, University of Oxford, No. 13. 144 p.

Hodge G.R. and Dvorak W.S. 1999. Genetic parameters and provenance variation of *Pinus tecunumanii* in 78 international trials. For. Genet. 6: 157–180.

INEGI 1980. Atlas nacional del medio físico. Carta de climas. Instituto Nacional de Estadística, Geografía e Informática, México, DF.

Kietzka J.E. 1988. *Pinus maximinoi*: a promising species in South Africa. South Afric. For. J. 145: 33–38.

Littell R.C., Milliken G.A., Stroup W.W. and Wolfinger R.D. 1996. SAS system for mixed models. SAS Institute Inc., Cary, North Carolina, 633 p.

López U.J. 1986. Características de la progenie de plantaciones jóvenes de *Pinus greggii* Engelm. B.Sc. Thesis, Univ. Autón. Chapingo, Chapingo, México, 71 p.

López A.J.L., Vargas H.J.J., Ramírez H.C. and López U.J. 1999. Variación intraespecífica en el patrón de crecimiento en altura del brote terminal en *Pinus greggii* Engelm. Revista Chapingo, Serie Ciencias Forestales 5: 133–140.

López U.J., Mendoza H.A.J., Jasso M.J., Vargas H.J.J. and Gómez G.A. 2000. Variación morfológica de plántulas e influencia del pH del agua de riego en doce poblaciones de *Pinus greggii* Engelm. Madera y Bosques 6: 81–94.

Magnussen S. and Yeatman C.W. 1990. Predictions of genetic gain from various selection methods in open pollinated *Pinus banksiana* progeny trials. Silvae Gen. 39: 140–153.

PRONARE 2000. Evaluación de la Reforestación 1999 del Programa Nacional de Reforestación. SEMARNAP, México, DF, n.p.

Ramírez H.C., Vargas H.J.J., Jasso M.J., Carrillo C.G. and Guillén A.H. 1997. Variación isoenzimática en diez poblaciones naturales de *Pinus greggii* Engelm. Agrociencia 31: 223–229.

Rehfeldt G.E. 2000. Genes, climate and wood. Leslie L. Schaffer Lectureship in Forest Science. 2 February 2000 The University of British Columbia, Vancouver, BC, 15 p.

Riemenschneider D.E. 1988. Heritability, age–age correlations, and inferences regarding juvenile selection in jack pine. For. Sci. 34: 1076–1082.

Romero G.Y.E. 1993. Análisis del crecimiento de *Pinus patula* Sch. *et* Cham. en diferentes niveles de competencia intraespecífica, en Huayacocotla, Ver. Masters Thesis, División de Ciencias Forestales, Universidad Autónoma Chapingo, 192 p.

Salazar G.J.G., Vargas H.J.J., Jasso M.J., Molina G.J.D., Ramírez H.C. and López U.J. 1999. Variación en el patrón de crecimiento en altura de cuatro especies de *Pinus* en edades tempranas. Madera y Bosques 5: 19–34.

SAS Institute Inc. 1999. SAS OnlineDoc®, Version 8, Cary, NC.

Sorensen F.C. 1997. Effects of sib mating and wind pollination on nursery seedling size, growth components, and phenology of Douglas-fir seed-orchard progenies. Can. J. For. Res. 27: 557–566.

van Wyk G. 1998. The status of tree breeding in South Africa. South Afric. For. J. 107: 54–59.

Vargas H.J.J. and Muñoz O.A. 1991. Potencial hídrico, transpiración y resistencia estomatal en plántulas de cuatro especies de *Pinus*. Agrociencia serie Recursos Naturales Renovables 1: 25–38.

Vázquez J. and Dvorak W.S. 1996. Trends in variances and heritabilities with stand development of tropical pines. Can. J. For. Res. 26: 1473–1480.

Velasco G.M.V. 2001. Ensayo de 13 procedencias de *Pinus greggii* Engelm. en dos localidades de la Mixteca Alta, Oaxaca. B.Sc. Thesis, División de Agronomía. Univ. Auton. Agraria Antonio Narro, 75 p.

Wright J.A., Osorio L.F. and Lambeth C.C. 1993. Development of a tree improvement program with *Pinus maximinoi* in Colombia. For. Ecol. Manage. 62: 313–322.
