# Supplementary material for: Provenance and family variations in early growth of Manchurian walnut (Juglans mandshurica Maxim.) and selection of superior families
Source: PLoS One. 2024 Mar 7;19(3):e0298918. doi: 10.1371/journal.pone.0298918 (PMC10919699; doi:10.1371/journal.pone.0298918)
Supplement: S2 File — (ZIP) [file pone.0298918.s005.zip › Provenance variation of seed traits of Juglans mandshurica in Changbai Mountains, northeastern China.pdf]

第 37 卷 第 12 期
2015 年 12 月

北 京 林 业 大 学 学 报
JOURNAL OF BEIJING FORESTRY UNIVERSITY

Vol. 37, No. 12
Dec., 2015

DOI: 10.13332/j.1000-1522.20150278

长白山区核桃楸结实性状种源变异分析

陈思羽[1] 杨 辉[2] 韩 姣[3] 张大伟[3] 赵珊珊[3] 张忠辉[3] 郭忠玲[1] 杨雨春[3]
(1 北华大学林学院 2 吉林省露水河林业局 3 吉林省林业科学研究院)

摘要: 以长白山 14 个核桃楸种源的结实性状为研究对象,采用主成分分析(PCA)法和方差分解分析研究了核桃楸种源结实性状的种源变异规律,分析了空间地理变异和地形因子对不同种源核桃楸结实性状的影响。结果表明:核桃楸不同种源的单株产量、干果质量、干果宽等差异显著,其中单株产量差异最为明显,其变异系数高达 36.12%,而干果质量、干果长和干果宽变异较小,变异系数的范围为 2.25% ~6.67%。PCA 分析表明,PC1 综合反映了核桃楸的果实产量、干果质量和树木高生长信息;而 PC2 则综合反映了果实表型性状和径生长信息,如干果长、干果宽和胸径。方差分解分析结果显示,空间变量对核桃楸单株产量、干果质量、干果长、干果宽、树高和胸径空间变异的解释量为 13% ~69%;地形变量对单株产量、干果宽、树高和胸径解释量范围为 19% ~24%;空间变量对 PC1 和 PC2 的解释量分别为 33% 和 24%,地形变量对 PC1 和 PC2 的解释量分别为 52% 和 66%。结实性状与地形变量相关分析表明,单株产量与坡度呈显著负相关($P<0.05$),干果宽与海拔呈显著正相关($P<0.05$)。聚类分析显示,种源组 II 中核桃楸单株产量最高,是最优的果实种源组;种源组 III 中核桃楸单株产量最低、平均胸径值最高,是最优的木材种源组。因此,在林业实践中应根据最终的培育目标来选取合适的种源。

关键词: 核桃楸;结实性状;种源变异;主成分分析;方差分解

中图分类号: S757.2 **文献标志码:** A **文章编号:** 1000-1522(2015)12-0032-09

CHEN Si-yu[1]; YANG Hui[2]; HAN Jiao[3]; ZHANG Da-wei[3]; ZHAO Shan-shan[3]; ZHANG Zhong-hui[3]; GUO Zhong-ling[1]; YANG Yu-chun[3]. **Provenance variation of seed traits of *Juglans mandshurica* in Changbai mountains, northeastern China.** *Journal of Beijing Forestry University* (2015) **37**(12) 32-40 [Ch, 23 ref.]

1 Forestry College of Beihua University, Jilin, Jilin, 132013, P. R. China;
2 Lushuihe Forestry Bureau of Jilin Province, Baishan, Jilin, 134506, P. R. China;
3 Forestry Academy of Jilin Province, Changchun, Jilin, 130033, P. R. China.

We investigated the seed traits of *Juglans mandshurica* from 14 provenances in Changbai Mountains, northeastern China. Principal component analysis (PCA) and analysis of variance were applied to analyze the variations of seed traits of *J. mandshurica* from different provenances, in order to reveal the effects of spatial geography and topography on the seed traits. The results showed that there were significant differences among provenances in the yield per plant, weight, length and width of dried fruit. The variation coefficient of yield per plant among provenances reached 36.12%, while the variations in the weight, length and width of dried fruit were relatively smaller, with the variation coefficient ranging from 2.25% to 6.67%. PCA analysis showed that PC1 reflected the fruit yield, dried fruit weight and characteristics of tree morphological, while PC2 expressed the fruit phenotypic traits, such as the length and width of dried fruit and the diameter at breast height. The variation partitioning analysis showed that spatial variables could explain 13% -69% of the variations in the yield per plant, weight, length, width of dried fruit, tree height and diameter at breast height (DBH). Topographic variables could explain 19% -24% of the variations in the yield per plant, width of dried fruit, tree height and DBH. The

收稿日期: 2015-08-02 **修回日期:** 2015-08-14

基金项目: 林业公益性行业科研专项(201204309)、吉林省科技厅计划发展项目(20100260)、吉林省林业厅项目(200905、2014-006)。

第一作者: 陈思羽。主要研究方向:森林培育。Email:33309797@qq.com 地址:132013 吉林省吉林市滨江东路 3999 号北华大学林学院。

责任作者: 杨雨春,博士,副研究员。主要研究方向:森林培育。Email:yang-yu-chun@163.com 地址:130033 吉林省长春市临河街 3528 号吉林省林业科学研究院。

本刊网址: http://j.bjfu.edu.cn; http://journal.bjfu.edu.cn

(C)1994-2019 China Academic Journal Electronic Publishing House. All rights reserved. http://www.cnki.net

spatial variables explained 33% and 24% of the variations of PC1 and PC2, respectively, and topographic variables explained 52% and 66% of the variations of PC1 and PC2, respectively. The correlation analysis represented that the yield per plant had significantly negative correlations with slope ($P < 0.05$) and width of dried fruit significantly positive correlations with elevation ($P < 0.05$). The cluster analysis showed that group II with the highest yield was the ideal group of fruit, while group III with the lowest yield but the highest DBH was the superior group of wood. Therefore, suitable provenances should be chosen according to the breeding goal in forestry practices.

Key words　*Juglans mandshurica*; seed trait; provenance variation; principal component analysis; variation partitioning

核桃楸(*Juglans mandshurica*)是珍贵的“东北三大硬阔”树种之一,属于国家 II 级珍稀树种,是中国珍稀濒危树种名录的三级保护植物; 主要分布于我国东北地区,同时在朝鲜、俄罗斯和日本等国也有分布[1-2]。核桃楸材质优良可作为优质用材树种[3],其果实也是粮油和药品的重要来源[4-7]。近年来,由于采伐和绿化大苗移植等原因,导致长白山区核桃楸资源的数量不断减少、质量不断下降。

了解树种内不同地理种源的变异,对制定合理的育种目标并充分利用其资源具有重要意义。目前长白山区核桃楸研究主要围绕着种群分布格局和种群数量动态展开[8-10]。核桃楸地理变异方面的研究发现,核桃楸的生长性状、适应性状和形态性状主要受经度影响,地理变异呈现由东北到西南冠幅变大、侧枝变粗、侧芽增多的变化趋势[11],而核桃楸材积则与经度表现出显著的负相关关系[12]; 坡度、坡向和坡位对核桃楸地理变异的影响不同,位于中上阳坡的核桃楸生长状况较好; 土壤中碱解氮和有效磷含量较高时,能够有效地促进核桃楸的生长[13]。庄倩倩等[14]分析不同地理位置核桃楸种实性状的形态指标差异时发现,不同采种点种子形态和苗期生长表现不同。尽管有关核桃楸地理变异方面的研究已有报道[11-12],但核桃楸种实性状地理变异方面的研究还鲜见报道,有待进一步加强。本文以长白山地区 14 个核桃楸地理种源的结实性状为研究对象,探讨了核桃楸结实性状的种源变异,分析了空间和地形变量对不同种源核桃楸结实性状的影响,筛选出核桃楸的最优种源地。研究结果将有助于进一步了解核桃楸结实性状的地理变异规律,为核桃楸珍贵资源的保护和利用提供理论依据。

1　材料与方法

1.1　研究样地位置

在长白山地区按 1°经纬度网格,选取 14 个具有代表性的核桃楸天然种源地(表 1)。本文中 14 个取样点的年均温范围为 2.7 ~ 4.9 ℃,≥10 ℃积温范围为 2 563 ~ 3 011 ℃,无霜期范围为 115 ~ 140 d,降水量范围为 572 ~ 896 mm,蒸发散范围为 615 ~ 700 mm(表 2)。

1.2　野外调查

2010—2012 年,在上述每个核桃楸天然种源林内分别建立 3 块 100 m × 100 m 标准样地,以核桃楸作为研究对象,在每个样地内选取至少 30 株核桃楸,记录胸径、树高等数据,同时测定单株产量、形态和果实表型等性状指标。

1.3　数据处理与分析

采用主成分分析建立核桃楸结实性状的主成分轴,在主成分排序图内添加聚类分析结果,从而划分不同核桃楸种源组。根据 14 个种源地的地理位置坐标,建立基于特征根的空间变量。利用邻体矩阵主坐标分析构建 PCNM(Principal coordinates of neighbour matrices)变量,从而实现在不同尺度上对空间结构进行识别[15]。Borcard[16]首先提出方差分解的概念和分解过程,量化两组或多组变量单独或共同解释响应变量的变差。Peres-Neto 等[17]提出使用校正 R^2 量化解释变量对响应变量的解释量。以核桃楸结实性状指标为响应变量 Y,以空间变量(X)和地形变量(W)为解释变量。空间变量采用向前选择过程筛选出显著的 PCNM 变量,地形变量则包括种源地海拔和坡度指标。本文所有计算均采用 R 软件实现,邻体矩阵主坐标分析利用 PCNM 包计算,方差分解利用 vegan 包计算,向前选择利用 packfor 包计算。

2　结果与分析

2.1　核桃楸结实性状种源变异

在 7 个结实性状中,除干果长指标在不同种源间差异不显著外,其余性状在不同种源间均差异显著($P < 0.01$,表 3),因此核桃楸结实性状在不同种源地之间存在广泛变异。不同种源地核桃楸单株产量差异明显,种源间变异系数高达 36.12%; 种子表型性状干果质量、干果长和干果宽变异相对较小,种

(C)1994-2019 China Academic Journal Electronic Publishing House. All rights reserved.　http://www.cnki.net

表1 核桃楸种源地概况

Tab. 1 Survey of *Juglans mandshurica* provenances

编号 Number	种源地 Provenance	林分组成 Stand composition	经度 Longitude	纬度 Latitude	海拔 Altitude/m	坡度 Slope/(°)
1	汪清大兴沟林业局周仁沟林场 Zhourengou Forest Farm of Daxingou Forestry Bureau in Wangqing County	4 核桃楸 *Juglans mandshurica* + 2 落叶松 *Larix gmelinii* + 2 红松 *Pinus koraiensis* + 1 枫桦 *Betula costata* + 1 杂 others	129°18′36″	43°24′36″	432	18.4
2	汪清林业局塔子沟林场 Tazigou Forest Farm of Wangqing Forestry Bureau in Wangqing County	3 核桃楸 *Juglans mandshurica* + 2 杨属 *Populus* spp. + 2 枫桦 *Betula costata* 2 + 白桦 *B. platyphylla* + 1 杂 others	130°07′48″	43°27′36″	690	40.8
3	珲春林业局山河林场 Shanhe Forest Farm of Hunchun Forestry Bureau	3 核桃楸 *Juglans mandshurica* + 2 椴属 *Tilia* spp. 2 + 白桦 *Betula platyphylla* + 1 蒙古栎 *Quercus mongolica* + 1 杨属 *Populus* spp.	130°35′24″	43°00′00″	183	5.0
4	大石头林业局东明林场 Dongming Forest Farm of Dashitou Forestry Bureau	3 核桃楸 *Juglans mandshurica* + 3 落叶松 *Larix gmelinii* + 2 蒙古栎 *Quercus mongolica* + 2 水曲柳 *Fraxinus mandshurica*	128°40′48″	43°27′01″	558	21.6
5	敦化市建设林场 Jianshe Forest Farm of Dunhua City	4 核桃楸 *Juglans mandshurica* + 3 杨属 *Populus* spp. + 1 红松 *Pinus koraiensis* + 1 蒙古栎 *Quercus mongolica*	127°51′00″	42°57′01″	580	19.0
6	蛟河实验管理局 Experimental and Management Bureau of Jiaohe	4 核桃楸 *Juglans mandshurica* + 2 榆属 *Ulmus* spp. 2 + 黄檗 *Phellodendron amurense* + 1 水曲柳 *Fraxinus mandshurica* + 1 槭属 *Acer* spp.	127°43′12″	44°01′12″	447	2.4
7	通化石湖公益村 Gongyi Village of Shihu Town in Tonghua County	6 核桃楸 *Juglans mandshurica* + 4 水曲柳 *Fraxinus mandshurica*	126°19′12″	41°28′48″	655	15.0
8	通化二密猪圈沟 Zhujuan Village of Ermi Town in Tonghua County	5 核桃楸 *Juglans mandshurica* + 5 蒙古栎 *Quercus mongolica*	125°50′24″	41°46′48″	484	35.0
9	柳河凉水河子林场 Liangshuihezi Forest Farm in Liuhe County	3 核桃楸 *Juglans mandshurica* + 2 槭属 *Acer* spp. + 2 椴属 *Tilia* spp. + 2 蒙古栎 *Quercus mongolica* + 1 枫桦 *Betula costata*	126°15′00″	42°10′12″	807	15.0
10	辉南三岔子半截沟 Banjiegou Village of Sanchazi Town in Huinan County	3 核桃楸 *Juglans mandshurica* + 2 水曲柳 *Fraxinus mandshurica* + 2 黄檗 *Phellodendron amurense* + 2 杨属 *Populus* spp. + 1 枫桦 *Betula costata*	126°36′00″	42°33′00″	523	5.0
11	临江闹枝小义和沟 Xiaoyihegou Village of Naozhi Town in Linjiang City	6 核桃楸 *Juglans mandshurica* + 4 落叶松 *Larix gmelinii*	127°01′12″	41°54′36″	527	24.0
12	临江六道沟 Liudaogou Town in Linjiang City	3 核桃楸 *Juglans mandshurica* + 3 蒙古栎 *Quercus mongolica* + 2 枫桦 *Betula costata* + 1 水曲柳 *Fraxinus mandshurica* + 1 黄檗 *Phellodendron amurense*	127°54′36″	41°37′12″	399	16.0
13	长白县十三道沟 Shisandaogou Town in Chanbai County	6 落叶松 *Larix gmelinii* + 3 核桃楸 *Juglans mandshurica* + 1 槭属 *Acer* spp.	127°48′36″	41°26′24″	713	24.0
14	抚松县露水河黎明林场 Liming Forest Farm of Lushuihe Forestry Bureau in Fusong County	4 核桃楸 *Juglans mandshurica* + 3 杨属 *Populus* spp. + 1 枫桦 *Betula costata* + 1 白桦 *B. platyphylla* + 1 杂 others	127°00′00″	42°39′00″	663	0.0

(C)1994-2019 China Academic Journal Electronic Publishing House. All rights reserved. http://www.cnki.net

表 2　研究区气候条件概况

Tab. 2　Summary of climatic conditions in the study area

编号 Number	种源地 Provenance	年均温 Annual mean temperature/℃	≥10 ℃积温 ≥10 ℃ accumulated temperature/℃	无霜期 Frostless period/d	降水量 Precipitation/mm	蒸发量 Evaporation/mm
1	汪清大兴沟林业局周仁沟林场 Zhourengou Forest Farm of Daxingou Forestry Bureau in Wangqing County	3.46	2 650	120	572	699
2	汪清林业局塔子沟林场 Tazigou Forest Farm of Wangqing Forestry Bureau in Wangqing County	4.42	2 819	128	567	700
3	珲春林业局山河林场 Shanhe Forest Farm of Hunchun Forestry Bureau	4.90	2 950	140	650	700
4	大石头林业局东明林场 Dongming forest farm of Dashitou forestry bureau	2.97	2 585	119	603	673
5	敦化市建设林场 Jianshe Forest Farm of Dunhua City	2.71	2 563	117	719	615
6	蛟河实验管理局 Experimental and Management Bureau of Jiaohe	3.00	2 683	126	747	654
7	通化石湖公益村 Gongyivillage of Shihu town in Tonghua County	5.14	3 011	138	896	615
8	通化二密猪圈沟 Zhujuangou Village of Ermi Town in Tonghua County	4.91	2 958	138	816	700
9	柳河凉水河林场 Liangshui Forest Farm in Liuhe County	3.72	2 767	129	804	675
10	辉南三岔子半截沟 Banjiegou Village of Sanchazi Town in Huinan County	3.09	2 815	122	759	689
11	临江闹枝小义和沟 Xiaoyihegou Village of Naozhi Town in Linjiang City	4.26	2 788	135	831	665
12	临江六道沟 Liudaogou Town in Linjiang City	4.38	3 000	126	788	600
13	长白县十三道沟 Shisandaogou Town in Chanbai County	3.51	2 840	115	752	600
14	抚松县露水河黎明林场 Liming Forest Farm of Lushuihe Forestry Bureau in Fusong County	3.12	2 673	120	761	628

源间变异系数在 2.25% ~6.67%。因此,长白山地区核桃楸结实性状在不同种源地之间遗传变异显著。

植物性状的相关性分析有助于确定不同性状间的关联性。由表 4 可知,单株产量与树高间显著正相关,相关系数高达 0.62($P<0.05$)。其他种实性状指标之间相关系数在 −0.51 ~0.32,但相关性均不显著($P>0.05$)。因此,树高是影响核桃楸单株产量的重要性状。

2.2　核桃楸结实性状主成分分析

主成分分析显示,前 2 个主成分轴的特征根值超过了平均特征根,根据 Kaiser-Guttman 准则保留 PC1 和 PC2 2 个轴(图 1)。PC1 和 PC2 解释了总方差 93.3% 变异,能够表达绝大部分数据结构信息。PC1 单独解释量为 56.1%,PC2 单独解释量为 37.8%。因此,前 2 个主成分综合反映了核桃楸的结实性状信息。PCA 分析图如图 2 所示,载荷系数可以近似看作原始性状与主成分轴之间的相关系数。PC1 与单株产量、干果质量、树高密切相关,综合反映了核桃楸的果实产量、质量和树木形态信息;PC2 则综合反映了干果长、干果宽和胸径

(C)1994-2019 China Academic Journal Electronic Publishing House. All rights reserved.　http://www.cnki.net

表3 核桃楸结实性状种源变异分析

Tab. 3 Analysis of provenance variances of seed traits of *J. mandshurica*

结实性状 Seed traits	极小值 Minimum value	极大值 Maximum value	平均值 Average value	标准差 Standard error	变异系数 Coefficient of variation/%	*F* 值 *F* value
单株产量 Yield per plant	0.92	3.48	2.15	0.78	36.12	50.20**
干果质量 Dried fruit weight	13.20	17.10	15.00	1.00	6.67	20.30**
干果长 Dried fruit length	4.28	4.74	4.57	0.10	2.25	4.58
干果宽 Dried fruit width	3.50	3.98	3.78	0.13	3.43	6.92**
树高 Tree height	5.60	20.80	11.50	3.40	29.60	16.80**
胸径 Diameter at breast height	17.00	33.50	22.40	3.70	16.40	16.40**

注：** 表示在 $P<0.01$ 水平差异显著，单株产量的极小值、极大值和平均值的单位为 kg，干果质量的极小值、极大值和平均值的单位为 g，干果长、干果宽、胸径的极小值、极大值和平均值的单位为 cm，树高的极小值、极大值和平均值的单位为 m。Notes: ** indicates significant difference at $P<0.01$, the unit of minimum, maximum and mean value of yield per plant is kg; the unit of minimum, maximum and mean value of dried fruit weight is g; the unit of minimum, maximum and mean value of dried fruit length, dried fruit width and diameter at breast height is cm; the unit of minimum, maximum and mean value of tree height is m.

表4 核桃楸结实性状之间的相关分析

Tab. 4 Correlation matrix between seed traits of *J. mandshurica*

结实性状 Seed traits	单株产量 Yield per plant	干果质量 Dried fruit weight	干果长 Dried fruit length	干果宽 Dried fruit width	树高 Tree height
干果质量 Dried fruit weight	0.29				
干果长 Dried fruit length	0.15	0.10			
干果宽 Dried fruit width	0.16	0.23	0.04		
树高 Tree height	0.62*	0.24	0.32	0.14	
胸径 Diameter at breast height	-0.12	0.21	-0.51	0.23	-0.17

注：* 表示在 $P<0.05$ 水平差异显著。Note: * indicates significant difference at $P<0.05$.

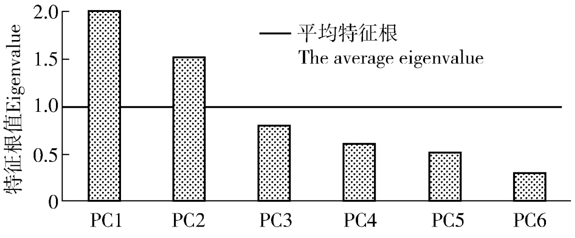

图1 通过 Kaiser-Guttman 准则选择 PCA 轴

Fig. 1 Selection of the PCA axis by Kaiser-Guttman criteria

信息(图2)。

2.3 核桃楸结实性状地理变异分析

采用邻体主坐标分析模拟14个种源地的空间结构，最终获得8个含正特征根的 PCNM 变量。本文采取不规则取样方法，PCNM 变量没有出现规律变化(图3)。方差分解分析表明空间变量对核桃楸单株产量、干果质量、干果长、干果宽、树高和胸径空间变异具有较高的解释能力，解释量范围为 13% ~ 69%(表5)。除干果质量、干果长外，其它性状均受中小尺度 PCNM 影响。地形变量主要影响单株产量、干果宽、树高和胸径，解释量在 19% ~24%。空间变量解释了 PC1 的 33% 变异，地形变量解释了 PC1 的 52% 变异；空间变量解释了 PC2 的 24% 变异，地形变量解释了 PC2 的 66% 变异(表5)。

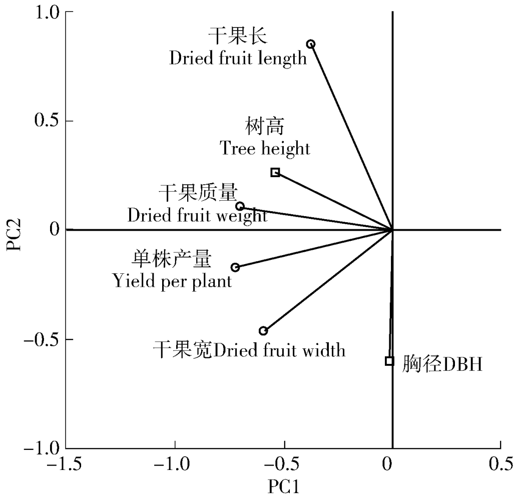

图2 主成分分析图

Fig. 2 Diagram of PCA

核桃楸结实性状与地形变量相关分析显示，核桃楸单株产量与坡度显著负相关($r=-0.56$, $P<0.05$)，干果宽与海拔显著正相关($r=0.58$, $P<0.05$)，树高与坡度负相关($r=-0.56$, $P=0.06$)，胸径与海拔正相关($r=0.49$, $P=0.07$)。因此，海拔和坡度是影响核桃楸结实性状的重要地形因子(图4)。

(C)1994-2019 China Academic Journal Electronic Publishing House. All rights reserved. http://www.cnki.net

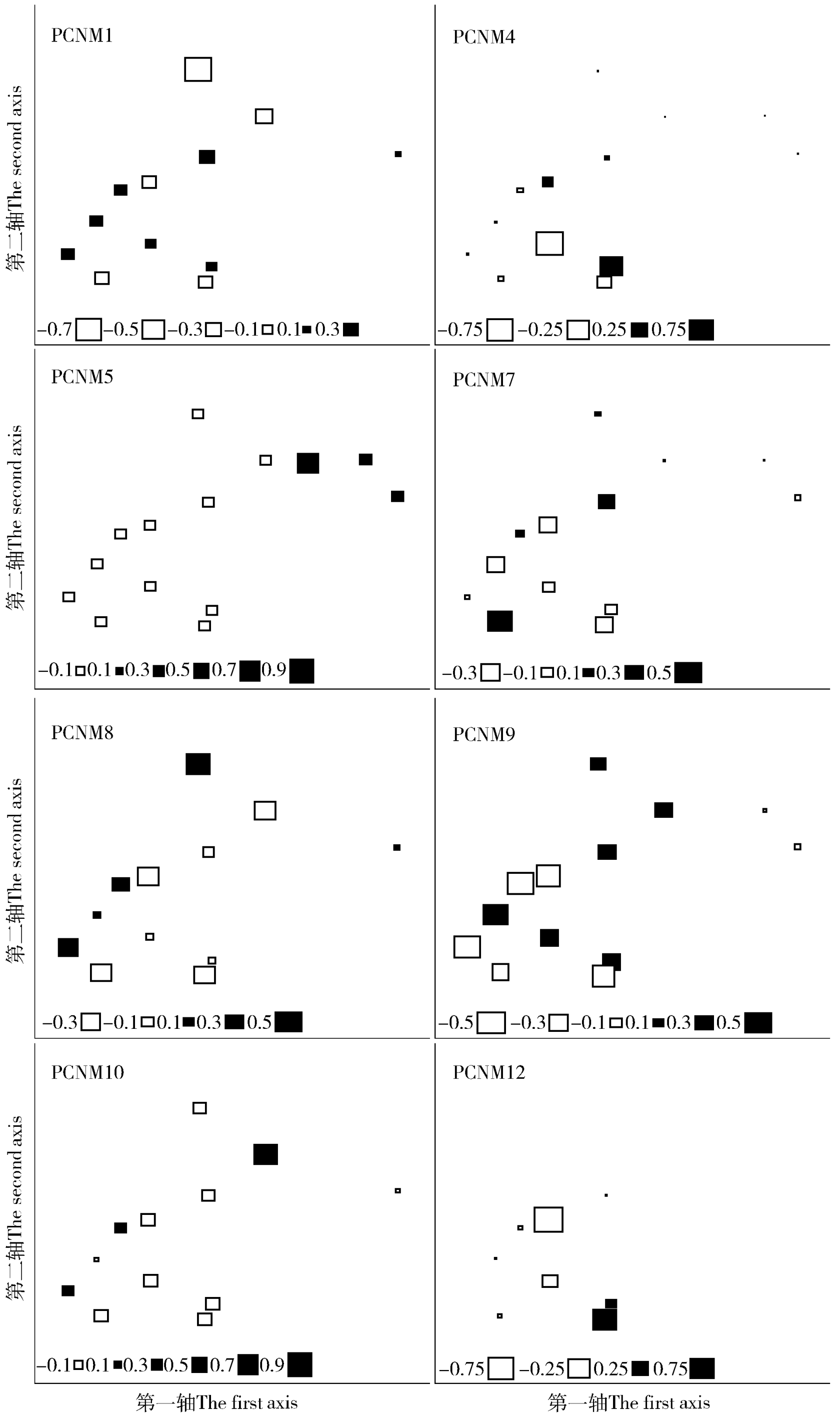

图 3　方差分解分析中涉及到的 8 个含正特征根的 PCNM 变量(数字表示为特征根值)

Fig. 3　Eight PCNM variables with positive eigenvalues in the variation partitioning analysis(The numbers indicate characteristic root value)

表 5　核桃楸结实性状方差分解分析

Tab. 5　Variation partitioning analysis of seed traits of *J. mandshurica*

结实性状 Seed traits	PCNM 选择 PCNM selection	校正 R^2 值 Corrected R^2 value	
		空间变量 Spatial variables	地形变量 Topographic variables
单株产量 Yield per plant	PCNM7	0. 13	0. 20
干果质量 Dried fruit weight	PCNM1, PCNM7, PCNM9, PCNM10	0. 64	0. 00
干果长 Dried fruit length	PCNM8, PCNM12	0. 69	0. 00
干果宽 Dried fruit width	PCNM7, PCNM10	0. 21	0. 20
树高 Tree height	PCNM10, PCNM12	0. 48	0. 24
胸径 Diameter at breast height	PCNM4, PCNM8	0. 40	0. 19
PC1	PCNM4, PCNM12	0. 33	0. 52
PC2	PCNM5, PCNM12	0. 24	0. 66

(C)1994-2019 China Academic Journal Electronic Publishing House. All rights reserved.　http://www.cnki.net

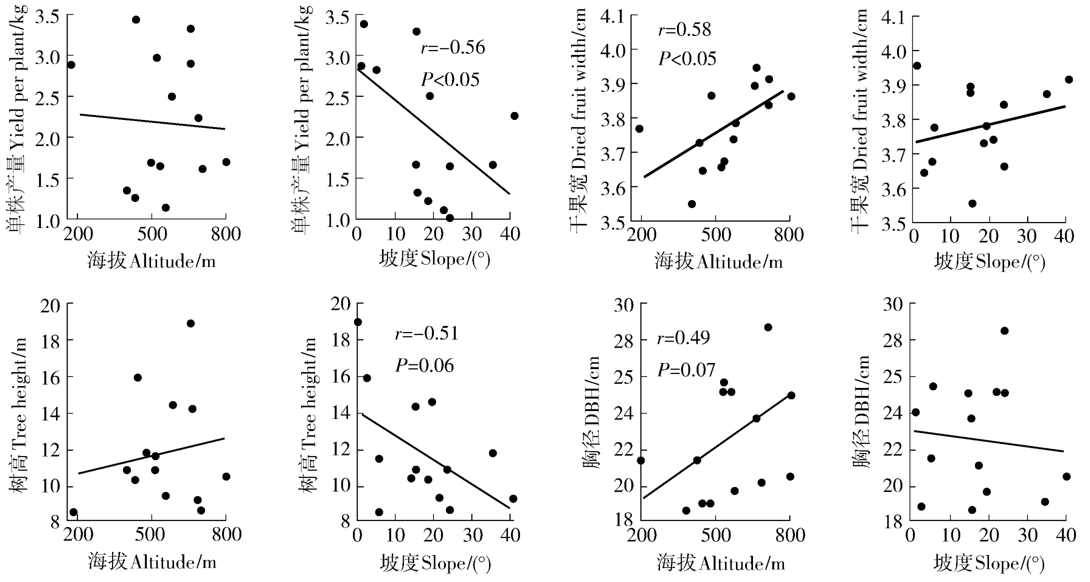

图4 核桃楸结实性状与地形变量关系

Fig. 4 Relationships between *J. mandshurica* seed traits and topographic variables

2.4 核桃楸结实性状聚类分析

不同种源核桃楸结实性状聚类分析表明，可将14个种源划分为3个种源组：组Ⅰ(1、2、8、9、11)、组Ⅱ(3、5、6、7、10、14)、组Ⅲ(4、12、13)(图5)。

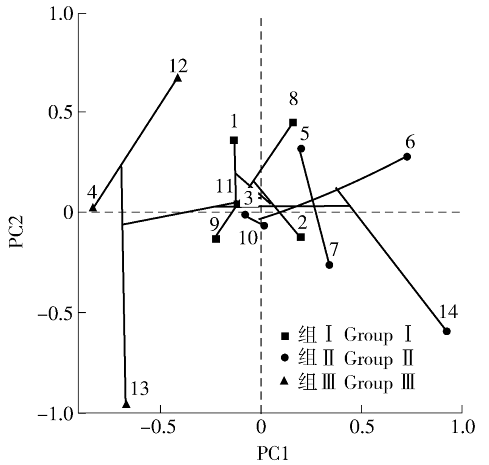

图5 核桃楸种源地聚类分析

Fig. 5 Dendrogram of cluster analysis of *J. mandshurica* provenance

由表6可知，种源组Ⅱ中核桃楸单株产量最高(平均为2.96 kg)、胸径最低(平均为21.85 cm)，是最优的果实种源组。种源组Ⅲ中核桃楸单株产量最低(平均为1.35 kg)、胸径最高(平均为24.09 cm)，是最优的木材种源组。

3 结论与讨论

近年来，植物性状已经成为生态学研究的热点，而研究植物果实或种子性状变异，可以揭示种源间的遗传规律及变异程度[18]。核桃楸作为长白山地区的重要用材树种和经济树种，研究其结实性状(包括产量、形态和果实表型等性状)的种源变异规律则显得更加必要。本文发现长白山地区不同种源核桃楸结实性状之间遗传变异显著。不同种源地的核桃楸单株产量种源间变异系数高达36.12%，干果质量、干果长和干果宽等种子表型性状的种源间变异系数的范围则为2.25%～6.67%。

帽儿山试验林场核桃楸种源试验研究表明，核桃楸的生长性状、适应性状和形态性状受经度和纬度影响显著，表现出以经向变异为主、纬向变异为辅的经纬双向渐变的趋势[19]。核桃楸生长性状呈现显著的西南—东北的变异趋势，越靠近分布区西南部的种源生长量越大，越靠近分布区东北部的种源生长量越

表6 不同种源组的核桃楸结实性状值(平均值±标准误差)

Tab. 6 Seed traits of *J. mandshurica* in different provenance groups (mean ± standard error)

种源组 Provenance	单株产量 Yield per plant/kg	干果质量 Dried fruit weight/g	干果长 Dried fruit length/cm	干果宽 Dried fruit width/cm	树高 Tree height/m	胸径 DBH/cm
组Ⅰ Group Ⅰ	1.68±0.35	15.10±0.82	4.64±0.36	3.81±0.11	10.61±0.90	22.07±2.77
组Ⅱ Group Ⅱ	2.96±0.35	14.81±0.75	4.56±0.36	3.75±0.10	13.01±2.88	21.85±2.75
组Ⅲ Group Ⅲ	1.35±0.26	14.69±1.45	4.44±0.08	3.71±0.14	9.73±1.09	24.09±4.99

(C)1994-2019 China Academic Journal Electronic Publishing House. All rights reserved. http://www.cnki.net

小; 生理生化性状与地理因子的相关性虽然未达到显著性水平,但也呈现出西南—东北的变异趋势[11]。本文中针对不同种源核桃楸结实性状的 PCA 分析表明,PC1 单独解释量为 56.1%,PC2 单独解释量为 37.8%,PC1 与单株产量、干果质量和树高密切相关,属于产量和形态性状因子; 空间变量解释了 PC1 的 33%变异,地形变量解释了 PC1 的52%变异。而 PC2 综合反映了干果长、干果宽和胸径的信息,属于果实表型性状和树木径生长因子; 空间变量解释了 PC2 的 24%变异,地形变量解释了 PC2 的 66%变异。空间结构分析表明,空间变量对核桃楸单株产量、干果质量、干果长、干果宽、树高和胸径空间变异的解释量范围为 13% ~69%。地形变量主要影响单株产量、干果宽、树高和胸径,解释量在 19% ~24%。因此,核桃楸结实性状具有显著的空间结构,而空间地理位置是影响其性状的重要因子。

地形通常对植物性状具有显著影响[20],海拔能够影响立地条件的气候变化,对光、热量、温度及降水量等生态因子产生显著的影响,进而影响植物生长发育和物质代谢[12]。相关研究表明,海拔对亚热带常绿阔叶林植物生理性状和形态性状产生影响[21],此外,坡向变化对其土壤养分和水分的影响也较大,从而影响植物性状变化。核桃楸结实性状与地形变量分析表明,单株产量和干果宽与坡度呈显著负相关,与海拔呈显著正相关。因此,海拔和坡度是影响核桃楸结实性状的主要地形因子。当然,由于树种不同,海拔对果实性状的影响亦不相同,海拔与紫椴果实的种子长和种子宽/长分别呈显著负相关和正相关关系[22],花楸果长则随海拔的上升略呈增加趋势,而对果实宽度无影响或影响较小[23]。

不同种源核桃楸结实性状聚类分析显示,种源组 II 中核桃楸单株产量最高,为最优的果实种源组; 种源组 III 中核桃楸单株产量最低、胸径值最高,为最优的木材种源组。因此,在林业实践中应根据最终的培育目标来选取合适的种源。

参 考 文 献

[1] 中国科学院中国植物志编辑委员会. 中国植物志: 第 21 卷[M]. 北京: 科学出版社, 1979: 32–33.
Editorial committee of Chinese journal of plant of Chinese academy of sciences. Flora of China: Twenty-first volume[M]. Beijing: Science Press. 1979: 32–33.

[2] 周以良. 中国东北植被地理[M]. 北京: 科学出版社, 1997: 147.
ZHOU Y L. Vegetation geography in Northeast China[M]. Beijing: Science Press, 1997: 147.

[3] 刘文华. 核桃楸的利用和苗木培育[J]. 中国林副特产, 2007(1): 44–45.
LIU W H. Using and seedling cultivation of *Juglans mandshurica*[J]. Forest By-Product and Speciaty in China, 2007(1): 44–45.

[4] 程翠林, 王振宇, 赵海田, 等. 核桃资源功能研究与开发现状[J]. 食品研究与开发, 2013, 34(13): 128–133.
CHENG C L, WANG Z Y, ZHAO H T, et al. Advances in development and functional research of walnut resources[J]. Food Research and Development, 2013, 34(13): 128–133.

[5] CARVALHO M, FERREIRA P L, MENDES V S, et al. Human cancer cell antiproliferative and antioxidant activities of *Juglans regia* L.[J]. Food and Chemical Toxicology, 2010, 48(1): 441–447.

[6] XU H L, YU X F, QU S C, et al. Juglone, isolated from *Juglans mandshurica* Maxim, induces apoptosis via down-regulation of AR expression in human prostate cancer LNCaP cells[J]. Bioorganic & Medicinal Chemistry Letters, 2013, 23(12): 3631–3634.

[7] SHARMA P, RAVIKUMAR G, KALAISELVI M, et al. In vitro antibacterial and free radical scavenging activity of green hull of *Juglans regia*[J]. Journal of Pharmaceutical Analysis, 2013, 3(4): 298–302.

[8] 阚彬彬, 王庆成, 吴文娟. 长白山核桃楸分布格局及其与伴生树种空间关联性的研究[J]. 安徽农业科学, 2014, 42(33): 11773–11777.
KAN B B, WANG Q L, WU W J. Study on the spatial pattern of *Juglans mandshurica* and its spatial correlation with accompanying species in Changbai Mountain[J]. Journal of Anhui Agriculture Science, 2014, 42(33): 11773–11777.

[9] 马万里, 荆涛, 罗菊春, 等. 长白山林区核桃楸种群分布格局研究[J]. 内蒙古师范大学学报, 2008, 37(2): 233–236.
MA W L, JIN T, LUO J C, et al. Spatially distributed pattern in *Juglans mandshurica* population from Changbai Mountain[J]. Journal of Inner Mongolia Normal University, 2008, 37(2): 233–236.

[10] 马万里, 罗菊春, 荆涛, 等. 长白山林区核桃楸种群数量动态变化的研究[J]. 植物研究, 2007, 27(2): 249–253.
MA W L, LUO J C, JIN T, et al. Study on dynamics of *Juglans mandshurica* population from Changbai Mountain[J]. Bulletin of Botanical Research, 2007, 27(2): 249–253.

[11] 杨书文, 刘桂丰, 王会仁, 等. 胡桃楸地理变异规律的再研究[J]. 东北林业大学学报, 1991, 19(专刊): 183–188.
YANG S W, LIU G F, WANG H R, et al. The further study on the Geographic variation of *Juglans mandshurica*[J]. Journal of Northeast Forestry University, 1991, 19(Special): 183–188.

[12] 张丽鹏,杨雨春,赵珊珊, 等. 环境因子对长白山区天然核桃楸林生长的影响[J]. 中国农学通报, 2014, 30(4): 34–41.
ZHANG L P, YANG Y C, ZHAO S S, et al. The impact of environmental factors on the growth of *Juglans mandshurica* in natural forest of Changbai Mountain[J]. Chinese Agricultural Science Bulletin, 2014, 30(4): 34–41.

[13] 陈永亮, 韩士杰, 周玉梅, 等. 胡桃楸、落叶松纯林及其混交林根际土壤有效磷特性的研究[J]. 应用生态学报, 2002, 13(7): 790–794.
CHEN Y L, HAN S J, ZHOU Y M, et al. Characteristics of available P in the rhizosphere soil in pure *Juglans mandshurica* and

(C)1994-2019 China Academic Journal Electronic Publishing House. All rights reserved. http://www.cnki.net

Larix gmelinii and their mixed plantation [J]. Chinese Journal of Applied Ecology, 2002, 13(7): 790-794.

[14] 庄倩倩，陈少鹏，刘洪章. 核桃楸不同地点种子形态及苗期生长的初步研究[J]. 吉林林业科技，2015, 44(3): 1-3, 20.
ZHUANG Q Q, CHEN S P, LIU H Z. The preliminary study of seed morphology and seedling growth of *Juglans mandshurica* from different regions of Jilin province [J]. Journal of Jilin Forestry Science and Technology, 2015, 44(3): 1-3, 20.

[15] BORCARD D, LEGENDRE P. All-scale spatial analysis of ecological data by means of principal coordinates of neighbour matrices [J]. Ecological Modelling, 2002, 153(1): 51-68.

[16] BORCARD D, LEGENDRE P, DRAPEAU P. Partialling out the spatial component of ecological variation [J]. Ecology, 1992, 73(3): 1045-1055.

[17] PERES-NETO P R, LEGENDRE P, DRAY S, et al. Variation partitioning of species data matrices: estimation and comparison of fractions [J]. Ecology, 2006, 87(10): 2614-2625.

[18] 刘军，张海燕，姜景民，等. 毛红椿种实和苗期生长性状地理种源变异[J]. 南京林业大学学报：自然科学版，2011, 35(3): 55-59.
LIU J, ZHANG H Y, JIANG J M, et al. Geographic variation of seed/fruit and seedling growing traits in *Toona ciliata* var. *pubescens* [J]. Journal of Nanjing Forestry University: Natural Science Edition, 2011, 35(3): 55-59.

[19] 杨书文，刘桂丰，张世英，等. 胡桃楸地理变异规律及最佳种源的初步选择[J]. 东北林业大学学报，1990, 18(专刊): 72-76.
YANG S W, LIU G F, ZHANG S Y, et al. A study on the geographic variation and preliminary selection for the best provenances of *Juglans mandshurica* [J]. Journal of Northeast Forestry University, 1990, 18(Special): 72-76.

[20] LIU J, TAN Y, SLIK J W F. Topography related habitat associations of tree species traits, composition and diversity in a Chinese tropical forest [J]. Forest Ecology and Management, 2014, 330: 75-81.

[21] 丁佳，吴茜，闫慧，等. 地形和土壤特性对亚热带常绿阔叶林内植物功能性状的影响[J]. 生物多样性，2011, 19(2): 158-167.
DING J, WU Q, YAN H, et al. Effects of topographic variations and soil characteristics on plant functional traits in a subtropical evergreen broad-leaved forest [J]. Biodiversity Science, 2011, 19(2): 158-167.

[22] 穆立蔷. 紫椴种群地理变异与环境相关性研究[D]. 哈尔滨：东北林业大学，2006.
MU L Q. Study on the Geographic variations of *Tilia amurensis* populations and its Correlation with Environments [D]. Harbin: Northeast Forestry University, 2006.

[23] 肖乾坤. 花椒树种子变异苗期生长变异及无性繁殖[D]. 北京：中国林业科学研究院，2010.
XIAO Q K. Genetie variation in seed morphology and seedling growth and vegetative propagation of *Sorbus pohuashanensis* (Hance) Hedl [D]. Beijing: Chinese Academy of Forestry, 2010.

（责任编辑 范 娟
责任编委 张春雨）

(C)1994-2019 China Academic Journal Electronic Publishing House. All rights reserved. http://www.cnki.net
