# Supplementary material for: Provenance and family variations in early growth of Manchurian walnut (Juglans mandshurica Maxim.) and selection of superior families
Source: PLoS One. 2024 Mar 7;19(3):e0298918. doi: 10.1371/journal.pone.0298918 (PMC10919699; doi:10.1371/journal.pone.0298918)
Supplement: S2 File — (ZIP) [file pone.0298918.s005.zip › Situation and recent trends on cultivation and breeding of Persian walnut in Iran.pdf]

Scientia Horticulturae 270 (2020) 109369

Contents lists available at ScienceDirect

Scientia Horticulturae

journal homepage: www.elsevier.com/locate/scihorti

Review

Situation and recent trends on cultivation and breeding of Persian walnut in Iran

Darab Hassani[a,*], Saadat Sarikhani[b], Raana Dastjerdi[a], Razie Mahmoudi[a], Asghar Soleimani[a], Kourosh Vahdati[b,*]

[a] Temperate Fruits Research Center, Horticultural Science Research Institute, Agricultural Research, Education and Extension Organizations, Karaj, Iran
[b] Department of Horticulture, College of Aburaihan, University of Tehran, Tehran, Iran

ARTICLE INFO

Keywords:
Walnut breeding
Superior genotypes
Germplasm evaluation
Commercial orchards

ABSTRACT

Persian walnut (*Juglans regia* L.) is one of the main nut crops in Iran. Walnut together with pistachio and almond have covered more than 800,000 ha (28.5 %) of Iran's orchards. After pistachio, walnut is the second important nut crop in term of production. In recent years, the demand for establishing walnut orchard has significantly increased. Walnut has a long history of cultivation and use and the country considered as one of the main walnut origin and distribution centers in the world. So, it is possible to find some walnut trees with more than 1000-years old in the traditional orchards. The walnut orchards could classify into the traditional and new orchards. In traditional orchards, walnut is usually cultivated on the borders of other fruit tree orchards. In recent years, new walnut orchards have been established using foreign ('Chandler' and 'Fernor') and Iranian commercial cultivars and supeior genotypes. Also, traditional orchards have either been replaced by new ones by top-working. Walnut breeding in Iran is being done by TFRC-HSRI and CEWIT. Germplasm evaluation, hybridization and omics studies are the main walnut breeding strategies. Iran's walnut breeding program initiated in 1983 and led to release of six commercial cultivars including 'Jamal', 'Damavand' (2010), 'Persia', 'Caspian', 'Chaldoran' and 'Alvand' (2019). In addition to cultivar breeding, rootstock program is currently going on, aiming to find appropriate clonal rootstocks.

1. Walnut situation

Iran is a vast country with an area of about 1.6 million km^2 in west Asia. There are two mountainous ranges (Alborz and Zagros) affected greatly the climate. The Alborz range separates the northern humid border of the Caspian Sea shores from the arid central plateau, while the Zagros mountains, divides the country into cold climate (from the northern west to the south) and hot desert climate (in the center and east). The climate is very variable so that there is around 20 °C of differences in annual averages temperature of warmest and coldest locations. The annual precipitation is also quite various which ranges from 50 mm (in the central deserts) to more than 1000 mm (in higher altitudes and Caspian Sea shores).

Agriculture is mainly located in north, west and northwest of the country where precipitation is annually more than 250 mm. Although, arable land is substantive 28.2 % (45.7 M ha); but only 10.9 % is suitable (very good, good and medium) for agriculture (Mesgaran et al., 2017). About 84 % of Iran's agriculture land is allocated to agronomy crops (e.g. wheat, barley etc.) and the rest to orchards (Anonymous, 2014). Due to multiple climates, a wide range of various horticultural and especially fruit crops are cultivated in Iran (Anonymous, 2017). As the 9th to 11th largest fruit producer in the world (2.8 M ha area and 21 M ton fruit production), Iran is one of the origin and diversity centers of many species of fruit trees(Vahdati et al., 2018). Nut crops include around 30 % of the total area devoted to fruit trees (Table 1). Pistachio, with almost 57 % of the total cultivated area of nut crops, is the most important nut crop of Iran. After pistachio, walnut is the second important nut crop, in term of production. In term of cultivated area, walnut is the third nut crop which occupies 5% of Iran's orchards (Table 1).

Persian walnut (*Juglans regia* L.) is among the oldest tree foods known to humanity which widely cultivated throughout the temperate and semiarid regions for edible nuts (Amiri et al., 2010; Vahdati et al., 2019b). Although, the origin center of this crop is almost obscure, but as its name implies, Persian walnut, it probably originated from ancient Persia which currently includes countries such as Iran, Afghanistan and Central Asia countries (Mahmoodi et al., 2019; Aradhya et al., 2017). So this species, in Iran, is among the old fruit tree species with a long

* Corresponding author.
E-mail addresses: d.hassani@areeo.ac.ir (D. Hassani), kvahdati@ut.ac.ir (K. Vahdati).

https://doi.org/10.1016/j.scienta.2020.109369
Received 12 December 2019; Received in revised form 11 March 2020; Accepted 13 March 2020
0304-4238/ © 2020 Elsevier B.V. All rights reserved.

Table 1
Fruit production and cultivated area in Iran.
Source: Anonymous (2017).

Species		Cultivated area (ha)			Production (T)
		Not Bearing [a]	Bearing [b]	Total [(a + b)]	
Pome fruits		35,827	243,362	279,190	4,036,055
Stone fruits		39,232	230,947	270,179	2,527,908
Subtropical fruits		142,885	674,452	817,337	7,725,363
Tropical fruits		3,437	10,032	13,468	157,641
Berries		19,148	297,575	316,723	3,272,742
Nuts	Pistachio	102,642	376,726	479,368	317,485
	Almond	31,246	150,649	181,896	147,511
	**Walnut**	**29,527**	**120,279**	**149,806**	**261,341**
	Hazelnut	3,201	21,692	24,893	25,208
	Total	166,616	669,347	835,963	751,545
Other		43,509	268,645	312,155	2,561,105
Grand Total		450,655	2,394,360	2,845,015	21,032,359

history of cultivation and use. According to FAO data, Iran with 9.1 % of the world's walnut production is the third leading walnut producer in the world. Also, Iran with 4.9 % of worldwide walnut orchards, is the 5th leading country in the world, in terms of area harvested (FAO, 2017). As Fig. 1 shows, walnut production and cultivated area has not been changed significantly over the past 10 years (2008–2017). In other words, traditional walnut orchards have been replaced by commercial orchards in the last decade which has not caused much change in production and area under cultivation (Fig. 1). It is expected that the walnut production will significantly increase in near future because of increasing yield per hectare in new orchards.

2. Walnut distribution and cultivation

Iran is one of the main origin of Persian walnut in the world. Remnants of the wild populations of Persian walnut are found in the Hyrcanian forest in north of Iran (Shamlu et al., 2018; Jafari Sayadi et al., 2012). Fig. 2 shows distribution of walnut in the different regions of Iran based on 2017 national statistics. Walnut cultivation is mainly concentrated on mountain hills and highlands of Zagros and Alborz ranges. However, the climate distribution of walnuts is very diverse in Iran. So that, walnut orchards are established from the sea level in Caspian Sea shores to areas with an altitude of more than 3,000 m above sea level in Rabor-Kerman and several other places. The average annual temperature of walnut cultivated areas ranged from less than 10 °C (in Khalkhal, Ardebil province) to more than 17 °C (in Sari Province). The absolute minimum and maximum temperature of these locations were −25 °C and 44.2 °C, respectively. In walnut cultivated areas, precipitation is mainly concentrated on cold months from October to February (Supplementary Table 1) (Hassani et al., 2019b).

As mentioned before, Iran's walnut orchards could be classified into traditional and new orchards. In addition to walnut orchards, walnut is cultivated on the roadsides and touristic places. There are also wild populations of walnuts in the Hyrcanian forests in north of Iran (Vahdati et al., 2014b). The major part of walnut trees in Iran are in traditional orchards which mainly established in the valleys of the highland areas (Fig. 3a) or on the border of fruit tree orchards (Fig. 3b). More than 90 % of walnut trees in traditional orchards are propagated by seed and it is easy to find walnut trees with more than hundreds years old (Fig. 3c, d). Although, existence of high genetic diversity in these orchards is invaluable for walnut breeders; but is not desirable for growers because of not being uniform trees which most of them have terminal bearing habit and low yield too.

In recent years, modern and commercial walnut orchards have been established in Iran, under the consultation of professional consultants. In addition, traditional orchards have either been replaced by new grafted trees or their cultivars have been replaced by topworking, under the instructions of the experts. The new walnut orchards have been established using foreign and Iranian commercial cultivars. Fig. 4 shows a grafted walnut orchard in Hamedan province and a commercial modern own-rooted tissue culture walnut orchard in Qazvin province. The most important walnut cultivars in new orchards included 'Chandler', 'Fernor' and new Iranian cultivars.

3. Walnut propagation

In general, walnut propagates by seed, budding, grafting and micropropagation. In Iran, walnuts have been traditionally propagated by seeds, however presently sexual propagation is only being used for production of rootstocks. Although, studies on propagation of walnuts by layering have been carried out (Vahdati and Khalighi, 2001; Vahdati et al., 2008); but in commercial level walnut is propagated by grafting and micropropagation. Among the different walnut grafting methods, chip, whip and bark grafting are commonly being used for walnut propagation in Iran (Ebrahimi et al., 2007; Rezaee et al., 2008). Also, studies have been done to investigate other walnut grafting methods (Soleimani et al., 2010; Dehghan et al., 2009; Dehgan et al., 2010; Raufi et al., 2017; Farsi et al., 2018; Sadeghi Majd et al., 2019). Numerous studies and consultation services have been done to improve the micropropagation of walnuts (Vahdati et al., 2004, 2017a; Asayesh et al., 2017a; Asayesh et al., 2017b) in Iran too.

4. Walnut research

4.1. Walnut breeding

The main walnut breeding objective in walnut is high yield. Traits related to yield and kernel quality including lateral bearing, nut and kernel weight, nut size, kernel percentage, shell thickness, kernel color and ease of kernel removal are among the primary walnut breeding goals. Moreover, regarding to global warming and climate change, some other breeding objectives have been considered. Late leafing date, earlier harvest, drought tolerance, high chilling requirement and disease resistance were some of the secondary traits (Dastjerdi and Hassani, 2009; Amiri et al., 2010; Sarikhani Khorami et al., 2014; Vahdati et al., 2019a).

Due to sexual propagation, there is a high genetic diversity in traditional walnut orchards which is an unimaginable potential for genetic improvement (Atefi, 1993; Vahdati, 2000). There are old manuscripts indicating that in the past, Iranian growers were using the seeds of the superior walnut trees to establish new orchards (Vahdati et al., 2014b), however Iran's walnut breeding program initiated with a joint project supported by Food and Agriculture Organization of United Nations (FAO) in 1980s (Hassani et al., 2014). The project was started by Jamal Atefi with selection of more than 110 superior walnut genotypes from different regions of Iran and planting their progeny in Temperate Fruits Research Center of Horticultural Science Research Institute (TFRC-HSRI)[1] in 1983 (Hassani et al., 2014). In the other words, exploration of walnut germplasm in the country and introducing foreign commercial cultivars ('Chandler', 'Pedro', 'Hartley', 'Lara', 'Serr', 'Vina', 'Franquette', 'Ronde de Montignac') were the first walnut breeding strategy in Iran (Atefi, 1990, 1993; Atefi, 1997; Hassani et al., 2014). After preliminary evaluation of the progenies, some superior genotypes were selected and grafted on seedling rootstocks. The grafted genotypes were planted in research orchards in Karaj, Shahrood, Mashhad and Urumia. Two walnut cultivars named 'Damavand' and 'Jamal' (Fig. 5) were released from this phase of walnut genetic improvement in 2010 (Hassani et al., 2012a, 2012b). Among the foreign cultivars, 'Chandler' as the main cultivar and 'Franquette' and 'Ronde de Montignac 'as its

[1] Ex Horticulture Department, Seed and Plant Improvement Institute (up to 2015).

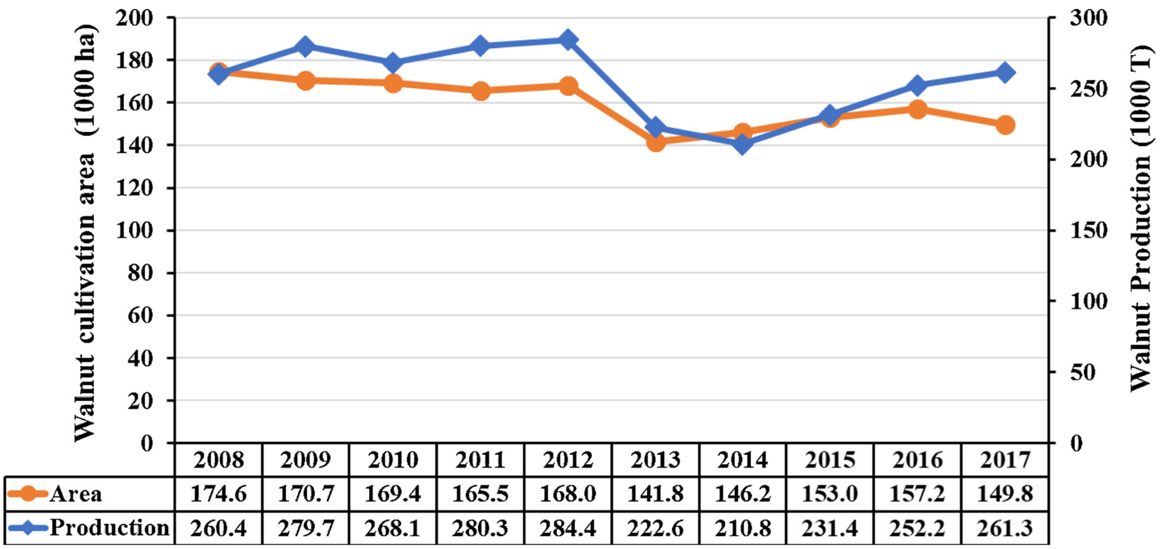

	2008	2009	2010	2011	2012	2013	2014	2015	2016	2017
Area	174.6	170.7	169.4	165.5	168.0	141.8	146.2	153.0	157.2	149.8
Production	260.4	279.7	268.1	280.3	284.4	222.6	210.8	231.4	252.2	261.3

Fig. 1. Trend of walnut cultivated area and production in Iran during 2008-2017 (Source: Iran's Agriculture Ministry (Anonymous, 2017)).

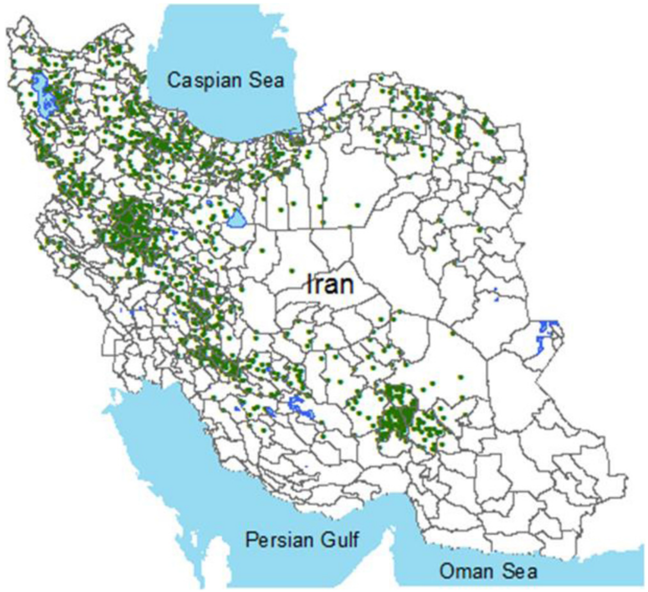

Fig. 2. The distribution map of walnut orchards in Iran (Each green point on the map is equal to 100 ha of walnut orchards).

appropriate pollinizers showed the most compatibilityin Karaj, Iran (Hassani et al., 2013, 2014).

Iran's walnut breeding program continued, in recent two decades, in TFRC-HSRI and Center of Excellence in Walnut Improvement and Technology (CEWIT), University of Tehran.

In TFRC-HSRI, three populations of walnut superior genotypes have been selected from different regions of Iran since 2000. The selected materials were vegetatively propagated and planted on the selection plots. The studies are in progress inside a national walnut program. In phase II of this project, the 39 walnut superior genotypes were evaluated during 2006–2018 and four new walnut cultivars including 'Persia', 'Caspian' (Fig. 6), 'Alvand' and 'Chaldoran' (Fig. 7) were released in March 2019 (Hassani et al., 2019a, 2019b, Hassani et al., 2020). Main pomological and phenological characteristics of Iranian walnut cultivars were presented in Table 2 and Fig. 8. In addition to selection, some targeted hybridization for important breeding traits especially lateral bearing and early harvest date has been done in TFRC-HSRI in 1996 and the progenies are currently being evaluated. Evaluation of pollen source on nut and kernel characteristics (Golzari et al., 2010, 2013; Golzari et al., 2016), pistillate flower abscission (PFA) susceptibility (Hassani et al., 2005), correlation study between main breeding traits (Mahmoodi et al., 2015), DNA content and ploidy levels (Mosivand et al., 2014) of superior walnut genotypes were some other breeding related studies of this program.

Walnut breeding program at CEWIT was started in 2003, aiming to release new walnut cultivars and rootstocks using both traditional and molecular tools. High yield, kernel quality, late leafing, early harvest for cultivars, dwarfing and drought stress tolerance for rootstocks, are the most important breeding objectives in CEWIT. Different breeding strategies including traditional (germplasm evaluation and targeted cross-breeding) and molecular (marker assisted selection, haploid production, gene transfer, metabolomics, genome sequencing, GWAS, GBS and transcriptomics) approaches were used to achieve the above-mentioned objectives. To release walnut commercial cultivars, walnut germplasms in different regions were evaluated to release new cultivars (Ebrahimi et al., 2010; Karimi et al., 2010, 2014; Vahdati et al., 2015; Khorami et al., 2018; Sarikhani and Vahdati, 2019). Currently, this project is ongoing and some superior genotypes have been identified and propagated vegetatively, which are in the stage of compatibility and stability evaluation. In addition to cultivar breeding, rootstock breeding program is currently going to release drought and salt tolerance and dwarf rootstocks. For this purpose, walnut germplasm from different regions are being evaluated and candidate genotypes are selected and planted in the Walnut Research Orchard at College of Aburaihan, University of Tehran. Compatibility evaluation and molecular studies of these selected genotypes is currently ongoing (Grouh et al., 2011; Vahdati and Mohseniazar, 2016; Vahdati et al., 2014a; Arab et al., 2019, 2020). To release drought-tolerant rootstocks, a targeted breeding program based on traditional and molecular breeding was conducted by CEWIT in collaboration with University of California, Davis. For this purpose, the novel Axiom® Walnut700 K SNP array has been applied to a total of 1278 trees in the walnut-breeding program at UC Davis and also a population of 95 walnut trees collected from different parts of Iran (Arab et al., 2019).

In addition to walnut breeding programs in TFRC-HSRI and CEWIT, studies have also been carried out especially on walnut germplasm to identify superior genotypes by other researchers too (Table 3).

4.2. Walnut pathology

Anthracnose *Ophiognomonia leptostyla* (Fr.) Sogonov; Anamorph: *Marssoniella juglandis* (Lib.) Höhn is the most serious fungal disease of

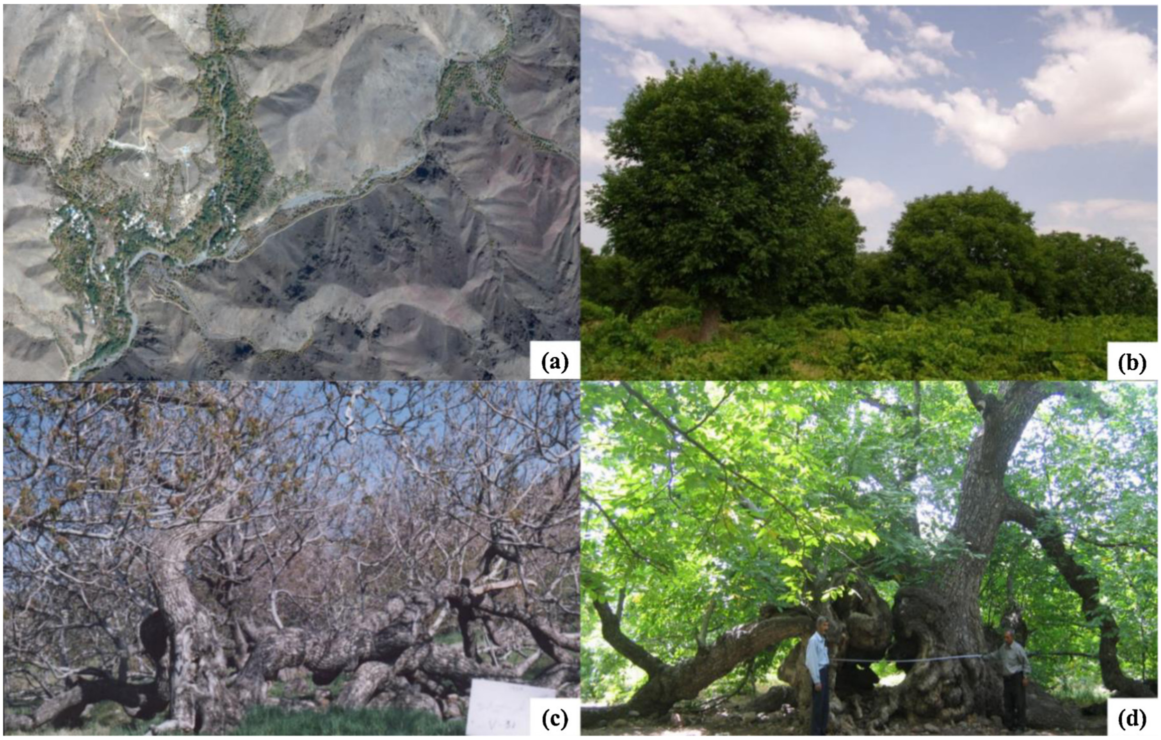

Fig. 3. Walnut trees in Hanza valley, Kerman (a). Walnut trees on the border of a vineyard in Ziaabad, Qazvin (b). Old walnut trees in Gugher and Rabor regions, Kerman (c and d).

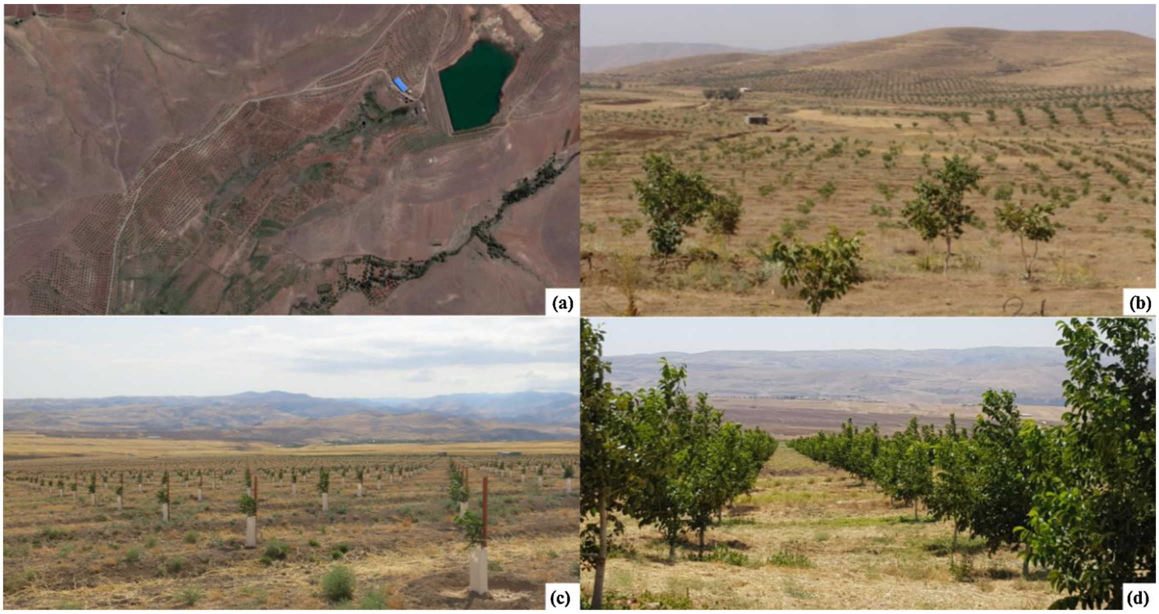

Fig. 4. A grafted walnut orchard in hilly areas in Geshani, Hamedan (a and b) and a commercial own-rooted modern tissue-culture ‘Chandler’ and ‘Fernor’ orchard in Kuhin, Qazvin (c and d).

walnut in Iran. Evaluation of relative resistant walnut genotypes to anthracnose disease in Kahriz walnut collection (Urumia, Iran) showed that the B21 genotype was susceptible and OR4 and T19 genotypes had a relative resistance in natural infection conditions (Rabieifar, 1997). Saremi and Amiri (2010) studied the response of different walnut clones in greenhouse to anthracnose. They reported ‘Alamoty’ and ‘Zeiabadi’ selected genotypes as resistant and moderately resistant to anthracnose, respectively (Saremi et al., 2003; Saremi and Amiri, 2010). Relative susceptibility of eleven walnut local genotypes to anthracnose has been investigated in greenhouse condition during 2006 – 2007. The results showed that there are significant differences among the fungal isolates and studied genotypes in number and size of spots (Dastjerdi and Hassani, 2009; Dastjerdi et al., 2009). The highest leaf infection percent occurred on ‘Z60′, ‘K72’ and ‘Hartley’. ‘Vina’ and ‘Ronde de Montignac’ showed the least leaf infection percent (Dastjerdi and Hassani, 2009). ‘Damavand’ and ‘Jamal’ as the first Iranian walnut cultivars showed low susceptibility to anthracnose (Hassani et al., 2012a, 2012b).

Blight is a bacterial disease caused by *Xanthomonas arboricola* pv. Juglandis. ‘Serr’, ‘Damavand’, ‘Jamal’ and ‘Z67’ were relatively

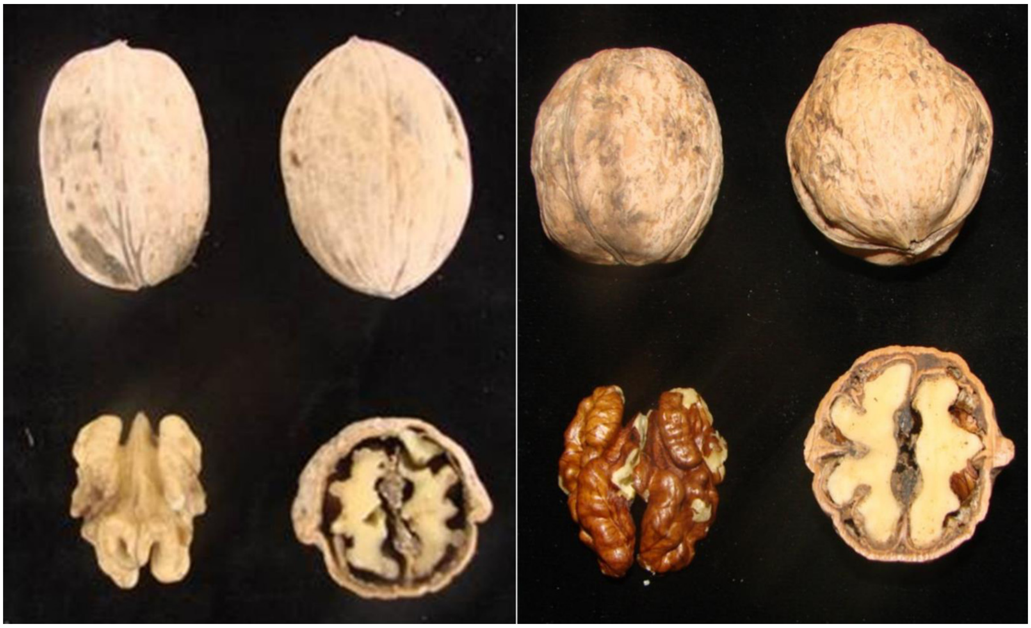

Fig. 5. Nut and kernel of early-leafing Persian walnut cultivars in Iran, namely 'Jamal' (left) and its pollinizer, 'Damavand' (right).

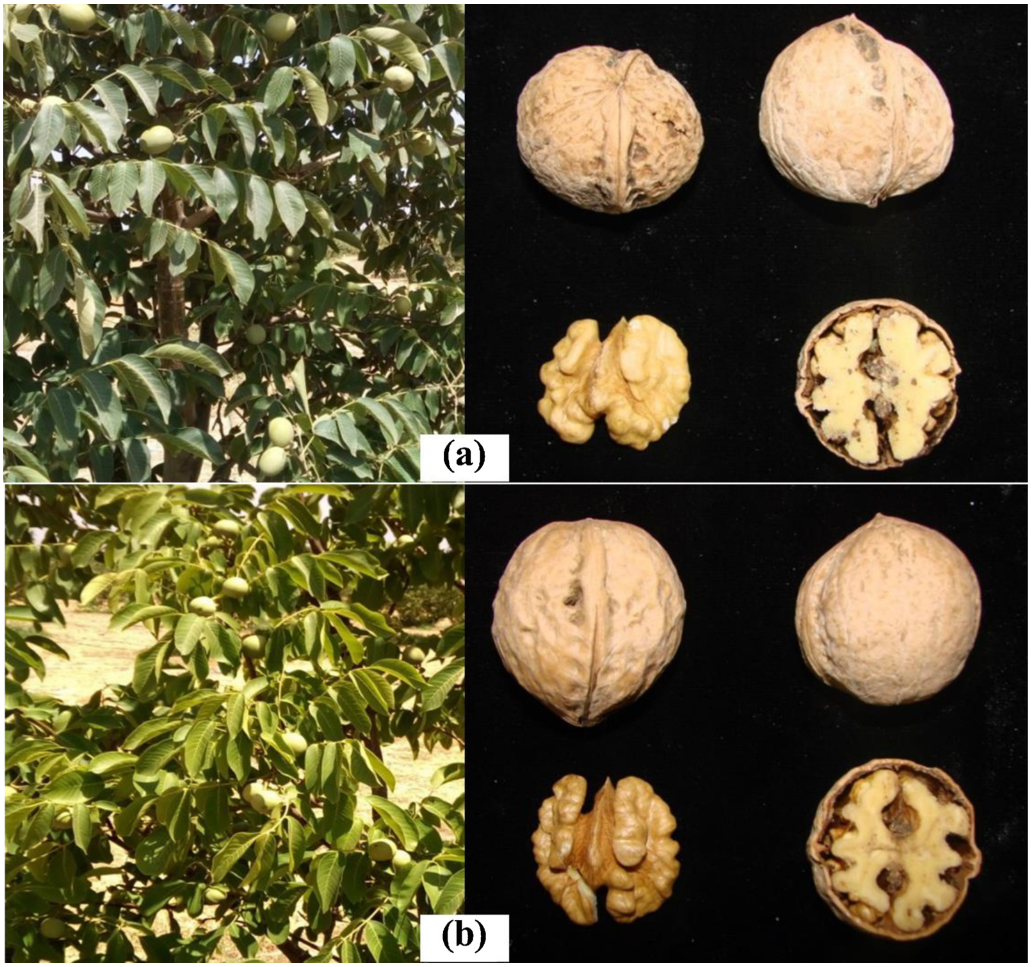

Fig. 6. Tree, nut and kernel of late-leafing walnut cultivars 'Persia' (a) and 'Caspian' (b).

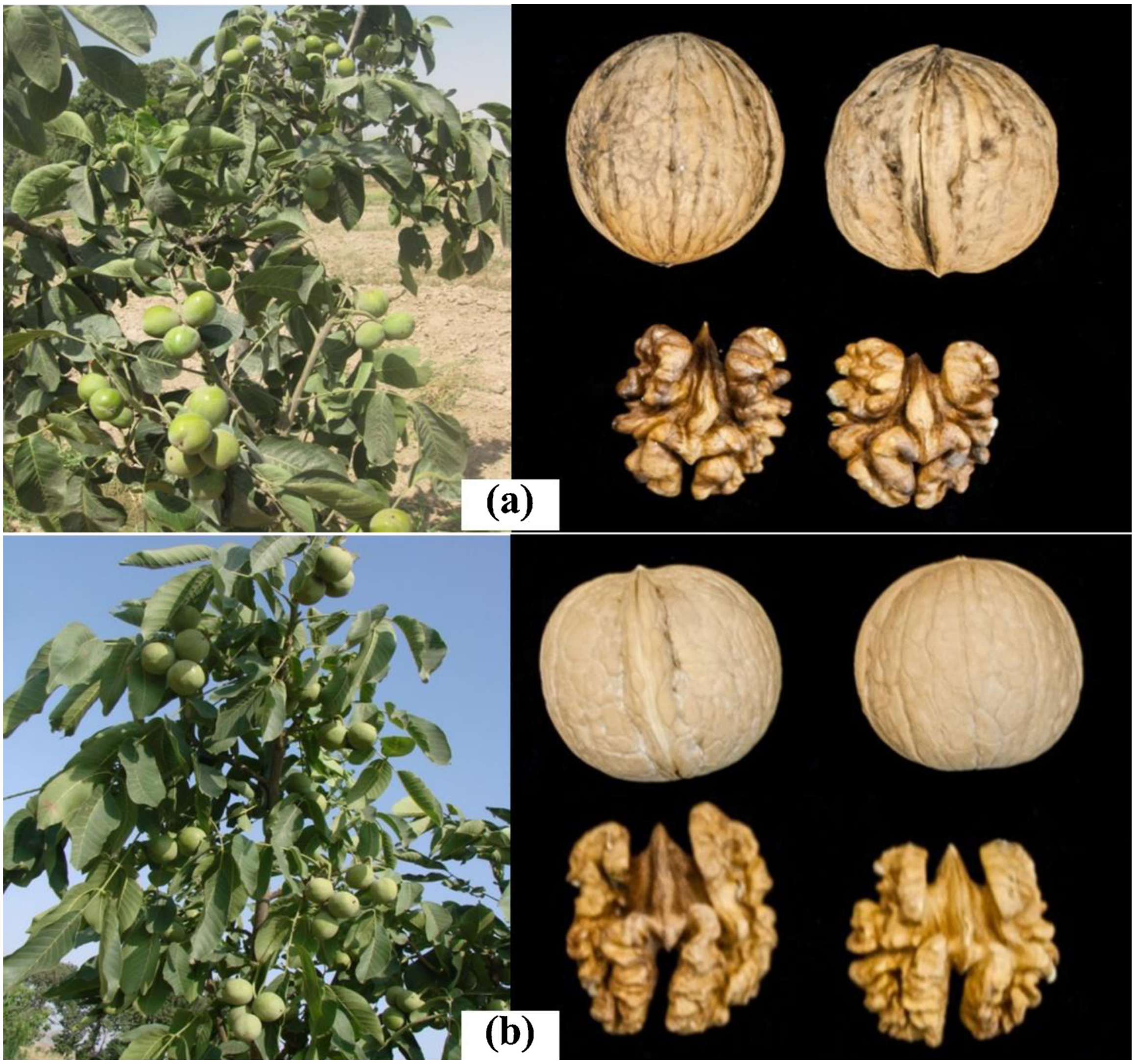

Fig. 7. Tree, nut and kernel of mid-leafing walnut cultivars 'Chaldoran' (a) and 'Alvand' (b).

(moderately) susceptible and 'Pedro', 'Chandler', 'Franquette' and 'Hartley' cultivars showed a relatively resistance to blight disease in Iran's condition (Silsepour et al., 2012; Hassani et al., 2012a, 2012b). Among Iranian walnut cultivars, 'Chaldoran' was tolerant, 'Alvand' was susceptible and 'Caspian' and 'Persia' were moderately susceptible to blight disease (Keshavarzi, 2019). The incidence of shallow-bark canker caused by *Brenneria* (*Erwinia*) *nigrifluens* on walnut trees also detected in Iran. Based on studies, 'Alvand' and 'Persia' were relatively susceptible cultivar to this disease (Keshavarzi, 2019).

The leopard moth borer (*Zeuzera pyrina* L. (Lepidoptera: Cossidae)) is an important pest which has been significantly developed in Iran's walnut orchards in recent years. Some walnut genotypes in TFRC-HSRI collection were evaluated for *Zeuzera*. The maximum and minimum number of larval entrance holes in walnut varieties was observed in 'Damavand' and 'Jamal', respectively. Therefore, 'Jamal' was recommended for further studies regarding the detection of resistance

Table 2
Main characteristics of Iran's walnut cultivars in compare to 'Chandler'.

Traits	Jamal	Damavand	Persia	Caspian	Chaldoran	Alvand	Chandler
Leafing date	Early to Med	Early to Med	Late	Late	Early to Med	Early to Med	Late
Dichogamy*	PR	PG	PG	PG	PR	PR	PR
Harvest date	Med to Late	Early	Medium	Medium	Early	Early	Late
Tree vigor	Vigorous	Vigorous	Moderate	Moderate	Moderate	Low	Moderate
Yield	Moderate	Moderate	High	High	High	High	High
Bearing habit	Intermediate	Intermediate	Lateral	Lateral	Lateral	Lateral	Lateral
Nut weight (g)	10.8	12	11.5	10.6	13.2	11.3	9.8
Kernel weight (g)	5.2	6.	6.5	6	7.6	5.5	4.1
Kernel percentage	48	50	57	57	58	49	42
Shell thickness	Medium	Medium	Thin	Thin	Medium	Thin	Medium
Kernel color	Amber	Amber	Light	Light	Amber	Amber	Light

* PR: Protandrous; PG: Protogynous.

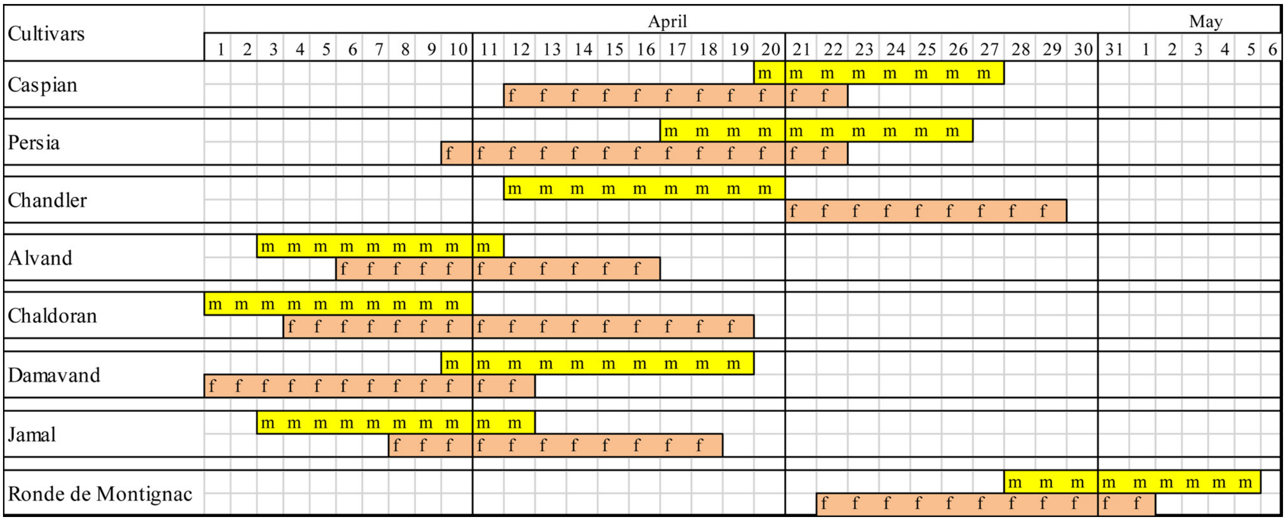

Fig. 8. Pollen shedding and pistillate receptivity period of some Persian walnut cultivars compared to 'Chandler'.

mechanisms (Hosseini Gharalari and Kolyai, 2014).

5. Conclusion

In conclusion, Iran as one of the leading walnut producer in the world has a long history in walnut cultivation and improvement. In the past, walnut trees were propagated by seed and planted on the border of other fruit orchards as the orchards boundaries. But, in the last decades, traditional orchards have been replaced by new ones or their cultivars have been changed by top-working. Walnut breeding programs and development of vegetative propagation methods have played a key role in the development of new walnut orchards. Iran's walnut breeding program primarily started as a joint project supported by FAO in TFRC-HSRI in 1980s. Due to high genetic diversity in traditional orchards, germplasm evaluation for identifying superior genotypes was the main breeding strategy to release new walnut cultivars and rootstocks. Currently, this project is going on inTFRC-HSRI and CEWIT. Walnut breeding programs led to release of six cultivars named 'Jamal', 'Damavand', 'Persia', 'Caspian', 'Chaldoran' and 'Alvand'. Other candidate have been identified as superior genotypes which are in the stage of compatibility test. In addition to cultivar breeding, a joint project between CEWIT and University of California-Davis is currently going on to understand the mechanisms of drought tolerance and introduce drought tolerant walnut rootstocks. Due to the significant development of new commercial walnut orchards, it is predicted that not only Iran will maintain the current status of walnut production, but also its position in the world walnut trade will improve significantly.

Declaration of Competing Interest

The authors declare that they have no known competing financial interests or personal relationships that could have appeared to influence the work reported in this paper.

Acknowledgements

We appreciate Temperate Fruits Research Center of Horticultural Science Research Institute (TFRC-HSRI), University of Tehran, Center for Progress and Development of Iran, Iran National Science Foundation (INSF) and Iranian Center of Excellence for Walnut Improvement and Technology for their supports.

Table 3
Literature review on walnut germplasm evaluation in different regions of Iran.

Provinces	References
Alborz	Eskandari et al., 2005; Ebrahimi et al., 2016; Mahmoodi et al., 2016, 2014; Mahmoodi et al., 2019
Ardabil	Ebrahimi et al., 2016
Azerbaijan, East	Ebrahimi et al., 2010; Mahmoodi et al., 2019
Azerbaijan, West	Eskandari et al., 2005; Rezaei et al., 2008
Chaharmahaal and Bakhtiari	Mosivand et al., 2012; Mousavi et al., 2015
Fars	Sarikhani Khorami et al., 2012, 2013; Sarikhani et al., 2018; Khadivi-Khub et al., 2015; Khadivi et al., 2019
Gilan	Ahandani et al., 2014
Golestan	Shamlu et al., 2018; Karamatlo et al., 2016
Hamadan	Eskandari et al., 2005; Khadivi et al., 2019; Rezaei et al., 2018; Zare-Rashnoodi et al., 2017
Ilam	Zare-Rashnoodi et al., 2017; Vahdati et al., 2017b
Kerman	Amiri et al., 2010; Mahmoodi et al., 2019
Kermanshah	Zare-Rashnoodi et al., 2017; Chorush and Arzani, 2018
Khorasan	Eskandari et al., 2005
Kohgiluyeh and Boyer-Ahmad	Khadivi et al., 2019
Kurdistan	Ebrahimi et al., 2016
Lorestan	Zare-Rashnoodi et al., 2017; Mohammadi et al., 2015
Markazi	Ghasemi et al., 2012
Qazvin	Eskandari et al., 2005; Mahmoodi et al., 2019
Semnan	Eskandari et al., 2005; Akhiani et al., 2017; Mahmoodi et al., 2019
Yazd	Arzani et al., 2008
Zanjan	Ebrahimi et al., 2015; Khadivi et al., 2019

Appendix A. Supplementary data

Supplementary material related to this article can be found, in the online version, at doi:https://doi.org/10.1016/j.scienta.2020.109369.

References

Ahandani, E.A., Ramandi, H.D., Sarmad, J., Samani, M.A., Yavari, A., Ahandani, R.A., 2014. Evaluation of morphological diversity among some Persian walnut accessions (*Juglans regia* L.) in Guilan, Northern Iran. Int. J. Plant Biol. Res. 2 (3), 1015.

Akhiani, S., Afshari, H., Parvaneh, T., 2017. Evaluation of some phenological and pomological characteristics of selected walnut genotypes from Shahroud-Iran. J. Nuts 8 (1), 21–30. https://doi.org/10.22034/jon.2017.530389.

Amiri, R., Vahdati, K., Mohsenipoor, S., Mozaffari, M.R., Leslie, C., 2010. Correlations between some horticultural traits in walnut. HortScience 45, 1690–1694. https://doi.org/10.21273/HORTSCI.45.11.1690.

Anonymous, 2014. Agriculture in Iran (National Census of Agriculture). Statistical Center of Iran.

Anonymous, 2017. Statistics of Agricultural Products in Iran; Volume II: Horticultural Crops. Planning and Economic Affair, Iran's Ministry of Agriculture-Jahad.

Arab, M.M., Marrano, A., Abdollahi-Arpanahi, R., Leslie, C.A., Askari, H., Neale, D.B., Vahdati, K., 2019. Genome-wide patterns of population structure and association mapping of nut-related traits in Persian walnut populations from Iran using the Axiom *J. regia* 700K SNP array. Sci. Rep. 9 (1), 6376. https://doi.org/10.1038/s41598-019-42940-1.

Arab, M.M., Marrano, A., Abdollahi-Arpanahi, R., Leslie, C.A., Cheng, H., Neale, D.B., Vahdati, K., 2020. Combining phenotype, genotype and environment to uncover genetic components underlying water use efficiency in Persian walnut. J. Exp. Bot. 71 (3), 1107–1127. https://doi.org/10.1093/jxb/erz467.

Aradhya, M., Velasco, D., Ibrahimov, Z., Toktoraliev, B., Maghradze, D., Musayev, M., Bobokashvili, Z., Preece, J.E., 2017. Genetic and ecological insights into glacial refugia of walnut (*Juglans regia* L.). PLoS One 12 (10), e0185974. https://doi.org/10.1371/journal.pone.0185974.

Arzani, K., MansouriArdakan, H., Vezvaei, A., Roozban, M.R., 2008. Morphological variation among Persian walnut (*Juglans regia*) genotypes from central Iran. N. Z. J. Crop Hortic. 36 (3), 159–168. https://doi.org/10.1080/01140670809510232.

Asayesh, Z.M., Vahdati, K., Aliniaeifard, S., Askari, N., 2017a. Enhancement of *ex vitro* acclimation of walnut plantlets through modification of stomatal characteristics *in vitro*. Sci. Hortic. 220, 114–121. https://doi.org/10.1016/j.scienta.2017.03.045.

Asayesh, Z.M., Vahdati, K., Aliniaeifard, S., 2017b. Investigation of physiological components involved in low water conservation capacity of in vitro walnut plants. Sci. Hortic. 224, 1–7. https://doi.org/10.1016/j.scienta.2017.04.023.

Atefi, J., 1990. Preliminary research of Persian walnut and correlation between pair characters. Acta Hortic. 284, 97–104. https://doi.org/10.17660/ActaHortic.1990.284.12.

Atefi, J., 1993. Evaluation of walnut genotypes in Iran. Acta Hortic. 311, 24–33. https://doi.org/10.17660/ActaHortic.1993.311.2.

Atefi, J., 1997. Study on phonological and pomological characters on walnut promising clones in Iran. Acta Hortic. 442, 101–108. https://doi.org/10.17660/ActaHortic.1997.442.13.

Chorush, Z.S., Arzani, K., 2018. Evaluation of diversity of walnut promising genotypes in Kermanshah province according to oil properties and determine the correlation of these traits with some morphological and ecological characters. Iran. J. Hortic. Sci. 48 (4), 887–897. https://doi.org/10.22059/ijhs.2018.212758.1054.

Dastjerdi, R., Hassani, D., 2009. Response of walnut genotypes to *Gnomonia leptostyla* (Fr.) Ces. & de Note. in greenhouse. Seed Plant Improve J. 25 (3), 433–449. https://doi.org/10.22092/spij.2017.110992.

Dastjerdi, R., Hassani, D., Javan Nikkhah, M., 2009. Study on some characteristics, assessment of pathogenicity and diversity in *Gnomonia leptostyla* isolates, causal agent of walnut anthracnose in Iran. Iran J. Plant Path. 45 (1), 61–73.

Dehgan, B., Vahdati, K., Rezaee, R., Hassani, D., 2010. Mature walnut grafting (topworking) as affected by grafting cover and scion cultivar. Acta Hortic. 861, 353–360. https://doi.org/10.17660/ActaHortic.2010.861.48.

Dehghan, B., Vahdati, K., Rezaee, R., Hassani, D., 2009. Persian walnut (*Juglans regia* L.) grafting as influenced by different bench grafting methods and scion cultivars. J. Appl. Hortic. 11, 56–58.

Ebrahimi, A., Vahdati, K., Fallahi, E., 2007. Improved success of Persian walnut grafting under environmentally controlled conditions. Int. J. Fruit Sci. 6 (4), 3–12. https://doi.org/10.1300/J492v06n04_02.

Ebrahimi, A., Fatahi, M.R., Zamani, Z., Vahdati, K., 2010. An investigation on genetic diversity of 608 Persian walnut accessions for screening of some genotypes of superior traits. Iran. J. Hortic. Sci. 40 (4), 83–94.

Ebrahimi, A., Khadivi-Khub, A., Nosrati, Z., Karimi, R., 2015. Identification of superior walnut (*Juglans regia* L) genotypes with late leafing and high kernel quality in Iran. Sci. Hortic. 193, 195–201. https://doi.org/10.1016/j.scienta.2015.06.049.

Ebrahimi, A., Zarei, A., Lawson, S., Woeste, K.E., Smulders, M.J.M., 2016. Genetic diversity and genetic structure of Persian walnut (*Juglans regia*) accessions from 14 European, African, and Asian countries using SSR markers. Tree Genet. Genomes 12 (6), 114. https://doi.org/10.1007/s11295-016-1075-y.

Eskandari, S., Hassani, D., Abdi, A., 2005. Investigation on genetic diversity of Persian walnut and evaluation of promising genotypes. Acta Hortic. 705, 159–166. https://doi.org/10.17660/ActaHortic.2005.705.18.

FAO, 2017. FAO Statistical Data. Food and Agriculture Organization of the United Nations. http://www.fao.org/faostat/en/#data/QC).

Farsi, M., Fatahimoghadam, M.R., Zamani, Z., Hassani, D., 2018. Effects of scion cultivar, rootstock age and hormonal treatment on minigrafting of Persian walnut. Int. J. Hortic. Sci. Technol. 5 (2), 185–197. https://doi.org/10.22059/ijhst.2018.255460.233.

Ghasemi, M., Arzani, K., Hassani, D., 2012. Evaluation and identification of walnut (*Juglans regia* L.) genotypes in Markazi. Crop Breed. J. 2 (2), 119–124. https://doi.org/10.22092/cbj.2012.100429.

Golzari, M., Rahemi, M., Vahdati, K., Hassani, D., 2010. Effect of pollen source on Persian walnut characteristics (*Juglans regia* L.). Acta Hortic. 861, 99–104. https://doi.org/10.17660/ActaHortic.2010.861.12.

Golzari, M., Rahemi, M., Hassani, D., Vahdati, K., Mohammadi, N., 2013. Protein content, fat and fatty acids of kernel in some Persian walnut (*Juglans regia* L.) cultivars affected by kind of pollen. Iran. J. Food Sci. Technol. 38 (10), 21–31.

Golzari, M., Hassani, D., Rahemi, M., Vahdati, K., 2016. Xenia and metaxenia in Persian walnut (*Juglans regia* L.). J. Nuts 7 (2), 101–108. https://doi.org/10.22034/jon.2016.527095.

Grouh, M.S.H., Vahdati, K., Lotfi, M., Hassani, D., Biranvand, N.P., 2011. Production of haploids in Persian walnut through parthenogenesis induced by gamma-irradiated pollen. J. Am. Soc. Hortic. Sci. 136 (3), 198–204. https://doi.org/10.21273/JASHS.136.3.198.

Hassani, D., Eskandari, S., Jarrahi, K., 2005. Pistillate flower abscission of walnut genotypes. Acta Hortic. 705, 257–260. https://doi.org/10.17660/ActaHortic.2005.705.31.

Hassani, D., Haghjooyan, R., Soleimani, A., Atefi, J., Loni, A., 2013. Evaluation of some walnut cultivars and selections in Iran. Acta Hortic. 981, 59–64. https://doi.org/10.17660/ActaHortic.2013.981.4.

Hassani, D., Atefi, J., Haghjooyan, R., Dastjerdi, R., Keshavarzi, M., Mozaffari, M.R., Soleimani, A., Rahmanian, A.R., Nematzadeh, F., Malmir, A., 2012a. Damavand, a new walnut cultivar as a pollinizer for Iranian walnut cultivars and genotypes. Seed Plant Improve J. 28–1, 529–531. https://doi.org/10.22092/spij.2017.111125.

Hassani, D., Atefi, J., Haghjooyan, R., Dastjerdi, R., Keshavarzi, M., Mozaffari, M.R., Soleimani, A., Rahmanian, A.R., Nematzadeh, F., Malmir, A., 2012b. Jamal, a new Persian walnut cultivar for moderate-cold areas of Iran. Seed Plant Improve J. 28–1, 523–525. https://doi.org/10.22092/spij.2017.111124.

Hassani, D., Dastjerdi, R., Haghjooyan, R., Soleimani, A., Keshavarzi, M., Atefi, J., Mozaffari, M., Rezaee, R., Fahadan, A., Rahmanian, A., 2014. Genetic improvement of Persian walnut (*Juglans regia* L.) in Iran. Acta Hortic. 1050, 95–102. https://doi.org/10.17660/ActaHortic.2014.1050.11.

Hassani, D., Mozaffari, M.R., Dastjerdi, R., Keshavarzi, M., Soleimani, A., Rezaee, R., Atefi, J., 2019a. Chaldoran, Alvand, Caspian and Persia as Four New Iranian Walnut Cultivars. National Cultivars Release Reports. Seed and Plant Certification and Registration Institute, pp. 37.

Hassani, D., Mozaffari, M.R., Soleimani, A., Dastjerdi, R., Rezaee, R., Keshavarzi, M., Vahdati, K., Fahadan, A., Atefi, J., 2020. Four New Persian Walnut Cultivars of Iran: Persia, Caspian, Chaldoran and Alvand. HortScience In press.

Hassani, D., Vahdati, K., Dastjerdi, R., Mahmoudi, R., Soleimani, A., 2019b. Persian walnut, the situation and recent trends on it's cultivation in Iran. Giornate Tecgniche Nazionali sul Noce, Nocicoltura da frutto: Innovazione e Sostenibilita'. Bologna, 20-21 Sep. 2019.

Hosseini Gharalari, A., Kolyai, R., 2014. Screening walnut and apple trees against Leopard moth, *Zeuzera pyrina* (Lep.: Cossidae). Appl. Entomol. Phytopathol. 81 (2), 11–15. https://doi.org/10.22092/jaep.2014.100379.

Jafari Sayadi, M.H., Vahdati, K., Mozafari, J., Mohajer, M.R.M., Leslie, C.A., 2012. Natural Hyrcanian populations of Persian walnut (*Juglans regia* L.) in Iran. Acta Hortic. 948, 97–101. https://doi.org/10.17660/ActaHortic.2012.948.10.

Karamatlo, I., Sharifani, M., Sabouri, H., 2016. Evaluation of genetic diversity in some walnut (*Juglans regia* L.) gentypes using morphological markers. J. Crop Prod. Process. 6 (20), 13–24.

Karimi, R., Ershadi, A., Vahdati, K., Woeste, K., 2010. Molecular characterization of Persian walnut populations in Iran with microsatellite markers. HortScience 45 (9), 1403–1406. https://doi.org/10.21273/HORTSCI.45.9.1403.

Karimi, R., Ershadi, A., Ehteshamnia, A., Sharifani, M., Rasouli, M., Ebrahimi, A., Vahdati, K., 2014. Morphological and molecular evaluation of Persian walnut populations in northern and western regions of Iran. J. Nuts 5 (2), 21–31. https://doi.org/10.22034/jon.2014.515686.

Keshavarzi, M., 2019. Evaluation of the resistance of walnut genotypes to *Xanthomonas campestris* pv. Juglandis and *Brenneria (Erwinia) nigrifluens*. Research Report of Project. HSRI, AREEO.

Khadivi, A., Montazeran, A., Rezaei, M., Ebrahimi, A., 2019. The pomological characterization of walnut (*Juglans regia* L.) to select the superior genotypes–an opportunity for genetic improvement. Sci. Hortic. 248, 29–33. https://doi.org/10.1016/j.scienta.2018.12.054.

Khadivi-Khub, A., Ebrahimi, A., Mohammadi, A., Kari, A., 2015. Characterization and selection of walnut (*Juglans regia* L.) genotypes from seedling origin trees. Tree Genet. Genomes 11 (3), 54. https://doi.org/10.1007/s11295-015-0882-x.

Khorami, S.S., Arzani, K., Karimzadeh, G., Shojaeiyan, A., Ligterink, W., 2018. Genome size: a novel predictor of nut weight and nut size of walnut trees. HortScience 53 (3), 275–282. https://doi.org/10.21273/HORTSCI12725-17.

Mahmoodi, R., Hassani, D., Amiri, M.E., Jaffaraghaei, M., 2014. Comparison of nut characteristics and yield of some selected Persian walnut (*Juglans regia* L.). Seed Plant Improve J. 30–1 (2), 442–456. https://doi.org/10.22092/spij.2017.111222.

Mahmoodi, R., Hassani, D., Amiri, M.E., Jaffaraghaei, M., Vahdati, K., 2015. Relationship between some traits and nut production in walnut cultivars and genotypes. J. Crop Prod. Process. 4 (13), 63–74.

Mahmoodi, R., Hassani, D., Amiri, M.E., Jaffaraghaei, M., 2016. Phenological and pomological characteristics of five promised walnut genotypes in Karaj, Iran. J. Nuts 7

(1), 1–8. https://doi.org/10.22034/jon.2016.522944.
Mahmoodi, R., Dadpour, M.R., Hassani, D., Zeinalabedini, M., Vendramin, E., Micali, S., Nahandi, F.Z., 2019. Development of a core collection in Iranian walnut (*Juglans regia* L.) germplasm using the phenotypic diversity. Sci. Hortic. 249, 439–448. https://doi.org/10.1016/j.scienta.2019.02.017.
Mesgaran, M.B., Madani, K., Hashemi, H., Azadi, P., 2017. Iran's land suitability for agriculture. Sci. Rep. 7 (1), 7670. https://doi.org/10.1038/s41598-017-08066-y.
Mohammadi, A., Vahdati, K., Abdousi, V., Najafabadi, F., Mahmoudi, M.J., 2015. Identification of some walnut genotypes in Lorestan Province of Iran and selection of 54 superior genotypes. J. Crop Prod. Process. 5 (15), 25–36.
Mosivand, M., Hassani, D., Payamnour, V., Jaffaraghaei, M., 2012. Comparison of tree, nut, and kernel characteristics in several walnut species and inter-specific hybrids. Crop Breed. J. 3 (1), 25–30. https://doi.org/10.22092/cbj.2013.100447.
Mosivand, M., Payamnoor, V., Hassani, D., Jaffaraghaei, M., 2014. Study of ploidy level of Walnut species and inter-specific hybrids by flow cytometry. Wood For. Sci. Technol. 21 (3), 183–194.
Mousavi, S.A., Tatari, M., Moradi, H., Hassani, D., 2015. Evaluation of genetic diversity among the superior walnut genotypes based on pomological and phenological traits in Chahar Mahal va Bakhtiari province. Seed Plant Improve J. 31 (2), 365–389. https://doi.org/10.22092/spij.2017.111264.
Rabieifar, A., 1997. Investigation of Relative Resistance of Iranian Walnut Genotypes to *Gnomonia leptostyla*. The Final Report. Iranian Research Institute of Plant Protection, pp. 7.
Raufi, A., Vahdati, K., Karimi, S., Roozban, M.R., 2017. Optimizing early grafting of Persian walnut by evaluating different rootstocks, covering materials and grafting methods. J. Nuts 8 (2), 97–106. https://doi.org/10.22034/jon.2017.536238.
Rezaee, R., Vahdati, K., Grigoorian, V., Valizadeh, M., 2008. Walnut grafting success and bleeding rate as affected by different grafting methods and seedling vigour. J. Hortic. Sci. Biotechnol. 83 (1), 94–99. https://doi.org/10.1080/14620316.2008.11512352.
Rezaei, R., Hassani, G., Hassani, D., Vahdati, K., 2008. Morphobiological characteristics of some newly selected walnut genotypes from seedling collection of Kahriz-Orumia. J. Hortic. Sci. Technol. 9, 205–214.
Rezaei, Z., Khadivi, A., ValizadehKaji, B., Abbasifar, A., 2018. The selection of superior walnut (*Juglans regia* L.) genotypes as revealed by morphological characterization. Euphytica 214 (4), 69. https://doi.org/10.1007/s10681-018-2153-z.
Sadeghi Majd, R., Vahdati, K., Roozban, M.R., Arab, M., 2019. Exploring combinations of graft cover and grafting method in commercial walnut cultivars. Int. J. Fruit Sci. 19 (4), 359–371. https://doi.org/10.1080/15538362.2018.1535355.
Saremi, H., Amiri, M.E., 2010. Evaluation of resistance to anthracnose (*Marssonina juglandis*) among diverse Iranian clones of walnut (*Juglans regia* L.). J. Food Agric. Environ. 8 (2), 375–378.
Saremi, H., Razaz Hashemi, S.R., Jafari, H., 2003. Study on walnut anthracnose disease at the northwest of Iran. J. Agric. Sci. Nat. Resour. 9 (4), 141–153.
Sarikhani, S., Vahdati, K., 2019. Determination of Persian walnut yield components and its correlation with phenological, morphological and biochemical traits. Iran. J. Hortic. Sci. https://doi.org/10.22059/ijhs.2018.260251.1474.
Sarikhani, S., Arzani, K., Karimzadeh, G., Shojaeiyan, A., 2018. Morphological characteristics, protein contents and fatty acids composition of some walnut (*Juglans regia* L.) Superior Genotypes in the North of Fars Province. Seed Plant Prod. J. 33 (2), 163–184. https://doi.org/10.22092/sppj.2018.116411.
Sarikhani Khorami, S., Arzani, K., Roozban, M.R., 2012. Identification and selection of twelve walnut superior and promising genotypes in Fars Province, Iran. Seed Plant Improve J. 28 (2), 277–296. https://doi.org/10.22092/spij.2017.111107.
Sarikhani Khorami, S., Arzani, K., Roozban, M.R., Mirsoleymani, M., 2013. Evaluation of morphological, phenological and pomological diversity of some Persian walnut (*Juglans regia* L.) genotypes north of Fars Province. Iran. J. Hortic. Sci. 44 (3), 301–313. https://doi.org/10.22059/ijhs.2013.36001.
Sarikhani Khorami, S., Arzani, K., Roozban, M.R., 2014. Correlations of certain high-heritability horticultural traits in Persian walnut (*Juglans regia* L.). Acta Hortic. 1050, 61–68. https://doi.org/10.17660/ActaHortic.2014.1050.6.
Shamlu, F., Rezaei, M., Lawson, S., Ebrahimi, A., Biabani, A., Khan-Ahmadi, A., 2018. Genetic diversity of superior Persian walnut genotypes in Azadshahr, Iran. Physiol. Mol. Biol. Plants 24 (5), 939–949. https://doi.org/10.1007/s12298-018-0573-9.
Silsepour, L., Keshavarzi, M., Hassani, D., Hashemi, M., 2012. Reaction of some walnut cultivars and genotypes to bacterial blight disease caused by *Xanthomonas arboricola* pv. *Juglandis* in greenhouse. Seed Plant Improve J. 28 (3), 395–405. https://doi.org/10.22092/spij.2017.111115.
Soleimani, A., Rabiei, V., Hassani, D., 2010. Effect of different techniques on walnut (*J. regia* L.) grafting. J. Food Agric. Environ. 8 (29), 544–546.
Vahdati, K., 2000. Walnut situation in Iran. Nucis Newsletter 9, 32–33.
Vahdati, K., Khalighi, A., 2001. Persian walnut stooling in Iran. Acta Hortic. 544, 527–530. https://doi.org/10.17660/ActaHortic.2001.544.74.
Vahdati, K., Mohseniazar, M., 2016. Early bearing genotypes of walnut: a suitable material for breeding and high density orchards. Acta Hortic. 1139 (2), 101–106. https://doi.org/10.17660/ActaHortic.2016.1139.18.
Vahdati, K., Leslie, C., Zamani, Z., McGranahan, G., 2004. Rooting and acclimatization of in vitro-grown shoots from mature trees of three Persian walnut cultivars. HortScience 39 (2), 324–327. https://doi.org/10.21273/HORTSCI.39.2.324.
Vahdati, K., Rezaee, R., Grigoorian, V., Valizadeh, M., Motallebi Azar, A., 2008. Rooting ability of Persian walnut as affected by seedling vigour in response to stool layering. J. Hortic. Sci. Biotechnol. 83 (3), 334–338. https://doi.org/10.1080/14620316.2008.11512388.
Vahdati, K., Hassani, D., Rezaee, R., 2014a. Behavior of some early mature and dwarf Persian walnut trees in Iran. Acta Hortic. 1050, 189–196. https://doi.org/10.17660/ActaHortic.2014.1050.24.
Vahdati, K., Hassani, D., Rezaee, R., Jafari Sayadi, M.H., Sarikhani Khorami, S., 2014b. Walnut footprint in Iran. In: In: Avanzato, D., McGranahan, G.H., Vahdati, K., Botu, M., Iannamico, L., Assche, J.V. (Eds.), Following Walnut Footprints (Juglans regia L.) Cultivation and Culture, Folklore and History, Traditions and Uses. Scripta Horticulturae 17. pp. 187–201.
Vahdati, K., Pourtaklu, S.M., Karimi, R., Barzehkar, R., Amiri, R., Mozaffari, M., Woeste, K., 2015. Genetic diversity and gene flow of some Persian walnut populations in southeast of Iran revealed by SSR markers. Plant Syst. Evol. 301 (2), 691–699. https://doi.org/10.1007/s00606-014-1107-8.
Vahdati, K., Asayesh, Z.M., Aliniaeifard, S., Leslie, C.A., 2017a. Improvement of ex vitro desiccation through elevation of CO_2 concentration in the atmosphere of culture vessels during in–vitro growth. HortScience 52 (7), 1006–1012. https://doi.org/10.21273/HORTSCI11922-17.
Vahdati, K., Hajinia, Z., Sarikhani Khorami, S., 2017b. Quest for walnut superior genotypes with low chilling requirements in west of Iran. In: First International and 10th National Horticultural Science Congress of Iran. 4–7 September 2017; Tarbiat Modares University, Tehran, Iran.
Vahdati, K., Sarikhani Khorami, S., Arab, M.M., 2018. Walnut: a potential multipurpose nut crop for reclaiming deteriorated lands and environment. Acta Hortic. 1190, 95–100. https://doi.org/10.17660/ActaHortic.2018.1190.16.
Vahdati, K., Arab, M.M., Sarikhani, S., Sadat Hosseini, M., Leslie, C.A., Brown, P.J., 2019a. Advances in walnut breeding strategies. In: Al-Khayri, J.M., Jain, S.M., Johnson, D.V. (Eds.), Advances in Plant Breeding Strategies: Nut and Beverage Crops. Springer Press.
Vahdati, K., Massah Bavani, A.R., Khosh-Khui, M., Fakour, P., Sarikhani, S., 2019b. Applying the AOGCM-AR5 models to the assessments of land suitability for walnut cultivation in response to climate change: a case study of Iran. PLoS One 14 (6), e0218725. https://doi.org/10.1371/journal.pone.0218725.
Zare-Rashnoodi, N., Erfani-Moghadam, J., Fazeli, A., 2017. Evaluation of some walnut genotypes in the west of Iran using fruit characteristics and RAPD marker. Iran. J. Plant Biol. 9 (3), 1–18. https://doi.org/10.22108/ijpb.2017.100338.
