# Supplementary material for: Provenance and family variations in early growth of Manchurian walnut (Juglans mandshurica Maxim.) and selection of superior families
Source: PLoS One. 2024 Mar 7;19(3):e0298918. doi: 10.1371/journal.pone.0298918 (PMC10919699; doi:10.1371/journal.pone.0298918)
Supplement: S2 File — (ZIP) [file pone.0298918.s005.zip › Studies on clone selection and silviculture effect for sawlog of Eucalyptus grandis × urophylla.pdf]

硕士学位
论　　文

尾巨桉中大径材优良无性系选择与栽培措施影响研究

李 昌 荣

廣西大學

二〇〇七年六月

中国知网　https://www.cnki.net

分类号 ____________

UDC ____________

硕士学位论文

尾巨桉中大径材优良无性系选择与栽培措施影响研究

李昌荣

学科专业：森林培育

指导教师：项东云 教授级高工
梁 机 副教授

论文答辩日期：2007年6月9日 学位授予日期：2007年6月

答辩委员会主席：何新华教授

论文评阅人：白嘉雨研究员、黄寿先教授

中国知网 https://www.cnki.net

广西大学学位论文原创性声明和使用授权说明

原创性声明

本人声明：所呈交的学位论文是在导师指导下完成的，研究工作所取得的成果和相关知识产权属广西大学所有，本人保证不以其它单位为第一署名单位发表或使用本论文的研究内容。除已注明部分外，论文中不包含其他人已经发表过的研究成果，也不包含本人为获得其它学位而使用过的内容。对本文的研究工作提供过重要帮助的个人和集体，均已在论文中明确说明并致谢。

论文作者签名：[illegible]　　　　2007年 6 月 18 日

学位论文使用授权说明

本人完全了解广西大学关于收集、保存、使用学位论文的规定，即：

按照学校要求提交学位论文的印刷本和电子版本；

学校有权保存学位论文的印刷本和电子版，并提供目录检索与阅览服务；

学校可以采用影印、缩印、数字化或其它复制手段保存论文；

在不以赢利为目的的前提下，学校可以公布论文的部分或全部内容。

请选择发布时间：

☐即时发布　　　　☐解密后发布

（保密论文需注明，并在解密后遵守此规定）

论文作者签名：[illegible]　导师签名：[illegible]　2007 年 6 月 18 日

中国知网 https://www.cnki.net

尾巨桉中大径材优良无性系选择与栽培措施影响研究

摘　要

本研究以尾巨桉（*E.urophylla*×*E.grandis*）无性系为研究对象，开展了9个性状的遗传变异分析，进行了中大径材优良无性系多性状综合选择研究，同时对栽培密度和施肥措施对尾巨桉生长和木材材性影响的探讨。得到如下结论：

（1）尾巨桉的生长、形质、材性性状在无性系之间差异显著。25个尾巨桉无性系，单株材积大于总体平均值的无性系有11个，其中生长表现最好的是无性系DH33-27，其15年生时的胸径、树高、单株材积、枝下高分别为25.0㎝、32.8m、0.7722m^3、20.1m；形质性状最好的是无性系DH32-28；外部木材密度指标值最优的无性系是DH15-3，螺旋纹理倾角最优的无性系是DH33-20。

（2）单株材积与胸径、树高呈极紧密的正相关，相关系数超过了0.9，另外，胸径、树高和枝下高、分枝、干形之间的相关性达中等偏上，相关系数达0.6；Pilodyn（外部木材密度指标值）与胸径、单株材积、枝下高、干形、分枝、树皮厚度呈弱度正相关，而与树高呈弱度负相关，与SG（纤维倾角）呈中度负相关；BT（树皮厚度）与胸径、树高、单株材积、枝下高、干形、分枝、纤维倾角呈弱度正相关，与Pilodyn呈弱度负相关；SG除了与BT呈轻度正相关之外，与其他7个性状呈中度负相关。

（3）各性状遗传分析表明：各个性状的表型变异系数在 7.92%～56.19%，遗传变异系数在 7.22%～47.17%，遗传变异系数和表型变异系数最大的都是纤维倾角（SG），分别为 47.17%、56.19%，最小的是 Pilodyn（活立木外部密度指标值），分别为 7.22%、7.92%；各个性状无性系重复力在 0.7263～0.9480，最大的是 Pilodyn，最小的是分枝（Br）。

（4）应用主成分分析和简单指数选择法对尾巨桉无性系进行多性状综合选择，聚类分析把25个无性系归为10类；简单指数选择法选择出综合性状表现优良的7个无性系，其胸径、树高、单株材积、枝下高、干形、分枝、外部木材密度指标值、树皮厚度、纤维倾角的遗传增益分别为15.52%、9.89%、39.51%、24.65%、24.65%、30.89%、25.63%、8.66%、24.67%、58.37%，无性系重复力分别为0.8980、0.8395、0.9166、0.8583、0.8043、0.7263、0.9480、0.9070、0.9036。

（5）不同的密度处理对尾巨桉平均胸径、蓄积量的生长影响差异显著，树高在4.2a、6.3a、7.3a、8.3a、9.2a、12.5a上差异显著。尾巨桉13年生时的林分结构分析：DBH≥26㎝的大径材只有密度处理5（株行距：5m×3m）有10%，20㎝≤DBH＜26㎝也就是中径材，随着密度的减少而增大，密度处理5所占的比例最大，几乎达到了80%；随着密度的减小，林分中树高大于等于30m的林木百分比逐渐增大，密度处理5的林分中树高大于等于30m的林木所占的比例最大，达到了70%。过密的栽培密度不利于尾巨桉生长量生长，667株/hm^2和883株/hm^2这两种密度是比较适合尾巨桉中大径材培育的。不同的密度

中国知网　https://www.cnki.net

处理对尾巨桉材性除了树皮厚度影响差异显著外，其余的不显著，对原木A段的木材缺陷影响不显著，对原木B段只有尖削度在5%水平上影响显著，其余不显著。

（6）不同施肥处理对胸径生长影响差异不显著，对树高生长影响只有在2.3a时在5％水平上显著，对蓄积量生长影响也是只有在2.3a时在5％水平上显著。不同的施肥处理对尾巨桉材性除了木材强度影响差异显著外，其余的不显著。

关键词：尾巨桉；无性系；遗传变异；多性状选择；中大径材；栽培密度；施肥；材性

中国知网 https://www.cnki.net

STUDIES ON CLONE SELECTION AND SILVICULTURE EFFECT FOR SAWLOG OF *E. UROPHYLLA*×*E. GRANGDIS*

ABSTRACT

The paper choses *E.urophylla* × *E.grandis* clones as the research object, genetic variance analysis of 9 characters and synthetic selection of multiple characters were undertaken, at the same time, the paper probed into the effects of growth and wood quality of *E.urophylla* × *E.grandis* with spacing and fertilizer treatments. Results are as follows:

(1) There were significant differences in growth, form and wood quality characters among clones. Single volume of 11 clones were bigger than the total mean values of 25 clones, the clone DH33-27 was of the best growth, its 15-year-old DBH, height, single volume and branch height was 25.0 cm, 32.8m, 0.7722m^3, 20.1m; the clone DH32-28's form quality were the best; for wood quality, the clone DH15-3 was the biggest outer wood density, the clone DH33-20 was the smallest spiral grain.

(2) Single volume and DBH, H showed strong positive relationships, the coefficient of relationship was more than 0.9, the relationship between DBH、height and branch height、branch、stem form was significant positive correlation, and the coefficient of relationship was more than 0.6; Pilodyn and DBH、volume、branch height、stem form、branch、bark thickness showed feebleness positive relationships，Pilodyn and H showed feebleness negative relationships，and spiral grain showed negative relationships; the relationship between bark thickness and DBH、H、volume、branch height、stem form、branch、spiral grain showed feebleness positive relationships，and Pilodyn showed feebleness negative relationships; spiral grain and DBH、H、volume、branch height、branch、stem form、Pilodyn showed negative relationships，and bark thickness showed positive relationships.

(3) Phenotype coefficient variance of 9 characters were 7.92%～56.19%，genotype coefficient variance were 7.22%～47.17%, clone repeatability were 0.7263～0.9480; Spiral grain has the biggest coefficient variance, Pilodyn has the smallest coefficient variance and the biggest clone repeatability, branch has the smallest clone repeatability.

(4) Synthetic selection of multiple characters for *E.urophylla* × *E.grandis* clones by the methods of principal component selection and index selection, 25 clones were separated into 10 clusters by principal component selection, picked out 7 clones with superior Synthetic characters, genetic gain of 9 characters were 15.52%, 9.89%, 39.51%, 24.65%, 24.65%, 30.89%, 25.63%, 8.66%, 24.67%, 58.37%, clone repeatability of 9 characters were 0.8980，0.8395，0.9166，0.8583，0.8043，0.7263，0.9480，0.9070，0.9036。

(5) There were significant differences in mean values of DBH and growing-stock among

中国知网 https://www.cnki.net

differences spacing treatments, in 4.2a、6.3a、7.3a、8.3a、9.2a、12.5a year had a significant differences in height. Stand structure analysis of 13 years old *E.urophylla*×*E.grandis* , DBH≥26㎝ in spacing 5 stand there was 10%, in 20㎝≤DBH＜26㎝ class, spacing 5(5m×3m) with the largest percent, nearly 80%; height of the stand, in 30m≤H class, spacing 5 with the largest percent, more than 70%.Bigger spacing was not good for the growth of *E.urophylla* × *E.grandis*, the spacing of 667stems/hm^2 and 883 stems/hm^2 was fit for *E.urophylla* × *E.grandis* middle/big diameter wood planting. There were significant differences only in bark thickness of *E.urophylla* × *E.grandis* wood quality. There were significant differences only in bark thickness of *E.urophylla* × *E.grandis* wood quality among 6 spacing treatments. There were not significant difference among 6 spacing treatments for A log; for B log, there were significant difference (a=0.05) .

（6）There was not significant difference in DBH among differences fertilizer treatments, only in 2.3 years old, there were significant differences in height and growing-stock (a=0.05). There were significant differences only in wood strength of *E.urophylla* × *E.grandis* wood quality.

KEY WORDS: *E.urophylla*×*E.grandis*; clones; genetic variance; multiple characters selection ; middle/large timber; plant spacing; fertilizer; wood quality

中国知网 https://www.cnki.net

目录

第一章 前言……1
1.1 引言……1
1.1.1 桉树的天然分布与木材特性……1
1.1.2 桉树引种的状况概要……2
1.1.3 尾巨桉简介……2
1.2 国内外研究现状综述……3
1.2.1 桉树无性系选择育种的研究进展……3
1.2.1.1 桉树无性系生长量选择育种的研究进展……3
1.2.1.2 桉树木材材质选择育种的研究进展……4
1.2.1.3 桉树生长性状与材质性状的相关性研究……5
1.2.1.4 多性状综合选择的研究进展……6
1.2.2 桉树中大径材栽培技术的研究进展……6
1.2.2.1 国外研究进展……6
1.2.2.2 国内研究进展……7
1.2.3 栽培措施对桉树木材性质影响的研究……7
1.2.3.1 栽培密度对桉树木材性质影响……7
1.2.3.2 施肥处理对桉树木材性质影响……8
1.2.4 木材缺陷的研究进展……8
1.2.4.1 节子……9
1.2.4.2 裂纹……9
1.2.4.3 弯曲……9
1.2.4.4 尖削……10
1.2.5 无损检测技术的研究进展……10
1.2.5.1 国外无损检测技术的研究进展……10
1.2.5.2 国内无损检测技术的研究进展……11
1.3 本研究的目的和意义……11
第二章 研究材料与方法……13
2.1 尾巨桉无性系生长与材性遗传变异试验材料与方法……13
2.1.1 试验地概况……13
2.1.2 试验设计……13
2.1.3 试验方法……13
2.1.3.1 生长性状的测定方法……13
2.1.3.2 材质性状的测定方法……14
2.1.4 数据处理……15
2.1.4.1 各性状平均值、单株材积、标准差的计算公式……15
2.1.4.2 主要遗传参数估算公式……16
2.2 尾巨桉栽培密度与施肥试验材料与方法……16

中国知网 https://www.cnki.net

2.2.1 试验地概况 16
2.2.2 试验设计 16
2.2.3 试验方法 18
2.2.3.1 生长性状的测定方法 18
2.2.3.2 Pilodyn、纤维倾角测定法 18
2.2.4 数据处理 19
第三章 结果与分析 20
3.1 尾巨桉无性系生长、材质性状遗传变异试验结果与分析 20
3.1.1 生长性状、形质性状和材性性状差异分析 20
3.1.2 性状遗传参数的估算 23
3.1.3 性状间的相关分析 23
3.1.4 主成分分析 24
3.1.5 简单选择指数法 28
3.1.6 尾巨桉 5 个无性系原木木材缺陷比较 30
3.2 尾巨桉密度试验结果与分析 32
3.2.1 尾巨桉生长过程分析 32
3.2.2 尾巨桉 13 年生时的林分结构分析 36
3.2.3 不同栽培密度对 13 年生尾巨桉材质的影响 38
3.3 尾巨桉施肥试验结果与分析 44
3.3.1 林分生长差异性 44
3.3.2 尾巨桉 13 年生林分结构分析 44
3.3.3 不同施肥措施对 13 年生尾巨桉材质的影响 46
第四章 结论与讨论 48
4.1 结论 48
4.2 讨论 50
参考文献 51
附图 57
致 谢 59
攻读学位期间发表论文情况 60
导师简介 60

中国知网 https://www.cnki.net

第一章 前言

1.1 引言

1.1.1 桉树的天然分布与木材特性

桉树是指包括杯果木属（*Angophora*）、伞房花属（*Corymbia*）和桉树属（*Eucalyptus*）共3个属的所有树种，共有800多个种与100多个变种。桉树天然分布于澳大利亚大陆及华莱士线（Wallace's Line）以东，大洋洲大陆附近的太平洋岛屿，在北纬7°0′～南纬43°39′之间（如图1-1所示），但绝大部分分布在澳大利亚大陆,只有剥桉（*Eucalyptus degulpta*）分布在巴布亚新几内亚和菲律宾棉兰老岛等岛屿，在北纬9°0′～南纬11°0′之间；尾叶桉（*Eucalyptus urophylla*）分布在东帝汶和印度尼西亚群岛东部的一些岛屿上，在北纬8°0′～南纬10°0′之间。在天然分布区从热带到温带，从海平面到海拔3000m以上高山，从滨海到内地，年降水量在200～4000mm的地区都有桉树生长[1,2]。

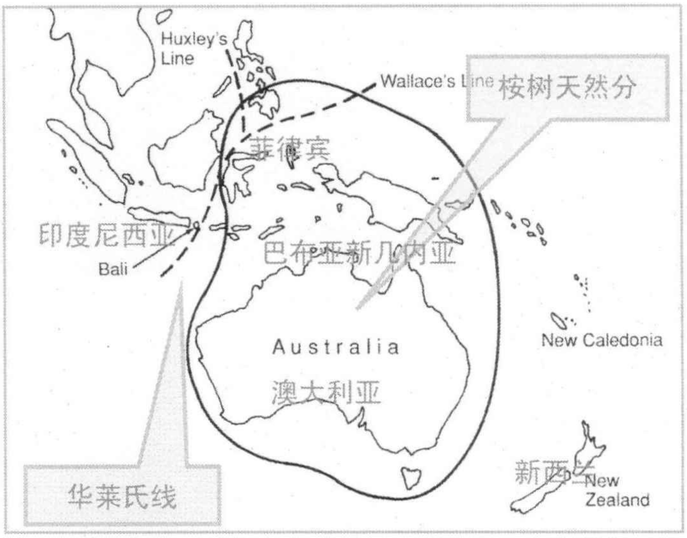

图 1-1. 桉树天然分布区域示意图（Ken. E, 1993）
Fig1-1. Nature distributiion area of *Eucalyptus*

桉树树种多样、生长快，耐贫瘠、抗逆性强，经济价值高。桉树用途广泛，可作为防护林和道路两旁的绿化行道树，多数是世界著名的硬木资源和造纸优质材料，其木材工业特性好，坚韧耐腐，是最常用的制造纸浆的主要原料, 亦可作为人造板、包装、建筑、矿井支柱、家具等用材，另外桉树叶可提取桉油，树皮可提取鞣质[2]。

桉树不但是优良的速生纸浆材树种，而且还是珍贵的锯材树种。如赤桉（*E. camaldulesis*）、王桉（*E. regnans*）、斑皮桉（*E. maqulata*）、大花序桉（*E. cloeziana*）、柠檬桉（*E. citrodora*）、斜叶桉（*E. obliqua*）、白桃花心桉（*E. acmenoides*）、异色桉（*E. divirsicolor*）、粗皮桉（*E. pelleta*）、邓恩桉（*E. dunnii*）、巨桉（*E. grandis*）和柳桉（*E. saligna*）等树种，是重要的硬阔材树种。它们的木材红色、深粉红色或米黄色，结构紧密，握钉性强，纹理美观，坚硬耐久，适用于家具制造、室内装饰、细木工、镶木地板、雕刻、单板、胶合板及建筑用材等[3]。在澳大利亚，桉树小径材的价格很低，为 20 澳元/m^3，而桉树大径材为 200 澳元/m^3 以上，若加工成板材，则高达 1000 澳元/m^3 [4]。澳大利亚是桉木锯材产量最大的国家之一，其生产技术也最成熟。在澳大利亚用桉木制作家具、木地板、房屋构架等比较普遍。近年来，澳大利亚开始实施大花序桉、弹丸桉、斑皮桉类、蓝桉和亮果桉等硬木树种的改良计划，以提高桉树人工林大径材质

中国知网 https://www.cnki.net

量。

1.1.2 桉树引种的状况概要

20世纪中后期，桉树人工林在全世界得到了迅速的发展。全世界共有58个国家和地区引种桉树并实施商业性桉树人工林栽培，另有50个国家和地区进行了引种栽培试验。据联合国粮农组织的资料[5]估计，全世界现有桉树人工林面积达1786万hm^2。在热带地区，桉树人工林占世界热带人工林总面积的25%[2]。有关资料表明[6]，印度桉树人工林栽培面积已达480万hm^2；其次为巴西占400万hm^2。每年全世界由桉树人工林提供的桉树木材生产量达15000～18000万m^3[2]。

巴西、南非、智利、中国、印度等国是桉树引进、改良和人工林栽培较为成功的国家，桉树商业栽培面积超过50万hm^2。泰国、缅甸、越南、西班牙、葡萄牙、马达加斯加、摩洛哥、乌拉圭、安哥拉、美国等国为桉树人工林栽培较多的国家，桉树商业栽培面积超过10万hm^2。巴西桉树人工林生长量高达40～70m^3/hm^2（无性系），5～7年皆伐，年产纤维材600万m^3，仅用不到1%的国土面积提供了全国30%的用材；南非桉树人工林生长量达30～50m^3/hm^2，10年皆伐，收获量210～350m^3/hm^2。

中国引种桉树开始于 1890 年，已有 100 多年历史，20 世纪 50 年代中期开始大面积造林[7]；华南地区于 20 世纪 80 年代初开展大量引种、改良和栽培技术研究，目前桉树已经遍布我国南方 17 个省、自治区的 600 多个县；据不完全统计，栽培面积 150 多万 hm^2，仅次于巴西和印度，居世界第三位；在引种栽培桉树的省、市、自治区中，广东、广西、海南和云南南部、福建南部是桉树栽培重点省区[2,8,9]。

1.1.3 尾巨桉简介

尾叶桉（*E.urophylla*），自然分布区以印度尼西亚东部岛屿和帝汶岛为主，南纬8～16°，海拔高达3000m。夏雨型，年降水量1000～1500mm。最热月平均气温29℃，最冷月平均温度8～12℃，不耐霜冻。目前已被南美、非洲以及澳大利亚等一些低纬度国家普遍引种栽培，生长表现优异。我国广东、广西、海南作为主要栽培树种，以尾叶桉作为母本与巨桉、赤桉、细叶桉杂交，表现出杂种优势。其木材紫红色，坚硬耐磨，广泛用于重型结构和桥梁用材。

巨桉（*E.grandis*）原产澳大利亚东部沿海。地理位置南纬22～32°，海拔0～300m，夏雨型，年降水量1000～1700mm，最热月温度29℃，最冷月平均温度5～6℃。性喜温湿肥沃土壤。世界上很多国家引种栽培，我国广东、广西等地均有种植，四川作为主要栽培树种。其木材桃红色，结构粗，纹理直，易开裂，可供矿柱、建筑、包装箱板以及纸浆等用。

尾巨桉（*E.urophylla*×*E.grandis*）是广西东门林场在尾叶桉和巨桉的优良个体选择的基础上，通过人工控制授粉获得的杂交种。尾巨桉、巨尾桉杂交种具有更高的速生性，不耐霜冻，适应热带、亚热带地区栽培，幼林期生长迅速，一般 1 年郁闭，3 年成材，年平均高生长 3～6m，年平均胸径生长 2.5～3.5cm，10 年生以后生长速度下降，有利于

中国知网 https://www.cnki.net

短周期纤维原料林栽培。萌芽和无性繁殖能力较强，但存在个体间差异，利于无性系选育和繁殖，适合萌芽更新[10]。

尾巨桉干形通直，出材率高，材质好，纹理直，耐瘠薄，速生丰产，轮伐期短，具短时耐低温能力，有较好的木材稳定性和耐光性[11,12]，是优良的建筑用材和纸浆材树种，有望在家具和室内装饰材料上发挥作用。近年来，该品种在广东、广西的部分地区得到广泛推广，很受欢迎。

1.2 国内外研究现状综述

1.2.1 桉树无性系选择育种的研究进展

1.2.1.1 桉树无性系生长量选择育种的研究进展

近几十年来，随着人工林集约经营强度的提高，特别是木材加工和制浆造纸等工业原料的需求，短周期工业原料林的大规模发展，对林木遗传改良提出了迫切需求，由于经过改良后，无性系林分单位面积产量高，增产效果显著，林相整齐，便于集约经营，产品性质一致，因此，无性系在商品林业中的应用，开始受到广泛的关注。

桉树具有速生、丰产和用途广泛等优点，近 20 年来被广泛用作纸浆原料，经济效益显著，人工造林面积迅速扩大，推动了林木遗传改良事业的发展。桉树无性系育种和无性系造林在近 20 多年有了快速发展，在巴西、南非、刚果和我国南方都取得了显著的成效[13]。刚果 1978 年开始大规模用高产杂种无性系造林，到 1987 年无性系人工林面积已达到 23000 hm^2。1970 年刚果细叶桉和尾叶桉每公顷年生长量仅为 12 m^3，1987 年无性系人工林每公顷年生长量提高到 20～25 m^3，最好的无性系每公顷年生长量达 30～38 m^3。巴西阿拉克鲁兹（Aracruz）公司建立的原料林已大规模使用无性系造林。该公司无性系育种的原始材料主要是巨桉和尾叶桉的杂种，该公司从引种第一代的 36000 hm^2 林分中，选出 5000 株候选优树，再从中筛选出 150 个无性系，经无性系测定，根据生长、抗性、干形、扦插成活率、木材比重、得浆率等性状最终选出 31 个最优无性系用于生产。该公司还用巨桉和尾叶桉的杂种后代无性系造林，年生长量为 50 m^3/hm^2，经过第二轮无性系的精选，年生长量提高到 80 m^3/hm^2。据报道，在高度集约经营条件下，创造了年生长量高达 120 m^3/hm^2 的记录。自 90 年代以来，在我国广西、广东、海南一带，桉树无性系造林得到了大规模的发展，除了难以无性繁殖的桉树种以外，绝大部分都是应用无性系造林。广西东门林场从尾叶桉×巨桉 F_1 代中选出无性系，1 年生每公顷生长量为 16 m^3，3 年生的无性系林分年平均生长量为 43 m^3，6 年生时每公顷蓄积量可达 315 m^3。广西原有的桉树林，平均每公顷蓄积量仅为 27 m^3，采用无性系大面积造林后，平均每公顷蓄积量已增至 90 m^3，提高了 3 倍多。

东门项目期间[14]，东门林场对38个树种和81个种源进行选育，选择出尾叶桉等16个优良树种，其蓄积生长量都高于东门原传统当家树种窿缘桉，最高达237%，尾叶桉、大花序桉等5个树种的优良种源11个，这些种源的年均蓄积大多在15m^3/ (hm^2.a) 以上，

中国知网 https://www.cnki.net

最高为大花序桉的种源达22.53$m^3/(hm^2.a)$。通过进行子代试验，选择出了230个家系，其6～7年生年蓄积生长量在22.5m^3/hm^2 以上，在这些优良家系中，年蓄积生长量45m^3/hm^2以上的家系有14个。在无性系试验中，筛选出来的无性系年蓄积生长量都在30$m^3/(hm^2.a)$以上，最优无性系DH33-27（东门尾巨桉杂种）8.5年生年蓄积为60.29$m^3/(hm^2.a)$。

白嘉雨先生[15]在回顾我国桉树遗传育种及发展前景时认为我国经过20年的引种和改良育种研究后，新建的工业用材林取得了20m^3/hm^2的持续高生长，肯定了桉树改良的成功，同时指出下一步的研究重点是优良繁殖材料的再选择，在完成第2轮选择与育种的基础上开始进入第3轮选择，不断提高繁殖材料的遗传质量。

1.2.1.2 桉树木材材质选择育种的研究进展

国内外许多研究表明[16]，桉树人工林的木材性质在种间和种内的变异性都很大，因此不同的树种可以适合不同的产品和市场的需要，要充分利用桉树人工林木材资源，应加强对不同桉树人工林树种的木材性质的研究。澳大利亚、巴西和南非等桉树种植大国首先加强了对不同桉树人工林树种的木材性质，特别是与加工性质密切相关的密度和生长应力的研究，通过比较分析，在一定的材性范围内挑选人工林树种，进行定向培育，把速生、丰产、优质放到了同等重要的位置。

（1）木材密度

木材基本密度是木材质量的最基本性质，它与木材的物理力学性质及加工性能有密切的关系。桉树的密度差异极大，也没有一致的规律，但较一致的观点是, 桉树木材的密度变异主要是基于纤维细胞壁厚的变异[17]。桉树密度从0.45g/cm^3～1.0g/cm^3不等[18]。有文献报道[19]，基本密度0.65g/cm^3以下的桉树随着密度的增加，干燥时皱缩也显著增加，基本密度大于0.65g/cm^3时桉树不易发生皱缩。国内外大量的实验与报道表明：木材的力学强度与木材密度是明显的正相关关系，木材密度越大，则其力学强度越高。通过对澳大利亚20～40年树龄不同树种的桉树人工林木材性质的研究发现[20]，桉树生长速度与木材基本密度之间没有显著的相关性，同时表明桉树的树龄对其木材的质量的影响程度比桉树的生长速度要明显，采伐的木材树龄越小，密度越小。巴西[21]通过对巨桉的改良，将木材密度从0.48g/cm^3提高到0.52g/cm^3。对639株王桉木材密度的研究结果认为,木材的密度与种源有密切的关系，木材密度有明显遗传性。因此，可以通过选种和育种来改善木材的质量[17]。

（2）生长应力

生长应力是立木细胞在生长过程中于形成层部分形成的，存在于所有的树木中[22]，木材中因有生长应力而导致应变。高生长应力是桉树，特别是低龄桉树人工林木材的显著特征。4年生昆士兰桉(*E.cloeziana*)的表面径向生长应力与其高度、胸径和边材密度呈显著正相关。Chafe[23]通过比较8年生的人工林亮果桉和36年生次生林王桉在1.3m高处的生长应变，认为这2种桉木生长应变和树木直径成正相关关系，但Kubler[22]认为，桉树木材生长应变或应力与树木的胸高直径之间的关系随个体环境的变化而变化。Yang[24]比较分析了3个种源人工林蓝桉木材生长应变的关系，她认为南澳洲的Johnston Block种

中国知网 https://www.cnki.net

源的蓝桉木材生长应变较高，而King Islang种源的木材生长应变明显比Jeeralang和Southeast of Tasmania种源低。

桉树人工林的生长速度对生长应力略有影响，细胞壁微纤丝角可能是控制生长应力，以及影响生长应力和干燥应力之间关系的主要因子。这些研究表明，生长应变和应力可以通过选育措施来改变，人们可以采取一定的育种方法培育出生长应力较小的树种，生产出高质量的原木和锯材。在巴西和南非，已通过遗传选育和栽培管理措施成功地降低了桉树的生长应力。桉树的试验[19]表明栽培措施可以采用延长轮伐期，在生长期内使桉树尽可能地快速生长成较大的直径来释放径向生长应力；采伐之前可用环状剥皮、毒杀或化学落叶法，使树木在采伐前的生长期不再增加生长应力，就会把树皮下树木中原生长应力2/3释去；原木用水贮存或用水喷数月也会使原木中的应力减少到在加工时不造成损失，桉木贮存一年可把应力水平减少20%。

1.2.1.3 桉树生长性状与材质性状的相关性研究

桉树生长性状与材质性状的相关关系早已引起人们的兴趣，关于这方面的报道也较多。王豁然等[25]在研究巨桉种源生长与材性变异的结果表明：不同地理种群的单株材积与木材基本密度不呈规律性的相关变化。徐建民等[26]在研究尾叶桉家系综合选择的结果表明：3 个生长性状（胸径、树高、材积）除与基本密度、材积与保存率无显著相关外，与其它性状呈现紧密至极紧密的表型正相关和遗传正相关；基本密度与抗风指标、保存率与树高、胸径也呈一般的和紧密的表型和遗传正相关；基本密度与其它性状（抗风指标除外）的表型和遗传相关系数均为负值，但系数数值很低。李淡清[27]的研究表明：蓝桉的纤维长度与木材密度、树高、胸径都呈正相关，与木材密度的相关系数达到了 1，与树高在 0.01 水平上相关, 与胸径的相关也极显著（即在 0.01 水平），而木材密度与树高和胸径呈负相关，与树高虽未达显著水平, 但与胸径的相关系数却达到-1；直干桉的纤维长度与木材密度和树高的遗传相关都为正相关，但它与木材密度的相关不显著, 与树高却极其显著。郑白等[28]在研究东门桉树制浆造纸潜力及其材性变异中得出：不同无性系间木材密度与生长量之间存在一定的负相关, 但未达到显著性水平。莫晓勇等[29]对雷州半岛 27 个桉树无性系 5 个性状间的表型及遗传相关研究结果表明：3 年生桉树无性系性状间的树高与胸径、叶量，胸径与叶量均存在显著的表型及遗传相关且为正相关，树高与胸径间的表型相关系数为 0.92，遗传相关系数达 0.93，树高与叶量间的相关系数分别为 0.56 和 0.58。胸径与叶量的表型及遗传相关系数分别为 0.44 和 0.52。树皮厚度和胸径的表型及遗传相关未达显著水平，而树皮厚度与树高、叶量及木材密度与树高、胸径、叶量、树皮厚度间的表型及遗传相关系数较小，说明这些性状间不因另一些性状的改变而发生较大的变化。陆钊华、徐建民等[30]对韦塔桉种源的研究结果表明：树高、胸径、材积等生长性状与分枝性状、枝下高及冠幅的表型和遗传相关均呈较紧密至紧密的正相关关系；而干形、保存率与其他性状的表型和遗传相关不显著；保存率与所有性状间均表现为负相关关系。

中国知网 https://www.cnki.net

1.2.1.4 多性状综合选择的研究进展

林木品种的经济价值多由多个性状共同决定的。优良品种是多个优良性状的有机结合，在林木改良方案中，往往要求同时改良几个性状，因此，必须考虑采用多性状选择。多性状选择主要有3种方法[13]：（1）单项排列选择法。在一定时间内，改良某一性状，直到达到所希望的要求符合时为止。如此一个性状一个性状依次选择改良。该法一般很少应用，主要因为改良的过程时间太长。（2）独立挑选法。对所需改良的性状同时进行选择时，给每个性状规定一个最低标准值，如果个体达到这些标准值就可入选。该方法在林木改良工作中被广泛应用。（3）指数选择法。就是把所有有价值的性状信息综合成一个简单的指数值，选择时把它作为单个性状值一样来看待。从理论上讲，指数选择法的选择效果最好。

多性状综合选择是缩短遗传测试时间、加速育种进程的重要手段，可大大提高性状改良效率，带来极为明显的经济效益。早期我国多数树种的遗传改良主要采用独立挑选法，对多性状的改良基于单一性状的改良。近年来对多性状综合选择的研究开始加强，多性状综合指数选择方法在杨树[31-35]、松树[36-39]、锥栗[40]、杉木[41,42]、泡桐[43]等树种上有一定的应用。

一些学者也对桉树综合选择进行研究。方玉霖[44]采用主成分遗传距离原理，对桉树进行多性状综合选择，选择出的材料树高遗传增益为10.99%，胸径为36.12%，材积为95.27%，选择效果明显；徐建民等[45]运用指数选择法对尾叶桉44个家系进行多性状综合选择出25个优良家系；Xiaoyong Mo等[46]通过产量及与产量相关的性状，运用无约束指数选择法对27个桉树无性系作综合评价；姚庆端[47]对桉树优良无性系制浆造纸性能与适应性的研究中，应用主成分聚类分析方法就生长性状、材性性状、抗逆性等13个指标进行多性状综合选择，选择出6个适合闽南山地发展的无性系；陆钊华等[48]对韦塔桉(*E.wetarensis*)种源也开展了多性状综合评价研究。

1.2.2 桉树中大径材栽培技术的研究进展

1.2.2.1 国外研究进展

澳大利亚联邦政府重视和促进桉树人工林的培育，进行中、大径材与纸浆材混合经营技术研究，取得了突破性的进展。在澳大利亚，桉树纸浆材的价格很低，只有20澳元/m^3，而桉树锯材的价格有100澳元/m^3。因此对于许多农场来说，培育桉树大径材比纸浆材更有吸引力了。在维多利亚有一个蓝桉和巨桉的试验林[49]，该试验林1990年造林，试验内容包括造林密度（3种造林密度：4400株/ hm^2 、2200株/hm^2和1000株/hm^2）、间伐和修枝等内容，试验的目的就是培育桉树大径材。该试验林在4年生时进行了间伐和修枝。各间伐一半作纸浆材，然后再选400株/hm^2，生长好、树干通直的优树进行修枝，修枝高度为6米。在8年生时进行第二次间伐，间伐材可用作纸浆材，只保留哪些经过修枝的优树。到2001年，胸径最大的已高达50cm，一般的都有40cm左右，年平均胸径生长量高达4～5cm，到15年生时，林木胸径可望达到60～75cm，成为真正的大径材桉树。

中国知网 https://www.cnki.net

南非[50]生长轮伐期为30年的巨桉以2.5m×3m为初值密度，培育过程中经过了6次疏伐，最终保留密度为100株/ hm^2，胸径可达到60cm左右。培育桉树大径材林木需要的营养空间最大，Medhurst J.L 等[51]研究认为轮伐期在20到25年的亮果桉适宜最终保留密度为200～300株/ hm^2，使每一株桉树的占地面积为33～50m^2，也就是株行距在3m×10m到5m×10m的范围之间。

在巴西[52]，为了大规模生产实木制品，培育大径级原木的桉树人工林还处于初始阶段。但是，一些成功的实验包括桉树新品种的选择和培育、营林（初植密度控制、间伐控制、修枝控制、轮伐期等）以及结合营林措施尽量降低生长应力对木材加工和利用的影响。在桉树人工林营林经验和技术的基础上，提出了桉树人工林大径级原木的培育模式：初植密度为1100株/ hm^2，株行距为3m×3m；第3年，伐去50%的树冠，树干5m以下部位全部整枝；第4年，树干7～8m以下部位全部整枝；第6年，间伐掉60%的林木，种植密度降低到440株/ hm^2；第9～10年，又间伐掉50%的林木，种植密度降低到220株/ hm^2；第15、16年皆伐。

1.2.2.2 国内研究进展

在一段时期内，桉树的研究主要集中在短周期工业用材林上，但是随着天然林的禁伐和锯材价格的提高，人们开始对桉树中大径材进行研究。项东云[53]1999年指出广西已有丰富的桉树中大径材培育的种质资源基础，而且已经有了30多年培育柠檬桉中大径材的技术经验，培育桉树中大径材可避免部分地区桉树人工林效益低的问题。陈少雄[4]认为大力发展桉树大径材人工林是一条快速缓解我国用材紧张的新途径，提出桉树大径材培育的技术要点，介绍了几种值得作为大径材培育的桉树树种，并对桉树大径材培育进行简单的经济效益分析，得出投入产出比为1:30，经济效益十分显著。林国金[54]2005年在闽南地区开展桉树大径材培育技术试验结果表明，在树种选择上，以柳桉为主，其生长表现优越，适应性较好，造林密度以1650株/hm^2为佳，能取得较好的经济效益，初步可在闽南地区经中试后推广应用。潘开平等[55]在十五计划项目期间进行了良种桉中大径材与纸浆材复合经营试验研究，探讨良种桉中大径材与纸浆材复层林经营的可行性及其经济效益情况。黄锡泽等[56]对尾巨桉人工林栽培密度进行研究，根据研究结果指出，如培育大中径材，种植密度应小于1000株/hm^2 。

1.2.3 栽培措施对桉树木材性质影响的研究

1.2.3.1 栽培密度对桉树木材性质影响

在众多关于大径材培育的文献中，无一例外地首要考虑密度控制[57]。不同的造林密度对林木的生长速度及木材的材质有不同的影响，主要是改变了树木之间对阳光、养分等相互竞争的空间。在造林密度对桉树木材纤维的影响方面研究表明，造林密度对木材纤维的长度无显著性影响，纤维宽度则有随造林密度增加而增大的趋势，二者之间的相关系数达显著水平[58,59] 。鲍甫成等[18]1998年在研究尾叶桉种植密度对材性的影响时得出，尾叶桉不同种植密度，纤维长度和宽度无显著差异，生长轮宽度无显著差异，纤维

中国知网 https://www.cnki.net

微纤丝角差异极显著，并且由髓心向外，微纤丝角有减少的趋势。种植密度还影响木材力学性质，其中抗弯弹性模量在1 %水平上差异显著，而顺纹抗压强度差异显著。所以，人工栽培的尾叶桉作为承重结构和桥梁用材，强度要求高，造林时则可以用比较低的种植密度或进行强度疏伐。

桉树纸浆材优化栽培模式研究协作组的研究表明，随着种植密度的增加，木材基本密度有所减少，但其差异未达到显著水平。此结论在朱林峰[58]、黄宝灵[59]和鲍甫成等[18]的试验中得到证实。国家林业局调查规划设计院的调查结果表明，雷州林业局种植的 4a 生尾叶桉木材的材性与造林密度有较密切的关系，尾叶桉的基本密度随着造林密度的增加而减少，但变幅不大，只有 3.4 %，木材纤维长度和宽度随着造林密度的增加而增加。

1.2.3.2 施肥处理对桉树木材性质影响

施肥是人工林速生丰产的关键技术措施之一，对培育桉树大径材更为重要。罗建举等[60]和方文彬等[61]在国家“八五”科技攻关专题中，研究了尾叶桉木材纤维形态及物理力学性能的影响，结果表明施肥可显著增大纤维宽度、降低木材密度、减小木材的干缩差异、提高木材品质系数，而对纤维没有显著影响。罗真付等[62]对不同施肥处理下 6a 生尾叶桉单株材积、年轮宽度、密度的影响结果表明，从髓心向外，尾叶桉年轮宽度呈逐渐减少趋势，基本密度呈逐渐上升趋势，各施肥处理与对照材基本密度无显著差异。潘彪等[63]研究认为施肥处理显著加快尾叶桉生长速度，对木材密度影响甚微，对纤维长度没有显著的影响，使纤维宽度显著增加，但并非施肥量越多，生长速度越快，纤维宽度越宽。鲍甫成等[18]研究得出，施肥处理对尾叶桉纤维长度没有显著性影响，对纤维宽度具有显著增大作用，尤以施用磷肥或氮磷钾及微量元素混施者效果为佳，不同施肥处理对尾叶桉木材微纤丝角有显著影响，施加氮肥时木材微纤丝角明显增大；而施加氮肥＋磷肥或氮磷钾及微量元素混施时会使木材微纤丝角减小。施肥处理对木材密度具有显著性降低的效应，对力学强度的影响有正效应，也有负效应，施肥处理可以提高人工林尾叶桉木材品质系数和降低木材干缩比，对木材性质有一定程度的改善。

1.2.4 木材缺陷的研究进展

对木材缺陷有种种定义。Brown 认为，木材缺陷是降低木材商用价值的非正常和不规则部分。Panshin[64]所述，木材缺陷指的是对木材适合某一特殊用途的质量缺损或偏离。我国高等林业院校木材学教科书[65]认为，木材组织，由于结构不正常或者受到机械损伤及发生病虫害，致使材质受到影响，减低了木材的工业价值，甚至使木材完全不能使用。这些与正常木材不同，而又影响材质的改变，都称为“木材缺陷”。我国国家标准（1984）对木材缺陷的定义是：凡呈现在木材上能降低其质量，影响其使用的各种缺点，均为木材缺陷。

有材料指出[66]，木材净收益的80％～90％体现在10～14m长的下部树干上。因此，木材缺陷在木材质量上有着重大的意义。

中国知网 https://www.cnki.net

1.2.4.1 节子

在树干或主枝木材中包含的枝条部分，称为节子。节子是决定木材等级的主要因子，是造成木材降等的主要缺陷。

节子对木材弹性有一定的影响。Baumann R.测定木材弹性模量结果表明：对于含木节弯曲时测定的纵向弹性模量，松木降低 7.2%；柚木弹性模量降低 38.3%。Ylinen 松木木节处的弹性柔量约为周围木材的 7 倍。节子对抗拉强度也有影响。Kunesh 和 Johnson[67]的研究表明：节子的大小和位置对木材顺纹抗拉强度均有显著的影响，木材顺纹抗拉强度随节子尺寸增加而减小。戴澄月等[68]在研究兴安落叶松木节尺寸对抗弯强度的影响表明：兴安落叶松木节受弯构件承载能力的影响，不论抗弯强度或抗弯弹性模量均随节径比的增大而明显降低。魏亚等[69]进行 5 种针叶树材木节对顺纹抗压强度影响试验表明：木节对纵压强度的影响是随着木节率的加大而增加的，一般活节的影响较小，死节影响较大。

1.2.4.2 裂纹

木材纤维和纤维之间的分离所形成的裂隙称为裂纹，它可在树木生长期间或伐倒后形成。裂纹是使锯材降等的第二或第三位因素。裂纹破坏木材的完整性，降低木材的强度。

裂纹对木材力学强度的影响随着机械荷重的性质、作用力、方向和裂纹大小等因子而不同。魏亚[69]进行 11 种阔叶树材裂纹对弯曲强度影响的试验表明：垂直于加力方向的裂纹，对弯曲强度有影响，若裂纹在材身的一侧，且其长度不超过试件全长之半者，则影响较小；若裂纹在材身两侧开裂，其长度超过试件全长一半以上者，则影响较大。同时他对榆木进行了研究，结果为：裂纹长度在材端的 1/2 以内时，水平径裂的静曲强度均为完整材的 96%以上；垂直径裂为完整材的 94%以上。但当材端贯通裂，而材身裂纹延长至材长的 1/2 以上时，则其静曲强度显然有所降低，水平径裂最低有达完整材强度的 61%；垂直径裂则降低为完整材的 85%。

1.2.4.3 弯曲

树干形状缺陷，是树干在生长过程中，受树木生长特性和环境条件的影响，而形成的不正常形状。弯曲在确定木材的用途方面常有决定性的意义。

弯曲特别降低木材的纵向强度。别列雷金[70]研究认为，弯曲将使木材易于失去稳定性，并引起纵曲临界负荷降低，弯曲度>0.008，利用直轴支柱的公式就会有误差。研究证明：弯曲对锯材的总出材率有很大的影响，弯曲度每增加 1%，则出材率减少 10%，弯曲所引起的出材率的降低是呈直线增长的，弯曲度在 0.3%以下时，对出材率没有任何影响。原木弯曲不仅影响出材率，而且对成材的尺寸也有很大的影响，弯曲原木在以腹背下锯法锯割时，对板材宽窄影响最大。

檀庆忠等[71]在闵东桉树引种抗逆性的早期选择研究中认为，桉树品系和海拔两个因素对树干弯曲指数有显著影响。李淡清等[72]在研究直干桉生长性状的遗传效应分析时得

中国知网 https://www.cnki.net

出：弯曲度在直杆桉组合间差异显著。他[73]在研究蓝桉时认为弯曲度的狭义遗传力为0.35，选择育种效果较好。潘惠新等[74]研究美洲黑杨得出，美洲黑杨干形弯曲度无性系间差异达到极显著水平，弯曲度的遗传力为 0.4179，弯曲度的变异最大。

1.2.4.4 尖削

尖削就是树干粗细、原木直径或毛边成材的宽度，在全长范围内自大头至小头超过正常的递减程度。一般来讲，在密度比较大的林分中，林木为了争夺阳光，占据较大的营养空间，呈现为高向生长旺盛、树干长得通直，圆满，尖削度小；而在密度稀疏的林分中，树干往往比较弯曲，尖削度大，并且容易产生斜向纹理，降低木材质量。另外，人工林的密度不同，自然整枝出现的时间和强度也不相同，因而影响树干节子的数量、粗度和分布，使木材的质量发生改变。一般密度大的人工林，自然整枝出现的时间早，强度大，活枝下高增加；而密度小的人工林，则会推迟自然整枝出现的时间，形成的侧枝过于粗大，节子量多，活枝下高减少，侧枝低垂。因此，通过调节密度，可减少节子的数量，降低侧枝粗度，有利于形成无节或少节的良材。

余雪标等[75]在研究不同连载代次桉树木材主要性质时认为：随着连载代次的增加，树干尖削度逐渐下降。陈章水[76]在研究泡桐时指出，为了改变泡桐尖削度大的问题，人工林要强调合理栽培密度。肖祥希[77]在研究福建柏修枝时得出：修枝降低了福建柏人工林的尖削度，以 6 年生时修枝对尖削度的降低和提高材积增长速率最好。吴际友等[78]的研究也认为修枝能降低木材的尖削度。

1.2.5 无损检测技术的研究进展

1.2.5.1 国外无损检测技术的研究进展

美国、日本、德国、加拿大等国家对木材无损检测技术十分重视，而且已经将应力波、超声波、射线、微波等无损检测技术应用到木材物理力学性质、树木生长特性、木材缺陷及木结构建筑和古树古木的保护等方面。

利用无损检测技术对活立木进行早期预测不仅可以快速无损地检测到木材的物理力学性质，对人工林优质树种选择及培育也有非常重要的意义。日本对活立木无损检测技术的研究很深入，主要是利用应力波方法对日本扁柏和柳杉人工林进行材质评估，研究了应力波传播速度与树龄、胸高直径、密度以及含水率之间的关系，并分析了活立木的应力波传播速度与伐倒后原木和方材的弹性模量的相关关系。一系列的研究表明：同一树种，树龄、密度和含水率对应力波的传播速度有一定的影响，但是影响不大，而活立木的波速与原木及方材的弹性模量之间有很大的相关性。由此得出结论：波速是评估活立木机械性质的有效参数。另外，利用应力波的传播速度不仅可以评估活立木的机械性质，还可以追踪探测活立木木材性质的变化，进而确定如何培育社会需求和满意的木材。

活立木的密度也是木材性质中的一个重要因素，传统方法主要是利用生长锥钻取的锥芯测其基本密度，但是对活立木有一定的破坏性，并且消耗时间长。70年代中一种叫

中国知网 https://www.cnki.net

Pilodyn的快速测定活立木木材密度的仪器开发成功，它是一个带有2.5 mm 粗针头的弹簧推射装置，通过一定压力使针头射入立木树干一定部位，用其显示的深度值与木材密度相关的关系，来推定所测立木的木材密度，首先在辐射松成功利用，这种仪器在预测树木的外测密度比较有效[79,80]。在加拿大，Hall[81]在18 个不同林龄的白云杉、黑云杉和北美落叶松中大量试用，并与传统排水法所测的结果相比较以检验其可用性和范围。结果看到Pilodyn的示度值与实测木材密度间相关系数因树种变动在- 0.451～- 0.564 间，表明这一工具的适用价值。近年来此法已经开始应用于阔叶树，甚至木材密度极高的树种上。在主要制浆材桉树的遗传研究中已广为使用，研究表明Pilodyn测定值与实测木材密度的表型相关在0.7 以上，受立地影响不大，在尾叶桉上直接测定的木材密度的遗传力为0.71，用Pilodyn法测得的为0.64，均在强度遗传控制下，证明按照Pilodyn测定的结果进行木材密度的选择是可靠的[82,83]。

1.2.5.2 国内无损检测技术的研究进展

近几年来，我国木材无损检测技术也有一定的研究和发展，但是只是在木材缺陷检测、木材物理性质、木材保护和树木生长特性等方面进行了初步的研究，这些研究也只是处于基础理论和实验阶段。

王志同等[84]人用应力波无损检测技术检测了中密度纤维板弹性模量。东北林业大学的胡英成等[85]人利用振动法对人造板动态弹性模量进行无损检测的研究。林文树等[86]采用超声波和应力波分别对木材试件进行了检测，结果表明：木材密度、孔洞大小及数量对两种波传播参数及动态弹性模量都有不同程度的影响。通过两种波的对比研究，得知二者对木材内部缺陷检测的灵敏度和准确度存在差异。杨学春等[87]简要叙述了应力波检测技术的原理以及在木材性质检测方面的研究进展，在此基础上提出用于木材性质检测的应力波技术的发展趋势。陈清波等[88]人通过对多株树分别采用FAKOPP(应力波速度测度机)和敲打法2种不同方法测量同一树干的杨氏弹性模量，并对2种测定数据进行相关性分析，检验用FAKOPP测量立木状态下树干弹性模量的准确性，结果表明：两者具有很强相关性，说明利用FAKOPP的方法能准确评价树干杨氏弹性模量大小。

1.3 本研究的目的和意义

随着天保工程的实施，天然林已经全部停止砍伐，使得我国的木材供需市场矛盾更加的突出。根据预测[89]到2015年我国木材需求量约为3.4亿m^3，缺口高达1.5亿m^3。从木材结构上看，天然林禁伐后，受影响最大的是大径材的供应。而随着我国人民生活水平的提高，对大径材的需求只会越来越大，依靠进口大径材虽然可解决部分需求，但这不是解决问题的根本办法，主要原因是进口材价格昂贵、资源也有限并且出口国逐步限制出口与采伐天然林，因此必须立足国内进行人工培育大径材，特别是培育一些生长快、木材质量好的用材树种。从目前看，桉树正是能够满足这一要求的最佳树种！桉树中大径材培育时间在8～12年，具有周期短、见效快、效率高等优势，我国一些传统珍贵硬

中国知网 https://www.cnki.net

木如红榉、橡木、红松等的培育时间长达30年以上。因此，大力发展桉树中大径材人工林是一条快速缓解我国用材紧张的新途径[4,90,91]。我国对桉树的引种和栽培已有100多年的历史，取得了巨大的成就。

尾巨桉干形通直，出材率高，材质好，纹理直，耐瘠薄，速生丰产，轮伐期短，是优良的建筑用材和纸浆材树种，有望在家具和室内装饰材料上发挥作用。随着国民经济建设和人民生活水平的不断提高，市场对木材的需求愈来愈大，而且对材种和材质的要求也是越来越高。以小径材种的市场需求比重减少，锯材及其产品在木材需求数量上占有越来越大的份额为特征，是林业木材生产发展的主要方向。桉树是优良的实木利用树种，目前在国内桉树种植是以培育短周期工业用材林为主，我国仍然缺乏对桉树中大径材人工林及其良种的培育[92]。应以市场为向导，在培育短周期工业纸浆材的同时，瞄准国内外市场，培育桉树中大径材良种资源，实行中、大径材与纸浆材混合经营模式，培育中大径材人工林[91]。为探索我国桉树人工林中大径材的育种和栽培潜力。

很多研究表明，桉树锯材材性是受到高水平的遗传控制和培育措施的影响。所以，通过本课题的研究，选择出材性和生长性状兼优，适合作为中大径材培育的优良无性系是有可能的，同时对培育尾巨桉中大径材的栽培技术措施进行探讨，以求得适合桉树中大径材培育的栽培技术。缩短轮伐期，提高桉树人工林的经济效益和社会效益，满足国内市场对中大径材的需求，实现林业的可持续发展都有重要的意义。

本研究得到澳大利亚国际农业研究中心（ACIAR）中、越、澳技术合作项目“通过遗传改良与栽培技术提高桉树木材价值的研究（NO.FST/1999/095）”以及广西壮族自治区林业局科技项目“桉树中大径材良种培育与高产栽培技术研究”的支持。

中国知网 https://www.cnki.net

第二章 研究材料与方法

2.1 尾巨桉无性系生长与材性遗传变异试验材料与方法

2.1.1 试验地概况

本试验是广西东门林场比较系统的桉树无性系试验林，试验区为 73 试验（Expt73），设在雷卡分场 13 林班 13～15 经营班。气候条件：年平均气温 21.2～22.3℃极端最高温度为 38～41℃，极端最低温度为-0.1～1.9℃；年无霜期 346 天，年降雨量 1000～1300 ㎜，降雨主要集中在夏季，年蒸发量 1192～1704 ㎜，相对湿度为 74～83%。土壤为东门地区典型的砖红壤性红壤。前作为柠檬桉，土壤肥力中等，pH 值为 4.5～5.5，坡度为 1～5°。1990 年皆伐柠檬桉后，拔桩、平土，再用带翅犁松土两次，整地深度为 25～30 ㎝。

造林情况：无性系材料来源于东门林场桉树人工杂交试验 50A 和美国佛罗里达杂交桉试验 25 等，试验苗木为 1990 年 5～9 月扦插。于 1991 年 3 月 17 日定植，外围于 4 月 4 日种植，4 月 2 日调查试区死亡株数并同时补植。基肥：第一、二区组于定植前一周施钙镁磷肥 100 克/株作基肥(即 P21.25kg/hm^2)。追肥：4 月中旬施南宁产复合肥 400 克/株(即 N50P50K50kg/hm^2 有效元素)。

2.1.2 试验设计

试区面积 1.92hm^2。采用随机区组设计，60 个无性系，4 个重复，单行小区每行 10 株；株行距 4×2m，区组间距 4m；另外雷林一号桉等枝叶稀疏型树种的 10 个无性系列入一组，组内随机排列。

2.1.3 试验方法

2006 年 9 月份对 60 个无性系 4 个重复进行调查，由于人为等的破坏，有些无性系已经不存在或者数量非常的少，因此本次试验只测定存活 4 株或 4 株以上的无性系。一共测定尾巨桉无性系 25 个，其他杂种的无性系 19 个，分别为巴西巨尾桉 5 个、东门林场巨桉 5 个、东门林场巨尾桉 6 个、东门林场尾赤桉 3 个。同时砍伐 DH32-28、DH33-27、DH32-26、DH15-3、DH61-1 五个无性系一共 20 棵树，每个无性系 4 株。

2.1.3.1 生长性状的测定方法

胸径进行每木调查，胸径用胸径围尺测量，单位为㎝；树高、枝下高选择 4 株能代表这个无性系水平的平均木进行测量，树高、枝下高用瑞典生产的红外线测高仪进行测量，单位为 m。

形质指标的确定：对每个无性系的 4 株测定高的平均木分别进行观察，然后根据等级进行打分。干形指标分 5 个等级：Ⅰ级：主干通直圆满，得 5 分；Ⅱ级：主干直、不圆满，得 4 分；Ⅲ级：主干稍有弯曲、圆满，得 3 分；Ⅳ级：主干稍弯曲、不圆满，得

中国知网 https://www.cnki.net

2 分；Ⅴ级：主干有两个以上弯曲，得 1 分。分枝指标分 4 个等级：Ⅰ级：侧枝小、树冠匀称，得 4 分；Ⅱ级：侧枝中等、树冠匀称，得 3 分；Ⅲ级：侧枝中等、树冠不匀称，得 2 分；Ⅳ级：有大枝、树冠不匀称，得 1 分。

2.1.3.2 材质性状的测定方法

2.1.3.2.1 Pilodyn 测定法

活立木木材密度测定的常规方法是在胸高位置上用生长锥钻取木心，然后用排水法测定其基本密度，现在用一种快捷测定活立木木材密度的方法，用 Pilodyn 仪器测定。Pilodyn 木材检测仪是用来测量活立木及木材建设，如电线杆及水底打桩的木材密度与强度的有用工具。

原理：Pilodyn 是丹麦一家公司研制生产的原本专门用于进行电杆安全检测的一种无损检测仪器，目前广泛用于古建筑木结构和古木保护的检测，在活立木密度检测中也得到了一定的应用。其工作原理是预先将弹簧压缩，有了能量，将连在弹簧前端直径为 2 ㎜的钢针打入木材中（见附图 2-4），钉子射入的深度与木材密度密切相关。木材密度越大，则射入深度越浅；反之，射入深度大。同时通过测定木构件表面硬度，确定木材表层一定深度是否产生缺陷或木材是否发生腐朽。通过简单、无损的方法可快速及客观的检测到不可见的软腐病，并确定与此相关的强度降级。使用 Pilodyn 仪器测定的影响非常轻微，不会对树木造成破坏，所使用的是无损的检测方法。本次用的仪器型号是 Pilodyn 6J（见附图 2-1）。

Pilodyn 使用方法：

〔1〕 在活力木 1.3m 处的东南西北四个方向上用凿子凿开大约 5 ㎝×5 ㎝的窗口，刚好到木质部为宜，如图 2-2 所示，同时用钢尺测定树皮厚度，单位为㎝。

〔2〕 把弹簧压紧，仪器前端的两个支撑脚放在窗口里固定好位置，按下触发盖，把钢针射入木质部里，然后读取数据。

〔3〕 同样的方法分别在其他三个方向进行测定。

2.1.3.2.2 纤维倾斜角度测定

螺旋纹理（spiral grain）是指在活树中木材纤维围绕树轴呈螺旋形排列，左旋或右旋。螺旋纹理的走向与垂直方向形成一个角度，就是纹理倾角，也叫纤维倾角。在树干的不同高度，在同一高度离髓心的不同部位，纤维的倾角不同。其成因目前尚不清楚，但有证据表明与个别树木的遗传特性有关。虽然在活树上通常不容易见到，但螺旋纹理常可从树木表面产生的干燥裂纹的方向，或已经失去树皮的原木上观察到。螺旋纹理对木材物理、力学性质的影响很显著，螺旋纹理的存在降低了木材的强度，干燥时容易发生翘曲，对重要木结构件来说，这是一种严重缺陷，要限制使用。具有螺旋纹理的原木在制材后，会产生斜纹理板材。

纤维倾斜角度测定仪用来测定木材纤维倾角，以木材纹理走向为依据，以水平方向为参照，进而得到一个角度。以垂直方向的纤维倾角为 0°。

中国知网 https://www.cnki.net

纤维倾斜角度测定仪测定方法：

① 仪器准备。仪器：沿纹理滑动的划针、主设备。 探针去保护帽。

② 开窗。位置在树干 1.30m 处四个方向切开树皮，深度至木质部可见木材纤维走向。

③ 划针。沿待测木材纤维走向划动，注意划动时一定要手握下部转轴而不可握连杆，否则极易造成人为干扰。

④ 测定。主设备两探针对准压在划出的纹理线上，固定探针，转动显示屏连接轴至水平仪水平，读数。如附图 2-5。

⑤ 数据整理记录。

2.1.3.2.3 原木质量（缺陷）的测定方法

野外工作：对选定的活立木用油漆标出南北向，将活立木伐倒，从端部的锯口开始量取两段原木，根部为 A 段原木，另外一段为 B 段原木，每段长约 1.37m，剥皮，在原木上作好南北方向和 AB 段原木标记。

室内工作：对每一根原木进行木材缺陷指标的测定，主要是数节子的数量，测量弯曲度，大头和小头的直径，端部裂纹，木材腐朽等，做好记录。主要工具是钢尺。

2.1.4 数据处理

数据采用 SPSS（Statistical Product and Service Solutions）软件和 SAS（Statistical Analysis System）软件进行统计分析，并参考马育华[93]1982 年编的数量遗传学基础知识计算各性状遗传参数以及方差分析等，应用主成分距离分析法、简单指数选择法对尾巨桉无性系进行综合选择。

2.1.4.1 各性状平均值、单株材积、标准差的计算公式

$$\overline{X}=\frac{\sum_{i=1}^{n}x_i}{n} \quad\cdots\cdots（2-1）$$

$$S=\sqrt{\frac{\sum_{i=1}^{n}\left(X_i-\overline{X}\right)^2}{n-1}} \quad\cdots\cdots（2-2）$$

公式中：$\overline{X}$ 表示性状的平均值；S 表示性状表型标准差；

$$V=C_0\times D^{[C_1-C_2(D+H)]}\times H^{[C_3+C_4(D+H)]} \quad\cdots\cdots（2-3）$$

单株材积采用广西林业勘查设计院研制的广西速生桉单株材积计算公式。

V 为单株材积；D 为胸径；H 为树高。

C_0=0.000109154150；C_1=1.87892370；C_2=0.00569185503；

C_3=0.65259805；C_4=0.00784753507。

中国知网 https://www.cnki.net

2.1.4.2 主要遗传参数估算公式

变异系数：$C.V=\frac{S}{\overline{X}}\times 100\%$ ……………………………………………… (2-4)

无性系重复力：$R_I=\frac{\sigma_b^2}{\left(\sigma_b^2+\frac{\sigma_w^2}{r}\right)}$ ……………………………………………… (2-5)

选择响应：$R=i\times h^2\times\sigma_p$ ……………………………………………… (2-6)

遗传增益：$\Delta G=\frac{i\times h^2\times\sigma_p}{\overline{X}}\times 100\%$ ……………………………………………… (2-7)

公式中：$\sigma_b{}^2$是无性系方差分量；$\sigma_w{}^2$是机误方差分量；r 是重复次数；i 是选择强度；h^2是遗传力；σ_p是性状表型标准差。

简单指数方程：$I=\frac{w_1h_1{}^2\left(X_1-\overline{X}\right)}{\sigma_1}+\frac{w_2h_2{}^2\left(X_2-\overline{X}\right)}{\sigma_2}+\cdots+\frac{w_ih_i{}^2\left(X_i-\overline{X}\right)}{\sigma_i}$ ……… (2-8)

其中 I 表示指数值，1，2，3………i 表示参与选择的性状，w 表示性状的经济权重，X_i表示各个无性系的性状平均值，h^2表示性状遗传力，$\overline{X}$表示性状平均值，σ_i表示性状的标准差。

2.2 尾巨桉栽培密度与施肥试验材料与方法

2.2.1 试验地概况

试验区为 87 试验(Expt87)，位于广西东门林场华侨分场 21 林班，年平均气温 21.2～22.3℃，极端最高温为 38～41℃，极端最低温为-0.1～1.9℃，年降雨量为 1000～1300mm，土壤为砖红壤性红壤，前作为柠檬桉，土壤肥力中等，pH 值为 4.5～5.5，向南坡，坡度 5～10 度。机耕带根全垦整地，深度 30～35cm。苗木为无性系扦插苗（营养杯），苗高 15～25cm，树种为东门桉树杂交种（尾巨桉无性系 DH32-13）。以钙镁磷作基肥，100g/株。1993 年 4 月 2 日定植。追肥：分两次进行，第一次于定植后两个月内进行，第二次 1996 年 6 月进行。

2.2.2 试验设计

试验区面积为 6.67 公顷，试验为裂区设计，设两个因子(密度、施肥量)，每个因子设 6 个水平，密度因子作主区，肥料因子作副区，4 个重复，144 个小区。密度、施肥各处理水平见（表 2-1、表 2-2）。

中国知网 https://www.cnki.net

表 2-1　尾巨桉 6 种密度试验设计

Tab.2-1　6 Spacing treatments design for *E.urophylla* × *E.grandis*

处理号	株数/hm^2	行距（m）	株距（m）
1	2222	3	1.5
2	1667	3	2
3	1250	4	2
4	883	4	3
5	667	5	3
6	1250	6.0×2.0	2

表 2-2　尾巨桉 6 种施肥试验设计

Tab.2-2　6 apply fertilizer treatments design for E.urophylla × E.grandis

处理号	总施肥量（kg）	第 1 次追肥（kg）	第 2 次追肥（kg）
1	N300P200K200	N150P100K100	N150P100K100
2	N200P100K150	N100P50K50	N100P50K100
**3**	**N200P150K100**	**N100P50K50**	**N100P100K50**
4	N150P150K100	N100P100K50	N50P50K50
5	N100P50K50	N50P0K50	N50P50K0
6	N100P150K150	N50P50K50	N50P100K100

表 2-3　测定方案

Tab.2-3　Standing trees assessments per repetition

Experiment87 施肥处理		密度处理					
		1	2	3	4	5	6
		3.0 x 1.5	3.0x2.0	**4.0x2.0**	4.0x3.0	5.0x3.0	6.0x2.0x2.0
1	N300P200K200			**24**			
2	N200P100K150			**24**			
**3**	**N200P150K100**	**24**	**24**	**24**	**15**	**10**	**16**
4	N150P150K100			**24**			
5	N100P50K50			**24**			
6	N100P150K150			**24**			

但是本次试验没有考虑密度和施肥的互作效应，只是在同一个密度处理 3 水平上测定不同施肥处理的各个小区，和测定同一个施肥处理 3 水平上不同密度处理的各个小区，一共 44 个小区，测定方案见（表 2-3）。另外，在施肥处理 3 水平上选定 6 种密度处理林分的活立木，选择的原则是按胸径分大中小选 1 株，伐倒进行原木缺陷的测定，第一重复选了 16 株树，第二重复选了 19 株树，第三重复选了 19 株树，第四重复选了 15 株树，一共 69 株树。

中国知网　https://www.cnki.net

2.2.3 试验方法

2.2.3.1 生长性状的测定方法

从 1994 年到 2005 年一直测定其树高、胸径，所以这些数据可以用来分析连年生长情况。2006 年 9 月份对 44 个小区进行每木调查，胸径用测径卡尺，分别测定行距方向上的胸径和株距方向上的胸径，单位为cm；树高、枝下高用瑞典生产的测高仪进行测定，单位为 m。

2.2.3.2 Pilodyn、纤维倾角测定法

Pilodyn 测定法跟 2.1.3.2.1 的测定方法一样；纤维倾角测定方法同 2.1.3.2.2。

2.2.3.3 FAKOPP 测定

FAKOPP 是一种应力波仪器。该仪器用于活立木、原木和锯材的检测，可以检测树木中的空洞、腐朽、裂纹、应力以及评估原木的硬度。FAKOPP”是匈牙利单词“fa”和“kopp”’的组合，意思是“树木”和“敲击”。

在木材利用方面，木材的强度与材内腐烂程度是评价木材好坏的重要指标。在开展材质育种时必须在测定林中做大量的材质检测调查，能在活立木状况下进行简便测量的手段就显得尤为重要。而本次用来测定的声波测试仪 FAKOPP 就是非常简便快捷的测定仪器。在活立木测定时主要测两个方向，即平行和垂直于行的方向。

（1）测定原理

应力波是指物质受敲打之后，因内应力作用而产生的可在物质内传播的机械波。应力波测定技术是基于应力波通过被测材料的速度与被测材料密度、弹性模量的物理关系而建立起来的。给发信端探测器一定冲击力，由该冲击力产生的应力波向接收方探测器传播，测量其传播时间。测量值为时间（micro sec.)，将其代入换算公式中就可算出应力波传播速度 m/s。应力波传播速度（m/s）=探测器距离（m）/（测量时间（micro sec.）$/10^6$)，此处动态弹性模量与轴向应力波传播速度之间的关系如下式所示：

$$E=\rho V_e^2$$

$$V_e=\frac{L}{S}$$

其中 V_e：音速；E：弹性模量；ρ：密度；L:传播距离；T：传播时间

当木材发生腐朽或虫蛀时,垂直于木材纹理方向的传播速度急速增加。若应力波传播速度增加 30% ，表明木材强度损失已达到 50%；若应力波传播速度增加 50% ,则意味着木材遭到了严重损害；横向（径向或弦向）是探测腐朽的最佳途径。在进行无损检测时,常以健康材应力波的传播速度为标准来判断分析被检测材是否腐朽。

（2）FAKOPP 测定方法：

① 仪器整理。将 START 探测器插入 START 探测器用接头孔中。将 STOP 探测

中国知网 https://www.cnki.net

器插入 STOP 探测器用接头孔中。

② 仪器固定。将 START 探测器与 STOP 探测器分别固定在测量位置。固定方法为：用锤子轻轻敲打探头部位，沿 45°角将其敲入树干，深度为 2cm，探头部位朝下；然后将 START 探头固定在距地面 0.30m 处的树干上，此时探头部分朝上，与树干呈 45°角。上下两部件间矩 1.50m（为了使用方便，通常会做好标定距离悬线）。如附图 2-6，2-7，2-8。

③ 测定。打开电源开关，液晶画面上出现“8888”的读数，用锤子轻轻敲打 START 探头，力度约为 100g 至 200g 左右。第一次击打显示一个相对较大的数据（估计此值是消除噪音），而后连续敲击 5 次，记下测量值。按下 RESET 按钮。分别测定株向和行向两方向的数据。如附图 2-9。

④ 数据整理：记录、输入、整理、利用。

注意：a. 敲击时要用力均匀而稳定，这样可以减少实验的人为误差；b. 仪器要避开节子。

2.2.3.4 原木质量（缺陷）测定方法

方法同 2.1.3.2.3 的方法一致。

2.2.4 数据处理

数据采用 SPSS（Statistical Product and Service Solutions）软件和 SAS（Statistical Analysis System）软件进行统计分析。

中国知网 https://www.cnki.net

第三章 结果与分析

3.1 尾巨桉无性系生长、材质性状遗传变异试验结果与分析

3.1.1 生长性状、形质性状和材性性状差异分析

从 25 个尾巨桉无性系 9 个性状方差分析（见表 3-1）结果表明：25 个尾巨桉无性系在生长性状（胸径 DBH、树高 H、单株材积 Vol、枝下高 BH）、形质性状（干形 SF、分枝 Br）、材性性状（木材外部密度指标值 Pilodyn、树皮厚度 BT、纹理倾角 SG）间差异极显著（显著水平 a=0.01）；在这 9 个性状中，F 值最大的是 Pilodyn，为 20.66，F 值最小的是分枝（Br）为 4.04，胸径、树高、单株材积、枝下高、树皮厚度、纹理倾角的 F 值在 10 左右。各性状之间的差异性为进一步无性系筛选提供了依据。

表 3-1 尾巨桉无性系 9 个性状方差分析

Tab.3-1 Variance analysis for 9 characters of *E.urophylla*×*E.grandis* clones

性状	差异源	df	SS	MS	F	性状	差异源	df	SS	MS	F
	无性系间	24	640.09	26.67	11.24^{**}		无性系间	24	21.78	0.91	4.04^{**}
DBH	重复间	3	33.08	11.03	4.65^{**}	Br	重复间	3	2.48	0.83	3.68^{*}
	误差	72	170.89	2.37			误差	72	16.16	0.22	
	总计	99	844.06				总计	99	40.42		
	无性系间	24	599.71	24.99	8.67^{**}		无性系间	24	118.83	4.95	20.66^{**}
H	重复间	3	93.43	31.14	10.81^{**}	Pilodyn	重复间	3	2.07	0.69	2.88^{*}
	误差	72	207.40	2.88			误差	72	17.25	0.24	
	总计	99	900.54				总计	99	138.15		
	无性系间	24	2.18	0.09	13.51^{**}		无性系间	24	151.82	6.33	12.11^{**}
Vol	重复间	3	0.08	0.03	4.16^{**}	BT	重复间	3	6.51	2.17	4.16^{**}
	误差	72	0.48	0.01			误差	72	37.60	0.52	
	总计	99	2.75				总计	99	195.93		
	无性系间	24	1090.09	45.42	8.98^{**}		无性系间	24	1223.05	50.96	10.54^{**}
BH	重复间	3	118.43	39.48	7.80^{**}	SG	重复间	3	20.41	6.80	1.41
	误差	72	364.31	5.06			误差	72	348.13	4.84	
	总计	99	1572.83				总计	99	1591.58		
	无性系间	24	56.63	2.36	5.54^{**}						
SF	重复间	3	3.95	1.32	3.09^{*}						
	误差	72	30.68	0.43							
	总计	99	91.26								

注：无性系间 $F_{0.05}$=1.67，$F_{0.01}$=2.06；重复间 $F_{0.05}$=2.73， $F_{0.01}$=4.06；‘*’‘**’分别表示在 5%和 1%水平上差显著。

中国知网 https://www.cnki.net

表 3-2　尾巨桉 25 个无性系生长性状和材质性状的平均值

Tab.3-2　Mean values of growth and wood quality of 25 E.urophylla × E.grandis clones

Clone	DBH (cm)	H (m)	Vol (m^3)	BH (m)	SF	Br	Pilodyn	BT (mm)	SG
DH33-27	25.0	32.8	0.7722	20.1	3.0	2.2	16.1	5.8	4.4
DH32-28	24.9	33.3	0.7715	19.3	4.4	3.4	16.2	8.3	3.6
DH33-9	22.6	33.5	0.6499	17.9	3.1	2.5	15.1	4.4	3.8
DH32-26	22.4	32.4	0.6153	21.2	4.0	2.5	14.2	7.1	7.4
DH30-7	22.2	30.4	0.5608	14.7	3.4	2.1	15.9	7.8	7.0
DH33-20	21.5	33.0	0.5963	19.1	3.9	2.7	15.3	5.1	3.1
DH32-11	21.4	31.3	0.5442	17.9	2.9	2.1	16.3	6.9	3.8
DH30-1	20.7	30.8	0.5016	16.5	3.1	2.5	14.3	4.3	5.3
DH29-7	20.5	31.0	0.4986	17.5	3.1	1.9	15.5	5.5	7.9
DH32-30	19.8	31.0	0.4656	17.2	3.6	2.6	14.9	5.9	8.7
DH17-2	19.8	29.9	0.4653	11.0	1.8	1.6	13.1	7.7	9.7
DH26-2	19.8	29.8	0.4395	17.1	2.2	1.9	14.8	4.2	3.2
DH32-22	19.5	30.0	0.4374	19.9	3.8	2.5	15.0	6.3	3.9
DH32-21	19.1	27.3	0.3699	14.4	3.1	2.3	17.1	6.5	9.5
DH32-25	18.3	29.3	0.3724	16.9	3.7	2.4	15.0	5.2	6.3
DH29-10	18.2	32.2	0.4241	18.7	3.2	2.5	12.8	5.5	10.7
DH16-8	18.2	28.2	0.3527	17.2	2.4	1.9	15.6	6.8	5.8
DH29-8	18.2	25.2	0.3053	16.8	1.9	1.8	15.5	5.7	5.1
DH16-6	18.1	29.4	0.3699	18.3	2.2	1.6	15.0	6.8	6.4
DH15-3	17.9	26.5	0.3182	12.8	2.3	1.5	12.6	6.1	16.2
DH15-1	17.7	27.6	0.3284	12.7	1.9	1.6	13.9	8.2	16.0
DH30-5	17.5	27.1	0.3124	6.5	2.8	1.5	15.1	5.0	6.4
DH33-32	16.1	26.9	0.2674	13.8	2.6	2.0	16.1	3.8	8.1
DH13-3	15.9	27.3	0.2666	13.7	1.9	1.5	14.6	6.4	6.3
DH17-5	14.8	25.6	0.2149	12.2	1.6	1.8	16.0	4.8	11.4
总均值	19.6	29.7	0.4488	16.1	2.9	2.1	15.0	6.0	7.2

由于数据众多，在多重比较表中只列出前 12 个无性系的 9 个性状，多重比较表(表 3-3)结合生长性状和材质性状平均值表(表 3-2)可以看出：单株材积大于总体平均值的无性系有 11 个，其中生长性状最优的是无性系 DH33-27，其胸径、树高、单株材积、枝下高分别为 25.0 cm、32.8m、0.7722m^3、20.1m，是平均值的 127.7%、110.5%、172.1%、125.1%，是最差无性系 DH17-5 的 169.7%、128.4%、359.3%、165.6%。干形指标大于总体平均值的无性系有 13 个，干形最好的是 DH32-28，分枝指标大于总体平均值的无性系有 11 个，分枝指标最好的无性系是 DH32-28。在材质方面，木材外部密度指标值优于总体平均值的无性系有 9 个，外部密度指标值最优的无性系是 DH15-3，为 12.6，是最大的 73.5%；树皮厚度小于总体平均值的无性系有 13 个，其中最小的是无性系 DH33-32，仅为 3.8mm；纤维（纹理）倾角优于总体平均值的无性

中国知网 https://www.cnki.net

表 3-3　尾巨桉无性系 9 个性状邓肯多重比较（a=0.01）

Tab.3-3　Mean values of 9 characters among *E.urophylla* × *E.grandis* clones with Duncan's multiple range test（a=0.01）

Clone	DBH	Duncan	Clone	H	Duncan	Clone	VOL	Duncan	Clone	Pilodyn	Duncan	Clone	BH
DH33-27	25.0	A	DH33-9	33.5	A	DH33-27	0.7722	A	DH32-21	17.1	A	DH32-26	21.2
DH32-28	24.9	B A	DH32-28	33.3	A	DH32-28	0.7715	A	DH32-11	16.3	B A	DH33-27	20.1
DH33-9	22.6	B A C	DH33-20	33.0	A	DH33-9	0.6499	B A	DH32-28	16.2	B A C	DH32-22	19.9
DH32-26	22.4	B A C	DH33-27	32.8	A	DH32-26	0.6153	B A C	DH33-32	16.1	B A C	DH32-28	19.3
DH30-7	22.2	B A C	DH32-26	32.4	B A	DH33-20	0.5963	B D A C	DH33-27	16.1	B A C	DH33-20	19.1
DH33-20	21.5	B D C	DH29-10	32.2	B A	DH30-7	0.5608	B D C	DH17-5	16.0	B A C	DH29-10	18.7
DH32-11	21.4	E D C	DH32-11	31.3	B A C	DH32-11	0.5442	B D E C	DH30-7	15.9	B A C	DH16-6	18.3
DH30-1	20.7	E F D C	DH29-7	31.0	B A C	DH30-1	0.5016	F B D E C	DH16-8	15.6	B D E C	DH33-9	17.9
DH29-7	20.5	E F D C	DH32-30	31.0	B A C	DH29-7	0.4986	F B D E C	DH29-7	15.5	B D E C	DH32-11	17.9
DH32-30	19.8	E F D C	DH30-1	30.8	B D A C	DH32-30	0.4656	F B D E C G	DH29-8	15.5	B D E C	DH29-7	17.5
DH17-2	19.8	E F D C	DH30-7	30.4	B D A C	DH17-2	0.4653	F B D E C G	DH33-20	15.3	B D E C	DH32-30	17.2
DH26-2	19.8	E F D C	DH32-22	30.0	B D A C	DH26-2	0.4395	F H D E C G	DH30-5	15.1	F D E C	DH16-8	17.2

续表

Duncan	Clone	Br	Duncan	Clone	SF	Duncan	Clone	BT	Duncan	Clone	SG	Duncan
A	DH32-28	3.4	A	DH32-28	4.4	A	DH32-28	8.3	A	DH15-3	16.2	A
B A	DH33-20	2.7	B A	DH32-26	4.0	B A	DH15-1	8.2	A	DH15-1	16.0	A
B A C	DH32-30	2.6	B A C	DH33-20	3.9	B A	DH30-7	7.8	B A	DH17-5	11.4	B
B D A C	DH32-22	2.5	B D A C	DH32-22	3.8	B A	DH17-2	7.7	B A	DH29-10	10.7	C B
B D A C	DH32-26	2.5	B D A C	DH32-25	3.7	B A C	DH32-26	7.1	B A C	DH17-2	9.7	C B D
B D A C	DH29-10	2.5	B D A C	DH32-30	3.6	B A C	DH32-11	6.9	B D A C	DH32-21	9.5	C B D
E B D A C	DH33-9	2.5	B D A C	DH30-7	3.4	B D A C	DH16-6	6.8	B D A C	DH32-30	8.7	C E B D
E B D A C	DH30-1	2.5	B D A C	DH29-10	3.2	E B D A C	DH16-8	6.8	E B D A C	DH33-32	8.1	F C E B D
E B D A C	DH32-25	2.4	B D A C	DH33-9	3.1	E B D A C F	DH32-21	6.5	E B D F C	DH29-7	7.9	F C E B D
E B D A C F	DH32-21	2.3	B D A C	DH30-1	3.1	E B D A C F	DH13-3	6.4	E B D F C G	DH32-26	7.4	F C E B D
E B D A C F	DH33-27	2.2	B D C	DH32-21	3.1	E B D A C F	DH32-22	6.3	E B D F C G	DH30-7	7.0	F C E B D
E B D A C F	DH32-11	2.1	B D C	DH29-7	3.1	E B D A C F	DH15-3	6.1	E B D F C G	DH16-6	6.4	F C E D

中国知网 https://www.cnki.net

系有 16 个，其中最优的是无性系 DH33-20，为 3.1。

3.1.2 性状遗传参数的估算

无性繁殖植物的原株与分株之间不存在亲子关系，只是原株与繁殖体的关系，同一无性系不同个体具有相同的基因型，过去在林业上把无性系的变异分析归为遗传力，这是不确切的。从上个世纪八十年代后期开始，美国林木遗传学家孔繁浩教授和日本林木育种专家古越隆信教授在他们的著作中都提到了林木无性系的变异应该叫重复力。重复力（Repeatability）是指同一基因型的生物个体在不同时间或不同地点的表型持续稳定的程度，无性繁殖植物的重复力与有性繁殖植物的遗传力具有类似的意义。

从表 3-4 可知：各个性状的表型变异系数在 7.92%～56.19%，遗传变异系数在 7.22%～47.17%，各个性状的变异系数差异很大，遗传变异系数和表型变异系数最大的都是纤维倾角（SG），分别为 47.17%、56.19%，最小的是 Pilodyn（活立木外部密度指标值），分别为 7.22%、7.92%，另外，变异系数比较大的还有 SF（干形）、Vol（材积）；各个性状无性系重复力在 0.7263～0.9480，无性系重复力的水平非常的高，最大的是 Pilodyn，达到了 0.9480，最小的是 Br（分枝），也达到了 0.7263。Pilodyn 的重复力最大，而变异系数最小，其稳定性最好。

表 3-4　尾巨桉无性系 9 个性状遗传参数分析结果

Tab. 3-4　Genetic analysis of 9 characters of E.urophylla×E.grandis clones

遗传参数	DBH	H	Vol	BH	SF	Br	Pilodyn	BT	SG
总平均值	19.6	29.7	0.4488	16.1	2.9	2.1	15.0	6.0	7.2
标准差	2.9199	3.016	0.1668	3.9859	0.9601	0.6389	1.1813	1.4068	4.0096
表型变异系数%	14.83	9.77	37.15	24.13	45.22	21.89	7.92	23.35	56.19
遗传变异系数%	12.58	7.92	32.34	19.69	32.97	14.39	7.22	20.03	47.17
无性系重复力	0.8980	0.8395	0.9166	0.8583	0.8043	0.7263	0.9480	0.9070	0.9036

3.1.3 性状间的相关分析

从各个性状的相关分析（见表 3-5）可知：单株材积与胸径、树高呈极紧密的正相关，相关系数超过了 0.9，通过对胸径、树高的改良可以显著地提高单株材积水平，另外，胸径与树高和枝下高、分枝、干形之间的相关性达中等偏上，相关系数达 0.6 左右，相关性极显著，对胸径和树高的改良有望同时实现对枝下高、干形、分枝的改良；Pilodyn 与胸径、单株材积、枝下高、干形、分枝、树皮厚度呈弱度正相关，而与树高呈弱度负相关，与 SG 呈中度负相关，相关性在 5%水平上显著；BT（树皮厚度）与胸径、树高、单株材积、枝下高、干形、分枝、纤维倾角呈弱度正相关，与 Pilodyn 呈弱度负相关；SG 除了与 BT 呈轻度正相关之外，与其他 7 个性状呈中度负相关，相关性在 5%水平上

中国知网 https://www.cnki.net

显著。Pilodyn（活立木外部木材密度指标值）与生长性状之间呈轻度正相关或负相关，这就可以选择生长迅速的无性系而不会导致木材密度的显著降低或者升高，通过生长与材性联合选择，有望选择出生长迅速、材质优良的无性系。

表 3-5　尾巨桉无性系 9 个性状相关关系

Tab. 3-5　Cerrelation coefficients between 9 characters of *E.urophylla*×*E.grandis* clones

相关系数	DBH	H	Vol	BH	SF	Br	Pilodyn	BT	SG
DBH	1								
H	0.835**	1							
Vol	0.982**	0.902**	1						
BH	0.590**	0.676**	0.619**	1					
SF	0.650**	0.706**	0.668**	0.581**	1				
Br	0.638**	0.717**	0.683**	0.657**	0.886**	1			
Pilodyn	0.191	-0.031	0.159	0.142	0.209	0.252	1		
BT	0.268	0.101	0.222	0.052	0.069	0.027	-0.071	1	
SG	-0.490*	-0.470*	-0.497*	-0.493*	-0.426*	-0.445*	-0.486*	0.226	1

注：‘*’‘**’分别表示在 5%和 1%水平上差异显著。

3.1.4 主成分分析

主成分分析是把原来多个指标化为少数几个相互独立综合指标的一种多元统计方法。在进行主成分分析时，实际数据的单位不一致，给分析带来不便，故先将数据进行标准化变换。运用计算协方差矩阵法对 25 个无性系 9 个性状的平均值进行计算，得出特征向量、特征根、贡献率、累积贡献率，如表 3-6 所示。

根据累积贡献率大于 85%为原则确定主成分。前 4 个主成分累积贡献率已达到 88.97%，取前 4 个主成分已经能代表 88.97%的信息，故选前 4 个主成分即可，根据计算得到的特征向量，可以写出第 1 至第 4 主成分方程：

$$y_1=0.4025x_1+0.4026x_2+0.4134x_3+0.3465x_4+0.3729x_5+0.3825x_6+0.1263x_7+0.0550x_8-0.2840x_9$$

$$y_2=0.1601x_1+0.1766x_2+0.1610x_3-0.0238x_4+0.0123x_5-0.0491x_6-0.5725x_7+0.5871x_8+0.4917x_9$$

$$y_3=0.1613x_1-0.2140x_2+0.0771x_3-0.1603x_4-0.1096x_5-0.1404x_6+0.6392x_7+0.6710x_8-0.0769x_9$$

$$y_4=-0.2953x_1-0.2131x_2-0.2882x_3+0.0069x_4+0.5498x_5+0.5246x_6+0.1880x_7+0.1042x_8+0.4006x_9$$

从 4 个主成分方程结构式可以看出：y_1 的 x_1、x_2、x_3 的系数较大，y_1 的值受到胸径、树高、材积的影响最大，即胸径、树高、材积越大，y_1 值也越大，可以把第一主成分称为生长量因子；y_2 的结构式中活立木外部密度指标值 x_7、树皮厚度 x_8、纤维倾角 x_9 的系数较大，但是活立木外部密度指标值 x_7 的系数为负值，树皮厚度 x_8、纤维倾角 x_9 的系数为正值，表明 y_2 值越小，活立木外部密度指标值越大，即外部木材密度越小，y_2

中国知网 https://www.cnki.net

值越大，树皮厚度、纤维倾角越大，可以把第二主成分称为第一材性因子（树皮厚度和纤维倾角）；y_3 的结构式中活立木外部密度指标值 x_7、树皮厚度 x_8 系数较大，y_3 值的大小受到活立木外部密度指标值和树皮厚度的影响最大，因此第三主成分也可称为第二材性因子（活立木外部密度指标值和树皮厚度）；y_4 的结构式中干形 x_5、分枝 x_6 的系数最大，y_4 值的大小受到干形 x_5、分枝 x_6 的影响最大，因此，第四主成分可称为形质因子（干形和分枝）。

把无性系各个性状经过标准化的数据代入上述 4 种主成分方程，所得到的 y 值如表 3-7 所示。第一主成分值最大的是 DH32-28 无性系，排前 5 位的无性系为 DH32-28、DH33-27、DH33-20 、DH32-26、DH33-9，这几个无性系生长表现非常理想，生长迅速，生长量大，如果单单考虑生长量方面的因素，这 5 个无性系是最优的；第二主成分是材树皮厚度和纤维倾角因子，主成分值大的无性系树皮比较厚且纤维倾角较大，但是木材外部密度指标值小，主成分值排前 5 位的无性系是 DH15-1、DH17-2、DH15-3、DH32-26、DH29-10；第三主成分是活立木外部密度指标值和树皮厚度因子，主成分值大的活立木外部密度指标值较大，树皮较厚，前 5 位无性系依次为 DH30-7、DH32-21、DH32-28、DH32-11、DH16-8；第四主成分是形质因子，主成分值大，干形和分枝好，主成分值排前 5 位的无性系是 DH32-21、DH32-25、DH32-30、DH32-28、DH33-32。

表 3-6　主成分分析的特征向量和特征根表

Tab. 3-6　Eigenvectors and Latent root of principal component analysis

特征向量	PRIN1	PRIN2	PRIN3	PRIN4
X1 胸径（DBH）	0.4025	0.1601	0.1613	-0.2953
X2 树高（H）	0.4026	0.1766	-0.2140	-0.2131
X3 单株材积（Vol）	0.4134	0.1610	0.0771	-0.2882
X4 枝下高（BH）	0.3465	-0.0238	-0.1603	0.0069
X5 干形（SF）	0.3729	0.0123	-0.1096	0.5498
X6 分枝（Br）	0.3825	-0.0491	-0.1404	0.5246
X7 外部密度（Pilodyn）	0.1263	-0.5725	0.6392	0.1880
X8 树皮厚度（BT）	0.0550	0.5871	0.6710	0.1042
X9 纤维倾角（SG）	-0.2840	0.4917	-0.0769	0.4006
特征根	5.0031	1.4067	0.9504	0.6471
贡献率（%）	0.5559	0.1563	0.1056	0.0719
累积贡献率（%）	0.5559	0.7122	0.8178	0.8897

以这 4 个主成分值为依据，采用最短距离法进行系统聚类分析，得到主成分遗传距离聚类结果树状图（见图 3-1），如果阈值取 2.1，则把 25 个无性系分为 10 类，每一类的无性系、第一到第四主成分值的平均值见表 3-8。

从聚类结果表 3-8 结合表 3-1 和表 3-7 可以知道：第 I 类，生长量比较小，生长表现不好，而且形质指标也不好，外部木材密度指标值和树皮厚度属于中等水平；第 II 类，这一类无性系最多，是比较综合的，生长表现比较好，树皮厚度和纤维倾角较小，在此

中国知网　https://www.cnki.net

类中还可以根据需要再进一步的选择；第III类，此类无性系树皮厚度较小、而且外部密度指标值小（即木材密度较大），材质较好；第Ⅳ类，生长表现不理想，形质、材质较差；第Ⅴ类，生长表现非常理想，外部木材密度指标值小，纤维倾角属于中等水平，但是树皮较厚；第Ⅵ类，生长表现很不理想，树皮薄，纤维倾角大，形质指标差；第Ⅶ类，生长表现不理想，树皮厚度和纤维倾角较小；第Ⅷ类，生长表现非常的不理想，树皮厚，纤维倾角大，但是外部木材密度指标值最小；第Ⅸ类，生长表现不好，树皮厚，纤维倾角大；第Ⅹ类，生长表现是最好的，而外部木材密度指标值较大，纹理倾角很小，形质指标较好。

表 3-7 尾巨桉无性系前 4 个主成分值

Tab. 3-7 Score of first to fourth principal component of *E.urophylla*×*E.grandis* clones

第一主成分		第二主成分		第三主成分		第四主成分	
无性系	y_1值	无性系	y_2值	无性系	y_3值	无性系	y_4值
DH32-28	5.0114	DH15-1	2.5482	DH30-7	1.6462	DH32-21	1.4487
DH33-27	3.1672	DH17-2	2.3029	DH32-21	1.5624	DH32-25	1.0738
DH33-20	2.8808	DH15-3	2.2022	DH32-28	1.4369	DH32-30	1.0409
DH32-26	2.6939	DH32-26	1.4885	DH32-11	1.2414	DH32-28	0.9776
DH33-9	2.4939	DH29-10	1.4480	DH16-8	0.8645	DH33-32	0.7701
DH32-11	1.4679	DH32-28	0.7867	DH15-1	0.7885	DH32-22	0.7547
DH32-22	1.4474	DH30-7	0.7315	DH29-8	0.5828	DH29-10	0.6374
DH30-7	1.1353	DH32-30	0.3152	DH33-27	0.5786	DH17-5	0.6318
DH32-30	1.0194	DH16-6	0.1140	DH16-6	0.4617	DH15-1	0.3404
DH30-1	0.9654	DH29-7	-0.1599	DH13-3	0.2975	DH32-26	0.3381
DH29-7	0.5494	DH33-27	-0.1664	DH17-2	0.2859	DH15-3	0.2293
DH29-10	0.3003	DH13-3	-0.2439	DH17-5	0.2168	DH33-20	0.1055
DH32-25	0.2684	DH32-22	-0.3518	DH30-5	0.1927	DH30-7	0.0499
DH26-2	-0.1644	DH32-11	-0.3934	DH29-7	-0.0846	DH16-8	-0.1030
DH32-21	-0.5378	DH16-8	-0.4067	DH32-22	-0.2634	DH30-5	-0.2025
DH16-8	-0.8198	DH30-1	-0.5190	DH32-26	-0.4011	DH30-1	-0.2224
DH16-6	-0.9387	DH33-9	-0.6381	DH33-32	-0.4806	DH29-7	-0.2796
DH17-2	-1.7311	DH33-20	-0.6906	DH32-30	-0.5695	DH29-8	-0.4149
DH29-8	-1.8192	DH32-25	-0.7030	DH32-25	-0.7729	DH13-3	-0.5587
DH33-32	-2.0459	DH32-21	-0.8188	DH33-20	-0.8092	DH32-11	-0.6314
DH30-5	-2.6699	DH30-5	-0.9558	DH26-2	-0.9209	DH16-6	-0.7460
DH13-3	-2.6923	DH29-8	-1.2384	DH33-9	-1.0343	DH33-9	-0.9911
DH15-1	-2.9972	DH26-2	-1.2785	DH15-3	-1.0509	DH17-2	-1.3526
DH15-3	-3.3120	DH17-5	-1.2982	DH30-1	-1.4872	DH26-2	-1.3999
DH17-5	-3.6725	DH33-32	-2.0748	DH29-10	-2.2813	DH33-27	-1.4958

中国知网 https://www.cnki.net

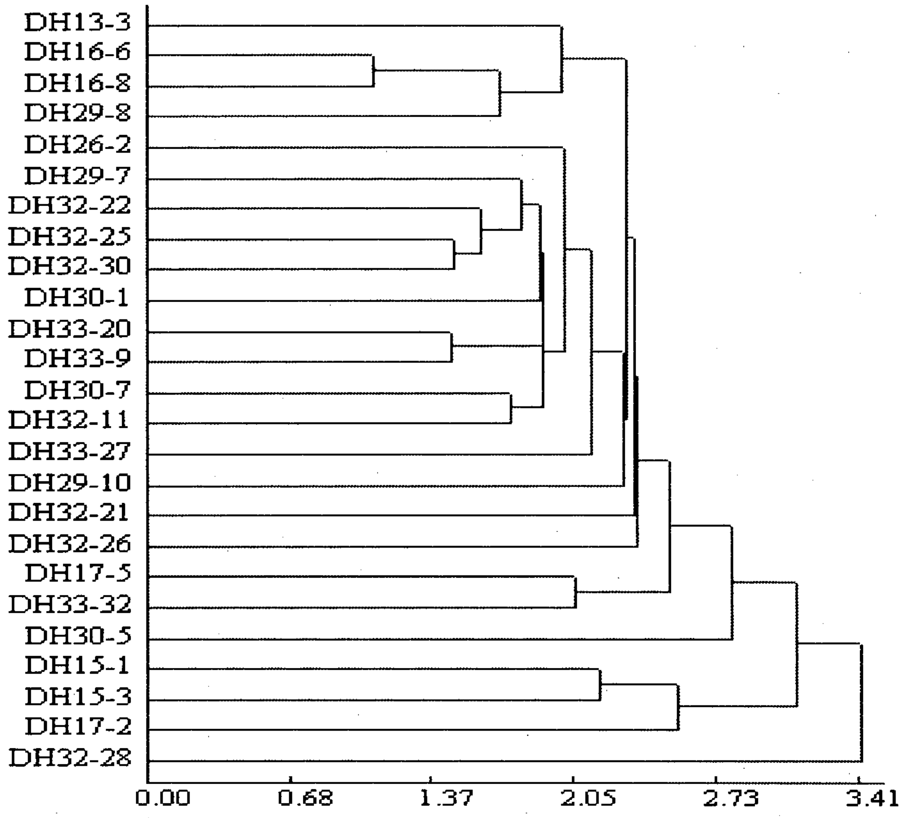

图 3-1　　聚类树状图

Fig.3-1　　Dendrogram of cluster

表 3-8　　聚类结果以及主成分平均值

Tab. 3-8　　Cluster result and the mean values of principal component

分类	无性系	y_1值	y_2值	y_3值	y_4值
I	DH13-3、DH16-8、DH16-6、DH29-8	-1.5675	-0.4438	0.5516	-0.4557
II	DH33-27、DH33-20、DH33-9、DH32-11、DH32-22、DH30-7、DH32-30、DH30-1、DH29-7、DH32-25、DH26-2	1.3846	-0.3504	-0.2251	-0.1814
III	DH29-10	0.3003	1.4480	-2.2813	0.6374
IV	DH32-21	-0.5378	-0.8188	1.5624	1.4487
V	DH32-26	2.6939	1.4885	-0.4011	0.3381
VI	DH17-5、DH33-32	-2.8592	-1.6865	-0.1319	0.7010
VII	DH30-5	-2.6699	-0.9558	0.1927	-0.2025
VIII	DH15-1、DH15-3	-3.1546	2.3752	-0.1312	0.2849
IX	DH17-2	-1.7311	2.3029	0.2859	-1.3526
X	DH32-28	5.0114	0.7867	1.4369	0.9776

中国知网　https://www.cnki.net

3.1.5 简单选择指数法

选择指数法是根据选育目标将所要选择的家系性状按其相对重要性、遗传力、经济价值和相互之间的遗传相关关系进行适当加权，由此合并成一个总的指数，然后再把其与各性状都有关系的指数作为单项指标进行选择的一种方法[94]。本次研究采用简单加权系数法对 25 个尾巨桉无性系 9 个性状进行综合选择。简单加权系数法的公式为：

$$I = \frac{w_1 h_1^2 \left(X_1 - \overline{X}\right)}{\sigma_1} + \frac{w_2 h_2^2 \left(X_2 - \overline{X}\right)}{\sigma_2} + \cdots + \frac{w_i h_i^2 \left(X_i - \overline{X}\right)}{\sigma_i} \quad \cdots\cdots\cdots\cdots\cdots\cdots\cdots\cdots\ (2\text{-}8)$$

其中 X_1、X_2……X_9 为胸径 DBH、树高 H、单株材积 Vol、枝下高 BH、干形 SF、分枝 Br、活立木外部密度指标值 Pilodyn、树皮厚度 BT、纹理倾角 SG，W 为性状的经济权重，$W = W_1 + W_2 \ldots\ldots + W_9 = 1$，Zobel[94]认为生长量是林木育种的主要程序，而且中大径材的用途主要是锯材，因此也要考虑材性、干形因子，同时由于树皮厚度会影响到材种的出材率，纤维倾角的大小会影响到木材强度，纤维倾角越大，木材强度越低，木材外部密度指标值越大，实际密度越小，所以树皮厚度、纤维倾角和木材外部密度指标值是负向选择，经过综合考虑，把各性状的经济权重分别假定为：$W_1=0.3$；$W_2=0.3$；$W_3=0.35$；$W_4=0.3$；$W_5=0.25$；$W_6=0.25$；$W_7=-0.3$；$W_8=-0.2$；$W_9=-0.25$；$\overline{X}$ 表示某性状组内总平均值，h^2 表示各性状的无性系重复力，σ 表示各性状的标准差，各性状的平均值、标准差、无性系重复力（见表 3-4）。把各个参数和数据代入公式得到指数值 I（见表 3-9）。

表 3-9 25 个尾巨桉无性系指数值

Tab. 3-9 Index selection value of 25 E.urophylla×E.grandis clones

排名	无性系	指数值	排名	无性系	指数值
1	DH32-28	1.9051	14	DH30-7	0.1272
2	DH33-27	1.6400	15	DH17-2	-0.5118
3	DH33-9	1.6279	16	DH16-6	-0.5164
4	DH33-20	1.6089	17	DH16-8	-0.6821
5	DH32-26	1.5308	18	DH29-8	-0.9952
6	DH30-1	0.9859	19	DH32-21	-1.1008
7	DH29-10	0.7821	20	DH15-3	-1.1319
8	DH32-22	0.7138	21	DH33-32	-1.1786
9	DH32-30	0.4793	22	DH13-3	-1.3030
10	DH32-11	0.3891	23	DH30-5	-1.3313
11	DH26-2	0.3774	24	DH15-1	-1.6698
12	DH29-7	0.2843	25	DH17-5	-2.1813
13	DH32-25	0.1505			

根据王明庥[96]和沈熙环[97]在指数选择中入选率与选择强度的研究，本试验以 30% 的入选率，选择强度为 1.16，按 I 指数值从高到低对 25 个尾巨桉无性系进行指数选择，一共选出 7 个优良无性系，这些优良无性系的各性状的平均值、遗传增益等遗传参数列

中国知网 https://www.cnki.net

于表 3-10。

从表 3-10 可知：简单加权系数法多性状综合选择出 7 个优良尾巨桉无性系，这 7 个无性系分别为：DH32-28、DH33-27、DH33-9、DH33-20、DH32-26、DH30-1、DH29-10；这 7 个无性系 9 个性状的平均值比总体平均值好，DBH、H、Vol、BH、SF、Br 比总体平均值分别大 13.27%、9.7%、37.86%、18.01%、20.69%、23.81%，Pilodyn、BT、SG 分别比总体平均值小 1.33%、3.33%、23.61%；9 个性状的遗传增益分别为 15.52%、9.89%、39.51%、24.65%、24.65%、30.89%、25.63%、8.66%、24.67%、58.37%，遗传增益最大的是螺旋纹理，其次是单株材积，最小的是外部木材密度指标值，其次是树高，可以看出，对螺旋纹理和单株材积的选择改良的遗传增益是很理想的，而对树高和外部木材密度的改良效果没有那么显著。

表 3-10　7 个优良尾巨桉无性系各个性状的平均值以及遗传参数

Tab.3-10　The mean value and genetic parameters of 9 characters for 7 superior *E.urophylla*×*E.grandis* clones

性状	DBH	H	Vol	BH	SF	Br	Pilodyn	BT	SG
DH32-28	24.9	33.3	0.7715	19.3	4.4	3.4	16.2	8.3	3.6
DH33-27	25.0	32.8	0.7722	20.1	3.0	2.2	16.1	5.8	4.4
DH33-9	22.6	33.5	0.6499	17.9	3.1	2.5	15.1	4.4	3.8
DH33-20	21.5	33.0	0.5963	19.1	3.9	2.7	15.3	5.1	3.1
DH32-26	22.4	32.4	0.6153	21.2	4.0	2.5	14.2	7.1	7.4
DH30-1	20.7	30.8	0.5016	16.5	3.1	2.5	14.3	4.3	5.3
DH29-10	18.2	32.2	0.4241	18.7	3.2	2.5	12.8	5.5	10.7
平均值	22.2	32.6	0.6187	19.0	3.5	2.6	14.8	5.8	5.5
总体平均值	19.6	29.7	0.4488	16.1	2.9	2.1	15.0	6.0	7.2
遗传增益(%)	15.52	9.89	39.51	24.65	30.89	25.63	8.66	24.67	58.37

7 个优良尾巨桉无性系又各有其特点：DH32-28 的指数值最大，也就是它的综合性状表现最优良的，从各性状的平均值不难看出，这个无性系生长表现非常的理想，生长量很大，而且干形、分枝很好，外部木材密度指标值比总体平均值大 8%，即木材密度偏小，纤维倾角很小，即螺旋纹理比较直，树皮有点厚，影响木材的出材率；DH33-27 的加权值仅次于 DH32-28，生长表现优于 DH32-28 无性系，生长迅速，生长量大，只是形质指标没有那么好，但是树皮比较薄，纤维倾角较小，纹理直，只是 Pilodyn 较平均值的大；DH33-20 和 DH33-9 各个性状平均值很相近，生长表现没有前面两个无性系那么好，树皮厚度和螺旋纹理却是非常的理想，是这 7 个无性系当中表现最好的，外部木材密度指标值也接近平均水平，分别为 15.3、15.1，只比总体平均值 15.0 大一点点；DH32-26 的生长表现还是很好的，枝下高非常的高，达到了 21.5m，干形和分枝很好，外部木材密度指标值低于平均水平，而树皮厚度和螺旋纹理倾角略比平均值高；DH30-1 的生长表现属于中上，枝下高是 7 个无性系里面最低的，形质指标不是很好，但是材质指标比较理想，外部木材密度指标值为 14.3，比总平均值小了 4.7%，树皮厚度和螺旋

中国知网 https://www.cnki.net

纹理倾角分别为 4.3、5.3，比平均值低 30%、26.4%；DH29-10 的生长表现是 7 个无性系里面最差的，形质指标还可以，木材外部密度指标值是最小的，比平均值小 14.7%，树皮厚度也比平均值小，但是螺旋纹理倾角却是最大的，为 10.7，比平均值大 48.6%。总之，这 7 个无性系是 25 个无性系里综合表现最好的，但是各个无性系又有其自身的优缺点，应当根据生产和培育目标出发来选择合适的无性系。

3.1.6 尾巨桉 5 个无性系原木木材缺陷比较

木材缺陷能减低木材强度，降低出材率，影响加工和装饰质量或外观，降低木材的商品价值。因此，选择木材缺陷比较低的无性系性对尾巨桉中大径材的推广利用是很有意义的。

节子是木材中最普遍存在的一种自然缺陷，是决定木材等级的主要因子，是造成木材降等的主要缺陷，节子对木材弹性、顺纹抗拉强度、抗压强度[69]等有影响，可见节子对材质的影响甚大。我国确定木材等级的一些标准中规定，如特级原木[98]、铁路货车锯材[99]、载重汽车锯材[100]、罐道木[101]和机台木[102]等标准的缺陷允许限度中规定的第一项就是节子，针、阔叶树加工用原木分等，针、阔叶树锯材分等和枕木等标准中，分等的第一个依据也是节子。DH32-28、DH15-3、DH61-1 三个无性系的原木段死节个数一样，A 段 2 个，B 段 3 个，DH33-27、DH32-26 的死节少，最多的原木段有 2 个。活节只有 DH15-3 比较多，两根原木都是 4 个。根据国家阔叶树加工用原木分等标准[103]，就节子对五个无性系进行分等，DH33-27、DH32-26 为Ⅰ等，DH32-28、DH15-3、DH61-1 为Ⅱ等。

表 3-11　尾巨桉 5 个无性系原木段的木材缺陷指标平均值表

Tab.3-11　The mean value of wood defects for 5 *E.urophylla*×*E.grandis* clones

Clone		DH32-28		DH33-27		DH32-26		DH61-1		DH15-3	
Log segment		A	B	A	B	A	B	A	B	A	B
LED (cm)		25.5	21.8	22.8	21.8	22	20	19.2	16.1	17.9	15.1
SED (cm)		22.3	20.7	21.7	20.6	19.9	18.9	16.5	15.7	14.6	13.9
Length (m)		1.37	1.35	1.37	1.36	1.36	1.36	1.37	1.36	1.38	1.36
Sweep (%)		0.18	0	1.18	0	1.23	0.11	0.98	0.77	1.74	0.57
Taper (%)		2.38	0.85	0.87	0.86	1.53	0.8	2.01	0.25	2.41	0.9
Dead Branch(#)	>25mm	1	2	1	1	0	1	1	2	1	2
	<25mm	1	1	0	0	0	0	1	1	1	1
Green Branch(#)	>25mm	1	0	0	0	0	0	0	0	2	1
	<25mm	1	0	0	0	0	0	0	0	2	3
Split index	Butt	0.86	1.45	2.68	3.58	0.96	1.23	1.34	3.37	0.4	0.58
	Top	2.13	2.57	2.38	5.96	1.29	2.58	3.91	2.24	0.59	0.55

弯曲是木材中常见的木材缺陷。弯曲特别降低木材的纵向强度，因此在电杆、桅杆、建筑原木、矿柱及类似用途中，弯曲度受到限制，特别是多向弯曲，降低强度较单向的大。弯曲对锯材的总出材率有很大的影响，弯曲度每增加 1%，则出材率减少 10%[104]。

中国知网 https://www.cnki.net

原木弯曲不仅影响出材率，而且对出材的尺寸也有很大的影响，弯曲原木在以腹背下锯法锯割时，对板材宽度变窄影响最大，弯曲原木同时也是锯材中产生人为斜纹的原因之一。从测定的原木弯曲度可知：DH32-28 最好，非常的通直，弯曲度只有 0.18%，其次是 DH33-27，最差的是 DH15-3。根据国家阔叶树加工用原木分等标准[103]，DH32-28、DH33-27、DH32-26、DH61-1 都为Ⅰ级，DH15-3 为Ⅱ级。

尖削度也是木材缺陷之一。一般认为，尖削度会随着径级的增加而提高，原木根部的尖削度大，梢部次之，中段最小。一般尖削度不足 0.5%的，为小尖削，自 0.5 至 1%的，为中等尖削；超过 1%的，为大尖削。A 段原木的尖削度，只有 DH33-27 不超过 1%，为中等尖削，其余的为大尖削。B 段原木，DH61-1 为小尖削，其余为中等尖削。

裂纹是使锯材降等的第二或第三位因素。裂纹破坏木材的完整性，降低木材的强度；在不良的保管条件下，裂隙还能成为变色菌或腐朽菌侵入木材的通路，引起木材的变色和腐朽。裂纹对木材力学强度的影响随着机械荷重的性质、作用力的方向和裂纹大小等因子而不同。从表 3-11 可知：原木段的底部端裂系数一般都比顶部端裂系数大，也就是顶部的端裂程度要比底部的大，端裂系数最大的是 DH33-27，最小的是 DH15-3。

尾巨桉中大径材作为锯材利用，木材缺陷是必须考虑的因素。从本次的试验调查来看，DH32-28、DH33-27、DH32-26 是生长表现非常好的无性系，而且树干通直圆满，节子少，弯曲度低，但是伐倒原木的端裂系数较大，这可能与桉树自身的生长应力有关，如果能通过栽培措施控制或者降低木材缺陷，改进锯材的加工工艺，DH32-28、DH33-27、DH32-26 无性系不仅在纸浆材方面，而且在锯材方面也是很有前景的。

中国知网　https://www.cnki.net

3.2 尾巨桉密度试验结果与分析

3.2.1 尾巨桉生长过程分析

从 2.3 年到 12.5 年 10 个生长阶段观测的生长量数据众多，故只列出了各个密度处理在 10 个生长阶段的树高、胸径、蓄积量的平均值（见表 3-12）。

表 3-12 尾巨桉 6 种密度处理平均树高、胸径、蓄积量生长过程

Tab.3-12 Growth of averge H,DBH,Stock with 6 spacing treatments of *E.urophylla*×*E.grandis*

项目	密度	生长过程（年）									
		2.3a	3.1a	3.5a	4.2a	5.2a	6.3a	7.3a	8.3a	9.2a	12.5a
树高(m)	1	11.4	14.0	15.4	16.2	17.9	19.7	20.9	21.5	21.9	23.0
	2	11.8	14.3	15.5	16.9	18.6	20.7	22.3	22.9	23.4	24.7
	3	11.6	14.5	15.8	17.0	19.8	22.1	24.0	24.5	24.9	26.2
	4	11.8	14.8	16.1	17.3	19.6	23.2	24.5	25.1	26.3	28.0
	5	11.1	14.2	15.7	17.7	19.7	22.7	24.3	25.5	26.3	28.1
	6	11.1	13.9	15.1	16.5	18.7	21.0	22.6	23.0	23.6	25.3
胸径(cm)	1	8.0	9.2	9.8	10.4	11.3	11.9	12.3	12.7	13.1	14.2
	2	8.6	9.9	10.4	11.2	12.0	12.7	13.1	13.6	14.1	15.3
	3	9.1	10.7	11.6	12.4	13.5	14.2	14.7	15.2	15.8	16.9
	4	10.3	12.0	13.0	14.0	15.5	16.6	17.2	18.4	18.6	20.1
	5	10.3	12.5	13.5	14.7	16.7	17.7	18.5	19.4	20.2	22.0
	6	8.8	10.4	11.1	11.9	13.1	13.9	14.6	15.2	15.7	16.9
蓄积量(m^3/hm^2)	1	68.0	105.3	129.3	151.6	194.9	239.4	270.2	295.6	318.9	389.5
	2	59.6	91.9	109.5	135.9	170.0	211.6	245.3	268.2	296.0	365.3
	3	49.4	80.8	101.2	124.2	168.3	208.9	245.5	268.1	291.6	352.2
	4	44.1	71.5	90.4	111.1	152.1	207.6	235.6	275.2	295.3	369.6
	5	31.6	55.8	71.2	93.6	131.9	171.0	200.7	232.5	259.8	328.7
	6	44.1	73.4	89.3	111.6	150.8	190.5	225.3	248.2	271.4	336.1

（1）胸径生长过程

从胸径生长过程表（见表 3-12）和年平均生长曲线图（图 3-2）可以看出：平均胸径是不断的在生长，但是不同的生长时期生长快慢不一样，而且不同的密度处理生长的快慢也是不一样的；前 2.3 年是胸径生长最快的，在第 2.3 年时胸径年平均生长量达到最大，年平均生长量都超过了 3.5cm，而密度处理 4 和 5 则达到了 4.5cm，从 2.3 年以后，胸径年平均生长量开始平缓的下降，密度处理 1、2、3、6 在 6.3 年时胸径年平均生长量仍然超过 2.0cm，然后逐渐下降，而密度处理 4 和 5 到 9.2 年时胸径仍然保持较快生长，年平均生长量超过 2.0cm；从开始至 12.5a，密度处理 4 和 5 的年平均生长量都是最快的，6 种密度处理胸径年平均生长量快慢依次为：密度 5、密度 4、密度 3、密度 6、密度 2、密度 1，相同的栽培密度不同的株行距配置密度处理 3 和 6 的平均生长曲线在 5.2 年之前，密度 3 比密度 6 年平均生长量大，5.2 年之后两条曲线几乎是重叠在一起的。

中国知网 https://www.cnki.net

表 3-13　13 年生尾巨桉 6 种密度原木段的木材缺陷指标平均值表

Tab.3-13　Mean values of wood quality with 6 spacing treatments and 4 repetitions of 13 years old *E.urophylla×E.grandis*

密度处理号		1		2		3		4		5		6	
原木段		A	B	A	B	A	B	A	B	A	B	A	B
LED (cm)		18.1	16.1	18.0	16.2	18.5	16.6	20.2	18.2	22.8	20.2	19.1	17.2
SED (cm)		16.2	15.8	16.4	15.9	16.6	16.2	18.3	17.6	20.0	19.6	17.3	16.6
Length (m)		1.37	1.35	1.36	1.35	1.37	1.36	1.37	1.35	1.37	1.36	1.39	1.35
Sweep (%)		1.36	0.19	1.22	0.25	1.42	0.31	1.38	0.51	2.03	0.44	1.29	0.40
Taper (%)		1.11	0.12	1.17	0.23	1.32	0.26	1.34	0.24	1.60	0.20	1.16	0.61
Dead Branch(＃)	>25mm	0	0	0	1	0	0	0	0	0	0	0	0
	<25mm	0	0	1	1	0	0	0	0	0	0	0	0
Green Branch(＃)	>25mm	0	0	0	0	0	0	0	0	0	0	0	0
	<25mm	0	0	0	0	0	0	0	0	0	0	0	0
Split index	Butt	1.60	0.90	1.05	0.73	1.10	0.71	1.73	0.67	1.02	0.80	1.10	0.91
	Top	1.28	1.03	1.08	1.18	0.75	0.98	1.31	1.20	0.75	1.33	1.28	1.16

中国知网　https://www.cnki.net

从连年生长曲线图（见图 3-3）可知：胸径连年生长量的高峰期出现在第 2.3 年，6 种密度处理林分的胸径连年生长量都达到了 3.5cm，密度处理 4 和 5 则达到了 4.5cm，随后连年生长量急剧下降，到 3.1 年时，只有密度处理 5 连年生长量仍然保持在 2.5cm 以上，密度处理 3、4、6 下降到 2.0cm，而密度处理 1、2 则下降到 1.5cm；密度处理 4 在 3.5、5.2、8.3 年时出现了三个连年生长高峰期，而在 9.2 年时出现了低谷期；密度处理 5 在 3.5、5.2、9.2 年时也出现了三个连年生长高峰期；从总体上看，密度处理 4 和 5 的连年生长量除了密度处理 4 在 9.2 年时最低之外，都比其他密度处理大。

因此，从上述可知：密度越大，胸径的年平均生长和连年生长越慢，反之则生长快，过大的栽培密度造成林木间的竞争过于激烈，抑制尾巨桉胸径的生长，不利于胸径的快速生长，因此，要培育中大径材，选择合适的栽培密度优为重要，而且在尾巨桉中后期生长过程中应考虑追加施肥，对胸径的生长是有促进作用的。从上述对 6 种密度处理的分析可知 667 株/hm^2 和 883 株/hm^2 这两种密度是比较合适培育尾巨桉中大径材。

从胸径方差分析表（3-14）可以知道：6 种密度处理间的历年胸径在 1%水平上差异显著，F 值在 5.2 年时最大，然后逐渐减小；不同的栽培密度对胸径的生长影响显著，而且这种影响自幼龄林开始就存在，到 12.5 年时影响仍然显著。

（2）树高生长过程

从平均树高生长过程表（见表 3-12）和树高年平均生长曲线图（见图 3-4）可以看出：平均树高一直不断的生长，不同的生长时期生长快慢不一样，而且不同的密度处理生长的快慢也是不一样；树高在 2.3 年之前生长最快，树高年平均生长量在 2.3 年时达到最大，随后进入平缓的生长时期，年平均生长量逐渐下降；6 种密度处理的树高年平均生长曲线差异不是很大，重叠的程度比较高，但是还是可以看出，密度处理 4 和 5 比其他的密度处理年平均生长量要大些。

从连年生长曲线图（见图 3-5）可知：树高连年生长量在 2.3 年时达到了最大值，密度处理 4 连年生长量超过了 6.0m，其他的密度处理也达到了 6.0m 或接近 6.0m，随后连年生长量急剧下降；密度处理 4 的连年生长曲线波动最大，在 6.3 年和 9.2 年出现了两个很明显的生长高峰期，在 6.3 年时，密度处理 4 的连年生长量仍然达到 3.4m。

对历年树高进行方差分析（见表 3-14），结果表明：不同的密度处理对尾巨桉前 3 年的树高生长影响差异不显著，到 4.2 年时在 5%水平上影响差异显著，在 5.2 年时影响又不显著，从 6.3 年到 12.5 年在 1%水平上影响差异显著；F 值的总体趋势是逐渐升高的。从上述可知，栽培密度对尾巨桉树高的初期生长没有显著影响。

（3）蓄积量生长过程

从平均蓄积量生长过程表（见表 3-12）和蓄积量年平均生长曲线图（图 3-6）可以看出：不同密度之间年平均蓄积量生长的快慢比较明显，从 2.3 年到 12.5 年，无论在哪一个生长阶段年平均蓄积量都是密度处理 1 最大，密度处理 5 最低；在 2.3 年时，密度 1 的年平均蓄积量达到了 30.0m^3/hm^2，而密度处理 5 还不到 15.0 m^3/hm^2，密度 1 的年平均蓄积量是密度 5 的 2 倍多；前 3.5 年，密度处理 1 的年平均蓄积量生长最快，3.5 年之后生长速度开始变缓，4.2 年时还略有下降，到 6.3 年时，年平均蓄积量生长达到了最大，

中国知网 https://www.cnki.net

表 3-14　尾巨桉 6 种密度处理胸径、树高、蓄积量历年方差分析

Tab. 3-14　Variability analysis of DBH,H,Stock by 6 spacing treatments of *E.urophylla*×*E.grandis*

时间(年)	变异来源	df	树高			胸径			蓄积量		
			SS	MS	F 值	SS	MS	F 值	SS	MS	F 值
2.3a	施肥间	5	2.0	0.4	1.5	17.6	3.5	50.9**	3293.1	658.6	37.9**
	区组间	3	0.6	0.2	0.7	0.5	0.2	2.2	18.4	6.1	0.4
	误　差	15	4.1	0.3		1.0	0.1		260.8	17.4	
	总　和	23	6.7			19.1			3572.4		
3.1a	施肥间	5	2.0	0.4	1.4	31.4	6.3	98.6**	5931.7	1186.3	44.0**
	区组间	3	1.9	0.6	2.2	0.9	0.3	4.5*	346.0	115.3	4.3*
	误　差	15	4.3	0.3		1.0	0.1		404.8	27.0	
	总　和	23	8.2			33.2			6682.5		
3.5a	施肥间	5	2.4	0.5	1.3	43.2	8.6	91.8**	7926.2	1585.2	29.4**
	区组间	3	3.0	1.0	2.8	1.9	0.6	6.8**	907.7	302.6	5.6**
	误　差	15	5.4	0.4		1.4	0.1		809.8	54.0	
	总　和		10.8			46.5			9643.8		
4.2a	施肥间	5	6.2	1.2	3.0*	54.6	10.9	119.1**	8427.7	1685.5	22.6**
	区组间	3	4.2	1.4	3.3	3.0	1.0	10.9**	1188.9	396.3	5.3*
	误　差	15	6.2	0.4		1.4	0.1		1118.0	74.5	
	总　和	23	16.6			59.0			10734.6		
5.3a	施肥间	5	11.7	2.3	2.8	86.3	17.3	143.7**	9191.5	1838.3	15.1**
	区组间	3	6.6	2.2	2.6	1.8	0.6	4.9*	2253.4	751.1	6.2**
	误　差	15	12.7	0.8		1.8	0.1		1827.0	121.8	
	总　和	23	31.1			89.9			13272.0		
6.3a	施肥间	5	36.0	7.2	9.0**	98.9	19.8	77.5**	10329.1	2065.8	10.3**
	区组间	3	3.3	1.1	1.4	2.3	0.8	3.0	2288.5	762.8	3.8*
	误　差	15	12.0	0.8		3.8	0.3		3014.9	201.0	
	总　和	23	51.3			105.0			15632.5		
7.3a	施肥间	5	40.0	8.0	13.8*	111.9	22.4	81.1**	10750.4	2150.1	8.7**
	区组间	3	2.2	0.7	1.3	2.4	0.8	2.9	3033.6	1011.2	4.1*
	误　差	15	8.7	0.6		4.1	0.3		3687.0	245.8	
	总　和	23	50.9			118.4			17471.1		
8.3a	施肥间	5	47.2	9.4	14.9*	140.9	28.2	69.9**	9429.5	1885.9	4.3*
	区组间	3	2.4	0.8	1.3	2.1	0.7	1.7	1989.3	663.1	1.5
	误　差	15	9.5	0.6		6.0	0.4		6520.3	434.7	
	总　和	23	59.1			149.0			17939.1		
9.2a	施肥间	5	60.7	12.1	19.6*	145.4	29.1	63.9**	8465.6	1693.1	3.4*
	区组间	3	3.5	1.2	1.9	4.4	1.5	3.2	5755.7	1918.6	3.9*
	误　差	15	9.3	0.6		6.8	0.5		7465.7	497.7	
	总　和	23	73.5			156.6			21687.0		
12.5a	施肥间	5	78.7	15.7	35.5*	174.8	35.0	63.5**	10231.6	2046.3	3.0*
	区组间	3	5.4	1.8	4.1*	4.7	1.6	2.8	10492.2	3497.4	5.1*
	误　差	15	6.7	0.4		8.3	0.6		10330.7	688.7	
	总　和	23	90.8			187.7			31054.5		

注：密度 $F_{0.05}$=2.90，$F_{0.01}$=4.56；区组 $F_{0.05}$=3.29，　$F_{0.01}$=5.41；'*''**'分别表示在 5%和 1%水平上差异显著。

中国知网　https://www.cnki.net

接近 40.0 m^3/hm^2，随后开始平缓下降；密度处理 2、3、6 年平均蓄积量在 7.3 年时达到最大；密度处理 4 的年平均蓄积量在 8.3 年时达到最大；密度处理 5 在 9.2 年时达到最大；在 3.5 年之前，6 种密度处理林分的年平均蓄积量相差很大，3.5 年之后之间的差距越来越小。

从连年生长曲线图（见图 3-7）可知：密度处理 1、2、3、5、6 的林分蓄积量连年生长量在 3.5 年生时达到最大，而密度处理 4 在 6.3 年时才达到最大，相差了将近三年；密度处理 1 的蓄积量连年生长量达到最大时超过了 60.0 m^3/hm^2，密度处理 4 超过了 50.0 m^3/hm^2，最小的为密度处理 5，仅为 38.6 m^3/hm^2；密度处理 4 的林分蓄积量连年生长曲线波动最大，3.5 年时出现了生长的一个高峰，然后在 4.2 年时进入了低谷，在 6.3 年时连年生长量达到了最高峰，7.3 年时又是一个低谷，8.3 年时又出现一个生长高峰。

对历年平均蓄积量进行方差分析（见表 3-14），结果表明：不同的密度处理对尾巨桉平均蓄积量的生长影响差异显著，从 2.3a 到 7.3a 在 1%水平上影响差异显著，从 8.3a 到 12.5a 在 5%水平上影响差异显著；F 值从 2.3a 到 12.5a 是逐渐减小的。根据方差分析得知：密度处理对尾巨桉平均蓄积量的生长影响随着时间的增长而减小。

3.2.2 尾巨桉 13 年生时的林分结构分析

通过对 13 年生尾巨桉林分树高、胸径的结构进行分析，以便了解各个密度处理林分中达到中大径材的林木所占的比例。尾巨桉胸径分级根据中大径材标准和现在桉树木材市场分级价格进行，大径材：尾径≥26 ㎝，中径材：20 ㎝≤尾径＜26 ㎝，桉树木材市场价格：尾径≥20 ㎝为 720 元，14 ㎝≤尾径＜20 ㎝为 680 元，所以本文把尾巨桉林分胸径分为≥26 ㎝，20 ㎝≤DBH＜26 ㎝，14 ㎝≤DBH＜20 ㎝三级进行分析。树高分为≥30m，25m≤H＜30m，20m≤H＜25m 三个级别进行分析。

（1）林分胸径结构

从图 3-8 可以看出：DBH≥26 ㎝也就是达到大径材标准的林木只有密度处理 5 的林分有，但是也是非常的少，还不到 10%；20 ㎝≤DBH＜26 ㎝也就是中径材，随着密度的减少而增大，密度处理 4 和 5 中径材所占的比例远远比其它 4 个密度大，密度 5 所占的比例最大，几乎达到了 80%，最少的是密度 1，还不足 10%；14 ㎝≤DBH＜20 ㎝径级，以相同栽培密度不同株行距配置的密度处理 6 和 3 为中心，林木所占比例随着密度的减少或者增大而减小。从上述可知密度越大，林分中达到中大径材的比例越大，反之则少。因此，要培育中大径材，本研究中的 667 株/hm^2 和 883 株/hm^2 两种栽培密度是比较适合的。

（2）林分树高结构

从图 3-9 可以看出：随着密度的减小，林分中树高大于等于 30m 的林木百分比逐渐增大，密度处理 5 的林分中树高大于等于 30m 的林木所占的比例最大，达到了 70%，而密度处理 1 最小，不足 10%；树高在 25m≤H＜30m，20m≤H＜25m 这两个级别里，林木百分比随着密度的减小而减少的趋势。在这 6 种密度里，密度越小，树高大于 30m 的林木所占的比例越大。因此，较小的栽培密度有利于树高的生长。

中国知网 https://www.cnki.net

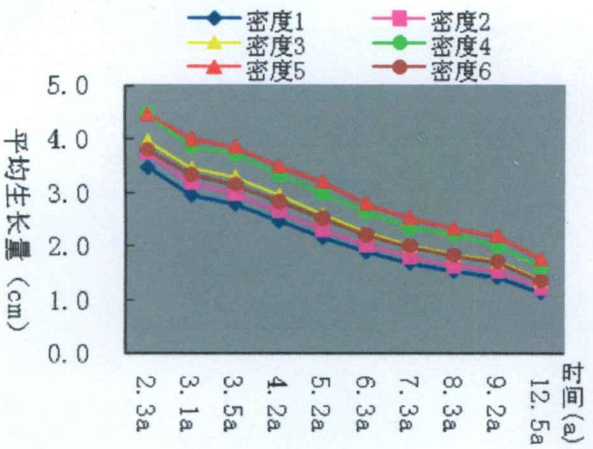

图 3-2 尾巨桉 6 种密度处理胸径平均生长曲线

Fig. 3-2 Curve of DBH mean increment by 6 spacing treatments of *E. urophylla × E. grandis* clones

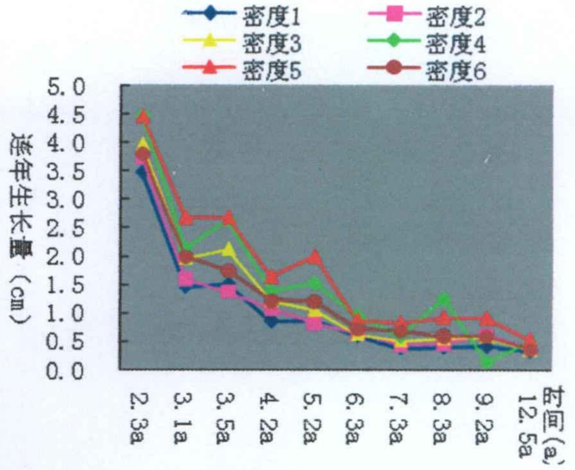

图 3-3 尾巨桉 6 种密度处理胸径连年生长曲线

Fig. 3-3 Curve of DBH annul increment by 6 spacing treatments of *E. urophylla × E. grandis* clones

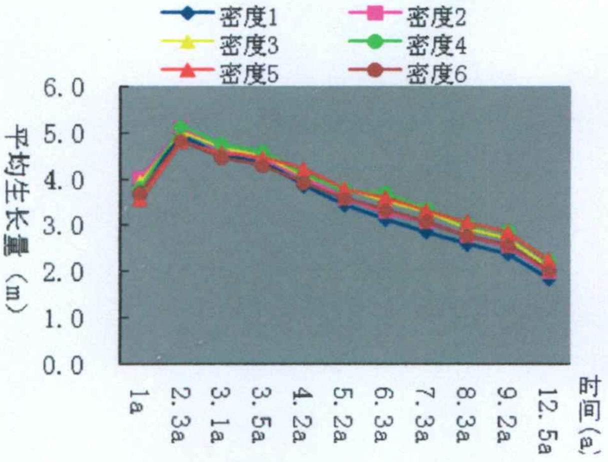

图 3-4 尾巨桉 6 种密度处理树高平均生长曲线

Fig. 3-4 Curve of H mean increment by 6 spacing treatments of *E. urophylla × E. grandis* clones

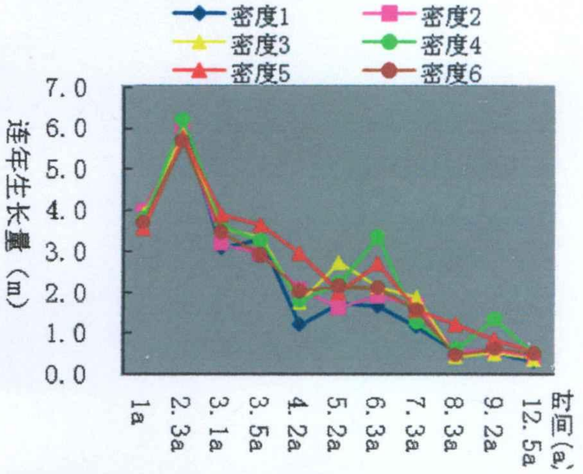

图 3-5 尾巨桉 6 种密度处理树高连年生长曲线

Fig. 3-5 Curve of H annul increment by 6 spacing treatments of *E. urophylla × E. grandis* clones

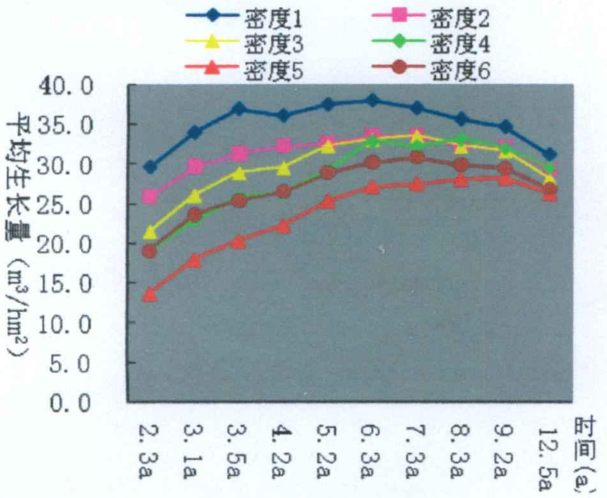

图 3-6 尾巨桉 6 种密度处理蓄积量平均生长曲线

Fig. 3-6 Curve of stock mean increment by 6 spacing spacing treatments of *E. urophylla × E. grandis* clones

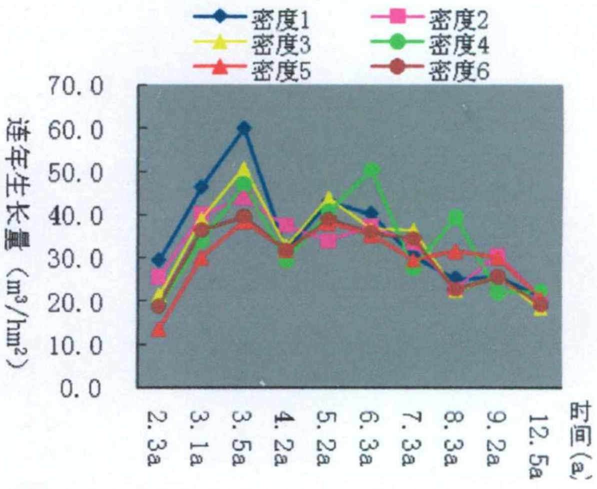

图 3-7 尾巨桉 6 种密度处理蓄积量连年生长曲线

Fig. 3-7 Curve of stock annul increment by 6 treatments of *E. urophylla × E. grandis* clones

中国知网 https://www.cnki.net

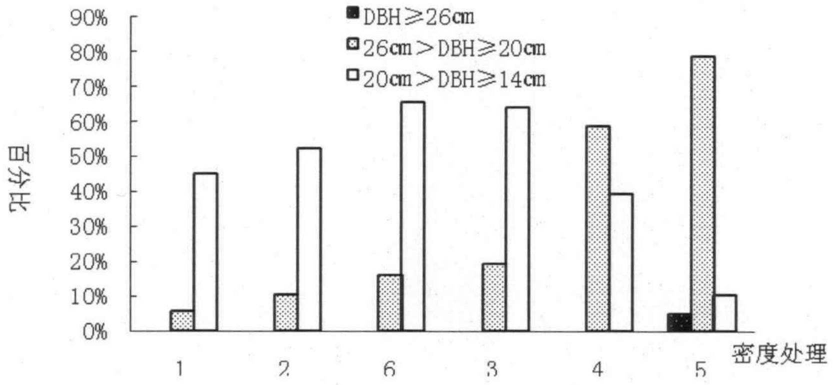

图 3-8　13 年生尾巨桉不同密度处理林分胸径结构图

Fig. 3-8　chart of DBH structure by 6 spacing treatments of 13-year-old *E. urophylla × E. grandis*

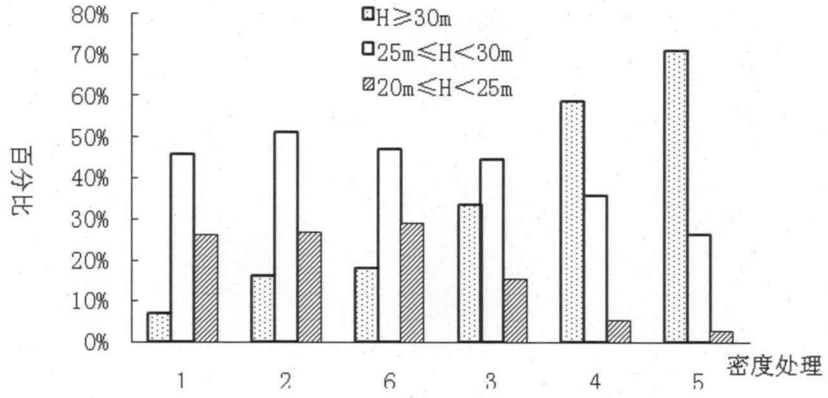

图 3-9　13 年生尾巨桉不同密度处理林分树高结构图

Fig. 3-9　chart of H structure by 6 spacing treatments of 13-year-old *E. urophylla × E. grandis*

3.2.3 不同栽培密度对 13 年生尾巨桉材质的影响

（1）不同栽培密度对尾巨桉树皮厚度的影响

从 13 年生尾巨桉树皮厚度方差分析表 3-16 可知：6 种密度处理间的树皮厚度差异极显著，区组间差异显著。说明不同的栽培密度对树皮厚度生长有显著影响。树皮最厚

表 3-15　13 年生尾巨桉 6 种密度处理生长性状和材质性状平均值

Tab. 3-15　Mean values of growth and quality wood characters with 6 spacing treatments of 13 years old *E.urophylla×E.grandis*

密度处理	胸径 (cm)	树高 (m)	蓄积量 (m^3/hm^2)	木材外部密度 Pilodyn	树皮厚度 (mm)	木材强度 FAKOPP(m/s)	螺旋纹理 spiral grain
1	14.5	24.4	434.4	16.6	5.1	3524.4	5.0
2	15.5	26.4	407.2	16.3	5.1	3447.4	4.0
3	17.2	28.0	396.7	16.3	6.6	3571.6	4.5
4	20.5	30.2	422.8	16.5	6.5	3571.8	3.7
5	22.2	31.3	387.6	16.9	7.9	3588.9	4.5
6	17.2	26.4	367.5	17.0	5.9	3566.8	4.9

中国知网　https://www.cnki.net

的是密度处理5，达到7.9mm，密度处理1和2最小，仅为5.1mm，树皮厚度随着密度的增大而减小的趋势。

表3-16　13年生尾巨桉不同栽培密度树皮厚度方差分析

Tab. 3-16　Variability analysis of bark thickness by 6 spacing treatments of 13 years old *E.urophylla×E.grandis*

差异源	df	SS	MS	F
密度间	5	22.030	4.406	5.234**
区组间	3	9.381	3.127	3.715*
误差	15	12.627	0.842	
总计	23	44.037		

注：密度 $F_{0.05}$=2.90，$F_{0.01}$=4.56；区组 $F_{0.05}$=3.29，$F_{0.01}$=5.41；'*''**'分别表示在5%和1%水平上差异显著。

表3-17　树皮厚度邓肯多重比较

Tab. 3-17　Multi-comparision by Duncan of bark thinckness

密度处理	株行距	平均值	a=0.05	a=0.01
5	5m×3m	7.9	a	A
3	4m×2m	6.6	ab	AB
4	4m×3m	6.5	ab	AB
6	6m×2m×2m	5.9	ab	B
2	3m×2m	5.2	b	B
1	3m×1.5m	5.1	b	B

表3-18　13年生尾巨桉不同栽培密度的外部木材密度指标值方差分析

Tab. 3-18　Variability analysis of Pilodyn by 6 spacing treatments of 13 years old *E.urophylla×E.grandis*

差异源	df	SS	MS	F
密度间	5	1.484	0.297	1.688
区组间	3	0.200	0.067	0.380
误差	15	2.636	0.176	
总计	23	4.320		

注：密度 $F_{0.05}$=2.90，$F_{0.01}$=4.56；区组 $F_{0.05}$=3.29，$F_{0.01}$=5.41

（2）不同栽培密度对尾巨桉外部木材密度指标值的影响

加拿大的Hall[81]研究结果看到Pilodyn的示度值与实测木材密度间相关系数因树种变动在-0.451～-0.564 间，表明这一工具的适用价值。在主要制浆材桉树的遗传研究中表明Pilodyn测定值与实测木材密度的表型相关在0.7 以上，受立地影响不大，在尾叶桉上直接测定的木材密度的遗传力为0.71，用Pilodyn法测得的为0.64，均在强度遗传控制下，证明按照Pilodyn测定的结果进行木材密度的选择是可靠的[82,83]。

故本次用无损检测仪器 Pilodyn 所测得到的数据在很大程度上反映了真实的木材密度，本次研究不同的栽培密度对这个指标值的影响，也就是对木材密度的影响。

中国知网　https://www.cnki.net

对测定所得到的数据进行方差分析，结果如表 3-18 所示。从方差分析可知：不同栽培密度对活立木外部密度指标值的影响差异不显著。从表 3-13 可知：活立木外部密度指标值最大的是密度处理 6，其次是密度处理 5，最小的是密度 2，它们的值分别为 16.98、16.88、16.32。

（3）不同栽培密度对尾巨桉木材强度指标值的影响

木材强度是评定木材品质和用途的重要因素，而木材密度和木材强度有着密切的关系。木材密度是木材胞壁实质相对量的一种度量，所以木材密度是预估木材强度的最可靠的指标。一般说来，对各树种木材密度和强度间的关系可以表达如下方程[92]：

$$S=K(G)^n$$

式中：S：木材强度性质； K：随每种强度而不同的比例常数； G：木材密度；n：确定密度和强度关系曲线形状的指数。

由此可知，木材密度是决定木材强度的第一位因素，木材强度与木材密度间的关系是直接的。用 FAKOPP 测定得到的数据是在 1.5m 距离的传播时间，然后换算成传播速度 m/s，在同一传播距离，传播速度越快，说明活立木的密度越小，木材强度就越低，反之则高。因此我们只要比较不同的传播速度就可以知道活立木的木材密度和强度的大小了。

用 FAKOPP 测得到的数据换算成传播速度，然后进行方差分析（见表 3-19），结果表明：不同的栽培密度对应力波在尾巨桉活力木中的传播速度差异影响不显著，也就是不同的栽培密度间的木材强度差异不显著。

从表 3-13 可知：应力波在密度处理 5 的传播速度最快，为 3588.9m/s，也就是木材强度最低；应力波在密度处理 2 的传播速度最慢，为 3447.4m/s，也就是木材强度最高。

表 3-19 13 年生尾巨桉不同栽培密度的木材强度指标值方差分析

Tab. 3-19 Variability analysis of FAKOPP by 6 spacing treatments of 13 years old *E.urophylla×E.grandis*

差异源	df	SS	MS	F
密度间	5	98033.20	19606.64	2.12
区组间	3	148086.10	49362.03	5.33
误差	15	138899.17	9259.94	
总计	23	385018.47		

注：密度 $F_{0.05}$=2.90，$F_{0.01}$=4.56；区组 $F_{0.05}$=3.29， $F_{0.01}$=5.41

（4）不同栽培密度对尾巨桉螺旋纹理（spiral grain）的影响

螺旋纹理（spiral grain）是指在活树中木材纤维围绕树轴呈螺旋形排列，左旋或右旋。螺旋纹理对木材物理、力学性质的影响很显著，螺旋纹理的存在降低了木材的强度，干燥时容易发生翘曲，对重要木结构件来说，这是一种严重缺陷，要限制使用。具有螺旋纹理的原木在制材后，会产生斜纹理板材。因此螺旋纹理对材质的好坏有重要影响。

根据纤维倾角仪器测定得到的倾角数据经整理后，对其进行方差分析，结果见表

中国知网 https://www.cnki.net

3-20。方差分析结果显示：不同栽培密度对螺旋纹理的影响差异不显著，说明螺旋纹理受外界影响较小，受到遗传控制大些。从表 3-13 可知：螺旋纹理倾角最大的是密度处理 1，其次是密度处理 6，最小的是密度处理 4，它们值分别为 5.01、4.91、3.73。

表 3-20　13 年生尾巨桉不同栽培密度的螺旋纹理方差分析

Tab. 3-20　Variability analysis of spiral grain by 6 spacing treatments of 13 years old *E.urophylla*×*E.grandis*

差异源	df	SS	MS	F
密度间	5	4.969	0.994	1.001
区组间	3	1.622	0.541	0.544
误差	15	14.898	0.993	
总计	23	21.489		

注：密度 $F_{0.05}$=2.90，$F_{0.01}$=4.56；区组 $F_{0.05}$=3.29，$F_{0.01}$=5.41

（5）不同栽培密度对尾巨桉木材缺陷的影响

木材缺陷能减低木材强度，降低出材率，影响加工和装饰质量或外观，降低木材的商品价值，因此，木材缺陷对木材的利用有着很大的影响。本文开展不同的栽培密度对木材缺陷的影响是很有必要的。

表 3-21　13 年生尾巨桉不同栽培密度间原木段 A、B 木材缺陷指标值方差分析

Tab.3-21　Variability analysis of wood defects by 6 spacing treatments of 13 years old *E.urophylla*×*E.grandis*

性状		差异源	df	A			B		
				SS	MS	F	SS	MS	F
Sweep		密度间	5	1.71	0.34	2.39	0.29	0.06	1.24
		区组间	3	2.81	0.94	6.56**	0.08	0.03	0.59
		误差	15	2.14	0.14		0.70	0.05	
		总计	23	6.67			1.07		
Taper		密度间	5	0.66	0.13	0.52	0.58	0.12	2.99*
		区组间	3	2.67	0.89	3.50*	0.78	0.26	6.69**
		误差	15	3.81	0.25		0.58	0.04	
		总计	23	7.14			1.94		
Split index	Bottom	密度间	5	1.94	0.39	0.71	0.20	0.04	0.37
		区组间	3	3.05	1.02	1.85	0.43	0.14	1.29
		误差	15	8.23	0.55		1.66	0.11	
		总计	23	13.22			2.29		
	Top	密度间	5	1.39	0.28	0.73	0.32	0.06	0.19
		区组间	3	1.77	0.59	1.55	0.60	0.20	0.60
		误差	15	5.73	0.38		5.02	0.33	
		总计	23	8.89			5.94		

注：密度 $F_{0.05}$=2.90，区组 $F_{0.05}$=3.29，$F_{0.01}$=5.41；'*''**'分别表示在 5%和 1%水平上差异显著。

中国知网 https://www.cnki.net

从表 3-13 可知，原木段 A 的弯曲度、尖削度都比 B 段的大，弯曲度最大的是密度处理 5 的 A 段原木，达到了 2.03%，最小的是密度处理 1 的 B 段原木，为 0.19%，尖削度最大的也是密度处理 5 的 A 段原木，为 1.60%，最小的是密度处理 1 的 B 段原木，仅为 0.12%；随着密度的增大，原木弯曲度和尖削度有增加的趋势；死节和活节比较少，只有密度处理 2 有少数几个；原木端裂没有比较明显的规律，端裂系数变动不大。

对原木 A、B 段的弯曲度、尖削度、原木两端的端裂程度指数进行方差分析，方差分析结果表明：不同的栽培密度措施对原木 A 段的木材缺陷影响不显著，对原木 B 段只有尖削度在 5%水平上影响显著，其余不显著。

表 3-22 13 年生尾巨桉 6 种栽培密度的原木 A 段与 B 段间木材缺陷指标值方差分析

Tab. 3-22 Variability analysis between log A and log B by 6 spacing treatments of 13 years old *E.urophylla×E.grandis*

密度	差异源	df	Taper			Sweep			Split index		
			SS	MS	F	SS	MS	F	SS	MS	F
1	密度间	1	2.77	2.77	18.06*	1.96	1.96	16.21*	0.45	0.45	3.60
	区组间	3	0.28	0.09	0.60	0.19	0.06	0.53	2.83	0.94	7.51
	误差	3	0.46	0.15		0.36	0.12		0.38	0.13	
	总计	7	3.5			2.51			3.66		
2	密度间	1	1.85	1.85	8.44	1.79	1.79	8.03	0.02	0.02	0.17
	区组间	3	0.78	0.26	1.19	0.63	0.21	0.94	0.77	0.26	1.84
	误差	3	0.66	0.22		0.67	0.22		0.42	0.14	
	总计	7	3.29			3.08			1.21		
3	密度间	1	2.46	2.46	32.38*	2.25	2.25	18.80*	0.01	0.01	0.13
	区组间	3	0.14	0.05	0.60	0.90	0.30	2.50	0.48	0.16	1.76
	误差	3	0.23	0.08		0.36	0.12		0.27	0.09	
	总计	7	2.82			3.50			0.77		
4	密度间	1	1.53	1.53	4.45	2.41	2.41	8.07	0.69	0.69	1.44
	区组间	3	0.60	0.20	0.58	1.42	0.47	1.59	2.99	1.00	2.08
	误差	3	1.03	0.34		0.90	0.30		1.44	0.48	
	总计	7	3.16			4.73			5.13		
5	密度间	1	5.05	5.05	40.51**	3.93	3.93	11.05*	0.07	0.07	3.23
	区组间	3	0.35	0.12	0.92	0.27	0.09	0.26	0.69	0.23	11.30*
	误差	3	0.37	0.12		1.07	0.36		0.06	0.02	
	总计	7	5.77			5.27			0.81		
6	密度间	1	1.61	1.61	12.48*	0.59	0.59	2.02	0.05	0.05	3.64
	区组间	3	0.46	0.15	1.19	0.20	0.07	0.22	0.17	0.06	3.95
	误差	3	0.39	0.13		0.88	0.29		0.04	0.01	
	总计	7	2.46			1.67			0.26		

注：密度 $F_{0.05}$=10.13，$F_{0.01}$=34.12；区组 $F_{0.05}$=9.28；'*''**'分别表示在 5%和 1%水平上差异显著。

中国知网 https://www.cnki.net

同时也探讨了同一密度处理里的原木 A、B 段在木材缺陷上的差异性，方差分析结果表明：同一密度的原木 A、B 段的原木端裂程度指数差异不显著；密度 1、3、5 的原木 A、B 段的弯曲度差异在 5%水平上差异显著，密度 2、4、6 则差异不显著；密度 1、3、6 的原木 A、B 段的尖削度在 5%水平上差异显著，密度 5 在 1%水平上差异显著，密度 2 和 4 差异不显著。

造成木材缺陷的原因是很复杂的，木材是树木的一部分，必然具有树木构造上及其生长条件上带来的一些能降低其质量、影响其使用的缺陷，树木在其生活期间也可能遭受各种病害或者损伤，所以这些内部外部原因都会造成木材缺陷。树干中节子的数目、大小和类型是与树种有关的，也就是它反映了树木的遗传生长特性，同时还与树木生长时的环境条件、林分组成和密度有关，一般来讲，在密度比较大的林分中，林木为了争夺阳光，占据较大的营养空间，呈现为高向生长旺盛、树干长得通直，圆满，尖削度小；而在密度稀疏的林分中，树干往往比较弯曲，尖削度大，并且容易产生斜向纹理，降低木材质量。弯曲是木材常见的一个主要缺陷，形成弯曲的原因如：由于顶牙死亡，由侧枝代替，或由于日照度的改变，或由于树木在斜坡上生长等，所以，树干弯曲的产生与树种的固有习性以及生长的环境都有密切的关系。端裂形成的原因主要是树木伐倒时与地面发生发生冲击或者是树木内部有内应力。栽培措施，特别是密度措施对控制一些木材缺陷的形成是有积极作用的，但是栽培密度对木材缺陷具体如何影响和影响程度还要作更多更深入的研究。

中国知网　https://www.cnki.net

3.3 尾巨桉施肥试验结果与分析

3.3.1 林分生长差异性

从 2.3 年到 12.5 年 10 个生长阶段观测的生长量数据众多，故只列出了各个施肥处理在 10 个生长阶段的树高、胸径、蓄积量的平均值（见表 3-23）。

表 3-23　尾巨桉 6 种施肥处理平均树高、胸径、蓄积量生长过程

Tab. 3-23　Growth of averge H, DBH, Stock with 6 fertiliser treatments of *E.urophylla*×*E.grandis*

项目	密度	生长过程（年）									
		2.3a	3.1a	3.5a	4.2a	5.3a	6.3a	7.3a	8.3a	9.2a	12.5a
树高 (m)	1	12.3	14.8	16.0	17.9	19.5	22.0	23.8	24.2	24.8	26.2
	2	11.0	13.6	15.0	16.6	18.5	20.6	22.5	23.3	24.0	25.5
	3	11.6	14.5	15.7	17.0	19.8	22.1	24.0	24.5	24.9	26.2
	4	11.6	14.2	15.3	17.0	19.2	21.5	23.4	23.8	24.2	25.7
	5	10.8	13.7	14.9	16.7	18.5	21.1	23.0	23.6	24.4	25.5
	6	12.0	14.7	16.0	17.6	19.7	21.8	23.6	24.1	24.6	26.4
胸径 (cm)	1	9.7	11.2	12.0	12.7	13.8	14.6	15.1	15.6	16.2	17.3
	2	8.9	10.3	11.1	11.9	13.1	13.9	14.5	15.1	15.8	17.1
	3	9.1	10.7	11.6	12.4	13.5	14.2	14.7	15.2	15.8	16.9
	4	9.1	10.6	11.3	12.2	13.3	14.1	14.6	15.1	15.6	16.7
	5	8.6	10.4	11.2	12.0	13.2	14.0	14.5	15.2	15.7	16.8
	6	9.4	10.9	11.6	12.4	13.5	14.4	14.9	15.4	16.0	17.3
蓄积量 (m^3/hm^2)	1	58.1	90.1	109.4	137.3	173.8	220.0	254.5	275.9	303.6	367.3
	2	44.7	71.7	89.2	112.3	150.0	187.5	221.5	249.0	279.2	345.4
	3	49.4	80.8	100.7	124.2	168.3	208.9	245.5	268.1	291.6	352.2
	4	48.8	78.1	94.9	119.2	158.4	200.7	234.7	255.4	275.4	335.5
	5	41.9	72.2	90.3	115.4	151.5	194.8	228.6	255.2	281.4	337.6
	6	54.2	84.5	103.9	129.1	167.8	210.2	246.2	268.6	295.7	368.8

从历年方差分析表（3-24）可以得出：施肥处理对胸径生长影响差异不显著，区组间从 3.5a 开始差异显著；施肥处理对树高生长影响只有在 2.3a 时在 5％水平上显著，区组间从 4.2a 开始影响差异显著；施肥处理对蓄积量生长影响也是只有在 2.3a 时在 5％水平上显著，区组间的差异从 6.3a 开始。

从上述可知：2.3 年生时树高、蓄积量在 5％水平上显著，说明前期的施肥 2.3 年生时在树高、蓄积量表现出了差异，对后期的生长没有显著的影响。

3.3.2 尾巨桉 13 年生林分结构分析

（1）林分胸径结构

从胸径分级百分比图 3-10 来看：施肥处理整体林分的中大径材的比例都比较小，主要集中在 14 ㎝～20 ㎝这一个径级里；6 种施肥处理林分的中大径材的百分比差异

中国知网　https://www.cnki.net

表 3-24　尾巨桉 6 种施肥处理胸径、树高、蓄积量历年方差分析

Tab. 3-24　Variability analysis of DBH,H,Stock by 6 fertiliser treatments of *E.urophylla*×*E.grandis*

年	变异来源	df	树高			胸径			蓄积量		
			SS	MS	F 值	SS	MS	F 值	SS	MS	F 值
2.3a	施肥间	5	6.43	1.29	3.55*	3.01	0.60	2.47	668.63	133.73	3.36*
	区组间	3	0.14	0.05	0.13	1.77	0.59	2.43	144.74	48.25	1.21
	误　差	15	5.43	0.36		3.65	0.24		597.47	39.83	
	总和	23	12.00			8.43			1410.84		
3.1a	施肥间	5	5.14	1.03	2.73	2.20	0.44	1.79	927.65	185.53	2.32
	区组间	3	0.56	0.19	0.49	2.38	0.79	3.23	554.53	184.84	2.31
	误　差	15	5.65	0.38		3.68	0.25		1201.89	80.13	
	总和	23	11.35			8.27			2684.07		
3.5a	施肥间	5	5.08	1.02	2.17	2.04	0.41	1.53	1148.17	229.63	1.93
	区组间	3	2.18	0.73	1.55	3.94	1.31	4.92*	1445.79	481.93	4.05*
	误　差	15	7.04	0.47		4.00	0.27		1784.61	118.97	
	总和	23	14.30			9.98			4378.57		
4.2a	施肥间	5	5.53	1.11	2.35	1.84	0.37	1.18	1588.64	317.73	1.76
	区组间	3	8.19	2.73	5.81**	4.94	1.65	5.27*	1479.89	493.30	2.74
	误　差	15	7.05	0.47		4.69	0.31		2701.44	180.10	
	总和	23	20.77			11.47			5769.97		
5.3a	施肥间	5	6.31	1.26	1.40	1.30	0.26	0.68	1696.97	339.39	0.82
	区组间	3	9.05	3.02	3.34*	4.18	1.39	3.61*	3904.84	1301.61	3.16
	误　差	15	13.56	0.90		5.80	0.39		6183.21	412.21	
	总和	23	28.92			11.29			11785.03		
6.3a	施肥间	5	6.54	1.31	2.45	1.26	0.25	0.57	2320.89	464.18	0.81
	区组间	3	12.51	4.17	7.80**	8.68	2.89	6.54**	11684.66	3894.89	6.83**
	误　差	15	8.02	0.53		6.64	0.44		8551.00	570.07	
	总和	23	27.07			16.57			22556.55		
7.3a	施肥间	5	5.96	1.19	1.46	1.15	0.23	0.49	2517.29	503.46	0.59
	区组间	3	12.50	4.17	5.09**	8.98	2.99	6.38**	15843.09	5281.03	6.14**
	误　差	15	12.28	0.82		7.04	0.47		12907.82	860.52	
	总和	23	30.75			17.17			31268.21		
8.3a	施肥间	5	3.55	0.71	0.97	0.80	0.16	0.29	1717.54	343.51	0.31
	区组间	3	12.01	4.00	5.47**	9.72	3.24	5.86**	18229.05	6076.35	5.44**
	误　差	15	10.98	0.73		8.29	0.55		16743.41	1116.23	
	总和	23	26.55			18.82			36689.99		
9.2a	施肥间	5	2.25	0.45	0.49	1.08	0.22	0.37	2070.62	414.12	0.27
	区组间	3	12.39	4.13	4.47*	9.39	3.13	5.33*	21379.78	7126.59	4.69*
	误　差	15	13.86	0.92		8.82	0.59		22777.44	1518.50	
	总和	23	28.50			19.29			46227.84		
12.5a	施肥间	5	3.37	0.67	0.54	1.12	0.22	0.27	4675.30	935.06	0.33
	区组间	3	29.81	9.94	7.93**	12.56	4.19	5.11*	45948.90	15316.30	5.39*
	误　差	15	18.80	1.25		12.28	0.82		42596.58	2839.77	
	总和	23	51.98			25.96			93220.79		

注：密度 $F_{0.05}$=2.90；区组 $F_{0.05}$=3.29， $F_{0.01}$=5.41；'*''**'分别表示在 5%和 1%水平上差异显著。

中国知网 https://www.cnki.net

很小，最大的是施肥处理 6，还不足 30%，最小的是施肥处理 1，10%多一点；在 14 cm～20 cm这一个径级里，施肥处理 1 的百分比最大，达到了 70%。

（2）林分树高结构

从树高结构图 3-11 可以看出：整个林分绝大部分的林木树高都在 25m 以上；树高大于 30m 的林木百分比最大的是施肥处理 6，超过了 40%，其它处理百分比从大到小依次是 1、3、4、2、5；树高在 20m－25m 这个级别里，百分比最大的是施肥处理 5，超过了 60%，最小的是施肥处理 6，还不足 40%。

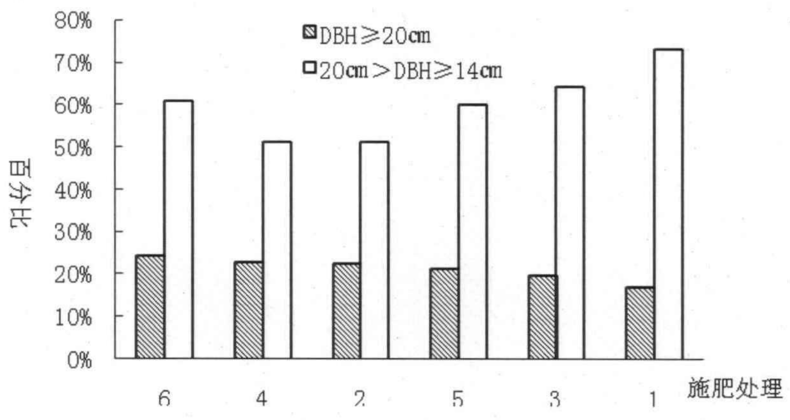

图 3-10 13 年生尾巨桉不同施肥处理林分胸径结构图

Fig. 3-10 chart of DBH structure by 6 fertiliser treatments of 13 years old *E. urophylla × E. grandis*

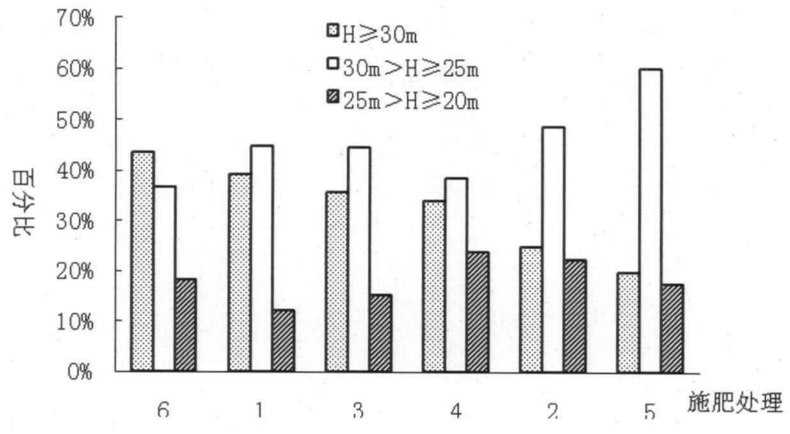

图 3-11 13 年生尾巨桉不同施肥处理林分树高结构图

Fig. 3-11 chart of H structure by 6 fertiliser treatments of 13 years old *E. urophylla × E. grandis*

3.3.3 不同施肥措施对 13 年生尾巨桉材质的影响

（1）不同施肥措施对尾巨桉树皮厚度的影响

从表 3-25 可知：平均树皮厚度施肥处理 3 最厚，施肥处理 1 最薄；平均木材外部密度指标值，施肥处理 2 最小，为 16.27，最大的是处理 5，达到了 17.14；应力波在施

中国知网 https://www.cnki.net

肥处理 1 的传播速度最快，为 3640.5m/s，也就是木材强度最低，应力波在施肥处理 2 的传播速度最慢，为 3469.9m/s，也就是木材强度最高；螺旋纹理倾角最大的是施肥处理 6，其值为 4.59，最小的为施肥处理 3.91，其值为 3.91。

表 3-25 13 年生尾巨桉 6 种施肥处理生长性状和材性性状的平均值

Tab.3-25 Mean values of growth and wood characters with 6 fertiliser treatments of 13 years old *E.urophylla×E.grandis*

密度处理	胸径 (cm)	树高 (m)	蓄积量 (m^3/hm^2)	木材外部密度 Pilodyn	树皮厚度 (mm)	木材强度 FAKOPP(m/s)	螺旋纹理 spiral grain
1	17.5	28.1	410.9	16.54	5.49	3640.5	4.33
2	17.2	27.4	397.4	16.27	5.73	3469.9	4.01
3	17.2	28.0	396.7	16.33	6.64	3540.9	4.52
4	16.9	27.6	376.2	16.93	5.91	3571.8	3.91
5	17.1	27.5	382.2	17.14	6. 54	3604.9	4.48
6	17.5	28.1	415.1	16.62	5.69	3632.8	4.59

从方差分析表 3-26 表明：不同的施肥措施对 13 年生尾巨桉树皮厚度、木材外部密度指标值、螺旋纹理的影响差异不显著；不同施肥处理对应力波在尾巨桉活立木的传播速度的影响差异在 5%水平上差异显著，也就是不同施肥处理间的木材强度差异显著。

表 3-26 13 年生尾巨桉不同施肥措施立木材性指标方差分析

Tab.3-26 Variability analysis of BT, Pilodyn, FAKOPP, SG by 6 fertiliser treatments of 13 years old *E.urophylla×E.grandis* living trees

项目	差异源	df	SS	MS	F
Pilodyn	施肥间	5	2.3414	0.4683	2.46
	区组间	3	2.0742	0.6914	3.63
	误差	15	2.8581	0.1905	
	总计	23	7.2737		
Bark thickness	施肥间	5	4.5555	0.9111	1.15
	区组间	3	0.2695	0.0898	0.11
	误差	15	11.8474	0.7898	
	总计	23	16.6724		
FAKOPP	施肥间	5	1199.6864	239.9373	3.19*
	区组间	3	79.0575	26.3525	0.35
	误差	15	1127.2984	75.1532	
	总计	23	2406.0423		
Spiral grain	施肥间	5	1.5901	0.3180	1.15
	区组间	3	8.2089	2.7363	9.86
	误差	15	4.1632	0.2775	
	总计	23	13.9622		

注：$F_{0.05}$=2.90，‘*’表示在 5%水平上的显著

中国知网 https://www.cnki.net

第四章 结论与讨论

4.1 结论

本研究是以尾巨桉无性系为研究对象，这些尾巨桉无性系试验年龄达到了 10 年以上，以这些材料来研究尾巨桉无性系中大径材的选育和栽培技术是比较合适的，开展了生长、形质、材质性状的遗传分析，通过方差分析和多重比较，分析了不同无性系之间的差异，通过相关分析找出他们之间的相关性，用主成分分析法和简单指数选择法对 25 个尾巨桉无性系进行多性状综合选择，最后对 6 种密度处理和 6 种施肥处理的栽培措施进行了分析，得到的结论如下：

（1）通过对生长、形质、材性性状差异性分析表明：生长、形质、材性性状 25 个尾巨桉无性系之间差异性达到极显著水平（a=0.01）。

（2）从邓肯多重比较和 25 个无性系 9 个性状的平均值可以知道：单株材积大于总体平均值的无性系有 11 个，其中生长性状最优的是无性系 DH33-27，其胸径、树高、单株材积、枝下高分别为 25.0 ㎝、32.8m、0.7722m^3、20.1m；干形指标大于总体平均值的无性系有 13 个，干形最好的是 DH32-28，分枝指标大于总体平均值的无性系有 11 个，分枝指标最好的无性系是 DH32-28；在材质方面，外部密度指标优于总体平均值的无性系有 9 个，外部密度指标最优的无性系是 DH15-3，树皮厚度优于总体平均值的无性系有 13 个，其中最优的是无性系 DH33-32，纤维（纹理）倾角优于总体平均值的无性系有 16 个，其中最优的是无性系 DH33-20。

（3）通过各性状遗传分析表明：各个性状的表型变异系数在 7.92%～56.19%，遗传变异系数在 7.22%～47.17%，遗传变异系数和表型变异系数最大的都是纤维倾角（SG），最小的是 Pilodyn（活立木外部密度指标值）；各个性状无性系重复力在 0.7263～0.9480，最大的是 Pilodyn，最小的是分枝（Br）。

（4）性状之间的相关分析表明：单株材积与胸径、树高呈极紧密的正相关，相关系数超过了 0.9，胸径与树高和枝下高、分枝、干形之间的相关系数达 0.6，相关性极显著；Pilodyn 与胸径、单株材积、枝下高、干形、分枝、树皮厚度呈弱度正相关，而与树高呈弱度负相关，与 SG（纤维倾角）呈中度负相关，相关性在 5%水平上显著；BT（树皮厚度）与胸径、树高、单株材积、枝下高、干形、分枝、纤维倾角呈弱度正相关，与 Pilodyn 呈弱度负相关；SG 除了与 BT 呈轻度正相关之外，与其他 7 个性状呈中度负相关，相关性在 5%水平上显著。

（5）主成分分析表明：取前 4 个主成分已经能代表 88.97%的信息，从 4 个主成分方程结构式来看，把第一主成分称为生长量因子，第二主成分称为第一材性因子（树皮厚度和纤维倾角），第三主成分称为第二材性因子（活立木外部密度指标值和树皮厚度），第四主成分称为形质因子。以这 4 个主成分值采用最短距离法进行系统聚类，当阈值取 2.1，则把 25 个无性系分为 10 类。

（6）采用简单选择指数法多性状综合选择：从 25 个尾巨桉无性系中选出了 7 个优

中国知网 https://www.cnki.net

良无性系，分别为：DH32-28、DH33-27、DH33-9、DH33-20、DH32-26、DH30-1、DH29-10；胸径、树高、单株材积、枝下高、干形、分枝、树皮厚度、木材外部密度指标值、纤维倾角的遗传增益分别为 15.52%、9.89%、39.51%、24.65%、24.65%、30.89%、25.63%、8.66%、24.67%、58.37%，遗传增益最大的是螺旋纹理和单株材积，最小的是树高和外部木材密度指标值。

（7）按国家阔叶树加工用原木分等标准[91]，Expt73. DH32-28、DH33-27、DH32-26、DH61-1、DH15-3 五个无性系就节子缺陷把原木分为三等，DH33-27、DH32-26 为Ⅰ等，DH32-28、DH15-3、DH61-1 为Ⅱ等；弯曲度，DH32-28、DH33-27、DH32-26、DH61-1 都为Ⅰ级，DH15-3 为Ⅱ级；端裂系数最大的是 DH33-27，最小的是 DH15-3。

（8）不同栽培密度措施尾巨桉生长过程分析：胸径和树高生长的年平均生长量和连年生长量的高峰值都出现在 2.3 年时；密度处理 1 年平均蓄积量生长到 6.3 年时达到了最大，密度处理 2、3、6 年平均蓄积量在 7.3 年时达到最大，密度处理 4 在 8.3 年时达到最大，密度处理 5 在 9.2 年时达到最大；密度处理 1、2、3、5、6 的林分蓄积量连年生长量在 3.5 年生时达到最大，而密度处理 4 在 6.3 年时才达到最大。总体来说，密度处理 4 和 5 的胸径和树高平均生长量和连年生长量是最快的，这种趋势在 12.5 年时明显减弱，密度处理 4 在 8.3 年时达到数量成熟龄，密度处理 5 在 9.2 年时从达到数量成熟龄。

（9）6 种密度处理间，胸径在 1%水平上差异显著；不同的密度处理对尾巨桉前 3 年的树高生长影响差异不显著，到 4.2 年时在 5%水平上影响差异显著，在 5.2 年时影响又不显著，从 6.3 年到 12.5 年在 1%水平上影响差异显著；不同的密度处理对尾巨桉平均蓄积量的生长影响差异显著。

（10）不同栽培密度措施尾巨桉 13 年生时的林分结构分析：DBH≥26 ㎝的大径材只有密度处理 5（株行距：5m×3m）有 10%，20 ㎝≤DBH＜26 ㎝也就是中径材，随着密度的减少而增大，密度 5 所占的比例最大，几乎达到了 80%，最少的是密度 1，还不足 10%；随着密度的减小，林分中树高大于等于 30m 的林木百分比逐渐增大，密度处理 5 的林分中树高大于等于 30m 的林木所占的比例最大，达到了 70%。因此，密度处理 5 也就是 667 株/hm^2（株行距：5m×3m）是这 6 种密度处理中最适合培育中大径材的。

（11）6 种密度处理间的树皮厚度差异极显著，区组间差异显著，树皮最厚的是密度处理 5，达到 7.9mm；不同栽培密度对活立木外部密度指标值、木材强度、纤维倾角的影响差异都不显著。

（12）对原木 A、B 段的弯曲度、尖削度、原木两端的端裂程度指数进行方差分析，结果表明：不同的栽培密度措施对原木 A 段的木材缺陷影响不显著，对原木 B 段只有尖削度在 5%水平上影响显著，其余不显著。同一密度处理里的原木 A、B 段方差分析结果表明：同一密度的原木 A、B 段的原木端裂程度指数差异不显著；密度 1、3、5 的原木 A、B 段的弯曲度差异在 5%水平上差异显著；密度 1、3、6 的原木 A、B 段的尖

中国知网 https://www.cnki.net

削度在5%水平上差异显著，密度5在1%水平上差异显著。

（13）施肥处理对胸径生长影响差异不显著，对树高生长影响只有在2.3a时在5%水平上显著，对蓄积量生长影响也是只有在2.3a时在5%水平上显著。说明前期的施肥2.3年生时在树高、蓄积量表现出了差异，对后期的生长没有显著的影响。施肥处理1对尾巨桉前2.3年的生长是最有利的。

（14）施肥处理整体林分的中大径材的比例都比较小，主要集中在14 ㎝～20 ㎝这一个径级里，6种施肥处理林分的中大径材的百分比差异很小；树高大于30m的林木百分比最大的是施肥处理6，超过了40%，树高在20m～25m这个级别里，百分比最大的是施肥处理5，超过了60%。

（15）不同的施肥措施对13年生尾巨桉树皮厚度、木材外部密度指标值、螺旋纹理的影响差异不显著，对应力波在尾巨桉活立木的传播速度的影响差异在5%水平上差异显著，也就是不同施肥处理间的木材强度差异显著。

4.2 讨论

（1）不同尾巨桉无性系的性状差异显著，说明可以对单个性状进行直接选择，根据培育目标选择生长、形质、材质均优的无性系，也可根据性状之间的相关性，对一个性状的改良同时实现对另一个性状的改良。Pilodyn与生长性状、形质性状呈弱度正相关或负相关，说明生长性状和形质性状得到改良时不会造成木材密度急剧下降。

（2）在众多的选择方法中，从理论上说，指数选择是较好的多性状综合选择方法，指数选择法除了考虑主成分的优点外，还考虑了各个性状的重要性的不同，从而确定不同的经济权重，比如要选择生长表现优良的无性系，这样胸径、树高、材积的经济权重就会加大，如果选择材性好的无性系，木材密度等材性性状的经济权重要加大，在用指数选择法时，选择性状不宜过多，性状多了经济权重就比较难以确定，经济权重确定的不适当也会造成选择效果不理想。因此，在进行多性状综合选择时应多用几种方法进行选择，通过比较，根据培育目标来确定优良无性系。

（3）本次研究运用了Pilodyn、FAKOPP、Spiral grain新的仪器进行材性指标的测定，虽然这些仪器不能直接测定材性的真实值，但是跟真实值有着密切的相关性的，在一定程度上代表了真实值，是真实值的反映指标。由于他们测定的快捷、方便，在今后的材性研究工作中应用越来越广泛，应该多研究不同树种，不同无性系真实值与测定值之间的相关性，才能使这方面的研究更加完善、科学。

（4）培育中大径材，合适的栽培密度是非常重要的，较小的栽培密度有利于生长量的生长，但是过疏的栽培密度不利于早期生长和造成土地的浪费，应该跟间伐结合起来，但是间伐时间，间伐强度都有待进一步的研究。同时，应该加强尾巨桉中后期追加施肥，施肥的时间和量有待进一步的研究。

（5）栽培措施对材性的影响是比较复杂的，需要进行更多的试验，采集更多的数据，并跟伐倒样木取样测定结合起来进行研究。

中国知网 https://www.cnki.net

参考文献

[1] 徐建民, 白嘉雨, 陆钊华. 华南地区桉树可持续遗传改良与育种策略[J]. 林业科学研究, 2001, 14(6): 587-597

[2] 项东云, 陈健波, 叶露, 等. 广西桉树人工林发展现状、问题与对策[J]. 广西林业科学, 2006, 35(4): 195-201

[3] W.E.西里斯, A.G.布朗主编. 王豁然等译. 桉树培育与利用[M]. 北京: 中国林业出版社. 1984

[4] 陈少雄. 桉树大径材培育—桉树培育的新方向[J]. 桉树科技, 2002, (1): 6-10

[5] FAO. Tree planting practices in African savannas. FAO Forestry Development Paper No.19. Rome. 1974

[6] FAO. 世界森林状况. 罗马: 英国牛津文字出版社设计出版, 1997

[7] 祁述雄主编. 中国桉树[M]. 北京: 中国林业出版社. 1989

[8] 谢耀坚. 科学发展桉树, 为我国南方新农村建设作贡献[J]. 广西林业科学, 2006, 35(4): 268-270

[9] 余超凡, 陈健波, 骆栋卿. 广西桉树大径材培育前景[J]. 广西林业科学，2006, 35(3): 168-170

[10] 朱积余, 廖培来主编. 广西名优经济树种[M]. 北京: 中国林业出版社, 2006

[11] 周少英, 徐峰. 广西东门林场五种桉树木材干缩和变形特性研究[C]. 摘自《中国林学会木材科学分会第九次学术研讨会论文集》, 2004, 2-6

[12] 江泽慧著. 中国现代林业[M]. 北京: 中国林业出版社, 2000, 14-71

[13] 王明庥主编. 林木遗传育种学[M]. 北京: 中国林业出版社. 2001

[14] 申文辉, 李宏伟, 黄锡泽, 等. 广西东门桉树无性系选育研究[J]. 广西林业科学, 2004, 33(3): 111-114

[15] 白嘉雨. 桉树遗传育种的回顾及发展前景[J]. 广西林业科学, 2006, 35(4): 221-226

[16] IUFRO.The Future of Eucalyptus or Wood Products[R].IUFRO Conference Proceedings. Tasmania, 2000

[17] 江泽慧, 费本华，王喜明, 等. 桉树木材干燥特性与工艺及其皱缩研究现状[J]. 木材工业, 2002, 16(4): 3-6

[18] 鲍甫成, 江泽慧. 中国主要人工林树种木材性质[M]. 北京: 中国林业出版社. 1998

中国知网 https://www.cnki.net

[19] 翁继生，李维甫．桉树的加工利用[J]．建筑人造板，2001(2): 34-37

[20] ZobelB J and van Buijtenen J P.Wood variation.It's causes and contro1. Springer-Verlag. New York.1989

[21] 盛炜彤．国外工业人工林培育的目标及技术途径[J]，世界林业研究，1992，(4): 75-82

[22] Kubler H.Growth stresses in trees and related wood properties.Forestry Abstracts. 1987, 48: 131-189

[23] Chafe S C.Peripheral growth stress and tree diameter in Eucalyptus.Institute of Wood Science Journa1. 1995, 13: 523-525

[24] Yang J L.Growth strain in three provenances of plantation growth Eucalyptus globules. Australia Forestry. 2001, 6-1(4): 248-256

[25] 王豁然，柴修武，郑勇奇，等．不同种源巨桉生长与材性变异[J].林业科学研究，1994, 7(2): 199-202

[26] 徐建民，白嘉雨，甘四明．尾叶桉家系综合选择的研究[J]．林业科学研究，1996, 9(6): 561-567

[27] 李淡清，刘金凤，张必福，等．蓝桉、直干桉主要材性和生长性状的遗传参数和遗传增益测算[J]．云南林业科技，2001，(2): 1-6

[28] 郑白，李琼初，黄锡泽，等．桉树制浆造纸潜力及其材性变异分析[J]．广西林业科学，2002, 31(4): 181-185

[29] 莫晓勇，彭仕尧，龙腾，等．雷州半岛桉树无性系选择及其早期综合评价[J]．林业科学研究，2003, 16(3): 293-298

[30] 陆钊华，徐建民，卢国桓，等．韦塔桉种源多性状综合评价及育种值的估算[J].林业科学研究，2004, 17(2): 220-225

[31] 王克胜，卞学瑜，佟永昌，等．杨树无性系生长与材性的遗传变异及多性状选择[J].林业科学，1996, 32(2): 111-117

[32] 王克胜，卞学瑜，李淑梅，等．杨树优良无性系多性状选择方法研究[J]．林业科技通讯，1995(1): 16-18

[33] 任建中，刘长青，汪清锐，等．杨树纸浆材优良无性系选择方法的研究[J]．北京林业大学学报，2003, 25(4): 25-29

[34] 李善文，姜岳忠，王桂岩，等．黑杨派无性系生长与材性联合选择[J]．林业科学，2005, 41(2): 53-58

[35] 管兰华，潘惠新，黄敏仁，等．美洲黑杨×欧美杨 F1 无性系的多性状联合选择[J].

中国知网 https://www.cnki.net

南京林业大学学报, 2005, 29(2): 6-10

[36] 姜景民, 孙海菁, 刘昭息. 火炬松纸浆材优良家系多性状选择[J]. 林业科学研究, 1996, 9(5): 455-460

[37] 曾令海, 王以珊, 阮梓材. 高脂马尾松优良家系的多性状综合选择[J]. 广东林业科技, 1998, 14(2): 1-8

[38] 孙晓梅, 张守攻, 李时元, 等. 日本落叶松纸浆材优良家系多性状联合选择[J]. 林业科学, 2005, 41(4): 48-54

[39] 刘永红, 杨培华, 樊军锋, 等. 油松优良家系多性状选择方法研究[J]. 西北农林科技大学学报, 2006, 34(12): 115-119

[40] 梁一池, 黄铭利. 锥栗无性系多性状综合选择的研究[J]. 中南林学院学报, 1996, 16(1): 50-55

[41] 郑郁善, 洪伟, 吴摧溪. 杉木种子园优良无性系综合选择研究[J]. 生物数学学报, 1997, 12(5): 599-605

[42] 夏根清, 吴毅聪, 徐金良, 等. 杉木造林优良种源多性状选择研究[J]. 浙江林业科技, 1999, 19(5): 1-5

[43] 杨途熙, 魏安智, 杨焕叶, 等. 泡桐无性系数量性状的遗传分析和指数选择研究[J]. 西北植物学报, 1997, 17(3): 374-381

[44] 方玉霖. 桉树多性状综合选择的研究[J]. 福建林学院学报, 1995, 15(2): 184-188

[45] 徐建民, 白嘉雨, 甘四明. 尾叶桉家系综合选择的研究[J]. 林业科学研究, 1996, 9(6): 561-567

[46] Xiaoyong Mo,shiyao Peng,Teng Long,et al. Important traits and combined evaluation of Eucalyptus clones.In:Run-peng Wed.Eucalyptus plantations,World scientific, 2003

[47] 姚庆端. 桉树优良无性系制浆造纸性能与适应性的研究[J]. 福建林学院学报, 2004, 24(4): 316-322

[48] 陆钊华, 徐建民, 卢国桓, 等. 韦塔桉种源多性状综合评价及育种值的估算[J].林业科学研究, 2004, 17(2): 220-225

[49] 杨民胜, 刘效章, 陈少雄. “桉树珍贵用材树种引进”课题组赴澳采购及考察报告[J]. 桉树科技, 2001(2): 1-8

[50] M.R.Jacobs. 桉树栽培[M]. 罗马: 联合国粮食及农业组织, 1979

[51] Medhurst J.L.etal. Early-age and Later-age Thinning Affects Growth, Dominance and Intraspecific Competition in Eucalyptus intense Plantations. In: Canadian journal of

中国知网 https://www.cnki.net

forest research. Can.j.for.res.Feb 2001.v.31(2)p.187-197

[52] 殷亚方，杨民胜，王丽娟，等．巴西桉树人工林资源及其实木加工利用[J]．世界林业研究, 2005, 18(1): 60-64

[53] 项东云，郑白，周维，等．广西桉树育种研究概述[J]．广西林业科学，1999，28(2): 71-80

[54] 林国金．闽南桉树大径材培育技术[J]．林业实用技术, 2005(6): 20

[55] 潘平开，申文辉，周国福，等．良种桉中大径材复层林经营试验研究[J]．广西林业科学，2005, 34(3): 116-119

[56] 黄锡泽，周国福，李宏伟，等．尾巨桉人工林栽培密度研究[J]．广西林业科学, 2005, 34(1): 5-7

[57] 杨先锋，叶金山．关于杉木大径材定向培育几项措施的初步探讨.江西林业科技[J], 2001, (2): 32-34

[58] 朱林峰，方文彬．初植密度对尾叶桉木材材性影响规律的研究[J]．世界林业研究, 1995, (8): 327-335

[59] 黄宝灵，吕成群．不同造林密度对尾叶桉生长、产量及材性的影响[J]．林业科学, 2000, (1): 81-90

[60] 罗建举，曹琳，杨建林，等．施肥处理对尾叶桉木材化学成分含量的影响[J]．林业科学, 1998, 34(5): 96-102

[61] 方文彬．人工林尾叶桉施肥与未施肥林材性的差异[J].世界林业研究，1995(8): 344-349

[62] 罗真付，徐永吉，潘彪，等．施肥处理对尾叶桉生长量和木材密度的影响[J]．南京林业大学学报, 1999, 23(5): 31-34

[63] 潘彪，徐永吉，李贻铨，等．施肥处理对尾叶桉无性系纸浆材生长和材性的影响[J]．南京林业大学学报, 2004, 28(5): 11-14

[64] Panshin, A.J.,and C.de Zeeuw,1980,Textbook of wood Technology,McGraw-Hill Book Company

[65] 南京林学院．高等林业院校木材学试用教科书．农业出版社. 1961

[66] 汉斯.迈耶尔著．造林学[M]．第二分册．肖承刚，郑慧莹译．北京:中国林业出版社, 1989

[67] Kunesh,R.H.,and J.W.Johnson,1972,Effect of single knots on tensile strength of 2-by 8-inch Douglas-fir dimension lumber. Forest Products Journal, 22(1): 32-35

中国知网　https://www.cnki.net

[68] 戴澄月. 兴安落叶松木节对受弯构件承载能力影响的研究[J]. 东北林学院学报, 1981, 81(2): 41-47

[69] 魏亚, 黄达章, 白同仁. 木材性质的研究（一）[M]. 林业出版社, 1957

[70] 别列雷金. 木材缺点对材性的影响[M]. 中国林业出版社, 1957

[71] 檀庆忠, 黎维英, 陈致旺. 闵东桉树引种抗逆性的早期选择[J]. 林业科技开发, 2006, 20(3): 45-48

[72] 李淡清,刘永平,郑行生, 等. 直干桉生长性状的遗传效应分析[J]. 林业科学, 2003, 39(2): 52-57

[73] 李淡清, 刘永平, 曾德贤, 等. 蓝桉 6×6 全双列交配生长性状的遗传效应分析[J]. 遗传学报, 2002, 29(9): 835-840

[74] 潘惠新, 黄敏仁, 李火根, 等. 美洲黑杨新无性系干形性状遗传变异初步研究[J].南京林业大学学报, 1999, 23(5): 1-6

[75] 余雪标, 杨敬华, 吕春艳, 等. 不同连载代次桉树木材主要性质的研究[J]. 林业科学, 1999, 35(5): 94-98

[76] 陈章水. 泡桐原木削度分析. 研究报告, 1984 b , (2): 58-63

[77] 肖祥希. 修枝对福建柏林分生长及无节材形成的影响[J]. 林业科学研究, 2005, 18(1): 22-26

[78] 吴际友, 童方平, 龙应忠, 艾文胜. 火炬松纸浆材优良家系人工林修枝效应[J]. 林业科技开发, 2006, 20(2): 36-38

[79] Cown D.J. Comparison of the Pilodyn and torsi meter methods for the rapid assessment of wood density in living trees. New Zealand J.For. Sci. 1978, (8): 384-391

[80] Taylor F.W. Rapid determination of southern pine specific gravity with a Pilodyn tester. For.Sci. 1981, 27: 59-61

[81] Hall J P et al. Using the Pilodyn tester in selecting plus trees. NJA F, 1988, (5)：170-171

[82] Mac Donald A C et al. Genetic variation for growth and wood density in Eucalyptus globules ssp. globulus inTasmania (Australia). Silvae Genetics, 1997, 46 (4) : 236-241

[83] Wei X et al. Genetic control of wood density and bark thickness and their relationships with growth traits of Eucalyp tusurophylla in South East China. Silvae Genetics, 1997, 46 (4) : 245-250

[84] 王志同, 曹志强, 袁卫国. 用应力波非破损检测技术检测中密度纤维板弹性模量的研究[J].木材工业. 1995, 9(5): 17-21

中国知网 https://www.cnki.net

[85] 胡英成, 顾继友, 王逢瑚. 木材及人造板物理力学性能无损检测技术研究的发展与展望[J]. 世界林业研究. 2002, 15(4): 39-46

[86] 林文树, 杨慧敏, 王立海. 超声波与应力波在木材内部缺陷检测中的对比研究[J]. 林业科学, 2005, 30(2): 39-41

[87] 杨学春, 王立海. 应力波技术在木材性质检测中的研究进展[J]. 森林工程, 2002, 18(6): 11-12

[88] 陈清波, 陈红林, 藤泽义武. 利用 FAKOPP 评价树干杨氏弹性模量可行性研究[J]. 湖北林业科技. 2007, (143): 13-15

[89] 徐大平, 张宁南. 桉树人工林生态效应研究进展[J]. 广西林业科学, 2006, 35(4): 179-187

[90] 郑镜明, 王洪峰. 论我国南方发展珍优阔叶树大径材人工林[J]. 热带林业, 2002，30(2): 41-45

[91] 项东云. 新世纪广西桉树人工林可持续发展策略探讨[J], 广西林业科学, 2002, 31(3): 114-121

[92] 赵荣军, 费本华, 江泽慧, 等. 桉树人工林木材颜色和耐光性[C]. 摘自《中国林学会木材科学分会第九次学术研讨会论文集》, 2004, 2-13

[93] 马育华. 植物育种的数量遗传学基础[M]. 南京: 江苏科技出版社, 1982, 224-375

[94] 刘永红, 杨培华, 樊军锋. 油松优良家系多性状选择方法研究. 西北农林科技大学学报 (自然科学版) [J]. 2006, 34(12): 115-119

[95] Zobel BJ. Talber J. Applied Forest Tree Improvement.John Wiley & Sons,1984

[96] 王明庥主编. 林木育种学概论[M]. 北京: 中国林业出版社. 1988

[97] 沈熙环编著. 林木育种学[M]. 北京: 中国林业出版. 1988

[98] 国家标准（GB4812-84）, 特级原木

[99] 国家标准（GB4818-84）, 铁路货车锯材

[100] 国家标准（GB4819-84）, 载重汽车锯材

[101] 国家标准（GB4820-84）, 罐道木

[102] 国家标准（GB4821-84）, 机台木

[103] 国家标准（GB4813-84）, 阔叶树加工用原木分等

[104] 尹思慈编著. 木材品质和缺陷[M]. 北京: 中国林业出版社. 1990

中国知网 https://www.cnki.net

附图

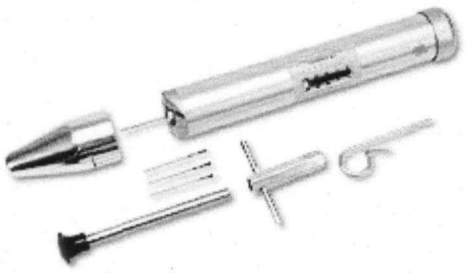

图 2-1　Pilodyn 6J 仪器

Fig.2-1　Pilodyn 6J equipment

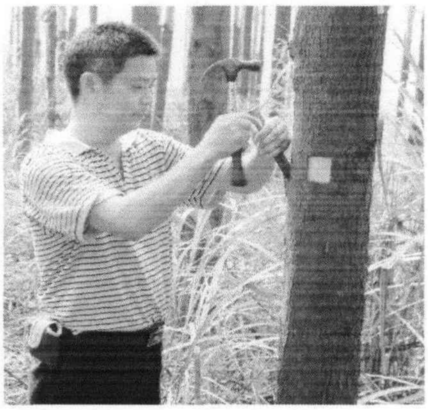

图 2-2 在胸高处开窗口

Fig.2-2　Cut windows in breast height of living trees

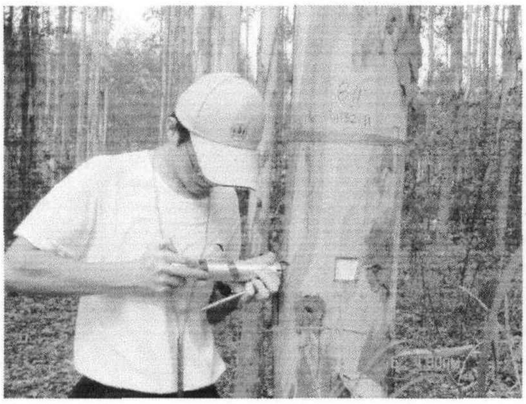

图 2-3　用 Pilodyn 进行测定

Fig.2-3　Measuring in living trees

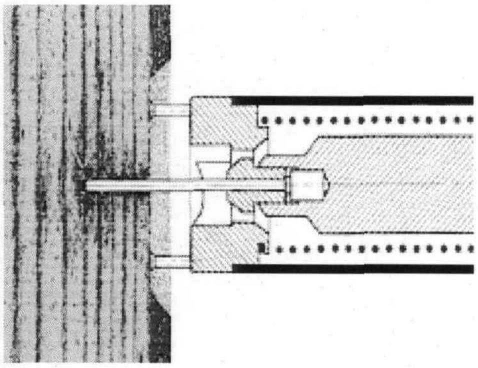

图 2-4　钢针射入立木示意图

Fig.2-4　The pin shot into the wood

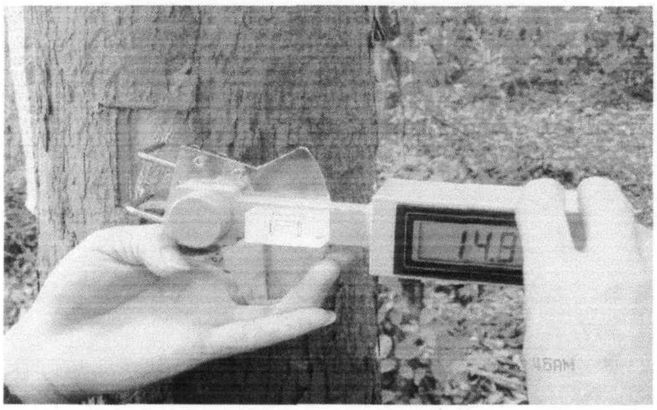

图 2-5　纤维倾角测定

Fig.2-5　Measuring spiral grain in living trees

中国知网　https://www.cnki.net

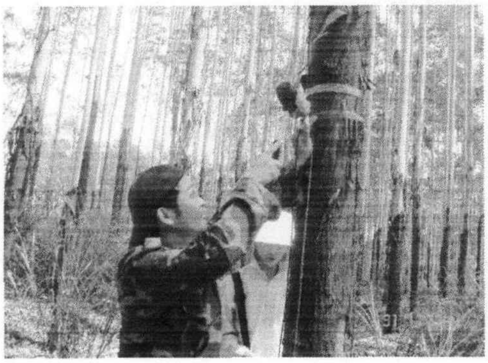

图 2-6 把 Stop 传感器打入活立木上端
Fig.2-6 Knock the Stop transducer into living trees

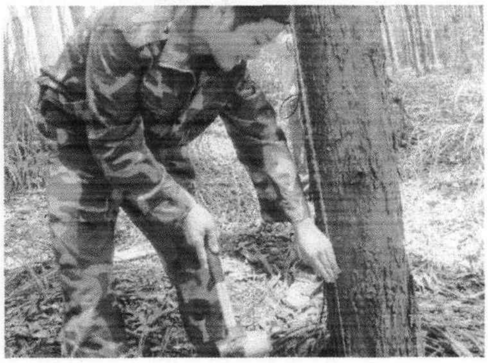

图 2-7 把 Start 传感器打入活立木下端
Fig.2-7 Knock the Start transducer into living trees

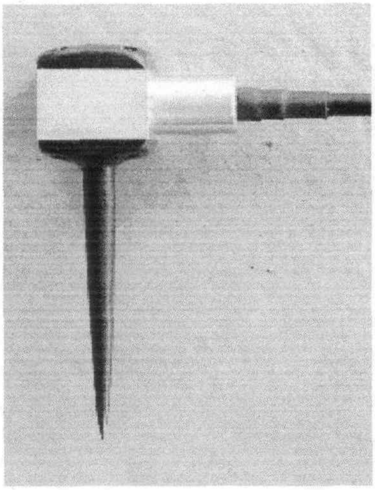

图 2-8 Start & Stop 传感器
Fig.2-8 Start & Stop transducer

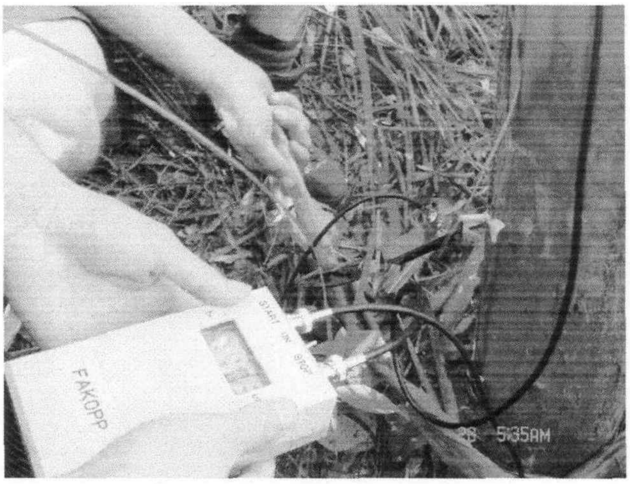

图 2-9 敲击 Start 传感器进行测量
Fig.2-9 Knock the start transducer to testing

中国知网 https://www.cnki.net

致 谢

在我论文完成之际，我要感谢三年硕士生活赐予我的一切。有成功和喜悦，也有失败和悲伤，但是正是这一切让我的思想逐渐成熟。三年的硕士生活，老师的关心和教诲，同学的帮助和鼓励，朋友的理解和安慰，家人的支持和关爱。总之，我要感谢三年生活给我带来的点点滴滴，它们将是我一生的财富。

首先我要感谢我的两位导师，能够成为桉树专家项东云教授级高工和梁机副教授的学生是我一生的荣幸，他们一丝不苟的作风，严谨求实的态度，踏踏实实的精神，不仅授予我知识，而且教我做人，使我终身受益。特别感谢项老师，他在完成博士论文的关键时候，晚上加班帮我们修改、排版论文，花费了巨大了精力，对导师的感激之情是无法用言语表达的，在这里我要对我的导师说声“谢谢”。

感谢广西林科院、国家林业局中南速生材繁育实验室的陈健波高工、申文辉工程师以及叶露、唐庆兰、张照远和梁萍在外业工作中无私的帮助和论文撰写过程中的悉心指导。非常感谢翟新翠师姐，龙腾周师弟给予的帮助和鼓励。

衷心感谢广西东门林场李宏伟场长、林科所的周国福所长、兰俊副所长、陈东林工程师和吴兵等人和国家桉树中心的陈少雄主任、彭彦主任、吴志华、周群英在外业和内业工作中提供的帮助。同时感谢澳大利亚ACIAR项目的专家Michael Henson博士、Kevin Harding博士、Steve Boyton先生、John Simpson博士、Russell Washusen博士和Richard Northway先生的指导和帮助。

最后向三年来所有关心和帮助我的领导、老师、同学和朋友，表示最衷心的谢意！感谢对论文进行评审并提出宝贵建议的各位专家！

中国知网 https://www.cnki.net

攻读学位期间发表论文情况

李昌荣，项东云，周国福等. 栽培密度与施肥措施对尾巨桉中大径材生长的影响. 广西林业科学，2007，36（1）：31-35

导师简介

项东云，男，壮族，1960 年 1 月出生，广西扶绥人，中共党员，1983 年 7 月毕业于广西农学院林学分院林学系，南京林业大学林木育种博士研究生，教授级高工。1983 年 8 月 1995 年 10 于在广西东门林场工作，参加中澳技术合作东门桉树示范林项目；1995 年 12 月到广西林科院工作，现任院长、国家林业局中南速生材繁育实验室主任，中国林学会理事、桉树专业委员会常务委员、林木遗传育种分会委员、树木引种驯化委员会常务委员，广西林学会常务理事、桉树专业委员会主任、林木遗传育种专业委员会常务委员和广西大学兼职教授。

长期以来从事桉树遗传改良和栽培技术研究，主持和主要参加完成国家、省（部）级科研课题 11 项。获国家科技进步奖三等奖 1 项，省（部）级科技进步奖二等奖 5 项、三等奖 3 项。发表科技论文 60 篇，合著出版专著 5 部，多次参加 IUFRO 和 FAO 的学术讨论会。现主持和主要参加包括 ITTO、中澳越技术合作项目和国家林业局 948 项目等科技项目 6 项。1998 年被确定为广西“十百千人才工程”第二层次人选；1999 年获广西优秀专家；2003 年被人事部、国家林业局评为全国林业系统先进工作者；2005 年获得广西林业科技重奖集体和个人；为广西林木育种和丰产林栽培等领域的学科带头人和专家。

中国知网 https://www.cnki.net
