# Supplementary material for: Provenance and family variations in early growth of Manchurian walnut (Juglans mandshurica Maxim.) and selection of superior families
Source: PLoS One. 2024 Mar 7;19(3):e0298918. doi: 10.1371/journal.pone.0298918 (PMC10919699; doi:10.1371/journal.pone.0298918)
Supplement: S2 File — (ZIP) [file pone.0298918.s005.zip › Study on genetic variation law of Juglans mandshurica provenances.pdf]

胡桃楸种源遗传变异规律的研究

夏德安[1],许忠志[2],侯丹[1],周佳[1],王会仁[1]
(1. 东北林业大学林木遗传育种国家重点实验室,黑龙江哈尔滨 150040;2. 辽宁省林业种苗管理总站,辽宁沈阳 110006)

摘要　采用方差分析、相关分析等统计方法研究了胡桃楸种源试验林的遗传变异和优良种源的选择。结果表明,胡桃楸种源间的树高、胸径、材积等生长性状的差异都达到显著或极显著水平;胡桃楸的主要性状与经度、纬度、海拔的相关系数都未达到显著水平,呈现随机变异;胡桃楸生长性状与气候因子的相关系数均未达到显著水平,但从相关系数看,年均温、年积温、日照常数和年降水量对生长有一定影响,表现为随着年均温、年积温、日照常数和年降水量的增加,树高、胸径、材积等生长性状的取值增大;舒兰是优良种源,材积比其他种源高出64.03%。

关键词　胡桃楸;地理变异;种源
中图分类号　S792.132　**文献标识码**　A　**文章编号**　0517 - 6611(2014)31 - 10956 - 03
DOI:10.13989/j.cnki.0517-6611.2014.31.047

Study on Genetic Variation Law of *Juglans mandshurica* Provenances

XIA De-an[1], XU Zhong-zhi[2], HOU Dan[1] et al　(1. State Key Laboratory of Tree Genetics and Breeding, Northeast Forestry University, Harbin, Heilongjiang 150040; 2. Liaoning Province Forestry Seed Management Station, Shenyang, Liaoning 110006)

Abstract　The genetic variation and provenance selection were studied using analysis of variance, correlation analysis and other methods in Juglans mandshurica provenance experimental forest. The results showed that the height, DBH and volume among Juglans mandshurica provenance are significant or very significant level. The correlation coefficients between the main characters of Juglans mandshurica and the longitude, latitude, elevation aren't significant and their variations are random. The correlation coefficients between growth trait of Juglans mandshurica and climate factors are not significant. According to the correlation coefficient, mean annual temperature, annual accumulated temperature, sunshine duration and annual precipitation have impact on growth. With the increasing in mean annual temperature, annual accumulated temperature, sunshine and annual precipitation, these traits of value increase. Shulan is the optimal provenance, its volume 64.03% higher than other provenance.

Key words　*Juglans mandshurica*; Geographical variation; Provenances

胡桃楸(*Juglans mandshurica* Maxim)属于胡桃科(Juglandaceae)胡桃属(*Juglans*)。胡桃属约20种,分布于北温带,我国有5种1变种。胡桃楸与水曲柳、黄菠萝并称“东北三大硬阔”,是国家Ⅱ级珍稀树种和我国珍稀濒危树种的三级保护植物[1]。其木材有光泽,刨面光滑,纹理美观,并有坚韧不裂、耐腐蚀等优点,被广泛用于军工、建筑、家具、车辆、舰船和乐器等。其种仁含油率为40% ~63%,营养丰富,可榨油,是很有发展前途的木本油料植物[2];其果壳可制活性炭;其树皮含单宁,可制栲胶。

目前,由于过量采伐,胡桃楸天然林大树接近枯竭。进行胡桃楸种源试验,对于收集与保存资源、保护生物多样性、维护生态平衡具有重大的理论指导意义。同时,通过种源试验选择优良种源,可为林业生产提供良种、提高胡桃楸林分的生长量,为以后的植树造林以及森林的经营提供更加便利的条件。鉴于此,笔者采用方差分析、相关分析等统计方法研究了胡桃楸种源试验林的遗传变异和优良种源的选择,旨在为林业生产提供参考。

1　材料与方法

1.1　试验地概况　试验地点位于黑龙江省尚志市东北林业大学试验林场,地理坐标为45°24′N、127°30′E。

1.2　胡桃楸种源试验　于1985年开始进行胡桃楸种源试验,采种点有8个,分别是辽宁省的宽甸,吉林省的临江、汪清、舒兰,黑龙江省的东方红、桦南、帽儿山、带岭。1985年秋季分别从各点采集种子,翌春在帽儿山林场直播造林。采用随机完全区组设计,5次重复,双行小区,每小区50穴,每穴播5粒种子,试验地周围有保护行。

1.3　调查方法　2009年秋季对试验林进行调查。每个区组每个种源选取10株具有代表性的树木作为调查样株。每株样株分别调查树高、胸径、冠幅、枝下高、一级侧枝数(简称侧枝数)和分枝角6个性状,并利用树高与胸径的调查数据计算材积。

1.4　数据处理　利用统计软件对调查数据进行处理,计算出平均数、标准差及变异系数,用方差分析研究种源之间的差异显著性,用相关分析研究性状与环境因子的相互关系,用选择指数法选择优良种源。

2　结果与分析

2.1　各种源的基本情况　由表1可知,8个种源树高平均值为9.618 m,变化幅度在8.968 0 ~10.816 0 m;胸径平均值为8.595 0 cm,变化幅度在7.741 0 ~10.184 0 cm;材积平均值为0.032 0 m^3,变化幅度在0.024 6 ~0.048 6 m^3;冠幅平均值为4.590 0 m,变化幅度在4.256 0 ~5.513 0 m;枝下高平均值为7.112 0 m,变化幅度在6.691 0 ~7.726 0 m;侧枝数平均值为10.500 0个,变化幅度在9.370 0 ~11.880 0个;分枝角平均值为36.304 0°,变化幅度在32.860 0 ~38.300 0°。

从变异系数来看,树高、胸径、冠幅、枝下高、侧枝数及分枝角6个性状的变异系数要么较小,要么十分接近,这有可能是受相似的环境因素影响造成的。材积的变异系数较大,大致在45.30% ~71.20%,这有可能是植株本身及外界环境共同造成的结果。

作者简介　夏德安(1962 -),男,黑龙江哈尔滨人,副教授,硕士,从事林木遗传育种研究。
收稿日期　2014-09-26

(C)1994-2021 China Academic Journal Electronic Publishing House. All rights reserved.　http://www.cnki.net

表 1　帽儿山试验点胡桃楸种源情况

种源	项目	树高//m	胸径//cm	材积//m^3	冠幅//m	枝下高//m	侧枝数//个	分枝角//°
带岭	平均数	8.968 0	7.869 0	0.024 6	4.307 0	6.691 0	9.970 0	36.470 0
	标准差	0.773 0	1.972 0	0.012 9	0.955 0	0.632 0	2.960 0	5.580 0
	变异系数	0.086 0	0.251 0	0.523 0	0.222 0	0.094 0	0.297 0	0.153 0
东方红	平均数	9.467 0	8.083 0	0.026 9	4.531 0	7.071 0	10.530 0	38.270 0
	标准差	0.733 0	1.746 0	0.012 9	0.921 0	0.762 0	2.430 0	9.160 0
	变异系数	0.077 0	0.216 0	0.481 0	0.203 0	0.108 0	0.231 0	0.239 0
桦南	平均数	9.287 0	7.922 0	0.025 3	4.256 0	6.923 0	9.700 0	37.330 0
	标准差	0.929 0	1.704 0	0.011 5	0.884 0	0.848 0	2.820 0	5.380 0
	变异系数	0.100 0	0.215 0	0.453 0	0.208 0	0.123 0	0.290 0	0.144 0
宽甸	平均数	10.345 0	9.440 0	0.040 4	4.888 0	7.726 0	11.220 0	38.200 0
	标准差	1.302 0	2.357 0	0.022 1	0.972 0	0.838 0	3.050 0	8.000 0
	变异系数	0.126 0	0.250 0	0.547 0	0.199 0	0.108 0	0.272 0	0.209 0
临江	平均数	9.304 0	8.388 0	0.028 7	4.552 0	6.786 0	9.960 0	33.200 0
	标准差	0.829 0	2.010 0	0.014 7	1.002 0	0.910 0	2.520 0	5.600 0
	变异系数	0.089 0	0.240 0	0.512 0	0.220 0	0.134 0	0.253 0	0.169 0
帽儿山	平均数	9.934 0	9.135 0	0.035 5	4.719 0	7.262 0	11.880 0	35.800 0
	标准差	0.933 0	2.017 0	0.017 4	0.708 0	0.507 0	5.860 0	6.340 0
	变异系数	0.094 0	0.221 0	0.491 0	0.150 0	0.070 0	0.493 0	0.177 0
舒兰	平均数	10.816 0	10.184 0	0.048 6	5.153 0	7.676 0	11.340 0	38.300 0
	标准差	0.913 0	2.541 0	0.029 1	1.041 0	0.754 0	2.510 0	8.060 0
	变异系数	0.084 0	0.250 0	0.598 0	0.202 0	0.098 0	0.222 0	0.210 0
汪清	平均数	9.304 0	7.741 0	0.026 0	4.314 0	6.763 0	9.370 0	32.860 0
	标准差	0.975 0	2.447 0	0.018 5	0.972 0	0.828 0	3.250 0	6.610 0
	变异系数	0.105 0	0.316 0	0.712 0	0.225 0	0.122 0	0.347 0	0.201 0

2.2　各性状方差分析　对各个性状进行了方差分析，帽儿山试验点不同种源间树高、胸径、材积、冠幅及枝下高的差异皆达到极显著水平，说明不同种源间树高、胸径、材积、冠幅及枝下高的遗传差异较突出，选择潜力很大; 不同种源间侧枝数的差异达到显著水平，说明不同种源间侧枝数的遗传差异较突出，选择潜力较大; 不同种源间分枝角的差异未达到显著水平，说明不同种源间分枝角的遗传差异不大，选择潜力很小。

2.3　胡桃楸生长性状的地理变异规律　为了了解生长性状与地理因子的关系，计算二者之间的相关系数。由表 2 可知，没有一个相关系数达到统计上的显著水平，说明胡桃楸种源的生长性状与地理因子没有明显关系，呈现的是随机变异规律。

表 2　胡桃楸生长性状与地理坐标的相关关系

地理位置	项目	树高	胸径	材积	冠幅	枝下高	侧枝数	分枝角
纬度	相关系数	−0.386	−0.380	−0.396	−0.416	−0.374	−0.117	0.303
	显著水平	0.354	0.353	0.332	0.305	0.361	0.782	0.466
经度	相关系数	−0.552	−0.656	−0.635	−0.582	−0.544	−0.445	0.033
	显著水平	0.156	0.078	0.091	0.130	0.163	0.269	0.938
海拔	相关系数	−0.256	−0.192	−0.239	−0.041	−0.233	0.084	0.074
	显著水平	0.540	0.649	0.569	0.923	0.579	0.842	0.862

2.4　胡桃楸性状与气候因子的关系　为了了解生长性状与气候因子的关系，计算二者之间的相关系数。由表 3 可知，胡桃楸种源的生长性状与气候因子的相关系数都没有达到显著水平。但是有些性状与气候因子的相关系数较大。树高、胸径与年均温、年积温、日照时数有一定的关系，相关系数都是正数，说明随着年均温、年积温、日照时数的增大，树高和胸径增大。冠幅与年均温、年积温、日照时数、年降水量有一定的关系，相关系数都是正数，说明随着年均温、年积温、日照时数、年降水量的增大，冠幅增大。枝下高与年均温、年积温有一定的关系，相关系数都是正数，说明随着年均温、年积温的增大，枝下高增大。

2.5　优良种源的选择　在选择优良种源时，往往选材积大、枝下高比较高、侧枝数少、分枝角接近 90°的种源，但将单一或少数几个性状作为选择标准往往误差大且不方便。为了避免上述问题，该研究定义一个选择指数(Zi) 作为选择最佳种源的标准，即分别对各种源的材积(V) 赋予权重 0.80、枝下高(Hz) 赋予权重 0.10、侧枝数的相反数(Cz) 赋予权重 0.05及分枝角(Fz) 赋予权重 0.05，选择最大 Zi 值的种源作为优良种源。Zi 计算公式如下:

$$Zi = 0.80V + 0.10Hz + 0.05Cz + 0.05Fz$$

(C)1994-2021 China Academic Journal Electronic Publishing House. All rights reserved.　http://www.cnki.net

从表 4 可知，Zi 的最大值为 2.154 5，对应的种源是舒兰种源。因此，将舒兰种源选为优良种源，其次是宽甸种源。

舒兰种源树高（10.816 0 m）比其他 7 个种源树高平均值（9.516 0 m）高出 13.67%；胸径（10.184 0 cm）比其他 7 个种源胸径平均值（8.368 0 cm）高出 21.70%；材积（0.048 6 m^3）比其他 7 个种源材积平均值（0.029 6 m^3）高出 64.03%；枝下高（7.676 0 m）比其他 7 个种源枝下高平均值（7.032 0 m）高出 9.16%；侧枝数（11.340 0 个）在 8 个种源中数目一般；分枝角（38.300 0°）比其他 7 个种源分枝角平均值（36.020 0°）高出 6.33%。总之，舒兰种源材积大、枝下高比较高、侧枝数较少、分枝角较大。可见，利用选择指数（Zi）选择舒兰为优良种源是有意义的。

表 3　胡桃楸生长性状与气候因子的相关关系

气候因子	项目	树高	胸径	材积	冠幅	枝下高	侧枝数	分枝角
年均温	相关系数	0.560	0.506	0.538	0.516	0.572	0.163	0.002
	显著水平	0.149	0.200	0.169	0.190	0.138	0.700	0.996
1 月均温	相关系数	-0.112	-0.162	-0.154	-0.167	0.040	-0.151	-0.261
	显著水平	0.792	0.701	0.716	0.693	0.925	0.721	0.532
7 月均温	相关系数	-0.417	-0.423	-0.483	-0.435	-0.240	-0.251	-0.197
	显著水平	0.305	0.296	0.226	0.281	0.567	0.548	0.640
年积温	相关系数	0.553	0.574	0.564	0.509	0.513	0.272	-0.098
	显著水平	0.155	0.137	0.145	0.197	0.193	0.515	0.817
绝对湿度	相关系数	0.033	0.123	0.073	0.223	0.157	0.379	0.315
	显著水平	0.939	0.771	0.864	0.596	0.711	0.355	0.448
相对湿度	相关系数	0.226	0.343	0.295	0.452	0.164	0.432	-0.279
	显著水平	0.590	0.405	0.479	0.261	0.698	0.285	0.504
年降水量	相关系数	0.451	0.507	0.468	0.567	0.490	0.409	-0.074
	显著水平	0.262	0.199	0.242	0.143	0.218	0.314	0.863
日照时数	相关系数	0.603	0.581	0.607	0.473	0.484	0.352	-0.180
	显著水平	0.113	0.131	0.111	0.236	0.224	0.393	0.669

表 4　胡桃楸最佳种源选择指数

种源	Zi	种源	Zi
带岭	2.013 8	临江	1.863 6
东方红	2.115 6	帽儿山	1.950 6
桦南	2.094 0	舒兰	2.154 5
宽甸	2.153 9	汪清	1.871 6

3　结论与讨论

3.1　结论　方差分析表明，胡桃楸种源间的树高、胸径、材积、冠幅及枝下高的生长性状的差异都达到极显著水平，不同种源间侧枝数的差异达到显著水平。对胡桃楸种源的地理变异研究结果表明，胡桃楸的主要性状与经度、纬度、海拔的相关系数都未达到显著水平，呈现随机变异的规律。胡桃楸生长性状与气候因子的相关系数均未达到显著水平。但从相关系数看，年均温、年积温、日照常数和年降水量对生长有一定影响，表现为随着年均温、年积温、日照时数和年降水量的增加，生长性状的取值增大。舒兰是优良种源，胸径比其他种源高出 21.70%，材积比其他种源高出 64.03%。

3.2　讨论　对单一地区即帽儿山地区的胡桃楸进行种源试验研究，可为帽儿山地区及其相似地区提供优良种源参考。该试验还研究了树高、胸径与气候因子的关系，当优良种源不足时，可根据相关性的规律从其他地区调种。例如树高与纬度呈负相关，调种时可从纬度低的地区调种。

8 个种源胡桃楸的胸径变异系数为 21.50% ~31.60%，与褚宪丽等[2]对青山林场胡桃楸种源家系试验林胸径调查所得的变异系数（25.58% ~28.77%）相当，说明胸径的变异与种源地和试验地的关系不大，但具体的关系还需进一步试验验证。

从 8 个种源中选择出舒兰为最佳种源，与杨书文等[3-4]通过分布在东北地区胡桃楸的种源研究所得的“舒兰种源为最佳种源”的结论一致。

该研究中，胡桃楸种源的性状与地理因子没有明显的关系，呈现的是随机变异的规律，与杨书文[3-4]等研究得到的“东北地区胡桃楸的地理变异规律”不符。这可能与调查时胡桃楸树龄的大小有关系。虽然该研究中胡桃楸种源的性状与地理因子没有明显的关系，但相关系数大多为负值，与马建路[5]研究得出的“胡桃楸最适生长立地是阴坡的下坡位”的结论相似。

参考文献

[1] 王东娜，牟长城，冯富娟. 胡桃楸 ISSR-PCR 反应体系的建立及优化[J]. 实验室研究与探索，2010，29(11)：18-22.
[2] 褚宪丽，朱航勇，张含国，等. 胡桃楸种源家系变异与选择[J]. 东北林业大学学报，2010，38(11)：5-7.
[3] 杨书文，刘桂丰，张世英，等. 胡桃楸地理变异规律及最佳种源的初步选择[J]. 东北林业大学学报，1990(S2)：72-76.
[4] 刘桂丰，杨书文. 胡桃楸种源的初步区划及最佳种源选择[J]. 东北林业大学学报，1991，19(S2)：189-195.
[5] 马建路. 水曲柳、胡桃楸、黄波罗和紫椴适生立地条件的对比研究[J]. 林业资源管理，1996(4)：52-56.

(C)1994-2021 China Academic Journal Electronic Publishing House. All rights reserved. http://www.cnki.net
