# Supplementary material for: Provenance and family variations in early growth of Manchurian walnut (Juglans mandshurica Maxim.) and selection of superior families
Source: PLoS One. 2024 Mar 7;19(3):e0298918. doi: 10.1371/journal.pone.0298918 (PMC10919699; doi:10.1371/journal.pone.0298918)
Supplement: S2 File — (ZIP) [file pone.0298918.s005.zip › Study on seed morphology, quality and seedling growth of Juglans mandshurica from different provenances.pdf]

尤海舟,郭福忠,金长谦,等. 胡桃楸不同种源种子形态、质量及苗期生长研究[J]. 江苏农业科学,2020,48(2):155 - 158.
doi:10.15889/j.issn.1002 - 1302.2020.02.028

# 胡桃楸不同种源种子形态、质量及苗期生长研究

尤海舟[1,2],郭福忠[3],金长谦[3],王立方[3],孟宪聪[3],毕 君[1,2]
(1. 河北省林业科学研究院,河北石家庄 050061; 2. 河北小五台山森林生态系统定位研究站,河北涿鹿 075600;
3. 河北省承德市滦平国有林场管理处,河北承德 068250)

摘要:收集胡桃楸自然分布区内 5 个种源的种子,分别为黑龙江铁力种源、河北滦平种源、河北平泉种源、河北武安种源和河北易县种源,调查其果实形态差异,并在河北滦平进行播种育苗,研究胡桃楸不同种源种子质量及苗期生长变异规律,结果发现: 胡桃楸果实表现出丰富的变异类型,在种子直径、纵径和侧径 3 个方面存在显著差异($P<0.05$);胡桃楸种子质量差异明显、存在广泛变异,其种子百粒质量、场圃发芽率、发芽势、发芽指数等 4 个性状变异系数的变化范围分别为 0.90% ~9.39%、10.59% ~18.71%、10.58% ~30.41%、16.34% ~31.59%。试验点胡桃楸 2 年生株高、基径生长量差异显著,武安种源和易县种源苗期生长表现较好,株高、基径在种源间及种源内变异系数差异十分明显,其变异系数的变化范围分别为 19.52% ~33.50%、20.65% ~31.38%。

关键词:胡桃楸; 种源; 种子形态; 种子质量; 苗期生长量
中图分类号: S792.132.01 文献标志码: A 文章编号:1002 - 1302(2020)02 - 0155 - 04

胡桃楸(*Juglans mandshurica*),别称核桃楸,胡桃科(Juglandaceae)核桃属(*Juglans*)落叶高大乔木,广泛分布于我国东北、华北地区,胡桃楸材质坚硬、花纹美丽,主干通直,根系发达,生长迅速,易于萌芽和种子更新,近年来在冀北山地造林中开始推广应用,是该区重要的乡土阔叶树种,也是列入国家和地方重点保护的珍贵树种。由于过量采伐,胡桃楸天然林大树接近枯竭。近些年,关于胡桃属苗木繁育及培育技术研究有大量报道[1-3],遗传改良方面也有一些研究被报道[4-7]。植物遗传因素和外界环境条件相互作用决定了个体的表现类型,当一个物种的遗传基础较窄时其表型就有可能会随着外界环境条件的变化而发生变化[8]。胡桃楸种源间生长性状、适应性状等存在显著差异[9-10]。李广玉等对胡桃楸 1 年生苗高生长变异的分析结果表明,胡桃楸种源间和种源内部存在丰富的变异[11]。

以上研究种源大多来自东北地区,华北地区关于胡桃楸种源遗传变异的研究报道甚少。因此,本研究通过收集胡桃楸自然分布区内不同种源种子,调查其果实形态差异,播种育苗,进行发芽率等种子质量性状测定,并对 2 年生幼苗进行生长观测,研究胡桃楸不同种源种子质量及苗期生长变异规律,以期为胡桃楸种源选择、优质苗木培育和遗传改良提供理论依据。

## 1 材料与方法

### 1.1 研究区概况

三峰山省级森林公园位于河北省滦平县西南部的于营子国有林场,地处首都密云水库的上游,地理位置为 116°56′54″~117°03′03″E,40°41′24″~40°44′44″N。本区属燕山中段山脉,地势南高北低,形成中低山地貌,海拔 350 ~1 180 m,气候属中温带向暖温带过渡、半干旱半湿润大陆性季风型燕山山地气候,年均气温 7.7 ℃,全年无霜期约 150 d,降水量 450 mm,70% ~80% 的降水量集中在 6—8 月。土壤以棕壤和褐土为主,土层厚度 30 ~60 cm。森林植被以阔叶次生落叶林为主,主要乔木树种有蒙古栎(*Quercus mongolica*)、椴树属(*Tilia*)、山杨(*Populus davidiana*)、桦木属(*Betula*)、胡桃楸(*Juglans mandshurica*)、槭属(*Acer*)、侧柏(*Platycladus orientalis*)、油松(*Pinus tabuliformis*)等,部分为人工更新的油松人工林和落叶松人工林,森林覆被率约 89%。

收稿日期:2018 - 10 - 10
基金项目:河北省林业科技计划(编号:1409457);河北省重点研发项目(编号:2018106451 - 2);河北省科技计划(编号:16226813D)。
作者简介:尤海舟(1983—),女,河北保定人,硕士,工程师,主要从事森林生态方面研究。E - mail:595802563@qq.com。
通信作者:毕 君,博士,正高级工程师,主要从事森林生态、森林培育等方面研究。E - mail:1102995220@qq.com。

1.2　采种

供试材料种源地共 5 个,详见表 1,各种源地选择不少于 10 株植株健壮、无病虫害、结实量大的母树,于 2015 年秋季当果皮由绿变黄并开裂时进行采种,每株采种不少于 30 粒,冬季沙藏,2016 年春季采用变温处理种子后进行播种育苗。各种源地主要环境因子见表 1。

表 1　胡桃楸种源地主要环境因子

| 种源地 | 经度 | 纬度 | 年均温度(℃) | 有效积温(℃) | 日照时数(h) | 年均降水量(mm) | 无霜期(d) |
|---|---|---|---|---|---|---|---|
| 黑龙江铁力 | 127°20′E | 45°20′N | 2.8 | 2 550 | 1 114.2 | 723.8 | 125 |
| 河北滦平 | 117°23′E | 40°55′N | 7.7 | 3 296 | 1 606.0 | 351.1 | 151 |
| 河北平泉 | 118°41′E | 41°02′N | 7.3 | 2 850 | 1 617.0 | 542.0 | 135 |
| 河北武安 | 114°10′E | 36°41′N | 12.2 | 4 880 | 1 522.0 | 560.0 | 196 |
| 河北易县 | 115°08′E | 39°19′N | 11.9 | 4 266 | 1 558.0 | 544.8 | 185 |

1.3　种子性状测定及育苗试验

首先每个种源选取 30 粒种子进行种子纵径、横径与侧径的形态测量,然后对各种源 300 粒种子的百粒质量、场圃发芽率、发芽势、发芽指数分别进行测定。种子百粒质量的测定参照《林木种子规程》(2000 年)的规定进行。于 2016 年春季在河北省滦平县西南部的于营子国有林场试验点进行垄式、分种源播种,每 100 粒重复 1 次,各种源重复 3 次,根据发芽的粒数统计发芽率,场圃发芽率 = 正常发芽种子粒数/参试种子总粒数 ×100%,发芽势 = 达到高峰时正常发芽种子粒数/参试种子总粒数 ×100%,发芽指数 = $\sum(G_t/D_t)$,其中 $G_t$ 为不同发芽时间的发芽率,$D_t$ 为不同发芽试验时间,以 d 计。

待出苗较整齐后,按苗圃常规管理进行适时浇水、除草、松土,促进苗木生长。2017 年 10 月,每个种源内随机选取苗木 100 株,用钢卷尺和游标卡尺测定 2 年生播种苗的株高和基径。

1.4　数据处理

对 5 个种源胡桃楸种子的百粒质量、种子纵径、种子横径与种子侧径分别进行单因素方差分析和多重比较。变异系数的计算公式:变异系数 = 标准偏差/平均值 ×100%。

对不同种源胡桃楸 2 年生苗木生长指标与种子形态、百粒质量进行相关性分析;并对种源地与试验地的苗木生长差异与其主要环境因子差异进行相关性分析。

## 2　结果与分析

2.1　不同种源胡桃楸种子形态分析

如表 2 所示,测得黑龙江铁力种源果核平均纵横侧径为 43.66 mmc ×28.87 mm ×29.23 mm,相对值为 1.00∶0.66∶0.67,果核为大粒型,长条形,顶端尖状突出,花纹深,棱突起,平均粒质量 9.567 g;测得滦平种源果核平均纵横侧径为 32.80 mm ×23.89 mm ×23.90 mm,相对值为 1.00∶0.73∶0.73,果核为小粒型,短圆柱形,顶部钝尖,棱突出,花纹浅,平均粒质量 6.167 g;测得平泉种源果核平均纵横侧径为 32.88 mm ×23.94 mm ×23.91 mm,相对值为 1.00∶0.73∶0.73,果核为短圆柱形,顶部钝尖,棱不突出,花纹浅,平均粒质量 5.692 g;测得武安种源果核平均纵横侧径为 34.01 mm ×25.31 mm ×24.83 mm,相对值为 1.00∶0.74∶0.73,果核为短圆柱形或球形,顶部尖状突出,棱突出,花纹细碎且深裂,粗糙感最明显,平均粒质量 6.506 g;测得易县种源果核平均纵横侧径为 31.48 mm ×24.89 mm ×25.02 mm,相对值为 1.00∶0.79∶0.80,果核小粒型,短圆柱形或球形,顶部尖状突出,棱突出,花纹细碎且裂较深,粗糙感明显,平均粒质量 6.292 g。综上可知,黑龙江种源与其他 4 个种源胡桃楸种子形态差异最大,平泉、滦平种源胡桃楸种子形态特征最为接近。方差分析结果表明,铁力种源种子纵径、横径和侧径显著高于其他种源($P<0.05$);其他 4 个种源种子间仅武安种子横径显著大于其他 3 个种源的种子($P<0.05$),这 4 个种源间种子纵径和侧径差异均不显著($P>0.05$)。

2.2　不同种源胡桃楸种子质量分析

从表 3 可以看出,不同种源胡桃楸种子百粒质量均值为 684.5 g,黑龙江铁力种源百粒质量最大,超出最小值 68.08%;不同种源胡桃楸种子发芽率平均值为 53.95%,武安种源发芽率最大,超出最小值 26.45%;发芽势平均为 24.36%,武安种源发芽势最大,超出最小值的 42.50%;发芽指数的平均值

表 2　不同种源胡桃楸种子形态特征

| 种源地 | 种子纵径 (mm) | 种子横径 (mm) | 种子侧径 (mm) |
|---|---|---|---|
| 铁力 | 43.66 ±5.30a | 28.87 ±1.89a | 29.23 ±2.81a |
| 滦平 | 32.80 ±3.20b | 23.89 ±1.74c | 23.90 ±2.00b |
| 平泉 | 32.88 ±3.37b | 23.94 ±2.34c | 23.91 ±2.57b |
| 武安 | 34.01 ±4.10b | 25.31 ±1.62b | 24.83 ±1.81b |
| 易县 | 31.48 ±5.29b | 24.89 ±2.0bc | 25.02 ±2.24b |

注: 同列数据后不同小写字母表示不同种源间差异达显著水平 ($P<0.05$)。下同。

为 10.48%，滦平种源发芽指数最大，超出最小值 19.63%。

种源间的平均百粒质量变异系数为 43.32%，种源内的变异系数范围为 0.90% ~9.39%，其中易县种源内百粒质量变异系数最大，滦平种源内百粒质量变异系数最小。种源间的平均发芽率变异系数为 13.23%，种源内的变异系数范围为 10.59% ~ 18.71%，其中滦平种源内发芽率变异系数最大，易县种源内发芽率变异系数最小。种源间的平均发芽势变异系数为 17.42%，种源内的变异系数范围为 10.58% ~30.41%，其中平泉种源内发芽势变异系数最大，易县种源内发芽势变异系数最小。种源间的平均发芽指数变异系数为 19.47%，种源内的变异系数范围为 16.34% ~31.59%，其中平泉种源内发芽指数变异系数最大，武安种源内发芽指数变异系数最小。发芽指数的变异系数普遍大于其他指标，而差异越大越有利于进行早期选择。

表 3　不同种源胡桃楸种子的质量性状

| 种源地 | 百粒质量 (g) | 变异系数 (%) | 发芽率 (%) | 变异系数 (%) | 发芽势 (%) | 变异系数 (%) | 发芽指数 (%) | 变异系数 (%) |
|---|---|---|---|---|---|---|---|---|
| 铁力 | 956.7 ±70.0 | 7.32 | 50.01 ±6.68 | 13.39 | 20.33 ±3.34 | 16.44 | 10.25 ±2.54 | 24.78 |
| 滦平 | 616.7 ±5.8 | 0.90 | 52.66 ±9.87 | 18.71 | 27.45 ±4.77 | 17.35 | 11.58 ±2.47 | 21.30 |
| 平泉 | 569.2 ±39.7 | 7.01 | 48.57 ±5.50 | 11.56 | 20.88 ±6.35 | 30.41 | 10.89 ±3.44 | 31.59 |
| 武安 | 650.6 ±20.2 | 3.11 | 63.24 ±7.54 | 11.90 | 28.97 ±3.57 | 12.32 | 9.68 ±1.58 | 16.34 |
| 易县 | 629.2 ±59.1 | 9.39 | 55.35 ±5.87 | 10.59 | 24.11 ±2.55 | 10.58 | 10.02 ±2.11 | 21.17 |
| 平均 | 684.5 ±296.5 | 43.32 | 53.95 ±6.66 | 13.23 | 24.36 ±3.15 | 17.42 | 10.48 ±2.04 | 19.47 |

由表 4 方差结果可知，种子百粒质量和发芽指数在种源间差异极显著，发芽率和发芽势在种源间差异显著; 种子发芽势和发芽指数在种源内差异显著，百粒质量和发芽率在种源内差异不显著。说明胡桃楸种子质量差异明显、存在广泛变异。

表 4　不同种源间胡桃楸种子质量性状的方差分析

| 性状 | 均方 | | $F$ 值 | |
|---|---|---|---|---|
| | 种源间 | 种源内 | 种源间 | 种源内 |
| 百粒质量 | 789 176.133 | 12 105.353 | 4.987** | 0.458 |
| 发芽率 | 587.554 | 114.241 | 2.804* | 0.551 |
| 发芽势 | 154.787 | 22.455 | 2.848* | 0.724* |
| 发芽指数 | 28.342 | 7.987 | 3.356** | 0.796* |

注: ** 表示差异极显著 ($P<0.01$)，* 表示差异显著 ($P<0.05$)。

### 2.3　不同种源胡桃楸 2 年生苗木生长表现

对 5 个种源胡桃楸种子 2 年生苗木株高、基径生长量分析 (表 5) 表明，武安种源苗木株高、基径生长量最大，易县种源次之，其次为平泉和滦平种源，铁力种源苗木株高、基径生长量最小。方差分析表明，武安种源苗木基径生长量显著大于滦平、平泉和铁力种源 ($P<0.05$)，易县种源基径生长量显著大于铁力种源 ($P<0.05$)，其他种源间差异不显著 ($P>0.05$)；武安种源和易县种源株高显著大于铁力种源 ($P<0.05$)，其他种源间差异不显著 ($P>0.05$)。

种源间的平均基径变异系数为 26.54% (表 5)，种源内的变异系数范围为 19.52% ~33.50%，其中平泉种源内变异系数最大，滦平种源内变异系数最小。种源间的平均株高变异系数为 28.33%，种源内的变异系数范围 20.65% ~31.38%，其中平泉种源内变异系数最大，铁力种源内变异系数最小。可知，不同种源间的株高生长量差异大于地径生长量，变异系数越大，说明这些种源株高、基径等性状的稳定性相对较低，选择潜力大。

## 3　结论与讨论

本研究通过种质资源调查发现，胡桃楸果实类型表现出丰富的变异类型，5 个种源地核桃楸果实类型皆不相同，种子直径、纵径和侧径等方面存在显著差异 ($P<0.05$)。说明，胡桃楸种群具有丰富的遗传多样性[12]，胡桃楸果实的大小、形状、颜色等性状与其生长的立地条件、气候条件、土壤条件、树木生理年龄以及所处的生境条件等都会存在一定关系[13]。遗传基础和环境条件是决定个体表现型的 2 个要素[14]。

表 5 不同种源 2 年生苗木株高与基径生长量

| 种源 | 基径(mm) | 变异系数(%) | 株高(cm) | 变异系数(%) |
| --- | --- | --- | --- | --- |
| 铁力 | 21.07 ±4.60c | 21.85 | 98.67 ±20.37b | 20.65 |
| 滦平 | 24.78 ±4.84bc | 19.52 | 120.48 ±30.76ab | 25.53 |
| 平泉 | 23.91 ±7.34bc | 33.50 | 126.59 ±39.72ab | 31.38 |
| 武安 | 27.21 ±5.43a | 19.95 | 149.78 ±32.55a | 21.73 |
| 易县 | 25.93 ±5.77ab | 22.24 | 141.43 ±34.92a | 24.69 |
| 平均值 | 23.022 ±6.11 | 26.54 | 119.77 ±33.93 | 28.33 |

由于种子形态的稳定性,种子性状的变异性研究在林木遗传育种上具有重要价值[15]。种子质量在反映种子品质和变异规律方面被证明是有效的[16]。种子的质量、大小等表型指标与发芽率呈现不同程度的相关性,并且影响到苗木的质量。场圃发芽率、发芽势和发芽指数等代表种子质量的指标,综合反映了种子活力的大小。方差分析结果表明,胡桃楸种子质量性状在种源间均达到显著、极显著差异水平,发芽势和发芽指数在种源内达到显著差异水平。

根据余诚棋等对杉木、火炬松、巨桉等树种的研究表明,在苗圃选择超级苗木是有遗传增益的,早期选择具有一定的可靠性[17]。根据刘桂丰等的研究,胡桃楸各年份树高间的相关系数均达显著或极显著水平,说明幼龄期的胡桃楸就有代表性[18]。因此苗期生长观测是早期选择的重要途径之一,可了解各种源苗之间差异性,从而选育出适宜当地造林苗木[7]。本研究胡桃楸 2 年生株高、基径在种源间及种源内变异系数差异十分明显,种源间株高的变异系数高达 26.54%,基径高达 28.33%,种源内性状间的变异系数差异同样明显。充分说明胡桃楸苗期的分化大,选择的潜力大,有利于新品种的选育和种质创新[19]。

遗传差异是早期选择的基础,差异越大越有利于进行早期选择。在进行早期选择时,适宜的指标应选择生长差异较大的性状指标[20],说明应以发芽指数和苗高生长量为主,以发芽势、发芽率和基径生长量为辅进行胡桃楸早期选择。另外,有研究表明,随着树龄增加,种源内分化加剧,种源间变异幅度下降[5]。本试验仅为种源试验的初步结论,将继续关注各种源苗木的生长情况。

**参考文献:**

[1] 杜香莉,郭军战,冯 汀. 我国核桃资源的综合利用研究[J]. 西北林学院学报,2003,18(3):82-85.
[2] 范成民,董丽芬,朱 帜,等. 核桃芽苗砧嫁接方法研究[J]. 西北林学院学报,2008,23(4):109-111.
[3] 李保国,齐国辉,郭素平,等. 河北省太行山中南部核桃栽植时期及栽植技术研究[J]. 西北林学院学报,2006,21(4):83-84,92.
[4] 高绍棠,曹玉美,尹卫东,等. 淳化泥河沟试区核桃引种小结[J]. 西北林学院学报,1993,8(2):58-66.
[5] 张含国,邓继峰,张 磊,等. 胡桃楸种源家系变异规律及家系选择研究[J]. 西北林学院学报,2011,26(2):91-95.
[6] 庄倩倩,陈少鹏,刘洪章. 核桃楸不同地点种子形态及苗期生长的初步研究[J]. 吉林林业科技,2015,44(3):1-4.
[7] 赵珊珊,张大伟,许延国,等. 核桃楸不同种源苗期生长的研究[J]. 吉林林业科技,2016,45(1):4-9.
[8] 陈晓阳,沈熙环. 林木育种学[M]. 北京:高等教育出版社,2005:35-37.
[9] 杨书文,刘桂丰,赵克尊,等. 胡桃楸早期选择的初步研究[J]. 东北林业大学学报,1990,19(增刊 2):77-82.
[10] 杨书文,刘桂丰,王会仁,等. 胡桃楸地理变异规律的再研究[J]. 东北林业大学学报,1991,19(增刊 2):183-188.
[11] 李广玉,张学政,张含国,等. 胡桃楸苗木高生长种源家系变异规律初报[J]. 林业科技,2004,19(4):1-3.
[12] 宋佳兴,李 吉,果 冲,等. 辽东山区胡桃楸种质资源果实变异类型的筛选[J]. 分子植物育种,2017,15(9):3798-3802.
[13] 岁立云,刘晓敏,李周岐,等. 山桐子果实性状的自然变异及类型划分[J]. 西北农林科技大学学报(自然科学版),2009,37(8):115-120.
[14] 袁显磊,祁永会,刘忠玲,等. 核桃楸种源选择试验及其环境因子的影响[J]. 植物研究,2013,33(4):468-476.
[15] 魏志刚,高玉池,杨传平,等. 引种盐松不同种源种子表型性状和发芽特性[J]. 东北林业大学学报,2009,37(11):7-10.
[16] 姚淑均. 滇楸优树及其子代苗期性状遗传变异研究[D]. 北京:中国林业科学研究院,2013:49-61.
[17] 余诚棋,杨万霞,方升佐,等. 青钱柳种源间苗期性状变异分析[J]. 南京林业大学学报(自然科学版),2010,34(1):34-38.
[18] 刘桂丰,杨书文,李俊涛,等. 胡桃楸种源的初步区划及最佳种源选择[J]. 东北林业大学学报,1991,19(增刊 2):189-196.
[19] 袁显磊. 核桃楸优良种源和家系早期选择及苗期环境因子影响评价[D]. 哈尔滨:东北林业大学,2013:10-17.
[20] 蔡永立,王希华,宋永昌,等. 中国东部亚热带青冈果实形态变异的研究[J]. 生态学报,1999,19(4):581-586.
