# Supplementary material for: Provenance and family variations in early growth of Manchurian walnut (Juglans mandshurica Maxim.) and selection of superior families
Source: PLoS One. 2024 Mar 7;19(3):e0298918. doi: 10.1371/journal.pone.0298918 (PMC10919699; doi:10.1371/journal.pone.0298918)
Supplement: S2 File — (ZIP) [file pone.0298918.s005.zip › Study on seed traits and seedling growth characteristics of Juglans mandshurica Maxim.pdf]

不同种源核桃楸种子性状及幼苗生长特性研究

逄宏扬[1]，　李红莉[1]，　龙作义[1]，　祁永会[2]
(1.黑龙江省牡丹江林业科学研究所，　黑龙江 牡丹江 157010；
2.黑龙江省林业科学研究所，　哈尔滨 150081)

Study on Seed Traits and Seedling Growth Characteristics of *Juglans mandshurica* Maxim. from Different Provenances

PANG Hongyang[1], LI Hongli[1], LONG Zuoyi[1], QI Yonghui[2]

摘　要:对5个种源核桃楸种子性状及苗期生长特性进行分析,结果表明,不同种源间核桃楸果实及种子性状存在着丰富的遗传变异,以种仁和种子重量的变异最大,变异系数高达25%以上,而果实及种子的横径与侧径、壳厚的变异较小;以双鸭山青山和方正星火的果实及种子三径较大,种子及仁重量大,出仁率高,壳厚薄。不同种源间苗高、地径和叶片生长均差异显著,种源间生长性状的变异较大。种子重量与仁重、种子三径呈极显著正相关关系,与壳厚呈显著负相关关系;但种子性状与苗高、地径、叶面积等相关性不大。通过聚类分析可将5个种源划分为3组,其中双鸭山青山和方正星火种源的种子三径大,种子及仁重量较大,苗高、地径及叶片生长较大。

关键词:　核桃楸;种源;种子性状;生长性状
DOI 编码:　10.16590/j.cnki.1001-4705.2019.09.089
中图分类号:　S 792.132　　文献标志码:　A
文章编号:　1001-4705(2019)09-0089-04

核桃楸(*Juglans mandshurica* Maxim.)又名胡桃楸,属于胡桃科(Juglandaceae)核桃属(*Juglans*)落叶乔木,是东北地区三大珍贵硬阔叶树种之一[1]。其木材材质坚硬,致密,纹理通直,耐腐,可用作建筑、军工、车辆装修、船舶和家俱等的材料[2];果实营养价值高,含油率高,是极佳的森林食品,是东北地区具有开发前途的优良干果树种[3];核桃楸还有一定的药用价值,青果、枝皮及种仁均可入药[4],具有抗癌、抗肿瘤、降血脂、养胃温肾润肠等功效[5-7]。有学者对核桃楸种子性状进行了研究[8-9],但对不同种源种子性状及苗期生长差异的研究报道较少。本试验通过研究不同种源核桃楸种子性状及苗期生长差异分析,探讨种子性状与幼苗生长的关系,为早期种源选择提供理论依据。

1　材料与方法

1.1　材料来源

试验材料来源于海林林业局三部落林场、绥阳林业局寒葱河林场、双鸭山林业局青山林场、方正林业局星火林场、兴隆林业局蚂螂河林场等地的核桃楸种子。

表1　不同种源核桃楸编号及种源地情况

编号	采种地点	简称	分布情况
1号	海林林业局三部落林场	海林三部落	带状
2号	绥阳林业局寒葱河林场	绥阳寒葱河	块状
3号	双鸭山林业局青山林场	双鸭山青山	块状
4号	方正林业局星火林场	方正星火	块状
5号	兴隆林业局蚂螂河林场	兴隆蚂螂河	带状

1.2　育苗试验地概况

试验地设在黑龙江省海林林业局三部落苗圃。该

收稿日期:2019-04-20
基金项目:黑龙江省森林工业总局应用研究项目(sgzjY 2015027);黑龙江省科技厅科技攻关项目(GZ 13 B 010);黑龙江省牡丹江市科技攻关项目(G 2014 n 0012)和“十三五”国家重点研发计划专项(2017 YFD 0600605-02)。
作者简介:逄宏扬(1983—),男,硕士,高级工程师,主要从事经济林培育工作;E-mail:phy615@163.com。
通讯作者:李红莉(1981—),女,硕士,高级工程师,主要从事经济林培育工作;E-mail:lihonli0916@163.com。

[36]韩坤龙,崔子恒,付博,等.栽培模式和复合肥类型对隆平206农艺性状及干物质积累的影响[J].广西植物,2017,37(3):400-406.
[37]程相文,张守林,秦贵文,等.耐密高产玉米杂交种浚单29(浚009)的选育及高产栽培技术要点[J].种子,2011,30(10):100-101.
[38]霍焕霞.玉米品种豫禾988种植建议[J].农家参谋(种业大观),2014(08):35.
[39]杨国航,白琼岩,张春原,等.玉米抗旱品种筛选鉴定研究[J].种子,2009,28(09):86-88.
[40]刘松涛,刘过,晋宏宇,等.河北省不同生态区夏玉米丰产,稳产性品种筛选研究[J].作物杂志,2018(2):56-60.

(C)1994-2020 China Academic Journal Electronic Publishing House. All rights reserved.　http://www.cnki.net

地位于北纬 44°41′51、东经 129°14′39″,年平均气温 3.3 ℃,年最高温度 32.3 ℃,最低温度 −30.0 ℃,无霜期 125 d,降水量 723.4 mm,蒸发量 853.3 mm,≥10 ℃有效积温 2 400,日照时数 1 981.8 h,相对湿度 73%。试验土壤为沙壤土,有机质含量 6.6%,速效氮含量 0.3%、速效磷含量 0.24%和速效钾含量 0.42%。

1.3 试验方法

采集不同种源的果实 5 kg,每个种源随机抽取 50 粒,在实验室用电子游标卡尺测量果实的纵径、横径、侧径,坚果的纵径、横径和侧径,用电子天平称量各种源种子的带皮重和坚果干重。对坚果进行去壳处理,测量种仁质量,计算出仁率;在种壳碎片中挑选出较大碎片测量其壳厚,计算平均值。种子经沙藏变温处理后第 2 年播种育苗,随机区组排列,各种源区内随机选 20 株苗木作为生长量测定的固定株,于 9 月上旬测定苗高、地径(主干距地面 10 cm 处的直径)、叶长、叶宽、叶面积等生长指标。

1.4 数据处理

采用 Excel 2007 软件进行数据整理,SPSS 17.0 统计软件进行方差分析和相关分析,采用 DPS 数据处理系统 v 17.10 进行聚类分析。

2 结果与分析

2.1 不同种源核桃楸种子性状变异分析

从表 2 可以看出,5 个不同种源的核桃楸果实及种子性状均有一定的差异。5 个种源果实三径以 3 号、5 号和 4 号种源的较大,三径分别为 5.61~6.03 cm、3.83~3.93 cm 和 3.72~3.79 cm,其次为 2 号,而 1 号的三径较小。种子三径的范围分别为 4.17~5.17 cm、2.59~2.92 cm 和 2.57~2.94 cm,5 个种源大小依次为 3 号>5 号>4 号>2 号>1 号。不同种源的种子及种仁重量与果实及种子三径的表现有所不同,种子重量以 4 号的最大,平均为 11.22 g;其次为 5 号和 3 号,分别比前者小 10.16%和 10.34%;而 2 号和 1 号的较小,分别比 4 号小 27.80%和 36.10%。种仁重量的范围在 1.24~2.04 g 之间,5 个种源大小依次为 4 号>3 号>5 号>2 号>1 号。各种源的出仁率表现与种仁质量有所不同,以 3 号的出仁率最大,平均为 18.68%,其他种源依次为 4 号>1 号>5 号>2 号,分别比 3 号少 0.46%、1.37%、1.89%和 2.15%。5 个种源的果壳厚度在 5.04~5.19 mm 之间,各种源间相差不大,2 号和 5 号种源的果壳较厚,3 号和 4 号的较薄。方差分析结果表明,各种源在果实三径、种子三径、种子及种

表 2 不同种源核桃楸种子性状

种源编号	果实三径/cm			种子三径/cm			重量/g		出仁率/%	壳厚/mm
	纵径	横径	侧径	纵径	横径	侧径	种子重	仁重		
1 号	4.94±0.72 d	3.54±0.40 b	3.47±0.38 c	4.17±0.67 d	2.61±0.25 c	2.58±0.33 c	7.17±2.81 d	1.24±0.50 c	17.31±1.59 bc	5.11±0.21 ab
2 号	5.29±0.55 c	3.63±0.33 b	3.59±0.30 bc	4.49±0.44 c	2.59±0.23 c	2.57±0.22 c	8.10±1.50 c	1.34±0.32 c	16.53±2.25 c	5.19±0.22 a
3 号	6.03±0.69 a	3.83±0.28 a	3.72±0.29 ab	5.17±0.59 a	2.75±0.13 b	2.74±0.16 b	10.06±1.64 b	1.87±0.38 a	18.68±2.31 a	5.04±0.35 b
4 号	5.61±0.34 b	3.93±0.36 a	3.79±0.33 a	4.69±0.26 bc	2.92±0.18 a	2.94±0.27 a	11.22±1.67 a	2.04±0.44 a	18.22±2.94 ab	5.07±0.17 b
5 号	5.78±0.49 ab	3.84±0.25 a	3.72±0.34 ab	4.90±0.47 b	2.91±0.23 a	2.91±0.22 a	10.08±1.94 b	1.69±0.40 b	16.79±2.41 c	5.15±0.18 ab

注:数值后同列不同小写字母表示 0.05 水平上差异显著。下同。

表 3 不同种源核桃楸种子性状变异及方差分析

性状	最大值	最小值	极差	平均值	变异系数/%		F 值
					种源间	种源内	
果实纵径/cm	7.50	3.61	3.89	5.48	12.69	6.07~14.52	24.688**
果实横径/cm	4.55	2.83	1.72	3.72	9.47	6.58~11.20	10.584**
果实侧径/cm	5.52	2.48	3.04	3.64	9.38	7.67~11.05	6.658**
种子纵径/cm	6.40	2.88	3.52	4.64	13.29	5.59~16.09	25.519**
种子横径/cm	3.57	2.05	1.52	2.72	9.34	4.73~9.54	24.695**
种子侧径/cm	3.94	2.10	1.84	2.71	10.57	5.99~12.86	22.345**
种子重量/g	15.69	3.39	12.30	8.99	26.70	14.91~39.14	28.726**
仁重量/g	3.28	0.57	2.71	1.56	31.46	20.06~40.18	29.134**
出仁率/%	23.08	8.06	15.02	17.30	13.76	9.19~16.12	7.447**
壳厚/mm	5.70	4.30	1.40	5.13	4.62	3.42~6.88	3.250*

注:** 表示在 0.01 水平上差异显著,* 表示在 0.05 水平上差异显著。下同。

(C)1994-2020 China Academic Journal Electronic Publishing House. All rights reserved. http://www.cnki.net

仁质量、出仁率和壳厚等性状方面差异达到显著水平或极显著水平。

对不同种源核桃楸果实及种子性状进行方差分析(见表2、表3),结果表明,果实及种子主要性状在种源间及种源内均存在着丰富的变异,各性状在种源间存在极显著差异。种源间的变异以种仁和种子重量最大,变异系数分别为31.46%和26.70%,其次为出仁率、种子纵径、果实纵径和种子侧径,变异系数均大于10%,而其他性状的变异较小,变异系数在9%以下。

各性状在种源内的变异也较大。种源内也以种仁重量的变异最大,变异范围为20.06%~40.18%,各种源的变异系数均在20%以上;种子重量变异范围在14.91%~39.14%之间,其中1号种源的变异系数最大,其次为5号和2号,变异系数接近20%,其他2个种源在15%左右。出仁率变异范围在9.19%~16.12%之间,其中4号和5号的变异系数较大,1号的变异系数较小。果实三径及种子三径以1号的变异最大,变异系数约为10%以上,其他种源在5%~10%之间。而壳厚的变异最小,范围在3.42%~6.88%之间,其中3号种源的变异系数较大,4号和5号的变异系数较小。

2.2 不同种源核桃楸苗期生长差异分析

由表4可知,不同种源间苗木的生长也存在明显的差异。5个种源中以1号、3号和4号的苗较高,平均28.0 cm左右,显著地高于其他2个种源,三者间差异不显著。这3个种源苗高的变异幅度也较大,以4号的变异系数最大,为34.59%;其次为3号和1号,变异系数分别为30.46%和29.84%;而2号和5号的苗高较小,变幅不大,变异系数分别为28.76%和20.38%。地径生长方面,以3号、4号和5号的较大,平均0.73~0.79 cm,1号和2号的较小,约为0.66~0.69 cm;5个种源中以1号地径的变异系数最大,高达34.22%,3号的最小,变异系数为16.44%,其他几个种源变异系数均在22%以上。5个种源叶片形状、长宽比和叶面积等存在一定的差异,3号种源叶片长度最大,宽度最小,叶形较长;4号、5号和2号种源叶长13.26~13.98 cm,叶宽4.99~5.35 cm,长宽比2.57~2.68;而1号种源叶长最小,叶宽最大,长宽比最大。各种源叶长、叶宽及长宽比的变异系数范围分别为9.23%~17.57%、13.28%~18.42%和11.57%~23.71%,以1号的叶片的变异系数较大,4号的变异系数较小。叶面积以3号的最大,平均为58.23 cm^2;其次为1号和4号,分别比前者少5.01%和5.17%;再次为2号和5号,平均50.00 cm^2 左右。3号种源叶面积的变异系数较小,而其他几个种源的变异系数均较大,平均约为20.0%。方差分析结果表明,不同种源间核桃楸苗木的苗高、地径、叶长、叶宽、长宽比及叶面积生

表4　不同种源核桃楸苗木生长性状及多重比较

种源	苗高		地径		叶面积		叶长		叶宽		长宽比	
	均值/cm	变异系数/%	均值/cm	变异系数/%	均值/cm^2	变异系数/%	均值/cm	变异系数/%	均值/cm	变异系数/%	均值/cm	变异系数/%
1号	28.68 a	29.94	0.69 bc	34.22	55.31 ab	19.66	12.39 c	17.57	5.52 a	18.42	2.29 c	20.52
2号	20.29 b	28.76	0.66 c	25.11	50.92 bc	20.05	13.26 b	13.00	4.99 bc	13.37	2.68 b	14.56
3号	28.10 a	30.46	0.79 a	16.44	58.23 a	9.30	14.22 a	14.50	4.80 c	13.28	3.02 a	23.71
4号	27.40 a	34.59	0.76 ab	22.13	55.22 ab	20.76	13.98 ab	9.23	5.35 a	13.51	2.64 b	11.57
5号	20.10 b	20.38	0.73 abc	29.43	49.88 c	20.01	13.36 b	9.69	5.26 ab	14.29	2.57 b	13.95

表5　不同种源核桃楸种子性状及幼苗生长的相关分析

性状	种子重量	仁重	出仁率	种子纵径	种子横径	种子侧径	壳厚	苗高	地径	叶面积
种子重量	1									
仁重	0.889**	1								
出仁率	0.099	0.531**	1							
种子纵径	0.690**	0.634**	0.111	1						
种子横径	0.754**	0.677**	0.094	0.427**	1					
种子侧径	0.727**	0.622**	0.028	0.426**	0.765**	1				
壳厚	−0.158*	−0.155*	−0.052	−0.058	−0.027	0.004	1			
苗高	0.046	0.100	0.132*	−0.041	0.017	0.028	−0.044	1		
地径	−0.015	0.039	0.127	0.081	−0.061	0.023	−0.036	0.237**	1	
叶面积	0.055	0.072	0.047	0.099	−0.025	−0.004	−0.016	0.062	0.021	1

注:相关系数为Pearson相关系数。

(C)1994-2020 China Academic Journal Electronic Publishing House. All rights reserved.　http://www.cnki.net

长差异显著,苗高、地径和叶片性状的遗传变异明显。

2.3 核桃楸种子性状及幼苗生长的相关性分析

对核桃楸种子性状及幼苗生长性状进行相关分析(见表 5),结果表明,种子重量与仁重、种子三径呈极显著正相关,相关系数分别为 0.889、0.690、0.754 和 0.727;与壳厚呈显著负相关。仁重也与种子重量、三径呈极显著正相关,与壳厚呈显著负相关。出仁率与仁重呈极显著正相关,相关系数为 0.531。种子性状与幼苗生长性状具有一定的相关性,但相关性不大,相关系数较小,其中苗高与出仁率呈显著正相关,相关系数为 0.132。

2.4 种源间的聚类分析

对 5 个种源核桃楸的种子三径、种子重量、仁重、出仁率、苗高、地径等种子及幼苗生长主要性状采用欧氏距离类平均法进行聚类分析,聚类结果如图 1 所示。结果表明,5 个种源可在阈值 6.0 左右划分为 3 组,第 1 组为兴隆蚂螂河和绥阳寒葱河种源,其特点表现为种子三径、种子重量、仁重和出仁率中等,苗高、地径和叶片生长较大;第 2 组为双鸭山青山和方正星火种源,表现为种子三径、种子重量、仁重和出仁率大,苗高、地径和叶片生长大;第 3 组为海林三部落种源,表现为种子三径小,种子和仁重量小,出仁率中等,苗高和叶片生长中等,地径生长较小。

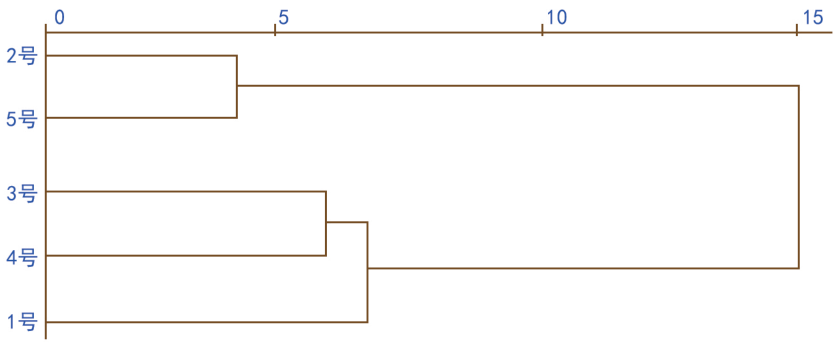

图 1 种源间核桃楸种子及幼苗生长性状的聚类分析

3 结论与讨论

核桃楸在我国东北地区资源丰富,其种群地理分布区域为温带针阔混交林和阔叶林区域,黑龙江省的完达山脉和小兴安岭是核桃楸的最适生长区域[10]。通过对黑龙江主要适生区的核桃楸资源进行调查,根据果实种子大、出仁率高、树体生长快等优良资源选择标准收集各个地区的核桃楸资源,将收集的资源进一步开展种源试验。同一树种因环境的变化会在不同产地发生遗传变异,这种变异会表现在种子及苗木生长过程中。本试验以 5 个种源的核桃楸资源为材料,研究其地理变异,筛选优良种源。结果表明,不同种源间核桃楸果实及种子性状存在着丰富的遗传变异,种源间和种源内的变异均以种仁和种子重量最大,变异系数高达 25%以上,而果实及种子的横径与侧径、壳厚变异较小,属于相对稳定的性状;各性状在种源间及种源内的差异达到极显著水平。这与成文博等[11]的研究结果基本一致。5 个种源中以双鸭山青山和方正星火的果实及种子三径较大,种子及仁重量大,出仁率高,壳厚薄。不同种源间苗木的生长也存在着明显地差异,苗高、地径和叶面积均差异显著。种源间苗高、地径和叶片形状变异较大,生长性状的遗传变异明显。总体来看,以双鸭山青山和方正星火种源的苗木生长较快。核桃楸不同种源种子性状和苗木生长性状间的相关性分析结果表明,种子重量与仁重、种子三径呈极显著正相关,与壳厚呈显著负相关;但种子性状与苗高、地径、叶面积等相关性不大,其中苗高与出仁率呈显著正相关。对 5 个种源核桃楸种子及幼苗生长性状进行聚类分析,可将 5 个种源划分为 3 组,双鸭山青山和方正星火种源的种子三径大,种子及仁重量较大,苗高、地径及叶片生长较大,为优良种源。

参考文献:

[1]周以良,董世林,聂少茎.黑龙江树木志[M].哈尔滨:黑龙江科学技术出版社,1986:172-176.
[2]李新久.核桃楸的育苗造林技术[J].中国林副特产,2003(2):39.
[3]刘文华.核桃楸的利用和苗木培育[J].中国林副特产,2007(1):44-45.
[4]陈翠英.核桃楸的经济价值与繁育技术[J].河北林业科技,2003(5):49.
[5]庄倩倩,陈少鹏,刘洪章.核桃楸不同地点种子形态及苗期生长的初步研究[J].吉林林业科技,2015(3):1-3.
[6]沈广志,邹桂华,梁婷,等.核桃楸的化学成分研究进展[J].中国实验方剂学杂志,2015,21(17):219-224.
[7]朱红波,赵云,林士杰,等.核桃楸资源研究进展[J].中国农学通报,2011(25):1-4.
[8]曾栋,张海啸,张含国,等.核桃楸果实及种子变异规律分析[J].林业科技通讯,2016(3):3-7.
[9]李程,张姣,李寒,等.核桃楸种子性状的初步研究[J].种子,2017,36(01):15-18.
[10]马万里,罗菊春,荆涛,等.珍贵树种核桃楸的生态学问题及培育前景[J].内蒙古师范大学学报,2005,34(4):489-492.
[11]成文博,徐贵军,王浩,等.不同种源的核桃楸种子性状变异及聚类分析[J].辽宁林业科技,2016(5):33-35.

(C)1994-2020 China Academic Journal Electronic Publishing House. All rights reserved. http://www.cnki.net
