# Supplementary material for: Provenance and family variations in early growth of Manchurian walnut (Juglans mandshurica Maxim.) and selection of superior families
Source: PLoS One. 2024 Mar 7;19(3):e0298918. doi: 10.1371/journal.pone.0298918 (PMC10919699; doi:10.1371/journal.pone.0298918)
Supplement: S2 File — (ZIP) [file pone.0298918.s005.zip › Study on the growth of young trees in mixed forests between hard-wood and conifer species.pdf]

第19卷 水胡黄椵专刊 东北林业大学学报 Vol.19 Sp. Iss.
1991年 4月 JOURNAL OF NORTHEAST FORESTRY UNIVERSITY Apr. 1991

水曲柳、胡桃楸、黄波罗、紫椴与不同针叶树种混交生长的探讨

胡润田 鞠永贵 张世英
（东北林业大学）

【摘 要】
对5年生针阔混交林调查结果证明，在帽儿山地区以水曲柳、胡桃楸与落叶松混交效果最好，但黄波罗、紫椴不应与落叶松混交、以免被压。

主题词：混交林；混交类型；生长

研究水曲柳（*Fraxinus mandshurica* Rupr.）、胡桃楸（*Juglans mandshurica* Maxim）、黄波罗（*Phellodendron amurense* Rupr.）、紫椴（*Tilia amurensis* Rupr.）与落叶松（*Larix gmelini*（Rupr.）Rupr）、红皮云杉（*Picca koraiensis* Nakai）、红松（*Pinus koraiensis* Sieb. et Zucc）混交林，力图从不同混交方式中，选出生长最好的类型，用以指导今后培育针阔混交林工作。

1 试验地概况

帽儿山地区是黑龙江省东部较典型的天然次生林区，面积 26 507 ha，森林覆盖率为70.5 %，土壤为暗棕色森林土，平均海拔 350 m 左右，年平均气温 2.8℃，年平均降雨量 723.8 mm，无霜期 130 d。

试验地位于帽儿山实验林场水胡黄椵丰产林基地内，每个混交类型试验分别在强度疏伐、全面皆伐、带状皆伐三种不同采伐迹地上进行。每个类型的针阔混交比（行数比）有 6 阔 2 针，4 阔 2 针，3 阔 5 针三种。造林所用苗木是本场苗圃培育的，苗龄型为：水曲柳、胡桃楸 2－0、黄波罗、紫椴为 1－0，均为 I 级苗。同时设各树种纯林对照区。造林时穴状整地，株行距1.5 m×1.5 m，按 3，2，1，1，1 顺序抚育管理。

2 研究方法

材料取自 1986 年营造的不同混交类型 5 年生幼林的调查结果，共调查混交类型 12 个，每个类型重复 3 次和 4 块纯林对照。1990 年 10 月 22 日至 27 日对各混交类型的水

胡黄椴及其纯林，用典型抽样法设100 m^2的临时标准地，进行每木地径、全高调查，然后统计分析。

3 研究结果

3.1 水、胡、黄、椴与落、云、红、混交林生长的方差分析

水曲柳与落叶松混交，水曲柳树高平均生长量最大；水曲柳与红松混交，水曲柳地径平均生长量最大（见表1—4）。

表1 水曲柳与落、云、红混交地径生长量的统计

试验地别	重复试验数据			试验区地径总和	平均
水落混	1.519	1.674	1.594	4.787	1.595
水云混	1.329	1.206	1.226	3.761	1.254
水红混	1.708	1.55	1.627	4.885	1.628 *
总和	4.556	4.43	4.447	13.433	
区组平均	1.519	1.48	1.482		1.493

表2 水曲柳与落、云、红混交地径方差分析

变异来源	自由度	平方和	方差	F计	F理论
混交种类	2	0.258 4	0.129 2	0.129 2/0.007 52	$F_{0.05}(2,4)=6.94$
区组	2	0.003 1	0.001 55	=17.17*	$F_{0.01}(2.4)=18$
机误	4	0.030 1	0.007 52		

表3 水曲柳与落、云、红混交树高生长量的统计

试验地别	重复试验数据			试验区树高总和	平均
水落混	78.56	86.54	73.76	238.77	79.59*
水云混	57.82	59.71	63.75	181.28	60.43
水红混	87.11	80.5	68.65	236.26	78.753
总和	223.49	226.66	206.16	656.31	
区组平均	74.497	75.553	68.72		72.92

表4 水曲柳与落、云、红混交树高方差分析

变异来源	自由度	平方和	方差	F计	F理论
混交种类	2	703.800 8	351.90	351.9/48.56	$F_{0.05}(2,4)=6.94$*
区组	2	81.183 6	40.59	=7.25*	$F_{0.01}(2,4)=18$
机误	4	194.238 3	48.56		

胡桃楸与落叶松混交，胡桃楸平均地径、树高生长量最大（见表5—8）

表 5　胡桃楸与落、云、红混交地径生长量的统计

试验地别	重复试验数据			试验区地径总和	平均
胡落混	2.449	2.571	2.70	7.72	2.57*
胡云混	2.11	2.21	2.32	6.64	2.21
胡红混	1.11	1.78	1.23	4.12	1.373
总和	5.669	6.561	6.25	18.48	
区组平均	1.889	2.187	2.083		2.053

表 6　胡桃楸与落、云、红混交地径方差分析

变异来源	自由度	平方和	方差	F计	F理论
混交种类	2	2.275	1.1376	1.1376/0.043	F 0.05(2,4)=6.94
区组	2	0.1367	0.0683	=26.45**	F 0.01(2,4)=18
机误	4	0.1721	0.043		

表 7　胡桃楸与落、云、红混交高生长量的统计

试验地别	重复试验数据			试验区树高总和	平均
胡落混	97.45	92.83	108.47	298.75	99.58*
胡云混	77.78	81.67	85.75	245.2	81.73
胡红混	43.03	59.1	53.3	155.43	51.81
总和	218.26	233.6	247.52	699.38	
区组平均	72.753	77.86	82.51		77.71

表 8　胡桃楸与落、云、红混交高生长量方差分析

变异来源	自由度	平方和	方差	F计	F理论
混交种类	2	3493.317	1748.158	1748.158/37.6367	F 0.05(2,4)=6.94
区组	2	142.8047	71.4024	=46.44**	F 0.01(2,4)=18
机误	4	150.5469	37.6367		

黄波罗与红松混交黄波罗平均地径、树高生长量最大（见表 9—12）。

表 9　黄波罗与落、云、红混交地径生长量的统计

试验地别	重复试验数据			试验区地径总和	平均
黄落混	1.42	1.51	1.62	4.55	1.52
黄云混	1.98	2.07	2.1	6.15	2.05
黄红混	2.18	2.29	2.04	6.51	2.17 *
总和	5.58	5.87	5.76	17.21	
区组平均	1.86	1.96	1.92		19.12

表 10　黄波罗与落、云、红混交地径方差分析

变异来源	自由度	平方和	方差	F 计	F 理论
混交种类	2	0.725 6	0.362 8	0.362 8/0.011 2	F 0.05(2,4)=6.94
区组	2	0.014 3	0.071 4	=32.39**	F 0.01(2,4)=18
机误	4	0.044 9	0.011 2		

表 11　黄波罗与落、云、红混交树高生长量的统计

试验地别	重复试验数据			试验区树高总和	平均
黄落混	105.3	110.57	116.09	331.96	110.65
黄云混	114.22	119.93	115.93	350.08	116.69
黄红混	126.86	123.2	129.86	379.92	126.64 *
总和	346.38	353.7	361.88	1 061.96	
区组平均	115.46	117.9	12.063		117.99

表 12　黄波罗与落、云、红混交树高方差分析

变异来源	自由度	平方和	方差	F 计	F 理论
混交种类	2	390.984	195.492	195.492/14.393	F 0.05(2,4)=6.94
区组	2	40.094	20.046 8	=13.58 *	F 0.01(2,4)=18
机误	4	57.57	14.393		

紫椴与红松混交紫椴平均地径、树高生长量最大（见表13—16）。

表 13　紫椴与落、云、红混交地径生长量的统计

试验地别	重复试验数据			试验区地径总和	平均
椴落混	1.5	1.57	1.66	4.73	1.58
椴云混	1.49	1.55	1.67	4.71	1.57
椴红混	1.56	1.67	1.76	4.99	1.663*
总和	4.55	4.79	5.09	14.43	
区组平均	1.516	1.596	1.696		1.603

表 14　紫椴与落、云、红混交地径方差分析

变异来源	自由度	平方和	方差	F 计	F 理论
混交种类	2	0.016 3	0.008 1	0.008 1/0.000 23	F 0.05(2,4)=6.94
区组	2	0.048 8	0.024 4	=35.21**	F 0.01(2,4)=18
机误	4	0.000 93	0.000 23		

表 15 紫椴与落、云、红混交树高生长量的统计

试验地别	重复试验数据			试验区树高总和	平均
椴落混	79.83	73.31	76.99	230.13	76.71
椴云混	84.49	86.72	83.15	254.36	84.787
椴红混	87.51	92.23	97.51	277.25	92.42*
总和	251.83	252.26	257.65	761.74	
区组平均	83.943	84.087	85.88		86.64

表 16 紫椴与落、云、红混交树高方差分析

变异来源	自由度	平方和	方差	F 计	F 理论
混交种类	2	370.1445	185.0723	185.0723/17.7295	$F_{0.05}(2,4)=6.94$
区组	2	7.0156	3.5078	=10.44 *	$F_{0.01}(2,4)=18$
机误	4	70.918	17.7295		

3.2 水、胡、黄、椴混交林与纯林生长比较分析

混交水曲柳、胡桃楸、黄波罗、紫椴与对照的比较结果（见表17）。

表 17 纯林对照区 5 a 间地径树高平均生长量

对照区树种	地径(cm)	全高(cm)
水曲柳	1.14	65.33
胡桃楸	1.80	65.13
黄波罗	2.03	115.46
椴树	1.65	85.94

注：此材料取自抚育方式中纯林对照区数据。

（1）水曲柳与落叶松混交是一个比较好的针阔混交类型，混交水曲柳平均地径、平均树高比对照区地径大 0.46 cm，树高大 14.26 cm；水曲柳与红松混交水曲柳地径生长量大于对照区 0.488 cm，高生长稍慢，大于对照区 13.42 cm；水曲柳与云杉混交，水曲柳地径和高生长量不理想，地径大于对照区 0.114 cm，树高为 -4.90 cm，混交优势不明显，而且水曲柳发生疣纹蝙蝠蛾虫害占 12% 左右。

（2）胡桃楸与落叶松混交，胡桃楸地径和高生长量比纯林对照大，地径大 0.77 cm，树高大 34.45 cm，并生长健壮；胡桃楸与云杉混交，胡桃楸地径和树高比对照地径大 0.41 cm，树高大 16.60 cm。胡桃楸与红松混交，胡桃楸的地径和树高生长均低于对照区的生长量。

（3）黄波罗和红松混交，黄波罗地径和树高生长量都比纯林对照高，地径大 0.14 cm，树高大 11.18 cm；黄波罗和云杉混交与纯林对照相比优势不明显，地径大 0.02 cm，树高大 1.23 cm；黄波罗与落叶松混交，黄波罗地径、树高生长量都低于对照区。

（4）紫椴与红松混交，紫椴地径和树高生长量比纯林对照高，地径大 0.013 cm，树高大 6.84 cm；椴树与云杉、落叶松混交，椴树地径和树高小于对照区。

4 结论与建议

4.1 从造林几年后的生长观察分析，水曲柳、胡桃楸与落叶松混交地径和树高生长较明显，是最好的混交组合。

4.2 黄波罗和紫椴和红松混交生长较好，但与落叶松混交常常受压、生长不良。

4.3 由于云杉幼年生长较慢，这4个阔叶树种与云杉混交，生长都较好，但需继续观察。

参考文献

1 俞新妥主编．混交林营造原理及技术．北京：中国林业出版社，1989

STUDY ON THE GROWTH OF YOUNG TREES IN MIXED FORESTS BETWEEN HARDWOODS AND CONIFER SPECIES

Hu Runtian　Ju Yonggui　Zhang Shiying

(Northeast Forestry University)

ABSTRACT

In this paper, the growth of young trees in mixed forests between hardwoods (Manchurian ash, Manchurican walnut, Amur corktree, and Amur linden) and conifer species (Korean pine, Larch, Scotch pine, and Spruce) were studied by using the method of statistics. The results show that Ash and Walnut grow well with the mixture species of Larch. But the growth of Corktree and Linden are not good due to the shading press of Larch, therefore, Corktree and Linden can not be the mixture species with Larch.

Descriptors: Mixed forest; Mixture type; Growth
