# Supplementary material for: Provenance and family variations in early growth of Manchurian walnut (Juglans mandshurica Maxim.) and selection of superior families
Source: PLoS One. 2024 Mar 7;19(3):e0298918. doi: 10.1371/journal.pone.0298918 (PMC10919699; doi:10.1371/journal.pone.0298918)
Supplement: S2 File — (ZIP) [file pone.0298918.s005.zip › Suitable distribution area of drought-resistant afforestation tree species in north China based on MaxEnt model.pdf]

DOI:10.12171/j.1000−1522.20210527

基于 MaxEnt 模型的北方抗旱造林树种适宜区分布

刘佳琪　魏广阔　史常青　赵廷宁　钱云楷
(北京林业大学水土保持学院，北京 100083)

摘要:【目的】樟子松、油松、山桃和山杏作为中国北方半干旱半湿润气候区的常用造林树种，具备抗旱耐寒特性和保持水土的功能，研究其适宜空间分布对中国北方植被恢复具有指导作用。【方法】以半干旱半湿润气候区的樟子松、油松、山桃和山杏为研究对象，获取树种地理分布点位数据和与树种生态学相关的 24 个环境因子（地形、土壤和气象），基于协同克里金插值法，将限制因子叠加法与最大熵模型（MaxEnt）相结合，研究 4 类树种适宜区分布。【结果】（1）4 类树种 MaxEnt 模型预测精度达到准确水平（AUC > 0.90）。（2）影响樟子松分布的主导因子依次为土壤类型、最冷月均温和最冷月平均风速；油松的主导因子依次为高程、年均气温标准差、土壤类型、年降水量；山桃的主导因子依次为最暖月均温、高程、年极端最低气温、年均降水量标准差、坡度、土壤类型；山杏的主导因子依次为高程、土壤类型、最暖月平均降水量、湿润系数、最暖月均温。（3）樟子松中高适宜区主要分布于内蒙古、黑龙江、吉林等地，油松、山桃和山杏主要分布在山西、陕西、甘肃、河北、内蒙古等地。【结论】MaxEnt 模型模拟结果，可准确反映 4 类树种的适宜区分布情况，结果可为我国半干旱半湿润区绿化造林提供适地适树的科学指导。

关键词: 适宜区；最大熵模型；樟子松；油松；山桃；山杏
中图分类号: S725.1　**文献标志码:** A　**文章编号:** 1000−1522(2022)07−0063−15
引文格式: 刘佳琪，魏广阔，史常青，等. 基于 MaxEnt 模型的北方抗旱造林树种适宜区分布 [J]. 北京林业大学学报，2022，44(7)：63−77. Liu Jiaqi, Wei Guangkuo, Shi Changqing, et al. Suitable distribution area of drought-resistant afforestation tree species in north China based on MaxEnt model［J］. Journal of Beijing Forestry University, 2022, 44(7): 63−77.

Suitable distribution area of drought-resistant afforestation tree species in north China based on MaxEnt model

Liu Jiaqi　Wei Guangkuo　Shi Changqing　Zhao Tingning　Qian Yunkai
(School of Soil and Water Conservation, Beijing Forestry University, Beijing 100083, China)

Abstract:［**Objective**］*Pinus sylvestris* var. *mongolica*, *Pinus tabuliformis*, *Amygdalus davidiana* and *Armeniaca sibirica* are commonly used afforestation tree species in semi-arid and semi-humid areas, which have drought-resistant and cold-resistant characteristics and the function of soil and water conservation. Studying their suitable spatial distribution can guide the vegetation restoration in northern China.［**Method**］Based on the ecological characteristics of tree species, with the data of tree species distribution and 24 environmental variables (topography, soil and meteorology), based on the Co-Kriging method, the limiting factor superposition method and the maximum entropy model (MaxEnt) were combined to study the distribution of the suitable areas of 4 tree species.［**Result**］(1) The prediction accuracy of MaxEnt model of four tree species reached the accurate level (AUC > 0.90). (2) The dominant factors affecting the distribution of *Pinus sylvestris* var. *mongolica* were ordered as soil type, average temperature in the coldest month and average wind speed in the coldest month. The dominant factors of *Pinus tabuliformis* were ordered as

收稿日期: 2021−12−13　**修回日期:** 2022−02−23
基金项目: 半干旱粟钙土区抗旱防沙造林综合技术研究（2015HXFWSBXY014）。
第一作者: 刘佳琪。主要研究方向：生态修复。Email：1748620425@qq.com　地址：100083 北京市海淀区清华东路 35 号北京林业大学水土保持学院。
责任作者: 史常青，博士，副教授。主要研究方向：林业生态工程。Email：scqbj@126.com　地址：同上。
本刊网址: http://j.bjfu.edu.cn；http://journal.bjfu.edu.cn

(C)1994-2022 China Academic Journal Electronic Publishing House. All rights reserved.　http://www.cnki.net

elevation, standard deviation of annual average temperature, soil type and annual precipitation; the dominant factors of *Amygdalus davidiana* were ordered as the average temperature of the warmest month, elevation, annual extreme minimum temperature, standard deviation of annual precipitation, slope and soil type; the dominant factors of *Prunus armeniaca* were elevation, soil type, average precipitation in the warmest month, wetting coefficient and average temperature in the warmest month in turn. (3) The middle and high suitable areas of *Pinus sylvestris* var. *mongolica* were mainly distributed in Inner Mongolia of northern China, Heilongjiang and Jilin provinces of northeastern China, *Pinus tabuliformis*, *Amygdalus davidiana* and *Armeniaca sibirica* were mainly distributed in Shanxi, Hebei provinces and Inner Mongolia of northern China, Shaanxi, Gansu provinces of northwestern China, [**Conclusion**] In this study, MaxEnt model can accurately reflect the distribution of four tree species, and the results can provide scientific guidance for the afforestation in the semi-arid and semi-humid climate regions of China.

Key words: suitable area; MaxEnt model; *Pinus sylvestris* var. *mongolica*; *Pinus tabuliformis*; *Amygdalus davidiana*; *Armeniaca sibirica*

早期受数据精度及方法限制，人们多以气候区划[1]或立地划分[2−3]作为造林指导，由于未考虑树种适宜区分布，在气候变化和人为扰动下，部分林地多年后出现林木生长缓慢、生态功能衰退、面积收缩等现象[4]，对此学者引入物种分布模型（species distribution model，SDM）开展物种适生区分布研究，该模型可通过现存生物气候变量，预测物种分布及所受环境的影响[5]。因最大熵模型（MaxEnt）在少量物种分布数据下，仍有较高的物种空间分布模拟准确性[6]，因此，该模型广泛应用于生态学、生物地理学[7−9]和濒危动植物保护[10−11]等领域。

半干旱半湿润气候区为我国北方典型气候区，该区域的造林树种需具备抗旱耐寒和抗风性强等特性，樟子松（*Pinus sylvestris* var. *mongolica*）、油松（*Pinus tabuliformis*）、山桃（*Amygdalus davidiana*）和山杏（*Armeniaca sibirica* ）为我国北方常用造林树种，具备以上优良特性和保持水土功能，且经济和景观价值较高。目前针对樟子松、油松的研究多集中于生态学特性、生长规律、育苗管理和造林技术等方面[12−13]，对山桃、山杏的研究多偏向生态特性、种质资源和栽培技术[14−16]，但对于 4 类树种适宜区分布的研究较少，现存研究多针对某行政区域内的单一树种[17−19]，尚无气候区尺度的研究。在气象数据插值方面，多数学者利用世界气象数据库（Worldclim）中已插值的气象数据[20]，或采用普通克里金法[21]进行插值，忽略了站点经纬度和海拔对气象数据的影响[21−24]，本研究基于树种的生态学特性，综合气象、土壤、地形等多种环境因素，将经纬度和高程作为协变量对气象数据进行协同克里金（Co-Kriging）插值，将限制因子空间叠加与 MaxEnt 法相结合，对半干旱半湿润气候区内樟子松、油松、山桃和山杏的适宜区分布进行研究，以期为国土绿化中造林项目的实施提供理论依据，发挥树种生态经济效益。

1 研究区概况

我国半干旱半湿润气候区（18°25′ ~ 51°46′E、78°24′ ~ 128°36′N）[25−26]，属于温带大陆性季风气候，年均降水量 200 ~ 800 mm，地貌复杂，海拔变化大，包含内蒙古高原中东部、东北平原、华北平原、青藏高原与黄土高原大部分地区，占我国国土面积约 37%，总面积 410.42 万 km^2（图 1）。

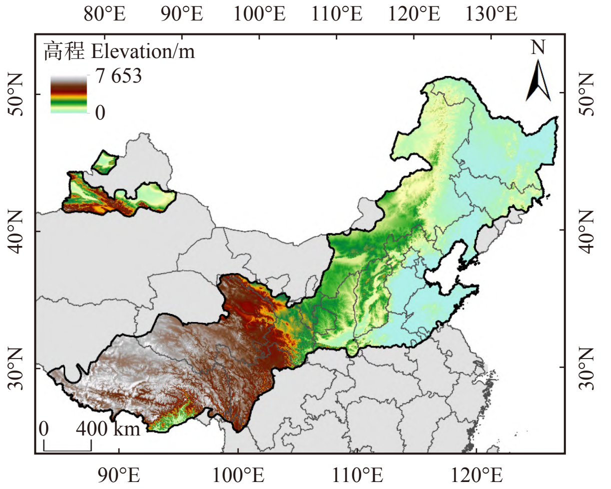

底图审图号：GS(2021)5448 号。下同。Base drawing review No.: GS(2021)5448. The same below.

图 1 半干旱半湿润气候区分布

Fig. 1 Distribution of semi-arid and semi-humid climate zone

2 材料与方法

2.1 数据来源

2.1.1 树种分布数据

通过《中国植物志》[27]了解树种的生态学特征，

(C)1994-2022 China Academic Journal Electronic Publishing House. All rights reserved. http://www.cnki.net

确定研究区内樟子松、油松、山桃和山杏的主要分布地区，根据树种拉丁文学名在中国国家标本资源平台和中国数字植物标本馆获取其标本采集地信息和分布点坐标，查阅文献及各省《植物志》，确保树种分布点为天然林地或人工引种多年且成活率高的区域，剔除重复及距离过近点位后，得到 101 条樟子松、242 条油松、109 条山桃、104 条山杏分布点数据，每条数据包括树种名称、经度和纬度。

2.1.2 环境数据

气象数据源自中国气象数据网站 1 104 个气象站点（研究区内部 977 个、外部 127 个）的 1981—2010 年中国地面累年值年值数据集，共 18 个气象因子；土壤类型数据源自资源环境科学与数据中心；土壤 pH 数据源自联合国粮农组织世界土壤数据库；地形数据源自地理空间数据云，经 ArcGIS 提取坡度、坡向数据。共计 24 个环境因子（表 1）。

表 1 环境因子

Tab. 1 Environmental factors

类型 Type	变量 Variable	描述 Description	单位 Unit
地形 Terrain	Ele	高程 Elevation	m
	Slo	坡度 Slope	(°)
	Asp	坡向 Aspect	
土壤 Soil	Soil-type	土壤类型 Soil type	
	Soil-pH-t	0 ~ 30 cm土壤pH Soil pH in 0−30 cm	
	Soil-pH-s	30 ~ 100 cm土壤pH Deep soil pH in 30−100 cm	
气象 Meteorology	PRE1	最暖月平均降水量 Mean precipitation in the warmest month	mm
	PRE2	最冷月平均降水量 Mean precipitation in the coldest month	mm
	PRE3	年降水量 Annual precipitation	mm
	PRE4	累年年最多降水量 Cumulative annual maximum precipitation	mm
	PRE5	年均降水量标准差 SD of mean annual precipitation	mm
	TEM1	年日均温 Annual mean temperature	℃
	TEM2	最暖月均温 Mean temperature of the warmest month	℃
	TEM3	最冷月均温 Mean temperature the coldest month	℃
	TEM4	年平均气温 Annual mean temperature	℃
	TEM5	气温年较差 Annual range of temperature	℃
	TEM6	年极端最高气温 Annual extreme maximum temperature	℃
	TEM7	年极端最低气温 Annual extreme minimum temperature	℃
	TEM8	年均气温日较差 Annual mean daily temperature range	℃
	TEM9	年均气温标准差 SD of mean annual temperature	℃
	GDD5	≥ 5 ℃积温 ≥ 5 ℃ accumulated temperature	℃·d
	Wind	最冷月平均风速 Mean wind speed of the coldest month	m/s
	HUM	年平均相对湿度 Annual average relative humidity	%
	Wet	湿润系数 Humid coefficient	

避免站点经纬度和高程对气象因子的影响，采用协同克里金法（Co-kriging）插值，该方法是普通克里金法的拓展，将单一属性拓展至 2 个及以上协同区域化属性[28]。公式如下：

$$Z_0 = \sum_{i=1}^{n} \alpha_i x_i + \sum_{i=1}^{m} \beta_i y_i \tag{1}$$

式中：Z_0为估算点气象因子的预测值；x_i为主要变量的实测值；y_i为二级变量的实测值；α_i和β_i为插值对象对估算点气象因子的权重。

将气象因子作为主要变量，经度、纬度和高程为协变量，对 18 个气象因子进行插值，为确保边缘数据的准确[29]，插值包含区域内 977 个及周边 127 个气象站点，精度检验的交叉验证 R^2 均大于 90%（达到准确水平）。裁剪后进行投影转换，统一分辨率 1 km。

2.2 可能适宜区筛选

考虑树种生态学特征，采用空间叠置法对树种

(C)1994-2022 China Academic Journal Electronic Publishing House. All rights reserved. http://www.cnki.net

的地理限制因素叠加，筛选可能适宜区。樟子松适生于海拔 500 ~ 2 000 m、年降水量为 350 ~ 900 mm 的山区[30]，喜酸、中性土壤，能够在−40 ~ −50 ℃ 的极端低温下生存；油松生长于海拔 100 ~ 2 600 m、年降水量为 400 ~ 968 mm 地区[31−32]，能够生存在−25 ℃ 的极端低温条件下；山桃生长于海拔 800 ~ 3 200 m、年降水量 100 ~ 1 700 mm、年均温 8 ~ 25 ℃ 的地区，耐受的极端低温达到−26 ℃，耐盐碱[33]。山杏分布于海拔 700 ~ 2 000 m、年降水量 200 ~ 600 mm、年均温 4 ~ 12 ℃ 地区，耐−40℃ 的极端低温。根据以上特性，各树种适宜区模型如下：

$$SR_Z = Ele \cap PRE3 \cap TEM7 \cap Soil\text{-}pH\text{-}t \cap Soil\text{-}pH\text{-}s \quad (2)$$

$$SR_Y = Ele \cap PRE3 \cap TEM7 \quad (3)$$

$$SR_T = Ele \cap PRE3 \cap TEM4 \cap TEM7 \quad (4)$$

$$SR_X = Ele \cap PRE3 \cap TEM4 \cap TEM7 \quad (5)$$

式中：SR_Z、SR_Y、SR_T 和 SR_X 分别为樟子松、油松、山桃和山杏的可能适生单元，当 SR = 1，为树种可能适宜空间单元，当 SR = 0，为不适宜单元；各树种的限制因子若满足表 2 中适宜范围，则取值为 1，反之取值为 0，结果见图 2。

表 2 限制因子适宜区范围

Tab. 2 Suitable range of limiting factors

树种<br>Tree species	限制因子<br>Limiting factor	可能适宜区范围<br>Possible suitable area range
樟子松<br>*Pinus sylvestris* var. *mongolica*	Ele/m	[500, 2 000]
	PRE3/mm	[350, 900]
	TEM7/℃	[−50, 25]
	Soil-pH-t	（5, 8]
	Soil-pH-s	（5, 8]
油松<br>*Pinus tabuliformis*	Ele/m	[100, 2 600]
	PRE3/mm	[400, 968]
	TEM7/℃	[−25, 25]
山桃<br>*Amygdalus davidiana*	Ele/m	[800, 3 200]
	PRE3/mm	[100, 1 700]
	TEM4/℃	[8, 25]
	TEM7/℃	[−26, 25]
山杏<br>*Armeniaca sibirica*	Ele/m	[700, 2 000]
	PRE3/mm	[200, 600]
	TEM4/℃	[4, 12]
	TEM7/℃	[−40, 25）

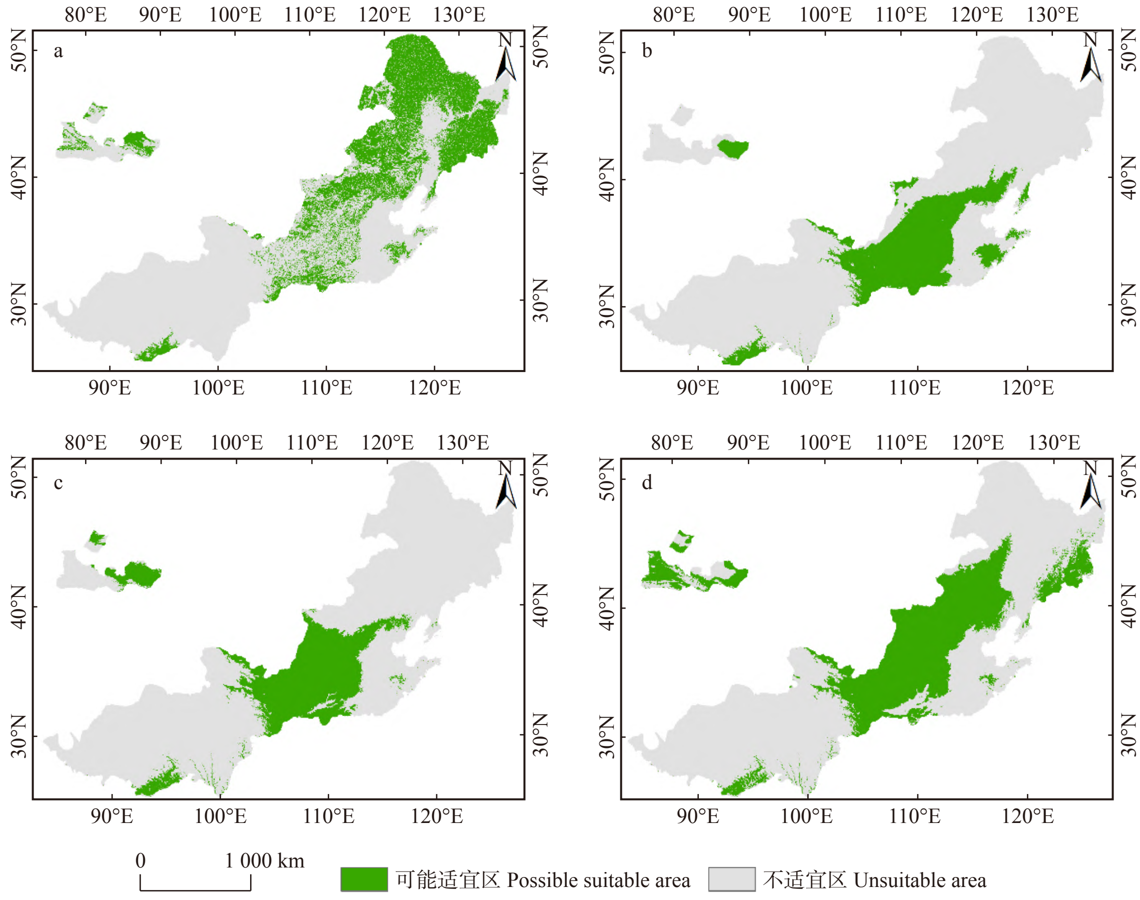

图 2 樟子松(a)、油松(b)、山桃(c)和山杏(d)可能适宜区分布图

Fig. 2 Distribution map of possible suitable areas of *Pinus sylvestris* var. *mongolica* (a), *Pinus tabuliformis* (b), *Amygdalus davidiana* (c) and *Armeniaca sibirica* (d)

(C)1994-2022 China Academic Journal Electronic Publishing House. All rights reserved. http://www.cnki.net

2.3　MaxEnt 模型构建

最大熵模型（MaxEnt）于 1957 年由 Jaynes[34]首次提出，因其在物种分布模拟、主导因子筛选中效果显著，被生态学者广泛使用。在树种的可能适宜区基础上，利用 MaxEnt 模型模拟树种适宜区的精准分布。将树种分布数据和环境因子导入模型，随机选取 75% 分布数据作为训练数据，25% 为检验数据，设置 10 次重复。根据受试者工作特征曲线（ROC）评价预测模型精度，ROC 曲线下方面积（即 AUC 值）为模型预测结果准确度，AUC 越大则越准确，划分标准为：0.50 ~ 0.60，失败；0.60 ~ 0.70，较差；0.70 ~ 0.80，一般；0.80 ~ 0.90，好；0.90 ~ 1.00，非常好。

2.4　适宜区等级划分

将模拟结果导入 ArcGIS 进行栅格转换，得到树种在环境因子中的存在概率（B），取值 0 ~ 1。参考车乐[35]的分级标准，划分为 4 个等级：不适宜区（$0 \leqslant B < 0.10$）、低适宜区（$0.10 \leqslant B < 0.30$）、中适宜区（$0.30 \leqslant B < 0.50$）与高适宜区（$0.50 \leqslant B \leqslant 1.00$）。

2.5　主要环境因子筛选

为避免由气象因子间多重共线性导致 MaxEnt 模型过度拟合，需对气象因子进行相关性分析，根据贡献率对强相关的两因子进行择优筛选。刀切法（Jackknife）可确定各因子对树种适宜区分布的贡献率，提供筛选依据。本研究将 Pearson 相关性分析与刀切法相结合，将 18 个气象因子导入 SPSS 进行 Pearson 相关性分析（图 3），相关性显著（$P < 0.05$）下，对强相关$|R| \geqslant 0.80$ 的两因子进行筛选，保留贡献率较高的气象因子，同地形、土壤因子作为主要环境因子（表 3），用于 MaxEnt 模型模拟。

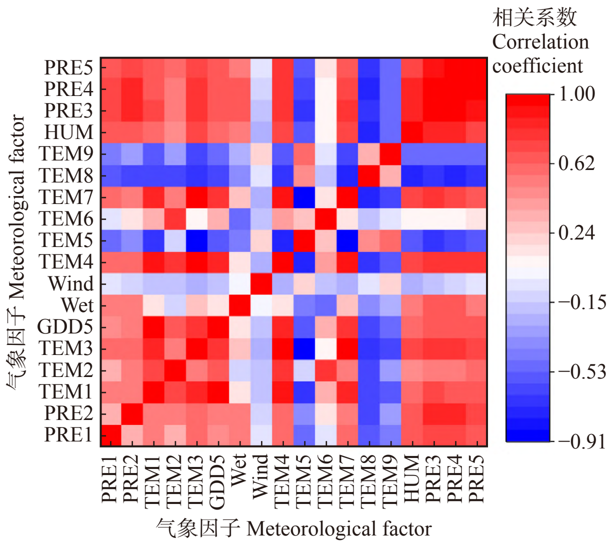

图 3　气象因子相关性分析

Fig. 3　Correlation analysis of meteorological factors

3　结果与分析

3.1　MaxEnt 模型精度评价

4 类树种 ROC 曲线评价结果显示（图 4）：各树种训练数据集 AUC 值均大于 0.90，达“非常好”水平；检验数据集 AUC 值均大于 0.80，达“好”水平。表明利用 MaxEnt 模型模拟樟子松、油松、山桃和山

表 3　树种主要环境因子

Tab. 3　Main environmental factors of tree species

樟子松 *Pinus sylvestris* var. *mongolica*	油松 *Pinus tabuliformis*	山桃 *Amygdalus davidiana*	山杏 *Armeniaca sibirica*
Ele	Ele	Ele	Ele
Slo	Slo	Slo	Slo
Asp	Asp	Asp	Asp
Soil-pH-T	Soil-pH-T	Soil-pH-T	Soil-pH-T
Soil-pH-S	Soil-pH-S	Soil-pH-S	Soil-pH-S
Soil-type	Soil-type	Soil-type	Soil-type
PRE1	PRE1	PRE1	PRE1
PRE2	PRE2	PRE2	PRE2
PRE3	PRE3	PRE5	PRE3
TEM1	TEM1	TEM2	TEM1
TEM2	TEM2	TEM6	TEM2
TEM3	TEM5	TEM7	TEM3
TEM6	TEM6	TEM8	TEM6
TEM8	TEM7	TEM9	TEM8
TEM9	TEM8	GDD5	TEM9
Wet	TEM9	Wet	Wet
Wind	Wet	Wind	Wind
HUM	Wind	HUM	HUM
	HUM		

(C)1994-2022 China Academic Journal Electronic Publishing House. All rights reserved.　http://www.cnki.net

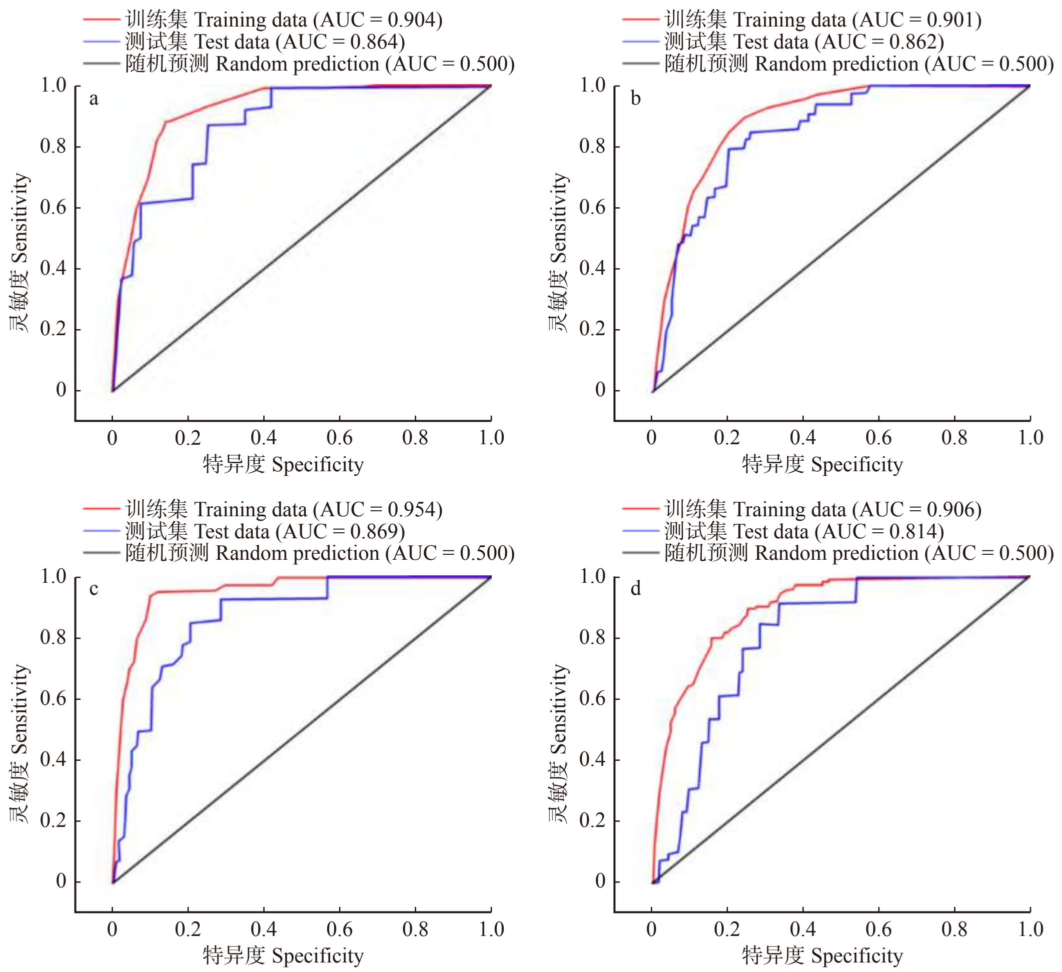

AUC 为 ROC 曲线下方的面积。AUC is area under the curve of ROC.

图 4 樟子松(a)、油松(b)、山桃(c)和山杏(d)的受试者工作特征曲线

Fig. 4 Receiver operating characteristic curves of *Pinus sylvestris* var. *mongolica* (a), *Pinus tabuliformis* (b), *Amygdalus davidiana* (c) and *Armeniaca sibirica* (d)

杏适宜区分布有较高的准确性，可利用该模型进行后续研究。

3.2 主导环境因子筛选与分析

经 MaxEnt 模型迭代运算和归一化处理后，得到影响树种分布的各环境因子贡献率，筛选出累积贡献率 > 80% 的环境因子为各树种的主导环境因子。

由图 5 可知，影响樟子松的主导因子依次为土壤类型(Soil-type)、最冷月均温(TEM3)、最冷月平均风速(Wind)，其累积贡献率为 80.60%；影响油松的主导因子依次为高程(Ele)、年均气温标准差(TEM9)、土壤类型(Soil-type)和年降水量(PRE3)，累积贡献率达 80.90%；山桃的主导因子依次为最暖月均温(TEM2)、高程(Ele)、年极端最低气温(TEM7)、年均降水量标准差(PRE5)、坡度(Slope)和土壤类型(Soil-type)，累积贡献率达 85.40%；影响山杏的主导因子依次为高程(Ele)、土壤类型(Soil-type)、最暖月平均降水量(PRE1)、湿润系数(Wet)和最暖月均温(TEM2)，其累积贡献率达 82.30%。根据结果可知绝大部分树种的分布主要受土壤类型和高程的影响，且两因子的平均贡献率高达 22.80% 和 23.15%(> 20%)，明显高于其他环境因子，表明土壤和地形两类因子对模型的构建具有重要意义，在环境因子选择时应充分考虑这两类因子。

经 MaxEnt 模拟得到各环境因子与树种分布概率的响应曲线，该曲线反映树种在各环境因子下的

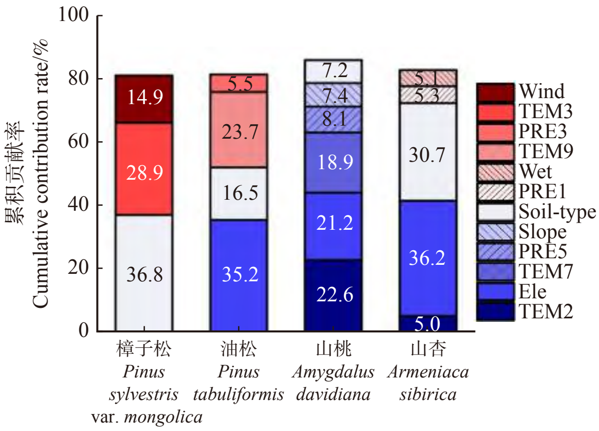

图 5 主导因子累积贡献率

Fig. 5 Cumulative contribution rates of dominant factors

(C)1994-2022 China Academic Journal Electronic Publishing House. All rights reserved. http://www.cnki.net

适宜分布程度[36]。定义分布概率 $B > 0.50$ 为显著响应区间，即树种适宜分布的环境阈值。定义 B 的峰值点为树种最适分布的环境因子取值；定义分布概率 $B > 0.70$ 为树种最适分布的土壤类型。

分析可知，樟子松最适分布于最冷月均温为−27 ℃，最冷月风速为 3.25 m/s，土壤类型为棕壤土、黑钙土的地区（图 6a ~ c）；油松最适生长于海拔 1 900 m，年均气温标准差 0.56 ~ 0.70 ℃，年均降水量 670 mm，土壤类型为棕壤土、褐土、灰褐土、栗褐土的地区（图 6d ~ g）；山桃在最暖月均温 26 ℃，海拔 1 100 m，

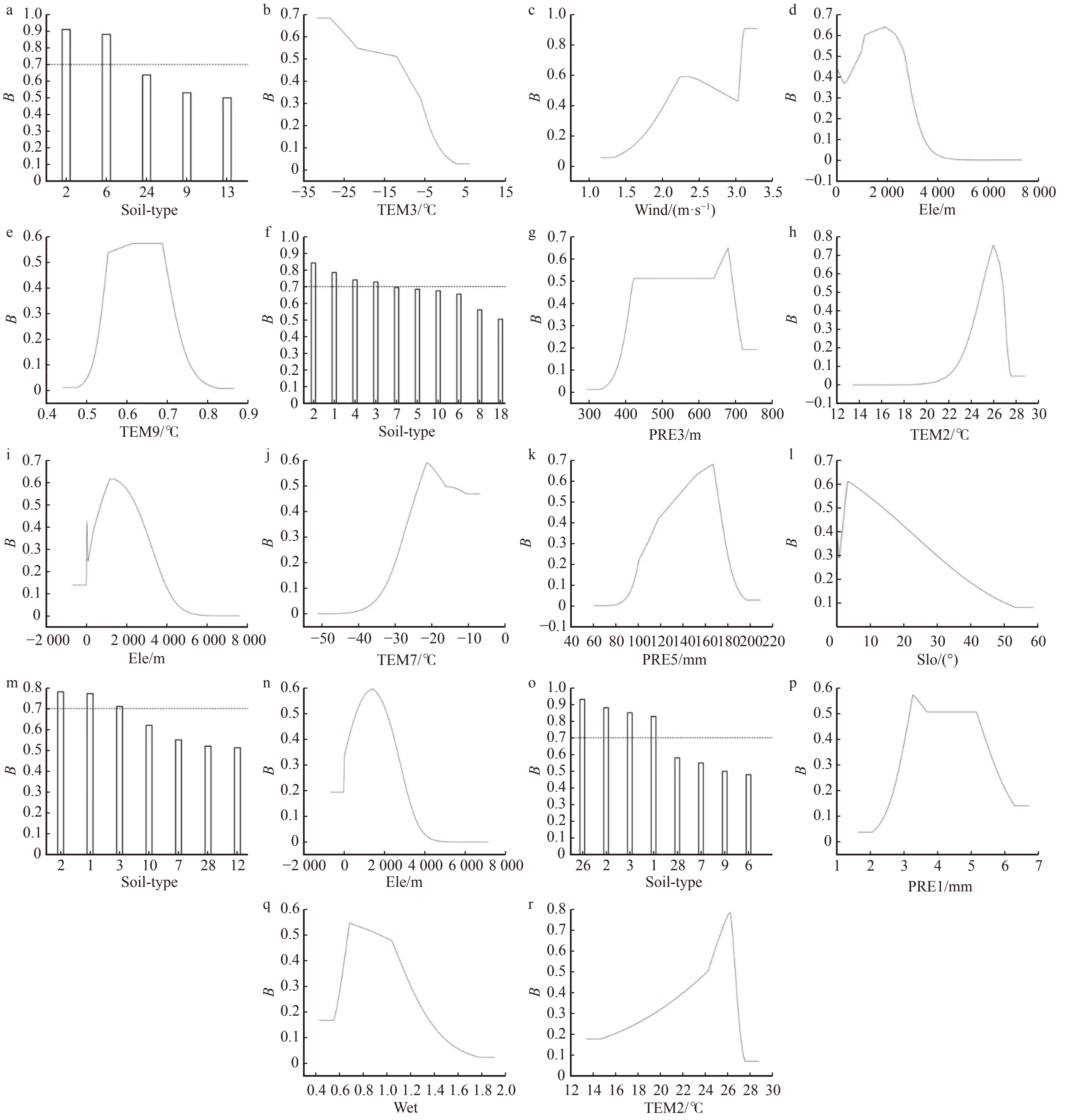

图 a~c、d~g、h~m 和 n~r 分别为樟子松、油松、山桃和山杏主导环境因子；a、f、m 和 o 中，1 为褐土；2 为棕壤土；3 为栗褐土；4 为灰褐土；5 为石质土；6 为黑钙土；7 为粗骨土；8 为新积土；9 为栗钙土；10 为黄棕壤；12 为潮土；13 为白浆土；18 为黄绵土；24 为风沙土；26 为黑土；28 为草甸土；B 为存在概率。In the figure, a−c、d−g、h−m and n−r are dominant environmental factors of *Pinus sylvestris* var. *mongolica*, *Pinus tabuliformis*, *Amygdalus davidiana* and *Armeniaca sibirica*, respectively. In figure a, f, m and o, 1 is cinnamon soil; 2 is brown soil; 3 is chestnut cinnamon soil; 4 is grey cinnamon soil; 5 is stony soil; 6 is chernozem; 7 is coarse bone soil; 8 is newly accumulated soil; 9 is chestnut soil; 10 is yellow brown soil; 12 is fluvo-aquic soil; 13 is albic soil; 18 is loessial soil; 24 is aeolian sandy soil; 26 is black soil; 28 is meadow soil. B is probability of existence.

图 6　主导环境因子响应曲线

Fig. 6　Response curves of dominant environmental factors

(C)1994-2022 China Academic Journal Electronic Publishing House. All rights reserved.　http://www.cnki.net

年极端最低气温−21 ℃，年均降水量标准差 1.30 ~ 1.70 mm，坡度 3° ~ 10°，土壤类型为棕壤土、褐土、栗褐土的地区最适宜分布（图 6h ~ m）；山杏最适生长于海拔 1 450 m，最暖月平均降水量 340 mm，湿润系数 0.70，最暖月均温 26.20 ℃，土壤类型为黑土、棕壤土、栗褐土、褐土的地区（图 6n ~ r）。

3.3 适宜区分布

经 ArcGIS 空间分析得到 4 类树种高、中、低和不适宜区的分布面积及比例（表 4），可知研究区内山杏和油松中高适宜区分布面积最广，面积及占比分别为 652 817.70 km^2（15.91%）和 617 382.00 km^2（15.04%）；其次是樟子松，为 598 442.00 km^2（14.58%）；山桃分布范围最小，为 357 961.90 km^2（8.72%）。

半干旱半湿润气候区 4 类树种适宜区分布见图 7，统计各树种中高适宜区的主要分布省份情况（图 8），樟子松中高适宜区主要分布于内蒙古自治区北部呼伦贝尔市海拉尔一带至西部鄂尔多斯市，面积 317 585 km^2，占樟子松中高适宜区总面积的 52.34%；黑龙江省大兴安岭山区，面积 135 680 km^2，占 21.76%；吉林省中部和东部，面积 80 872 km^2，占

表 4 树种的适宜分布区面积

Tab. 4 Suitable distribution area of tree species

树种<br>Tree species	高适宜区<br>High suitable area		中适宜区<br>Moderate suitable area		低适宜区<br>Low suitable area		不适宜区<br>Unsuitable area	
	面积<br>Area/km^2	占比<br>Proportion/%	面积<br>Area/km^2	占比<br>Proportion/%	面积<br>Area/km^2	占比<br>Proportion/%	面积<br>Area/km^2	占比<br>Proportion/%
樟子松<br>*Pinus sylvestris* var. *mongolica*	231 997.00	5.65	366 445.00	8.93	579 222.00	14.11	2 926 535.00	71.31
油松<br>*Pinus tabuliformis*	337 415.00	8.22	279 967.00	6.82	224 080.00	5.46	3 262 737.00	79.50
山桃<br>*Amygdalus davidiana*	195 493.00	4.76	162 468.90	3.96	318 831.50	7.77	3 427 405.60	83.51
山杏<br>*Armeniaca sibirica*	341 497.80	8.32	311 319.90	7.59	672 050.90	16.37	2 779 330.40	67.72

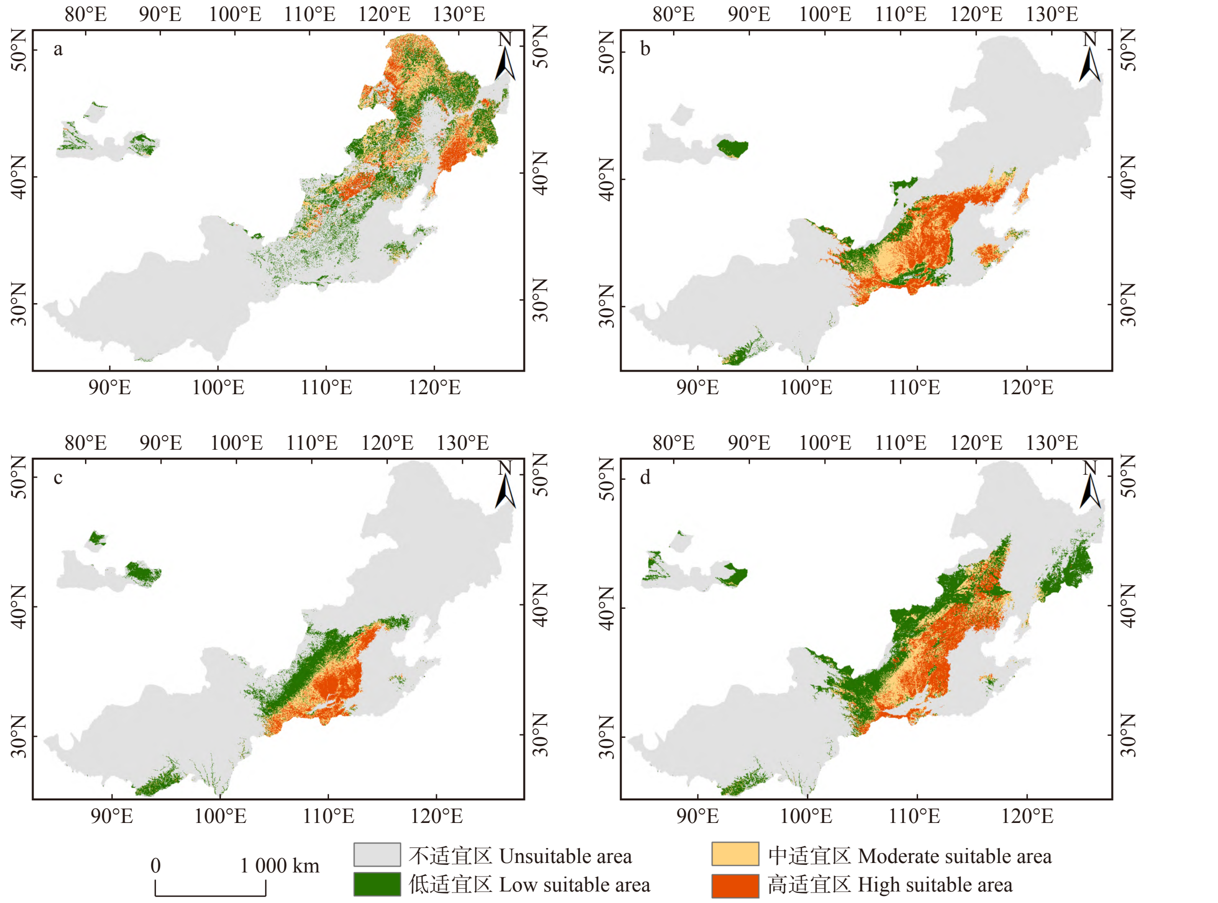

图 7 樟子松(a)、油松(b)、山桃(c)和山杏(d)适宜分布区

Fig. 7 Suitable distribution areas of *Pinus sylvestris* var. *mongolica* (a), *Pinus tabuliformis* (b), *Amygdalus davidiana* (c) and *Armeniaca sibirica* (d)

(C)1994-2022 China Academic Journal Electronic Publishing House. All rights reserved. http://www.cnki.net

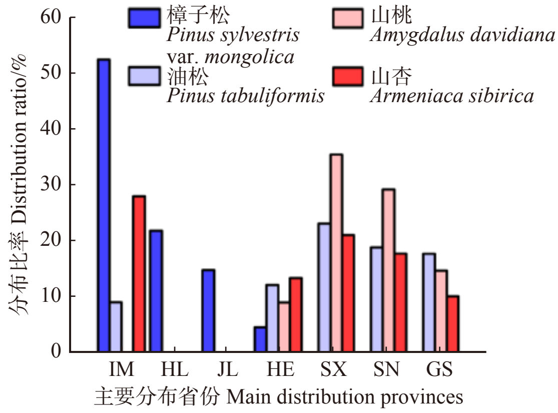

IM.内蒙古自治区；HL.黑龙江省；JL.吉林省；HE.河北省；SX.山西省；SN.陕西省；GS.甘肃省。IM, Inner Mongolia Autonomous Region; HL, Heilongjiang Province; JL, Jilin Province; HE, Hebei Province; SX, Shanxi Province; SN, Shaanxi Province; GS, Gansu Province.

图 8　樟子松、油松、山桃和山杏中高适宜区各省份占比

Fig. 8　Distribution ratio of provinces in middle and high suitable areas of *Pinus sylvestris* var. *mongolica*, *Pinus tabuliformis*, *Amygdalus davidiana* and *Armeniaca sibirica*

14.71%，以上 3 省（自治区）樟子松中高适宜区占研究区内樟子松中高适宜区的 88.81%（> 80%）。油松主要分布于山西省，面积 145 595 km^2，占油松中高适宜区总面积的 22.99%；陕西省中北部，面积 114 819 km^2，占 18.76%；甘肃省南部，面积 104 568 km^2，占 17.60%；河北省北部，面积 77 804 km^2，占 12.03%；内蒙古自治区中部，面积 51 169 km^2，占 8.94%，以上 5 省（自治区）中高适宜区分布达 80.32%（> 80%）。山桃分布于山西省中南部，面积 121 713 km^2，占山桃中高适宜区总面积的 35.40%；陕西省中部，面积 97 851 km^2，占 29.13%；甘肃省南部，面积 47 621 km^2，占 14.61%；河北省，面积 30 957 km^2，占 8.90%，以上 4 省分布达 88.04%（> 80%）。山杏分布于内蒙古中南部，面积 171 994 km^2，占山杏中高适宜区总面积的 27.87%；山西省，面积 132 483 km^2，占 20.93%；陕西中北部，面积 108 846 km^2，占 17.65%；河北北部，面积 84 347 km^2，占 13.26%；甘肃省东南部，面积 60 667 km^2，占 10.02%，以上 5 省（自治区）山杏中高适宜区分布达 89.73%（> 80%）。各树种中高适宜区最高分布省份的县级分布情况如表 5 和图 9 所示，在利用 4 类树种造林绿化时可着重考虑

表 5　4 类树种最高适生省份的县级分布

Tab. 5　County-level distribution of the provinces with the highest suitability of four tree species

树种<br>Tree species	省级行政区<br>Provincial-level administrative region	地市级行政区<br>Prefecture-level administrative region	县级行政区<br>County-level administrative region
樟子松<br>*Pinus sylvestris* var. *mongolica*	内蒙古自治区<br>Inner Mongolia Autonomous Region	呼和浩特市<br>Hohhot City	全市<br>Whole City
		赤峰市<br>Chifeng City	林西县、阿鲁科尔沁旗、巴林左旗、巴林右旗、克什克腾旗、翁牛特旗<br>Linxi County, Alukhorqin Banner, Balinzuo Banner, Balinyou Banner, Hexigten Banner, Ongniud Banner
		通辽市<br>Tongliao City	扎鲁特旗<br>Jarud Banner
		鄂尔多斯市<br>Ordos City	准格尔旗、乌审旗、伊金霍洛旗<br>Jungar Banner, Wushen Banner, Ejin Horo Banner
		呼伦贝尔市<br>Hulunbeier City	满洲里市、阿荣旗、新巴尔虎左旗、鄂伦春自治旗、鄂温克族自治旗<br>Manzhouli City, Arong Banner, Xinbaerhuzuo Banner, Oroqen Autonomous Banner, Ewenki Autonomous Banner
		乌兰察布市<br>Wulanchabu City	丰镇市、卓资县、化德县、商都县、兴和县、察哈尔右翼中旗、察哈尔右翼后旗<br>Fengzhen City, Zhuozi County, Huade County, Shangdu County, Xinghe County, Chahar Wing Right Middle Banner, Chahar Wing Right Back Banner
		兴安盟<br>Hinggan League	突泉县、科尔沁右翼前旗、科尔沁右翼中旗<br>Tuquan County, Horqin Right Front Banner, Horqin Right Middle Banner
		锡林郭勒盟<br>Xilinguole League	锡林浩特市、多伦县、镶黄旗、正镶白旗、正蓝旗<br>Xilinhot City, Duolun County, Xianghuang Banner, Zhengxiangbai Banner, Zhenglan Banner
油松<br>*Pinus tabuliformis*	山西省<br>Shanxi Province	太原市 Taiyuan City	全市 Whole city
		阳泉市 Yangquan City	全市 Whole city
		长治市 Changzhi City	全市 Whole city
		晋城市 Jincheng City	全市 Whole city
		朔州市 Shuozhou City	全市 Whole city
		晋中市 Jinzhong City	全市 Whole city
		忻州市 Xinzhou City	全市 Whole city
		吕梁市 Lüliang City	全市 Whole city
		运城市 Yuncheng City	绛县、夏县 Jiang County, Xia County

(C)1994-2022 China Academic Journal Electronic Publishing House. All rights reserved.　http://www.cnki.net

表 5(续)

Tab.5 (continued)

树种<br>Tree species	省级行政区<br>Provincial-level administrative region	地市级行政区<br>Prefecture-level administrative region	县级行政区<br>County-level administrative region
山桃<br>*Amygdalus davidiana*	山西省<br>Shanxi Province	阳泉市<br>Yangquan City	全市<br>Whole city
		长治市<br>Changzhi City	全市<br>Whole city
		晋城市<br>Jincheng City	全市<br>Whole city
		晋中市<br>Jinzhong City	全市<br>Whole city
		运城市<br>Yuncheng City	全市<br>Whole city
		临汾市<br>Linfen City	全市<br>Whole city
		吕梁市<br>Lüliang City	全市<br>Whole city
		太原市<br>Taiyuan City	古交市、清徐县、阳曲县、娄烦县<br>Gujiao City, Qingxu County, Yangqu County, Loufan County
		大同市<br>Datong City	广灵县、灵丘县、浑源县<br>Guangling County, Lingqiu County, Hunyuan County
山杏<br>*Armeniaca sibirica*	内蒙古自治区<br>Inner Mongolia Autonomous Region	呼和浩特市<br>Hohhot City	托克托县、清水河县、武川县<br>Tuoketuo County, Qingshuihe County, Wuchuan County
		赤峰市<br>Chifeng City	林西县、宁城县、巴林左旗、巴林右旗、克什克腾旗、翁牛特旗、喀喇沁旗、敖汉旗<br>Linxi County, Ningcheng County, Balinzuo Banner, Balinyou Banner, Hexigten Banner, Ongniud Banner, Harqin Banner, Aohan Banner
		通辽市<br>Tongliao City	霍林郭勒市、库伦县、奈曼旗、扎鲁特旗、科尔沁右翼中旗<br>Huolinguole City, Kulun County, Naiman Banner, Jarud Banner, Horqin Right Middle Banner
		鄂尔多斯市<br>Ordos City	达拉特旗、准格尔旗、伊金霍洛旗<br>Datuk Banner, Jungar Banner, Ejinhoro Banner
		乌兰察布市<br>Ulanqab City	丰镇市、卓资县、化德县、商都县、兴和县、凉城县、察哈尔右翼中旗、察哈尔右翼后旗<br>Fengzhen City, Zhuozi County, Huade County, Shangdu County, Xinghe County, Liangcheng county, Chahar Right Middle Banner, Chahar Right Back Banner
		兴安盟<br>Hinggan League	乌兰浩特市、突泉县、扎赉特旗、科尔沁右翼前旗<br>Wulanhaote City, Tuquan County, Jalaid Banner, Horqin Right Front Banner
		锡林郭勒盟<br>Xilinguole League	多伦县、西乌珠穆沁旗、太仆寺旗、镶黄旗、正镶白旗、正蓝旗<br>Duolun County, West Ujimqin Banner, Taipusi Banner, Xianghuang Banner, Zhengxiangbai Banner, Zhenglan Banner

表内县域。

4 讨 论

4.1 主导环境因子分析

樟子松分布受气象因素中最冷月均温的影响最为显著，最适区间为−30 ~ −21 ℃，与吴祥云等[37]得出的樟子松耐寒且对地温较敏感的结果一致；其次受最冷月平均风速影响较显著，赵哈林等[38]得出樟子松适宜分布在风速大于 3.10 m/s 的地区，随风速增加，水分利用率上升；风速 > 18 m/s 时，水分利用率下降，因此适当高风速利于樟子松生长，与本研究结果相同。油松分布受高程、土壤类型、年气温标准差和年降水量影响最为显著，在升幅为 0.50 ℃/10 a[39]的全球变暖趋势下，郑景云等[40]指出在 1951—2001 年历史气象下我国亚热带和暖温带北界均有北移趋势，吕振刚等[19]证明在 2040—2069 年未来气候变化下，油松适宜区向高海拔转移，低海拔(< 1 000 m)地区的油松逐渐消失，因为海拔重塑了区域气候，间接改变地区的水热条件，进而影响树种的生理生化特性[41]，导致油松适宜区向高海拔高纬度迁移。众多研究表明，大气环流模式可模拟物种分布，气温模拟效果优于降水[42]，与本研究得出年均气温标准差对油松分布的贡献率(23.70%)高于年降水量(5.50%)的结果相符。山桃分布受气象因子中最暖月均温影响最为显著，适宜区间为 25 ~ 27 ℃，其次是年极端最低气温，区间为 −22.50 ~ −16.50 ℃，与贾光林等[33]利用 TCMGIS 提取生态因子得出山桃生态因子适宜阈值相符，其研究还得出山桃易受干旱、高温影

(C)1994-2022 China Academic Journal Electronic Publishing House. All rights reserved. http://www.cnki.net

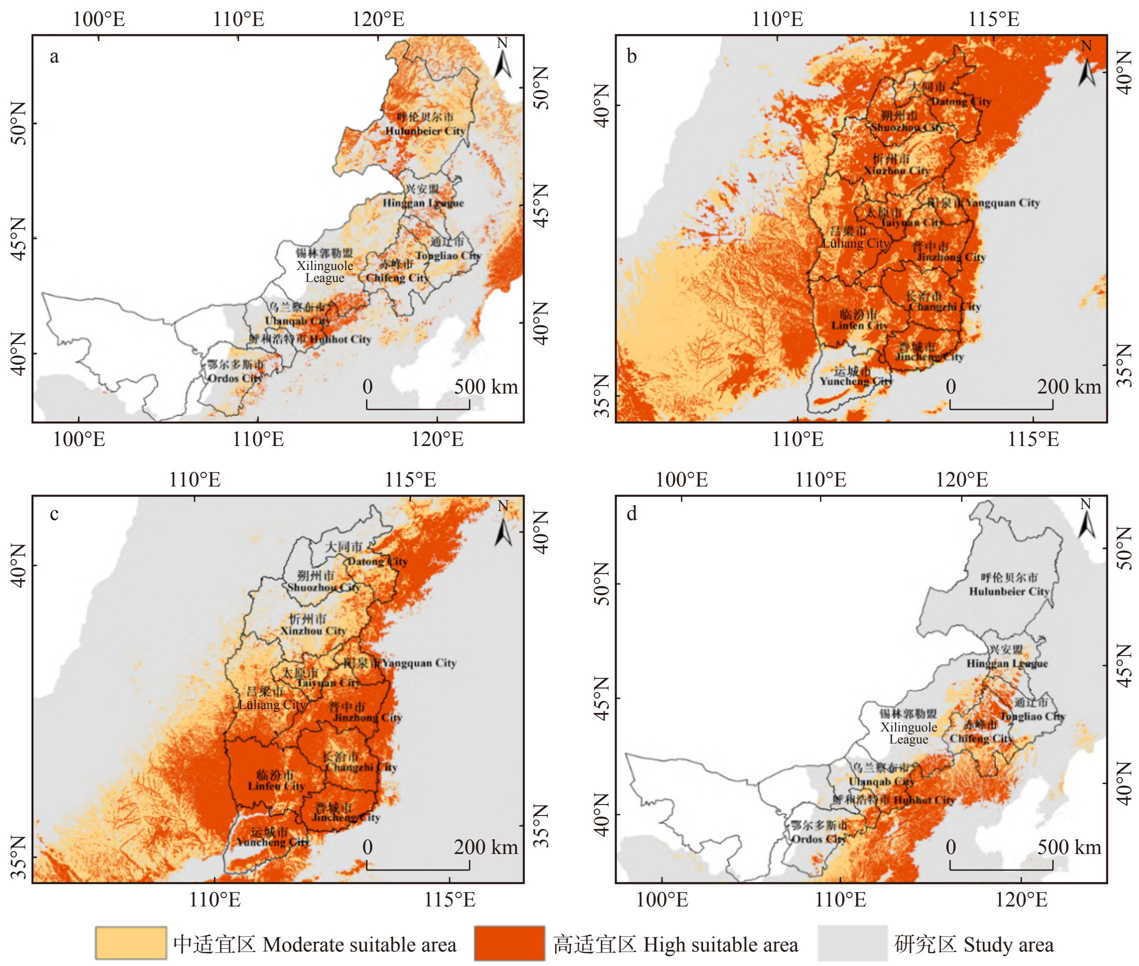

图 9　樟子松(a)、油松(b)、山桃(c)和山杏(d)最高适生省份中高适宜区地级分布

Fig. 9　Distribution of *Pinus sylvestris* var. *mongolica* (a), *Pinus tabuliformis* (b), *Amygdalus davidiana* (c) and *Armeniaca sibirica* (d) at prefecture level in middle and high suitable areas of the highest suitable provinces

响，因此山桃栽培需选择水分、温度适宜的地区，验证本文结果。山杏分布受高程、土壤类型、湿润系数、最暖月平均降水量和最暖月均温的影响最为显著，适宜高程为 700～2 000 m，超过 1 450 m 时分布概率下降，与张家琛等[43]通过野外勘测对阜平县山杏地形适宜性研究结果相符。由于山杏为喜温树种[44]，对本研究得出的最暖月降水量及温度两因子较为敏感，针对山杏苗木的栽培应以温度调控和水分管理工作为主。由于油松、山杏和山桃均受海拔影响显著，且树种的物候期与海拔梯度有关，可发挥不同树种在垂直尺度上的物候差异，提升山地树种的观赏价值，造林绿化同时提升景观效益。

4.2　适宜区分布情况

樟子松为我国高寒地带主要树种，中高适宜区集中分布在针叶林植被覆盖区，位于我国东北和华北与天然分布区自然气候相似地区，与李蒙蒙等[45]针对樟子松分布研究一致。但水热条件充裕的甘肃南部不宜引种，由于该地水热过高易引发涝害次生胁迫，导致根系缺氧，过量活性氧自由基引发呼吸代谢紊乱[46]，且影响土壤氮素矿化过程[47]。除气候和土地条件以外，造林密度也影响樟子松的引种成效，造林密度过高易加大蒸腾导致植物缺水，过低则难达到防护效果[48]，冯奥哲等[49]研究得出沙地樟子松造林密度影响土壤无机氮空间分布，造林密度 1 000 株/hm^2 时，土壤碳有效性最优。油松分布结果与张雷等[50]使用多种模型研究油松的适宜分布区基本一致，全球变暖下，基于未来气候数据(Worldclim)，Wang[51]等研究指出，2050 年油松适宜海拔将大幅升高(增加 1 100 m)；2100 年我国西、北部干旱加重，油松为耐旱树种且长速快[52]，适宜区有向西、北部地区扩散的趋势，可考虑提升适应性森林资源管理机制，将油松适度向西、北部迁移同时，综合考虑病虫害[53]和种间关系[54]等因素，确定油松引种区域。山桃在山西、陕西大部分地区虽适宜，但因土地利用现状，存在大量未开发的荒地，因此造林前需考虑当地国土空间规划，综合自然、社会因素限制，科学开展造林作业。油松、山桃和山杏作为喜温树种和景观树种，全球变暖下适宜区将逐渐扩大，可适当扩大造林规模，最大化发挥气候优势与规模效应；同时 3 类树种适生区多与北方贫困山区重叠，适

(C)1994-2022 China Academic Journal Electronic Publishing House. All rights reserved.　http://www.cnki.net

度加大生态经济林建设，可帮助北方地区脱贫致富，提升景观与经济效益。

为保证造林工作的科学实施，应在本研究提出树种县级中高适宜分布区域基础上，考虑局地小气候、土壤理化性质、种间关系、树种适应环境的进化能力、社会经济和人为影响[55]，针对低适宜区，结合病虫害防治、密度控制、林种结构调整和造林整地等技术提高适宜度；针对不适宜区，应尽量避免引种，降低人力、财力和物力的浪费。

5 结　论

本研究模拟了自然条件(地形、气候和土壤)下樟子松、油松、山桃和山杏的适宜区分布，划分了半干旱半湿润区 4 类树种的适宜等级，利用 MaxEnt 模型模拟得到樟子松、油松、山桃和山杏分布的 AUC 值均大于 0.90，表明该模型对本研究具有很好的适用性。研究得出，樟子松除土壤类型外，对最冷月均温和最冷月平均风速两种低温环境因子较为敏感，由于其具有抗风耐寒特性，在我国东北的内蒙古、黑龙江、吉林的高寒地区均适宜引种；油松受高程、气温标准差、土壤类型和年均降水量影响显著，随着全球变暖，油松具有向高海拔高纬度地区转移的趋势，当前气候背景下我国华北的山西、河北、内蒙古地区和西北的陕西、甘肃等地为油松适宜引种区，今后需结合未来气候调整适宜区范围；山桃受最暖月均温、高程、年极端最低气温、年降水量标准差、坡度和土壤类型影响显著，适宜引种区主要分布于山西、陕西、甘肃、河北等地；山杏受高程、土壤类型、最暖月均降水量、湿润指数和最暖月均温的影响最为显著，内蒙古、山西、陕西、河北、甘肃等地为适宜引种区。喜温树种油松、山桃和山杏的适宜区多与北方贫困山区重叠，全球变暖背景下适宜分布区将逐渐扩大，可考虑适宜区内适度建设经济林，连片引种栽培，产生规模效应，发挥林木防风固沙、保持水土和涵养水源功能的同时，推动半干旱半湿润区林木产业生态扶贫工程，实现区域生态景观和社会经济共同发展。

参 考 文 献

[1] 程林仙, 王万瑞, 仁宗启, 等. 陕北仁用杏气候适宜性区划[J]. 西北林学院学报, 2001, 16(2): 18−21.
Cheng L X, Wang W R, Ren Z Q, et al. Climatic adaptability division for apricot in northern Shaanxi[J]. Journal of Northwest Forestry University, 2001, 16(2): 18−21.

[2] 赖文豪, 席沁, 武海龙, 等. 内蒙古兴和县低山丘陵立地类型划分与林草适宜性评价[J]. 浙江农林大学学报, 2018, 35(2): 331−339.
Lai W H, Xi Q, Wu H L, et al. Site classification type and vegetation suitability evaluation for hilly land in Xinghe, Inner Mongolia[J]. Journal of Zhejiang A&F University, 2018, 35(2): 331−339.

[3] 闫烨琛. 大清河流域山丘区立地类型划分与评价 [D]. 北京: 北京林业大学, 2020.
Yan Y C. Classification and evaluation of site types in hilly areas of Daqing River Basin [D]. Beijing: Beijing Forestry University, 2020.

[4] 周立江. 低效林评判与改造途径的探讨[J]. 四川林业科技, 2004, 25(1): 16−21.
Zhou L J. Discussion on judgment and rebuilding approaches of low-efficiency forest[J]. Journal of Sichuan Forestry Science and Technology, 2004, 25(1): 16−21.

[5] 张明珠, 叶兴状, 刘益鹏, 等. 基于 SSPs 预测格木在中国的潜在地理分布[J]. 北京林业大学学报, 2022, 44(4): 54−65.
Zhang M Z, Ye X Z, Liu Y P, et al. Predicting the potential geographical distribution of *Erythrophleum fordii* in China based on SSPs[J]. Journal of Beijing Forestry University, 2022, 44(4): 54−65.

[6] Ahmed S E, Mcinerny G, O'Hara K, et al. Scientists and software-surveying the species distribution modelling community[J]. Diversity & Distributions, 2015, 21(3): 258−267.

[7] 郭虹扬, 史明昌, 杨建英, 等. 白洋淀大清河流域油松精准适宜性空间分布[J]. 浙江农林大学学报, 2021, 38(6): 1−9.
Guo H Y, Shi M C, Yang J Y, et al. Precise spatial distribution of suitability of *Pinus tabulaeformis* in Daqing River Basin, Baiyangdian[J]. Journal of Zhejiang A&F University, 2021, 38(6): 1−9.

[8] 张春华, 和菊, 孙永玉, 等. 基于 MaxEnt 模型的紫椿适生区预测[J]. 北京林业大学学报, 2017, 39(8): 33−41.
Zhang C H, He J, Sun Y Y, et al. Distributional change in suitable areas for *Toona sureni* based on MaxEnt model[J]. Journal of Beijing Forestry University, 2017, 39(8): 33−41.

[9] Sharifian S, Kamrani E, Saeedi H. Global future distributions of mangrove crabs in response to climate change[J]. Wetlands, 2021, 41(8): 1−14.

[10] 黄睿智, 于涛, 赵辉, 等. 气候变化背景下濒危植物梓叶槭在中国适生分布区预测[J]. 北京林业大学学报, 2021, 43(5): 33−43.
Huang R Z, Yu T, Zhao H, et al. Prediction of suitable distribution area of endangered plant *Acer catalpa* in China under the background of climate change[J]. Journal of Beijing Forestry University, 2021, 43(5): 33−43.

[11] 刘维, 赵儒楠, 圣倩倩, 等. 矮牡丹在中国的地理分布及潜在分布区预测[J]. 北京林业大学学报, 2021, 43(12): 83−92.
Liu W, Zhao R N, Sheng Q Q, et al. Geographical distribution and potential distribution area prediction of *Paeonia jishanensis* in China[J]. Journal of Beijing Forestry University, 2021, 43(12): 83−92.

(C)1994-2022 China Academic Journal Electronic Publishing House. All rights reserved. http://www.cnki.net

[12] 满多清, 孙坤, 刘世增, 等. 干旱荒漠区樟子松幼苗的抗逆性分析[J]. 甘肃农业大学学报, 2004, 39(5): 543−547.
Man D Q, Sun K, Liu S Z, et al. A research on seedling resistance of *Pinus sylvestris* var. *mongolica* in arid desert area[J]. Journal of Gansu Agricultural University, 2004, 39(5): 543−547.

[13] Xi Q, Lai W H, Cui Y Y, et al. Effect of yeast extract on seedling growth promotion and soil improvement in afforestation in a semiarid chestnut soil area[J/OL]. Forests, 2019, 10(1): 76[2021-12-20]. https://doi.org/10.3390/f10010076.

[14] Zhebentyayeva T, Reighard G, Gorina V, et al. Simple sequence repeat (SSR) analysis for assessment of genetic variability in apricot germplasm[J]. Theoretical and Applied Genetics, 2003, 106(3): 435−444.

[15] 于笑, 纪若璇, 常远, 等. 四种抗旱植物在不同区域的生长稳定性[J]. 应用生态学报, 2021, 32(12): 4212−4222.
Yu X, Ji R X, Chang Y, et al. Growth stability of four drought resistant plant species in different regions[J]. Chinese Journal of Applied Ecology, 2021, 32(12): 4212−4222.

[16] 张淑勇, 周泽福, 张光灿, 等. 水分胁迫下天然次生灌木山桃和山杏光合气体交换特征[J]. 西北植物学报, 2008, 28(12): 2492−2499.
Zhang S Y, Zhou Z F, Zhang G C, et al. Gas exchange characteristics of natural secondary shrubs *Prunus davidiana* and *Prunus sibirica* under different water stresses[J]. Acta Botanica Boreali-Occidentalia Sinica, 2008, 28(12): 2492−2499.

[17] Garzón M, Blazek R, Neteler M, et al. Predicting habitat suitability with machine learning models: the potential area of *Pinus sylvestris* L. in the Iberian Peninsula[J]. Ecological Modelling, 2006, 197(3−4): 383−393.

[18] 张晨星, 张炜, 徐晶晶, 等. 基于 GIS 和最大熵模型的河北省油松适宜性分布分析[J]. 地理与地理信息科学, 2020, 36(6): 18−25.
Zhang C X, Zhang W, Xu J J, et al. Analysis on suitability distribution of *Pinus tabulaeformis* in Hebei Province based on GIS and MaxEnt model[J]. Geography and Geo-Information Science, 2020, 36(6): 18−25.

[19] 吕振刚, 李文博, 黄选瑞, 等. 气候变化情景下河北省 3 个优势树种适宜分布区预测[J]. 林业科学, 2019, 55(3): 13−21.
Lü Z G, Li W B, Huang X R, et al. Predicting suitable distribution area of three dominant tree species under climate change scenarios in Hebei Province[J]. Scientia Silvae Sinicae, 2019, 55(3): 13−21.

[20] Vasquez V L, de Lima A A, dos Santos A P, et al. Influence of spatial extent on habitat suitability models for primate species of Atlantic forest[J/OL]. Ecological Informatics, 2021, 61: 101179 [2022−02−10]. https://doi.org/10.1016/j.ecoinf.2020.101179.

[21] 李昂. 应用 ArcGIS 软件和最大熵模型分析樟子松潜在分布及其气候适宜性 [D]. 沈阳: 沈阳农业大学, 2016.
Li A. Using ArcGIS software and maximum entropy model to analyze the potential distribution and climate suitability of *Pinus sylvestris* var. *mongolica* [D]. Shenyang: Shenyang Agricultural University, 2016.

[22] 唐燕, 赵儒楠, 任钢, 等. 基于 MaxEnt 模型的中华枸杞潜在分布预测及其重要影响因子分析[J]. 北京林业大学学报, 2021, 43(6): 23−32.
Tang Y, Zhao R N, Ren G, et al. Prediction of potential distribution of *Lycium chinense* based on MaxEnt model and analysis of its important influencing factors[J]. Journal of Beijing Forestry University, 2021, 43(6): 23−32.

[23] 王爱君, 路东晔, 张国盛, 等. 基于 MaxEnt 模拟欧亚大陆气候变化下叉子圆柏的潜在分布[J]. 林业科学, 2021, 57(8): 43−55.
Wang A J, Lu D Y, Zhang G S, et al. Potential distribution of *Juniperus sabina* under climate change in Eurasia continent based on MaxEnt model[J]. Scientia Silvae Sinicae, 2021, 57(8): 43−55.

[24] Zhou Y, Zhang Z, Zhu B, et al. MaxEnt modeling based on CMIP6 models to project potential suitable zones for *Cunninghamia lanceolata* in China[J/OL]. Forests, 2021, 12(6): 752[2022−02−10]. https://doi.org/10.3390/f12060752.

[25] 赵宇铭, 邱新法, 朱晓晨, 等. 1971—2010 年中国干湿区降雨资源变化特征分析[J]. 长江科学院院报, 2019, 36(5): 34−41.
Zhao Y M, Qiu X F, Zhu X C, et al. Characteristics of rainfall amount variations in wet and drv partitions of China from 1971 to 2010[J]. Journal of Yangtze River Scientific Research Institute, 2019, 36(5): 34−41.

[26] 祖力卡尔·海力力, 赵廷宁, 姜群鸥. 西北干旱荒漠区边界范围及变化分析[J]. 干旱区地理, 2021, 44(6): 1635−1643.
Zulikar H, Zhao T N, Jiang Q O. Boundary scope and change of arid desert area in northwest China[J]. Arid Land Geography, 2021, 44(6): 1635−1643.

[27] 中国植物志编辑委员会. 中国植物志 [M]. 北京: 科学出版社, 1978.
Editorial Board of Flora of China. Flora of China[M]. Beijing: Science Press, 1978.

[28] 阳宽达, 谢红霞, 隋兵, 等. 基于 GIS 的降雨空间插值研究: 以湖南省为例[J]. 水土保持研究, 2020, 27(3): 134−138.
Yang K D, Xie H X, Sui B, et al. Research on spatial interpolation of rainfall based on GIS: a case study of Hunan Province[J]. Research of Soil and Water Conservation, 2020, 27(3): 134−138.

[29] 李宗梅, 张增祥, 赵晓丽, 等. 全国干湿分布区动态变化研究[J]. 地球与环境, 2017, 45(4): 420−433.
Li Z M, Zhang Z X, Zhao X L, et al. Study on the dynamic change of dry and wet distribution areas in China[J]. Earth and Environment, 2017, 45(4): 420−433.

[30] 赵兴梁, 李万英. 樟子松 [M]. 北京: 农业出版社, 1963: 154.
Zhao X L, Li W Y. *Pinus sylvestris* var. *mongolica* [M]. Beijing: Agriculture Press, 1963: 154.

[31] 徐化成. 油松 [M]. 北京: 中国林业出版社, 1993.

(C)1994-2022 China Academic Journal Electronic Publishing House. All rights reserved.　http://www.cnki.net

Xu H C. *Pinus tabuliformis* [M]. Beijing: China Forestry Publishing House, 1993.

[32] 吴征镒. 中国植被 [M]. 北京: 科学出版社, 1980.

Wu Z Y. Vegetation in China [M]. Beijing: Science Press, 1980.

[33] 贾光林, 王珍, 李家春, 等. 山桃仁产地适宜性分析[J]. 湖北农业科学, 2011, 50(18): 3778−3780.

Jia G L, Wang Z, Li J C, et al. Regional suitability evaluation of *Prunus daviadiana*[J]. Hubei Agricultural Sciences, 2011, 50(18): 3778−3780.

[34] Jaynes E T. Information theory and statistical mechanics[J]. Physical Review, 1957, 106(4): 343–369.

[35] 车乐, 曹博, 白成科, 等. 基于 MaxEnt 和 ArcGIS 对太白米的潜在分布预测及适宜性评价[J]. 生态学杂志, 2014, 33(6): 1623−1628.

Che L, Cao B, Bai C K, et al. Predictive distribution and habitat suitability assessment of *Notholirion bulbuliferum* based on MaxEnt and ArcGIS[J]. Chinese Journal of Ecology, 2014, 33(6): 1623−1628.

[36] 古丽米拉·克孜尔别克, 邱琴, 海拉提·克孜尔别克. 基于 MaxEnt 模型的阿勒泰金莲花潜在适生区预测[J]. 江苏农业科学, 2021, 49(4): 82−87.

Gulimilla K, Qiu Q, Hailati K. Prediction of potential suitable area of *Trollius altaicus* based on MaxEnt model[J]. Jiangsu Agricultural Sciences, 2021, 49(4): 82−87.

[37] 吴祥云, 姜凤岐, 李晓丹, 等. 樟子松人工固沙林衰退的规律和原因[J]. 应用生态学报, 2004, 15(12): 2225−2228.

Wu X Y, Jiang F Q, Li X D, et al. Decline regularity and causes of *Pinus sylvestris* var. *mongolica* plantation on sandy land[J]. Chinese Journal of Applied Ecology, 2004, 15(12): 2225−2228.

[38] 赵哈林, 李瑾, 周瑞莲, 等. 不同强度净风频繁吹袭对樟子松(*Pinus sylvestris* var. *mongolica*) 幼苗光合蒸腾特征的影响[J]. 生态学报, 2017, 37(5): 1431−1437.

Zhao H L, Li J, Zhou R L, et al. Effects of wind frequency on the rates of photosynthesis and transpiration in *Pinus sylvestris* var. *mongolica* seedlings[J]. Acta Ecologica Sinica, 2017, 37(5): 1431−1437.

[39] 肖敏, 胡卓玮, 董琳. 基于 MaxEnt 模型的油松潜在地理分布研究[J]. 地理空间信息, 2017, 15(6): 34−37.

Xiao M, Hu Z W, Dong L. Potential geographical distribution of *Pinus Tabuliformis* based on MaxEnt model[J]. Geospatial Information, 2017, 15(6): 34−37.

[40] 郑景云, 尹云鹤, 李炳元. 中国气候区划新方案[J]. 地理学报, 2010, 65(1): 3−12.

Zheng J Y, Yin Y H, Li B Y. A new scheme for climate regionalization in China[J]. Acta Geographica Sinica, 2010, 65(1): 3−12.

[41] Wang, Jr, Hawkins, et al. Photosynthesis, water and nitrogen use efficiencies of four paper birch (*Betula papyrifera*) populations grown under different soil moisture and nutrient regimes[J]. Forest Ecol Manage, 1998, 112(3): 233−244.

[42] Ribes A, Azas J M, Planton S. A method for regional climate change detection using smooth temporal patterns[J]. Climate Dynamics, 2010, 35(2): 391−406.

[43] 张家琛, 党怡雯, 陈亚恒. 基于 GIS 的阜平县北流河区域山杏生态适宜性区划研究[J]. 西南林业大学学报(自然科学), 2021, 41(5): 98−104.

Zhang J C, Dang Y W, Chen Y H. Ecological suitability regionalization of *Armeniaca sibirica* in Beiliuhe region of Fuping County based on GIS[J]. Journal of Southwest Forestry University (Natural Sciences), 2021, 41(5): 98−104.

[44] 张山清, 吉春容, 普宗朝. 气候变暖对新疆杏种植气候适宜性的影响[J]. 中国农业资源与区划, 2019, 40(9): 131−141.

Zhang S Q, Ji C R, Pu Z C. Impact of climate warming on climate suitability of apricot planting in Xinjiang[J]. Chinese Journal of Agricultural Resources and Regional Planning, 2019, 40(9): 131−141.

[45] 李蒙蒙, 丁国栋, 高广磊, 等. 樟子松 (*Pinus sylvestris* var. *mongholica*) 在中国北方 10 省 (区) 引种的适宜性 [J]. 中国沙漠, 2016, 36(4): 1021−1028.

Li M M, Ding G D, Gao G L, et al. Introduction suitability of *Pinus sylvestris* var. *mongholica* in 10 northern provinces of China [J]. Journal of Desert Research 2016, 36(4): 1021−1028.

[46] 喻方圆, 徐锡增. 植物逆境生理研究进展[J]. 世界林业研究, 2003, 16(5): 6−11.

Yu F Y, Xu X Z. A review on plant stress physiology[J]. World Forestry Research, 2003, 16(5): 6−11.

[47] Zhao Q, Zeng D H, Fan Z P. Nitrogen and phosphorus transformations in the rhizospheres of three tree species in a nutrient-poor sandy soil[J]. Applied Soil Ecology, 2010, 46(3): 341−346.

[48] 赵晓彬, 刘光哲. 沙地樟子松引种栽培及造林技术研究综述[J]. 西北林学院学报, 2007, 22(5): 86−89.

Zhao X B, Liu G Z. A review of studies of introduction cultivates and afforestation technology on *Pinus sylvestris* var. *mongolica* in sandy area[J]. Journal of Northwest Forestry University, 2007, 22(5): 86−89.

[49] 冯奥哲, 孔涛, 孙溥璠, 等. 沙地不同密度樟子松人工林土壤矿化氮质量分数与矿化特征[J]. 东北林业大学学报, 2021, 49(10): 96−103.

Feng A Z, Kong T, Sun P F, et al. Soil mineralized nitrogen content and mineralization characteristics of *Pinus sylvestris* var. *mongolica* plantations with different densities in sandy land[J]. Journal of Northeast Forestry University, 2021, 49(10): 96−103.

[50] 张雷, 刘世荣, 孙鹏森, 等. 气候变化对物种分布影响模拟中的不确定性组分分割与制图: 以油松为例[J]. 生态学报, 2011, 31(19): 5749−5761.

(C)1994-2022 China Academic Journal Electronic Publishing House. All rights reserved. http://www.cnki.net

Zhang L, Liu S R, Sun P S, et al. Partitioning and mapping the sources of variations in the ensemble forecasting of species distribution under climate change: acase study of *Pinus tabulaeformis*[J]. Acta Ecologica Sinica, 2011, 31(19): 5749−5761.

[51] Wang T, Wang G, Innes J, et al. Climatic niche models and their consensus projections for future climates for four major forest tree species in the Asia-Pacific region[J]. Forest Ecology & Management, 2016, 360: 357−366.

[52] Fettig C J, Reid M L, Bentz B J, et al. Changing climates, changing forests: a western north American rerspective[J]. Journal of Forestry, 2013, 111(3): 214−228.

[53] Poland T M, Mccullough D G. Emerald A B: invasion of the urban forest and the threat to north America's Ash resource[J]. Journal of Forestry, 2006, 104(3): 118−124.

[54] 裴顺祥, 法蕾, 杜满义, 等. 种间关系对中条山油松人工林天然更新及群落稳定性的影响[J]. 林业科学研究, 2022, 35(1): 150−157.

Pei S X, Fa L, Du M Y, etl. Effects of interspecific relationships on natural regeneration and community stability of *Pinus tabulaeformis* plantation in Zhong tiao Mountain[J]. Forestry Research, 2022, 35(1): 150−157.

[55] Yang X Q, Kushwaha S, Saran S, et al. Maxent modeling for predicting the potential distribution of medicinal plant, *Justicia adhatoda* L. in Lesser Himalayan foothills[J]. Ecological Engineering, 2013, 51: 83−87.

（责任编辑　孟　瑶
责任编委　孟　平）

(C)1994-2022 China Academic Journal Electronic Publishing House. All rights reserved.　http://www.cnki.net
