# Supplementary material for: Provenance and family variations in early growth of Manchurian walnut (Juglans mandshurica Maxim.) and selection of superior families
Source: PLoS One. 2024 Mar 7;19(3):e0298918. doi: 10.1371/journal.pone.0298918 (PMC10919699; doi:10.1371/journal.pone.0298918)

Accepted Manuscript

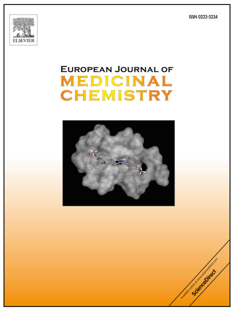

Synthesis and anticancer activity of some novel 5,6-fused hybrids of juglone based 1,4-naphthoquinones

Uppuluri Venkata Mallavadhani, Chakka Vara Prasad, Shweta Shrivastava, V.G.M. Naidu

PII: S0223-5234(14)00531-5

DOI: 10.1016/j.ejmech.2014.06.012

Reference: EJMECH 7055

To appear in: *European Journal of Medicinal Chemistry*

Received Date: 4 February 2014

Revised Date: 1 June 2014

Accepted Date: 8 June 2014

Please cite this article as: U.V. Mallavadhani, C.V. Prasad, S. Shrivastava, V.G.M. Naidu, Synthesis and anticancer activity of some novel 5,6-fused hybrids of juglone based 1,4-naphthoquinones, *European Journal of Medicinal Chemistry* (2014), doi: 10.1016/j.ejmech.2014.06.012.

This is a PDF file of an unedited manuscript that has been accepted for publication. As a service to our customers we are providing this early version of the manuscript. The manuscript will undergo copyediting, typesetting, and review of the resulting proof before it is published in its final form. Please note that during the production process errors may be discovered which could affect the content, and all legal disclaimers that apply to the journal pertain.

GRAPHICAL ABSTRACT

Synthesis and anticancer activity of some novel 5,6-fused hybrids of juglone based 1,4-naphthoquinones

Uppuluri Venkata Mallavadhani[a*], Chakka Vara Prasad[a], Shweta Shrivastava[b], V.G.M. Naidu[b]

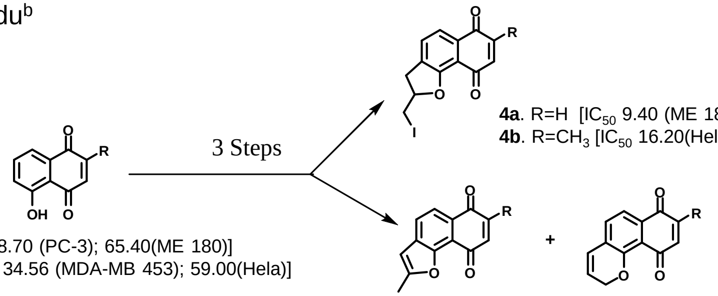

4a. R=H [IC_{50} 9.40 (ME 180)]
4b. R=CH_3 [IC_{50} 16.20(Hela)]

1a. R=H [IC_{50} 88.70 (PC-3); 65.40(ME 180)]
1b. R=CH_3 [IC_{50} 34.56 (MDA-MB 453); 59.00(Hela)]

5a. R=H [IC_{50} 15.00(ME 180)]
5b. R=CH_3 [IC_{50} 28.80(MCF 7)]

6a. R=H [IC_{50} 5.70 (PC-3)]
6b. R=CH_3 [IC_{50} 10.90 (MDA-MB 453)]

ACCEPTED MANUSCRIPT

Revised

Synthesis and anticancer activity of some novel 5,6-fused hybrids of juglone based 1,4-naphthoquinones

Uppuluri Venkata Mallavadhani[a]*, Chakka Vara Prasad[a], Shweta Shrivastava[b], V.G.M. Naidu[b]

[a] Natural Products Chemistry Division, CSIR-Indian Institute of Chemical Technology, Hyderabad 500007, India

[b] Department of Pharmacology & Toxicology, National Institute of Pharmaceutical Education& Research, Hyderabad 500037, India

ABSTRACT

Six novel 5,6-fused hybrids such as dihydrobenzofuran-quinone (**4a** and **4b**), benzofuran-quinone (**5a** and **5b**) and chromene-quinone (**6a** and **6b**) of juglone based 1,4-naphthoquinones were synthesized by employing a three step protocol with the cyclisation of *o*-allylphenol as the key step. The anticancer activity of the newly synthesized compounds was evaluated *in vitro* against seven human cancer cell lines including cervix (ME-180 and HeLa), breast (MCF-7, MDA-MB-453 and MDA-MB-231), prostate (PC-3) and colon (HT-29) by using MTT assay. The screening results showed that majority of the synthesized compounds exhibited significant anticancer activity. In particular, compounds **6a** and **6b** showed potent activities than the standard drug etoposide against prostate and breast cancer cell lines respectively. Flow cytometric analysis revealed that compounds **6a** and **6b** induced apoptosis and arrested the cell cycle at G2/M phase in PC-3 and MDA-MB-453 cells respectively.

Keywords: Juglone, plumbagin, anticancer activity, apoptosis, cell cycle arrest

*Corresponding author Tel: +91-4027191882; Fax: +91-4027191882

E. Mail address: uvmavadani@yahoo.com (U. V. Mallavadhani)

1. Introduction

Quinones are widespread in nature as secondary metabolic products and possess a variety of pharmacological properties such as antibacterial, antifungal, antioxidant, anti-inflammatory and anticancer activity [1]. Interestingly many clinically used cancer chemotherapeutic drugs such as doxorubicin, daunorubicin, mitoxantrone, mitomycin, saintopin contain quinone moiety (Fig.1). Thus in order to search for novel biologically active lead molecules having quinone moiety, continuous efforts are under way to synthesize diverse hybrid molecules. Recently, naturally occurring hydroxynaphthoquinones such as juglone (5-hydroxy-1,4-naphthoquinone) and plumbagin (5-hydroxy-2-methyl-1,4-naphthoquinone) (Fig.2) have attracted considerable attention due to their interesting biological activities including anticancer activity [2-8]. Juglone is present in *Juglandaceae* species (*ver*: Black walnut, Butternut, European walnut) [9] and exhibits cytotoxic properties against various cancer cell lines [10,11]. Plumbagin is present in *Plumbaginaceae* (Plumbago), *Droseraceae* (Drosera) and *Ebenaceae* (Diospyros) species [12,13] and reports have shown that *Plumbaginaceae* species are particularly rich in plumbagin. Among *Plumbaginaceae* species, *Plumbago zeylanica* (commonly known as *Chitraka*) has been extensively used in Indian traditional system of medicine for the treatment of a number of diseases including cancer [14]. Plumbagin has been shown to exert antiproliferative and anticancer activities against various cancer cell lines [15-17] as well as *in vivo* in animal models [18,19]. In spite of such promising activities these compounds exert some toxic side effects [20]. Due to this, they still remained as highly useful natural scaffolds to synthesize diverse chemical structures with a view to maximize anticancer activity and minimize toxicity. Both juglone and plumbagin have two important components such as

1,4-quinone moiety and an aryl ring with a phenolic hydroxyl group at C-5 position. The quinone moiety and the phenolic functionality are amenable for a wide range of chemical transformations to synthesize diverse New Chemical Entities to identify potent lead molecules. Literature search revealed that mostly the quinone moiety of both the scaffolds has been exploited and reported some C-2 and C-3 analogues [21-25]. The phenolic hydroxyl has so far been utilized only to make some ester derivatives [26,27]. But still there exists excellent scope and opportunities in the aryl ring side. Novel hybrids can be synthesized by utilizing the phenolic hydroxyl to make some cyclic ethers such as furan or pyran rings by keeping the quinone moiety intact as this is the key function in both the compounds. These benzofuran (benzopyran)–quinone hybrids are expected to exhibit enhanced anticancer activity with less toxicity. In this connection it is to mention here that some furaquinocins (Fig. 3) isolated from the fermentation broth *streptomyces* sp. KO-3988 have been reported to exhibit *in vitro* cytotoxicity [28]. With this background in view, we have synthesized some novel 5,6-fused hybrids from juglone and plumbagin and evaluated for their anticancer potential. We herein report their synthesis and anticancer activity.

2. Results and discussions

2.1. Chemistry

The synthetic protocols followed to synthesize the target compounds are illustrated in scheme 1. Treatment of juglone (**1a)** and plumbagin (**1b)** with allyl bromide and silver(I) oxide in dichloromethane afforded the corresponding allyl ethers **2a** and **2b** in 79% and 75% yields respectively. These allyl ethers (**2a** and **2b**) on Claisen rearrangement in diphenyl ether at 160 ^{o}C gave *o*-allyl phenols **3a** and **3b** in 60% and 62% yields

respectively. The O-allyl (**2a and 2b**) and C-allyl (**3a and 3b**) compounds can be easily distinguished by their spectral data. Compounds **2a** and **2b** showed the characteristic peaks in ^{1}H NMR spectra at δ 4.73 and 4.72 ppm respectively indicating the protons of O-$\underline{CH_2}$-CH=CH_2, whereas the compounds **3a** and **3b** showed peaks at δ 3.5, 12.3 and 3.48, 12.36 ppm corresponding to Ar-$\underline{CH_2}$-CH=CH_2 and -OH protons respectively. Similarly the ^{13}C NMR spectra of compounds **2a** and **2b** showed diagnostic peaks at δ 69.5 and 69.7 ppm, whereas **3a** and **3b** showed peaks at δ 33.6 and 33.6 ppm respectively. The *o*-allyl phenols (**3a** and **3b**) can undergo cyclisation either at the central carbon of allylic chain to form a 5-membered cyclic ether (benzofuran) or at the terminal carbon to form a 6-membered cyclic ether (benzopyran). In the present work compounds **3a** and **3b** when treated with iodine in the presence of $SnCl_4$ in CH_2Cl_2 underwent regioselective cyclisation to afford the corresponding dihydrobenzofurans **4a** and **4b** in 40% and 43% yields respectively. Compounds **4a** and **4b** showed the characteristic peaks of dihydrobenzofuran in their ^{1}H NMR spectra at δ 3.16, 3.42-3.52, 3.6 and 3.14, 3.38-3.53, 3.6 ppm respectively. Similarly their ^{13}C NMR spectra showed the characteristic peaks of **4a** and **4b** at δ 8.2, 35.1, 83.6 and 8.2, 35.1, 83.6 ppm respectively. Interestingly, compounds **3a** and **3b** reacted differently when treated with $PdCl_2$ in the presence of $Cu(OAc)_2$, H_2O, LiCl in DMF medium and afforded mixture of corresponding benzofurans (**5a, 5b**) and benzopyrans (chromenes) (**6a, 6b**). In case of compound **3a**, the yield of benzopyran **6a** (36%) is more than that of benzofuran **5a** (25%). whereas in case of compound **3b**, it is reverse with benzofuran **5b** (31%) is more than that of benzopyran **6b** (26%). The benzofurans **5a** and **5b** showed the characteristic peaks in their ^{1}H NMR at δ 2.61, 6.51 and 2.6, 6.49 ppm respectively.

The ^{13}C NMR spectra of compounds **5a** and **5b** showed the characteristic peaks at δ 14.5, 103, 151 and 14.4, 102.9, 150.7 ppm respectively. Similarly benzopyrans **6a** and **6b** (Table 1) showed the characteristic peaks in their ^{1}H NMR at δ 5.1, 6.0, 6.47 and 5.08, 6.0, 6.46 ppm respectively, Whereas their ^{13}C NMR spectra showed the characteristic peaks at δ 66.4, 120.2 and 66.3, 120.3 ppm respectively.

2.2. Biology

2.2.1. In vitro Cytotoxicity

The *in vitro* cytotoxicity of the synthesized compounds **(2–6)** was studied using cervix (ME-180 and HeLa), breast (MCF-7, MDA-MB-453 and MDA-MB-231), prostate (PC-3) and colon (HT-29) cancer cell lines by employing MTT assay [29]. Doxorubicin and etoposide along with compounds **1a** and **1b** were taken as reference standards in this study and the results are reported in terms of IC_{50} values (Table 2). From the IC_{50} values, it is clear that majority of the synthesized compounds showed significant anticancer activity. The efficacy order of the synthesized compounds, which showed potent and higher activities than the parent compounds is:

ME 180: **4a** > **5a**; MCF-7: **3b** = **6b** > **4a**; Hela: **4b** > **4a**; MDA-MB-453: **6b** > **4a ~ 4b**; MDA-MB-231: **6b** > **4a**; PC-3: **6a** > **4a** > **3b** > **5a**; HT-29: **6a** > **4a** > **2a**.

For each cell line the highly active analogues are: ME 180: **4a** (IC_{50} 9.4 μM); MCF-7: **3b** (IC_{50} 11.6 μM); HeLa: **4b** (IC_{50} 16.2 μM); MDA-MB-453: **6b** (IC_{50} 10.9 μM); MDA-MB-231: **6b** (IC_{50} 18.31 μM); PC-3: **6a** (IC_{50} 5.7 μM); HT-29: **6a** (IC_{50} 27.8 μM).

Compound **4a** (IC_{50} 9.4 μM) showed almost equal activity as that of etoposide and 6.9 fold more activity than parent compound **1a** against ME 180 cell line. Compounds **3b** (IC_{50} 11.6 μM) and **6b** (IC_{50} 11.7 μM) showed almost equal activity as that of parent

compound **1b** as well as doxorubicin and 2-fold more activity than etoposide against MCF-7 cell line. Compound **6b** (IC_{50} 10.9, 18.31 µM) exhibited 1.1, 1.3 folds more activity than etoposide and 3.1, 2.7 folds more activity than parent compound **1b** against MDB-MB-453 and MDA-MB-231 cell lines respectively. The highest anticancer activity was exhibited by compound **6a** (IC_{50} 5.7 µM) against PC-3 cell line, with a significant increase of 15.5 fold than the parent compound **1a** and 2.5 fold than etoposide. Close observation of these results suggests that the chromene hybrids **6a** and **6b** are very effective against prostate (PC-3) and breast (MDA-MB-453 and MDA-MB-231) cancer cell lines respectively. Whereas, the dihydrobenzofuran hybrids (**4a, 4b**) are effective against cervix cancer cell lines (ME-180 and HeLa). In case of colon cancer cell line (HT-29), such selectivity was not observed. Interestingly in juglone (**1a**) series the activity is increased on chemical modification and attained maximum for compound **6a** (IC_{50} 27.8 µM), surprisingly in plumbagin (**1b**) series moderate activity was found for compound **6b** (IC_{50} 34.6 µM) against HT-29 cell line. Based on these results, the most potent chromene hybrids **6a** and **6b** were taken up further for apoptosis and cell cycle studies in PC-3 and MDB-MB-453 cells respectively.

2.2.2. Morphological observation by Acridine Orange and Ethidium Bromide (AO/EB) staining and Cell cycle analysis:

The morphological abnormalities induced by compound **6a** in PC-3 cells and compound **6b** in MDA-MB-453 were studied under fluorescence microscopy using AO/EB staining technique. AO permeates the intact cell membrane and stains the nuclei green, whereas EB is taken up only when the membrane integrity is deteriorated, and stains the nucleus red. Intact cells therefore exhibit homogeneous green nuclei, whereas

apoptotic cells show condensed or fragmented chromatin. Late apoptotic or necrotic cells have orange to red nuclei. Compared with spontaneous apoptosis observed in control cells (early apoptotic 4.19%, 0% late apoptotic and 0% necrotic cells). Fluorescence microscopic images of cells treated with 6 µM of compound **6a** (Fig. 4) and 5, 10 µM of compound **6b** (Fig. 5) clearly showed the morphological changes such as cell shrinkage, membrane blebbing, chromatin condensation with destructive fragmentation of the nucleus, suggesting that the compounds **6a, 6b** induced cell death in prostate cancer cells and breast cancer cells by apoptosis respectively.

The effects of compounds **6a** and **6b** on cell cycle progression were examined by propidium iodide staining method. When the PC-3 cells were treated with 3 and 6 µM of compound **6a** for 48 h resulted in a marked accumulation of cells in G2/M-phase (1.6 fold) and a slight reduction in G1 Phase as shown in Fig. 6. These results indicated that compound **6a** inhibited the growth of the cancer cells by inhibiting the cell cycle via G2/M-phase arrest, whereas when the MDA-MB-453 cells were treated with 5 and 10 µM of compound **6b** for 48 h as shown in Fig. 7, exposure of cancer cells to compound **6b** resulted in an increase in the G2/M phase population from 23.5 to 55.9 %, an increase from 5.9 to 21.5 % cells in S phase was also found. There was a decrease of population from 52.6 to 10.5 % in G1 phase in cells treated with compound **6b** compared with the untreated cells. Hence, compound **6b** exerted proliferation-inhibitory effects on the MDA-MB-453 cells via S phase and G2/M phase arrest in a concentration-dependent manner.

3. Conclusion

In conclusion, six novel 5,6-fused hybrids from juglone and plumbagin have been synthesized by employing a three step protocol with cyclisation of *o*-allylphenol as the

key step and evaluated for their anti-cancer activity against seven human cancer cell lines. The results of *in vitro* anticancer activity indicated that hybrids **4a**, **6a** and **6b** were more potent than the standard etoposide and parent compounds against MCF-7, PC-3 and MDA-MB 453 cancer cell lines respectively. The mechanism studies showed that the anticancer activity of hybrids **6a** and **6b** could be attributed to the induction of cell cycle arrest and apoptosis in prostate (PC-3) and breast (MDA-MB 453) cancer cell lines. Fused dihydrobenzofuran or benzopyran moiety seems to be important for enhanced anticancer activity of juglone and plumbagin.

4. **Experimental section**

4.1 *Chemistry*

Solvents were distilled before use. All reagents were purchased from commercial sources (Sigma-Aldrich, Alfa Aesar). All reactions were monitored by silica gel 60 F_{254} glass TLC plates (Merck) with UV irradiation at 254 nm for visualization. Column chromatography was carried on silica gel (60-120 or 100-200 mesh). Melting points were determined on a Buchi melting point apparatus and are uncorrected. IR spectra were recorded on Nicollet 740 FTIR spectrophotometer using KBr pellets. ^{1}H and ^{13}C NMR spectra were recorded on Bruker 300 MHz and Varian 500 MHz in $CDCl_3$ with TMS as internal standard. Chemical shifts were expressed as δ values in parts per million (ppm) and coupling constants (*J*) in Hertz (Hz). HRMS spectra were recorded on Agilent-ESI QTOF and JEOL mass spectrometers.

4.1.1 Starting materials

Commercially available Juglone (**1a**) was used without any further purification. Plumbagin was isolated from the roots of *Plumbago zeylanica* and used in this work.

ACCEPTED MANUSCRIPT

4.1.1.1 Isolation of Plumbagin (**1b**)

Plumbago zeylanica roots were collected from the medicinal plant garden of IMMT, Bhubaneswar. Roots were cut into small pieces, shade-dried and powdered in a pulveriser. The powdered root material (2.7 kg) was packed in an aspiratory bottle and macerated with acetone (6 L) at room temperature for 24hrs. This process was repeated thrice and the combined extract was concentrated under reduced pressure. The resultant dark brown residue was purified by column chromatography on silica gel using hexane-ethyl acetate (9:1) as eluent to yield a solid, which on recrystallization from hexane-acetone mixture afforded plumbagin **1b** (4.01 g, yield 0.14%) as orange coloured crystals, mp 78-80 ^{o}C. The identity of this compound was confirmed by its spectral data (IR, ^{1}H NMR, ^{13}C NMR and HRMS) and by comparing the data published in literature [30]

4.1.2 Chemical synthesis

4.1.2.1. Preparation of 5-(allyloxy)-naphthalene-1,4-dione (**2a**)

Silver (I) oxide (1.0 g, 4.3 mmol) and allyl bromide (0.5 mL, 5.7 mmol) were added to a stirred solution of **1a** (0.5 g, 2.8 mmol) in CH_2Cl_2 (8 mL), and stirring was continued for 20 h. Additional portions of silver oxide (0.68 g, 2.9 mmol) and allyl bromide (0.23 mL, 2.6 mmol) were added and stirring was continued for 15 h. The reaction mixture was filtered through Celite. The filtrate was washed with CH_2Cl_2, and concentrated under reduced pressure. The crude product was purified by column chromatography on silica gel using hexane/ethyl acetate to afford **2a** (0.48 g, Yield 79%) as yellow coloured solid; mp 54-56 ^{o}C; HRMS (ESI): m/z calcd for $C_{13}H_{10}O_3Na$ $(M+Na)^+$ 237.0522, found 237.0517, and NMR spectra were compared with published data [31]

4.1.2.2. Preparation of 5-(allyloxy)-2-methylnaphthalene-1,4-dione (**2b**)

Silver (I) oxide (0.92 g, 3.9 mmol) and allyl bromide (0.46 mL, 5.3 mmol) were added to a stirred solution of **1b** (0.5 g, 2.6 mmol) in CH_2Cl_2 (8 mL), and stirring was continued for 20 h. Additional portions of silver oxide (0.62 g, 2.7 mmol) and allyl bromide (0.21 mL, 2.4 mmol) were added and stirring was continued for 15 h. Purification was done as above mentioned procedure to afford **2b** (0.45 g, Yield 75%) as yellow coloured solid; mp 88-90 ^oC; R_f 0.45 (hexane/ethyl acetate 3:1); IR (KBr, cm^{-1}): 3040, 2986, 2923, 2856, 1659, 1631, 1581; ^1H NMR (500 MHz, $CDCl_3$): δ 2.13 (3H, d, J = 1.51 Hz), 4.72 (2H, dt, J = 5.28 , 2.2 Hz), 5.37 (1H, dq, J = 10.5, 1.51 Hz), 5.65 (1H, dq, J = 17.3, 1.51 Hz), 6.0-6.16(1H, m, J = 10.5, 5.28, Hz), 6.73(1H, d, J = 1.51 Hz), 7.25 (1H, d, J = 8.3 Hz), 7.62 (1H, t, J = 8.3 Hz), 7.75 (1H, d, J = 6.7 Hz); ^{13}C NMR (125 MHz, $CDCl_3$): δ 15.7, 69.7, 117.8, 119.1, 119.4, 120.2, 131.9, 134.3, 137.7, 145.2, 158.2, 184, 185.6; HRMS (ESI): m/z calcd for $C_{14}H_{12}O_3Na$ $(M+Na)^+$ 251.0678, found 251.0672

4.1.2.3. Preparation of 6-allyl-5-hydroxynaphthalene-1,4-dione (**3a**)

Compound **2a** (0.4 g, 1.86 mmol) was dissolved in diphenyl ether (6 mL) and the reaction mixture was heated at 160 ^oC for 24 h. After cooling to room temperature, reaction mixture was directly purified by column chromatography on silica gel using hexane/ethyl acetate as eluent to afford **3a** (0.24 g, Yield 60%) as orange coloured solid; mp 86-88 ^oC; R_f 0.39 (hexane/ethyl acetate 19:1); IR (KBr, cm^{-1}): 3447, 3062, 2904, 1662, 1638, 1593; ^1H NMR (500 MHz, $CDCl_3$): δ 3.5 (2H, d, J = 6.0 Hz), 5.1-5.16 (1H, m), 5.16 (1H, s), 5.92-6.0 (1H, m), 6.93 (2H, s), 7.51 (1H, d, J = 7.5 Hz), 7.58 (1H, d, J = 7.5 Hz), 12.3 (1H, s) ; ^{13}C NMR (125 MHz, $CDCl_3$): δ 33.6, 114.3, 117.1, 118.9,

129.9, 134.6, 136.1, 136.8, 138.4, 139.6, 159.4, 184.1, 190.5; HRMS (EI): m/z calcd for $C_{13}H_{10}O_3$ M^+ 214.0629, found 214.0625

4.1.2.4. Preparation of 6-allyl-5-hydroxy-2-methylnaphthalene-1,4-dione (**3b**)

Compound **2b** (0.4 g, 1.75 mmol) was dissolved in diphenyl ether (6 mL) and the reaction mixture was heated at 160 ^oC for 24 h. Purification was done as above mentioned procedure to afford **3b** (0.24 g, Yield 62%) as orange coloured solid; mp 99-101 ^oC; R_f 0.35 (hexane/ethyl acetate 19:1); IR (KBr, cm^{-1}): 3448, 2983, 2905, 1644, 1607, 1476; ^1H NMR (500 MHz, $CDCl_3$): δ 2.18 (3H, s), 3.48 (2H, d, *J* = 6.6 Hz), 5.11 (1H, *J* = 3.7 Hz), 5.16 (1H, s), 5.92-6 (1H, m), 6.78 (1H, s), 7.46 (1H, d, *J* = 7.7 Hz), 7.58 (1H, d, *J* = 7.7 Hz), 12.36 (1H, s); ^{13}C NMR (125 MHz, $CDCl_3$): δ 16.3, 33.6, 114.4, 116.9, 119, 130.1, 134.7, 135.2, 135.5,136.3, 149.5, 159.1, 184.5, 190.4 ; HRMS (ESI): m/z calcd for $C_{14}H_{13}O_3$ $(M+H)^+$ 229.0859, found 229.0859

4.1.2.5. Preparation of 2,3-dihydro-2-(iodomethyl)-naphtho[1,2-b]furan-6,9-dione (**4a**)

A mixture of compound **3a** (0.1 g, 0.46 mmol), $SnCl_4$ (0.027 mL, 0.23 mmol) and iodine (0.11 g, 0.46 mmol) in CH_2Cl_2 (4 mL) was stirred at room temperature for 12 h. The reaction mixture was treated with slush and then washed with 5 % $Na_2S_2O_3$ (20 mL), water (10 mL) and dried over anhydrous Na_2SO_4. After evaporation of the solvent under reduced pressure, the residue was purified by column chromatography on silica gel using hexane/ethyl acetate as eluent to afford **4a** (62 mg, yield 40%) as yellow coloured solid; mp 165-167 ^oC; R_f 0.48 (hexane/ethyl acetate 1.8:1); IR (KBr, cm^{-1}): 2921, 2851, 1655, 1585, 1433; ^1H NMR (500 MHz, $CDCl_3$): δ 3.16 (1H, dd, *J* = 16.9, 6.0 Hz), 3.42-3.52 (2H, m, *J* = 16.9, 10.1 Hz), 3.6 (1H, dd, *J* = 10.1, 3.3 Hz), 5.1-5.19 (1H, m), 6.87 (1H, d, *J* = 10.1 Hz), 6.9(1H, d, *J* = 10.1 Hz), 7.5 (1H, d, *J* = 7.5 Hz), 7.68 (1H,d, *J* = 7.5

Hz) ; ^{13}C NMR (125 MHz, $CDCl_3$): δ 8.2, 35.1, 83.6, 114.9, 120.6, 129.7, 132, 135.7, 137.7, 139.5, 158.8, 183.7, 184.4 ; HRMS (ESI): m/z calcd for $C_{13}H_9O_3INa$ $(M+Na)^+$ 362.9488, found 362.9484

4.1.2.6. Preparation of 2,3-dihydro-2-(iodomethyl)-7-methylnaphtho[1,2-b]furan-6,9-dione (**4b**)

A mixture of compound **3b** (0.1 g, 0.43 mmol), $SnCl_4$ (0.025 mL, 0.19 mmol) and iodine (0.11 g, 0.43 mmol) in CH_2Cl_2 (4 mL) was stirred at room temperature for 12 h. Workup and purification was done as above mentioned procedure to afford **4b** (65 mg, yield 43%) as yellow coloured solid; mp 143-145 ^oC; R_f 0.38 (hexane/ethyl acetate 1.8:1); IR (KBr, cm^{-1}): 2954, 1658, 1620, 1590, 1437; ^1H NMR (500 MHz, $CDCl_3$): δ 2.15 (3H, s), 3.14 (1H, dd, J = 17.9, 6.0 Hz), 3.38-3.53 (2H, m), 3.6 (1H, dd, J = 10. 3.3 Hz), 5.12-5.21 (1H, m), 6.75 (1H, s), 7.47 (1H, d, J = 7.7 Hz), 7.7 (1H, d, J = 7.7 Hz) ; ^{13}C NMR (125 MHz, $CDCl_3$): δ 8.2, 16.2, 35.1, 83.6, 115.3, 120.8, 129.3,132.3, 135.3, 136.6, 147, 158.6, 183.8, 184.9 ; HRMS (ESI): m/z calcd for $C_{14}H_{11}O_3INa$ $(M+Na)^+$ 376.9645, found 376.9647

4.1.2.7. Preparation of compounds (**5a** and **6a**)

To a solution of $Cu(OAc)_2.H_2O$ (0.56 g, 2.8 mmol) in DMF (3 mL), compound **3a** (0.2 g, 0.93 mmol), $PdCl_2$ (3 mg, 2 mol %), LiCl (0.11 g, 2.7 mmol), water (0.5 mL) were added and the solution was stirred at room temperature for 3 h. The reaction mixture was treated with slush and extracted with ether. The organic layer was dried over anhydrous Na_2SO_4. After the evaporation of solvent under reduced pressure, the residue was purified by column chromatography on silica gel using hexane/ethyl acetate as eluent to afford **5a** (50 mg, yield 25%) and **6a** (72 mg, yield 36%).

4.1.2.7.1. 2-methylnaphtho[1,2-b]furan-6,9-dione (**5a**)

Red coloured solid; mp 208-210 ^{o}C; R_f 0.42 (hexane/ethyl acetate 3:1); IR (KBr, cm^{-1}): 3114, 2922, 1659, 1586, 1305; ^{1}H NMR (500 MHz, $CDCl_3$): δ 2.61 (3H, s), 6.51 (1H, s), 6.9(1H, d, J = 9.8 Hz), 6.94 (1H, d, J = 9.8 Hz), 7.78 (1H, d, J = 8.3 Hz), 7.96 (1H, d, J = 8.3 Hz); ^{13}C NMR (125 MHz, $CDCl_3$): δ 14.5, 103, 116.4, 121.4, 125.3, 127.4, 136.7, 138.1, 138.5, 151, 162, 184.5, 184.9; HRMS (ESI): m/z calcd for $C_{13}H_9O_3$ $(M+H)^+$ 213.0546, found 213.0544

4.1.2.7.2. 2H-benzo[h]chromene-7,10-dione (**6a**)

Red coloured solid; mp 162-164 ^{o}C; R_f 0.38 (hexane/ethyl acetate 1.8:1); IR (KBr, cm^{-1}): 3059, 2924, 2855, 1657, 1607, 1563, 1292; ^{1}H NMR (500 MHz, $CDCl_3$): δ 5.10 (2H, dd, J = 3.3, 1.8 Hz), 6.00 (1H, td, J = 10.0, 3.5 Hz), 6.47 (1H, td, J = 10.0, 1.8 Hz), 6.85 (2H, s), 7.24 (1H, d, J = 7.7 Hz), 7.64 (1H, d, J = 7.7 Hz) ; ^{13}C NMR (125 MHz, $CDCl_3$): δ 66.4, 120.2, 123.6, 125.4, 128.5, 131, 132.7, 136.7, 140.7, 154.7, 184, 184.4 ; HRMS (ESI): m/z calcd for $C_{13}H_7O_3$ $(M-H)^+$ 211.0389, found 211.0386

4.1.2.8. Preparation of compounds (**5b** and **6b**)

To a solution of $Cu(OAc)_2.H_2O$ (0.52 g, 2.6 mmol) in DMF (3 mL), compound **3a** (0.2 g, 0.87 mmol), $PdCl_2$ (3 mg, 2 mol %), LiCl (0.11 g, 2.6 mmol), water (0.5 mL) were added and the solution was stirred at room temperature for 3 h. Workup and purification was done as above mentioned procedure to afford **5b** (62 mg, yield 31%) and **6b** (52 mg, yield 26%)

4.1.2.8.1. 2,7-dimethylnaphtho[1,2-b]furan-6,9-dione (**5b**)

Red coloured solid; mp 126-128 ^{o}C; R_f 0.43 (hexane/ethyl acetate 3:1); IR (KBr, cm^{1}): 2920, 1657, 1623, 1592, 1263; ^{1}H NMR (500 MHz, $CDCl_3$): δ 2.19 (3H, s), 2.6 (3H,s),

6.49(1H, s), 6.78 (1H, s), 7.74 (1H, d, *J* = 8.12 Hz), 7.97 (1H, d, *J* = 8.12 Hz) ; ^{13}C NMR (125 MHz, $CDCl_3$): δ 14.4, 16.2, 102.9, 116.5, 121.4, 124.7, 127.6, 135.4, 136.3, 147.3, 150.7, 161.7, 184.3, 185.2 ; HRMS (ESI): m/z calcd for $C_{14}H_{11}O_3$ $(M+H)^+$ 227.0702, found 227.0698

4.1.2.8.2. 8-methyl-2H-benzo[h]chromene-7,10-dione (**6b**)

Red coloured solid; mp 147-149 ^{0}C; R_f 0.39 (hexane/ethyl acetate 1.8:1); IR (KBr, cm^{-1}): 2922, 1653, 1562, 1467, 1426, 1358, 1253; ^{1}H NMR (500 MHz, $CDCl_3$): δ 2.12 (3H, d, *J* = 1.5 Hz), 5.08 (2H, dd, *J* = 3.02, 2.26 Hz), 6.00 (1H, td, *J* = 9.8, 3.02 Hz), 6.46 (1H, dt, *J* = 9.8, 2.26 Hz), 6.71 (1H, d, *J* = 1.5 Hz), 7.21 (1H, d, *J* = 7.5 Hz), 7.65 (1H, d, *J* = 7.5 Hz); ^{13}C NMR (125 MHz, $CDCl_3$): δ 15.8, 66.3, 120.3, 123.6, 125.2, 128.2, 130.7, 133, 137.6, 145.8, 154.3, 184, 184.9 ; HRMS (ESI): m/z calcd for $C_{14}H_9O_3$ $(M-H)^+$ 225.0546, found 225.0541

4.2. *Biology*

PC-3 (Prostate cancer), ME-180, HeLa (Cervix cancer) and HT-29 (Colon cancer) were obtained from National center for Cell science (NCCS), Pune, India. MCF-7, MDA-MB-231, MDA-MB-453 (Breast cancer) were Kind gift samples by Dr. Radha, Centre for Cellular and Molecular Biology, Hyderabad, A.P. PC-3 cells was cultured in RPMI, ME-180 was cultured in MEM, HeLa, HT-29, MCF-7, MDA-MB-231, MDA-MB-453 cells were cultured in DMEM and all media were supplemented with 10% fetal bovine serum, penicillin-streptomycin. The cells were maintained in a humidified incubator at 37 ^{0}C with 5% CO_2. Cell lines were sub cultured by enzymatic digestion with 0.25% trypsin/1mM EDTA solution when they reached approximately 70-80% confluency. DMEM, MTT [3-(4, 5- dimethylthiazol-2-yl)-2, 5-diphenyl tetrazolium bromide], Trypsin,

EDTA, Acridine orange, Ethidium bromide were purchased from Sigma Chemicals Co (st.Louis, MO), Fetal bovine serum were purchased from Gibco, USA, Muse^{TM} Cell Cycle reagent from Milliopore, 6 well flat bottom tissue culture plates were purchased from Tarson.

4.2.1. Cytotoxicity screening using MTT assay

MTT assay was performed according to the method of Naidu et al., [29]. MTT assay is a standard colorimetric assay for measuring cellular proliferation. MTT is a tetrazolium salt, which is yellow in color and is photosensitive. MTT [3-(4, 5- dimethylthiazol-2-yl)-2, 5-diphenyl tetrazolium bromide] is taken by the living cells and reduced by a mitochondrial dehydrogenase enzyme to a purple formazan product that is impermeable to the cell membrane. Solubilisation with solvents like DMSO leads to liberation of product and amount of purple formazan product is directly related to the cell viability. $1x10^4$ Cells (counted by Trypan blue exclusion dye method)) in 96- well plates were incubated with series of concentrations of compounds for 48 h at 37 ^{0}C in DMEM with 10% FBS medium. Then the above media was replaced with 90 µl of fresh serum free media and 10 µl of MTT reagent (5mg/ml) and plates were incubated at 37 ^{0}C for 4h, there after the above media was replaced with 200 µl of DMSO and incubated at 37 ^{0}C for 10 min. The absorbance at 570 nm was measured on a spectrophotometer (spectra max, Molecular devices, USA). IC_{50} values were determined from plot: % cell viability (from control) versus concentration.

4.2.2. Assessment of cell morphology:

Characteristic apoptotic morphological changes were assessed by fluorescent microscopy using acridine orange/ethidium bromide (AO/EB) dual staining [32]. PC-3

cells and MDA-MB-453 cells were grown in 6-well plates (1×10^6 cells/well) and were treated with compound **6a** (3 and 6 µM) and **6b** (5 and 10 µM) respectively for 48 h. After washing once with phosphate-buffered saline, the cells were stained with 100 µl of a mixture (1:1) of acridine orange ethidium bromide for 5 min in the dark. The cells were immediately washed once with phosphate-buffered saline and viewed under a Nikon inverted fluorescent microscope (TE-Eclipse 300, Nikon, Japan) at 200X magnification. To differentiate the various forms of cell death, two thousand cells were counted and the numbers of viable, early apoptotic, late apoptotic and necrotic cells were represented as % of cells.

4.2.3. Cell cycle analysis:

Cell cycle distribution and measurement of the percentage of apoptotic cells were performed by flow cytometry [33]. PC-3 and MDA-MB-453 cells were plated in six well plates at a density of 5×10^6 cells/well. After 48 h of treatment, cells were harvested by trypsinization and washed twice with PBS. Cells were then gently fixed with 70% ice cold ethanol at −20 ℃ for 3 h and resuspended in M use^{TM} Cell Cycle reagent and incubated at 37 ℃ for 30 min as per instruction ma nual. Following this, DNA content was analyzed on a flow cytometer (MUSE^{TM} Cell Cycle Analyzer, Millipore, USA).

Acknowledgements

We are thankful to Director, CSIR- IICT for support and encouragement. We are also thankful to CSIR, India for providing fellowship to CVP and partial funding under the Network project ORIGIN (CSC 0108).

References

[1] N.E. Najjar, H.G. Muhtasib, R.A. Ketola, P.Vuorela, A. Urtti, H. Vuorela, Phytochem. Rev. 10 (2011) 353-370

[2] K.S. Shin, S. Lee, B. Cha, Plant Pathol. J. 23 (2007) 113-115

[3] C. Sunil, V. Duraipandiyan, P. Agastian, S. Ignacimuthu, Food Chem. Toxicol. 50 (2012) 4356-4363

[4] B.R. Acharya, B. Bhattacharyya, G. Chakrabarti, Biochemistry 47 (2008) 7838-7845

[5] J.J. Inbaraj, C.F. Chignell, Chem. Res. Toxicol. 17 (2004) 55-62

[6] P.B. Bescos, S.M. Aragon, K.L.J. Aliaga, A. Ortega, M.T. Molina, E. Buxaderas, G. Orellana, A.G. Csaky, Biochem. Biophys. Res. Commun. 400 (2010) 169–174

[7] L. Lai, J. Liu, D. Zhai, Q. Lin, L. He, Y. Dong, J. Zhang, B. Lu, Y. Chen, Z. Yi, M. Liu, Br. J. Pharmacol.165 (2012) 1084–1096

[8] S. Sharma, B.K. Sharma, Y.S. Prabhakar, Eur. J. Med. Chem. 44 (2009) 2847-2853

[9] A. Thakur, J. Med. Plants Res. 5 (2011) 5324-5330

[10] H. Xu, X. Yu, S. Qu, D. Sui, Bioorg. Med. Chem. Lett. 23 (2013) 3631-363

[11] K.B. Aithal, S.M.R. Kumar, N.B. Rao, N. Udupa, S.B.S. Rao, Cell Biol. Int. 33 (2009) 1039-1049

[12] U.V. Mallavadhani, G. Sahu. J. Muralidhar, Pharm. Biol. 40 (2002) 508–511

[13] S. Padhye, P. Dandawate, M. Yusufi, A. Ahmad, F.H. Sarkar, Med. Res. Rev. 32 (2012) 1131-1158

[14] Y.D. Mandavkar, S.S. Jalapure, Afr. J. Pharm. Pharacol. 5 (2011) 2738-2747

[15] S. Nair, R.R.K. Nair, P. Srinivas, G. Srinivas, M.R. Pillai, Mol. Carcinog. 47 (2008) 22–33

[16] A. Kawiak, J.Z. Pankau, E. Lojkowska, J. Nat. Prod. 75 (2012) 747−751

[17] J. Sun, R.J. McKallip, Leukemia Res. 35 (2011) 1402– 1408

[18] S. Sinha, K. Pal, A. Elkhanany, S. Dutta, Y. Cao, G. Mondal, S. Iyer, V. Somasundaram, F.J. Couch, V. Shridhar, R. Bhattacharya, D. Mukhopadhyay, P. Srinivas, Int. J. Cancer 132 (2013) 1201–1212

[19] Y.L. Hsu, C.Y. Cho, P.L. Kuo, Y.T. Huang, C.C. Lin, J. Pharmacol. Exp. Ther. 318

(2006) 484–494
[20] P. Seshadri, A. Rajaram, R. Rajaram, Free Radical Biol. Med. 51 (2011) 2090–2107
[21] P. Dandawate, E. Khan, S. Padhye, H. Gaba, S. Sinha, J. Deshpande, K.V. Swamy, M. Khetmalas, A. Ahmad, F.H. Sarkar, Bioorg. Med. Chem. Lett. 22 (2012), 3104-3108
[22] D. Bhasin, S.N. Chettiar, J.P. Etter, M. Mok, P.K. Li, Bioorg. Med. Chem. 21 (2013) 4662-4669
[23] L.S. Chemin, E. Buisine, V. Yardley, S. Kohler, M.A. Debreu, V. Landry, C. Sergheraert, S.L. Croft, R.L.K. Siegel, E.D. Charvet, J. Med. Chem. 44 (2001) 548-565
[24] D.W. Gammon, D.J. Steenkamp, V. Mavumengwana, M.J. Marakalala, T.T. Mudzunga, R. Hunter, M. Munyololo, Bioorg. Med. Chem. 18 (2010) 2501–2514
[25] J. Zhou, L. Duan, H. Chen, X. Ren, Z. Zhang, F. Zhou, J. Liu, D. Pei, K. Ding, Bioorg. Med. Chem. Lett. 19 (2009) 5091–5094
[26] S. Maruo, I. Kuriyama, K. Kuramochi, K. Tsubaki, H. Yoshida, Y. Mizushina, Bioorg. Med. Chem.19 (2011) 5803–5812
[27] R. Mathew, A.K. Kruthiventi, J.V. Prasad, S.P. Kumar, G. Srinu, D. Chatterji, Chem. Biol. Drug Des. 76 (2010) 34–42
[28] T. Saito, T. Suzuki, M. Morimoto, C. Akiyama, T. Ochiai, K. Takeuchi, T. Matsumoto, K. Suzuki, J.Am.Chem. Soc. 120 (1998) 11633-11644
[29] V.G.M. Naidu, U.M. Bandari, A.K. Giddam, K.R.D. Babu, J.Ding, K.S. Babu, B. Ramesh, R.R. Pragada, P. Gopalakrishnakone, Asian Pac. J. Trop. Med. 6 (2013) 337-345
[30] C. Bothiraja, P.P. Joshi, G.Y. Dama, A.P. Pawar, Eur. J. Integr. Med. 3 (2011) 39–42
[31] D.L.J. Clive, S.P. Fletcher, D. Liu, J. Org. Chem. 69 (2004) 3282-3293
[32] D. Ribble, N.B. Goldstein, D.A. Norris, Y.G. Shellman, BMC Biotechnol. 5 (2005) 5-12
[33] E.-J. Lee, S.-Y. Oh, M.-K. Sung, Food Chem. Toxicol. 50 (2012) 4136-4143

ACCEPTED MANUSCRIPT

List of Captions

Fig. 1. Examples of potent anticancer drugs having quinone moiety.

Fig. 2. Structure of juglone (5-hydroxy-1,4-naphthoquinone) and plumbagin (5-hydroxy-2-methyl-1,4-naphthoquinone).

Fig. 3. Structure of some furaquinocins.

Fig. 4. Morphological changes in PC-3 cells treated with and without compound **6a** for 48 h.

Fig. 5. Morphological changes in MDA-MB-453 cells treated with and without compound **6b** for 48 h.

Fig. 6. Effect of compound **6a** on cell cycle progression of prostate cancer cells.

Fig. 7. Effect of compound **6b** on cell cycle progression of breast cancer cells.

Scheme 1. Synthesis of compounds **2a,b** – **6a,b**.

Table 1. ^{1}H NMR data of compounds **6a** and **6b** in $CDCl_3$.

Table 2. Anticancer activity (IC_{50}, μM) of compounds **1a,b-6a,b** against seven human cancer cell lines.

ACCEPTED MANUSCRIPT

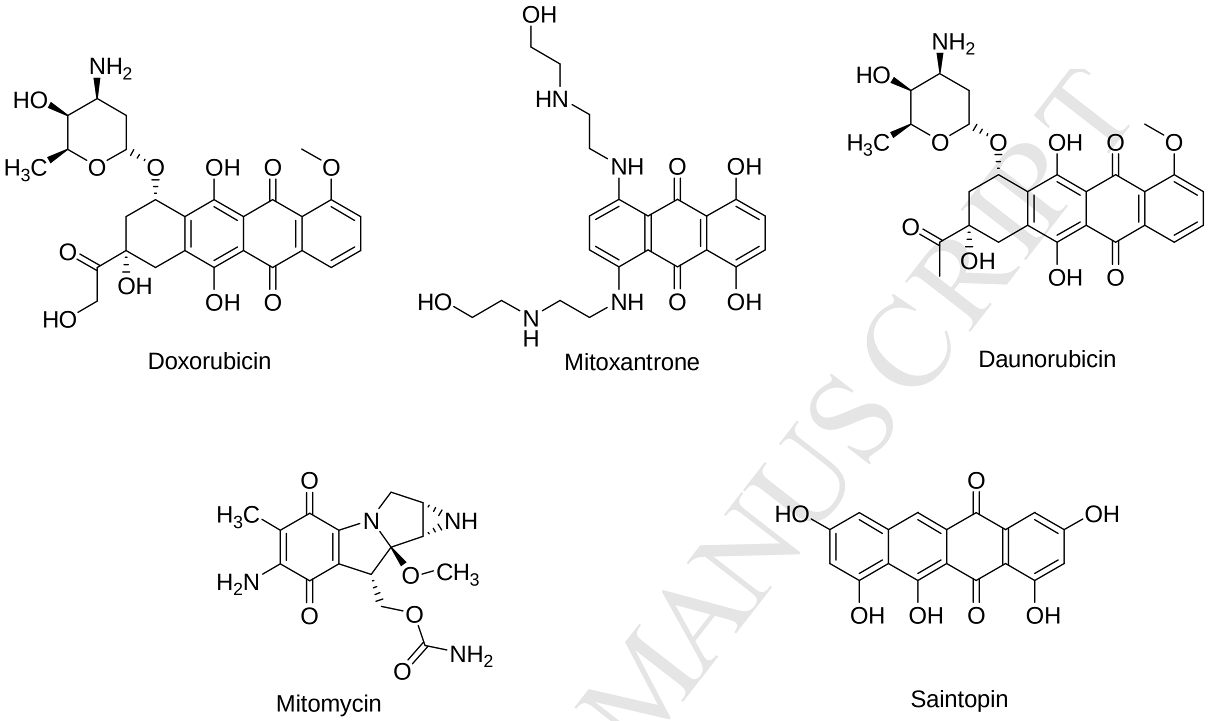

Fig. 1. Examples of potent anticancer drugs having quinone moiety

ACCEPTED MANUSCRIPT

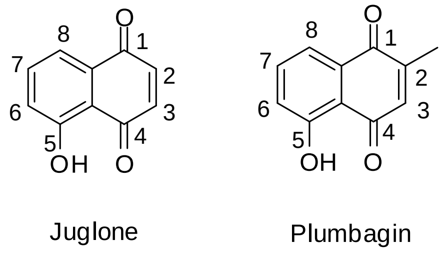

Fig. 2. Structure of juglone (5-hydroxy-1,4-naphthoquinone) and plumbagin (5-hydroxy-2-methyl-1,4-naphthoquinone)

ACCEPTED MANUSCRIPT

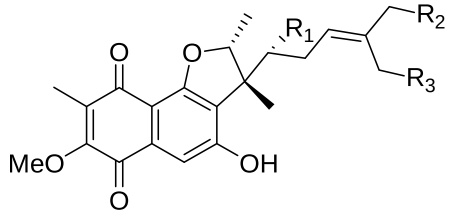

		R_1	R_2	R_3
furaquinocin	A	OH	H	OH
	B	OH	OH	H
	C	H	H	H
	D	OH	H	H
	F	H	OH	H
	H	OH	OH	OH

Fig. 3. Structure of some furaquinocins

ACCEPTED MANUSCRIPT

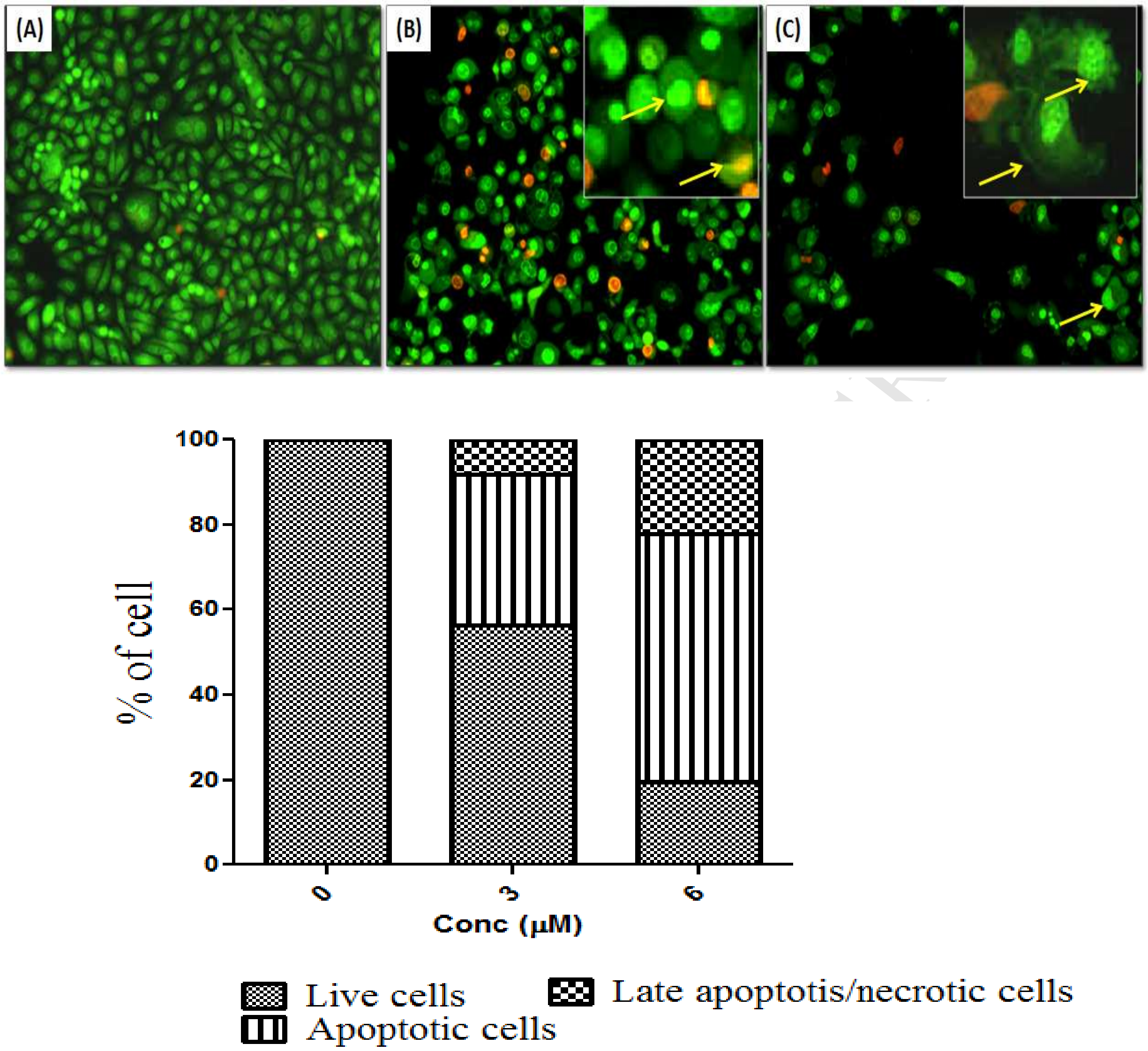

Fig. 4. Morphological changes in PC-3 cells treated with and without compound **6a** for 48 h. (A) PC-3 control cells showed green live cells with intact membrane and nuclei; (B) cells treated with 3 μM showed early signs of apoptosis characterized by condensed chromatin, cell membrane blebbing, and destructive fragmentation of the nuclei and (C) cells treated with 6 μM showed late apoptotic cells/ necrotic cells characterised with orange-red staining of the nucleus after treatment.

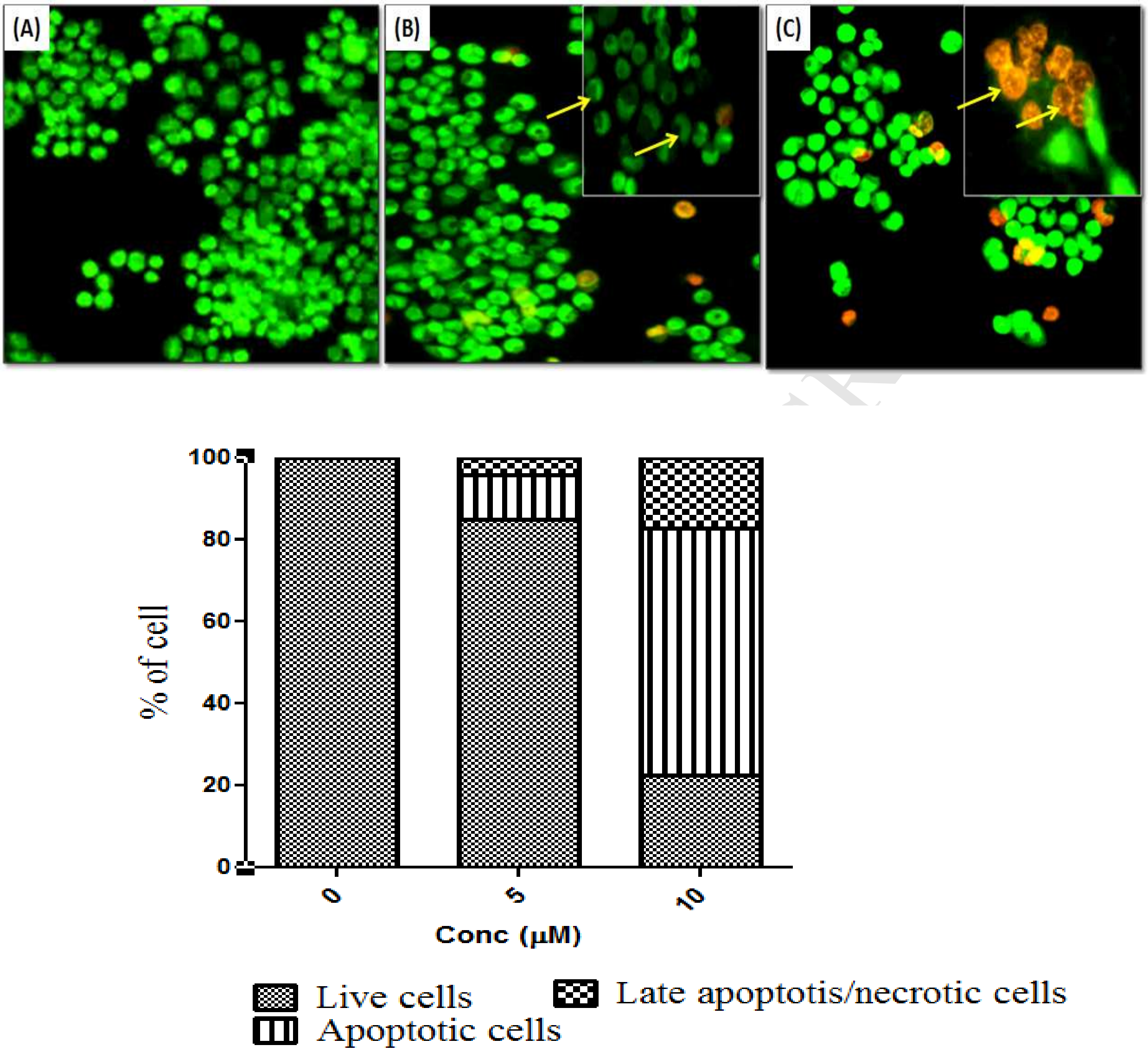

Fig. 5. Morphological changes in MDA-MB-453 cells treated with and without compound **6b** for 48 h. (A) MDA-MB-453 control cells showed green live cells with intact membrane and nuclei; (B) cells treated with 5 µM showed early signs of apoptosis characterized by condensed chromatin, cell membrane blebbing, and destructive fragmentation of the nuclei and (C) cells treated with 10 µM showed late apoptotic cells/ necrotic cells characterised with orange-red staining of the nucleus after treatment.

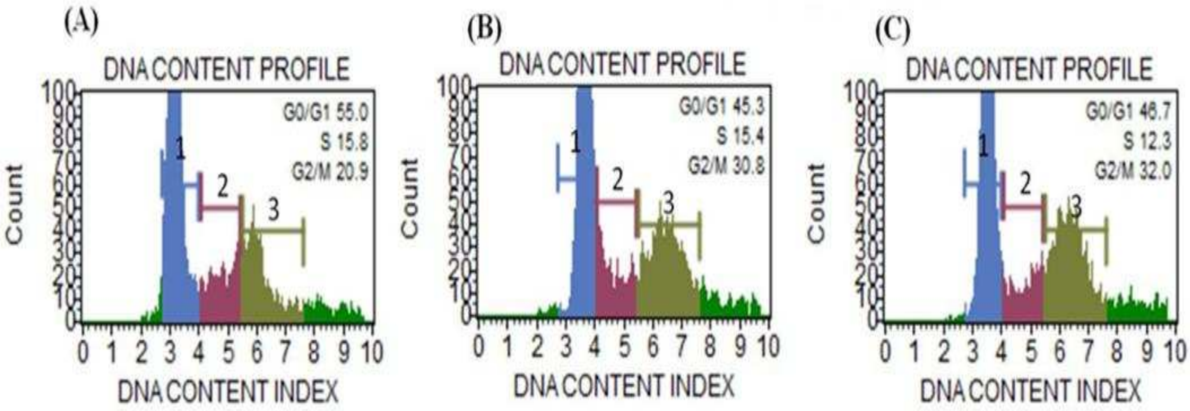

Fig. 6. Effect of compound **6a** on cell cycle progression of prostate cancer cells, (A) PC-3 control cells; (B, C) PC-3 cells treated with 3 and 6µM for 48 h followed by analysis of cell cycle distribution using propidium iodide cell staining method. All assays were done in duplicate.1:G0/G1 Phase, 2: S Phase, 3: G2/M Phase.

ACCEPTED MANUSCRIPT

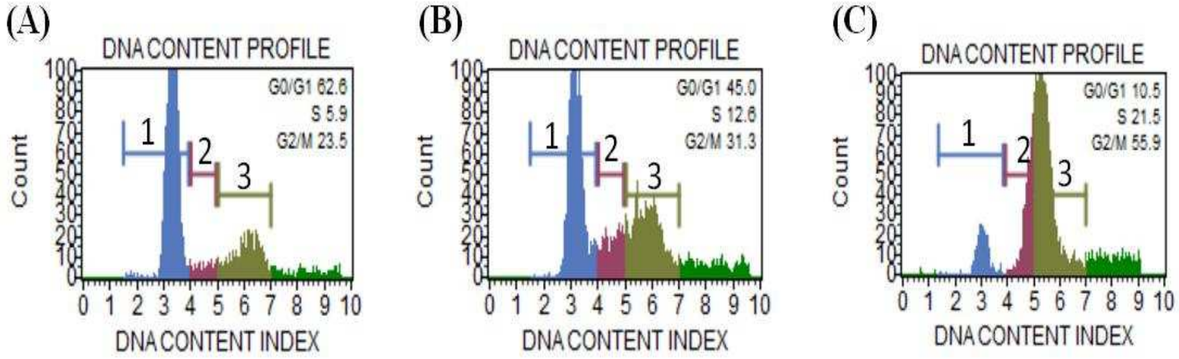

Fig. 7. Effect of compound **6b** on cell cycle progression of breast cancer cells, (A) MDA-MB-453 control cells; (B, C) MDA-MB-453 cells treated with 5 and 10 µM for 48 h followed by analysis of cell cycle distribution using propidium iodide cell staining method. All assays were done in duplicate. 1:G0/G1 Phase, 2: S Phase, 3: G2/M Phase.

ACCEPTED MANUSCRIPT

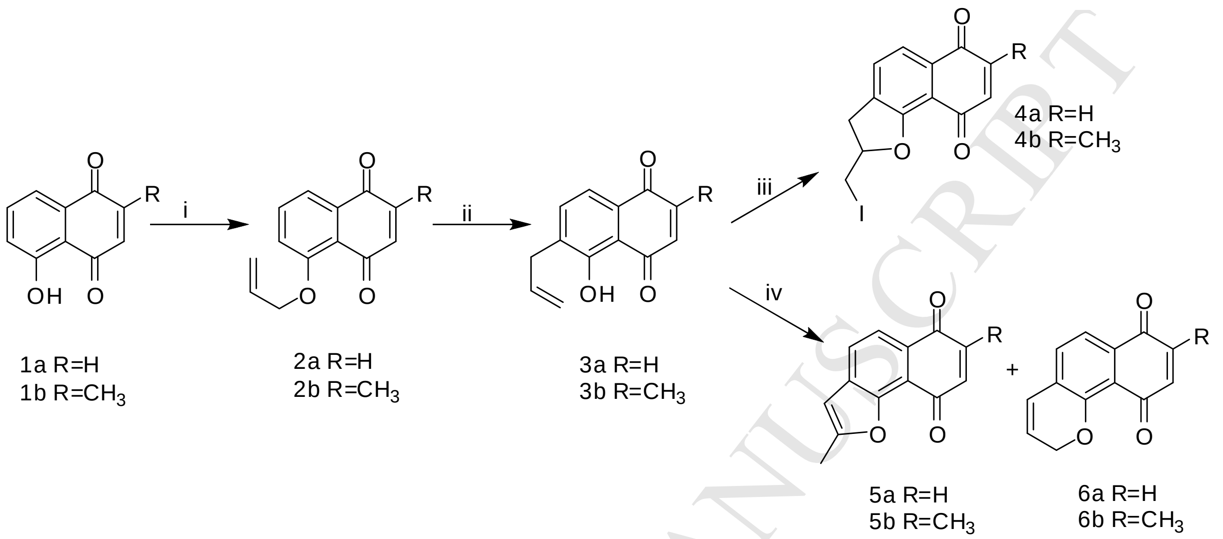

Scheme 1. Reagents and conditions: (i) allyl bromide, Ag_2O, CH_2Cl_2, rt, 20 h; (ii) diphenyl ether, 160 ^oC, 24 h; (iii) iodine, $SnCl_4$, CH_2Cl_2, 12 h; (iv) $PdCl_2$, $Cu(OAc)_2.H_2O$, LiCl, DMF, rt, 3 h

ACCEPTED MANUSCRIPT

ACCEPTED MANUSCRIPT

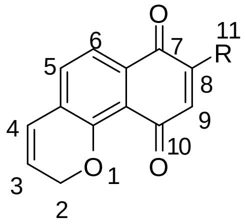

6a R=H
6b R=CH_3

Table 1

^{1}H NMR data of compounds **6a** and **6b** in $CDCl_3$

No	Compound **6a**	Compound **6b**
	δ_H ppm (*J* in Hz)	δ_H ppm (*J* in Hz)
1	-	-
2	5.10 dd (3.3, 1.8)	5.08 dd (3.02, 2.26)
3	6.00 td (10.0, 3.5)	6.00 td (9.8, 3.02)
4	6.47 td (10.0, 1.8)	6.46 td (9.8, 2.26)
5	7.64 d (7.7)	7.65 d (7.5)
6	7.24 d (7.7)	7.21 d (7.5)
7	-	-
8	6.85 s	-
9	6.85 s	6.71 d (1.5 Hz)
10	-	-
11	-	2.12 d (1.5 Hz)

ACCEPTED MANUSCRIPT

ACCEPTED MANUSCRIPT

Table 2

Anticancer activity (IC_{50}, μM) of compounds **1a,b**-**6a,b** against seven human cancer cell lines

Compounds	IC_{50} ±S.D (μM)						
	ME180	MCF-7	HeLa	MDA-MB-453	MDA-MB-231	PC-3	HT-29
**1a**	65.4±1.82	16.5±1.21	93.7±2.73	27.52±8.45	75.76±2.51	88.7±3.11	>100
**1b**	43.4±0.27	12.2+1.12	59.0±4.75	34.56±1.37	51.12±4.64	17.19±0.17	26.2+2.43
**2a**	>100	24.0±1.06	>100	37.8±0.33	>100	22.3±2.78	32.3±0.48
**2b**	46.7±3.08	14.7±1.08	78.0±6.07	29.29±0.56	36.10±0.40	34.2±2.73	33.7±2.51
**3a**	82.9±3.48	58.2±3.45	>100	53.7±5.67	>100	92.9±3.48	>100
**3b**	60.8±2.01	11.6±2.10	60.8±0.55	39.02±0.43	62.93±1.81	16.8±1.21	63.0±3.45
**4a**	9.4±0.66	13.4±1.06	27.4±1.43	20.84±1.28	29.25±0.78	12.7±1.66	31.3±2.71
**4b**	31.2±4.29	33.8±1.91	16.2±0.47	21.55±3.4	33.20±0.98	26.8±4.15	48.4±2.87
**5a**	15.0±0.27	24.3±0.18	51.2±2.29	25.23±0.65	38.94±1.82	18.2±2.17	57.9±3.18
**6a**	45.5±2.33	23.2±5.25	59.6±3.28	37.25±4.52	45.60±1.90	5.7±1.53	27.8±4.18
**5b**	44.1±0.97	28.8±1.85	55.2±2.87	32.65±1.8	43.96±1.21	31.1±4.77	60.1±3.85
**6b**	25.9±1.90	11.7±1.71	>100	10.9±0.7	18.31±2.49	24.5±2.18	34.6±1.03
Doxorubicin	0.39± 0.01	10.9±1.76	0.36± 0.02	1.99±1.75	1.69±0.56	0.19±0.01	1.76±0.23
Etoposide	8.9±0.3	23.9±0.3	4.71±1.4	12.5±0.85	24.22±2.94	14.4±3.23	21.45±3.87

Synthesis and anticancer activity of some novel 5,6-fused hybrids of juglone based 1,4-naphthoquinones

Highlights

- Some novel 5,6-fused hybrids of 1,4-naphthoquinones were synthesized.
- Cyclisation of *o*-allyl phenol is the key step in the synthesis.
- The synthesized compounds showed significant anti-cancer activity
- Compounds **6a** and **6b** induced apoptosis in PC-3 and MDA-MB-453 cells
- Both the compounds arrested the cell cycles at G2/M phase.

ACCEPTED MANUSCRIPT

ACCEPTED MANUSCRIPT

Synthesis and anticancer activity of some novel 5,6-fused hybrids of juglone based 1,4-naphthoquinones

Uppuluri Venkata Mallavadhani[a]*, Chakka Vara Prasad[a], Shweta Shrivastava[b], V.G.M. Naidu[b]

[a] Natural Products Chemistry Division, CSIR-Indian Institute of Chemical Technology, Hyderabad 500007, India

[b] Department of Pharmacology & Toxicology, National Institute of Pharmaceutical Education& Research, Hyderabad 500037, India

^{1}H NMR, ^{13}C NMR & HRMS spectra of compound **2b**	2-4
^{1}H NMR, ^{13}C NMR & HRMS spectra of compound **3a**	5-7
^{1}H NMR, ^{13}C NMR & HRMS spectra of compound **3b**	8-10
^{1}H NMR, ^{13}C NMR & HRMS spectra of compound **4a**	11-13
^{1}H NMR, ^{13}C NMR & HRMS spectra of compound **4b**	14-16
^{1}H NMR, ^{13}C NMR & HRMS spectra of compound **5a**	17-19
^{1}H NMR, ^{13}C NMR & HRMS spectra of compound **6a**	20-22
^{1}H NMR, ^{13}C NMR & HRMS spectra of compound **5b**	23-25
^{1}H NMR, ^{13}C NMR & HRMS spectra of compound **6b**	26-28

^{1}H NMR

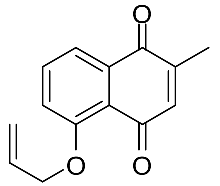

Compound 2b

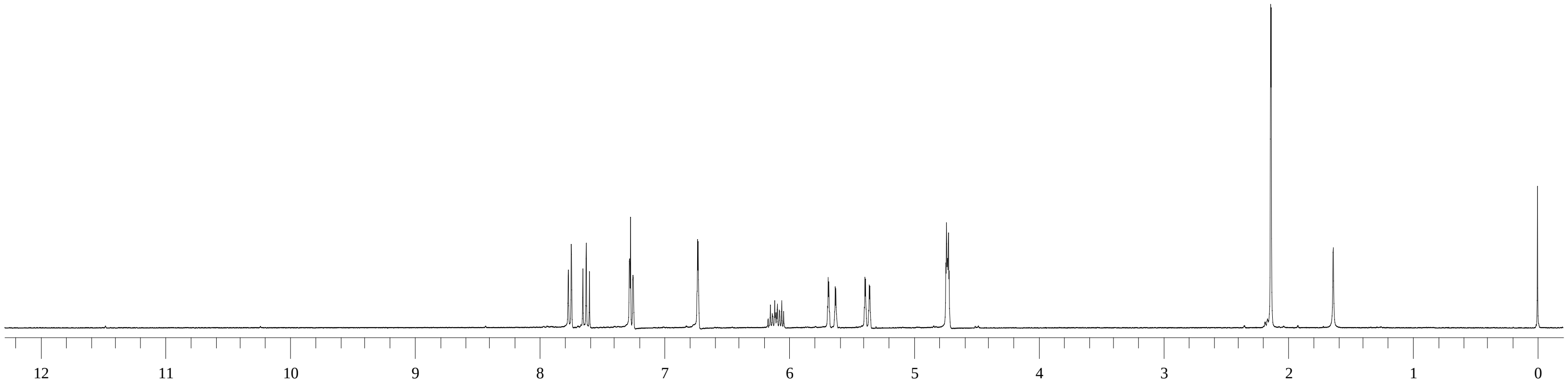

^{13}C NMR

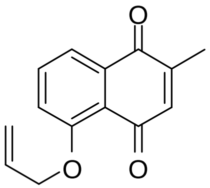

Compound 2b

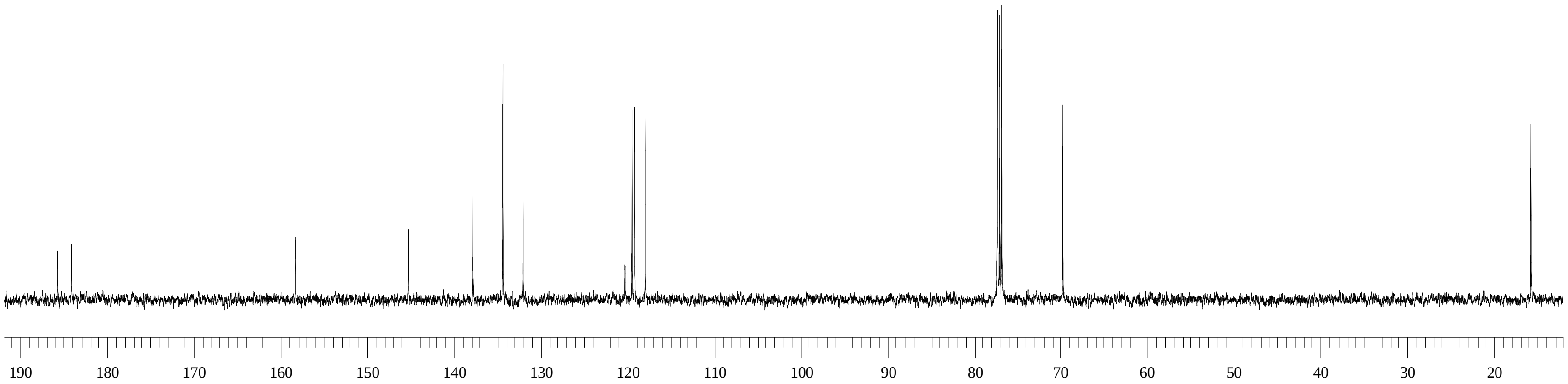

HRMS

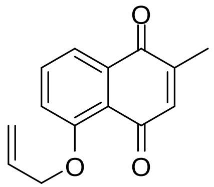

Compound 2b

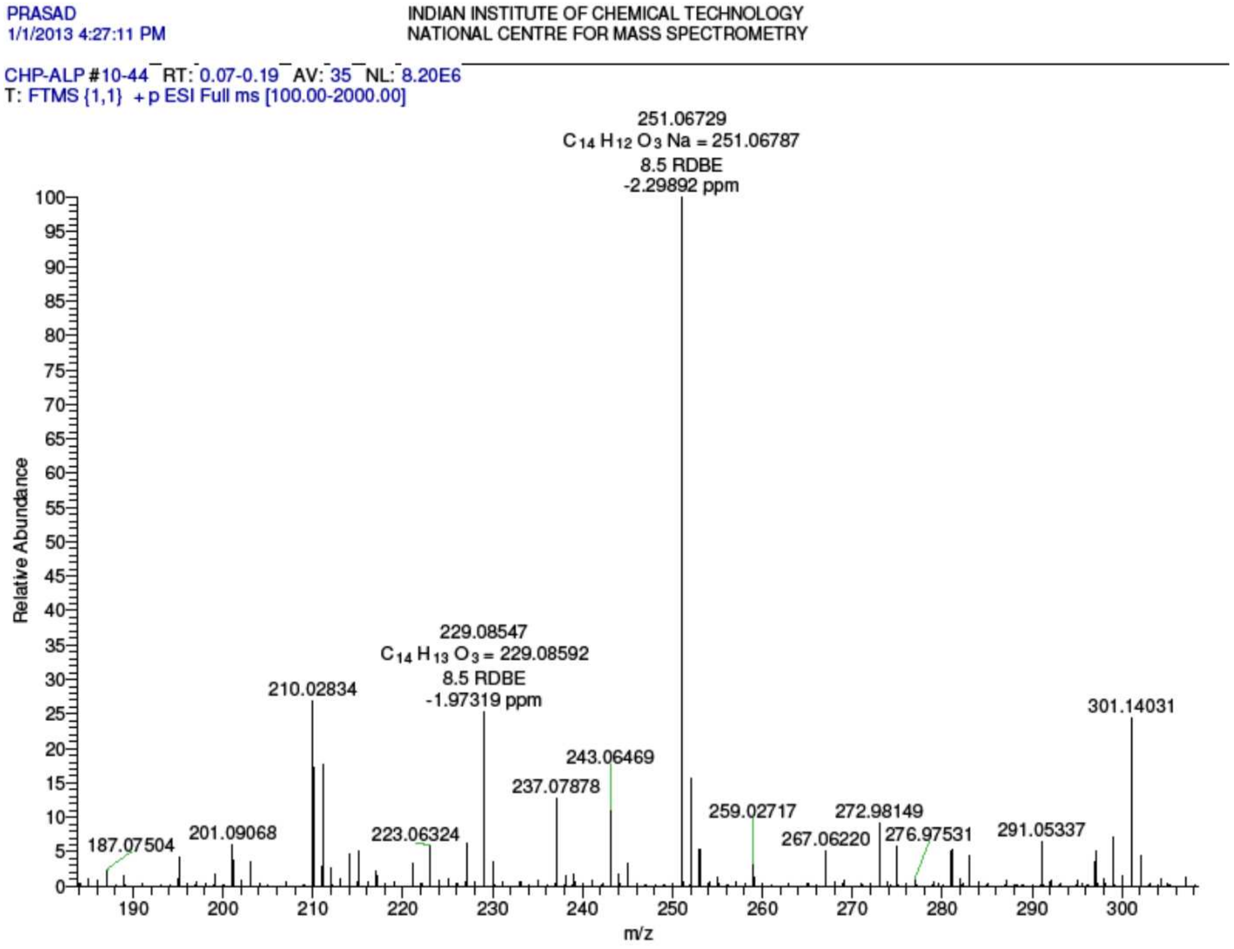

ACCEPTED MANUSCRIPT

^{1}H NMR

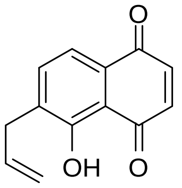

Compound 3a

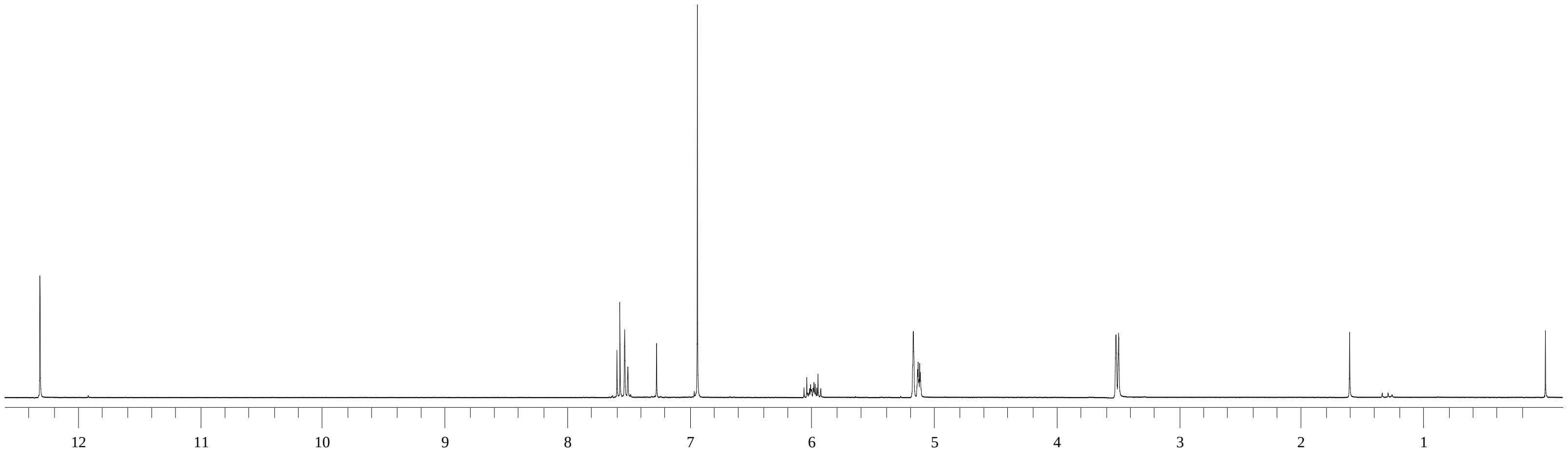

ACCEPTED MANUSCRIPT

^{13}C NMR

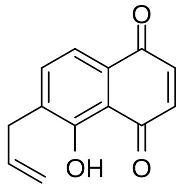

Compound 3a

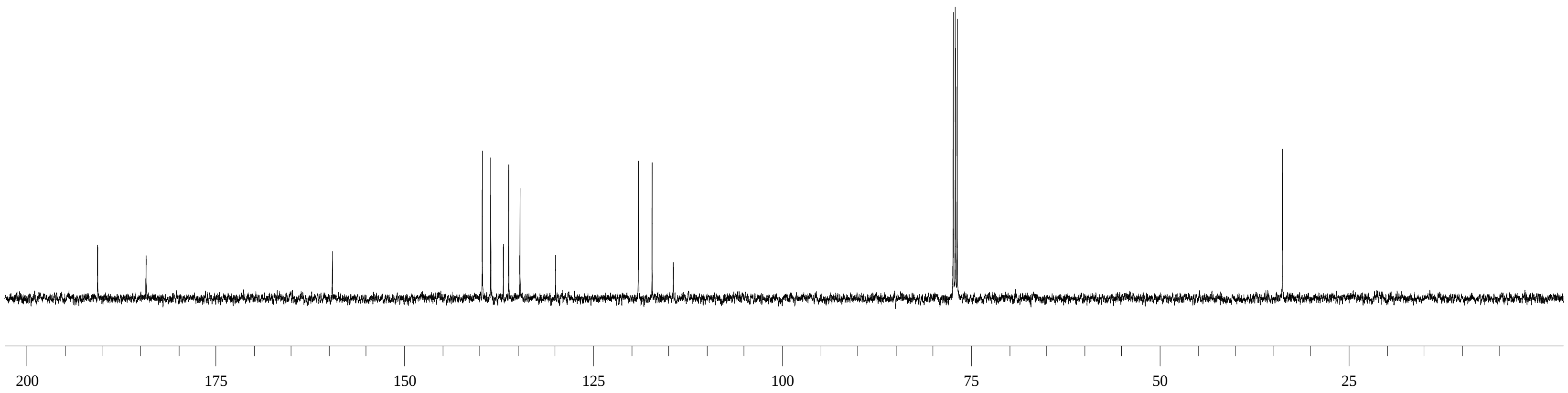

HRMS

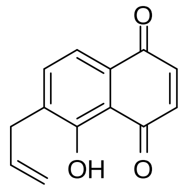

Compound 3a

CSIR-INDIAN INSTITUTE OF CHEMICAL TECHNOLOGY
NATIONAL CENTRE FOR MASS SPECTROMETRY

Data:CHP-CLJ
Sample Name:CH PRASAD CHP-CLJ, EIMS-DIP
Description:
Ionization Mode:EI+
History:Determine m/z[Peak Detect[Centroid,1,Area];Smooth[3]];Correct Base[];Average(MS[1] 1.18..1.35)

Acquired:26-06-2013 17:23:04
Operator:JEOL
Mass Calibration data:EIPOSCAL7112012
Created:26-06-2013 17:33:17
Created by:JEOL

Charge number:1
Element:^{12}C:8 .. 14, ^{1}H:0 .. 23, ^{14}N:0 .. 0, ^{16}O:0 .. 3

Tolerance:5.00(ppm)

Unsaturation Number:-1.5 .. 50.0 (Fraction:Both)

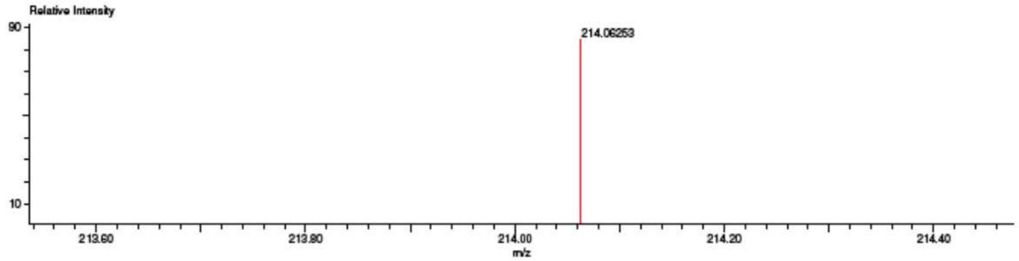

Mass	Intensity	Calc. Mass	Mass Difference (mmu)	Mass Difference (ppm)	Possible Formula	Unsaturation Number
214.06253	546085.64	214.06299	-0.46	-2.17	$^{12}C_{13}{}^{1}H_{10}{}^{16}O_{3}$	9.0

^{1}H NMR

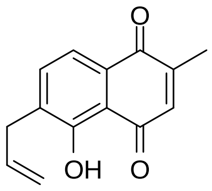

Compound 3b

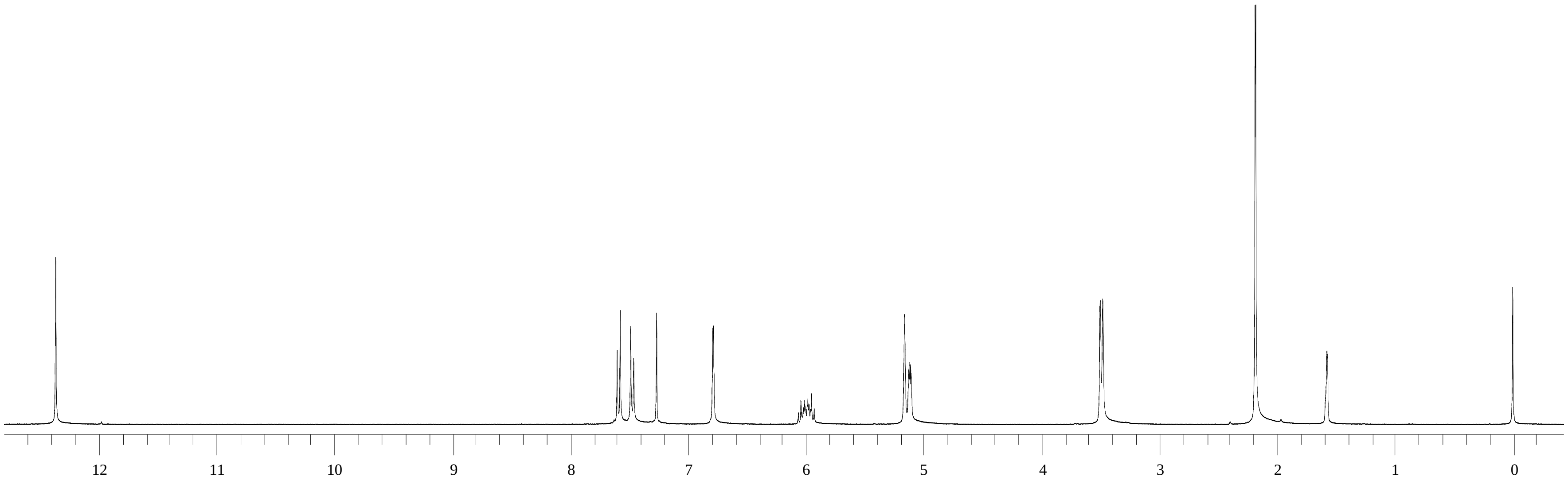

^{13}C NMR

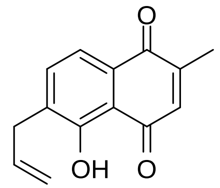

Compound 3b

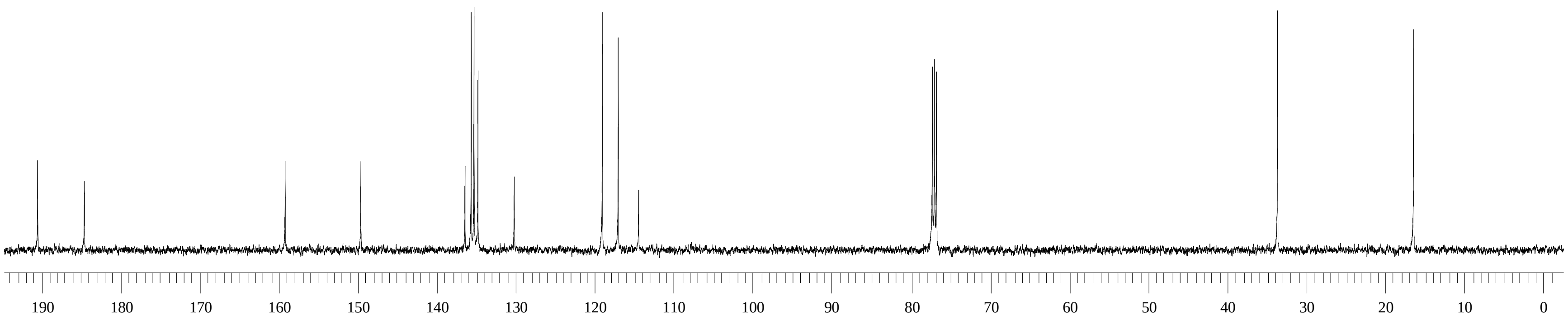

HRMS

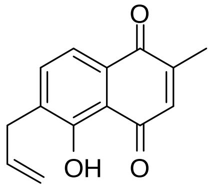

Compound 3b

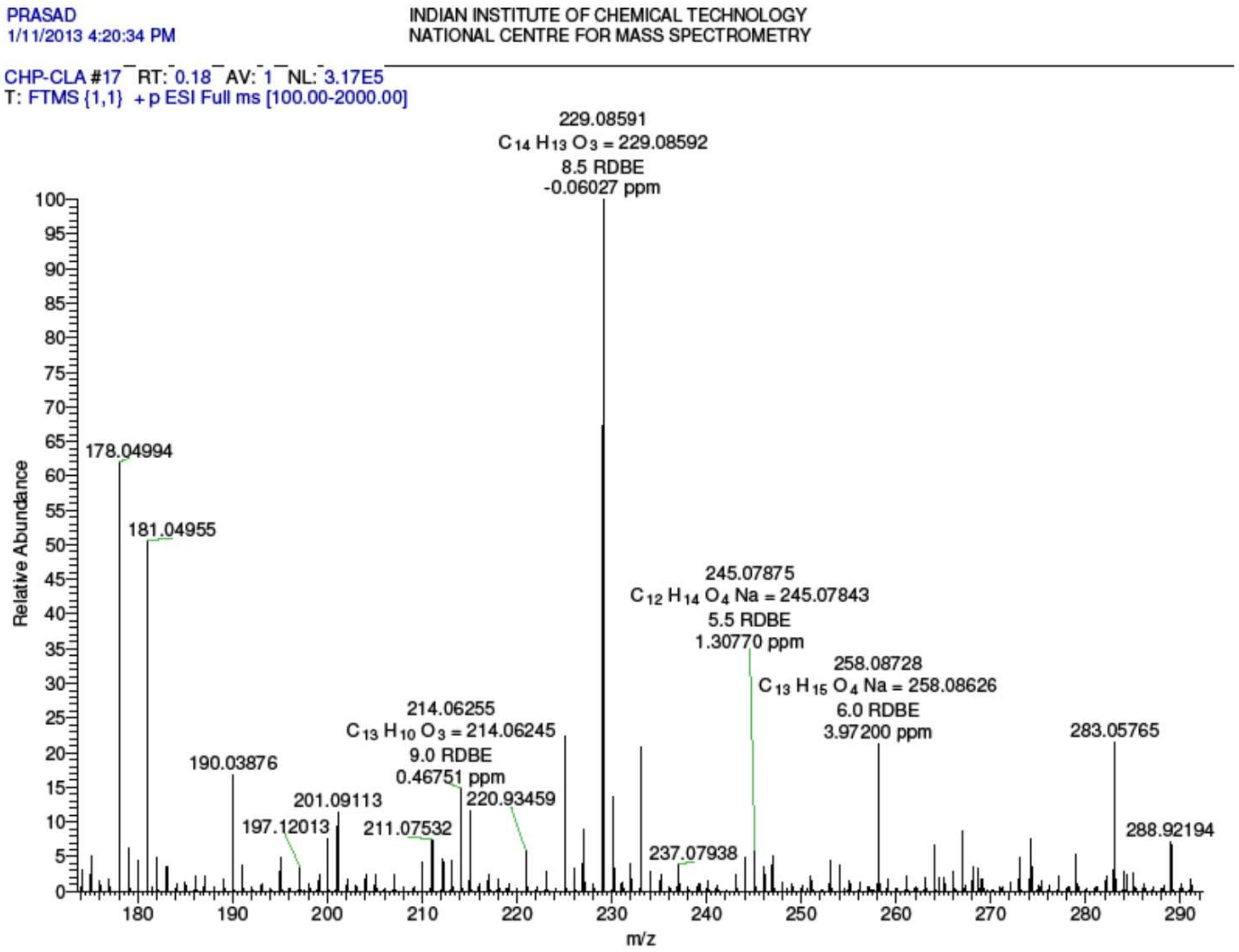

^1H NMR

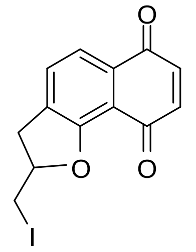

Compound 4a

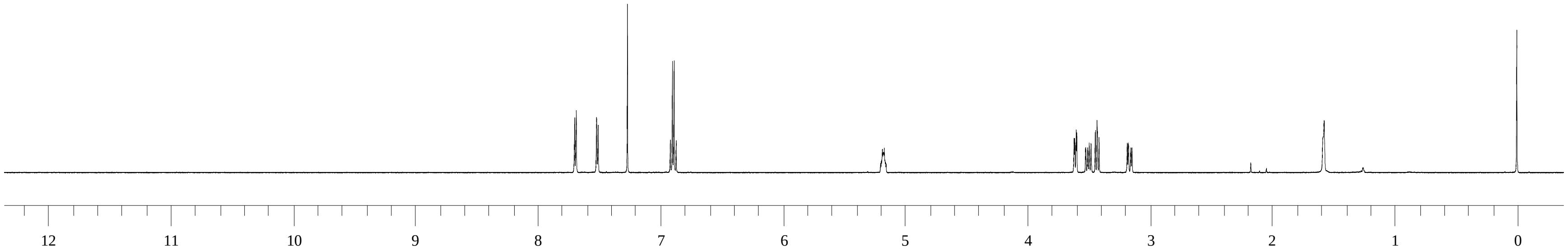

^{13}C NMR

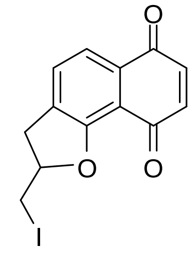

Compound 4a

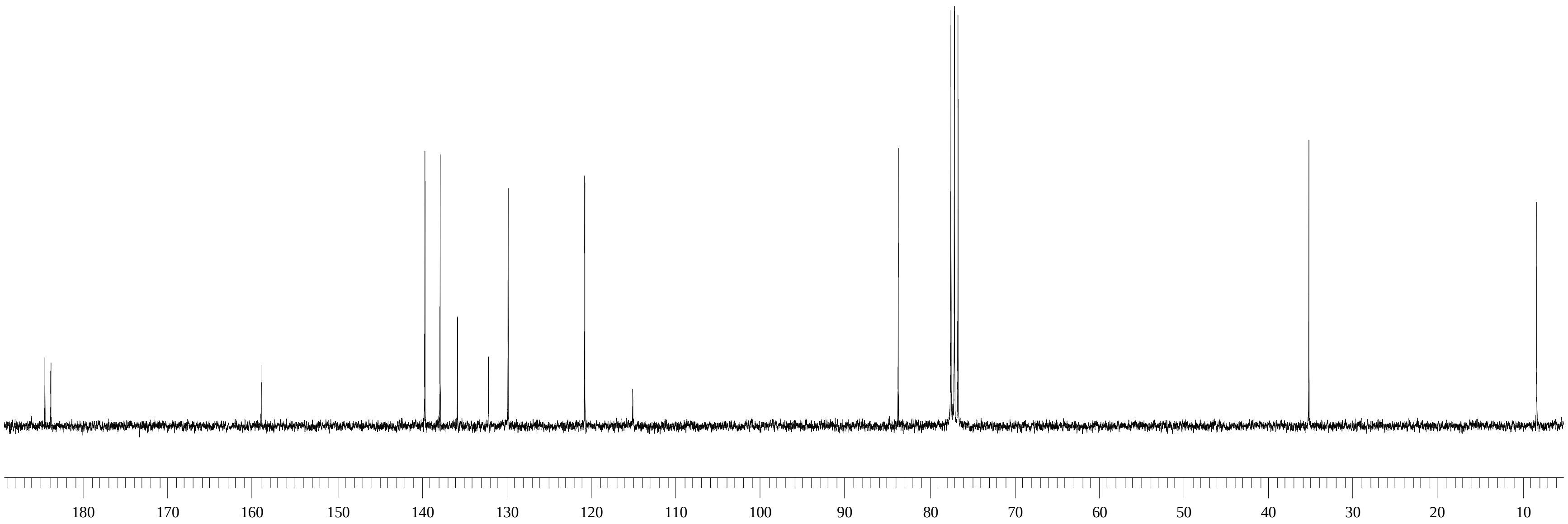

HRMS

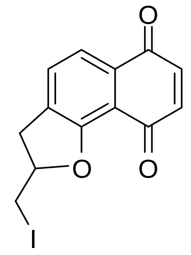

Compound 4a

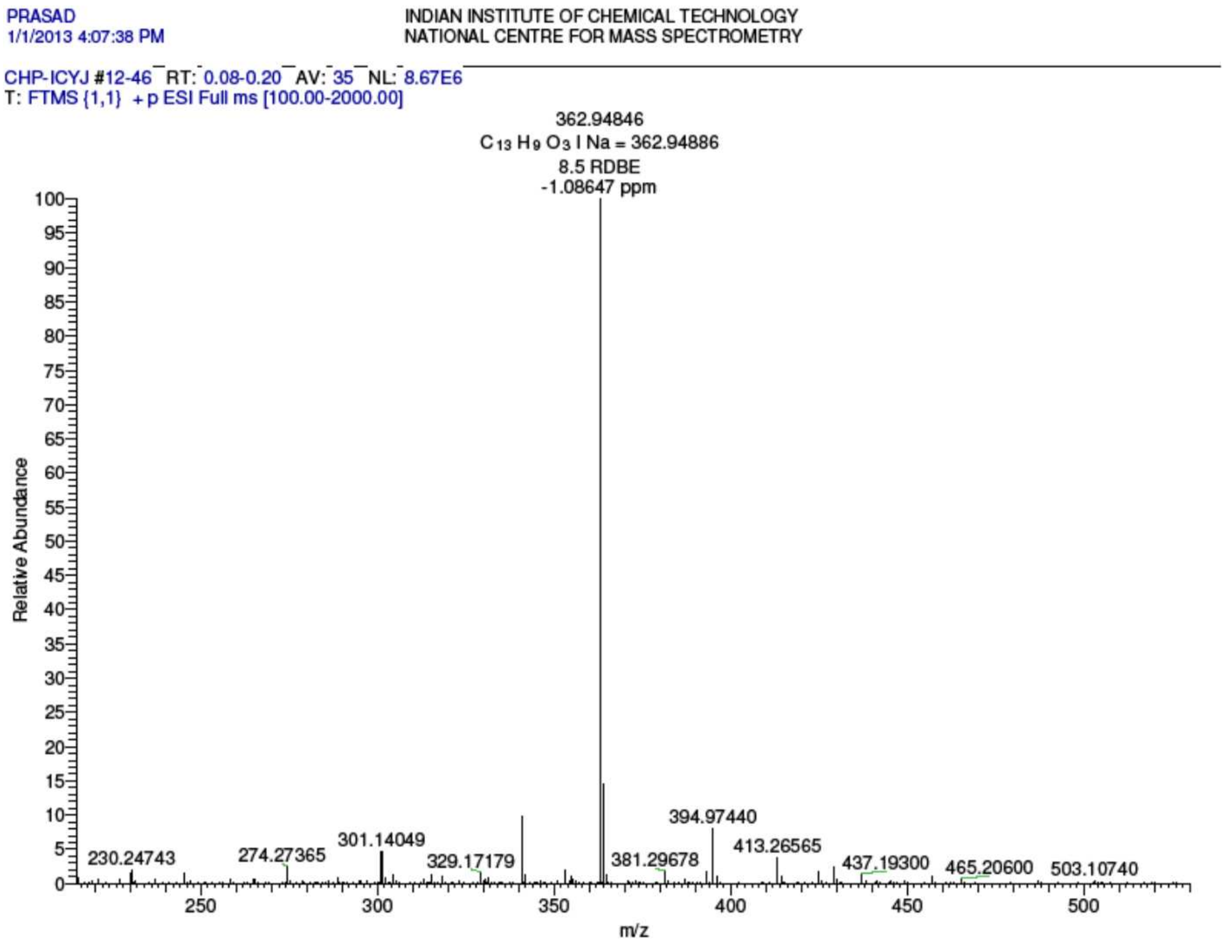

^{1}H NMR

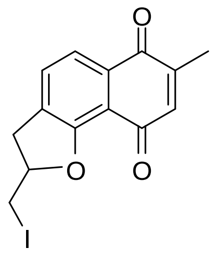

Compound 4b

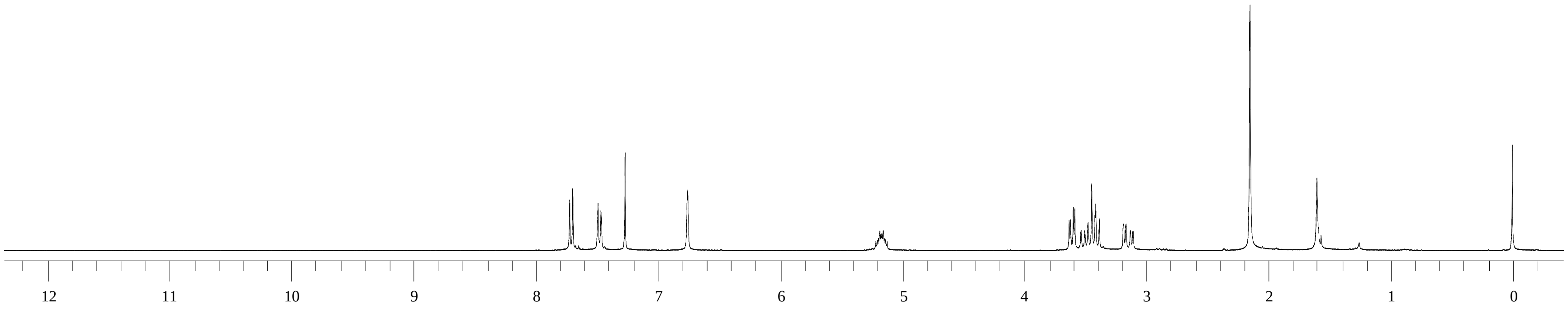

ACCEPTED MANUSCRIPT

^{13}C NMR

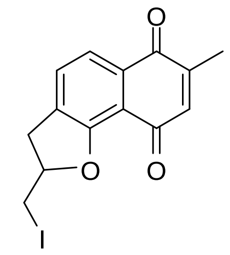

Compound 4b

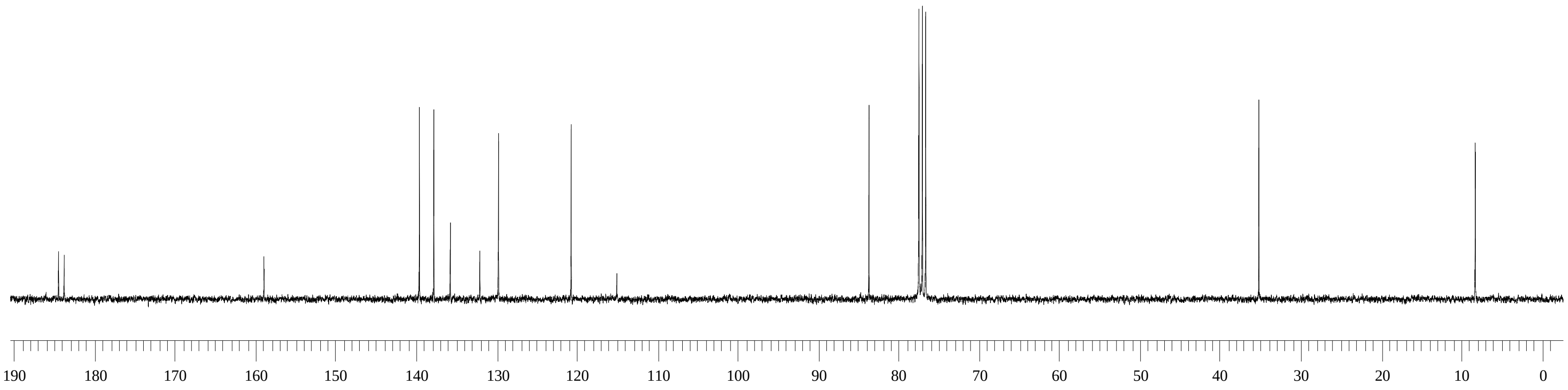

HRMS

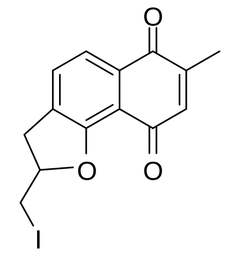

Compound 4b

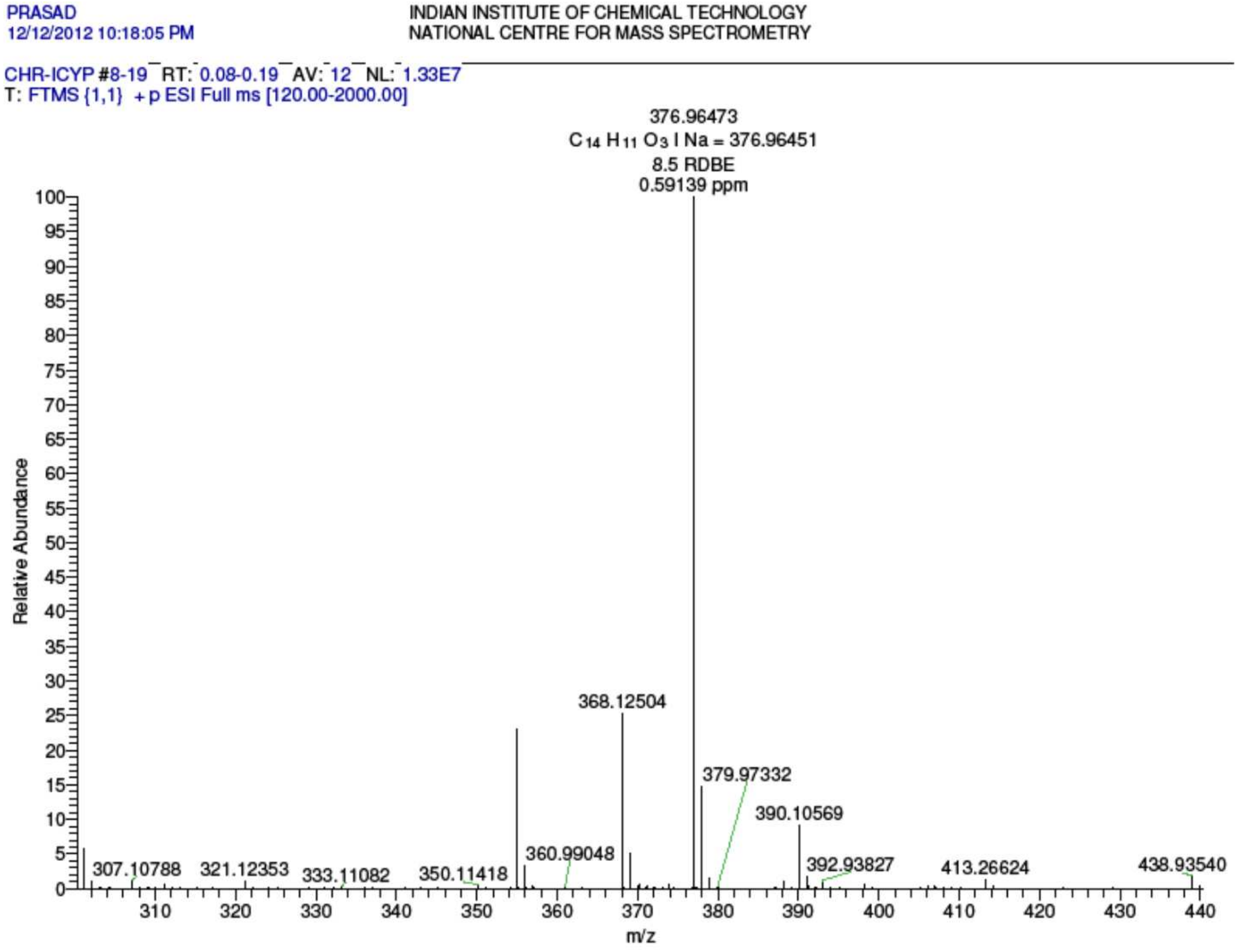

^{1}H NMR

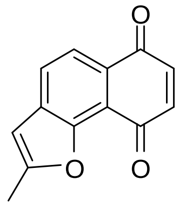

Compound 5a

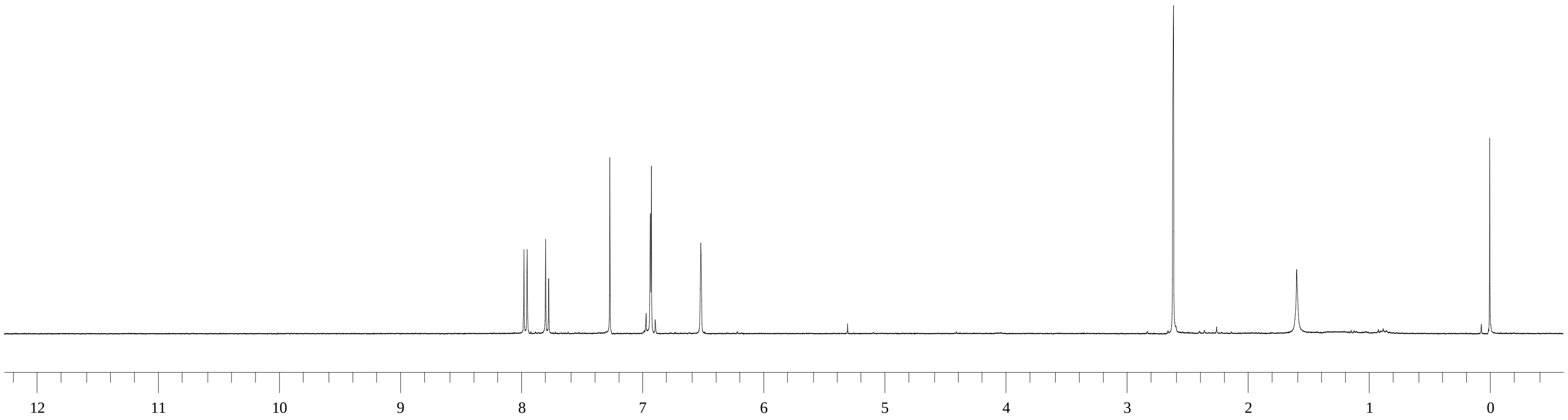

^{13}C NMR

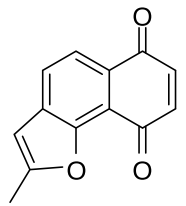

Compound 5a

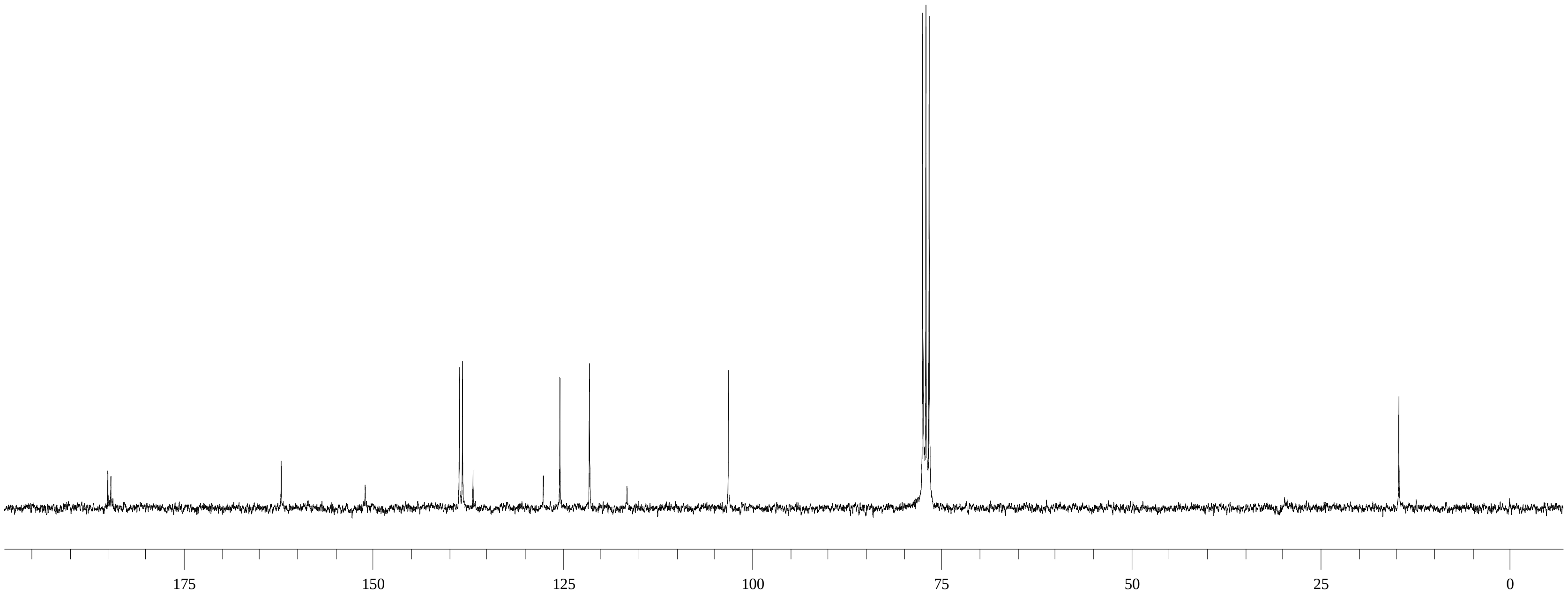

HRMS

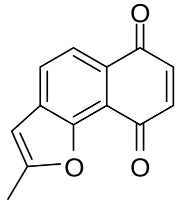

Compound 5a

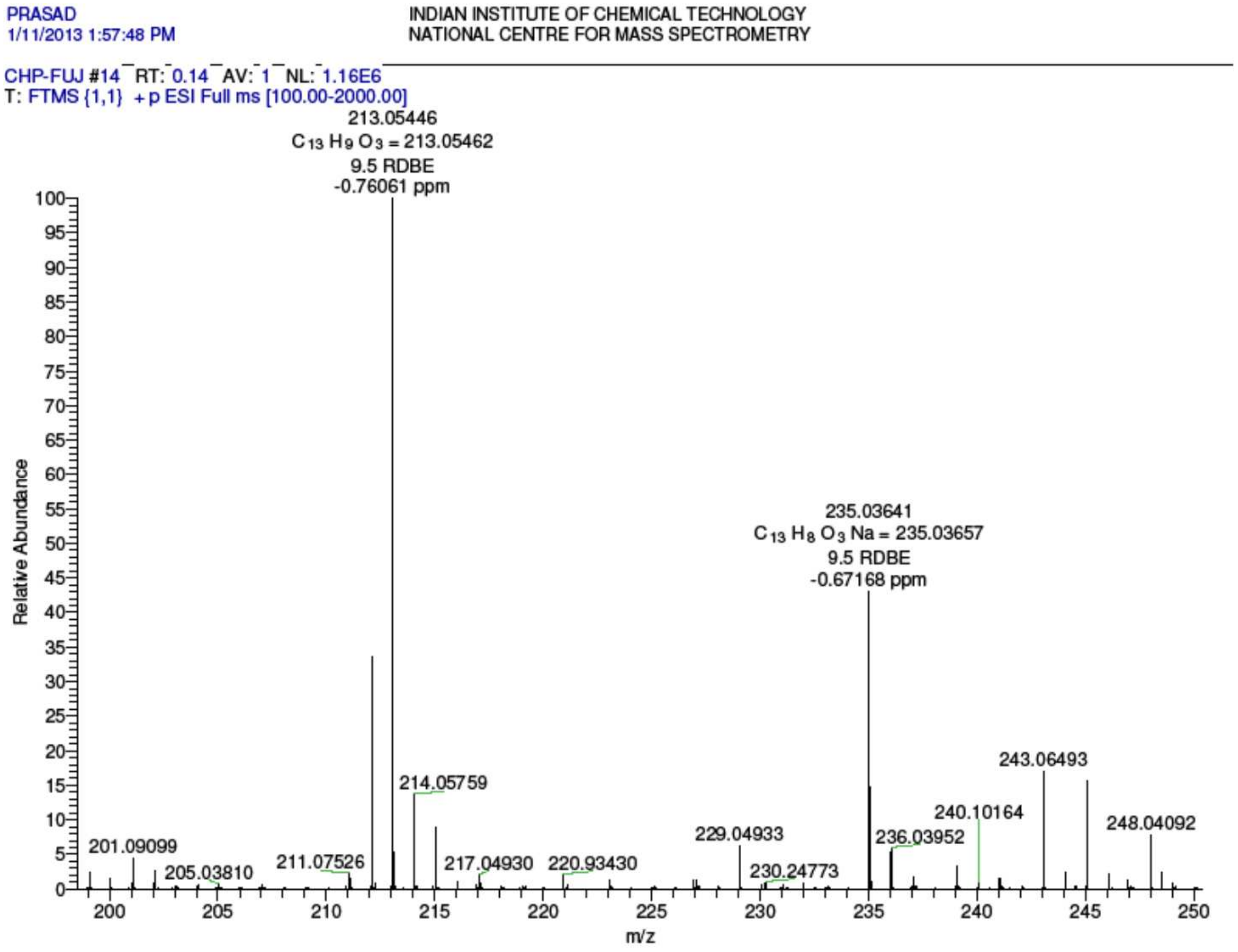

^{1}H NMR

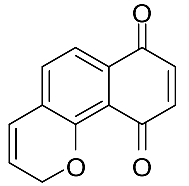

Compound 6a

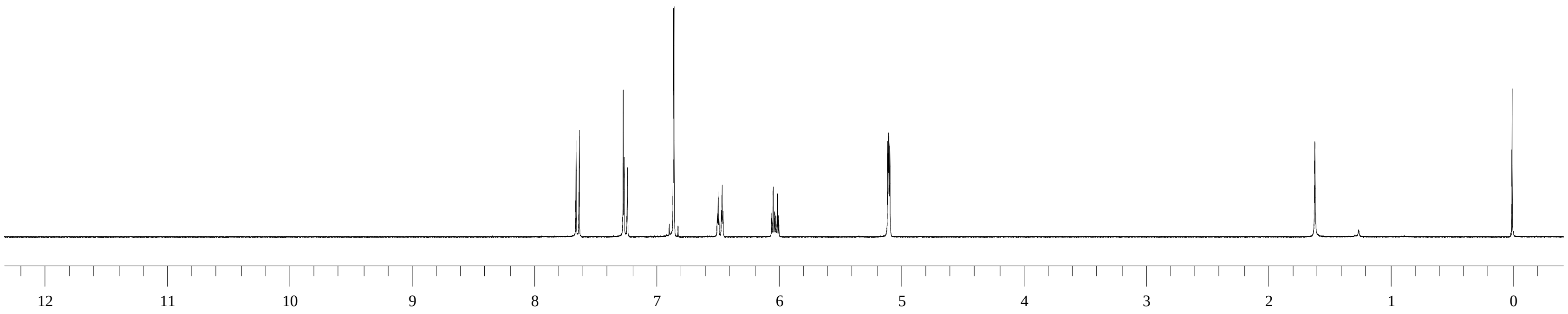

^{13}C NMR

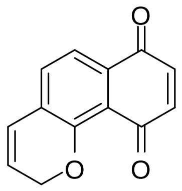

Compound 6a

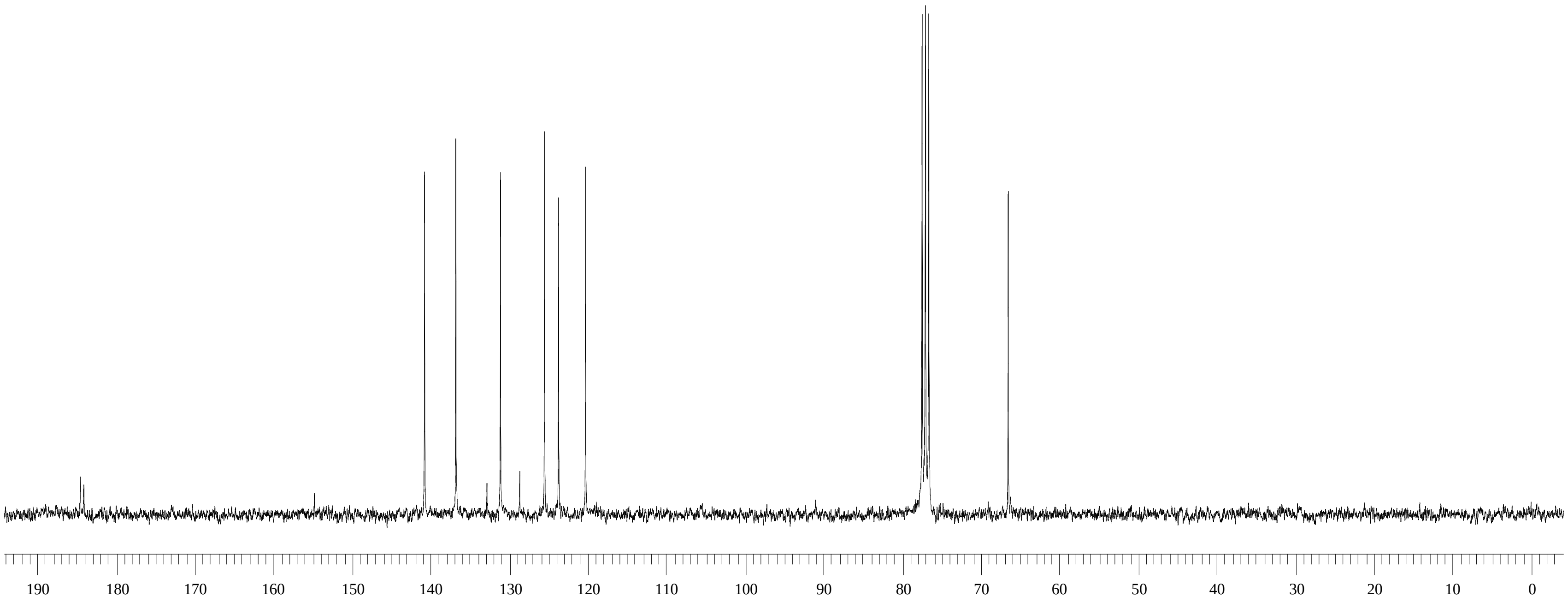

HRMS

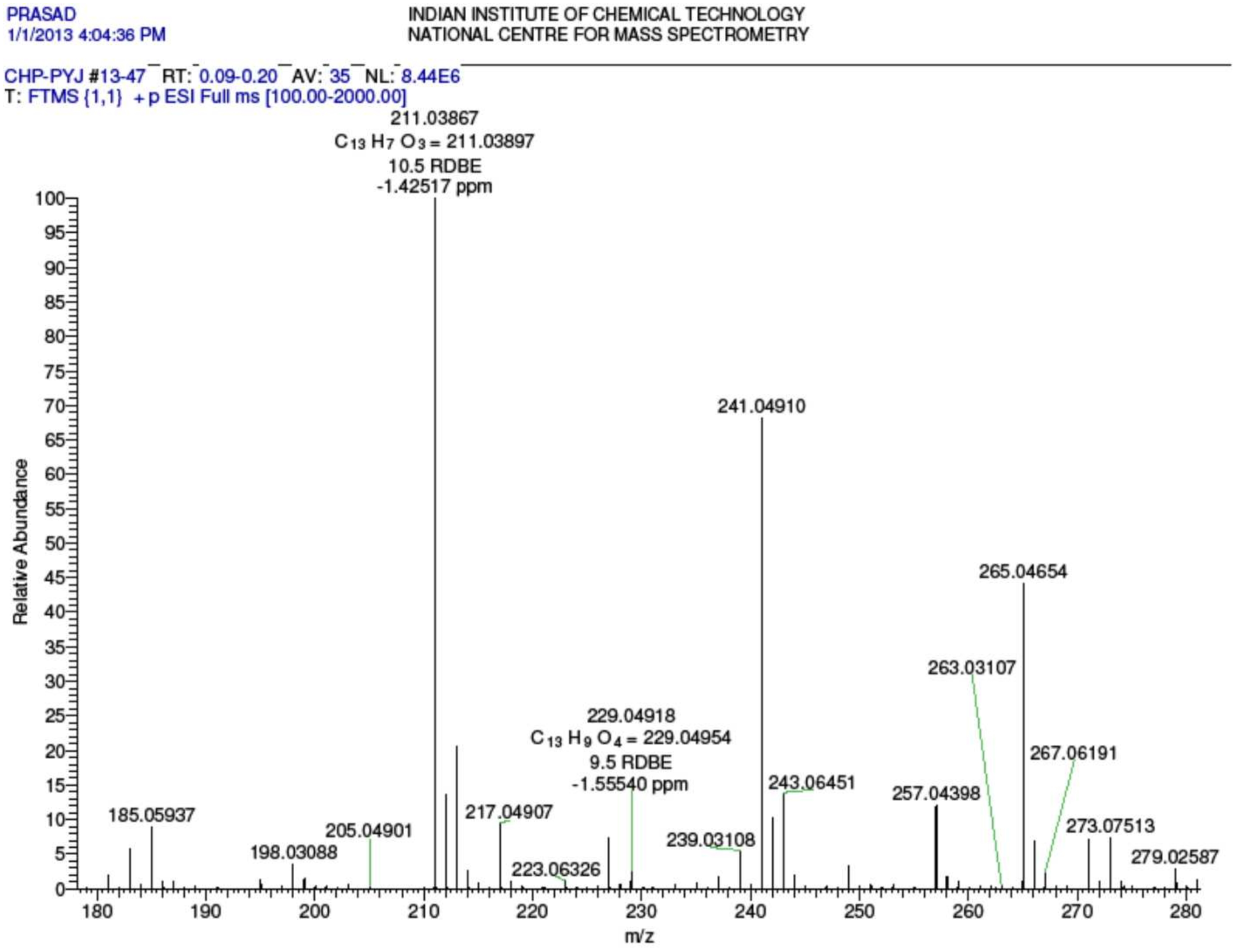

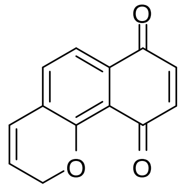

Compound 6a

^{1}H NMR

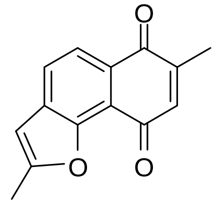

Compound 5b

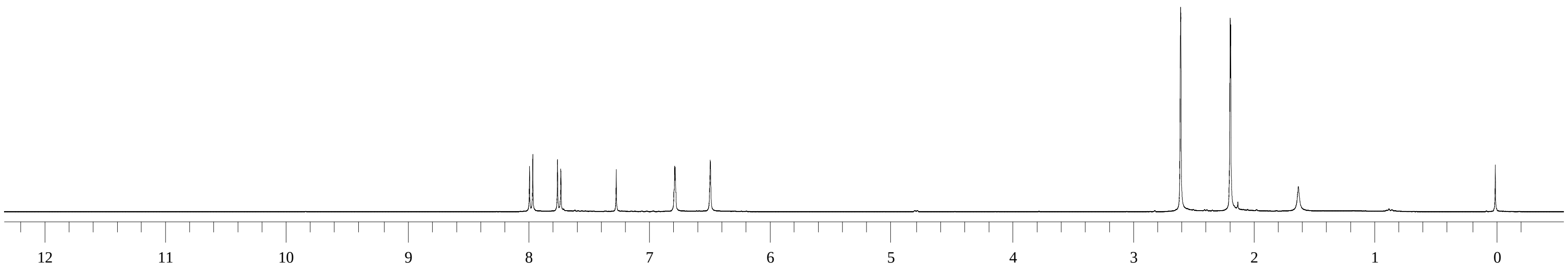

^{13}C NMR

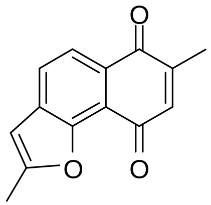

Compound 5b

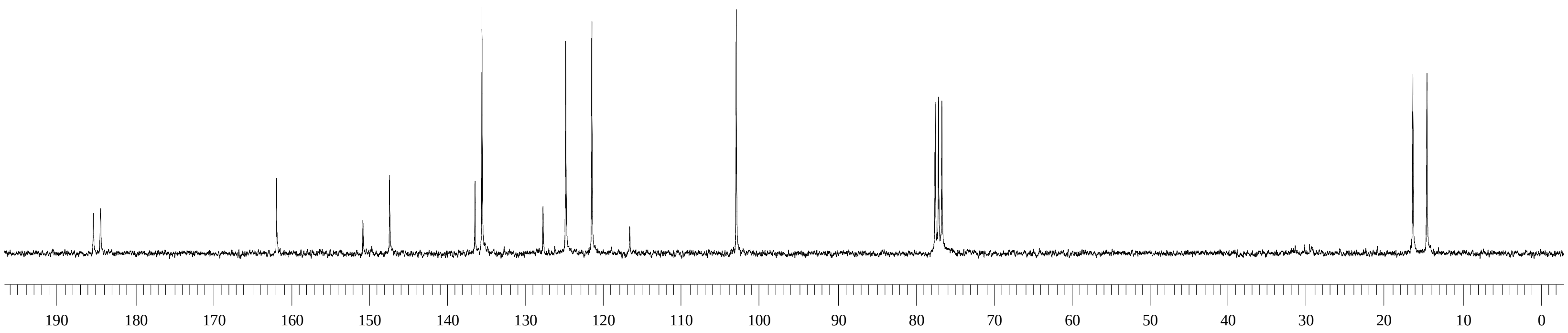

HRMS

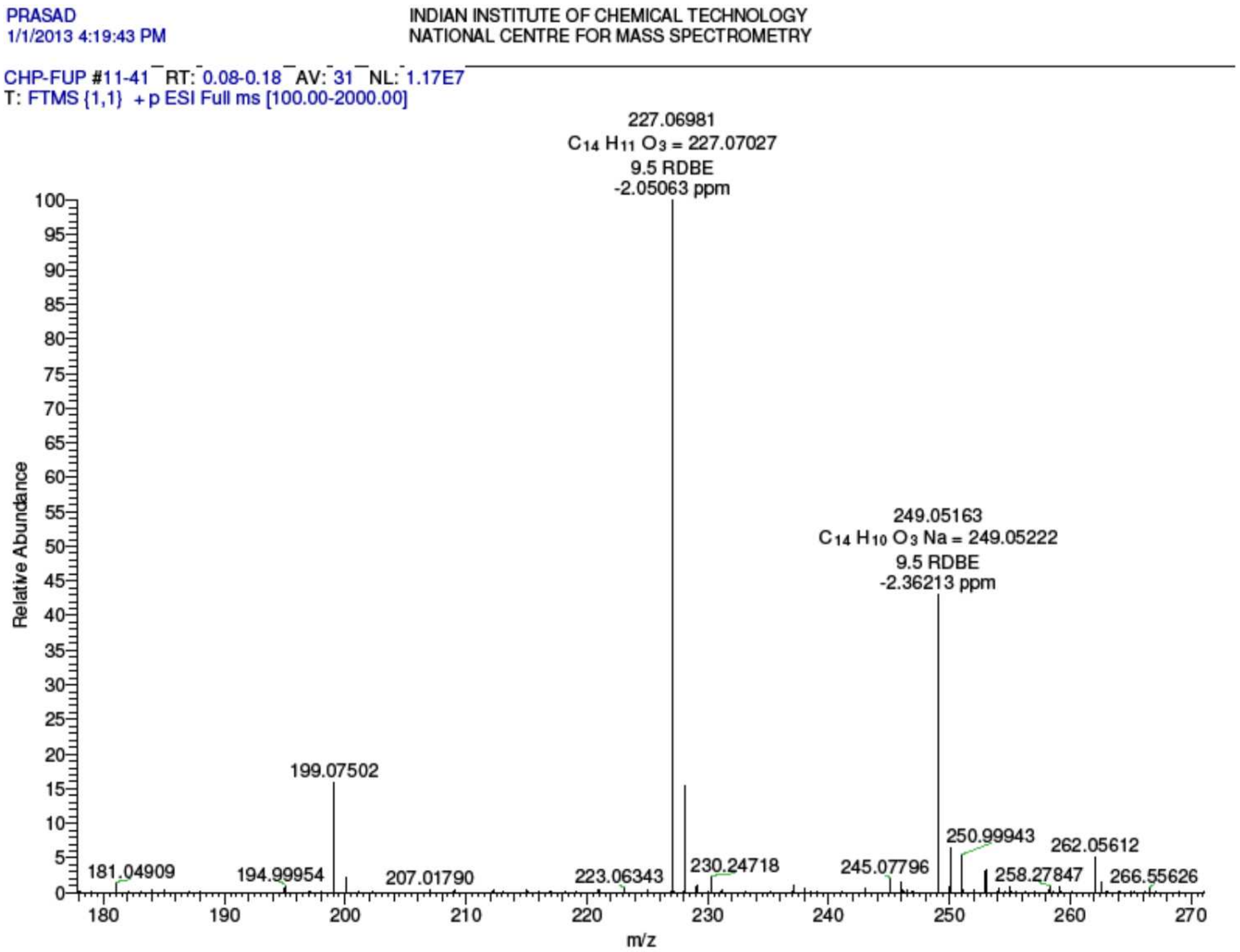

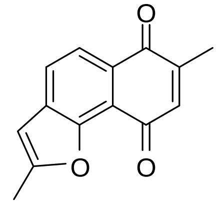

Compound 5b

^{1}H NMR

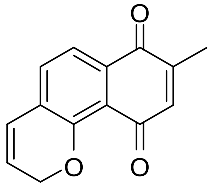

Compound 6b

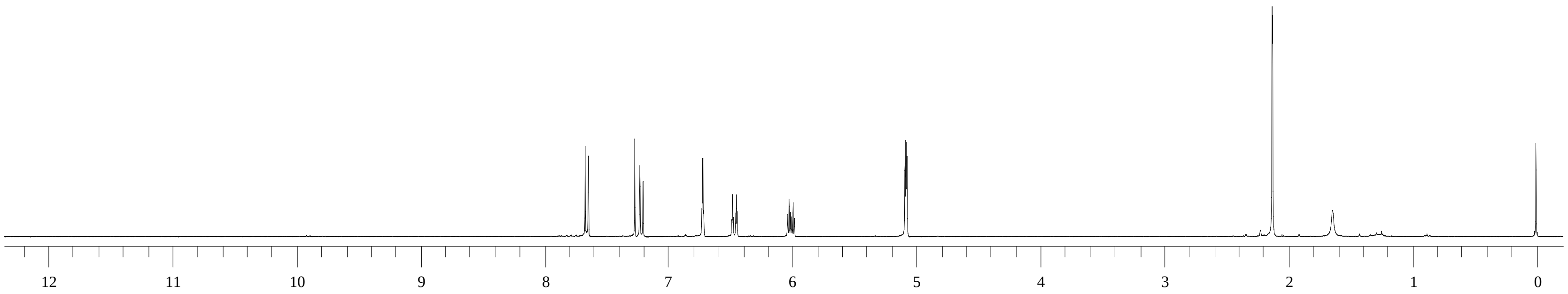

ACCEPTED MANUSCRIPT

^{13}C NMR

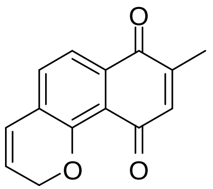

Compound 6b

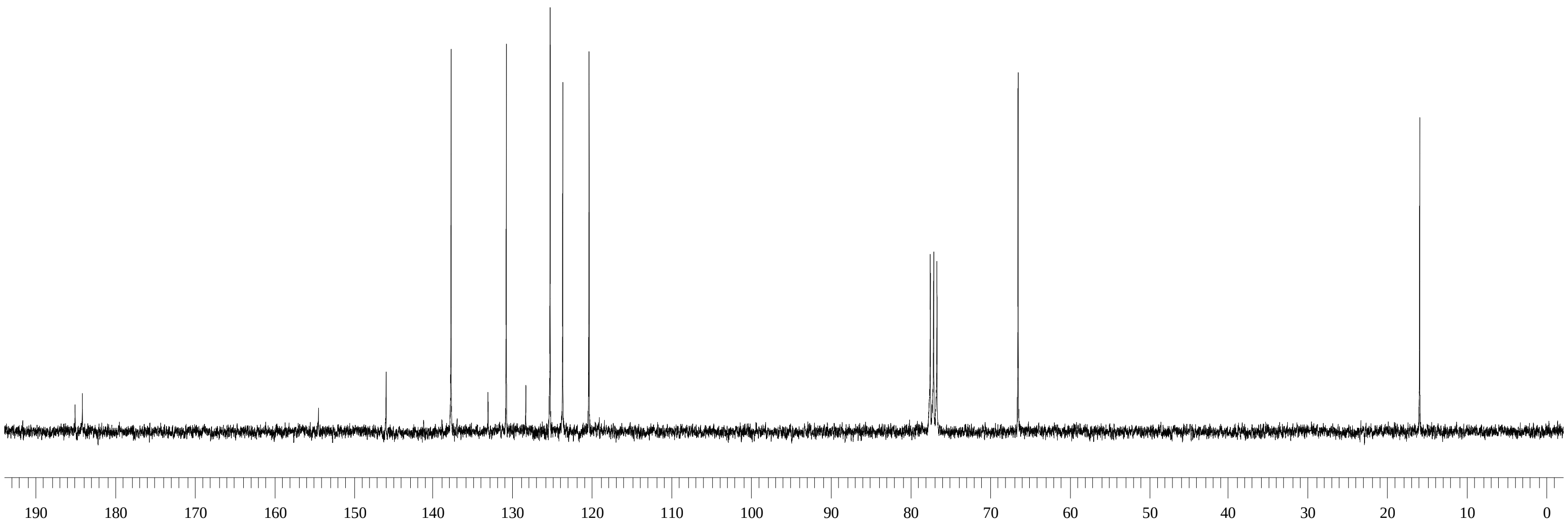

HRMS

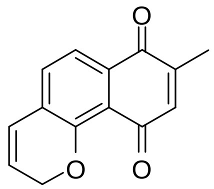

Compound 6b

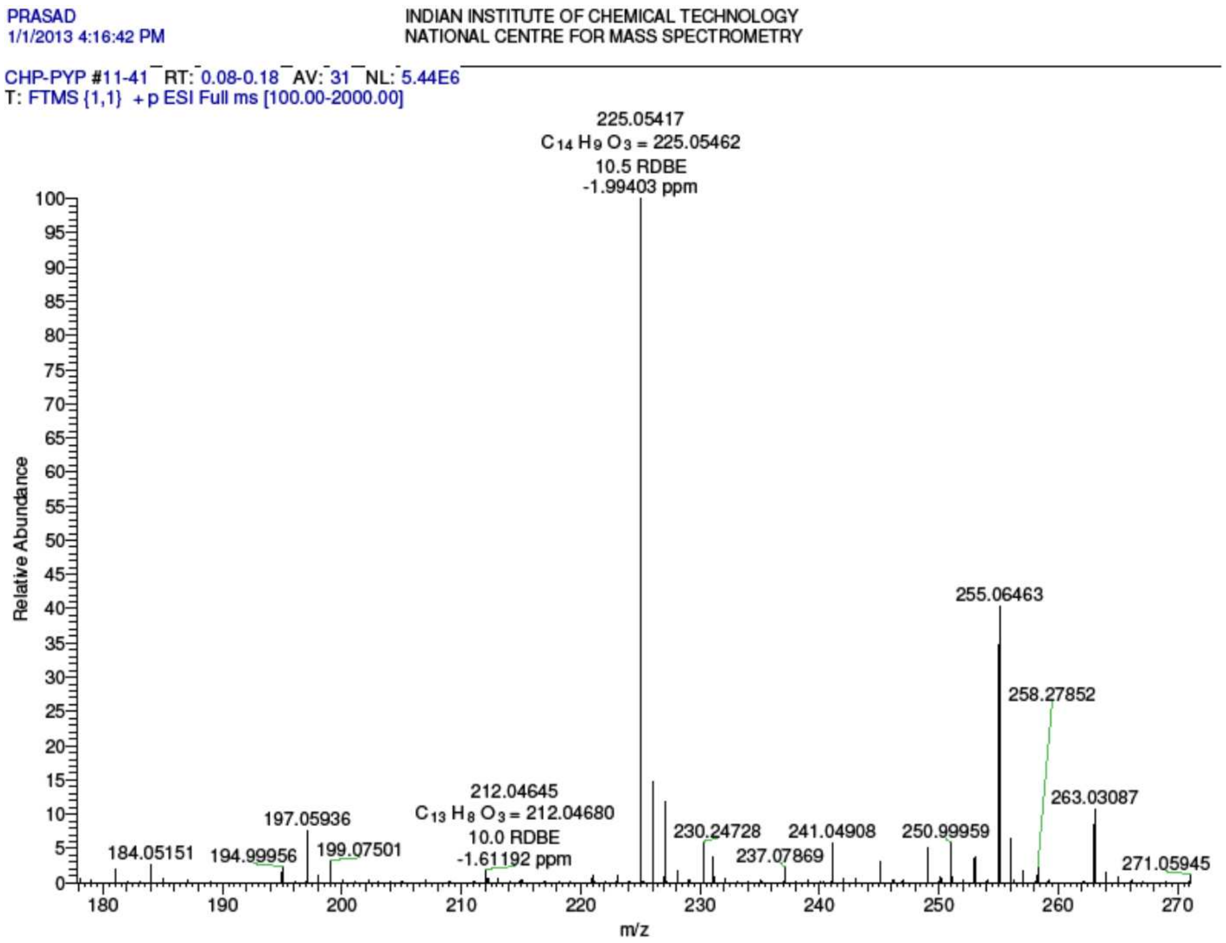

Supplement: S2 File — (ZIP) [file pone.0298918.s005.zip › Synthesis and anticancer activity of some novel 5, 6-fused hybrids of juglone based 1, 4-naphthoquinones.pdf]
