# Supplementary material for: Provenance and family variations in early growth of Manchurian walnut (Juglans mandshurica Maxim.) and selection of superior families
Source: PLoS One. 2024 Mar 7;19(3):e0298918. doi: 10.1371/journal.pone.0298918 (PMC10919699; doi:10.1371/journal.pone.0298918)
Supplement: S2 File — (ZIP) [file pone.0298918.s005.zip › The PCA index for measuring functional diversity and its application to Juglans mandshurica communities in the Beijing mountains, China.pdf]

International Journal of Biomathematics
Vol. 10, No. 7 (2017) 1750097 (13 pages)
© World Scientific Publishing Company
DOI: 10.1142/S1793524517500978

World Scientific
www.worldscientific.com

Int. J. Biomath. 2017.10. Downloaded from www.worldscientific.com
by UNIVERSITY OF GOTHENBURG on 10/20/17. For personal use only.

The PCA index for measuring functional diversity and its application to *Juglans mandshurica* communities in the Beijing mountains, China

Naiqi Song

School of Chinese Materia Medica
Beijing University of Chinese Medicine
Beijing 100102, P. R. China
Songnaiqi2007@126.com

Jin-Tun Zhang* and Fenggu Zhao

College of Life Sciences
Beijing Normal University
Beijing 100875, P. R. China
**Zhangjt@bnu.edu.cn*

Received 6 June 2016
Accepted 19 May 2017
Published 14 July 2017

Methods for measuring functional diversity are essential for functional studies of plant communities. A useful method, the PCA index, based on principal component analysis ordination of functional trait data was introduced and applied to functional diversity analysis of *Juglans mandshurica* communities in the Beijing Mountains. Thirty-five $10\,\mathrm{m} \times 10\,\mathrm{m}$ quadrats were established in *Juglans mandshurica* communities. Species composition, functional traits and environmental factors were measured and recorded. The four common indices, FAD, MFAD, FDp and FDc, were used and compared with the PCA index in the analysis. The results showed that the PCA index was successful in quantifying functional diversity and describing its relationships with environmental variables; therefore, it was an effective index in functional diversity analyses. Functional diversity in *Juglans mandshurica* communities varied widely. Elevation and aspect were the key factors affecting functional diversity in communities. Functional diversity increased with elevation increases and with the change in aspect from North to South. Functional diversity was significantly correlated with species richness and heterogeneity.

Keywords: Functional diversity; functional traits; principal component analysis; functional diversity index; environmental variable.

Mathematics Subject Classification 2010: 80A25, 62H25

*Corresponding author.

Int. J. Biomath. 2017.10. Downloaded from www.worldscientific.com
by UNIVERSITY OF GOTHENBURG on 10/20/17. For personal use only.

1. Introduction

Functional diversity (FD) is recognized as an important driver of ecosystem functions and thus governs the adaptability of ecosystems in the face of environmental change and disturbance [1]. Species with special functional traits are a result of long-term interaction with environmental variables. Functional traits are the main ecological attributes by which different species and their communities influence ecological processes [2]. Therefore, FD was defined as the value and scope of species' functional traits in a community [3, 4]. This concept emphasizes differences of functions among species in ecosystems [5, 6].

Recently, an increasing number of methods have been proposed to quantify FD in communities or ecosystems [7]. These methods are different in mathematical properties, their features, their emphasis on location, the use of single trait, or a group or multiple traits and the inclusion of relative abundances [8]. Walker *et al.* [9] put forward an index for measuring FD by combining the number of various traits. The later progresses are based on functional distances of species in trait space. Functional attribute diversity (FAD) is the sum of all functional distances among pairs of species in a community [6]. Rao [10] applied quadratic entropy to calculate FD index (Rao's index) and attempted to refer to species relative abundance with species functional traits. Petchey and Gaston [5] suggested the use of a dendrogram produced by clustering to obtain FD values using multivariate trait profiles. Casanoves *et al.* [8] developed the software (FDivesity) for these methods. Zhang *et al.* [6] introduced the fuzzy equivalence clustering index to FD studies, based on fuzzy mathematical theory. Song and Zhang [11] applied a self-organizing feature map index based on artificial neural network theory to FD analysis. Podani *et al.* [2] proposed a new approach to the measurement of FD based on information theory. These new methods will promote the study of FD.

Principal component analysis (PCA) is the most effective eigenanalysis-based ordination method [12, 13]. Ordination primarily endeavors to represent sample and species relationships as faithfully as possible in a low-dimensional space. Therefore, the spatial distances among species in PCA ordination space based on the functional trait matrix should faithfully summarize the FD in a community. Therefore, FD index based on PCA may provide more precise and effective result.

Juglans mandshurica Maxim (Juglandaceae) is an endangered tree species and became a nationally protected plant species since 1980 in China, and its natural distribution range is mainly limited to Northeastern China and Northern China. This plant became endangered due to frequently disturbance from human activities before 1980 when nature reserves were set up [14]. The need for conservation of this plant and its survival communities is important. The variation of functional trait diversity and its attribute factors are significant in the conservation of *Juglans mandshurica* populations and forests [15]. This study aimed: (1) to apply and test the PCA index in quantifying FD of plant communities; and (2) to assess

the relationships between FD and environmental variables in *Juglans mandshurica* communities.

2. Materials and Methods

2.1. *Study area*

The study sites include Dongling Mountain and Wuling Mountain, which are the main distribution areas of *Juglans mandshurica* communities in Beijing. Dongling Mountain is located at 115°26′–115°40′ E, 40°00′–40°05′ N. This mountain is an extension of the Xiaowutai Mountain Range (Fig. 1). The climate of this area is typical warm temperate monsoon climate. It has an average annual precipitation of 500–650 mm and a annual mean temperature of 7°C. The monthly mean temperature is −7.8°C in January and 21.1°C in July [16]. The elevation varies from 800 m asl to 2303 m asl. Wuling Mountain is located at 117°17′–117°35′ E, 40°29′–40°36′ N. It is the main peak of the Yanshan Mountain Range (Fig. 1). This mountain also has a typical warm temperate monsoon climate with a mean annual precipitation of 550–750 mm and an annual average temperature of 7.6°C. The monthly average temperature is −15.6°C in January and 17.6°C in July [16]. The elevation changes between 800 m asl and 2118 m asl. Several soils, such as grey cinnamon soil, mountain grey soil, brown forest soil and meadow soil are present from the low area to the upland in the Dongling and Wuling mountains [17]. Vegetation mainly includes secondary scrublands, secondary forests and mountain meadows from the bottom of the mountains to the summits.

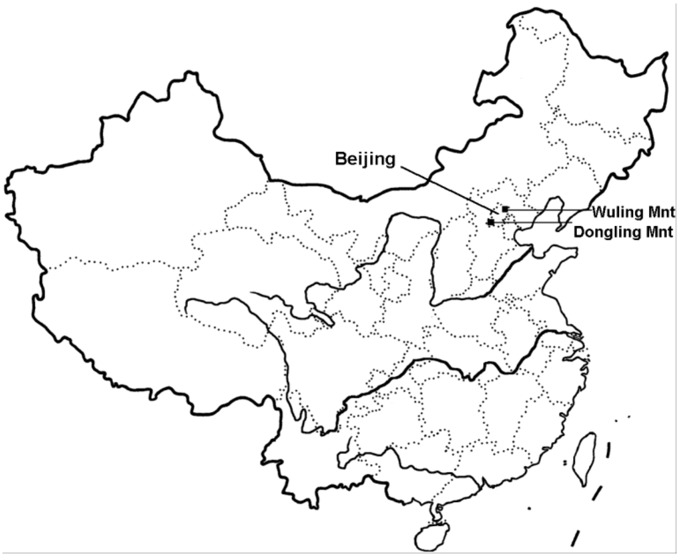

Fig. 1. The geographical location of Dongling Mountain and Wuling Mountain in Beijing, China.

Int. J. Biomath. 2017.10. Downloaded from www.worldscientific.com by UNIVERSITY OF GOTHENBURG on 10/20/17. For personal use only.

2.2. *Sampling*

Based on a comprehensive field survey, 35 *Juglans mandshurica* forest communities or forest patches were located in the Dongling and Wuling Mountains. One standard quadrat in each forest community or forest patch was set up and there were 35 sampling quadrats of *Juglans mandshurica* forest communities in total. The quadrat size was $10\,\text{m} \times 10\,\text{m}$. Species name, cover, height, basal area and individual abundance for tree species, and name, cover, individual or tussock abundance and height for shrubs and herbs were recorded in each quadrat [13]. Plant heights were measured by using a clinometer for trees and a steel tape ruler for shrubs and herbs. One hundred and seventy-two plant species in total were recorded in the 35 sampling quadrats.

Ten plant functional traits were used to reflect plant species functions in the forest community (Table 1) [11]. The traits of photosynthetic pathway, nitrogen-fixing capacity, seed dispersal, and pollination syndrome were identified from local flora [18], whilst growth type, life-form, leaf hair, leaf shape, plant height, flowering period length, and initial flowering date were measured and recorded *in situ*. For calculating functional diversity index, a data matrix of plant functional traits by species in a sampling quadrat was constructed, and there were in total 35 data matrices for 35 quadrats. To avoid scale effects, all plant trait data were standardized before calculating functional diversity values [6].

Elevation, slope and aspect were recorded for each quadrat. Elevation was measured by using a GPS; the slope and aspect were measured by using a compass meter [19]. Elevation and slope were record values, while the aspect values were transformed from 1 to 8 in the way described in Zhang *et al.* [19] The greater the value is, the more sunlight availability there is.

Int. J. Biomath. 2017.10. Downloaded from www.worldscientific.com
by UNIVERSITY OF GOTHENBURG on 10/20/17. For personal use only.

Table 1. Plant functional traits and their values in *Juglans mandshurica* communities in the Beijing Mountains, China.

Functional trait type	Functional traits and values
Nitrogen-fixing	0 No nitrogen-fixing, 1 Elaeagnaceae nitrogen-fixing, 2 Leguminosae nitrogen-fixing
Photosynthesis pathway	1 Crassulacean acid metabolism (CAM) pathway, 2 C3 pathway, 3 Between C3 and C4 pathway, 4 C4 pathway
Seed dispersal	1 Authochory (self-dispersal), 2 Barochory (dispersal by gravity), 3 Anemochory (wind dispersal), 4 Zoochory (animal dispersal)
Pollination syndrome	1 Anemophilous, 2 Entomophilous
Life-form and growth type	1 Tree, 2 Shrubs, 3 Woody vine, 4 Perennial herb, 5 Biennial herb, 6 Annual herb
Leaf shape	1 Coniferous, 2 Broad leaf
Leaf hair	0 No hair on back surface, 1 With hairs on back surface
Plant height	Measured value in meter
Initial flowering date	First month of flowering
Flowering period length	Number of flowering months

2.3. Data analysis

2.3.1. Principal component analysis (PCA index)

Mathematically, PCA transforms the species data into a new coordinate system so that the largest variance of the dataset can be demonstrated by the first principal component (first axes), the second largest variance by the second component (second axes), and so on.

For a data matrix, $\mathbf{X}$, with zero mean, each row (total N rows) represents a plant species, and each column (total P columns) represents a quadrat.

The PCA transformation can be showed by a set of vectors (p-dimensional), $\mathbf{W}_{(k)} = (w_1, \ldots, w_p)_{(k)}$, that change each row vector $\mathbf{X}_{(i)}$ of $\mathbf{X}$ to an ordination score vector (principal component scores) $\mathbf{t}_{(i)} = (t_1, \ldots, t_p)_{(i)}$, got from $t_{k(i)} = \mathbf{X}_{(i)} \cdot \mathbf{W}_{(k)}$.

The first ordination vector $\mathbf{w}_{(1)}$ thus must satisfy

$$\mathbf{w}_{(1)} = \underset{\|\mathbf{w}\|=1}{\arg\max} \left\{ \sum_i (t_1)^2_{(i)} \right\} = \underset{\|\mathbf{w}\|=1}{\arg\max} \sum_i (\mathbf{X}_{(i)} \cdot \mathbf{w})^2. \tag{2.1}$$

The matrix form is

$$\mathbf{w}_{(1)} = \underset{\|\mathbf{w}\|=1}{\arg\max} \left\{ \|\mathbf{X}\mathbf{w}\|^2 \right\} = \underset{\|\mathbf{w}\|=1}{\arg\max} \{\mathbf{w}^T \mathbf{X}^T \mathbf{X}\mathbf{w}\} \tag{2.2}$$

and

$$\mathbf{w}_{(1)} = \arg\max \left\{ \frac{\mathbf{W}^T \mathbf{X}^T \mathbf{X}\mathbf{w}}{\mathbf{w}^T \mathbf{w}} \right\}. \tag{2.3}$$

For a symmetric matrix $\mathbf{X}^T\mathbf{X}$, the possible quotient's largest value is the maximum eigenvalue. The first component of a data vector $\mathbf{x}_{(i)}$ may then be obtained as a ordination score $t_{1(i)} = \mathrm{x}_i \cdot \mathrm{w}_{(1)}$ in the coordinate system. The kth-component can be found

$$\hat{\mathbf{X}}_{k-1} = \mathbf{X} - \sum_{s=1}^{k-1} \mathbf{X}\mathbf{w}_{(s)} \mathbf{w}^T_{(s)} \tag{2.4}$$

and then

$$\mathbf{W}_{(k)} = \underset{\|\mathbf{w}\|=1}{\arg\max} \left\{ \left\| \hat{\mathbf{X}}_{k-1}\mathbf{w} \right\|^2 \right\} = \arg\max \left\{ \frac{\mathbf{W}^T \hat{\mathbf{X}}^T_{k-1} \hat{\mathbf{X}}_{k-1}\mathbf{w}}{\mathbf{w}^T\mathbf{w}} \right\}. \tag{2.5}$$

The results will provide the remaining eigenvectors of $\mathbf{X}^T\mathbf{X}$ in the transformed coordinate system, the kth-ordination axes (kth-principal component) of a data vector $\mathbf{x}_{(i)}$ can be defined as a score $t_{k(i)} = \mathrm{x}_i \cdot \mathrm{w}_{(k)}$, or as the corresponding vector in the ordination space, $\{\mathrm{x}_{(i)} \cdot \mathrm{w}_{(k)}\}\mathrm{w}_{(k)}$, where $\mathbf{w}_{(k)}$ is the kth-eigenvector. The whole principal components decomposition of $\mathbf{X}$ can therefore be obtained from

$$\mathbf{T} = \mathbf{X}\mathbf{W}, \tag{2.6}$$

where $\mathbf{W}$ is a matrix ($p \times p$) composed by the eigenvectors of $\mathbf{X}^T\mathbf{X}$.

Int. J. Biomath. 2017.10. Downloaded from www.worldscientific.com
by UNIVERSITY OF GOTHENBURG on 10/20/17. For personal use only.

Taking the first two principal components to calculate functional distances between species,

$$d_{jk} = \sqrt{(x_j - x_k)^2 + (y_j - y_k)^2}, \tag{2.7}$$

where d_{jk} is a measurement of functional distance between plant species j and k in PCA ordination space, x_j and x_k are ordination scores of species j and k on the x-axes (the first component), and y_j and y_k are ordination scores of species j and k on the y-axes (the second component).

The PCA FD index is

$$\mathrm{PCA} = \frac{1}{2} \sum_{j=1,k=1}^{N} d_{jk} \tag{2.8}$$

($j = k = 1, 2, \ldots, N =$ species number in a quadrat).

Usually the first two components can explain over 60% ecological variance for plant trait matrix [8], and we take two PCA axes in the FD calculation.

2.3.2. *Compared indices*

***Functional attribute diversity* (FAD)**

FAD aimed at estimating the dispersion of species in trait space as the sum of the pairwise species distances [9]:

$$\mathrm{FAD} = \sum_{i,j} d_{ij}, \tag{2.9}$$

where d_{ij} is the functional distance between plant species i and j in the functional trait space.

***Modified functional attribute diversity* (MFAD)**

For a data matrix of species by traits, we defined the functional species first. The functional species refer to species with the same traits values. The entity number in the data matrix will be reduced from species number to functional species number:

$$\mathrm{MFAD} = \frac{\sum_{i,j}^{P} d_{ij}}{P}, \tag{2.10}$$

where d_{ij} is the distance between functional species "i" and "j", and P is the number of functional species.

***Functional diversity based on dendrogram* (FDp and FDc)**

The total branch length of the dendrogram produced by clustering based on functional trait data matrix can reflect FD [20]. FDp is a plot-based functional index recalculating the dendrogram for each plot (quadrat), but the desirable property of "set monotonicity" does not hold in calculation. FDc is a community-based index corrected the lack of monotonicity [21].

Int. J. Biomath. 2017.10. Downloaded from www.worldscientific.com
by UNIVERSITY OF GOTHENBURG on 10/20/17. For personal use only.

Int. J. Biomath. 2017.10. Downloaded from www.worldscientific.com
by UNIVERSITY OF GOTHENBURG on 10/20/17. For personal use only.

2.3.3. *Species diversity*

Three-species diversity indices, one for species richness, one for species heterogeneity and one for species evenness, were used here [22]:

$$\begin{aligned}&\text{Species number (as a richness index)}: S,\\&\text{Shannon–Wiener heterogeneity index}: H' = -\sum P_i \ln P_i,\end{aligned} \tag{2.11}$$

$$\text{Pielou evenness index}: E = \frac{H^{'}}{\ln(S)}, \tag{2.12}$$

where P_i is the relative importance value of species i, $P_i = N_i/N$, N_i the importance value of species i, N the sum of importance values for all species in a quadrat, and S the number of species present in a quadrat.

3. Results

The PCA FD index was calculated by CANOCO 4.5 [12] and Excel. Other indices, namely FAD, MFAD, FDp and FDc, were calculated by use of the FDiversity software [8]. Euclidean distance measurement was used in the calculations. Functional diversity varied greatly in *Juglans mandshurica* communities in the Beijing Mountains. For example, PCA varied from 78.8 to 352.1, FAD from 44.79 to 294.03, MFAD from 2.99 to 7.74, FDp from 4.78 to 10.21, and FDc from 4.89 to 10.22. FD was rich in *Juglans mandshurica* communities according to index values.

The variation pattern of FD usually depends on the variation of environmental factors [4, 23]. PCA FD index was significantly correlated with elevation and aspect in *Juglans mandshurica* communities in the Beijing Mountains (Fig. 2), but its correlation with slope was insignificant. All other common indices showed similar relations with these environmental factors as PCA. FD in *Juglans mandshurica* communities showed a positive and nonlinear relation with altitude and slope aspect. Elevation and aspect were key factors affecting FD in *Juglans mandshurica* communities.

The PCA index, as a technique measuring for FD, could calculate FD successfully and provide reasonable results, i.e. quantifying the FD and its variation, and successfully describing the relationships between FD and environmental variables in *Juglans mandshurica* communities. This suggested that the PCA index is useful for functional diversity studies.

The PCA index was significantly correlated with species richness (S) and species heterogeneity (H′), but not significantly correlated with evenness (E) (Fig. 3). The other four common indices were also significantly correlated with species richness. All correlations of FD with species diversities were positive. Their relations were nonlinear which suggested that the PCA index showed the difference between FD and species diversity and was effective in the study of FD.

The PCA index results were significantly and positively correlated with the other four common methods, FAD, MFDA, FDp and FDc in *Juglans mandshurica*

Int. J. Biomath. 2017.10. Downloaded from www.worldscientific.com
by UNIVERSITY OF GOTHENBURG on 10/20/17. For personal use only.

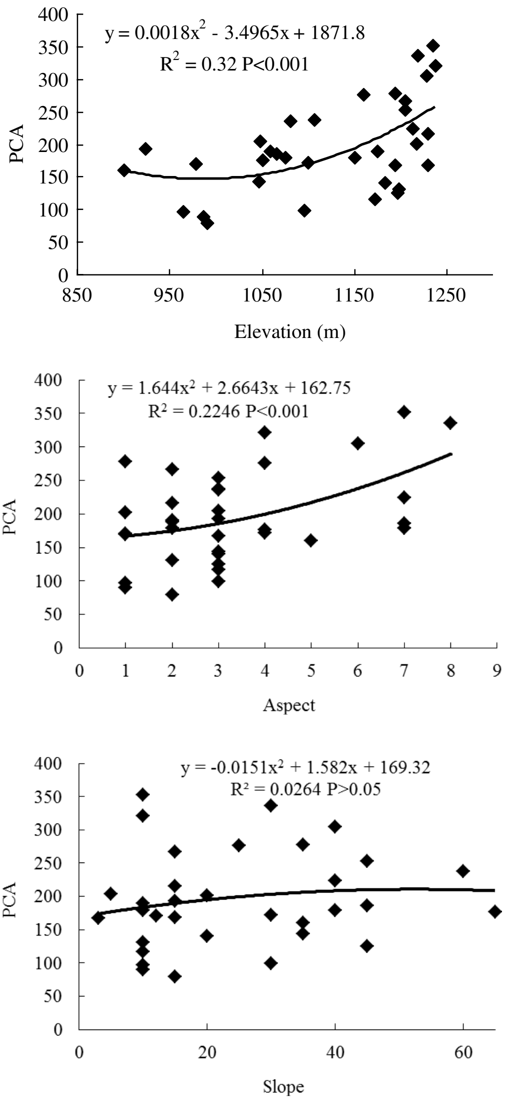

Fig. 2. Regression analysis between PCA FD index and environmental variables in *Juglans mandshurica* communities in the Beijing Mountains, China.

communities (Fig. 4). Their relationships were also nonlinear. This further suggested that the PCA index was an effective method in FD studies in plant communities.

4. Discussion

Methods for measuring FD are of importance in functional studies of plant communities [23, 24]. Many researches attempted to develop new methods to quantify FD

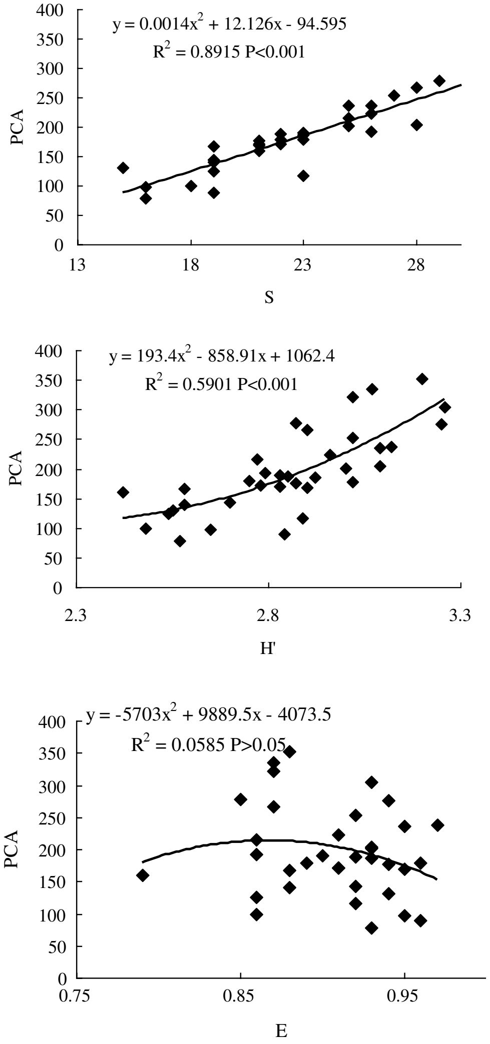

Fig. 3. Regression analysis between PCA FD index and species richness (S), heterogeneity (H′) and evenness (E) in *Juglans mandshurica* communities in the Beijing Mountains, China.

in communities [11, 21]. The PCA index was successful in describing the magnitude and variation of FD of *Juglans mandshurica* communities, which showed that the PCA index was fully applicable in FD analyses in plant communities. Significant relationships of the PCA index with FAD, MFAD, FDc and FDp indices also confirmed that the PCA index is a fully useful technique in studying FD in forest communities [7, 16]. The PCA index was measured in PCA ordination space of functional traits. It is a multi-dimensional and eigenvector ordination method and theoretically different from other indices [25]. Although the ordination was based on a linear model, the data standardization could improve the model efficiency [12, 13].

Int. J. Biomath. 2017.10. Downloaded from www.worldscientific.com
by UNIVERSITY OF GOTHENBURG on 10/20/17. For personal use only.

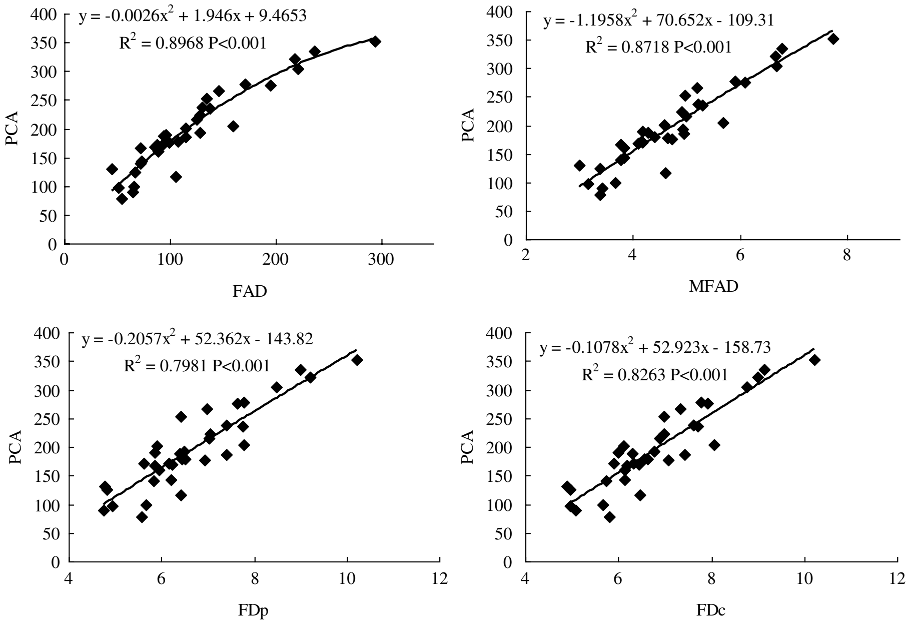

Fig. 4. Regression analysis between PCA FD index and other FD indices in *Juglans mandshurica* communities in the Beijing Mountains, China. FAD, MFAD, FDp and FDc refer to functional attribute diversity, modified functional attribute diversity, plot-based FD and community-based FD, respectively.

Therefore, the PCA index can manage significant amounts of data information and should have advantages in studying nonlinear relations in complex ecosystems. These advantages of the PCA index need to be further tested by more applications in various communities in different regions.

FD values in *Juglans mandshurica* communities in the Beijing Mountains varied greatly, which showed that FD index is one of suitable indicators for describing relations of community composition, structure, function and environmental factors in forests [26]. FD showed significant correlations with elevation and aspect, which was consistent with the former research results that altitude and aspect were the key environmental variables influencing community structure, composition, diversity and distribution in the Beijing Mountains [23]. The same conclusion that elevation and aspect were key environmental variables affecting FD in *Juglans mandshurica* communities can be obtained. FDs increased gradually with elevation increase and with the change in slope direction from north to south. The increase of FD with elevation seems to be due to the increasing precipitation along elevation gradient. Water conditions and precipitation are the key factors for determining the distribution of plant communities in these mountains [23]. The increase of FD with the slope direction change from north to south seems to be due to the increasing light availability along the aspect gradient [27]. The correlation of FD with slope was not significant because *Juglans mandshurica* communities usually occurred on hills with

Int. J. Biomath. 2017.10. Downloaded from www.worldscientific.com
by UNIVERSITY OF GOTHENBURG on 10/20/17. For personal use only.

gentle slope close to the valley bottom and their slope did not change significantly [25, 28]. The relationships between FD and environmental variables were described clearly by the PCA index and proved by four other common indices, which further showed that the PCA index is useful in FD studies [2, 28].

FD and species diversity of*Juglans mandshurica* communities had the same pattern in response to environmental gradients, because they were significantly related to each other. The PCA index and other common FD indices were positively correlated with species richness and heterogeneity (Shannon–Wiener index), and they were negatively correlated with evenness. These results are consistent with other studies [1, 4, 6]. Theoretically, PCA ordination of functional trait data was independent of species richness, and so the correlation of the PCA index with species richness was ecologically meaningful [17]. This requires further study in the future [23].

The PCA index showed positive and nonlinear correlations with the other four common indices in FD analysis of *Juglans mandshurica* communities, which suggested that they provided similar results in quantifying FD, but they were independent of each other and should not to be replaced with each other [28, 29]. Several indices used and compared in a similar study may be more useful [4, 30]. The PCA index should be applied and its efficiency tested in more cases in the future [2].

Int. J. Biomath. 2017.10. Downloaded from www.worldscientific.com
by UNIVERSITY OF GOTHENBURG on 10/20/17. For personal use only.

Acknowledgments

The study was financially supported by the National Natural Science Foundation of China (Grant No. 31170494) and the Specialized Research Fund for the Doctoral Program of Higher Education (Grant No. 20120003110024).

References

[1] S. S. Díaz, F. Lavorel, F. de Bello, F. Quétier, K. Grigulis and T. M. Robson, Incorporating plant functional diversity effects in ecosystem service assessments, *Proc. Natl. Acad. Sci. USA* **104** (2007) 20,684–20,689.

[2] J. Podani, C. Ricotta, J. G. Pausas and D. Schmera, Combinatorial functional diversity: An information theoretical approach, *Commun. Ecol.* **14** (2013) 180–188.

[3] D. Tilman, P. Reich, J. Knops, D. Wedin, T. Mielke and C. L. Lehman, Diversity and productivity in a long-term grassland experiment, *Science* **294** (2001) 843–845.

[4] F. de Bello, The quest for trait convergence and divergence in community assembly: Are null-models the magic wand? *Global Ecol. Biogeograp.* **21** (2012) 312–317.

[5] O. Petchey and K. Gaston, Functional diversity (FD), species richness and community composition, *Ecol. Lett.* **5** (2002) 402–411.

[6] J.-T. Zhang, L. H. Fan and M. Li, Functional diversity in plant communities: Theory and analysis methods, *African J. Biotechnol.* **11** (2012) 1014–1022.

[7] N. W. H. Mason, F. de Bello, D. Mouillot, S. Pavoine and S. Dray, A guide for using functional diversity indices to reveal changes in assembly processes along ecological gradients, *J. Veget. Sci.* **24** (2013) 794–806.

[8] F. Casanoves, L. Pla, J. Di Rienzo and S. Díaz, FDiversity: A software package for the integrated analysis of functional diversity, *Meth. Ecol. Evol.* **2** (2011) 233–237.

[9] B. H. Walker, A. Kinzig and J. L. Langridg, Plant attribute diversity, resilience, and ecosystem function: The nature and significance of dominant and minor species, *Ecosystems* **2** (1999) 95–113.

[10] C. R. Rao, Diversity and dissimilarity coefficients: A unified approach, *Theor. Popul. Biol.* **21** (1982) 24–43.

[11] N. Q. Song and J.-T. Zhang, An index for measuring functional diversity in plant communities based on neural network theory, *J. Appl. Math.* **2013** (2013) 320905, 6 pp.

[12] C. J. F. ter Braak and P. Šmilauer, CANOCO Reference Manual and User's Guide to *Canoco for Windows*, Software for Canonical Community Ordination (version 4.5). Centre for Biometry Wageningen (Wageningen, NL) and Microcomputer Power (Ithaca, New York, USA, 2002), 352 pp.

[13] J.-T. Zhang, Quantitative Ecology, 2nd edn. (Science Press, Beijing, 2011) (in Chinese).

[14] J. F. Li and Y. L. Gu, Chemical components of tree bark of *Juglans mandshurica*, *J. Jiamusi Med. College* **17** (1994) 1–3.

[15] J.-T. Zhang, D. L. Wu, B. Zhang, Y. Xu, S. T. Huangand and F. G. Zhao, *Ecological Study of Suvival Communities for Several Endangered and Protective Plant Species in Beijing, China* (Science and Technology Press, Beijing, 2013) (in Chinese).

[16] J.-T. Zhang, N. Q. Song and L. H. Fan, Evaluation of nine distance-based measures of functional diversity applied to forest communities, *Ann. Forest Res.* **56** (2013) 43–52.

[17] Y. Z. Huo, *Beijing Physical Geography* (Beijing Normal University Press, Beijing, 1989).

[18] S. Y. He, *Flora of Beijing* (Beijing People's Press, Beijing, 1992).

[19] J.-T. Zhang, B. Zhang, M. Li and X. Zhu, Functional diversity and conservation of *Phellodendron amurense* communities in the Dongling Mountain of Beijing, China, *Botanical Sci.* **91** (2013) 505–513.

[20] O. Petchey and K. Gaston, Functional diversity: Back to basics and looking forward, *Ecol. Lett.* **9** (2006) 741–758.

[21] J. Podani and D. Schmera, On dendrogram-based measures of functional diversity, *Oikos* **115** (2006) 179–185.

[22] E. C. Pielou, *Ecological Diversity* (Wiley and Sons, London, 1975).

[23] J.-T. Zhang, M. Li and E. B. Nie, Pattern of functional diversity along an altitudinal gradient in the Baihua Mountain Reserve of Beijing, China, *Brazilian J. Botany* **37** (2014) 37–45.

[24] K. Suding, S. Lavorel, F. Chapin III, J. H. C. Cornelissen, S. Díaz, E. Garnier, D. Goldberg, D. U. Hooper, S. T. Jackson and M. L. Navas, Scaling environmental change through the community-level: A trait-based response-and-effect framework for plants, *Global Change Biol.* **14** (2008) 1125–1140.

[25] F. de Bello, W. Thuiller, J. Lepš, P. Choler, J. Clement, P. Macek, M.-T. Sebastia and S. Lavorel, Partitioning of functional diversity reveals the scale and extent of trait convergence and divergence, *J. Veget. Sci.* **20** (2009) 475–486.

[26] E. Laliberte and P. Legendre, A distance-based framework for measuring functional diversity from multiple traits, *Ecology* **91** (2010) 299–305.

[27] G. Austrheim, Plant diversity patterns in semi-natural grasslands along an elevational gradient in southern Norway, *Plant Ecol.* **161** (2002) 193–205.

[28] D. Mouillot, W. H. N. Mason, O. Dumay and B. J. Wilson, Functional regularity: A neglected aspect of functional diversity, *Oecologia* 142 (2005) 353–359.

Int. J. Biomath. 2017.10. Downloaded from www.worldscientific.com
by UNIVERSITY OF GOTHENBURG on 10/20/17. For personal use only.

[29] N. Mason, D. Mouillot and W. Lee, Functional richness, functional evenness and functional divergence: The primary components of functional diversity, *Oikos* **111** (2005) 112–118.

[30] S. Villéger, N. W. H. Mason and D. Mouillot, New multidimensional functional diversity indices for a multifaceted framework in functional ecology, *Ecology* **89** (2008) 2290–2301.

Int. J. Biomath. 2017.10. Downloaded from www.worldscientific.com by UNIVERSITY OF GOTHENBURG on 10/20/17. For personal use only.
