# Supplementary material for: Provenance and family variations in early growth of Manchurian walnut (Juglans mandshurica Maxim.) and selection of superior families
Source: PLoS One. 2024 Mar 7;19(3):e0298918. doi: 10.1371/journal.pone.0298918 (PMC10919699; doi:10.1371/journal.pone.0298918)
Supplement: S2 File — (ZIP) [file pone.0298918.s005.zip › The progress of Juglans mandshurica Maxim.pdf]

中国农学通报　2011,27(25):1-4
Chinese Agricultural Science Bulletin

核桃楸资源研究进展

朱红波[1],赵　云[2],林士杰[2],杨雨春[2],赵珊珊[2],孙立国[3],姜英杰[4],徐炳芳[5],梁宏玉[5],耿丽红[6]
([1]吉林省林业调查规划院,长春 130022;[2]吉林省林业科学研究院,长春 130033;[3]辉南县森林经营局四岔林场,吉林通化 135115;[4]通化县林业局,吉林通化 134100;[5]长春北方绿化中心,长春 130119;[6]东丰县林业局,吉林东丰 136300)

摘　要:从地理分布、群落特征、生长特性、遗传改良、育种造林技术等方面对核桃楸的研究进展进行了综述,总结了核桃楸作为用材树种、干果树种、药用植物、绿化树种的经济价值,在此基础上,探讨了核桃楸资源生产中存在的问题和今后研究的发展方向,以实现资源的合理利用和可持续发展。

关键词:核桃楸;资源;对策
中图分类号:S72　　**文献标志码:**A　　**论文编号:**2011-1382

The Progress of *Juglans mandshurica* Maxim.

Zhu Hongbo[1], Zhao Yun[2], Lin Shijie[2], Yang Yuchun[2], Zhao Shanshan[2], Sun Liguo[3], Jiang Yingjie[4], Xu Bingfang[5], Liang Hongyu[5], Geng Lihong[6]
([1]*Jilin Provincial Institute of Forest Inventory and Planning*, Changchun 130022; [2]*Jilin Provincial Academy of Forestry Science*, Changchun 130033; [3]*Sicha Forest Farm, Huinan Forest Management Bureau of Jilin Province*, Tonghua Jilin 135115; [4]*Forestry Bureau of Tonghua County*, Tonghua Jilin 134100; [5]*North Gardening Centre of Changchun City*, Changchun 130119; [6]*Forestry Bureau of Dongfeng County*, Dongfeng Jilin 136300)

Abstract: In this paper, the research advances of *Juglans mandshurica* Maxim. were summarized, including the aspects of distribution, community characteristic, growth characteristics, genetic improvement, plant breeding and forestation. Economic values of *Juglans mandshurica* Maxim. were concluded as lumber trees, dry fruit trees, medicinal plant, landscaping trees. Some questions about the production were discussed and the future development direction was proposed. Thus the reasonable exploitation and the sustainable development of plant resources would come true.

Key words: *Juglans mandshurica* Maxim.; resources; countermeasure

0 引言

核桃楸(*Juglans mandshurica* Maxim.),又称胡桃楸、山核桃,是胡桃科(juglandaceae)胡桃属(*Juglans*)落叶阔叶乔木,与水曲柳、黄菠萝并称“东北三大硬阔”,是国家Ⅱ级珍稀树种和中国珍稀濒危树种的三级保护植物[1],是很有发展前途的木本油料植物[2]。其材质坚硬致密,弹性好,易加工,为优良的军用细木工和家具用材;果肉及树皮含鞣质;种子富含脂肪,营养丰富,可食用或供工业用;树皮及叶可药用,具有消热解毒、抗癌等作用。世界化石能源资源日益枯竭,环境污染和全球气候变暖威胁增加,引起人类对生物质能源的重视[3-5]。

1 研究现状

1.1 地理分布及群落特征

中国是胡桃属的分布中心之一,原产中国的有5种:核桃(*J. regia*)、铁核桃(*J. sigillata*)、核桃楸(*J. marulshurica*)、麻核桃(*J. hyeiensis*)、野核桃(*J. cathayensis*)。核桃楸在中国主要分布于小兴安岭、

基金项目:吉林省科技厅计划发展项目“长白山区珍稀野生核桃楸果材兼用林选育研究”(20100260);吉林省财政厅资助项目“核桃楸种源区划、优势种质选择及无性繁育技术研究”;吉林省林业厅项目“核桃楸良种选育技术研究”。

第一作者简介:朱红波,男,1976年出生,吉林长春人,工程师,从事林木种质资源调查研究。通信地址:130022 吉林省长春市南关区亚泰大街3698号,吉林省林业调查规划院资源二室,Tel:0431-85850461,E-mail:linshijie1979@126.com。

通讯作者:杨雨春,男,1976年出生,吉林长春人,副研究员,博士,主要从事林木遗传育种研究。通信地址:130033 吉林省长春市临河街浦东路3528号,吉林省林业科学研究院,Tel:0431-85850461,E-mail:jllkyyangyuchun@yahoo.com。

收稿日期:2011-05-12,**修回日期:**2011-07-30。

(C)1994-2020 China Academic Journal Electronic Publishing House. All rights reserved.　http://www.cnki.net

完达山脉、长白山区及辽宁东部，华北地区也有零星分布，向北延伸至大兴安岭与俄罗斯远东地区，往东南到朝鲜北部和日本。核桃楸种群地理分布区域是温带针阔混交林和阔叶林区域[6]。从分布范围和数量上看，长白山地区和小兴安岭是核桃楸的最适生长区域[7]。

由于开发历史早，原生的阔叶林已不多见，核桃楸种群的数量下降，而且对植物种群的分布格局产生了影响。马万里[8-9]运用多个聚集强度指数对长白山核桃楸林分布格局进行研究表明，核桃楸幼苗、幼树及成株的分布格局均呈现聚集分布，进一步对长白山林区核桃楸种群数量动态变化的研究表明，核桃楸种群表现出衰退型的年龄结构特点，Leslie模型预测显示，核桃楸幼苗数量和种群总数量在今后35年基本呈下降趋势，因此，促进核桃楸的天然更新、加强幼苗幼树的抚育工作对长白山地区核桃楸种群的发展至关重要。林大影等[10]通过对北京雾灵山自然保护区核桃楸群落及其自然生境进行研究，进而分析在核桃楸群落中，不同物种之间及不同物种在主林层、演替层和更新层3个层次上复杂的种间关系，为制定该物种的保护措施提供理论依据。

1.2 生长特性

核桃楸种群是东北植被区阔叶红松混交林群落的主要伴生种群及人工天然复合群落的优良混交组分。综合土壤温度、土壤含水率、林内乔木树种组成以及树木长势来看，胡桃楸适合生长在土壤温度较高、含水率偏低的中坡位[11]。核桃楸起源不同生长规律上差异较大，萌生林高、径生长速生期均为1~15年，人工林高生长速生期为1~25年，径生长速生期为5~35年[12]。根据胡桃楸天然林和人工林优势木的树干解析材料分析，初步确定胡桃楸最佳早期选择龄天然林为15年，人工林为14年的结论[13]。人工林个体幼、成年龄生长变异的相关从不密切趋于密切的转折点为14年，人工林早期测定以胸径为主要选择指标为好[14]。

对辽东山区次生林核桃楸、水曲柳和黄菠萝对生长光环境的适应性研究表明，核桃楸具强阳性树种的特点[15]。王凯等[16]研究光环境对胡桃楸幼苗生长与光合作用的影响表明，在春季，胡桃楸幼苗对光反应不敏感，夏季和秋季随着光强的增加，100%和60%自然光处理的幼苗相对生长率差异不显著，但是随着光强下降，相对生长率显著下降，并提出胡桃楸幼苗对不同的光环境表现出较强的适应性和可塑性，掌握不同光照处理条件下胡桃楸幼苗光合特性的季节变化及其对不同光环境的适应能力，可以为胡桃楸的天然更新和人工林经营管理提供科学参考。

1.3 遗传改良研究

杨书文等[17-18]通过分布在东北地区核桃楸的种源研究，揭示了胡桃楸地理变异规律，将东北地区的胡桃楸划分为4个种源区，即长白山完达山种源区、吉林中部浅山种源区、辽宁东部种源区、小兴安岭松花江地区种源区，确定了舒兰种源为最佳种源。

李广玉等[19]对林口县青山林场胡桃楸1年生苗高生长量调查结果表明，铁力、穆棱和迎春3种源间和种源内部存在丰富的变异，变异系数范围在26.0%~32.7%。张含国等[20]选择黑龙江省林口县青山林场3个种源，122个家系7年生胡桃楸子代林测定研究，种源树高、胸径变异系数分别为32.17%、43.25%，种源间、家系间差异达显著或极显著水平，以3年生树高、胸径为主要指标，从参试的家系中初步选出13个优良家系，树高、胸径分别超过家系均值44.8%、46.9%。褚宪丽等[21]对青山林场15年生胡桃楸3种源45个家系试验林的树高、胸径和分枝情况调查研究，进行种源及家系的变异情况分析，结果表明，总体树高的变异系数为35.60%~45.35%，胸径变异系数为25.58%~28.77%，从45个家系中初步选出9个优良家系，入选率为20%，树高、胸径遗传增益分别为13.33%和17.02%，选择效果非常明显。苏喜廷等[22]开展了胡桃楸无性系种子园建立技术、嫁接技术、散粉规律、不同果型等研究工作，取得了良好效果。提出核桃楸果核类型差异很大，为生产木材而建立无性系种子园，优树选择或营造用材林育苗时，应偏重于长核型；圆核型树木单株适合于建立以生产果仁为主要目的的木本油料经济林。

1.4 育种造林技术研究

核桃楸是中国东北地区主要造林树种和重要工业用材树种，多年来由于大量的采伐和利用，核桃楸种群的数量和质量逐年下降，面积蓄积大幅度减少。为了及时挽救和恢复核桃楸这一珍贵资源，开展了一系列核桃楸育种造林技术方面的研究。

核桃楸一般采用播种育苗，播种前对种子水选选出大而重，种仁饱满，无病虫害的种子，当年11月混沙层积，翌年4月上旬播种，按行距30 cm开沟，沟深5~7 cm，采用点播，覆土3~5 cm。6月中旬追尿素1次，每hm^2用量225 kg，8月下旬追施1次过磷酸钙，每公顷追施300 kg，天气持续干旱时要对苗木适时浇灌。与传统的育种方法比较，生物技术具有独特的优势，能快速繁殖一些优良品种并进行脱毒，张建瑛等[23]认为外植体最佳取材时期为5—6月。胚性愈伤组织诱导的最适培养基为MS+1.0 mg/L 2,4-D+0.5 mg/L 6-BA；体细胞胚的诱导、发育和分化的适宜的培养基为附加蔗糖

(C)1994-2020 China Academic Journal Electronic Publishing House. All rights reserved. http://www.cnki.net

60 g/L、水解酪蛋白700 mg/L MS基本培养基。王彦清等[24]提出取中期胚做试材为宜。KT-2,4-D组合，对诱导体胚发生有显著作用。中期胚接种于H+2.0 mg/L KT+0.3 mg/L 2,4-D+3%蔗糖培养基，体胚发生诱导率可达30%，具有实际应用价值。

赵荣慧等[25]对核桃楸人工林适生条件的研究表明，土层厚度对树高生长的贡献最大，土壤湿度与有机质含量稍次，坡位和坡向因子的偏相关系数较小，在核桃楸人工林生长中居次要地位。而邹学忠[26]的研究表明，在立地因子中，坡向的影响最大，坡形和坡位的影响次之。在坡向因子中，阴坡树木的生长最好，阳坡的最差，半阴半阳坡的居中在坡形因子中凹形坡树末生长最好，线形坡上好于凸形坡的，凸形坡的最差坡位因子中。最适于胡桃楸生长的立地是阴坡的下坡位[27]，不宜在坡地营造，特别是山腹上部，生长不良，28年生，胸径只有5.9~8.3 cm，树高5.6~8.9 m，树矮槛多，干形不良，绝大部分已成为“小老树”[28]。沈培元[29]进行了核桃楸林分立地类型的分类的研究，可以以此为依据预测不同立地类型核桃楸林各个发育时期生育状况。陈永亮等[30-31]研究胡桃楸与落叶松混交的试验证明，胡落混交林对胡桃楸的生长具有明显的促进作用，混交林中胡桃楸的优势木高、胸径和单株材积分别为纯林的133%、187%和471%，单位面积蓄积量是纯林的1.69倍。同时，胡桃楸与落叶松混交林中胡桃楸根际土的有效磷含量相对于胡桃楸纯林显著提高。王政权等[32]采用沙培方法，在温室中研究氮和磷对胡桃楸幼苗根系、叶和当年高生长的影响，结果证实，氮和磷均能影响细根的生长，尤其是磷对提高细根生物量具有明显的作用。

2 开发利用价值

核桃楸的木材质地细韧，色泽淡雅，纹理致密，可经受剧烈震动而不易变形，因此用途广、经济价值高，是难得的军工国防器械用材。木材经打磨后光泽宜人，宜于制作高级包装箱及优质乐器。白波等[33]研究了核桃楸薄木刨切工艺，王喜明等[34]研究了核桃楸小径材干燥工艺的研究，翟冰云[35]观察了胡桃楸木材超微构造，这些研究为核桃楸木材加工与利用，提供了科学依据。

核桃楸的结实时间长，调查表明，在自然状态下，树木成熟龄为150年，寿命可达250年，30~100年结实量多，20年生平均树高可达14.3 m，平均胸径可达13.3 cm[36]。种仁营养丰富，脂肪含量60%~75%，蛋白质近15%~20%，还含有P、Ca、Fe、Zn，胡萝卜素、抗坏血酸等矿质元素、维生素物质，对人体具有良好的保健作用；种子油含肉寇酸、棕榈酸、硬脂酸、油酸、亚油酸等多种物质，为高级食用油。核桃楸是东北地区具有开发前途的优良干果树种，种壳还可作活性炭和观赏工艺品[36-37]。

核桃楸树皮及叶可药用，具有消热解毒抗癌等作用[38]。用胡桃皮、胡桃枝作为抗癌民间验方，历史源远。胡桃楸提取液中的有效成分对体外培养的肿瘤细胞有直接的细胞毒杀作用[39]，Hirakawa等[40-44]从核桃楸皮中分离得到黄酮醇等6种化合物，从鲜皮中分离得到萘醌，从根中分离得到二芳基庚酸等6种化合物，从鲜果中分离得到4种新的α-四氢萘酮对映体，从未熟果中分离得到胃毒活性物。它还是重要的滋补中药，可治慢性阑尾炎、高血压、腰腿酸疼、止咳消肿等。

核桃楸枝干粗壮，颇具阳刚之气；叶长大而美，亦具美人之姿，果缀枝头青绿可人，可与碧桃并提，单植赏叶，丛植赏荫，是东北地区极具观赏价值的乡土树种。同时可与许多针阔叶树种组成混交林，形成复合结构的森林生态系统，对提高整个林分的涵养水源、保持水土、防止环境恶化等有很大意义和作用。

3 核桃楸生产应用中存在的问题和发展方向

胡桃楸作为优良的硬阔叶能源和用材树种在中国栽培利用已有悠久的历史，以其生态效益，经济效益和社会效益显著而被群众所喜欢。但由于开发利用较早，种子库低效，系统化、规模化的生产基地缺乏，资源收集和保护、推广滞后等多项原因使其分布范围及种群数量明显减少，如不及时给予重视，宝贵的胡桃楸基因资源将迅速消失。针对目前现状，今后应重点开展以下研究：(1)优势树种筛选，定向优质壮苗培育技术研究；(2)果材兼用林栽培技术体系研究与示范；(3)高效加工利用配套技术研究。总之，胡桃楸资源具有广阔的开发利用前景，加强对这一资源的综合研究，有利于加快这一多用途树种的产业化和低产低效林分的改造，积极实施规模化生产，向“品种+科研+基地+市场+服务”模式深化，把胡桃楸产业化提升到一个新的水平。

参考文献

[1] 王东娜,牟长城,冯富娟.胡桃楸ISSR-PCR反应体系的建立及优化[J].实验室研究与探索,2010,29(11):18-22.

[2] 褚宪丽,朱航勇,张含国,等.胡桃楸种源家系变异与选择[J].东北林业大学学报,2010,38(11):5-7.

[3] 方升佐.关于加速发展中国生物质能源的思考[J].北京林业管理干部学院学报,2005 2:30-34.

[4] Shao H, Chu L. Resource evaluation of typical energy plants and

(C)1994-2020 China Academic Journal Electronic Publishing House. All rights reserved. http://www.cnki.net

possible functional zone planning in China[J]. Biomass and Bioenergy,2008,32: 283-288.
[5] Fang S Z, Xue J H, Tang L Z. Biomass production and carbon sequestration potential in poplar plantations with different management patterns[J].Journal of Environment Management,2007, 85:672-679.
[6] 马万里,罗菊春,荆涛,等.珍贵树种核桃楸的生态学问题及培育前景[J].内蒙古师范大学学报,2005,34(4):489-492.
[7] 赵光仪,田兴军,吴振河,等.黄波罗、核桃楸、水曲柳分布北限论析[J].东北林业大学学报,1991,19(水胡黄椴专刊):290-295.
[8] 马万里,荆涛,罗菊春,等.长白山林区核桃楸种群分布格局研究[J].内蒙古师范大学学报,2008,37(2):233-236.
[9] 马万里,罗菊春,荆涛,等.长白山林区核桃楸种群数量动态变化的研究[J].植物研究,2007,27(2):249-253.
[10] 林大影,鲜冬娅,邢韶华,等.北京雾灵山自然保护区核桃楸群落的优势种种间联结分析[J].北京林业学学报,2008,3(5):154-158.
[11] 梁淑娟,潘攀,孙志虎.坡位对水曲柳及胡桃楸生长的影响[J].东北林业大学学报,2005,33(3):18-19.
[12] 赵荣慧,胡承海,何福广,等.核桃楸生长规律的研究[J].辽宁林业科技,1985(2)：15-21.
[13] 杨书文,刘桂丰,赵克尊,等.胡桃楸早期选择的初步研究[J].东北林业大学学报,1990,18(S2):77-82.
[14] 孙丽敏,侯旭光.核桃楸的生长变异及其早期测定的研究[J].林业科技通讯,1997(11):12-15.
[15] 孙一荣,朱教君,于立忠,等.不同光强下核桃楸、水曲柳和黄菠萝的光合生理特征[J].林业科学,2009,45(9):29-35.
[16] 王凯,朱教君,于立忠,等.光环境对胡桃楸幼苗生长与光合作用的影响[J].应用生态学报,2010,21(4):821-826.
[17] 杨书文,刘桂丰,张世英,等.胡桃楸地理变异规律及最佳种源的初步选择[J].东北林业大学学报,1990(S2):72-76.
[18] 刘桂丰,杨书文.胡桃楸种源的初步区划及最佳种源选择[J].东北林业大学学报,1991,19(S2):189-195.
[19] 李广玉,张学政,张含国,等.胡桃楸苗木高生长种源家系变异规律初报[J].林业科技,2004,29(4):1-3.
[20] 张含国,邓继峰,张磊,等.胡桃楸种源家系变异规律及家系选择研究[J].西北林学院学报,2011,26(2):91-95.
[21] 褚宪丽,朱航勇,张含国,等.胡桃楸种源家系变异与选择[J].东北林业大学学报,2010,38(11):5-7.
[22] 苏喜廷,姚盛智,陈志诚,等.胡桃楸遗传改良中几项技术的试验研究[J].林业科技,2008,33(1):8-9.
[23] 张建瑛,姜思佳,刑亚娟,等.胡桃楸胚性愈伤组织诱导与体细胞胚胎发生[J].植物研究,2010,30(1):22-26.
[24] 王彦清,吴克贤,张泉. 胡桃楸体细胞胚胎发生的研究[J].林业科技,2000,25(2):8-9.
[25] 赵荣慧,胡承海,敖永长. 核桃楸人工林适生条件的研究[J].沈阳农业大学学报,1986,17(1):10-16.
[26] 邹学忠,阎忠林,朴素梅,等.不同立地条件水曲柳、核桃楸、紫椴造林的研究[J].林业科技通讯,1995(3):16-18.
[27] 马建路.水曲柳、胡桃楸、黄波罗和紫锻适生立地条件的对比研究[J].林业资源管理,1996(4):52-56.
[28] 李谷景.胡桃楸人工林的生长效应[J].吉林林业科技,1980(1): 19-22.
[29] 沈培元.核桃楸林分立地类型的分类[J].辽宁林业科技,1987,159 (3):27-32.
[30] 陈永亮,韩士杰,周玉梅,等.胡桃楸、落叶松纯林及其混交林根际土壤有效磷特性的研究[J].应用生态学报,2007,13(7):790-794.
[31] 郭景然,张侃. 落叶松对胡桃楸幼苗存活及生长的影响[J]. 林业科技情报,2009,41(4):20-21.
[32] 王政权,张彦东,王庆成,等.氮、磷对胡桃楸幼苗根系生长的影响[J].东北林业大学学报,1999,27(1):1-4.
[33] 白波,高岩.核桃楸薄木刨切工艺[J].林业机械,1995(2):28-29.
[34] 王喜明,吴永泰.核桃楸小径材干燥工艺的研究[J].内蒙古林业科技,1990(2):43-46.
[35] 翟冰云,李舫,曹雪艳.胡桃楸木材超微构造观察[J].林业科技,1995, 20(2):59-60.
[36] 刘文华.核桃楸的利用和苗木培育[J].中国林副特产,2007,86(1): 44-45.
[37] 祁爽,祁国金.核桃楸早实丰产栽培技术[J]. 中国林副特产,99(2): 42-43.
[38] Kim S H, Lee K S, Son J K, et al. Cytotoxoe compounds from the roots of *Julans mandshuroca*[J].J Nat prod,1998,61(5):643-645.
[39] 王春玲,包永明.胡桃楸的抗肿瘤活性研究[J].中草药,2003,25(8): 22-24.
[40] Hirakawa K, Ogiue E, Motoyoshiya J, et al. Naphthoquinones from Juglandaceae[J].Phytochemistry, 1986,25(6): 1494-1495.
[41] Lee E B, Hyun J N, Li D W, et al. Gastroprotective activity of the unripe fruit extract of *Juglans mandshurica* in rats[J].Natural Product Sciences, 2001,7(3): 87-89.
[42] Li G, Xu M L, Choi H G, et al. Fournew diarylheptanoids from the roots of *Juglans mandshurica*[J].Chemical & Pharmaceutical Bulletin, 2003,51(3): 262-264.
[43] Machida K, Matsuoka E, Kasahara T, et al. Studies on the constituents of Juglans species. I. Structural determination of (4S)-and (4R)-4-hydroxy-al-pha-tetralone derivatives from the fruit of *Juglansmandshurica* Maxim. var. *sieboldiana* Makino[J]. Chemical & Pharmaceutical Bulletin, 2005,53(8): 934-93.
[44] Min B S, Lee S Y, Kim J H, et al. Anti-complement activity of constituents from the stem-bark of *Juglans mandshurica*[J]. Biological & Pharmaceutical Bulletin, 2003,26(7): 1042-1044.

(C)1994-2020 China Academic Journal Electronic Publishing House. All rights reserved. http://www.cnki.net
