# Supplementary material for: Provenance and family variations in early growth of Manchurian walnut (Juglans mandshurica Maxim.) and selection of superior families
Source: PLoS One. 2024 Mar 7;19(3):e0298918. doi: 10.1371/journal.pone.0298918 (PMC10919699; doi:10.1371/journal.pone.0298918)
Supplement: S2 File — (ZIP) [file pone.0298918.s005.zip › The researches on seedling characters difference of Fokienia hodginsii from different provenances and excellent provenance selection.pdf]

分类号：S722　　　　　　　　　　　　　　　　　　　　单位代码：10389
密　级：　　　　　　　　　　　　　　　　　　　　　　学　　号：3130422002

福建农林大学硕士专业学位论文

不同种源福建柏苗期性状差异性研究及优良种源选择

学位类别：林业硕士专业学位

专业领域：

研究方向：森林培育

学生姓名：陈澜

指导教师：郑郁善　教授

完成时间：二〇一五年四月

中国知网　https://www.cnki.net

分类号：S722　　　　　　　　　　　　　　　　　　　　单位代码：10389
密　级：　　　　　　　　　　　　　　　　　　　　　　学　　号：3130422002

福建农林大学硕士专业学位论文

不同种源福建柏苗期性状差异性研究及优良种源选择

学位类别：林业硕士专业学位

专业领域：

研究方向：森林培育

学生姓名：陈澜

指导教师：郑郁善　教授

完成时间：二〇一五年四月

中国知网　https://www.cnki.net

The Thesis of FAFU's Professional Master Student

The researches on seedling characters difference of *Fokienia hodginsii* from different provenances and excellent provenance selection

Degree category: Master of forestry

Professional field:

Research area: Silciculture

Student name: Chen Lan

Supervisor: Prof. Zheng Yushan

Submitted time:April, 2014

Fujian Agriculture and Forestry University(FAFU)

中国知网 https://www.cnki.net

中国知网 https://www.cnki.net

目 录

摘要 I
关键词 II
Abstract III
Keywords V
1. 前言 1
1.1 福建柏种源试验综述 1
1.1.1 福建柏种源种子品质差异试验 1
1.1.2 福建柏种源苗期试验 1
1.1.2.1 幼苗生长性状和生物量 1
1.1.2.2 幼苗生长节律 2
1.1.2.3 幼苗根系性状 2
1.1.3 福建柏的种源幼林期试验 3
1.1.3.1 生长性状 3
1.1.3.2 地理变异规律 3
1.1.4 开花结实 4
1.2 近年来其他植物种源试验研究情况 4
1.2.1 生长节律、生长模型拟合 4
1.2.2 光合特性 5
1.2.3 胁迫下的研究 5
1.2.4 其他方面的研究 5
1.3 小结 5
2.试验地概况、研究内容、材料和方法 8
2.1 试验地概况 8
2.2 研究内容 8
2.2.1 福建柏各种源球果和种子性状比较试验 8
2.2.2 福建柏地理种源苗期试验 8
2.3 试验材料 8
2.4 研究方法 9
2.4.1 福建柏各种源球果和种子性状比较试验 9
2.4.1.1 球果和种子指标测量方法 9
2.4.1.2 球果处理 9
2.4.2 福建柏地理种源苗期试验 9
2.4.2.1 福建柏种子发芽试验方法 9
2.4.2.2 试验设计和苗期管理 10
2.4.2.3 福建柏各种源苗生长节律试验方法 10
2.4.2.4 福建柏各种源苗高地径和生物量指标试验方法 10
2.4.2.5 福建柏各种源苗根系指标试验方法 10
2.4.2.6 福建柏各种源苗叶绿素含量测量方法 10
2.4.2.7 福建柏各种源苗可溶性糖含量测量方法 10
2.4.2.8 福建柏各种源苗可溶性蛋白含量测量方法 11
2.4.2.9 福建柏各种源苗硝酸还原酶活性测定方法 11
2.4.2.10 统计方法 11
3.结果与分析 11

中国知网 https://www.cnki.net

3.1 福建柏各种源球果和种子性状比较试验结果分析......11
3.1.1 不同种源福建柏球果性状比较分析......12
3.1.2 不同种源福建柏种子千粒重比较分析......13
3.1.3 不同种源福建柏球果和种子性状间相关性分析......14
3.2 福建柏地理种源苗期试验结果分析......15
3.2.1 不同种源福建柏发芽情况比较分析......15
3.2.2 不同种源福建柏生长节律比较分析......16
3.2.2.1 苗高地径生长趋势变化......16
3.2.2.2 苗高地径 Logistic 方程拟合结果分析......20
3.2.3 不同种源福建柏苗高地径和生物量比较分析......22
3.2.3.1 苗高地径差异分析......22
3.2.3.2 生物量差异分析......23
3.2.3.3 苗高地径和生物量相关性分析......26
3.2.4 不同种源福建柏根系性状比较分析......27
3.2.4.1 根系性状差异分析......27
3.2.4.2 根系性状间相关性分析......28
3.2.5 不同种源福建柏生理指标比较......29
3.2.5.1 生理指标差异分析......29
3.2.5.2 叶绿素含量比较分析......30
3.2.6 福建柏地理种源变异规律......31
3.2.7 福建柏种源性状聚类分析......33
3.2.8 福建柏优良种源选择......34
3.2.8.1 各性状的主成分分析......34
3.2.8.2 各种源的综合得分......36
4.结论与讨论......37
4.1 结论......37
4.1.1 不同种源福建柏球果和种子性状比较......37
4.1.2 不同种源福建柏发芽情况比较......38
4.1.3 不同种源福建柏生长节律比较......38
4.1.4 不同种源福建柏苗高地径和生物量比较......38
4.1.5 不同种源福建柏根系性状比较......39
4.1.6 不同种源福建柏生理指标比较......39
4.1.7 福建柏地理种源变异规律......39
4.1.8 福建柏种源性状聚类分析......39
4.1.9 福建柏优良种源选择......39
4.2 讨论......40
4.2.1 球果和种子指标和生长指标......40
4.2.2 生长曲线的拟合......40
4.2.3 福建柏苗期地理种源变异规律差异......40
4.2.4 各指标高低和综合得分的差异......40
4.2.5 最优种源和根系性状与生理性状的关系......41
4.2.6 苗期试验种源评价选择指标......41
参考文献......42
附　录......46
致　谢......48

中国知网 https://www.cnki.net

不同种源福建柏苗期性状差异性研究及优良种源选择

摘 要

福建柏[*Fokienia hodgirtsii*（Dunn）Henry et Thomas]是国家第一批珍稀濒危二级保护植物，珍贵用材树种，是继杉木后又一值得推广的优良树种。本试验播种材料选自福建、广东、广西 3 省 17 个种源地的福建柏种子，采用方差分析，多重比较，相关性分析，Logistic 曲线方程拟合，主成分分析几种数据处理方式对不同种源一年生福建柏苗苗期性状差异性进行研究比较，选出各方面性状皆兼备的优良种源，旨在为福建柏的幼林期试验提供实践指导和理论支持，也为今后进一步开展福建柏优良种源选择和育苗工作奠定良好基础。主要研究结果如下：

1.福建柏种源间球果性状差异达到极显著水平，经多重比较，其中广东韶关曲江种源的球果长、宽、重各项指标与其他种源相比差异最大，在 0.01 水平下最大达到 F；球果长、宽、长×宽、长/宽、重各指标表现最佳的是福建龙岩长汀种源，分别达到 1.94cm、1.87cm、3.63、1.04 和 3.63g。

2. 福建柏种源间种子性状差异达到极显著水平，经多重比较，广西柳州柳北种源的种子千粒重与其他种源相比差异较大，在 0.01 水平下达到 F；种子千粒重最大为广西来宾金秀种源，达到 0.9506g；福建龙岩长汀种源种子千粒重种内变化较大，变异系数达 22.26%。

3.球果和种子性状相关性分析结果显示：球果和种子各性状间总体上显著相关，相关系数最大达到 0.956，球果重随球果长和宽的增大而增大，而千粒重则呈现相反规律。

4.对福建柏各种源种子进行催芽试验后得到不同种源种子发芽率存在很大差异的结论。福建龙岩的两个种源和福建泉州的两个种源发芽率相对较高，均超过 40%，其中福建泉州安溪种源和福建泉州永春种源超过 50%，发芽率最高的是福建泉州安溪种源，达到 59.22%。

5.对不同种源福建柏生长节律的比较研究结果显示：苗高生长基本呈现“慢-快-慢-快-慢”变化趋势，4-5 月份出现第一次生长小高峰，7-8 月到达第二次高峰，10 月后开始缓慢；地径生长总体呈现“慢-快-慢”趋势，各种源生长 6 月前规律不大相同，7-8 月出现一次生长高峰，8 月后进入缓慢生长。用 Logistic 曲线方程对苗高地径的拟合结果表明福建柏苗高地径生长过程拟合并不适用该方程。

6.福建柏种源间苗高地径中仅苗高呈现显著差异，苗高最大为广西柳州柳北种源，达到25.06cm，地径最大为福建福州永泰种源，达到2.85mm；苗高和地径变异系数变动幅度很相近，广东韶关曲江种源苗高地径变异最大，苗高地径的变异系数分别达到20.99%和29.90%，说明其种内生长最不平均。

7. 福建柏种源间生物量总体上呈现显著差异，广西柳州柳北种源的生物量积累情况与其他种源相比差异较大；生物量积累最大的为广西柳州柳北种源，总鲜重达到6.18g，总干重达到2.23g，地上鲜重达到5.58g，地上干重达到1.94g，地下鲜重达到0.61g，地下干重达到0.29g。

8. 生物量相关性分析结果表明福建柏种源苗生物量在地上地下部分空间分配上呈现高度相关性，相关系数最大达到0.996，可通过测定某部分生物量推测出其他部分的生物量情况。

中国知网 https://www.cnki.net

9.福建三明莘口种源苗期生物量在地下积累最大，地下鲜重/地上鲜重和地下干重/地上干重分别达到0.192和0.273；地上部分含水量最高为福建泉州永春种源，干鲜比为0.306；地下部分含水量最高为福建莆田仙游种源，干鲜比为0.460；整株含水量最高为福建宁德古田种源，干鲜比为0.328。福建泉州永春种源在种源内地上地下部分生物量分配和干鲜比差异很大，变异系数达到61.52%。

10.福建柏根系性状方面，不同种源间根系性状差异均达到极显著水平，根长最大达177.09cm，根表面积最大达34.42 cm^2，平均根系直径最大达0.68mm，根体积最大达0.54 cm^3，根尖数达159，其中福建三明莘口种源的根长、根表面积和根体积指标均为最大，其根系情况较其他种源更好；不同种源根系性状间相关性很大，相关系数最大达0.975，在根系分析中，可以通过一种指标推测出其他指标的情况。

11.在叶绿素、可溶性糖、可溶性蛋白、硝酸还原酶活性四种生理指标中，仅种源间叶绿素差异达到显著水平，其中叶绿素a含量、叶绿素b含量和叶绿素总含量最高的均为福建三明莘口种源，分别达到1181.74$mg \cdot g^{-1}$，435.03$mg \cdot g^{-1}$，1616.77$mg \cdot g^{-1}$，且不同种源间种内变异幅度很大。

12.结合福建柏各性状和各种源地地理气候因子进行相关性分析，结果显示：最大相关系数为0.688，球果长和球果重都随经度增大而增大；生物量积累量随年无霜期的加长而增大，苗高和总干重随海拔的上升而减小，可溶性糖含量随经纬度的增大而升高。

13.对福建柏种源四种性状进行聚类分析，得到以下结果：球果和种子性状方面，评价最高的一类包括福建省龙岩、福州、莆田、泉州几地共计 9 个种源；苗高地径和生物量情况方面，评价最高的是广西柳州柳北种源；根系性状方面，评价最高的一类包括广东省、福建省龙岩、福州、南平、莆田、泉州、漳州和三明几地共计 15 个种源；生理指标含量方面，评价最高的是福建福州罗源种源。

14. 福建柏优良种源选择的结果表明：利用主成分分析法提取涵盖所有性状 88.778%的信息的前 6 个主成分，最高分 207.9150，最低分 95.9367，相差 111.9783 分，选出前 5 名优良种源分别是：福建三明莘口、广西柳州柳北、福建泉州安溪、福建三明尤溪、广东韶关曲江，排名比较靠后的几个种源是福建福州闽侯、福建莆田仙游、福建龙岩上杭、福建泉州永春、广西来宾金秀。

关键词：福建柏；球果与种子性状；苗期试验；优良种源选择

中国知网 https://www.cnki.net

The researches on seedling characters difference of *Fokienia hodginsii* from different provenances and excellent provenance selection

Abstract

Fokienia hodgirtsii is the first batch of rare and endangered national secondary protected plants, precious timber tree species, and a fine tree species to be promoted after *Cunninghamia lanceolata*. The experiment seeding material is *Fokienia hodgirtsii* seed selected from 17 provenances of Fujian, Guangdong, Guangxi provinces. The experiment use variance analysis, multiple comparison, correlation analysis, Logistic curve equation fitting of several data processing methods to study and compare seedling traits differences of *Fokienia hodgirtsii* annual seedling from different provenances, and select the traits are all aspects of both the excellent provenance. Aimed at providing practical guidance and theoretical support for young period test, also for lay a good foundation for the future further *Fokienia hodgirtsii* excellent provenance selection and nursery work. The main results are as follows:

1.The *Fokienia hodgirtsii* cone traits from different provenances achieve significant difference, length, width, weight on cones of Guangdong Shaoguan Qujiang provenance has a big difference with other provenance by means of multiple comparisons, maximum reach F under the 0.01 level; The cone indexes in terms of length, width, length×width, length/width, weight of Fujian Longyan Changting provenance is the best in all provenances, respectively reach 1.94cm, 1.87cm, 3.63, 1.04 and 3.63g.

2.The *Fokienia hodgirtsii* seed traits from different provenances achieve significant difference, Guangxi Liuzhou Liubei pronenance has a big difference with other provenances by multiple comparisons, maximum reach F under the 0.01 level; the weight of 1000-seeds of Guangxi Laibin Jinxiu provenance is the best in all provenances, reach 0.9506g; the weight of 1000-seeds of Fujian Longyan Changting has relatively large change within provenance, CV reach 22.26%.

3.The correlation analysis results of *Fokienia hodgirtsii* cone and seed traits show that: As a whole, there is significant correlation between cone and seed traits, the coefficient of association reach 0.956, the weight of cone increases with length of cone increasing, while the weight of 1000-seeds presents opposite rule.

4. *Fokienia hodgirtsii* seed accelerating germination test come to a conclusion—there are considerable differences in seed germination rate from different provenances. Germination rate of two provenances from Fujian Longyan and two provenances from Fujian Quanzhou are relatively high, also exceed 40%, Fujian Quanzhou Anxi provenance and Fujian Quanzhou Yongchun provenance also exceed 50%, the germination rate of Fujian Quanzhou Anxi provenance is the highest in all provenances, reach 59.22%.

中国知网 https://www.cnki.net

5. The comparative research results of *Fokienia hodgirtsii* growth rhythm show that: Seedling height growth basic presents a trend of "slow-fast-slow-fast-slow", appeared the first small peak from April to May, reached the second peak from July to August, began to slowly after October; diameter growth basic presents a trend of "slow-fast-slow", each provenance growth rhythm is quite different before June, appeared the first small peak from July to August, began to slowly after August. The results using Logistic curve equation to fit seedling height and diameter show that this equation is not suitable for fitting *Fokienia hodgirtsii* seedling height and diameter.

6. Only seedling height of *Fokienia hodgirtsii* from different provenances present significantly different in height and diameter. The highest seedling height is Guangxi Liuzhou Liubei provenance, reach 25.06cm, the largest seedling diameter is Fujian Fuzhou Yongtai provenance, reach 2.85mm, the CV of height and diameter amount of variation is very close. The height and diameter variation of Guangdong Shanguan Qujiang provenance is the largest, the CV of height and diameter respectively 20.99% and 29.90%, it show that growth within provenance is overbalance.

7. As a whole, biomass of *Fokienia hodgirtsii* from different provenances present significantly different, biomass accumulation of Guangxi Liuzhou Liubei provenance has a big difference with other provenances. the most biomass accumulation is Guangxi Liuzhou Liubei provenance, the total fresh weight reach 6.18g, the total dry weight reach 2.23g, the fresh weight on the ground reach 5.58g, the dry weight on the ground reach 1.94g, the fresh weight under the ground reach 0.61g, the dry weight under the ground reach 0.29g.

8. The results of biomass correlation show that biomass space allocation of *Fokienia hodginsii* provenance present high correlation on the ground and under the ground, the coefficient of association reach 0.996, so it is believed that we can speculate other part of biomass by measuring a part of biomass.

9. The seedling most biomass accumulation of Fujian Sanming Xinkou provenance is under the ground, the fresh weight under the ground/the fresh weight on the ground and the dry weight under the ground/the dry weight on the ground respectively reach 0.192 and 0.273; The most moisture content on the ground is Fujian Quanzhou Yongchun provenance, the dry-fresh radio reach 0.306; the most moisture content under the ground is Fujian Putian Xianyou provenance, the dry-fresh radio reach 0.460; the most moisture content of whole plant is Fujian Ningde Gutian provenance, the dry-fresh radio reach 0.328. The biomass allocation on the ground and under the ground and dry-fresh radio of Fujian Quanzhou Yongchun provenance has a big difference within provenance, the largest CV reach 61.52%.

10.In terms of *Fokienia hodgirtsii* root traits, the maximum root length reach 177.09cm, the maximum

中国知网 https://www.cnki.net

root superficial area reach 34.42cm^2, the maximum root average diameter reach 0.68mm, the maximum root volume reach 0.54cm^3, the maximum root tips reach 159, the the root length and root superficial area and root volume of Fujian Sanming Xinkou both are the maximum, it's root condition is better than other provenance. The correlation between root traits from different provenance is great, the coefficient of association reach 0.975, it can speculate other indexes by one index in analysis of root system.

11. In four physiological indexes—the chlorophyll, soluble sugar, soluble protein, nitrate reductase activity, only difference of chlorophyll between provenance reached significant level in physical signs, of which the highest content of chlorophyll a and chlorophyll b and total chlorophyll both are Fujian Sanming Xinkou provenance, respectively reach 1181.74$mg{\cdot}g^{-1}$, 435.03$mg{\cdot}g^{-1}$, 1616.77$mg{\cdot}g^{-1}$, moreover, the amount of variation within provenance between different provenance is great.

12. The results of correlation analysis in combination with *Fokienia hodgirtsii* every traits and geographical climate factors show that: the maximum coefficient of association reach 0.688, the length of cone and weight of cone both increase with longitude increasing; biomass accumulation increases with annual frost-free season lengthening, seedling height and total dry weight decrease with altitude rising, the content of soluble sugar increases with the increase of latitude and longitude.

13. The results of cluster analysis of *Fokienia hodgirtsii* provenance four traits show that: In terms of phenotypic traits on seeds and cones, the highest evaluation includes some places of longyan, fuzhou, putian, quanzhou in Fujian province to sum to 9 provenances; in terms of seedling height and diameter and biomass condition, the highest evaluation is Guangxi Liuzhou Liubei provenance; in terms of root traits, the highest evaluation includes some places of Guangdong province, longyan, fuzhou, nanping, putian, quanzhou, zhangzhou and sanming in Fujian province to sum to 15 provenances; in terms of physical signs, the highest evaluation is Fujian Fuzhou Luoyuan provenance.

14. The results of *Fokienia hodgirtsii* excellent provenance selection show that: Principal component analysis is utilized to extract the first 6 principal components to cover all traits 88.778% of the information, the highest score is 207.9150, the lowest score is 95.9367, differ 111.9783, and select the top five excellent provenance respectively is Fujian Sanming Xinkou, Guangxi Liuzhou Liubei, Fujian Quanzhou Anxi, Fujian Sanming Youxi, Guangdong Shaoguan Qujiang, a few poorly provenance are Fujian Fuzhou Minhou, Fujian Putian Xianyou, Fujian Longyan Shanghang, Fujian Quanzhou Yongchun, Guangxi Laibin Jinxiu.

Keywords: *Fokienia hodgirtsii*; Cone and seed traits; Seedling test; Excellent provenance selection

中国知网 https://www.cnki.net

1. 前言

1.1 福建柏种源试验综述

福建柏[*Fokienia hodgirtsii*（Dunn）Henry et Thomas]，别名建柏、滇柏、杜柴等，国家第一批珍稀濒危二级保护植物[1-6]，柏科福建柏属的唯一植物，为中国特有的单属种植物，人们称之为福建的"植物名片"[7]。福建柏是我国珍贵的用材树种，近年来，南方许多省营造杉木林带来的杉天牛、杉梢卷叶蛾等病虫害蔓延，已经严重威胁到森林生态系统的生产力和稳定性，试验表明福建柏的各项木材物理性质都优于杉木，是杉木二代更新值得推广的优良树种[8-12]。福建柏在我国分布范围广泛，但是野生种群个体数量少，主要以混交林形式存在，近年来由于对福建柏资源不合理的开发利用，使福建柏残存林分和珍稀古树越发罕见，福建柏资源日渐枯竭，岌岌可危，因此对这一优质用材资源的保护十分紧迫[12-14]。

种源试验是林木育种工作的重要前提和有力基础。由于大部分树种都普遍存在地理变异，不同种源的种子形成的人工林在生长和稳定性上都存在很大差异，需在种源选择的基础上进行单株选择和林分选择。福建柏是国家"十一五"科技攻关项目推广的重要造林树种之一，福建柏种源试验为今后进一步开展福建柏优良种源选择和育苗工作奠定了良好基础，同时对保护福建柏这一珍稀树种的优良种质基因资源也具有重要意义。关于福建柏在种源方面的研究成果主要来自国内，下文主要对近年来在福建柏种源试验上取得的丰硕成果进行阐述和讨论。

1.1.1 福建柏种源种子品质差异试验

种子品质研究是苗期试验的基础，种子品质对苗期生长和造林培育工作的开展具有重要意义。曾志光等[15]和李晓储等[16]在对福建柏种源试验种子室内品质测定和方差分析表明，不同种源之间发芽率、千粒重等都存在极显著差异，其中福建德化种源表现最好。发芽率和千粒重只是种子品质的一小方面，不同种源福建柏的球果表型性状，如球果外形、长宽比、重量乃至球果开裂后的苞鳞数等也许都存在显著差异，还有福建柏种子是否含有某些特殊成分也值得研究和开发。

1.1.2 福建柏种源苗期试验

1.1.2.1 幼苗生长性状和生物量

福建柏是用材树种，树干力学性质本应是最合适反映其生长好坏的指标，但由于在幼苗时期茎部生长不完全，不具备测量力学性质的条件，幼苗生长性状和生物量情况就是最能直接反映苗木生长状况的指标。幼苗生长性状和生物量情况分析主要通过方差分析、相关性分析、遗传参数估计、多重比较和聚类分析等统计方法得到结果[16-25]。杨宗武等[17-18]、郑仁华等[19]在对全国6个省福建柏种源的苗期生长性状和生物量情况研究表明苗高、地径、总鲜重、总干重、地上部分干（鲜）重、地下部分干（鲜）重、地上部分干（鲜）重/地下部分干（鲜）重、侧枝数、主根长、侧根数等指标在种源间基本都存在显著或极显著差异，且差异主要来自遗传因素的制约，与环境关系不大；各种源苗木地上部分干（鲜）重、地下部分干（鲜）重、地上部分干（鲜）重/地下部分干（鲜）重等指

中国知网 https://www.cnki.net

标显著相关，说明生物量在各器官、地上和地下空间结构的分配上具有高度相关性，这与廖纯茂等[20]关于各生物量指标差异显著的结论相同。郑仁华等[21]对福建省和湖南省福建柏优树子代苗期性状遗传变异的研究表明种内遗传变异丰富，且苗高较地径更适合作为苗期选择的依据。侯伯鑫等[22]、李振军等[23]在不同地理种源试验基础上对优树家系苗期生长性状和生物量各指标的分析研究表明，种内各性状差异显著，且秋稍高可作为苗期选择的依据。

杨宗武等[18]在对苗高、地径遗传变异研究中发现苗高各系数均高于地径，说明苗高受遗传因素影响大于地径，以苗高作为选择依据比地径更有说服力。侯伯鑫等[22]认为在进行种源间差异性分析时，由于种源间极显著差异会掩盖有些种源间不显著差异，因此单靠方差分析不能说明种源两两之间差异显著，需进行多重比较。张新华[24]研究了 14 个生长和生物量指标，发现侧枝数和干鲜重两个指标遗传力较其他 12 个指标低，曾志光等[25]发现各种源苗高与主根长、侧根数与侧枝数呈正相关，鲜重分别与苗高和地径存在相关。李晓储等[16] 把 2 年生福建柏苗和杉木苗进行对比后发现两者地上部分的生物量相近，但是地上部分/地下部分较杉木苗大。林峰[26]也得出了类似结论。在生长性状和生物量研究上，大部分集中于苗高地径和干鲜重的宏观比较，指标较单一，可以进行一些分子水平上的微观研究，另外统计分析方法还有待进一步多样化。

1.1.2.2 幼苗生长节律

苗木生长节律可以直观看出苗高地径随时间变化的规律，从而为不同生长周期应采取何种育苗措施提供参考。李振军等[23]研究表明，各种源苗的苗高生长大致趋势都是从 8 月份开始加快，10 月以后逐渐减慢；8-10 月间各种源苗苗高差异明显，尤其表现优良的家系或种源差异更明显；在地径生长上，不同种源差异显著，5-8 月是差异最显著时期。曾志光等[25]和李晓储等[16]发现各种源福建柏年苗高生长出现 2 次高峰，基本于 5-7 月和 9-10 月出现，8 月是高温季节,所以生长趋于迟缓。

侯伯鑫等[22]对福建柏种源生长节律研究中发现了一个生长规律：在生长过程中，各种源表现差异很大，幼苗总体表现和各月表现基本呈正相关，且这些差异和种源本身的遗传特性有关。侯伯鑫等[22]和廖纯茂等[20]研究表明，优秀种源的秋梢都主梢明显且长而粗壮，秋梢高可以作为种源选择的重要依据之一，另外，廖纯茂等[20]发现 8 月下旬的径粗生长量可作为种源苗期选择的另一重要依据。郑仁华等[21]发现不同时期苗高的表型相关系数和遗传相关系数达极显著水平，也验证了杨宗武等[18]苗高各系数高于地径的结论。目前关于生长节律方面的研究还比较简单，基本都是通过绘制折线图看生长走向得出生长趋势，统计分析方法比较单一，可借鉴其他植物种源试验在生长节律方面采用的类似 Logistic 拟合方程的分析方法，以期得到一些新结论。

1.1.2.3 幼苗根系性状

在植物的生长发育过程中，根系吸收土壤水分和营养物质的能力对植物生长发育产生很大影响，对根系性状和根系遗传特性的研究对后期优良种源的选择、造林培育和提高林分生产力很有意义。福建柏主根不明显，侧根较发达，属浅根性树种，李晓储等[16]研究发现，2 年生福建柏留床苗平均

中国知网 https://www.cnki.net

根系深 32.67cm，在相同育苗方式下培育的 2 年生杉木苗平均根深 21.67cm，可见福建柏苗根系分布深度还是明显超过杉木苗，这与杨宗武等[9]得出的在板结粘重的土壤中，福建柏根系穿透能力大大超过杉木根系的结论大致相符。杨宗武等[18]认为采用容器育苗和大田育苗会对根系生长造成不同的影响；同是大田育苗的情况下，在比较遮阴，土壤疏松，苗期管理较好的圃地中生长的苗，根茎较粗且侧根数多；而采用容器育苗，苗木根系被大量束缚在有限空间里，也萌生出许多侧根。现在福建柏种源试验中大多选择根深和根数作为根系性状的指标，对根系遗传特性方面的研究甚少，可以使用一些专门的根系分析仪器测量一些更深入的指标，或进行微观分析，对根系性状进行更全面的研究。

1.1.3 福建柏的种源幼林期试验

种源苗期试验只是种源试验的初级阶段，在时间允许的情况下将前期培育出的种源苗进行幼林期造林试验才是完整的种源试验，才具生产实践意义。苗期试验和幼林期试验具有一定联系，侯伯鑫等[27-28]在进行幼林期试验时发现苗期生长良好的种源苗在幼林期一般也表现良好，少数不符合的也许和后天的环境管理及栽培措施有关。

1.1.3.1 生长性状

幼林期试验主要以树高、胸径、材积等生长性状作为分析评价的指标，以木材速生丰产为目的的用材林还常选择材积遗传增益作为综合评价指标。侯伯鑫等[27-29]进行福建柏地理种源幼林期试验时发现不同种源树高、胸径、材积均存在受遗传因素影响的显著差异，其中胸径差异更大，说明胸径较树高更适合作为选择依据；3 种地理条件下（山区、半山区、丘陵区），种源×地点交互作用显著，3 个生长性状在山区的表现优于在半山区和丘陵区的表现。余格非等[30]参照福建柏优树国家标准，在优良种源和家系林分中，选择出许多优树。林峰等[31]对 12 个地理种源 4 年生的幼林的多年相关性检验结果表明：2 年生、3 年生、4 年生的种源林生长性状均高度相关且相关系数呈增大趋势。郑仁华等[32]对 5 年生种源林的调查发现这些生长性状在福建柏种内存在丰富遗传变异，且具有中等以上的遗传力。虽然树高胸径是反映幼林期树木生长好坏最直接的指标，但作为用材树种，木材的物理力学性质也从另一方面体现了生长的质量差异，可以将树高胸径和木材物理力学性质结合分析，或许更具实用性。

1.1.3.2 地理变异规律

研究各性状和地理气候因子的相关性有助于发现种源地理变异规律，为苗木选择具有最适地理气候条件的造林地提供帮助。侯伯鑫等[28]发现种源幼林期生物量随着纬度增大、年最低温度降低、年无霜期天数减少相应下降，树高、胸径 2 个性状呈现出一种以纬向渐变为主的地理变异规律。这与杨宗武等[15]关于福建柏种源苗期生长性状地理变异规律的研究结果相同。但林峰等[31]发现在所有因子中，仅地径和年平均温极显著相关，与海拔和年日照时数显著相关；而郑仁华等[32]则认为主要生

中国知网 https://www.cnki.net

长性状和地理气候因子并不相关，均未达到显著水平。可见在福建柏种源地理气候变异上得到的结论众说纷纭，还有待进一步研究。

1.1.4 开花结实

福建柏是雌雄同株的单性花树种。目前关于研究福建柏地理种源开花结实的试验较少，侯伯鑫等[33]在 3 种造林地的开花结实试验表明秋花期、球果成熟期、种子散落期 3 个指标山区比半山区早，半山区比丘陵区早，高海拔地区比低海拔地区早；地点和海拔高度的不同，会造成种源开花结实的物候期不同；方差分析表明果实和种子的主要指标差异显著或极显著。可见不同种源花期及果期变异与适生区的地点、海拔、温度等地理气候因子紧密相关；侯伯鑫等[33]还认为福建柏一年两次花期，有效花期在秋季，这与其他文献报道福建柏花期在晚春（3～4 月）有所差异，还有待今后进一步验证[4]、[6]、[13]、[34]。

1.2 近年来其他植物种源试验研究情况

种源试验的历史可以追溯到大约 1745 年，法国人 H.L.杜蒙索首次进行了不同来源欧洲赤松的种子栽培试验。1821 年，法国人德维尔莫兰在巴黎同一块土地上进行了不同种源欧洲赤松后代在冠形、干形、树皮、分枝、针叶、芽、球果等方面差异的研究。此后，种源试验多以国际合作的形式得到了快速发展。例如，国际林业研究组织联盟在 1907 年组织了第 1 次欧洲松国际种源试验，31 年后又组织了第二次欧洲赤松和欧洲云杉的国家协作试验。刚开始的种源试验研究内容主要是揭示地理变异的存在；三四十年代重点在于揭示地理变异规律；到八九十年代，则开始强调种源选择和林木育种需要的结合。中国种源试验到 50 年代后期才开展起来，研究树种主要有马尾松、杉木、湿地松、白榆、柚木等。

1.2.1 生长节律、生长模型拟合

近 5-6 年来，国内其他植物在苗期种源试验研究中取得了一系列成果，首先在研究生长节律、生长曲线拟合方面，大部分人选择使用 Logistic 曲线方程对不同种源苗高、地径生长过程进行拟合，得到显著效果。杨志玲等[35]对不同种源厚朴苗期生长模型进行拟合，发现苗高地径的生长规律，并通过计算测得物候期与生长特征参数，得到它们之间的线性相关关系，从而得到应该在幼苗线性生长期加强水肥管理的结论。周正立等[36]对柠条锦鸡儿、小叶锦鸡儿不同种源苗高与地径生长过程进行拟合，将生长时期划分为生长初期、速生期、硬化期 3 个时期。李爱萍[37]通过对定植于库布齐沙漠东北缘的 7 个樱桃圆柏种源的苗高生长与物候节律连续 3 年的观测，得出种源间苗高、春季萌动率、停止生长率和叶变色率等物候期存在差异的结论。黄志玲等[38]将红锥种源苗期生长期划分为生长初期、速生前期、速生期、生长后期 4 个时期。王旭军等[39]和李秋荔等[40]分别模拟出了红榉种源和中国马褂木种源苗期“S”型生长曲线，并对苗期生长期进行了划分。叶金山等[41]还结合红楠种源苗期生长节律和生物量差异，选出了表现最好的九连山种源。

中国知网 https://www.cnki.net

1.2.2 光合特性

光合特性测量的指标主要包括光合速率、气孔导度、净光合速率、蒸腾速率、光饱和点、光补偿点、胞间 CO_2 浓度、水分利用效率等，赵勋等[42]、惠利省等[43]、杨万霞[44]、赵勋[45]等人通过测量这些指标，分别对不同种源白花树、马褂木、青钱柳、越南安息香的光响应特性和光合特性差异进行分析，发现了种源间一系列光合特性的差异性。

1.2.3 胁迫下的研究

在胁迫条件下的种源试验，不同于平时在正常水肥条件下进行的种源试验，它通过设计一种或几种极端的环境条件下的种源试验，最坏情况充分考虑在内，对现实生产更具意义。刘鹏[46]对干旱胁迫下不同种源喜树的生理生化特性进行研究，得出干旱胁迫限制喜树生长，影响喜树苗期苗高和生物量积累和分配的结论；金雅琴[47]对乌桕实生苗进行干旱胁迫的种源试验后发现 SOD 活性、POD 活性、MDA 含量等生理生化指标均存在显著差异。

1.2.4 其他方面的研究

在其他植物的苗期种源试验上，先后有杜超群等[48]对不同种源枫香的研究、李文刚等[49]对不同地理种源麻疯树的研究、陈素传等[50]对不同种源栓皮栎的研究与初步选择、祝旭加等[51]对不同种源挪威云杉及家系苗期生长适应性的聚类分析、刘霞等[52]对文冠果苗期种源试验、金雅琴等[53]对不同种源乌桕苗期试验等等，都成效显著。

通过阅读文献，还发现很多博硕士研究生也纷纷选择把种源试验作为自己的毕业论文试验。陈隆升[54]对不同种源黄连木种子品质与苗期生长特性进行研究，初步选出了最适合在江苏南部作为生物质能源林种植的优良种源。李秋荔[55]对 8 个中国马褂木种源的种子形态、苗期生长性状、生物量、叶片形态、各器官营养元素含量、造林一年后的成活率等进行分析和比较研究，选择出优良的马褂木种源。张锁[56]在对油松不同种源和家系苗期性状的遗传变异分析中，通过一系列数量遗传分析和主成分分析，选出了综合性状表现最好的油松家系。除此之外，还有袁显磊[57]对核桃楸优良种源和家系进行了早期选择及苗期环境因子影响评价；王玉[58]进行了刺槐种源试验和无性系苗期选择等等，都取得了不错的成果。

1.3 小结

目前福建柏种源试验研究已相对完善，但仍然存在一些问题：可以进一步完善福建柏种源间种实表型差异的研究，如分析球果外形、长宽比、重量和苞鳞数等差异，关于福建柏种子是否含有某些特殊成分也值得研究和开发；在生长性状和生物量情况研究上，大部分学者集中于对苗高地径和干鲜重的比较，指较为单一，可以进行一些在分子水平上的微观分析，采用多样化的统计分析方式对种源鉴定进行多角度分析；关于生长节律方面的研究比较简单，基本都是通过绘制折线图看生长走向得出生长趋势，统计分析方法比较单一，可以使用 Logistic 拟合方程等进行生长模型拟合，计

中国知网 https://www.cnki.net

算苗期物候参数和生长参数等再进行差异性研究可能会得到一些新结论；有关根系性状的研究大都选择根长和根数作为研究指标，对于根系遗传特性方面的研究甚少，建议可以使用根系扫描仪等专门分析根系的仪器，测量根系表面积，根平均直径，根体积，根间数等更深入的指标，对根系性状进行更全面的研究；作为用材树种，用木材物理力学性质（如抗弯强度、端面硬度、气干密度等）体现福建柏幼林期生长质量差异可能更有说服力，可以将树高胸径和木材物理力学性质结合分析，或许更具实用性；许多专家学者对福建柏种源地理气候变异规律研究得出的结果都存在分歧，或许跟选择的指标不同有关，也可以尝试选择新的指标，这些都有待今后进一步分析探讨。

随着福建柏种源试验研究的不断进步，已取得诸多成果，为福建柏良种选育和森林培育工作提供了很多建设性的意见，但是关于研究福建柏种源苗木各生理指标差异和光合特性差异的试验还涉及很少，细胞分子水平的种源试验更是几乎空白，而且平常的福建柏种源试验只是在普通环境下进行，在各种胁迫条件下的种源试验结果如何也十分值得研究。福建柏是集经济价值、药用价值、生态价值、观赏价值为一体的珍稀乡土树种，具有广阔的研究前景，种源试验又是林木育种的重要基础，所以应进一步完善对福建柏种源的研究[59]。

本项目的研究目的在于通过福建柏苗期种源试验，研究来自福建、广东、广西三省 17 个种源地的福建柏球果和种子性状、苗期生长节律、苗期苗高、地径、根系性状、生物量情况、生理指标情况、地理变异规律这几块内容，发现福建柏种源间差异，从而以此为基础选择出福建柏优良种源，旨在为今后福建柏良种选育工作提供帮助。

通过福建柏苗期种源试验和地理变异规律研究，对不同种源地福建柏幼苗生长差异性、地理变异格式、变异大小、变异与生态环境及进化因素关系等等进行分析，初步选择出适合我省种植的福建柏优良种源，并以此为基础为不同地区确定造林用的适宜种源，或为一定地域范围（如一个国家或一个树种的分布区）确定种子区划方案和用种规则。图 1-4-1 为本研究技术路线图。

中国知网 https://www.cnki.net

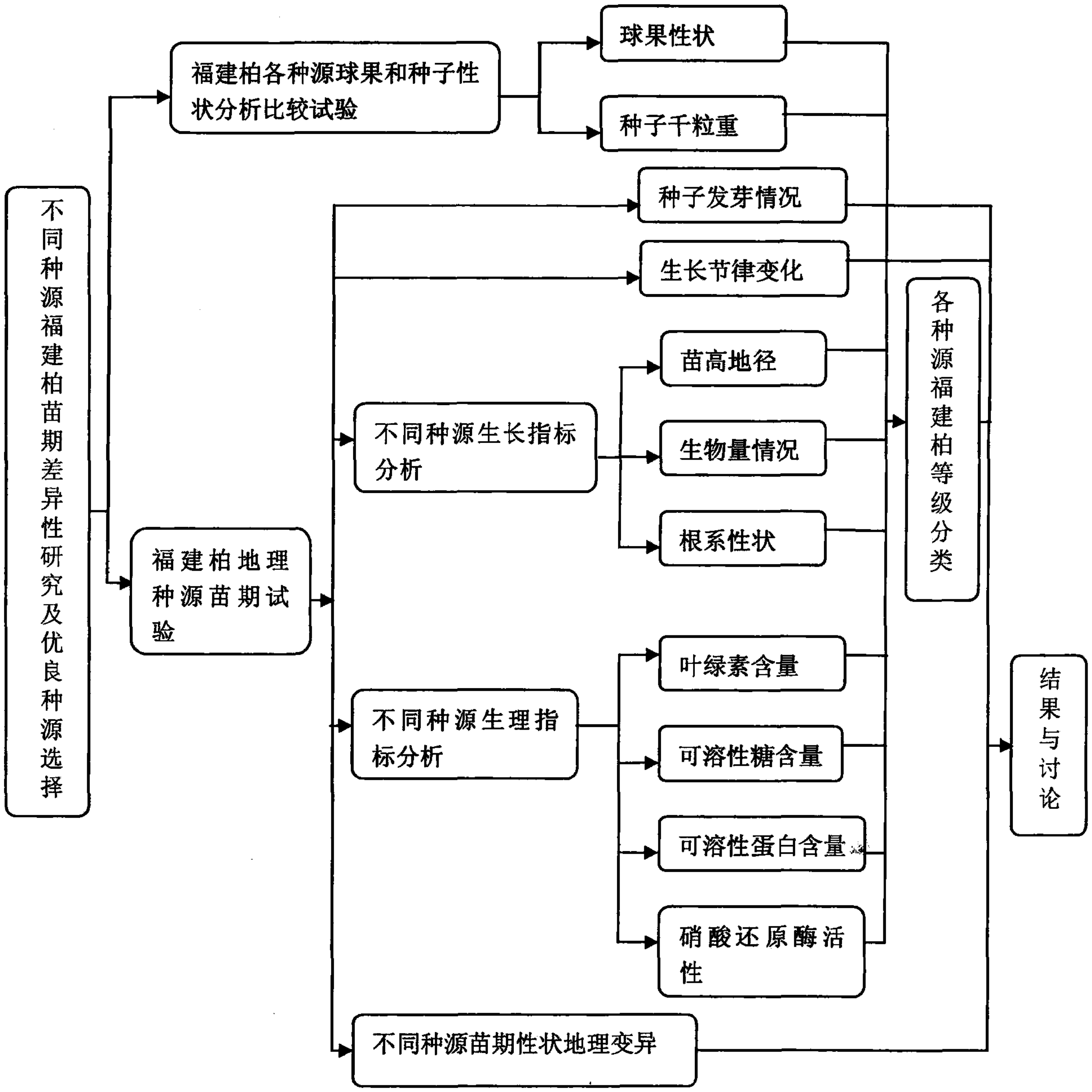

图 1-4-1 技术路线图

Fig.1-4-1 Technology Roadmap

中国知网 https://www.cnki.net

2.试验地概况、研究内容、材料和方法

2.1 试验地概况

本试验育苗地位于福建省泉州市安溪县湖头镇白濑国有林场苗圃内。白濑国有林场位于安溪县西北部的湖头镇，东经 118°00′，北纬 25°18′，海拔 200～800m，坡度 20～40°。属亚热带季风气候，年平均气温 19.5℃，最高 37℃，最低 0℃，年平均降水量 1800mm，无霜期 330 天，年日照时数约 1875h。森林覆盖率达到 67.5%，森林资源十分丰富。林场属于戴云山系，地处晋江西溪上游，气候温和，雨量充沛，土壤以红壤为主，局部地区分布有黄红壤，土层较厚，土壤理化性能良好，植被以五节芒、芒萁骨群落为主。白濑林场于 1959 年创立，总面积 2187.933hm^2，用材林和生态林分别占总面积的 45.4%和 54.6%。2002 年建成全国首个福建柏优良种质资源库，还有福建柏种子园、母树林、试验林等各类基地共 230.067hm^2。从林场的七冶西山的山脚至山顶，依次是苗圃，柑橘园，杉木、福建柏混交林，整个林场形成一个巨大的立体生态林网。

2.2 研究内容

2.2.1 福建柏各种源球果和种子性状比较试验

收集来自 17 个种源地福建柏球果，主要通过测量球果长、球果宽、球果长×宽、球果长/宽、球果重、种子千粒重这几项指标分析福建柏各种源球果和种子性状间的差异性和相关性。

2.2.2 福建柏地理种源苗期试验

将 17 个种源地福建柏种子在同一块试验地种下进行育苗试验，比较苗期各性状间的差异并选出优良种源，主要分为以下几部分试验：（1）生长节律变化情况；（2）生长性状和生物量情况；（3）根系性状情况；（4）生理指标情况；（5）苗期地理种源变异规律；（6）种源分类；（7）优良种源选择。

2.3 试验材料

2013 年 9-10 月份收集来自福建、广东、广西 3 省 17 个种源地的福建柏球果，球果经晒干开裂后收集种子，各种源的地理气象因子概况见表 2-3-1。

中国知网 https://www.cnki.net

表 2-3-1 福建柏各种源地地理气象因子

Tab.2-3-1 The geographical and meteorological factors of *Fokienia hodgirtsii* provenances

编号	种源地	北纬	东经	海拔（m）	年均温（℃）	年无霜期（d）	年日照时数（h）	年降水量（ml）
	广东韶关	24°31′	3°21′	470	20.20	310	699	900
2	广西金秀	24°07′	0°11′	800	17.00	283	1269	648
3	广西柳州	24°27′	09°22′	00	18.75	320	1410	643
4	福建上杭	26°38′	118°00′	700	20.10	277	801	646
5	福建长汀	25°39′	116°08′	800	8.30	260	1792	687
6	福建闽侯	26°10′	9°05′	205	17.15	250	959	674
7	福建闽清	26°11′	8°41′	600	9.70	269	644	650
8	福建罗源	26°29′	9°33′	360	9.50	280	558	652
9	福建永泰	25°18′	6°18′	780	17.35	300	755	700
10	福建古田	26°32′	8°51′	753	8.50	276	1895	750
1	福建南平	26°38′	8°00′	350	18.40	275	820	731
12	福建仙游	25°28′	8°44′	200	20.30	318	848	1610
13	福建安溪	24°52′	17°42′	608	7.00	260	1857	1800
14	福建永春	25°36′	8°17′	500	20.40	320	1886	850
15	福建华安	25°02′	17°53′	330	21.30	320	2000	1620
16	福建莘口	26°10′	7°28′	222	8.20	270	800	1700
17	福建尤溪	26°11′	8°16′	121	8.90	312	762	1700

2.4 研究方法

2.4.1 福建柏各种源球果和种子性状比较试验

2.4.1.1 球果和种子指标测量方法

球果选择采用随机取样法，各种源随机选取 20 颗，用数显游标卡尺测量球果长和宽，精确到 0.01mm，在 Excel 上计算出长×宽、长/宽的值；用电子天平称果重，精确到 0.01g。测量种子千粒重采取百粒法，每个种源地种子随机挑选 100 粒，3 个重复，将得到的值乘以 10 后取平均得到种子千粒重。将球果长、宽、长×宽、长/宽、球果重、种子千粒重 5 种指标进行方差分析和相关性分析。

2.4.1.2 球果处理

将从各地收集来的球果平铺于高为 3cm 的无盖纸盒内，晴天放于阳光下曝晒，注意防风以免吹走种子；阴雨天放于室内阴干，注意球果不要重叠以免发霉。待球果基本开裂完全后收集种子于种子袋内，置于 4℃冰箱内冷藏备用。

2.4.2 福建柏地理种源苗期试验

2.4.2.1 福建柏种子发芽试验方法

浸种消毒：播种前种子先用 60℃温水处理 1h，再用 0.1%的高锰酸钾溶液浸种消毒 2h 后洗净，最后用 500mg·L^{-1} 赤霉素溶液浸种 12h 后备用。

催芽：培养皿内铺好 2cm 左右厚的细沙（细沙经过高温杀菌），将处理好的种子放于沙床培养

中国知网 https://www.cnki.net

皿中，轻盖细沙覆盖种子，在保持温度25℃，光照80%培养箱环境中培养，每日喷水，保持湿度在80%。待种子发芽（以露出小白点为标准）后，统计各种源种子发芽率。

发芽率=（发芽种子数/试验种子总数）×100%

2.4.2.2 试验设计和苗期管理

2014年3月3日在田间试验采取完全随机区组设计，17个处理，种源随机排列，重复3次。苗床宽2m，高50cm，苗床间距20cm，条播育苗，条距10cm。育苗期间各种源苗采用统一的苗期管理措施[60]。

2.4.2.3 福建柏各种源苗生长节律试验方法

每个种源地选取20株苗作为测量标准株，从3月份开始，每月15日观测并记录各种源苗的苗高，地径，至10月份观察结束。苗高用钢卷尺测量，精确到0.1cm，地径用数显游标卡尺测量，精确到0.01mm，制作生长折线图，分析生长节律，并用Logistic方程拟合建立生长模型，比较拟合效果。

2.4.2.4 福建柏各种源苗高地径和生物量指标试验方法

11月份从每个种源地选出5株完整苗木作为标准株。

生长性状测定：苗高用钢卷尺测量，精确到0.1cm，地径用数显游标卡尺测量，精确到0.01mm。

生物量测定：用剪刀将苗剪成地上地下两部分，分别称鲜重，在65℃烘箱中杀青72h后称干重，都精确到0.001g。

2.4.2.5 福建柏各种源苗根系指标试验方法

11月份从每个种源地选出5株标准株，剪取地下部分，用流水法冲洗根系后阴干，用Epson V700根系扫描仪扫描根系得到形态结构图像，用LA2400 WinRHIZO Pro根系分析软件分析根系图像，得到根长、根表面积、根平均直径、根体积、根尖数几项根系指标。

2.4.2.6 福建柏各种源苗叶绿素含量测量方法

采用丙酮乙醇法[61]，按照丙酮：乙醇：水=4.5:4.5:1体积比配成提取液提取叶绿素。提取后分别在663nm和645nm下测吸光值。

叶绿素a（$mg\cdot g^{-1}$）=（12.7A663-2.69A645）×V/W

叶绿素b（$mg\cdot g^{-1}$）=（22.9A645-4.68A663）×V/W

叶绿素总（$mg\cdot g^{-1}$）=叶绿素a+叶绿素b

式中：V-提取液总体积，ml；　　W-重量，g。

2.4.2.7 福建柏各种源苗可溶性糖含量测量方法

采用蒽酮比色法[62]。称取鲜样0.1g剪碎于试管中，加10ml超纯水，在100℃沸水浴中煮沸20min，取出冷却，过滤到25ml容量瓶里定容。取待测样品液1ml（0.5ml提取液+0.5ml超纯水）加蒽酮试

中国知网 https://www.cnki.net

剂5ml，显色反应后在620nm下测吸光值，重复3次。

$$可溶性糖含量（\%）=\frac{\frac{C}{V_1}\times V_T}{W\times 10^6}\times 100\%$$

式中：C-查标准曲线值，μg；　　　V_T-样品提取液总体，ml；

V_1-显色时取样品液量，ml；　　　W-样品重，g。

2.4.2.8 福建柏各种源苗可溶性蛋白含量测量方法

采用考马斯亮蓝法[62]。称取鲜样0.1g于研钵中，加4ml预冷的磷酸缓冲液在冰浴上研磨成浆，匀浆全部转入5ml离心管，在4℃下，10000 $r\cdot nm^{-1}$离心20min得上清液。取待测样品液1ml（0.5ml提取上清液+0.5ml超纯水）于试管中加入5ml考马斯亮蓝试剂，摇匀放置5-20min后在595nm下测吸光值，重复3次。

$$可溶性蛋白含量(mg/g)=\frac{C\times V_T}{V_S\times W_F\times 1000}$$

式中：C-查标准曲线值，$\mu g\cdot ml^{-1}$；　　　V_T-提取液总体积, ml;

W_F-样品鲜重, g 。　　　V_S-测定时加样量, ml。

2.4.2.9 福建柏各种源苗硝酸还原酶活性测定方法

采用离体法[62]。称取鲜样0.5g于研钵中，加4ml预冷的提取缓冲液在冰浴上研磨成浆，匀浆全部转入5ml离心管，在4℃下，4000$r\cdot nm^{-1}$离心20min得上清液。取上清液0.4ml于试管中加0.2ml NADH溶液和1.4ml0.1$mol\cdot L^{-1}$硝酸钾磷酸溶液，混匀在25℃水浴中保温30min。保温结束立即加1ml磺胺溶液终止酶反应，再加1ml萘基乙烯胺溶液显色15min后于4000$r\cdot nm^{-1}$下离心5min，取上清液在540nm下测吸光度，重复3次。

$$酶活性（\mu g\cdot g^{-1}\cdot h^{-1}）=\frac{X\times\frac{V_1}{V_2}}{W\times t}$$

式中：X-查标曲值，μg；　　　V1-提取酶时加入的缓冲液的体积，ml；
V2-酶反应时加入的粗酶体积，ml；W-样品重量，g；　t-反应时间，h。

2.4.2.10 统计方法

使用SPSS16.0和DPS数据处理软件对数据进行方差分析，多重比较，相关性分析，Logistic方程拟合，聚类分析和主成分分析等[63-64]。

3.结果与分析

3.1 福建柏各种源球果和种子性状比较试验结果分析

植物表型性状是基因型对环境选择的表现,可以在很大程度上反映植物对环境变化的适应情况。球果和种子表型指标是苗木质量优劣的基础，具有遗传稳定性，但在实际生产研究中，由于不同地理位置及生长环境的差异，球果和种子的表型性状在种源间易产生较大变异，球果和种子的表型性

中国知网 https://www.cnki.net

状决定了物种扩散能力和种群分布格局，在良种选育、种质资源收集、遗传改良等方面具有重要意义[65-68]。

3.1.1 不同种源福建柏球果性状比较分析

对不同种源福建柏球果性状进行方差分析，结果如表 3-1-1 所示，球果长、宽、长×宽、长/宽、重 5 项球果指标 F 值最大达到 28.5280，所有 p 值<0.01，均达到极显著水平。

表 3-1-1 福建柏不同种源球果性状方差分析

Tab.3-1-1 The variance analysis of *Fokienia hodgirtsii* cone traits from different provenances

项目	平方和	自由度	均 方	F 值	p 值
长	5.4004	16	0.3375	12.1020	0.0001**
宽	6.2754	16	0.3922	20.3410	0.0001**
长×宽	59.9819	16	3.7489	15.8890	0.0001**
长/宽	0.9972	16	0.0623	7.3960	0.0001**
重	154.6970	16	9.6686	28.5280	0.0001**

**代表 p<0.01 达到极显著水平

方差分析显示的是所有种源的总体差异，为避免个别种源间的较大差异掩盖了其他种源间的较小差异，需进行 Duncan 多重比较，进一步比较不同种源间两两差异。从表 3-1-2 可知，不同种源球果性状存在很大差异，在 0.01 极显著水平下，长最大达到 F，宽最大达到 H，长×宽最大达到 G，长/宽最大达到 E，重最大达到 G，且最大差异的指标均来自 1 号种源，可见 1 号种源与其他种源相比差异较大。

17 个种源中，球果长最大的 12 号种源，达到 1.95cm，5 号、17 号、14 号紧随其后，都达到 1.9cm 之上；最小为 1 号种源，为 1.43cm。球果宽最大为 17 号种源，达到 1.94cm，5 号和 12 号也均超过 1.85cm，最小为 1 号，仅 1.38cm。17 号种源长×宽的值最大，达到 3.73cm，5 号和 12 号也达到 3.63cm，最小为 1 号，仅有 1.99cm。长/宽最大为 10 号种源，为 1.17cm，最小为 16 号，仅有 0.97cm。果重最大为 5 号种源，有 3.63g，紧随其后的是 12 号，也有 3.38g，其余种源都在 3.2g 以下，最小为 1 号，为 1.08g。综上比较，球果较为饱满大颗的为 5 号和 12 号，17 号虽然长×宽最大，但球果呈宽扁形，果重也比 5 号和 12 号小，而 1 号种源，各方面指标均为最小。

中国知网 https://www.cnki.net

表 3-1-2 福建柏不同种源球果性状 Duncan 多重比较($\bar{x}$ ±SD)

Tab.3-1-2 Duncan multiple comparison of *Fokienia hodgirtsii* cone traits from different provenances ($\bar{x}$ ±SD)

编号	长(cm)	宽(cm)	长×宽	长/宽	重(g)
1	1.43±0.14Fe	1.38±0.10Hi	1.99±0.31Gg	1.03±0.06BCDEbcdef	1.08±0.17Gh
2	1.70±0.13DEd	1.55±0.13Gh	2.65±0.37Ff	1.10±0.09ABb	1.66±0.39Fg
3	1.73±0.22CDEcd	1.62±0.12EFGfgh	2.83±0.53DEFef	1.07±0.10BCDbcd	1.69±0.41Fg
4	1.86±0.17ABCab	1.82±0.16BCbcd	3.40±0.58ABCabc	1.02±0.06BCDEcdef	2.86±0.72BCcd
5	1.94±0.12Aa	1.87±0.12ABab	3.63±0.32ABab	1.04±0.09BCDEbcde	3.63±0.74Aa
6	1.76±0.16BCDEbcd	1.75±0.15BCDde	3.09±0.52CDEFcde	1.01±0.08BCDEdef	1.97±0.40EFfg
7	1.87±0.18ABCab	1.81±0.13BCDbcd	3.40±0.54ABCabc	1.04±0.06BCDEbcdef	2.65±0.70CDde
8	1.87±0.18ABCab	1.61±0.14EFGgh	3.04±0.53CDEFde	1.16±0.07Aa	2.32±0.60DEef
9	1.66±0.17Ed	1.69±0.10DEFefg	2.81±0.41DEFef	0.98±0.08DEef	1.96±0.38EFfg
10	1.83±0.12ABCDabc	1.58±0.16FGh	2.90±0.44DEFef	1.17±0.08Aa	1.93±0.52EFfg
11	1.72±0.16CDEcd	1.61±0.12EFGgh	2.77±0.44EFef	1.07±0.07BCbcd	1.92±0.49EFfg
12	1.95±0.19Aa	1.86±0.24ABabc	3.63±0.68ABab	1.07±0.21BCbcd	3.38±0.66ABab
13	1.83±0.13ABCDabc	1.71±0.24CDEef	3.12±0.37CDEcde	1.07±0.07Bbcd	2.94±0.54BCcd
14	1.91±0.17ABa	1.76±0.15BCDcde	3.38±0.53ABCbc	1.09±0.08ABbc	3.02±0.86BCbcd
15	1.85±0.17ABCDab	1.75±0.14BCDde	3.26±0.49BCDcd	1.06±0.09BCDbcd	3.16±0.68ABCbc
16	1.72±0.21CDEcd	1.77±0.13BCDcde	3.07±0.57CDEFcde	0.97±0.09Ef	2.25±0.72DEf
17	1.91±0.17ABa	1.94±0.10Aa	3.73±0.48Aa	0.98±0.07CDEef	2.71±0.51CDd

注：同一列中不同大小写字母分析表示 0.01 水平和 0.05 水平差异显著

3.1.2 不同种源福建柏种子千粒重比较分析

对不同种源福建柏种子千粒重进行方差分析，结果如表 3-1-3 所示，不同种源千粒重差异很大，F 值为 12.8370，p 值<0.01，达到极显著水平。

表 3-1-3 福建柏不同种源种子千粒重方差分析

Tab.3-1-3 The variance analysis of *Fokienia hodgirtsii* weight of 1000-seeds from different provenances

变异来源	平方和	自由度	均 方	F 值	p 值
处理间	0.8215	16	0.0513	12.8370	0.0001**
处理内	0.1360	34	0.0040		
总变异	0.9575	50			

**代表 p<0. 01 达到极显著水平

对不同种源种子千粒重进行 Duncan 多重比较，从表 3-1-4 可知，在 0.01 极显著水平下达到 F，可见不同种源间种子千粒重差异较大，但从后面字母情况可知 5 号、7 号、9 号、11 号、15 号、16 号几个种源间的差异较小，3 号多重比较结果为 Fg，和其他种源相比差异很大。2 号种源种子千粒重最大，达到 0.9506g，最小为 3 号种源，仅有 0.4046g。变异系数方面，种源间的变异系数相差很

中国知网 https://www.cnki.net

大，从 0.95%～22.26%变化，最大为 5 号种源，最小为 14 号种源，可见 5 号种源内种子千粒重变异很大。

表 3-1-4 福建柏不同种源种子千粒重 Duncan 多重比较及变异系数

Tab.3-1-4 Duncan multiple comparison and CV of *Fokienia hodgirtsii* weight of 1000-seeds from different provenances

种源编号	$\bar{x}$ ±SD（g）	CV（%）
1	0.5963±0.1202CDEcde	20.16
2	0.9506±0.0613Aa	6.45
3	0.4046±0.0225Fg	5.56
4	0.6398±0.0688BCDcd	10.75
5	0.5247±0.1168CDEFdef	22.26
6	0.4882±0.0539DEFefg	11.04
7	0.5321±0.0654CDEFdef	12.29
8	0.6040±0.1313CDEcde	21.74
9	0.5401±0.0113CDEFdef	2.09
10	0.4524±0.0211EFfg	4.66
11	0.5529±0.0441CDEFdef	7.98
12	0.6441±0.0232BCDcd	3.60
13	0.6754±0.0069BCc	1.02
14	0.7902±0.0075Bb	0.95
15	0.5314±0.0222CDEFdef	4.18
16	0.5468±0.0495CDEFdef	9.05
17	0.4870±0.0202DEFefg	4.15

注：同一列中不同大小写字母分析表示 0.01 水平和 0.05 水平差异显著

3.1.3 不同种源福建柏球果和种子性状间相关性分析

对所有球果和种子性状进行相关性分析后得到如表 3-1-5 结果，不同种源福建柏球果和种子性状间呈现较大相关性，球果长除与千粒重呈负相关外，与宽、长×宽、重均呈极显著相关，相关系数分别为 0.787、0.931 和 0.857，与长/宽呈较小正相关，重除与长 / 宽呈负相关外，与长、宽、长×宽呈极显著正相关，其中重与宽的相关系数为 0.816，与长×宽的相关系数为 0.888，与千粒重呈较小正相关。而千粒重除与长/宽、重成较小正相关，与其他性状均呈负相关，说明果重基本随长和宽的增大而增大，而千粒重则呈现相反规律。

中国知网 https://www.cnki.net

表3-1-5 福建柏不同种源球果和种子性状间相关性分析

Tab.3-1-5 Correlation analysis of *Fokienia hodgirtsii* cone and seed traits from different provenances

性状	果长	果宽	果长×宽	果长/宽	果重
果宽	0.787**				
果长×宽	0.931**	0.956**			
果长/宽	0.209	-0.437	-0.157		
果重	0.857**	0.816**	0.888**	-0.051	
种子千粒重	-0.018	-0.160	-0.098	0.232	0.057

注：**代表在0.01水平下极显著相关，*代表在0.05水平下显著相关

3.2 福建柏地理种源苗期试验结果分析

3.2.1 不同种源福建柏发芽情况比较分析

发芽率是衡量种子质量优劣最重要的指标，发芽率与苗期出苗率和苗高等指标息息相关，发芽率高的种子大部分出苗速度快且苗高大，因此研究不同种源种子发芽率的高低对良种选择具有重要意义。

由于不同种源球果的采果量不同，导致得到的种子数量也存在差异，因此参与发芽试验的各种源种子数也有所不同。从表 3-2-1 可知，发芽率最高为 13 号种源，达到 59.22%，最低为 1 号种源，仅有 15.75%，最高最低发芽率相差 43.47%；超过 50%的仅为 13 号和 14 号两个种源，超过 40%的有 6 个种源，低于 20%也有 6 个种源，说明不同种源福建柏种子发芽率存在很大差异。

中国知网 https://www.cnki.net

表 3-2-1 福建柏不同种源发芽率

Tab.3-2-1 The germination rate of *Fokienia hodgirtsii* from different provenances

种源编号	总数	发芽数	发芽率（%）
1	254	40	15.75
2	231	97	41.99
3	286	58	20.28
4	216	91	42.13
5	205	98	47.8
6	302	51	16.89
7	160	71	44.38
8	192	78	40.63
9	244	46	18.85
10	279	44	15.77
11	304	84	27.63
12	347	168	48.41
13	385	228	59.22
14	261	150	57.47
15	231	89	38.53
16	171	34	19.88
17	310	54	17.42

3.2.2 不同种源福建柏生长节律比较分析

苗期生长节律反应了不同种源苗期生长趋势变化规律，通过将整个生长过程划分为不同生长阶段，为不同阶段提供不同的水肥管理措施，以达到加快线性生长量的目的。利用Logistic方程对苗期生长曲线进行拟合，还可以得到不同种源苗物候期参数和生长参数等相关指标。

3.2.2.1苗高地径生长趋势变化

绘制各种源3-10月份苗高折线图进行地径生长节律分析，由图3-2-1至图3-2-4可知，各种源苗高生长趋势均十分相似，基本呈现“慢-快-慢-快-慢”变化趋势，在生长过程中，从3月份开始，苗高生长还较为缓慢，4-5月份时开始出现第一次生长小高峰，从7月份开始，生长迅速加快，到达第二次高峰，8月结束，10月份之后又开始缓慢生长。最初生长快的种源到后期苗高并不一定为最大，而生长慢的到后期苗高也不一定小，可见在生长前后苗高变化并不存在相关性。

中国知网 https://www.cnki.net

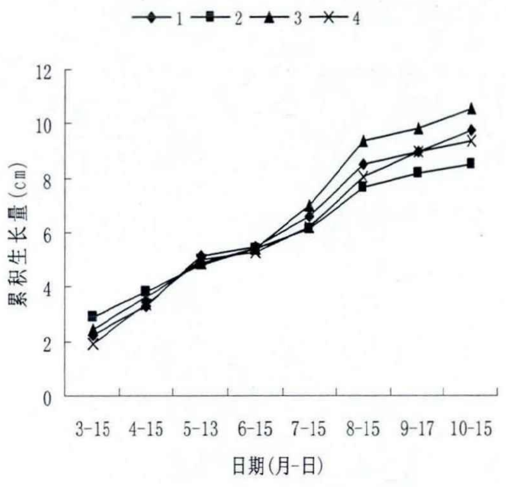

图3-2-1 1-4号种源苗高生长趋势比较
Fig.3-2-1 The comparison of height growth tendency of 1-4 provenances

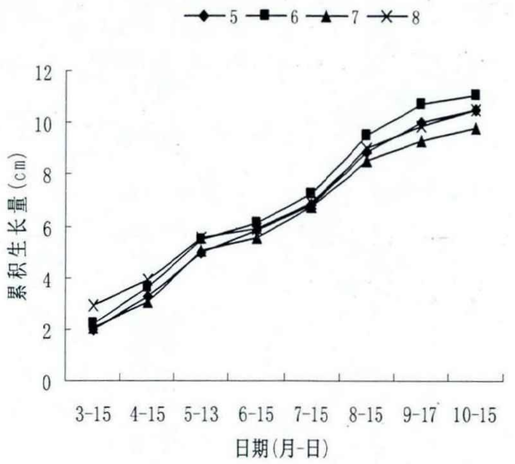

图3-2-2 5-8号种源苗高生长趋势比较
Fig.3-2-2 The comparison of height growth tendency of 5-8 provenances

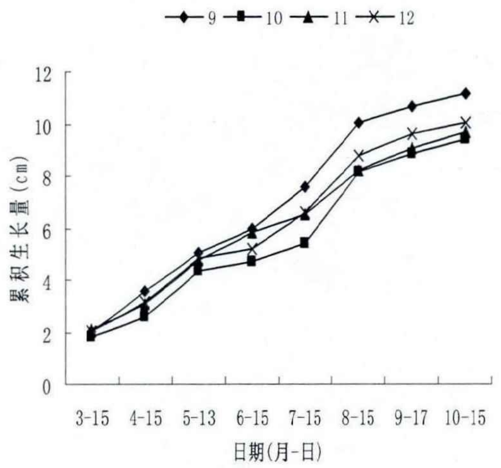

图3-2-3 9-12号种源苗高生长趋势比较
Fig.3-2-3 The comparison of height growth tendency of 9-12 provenances

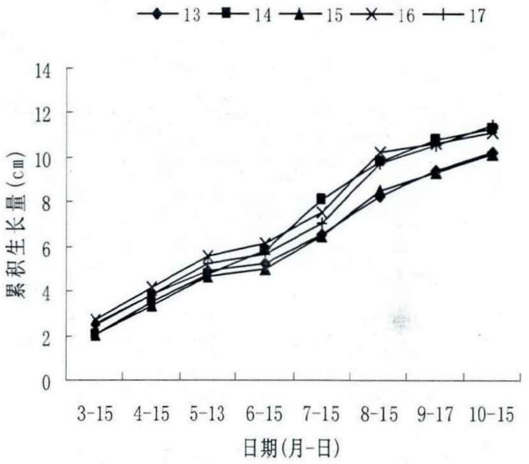

图3-2-4 13-17号种源苗高生长趋势比较
Fig.3-2-4 The comparison of height growth tendency of 13-17 provenances

绘制各种源3-10月份地径折线图进行地径生长节律分析，由图3-2-5至图3-2-8可知，总体呈现“慢-快-慢”趋势，但各种源间的生长趋势并不如苗高相似。在6月份之前，不同种源生长规律都大不相同，但从7月份开始，除了5号种源，其他种源地径生长均迅速加快，出现一个高峰期，到了后期，不同种源进入缓慢生长期的时间也出现差异，大部分种源从8月开始，但5号和10号种源从9月开始。可见，不同种源福建柏一年生苗地径生长节律存在一定差异，由于生长初期苗木茎部幼嫩且细，给测量带来困难，导致存在一定测量误差。

中国知网 https://www.cnki.net

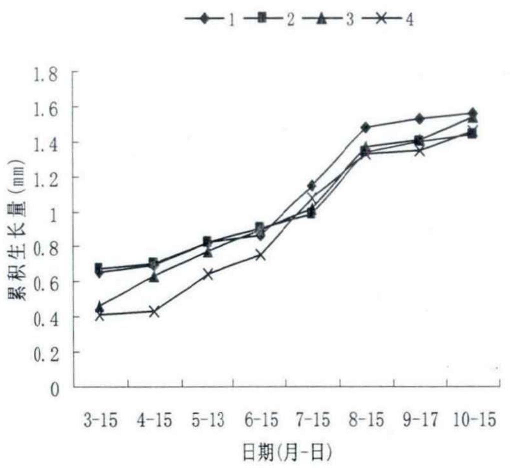

图3-2-5 1-4号种源地径生长趋势比较

Fig.3-2-5 The comparison of diameter growth tendency of 1-4 provenances

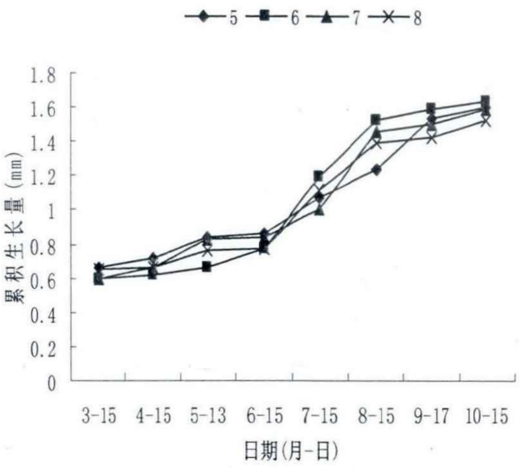

图3-2-6 5-8号种源地径生长趋势比较

Fig.3-2-6 The comparison of diameter growth tendency of 5-8 provenances

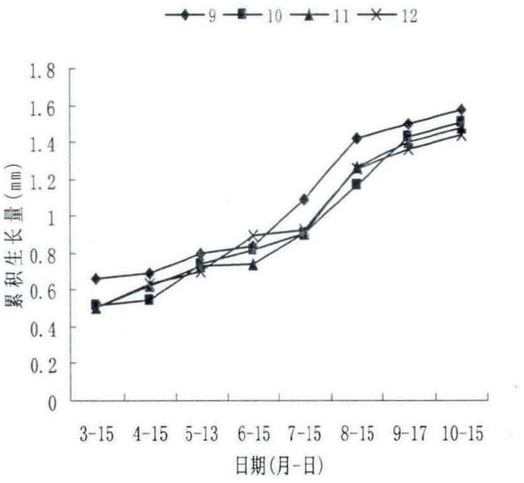

图3-2-7 9-12号种源地径生长趋势比较

Fig.3-2-7 The comparison of diameter growth tendency of 9-12 provenances

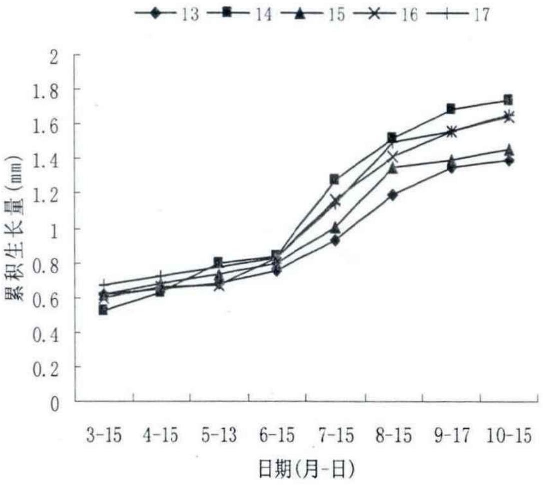

图3-2-8 13-17号种源地径生长趋势比较

Fig.3-2-8 The comparison of diameter growth tendency of 13-17 provenances

中国知网 https://www.cnki.net

从表3-2-2苗高净生长量结果来看，与前文分析的生长趋势基本相符，各种源7-8月净生长量均值达到2.105cm，为全年最大值，5-6月和9-10月净生长量偏小。7-8月净生长量中10号最大（2.725cm），2号最小（1.545cm），达到2cm以上的种源有10个，但也有个别不同，比如7号和14号7-8月的净生长量就分别小于4-5月和6-7月。

表3-2-2 不同种源福建柏3-10月苗高净生长量

Tab.3-2-2 Height net growth of *Fokienia hodgirtsii* from different provenances from March to October

编号	3月-4月	4月-5月	5月-6月	6月-7月	7月-8月	8月-9月	9月-10月
1	1.070	1.800	0.360	1.150	1.885	0.470	0.785
2	0.985	0.955	0.615	0.700	1.545	0.505	0.300
3	1.195	1.265	0.505	1.590	2.380	0.435	0.775
4	1.415	1.670	0.240	0.975	1.805	0.945	0.415
5	1.230	1.705	0.890	1.005	2.035	1.145	0.520
6	1.400	1.850	0.680	1.09	2.290	1.200	0.350
7	0.940	2.030	0.445	1.25	1.745	0.78	0.530
8	0.940	1.680	0.330	1.01	2.145	0.835	0.645
9	1.540	1.445	0.940	1.600	2.430	0.630	0.510
10	0.770	1.760	0.365	0.705	2.725	0.655	0.560
11	0.960	1.710	1.020	0.68	1.705	0.880	0.615
12	1.065	1.700	0.365	1.365	2.195	0.830	0.415
13	1.295	1.075	0.290	1.360	1.695	1.125	0.825
14	1.455	1.240	1.085	2.29	1.740	0.975	0.475
15	1.290	1.29	0.360	1.435	2.040	0.820	0.855
16	1.455	1.345	0.640	1.345	2.740	0.350	0.515
17	1.370	1.425	0.390	1.390	2.690	0.875	0.890
$\bar{x}$	1.199	1.526	0.560	1.232	2.105	0.791	0.587

从表3-2-3地径生长量结果来看，大体上7-8月地径均值最大，达到0.3097mm，6-7月也有0.2325mm，6-7月净生长量从0.0320mm（12号）～0.4450mm（14号）变化，7-8月份从0.1650mm（5号）～0.4530mm（7号），可见不同种源地径生长量变化仍存在很大差异。

中国知网 https://www.cnki.net

表3-2-3 不同种源福建柏3-10月地径净生长量

Tab.3-2-3 Diameter net growth of *Fokienia hodgirtsii* from different provenances from March to October

编号	3月-4月	4月-5月	5月-6月	6月-7月	7月-8月	8月-9月	9月-10月
1	0.0430	0.1275	0.0475	0.2830	0.3320	0.0445	0.0350
2	0.0250	0.1265	0.0750	0.0860	0.3505	0.0595	0.0460
3	0.1725	0.1360	0.1260	0.1170	0.3575	0.0380	0.1315
4	0.0235	0.2160	0.1090	0.3205	0.2505	0.0200	0.1110
5	0.0520	0.1205	0.0325	0.2065	0.1650	0.2985	0.0640
6	0.0160	0.0460	0.1155	0.4170	0.3300	0.0665	0.0430
7	0.0665	0.1580	0.0130	0.1680	0.4530	0.0480	0.0900
8	0.0190	0.0890	0.0140	0.3420	0.2830	0.0325	0.0920
9	0.0310	0.1020	0.0455	0.2530	0.3315	0.0750	0.0795
10	0.0320	0.1945	0.0810	0.0880	0.2535	0.2690	0.0735
11	0.1155	0.1075	0.0110	0.1590	0.3655	0.1385	0.0700
12	0.1290	0.0655	0.1980	0.0320	0.3335	0.1040	0.0795
13	0.0320	0.0305	0.0710	0.1730	0.2700	0.1575	0.0405
14	0.1070	0.1650	0.0450	0.4450	0.2370	0.1710	0.0440
15	0.0660	0.0520	0.0595	0.2130	0.3430	0.0390	0.0630
16	0.0685	0.0095	0.1590	0.3355	0.2530	0.1375	0.0895
17	0.0560	0.0530	0.0480	0.3145	0.3570	0.0580	0.0955
$\bar{x}$	0.0620	0.1058	0.0736	0.2325	0.3097	0.1034	0.0734

3.2.2.2 苗高地径 Logistic 方程拟合结果分析

用 Logistic 曲线方程对不同种源福建柏苗高地径变化折线图进行拟合，得到如表 3-2-4 和表 3-2-5 结果，不论苗高还是地径，各种源 P 值均<0.01，达到极显著结果，苗高决定系数 R^2 最大达到 0.963，最小也有 0.920，地径决定系数 R^2 最小为 0.921，最大也有 0.981，可见拟合效果不错，但是计算出的拟合方程参数却并不理想，尤其苗木生长极限 k 值，不可能为负数，通过查阅其他文献可知，决定系数一般达到 0.99 以上才能计算出正确的 k 值[36-42]。因此使用 Logistic 方程对福建柏苗高地径生长过程进行拟合并不合适。

中国知网 https://www.cnki.net

表 3-2-4 不同种源福建柏苗高生长 Logistic 方程拟合结果

Tab.3-2-4 The Logistic equation fitting result of *Fokienia hodgirtsii* height growth from different provenances

编号	拟合方程参数			统计量		
	k	a	b	R^2	F	P
1	4.895	0.440	0.817	0.927	75.977	0.0001
2	-70.835	0.357	0.858	0.954	123.676	0.0001
3	4.537	0.428	0.812	0.953	122.336	0.0001
4	8.194	0.506	0.805	0.938	90.035	0.0001
5	8.072	0.500	0.793	0.944	100.341	0.0001
6	3.554	0.439	0.801	0.924	72.849	0.0001
7	4.7154	0.476	0.805	0.920	69.379	0.0001
8	5.355	0.3600	0.834	0.957	133.12	0.0001
9	2.298	0.465	0.792	0.924	72.451	0.0001
10	3.829	0.575	0.793	0.940	94.358	0.0001
11	6.836	0.483	0.807	0.943	99.436	0.0001
12	4.649	0.489	0.801	0.937	89.888	0.0001
13	4.813	0.404	0.827	0.963	154.924	0.0001
14	17.996	0.474	0.788	0.926	74.624	0.0001
15	4.494	0.481	0.805	0.948	108.635	0.0001
16	4.658	0.367	0.822	0.940	93.813	0.0001
17	5.030	0.421	0.807	0.954	124.409	0.0001

中国知网 https://www.cnki.net

表 3-2-5 不同种源福建柏地径生长 Logistic 方程拟合结果

Tab.3-2-5 The Logistic equation fitting result of *Fokienia hodgirtsii* diameter growth from different provenances

编号	拟合方程参数			统计量		
	k	a	b	R^2	F	P
1	-0.263	1.856	0.865	0.951	116.131	0.0001
2	-0.186	1.746	0.885	0.946	105.05	0.0001
3	-0.194	2.287	0.844	0.964	163.014	0.0001
4	-0.438	2.987	0.816	0.946	106.069	0.0001
5	-0.258	1.819	0.873	0.979	277.264	0.0001
6	-0.430	2.238	0.839	0.921	70.354	0.0001
7	-0.324	1.994	0.859	0.952	117.904	0.0001
8	-0.464	1.932	0.868	0.923	71.986	0.0001
9	-0.306	1.869	0.867	0.955	125.894	0.0001
10	-0.309	2.353	0.85	0.981	317.737	0.0001
11	-0.421	2.288	0.853	0.966	169.918	0.0001
12	-0.197	2.222	0.856	0.974	226.215	0.0001
13	-0.410	2.020	0.875	0.947	106.47	0.0001
14	-0.343	2.269	0.829	0.960	145.859	0.0001
15	-0.371	1.963	0.871	0.949	111.861	0.0001
16	-0.316	2.139	0.846	0.952	119.844	0.0001
17	-0.339	1.870	0.863	0.939	92.014	0.0001

3.2.3 不同种源福建柏苗高地径和生物量比较分析

苗高地径和干鲜重等生物量情况历来是评价苗木质量好坏的首选标准，具有直观明了，简便易得等特点，研究不同种源苗苗高地径和生物量差异情况是福建柏优良种源选择的重要依据。

3.2.3.1 苗高地径差异分析

对不同种源福建柏苗高地径进行方差分析，由表 3-2-6 可知，苗高 p 值<0.01，F 值为 5.3210，达到极显著水平，地径 p 值>0.05，F 值仅为 0.9000，未达到显著水平。

表 3-2-6 福建柏不同种源苗高地径方差分析

Tab.3-2-6 The variance analysis of *Fokienia hodgirtsii* height and diameter from different provenances

项目	平方和	自由度	均 方	F 值	p 值
苗高	394.9181	16	24.6824	5.3210	0.0001**
地径	2.6713	16	0.1670	0.9000	0.5725

注：**代表 p<0.01 达到极显著水平

通过苗高地径多重比较，得到结果如表 3-2-7 所示，在 0.01 极显著水平下，苗高最大只到 C，其中 11 号、12 号、17 号、18 号之间差异很小，5 号、13-16 号之间差异很小；地径在种源两两之间差异不明显。苗高最大的 3 号种源达到 25.06cm，最小的 1 号种源为 15.20cm，在 20cm 以上的种

中国知网 https://www.cnki.net

源共有 3 个；地径最大的 9 号种源为 2.85mm，最小的 10 号种源为 2.25mm，可见苗高地径在种源间变化并不大。苗高和地径变异系数变动幅度很相近，苗高从 6.42%～20.99%变化，地径从 6.12%～29.90%变化，其中 1 号种源苗高地径变异最大，可见 1 号种源种内生长最不平均。

表 3-2-7 福建柏不同种源苗高地径 Duncan 多重比较及变异系数

Tab.3-2-7 Duncan multiple comparison and CV of *Fokienia hodgirtsii* height and diameter from different provenances

编号	苗高(cm)		地径(mm)	
	$\bar{x}$ ±SD	CV（%）	$\bar{x}$ ±SD	CV（%）
1	15.20±3.19Ce	20.99	2.35±0.7Aa	29.90
2	15.76±2.34Cde	14.86	2.39±0.15Aa	6.12
3	25.06±1.68Aa	6.70	2.65±0.40Aa	15.24
4	20.02±1.77Bb	8.86	2.35±0.30Aa	12.67
5	17.04±1.55BCbcde	9.10	2.67±0.45Aa	16.83
6	20.14±2.47Bb	12.25	2.45±0.65Aa	26.41
7	16.76±2.70BCcde	16.10	2.62±0.25Aa	9.66
8	18.50±1.57BCbcd	8.46	2.41±0.30Aa	12.52
9	19.36±3.28BCbc	16.95	2.85±0.52Aa	18.24
10	16.64±1.74BCcde	10.43	2.25±0.22Aa	9.77
11	18.58±1.49BCbcd	8.01	2.46±0.54Aa	22.16
12	18.82±1.52BCbcd	8.06	2.51±0.16Aa	6.21
13	17.22±1.20BCbcde	6.95	2.77±0.20Aa	7.33
14	17.92±3.22BCbcde	17.97	2.80±0.64Aa	22.67
15	17.22±1.98BCbcde	11.47	2.46±0.45Aa	18.28
16	18.00±1.16BCbcde	6.42	2.60±0.42Aa	16.26
17	18.60±1.92BCbcd	10.31	2.79±0.38Aa	13.70

注：同一列中不同大小写字母分别表示 0.01 水平和 0.05 水平差异显著

3.2.3.2 生物量差异分析

对不同种源福建柏生物量进行方差分析，由表 3-2-8 可知，除地下干重/地下鲜重和侧枝数两个指标 p 值＞0.05，差异不显著，其余指标中地上干重/地上鲜重 0.01＜p 值＜0.05，差异显著，剩余指标 p 值＜0.01，均达到极显著差异。

中国知网 https://www.cnki.net

表 3-2-8 福建柏不同种源生物量方差分析

Tab.3-2-8 The variance analysis of *Fokienia hodgirtsii* biomass from different provenances

项目	平方和	自由度	均 方	F 值	p 值
总鲜重	74.8267	16	4.6767	2.9350	0.0010**
总干重	8.1614	16	0.5101	2.6730	0.0026**
地上鲜重	63.9057	16	3.9941	3.0150	0.0008**
地上干重	6.8611	16	0.4288	2.7520	0.0019**
地下鲜重	0.9907	16	0.0619	2.7750	0.0018**
地下干重	0.1863	16	0.0116	2.9350	0.0010**
地下鲜/地上鲜	0.0685	16	0.0043	5.2690	0.0001**
地下干/地上干	0.1269	16	0.0079	2.9810	0.0009**
地上干/地上鲜	0.0372	16	0.0023	2.0080	0.0248*
地下干/地上鲜	0.1142	16	0.0071	0.9480	0.5212
总干/总鲜	0.0361	16	0.0023	3.5850	0.0001**
侧枝数	15.1059	16	0.9441	0.9730	0.4953

注：**代表 $p<0.01$ 达到极显著水平，*代表 $p<0.05$ 达到显著水平

为进一步比较福建柏不同种源间生物量两两差异情况，在方差分析的基础上进行 Duncan 多重比较，得到结果如表 3-2-9 所示，在 0.01 极显著水平下除了地上干重达到 B，其他指标字母也只到 C，说明差异不明显；总鲜重中，3 号和 6 号种源与其他种源相差较大，总干重中，3 号、9 号、10 号、16 号、17 号与其他种源相差较大，地上鲜重中，3 号和 6 号与其他种源相差大；地上干重中，仅 3 号与其他种源都不一样，地下鲜重中，3 号、6 号、10 号和 17 号与其他种源相差大，地下干重中，2 号、9 号、10 号、11 号和 13 号与其他种源相差大。可见 3 号种源与其他种源相比，差异较大。

在所有指标中，除了来自 17 号种源最大的地下干重是 0.30g 外，总鲜重最大为 6.18g，总干重最大为 2.23g，地上鲜重最大为 5.58g，地上干重最大为 1.94g，地下鲜重最大为 0.61g，这些指标均来自 3 号种源；总鲜重和地上鲜重最小的均为 6 号种源，值分别为 2.96g 和 2.60g，而最小总干重（1.06g）、地上干重（0.94g）、地下鲜重（0.24g）、地下干重（0.13g）均来自 10 号种源。综上可知，在所有种源中，3 号种源在生物量积累上占有绝对优势，6 号种源和 10 号种源则表现较差。

中国知网 https://www.cnki.net

中国知网 https://www.cnki.net

表 3-2-9 福建柏不同种源生物量 Duncan 多重比较（$\bar{x}$ ±SD）

Tab.3-2-9 Duncan multiple comparison of *Fokienia hodgirtsii* biomass from different provenances（$\bar{x}$ ±SD）

编号	总鲜重（g）	总干重（g）	地上鲜重（g）	地上干重（g）	地下鲜重（g）	地下干重（g）
1	4.21±2.47ABCbc	1.57±0.90ABCbcd	3.78±2.16ABCbc	1.34±0.78ABbc	0.44±0.31ABCabcd	0.24±0.13ABCabc
2	3.22±1.53BCc	1.19±0.50BCd	2.94±1.44BCc	1.05±0.47Bc	0.28±0.09BCd	0.15±0.03BCcd
3	6.18±1.44Aa	2.23±0.49Aa	5.58±1.39Aa	1.94±0.46Aa	0.61±0.11Aa	0.29±0.03Aa
4	3.10±0.80BCc	1.28±0.27BCd	2.69±0.78BCc	1.04±0.25Bc	0.41±0.03ABCabcd	0.24±0.03ABCabc
5	3.09±1.12BCc	1.19±0.0.34BCd	2.71±0.98BCc	0.99±0.30Bc	0.38±0.15ABCbcd	0.21±0.06ABCabcd
6	2.96±1.12Cc	1.19±0.42BCd	2.60±0.96Cc	0.99±0.35Bc	0.36±0.16ABCcd	0.20±0.08ABCabcd
7	3.27±0.68BCc	1.27±0.23BCd	2.86±0.64BCc	1.06±0.22Bc	0.42±0.10ABCabcd	0.21±0.04ABCabcd
8	4.01±0.71ABCbc	1.45±0.28ABCbcd	3.58±0.65ABCbc	1.23±0.24ABbc	0.44±0.09ABCabcd	0.22±0.05ABCabcd
9	5.51±1.59ABab	1.93±0.51ABCabc	4.96±1.47ABab	1.66±0.46ABab	0.56±0.22ABabc	0.27±0.07ABa
10	3.25±0.99BCc	1.06±0.32Cd	3.02±0.92BCc	0.94±0.29Bc	0.24±0.08Cd	0.13±0.04Cd
11	4.26±1.53ABCbc	1.51±0.52ABCbcd	3.94±1.38ABCabc	1.33±0.45ABbc	0.32±0.16ABCd	0.17±0.07ABCbcd
12	4.74±0.72ABCabc	1.68±0.33ABCabcd	4.29±0.73ABCabc	1.47±0.31ABabc	0.46±0.05ABCabcd	0.21±0.04ABCabcd
13	4.54±0.71ABCabc	1.66±0.25ABCabcd	3.98±0.69ABCabc	1.40±0.24ABabc	0.57±0.15ABabc	0.26±0.02ABab
14	3.94±1.17ABCbc	1.45±0.33ABCbcd	3.54±1.07ABCbc	1.05±0.46Bc	0.40±0.11ABCabcd	0.20±0.02ABCabcd
15	4.26±1.55ABCbc	1.55±0.46ABCbcd	3.89±1.36ABCbc	1.34±0.38ABbc	0.38±0.19ABCbcd	0.21±0.09ABCabcd
16	3.49±0.43BCc	1.34±0.20BCcd	2.93±0.33BCc	1.05±0.14Bc	0.57±0.19ABabc	0.29±0.08Aa
17	5.54±1.28ABab	2.00±0.50ABab	4.95±1.22ABab	1.71±0.45ABab	0.59±0.11Aab	0.30±0.07Aa

注：同一列中不同大小写字母分别表示 0.01 水平和 0.05 水平差异显著

从表 3-2-10 结果看，地下鲜重/地上鲜重变化范围在 0.079（10 号）～0.192（16 号），地下干重/地上干重在 0.128（11 号）～0.273（16 号），说明相对于其他种源，16 号种源生物量在地下积累较多。地上干重/地上鲜重变化范围在 0.306（14 号）～0.391（4 号），地下干重/地下鲜重在 0.460（12 号）～0.584（1 号），总干重/总鲜重在 0.328（10 号）～0.416（4 号），说明地上部分含水量最高为 14 号种源，地下部分含水量最高为 12 号种源，整株含水量最高为 3 号种源。

从变异系数看，各指标在种源内变异最高的依次为 7 号（29.13%）、14 号（61.62%）、14 号（37.11%）、5 号（33.31%）、5 号（9.55%），可知 14 号和 5 号在种源内地上地下部分生物量分配和干鲜比差异很大。

表 3-2-10 福建柏不同种源生物量干鲜重比比较

Tab.3-2-10 The comparison of dry-fresh radio of biomass about *Fokienia hodgirtsii* from different provenances

编号	地下鲜重/地上鲜重		地下干重/地上干重		地上干重/地上鲜重		地下干重/地下鲜重		总干重/总鲜重	
	$\bar{x}$	CV（%）	$\bar{x}$	CV（%）	$\bar{x}$	CV（%）	$\bar{x}$	CV（%）	$\bar{x}$	CV（%）
1	0.112	16.55	0.186	13.94	0.349	7.34	0.584	13.85	0.372	6.56
2	0.102	18.46	0.155	23.00	0.363	6.56	0.546	9.88	0.381	7.23
3	0.113	21.29	0.153	12.43	0.348	3.08	0.478	14.44	0.360	3.55
4	0.161	19.67	0.236	18.88	0.391	4.73	0.575	6.38	0.416	4.45
5	0.140	8.15	0.216	32.79	0.368	6.11	0.573	33.31	0.394	9.55
6	0.138	11.25	0.203	7.50	0.381	3.96	0.564	9.98	0.403	4.64
7	0.150	29.13	0.209	29.77	0.371	4.21	0.519	11.51	0.390	5.49
8	0.122	16.34	0.177	14.21	0.344	6.59	0.506	19.85	0.362	7.94
9	0.114	32.57	0.164	20.95	0.338	7.45	0.507	19.01	0.354	8.35
10	0.079	12.98	0.136	20.70	0.312	5.33	0.539	13.76	0.328	6.21
11	0.080	17.17	0.128	13.71	0.339	3.30	0.546	9.07	0.355	3.72
12	0.109	23.40	0.145	21.42	0.341	7.79	0.460	20.34	0.352	9.38
13	0.146	27.50	0.186	16.17	0.353	7.45	0.469	23.45	0.366	9.25
14	0.114	9.75	0.233	61.52	0.306	37.11	0.518	16.59	0.374	6.82
15	0.094	16.42	0.151	18.15	0.349	5.48	0.557	7.68	0.368	5.38
16	0.192	32.12	0.273	22.38	0.360	7.26	0.522	10.18	0.384	7.55
17	0.123	21.96	0.178	25.45	0.343	3.50	0.501	18.95	0.360	4.08

3.2.3.3 苗高地径和生物量相关性分析

对不同种源所有生长性状和生物量指标进行相关性分析，结果如表 3-2-11 所示，除侧枝数与苗高呈现负相关外，所有指标间均呈不同程度正相关，正相关系数从 0.229～0.996 变化。

苗高与其他性状间正相关偏弱，仅与总鲜重、总干重、地上鲜重、地上干重呈显著相关，其他则未达到显著水平。地径除了与地下干重的正相关未达到显著水平，与其他指标均达到显著水平，与地下鲜重还达到极显著水平，相关系数为 0.683。侧枝数与地径呈显著正相关，相关系数为 0.596，

中国知网 https://www.cnki.net

与其他性状均未达到显著水平。

极显著正相关集中在干鲜重生物量指标间，其中总鲜重分别与总干重、地上干重、地上鲜重、地下鲜重极显著相关，相关系数从 0.712～0.984 变化，和地下干重显著相关，相关系数为 0.600；总干重分别与地上鲜重、地上干重、地下鲜重、地下干重极显著相关，相关系数从 0.703～0.982 变化；地上鲜重分别与地上干重和地下鲜重极显著相关，相关系数从 0.649～0.975 变化，和地下干重显著相关，相关系数为 0.532；地下干重分别与地下鲜重和地下干重极显著相关，相关系数从 0.622～0.714 变化；地下鲜重和地下干重极显著相关，相关系数达 0.962。可见福建柏种源苗生物量在地上地下部分空间分配上呈现高度相关性，通过测定出某部分生物量能推测出其他部分的生物量情况。

表3-2-11 福建柏不同种源苗高地径和生物量相关性分析

Tab.3-2-11 The correlation analysis between *Fokienia hodgirtsii* height ,diameter and biomass from different provenances

	苗高	地径	总鲜重	总干重	地上鲜重	地上干重	地下鲜重	地下干重
地径	0.229							
总鲜重	0.524*	0.522*						
总干重	0.572*	0.559*	0.984**					
地上鲜重	0.508*	0.483*	0.996**	0.970**				
地上干重	0.555*	0.451	0.981**	0.982**	0.975**			
地下鲜重	0.477	0.683**	0.712**	0.786**	0.649**	0.714**		
地下干重	0.458	0.596*	0.600*	0.703**	0.532*	0.622**	0.962**	
侧枝数	-0.176	0.519*	0.332	0.352	0.315	0.380	0.368	0.338

注：**代表在0.01水平下极显著相关，*代表在0.05水平下显著相关

3.2.4 不同种源福建柏根系性状比较分析

根系是吸收土壤营养物质和水分的重要器官，对植物生长发育有重要意义，根系指标的高低，直接影响苗木成活率及林分生产力水平，因此对不同种源苗根系性状的分析结果可作为苗木质量评价的重要依据[69]。

3.2.4.1 根系性状差异分析

对不同种源福建柏根系指标进行方差分析，由表 3-2-12 结果可知，福建柏根系性状不同种源间差异显著，根长 0.01＜p 值＜0.05，达到显著水平，其余根系指标 p 值＜0.01，均达到极显著水平。

中国知网 https://www.cnki.net

表 3-2-12 福建柏不同种源根系性状方差分析

Tab.3-2-12 The variance analysis of *Fokienia hodgirtsii* root traits from different provenances

项目	平方和	自由度	均 方	F 值	p 值
根长	47414.6670	16	2963.4167	2.0390	0.0223*
根表面积	2192.3339	15	137.0209	2.7670	0.0018**
平均根直径	0.1300	16	0.0081	4.5290	0.0001**
根体积	0.7039	16	0.0440	3.4810	0.0002**
根尖数	49579.3882	16	3098.7118	2.6290	0.0030**

注：**代表 $p<0.01$ 达到极显著水平，*代表 $p<0.05$ 达到显著水平

对不同种源福建柏根系指标进行比较分析，结果如表 3-2-13 所示，根长和根表面积最大的为 16 号种源，根长达 177.09cm，根表面积达 34.42cm^2，最小的均为 2 号种源，根长为 78.13cm，根表面积为 15.86 cm^2，平均根系直径最大为 17 号（0.68mm），最小为 11 号（0.53mm），种源间变异幅度小；根体积最大为 16 号（0.54 cm^3），最小为 10 号（0.23 cm^3），根尖数最大和最小的分别为 3 号和 2 号种源，变化范围在 54～159。综上分析根系指标情况可知，16 号种源根系情况较其他种源更良好。

表 3-2-13 福建柏不同种源根系性状比较（$\bar{x}$±SD）

Tab.3-2-13 The comparison of *Fokienia hodgirtsii* root traits from different provenances（$\bar{x}$±SD）

编号	根长(cm)	根表面积(cm^2)	平均根直径(mm)	根体积(cm^3)	根尖数
1	148.90±63.70	28.63±12.92	0.61±0.05	0.44±0.22	129±60.75
2	78.13±18.79	15.86±3.87	0.65±0.03	0.26±0.07	54±13.08
3	162.23±34.05	30.39±5.60	0.60±0.05	0.46±0.08	159±29.18
4	142.05±20.76	25.83±3.72	0.58±0.04	0.38±0.07	123±31.75
5	126.30±31.76	23.13±5.97	0.58±0.03	0.34±0.09	102±35.90
6	127.23±33.47	21.70±5.72	0.54±0.03	0.30±0.08	126±36.51
7	155.23±14.37	26.93±2.20	0.55±0.03	0.37±0.04	132±26.44
8	133.82±24.13	24.89±4.77	0.59±0.02	0.37±0.08	109±26.41
9	156.58±45.91	29.89±8.50	0.61±0.03	0.45±0.13	136±42.16
10	99.02±26.68	16.86±4.43	0.54±0.03	0.23±0.06	86±30.20
11	129.01±65.80	20.90±9.55	0.53±0.06	0.27±0.11	99±31.77
12	128.52±19.78	25.19±4.66	0.62±0.03	0.39±0.09	99±14.32
13	168.23±14.54	33.26±1.89	0.63±0.04	0.52±0.04	129±23.43
14	135.75±19.13	24.23±3.56	0.57±0.04	0.35±0.06	102±12.52
15	139.98±30.39	24.78±7.60	0.56±0.08	0.36±0.16	109±36.26
16	177.09±62.08	34.42±11.52	0.62±0.05	0.54±0.18	136±59.98
17	149.45±53.81	31.29±10.35	0.68±0.05	0.52±0.16	143±27.82

3.2.4.2 根系性状间相关性分析

对所有根系性状进行相关性分析，结果如表 3-2-14 所示，从中可得，相关系数最大达到 0.975，

中国知网 https://www.cnki.net

在所有根系指标间，除根长和根体积、根表面积和根体积、根直径和根尖数正相关系数较小，未达到显著水平，其余各指标间均达到极显著正相关。其中根长和根表面积的相关系数为 0.950，与根体积的相关系数为 0.857，与根尖数的相关系数为 0.891；根表面积与根体积的相关系数为 0.975，与根尖数的相关系数为 0.846；根直径与根尖数的相关系数为 0.659；根体积与根尖数的相关系数为 0.763。说明不同种源根系指标间相关性很大，在根系分析中，可通过一种指标推测出其他指标的情况。

表 3-2-14 福建柏不同种源根系性状间相关性分析

Tab.3-2-14 The correlation analysis of *Fokienia hodgirtsii* root traits from different provenances

	根长	根表面积	根直径	根体积
根表面积	0.950**			
根直径	0.189	0.481		
根体积	0.857**	0.975**	0.659**	
根尖数	0.891**	0.846**	0.165	0.763**

注：**代表在0.01水平下极显著相关，*代表在0.05水平下显著相关

3.2.5 不同种源福建柏生理指标比较

在森林培育研究进程中，大都采用直观的表型指标和生长指标等形态指标作为苗木质量评价的标准，但这些指标却不能反映出苗木内在生命活动状况，选择一些生理指标作为良种选择的标准可以反映苗木生命活动的强弱，对良种选择同样具有重要意义。叶绿素是光合作用的重要色素，叶绿素含量高低直接影响植物光合作用过程，影响植物积累养分的过程[70]。可溶性糖在植物体内具有储存能量、充当物质转移介质、结构物质和功能物质的作用[71]。可溶性蛋白与植物生长发育有很大关系[71]。硝酸还原酶在植物氮素同化过程中起关键作用，可有效避免植物在向土壤吸收氮元素过程中产生过多对植物有害的亚硝酸盐，从而保护植物，提高氮素同化效率[72]。

3.2.5.1 生理指标差异分析

表 3-2-15 是各生理指标的方差分析结果，从中可以看出，各生理指标差异并不显著，只有叶绿素 a 和叶绿素总 0.01＜p 值＜0.05，达到显著水平，其余指标 p 值＞0.05，均未达到显著水平。

表 3-2-15 福建柏不同种源生理指标方差分析

Tab.3-2-15 The variance analysis of *Fokienia hodgirtsii* physiological indexs from different provenances

项目	平方和	自由度	均 方	F 值	p 值
叶绿素 a	1597642.5060	16	99852.6566	2.2170	0.0252*
叶绿素 b	207101.3603	16	12943.8350	1.8360	0.0673
叶绿素总	2826997.6579	16	176687.3536	2.3100	0.0199*
可溶性糖	208.7243	16	13.0453	1.2400	0.2899
可溶性蛋白	13.9457	16	0.8716	1.6160	0.1175
硝酸还原酶活性	237910.7250	16	14869.4230	1.0520	0.4330

注：**代表 p＜0.01 达到极显著水平，*代表 p＜0.05 达到显著水平

中国知网 https://www.cnki.net

3.2.5.2 叶绿素含量比较分析

根据方差分析结果，选取差异达到显著水平的叶绿素含量进行种源间比较分析，得到结果如图3-2-9和表3-2-16，从中可知，叶绿素a含量、叶绿素b含量和叶绿素总含量最高的均为16号种源，分别达到1181.74mg·g^{-1}，435.03mg·g^{-1}，1616.77mg·g^{-1}，而含量最低的也均为4号种源，仅有563.54 mg·g^{-1}, 188.15 mg·g^{-1}, 751.69 mg·g^{-1}，最高种源和最低种源差异甚大。叶绿素a的变异范围在8.81%～46.82%，叶绿素b在6.28%～52.59%，叶绿素总在7.72%～47.24%，可见不同种源间种内变异幅度很大。

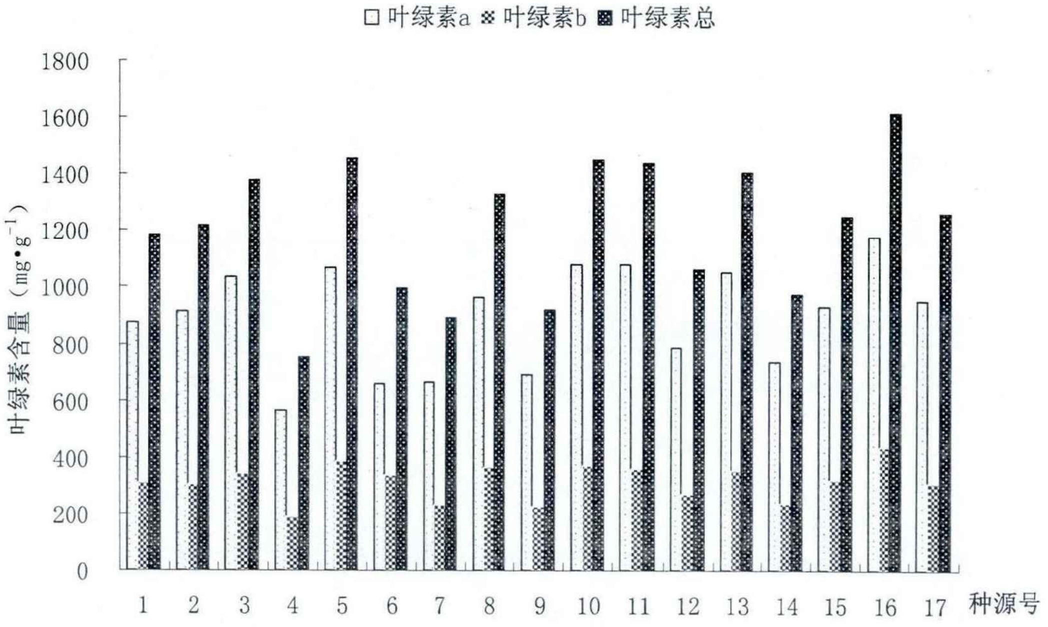

图 3-2-9 福建柏不同种源叶绿素含量比较

Fig.3-2-9 The comparison of *Fokienia hodgirtsii* chlorophyll content from different provenances

中国知网 https://www.cnki.net

表 3-2-16 福建柏不同种源叶绿素含量比较

Tab.3-2-16 The comparison of *Fokienia hodgirtsii* chlorophyll content from different provenances

编号	叶绿素 a（mg·g^{-1}）		叶绿素 b（mg·g^{-1}）		叶绿素总（mg·g^{-1}）	
	$\bar{x}$	CV（%）	$\bar{x}$	CV（%）	$\bar{x}$	CV（%）
1	875.70	20.20	309.73	18.60	1185.43	19.78
2	914.23	46.82	305.76	48.51	1219.99	47.24
3	1035.24	35.17	344.42	35.40	1379.66	35.15
4	563.54	19.76	188.15	21.29	751.69	20.14
5	1069.25	8.81	385.93	6.28	1455.18	8.14
6	656.94	33.35	337.77	52.59	994.70	7.72
7	663.08	29.61	230.34	40.26	893.42	32.16
8	965.02	11.09	362.99	7.97	1328.01	9.42
9	692.84	20.36	225.22	29.74	918.06	22.55
10	1078.97	21.91	372.12	18.42	1451.09	20.89
11	1078.53	17.29	362.06	20.87	1440.59	18.16
12	787.60	20.21	273.55	16.94	1061.15	19.27
13	1051.23	15.85	353.13	21.31	1404.36	17.22
14	734.97	24.07	238.90	24.68	973.88	24.03
15	929.50	15.42	320.58	13.05	1250.08	14.79
16	1181.74	21.79	435.03	17.43	1616.77	20.54
17	951.52	13.92	312.47	16.98	1263.98	14.68

3.2.6 福建柏地理种源变异规律

通过不同种源福建柏生长性状、生理性状和种源地地理气象因子相关性分析，可获悉各性状受不同地理气象因子的影响情况，从而得到福建柏种源地理变异规律，为福建柏种源选择合适的育苗环境提供帮助。

从表 3-2-17 中各性状和地理气象因子相关性分析得知，总体相关性程度不高，最大相关系数为 0.688，仅个别指标达到显著相关。其中球果宽、种子千粒重、地径、地下鲜重、地下干重、侧枝数、根长、根表面积、根平均直径、根体积、根尖数、叶绿素含量、可溶性蛋白和硝酸还原酶活性这 14 个指标与各地理气象因子的相关性均未达到显著水平，可认为它们之间相关性不大甚至不相关。

球果和种子指标中，在所有地理气象因子中，球果长和球果重只与经度于 0.05 水平下达到正相关，相关系数分别为 0.536 和 0.503。

苗高只与海拔于 0.05 水平下达到显著负相关，相关系数为-0.500。

生物量指标中，总鲜重与年无霜期于 0.01 水平下达到极显著正相关，相关系数为 0.667；总干重与海拔于在 0.05 水平下达到显著负相关，相关系数为-0.491，与年无霜期于 0.01 水平下达到极显著正相关，相关系数为 0.647；地上鲜重与年无霜期于 0.01 水平下达到极显著正相关，相关系数为 0.668；地上干重与年无霜期于 0.01 水平下达到极显著正相关，相关系数为 0.610。

中国知网 https://www.cnki.net

生理指标中，仅可溶性糖含量与经度和纬度于 0.05 水平下达到显著正相关，相关系数分别为 0.596 和 0.448。

综上可知，在所有地理气象因子中，经纬度、海拔和年无霜期对各生长和生理指标有不同程度的影响。球果长和球果重受经度影响较大，总干鲜重和地上干鲜重受到年无霜期影响较大，总干重和苗高受海拔影响较大，可溶性糖含量受经纬度影响较大。得到种源地理变异规律为：球果长和球果重都随经度增大而增大；生物量积累量随年无霜期加长而增大，苗高和总干重随海拔上升而减小，可溶性糖含量随经纬度增大而升高。

表 3-2-17 福建柏不同种源各性状和地理气象因子相关性分析

Tab.3-2-17 The correlation analysis between geographical and meteorological factors and *Fokienia hodgirtsii* each traits from different provenances

项目	北纬	东经	海拔	年均温	年无霜期	年日照时数	年降水量
球果长	0.425	0.536*	-0.047	0.230	-0.054	0.281	-0.444
球果宽	0.386	0.478	-0.179	0.121	-0.053	0.378	-0.448
球果重	0.224	0.503*	0.046	0.328	0.000	0.453	-0.296
种子千粒重	-0.367	-0.205	0.390	-0.056	0.043	-0.322	0.158
苗高	0.040	-0.215	-0.500*	-0.064	0.204	-0.146	-0.392
地径	-0.174	0.012	-0.090	-0.195	0.191	0.064	0.132
总鲜重	-0.359	-0.298	-0.452	0.045	0.667**	-0.171	0.004
总干重	-0.372	-0.323	-0.491*	0.063	0.647**	-0.187	-0.014
地上鲜重	-0.364	-0.307	-0.434	0.056	0.688**	-0.175	0.003
地上干重	-0.382	-0.354	-0.468	0.020	0.610**	-0.227	-0.092
地下鲜重	-0.205	-0.137	-0.443	-0.073	0.279	-0.096	0.011
地下干重	-0.127	-0.127	-0.444	-0.004	0.239	-0.057	0.008
侧枝数	-0.186	-0.007	0.052	-0.105	-0.003	0.081	-0.074
根长	0.025	0.117	-0.387	0.118	0.101	0.201	0.145
根表面积	-0.109	0.014	-0.381	0.044	0.178	0.083	0.147
根平均直径	-0.446	-0.354	-0.122	-0.219	0.280	-0.408	-0.011
根体积	-0.195	-0.056	-0.365	-0.009	0.227	0.001	0.140
根尖数	0.048	0.005	-0.473	0.048	0.126	0.102	0.052
叶绿素含量	-0.057	-0.160	-0.203	-0.284	-0.163	-0.073	0.113
可溶性糖含量	0.596*	0.488*	-0.237	0.379	0.021	0.193	-0.101
可溶性蛋白含量	-0.199	-0.278	0.028	0.078	0.218	-0.149	0.008
硝酸还原酶活性	0.029	0.073	0.258	-0.198	-0.199	-0.318	-0.181

注：**代表在0.01水平下极显著相关，*代表在0.05水平下显著相关

中国知网 https://www.cnki.net

3.2.7 福建柏种源性状聚类分析

本试验采用欧式距离的最短距离法对 17 个福建柏种源进行聚类分析，每组数据均经过标准化处理生成四种聚类分析树状图。

对不同种源球果和种子性状进行聚类分析（图 3-2-10），在距离为 1.36 时，将 17 个种源分成 4 类：第一类仅 1 号种源，此类球果和种子性状评价最低；第二类有 3 号、10 号、6 号、16 号、9 号、11 号共计 6 个种源，此类球果和种子性状评价较高；第三类有 4 号、7 号、15 号、13 号、5 号、12 号、17 号、14 号、8 号共计 9 个种源，此类球果和种子性状评价最高；第四类只有 2 号种源，此类球果和种子性状评价较低。

对不同种源苗高地径和生物量情况进行聚类分析（图 3-2-11），在距离为 2.74 时，将 17 个种源分成 3 类：第一类有 1 号、4 号、6 号、8 号、12 号、11 号、15 号、5 号、7 号、14 号、16 号、2 号和 10 号共计 13 个种源，此类苗高地径和生物量评价最低；第二类有 9 号、17 号和 13 号这 3 个种源，此类苗高地径和生物量评价中等；第三类仅 3 号种源，此类苗高地径和生物量评价最高。

对不同种源根系性状进行聚类分析（图 3-2-12），在距离为 1.6 时，将 17 个种源分成 3 类：第一类有 1 号、9 号、3 号、4 号、5 号、14 号、15 号、8 号、12 号、7 号、13 号、16 号、6 号、11 号、17 号共计 15 个种源，此类根系性状评价最高；第二类仅 10 号种源，此类根系性状评价中等，第三类仅 2 号种源，此类根系性状评价最低。

对不同种源生理指标含量进行聚类分析（图 3-2-13），在距离为 1.93 时，将 17 个种源分成 3 类：第一类有 1 号、6 号、12 号、15 号、7 号、9 号、14 号、10 号、17 号、13 号、2 号、11 号、3 号、5 号、16 号共计 15 个种源，此类生理指标评价中等；第二类只有 4 号种源，此类生理指标评价最低；第三类只有 8 号种源，此类生理指标评价最高。

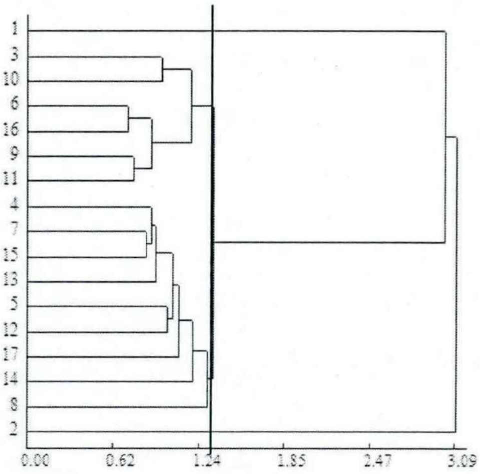

图 3-2-10 球果和种子性状的聚类分析

Fig.3-2-10 Cluster analysis of cone and seed traits

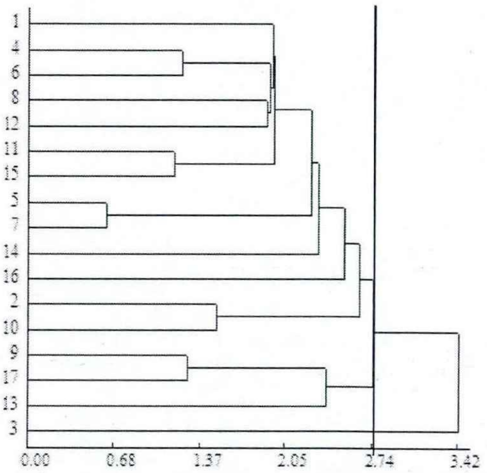

图 3-2-11 苗高地径和生物量的聚类分析

Fig.3-2-11 Cluster analysis of height , diameter and biomass

中国知网 https://www.cnki.net

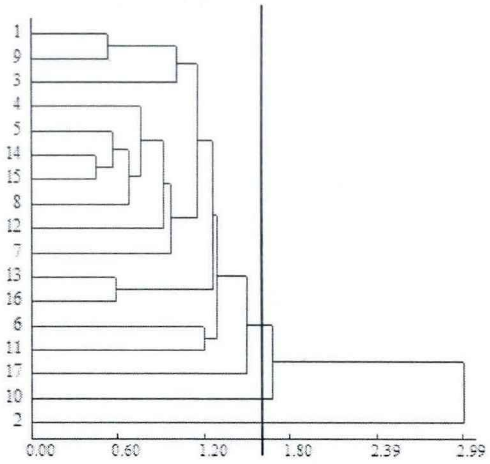

图 3-2-12 根系性状的聚类分析

Fig.3-2-12 Cluster analysis of root traits

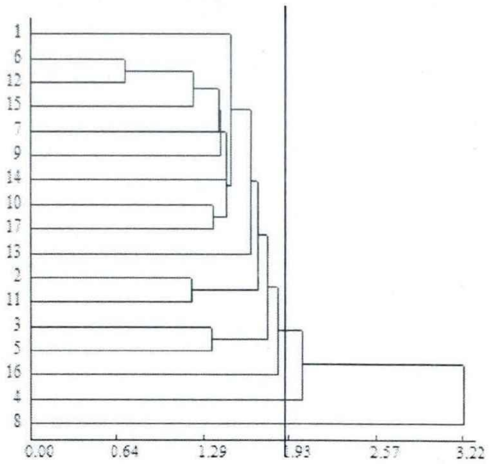

图 3-2-13 生理指标含量的聚类分析

Fig.3-2-13 Cluster analysis of physiological indexes content

3.2.8 福建柏优良种源选择

3.2.8.1 各性状的主成分分析

对福建柏种源 22 个性状进行主成分分析，得到结果如表 3-2-18 所示，以特征值大于 1 为标准提取 6 个主成分，累计贡献率达到 88.778%，意味着这 6 个主成分可以涵盖所有性状 88.778%的信息，起到高度概括的效果，因此选取前 6 个主成分进行分析。

表3-2-18 解释总方差

Tab.3-2-18 Total variance explained

成分	初始特征值			提取的平方和载荷量		
	特征值	贡献率%	累计贡献率%	特征值	贡献率%	累计贡献率%
1	9.238	41.990	41.990	9.238	41.990	41.990
2	3.230	14.682	56.672	3.230	14.682	56.672
3	2.256	10.253	66.924	2.256	10.253	66.924
4	2.057	9.349	76.273	2.057	9.349	76.273
5	1.467	6.667	82.940	1.467	6.667	82.940
6	1.284	5.838	88.778	1.284	5.838	88.778

将各因子的指标负荷量排列成成分矩阵，得到如表 3-2-19 结果，可以看出，主成分 1 中，苗高（0.514）、地径（0.679）、总鲜重（0.820）、总干重（0.882）、地上鲜重（0.770）、地上干重（0.828）、地下鲜重（0.965）、地下干重（0.930）、根长（0.826）、根表面积（0.896）、根体积（0.894）、根尖数（0.850）分别都是 6 个主成分里最高的，说明第 1 主成分可以代表这 12 个变量。主成分 2 中，球果和种子性状里的球果长（0.883）、球果宽（0.902）、球果重（0.916）分别是 6 个主成分里最高的，说明第 2 主成分可以代表这 3 个变量。主成分 3 中，生理指标里的可溶性蛋白（0.608）和硝

中国知网 https://www.cnki.net

酸还原酶活性（0.419）是 6 个主成分里最高的，说明第 3 主成分可以代表这 2 个变量。主成分 4 中，种子千粒重（0.741）和根平均直径（0.640）是 6 个主成分里最高的，说明第 4 主成分可以代表 2 个变量。主成分 5 中，叶绿素含量（0.765）和可溶性糖含量（0.671）是 6 个主成分里最高的，说明第 5 主成分可以代表这个变量。主成分 6 中，侧枝数（0.493）和叶绿素含量（0.820）是 6 个主成分里最高的，说明第 6 主成分可以代表这 2 个变量。

分析可知，第 1 主成分涵盖了除了根平均直径外所有的生长指标，第 2 主成分代表了除种子千粒重外所有球果和种子指标，第 3 主成分则代表了多数生理指标，而第 4 主成分是对第 1 和第 2 主成分的补充，第 5 主成分是对第 3 主成分的补充，第 6 主成分是对第 1 和第 3 主成分的补充。

表3-2-19 福建柏种源各指标的成分矩阵

Tab.3-2-19 Component Matrix of each indexs of *Fokienia hodgirtsii*

指标	主成分					
	1	2	3	4	5	6
球果长	-0.084	0.883	0.300	-0.114	0.254	-0.055
球果宽	0.262	0.902	0.084	-0.043	-0.053	-0.113
球果重	0.041	0.916	0.187	0.099	0.015	0.004
种子千粒重	-0.397	-0.043	0.172	0.741	0.085	-0.397
苗高	0.514	-0.054	0.162	-0.613	0.160	-0.343
地径	0.679	0.362	0.299	0.221	-0.157	-0.003
总鲜重	0.820	-0.275	0.434	-0.071	0.169	-0.007
总干重	0.882	-0.235	0.357	-0.059	0.114	-0.074
地上鲜重	0.770	-0.306	0.485	-0.091	0.170	0.001
地上干重	0.828	-0.307	0.404	-0.078	0.112	-0.013
地下鲜重	0.965	0.060	-0.121	0.109	0.101	-0.064
地下干重	0.930	0.068	-0.285	0.062	0.037	-0.090
侧枝数	0.462	0.262	0.248	0.352	-0.387	0.493
根长	0.826	0.137	-0.401	-0.046	-0.141	0.058
根表面积	0.896	0.116	-0.359	0.153	-0.017	0.036
根平均直径	0.522	-0.029	0.054	0.640	0.301	-0.101
根体积	0.894	0.094	-0.305	0.286	0.071	0.029
根尖数	0.850	0.022	-0.337	-0.313	-0.174	-0.035
叶绿素含量	0.053	-0.131	-0.045	0.036	0.435	0.820
可溶性糖含量	-0.173	0.432	-0.244	-0.349	0.671	0.082
可溶性蛋白含量	0.014	0.137	0.608	-0.313	-0.440	0.188
硝酸还原酶活性	-0.474	-0.039	0.419	0.227	0.344	0.023

中国知网 https://www.cnki.net

由表3-2-19中各指标的载荷量可以得到各主成分得分的线性方程，用X_1、X_2、X_3、X_4、X_5、X_6、X_7、X_8、X_9、X_{10}、X_{11}、X_{12}、X_{13}、X_{14}、X_{15}、X_{16}、X_{17}、X_{18}、X_{19}、X_{20}、X_{21}、X_{22}分别表示球果长、球果宽、球果重、种子千粒重、苗高、地径、总鲜重、总干重、地上鲜重、地上干重、地下鲜重、地下干重、侧枝数、根长、根表面积、根平均直径、根体积、根尖数、叶绿素含量、可溶性糖含量、可溶性蛋白含量和硝酸还原酶活性，用Y_1、Y_2、Y_3、Y_4、Y_5、Y_6分别表示第1、第2、第3、第4、第5和第6主成分，得到如下线性方程：

$$Y_1=-0.084X_1+0.262X_2+0.041X_3-0.397X_4+0.514X_5+0.679X_6+0.820X_7+0.882X_8+0.770X_9+0.828X_{10}+0.965X_{11}+0.930X_{12}+0.462X_{13}+0.826X_{14}+0.896X_{15}+0.522X_{16}+0.894X_{17}+0.850X_{18}+0.053X_{19}-0.173X_{20}+0.014X_{21}-0.474X_{22}$$

$$Y_2=0.883X_1+0.902X_2+0.916X_3-0.043X_4-0.054X_5+0.362X_6-0.275X_7-0.235X_8-0.306X_9-0.307X_{10}+0.060X_{11}+0.068X_{12}+0.262X_{13}+0.137X_{14}+0.116X_{15}-0.029X_{16}+0.094X_{17}+0.022X_{18}-0.131X_{19}+0.432X_{20}+0.137X_{21}-0.039X_{22}$$

$$Y_3=0.300X_1+0.084X_2+0.187X_3+0.172X_4+0.162X_5+0.299X_6+0.434X_7+0.357X_8+0.485X_9+0.404X_{10}-0.120X_{11}-0.285X_{12}-0.248X_{13}-0.401X_{14}-0.359X_{15}+0.054X_{16}-0.305X_{17}-0.337X_{18}-0.045X_{19}-0.224X_{20}+0.608X_{21}+0.419X_{22}$$

$$Y_4=-0.114X_1-0.043X_2+0.099X_3+0.741X_4-0.613X_5+0.221X_6-0.071X_7-0.059X_8-0.091X9-0.078X_{10}+0.109X_{11}+0.062X_{12}+0.352X_{13}-0.046X_{14}+0.153X_{15}+0.640X_{16}+0.286X_{17}-0.313X_{18}+0.036X_{19}-0.349X_{20}-0.313X_{21}+0.227X_{22}$$

$$Y_5=0.254X_1-0.053X_2+0.015X_3+0.085X_4+0.160X_5-0.157X_6+0.169X_7+0.114X_8+0.170X_9+0.112X_{10}+0.101X_{11}+0.037X_{12}-0.387X_{13}-0.141X_{14}-0.017X_{15}+0.301X_{16}+0.071X_{17}-0.174X_{18}+0.435X_{19}+0.671X_{20}-0.440X_{21}+0.344X_{22}$$

$$Y_6=-0.055X_1-0.113X_2+0.004X_3-0.397X_4-0.343X_5-0.003X_6-0.007X_7-0.074X_8+0.001X_9-0.013X_{10}-0.064X_{11}-0.090X_{12}+0.493X_{13}+0.058X_{14}+0.036X_{15}-0.101X_{16}+0.029X_{17}-0.035X_{18}+0.820X_{19}+0.082X_{20}+0.188X_{21}+0.023X_{22}$$

3.2.8.2 各种源的综合得分

由于各主成分的贡献率不同，因此选取它们的贡献率作为计算综合得分的权重，所以这6个主成分的权重分别为：41.990%、14.682%、10.253%、9.349%、6.667%、5.838%。综合评价得分计算公式为：

$$W=41.990\%\times Y_1+14.682\%\times Y_2+10.253\%\times Y_3+9.349\%\times Y_4+6.667\%\times Y_5+5.838\%\times Y_6$$

经过计算后得到福建柏各种源综合得分和排名情况如表3-2-20：最高得分为207.9150，最低得分为95.9367，相差111.9783。根据综合得分选出前5名优良种源是：福建三明莘口、广西柳州柳北、

中国知网 https://www.cnki.net

福建泉州安溪、福建三明尤溪、广东韶关曲江，得分分别为：207.9150、194.4600、182.4412、179.5356、165.3299。排名比较靠后的几个种源是福建福州闽侯、福建莆田仙游、福建龙岩上杭、福建泉州永春、广西来宾金秀。

表 3-2-20 福建柏种源综合得分和排名

Tab.3-2-20 Comprehensive scores and rangkings of *Fokienia hodgirtsii* provenances

编号	种源地	综合得分	排名
1	广东韶关曲江	165.3299	5
2	广西来宾金秀	95.9367	17
3	广西柳州柳北	194.4600	2
4	福建龙岩上杭	127.2079	15
5	福建龙岩长汀	157.0483	6
6	福建福州闽侯	131.4919	13
7	福建福州闽清	133.1809	12
8	福建福州罗源	135.4303	11
9	福建福州永泰	137.9515	10
10	福建宁德古田	144.5179	9
11	福建南平延平	150.3028	8
12	福建莆田仙游	130.2753	14
13	福建泉州安溪	182.4412	3
14	福建泉州永春	121.2157	16
15	福建漳州华安	152.5312	7
16	福建三明莘口	207.9150	1
17	福建三明尤溪	179.5356	4

4.结论与讨论

4.1 结论

4.1.1 不同种源福建柏球果和种子性状比较

对不同种源福建柏球果和种子性状的方差分析结果显示各种源的球果和种子指标在0.01 水平下均达到极显著差异。

多重比较结果显示广东韶关曲江种源球果性状与其他种源相比差异较大，广西柳州柳北种源种子千粒重与其他种源相比差异较大，在 0.01 水平下最大均达到 F。球果长、宽、长×宽、长/宽、重各指标表现最佳的是福建龙岩长汀种源，分别达到 1.94cm、1.87cm、3.63、1.04 和 3.63g。种子千粒重最高为广西来宾金秀种源，达到 0.9506g。变异系数方面，福建龙岩长汀种源种子千粒重种内变化较大，变异系数达 22.26%。

对所有球果和种子性状相关性分析结果显示，球果和种子性状间总体上呈显著相关，相关系数最大达到 0.956，只有长/宽与千粒重和其他性状间的相关性偏小，球果重基本随球果长宽的增大而增大，而千粒重则呈现相反规律。

中国知网 https://www.cnki.net

4.1.2 不同种源福建柏发芽情况比较

对福建柏不同种源的种子发芽率进行分析得出，发芽率从 15.75%～59.22%不等，不同种源福建柏种子发芽率存在很大差异。福建龙岩的两个种源和福建泉州的两个种源发芽率相对较高，均超过 40%，其中福建泉州安溪种源和福建泉州永春种源超过 50%，发芽率最高的是福建泉州安溪种源，达到 59.22%。

4.1.3 不同种源福建柏生长节律比较

对苗高生长趋势和各月净生长量值分析显示，各种源苗高生长基本呈现“慢-快-慢-快-慢”变化趋势，3 月份开始，生长较为缓慢，4-5 月份时开始出现第一次生长小高峰，7-8 月到达第二次高峰，净生长量达到全年最大值，其中福建宁德古田种源最大，10 月份之后开始进入缓慢生长阶段。通过苗高前后生长情况比较，发现前后生长并不存在相关性。

对地径生长趋势和各月净生长量值分析显示，总体呈现“慢-快-慢”变化趋势，但各种源间生长趋势并不如苗高相似，6 月前规律大不相同，7-8 月出现一次生长高峰，净生长量达到全年最大值，其中福建福州闽清最大，8 月之后开始进入缓慢生长阶段。

用 Logistic 曲线方程对不同福建柏苗高地径生长过程的拟合结果显示拟合效果并不理想，虽然各种源的 P 值均＜0.01，但决定系数 R^2 没有达到 0.99 以上，说明福建柏苗高地径生长过程并不适合用 Logistic 曲线方程进行拟合。

4.1.4不同种源福建柏苗高地径和生物量比较

对不同种源福建柏苗高地径和生物量的方差分析结果显示，除地径、地下干重/地下鲜重和侧枝数差异未达到显著水平，其他指标均达到极显著差异。

多重比较结果显示，苗高地径在种源两两之间差异显著性不强，广西柳州柳北种源的生物量积累情况与其他种源相比差异较大。苗高最大为广西来宾金秀种源，达到25.06cm，其次是福建福州闽侯种源，地径最大为福建福州永泰种源，达到2.85mm，其次是福建泉州永春种源。生物量积累最大的为广西柳州柳北种源，总鲜重达到6.18g，总干重达到2.23g，地上鲜重达到5.58g，地上干重达到1.94g，地下鲜重达到0.61g，地下干重达到0.29g。从变异系数看，苗高和地径变异系数变动幅度很相近，其中广东韶关曲江种源苗高地径变异最大，苗高地径的变异系数分别达到20.99%和29.90%，可见其种内生长最不平均。

对不同种源干鲜重比的比较分析结果显示，福建三明尤溪种源生物量在地下积累最大，地下鲜重/地上鲜重和地下干重/地上干重分别达到0.192和0.273。地上部分含水量最高为福建泉州永春种源，干鲜比为0.306；地下部分含水量最高为福建莆田仙游种源，干鲜比为0.460；整株含水量最高为福建宁德古田种源干鲜比为0.328。从变异系数看，福建泉州永春种源在种源内地上地下部分生物量分配和干鲜比差异很大，变异系数达到61.52%。

中国知网 https://www.cnki.net

对不同种源所有生长性状和生物量指标的相关性分析结果显示，所有指标间基本呈现不同程度正相关，相关系数最大达到 0.996，极显著正相关性集中在干鲜重生物量指标间，可见福建柏种源苗生物量在地上地下部分空间分配上呈现高度相关性，可通过测定某部分生物量推测出其他部分的生物量情况。

4.1.5 不同种源福建柏根系性状比较

对不同种源福建柏根系指标的方差分析结果显示，各根系性状差异均达到极显著水平。根长最大达 177.09cm，根表面积最大达 34.42 cm^2，平均根系直径最大达 0.68mm，根体积最大达 0.54 cm^3，根尖数达 159，其中福建三明莘口种源的根长、根表面积和根体积指标均为最大，其根系情况较其他种源更好。对所有根系性状间的相关性分析结果显示，不同种源根系指标间相关性很大，相关系数最大达 0.975，在根系分析中，可以通过一种指标推测出其他指标的情况。

4.1.6 不同种源福建柏生理指标比较

对不同种源福建柏生理指标的方差分析结果显示，各生理指标差异并不显著，仅种源间叶绿素差异达到显著水平。选取差异达到显著水平的叶绿素含量进行种源间比较分析得，叶绿素 a 含量、叶绿素 b 含量和叶绿素总含量最高的均为福建三明莘口种源，分别达到 1181.74$mg \cdot g^{-1}$，435.03$mg \cdot g^{-1}$，1616.77$mg \cdot g^{-1}$，且不同种源间种内变异幅度很大。

4.1.7 福建柏地理种源变异规律

结合福建柏各性状和各种源地地理气候因子相关性分析后得最大相关系数为 0.688，在所有地理气象因子中，经纬度、海拔和年无霜期对各生长和生理指标均有不同程度影响。球果长和球果重受经度的影响较大，总干鲜重和地上干鲜重受到年无霜期的影响最大，总干重和苗高受海拔影响较大，可溶性糖含量受经纬度影响较大。得到的种源地理变异规律为：球果长和球果重都随经度增大而增大；生物量积累量随年无霜期加长而增大，苗高和总干重随海拔上升而减小，可溶性糖含量随经纬度增大而升高。

4.1.8 福建柏种源性状聚类分析

对 17 个种源 22 个性状进行聚类分析，根据球果和种子性状分成 4 类，评价最高的一类包括福建省龙岩、福州、莆田、泉州几地共计 9 个种源；根据苗高地径和生物量分成 3 类，评价最高的是广西柳州柳北种源，评价中等的一类包括福建省福州、泉州、三明几地共计 3 个种源；根据根系性状分成 3 类，评价最高的一类包括广东省、福建省龙岩、福州、南平、莆田、泉州、漳州和三明几地共计 15 个种源；根据生理指标含量分成 3 类，评价最高的是福建福州罗源种源，评价中等的一类包括除福建龙岩上杭种源和福建福州罗源种源之外的其他种源。

4.1.9 福建柏优良种源选择

对福建柏各种源 22 个性状进行主成分分析，提取 6 个主成分，涵盖所有性状 88.778%的信息，

中国知网 https://www.cnki.net

以每个主成分贡献率作为权重，经过计算后得到福建柏各种源综合得分和排名，最高得分为207.9150，最低得分为 95.9367，相差 111.9783。选出前 5 名的优良种源分别为：福建三明莘口、广西柳州柳北、福建泉州安溪、福建三明尤溪、广东韶关曲江，排名比较靠后的几个种源是福建福州闽侯、福建莆田仙游、福建龙岩上杭、福建泉州永春、广西来宾金秀。

4.2 讨论

4.2.1 球果和种子指标和生长指标

在前文对球果和种子指标与生长指标的分析研究中，可知球果和种子指标中表现良好的种源并不一定是后期生长指标表现优秀的种源，说明这两种指标之间可能并不存在必然联系。虽说果实和种子是每一种植物生长的基础，但是这其中涉及到其他方方面面的指标，比如种子含水率、糖和蛋白质等有机营养成分、种子生活力等种子品质指标等[54]、[73]，由于种子数量有限等原因，所以没能完成这些生理指标的测量，有待今后进一步试验。

4.2.2 生长曲线的拟合

用 Logistic 曲线方程对苗高地径生长过程的拟合研究显示多种植物采用此方程拟合都取得了很好效果，计算出的决定系数 R^2 均达到 0.99 以上，并应用方程参数 a、b 和 k 计算出生长极限、物候期参数和生长期参数各种指标[35-41]，而使用 Logistic 曲线方程对福建柏不同种源苗高地径生长过程进行拟合后发现 k 值＜0 或 a、b 无法算出等情况，可能存在两方面原因，一方面由于福建柏的生长过程不适合使用该方程拟合，另一方面由于试验周期不够长导致得到的数值不够全面，这有待今后进行试验验证。

4.2.3 福建柏苗期地理种源变异规律差异

在苗期地理种源变异规律研究中，得到球果长和球果重都随经度增大而增大；生物量积累量随年无霜期加长而增大，苗高和总干重随海拔上升而减小，可溶性糖含量随经纬度增大而升高的规律。其中生物量积累量随年无霜期加长而增大的结论与侯伯鑫[28]、杨宗武等人[15]相同，关于福建柏球果形态，可溶性糖含量与地理气象因子关系的研究还偏少。而在本研究中发现，地径和各地理气象因子间的相关性并不显著，这和林峰[31]的研究结论有些不同，可见关于福建柏种源地理气候变异规律还有待进一步研究探讨。

4.2.4 各指标高低和综合得分的差异

在对各种源进行综合得分计算后，得到排在前几位的福建三明莘口种源、广西柳州柳北种源、福建泉州安溪种源和福建三明尤溪种源等在前面各指标的评价中并没有太多突出表现，而是表现的比较平均，在计算总分时将所有指标纳入考量范围后得出，只有在球果和种子、生长、生理各方面都兼备的种源才是最优种源，因此我们不能仅靠单一指标就判断该种源是否优良，而是要从多方面进行考量。

中国知网 https://www.cnki.net

4.2.5 最优种源和根系性状与生理性状的关系

最后选出的最优种源是福建三明莘口种源，在经过仔细研究后发现，该种源的根系指标和生理指标表现较苗高地径和生物量指标更突出，这和以往人们通常采用苗高地径和生物量作为评价种源质量优良的做法有些出入，苗高地径和生物量是进行种源优良评价的基础，但是仅以它们作为评价标准略显单薄，还应结合根系性状和叶绿素等生理指标等生理指标评价种源地下情况和生命力情况，从而为良种选择做出更为全面的评价。

4.2.6 苗期试验种源评价选择指标

福建柏是用材树种，进行良种选择时，本应选择木材物理力学性质，例如木材抗弯强度、端面硬度、气干密度等指标对树干优劣进行评价更有说服力[11]，但由于苗期试验采用的是一年生幼苗，苗木的茎部细嫩，木质化程度低，物理性质指标难以测量，种源试验是一个长期的过程，绝不能一蹴而就，苗期试验只是基础，应在今后幼林期的造林试验中将苗期性状与幼林期树高胸径和木材物理力学性质加以结合分析，以期得到更加准确的结论。

中国知网 https://www.cnki.net

参考文献

[1]国家林业局,农业部.国家重点保护野生植物名录(第一批)[J].中华人民共和国国务院公报,2000,(13):39-47.

[2]国家环保局,中国科学院植物研究所.中国珍稀濒危保护植物名录[M].北京:科学出版社,1987:200-203.

[3]傅立国.中国植物红皮书——稀有和濒危植物(第 1 卷)[M].北京:科学出版社,1991:125-126.

[4]中国树木志编辑委员会.中国树木志[M].北京:中国林业出版社,1983:334-345.

[5]郑万钧.中国树木学（上册）[M].南京:江苏人民出版社,1961:244-245.

[6]郑万钧,傅立国.中国植物志（第 7 卷）[M].北京:科学出版社,1978:328-337.

[7]刘伟强,候伯鑫,林峰等.福建柏栽培技术[J].湖南林业科技,2013,40(3):65-67.

[8]赵青毅.福建柏森林培育研究进展[J].亚热带植物科学,2005,34(3):78-81.

[9]侯柏鑫,林峰,余格非等.福建柏资源分布的研究[J].中国野生植物资源,2005,24(1):58-64.

[10]黄树军.福建柏材用材林优良种质选育的研究[D].福州:福建农林大学,2014.

[11 陈祖松.福建柏人工林木材物理力学性质的试验研究[J].福建林学院学报,1999,19(3):223-226.

[12]黄晓东.杉木、福建柏混交林林木生长分析[J].林业勘察设计,2014,(2):110-116.

[13]中国树木志编辑委员会.中国主要树种造林技术[M].北京:中国林业出版社,1981:387-398.

[14]池上评.福建柏人工林大中径材经营模式的研究[D].福州:福建农林大学,2014.

[15]杨宗武,郑仁华,侯伯鑫等.福建柏苗期生物量种源间遗传变异及其综合评价的研究[J].林业科学研究,2003,16(1):39-44.

[16]杨宗武,郑仁华,肖祥希等.福建柏种源苗期生长和根系性状的遗传变异[J].南京林业大学学报:自然科学版,2001,25(3):26-30.

[17]郑仁华,杨宗武,梁鸿燊木等.福建柏地理种源苗期试验的研究[J].福建林学院学报,2001,21(1):40-44.

[18]廖纯茂,李明贵,何晓红等.福建柏种源试验苗期选择研究[J].湖南林业科技,1998,25(3):5-11.

[19]郑仁华,杨宗武,施季森等.福建柏优树子代苗期性状遗传变异和生长节律研究[J].林业科学,2003,39(1):179-183.

[20]侯伯鑫,程政红,曾万明等.福建柏地理种源试验苗期研究[J].湖南林业科技,2000,27(2):1-5.

[21]李振军,张新华,饶逢春等.福建柏地理种源优树家系苗期试验[J].湖南林业科技,2003,30(1):65-67.

[22]张新华.福建柏优树家系苗期生长性状遗传变异和选择研究[J].湖南林业科技,2003,30(4):19-21.

[23]曾志光,肖复明,王城辉等.福建柏种源试验苗期选择初报[J].江西林业科技,1998,(4):2-5.

[24]李晓储,黄利斌,施士争等.福建柏引种苗期研究初报[J].江苏林业科技,1999,26(3):2-5.

中国知网 https://www.cnki.net

[25]林峰.福建柏地理种源试验及培育技术研究[D].长沙:中南林学院,2004.

[26]曾志光,肖复明,包国华等.福建柏种源种子品质分析报告[J].江西林业科技,1999,(1):2-3.

[27]侯伯鑫,林峰,余格非等.福建柏地理种源湖南试点幼林期结果初报[J].林业科学研究,2006,19(1):21-26.

[28]侯伯鑫,林峰,程政红等.福建柏地理种源遗传变异及早期选择研究[J].植物遗传资源学报,2004,5(2):179-184.

[29]侯伯鑫,林峰,余格非等.福建柏地理种源试验幼林期综合评价[J].南京林业大学学报:自然科学版,2006,30(3):41-46.

[30]余格非,侯伯鑫,林峰等.福建柏优良种源和家系优树选择研究[J].湖南林业科技,2005,32(4):26-28.

[31]林峰,侯伯鑫,程政红等.福建柏幼林期种源遗传变异及早期选择研究[J].湖南林业科技,2004,31(4):18-21.

[32]郑仁华.幼龄福建柏种源生长性状的遗传变异和种源选择[J].南京林业大学学报:自然科学版,2005,29(5):8-12.

[33]侯伯鑫,林峰,余格非等.福建柏地理种源开花与结实变异规律的研究[J].植物遗传资源学报,2005,6(2):163-167.

[34]侯伯鑫,林峰,余格非等.福建柏开花与结实物候期的研究[J].中国野生植物资源,2006,25(1):43-47.

[35]杨志玲,杨旭,谭梓峰等.厚朴不同种源苗期生长模型的拟合[J].西北农林科技大学学报(自然科学版),2011,39(4):60-68.

[36]周正立,于军,陈加利等.柠条锦鸡儿和小叶锦鸡儿不同种源苗期生长动态研究[J].西北林学院学报,2011,26(6):74-79.

[37]李爱平.樱桃圆柏不同种源苗期高生长与物候节律的研究[J].内蒙古林业科技,2011,37(3):13-15.

[38]黄志玲,郝海坤,庞世龙等.红锥种源苗期生长节律研究[J].林业科技开发,2012,26(3):24-28.

[39]王旭军,张日清,许忠坤等.不同种源红榉苗期生长节律的研究[J].中南林业科技大学学报,2013,33(7):31-34.

[40]李秋荔,黄寿先,李志先等.中国马褂木不同种源苗期生长规律研究[J].广西植物,2012,32(3):355-361.

[41]叶金山,李小林,朱恒等.红楠种源苗期生长特性分析[J].江西林业科技,2013,(2):5-6.

[42]赵勋,李因刚,柳新红等.白花树不同种源苗期光响应特性研究[J].浙江林业科技,2011,31(1):1-6.

[43]惠利省,徐立安,王章荣.马褂木不同种源苗期光合特性差异分析[J].安徽农业科学,2010,38(6):3222-3224.

[44]杨万霞.不同种源青钱柳苗期生长及叶药用成分含量的差异性研究[D].南京:南京林业大学,2013.

中国知网 https://www.cnki.net

[45]赵勋.越南安息香不同种源苗期光合特性研究[D].临安:浙江农林大学,2011.

[46]刘鹏.干旱胁迫下不同喜树种源苗期生理生化特性的研究[D].临安:浙江农林大学,2011.

[47]金雅琴.乌柏实生苗培育及耐盐抗旱生理研究[D].南京:南京林业大学,2012.

[48]杜超群,许业洲,胡兴宜等.枫香不同种源苗期生长差异研究[J].湖北林业科技,2009,(5):17-20.

[49]杜鹏珍,廖绍波,孙冰等.班克木属 15 个种和种源苗期生长特征的研究[J].热带作物学报,2014,35(4):644-648.

[50]陈素传,王陆军,蔡新玲等.栓皮栎不同种源苗期变异与初步选择[J].林业科技开发,2012,26(4):22-26.

[51]祝旭加,潘丽丽,孙岳胤等.挪威云杉种源及家系苗期生长适应性的聚类分析[J].防护林科技,2012,(6):19-22.

[52]刘霞,孙冲,有详亮等.文冠果苗期种源试验[J].昆明:西南林业大学学报,2013,33(6):51-55.

[53]金雅琴,李冬林,倪利清.乌柏不同种源苗期试验初报[J].江苏林业科技,2009,36(5):1-5.

[54]陈隆升.不同种源黄连木种子品质与苗期生长特性研究[D].南京:南京林业大学,2009.

[55]李秋荔.马褂木种源苗期性状变异研究[D].南宁:广西大学,2012.

[56]张锁.油松不同种源和家系苗期性状的遗传变异分析[D].杨凌:西北农林科技大学,2010.

[57]袁显磊.核桃楸优良种源和家系早期选择及苗期环境因子影响评价[D].哈尔滨:东北林业大学,2013.

[58]王玉.刺槐（*Robinia pseudoacacia L.*)种源试验和无性系苗期选择[D].保定:河北农业大学,2011.

[59] 黄树军,荣俊冬,张龙辉等.福建柏研究综述[J].福建林业科技,2013,40(4):236-242.

[60]侯伯鑫,杨国东,余格非等. 福建柏圃地育苗技术[J].林业实用技术,2006,(12):19-20.

[61]陈福明.混合液测定叶绿素含量的研究[J].浙江林业科技,1984,2(1):19-23.

[62]王学奎.植物生理生化实验原理和技术（第 2 版）[M].北京:高等教育出版社,2006:219-221.

[63]宋志刚,谢蕾蕾,何旭东.SPSS16 实用教程（第一版）[M].北京:人民邮电出版社,2008:132-136,182-185.

[64]唐启义.DPS 数据处理系统—实验设计、统计分析及数据挖掘（第 2 版）[M].北京:科学出版社,2010:75-86,719-725,761-771.

[65]郑仁华,黄德龙,李金良等.福建柏优树选择及种实表型变异研究[J].福建林业科技,2004,31:1-6.

[66]刁松锋,邵文豪,姜景民等.基于种实性状的无患子天然群体表型多样性研究[J].生态学报,2014,34(6):1451-1460.

[67]黄勇,姚小华,王开良等.小果油茶种实表型性状遗传多样性研究[J].安徽农业大学学报,2011,38(5):698-707.

中国知网 https://www.cnki.net

[68]靳高中,任华东,姚小华等.滇西腾冲红花油茶天然居群种实表型性状变异分析[J].南京林业大学学报:自然科学版,2013,37(6):53-58.

[69]宋维峰,王希群. 林木根系研究综述[J].西南林学院学报,2007,27(5):8-13.

[70]黄秋婵,韦友欢,韦方立等.三种夹竹桃科植物中叶绿素含量的比较分析[J].湖北农业科学,2011,50(16):3392-3394.

[71]May Sandar Kyaing,顾立江,程红梅.植物中硝酸还原酶和亚硝酸还原酶的作用[J].生物技术进展,2011,1(3):159-164.

[72]吴青君,龚佑辉,徐宝云.西花蓟马主要寄主植物可溶性糖和蛋白质含量测定[J].中国蔬菜,2007,(10):20-22.

[73]孙鸿有,郑勇平,付顺华,邵晓根,董汝湘,徐高健,蔡克孝.杉木不同世代、类型种子园种子品质改良效果的研究[J].南京林业大学学报:自然科学版,2003,27(2):40-44.

中国知网 https://www.cnki.net

附 录

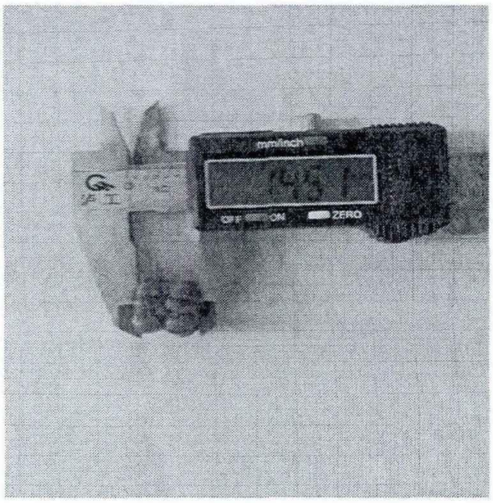

测量球果长

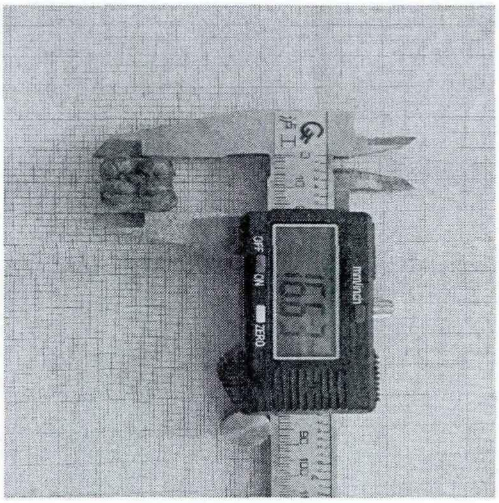

测量球果宽

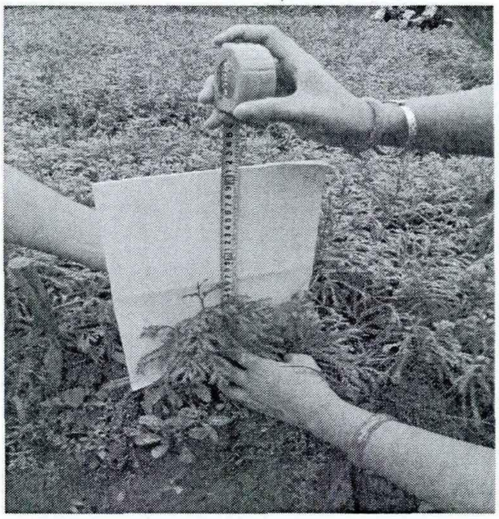

测量苗高

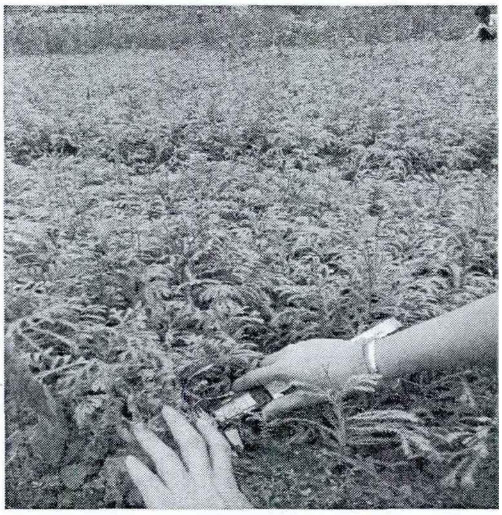

测量地径

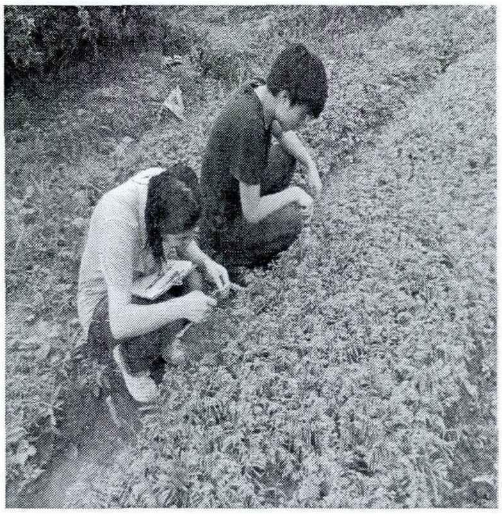

田间测量

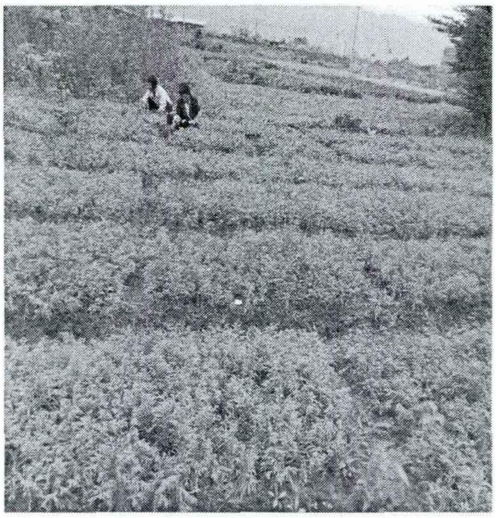

试验苗圃地

中国知网 https://www.cnki.net

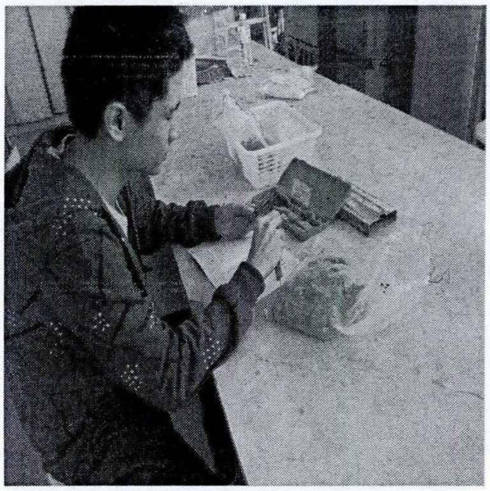

测量球果表型指标

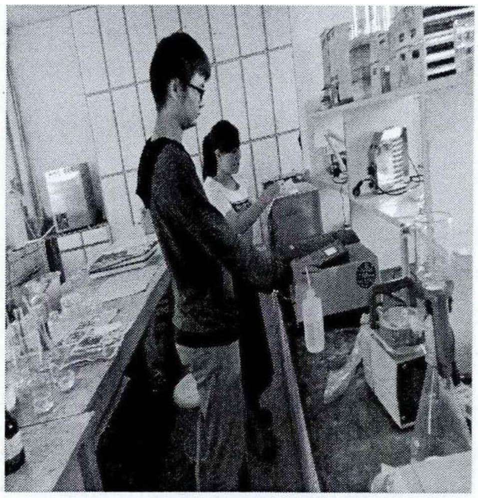

测量吸光值

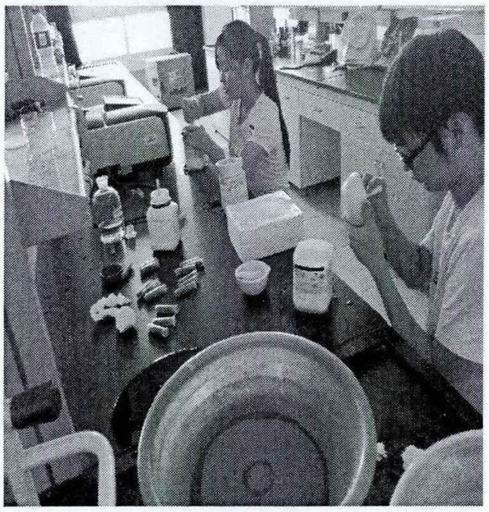

研磨样品

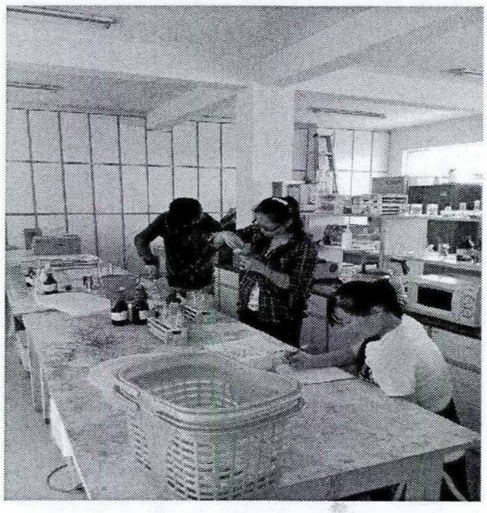

添加样品到比色皿

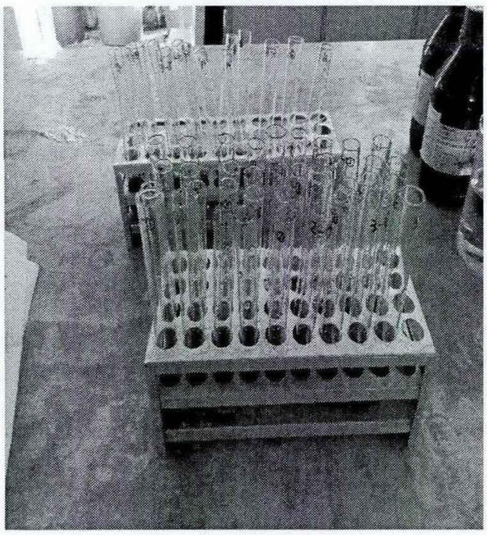

待测样品

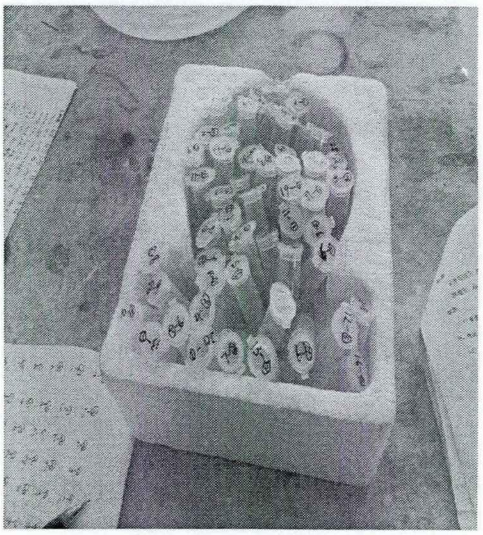

离心样品

中国知网 https://www.cnki.net

致 谢

光阴似箭，岁月如梭，两年的研究生生活即将结束。经历了找工作的喧嚣与坎坷，我深深体会到了写作论文时的那份宁静与思考。回首两年的求学历程，对那些引导我、帮助我、激励我的人，我心中充满了感激。

首先要感谢我的导师郑郁善教授，论文从定题到写作到定稿，倾注了郑老师大量的心血。在我攻读硕士研究生期间，深深受益于郑老师的关心、爱护和谆谆教导。他作为老师，点拨迷津，让人如沐春风；作为长辈，关怀备至，让人感念至深。能师从郑老师，我为自己感到庆幸。我还要感谢我的师母郑林老师，师母的和蔼可亲让我倍感亲切，感谢师母在我读研期间对我的所有关心和帮助。在此谨向郑老师和师母表示我最诚挚的敬意和感谢！

还要感谢所有教导过我、关心过我的老师，陈礼光老师、荣俊冬老师、陈凌艳老师和何天友老师，你们为我的学业倾注了大量心血，在论文的写作中给予了我许多指导与建议，并在实验过程中为我提供设备上的帮助，谨在此表示衷心的感谢。

同时，我要感谢一直关心与支持我的同学朋友们！我要感谢我的舍友们，感谢你们的一直以来的鼓励和支持，感谢工业原料林研究所的所有同门：王汉琪师姐、周少卿师兄、李单琦师兄、郑晶晶师姐和魏健康师兄，是你们在实验过程中为我答疑解惑；我的同学和师弟师妹们：许雅明、谢德金、卫梅、谢燕燕、丁鹏、蔡昕航、陆宁、余勇、田婷婷、吴玉香、罗睿、王舒、黄佳榕、姚旺、沈少炎、祁潇勇、万娟，感谢你们对我实验提供的所有帮助，没有你们就无法顺利完成实验。两年时光即将画上句号，我们朝夕相处，共同进步，感谢你们给予我的所有关心和帮助。同窗之谊，我将终生难忘！

在此要感谢我生活学习了六年的母校——福建农林大学，母校给了我一个宽阔的学习平台，让我不断吸取新知，充实自己。

最后需要特别感谢的是我的父母。父母的养育之恩无以为报，他们是我十多年求学路上的坚强后盾，在我面临人生选择的迷茫之际，为我排忧解难，他们对我无私的爱与照顾是我不断前进的动力。

中国知网 https://www.cnki.net
