# Supplementary material for: Provenance and family variations in early growth of Manchurian walnut (Juglans mandshurica Maxim.) and selection of superior families
Source: PLoS One. 2024 Mar 7;19(3):e0298918. doi: 10.1371/journal.pone.0298918 (PMC10919699; doi:10.1371/journal.pone.0298918)
Supplement: S2 File — (ZIP) [file pone.0298918.s005.zip › The selection of superior walnut (Juglans regia L.) genotypes as revealed by morphological characterization.pdf]

Euphytica (2018)214:69
https://doi.org/10.1007/s10681-018-2153-z

# The selection of superior walnut (*Juglans regia* L.) genotypes as revealed by morphological characterization

**Zahra Rezaei · Ali Khadivi · Babak ValizadehKaji · Ahmadreza Abbasifar**

Received: 7 December 2017 / Accepted: 10 March 2018
© Springer Science+Business Media B.V., part of Springer Nature 2018

**Abstract** Walnut (*Juglans regia* L.), is one of the main economic fruit species in the world and is rich in nutritious substances. In order to obtain superior walnut genotypes, a set of seedling walnut genotypes were evaluated and their results were recorded. The range of nut length was between 25.57 and 47.77 mm. Also, the ranges of nut weight, kernel weight and kernel percentage varied from 5.35 to 21.31 g, 2.49 to 11.15 g and 37.27 to 66.29%, respectively. Simple correlation analysis showed significant positive and negative correlations between some of the measured traits. Principal component analysis reduced the evaluated traits into the seven main components, which explained 71.71% of the total variance. Bi-plot analysis performed using the first two components, distributed the genotypes into four sides of the plot. Based on the present results, a high variation was observed among the trees, indicating a high potential of the studied germplasm to select the superior genotypes. According to the present research, 57 genotypes with having the highest nut weight and kernel weight, as well as bright color of the kernel, were recognized as superior genotypes and can be used to establish the new orchards or improve cultivars.

**Keywords** Walnut · Superior genotype · Nut weight · Kernel weight · Kernel percentage · Kernel color

## Introduction

Walnut (*Juglans regia* L.), known as Persian walnut, has been planted primarily for nut production since ancient times and is one of the main nut crops in the world. It is a diploid fruit tree species (Vinson and Cai 2012; Hayes et al. 2015; Tsoukas et al. 2015; Pollegioni et al. 2015) and is native to the mountainous regions of central Asia but is now the most widespread tree nut in the world (Bayazit et al. 2007; Chen et al. 2014; Pollegioni et al. 2015). In the Persian walnut, male and female flowers are formed separately on the same tree and thus it is monoecious (McGranahan and Leslie 1990). Walnut is self-compatible, but it is protandrous and in some cases is protogynous (Mert 2010) and thus the rate of cross-pollination might be high because the pollen shedding periods do not completely overlap the female receptivity (Luza and Polito 1988). Thus, considerable variation and genetic heterozygosity have been maintained in phenological and pomological characters in the natural populations

Z. Rezaei · A. Khadivi (✉) · B. ValizadehKaji · A. Abbasifar
Department of Horticultural Sciences, Faculty of Agriculture and Natural Resources, Arak University, Arak 38156-8-8349, Iran
e-mail: a-khadivi@araku.ac.ir

Published online: 15 March 2018

of walnut such as nut shape and size, shell thickness, kernel color, kernel taste and kernel oil content (McGranahan and Leslie 1990; Korac et al. 1997; Solar et al. 2002; Zeneli et al. 2005).

It has been reported that walnut is originated from Iran (McGranahan et al. 1998; Bayazit et al. 2007). Thus, this country is rich in this nut crop. Furthermore, seed has been extensively used for walnut propagation and therefore a wide range of phenotypical and pomological variabilities is presented among different populations and accessions which can be used in breeding programs.

The spontaneous flora is an important source of genetic diversity for walnut because of open-pollination (Cosmulescu et al. 2010; Cosmulescu and Botu 2012). The wide genetic variation in different geographical regions offers the opportunity to select perspective genotypes that can be used in breeding programs or used directly in culture, thus avoiding genetic erosion. Valuable genotypes have been identified in local populations from different countries including Romania (Cosmulescu and Botu 2012; Cosmulescu 2013; Tsampas and Botu 2013), India (Pandey and Tripathi 2007; Sharma et al. 2014; Angmo et al. 2015), Turkey (Keles et al. 2014), Iran (Khadivi-Khub and Ebrahimi 2015; Ebrahimi et al. 2015) and Pakistan (Hussain et al. 2016). With regards to genetic diversity, this factor is important in walnut as it offers the chance to select superior genotypes, adapted to climate conditions in the home area. Local walnuts with high variation are important to breeding programs because walnut has low biological plasticity. Analysis of diversity in walnut using non-hierarchical Euclidean cluster analysis has been applied in order to identify the best parent combinations in breeding programs and to increase the chance to get valuable elites (Attar et al. 2014).

A large number of the native genotypes in Iran possess high genetic diversity in yield, nut and kernel characters, late bud-breaking, late flowering, winter hardiness, and tolerance to disease (Khadivi-Khub 2014). This high genetic variability in walnut throughout the country presents suitable opportunities to obtain new cultivars directly or to use them in cross breeding activity. Thus, the aims of the present study were to study phenological and pomological diversity in seedling genotypes of walnut and to select the superior genotypes among several populations of walnut collected from Malayer region in Iran for direct cultivation or use them as parents in breeding programs. Further information on the nature and degree of genetic diversity found in walnuts could help to identify elite trees for genetic improvement through hybridization.

## Materials and methods

### Plant material

In the current study, 1000 walnut genotypes were selected from the five different parts of Malayer region in Iran including Samen, Avarzaman, Malayer, Jokar, and Zangeneh. Geographical coordinates and altitude corresponding to each surveyed area are presented in Table 1. The selection of the studied genotypes were based on the important pomological traits.

### Evaluation methods

The selected genotypes were evaluated in terms of 30 phenological and pomological traits based on the evaluation guide of walnut trees (IPGRI 1994). For this purpose, the visits were took place at several stages including time of leafing and flowering, and also at harvest time from the target areas. Phenological traits are largely influenced by environmental conditions. Therefore, they are usually evaluated in comparison with the reference standard (McGranahan and Forde 1985). The genotype that was the leaf earliest, was considered as the reference standard and the other genotypes were evaluated compared to that genotype (Zeneli et al. 2005). The time of leafing was considered when more than 50.00% of the terminal buds were opened and the bud scales were split and green leaves were visible (IPGRI 1994). The genotypes with minimum 6 days overlapping in their pistil receptivity and pollen shedding period, were considered to be homogamous (Yarilgac et al. 2001; Arzani et al. 2008). The harvesting date was considered when almost all of the hulls (95.00%) were easily and completely separated from hard shell (Westwood 1993). Regarding the flowering habit, the absence of flowering on lateral buds, the formation of less than 25.00% of female flowers on lateral buds, the formation of between 25.00 and 50.00% of female flowers on lateral buds and the formation of more than 50.00% of female flowers on lateral bud, respectively, were

**Table 1** Geographical data of the studied localities with sampling sizes

| Collection site | Code | Sample size | Longitude (E) | Latitude (N) | Altitude (m) |
|---|---|---|---|---|---|
| Samen | S | 300 | 48°42′36″ | 34°11′21″ | 1908 |
| Avarzaman | A | 250 | 48°31′16″ | 34°16′45″ | 1680 |
| Malayer | M | 100 | 48°51′57″ | 34°17′45″ | 1790 |
| Jokar | J | 150 | 41°41′13″ | 34°23′18″ | 1764 |
| Zanganeh | Z | 200 | 48°01′33″ | 34°09′54″ | 2014 |

named as terminal, terminal-lateral, lateral-terminal and lateral flowering habits (IPGRI 1994).

Measurements of each nut and kernel trait were based on 30 replicates and the mean values were used. The kernel weight and nut weight were measured using an electronic balance with a 0.01 g precision. The kernel percentage was also calculated from the ratio of kernel weight to nut weight: "kernel weight/ nut weight × 100". The nut width, nut length, and shell thickness were measured using a digital caliper. Characteristics such as shell texture, shell shape, shell hardness, shell color, ease of kernel removal from nut, kernel color, kernel filled, and kernel plumpness were evaluated qualitatively and based on IPGRI descriptions (IPGRI 1994).

### Statistical analysis

The differences found between the studied genotypes ($P < 0.01$) were analyzed by SAS software (SAS Inst. 1990) using one-way ANOVA. The method used to discriminate among means was Duncan's test (Multiple Range Test). The parameters including mean, minimum value, maximum value, standard deviation (SD) and coefficient of variation (CV %) were determined. Coefficients of variation (CV %) were determined as indicators of variability. Frequency and percentage distribution of the measured characters were determined for qualitative characters. The Pearson correlation coefficients were used to determine correlations between the characters using SPSS software. Principal component analysis (PCA) was applied to investigate the relationships among the genotypes using SPSS statistics software. Mean values were used to create a correlation matrix from which standardized principal component (PC) scores were extracted. The PCA was performed based on the covariance matrix of the coefficients. To avoid the effects due to scaling differences, mean of each character was normalized prior to cluster analyses using Z scores. To better understand the patterns of variation among the genotypes, the distance matrix generated from morphological data was used as input data for cluster analysis using PAST statistics software (Hammer et al. 2001). In addition, a bi-plot was created using PAST statistics software according to the PC1 and PC2.

## Results and discussion

### Genotype characterization

Out of the 1000 selected genotypes, 574 genotypes did not produce fruit properly due to different conditions including very-early flowering and then spring frost, and thus were excluded from the study. Finally, 426 genotypes were used in the final evaluations. The results of analysis of variance showed that the studied genotypes had significant differences among all the traits, which is due to diversity in the measured traits. Therefore, it was possible to select the genotypes for different values of a trait.

The flowering type had the highest coefficient of variation (CV = 71.18%), while the lowest CV was given by kernel percentage (8.20%) (Table 2). The traits with a high coefficient of variation have a wider range of attribute qualities, which provides a larger range for that attribute. Khadivi-Khub and Ebrahim (2015) reported the highest CV (31.10%) for shell thickness and the lowest CV for the nut diameter (8.60%). In another study, Khadivi-Khub et al. (2015a) reported the highest CV for the flowering date (104.20%) and the lowest CV for the nut length (9.87%) and also the nut diameter (9.20%).

Leafing time is an important trait and the difference between genotypes for this trait is due to differences in genetic and environmental conditions. The selection of late leafing cultivars is one of the major breeding goals in walnut. Late leafing genotypes can have two

**Table 2** Descriptive statistics for the morphological characters in the studied genotypes of walnut

| No | Character | Abbreviation | Unit | Min | Max | Mean | SD | CV (%) |
|---|---|---|---|---|---|---|---|---|
| 1 | Leafing date | LDa | Date | 7-Apr | 2-May | – | – | – |
| 2 | First male flowering date | FiMFlDa | Date | 9-Apr | 3-May | – | – | – |
| 3 | First female flowering date | FiFFlDa | Date | 11-Apr | 3-May | – | – | – |
| 4 | Last male flowering date | LaMFlDa | Date | 19-Apr | 7-May | – | – | – |
| 5 | Last female flowering date | LaFFlDa | Date | 22-Apr | 8-May | – | – | – |
| 6 | Female flower number | FeFlNo | Number | 1 | 15 | 5.83 | 2.86 | 49.01 |
| 7 | Male flower number | MaFlNo | Number | 5 | 30 | 14.62 | 5.86 | 40.11 |
| 8 | Flowering habit | FlHab | Code | 1 | 9 | 5.61 | 1.41 | 25.17 |
| 9 | Flowering type | FlTy | Code | 1 | 5 | 1.19 | 0.85 | 71.18 |
| 10 | Ripening date | RiDa | Date | 18-Sep | 2-Oct | – | – | – |
| 11 | Nut diameter | NutDi | mm | 24.87 | 43.59 | 32.52 | 3.19 | 9.80 |
| 12 | Nut length | NutLe | mm | 25.57 | 47.77 | 36.34 | 4.86 | 13.36 |
| 13 | Nut weight | NutWe | g | 5.35 | 21.31 | 12.48 | 3.06 | 24.54 |
| 14 | Shell thickness | SheTh | mm | 0.72 | 2.46 | 1.51 | 0.28 | 18.31 |
| 15 | Shell hardness | SheHar | Code | 1 | 9 | 5.44 | 1.78 | 32.76 |
| 16 | Shell texture | SheTe | Code | 1 | 9 | 4.07 | 2.08 | 51.13 |
| 17 | Shell color | SheCo | Code | 1 | 9 | 3.96 | 1.64 | 41.31 |
| 18 | Shell seal | SheSea | Code | 1 | 7 | 3.12 | 2.10 | 67.31 |
| 19 | Shell surface serration | SheSer | Code | 1 | 7 | 2.97 | 1.54 | 51.95 |
| 20 | Shell retention | SheRet | Code | 1 | 7 | 3.07 | 1.28 | 41.69 |
| 21 | Shell cover | SheCov | Code | 1 | 7 | 3.07 | 1.28 | 41.69 |
| 22 | Ease of kernel removal from nuts | EKerRe | Code | 1 | 9 | 4.06 | Code | 46.23 |
| 23 | Kernel weight | KerWe | g | 2.49 | 11.15 | 6.39 | 1.69 | 26.49 |
| 24 | Kernel percentage | KerPer | % | 37.27 | 66.29 | 51.12 | 4.19 | 8.20 |
| 25 | Kernel color | KerCo | Code | 1 | 9 | 4.27 | 2.60 | 60.77 |
| 26 | Kernel vein | KerVe | Code | 1 | 5 | 1.66 | 0.97 | 58.49 |
| 27 | Kernel shape | KerSha | Code | 1 | 9 | 4.75 | 3.14 | 66.19 |
| 28 | Kernel filled | KerFil | Code | 1 | 7 | 5.65 | 1.45 | 25.66 |
| 29 | Kernel plumpness | KerPlu | Code | 1 | 7 | 5.43 | 1.42 | 26.06 |
| 30 | Kernel shriveling | KerShri | Code | 1 | 7 | 2.32 | 1.34 | 57.59 |

applications. Firstly, they can be used as a suitable parent for transferring this attribute to improve cultivars in breeding programs. Secondly, they can be used as commercial cultivars if they are superior in terms of quantity and quality of fruit. Although flowering of walnut in different regions are affected by the weather, but this difference is also indicative of the genetic diversity of the walnut. Late leafing trait in walnuts has a high heritability (0.80) (Hansche et al. 1972). The range of leafing date in the studied genotypes was from April 07 to May 02. The earliest genotypes to leafing were included Malayer-552, Malayer-554, Malayer-559, Malayer-560, Malayer-585, Malayer-597, Malayer-608, Malayer-619, Malayer-640, and Malayer-645. The genotypes including Avarzaman-302, Avarzaman-304, Avarzaman-305, Avarzaman-314, Avarzaman-315, Avarzaman-322, Avarzaman-397, Avarzaman-492, Zanganeh-803, Zanganeh-815, Zanganeh-820, Zangeneh-823, Zanganeh-834, Zanganeh-850, Zanganeh-851, Zanganeh-861, Zanganeh-877, Zanganeh-955, Zanganeh-960, Zangeneh-961, Zangeneh-979 and Zangeneh-981 were late-leafing genotypes. The range of male flowering date in the genotypes was from April 19 to May 07, while the

female flowering date varied from April 22 to May 08. The positive relationships between leafing and flowering times can be used to develop resistant cultivars to frost damage (Khadivi-Khub et al. 2015a). In a research, Khadivi-Khub et al. (2015b) reported a significant positive relationships between leafing and flowering times. The positive and significant correlations between the leafing and flowering dates with the characters related to the nut and the kernel can be useful in selecting and breeding of the walnut germplasm (Khadivi-Khub 2014).

Nowadays, late leafing, early maturity, high yield and kernel quality are the most important goals in walnut. Later emergence of leaves in the spring even for a few days can play a significant role in reducing the damages due to spring frost and reducing the damage to bacterial disease (Forde 1979). The number of female flowers in the Samen-66, Avrazaman-308, Avrazaman-356, Samen-119, Samen-120, Samen-65, Samen-146, Samen-32, Samen-33, Samen-60, Samen-63 and Samen-64 genotypes was higher than the rest of the genotypes. The number of male flowers was also higher in the genotypes including Avarzaman-399, Avarzaman-329, Avarzaman-327, Avarzaman-252, Avarzaman-322, Avarzaman-321, Avarzaman-333, Avarzaman-333, Avarzaman-308 and Avarzaman-305, respectively, than the remaining genotypes. In the genotypes studied, protandry was the dominant phenomenon (96.20% of genotypes), which is consistent with most of the reports about the protandry in walnut (Forde and McGranahan 1996; Khadivi-Khub et al. 2015b).

Based on the results of the present study, 34.27% of the genotypes were mostly terminal flowering and their lateral flowering was less than 25.00%. Also, 52.34% of the genotypes had 25.00 and 50.00% lateral flowering (moderate). In a report among 31 walnut genotypes in Karaj region in Iran, 22.60% of genotypes had less than 50.00% lateral flowering and 16.10% of genotypes had more than 50.00% lateral flowering. Among them, 16.00% of genotypes were mostly terminal flowering (Norouzi et al. 2013). Karadag and Akca (2011) argued that the lateral fruiting determines the potential production. On the other hand, Norouzi et al. (2013) reported that trees with lateral fruitfulness are more susceptible to bacterial blight. The genotypes with lateral fruitfulness were often early fruitfulness and early ripened, and faster lose their leaves and, in general, they have more crops compared to those with terminal fruitfulness. The early-fruitfulness in walnut is controlled by polygene (gene clusters), and is a heritable quantitative character with phenomenon of segregation (Erturk and Dalkilic 2011).

The most important traits in walnut breeding programs are related to fruit (Sharma and Sharma 1998). High variability in fruit characteristics indicates the high potential of genotypes in the region for choosing superior genotypes based on the objectives of the breeding program (Cosmulescu and Botu 2012). Ripening date ranged from September 18 to October 02. An ideal nut for walnut should have a clean, tight, thin and well-sealed skin and weighed by 12–18 g. The kernel should be easily detachable from the shell. The kernel color should be uniform and clear, the ideal kernel weight is 6.00–10.00 g or at least 50.00% of the total weight of the nut (Khadivi-Khub 2014). Nut and kernel quality is strongly influenced by the genotype, the environment and their interaction (McGranahan and Leslie 2012). In breeding programs, genotypes with a kernel percentage of over 50.00% are highly desirable (Germain 1997). Nut weight varied from 5.35 to 21.31 g, with the highest nut weight belonging to the Samen-287 genotype, and the range of kernel weight varied from 2.49 to 11.15 g (Table 2). The Samen-243 genotype had the highest kernel weight. These values were higher than that of reported by Khadivi-Khub and Ebrahimi (2015) (19.00 g for nut weight and 9.25 g for kernel weight). The rates of variation index in nut weight and kernel weight were 24.54 and 26.49%, respectively, which indicates that the differences in kernel weight are higher than nut weight. The range of nut diameter was from 24.87 to 43.59 mm, and the Samen-72 genotype had the highest nut diameter. The range of nut length was from 25.57 to 47.77 mm, and the Samen-236 genotype had the longest nut length. Mosivand et al. (2013) reported that the highest and lowest nut diameter was 30.20 mm 14.80 mm, respectively in Iranian walnut. Khadivi-Khub et al. (2015b) reported a range of 25.00–47.00 mm for nut length and 24.00–48.00 mm for nut diameter. The pictures of kernels belonging to several the studied superior genotypes are presented in Fig. 1.

In breeding programs of walnut, increasing kernel percentage is one of the most important priorities. The kernel percentage is influenced by length, diameter and weight of the nut, and there are significant

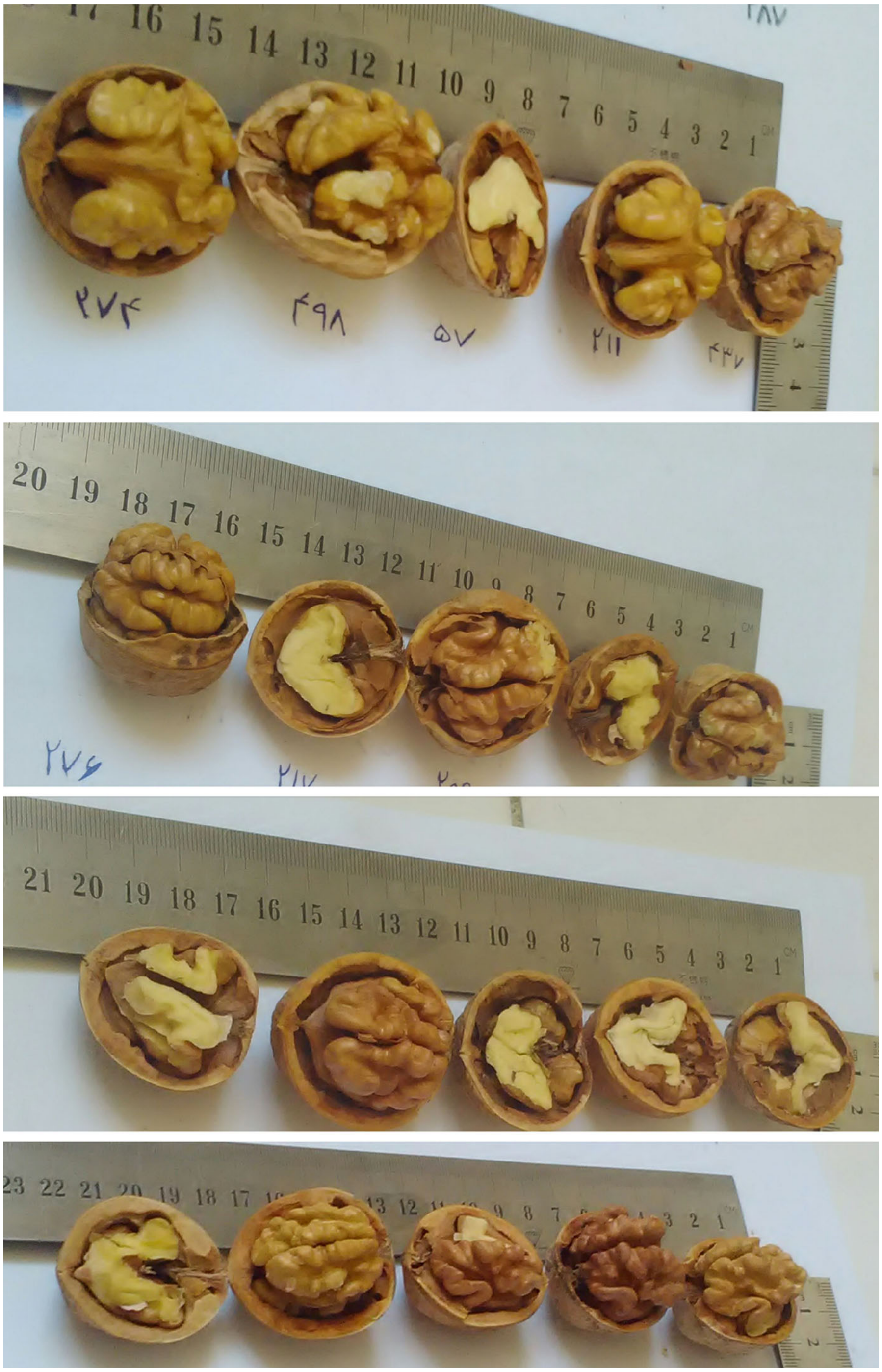


**Fig. 1** The pictures of kernels belonging to several the studied superior genotypes of walnut

correlations between these traits (Cosmulescu and Botu 2012). The range of kernel percentage varied from 37.27 to 66.29%, and Samen-276 genotype had the highest kernel percentage. The kernel percentage is considered as an indicator of the economic performance of walnut trees (Bayazit and Sumbul 2012). The highest percentage of kernel observed in the

Samen-276 genotype (66.29%) was higher than that of reported by Zeneli et al. (2005) (63.80%).

The shell seal is one of the most important traits in the storage of walnut nut (McGranahan and leslie 1990). Nuts with wide shell seal are attacked by insects, and they are damaged by fungi and mildew during storage (Khadivi-Khub 2014). The degree of diversity index obtained in this trait was 67.31%, which indicates that there is a significant difference between the genotypes in terms of opening or closure of the aperture. The Avarzaman-365 had the lowest shell thickness (0.72 mm), and the adhesion of the shell to the kernel was very weak and the kernel was easily separated from the shell in this genotype. The genotypes including Zangeneh-982, Joker-745, Jokar-666, Avarzaman-546, Avarzaman-468, Avarzaman-437, Avarzaman-414, Avarzaman-385, Avarzaman-365, Samen-294, Avarzaman-310, Samen-199 and Samen-81 had low values for shell thickness. The Avarzaman-336 had the highest shell thickness (2.46 mm) and the kernel was hardly separated from the shell in this genotype. The genotypes including Zangeneh-888, Joker-789, Avarzaman-336, Samen-244, Samen-30 and Samen-50 were also had higher shell thickness than the rest of the genotypes. The optimum shell thickness is recommended between 0.70 and 1.50 mm in the promising genotypes and commercial cultivars (Akca and Ozongun 2004; Asma 2012). In another study on the superior walnut of Iran, the shell thickness in various genotypes was reported to be 0.40–1.40 mm (Arzani et al. 2008).

The bright color of the kernel usually has higher economic value than other colors, which is an important factor in the selection of new cultivars (McGranahan et al. 1998). The bright color of the kernel is one of the most important indicators in determining the quality of walnut and its market, and the brightness color varies in different cultivars. The mean for this attribute was 4.27 and the CV was 60.77% which indicated that most of the genotypes were bright to amber. The status of the kernel color is important in the breeding studies. Investigating the effects of genotype and environment on the kernel color and how the kernel color is changed in different environments and also comparing the kernel color of a genotype in different areas is useful. The interest kernel color for the Iranian people are bright, but the American people like the amber kernel (McGranahan et al. 1998). According to Atefi (1990), in order to obtain kernel light-colored cultivars, they should be planted in highlands and in low-light regions. In a study in Turkey, the percentage of bright kernels varied from 28.15 to 41.61% (Bayazit and Sumbul 2012). Ease of kernel removing from nuts and the kernel color are important traits to be used to select the superior genotypes in the walnut (Sharma and Sharma 2001; Aslantas 2006; Cosmulescu and Botu 2012).

## Correlations among the traits

Correlation coefficients analysis indicated positive or negative correlations between some of the measured traits (Table 3). The correlation between traits is used to investigate and establish a logical and meaningful relationship between them. Creating a relationship between multiple attributes can pave the way for examining the attributes that may be difficult to measure. Therefore, a high correlation can be used to estimate other traits. Knowing the relationship between the characters related to the nut, the kernel, and other traits can guide the selection of the appropriate options for walnut breeding programs. In addition, a positive correlation between different traits shows that the improvement of a trait can improve the other trait (Yucel et al. 2009). In the present study, the number of female flowers was positively and significantly correlated with the number of male flowers ($r = 0.77$) and kernel percentage ($r = 0.13$), while it had a negative and significant correlation with the flowering habit ($r = -0.12$). The number of male flowers showed positive and significant correlations with the kernel percentage ($r = 0.10$) and the kernel color ($r = 0.10$). The positive correlations between the characters related to the nut and the kernel suggest that improving kernel quality can improve the commercial quality of the walnut crop.

The flowering habit had a positive and significant correlation with flowering type ($r = 0.28$). The flowering type had a positive correlation with kernel shriveling ($r = 0.18$), while it had a negative and significant correlation with kernel percentage ($r = -0.24$). Nut diameter showed positive and significant correlations with nut length ($r = 0.71$), nut weight ($r = 0.81$), kernel weight ($r = 0.80$), kernel percentage ($r = 0.17$), kernel filled ($r = 0.20$), and kernel plumpness ($r = 0.24$), while it had significant negative correlations with ease of kernel removing from nuts ($r = -0.11$) and kernel shriveling

**Table 3** Bivariate correlations among the morphological characters in the studied genotypes of walnut

| Character | FeFlNo | MaFlNo | FlHab | FlTy | NutDi | NutLe | NutWe | SheTh | SheHar | SheTe | SheCo | SheSea |
|---|---|---|---|---|---|---|---|---|---|---|---|---|
| FeFlNo | 1.00 | | | | | | | | | | | |
| MaFlNo | 0.77** | 1.00 | | | | | | | | | | |
| FlHab | − 0.12* | − 0.06 | 1.00 | | | | | | | | | |
| FlTy | 0.06 | 0.06 | 0.28** | 1.00 | | | | | | | | |
| NutDi | 0.00 | − 0.06 | − 0.16** | − 0.01 | 1.00 | | | | | | | |
| NutLe | − 0.08 | − 0.11* | − 0.05 | 0.04 | 0.71** | 1.00 | | | | | | |
| NutWe | 0.02 | − 0.05 | − 0.10* | − 0.01 | 0.81** | 0.77** | 1.00 | | | | | |
| SheTh | − 0.05 | − 0.05 | 0.34** | 0.03 | 0.01 | 0.10* | 0.17** | 1.00 | | | | |
| SheHar | − 0.06 | − 0.05 | 0.28** | 0.01 | 0.04 | 0.15** | 0.18** | 0.88** | 1.00 | | | |
| SheTe | − 0.16** | − 0.10* | 0.09 | − 0.03 | 0.05 | 0.04 | − 0.01 | 0.23** | 0.25** | 1.00 | | |
| SheCo | − 0.01 | − 0.01 | − 0.04 | − 0.05 | 0.07 | − 0.05 | 0.02 | 0.18** | 0.18** | 0.39** | 1.00 | |
| SheSea | − 0.08 | − 0.09 | 0.05 | 0.01 | 0.29** | 0.15** | 0.27** | 0.09 | 0.10* | 0.15** | 0.22** | 1.00 |
| SheSer | − 0.15** | − 0.09 | 0.21** | 0.08 | 0.02 | 0.03 | 0.12* | 0.50** | 0.54** | 0.19** | 0.17** | 0.05 |
| SheRet | − 0.16** | − 0.13** | 0.31** | 0.06 | − 0.01 | 0.08 | 0.05 | 0.59** | 0.61** | 0.21** | 0.13** | 0.06 |
| SheCov | − 0.16** | − 0.13** | 0.31** | 0.06 | − 0.01 | 0.08 | 0.05 | 0.59** | 0.61** | 0.21** | 0.13** | 0.06 |
| EKerRe | − 0.11* | − 0.05 | 0.32** | 0.02 | − 0.11* | − 0.05 | − 0.01 | 0.50** | 0.52** | 0.19** | 0.16** | 0.04 |
| KerWe | 0.06 | − 0.01 | − 0.33** | − 0.08 | 0.80** | 0.73** | 0.95** | 0.02 | 0.06 | − 0.04 | 0.02 | 0.24** |
| KerPer | 0.13** | 0.10* | − 0.76** | − 0.24** | 0.17** | 0.05 | 0.96* | − 0.49** | − 0.38** | − 0.11* | 0.00 | − 0.02 |
| KerCo | 0.09 | 0.10* | 0.07 | 0.06 | 0.15** | 0.08 | 0.00 | 0.02 | 0.03 | 0.09 | 0.23** | 0.11* |
| KerVe | 0.05 | 0.07 | 0.05 | 0.03 | 0.11* | 0.05 | 0.04 | 0.08 | 0.07 | 0.03 | 0.15** | 0.10* |
| KerSha | − 0.08 | − 0.07 | 0.02 | 0.03 | − 0.07 | 0.41** | 0.08 | 0.08 | 0.13** | 0.15** | 0.00 | − 0.06 |
| KerFil | − 0.01 | − 0.05 | − 0.31** | − 0.05 | 0.20** | 0.14** | 0.29** | − 0.08 | − 0.05 | − 0.05 | − 0.02 | 0.02 |
| KerPlu | 0.05 | 0.00 | − 0.34** | − 0.12* | 0.24** | 0.19** | 0.34** | − 0.05 | − 0.04 | − 0.10* | − 0.01 | 0.07 |
| KerShri | − 0.06 | 0.01 | 0.45** | 0.18** | − 0.19** | − 0.15** | − 0.20** | 0.12* | 0.09 | 0.16** | 0.02 | 0.00 |

| Character | SheSer | SheRet | SheCov | EKerRe | KerWe | KerPer | KerCo | KerVe | KerSha | KerFil | KerPlu | KerShri |
|---|---|---|---|---|---|---|---|---|---|---|---|---|
| FeFlNo | | | | | | | | | | | | |
| MaFlNo | | | | | | | | | | | | |
| FlHab | | | | | | | | | | | | |
| FlTy | | | | | | | | | | | | |
| NutDi | | | | | | | | | | | | |
| NutLe | | | | | | | | | | | | |
| NutWe | | | | | | | | | | | | |

**Table 3** continued

| Character | SheSer | SheRet | SheCov | EKerRe | KerWe | KerPer | KerCo | KerVe | KerSha | KerFil | KerPlu | KerShri |
|---|---|---|---|---|---|---|---|---|---|---|---|---|
| SheTh | | | | | | | | | | | | |
| SheHar | | | | | | | | | | | | |
| SheTe | | | | | | | | | | | | |
| SheCo | | | | | | | | | | | | |
| SheSea | | | | | | | | | | | | |
| SheSer | 1.00 | | | | | | | | | | | |
| SheRet | 0.45** | 1.00 | | | | | | | | | | |
| SheCov | 0.45** | 1.00** | 1.00 | | | | | | | | | |
| EKerRe | 0.66** | 0.49** | 0.49** | 1.00 | | | | | | | | |
| KerWe | 0.03 | − 0.08 | − 0.08 | − 0.12* | 1.00 | | | | | | | |
| KerPer | − 0.25** | − 0.40** | − 0.40** | − 0.34** | 0.40** | 1.00 | | | | | | |
| KerCo | − 0.02 | 0.02 | 0.02 | 0.02 | − 0.02 | − 0.09 | 1.00 | | | | | |
| KerVe | 0.01 | 0.07 | 0.07 | 0.05 | 0.02 | − 0.06 | 0.54** | 1.00 | | | | |
| KerSha | 0.03 | 0.11* | 0.11* | 0.02 | 0.07 | − 0.04 | − 0.03 | − 0.02 | 1.00 | | | |
| KerFil | − 0.05 | − 0.11* | − 0.11* | − 0.11* | 0.36** | 0.31** | − 0.38** | − 0.20** | − 0.01 | 1.00 | | |
| KerPlu | − 0.04 | − 0.12* | − 0.12* | − 0.18** | 0.43** | 0.35** | − 0.33** | − 0.18** | 0.00 | 0.80** | 1.00 | |
| KerShri | 0.10* | 0.18** | 0.18** | 0.19** | − 0.34** | − 0.47** | 0.17** | 0.15** | − 0.01 | − 0.49** | − 0.48** | 1.00 |

For explanation of the measured character symbols, see Table 2

*Correlation is significant at the 0.05 level

**Correlation is significant at the 0.01 level

($r = -0.19$). A positive and significant correlation between length and diameter of nut has been reported in a number of Turkish genotypes (Karadag and Akca 2011). These results were also consistent with the results of Sharma and Shrama (2001) and Arzani et al. (2008). Nut length showed positive and significant correlations nut weight ($r = 0.77$), shell seal ($r = 0.15$), kernel weight ($r = 0.73$), kernel shape ($r = 0.41$), kernel filled ($r = 0.14$) and kernel plumpness ($r = 0.19$), while there was a significant negative correlation between nut length and kernel shriveling ($r = -0.15$). Nut weight showed positive correlations with shell thickness ($r = 0.17$), shell hardness ($r = 0.18$), shell seal ($r = 0.27$), kernel weight ($r = 0.95$), kernel percentage ($r = 0.96$), kernel filled ($r = 0.29$), and kernel plumpness ($r = 0.34$), while it had a negative correlation with kernel shriveling ($r = -0.20$). It has been previously indicated a positive correlation between nut weight and kernel weight (Eskandar et al. 2005; Amiri et al. 2010; Ghasemi et al. 2012; Sarikhani Khorami et al. 2014). The shell thickness showed positive and significant correlations with shell hardness ($r = 0.88$), shell texture ($r = 0.23$), shell surface serration ($r = 0.50$), ease of kernel removing from nuts ($r = 0.50$), kernel shriveling ($r = 0.12$), and shell retention ($r = 0.59$), and also had a negative correlation with kernel percentage ($r = -0.49$). Khadivi-Khub and Ebrahimi (2015) also pointed a negative correlation between the shell thicknesses and the kernel percentage. Shell hardness showed positive and significant correlations with shell texture ($r = 0.25$), shell color ($r = 0.18$), shell seal ($r = 0.10$), and shell surface serration ($r = 0.54$).

The ease of kernel removing from nuts was positively correlated with kernel shriveling ($r = 0.19$), shell retention ($r = 0.49$) and shell cover ($r = 0.49$), and had negative correlations with kernel filled ($r = -0.11$) and kernel plumpness ($r = -0.18$). Kernel weight showed positive correlations with kernel percentage ($r = 0.40$), shell seal ($r = 0.24$), kernel filled ($r = 0.36$) and kernel plumpness ($r = 0.43$) and had negative correlations with flowering habit ($r = -0.33$), ease of kernel removing from nuts ($r = -0.12$) and kernel shriveling ($r = -0.34$). The kernel percentage showed positive and significant correlations with kernel filled ($r = 0.31$) and kernel plumpness ($r = 0.35$). In addition, it had significant negative correlations with shell hardness ($r = -0.38$), shell surface serration ($r = -0.25$), shell retention ($r = -0.40$), ease of kernel removing from nuts ($r = -0.34$), and kernel shriveling ($r = -0.47$). The negative correlation of kernel percentage with the shell thickness has also been reported by other researchers (Sharma and Sharma 2001; Arzani et al. 2008; Ebrahimi et al. 2011; Cosmulescu and Botu 2012; Norouzi et al. 2013; Sarikhani Khorami et al. 2014). The kernel color had positive correlations with shell color ($r = 0.23$), shell seal ($r = 0.11$) and kernel shriveling ($r = 0.17$), and had negative and significant correlations with the kernel filled ($r = -0.38$) and kernel plumpness ($r = -0.33$). Kernel vein had positive correlations with shell color ($r = -0.15$) and kernel shriveling ($r = -0.15$), while it showed negative correlations with kernel filled ($r = -0.20$) and kernel plumpness ($r = -0.18$). Kernel shape showed positive and significant correlations with shell hardness ($r = 0.13$), shell texture ($r = 0.15$), shell retention ($r = 0.11$) and shell cover ($r = 0.11$).

## Principal component analysis (PCA)

The PCA method is a powerful multivariate statistical method which can put the number of evaluated attributes into effective groups. Using PCA, various traits can be placed in the factors or components, each containing several traits. This analysis can clarify the main differences between the genotypes studied and also reduce the amount of data. The relative variance of each component indicates the importance of the component in the variance of the studied traits and is expressed as percentages. The first component justifies the maximum amount of variance, and the subsequent components justify the remaining variances after the first component. In the current study, PCA could describe the evaluated traits as the seven main components (Table 4). In total, seven main and independent components with loading values more than 1.00 were able to justify 71.71% of total variance. The first component (PC1) was correlated with six traits including shell thickness, shell hardiness, shell surface serration, ease of kernel removing from nuts, shell retention and shell cover which explained 17.63% of the contribution of variance. In PC2, the traits including nut diameter, nut length, nut weight, and kernel weight were found, which accounted for 15.62% of the variance. Traits including kernel color,

**Table 4** Eigenvectors of principal component axes from PCA for the morphological characters in the studied genotypes of walnut

| Character | Component | | | | | | |
|---|---|---|---|---|---|---|---|
| | 1 | 2 | 3 | 4 | 5 | 6 | 7 |
| Female flower number | − 0.09 | 0.01 | 0.04 | − 0.02 | 0.93** | − 0.06 | − 0.03 |
| Male flower number | − 0.06 | − 0.06 | 0.07 | 0.02 | 0.92** | − 0.03 | − 0.02 |
| Flowering habit | 0.31 | − 0.11 | 0.10 | 0.79** | − 0.09 | − 0.04 | − 0.02 |
| Flowering type | − 0.05 | 0.08 | − 0.08 | 0.63** | 0.16 | 0.01 | 0.02 |
| Nut diameter | − 0.03 | 0.90** | 0.08 | − 0.06 | − 0.04 | 0.08 | − 0.11 |
| Nut length | 0.05 | 0.84** | 0.03 | 0.03 | − 0.08 | − 0.05 | 0.41 |
| Nut weight | 0.12 | 0.95** | − 0.09 | − 0.03 | 0.02 | 0.00 | 0.00 |
| Shell thickness | 0.83** | 0.08 | 0.00 | 0.14 | 0.06 | 0.11 | 0.03 |
| Shell hardness | 0.85** | 0.11 | 0.00 | 0.07 | 0.05 | 0.12 | 0.08 |
| Shell texture | 0.19 | − 0.05 | 0.02 | 0.07 | − 0.11 | 0.75** | 0.26 |
| Shell color | 0.15 | − 0.01 | 0.13 | − 0.13 | 0.06 | 0.79** | − 0.06 |
| Shell seal | 0.01 | 0.36 | 0.04 | 0.15 | − 0.12 | 0.47** | − 0.31 |
| Shell surface serration | 0.71** | 0.03 | − 0.05 | 0.05 | − 0.06 | 0.12 | − 0.10 |
| Shell retention | 0.84** | 0.00 | 0.08 | 0.08 | − 0.11 | − 0.02 | 0.11 |
| Shell cover | 0.84** | 0.00 | 0.08 | 0.08 | − 0.11 | − 0.02 | 0.11 |
| Ease of kernel removal from nuts | 0.72** | − 0.11 | 0.04 | 0.11 | − 0.03 | 0.10 | − 0.09 |
| Kernel weight | − 0.02 | 0.92** | − 0.12 | − 0.25 | 0.05 | 0.01 | 0.00 |
| Kernel percentage | − 0.42 | 0.14 | − 0.12 | − 0.73** | 0.11 | 0.04 | 0.00 |
| Kernel color | − 0.03 | 0.13 | 0.79** | − 0.01 | 0.11 | 0.18 | − 0.05 |
| Kernel vein | 0.07 | 0.14 | 0.69** | − 0.10 | 0.08 | 0.07 | − 0.11 |
| Kernel shape | 0.06 | 0.09 | − 0.03 | 0.02 | − 0.04 | 0.07 | 0.91** |
| Kernel filled | − 0.05 | 0.28 | − 0.72** | − 0.30 | 0.04 | 0.06 | − 0.10 |
| Kernel plumpness | − 0.05 | 0.34 | − 0.67** | − 0.35 | 0.10 | 0.05 | − 0.11 |
| Kernel shriveling | 0.10 | − 0.22 | 0.37 | 0.59** | − 0.07 | 0.07 | 0.01 |
| Total | 4.23 | 3.75 | 2.32 | 2.28 | 1.86 | 1.52 | 1.26 |
| % of variance | 17.63 | 15.62 | 9.65 | 9.49 | 7.73 | 6.34 | 5.24 |
| Cumulative % | 17.63 | 33.25 | 42.90 | 52.39 | 60.13 | 66.46 | 71.71 |

**Eigenvalues $\geq$ 0.47 are significant

kernel vein, kernel filled and kernel plumpness were in the PC3 and included 9.65% of the variance. These components played a major role in distinguishing the genotypes studied.

The PC4 included the flowering habit, type of flowering, kernel percentage and kernel shriveling which explained 9.49% of the variance. In PC5, number of female flowers and number of male flowers were found and explained 7.73% of variance, and the PC6 explained 6.34% of the variance and correlated with shell texture, shell color and shell seal. In PC7, nut shape was only found and explained 5.24% of variance.

## Bi-plot analysis

Bi-plot is used for the two-dimensional distribution of genotypes and accumulation of genotypes in a region of the plot indicates their similarity. Bi-plot analysis was performed using PC1 and PC2 which accounted for 33.25% of the variance (Fig. 2). Genotypes that were in close proximity were more similar in terms of effective traits in PC1 and PC2 and were placed into the same group. The genotypes including Samen-1, Samen-52, Samen-81, Samen-73, Samen-3, Samen-122, Samen-65, Samen-148, Samen-165, Samen-104, Samen-136, Samen-135, Samen-177, Samen-284,

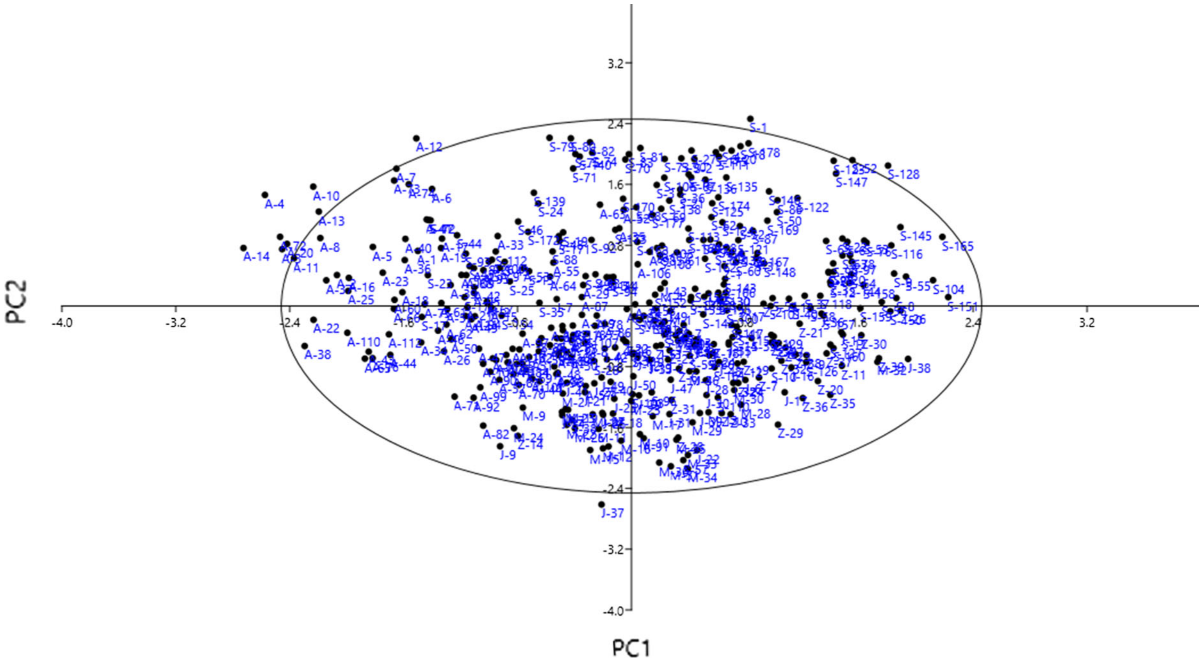


**Fig. 2** Two-dimensional plot of the first two principal components (PC1 and PC2) based on the morphological characters for the studied genotypes of walnut

Avarzaman-378, Avarzaman-303, Samen-152, Samen-122, Samen-147, Samen-128, Samen-178, Samen-90, Samen-169, Samen-65, Samen-116 and Malayer-559 were grouped into one group. In addition, according to the population analysis (Fig. 3), the studied populations were placed into four groups. The Jokar and Malayer populations were placed into the same group and showed the highest similarity. The geographical distance is low among the studied populations and gene flow may occur among them.

## Conclusions

The results showed that there is a relatively high variation among the studied genotypes of walnut, especially in qualitative and quantitative traits of fruit such as nut weight, kernel weight, kernel color, nut shape and ease of kernel removing from nuts. There were significant positive correlations between nut weight, kernel weight and kernel percentage. Two key characteristics including nut weight and kernel weight are the main traits to produce suitable populations or

**Fig. 3** Bi-plot for the five studied populations of walnut based on the morphological characters

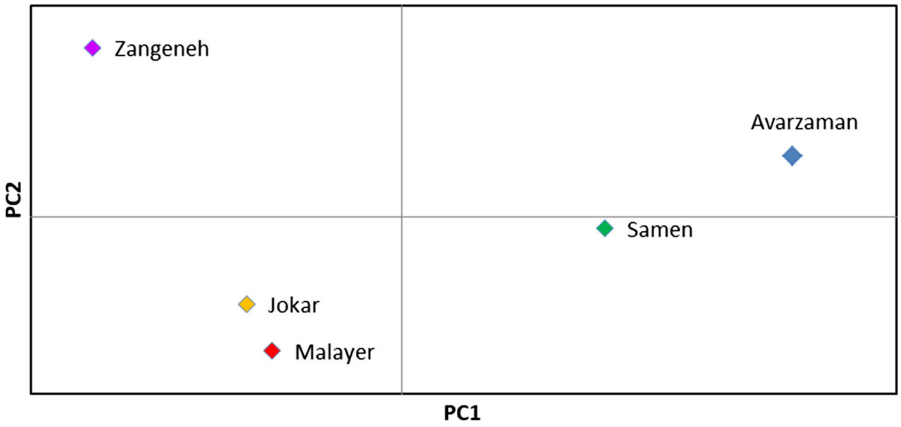

improve cultivars in walnut which should be considered in breeding programs and should focus on reducing shell weight and shell thickness, and increasing nut weight and kernel weight. According to the present research, genotypes Samen-1, Samen-12, Samen-19, Samen-25, Samen-36, Samen-3, Samen-37, Avarzaman-374, Avarzaman-372, Avarzaman-513, Samen-95, Avarzaman-536, Samen-70, Samen-219, Samen-299, Samen-294, Samen-274, Samen-169, Samen-66, Samen-45, Samen-245, Samen-72, Samen-177, Samen-130, Samen-198, Samen-258, Samen-276, Samen-33, Samen-119, Avarzaman-434, Samen-21, Samen-259, Samen-267, Samen-277, Samen-78, Samen-268, Samen-258, Samen-236, Samen-235, Samen-85, Samen-88, Samen-9, Samen-4, Samen-2, Samen-74, Samen-33, Samen-6, Samen-23, Jokar-767, Avarzaman-498, Avarzaman-437, Samen-11, Samen-57, Samen-193 and Samen-145 with having the highest nut weight and kernel weight, as well as bright color of the kernel, are recognized as superior genotypes and can be used to establish new orchards or improve cultivars. It is necessary to use the best-known genotypes of this research in future walnut breeding programs to introduce new cultivars and revitalize traditional walnut orchards to commercialize them. The rate of grafting in these genotypes as a scion can be studied and the genotypes examined may be investigated in terms of the value and composition of kernel oil.

## References


Akca YS, Ozongun S (2004) Selection of late leafing, late flowering, laterally fruitful walnut (*Juglans regia* L.) types in Turkey. R Soc N Z J Crop Hortic 32:337–342

Amiri R, Vahdati K, Mohsenipoor S, Mozaffari MR, Leslie C (2010) Correlations between some horticultural traits in walnut. Hortic Sci 45:1690–1694

Angmo S, Diengngan S, Hasan MA (2015) Characterization of walnut (*Juglans regia* L.) genotypes in Ladakh Region of India. Int J Agric Environ Biotechnol 8:265–270

Arzani K, Mansouri Ardakan H, Vezvaei A, Roozban MR (2008) Morphological variation among Persian walnut (*Juglans regia* L.) genotypes from central Iran. N Z J Crop Hortic Sci 36:159–168

Aslantas R (2006) Identification of superior walnut (*Juglans regia* L.) genotypes in north-eastern Anatolia, Turkey. N Z J Crop Hortic Sci 34:231–237

Asma BM (2012) Pomological and phenological characterization of promising walnut (*Juglans regia* L.) genotypes from Malatya, Turkey. Acta Sci Pol Horturum Cultus 11(4):169–178

Atefi J (1990) Preliminary research of Persian walnut and correlation between pair characters. Acta Hort 284:97–104

Attar SK, Kumar K, Jha SK (2014) Diversity analysis in Persian walnut (*Juglans regia* L.) trees of Shimla Hills. Indian Forester 140:789–792

Bayazit S, Sumbul A (2012) Determination of fruit quality and fatty acid composition of Turkish walnut (*Juglans regia*) cultivars and genotypes grown in subtropical climate of eastern Mediterranean region. Int J Agric Biol 14(3):419–424

Bayazit S, Kazan K, Golbitti S, Cevik V, Ayanogla H, Ergul A (2007) AFLP analysis of genetic diversity in low chill requiring walnut (*Juglans regia* L.) genotyping from Hatay Turkey. Sci Hortic 111:394–398

Chen L, Ma Q, Chen Y, Wang B, Pei D (2014) Identification of major walnut cultivars grown in China based on nut phenotypes and SSR markers. Sci Hortic 168:240–248

Cosmulescu S (2013) Phenotypic diversity of walnut (*Juglans regia* L.) in Romania - opportunity for genetic improvement. South-west J Hortic Biol Environ 4:117–126

Cosmulescu S, Botu M (2012) Walnut biodiversity in South-Western Romania-resource for perspective cultivars. Pak J Bot 44(1):307–311

Cosmulescu S, Botu M, Trandafir I (2010) Mineral composition and physical characteristics of walnut (*Juglans regia* L.) cultivars originating in Romania. Selçuk Tarım ve Gıda Bilimleri Dergisi 24:33–37

Ebrahimi A, Fattahi Moghaddam MR, Zamani Z (2011) Analysis of genetic diversity among some Persian walnut genotypes (*Juglans regia* L.) using morphological traits and SSRs markers. Sci Hortic 130:146–151

Ebrahimi A, Khadivi-Khub A, Nosrati Z, Karimi R (2015) Identification of superior walnut (*Juglans regia*) genotypes with late leafing and high kernel quality in Iran. Sci Hortic 193:195–201

Erturk U, Dalkilic Z (2011) Determination of genetic relationship among some walnut (*Juglans regia* L.) genotypes and their early-bearing progenies using RAPD markers. Romanian Biotechnol Lett 16(1):5944–5952

Eskandar S, Hassani D, Abdi A (2005) Investigation on genetic diversity of Persian walnut and evaluation of promising genotypes. Acta Hort 705:159–163

Forde HI (1979) Walnut. In: Janick J, Moore JN (eds) Advances in fruit breeding. Purdue University Press, West Lafeyette, pp 439–455

Forde HI, Mcgranahan GH (1996) Walnuts. In: Fruit breeding, Nuts, vol III. Pordue University Press, pp 241–273

Germain E (1997) Genetic improvement of the Persian walnut (*Juglans regia* L.). Acta Hort 442:21–31

Ghasemi M, Arzani K and Hassani D (2012). Evaluation and identification of walnut (*Juglans regia* L.) genotypes in Markazi province of Iran. Crop Breed J 2(2):119–124

Hammer Ø, Harper DAT, Ryan PD (2001) PAST: paleontological statistics software package for education and data analysis. Palaeontol Electron 4(1):9. http://palaeoelectronica.org/2001_1/past/issue1_01.htm

Hansche PE, Beres V, Fordde HI (1972) Estimates of quantitative genetic properties of walnut and their implications for cultivar improvement. J Am Soc Hort Sci 97:279–285

Hayes D, Angove MJ, Tucci J, Dennis C (2015) Walnuts (*Juglans regia*) chemical composition and research in human health. Crit Rev Food Sci 56:1231–1241

Hussain I, Sulatan A, Shinwari ZK, Raza G, Ahmed K (2016) Genetic diversity based on morphological traits in walnut (*Juglans regia* L.) Landraces from Karakoram region-I. Pak. J Bot 48:653–659

IPGRI (1994) Descriptors for walnut (*Juglans* spp.). International Plant Genetic Resources Institute, Rome

Karadag H, Akca Y (2011) Phenological and pomological properties of promising walnut (*Juglans regia* L.) genotypes from selected native population in Amasya Province. Afr J Biotech 10:16763–16768

Keles H, Akca Y, Ercisli S (2014) Selection of promising walnut genotypes (*Juglans regia* L.) from Inner Anatolia. Acta Sci Pol. Hortorum Cultus 13:167–173

Khadivi-Khub A (2014) Genetic divergence in seedling trees of Persian walnut for morphological characters in Markazi province from Iran. Braz J Bot 37(3):273–281

Khadivi-Khub A, Ebrahimi A (2015) The variability in walnut (*Juglans regia* L.) germplasm from different regions in Iran. Acta Physiol Plant 37:57

Khadivi-Khub A, Ebrahimi A, Mohammadi A, Kari A (2015a) Characterization and selection of walnut (*Juglans regia* L.) genotypes from seedling origin trees. Tree Genet Genomes 11:54

Khadivi-Khub A, Ebrahimi A, Sheibani F, Esmaeili A (2015b) Phenological and pomological characterization of Persian walnut to select promising trees. Euphytica 205:557–567

Korac M, Cerovic S, Golosin B, Miletic R (1997) Collecting, evaluation and utilization of walnut (*Juglans regia* L.) in Yugoslavia. Plant Genet Resour Newsl 111:72–74

Luza JG, Polito VS (1988) Microsporogenesis and anther differentiation in *Juglans regia* L.; a developmental basis for heterodichogamy in walnut. Bot Gaz 149:30–36

McGranahan GH, Forde HI (1985) Relationship between clone age and selection trait expression in mature walnuts. J Am Soc Hortic Sci 110:692–696

McGranahan G, Leslie C (1990) Walnuts (*Juglans*). In: Moore JN, Balington JR (eds) Genetic resources of temperate fruit and nut crops, vol 2. Wageningen, The Netherlands, pp 907–951

McGranahan GH, Leslie C (2012) Walnut. In: Badenes ML, Byrne DH (eds) Fruit breeding. Springer, New York

McGranahan GH, Charles A, Leslie CA, Philips HA, Dandaker A (1998) Walnut propagation. In: Ramos D (ed) Walnut Production Manual. University of California, DANR Publication, Davis, pp 71–83

Mert C (2010) Anther and pollen morphology and anatomy in walnut (*Juglans regia* L.). Hort Sc 45:757–760

Mosivand M, Hassani D, Payamnour V, Jafar Aghaei M (2013) Comparison of tree, nut, and kernel characteristics in several walnut species and inter-specific hybrids. Crop Breed J 3(1):25–30

Norouzi R, Heidari S, Mohammadi AAS, Shahi-Garahlar A (2013) Estimation of phenotypical and morphological differentiation among some selected Persian walnut (*Juglans regia* L.) accessions. Int J Agronomy Plant Prod 4:2438–2445

Pandey G, Tripathi AN (2007) Estimation of genetic divergence in walnut (*Juglans regia* L.). Ind J Hort 64:399–401

Pollegioni P, Woeste KE, Chiocchini F, Del Lungo S, Olimpieri I, Tortolano V, Clark J, Hemery GE, Mapelli S, Malvolti ME (2015) Ancient humans influenced the current spatial genetic structure of common walnut populations in Asia. PLoS One 10:e0135980

Sarikhani Khorami S, Arzani K, Roozban MR (2014) Correlation of certain high-heritability horticultural traits in Persian walnut (*Juglans regia* L.). Acta Hort 1050:61–68

SAS® Procedures (1990) Version 6, 3rd edn. SAS Institute, Cary, NC

Sharma SD, Sharma OC (1998) Studies on the variability in nuts of seedlings walnut (*Juglans regia* L.) in relation to the tree age. Fruit Var J 52:20–23

Sharma OC, Sharma SD (2001) Genetic divergence in seedling trees of Persian walnut (*Juglans regia* L.) for various metric nut and kernel characters in Himachal Pradesh. Sci Hortic 88:163–171

Sharma RM, Kour K, Singh B, Yadav S, Kotwal N, Rana JC, Anand R (2014) Selection and characterization of elite walnut (*Juglans regia* L.) clone from seedling origin trees in North Western Himalayan region of India. Aust J Crop Sci 8:257–262

Solar A, Ivancic A, Stampar F, Hudina M (2002) Genetic resources of walnut (*Juglans regia* L.) improvement in Solvenia: evaluation of the largest collection of local accessions. Genet Resour Crop Evol 49(5):191–501

Tsampas T, Botu M (2013) Study on genetic variability of common walnut (*Juglans regia* L.) from Northern Oltenia and Epirus. South-west J Hortic Biol Environ 4:127–136

Tsoukas MA, Ko BJ, Witte TR, Dincer F, Hardman WE, Mantzoros CS (2015) Dietary walnut suppression of colorectal cancer in mice: mediation by miRNA patterns and fatty acid incorporation. J Nut Biochem 26:776–783

Vinson JA, Cai Y (2012) Nuts, especially walnuts, have both antioxidant quantity and efficacy and exhibit significant potential health benefits. Food Function 3:134–140

Westwood MN (1993) Temperate-zone pomology: physiology and culture, 3rd edn. Timber Press, Portland

Yarilgac T, Koyuncu F, Koyuncu MA, Kazankaya A, Sen SM (2001) Some promising walnut selections (*Juglans regia* L.). Acta Hortic 544:93–100

Yucel C, Baloch FS, Ozkan H (2009) Genetic analysis of some physical properties of bread wheat grain (*Triticum aestivum* L. em Thell). Turk J Agric For 33:525–535

Zeneli G, Kola H, Maxhum D (2005) Phenotypic variation in native walnut populations of Northern Albania. Sci Hort 105:91–100
