# Supplementary material for: Provenance and family variations in early growth of Manchurian walnut (Juglans mandshurica Maxim.) and selection of superior families
Source: PLoS One. 2024 Mar 7;19(3):e0298918. doi: 10.1371/journal.pone.0298918 (PMC10919699; doi:10.1371/journal.pone.0298918)
Supplement: S2 File — (ZIP) [file pone.0298918.s005.zip › Tree crown complementarity links positive functional diversity and aboveground biomass along large-scale ecological gradients in tropical forests.pdf]

Science of the Total Environment 656 (2019) 45–54

Contents lists available at ScienceDirect

Science of the Total Environment

journal homepage: www.elsevier.com/locate/scitotenv

Tree crown complementarity links positive functional diversity and aboveground biomass along large-scale ecological gradients in tropical forests

Arshad Ali [a], Si-Liang Lin [a], Jie-Kun He [a], Fan-Mao Kong [b], Jie-Hua Yu [a], Hai-Sheng Jiang [a,*]

[a] Spatial Ecology Lab, School of Life Sciences, South China Normal University, Guangzhou 510631, Guangdong, China
[b] Guangzhou Qimao Ecological Technology Co., Ltd., Guangzhou 510631, Guangdong, China

HIGHLIGHTS

- Tree crown complementarity enhanced aboveground biomass
- Tree crown complementarity and functional diversity promoted each other
- Crown complementarity effect increased with soil fertility and climatic water availability
- Soil fertility enhanced functional diversity
- Structurally-complex stand can increase species coexistence and aboveground biomass

GRAPHICAL ABSTRACT

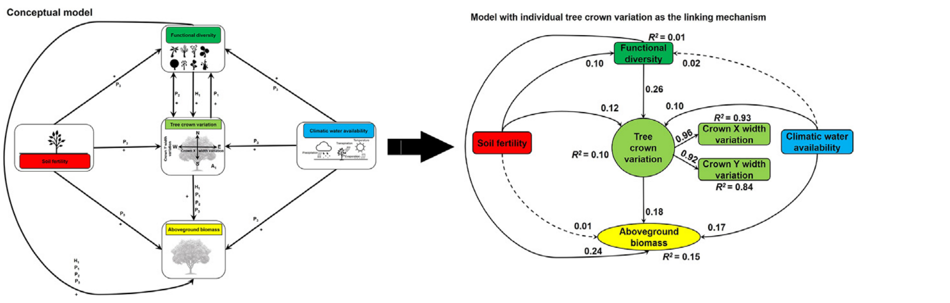

ARTICLE INFO

Article history:
Received 6 October 2018
Received in revised form 22 November 2018
Accepted 23 November 2018
Available online 26 November 2018

Editor: Elena PAOLETTI

Keywords:
Biodiversity
Climatic water availability
Tree crown variations
Ecosystem functioning
Niche differentiation
Soil fertility

ABSTRACT

Most of the previous studies have shown that the relationship between functional diversity and aboveground biomass is unpredictable in natural tropical forests, and hence also contrary to the predictions of niche complementarity effect. However, the direct and indirect effects of functional diversity on aboveground biomass via tree crown complementarity in natural forests remain unclear, and this potential ecological mechanism is yet to be understood across large-scale ecological gradients. Here, we hypothesized that tree crown complementarity would link positive functional diversity and aboveground biomass due to increasing species coexistence through efficient capture and use of available resources in natural tropical forests along large-scale ecological gradients. We quantified individual tree crown variation, functional divergence of tree maximum height, and aboveground biomass using data from 187,748 trees, in addition to the quantifications of climatic water availability and soil fertility across 712 tropical forests plots in Hainan Island of Southern China. We used structural equation modeling to test the tree crown complementarity hypothesis. Aboveground biomass increased directly with increasing functional diversity, individual tree crown variation and climatic water availability. As such, functional diversity enhanced individual tree crown variation, thereby increased aboveground biomass indirectly via individual tree crown variation. Additional positive effects of climatic water availability and soil fertility on aboveground biomass were accounted indirectly via increasing individual tree crown variation and/or functional diversity. This study shows that tree crown complementarity mediates the positive effect of functional diversity on aboveground biomass through light capture and use along large-scale ecological gradients in natural forests. This study also mechanistically shows that tree crown complementarity increases species coexistence through maintenance of functional diversity, which in turn enhances aboveground biomass in natural tropical forests.

* Corresponding author.
E-mail addresses: arshadforester@m.scnu.edu.cn (A. Ali), linsiliang@m.scnu.edu.cn (S.-L. Lin), jiekunhe@m.scnu.edu.cn (J.-K. He), jhs@scnu.edu.cn (H.-S. Jiang).

https://doi.org/10.1016/j.scitotenv.2018.11.342
0048-9697/© 2018 Elsevier B.V. All rights reserved.

Hence, managing natural forests with the aim of increasing tree crown complementarity holds promise for enhancing carbon storage while conserving biodiversity in functionally-diverse communities.

© 2018 Elsevier B.V. All rights reserved.

1. Introduction

Exploring the multivariate relationships between abiotic (i.e. climate and soils) and biotic (i.e. biodiversity, tree crown variation and stand structure) drivers of aboveground biomass is important for understanding ecosystem productivity (Ali et al., 2019; Michaletz et al., 2018; Poorter et al., 2017; Ratcliffe et al., 2017). In forest ecosystems, the multivariate relationships among environmental factors, biodiversity and ecosystem functioning such as aboveground biomass and productivity are well documented under the niche complementarity and mass ratio hypotheses (Ali and Yan, 2017a; Poorter et al., 2017; Prado-Junior et al., 2016; Yuan et al., 2018). Yet, few studies provide empirical evidence of the tree crown complementarity mechanism that underlies the positive effects of biodiversity on aboveground biomass through the partitioning of vertical physical space among component species and interacting individuals (Jucker et al., 2015; Sapijanskas et al., 2014; Williams et al., 2017), and this ecological mechanism along large-scale ecological gradients is yet to be understood (Fig. 1).

A number of ecological mechanisms have been put forward to explain why the relationship between biodiversity and aboveground biomass might be context-dependent in natural forests (Ratcliffe et al., 2017). Here, in addition to the niche complementarity and resource availability effects (Ali et al., 2019; Poorter et al., 2017; Ratcliffe et al., 2017), we focus on the tree crown complementarity effect for explaining aboveground biomass in natural forests along large-scale ecological gradients (Fig. 1). For example, species complementarity in space may explain the positive effects of biodiversity on aboveground biomass by describing how much the coexisting species, having a variety of functional traits, can efficiently utilize the available resources in space such as light (Tilman, 1997; Williams et al., 2017; Yachi and Loreau, 2007). As such, variations in plant architectural traits (e.g. crown widths of individual trees) has been recognized to enhance light capture and use within a community through high canopy packing and leaf area index, and hence may also provide explanation for aboveground biomass (Jucker et al., 2015; Schmid and Niklaus, 2017; Seidel et al., 2013; Williams et al., 2017). In this context, the tree crown complementarity hypothesis predicts that biodiversity enhances ecosystem functioning through light capture and use by the coexisting species and interacting individuals, i.e., the complementarity use of vertical physical space among trees increases productivity due to variations in tree crown architecture among and within species in a community (Schmid and Niklaus, 2017). We, therefore, anticipate that greater individual tree crown variation, i.e., the crown widths variations among and within species, is associated with greater crown complementarity in complex natural tropical forests (A_1 in Fig. 1).

It is theoretically well-understood that niche complementarity can be quantified by among or between and within component species or interacting individuals within a community (de Bello et al., 2011; Mason et al., 2005; Yuan et al., 2018). Consequently, previous empirical studies have suggested that niche complementarity is a mechanism for species coexistence, which, in turn, leading to the positive relationships between biodiversity and ecosystem functioning due to the niche or resource partitioning (Loreau and Hector, 2001; Tilman, 1997). For example, variations in plant physiological, architectural, chemical and morphological functional traits within and among species help link community-scale properties (e.g. niche complementarity) and ecosystem-scale functioning (e.g. aboveground biomass) (Ali et al., 2017; Conti and Díaz, 2013; Lutz et al., 2018; Prado-Junior et al.,

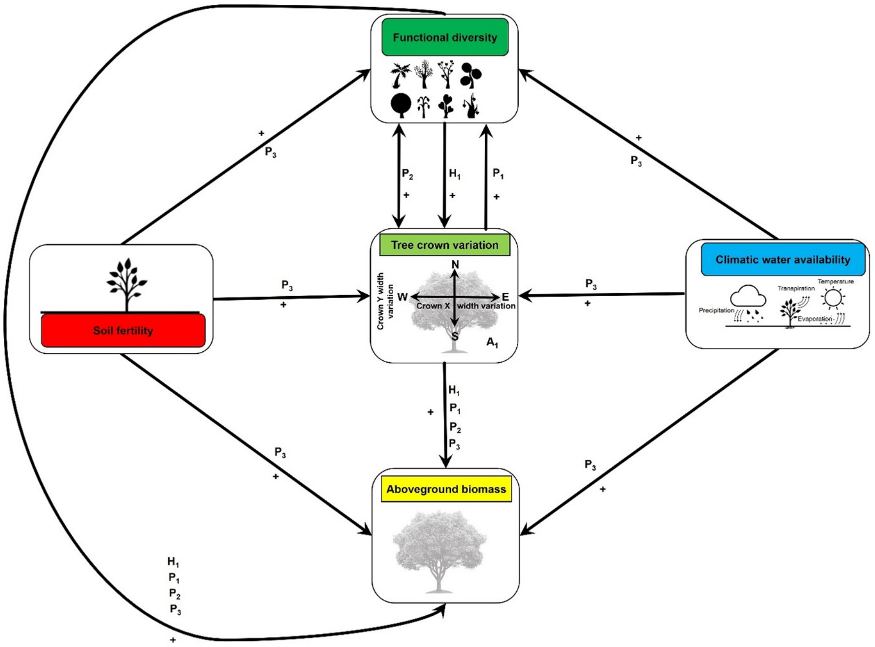

Fig. 1. A conceptual model for linking multivariate relationships among climatic water availability, soil fertility, functional diversity, individual tree crown variation, and aboveground biomass in tropical forests of Hainan Island in Southern China. Functional diversity is characterized by the functional divergence of a single trait (i.e. tree maximum height), whereas individual tree crown variation is attributed to a latent variable which incorporated X (east to west direction) and Y (north to south direction) widths of the individual tree crowns. For each hypothesized path, the expected anticipation (A_1), hypothesis (H_1) and predictions (P_1, P_2, and P_3) along with expected positive (+) effects are shown in the conceptual model (see introduction section for detail).

2016). Furthermore, it is expected that individual tree crown variation is also important for species coexistence through greater variation in tree maximum height for efficient light capture and use (Jucker et al., 2015; Williams et al., 2017). For example, niche partitioning or differentiation requires a variety of coexisting species having greater variation in functional or architectural traits that enable them to capture and utilize available resources in different ways (Díaz and Cabido, 2001; Ratcliffe et al., 2017).

At both global and regional scales, climate and soils have important influences on biodiversity, stand structural complexity and aboveground biomass (Ali et al., 2019; Paquette and Messier, 2011; Poorter et al., 2017; Ratcliffe et al., 2017). For example, along large-scale climatic gradients, the strongest positive relationships between biodiversity and ecosystem functioning have been reported under less favorable conditions for plant growth (Paquette and Messier, 2011; Ratcliffe et al., 2016). However, it is increasingly being reported that high climatic water availability increases the species distribution patterns (Toledo et al., 2012), and thereby high ecosystem functioning due to the greater length of growing season in natural forests (Ali et al., 2018; Poorter et al., 2017). In addition, the resource availability hypothesis predicts that greater niche partitioning is expected to be greater in resource-limited environments (Harpole et al., 2016), whereas the soil fertility hypothesis predicts that plant growth requires high soil nutrients (Quesada et al., 2012). However, it is increasingly being reported that forest communities with nutrient-poor soils have been found to show stronger positive biodiversity effects on aboveground biomass or productivity than forest communities on nutrient-rich soils in natural forests (Ali and Yan, 2017a; Chiang et al., 2016; Prado-Junior et al., 2016). Therefore, when the resources are limited it is predicted that positive relationships between biodiversity and ecosystem functioning are becoming stronger in functionally-diverse (e.g. phenological differences) and structurally-complex (e.g. heterogeneity in rooting or crown architectures and shade tolerance) forest communities (Jucker et al., 2015; Ratcliffe et al., 2017; Sapijanskas et al., 2014).

This study attempts to test the tree crown complementarity mechanism based on multivariate relationships among functional diversity, individual tree size variation and aboveground biomass along climate and soils gradients in tropical forests. To address the aim of the study, we used forest inventory data from 187,748 trees, in addition to climatic water availability and soil fertility across 712 plots in tropical forests in Hainan Island of Southern China. Specifically, we test the following hypothesized paths using structural equation models (SEMs). First, functional divergence in tree maximum height would have positive effects on aboveground biomass via increasing individual tree crown variation (H_1 in Fig. 1), probably due to the increasing niche differentiation and tree crown complementarity effects (Jucker et al., 2015; Williams et al., 2017). Second, greater individual tree crown variation would result in high functional divergence in tree maximum height, which in turn would exert a positive effect on aboveground biomass (P_1 in Fig. 1), probably due to the efficient capture and use of available resources by component species within a community (Conti and Díaz, 2013; Yachi and Loreau, 2007). Third, both functional divergence in tree maximum height and individual tree crown variation would provide positive feedback to each other for higher aboveground biomass (P_2 in Fig. 1), probably due to the variations in both phenological and crown architecture traits (Jucker et al., 2015; Sapijanskas et al., 2014). Fourth, climatic water availability and soil fertility would enhance aboveground biomass directly or indirectly via functional diversity and individual tree crown variation (P_3 in Fig. 1), under the predictions of soil fertility and length of growing seasons for plant growth (Ali et al., 2019; Poorter et al., 2017; Quesada et al., 2012).

To test the hypothesized paths, we designed four SEMs based on a hierarchical approach. For example, we first tested the strength and direction of aboveground biomass responses to functional divergence in tree maximum height, and then used tree crown complementarity as a linking mechanism based on the interrelationships between functional divergence in tree maximum height and individual tree crown variation (H_1, P_1 and P_2 in Fig. 1) for explaining aboveground biomass while considering for climate and soils (P_3 in Fig. 1). Based on the hierarchical approach in SEM, we mainly hypothesize that tree crown complementarity is a potential ecological mechanism for linking positive functional diversity and aboveground biomass due to increasing species coexistence through efficient capture and use of available resources in natural tropical forests along large-scale ecological gradients.

2. Materials and methods

2.1. Study area and forest inventory data

We studied tropical forests including rainforest and tropical monsoon forest (spanned a geographical area from 18°10′–20°10′ N in latitude, 108°37′–111°03′E in longitude) along climate and soils gradients in Hainan Island of Southern China (Ali et al., 2018). The study area has distinct dry and wet seasons including typhoons. In this study, we used biophysical data from 712 plots distributed randomly across the Hainan Island of Southern China (Fig. 2).

Prior to 1950s, the area occupied by forests on Hainan Island was largely natural, whereas the non-forested area was mainly farmland. In the history, forest area had been subjected to both anthropogenic and natural disturbances, and hence decreased considerably due to logging for timber, windthrow through typhoons, artificial plantations, and residential expansion, with natural forest cover, reached to a minimum level in the 1980s. In response to this dramatic loss of the natural forest cover due to the variable intensities of the human disturbances, the Hainan Provincial Government implemented logging ban rules in the natural forests in 1994. The secondary forests in the region have been protected from human activities for more than four decades, whereas the old growth forests have been protected for centuries. Consequently, the studied region contained both secondary and old growth forests. Most of our studied forest plots are recovered naturally from logging and also included old growth forests, with no visible anthropogenic disturbances for more than four decades (Ali et al., 2018; Lin et al., 2017).

In each plot, during April 2009 – August 2017, all individual trees having a diameter at breast height (DBH) ≥ 3 cm were separately measured for both DBH and height, and recognized to species-level. For the identification of species in Latin names, Chinese Flora Database (http://foc.eflora.cn/) was used. This forest inventory resulted in a total of 187,748 stems belonging to 994 identified species, 379 genera and 105 families across 712 plots. The size of plot ranged from 0.035 to 0.25 ha (average plot size 0.16 ha, i.e. 1600 m^2 or approximately 40 m × 40 m) where most of the plots were square in shape including 380 plots (50 m × 50 m; 0.25 ha), 322 plots (25 m × 25 m; 0.0625 ha), 7 plots (20 m × 20 m; 0.04 ha), 1 plot (30 m × 30 m; 0.09 ha), and only two of them were almost rectangular in shape including 1 plot (30 m × 50 m; 0.15 ha) and 1 plot (10 m × 35 m; 0.035). Note that we used 907 plots in our previous studies (Ali et al., 2018; Ali et al., 2019) but used 712 plots in this study because crown dimensions data were missing for the remaining plots. More details about the study area in the Supplementary text S1 (Appendix A) and in our previous study (Ali et al., 2018).

2.2. Variables used in the analyses

Our conceptual model (Fig. 1) included five biophysical variables: climatic water availability, soil fertility, functional diversity, individual tree crown variation and aboveground biomass.

2.2.1. Climatic water availability

We used climatic moisture index (CMI) to represent overall water availability. For example, higher values of climatic moisture index indicate greater water availability for plants (Hogg, 1997; Poorter et al., 2017). The CMI for each plot was quantified as the difference between

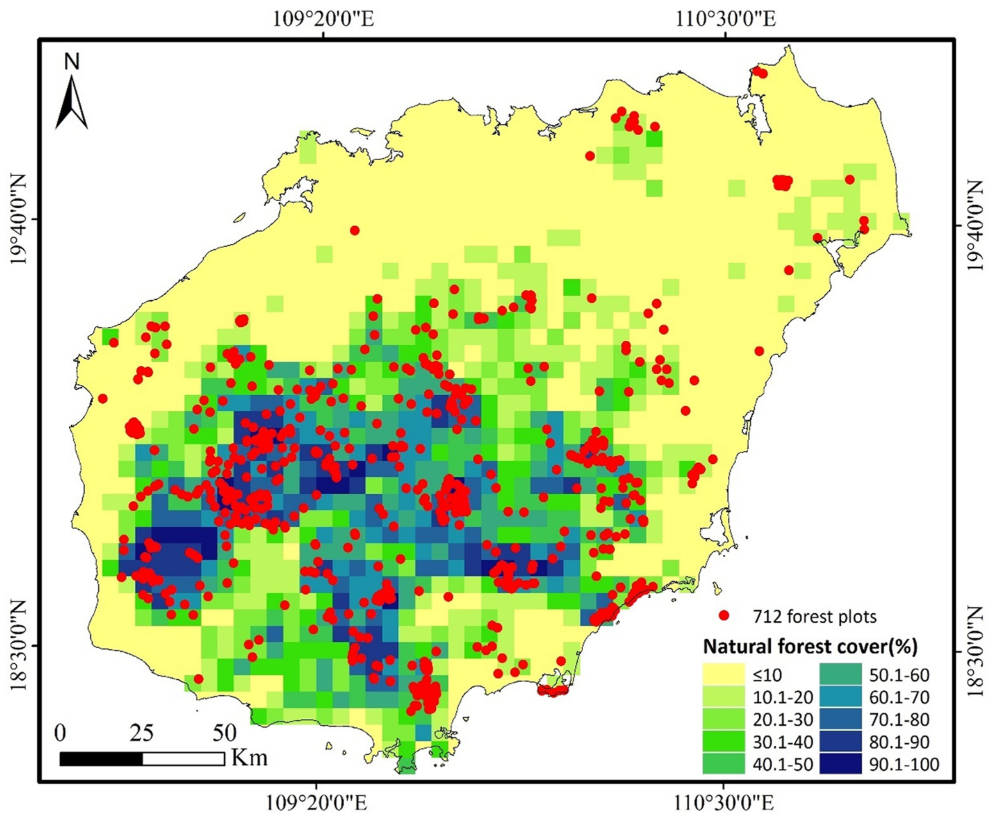

Fig. 2. Map of vegetation cover in Hainan Island of Southern China, with the location of the 712 study plots. The amount of natural forest cover is indicated in the background. For more information about the map and study area, see Lin et al. (2017).

mean annual precipitation and mean annual potential evapotranspiration, where mean annual precipitation and mean annual potential evapotranspiration for each plot was extracted from a world climate dataset (http://www.worldclim.org/) using geographical location of the plot (Ali et al., 2018).

2.2.2. Soil fertility

Soil total exchangeable bases (soil TEB; the sum of base cations Ca^{2+}, Mg^{2+}, K^{+} and Na^{+}, in cmol kg^{-1}) of the topsoil (0–30 cm) and subsoil (30–100 cm) were obtained from the Harmonized World Soil Database (FAO et al., 2012). We used a mean value of soil total exchangeable bases of the topsoil and subsoil, as an indicator of soil fertility, for each plot to better represent the available soil nutrients for plant growth (Ali et al., 2018; Poorter et al., 2017). We acknowledge that soil fertility data derived from the Harmonized World Soil Database may under- or over-estimated the effects of soil fertility or nutrients on functional diversity, individual tree crown variation and aboveground biomass in natural forests. However, this data has been confirmed by a recent study using locally available data for some plots (Poorter et al., 2017), and almost found the similar results that climate, soils and biodiversity determines aboveground biomass in tropical forests across large-scale ecological gradients (Ali et al., 2018; Ali et al., 2019).

2.2.3. Functional diversity

For the quantification of functional diversity, we used functional divergence of tree maximum height. We selected the functional divergence index because it reflects the realized degree of niche differentiation to which relative species' abundance in niche space maximizes trait divergence within the plot (Mason et al., 2005). Specifically, forest stands with high functional divergence may have high ecosystem functioning because of more efficient utilization of resources (Ali et al., 2017; Conti and Díaz, 2013). We, therefore, anticipated that functional divergence of tree maximum height would be closely related to the tree crown complementarity mechanism. As such, in the SEMs, we used tree crown complementarity as a linking mechanism for functional diversity (based on the tree maximum height) and aboveground biomass, which may be more informative for how vertical physical space was occupied by tree crowns for higher biodiversity and ecosystem functioning (Jucker et al., 2015). We, therefore, used plant or tree maximum height as an important functional trait for tree demographic process and functional strategies under the light partitioning or space-use effect (Poorter and Markesteijn, 2008; Wright et al., 2010), and hence a potential driver for ecosystem functioning (Ali et al., 2017; Conti and Díaz, 2013). Species' maximum tree height (m) was calculated as the upper 95-percentile tree height for those trees whose height was equal to or >10% of the observed maximum tree height of a population (King et al., 2006; Prado-Junior et al., 2016). Functional divergence (Eq. (1)) was quantified as the variance in tree maximum height values weighted by the species' relative basal area in each plot (Mason et al., 2005). Functional diversity (i.e. functional divergence of tree maximum height) was calculated using the *FD* package (Laliberté and Legendre, 2010).

$$\mathrm{FDvar} = 2/\pi \arctan\,(5V) \text{and } V = \sum_{i=1}^{s} p_i (\ln x_i - \ln x)^2 \qquad (1)$$

where $FDvar_x$ is the functional divergence of trait x, s is the number of species in the plot, p_i is the relative basal area of ith species in the plot $p_i = a_i / \sum_{n=1}^{s} a_i$, and $\ln x = \sum_{i=1}^{s} p_i \ln x_i$. FDvar values range between 0 and 1 (Ali et al., 2017).

2.2.4. Individual tree crown variation

For the quantification of tree crown complementarity or partitioning of canopy space, we used variations in the widths of individual tree crowns among and within species within each plot as a potential

indicator for individual tree crown variation. Tree crown complementarity or canopy packing characterizes the extent of the true crown differentiation via positive interacting individuals in vertical physical space (Williams et al., 2017; Yachi and Loreau, 2007). The coefficient of variation of tree crown (Eq. (2)), the ratio of the standard deviation of all tree crown measurements to the mean tree crown, was used to quantify individual tree crown variation within each plot, expressed as a percentage (Ali and Yan, 2017a).

$$CV_j = \frac{s_j}{\overline{x}_j} \times 100 \tag{2}$$

where CV_j is the individual tree crown variation, s_j is the standard deviation of all tree crown width (either X or Y width) measurements within *j*th plot, i.e. $s_j = \sqrt{\frac{\sum (x_j - \overline{x}_j)^2}{n_i - 1}}$, $\overline{x}_j$ is the mean tree crown width (either X or Y width) of the *j*th plot, i.e. $\overline{x}_j = \frac{\sum_{i=1}^{n} x_i}{n_i}$, and x_j is the value of each individual tree crown width (either X or Y width) in the *j*th plot being averaged.

It is noteworthy to mention that tree crown depth for individual trees was not assessed or available, and hence we calculated the coefficient of variation for each width of the crown, separately, i.e., X-width (east to west direction) and Y-width (north to south direction). In SEMs, we used a latent variable for characterizing the individual tree size variation, which incorporated the coefficient of variation for both X and Y widths of the tree crowns within each plot.

2.2.5. Aboveground biomass

For the estimation of aboveground biomass of each individual trees, we used the best-fit pantropical allometric equation, $AGB = 0.0673 \times (\rho \times DBH^2 \times H)^{0.976}$, which is based on tree DBH, height and species' wood density (Chave et al., 2014). We collected wood densities of all studied species in the wood density databases (Reyes et al., 1992; Zanne et al., 2009), following a previous critical study (Jucker et al., 2018).

2.3. Statistical analyses

In this study, we first constructed an SEM without individual tree crown variation for the purpose to evaluate the known theoretical multivariate relationships among climatic water availability, soil fertility, functional diversity and aboveground biomass. We, then, tested three SEMs based on our three hypothesized paths between individual tree crown variation and functional diversity in the conceptual model (Fig. 1). Indicators for the best fit of SEM to the data critically included a nonsignificant Chi-square (χ^2) test statistic ($P > 0.05$), standardized root mean square residual (SRMR) < 0.08, and both goodness of fit index (GFI), comparative fit index (CFI) > 0.95. We critically used the χ^2 test for the selection of SEM. The relative contribution of each

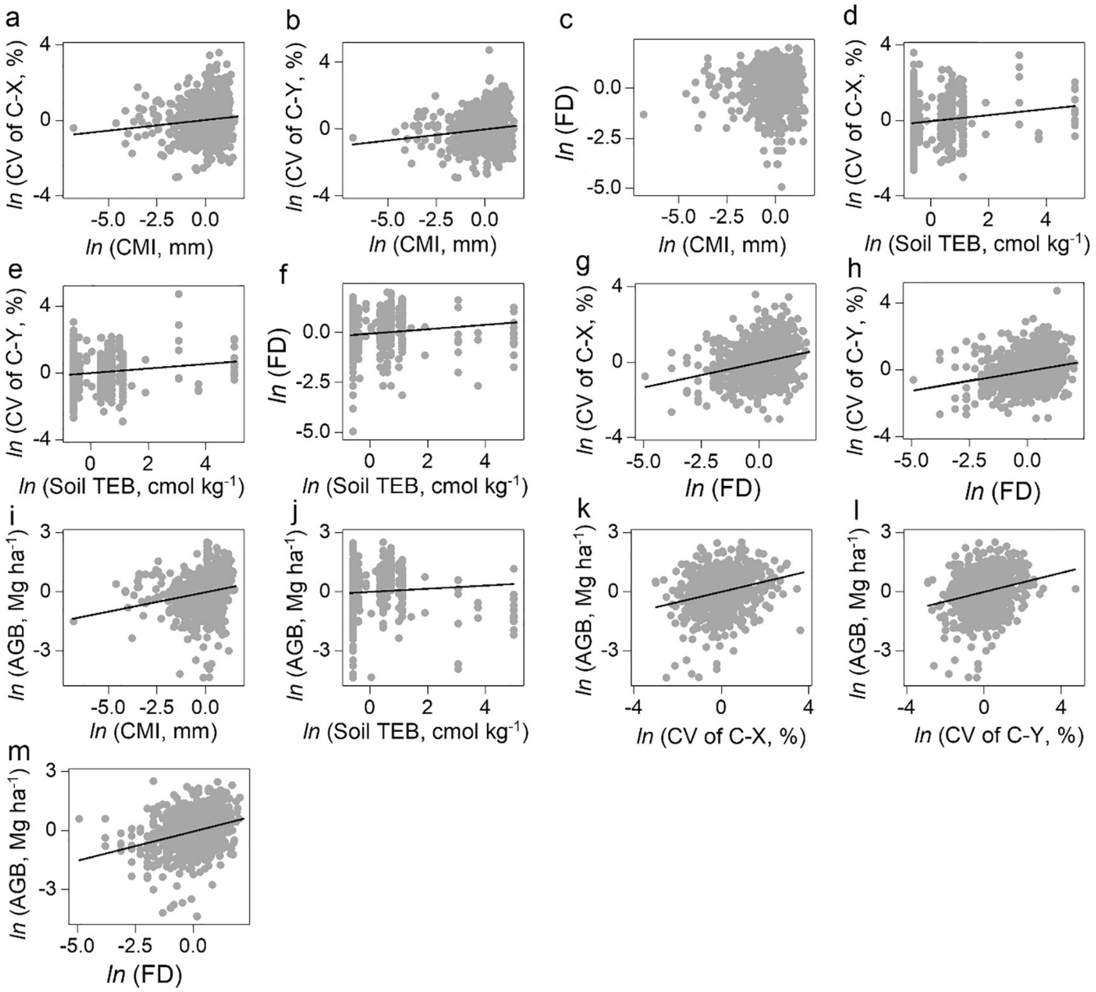

Fig. 3. Bivariate relationships between climatic water availability, soil fertility, functional diversity, individual tree crown variation, and aboveground biomass (see Table S2). Fitted regression is significant at $P < 0.05$ and the relationships without fitted lines are non-significant at $P > 0.05$ ($n = 712$). Abbreviations: CV of C-X, coefficient of variation of tree crown X-width variation; CV of C-Y, coefficient of variation of tree crown Y-width variation; FD, functional diversity; CMI, climatic moisture index; Soil TEB, soil total exchangeable bases; AGB, aboveground biomass.

Table 1
Model-fit statistics of structural equation models (SEMs) for linking multivariate relationships among climatic water availability, soil fertility, functional diversity, individual tree crown variation (a latent variable of crown X and Y widths), and aboveground biomass in tropical forests of Hainan Island in Southern China.
Abbreviations: CFI, comparative fit index; GFI, goodness-of-fit index; SRMR, standardized root mean square residual; *df*, degree of freedom; χ^2, Chi-square test; R^2, coefficient of determination (for aboveground biomass; a variable of interest in this study).

Hypothesized model	*df*	Model fit statistics summary						Model remarks	SEM
		CFI	GFI	SRMR	R^2	χ^2	*P*-value		
Model without individual tree crown variation	0	1.000	1.000	0.000	0.12	0.00	NA	Saturated	Fig. 4a
Model with individual tree crown variation as the linking mechanism	3	1.000	0.998	0.005	0.15	3.33	0.343	Accepted	Fig. 4b
Model with effect of individual tree crown variation on functional diversity	3	1.000	0.998	0.005	0.15	3.33	0.343	Accepted	Fig. 4c
Model with feedback between functional diversity and individual tree crown variation	3	1.000	0.998	0.005	0.15	3.33	0.343	Accepted	Fig. 4d

predictor to the explained variance in the response variable (i.e. aboveground biomass) was calculated as the ratio between the beta coefficient of a given predictor and the sum of beta coefficients of all predictors, and expressed in %. In order to keep consistency with our conceptual model and tested SEMs, we used the total standardized effect or beta coefficient (i.e. direct + indirect effects) of a given predictor, and those cases where there are no indirect effects, we then used the direct effects only. For the complementarity results to SEMs, we also evaluated the bivariate relationship for each hypothesized path, as shown in our conceptual model (Fig. 1). The matrix of Pearson's correlation coefficients between pairs of variables used in the SEMs is shown in Fig. S1. The SEMs were employed using the *lavaan* package (Rosseel, 2012).

In addition, the Spearman's rho revealed no or very weak correlation for plot sizes with each of aboveground biomass ($r = 0.08$), functional diversity ($r = 0.04$), and individual tree crown variation ($r = -0.15$), indicating that plot size heterogeneity had no strong influences on the multivariate relationships among these variables. The possible assumptions of normality and linearity of the tested variables were assessed and transformed by natural-logarithm function while standardizing at the mean of zero (Zuur et al., 2009). The summary of variables used in SEMs is presented in Table S1. All ecological and statistical analyses were conducted in R 3.4.2 (R Development Core Team, 2017).

3. Results

The simple bivariate regressions relationships showed that individual tree crown (X and Y widths) variation and aboveground biomass increased significantly with climatic water availability and soil fertility. As such functional diversity increased significantly with soil fertility but not with climatic water availability. Aboveground biomass increased significantly with individual tree crown variation and functional diversity, and that functional diversity had significant positive relationships with individual tree crown (X and Y widths) variation (Fig. 3; Table S2).

The model-fit statistics (Table 1) showed that the SEM without individual tree crown variation as a linking mechanism was saturated, but functional diversity ($\beta = 0.29$, $P < 0.001$), climatic water availability ($\beta = 0.19$, $P < 0.001$) and soil fertility ($\beta = 0.03$, $P = 0.433$) had explained 12% of the variation in aboveground biomass (Fig. 4a; Table 2). Soil fertility had a positive direct effect on functional diversity ($\beta = 0.10$, $P = 0.009$), and hence a positive indirect effect on aboveground biomass via functional diversity (Table 2). Functional diversity was not significantly related to climatic water availability, and hence a

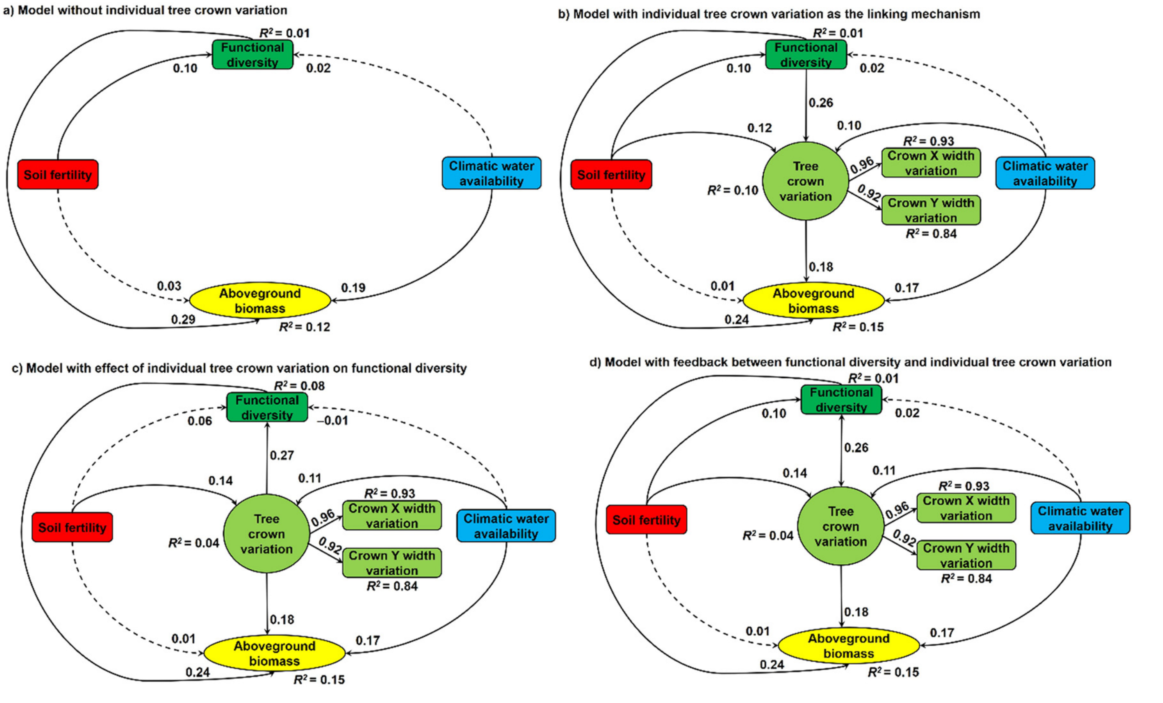

Fig. 4. Structural equation models for linking multivariate relationships among climatic water availability, soil fertility, functional diversity, individual tree crown variation (a latent variable of crown X and Y widths), and aboveground biomass in tropical forests. Solid arrows represent significant paths ($P < 0.05$) and dashed arrows represent non-significant paths ($P > 0.05$). For each path, the standardized regression coefficient is shown (see Table 2 for statistics).

Table 2
The direct, indirect, and total standardized effects of climatic water availability, soil fertility, functional diversity, individual tree crown variation (a latent variable of crown X and Y widths) on aboveground biomass, based on the structural equation models (SEMs). The SEMs are presented in Fig. 4. Significant effects ($P < 0.05$) are indicated in bold.

Predictor	Pathway to aboveground biomass	Model 4a		Model 4b		Model 4c		Model 4d	
		Effect	*P*-value	Effect	*P*-value	Effect	*P*-value	Effect	*P*-value
Climatic water availability	Direct effect	0.19	**<0.001**	0.17	**<0.001**	0.17	**<0.001**	0.17	**<0.001**
	Indirect effect via functional diversity	0.00	0.688	0.00	0.689	−0.01	0.719	0.00	0.689
	Indirect effect via individual tree crown variation	–	–	0.02	**0.016**	0.02	**0.016**	0.02	**0.016**
	Total effect	0.19	**<0.001**	0.19	**<0.001**	0.18	**<0.001**	0.19	**<0.001**
Soil fertility	Direct effect	0.03	0.433	0.01	0.838	0.01	0.838	0.01	0.838
	Indirect effect via functional diversity	0.03	**0.013**	0.02	**0.015**	0.02	0.109	0.02	**0.015**
	Indirect effect via individual tree crown variation	–	–	0.02	**0.009**	0.03	**0.004**	0.03	**0.004**
	Total effect	0.06	0.130	0.05	0.159	0.05	0.198	0.06	0.130
Functional diversity	Direct effect	0.29	**<0.001**	0.24	**<0.001**	0.24	**<0.001**	0.24	**<0.001**
	Indirect effect via individual tree crown variation	–	–	0.05	**<0.001**	–	–	–	–
	Total effect	0.29	**<0.001**	0.29	**<0.001**	0.24	**<0.001**	0.24	**<0.001**
Individual tree crown variation	Direct effect	–	–	0.18	**<0.001**	0.18	**<0.001**	0.18	**<0.001**
	Indirect effect via functional diversity	–	–	–	–	0.06	**<0.001**	–	–
	Total effect	–	–	0.18	**<0.001**	0.24	**<0.001**	0.18	**<0.001**

non-significant indirect effect of climatic water availability on aboveground biomass via functional diversity (Fig. 4a; Table 2). This result suggested that aboveground biomass increased directly with functional diversity and climatic water availability, and that functional diversity was directly influenced by soil fertility (Fig. 5a).

The full SEM including individual tree crown variation as a linking mechanism had a good fit to the data (Table 1), explained for 15% of the variation in aboveground biomass, and provided support to the tree crown complementarity effect. Functional diversity had a strongest positive direct effect on aboveground biomass ($\beta = 0.24$, $P < 0.001$), followed by individual tree crown variation ($\beta = 0.18$, $P < 0.001$) and climatic water availability ($\beta = 0.17$, $P < 0.001$) (Fig. 4b; Table 2). As such, the direct effect of functional diversity on individual tree crown variation ($\beta = 0.26$, $P < 0.001$) and indirect effect on aboveground biomass via individual tree crown variation ($\beta = 0.05$, $P < 0.001$) were significantly positive (Table 2; Fig. 4b). While the positive direct effect of climatic water availability on aboveground biomass remained, the additional positive effect of climatic water availability was accounted indirectly via increasing individual tree crown variation (Table 2; Fig. 4b). Similarly, the non-significant direct effect of soil fertility on aboveground biomass remained, the additional positive effect of soil fertility was accounted indirectly via increasing individual tree crown variation, as well as via functional diversity (Table 2; Fig. 4b). This result suggested that individual tree crown variation is a potential ecological mechanism linking positive functional diversity and aboveground biomass while accounting for the effects of soil fertility and climatic water availability (Fig. 5b).

An alternative SEM (Fig. 4c) with the effect of individual tree crown variation on functional diversity had a similar good fit to the data (Table 1) and accounted for 15% of the variation in aboveground biomass. Like the SEM in Fig. 4b, functional diversity, individual tree crown variation and climatic water availability had strong positive direct effects on aboveground biomass. Individual tree crown variation had a positive direct effect on functional diversity, and hence indirect positive effect on aboveground biomass via functional diversity (Fig. 4c; Table 2). In this alternative SEM, it was noted that the strength and magnitude for the effects of soil fertility and climatic water availability on individual tree crown variation, functional diversity and aboveground biomass were slightly changed (Fig. 4c; Table 2). As such, another alternative SEM (Fig. 4d), having positive feedback between individual tree crown variation and functional diversity, had also a similar good fit to the data (Tables 1and 2). These results from alternative models suggested that individual tree crown variation and functional diversity are simultaneously maintaining each other for enhanced aboveground biomass while providing positive responses (in most cases) to the climatic water availability and soil fertility in tropical forests (Fig. 5c and d).

4. Discussion

This study reveals that tree crown complementarity in natural tropical complex forests is strongly and positively related to both functional diversity and aboveground biomass, as well as to soil fertility and climatic water availability across large-scale ecological gradients. These findings provide new insights for the tree crown complementarity mechanism by which component species having a variety of functional traits (e.g. tree maximum height in this study) cause greater resource capture and utilization for higher species coexistence in niche space through maintenance of functional diversity within the community (Ali et al., 2016; Jucker et al., 2015; Morin et al., 2011; Wright, 2002).

Previous studies in natural forests have showed that species richness does (Jucker et al., 2015) or does not (Seidel et al., 2013) explain canopy packing, whereas a recent experimental study has suggested that tree crown complementarity through resource partition in space is crucial for high aboveground biomass in young tree communities (Williams et al., 2017). We acknowledge that our full models explained only 15% of the variation in aboveground biomass in relation to the functional diversity and individual tree crown variation while accounting for the direct and indirect effects of climatic water availability and soil fertility. This result implies that even though there is a functional diversity effect, it is not a major driver of aboveground biomass variation as shown by previous several studies in natural forests (Conti and Díaz, 2013; Prado-Junior et al., 2016; Yuan et al., 2018). However, we show that climate and soils are important for biotic factors (Fig. 4) (this study; Ali et al., 2018; Ali et al., 2019) but functional diversity and individual tree crown variation relative to climate and soils explained most of the variation in aboveground biomass (Fig. 5). Therefore, this study suggests that tree crown complementarity acting as a potential ecological mechanism where the observed strong positive relationship between biodiversity and aboveground biomass exists.

The main originality of this study determines that a positive relationship between functional diversity and aboveground biomass is mediated via increasing individual tree crown variation through efficient capture and utilization of available resources along large-scale ecological gradients. Specifically, in complex species-rich and structurally-complex tropical forests, crown complementarity increases aboveground biomass, and that crown complementarity is positively explained by the functional diversity, climatic water availability and soil fertility. In this study, the quantification approach for tree crown complementarity in species-rich forests was variation in crown widths

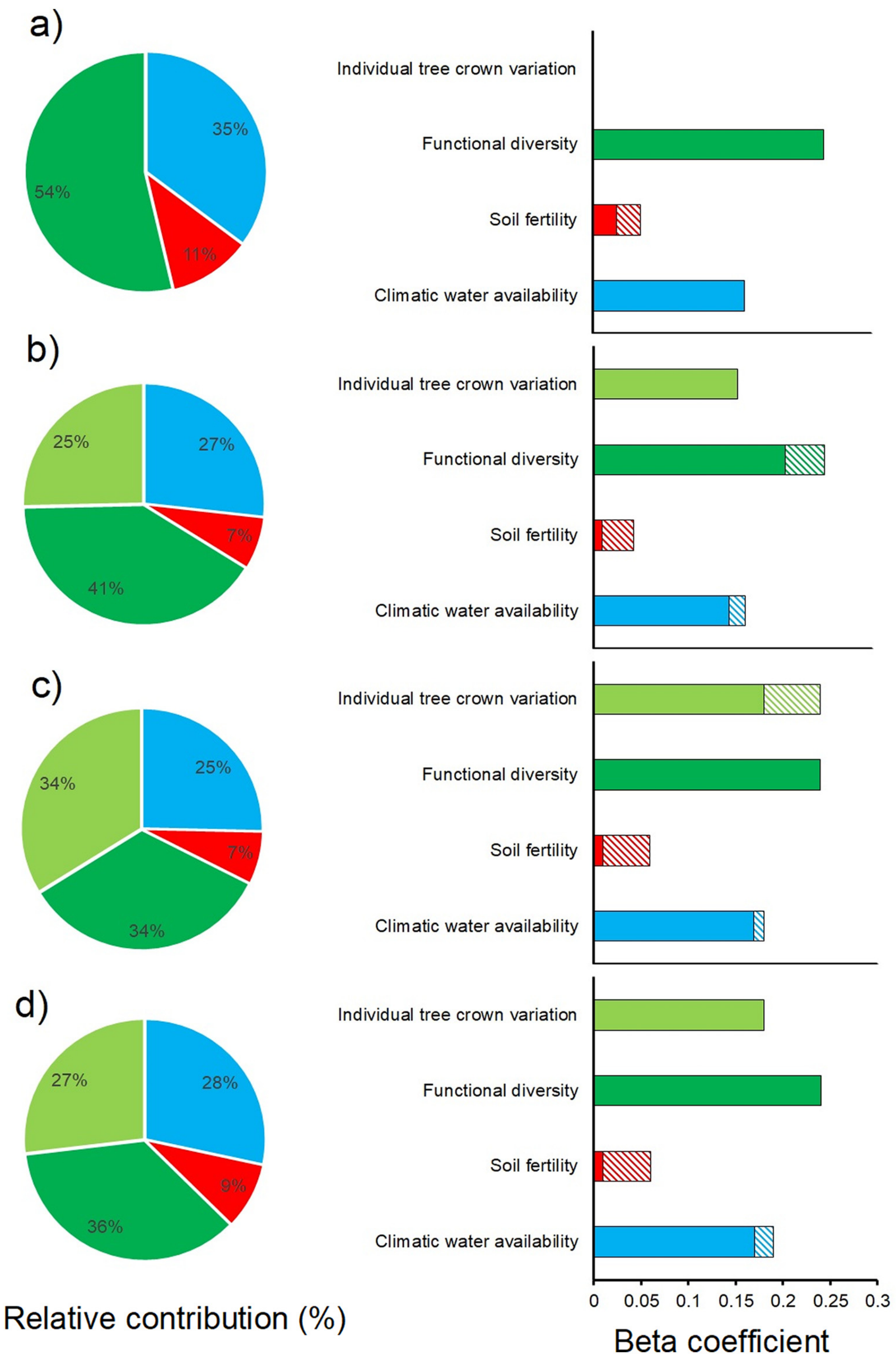

Fig. 5. Beta coefficients and relative contribution (in %) of predictors on aboveground biomass, in accordance with SEMs in Fig. 4. The solid filled bars indicate the direct effect and the streak filled bars indicate the indirect effect (summarized indirect effect, in case of multiple paths) of a given predictor on aboveground biomass. The relative contribution in a pie-chart indicates the amount of variance explained by a given predictor of aboveground biomass, within a given SEM.

among and within component species and interacting individuals (Ali and Mattsson, 2017; Jucker et al., 2015; Williams et al., 2017). Although our approach of quantifying individual tree crown variation is relatively simple, its strong positive relationship with functional diversity is interesting. This relationship is stronger than the relationship between individual tree crown variation and aboveground biomass, or functional diversity and aboveground biomass. These positive relationships among individual tree crown variation, functional diversity and aboveground biomass might be the result of increased light capture and utilization by the component species and interacting individuals (Reich, 2012; Sapijanskas et al., 2014; Schmid and Niklaus, 2017; Yachi and Loreau, 2007). Our finding suggests that tree crown complementarity links positive functional diversity and aboveground biomass probably due to the high light interception through greater canopy space and

higher leaf area, and/or light-use efficiency due to the unequal responses of trees for light in the canopy (Jucker et al., 2015; Loreau, 1998; Onoda et al., 2014; Williams et al., 2017).

This study shows that functional divergence of tree maximum height, an ecological index of functional diversity, enhances individual tree crown variation in species-rich forests. Functional dissimilarity among tree maximum heights may first make vertical stratification within the stand (Ali et al., 2017; Ruiz-Benito et al., 2014), especially in species-rich and structurally-complex forests like ours, and increase light capture (Ali et al., 2016; Yachi and Loreau, 2007). Functional divergence of tree maximum height may then allow light partitioning within the stand because variation in tree heights aligns with tree growth rate, shade tolerance and functional strategy (Ali et al., 2016; Pretzsch, 2014; Reich, 2012), and hence increase overall light-use efficiency (Yachi and Loreau, 2007). These findings suggest that a multilayered stand structure, having a species mixture of both shade tolerant and intolerant with contrasting functional strategies for growth, can increase species coexistence and aboveground biomass in natural forests (Hardiman et al., 2013; Morin et al., 2011), and hence provide support to the tree crown complementarity effect (Williams et al., 2017). This study supports the empirical evidence that functional diversity enhances space use (Williams et al., 2017), and the underlying ecological mechanism seems to be dependent on the functional strategies of individual trees in responses to available resources and interacting neighbors (Forrester, 2014; Jucker et al., 2015).

The tree crown complementarity, i.e., the partitioning of aboveground space and light, is a universal underlying mechanism for linking positive biodiversity and aboveground biomass in both experimental and natural systems, including forests (Hardiman et al., 2013; Jucker et al., 2015; Schmid and Niklaus, 2017; Williams et al., 2017). However, many other abiotic factors such as soil fertility and climatic water availability may modulate the relationships among biodiversity, individual tree crown variation and aboveground biomass along large-scale ecological gradients. As expected, we found that individual tree crown variation increases with climatic water availability and soil fertility, and provides a new prospect that the tree crown complementarity effect increases through efficient capture and utilization of resources, which in turn leads to higher aboveground biomass (Ali et al., 2018). Moreover, we found that functional diversity increases with soil fertility but not with climatic water availability, suggesting species coexistence due to the high variation in tree maximum heights and crown complementarity on nutrient-rich soils that increases functional diversity and hence higher aboveground biomass at the community level (Ali and Mattsson, 2017; Poorter et al., 2017; Ratcliffe et al., 2017).

In addition, our alternative models (Fig. 4c and d) show a significant positive effect of individual tree crown variation on functional diversity and a positive feedback between them, indicating that tree crown complementarity is critical to the coexistence of functionally-diverse species, and hence high functional diversity and aboveground biomass in natural complex forests. This provides a new insight into the tree crown differentiation as an ecological underlying mechanism for the positive effect of biodiversity on aboveground biomass, and as a species coexistence mechanism for the improvement of functional diversity in natural forests (Jucker et al., 2015; Seidel et al., 2013; Williams et al., 2017; Wright, 2002). Alternatively, the greater tree crown variation could result from greater resource availability, e.g., larger trees with bigger crowns can occur where there are more resources (Ali and Yan, 2017b; Lutz et al., 2018).

Lastly, based on the limitations of this study, we recommend the inclusion of some other well-documented factors into our conceptual model for further better understanding. We did not assess the crown depth for each individual trees, which may limit the tree crown complementarity or canopy packing in term of crown volume, so testing the canopy packing approach developed by Jucker et al. (2015) is needed to further validate our findings. We also recommend to include the effects of stand age, historical disturbance intensities and other micro- and macro-environmental factors into our conceptual model that might influence tree crown complementarity for linking biodiversity and aboveground biomass in natural complex forests. We recommend to include all the above-suggested factors into our conceptual model because datasets for these factors were not yet available in our study area. Nevertheless, this study provides strong support to the tree crown complementarity for linking positive functional diversity and aboveground biomass along climate and soils gradients in tropical forests.

5. Conclusions

This study, for the first time, attempts to mechanistically demonstrate the multivariate relationships among climate, soils, functional diversity, tree crown complementarity and aboveground biomass in natural forests. Together with other ecological mechanisms, such as niche differentiation, facilitation, species coexistence, plant–soil and plant–climate feedbacks, this study shows that tree crown complementarity is a potential underlying ecological mechanism for linking the positive effect of functional diversity on aboveground biomass in natural forests. More importantly, this study also shows that favorable climate conditions and high soil fertility tend to increase tree crown complementarity at the community level, which in turn links positive functional diversity and aboveground biomass in structurally-complex and species-rich natural tropical forests. Therefore, at the community level, increasing individual tree crown variation, light partitioning and stand structural complexity would be sustainable management strategies for enhancing the wood productivity, carbon sequestration and more importantly for the biodiversity conservation across large-scale ecological gradients in natural complex forests. We conclude that maintaining the structure of an individual tree crown at the community level offers a simple solution towards positive biodiversity effect on forest functioning.

Data availability

More detail about the dataset is available upon reasonable request to the authors.

Contribution of the co-authors

AA and HSJ designed the study. SLL, JKH, FMK, JHY and HSJ collected field data. AA complied and analyzed the data and wrote the manuscript. SLL, JKH and HSJ contributed critically to the draft. All coauthors gave final approval for publication. The authors declare that they have no conflict of interest.

Acknowledgments

This work is supported by China Postdoctoral Science Foundation (Grant No. 2018M643117, awarded to Dr. Arshad Ali). We greatly acknowledge the support from Wildlife Protection, Nature reserves, Forest Farms and Forestry Bureaus of Hainan Province. Authors would like to thank Qing Chen, Huanqiang Chen, Shuo Sun and Yiwen Liang for their support during the fieldwork campaigns. We thank Chundong Wang, Lin Fang, Yanni Mo for their support related to the fieldwork and administrative communication. We also thank Hua Zhu and Qiang Xie for their advice regarding the field surveys.

Appendix A. Supplementary data

Supplementary data to this article can be found online at https://doi.org/10.1016/j.scitotenv.2018.11.342.

References

Ali, A., Mattsson, E., 2017. Disentangling the effects of species diversity, and intraspecific and interspecific tree size variation on aboveground biomass in dry zone homegarden agroforestry systems. Sci. Total Environ. 598, 38–48.

Ali, A., Yan, E.-R., 2017a. The forest strata-dependent relationship between biodiversity and aboveground biomass within a subtropical forest. For. Ecol. Manag. 401, 125–134.

Ali, A., Yan, E.-R., 2017b. Functional identity of overstorey tree height and understorey conservative traits drive aboveground biomass in a subtropical forest. Ecol. Indic. 83, 158–168.

Ali, A., Yan, E.-R., Chen, H.Y.H., Chang, X.S., Zhao, Y.-T., Yang, X.-D., et al., 2016. Stand structural diversity rather than species diversity enhances aboveground carbon storage in secondary subtropical forests in eastern China. Biogeosciences 13, 4627–4635.

Ali, A., Yan, E.-R., Chang, S.X., Cheng, J.-Y., Liu, X.-Y., 2017. Community-weighted mean of leaf traits and divergence of wood traits predict aboveground biomass in secondary subtropical forests. Sci. Total Environ. 574, 654–662.

Ali, A., Lin, S.-L., He, J.-K., Kong, F.-M., Yu, J.-H., Jiang, H.-S., 2018. Climatic water availability is the main limiting factor of biotic attributes across large-scale elevational gradients in tropical forests. Sci. Total Environ. 647, 1211–1221.

Ali, A., Lin, S.-L., He, J.-K., Kong, F.-M., Yu, J.-H., Jiang, H.-S., 2019. Climate and soils determine aboveground biomass indirectly via species diversity and stand structural complexity in tropical forests. For. Ecol. Manag. 432, 823–831.

de Bello, F., Lavorel, S., Albert, C.H., Thuiller, W., Grigulis, K., Dolezal, J., et al., 2011. Quantifying the relevance of intraspecific trait variability for functional diversity. Methods Ecol. Evol. 2, 163–174.

Chave, J., Rejou-Mechain, M., Burquez, A., Chidumayo, E., Colgan, M.S., Delitti, W.B., et al., 2014. Improved allometric models to estimate the aboveground biomass of tropical trees. Glob. Chang. Biol. 20, 3177–3190.

Chiang, J.-M., Spasojevic, M.J., Muller-Landau, H.C., Sun, I.-F., Lin, Y., Su, S.-H., et al., 2016. Functional composition drives ecosystem function through multiple mechanisms in a broadleaved subtropical forest. Oecologia 182, 829–840.

Conti, G., Díaz, S., 2013. Plant functional diversity and carbon storage - an empirical test in semi-arid forest ecosystems. J. Ecol. 101, 18–28.

R Development Core Team, 2017. R Version 3.4.2. R Foundation for Statistical Computing, Vienna, Austria.

Díaz, S., Cabido, M., 2001. Vive la difference: plant functional diversity matters to ecosystem processes. Trends Ecol. Evol. 16, 646–655.

Forrester, D.I., 2014. The spatial and temporal dynamics of species interactions in mixed-species forests: from pattern to process. For. Ecol. Manag. 312, 282–292.

Hardiman, B.S., Gough, C.M., Halperin, A., Hofmeister, K.L., Nave, L.E., Bohrer, G., et al., 2013. Maintaining high rates of carbon storage in old forests: a mechanism linking canopy structure to forest function. For. Ecol. Manag. 298, 111–119.

Harpole, W.S., Sullivan, L.L., Lind, E.M., Firn, J., Adler, P.B., Borer, E.T., et al., 2016. Addition of multiple limiting resources reduces grassland diversity. Nature 537, 93.

Hogg, E.H., 1997. Temporal scaling of moisture and the forest-grassland boundary in western Canada. Agric. For. Meteorol. 84, 115–122.

FAO, IIASA, ISRIC, ISSCAS, JRC, 2012. Harmonized World Soil Database (Version 1.2). FAO and IIASA, Rome, Italy and Laxenburg, Austria.

Jucker, T., Bouriaud, O., Coomes, D.A., Baltzer, J., 2015. Crown plasticity enables trees to optimize canopy packing in mixed-species forests. Funct. Ecol. 29, 1078–1086.

Jucker, T., Bongalov, B., Burslem, D.F.R.P., Nilus, R., Dalponte, M., Lewis, S.L., et al., 2018. Topography shapes the structure, composition and function of tropical forest landscapes. Ecol. Lett. 21, 989–1000. https://doi.org/10.1111/ele.12964.

King, D.A., Davies, S.J., Noor, N.S.M., 2006. Growth and mortality are related to adult tree size in a Malaysian mixed dipterocarp forest. For. Ecol. Manag. 223, 152–158.

Laliberté, E., Legendre, P., 2010. A distance-based framework for measuring functional diversity from multiple traits. Ecology 91, 299–305.

Lin, S., Jiang, Y., He, J., Ma, G., Xu, Y., Jiang, H., 2017. Changes in the spatial and temporal pattern of natural forest cover on Hainan Island from the 1950s to the 2010s: implications for natural forest conservation and management. PeerJ 5, e3320.

Loreau, M., 1998. Biodiversity and ecosystem functioning: a mechanistic model. Proc. Natl. Acad. Sci. 95, 5632–5636.

Loreau, M., Hector, A., 2001. Partitioning selection and complementarity in biodiversity experiments. Nature 412, 72–76.

Lutz, J.A., Furniss, T.J., Johnson, D.J., Davies, S.J., Allen, D., Alonso, A., et al., 2018. Global importance of large-diameter trees. Glob. Ecol. Biogeogr. 27, 849–864.

Mason, N.W., Mouillot, D., Lee, W.G., Wilson, J.B., 2005. Functional richness, functional evenness and functional divergence: the primary components of functional diversity. Oikos 111, 112–118.

Michaletz, S.T., Kerkhoff, A.J., Enquist, B.J., 2018. Drivers of terrestrial plant production across broad geographical gradients. Glob. Ecol. Biogeogr. 27, 166–174.

Morin, X., Fahse, L., Scherer-Lorenzen, M., Bugmann, H., 2011. Tree species richness promotes productivity in temperate forests through strong complementarity between species. Ecol. Lett. 14, 1211–1219.

Onoda, Y., Saluñga, J.B., Akutsu, K., Si, Aiba, Yahara, T., Anten, N.P., 2014. Trade-off between light interception efficiency and light use efficiency: implications for species coexistence in one-sided light competition. J. Ecol. 102, 167–175.

Paquette, A., Messier, C., 2011. The effect of biodiversity on tree productivity: from temperate to boreal forests. Glob. Ecol. Biogeogr. 20, 170–180.

Poorter, L., Markesteijn, L., 2008. Seedling traits determine drought tolerance of tropical tree species. Biotropica 40, 321–331.

Poorter, L., van der Sande, M.T., Arets, E.J.M.M., Ascarrunz, N., Enquist, B., Finegan, B., et al., 2017. Biodiversity and climate determine the functioning of Neotropical forests. Glob. Ecol. Biogeogr. 26, 1423–1434.

Prado-Junior, J.A., Schiavini, I., Vale, V.S., Arantes, C.S., Sande, M.T., Lohbeck, M., et al., 2016. Conservative species drive biomass productivity in tropical dry forests. J. Ecol. 104, 817–827.

Pretzsch, H., 2014. Canopy space filling and tree crown morphology in mixed-species stands compared with monocultures. For. Ecol. Manag. 327, 251–264.

Quesada, C.A., Phillips, O.L., Schwarz, M., Czimczik, C.I., Baker, T.R., Patiño, S., et al., 2012. Basin-wide variations in Amazon forest structure and function are mediated by both soils and climate. Biogeosciences 9, 2203–2246.

Ratcliffe, S., Liebergesell, M., Ruiz-Benito, P., Madrigal González, J., Muñoz Castañeda, J.M., Kändler, G., et al., 2016. Modes of functional biodiversity control on tree productivity across the European continent. Glob. Ecol. Biogeogr. 25, 251–262.

Ratcliffe, S., Wirth, C., Jucker, T., der Plas, F., Scherer-Lorenzen, M., Verheyen, K., et al., 2017. Biodiversity and ecosystem functioning relations in European forests depend on environmental context. Ecol. Lett. 20, 1414–1426.

Reich, P.B., 2012. Key canopy traits drive forest productivity. Proc. R. Soc. B Biol. Sci. 279, 2128–2134.

Reyes, G., Brown, S., Chapman, J., Lugo, A.E., 1992. Wood Densities of Tropical Tree Species. Gen. Tech. Rep. SO-88. 15. US Dept of Agriculture, Forest Service, Southern Forest Experiment Station, New Orleans, LA, p. 88.

Rosseel, Y., 2012. Lavaan: an R package for structural equation modeling. J. Stat. Softw. 48, 1–36.

Ruiz-Benito, P., Gómez-Aparicio, L., Paquette, A., Messier, C., Kattge, J., Zavala, M.A., 2014. Diversity increases carbon storage and tree productivity in Spanish forests. Glob. Ecol. Biogeogr. 23, 311–322.

Sapijanskas, J., Paquette, A., Potvin, C., Kunert, N., Loreau, M., 2014. Tropical tree diversity enhances light capture through crown plasticity and spatial and temporal niche differences. Ecology 95, 2479–2492.

Schmid, B., Niklaus, P.A., 2017. Biodiversity: complementary canopies. Nat. Ecol. Evol. 1, 0104.

Seidel, D., Leuschner, C., Scherber, C., Beyer, F., Wommelsdorf, T., Cashman, M.J., et al., 2013. The relationship between tree species richness, canopy space exploration and productivity in a temperate broad-leaf mixed forest. For. Ecol. Manag. 310, 366–374.

Tilman, D., 1997. The influence of functional diversity and composition on ecosystem processes. Science 277, 1300–1302.

Toledo, M., Peña-Claros, M., Bongers, F., Alarcón, A., Balcázar, J., Chuviña, J., et al., 2012. Distribution patterns of tropical woody species in response to climatic and edaphic gradients. J. Ecol. 100, 253–263.

Williams, L.J., Paquette, A., Cavender-Bares, J., Messier, C., Reich, P.B., 2017. Spatial complementarity in tree crowns explains overyielding in species mixtures. Nat. Ecol. Evol. 1, 0063.

Wright, J.S., 2002. Plant diversity in tropical forests: a review of mechanisms of species coexistence. Oecologia 130, 1–14.

Wright, S.J., Kitajima, K., Kraft, N.J., Reich, P.B., Wright, I.J., Bunker, D.E., et al., 2010. Functional traits and the growth-mortality trade-off in tropical trees. Ecology 91, 3664–3674.

Yachi, S., Loreau, M., 2007. Does complementary resource use enhance ecosystem functioning? A model of light competition in plant communities. Ecol. Lett. 10, 54–62.

Yuan, Z., Wang, S., Ali, A., Gazol, A., Ruiz-Benito, P., Wang, X., et al., 2018. Aboveground carbon storage is driven by functional trait composition and stand structural attributes rather than biodiversity in temperate mixed forests recovering from disturbances. Ann. For. Sci. 75, 67.

Zanne, A.E., Lopez-Gonzalez, G., Coomes, D.A., Ilic, J., Jansen, S., Lewis, S.L., et al., 2009. Data from: towards a worldwide wood economics spectrum. Dryad Data Repository.

Zuur, A., Ieno, E.N., Walker, N., Saveliev, A.A., Smith, G.M., 2009. Mixed Effects Models and Extensions in Ecology With R. Springer, New York.
