# Supplementary material for: Provenance and family variations in early growth of Manchurian walnut (Juglans mandshurica Maxim.) and selection of superior families
Source: PLoS One. 2024 Mar 7;19(3):e0298918. doi: 10.1371/journal.pone.0298918 (PMC10919699; doi:10.1371/journal.pone.0298918)
Supplement: S2 File — (ZIP) [file pone.0298918.s005.zip › Variance analyses on growth traits of Larix kaempferi in different seed sources.pdf]

DOI：10.13332/j.1000-1522.20170478

日本落叶松不同种源及家系生长性状变异分析

潘艳艳[1,2] 梁德洋[1] 郭婧[1] 王芳[1] 王福维[3] 李树春[3] 赵曦阳[1]

（1.林木遗传育种国家重点实验室，黑龙江 哈尔滨 150040；2.吉林省林业科学研究院，吉林 长春 130033；
3.吉林省林木种苗管理站，吉林 长春 130022）

摘要：【**目的**】研究日本落叶松不同种源及家系生长性状变异，为日本落叶松遗传改良提供可靠依据。【**方法**】本文以4个种源的169个日本落叶松半同胞家系为材料，对不同树龄日本落叶松的树高和胸径进行方差分析、遗传参数分析、相关性分析以及家系选择。【**结果**】各指标在种源和家系间均达极显著差异水平（$P<0.01$），各家系间的树高和胸径的表型变异系数（4.00% ~37.37%和24.89% ~26.48%）和遗传变异系数（1.94% ~20.93%和8.93% ~13.68%）均随树龄的增加而上升。树高和胸径的家系遗传力变化范围分别为0.881 ~0.972和0.877 ~0.879，单株遗传力变化范围分别为0.406 ~0.664和0.301 ~0.410。各性状变异系数及遗传力均属于较高水平，有利于优良家系的评价选择。相关分析表明种源内不同树龄树高和胸径相关性均达到极显著正相关。利用多性状综合评价法，以10%的入选率进行优良家系选择，17个入选家系的树高和胸径平均值分别为11.10 m和13.82 cm，现实增益分别为11.68%和17.98%，遗传增益分别为11.35%和15.80%。以5%的入选率进行优良家系内优良单株的选择，25个入选单株的树高和胸径平均值分别为12.58 m和17.72 cm，遗传增益分别为8.54%和12.50%。【**结论**】在本研究中，种源和家系间均存在丰富的变异，可以进行优良种源、家系和单株的选择，为建立改良种子园和二代种子园提供育种材料，也为日本落叶松的遗传改良提供理论依据。

关键词：日本落叶松；种源；家系；遗传变异；综合评价

中图分类号：S791.223 **文献标志码**：A **文章编号**：1000-1522(2018)11-0019-09

引文格式：潘艳艳，梁德洋，郭婧，等. 日本落叶松不同种源及家系生长性状变异分析[J]. 北京林业大学学报，2018，40(11)：19-27. Pan Yanyan, Liang Deyang, Guo Jing, et al. Variance analyses on growth traits of *Larix kaempferi* in different seed sources[J]. Journal of Beijing Forestry University, 2018, 40(11): 19-27.

Variance analyses on growth traits of *Larix kaempferi* in different seed sources

Pan Yanyan[1,2] Liang Deyang[1] Guo Jing[1] Wang Fang[1] Wang Fuwei[3] Li Shuchun[3] Zhao Xiyang[1]

(1. State Key Laboratory of Tree Genetics and Breeding, Northeast Forestry University, Harbin 150040, Heilongjiang, China;
2. Jilin Provincial Academy of Forestry Sciences, Changchun 130033, Jilin, China;
3. Tree Seedling Management Station of Jilin Province, Changchun 130022, Jilin, China)

Abstract: [**Objective**] To determine the genetic variation of growth traits of different seed sources and families of *Larix kaempferi* could provide basis for the genetic improvement of *Larix kaempferi*. [**Method**] 169 *Larix kaempferi* half-sib families in 4 seed sources were taken as materials, the ANOVA analysis, genetic parameter analysis, correlation analysis and family selection were carried out for the tree height and DBH in different growth years. [**Result**] The results of ANOVA showed that all the traits were significantly different ($P<0.01$) among varied families and seed sources. The phenotype variation coefficients of tree height and DBH ranged from 4.00% to 37.37% and 24.89% to 26.48%, and

收稿日期：2017-12-29 **修回日期**：2018-04-20

基金项目："十三五"国家重点研发计划项目(2017YFD0600402)。

第一作者：潘艳艳。主要研究方向：落叶松遗传改良研究。Email：zhaoxyphd@163.com 地址：150040 黑龙江省哈尔滨市香坊区和兴路51号。

责任作者：赵曦阳，博士，副教授。主要研究方向：白桦、红松和落叶松遗传改良。Email：zhaoxyphd@163.com 地址：同上。

本刊网址：http://j.bjfu.edu.cn；http://journal.bjfu.edu.cn

genetic variation coefficients of tree height and DBH ranged from 1.94% to 20.93% and 8.93% to 13.68%, respectively, which all decreased with the increase of time. The family heritability of tree height and DBH of families ranged from 0.881 to 0.972 and 0.877 to 0.879, respectively. The high variation coefficients and high heritability were beneficial to evaluate and select the elite families and plants. Correlation analysis results showed that there existed significantly positive correlation among all traits in different seed sources. By the multiple-traits comprehensive method, when evaluated the families with the selected rate of 10%, 17 families were selected as elite families. The average of tree height and DBH of the elite families were 11.10 m and 13.82 cm and the genetic gains were 11.35% and 15.80%, respectively. When evaluated with the selected rate of 5%, 25 plants were selected as elite trees. The average of tree height and DBH of the elite single plants were 12.58 m and 17.72 cm, and the genetic gains were 8.54% and 12.50%, respectively. [**Conclusion**] In this study, the abundant variations among seed sources and families are beneficial to select the elite seed sources, families and single trees, respectively. The elite families and single trees can provide superior materials for the establishment of improved seed orchard and the second generation seed orchard. The study can provide basis for the genetic improvement of *Larix kaempferi*.

Key words: *Larix kaempferi*; seed source; family; genetic variation; comprehensive evaluation

日本落叶松(*Larix kaempferi*)为松科(Pinaceae)落叶松属(*Larix* Mill)植物,原产日本,已经成功引入我国100多年,全国已有14个省市引种造林,总面积已达30万 hm^2,在东北地区已成为重要的造林树种[1]。由于日本落叶松具有生长速度快、材质好、抗性强和适应能力强等优点,被广泛应用在建筑、家具制造、造纸和生物燃料等方面[2],已成为部分地区主要用材和绿化树种[3],具有很高的经济和生态价值[4]。目前对日本落叶松的研究主要集中在种源选择[5]、家系选择、无性系选择[6]、木材性状[7-8]、光合特性[9]、遗传变异[10]和分子标记[11]等方面。

种源及优良家系的评价选择是林木育种的重要方法[12],也是林木遗传改良最基础的工作[13],其结果可为选育地区提供优质、高产、稳定的应用材料,对于实现适地适种源具有重要意义[14-15]。20世纪50年代,落叶松种源试验就已经开展,但主要以华北落叶松、长白落叶松及兴安落叶松为主,选育的优良材料在生产中产生了巨大的经济效益[16]。日本落叶松虽然引种多年,对其种源及家系评价选择的较多,如朱于勤等[17]以16年生日本落叶松原生种质资源试验林为研究对象,通过生长性状分析比较发现不同种源、林分的生长性状差异极显著,并选择出4个最适合在中北亚热带高山区栽植的优良林分,但对种子园内种源、家系联合评价研究较少,尤其是利用优良家系、单株评价回向及前向选择的研究尚未见报道。本研究以吉林省柳河五道沟落叶松国家良种基地的4个种源及种源内169个日本落叶松半同胞家系为材料,对其不同树龄树高和胸径进行调查分析,并对最佳种源、优良家系及优良单株进行评价选择,为落叶松良种选择提供基础,也为其他树种种子园升级改建提供依据。

1 材料与方法

1.1 试验林和试验材料

试验林位于吉林省五道沟落叶松国家良种基地(41°54′ N,125°17′ E),属于我国落叶松东北栽植区,该地气候属北温带大陆性季风气候,年平均气温5.5 ℃,年均日照2 479 h,年均降水量736 mm,无霜期约145 d,土壤母质较粗,腐殖质较多,肥力较高,适于发展林业生产。试验材料包括东丰(A)、临江(B)、通化(C)及兰山(D)4个种源,各种源分别按不同批次直接从日本引种,后五道沟落叶松国家良种基地从各种源引种,各种源包括不同数量的家系,共计169个半同胞家系,由种子园无性系自由授粉获得,具体见表1。于2005年春季利用2年生苗木进行定植,试验设计采用完全随机区组设计,6株小区,5次重复,株行距2 m×2 m,外设2行保护行。

1.2 生长性状的测定

分别于2007(4年生)、2011(8年生)及2014年(11年生)秋季对试验林中所有单株树高和胸径进行测量。2007年树高利用塔尺进行测量,2011和2014年树高利用Vtrtex Ⅳ超声波测高测距仪直接测定,测量精度为±0.1 m,胸径利用胸径尺测定,测量精度为±0.1 cm。

1.3 统计分析方法

种源、种源内家系的树高和胸径方差分析线性模型[18]为:

$$X_{kijl} = \mu + B_k + \alpha_i + \beta_{j(i)} + \alpha B_{ik} + \beta B_{j(i)k} + e_{kijl} \quad (1)$$

表 1　参试 4 个种源 169 个日本落叶松家系号

Tab. 1　No. of 169 *Larix kaempferi* families in 4 testing seed sources

种源<br>Seed source	数量<br>Number	家系号<br>Family No.
A	50 个家系<br>50 families	A001、A002、A003、A004、A005、A006、A007、A008、A009、A011、A012、A013、A014、A015、A016、A017、A018、A019、A020、A021、A023、A025、A029、A032、A041、A043、A060、A061、A062、A063、A064、A065、A067、A068、A070、A071、A072、A074、A077、A078、A079、A080、A081、A082、A083、A084、A085、A086、A089、A090
B	36 个家系<br>36 families	B101、B102、B103、B104、B105、B106、B107、B108、B109、B110、B113、B114、B115、B116、B117、B118、B119、B120、B121、B122、B123、B124、B125、B126、B128、B130、B131、B133、B134、B135、B136、B137、B139、B141、B142、B146
C	53 个家系<br>53 families	C201、C203、C206、C208、C209、C212、C214、C215、C216、C217、C218、C220、C222、C223、C225、C227、C230、C232、C233、C236、C237、C238、C239、C254、C256、C258、C261、C266、C267、C269、C270、C271、C272、C273、C274、C275、C279、C282、C284、C285、C286、C287、C288、C289、C291、C292、C295、C296、C297、C298、C299、C204、C202
D	30 个家系<br>30 families	D301、D302、D303、D304、D306、D307、D308、D309、D310、D311、D312、D313、D314、D315、D316、D317、D318、D319、D320、D321、D322、D323、D324、D325、D326、D327、D328、D329、D330、D331

注：A 为东丰，B 为临江，C 为通化，D 为兰山。Notes：A stands for Dongfeng, B stands for Linjiang, C stands for Tonghua, D stands for Lanshan.

式中：X_{kijl}为第 k 区组第 i 种源（或家系群）内第 j 家系第 l 小区单株观测值；μ 为试验平均值；B_k为第 k 区组效应值（固定）；α_i为第 i 种源（家系群）效应值（固定）；$\beta_{j(i)}$为第 i 种源（家系群）中第 j 家系效应值（随机）；αB_{ik}为区组与种源（或家系群）互作效应值（固定）；$\beta B_{j(i)k}$为区组与第 i 种源（家系群）中第 j 家系互作效应值；e_{kijl}为包括单株变异的剩余项。

家系遗传力（H^2）采用公式[19]：

$$H^2 = \sigma^2\beta_{j(i)} / (\sigma^2\beta_{j(i)} + \sigma^2\beta B_{j(i)k}/n + e_{ijkl}/nB) \quad (2)$$

单株遗传力（h^2）采用公式：

$$h^2 = \sigma^2\beta_{j(i)} / (\sigma^2\beta_{j(i)} + \sigma^2\beta B_{j(i)k} + e_{ijkl}) \quad (3)$$

式中：σ^2 为方差分量，n 为评价性状的个数，B 为区组数量。

表型变异系数（PCV）和遗传变异系数（GCV）采用公式：

$$\mathrm{PCV} = \frac{\sigma_p^2}{\overline{X}} \times 100 \quad (4)$$

$$\mathrm{GCV} = \frac{\sigma_\gamma^2}{\overline{X}} \times 100 \quad (5)$$

式中：σ_γ^2 为遗传变异方差，σ_p^2 为表型变异方差，$\overline{X}$ 为各性状的总体平均值。

采用布雷津多性状综合评定法对家系进行综合评定[20]，具体公式为：

$$Q_i = \sqrt{\sum_{j=1}^{n} \overline{X}_{ij} / X_{j\max}} \quad (6)$$

式中：Q_i 为综合评价值，$\overline{X}_{ij}$ 为某一性状的平均值，$X_{j\max}$为某一性状的最优值。

遗传增益（ΔG_1）估算公式[20]：

$$\Delta G_1 = h^2 W / \overline{X} \quad (8)$$

现实增益（ΔG_2）采用公式：

$$\Delta G_2 = W / \overline{X} \quad (9)$$

式中：W 为选择差。

2　结果与分析

2.1　各性状方差分析结果

4 个种源、169 个日本落叶松半同胞家系各性状方差分析结果见表 2。不同树龄条件下，各性状区组间、种源间、家系间以及各交互作用间均达到极显著差异水平（$P < 0.01$）。

2.2　种源、家系各生长指标平均值

多重比较分析结果表明，不同树龄日本落叶松的树高和胸径 4 个种源两两之间也存在一定差异（表 3）。4 个种源树高和胸径平均值见图 1 和表 3，不同树龄所有家系树高和胸径均值变化范围分别为 1.60 ~ 10.08 m 和 7.46 ~ 12.03 cm。2007—2011 年，种源 B 的树高生长速率最快，平均值变化范围为 1.49 ~ 7.04 m，种源 A、C 和 D 的生长速率相近；2011—2014 年，种源 D 的树高生长速率最快，平均值变化范围为 6.19 ~ 10.44 m；2014 年，种源 A 树高平均值在 4 个种源中最低为 9.76 m，种源 D 树高平均值在 4 个种源中最高，达到 10.44 m，是种源 A 树高平均值(9.76 m)的 1.06 倍；2014 年，4 个种源中种源 C 的胸径平均值最大，达到 12.56 cm，是胸径平均值最小种源 B(11.06)的 1.14 倍。本研究中种源 C 和种源 D 11 年生树高和胸径平均值高于总平均值，说明种源 C 和种源 D 的生长较快，可以考虑作为优良种源。种源内各家系树高平均值分析见图 2，各家系在测量年份间生长速率均呈现先快后慢的趋势。在 2014 年，种源 A 中家系 A78 的树高平均值最大，为 10.61 m，是树高平均值最小家系 A43 (8.23 m)的 1.29 倍；种源 B 中，家系 B135 的树高平均值最大为 10.93 m，是树高平均值最小家系

表2 不同性状方差分析

Tab. 2 ANOVA of different traits

性状 Trait	变异来源 Variance source	自由度 df	均方 MS	F	σ^2
4 年生树高 Height of 4 years old tree	区组 Block	4	49.613	1 498.705**	0.046
	种源 Seed source	3	234.275	216.898**	0.173
	家系/种源 Family/seed source	176	1.080	6.775**	0.031
	区组×种源 Block×seed source	12	1.354	40.906**	0.005
	区组×家系/种源 Block×family/seed source	704	0.159	4.816**	0.021
	机误 Error	4 500	0.033		
8 年生树高 Height of 8 years old tree	区组 Block	4	105.896	326.836**	0.098
	种源 Seed source	3	3 267.151	124.172**	2.391
	家系/种源 Family/seed source	176	26.311	27.787**	0.845
	区组×种源 Block×seed source	12	17.011	52.503**	0.062
	区组×家系/种源 Block×family/seed source	704	0.947	2.922**	0.104
	机误 Error	4 500	0.324		
8 年生胸径 DBH of 8 years old tree	区组 Block	4	349.173	293.206**	0.322
	种源 Seed source	3	5 507.778	241.926**	4.065
	家系/种源 Family/seed source	176	22.766	8.133**	0.666
	区组×种源 Block×seed source	12	57.188	48.021**	0.207
	区组×家系/种源 Block×family/seed source	704	2.799	2.350**	0.268
	机误 Error	4 500	1.191		
11 年生树高 Height of 11 years old tree	区组 Block	4	837.612	109.397**	0.768
	种源 Seed source	3	7 094.938	109.000**	5.209
	家系/种源 Family/seed source	176	65.091	36.206**	2.110
	区组×种源 Block×seed source	12	233.517	30.499**	0.837
	区组×家系/种源 Block×family/seed source	704	1.798	0.235**	0.135
	机误 Error	4 500	7.657		
11 年生胸径 DBH of 11 years old tree	区组 Block	4	1 173.197	760.764**	1.085
	种源 Seed source	3	14 013.099	251.314**	10.344
	家系/种源 Family/seed source	176	55.759	8.603**	1.643
	区组×种源 Block×seed source	12	129.095	83.712**	0.472
	区组×家系/种源 Block×family/seed source	704	6.481	4.203**	0.823
	机误 Error	4 500	1.542		

注：** 表示差异极显著($P<0.01$)。Note: ** means the difference is very significantly different($P<0.01$).

表 3 4 个种源树高和胸径平均值分析表

Tab. 3 Average values of height and DBH of 4 seed sources

种源 Seed source	4 年生树高 Height of 4 years old tree/m	8 年生树高 Height of 8 years old tree/m	8 年生胸径 DBH of 8 years old tree/cm	11 年生树高 Height of 11 years old tree/m	11 年生胸径 DBH of 11 years old tree/cm
A	1. 64 ±0. 27 ab	6. 27 ±0. 88 c	7. 08 ±1. 66 c	9. 76 ±1. 06 d	11. 93 ±2. 31 b
B	1. 49 ±0. 32 c	7. 04 ±0. 92 a	7. 65 ±1. 52 b	10. 02 ±1. 03 c	11. 06 ±2. 09 c
C	1. 62 ±0. 39 b	6. 50 ±0. 74 b	7. 92 ±1. 45 a	10. 22 ±0. 96 b	12. 56 ±2. 07 a
D	1. 66 ±0. 31 a	6. 19 ±0. 89 d	7. 06 ±1. 49 c	10. 44 ±1. 00 a	12. 42 ±2. 02 a
均值 Mean	1. 60 ±0. 34	6. 49 ±0. 90	7. 46 ±1. 58	10. 08 ±1. 04	12. 03 ±2. 21

注：不同字母（a、b、c、d）代表差异极显著（$P<0.01$）。Note: different letters (a, b, c, d) represent extremely significant difference ($P<0.01$).

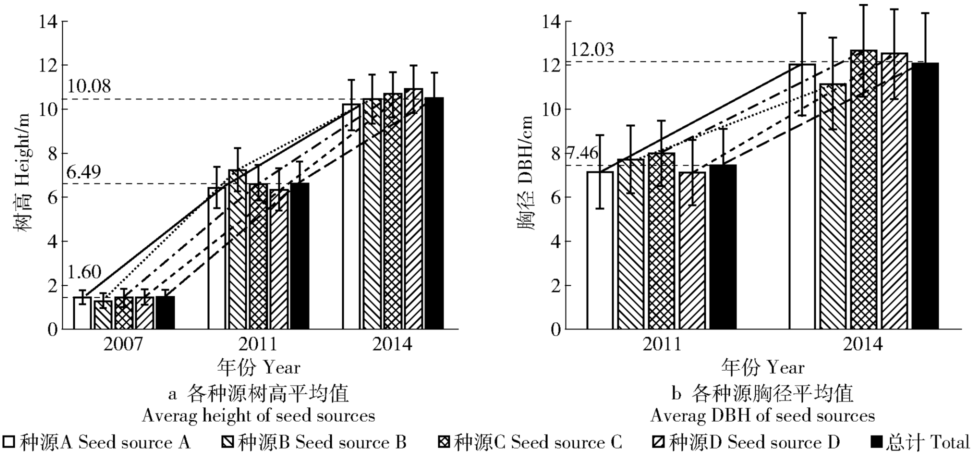

图 1 4 种源树高、胸径平均值

Fig. 1 Averag height and DBH of 4 seed sources

B126(9. 10 m)的 1. 20 倍；种源 C 中，家系 C272 的树高平均值最大为 11. 87 m，是树高最小家系 C295 平均值(9. 30 m)的 1. 28 倍；种源 D 中，家系 D318 的树高平均值最大为 11. 82 m，是家系树高最小 D323 平均值(9. 47 m)的 1. 25 倍。

2. 3 家系间遗传和变异参数

169 个家系树高和胸径遗传变异参数见表 4。树高和胸径的表型变异系数变化范围分别为 4. 00% ~37. 37% 和 24. 89% ~26. 48%；树高和胸径遗传变异系数范围分别为 1. 94% ~20. 93% 和 8. 93% ~13. 68%。由数据可知，树高和胸径表型变异系数和遗传变异系数均随着树龄的增加呈上升的趋势。树高和胸径的家系遗传力变化范围分别为 0. 881 ~0. 972 和 0. 877 ~0. 879，单株遗传力变化范围分别为 0. 406 ~0. 664 和 0. 301 ~0. 410，属于高遗传力。高变异、高遗传力有利于家系和单株的评价选择。

2. 4 多性状综合评价

根据 2014 年的树高和胸径值，以 10% 的入选率，利用多性状综合评价法进行优良家系的评价选择，17 个家系（D318、C272、D312、C222、C209、D308、D303、C269、C274、C266、D325、D320、C271、B124、B135、D310 和 D324）入选为优良家系（表 5），入选家系的树高与胸径平均值分别为 11. 10 m 和 13. 82 cm，分别比总平均值高 10. 98% 和 16. 90%，现实增益分别为 11. 68% 和 17. 98%，遗传增益分别为 11. 35% 和 15. 80%。以 5% 的入选率，根据 2014 年的树高和胸径在优良家系内进行优良单株的选择，结果见表 6，25 个单株被选作优良单株，入选单株的树高和胸径平均值分别为 12. 58 m 和 17. 72 cm，遗传增益分别为 8. 54% 和 12. 50%。

3 讨论与结论

在林木育种研究中，方差分析是评估变异幅度的重要方法[21]。本研究中各指标在种源间、家系间和家系与种源的交互作用间差异均达到极显著水平（$P<0.01$），各家系间差异较大是因为区组面积较大，不同区组间环境对家系的生长产生一定影响，同时由遗传方差可知，各家系受较强的遗传因素影响，这与 Nakada[22] 对杂种落叶松变异分析研究结果一致，表明进行优良家系的评价选择是有必要的[23]。遗传和变异是林木育种研究的主要内容[24]，也是选择和评价优良家系的重要参数[25]。在树木的生长过程中，全面理解和掌握各遗传参数随时间的动态

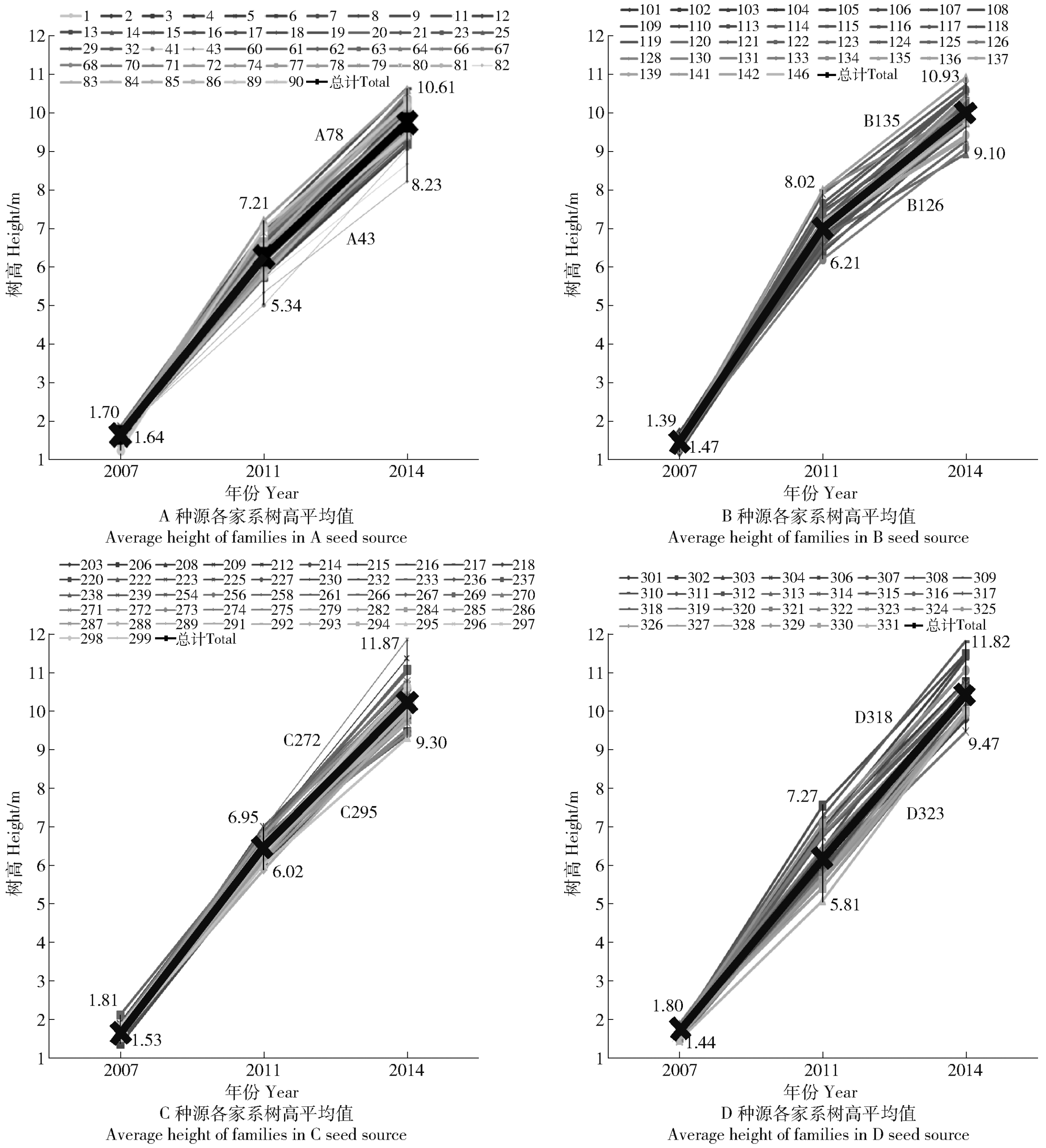

A 种源各家系树高平均值 Average height of families in A seed source

B 种源各家系树高平均值 Average height of families in B seed source

C 种源各家系树高平均值 Average height of families in C seed source

D 种源各家系树高平均值 Average height of families in D seed source

图2　4 个种源内家系树高平均值分析

Fig. 2　Average height of each family in 4 seed sources

表4　家系间各性状遗传变异参数

Tab. 4　Genetic and variation parameters of each traits of families

性状 Trait	变幅 Range	标准差 SD	表型变异系数 Phenotype variation coefficient	遗传变异系数 Genetic variation coefficient	家系遗传力 Family heritability	单株遗传力 Heritability of single tree
4 年生树高 Height of 4 years old tree	1. 20(B110) ~2. 11(C269)	0. 34	4. 00	1. 94	0. 881	0. 406
8 年生树高 Height of 8 years old tree	4. 00(A41) ~9. 00(B135)	0. 90	18. 01	13. 02	0. 964	0. 664
8 年生胸径 DBH of 8 years old tree	4. 62(A41) ~12. 00(D318)	1. 58	24. 89	8. 93	0. 877	0. 301
11 年生树高 Height of 11 years old tree	8. 23(A43) ~13. 20(C272)	1. 04	37. 37	20. 93	0. 972	0. 652
11 年生胸径 DBH of 11 years old tree	8. 33(B126) ~15. 23(D318)	2. 21	26. 48	13. 68	0. 879	0. 410

表 5 不同家系 Q_i 值分析表

Tab. 5 Q_i value analysis of different families

家系号 Family No.	Q_i 值 Q_i value	家系号 Family No.	Q_i 值 Q_i value	家系号 Family No.	Q_i 值 Q_i value	家系号 Family No.	Q_i 值 Q_i value	家系号 Family No.	Q_i 值 Q_i value
D318	1.311 5	C208	1.195 3	C287	1.177 8	A021	1.158 0	A090	1.154 1
C272	1.275 8	A015	1.195 3	B101	1.175 8	A070	1.158 0	D313	1.154 0
D312	1.275 8	A063	1.193 5	C296	1.175 8	A084	1.158 0	B205	1.150 1
C222	1.274 0	A080	1.193 4	A006	1.175 8	A013	1.158 0	D311	1.150 1
C209	1.272 1	C267	1.193 4	B120	1.169 9	C230	1.157 9	D330	1.150 1
D308	1.272 0	B119	1.193 4	C217	1.169 9	C238	1.155 9	B137	1.150 1
D303	1.264 9	C292	1.193 4	A060	1.166 0	B107	1.155 9	A068	1.150 1
C269	1.264 7	C232	1.193 4	C256	1.166 0	C204	1.155 9	B136	1.142 0
C274	1.253 9	A061	1.193 3	D328	1.166 0	C202	1.155 9	A081	1.142 0
C266	1.250 2	B108	1.193 3	D314	1.166 0	C279	1.155 9	C261	1.142 0
D325	1.242 9	C299	1.189 6	D319	1.166 0	A062	1.155 9	A029	1.141 9
D320	1.239 0	A086	1.189 6	B109	1.166 0	C291	1.155 9	A025	1.141 9
C271	1.235 2	B142	1.189 6	C275	1.166 0	D326	1.155 9	D316	1.137 8
B124	1.231 5	D321	1.181 6	A019	1.166 0	D309	1.155 9	A072	1.137 8
B135	1.224 1	C227	1.181 6	D306	1.166 0	B218	1.155 9	A001	1.137 8
D310	1.220 2	C270	1.179 7	D331	1.166 0	C288	1.155 9	B126	1.137 8
D324	1.216 4	C223	1.178 0	C216	1.166 0	C289	1.155 9	D323	1.137 8
B106	1.215 4	A008	1.177 5	B146	1.165 4	B131	1.155 9	C220	1.137 1
C203	1.214 3	C236	1.176 4	A071	1.165 4	C212	1.155 8	A074	1.137 1
B121	1.214 1	D301	1.176 4	C206	1.165 4	C298	1.155 8	A085	1.137 1
⋮	⋮	⋮	⋮	⋮	⋮	⋮	⋮	⋮	⋮
C258	1.196 4	A011	1.177 8	A064	1.162 0	B123	1.154 1	A032	1.105 0

表 6 不同单株 Q_i 值分析表

Tab. 6 Q_i value analysis of different single plants

区组 Block	家系号 Family No.	单株 Plant	Q_i 值 Q_i value	区组 Block	家系号 Family No.	单株 Plant	Q_i 值 Q_i value	区组 Block	家系号 Family No.	单株 Plant	Q_i 值 Q_i value
1	D318	1	1.408 8	2	D320	3	1.351 5	1	C269	1	1.326 0
1	D318	2	1.408 8	1	C272	2	1.351 0	1	C269	2	1.326 0
1	C266	1	1.408 8	4	C272	4	1.348 4	4	C269	1	1.326 0
1	C266	2	1.408 8	3	D312	1	1.348 4	4	C269	2	1.326 0
1	D318	3	1.390 0	2	D318	4	1.344 0	1	C271	2	1.325 7
1	D318	4	1.390 0	2	D312	1	1.343 2	5	C271	1	1.325 7
1	D318	5	1.390 0	2	D318	5	1.343 1	3	D318	3	1.323 3
1	D318	6	1.390 0	1	C272	3	1.343 1	2	D312	4	1.323 3
2	D318	1	1.373 7	4	C272	5	1.340 4	1	C209	3	1.323 3
2	D318	2	1.371 0	2	D312	2	1.340 2	1	C209	4	1.323 3
2	D318	3	1.371 0	2	D312	3	1.340 2	⋮	⋮	⋮	⋮
1	C209	1	1.371 0	1	D308	4	1.337 6	⋮	⋮	⋮	⋮
4	C209	1	1.370 8	1	D308	5	1.337 6	⋮	⋮	⋮	⋮
1	C271	1	1.370 8	1	D308	6	1.337 6	⋮	⋮	⋮	⋮
4	C271	6	1.365 2	2	D308	1	1.337 6	⋮	⋮	⋮	⋮
1	D325	1	1.357 2	1	C274	2	1.337 6	⋮	⋮	⋮	⋮
1	D325	2	1.357 2	3	C274	6	1.337 4	⋮	⋮	⋮	⋮
1	D320	3	1.357 1	2	D318	6	1.334 8	5	D310	6	1.136 3
1	C274	1	1.356 9	1	C222	1	1.334 7	3	B124	4	1.133 1
3	C274	5	1.356 9	1	C222	2	1.334 6	5	D324	2	1.133 1
1	C272	1	1.354 3	3	C222	2	1.334 6	5	D324	3	1.133 1
4	C272	3	1.354 3	3	C222	3	1.334 6	3	D310	2	1.133 1
1	C209	2	1.351 6	5	C222	3	1.334 6	5	D324	5	1.116 1
4	C209	2	1.351 6	5	C222	4	1.334 6	5	D310	5	1.115 9
2	D320	2	1.351 6	2	D320	4	1.334 6	1	D320	2	1.102 8

变化规律,对林木育种策略制定具有十分重要的作用[26]。遗传变异系数是衡量相关生长性状遗传变异潜力的有效指标,变异系数的大小反映群体的变异程度[27]。本研究中,4 年生落叶松树高和胸径变异系数小,可能是由于树龄小,个体间差异不明显。树高和胸径的表型变异系数和遗传变异系数均随树龄的增加呈上升趋势,且遗传变异系数占表型变异系数的比例也随着树龄的增加而变大,这与杜超群等[28]对日本落叶松无性系与家系变异分析的研究结论一致,说明随着树龄的增加,家系个体间的差异受环境因素影响越来越小,受遗传因素影响越来越大。本研究还发现,胸径的表型变异系数均高于树高的表型变异系数,这与白天道[29]对马尾松研究结果相一致,说明在此树龄条件下,胸径性状的选择潜力要高于树高性状,胸径更适宜作为日本落叶松选择的因子,杨秀艳等[30]在对日本落叶松家系的研究中也有此发现。本研究中,各性状家系遗传力保持较高水平,表明各性状均具有较高遗传力,对其进行选择可获得较大的遗传增益[31]。从不同树龄各家系生长性状平均值来看,早期树高生长快的家系后期生长也较快,说明日本落叶松家系生长比较稳定,为日本落叶松家系的早期选择提供依据[32]。

综合评价可以对多个性状同时进行评价,选育出的优良家系和单株更稳定[33]。为考虑群体的遗传多样性及遗传增益,在进行家系选择中家系遗传力较高时采用较高的入选率,反之采用较低的入选率[34]。本研究在对家系和单株评价过程中,初步选出 17 个优良家系和 25 个优良单株。在林木育种中,遗传增益是衡量选择效果的最重要的参数之一,它反映了下一代比亲本可能增加的收获量[35]。本研究中入选家系树高和胸径的遗传增益分别为 11.35% 和 15.80%,入选单株遗传增益分别为 8.54% 和 12.50%,入选的优良家系和单株均表现出明显的生长优势。同时优良家系的遗传增益比优良单株的遗传增益大,说明家系选择能更大限度的提高选择效果。选择的优良家系和优良单株可以尝试在生产中推广应用,回向选择的优良亲本可以为改良种子园的营建提供基础,前向选择获得的优良单株可以为 2 代种子园提供建园材料[36],由于本研究中部分优良单株来源于相同家系,在种子园营建时应避免相邻栽植。

木材性状也是评价落叶松的重要因素,本研究只对日本落叶松的生长性状进行了测定分析,下一步将进行木材性状的连年测定分析,联合生长性状和木材性状对日本落叶松家系进行评价选择,以期能够培育出生长迅速,材性优良的日本落叶松家系。

参 考 文 献

[1] 王亚南,王军辉,张守攻,等. 日本落叶松种源对树高生长模型参数的影响[J]. 东北林业大学学报,2016,44(9):1-4.
Wang Y N, Wang J H, Zhang S G, et al. Parameters of height-age models for *Larix kaempferi* provenances [J]. Journal of Northeast Forestry University, 2016,44(9):1-4.

[2] Fukatus E, Tsubomura M, Fujisawa Y, et al. Genetic improvement of wood density and radial growth in *Larix kaempferi*: results from a diallel mating test[J]. Annals of Forest Science, 2013, 70(5): 451-459.

[3] 董健,尤文忠,黄国学,等. 日本落叶松良种选育的现状及发展对策[J]. 辽宁林业科技,2003(6):27-29.
Dong J, You W Z, Huang G X, et al. Present situation and development strategy of *Larix kaempferi* seed breeding[J]. Journal of Liaoning Forestry Science & Technology, 2003(6):27-29.

[4] Paques L E, Miller F, Rozenberg P. Selection perspectives for genetic improvement of wood stiffness in hybrid larch (*Larix* × *eurolepis* Henry)[J]. Tree Genetics & Genomes, 2010, 6(1): 83-92.

[5] Nagemitsu T, Nagesaka K, Youshimaru H, et al. Provenance tests for survival and growth of 50-year-old Japanese larch (*Larix kaempferi*) trees related to climatic conditions in central Japan [J]. Tree Genetics & Genomes, 2014, 10(1):87-99.

[6] Pan Y, Li S, Wang C, et al. Early evaluation of growth traits of *Larix kaempferi* clones[J]. Journal of Forestry Research, 2018, 29(4): 1031-1039

[7] Cáceres C B, Hernández R E, Fortin Y, et al. Wood density and extractive content variation among Japanese larch (*Larix kaempferi* [Lamb.] Carr.) progenies/provenances trials in eastern Canada [J]. Wood & Fiber Science Journal of the Society of Wood Science & Technology,2017,49(4):363-372.

[8] 朱景乐,王军辉,张守攻,等. Pilodyn 在日本落叶松活立木材性指标预测中的应用[J]. 林业科学研究,2009, 22(1): 75-79.
Zhu J L, Wang J H, Zhang S G, et al. Using the Pilodyn to assess wood traits of standing trees *Larix kempferi*[J]. Forest Research, 2009, 22(1): 75-79.

[9] Watanabe M, Watanabe Y, Kitaoka S, et al. Growth and photosynthetic traits of hybrid larch F_1 (*Larix gmelinii* var. *japonica* × *L. kaempferi*) under elevated CO_2 concentration with low nutrient availability[J]. Tree Physiology, 2011, 31(9): 965-975.

[10] Han H, Sun X, Xie Y, et al. Transcriptome and proteome profiling of adventitious root development in hybrid larch (*Larix kaempferi* × *Larix olgensis*) [J]. BMC Plant Biology, 2014, 14(1): 305.

[11] 汪成成. 利用 SRAP 标记分析日本落叶松 2 代优树的遗传多样性[J]. 辽宁林业科技, 2017(2): 19-24,37.
Wang C C. Analysis of genetic diversity of second-generation tree of *Larix kaempferi* by SRAP [J]. Liaoning Forestry Science & Technology, 2017(2): 19-24,37.

[12] Kaushik N, Deswal R P, Malik S, et al. Genetic variation and heritability estimation in *Jatropha curcas* L. progenies for seed yield and vegetative traits [J]. Journal of Applied and Natural Science, 2015, 7(2): 567-573.

[13] Zas A R, Merlo E, Fernández L J. Genetic parameter estimates for Maritime pine in the Atlantic coast of North-west Spain [J].

International Journal of Forest Genetics, 2004, 11(1): 45-53.

[14] Pinyopusarerk K, Williams E R. Range-wide provenance variation in growth and morphological characteristics of *Casuarina equisetifolia* grown in Northern Australia[J]. Forest Ecology and Management, 2000, 134(1-3): 219-232.

[15] Vizcaíno P N, Ibáñez I, Benito G M, et al. Climate and population origin shape pine tree height-diameter allometry[J]. New Forests, 2017, 48(3): 363-379.

[16] 李艳霞. 长白落叶松优树子代生长与材质的遗传及多性状联合选择[D]. 哈尔滨:东北林业大学, 2012.

Li Y X. The genetic variation and multi-trait associated selection of the growth traits and material quality traits of the excellent progenies of *Larix olgensis* [D]. Haerbin: Northeast Forestry University, 2012.

[17] 朱于勤,侯义梅. 日本落叶松原生种质资源及优良林分选择[J]. 湖北林业科技,2015(4):9-11.

Zhu Y Q, Hou Y M. Selection of provenance and superior stand of *Larix kaempferi* [J]. Hubei Forestry Science and Technology, 2015(4):9-11.

[18] 陈晓阳,沈熙环. 林木育种学[M]. 北京:高等教育出版社, 2005.

Chen X Y, Shen X H. Forest tree breeding[M]. Beijing: Higher Education Press, 2005.

[19] 续九如. 林木数量遗传学[M]. 北京:高等教育出版社,2006: 31,55.

Xu J R. Quantitative genetics in forestry[M]. Beijing: Higher Education Press, 2006:31,55.

[20] 朱之悌. 林木遗传学基础[M]. 北京: 中国林业出版社,1989: 190-191.

Zhu Z T. Foundation of tree genetic[M]. Beijing: China Forestry Publishing House, 1989:190-191.

[21] Safavi S A, Pourdad S A, Mohmmad T, et al. Assessment of genetic variation among safflower (*Carthamus tinctorius* L.) accessions using agro-morphological traits and molecular markers [J]. Journal of Food Agriculture & Environment,2010, 8(3): 616-625.

[22] Nakada R, Fujisawa Y, Taniguchi T. Variations of wood properties between plus-tree clones in *Larix kaempferi* (Lamb.) Carrière [J]. Bulletin of the Forest Tree Breeding Center (Japan), 2005, 21: 85-105.

[23] 许鲁平. 枫香优良家系和单株选择研究[J]. 福建林业科技, 2002,29(2): 26-30.

Xu L P. Studies on the selection of *Liquidambar formosana* fine families and individuals[J]. Journal of Fujian Forestry Science and Technology, 2002,29(2):26-30.

[24] Wright J. Introduction to forest genetics [M]. New York: Academic Press, 1976: 427-438.

[25] Weng Y H, Lu P, Adams G W, et al. Genetic parameters of growth and stem quality traits for jack pine second-generation progeny tested in New Brunswick [J]. Canadian Journal of Forest Research, 2015,45(1):36-43.

[26] Pliura A, Zhang S Y, Mackay J, et al. Genotypic variation in wood density and growth traits of poplar hybrids at four clonal trials[J]. Forest Ecology and Management, 2007, 238(1-3): 92-106.

[27] Lepoittevin C, Rousseau J P, Guillemin A, et al. Genetic parameters of growth, straightness and wood chemistry traits in *Pinus pinaster* [J]. Annals of Forest Science, 2011, 68(4): 873-884.

[28] 杜超群,许业洲,孙晓梅,等. 鄂西亚高山区日本落叶松无性系生长性状变异分析与早期选择[J]. 华中农业大学学报, 2015, 34(3):19-23.

Du C Q, Xu Y Z, Sun X M, et al. Variation of growth traits and early selection of *Larix kaempferi* clones in sub-alpine area of western Hubei Province [J]. Journal of Huazhong Agricultural University, 2015,34(3):19-23.

[29] 白天道,徐立安,王章荣,等. 马尾松实生种子园自由授粉子代测定及亲本家系选择增益估算化[J]. 林业科学研究,2012,25(4):449-455.

Bai T D, Xu L A, Wang Z R, et al. Estimation of parents genetic gain by open-pollinated progeny test of seedling seed orchard of Masson pine[J]. Forest Research, 2012, 25(4): 449-455.

[30] 杨秀艳,张守攻,孙晓梅,等. 北亚热带高山区日本落叶松自由授粉家系遗传测定与二代优树选择[J]. 林业科学,2010,46(8):45-50.

Yang X Y, Zhang S G, Sun X M, et al. Genetic test of open-pollinated *Larix kaempferi* families and selection for the second generation elite trees in northern sub-tropical alpine area [J]. Scientia Sillvae Sinicae, 2010, 46(8):45-50.

[31] 梁德洋,金允哲,赵光浩,等. 50 个红松无性系生长与木材性状变异研究[J]. 北京林业大学学报,2016,38(6):51-59.

Liang D Y, Jin Y Z, Zhao G H, et al. Variance analyses of growth and wood characters of 50 *Pinus koraiensis* clones[J]. Journal of Beijing Forestry University, 2016,38(6):51-59.

[32] 邓继峰,张含国,张磊,等. 17 年生杂种落叶松遗传变异及优良家系选择[J]. 东北林业大学学报,2010,38(1):8-11.

Deng J F, Zhang H G, Zhang L, et al. Genetic variation of 17-year-old hybrid larch and its superior family selection[J]. Journal of Northeast Forestry University, 2010, 38(1):8-11.

[33] 韩泽群,姜波. 加工番茄品种多性状综合评价方法研究[J]. 中国农业科学,2014,47(2):357-365.

Han Z Q, Jiang B. A study on comprehensive evaluation of the tomato varieties multiple traits processing[J]. Scientia Agricultura Sinica, 2014,47(2):357-365.

[34] 潘琼荣. 马尾松自由授粉 18 年生子代生长状况比较及家系选择[J]. 福建农林大学学报(自然科学版),2014,43(6):592-595.

Pan Q R. Comparisons of the growth of open-pollinated progeny of 18 year-old *Pinus massoniana* seed orchard and its family selection [J]. Journal of Fujian Agriculture and Forestry University (Natural Science Edition), 2014, 43(6): 592-595.

[35] Ruotsalainen S, Lindgren D. Predicting genetic gain of backward and forward selection in forest tree breeding[J]. Silvae Genetica, 1998, 47(1): 42-50.

[36] 苏顺德,黄德龙,魏永平,等. 马尾松自由授粉家系产脂力遗传变异及选择[J]. 福建林业科技,2017,44(2):1-6,50.

Su S D, Huang D L, Wei Y P, et al. Genetic variation and selection of the resin-yielding capacity of open-pollinated families of Masson pine[J]. Journal of Fujian Science and Technology, 2017, 44(2):1-6,50.

(责任编辑　崔艳红
责任编委　康向阳)
