# Supplementary material for: Provenance and family variations in early growth of Manchurian walnut (Juglans mandshurica Maxim.) and selection of superior families
Source: PLoS One. 2024 Mar 7;19(3):e0298918. doi: 10.1371/journal.pone.0298918 (PMC10919699; doi:10.1371/journal.pone.0298918)
Supplement: S2 File — (ZIP) [file pone.0298918.s005.zip › Variance analysis of growth characteristics of 30 Pinus koraiensis half-sib families.pdf]

依据生长性状对红松半同胞家系的评价选择1)

王璧莹　赵曦阳
(林木遗传育种国家重点实验室(东北林业大学),哈尔滨,150040)

王洪武　姜国云　沈光　王连奎　李焱龙　　林健　王忠良
(吉林省三岔子林业局林木良种基地管理中心)　　(吉林省临江林业局)

摘　要　以吉林省江源市三岔子种子园的 30 个 24 年生红松半同胞家系为材料,对其树高、地径、胸径、3 m 径和材积等 9 个生长性状进行测定,结果表明:不同区组间、不同家系间各性状差异均达到极显著水平;各性状表型变异系数变化范围为 5.28%~44.44%;各性状遗传力均超过 0.50,其中树高、地径、胸径、3 m 径、材积和第 6 轮枝高的遗传力均超过 0.80,属于高遗传力;高变异、高遗传力有利于家系的评价选择。相关性分析结果表明,各性状间相关关系均达到极显著正相关水平。利用综合评价法,初步选出 97 和 106 这两个家系,入选家系树高、地径、胸径、3 m 径和材积分别比总平均值高 7.00%、18.60%、17.70%、22.90%和 40.00%,遗传增益分别为 6.04%、15.49%、15.84%、20.43%和 35.68%。初步选出的优良家系可以为红松改良种子园及二代种子园提供材料。

关键词　红松;半同胞家系;生长性状;遗传力;遗传增益
分类号　S722.5

Variance Analysis of Growth Characteristics of 30 *Pinuskoraiensis* Half-sib Families//Wang Biying, Zhao Xiyang (State Key Laboratory of Tree Genetics and Breeding, Northeast Forestry University, Harbin 150040, P. R. China); Wang Hongwu, Jiang Guoyun, Shen Guang, Wang Liankui, Li Yanlong (Sanchazi Forestry Bureau of Jilin Province); Lin Jian, Wang Zhongliang (Linjiang Forestry Bureau of Jilin Province) //Journal of Northeast Forestry University, 2019, 47(4): 8-11, 20.

Thirty 24-year *Pinuskoraiensis* half-sibfamilies from the Sanchazi Seeds Orchard of Jiangyuan City, Jilin Province were taken as materials. Tree height, ground diameter, diameter of breast height, diameter of stem at 3-m height, volume and four other characters were measured and analyzed. The characters in different blocks and different families reached a very significant level. The coefficients of phenotypic variation of differenttraits were in 5.28%-44.44%. The heritability of all the traits was more than 0.50. The heritability of tree height, ground diameter, diameter of breast height, diameter of stem at 3-m height, volume and under the sixth branch height were higher than 0.80, which were high heritability. High variation and high heritability were beneficial to the selection of elite families. By correlation analysis, the correlations between all traits reached a very significant positive correlation level. By the method of multiple-traits comprehensive, family 97 and 106 were selected as elite families. The average of tree height, ground diameter, diameter at breast height, diameter of stem at 3-m height and volume were higher than the total average 7.00%, 18.60%, 17.70%, 22.90% and 40.00%, and the genetic gains were 6.04%, 15.49%, 15.84%, 15.49% and 15.84%, respectively. In this study, the elite families could provide materials for the improved seed orchard and the second generation seed orchard.

Keywords　*Pinuskoraiensis*; Half-sib families; Growth character; Heritability; Genetic gain

DOI:10.13759/j.cnki.dlxb.2019.04.002

红松(*Pinus koraiensis*)为松科(Pinaceae)松属(*Pinus* Linn)乔木,是寒温带针阔混交林的重要组成树种,国家一级重点保护植物[1-2],在我国主要分布于长白山及其北部的张广才岭、老爷岭、完达山和小兴安岭[3],是中国东北林区自然演替顶级群落植被建群树种[4]。红松作为珍贵经济树种,具有耐寒性强、树干通直、材质轻软,结构细腻,纹理密直通达,耐腐蚀性强等优良特性[5],是建筑、桥梁、枕木和家具制作的上等木料。综上,红松具有较高的生态价值和经济价值[6]。

目前对红松的研究主要集中在种源选择[7]、无性系评价[8]、种子园建设[9]和分子育种[10]等方面,在常规育种方面利用生长或种实性状对家系进行评价选择,但一般是利用单个性状对家系进行选择,综合多个性状进行选择的研究较少。本研究以吉林省江源市三岔子种子园的 30 个红松半同胞家系为材料,对其生长性状进行测定分析,利用多性状对各家系进行综合评价及初步选择,为红松优良家系评价提供理论基础。

1　材料与方法

试验林位于吉林省江源市三岔子种子园,该基地年平均气温 2.5 ℃,无霜期 110 d,年降水量 755 mm,平均年日照时间 2 300 h。

1) 国家重点研发计划项目(2017YFD0600601)。

第一作者简介:王璧莹,女,1995 年 5 月生,林木遗传育种国家重点实验室(东北林业大学),硕士研究生。E-mail:449017740@qq.com。

通信作者:赵曦阳,林木遗传育种国家重点实验室(东北林业大学),副教授。E-mail:zhaoxyphd@163.com。

收稿日期:2018 年 6 月 23 日。
责任编辑:潘　华。

(C)1994-2019 China Academic Journal Electronic Publishing House. All rights reserved.　http://www.cnki.net

试验材料包括 29 个红松半同胞家系和一个对照家系，1993 年采集半同胞家系种子，次年育苗，于 1998 年营建实生苗子代测定林。试验设计采用完全随机区组设计，5 个区组，6 株小区，株行距为 2 m×3 m。

于 2017 年底对各家系树高（H）、地径（D_G）、胸径（H_{DB}）、3 m 径（H_{DM}）、材积（V）、分支角（A_B）、枝下高（H_{FB}）、第 6 轮枝高（H_{SB}）和轮枝数（N_{WB}）进行测定。利用 Vertex Laser 测高仪及围尺测定单株树高和胸径。通过公式（1）计算得到单株材积[11]。

$$V=0.193\,283\,21D^2H+0.007\,734\,35DH+0.821\,419\,15D^2。\quad (1)$$

式中：V 为材积，m^3；D 为胸径，m；H 为树高，m。所有数据利用 SPSS 软件进行分析。

方差分析模型为：

$$X_{ijk}=\mu+B_i+F_j+BF_{ij}+e_{ijk}。\quad (2)$$

式中：μ 为总体平均值，B_i 为家系效应，F_j 为区组效应，BF_{ij}为家系和区组之间的交互，e_{ijk}为环境误差。

根据续九如[12]的方法估算家系遗传力：

$$h^2=1-1/F。\quad (3)$$

式中：F 为方差分析值。

表型变异系数[12]采用公式：

$$V_{PC}=\frac{\sqrt{\sigma_p^2}}{\bar{X}}\times100\%。\quad (4)$$

遗传变异系数[12]公式：

$$V_{GC}=\frac{\sqrt{\sigma_p^2}}{\bar{X}}\times100\%。\quad (5)$$

式中，σ_p^2 = 表型变异方差，σ_g^2 = 遗传变异方差，$\bar{X}$ 为各性状的总体平均值。

表型相关系数（r_{pn}）采用公式[13]：

$$r_{pn}=\frac{C_{ovp12}}{\sqrt{\sigma_{p1}^2、\sigma_{p2}^2}}。\quad (6)$$

式中：C_{ovp12}为两个性状的表型协方差，σ_{p1}^2、σ_{p2}^2分别为两性状的表型方差。

一般配合力（A_{GC}）采用公式[14]：

$$A_{GC}=X_i-\bar{X}。\quad (7)$$

式中：X_i 为某个亲本若干交配组合子代平均值，$\bar{X}$ 为子代总平均值。

采用布雷津多性状综合评定法对家系进行综合评定[13]，采用公式为：

$$Q_i=\sqrt{\sum_{j=1}^{n}a_i}，a_i=X_{ij}/X_{j\max}。\quad (8)$$

式中：Q_i 为综合评价值，X_{ij}为某一性状的平均值，$X_{j\max}$为某一性状的最优值，n 为评价性状的数量。

遗传增益估算采用公式[15]：

$$\Delta G=(h^2W/\bar{X})\times100\%。\quad (9)$$

式中：W 为选择差，h^2 为性状的遗传力，$\bar{X}$ 为某一性状的平均值。

2　结果与分析

2.1　各性状方差分析

结果表明不同变异来源间各性状差异均达极显著水平（$P<0.01$），说明各性状之间差异明显，对家系进行评价选择是有意义的（表 1）。

表 1　30 个红松半同胞家系各性状方差分析

性　状	变异来源	平方和	自由度	均方	F
树高	家系	105.23	29	3.63	7.20
	区组	39.36	4	9.84	19.53
	家系×区组	227.06	116	1.96	3.89
地径	家系	1 507.37	29	51.98	8.44
	区组	703.27	4	175.82	28.55
	家系×区组	3 849.96	116	33.19	5.39
胸径	家系	1 093.61	29	37.71	9.32
	区组	155.24	4	38.81	9.59
	家系×区组	2 730.66	116	23.54	5.82
3 m 径	家系	1 043.01	29	35.97	9.20
	区组	460.71	4	115.18	29.46
	家系×区组	2 643.04	116	22.78	5.83
材积	家系	0.06	29	0	9.28
	区组	0.01	4	0	8.62
	家系×区组	0.13	116	0	5.31
分支角	家系	936.81	29	32.30	2.58
	区组	302.34	4	75.58	6.03
	家系×区组	5 570.36	116	48.02	3.83
枝下高	家系	2.66	29	0.09	2.24
	区组	6.14	4	1.54	37.46
	家系×区组	14.81	116	0.13	3.11
第 6 轮枝高	家系	35.89	29	1.24	5.67
	区组	10.53	4	2.63	12.07
	家系×区组	84.60	116	0.73	3.34
轮枝数	家系	52.53	29	1.81	1.79
	区组	106.74	4	26.69	26.44
	家系×区组	260.58	116	2.25	2.23

2.2　红松半同胞家系家系间性状变异参数

30 个红松家系树高、地径和胸径等 9 个性状变异参数分析见表 2。所有家系树高平均值为 6.84 m，变幅为 3.90～11.80 m；地径的平均值为 16.28 cm，变幅为 6.50～28.40 cm；胸径的平均值为 12.91 cm，变幅为 4.80～21.10 cm；3 m 径的平均值为 10.25 cm，变幅为 2.30～18.60 cm；材积的平均值为 0.045 m^3，变幅为 0.005～0.122 m^3；分支角的平均值为 80.37°，变幅为 60.00°～87.50°；枝下高的平均值为 1.78 m，变幅为 0.90～3.40 m；第 6 轮枝高的平均值为 4.08 m，变幅为 1.40～8.00 m；轮枝数的平均值为 5.76，变幅为 2.60～10.00。

各性状表型变异系数变化范围为 5.28%～44.44%，其中材积的表型变异系数超过 40%，轮枝数的表型变异系数为 5.28%；各性状的遗传变异系数变化范

(C)1994-2019 China Academic Journal Electronic Publishing House. All rights reserved.　http://www.cnki.net

围为 2.92%~31.43%；各性状的遗传力较高，除轮枝数外遗传力均超过 0.50。

表 2 30 个红松半同胞家系各性状平均值及变异系数

数 值	树高/m	地径/cm	胸径/cm	3 m 径/cm	材积/m^3	分支角/(°)	枝下高/m	第 6 轮枝高/m	轮枝数
平均值	6.84	16.28	12.91	10.25	0.05	80.37	1.78	4.08	5.76
变幅	3.90~11.80	6.50~28.40	4.80~21.10	2.30~18.60	0.01~0.12	60.00~87.50	0.90~3.40	1.40~8.00	2.60~10.00
标准差	0.91	3.45	2.79	2.81	0.02	4.25	0.25	0.57	1.17
表型变异系数%	13.35	21.17	21.64	27.38	44.44	5.28	13.82	14.04	13.35
遗传变异系数%	8.39	14.69	15.02	19.43	31.43	2.92	7.85	8.14	10.42
遗传力	0.86	0.88	0.89	0.89	0.89	0.61	0.55	0.82	0.44

2.3 不同性状相关性分析

各性状相关系数见表 3。由表 3 可知，树高与其他性状间均达极显著正相关，其中与材积之间的相关系数最高，为 0.760；地径与其他各性状均达极显著正相关，其中与胸径、3 m 径和材积之间相关系数均大于 0.800；胸径与其他各性状均达极显著正相关，其中与材积之间的相关系数高达 0.977；3 m 径与其他各性状均达极显著正相关，其中与胸径之间的相关系数最高，为 0.914；材积与其他各性状均达极显著正相关，其中与地径、胸径和 3 m 径之间的相关系数均达 0.900 以上；分支角与其他各性状均达极显著正相关，其中与胸径之间的相关系数最高，为 0.176；枝下高与其他各性状均达极显著正相关，其中与第 6 轮枝高之间的相关系数最高，为 0.523；第 6 轮枝高与其他各性状均达极显著正相关，其中与枝下高之间的相关系数最高；轮枝数与其他各性状均达极显著正相关，其中与地径之间的相关系数最高，为 0.419。

表 3 30 个红松半同胞家系各性状相关性

性 状	树高	地径	胸径	3 m 径	材积	分支角	枝下高	第 6 轮枝高
地径	0.660**							
胸径	0.686**	0.940**						
3 m 径	0.702**	0.884**	0.914**					
材积	0.760**	0.917**	0.977**	0.908**				
分支角	0.105**	0.153**	0.176**	0.169**	0.171**			
枝下高	0.187**	0.096**	0.141**	0.087**	0.166**	0.164**		
第六轮枝高	0.308**	0.202**	0.206**	0.189**	0.224**	0.120**	0.523**	
轮枝数	0.297**	0.419**	0.344**	0.384**	0.348**	0.094**	0.112**	0.231**

注：**表示相关性极显著(P<0.01)。

2.4 红松家系材积一般配合力

红松家系材积一般配合力计算结果见表 4。材积一般配合力变化范围为-0.013 3(家系 89)~0.024 0(家系 97)，对材积来说，家系 97 和家系 106 一般配合力较高(0.024 0 和 0.010 3)，表明利用这些家系的亲本作为杂交母本，子代材积遗传改良潜力较大。

表 4 30 个红松半同胞家系材积一般配合力

家系	材积	家系	材积
97	0.024 0	102	−0.001 9
106	0.010 3	95	−0.002 0
ck	0.010 2	99	−0.002 7
90	0.008 5	98	−0.004 5
81	0.007 6	105	−0.005 9
110	0.007 4	108	−0.006 1
79	0.006 6	107	−0.006 2
104	0.006 5	94	−0.006 5
96	0.005 5	77	−0.006 7
91	0.005 4	83	−0.006 8
84	0.002 1	111	−0.007 3
100	0.001 7	78	−0.008 4
101	0.001 0	86	−0.009 3
88	0.000 8	109	−0.011 3
87	0.000 4	89	−0.013 3

2.5 多性状综合评价

多性状综合评价结果见表 5。利用综合评价法对 30 个家系进行评价选择，家系 97 和 106 入选，入选家系树高平均值为 7.32 m，比总平均值高 0.48 m，遗传增益为 6.04%；地径的平均值为 19.14 cm，比总平均值高 2.86 cm，遗传增益为 15.49%；胸径的平均值为 15.20 cm，比总平均值高 2.29 cm，遗传增益为 15.84%；3 m 径的平均值为 12.60 cm，比总平均值高 2.35 cm，遗传增益为 20.43%；材积的平均值为 0.063 m^3，比总平均值高 0.018 m^3，遗传增益为 35.68%。

3 结论与讨论

遗传和变异是林木育种的主要内容[16]。本研究中 30 个红松半同胞家系区组间、家系间和区组与家系交互作用中各性状方差分析均达到极显著水平，说明不同家系间具有显著差异，对这些家系进行优良家系的评价选择是有意义的。变异系数可以反应不同家系间各指标的变异情况，变异系数越大，表明变异越大，越有利于优良家系的评价选择[17]，本

(C)1994-2019 China Academic Journal Electronic Publishing House. All rights reserved. http://www.cnki.net

研究中各性状遗传变异系数均占表型变异系数的50%以上，表明变异很大一部分是由遗传控制的。表型变异系数变化范围为 5.28% ~ 44.44%；表型变异系数最大的指标为材积，这与徐悦丽等[18]在对长白落叶松的研究结果相近，说明利用材积指标评价选择遗传改良潜力较大。

表 5　30 个红松半同胞家系多性状综合评价

家系	综合评价值	家系	综合评价值
97	1.83	95	1.64
106	1.75	102	1.63
ck	1.73	99	1.62
110	1.71	98	1.61
90	1.71	108	1.59
81	1.70	107	1.58
79	1.70	111	1.57
91	1.69	94	1.57
104	1.69	77	1.57
96	1.69	83	1.57
101	1.66	78	1.57
100	1.65	105	1.56
84	1.65	86	1.54
88	1.65	109	1.52
87	1.65	89	1.51

遗传力是亲本某一性状遗传给子代的能力，可以体现性状的稳定程度，其值越大则性状越稳定，受环境作用越小[19]。本研究中 30 个半同胞家系各指标遗传力除轮枝数外均超过 0.50，表明各性状在不同家系内均能稳定遗传。其中树高、胸径和材积的遗传力均高于谭建晖等[20]对马尾松的研究，高变异、高遗传力有利于家系的评价选择。

相关性分析可以体现各变量之间的关系[21]，本研究中红松半同胞家系间各性状相关性均达极显著正相关水平，其中材积与胸径的相关系数最高，表明材积与胸径存在极强的相关性；树高、地径、胸径、3 m 径和材积之间的相关系数均大于 0.6，表明生长指标之间存在相互制约的关系；分支角与树高和胸径之间存在极强相关性，这可能是由于分支角越大，接受光线越多，光合作用越强，使树木生长发育旺盛[22]。

一般配合力是评价亲本利用价值的重要指标[23]。通过子代测定数据可以选择出优良的亲本，在作物育种中配合力被广泛应用，已经取得了较高的效果[24]。本研究中对材积进行一般配合力分析，选出 97 和 106 两个家系。多性状综合评价可以对多个性状同时进行评价，选出的家系更稳定[25]。本研究以树高、地径、胸径、3 m 径和材积为标准对 30 个红松家系进行综合评价，选出了 97 和 106 两个优良家系。利用综合评价所选出的家系与利用一般配合力所选出的亲本相同，表明配合力高的亲本可以产生相应性状表现好的子代。遗传增益是评价育种效果的关键参数，可以表明育种群体某指标超出现有群体的多少，体现育种成效[26]。本研究中入选的优良家系树高、地径、胸径、3 m 径和材积的遗传增益范围为 6.04% ~ 35.68%；入选的家系对不同性状改良程度不同，材积的遗传增益最高，为 35.68%，这与梁德洋等[26]对红松的研究结果相似。

随着林木遗传改良研究深入，建立高世代种子园是种子园发展的必然趋势[28]。本研究中家系 97 和 106 较为优良，可以考虑其作为 1.5 代种子园建园材料，也可以利用其家系中的优良单株营建 2 代种子园。本研究只对红松的生长性状进行了测量分析，未来将会对家系的木材性状和种实性状进行测量分析，联合生长性状、木材性状和种实性状对红松家系进行评价选择，以期能够培育生长快，材性好、结实多的红松家系。

参　考　文　献

[1] 马建路，庄丽文，陈动，等. 红松的地理分布[J]. 东北林业大学学报，1992，20(5)：40-48.
[2] 司洪生. 关于我国红松分类与分布的探讨[J]. 林业实用技术，2015(8)：3-5.
[3] 刘德栋. 我国红松良种选育研究进展[J]. 防护林科技，2017(3)：96-99.
[4] 曹焱. 红松合子胚愈伤组织诱导及不定芽发生的研究[D]. 哈尔滨：东北林业大学，2009.
[5] 屈红军，侯庆娟，张冉，等. 红松球果产量的影响因子及预测方法[J]. 种子，2015，34(1)：71-73.
[6] 王卫，史绍林，刘洋. 人工红松生殖生长研究现状与展望[J]. 防护林科技，2015(3)：97-98.
[7] WANG H M，XIA D A，WANG W J，et al. Genetic variations of wood properties and growth characters of Korean pines from different provenances[J]. Journal of Forestry Research，2002，13(4)：277-280.
[8] 王洪梅. 红松种子园优良无性系遗传结构研究[D]. 哈尔滨：东北林业大学，2006.
[9] 孙文生. 红松种子园优质高产经营技术研究[D]. 北京：北京林业大学，2006.
[10] 张振，张含国，周宇，等. 红松多无性系群体的种实性状变异研究[J]. 北京林业大学学报，2015，37(2)：67-78.
[11] 赵曦阳，李颖，赵丽，等. 不同地点白杨杂种无性系生长和适应性表现分析和评价[J]. 北京林业大学学报，2013，35(6)：7-14.
[12] 续九如. 林木数量遗传学[M]. 北京：高等教育出版社，2006.
[13] 解孝满，李景涛，赵合娥，等. 柳树无性系苗期遗传测定与选择[J]. 江苏林业科技，2008，35(3)：6-9.
[14] 陈晓阳，沈熙环. 林木育种学[M]. 北京：高等教育出版社，2005.
[15] 朱之悌. 林木遗传学基础[M]. 北京：中国林业出版社，1989.
[16] MWASE W F，SAVILL P S，HEMERY G. Genetic parameter estimates for growth and form traits in common ash (*Fraxinus excelsior* L.) in a breeding seedling orchard at Little Wittenham in England[J]. New Forests，2008，36(3)：225-238.
[17] BOGDAN S，KATICICTRUPCEVIC I，KAJBA D. Genetic variation in growth traits in a *Quercus robur* L. Open-pollinated progeny test of the Slavonian provenance.[J]. Silvae Genetica，2004，53(5/6)：198-201.
[18] 徐悦丽，张含国，姚宇，等. 长白落叶松群体遗传变异及优良家系选择的研究[J]. 植物研究，2012，32(3)：284-289.
[19] MANIEE M，KABRIZI D，MOHAMMADI R. Genetic variability of some Morpho-Physiological traits in durum wheat (var.)[J]. African Journal of Biotechnology，1998，9(30)：4687-4691.DOI：10.1186/1475-2859-9-60.(下转 20 页)

(C)1994-2019 China Academic Journal Electronic Publishing House. All rights reserved.　http://www.cnki.net

[30] 谷彦冰,冀志蕊,迟福梅,等.桃 *WRKY* 基因家族全基因组鉴定和表达分析[J].遗传,2016,38(3):254-270.
[31] DONG J, CHEN C, CHEN Z. Expression profiles of the *Arabidopsis WRKY* gene superfamily during plant defense response[J]. Plant Molecular Biology,2003,51(1):21-37.
[32] HE H, DONG Q, SHAO Y, et al. Genome-wide survey and characterization of the *WRKY* gene family in *Populus trichocarpa*[J]. Plant Cell Reports,2012,31(7):1199-1217.
[33] ROSS C A, LIU Y, SHEN Q J. The *WRKY* gene family in rice (*Oryza sativa*)[J]. Journal of Integrative Plant Biology,2010,49(6):827-842.
[34] LI L, MU S, CHENG Z, et al. Characterization and expression analysis of the *WRKY* gene family in moso bamboo[J]. Scientific Reports,2017,7(1):1-16.
[35] LIU J J, EKRAMODDOULLAH A K. Identification and characterization of the *WRKY* transcription factor family in *Pinus monticola*[J]. Genome,2009,52(1):77-88.
[36] JIN J, TIAN F, YANG D C, et al. PlantTFDB 4.0: toward a central hub for transcription factors and regulatory interactions in plants[J]. Nucleic Acids Research,2017,45(Database issue):1040-1045.
[37] XIA H, ZHANG L, WU G, et al. Genome-Wide Identification and characterization of microRNAs and target Genes in *Lonicera japonica*[J]. PLoS One,2016,11(10).DOI: 10.1371/journal.pone.0164140.
[38] GIACOMELLI J I, WEIGEL D, CHAN R L, et al. Role of recently evolved miRNA regulation of sunflower Ha *WRKY*6 in response to temperature damage[J]. The New Phytologist,2012,195(4):766-773.
[39] GAO J, PENG H, HE X J, et al. Molecular phylogenetic characterization and analysis of the *WRKY* transcription factor family responsive to *Rhizoctonia solani* in maize[J]. Maydica,2014,59(1):32-41.
[40] KARANJA B K, FAN L, XU L, et al. Genome-wide characterization of the *WRKY* gene family in radish (*Raphanus sativus* L.) reveals its critical functions under different abiotic stresses[J]. Plant Cell Reports,2017,36(11):1757-1773.
[41] ZHANG Q, LI Y, ZHANG Y, et al. Md-miR156ab and Md-miR395 Target WRKY transcription factors to influence apple resistance to leaf spot disease[J]. Frontiers in Plant Science,2017,8.DOI: 10.3389/fpls.2017.00526.
[42] LIN Y C, LI W, CHEN H, et al. A simple improved-throughput xylem protoplast system for studying wood formation[J]. Nature Protocols,2014,9(9):2194-2205.DOI: 10.1038/nprot.2014.147.
[43] JIN J, HE K, TANG X, et al. An arabidopsis transcriptional regulatory map reveals distinct functional and evolutionary features of novel transcription factors[J]. Molecular Biology & Evolution,2015,32(7):1767-1773.
[44] BAILEY T L, WILLIAMS N, MISLEH C, et al. MEME: discovering and analyzing DNA and protein sequence motifs[J]. Nucleic Acids Research,2006,34(Web Server issue):369-373.
[45] WAN L C, FENG W, GUO X, et al. Identification and characterization of small non-coding RNAs from Chinese fir by high throughput sequencing[J]. BMC Plant Biology,2012,12(1):146-161.
[46] BAO W, QU Y, SHAN X, et al. Screening and validation of housekeeping genes of the root and cotyledon of *Cunninghamia ianceolata* under abiotic stresses by using quantitative Real-Time PCR[J]. International Journal of Molecular Sciences,2016,17(8):1198-1215.
[47] LIVAK K J, SCHMITTGEN T D. Analysis of relative gene expression data using real-time quantitative PCR and the 2(-Delta Delta C(T)) Method[J]. Methods,2001,25(4):402-408.
[48] LI H L, GUO D, YANG Z P, et al. Genome-wide identification and characterization of WRKY gene family in *Hevea brasiliensis*[J]. Genomics,2014,104(1):14-23.
[49] CANNON S B, MITRA A, BAUMGARTEN A, et al. The roles of segmental and tandem gene duplication in the evolution of large gene families in *Arabidopsis thaliana*[J]. BMC Plant Biology,2004,4(1):10-31.
[50] ULKER B, SOMSSICH I E. WRKY transcription factors: from DNA binding towards biological function[J]. Current Opinion in Plant Biology,2004,7(5):491-498.
[51] 高国庆,储成才,刘小强,等.植物 *WRKY* 转录因子家族研究进展[J].植物学报,2005,22(1):11-18.

(上接 11 页)

[20] 谭健晖,冯源恒,黄永利,等.26 年生马尾松初级种子园半同胞子代变异及家系选择[J].南京林业大学学报(自然科学版),2017,41(3):189-192.
[21] 王有和,王振明.帽儿山地区二年生红松苗地理变异规律及最佳种源的选择[J].林业科技,1995,20(3):15-16.
[22] 费世民,蔡小虎,何亚平,等.生物柴油植物麻疯树群体构型对资源性环境因素的反应[J].四川林业科技,2009,30(5):1-8.
[23] MAHGOUB G M A, DIVIDICH J L, ROOKE J A, et al. Partitioning of general and specific combining ability effects for estimating maternal and reciprocal effects[J]. Journal of Agricultural Science,2011,62(3):213-222.DOI: 101.1017/S0021859609990268.
[24] MOTEERLE L M, ALESSANDRO L E B, SCAPIM C A, et al. Combining ability of popcorn lines for seed quality and agronomic traits[J]. Euphytica,2012,185(3):337-347.
[25] 王庆成,王政权,李国江,等.苗木规格质量与造林成活率、保存率及幼林生长的关系[J].东北林业大学学报,1991,19(S1):16-24.
[26] 梁德洋,金允哲,赵光浩,等.50 个红松无性系生长与木材性状变异研究[J].北京林业大学学报,2016,38(6):51-59.
[27] 翁玉榛.杉木第二代种子园自由授粉子代遗传变异及优良家系选择[J].南京林业大学学报(自然科学版),2008,32(1):15-18.

(C)1994-2019 China Academic Journal Electronic Publishing House. All rights reserved. http://www.cnki.net
