# Supplementary material for: Provenance and family variations in early growth of Manchurian walnut (Juglans mandshurica Maxim.) and selection of superior families
Source: PLoS One. 2024 Mar 7;19(3):e0298918. doi: 10.1371/journal.pone.0298918 (PMC10919699; doi:10.1371/journal.pone.0298918)
Supplement: S2 File — (ZIP) [file pone.0298918.s005.zip › Variation among provenances and families of Juglans mandshurica and preliminary selection.pdf]

DOI:10.13759/j.cnki.dlxb.2010.11.014

第38卷 第11期
2010年11月

东北林业大学学报
JOURNAL OF NORTHEAST FORESTRY UNIVERSITY

Vol. 38 No. 11
Nov. 2010

# 胡桃楸种源家系变异与选择[1)]

**褚宪丽** （林口县林业局，林口，157600）
**朱航勇** （七台河市园林管理局）
**张含国　张　磊　张　振** （林木遗传育种与生物技术教育部重点实验室（东北林业大学））

**摘　要**　通过研究青山林场15年生胡桃楸3种源、45个家系试验林，分析种源及家系的变异情况，并对其进行初步选择。总体树高的变异系数为35.60%～45.35%，平均值为40.98%，胸径变异系数为25.58%～28.77%，平均值为27.70%，树高变异较胸径变异大，其中宝龙店种源的变异最大，牡丹峰种源的变异最小，牡丹峰种源生长性状与其他种源相比差异显著且保存率最高，为优良种源。从45个家系中初步选出DC1、DC3、DC4、DC5、DC7、DC10、DC13、DC14、LQ7九个优良家系，入选率为20%，树高、胸径遗传增益分别为13.33%和17.02%，入选家系的树高、胸径的平均值分别为4.18 m、3.69 cm，分别超过树高、胸径平均值的24.40%、16.40%。若只对牡丹峰种源内优良家系进行选择，DC1、DC3、DC4、DC10、DC13、DC14树高、胸径较大，与其他家系差异显著，为优良家系，胸径遗传增益为15.58%。

**关键词**　家系；胡桃楸；种源；选择；变异

**分类号**　S722.3+1；S722.3+3；S792.132

**Variation Among Provenances and Families of *Juglans mandshurica* and Preliminary Selection**/Chu Xianli（the Forestry Bureau of Linkou City, Heilongjiang Province, Linkou 157600, P. R. China）; Zhu Hangyong（Landscape Architecture Bureau of Qitaihe, Heilongjiang Province）; Zhang Hanguo, Zhang Lei, Zhang Zhen（Key Laboratory of Forest Tree Genetic Improvement and Biotechnology（Northeast Forestry University）, Ministry of Education）//Journal of Northeast Forestry University. -2010, 38(11). -5～6, 14

A study was conducted to explore the variation among provenances and families of *Juglans mandshurica* using the experimental forests from three provenances and 45 families in Qinshan Forest Farm in Linkou County, Heilongjiang Province. The superior provenances and families were preliminarily selected. The coefficients of variation of tree height among the three provenances ranged from 35.60% to 45.35%, with an average of 40.98%; those of diameter at breast height（DBH）were between 25.58% and 28.77%, with an average of 27.70%. The variation in tree height was larger than that in DBH, and Baolongdian provenance exhibited the maximum variation and Mudanfeng provenance showed the minimum one. The growth traits of Mudanfeng were significantly different from those of the others and its survival rate was also the maximum, so it was a superior provenance and the genetic gains of tree height and DBH were 44.52% and 36.20%, respectively. Nine superior families were preliminarily selected from 45 families, which were DC1, DC3, DC4, DC5, DC7, DC10, DC13, DC14 and LQ7, with a selection rate of 20%, and the genetic gains of tree height and DBH were 13.33% and 17.02%, respectively. The average tree height and DBH of the trees from the nine families were 4.18m and 3.69cm, which were 24.40% and 16.40% higher than the population mean. DC1, DC3, DC4, DC10, DC13 and DC14 were superior families in Mudanfeng provenance, and the genetic gain of DBH was 15.58%.

**Keywords**　Families; *Juglans mandshurica*; Provenances; Selection; Variations

胡桃楸（*Juglans mandshurica* Maxim）为胡桃科胡桃属植物，主要分布于我国东北及华北的海拔400～1 000 m的山坡或向阳沟谷中，是东北地区最珍贵的用材树种，其材质坚硬致密、弹性好、易加工，为优良的军用、细木工和家具用材；果肉及树皮含鞣质；种子富含脂肪，营养丰富，可食用或供工业用，是很有发展前途的木本油料植物[1-3]，树皮及叶可药用，具有消热解毒、抗癌等作用[4-5]。由于过量采伐，胡桃楸天然林接近枯竭。它是第三纪子遗植物，渐危种，已列为国家三级保护植物[6-7]。因此需要大规模营造人工林满足市场对胡桃楸的各类需求。但是由于各种因素，对胡桃楸遗传改良的研究报道较少。对胡桃楸苗期地理种源变异规律、种源选择、早晚相关性进行研究表明，生长性状、适应性状等种源间存在显著差异，初步确定胡桃楸最佳早期选择年龄，天然林为15 a，人工林为14 a[8-13]。文中通过对15年生的胡桃楸试验林进行研究，分析种源和家系的变异规律并初步选择出优良家系及种源，用于林口及周边相似地区造林。

## 1　材料与方法

1993年秋季在五常宝龙店、牡丹江市牡丹峰自然保护区、林口青山林场选优收集种子，每个种源采集15个家系；1994年6月在青山林场直播造林，株距2.0 m、行距3.0 m，巢式完全随机区组设计单株小区，共设置50个区组；试验林沿山坡自上向下设置，区组间无保护行。于2009年10月对试验林中保存率较好的31个区组进行每木检尺，主要调查树高胸径和分枝情况。

对试验进行整理后，数据按照巢式设计进行方差分析、Tukey法检验等，应用Mini Tab15.0中文版统计软件进行分析。

## 2　结果与分析

### 2.1　胡桃楸种源变异分析与优良种源选择

对胡桃楸各种源生长性状调查结果表明（表1）：种源间、种源内存在丰富的变异，种源内树高的变异系数为35.60%～45.35%，平均值为40.98%，胸径变异系数为25.58%～28.77%，平均值为27.70%，树高变异较胸径变异大。

方差分析表明（表2），3个种源树高、胸径差异极显著。其中牡丹峰种源生长较快、青山种源生长次之、宝龙店种源最差（表1），最优与最差种源胸径相差28%，树高相差41%；各种源适应

---

1）黑龙江省科技攻关重点项目（GB08B203）。
第一作者简介：褚宪丽，女，1956年6月生，林口县林业局，助理工程师。
通信作者：张含国，男，林木遗传育种与生物技术教育部重点实验室（东北林业大学），教授。
收稿日期：2010年3月3日。
责任编辑：任　俐。

?1994-2016 China Academic Journal Electronic Publishing House. All rights reserved.　http://www.cnki.net

性相差极大,牡丹峰种源的保存率(74.19%)远强于宝龙店种源保存率(38.92%),超过青山种源的保存率(62.80%)。

表1 胡桃楸种源生长性状统计因子

| 性状 | 种源 | 株数/株 | 平均值/cm | 平均值标准误/cm | 标准差/cm | 变异系数/% | 95%置信区间 | | 保存率/% |
|---|---|---|---|---|---|---|---|---|---|
| | | | | | | | 上限 | 下限 | |
| 胸径 | 宝龙店 | 181 | 2.73 | 0.058 | 0.78 | 28.74 | 2.62 | 2.85 | 38.92 |
| | 牡丹峰 | 345 | 3.50 | 0.048 | 0.90 | 25.58 | 3.41 | 3.59 | 74.19 |
| | 青 山 | 292 | 3.04 | 0.051 | 0.87 | 28.77 | 2.94 | 3.14 | 62.80 |
| 树高 | 宝龙店 | 181 | 278 | 0.094 | 1.26 | 45.35 | 2.60 | 2.97 | 38.92 |
| | 牡丹峰 | 345 | 392 | 0.075 | 1.40 | 35.60 | 3.77 | 4.07 | 74.19 |
| | 青 山 | 292 | 334 | 0.082 | 1.40 | 42.00 | 3.18 | 3.51 | 62.80 |

表2 生长性状联合方差分析

| 性状 | 来源 | 自由度 | 平方和 | 调整平方和 | 均方 | $F$ | $P$ |
|---|---|---|---|---|---|---|---|
| 胸径 | 区组 | 30 | 62.52 | 52.84 | 1.76 | 2.54 | 0 |
| | 种源 | 2 | 68.46 | 68.66 | 34.33 | 49.58 | 0 |
| | 家系(种源) | 42 | 41.33 | 41.32 | 0.99 | 1.42 | 0.04 |
| | 误差 | 743 | 514.47 | 514.46 | 0.70 | | |
| | 合计 | 817 | 686.78 | | | | |
| 树高 | 区组 | 30 | 172.81 | 137.80 | 4.60 | 2.65 | 0 |
| | 种源 | 2 | 126.42 | 125.10 | 62.55 | 36.14 | 0 |
| | 家系(种源) | 42 | 104.24 | 104.24 | 2.46 | 1.43 | 0.04 |
| | 误差 | 743 | 1 285.91 | 1 285.91 | 1.74 | | |
| | 合计 | 817 | 1 689.38 | | | | |

区组间差异极显著,表明胡桃楸生长受环境条件影响较大,对生长性状进行回归分析表明,沿坡每上升100 m树高平均增加0.15 m,胸径平均增加0.045 cm。3个种源中,宝龙店种源生长性状与坡位呈负相关,其他两个种源生长性状与坡位呈正相关。对回归方程进行方差分析(表3),结果表明回归方程有效。对3个种源进行Tukey法多重比较(表4),3个种源之间差异显著,种源的树高及胸径遗传力分别为0.98、0.97(遗传力估算方法为$1-1/F$)。牡丹峰种源生长性状、保存率均优于其他两个种源,且经Tukey法检验与其他两个种源差异极显著。因此牡丹峰种源为3个种源中的最佳种源。

表3 生长性状回归方程方差检验

| 性状 | 来源 | 自由度 | 平方和 | 均方 | $F$ | $P$ |
|---|---|---|---|---|---|---|
| 树高 | 回归 | 1 | 42.646 | 42.646 | 21.13 | 0 |
| | 残差误差 | 816 | 1 646.734 | 2.018 | | |
| | 合计 | 817 | 1 689.380 | | | |
| 胸径 | 回归 | 1 | 3.988 | 3.988 | 4.77 | 0.03 |
| | 残差误差 | 816 | 682.770 | 0.837 | | |
| | 合计 | 817 | 686.758 | | | |

表4 胡桃楸种源生长性状Tukey法多重比较

| 种源 | 胸径/cm | 显著水平 | | 树高/m | 显著水平 | |
|---|---|---|---|---|---|---|
| | | 5% | 1% | | 5% | 1% |
| 牡丹峰 | 3.50±0.90 | a | A | 3.92±1.40 | a | A |
| 青 山 | 3.04±0.87 | b | B | 3.34±1.40 | b | B |
| 宝龙店 | 2.73±0.78 | c | C | 2.78±1.26 | c | C |

注:小写字母表示5%显著水平分组;大写字母表示1%显著水平分组。

## 2.2 胡桃楸家系间变异分析及优良家系选择

由于种源内存在丰富的遗传变异,即证明家系间及家系内也存在较大的变异,牡丹峰家系内树高的变异系数为27.68%~44.28%,平均值为35.39%,胸径的变异系数为18.14%~32.70%,平均值为25.11%;宝龙店种源家系内树高的变异系数为25.53%~60.12%,平均值为45.27%,胸径的变异系数为20.33%~37.29%,平均值为28.78%;青山种源家系内树高的变异系数为31.10%~62.25%,平均值为41.55%,胸径的变异系数为19.16%~39.94%,平均值为28.24%。

对3个种源内家系间生长性状进行方差分析,45个家系间差异显著,家系树高、胸径遗传力为0.67、0.72。树高前10名中有牡丹峰种源8个家系,胸径前10名中有牡丹峰9个家系;而树高、胸径后10名中宝龙店有8个家系、青山种源有2个家系。对45个家系树高、胸径进行Tukey法多重比较,初步筛选出DC1、DC3、DC4、DC5、DC7、DC10、LQ7、DC13、DC14九个家系,入选率为20%,树高、胸径遗传增益分别为13.33%和17.02%。入选家系的树高、胸径的平均值分别为4.18 m、3.69 cm,分别超过树高、胸径平均值(3.36 m、3.17 cm)的24.40%、16.40%。

宝龙店种源家系间树高、胸径差异不显著($P$值分别为0.569、0.784);而青山种源家系间树高差异显著、胸径差异不显著(树高、胸径的$P$值分别为0.015、0.096);牡丹峰生长性状的方差分析结果表明(表5):树高差异不显着,胸径差异显著,区组间差异显著,胸径的遗传力0.44。通过对种源内家系树高、胸径进行Ducan法多重比较的结果进行分析(表6、表7)初步筛选出DC1、DC3、DC4、DC10、DC13、DC14六个优良家系,入选率为40%。入选家系树高、胸径的平均值分别为4.17 m、3.74 cm,分别超过种源树高、胸径平均值(3.92 m、3.50 cm)的6.86%、6.38%。种源内家系选择树高、胸径的遗传增益分别为19.32%和15.58%。

表5 牡丹峰种源生长性状联合方差分析

| 性状 | 来源 | 自由度 | 平方和 | 调整平方和 | 均方 | $F$ | $P$ |
|---|---|---|---|---|---|---|---|
| 胸径 | 家系 | 14 | 18.92 | 18.17 | 1.30 | 1.77 | 0.04 |
| | 区组 | 30 | 36.19 | 36.19 | 1.21 | 1.64 | 0.02 |
| | 误差 | 300 | 220.57 | 220.57 | 0.74 | | |
| | 合计 | 344 | 275.68 | | | | |
| 树高 | 家系 | 14 | 31.53 | 30.34 | 2.17 | 1.21 | 0.26 |
| | 区组 | 30 | 102.87 | 102.87 | 3.43 | 1.92 | 0 |
| | 误差 | 300 | 535.50 | 535.50 | 1.79 | | |
| | 合计 | 344 | 669.90 | | | | |

表6 牡丹峰种源家系树高Ducan法检验

| 家系 | 树高/m | 显著水平 | | 家系 | 树高/m | 显著水平 | |
|---|---|---|---|---|---|---|---|
| | | 5% | 1% | | | 5% | 1% |
| DC1 | 4.51±1.58 | a | A | DC14 | 3.85±1.64 | abc | A |
| DC4 | 4.49±1.53 | ab | A | DC12 | 3.70±1.53 | abc | A |
| DC10 | 4.14±1.37 | abc | A | DC15 | 3.69±1.36 | abc | A |
| DC3 | 4.13±1.19 | abc | A | DC6 | 3.64±1.21 | bc | A |
| DC7 | 4.09±1.34 | abc | A | DC2 | 3.59±1.44 | c | A |
| DC5 | 4.07±1.51 | abc | A | DC8 | 3.57±1.08 | c | A |
| DC9 | 3.97±1.34 | abc | A | DC11 | 3.51±1.56 | c | A |
| DC13 | 3.98±1.10 | abc | A | | | | |

注:小写字母表示5%显著水平分组;大写字母表示1%显著水平分组。

表7 牡丹峰种源家系胸径Ducan法检验

| 家系 | 胸径/cm | 显著水平 | | 家系 | 胸径/cm | 显著水平 | |
|---|---|---|---|---|---|---|---|
| | | 5% | 1% | | | 5% | 1% |
| DC1 | 4.00±0.98 | a | A | DC9 | 3.53±0.87 | bcdefg | A |
| DC3 | 3.82±0.69 | ab | A | DC2 | 3.39±0.80 | cdefg | A |
| DC10 | 3.71±0.81 | abc | A | DC8 | 3.33±0.78 | defg | A |
| DC4 | 3.68±0.95 | abcd | A | DC6 | 3.28±0.77 | efg | A |
| DC13 | 3.63±0.74 | abcde | A | DC12 | 3.20±1.05 | fg | A |
| DC14 | 3.60±1.09 | abcde | A | DC15 | 3.20±0.84 | fg | A |
| DC5 | 3.57±0.99 | bcdef | A | DC11 | 3.16±0.94 | g | A |
| DC7 | 3.55±0.86 | bcdefg | A | | | | |

注:小写字母表示5%显著水平分组;大写字母表示1%显著水平分组。(下转14页)

?1994-2016 China Academic Journal Electronic Publishing House. All rights reserved. http://www.cnki.net

[20] 张金屯.植被数量生态学方法[M].北京:中国科学技术出版社,1995.
[21] 马克平,刘玉明.生物群落多样性的测度方法I:α多样性的测度方法(下)[J].生物多样性,1994,2(4):231-239.
[22] Magurran A E. Ecological diversity and its measurements[M]. New Jersey: Princeton University Press,1988.
[23] Whittaker R H. Evolution and measurement of species diversity[J]. Taxon,1972,21(2/3):213-251.
[24] Hurlbert S H. The nonconcept of species diversity: A critique and alternative parameters[J]. Ecology,1971,52(4):577-586.
[25] Lepš J, Šmilauer P. Multivariate analysis of ecological data using CANOCO[M]. Cambridge: Cambridge University Press,2003.
[26] 赵淑清,方精云,朴世龙,等.大兴安岭呼中地区白卡鲁山植物群落结构及其多样性研究[J].生物多样性,2004,12(1):182-189.
[27] Gao Junfeng, Zhang Yunxiang. Distributional patterns of species diversity of main plant communities along altitudinal gradient in secondary forest region, Guandi Mountain, China[J]. Jouranl of Forestry Research,2006,17(2):111-115.
[28] 沈泽昊,刘增力,方精云.贡嘎山海螺沟冷杉群落物种多样性与群落结构随海拔的变化[J].生物多样性,2004,12(2):237-244.
[29] Ojeda F, Maraiñon and Airoyo J. Plant diversity patterns in the Aljibe Mountains(*S. Spain*): a comprehensive account[J]. Biodiversity and Conservation,2000,9(9):1323-1343.
[30] 陈廷贵,张金屯.山西关帝山神尾沟植物群落物种多样性与环境关系的研究I.丰富度、均匀度和物种多样性指数[J].应用与环境生物学报.2000,6(5):406-411.
[31] 岳明,张林静,党高弟,等.佛坪自然保护区植物群落物种多样性与海拔的梯度关系[J].地理科学,2002,22(3):349-354.
[32] 赵淑清,方精云,宗占江,等.长白山北坡植物群落组成、结构及物种多样性的垂直分布[J].生物多样性,2004,12(1):164-173.
[33] Ohlemueller R, Wilson J B. Vascular plant species richness along latitudinal and altitudinal gradients: a contribution from New Zealand temperate rainforests[J]. Ecology Letters,2002,4:262-266.
[34] 方精云.探索中国山地植物多样性的分布规律[J].生物多样性,2004,12(1)1-4.
[35] 史作民,刘世荣,程瑞梅,等.河南宝天曼植物群落数量分类与排序[J].林业科学,2000,36(6):20-27.
[36] 欧光龙,彭明春,和兆荣,等.高黎贡山北段植物群落TWINSPAN数量分类研究[J].云南植物研究,2008,30(6):679-687.
[37] 胡刚,梁士楚,张忠华,等.桂林岩溶石山青冈栎群落的数量分析[J].生态学杂志,2007,26(8):1177-1181.
[38] 刘鸿雁,曹艳丽,田军,等.山西五台山高山林线的植被景观[J].植物生态学报,2003,27(2):263-269.
[39] Lenoir J, Gégout J C, Marquet P A, et al. A significant upward shift in plant species optimum elevation during the 20th century[J]. Science,2008,320:1768-1771.


(上接6页)

## 3 结论与讨论

不同种源内存在丰富遗传变异,其中宝龙店种源内变异幅度最大,本地种源次之,最差的是牡丹峰种源。生长受环境条件影响较大,每沿坡上升100 m树高增加0.15 m、胸径增加0.045 cm。坡上有利于胡桃楸的生长。种源间适应性差异显著,牡丹峰种源保存率较最差的宝龙店种源高85.50%,且优于青山种源。各种源内家系内存在丰富的遗传变异,其中宝龙店种源的遗传变异最丰富(树高、胸径的变异系数分别为45.35%、28.74%)。

通过对各种源生长性状和适应性的分析,选择出牡丹峰种源为最优种源,适宜在青山及其附近地区造林,牡丹峰种源树高较宝龙店种源高41.00%,胸径大28.20%。对全部家系进行选择,初步选择出DC1、DC3、DC4、DC5、DC7、DC10、LQ7、DC13、DC14九个优良家系,入选率为20%。树高、胸径遗传增益13.33%和17.02%。入选家系的树高、胸径的平均值为4.18m、3.69cm,超过种源胸径、树高平均值的24.40%、16.40%。若单独对牡丹峰种源内各家系的生长性状分析,选择出DC1、DC3、DC4、DC10、DC13、DC14六个家系为优良家系,适宜在青山及周边地区造林使用。入选家系树高、胸径的平均值分别为4.17 m、3.74 cm,分别超过种源树高、胸径的平均值6.86%、6.38%。种源内家系选择树高、胸径的遗传增益为19.32%和15.58%。比较两种选择结果,在牡丹峰种源内选择优良家系作为林口及其周边相似地区推广应用为好。

对于本研究涉及的胡桃楸总体而言,生长性状与坡位呈正相关,这与梁淑娟、王庆成的研究是一致的[14],但是其中宝龙店种源生长却与坡位呈负相关,这可能是宝龙店种源距试验地相对较远、气候差异大,或者是种源本身特异性造成的,有待于进一步研究。

### 参 考 文 献


[1] 郑万钧.中国树木志[M].北京:中国林业出版社,1983.
[2] 周以良.黑龙江树木志[M].哈尔滨:黑龙江科学技术出版社,1986.
[3] 中国科学院中国植物志编委会.中国植物志:第20卷,第2分册[M].北京:科学出版社,1984.
[4] Kim S H, Lee K S, Son J K, et al. Cytotoxic compounds from the roots of Julans mandshurica[J]. J Natl Prod,1998,61(5):643-645.
[5] Georgoulias V, Crown J P. Increasing options in cancer therapy: current status and future prospects[J]. Anticancer Drugs,1999,10(1):1-3.
[6] 宋朝枢.中国珍稀濒危保护植物[M].北京:中国林业出版社,1989.
[7] 秦瑞明.黑龙江省稀有濒危植物[M].哈尔滨:东北林业大学出版社,1993.
[8] 杨书文,刘桂丰,赵克尊.胡桃楸早期选择的初步研究[J].东北林业大学学报,1990,19(育种专刊):77-82.
[9] 杨书文,刘桂丰,王会仁,等.胡桃楸地理变异规律的再研究[J].东北林业大学学报,1991,19(育种专刊):183-188.
[10] 刘桂丰,杨书文,李俊涛,等.胡桃楸种源的初步区划及最佳种源选择[J].东北林业大学学报,1991,19(育种专刊):189-196.
[11] 孙丽敏,侯旭光.核桃楸的生长变异及其早期测定的研究[J].林业科技通讯,1997(11):12-15.
[12] 孙丽敏,侯旭光,刘殿辉.核桃楸早期测定的研究[J].防护林科技,2004(4):18-19,39.
[13] Xia De'an, Zhu Hong, Wang Huiren, et al. Provenance trials of *Juglans mandshurica*[J]. Journal of Forestry Research,1997,8(3):156-159.
[14] 梁淑娟,潘攀,孙志虎,等.坡位对水曲柳及胡桃楸生长的影响[J].东北林业大学学报,2005,33(3):18-19.


?1994-2016 China Academic Journal Electronic Publishing House. All rights reserved. http://www.cnki.net
