# Supplementary material for: Provenance and family variations in early growth of Manchurian walnut (Juglans mandshurica Maxim.) and selection of superior families
Source: PLoS One. 2024 Mar 7;19(3):e0298918. doi: 10.1371/journal.pone.0298918 (PMC10919699; doi:10.1371/journal.pone.0298918)
Supplement: S2 File — (ZIP) [file pone.0298918.s005.zip › Variation analysis and early selection of Juglans mandshurica provenance and family.pdf]

核桃楸种源及家系变异分析与早期选择研究

代伟昭
(五常宝龙店种子林场，黑龙江 哈尔滨 150211)

摘要: 以 7a 生核桃楸子代测定林作为试验对象，对其树高、胸径等生长性状进行每木测定，探索其遗传变异规律，筛选出性状优良、对当地适应性好的家系，为后期造林提供理论基础。结果表明: 各种源、家系的树高、胸径存在较丰富变异，树高变异系数小于胸径; 以 20% 的入选率，初步选出清原、五常为优良种源; 清原 9、万人欢 6、万人欢 15、清原 2 为优良家系。

关键词: 核桃楸; 种源; 生长性状; 遗传变异; 家系选择

中图分类号: S792. 132; S722 **文献标识码:** A **文章编号:** 1673 - 4505 (2023) 02 - 0058 - 05

Variation Analysis and Early Selection of Juglans Mandshurica Provenance and Family

DAI Weizhao
(Wuchang Baolongdian Seed Forest Farm, Harbin 150211)

Abstract: Taking the 7 - year - old Juglans mandshurica progeny test forest as the test object, the growth characteristics such as tree height and DBH were measured per tree, and its genetic variation rules were explored, and the families with good characters and good adaptability to local areas were screened, which provided a theoretical basis for later afforestation. The results showed that there were abundant variations in tree height and DBH of various sources and families, and the variation coefficient of tree height was smaller than DBH; With 20% selection rate, Qingyuan and Wuchang were selected as excellent provenances; Qingyuan 9, Wanrenhuan 6, Wanrenhuan 15, Qingyuan 2 are excellent families.

Key words: Juglans Mandshurica; Provenance; Growth Traits; Genetic Variation; Family Selection

核桃楸 (*Juglans mandshurica Maxim.*) 属胡桃科 (Juglandaceae) 胡桃属 (Juglans) 落叶阔叶乔木，与水曲柳、黄波萝并称 “东北三大硬阔”，被列为国家Ⅱ级珍稀树种和珍稀濒危树种的三级保护植物[1]。核桃楸树干通直圆满，材质坚硬致密，木材弹性好、易加工，被广泛用于军工、船舶、家具、居室装修及乐器制造等方面，是优良的用材树种[2]。林木幼龄期选择是根据林木优良性状在早、晚龄间存在的正相关关系，针对其幼龄期的性状表现，进行优良个体选择[3]。近年来，国内外学者已对多树种早期选择进行研究，并取得诸多进展。在核桃楸方面，刘桂丰等[4]和颜廷武等[5]的研究表明，幼龄期的核桃楸已具有代表性，核桃楸的早期选择是十分可行的。由于核桃楸用途广泛，导致人为的过度采伐，其自然资源遭到严重破坏，导致野生核桃楸生存现状堪忧，核桃楸的良种选育工作更显意义重大。

收稿日期: 2022 - 11 - 28.

作者简介: 代伟昭(1988 -),男,哈尔滨五常人,助理工程师. 从事林业管理和技术指导工作. Email: 623510892@ qq. com

(C)1994-2023 China Academic Journal Electronic Publishing House. All rights reserved. http://www.cnki.net

1　试验地概况

1.1　试验地简介

试验地位于五常市宝龙店种子林场，五常市宝龙店种子林场 1962 年建场，前身为宝龙店林场，1965 年被黑龙江省林业厅划定为三大硬阔天然母树林林场，1977 年建立初级无性系种子园，同时更名为五常宝龙店种子林场，开始进行林木良种基地建设。2001 年对良种基地进行改扩建，确定为省级林木良种基地; 2012 年被国家林业局命名为国家重点林木良种基地; 2015 年对良种基地基础设施进行维修和改建，经过多年的建设和发展，基地以水曲柳、核桃楸、黄菠萝三个树种进行良种培育和高世代种子园区的方向深入研究来提高良种生产和可持发展。

1.2　地理位置

试验地位于黑龙江省东南部，距五常市 45km，地理坐标为东经 127°38′ ~ 129°55′，北纬 44°50′ ~ 44°58′。北与省森工集团长岗实验林场接壤，东与尚志国有林场管理局老街基林场相连，西与小山子镇相连，南与胜利林场毗邻。气候水文方面，属于寒温带大陆性季风气候，冬季多为蒙古干旱西北风，气候干燥寒冷; 夏季受海洋性东南季风影响，气候温暖多雨。年降水量 620.9mm。水资源丰富，属于松花江水系。西部主要河流是小苇沙河，由东南向西北流入松花江的第二支流牤牛河，东部主要河流为小泥河，由东向西北流入松花江第三支流大泥河。

2　试验材料与方法

2.1　试验材料

1） 本试验种子于 2014 年采自 8 个种源，种源地分别为亚布力林业局、七台河林业局、宾县万人欢、吉林三岔子林业局、宾县大泉子、清原大姑家、穆棱林业局、山河屯林业局。

2） 22 个核桃楸家系包括宾县大泉子 29、宾县大泉子 30、牡丹江穆棱 7、牡丹江穆棱 8、牡丹江穆棱 9、七台河 1、七台河 2、七台河 3、七台河 6、七台河 7、七台河 8、清原 2、清原 9、三岔子 3、万人欢 5、万人欢 6、万人欢 8、万人欢 15、五常山河 15、五常山河 16、亚布力 4 和对照家系。

3） 2014 年秋季播种，2016 年春季定植，试验采用完全随机区组设计，4 次重复。其中: 1、2、3 次重复每个家系栽植 9 株，4 次重复每个家系 10 株，株行距 2m × 2m。

2.2　统计分析方法

1） 所有数据利用 SPSS 软件进行分析。其中树高和胸径方差分析线性模型为:

$$Y_{ij} = \mu + B_i + P/F + Pi_{ji}/Fi_{ji} + M_{eiij}$$

式中: μ: 为总体平均值;

B_j: 为区组效应;

P/F: 为种源/家系效应;

Pi_{ji}/Fi_{ji}: 为区组与种源/家系的交互作用;

M_{eiij}: 为机误。

2） 家系遗传采用公式:

$$h^2 = 1 - 1/F$$

式中: F: 为方差分析的 F 值[5]。

3） 表型变异系数采用公式:

$$PCV = S/\bar{X} \times 100\%$$

式中: S: 为表型标准差;

$\bar{X}$: 为某一性状群体平均值[6]。

4）采用布雷津多性状综合评定法对种源、家系进行综合评定，具体公式为:

$$Q_i = \sqrt{\sum_{j=1}^{n} ai}, \quad ai = X_{ij}/X_{jmax}$$

式中: Q_i: 为综合评价值;

X_{ij}: 为某一性状的平均值;

X_{jmax}: 为某一性状的最优值;

n: 为评价指标的个数[7]。

5） 遗传增益估算利用公式:

$$\triangle G = h^2 W/\bar{X}$$

式中: W: 为选择差;

h^2: 为性状的遗传力;

$\bar{X}$: 为某一性状的平均值[8]。

3　结果与分析

3.1　种源、家系显著性分析

1） 核桃楸种源、家系除胸径在种源* 区组水

(C)1994-2023 China Academic Journal Electronic Publishing House. All rights reserved.　http://www.cnki.net

平差异分析:

显著水平 (0.01 < P < 0.05), 其余各性状在各变异来源间方差分析均达极显著差异水平 (P < 0.01), 说明核桃楸各生长性状在家系间具有较大差异, 具有较大改良潜力 (表 1, 表 2)。

表 1　8 个核桃楸种源树高、胸径方差分析

Tab. 1　Variance Analysis of Tree Height and DBH of 8 *Juglans Mandshurica* Provenances

性　状	变异来源	Ⅲ类平方和	自由度	均方	F	显著性
树高	种源	39.025	8	4.878	7.813	0.000
	区组	26.237	3	8.746	14.007	0.000
	种源 * 区组	29.745	23	1.293	2.071	0.002
胸径	种源	51.165	8	6.396	5.576	0.000
	区组	41.061	3	13.687	11.933	0.000
	种源 * 区组	46.581	23	2.025	1.766	0.015

表 2　22 个核桃楸家系树高、胸径方差分析

Tab. 2　Variance Analysis of Tree Height and DBH of 22 *Juglans Mandshurica* Families

性　状	变异来源	平方和	自由度	均方	F	Sig.
树高	家系	45.049	21	2.145	5.425	0.000
	区组	14.519	3	4.84	12.24	0.000
	家系 * 区组	45.204	59	0.766	1.938	0.000
胸径	家系	62.048	21	2.955	3.459	0.000
	区组	28.004	3	9.335	10.927	0.000
	家系 * 区组	97.505	59	1.653	1.935	0.000

2) 22 个核桃楸家系树高、胸径指标遗传变异情况分析:

各家系树高平均值 3.07m, 变幅达到 1.40 ~ 4.70m; 胸径平均值 3.33cm, 最小值 1.10cm, 最大值 5.60cm。树高变异系数为 23.45%, 胸径变异系数为 30.96%, 胸径变异系数大于树高变异系数。两个性状均为高遗传力, 树高、胸径遗传力分别为 0.82 与 0.71 (表 3)。

表 3　家系树高、胸径遗传变异参数

Tab. 3　Genetic Variation Parameters of Family Tree Height and DBH

性状	平均值/m	变幅/m	标准差/cm	变异系数/%	遗传力
树高	3.07	1.40 ~ 4.70	0.72	23.45	0.82
胸径	3.33	1.10 ~ 5.60	1.03	30.96	0.71

3.2　种源、家系树高与胸径变异分析

8 个种源与 22 个核桃楸家系树高、胸径均值情况分析:

1) 最高种源为清原, 树高达到 3.54m, 超过最低种源牡丹江 (2.70m) 31.11%, 比所有参试种源平均值大 16.89%; 三岔子树高变异最小, 为 19.22%。变异最大的种源是七台河, 变异系数达到 30.16% (表 4)。

2) 胸径最大种源为清原 (4.04cm), 是最小值宾县大泉子 (2.97cm) 的 1.36 倍, 超过平均值 (3.35cm) 20.60%; 胸径变异系数最大的种源为对照种源, 达到 37.86%, 三岔子种源 (25.70%) 变异最小 (表 4)。

(C)1994-2023 China Academic Journal Electronic Publishing House. All rights reserved.　http://www.cnki.net

表 4　各家系树高、胸径变异

Tab. 4　Variation of Tree Height and DBH of Each Family

种　源	树高/m			胸径/cm		
	平均值	标准偏差	变异系数/%	平均值	标准偏差	变异系数/%
对照	2.91	0.78	26.75	3.20	1.21	37.86
宾县大泉子	3.06	0.82	26.85	3.27	0.98	29.97
牡丹江	2.70	0.71	26.09	2.97	0.96	32.22
七台河	2.86	0.86	30.16	3.20	1.19	37.34
清原	3.54	0.94	26.53	4.04	1.24	30.65
三岔子	3.36	0.64	19.22	3.34	0.86	25.70
万人欢	3.06	0.83	27.17	3.41	1.11	32.43
五常	3.31	0.93	28.18	3.69	1.28	34.73
亚布力	2.94	0.77	26.10	3.37	0.96	28.33

3）最高家系为清原 9，树高达到 3.68m，超过最低家系牡丹江穆棱 9（2.72m）35.29%，大于试验家系平均值 19.87%；家系万人欢 6 树高变异最小，为 15.87%；变异最大的家系为七台河 7，变异系数达到 27.82%（表 5）。

4）胸径最大家系清原 9（4.18cm），是最小值万人欢 5（2.61cm）的 1.60 倍，平均值为 3.33m，超过平均值 25.5 个百分点；胸径变异系数最大的家系为七台河 6，达到 36.41%，家系清原 9 变异最小，为 19.99%（表 5）。

表 5　各家系树高、胸径变异

Tab. 5　Variation of Tree Height and DBH of Each Family

家　系	树高/m			胸径/cm		
	平均值	标准偏差	变异系数/%	平均值	标准偏差	变异系数/%
宾县大泉子 29	3.30	0.67	20.19	3.35	0.88	26.27
宾县大泉子 30	2.99	0.64	21.51	3.18	0.95	29.82
对照	2.95	0.76	25.66	3.27	1.16	35.60
牡丹江穆棱 7	2.88	0.61	21.23	3.09	0.92	29.93
牡丹江穆棱 8	2.80	0.63	22.47	2.99	0.75	25.11
牡丹江穆棱 9	2.72	0.63	23.23	3.06	1.00	32.68
七台河 1	3.23	0.65	20.18	3.53	1.05	29.73
七台河 2	3.16	0.51	16.08	3.37	0.91	27.16
七台河 3	2.84	0.73	25.73	3.12	1.07	34.50
七台河 6	2.43	0.59	24.26	2.70	0.98	36.41
七台河 7	2.88	0.80	27.82	3.23	1.17	36.34
七台河 8	3.14	0.72	23.06	3.48	1.10	31.60
清原 2	3.37	0.79	23.41	3.67	1.18	32.06
清原 9	3.68	0.64	17.29	4.18	0.83	19.99
三岔子 3	3.31	0.60	18.06	3.30	0.82	24.98
万人欢 15	3.34	0.69	20.64	3.72	0.99	26.74
万人欢 5	2.74	0.74	27.10	2.61	0.83	31.69
万人欢 6	3.28	0.52	15.87	3.86	0.89	23.13
万人欢 8	2.95	0.71	24.12	3.21	0.95	29.47
五常山河 15	2.91	0.64	22.01	3.18	0.99	30.99
五常山河 16	3.36	0.81	24.10	3.61	1.15	31.78
亚布力 4	3.02	0.67	22.12	3.37	0.96	28.33

(C)1994-2023 China Academic Journal Electronic Publishing House. All rights reserved.　http://www.cnki.net

3.3 核桃楸优良种源、家系初步选择

利用树高、胸径2个性状对试验林所含8个种源、22个核桃楸家系进行综合评价，获得的各家系 Q_i 值分析:

1）以20%的入选率对8个种源、22个家系进行筛选评价，种源清原、五常入选，家系清原9、万人欢6、万人欢15、清原2入选。入选的2个种源树高平均值为3.43m，比总平均值高0.40m；胸径平均值为3.87cm，比总平均值高0.52cm。

2）入选的4个家系树高平均值为3.42m，比总平均值高0.35m，4个家系遗传增益分别为16.28%（清原9）、5.84%（万人欢6）、7.22%（万人欢15）、7.99%（清原2）；胸径平均值为4.86cm，比总平均值高0.53cm，遗传增益分别为16.28%（清原9）、5.84%（万人欢6）、8.32%（万人欢15）、7.23%（清原2）（表6）。

表6 8个种源、22个核桃楸家系综合评价

Tab. 6 Comprehensive Evaluation of 8 Provenances and 22 *Juglans mandshurica* Maxim. Families

种源	Qi值	家系	Qi值	家系	Qi值
清原	1.214607	清原9	1.235901	对照	1.101059
五常	1.167376	万人欢6	1.178306	宾县大全子30	1.097163
三岔子	1.144936	万人欢15	1.172108	万人欢8	1.095844
万人欢	1.122223	清原2	1.17078	七台河7	1.090525
宾县大泉子	1.111516	五常山河16	1.166453	五常山河15	1.089511
亚布力	1.108608	七台河1	1.148472	牡丹江穆棱7	1.07865
对照	1.091615	宾县大全子29	1.140426	七台河3	1.077475
七台河	1.086321	三岔子3	1.137353	牡丹江穆棱8	1.062893
牡丹江	1.051384	七台河8	1.135493	牡丹江穆棱9	1.061239
		七台河2	1.128944	万人欢5	1.024292
		亚布力4	1.115866	七台河6	0.999409

4 结论

1）8个种源、22个核桃楸家系间树高、胸径生长性状差异在大部分变异来源之间均达到极显著水平，具有较大的改良潜力。

2）各家系树高变异系数在15.87%～27.82%范围内，胸径变异系数在19.99%～36.41%范围内。

3）利用树高、胸径2个生长性状对参试8个种源、22个核桃楸家系进行早期评价，初步筛选出清原、五常为优良种源，清原9、万人欢6、万人欢15、清原2为优良家系。

参考文献:

[1] 王东娜，牟长城，冯富娟．胡桃楸ISSR－PCR反应体系的建立及优化［J］．实验室研究与探索，2010，29（11）：18－22，37.

[2] 芦贤博，徐连峰，庞忠义，等．胡桃楸种源家系幼龄期生长变异及选择研究［J］．林业科学研究，2022，35（1）：20－30.

[3] 李佳娜，高瑞馨．我国胡桃楸的遗传育种研究进展［J］．安徽农业科学，2020，48（17）：4－7..

[4] 刘桂丰，杨书文，李俊涛，等．胡桃楸种源的初步区划及最佳种源选择［J］．东北林业大学学报，1991（S2）：189－196.

[5] 张晓林，刘超，刘剑，等．核桃楸研究现状及育种策略［J］．吉林林业科技，2019，48（1）：14－17.

[6] 续九如．林木数量遗传学［M］．北京，中国林业出版社，2006.

[7] 解孝满，李景涛，赵合娥．柳树无性系苗期遗传测定与选择［J］．江苏林业科技，2008，35（3）：6－14.

[8] 朱之悌．林木遗传学基础［M］．北京，中国林业出版社，1989.

责任编辑：杨吉江 许易真
校　　对：王 卉 周 玉

(C)1994-2023 China Academic Journal Electronic Publishing House. All rights reserved. http://www.cnki.net
