# Supplementary material for: Provenance and family variations in early growth of Manchurian walnut (Juglans mandshurica Maxim.) and selection of superior families
Source: PLoS One. 2024 Mar 7;19(3):e0298918. doi: 10.1371/journal.pone.0298918 (PMC10919699; doi:10.1371/journal.pone.0298918)
Supplement: S2 File — (ZIP) [file pone.0298918.s005.zip › Variation analysis and selection of Pinus koraiensis half-sib families.pdf]

植物研究 2019,39(4):557~567
Bulletin of Botanical Research

红松半同胞家系变异分析及选择研究

张秦徽[1] 王洪武[2] 姜国云[2] 沈 光[2] 王连奎[2] 李焱龙[2] 王 雷[3]
王立祥[3] 李月季[3] 李 蕊[3] 赵曦阳[1*]

(1. 林木遗传育种国家重点实验室东北林业大学,哈尔滨 150040; 2. 吉林省三岔子林业局林木良种基地管理中心,白山 134702; 3. 白城市国有林场总场,白城 137000)

摘 要 为评价和筛选优质红松种质资源,以吉林省三岔子林业局国家红松良种基地的53个28年生红松子代半同胞家系为材料,对其生长性状(树高、地径、胸径、三米径、枝下高和第六轮枝高)及形质性状(分枝角、通直度和轮枝数)进行测定。方差分析结果表明,除部分形质性状外,大部分生长性状在各变异来源间均达极显著差异($P<0.01$);各性状表型变异系数变化范围为6.97%~37.39%,遗传变异系数变化范围为1.76%~26.75%;各性状家系遗传力变化范围为0.136~0.746;单株遗传力变化范围为0.031~0.827,个别性状遗传力较低。相关性分析结果表明,除分枝角外,其余各性状间大部分呈极显著正相关($r>0.073$)。一般配合力分析结果表明,不同性状一般配合力高的家系差异较大,难以进行联合筛选,需进一步进行分析。主成分分析结果表明,三个主成分的累计贡献率达72.31%,表明三个主成分包含了供试家系生长及形质性状的大部分信息。树高、地径、胸径、三米径和材积对主成分Ⅰ的贡献较大,且描述红松的生长性状,因此可作为选择优良家系的评价指标。利用多性状综合评价法对家系及单株进行选择,以家系材积现实增益超过35%为标准,可筛选出5个优良家系,入选率为10%,入选的优良家系树高、地径、胸径、三米径和材积平均值分别为7.90 m、25.02 cm、19.21 cm、16.23 cm和0.1074 $kg\cdot m^{-3}$,现实增益分别为3.94%、14.71%、17.26%、19.34%和39.48%。对优良家系内的单株进行选择,按单株材积遗传增益超过100%为标准,可筛选出6株优良单株,入选率为4%,入选单株树高、地径、胸径、三米径和材积平均值比总平均值分别高1.60 m、8.54 cm、7.04 cm、6.69 cm和0.097 $kg\cdot m^{-3}$,遗传增益分别为5.99%、29.92%、35.53%、37.06%和102.04%。所选优良家系及单株可为良种审定提供基础,也可以为种子园的营建、改建提供材料。

关键词 红松;半同胞家系;生长性状;遗传增益;家系选择;单株选择

中图分类号: S791.247 **文献标志码:** A **doi:** 10.7525/j.issn.1673-5102.2019.04.010

Variation Analysis and Selection of *Pinus koraiensis* Half-sib Families

ZHANG Qin-Hui[1] WANG Hong-Wu[2] JIANG Guo-Yun[2] SHEN Guang[2] WANG Lian-Kui[2] LI Yan-Long[2]
WANG Lei[3] WANG Li-Xiang[3] LI YUE-Ji[3] LI Rui[3] ZHAO Xi-Yang[1*]

(1. State Key Laboratory of Tree Genetics and Breeding, Northeast Forestry University, Harbin 150040; 2. Sanchazi Forestry Bureau Forest Improved Seed Base Management Center, Baishan 134702; 3. Baicheng State-owned Forest Farm, Baicheng 137000)

Abstract In order to evaluate and select superior germplasm of *Pinus koraiensis*, with the 28-year-old 53 half-sib *P. koraiensis* families in Sanchazi forestry bureau, Jilin Province as materials, we measured the growth traits (tree height, basal diameter, diameter at breast height, diameter at 3 m, tree height under the first branch and tree height under the sixth branch) and shape traits (branch angle, stem straightness degree and branch number per node). Variance analysis showed that except for some of the shape traits, most of the growth traits showed

基金项目:国家重点研发计划项目(2017YFD0600601)
第一作者简介:张秦徽(1995—),男,硕士研究生,主要从事林木遗传改良研究。
* 通信作者:E-mail:zhaoxyphd@163.com
收稿日期:2019-01-17
Foundation item: National Key Research and Development Program of China(2017YFD0600601)
First author introduction: ZHANG Qin-Hui(1995—), male, master, mainly engaged in tree genetic and improvement.
* Corresponding author: E-mail: zhaoxyphd@163.com
Received date: 2019-01-17

(C)1994-2019 China Academic Journal Electronic Publishing House. All rights reserved. http://www.cnki.net

strongly significant differences among the sources of variation($P<0.01$). The phenotypic coefficient of variation of all the traits ranged in 6.97% −37.39%. The genotypic coefficient of variation of all the traits ranged in 1.76% −26.75%; The family heredity of all the traits ranged from 0.031 −0.827; The single heredity of all the traits ranged in 0.019 −0.864, lower heritability of a few traits. Correlation analysis results showed that there were significantly positive correlations($r>0.073$) of the most traits except for branch angle. The results of general combining ability analysis showed that the families with high general combining ability of different traits had great differences, which was difficult to carry out combined screening and further analysis was needed. The results of principal component analysis showed that the cumulative contribution of the three principal components was 72.31%, which indicated that the three principal components contained most information of the growth and shape traits of the tested families. Tree height, basal diameter, diameter at breast height, diameter at 3 m height and volume contributed greatly to principal component Ⅰ, and described the growth characters of *P. koraiensis*, so it can be used as an evaluation index for selecting excellent families. Selection of family and single plant by multiple-trait comprehensive evaluation, based on the real gain of family volume over 35%, 5 excellent families were selected, and the selection rate was 10%, average tree height, basal diameter, diameter at breast height, diameter at 3 m height and volume were 7.90 m, 25.02 cm, 19.21 cm, 16.23 cm and 0.107 4 kg·m^{-3}, and the real gains were 3.94%, 14.71%, 17.26%, 19.34% and 39.48%, respectively. Selecting single plants within the excellent families, according to the standard of genetic gain over 100% of single plant volume, 6 excellent individual plants were selected, and the selection rate was 4%, the tree height, basal diameter, diameter at breast height, diameter at 3 m height and volume mean values of the single plants were 1.60 m, 8.54 cm, 7.04 cm, 6.69 cm and 0.097 kg·m^{-3} higher than the total average, and the genetic gains were 5.99%, 29.92%, 35.53%, 37.06% and 102.04%, respectively. The selected excellent families and individuals can provide the basis for validation of improved varieties and provide materials for the construction or reconstruction of the orchard.

Key words *Pinus koraiensis*; half-sib families; growth traits; genetic gains; family selection; individual selection

红松(*Pinus koraiensis*)系五针松，为松科(Pinaceae)松属(*Pinus*)常绿乔木，国家二级重点保护野生植物[1]，中国优质用材树种，主要分布于长白山及其北部的张广才岭、老爷岭、完达山和小兴安岭[2]。红松树形高大，干形通直，材质优良，易加工，广泛应用于家具制造、建筑等[3]，是东北地区重要的用材树种；松籽味美，富含人体所需的多种营养物质及维生素，具有较高的食用和药用价值[4]，因此也是我国重要的经济树种[5]。自“六五”开始，红松就被列为国家科技攻关计划课题支持树种，科研工作者们在优良种源选择[6]、林分选择[7]、无性繁殖[8]、种子园营建[9]、光合生理[10]、分子标记[11]等方面进行了广泛研究，并取得一定进展。

林木种子园是为生产优良种子、按人为设计要求所营建的特种人工林，最早可追溯到1880年[12]。我国红松种子园营建工作始于20世纪80年代，按种源区划选择种源或优树，以穗条嫁接的方式在红松分布区内营建了20多个红松无性系种子园，为后期红松的遗传改良提供了物质基础[13]。由于红松生长相对较慢，这些种子园目前大都处在初级种子园阶段，存在遗传增益低、种子产量不稳定等问题，不能满足东北林区生产用种的实际需要[14]，因而需进行子代测定以及优良家系和单株选择来建立改良代种子园和二代种子园，以实现对红松的遗传改良。

研究生长性状的遗传变异是筛选优良群体及个体的重要内容。林木树种的生长性状研究较多，国内外均有报道。如Doran等人[15]用17个杨树无性系进行对比试验，对4年、6年和8年生的树高、胸径和材积进行了分析，估算遗传参数，进而筛选出应用前景较好的杨树无性系；张正刚等人[16]对7年生日本落叶松自由授粉家系的树高、胸径和冠幅等生长性状进行了测定，研究不同家系遗传变异，筛选出二代优树群体，作为日本落叶松高世代种子园的建园材料。本研究以吉林省三岔子林业局国家红松良种基地的53个28年生红松半同胞家系为材料，对其生长性状和形质性状的遗传变异进行分析评价，并选出优良家系和单株，以期为改良代种子园的营改建提供理论依据及造林生产应用提供优良种质材料，也为红松育种群体的建立及推广提供参考。

(C)1994-2019 China Academic Journal Electronic Publishing House. All rights reserved. http://www.cnki.net

1 材料与方法

1.1 试验地点与材料

子代测定林位于吉林省三岔子林业局国家红松良种基地(E126°56′,N41°95′),该地海拔 520 m,年均温 2.5℃,年降水量 725.5 mm,年日照时数 2 300 h,无霜期 110 d。试验材料共 53 个家系,其中 52 个家系来源于吉林省露水河林业局天然林母树林中的红松优树种子,1990 年春季育苗,培育 4 年,苗龄型为 S2-2,1995 年春季选择试验地营建子代测定林。利用当地种子作为对照,具体编号见表 1。试验地采用完全随机区组设计,6 株小区,5 个区组,株行距为 2 m×3 m,周边设置保护行。

表 1 不同家系编号

Table 1 Family number

子代来源 Progeny source	编号 Number									
52 个半同胞家系 52 Half-sib families	10	49	65	77	82	83	84	85	87	88
	90	91	92	93	94	96	97	98	100	101
	102	103	105	106	108	109	110	111	112	113
	114	115	117	118	120	122	125	129	130	132
	133	134	135	136	137	138	140	143	144	145
	148	163								
对照 CK	1									

1.2 试验方法

于 2017 年 10 月对 28 年生的红松半同胞子代测定林的树高、地径、胸径、三米径、枝下高和第六轮枝高等生长性状及通直度、分枝角和轮枝数等形质性状进行全林调查。利用 Vertex Ⅳ测高仪测量树高、枝下高和第六轮枝高; 利用测树钢围尺测量地径、胸径和三米径; 利用量角器测量分枝角; 利用分级法[17]确定通直度。

利用实验形数法[18]计算材积(V):

$$V = (H + 3) g_{1,3} f \quad (1)$$

式中: 红松平均实验形数 f 为 0.33,H 为树高,d 为胸径,$g_{1.3}$ 为胸高处横断面积。

方差分析线性模型为:

$$X_i = \mu + B_i + F + Fi_{ji} + M_{eiji} \quad (2)$$

式中:μ 为总体平均值,B_i 为区组效应,F 为家系效应,Fi_{ji} 为区组和家系的交互作用,M_{eiji} 为机误。

遗传力采用公式[19~20]:

家系遗传力(H^2):

$$H^2 = \frac{\sigma_F^2}{\sigma_F^2 + \sigma_{FB}^2/B + \sigma_e^2/NB} \quad (3)$$

家系内单株狭义遗传力(h_N^2):

$$h_N^2 = \frac{3\sigma_F^2}{\sigma_{FB}^2 + \sigma_e^2} \quad (4)$$

式中: σ_F^2 为家系的方差分量,σ_{FB}^2 为家系与区组交互作用的方差分量,σ_e^2 为机误的方差分量,B 为区组数,N 为区组内重复。

变异系数采用公式[21]:

表型变异系数(PCV):

$$PCV = \frac{\sqrt{\sigma_p^2}}{\bar{X}} \times 100\% \quad (5)$$

遗传变异系数(GCV):

$$GCV = \frac{\sqrt{\sigma_g^2}}{\bar{X}} \times 100\% \quad (6)$$

式中: σ_p^2 为性状的表型方差分量,σ_g^2 为性状的遗传方差分量,$\bar{X}$ 为性状的平均值。

采用布雷金多性状综合评价法[22]对家系及家系内单株进行综合评定,采用公式:

$$Q_i = \sqrt{\sum_{j=1}^{n} a_i} \quad (7)$$

式中: $a_i = X_{ij}/X_{jmax}$,X_{ij} 为某一性状的平均值,X_{jmax} 为某一性状的最优值。

家系现实增益估算公式[23]:

$$\Delta G_r = S/\bar{X} \times 100\% \quad (8)$$

式中: S 为选择差,$\bar{X}$ 某一性状的平均值。

单株遗传增益估算公式[24]:

$$\Delta G = (R/\bar{X}) \times 100\%, R = h^2 \cdot S \quad (9)$$

式中: R 为选择响应,S 为选择差,$\bar{X}$ 表示总体平均值。

一般配合力采用公式[25]:

$$g = x - \mu \quad (10)$$

式中,g 为亲本的一般配合力,x 为亲本的某个交配组合在某个性状的子代平均值,μ 为这个性状所有组合的子代总平均值。

所有数据利用 SPSS19.0 软件进行分析[26]。

2 结果与分析

2.1 各性状方差分析

53 个家系各性状方差分析结果见表 2。结果显示,通直度在家系间、分枝角在区组间及家系和区组的交互作用间、枝下高在家系间以及轮枝数在区组间的差异未达显著水平($P > 0.05$); 第六轮枝高在家系间以及轮枝数在家系和区组的交互作用间的差异显著($0.01 < P < 0.05$),除此之外的各生长性状在各变异来源间的差异均达极显著水平($P < 0.01$)。

(C)1994-2019 China Academic Journal Electronic Publishing House. All rights reserved. http://www.cnki.net

表2 不同家系各性状方差分析表

Table 2 Variance analysis of different traits among families

性状 Traits	变异来源 Variance source	*df*	*MS*	*F*	性状 Traits	变异来源 Variance source	*df*	*MS*	*F*
树高 Tree height	家系 Family	52	3.514	1.783**	通直度 Stem straightness degree	家系 Family	52	0.096	1.157
	区组 Block	4	35.345	17.932**		区组 Block	4	0.365	4.525**
	家系×区组 Family×Block	208	1.971	7.739**		家系×区组 Family×Block	208	0.083	2.568**
地径 Basal diameter	家系 Family	52	80.683	3.757**	分枝角 Branch angle	家系 Family	52	70.501	2.249**
	区组 Block	4	86.995	4.051**		区组 Block	4	33.062	1.055
	家系×区组 Family×Block	208	21.477	4.295**		家系×区组 Family×Block	208	31.348	1.132
胸径 Diameter at breast height	家系 Family	52	60.532	3.939**	枝下高 Tree height under the first branch	家系 Family	52	0.336	1.336
	区组 Block	4	93.515	6.085**		区组 Block	4	1.478	5.872**
	家系×区组 Family×Block	208	15.368	4.415**		家系×区组 Family×Block	208	0.252	6.787**
三米径 Diameter at 3 m height	家系 Family	52	51.495	3.672**	第六轮枝高 Tree height under the sixth branch	家系 Family	52	2.607	1.510*
	区组 Block	4	135.865	9.687**		区组 Block	4	9.64	5.582**
	家系×区组 Family×Block	208	14.025	4.424**		家系×区组 Family×Block	208	1.727	6.884**
材积 Volume	家系 Family	52	0.007	3.928**	轮枝数 Branch number per node	家系 Family	52	5.355	3.834**
	区组 Block	4	0.017	9.542**		区组 Block	4	3.079	2.205
	家系×区组 Family×Block	208	0.002	4.284**		家系×区组 Family×Block	208	1.397	1.253*

表3 不同家系各性状变异系数

Table 3 Variation parameters of different traits among families

性状 Traits	平均值 Mean	变幅 Range	标准差 SD	表型变异系数 Phenotypic coefficient of variation	遗传变异系数 Genotypic coefficient of variation	家系遗传力 Family heredity	单株遗传力 Single heredity
树高 Tree height	7.60	4.80~11.00	0.82	10.80	8.52	0.439	0.285
地径 Basal diameter	21.81	12.70~32.80	3.14	14.38	10.09	0.734	0.764
胸径 Diameter at breast height	16.38	7.50~24.80	2.67	16.30	11.66	0.746	0.827
三米径 Diameter at 3 m height	13.60	5.00~21.60	2.55	18.75	13.43	0.728	0.753
材积 Volume	0.077 0	0.012 7~0.190 3	0.028 8	37.39	26.75	0.745	0.810
通直度 Stem straightness degree	3.94	1~5	0.78	19.87	9.89	0.136	0.031
分枝角 Branch angle	78.08	48~90	5.44	6.97	1.77	0.555	0.138
枝下高 Tree height under the first branch	2.11	1.00~4.00	0.28	13.30	9.67	0.252	0.116
第六轮枝高 Tree height under the sixth branch	4.54	3.00~9.00	0.74	16.25	11.94	0.338	0.177
轮枝数 Branch number per node	5.56	2.40~11.20	1.14	20.46	7.64	0.739	0.341

2.2 各性状遗传变异分析

53 个家系树高、地径和胸径等 5 个生长性状及通直度、分枝角和轮枝数等形质性状变异参数见表 3。所有家系树高平均值为 7.60 m，变幅为 4.80~11.00 m；地径的平均值为 21.81 cm，变幅为 12.70~32.80 cm；胸径的平均值为 16.38 cm，变幅为 7.50~24.80 cm；三米径的平均值为 13.60 cm，变幅为 5.00~21.60 cm；材积的平均值为 0.077 0 m^3，变幅为 0.0127~0.1903 m^3；通直度的平均值为 3.94，变幅为 1~5；分枝角平均值为 78.08°，变幅为 48°~90°；枝下高平均值为 2.11 m，变幅为 1.00~4.00 m；第六轮枝高平均值为 4.54 m，变幅为 3.00~9.00 m；轮枝数的平均值为 5.56 个，变幅为 2.40~11.20 个。

(C)1994-2019 China Academic Journal Electronic Publishing House. All rights reserved. http://www.cnki.net

各性状表型变异系数变化范围为 6.97% ~ 37.39%，除分枝角外，其余各性状表型变异系数均超过 10%；其中材积的表型变异系数最大，为 37.39%。遗传变异系数变化范围为 1.77% ~ 26.75%。除通直度、枝下高和第六轮枝高外，其余各性状的家系遗传力较高，部分性状遗传力超过 0.7。从单株遗传力来看，地径、胸径、三米径和材积的单株遗传力较大，均超过 0.7。

2.3 各性状相关性分析

各性状间相关系数见表 4。由表 4 可知，从生长性状来看，树高、地径、胸径、三米径和材积各性状之间均达极显著正相关水平，其中地径、胸径、三米径和材积各性状间相关性较高，相关系数均超过 0.9。从形质性状来看，通直度与分枝角达正相关(0.010)，与轮枝数达极显著正相关(0.219)；分枝角和轮枝数呈正相关(0.036)。对比生长性状和形质性状，通直度与树高、地径、胸径、三米径和材积均呈正相关关系，其中与树高和三米径达极显著正相关水平，与材积达显著正相关水平(0.054)；分枝角与地径、胸径、三米径和材积表现为负相关，与树高表现为正相关(0.006)；轮枝数与树高、地径、胸径、三米径和材积均表现为极显著正相关水平。

2.4 各性状一般配合力分析

各性状一般配合力见表 5。由表 5 可知，树高一般配合力变化范围为 -0.598 6 ~ 0.971 4，地径一般配合力变化范围为 -4.462 5 ~ 5.267 5，胸径一般配合力变化范围为 -3.466 4 ~ 4.414 0，三米径一般配合力变化范围为 -3.283 6 ~ 4.066 4，材积一般配合力变化范围为 -0.030 6 ~ 0.054 7，通直度一般配合力变化范围为 -0.602 8 ~ 0.497 2，分枝角一般配合力变化范围为 -6.201 9 ~ 6.498 1，枝下高一般配合力变化范围为 -0.167 2 ~ 0.269 6，第六轮枝高一般配合力变化范围为 -0.398 9 ~ 0.907 8，轮枝数平均值一般配合力变化范围为 -1.095 7 ~ 1.017 6。其中家系 115 各性状一般配合力均较高，其亲本可选作优良亲本材料。

2.5 各性状主成分分析

各性状主成分分析结果见表 6。由表 6 可知，主成分Ⅰ特征值为 4.36，贡献率为 43.56%，树高、地径、胸径、三米径和材积等性状特征值较高，分别为 0.74、0.93、0.96、0.95 和 0.98；主成分Ⅱ特征值为 1.74，贡献率为 17.39%，枝下高和第六轮枝高等性状特征值较高，分别为 0.78 和 0.84；主成分Ⅲ特征值为 0.14，贡献率为 11.36%，通直度和轮枝数平均值等性状特征值较高，分别为 0.71 和 0.69。三个主成分的累计贡献率为 72.31%，包含了供试家系生长及形质性状的大部分信息。

表 4 不同家系各性状相关性分析

Table 4 Correlation coefficients among different traits among families

	树高 Tree height	地径 Basal diameter	胸径 Diameter at breast height	三米径 Diameter at 3 m height	通直度 Stem straightness degree	材积 Volume	分支角 Branch angle	枝下高 Tree height under the first branch	第六轮枝高 Tree height under the sixth branch
地径 Basal diameter	0.546**								
胸径 Diameter at breast height	0.575**	0.941**							
三米径 Diameter at 3 m height	0.586**	0.908**	0.960**						
通直度 Stem straightness degree	0.187**	0.013	0.018	0.081**					
材积 Volume	0.703**	0.921**	0.973**	0.936**	0.054*				
分枝角 Branch angle	0.006	-0.015	-0.015	-0.015	0.010	-0.013			
枝下高 Tree height under the first branch	0.268**	0.052*	0.073**	0.114**	0.059*	0.109**	0.045		
第六轮枝高 Tree height under the sixth branch	0.333**	0.076**	0.079**	0.112**	0.133**	0.129**	0.102**	0.620**	
轮枝数 Branch number per node	0.082**	0.146**	0.106**	0.117**	0.219**	0.109**	0.036	0.051*	0.186**

注: **极显著相关水平($P<0.01$); * 显著相关水平($P<0.05$)

Note: **means correlation is significant at the 0.01 level; * means correlation is significant at the 0.05 level

(C)1994-2019 China Academic Journal Electronic Publishing House. All rights reserved. http://www.cnki.net

表 5 各家系不同性状一般配合力

Table 5 General combining ability values of different traits among families

家系 Family	树高 Tree height	家系 Family	地径 Basal diameter	家系 Family	胸径 DB	家系 Family	三米径 Diameter at 3 m height	家系 Family	材积 Volume	家系 Family	通直度 Stem straightness degree	家系 Family	分枝角 Branch angle	家系 Family	枝下高 Tree height under the first branch	家系 Family	第六轮枝高 Tree height under the sixth branch	家系 Family	轮枝数平均值 Branch number per node
115	0.971 4	115	5.267 5	115	4.41 4	115	4.066 4	115	0.054 7	132	0.497 2	49	6.498 1	49	0.269 6	135	0.907 8	49	1.017 6
120	0.731 4	117	3.420 8	117	3.020 7	117	2.853 3	117	0.031 1	106	0.397 2	96	6.364 8	130	0.246 3	49	0.627 8	115	0.657 6
144	0.614 8	133	2.607 5	138	2.504 7	133	2.223 5	138	0.030 1	140	0.330 6	84	5.764 8	148	0.246 3	120	0.574 5	10	0.644 3
138	0.588 1	138	2.447 5	133	2.367 4	138	2.186 4	133	0.021 4	163	0.330 6	135	5.131 4	135	0.233 3	103	0.514 5	77	0.570 9
134	0.488 1	112	2.290 8	112	1.824 1	112	1.826 4	137	0.015 1	148	0.263 9	90	4.931 4	90	0.143 5	148	0.464 5	132	0.544 3
·	·	·	·	·	·	·	·	·	·	·	·	·	·	·	·	·	·	·	·
·	·	·	·	·	·	·	·	·	·	·	·	·	·	·	·	·	·	·	·
·	·	·	·	·	·	·	·	·	·	·	·	·	·	·	·	·	·	·	·
88	−0.391 9	102	−1.652 5	103	−1.332 6	102	−1.263 6	132	−0.014 3	125	−0.269 4	111	−4.335 2	163	−0.137 5	113	−0.335 5	109	−0.529 1
91	−0.415 2	87	−1.935 8	105	−1.376 9	103	−1.440 3	105	−0.014 4	88	−0.336 1	112	−5.235 2	145	−0.143 7	136	−0.348 9	92	−0.689 1
94	−0.481 9	132	−1.939 2	87	−2.042 6	87	−1.583 6	87	−0.020 5	110	−0.336 1	134	−5.468 6	77	−0.147 3	110	−0.368 9	110	−0.749 1
87	−0.515 2	91	−3.769 2	91	−3.456 5	106	−3.127 1	91	−0.030 3	111	−0.469 4	118	−5.768 6	102	−0.153 7	91	−0.375 5	91	−0.842 4
111	−0.598 6	106	−4.462 5	106	−3.466 4	91	−3.283 6	106	−0.030 6	101	−0.602 8	117	−6.201 9	114	−0.167 2	145	−0.398 9	111	−1.095 7

注:GCA. 一般配合力

Note: GCA. General combining ability

(C)1994-2019 China Academic Journal Electronic Publishing House. All rights reserved. http://www.cnki.net

表 6　各性状主成分分析

Table 6　Principal component analysis of different traits

主要成分因子 Principal component factor	主成分Ⅰ Component Ⅰ	主成分Ⅱ Component Ⅱ	主成分Ⅲ Component Ⅲ
特征值 Eigenvalue	4.36	1.74	1.14
贡献率 Contribution(%)	43.56	17.39	11.36
累计贡献率 Cumulative contribution(%)	43.56	60.95	72.31
树高 Tree height	0.74	0.25	-0.04
地径 Basal diameter	0.93	-0.20	0.00
胸径 Diameter at breast height	0.96	-0.20	-0.03
三米径 Diameter at 3 m height	0.95	-0.14	0.00
材积 Volume	0.98	-0.13	-0.03
通直度 Stem straightness degree	0.11	0.34	0.71
分枝角 Branch angle	0.00	0.20	-0.02
枝下高 Tree height under the first branch	0.21	0.78	-0.36
第六轮枝高 Tree height under the sixth branch	0.24	0.84	-0.18
轮枝数平均值 Branch number per node	0.18	0.29	0.69

表 7　不同家系 Q_i 值

Table 7　Q_i value of different families

家系 Family	Q_i 值 Q_i value	家系 Family	Q_i 值 Q_i value	家系 Family	Q_i 值 Q_i value
115	2.24	122	1.98	94	1.90
117	2.13	65	1.98	98	1.90
138	2.11	101	1.98	114	1.90
133	2.08	130	1.98	88	1.90
112	2.05	108	1.98	85	1.90
120	2.05	82	1.98	109	1.89
137	2.04	92	1.96	111	1.89
136	2.04	100	1.95	97	1.88
129	2.04	113	1.94	145	1.88
134	2.03	143	1.94	105	1.88
90	2.03	148	1.94	102	1.88
144	2.02	84	1.94	103	1.88
118	2.02	110	1.93	132	1.87
135	2.01	125	1.93	10	1.87
93	2.00	49	1.93	87	1.83
1	1.99	77	1.92	91	1.75
140	1.99	163	1.91	106	1.75
96	1.99	83	1.91		

2.6　优良家系选择

依据主成分分析结果，以树高、地径、胸径、三米径和材积等生长性状为选择指标对 53 个半同胞家系进行综合评价，各家系 Q_i 值见表 7。按家系材积现实遗传增益在 35% 以上，兼顾树高、地径、胸径和三米径对家系进行评价选择，家系 115、117、138、133 和 112 入选，入选率为 10%，入选家系树高、地径、胸径、三米径和材积等性状平均值分别为 7.90 m、25.02 cm、19.21 cm、16.23 cm 和 0.107 4 kg · m^{-3}，现实增益分别为 3.94%、14.71%、17.26%、19.34%和 39.48%。

2.7　单株选择

依据主成分分析结果，以树高、地径、胸径、三米径和材积等生长性状为选择指标对优良家系内的单株进行评价选择，各单株 Qi 值见表 8。以单株材积遗传增益超过 100%为标准，兼顾树高、地径、胸径和三米径对优良家系内单株进行评价选择，共获得 6 株优良单株，入选率为 6%，入选优良单株树高、地径、胸径、三米径和材积平均值比总平均值分别高 1.60 m、8.54 cm、7.04 cm、6.69 cm 和 0.097 kg · m^{-3}，单株遗传增益分别为 5.99%、29.92%、35.53%、37.06%和 102.04%。

表 8　不同单株 Q_i 值

Table 8　Q_i value of different single plants

区组 Block	家系 Family	株号	Q_i
4	115	2	2.21
5	138	3	2.16
3	138	1	2.15
5	115	2	2.14
5	133	6	2.13
5	117	2	2.13
1	115	2	2.12
1	115	4	2.12
1	138	1	2.12
5	115	5	2.12
.	.	.	.
.	.	.	.
.	.	.	.
4	138	2	1.67
2	133	4	1.67
4	138	3	1.65
2	112	3	1.65
4	112	4	1.65
2	138	4	1.64
3	112	1	1.63
4	112	1	1.63
1	133	2	1.61
1	133	6	1.60

(C)1994-2019 China Academic Journal Electronic Publishing House. All rights reserved.　http://www.cnki.net

3 讨论

遗传和变异是林木育种研究的主要内容[27]，对变异来源、特点和规律的认识是进行树种改良的重要基础[28]。本研究对 28 年生的红松优树子代半同胞家系的方差分析结果显示，家系间大部分性状均达极显著差异水平，表明母树林后代具有较大的分化[29]，与王顺安等[30]对日本落叶松的子代分析结果一致，有必要进一步进行优良家系的评价选择。

变异系数是衡量群体变异程度大小的指标，它能反映树体各性状的遗传变异能力，包括表型变异系数和遗传变异系数[21]。变异程度高，有利于进行优良家系选择[31]。本研究中材积表型变异系数最大，达到 37.39%；胸径表型变异系数为 16.30%；树高的表型变异系数较小，为 10.80%，这与李自敬对 23 年生长白落叶松研究结果较为相似[32]，表明以材积作为选择指标进行家系选择更具有选择潜力。三个性状的遗传变异系数占表型变异系数的比例较大，均超过 70%，比马尾松（*Pinus massoniana*）研究结果高[33]，说明家系间变异受遗传因素的影响较大，为优良家系的选择提供了基础。

遗传力表示亲本某一性状遗传给子代能力的大小，是估算遗传增益的重要参数[34]。本研究中树高、胸径和材积家系遗传力均高于宋云平等人对 27 年生红松的研究结果[35]，部分性状的家系遗传力超过 0.7，属高遗传力，表明所选材料各性状能较为稳定遗传，有利于进行优良家系的评价选择[36]。本研究中各性状的单株遗传力变化范围为 0.031～0.827，其中树高单株遗传力为 0.285，与张振等对 25 年和 27 年生的红松研究结果相似[37]；胸径和材积的单株遗传力达到 0.8 以上，比侯义梅对 15 年生的日本落叶松研究结果偏高[38]。单株遗传力较高，可在家系内进行优良单株的筛选，为二代种子园的营建提供建园材料。本研究中各性状家系遗传力均高于单株遗传力，比 Magnussen 对白云杉（*Picea glauca*）的研究结果偏高[39]，表明各性状在单株水平上的遗传能力弱于家系，家系在各性状的遗传差异具有更稳定的遗传能力。

林木改良过程中，通常希望综合性状得到改良，所以性状之间相关性至关重要[40]，相关性分析可以反映各性状之间存在的联系，为多性状联合控制育种提供参考[41]。本研究中的相关性分析结果显示，从生长性状来看，树高、胸径和材积等性状之间均达极显著正相关水平，这与 Liang 等对 32 年红松无性系研究结果相似[42]，其中胸径与材积相关性较大，相关系数超过 0.9，表明树高和胸径不是独立遗传的两个性状。从形质性状来看，通直度与分枝角呈正相关，表明树干的生长势强于树枝，树枝侧向生长。结合生长性状和形质性状，通直度与树高、三米径达极显著正相关水平，与材积达显著正相关水平，为长势和干型优良的优树选择提供依据。

配合力是林木育种工作的研究内容之一，其大小与具体性状有关，反映亲本优良性状传递给子代的相对能力[43]。本研究对 53 个半同胞家系各性状进行一般配合力分析，除家系 115 各性状一般配合力均较高，其亲本可作为优良亲本材料外，不同性状一般配合力高的家系差异较大，难以进行联合筛选，因而需结合主成分分析进一步对家系进行评价选择。

育种目标决定育种方向。红松是东北地区特有的经济用材树种，但因其生长缓慢，使得红松种子园升级换代较慢。为选育高生长、高出材量的优质红松资源，本研究通过主成分分析研究发现主成分 I 中的树高、地径、胸径、三米径和材积等生长性状特征值较高，贡献率较大，且各性状间相关性较高，因此可作为筛选优质红松资源的评价指标。在进行家系选择时，入选率越大，遗传多样性丰富度越高，但遗传增益越小[44]。本研究以家系材积现实遗传增益在 35% 以上为期望目标，对家系进行评价选择，入选率为 10%，入选家系树高、地径、胸径、三米径和材积等性状现实增益分别为 3.94%、14.71%、17.26%、19.34% 和 39.48%，与梁德洋等人的研究结果相似[45]，表明利用表型选择对红松进行遗传改良具有较好的效果，入选的家系在生长上存在明显的优势，若用优良家系进行生产造林，可减少林分工艺成熟时间，进而提高经济效益[41]。另一方面，筛选出的优良家系同时反映出对应亲本的优良特性，可进一步筛选出优良亲本，为 1.5 代种子园的营建提供理论支持。在优良家系选择的同时，还可进行优良单株的筛选。利用多性状综合评价法对优良家系内单株进行评价选择，按单株期望遗传增益在 100% 以上为标准对家系内单株进行筛选，获得 6 株优良单株，入选率为 4%，入选单株树高、地径、

(C)1994-2019 China Academic Journal Electronic Publishing House. All rights reserved. http://www.cnki.net

胸径、三米径和材积等性状的平均值分别比总平均值高 1.60 m、8.54 cm、7.04 cm、6.69 cm 和 0.097 kg · m^{-3}，遗传增益分别为 5.99%、29.92%、35.53%、37.06%和 102.04%，高于张振对红松[36]和林能庆对马尾松[46]的研究结果，具有较大的遗传改良潜力，可作为红松优良种质材料推广使用。

4 结论

随着林木改良工作日渐深入，在大力营建高世代种子园的同时，对初级种子园的改良和升级亦在进行。本研究的材料存在丰富的变异，所选的 5 个优良家系和 6 个优良单株生长及干型优势明显，各性状相关性强，遗传力高，家系间和家系内的个体间有较大的选择潜力，有利于红松的良种选育，为推广造林应用提供物质基础; 根据子代测定结果可对亲本无性系进行重新选择，为初级种子园的去劣疏伐、1.5 代种子园的改良营建提供理论参考; 在优良家系内选出优良单株，可为二代种子园的营建提供建园材料; 通过筛选得到的一般配合力高的亲本若进行杂交实验，还会提高子代杂种优势的机率，优良的个体也可用于无性化推广应用。

参 考 文 献

1. 程春香，毛子军，靳世波，等. 小兴安岭北部原始阔叶红松林红松结实气候敏感性及其种子年机制探讨[J]. 植物研究，2017，37(1): 118 – 127.
Cheng C X，Mao Z J，Jin S B，et al. Sensitivity of fruiting for *Pinus koraiensis* to climate change and mechanisms of masting in the original broad-leaved Korean Pine forest in North Xiaoxing'an mountain，China[J]. Bulletin of Botanical Research，2017，37(1): 118 – 127.
2. 刘德栋. 我国红松良种选育研究进展[J]. 防护林科技，2017(3): 96 – 99，116.
Liu D D. Research progress on breeding of *Pinus koraiensis* in China[J]. Protection Forest Science and Technology，2017(3): 96 – 99，116.
3. Lim T K. *Pinus koraiensis*[M]. //Lim T K. Edible medicinal and non-medicinal plants. Dordrecht: Springer，2012.
4. 郭景瑞，周鑫. 赤霉素诱导对红松幼树开花结实的影响[J]. 林业勘查设计，2013(2): 82 – 83.
Guo J R，Zhou X. The effect of GA for the flowering of fruiting of *Pinus koraiensis*[J]. Forest Investigation Design，2013(2): 82 – 83.
5. 曹世刚. 清河城红松种子园高产结实无性系选择的研究[J]. 防护林科技，2015(5): 61 – 62，67.
Cao S G. Selection of high-yielding clones of Qinghecheng *Pinus koraiensis* seed orchard[J]. Protection Forest Science and Technology，2015(5): 61 – 62，67.
6. Wang H M，Xia D A，Wang W J，et al. Genetic variations of wood properties and growth characters of Korean pines from different provenances[J]. Journal of Forestry Research，2002，13(4): 277 – 280.
7. 党常顺，杜明广，秦桂珍，等. 红松人工林改为坚果园的林分选择[J]. 中国林副特产，1998(4): 5 – 7.
Dang C G，Du M G，Qin G Z，et al. The stand selection of the *Pinus koraiensis* plantation to the nut garden[J]. Quarterly of Forest By-Product and Speciality in China，1998(4): 5 – 7.
8. 张凌梅. 优良红松种苗繁殖技术[J]. 林业科技通讯，2015(12): 31 – 32.
Zhang L M. Excellent *Pinus koraiensis* seedlings breeding techniques[J]. Practical Forestry Technology，2015(12): 31 – 32.
9. 孙志学. 红松种子园的营建与经济效益[J]. 现代园艺，2013(10): 35.
Sun Z X. The construction and economic benefit of *Pinus koraiensis* seed orchard[J]. Xiandai Horticulture，2013(10): 35.
10. 孙一荣，朱教君，于立忠，等. 不同光环境对红松幼苗光合生理特征的影响[J]. 生态学杂志，2009，28(5): 850 – 857.
Sun Y R，Zhu J J，Yu L Z，et al. Photosynthetic characteristics of *Pinus koraiensis* seedlings under different light regimes[J]. Chinese Journal of Ecology，2009，28(5): 850 – 857.
11. 陈家媛，靖晶，高嵩，等. 草河口林场红松人工林遗传多样性的 ISSR 分析[J]. 植物研究，2009，29(5): 633 – 636.
Chen J Y，Jing J，Gao S，et al. Analysis of genetic diversity of *Pinus koraiensis* plantation in Caohekou forest farm by ISSR marker[J]. Bulletin of Botanical Research，2009，29(5): 633 – 636.
12. 王昊. 林木种子园研究现状与发展趋势[J]. 世界林业研究，2013，26(4): 32 – 37.
Wang H. Research progress and development trend of tree seed orchard[J]. World Forestry Research，2013，26(4): 32 – 37.
13. 孙文生. 红松种子园优质高产经营技术研究[D]. 北京: 北京林业大学，2006.
Sun W S. Study on management techniques of Korean Pine seed orchard for high genetic quality and ample production of seeds[D]. Beijing: Beijing Forest University，2006.

(C)1994-2019 China Academic Journal Electronic Publishing House. All rights reserved. http://www.cnki.net

14. 刘宏伟，孙美欧，王国义. 红松二代无性系种子园建立技术的研究[J]. 林业科技，2015，40(6)：13－15.
Liu H W，Sun M O，Wang G Y. Study on the establishment techniques for second generation clonal seed orchard of *Pinus koraiensis*[J]. Forestry Science & Technology，2015，40(6)：13－15.

15. Doran J，Bush D，Page T，et al. Variation in growth traits and wood density in whitewood(*Endospermum medullosum*)：a major timber species in Vanuatu[J]. International Forestry Review，2012，14(4)：476－485.

16. 张正刚，马建伟，靳新春，等. 日本落叶松自由授粉家系子代测定林分析与选择研究[J]. 西北林学院学报，2013，28(4)：74－79.
Zhang Z G，Ma J W，Jin X C，et al. Analysis and selection of progeny test forest of the open pollinated family of Japanese larch[J]. Journal of Northwest Forestry University，2013，28(4)：74－79.

17. Zhao X Y，Bian X Y，Liu M R，et al. Analysis of genetic effects on a complete diallel cross test of *Betula platyphylla*[J]. Euphytica，2014，200(2)：221－229.

18. 邓继峰，张含国，张磊，等. 17年生杂种落叶松遗传变异及优良家系选择[J]. 东北林业大学学报，2010，38(1)：8－11.
Deng J F，Zhang H G，Zhang L，et al. Genetic variation of 17-year-old hybrid larch and its superior family selection[J]. Journal of Northeast Forestry University，2010，38(1)：8－11.

19. 续九如. 林木数量遗传学[M]. 北京：高等教育出版社，2006.
Xu J R. Quantitative genetics in forestry[M]. Beijing：Higher Education Press，2006.

20. 赵奋成，郭文冰，钟岁英，等. 基于针刺仪测定技术的湿地松木材密度间接选择效果[J]. 林业科学，2018，54(10)：172－179.
Zhao C F，Guo W B，Zhong S Y，et al. Effects of indirect selection on wood density based on resistograph measurement of slash pine[J]. Scientia Silvae Sinicae，2018，54(10)：172－179.

21. Metougui M L，Mokhtari M，Maughan P J，et al. Morphological variability，heritability and correlation studies within an Argan Tree Population(*Argania spinosa*(L.) Skeels) Preserved in situ[J]. International Journal of Agriculture and Forestry，2017，7(2)：42－51.

22. Wang F，Zhang Q H，Tian Y G，et al. Comprehensive assessment of growth traits and wood properties in half-sib *Pinus koraiensis* families[J]. Euphytica，2018，214(11)：202.

23. 叶代全. 枫香优树自由授粉子代测定与速生优良家系选择[J]. 中南林业科技大学学报，2011，31(8)：79－82.
Ye D Q. Open pollination progeny test and excellent family selection of *Liquidambar formosana*[J]. Journal of Central South University of Forestry & Technology，2011，31(8)：79－82.

24. 覃敏，尹光天，杨锦昌，等. 米老排种源家系生长性状变异分析及早期选择[J]. 植物研究，2017，37(1)：139－146.
Qin M，Yin G T，Yang J C，et al. Growth traits variation and early selection of *Mytilaria laosensis* provenances and families[J]. Bulletin of Botanical Research，2017，37(1)：139－146.

25. 王虹，师尚礼，张旭业，等. 紫花苜蓿多元杂交后代产量和品质一般配合力分析及遗传参数的估算[J]. 草业学报，2016，25(3)：126－134.
Wang H，Shi S L，Zhang X Y，et al. Determination of general combining ability and estimation of genetic parameters for yield and quality in alfalfa[J]. Acta Prataculturae Sinica，2016，25(3)：126－134.

26. 李玉光，杜宏巍，黄永生. SPSS 19.0 统计分析入门与提高[M]. 北京：清华大学出版社，2014.
Li Y G，Du H W，Huang Y S. Introduction and improvement of statistical analysis of SPSS 19.0[M]. Beijing：Tsinghua University Press，2014.

27. Mwase W F，Savill P S，Hemery G. Genetic parameter estimates for growth and form traits in common ash(*Fraxinus excelsior* L.) in a breeding seedling orchard at Little Wittenham in England[J]. New Forests，2008，36(3)：225－238.

28. White T L，Adams W T，Neale D B. Forest genetics[M]. London：CABI Publishing，2007.

29. 刘宏伟，王国义. 兴安落叶松种子园半同胞子代测定及优良家系选择[J]. 林业科技，2016，41(5)：12－14.
Liu H W，Wang G Y. Fine families selectiong in half-sid progeny test of *Larix gmelinii*[J]. Forestry Science & Technology，2016，41(5)：12－14.

30. 王顺安，向金莲. 日本落叶松子代测定林调查分析[J]. 湖北民族学院学报：自然科学版，2009，27(3)：275－278.
Wang S A，Xiang J L. Investigation and analysis on progeny testing forest of *Larrix kaempfri*(lamb.) Carr.[J]. Journal of Hubei University for Nationalities：Natural Science Edition，2009，27(3)：275－278.

31. 尹绍鹏，赵国辉，夏辉，等. 长白落叶松半同胞子代测定研究[J]. 西南林业大学学报，2016，36(1)：63－68.
Yin S P，Zhao G H，Xia H，et al. Stacly on progeny test of half-sibs families in *Larix olgensis*[J]. Journal of Southwest Forestry University，2016，36(1)：63－68.

(C)1994-2019 China Academic Journal Electronic Publishing House. All rights reserved. http://www.cnki.net

32. 李自敬,李雪峰,张含国,等. 长白落叶松优良家系选择的研究[J]. 林业科技,2008,33(4):1-4.
Li Z J,Li X F,Zhang H G,et al. Study on selection of superior families of *Larix olgensis* [J]. Forestry Science & Technology,2008,33(4):1-4.
33. Ji K S,Fan M L,Xu L A. Variation analysis and fine family selection on half-sib progenies from clonal seed orchard of *Pinus massoniana* [J]. Frontiers of Forestry in China,2007,2(3):340-346.
34. 卢超,高明博,焦小钟,等. 几个小麦亲本主要农艺性状的配合力评价及遗传力分析[J]. 麦类作物学报,2010,30(6):1023-1028.
Lu C,Gao M B,Jiao X Z,et al. Combining ability and heritability analysis of main agronomic traits in wheat [J]. Journal of Triticeae Crops,2010,30(6):1023-1028.
35. 宋云平,闫朝福,刘洪志,等. 苇河红松二代种子园自由授粉子代评选[J]. 四川林业科技,2015,36(6):74-76.
Song Y P,Yan C F,Liu H Z,et al. Open pollination progeny selection of the second generation seed orchard of *Pinus koraiensis* in Weiher [J]. Journal of Sichuan Forestry Science and Technology,2015,36(6):74-76.
36. Zhao X Y,Li Y,Zheng M,et al. Comparative analysis of growth and photosynthetic characteristics of(*Populus simonii* × *P. nigra*) × (*P. nigra* × *P. simonii*) hybrid clones of different ploidides [J]. PLoS One,2015,10(4):e0119259.
37. 张振,张含国,张磊. 红松自由授粉子代家系生产力年度变异与家系选择[J]. 植物研究,2016,36(2):305-309.
Zhang Z,Zhang H G,Zhang L. Age variations in productivity and family selection of open-pollinated families of Korean pine(*Pinus koraiensis*) [J]. Bulletin of Botanical Research,2016,36(2):305-309.
38. 侯义梅,李时元,杨年友. 日本落叶松自由授粉家系子代测定林研究[J]. 湖北林业科技,2006(1):4-7.
Hou Y M,Li S Y,Yang N Y. Research on provenance test of open-pollinated *Larix kaempferi* family [J]. Hubei Forestry Science and Technology,2006(1):4-7.
39. Magnussen S. Growth differentiation in White spruce crop tree progenies [J]. Silvae Genetica,1993,42(4-5):258-266.
40. 林思京. 25 年生马尾松生长和木材基本密度家系变异与选择[J]. 林业科学研究,2010,23(6):804-808.
Lin S J. Growth and wood density of 25-year-old Masson's pine: inter-family variation and selection [J]. Forest Research,2010,23(6):804-808.
41. 贾庆彬,张含国,张磊,等. 杂种落叶松家系变异分析与优良家系选择[J]. 东北林业大学学报,2016,44(4):1-7.
Jia Q B,Zhang H G,Zhang L,et al. Variation analysis of hybrid larch families and superior families selection [J]. Journal of Northeast Forestry University,2016,44(4):1-7.
42. Liang D Y,Ding C P,Zhao G H,et al. Variation and selection analysis of *Pinus koraiensis* clones in northeast China [J]. Journal of Forestry Research,2018,29(3):611-622.
43. 周志春,金国庆,秦国峰,等. 马尾松纸浆材重要经济性状配合力及杂种优势分析[J]. 林业科学,2004,40(4):52-57.
Zhou Z C,Jin G Q,Qin G F,et al. Analysis on combining ability and heterosis of main economic traits of *Pinus massoniana* for pulp production [J]. Scientia Silvae Sinicae,2004,40(4):52-57.
44. 潘琼蓉. 马尾松自由授粉 18 年生子代生长状况比较及家系选择[J]. 福建农林大学学报: 自然科学版,2014,43(6):592-595.
Pan Q R. Comparisons of the growth of open-pollinated progeny of 18 year-old *Pinus massoniana* seed orchard and its family selection [J]. Journal of Fujian Agriculture and Forestry University: Natural Science Edition,2014,43(6):592-595.
45. 梁德洋,金允哲,赵光浩,等. 50 个红松无性系生长与木材性状变异研究[J]. 北京林业大学学报,2016,38(6):51-59.
Liang D Y,Jin Y Z,Zhao G H,et al. Variance analyses of growth and wood characteristics of 50 *Pinus koraiensis* clones [J]. Journal of Beijing Forestry University,2016,38(6):51-59.
46. 林能庆. 闽西马尾松优树子代测定及优良单株选择[J]. 南京林业大学学报: 自然科学版,2013,37(5):31-34.
Lin N Q. Plus tree selection and progenies test of *Pinus massoniana* in western Fujian province [J]. Journal of Nanjing Forestry University: Natural Sciences Edition,2013,37(5):31-34.

(C)1994-2019 China Academic Journal Electronic Publishing House. All rights reserved. http://www.cnki.net
