# Supplementary material for: Provenance and family variations in early growth of Manchurian walnut (Juglans mandshurica Maxim.) and selection of superior families
Source: PLoS One. 2024 Mar 7;19(3):e0298918. doi: 10.1371/journal.pone.0298918 (PMC10919699; doi:10.1371/journal.pone.0298918)
Supplement: S2 File — (ZIP) [file pone.0298918.s005.zip › Variation analysis of growth traits and coning quantity of Pinus sylvestris var. mongolica clones.pdf]

植物研究 2020,40(2):217~223
Bulletin of Botanical Research

樟子松无性系生长性状与结实量变异研究

李嘉琪[1] 韩喜东[2] 马盈慧[2] 李月季[2] 王立祥[2] 韩喜田[2] 刘 志[2] 李海民[2] 赵曦阳[1*]

(1.东北林业大学林木遗传育种国家重点实验室,哈尔滨 150040;2.白城市国有林总场,白城 137000)

摘 要 为选育高产、优质的樟子松种质资源,本研究以吉林省白城市林木种子园的304个樟子松无性系为材料,对其生长性状(树高、地径、胸径、3 m处直径、冠幅、分枝角度和侧枝粗度)和结实性状(2015、2016和2017年的结实量)进行调查分析。方差分析结果表明除冠幅外无性系间各性状差异均达极显著水平($P<0.01$);各指标表型变异系数的变化范围为3.79%~65.22%,重复力变化范围为0.24~0.70;相关性分析结果表明除侧枝粗度与3 m处直径相关未达到显著水平外,树高、胸径、地径、冠幅与侧枝粗度间相关均达极显著水平,不同树龄的结实量与大部分生长性状相关未达显著水平。依据生长性状,以5%的入选率对无性系进行综合评价,15个无性系入选,入选无性系在树高、地径、胸径、3 m处直径、分枝角度和侧枝粗度等指标的遗传增益分别为5.47%、4.48%、15.18%、11.78%、2.38%和6.66%;依据3年结实量,以5%的入选率对无性系进行综合评价,15个无性系入选,入选无性系在2015年、2016年和2017年的平均结实量的遗传增益分别为2.89%、46.32%和13.88%。该研究为樟子松种子园优良无性系的选择提供材料,也为吉林西部樟子松良种选育提供基础。

关键词 樟子松;无性系;生长性状;结实量;遗传增益

中图分类号 S791.253 **文献标志码** A **doi** 10.7525/j.issn.1673-5102.2020.02.008

Variation Analysis of Growth Traits and Coning Quantity of *Pinus sylvestris* var. *mongolica* Clones

LI Jia-Qi[1] HAN Xi-Dong[2] MA Ying-Hui[2] LI Yue-Ji[2] WANG Li-Xiang[2]
HAN Xi-Tian[2] LIU Zhi[2] LI Hai-Min[2] ZHAO Xi-Yang[1*]

(1. State Key Laboratory of Tree Genetics and Breeding, Northeast Forestry University, Harbin 150040; 2. State Forest Farm of Baicheng City, Baicheng 137000)

Abstract In order to obtain high yield and excellent quality resources of *Pinus sylvestris* var. *mongolica*, 304 *P. sylvestris* var. *mongolica* clones from the forest tree seed orchard in Baicheng City of Jilin Province were taken as materials, growth traits (tree height, basal diameter, diameter at breast height, diameter at 3 m height, canopy, branch angle and lateral branch thickness) and coning quantity characteristics (coning quantity of 2015, 2016 and 2017) of different clones were investigated. By variance analysis, all the traits were significant different ($P<0.01$) except for canopy. The coefficients of phenotypic variation of all the traits ranged from 3.79%-65.22%. Repeatability range from 0.24-0.70; By correlation analysis, the existed significantly positive correlation among all the growth traits (0.181-0.896) except for lateral branch thickness with diameter at 3 m height (0.082). Most of growth traits with the different age of coning quantity was not significantly correlation By growth traits, with the selected rate by 5%, 15 clones were selected as excellent clones, the genetic gains of height, basal diameter, diameter at 3 m height, branch angle and lateral branch thickness of the selected clones were 5.47%, 4.48%, 15.18%, 11.78%, 2.38% and 6.66%, respectively. When

基金项目:中央高校基本科研业务费专项资金项目(2572017DA02)
第一作者简介:李嘉琪(1992—),女,硕士研究生,主要从事林木常规育种方面的研究。
* 通信作者:E-mail:zhaoxyphd@163.com
收稿日期:2019-07-07
Foundation item:The Fundeamental Research Funds for the Central Universities(2572017DA02)
First author introduction:LI Jia-Qi(1992—),female,master,mainly engaged in tree genetic and improvement.
* Corresponding author:E-mail:zhaoxyphd@163.com
Received date:2019-07-07

evaluated clones by coning quantity characteristics, with the selected rate by 5%, 15 clones were selected as excellent clones, the genetic gains of coning quantity of 2015, 2016 and 2017 of the selected clones were 2.89%, 46.32% and 13.88%, respectively. This study provides materials for the selection of excellent clones of *P. sylvestris* var. *mongolica* seed orchard, and also provides a basis for the breeding of *P. sylvestris* var. *mongolica* for west of Baicheng, Jilin Province.

Key words *Pinus sylvestris* var. *mongolica*; clones; growth traits; coning yield characteristics; genetic gain

樟子松(*Pinus sylvestris* var. *mongolica*)为松科(Pinaceae)松属(*Pinus*)植物,是我国乃至世界的沙地稀少珍贵树种,其天然分布区主要位于我国大兴安岭和大兴安岭西麓的呼伦贝尔草原红花尔基沙地[1]。樟子松具有耐干旱、耐瘠薄、生长快、木材产量高、适应性强等特点,在沙壤土,粘质盐碱地,砾质粗沙土都能生长[2]。樟子松根系发达,具有较强的防止水土流失、防风固沙能力,是治沙造林的先锋树种[3]。其树干通直,材质优良,耐腐蚀力强、易干燥、易加工,是良好的建筑、造船、家具、木纤维工业原料等用材[4~5]。樟子松是"三北"防护林建设中的主要造林树种,同时也是东北山地人工林建设中三大针叶造林树种(红松、落叶松、樟子松)之一[6],具有极高的生态效益、经济效益和社会效益[7]。

林木种子园是生产培育优良的种子,并且按照人为设计要求而营建的特种人工林[8]。随着林木遗传改良的深入,樟子松的生态价值、经济价值和社会价值不断被发掘,营建高世代樟子松种子园对其树种的开发和利用具有重要意义[9]。我国在20世纪就已经开始对樟子松进行科学研究,主要集中在种源选择[10]、开花结实[11]、引种驯化[12]和土壤改良[13]等方面,对樟子松种子园无性系多性状联合评价选择的研究较少[14],极大影响樟子松种子园的升级换代。本研究以吉林省白城市青山林场的304个樟子松为材料,对其生长及结实性状进行调查研究,初步对不同性状进行综合评价及选择,为吉林省西部樟子松遗传改良及种子园的改建与升级提供基础。

1 材料与方法

1.1 试验地点与材料

试验林位于吉林省白城市洮北区青山镇境内(122°51′E、45°38′N),该地区年平均气温4.2℃,最高气温38.6℃,最低气温-36℃,生长期为140 d左右,年平均降雨量为452 mm,年蒸发量为1 581 mm。樟子松种子园于1984年建成,包括304个樟子松无性系,采用髓心形成层贴接法嫁接,采用完全随机设计,9个大区,每个大区内13个小区,每个小区内304个无性系随机排列(单株小区),株行距5 m×5 m。

1.2 试验方法

于2017年在种子园内选择前1~9个小区,每个小区内全林调查,每个无性系测定9株(单株缺失的顺延到其他小区),对各单株进行树高、胸径、地径、3 m处直径、冠幅、侧枝粗度和分枝角度进行测定,其中树高利用VtrtexⅣ超声波测高测距仪测定,胸径、地径和3 m处直径利用胸径尺测定,利用米尺和游标卡尺测定冠幅和侧枝粗度,利用量角器测定分枝角度;

于2015、2016和2017年对1~6个小区内的各无性系单株进行结实量的调查(单株缺失的顺延到其他小区),具体方法采用收集全株球果数量进行调查(其中3年均结实的只有284个无性系)。

1.3 统计方法

所有数据利用SPSS19.0软件进行分析[15]。其中树高、胸径、地径、3 m处直径、冠幅、分枝角度、侧枝粗度和结实量性状的方差分析线性模型为[16]:

$$X_{ijk} = \mu + C_i + B_j + e_{ijk} \quad (1)$$

式中:μ为总体平均值;C_i为无性系效应;B_j为区组效应;e_{ijk}为环境误差;

根据续九如[17]的方法计算无性系重复力:

$$R = 1 - 1/F \quad (2)$$

式中:F为方差分析的F值。

表型变异系数[18]:

$$PCV = SD/\bar{X} \times 100 \quad (3)$$

式中:SD为表型标准差,$\bar{X}$为某一指标群体平均值。

表型相关分析采用公式[19]:

$$r_{p_{12}} = \frac{Cov_{p_{12}}}{\sqrt{\sigma_{p_1}^2,\sigma_{p_2}^2}} \quad (4)$$

式中:$Cov_{p_{12}}$为2个指标间的表型协方差;$\sigma_{p_1}^2$、$\sigma_{p_2}^2$分别为2个指标间的表型方差(结实量与生长性状的相关分析利用284个无性系进行)。

采用布雷津多性状综合评定法对无性系进行综合评定，具体公式为[20]：

$$Q_i = \sqrt{\sum_{j=1}^{n} ai},\quad ai = X_{ij}/X_{j\max} \qquad (5)$$

式中：Q_i 为综合评价值；X_{ij} 为某一指标的平均值；$X_{j\max}$ 为某一指标的最优值；n 为评价指标的个数。

遗传增益估算[21]：

$$\triangle G = WR/\bar{X} \qquad (6)$$

式中：W 为选择差；R 为性状的遗传力；$\bar{X}$ 为某一指标的平均值。

2　结果与分析

本研究中，各无性系树高、冠幅、地径等指标平均值相对较高，不同无性系间各指标变幅相对较大，可进一步对无性系进行分析评价。

2.1　304 个樟子松无性系各指标方差分析

各指标方差分析见表 1，除冠幅在无性系间及 2016 年总结实量在区组间外，其余变异来源差异均达显著水平（$P<0.05$）。

2.2　304 个樟子松无性系各指标变异参数分析

樟子松无性系各测定指标遗传变异参数见表 2。所有无性系树高平均值为 11.11 m，变幅为 4.00～14.50 m，最大值是最小值的 3.63 倍；冠幅的平均值为 6.03 m，变幅为 4.00～10.57 m，最大值是最小值的 2.64 倍；地径的平均值为 30.04 cm，变幅为 19.98～44.78 cm，最大值是最小值的 2.24 倍；胸径的平均值为 26.29 cm，变幅为 19.78～39.00 cm，最大值是最小值的 1.97 倍；3 m 处直径的平均值为 23.59 cm，变幅为 15.67～34.27 cm，最大值是最小值的 2.19 倍；分枝角度的平均值为 50.53°，变幅为 30.00°～70.00°，最大值是最小值的 2.33 倍；侧枝粗度的平均值为 4.95 cm，变幅为 3.86～7.00 cm，最大值是最小值的 1.81 倍；2015 年结实量的平均值为 1.89，变幅为 1～3，最大值是最小值的 3 倍；2016 年结实量平均值为 65.22，变幅为 0～146，最大值为最小值的 146 倍；2017 年结实量平均值为 22.99，变幅为 15～55，最大值是最小值的 3.67 倍。各指标表型变异系数变化范围为 3.79%～65.22%。其中冠幅、侧枝粗度和 2016 年结实量的表型变异系数均超 30%。分枝角度的表型变异系数低于 10%。各指标的重复力均较高，除冠幅和地径外重复力均超 0.30。

表 1　樟子松无性系各指标方差分析

Table 1　Variance analysis of different traits among *P. sylvestris* var. *mongolica* clones

性状 Traits	种源 Origin	平方和 SS	自由度 *df*	均方 MS	*F*	Sig.
树高 Height	无性系 Clone	2 980.26	303	9.840	2.28	0.000
	区组 Block	3 989.43	8	498.680	115.81	0.000
冠幅 Canopy	无性系 Clone	3 894.28	303	12.850	1.32	0.991
	区组 Block	4 383.90	8	547.990	34.36	0.000
地径 Basal diameter	无性系 Clone	24 145.57	303	79.690	1.32	0.000
	区组 Block	43 591.99	8	5 449.000	90.33	0.000
胸径 Diameter at breast height	无性系 Clone	17 270.45	303	57.000	3.36	0.000
	区组 Block	29 697.34	8	3 712.170	218.93	0.000
3 m 处直径 Diameter at 3 m height	无性系 Clone	20 056.30	303	66.190	1.96	0.000
	区组 Block	39 619.11	8	4 952.390	146.74	0.000
分枝角度 Branch angle	无性系 Clone	75 076.53	303	247.780	1.81	0.000
	区组 Block	66 832.05	8	8 354.010	60.87	0.000
侧枝粗度 Lateral branch thickness	无性系 Clone	948.84	303	3.130	1.77	0.000
	区组 Block	946.60	8	118.330	66.69	0.000
2015 年结实量 Coning quantity of 2015	无性系 Clone	170.43	283	0.602	1.746	0.000
	区组 Block	4.02	5	0.804	2.363	0.038
2016 年结实量 Coning quantity of 2016	无性系 Clone	155 206.31	283	548.432	2.669	0.000
	区组 Block	1 506.99	5	301.399	1.500	0.186
2017 年结实量 Coning quantity of 2017	无性系 Clone	14 951.97	283	52.834	3.343	0.000
	区组 Block	1 236.19	5	247.239	16.826	0.000

注：生长性状测定 304 个无性系，3 年同时结实的无性系只有 284 个。

Note：the growth traits were determined in 304 clones，and only 284 clones had clones at the same time for 3 years.

表 2 樟子松无性系各指标遗传变异参数分析

Table 2 Genetic and variation parameters of different traits in *P. sylvestris* var. *mongolica* clones

性状<br>Traits	平均值<br>Average	变幅<br>Range	标准差<br>SD	重复力<br>R	表型变异系数<br>PCV
树高 Height(m)	11.11	4.00~14.50	1.83	0.56	16.53
冠幅 Canopy(m)	6.03	4.00~10.57	2.41	0.24	41.33
地径 Basal diameter(cm)	30.04	19.98~44.78	8.58	0.24	28.57
胸径 Diameter at breast height(cm)	26.29	19.87~39.00	5.29	0.70	20.13
3 m 处直径 Diameter at 3 m height(cm)	23.59	15.67~34.27	6.92	0.49	29.34
分枝角度 Branch angle(°)	50.53	30.00~70.00	1.69	0.45	3.79
侧枝粗度 Lateral branch thickness	4.95	3.86~7.00	1.50	0.43	30.19
2015 年结实量(个)Coning quantity of 2015	1.89	1.00~3.00	0.61	0.32	41.21
2016 年结实量(个)Coning quantity of 2016	65.22	0.00~146.00	15.56	0.59	65.22
2017 年结实量(个)Coning quantity of 2017	22.99	15.00~55.00	4.59	0.50	22.99

表 3 生长性状与结实量相关性分析

Table 3 Correlation coefficients among growth traits and coning quantity traits

性状<br>Traits	树高<br>Height(m)	冠幅<br>Canopy(m)	地径<br>Basal diameter<br>(cm)	胸径<br>Diameter at breast height<br>(cm)	3 m 处直径<br>Diameter at 3 m height<br>(cm)	分枝角度<br>Branch angle<br>(°)	侧枝粗度<br>Lateral branch thickness	2015 年结实量(个)<br>Coning quantity of 2015	2016 年结实量(个)<br>Coning quantity of 2016
冠幅 Canopy(m)	0.572**								
地径 Basal diameter(cm)	0.661**	0.569**							
胸径<br>Diameter at breast height(cm)	0.712**	0.602**	0.854**						
3 m 处直径<br>Diameter at 3 m height(cm)	0.590**	0.590**	0.896**	0.862**					
分枝角度 Branch angle(°)	-0.064	0.100	0.102	-0.039	0.092				
侧枝粗度<br>Lateral branch thickness	0.263**	0.181**	0.191**	0.335**	0.082	-0.113*			
2015 年结实量<br>Coning quantity of 2015	0.029	0.116*	0.185**	0.122*	0.157**	-0.045	0.141*		
2016 年结实量<br>Coning quantity of 2016	0.029	0.032	-0.062	0.009	-0.106	-0.045	0.173**	0.080	
2017 年结实量<br>Coning quantity of 2017	0.093	-0.042	-0.110	0.008	-0.169**	-0.154**	0.275**	0.036	0.150**

2.3 304 个樟子松无性系各指标相关性分析

各指标相关系数见表 3，从生长性状来看，树高、胸径、地径、冠幅及 3 m 处直径之间达极显著正相关水平(0.181~0.896)，侧枝粗度除了与 3 m 处直径相关未达显著水平外($r=0.082$)，与其他生长性状相关均达极显著水平，分枝角度与其他性状相关均未达显著水平；从结实量与生长性状来看，3 年结实量均与侧枝粗度呈显著正相关水平，2015 年结实量与冠幅、地径、3 m 处直径、胸径和侧枝粗度呈显著正相关水平，2017 年结实量与 3 m 处直径、分枝角度显著负相关，与侧枝粗度显著正相关；从不同树龄结实量来看，2016 年与 2017 年结实量极显著正相关($r=0.150$)。

2.4 304 个樟子松亲无性系多性状综合评价

本文的目的是从樟子松无性系中分别选出生长优良或结实量大的无性系，因此本研究分别以生长性状和结实量对亲本无性系进行多性状综合评价，以不同性状评价樟子松无性系获得的各无性系 Q_i 值见表 4，依据生长性状，以 5% 的入选率对无性系进行多性状综合评价，PS244、PS132、PS131、PS123 和 PS288 等 15 个无性系入选，入选无性系在树高、地径、胸径、3 m 处直径、分枝角度和侧枝粗度等各生长性状的遗传增益分别为 5.47%、4.48%、15.18%、11.78%、2.38% 和 6.66%。依据结实量，以 5% 的入选率对无性系进行多性状综合评价，PS229、PS208、PS301、PS288 和 PS343 等 15 个无性系入选，入选无性系在 2015 年结实量、2016 年结实量和 2017 年结实量的遗传增益分别为 2.89%、46.32% 和 13.88%。

表 4　以不同性状为标准的樟子松无性系多性状综合评价

Tabel 4　Q_i values of *P. sylvestris* var. *mongolica* clones based on the different traits by comprehensive evaluation methods

生长性状 Growth traits				结实性状 Coning quantity			
无性系 Clone	Q_i	无性系 Clone	Q_i	无性系 Clone	Q_i	无性系 Clone	Q_i
PS244	2.46	PS097	2.18	PS229	2.83	PK088	2.15
PS132	2.44	PS186	2.18	PS208	2.75	PK268	2.15
PS131	2.42	PS007	2.18	PS301	2.67	PK009	2.15
PS123	2.40	PS178	2.18	PS288	2.66	PK196	2.15
PS288	2.38	PS087	2.18	PS343	2.64	PK066	2.15
PS118	2.38	PS182	2.18	PS222	2.62	PK160	2.15
PS135	2.37	PS296	2.18	PS255	2.61	PK124	2.14
PS082	2.36	PS280	2.18	PS164	2.59	PK070	2.14
PS170	2.36	PS124	2.18	PS266	2.59	PK181	2.14
PS187	2.36	PS258	2.18	PS192	2.59	PK062	2.14
PS046	2.35	PS026	2.18	PS330	2.56	PK274	2.14
PS158	2.34	PS088	2.18	PS338	2.56	PK217	2.14
PS116	2.34	PS228	2.18	PS324	2.51	PK006	2.14
PS304	2.34	PS218	2.18	PS258	2.50	PK044	2.14
PS119	2.34	PS292	2.17	PS226	2.49	PK252	2.14
PS127	2.34	PS197	2.17	PS031	2.44	PK287	2.13
□	□	□	□	□	□	□	□
□	□	□	□	□	□	□	□
PS316	2.19	PS294	2.01	PS323	2.16	PK086	1.75
PS020	2.19	PS291	2.01	PS248	2.16	PK652	1.74
PS185	2.19	PS235	2.00	PS059	2.15	PK054	1.7
PS237	2.19	PS248	2.00	PS132	2.15	PK072	1.54
PS278	2.19	PS224	2.00				
PS317	2.19	PS232	1.99				
PS006	2.19	PS324	1.99				
PS009	2.19	PS331	1.95				
PS089	2.18	PS338	1.93				

3　讨论

方差分析是在育种研究中评价变异程度的一个重要方法[22]。在本研究中,304 个无性系间除冠幅外各性状均达极显著差异水平($P<0.01$),各区组间,2015 年结实量达显著水平($P=0.038$),2016 年结实量未达显著差异水平($P=0.186$),其他指标均达极显著差异水平($P<0.01$),说明本研究中对樟子松无性系的评价和选择是具有可行性和必要性的。

遗传和变异是林木育种研究的主要内容[23]。在本研究中,各指标表型变异系数变化范围为 3.79% ~65.22%。这与夏德安[7]对樟子松的研究相似,其中冠幅、侧枝粗度和 2016 年结实量的表型变异系数超过 30%。分枝角度的表型变异系数低于 10%。各指标的重复力较高,除冠幅(0.24)和地径(0.24)外重复力均超 0.30,这与刘文线[24]对樟子松的研究相似。重复力可以体现性状的稳定程度,重复力越大性状越稳定,受环境作用越小,选择的效果越好[25]本研究中,各性状具有高变异、高重复力有利于樟子松无性系的评价选择。

相关性分析可以体现各变量之间的关系[26]。在本研究中,从生长性状来看,除分枝角度与其他生长性状相关未达显著水平外,其他生长性状均达到极显著正相关水平(0.181 ~0.896),这说明在樟子松各生长性状间存在着一定的相互作用。从结实量来看,2015 年结实与地径和 3 m 处直径呈极显著正相关($r=0.185$,$r=0.122$,$r=0.157$),与冠幅、胸径和侧枝粗度达到显著水平($r=$

0.116,r=0.122,r=0.141)。2016 年结实与侧枝粗度达到极显著正相关(r=0.173),其他均未达到显著水平。2016 与 2017 年结实和侧枝粗度呈极显著正相关,与 3 m 处直径和分枝角度呈极显著负相关,其他未与 2017 年结实达到显著水平。根据孙洪志[27]在对沙地樟子松的结实规律的研究中提到,樟子松的结实与林分密度,林木生长发育的不同,在结实上有所差异,同时樟子松的结实量存在丰欠年的状况,可以通过以往的樟子松的结实量推测出未来樟子松的结实量情况。

多性状综合评价的方法有很多,不同的评价方法有不同的优点[28]。本研究利用布雷津多性状综合评价法,利用不同性状标准化处理后的数据计算 Q_i 值,计算结果更有意义。本研究分别以生长性状和结实量对无性系进行多性状综合评价,依据生长性状,以 5% 的入选率对无性系进行多性状综合评价,无性系 PS244、PS132、PS131、PS123 和 PS288 等 15 个无性系入选,入选无性系在树高、地径、胸径、3 m 处直径、分枝角度和侧枝粗度等各生长性状的遗传增益分别为 5.47%、4.48%、15.18%、11.78%、2.38% 和 6.66%。依据结实量,以 5% 的入选率对无性系进行多性状综合评价,无性系 PS229、PS208、PS301、PS288 和 PS343 等 15 个无性系入选,入选无性系在 2015 年结实量、2016 年结实量和 2017 年结实量的遗传增益分别为 2.89%、46.32% 和 13.88%。其中无性系 PS288 同时具有优良的生长性状和结实量性状,可以将其作为优良无性系进行大量扩繁,以获得在生长性状和结实量两个方面同时优良的无性系。

参 考 文 献

[1] 刘桂丰,褚延广,时玉龙,等. 17 年生帽儿山地区樟子松种源试验[J]. 东北林业大学学报,2003,31(4):1-3.

Liu G F,Chu Y G,Shi Y L,et al. The provenance test of 17-year-old *Pinus sylvestris* var. *mongolica* at Maoershan area[J]. Journal of Northeast Forestry University,2003,31(4):1-3.

[2] 谢瑞芬,王虎林. 樟子松沙地抗旱造林关键技术研究[J]. 山西农经,2018(17):64.

Xie R F,Wang H L. Key Technologies of drought-resistant afforestation with *Pinus sylvestris* var. *mongolica* in sandland[J]. Shan Xi Agricultural Economy,2018(17):64.

[3] 苗禹博,朱晓梅,李志娟,等. 不同世代樟子松育种资源遗传评价[J]. 北京林业大学学报,2017,39(12):71-78.

Miao Y B,Zhu X M,Li Z J,et al. Genetic evaluation of breeding resources of *Pinus sylvestris* var. *mongolica* from different improved generations[J]. Journal of Beijing Forestry University,2017,39(12):71-78.

[4] 火树华. 树木学[M]. 北京:中国林业出版社,1992.

Huo S H. Dendrology[M]. Beijing:China Forestry Publishing House,1992.

[5] 刘平. 樟子松的生长规律及影响因子的研究[D]. 保定:河北农业大学,2009.

Liu P. Study on the growth law of *Pinus sylvestris* var. *mongolica* and the influential factors[D]. Baoding:Agricultural University of Hebei,2009.

[6] 石艳丽. 沙地樟子松球果产量测报的研究[D]. 哈尔滨:东北林业大学,2005.

Shi Y L. Study of cone production prediction for scotch pine in the sandy area[D]. Harbin:Northeast Forestry University,2005.

[7] 夏德安,杨书文,安扎布,等. 樟子松天然林生长性状早期选择的研究[J]. 东北林业大学学报,1990,18(S2):89-93.

Xia D A,Yang S W,An Z B,et al. Growth variation and early selection of *Pinus sylvestris* var. *mongolica* from natural forests[J]. Journal of Northeast Forestry University,1990,18(S2):89-93.

[8] 王昊. 林木种子园研究现状与发展趋势[J]. 世界林业研究,2013,26(4):32-37.

Wang H. Research progress and development trend of tree seed orchard[J]. World Forestry Research,2013,26(4):32-37.

[9] 贯春雨,王福森,李树森,等. 樟子松第二代无性系种子园建立与经营管理技术[J]. 防护林科技,2017(1):126-127.

Guan C Y,Wang F S,Li S S,et al. Establishment and management technology of the second generation clonal seed orchard of *Pinus sylvestris*[J]. Protection Forest Science and Technology,2017(1):126-127.

[10] 秦泗华,扬传平,张鹏,等. 樟子松种内的地理变异以及最佳种源的初步选择[J]. 东北林业大学学报,1990,18(S2):40-48.

Qin S H,Yang C P,Zhang P,et al. Intraspecific geographic variation and primary selection of the best provenance of *Pinus sylvestris* var. *mongolica*[J]. Journal of Northeast Forestry University,1990,18(S2):40-48.

[11] 刘恩海,昝德平,刘兴刚,等. 樟子松开花结实规律的研究(I)——樟子松球果变异与种子预测方法的关系[J]. 林业科技,1995,20(2):19-21,16.

Liu E H,Jiu D P,Liu X G,et al. The laws of blossoming and fruiting in *Pinus sylvestris* [J]. Forestry Science & Technology,1995,20(2):19-21,16.

[12] 兰士波. 樟子松引种驯化及遗传效应综合评价[J]. 安徽农业科学,2014,42(27):9412-9414,9417.
Lan S B. Introduction,domestication and comprehensive evaluation on heredity effects of *Pinus sylvestris* [J]. Journal of Anhui Agricultural Sciences,2014,42(27):9412-9414,9417.

[13] 朱弘,宋金辉,王红梅. 樟子松种子园土壤改良与母树生长的关系[J]. 防护林科技,2011(2):36-37.
Zhu H,Song J H,Wang H M. Relationship between soil improvement & growth of mother trees of *Pinus sylvestris* var. *mongolica* seed orchard [J]. Protection Forest Science and Technology,2011(2):36-37.

[14] Watson P,Bradley M. Canadian pulp fibre morphology: superiority and considerations for end use potential[J]. The Forestry Chronicle,2009,85(3):401-408.

[15] 李玉光,杜宏巍,黄永生. SPSS 19.0 统计分析入门与提高[M]. 北京:清华大学出版社,2014.
Li Y G,Du H W,Huang Y S. Introduction and improvement of statistical analysis of SPSS 19.0[M]. Beijing: Tsinghua University Press,2014.

[16] Hansen J,Roulund H. Genetic parameters for spiral grain,stem form,pilodyn and growth in 13 years old clones of Sitka Spruce(*Picea sitchensis*(Bong.) Carr.) [J]. Silvae Genetica,1997,46:107-113.

[17] 续九如. 林木数量遗传学[M]. 北京:高等教育出版社,2006.
Xu J R. Quantitative genetics in forestry[M]. Beijing: Higher Education Press,2006.

[18] Hai P H,Jansson G,Harwood C,et al. Genetic variation in growth,stem straightness and branch thickness in clonal trials of *Acacia auriculiformis* at three contrasting sites in Vietnam[J]. Forest Ecology and Management,2008,255(1):156-167.

[19] 梁德洋,金允哲,赵光浩,等. 50 个红松无性系生长与木材性状变异研究[J]. 北京林业大学学报,2016,38(6):51-59.
Liang D Y,Jin Y Z,Zhao G H,et al. Variance analyses of growth and wood characteristics of 50 *Pinus koraiensis* clones[J]. Journal of Beijing Forestry University,2016,38(6):51-59.

[20] Liu M R,Yin S P,Si D J,et al. Variation and genetic stability analyses of transgenic *TaLEA* poplar clones from four different sites in China[J]. Euphytica,2015,206(2):331-342.

[21] 赵曦阳,李颖,赵丽,等. 不同地点白杨杂种无性系生长和适应性表现分析和评价[J]. 北京林业大学学报,2013,35(6):7-14.
Zhao X Y,Li Y,Zhao L,et al. Analysis and evaluation of growth and adaptive performance of white poplar hybrid clones in different sites[J]. Journal of Beijing Forestry University,2013,35(6):7-14.

[22] Safavi S A,Pourdad S A,Taeb M,et al. Assessment of genetic variation among safflower(*Carthamus tinctorius* L.) accessions using agro-morphological traits and molecular markers[J]. Journal of Food,Agriculture and Environment,2010,8:616-625.

[23] Mwase W F,Savill P S,Hemery G E. Genetic parameter estimates for growth and form traits in common ash (*Fraxinus excelsior* L.) in a breeding seeding orchard at little wittenham in England[J]. New Forests,2008,36(3):225-238.

[24] 刘文线. 樟子松半同胞子代遗传增益分析[J]. 林业勘察设计,2013(4):12-14.
Liu W X. Genetic gain of analysis on half-sib progenies of *Pinus sylvestris* L. [J]. Forestry Prospect and Design,2013(4):12-14.

[25] 吴裕,毛常丽. 树木育种学中遗传力、重复力和遗传增益的概念及思考[J]. 热带农业科技,2012,35(1):47-50.
Wu Y,Mao C L. Simple introduction to heritability,repeatability and genetic gain in percent in tree breeding [J]. Tropical Agricultural Science & Technology,2012,35(1):47-50.

[26] Pang F H,Yang J W,Pang Z L,et al. The correlation analysis between *Populus simonii* ecophysiological indexes and environmental factors[J]. Acta Ecologica Sinica,2010,30(12):3188-3197.

[27] 孙洪志,石丽艳. 沙地樟子松的结实规律[J]. 东北林业大学学报,2004,32(4):6-8.
Sun H Z,Shi L Y. The law of seed harvest of scotch pine in the sandy area[J]. Journal of Northeast Forestry University,2004,32(4):6-8.

[28] 骆汝九,胡治球,宋雯,等. 多性状综合评定的秩和差测验方法[J]. 中国农业科学,2010,43(10):2008-2015.
Luo R J,Hu Z Q,Song W,et al. A rank-sum-difference testing method for multi-trait comprehensive ranking [J]. Scientia Agricultura Sinica,2010,43(10):2008-2015.
