# Supplementary material for: Provenance and family variations in early growth of Manchurian walnut (Juglans mandshurica Maxim.) and selection of superior families
Source: PLoS One. 2024 Mar 7;19(3):e0298918. doi: 10.1371/journal.pone.0298918 (PMC10919699; doi:10.1371/journal.pone.0298918)
Supplement: S2 File — (ZIP) [file pone.0298918.s005.zip › Variation analysis on growth traits of 39 Juglans mandshurica families.pdf]

DOI:10.16115/j.cnki.issn.1005－7129.2020.06.001
文章编号:1005－7129(2020)06－0001－04 中图分类号:S792.132 文献标识码:A

# 39个核桃楸家系苗期生长性状变异分析

韩玉霞[1,2],吴 琳[1,2],于 琪[1,2],陈 华[1,2],杨雨春[3],王 芳[3],王学春[4],李 佳[5]
(1.吉林省露水河林业局,吉林 白山 134300;2.吉林森工露水河遗传育种国家长期科研基地,吉林 白山 134300;3.吉林省林业科学研究院,吉林 长春 130033;4.桦甸市常山林场,吉林 吉林 132419;5.长春市九台区气象局,吉林 长春 130500)

**摘要:** 以吉林省白山市露水河林业局清水河林场内39个4年生核桃楸家系为材料,对其树高、地径进行调查并分析。结果表明:树高和地径在不同家系间、不同区组间、不同区组×家系间均达到极显著差异($P<0.01$)水平。所有核桃楸家系的树高平均值为0.71 m,地径平均值为1.87 cm;树高与地径的变异系数分别为36.62 %和36.36 %;树高与地径的遗传力分别为0.80和0.81。相关分析结果表明,树高与地径呈极显著正相关关系。利用综合评价法,以10 %的入选率选出优良家系4个。
**关键词:** 核桃楸;家系;生长性状;变异

# Variation Analysis on Growth Traits of 39 *Juglans mandshurica* Families

HAN Yuxia[1,2], WU Lin[1,2], YU Qi[1,2], CHEN Hua[1,2], YANG Yuchun[3], WANG Fang[3], WANG Xuechun[4], LI Jia[5]
(1. Lushuihe Forestry Bureau of Jilin Province, Baishan 134300, China; 2. Jilin Forest Industry Lushuihe Genetic Breeding National Long－term Scientific Research Base, Baishan 134300, China; 3. Jinlin Provincial Academy of Forestry Science, Changchun 130033, China; 4. Changshan Forest Center of Huadian City, Jilin 132419, China; 5. Meteorological Bureau of Jiutai Area Changchun City, Changchun 130500, China)

**Abstract:** In this study, 39 4－year－old *Juglans mandshurica* families in Qingshui Forest Farm, Lushuihe Forestry Bureau, Baishan city, Jilin Province were taken as materials, tree height and ground diameter were investigated and analyzed. The results showed that tree height and ground diameter were all significantly different between different families, between different groups, and between different groups × families ($P<0.01$). The mean of tree height and ground diameter of all *J. mandshurica* families were 0.71 m and 1.87 cm, respectively. The variation coefficients of tree height and ground diameter were 36.62 % and 36.36 %, respectively. The heritability of tree height and ground diameter were 0.80 and 0.81, respectively. The correlation analysis results showed that there was an extremely significant positive correlation between the tree height and ground diameter. Using the method of comprehensive evaluation, 4 excellent families were selected with a selection rate of 10 %.
**Keywords:** *Juglans mandshurica*; families; growth traits; variation

核桃楸(*Juglans mandshurica*)是胡桃科(Juglandaceae)胡桃属(*Juglans*)阔叶乔木,与水曲柳(*Fraxinus mandshurica*)、黄檗(*Phellodendron amurense*)并称为“东北三大硬阔”树种[1],主要分布于我国东北及华北地区[2]。其不仅木材优良[3],而且是珍贵的木本油料树种之一[4]。

目前我国核桃楸良种选育研究主要以种源选择与家系选择为主要方向[5]。褚宪丽等通过对青山林场45个15年生的核桃楸家系试验

收稿日期:2020—10—31
基金项目:“十三五”科技攻关项目子课题(2016YFD0600605－02);吉林省科技攻关项目(20160203010NY);吉林省林业科技项目(2014－006)
作者简介:韩玉霞(1968—),女,吉林白山人,高级工程师,主要从事林木遗传育种工作.
通讯作者简介:王芳(1988—),女,山西晋城人,助理研究员,主要从事林木遗传育种工作.



(C)1994-2022 China Academic Journal Electronic Publishing House. All rights reserved. http://www.cnki.net

林的生长性状进行调查分析，最终选出 9 个优良家系[6]。本文以吉林省白山市露水河林业局清水河林场的 39 个 4 年生核桃楸家系为材料，对其树高和地径进行每木调查，分析各性状的变异参数，利用多性状综合评价法选出优良家系，为核桃楸的良种选育提供基础。

## 1 材料与方法

### 1.1 材料来源

试验材料包括 39 个核桃楸家系(JM1 – JM39)，种源主要来源于东北地区，2014 年进行春季播种(种子经过 2013 年整个冬季的混沙层积处理)，2016 年春季造林，试验林位于吉林省白山市抚松县清水河林场，造林采用随机区组设计，8 株小区，4 次重复，株行距为 3.0 m ×3.0 m。

### 1.2 试验方法

2018 年 10 月，对 39 个 4 年生核桃楸家系幼苗的树高和地径进行调查，树高利用卷尺测定，地径使用游标卡尺测定。

### 1.3 数据分析

利用 SPSS19.0 统计软件对数据进行分析，树高和地径的方差分析模型如下：

$$X_{ijk} = \mu + a_i + b_j + ab_{ij} + e_{ijk}$$

式中：$X_{ijk}$ 表示家系 $i$ 在区组 $j$ 中的核桃楸单株 $k$ 的表型；$\mu$ 表示总体平均值；$a_i$ 表示家系效应；$b_j$ 表示区组效应；$ab_{ij}$ 表示家系与区组互作效应；$e_{ijk}$ 表示环境误差。

相关分析采用的计算公式如下[7]：

$$r_{p_{12}} = Cov_{p_{12}} / \sqrt{\sigma_{p_1}^2 \sigma_{p_2}^2}$$

式中：$r_{p_{12}}$ 表示性状 1 与性状 2 间的表型相关系数；$Cov_{p_{12}}$ 表示性状 1 与性状 2 之间的协方差；$\sigma_{p_1}^2$ 与 $\sigma_{p_2}^2$ 分别表示性状 1 与性状 2 的表型方差。

多性状综合评价计算公式如下[8]：

$$Q_i = \sqrt{\sum_{j=1}^{n} a_i}，其中\ a_i = X_{ij} / X_{j\max}$$

式中：$Q_i$ 表示多性状综合评价值；$X_{ij}$ 表示家系 $i$ 中性状 $j$ 的平均值；$X_{j\max}$ 表示性状 $j$ 的最大值；$n$ 表示需要评价的性状个数。

## 2 结果

### 2.1 方差分析

39 个核桃楸家系子代幼苗的各生长性状的方差分析结果见表 1。树高和地径这两个生长性状在不同家系间、不同区组间、不同区组 × 家系均达到极显著差异($P < 0.01$)。

表 1 各性状的方差分析

Tab. 1 Variance analysis of each trait

| 性状 | 变异来源 | *df* | *MS* | *F* | *Sig.* |
|---|---|---|---|---|---|
| 树高 | 区组 | 3 | 0.733 | 12.921 | 0.000 |
| | 家系 | 38 | 0.280 | 4.941 | 0.000 |
| | 区组 × 家系 | 114 | 0.112 | 1.969 | 0.000 |
| 地径 | 区组 | 3 | 2.905 | 7.504 | 0.000 |
| | 家系 | 38 | 2.051 | 5.298 | 0.000 |
| | 区组 × 家系 | 114 | 0.851 | 2.198 | 0.000 |

### 2.2 核桃楸家系各性状的变异参数分析

39 个核桃楸家系子代的树高和地径的变异参数见表 2。所有核桃楸家系的树高平均值为 0.71 m，变幅为 0.53 ~ 0.87 m；地径平均值为 1.87 cm，变幅为 1.21 ~ 2.32 cm。树高与地径的变异系数相似，分别为 36.62 % 和 36.36 %。树高与地径的遗传力均较高，分别为 0.80 和 0.81。

表 2 各性状平均值及变异系数

Tab. 2 Mean value and variation coefficient of each trait

| 性状 | 平均值 | 变幅 | 标准差 | 变异系数 | 遗传力 |
|---|---|---|---|---|---|
| 树高 | 0.71 m | 0.53 ~ 0.87 m | 0.26 | 36.62 % | 0.80 |
| 地径 | 1.87 cm | 1.21 ~ 2.32 cm | 0.68 | 36.36 % | 0.81 |

### 2.3 核桃楸家系各性状的平均值分析

39 个核桃楸家系的各性状的平均值见表 3。家系 JM1、JM3、JM9、JM12 和 JM38 的树高较高，均超过了 0.80 m，而家系 JM22、JM26、JM28 和 JM32 的树高较小，均小于 0.60 m。家系 JM9、JM12、JM34、JM36 和 JM38 的地径较粗，均超过 2.10 cm，而家系 JM16、JM22、JM23、JM26、JM28 和 JM32 的地径均小于 1.60 cm。



(C)1994-2022 China Academic Journal Electronic Publishing House. All rights reserved. http://www.cnki.net

表 3　各家系中不同性状的平均值

Tab. 3　The mean values of different growth traits for each family

| 家系 | 树高/m | 地径/cm | 家系 | 树高/m | 地径/cm |
|---|---|---|---|---|---|
| JM1 | 0.83 ±0.35 | 1.95 ±0.61 | JM21 | 0.68 ±0.24 | 1.82 ±0.58 |
| JM2 | 0.74 ±0.20 | 2.02 ±0.49 | JM22 | 0.53 ±0.14 | 1.21 ±0.38 |
| JM3 | 0.84 ±0.35 | 2.01 ±0.67 | JM23 | 0.61 ±0.21 | 1.54 ±0.51 |
| JM4 | 0.68 ±0.23 | 1.92 ±0.58 | JM24 | 0.79 ±0.34 | 2.09 ±0.81 |
| JM5 | 0.73 ±0.21 | 1.98 ±0.67 | JM25 | 0.70 ±0.22 | 1.88 ±0.73 |
| JM6 | 0.65 ±0.24 | 1.87 ±0.62 | JM26 | 0.56 ±0.19 | 1.52 ±0.53 |
| JM7 | 0.64 ±0.26 | 1.76 ±0.69 | JM27 | 0.65 ±0.27 | 2.02 ±0.75 |
| JM8 | 0.67 ±0.24 | 1.85 ±0.51 | JM28 | 0.56 ±0.19 | 1.55 ±0.51 |
| JM9 | 0.87 ±0.20 | 2.21 ±0.57 | JM29 | 0.60 ±0.22 | 1.67 ±0.73 |
| JM10 | 0.73 ±0.28 | 1.81 ±0.75 | JM30 | 0.75 ±0.25 | 1.91 ±0.52 |
| JM11 | 0.69 ±0.31 | 1.84 ±0.83 | JM31 | 0.71 ±0.27 | 1.78 ±0.62 |
| JM12 | 0.82 ±0.23 | 2.32 ±0.89 | JM32 | 0.55 ±0.19 | 1.44 ±0.68 |
| JM13 | 0.68 ±0.21 | 1.90 ±0.50 | JM33 | 0.73 ±0.24 | 2.04 ±0.75 |
| JM14 | 0.73 ±0.22 | 1.74 ±0.51 | JM34 | 0.78 ±0.27 | 2.11 ±0.73 |
| JM15 | 0.74 ±0.26 | 1.73 ±0.58 | JM35 | 0.77 ±0.22 | 2.10 ±0.69 |
| JM16 | 0.64 ±0.21 | 1.54 ±0.60 | JM36 | 0.71 ±0.24 | 2.17 ±0.75 |
| JM17 | 0.76 ±0.24 | 1.98 ±0.73 | JM37 | 0.76 ±0.25 | 2.00 ±0.57 |
| JM18 | 0.75 ±0.28 | 1.85 ±0.70 | JM38 | 0.83 ±0.28 | 2.12 ±0.75 |
| JM19 | 0.71 ±0.27 | 1.82 ±0.63 | JM39 | 0.73 ±0.25 | 1.94 ±0.53 |
| JM20 | 0.62 ±0.28 | 1.82 ±0.85 | | | |

## 2.4 表型相关分析

树高与地径的表型相关分析见表4。核桃楸家系的树高与地径呈极显著正相关关系(0.711)。

表 4　树高与地径的表型相关分析

Tab. 4　Correlation analysis between tree height and ground diameter

| 性状 | 地径 |
|---|---|
| 树高 | 0.711** |

注: ** 表示在 0.01 水平(双侧)上显著相关。

## 2.5 多性状综合评价

利用树高与地径两性状对核桃楸家系进行综合评价,评价结果 $Q_i$ 值见表5。以10%的入选率对39个核桃楸家系进行评价选择,家系JM9、JM12、JM38和JM3入选。入选的4个家系树高的平均值为0.84 m,比总体平均值提高0.13 m,遗传增益为14.65%;地径的平均值为2.16 cm,比总体平均值提高0.29 cm,遗传增益为12.68%。

表 5　各家系的多性状综合评价

Tab. 5　Comprehensive evaluation of multiple traits for each family

| 家系 | $Q_i$ 值 | 家系 | $Q_i$ 值 |
|---|---|---|---|
| JM9 | 1.40 | JM4 | 1.27 |
| JM12 | 1.39 | JM13 | 1.27 |
| JM38 | 1.37 | JM19 | 1.27 |



(C)1994-2022 China Academic Journal Electronic Publishing House. All rights reserved.　http://www.cnki.net

表5(续)

| 家系 | $Q_i$ 值 | 家系 | $Q_i$ 值 |
|---|---|---|---|
| JM3 | 1.36 | JM15 | 1.26 |
| JM24 | 1.35 | JM14 | 1.26 |
| JM34 | 1.34 | JM31 | 1.26 |
| JM1 | 1.34 | JM11 | 1.26 |
| JM35 | 1.34 | JM21 | 1.25 |
| JM36 | 1.33 | JM8 | 1.25 |
| JM37 | 1.32 | JM6 | 1.24 |
| JM17 | 1.32 | JM20 | 1.22 |
| JM2 | 1.31 | JM7 | 1.22 |
| JM33 | 1.31 | JM29 | 1.19 |
| JM5 | 1.30 | JM16 | 1.18 |
| JM30 | 1.30 | JM23 | 1.17 |
| JM39 | 1.30 | JM28 | 1.14 |
| JM18 | 1.29 | JM26 | 1.14 |
| JM25 | 1.27 | JM32 | 1.12 |
| JM27 | 1.27 | JM22 | 1.07 |
| JM10 | 1.27 | | |

## 3 结论

不同家系间存在丰富的变异,方差分析结果表明,不同核桃楸家系间的树高与地径差异均极显著,这为优良家系的选择提供了基础[9]。变异系数和遗传力是林木数量遗传学中家系评价选择的两个重要指标,分别表示各性状的变异程度和遗传能力[10]。本研究中树高与地径的变异系数均约为36.00 %,遗传力均约为0.80,属于高的变异和遗传力。相关分析为综合评价指标的选择提供依据[8],本研究中树高与地径呈极显著正相关关系,因此,将树高与地径结合进行多性状综合评价,最终以10 %的入选率筛选出4个优良家系。本研究结果将为核桃楸良种选育提供基础,并为当地以及周边相似地区造林提供材料。

参考文献


[1] 王宇. 东北地区胡桃楸遗传多样性SRAP研究[D]. 哈尔滨: 东北林业大学,2007.

[2] 张含国,邓继峰,张磊,等. 胡桃楸种源家系变异规律及家系选择研究[J]. 西北林学院学报,2011(2):91-95.

[3] 朱力国,张军,徐福成. 黑河胡桃楸9个家系变异规律初报[J]. 中国林副特产,2018,157(6):45-48.

[4] 于阳阳. 东北核桃楸种仁蛋白提取及降血压肽制备的研究[D]. 哈尔滨: 东北林业大学,2012.

[5] 冯健,陆爱君. 我国核桃楸遗传育种研究进展[J]. 安徽农业科学,2014(30):10721-10723.

[6] 褚宪丽,朱航勇,张含国,等. 胡桃楸种源家系变异与选择[J]. 东北林业大学学报,2010,38(11):5-6.

[7] Wang F, Zhang QH, Tian YG, et al. Comprehensive assessment of growth traits and wood properties in half-sib *Pinus koraiensis* families [J]. *Euphytica*, 2018, 214(11): 202-217.

[8] 王芳,王元兴,王成录,等. 红松优树半同胞子代家系生长、结实及抗病虫能力的变异特征[J]. 应用生态学报,2019,5(30):1679-1686.

[9] 张秦徽,王洪武,姜国云,等. 红松半同胞家系变异分析及选择研究[J]. 植物研究,2019,39(4):557-567.

[10] 王芳,陆志民,王元兴,等. 233个红松优树半同胞家系的生长性状变异研究[J]. 吉林林业科技,2020,49(2):1-4.


(本篇专家编审:陈建军)



(C)1994-2022 China Academic Journal Electronic Publishing House. All rights reserved. http://www.cnki.net
