# Supplementary material for: Provenance and family variations in early growth of Manchurian walnut (Juglans mandshurica Maxim.) and selection of superior families
Source: PLoS One. 2024 Mar 7;19(3):e0298918. doi: 10.1371/journal.pone.0298918 (PMC10919699; doi:10.1371/journal.pone.0298918)
Supplement: S2 File — (ZIP) [file pone.0298918.s005.zip › Variation and selection of seedling growth traits among Juglans mandshurica provenances and families.pdf]

2022, 35(1): 20-30
http://www.lykxyj.com

林 业 科 学 研 究
Forest Research

DOI:10.13275/j.cnki.lykxyj.2022.01.003

胡桃楸种源家系幼龄期生长变异及选择研究

芦贤博[1]，徐连峰[2]，庞忠义[3]，刘继锋[4]，温宝阳[2]，裴晓娜[5,6]，
赵　岭[2]，王学刚[4]，赵曦阳[1*]

(1. 林木遗传育种国家重点实验室东北林业大学，黑龙江 哈尔滨　150040；2. 黑龙江省林科院齐齐哈尔分院，黑龙江 齐齐哈尔　161005；3. 国有新民市机械林场，辽宁 沈阳　110300；4. 宾县万人欢林场，黑龙江 哈尔滨　150411；5. 国家林业和草原局哈尔滨林业机械研究所，黑龙江 哈尔滨，150086；6. 中国林科院寒温带林业研究中心，黑龙江 哈尔滨，150086)

摘要:［目的］通过分析胡桃楸种源与家系间生长性状的遗传变异规律，对其进行初步选择，以推进胡桃楸良种选育工作。［方法］对万人欢林场胡桃楸 4 个种源内的 28 个家系（6 a）生长性状（树高、树高年均增长量、地径、冠幅、通直度、分枝角、侧枝数和尖削度）进行了测定分析。［结果］方差分析结果表明，大部分性状在各变异来源间均达到极显著差异（$p < 0.01$）；不同性状的表型变异系数和遗传变异系数变化范围分别为 14.049%～41.519% 和 8.754%～23.873%；家系遗传力和单株遗传力的变化范围分别为 0.284～0.797 和 0.110～0.886；相关性分析结果表明，除尖削度与分枝角、尖削度与侧枝数以及通直度与冠幅之间的相关性未达显著水平外，其余各性状之间均达到显著水平（$p < 0.05$）。一般配合力分析结果表明，以 20% 的入选率，每个性状均筛选出 6 个优良亲本。主成分分析结果表明，3 个主成分的累计贡献率达 73.264%，表明 3 个主成分包含了大部分测定性状信息。根据主成分综合得分，初步选出 1 个优良种源（万人欢）与 3 个优良家系（WRH3、WRH5、QTH2）及 10 个优良单株，入选种源树高、树高年均增长量、地径及冠幅现实增益分别为 3.109%、5.014%、2.193%、1.040%；入选家系相同性状的遗传增益分别为 5.835%、5.410%、7.908%、6.069%，入选单株相同性状的遗传增益分别为 26.74%、24.11%、23.91% 和 26.53%。［结论］胡桃楸的生长性状在种源和家系间存在丰富的变异，初步筛选的优良种源、亲本及家系和单株可以为胡桃楸良种选育与应用提供基础。

关键词: 胡桃楸；半同胞家系；种源选择；家系选择；遗传增益
中图分类号: S722.3+1; S792.132　　**文献标志码:** A　　**文章编号:** 1001-1498(2022)01-0020-11

胡桃楸（*Juglans mandshurica* Maxim.）为胡桃科（Juglandaceae）胡桃属（*Juglans*）落叶乔木。在我国主要分布于东北地区的小兴安岭、完达山脉、长白山区和辽宁东部[1]，是我国Ⅱ级珍稀树种和中国珍稀濒危树种的三级保护植物[2]，与黄菠萝、水曲柳并称为“东北三大硬阔”。胡桃楸树干通直圆满，材质坚硬致密，木材弹性好、易加工，被广泛用于军工、船舶、家具、居室装修及乐器制造等方面，是优良的用材树种[3]；其果实果仁营养丰富，含油量高，可制作油料和保健食品，也可作为一些特色食品的添加剂原料；胡桃楸还具有一定的药用价值，其青果、枝皮及种仁均可入药[4-5]；并且，其新鲜根皮、枝皮、青果皮中均含有药用价值较高的化学物质——胡桃醌[6]，具有极强的抗菌[7-8] 及抗肿瘤作用[9]，目前在临床医学及医药化学领域中得到广泛应用。

林木幼龄期选择是根据林木优良性状在早、晚龄间存在的正相关关系,针对其幼龄期的性状表现，进行优良个体选择[10]。自 20 世纪 80 年代以来，国内外学者已对多个树种进行幼龄期选择研

收稿日期：2021-03-27　　修回日期：2021-09-24
基金项目：林业公益性行业科研专项：东北乡土木本粮油树种良种选育及利用研究（201304704）
* 通讯作者: 赵曦阳. E-mail：zhaoxyphd@163.com

究，并取得重大进展[11]。在胡桃楸方面，刘桂丰等[12]和颜廷武等[13]的研究表明，幼龄期的胡桃楸已具有代表性，因此，对胡桃楸进行幼龄期选择是可行且有效的。育种专家已经对胡桃楸进行种源区划，并对少数种源、家系进行初步评价[14]。但是存在选育的良种少，繁殖系数低，很难满足东北林区造林需求等问题，并且由于胡桃楸用途广泛，导致人为的过度采伐，野生胡桃楸资源遭到严重破坏，近年来在林业生产上虽然对胡桃楸进行了大面积人工栽培，但多利用现有的普通胡桃楸造林，在生长和木材产量方面仅表现出较低的增益，因此，胡桃楸的良种选育工作意义重大，不同生态区、不同用途良种选育迫在眉睫。本研究以黑龙江省不同种源内的 28 个胡桃楸半同胞家系为材料，对其 6 年生生长性状的遗传变异进行分析，初步筛选优良亲本、优良家系和单株，为胡桃楸用材林的良种选育提供基础。

1　材料与方法

1.1　试验地点与试验材料

试验地位于黑龙江省宾县万人欢林场（128°04′29″～128°19′13″E，45°44′20″～45°57′18″N），该地平均海拔 340 m，年平均温度 2.4℃，年降水量 600～800 mm，无霜期 130 d 左右，植物生长期 140 d 左右，年日照时数 2 560 h。

试验材料包括黑龙江省万人欢、七台河、牡丹江、宝清等 4 个种源的 28 个胡桃楸半同胞家系，其中，万人欢种源有 14 个家系，七台河种源有 7 个家系，牡丹江种源有 4 个家系，宝清种源有 3 个家系。不同种源的地理气象因子概况见表 1。2015 年春季育苗，2016 年定植。采用随机完全区组试验设计，3 次重复，12 株小区，株行距为 3 m×4 m，周围设置保护行。

表 1　胡桃楸不同种源地理气象因子

Table 1　The geographical and meteorological factors of different *J. mandshurica* provenances

种源 Provenances	北纬 North latitude	东经 East longitude	海拔 Altitude/m	年均温 Average annual temperature/℃	年无霜期 Frost-free period/d	年日照时数 Annual sunshine hours/h	年降水量 Annual recipitation/mm
宝清 Baoqing	46°45′	132°48′	83	3.2	145	2 491	548.6
牡丹江 Mudanjiang	44°60′	129°58′	230	4.3	126	2 305	540.0
七台河 Qitaihe	45° 45'	130° 35'	235	4.1	152	2 484	518.5
万人欢 Wanrenhuan	45°50′	128°12′	340	2.4	130	2 560	700.0

1.2　试验方法

于 2020 年 11 月对 6 年生的胡桃楸半同胞家系的树高、地径、冠幅、分枝角、通直度、侧枝数等性状进行测量。利用塔尺测量树高；利用围尺测量地径；利用钢卷尺测量冠幅（分别测量东西和南北两个方向后取平均值）；利用量角器测量分枝角；利用分级法确定通直度[15]，具体见表 2；利用地径和树高比值计算尖削度计算单株尖削度[16]；连续测定近 3 年树高生长量，利用平均值计算树高年均生长量。

表 2　通直度调查评判标准及分值

Table 2　The research criteria and score of stem straightness degree

性状 Trait	分值 Scores				
	1	2	3	4	5
通直度 Stem straightness degree	树干有2 段以上明显弯曲	树干有2段以上稍微弯曲或1段明显弯曲	树干有2段以上稍微弯曲或1段明显弯曲	树干有1段稍弯曲	完全通直

1.3　统计分析方法

本研究所有数据均使用 SPSS 22.0 软件和 EXCEL 软件进行分析。方差分析线性模型为[17]：

$$X_i = \mu + B_i + P/F + Pi_{ji}/Fi_{ji} + M_{eiji}$$

式中：μ为总体平均值，B_i为区组效应，P/F为种源/家系效应，Pi_{ji}/Fi_{ji}为区组与种源/家系的交互作

用，M_{eiji}为机误。

家系遗传力（H^2）计算公式[18]：

$$H^2 = \frac{\sigma_F^2}{\sigma_F^2 + \sigma_{FB}^2/B + \sigma_e^2/NB}$$

式中：σ_F^2为家系的方差分量，σ_{FB}^2为家系与区组交互作用的方差分量，σ_e^2为机误的方差分量，B为区组数，N为区组内重复。

单株狭义遗传力（h_N^2）计算公式[19]：

$$h_N^2 = \frac{4\sigma_F^2}{\sigma_F^2 + \sigma_{FB}^2 + \sigma_e^2}$$

式中：σ_F^2为家系的方差分量，σ_{FB}^2为家系与区组交互作用的方差分量，σ_e^2为机误的方差分量。

表型变异系数（PCV）与遗传变异系数（GCV）计算公式分别为[20]：

$$PCV = \frac{\sqrt{\sigma_P^2}}{\bar{X}} \times 100\%;\ GCV = \frac{\sqrt{\sigma_g^2}}{\bar{X}} \times 100\%$$

式中：σ_P^2为性状的表型方差分量，σ_g^2为性状的遗传方差分量，$\bar{X}$为性状的平均值。

种源现实增益估算公式[21]：

$$\Delta G_r = S / \bar{X} \times 100\%$$

式中：S为选择差，$\bar{X}$为某一性状的平均值。

家系及单株遗传增益估算公式[22]：

$$\Delta G = \left(R/\bar{X}\right) \times 100\%;\ R = h^2 \cdot S$$

式中：R为选择响应，S为选择差，$\bar{X}$为总体平均值。

表型相关分析计算公式[23]：

$$r_{p12} = \frac{Cov_{p12}}{\sqrt{\sigma_{p1}^2 \sigma_{p2}^2}}$$

式中：Cov_{p12}为 2 个性状的表型协方差，σ_{p1}^2、σ_{p2}^2为 2 性状的表型方差。

一般配合力计算公式[24]：

$$g = x - \mu$$

式中：g为亲本的一般配合力，x为亲本的某个交配组合在某个性状的子代平均值，μ为这个性状所有组合的子代总平均值。

主成分值和综合得分计算公式分别为[25]：

$$Y_i = \sum_{j=1}^{n} \alpha_{ij} X_j \,(j = 1,2,3,\cdots n)$$

$$W = \sum_{i=1}^{p} Y_i \omega_i \,(i = 1,2,3,\cdots p)$$

式中：Y_i为第i主成分值，α_{ij}为主成分 i 内性状 j的特征值，X_j为性状 j 的平均值，W为综合得分值，ω_i为第 i主成分贡献率，n为性状个数，p为提取主成分个数。

2 结果与分析

2.1 各性状方差分析

对胡桃楸各种源及各家系不同生长性状进行方差分析，结果见表 3、4。除侧枝数外，其余各性状在种源间的差异均达到显著水平（$p < 0.05$）；通直度、侧枝数和尖削度在区组间以及通直度在区组 × 种源间的差异未达显著水平（$p > 0.05$）；分枝角在种源间和侧枝数、尖削度在区组 × 种源间的差异显著（$0.01 < p < 0.05$）；除此外，各性状在其他变异来源间的差异均达到极显著水平（$p < 0.01$）。不同家系各性状方差分析表明：除通直度、侧枝数及尖削度等性状在区组间的差异不显著外（$p > 0.05$），各性状在各变异来源间的差异均达显著水平（$p < 0.05$）。因此，胡桃楸的生长性状在种源及家系间具有较大遗传差异，对优良种源及优良家系的选择具有较大改良潜力。

2.2 各性状遗传变异分析

胡桃楸 28 个家系各生长性状遗传变异参数见表 5。所有家系树高平均值为 2.565 m，变幅为 2.042～3.046 m；树高年增长量的平均值为 0.770 m，变幅为 0.598～0.931 m；地径的平均值为 6.135 cm，变幅为 4.772～7.439 cm；冠幅的平均值为 1.776 m，变幅为 1.206～2.263 m；分枝角平均值为 50.129°，变幅为 44.792°～57.833°；通直度平均值为 3.816，变幅为 2.972～4.278；侧枝数平均值为 2.356 个，变幅为 1.810～2.704 个；尖削度平均值为 2.411，变幅为 2.209～2.667。

各性状表型变异系数变化范围为 14.049%～41.519%，除尖削度外，其余各性状表型变异系数均超过 20%。各性状遗传变异系数变化范围为 8.754%～23.873%，出尖削度外，其余各性状遗传变异系数均超过 10%。从家系遗传力来看，除通直度外，其余各性状的家系遗传力较高；从单株遗传

表 3　不同种源各性状方差分析表

Table 3　Variance analysis of different traits among provenances

性状 Traits	变异来源 Variance sources	*df*	*MS*	*F*	性状 Traits	变异来源 Variance sources	*df*	*MS*	*F*
树高 Tree height	区组 Block	2	28.500	107.179**	分枝角 Branch angle	区组 Block	2	798.057	6.841**
	种源 Provenance	3	5.063	19.040**		种源 Provenance	3	365.812	3.136 *
	区组 × 种源 Block × Provenance	6	1.665	6.260**		区组 × 种源 Block × Provenance	6	513.313	4.400**
树高年增长量 Annual height increaset	区组 Block	2	3.212	129.016**	通直度 Stem straightness degree	区组 Block	2	0.766	1.010
	种源 Provenance	3	0.818	32.865**		种源 Provenance	3	8.238	10.869**
	区组 × 种源 Block × Provenance	6	0.066	2.653*		区组 × 种源 Block × Provenance	6	1.457	1.923
地径 Basal diameter	区组 Block	2	158.255	89.019**	侧枝数 Lateral branch number	区组 Block	2	1.472	1.968
	种源 Provenance	3	18.857	10.607**		种源 Provenance	3	0.323	0.432
	区组 × 种源 Block × Provenance	6	5.079	2.857**		区组 × 种源 Block × Provenance	6	1.990	2.662*
冠幅 Crown diameter	区组 Block	2	31.601	69.050**	尖削度 Taperingness	区组 Block	2	0.078	0.708
	种源 Provenance	3	2.355	5.145**		种源 Provenance	3	1.005	9.066**
	区组 × 种源 Block × Provenance	6	2.352	5.140**		区组 × 种源 Block × Provenance	6	0.304	2.741*

注：**表示差异极显著（$p < 0.01$）；*表示差异显著（$p < 0.05$）。下同。
Notes: ** difference is significant at the 0.01 level; * difference is significant at the 0.05 level.The same below.

表 4　不同家系各性状方差分析表

Table 4　Variance analysis of different traits among families

性状 Traits	变异来源 Variance sources	*df*	*MS*	*F*	性状 Traits	变异来源 Variance sources	*df*	*MS*	*F*
树高 Tree height	区组 Block	2	30.038	191.633**	分枝角 Branch angle	区组 Block	2	345.805	3.619*
	家系 Family	27	2.552	16.282**		家系 Family	27	483.204	5.058**
	区组 × 家系 Block × Family	54	1.413	9.011**		区组 × 家系 Block × Family	54	352.694	3.692**
树高年增长量 Annual height increase	区组 Block	2	3.869	242.242**	通直度 Stem straightness degreedegree	区组 Block	2	1.370	2.092
	家系 Family	27	0.240	15.026**		家系 Family	27	4.326	6.604**
	区组 × 家系 Block × Family	54	0.119	7.432**		区组 × 家系 Block × Family	54	1.227	1.873**
地径 Basal diameter	区组 Block	2	177.571	171.221**	侧枝数 Lateral branch number	区组 Block	2	0.858	1.227
	家系 Family	27	16.018	15.445**		家系 Family	27	1.595	2.281**
	区组 × 家系 Block × Family	54	8.647	8.338**		区组 × 家系 Block × Family	54	1.271	1.818**
冠幅 Crown diameter	区组 Block	2	35.209	104.693**	尖削度 Taperingness	区组 Block	2	0.139	1.706
	家系 Family	27	2.643	7.857**		家系 Family	27	0.635	7.801**
	区组 × 家系 Block × Family	54	1.758	5.226**		区组 × 家系 Block × Family	54	0.424	5.209**

力来看，除冠幅、分枝角、侧枝数和尖削度外，其余各性状的单株遗传力较高。

2.3　各性状相关性分析

对所有家系生长性状进行相关性分析，结果见表 6。各性状中，除尖削度与分枝角（−0.056）、尖削度与侧枝数（0.049）以及通直度与冠幅（−0.009）之间的相关性不显著外（$p > 0.05$），其余各性状之间均达显著水平（$p < 0.05$），其中，树高与树高年增长量以及树高与地径之间相关性较高，相关系数均在 0.80 以上。树高与尖削度之间呈极显著负相关，相关系数为−0.243。此外，尖削度与树高年增长量、通直度之间均达极显著负

表 5 不同家系各性状变异参数

Table 5 Variation parameters of different traits families

性状<br>Traits	平均值<br>Mean	变幅<br>Range	标准差<br>SD	表型变异系数<br>*PCV*/%	遗传变异系数<br>*GCV*/%	家系遗传力<br>Family heritability	单株遗传力<br>Single heritability
树高 Tree height	2.565 m	2.042～3.046 m	0.590	22.989	16.940	0.553	0.642
树高年增长量 Annual height increase	0.770 m	0.598～0.931 m	0.187	24.345	17.201	0.495	0.743
地径 Basal diameter	6.135 cm	4.772～7.439 cm	1.482	24.164	17.733	0.540	0.659
冠幅 Crown diameter	1.776 m	1.206～2.263 m	0.737	41.519	23.873	0.665	0.359
分枝角 Branch angle	50.129 °	44.792～57.833 °	11.150	22.242	11.143	0.730	0.233
通直度 Stem straightness degree	3.816	2.972～4.278	0.886	23.223	14.427	0.284	0.886
侧枝数 Lateral branch number	2.356	1.810～2.704	0.868	36.860	11.389	0.797	0.110
尖削度 Taperingness	2.411	2.209～2.667	0.339	14.049	8.754	0.668	0.355

表 6 不同家系各性状相关性分析

Table 6 Correlation coefficients among different traits among families

性状<br>Traits	树高<br>Tree height	树高年增长量<br>Annual height increase	地径<br>Basal diameter	冠幅<br>Crown diameter	分枝角<br>Branch angle	通直度<br>Stem straightness degree	侧枝数<br>Lateral branch number
树高年增长量 Annual height increase	0.888**						
地径 Basal diameter	0.827**	0.690**					
冠幅 Crown diameter	0.718**	0.590**	0.741**				
分枝角 Branch angle	0.191**	0.187**	0.144**	0.107**			
通直度 Stem straightness degree	0.149**	0.130**	0.064*	−0.009	0.161**		
侧枝数 Lateral branch number	0.242**	0.165**	0.270**	0.236**	0.148**	0.071*	
尖削度 Taperingness	−0.243**	−0.292**	0.326**	0.085**	−0.056	−0.131**	0.049

相关，相关系数分别为−0.292、−0.131。

2.4 各性状一般配合力分析

28 个胡桃楸半同胞家系各性状一般配合力见表 7、8。树高一般配合力变化范围为−0.523～0.481，其中，较高的家系为 WRH2、WRH3 和 QTH8；树高年增长量一般配合力变化范围为−0.173～0.161，其中，较高的家系为 WRH2、WRH3 和 WRH13；地径一般配合力变化范围为−1.363～1.304，其中，较高的家系为 QTH2、WRH3 和 WRH6；冠幅一般配合力变化范围为 −0.569～0.487，其中，较高的家系为 QTH2、BQ3 和 QTH8；分枝角一般配合力变化范围为 −5.239～7.803，其中，较高的家系为 WRH5、WRH14和 WRH3；通直度一般配合力变化范围为−0.844～0.461，其中，较高的家系为 WRH8、WRH5 和 QTH1；侧枝数一般配合力变化范围为−0.546～0.348，其中，较高的家系 WRH2、WRH13 和 MDJ3；尖削度一般配合力变化范围为−0.202～0.256，其中，较高的家系为 MDJ7、WRH6和 QTH10。

2.5 各性状主成分分析

对胡桃楸的各性状进行主成分分析，分析结果见表 9。以特征值大于 1 为标准提取出 3 个主成分，累计贡献率达到 73.264%，说明选出来的 3 个主成分可以涵盖所有性状 73.264% 的信息，具有高度概括的效果，故选取前 3 个主成分进行分析。

由表 9 可知：主成分Ⅰ 中，树高（0.956）、树高年增长量（0.878）、地径（0.892）及冠幅（0.824）分别在 3 个主成分中的特征值最高，故主成分Ⅰ 可以代表这 4 个性状。主成分Ⅱ 中，尖削度（0.879）在 3 个主成分中的特征值最高，故主成分Ⅱ 可以代表该性状。在主成分Ⅲ 中，分枝角（0.622）、通直度（0.482）、侧枝数（0.526）在 3 个主成分中的特征值最高，故主成分Ⅲ 可以代表这 3 个性状。

由表 9 中各性状的载荷量可以得到主成分的线性方程，用 X_1、X_2、X_3、X_4、X_5、X_6、X_7、X_8 分别表示树高、树高年增长量、地径、冠幅、分枝角、通直度、侧枝数、尖削度；用 Y_1、Y_2、Y_3 分

表 7　树高、树高年增长量、地径、冠幅的一般配合力

Table 7　General combining ability values of tree height, annual height increase, basal diameter and crown diameter

家系 Families	树高 Tree height	家系 Families	树高年增长量 Annual height increase	家系 Families	地径 Basal diameter	家系 Families	冠幅 Crown diameter
WRH2	0.481	WRH2	0.161	QTH2	1.304	QTH2	0.487
WRH3	0.377	WRH3	0.107	WRH3	1.276	BQ3	0.399
QTH8	0.372	WRH13	0.097	WRH6	0.737	QTH8	0.356
WRH15	0.350	WRH15	0.089	WRH15	0.721	WRH2	0.356
QTH2	0.317	WRH14	0.083	BQ3	0.715	WRH15	0.287
BQ3	0.298	QTH2	0.078	WRH2	0.585	WRH10	0.276
WRH14	0.245	WRH5	0.067	QTH8	0.557	WRH11	0.256
WRH13	0.176	BQ3	0.056	WRH10	0.549	WRH3	0.204
WRH5	0.117	WRH6	0.048	WRH13	0.212	WRH13	0.146
WRH6	0.088	QTH8	0.036	QTH6	0.201	QTH10	0.116
QTH7	0.081	QTH7	0.035	QTH10	0.149	WRH14	0.110
WRH10	0.021	WRH10	0.027	WRH5	0.115	QTH6	0.062
WRH11	0.012	WRH11	0.022	QTH7	0.096	WRH6	0.052
MDJ3	−0.001	WRH7	0.014	WRH4	0.090	QTH7	0.020
QTH6	−0.029	WRH4	0.000	WRH14	0.071	MDJ14	−0.030
WRH4	−0.042	QTH6	−0.003	QTH1	−0.010	BQ5	−0.062
QTH10	−0.059	MDJ3	−0.024	WRH11	−0.079	MDJ3	−0.079
WRH7	−0.085	BQ5	−0.032	MDJ3	−0.132	QTH1	−0.090
BQ5	−0.088	QTH10	−0.032	MDJ14	−0.338	WRH4	−0.114
QTH1	−0.097	WRH	−0.034	WRH7	−0.365	BQ1	−0.173
WRH8	−0.157	QTH1	−0.041	MDJ7	−0.499	WRH12	−0.183
MDJ14	−0.182	WRH8	−0.044	WRH	−0.565	WRH5	−0.205
WRH	−0.197	BQ1	−0.074	BQ5	−0.671	WRH7	−0.213
WRH12	−0.270	MDJ14	−0.095	WRH8	−0.729	MDJ7	−0.274
BQ1	−0.309	WRH12	−0.096	WRH12	−0.735	WRH8	−0.348
MDJ7	−0.438	QTH3	−0.136	BQ1	−0.935	WRH	−0.364
QTH3	−0.458	MDJ7	−0.137	MDJ9	−0.960	MDJ9	−0.421
MDJ9	−0.523	MDJ9	−0.173	QTH3	−1.363	QTH3	−0.569

别表示主成分Ⅰ、Ⅱ、Ⅲ，得到如下线性方程：

$$Y_1 = 0.956X_1 + 0.878X_2 + 0.892X_3 + 0.824X_4 + 0.270X_5 + 0.160X_6 + 0.359X_7 - 0.061X_8$$

$$Y_2 = -0.149X_1 - 0.245X_2 + 0.360X_3 + 0.245X_4 - 0.282X_5 - 0.491X_6 + 0.119X_7 + 0.879X_8$$

$$Y_3 = -0.161X_1 - 0.219X_2 + 0.027X_3 - 0.111X_4 + 0.622X_5 + 0.482X_6 + 0.526X_7 + 0.329X_8$$

2.6　各种源综合得分

由于各主成分的贡献率不同，因此，选取它们的贡献率作为计算综合得分的权重，由表 9 可知：这 3 个主成分的权重分别为：42.398%、17.219%、13.646%。综合评价得分计算公式为：

$$W = 42.398\% \times Y_1 + 17.219\% \times Y_2 + 13.646\% \times Y_3$$

经过计算后得到胡桃楸各种源的综合得分以及排名情况见表 10。各种源中，综合得分变化范围为 12.362～13.319，按树高、树高年增长量和地径现实增益在 2% 以上，兼顾其他性状对种源进行评

表 8 分枝角、通直度、侧枝数、尖削度的一般配合力

Table 8 General combining ability values of branch angle, stem straightness degree, lateral branch number and taperingness

家系 Families	分枝角 Branch angle	家系 Families	通直度 Stem straightness degree	家系 Families	侧枝数 Lateral branch number	家系 Families	尖削度 Taperingness
WRH5	7.803	WRH8	0.461	WRH2	0.348	MDJ7	0.256
WRH14	5.289	WRH5	0.434	WRH13	0.343	WRH6	0.196
WRH3	5.164	QTH1	0.378	MDJ3	0.292	WRH10	0.186
WRH4	3.789	WRH6	0.295	QTH2	0.271	QTH2	0.180
BQ5	3.789	QTH2	0.295	QTH1	0.246	QTH1	0.132
WRH10	3.719	WRH13	0.295	WRH12	0.200	QTH10	0.126
QTH7	3.303	QTH8	0.267	WRH10	0.163	MDJ9	0.125
QTH6	3.066	WRH7	0.211	WRH11	0.153	WRH3	0.117
QTH10	3.011	QTH10	0.211	QTH8	0.075	WRH4	0.110
BQ3	2.789	WRH3	0.184	MDJ14	0.061	QTH6	0.109
QTH2	1.233	WRH10	0.128	BQ3	0.040	MDJ14	0.063
WRH13	0.525	WRH14	0.100	WRH4	0.014	QTH7	0.006
WRH7	0.400	WRH15	0.072	WRH3	0.010	BQ3	−0.002
QTH1	−0.781	MDJ3	0.072	WRH15	0.010	WRH12	−0.028
MDJ7	−0.836	MDJ9	0.072	QTH7	−0.013	WRH	−0.028
MDJ3	−1.045	QTH7	0.045	BQ5	−0.018	WRH15	−0.059
WRH2	−1.406	BQ1	0.045	WRH5	−0.037	WRH11	−0.066
WRH12	−1.447	WRH12	0.017	QTH10	−0.050	WRH5	−0.083
WRH6	−1.809	WRH2	−0.011	QTH6	−0.055	QTH3	−0.084
WRH15	−2.711	MDJ7	−0.039	WRH6	−0.064	WRH13	−0.093
QTH8	−2.975	QTH3	−0.039	MDJ7	−0.106	BQ1	−0.098
WRH8	−3.350	QTH6	−0.066	MDJ9	−0.111	WRH7	−0.105
MDJ14	−3.600	WRH11	−0.205	WRH7	−0.199	MDJ3	-0.107
WRH11	−4.170	BQ3	−0.483	WRH	−0.231	QTH8	−0.125
MDJ9	−4.684	MDJ14	−0.594	BQ1	−0.240	WRH8	−0.143
BQ1	−4.725	WRH	−0.594	WRH14	−0.277	BQ5	−0.188
QTH3	−5.100	BQ5	−0.705	QTH3	−0.277	WRH14	−0.196
WRH	−5.239	WRH4	−0.844	WRH8	−0.546	WRH2	−0.202

价选择，万人欢种源入选为优良种源，其树高、树高年均增长量、地径及冠幅现实增益分别为 3.109%、5.014%、2.193% 和 1.040%。

2.7 优良家系及单株综合得分

根据主成分分析的结果，对所有家系及单株进行综合得分计算，根据各家系及各单株综合得分情况，以 10% 和 1% 的入选率，分别对家系与单株进行筛选，共获得 3 个优良家系（WRH3、WRH5、QTH2）与 10 株优良单株，入选家系及单株综合得分及排名情况见表 11、12。入选家系树高、树高年增长量、地径及冠幅的平均值分别为 2.835 m、0.854 m、7.033 cm 和 1.938 m，遗传增益分别为 5.835%、5.410%、7.908% 和 6.069%；入选优良单株树高、树高年增长量、地径及冠幅平均值比总体平均值分别高 1.068 m、0.250 m、2.225 cm 和 1.312 m，单株遗传增益分别为 26.74%、24.11%、23.91% 和 26.53%。

表 9　各性状主成分分析

Table 9　Principal component analysis of different traits

主要成分因子 Principal component factors	主成分 I Component Ⅰ	主成分 II Component Ⅱ	主成分III Component III
特征值 Eigenvalue	42.398	17.219	13.646
贡献率 Contribution/%	42.398	59.617	73.264
累计贡献率 Cumulative contribution/%	3.392	1.378	1.092
树高 Tree height	0.956	−0.149	−0.161
树高年增长量 Annual height increase	0.878	−0.245	−0.219
地径 Basal diameter	0.892	0.360	0.027
冠幅 Crown diameter	0.824	0.245	−0.111
分枝角 Branch angle	0.270	−0.282	0.622
通直度 Stem straightness degree	0.160	−0.491	0.482
侧枝数 Lateral branch number	0.359	0.119	0.526
尖削度 Taperingness	−0.061	0.879	0.329

表 10　各种源综合得分和排名

Table 10　Comprehensive score and ranking of provenances

种源 Provenances	综合得分 Comprehensive scores	排名 Rankings
万人欢 Wanrenhuan	13.319	1
七台河 Qitaihe	13.291	2
宝清 Baoqing	13.252	3
牡丹江 Mudanjiang	12.362	4

表 11　优良家系综合得分和排名

Table 11　Comprehensive score and ranking of excellent families

家系 Families	综合得分 Comprehensive scores	排名 Rankings
WRH3	14.893	1
WRH5	14.484	2
QTH2	14.468	3

表 12　优良单株综合得分和排名

Table 12　Comprehensive score and ranking of excellent individual plants

区组 Block	种源 Provenances	家系 Families	株号 Plant number	综合得分 Comprehensive scores	排名 Rankings
1	七台河 Qitaihe	QTH1	9	19.908	1
1	七台河 Qitaihe	QTH7	3	19.514	2
3	万人欢 Wanrenhuan	WRH6	1	19.322	3
3	万人欢 Wanrenhuan	WRH2	9	19.117	4
1	万人欢 Wanrenhuan	WRH15	5	19.026	5
1	宝清 Baoqing	BQ5	4	18.664	6
2	七台河 Qitaihe	QTH6	2	18.661	7
3	万人欢 Wanrenhuan	WRH5	2	18.521	8
1	万人欢 Wanrenhuan	WRH14	1	18.377	9
1	万人欢 Wanrenhuan	WRH4	5	18.376	10

3　讨论

遗传和变异是林木育种工作的主要研究内容[26]，对变异来源、特点以及规律的认识是进行林木良种选择工作的重要基础[27]。本研究通过对不同性状种源间及家系间差异分析发现，各性状在胡桃楸种源间以及家系间均存在丰富的遗传变异，这与褚宪丽等[28]对胡桃楸 3 个种源、45 个家系的分析结果一致，表明不同种源、半同胞家系间各生长指标间存在较大差异，有利于优良家系与单株的选择。

变异系数的大小反映植物的变异程度，它能体现林木各性状的遗传变异能力，包括遗传变异系数

和表型变异系数[21]。遗传变异系数越大，表明该家系在该性状中可被利用的改良潜力越大[29]。本研究中，各性状的表型变异系数变化范围为 14.049%～41.519%，与袁显磊等[30]、周彦超[31] 对胡桃楸的研究结果相近，变化幅度较大，说明胡桃楸各表型性状间存在丰富的变异。此外，冠幅与侧枝数的表型变异系数最高，表明以冠幅与侧枝数作为选优指标的潜力更大。树高、树高年增长量以及地径 3 个性状的遗传变异系数占表型变异系数的比例较大，均超过了 70%，与解懿妮等[32] 对桉树（*Eucalyptus S.* T. Blake）的研究结果相似，说明胡桃楸家系间的变异受遗传因素的影响较大。遗传力表示亲本某一性状遗传给子代的能力，遗传力的高低表明该性状稳定遗传给后代受到环境影响的大小[33]。本研究中，28 个胡桃楸半同胞家系各性状遗传力变化范围为 0.284～0.797，除通直度外，各性状遗传力均超过 0.45，属于中等遗传力水平[34]，与翟文继等[35] 对楸树（*Catalpa bungei* C. A. Mey.）的研究结果相似，表明不同家系的胡桃楸生长性状受到了环境与遗传的综合控制；其中，侧枝数与分枝角的遗传力最高，说明侧枝数与分枝角在胡桃楸家系间的优良表现受环境影响较小，能够稳定遗传给子代。

相关性分析可以反映各性状之间存在的联系，对了解不同性状之间的关系具有重要作用[36-37]。本研究中，从各性状的相关性分析可以看出，胡桃楸各性状之间的相关性较高，其中，树高、树高年增长量、地径、冠幅以及分枝角等性状间均达极显著正相关；而树高与地径决定树木长势情况，树高与地径之间达极显著正相关，且相关系数较高，为 0.827，与赵曦阳等[38] 对白杨派杨树无性系的研究结果相似，研究结果为长势优良的胡桃楸综合评价选择提供依据。

一般配合力指在一个杂交群体中，某个亲本杂交组合的子代平均值与子代总平均值的离差[39]。一般配合力通常被认为是选择理想亲本和发展优良杂交的先决条件[40]。本研究对 28 个半同胞家系各性状的一般配合力进行了分析，以 20% 的入选率，各性状均筛选出 6 个亲本，根据树高、树高年增长量与地径作为木材产量的指标，综合筛选出 4 个优良亲本（WRH2、WRH3、WRH15 和 QTH2）；根据冠幅、分枝角以及侧枝数（注，各指标取所得参数相反数进行筛选）作为种植密度的指标，综合筛选出 2 个优良亲本（WRH、QTH3）；根据通直度与尖削度作为干形的指标，综合筛选出 3 个优良亲本（QTH1、QTH2 和 WRH6）。所选家系的亲本可以作为未来不同育种目标的杂交育种的首选亲本。

主成分分析法既能把握植株的综合性状表现，又能简化选优指标，简化选择程序，克服人为主观误差，选择结果准确、客观[41]。本研究对胡桃楸 8 个生长性状进行了主成分分析，提取出 3 个主成分，主成分Ⅰ中树高、树高年均增长量、地径和冠幅特征值较高，可代表生长量相关信息；主成分Ⅱ中尖削度特征值较高，可代表干形相关信息；主成分Ⅲ中分枝角和侧枝数平均值特征值较高，可代表分枝相关信息。依据主成分分析结果，筛选出 1 个优良种源（万人欢），其树高、树高年均增长量及冠幅现实增益分别为 3.109%、5.014%、2.193% 和 1.040%；筛选出 3 个优良家系（WRH3、WRH5、QTH2），入选家系树高、树高年增长量、地径及冠幅等性状遗传增益分别为 5.835%、5.410%、7.908% 和 6.069%。与胡文杰等[42] 对 9 年生枫香（*Liquidambar formosana* Hance）的研究结果相似。其中，QTH2 既是优良家系所选材料，又是优良亲本所选材料，表明 QTH2 性状表现优良，可进一步作为胡桃楸优良材料用于推广应用。在优良家系选择的同时，还筛选出优良单株，以 1% 的入选率，共获得 10 株优良单株，入选优良单株在树高、树高年增长量、地径及冠幅的平均值比总体平均值分别高 1.068 m、0.250 m、2.225 cm 和 1.312 m，单株遗传增益分别为 26.74%、24.11%、23.91% 和 26.53%。

4 结论

育种目标决定育种方法，胡桃楸主要作为果材兼用树种，对胡桃楸的评价应从多方面、多角度进行。但因其有性繁殖周期长、扦插和嫁接等无性繁殖成活率低等问题，使得胡桃楸研究进展仍较为缓慢。本研究以胡桃楸良种选育为目的，综合多个性状，结合主成分分析法，对胡桃楸种源、家系及单株进行评价与选择，共筛选出 1 个优良种源、3 个优良家系和 10 个优良单株，入选材料各性状之间相关性强，遗传力高，种源间以及家系间有较大的选择潜力，所选材料优势明显，在胡桃楸良种选育与推广应用中可重点考虑。

参考文献：

[1] 中国农业百科全书总委员会林业卷委员会, 中国农业百科全书部.

中国农业百科全书: 林业卷. 上[M]. 农业出版社, 1989.
[2] 王东娜, 牟长城, 冯富娟. 胡桃楸ISSR-PCR反应体系的建立及优化[J]. 实验室研究与探索, 2010, 29（11）：18-22.
[3] 李佳娜, 高瑞馨. 我国胡桃楸的遗传育种研究进展[J]. 安徽农业科学, 2020, 48（17）：4-7.
[4] Joolka N K, Sharma S K. Selection of superior Persian walnut (*Juglans regia* L.) strains from a population of seedling origin[J]. Acta Horticulturae, 2005,12(696): 75-78.
[5] 陈翠英. 核桃楸的经济价值与繁育技术[J]. 河北林业科技, 2003（4）：49-49.
[6] 许绍惠, 许　泓. 胡桃属植物毒性成分及其应用[J]. 沈阳农业大学学报, 1990, 21（2）：167-170.
[7] Oliveira I, Sousa A, Ferreira I C F R, *et al*. Total phenols, antioxidant potential and antimicrobial activity of walnut (*Juglans regia* L.) green husks[J]. Food and Chemical Toxicology, 2008, 46(7): 2326-2331.
[8] 吕海宁, 张克诚, 崔增杰, 等. 核桃青皮体外抑菌活性的研究[J]. 华西药学杂志, 2011, 26（2）：150-152.
[9] 李智博. 胡桃楸活性成分分离解析及抗肿瘤作用的研究[D]. 大连：大连理工大学, 2008.
[10] 马常耕. 世界加速林木育种轮回研究的现状[J]. 世界林业研究, 1996, 9（6）：16-24.
[11] 袁显磊. 核桃楸优良种源和家系早期选择及苗期环境因子影响评价[D].哈尔滨：东北林业大学, 2013.
[12] 刘桂丰, 杨书文, 李俊涛, 等. 胡桃楸种源的初步区划及最佳种源选择[J]. 东北林业大学学报, 1991, 19（S2）：189-196.
[13] 张晓林, 刘　超, 刘　剑, 等. 核桃楸研究现状及育种策略[J]. 吉林林业科技, 2019, 48（1）：14-17.
[14] 颜廷武, 于世河, 王骞春, 等. 辽宁地区核桃楸半同胞家系间苗期生长差异分析[J]. 辽宁林业科技, 2020（6）：13-15.
[15] Zhao X Y, Bian X, Liu M, *et al*. Analysis of genetic effects on a complete diallel cross test of *Betula platyphylla*[J]. Euphytica, 2014, 200(2): 221-229.
[16] 欧建德, 吴志庄, 康永武. 杉莲混交林中乳源木莲生长形质、空间利用能力的混交比例效应[J]. 南京林业大学学报：自然科学版, 2020, 44（1）：089-96.
[17] Liang D Y, Wang B Y, Song S L, *et al*. Analysis of genetic effects on a complete diallel cross test of *Pinus koraiensis*[J]. Euphytica, 2019, 215(5): 1-12.
[18] 续九如. 林木数量遗传学[M]. 北京：高等教育出版社, 2006.
[19] Kaviriri D K, Liu X, Fan Z, *et al*. Genetic variation in growth and cone traits of *Pinus koraiensis* half-sib families in northeast China[J]. Phyton, 2019, 88(3): 57-69.
[20] Metougui M L, Mokhtari M, Maughan P J, *et al*. Morphological variability, heritability and correlation studies within an argan tree population (*Argania spinosa* (L.) Skeels) preserved in situ[J]. International Journal of Agriculture and Forestry, 2017, 7(2): 42-51.
[21] Wang F, Zhang Q H, Tian Y G, *et al*. Comprehensive assessment of growth traits and wood properties in half-sib *Pinus koraiensis* families[J]. Euphytica, 2018, 214(11): 1-15.
[22] 王　虹, 师尚礼, 张旭业, 等. 紫花苜蓿多元杂交后代产量和品质一般配合力分析及遗传参数的估算[J]. 草业学报, 2016, 25（3）：126-134.
[23] 解孝满, 李景涛, 赵合娥, 等. 柳树无性系苗期遗传测定与选择[J]. 江苏林业科技, 2008, 35（3）：6-9.
[24] 王　虹, 师尚礼, 刘正璟. 紫花苜蓿多元杂交后代形态学特征及变异分析[J]. 草原与草坪, 2014, 34（5）：21-25.
[25] 陈　澜. 不同种源福建柏苗期性状差异性研究及优良种源选择[D]. 福州：福建农林大学, 2015.
[26] Mwase W F, Savill P S, Hemery G. Genetic parameter estimates for growth and form traits in common ash (*Fraxinus excelsior* L.) in a breeding seedling orchard at Little Wittenham in England[J]. New Forests, 2008, 36(3): 225-238.
[27] White T L, Adams W T, Neale D B. Forest genetics [M]. London: CABI, 2007.
[28] 褚宪丽, 朱航勇, 张含国, 等. 胡桃楸种源家系变异与选择[J]. 东北林业大学学报, 2010, 38（11）：5-6.
[29] 贾　晨, 辜云杰, 夏　川, 等. 枫香半同胞家系子代测定及优良家系选择[J]. 西部林业科学, 2014, 43（5）：122-128.
[30] 袁显磊, 祁永会, 刘忠玲, 等. 核桃楸种源选择试验及其环境因子的影响[J]. 植物研究, 2013, 33（4）：468-476.
[31] 周彦超. 辽东地区胡桃楸优树选择与果核表型多样性研究[D]. 沈阳：沈阳农业大学, 2019.
[32] 解懿妮, 莫晓勇, 彭仕尧, 等. 粤西21个桉树无性系早期性状遗传变异分析和无性系综合选择[J]. 南京林业大学学报：自然科学版, 2018, 42（3）：73-80.
[33] Maniee M, Kahrizi D, Mohammadi R. Genetic variability of some Morpho-Physiological traits in Durum Wheat (*Triticum turgidum var*. Durum.)[J]. African Journal of Biotechnology, 1998, 9(30): 4687-4691.
[34] Zhang H, Zhang Y, Zhang D, *et al*. Progeny performance and selection of superior trees within families in *Larix olgensis*[J]. Euphytica, 2020, 216(3): 1-10.
[35] 翟文继, 麻文俊, 王秋霞, 等. 楸树苗期优良家系及单株的配合选择[J]. 西北林学院学报, 2012, 27（3）：68-71.
[36] 庞志慧, 舒凤梅. 红松天然林优树子代测定综合分析[J]. 林业科技通讯, 1997（4）：17-18.
[37] 王有和, 王振明. 帽儿山地区二年生红松苗地理变异规律及最佳种源的选择[J]. 林业科技, 1995, 20（3）：15-16.
[38] 赵曦阳, 马开峰, 沈应柏, 等. 白杨派杂种无性系植株早期性状变异与选择研究[J]. 北京林业大学学报, 2012, 34（2）：45-51.
[39] 陈晓阳, 沈熙环. 林木育种学[M]. 北京：高等教育出版社, 2005.
[40] Chen X, Zhao B, Ji C, *et al*. Transcriptome profiling analysis of two contrasting barley genotypes in general combining ability for yield traits[J]. Brazilian Journal of Botany, 2021, 44(1): 117-123.
[41] 贾春红, 张　利, 魏　晓, 等. 主成分分析法在毛叶木姜子优树选择中的应用[J]. 中药材, 2016, 39（5）：943-947.
[42] 胡文杰, 庞宏东, 胡兴宜, 等. 9年生枫香种源变异及优良种源选择[J]. 中南林业科技大学学报, 2019, 39（3）：40-46.

Variation and Selection of Seedling Growth Traits Among *Juglans mandshurica* Provenances and Families

LU Xian-bo[1], *XU Lian-feng*[2], *PANG Zhong-yi*[3], *LIU Ji-feng*[4], *WEN Bao-yang*[2], *PEI Xiao-na*[5,6], *ZHAO Ling*[2], *WANG Xue-gang*[4], *ZHAO Xi-yang*[1]

(1. State Key Laboratory of Tree Genetics and Breeding, Northeast Forestry University, Harbin 150040, Heilongjiang, China; 2. Qiqihar Branch of Heilongjiang Academy of Forestry, Qiqihar 161005, Heilongjiang, China; 3. State-owned Xinmin Mechanized Forest Farm, Shenyang 110300, Liaoning, China; 4. Wanrenhuan Forest Farm of Binxian County, Harbin 150411, Heilongjiang, China; 5. Harbin Forestry Machinery Research Institute of the National Forestry and Grassland Administration, Harbin 150086, Heilongjiang, China; 6. Research Center of Cold Temperate Forestry, Chinese Academy of Forestry, Harbin 150086, Heilongjiang, China)

Abstract: [**Objective**] To analyze and select the genetic variation of growth traits among provenances and families of *Juglans mandshurica*, in order to promote the selection and breeding of *J. mandshurica* superior varieties. [**Method**] In this study, the growth characteristics (tree height, annual growth of tree height, ground diameter, crown width, straightness, branch angle, number of side branches and taperingness) of 28 *J. mandshurica* families (6-years-old) within 4 provenances at Wanrenhuan Forest Farm were measured and analyzed. [**Result**] The results of analysis of variance showed that most of the traits reached extremely significant differences among various sources ($P < 0.01$). The phenotypic and genetic coefficient of variation of these traits ranged from 14.049% to 41.519% and from 8.754% to 23.873%, respectively. The family heritability and individual heritability were ranged from 0.284 to 0.797 and from 0.110 to 0.886, respectively. The results of correlation analysis showed that the correlation of almost all traits reached a significant level ($P < 0.05$), except the correlations between sharpness and branch angle, between sharpness and number of side branches, and between straightness and crown width. The results of general combining ability analysis showed that with a selection rate of 20%, 6 excellent parents were screened according to different traits. The results of principal component analysis (PCA) showed that the cumulative contribution rate of the three principal components reached 73.264%, indicating that the three principal components contained most information of the measured trait. According to the comprehensive scores of PCA, one excellent provenance, three excellent families and 10 excellent individual plants were initially selected. The realistic gains of tree height, average annual increase in tree height, ground diameter and crown width of selected provenance were 3.109%, 5.014%, 2.193%, and 1.040%, respectively. The genetic gains of the 4 traits of the families selected were 5.835%, 5.410%, 7.908%, and 6.069%, respectively. The genetic gains of the 4 traits of selected individuals were 26.74%, 24.11%, 23.91% and 26.53%, respectively. [**Conclusion**] The growth traits of *J. mandshurica* have rich variations within provenances and families. The excellent provenances, parents, families and individuals preliminary selected could lay a basis for the selection and application of *J. mandshurica* varieties.

Keywords: *Juglans mandshurica*; half-sib family; provenance selection; family selection; genetic gain

（责任编辑：张 研）
