# Supplementary material for: Provenance and family variations in early growth of Manchurian walnut (Juglans mandshurica Maxim.) and selection of superior families
Source: PLoS One. 2024 Mar 7;19(3):e0298918. doi: 10.1371/journal.pone.0298918 (PMC10919699; doi:10.1371/journal.pone.0298918)
Supplement: S2 File — (ZIP) [file pone.0298918.s005.zip › Variation regularity among provenances of Juglans mandshurica and family selection.pdf]

西北林学院学报 2011, 26(2): 91~95
Journal of Northwest Forestry University

胡桃楸种源家系变异规律及家系选择研究

张含国, 邓继峰, 张 磊, 徐悦丽

(东北林业大学 林木遗传育种与生物技术教育部重点实验室, 黑龙江 哈尔滨 150040)

摘 要: 利用黑龙江省林口县青山林场 3 个种源 122 个家系 7 a 生胡桃楸子代林材料, 分析种源、家系变异规律并对家系进行初步选择。种源树高、胸径变异系数分别为 32.17 %、43.25 %, 穆棱种源树高变异最大, 铁力种源胸径变异最大, 迎春种源生长性状变异都小。铁力、穆棱及迎春种源家系间树高平均变异系数分别为 4.00 %~41.81 %、12.37 %~45.40 %及 2.76 %~38.41 %, 3 个种源 7 a 生与 1 a 生相比家系变异幅度分别缩减 4.04 %、2.57 %及 4.71 %。种源间、家系间差异达显著或极显著水平, 铁力、穆棱种源 7 a 生高生长快慢家系差距较 1 a 生分别缩小 65 %、5.4 %, 迎春种源则扩大 67.7 %, 入选 13 个家系中迎春种源 8 个; 铁力、穆棱分别 4 个; 树高、胸径分别超过家系均值为 44.8 %、46.9 %。

关键词: 胡桃楸; 种源; 家系; 变异; 选择
中图分类号: S722.132.05 文献标志码: A 文章编号: 1001-7461(2011)02-0091-05

Variation Regularity among Provenances of *Juglans mandshurica* and Family Selection

ZHANG Han-guo, DENG Ji-feng, ZHANG Lei, XU Yue-li

(Key Laboratory of Forest Tree Genetic Improvement and Biotechnology, Ministry of Education, Northeast Forestry University, Harbin, Heilongjiang 150040, China)

Abstract: By using 122 families of, 7-year-old *Juglans mandshurica* progeny forest belonging to 3 provenances (Muling, Tieli, and Yingchun) in Linkou County, Heilongjiang Province as materials, variation regularities of provenances and families were investigated, and a preliminary family selection was conducted. The coefficients of variation on height, diameter at breast height (DBH) were 32.17 % and 43.25 % respectively. Those from Muling provenance had the maximum variation of tree height, those from Tieli provenance exhibited the maximum variation on DBH, and those from Yingchun provenance had minimum variation in all the growth characters. The average variation coefficients of tree height from 3 provenances were between 4.00 % to 41.81 %, 12.37 % to 45.40% and 2.76 % to 38.41 %, respectively. Compared to one year-old progeny, the family variation amplitude of seven year-old progeny from 3 provenances decreased by 4.04 %, 2.57 % and 4.71 %, respectively. Differences in variations among provenances and families were significant or most significant. Compared to one year-old progeny, the gaps between fast and slow growing families of 7-year-old progeny from Tieli and Muling proven seven year-old progeny from Tieli, Muling were reduced by 65 % and 5.4 % respectively, from Yingchun however, increased by increased by 67.7 %. Among 13 families selected, 8 were from Yingchun, 4 from Tieli and Muling, respectivelyly. The tree height and *DBH* for those selected 44.8 % and 46.9 % more than the average ones in their families.

Key words: *Juglans mandshurica*; provenance; family; variation; selection

收稿日期: 2010-02-01 修回日期: 2010-03-24
基金项目: 黑龙江省科技攻关重点项目(GB08B203)。
作者简介: 张含国, 男, 教授, 从事林木遗传改良教学与研究。

(C)1994-2020 China Academic Journal Electronic Publishing House. All rights reserved. http://www.cnki.net

胡桃楸(*Juglans mandshurica*)主要分布于我国东北及华北海拔在 400～1 000 m 的山坡或向阳沟谷中,属第三纪孑遗植物,已列为国家三级保护树种。其不仅木材优良,而且还是药用和木本油料植物。由于过量采伐,胡桃楸天然林大树接近枯竭,人工造林较少。关于胡桃属苗木繁育及培育技术研究已有报道[1-3],遗传改良方面研究较少[4]。胡桃楸苗期地理种源变异规律、种源选择、早晚相关进行研究表明,生长性状、适应性状等种源间存在显著差异,初步确定胡桃楸最佳早期选择龄天然林为 15 a,人工林为 14 a[5-9]。对林口县青山林场胡桃楸 1 a 生苗高生长变异进行了分析,胡桃楸种源间和种源内部存在丰富的变异,127 个家系间苗高差异极显著,苗高前 10 名家系中有铁力种源 9 个家系、穆棱种源 1 个家系,前后 10 名家系高生长相差 138%,种源内家系间存在丰富的遗传变异,变异系数在 11.7 %～48.2 %之间[10]。本研究主要是对胡桃楸种源间、种源内家系间变异进行了研究,同时进行优良家系的初步选择,以提高家系的利用效率。

1 材料与方法

1.1 试验材料

2002 年秋季在铁力(T)、穆棱(M)和迎春(Y)林业局收集胡桃楸种子,采种单株间距离在 50 m 以上,每个地理种源 40 株。种子采到后在林口县青山林场对单株种子进行变温处理,2003 年 5 月单株播种育苗,2004 年春季造林,株行距 1.5 m× 2.0 m,面积 0.6 hm^2,试验地为暗棕壤。试验采用完全随机设计,小区 15～25 株。2009 年 9 月调查各种源、家系的树高和胸径。

1.2 统计分析方法

方差分析采用 SPSS16.0 的 Compare Means 中的 One-Way ANOVA 软件,相关性分析用 Correlate 中的 Bivariate 软件[11]。

2 结果与分析

2.1 胡桃楸种源变异分析

核桃楸 7 a 生树高、胸径种源间与种源内相比变异稍小(表 1),树高变异系数为 29.64 %～33.87 %,平均为 32.17 %,穆棱种源变异稍大,迎春种源变异稍小,二者相差 4.23 %;胸径变异系数为38.99 %～44.95 %,平均 43.25 %,铁力种源变异稍大,迎春种源变异较小,二者相差 5.96 %。

7 a 生树高同 1 年生苗高变异系数(28.7 %)相比增加了 3.47 %,种内分化更加明显;最大与最小变异幅度下降了 2.47 %,说明种源间差异有所缩小。穆棱种源变异系数由第 2 位上升到第 1 位,迎春种源仍然变异最小。

表 1 胡桃楸种源树高、胸径变异分析

Table 1 Analysis of variation on height, *DBH* of *J. mandshurica* from three provenances

种源		株数	平均值	标准差	变异系数 /%	标准误	95% 置信区间	
							下限	上限
铁力	*H*/m	1 263	1.629	0.487	29.89	0.014	1.602	1.655
	D/cm	902	1.498	0.674	44.95	0.022	1.454	1.542
穆棱	*H*/m	808	1.528	0.517	33.87	0.018	1.492	1.563
	D/cm	502	1.584	0.650	41.01	0.029	1.527	1.641
迎春	*H*/m	434	1.933	0.573	29.64	0.028	1.879	1.987
	D/cm	376	1.852	0.722	38.99	0.037	1.779	1.925
和与均值	*H*/m	2 505	1.649	0.530	32.17	0.011	1.628	1.670
	D/cm	1 780	1.597	0.691	43.25	0.016	1.565	1.630

注:*H*:树高;*D*:胸径

方差分析表明,胡桃楸 3 个种源间树高、胸径差异极显著(表 2),迎春种源树高、胸径均排在第 1 位,分别为 1.93 m、1.85 cm,与最差种源相比分别超出 26.51 %、23.3 %。

表 2 胡桃楸种源树高、胸径方差分析

Table 2 Analysis of variance on height, *DBH* of *J. mandshurica* frpom three provenance

变异来源	平方和 *SS*	自由度 *df*	均方 *MS*	*F* 值	*P* 值
树高/m	47.475	2	23.738	90.373	0.000
胸径/cm	33.338	2	16.669	36.305	0.000

7 a 生树高同 1 a 生苗高相比,最优与最差种源相比仅高出 1.51 %,几乎没有变化。迎春种源由高生长第 2 位上升到第 1 位,穆棱种源第 3 位的位置没有变化。

2.2 家系变异及选择研究

2.2.1 铁力种源家系变异规律及选择 铁力种源 53 个家系间存在较大的变异,树高平均变异系数为 29.89 %,T47、T030、T53 家系树高变异较小,变异系数分别为 4.00 %、12.49 %、13.09 %;T43、T022、T005 家系变异较大,变异系数分别为 41.81 %、37.21 %、36.46 %,变异系数最大值与最小值

(C)1994-2020 China Academic Journal Electronic Publishing House. All rights reserved. http://www.cnki.net

相差32.46 %。1 a 生变异系数最大值与最小值相差36.5 %,2 个年度变异幅度仅相差 4.04 %。

胸径 51 个家系平均变异系数为 44.95 %,T47、T031、T40 家系胸径变异较小,变异系数分别为15.03 %、16.38 %、17.83 %,T018(20)、T022(10)、T42(24)号家系变异较大,变异系数分别为70.62 %、63.41 %、63.37 %,变异系数最大值与最小值相差 55.59 %。

家系间树高、胸径存在极显著差异(表 3)。53 个家系树高平均值为 1.63 m,T42、T44、T54、T47、T27 家系生长较快,树高分别为 2.68、2.50、2.43、2.35、2.13 m;T38、T13、T40、T46、T024 家系生长较慢,树高分别为 1.24、1.34、1.36、1.37、1.38 m。树高生长最快家系超过生长最慢家系 116.1 %,7 a 生高生长差距较 1 a 生差距缩小 65 %,说明家系间的差距在缩小。

表 3　铁力种源内家系间树高、胸径方差分析

Table 3　Analysis of variance on three height, *DBH* of *J. mandshurica* among families from Tieli provenance

变异来源	平方和 *SS*	自由度 *df*	均方 *MS*	*F* 值	*P* 值
树高/m	49.540	52	0.953	4.621	0.000
胸径/cm	70.620	50	1.412	3.552	0.000

51 个家系胸径平均值为 1.50 cm,T44、T47、T54、T42、T49 家系生长较快,胸径分别为 2.63、2.53、2.47、2.27、2.08 cm;T53、T13、T024、T033、T018 家系生长较慢,胸径分别为 0.85、0.98、0.99、1.05、1.10 cm,生长最快家系超过生长最慢家系 209.4 %。

选出生长较快家系的 10 个,树高分别为 T42、T44、T54、T47、T27、T030、T010、T028、T41、T52 家系,胸径分别为 T44、T 47、T54、T42、T49、T030、T010、T005、T028、T015 家系。综合树高与胸径生长情况,初选家系确定为 7 个,入选率分别为 13.0 %、13.8 %,家系为 T42、T44、T54、T47、T030、T010、T028。7 个初选家系树高、胸径平均值分别为 2.46 m 和 2.24 cm,超过家系均值分别为 50.9 %、49.3 %。

2.2.2　*穆棱种源家系变异规律及选择*　穆棱种源家系间存在较大变异,36 个家系树高平均变异系数为33.87 %,M33、M30、M23 家系变异较小,变异系数分别为 12.37 %、12.49 %、18.09 %;M10、M5、M34 家系变异较大,变异系数分别为 45.40 %、40.36 %、39.64 %,变异系数最大值与最小值相差33.03 %。1 a 生变异系数最大值与最小值相差35.6 %,7 a 生变异幅度仅下降了 2.57 %。33 个家系胸径平均变异系数为 41.05 %,M16、M33、M13 家系变异较小,变异系数分别为 11.48 %、21.86 %、24.51 %;M25、M7、M10 家系变异较大,变异系数分别为 64.62 %、61.02 %、57.70 %,变异系数最大值与最小值相差 53.14 %。

36 个家系间树高、胸径存在极显著差异(表 4)。树高平均值为 1.53 m,M20、M30、M33、M10、M14 家系生长较快,树高分别为 2.06、2.02、1.87、1.85、1.81 m;M2、M16、M11、M34、M8 家系生长较慢,树高分别为 0.95、1.02、1.07、1.11、1.12 m。树高生长最快家系超过生长最慢家系 116.8 %,7 a 生高生长差距较 1 a 生差距缩小 5.4 %,说明家系间的差距稍微缩小。

表 4　穆棱种源内家系间树高、胸径方差分析

Table 4　Analysis of variance on three height, *DBH* of *J. mandshurica* among families from Muling provenance

变异来源	平方和 *SS*	自由度 *df*	均方 *MS*	*F* 值	*P* 值
树高/m	52.886	35	1.511	7.149	0.000
胸径/cm	28.725	32	0.898	2.291	0.000

33 个家系胸径平均值为 1.59 cm,M20、M5、M12、M10、M2 家系生长较快,胸径分别为 2.29、2.06、2.03、1.84、1.78 cm;M8、M16、M32、M7、M35 家系生长较慢,分别为 0.86、1.08、1.16、1.31、1.36 cm,生长最快家系 M20 超过生长最慢家系M35 166.3 %。

选出生长较快家系的 7 个,树高分别为 M20、M30、M33、M10、M14、M23、M3 家系,胸径分别为M20、M5、M12、M10、M2、M17、M6 家系。综合树高与胸径生长情况,初选家系确定为 5 个,入选率分别为 13.9 %、15.2 %,家系为 M20、M30、M10、M5、M12。5 个初选家系树高、胸径平均值分别为1.86、1.94 cm,超过家系均值分别为 21.6 %、22.0 %。

2.2.3　*迎春种源家系变异规律及选择*　迎春种源33 个家系间存在较大变异,家系树高平均变异系数为 29.64 %,Y14、Y13、Y30 家系变异较小,变异系数分别为 2.76 %、8.21 %、14.91 %;Y10、Y27、Y22 家系变异较大,变异系数分别为 38.41 %、33.65 %、32.73 %,变异系数最大值与最小值相差 30.89 %。1 a 生变异系数最大值与最小值相差 35.6 %,7 a 生变异幅度下降了 4.71 %。

家系胸径平均变异系数为 38.99 %,Y14、Y12、Y20 家系变异较小,变异系数分别为 3.72 %、12.01 %、12.92 %;Y22、Y29、Y15 家系变异较大,变异系数分别为 53.24 %、51.30 %、48.32 %,变异系数最大值与最小值相差 49.52 %。

(C)1994-2020 China Academic Journal Electronic Publishing House. All rights reserved.　http://www.cnki.net

家系间树高、胸径存在极显著差异(表 5)。树高平均值为 1.93 m, Y14、Y19、Y30、Y22、Y21 家系生长较快,树高分别为 3.13 、2.39 、2.36 、2.28 、2.27 m;Y12、Y10、Y2、Y20、Y13 家系生长较慢,树高分别为 1.19 、1.31 、1.40 、1.40、1.45 m。树高生长最快家系超过生长最慢家系 163.0 %,7 a 生高生长差距较 1 a 生差距扩大 67.7 %,说明家系间生长差距在扩大。

表 5 迎春种源内家系间树高、胸径方差分析

Table 5 Analysis of variance on three height, *DBH* of *J. mandshurica* among families from Yingchun provenance

变异来源	平方和 *SS*	自由度 *df*	均方 *MS*	*F* 值	*P* 值
树高/ m	38.250	32	1.195	4.615	0.000
胸径/ cm	34.618	32	1.082	2.305	0.000

胸径平均值为 1.85 cm, Y14、Y30、Y4、Y19、Y15 家系生长较快,胸径分别为 3.19 、2.41 、2.33 、2.22 、2.18 cm;Y12、Y13、Y10、Y2、Y8 家系生长较慢,分别为 0.78 、0.99 、1.33 、1.37 、1.41 cm,生长最快家系超过生长最慢家系 309.0 %。

选出生长较快家系的 8 个,树高分别为 Y14、Y19、Y30、Y22、Y21、Y15、Y25 家系,胸径分别为 Y14、Y30、Y4、Y19、Y15、Y31、Y21 家系。综合树高与胸径生长情况,初选家系确定为 5 个,入选率为 15.2 %,家系为 Y14、Y19、Y30、Y15、Y4 。5 个初选家系树高、胸径平均值分别为 2.44 m、2.47 cm,超过家系均值分别为 26.4 %、33.5 %。

2.2.4 家系总体变异规律及家系选择 将所有家系汇总分析,而不考虑种源,则家系间树高变异系数为 2. 76 % ~ 45. 40 %,最高与最低家系相差 42.64 %。树高变异较小的家系为 Y14、T47、Y13、M13、M30,变异系数分别为 2.76 %、4.00 %、8.21 %、12.37 %、12.49 %;变异较大的家系 M10、T43、M5、M34、M1,变异系数分别为 45. 40 %、41.81 %、40.36 %、39.64 %、38.59 %。变异较大、较小的家系穆棱种源居多,由此也证明穆棱种源树高变异较大。

家系间胸径变异系数为 3.72 % ~70.62 %,最高与最低家系相差 66.90 %。胸径变异较小的家系为 Y14、M16、Y20、T47、T031,变异系数分别为 3.72 %、11.48 %、12.92 %、15.03 %、16.38 %;变异较大的家系 T018、M25、T022、T42、T026,变异系数分别为 70.62 %、64.62 %、63.41 %、63.37 %、61.19 %。变异较大、较小的家系铁力种源居多,由此也证明该种源胸径变异较大。

122 个家系间树高差异达极显著水平(表 6)。树高平均值为 1. 65 m, Y14、T42、T44、T54、Y19 家系生长较快,树高分别为 3.13 、2.68 、2.50 、2.43 、2.39 m,平均为 2.63 m;M2、M16、M11、M34、M8 家系生长较慢,树高分别为 0.95、1.02 、1.07 、1.11 、1.12 m,平均为 1.05 m,生长较快的前 5 个家系较生长较慢的后 5 个家系大 149.2 %。

表 6 胡桃楸家系树高、胸径方差分析

Table 6 Analysis of variance on three height, *DBH* of familes of *J. mandshurica*

变异来源	平方和 *SS*	自由度 *df*	均方 *MS*	*F* 值	*P* 值
树高/ m	188.151	121	1.555	7.174	0.000
胸径/ cm	166.241	115	1.446	3.516	0.000

116 个家系胸径差异极显著,胸径平均值为 1.60 cm, Y14、T44、T47、T54、Y30 家系生长较快,胸径分别为 3.19 、2.63 、2.53 、2.47 、2.41 cm,平均为 2.65 cm;Y32、M8、T13、T024、Y13 家系生长较慢,胸径分别为 0.85 、0.86 、0.98 、0.99 、0.99 cm,平均为 0.93 cm。生长较快的前 5 个家系较生长较慢的后 5 个家系大 183.3 %。

选出生长较快家系的 15 个,树高分别为 Y14、T42、T44、T54、Y19、Y30、T47、Y22、Y21、Y15、Y25、Y4、Y31、T27、Y5 家系,胸径分别为 Y14、T44、T47、T54、Y30、Y4、M20、T42、Y19、Y15、T49、Y31、M5、Y21、T005 家系。综合树高与胸径生长情况,入选家系确定为 13 个,入选率分别为 10.7 %、11.3 %,家系为 Y14、T44、T47、T54、Y30、T42、Y19、Y4、M20、Y15、Y31、Y22、Y21。13 个家系树高、胸径平均值分别为 2.39 m、2.35 cm,超过家系均值为 44.8 %、46.9 %。入选家系迎春种源居多为 8 个,铁力、穆棱分别仅有 4 个和 1 个。

用 Pearson 法进行相关分析,结果表明,7 年生树高与胸径相关极显著,相关系数为 0.833;1 年生苗高与 7 年生树高相关性不显著。

3 结论与讨论

种源变异规律。核桃楸 7 a 生树高变异系数平均为 32.17 %,穆棱种源变异稍大,迎春种源变异稍小,二者相差 4.23 %;胸径变异系数平均 43.25 %,铁力种源变异稍大,迎春种源变异较小,二者相差 5.96 %。7 a 生树高同 1 年生苗高变异系数相比增加了 3.47 %,个体间分化更加明显;最大与最小变异幅度下降了 2.47 %,说明种源间差异有所缩小。种源间树高、胸径差异极显著,迎春种源树高、胸径均排在第 1 位,超出最差种源分别为 26.51 %、23.3 %。

家系变异规律。122 个家系间树高变异系数为 2.76 % ~45.40 %,穆棱种源家系变异较丰富;家

(C)1994-2020 China Academic Journal Electronic Publishing House. All rights reserved. http://www.cnki.net

系间胸径变异系数为 3.72 %~70.62 %,铁力种源家系变异较丰富。铁力种源家系间树高平均变异系数为 29.89 %,胸径平均变异系数为 44.95 %。穆棱种源家系树高平均变异系数为 33.87 %,胸径平均变异系数为 41.05 %。迎春种源家系树高平均变异系数为 29.64 %,胸径平均变异系数为 38.99 %。

优良家系选择。铁力种源树高、胸径生长最快家系分别超过生长最慢家系 116.1 %、209.4 %,7 a 生高生长差距较 1 a 生差距缩小 65 %,5 个初选家系其树高、胸径超过家系均值分别为 50.9 %、49.3 %。穆棱种源树高、胸径生长最快家系分别超过生长最慢家系 116.8 %、166.3 %,7 a 生高生长差距较 1 a 生差距缩小 5.4 %,5 个初选家系其树高、胸径超过家系均值分别为 21.6 %、22.0 %。迎春种源树高、胸径生长最快家系分别超过生长最慢家系 163.0 %、309.0 %,7 a 生高生长差距较 1 a 生差距扩大 67.7 %,5 个初选家系其树高、胸径超过家系均值分别为26.4 %、33.5 %。不分种源情况下122 个家系间树高、胸径差异亦达极显著水平,综合树高与胸径生长情况,入选迎春种源 8 个,铁力、穆棱分别 4 个和 1 个共 13 个家系,超过家系均值分别为 44.8 %、46.9 %。

参考文献:

[1] 杜香莉,郭军战,冯汀.我国核桃资源的综合利用研究[J].西北林学院学报,2003,18(3):82-85.
DU X L,GUO J Z,FENG T. On comprehensive utilization of walnut resources in China[J]. Journal of Northwest Foretry University,2003,18(3):82-85.(in Chinese)

[2] 范成民,董丽芬,朱帜,等.核桃芽苗砧嫁接方法研究[J].西北林学院学报,2008,23(4):109-111.
FAN C M,DONG L F,ZHU Z,*et al*. Nurse seed grafting methods of *Juglans regia*[J]. Journal of Northwest Foretry University,2008,23(4):109-111.(in Chinese)

[3] 李保国,齐国辉,郭素平,等.河北省太行山中南部核桃栽植时期及栽植技术研究[J].西北林学院学报,2006,21(4):83-84.
LI B G,QI G H,GUO S P,*et al*. A study on planting period and technique of walnut(*Juglans regia* L.) in Central-south of Taihang Mountains in Hebei Province[J]. Journal of Northwest Foretry University,2006,21(4):83-84.(in Chinese)

[4] 高绍棠,曹玉美,尹卫东,等.淳化泥河沟试区核桃引种小结[J].西北林学院学报,2006,8(2):58-66.
GAO S T,CAO Y M,YIN W D,*et al*. Introduetion of walnut in Chunhua County[J]. Journal of Northwest Foretry University,2006,8(2):58-66.(in Chinese)

[5] 杨书文,刘桂丰,赵克尊.胡桃楸早期选择的初步研究[J].东北林业大学学报,1990,19(S):76-81.
YANG S W,LIU G F,ZHAO K Z. A preliminary study on the early selection of *Juglans mandshurica*[J]. Journal of Northeast Forestry University,1990,19(S):76-81.(in Chinese)

[6] 杨书文,刘桂丰.胡桃楸地理变异规律的再研究[J].东北林业大学学报,1991,19(S):183-188.
YANG S W,LIU G F. The further study on the geographic variation of *Juglans mandshurica*[J]. Journal of Northeast Forestry University,1991,19(S):183-188.(in Chinese)

[7] 刘桂丰,杨书文.胡桃楸种源的初步区划及最佳种源选择[J].东北林业大学学报,1991,19(S):189-195.
LIU G F,YANG S W. The primary division and selection for the optimal provenance of *Juglans mandshurica*[J]. Journal of Northeast Forestry University,1991,19(S):189-195.(in Chinese)

[8] 孙丽敏,侯旭光.核桃楸的生长变异及其早期测定的研究[J].林业科技通讯,1997(11):12-15.

[9] 孙丽敏,侯旭光,刘殿辉.核桃楸早期测定的研究[J].防护林科技,2004(4):18-19,39.
SUN L M,HOU X G,LIU D H. Study on early determination of *Juglans mandshurica*[J]. Protection Forest Science and Technology,2004,(4):18-19,39.(in Chinese)

[10] 李广玉,张学政,张含国,等.胡桃楸苗木高生长种源家系变异规律初报[J].林业科技,2004,29(4):1-3.
LI G Y,ZHANG X Z,ZHANG H G,Variation regularity on seedling height growth of *Juglans mandshurica* in different germ plasm resources and families[J]. Forestry Science & Technolo,2004,29(4):1-3.(in Chinese)

[11] 宋志刚,谢蕾蕾,何旭洪.SPSS16 实用教程[M].北京:人民邮电出版社,2008:112-117,132-140.

[12] XIA D A,ZHU H,WANG H R,*et al*. Provenance trials of *Juglans mandshurica*[J]. Journal of Forestry Research,1997,8(3):156-159.

(C)1994-2020 China Academic Journal Electronic Publishing House. All rights reserved. http://www.cnki.net
