# Supplementary material for: Provenance and family variations in early growth of Manchurian walnut (Juglans mandshurica Maxim.) and selection of superior families
Source: PLoS One. 2024 Mar 7;19(3):e0298918. doi: 10.1371/journal.pone.0298918 (PMC10919699; doi:10.1371/journal.pone.0298918)
Supplement: S2 File — (ZIP) [file pone.0298918.s005.zip › Variation regularity on seedling height growth of Juglans mandshurica in different provenances and families.pdf]

第29卷 第4期 林 业 科 技 Vol. 29 No. 4
2004年7月 FORESTRY SCIENCE & TECHNOLOGY July 2004

文章编号：1001－9499(2004)04－0001－03

胡桃楸苗木高生长种源家系变异规律初报*

李广玉[1] 张学政[1] 张含国[2] 兰士波[2] 康迎昆[2]
(1. 黑龙江省铁力林业局，铁力 152500；2. 黑龙江省林业科学研究所，哈尔滨 150040)

摘要：对林口县青山林场胡桃楸1年生苗高生长变异进行了分析。胡桃楸种源间和种源内部存在丰富的变异，变异系数范围在26.0%～32.7%之间，平均值为28.7%。3个种源间苗高差异极显著，其中，铁力种源高生长较快；穆棱种源高生长最差；最优种源与最差种源高生长相差25%。127个家系间苗高差异极显著，苗高前10名家系中有铁力种源9个家系、穆棱种源1个家系，前后10名家系高生长相差138%，种源内家系间存在丰富的遗传变异，变异系数在11.7%～48.2%之间。

关键词：胡桃楸；苗高；种源；家系；变异
中图分类号：S 718.46 文献标识码：A

胡桃楸（*Juglans mandshurica* Maxim.）主要分布于黑龙江省东部小兴安岭、完达山及张广才岭等地，多与其他树种混生，蓄积较少，为黑龙江省珍贵树种之一。其材质坚硬致密、弹性好、易加工，为优良的军用、细木工和家具用材；果肉及树皮含鞣质；种子富含脂肪，营养丰富，可食用或供工业用，是很有发展前途的木本油料植物[1～3]。由于过量采伐，胡桃楸天然林大树接近枯竭。它是第三纪孑遗植物，渐危种，已列为国家三级保护植物[4,5]。了解胡桃楸树种、种源和家系变异规律，对收集与保存资源，保护生物多样性，维护生态平衡，具有较大的理论指导意义。现就胡桃楸种源间、种源内家系间变异的研究结果介绍如下。

1 材料与方法

1.1 试验材料

2002年秋季在铁力、穆棱和迎春林业局收集胡桃楸种子，采种单株间距离在50 m以上，每个地理种源40株。种子采到后在林口县青山林场对单株种子进行变温处理，2003年5月单株播种育苗，苗木管理同当地生产用苗，当年11月调查各种源、家系的苗高生长。

1.2 统计分析方法

数据分析主要是采用方差分析、LSR检验等，方差分析、LSR检验采用SPSS10.0的General Linear Model中的Univariate软件[8]。

2 结果与分析

2.1 胡桃楸种源变异分析

对当年胡桃楸苗木高生长量进行调查的结果（表1）表明，种源间和种源内都存在丰富的变异，变异系数在26.0%～32.7%之间，平均值为28.7%。方差分析表明，胡桃楸3个种源间苗高差异极显著（表2），其中铁力（25.0 cm）种源高生长较快，迎春（22.3 cm）种源高生长居中，穆棱种源高生长最差（20.0 cm），最优种源与最差种源高生长相差25%。

表1 胡桃楸各种源苗高性状统计因子

家系	株数	平均值(cm)	标准差	变异系数(%)	标准误	95%下限置信区间	
						下限	上限
穆棱	657	19.990	5.4655	27.3	0.268	19.465	20.515
铁力	1 024	25.023	8.1725	32.7	0.215	24.602	25.443
迎春	644	22.280	5.7818	26.0	0.270	21.750	22.811
总和	2 325						

表2 不同种源胡桃楸苗高方差分析

变异来源	平方和SS	自由度df	均方MS	F值	P值
处理	10 416.184	2	5 208.092	110.525	0.000
误差	109 416.016	2 322	47.121		
总和	1 332 800.650	2 325			

* 黑龙江省科技厅和黑龙江省森林工业总局项目资助

(C)1994-2019 China Academic Journal Electronic Publishing House. All rights reserved. http://www.cnki.net

2.2　胡桃楸种源内家系间变异分析

127 个胡桃楸家系苗高差异极显著（表 3），苗高前 10 名家系中有铁力种源 9 个家系（34.0 cm）、穆棱种源 1 个家系（31.6 cm），最优家系铁力 12 号高为 36.1 cm，生长量最弱家系穆棱 34 号为 11.4 cm，二者相差 217%。后 10 名家系中有穆棱种源 5 个家系（13.1 cm）、迎春种源 2 个家系（14.4 cm）、铁力种源 3 个家系（14.0 cm）。前 10 名家系比后 10 名家系高生长快 138%。

表 3　不同家系胡桃楸苗高方差分析

变异来源	平方和 SS	自由度 df	均方 MS	F 值	P 值
处理	65 331.591	126	518.505	20.911	0.000
误差	54 500.609	2 198	24.796		
总和	1 332 800.650	2 325			

2.3　铁力种源家系变异分析

铁力种源内有 54 个家系共 1024 个单株，家系内存在丰富的遗传变异（图 1），变异系数在 11.7%～48.2%之间，其中，42（48.2%），43（41.3%）号家系内变异较大，40（11.7%），47（13.0%），16（14.3%）号家系变异较小。方差分析表明，胡桃楸家系间苗高差异极显著（表 4），其中，12，27，16 号家系高生长较快，平均值为 35.65 cm；53，47，42 号家系高生长最差，平均值为 14.3 cm；3 个生长快的家系超过生长慢家系数值的 149.3%，生长最快的 12 号家系较生长最慢的 53 号家系高生长快 181.1%。

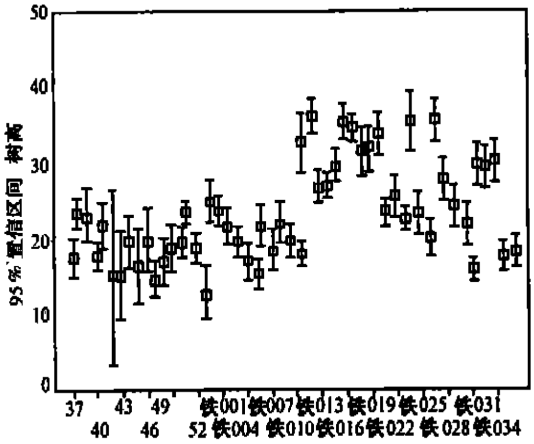

图 1　铁力种源不同家系苗高生长分布

表 4　铁力种源胡桃楸不同家系苗高方差分析

变异来源	平方和 SS	自由度 df	均方 MS	F 值	P 值
处理	39 234.633	53	740.276	24.684	0.000
误差	29 090.476	970	29.990		
总和	709 480.630	1 024			

2.4　穆棱种源家系变异分析

穆棱种源内有 36 个家系 657 个单株，家系内存在丰富的遗传变异（图 2），变异系数在 12.6%～48.2%之间，其中，穆 30（30.6%）、穆 17（29.9%）、穆 2（26.8%）家系内变异较大，穆 3（12.6%）、穆 16（13.8%）、穆 18（15.9%）号家系内变异较小。方差分析表明，穆棱种源内胡桃楸家系间苗高差异极显著（表 5），其中，穆 6、穆 7、穆 11 号家系高生长较快，平均值为 25.0 cm；穆 34、穆 24、穆 17 号家系高生长最差，平均值为 12.1 cm；3 个生长快的家系超过生长慢家系数值的 106.6%，生长最快的穆 6 号家系较生长最慢的穆 34 号家系高生长快 122.2%。

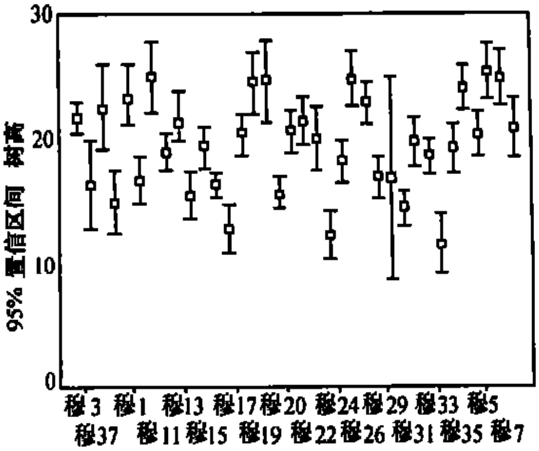

图 2　穆棱种源不同家系苗高生长分布

表 5　穆棱种源胡桃楸不同家系苗高方差分析

变异来源	平方和 SS	自由度 df	均方 MS	F 值	P 值
处理	8 339.744	35	238.278	13.146	0.000
误差	11 256.270	621	18.126		
总和	282 132.080	657			

铁力生长快的前 3 个家系平均值为 35.65 cm，生长快的 3 个家系均值超过生长慢家系数值的 149.3%；穆棱生长快的前 3 个家系平均值为 25.00 cm，生长快的 3 个家系均值超过生长慢家系数值的 106.6%。生长较快的铁力种源变异系数为 11.7%～48.2%；生长较慢的穆棱种源变异系数为 12.6%～48.2%。生长快与生长慢的种源家系，其生长量绝对值存在很大差异，但相对值没有明显的差异，家系间都存在丰富的遗传变异。

3　结果与分析

3.1　胡桃楸种源间和种源内都存在丰富的变异，变异系数范围在 26.0%～32.7%之间，平均值为

(C)1994-2019 China Academic Journal Electronic Publishing House. All rights reserved.　http://www.cnki.net

28.7％。3 个种源间苗高差异极显著，其中，铁力（25.0 cm）种源高生长较快，穆棱种源高生长最差（20.0 cm），最优种源与最差种源高生长相差 25％。

3.2　127 个家系间苗高差异极显著，苗高前 10 名家系中有铁力种源 9 个家系、穆棱种源 1 个家系，最优家系铁力 12 号高达为 36.1 cm。后 10 名家系中有穆棱种源 5 个家系、迎春种源 2 个家系、铁力种源 3 个家系，前者比后者高生长快 138％。

3.3　铁力种源内有 54 个家系，家系间存在丰富的遗传变异，变异系数在 11.7％～48.2％之间，其中，42，43 号家系内变异较大，40，47，16 号家系内变异较小。家系间苗高差异极显著，其中，12，27，16 号家系高生长较快，平均值为 35.65 cm；53，47，42 号家系高生长最差，3 个生长快的家系超过生长慢家系数值的 149.3％。

3.4　穆棱种源内有 **36** 个家系 **657** 个单株，家系内存在丰富的遗传变异，变异系数在 12.6％～48.2％之间，其中，穆 30、穆 17、穆 2 家系内变异较大，穆 3、穆 16、穆 18 号家系内变异较小。穆棱种源内胡桃楸家系间苗高差异极显著，其中，穆 6、穆 7、穆 11 号家系高生长较快，平均值为 25.0 cm；穆 34、穆 24、穆 17 号家系高生长最差，平均值为 12.1 cm；3 个生长快的家系超过生长慢家系数值的 106.6％，生长最快的穆 6 号家系较生长最慢的穆 34 号家系高生长快 122.2％。

参 考 文 献

[1]　郑万均．中国树木志［M］．北京：中国林业出版社，1983

[2]　周以良等．黑龙江树木志［M］．哈尔滨：黑龙江科学技术出版社，1986

[3]　中国科学院中国植物志编辑委员会．中国植物志（第二十卷第二分册）［M］．北京：科学出版社，1984

[4]　宋朝枢等．中国珍稀濒危保护植物［M］．北京：中国林业出版社，1989

[5]　秦瑞明等．黑龙江省稀有濒危植物［M］．哈尔滨：东北林业大学出版社，1993

[6]　杨书文，刘桂丰等．胡桃楸地理变异规律的再研究［J］．东北林业大学学报，1991，19（育种专刊）：183～188

[7]　刘桂丰，杨书文等．胡桃楸种源的初步区划及最佳种源选择［J］．东北林业大学学报，1991，19（育种专刊）：189～195

[8]　刘先勇，袁长迎等．SPSS10.0 统计分析软件与应用［M］．北京：国防工业出版社，2002

第 1 作者简介：李广玉（1964—），男，1987 年毕业于东北林业大学，工程师，主要从事营林生产和管理。

收稿日期：2004—02—10

Variation Regularity on Seedling Height Growth of *Juglans mandshurica* in Different Germ plasm Resources and Families

LI Guangyu

（*Tieli Forestry Bureau of Heilongjiang Province*，*Tieli* 152500）

Abstract　The height growth variation of *Juglans mandshurica* was analyzed. The studied materials are one year old seedlings in Qingshan Forest Farm of Linkou County. The study shows that there are rich variations within and among germ plasm resources, which the variations parameter fluctuates from 26.0％ to 32.7％ and the average level is 28.7％. The differences of seedling height growth among three germ plasm resources reach the extreme marked level. The height growth of Tieli germ plasm resource is the fastest and that of the Muling germ plasm resource is the slowest. The difference between the two families is 25％. There are extreme marked variations on seedling height among 127 families. In the 10 highest families, there are 9 families from Tieli and 1 from Muling. The difference between the 10 highest and lowest families is 138％. There are abundant of variations between the families in germ plasm resources and the variation parameter is from 11.7％ to 48.2％.

Key words　*Juglans mandshurica*；Seedling height；Germ plasm resources；Family；Variation

(C)1994-2019 China Academic Journal Electronic Publishing House. All rights reserved.　http://www.cnki.net
