# Supplementary material for: Provenance and family variations in early growth of Manchurian walnut (Juglans mandshurica Maxim.) and selection of superior families
Source: PLoS One. 2024 Mar 7;19(3):e0298918. doi: 10.1371/journal.pone.0298918 (PMC10919699; doi:10.1371/journal.pone.0298918)
Supplement: S2 File — (ZIP) [file pone.0298918.s005.zip › Within‐ and between‐population variations in seed and seedling traits of Juglans mandshurica.pdf]

J. For. Res.
https://doi.org/10.1007/s11676-021-01381-1

ORIGINAL PAPER

Within- and between-population variations in seed and seedling traits of *Juglans mandshurica*

Qinhui Zhang[1] · Shihe Yu[2] · Xiaona Pei[3,4] · Qianchun Wang[2] · Aijun Lu[2] · Ying Cao[5] · Mulualem Tigabu[6] · Jian Feng[2] · Xiyang Zhao[1]

Received: 18 February 2021 / Accepted: 6 June 2021
© Northeast Forestry University 2021

Abstract In order to quantify within- and between-population variation in seed and seedling traits of *Juglans mandshurica* and reveal the relationship among genetic and environmental variations and select elite families, samples of 50 *J. mandshurica* families from five natural populations in Liaoning Province, northeast China, were taken to measure seed and seedling traits. The results show that all seed traits varied significantly among families within the population, but only kernel weight and kernel rate showed significant variations among populations. Average values of single seed weight, length, width, lateral diameter, and average size, and kernel weight and rate were 10.1 g, 43.0 mm, 29.2 mm, 28.1 mm, 33.4 mm, and 2.2 g and 22.5%, respectively. Significant variations were observed in seedling height and root collar diameter among families and interaction between families and blocks, but the block effects on height and root collar diameter were insignificant. Average values of height and root collar diameter were 94.0 cm and 8.7 mm, respectively. Family heritability of traits ranged from 0.6 gm (kernel weight) to 0.9 mm (seedling height). Correlation analysis showed a strong relationship among seed traits but a weak correlation between seed and seedling traits. Cluster analysis grouped the five natural populations of *J. mandshurica* into three significant clusters with different characteristics. The general combining ability analysis showed that most traits in one family (WD11) were higher, suggesting parental traits were excellent for selecting elite parent clones. Using the comprehensive evaluation method, five families with better seed traits and five families with better seedling traits were selected as elite materials with 10% selection rate. The genetic gains of these elite materials for seed weight, kernel weight, average seed size, kernel rate, seedling height, and root collar diameter were 13.1%, 10.3%, 4.1%, 2.4%, 29.7%, and 21.1%, respectively.

Project funding: This study was financially supported by Liaoning Academy of Agricultural Sciences Subject Construction Program Project (NO. 2019DD217032).

The online version is available at http://www.springerlink.com.

Corresponding editor: Yu Lei

Supplementary Information The online version contains supplementary material available at https://doi.org/10.1007/s11676-021-01381-1.

✉ Jian Feng
lnlkyfj@163.com

Xiyang Zhao
zhaoxyphd@163.com

[1] State Key Laboratory of Tree Genetics and Breeding, Northeast Forestry University, Harbin 150040, People's Republic of China

[2] Liaoning Academy of Forest Science, Shenyang 110032, People's Republic of China

[3] State Administration of Forestry and Grassland, Harbin Research Institute of Forestry Machinery, Harbin 150086, People's Republic of China

[4] Research Center of Cold Temperate Forestry, CAF, Harbin 150086, People's Republic of China

[5] Forestry Development and Service Center of Liaoning, Shenyang 110032, People's Republic of China

[6] Southern Swedish Forest Research Centre, Swedish University of Agricultural Sciences, PO Box 49, 230 53 Alnarp, Sweden

Published online: 25 August 2021

Keywords General combining ability · Heritability · Comprehensive evaluation · Genetic gain

Introduction

Juglans mandshurica Maxim. is a deciduous species of the Juglandaceae family and one of the most important hardwood species in northeast China. It is widely distributed in the Changbai Mountains, and the Greater and Lesser Khingan regions in northeast China (Yuan et al. 2013). The wood of *J. mandshurica* has high commercial value because of its aesthetic appearance, straight texture, high density, mechanical strength, rich elasticity, and corrosion resistance, and is widely used in the production of high-quality materials such as high-grade furniture, sports equipment, and musical instruments (Zhang et al. 2017; Zhu et al. 2018). The fruit has high nutritional value and rich in oils, proteins, mineral elements and vitamins. The kernels can be eaten directly or as a specialty food additive and have significant economic value (Yu 2012). The shape of the kernel is unique and so it also has good economic benefits in dried fruit and antique walnut (Song et al. 2017). *J. mandshurica* also has high medicinal values due to the presence of juglone, an allelopathic compound, in bark, leaves, and the fruit pericarp, is used to treat cancer, reduce blood lipids, acts as an antioxidation, and used in anti-inflammation treatments (Aithal et al. 2009; Silva-Belmares et al. 2014; Vardhini 2014; Wu and Sun 2019). In recent years, the demand for *J. mandshurica* products has been increasing in China but natural sources of the species are scarce due to anthropogenic disturbances. To protect the remaining germplasm resources and develop improved varieties for production, studies on variation in seed and seedling traits in natural populations are necessary.

Phenotypic traits are the embodiment of genetic and environmental diversity as well as interactions between them (Zhao and Si 2016). A thorough evaluation of phenotypic traits has significant importance for preserving and developing elite *J. mandshurica* germplasm resources. Phenotypic traits have previously been studied using select populations and a limited number of families (Chu et al. 2010; Zhang et al. 2011, 2017), but differences persist in character performances in different regions due to differences in environments. Therefore, seed and seedling traits were characterized for five natural populations and 50 *J. mandshurica* families from five regions of Liaoning Province. The objectives were to: (1) quantify variations within- and between-populations in seed and seedling traits; (2) analyze interactions between heredity and environment; (3) explore the relationship between seed and seedling traits; and, (4) select elite families according to these traits. This study will provide information for the selection of elite cultivars and the protection of the genotypes in natural forests of *J. mandshurica*.

Table 2 Names of different families

Populations	Names				
DBG	DBG1	DBG2	DBG3	DBG4	DBG5
	DBG6	DBG7	DBG8	DBG9	DBG10
DGJ	DGJ4	DGJ8	DGJ10	DGJ12	DGJ13
	DGJ18	DGJ20	DGJ21	DGJ22	DGJ25
JC	JC2	JC3	JC7	JC9	JC12
	JC16	JC17	JC22	JC25	JC32
KD	KD8	KD9	KD10	KD11	KD13
	KD15	KD16	KD18	KD19	KD21
WD	WD2	WD3	WD4	WD5	WD6
	WD7	WD8	WD9	WD10	WD11

Materials and methods

Plant materials

The seed samples of individual trees (n = 50) were collected in 2013 from five populations in Liaoning Province (Tables 1 and

Table 1 The main geographical and climatic conditions of the sampled populations in *J. mandshurica*

Population	Longitude (E)	Latitude (N)	Climate	Elevation (m)	Annual temperature (°C)	Annual rainfall (mm)	Frost-free days (d)
Dabiangou (DBG)	124°05′	42°01′	Temperate continental monsoon climate	442	5.0	750	123
Dagujia (DGJ)	125°48′	52°45′	Middle temperate continental monsoon climate	300	6.0	650	128
Jianchang (JC)	124°29′	41°15′	Northern temperate continental monsoon climate	376	4.8	850	150
Kuandian (KD)	125°21′	40°43′	Southern temperate continental monsoon climate	500	7.0	1093	129
Wendao (WD)	123°39′	41°14′	Humid continental climate	208	5.2	810	140

2). Ten trees were collected for each population and ten seeds/tree collected for characterizing phenotypic traits. All sample trees were healthy, mature indigenous specimens. The following year, seeds were planted in a randomized block design with five blocks of six trees. Different families were randomly planted in row plots at 3.0 m×4.0 m.

Seed trait measurements

These traits were measured in November 2013. Seed weight (SWt, W_{St}), kernel weight (KW, W_k), seed length (SL, L_s), seed width (SW, W_s), lateral diameter (LD, D_l), average seed size (ASS, S_{as}), and kernel rate (KR, R_k) were determined. Seed weight and kernel weight were measured using an electronic balance. L_s, W_s, and D_l were measured using a vernier caliper. The average seed size (ASS, S_{as}) was calculated as:

$$S_{as} = \frac{L_s+W_s+D_l}{3} \tag{1}$$

was calculated as

$$R_k = \frac{W_k}{W_{st}} \times 100\% \tag{2}$$

Seedling trait measurements

Two-year-old seedlings were measured in October 2015. Seedling height (SH, H_s) and root collar diameter (RCD, D_{rc}) were determined for each seedling. H_s was measured using a sliding ruler, while D_{rc} were measured with a Vernier caliper.

Statistical analysis

All data were analyzed using data processing system (DPS) version 18.10 and SPSS Statistical Package (SPSS version 26.0, IBM Corp., Armonk, NY, USA). The following linear model was used to analyze seed traits (Ji et al. 2013):

$$Y_{ijk} = \mu + \tau_i + \delta_{j(i)} + \varepsilon_{k(ij)} \tag{3}$$

where Y_{ijk} is the observed value of seed k in family j growing in population i; μ is the overall mean; τ_i is the fixed effect of population i, $\delta_{j(i)}$ is the random effect of family j within population i, and $\varepsilon_{k(ij)}$ is the random error.

The following linear model was used for joint analysis of families and blocks of seedling traits (Pan et al. 2018):

$$X_{ijkl} = \mu + B_k + P_i + F_{j(i)} + BP_{ik} + BF_{j(i)k} + e_{ijkl} \tag{4}$$

where X_{ijkl} is the observed value of an individual tree l in family j within population i growing in block k, μ is the family mean, B_k is the fixed effect of block k, P_i is the fixed effect of population i, $F_{j(i)}$ is the random effect of family j within population i, BP_{ik} is the fixed interactive effect of block k and population i, $BF_{j(i)k}$ is the random interactive effect of block k and family j within population i, and e_{ijkl} is the random error.

The phenotypic differentiation coefficient (V_{ST}) was calculated using the following formula (Wei et al. 2020):

$$V_{ST} = \left(\frac{\delta^2_{\frac{t}{s}}}{\delta^2_{\frac{t}{s}} + \delta^2_s} \right) \times 100\% \tag{5}$$

where s is the number of populations, t is the number of plants in populations, $\delta^2_{t/s}$ is the variance component among populations, and δ^2_s is the variance component within populations.

The coefficient of variation (CV) was calculated using the following formula (Munilla and Guitián 2014):

$$CV = SD/\overline{X} \tag{6}$$

where $\overline{X}$ and SD are the phenotypic mean and standard deviation of the trait, respectively.

Broad-sense heritability (h^2) among families for each trait was calculated according to Xu (2006) as:

$$h^2 = 1 - \frac{1}{F} \tag{7}$$

where F is the F-test value in the ANOVA.

The phenotypic correlation between traits x and y was calculated according to Han et al. (2017):

$$r_P(x,y) = \frac{Cov_P(x,y)}{\sqrt{\sigma^2_{P_x} \cdot \sigma^2_{P_y}}} \tag{8}$$

where $Cov_P(x,y)$ is the phenotypic covariance between traits x and y, $\sigma^2_{P_x}$ and $\sigma^2_{P_y}$ are the phenotypic variance component for trait x and y, respectively.

General combining ability (GCA) of each trait among different families was calculated according to Wang et al. (2016):

$$g = x - \mu \tag{9}$$

where g is the parent's general combining ability, x is the mean value of the offspring of a specific parental combination in a specific trait, and μ is the total mean value of all combinations of this trait.

Comprehensive evaluations of different families were calculated as described by Zhao et al. (2016):

$$Q_\text{i} = \sqrt{\sum_{j=1}^{n} a_i} \tag{10}$$

where $a_i = X_{ij}/X_{jmax}$; the Q_i value is the comprehensive evaluation value of each family; X_{ij} is the mean value of

one trait. X_{jmax} is the maximum value of that trait; n is the number of traits;

Genetic gain was estimated using the formula of Silva et al. (2008):

$$\Delta G = \frac{h^2 \Delta S}{\overline{X}} \times 100\% \quad (11)$$

where ΔG is the genetic gains, h^2 and $\overline{X}$ are the heritability and mean value of a given trait, respectively. ΔS is the selection differential.

Results

Variations in seed and seedling traits

All seed traits varied significantly among families within the populations but only kernel weight and rate showed significant variations among populations (Table 3). Similarly, seedling height and root collar diameter showed significant variation among populations, families, interaction between block and population and interaction between block and families within populations (Table 4), but the block effects for these seedling traits were insignificant. Descriptive statistics for seed and seedling traits are presented in Table 4. The mean seed weight and kernel weight of all families were 10.1 g and 2.2 g, respectively, and the maximum values were 1.8- and 1.7-fold the minimum values, respectively. The mean seed length, seed width, lateral diameter, and average seed size were 43.0 cm, 29.2 cm, 28.1 cm, and 33.4 cm, respectively, and the maximum values were 1.5-, 1.5-, 1.4-, and 1.3-fold the minimum values, respectively (Table 4). The mean kernel rate was 22.5%, and the maximum value was 1.8- fold the minimum value. The mean seedling height and root collar diameter were 0.9 m and 8.7 mm, respectively, and the maximum values were 2.7- and 2.1-fold the minimum values, respectively. The *CV*s of all traits ranged from 6.4% (average seed size) to 34.2% (root collar diameter), among which the *CV*s of seed length, seed width, lateral diameter, and average seed size were lower and did not surpass 10%, but the *CV*s of seedling height and root collar diameter were higher than 30% (Fig. 1). The broad sense heritability, h^2, for kernel weight (0.56) and kernel rate (0.69) was moderate, and for other traits was high, ranging from 0.87 to 0.95 (Table 5). The mean values of each trait for different populations are shown in Fig. 2. The DBG population showed the largest seed weight (10.7 g ± 0.4 g), seed width (29.8 mm ± 0.4 mm), seedling height (1.1 m ± 0.2 m), and root collar diameter (10.2 mm ± 2.0 mm). The DGJ population showed the largest kernel weight (2.37 g ± 0.15 g), seed length (44.50 mm ± 2.98 mm), lateral diameter (28.66 mm ± 1.76 mm), and

Table 4 ANOVA analysis of different seedling traits of *J. mandshurica*

Traits	Variance source	*df*	MS	*F*	δ^2
SH	Block	4	0.059	2.043	0.030
	Provenance	4	4.984	3.746**	3.686
	Family/Provenance	45	1.331	21.993**	1.270
	Block × Provenance	16	0.190	6.612**	0.162
	Block × Family/Provenance	180	0.060	2.102**	0.032
	Error	1250	0.029		0.029
RCD	Block	4	6.196	0.955	0.106
	Provenance	4	211.726	4.035**	162.335
	Family/Provenance	45	52.478	5.719**	43.302
	Block × Provenance	16	20.510	3.160**	14.420
	Block × Family/Provenance	180	9.176	1.414**	3.086
	Error	1250	6.090		6.090

**Significant at 1% level; SH and RCD stand for seedling height and root collar diameter, respectively.

Table 3 ANOVA analysis of different seed traits of *J. mandshurica*

Traits	MS(*df*)			*F* value	
	Among populations	Within population	Random errors	Among populations	Within population
SWt	20.58(4)	11.75(45)	1.75(450)	1.75	6.73**
KW	1.27(4)	0.41(45)	0.18(450)	3.08*	2.27**
SL	163.78(4)	74.42(45)	9.73(450)	2.20	7.65**
SW	34.27(4)	26.56(45)	3.34(450)	1.29	7.95**
LD	14.96(4)	25.20(45)	2.30(450)	0.59	10.96**
ASS	45.09(4)	21.85(45)	2.48(450)	2.06	8.83**
KR	140.87(4)	46.19(45)	14.38(450)	3.05*	3.21**

*Significant at 5% level and ** significant at 1% level SWt, KW, SL, SW, LD, ASS and KR stand for seed weight, kernel weight, seed length, seed width, lateral s eed diameter, average seed size and kernel rate, respectively.

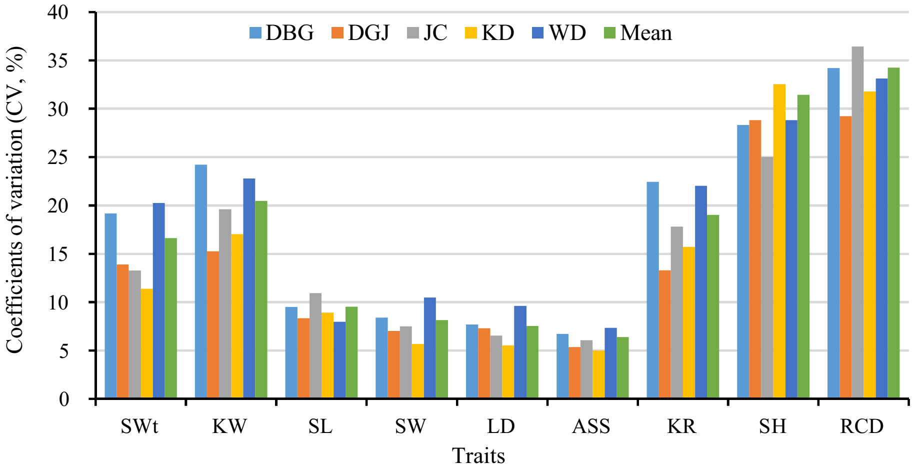

Fig. 1 Coefficients of variation (CV, %) of seed and seedling traits among populations. For traits, SWt, KW, SL, SW, LD, ASS, KR, SH and RCD stand for seed weight, kernel weight, seed length, seed width, lateral seed diameter, average seed size, kernel rate, seedling height, and root collar diameter, respectively

Table 5 Descriptive statistics for seed and seedling traits for *J. mandshurica* families

Traits	Mean	Min	Max	SD	CV	h^2
SWt	10.07	7.74	14.08	1.67	16.61	0.85
KW	2.25	1.79	3.02	0.46	20.45	0.56
SL	42.96	34.88	51.45	4.10	9.54	0.87
SW	29.25	23.15	34.57	2.38	8.15	0.87
LD	28.05	24.67	33.41	2.11	7.53	0.91
ASS	33.42	30.01	38.06	2.14	6.39	0.89
KR	22.47	17.25	31.35	4.27	19.02	0.69
SH	0.94	0.56	1.52	0.29	31.44	0.95
RCD	8.71	6.20	13.14	2.98	34.25	0.83

SWt, KW, SL, SW, LD, ASS, KR, SH and RCD stand for seed weight (g), kernel weight (g), seed length (mm), seed width (mm), lateral seed diameter (mm), average seed size (mm), kernel rate (%), seedling height (m), and root collar diameter (mm) respectively.

average seed size (34.22 mm ± 1.83 mm). The WD population had the highest kernel rate (23.32% ± 3.15%). The KD population showed the lowest seed weight (9.5 g ± 0.7 g), kernel weight (2.1 g ± 0.1 g), seed length (41.39 mm ± 3.24 mm), seed width (28.43 mm ± 0.98 mm), lateral diameter (27.73 mm ± 0.60 mm), average seed size (32.52 mm ± 1.62 mm), and seedling height (0.74 m ± 0.14 m). The DBG population showed the lowest lateral diameter (27.73 mm ± 1.03 mm), and kernel rate (20.5% ± 1.6%), while the JC population had the lowest root collar diameter (8.10 mm ± 0.82 mm).

Phenotypic differentiation among natural populations

The proportion of different variance components to the total variation was analyzed, and phenotypic differentiation coefficients calculated. The variance component among populations accounted for 32.53% of the total variation, the variance component within populations accounted for 33.98% of the total variation, and the random error accounted for 22.62% (Table 6). The variance component for seed length and average seed size among populations was greater than 40% while that of lateral diameter was lower than 20%. The variance component for seed width and lateral diameter within populations was higher than 40%, whereas it was lower than 20% for kernel weight and kernel rate. The phenotypic differentiation coefficient ranged from 21.71% (lateral diameter) to 68.75% (kernel weight), and the mean phenotypic differentiation coefficient of all traits was 50.31% among populations and 49.69% within populations (Fig. 3).

Correlation analysis

The correlation coefficients between different traits are shown in Fig. 4. Seed weight had a significantly positive correlation with kernel weight, seed length, seed width, lateral diameter, and average seed size. Kernel weight was significantly positively correlated with seed length, seed width, average seed size, and kernel rate. Average seed size was significantly positively correlated with seed length, seed width and lateral diameter. The correlation between the seed width and lateral diameter was significant and positive, but seed

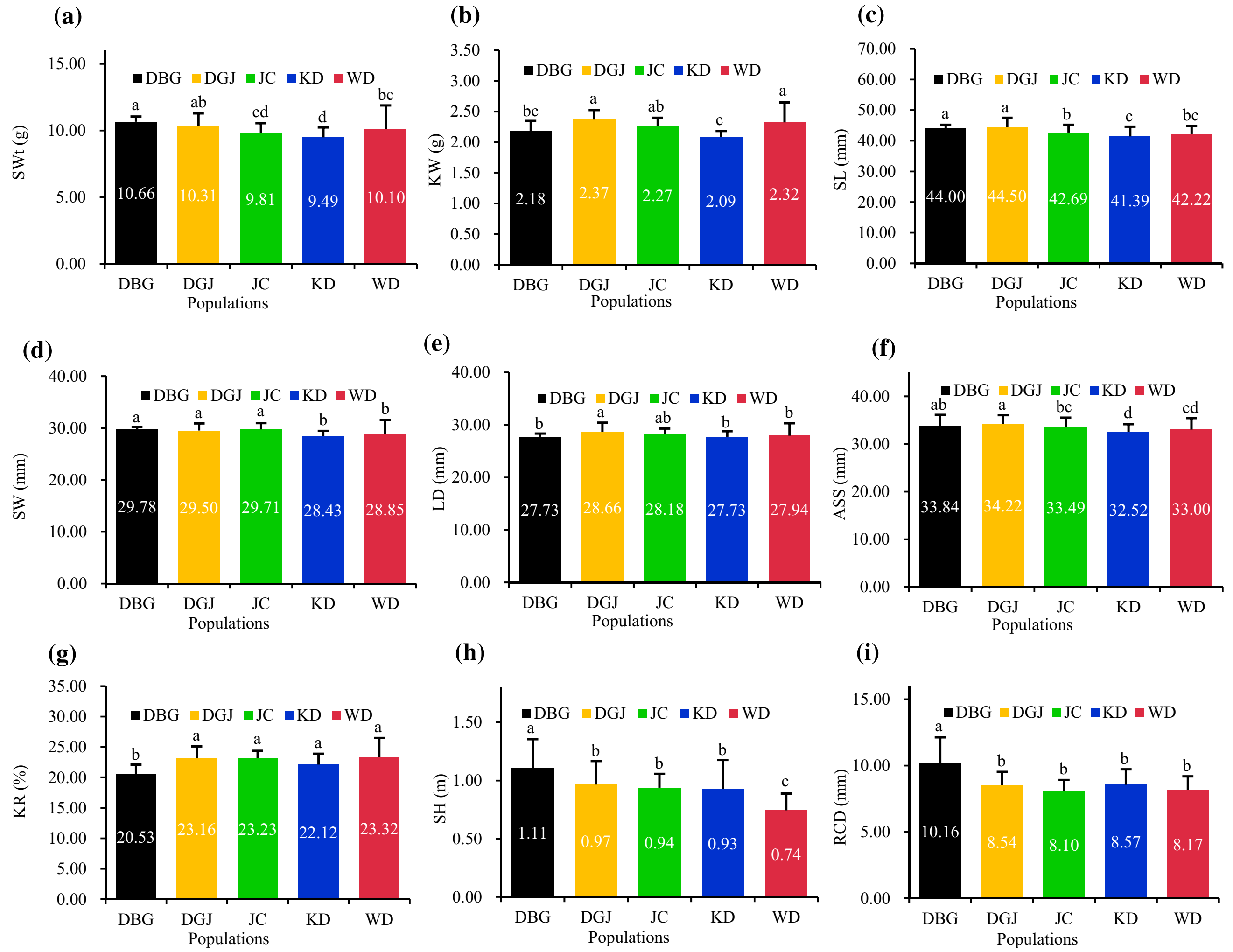

Fig. 2 Mean values of different traits among different populations. Bars followed by the same letter are not significantly different. For traits, SWt, KW, SL, SW, LD, ASS, KR, SH and RCD stand for seed weight, kernel weight, seed length, seed width, lateral seed diameter, average seed size, kernel rate, seedling height, and root collar diameter, respectively

Table 6 Variance components for seed traits among/within populations in *J. mandshurica*

Traits	Variance component			Variance components percentage (%)		
	Among populations ($\delta^2_{t/s}$)	Within populations (δ^2_s)	Random errors (δ^2_{se})	Among populations ($P_{t/s}$)	Within populations (P_s)	Random errors (P_{se})
SWt	1.8838	2.0011	1.7450	33.39	35.52	31.08
KW	0.1085	0.0460	0.1814	32.35	14.71	52.94
SL	15.4046	12.9364	9.7336	40.45	33.99	25.56
SW	3.0927	4.6438	3.3402	27.91	41.92	30.17
LD	1.2659	4.5810	2.2996	15.58	56.20	28.22
ASS	4.2610	3.8744	2.4754	40.15	36.48	23.37
KR	12.6497	6.3628	14.3770	37.89	19.05	43.07
Mean	5.5237	4.9208	4.8789	32.53	33.98	33.49

SWt, KW, SL, SW, LD, ASS, and KR stand for seed weight, kernel weight, seed length, seed width, lateral seed diameter, average seed size, and kernel rate, respectively.

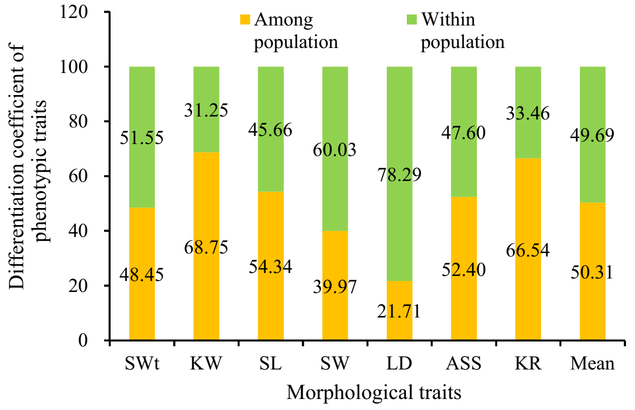

Fig. 3 The differentiation coefficient of morphological traits among/within populations in *J. mandshurica.* For traits, SWt, KW, SL, SW, LD, ASS, and KR stand for seed weight, kernel weight, seed length, seed width, lateral seed diameter, average seed size, and kernel rate, respectively

width was significantly negatively correlated with kernel rate (0.437). There was a weak correlation between average seed size and kernel rate (0.336). Significant positive correlation was also observed between seedling height and root collar diameter (0.784). However, there was no significant correlation between seed and seedling straits.

The correlation coefficients between seed traits and geoclimatic factors are shown in Fig. 5. Kernel weight was significantly negatively correlated with elevation (– 0.889); annual rainfall had a significantly negative correlation with seed length (– 0.906) and average seed size (– 0.910) while there was no significant correlation between other seed traits and ecological factors.

Cluster analysis of different populations

The Between-groups Linkage of Euclidean distance was used to cluster all the traits of five natural populations of *J. mandshurica* (Fig. 6). According to the cluster analysis, the natural population of *J. mandshurica* could be divided into three major clusters when the genetic distance was 7. The first cluster included the population DBG, chracterized by families with large seed weight, seed width, seedling height, and root collar diameter. The second cluster included the population DGJ and this population was characterized by families with large kernel weight, seed length, lateral diameter, average seed size, and kernel rate. The third cluster included JC, WD, and KD populations with characteristics of low seed weight, seed length, seed width, average seed size, seedling height, and root collar diameter.

General combining ability

The general combining ability (GCA) values of different traits among families are shown in Table S1. The GCAs for seed weight ranged from – 2.333 (WD10) to 4.007 (WD11), for kernel weight, from – 0.457 (WD7) to 0.774 (WD11), for seed length, from – 8.078 (KD16) to 8.491 (DGJ4), for seed width, from – 6.104 (WD10) to 5.315 (WD11), for lateral diameter, from – 3.383 (WD10) to 5.360 (WD11), for average seed size from – 3.407 (WD16) to 4.639 (WD11), for kernel rate from – 5.223 (DBG10) to 8.877 (WD10), for seedling height, from – 0.373 (WD8) to 0.585 (KD10), and for root collar diameter, from – 2.505 (WD7) to 4.432 (DBG5).

Fig. 4 Correlation coefficients among different traits in *J. mandshurica* families. *correlation is significant at 5% level; **correlation is significant at 1% level; Numbers are the correlation coefficients among different traits

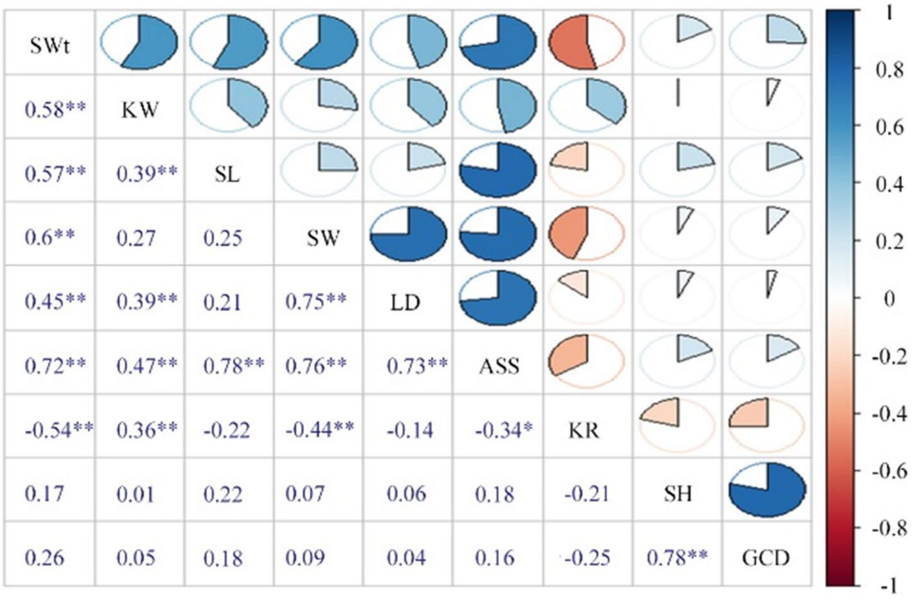

Fig. 5 Correlations between seed traits and geo-climatic factors in *J. mandshurica* families. LOG: Longitude; LAT: Latitude; ELE: Elevation; ATE: Annual temperature; ARA: Annual rainfall; FFD: Frost-free days. *correlation is significant at 5% level; Numbers are the correlation coefficients between seed traits and geo-climatic factors

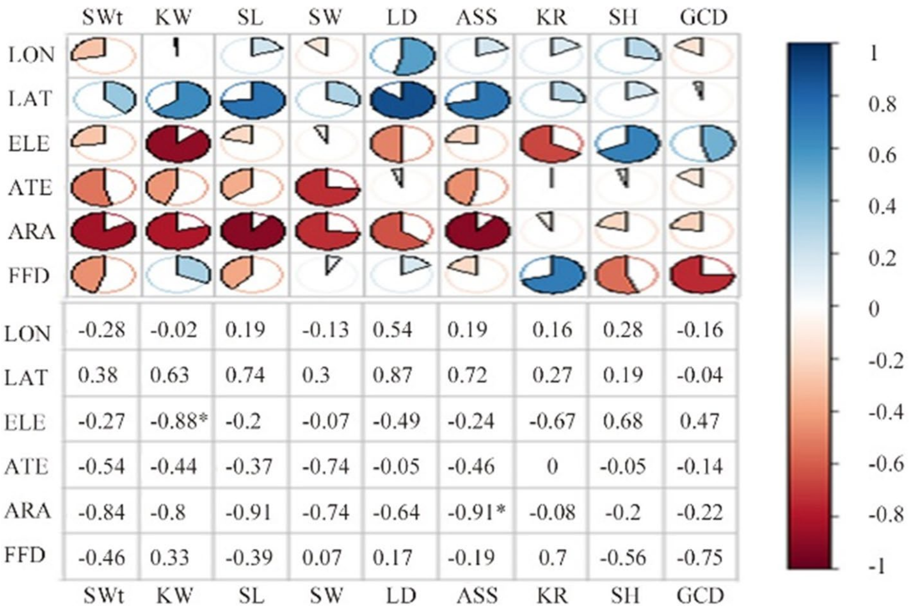

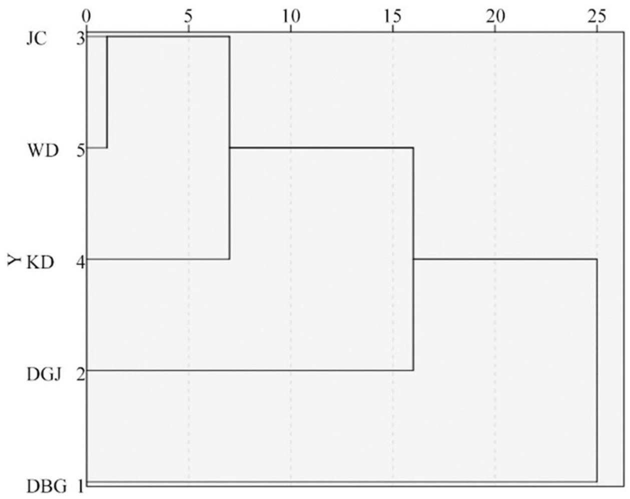

Fig. 6 Cluster analysis of the seed and seedling traits for *J. mandshurica* in different natural populations JC (Jianchang), WD (Wendao), KD (Kuandian), DGJ (Dagujia) and DBG (Dabiangou)

Table 7 Genetic gains for seed weight (SWt), kernel weight (KW), average seed size (ASS), kernel rate (KR), seedling height (SH) and root collar diameter (RCD)

The elite families with good seed performance		The elite families with good seedling performance	
Traits	Genetic gains (%)	Traits	Genetic gains (%)
SWt	12.82	SH	29.72
KW	10.25	RCD	21.05
ASS	4.08		
KR	2.42		

Selection of elite family

Based on the results of correlation analysis, seed weight, kernel weight, average seed size and kernel rate were selected as the comprehensive evaluation indices of seed traits. The comprehensive evaluation value, Q_i, of seed and seedling traits are shown in Table S2. Family WD11 had the largest Q_i value (1.918), followed by families DGJ8 (1.841), DGJ4 (1.824), WD9 (1.795), and JC17 (1.790), while the family WD7 had the lowest Q_i value (1.633). Comprehensive evaluation for seedling traits showed that family DBG5 had the highest Q_i value (1.391), followed by families KD10 (1.370), DBG8 (1.311), DBG7 (1.312) and DBG1 (1.297); the family KD7 had the lowest Q_i value (0.949).

Genetic gains

Based on the Q_i values in Table S1 and a selection rate of 10%, families WD11, DGJ8, DGJ4, WD9, and JC11 were selected as elite families for seed traits, and the genetic gains in seed weight, kernel weight, average seed size and kernel rate of these elite families were 12.82%, 10.25%, 4.08%, and 2.42%, respectively (Table 7). Families DBG5, KD10, DBG8, DBG7 and DBG1 were selected as elite families according to seedling traits, and the genetic gains in seedling height and root collar diameter of these elite families were 29.72% and 21.05%, respectively (Table 7).

Discussion

Genetic variation is the driving force of evolution, enabling organisms to overcome environmental challenges (Cardoso et al. 2015). Thus, assessment of genetic variation within and among populations is an essential process for the effective conservation of forest genetic resources (Papi et al. 2012). In natural environments, different populations often produce significant variations due to the interaction of genetic and environmental diversity (Ming and Gu 2006). In this study, there were significant differences in all seed and seedling traits within-population, while there existed significant differences in kernel weight and kernel rate between populations. The heritability of the seed traits, such as W_{st}, L_s, and W_s were as high as 0.85, 0.87 and 0.87, indicating that the variation of these traits can be stably inherited, which indicates that detecting genetic gain by observing phenotypic variations makes sense. In addition, the variance component of seed traits among and within populations were 32.53% and 33.98%, respectively. Furthermore, the mean phenotypical differentiation coefficients were 50.31% and 49.69%, respectively. This indicates that the genetic differentiation among populations was similar to within populations, and the primary phenotypic sources variation of *J. mandshurica* in natural populations included both among and within populations. The phenotypic coefficients observed in our study were lower than those observed in *Amygdalus mira* (Koehne) Yu et Lu (Wei et al. 2020), *Carya dabieshanensis* M.C Liu & Z.J. Li (Zhang et al. 2020), *Liquidambar formosana* Hance (He et al. 2018), and *Sophora japonica* L. (Sun et al. 2011), but higher than that of some coniferous species (Loha et al. 2009; Li et al. 2013; Chen et al. 2015; Deng et al. 2017). This discrepancy reflects the complexity of genotypes by environment interaction and the universality of phenotypic variation, resulting from a different environmental selection (Aragão et al. 2015). The mean phenotypic differentiation coefficients of kernel weight and kernel rate were more than 60%, which indicates that the phenotypic variation of these two traits mainly came from among populations (Zhang et al. 2008). The diversity variation among populations reflects the population's adaptation in different environments; larger phenotypic differentiation coefficient means wider adaptation range.

In this study, family variation accounted for more than 30% of the total phenotypic variation for seed traits (Table 5), suggesting the presence of adequate genetic variability in the present material, as observed in other studies (Zhang et al. 2014a). Generally, variations in seed size are the results of environmental influences during seed development combined with genetic variability. This study also confirms that seed size was more under genetic influence than environmental influence. This is further confirmed from lower CVs for seed traits than for seedling traits, which were relatively higher compared with studies of Chen et al. (2015) and Zhang et al. (2014b). This in turn indicates that seed traits are more stable than seedling traits that have higher *CV*s, although a high degree of variability in seedling traits has relatively great selection potential.

Heritability reflects the relative role of genetics and environment in the expression of various traits, and is also useful for ranking the importance of each trait in crossbreeding programs (Jaisankar et al. 2014). In this study, the h^2 for of seed weight, seed length, seed width, lateral diameter, average seed size, seedling height and root collar diameter were all higher than 0.8, suggesting the variation in these traits was greatly controlled by genetic factors, and it is more accurate to use these traits as selection indicators. The heritability values found in our study were higher than that observed in *Pinus wallichiana* A. B. Jacks (Singh and Thapliyal 2012), indicating that the selection of elite families is feasible, and the selection intensity can be increased appropriately. Strong and intermediate genetic control is favorable for selection in breeding programs as it allows the use of small numbers of families to achieve high genetic gains (Munthali et al. 2012), providing a reliable guarantee for the genetic improvement of *J. mandshurica*. As a whole, the pattern of genetic variations in seed and seedling traits observed in this study were consistent with previous studies made on several tropical tree species (Zhang et al. 2014b, 2015, 2020; Chen et al. 2015; Xu et al. 2016; Wei et al. 2020).

Strong positive correlations were found between seed weight, kernel weight, and other seed size traits, which was similar to the results by Li et al. (2017). The strong linkage between seed traits could provide a basis for multiple-trait selection (Li et al. 2018). Besides, this study found that a strong negative correlation between kernel rate and seed width and average seed size, which was different from other studies (Xiong et al. 2017; Zhang et al. 2014a; Wang et al. 2012). This could be the reason for some seeds with higher weight and size but lower kernel content due to seed coat thickness. Seeds with increasing thickness during maturation are more influenced by environmental factors during late growth and are prone to measurement errors, which may have contributed to this negative correlation. There was also a significant positive correlation between seedling height and root collar diameter, which was similar to numerous other species (Wu et al. 2018; Bai et al. 2019). In this study, seed phenotypic traits had weak correlation with seedling traits. Similar results were found with *Cordia africana* Lam. (Loha et al. 2006). This suggests that any single trait could not be employed for selecting elite materials for tree improvement programs.

Phenotype differences are the result of interactions between genes and the environment, and environmental factors play crucial roles in shaping plant phenotypes (Nicotra et al. 2010). The strong negative correlation between kernel

weight and elevation in this study was similar to the findings of Diao et al. (2014) for *Sapindus mukorossi* Gaertn. It may be due to decreases in atmospheric pressure and oxygen as elevation increases, affecting seed development of *J. mandshurica* (Hou et al. 2017). Annual rainfall was strongly negatively correlated with seed length and average seed size, contrary to the study of plant communities by Wang (2015). Seed size was significantly negatively correlated with rainfall in deserts and grasslands, while size was significantly positively correlated with rainfall in forest species. This indicates that factors influencing seed size are different in different vegetation types, and the reproductive strategies of plants are also different. The rest of the environmental factors were not significantly correlated with seed traits, indicating that these had less influence on seed traits of *J. mandshurica*, which was also an external manifestation of environmental adaptation in various parts of the seed. In summary, the variation in selected seed traits in natural populations of *J. mandshurica* is correlated with environmental and geographic factors, reflecting a clear trend in geographic variation. This is further evidenced from the cluster analysis in which the natural populations of *J. mandshurica* were divided into the major clusters. The first cluster included the DBG population of Liaoning province with usually the highest seed weights, seed widths, seedling heights and root collar diameters, which could be used to screen elite germplasm resources for breeding improvement of *J. mandshurica*. The second cluster contained population GDJ, with the highest seedling growth; growth in this population was rapid, which could provide materials for the study of fast-growing genotypes for timber production.

Using the general combining ability analysis, family WD11 has the highest value for most traits. The results showed that the additive effect of genes controlled the inheritance of growth traits in this material. General combining ability is an essential parameter in tree improvement programs that reflects the ability of parents to transmit valuable traits to their offspring, and is often used to screen for elite parents or cross combinations to provide theoretical guidance for seed orchard construction (Zhou et al. 2004; Biabani et al. 2012). However, GCA values for different traits varied widely among the families, so it was necessary to use a comprehensive evaluation to choose elite families.

In tree breeding, comprehensive evaluation is a good analytical method for selecting elite families or clones (Liang et al. 2018). There are several methods of comprehensive evaluation, such as the optimal liner breeding value prediction method (White and Hodge 1988). Different methods have their appropriate territoriality, so it is necessary to choose an applicable method for data analysis (Chen et al. 2004). In this study, Q_i values of different *J. mandshurica* families were calculated for multiple-trait comprehensive evaluation, and elite families were selected based on the results of correlation analysis. When families were evaluated according to their seed traits, families WD11, DGJ18, DGJ4, WD9 and JC17 were considered elite (genetic gains in seed weight, kernel weight, average seed size and kernel rate were 12.82%, 10.25%, 4.08% and 2.42%, respectively). Similar results have been reported for *Carya illinoensis* K. Koch (Li et al. 2011). The selected families have better seed characters and economic benefits and are suitable for seed production. When evaluated based on seedling traits, families DBG5, KD10, DBG8, DBG7, and DBG1 were considered elite (genetic gains in seedling height and root collar diameter were 29.72% and 21.25%), higher than the results by Li and Liu (2014). These elite families had good growth performances which could provide superior material for timber production. Breeding objectives determine breeding methods, and *J. mandshurica* is an excellent species for both nut and timber production, and its premium materials should be selected from multiple perspectives. These materials have high potential for improvement, and their genetic quality for major economic traits and their flowering and fruiting selection should be studied in the next step to obtain elite materials with higher yield and quality. Studies have shown that the juvenile period of *J. mandshurica* was representative and that a one-year-old seedling can predict the growth of s six-year-old seedling (Yuan 2013). Therefore, this study has practical implications for early selection using two-year-old seedlings of *J. mandshurica*.

Conclusion

Juglans mandshurica is a species with considerable economic value and has been extensively studied in medicine because of its juglone component, while research on conventional breeding has not been studied as much. Besides, *J mandshurica* is a heterodichogamous plant, which has always been propagated by seedlings and has been wild or semi-wild for a long time, resulting in lower improved varieties and benefits. Coupled with over-logging in natural forests, natural resources of *J. mandshurica* are near depletion. Therefore, within- and between-population variations in seed and seedling traits were analyzed in this study. The results confirmed the existence of good variation in all seed and seedling traits among families within a population. Several families with elite seed traits or seedling traits were selected, and will contribute to the genetic improvement of *J. mandshurica* and the collection, preservation, evaluation, and utilization of germplasm resources.

Author contributions QHZ and XYZ conceived and designed the research. JF conducted the experiments. SHY, QHW and AJL collected data, XNP and YC analyzed the data. QHZ wrote the manuscript. MT

provided expert knowledge and revision of the manuscript. All authors read and approved the manuscript.

References

Aithal KB, Kumar SMR, Rao NB, Udupa N, Rao SBS (2009) Juglone, a naphthoquinone from walnut, exerts cytotoxic and genotoxic effects against cultured melanoma tumor cells. Cell Biol Int 33(10):1039–1049

Aragão FAS, Nunes GHS, Queiróz MA (2015) Genotype x environment interaction of melon families based on fruit quality traits. Crop Breed Appl Biotechnol 15(2):79–86

Bai FY, Kang N, Zhang PD, Kang XY (2019) Selection of female parents with high fertility and high combining abilities for crossbreeding *Populus tomentosa*. J For Res 30(2):445–450

Biabani A, Rafii MY, Saleh G, Shabanimofrad M, Latif MA (2012) Combining ability analysis and evaluation of heterosis in *Jatropha curcas* L. F_1-Hybrids. Aust J Crop Sci 6(6):1030–1036

Cardoso JGR, Andersen MR, Herrgard MJ, Sonnenschein N (2015) Analysis of genetic variation and potential applications in genome-scale metabolic modeling. Front Bioeng Biotech 3(13):1–12

Chen YT, Chen GH, Li MJ (2004) Classification and research progress of comprehensive evaluation methods. J Manag Sci China 7(2):72–82

Chen SY, Yang H, Han J, Zhang DW, Zhao SS, Zhang ZH, Guo ZL, Yang YC (2015) Provenance variation of seed traits of *Juglans mandshurica* in Changbai mountains, northeastern China. J Beijing For Univ 37(12):32–40

Chu XL, Zhu HY, Zhang HG, Zhang L, Zhang Z (2010) Variation among provenances and families of *Juglans mandshurica* and preliminary selection. J Northeast For Univ 38(11):5–6

Deng LL, Zhu X, He RX, Xu YL, Wang DW, Li W, Cai NH (2017) Compare on the cones and seeds phenotypic variations between the different type of trunk types of *Pinus yunnanensis* Franch. Seed 36(3):4–9

Diao SF, Shao WH, Jiang JM, Dong RX, Sun HG (2014) Phenotypic diversity in natural populations of *Sapindus mukorossi* based on fruit and seed traits. Acta Ecol Sin 36(6):1451–1460

Han Q, Zhong CL, Zhang Y, Jiang QB, Chen Y, Chen Z, Khongsak P (2017) Genetic variation and selection of *Casuarina junghuhniana* provenances at Lingao, Hainan. For Res 30(4):595–603

He QH, Yang SZ, Li YG, Shen X, Liu XH (2018) Phenotypic variations in seed and fruit traits of *Liquidambar formosana* populations. Chin J Plant Ecol 42(7):752–763

Hou WH, Wang JL, Dan B, Hu D (2017) Phenotypic correlation analysis of *Hullessbarley* kernel traits form Tibet Plateau region under the condition of different ecological environment. J Nucl Agric Sci 31(10):2063–2071

Jaisankar I, Sankaran M, Singh DR, Damodaran V (2014) Genetic variability and divergence studies in pod and seed traits of *Pongamia pinnata* (L.) Pierre. accessions in Bay Islands. J For Res 25(2):351–358

Ji MF, Zhang XW, Han J, Ding DL (2013) Phenotypic diversity of cone and seed in natural *Pinus tabuliformis* populations in China. Acta Bot Boreali-Occident Sin 33(9):1898–1905

Li YN, Liu HW (2014) Preliminary report of *Juglans mandshurica* provenance. Inner Mongolia Forest Investig Des 37(6):88–89

Li YR, Liu YZ, Zhai M, Wu WL, Pan WB, Du J (2011) A comparative study on nut characteristics varieties and improvement of *Carya illinoensis*. J Jiangsu For Sci Technol 38(3):6–11

Li C, Zhang J, Li H, Zhang XM, Gu YH, Huang KB, Chu C, Zhao Y (2017) Preliminary study on seed traits of *Juglans mandshurica*. Seed 36(1):15–18

Li YL, Dong MM, Chen H, Li Y, Zheng GH, Gu GS, Zheng FY (2018) Variation and selection of important quantitative traits in *Castanea henryi* cultivars. J Southwest For Univ 38(3):36–43

Li SF, Su JR, Liu WD, Lang XD, Zhang ZJ, Su L, Jia CXZ, Yang HJ (2013) Phenotypic variation in cones and natural *Pinus kesiya* var. *langbianensis* populations in Yunnan province, China. Chin J Plant Ecol 37(11):998–1009

Liang DY, Ding CJ, Zhao GH, Leng WW, Zhang M, Qu ZXY (2018) Variation and selection analysis of *Pinus koraiensis* clones in northeast China. J For Res 29(3):611–622

Loha A, Tigabu M, Teketay D, Lundkvist K, Fries A (2006) Provenance variation in seed morphometric traits, germination, and seedling growth of *Cordia africana* Lam. New For 32(1):71–86

Loha A, Tigabu M, Fries A (2009) Genetic variation among and within populations of *Cordia africana* in seed size and germination responses to constant temperatures. Euphytica 165:189–196

Ming J, Gu WC (2006) Phenotypic variation of *Syringa oblata* Lindl. For Res 19(2):199–204

Munilla I, Guitián J (2014) Long-term individual-level variation of reproductive features in *Sorbus aucuparia*, a fleshy-fruited tree. Trees 28(5):1489–1496

Munthali CRY, Chirwa PW, Akinnifesi FK (2012) Genetic variation among and within provenances of *Adansonia digitata* L. (Baobab) in seed germination and seedling growth from selected natural populations in Malawi. Agroforestry Syst 86(3):419–431

Nicotra AB, Atkin OK, Bonser SP, Davidson AM, Finnegan EJ, Mathesius U, Poot P, Purugganan MD, Richards CL, Valladares F, Kleunen M (2010) Plant phenotypic plasticity in a changing climate. Trends Plant Sci 15(12):684–692

Pan YY, Liang DY, Guo J, Wang F, Wang FW, Li SC, Zhao XY (2018) Variance analyses on growth traits of *Larix kaempferi* in different seed sources. J Beijing For Univ 40(11):19–27

Papi RM, Spanos KA, Kyriakidis DA (2012) Genetic variation of *Fraxinus angustifolia* natural populations in Greece based on nuclear and chloroplast microsatellite markers. Eur J For Res 131(4):1151–1161

Silva FFD, Pereira MG, Ramos HCC, Junior PCD, Pereira TNS, Gabriel APC, Viana AP, Ferreguetti GA (2008) Selection and estimation of the genetic gain in segregating generations of papaya (*Carica papaya* L.). Crop Breed Appl Biotechnol 8(1):1–8

Silva-Belmares SY, Saenz-Galindo A, Garcia J, López-López LI, Vaquera Garcia JJ, Saenz-Galindo A, Silva-Belmares SY (2014) Ultrasonic and microwave assisted synthesis of nitrogen-containing derivatives of juglone as potential antibacterial agents. Lett Org Chem 11(8):573–582

Singh O, Thapliyal M (2012) Variation in cone and seed characters in blue pine (*Pinus wallichiana*) across natural distribution in western Himalayas. J For Res 23(2):235–239

Song JX, Li J, Guo C, Zhou YC, Wang H, Zhang LJ (2017) Selection of fruit variation type of *Juglans mandshurca* germplasm resources in the eastern Liaoning mountain region. Mol Plant Breed 15(9):3798–3802

Sun RX, Zhen YQ, Zhang CH, He Y, Zong YC, Yu XD (2011) Study on the seed phenotypic variation of *Sophora japonica* L. in different populations. J Hebei Agr Univ 34(3):65–70

Vardhini SRD (2014) Exploring the antiviral activity of juglone by computational method. J Recept Signal Transduct 34(6):456–457

Wang ZM, Chen JY, Wu ZH (2012) Analysis on nut traits of plus tree selection of cold resistant walnut in Zhaotong city. Seed 32(2):67–70

Wang H, Shi SL, Zhang XY, Zhang L (2016) Determination of general combining ability and estimation of genetic parameters for yield and quality in alfalfa. Acta Pratacul Sin 25(3):126–134

Wang GX (2015) Shift of seed mass spectrum and seed dispersal spectrum along longitude gradient in Inner Mongolia plateau. Master's Thesis of Northwest University

Wei LP, Han YY, Da BQ, Gong WF, Deng GF, Hu J (2020) Analysis on phenotypic variation and germplasm resource selection of wild *Amygdalus mira* in Tibet of southwestern China. J Beijing For Univ 42(7):48–57

White TL, Hodge GR (1988) Best linear prediction of breeding values in a forest tree improvement program. Theor Appl Genet 76(5):719–727

Wu M, Sun ML (2019) Extraction of total anthraquinones from green peel of *Juglans mandshurica* Maxim and antioxidant activity. Forest Eng 35(3):48–53

Wu QK, Wu YL, Yu FY (2018) Variation of seedling growth in different families of *Styrax tonkinensis*. J Nanjing For Univ (Nat Sci Ed) 42(2):191–196

Xiong LQ, Xiao LJ, Geng SX, Luo Y, Ning DL (2017) The difference of high yield and nuts quality of *Juglans sigilltata* cv. Lushui 1 at different elevations. J Southwest For Univ 37(6):30–35

Xu YL, Cai NH, Chen S, Wang DW, Duan AA, Kang XY (2016) Study on the phenotypic differentiation of needle traits. J Southwest For Univ 36(5):1–9

Xu JR (2006) Tree quantitative genetics. Higher Education, Beijing, pp 34–51.

Yu YY (2012) The protein extracted and antihypertensive peptides preparation from the kernel of *Juglans mandshurica* Maxim. Master's Thesis of Northeast Forestry University

Yuan XL, Qi YH, Liu ZL, Zhou ZJ, Mao ZJ (2013) Provenance selection of *Juglans mandshurica* Maxim. and the effects of environmental factors. Bull Bot Res 33(4):468–476

Yuan XL (2013) The early *Juglans mandshurica* Maxim. excellent provenance and pedigree selection and seedling environmental factors impact assessment. Master's Thesis of Northeast Forestry University

Zhang CX, Ming J, Liu C, Zhao HT, Wang CC, Shan HC, Ren JF, Zhou X, Mu D (2008) Phenotypic variation of natural populations in *Lilium regale* Wilson. Acta Hortic Sin 35(8):1183–1188

Zhang HG, Deng JF, Xu YL (2011) Variation regularity among provenances of *Juglans mandshurica* and family selection. J Northwest For Univ 26(2):91–95

Zhang H, Dong LM, Zeng YR, Liu HK, Xia GH (2014a) Phenotypic variation of fresh nuts, seeds, and seedlings traits among open-pollinated families of *Carya cathayensis* from natural population. China Forest Sci Technol 28(3):97–99

Zhang Z, Zhang L, Zhang HG, Liu L, Jiang Y (2014b) Variation in nutrition compositions and morphology characteristics of pine-nuts in Korean pine (*Pinus koraiensis*) seed orchard of Tieli. Bull Bot Res 34(3):356–363

Zhang Z, Zhang HG, Zhou Y, Liu L, Yu HY, Wang X, Feng WJ (2015) Variation of seed characters in Korean pine (*Pinus koraiensis*) multi-clonal populations. J Beijing For Univ 37(2):67–78

Zhang HX, Li AQ, Zhang HG, Zhang L, Zhang JB (2017) *Juglans mandshurica* fruits and seed variation and plus tree selection. J Northeast For Univ 45(3):1–7

Zhang SM, Xi JW, Hong JY, Xia GH, Li Y, Huang XZ, Zhu ZF, Huang JQ (2020) A Study on phenotypic diversity of fruit and leaf traits in *Carya dabieshanensis*. For Res 40(7):48–57

Zhao MJ, Si JH (2016) Phenotypic traits analysis of local walnut. J Qinghai Univ 34(6):20–25

Zhao XY, Xia H, Wang XW, Wang C, Liang DY, Li KL, Liu GF (2016) Variance and stability analyses of growth characters in half-sib *Betula platyphylla* families at three different sites in China. Euphtyica 208(1):173–186

Zhou ZC, Jin GQ, Qin GF, Zhang JM, Luo XH (2004) Analysis on combining ability and heterosis of main economic traits of *Pinus massoniana* for pulp production. Sci Silvae Sin 40(4):51–56

Zhu LG, Zhang J, Xu FC (2018) Variation regularity on 9 families of *Juglans mandshurica* in Heihe. For By-Prod Special China 157(6):45–46.

Publisher's Note Springer Nature remains neutral with regard to jurisdictional claims in published maps and institutional affiliations.
