# Supplementary material for: Provenance and family variations in early growth of Manchurian walnut (Juglans mandshurica Maxim.) and selection of superior families
Source: PLoS One. 2024 Mar 7;19(3):e0298918. doi: 10.1371/journal.pone.0298918 (PMC10919699; doi:10.1371/journal.pone.0298918)
Supplement: S2 File — (ZIP) [file pone.0298918.s005.zip › Xylem Anomalies as Indicators of Maladaptation to Climate in Forest Trees Implications for Assisted Migration.pdf]

frontiers in Plant Science

ORIGINAL RESEARCH
published: 27 February 2020
doi: 10.3389/fpls.2020.00208

Xylem Anomalies as Indicators of Maladaptation to Climate in Forest Trees: Implications for Assisted Migration

Jaime Sebastian-Azcona, Uwe Hacke and Andreas Hamann**

Department of Renewable Resources, University of Alberta, Edmonton, AB, Canada

Xylem anomalies that are caused by unusual climate events have long been used to aid cross-dating in tree ring research. Here, we analyzed a range of xylem anomalies in a 39-year-old common garden experiment of white spruce (*Picea glauca* [Moench] Voss) in central Alberta, Canada, designed to investigate local adaptation. We extracted wood cores from trees representing 24 provenances covering much of the species range across the Canadian boreal forest. Using a double stain and light microscopy analysis, four xylem anomalies and their causes could be distinguished: (1) frost rings indicate issues with synchronizing the onset of growth with the start of the growing season, and were prevalent in young trees; (2) light rings represent thin cell walls caused by an insufficient growing season length, most prevalent in southern sources; (3) blue rings were caused by a failure to complete lignification of new wood due to an early end of the growing season; and (4) double rings represent density fluctuations due to drier than normal summers. Local provenances showed the least amount of xylem anomalies, indicating that they are correctly adapted to the environment in which they occur. In contrast, trees moved to the test site from other climate regions showed various types of xylem anomalies depending on their origin. In particular, populations originating from warmer regions showed an increased presence of latewood anomalies, consistent with a more extensive use of the growing season in the fall. We conclude that xylem anomalies may serve as a sensitive early indicator of maladaptation to climate before populations experience tree dieback or mortality. They may therefore be useful to monitor the health of natural populations, or to evaluate the success of assisted migration in reforestation to address climate change.

Keywords: white spruce, xylem anomalies, tree rings, climate change, provenance trials, assisted migration

OPEN ACCESS

Edited by:
Jian-Guo Huang,
South China Botanical Garden,
Chinese Academy of Sciences, China

Reviewed by:
Shaokang Zhang,
Chinese Academy of Sciences, China
Feng Chen,
Yunnan University, China

****Correspondence:***
Jaime Sebastian-Azcona
jaime4@ualberta.ca
Andreas Hamann
andreas.hamann@ualberta.ca

Specialty section:
This article was submitted to Functional Plant Ecology, a section of the journal Frontiers in Plant Science

Received: *15 May 2019*
Accepted: *11 February 2020*
Published: *27 February 2020*

Citation:
Sebastian-Azcona J, Hacke U and Hamann A (2020) Xylem Anomalies as Indicators of Maladaptation to Climate in Forest Trees: Implications for Assisted Migration. Front. Plant Sci. 11:208. doi: 10.3389/fpls.2020.00208

INTRODUCTION

Much of the biomass of a tree is produced by the vascular cambium. In boreal regions, the period of cambial activity is limited by cold temperatures. Trees must balance the need to fully utilize the short growing season with the risk of exposing the vascular cambium to frost damage. An overly conservative strategy would limit growth; an overly aggressive expansion of the growing season

could lead to frost damage through late spring and early fall frost events (Loehle, 1998; Howe et al., 2003).

As evidenced by tree ring studies, the cambium is not immune to frost damage. "Frost rings" have been found in trees growing near the timberline, in boreal regions, and in Patagonia (Schweingruber et al., 2007; Waito and Conciatori, 2013; Molina et al., 2016). A severe frost ring contains tracheids with thin and unlignified cell walls in the earlywood, followed by a zone of collapsed cells (Glerum and Farrar, 1966; Lee et al., 2007). In addition to frost rings, which are usually found in the earlywood, extreme climate events may also cause xylem anomalies in the latewood (Wimmer, 2002). Among these are light, double, and blue rings (see later). Xylem anomalies such as these have been widely observed in natural populations, but they are usually just used as an aid for cross-dating multiple cores from the same region, or to date notable historic events, such as volcanic eruptions (Bräuning et al., 2016). However, such anomalies may also be useful tools to detect maladaptation of tree populations in the context of climate change. For instance, if certain populations were particularly susceptible to the formation of spring frost rings, then this could indicate that cambial activity begins too early in the spring.

Genetic adaptation of forest trees to local environments is normally studied with provenance trials. These are transplant experiments where seed sources from a range of locations (*provenances*) are planted in a common garden trial with a systematic experimental design to quantify genetic population differentiation (Matyas, 1996). The performance characteristics of tree populations at the test site can be used to guide seed transfer to address climate change (McLachlan et al., 2007; Pedlar et al., 2012; Aitken and Whitlock, 2013).

In this present study, we used a long-term provenance trial in Alberta, Canada to analyze xylem anomalies in 39-year old white spruce (*Picea glauca* [Moench] Voss) trees representing six ecozones. We hypothesized that xylem anomalies can be viewed as symptoms of maladaptation to local growing conditions, and that they can be used as a tool to guide seed transfer in the light of climate change. Specifically, we will interpret xylem anomalies in the light of the climate differences between the source environment of the provenance, and the test environment at the planting site. We expect that subjecting seed sources to new climate conditions will leave signature xylem anomalies that can indicate the type of maladaptation. A broader context of this research is to infer the vulnerability of different tree populations to climate conditions and extreme events when moved to new location in assisted migration efforts, or to monitor the health of natural populations under climate change.

MATERIALS AND METHODS

Plant Material

Samples were collected in a white spruce provenance trial in central Alberta, Canada (55°17′N, 113°10′W). The trial site is located in the central boreal plains of western Canada, and supports a boreal mixedwood ecosystem on a clay-loam gray luvisol soil. The test site climate is cool and dry, with a mean annual temperature of 0.5°C and mean annual precipitation of 491 mm. Additional climate variables are provided in **Supplementary Table S1**. Trees from 43 provenances across the Canadian species range were planted in 1982 as 4-year-old seedlings. The experimental design is a randomized complete block design with five blocks and five-tree row plots planted in each block with a 2.5 × 2.5 m spacing. We selected a subset of 24 provenances, avoiding multiple samples from local clusters of provenances, for anatomical analyses representing six different Canadian ecozones (**Figure 1** and **Supplementary Table S1**). We extracted a wood core from one tree of average height and DBH from each row plot, i.e. one core per block and provenance of the sampling design. Five cores did not have the sufficient quality for anatomical thin sections, resulting in a total of 115 cores analyzed.

Section Preparation

To study the presence of climate-induced xylem anomalies, we obtained thin sections from tree cores to be analyzed with light microscopy. Tree cores of 5 mm diameter were taken from each selected tree approximately 50 cm above ground with an increment borer from bark to pith, covering as much of the tree life history as possible. Samples were collected during the summer of 2017, with 2016 being the last complete year used for analyses. Tree cores were air dried immediately after collection. After 3 months of storage, the cores were sanded with progressively finer grit sizes (P240, P320, and P800) for a complementary ring width study (Sang et al., 2019). The cores were split in approximately 5 cm long segments to facilitate sectioning the whole core without breaking the section. We made sure that the cut was at the transition zone between two rings so that the accurate dating of the rings in each segment would be possible. Then sections of 15–20 μm thickness were obtained using a GSL-1 microtome (Gärtner et al., 2014). For every core, we discarded the first few sections to avoid sampling tissue that could have been damage by the sanding process. The selected sections were stained using a 1:1 mix of safranin and astra blue solution (Gärtner and Schweingruber, 2013). The safranin solution contained 0.8 g of safranin in 100 ml of distilled water, and the astra blue solution consisted of 0.5 g of astra blue dissolved in 100 ml of distilled water and 2 ml acetic acid. The double stain was applied for 5 min. Samples were then washed with distilled water, followed by 70 and 99% ethanol. Finally, the stained samples were mounted for microscopy in glycerol. The sections were analyzed within a month of their preparation so a more permanent mounting media was not considered necessary.

Tree Ring Analyses

The presence of frost, light, blue and double rings was analyzed using a light microscope (Leica DM3000) at 100x and 200x magnifications. Frost rings were classified according to their intensity (low, medium and severe) and position in the ring, initial, and spring as indicated in **Figures 2a,b**. Low intensity frost rings consisted of only a few rows of tracheids that were slightly deformed (**Figure 2a**), while

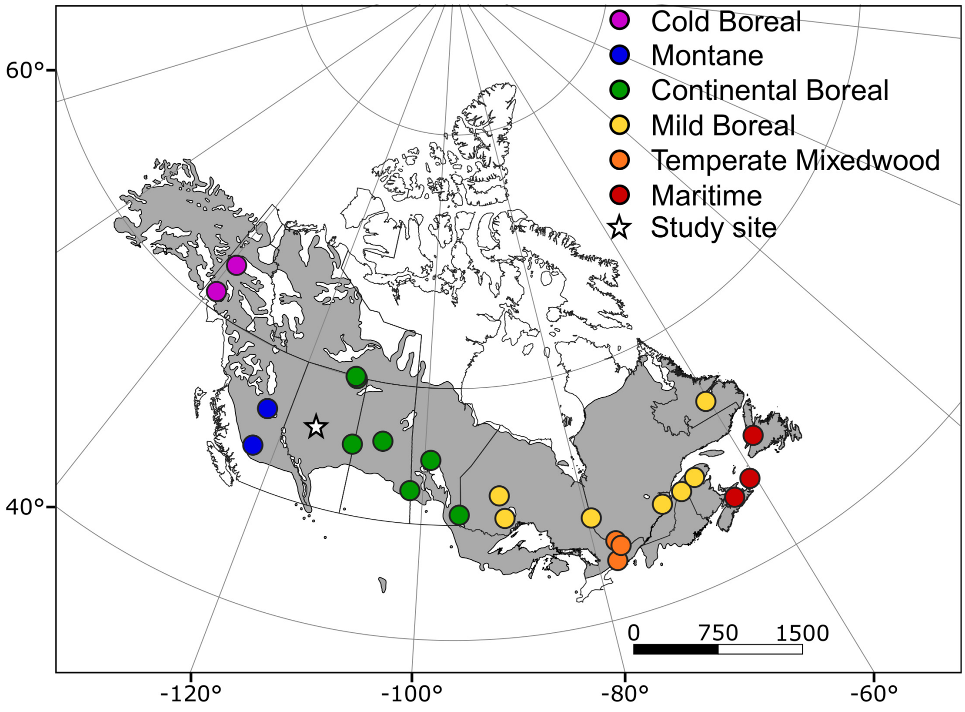

FIGURE 1 | Locations of origin of the different provenances included in this study (circles) and plantation site (star). Trees were planted in 1982 as 4-year-old seedlings. The distribution of white spruce across North America is displayed in gray.

medium intensity frost rings had several rows with clearly damaged tracheids. Severe frost rings showed extremely distorted tracheids usually preceded by unlignified (blue) cells (**Figure 2b**). The position depended on whether the damaged xylem was observed within the first three tracheid rows of the year (*initial* frost rings), likely caused by damage in the cambium prior to the onset of xylem formation (**Figure 2a**), or after the third tracheid row (*spring* frost rings) which corresponds to damage induce by a late spring frost event (**Figure 2b**).

The severity of blue rings was evaluated with three intensity categories: low intensity rings consisted of patches of unlignified (blue stained) cells in the last few tracheid rows, medium intensity rings had their last one or two tracheid rows completely unlignified and the severe blue rings had three or more unlignified tracheid rows. For light and double rings, we assigned either low or severe intensity, according to how clear and unambiguously they could be identified under the microscope. For consistency, this score was assigned by the same observer for all cores.

Climate Data

All the climate data used in this study was generated by the software ClimateNA v5.21 (Wang et al., 2016). To explore the relationship between the occurrence of the different ring anomalies and the climate of that year, monthly, seasonal and yearly variables were extracted for the duration of the trial, from 1982 to 2016. To analyze the association of the climate of origin of the different provenances, we used climate normal data for the 1961–1990 period, a common reference period for the climate condition prior to a strong anthropogenic warming signal. The climate variables used in this study were: mean annual temperature (MAT), mean warmest month temperature (MWMT), mean coldest month temperature (MCMT), mean annual precipitation (MAP), climate moisture index calculated as MAP minus the Hargreaves reference evaporation (CMI), the length of the frost-free period (FFP), day of year at which the frost-free period begins (bFFP) and ends (eFFP). We also included latitude as a proxy for the day length regime at the origin of the provenance.

Statistical Analyses

To investigate differences among populations, we used generalized linear mixed models. Dependent variables were the average presence ratio of xylem anomalies, as well as the intensity of xylem anomalies. We chose the binomial family to represent the error distribution of the presence ratio, and a Poisson distribution to represent the error of the intensity data. Ecozones, representing different populations (**Figure 1**), were treated as fixed effects, and blocks and provenance within ecozones were treated as random effects. We used the functions *glmer* and *lmer* from the package *lme4* (Bates et al., 2015) for the R programming environment (R Core Team, 2017). Least square means of ecozones and provenances were extracted from the mixed model

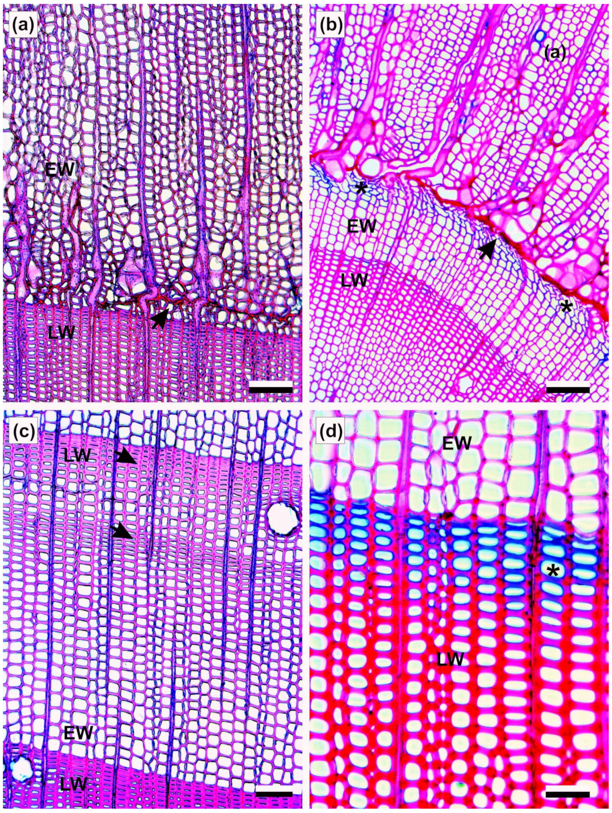

FIGURE 2 | Sections of tree rings showing different xylem anomalies. The safranin-astra blue double stain distinguishes between lignified (red) and unlignified cells (blue). **(a)** The arrow points to a low intensity frost ring right at the start of the tree ring (Initial frost ring), showing tracheids and ray parenchyma cells with abnormal shapes in the early wood. **(b)** The arrow points to a high intensity frost ring occurring during the growing season (Spring frost ring). The severe spring frost damage stopped the lignification of some tracheids (stars) and induced the collapse and malformation of tracheids and parenchyma cells. **(c)** Double ring with two bands of latewood-like tracheids (labeled by arrows) caused by dry conditions in late summer and a subsequent spike in precipitation. **(d)** Blue ring showing unlignified cells in the latewood of a tree ring due to low temperatures. Scale bars in **(a–c)** are 100 and 50 μm in **(d)**. EW = early wood, LW = late wood.

result object with the *emmeans* function of the *emmeans* package (Lent, 2016). Statistical pairwise comparisons among ecozones with a Tukey adjustment for multiple inference were implemented with the *CLD* function, also included with the *emmeans* package.

Associations of monthly climate variables at the planting site with xylem anomalies were analyzed with a generalized linear mixed model, where block and provenance were random effects. To facilitate the interpretation of these models, we calculated a marginal pseudo-R^2 with the r.*squaredGLMM* function the *MuMIn* package (Barton, 2018). The pseudo-R^2 represents the deviance explained by the fixed effects of the model, which in this case are the monthly climate variables.

Associations of climate normal variables at the provenance origins with xylem anomalies were analyzed with Pearson correlations and fitting linear regressions. Square root or logarithmic transformations were performed to correct non-linear relationships. Correction for multiple comparisons was performed using the Holm's method implemented with the *p.adjust* function of the R base package.

RESULTS

How Common Are Xylem Anomalies at the Site and Are Young Trees More Susceptible to Their Formation Than Older Trees?

Our samples included all four types of xylem irregularities (**Figures 2**, **3**). Of the xylem anomalies studied, frost rings were the most common; they were present in 13.7% of all tree rings. Almost all frost rings were formed prior to 2000 (**Figure 3**); i.e. in the first decade of a tree's life, while trees were small. Anatomically, frost rings consisted of severely deformed tracheids and bent rays in the earlywood. In some cases, tracheid walls were stained blue, indicating a low degree of lignification in these cell walls. Many cells were abnormally enlarged. These frost wounds were located at different distances from the growth ring border (**Figures 2a,b**), depending on the time of the frost event.

Light rings were detected in 7.6% of the rings, followed by double rings with a 4.2% occurrence rate. Light and double rings appeared throughout the lifetime of the trees (**Figure 3**), i.e. their formation was independent of a tree's age or size. Light rings had latewood tracheids with thinner cell walls than in normal rings. Double rings were characterized by two latewood bands per rings, interrupted by a layer of wider earlywood-like tracheids.

The least common type of xylem anomaly were blue rings. We detected blue rings in only 1.6% of the rings. In all of these cases they occurred in rings formed prior to 2001. Blue rings were characterized by continuous rows of latewood tracheids whose walls were stained blue (**Figure 2d**). The shape or cell wall thickness of these cells did not necessarily differ from "normal" (red stained) tracheids in their proximity.

What Climate Factors Trigger the Formation of Xylem Anomalies?

We next studied the climate variables at the common garden site that could be linked with the occurrence of xylem anomalies. The formation of frost rings was generally linked to spring temperatures (March to May), but the formation of initial *versus* spring frost rings was caused by unique temperature patterns (**Figure 4**). Initial frost rings were associated to cold minimum temperatures in March (pseudo-R^2 = 0.12, $p < 0.001$). By contrast, spring frost rings were associated with *mild* temperatures in April (R^2 = 0.38, $p < 0.001$) and May (**Figure 4**).

As shown in **Figures 4**, **5**, a high percentage of light rings was formed in years that were characterized by cold temperatures at the beginning *and* end of the growing season (see large pink circles in **Figure 5** for 1996, 2000, and 2004).

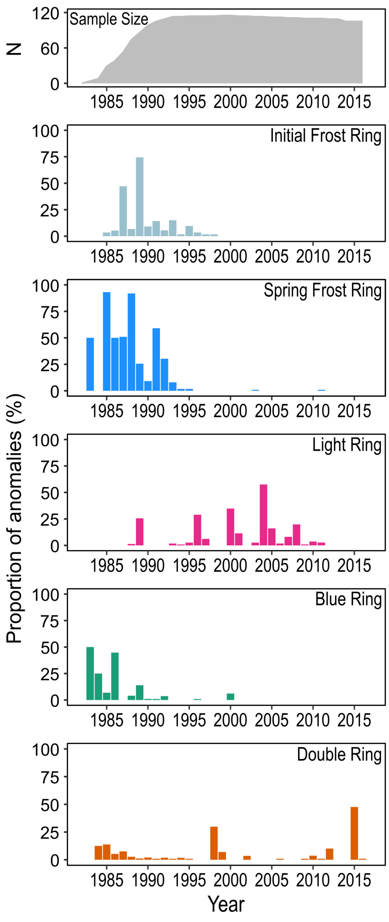

FIGURE 3 | Proportion of tree ring anomalies (initial and spring frost rings, light rings, blue rings, and double rings) formed in 1983–2016. The gray area in the top figure shows the sample size for each year. Sample size was reduced closer to the tree pith because the corer often missed the earliest rings close to the pith.

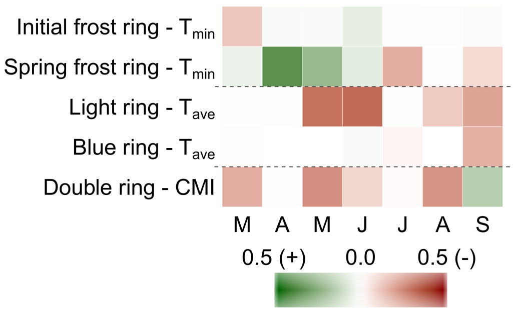

FIGURE 4 | Relationship between the intensity of xylem anomalies and monthly climate variables at the common garden site. The values represent the marginal pseudo-R^2 of the generalized linear mixed models of the climate variables that better explained the occurrence of the different anomalies (minimum temperatures for initial and spring frost rings, average temperatures for light and blue rings and climate moisture index for double rings). The symbol indicates whether the relationship is positive or negative. Capital letters represent months for the period from March to September.

A cold beginning of the growing season alone (e.g. 2009 in **Figure 5A**) or cold end of the growing season alone (e.g. 1992 in **Figure 5B**) did not induce the formation of light rings. The monthly variables that were most related to the presence of light rings were high average temperatures in May (R^2 = 0.30, p < 0.001), June (R^2 = 0.31, p < 0.001), and September (R^2 = 0.19, p < 0.001).

Blue rings were associated with cool temperatures in September (R^2 = 0.17, p < 0.001); dry conditions in August also contributed to their formation (R^2 = 0.12, p < 0.001). Finally, the formation of double rings was favored by warm and dry summers characterized by highly negative climate moisture indices, combined with a sudden increase in precipitation in September. The highest percentage of double rings was observed in 1998 and 2015 (**Figure 3**); years with a very dry summer causing the formation of a narrow band of latewood tracheids, followed by a sudden increase in precipitation in September, which led to the transient formation of wider tracheids (**Figures 2c**, **4**).

Do Provenances From Different Ecozones Differ in Their Susceptibility to Xylem Anomalies, and If So, Can These Differences Be Linked With the Native Climate of the Provenances?

To infer local adaptations, we investigated differences in the frequency of the xylem anomalies in provenances representing six ecozones across Canada (**Table 1**). Provenances native to the ecozone in which the study site was located (Continental Boreal) showed a high resistance to all types of anomalies. The other ecozones showed contrasting vulnerabilities to the different anomalies. Provenances from the most northern ecozone (Cold Boreal) were the most vulnerable to spring frosts, but had very low instances of light, double, and blue rings. Provenances originating from southern ecozones showed a relatively low percentage of frost rings, but were susceptible to other xylem anomalies. Specifically, provenances from the Temperate Mixedwood and the Mild Boreal ecozones showed the highest proportion of light rings, while trees from the humid Maritime ecozone had the highest occurrence of blue and double rings (**Table 1**).

Correlations between the intensity of xylem anomalies and the climate of the source location of the provenance can aid the interpretation of how populations are adapted to local climate conditions. The latitude of the seed sources was negatively correlated with the intensity of light, blue, and double rings (**Table 2**). Mean annual temperature and the end of the frost free period were positively correlated with these latewood anomalies, meaning that these anomalies were more prevalent in provenances from regions with warm and long growing seasons. Provenances that showed a higher percentage of blue rings usually came from areas with a late end of the growing season, relatively mild winters and high precipitation levels (**Table 2**).

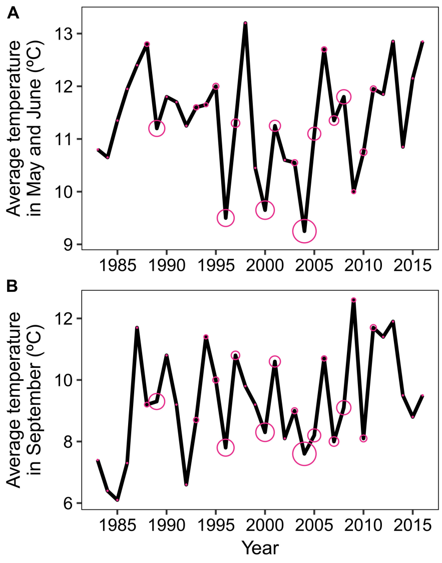

FIGURE 5 | The formation of light rings was linked to low temperatures at the beginning and end of the growing season. The graph shows average temperatures in May and June **(A)** and September **(B)**. The pink circles represent the proportion of light rings in each year, from 0% (e.g. 1985) to 58% (2004). The three years with the highest presence of light rings (1996, 2000, and 2004) correspond to the three coldest May and June temperatures of the study period and below-average September temperatures.

DISCUSSION

Frost Rings

We detected a range of xylem anomalies in the common garden trial, including frost rings. These were mostly related to temperatures between March and June, depending on the position of the frost ring. Initial frost rings that appeared right at the beginning of the tree ring were associated with low temperatures in March, i.e. before the beginning of the growing season. This indicates that the cambium can be damaged by a severe frost before the tree starts that year's growth, so the very first cells produced by the tree will be damaged.

Spring frost rings on the other hand were related to *high* temperatures in April and May. We suggest that these higher temperatures will advance the start of the growing season for that year, making trees more vulnerable to a late spring frost. A similar observation was made by Montwé et al. (2018) who reported that a "false spring" in April contributed to the formation of these frost rings in *Pinus contorta* trees.

Frost rings appeared almost exclusively in the first 15 years after planting although favorable climate conditions for their formation persisted in subsequent decades. This agrees with previous reports that frost rings are often restricted to smaller trees and stems characterized by small diameters and thin bark (Gurskaya and Shiyatov, 2006; Waito and Conciatori, 2013; Molina et al., 2016). A possible explanation for the higher resistance of bigger trees to frost damage is that thicker stems, especially if accompanied by a thicker bark, are able to accumulate more heat so that they can maintain temperatures above freezing in the stem even if outside temperatures drop below 0°C (Molina et al., 2016). In addition, a stand comprised of small saplings will experience more dynamic temperature variations than a closed stand formed by larger trees (Krasowski and Simpson, 2001).

Latewood Anomalies

Light and double rings formed in response to low temperatures and dry conditions, respectively. Low temperatures at the beginning of the growing season can limit carbon assimilation reducing the amount of resources available for xylogenesis, while low temperatures at the end of the growing season may restrict carbon mobilization and deposition rates in the cell wall (Deslauriers et al., 2009; Castagneri et al., 2017). Double rings appear when there is an environmental constraint to xylem formation within the growing season leading to the formation of a band of latewood-like cells. This constraint is usually caused by summer droughts (Hoffer and Tardif, 2009; De Micco et al., 2016). Trees that are able to expand their growing season for a longer period can take advantage of the good growing conditions in late summer and form a double ring (Pacheco et al., 2015). The fact that these xylem anomalies were more frequent in provenances originating from lower latitudes (**Table 2** and **Supplementary Figure S1**) is consistent with a more extensive use of the growing season and a later onset of cold hardiness in these trees at our study site (Sebastian-Azcona et al., 2019).

Blue rings are formed as a result of incomplete lignification. As such, rings can be linked with low temperatures at the end of the growing season (Gindl et al., 2000; Donaldson, 2001), but lignification is also affected by other factors including water availability (Donaldson, 2002). Piermattei et al. (2015) observed

TABLE 1 | The frequency (in percent) of tree ring anomalies in trees representing six Canadian ecozones.

Population	Initial frost ring	Spring frost ring	Light ring	Blue ring	Double ring
Cold Boreal	6.2 (1.4)[ab]	12.8 (2.0)[a]	1.8 (0.8)[a]	0.0 (0.0)[abc]	1.1 (0.6)[a]
Montane	10.3 (2.0)[b]	8.6 (1.8)[a]	6.4 (1.6)[abc]	0.9 (0.6)[abc]	2.5 (1.0)[ab]
Continental Boreal	3.2 (0.6)[a]	7.9 (0.9)[a]	5.7 (0.8)[ab]	0.3 (0.2)[a]	3.3 (0.6)[a]
Mild Boreal	4.7 (0.7)[a]	8.2 (0.9)[a]	9.7 (1.1)[c]	1.6 (0.4)[ab]	4.5 (0.6)[ab]
Temperate Mixedwood	5.6 (1.1)[ab]	7.4 (1.2)[a]	11.0 (1.6)[c]	3.1 (0.8)[bc]	5.2 (1.0)[ab]
Maritime	5.8 (1.2)[ab]	10.6 (1.6)[a]	7.2 (1.4)[bc]	4.7 (1.1)[c]	7.0 (1.3)[b]

Standard errors of the estimates are given in parenthesis. Mean estimates for ecozones with the same letter were not significantly different at $p < 0.05$, using a Tukey adjustment for multiple comparisons.

TABLE 2 | Pearson correlation between the intensity of different tree ring disturbances and the climate of origin of the seed sources.

	Initial frost ring	Spring frost ring	Light ring	Blue ring	Double ring
Latitude	−0.13	0.30	**−0.77**	**−0.78**	**−0.68**
Mean annual temperature	0.24	−0.20	**0.61**	**0.75**	**0.57**
Mean warmest month temperature	−0.32	−0.28	0.53	0.45	0.53
Mean coldest month temperature	0.44	0.23	0.35	**0.72**	0.43
Mean annual precipitation	0.30	0.01	0.51	**0.72**	−0.45
Beginning of frost free period	0.29	0.42	−0.35	−0.38	−0.39
End of frost free period	0.02	−0.22	**0.62**	**0.69**	**0.59**
Frost free period	−0.12	−0.30	0.54	**0.59**	0.55

Significant correlations with $p < 0.05$ after a Holms adjustment for multiple comparisons are indicated in bold.

a high proportion of blue rings in years with low temperatures at the end of the growing season. In our study, the occurrence of blue rings was correlated with low temperatures in September and low precipitation in August.

The percentage of blue rings in our study was lower than in studies on *Pinus contorta* (Montwé et al., 2018; 23% blue rings) and *Pinus nigra* (Piermattei et al., 2015). This may be related to differences in the "life strategies" of pines and spruces (sensu Cuny et al., 2012), and to the inherently short growing season at our boreal planting site. Similar to light and double rings, blue rings were more intense in provenances from southern latitudes. However, blue rings were also more intense in provenances coming from wetter regions, suggesting that a lack of adaptation to drought might favor the formation of blue rings. Our finding that blue rings were primarily formed in the first 10 years of the trees' life agrees with the data of Montwé et al. (2018), who observed significantly lower severity of blue rings with increasing diameter. Montwé et al. (2018) suggested that this "may be due to their smaller size and lower heat absorption, . . ., and their proximity to the soil surface, which is colder due to radiative cooling."

CONCLUSION

The analysis supports that xylem anomalies can serve as an early indicator of maladaptation to climate before populations experience dieback or mortality. Local provenances collected near the planting site showed the least overall amount of xylem anomalies. This indicates that they are correctly adapted to the environment in which they occur, and that xylem anomalies should be at a minimum when planting stock is correctly matched to their optimal growing conditions.

By contrast, provenances from southern regions with longer and warmer growing conditions showed an increased presence of latewood anomalies. These southern populations showed thin and unlignified cell walls in years when cold temperatures arrived before they could finish the last stages of xylem formation, indicating a potential maladaptation to the shorter growing seasons in the study site. When such anomalies are observed in assisted migration schemes to address climate change, it would indicate that seed sources have been moved too far poleward or upward in elevation.

Xylem anomalies may also be useful to monitor the health of natural populations. For example, in this study double-rings were an early sign of growth cessation due to summer drought observed in populations adapted to cooler and wetter environments. Under climate change toward warmer and drier conditions, this xylem anomaly would serve as an early warning sign of maladaptation before dieback and tree mortality occur.

DATA AVAILABILITY STATEMENT

The raw data supporting the conclusions of this article will be made available by the authors, without undue reservation, to any qualified researcher.

AUTHOR CONTRIBUTIONS

AH, UH, and JS-A conceived the study and designed the methodology. JS-A collected the data and led the writing of the manuscript. All authors analyzed the data and contributed to draft versions of the manuscript.

FUNDING

Funding was provided by the Natural Sciences and Engineering Research Council of Canada (NSERC) with an NSERC Strategic grant STPGP-494071 to AH and UH and an NSERC Discovery grant to UH.

ACKNOWLEDGMENTS

We are grateful to Dante Castellanos Acuña for assistance with field work. This study would not have been possible without generous in-kind contributions and sharing of provenance trial measurements by our supporting organization, the Government of Alberta.

SUPPLEMENTARY MATERIAL

The Supplementary Material for this article can be found online at: https://www.frontiersin.org/articles/10.3389/fpls.2020.00208/full#supplementary-material

REFERENCES

Aitken, S. N., and Whitlock, M. C. (2013). Assisted gene flow to facilitate local adaptation to climate change. *Ann. Rev. Ecol. Evol. Syst.* 44, 367–388.

Barton, K. (2018). *MuMIn: Multi-Model Inference. R Package Version 0.12.2/r18.*

Bates, D., Mächler, M., Bolker, B., and Walker, S. (2015). Fitting linear mixed-effects models using lme4. *J. Statist. Softw.* 67, 1–48.

Bräuning, A., De Ridder, M., Zafirov, N., García-González, I., Dimitrov, D. P., and Gärtner, H. (2016). Tree-ring features: indicators of extreme event impacts. *IAWA J.* 37, 206–231.

Castagneri, D., Fonti, P., von Arx, G., and Carrer, M. (2017). How does climate influence xylem morphogenesis over the growing season? Insights from long-term intra-ring anatomy in Picea abies. *Ann. Bot.* 119, 1011–1020. doi: 10.1093/aob/mcw274

Cuny, H. E., Rathgeber, C. B. K., Lebourgeois, F., Fortin, M., and Fournier, M. (2012). Life strategies in intra-annual dynamics of wood formation: example of three conifer species in a temperate forest in north-east France. *Tree Physiol.* 32, 612–625. doi: 10.1093/treephys/tps039

De Micco, V., Campelo, F., De Luis, M., Bräuning, A., Grabner, M., Battipaglia, G., et al. (2016). Intra-annual density fluctuations in tree rings: how, when, where, and why? *IAWA J.* 37, 232–259.

Deslauriers, A., Giovannelli, A., Rossi, S., Castro, G., Fragnelli, G., and Traversi, L. (2009). Intra-annual cambial activity and carbon availability in stem of poplar. *Tree Physiol.* 29, 1223–1235. doi: 10.1093/treephys/tpp061

Donaldson, L. A. (2001). Lignification and lignin topochemistry—an ultrastructural view. *Phytochemistry* 57, 859–873.

Donaldson, L. A. (2002). Abnormal lignin distribution in wood from severely drought stressed Pinus radiata trees. *IAWA J.* 23, 161–178.

Gärtner, H., Lucchinetti, S., and Schweingruber, F. H. (2014). New perspectives for wood anatomical analysis in dendrosciences: the GSL1-microtome. *Dendrochronologia* 32, 47–51.

Gärtner, H., and Schweingruber, F. H. (2013). *Microscopic Preparation Techniques for Plant Stem Analysis*. Remagen: Verlag Dr. Kessel.

Gindl, W., Grabner, M., and Wimmer, R. (2000). The influence of temperature on latewood lignin content in treeline Norway spruce compared with maximum density and ring width. *Trees* 14, 409–414.

Glerum, C., and Farrar, J. L. (1966). Frost ring formation in the stems of some coniferous species. *Can. J. Bot.* 44, 879–886.

Gurskaya, M. A., and Shiyatov, S. G. (2006). Distribution of frost injuries in the wood of conifers. *Russ. J. Ecol.* 37, 7–12.

Hoffer, M., and Tardif, J. C. (2009). False rings in jack pine and black spruce trees from eastern Manitoba as indicators of dry summers. *Can. J. Forest Res.* 39, 1722–1736.

Howe, G. T., Aitken, S. N., Neale, D. B., Jermstad, K. D., Wheeler, N. C., and Chen, T. H. H. (2003). From genotype to phenotype: unraveling the complexities of cold adaptation in forest trees. *Can. J. Bot.* 81, 1247–1266.

Krasowski, M. J., and Simpson, D. G. (2001). "Frost-related problems in the establishment of coniferous forests," in *Conifer Cold Hardiness*, eds F. J. Bigras, and S. J. Colombo, (Dordrecht: Springer), 253–285.

Lee, K. H., Singh, A. P., and Kim, Y. S. (2007). Cellular characteristics of a traumatic frost ring in the secondary xylem of *Pinus radiata*. *Trees* 21, 403–410.

Lent, R. V. (2016). Least-squares means: the R package lsmeans. *J. Statist. Softw.* 69, 1–33.

Loehle, C. (1998). Height growth rate tradeoffs determine northern and southern range limits for trees. *J. Biogeogr.* 25, 735–742.

Matyas, C. (1996). Climatic adaptation of trees: rediscovering provenance tests. *Euphytica* 92, 45–54.

McLachlan, J. S., Hellmann, J. J., and Schwartz, M. W. (2007). A framework for debate of assisted migration in an era of climate change. *Conserv. Biol.* 21, 297–302.

Molina, J. G. A., Hadad, M. A., Domínguez, D. P., and Roig, F. A. (2016). Tree age and bark thickness as traits linked to frost ring probability on *Araucaria araucana* trees in northern Patagonia. *Dendrochronologia* 37, 116–125.

Montwé, D., Isaac-Renton, M., Hamann, A., and Spiecker, H. (2018). Cold adaptation recorded in tree rings highlights risks associated with climate change and assisted migration. *Nat. Commun.* 9:1574. doi: 10.1038/s41467-018-04039-5

Pacheco, A., Camarero, J. J., and Carrer, M. (2015). Linking wood anatomy and xylogenesis allows pinpointing of climate and drought influences on growth of coexisting conifers in continental Mediterranean climate. *Tree Physiol.* 36, 502–512. doi: 10.1093/treephys/tpv125

Pedlar, J. H., McKenney, D. W., Aubin, I., Beardmore, T., Beaulieu, J., Iverson, L., et al. (2012). Placing forestry in the assisted migration debate. *Bioscience* 62, 835–842.

Piermattei, A., Crivellaro, A., Carrer, M., and Urbinati, C. (2015). The "blue ring": anatomy and formation hypothesis of a new tree-ring anomaly in conifers. *Trees* 29, 613–620.

R Core Team, (2017). *R: A Language and Environment for Statistical Computing*. Vienna: R Foundation for Statistical Computing.

Sang, Z., Sebastian-Azcona, J., Hamann, A., Menzel, A., and Hacke, U. (2019). Adaptive limitations of white spruce populations to drought imply vulnerability to climate change in its western range. *Evol. Appl.* 12, 1850–1860. doi: 10.1111/eva.12845

Schweingruber, F. H., Börner, A., and Schulze, E.-D. (2007). *Atlas of Woody Plant Stems: Evolution, Structure, and Environmental Modifications*. Berlin: Springer.

Sebastian-Azcona, J., Hamann, A., Hacke, U. G., and Rweyongeza, D. (2019). Survival, growth and cold hardiness tradeoffs in white spruce populations: implications for assisted migration. *Forest Ecol. Manag.* 433, 544–552.

Waito, J., and Conciatori, F. (2013). Frost rings and white earlywood rings in *Picea mariana* trees from the boreal plains, central Canada. *IAWA J.* 34, 71–87.

Wang, T., Hamann, A., Spittlehouse, D., and Carroll, C. (2016). Locally downscaled and spatially customizable climate data for historical and future periods for North America. *PLoS One* 11:e0156720. doi: 10.1371/journal.pone.0156720

Wimmer, R. (2002). Wood anatomical features in tree-rings as indicators of environmental change. *Dendrochronologia* 20, 21–36. doi: 10.1093/treephys/tpy061

Conflict of Interest: The authors declare that the research was conducted in the absence of any commercial or financial relationships that could be construed as a potential conflict of interest.

Copyright © 2020 Sebastian-Azcona, Hacke and Hamann. This is an open-access article distributed under the terms of the Creative Commons Attribution License (CC BY). The use, distribution or reproduction in other forums is permitted, provided the original author(s) and the copyright owner(s) are credited and that the original publication in this journal is cited, in accordance with accepted academic practice. No use, distribution or reproduction is permitted which does not comply with these terms.
